# Global, regional, and national disability-adjusted life-years (DALYs) for 359 diseases and injuries and healthy life expectancy (HALE) for 195 countries and territories, 1990–2017: a systematic analysis for the Global Burden of Disease Study 2017

**DOI:** 10.1016/S0140-6736(18)32335-3

**Published:** 2018-11-10

**Authors:** GBD 2017 DALYs and HALE Collaborators

## Abstract

**Background:**

How long one lives, how many years of life are spent in good and poor health, and how the population’s state of health and leading causes of disability change over time all have implications for policy, planning, and provision of services. We comparatively assessed the patterns and trends of healthy life expectancy (HALE), which quantifies the number of years of life expected to be lived in good health, and the complementary measure of disability-adjusted life-years (DALYs), a composite measure of disease burden capturing both premature mortality and prevalence and severity of ill health, for 359 diseases and injuries for 195 countries and territories over the past 28 years.

**Methods:**

We used data for age-specific mortality rates, years of life lost (YLLs) due to premature mortality, and years lived with disability (YLDs) from the Global Burden of Diseases, Injuries, and Risk Factors Study (GBD) 2017 to calculate HALE and DALYs from 1990 to 2017. We calculated HALE using age-specific mortality rates and YLDs per capita for each location, age, sex, and year. We calculated DALYs for 359 causes as the sum of YLLs and YLDs. We assessed how observed HALE and DALYs differed by country and sex from expected trends based on Socio-demographic Index (SDI). We also analysed HALE by decomposing years of life gained into years spent in good health and in poor health, between 1990 and 2017, and extra years lived by females compared with males.

**Findings:**

Globally, from 1990 to 2017, life expectancy at birth increased by 7·4 years (95% uncertainty interval 7·1–7·8), from 65·6 years (65·3–65·8) in 1990 to 73·0 years (72·7–73·3) in 2017. The increase in years of life varied from 5·1 years (5·0–5·3) in high SDI countries to 12·0 years (11·3–12·8) in low SDI countries. Of the additional years of life expected at birth, 26·3% (20·1–33·1) were expected to be spent in poor health in high SDI countries compared with 11·7% (8·8–15·1) in low-middle SDI countries. HALE at birth increased by 6·3 years (5·9–6·7), from 57·0 years (54·6–59·1) in 1990 to 63·3 years (60·5–65·7) in 2017. The increase varied from 3·8 years (3·4–4·1) in high SDI countries to 10·5 years (9·8–11·2) in low SDI countries. Even larger variations in HALE than these were observed between countries, ranging from 1·0 year (0·4–1·7) in Saint Vincent and the Grenadines (62·4 years [59·9–64·7] in 1990 to 63·5 years [60·9–65·8] in 2017) to 23·7 years (21·9–25·6) in Eritrea (30·7 years [28·9–32·2] in 1990 to 54·4 years [51·5–57·1] in 2017). In most countries, the increase in HALE was smaller than the increase in overall life expectancy, indicating more years lived in poor health. In 180 of 195 countries and territories, females were expected to live longer than males in 2017, with extra years lived varying from 1·4 years (0·6–2·3) in Algeria to 11·9 years (10·9–12·9) in Ukraine. Of the extra years gained, the proportion spent in poor health varied largely across countries, with less than 20% of additional years spent in poor health in Bosnia and Herzegovina, Burundi, and Slovakia, whereas in Bahrain all the extra years were spent in poor health. In 2017, the highest estimate of HALE at birth was in Singapore for both females (75·8 years [72·4–78·7]) and males (72·6 years [69·8–75·0]) and the lowest estimates were in Central African Republic (47·0 years [43·7–50·2] for females and 42·8 years [40·1–45·6] for males). Globally, in 2017, the five leading causes of DALYs were neonatal disorders, ischaemic heart disease, stroke, lower respiratory infections, and chronic obstructive pulmonary disease. Between 1990 and 2017, age-standardised DALY rates decreased by 41·3% (38·8–43·5) for communicable diseases and by 49·8% (47·9–51·6) for neonatal disorders. For non-communicable diseases, global DALYs increased by 40·1% (36·8–43·0), although age-standardised DALY rates decreased by 18·1% (16·0–20·2).

**Interpretation:**

With increasing life expectancy in most countries, the question of whether the additional years of life gained are spent in good health or poor health has been increasingly relevant because of the potential policy implications, such as health-care provisions and extending retirement ages. In some locations, a large proportion of those additional years are spent in poor health. Large inequalities in HALE and disease burden exist across countries in different SDI quintiles and between sexes. The burden of disabling conditions has serious implications for health system planning and health-related expenditures. Despite the progress made in reducing the burden of communicable diseases and neonatal disorders in low SDI countries, the speed of this progress could be increased by scaling up proven interventions. The global trends among non-communicable diseases indicate that more effort is needed to maximise HALE, such as risk prevention and attention to upstream determinants of health.

**Funding:**

Bill & Melinda Gates Foundation.

## Introduction

Understanding global trends in the health status of populations and changes in the leading causes of disease burden over time is crucial to tracking progress towards the Sustainable Development Goal to ensure healthy lives and promote wellbeing for all at all ages.^1^ Robust assessment of these trends requires objective and comparable measures of population health that can help countries identify priorities and address challenges to achieving this goal. The Global Burden of Diseases, Injuries, and Risk Factors Study (GBD) 2017, the third annual update in the series, uses all available up-to-date epidemiological data and improved standardised methods to provide a comparative assessment of health loss across 359 diseases and injuries and 73 age and sex groups for 195 countries and territories. The availability of GBD 2017 data for years of life lost (YLLs) because of premature mortality and years lived with disability (YLDs) provides an opportunity to assess trends in population health over the past 28 years by analysing two complementary summary measures: healthy life expectancy (HALE), which quantifies the number of years expected to be lived in good health, and disability-adjusted life-years (DALYs), which quantifies the health loss due to specific diseases and injuries. HALE provides a snapshot of overall population health and DALYs are useful for quantifying and ranking disease burden due to specific causes. DALYs can be utilised to help decision makers and the public understand the leading causes of health burden and whether improvement occurs over time.

The continuing trend of increasing life expectancy and decreasing mortality because of improvements in living conditions, income per capita, education, and medical practices is well known and understood.^2^–^5^ Previous GBD papers have reported that increases in HALE have been slower than increases in life expectancy, resulting in more years of poor health, and suggesting an absolute expansion of morbidity.^6^–^9^ However, details of how many of the additional years of life gained are spent in good health versus poor health across countries and sociodemographic groups have not been well characterised. As people live longer, such information becomes increasingly relevant for policy development, health systems planning, and resource allocation, the effects of which cannot be understated for population health. The estimates herein provide insight into the importance of access to services and appropriate health care, and the potential societal burden of caregiving and excess health-care expenditure for years lived in poor health.^10^

In this study, we present GBD 2017 results for HALE and DALYs by age and sex from 1990 to 2017 for 195 countries and territories. GBD 2017 includes new morbidity and mortality data (epidemiological surveillance data, disease registry data, scientific literature sources, survey sources, verbal autopsy studies, vital registration systems, cancer registries, complete birth history sources, summary birth history sources, and sibling history surveys); refined methods; and new estimations at the subnational level for Ethiopia, Iran, Norway, and Russia, and stratified by ethnicity for New Zealand. Also, the disaggregation of larger cause categories (eg, diabetes) has allowed separate estimation for several additional diseases (eg, type 1 and type 2 diabetes). GBD 2017 provides a complete reanalysis of all available data by country from 1990 to 2017, and thus supersedes all previously published GBD estimations of HALE and DALYs.

## Methods

### Overview

The GBD study comprehensively and systematically quantifies the comparative magnitude of health loss due to diseases and injuries by age, sex, and location over time. We estimated all-cause and cause-specific mortality using the following key principles: identification of all data sources that are available, assessment of the quality of the data and correction for known bias, application of highly standardised analytical procedures, and assessment of model performance using cross-validation analysis. We used similar principles to identify, enhance comparability, and analyse data to estimate the incidence, prevalence, and YLDs of diseases and injuries.^7^ Using the GBD 2017 results for YLLs and YLDs, we calculated DALYs for 359 diseases and injuries.^11^,^12^ We used age-specific mortality and YLDs per person to calculate HALE, defined as the average number of years that a person at a given age can expect to live in good health, taking into account mortality and loss of functional health.^13^ Additional details for computing HALE can be found in appendix 1. We calculated years lived in poor health (ie, years lived with functional health loss) as life expectancy minus HALE. Estimations for GBD 2017 cover the period 1990 to 2017 for 195 countries and territories. We did analyses using Python versions 2.7.12 and 2.7.3, Stata version 13.1, and R version 3.2.2.

For this study, we followed the Guidelines for Accurate and Transparent Health Estimates Reporting (GATHER),^14^ which include recommendations on documentation of data sources, estimation methods, and statistical analysis (appendix 1). Interactive online tools are available to explore GBD 2017 data sources in detail using our online sourcing tool, the Global Health Data Exchange. Data before and after adjustments and the fit of the model to the data for causes of death and non-fatal outcomes can be explored with the available data visualisation tool.

### Cause and location hierarchies

In GBD 2017, as in previous GBDs, causes of mortality and morbidity are structured using a four-level classification hierarchy to produce results that are mutually exclusive and collectively exhaustive. GBD 2017 estimates 359 causes of DALYs, 77 of which are a source of disability but not a cause of death (eg, attention-deficit hyperactivity disorder, headache disorders, low back pain, and neck pain), and five of which are causes of death but not sources of morbidity (sudden infant death syndrome, aortic aneurysm, late maternal deaths, indirect maternal deaths, and maternal deaths aggravated by HIV/AIDS). In the GBD hierarchy, the number of mutually exclusive and collectively exhaustive fatal and non-fatal causes in each level for which GBD estimates is three at Level 1, 22 at Level 2, 169 at Level 3, and 293 at Level 4. The full GBD cause hierarchy, including corresponding International Classification of Diseases (ICD)-9 and ICD-10 codes and detailed cause-specific methods, is in GBD 2017 publications on cause-specific mortality^11^ and non-fatal health outcomes^12^ in the corresponding appendices.

GBD 2017 includes 195 countries and territories that are grouped into 21 regions on the basis of epidemiological similarities and geographical proximity.^15^ For the purposes of statistical analyses, we further grouped regions into seven super-regions (central Europe, eastern Europe, and central Asia; high income; Latin America and Caribbean; north Africa and Middle East; south Asia; southeast Asia, east Asia and Oceania; and sub-Saharan Africa). Each year, GBD includes subnational analyses for a few new countries and continues to provide subnational estimates for countries that were added in previous cycles. Subnational estimation in GBD 2017 includes five new countries (Ethiopia, Iran, New Zealand, Norway, and Russia) and countries previously estimated at subnational levels (GBD 2013: China, Mexico, and the UK [regional level]; GBD 2015: Brazil, India, Japan, Kenya, South Africa, Sweden, and the USA; and GBD 2016: Indonesia and the UK [local government authority level]). All analyses are at the first level of administrative organisation within each country except for New Zealand (by Māori ethnicity), Sweden (by Stockholm and non-Stockholm), and the UK (by local government authorites). All subnational estimates for these countries were incorporated into model development and evaluation as part of GBD 2017. To meet data use requirements, we present all subnational estimates excluding those pending publication (Brazil, India, Japan, Kenya, Mexico, Sweden, the UK, and the USA); these results are presented in appendix tables and figures (appendix 2). Subnational estimates for countries with populations larger than 200 million people (as measured according to our most recent year of published estimates) that have not yet been published elsewhere are presented wherever estimates are illustrated with maps but are not included in data tables.

### Estimation of mortality and non-fatal health loss

We estimated age-specific mortality using data from vital registration systems, sample registration systems, household surveys, censuses, and demographic surveillance sites.^13^ We estimated cause-specific mortality and YLLs using the GBD cause of death database, composed of vital registration and verbal autopsy data, survey and census data for injuries and maternal mortality, surveillance data for maternal and child mortality, cancer registries, and police records for interpersonal violence and road injuries.^11^ The quality and comparability of the cause of death data were evaluated and improved through several steps, including adjustment of data from vital registration systems for incompleteness, conversion of causes found in the original data to the GBD 2017 cause list, and redistribution of deaths assigned to ICD codes that cannot be underlying causes of death. Detailed methods for each step are available in the appendix of the GBD 2017 causes of death paper.^11^ We estimated cause-specific mortality using standardised modelling processes, most commonly the Cause of Death Ensemble model (CODEm), which uses a covariate selection algorithm to generate several plausible combinations of covariates that are then run through four model classes—namely, mixed effects linear models and spatiotemporal Gaussian process regression models for cause fractions and death rates. For a given cause, we categorised covariates into three groups on the basis of the following criteria: evidence of proximal or causal association (Level 1), strong evidence for an association but without adequate evidence of a causal link (Level 2), and covariates that are distal in the causal pathway and therefore might be mediated by other factors in Levels 1 or 2 (Level 3).^16^ The programme then selects an ensemble of models that performs best on out-of-sample predictive validity tests for each cause of death. Ensemble models have been shown to produce smaller errors in estimated cause-specific mortality and more accurate trends than single-component models.^16^ Additional detail, including model specifications and data availability for each cause-specific model, can be found in the appendices of the GBD 2017 causes of death^11^ and mortality^13^ publications. We calculated YLLs from the sum of each death multiplied by the standard life expectancy at each age. The standard life expectancy was taken from the lowest observed risk of death for each 5-year age group in all populations greater than 5 million people. For consistency across all fatal and non-fatal estimates in GBD 2017, we calculated our own population and fertility estimates.^17^ We then used the GBD world population age standard to calculate age-standardised rates for cause-specific deaths and YLLs. The GBD world population age standard and the standard life expectancies are available in the appendix of the GBD 2017 mortality publication.^13^

Changes we have implemented since GBD 2016 for cause-specific mortality include the addition of important sources of new mortality data (detailed at the beginning of this section) and the expansion of the GBD location hierarchy, refinements in the calculation of Socio-demographic Index (SDI), and disaggregation of specific causes into subgroupings to provide additional detail. We estimated the following specific causes separately for the first time: invasive non-typhoidal salmonella disease; liver cancer due to non-alcoholic steatohepatitis (NASH); cirrhosis due to non-alcoholic steatohepatitis; myelodysplastic, myeloproliferative, and other haemopoietic neoplasms; benign and in-situ intestinal neoplasms; benign and in-situ cervical and uterine neoplasms; other benign and in-situ neoplasms; subarachnoid haemorrhage; non-rheumatic valvular heart disease; non-rheumatic calcific aortic valve disease; non-rheumatic degenerative mitral valve disease; other non-rheumatic valve diseases; gastro-oesophageal reflux disease; type 1 diabetes; type 2 diabetes; chronic kidney disease due to type 1 diabetes; chronic kidney disease due to type 2 diabetes; poisoning by carbon monoxide; and poisoning by other means. Specific data sources are available in the appendices of the GBD 2017 non-fatal diseases and injuries^12^ and causes of death^11^ publications. Additional information on data sources used can be found in our online source tool.

For estimation of non-fatal health loss, we most commonly used the Bayesian meta-regression tool DisMod-MR 2.1, which synthesises variable data sources to produce internally consistent estimates of incidence, prevalence, remission, and excess mortality.^18^ If DisMod-MR 2.1 did not capture the complexity of the disease, or if incidence and prevalence needed to be calculated from other data, we used custom models; detailed methods for each cause are in the appendices of the GBD 2017 non-fatal diseases and injuries publication.^12^

We estimated each non-fatal sequela separately and assessed the occurrence of comorbidity for each age group, sex, location, and year separately using a micro-simulation framework.^12^ Disability estimated for co-morbid conditions was distributed to each contributing cause during the comorbidity estimation process. Although the distribution of sequelae and the severity and cumulative disability per case of a condition might be different by age, sex, location, and year, previous studies have found that disability weights do not substantially vary between locations, income per capita, or levels of educational attainment.^19^,^20^ Additional details, including model specifications, data availability, data adjustments to enhance comparability for each cause-specific model, and the development of disability weights by cause and their use in the estimation of non-fatal health loss, are available in the appendices of the GBD 2017 non-fatal diseases and injuries publication.^12^

### Estimation of DALYs, HALE, and corresponding uncertainty

To calculate HALE, we used the following inputs from GBD 2017: age-specific mortality rates; estimates of the prevalence of sequelae by age, sex, location, and year; and disability weights for all unique health states. We used the method originally developed by Sullivan^21^ to estimate HALE (appendix 1). We calculated DALYs as the sum of YLLs^11^ and YLDs^12^ for each location, year, age group, and cause, by sex.

We calculated 95% uncertainty intervals (UIs) on the basis of 1000 draws from the posterior distribution of each step in the estimation process using the 2·5th and 97·5th percentiles of the ordered 1000 values. We attributed the uncertainty associated with estimation of mortality and YLLs to multiple sources, including sample size variability in data sources, adjustment and standardisation methods applied to data, and model specifications. We attributed the uncertainty associated with estimation of YLDs to sampling error of data inputs, adjustment and standardisation methods applied to data, the uncertainty in coefficients from model fit, and the uncertainty of severity distributions and disability weights.

### Estimation of SDI and expected DALYs and HALE on the basis of SDI

The SDI is the geometric mean of three rescaled components: total fertility rate under age 25 years (ie, the number of births expected per woman aged 10–24 years), lag-distributed income per capita, and average educational attainment in populations aged 15 years or older. The methods we used to calculate the SDI are in appendix 1. SDI scores were scaled from 0 (lowest income, fewest years of schooling, and highest fertility) to 1 (highest income, most years of schooling, and lowest fertility). We estimated the association between SDI and cause-specific mortality using a generalised additive model with a Loess smoother on SDI; we then used this association to calculate expected YLLs. Expected YLDs were calculated on the basis of the relationship between SDI and YLD rates. We then calculated expected DALYs as the sum of expected YLLs and YLDs, and expected HALE using expected YLDs and expected life tables. All results are available both in appendix 2 and through our online visualisation tool.

### Role of the funding source

The funder of the study had no role in study design, data collection, data analysis, data interpretation, or writing of the report. All authors had full access to all the data in the study and had final responsibility for the decision to submit for publication.

## Results

### Levels and trends in life expectancy and HALE at birth

Globally, life expectancy at birth for both sexes combined increased by 7·4 years (95% UI 7·1–7·8), rising from 65·6 years (65·3–65·8) in 1990 to 73·0 years (72·7–73·3) in 2017 (appendix 2). The increase in life expectancy at birth was 7·3 years (6·9–7·7) for males (from 63·2 years [62·9–63·4] in 1990 to 70·5 years [70·1–70·8] in 2017) and 7·5 years (7·2–7·9) for females (from 68·0 years [67·8–68·3] in 1990 to 75·6 years [75·3–75·9] in 2017; table 1). During the same period, global HALE at birth for both sexes combined increased by 6·3 years (5·9–6·7), from 57·0 years (54·6–59·1) in 1990 to 63·3 years (60·5–65·7) in 2017 (appendix 2). The increase in HALE at birth was 6·2 years (5·8–6·7) for males (from 55·6 years [53·5–57·5] in 1990 to 61·8 years [59·4–64·0] in 2017) and 6·4 years (6·0–6·8) for females (from 58·4 years [55·7–60·8] in 1990 to 64·8 years [61·7–67·4] in 2017; table 1). The number of years lived in poor health from birth increased globally by 1·1 years (95% UI 0·9–1·4), in the low SDI countries by 1·5 years (1·2–1·9), and in the high SDI countries by 1·3 years (1·0–1·7; appendix 2).

**Figure 1:**
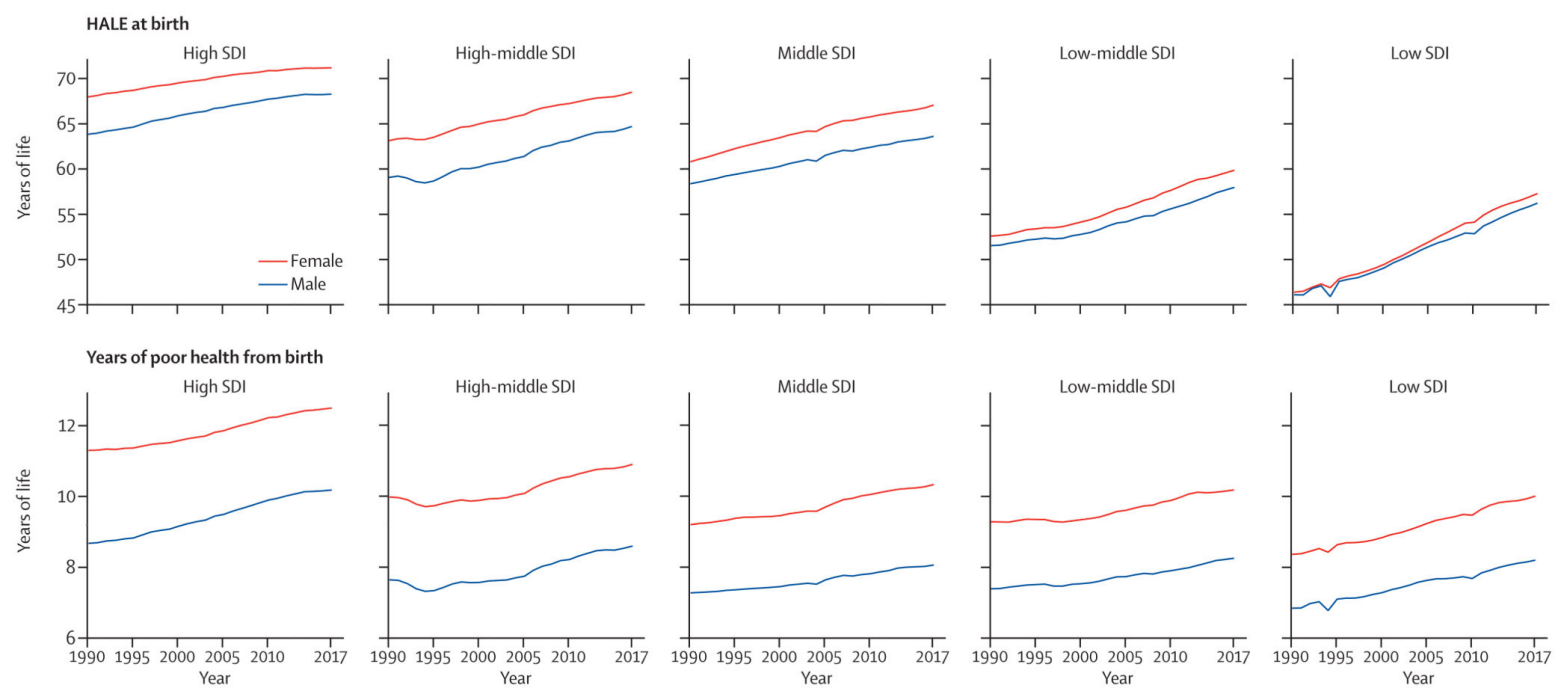
Trends of HALE at birth and years in poor health from birth by SDI quintile and sex, 1990–2017 HALE=healthy life expectancy. SDI=Socio-demographic Index.

The increases from 1990 to 2017 in life expectancy and HALE varied across SDI quintiles and countries, with the greatest increases in life expectancy and HALE seen in the low SDI quintile and the smallest increases seen in the high SDI quintile. The increase in life expectancy varied from 5·1 years (5·0–5·3) in high SDI countries to 12·0 years (11·3–12·8) in low SDI countries. HALE increased for both sexes in low SDI countries by 10·5 years (95% UI 9·8–11·2; by 10·1 years [9·3–10·9] for males and by 10·9 years [10·1–11·7] for females), and in high SDI countries by 3·8 years (3·4–4·1; by 4·4 years [4·0–4·8] for males and by 3·2 years [2·9–3·5] for females; table 1, figure 1; appendix 2). Increases in HALE in the middle SDI quintiles for both sexes follow a gradient, with increases of 6·8 years (6·3–7·4) for low-middle SDI, 5·7 years (5·3–6·2) for middle SDI, and 5·5 years (5·1–5·8) for high-middle SDI (figure 1; appendix 2).

**Figure 2:**
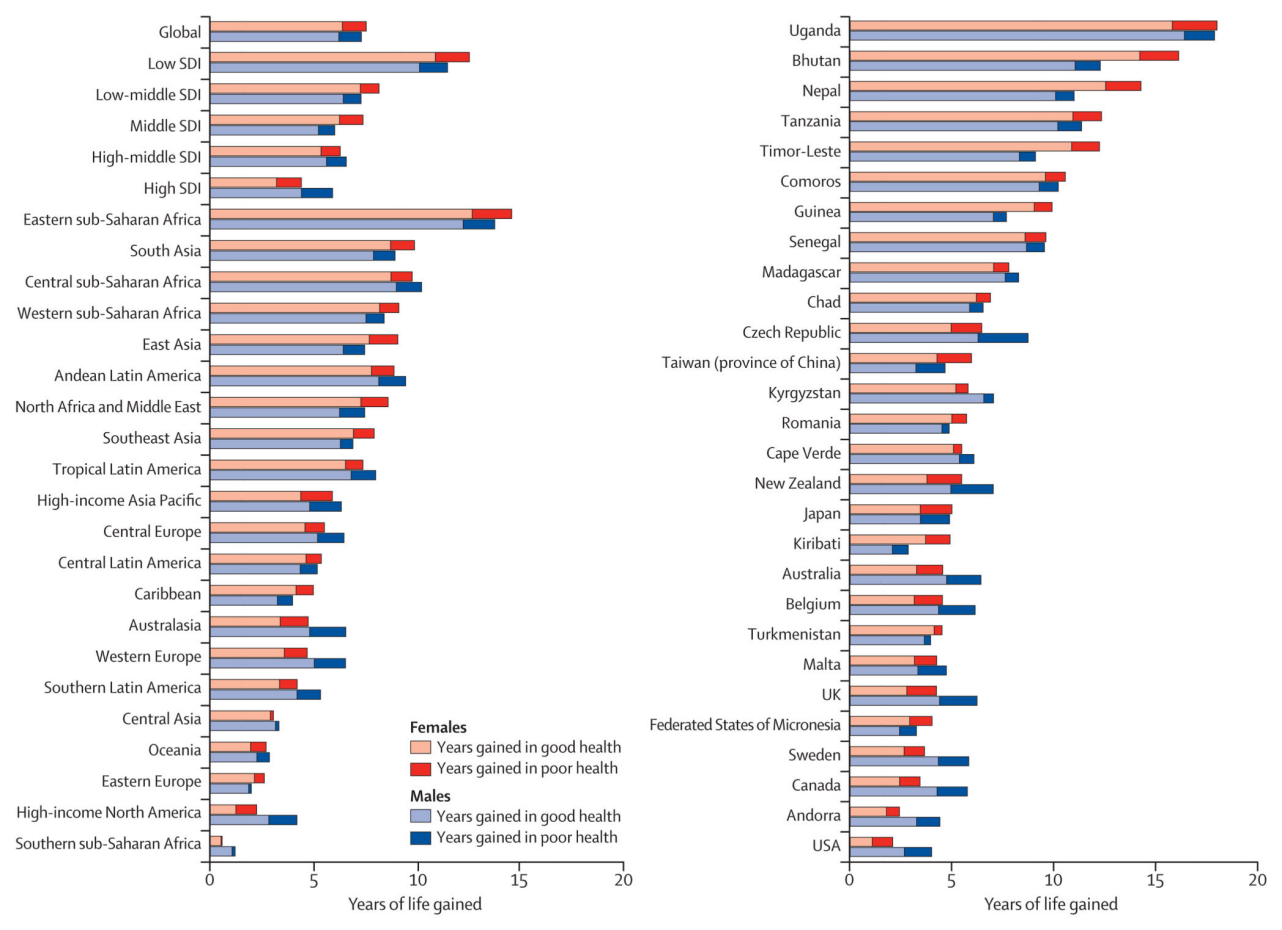
Years of life gained at birth by sex and functional health status for five SDI quintiles, 21 GBD regions, and 28 countries with the largest and smallest proportions of years spent in poor health between 1990 and 2017 GBD=Global Burden of Diseases, Injuries, and Risk Factors Study. SDI=Socio-demographic Index.

**Figure 3:**
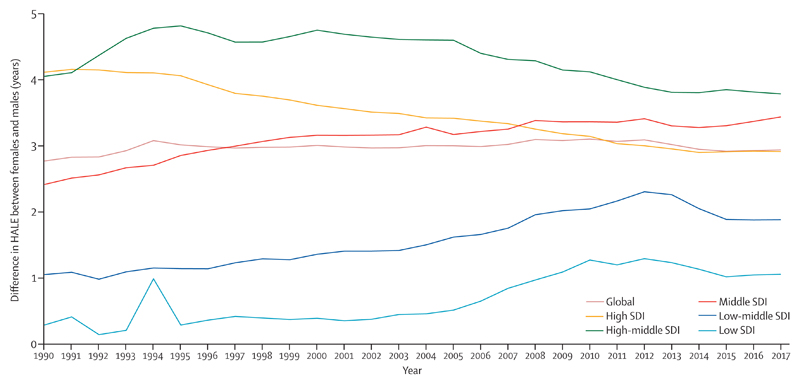
Difference in HALE at birth between males and females by SDI quintile, 1990–2017 HALE=healthy life expectancy. SDI=Socio-demographic Index.

Increases in HALE at birth between 1990 and 2017 were smaller than increases in life expectancy over the same period in all SDI quintiles, resulting in an increase in years lived in poor health. Of the additional years of life expected at birth, 26·3% (20·1–33·1) were spent in poor health in high SDI countries compared with 11·7% (8·8–15·1) in low-middle SDI countries (figure 2). The largest increase in years lived in poor health was for females in low SDI quintile countries, by 1·6 years (1·2–2·1), and the smallest increase was for males in middle SDI quintile countries, by 0·8 years (0·6–1·0; figure 1). Although males and females in all SDI quintiles had increases in HALE at birth, the increase was disparate between the sexes. In 1990, high SDI quintile countries had the largest gap in HALE between males and females, at 4·1 years (3·4–4·8; figure 3); this gap decreased to 2·9 years (2·3–3·5) by 2017. In 2017, the high-middle SDI quintile countries had the largest gap in HALE between males and females (3·8 years [3·1–4·5]); this gap had increased from 1990 to peak in 1995 at 4·8 years (4·5–5·2) and then decreased gradually thereafter. Low SDI quintile countries had the smallest gap in HALE between males and females in 2017 (figure 3). The difference in HALE at birth between the sexes during the period 1990–2017 increased in countries in the low SDI, low-middle SDI, and middle SDI quintiles. South Asia was the only region where males had higher HALE at birth than females did in 1990, and this trend lasted until 2000, after which point females had higher HALE at birth (appendix 2). In all other regions, females had higher HALE at birth than males did in all study years. Eastern Europe had the biggest difference in HALE at birth between females and males throughout the period 1990–2017, with a difference of 7·7 years (6·8–8·4) in 2017. North Africa and the Middle East had the smallest difference in HALE at birth between females and males in 1990, and south Asia had the lowest difference in 2017 (appendix 2).

At the country level, countries that had the largest increases in HALE at birth for both sexes combined between 1990 and 2017 were Eritrea (23·7 years [95% UI 21·9–25·6], Ethiopia (18·5 years [17·2–19·9], and Uganda (16·3 years [13·5–19·0]). The smallest increases were seen in Saint Vincent and the Grenadines (1·0 years [0·4–1·7]), South Africa (1·1 years [0·3–1·8]), and Uzbekistan (1·4 years [0·4–2·4]). Decreases in HALE at birth for both sexes combined occurred in Lesotho (5·0 years [3·1–6·8]), Swaziland (eSwatini; 2·3 years [0·4–4·3]), and Guam (1·2 years [0·5–1·9]). In 2017, the highest HALE at birth was estimated to be in Singapore for both females (75·8 years [72·4–78·7]) and males (72·6 years [69·8–75·0]; table 1). The lowest HALE at birth in 2017 for both males and females was estimated to be in the Central African Republic, at 47·0 years (43·7–50·2) for females and 42·8 years (40·1–45·6) for males. The largest differences in HALE at birth in 2017 between males and females were in Ukraine [8·7 years (7·5–9·8), Lithuania (7·8 years [6·6–8·9]), and Russia (7·4 years [6·6–8·1]).

### Trends in HALE for males and females at age 65 years

Globally, HALE at age 65 years for females was 11·9 years (95% UI 10·8–12·9) in 1990 and 13·7 years (12·4–14·9) in 2017, an increase of 1·8 years (1·6–2·0; table 2). For males, HALE at age 65 years increased by 1·7 years (1·5–1·9), from 10·3 years (9·4–11·1) to 12·0 years (10·9–12·9). At the SDI level, both males and females in the high SDI quintile had the largest increases in HALE at age 65, increasing from 13·9 years (12·7–15·1) in 1990 to 16·2 years (14·6–17·6) in 2017 for females, and increasing from 11·3 years (10·3–12·1) in 1990 to 13·8 years (12·6–15·0) in 2017 for males. The countries with the largest increases in HALE for females at age 65 years from 1990 to 2017 were the Maldives (by 7·0 years [6·3–7·8]), Singapore (by 4·9 years [4·4–5·5]), and Ethiopia (by 4·6 years [4·0–5·3]). For males, the largest increases were in Iraq (by 4·7 years [3·8–5·6]), the Maldives (by 4·5 years [4·0–5·1]), and South Korea (by 4·5 years [4·0–5·0]). In 2017, the largest difference between HALE at age 65 years between females and males was in Bermuda (17·7 years [16·2–19·2] for females, and 13·4 years [12·4–14·5] for males).

### Decomposition of years of life gained at birth and at age 65 years

Although life expectancy at birth increased for both males and females globally during the period 1990–2017, the proportion of additional years spent in poor health was 15·3% (95% UI 11·8–19·2) of the additional 7·5 years (7·2–7·9) for females and 14·9% (11·3–19·0) of the additional 7·3 years (6·9–7·7) for males, with substantial variations across SDI quintiles, regions, and countries (figure 2). The proportion of additional years spent in poor health varied from 11·1% (8·3–14·3) of the additional 8·2 years (7·5–8·8) among females in low-middle SDI countries, to 27·3% (20·8–34·4) of the additional 4·4 years (4·2–4·6) among females in high SDI countries. From 1990 to 2017, life expectancy at birth increased the most for both males and females in eastern sub-Saharan Africa compared with other regions (table 1), but of the additional years gained, 11·1% (8·3–14·3) of 13·8 years (12·8–14·8) for males and 13·1% (10·0–16·7) of 14·6 years (13·7–15·5) for females were spent in poor health. At the country level, 179 countries gained additional years of life from 1990 to 2017, and 16 did not (appendix 2). The most additional years of life were gained by males in Eritrea (28·6 years [25·7–31·4]), Ethiopia (21·1 years [19·5–22·8]), and Equatorial Guinea (18·6 years [15·1–21·8]). Of these years gained, the proportion spent in poor health was 11·3% (8·0–14·9) for Eritrea, 12·1% (9·2–15·6) for Ethiopia, and 13·7% (9·4–18·7) for Equatorial Guinea. Females gained the most additional years of life in Eritrea (22·4 years [19·7–25·4]), Ethiopia (21·5 years [20·0–23·2]), and Rwanda (19·5 years [17·1–22·1]). Of these years gained, the proportion spent in poor health was 10·4% (7·1–14·6) for Eritrea, 13·6% (10·2–17·4) for Ethiopia, and 16·1% (11·8–21·1) for Rwanda. Males spent over a third of the years of life gained in poor health in Belize (45·9% [5·5–155·6]), Ukraine (39·3% [14·1–128·7]), the Federated States of Micronesia (34·2% [7·0–55·9]), and the USA (33·6% [25·1–42·2]). Similarly, females spent over a third of their years of life gained in poor health in the USA (47·9% [35·2–61·6]), Northern Mariana Islands (40·7% [25·9–70·7]), and the UK (34·3% [25·8–43·6]; appendix 2).

Globally, between 1990 and 2017, life expectancy for females at age 65 years increased by 2·5 years (95% UI 2·4–2·6) and they spent 27·6% (21·2–35·0) of those years in poor health (appendix 2). Similarly, males at age 65 years gained 2·4 years (2·2–2·5) of life and spent 26·6% (20·1–34·0) of those years in poor health. Between 1990 and 2017, at the SDI quintile level, females in the high SDI quintile spent the largest proportion of years gained in poor health (29·9% [22·9–37·5] of 3·2 years [3·1–3·4] gained), and females in the low-middle SDI quintile spent the smallest proportion of years gained in poor health (23·8% [17·1–31·6] of 2·0 years [1·7–2·3] gained; appendix 2). Similarly, males in the high SDI quintile spent the largest proportion of additional years in poor health in the same period (30·0% [22·9–37·8] of 3·7 years [3·6–3·8] gained), and males in the low-middle SDI quintile spent the smallest proportion of additional years in poor health (23·7% [17·2–30·9] of 1·5 years [1·2–1·8] gained). At the country level, life expectancy between 1990 and 2017 for females at age 65 years increased the most in the Maldives (9·3 years [8·6–9·9]), Singapore (6·4 years [5·9–6·8]), and Iraq (6·2 years [5·0–7·4]), with the proportion of years spent in poor health being 24·4% (18·2–31·3) for those in the Maldives, 23·2% (17·4–29·7) for those in Singapore, and 28·5% (21·3–36·3) for those in Iraq (appendix 2). For males at age 65 years, life expectancy increased the most for Iraq (6·8 years [5·6–7·9]), South Korea (6·1 years [5·6–6·6]), and the Maldives (5·9 years [5·3–6·4]), with the proportion of those years spent in poor health being 30·3% (22·9–38·2) for Iraq, 26·1% (19·6–33·1) for South Korea, and 22·5% (16·6–29·4) for the Maldives (appendix 2).

### Decomposition of extra years lived by females compared with males in 2017

In 2017, females had longer life expectancy than males in 180 of 195 countries (appendix 2). Of these countries, the largest differences (>10 years) between female and male life expectancy at birth were in Ukraine (76·5 years [95% UI 75·8–77·2] for females and 64·7 years [63·9–65·4] for males), Lithuania (80·2 years [79·4–81·0] for females and 69·6 years [68·7–70·5] for males), Russia (77·2 years [77·1–77·4] for females and 66·8 years [66·6–66·9] for males), and Swaziland (eSwatini; 65·1 years [62·1–68·4] for females and 54·9 years [52·6–57·6] for males). For the other 15 countries, life expectancy was not significantly different between the sexes.

**Figure 4:**
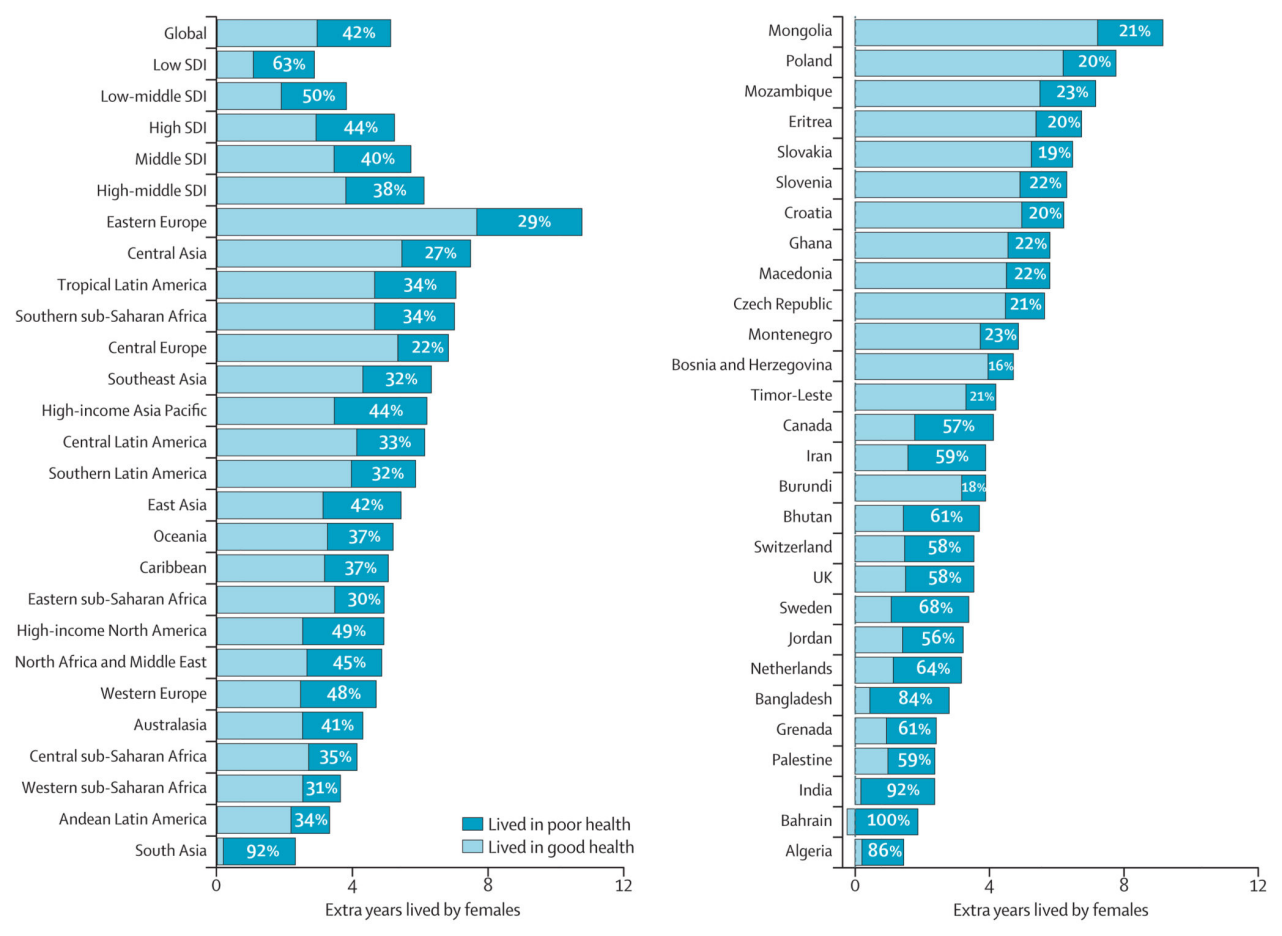
Extra years of life expected at birth in females compared with males by functional health status for five SDI quintiles, 21 GBD regions, and 28 countries with the largest and smallest percentages of years spent in poor health, 2017 GBD=Global Burden of Diseases, Injuries, and Risk Factors Study. SDI=Socio-demographic Index.

Globally, in 2017, females lived an additional 5·1 years (95% UI 4·9–5·3) compared with males, and of those extra years 57·5% (46·2–67·4) were spent in good health and 42·5% (32·6–53·8) in poor health (figure 4). The proportion of additional years spent in poor health varied a lot across SDI quintiles, regions, and countries. Compared with all other SDI quintiles, females in the high-middle SDI quintile lived the most extra years of life compared with males, living longer than males by 6·1 years; however, 37·8% (29·1–47·6) of those extra years were lived in poor health. At the regional level, the proportion of extra years lived in poor health among females in 2017 ranged from 21·9% (16·2–28·4) in central Europe to 92·2% (87·2–96·2) in south Asia (figure 4). Extra years lived by females varied from 11·9 years (10·9–12·9) in Ukraine to 1·4 years (0·6–2·3) in Algeria (appendix 2). Of the extra years lived, the proportion spent in poor health varied largely at the country level, with less than 20% of the additional years spent in poor health for females in Bosnia and Herzegovina, Burundi, and Slovakia, whereas females lived all extra years in poor health in Bahrain (1·6 years [0·2–3·1]; appendix 2).

Globally, at age 65 years, women lived an additional 2·7 years (95% UI 2·6–2·9) compared with men, but 63·9% (55·2–72·2) of those years were spent in good health and 36·1% (27·8–44·8) in poor health (appendix 2). Compared with other SDI quintiles, the difference in life expectancy was greatest between females and males in the high SDI quintile, with females living 3·3 years (3·1–3·5) longer than males, but 28·4% (21·3–36·1) of those years were spent in poor health. In the low SDI quintile, females are estimated to live the largest proportion of extra years in poor health compared with other SDI quintiles (58·0% [43·8–74·6] of 1·1 years [0·7–1·4]). For females aged 65 years in Iran and India, of the extra years lived compared with males, more than 60% of extra years were spent in poor health, 70·1% (53·5–88·2) for those in Iran and 63·4% (49·0–79·6) for those in India (appendix 2).

### Global levels and trends for DALYs

The global level of all-age all-cause DALYs in 2017 was 2·50 billion (95% UI 2·29–2·74). The estimated DALY counts and age-standardised rate of DALYs, and changes in these metrics between 2007 and 2017, are in table 3. The largest contribution to global DALYs was from non-communicable diseases, which, combined, accounted for 62·0% (60·3–63·8) of total DALYs, whereas communicable, maternal, neonatal, and nutritional (CMNN) causes accounted for 27·9% (26·4–29·4), and injuries 10·1% (9·7–10·5) of total DALYs.

For non-communicable diseases, global DALYs increased by 40·1% (95% UI 36·8–43·0), although age-standardised DALY rates decreased by 18·1% (16·0–20·2). Between 2007 and 2017, 12 causes at Level 2 of the GBD cause hierarchy (diabetes and kidney diseases, sense organ diseases, neurological disorders, neoplasms, musculoskeletal disorders, substance use disorders, cardiovascular diseases, chronic respiratory diseases, skin and subcutaneous diseases, mental disorders, digestive diseases, and self-harm and interpersonal violence) had significant increases in DALY counts. The greatest number of DALYs among Level 3 non-communicable disease causes in 2017 were estimated for ischaemic heart disease (170 million [167–174]), stroke (132 million [126–137]), and chronic obstructive pulmonary disease (81·6 million [76·0–86·8]) which, combined, accounted for 15·4% (14·2–16·4) of all-cause DALYs (table 3). By contrast with the overall decrease in DALYs during 2007–17 for many causes, total all-age DALYs from non-communicable diseases increased from 1·11 billion (1·00–1·23) in 1990 to 1·34 billion (1·19–1·51) in 2007, and continued to increase to 1·55 billion (1·38–1·75) in 2017 (appendix 2): a change of 20·7% (18·4–22·8) from 1990 to 2007, and 16·0% (15·1–16·9) from 2007 to 2017. At Level 4 between 1990 and 2007, the largest significant increases in total DALYs were estimated for HIV/AIDS and multidrug-resistant tuberculosis co-infection without extensive drug resistance (5329·0% [3143·5–8492·9]), multidrug-resistant tuberculosis without extensive drug resistance (700·6% [335·0–1,431·6]), and HIV/AIDS resulting in other diseases (454·3% [389·5–529·2]; appendix 2. From 2007 to 2017, the largest significant increases in DALYs were for liver cancer due to NASH (37·4% [32·8–42·9]), chronic kidney disease due to type 2 diabetes (34·3% [30·9–37·2]), and diabetes type 2 (34·0% [30·3–38·1]). Relative to changes in the population, the age-standardised DALY rate for non-communicable diseases decreased by 13·2% (11·7–14·7), from 24 017·4 DALYs (21 861·4–26 509·6) per 100 000 in 1990 to 20 852·2 DALYs (18 710·2–23 388·5) per 100 000 in 2007, and decreased by a further 5·6% (4·8–6·6) to 19 676·5 DALYs (17 509·8–22 177·9) per 100 000 in 2017. From 1990 to 2017, the greatest significant decreases in age-standardised DALY rates were for visceral leishmaniasis (97·8% [97·0–99·2]), maternal haemorrhage (78·7% [73·4–83·0]), and ascariasis (70·8% [66·0–75·3]). Significant increases in age-standardised DALY rates from 1990 to 2017 were observed for 17 Level 4 non-communicable diseases, with other benign and in-situ neoplasms (35·1% [7·8–81·7]), type 2 diabetes (28·2% [24·1–32·3]), and opioid use disorders (24·8% [20·1–31·0]) having the most pronounced changes.

Among CMNNs, at Level 3 of the GBD cause hierarchy the greatest contributors to global DALYs in 2017 were neonatal disorders (186 million [95% UI 175–197] DALYs), lower respiratory infections (106 million [100–112] DALYs), and diarrhoeal diseases (81·0 million [70·1–97·2] DALYs). Globally, total DALYs from all CMNNs decreased from 1·19 billion (1·14–1·23) in 1990 to 0·946 billion (0·910–0·987) in 2007, to 0·696 billion (0·660–0·740) in 2017, a decrease of 41·3% (38·8–43·5) from 1990 to 2017. In parallel, the age-standardised DALY rate for all CMNN causes decreased by 49·8% (47·9–51·6) from 1990 to 2017. For 2007–17, the largest decreases were for visceral leishmaniasis (by 63·9% [40·0–84·5]), HIV/AIDS and drug-susceptible tuberculosis co-infection (by 54·9% [51·0–57·9]), and maternal haemorrhage (52·4% [44·3–59·5]; table 3). Significant decreases of more than 50% in age-standardised DALY rates between 2007–17 were estimated for five Level 4 CMNN causes, specifically, visceral leishmaniasis (66·2% [44·3–86·0]), HIV/AIDS and drug-susceptible tuberculosis co-infection (59·8% [56·3–62·5]), HIV/AIDS and multidrug-resistant tuberculosis without extensive drug resistance co-infection (56·6% [40·1–69·1]), maternal haemorrhage (56·4% [49·1–62·8]), and HIV/AIDS resulting in other diseases (53·7% [51·1–56·1]). Against this general decreasing trend, seven CMNN causes at Level 4 were estimated to have Significant increases in age-standardised DALY rate between 1990 and 2017, specifically, HIV/AIDS and multidrug-resistant tuberculosis without extensive drug resistance co-infection (1805·0% [1016·3–3049·2]), HIV/AIDS resulting in other diseases (103·1% [76·3–134·9]), and cutaneous and mucocutaneous leishmaniasis (40·5% [7·0–106·0]).

**Figure 5:**
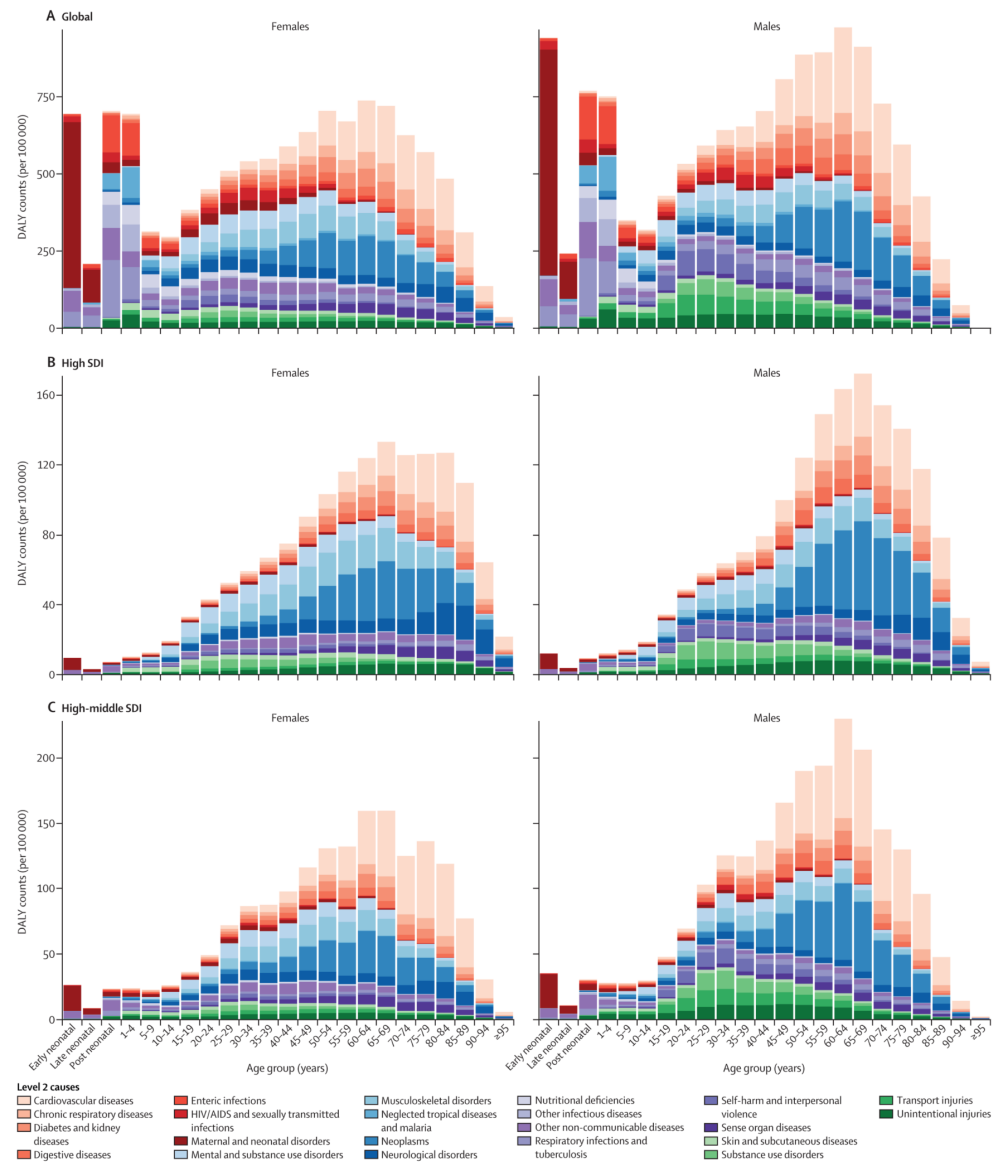

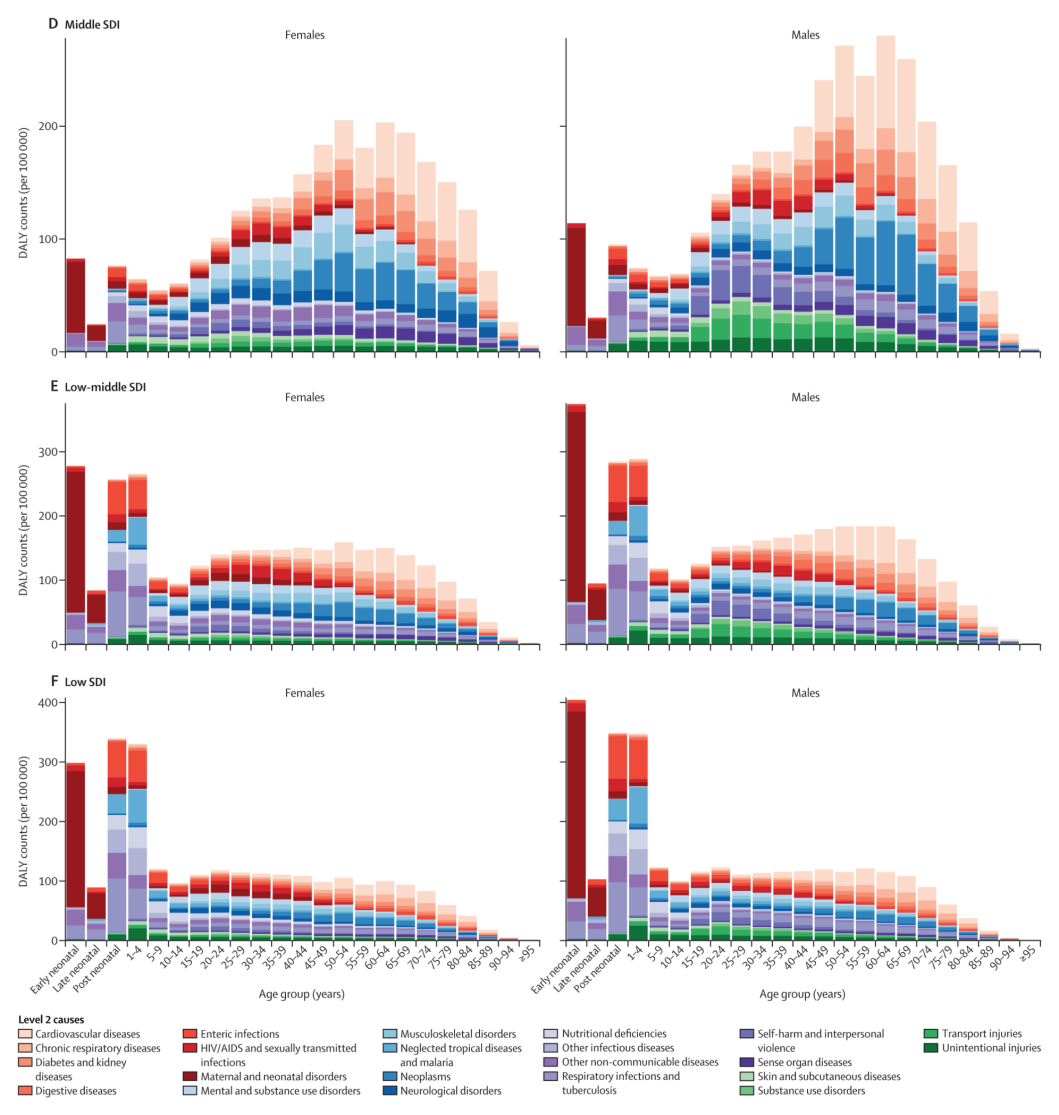
DALYs by Level 2 causes by age and sex, for global (A), high SDI (B), high-middle SDI (C), middle SDI (D), low-middle SDI (E), and low SDI (F), 2017 Scales in each panel are different. The early neonatal period is 0–6 days, the late neonatal period is 7–27 days, and the post neonatal period is 28–364 days. DALYs=disability-adjusted life-years. GBD=Global Burden of Diseases, Injuries, and Risk Factors Study. SDI=Socio-demographic Index.

In 2017, the top five leading causes of DALYs were communicable diseases (lower respiratory infections, malaria, diarrhoeal diseases, HIV/AIDS, and tuberculosis) and neonatal disorders (table 3). For low-middle SDI countries, both communicable and non-communicable diseases ranked highly as leading causes of DALYs, and the top five causes were neonatal disorders, lower respiratory infections, ischaemic heart disease, diarrhoeal diseases, and stroke (figure 5). In high SDI countries, non-communicable diseases (ischaemic heart disease, low back pain, stroke, lung cancer, and chronic obstructive pulmonary disease) were the leading causes of DALYs (figure 5). Younger age groups contributed more to total DALY counts in low SDI countries than did older age groups, and mostly from communicable diseases. In low SDI countries in 2017, 136 million (95% UI 130–143) DALYs were estimated for neonatal age groups to be from communicable diseases, with a further 18·9 million (17·2–21·0) from non-communicable diseases, and 2·95 million (2·60–3·26) from injuries. For low and low-middle SDI countries, the burden is more equal across age groups than in other SDI quintiles. The burden from DALYs in high-middle and high SDI countries occurs mostly from age 15 years and consists largely of non-communicable diseases (figure 5).

### Leading causes of DALYs and changes during 1990–2017

**Figure 6:**
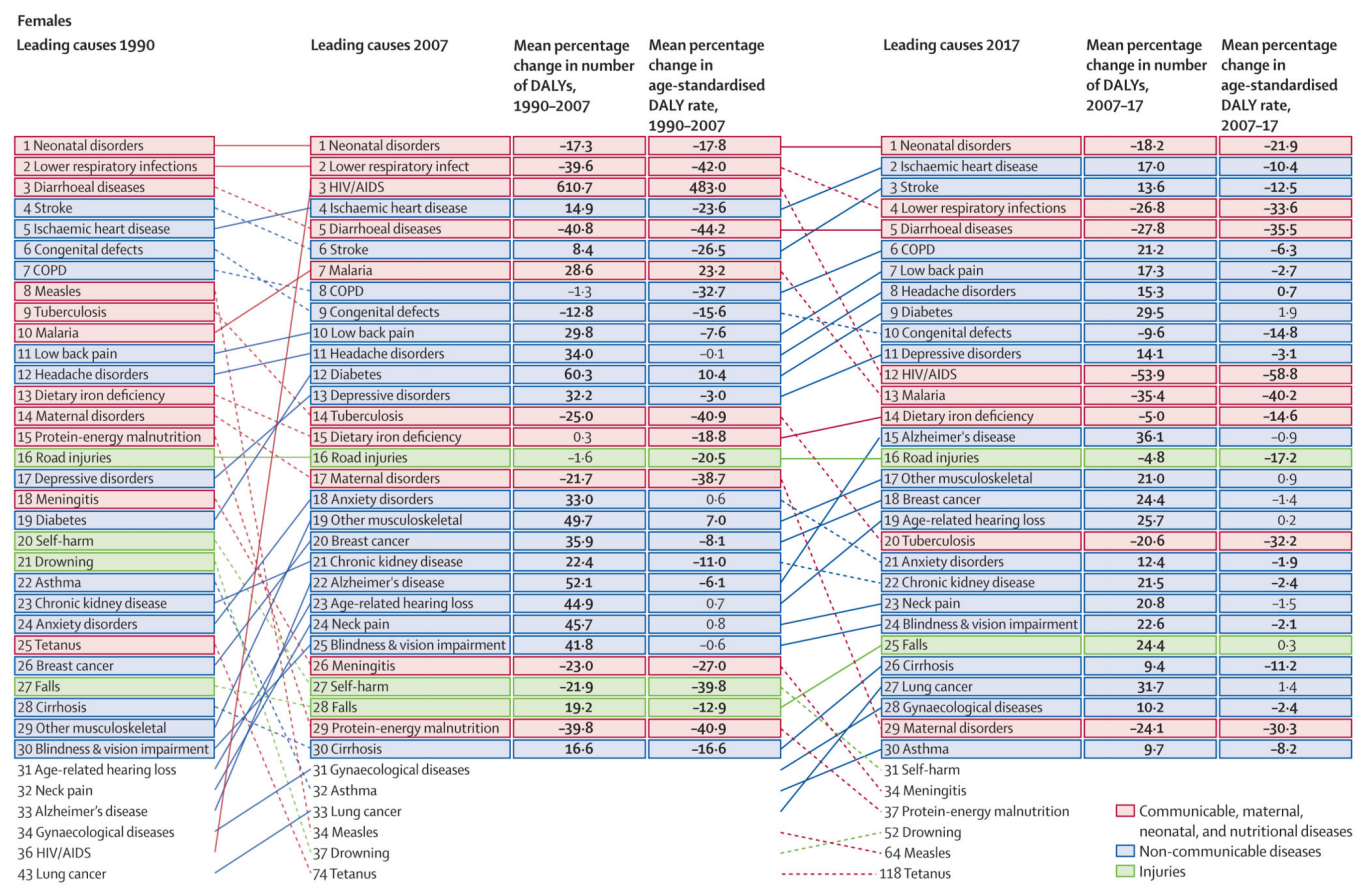

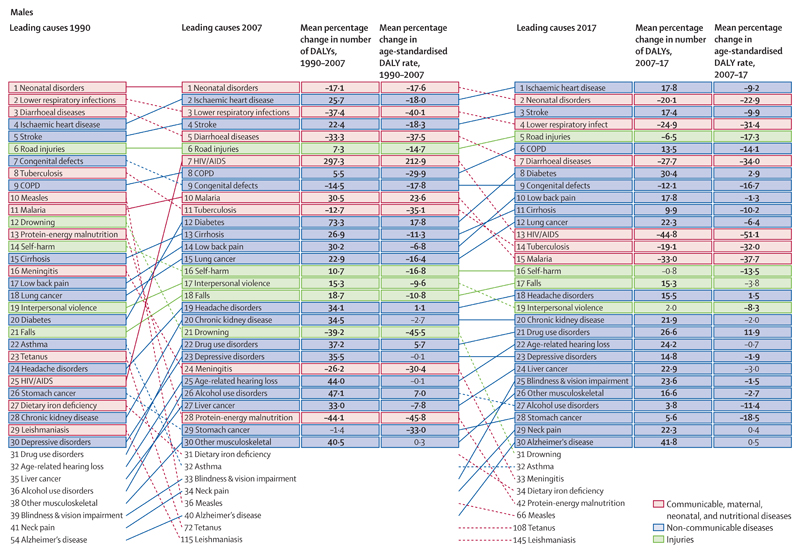
Leading 30 Level 3 causes of global DALYs for 1990, 2007, and 2017 with percentage change in number of DALYs and age-standardised DALY rates by sex Solid lines indicate increases and dashed lines indicate decreases in rank between periods. Significant changes are shown in bold. COPD=chronic obstructive pulmonary disease. DALYs=disability-adjusted life-years.

Globally in 2017, the leading causes of DALYs were predominantly CMNN and non-communicable causes for both men and women (figure 6). Of the top 30 causes of DALYs for men, four were injuries: road injuries (49·8 million [95% UI 47·3–52·1] DALYs), self-harm (22·9 million [20·9–24·0] DALYs), falls (21·0 million [17·6–24·9] DALYs), and interpersonal violence (19·8 million [17·8–21·0] DALYs; table 3). By contrast, two of the top 30 causes of DALYs for women were injuries: road injuries (18·0 million [16·6–19·4] DALYs) and falls (15·0 million [12·3–18·0] DALYs). Our results showed disparities in disease burden between males and females. Females are more likely than males to have a higher burden from disabling conditions such as most musculoskeletal disorders except for gout, iron-deficiency anaemia, and major depressive disorder. Iron-deficiency aneaemia is common especially at reproductive ages for females, and for boys and girls (aged 5–14 years) equally (appendix 2). Males are more likely than females to be affected by fatal conditions including different types of cancer, injuries, and ischaemic heart disease. Globally, the all-age DALY count of HIV/AIDS for females increased sharply from 1990 to 2007, rising from 36th to third leading cause of DALYs with an increase of 610·7% (548·5–680·6), then decreased from 2007 to 2017 by 53·9% (51·6–53·9), dropping to 12th leading cause of DALYs (figure 6). Similarly, the global age-standardised DALY rate for females increased from 1990 to 2007 by 483·0% (431·4–540·5), and then decreased from 2007 to 2017 by 58·8% (56·8–60·6; appendix 2). Males had similar patterns in all-age DALY counts and age-standardised DALY rates for HIV/AIDS during the same period. The all-age DALY count of HIV/AIDS for males increased by 297·3 (259·8–338·4) from 1990 to 2007, rising from the 25th leading cause of DALYs to seventh (figure 6; appendix 2). By 2017, all-age DALYs from HIV/AIDS for males dropped to 13th leading cause of DALYs, a decrease of 44·8% (42·8–46·6) between 2007 and 2017. Similarly, the age-standardised DALY rate of HIV/AIDS for males increased sharply from 1990 to 2007, with an increase of 212·9% (182·8–246·4), and then decreasing by 51·1% (49·0–52·6) in 2017. The global all-age DALY count of malaria for females increased from 1990 to 2007 by 28·6% (11·7–51·2), rising from the tenth leading cause of DALYs to the seventh leading cause of DALYs. Malaria age-standardised DALY rates for females also increased, with an increase of 23·2% (7·4–44·2) between 1990 and 2007, and then decreased from 2007 to 2017 by 40·2% (30·7–49·2). DALYs from malaria for males also increased from 1990 to 2007, increasing by 30·5% (12·9–55·7) from the 11th to the tenth leading cause of DALYs. Similarly, the age-standardised DALY rate for malaria in males increased between 1990 and 2007 by 23·6% (7·0–46·0), and then decreased by 37·7% (27·6–46·8) in 2017.

The leading 30 causes of DALYs for men and women varied between SDI quintiles (appendix 2). In the low SDI quintile countries, males and females had similar CMNN causes as the leading causes of DALYs in 2017, with the greatest total DALYs for both sexes being from neonatal disorders, lower respiratory infections, diarrhoeal diseases, and malaria for both sexes, and thereafter for females the fifth leading cause was congenital defects and for males was ischaemic heart disease. In the low SDI quintile, males had more injury sources of DALYs than females, whereas CMNN causes of DALYs were more common among the leading DALY causes for females than males. An epidemiological shift in sources of DALYs was evident in the leading causes for both men and women at middle to high SDI levels, which were dominated by non-communicable disease causes. In high SDI quintiles, two CMNN causes of DALYs were in the leading 30 causes for both men and women, lower respiratory infections and neonatal disorders, and the age-standardised DALYs rate for these causes decreased between 1990 and 2017, by 36·2% (95% UI 33·9–38·6) for lower respiratory infections and 30·1% (25·1–36·7) for neonatal disorders for men, and by 37·8% (35·8–39·9) for lower respiratory infections and 27·5% (22·3–34·1) for neonatal disorders for women (appendix 2).

### YLLs and YLDs composition by age, sex, and SDI

**Figure 7:**
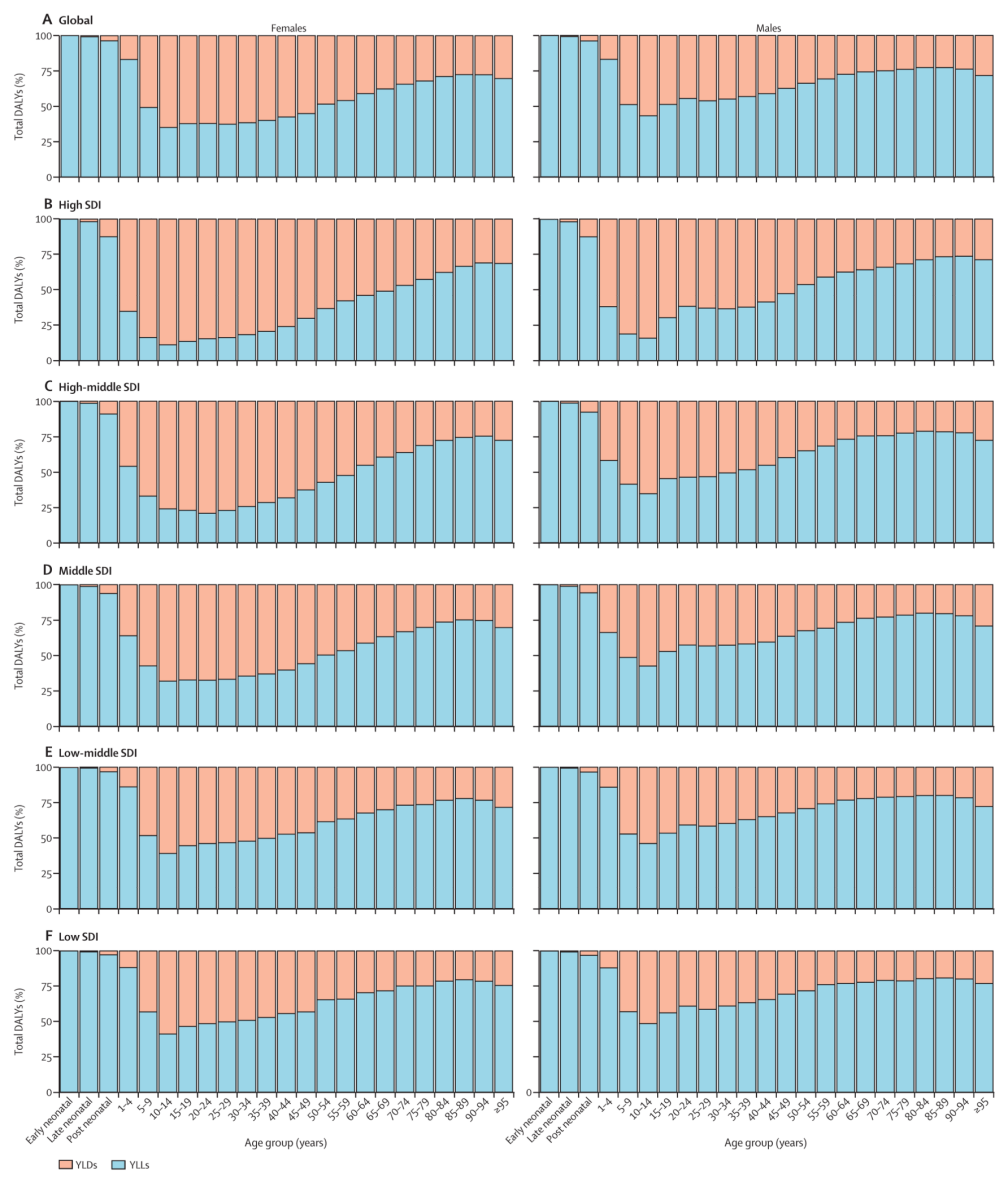
Contribution of YLLs and YLDs to DALYs by age and sex for global (A), high SDI (B), high-middle SDI (C), middle SDI (D), low-middle SDI (E), and low SDI (F), 2017 The early neonatal period is 0–6 days, the late neonatal period is 7–27 days, and the post neonatal period is 28–364 days. DALYs=disability-adjusted life-years. SDI=Socio-demographic Index. YLDs=years lived with disability. YYLs=years of life lost.

Globally in 2017, males had 1·34 billion (95% UI 1·24–1·46) DALYs and females had 1·16 billion (1·04–1·29) DALYs. For males, 29·4% (23·8–34·9) of DALYs were from YLDs and 70·6% (65·1–76·2) were from YLLs. For females, 39·4% (33·0–45·6) of DALYs were from YLDs and 60·6% (54·4–67·0) were from YLLs. A greater proportion of DALYs were from YLDs for females than for males, particularly for those older than 65 years (figure 7). Globally, among females YLDs were the larger fraction of DALYs for many age groups, whereas for males this was only the case for those aged 10–14 years. Across SDI quintiles, the composition of DALYs varied. Females aged 1–69 years in the high SDI quintile had more DALYs from YLDs than from YLLs, whereas in the low SDI quintile all age groups except those aged 10–29 years had more DALYs from YLLs than from YLDs. In all quintiles, women older than 65 years had more DALYs from YLDs than men older than 65 years did.

### Observed and expected values of HALE and DALYs based on SDI level

**Figure 8:**
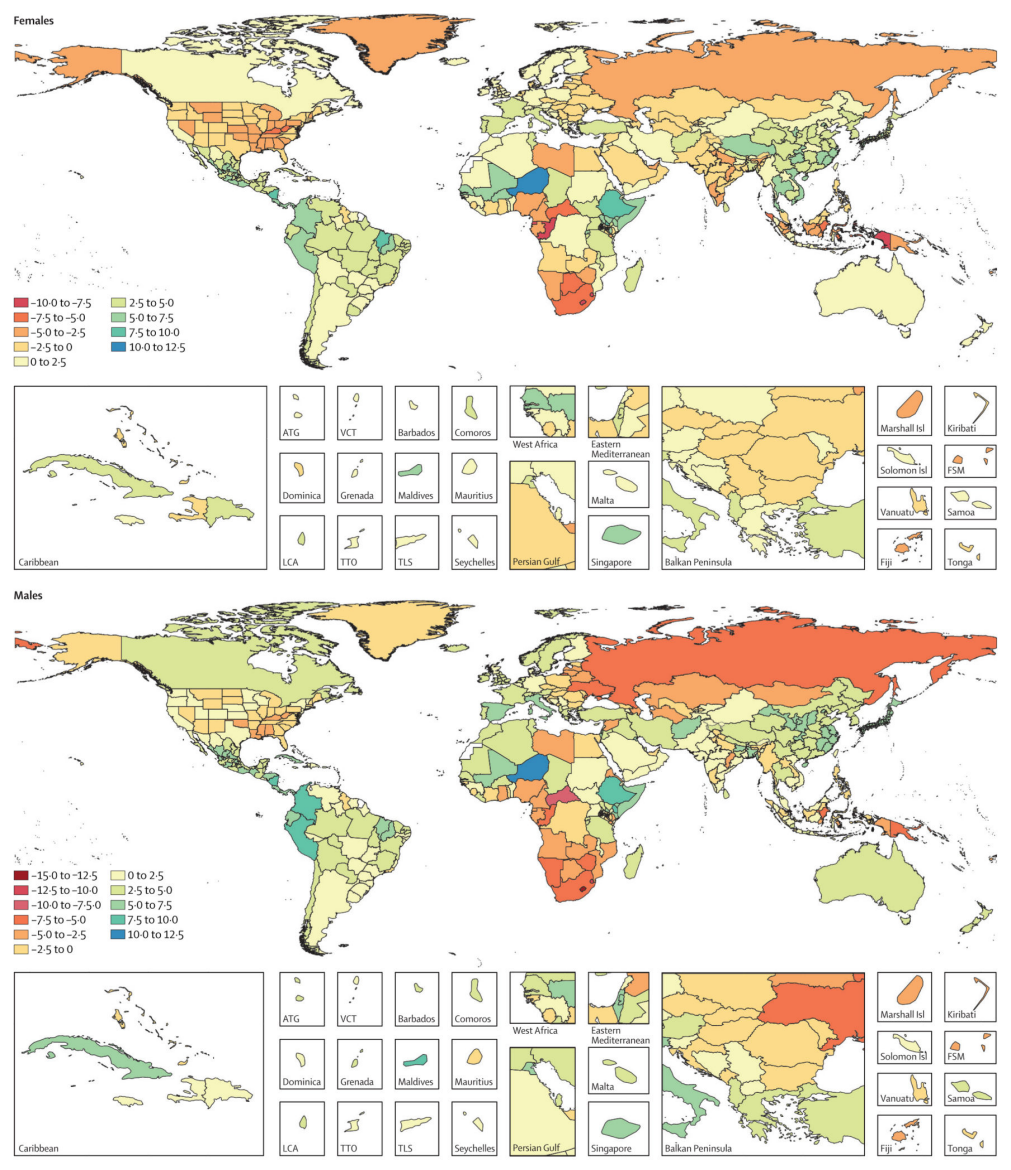
Difference in years between observed and expected HALE at birth on the basis of SDI for females and males, 2017 ATG=Antigua and Barbuda. FSM=Federated States of Micronesia. HALE=healthy life expectancy. Isl=Islands. LCA=Saint Lucia. SDI=Socio-demographic Index. TTO=Trinidad and Tobago. TLS=Timor-Leste. VCT=Saint Vincent and the Grenadines.

Maps of observed minus expected HALE can highlight which countries have worse or better HALE than expected based on SDI quintile, as shown in figure 8. The three countries with the greatest difference between expected HALE at birth and observed HALE at birth for males, with observed HALE much lower than expected on the basis of their SDI value, were Lesotho, with a difference of 13·4 years, Swaziland (eSwatini), with a difference of 11·7 years, and Central African Republic, with a differences of 7·9 years. For females, the countries with the greatest differences were Lesotho, with a difference of 10·0 years, Congo (Brazzaville), with a difference of 9·3 years, and Swaziland (eSwatini), with a difference of 8·1 years. The three countries that had the greatest difference between observed and expected HALE at birth for males, where HALE was much better than expected, were Niger, Nicaragua, and the Maldives, with differences of 11·9 years for Niger, 9·5 years for Nicaragua, and 9·2 years for the Maldives. For females, these countries were Niger, Nicaragua, and Ethiopia, with differences between observed and expected HALE at birth of 11·5 years for Niger, 8·3 years for Nicaragua, and 8·2 years for the Maldives. We saw a similar trend in countries having differences in observed and expected HALE at age 65 years for both males and females (appendix 2). The greatest differences in observed and expected HALE at age 65 years for males, where HALE was worse than expected, were seen in Lesotho (3·1 years), Swaziland (eSwatini; 2·8 years), and Papua New Guinea (2·3 years), and for females were seen in Congo (Brazzaville; 2·7 years), Papua New Guinea (2·6 years), and Marshall Islands (2·2 years). HALE at age 65 years was better than expected on the basis of SDI level for males in Peru (by 5·0 years), Colombia (by 5·0 years), and Nicaragua (by 4·9 years), and for females in Colombia (by 4·5 years), Peru (by 4·3 years), and Nicaragua (by 3·7 years).

Higher ratios of observed to expected age-standardised DALY rates indicate more DALYs experienced than expected on the basis of SDI (appendix 2). Males had the highest ratio of observed to expected age-standardised DALY rates in Lesotho (1·94), Swaziland (eSwatini; 1·86), and Central African Republic (1·60). Females had the highest ratio of observed to expected age-standardised DALY rates in Congo (Brazzaville; 1·67), Lesotho (1·62), and Equatorial Guinea (1·57). Alternatively, males in the Maldives, Niger, and Nicaragua experience DALYs at a much lower rate than expected, with observed to expected ratios of 0·54 in the Maldives, 0·56 in Niger, and 0·57 in Nicaragua. Females had low ratios of observed to expected age-standardised DALY rates in Niger, Nicaragua, and Ethiopia, with ratios of 0·57 in Niger, 0·58 in Nicaragua, and 0·62 in Ethiopia.

## Discussion

### Main findings

Globally, HALE at birth increased by 6·3 years (95% UI 5·9–6·7) and the number of years lived in poor health from birth increased by 1·1 years (0·9–1·4) during the period 1990–2017, with substantial variation across socio-demographic quintiles and countries. People in low SDI countries gained an additional 10·5 years (9·8–11·2) of life in good health and 1·5 years (1·2–1·9) in poor health from birth, whereas people in high SDI countries gained an additional 3·8 years (3·4–4·1) in good health and 1·3 years (1·0–1·7) in poor health from birth. Women are expected to live longer than men (both at birth and at age 65 years) in most countries, but the number of extra years lived and the proportions of extra years spent in poor health vary greatly across countries. In 2017, the global leading causes of DALYs were neonatal disorders, ischaemic heart disease, stroke, lower respiratory infections, and chronic obstructive pulmonary disease. Communicable diseases and neonatal disorders were the leading causes of DALYs in low SDI countries in 2017. Non-communicable diseases were the leading causes of DALYs in the remaining SDI quintiles in the same year.

### Sex disparities in health

Sex differences in HALE exist across SDI quintiles, with the smallest difference seen in low SDI countries. Although societal factors, biological factors, and men’s risk behaviours probably contribute to their shorter HALE,^22^,^23^ the smaller male–female gap in HALE in low SDI countries might be explained by the increased risk of mortality in both sexes due to the high occurrence of infectious diseases. Additionally, women are affected by pregnancy-related conditions, and maternal disorders were a leading cause of DALYs in low SDI countries in 2017. In several high SDI countries, the difference between male and female HALE has decreased over time, which could partly be attributable to the decreasing gap between the sexes in the prevalence of specific risk factors—eg, smoking and alcohol use.^24^,^25^

Our results show increased life expectancy and more years lived in poor health for women than men in most countries. This finding could be attributable to sex differences in the patterns of disease burden. For example, women are more likely than men to have a higher burden from disabling conditions (eg, most musculoskeletal disorders except for gout, iron-deficiency anaemia, and major depressive disorder), whereas men are more likely than women to be affected by fatal conditions including different types of cancer (eg, liver cancer, lung cancer, leukaemia, colorectal cancer, and pancreatic cancer), injuries, and ischaemic heart disease. Various explanations have been suggested for the sex difference in disease risk, including social norms (eg, heavy drinking is socially acceptable for men in Russian tradition), health-related beliefs and behaviours, and biological factors (eg, sex hormones).^22^,^26^

In 2017, the widest difference between male and female HALE at birth was observed in the eastern European region (7·7 years [95% UI 6·8–8·4]), where the age-standardised DALY rate from all causes among men was also 1·7 times higher than among women. A large proportion of DALYs in men in these countries was attributable to alcohol use and smoking, which led to high incidence and prevalence of cardiovascular disease and other leading causes of DALYs.^27^ Interventions aiming to reduce these risk factors could help decrease the difference. Ukraine and Lithuania had the biggest sex difference in HALE at birth globally (8·7 years [7·5–9·8] for Ukraine and 7·8 years [6·6–8·9] for Lithuania) in 2017. Age-standardised DALY rates due to cardiovascular diseases and alcohol use disorders were two times higher in both these countries for men than for women, whereas self-harm and drug use disorders were four or more times higher.

### Longevity and functional health status

With increasing life expectancy in almost all locations worldwide, the question of whether the years of life gained are spent in good health or poor health is increasingly relevant because of the associated policy implications, ranging from health-care provisions to extending retirement ages.

In the context of improving longevity, the age of retirement versus extending working life has been much debated. Some key questions need to be considered in this debate, including whether the increase in life expectancy is accompanied by an equivalent increase in years in good health, and whether the common causes of early retirement,^28^,^29^ such as mental disorders and musculoskeletal disorders, have improved over time. Our results showed that the additional years of life were accompanied by poor health to some extent, but varied widely across countries, and the burden from musculoskeletal disorders and mental disorders has not improved over time. A lack of progress in reducing the burden of these causes and other conditions associated with ageing, such as sense organ disorders and Alzheimer’s disease, might restrict the ability of older workers to contribute to the workforce.^30^

Globally, 12 Level 2 causes have had significant increases in DALY counts since 2007. Causes with the largest increases in DALY counts include musculoskeletal disorders, sense organ diseases, neurological disorders, diabetes and kidney diseases, and neoplasms. The large and increasing number of people living with these diseases necessitates careful planning by governments and health-care providers to ensure adequate funding and staff for treatment and rehabilitation services. Some of these diseases are expensive to manage.^31^ Despite the large amount of spending on health in some countries, such as India and Nigeria, the spending is inefficient because most of it is through out-of-pocket expenditures (ie, direct payments by individuals to health-care providers); additional health services could be provided if the money was consolidated.^32^

### Cross-national variation in health gains

Our results showed that substantial variations in health gains exist even between countries within the same SDI quintile. For example, between 1990 and 2017, the increase in HALE at birth among females in Singapore was more than six times greater than the increase among females in the USA (7·3 years [95% UI 6·6–7·9] *vs* 1·1 years [0·8–1·4]), and the females in Singapore spent a smaller proportion of the extra years in poor health (17·7% [12·7–23·1] *vs* 33·6% [25·1–42·2]). The comparatively small increase in HALE in the USA is probably due to inequalities in access to health care.^33^ The burden attributable to drug use disorders has also been increasing in the USA, with the age-standardised DALY rates due to drug use disorders almost three times higher in 2017 compared with 1990. This trend coincides with a sharp increase between 2015 and 2016 in the use of and deaths from synthetic opioids such as fentanyl, suggesting the need to improve harm-reduction efforts in the USA.^34^,^35^

Over the past 28 years, several countries in sub-Saharan Africa have had substantial increases in HALE. But at the same time, some countries in this region, especially those with major HIV epidemics, had a stagnation or reduction in HALE. Although HIV control efforts have resulted in a decrease in HIV-related mortality and an increase in life expectancy and HALE in the past decade, countries such as Lesotho and Swaziland (eSwatini) still have yet to catch up with the level of HALE in 1990.

In addition to examining improvements in health, we compared the observed HALE and disease burden with those expected based on development status to discern which countries are lagging behind and identify priority areas that need to be addressed to further improve HALE. In 2017, several countries, most of them from southern sub-Saharan Africa and eastern Europe, had higher disease burden and lower HALE than expected on the basis of their sociodemographic development. Our study also identified countries that outperformed expectations based on their development status (eg, in Niger and the Maldives, males had more than 9 years higher HALE at birth than expected based on the countries’ SDI). The approaches used in these countries to accelerate improvements in health (eg, the child survival programme in Niger^36^) could inform successful programmatic strategies for countries with poor performance with similar levels of development.

### Disease burden related to undernutrition and obesity

Although child and maternal undernutrition is the main risk factor for the leading causes of disease burden in low SDI countries (eg, lower respiratory infections, diarrhoea, and neonatal disorders), obesity remains a key driver of the leading causes of burden in high-middle and high SDI countries (eg, ischaemic heart disease, ischaemic stroke, diabetes, low back and neck pain).^27^ With changing diets and lifestyle over time, obesity-related diseases are emerging, especially in low-middle and middle SDI countries where diseases related to both undernutrition and obesity contribute substantially to the total burden of disease. Evidence suggests that the disease burden associated with undernutrition and obesity can potentially be reduced.^37^ For example, nutritional counselling, food supplementation, and conditional cash-transfer programmes have been shown to substantially reduce stunting and associated burdens of disease in settings with food insecurity.^37^ Randomised controlled trials have shown that a lifestyle intervention with a small amount of weight loss (5–7%) decreases the incidence of type 2 diabetes by 58% during a 3-year follow-up and by 34% during a 10-year follow-up.^38^–^40^ However, weight maintenance after weight loss is challenging and requires continuing support to help maintain diet, physical activity, and behavioural changes.^41^ For countries with a dual burden of malnutrition, an integrated approach addressing both undernutrition and obesity to simultaneously reduce the burden associated with both risk factors has been recommended.^42^,^43^

Dietary iron deficiency was the fifth leading cause of DALYs among women of reproductive age and the top leading cause of DALYs among children of both sexes at ages 5–14 years in 2017. Age-standardised DALY rates due to dietary iron deficiency were similar between boys and girls aged 5–14 years, but for adult women the rates were almost double those for men. Despite its high burden, iron deficiency remains commonly under-diagnosed and undertreated.^44^ Iron deficiency is not only a problem in low SDI countries, but also in high SDI countries, and reduction of iron deficiency among children and women of reproductive age, for example, is one of the objectives of the US Public Health Service’s Healthy People 2020 initiative.^45^ Various options are available to treat iron deficiency, ranging from iron supplementation to biofortification of crops.^46^,^47^ Although iron supplementation has been shown to be efficacious, challenges exist in terms of distribution, cost and compliance, and inability to tolerate it because of potential adverse gastrointestinal effects.^46^ Food fortification is highly cost-effective but can be a challenge in rural populations with little access to marketed fortified food.^47^ Biofortification of crops is considered to be a sustainable strategy to prevent iron deficiency, especially in settings with few resources; this strategy has been shown to improve iron status in iron-deficient individuals, but the longer-term effects on functional outcomes has yet to be determined.^48^

### Communicable diseases with increasing trends

A global shift has occurred from communicable to non-communicable causes of disease burden with sociodemo-graphic development. However, a few key exceptions exist among communicable diseases. In most tropical and subtropical countries, the burden of dengue has been increasing over time, in terms of both DALY counts and age-standardised DALY rates, with the highest number of DALYs occurring in south Asia. A dengue vaccine has been licensed for use in people aged 9–45 years living in several endemic settings;^49^ however, the vaccine efficacy varies from 76% in seropositive individuals to only 39% in seronegative individuals.^50^ In the absence of specific treatment for dengue, prevention and control measures are crucial. Top-down vector-control efforts have restricted effectiveness and sustainability, and innovative community-based vector-control strategies are being developed.^51^

All-age DALY counts and age-standardised DALY rates due to HIV/AIDS have been decreasing since 2007, but for adolescents (aged 15–19 years) age-specific DALY rates continued to increase after 2007. The HIV/AIDS YLL and YLD trends among adolescents also differ from the all-age trends. The all-age YLL rate increased after 1990, peaked around 2005, and then declined steadily afterwards^11^ as antiretroviral treatment became more widely available. Among adolescents, the YLL rate continued to increase after 2005, reaching a plateau since 2013. The all-age YLD rate due to HIV/AIDS has decreased slightly since 2007, but the adolescent YLDs rate has continued to increase.^12^ DALYs from HIV/AIDS are concentrated in sub-Saharan Africa. Adolescents have low rates of HIV testing and poor access to antiretroviral treatment, which might partly explain some of the increases in HIV/AIDS DALYs in this age group.^52^

### Causes with sharp decreases in DALYs

Since 1990, we have seen exceptional progress in many countries in reducing the burden from communicable diseases, especially vaccine-preventable diseases such as tetanus and measles. Much of this burden came from premature mortality, which has sharply decreased in the past 28 years. However, despite these interventions, DALYs due to these causes remain unnecessarily high in several low, low-middle, and middle SDI countries. Immunisation efforts have been helpful, but progress in immunisation coverage has slowed in the past decade; about 20 million children younger than 1 year, most of them in sub-Saharan Africa and south Asia, did not receive the measles vaccine and the three recommended doses of the diphtheria, tetanus, and pertussis vaccine in 2016.^53^ Conflict, inadequate investment in national immunisation programmes, and vaccine stock outages were among the reasons for the stalled progress in immunisation coverage.^53^

### Comparisons with GBD 2016

We have made substantial improvements in the estimation of mortality and life expectancy in GBD 2017 across our publications, including an independent estimation of population, a comprehensive update on fertility, adding a substantial amount of new data (from censuses, Demographic Surveillance Sites, and other sources), improvements to the GBD model life-table system, and enhancements to the modelling framework.^13^ We made changes to the GBD cause hierarchy, which can restrict comparisons to estimates from previous GBD iterations. For example, we have combined maternal and neonatal conditions at Level 2 of the cause hierarchy in GBD 2017 and reported these causes separately at Level 3 (rather than Level 2). Because of these changes, neonatal disorders now appear for the first time as the first leading cause of DALYs in both 1990 and 2017.

Estimates of DALYs due to congenital birth defects were higher in GBD 2017 than in GBD 2016 for two reasons.^9^ First, all data sources that captured only a small subset of congenital causes (eg, only congenital heart disease and neural tube defects) were excluded for GBD 2017, leading to increased mortality estimates. Second, for GBD 2017 we implemented an algorithm to empirically identify data sources for registration of birth defects and for administrations (ie, hospital and claims) that had systematic under-reporting by age, sex, and defect. This change led to exclusion of several sources and, in many cases, higher prevalence estimates. The estimated DALYs due to malaria were lower in GBD 2017 than in GBD 2016 because of newly included *Plasmodium* parasite rate survey data and updates to insecticide-treated bednet coverage, which decreased incidence estimates, particularly in Nigeria. The decrease in incidence was in agreement with additional verbal autopsy data for Nigeria, which resulted in a decrease in estimated YLLs and DALYs.

The global age-standardised DALY rate for drug use disorders in GBD 2017 was higher for the most recent decade than in GBD 2016, because of a better fit to the most recent years of cause of death data in the USA and the use of more appropriate covariates, including the prevalence of injecting drug use and an estimate of consumption (measured by import and domestic manufacture of scheduled substances, including opioids, expressed as daily doses per 1000 people per day). Estimates of DALYs due to acute hepatitis overall are very similar to GBD 2016, but our assessments of the relative contribution of different viruses changed. We used clinical administrative data sources to calculate location-specific and age-specific case-fatality ratios for acute hepatitis infection, pairing these ratios with incidence estimates of each virus. Previously all locations were assumed to have identical case-fatality ratios. This resulted in higher estimates of DALYs for acute hepatitis A, and lower DALY estimates for acute hepatitis B, C, and E. Finally, estimated DALYs due to HIV/AIDS among children younger than 5 years have increased in the GBD 2017 study because of methodological improvements. In countries with high-quality vital registration data, incidence among children was adjusted to produce mortality estimates that better align with recorded HIV/AIDS deaths. Additionally, we produced the paediatric estimates of HIV/AIDS mortality using the CD4 progression and CD4-specific mortality rates developed by UNAIDS.^54^

### Limitations

This study has several limitations. First, the GBD 2017 YLL and YLD analyses had limitations as described in the GBD 2017 cause of death,^11^ disease and injury,^12^ and mortality papers.^13^ Second, we assumed that uncertainty is independent between YLLs and YLDs because little empirical evidence exists to establish this correlation; however, this assumption could result in an under-estimation of the total uncertainty for DALYs. Third, HALE and DALY estimates are influenced by the availability of data for YLL and YLD estimations. Because of time lags in the reporting of health data by countries and their subsequent incorporation into the GBD estimation, recent changes in health states might not have been captured in our estimates. The scarcity of data for a particular location is reflected by wider uncertainty intervals. Fourth, although we have included several sources of uncertainty in our estimations, we have not been able to incorporate uncertainty into the covariates used by cause of death and non-fatal models. Fifth, SDI utility might be restricted in countries with high income inequality. The applicability of SDI could be enhanced in the future by taking into account social heterogeneity within countries. Finally, time trends for specific causes, such as cancer, might be influenced by changes in diagnostic technology, whereas in previous years under-reporting might have occurred when diagnostic tests were done infrequently.

### Conclusion

Understanding trends in the health status of the global population and changes in the leading causes of disease burden over time is needed to accurately inform policies and set priorities for action. Updating the GBD study on a regular basis provides an opportunity to assess the latest evidence and monitor these trends with time to understand where interventions are having an effect and how much they have affected the disease burden. Our results showed that, globally, enormous improvements in health have occurred over the past 28 years. Nevertheless, large inequalities in HALE, years lived in poor health, and disease burden exist across SDI quintiles and countries, and between sexes. Despite the progress made in reducing the total burden of disease, hundreds of millions of DALYs could still be averted and disparities could be minimised through targeted interventions ranging from the prevention of risk factors and extended vaccine coverage, to universal access to essential health services.

## Supplementary Material

Supplementary appendix 1

Supplementary appendix 2

## Figures and Tables

**Table 1:** Life expectancy and HALE at birth for 21 GBD regions and 195 countries and territories, by sex in 1990 and 2017

	Life expectancy at birth				HALE at birth			
	
	Females		Males		Females		Males	
	
	1990	2017	1990	2017	1990	2017	1990	2017
**Global**	**68·0 (67·8–68·3)**	**75·6 (75·3–75·9)**	**63·2 (62·9–63·4)**	**70·5 (70·1–70·8)**	**58·4 (55·7–60·8)**	**64·8 (61·7–67·4)**	**55·6 (53·5–57·5)**	**61·8 (59·4–64·0)**

Low SDI	54·8 (54·3–55·4)	67·3 (66·7–67·9)	53·0 (52·4–53·6)	64·5 (63·8–65·1)	46·4 (44·0–48·5)	57·3 (54·5–59·8)	46·1 (44·1–47·9)	56·2 (53·9–58·4)
Low-middle SDI	61·9 (61·5–62·3)	70·1 (69·5–70·7)	59·0 (58·5–59·4)	66·3 (65·7–66·9)	52·6 (50·0–55·0)	59·8 (56·9–62·4)	51·5 (49·3–53·5)	58·0 (55·6–60·1)
Middle SDI	70·0 (69·7–70·3)	77·4 (77·1–77·7)	65·7 (65·3–66·1)	71·7 (71·4–72·1)	60·8 (58·2–63·1)	67·0 (64·1–69·5)	58·4 (56·3–60·2)	63·6 (61·2–65·6)
High-middle SDI	73·1 (72·9–73·4)	79·4 (79·1–79·7)	66·8 (66·5–67·0)	73·3 (73·0–73·7)	63·1 (60·4–65·5)	68·5 (65·4–71·1)	59·0 (56·9–61·0)	64·7 (62·2–66·8)
High SDI	79·3 (79·3–79·3)	83·7 (83·5–83·9)	72·6 (72·5–72·6)	78·5 (78·3–78·6)	67·9 (64·6–70·7)	71·1 (67·6–74·2)	63·8 (61·3–66·0)	68·2 (65·4–70·8)

**Central Europe, eastern Europe, and central Asia**	**73·9 (73·8–74·0)**	**77·6 (77·4–77·7)**	**64·8 (64·7–64·9)**	**68·5 (68·3–68·7)**	**63·3 (60·3–65·9)**	**66·3 (63·2–69·1)**	**56·5 (54·2–58·6)**	**59·7 (57·2–62·0)**

Central Asia	71·8 (71·5–72·0)	74·8 (74·3–75·4)	64·1 (63·7–64·4)	67·4 (66·8–67·9)	61·9 (59·0–64·5)	64·8 (61·9–67·4)	56·3 (54·0–58·3)	59·4 (57·0–61·5)
Armenia	73·3 (72·8–73·8)	78·7 (78·2–79·1)	66·7 (66·2–67·2)	72·4 (72·0–72·8)	63·5 (60·7–66·0)	68·1 (65·0–70·7)	58·3 (55·9–60·5)	63·4 (60·7–65·7)
Azerbaijan	71·2 (70·4–71·9)	74·7 (73·7–75·7)	63·4 (62·6–64·3)	67·2 (66·2–68·2)	61·7 (59·0–64·1)	64·9 (61·9–67·6)	56·0 (53·7–58·1)	59·5 (57·2–61·6)
Georgia	73·9 (73·4–74·4)	77·3 (76·9–77·7)	65·9 (65·2–66·6)	68·4 (68·0–68·8)	64·6 (61·8–67·0)	67·2 (64·3–69·7)	58·4 (56·2–60·4)	60·4 (58·1–62·4)
Kazakhstan	73·3 (73·0–73·5)	76·4 (75·8–77·1)	63·4 (63·1–63·7)	67·5 (66·8–68·2)	62·9 (60·0–65·5)	66·1 (63·2–68·6)	55·5 (53·2–57·5)	59·3 (56·9–61·4)
Kyrgyzstan	70·5 (69·7–71·1)	76·3 (75·9–76·6)	62·0 (61·2–62·8)	69·1 (68·7–69·4)	60·8 (57·8–63·3)	66·0 (63·0–68·5)	54·3 (51·9–56·3)	60·9 (58·5–63·0)
Mongolia	64·0 (63·3–64·7)	73·7 (72·5–74·8)	58·6 (57·9–59·4)	64·5 (63·2–65·9)	55·8 (53·4–58·0)	64·0 (61·0–66·7)	51·7 (49·7–53·7)	56·7 (54·3–59·0)
Tajikistan	69·5 (68·9–70·1)	73·3 (72·1–74·5)	64·5 (63·8–65·1)	67·7 (66·3–68·9)	59·9 (57·2–62·4)	63·5 (60·6–66·1)	56·4 (54·0–58·5)	59·4 (56·8–61·8)
Turkmenistan	69·3 (68·8–69·9)	73·9 (72·7–74·9)	62·6 (62·0–63·1)	66·5 (65·4–67·7)	60·4 (57·8–62·7)	64·5 (61·7–67·1)	55·3 (53·2–57·3)	59·0 (56·6–61·2)
Uzbekistan	72·6 (72·2–72·9)	73·7 (72·2–75·3)	66·0 (65·6–66·4)	67·1 (65·5–68·6)	62·3 (59·3–64·9)	63·9 (60·9–66·8)	57·9 (55·4–60·0)	59·4 (56·8–61·8)
Central Europe	74·9 (74·9–75·0)	80·4 (80·2–80·7)	67·2 (67·1–67·2)	73·6 (73·3–73·9)	64·2 (61·1–66·9)	68·8 (65·5–71·7)	58·3 (55·8–60·5)	63·5 (60·6–66·0)
Albania	77·4 (77·0–77·7)	82·1 (79·9–84·3)	69·8 (69·5–70·2)	74·9 (72·8–77·1)	66·3 (63·0–69·1)	70·5 (67·0–73·9)	60·7 (58·0–63·1)	65·0 (61·8–68·2)
Bosnia and Herzegovina	76·6 (76·4–76·8)	79·1 (78·4–79·7)	70·5 (70·4–70·7)	74·3 (73·6–75·0)	65·6 (62·5–68·3)	67·7 (64·5–70·5)	61·1 (58·5–63·4)	63·7 (60·7–66·4)
Bulgaria	75·5 (75·4–75·7)	78·6 (77·9–79·2)	68·2 (68·1–68·3)	71·3 (70·6–72·1)	65·2 (62·2–67·8)	67·7 (64·7–70·5)	59·4 (56·9–61·6)	62·2 (59·5–64·5)
Croatia	76·3 (76·1–76·4)	81·6 (80·9–82·3)	68·7 (68·6–68·9)	75·4 (74·7–76·1)	65·7 (62·8–68·4)	69·9 (66·6–72·8)	59·9 (57·3–62·1)	64·9 (62·0–67·4)
Czech Republic	75·5 (75·4–75·6)	82·0 (81·3–82·6)	67·6 (67·5–67·6)	76·3 (75·6–77·0)	64·7 (61·6–67·4)	69·6 (66·0–72·7)	58·9 (56·3–61·1)	65·1 (61·9–68·0)
Hungary	73·9 (73·8–74·0)	80·2 (79·5–80·9)	65·3 (65·2–65·4)	73·2 (72·4–73·9)	62·9 (59·8–65·7)	68·3 (65·1–71·3)	56·6 (54·2–58·7)	63·1 (60·3–65·6)
Macedonia	74·5 (74·2–74·7)	79·7 (79·2–80·3)	69·6 (69·4–69·8)	73·9 (73·2–74·6)	64·1 (61·2–66·7)	68·4 (65·2–71·3)	60·5 (57·9–62·7)	63·9 (61·1–66·3)
Montenegro	77·5 (77·2–77·8)	78·9 (78·1–79·7)	71·1 (70·8–71·5)	74·1 (72·9–75·2)	66·7 (63·6–69·5)	67·9 (64·7–70·7)	61·8 (59·1–64·2)	64·1 (61·1–66·6)
Poland	75·8 (75·7–75·8)	81·8 (81·2–82·4)	66·8 (66·8–66·9)	74·1 (73·3–74·8)	65·0 (61·9–67·7)	69·9 (66·6–72·9)	58·1 (55·6–60·2)	63·7 (60·7–66·4)
Romania	73·2 (73·1–73·3)	79·0 (78·3–79·6)	66·7 (66·6–66·8)	71·5 (70·8–72·3)	62·6 (59·5–65·2)	67·6 (64·3–70·4)	57·4 (54·8–59·7)	61·9 (59·2–64·4)
Serbia	74·5 (74·3–74·6)	77·9 (77·2–78·5)	67·6 (67·4–67·7)	73·6 (72·9–74·2)	64·0 (60·9–66·5)	66·9 (63·8–69·6)	58·8 (56·4–61·0)	63·7 (60·8–66·2)
Slovakia	75·5 (75·3–75·6)	80·6 (79·9–81·3)	66·7 (66·6–66·8)	74·1 (73·4–74·8)	64·9 (61·8–67·5)	68·9 (65·7–71·8)	57·9 (55·4–60·1)	63·7 (60·7–66·3)
Slovenia	77·8 (77·6–78·0)	84·2 (83·5–85·0)	69·7 (69·6–69·9)	77·9 (77·2–78·7)	66·4 (63·3–69·3)	71·2 (67·5–74·4)	60·1 (57·4–62·5)	66·3 (63·0–69·2)
Eastern Europe	74·6 (74·6–74·7)	77·2 (77·1–77·4)	64·5 (64·5–64·6)	66·5 (66·3–66·7)	63·7 (60·7–66·4)	65·9 (62·7–68·6)	56·3 (54·0–58·4)	58·2 (55·8–60·3)
Belarus	75·7 (75·5–75·9)	78·8 (78·1–79·4)	66·1 (65·8–66·3)	69·0 (68·2–69·7)	64·7 (61·7–67·4)	67·3 (64·1–70·2)	57·7 (55·3–59·9)	60·3 (57·7–62·6)
Estonia	75·0 (74·7–75·2)	82·1 (80·7–83·5)	64·7 (64·5–64·9)	73·6 (72·0–75·3)	64·3 (61·2–66·9)	70·0 (66·5–73·4)	56·5 (54·1–58·7)	63·8 (60·8–66·6)
Latvia	74·7 (74·5–74·9)	79·8 (78·4–81·3)	64·6 (64·4–64·8)	70·1 (68·6–71·7)	63·9 (60·9–66·6)	68·0 (64·6–71·0)	56·3 (53·9–58·5)	60·9 (58·2–63·6)
Lithuania	76·2 (76·0–76·3)	80·2 (79·4–81·0)	66·4 (66·2–66·5)	69·6 (68·7–70·5)	65·2 (62·1–67·8)	68·1 (64·8–71·2)	57·8 (55·4–60·0)	60·4 (57·7–62·8)
Moldova	71·4 (71·2–71·7)	77·4 (77·0–77·9)	64·5 (64·2–64·7)	68·2 (67·8–68·7)	61·1 (58·1–63·6)	66·2 (63·1–69·0)	56·1 (53·6–58·2)	59·6 (57·2–61·8)
Russia	74·6 (74·6–74·6)	77·2 (77·1–77·4)	64·0 (64·0–64·0)	66·8 (66·6–66·9)	63·7 (60·6–66·4)	65·8 (62·6–68·6)	55·9 (53·6–58·0)	58·4 (56·0–60·5)
Ukraine	74·7 (74·5–74·9)	76·5 (75·8–77·2)	65·5 (65·3–65·7)	64·7 (63·9–65·4)	63·7 (60·6–66·4)	65·4 (62·1–68·1)	57·2 (54·8–59·3)	56·7 (54·4–58·8)

**High income**	**79·4 (79·4–79·4)**	**83·6 (83·4–83·7)**	**72·8 (72·7–72·8)**	**78·4 (78·2–78·6)**	**68·0 (64·7–70·8)**	**71·0 (67·5–74·0)**	**64·0 (61·4–66·1)**	**68·2 (65·3–70·7)**

Australasia	79·7 (79·6–79·8)	84·4 (83·4–85·4)	73·6 (73·5–73·6)	80·1 (79·1–81·2)	68·0 (64·7–70·9)	71·4 (67·7–74·6)	64·1 (61·4–66·4)	68·9 (65·7–71·8)
Australia	80·0 (79·9–80·1)	84·6 (83·4–85·7)	73·8 (73·7–73·9)	80·2 (78·9–81·5)	68·4 (65·1–71·3)	71·7 (68·0–74·9)	64·3 (61·6–66·7)	69·1 (65·8–72·0)
New Zealand	78·1 (77·9–78·3)	83·6 (83·0–84·2)	72·6 (72·4–72·8)	79·7 (79·0–80·3)	66·3 (62·9–69·3)	70·1 (66·1–73·3)	63·1 (60·2–65·4)	68·0 (64·6–71·0)
High-income Asia Pacific	81·0 (81·0–81·1)	86·9 (86·7–87·2)	74·4 (74·4–74·5)	80·8 (80·5–81·0)	70·1 (67·0–72·8)	74·5 (71·0–77·6)	66·3 (63·9–68·3)	71·1 (68·2–73·4)
Brunei	72·1 (71·5–72·8)	77·5 (76·6–78·4)	69·1 (68·5–69·8)	73·3 (72·3–74·4)	62·9 (60·2–65·3)	67·5 (64·5–70·0)	61·4 (59·2–63·4)	65·0 (62·4–67·3)
Japan	82·2 (82·2–82·2)	87·2 (87·0–87·4)	76·2 (76·2–76·2)	81·1 (80·8–81·3)	71·2 (68·0–73·9)	74·6 (71·1–77·8)	68·0 (65·6–70·0)	71·4 (68·6–73·8)
Singapore	78·8 (78·6–79·0)	87·6 (86·9–88·1)	73·5 (73·3–73·7)	81·9 (81·2–82·6)	68·5 (65·5–71·0)	75·8 (72·4–78·7)	65·6 (63·3–67·6)	72·6 (69·8–75·0)
South Korea	76·4 (76·3–76·5)	85·5 (84·9–86·1)	68·0 (67·9–68·1)	79·5 (78·7–80·3)	66·0 (62·9–68·7)	73·5 (70·0–76·5)	60·3 (58·1–62·2)	69·7 (67·0–72·1)
High-income North America	79·1 (79·1–79·2)	81·4 (81·1–81·7)	72·3 (72·3–72·3)	76·5 (76·2–76·8)	67·0 (63·6–69·9)	68·2 (64·7–71·3)	62·9 (60·2–65·1)	65·7 (62·7–68·3)
Canada	80·6 (80·5–80·6)	84·0 (83·4–84·6)	74·1 (74·0–74·2)	79·9 (79·2–80·5)	69·0 (65·6–71·8)	71·4 (67·7–74·5)	65·3 (62·7–67·5)	69·6 (66·6–72·3)
Greenland	69·0 (68·4–69·6)	77·2 (76·2–78·0)	62·1 (61·6–62·6)	70·8 (70·3–71·4)	58·5 (55·7–61·1)	65·3 (62·2–68·3)	54·4 (52·2–56·3)	62·2 (59·7–64·3)
USA	79·0 (79·0–79·0)	81·1 (80·8–81·4)	72·1 (72·1–72·1)	76·1 (75·8–76·4)	66·8 (63·4–69·7)	67·9 (64·3–71·0)	62·6 (59·9–64·9)	65·3 (62·3–67·9)
Southern Latin America	76·2 (76·1–76·2)	80·4 (79·3–81·3)	69·2 (69·1–69·2)	74·5 (73·3–75·5)	65·9 (62·9–68·5)	69·3 (66·2–72·1)	61·1 (58·8–63·2)	65·3 (62·5–67·9)
Argentina	75·9 (75·8–76·0)	79·7 (78·3–81·0)	68·9 (68·9–69·0)	73·6 (72·0–75·0)	65·9 (62·9–68·4)	68·9 (65·7–71·8)	61·0 (58·7–63·0)	64·7 (61·8–67·4)
Chile	76·4 (76·3–76·5)	82·1 (80·8–83·4)	69·9 (69·8–70·0)	77·2 (75·7–78·7)	65·7 (62·6–68·4)	70·2 (66·6–73·3)	61·5 (59·0–63·6)	67·1 (64·1–70·0)
Uruguay	76·8 (76·6–77·0)	80·4 (79·0–81·9)	69·4 (69·2–69·5)	73·5 (72·1–75·0)	66·6 (63·7–69·2)	69·5 (66·4–72·4)	61·5 (59·2–63·5)	64·8 (62·2–67·4)
Western Europe	79·5 (79·5–79·5)	84·2 (83·9–84·5)	73·0 (73·0–73·0)	79·5 (79·2–79·8)	68·2 (65·0–71·0)	71·8 (68·3–74·8)	64·3 (61·8–66·5)	69·4 (66·5–71·9)
Andorra	82·6 (80·8–84·6)	85·1 (83·6–86·7)	76·1 (74·7–77·3)	80·5 (79·4–81·7)	70·6 (67·0–74·1)	72·4 (68·6–75·8)	66·8 (63·9–69·5)	70·1 (67·1–72·8)
Austria	78·9 (78·8–79·0)	84·0 (83·4–84·6)	72·4 (72·3–72·5)	79·4 (78·8–80·1)	67·9 (64·7–70·6)	71·7 (68·2–74·8)	63·9 (61·4–66·0)	69·1 (66·2–71·8)
Belgium	79·3 (79·2–79·4)	83·8 (83·1–84·5)	72·7 (72·6–72·8)	78·9 (78·2–79·5)	67·8 (64·5–70·6)	70·9 (67·3–74·1)	63·9 (61·4–66·1)	68·3 (65·2–71·0)
Cyprus	78·3 (78·1–78·5)	85·2 (84·3–86·0)	73·6 (73·4–73·8)	78·5 (77·4–79·5)	67·2 (64·1–70·0)	72·8 (69·1–76·0)	64·9 (62·5–67·1)	68·8 (65·9–71·4)
Denmark	77·8 (77·6–77·9)	82·7 (81·9–83·4)	72·2 (72·1–72·4)	78·8 (78·1–79·5)	66·9 (63·7–69·5)	70·6 (67·2–73·6)	63·7 (61·2–65·8)	68·6 (65·6–71·3)
Finland	79·1 (78·9–79·3)	84·3 (83·6–84·9)	71·0 (70·9–71·2)	78·5 (77·8–79·2)	67·6 (64·2–70·5)	71·5 (67·9–74·8)	62·2 (59·6–64·4)	68·0 (64·9–70·7)
France	81·1 (81·0–81·1)	85·7 (85·1–86·3)	73·0 (72·9–73·0)	79·8 (79·2–80·4)	69·8 (66·5–72·6)	73·4 (69·9–76·5)	64·7 (62·2–66·8)	70·0 (67·2–72·5)
Germany	78·6 (78·5–78·6)	83·0 (81·8–84·2)	72·1 (72·1–72·2)	78·2 (76·9–79·5)	67·4 (64·2–70·2)	70·8 (67·2–74·1)	63·5 (61·0–65·7)	68·3 (65·2–70·9)
Greece	80·4 (80·3–80·5)	83·6 (83·0–84·2)	74·7 (74·6–74·8)	78·4 (77·8–79·1)	68·9 (65·6–71·8)	71·3 (67·8–74·4)	65·8 (63·3–68·0)	68·6 (65·8–71·1)
Iceland	80·2 (79·8–80·5)	85·9 (85·5–86·4)	75·6 (75·2–75·9)	79·8 (79·4–80·2)	68·5 (65·2–71·4)	73·1 (69·5–76·2)	66·3 (63·7–68·5)	69·6 (66·5–72·2)
Ireland	77·6 (77·4–77·8)	83·7 (82·9–84·4)	72·2 (72·1–72·4)	80·0 (79·3–80·7)	66·7 (63·6–69·5)	71·3 (67·7–74·4)	63·7 (61·2–65·9)	69·4 (66·3–72·0)
Israel	78·9 (78·8–79·1)	84·6 (83·9–85·2)	75·8 (75·6–76·0)	81·3 (80·6–81·9)	67·6 (64·4–70·4)	72·1 (68·6–75·2)	66·4 (63·7–68·7)	70·6 (67·6–73·4)
Italy	80·3 (80·2–80·4)	85·3 (84·7–85·9)	73·7 (73·7–73·8)	80·8 (80·2–81·4)	68·8 (65·6–71·7)	73·0 (69·5–76·1)	65·0 (62·5–67·2)	70·6 (67·7–73·3)
Luxembourg	78·9 (78·6–79·2)	83·3 (82·3–84·2)	71·8 (71·6–72·1)	80·0 (78·9–81·2)	67·0 (63·6–69·9)	70·4 (66·8–73·7)	62·7 (60·1–64·9)	69·0 (65·6–71·9)
Malta	78·8 (78·5–79·0)	83·0 (82·4–83·6)	74·2 (73·9–74·4)	78·9 (78·4–79·5)	67·7 (64·6–70·5)	70·9 (67·4–73·9)	65·3 (62·7–67·5)	68·7 (65·6–71·3)
Netherlands	80·1 (80·0–80·2)	83·1 (82·4–83·7)	73·8 (73·7–73·9)	79·9 (79·2–80·5)	68·4 (65·1–71·3)	70·7 (67·2–73·9)	64·9 (62·4–67·1)	69·6 (66·6–72·2)
Norway	80·0 (79·9–80·1)	84·2 (84·0–84·4)	73·4 (73·3–73·5)	80·5 (80·2–80·7)	68·1 (64·7–71·0)	71·1 (67·4–74·3)	64·0 (61·3–66·4)	69·3 (66·2–72·0)
Portugal	77·6 (77·5–77·7)	84·2 (83·6–84·8)	70·7 (70·5–70·8)	78·5 (77·9–79·2)	66·2 (62·9–69·0)	71·6 (68·0–74·7)	62·1 (59·6–64·2)	68·6 (65·6–71·2)
Spain	80·5 (80·4–80·6)	85·8 (85·3–86·3)	73·5 (73·4–73·5)	80·2 (79·7–80·8)	69·4 (66·3–72·1)	73·6 (70·0–76·7)	65·0 (62·5–67·1)	70·5 (67·7–72·9)
Sweden	80·5 (80·4–80·6)	84·2 (83·7–84·7)	74·9 (74·8–75·1)	80·8 (80·2–81·4)	68·8 (65·4–71·6)	71·4 (67·8–74·6)	66·0 (63·4–68·3)	70·4 (67·4–73·1)
Switzerland	81·1 (81·0–81·3)	85·7 (85·1–86·3)	74·4 (74·3–74·5)	82·1 (81·5–82·8)	69·0 (65·6–72·0)	72·7 (69·0–75·9)	65·1 (62·4–67·4)	71·2 (68·1–74·0)
UK	78·5 (78·4–78·5)	82·7 (82·6–82·8)	72·9 (72·9–73·0)	79·2 (79·0–79·3)	67·3 (64·0–70·0)	70·0 (66·5–73·1)	64·1 (61·6–66·3)	68·5 (65·5–71·1)
England	78·7 (78·7–78·7)	82·9 (82·8–83·0)	73·2 (73·1–73·2)	79·5 (79·4–79·6)	67·4 (64·2–70·2)	70·1 (66·5–73·2)	64·4 (61·8–66·5)	68·7 (65·6–71·3)
Northern Ireland	77·3 (77·0–77·6)	82·5 (81·5–83·4)	71·5 (71·3–71·7)	78·7 (77·7–79·8)	66·4 (63·3–69·0)	70·3 (67·0–73·5)	63·1 (60·6–65·2)	68·5 (65·5–71·3)
Scotland	76·8 (76·7–77·0)	81·2 (80·3–82·1)	71·2 (71·0–71·3)	76·9 (76·0–78·0)	65·8 (62·7–68·5)	69·3 (66·0–72·2)	62·5 (60·0–64·6)	66·8 (64·0–69·5)
Wales	78·6 (78·4–78·7)	82·5 (81·7–83·2)	72·9 (72·8–73·1)	78·3 (77·5–79·1)	67·3 (64·1–70·1)	70·4 (66·9–73·6)	64·1 (61·5–66·3)	68·1 (65·1–70·7)

**Latin America and Caribbean**	**72·5 (72·3–72·6)**	**78·9 (78·6–79·2)**	**66·2 (66·0–66·4)**	**72·8 (72·4–73·2)**	**62·6 (59·8–65·0)**	**68·3 (65·2–70·8)**	**58·6 (56·3–60·5)**	**64·2 (61·7–66·3)**

Andean Latin America	70·6 (70·0–71·2)	79·5 (78·4–80·6)	66·7 (66·1–67·3)	76·2 (74·9–77·4)	61·4 (58·8–63·8)	69·2 (66·1–72·0)	58·9 (56·5–60·9)	67·0 (64·3–69·6)
Bolivia	62·1 (60·8–63·3)	74·2 (72·1–76·6)	59·7 (58·5–61·0)	71·3 (68·8–73·9)	54·0 (51·5–56·3)	64·4 (61·2–67·7)	52·6 (50·2–54·6)	62·8 (59·8–65·8)
Ecuador	74·4 (74·1–74·6)	78·7 (77·5–79·9)	69·7 (69·5–70·0)	74·8 (73·3–76·1)	64·8 (62·0–67·3)	68·7 (65·6–71·4)	61·5 (59·0–63·5)	65·8 (63·0–68·2)
Peru	72·2 (71·3–73·2)	81·9 (80·1–83·7)	67·9 (66·9–68·9)	78·7 (76·8–80·8)	62·7 (59·8–65·3)	71·3 (68·1–74·5)	60·0 (57·6–62·2)	69·3 (66·2–72·4)
Caribbean	70·4 (69·9–70·8)	75·4 (74·4–76·4)	66·4 (65·9–66·8)	70·3 (69·3–71·4)	61·2 (58·5–63·6)	65·3 (62·5–67·9)	58·9 (56·7–60·9)	62·2 (59·7–64·5)
Antigua and Barbuda	77·9 (77·2–78·7)	78·7 (78·1–79·4)	70·8 (70·1–71·4)	75·3 (74·4–76·2)	67·3 (64·1–70·2)	68·1 (65·0–70·8)	62·6 (60·1–64·8)	66·4 (63·7–68·7)
The Bahamas	74·7 (74·3–75·1)	76·6 (75·4–77·9)	67·6 (67·2–68·0)	70·8 (69·6–72·1)	65·4 (62·7–67·7)	66·9 (63·9–69·5)	60·5 (58·5–62·3)	63·2 (60·7–65·5)
Barbados	76·1 (75·7–76·5)	78·6 (77·7–79·6)	71·2 (70·8–71·5)	75·5 (74·4–76·6)	66·7 (63·9–69·0)	68·6 (65·5–71·2)	63·8 (61·6–65·7)	67·2 (64·5–69·5)
Belize	73·8 (73·1–74·4)	77·4 (76·9–77·9)	70·3 (69·5–71·0)	71·2 (70·7–71·8)	64·0 (61·0–66·5)	67·0 (64·0–69·5)	62·4 (60·0–64·6)	63·1 (60·6–65·3)
Bermuda	78·3 (77·9–78·6)	85·7 (84·8–86·5)	69·7 (69·3–70·2)	77·1 (76·4–77·6)	68·5 (65·7–70·9)	74·3 (71·0–77·3)	62·5 (60·5–64·4)	68·5 (66·0–70·7)
Cuba	76·8 (76·6–76·9)	80·7 (79·3–82·1)	73·0 (72·9–73·1)	76·2 (74·6–77·7)	66·8 (63·9–69·4)	70·4 (67·4–73·2)	65·3 (63·0–67·2)	67·9 (65·4–70·5)
Dominica	75·3 (74·7–75·7)	75·4 (74·3–76·4)	70·4 (70·0–70·8)	70·4 (69·4–71·4)	65·6 (62·7–68·0)	65·8 (62·9–68·3)	62·7 (60·5–64·8)	62·6 (60·2–64·7)
Dominican Republic	74·4 (73·4–75·4)	76·8 (75·2–78·5)	69·6 (68·4–70·7)	69·8 (67·8–71·9)	64·6 (61·6–67·2)	66·8 (63·6–69·5)	61·5 (59·0–63·8)	62·0 (59·3–64·7)
Grenada	71·6 (71·0–72·1)	75·4 (74·7–76·2)	67·1 (66·6–67·6)	73·0 (72·3–73·6)	62·5 (59·8–64·9)	65·8 (62·9–68·3)	60·0 (57·9–61·9)	64·8 (62·2–66·9)
Guyana	69·0 (68·7–69·4)	72·2 (70·5–73·9)	62·4 (62·0–62·8)	66·4 (64·6–68·2)	59·6 (56·9–62·0)	62·4 (59·3–65·3)	54·9 (52·7–56·9)	58·6 (56·0–60·9)
Haiti	55·0 (53·6–56·5)	66·0 (63·3–68·8)	53·9 (52·3–55·5)	63·8 (61·4–66·4)	47·4 (44·9–49·8)	56·8 (53·6–59·9)	47·3 (44·9–49·5)	55·8 (52·7–58·6)
Jamaica	76·4 (75·7–77·1)	77·5 (75·4–79·4)	73·6 (73·0–74·3)	72·0 (69·8–74·1)	66·6 (63·5–69·0)	67·4 (64·2–70·3)	65·4 (62·9–67·5)	63·9 (61·0–66·6)
Puerto Rico	78·4 (78·2–78·6)	81·6 (80·9–82·3)	70·0 (69·8–70·1)	74·5 (73·7–75·4)	68·6 (65·8–71·1)	70·8 (67·7–73·6)	62·3 (60·0–64·1)	65·8 (63·1–68·1)
Saint Lucia	73·2 (72·8–73·6)	78·1 (77·2–78·9)	67·8 (67·4–68·2)	73·1 (72·2–74·0)	63·7 (61·1–66·2)	67·9 (64·9–70·6)	60·4 (58·3–62·3)	64·9 (62·5–67·0)
Saint Vincent and the Grenadines	72·9 (72·4–73·5)	75·4 (74·6–76·3)	69·1 (68·5–69·6)	69·6 (68·9–70·4)	63·5 (60·5–65·9)	65·5 (62·6–68·1)	61·4 (59·2–63·4)	61·7 (59·5–63·9)
Suriname	71·3 (70·5–72·2)	75·3 (74·0–76·6)	66·4 (65·4–67·4)	68·9 (67·2–70·7)	62·1 (59·4–64·5)	65·2 (62·2–68·0)	59·2 (56·9–61·3)	61·2 (58·7–63·7)
Trinidad and Tobago	72·5 (72·1–72·8)	77·6 (74·8–80·3)	67·5 (67·2–67·8)	71·1 (68·4–74·0)	63·0 (60·3–65·4)	67·2 (63·6–70·6)	60·1 (57·9–62·0)	63·0 (59·9–66·0)
Virgin Islands	76·2 (75·1–77·0)	78·8 (77·2–80·1)	69·0 (68·2–69·7)	69·5 (67·9–71·8)	66·9 (64·1–69·4)	69·0 (65·9–71·7)	61·9 (59·7–63·7)	62·2 (59·7–64·9)
Central Latin America	74·0 (73·9–74·2)	79·4 (79·0–79·8)	68·1 (67·9–68·3)	73·3 (72·8–73·8)	64·4 (61·8–66·8)	69·1 (66·1–71·6)	60·6 (58·4–62·4)	65·0 (62·5–67·1)
Colombia	74·8 (74·6–75·0)	82·7 (81·4–83·9)	68·1 (67·9–68·4)	77·4 (75·9–79·0)	65·1 (62·4–67·6)	72·1 (68·9–75·0)	60·7 (58·6–62·5)	68·7 (66·1–71·4)
Costa Rica	78·7 (78·5–79·0)	82·7 (81·9–83·4)	74·4 (74·2–74·6)	76·3 (75·5–77·1)	68·4 (65·4–71·0)	71·9 (68·8–74·4)	66·4 (64·1–68·3)	67·9 (65·2–70·0)
El Salvador	73·6 (73·2–73·9)	78·3 (76·0–80·4)	64·9 (64·5–65·2)	69·3 (66·7–72·0)	64·0 (61·4–66·5)	68·3 (65·0–71·3)	57·2 (55·0–59·2)	61·5 (58·4–64·3)
Guatemala	65·7 (65·3–66·2)	76·0 (74·5–77·4)	60·4 (59·9–60·9)	69·1 (67·4–70·8)	57·1 (54·5–59·3)	66·1 (63·1–69·0)	53·5 (51·4–55·3)	61·2 (58·7–63·7)
Honduras	71·2 (69·6–72·8)	75·0 (72·4–78·2)	66·6 (64·8–68·6)	72·9 (70·2–75·6)	61·7 (58·7–64·3)	65·3 (61·8–68·7)	59·0 (56·4–61·7)	64·6 (61·4–67·7)
Mexico	74·3 (74–74·6·0)	78·5 (78·2–78·8)	68·6 (68·2–68·9)	72·6 (72·3–72·9)	64·7 (62·1–67·0)	68·2 (65·3–70·7)	61·0 (58·9–62·9)	64·2 (61·8–66·3)
Nicaragua	74·1 (73·2–74·9)	80·6 (79·4–82·0)	69·7 (68·6–70·7)	76·9 (75·3–78·4)	63·5 (60·5–66·2)	69·8 (66·6–72·7)	61·2 (58·6–63·4)	67·8 (65·1–70·5)
Panama	78·1 (77·7–78·4)	81·7 (80·9–82·5)	73·8 (73·4–74·1)	77·0 (76·2–77·9)	67·8 (64·8–70·4)	70·9 (67·8–73·6)	65·7 (63·2–67·7)	68·1 (65·4–70·5)
Venezuela	75·1 (75·0–75·2)	79·6 (77·7–81·5)	69·2 (69·1–69·3)	71·2 (68·9–73·7)	65·5 (62·9–67·9)	69·3 (66·0–72·3)	61·8 (59·6–63·6)	63·4 (60·5–66·1)
Tropical Latin America	71·7 (71·4–72·0)	79·1 (78·8–79·3)	64·0 (63·7–64·4)	72·0 (71·8–72·3)	61·2 (58·2–63·8)	67·8 (64·6–70·5)	56·3 (54·0–58·3)	63·1 (60·5–65·3)
Brazil	71·6 (71·2–71·9)	79·1 (78·8–79·3)	63·8 (63·4–64·2)	72·0 (71·7–72·2)	61·1 (58·1–63·7)	67·7 (64·6–70·5)	56·1 (53·9–58·1)	63·1 (60·5–65·3)
Paraguay	76·4 (75·6–77·2)	78·9 (76·8–81·2)	72·3 (71·4–73·0)	73·4 (71·0–76·0)	65·5 (62·3–68·4)	67·9 (64·4–71·0)	63·3 (60·7–65·7)	64·4 (61·1–67·4)

**North Africa and Middle East**	**68·2 (67·9–68·6)**	**76·8 (76·4–77·3)**	**64·5 (64·1–64·9)**	**72·0 (71·5–72·5)**	**57·5 (54·5–60·2)**	**64·8 (61·4–67·7)**	**55·9 (53·5–58·1)**	**62·1 (59·4–64·5)**

Afghanistan	52·0 (49·9–54·3)	63·2 (60·6–65·8)	53·1 (51·0–55·2)	63·6 (61·3–65·9)	42·9 (40·0–45·9)	52·5 (49·2–56·2)	44·1 (41·1–46·8)	53·6 (50·4–56·5)
Algeria	73·2 (72·3–74·1)	78·5 (77·9–79·1)	70·3 (69·4–71·2)	77·0 (76·4–77·6)	61·9 (58·6–64·9)	66·6 (63·1–69·6)	60·9 (58·2–63·4)	66·4 (63·4–69·0)
Bahrain	71·9 (71·4–72·4)	80·4 (79·5–81·4)	69·4 (69·0–70·0)	78·8 (77·8–79·8)	60·9 (57·8–63·6)	67·6 (64·0–70·9)	60·5 (58·0–62·7)	67·8 (64·7–70·8)
Egypt	66·6 (66·1–67·1)	74·3 (72·9–75·8)	62·6 (62·1–63·0)	68·0 (66·6–69·3)	56·3 (53·3–58·9)	63·0 (59·8–66·0)	54·5 (52·1–56·6)	59·3 (56·7–61·7)
Iran	70·8 (70·1–71·4)	79·4 (79·3–79·5)	65·7 (65·0–66·3)	75·5 (75·4–75·6)	59·6 (56·4–62·4)	66·5 (63·0–69·7)	56·9 (54·5–59·3)	65·0 (62·1–67·5)
Iraq	67·6 (65·8–69·3)	78·8 (78·1–79·6)	64·4 (62·5–66·4)	74·8 (73·9–75·6)	56·6 (53·4–59·7)	65·7 (61·9–69·0)	54·8 (51·9–57·7)	63·3 (60·1–66·2)
Jordan	71·7 (70·3–73·0)	81·1 (79·8–82·3)	70·5 (69·0–71·9)	77·8 (76·3–79·2)	60·9 (57·8–63·7)	68·5 (65·0–71·7)	61·1 (58·3–63·8)	67·1 (63·8–70·0)
Kuwait	77·1 (76·9–77·4)	87·2 (86·7–87·7)	73·3 (73·1–73·6)	80·7 (80·0–81·3)	65·3 (62·0–68·2)	73·1 (69·1–76·6)	63·7 (61·0–66·1)	69·4 (66·1–72·2)
Lebanon	73·4 (72·2–75·0)	80·0 (79·4–80·7)	67·3 (66·1–68·8)	75·8 (75·1–76·4)	62·1 (58·9–65·2)	67·4 (63·9–70·5)	58·3 (55·8–60·7)	65·0 (62·0–67·7)
Libya	73·5 (71·7–75·2)	75·0 (73·3–76·9)	70·8 (69·0–72·6)	71·1 (69·4–73·2)	62·3 (59·0–65·4)	63·5 (60·0–66·7)	61·2 (58·1–63·9)	60·9 (58·1–64·1)
Morocco	66·2 (65·3–67·2)	74·7 (72·7–76·8)	67·1 (66·1–68·1)	73·2 (71·0–75·5)	56·0 (53·1–58·6)	63·3 (59·9–66·7)	57·8 (55·0–60·1)	63·0 (59·7–66·2)
Oman	71·4 (69·2–73·7)	79·4 (78·2–81·2)	66·9 (64·4–69·4)	75·5 (73·3–77·9)	59·6 (55·8–63·1)	66·9 (63·1–70·3)	57·8 (54·7–61·0)	65·0 (61·7–68·5)
Palestine	72·6 (70·7–74·5)	78·0 (77·3–78·9)	68·5 (66·7–70·7)	75·6 (74·7–76·4)	61·1 (57·6–64·3)	65·6 (62·1–68·7)	59·1 (56·0–62·1)	64·6 (61·6–67·4)
Qatar	72·8 (71·2–74·3)	81·7 (79·8–83·5)	70·7 (69·1–72·4)	79·6 (77·7–81·6)	61·7 (58·7–64·6)	68·7 (65·0–72·1)	61·0 (58·0–63·8)	68·1 (64·7–71·5)
Saudi Arabia	73·6 (71·6–75·8)	79·4 (78·0–80·2)	70·3 (68·0–72·5)	75·3 (73·9–76·6)	62·2 (58·8–65·5)	67·8 (64·4–70·7)	61·1 (57·9–64·2)	65·4 (62·7–68·2)
Sudan	59·9 (58·0–61·7)	72·0 (69·5–74·7)	57·4 (55·6–59·1)	68·8 (66·4–71·5)	50·5 (47·3–53·4)	60·9 (57·4–64·3)	49·6 (47·0–52·1)	59·4 (56·2–62·6)
Syria	72·3 (71·2–73·4)	75·0 (74·0–76·3)	67·7 (66·3–69·0)	65·5 (63·8–67·2)	61·2 (58·1–64·0)	63·5 (60·2–66·5)	59·1 (56·4–61·5)	56·7 (54·0–59·3)
Tunisia	74·5 (73·9–75·0)	80·7 (78·5–83·0)	70·8 (70·3–71·5)	76·1 (73·7–78·6)	63·5 (60·4–66·2)	69·0 (65·3–72·2)	61·4 (58·8–63·7)	65·8 (62·5–69·1)
Turkey	72·1 (71·3–72·8)	83·0 (82·0–84·0)	65·6 (64·8–66·3)	75·2 (74·1–76·3)	60·8 (57·5–63·6)	70·3 (66·8–73·6)	57·3 (55·0–59·4)	65·7 (62·9–68·2)
United Arab Emirates	73·0 (71·2–75·0)	76·9 (74·7–79·2)	70·2 (68·2–72·4)	71·7 (69·3–74·0)	62·1 (58·7–65·3)	65·6 (62·2–69·0)	60·8 (57·9–63·7)	62·0 (58·8–65·0)
Yemen	59·8 (57·3–62·6)	70·3 (67·6–72·7)	57·5 (55·0–60·1)	66·0 (63·6–68·3)	48·6 (45·0–52·3)	57·8 (54·1–61·7)	48·5 (45·3–51·7)	55·8 (52·6–59·1)

**South Asia**	**60·3 (59·7–61·0)**	**70·2 (69·7–70·7)**	**59·0 (58·4–59·5)**	**67·9 (67·4–68·4)**	**50·9 (48·2–53·3)**	**59·6 (56·7–62·1)**	**51·5 (49·3–53·4)**	**59·4 (57·1–61·5)**

Bangladesh	59·5 (58·5–60·6)	74·6 (73·1–76·0)	57·3 (56·4–58·3)	71·8 (70·3–73·3)	50·5 (47·9–52·9)	63·3 (60·1–66·3)	50·4 (48·3–52·3)	62·9 (60·2–65·2)
Bhutan	59·9 (57·8–62·1)	76·0 (73·9–78·1)	60·0 (57·9–62·3)	72·3 (69·8–74·8)	50·6 (47·6–53·7)	64·9 (61·6–68·2)	52·4 (49·6–55·0)	63·5 (60·4–66·4)
India	60·4 (59·6–61·1)	70·2 (69·5–70·8)	58·9 (58·3–59·6)	67·8 (67·2–68·3)	50·8 (48·1–53·2)	59·5 (56·5–62·1)	51·4 (49·2–53·4)	59·3 (56·9–61·4)
Nepal	59·0 (57·3–60·9)	73·3 (71·5–75·1)	57·7 (56·0–59·5)	68·7 (67·2–70·6)	49·7 (47·0–52·4)	62·3 (59·3–65·2)	50·0 (47·3–52·4)	60·1 (57·4–62·6)
Pakistan	61·6 (60·8–62·4)	67·4 (65·1–70·1)	61·7 (60·9–62·6)	66·3 (63·8–69·1)	52·5 (50·0–55·0)	57·8 (54·6–60·9)	54·1 (51·8–56·2)	58·2 (55·3–61·1)

**Southeast Asia, east Asia, and Oceania**	**69·9 (69·5–70·3)**	**78·6 (78·2–78·9)**	**65·8 (65·3–66·2)**	**72·9 (72·5–73·3)**	**61·1 (58·7–63·2)**	**68·4 (65·6–70·9)**	**58·8 (56·9–60·5)**	**65·0 (62·7–66·9)**

East Asia	70·8 (70·3–71·3)	79·9 (79·4–80·3)	67·0 (66·4–67·6)	74·5 (74·0–74·9)	62·0 (59·6–64·1)	69·7 (66·9–72·1)	60·1 (58·2–61·9)	66·6 (64·4–68·5)
China	70·7 (70·1–71·2)	79·9 (79·4–80·4)	66·9 (66·3–67·5)	74·5 (74·1–75·0)	61·9 (59·5–63·9)	69·7 (66·9–72·1)	60·0 (58·1–61·8)	66·6 (64·4–68·6)
North Korea	74·3 (72·1–76·5)	75·0 (72·9–77·2)	68·5 (66·7–70·7)	68·6 (67·1–70·2)	64·7 (61·8–67·6)	65·2 (62·3–68·0)	61·8 (59·2–64·3)	61·6 (59·2–64·0)
Taiwan (province of China)	77·3 (77·2–77·4)	83·3 (82·6–83·9)	72·1 (72·1–72·2)	76·8 (76·1–77·5)	67·5 (64·9–69·9)	71·8 (68·7–74·5)	64·9 (62·8–66·7)	68·2 (65·8–70·3)
Oceania	60·7 (58·8–62·6)	63·4 (61·1–65·5)	55·3 (53·5–57·3)	58·2 (55·9–60·6)	52·4 (49·7–54·9)	54·4 (51·4–57·0)	48·9 (46·5–51·2)	51·1 (48·3–53·8)
American Samoa	74·9 (74·2–75·8)	73·8 (72·9–74·8)	67·6 (66·9–68·4)	70·0 (68·5–71·7)	64·4 (61·5–67·0)	63·2 (60·1–66)	59·5 (57·0–61·6)	61·2 (58·5–63·8)
Federated States of Micronesia	65·5 (63·4–67·7)	69·6 (67·2–71·7)	61·7 (59·2–64·2)	65·0 (62·8–67·2)	56·8 (53·6–59·7)	59·7 (56·2–62·7)	54·4 (51·5–57·3)	56·9 (54·0–59·5)
Fiji	70·1 (68·7–71·7)	70·4 (68·4–72·5)	65·6 (64·0–67·0)	65·9 (64·2–67·7)	60·7 (57·8–63·3)	60·5 (57·3–63·6)	57·5 (54·9–59·9)	57·9 (55·2–60·3)
Guam	77·0 (76·4–77·5)	76·4 (75·3–77·5)	70·8 (70·3–71·3)	70·2 (69·2–71·3)	66·8 (63·9–69·4)	65·8 (62·6–68·6)	63·1 (60·8–65·1)	61·8 (59·2–64·1)
Kiribati	61·4 (60·0–62·6)	66·3 (63·9–68·9)	55·7 (54·5–57·0)	58·6 (56·2–61·0)	53·0 (50·3–55·4)	56·7 (53·6–59·8)	49·3 (47·2–51·3)	51·4 (48·7–54·1)
Marshall Islands	66·4 (65·7–67·0)	66·8 (64·5–69)	59·9 (59·2–60·6)	62·6 (60·6–64·6)	57·6 (55·0–59·9)	57·7 (54·7–60·7)	53·3 (51·4–55·1)	55·3 (52·7–57·9)
Northern Mariana Islands	76·0 (74·3–78·0)	79·2 (78·0–80·2)	72·9 (70·7–74·6)	73·6 (72·3–75·0)	66·0 (63·0–68·9)	68·0 (64·7–71·0)	64·6 (61·8–67·1)	64·6 (61·8–66·9)
Papua New Guinea	57·3 (55·0–59·9)	61·2 (58·6–63·9)	52·0 (49·7–54·5)	56·2 (53·6–59·2)	49·5 (46·7–52·2)	52·6 (49·5–55·3)	46·0 (43·3–48·6)	49·4 (46·4–52·4)
Samoa	73·8 (71·8–75·9)	74·5 (72·9–76·7)	68·1 (66·0–70·2)	71·3 (70·0–72·7)	64·0 (60·7–66·8)	64·0 (61·0–67·0)	60·1 (57·4–62·8)	62·5 (59·8–65·0)
Solomon Islands	63·5 (61·0–65·8)	67·5 (65·4–69·4)	59·9 (57·2–62·5)	64·1 (62·0–66·3)	55·1 (52·1–58·0)	58·0 (54·8–60·9)	53·1 (50·2–55·9)	56·5 (53·7–59·1)
Tonga	72·1 (71·2–73·1)	75·1 (73·3–77·2)	68·3 (67·6–69·1)	68·6 (66·7–70·1)	62·1 (59·2–64·7)	64·3 (61·0–67·5)	60·5 (58·0–62·6)	60·5 (57·7–62·8)
Vanuatu	65·8 (63·1–68·3)	67·8 (65·0–70·2)	59·7 (57·0–62·6)	62·1 (59·2–65·0)	56·5 (53·1–59·7)	57·9 (54·6–60·9)	52·9 (49·8–56·0)	54·9 (51·9–57·8)
Southeast Asia	67·8 (67·4–68·3)	75·8 (75·2–76·3)	62·6 (62·0–63·0)	69·4 (68·9–70·0)	58·9 (56·3–61·1)	65·8 (63·0–68·3)	55·2 (53·1–57·2)	61·5 (59·1–63·5)
Cambodia	59·8 (58·6–61·1)	72·7 (70·6–74·2)	55·3 (54·0–56·6)	66·8 (65·3–68·3)	51·4 (48·7–53·8)	62·6 (59·6–65·5)	48·4 (46·2–50·5)	58·7 (56·1–61·1)
Indonesia	65·4 (64·8–66·0)	73·9 (73·0–74·7)	62·4 (61·8–63·0)	69·2 (68·4–70·1)	56·8 (54·3–58·9)	64·0 (61·2–66·4)	55·0 (52·9–57·0)	61·4 (59·0–63·6)
Laos	54·3 (52·4–56·5)	70·3 (68·3–72·3)	49·6 (47·4–51·7)	65·0 (63·0–67·1)	47·4 (44·6–49·9)	61·3 (58·3–64·2)	44·3 (42·0–46·6)	57·8 (55·2–60·4)
Malaysia	73·7 (73·6–73·8)	77·3 (76·4–78·4)	69·2 (69·1–69·2)	72·4 (71·3–73·5)	64·5 (61·9–66·8)	67·7 (65·0–70·2)	61·6 (59·4–63·5)	64·4 (61·9–66·7)
Maldives	64·6 (64·1–65·0)	83·4 (82·6–84·1)	65·5 (64·9–66·1)	79·9 (79·2–80·6)	55·5 (52·9–57·8)	72·0 (68·7–74·9)	57·6 (55·3–59·7)	70·4 (67·7–72·9)
Mauritius	74·2 (73·9–74·5)	78·1 (77·2–79·0)	66·3 (66·1–66·5)	71·5 (70·6–72·5)	64·3 (61·4–66·8)	67·2 (63·9–70·0)	58·5 (56·2–60·5)	62·5 (59·9–64·8)
Myanmar	58·4 (56·1–60·8)	72·2 (70·3–74·2)	52·5 (50·0–54·9)	64·9 (63·2–66·7)	50·4 (47·7–53·4)	62·4 (59·4–65·4)	46·2 (43·5–48·8)	57·4 (55·1–59·8)
Philippines	71·4 (70·7–72·2)	73·1 (71·2–75·0)	64·6 (63·7–65·6)	66·6 (64·7–68·6)	61·7 (58·9–64·1)	63·5 (60·5–66·2)	56·5 (54·1–58·6)	58·7 (56·1–61·4)
Sri Lanka	74·8 (74·5–75·2)	81·1 (79·6–83·3)	65·6 (65·3–65·9)	73·8 (71·7–76·0)	64·8 (61·8–67·3)	70·6 (67·1–73·9)	58·2 (56·0–60·1)	65·2 (62·2–68·0)
Seychelles	75·6 (75·1–76·1)	77·7 (77·0–78·4)	66·1 (65·7–66·5)	70·1 (69·5–70·7)	66·3 (63·5–68·6)	67·9 (65·1–70·4)	59·3 (57·3–61·1)	62·4 (60·1–64·4)
Thailand	74·3 (73·8–74·8)	82·0 (80·9–83·1)	67·4 (66·7–68·1)	74·3 (72·9–75·9)	64·9 (62·1–67·2)	71·3 (68·2–74·1)	59·6 (57·4–61·7)	65·7 (63·0–68·3)
Timor-Leste	60·7 (58·8–62·9)	73·0 (71·3–74·8)	59·7 (58·0–61·4)	68·8 (67·3–70·7)	52·1 (49·2–55·0)	63·0 (59·8–65·9)	51·3 (48·6–54·0)	59·7 (56·8–62·5)
Vietnam	72·7 (71·4–74·3)	79·2 (77·8–80·9)	64·9 (63·5–66·5)	70·0 (68·3–71·2)	63·1 (60·2–66·0)	69·2 (66·2–72·3)	57·8 (55·2–60·2)	62·4 (60·0–64·6)

**Sub-Saharan Africa**	**55·7 (55·0–56·3)**	**66·2 (65·4–67·0)**	**51·6 (51·0–52·3)**	**61·7 (60·8–62·4)**	**47·6 (45·2–49·7)**	**56·8 (54·1–59·3)**	**44·8 (42·8–46·7)**	**53·7 (51·3–55·9)**

Central sub-Saharan Africa	54·6 (53·4–56·1)	64·4 (62·7–66·0)	50·1 (48·8–51·4)	60·3 (58·7–62·0)	46·0 (43·3–48·3)	54·7 (51·9–57·4)	43·1 (40·7–45·3)	52·1 (49·2–54·7)
Angola	50·6 (48·5–52·9)	66·7 (64·5–68·9)	45·5 (43·3–47·6)	61·7 (59·7–64·0)	43·0 (40·1–45·7)	56·8 (53·5–59·8)	39·4 (37·0–41·8)	53·3 (50·3–56·3)
Central African Republic	50·1 (48·4–51·8)	54·9 (52·0–58·0)	44·6 (42·9–46·3)	49·1 (46·5–51·7)	42·5 (39·9–44·7)	47·0 (43·7–50·2)	38·5 (36·3–40·8)	42·8 (40·1–45·6)
Congo (Brazzaville)	56·2 (54·2–58·1)	62·7 (60·2–65·6)	51·5 (49·4–53·5)	62·6 (60·4–64·8)	48·0 (45·2–50·7)	53·8 (50·7–56·9)	44·6 (42·2–47·2)	54·3 (51·5–57·3)
Democratic Republic of the Congo	56·0 (54·1–58·0)	64·3 (62·0–66·7)	51·8 (50·0–53·7)	60·4 (58·2–62·7)	46·9 (43·8–49·7)	54·6 (51·4–57·6)	44·4 (41·8–47·1)	52·0 (48·9–55·2)
Equatorial Guinea	50·8 (48·3–53·5)	66·4 (62·6–70·5)	45·6 (43·0–48·3)	64·3 (61·3–67·1)	43·2 (40·3–46·0)	56·9 (53·1–60·8)	39·5 (36·8–42·1)	55·6 (52·3–58·9)
Gabon	64·0 (62·3–65·7)	72·1 (69·8–74·4)	56·4 (54·8–58·0)	65·1 (63·3–66·7)	54·3 (51·3–57·2)	61·2 (57·7–64·4)	49·0 (46·5–51·4)	56·6 (53·8–59·1)
Eastern sub-Saharan Africa	52·8 (52·3–53·4)	67·4 (66·8–68·1)	48·8 (48·0–49·5)	62·5 (61·7–63·3)	45·7 (43·5–47·5)	58·3 (55·6–60·7)	42·6 (40·8–44·4)	54·9 (52·6–57·0)
Burundi	50·5 (48·7–52·4)	63·6 (61·3–65·9)	46·9 (45·0–48·8)	59·7 (57·3–62·2)	44·2 (41·8–46·5)	55·5 (52·7–58·3)	41·7 (39·6–43·9)	52·3 (49·3–55·4)
Comoros	59·4 (57·5–61·6)	70·0 (67·8–72·3)	56·9 (54·9–58·8)	67·1 (65·0–69·2)	51·4 (48·6–54·1)	61·0 (58·0–63·9)	49·6 (47·0–52·0)	58·9 (56·1–61·6)
Djibouti	61·4 (58·8–64·1)	68·9 (65·3–72·0)	58·9 (56·3–61·5)	66·0 (63·1–68·8)	53·7 (50·7–56·7)	60·3 (56·9–63·8)	52·2 (49·2–54·9)	58·5 (55·5–61·5)
Eritrea	43·6 (42·4–44·7)	65·9 (63·4–69·0)	30·6 (29·9–31·4)	59·2 (56·4–61·9)	37·0 (34·8–39·0)	57·0 (53·8–60·4)	26·2 (24·7–27·5)	51·6 (48·7–54·4)
Ethiopia	48·8 (47·8–49·9)	70·4 (69·3–71·5)	45·6 (44·4–46·8)	66·7 (65·6–67·7)	42·2 (40·0–44·1)	60·8 (57·9–63·4)	39·9 (37·8–41·7)	58·5 (55·9–60·8)
Kenya	63·2 (62·4–63·9)	68·8 (67·9–69·5)	60·6 (59·9–61·3)	63·2 (62·4–63·9)	55·0 (52·6–57·3)	59·7 (57·0–62·1)	53·4 (51·1–55·4)	55·7 (53·4–57·7)
Madagascar	57·0 (55·8–58·2)	64·8 (62·3–67·5)	53·9 (52·7–55·1)	62·2 (59·7–64·8)	49·3 (46·9–51·5)	56·3 (53·3–59·3)	47·0 (44·7–48·9)	54·6 (51·7–57·7)
Malawi	50·5 (48·9–52·0)	66·9 (64·9–69·0)	46·7 (44·9–48·4)	59·6 (57·9–61·5)	43·8 (41·4–45·9)	57·8 (54·7–60·6)	40·8 (38·4–43·0)	52·4 (50·0–54·8)
Mozambique	52·6 (51·3–54·1)	62·0 (59·4–64·5)	48·0 (46·7–49·4)	54·8 (52·7–57·0)	45·3 (42·8–47·5)	53·4 (50·2–56·6)	41·6 (39·4–43·6)	47·9 (45·2–50·4)
Rwanda	51·4 (50·1–52·7)	70·8 (69·1–72·7)	47·4 (46·1–48·7)	65·8 (64·0–67·6)	45·0 (42·9–47·0)	61·3 (58·3–64·2)	42·1 (40·2–44·0)	57·7 (55·1–60·4)
Somalia	52·4 (49·9–55·0)	60·6 (57·7–63·3)	48·0 (45·5–50·5)	56·5 (53·7–59·3)	45·3 (42·5–48·2)	52·7 (49·5–55·6)	42·4 (39·8–45·1)	49·9 (46·7–52·9)
South Sudan	53·5 (50·8–56·3)	61·8 (58·6–65·1)	49·3 (46·6–52·0)	56·9 (53·9–60·0)	44·9 (41·7–47·8)	52·3 (48·5–55·8)	42·4 (39·5–45·2)	49·2 (45·9–52·3)
Tanzania	56·5 (54·9–58·0)	68·9 (67·2–70·6)	53·2 (51·2–55·1)	64·6 (62·9–66·3)	48·8 (46·3–51·1)	59·7 (56·8–62·5)	46·6 (44·1–48·9)	56·8 (54·1–59·3)
Uganda	51·1 (48·8–53·4)	69·2 (67·2–71·1)	44·3 (40·3–48·4)	62·3 (60·5–64·2)	43·7 (40·9–46·6)	59·5 (56·5–62·4)	38·0 (34·4–41·8)	54·5 (52·0–57·0)
Zambia	52·1 (50·2–53·7)	66·3 (64·5–68·3)	49·3 (46·6–51·5)	60·4 (58·5–62·3)	45·3 (42·9–47·6)	57·4 (54·3–60·1)	43·6 (40·8–46·1)	53·1 (50·7–55·4)
Southern sub-Saharan Africa	67·9 (67·1–68·6)	68·5 (67·6–69·3)	60·3 (59·3–61·1)	61·5 (60·7–62·2)	57·8 (55·0–60·3)	58·3 (55·4–60·8)	52·6 (50·2–54·6)	53·6 (51·4–55·6)
Botswana	68·8 (66·3–71·3)	71·0 (68·8–72·5)	58·6 (55·2–61·3)	67·0 (64·1–69·2)	58·4 (55·2–61·5)	60·0 (56·8–63·0)	51·0 (47·8–54·0)	57·7 (54·4–60·7)
Lesotho	65·2 (63·5–67·0)	59·3 (56·3–62·7)	56·0 (54·3–57·6)	50·3 (48·1–52·7)	55·3 (52·2–58·1)	50·3 (46·8–53·6)	49·0 (46·6–51·1)	43·9 (41·3–46·4)
Namibia	65·4 (63·9–66·7)	70·7 (67·5–73·5)	58·2 (56·0–59·6)	62·3 (60·3–64·3)	55·8 (53·0–58·4)	60·1 (56·5–63·6)	50·5 (47·9–52·8)	54·2 (51·4–56·8)
South Africa	68·7 (68·1–69·4)	69·7 (68·6–70·6)	61·5 (60·8–62·1)	62·8 (62·0–63·6)	58·4 (55·7–60·9)	59·3 (56·3–61·9)	53·5 (51·3–55·5)	54·7 (52·5–56·8)
Swaziland (eSwatini)	66·5 (64·9–68·2)	65·1 (62·1–68·4)	57·9 (56·1–59·7)	54·9 (52·6–57·6)	56·7 (53·9–59·6)	55·1 (51·5–58·5)	50·7 (48·4–53·0)	47·8 (45·1–50·4)
Zimbabwe	64·7 (61·2–67·3)	64·4 (62·1–66·6)	57·5 (52·7–60·7)	58·1 (56·3–60·1)	55·4 (51·9–58·6)	55·1 (52·1–58·1)	50·4 (46·4–53·8)	51·1 (48·6–53·4)
Western sub-Saharan Africa	56·2 (55·0–57·4)	65·3 (63·6–66·9)	53·3 (52·0–54·5)	61·7 (60·2–62·9)	47·8 (45·1–50·1)	56·0 (53·0–58·7)	45·9 (43·5–48·1)	53·5 (50·9–56·0)
Benin	57·5 (56·0–58·9)	66·6 (64·2–69·1)	53·0 (51·4–54·4)	62·6 (60·1–65·0)	48·4 (45·5–51·2)	57·2 (54·1–60·5)	45·3 (42·6–47·8)	54·5 (51·5–57·5)
Burkina Faso	52·4 (51·0–53·8)	64·4 (62·6–66·3)	49·1 (47·5–50·8)	58·9 (56·9–61·0)	44·4 (41·8–46·8)	55·4 (52·5–58·2)	41·5 (38·8–44·0)	51·5 (48·8–54·1)
Cameroon	59·2 (58·0–60·4)	65·1 (62·7–67·8)	56·2 (55·0–57·4)	61·0 (58·6–63·5)	50·1 (47·4–52·6)	56·0 (52·6–59·0)	48·1 (45·6–50·3)	53·2 (50·2–56·1)
Cape Verde	73·5 (73·0–74·0)	79·0 (78·2–80·1)	66·4 (66·0–66·9)	72·5 (71·3–73·7)	63·0 (60·0–65·6)	68·1 (65·0–70·9)	58·2 (55·8–60·3)	63·6 (60·9–66·1)
Chad	54·7 (53·3–56·2)	61·6 (59·2–64·2)	52·0 (50·4–53·6)	58·6 (56·4–60·8)	46·2 (43·6–48·7)	52·5 (49·1–55·6)	44·8 (42·5–47·2)	50·7 (47·8–53·4)
Côte d’Ivoire	58·0 (55·5–59·9)	65·3 (62·8–67·7)	52·1 (48·7–54·4)	60·1 (57·8–62·3)	48·9 (45·7–51·7)	56·1 (52·8–59·1)	45·1 (41·5–47·7)	52·6 (49·9–55·3)
The Gambia	62·5 (60·4–64·8)	67·9 (65·6–70·2)	57·8 (55·5–60·2)	63·8 (62·0–65·8)	53·1 (49·9–56·2)	57·9 (54·6–61·0)	50·0 (47·1–53·0)	55·6 (52·9–58·2)
Ghana	60·7 (58·8–62·6)	68·4 (66·7–70·3)	57·8 (55·9–59·8)	62·6 (61·0–64·3)	52·1 (49·2–54·7)	59·3 (56·4–62·1)	50·3 (47·7–52·8)	54·8 (52·0–57·3)
Guinea	52·3 (50·8–53·8)	62·2 (60·3–64·2)	51·6 (50·1–53·0)	59·3 (57·2–61·4)	44·7 (42·2–47·1)	53·8 (50·9–56·4)	44·8 (42·6–46·9)	51·9 (49·1–54·5)
Guinea-Bissau	52·1 (49·9–54·5)	62·6 (60·3–64·9)	45·5 (43·3–47·6)	57·4 (55·1–59·7)	44·6 (41·7–47·4)	53·9 (50·8–56·8)	39·8 (37·5–42·2)	50·2 (47·4–52·8)
Liberia	51·3 (49·5–53·1)	65·1 (63·1–67·4)	46·8 (45·3–48·4)	63·7 (61·5–65·8)	43·4 (40·6–45·8)	54·7 (51·3–57·8)	39·9 (37·6–42·3)	54·0 (50·9–57·3)
Mali	49·5 (48·3–50·6)	63·0 (61·1–64·9)	49·2 (48·0–50·5)	61·0 (58·7–63·2)	42·0 (39·6–44·2)	53·9 (50·9–56·8)	42·1 (39·6–44·2)	52·5 (49·4–55·3)
Mauritania	60·2 (58·7–61·7)	71·0 (68·9–73·0)	59·0 (57·6–60·5)	70·0 (68·0–72·3)	51·8 (49·0–54·0)	61·2 (58·2–64·1)	51·4 (48·9–53·8)	60·7 (57·5–63·7)
Niger	47·4 (45·8–49·0)	63·6 (61·4–66·0)	45·8 (44·3–47·3)	61·1 (58·8–63·5)	40·7 (38·4–43·0)	54·9 (52·2–57·8)	40·0 (37·9–42·1)	53·5 (50·6–56·5)
Nigeria	57·1 (54·5–59·5)	65·8 (62·3–69·1)	54·0 (51·5–56·4)	62·8 (59·7–65·2)	48·5 (45·3–51·6)	56·2 (52·3–59·6)	46·5 (43·6–49·4)	54·0 (50·6–57·2)
São Tomé and Príncipe	65·6 (64·3–67·0)	71·8 (70·1–73·8)	62·5 (61·3–63·7)	68·1 (66·5–69·8)	56·5 (53·7–59·0)	62·3 (59·2–65·1)	54·4 (52·0–56·6)	59·6 (56·9–62·3)
Senegal	60·4 (59·0–61·8)	70·0 (68·3–71·9)	56·6 (55·4–57·8)	66·1 (64·5–67·9)	51·5 (48·7–54·1)	60·1 (57·2–62·9)	49·1 (46·6–51·3)	57·7 (54·9–60·3)
Sierra Leone	52·3 (50·4–54·3)	61·4 (59·4–63·7)	48·1 (46·3–50·0)	59·5 (57·2–61·7)	44·7 (41·9–47·2)	52·7 (49·9–55·5)	41·6 (39·3–43·7)	51·5 (48·5–54·3)
Togo	59·3 (57·7–60·9)	67·2 (65·0–69·6)	56·0 (54·3–57·7)	61·4 (59·1–63·8)	50·5 (47·7–53·2)	57·8 (54·6–60·9)	48·6 (46·1–51·0)	53·8 (51·0–56·7)

Data in parentheses are 95% uncertainty intervals. Super-regions, regions, and countries are listed alphabetically. GBD=Global Burden of Diseases, Injuries, and Risk Factors Study. HALE=healthy life expectancy. SDI=Socio-demographic Index.

**Table 2:** Life expectancy and HALE at age 65 years for 21 GBD regions and 195 countries and territories, by sex, in 1990 and 2017

	Life expectancy at age 65 years				HALE at age 65 years			
	Females		Males		Females		Males	
	1990	2017	1990	2017	1990	2017	1990	2017

**Global**	**16·2 (16·1–16·2)**	**18·7 (18·6–18·8)**	**13·6 (13·5–13·6)**	**15·9 (15·8–16·0)**	**11·9 (10·8–12·9)**	**13·7 (12·4–14·9)**	**10·3 (9·4–11·0)**	**12·0 (10·9–12·9)**

Low SDI	12·2 (12·0–12·5)	14·7 (14·5–15·0)	11·4 (11·1–11·6)	13·7 (13·4–13·9)	8·6 (7·7–9·5)	10·5 (9·5–11·5)	8·3 (7·5–9·1)	10·0 (9·1–10·9)
Low-middle SDI	13·8 (13·6–14·0)	15·8 (15·6–16·1)	12·6 (12·4–12·8)	14·1 (13·9–14·3)	9·9 (8·9–10·7)	11·4 (10·3–12·5)	9·3 (8·4–10·1)	10·5 (9·5–11·4)
Middle SDI	15·4 (15·3–15·6)	18·2 (18·0–18·4)	13·8 (13·7–13·9)	15·4 (15·2–15·6)	11·5 (10·5–12·4)	13·5 (12·3–14·6)	10·6 (9·8–11·4)	11·8 (10·8–12·7)
High-middle SDI	15·8 (15·6–15·9)	18·5 (18·3–18·8)	13·2 (13·0–13·3)	15·6 (15·4–15·9)	11·6 (10·5–12·5)	13·7 (12·4–14·8)	10·0 (9·2–10·8)	11·8 (10·8–12·8)
High SDI	18·7 (18·6–18·7)	21·9 (21·8–22·0)	14·9 (14·9–14·9)	18·6 (18·5–18·7)	13·9 (12·7–15·1)	16·2 (14·6–17·6)	11·3 (10·3–12·1)	13·8 (12·6–15·0)

**Central Europe, eastern Europe, and central Asia**	**15·9 (15·9–15·9)**	**18 (17·9–18·1)**	**12·5 (12·5–12·5)**	**14·0 (14·0–14·1)**	**11·4 (10·2–12·5)**	**12·9 (11·6–14·1)**	**9·0 (8·1–9·9)**	**10·1 (9·1–11·1)**

Central Asia	16·4 (16·3–16·5)	16·5 (16·2–16·8)	13·2 (13·1–13·2)	12·8 (12·5–13·0)	12·0 (10·8–13·1)	12·2 (10·9–13·2)	9·8 (8·9–10·6)	9·5 (8·6–10·4)
Armenia	15·7 (15·4–15·9)	17·3 (17·0–17·6)	13·2 (12·9–13·4)	14·6 (14·4–14·9)	11·4 (10·3–12·5)	12·7 (11·4–13·9)	9·7 (8·7–10·6)	10·8 (9·8–11·8)
Azerbaijan	16·9 (16·6–17·3)	16·4 (15·8–17·1)	13·6 (13·2–13·9)	11·9 (11·4–12·4)	12·5 (11·4–13·6)	12·1 (10·9–13·4)	10·2 (9·1–11·1)	8·9 (8·0–9·8)
Georgia	16·2 (15·9–16·5)	16·7 (16·5–17·0)	13·2 (12·9–13·6)	13·1 (12·9–13·3)	12·1 (11·0–13·2)	12·3 (11·1–13·4)	10·0 (9·0–10·8)	9·8 (8·8–10·6)
Kazakhstan	16·5 (16·4–16·5)	17·0 (16·6–17·4)	12·3 (12·2–12·3)	13·2 (12·9–13·6)	11·8 (10·6–12·9)	12·4 (11·2–13·5)	8·9 (8·0–9·7)	9·7 (8·7–10·6)
Kyrgyzstan	16·0 (15·6–16·4)	17·0 (16·8–17·3)	12·5 (12·1–12·8)	13·7 (13·5–13·9)	11·7 (10·5–12·8)	12·6 (11·4–13·7)	9·2 (8·3–10·1)	10·3 (9·4–11·2)
Mongolia	13·1 (12·8–13·4)	16·1 (15·4–16·9)	11·1 (10·8–11·4)	12·1 (11·5–12·8)	9·8 (8·9–10·7)	12·0 (10·7–13·1)	8·4 (7·6–9·1)	9·0 (8·1–9·9)
Tajikistan	16·9 (16·6–17·1)	17·1 (16·3–17·9)	14·4 (14·3–14·6)	13·9 (13·2–14·6)	12·4 (11·2–13·5)	12·7 (11·4–13·8)	10·8 (9·7–11·7)	10·5 (9·4–11·6)
Turkmenistan	15·4 (15·4–15·5)	16·4 (15·7–17·1)	12·7 (12·6–12·7)	13·3 (12·8–13·9)	11·5 (10·4–12·5)	12·3 (11·0–13·4)	9·6 (8·7–10·3)	10·1 (9·1–11·0)
Uzbekistan	17·0 (16·9–17·0)	15·8 (14·8–16·9)	14·2 (14·1–14·2)	12·0 (11·2–12·8)	12·5 (11·3–13·6)	11·7 (10·4–13·0)	10·7 (9·7–11·5)	9·1 (8·1–10·1)
Central Europe	15·9 (15·8–15·9)	19·0 (18·8–19·2)	12·6 (12·6–12·6)	15·3 (15·2–15·5)	11·2 (10·0–12·3)	13·5 (12·1–14·8)	8·9 (8·0–9·8)	10·8 (9·6–11·9)
Albania	19·3 (19·2–19·5)	20·6 (18·9–22·4)	13·7 (13·6–13·8)	15·6 (14·4–17·1)	14·1 (12·7–15·4)	15·1 (13·4–17·0)	10·0 (9·1–10·9)	11·4 (10·0–13·0)
Bosnia and Herzegovina	16·4 (16·3–16·6)	17·4 (16·9–17·9)	13·6 (13·6–13·7)	15·1 (14·6–15·5)	11·8 (10·6–12·9)	12·4 (11·1–13·6)	9·8 (8·8–10·7)	10·6 (9·4–11·7)
Bulgaria	16·0 (15·9–16·1)	18·0 (17·5–18·5)	12·8 (12·7–12·8)	14·2 (13·8–14·7)	11·6 (10·4–12·6)	13·0 (11·7–14·3)	9·2 (8·3–10·1)	10·2 (9·1–11·2)
Croatia	15·9 (15·8–16·0)	19·3 (18·8–19·8)	12·6 (12·6–12·7)	15·7 (15·3–16·2)	11·4 (10·1–12·5)	13·7 (12·2–15·1)	9·0 (8·0–9·8)	11·1 (9·8–12·2)
Czech Republic	15·3 (15·2–15·4)	19·8 (19·3–20·3)	11·6 (11·5–11·6)	16·3 (15·9–16·8)	10·6 (9·3–11·7)	13·9 (12·2–15·3)	8·1 (7·2–8·9)	11·2 (9·9–12·5)
Hungary	15·5 (15·4–15·5)	19·1 (18·6–19·5)	12·1 (12·1–12·1)	15·0 (14·6–15·5)	10·5 (9·3–11·7)	13·4 (11·9–14·8)	8·4 (7·4–9·2)	10·5 (9·3–11·6)
Macedonia	16·3 (16·2–16·5)	18·8 (18·5–19·2)	13·6 (13·5–13·7)	14·8 (14·3–15·2)	11·7 (10·5–12·8)	13·6 (12·2–14·9)	9·8 (8·8–10·7)	10·6 (9·4–11·6)
Montenegro	17·6 (17·4–17·7)	17·4 (16·8–18·0)	14·5 (14·4–14·7)	14·8 (14·0–15·5)	12·7 (11·5–14·0)	12·5 (11·2–13·8)	10·5 (9·4–11·5)	10·6 (9·4–11·7)
Poland	16·3 (16·3–16·4)	20·2 (19·7–20·7)	12·6 (12·6–12·6)	16·0 (15·5–16·4)	11·6 (10·3–12·7)	14·4 (12·8–15·8)	9·0 (8·0–9·8)	11·2 (10–12·5)
Romania	15·2 (15·2–15·2)	18·2 (17·8–18·7)	13·1 (13·0–13·1)	14·7 (14·3–15·1)	10·8 (9·7–11·9)	13·1 (11·7–14·4)	9·2 (8·2–10·2)	10·4 (9·3–11·5)
Serbia	15·9 (15·8–16·0)	16·5 (16·0–17·0)	12·4 (12·4–12·5)	14·6 (14·2–15·0)	11·4 (10·2–12·4)	11·8 (10·5–12·9)	8·9 (8·0–9·7)	10·4 (9·3–11·5)
Slovakia	15·8 (15·7–15·9)	19·0 (18·5–19·5)	12·2 (12·2–12·3)	15·4 (14·9–15·8)	11·2 (10·0–12·3)	13·5 (12·0–14·8)	8·6 (7·6–9·5)	10·7 (9·4–11·8)
Slovenia	17·2 (17·0–17·3)	21·6 (21·0–22·2)	13·4 (13·3–13·5)	17·5 (17·0–18·0)	12·0 (10·6–13·2)	15·1 (13·3–16·7)	9·3 (8·2–10·3)	12·1 (10·6–13·5)
Eastern Europe	15·9 (15·9–15·9)	17·7 (17·5–17·8)	12·3 (12·2–12·3)	13·4 (13·2–13·5)	11·4 (10·2–12·4)	12·7 (11·3–13·8)	9·0 (8·1–9·8)	9·8 (8·8–10·6)
Belarus	16·7 (16·6–16·8)	17·9 (17·5–18·4)	13·2 (13·2–13·2)	13·0 (12·7–13·4)	12·1 (10·9–13·2)	13·0 (11·7–14·2)	9·7 (8·7–10·6)	9·6 (8·6–10·5)
Estonia	15·8 (15·7–16·0)	20·4 (19·4–21·4)	12·1 (12·0–12·2)	15·9 (15·0–16·9)	11·5 (10·4–12·5)	14·9 (13·3–16·5)	8·8 (7·9–9·6)	11·6 (10·3–12·9)
Latvia	15·9 (15·8–16·0)	19·0 (18·0–20·0)	12·3 (12·2–12·3)	14·2 (13·4–15·1)	11·4 (10·2–12·5)	13·7 (12·2–15·1)	8·9 (8·0–9·7)	10·3 (9·1–11·4)
Lithuania	16·9 (16·8–17·0)	19·3 (18·8–19·8)	13·4 (13·3–13·4)	14·3 (13·8–14·7)	12·2 (11·0–13·3)	13·8 (12·3–15·2)	9·8 (8·8–10·6)	10·2 (9·1–11·3)
Moldova	14·9 (14·8–14·9)	17·9 (17·6–18·2)	12·6 (12·6–12·7)	13·6 (13·4–13·8)	10·8 (9·7–11·7)	13·1 (11·9–14·3)	9·3 (8·3–10·1)	10·1 (9·1–11·0)
Russia	15·9 (15·9–15·9)	17·7 (17·7–17·8)	12·0 (12·0–12·0)	13·6 (13·6–13·7)	11·3 (10·2–12·4)	12·6 (11·3–13·8)	8·8 (7·9–9·6)	9·9 (8·9–10·8)
Ukraine	15·8 (15·7–15·9)	17·2 (16·7–17·6)	12·5 (12·4–12·6)	12·5 (12·2–12·9)	11·3 (10·1–12·3)	12·5 (11·2–13·6)	9·2 (8·3–10·0)	9·2 (8·3–10·1)

**High income**	**18·8 (18·8–18·8)**	**21·9 (21·8–22·0)**	**15·0 (15·0–15·0)**	**18·6 (18·5–18·7)**	**14·1 (12·8–15·2)**	**16·3 (14·7–17·6)**	**11·4 (10·4–12·3)**	**13·9 (12·6–15·0)**

Australasia	18·9 (18·8–18·9)	22·2 (21·4–22·9)	15·1 (15·0–15·1)	19·4 (18·7–20·2)	14·0 (12·7–15·2)	16·3 (14·6–17·8)	11·3 (10·2–12·2)	14·3 (12·8–15·7)
Australia	19·0 (18·9–19·1)	22·3 (21·4–23·2)	15·1 (15·1–15·2)	19·4 (18·6–20·3)	14·2 (12·8–15·3)	16·4 (14·7–17·9)	11·3 (10·3–12·2)	14·4 (12·8–15·7)
New Zealand	18·2 (18·1–18·3)	21·7 (21·2–22·1)	14·8 (14·6–14·9)	19·2 (18·8–19·7)	13·4 (12·0–14·6)	15·5 (13·8–17·0)	11·0 (9·9–11·9)	13·9 (12·4–15·2)
High-income Asia Pacific	19·7 (19·7–19·8)	24·1 (24·0–24·3)	15·8 (15·8–15·9)	19·5 (19·4–19·7)	15·1 (13·8–16·2)	18·2 (16·6–19·7)	12·3 (11·3–13·1)	14·9 (13·6–16·0)
Brunei	13·8 (13·4–14·2)	17·8 (17·2–18·4)	12·5 (12·1–12·8)	15·3 (14·4–16·2)	10·3 (9·3–11·2)	13·5 (12·2–14·6)	9·4 (8·5–10·2)	11·6 (10·5–12·8)
Japan	20·3 (20·3–20·3)	24·4 (24·2–24·6)	16·4 (16·4–16·4)	19·7 (19·5–19·9)	15·6 (14·2–16·7)	18·5 (16·8–19·9)	12·8 (11·8–13·6)	15·1 (13·8–16·2)
Singapore	18·1 (18·0–18·3)	24·6 (24·1–25·0)	14·7 (14·6–14·8)	20·2 (19·7–20·7)	13·8 (12·6–14·9)	18·8 (17·1–20·2)	11·4 (10·5–12·2)	15·7 (14·4–16·9)
South Korea	16·6 (16·6–16·7)	22·6 (22·2–23·1)	12·4 (12·4–12·4)	18·5 (18·0–19·0)	12·5 (11·3–13·5)	17·0 (15·4–18·5)	9·4 (8·5–10·1)	13·9 (12·6–15·0)
High-income North America	19·2 (19·2–19·2)	20·8 (20·6–20·9)	15·3 (15·3–15·3)	18·2 (18·0–18·4)	14·1 (12·8–15·3)	14·9 (13·4–16·3)	11·3 (10·2–12·2)	13·1 (11·7–14·3)
Canada	19·6 (19·6–19·7)	22·1 (21·7–22·6)	15·5 (15·4–15·5)	19·5 (19·0–19·9)	14·8 (13·4–15·9)	16·5 (14·9–17·9)	11·8 (10·7–12·7)	14·6 (13·3–15·9)
Greenland	14·3 (14·0–14·6)	18·3 (17·6–18·9)	11·6 (11·4–11·8)	14·2 (14·0–15·0)	10·4 (9·4–11·3)	13·4 (12·1–14·6)	8·5 (7·7–9·3)	10·5 (9·5–11·5)
USA	19·1 (19·1–19·1)	20·6 (20·4–20·8)	15·3 (15·3–15·3)	18·1 (17·9–18·3)	14·1 (12·7–15·3)	14·7 (13·2–16·1)	11·2 (10·2–12·2)	12·9 (11·6–14·1)
Southern Latin America	17·5 (17·5–17·6)	19·7 (18·9–20·3)	14·0 (14·0–14·0)	16·3 (15·6–16·9)	13·3 (12·1–14·3)	14·9 (13·5–16·2)	10·7 (9·7–11·5)	12·3 (11·1–13·5)
Argentina	17·6 (17·5–17·6)	19·3 (18·4–20·2)	13·9 (13·9–13·9)	15·7 (14·8–16·5)	13·4 (12·2–14·4)	14·7 (13·3–16·1)	10·6 (9·7–11·4)	11·9 (10·7–13·2)
Chile	17·2 (17·1–17·3)	20·6 (19·7–21·6)	14·4 (14·4–14·5)	17·9 (17·0–18·9)	12·9 (11·8–14·0)	15·4 (13·9–16·9)	10·9 (9·9–11·8)	13·4 (12·1–14·8)
Uruguay	17·9 (17·8–18·0)	19·8 (18·9–20·8)	13·9 (13·8–14·0)	15·8 (14·9–16·7)	13·5 (12·3–14·6)	15·0 (13·5–16·4)	10·5 (9·6–11·4)	11·9 (10·7–13·1)
Western Europe	18·5 (18·5–18·5)	21·9 (21·6–22·1)	14·7 (14·7–14·7)	18·7 (18·5–18·9)	13·9 (12·7–15·0)	16·4 (14·9–17·7)	11·2 (10·3–12·1)	14·1 (12·9–15·2)
Andorra	20·7 (19·4–22·3)	22·4 (21·3–23·8)	16·6 (15·8–17·2)	19·4 (18·8–20·1)	15·5 (13·8–17·3)	16·8 (15·0–18·5)	12·7 (11·5–13·8)	14·7 (13·4–15·9)
Austria	17·9 (17·8–18·0)	21·6 (21·1–22·0)	14·5 (14·4–14·6)	18·5 (18·0–18·9)	13·5 (12·3–14·6)	16·1 (14·6–17·5)	11·1 (10·1–11·9)	13·9 (12·6–15·1)
Belgium	18·5 (18·4–18·6)	21·7 (21·2–22·2)	14·3 (14·2–14·3)	18·3 (17·8–18·7)	13·7 (12·5–14·9)	15·9 (14·4–17·4)	10·8 (9·8–11·6)	13·5 (12·1–14·7)
Cyprus	17·3 (17·2–17·5)	22·5 (21·8–23·1)	15·0 (14·9–15·1)	17·5 (16·8–18·2)	13·0 (11·8–14·0)	16·8 (15·2–18·3)	11·5 (10·5–12·3)	13·3 (12·0–14·5)
Denmark	17·8 (17·7–17·9)	20·7 (20·1–21·2)	14·1 (14·0–14·2)	18·0 (17·5–18·4)	13·3 (12·1–14·4)	15·5 (14·0–16·7)	10·8 (9·8–11·6)	13·6 (12·3–14·7)
Finland	17·9 (17·7–18·0)	21·8 (21·2–22·3)	13·9 (13·8–13·9)	18·2 (17·7–18·7)	13·4 (12·2–14·5)	16·2 (14·7–17·7)	10·5 (9·5–11·3)	13·6 (12·2–14·8)
France	19·9 (19·8–19·9)	23·5 (23·0–23·9)	15·5 (15·4–15·5)	19·5 (19·1–19·9)	15·1 (13·7–16·3)	17·8 (16·1–19·2)	11·9 (10·9–12·8)	14·9 (13·6–16·1)
Germany	17·7 (17·7–17·8)	20·8 (19·9–21·7)	14·1 (14·1–14·1)	17·8 (16·9–18·7)	13·3 (12·0–14·3)	15·5 (13·9–17·0)	10·7 (9·7–11·5)	13·4 (12·0–14·6)
Greece	18·9 (18·8–18·9)	21·3 (20·8–21·8)	15·7 (15·6–15·7)	18·3 (17·9–18·8)	14·2 (13·0–15·3)	16·0 (14·5–17·3)	12·0 (11·0–12·9)	13·8 (12·6–15·0)
Iceland	19·0 (18·8–19·3)	23·1 (22·7–23·5)	16·1 (15·9–16·3)	18·4 (18·1–18·7)	14·2 (12·9–15·4)	17·2 (15·6–18·6)	12·2 (11·1–13·1)	13·9 (12·5–15·0)
Ireland	16·8 (16·7–17·0)	21·2 (20·6–21·7)	13·4 (13·3–13·5)	18·7 (18·2–19·2)	12·7 (11·6–13·7)	15·8 (14·3–17·1)	10·2 (9·3–11·0)	14·0 (12·6–15·2)
Israel	18·0 (17·9–18·1)	21·9 (21·4–22·4)	16·4 (16·3–16·5)	19·9 (19·4–20·4)	13·3 (12·0–14·5)	16·2 (14·6–17·6)	12·3 (11·2–13·3)	14·9 (13·4–16·2)
Italy	19·0 (18·9–19·0)	22·5 (22·1–23·0)	15·2 (15·2–15·2)	19·2 (18·8–19·6)	14·3 (13·0–15·4)	17·0 (15·5–18·4)	11·6 (10·6–12·5)	14·6 (13·2–15·8)
Luxembourg	18·1 (17·9–18·2)	20·9 (20·2–21·6)	13·9 (13·8–14·1)	18·8 (18·0–19·6)	13·2 (11·9–14·4)	15·3 (13·8–16·8)	10·4 (9·4–11·2)	13·8 (12·4–15·2)
Malta	17·4 (17·2–17·6)	20·6 (20·1–21·1)	14·7 (14·5–14·8)	17·9 (17·6–18·3)	13·1 (11·8–14·2)	15·4 (13·9–16·7)	11·1 (10·1–12·0)	13·4 (12·1–14·6)
Netherlands	19·0 (18·9–19·1)	20·9 (20·5–21·4)	14·4 (14·3–14·4)	18·3 (17·9–18·8)	14·2 (12·9–15·4)	15·6 (14·1–17·0)	10·9 (10·0–11·8)	13·8 (12·5–15·0)
Norway	18·8 (18·7–18·8)	21·6 (21·4–21·7)	14·7 (14·6–14·7)	19·0 (18·8–19·1)	14·1 (12·7–15·2)	16·0 (14·4–17·3)	11·0 (10·0–11·9)	13·9 (12·6–15·2)
Portugal	17·2 (17·2–17·3)	21·7 (21·2–22·1)	14·1 (14·0–14·1)	18·3 (17·9–18·8)	12·8 (11·5–13·8)	16·1 (14·5–17·5)	10·6 (9·6–11·4)	13·8 (12·5–15·0)
Spain	19·1 (19·1–19·2)	23·0 (22·6–23·4)	15·5 (15·5–15·5)	19·1 (18·7–19·5)	14·5 (13·3–15·6)	17·6 (16·0–18·9)	11·9 (10·9–12·8)	14·6 (13·4–15·7)
Sweden	19·1 (19·1–19·2)	21·5 (21·1–21·9)	15·4 (15·4–15·5)	19·2 (18·8–19·6)	14·4 (13·2–15·6)	16·1 (14·6–17·4)	11·9 (10·8–12·7)	14·5 (13·2–15·7)
Switzerland	19·8 (19·7–19·9)	22·8 (22·4–23·3)	15·6 (15·5–15·7)	20·2 (19·8–20·7)	14·7 (13·2–15·9)	17·0 (15·4–18·5)	11·8 (10·7–12·7)	15·2 (13·8–16·5)
UK	17·8 (17·7–17·8)	20·8 (20·7–20·9)	14·1 (14·0–14·1)	18·5 (18·4–18·6)	13·4 (12·3–14·5)	15·4 (14·0–16·7)	10·8 (9·9–11·6)	13·9 (12·6–15·0)
England	17·9 (17·9–17·9)	20·9 (20·8–21·0)	14·2 (14·2–14·2)	18·6 (18·6–18·7)	13·6 (12·4–14·6)	15·4 (14·0–16·8)	10·9 (10·0–11·7)	13·9 (12·7–15·1)
Northern Ireland	16·9 (16·7–17·1)	20·7 (19·9–21·4)	13·3 (13·2–13·4)	18·3 (17·7–19·0)	12·8 (11·7–13·8)	15·6 (14·2–16·9)	10·2 (9·3–10·9)	13·8 (12·5–15·1)
Scotland	16·7 (16·6–16·8)	19·8 (19·2–20·5)	13·2 (13·1–13·2)	17·4 (16·8–18·1)	12·6 (11·5–13·5)	14·9 (13·5–16·2)	10·0 (9·2–10·8)	13·2 (12·0–14·4)
Wales	17·8 (17·7–17·9)	20·7 (20·1–21·2)	13·9 (13·8–14·0)	18·1 (17·6–18·6)	13·4 (12·2–14·5)	15·6 (14·0–16·9)	10·6 (9·7–11·4)	13·7 (12·4–14·9)

**Latin America and Caribbean**	**17·3 (17·2–17·3)**	**19·7 (19·6–19·9)**	**15·3 (15·3–15·4)**	**17·4 (17·2–17·5)**	**13·0 (11·8–14·0)**	**14·9 (13·6–16·1)**	**11·7 (10·7–12·6)**	**13·3 (12·2–14·3)**

Andean Latin America	17·8 (17·5–18·1)	20·2 (19·4–20·9)	16·2 (15·9–16·5)	18·8 (18·0–19·5)	13·5 (12·4–14·6)	15·4 (14·0–16·8)	12·4 (11·3–13·4)	14·4 (13·1–15·7)
Bolivia	13·6 (13·0–14·2)	16·6 (15·4–18·3)	12·9 (12·4–13·5)	14·9 (13·2–16·7)	10·3 (9·3–11·2)	12·6 (11·2–14·2)	9·7 (8·7–10·7)	11·4 (9·8–13·0)
Ecuador	18·1 (18·0–18·1)	19·5 (18·7–20·3)	16·4 (16·4–16·5)	18·4 (17·7–19·1)	13·8 (12·6–14·9)	14·9 (13·5–16·3)	12·5 (11·4–13·4)	14·0 (12·6–15·2)
Peru	19·2 (18·6–19·7)	21·7 (20·4–23·0)	17·2 (16·7–17·8)	20·2 (19·0–21·5)	14·6 (13·3–15·8)	16·7 (15·1–18·4)	13·3 (12·1–14·3)	15·6 (14·0–17·2)
Caribbean	17·1 (16·9–17·2)	18·9 (18·3–19·4)	15·4 (15·2–15·5)	16·4 (15·9–16·9)	13·1 (12·0–14·1)	14·4 (13·2–15·6)	12·0 (11·0–12·8)	12·7 (11·7–13·8)
Antigua and Barbuda	19·0 (18·6–19·5)	18·5 (18·1–19·1)	14·7 (14·4–15·1)	17·3 (16·8–17·8)	14·6 (13·2–15·8)	14·2 (12·8–15·3)	11·5 (10·5–12·4)	13·4 (12·1–14·4)
The Bahamas	17·9 (17·7–18·1)	18·5 (17·7–19·3)	14·5 (14·4–14·7)	16·3 (15·7–17·0)	13·8 (12·6–14·8)	14·2 (12·9–15·5)	11·4 (10·5–12·2)	12·8 (11·6–13·9)
Barbados	17·5 (17·3–17·7)	19·2 (18·6–19·9)	15·0 (14·8–15·1)	17·5 (16·9–18·2)	13·6 (12·5–14·6)	14·8 (13·5–16·0)	11·8 (10·9–12·7)	13·7 (12·5–14·8)
Belize	17·0 (16·6–17·5)	19·2 (18·9–19·5)	16·3 (15·9–16·7)	17·4 (17·2–17·7)	13·0 (11·8–14·1)	14·6 (13·2–15·7)	12·8 (11·7–13·7)	13·5 (12·4–14·5)
Bermuda	18·3 (18·0–18·6)	23·1 (22·5–23·8)	13·7 (13·4–14·0)	17·3 (16·8–17·6)	14·1 (12·9–15·2)	17·7 (16·2–19·2)	10·8 (9·9–11·6)	13·4 (12·4–14·5)
Cuba	17·8 (17·7–17·9)	19·7 (18·7–20·7)	15·9 (15·8–15·9)	17·2 (16·2–18·1)	13·7 (12·5–14·7)	15·1 (13·7–16·5)	12·5 (11·5–13·3)	13·5 (12·3–14·7)
Dominica	17·0 (16·7–17·2)	17·7 (17·1–18·4)	14·8 (14·6–15·0)	15·4 (15·0–15·9)	13·1 (12·0–14·1)	13·7 (12·5–14·9)	11·6 (10·7–12·4)	12·0 (11·1–13·0))
Dominican Republic	19·6 (19·0–20·2)	19·3 (18·4–20·3)	18·0 (17·4–18·6)	16·2 (15·2–17·4)	15·0 (13·7–16·2)	14·8 (13·4–16·1)	14 (12·8–15·1)	12·6 (11·4–13·9)
Grenada	15·0 (14·7–15·3)	16·7 (16·2–17·3)	12·6 (12·4–12·8)	16·9 (16·5–17·3)	11·5 (10·5–12·4)	12·8 (11·7–13·9)	9·9 (9·1–10·7)	13·1 (12·0–14·2)
Guyana	15·1 (15·0–15·3)	16·2 (15·2–17·2)	12·7 (12·6–12·8)	14·1 (13·2–15·0)	11·5 (10·5–12·4)	12·3 (11·0–13·5)	9·8 (8·9–10·5)	10·9 (9·8–11·9)
Haiti	11·4 (10·6–12·0)	13·7 (12·3–15·4)	11·6 (10·9–12·3)	13·0 (12·2–14·3)	8·6 (7·7–9·5)	10·4 (9·0–11·8)	8·9 (8·0–9·7)	10·0 (8·9–11·3)
Jamaica	18·1 (17·7–18·6)	18·5 (17·1–19·8)	16·0 (15·7–16·4)	15·6 (14·5–16·8)	14·0 (12·7–15·1)	14·2 (12·7–15·6)	12·6 (11·6–13·5)	12·2 (10·9–13·5)
Puerto Rico	18·7 (18·6–18·9)	21·6 (21·1–22·1)	15·9 (15·8–16·0)	18·7 (18·2–19·2)	14·5 (13·3–15·5)	16·5 (15·0–17·8)	12·4 (11·4–13·3)	14·4 (13·1–15·5)
Saint Lucia	16·1 (15·9–16·3)	18·8 (18·2–19·3)	13·4 (13·2–13·5)	17·0 (16·6–17·5)	12·3 (11·3–13·3)	14·4 (13·1–15·6)	10·5 (9·6–11·2)	13·2 (12·1–14·2)
Saint Vincent and the Grenadines	16·2 (15·9–16·5)	17·4 (16·9–17·9)	14·7 (14·5–14·9)	14·6 (14·3–15·0)	12·4 (11·3–13·4)	13·3 (12·1–14·4)	11·5 (10·5–12·3)	11·3 (10·3–12·2)
Suriname	16·4 (15·9–16·8)	18·2 (17·4–19·0)	14·7 (14·2–15·1)	15·1 (14·2–16·0)	12·6 (11·5–13·6)	13·8 (12·4–15·1)	11·5 (10·5–12·4)	11·7 (10·5–12·8)
Trinidad and Tobago	15·8 (15·6–15·9)	19·0 (17·3–20·9)	13·4 (13·3–13·5)	15·7 (14·3–17·3)	11·9 (10·9–12·9)	14·4 (12·6–16·2)	10·3 (9·5–11·1)	12·1 (10·6–13·6)
Virgin Islands	17·3 (16·5–17·9)	18·8 (17·7–19·6)	13·7 (13·4–14·0)	13·5 (12·8–15·4)	13·5 (12·3–14·6)	14·5 (13·1–15·8)	10·8 (10·0–11·6)	10·6 (9·5–12·2)
Central Latin America	17·6 (17·5–17·6)	19·9 (19·6–20·1)	16·0 (16·0–16·1)	17·8 (17·6–18·1)	13·2 (12·0–14·2)	15·0 (13·7–16·2)	12·3 (11·2–13·2)	13·6 (12·5–14·7)
Colombia	17·5 (17·4–17·5)	22·1 (21·1–23·0)	16·4 (16·4–16·5)	20·1 (19·2–21·0)	13·2 (12·1–14·3)	16·9 (15·3–18·4)	12·6 (11·6–13·6)	15·5 (14·2–17·0)
Costa Rica	18·8 (18·7–19·0)	21·5 (20·9–22·0)	16·8 (16·7–16·9)	18·1 (17·6–18·5)	14·2 (12·9–15·4)	16·3 (14·8–17·5)	13·0 (12·0–14·0)	14·0 (12·8–15·0)
El Salvador	18·5 (18·4–18·6)	19·0 (17·5–20·5)	16·4 (16·4–16·5)	16·6 (15·4–17·8)	14·0 (12·7–15·0)	14·5 (12·8–16·0)	12·6 (11·5–13·5)	12·7 (11·3–14·0)
Guatemala	15·0 (14·9–15·0)	18·4 (17·5–19·3)	13·8 (13·8–13·9)	16·1 (15·3–16·8)	11·2 (10·2–12·1)	13·8 (12·5–15·2)	10·6 (9·7–11·4)	12·3 (11·2–13·4)
Honduras	18·2 (17·3–19·1)	17·0 (15·7–19·3)	17·4 (16·4–18·4)	17·0 (15·7–18·4)	13·6 (12·2–14·9)	12·9 (11·3–14·8)	13·3 (12·1–14·6)	13·0 (11·6–14·5)
Mexico	17·6 (17·6–17·6)	19·1 (18·9–19·2)	16·0 (16·0–16·0)	17·3 (17·1–17·4)	13·2 (12·0–14·2)	14·3 (13·0–15·4)	12·3 (11·2–13·2)	13·1 (12·0–14·2)
Nicaragua	19·1 (18·6–19·6)	20·6 (19·7–21·5)	18·0 (17·4–18·6)	19·9 (18·9–20·7)	14·2 (12·8–15·4)	15·4 (13·8–16·9)	13·7 (12·4–14·8)	15·2 (13·8–16·6)
Panama	19·3 (19·2–19·5)	21·6 (21·1–22·2)	17·3 (17·2–17·5)	19·7 (19·2–20·2)	14·6 (13·3–15·8)	16·4 (14·9–17·7)	13·4 (12·2–14·4)	15·1 (13·7–16·3)
Venezuela	17·6 (17·6–17·7)	20·0 (18·7–21·3)	15·4 (15·4–15·5)	16·9 (15·7–18·1)	13·2 (12·1–14·3)	15·1 (13·5–16·6)	11·9 (10·9–12·7)	13·0 (11·6–14·4)
Tropical Latin America	17·0 (16·9–17·0)	19·7 (19·6–19·8)	14·5 (14·5–14·5)	16·9 (16·8–16·9)	12·6 (11·4–13·6)	14·8 (13·5–16·0)	11·0 (10·0–11·9)	12·8 (11·7–13·8)
Brazil	16·9 (16·9–17·0)	19·7 (19·7–19·8)	14·4 (14·4–14·5)	16·9 (16·8–16·9)	12·5 (11·4–13·6)	14·8 (13·5–16·0)	11·0 (10·0–11·8)	12·8 (11·7–13·8)
Paraguay	18·5 (18–19·0)	19·3 (17·8–20·8)	16·3 (15·8–16·7)	16·5 (15·1–18·0)	13·9 (12·6–15·1)	14·5 (12·9–16·1)	12·4 (11·2–13·4)	12·5 (11·0–14·0)

**North Africa and Middle East**	**15·4 (15·3–15·6)**	**18·6 (18·3–18·9)**	**13·8 (13·6–14·0)**	**15·8 (15·5–16·1)**	**11·0 (9·9–12·1)**	**13·4 (12·0–14·7)**	**10·3 (9·3–11·1)**	**11·7 (10·6–12·7)**

Afghanistan	10·4 (9·5–11·3)	12·1 (11·3–13·2)	10·7 (10·0–11·7)	12·5 (12·0–13·2)	7·2 (6·2–8·3)	8·4 (7·3–9·6)	7·8 (6·8–8·8)	9·0 (7·9–10·0)
Algeria	16·9 (16·3–17·4)	18·9 (18·5–19·3)	15·8 (15·3–16·3)	18·5 (18·1–18·9)	12·1 (10·7–13·3)	13·7 (12·2–15·0)	11·7 (10·5–12·8)	13·6 (12·3–14·9)
Bahrain	13·7 (13·4–14·0)	18·7 (17·9–19·4)	12·2 (12·0–12·6)	17·7 (17·0–18·4)	9·7 (8·6–10·7)	13·3 (11·8–14·7)	9·0 (8·1–9·8)	12·9 (11·5–14·2)
Egypt	14·7 (14·7–14·7)	16·2 (15·3–17·2)	11·8 (11·8–11·9)	12·0 (11·3–12·7)	10·5 (9·4–11·5)	11·6 (10·2–12·9)	8·8 (8·0–9·6)	8·9 (8·0–9·8)
Iran	16·3 (16·0–16·6)	18·6 (18·5–18·6)	15·1 (14·8–15·4)	17·4 (17·3–17·4)	11·9 (10·6–13·0)	13·4 (12·0–14·7)	11·5 (10·5–12·4)	13·0 (11·8–14·1)
Iraq	14·1 (13·1–15·1)	20·3 (19·8–20·9)	13·2 (12·3–14·2)	20·0 (19·4–20·5)	9·9 (8·7–11·1)	14·3 (12·7–15·8)	9·6 (8·4–10·7)	14·3 (12·7–15·8)
Jordan	15·2 (14·3–16·1)	19·9 (19·0–20·8)	15·6 (14·7–16·5)	18·2 (17·1–19·2)	11·0 (9·8–12·2)	14·4 (12·8–15·9)	11·6 (10·4–12·9)	13·5 (12·0–14·9)
Kuwait	18·7 (18·5–18·8)	24·7 (24·3–25·1)	17·9 (17·7–18·0)	20·2 (19·8–20·7)	13·5 (12·0–14·8)	17·7 (15·7–19·4)	13·2 (11·9–14·4)	14·8 (13·3–16·2)
Lebanon	16·6 (15·9–17·6)	19·0 (18·6–19·5)	13·3 (12·8–14·3)	16·7 (16·1–17·2)	12·0 (10·6–13·3)	13·7 (12·2–15·0)	9·9 (8·9–11·0)	12·3 (11·0–13·4)
Libya	16·8 (15·7–17·9)	16·6 (15·5–17·9)	15·9 (14·8–16·9)	15·4 (14·4–16·6)	12·0 (10·6–13·4)	11·8 (10·4–13·3)	11·6 (10·2–12·9)	11·2 (9·9–12·7)
Morocco	14·1 (13·6–14·6)	16·4 (15·1–17·7)	15·4 (14·9–16·0)	15·9 (14·5–17·4)	10·2 (9·0–11·2)	11·9 (10·5–13·5)	11·4 (10·3–12·5)	11·8 (10·2–13·3)
Oman	16·1 (14·8–17·4)	18·8 (18·2–20·0)	13·2 (11·9–14·7)	16·4 (15·0–18·1)	11·0 (9·4–12·5)	13·1 (11·5–14·7)	9·4 (8·1–10·9)	11·8 (10·3–13·6)
Palestine	16·0 (14·7–17·3)	17·4 (16·9–18·0)	14·0 (12·9–15·4)	16·6 (15·9–17·3)	11·4 (10·0–13·0)	12·4 (11·0–13·7)	10·4 (9·1–11·9)	12·2 (10·9–13·4)
Qatar	14·2 (13·2–15·3)	19·9 (18·5–21·4)	13·3 (12·3–14·3)	18·8 (17·5–20·2)	9·9 (8·6–11·1)	14·1 (12·2–15·9)	9·4 (8·1–10·7)	13·5 (11·8–15·2)
Saudi Arabia	17·3 (16·0–18·6)	19·1 (18·3–19·7)	15·4 (14·1–16·8)	16·6 (15·7–17·1)	12·3 (10·9–13·9)	13·9 (12·5–15·2)	11·4 (10–12·9)	12·3 (11·0–13·5)
Sudan	13·5 (12·6–14·5)	16·4 (14·9–18·0)	13·0 (12·1–13·8)	14·8 (13·3–16·5)	9·7 (8·4–10·9)	11·8 (10·3–13·4)	9·5 (8·5–10·6)	10·9 (9·4–12·5)
Syria	16·0 (15·4–16·4)	18·5 (18·0–19·2)	14·2 (13·5–15·0)	15·3 (14·0–16·6)	11·5 (10·4–12·7)	13·4 (11·9–14·7)	10·7 (9·6–11·8)	11·4 (10·1–12·8)
Tunisia	17·0 (16·7–17·3)	19·4 (17·8–21·2)	15·6 (15·3–15·9)	16·7 (15·2–18·4)	12·4 (11·2–13·5)	14·3 (12·5–16·0)	11·5 (10·4–12·5)	12·4 (10·8–14·0)
Turkey	17·2 (16·7–17·6)	21·9 (21·2–22·7)	13·9 (13·5–14·3)	16·3 (15·6–17·0)	12·3 (11·0–13·6)	16·0 (14·4–17·6)	10·5 (9·5–11·4)	12·2 (11·0–13·4)
United Arab Emirates	14·8 (13·6–16·2)	16·9 (15·4–18·5)	13·6 (12·5–15·0)	13·8 (12·4–15·3)	10·5 (9·1–12·0)	12·1 (10·6–13·8)	10·0 (8·7–11·3)	10·1 (8·7–11·5)
Yemen	13·2 (11·8–14·7)	15·6 (14·0–17·0)	12·1 (10·8–13·4)	13·6 (12·5–15·0)	8·8 (7·4–10·2)	10·7 (9·2–12·3)	8·5 (7·3–9·9)	9·7 (8·5–11·1)

**South Asia**	**13·1 (12·9–13·3)**	**15·2 (15·0–15·4)**	**12·1 (11·9–12·3)**	**14·2 (14·0–14·4)**	** 9·2 (8·2–10·1)**	**10·8 (9·7–11·8)**	** 8·9 (8·0–9·6)**	**10·4 (9·4–11·3)**

Bangladesh	14·3 (13·9–14·9)	17·5 (16·6–18·5)	12·5 (12·0–12·9)	16·2 (15·3–17·1)	10·3 (9·2–11·4)	12·5 (11·1–13·9)	9·4 (8·5–10·2)	12·1 (10·9–13·2)
Bhutan	13·5 (12·4–14·5)	17·9 (16·5–19·3)	13·5 (12·5–14·5)	15·5 (13·7–17·0)	9·7 (8·5–10·9)	13·3 (11·8–14·7)	10·1 (8·9–11·3)	11·8 (10·2–13·3)
India	12·9 (12·6–13·1)	15·0 (14·8–15·2)	11·7 (11·5–12·0)	13·9 (13·7–14·1)	9·0 (8·0–9·9)	10·6 (9·5–11·6)	8·6 (7·7–9·3)	10·2 (9·3–11·1)
Nepal	12·9 (12·0–13·9)	16·3 (15·3–17·4)	12·4 (11·6–13·4)	13·3 (12·9–14·5)	9·1 (8·0–10·2)	11·6 (10·5–12·9)	9·1 (8·0–10·1)	9·9 (8·9–11·0)
Pakistan	14·1 (13·7–14·5)	14·9 (13·5–16·4)	14·1 (13·7–14·6)	14·5 (13·1–15·9)	10·1 (9·0–11·0)	10·6 (9·2–12·2)	10·5 (9·5–11·4)	10·8 (9·5–12·2)

**Southeast Asia, east Asia, and Oceania**	**15·0 (14·9–15·2)**	**18·4 (18·1–18·6)**	**13·3 (13·1–13·4)**	**15·4 (15·2–15·7)**	**11·3 (10·3–12·1)**	**13·7 (12·5–14·8)**	**10·3 (9·6–11·0)**	**11·9 (11·0–12·8)**

East Asia	15·0 (14·8–15·2)	18·6 (18·3–18·9)	13·3 (13·2–13·5)	15·7 (15·4–16·0)	11·3 (10·4–12·2)	14·0 (12·7–15·1)	10·5 (9·8–11·2)	12·2 (11·3–13·1)
China	15·0 (14·8–15·1)	18·6 (18·3–18·9)	13·3 (13·2–13·5)	15·7 (15·4–16·0)	11·3 (10·4–12·1)	14·0 (12·7–15·0)	10·5 (9·7–11·2)	12·2 (11·3–13·1)
North Korea	16·5 (15·4–18·0)	16·7 (15·4–18·1)	13·2 (12·8–14·4)	13·3 (12·9–13·8)	12·3 (11·1–13·7)	12·3 (11·1–13·7)	10·3 (9·5–11·4)	10·3 (9·4–11·1)
Taiwan (province of China)	17·0 (16·9–17·1)	21·3 (20·8–21·8)	15·3 (15·2–15·3)	18·0 (17·6–18·4)	12·7 (11·6–13·7)	15·7 (14·3–17·0)	11·9 (11·0–12·7)	13·6 (12·6–14·7)
Oceania	11·8 (11·4–12·3)	12·7 (12·1–13·3)	10·4 (10·1–10·8)	11·0 (10·5–11·6)	8·5 (7·6–9·4)	9·0 (8·0–10·0)	7·7 (6·9–8·4)	8·0 (7·2–8·8)
American Samoa	16·4 (15·9–17·1)	15·9 (15·6–16·4)	13·0 (12·7–13·5)	13·9 (12·9–15·3)	11·8 (10·6–13·0)	11·4 (10·1–12·5)	9·6 (8·7–10·6)	10·2 (9·0–11·6)
Federated States of Micronesia	12·7 (12·1–13·6)	14·3 (13·2–15·0)	11·7 (10·8–12·7)	12·3 (11·8–12·8)	9·3 (8·2–10·3)	10·2 (8·9–11·3)	8·8 (7·7–9·9)	9·1 (8·1–10·0)
Fiji	14·5 (13·6–15·4)	14·4 (13·3–15·7)	12·6 (11·9–13·3)	12·1 (11·3–13·1)	10·6 (9·4–11·7)	10·4 (9·0–11·8)	9·4 (8·4–10·4)	8·9 (7·9–10·0)
Guam	18·0 (17·6–18·3)	17·8 (17·1–18·5)	14·3 (14·1–14·6)	15·6 (15·1–16·2)	13·3 (12·0–14·4)	12·9 (11·5–14·2)	11·0 (10·0–11·8)	11·7 (10·5–12·7)
Kiribati	12·0 (11·5–12·5)	13·0 (12·2–14·3)	10·4 (10·0–10·7)	10·7 (10·1–11·4)	8·7 (7·8–9·6)	9·2 (8·1–10·5)	7·7 (7·0–8·4)	7·8 (6·9–8·7)
Marshall Islands	13·0 (12·8–13·2)	13·2 (12·3–14·0)	10·3 (10·0–10·6)	11·1 (10·4–12·0)	9·6 (8·7–10·4)	9·6 (8·4–10·7)	7·8 (7·1–8·5)	8·3 (7·4–9·3)
Northern Mariana Islands	17·0 (16·2–18·2)	19·0 (18·2–19·7)	15·6 (14·0–16·4)	15·9 (14·8–16·8)	12·5 (11·2–13·8)	13·8 (12·3–15·2)	11·8 (10·4–13·0)	11·9 (10·5–12·9)
Papua New Guinea	10·5 (10·0–11·2)	11·6 (10·8–12·3)	9·4 (9·0–10·0)	10·2 (9·6–11·1)	7·5 (6·6–8·3)	8·1 (7·1–9·1)	6·9 (6·2–7·7)	7·5 (6·6–8·3)
Samoa	17·0 (15·9–18·4)	16·2 (15·3–17·7)	14·3 (13·3–15·2)	14·1 (13·8–14·7)	12·5 (11·0–14·0)	11·7 (10·4–13·2)	10·8 (9·7–11·9)	10·5 (9·5–11·5)
Solomon Islands	12·1 (11·4–12·8)	13·4 (12·7–14·0)	11·2 (10·4–12·2)	12·1 (11·4–12·8)	8·9 (8·0–9·9)	9·7 (8·6–10·7)	8·5 (7·6–9·5)	9·1 (8·1–10·0)
Tonga	15·0 (14·6–15·5)	16·6 (15·5–18·0)	13·1 (12·9–13·4)	13·5 (12·7–14·0)	10·9 (9·7–11·9)	11·9 (10·4–13·4)	9·9 (9·0–10·7)	10·1 (9·0–11·0)
Vanuatu	12·8 (12·0–13·6)	13·4 (12·6–14·4)	10·9 (10·2–11·8)	11·5 (10·6–12·4)	9·3 (8·2–10·3)	9·5 (8·4–10·6)	8·3 (7·4–9·2)	8·6 (7·6–9·5)
Southeast Asia	15·1 (14·8–15·3)	17·5 (17·2–17·9)	13·0 (12·8–13·2)	14·3 (14·0–14·6)	11·0 (9·9–12·0)	12·9 (11·6–14·0)	9·8 (8·9–10·6)	10·7 (9·8–11·6)
Cambodia	12·4 (11·8–13·1)	15·9 (14·7–16·8)	11·0 (10·5–11·6)	13·0 (12·7–13·2)	8·8 (7·7–9·8)	11·4 (10·1–12·7)	8·1 (7·2–8·9)	9·6 (8·6–10·4)
Indonesia	14·2 (13·8–14·5)	16·1 (15·6–16·6)	13·2 (12·9–13·5)	13·4 (13·0–14·0)	10·2 (9·2–11·2)	11·6 (10·5–12·7)	9·8 (8·9–10·7)	10·0 (9·1–11·0)
Laos	11·8 (10·9–13·0)	15·7 (14·5–16·8)	10·5 (9·7–11·5)	13·2 (12·4–14·3)	8·8 (7·8–10·0)	11·7 (10·4–13·0)	8·1 (7·2–9·0)	10·0 (9·0–11·2)
Malaysia	15·1 (15·0–15·1)	16·9 (16·2–17·6)	13·3 (13·3–13·3)	14·8 (14·1–15·5)	11·3 (10·3–12·2)	12·6 (11·4–13·8)	10·2 (9·3–11·0)	11·3 (10·3–12·4)
Maldives	12·4 (12·2–12·5)	21·6 (21·0–22·2)	13·3 (13·2–13·5)	19·2 (18·7–19·7)	8·9 (8·0–9·7)	15·9 (14·3–17·4)	10·0 (9·1–10·8)	14·6 (13·3–15·8)
Mauritius	15·8 (15·6–16·0)	18·4 (17·8–19·0)	12·3 (12·2–12·4)	15·3 (14·8–15·8)	11·7 (10·6–12·8)	13·4 (12·0–14·7)	9·2 (8·4–10·0)	11·3 (10·2–12·3)
Myanmar	12·5 (11·4–13·7)	16·3 (15·2–17·6)	10·5 (9·8–11·6)	12·8 (12·5–13·5)	8·8 (7·7–10·1)	11·7 (10·4–13·1)	7·7 (6·7–8·7)	9·5 (8·6–10·3)
Philippines	16·3 (15·9–16·8)	15·8 (14·6–16·9)	14·3 (13·8–14·7)	12·8 (11·8–13·9)	11·9 (10·7–12·9)	11·5 (10·2–12·8)	10·5 (9·5–11·5)	9·4 (8·3–10·6)
Sri Lanka	17·0 (16·8–17·3)	19·7 (18·8–21·5)	13·2 (13·0–13·3)	15·8 (14·7–17·0)	12·8 (11·6–13·8)	14·8 (13·2–16·7)	10·0 (9·2–10·8)	12·0 (10·7–13·3)
Seychelles	17·4 (17·0–17·7)	18·1 (17·7–18·6)	12·6 (12·4–12·8)	14·2 (13·9–14·5)	13·0 (11·8–14·2)	13·5 (12·2–14·7)	9·7 (8·8–10·4)	10·8 (9·8–11·7)
Thailand	17·1 (16·8–17·4)	21·7 (20·9–22·5)	14·6 (14·3–14·9)	18·5 (17·7–19·4)	12·8 (11·6–13·8)	16·3 (14·8–17·7)	11·0 (10·1–11·9)	14·1 (12·8–15·4)
Timor-Leste	13·4 (12·3–14·5)	16·3 (15·3–17·3)	13·7 (12·9–14·6)	13·8 (13·0–15·0)	9·5 (8·3–10·8)	11·7 (10·3–13·0)	9·9 (8·8–11·1)	10 ·0 (9·0–11·3)
Vietnam	16·4 (15·7–17·4)	18·8 (18·0–19·9)	12·7 (12·3–13·3)	13·9 (13·0–14·6)	12·2 (11·0–13·4)	14·0 (12·6–15·5)	9·7 (8·8–10·6)	10·6 (9·6–11·5)

**Sub-Saharan Africa**	**13·2 (12·9–13·6)**	**15·4 (15·1–15·8)**	**12·1 (11·8–12·4)**	**13·4 (13·1–13·7)**	**9·6 (8·6–10·4)**	**11·3 (10·2–12·4)**	**8·8 (7·9–9·6)**	**9·9 (8·9–10·8)**

Central sub-Saharan Africa	12·2 (11·5–12·8)	13·9 (12·9–14·8)	10·7 (10·1–11·2)	12·1 (11·6–12·9)	8·5 (7·5–9·5)	9·9 (8·7–11·0)	7·6 (6·7–8·5)	8·7 (7·7–9·8)
Angola	11·6 (10·6–12·8)	15·0 (13·7–16·3)	10·0 (9·3–11·0)	12·3 (11·7–13·4)	8·4 (7·3–9·5)	10·8 (9·4–12·2)	7·3 (6·4–8·2)	8·9 (7·9–10·1)
Central African Republic	10·4 (9·8–11·1)	11·4 (10·2–13·2)	8·9 (8·6–9·3)	9·7 (9·0–10·6)	7·4 (6·5–8·2)	8·2 (7·0–9·7)	6·3 (5·6–7·0)	7·0 (6·2–7·9)
Congo (Brazzaville)	11·0 (10·4–11·7)	12·8 (11·9–14·4)	9·9 (9·4–10·4)	12·7 (12·1–13·7)	7·8 (6·9–8·7)	9·3 (8·1–10·6)	7·1 (6·3–7·8)	9·2 (8·2–10·3)
Democratic Republic of the Congo	12·5 (11·6–13·5)	13·7 (12·4–15·0)	11·0 (10·2–12·0)	12·1 (11·4–13·3)	8·7 (7·6–9·8)	9·7 (8·5–11·0)	7·8 (6·8–8·9)	8·7 (7·5–10·0)
Equatorial Guinea	10·6 (9·5–11·8)	15·9 (13·3–18·8)	9·1 (8·5–10·0)	13·7 (12·3–15·3)	7·6 (6·6–8·7)	11·5 (9·5–13·7)	6·6 (5·9–7·5)	9·9 (8·6–11·3)
Gabon	13·5 (12·5–14·4)	16·3 (14·9–17·8)	10·8 (10·3–11·2)	12·9 (12·5–13·1)	9·6 (8·5–10·8)	11·7 (10·2–13·3)	7·8 (7·0–8·7)	9·4 (8·4–10·2)
Eastern sub-Saharan Africa	11·6 (11·4–11·8)	15·3 (15·0–15·6)	11·1 (10·9–11·3)	13·0 (12·8–13·3)	8·6 (7·7–9·3)	11·4 (10·3–12·3)	8·2 (7·4–8·9)	9·7 (8·8–10·6)
Burundi	10·2 (9·5–11·1)	13·2 (12·0–14·6)	9·3 (8·8–9·9)	12·0 (11·2–13·2)	7·8 (6·9–8·6)	10·0 (8·8–11·3)	7·0 (6·2–7·7)	9·0 (8·0–10·2)
Comoros	12·4 (11·4–13·5)	15·2 (13·9–16·5)	11·7 (10·8–12·7)	13·5 (12·6–14·6)	9·2 (8·1–10·3)	11·5 (10·1–12·8)	8·6 (7·5–9·6)	10·1 (8·9–11·3)
Djibouti	13·1 (11·7–14·6)	15·3 (13·3–17·3)	12·7 (11·4–14·0)	13·4 (12·2–15·0)	9·8 (8·5–11·2)	11·6 (9·9–13·4)	9·6 (8·3–10·9)	10·1 (8·9–11·6)
Eritrea	10·0 (9·4–10·6)	13·2 (12·2–14·7)	8·0 (7·8–8·3)	11·0 (10·3–12·1)	7·2 (6·4–8·0)	9·8 (8·6–11·2)	5·7 (5·0–6·3)	8·0 (7·1–9·1)
Ethiopia	10·0 (9·6–10·4)	16·0 (15·4–16·6)	9·5 (9·2–9·8)	14·1 (13·6–14·6)	7·3 (6·5–8·0)	11·9 (10·7–13)	6·9 (6·2–7·6)	10·5 (9·5–11·5)
Kenya	13·9 (13·5–14·2)	15·9 (15·5–16·3)	13·2 (13·0–13·5)	12·8 (12·5–13·0)	10·4 (9·4–11·2)	11·9 (10·8–12·9)	9·9 (8·9–10·8)	9·5 (8·6–10·3)
Madagascar	12·1 (11·6–12·6)	13·5 (12·1–15·0)	12·9 (12·3–13·4)	12·6 (11·6–14·0)	9·0 (8·1–9·8)	10·1 (8·8–11·5)	9·6 (8·6–10·5)	9·5 (8·4–10·8)
Malawi	12·1 (11·5–12·7)	15·8 (14·5–17·0)	11·2 (10·7–11·8)	12·3 (11·8–13·0)	9·0 (8·1–9·9)	11·8 (10·4–13·2)	8·3 (7·4–9·1)	9·1 (8·2–10·0)
Mozambique	12·4 (11·7–13·0)	14·0 (12·6–15·5)	11·0 (10·5–11·5)	11·2 (10·5–12·0)	9·1 (8·2–10·0)	10·4 (9·0–11·8)	8·0 (7·2–8·9)	8·2 (7·3–9·2)
Rwanda	10·4 (9·8–11·0)	16·0 (14·9–17·1)	9·5 (9·1–9·9)	13·1 (12·5–14·1)	7·8 (7·0–8·6)	12·1 (10·7–13·4)	7·2 (6·5–7·9)	9·9 (8·9–11·0)
Somalia	10·8 (9·7–12·0)	12·3 (10·9–13·8)	10·2 (9·2–11·3)	11·4 (10·4–12·7)	8·0 (7·0–9·2)	9·2 (8·0–10·6)	7·6 (6·6–8·7)	8·5 (7·5–9·7)
South Sudan	12·0 (10·8–13·4)	13·7 (11·9–15·3)	11·0 (9·9–12·2)	12·1 (10·8–13·4)	8·4 (7·2–9·6)	9·5 (8·0–11·0)	7·9 (6·8–9·0)	8·6 (7·3–9·9)
Tanzania	13·1 (12·5–13·9)	16·0 (15·0–16·8)	12·5 (11·8–13·1)	13·9 (13·0–14·6)	9·8 (8·8–10·7)	12·0 (10·8–13·2)	9·3 (8·3–10·2)	10·4 (9·3–11·5)
Uganda	12·0 (11·3–12·8)	16·1 (14·9–17·1)	12·1 (11·1–13·4)	12·7 (12·1–13·9)	8·7 (7·7–9·7)	12·0 (10·6–13·3)	8·7 (7·6–10·0)	9·4 (8·5–10·5)
Zambia	11·5 (11·0–12·0)	14·9 (13·8–16·2)	11·3 (10·8–12·0)	12·4 (12·0–13·2)	8·6 (7·8–9·4)	11·2 (9·8–12·5)	8·5 (7·6–9·4)	9·3 (8·4–10·2)
Southern sub-Saharan Africa	17·8 (17·6–18·1)	17·5 (17·2–17·7)	14·5 (14·3–14·8)	14·0 (13·8–14·2)	12·6 (11·4–13·8)	12·4 (11·2–13·5)	10·5 (9·5–11·5)	10·1 (9·0–11·0)
Botswana	15·8 (14·8–17·0)	16·3 (15·3–17·1)	11·9 (11·2–12·4)	14·8 (13·5–15·4)	11·2 (10·0–12·5)	11·5 (10·3–12·8)	8·5 (7·6–9·4)	10·5 (9·1–11·8)
Lesotho	14·4 (13·5–15·3)	13·5 (11·9–15·7)	10·8 (10·3–11·3)	10·0 (9·4–10·7)	10·1 (8·9–11·3)	9·4 (7·9–11·1)	7·8 (6·9–8·5)	7·0 (6·2–7·9)
Namibia	13·9 (13·2–14·7)	17·7 (16·1–19·2)	11·4 (11·0–11·7)	12·8 (12·4–13·1)	9·8 (8·8–10·9)	12·6 (11·0–14·3)	8·1 (7·2–8·9)	9·1 (8·1–10·0)
South Africa	18·9 (18·6–19·1)	18·3 (18·1–18·4)	15·7 (15·4–15·9)	14·6 (14·5–14·8)	13·3 (12–14·6)	13·0 (11·7–14·2)	11·3 (10·2–12·4)	10·5 (9·4–11·5)
Swaziland (eSwatini	14·0 (13·1–15·0)	15·1 (13·2–17·2)	10·9 (10·3–11·5)	10·8 (10·1–11·8)	9·9 (8·7–11·1)	10·6 (8·9–12·3)	7·8 (6·9–8·6)	7·6 (6·6–8·5)
Zimbabwe	15·3 (14·5–16·0)	13·7 (12·5–15·1)	12·3 (11·6–13·3)	11·6 (10·7–12·3)	10·9 (9·8–12·1)	9·8 (8·5–11·1)	9·0 (8·0–10·1)	8·4 (7·4–9·3)
Western sub-Saharan Africa	13·6 (12·9–14·4)	15·3 (14·4–16·2)	12·8 (12·1–13·5)	13·9 (13·2–14·5)	9·8 (8·7–10·8)	11·2 (9·9–12·4)	9·3 (8·2–10·2)	10·2 (9·1–11·2)
Benin	13·5 (12·8–14·3)	14·9 (13·5–16·3)	12·0 (11·4–12·6)	13·3 (12·1–14·4)	9·6 (8·5–10·7)	10·9 (9·5–12·4)	8·7 (7·6–9·7)	9·8 (8·5–11·1)
Burkina Faso	12·5 (11·9–13·1)	14·3 (13·4–15·3)	11·7 (11·1–12·3)	12·5 (11·7–13·3)	8·9 (7·9–9·9)	10·6 (9·4–11·7)	8·3 (7·2–9·2)	9·2 (8·2–10·3)
Cameroon	12·9 (12·3–13·6)	14·8 (13·2–16·3)	12·1 (11·6–12·7)	12·8 (11·7–14·1)	9·2 (8·2–10·2)	10·9 (9·4–12·3)	8·8 (7·8–9·6)	9·5 (8·2–10·7)
Cape Verde	18·8 (18·5–19·1)	19·7 (19·4–20·4)	16·1 (15·9–16·3)	17·4 (16·8–18·1)	14·0 (12·6–15·1)	14·8 (13·4–16·1)	12·2 (11·1–13·1)	13·2 (11·9–14·4)
Chad	13·0 (12·4–13·7)	13·8 (12·6–15·0)	12·2 (11·6–12·9)	12·8 (11·8–13·7)	9·3 (8·3–10·4)	9·9 (8·6–11·2)	8·8 (7·8–9·8)	9·3 (8·2–10·4)
Côte d’Ivoire	13·4 (12·6–14·2)	14·7 (13·4–16·0)	11·7 (11·0–12·5)	12·9 (11·8–13·9)	9·3 (8·1–10·4)	10·7 (9·4–12·2)	8·3 (7·3–9·3)	9·5 (8·3–10·6)
The Gambia	13·6 (12·5–14·9)	14·5 (13·2–15·8)	12·0 (10·9–13·2)	12·7 (12·3–13·3)	9·9 (8·7–11·2)	10·6 (9·2–12·0)	8·9 (7·8–10·1)	9·5 (8·5–10·4)
Ghana	13·3 (12·3–14·4)	15·2 (14·1–16·3)	12·6 (11·7–13·6)	12·4 (12·0–13·1)	9·8 (8·7–11·0)	11·3 (10·1–12·6)	9·3 (8·2–10·4)	9·3 (8·3–10·2)
Guinea	12·4 (11·8–13·0)	13·1 (12·1–14·2)	12·8 (12·2–13·4)	12·2 (11·4–13·2)	9·0 (8·1–10·0)	9·7 (8·6–10·8)	9·4 (8·6–10·3)	9·1 (8·0–10·2)
Guinea-Bissau	11·3 (10·4–12·5)	13·0 (11·8–14·5)	9·6 (8·9–10·5)	11·4 (10·6–12·1)	8·2 (7·1–9·3)	9·5 (8·2–10·7)	7·0 (6·1–7·9)	8·4 (7·4–9·3)
Liberia	12·6 (11·7–13·5)	14·1 (12·8–15·5)	12·5 (11·7–13·3)	13·7 (12·6–14·6)	8·7 (7·6–9·8)	10·0 (8·6–11·3)	8·9 (7·7–10)	9·8 (8·6–11·1)
Mali	11·5 (11·0–12·0)	14·5 (13·4–15·6)	11·8 (11·3–12·3)	14·1 (13·3–15·2)	8·2 (7·2–9·0)	10·5 (9·2–11·8)	8·4 (7·5–9·3)	10·3 (9·0–11·4)
Mauritania	12·2 (11·5–13·0)	15·5 (14·3–16·7)	12·4 (11·8–13·2)	15·1 (14·1–16·6)	8·8 (7·8–9·7)	11·3 (9·9–12·7)	9·0 (8·1–10·0)	11·0 (9·6–12·5)
Niger	12·6 (11·9–13·3)	14·2 (13·1–15·5)	12·0 (11·4–12·6)	13·4 (12·5–14·4)	9·3 (8·2–10·2)	10·5 (9·4–11·8)	8·8 (7·9–9·8)	10·0 (8·8–11·1)
Nigeria	14·3 (12·8–15·9)	16·4 (14·3–18·8)	13·5 (12·1–15·0)	15·2 (13·5–16·4)	10·3 (8·9–11·8)	12·0 (10·0–13·8)	9·8 (8·5–11·3)	11·1 (9·5–12·5)
São Tomé and Príncipe	15·9 (15·2–16·6)	15·3 (14·3–16·6)	14·3 (13·7–14·9)	13·6 (13·0–14·4)	11·7 (10·5–12·8)	11·4 (10·2–12·8)	10·6 (9·6–11·6)	10·1 (9·1–11·2)
Senegal	13·5 (12·9–14·2)	15·3 (14·2–16·3)	12·3 (11·7–12·9)	13·3 (12·5–14·2)	9·8 (8·7–10·9)	11·2 (9·9–12·4)	9·0 (8·0–9·9)	9·8 (8·6–10·9)
Sierra Leone	13·2 (12·2–14·2)	13·3 (12·2–14·5)	11·6 (10·8–12·4)	13·0 (12·1–13·9)	9·6 (8·5–10·7)	9·7 (8·5–10·9)	8·4 (7·5–9·3)	9·5 (8·2–10·6)
Togo	13·2 (12·4–14·1)	15·1 (13·7–16·5)	12·2 (11·5–13·0)	12·5 (11·7–13·6)	9·6 (8·5–10·7)	11·1 (9·7–12·6)	9·0 (8·0–10·0)	9·3 (8·2–10·4)

Data in parentheses are 95% uncertainty intervals. Super-regions, regions, and countries are listed alphabetically. GBD=Global Burden of Diseases, Injuries, and Risk Factors Study. HALE=healthy life expectancy. SDI=Socio-demographic Index.

**Table 3:** Global all-age DALYs and age-standardised DALY rates in 2017 with percentage changes between 2007 and 2017 for all causes, by sex

	All-age DALYs (thousands)						Age-standardised DALY rate (per 100 000)					
	2017			Percentage change, 2007–17			2017			Percentage change, 2007–17		
				
	Males	Females	Both	Males	Females	Both	Males	Females	Both	Males	Females	Both
**All causes**	**1 340 000** **(1 240 000 to 1 460 000)**	**1 160 000** **(1 040 000 to 1 290 000)**	**2 500 000** **(2 290 000 to 2 740 000)**	** −1·4%** **(−3·0 to 0·1)**	** −1·6%** **(−3·5 to 0·2)**	** −1·5%** **(−3·1 to 0·1)**	** 35 834·3** **(33 218·6 to 38 740·3)**	** 29 934·6** **(26 981·7 to 33 211·3)**	** 32 796·9** **(30 041·9 to 35 849·3)**	** −15·9%** (−17·2 to −14·6)*	** −16·7%** (−18·3 to −15·1)*	** −16·2%** (−17·6 to −14·8)*
**Communicable, maternal, neonatal, and nutritional diseases**	** 362 000** **(345 000 to 384 000)**	** 334 000** **(314 000 to 357 000)**	** 696 000** **(660 000 to 740 000)**	**−25·8%** (−27·9 to −23·6)*	**−27·0%** (−29·1 to −24·8)*	**−26·4%** (−28·4 to −24·3)*	** 10 106·6** **(9630·6 to 10 695·2)**	** 9601·9** **(9073·8 to 10 240·7)**	** 9853·3** **(9349·7 to 10 459·3)**	** −31·5%** (−33·4 to −29·4)*	** −32·9%** (−34·8 to −30·9)*	** −32·2%** (−34·0 to −30·3)*
** HIV/AIDS and sexually transmitted infections**	** 35 100** **(30 900 to 40 400)**	** 30 800** **(27 700 to 34 400)**	** 65 900** **(58 700 to 74 700)**	**−40·4%** (−43·4 to −37·0)*	**−50·0%** (−52·7 to −46·9)*	**−45·3%** (−48·0 to −42·0)*	** 918·3** **(799·5 to 1074·4)**	** 828·2** **(735·9 to 937·9)**	** 874·1** **(767·5 to 1006·2)**	** −46·1%** (−49·3 to −42·5)*	** −54·7%** (−57·4 to −51·6)*	** −50·5%** (−53·4 to −47·1)*

HIV/AIDS	28 300 (26 700 to 30 200)	26 100 (24 300 to 28 000)	54 400 (51 200 to 57 700)	−44·8% (−46·6 to −42·5)*	−53·9% (−55·8 to −51·6)*	−49·6% (−51·3 to −47·4)*	724·0 (683·0 to 771·1)	686·1 (638·2 to 735·8)	704·8 (662·6 to 747·9)	−51·1% (−52·6 to −49·0)*	−58·8% (−60·6 to −56·8)*	−55·2% (−56·8 to −53·3)*
HIV/AIDS and drug-susceptible tuberculosis co-infection	5810 (4180 to 7430)	5260 (3760 to 6760)	11 100 (7940 to 14 200)	−51·9% (−55·0 to −48·0)*	−57·8% (−60·8 to −53·9)*	−54·9% (−57·9 to −51·0)*	150·0 (108·3 to 191·6)	140·2 (100·6 to 179·4)	145·1 (104·4 to 185·3)	−57·2% (−59·9 to −53·7)*	−62·3% (−65·0 to −58·9)*	−59·8% (−62·5 to −56·3)*
HIV/AIDS and multidrug-resistant tuberculosis without extensive drug resistance co-infection	674 (405 to 1020)	589 (347 to 905)	1260 (755 to 1920)	−48·9% (−62·9 to −29·4)*	−54·2% (−68·1 to −36·0)*	−51·5% (−65·6 to −32·9)*	17·4 (10·5 to 26·5)	15·8 (9·3 to 24·2)	16·6 (9·9 to 25·3)	−54·4% (−67·0 to −37·1)*	−58·9% (−71·4 to −42·4)*	−56·6% (−69·1 to −40·1)*
HIV/AIDS and extensively drug-resistant tuberculosis co-infection	39·3 (23·8 to 58·0)	23·9 (14·9 to 35·3)	63·3 (38·8 to 93·3)	−8·1% (−25·0 to 13·5)	−13·5% (−32·8 to 10·9)	−10·2% (−28·3 to 11·9)	1·0 (0·6 to 1·5)	0·6 (0·4 to 0·9)	0·8 (0·5 to 1·2)	−19·0% (−33·9 to −0·1)*	−23·3% (−40·4 to −1·7)*	−20·7% (−36·7 to −1·1)*
HIV/AIDS resulting in other diseases	21 800 (19 500 to 24 300)	20 200 (18 000 to 22 600)	42 100 (37 800 to 46 700)	−42·4% (−45·1 to −39·2)*	−52·8% (−55·5 to −49·8)*	−47·9% (−50·5 to −44·9)*	555·5 (497·5 to 618·1)	529·5 (469·3 to 591·4)	542·3 (487·0 to 603·1)	−49·0% (−51·3 to −46·1)*	−57·8% (−60·2 to −55·2)*	−53·7% (−56·1 to −51·1)*
Sexually transmitted infections excluding HIV	6740 (2970 to 12 200)	4730 (2350 to 8070)	11 500 (5320 to 20 200)	−10·4% (−18·2 to −1·8)*	−6·8% (−14·2 to 1·2)	−8·9% (−16·1 to −1·0)*	194·3 (84·5 to 355)	142·1 (67·7 to 246·7)	169·3 (76·7 to 300·4)	−13·8% (−21·2 to −5·7)*	−11·7% (−18·4 to −4·7)*	−13·0% (−20·1 to −5·5)*
Syphilis	6160 (2430 to 11 600)	3750 (1460 to 7060)	9910 (3930 to 18 700)	−11·9% (−19·7 to −2·9)*	−10·4% (−18·5 to −1·7)*	−11·3% (−19·1 to −2·9)*	179·6 (70·8 to 339)	117·3 (45·6 to 221·1)	149·5 (59·1 to 283·2)	−14·8% (−22·3 to −6·1)*	−13·4% (−21·2 to −5·0)*	−14·3% (−21·8 to −6·2)*
Chlamydial infection	192 (112 to 336)	163 (107 to 266)	355 (219 to 606)	7·9% (4·0 to 11·6)*	8·3% (4·8 to 11·3)*	8·1% (5·1 to 10·5)*	4·8 (2·8 to 8·5)	4·1 (2·7 to 6·8)	4·5 (2·8 to 7·7)	−2·6% (−5·9 to 0·7)	−3·5% (−7·0 to −0·9)*	−3·0% (−5·9 to −0·8)*
Gonococcal infection	135 (77·5 to 244)	168 (132 to 229)	303 (216 to 468)	11·2% (7·2 to 16·0)*	−0·3% (−5·7 to 5·5)	4·5% (0·1 to 8·6)*	3·5 (2·0 to 6·2)	4·2 (3·3 to 5·8)	3·9 (2·7 to 6·0)	2·2% (−1·5 to 6·5)	−12·0% (−17·4 to −6·2)*	−6·1% (−10·7 to −2·1)*
Trichomoniasis	7·04 (2·77 to 14·8)	236 (94·7 to 508)	243 (97·6 to 524)	16·2% (12·8 to 19·5)*	16·0% (14·2 to 17·7)*	16·0% (14·2 to 17·7)*	0·2 (0·1 to 0·4)	6·0 (2·4 to 12·9)	3·1 (1·2 to 6·6)	1·7% (−1·0 to 4·3)	2·3% (1·2 to 3·4)*	2·2% (1·1 to 3·2)*
Genital herpes	98·4 (32·0 to 235)	149 (48·0 to 357)	247 (79·8 to 594)	21·2% (19·3 to 22·5)*	18·9% (17·0 to 20·4)*	19·8% (18·1 to 21·0)*	2·5 (0·8 to 5·9)	3·7 (1·2 to 8·8)	3·1 (1·0 to 7·4)	2·6% (1·7 to 3·5)*	0·9% (−0·1 to 1·9)	1·5% (0·9 to 2·3)*
Other sexually transmitted infections	147 (86·7 to 258)	269 (189 to 399)	416 (276 to 659)	8·0% (5·3 to 11·1)*	8·2% (5·2 to 11·0)*	8·1% (5·9 to 10·1)*	3·7 (2·2 to 6·6)	6·8 (4·8 to 10·2)	5·3 (3·5 to 8·4)	−1·0% (−3·5 to 1·7)	−3·5% (−6·6 to −0·8)*	−2·6% (−5·0 to −0·7)*

** Respiratory infections and tuberculosis**	** 88 800** **(84 000 to 94 100)**	** 71 100** **(66 900 to 75 600)**	** 160 000** **(152 000 to 168 000)**	**−22·1%** (−25·2 to −18·5)*	**−24·0%** (−27·4 to −20·4)*	**−22·9%** (−25·6 to −19·9)*	** 2434·0** **(2298·0 to 2579·8)**	** 2002·9** **(1881·7 to 2134·1)**	** 2209·6** **(2095·9 to 2327·9)**	** −30·7%** (−33·5 to −27·5)*	** −32·1%** (−35·3 to −28·8)*	** −31·4%** (−33·9 to −28·6)*

Tuberculosis	28 000 (26 400 to 29 700)	17 000 (16 100 to 18 200)	45 000 (42 800 to 47 300)	−19·1% (−23·4 to −15·2)*	−20·6% (−24·6 to −13·1)*	−19·7% (−22·7 to −16·0)*	714·5 (672·6 to 759·4)	437·3 (412·6 to 467·3)	572·8 (544·0 to 602·4)	−32·0% (−35·5 to −28·7)*	−32·2% (−35·5 to −25·8)*	−32·0% (−34·5 to −28·9)*
Latent tuberculosis infection	0 (0 to 0)	0 (0 to 0)	0 (0 to 0)	··	··	··	0 (0 to 0)	0 (0 to 0)	0 (0 to 0)	··	··	··
Drug-susceptible tuberculosis	24 800 (22 600 to 26 900)	15 100 (13 700 to 16 500)	39 900 (36 400 to 43 100)	−19·4% (−25·7 to −13·5)*	−21·4% (−28·2 to −13·0)*	−20·2% (−26·0 to −14·4)*	632·9 (576·3 to 686·5)	389·1 (354·0 to 424·5)	508·2 (463·4 to 548·7)	−32·2% (−37·4 to −27·4)*	−32·9% (−38·4 to −25·6)*	−32·4% (−37·2 to −27·6)*
Multidrug-resistant tuberculosis without extensive drug resistance	2910 (1710 to 4530)	1740 (971 to 2780)	4650 (2660 to 7220)	−18·3% (−49·4 to 25·3)	−15·0% (−49·6 to 33·5)	−17·1% (−49·1 to 27·5)	74·0 (43·5 to 115·1)	44·6 (25·3 to 70·5)	59·0 (34·0 to 91·4)	−31·3% (−57·4 to 4·8)	−27·3% (−56·5 to 13·6)	−29·8% (−56·6 to 7·6)
Extensively drug-resistant tuberculosis	304 (219 to 416)	143 (92·7 to 211)	447 (311 to 626)	2·0% (−23·9 to 38·3)	15·8% (−20·7 to 69·3)	6·0% (−22·7 to 45·8)	7·7 (5·5 to 10·5)	3·6 (2·3 to 5·3)	5·6 (3·9 to 7·8)	−14·4% (−36·2 to 16·1)	−1·6% (−32·3 to 43·4)	−10·7% (−34·8 to 22·8)
Lower respiratory infections	56 600 (52 800 to 60 400)	49 900 (46 600 to 53 000)	106 000 (100 000 to 112 000)	−24·9% (−29·1 to −20·4)*	−26·8% (−30·7 to −22·6)*	−25·8% (−29·1 to −22·1)*	1605·9 (1498·2 to 1716·6)	1453·6 (1354·5 to 1547·7)	1523·9 (1433 to 1610·4)	−31·4% (−35·3 to −27·4)*	−33·6% (−37·3 to −29·7)*	−32·5% (−35·6 to −29·1)*
Upper respiratory infections	3200 (1930 to 4960)	3140 (1910 to 4880)	6340 (3870 to 9820)	5·5% (−1·7 to 10·4)	6·8% (0·7 to 10·7)*	6·1% (0·7 to 9·9)*	84·5 (51·1 to 131·1)	84·7 (51·4 to 131·0)	84·6 (51·7 to 130·7)	−5·2% (−11·5 to −0·8)*	−4·2% (−9·5 to −0·5)*	−4·7% (−9·7 to −1·4)*
Otitis media	1090 (668 to 1730)	992 (608 to 1560)	2080 (1280 to 3290)	1·7% (−2·9 to 5·5)	2·3% (−2·2 to 5·9)	2·0% (−1·6 to 4·8)	29·1 (17·8 to 46·2)	27·3 (16·7 to 43·2)	28·2 (17·3 to 44·7)	−6·9% (−11·1 to −3·5)*	−6·8% (−10·9 to −3·6)*	−6·8% (−10·2 to −4·4)*

**Enteric infections**	**49 100** **(41 400 to 61 500)**	**46 100** **(38 700 to 57 600)**	**95 200** **(83 900 to 112 000)**	**−27·0%** (−35·6 to −16·6)*	**−27·0%** (−33·1 to −18·7)*	**−27·0%** (−32·5 to −20·5)*	**1377·7** **(1165·5 to 1708·6)**	**1319·4** **(1127·4 to 1605·6)**	**1350·2** **(1195·2 to 1579·1)**	**−33·2%** (−40·8 to −23·6)*	**−34·3%** (−39·8 to −27·0)*	**−33·6%** (−38·7 to −27·9)*

Diarrhoeal diseases	41 400 (33 900 to 53 400)	39 600 (32 100 to 50 200)	81 000 (70 100 to 97 200)	−27·7% (−37·5 to −15·6)*	−27·8% (−35·0 to −18·1)*	−27·8% (−33·8 to −20·3)*	1166·8 (958·5 to 1491·2)	1128·4 (932·6 to 1406·7)	1149 (1005·2 to 1359·9)	−34·0% (−42·8 to −22·8)*	−35·5% (−41·8 to −27·1)*	−34·7% (−40·1 to −28·1)*
Typhoid and paratyphoid	5350 (3070 to 8560)	4450 (2530 to 7300)	9800 (5580 to 15 800)	−25·4% (−30·4 to −20·8)*	−21·9% (−27·7 to −17·0)*	−23·8% (−29·3 to −19·4)*	146·6 (84·1 to 235·4)	128·8 (72·5 to 211·4)	137·9 (78·4 to 223·3)	−30·0% (−35·0 to −25·6)*	−27·0% (−32·6 to −22·3)*	−28·7% (−33·9 to −24·4)*
Typhoid fever	4620 (2590 to 7330)	3820 (2140 to 6220)	8440 (4730 to 13 500)	−26·6% (−32·2 to −21·9)*	−23·7% (−30·4 to −18·7)*	−25·3% (−31·0 to −20·8)*	126·6 (71·2 to 202·5)	110·5 (61·9 to 180·5)	118·8 (66·9 to 190·4)	−31·2% (−36·6 to −26·5)*	−28·7% (−35·0 to −23·9)*	−30·1% (−35·6 to −25·8)*
Paratyphoid fever	730 (336 to 1400)	633 (287 to 1220)	1360 (633 to 2630)	−16·3% (−25·7 to −5·7)*	−9·3% (−20·3 to 2·5)	−13·2% (−21·2 to −3·8)*	20·0 (9·1 to 38·3)	18·3 (8·3 to 35·2)	19·1 (8·9 to 36·7)	−21·5% (−30·4 to −11·6)*	−15·1% (−25·6 to −3·8)*	−18·7% (−26·5 to −9·8)*
Invasive non-typhoidal salmonella disease	2240 (1240 to 3850)	2020 (1150 to 3500)	4260 (2380 to 7380)	−16·0% (−25·9 to −3·1)*	−18·5% (−27·7 to −8·1)*	−17·2% (−25·7 to −6·8)*	63·0 (34·9 to 108·8)	60·3 (34·0 to 104·8)	61·7 (34·7 to 107·6)	−21·4% (−31·0 to −9·1)*	−23·8% (−32·6 to −13·9)*	−22·6% (−30·7 to −12·5)*
Other intestinal infectious diseases	45·5 (26·6 to 87·5)	59·9 (33·5 to 120)	105 (68·7 to 172)	−43·0% (−75·3 to 37·9)	−44% (−77·7 to 38·8)	−43·6% (−71·4 to 11·5)	1·3 (0·7 to 2·5)	1·8 (1·0 to 3·7)	1·5 (1·0 to 2·5)	−46·4% (−76·8 to 30·8)	−47·3% (−79·2 to 32·5)	−46·9% (−73·5 to 5·7)

** Neglected tropical diseases and malaria**	** 33 300** **(25 900 to 43 000)**	** 29 000** **(22 300 to 36 900)**	** 62 300** **(48 600 to 79 900)**	**−29·5%** (−37·2 to −21·1)*	**−30·0%** (−38·0 to −21·3)*	**−29·7%** (−37·2 to −21·2)*	** 917·6** **(710·8 to 1192·3)**	** 836·2** **(636·3 to 1073·7)**	** 877·6** **(679·5 to 1129·2)**	** −35·2%** (−42·5 to −27·4)*	** −36·2%** (−43·7 to −28·0)*	** −35·7%** (−42·7 to −27·7)*

Malaria	24 000 (16 800 to 32 800)	21 100 (14 800 to 28 400)	45 000 (31 700 to 61 000)	−33·0% (−42·5 to −22·3)*	−35·4% (−44·9 to −25·4)*	−34·2% (−43·1 to −23·5)*	672·3 (468·6 to 924·1)	626·4 (437·9 to 848·8)	649·8 (457·1 to 878·7)	−37·7% (−46·8 to −27·6)*	−40·2% (−49·2 to −30·7)*	−38·9% (−47·4 to −28·8)*
Chagas disease	138 (125 to 156)	94 (84·1 to 107)	232 (210 to 261)	−3·6% (−8·8 to 4·6)	−1·4% (−5·9 to 4·6)	−2·8% (−6·8 to 4·1)	3·6 (3·2 to 4·0)	2·2 (2·0 to 2·6)	2·9 (2·6 to 3·2)	−24·0% (−28·1 to −17·7)*	−22·7% (−26·2 to −18·0)*	−23·4% (−26·6 to −18·0)*
Leishmaniasis	442 (89·4 to 1700)	332 (109 to 1030)	774 (199 to 2720)	−55·4% (−68·9 to 28·3)	−46·5% (−67·3 to 36·1)	−52% (−68·2 to 32·5)	11·9 (2·3 to 46·7)	9·2 (2·8 to 29·6)	10·6 (2·6 to 38·2)	−59·0% (−71·0 to 12·7)	−52·1% (−69·9 to 20·1)	−56·3% (−70·6 to 16·5)
Visceral leishmaniasis	326 (0·657 to 1570)	184 (0·355 to 878)	511 (1·02 to 2440)	−63·9% (−85·7 to −41·3)*	−63·8% (−81·6 to −36·5)*	−63·9% (−84·5 to −40·0)*	8·9 (0 to 43·3)	5·4 (0 to 25·8)	7·2 (0 to 34·7)	−66·3% (−87·4 to −46·2)*	−66·1% (−83·1 to −41·3)*	−66·2% (−86·0 to −44·3)*
Cutaneous and mucocutaneous leishmaniasis	116 (74·8 to 171)	148 (96·6 to 216)	264 (172 to 389)	30·5% (20·3 to 43·1)*	32·3% (22·0 to 45·5)*	31·5% (21·2 to 44·2)*	3·0 (1·9 to 4·4)	3·8 (2·5 to 5·5)	3·4 (2·2 to 5·0)	14·4% (4·6 to 26·4)*	15·8% (5·8 to 28·1)*	15·3% (5·2 to 27·4)*
African trypanosomiasis	41·5 (7·46 to 160)	37·5 (7·06 to 142)	79·0 (15·4 to 287)	−80·7% (−95·9 to −22·4)*	−80·8% (−95·8 to −30·1)*	−80·7% (−95·6 to −27·3)*	1·1 (0·2 to 4·2)	1·0 (0·2 to 3·8)	1·1 (0·2 to 3·9)	−82·3% (−96·3 to −30·8)*	−82·3% (−96·2 to −33·5)*	−82·3% (−95·9 to −33·4)*
Schistosomiasis	693 (427 to 1180)	738 (447 to 1250)	1430 (876 to 2440)	−19·3% (−21·9 to −15·7)*	−19·8% (−22·5 to −17·1)*	−19·6% (−21·6 to −17·3)*	17·7 (10·9 to 30·0)	18·9 (11·5 to 31·9)	18·3 (11·2 to 30·9)	−29·3% (−31·8 to −26·2)*	−29·5% (−32·1 to −27·1)*	−29·4% (−31·4 to −27·4)*
Cysticercosis	671 (440 to 928)	937 (618 to 1300)	1610 (1050 to 2230)	3·7% (−1·7 to 8·8)	10·5% (5·5 to 15·1)*	7·6% (2·4 to 12·0)*	17·1 (11·2 to 23·5)	23·1 (15·2 to 32·1)	20·1 (13·2 to 27·8)	−13·0% (−17·5 to −8·9)*	−7·3% (−11·7 to −3·5)*	−9·8% (−14·0 to −6·1)*
Cystic echinococcosis	46·8 (32·0 to 65·0)	53·4 (37·2 to 73·4)	100 (72·8 to 139)	−22·7% (−46·2 to 7·7)	−17·7% (−39 to 7·2)	−20·1% (−36·7 to 0·6)	1·2 (0·8 to 1·7)	1·4 (1·0 to 1·9)	1·3 (1·0 to 1·8)	−32·5% (−53·1 to −6·3)*	−29·4% (−48·1 to −7·5)*	−30·8% (−45·2 to −13·2)*
Lymphatic filariasis	1110 (569 to 1830)	258 (151 to 394)	1360 (752 to 2160)	−40·8% (−52·3 to −30·0)*	−13·2% (−16·7 to −9·8)*	−37·0% (−48·7 to −26·4)*	28·1 (14·4 to 46·4)	6·6 (3·9 to 10·0)	17·4 (9·6 to 27·5)	−48·1% (−58·2 to −38·6)*	−24·2% (−27·5 to −21·2)*	−44·8% (−54·7 to −35·5)*
Onchocerciasis	719 (346 to 1270)	624 (299 to 1130)	1340 (639 to 2370)	3·2% (−15·4 to 19·2)	4·7% (−16·0 to 21·7)	3·9% (−15·1 to 19·9)	18·5 (8·9 to 32·7)	16·2 (7·6 to 29·3)	17·3 (8·2 to 30·5)	−8·6% (−26·0 to 6·0)	−7·3% (−26·6 to 8·3)	−8·0% (−25·8 to 6·7)
Trachoma	122 (80·3 to 174)	181 (120 to 253)	303 (202 to 425)	−0·7% (−11·7 to 10·5)	−8·5% (−16·5 to −0·8)*	−5·5% (−13·1 to 2·0)	3·4 (2·2 to 4·8)	4·2 (2·8 to 5·9)	3·8 (2·6 to 5·4)	−25·6% (−33·6 to −17·3)*	−30·1% (−35·9 to −24·1)*	−28·2% (−33·8 to −22·5)*
Dengue	1530 (754 to 2110)	1390 (651 to 1930)	2920 (1630 to 3970)	39·9% (4·2 to 73·1)*	42·1% (4 to 79·5)*	40·9% (13·9 to 67·9)*	41·1 (20·2 to 56·2)	38 (17·6 to 52·8)	39·6 (22·0 to 53·3)	26·1% (−6·1 to 56·2)	26·3% (−8·0 to 60·3)	26·2% (2·2 to 49·5)*
Yellow fever	222 (45·9 to 632)	92·3 (18·7 to 267)	314 (67·2 to 900)	−16·9% (−33·2 to 4·4)	−13·5% (−31·2 to 8·6)	−16·0% (−28·9 to 0)*	6·0 (1·2 to 17·0)	2·6 (0·5 to 7·6)	4·3 (0·9 to 12·4)	−22·3% (−37·9 to −1·8)*	−19·1% (−35·6 to 2·3)	−21·3% (−33·6 to −5·9)*
Rabies	441 (356 to 598)	193 (98·0 to 287)	634 (504 to 836)	−49·9% (−60·1 to −33·9)*	−54·8% (−70·4 to −34·7)*	−51·5% (−61·3 to −38·9)*	11·7 (9·4 to 16·1)	5·3 (2·7 to 8·1)	8·6 (6·8 to 11·5)	−54·4% (−63·8 to −39·4)*	−59·5% (−73·7 to −40·8)*	−56·2% (−65·1 to −44·3)*
Intestinal nematode infections	907 (569 to 1410)	1010 (632 to 1560)	1920 (1200 to 2980)	−32·4% (−38·3 to −27·2)*	−32·2% (−37·8 to −26·9)*	−32·3% (−37·1 to −27·7)*	24·2 (15·3 to 37·5)	27·8 (17·5 to 42·6)	25·9 (16·4 to 40·1)	−38·3% (−43·7 to −33·3)*	−38·2% (−43·6 to −33·2)*	−38·3% (−42·7 to −33·9)*
Ascariasis	427 (284 to 641)	434 (284 to 661)	861 (569 to 1290)	−37·8% (−46·7 to −28·4)*	−37·2% (−46·2 to −27·1)*	−37·5% (−45·0 to −29·7)*	11·6 (7·7 to 17·3)	12·3 (8·1 to 18·6)	12·0 (8·0 to 17·8)	−42·7% (−51·0 to −33·7)*	−42·4% (−50·9 to −32·6)*	−42·6% (−49·5 to −35·3)*
Trichuriasis	107 (59·8 to 178)	106 (59·6 to 177)	213 (120 to 354)	−23·1% (−29·4 to −16·0)*	−23·0% (−29·3 to −15·7)*	−23·1% (−29·3 to −15·8)*	2·8 (1·6 to 4·6)	2·8 (1·6 to 4·7)	2·8 (1·6 to 4·7)	−29·3% (−35·1 to −22·7)*	−29·2% (−35·0 to −22·5)*	−29·3% (−35·0 to −22·5)*
Hookworm disease	373 (218 to 603)	472 (289 to 740)	845 (510 to 1340)	−27·8% (−33·3 to −22·0)*	−28·9% (−34·6 to −23·2)*	−28·5% (−34·0 to −22·7)*	9·8 (5·7 to 15·8)	12·6 (7·7 to 19·8)	11·2 (6·7 to 17·8)	−34·7% (−39·7 to −29·3)*	−35·5% (−40·6 to −30·3)*	−35·2% (−40·2 to −30·0)*
Food-borne trematodiases	1110 (627 to 1890)	763 (432 to 1280)	1870 (1070 to 3150)	4·3% (0·9 to 8·0)*	15·0% (9·8 to 20·2)*	8·5% (4·8 to 12·0)*	27·7 (15·7 to 47·1)	19 (10·7 to 31·9)	23·4 (13·4 to 39·3)	−9·4% (−12·4 to −6·5)*	−1·0% (−5·3 to 3·2)	−6·2% (−9·1 to −3·5)*
Leprosy	22·1 (15·0 to 31·0)	9·46 (6·35 to 13·3)	31·5 (21·5 to 44·6)	−2·2% (−5·2 to 0·8)	1·0% (−2·7 to 4·8)	−1·3% (−3·7 to 1·1)	0·6 (0·4 to 0·8)	0·2 (0·2 to 0·3)	0·4 (0·3 to 0·6)	−21·2% (−23·6 to −18·9)*	−18·8% (−21·9 to −15·9)*	−20·4% (−22·4 to −18·5)*
Ebola virus disease	0·319 (0·238 to 0·361)	0·184 (0·161 to 0·210)	0·503 (0·459 to 0·552)	−97·5% (−97·8 to −97·2)*	−98·6% (−98·8 to −98·4)*	−98·1% (−98·3 to −97·9)*	0 (0 to 0)	0 (0 to 0)	0 (0 to 0)	−97·7% (−98·0 to −97·4)*	−98·7% (−98·9 to −98·5)*	−98·2% (−98·4 to −98·0)*
Zika virus disease	1·13 (0·657 to 2·09)	1·12 (0·529 to 2·71)	2·24 (1·27 to 4·66)	··	··	··	0 (0 to 0·1)	0 (0 to 0·1)	0 (0 to 0·1)	··	··	··
Guinea worm disease	0·000249 (0·000133 to 0·000398)	0·000301 (0·000161 to 0·000482)	0·000550 (0·000294 to 0·000883)	−99·5% (−99·7 to −99·3)*	−99·5% (−99·6 to −99·2)*	−99·5% (−99·6 to −99·3)*	0 (0 to 0)	0 (0 to 0)	0 (0 to 0)	−99·6% (−99·7 to −99·4)*	−99·5% (−99·7 to −99·3)*	−99·5% (−99·7 to −99·3)*
Other neglected tropical diseases	1130 (743 to 2330)	1200 (794 to 2070)	2340 (1560 to 4190)	−2·6% (−13·5 to 9·7)	−2·6% (−8·6 to 4·8)	−2·6% (−9·5 to 5·6)	31·6 (20·7 to 65·7)	34·0 (22·5 to 60·8)	32·8 (21·8 to 60·1)	−9·7% (−20·1 to 1·9)	−10·5% (−16·5 to −2·9)*	−10·1% (−16·7 to −2·3)*

**Other infectious diseases**	**29 500** **(25 200 to 34 700)**	**27 500** **(23 200 to 32 600)**	**57 100** **(48 800 to 67 300)**	**−30·8%** (−37·5 to −23·1)*	**−32·1%** (−38·7 to −24·3)*	**−31·4%** (−37·8 to −23·8)*	**824·0** **(703·3 to 971·5)**	**810·9** **(677·3 to 966·3)**	**816·8** **(693·8 to 970·0)**	**−35·9%** (−42·2 to −28·7)*	**−37·3%** (−43·5 to −29·9)*	**−36·6%** (−42·6 to −29·5)*

Meningitis	10 900 (9460 to 12 700)	9430 (7810 to 11 100)	20 400 (17 800 to 23 400)	−23·3% (−31·0 to −12·2)*	−25·6% (−32·7 to −15·3)*	−24·4% (−30·6 to −15·3)*	306·8 (264·8 to 356·4)	278·6 (232·0 to 327·4)	293 (254·3 to 337·3)	−28·4% (−35·6 to −17·9)*	−30·8% (−37·7 to −21·0)*	−29·6% (−35·5 to −21·1)*
Pneumococcal meningitis	1770 (1480 to 2130)	1310 (1080 to 1560)	3080 (2640 to 3600)	−18·5% (−27·9 to −4·6)*	−20·3% (−27·8 to −10·3)*	−19·2% (−26·5 to −8·9)*	49·4 (41·1 to 59·6)	38·0 (31·3 to 45·3)	43·9 (37·5 to 51·4)	−24·2% (−33·1 to −11·3)*	−26·3% (−33·5 to −16·7)*	−25·1% (−32·1 to −15·1)*
H *influenzae* type B meningitis	2530 (2110 to 3140)	2460 (2070 to 3010)	4990 (4310 to 5890)	−39·5% (−46·2 to −31·0)*	−41·6% (−48 to −33·1)*	−40·6% (−46·1 to −33·3)*	70·9 (59·0 to 88·1)	72·6 (60·7 to 88·6)	71·6 (61·6 to 85)	−43·8% (−50·0 to −35·6)*	−45·9% (−52·0 to −37·8)*	−44·9% (−50·1 to −38·0)*
Meningococcal infection	1280 (1050 to 1550)	1000 (819 to 1200)	2280 (1920 to 2720)	−33·5% (−41·4 to −22·0)*	−34·7% (−41·7 to −24·7)*	−34·0% (−40·3 to −25·6)*	36·2 (29·6 to 44·2)	30·1 (24·3 to 36·1)	33·2 (27·8 to 39·7)	−37·5% (−45·0 to −26·4)*	−38·9% (−45·5 to −29·4)*	−38·1% (−44·1 to −30·0)*
Other meningitis	5360 (4570 to 6270)	4660 (3810 to 5480)	10 000 (8600 to 11 500)	−10·5% (−19·7 to 3·3)	−11·9% (−20·8 to 0·8)	−11·1% (−18·5 to 0·6)	150·4 (128·2 to 175·7)	137·9 (112·6 to 162·9)	144·2 (123·6 to 166·2)	−16·3% (−25·1 to −3·1)*	−17·8% (−26·3 to −5·6)*	−17·0% (−24·2 to −5·9)*
Encephalitis	2620 (2310 to 3030)	2490 (2220 to 2810)	5110 (4540 to 5760)	−12·4% (−27·6 to 4·5)	−8·4% (−24·1 to 8·3)	−10·5% (−25·5 to 4·7)	71·6 (63·0 to 82·3)	70·2 (62·0 to 79·2)	70·8 (62·8 to 79·3)	−20·4% (−34·4 to −5·1)*	−17·5% (−32·3 to −2·3)*	−18·9% (−32·9 to −5·2)*
Diphtheria	155 (80·2 to 331)	143 (74·1 to 291)	299 (182 to 510)	−21·9% (−63·8 to 75·5)	−26·1% (−65·7 to 71·5)	−23·9% (−56·7 to 38·7)	4·4 (2·3 to 9·5)	4·4 (2·3 to 8·9)	4·4 (2·7 to 7·6)	−26·3% (−66·2 to 66·6)	−30·4% (−67·9 to 62·6)	−28·3% (−59·5 to 31·4)
Whooping cough	3540 (1790 to 6340)	4440 (2250 to 7740)	7980 (4020 to 14 100)	−24·1% (−54·8 to 33·4)	−22·5% (−54·3 to 35·1)	−23·2% (−54·5 to 35·0)	102·5 (51·9 to 183·6)	137·4 (69·6 to 239·7)	119·3 (60·1 to 210·7)	−27·7% (−56·9 to 27·1)	−26·4% (−56·6 to 28·3)	−27·0% (−56·8 to 28·4)
Tetanus	1400 (955 to 1930)	1050 (753 to 1410)	2450 (1740 to 3200)	−58·1% (−70·2 to −37·9)*	−60·9% (−72·2 to −42·0)*	−59·3% (−69·9 to −43·5)*	38·9 (26·6 to 54·3)	31·3 (22·5 to 42·2)	35·2 (25 to 46·4)	−61·0% (−72·2 to −42)*	−63·4% (−74·1 to −45·6)*	−62·1% (−72·1 to −47)*
Measles	4080 (1460 to 8860)	4080 (1480 to 8710)	8160 (2950 to 17 600)	−56·5% (−61·9 to −50·6)*	−57·2% (−62·6 to −51·6)*	−56·8% (−61·8 to −51·7)*	117·7 (42·1 to 255·6)	125·6 (45·7 to 268·5)	121·5 (44·0 to 261·7)	−58·8% (−63·9 to −53·2)*	−59·5% (−64·7 to −54·2)*	−59·1% (−63·8 to −54·3)*
Varicella and herpes zoster	559 (476 to 656)	586 (495 to 690)	1140 (985 to 1320)	−15·8% (−25·9 to −4·3)*	−12·3% (−23·2 to −1·6)*	−14·0% (−22·4 to −5·6)*	15·5 (13·3 to 18·1)	16·5 (14·0 to 19·3)	16·0 (13·9 to 18·4)	−24·1% (−33·0 to −13·7)*	−21·6% (−31·6 to −11·5)*	−22·8% (−30·2 to −15·5)*
Acute hepatitis	3240 (2150 to 3940)	2750 (1970 to 3390)	5990 (4580 to 6900)	−18·9% (−25·9 to −5·0)*	−21·0% (−29·2 to −12·3)*	−19·8% (−25·6 to −12·7)*	84·2 (55·8 to 102·9)	74·2 (52·5 to 92·2)	78·9 (60·1 to 91·2)	−29·0% (−35·1 to −16)*	−30·1% (−37·5 to −22·4)*	−29·5% (−34·8 to −23·1)*
Acute hepatitis A	697 (465 to 949)	801 (565 to 1050)	1500 (1130 to 1870)	−31·6% (−42·8 to −10·4)*	−32·7% (−44·1 to −19·5)*	−32·2% (−40·9 to −21·1)*	18·8 (12·6 to 25·4)	23·1 (16·3 to 30·4)	20·9 (15·7 to 26·2)	−36·9% (−47·4 to −16·2)*	−37·5% (−48·6 to −24·5)*	−37·2% (−45·5 to −26·3)*
Acute hepatitis B	2050 (1370 to 2460)	1470 (1040 to 1810)	3530 (2620 to 4120)	−11·2% (−20·0 to 1·6)	−10·5% (−21·0 to 1·4)	−10·9% (−18·2 to −2·0)*	52·4 (34·8 to 63·0)	38·0 (26·7 to 46·7)	45·1 (33·3 to 53·0)	−24·7% (−32·1 to −13·9)*	−24·0% (−33·0 to −13·7)*	−24·5% (−30·4 to −17·0)*
Acute hepatitis C	106 (54 to 187)	122 (64·9 to 207)	228 (124 to 383)	−30·5% (−44·8 to −1·8)*	−29·9% (−44·3 to −9·9)*	−30·2% (−42·2 to −14·7)*	3·0 (1·5 to 5·3)	3·7 (1·9 to 6·2)	3·3 (1·8 to 5·6)	−35·1% (−48·3 to −7·0)*	−34·6% (−48·4 to −15·3)*	−34·8% (−46·4 to −20·3)*
Acute hepatitis E	382 (226 to 507)	356 (247 to 459)	739 (517 to 935)	−24·5% (−36·5 to −10·1)*	−24·6% (−38·9 to −9·6)*	−24·6% (−34·1 to −13·6)*	9·9 (5·9 to 13·1)	9·5 (6·6 to 12·3)	9·7 (6·8 to 12·2)	−31·2% (−41·6 to −18·4)*	−30·9% (−43·9 to −17·5)*	−31·1% (−39·5 to −21·2)*
Other unspecified infectious diseases	2990 (2240 to 3420)	2580 (1980 to 3060)	5570 (4300 to 6430)	−6·3% (−13·2 to 4·5)	−10·5% (−15·7 to −5·8)*	−8·3% (−13·2 to −2·7)*	82·5 (61·4 to 94·3)	72·7 (55·3 to 85·8)	77·6 (59·3 to 89·5)	−14·1% (−20·6 to −4·3)*	−18·8% (−23·7 to −14·4)*	−16·4% (−21 to −11·2)*

** Maternal and neonatal disorders**	** 103 000** **(97 100 to 109 000)**	** 94 500** **(89 300 to 100 000)**	** 198 000** **(187 000 to 209 000)**	**−20·1%** (−23·3 to −16·5)*	**−19·0%** (−22·1 to −15·9)*	**−19·6%** (−22·5 to −16·5)*	** 2979·1** **(2808·2 to 3151·8)**	** 2839·4** **(2691·4 to 3000·0)**	** 2916·4** **(2761·0 to 3077·1)**	** −22·9%** (−26·1 to −19·4)*	** −22·9%** (−25·8 to −19·8)*	** −22·9%** (−25·8 to −19·9)*

Maternal disorders	··	11 800 (11 000 to 12 800)	11 800 (11 000 to 12 800)	··	−24·1% (−28·3 to −19·9)*	−24·1% (−28·3 to −19·9)*	··	305·5 (284·3 to 330·6)	151·2 (140·7 to 163·6)	··	−30·3% (−34·1 to −26·3)*	−30·5% (−34·3 to −26·5)*
Maternal haemorrhage	··	2230 (1910 to 2610)	2230 (1910 to 2610)	··	−52·4% (−59·5 to −44·3)*	−52·4% (−59·5 to −44·3)*	··	57·8 (49·6 to 67·4)	28·6 (24·5 to 33·4)	··	−56·3% (−62·8 to −48·9)*	−56·4% (−62·8 to −49·1)*
Maternal sepsis and other pregnancy-related infections	··	1250 (1080 to 1480)	1250 (1080 to 1480)	··	−27·8% (−39·6 to −15·1)*	−27·8% (−39·6 to −15·1)*	··	32·5 (27·8 to 38·4)	16·1 (13·8 to 19·0)	··	−33·4% (−43·7 to −21·8)*	−33·6% (−43·9 to −21·9)*
Maternal hypertensive disorders	··	1870 (1620 to 2190)	1870 (1620 to 2190)	··	−6·2% (−21·4 to 9·8)	−6·2% (−21·4 to 9·8)	··	48·9 (42·2 to 57·1)	24·2 (20·9 to 28·2)	··	−13·0% (−27·2 to 1·9)	−13·3% (−27·5 to 1·6)
Maternal obstructed labour and uterine rupture	··	1120 (901 to 1380)	1120 (901 to 1380)	··	−16·1% (−29·9 to −2·6)*	−16·1% (−29·9 to −2·6)*	··	28·8 (23·2 to 35·6)	14·3 (11·5 to 17·6)	··	−23·7% (−36·1 to −11·2)*	−23·9% (−36·3 to −11·5)*
Maternal abortive outcome	··	982 (827 to 1180)	982 (827 to 1180)	··	−8·7% (−23·9 to 8·8)	−8·7% (−23·9 to 8·8)	··	25·3 (21·3 to 30·5)	12·5 (10·5 to 15·1)	··	−16·4% (−30·2 to −0·5)*	−16·6% (−30·3 to −0·7)*
Ectopic pregnancy	··	606 (424 to 896)	606 (424 to 896)	··	−12·8% (−42·8 to 26·4)	−12·8% (−42·8 to 26·4)	··	15·7 (11·0 to 23·4)	7·8 (5·4 to 11·6)	··	−19·6% (−46·9 to 17·3)	−19·8% (−47·1 to 16·9)
Indirect maternal deaths	··	1930 (1690 to 2220)	1930 (1690 to 2220)	··	−6·1% (−19·2 to 6·8)	−6·1% (−19·2 to 6·8)	··	50·1 (43·9 to 57·5)	24·8 (21·7 to 28·5)	··	−13·6% (−25·5 to −2·0)*	−13·9% (−25·8 to −2·3)*
Late maternal deaths	··	195 (152 to 251)	195 (152 to 251)	··	−2·0% (−8·2 to 4·1)	−2·0% (−8·2 to 4·1)	··	5·1 (4·0 to 6·5)	2·5 (2·0 to 3·2)	··	−9·8% (−15·1 to −4·3)*	−10·1% (−15·4 to −4·5)*
Maternal deaths aggravated by HIV/AIDS	··	84·4 (53·0 to 114)	84·4 (53·0 to 114)	··	−26·7% (−33·6 to −19·2)*	−26·7% (−33·6 to −19·2)*	··	2·2 (1·4 to 2·9)	1·1 (0·7 to 1·4)	··	−34·1% (−40·4 to −27·3)*	−34·2% (−40·6 to −27·5)*
Other maternal disorders	··	1520 (1280 to 1810)	1520 (1280 to 1810)	··	−8·0% (−24·7 to 10·9)	−8·0% (−24·7 to 10·9)	··	39·2 (33·0 to 46·7)	19·4 (16·4 to 23·1)	··	−15·5% (−30·5 to 1·5)	−15·7% (−30·7 to 1·3)
Neonatal disorders	103 000 (97 100 to 109 000)	82 700 (77 800 to 88 200)	186 000 (175 000 to 197 000)	−20·1% (−23·3 to −16·5)*	−18·2% (−21·7 to −14·6)*	−19·3% (−22·4 to −15·9)*	2979·1 (2808·2 to 3151·8)	2533·9 (2389·9 to 2691·1)	2765·2 (2611·8 to 2922·7)	−22·9% (−26·1 to −19·4)*	−21·9% (−25·2 to −18·4)*	−22·5% (−25·5 to −19·2)*
Neonatal preterm birth	38 400 (35 200 to 42 800)	31 800 (28 500 to 36 000)	70 200 (64 400 to 77 200)	−20·9% (−26·6 to −15·1)*	−18·7% (−24·7 to −12·4)*	−19·9% (−25·3 to −14·4)*	1109·6 (1019·0 to 1236·1)	966·4 (871·4 to 1090·2)	1041·2 (958·9 to 1138·5)	−23·8% (−29·2 to −18·3)*	−22·7% (−28·4 to −17·0)*	−23·3% (−28·4 to −18·3)*
Neonatal encephalopathy due to birth asphyxia and trauma	32 200 (28 300 to 36 200)	24 300 (21 200 to 27 700)	56 500 (50 200 to 63 100)	−19·7% (−27·1 to −11·6)*	−18·4% (−25·6 to −11·3)*	−19·1% (−25·0 to −12·4)*	928·1 (817·0 to 1038·6)	742·9 (644·7 to 836·9)	838·9 (746·3 to 930·9)	−22·7% (−29·8 to −14·9)*	−22·2% (−29·0 to −15·4)*	−22·5% (−28·0 to −16·4)*
Neonatal sepsis and other neonatal infections	11 900 (10 100 to 14 600)	10 100 (7820 to 13 700)	22 000 (18 900 to 28 000)	−10·5% (−20·2 to 0·8)	−3·2% (−14·6 to 7·0)	−7·3% (−15·3 to 1·5)	343·3 (291·8 to 416·1)	306·1 (241·7 to 420·6)	325·4 (281·2 to 417·5)	−14·1% (−23·4 to −3·1)*	−7·8% (−18·9 to 1·8)	−11·3% (−19·0 to −2·7)*
Haemolytic disease and other neonatal jaundice	2890 (2480 to 3300)	2300 (1970 to 2660)	5190 (4610 to 5790)	−31·9% (−41·3 to −21·1)*	−32·1% (−42·3 to −18·4)*	−32·0% (−39·7 to −22·9)*	83·3 (71·5 to 95·2)	70·4 (60·1 to 81·8)	77·1 (68·4 to 86·0)	−34·5% (−43·6 to −24·2)*	−35·3% (−45·1 to −21·8)*	−34·9% (−42·3 to −26·0)*
Other neonatal disorders	17 600 (13 900 to 19 800)	14 300 (12 200 to 16 000)	31 800 (27 100 to 34 800)	−22·3% (−30·3 to −12·3)*	−23·0% (−31·3 to −13·2)*	−22·6% (−28·7 to −14·7)*	514·8 (407·8 to 579·1)	448·2 (384·7 to 503·4)	482·6 (410·4 to 527·5)	−24·4% (−32·2 to −14·7)*	−25·3% (−33·3 to −15·8)*	−24·8% (−30·6 to −17·1)*

** Nutritional deficiencies**	** 23 600** **(17 900 to 30 700)**	** 34 400** **(26 000 to 45 600)**	** 58 000** **(44 300 to 76 900)**	**−21·1%** (−27·5 to −14·0)*	**−14·0%** (−17·6 to −10·5)*	**−17·0%** (−21·4 to −12·6)*	** 655·9** **(499·1 to 849·1)**	** 964·9** **(731·6 to 1268·5)**	** 808·7** **(619·2 to 1065·8)**	** −27·3%** (−33·2 to −20·8)*	** −22·0%** (−25·4 to −18·7)*	** −24·3%** (−28·3 to −20·3)*

Protein-energy malnutrition	7770 (6790 to 9010)	8430 (7490 to 9310)	16 200 (14 500 to 18 000)	−36·5% (−43·9 to −22·8)*	−28·6% (−34·8 to −22·5)*	−32·7% (−38·3 to −24·7)*	222·6 (194·7 to 258·2)	254·9 (225·6 to 281·7)	238·0 (212·7 to 264·8)	−40·7% (−47·6 to −27·8)*	−33·5% (−39·3 to −27·7)*	−37·2% (−42·5 to −29·7)*
Iodine deficiency	886 (542 to 1370)	1170 (705 to 1870)	2060 (1250 to 3260)	−6·9% (−10·6 to −4·0)*	−7·0% (−10·1 to −4·0)*	−6·9% (−10·1 to −4·3)*	22·8 (14·0 to 35·4)	30·8 (18·5 to 48·9)	26·7 (16·2 to 42·3)	−17·6% (−20·8 to −14·9)*	−17·2% (−20·2 to −14·3)*	−17·4% (−20·1 to −14·9)*
Dietary iron deficiency	10 700 (7040 to 15 800)	19 300 (13 000 to 27 800)	30 000 (20 300 to 43 600)	−5·6% (−11·3 to 0·3)	−5·0% (−8·0 to −2·1)*	−5·2% (−8·3 to −1·9)*	291·9 (191·3 to 428·4)	514·3 (345·7 to 740·8)	403·0 (272·4 to 586·6)	−13·7% (−18·8 to −8·1)*	−14·6% (−17·3 to −12·0)*	−14·3% (−17·2 to −11·4)*
Other nutritional deficiencies	561 (479 to 693)	885 (751 to 1020)	1450 (1250 to 1640)	−32·3% (−40·1 to −14·3)*	−21·7% (−30·7 to −12·7)*	−26·2% (−33·4 to −17·7)*	15·3 (13·1 to 18·9)	23·8 (20·0 to 27·7)	19·5 (16·8 to 22·3)	−40·7% (−47·5 to −25·0)*	−32·7% (−40·8 to −24·8)*	−35·9% (−42·3 to −28·5)*

**Non-communicable diseases**	** 811 000** **(732 000 to 902 000)**	** 740 000** **(649 000 to 844 000)**	**1 550 000** **(1 380 000 to 1 750 000)**	** 15·7%** (14·5 to 16·9)*	** 16·4%** (15·3 to 17·4)*	** 16·0%** (15·1 to 16·9)*	** 21 329·9** **(19 298·6 to 23 679·8)**	** 18 204·5** **(15 923·1 to 20 796·3)**	** 19 676·5** **(17 509·8 to 22 177·9)**	** −6·2%** (−7·4 to −5·2)*	** −5·0%** (−6·1 to −4·1)*	** −5·6%** (−6·6 to −4·8)*
** Neoplasms**	** 131 000** **(128 000 to 135 000)**	** 102 000** **(99 300 to 105 000)**	** 234 000** **(229 000 to 238 000)**	** 20·2%** (17·8 to 22·6)*	** 20·2%** (17·2 to 23·1)*	** 20·2%** (18·3 to 22·0)*	** 3417·7** **(3337·7 to 3497·3)**	** 2454·6** **(2386·7 to 2517·4)**	** 2900·9** **(2842·8 to 2957)**	** −5·7%** (−7·6 to −3·9)*	** −4·5%** (−7·0 to −2·3)*	** −5·1%** (−6·6 to −3·7)*

Lip and oral cavity cancer	3490 (3240 to 3680)	1770 (1660 to 1880)	5250 (4980 to 5500)	27·3% (18·3 to 34·2)*	38·4% (30·6 to 47·1)*	30·8% (24·1 to 36·6)*	87·6 (81·5 to 92·4)	42·2 (39·6 to 45·0)	64·2 (60·9 to 67·2)	0·4% (−6·6 to 5·8)	9·5% (3·3 to 16·5)*	3·2% (−2·0 to 7·7)
Nasopharynx cancer	1520 (1440 to 1610)	563 (539 to 589)	2090 (2000 to 2170)	19·0% (13·2 to 25·4)*	16·9% (11·2 to 23·7)*	18·4% (14·0 to 23·1)*	38·0 (36·0 to 40·1)	13·6 (13·0 to 14·2)	25·4 (24·4 to 26·5)	−4·7% (−9·2 to 0·4)	−5·5% (−10·1 to −0·2)*	−4·9% (−8·5 to −1·2)*
Other pharynx cancer	2340 (1940 to 2540)	927 (859 to 990)	3260 (2820 to 3460)	34·5% (20·6 to 47·1)*	40·2% (30·1 to 49·4)*	36·1% (25·6 to 44·3)*	58·2 (48·3 to 63·4)	22·1 (20·4 to 23·6)	39·6 (34·3 to 42·0)	5·4% (−5·6 to 15·1)	10·5% (2·4 to 17·8)*	6·6% (−1·6 to 12·9)
Oesophageal cancer	7190 (6960 to 7420)	2590 (2480 to 2690)	9780 (9530 to 10 000)	11·2% (7·2 to 15·2)*	3·4% (−1·7 to 8·3)	9·0% (5·9 to 12·2)*	184·3 (178·3 to 190·1)	61·0 (58·5 to 63·4)	119·9 (116·9 to 123·0)	−14·5% (−17·5 to −11·4)*	−20·3% (−24·2 to −16·6)*	−16·2% (−18·5 to −13·7)*
Stomach cancer	12 200 (11 900 to 12 600)	6880 (6680 to 7080)	19 100 (18 700 to 19 600)	5·6% (2·4 to 9·2)*	4·5% (0·8 to 8·0)*	5·2% (2·8 to 7·8)*	317·8 (308·7 to 327·9)	163·0 (158·3 to 167·8)	235·9 (231·1 to 241·3)	−18·5% (−20·9 to −15·8)*	−18·1% (−21·1 to −15·4)*	−18·4% (−20·2 to −16·3)*
Colon and rectum cancer	10 700 (10 200 to 11 000)	8330 (8060 to 8580)	19 000 (18 500 to 19 500)	26·9% (20·9 to 31·5)*	21·6% (15·9 to 26·0)*	24·5% (20·0 to 28·3)*	280·3 (269 to 290·5)	196·3 (189·9 to 202·2)	235·7 (229·7 to 242·0)	−2·6% (−7·1 to 0·8)	−6·0% (−10·4 to −2·6)*	−4·1% (−7·5 to −1·2)*
Liver cancer	15 200 (14 300 to 16 300)	5530 (5320 to 5750)	20 800 (19 900 to 21 800)	22·9% (16·8 to 31·8)*	17·6% (12·0 to 25·0)*	21·4% (17·3 to 27·5)*	382·9 (360·9 to 408·8)	130·9 (125·9 to 136·2)	253·6 (243·2 to 266·2)	−3·0% (−7·7 to 4·0)	−8·2% (−12·5 to −2·5)*	−4·5% (−7·8 to 0·2)
Liver cancer due to hepatitis B	7580 (7030 to 8270)	1970 (1840 to 2110)	9550 (8920 to 10 300)	16·4% (10·2 to 26·6)*	9·2% (2·6 to 17·3)*	14·9% (9·9 to 22·1)*	187·3 (173·8 to 204·4)	47·0 (43·8 to 50·4)	115·8 (108·3 to 124·4)	−6·7% (−11·6 to 1·3)	−13·1% (−18·4 to −6·7)*	−8·2% (−12·1 to −2·5)*
Liver cancer due to hepatitis C	3160 (2880 to 3450)	1800 (1690 to 1910)	4960 (4600 to 5330)	29·5% (23·6 to 36·8)*	23·0% (18·0 to 29·5)*	27·1% (23·4 to 31·7)*	81·5 (74·7 to 88·7)	42·1 (39·6 to 44·7)	61·1 (56·9 to 65·6)	−1·0% (−5·4 to 4·6)	−6·1% (−9·9 to −1·1)*	−2·9% (−5·7 to 0·6)
Liver cancer due to alcohol use	2490 (2160 to 2910)	589 (514 to 688)	3080 (2680 to 3590)	29·0% (22·0 to 36·7)*	23·6% (18·4 to 30·0)*	27·9% (22·5 to 34·0)*	63·2 (55·2 to 73·3)	13·9 (12·1 to 16·2)	37·6 (32·9 to 43·8)	0·4% (−4·6 to 6·0)	−4·2% (−8·2 to 0·7)	−0·5% (−4·4 to 4·0)
Liver cancer due to NASH	956 (842 to 1070)	505 (451 to 560)	1460 (1300 to 1620)	40·7% (33·5 to 49·8)*	31·6% (25·4 to 39·2)*	37·4% (32·8 to 42·9)*	24·6 (21·8 to 27·6)	11·9 (10·6 to 13·2)	18·0 (16·1 to 20·0)	9·1% (3·7 to 16·1)*	1·6% (−3·1 to 7·3)	6·4% (3·0 to 10·6)*
Liver cancer due to other causes	1050 (930 to 1200)	670 (607 to 743)	1720 (1540 to 1920)	24·9% (17·6 to 34)*	16·0% (9·6 to 24·5)*	21·3% (16·2 to 27·7)*	26·3 (23·3 to 29·7)	16·0 (14·5 to 17·7)	21·1 (19·0 to 23·5)	0·1% (−5·3 to 7·2)	−8·1% (−13 to −1·5)*	−3·3% (−7·0 to 1·5)
Gallbladder and biliary tract cancer	1460 (1220 to 1620)	2020 (1710 to 2160)	3480 (3040 to 3710)	21·9% (15·8 to 27·9)*	22·0% (16·7 to 27·2)*	22·0% (18·0 to 26·4)*	38·7 (32·2 to 42·9)	47·5 (40·2 to 50·7)	43·2 (37·7 to 46·1)	−6·9% (−11·4 to −2·5)*	−6·4% (−10·5 to −2·4)*	−6·7% (−9·8 to −3·4)*
Pancreatic cancer	4990 (4830 to 5140)	4090 (4000 to 4190)	9080 (8890 to 9260)	33·6% (28·1 to 38·1)*	38·6% (35·7 to 41·4)*	35·8% (32·5 to 38·6)*	129·4 (125·4 to 133·2)	96·1 (94·0 to 98·4)	112·2 (110·0 to 114·4)	2·6% (−1·5 to 6·0)	6·0% (3·8 to 8·1)*	4·0% (1·5 to 6·1)*
Larynx cancer	2730 (2650 to 2820)	547 (523 to 570)	3280 (3190 to 3380)	16·9% (13·2 to 20·8)*	22·5% (17·1 to 28·0)*	17·8% (14·4 to 21·4)*	69·0 (66·9 to 71·1)	13·0 (12·4 to 13·5)	39·9 (38·8 to 41·0)	−9·6% (−12·3 to −6·6)*	−4·0% (−8·2 to 0·3)	−8·8% (−11·3 to −6)*
Tracheal, bronchus, and lung cancer	28 400 (27 500 to 29 200)	12 600 (12 200 to 13 000)	40 900 (40 000 to 41 900)	22·3% (18·0 to 26·0)*	31·7% (27·5 to 35·9)*	25·0% (21·9 to 27·8)*	734·1 (712 to 754·8)	295·7 (286·6 to 305·2)	503·1 (491·9 to 514·3)	−6·4% (−9·6 to −3·5)*	1·4% (−1·8 to 4·6)	−4·0% (−6·4 to −1·9)*
Malignant skin melanoma	905 (603 to 1130)	749 (584 to 966)	1650 (1330 to 1940)	17·3% (9·6 to 22·9)*	17·3% (11·1 to 25·5)*	17·3% (14·0 to 21·0)*	23·3 (15·4 to 28·9)	17·9 (14·0 to 23·1)	20·5 (16·5 to 24·0)	−6·2% (−12·0 to −1·6)*	−6·2% (−11·0 to 0·8)	−6·3% (−8·9 to −3)*
Non-melanoma skin cancer	929 (883 to 976)	400 (380 to 426)	1330 (1270 to 1400)	28·4% (25·3 to 30·9)*	34·4% (27·7 to 40·1)*	30·1% (26·5 to 32·8)*	24·9 (23·6 to 26·2)	9·4 (9·0 to 10·0)	16·6 (15·9 to 17·5)	−1·5% (−3·8 to 0·4)	3·7% (−1·4 to 8·1)	0·3% (−2·3 to 2·4)
Non-melanoma skin cancer (squamous-cell carcinoma)	928 (883 to 975)	399 (379 to 424)	1330 (1270 to 1390)	28·4% (25·3 to 30·9)*	34·5% (27·7 to 40·2)*	30·1% (26·5 to 32·8)*	24·8 (23·6 to 26·1)	9·4 (8·9 to 10·0)	16·6 (15·9 to 17·5)	−1·5% (−3·8 to 0·4)	3·7% (−1·4 to 8·1)	0·3% (−2·3 to 2·4)
Non-melanoma skin cancer (basal-cell carcinoma)	1·35 (0·512 to 2·86)	1·12 (0·433 to 2·33)	2·47 (0·945 to 5·20)	32·6% (24·7 to 39·4)*	28·6% (20·0 to 35·8)*	30·8% (23·1 to 37·0)*	0 (0 to 0·1)	0 (0 to 0·1)	0 (0 to 0·1)	−1·5% (−7·3 to 3·4)	−1·5% (−7·6 to 3·5)	−1·0% (−6·4 to 3·2)
Breast cancer	285 (273 to 299)	17 400 (16 600 to 18 400)	17 700 (16 900 to 18 700)	39·0% (31·5 to 46·1)*	24·4% (17·8 to 29·1)*	24·6% (18·1 to 29·3)*	7·3 (7·0 to 7·6)	414·7 (395·5 to 437·6)	216·3 (206·4 to 228·1)	7·9% (2·3 to 13·2)*	−1·4% (−6·5 to 2·4)	−1·2% (−6·3 to 2·5)
Cervical cancer	··	8060 (7530 to 8400)	8060 (7530 to 8400)	··	15·2% (9·5 to 19·2)*	15·2% (9·5 to 19·2)*	··	193·0 (180·2 to 201·2)	98·2 (91·7 to 102·4)	··	−7·1% (−11·8 to −3·9)*	−7·0% (−11·6 to −3·8)*
Uterine cancer	··	2140 (2060 to 2230)	2140 (2060 to 2230)	··	16·6% (13·3 to 20·8)*	16·6% (13·3 to 20·8)*	··	50·5 (48·5 to 52·5)	26·3 (25·2 to 27·3)	··	−9·8% (−12·4 to −6·7)*	−9·8% (−12·4 to −6·7)*
Ovarian cancer	··	4670 (4530 to 4830)	4670 (4530 to 4830)	··	29% (24·7 to 33·1)*	29·0% (24·7 to 33·1)*	··	110·9 (107·5 to 114·6)	57·1 (55·4 to 59·0)	··	1·3% (−2·0 to 4·5)	1·2% (−2·1 to 4·3)
Prostate cancer	7060 (6050 to 8350)	··	7060 (6050 to 8350)	29·7% (26·3 to 36·3)*	··	29·7% (26·3 to 36·3)*	201·3 (171·7 to 237·1)	··	90·0 (77·2 to 106·6)	−4·4% (−6·8 to 0·6)	··	−2·6% (−5·1 to 2·4)
Testicular cancer	375 (356 to 398)	··	375 (356 to 398)	2·7% (−1·4 to 8·0)	··	2·7% (−1·4 to 8·0)	9·5 (9·0 to 10·1)	··	4·7 (4·5 to 5·0)	−9·4% (−13·0 to −4·7)*	··	−9·2% (−12·9 to −4·5)*
Kidney cancer	2170 (2030 to 2240)	1120 (1030 to 1160)	3280 (3090 to 3390)	25·3% (20·3 to 29·6)*	19·2% (11·8 to 25·7)*	23·2% (18·5 to 27·3)*	56·5 (53·0 to 58·5)	27·2 (25·0 to 28·3)	41·1 (38·7 to 42·5)	−1·9% (−5·7 to 1·4)	−5·7% (−11·5 to −0·3)*	−3·2% (−6·9 to 0·1)
Bladder cancer	2710 (2600 to 2870)	889 (854 to 924)	3600 (3480 to 3770)	24·2% (21·1 to 27·3)*	20·6% (14·3 to 26·1)*	23·3% (20·6 to 26·0)*	74·2 (71·2 to 78·7)	20·9 (20·1 to 21·7)	45·3 (43·7 to 47·4)	−6·5% (−8·8 to −4·2)*	−7·7% (−12·5 to −3·4)*	−6·4% (−8·4 to −4·3)*
Brain and nervous system cancer	5030 (4200 to 5740)	3720 (2690 to 4210)	8740 (7650 to 9550)	24·2% (16·8 to 33·9)*	12·4% (−3·8 to 31·5)	18·9% (12·3 to 25·1)*	129·4 (108·3 to 147·9)	95·0 (68·6 to 107·9)	111·9 (97·8 to 122·5)	5·0% (−1·2 to 13·3)	−5·3% (−19·1 to 11·1)	0·4% (−5·3 to 5·7)
Thyroid cancer	468 (444 to 493)	665 (618 to 747)	1130 (1070 to 1230)	30·3% (21·6 to 36·6)*	19·6% (12·6 to 27·0)*	23·8% (18·6 to 29·6)*	12·1 (11·5 to 12·7)	16·0 (14·9 to 18·0)	14·1 (13·3 to 15·3)	3·7% (−3·1 to 8·6)	−3·8% (−9·6 to 2·4)	−0·9% (−5·1 to 3·8)
Mesothelioma	478 (462 to 494)	193 (181 to 208)	671 (648 to 693)	23·0% (15·9 to 29·0)*	16·7% (7·0 to 25·5)*	21·1% (13·9 to 27·4)*	12·5 (12·1 to 12·9)	4·6 (4·3 to 5·0)	8·3 (8·0 to 8·6)	−4·9% (−10·2 to −0·4)*	−7·0% (−14·2 to −0·6)*	−5·3% (−10·7 to −0·7)*
Hodgkin lymphoma	871 (705 to 1090)	508 (400 to 606)	1380 (1160 to 1620)	−6·0% (−9·6 to −2·3)*	−1·8% (−7·7 to 4·4)	−4·5% (−8·0 to −1·1)*	22·5 (18·1 to 28·2)	13·1 (10·3 to 15·6)	17·8 (14·9 to 20·9)	−18·3% (−21·5 to −15·1)*	−13·1% (−18·4 to −7·1)*	−16·6% (−19·5 to −13·4)*
Non-Hodgkin lymphoma	4260 (4070 to 4420)	2760 (2670 to 2860)	7020 (6790 to 7230)	23·2% (12·7 to 28·8)*	21·5% (14·5 to 27·8)*	22·5% (16·2 to 27·3)*	111·1 (106·2 to 115·4)	68·5 (66·1 to 71·2)	89·3 (86·1 to 92·2)	1·2% (−7·2 to 5·8)	−0·7% (−6·6 to 4·7)	0·4% (−4·9 to 4·6)
Multiple myeloma	1240 (1090 to 1460)	1080 (1030 to 1240)	2330 (2180 to 2610)	32·4% (23·6 to 38·6)*	29·3% (23·9 to 34·6)*	30·9% (26·1 to 35·0)*	32·4 (28·2 to 37·9)	25·5 (24·3 to 29·2)	28·8 (26·9 to 32·2)	1·7% (−4·8 to 6·3)	−0·5% (−4·5 to 3·6)	0·7% (−2·9 to 3·8)
Leukaemia	6820 (6010 to 7300)	5150 (4420 to 5630)	12 000 (10 700 to 12 800)	4·3% (0·4 to 9·0)*	0·6% (−8·4 to 6·0)	2·7% (−3·3 to 6·6)	181·0 (159·4 to 194·1)	134·2 (115·0 to 147·3)	156·8 (140·8 to 168·1)	−10·4% (−13·9 to −6·5)*	−13·5% (−21·6 to −8·5)*	−11·7% (−17 to −8·3)*
Acute lymphoid leukaemia	1580 (1360 to 1740)	1130 (923 to 1310)	2700 (2380 to 2990)	4·2% (−6·1 to 19·1)	7·8% (−13·8 to 18·0)	5·6% (−8·2 to 15·9)	42·2 (36·2 to 46·7)	31·1 (25·5 to 36·4)	36·7 (32·2 to 40·7)	−5·7% (−15·3 to 7·9)	−2·3% (−22·5 to 7·4)	−4·4% (−17·4 to 5·0)
Chronic lymphoid leukaemia	436 (401 to 473)	273 (245 to 296)	709 (667 to 755)	20·5% (15·0 to 26·7)*	20·4% (15·9 to 24·7)*	20·5% (16·4 to 24·5)*	11·8 (10·9 to 12·8)	6·5 (5·8 to 7·0)	8·9 (8·4 to 9·5)	−8·4% (−12·5 to −3·7)*	−7·1% (−10·6 to −3·7)*	−7·7% (−10·8 to −4·6)*
Acute myeloid leukaemia	1870 (1590 to 2020)	1350 (1180 to 1520)	3220 (2890 to 3440)	20·5% (11·1 to 29·1)*	10·9% (−6·1 to 19·9)	16·3% (4·5 to 24·6)*	49·2 (41·7 to 53·1)	34·5 (30·0 to 39·1)	41·7 (37·3 to 44·5)	1·9% (−5·8 to 9·1)	−5·9% (−20·6 to 2·1)	−1·4% (−11·2 to 5·8)
Chronic myeloid leukaemia	347 (309 to 383)	308 (259 to 358)	655 (595 to 713)	−1·8% (−6·1 to 3·1)	−1·4% (−7·5 to 3·6)	−1·6% (−5·0 to 1·7)	9·0 (8·0 to 9·9)	7·5 (6·3 to 8·7)	8·2 (7·4 to 8·9)	−20·3% (−23·7 to −16·4)*	−19·0% (−23·9 to −14·8)*	−19·6% (−22·4 to −17·0)*
Other leukaemia	2590 (2190 to 2920)	2090 (1700 to 2320)	4690 (4040 to 5120)	−6·0% (−11·4 to 1·5)	−9·7% (−19·9 to −2·4)*	−7·7% (−14·2 to −1·4)*	68·8 (58·1 to 77·3)	54·6 (44·1 to 60·7)	61·4 (53·0 to 67·0)	−18·9% (−23·4 to −12·6)*	−22·3% (−31·4 to −15·8)*	−20·5% (−26·1 to −15·1)*
Other malignant cancers	6300 (5620 to 6650)	5570 (5090 to 5960)	11 900 (11 000 to 12 400)	21·1% (14·3 to 27·3)*	23·1% (17·0 to 28·8)*	22·0% (15·8 to 27·0)*	165·0 (147·5 to 174·4)	142·4 (129·9 to 152·7)	153·2 (142·0 to 160·8)	2·0% (−3·7 to 7·1)	3·7% (−1·8 to 9·0)	2·7% (−2·5 to 7·0)
Other neoplasms	1290 (963 to 1690)	1190 (926 to 1540)	2480 (2080 to 2980)	35·6% (23·6 to 50·5)*	30·2% (20·3 to 43·3)*	33·0% (26·1 to 42·4)*	34·5 (25·4 to 45·1)	29·4 (23·1 to 38·1)	31·8 (26·6 to 38·2)	9·5% (−0·4 to 21·8)	6·0% (−2·3 to 17·3)	7·9% (2·1 to 16·2)*
Myelodysplastic, myeloproliferative, and other haemopoietic neoplasms	1180 (876 to 1550)	1060 (823 to 1380)	2240 (1880 to 2720)	36·2% (23·8 to 51·8)*	31·3% (22·3 to 44·4)*	33·9% (26·8 to 43·1)*	31·6 (23·2 to 41·4)	26·0 (20·3 to 33·8)	28·6 (23·9 to 34·6)	8·7% (−1·4 to 21·4)	5·4% (−2·4 to 16·6)	7·2% (1·4 to 15·3)*
Benign and in-situ intestinal neoplasms	0 (0 to 0)	0 (0 to 0)	0 (0 to 0)	-	-	-	0 (0 to 0)	0 (0 to 0)	0 (0 to 0)	-	-	-
Benign and in-situ cervical and uterine neoplasms	-	0 (0 to 0)	0 (0 to 0)	-	-	-	-	0 (0 to 0)	0 (0 to 0)	-	-	-
Other benign and in-situ neoplasms	111 (77·4 to 148)	126 (89·7 to 150)	237 (186 to 278)	29·1% (18·4 to 45·4)*	21·5% (5·6 to 39·3)*	25·0% (12·7 to 38·6)*	2·9 (2·0 to 3·9)	3·4 (2·4 to 4·1)	3·2 (2·5 to 3·7)	18·7% (8·9 to 33·9)*	10·6% (−4·1 to 26·8)	14·3% (3·0 to 27·0)*

** Cardiovascular diseases**	** 210 000** **(204 000 to 216 000)**	** 156 000** **(150 000 to 162 000)**	** 366 000** **(355 000 to 377 000)**	** 17·1%** (15·3 to 19·0)*	** 15·4%** (13·5 to 17·5)*	** 16·4%** (15·0 to 17·8)*	** 5588·6** **(5428·4 to 5743·1)**	** 3680·1** **(3541·4 to 3821·0)**	** 4597·9** **(4463·7 to 4734·2)**	** −9·3%** (−10·7 to −7·8)*	** −10·8%** (−12·4 to −9·3)*	** −9·9%** (−11·0 to −8·8)*

Rheumatic heart disease	4110 (3710 to 4630)	5280 (4710 to 5950)	9390 (8580 to 10 300)	−9·2% (−14·6 to −4·0)*	−3·1% (−8·6 to 2·4)	−5·9% (−10·6 to −2·2)*	106·9 (96·7 to 120·0)	130·3 (116·1 to 147·3)	118·7 (108·5 to 130·7)	−23·8% (−28·3 to −19·5)*	−19·6% (−24·3 to −14·9)*	−21·5% (−25·4 to −18·2)*
Ischaemic heart disease	105 000 (103 000 to 108 000)	65 300 (63 300 to 67 200)	170 000 (167 000 to 174 000)	17·8% (15·7 to 20·0)*	17·0% (14·6 to 19·4)*	17·5% (15·7 to 19·2)*	2776 (2715·3 to 2843·4)	1534·3 (1487·3 to 1580·5)	2132·1 (2093·7 to 2179·8)	−9·2% (−10·8 to −7·6)*	−10·4% (−12·3 to −8·6)*	−9·7% (−11·0 to −8·4)*
Stroke	72 200 (69 400 to 75 000)	59 900 (56 700 to 63 000)	132 000 (126 000 to 137 000)	17·4% (14·6 to 20·1)*	13·6% (10·9 to 16·4)*	15·7% (13·5 to 17·7)*	1924·7 (1848·8 to 1998·4)	1412·1 (1336·8 to 1485·8)	1657·2 (1587·4 to 1723·8)	−9·9% (−11·9 to −7·9)*	−12·5% (−14·6 to −10·4)*	−11·0% (−12·6 to −9·4)*
Ischaemic stroke	28 500 (26 400 to 30 600)	26 700 (24 400 to 29 000)	55 100 (50 900 to 59 400)	28·6% (25·0 to 32·2)*	20·8% (17·3 to 24·2)*	24·7% (21·8 to 27·5)*	790·0 (732·7 to 846·5)	625·2 (570·9 to 679·3)	702·8 (649·5 to 756·5)	−3·7% (−6·3 to −1·0)*	−8·1% (−10·8 to −5·6)*	−5·8% (−8·0 to −3·7)*
Intracerebral haemorrhage	37 400 (35 900 to 38 900)	27 200 (25 900 to 28 500)	64 500 (62 300 to 66 600)	10·7% (7·5 to 14·2)*	7·9% (4·4 to 11·6)*	9·5% (6·7 to 12·0)*	971·6 (933·9 to 1011·4)	641·7 (611·1 to 673·3)	800·3 (773·3 to 826·1)	−14·5% (−16·9 to −11·8)*	−16·4% (−19·1 to −13·6)*	−15·2% (−17·4 to −13·3)*
Subarachnoid haemorrhage	6350 (5680 to 7240)	6060 (5610 to 6780)	12 400 (11 600 to 13 700)	13·4% (7·6 to 24·3)*	10·9% (7·1 to 14·7)*	12·2% (8·5 to 17·3)*	163·1 (145·8 to 185·9)	145·2 (134·5 to 162·8)	154·1 (143·7 to 170·1)	−8·8% (−13·7 to −0·5)*	−12·0% (−15·0 to −9·0)*	−10·4% (−13·3 to −6·4)*
Hypertensive heart disease	7880 (5500 to 8620)	8660 (6730 to 9560)	16 500 (12 700 to 17 900)	39·5% (19·4 to 51·3)*	32·2% (19·7 to 45·7)*	35·6% (20·5 to 46·6)*	214·6 (149·8 to 234·1)	203·2 (158·1 to 224·5)	209·4 (160·5 to 226·3)	7·0% (−8·7 to 15·5)	0·9% (−8·4 to 10·8)	3·7% (−7·9 to 11·8)
Non-rheumatic valvular heart disease	1270 (1130 to 1400)	1260 (1100 to 1420)	2530 (2290 to 2770)	23·6% (19·2 to 27·7)*	23·7% (19·3 to 28)*	23·6% (20·6 to 26·5)*	36·0 (31·6 to 39·7)	29·6 (26·0 to 33·5)	32·7 (29·5 to 36·0)	−5·0% (−8·2 to −1·8)*	−5·6% (−8·4 to −2·2)*	−5·5% (−7·5 to −3·2)*
Non-rheumatic calcific aortic valve disease	793 (682 to 873)	720 (595 to 818)	1510 (1340 to 1650)	32·1% (26·1 to 36·8)*	30·5% (23·7 to 36·1)*	31·3% (26·4 to 35·6)*	22·9 (19·5 to 25·1)	16·8 (14·0 to 19·1)	19·8 (17·5 to 21·5)	−0·3% (−4·3 to 3·2)	−2·5% (−6·7 to 1·2)	−1·5% (−4·7 to 1·4)
Non-rheumatic degenerative mitral valve disease	408 (340 to 480)	466 (381 to 577)	874 (748 to 1010)	12·5% (7·0 to 19·9)*	16·4% (10·8 to 22·3)*	14·6% (9·8 to 19·8)*	11·2 (9·4 to 13·2)	11·1 (9·1 to 13·7)	11·2 (9·5 to 12·9)	−12·1% (−16·1 to −6·3)*	−9·1% (−13·0 to −4·7)*	−10·7% (−14·3 to −6·7)*
Other non-rheumatic valve diseases	71·5 (46·3 to 103)	70·6 (48·9 to 102)	142 (109 to 190)	7·2% (−4·1 to 29·7)	9·7% (−1·3 to 32·3)	8·5% (−1·8 to 27·8)	1·9 (1·2 to 2·8)	1·7 (1·2 to 2·5)	1·8 (1·4 to 2·4)	−13·3% (−23·3 to 7·0)	−11·0% (−20·1 to 7·4)	−12·2% (−21·0 to 4·7)
Cardiomyopathy and myocarditis	6530 (5880 to 7020)	3720 (3490 to 4010)	10 200 (9500 to 10 900)	−3·6% (−9·6 to 12·4)	−4·2% (−7·1 to −0·2)*	−3·8% (−8·1 to 6·4)	168·8 (152·5 to 181·2)	92·8 (86·9 to 100·2)	130·3 (121·0 to 138·8)	−19·9% (−24·7 to −6·8)*	−21·5% (−23·8 to −18·4)*	−20·6% (−24·1 to −12·5)*
Myocarditis	745 (625 to 894)	646 (561 to 723)	1390 (1220 to 1560)	6·2% (−2·2 to 17·6)	−4·3% (−10·6 to 5·0)	1·0% (−5·3 to 8·3)	19·9 (16·7 to 23·7)	16·7 (14·5 to 18·7)	18·3 (16·1 to 20·4)	−9·3% (−16·7 to 0·6)	−19·1% (−24·5 to −11·2)*	−14·2% (−19·6 to −7·2)*
Alcoholic cardiomyopathy	2330 (1990 to 2530)	660 (610 to 750)	2990 (2720 to 3210)	−26·9% (−32 to −0·2)*	−37·1% (−40 to −31·7)*	−29·4% (−33·5 to −11·1)*	57·6 (49·2 to 62·7)	15·9 (14·6 to 18·0)	36·4 (33·2 to 39·1)	−39·7% (−43·9 to −18·2)*	−49·0% (−51·3 to −44·6)*	−42·2% (−45·5 to −27·5)*
Other cardiomyopathy	3450 (3050 to 3800)	2420 (2160 to 2640)	5870 (5290 to 6390)	19·7% (12·7 to 25·7)*	11·7% (7·6 to 17·6)*	16·3% (11·6 to 20·3)*	91·4 (81·2 to 100·1)	60·3 (53·8 to 65·9)	75·6 (68·3 to 82·1)	−2·2% (−7·6 to 2·7)	−9·5% (−12·7 to −5·2)*	−5·3% (−8·9 to −2·1)*
Atrial fibrillation and flutter	2850 (2360 to 3460)	3120 (2690 to 3640)	5980 (5040 to 7090)	36·0% (33·1 to 38·5)*	35·8% (34·1 to 37·9)*	35·9% (34·3 to 37·8)*	84·0 (70·0 to 101·4)	72·7 (62·6 to 84·9)	78·0 (66·1 to 92·0)	−0·1% (−2·2 to 1·6)	1·1% (−0·1 to 2·6)	0·5% (−0·5 to 1·7)
Aortic aneurysm	2090 (1950 to 2250)	949 (907 to 1050)	3040 (2880 to 3190)	17·9% (12·9 to 23·6)*	21·6% (16·2 to 25·9)*	19·0% (14·5 to 23·6)*	56·0 (52·3 to 60·2)	22·3 (21·4 to 24·7)	38·2 (36·2 to 40·0)	−9·9% (−13·6 to −5·7)*	−6·6% (−10·6 to −3·2)*	−8·5% (−11·9 to −5·1)*
Peripheral vascular disease	759 (513 to 1190)	674 (395 to 1150)	1430 (970 to 2130)	40·6% (22·7 to 52·9)*	39·8% (23·3 to 55·6)*	40·2% (25·9 to 53·5)*	21·4 (14·4 to 33·9)	15·7 (9·2 to 26·7)	18·4 (12·5 to 27·6)	4·1% (−9·0 to 13·4)	4·1% (−8·1 to 15·4)	4·1% (−6·5 to 13·9)
Endocarditis	1160 (1090 to 1230)	1070 (920 to 1220)	2230 (2080 to 2420)	20·6% (11·0 to 26·2)*	13·6% (5·9 to 20·5)*	17·1% (9·3 to 22·3)*	30·8 (28·9 to 32·7)	27·3 (23·5 to 31·1)	29·0 (27·1 to 31·7)	0·8% (−6·7 to 5·2)	−5·2% (−11·4 to 0·6)	−2·3% (−8·6 to 2·0)
Other cardiovascular and circulatory diseases	6420 (5620 to 7410)	5730 (4950 to 6750)	12 200 (10 600 to 14 000)	15·3% (12·3 to 18·4)*	17·1% (14·2 to 21·3)*	16·2% (13·6 to 18·6)*	169·4 (148·6 to 195·5)	139·8 (120·4 to 163·9)	153·8 (134·7 to 176·5)	−7·5% (−9·9 to −5·2)*	−5·8% (−8·2 to −2·6)*	−6·7% (−8·7 to −5·0)*

** Chronic respiratory diseases**	** 58 600** **(54 900 to 62 300)**	** 53 700** **(49 000 to 57 900)**	** 112 000** **(105 000 to 120 000)**	** 10·7%** (8·5 to 13·3)*	** 18·9%** (14·8 to 23·6)*	** 14·5%** (12·3 to 17·2)*	** 1586·3** **(1489·1 to 1681·7)**	** 1293·2** **(1178·8 to 1399·0)**	** 1422·9** **(1326·6 to 1517·2)**	** −14·2%** (−16·1 to −12·1)*	** −6·0%** (−9·2 to −2·4)*	** −10·2%** (−12·0 to −8·1)*

Chronic obstructive pulmonary disease	43 300 (40 100 to 46 100)	38 300 (34 900 to 41 400)	81 600 (76 000 to 86 800)	13·5% (10·3 to 16·9)*	21·2% (14·9 to 26·5)*	17·0% (13·7 to 20·0)*	1181·3 (1097·1 to 1255·3)	906·4 (824·1 to 979·9)	1028·8 (960·4 to 1092·5)	−14·1% (−16·4 to −11·5)*	−6·3% (−11·1 to −2·3)*	−10·3% (−12·9 to −8·0)*
Pneumoconiosis	453 (421 to 489)	54·1 (45·7 to 63·1)	507 (472 to 548)	10·4% (4·3 to 17·1)*	11·8% (0·1 to 22·4)*	10·6% (5·2 to 16·6)*	12·1 (11·2 to 13·0)	1·3 (1·1 to 1·5)	6·3 (5·9 to 6·8)	−15·6% (−20·1 to −10·7)*	−12·3% (−21·5 to −4·1)*	−14·4% (−18·6 to −9·8)*
Silicosis	247 (220 to 272)	13·3 (8·66 to 17·0)	261 (233 to 286)	13·0% (0·3 to 24·2)*	17·8% (6·0 to 31·2)*	13·2% (1·2 to 24·1)*	6·5 (5·8 to 7·2)	0·3 (0·2 to 0·4)	3·2 (2·9 to 3·5)	−13·6% (−23·1 to −5·1)*	−7·6% (−17·0 to 3·0)	−12·3% (−21·6 to −3·9)*
Asbestosis	57·3 (41·7 to 67·9)	11·7 (8·38 to 15·0)	69·1 (52·2 to 81·4)	20·2% (12·3 to 31·3)*	14·7% (0·8 to 32·0)*	19·2% (11·8 to 30·4)*	1·6 (1·2 to 1·9)	0·3 (0·2 to 0·4)	0·9 (0·7 to 1·0)	−9·1% (−14·9 to −0·7)*	−10·5% (−21·3 to 3·4)	−8·5% (−14·1 to 0·3)
Coal worker pneumoconiosis	74·6 (63 to 92·9)	6·24 (4·00 to 8·12)	80·8 (69·0 to 100)	−0·6% (−10·0 to 12·9)	9·0% (−2·8 to 27·2)	0·1% (−8·8 to 12·9)	2·0 (1·7 to 2·5)	0·1 (0·1 to 0·2)	1·0 (0·9 to 1·3)	−24·3% (−31·2 to −14·5)*	−14·8% (−24·0 to −0·6)*	−23% (−29·8 to −13·3)*
Other pneumoconiosis	74·1 (61·6 to 94·6)	22·8 (17·6 to 28·8)	96·9 (81·8 to 117)	7·4% (−1·2 to 22·6)	8·1% (−3·9 to 19·7)	7·6% (0·6 to 20·5)*	2·0 (1·6 to 2·5)	0·5 (0·4 to 0·7)	1·2 (1·0 to 1·5)	−16·6% (−23·4 to −4·8)*	−15·0% (−24·6 to −5·9)*	−15·9% (−21·4 to −5·5)*
Asthma	11 000 (8470 to 14 100)	11 700 (9050 to 14 800)	22 800 (18 100 to 28 300)	−2·7% (−8·9 to 4·8)	9·7% (4·3 to 17·0)*	3·3% (−1·1 to 9·0)	290·1 (223·0 to 371·5)	297·4 (229·0 to 376·2)	293·3 (232·4 to 365·2)	−19·3% (−25·5 to −10·8)*	−8·2% (−13·6 to −0·9)*	−13·7% (−18·2 to −7·9)*
Interstitial lung disease and pulmonary sarcoidosis	1840 (1490 to 2390)	1530 (1160 to 2000)	3360 (2770 to 4080)	40·6% (25·8 to 49·7)*	39·5% (25·2 to 53·3)*	40·1% (31·5 to 47·8)*	49·8 (40·1 to 64·4)	36·2 (27·5 to 47·5)	42·3 (34·8 to 51·4)	7·9% (−3·0 to 14·7)	8·3% (−2·6 to 19·1)	8·3% (1·8 to 14·3)*
Other chronic respiratory diseases	2070 (1820 to 2360)	2010 (1740 to 2280)	4080 (3600 to 4560)	14·8% (9·5 to 22·0)*	21·3% (14·8 to 29·3)*	17·9% (13·2 to 24·5)*	53·0 (46·6 to 60·4)	51·9 (44·7 to 58·9)	52·1 (46·1 to 58·2)	−1·7% (−6·3 to 4·3)	6·0% (0·1 to 13·5)*	2·0% (−2·2 to 7·8)

** Digestive diseases**	** 52 800** **(48 500 to 58 200)**	** 32 500** **(29 100 to 36 800)**	** 85 300** **(78 000 to 94 500)**	** 9·9%** (6·7 to 16·1)*	** 10·9%** (8·1 to 14·4)*	** 10·3%** (7·5 to 14·3)*	** 1346·7** **(1237·4 to 1486·3)**	** 803·1** **(717·1 to 911·3)**	** 1070·8** **(979·3 to 1187·9)**	** −9·6%** (−12·2 to −4·5)*	** −8·9%** (−11·5 to −6·0)*	** −9·4%** (−11·7 to −6·0)*

Cirrhosis and other chronic liver diseases	28 800 (27 300 to 31 400)	12 600 (11 900 to 14 700)	41 400 (39 600 to 45 100)	9·9% (4·3 to 16·4)*	9·4% (2·9 to 15·5)*	9·8% (4·5 to 15·2)*	719·3 (683·0 to 784·9)	307·6 (288·5 to 359·6)	510·7 (487·6 to 557·1)	−10·2% (−14·8 to −4·9)*	−11·2% (−16·5 to −6·3)*	−10·6% (−14·9 to −6·3)*
Cirrhosis and other chronic liver diseases due to hepatitis B	9190 (8310 to 10 500)	3020 (2700 to 3600)	12 200 (11 100 to 13 900)	4·5% (−2·3 to 11·9)	3·9% (−4·3 to 13·3)	4·3% (−2·2 to 11·4)	229·5 (208·0 to 261·6)	72·9 (65·0 to 86·8)	150·1 (136·2 to 170·3)	−14·3% (−20 to −8·2)*	−15·8% (−22·4 to −8·4)*	−14·7% (−20·0 to −8·9)*
Cirrhosis and other chronic liver diseases due to hepatitis C	7160 (6450 to 8000)	3250 (2950 to 3700)	10 400 (9470 to 11 600)	13·0% (7·5 to 19·1)*	13·1% (6·0 to 20·0)*	13·0% (7·7 to 18·1)*	178·1 (160·8 to 198·8)	77·8 (70·7 to 88·4)	127·2 (115·9 to 141·8)	−8·5% (−12·9 to −3·7)*	−9·6% (−15·2 to −4·4)*	−8·9% (−13·1 to −4·9)*
Cirrhosis and other chronic liver diseases due to alcohol use	7660 (6980 to 8450)	2530 (2290 to 2980)	10 200 (9310 to 11 300)	13·0% (7·8 to 19·0)*	13·3% (7·2 to 22·4)*	13·1% (8·1 to 18·9)*	189·6 (173·1 to 209·1)	60·2 (54·5 to 70·8)	123·9 (113·2 to 138·1)	−8·8% (−13·2 to −4)*	−10·3% (−15·2 to −3·3)*	−9·3% (−13·4 to −4·8)*
Cirrhosis due to NASH	2110 (1920 to 2310)	1330 (1220 to 1470)	3430 (3150 to 3740)	24·3% (18·4 to 30·1)*	21·9% (15·4 to 27·6)*	23·4% (17·8 to 28·3)*	52·2 (47·8 to 57·3)	31·6 (29·0 to 35·0)	41·8 (38·5 to 45·5)	−0·6% (−5·2 to 4·2)	−4·1% (−9·2 to 0·3)	−2·0% (−6·4 to 1·9)
Cirrhosis and other chronic liver diseases due to other causes	2660 (2370 to 3000)	2500 (2220 to 2960)	5160 (4650 to 5760)	3·6% (−3·7 to 13·8)	2·5% (−4·5 to 11·5)	3·1% (−3·0 to 11·2)	70·0 (62·7 to 78·6)	65·2 (57·7 to 77·8)	67·7 (61·1 to 75·4)	−10·5% (−16·8 to −1·6)*	−11·8% (−18·2 to −3·9)*	−11·1% (−16·5 to −3·9)*
Upper digestive system diseases	10 200 (7880 to 13 400)	9640 (7080 to 13 200)	19 800 (15 100 to 26 600)	7·4% (2·3 to 12·3)*	12·7% (9·4 to 15·7)*	9·9% (6·0 to 13·4)*	261·0 (202·6 to 342·2)	237·4 (173·6 to 324·9)	248·7 (189·1 to 333·3)	−11·5% (−16·1 to −7·2)*	−6·6% (−9·7 to −3·8)*	−9·1% (−12·7 to −5·9)*
Peptic ulcer disease	3740 (3440 to 4200)	2610 (2270 to 2910)	6350 (5900 to 6870)	−7·2% (−12·8 to −1·0)*	0·7% (−4·3 to 6·9)	−4·1% (−8·5 to 0·9)	96·9 (89·1 to 108·9)	63·2 (55·0 to 70·7)	79·6 (73·9 to 86·2)	−25·4% (−29·9 to −20·5)*	−19·1% (−23·2 to −14·1)*	−22·9% (−26·5 to −18·9)*
Gastritis and duodenitis	3470 (2500 to 4700)	4000 (2750 to 5550)	7470 (5240 to 10 200)	16·4% (11·8 to 21·0)*	15·7% (12·8 to 18·7)*	16·1% (13·0 to 19·1)*	89·1 (64·1 to 120·1)	99·7 (68·4 to 138·5)	94·3 (66·4 to 129·1)	−2·0% (−6·0 to 1·9)	−2·7% (−5·3 to −0·3)*	−2·3% (−5·1 to 0·4)
Gastro-oesophageal reflux disease	2980 (1590 to 5020)	3040 (1630 to 5150)	6010 (3220 to 10 200)	20·3% (18·6 to 22·2)*	21·0% (19·3 to 22·8)*	20·7% (19·1 to 22·3)*	75·1 (40·1 to 126·9)	74·5 (40·1 to 126·9)	74·8 (40·1 to 126·9)	1·2% (0·1 to 2·3)*	1·4% (0·4 to 2·4)*	1·3% (0·6 to 2·0)*
Appendicitis	910 (813 to 1030)	947 (799 to 1050)	1860 (1680 to 2020)	−7·3% (−16·6 to 3·8)	−6·2% (−14·3 to 2·8)	−6·7% (−14·1 to 1·6)	23·9 (21·4 to 27)	25·0 (21·1 to 27·7)	24·4 (22·0 to 26·5)	−19% (−27·1 to −9·5)*	−17·3% (−24·8 to −9·0)*	−18·1% (−24·7 to −10·6)*
Paralytic ileus and intestinal obstruction	4080 (3230 to 4700)	3210 (2350 to 3650)	7290 (5910 to 8030)	5·9% (−4·9 to 17·8)	7·4% (−3·2 to 18·7)	6·6% (−2·9 to 15·5)	110·7 (88·4 to 127·2)	84·9 (62·3 to 96·2)	97·6 (79·4 to 107·4)	−8·9% (−18·0 to 1·0)	−8·2% (−17·1 to 1·5)	−8·6% (−16·7 to −0·8)*
Inguinal, femoral, and abdominal hernia	2490 (1860 to 3230)	989 (753 to 1250)	3480 (2610 to 4480)	13·3% (10·1 to 16·7)*	15·2% (11·9 to 19·7)*	13·9% (11·0 to 16·8)*	65·5 (49·1 to 84·6)	25·0 (18·9 to 31·8)	44·8 (33·6 to 57·5)	−5·3% (−8·0 to −2·6)*	−4·4% (−7·2 to −1·1)*	−5·0% (−7·3 to −2·7)*
Inflammatory bowel disease	885 (741 to 1050)	964 (751 to 1180)	1850 (1510 to 2230)	16·8% (8·4 to 23·2)*	15·8% (8·3 to 21·0)*	16·3% (8·8 to 21·4)*	23·0 (19·3 to 27·2)	23·5 (18·3 to 28·6)	23·2 (19·1 to 27·8)	−5·0% (−11·5 to 0·1)	−6·2% (−12·2 to −2·1)*	−5·6% (−11·5 to −1·9)*
Vascular intestinal disorders	806 (742 to 866)	791 (688 to 869)	1600 (1460 to 1700)	16·9% (9·5 to 28·2)*	18·6% (12·1 to 24·5)*	17·7% (11·0 to 24·8)*	21·9 (20·2 to 23·6)	18·6 (16·1 to 20·5)	20·4 (18·7 to 21·6)	−10·1% (−15·6 to −2·3)*	−9·4% (−14·4 to −4·9)*	−9·9% (−15·0 to −4·9)*
Gallbladder and biliary diseases	940 (882 to 1040)	1070 (933 to 1140)	2010 (1890 to 2130)	22·5% (15·6 to 31·7)*	15·3% (9·9 to 23·9)*	18·6% (13·6 to 25·3)*	25·7 (24·2 to 28·4)	25·8 (22·4 to 27·7)	25·7 (24·1 to 27·2)	−3·8% (−8·9 to 3·0)	−9·2% (−13·6 to −2·3)*	−6·8% (−10·8 to −1·7)*
Pancreatitis	2210 (1950 to 2470)	1050 (831 to 1210)	3250 (2890 to 3610)	14·7% (7·4 to 21·7)*	15·9% (8·8 to 22·6)*	15·1% (10·6 to 20·5)*	55·6 (49·2 to 62·2)	25·3 (20·0 to 29·3)	40·3 (35·9 to 44·7)	−5·2% (−11 to 0·5)	−6·9% (−12·5 to −1·5)*	−5·9% (−9·5 to −1·6)*
Other digestive diseases	1510 (1300 to 1750)	1210 (1050 to 1380)	2720 (2380 to 3110)	22·3% (11·7 to 34·6)*	18·8% (12·2 to 26·9)*	20·7% (12·9 to 28·4)*	40·2 (34·7 to 46·3)	30·0 (26·0 to 34·4)	35·0 (30·7 to 40·0)	−0·4% (−8·3 to 9·2)	−3·7% (−9·2 to 3·1)	−1·9% (−8·2

** Neurological disorders**	** 46 300** **(37 200 to 57 200)**	** 64 900** **(51 100 to 81 400)**	** 111 000** **(88 500 to 139 000)**	** 20·5%** (18·4 to 23·3)*	** 20·6%** (18·9 to 22·4)*	** 20·5%** (18·8 to 22·6)*	** 1275·1** **(1036·4 to 1554·8)**	** 1606·9** **(1256·7 to 2030·1)**	** 1444·4** **(1154 to 1792·7)**	** −0·1%** **(−1·9 to 2·0)**	** −0·2%** **(−1·5 to 1·2)**	** −0·2%** **(−1·6 to 1·4)**

Alzheimer’s disease and other dementias	11 700 (10 900 to 12 500)	18 900 (17 600 to 20 200)	30 500 (28 500 to 32 600)	41·8% (38·0 to 44·4)*	36·1% (32·8 to 38·2)*	38·3% (36·0 to 40·1)*	376·8 (352·1 to 404·4)	436·2 (407·7 to 466·2)	412·6 (385·7 to 440·3)	0·5% (−2·0 to 2·1)	−0·9% (−3·2 to 0·6)	−0·6% (−2·2 to 0·6)
Parkinson’s disease	3190 (2940 to 3500)	2390 (2200 to 2630)	5580 (5150 to 6080)	36·1% (31·2 to 39·3)*	31·0% (26·5 to 34·0)*	33·9% (29·7 to 36·4)*	94·0 (86·7 to 103·1)	55·9 (51·3 to 61·3)	72·5 (66·9 to 79·0)	0·2% (−3·4 to 2·5)	−0·7% (−4·0 to 1·6)	0·4% (−2·7 to 2·3)
Epilepsy	7910 (6200 to 10 100)	6880 (5220 to 8960)	14 800 (11 400 to 19 000)	4·3% (−3·3 to 15·0)	7·7% (−1 to 18·2)	5·8% (−2·0 to 15·9)	208·1 (162·8 to 266·7)	183·5 (139·3 to 239·7)	195·8 (151·4 to 251·8)	−6·4% (−13·2 to 3·2)	−3·8% (−11·4 to 5·4)	−5·2% (−12·2 to 3·7)
Multiple sclerosis	408 (354 to 459)	677 (579 to 781)	1080 (943 to 1240)	17·9% (9·3 to 21·6)*	17·4% (11·1 to 22·3)*	17·6% (10·2 to 21·9)*	10·1 (8·8 to 11·4)	16·3 (14·0 to 18·9)	13·3 (11·5 to 15·2)	−3·8% (−10·3 to −0·8)*	−4·5% (−9·1 to −0·4)*	−4·2% (−9·8 to −0·6)*
Motor neuron disease	508 (481 to 556)	371 (345 to 407)	879 (841 to 966)	27·9% (21 to 31·9)*	26·1% (20·0 to 31·1)*	27·2% (22·9 to 30·9)*	13·1 (12·4 to 14·3)	9·0 (8·3 to 9·8)	11·0 (10·5 to 12·0)	1·4% (−3·8 to 4·5)	−1·0% (−5·8 to 2·9)	0·3% (−3·1 to 3·3)
Headache disorders	20 500 (13 400 to 29 300)	33 900 (22 200 to 48 100)	54 300 (35 600 to 76 800)	15·5% (14·5 to 16·5)*	15·3% (14·4 to 16·2)*	15·4% (14·6 to 16·2)*	516·5 (338 to 738·7)	857·0 (561·5 to 1217·0)	686·5 (448·7 to 970·5)	1·5% (0·8 to 2·3)*	0·7% (0·1 to 1·3)*	1·0% (0·5 to 1·5)*
Migraine	17 300 (10 900 to 25 500)	29 900 (19 000 to 42 900)	47 200 (30 000 to 68 700)	15·5% (14·4 to 16·7)*	15·2% (14·2 to 16·2)*	15·3% (14·5 to 16·2)*	437·7 (273·7 to 645·5)	756·5 (480·9 to 1088·1)	596·8 (378·2 to 866)	1·7% (0·9 to 2·6)*	0·8% (0·1 to 1·5)*	1·1% (0·6 to 1·7)*
Tension-type headache	3110 (1740 to 5000)	3990 (2270 to 6230)	7100 (4040 to 11 200)	15·3% (14·1 to 16·7)*	15·8% (14·6 to 17·2)*	15·6% (14·5 to 16·9)*	78·8 (44·4 to 126·9)	100·5 (57·2 to 157·6)	89·7 (51·0 to 142·2)	0·5% (0 to 1·0)	0·1% (−0·4 to 0·6)	0·3% (−0·1
Other neurological disorders	2130 (1780 to 2570)	1830 (1480 to 2300)	3970 (3270 to 4850)	19·1% (10·1 to 28·5)*	22·4% (11·4 to 32·8)*	20·6% (12·5 to 29·7)*	56·4 (47·3 to 68)	49·1 (39·2 to 61·9)	52·7 (43·3 to 64·7)	6·3% (−1·9 to 14·6)	8·2% (−1·6 to 17·6)	7·2% (−0·3 to 15·3)

** Mental disorders**	** 56 200** **(41 800 to 72 700)**	** 66 500** **(49 700 to 85 700)**	** 123 000** **(91 600 to 158 000)**	** 13·4%** (12·7 to 14·0)*	** 13·6%** (12·9 to 14·4)*	** 13·5%** (12·9 to 14·1)*	** 1435·0** **(1065·8 to 1856·2)**	** 1681·8** **(1255·2 to 2169·0)**	** 1560·4** **(1165·1 to 2010·5)**	** −0·6%** (−1·1 to −0·2)*	** −1·5%** (−2·1 to −1·0)*	** −1·1%** (−1·5 to −0·7)*

Schizophrenia	6510 (4860 to 8020)	6140 (4610 to 7540)	12 700 (9480 to 15 600)	16·7% (15·4 to 18·2)*	17·7% (16·3 to 19·2)*	17·2% (16·1 to 18·3)*	161·6 (120·5 to 198·9)	151·3 (113·2 to 185·8)	156·4 (117·1 to 192·3)	−0·4% (−1·4 to 0·6)	−0·2% (−1·3 to 0·9)	−0·3% (−1·1 to 0·4)
Depressive disorders	16 800 (11 900 to 22 900)	26 300 (18 700 to 35 900)	43 100 (30 500 to 58 900)	14·8% (13·5 to 16·2)*	14·1% (12·8 to 15·5)*	14·3% (13·1 to 15·6)*	425·1 (300·8 to 579·9)	653·5 (462·2 to 892·4)	540·5 (382·4 to 737·8)	−1·9% (−2·8 to −1·0)*	−3·1% (−4·1 to −2·1)*	−2·6% (−3·5 to −1·8)*
Major depressive disorder	12 700 (8910 to 17 300)	20 100 (14 200 to 27 000)	32 800 (23 100 to 44 300)	13·2% (11·8 to 14·6)*	12·2% (10·7 to 13·7)*	12·6% (11·3 to 14·0)*	323·6 (225·9 to 439·5)	500·9 (352·5 to 676·7)	413·0 (289·8 to 558·9)	−2·7% (−3·8 to −1·8)*	−4·2% (−5·3 to −3·1)*	−3·6% (−4·6 to −2·7)*
Dysthymia	4030 (2690 to 5880)	6220 (4180 to 9090)	10 300 (6880 to 15 000)	20·2% (17·4 to 23·2)*	20·6% (17·7 to 23·5)*	20·4% (18·2 to 22·6)*	101·5 (67·9 to 148·0)	152·7 (102·4 to 222·6)	127·4 (85·4 to 185·6)	1·0% (−1·0 to 3·0)	0·6% (−1·4 to 2·8)	0·8% (−0·8 to 2·3)
Bipolar disorder	4490 (2820 to 6630)	4810 (3030 to 7100)	9290 (5870 to 13 700)	15·3% (14·0 to 16·8)*	15·2% (13·8 to 16·7)*	15·2% (14·0 to 16·6)*	113·4 (71·3 to 167·4)	121·6 (76·8 to 179·5)	117·5 (74·1 to 173·5)	1·5% (0·7 to 2·3)*	0·9% (0·1 to 1·8)*	1·2% (0·6 to 1·8)*
Anxiety disorders	10 200 (7200 to 13 500)	17 000 (12 000 to 22 500)	27 100 (19 200 to 36 100)	13·6% (12·2 to 15·1)*	12·4% (11·0 to 13·8)*	12·8% (11·7 to 14·0)*	259·2 (183·1 to 343·3)	431·9 (306·8 to 575·5)	345·7 (245·6 to 459·7)	0% (−1·1 to 1·1)	−1·9% (−2·9 to −0·7)*	−1·2% (−2·0 to −0·4)*
Eating disorders	1050 (662 to 1530)	2320 (1500 to 3370)	3370 (2170 to 4890)	20·7% (18·4 to 22·9)*	18·2% (16·4 to 19·8)*	18·9% (17·3 to 20·5)*	26·7 (16·8 to 39·1)	60·5 (39·2 to 87·6)	43·4 (27·9 to 62·9)	10·7% (8·8 to 12·4)*	8·9% (7·6 to 10·3)*	9·4% (8·2 to 10·5)*
Anorexia nervosa	143 (87·8 to 215)	585 (365 to 864)	728 (454 to 1080)	13·8% (9·4 to 18·2)*	13·4% (10·8 to 16·0)*	13·5% (11·1 to 15·7)*	3·7 (2·3 to 5·6)	15·5 (9·6 to 22·8)	9·5 (5·9 to 14·1)	6·7% (3·0 to 10·6)*	6·2% (3·9 to 8·5)*	6·1% (4·2 to 8·1)*
Bulimia nervosa	904 (559 to 1340)	1740 (1100 to 2620)	2640 (1660 to 3940)	21·9% (19·3 to 24·3)*	19·8% (17·8 to 21·9)*	20·5% (18·7 to 22·1)*	22·9 (14·2 to 33·9)	45·0 (28·6 to 67·8)	33·8 (21·3 to 50·5)	11·4% (9·3 to 13·2)*	9·9% (8·4 to 11·4)*	10·3% (9·0 to 11·6)*
Autism spectrum disorders	3590 (2460 to 4940)	1140 (771 to 1570)	4730 (3240 to 6520)	11·1% (10·4 to 11·9)*	12·4% (11·3 to 13·6)*	11·4% (10·8 to 12·1)*	94 (64·4 to 129·2)	30·4 (20·5 to 41·9)	62·4 (42·7 to 86·0)	−0·6% (−1·2 to 0·1)	0·5% (−0·5 to 1·6)	−0·3% (−0·8 to 0·3)
Attention-deficit/hyperactivity disorder	627 (383 to 996)	262 (158 to 416)	889 (543 to 1410)	6·6% (4·4 to 8·6)*	7·7% (5·3 to 10·0)*	6·9% (5·2 to 8·5)*	16·5 (10·0 to 26·2)	7·1 (4·3 to 11·3)	11·9 (7·3 to 18·9)	−0·8% (−2·9 to 1·1)	0% (−2·2 to 2·2)	−0·6% (−2·1 to 0·8)
Conduct disorder	4190 (2520 to 6630)	2250 (1330 to 3650)	6450 (3870 to 10 300)	4·1% (1·9 to 6·1)*	6·3% (4·0 to 8·7)*	4·8% (3·1 to 6·5)*	113·8 (68·5 to 179·9)	64·9 (38·2 to 105·5)	90·0 (54·0 to 143·9)	1·7% (−0·4 to 3·6)	3·7% (1·4 to 5·8)*	2·4% (0·9 to 3·8)*
Idiopathic developmental intellectual disability	2200 (1060 to 3740)	1840 (901 to 3080)	4050 (1940 to 6860)	0·6% (−2·0 to 2·0)	−0·6% (−3·9 to 1·3)	0% (−2·8 to 1·5)	58·0 (28·0 to 98·6)	50·4 (24·7 to 84·1)	54·2 (26·1 to 91·9)	−8·4% (−10·6 to −7·1)*	−9·1% (−12·0 to −7·5)*	−8·7% (−11·1 to −7·5)*
Other mental disorders	6640 (4330 to 9180)	4470 (3010 to 6140)	11 100 (7360 to 15 300)	17·2% (16·5 to 18·0)*	18·2% (17·3 to 19·1)*	17·6% (17·0 to 18·3)*	166·8 (108·9 to 230·4)	110·3 (74·2 to 151·5)	138·4 (91·6 to 191)	−0·1% (−0·7 to 0·5)	0·1% (−0·6 to 0·8)	0% (−0·4 to 0·5)

** Substance use disorders**	** 31 900** **(25 900 to 38 400)**	** 12 800** **(9930 to 15 700)**	** 44 700** **(35 900 to 54 100)**	** 15·9%** (13·5 to 18·1)*	** 20·8%** (18·9 to 23·3)*	** 17·3%** (15·2 to 19·3)*	** 797·5** **(645·9 to 960·9)**	** 320·9** **(248·9 to 395·7)**	** 559·2** **(448·2 to 677·9)**	** 0·9%** **(−1·3 to 2·9)**	** 5·8%** (4·2 to 8·0)*	** 2·3%** (0·5 to 4·0)*

Alcohol use disorders	13 300 (10 900 to 16 200)	4170 (3140 to 5420)	17 500 (14 100 to 21 600)	3·8% (0·8 to 6·1)*	9·4% (6·0 to 15·3)*	5·0% (2·4 to 7·4)*	329·7 (269·2 to 401·9)	104·0 (78·1 to 134·8)	216·4 (174·1 to 268·5)	−11·4% (−14·1 to −9·3)*	−5·9% (−9·1 to −0·8)*	−10·2% (−12·7 to −8·1)*
Drug use disorders	18 600 (14 500 to 22 900)	8590 (6660 to 10 700)	27 200 (21 100 to 33 600)	26·6% (23·5 to 29·9)*	27·3% (24·3 to 30·7)*	26·8% (24·2 to 29·6)*	467·7 (364·7 to 576·6)	217·0 (168·1 to 270·9)	342·8 (266·4 to 424·1)	11·9% (9·3 to 14·7)*	12·4% (10·1 to 15·2)*	12·1% (9·9 to 14·5)*
Opioid use disorders	14 800 (11 300 to 18 700)	6640 (4960 to 8460)	21 500 (16 300 to 27 100)	31·2% (27·6 to 34·9)*	29·8% (25·3 to 35·0)*	30·8% (27·5 to 34·4)*	372·9 (282·9 to 468·1)	167·4 (124·8 to 214·1)	270·5 (204·4 to 342·2)	15·6% (12·3 to 18·9)*	14·4% (10·7 to 18·5)*	15·3% (12·4 to 18·3)*
Cocaine use disorders	698 (531 to 900)	293 (215 to 391)	992 (747 to 1290)	17·2% (13·2 to 21·2)*	23·4% (14·9 to 35·6)*	19·0% (15·5 to 24·2)*	17·6 (13·3 to 22·7)	7·4 (5·4 to 9·9)	12·5 (9·4 to 16·3)	3·3% (−0·2 to 6·7)	8·7% (1·4 to 19·3)*	4·9% (1·9 to 9·3)*
Amphetamine use disorders	785 (501 to 1150)	399 (253 to 593)	1180 (757 to 1740)	4·0% (−0·4 to 8·4)	8·5% (2·7 to 16·7)*	5·5% (1·5 to 9·4)*	20·1 (12·7 to 29·5)	10·4 (6·5 to 15·5)	15·3 (9·7 to 22·6)	−4·1% (−8·2 to −0·5)*	0·6% (−4·4 to 7·4)	−2·5% (−6·2 to 0·4)
Cannabis use disorders	343 (217 to 510)	174 (110 to 257)	518 (329 to 766)	5·4% (2·7 to 8·3)*	2·6% (−0·1 to 5·4)	4·4% (2·2 to 6·6)*	8·8 (5·6 to 13·1)	4·5 (2·9 to 6·7)	6·7 (4·2 to 9·9)	−2·8% (−5·4 to −0·2)*	−5·6% (−8·0 to −3·3)*	−3·7% (−5·7 to −1·7)*
Other drug use disorders	1920 (1630 to 2260)	1090 (908 to 1300)	3010 (2550 to 3540)	12·9% (8·1 to 17·1)*	26·5% (14·5 to 41·6)*	17·5% (10·9 to 24·1)*	48·5 (41·0 to 57·2)	27·2 (22·6 to 32·6)	37·9 (32·1 to 44·7)	−0·1% (−4·5 to 3·3)	10·4% (0·1 to 22·9)*	3·5% (−2·2 to 8·9)
**Diabetes and kidney diseases**	** 54 000** **(46 900 to 62 700)**	** 50 000** **(43 100 to 58 000)**	** 104 000** **(90 100 to 121 000)**	** 27·1%** (24·6 to 29·7)*	** 26·6%** (24·1 to 29·2)*	** 26·9%** (24·7 to 29·2)*	** 1399·8** **(1218·1 to 1617·7)**	** 1197·7** **(1033·3 to 1390·8)**	** 1294·2** **(1122·0 to 1496·7)**	** 1·0%** **(−0·9 to 3·0)**	** 0·3%** **(−1·7 to 2·4)**	** 0·8%** **(−1·0 to 2·6)**
Diabetes mellitus	35 000 (28 500 to 42 700)	32 900 (26 800 to 39 900)	67 900 (55 400 to 82 600)	30·4% (27·1 to 34·1)*	29·5% (26·4 to 32·9)*	30·0% (26·9 to 33·3)*	898·7 (733·5 to 1095·5)	782·9 (638·0 to 951·0)	839 (685·1 to 1020·0)	2·9% (0·2 to 5·7)*	1·9% (−0·7 to 4·6)	2·5% (0 to 5·1)*
Type 1 diabetes mellitus	5270 (4760 to 5820)	5180 (4640 to 5700)	10 400 (9790 to 11 100)	13·7% (9·5 to 18·3)*	9·3% (4·0 to 14)*	11·5% (7·9 to 14·5)*	133·6 (120·6 to 147·8)	125·3 (112·6 to 138·1)	129·4 (121·3 to 137·6)	−7·7% (−11·4 to −4·0)*	−11·9% (−16·2 to −7·9)*	−9·8% (−12·8 to −7·2)*
Type 2 diabetes mellitus	29 700 (23 200 to 37 200)	27 700 (21 900 to 34 500)	57 400 (45 000 to 71 900)	33·9% (30·0 to 38·2)*	34·2% (30·3 to 38·4)*	34·0% (30·3 to 38·1)*	765·2 (599·9 to 955·7)	657·5 (518·1 to 820·8)	709·6 (557·2 to 888·3)	5·0% (1·9 to 8·2)*	5·0% (2·1 to 8·3)*	5·1% (2·3 to 8·1)*
Chronic kidney disease	18 900 (17 700 to 20 000)	16 900 (15 800 to 18 100)	35 800 (33 700 to 38 000)	21·9% (18·6 to 24·7)*	21·5% (18·7 to 24·1)*	21·7% (19·2 to 23·9)*	496·3 (465·5 to 524·8)	411·5 (384·8 to 439·6)	451·3 (424·9 to 478·3)	−2·0% (−4·6 to 0·2)	−2·4% (−4·6 to −0·3)*	−2·1% (−4·0 to −0·4)*
Chronic kidney disease due to type 1 diabetes mellitus	1590 (1280 to 1950)	1300 (1060 to 1560)	2890 (2370 to 3500)	18·5% (13·4 to 23·2)*	17·7% (13·3 to 22·2)*	18·2% (14·3 to 22·3)*	39·1 (31·6 to 48·0)	31·3 (25·7 to 37·7)	35·2 (28·9 to 42·6)	−1·9% (−5·5 to 1·4)	−3·4% (−6·3 to −0·4)*	−2·6% (−5·1 to −0·3)*
Chronic kidney disease due to type 2 diabetes mellitus	4280 (3700 to 4900)	3840 (3380 to 4340)	8120 (7120 to 9250)	34·2% (29·0 to 38·1)*	34·4% (31·2 to 37·6)*	34·3% (30·9 to 37·2)*	112·7 (97·7 to 128·8)	90·2 (79·2 to 102·0)	100·9 (88·4 to 114·8)	2·0% (−1·9 to 4·6)	2·4% (0·1 to 4·8)*	2·3% (−0·2 to 4·2)
Chronic kidney disease due to hypertension	4050 (3520 to 4550)	3300 (2900 to 3710)	7350 (6450 to 8220)	32·4% (27·5 to 36·2)*	32·3% (29·1 to 35·2)*	32·3% (29·0 to 35·0)*	110·2 (96·2 to 123·3)	78·0 (68·6 to 87·7)	92·7 (81·7 to 103·7)	1·4% (−2·1 to 4·0)	2·3% (−0·1 to 4·5)	2·1% (−0·3 to 4·1)
Chronic kidney disease due to glomerulonephritis	3570 (3160 to 4020)	3040 (2680 to 3420)	6600 (5860 to 7420)	14·7% (11·3 to 18·0)*	12·9% (9·4 to 16·4)*	13·8% (11·0 to 17·0)*	92·3 (81·9 to 103·7)	75·9 (67·1 to 84·9)	83·7 (74·4 to 93·7)	−3·6% (−5·9 to −1·4)*	−5·4% (−7·9 to −3)*	−4·4% (−6·3 to −2·4)*
Chronic kidney disease due to other and unspecified causes	5390 (4780 to 6060)	5470 (4800 to 6270)	10 900 (9660 to 12 200)	12·6% (9·2 to 16·1)*	14·0% (10·7 to 17·3)*	13·3% (10·4 to 16·2)*	142·1 (126·4 to 159·3)	136·1 (119·7 to 155·0)	138·8 (123·7 to 155·9)	−6·3% (−8·5 to −3·8)*	−5·9% (−8·4 to −3·4)*	−6·0% (−8·0 to −4·1)*
Acute glomerulonephritis	180 (165 to 201)	130 (117 to 145)	311 (285 to 339)	−3·9% (−10·5 to 7·0)	−7·6% (−13·0 to 0·5)	−5·5% (−10·3 to 2·2)	4·7 (4·3 to 5·3)	3·3 (2·9 to 3·7)	4·0 (3·6 to 4·3)	−19·2% (−24·8 to −10·5)*	−23·0% (−27·5 to −16·3)*	−20·9% (−25·0 to −15·0)*
**Skin and subcutaneous diseases**	** 20 900** **(14 200 to 30 700)**	** 23 200** **(15 800 to 33 500)**	** 44 100** **(29 900 to 64 200)**	** 13·7%** (12·9 to 15·1)*	** 13·7%** (13·0 to 14·9)*	** 13·7%** (13·0 to 14·7)*	** 553·3** **(375·4 to 812·0)**	** 618·0** **(418·6 to 893·5)**	** 585** **(396·2 to 851·8)**	** 0·9%** (0·2 to 2·1)*	** 0·7%** (0·1 to 1·5)*	** 0·8%** (0·2 to 1·6)*
Dermatitis	5100 (2980 to 8140)	6020 (3510 to 9590)	11 100 (6480 to 17 700)	12·3% (11·3 to 13·3)*	11·9% (11·1 to 12·8)*	12·1% (11·3 to 12·8)*	136·5 (79·3 to 217·8)	164·7 (94·8 to 263·5)	150·3 (87·0 to 240·3)	1·4% (0·5 to 2·4)*	0·9% (0 to 1·7)*	1·1% (0·4 to 1·8)*
Atopic dermatitis	4100 (2220 to 6820)	4900 (2670 to 8160)	9000 (4890 to 15 000)	11·9% (10·8 to 13·0)*	11·4% (10·4 to 12·4)*	11·6% (10·8 to 12·5)*	110·7 (59·8 to 184·4)	136·4 (74·0 to 227·2)	123·3 (66·8 to 205·2)	2·0% (0·9 to 3·2)*	1·4% (0·5 to 2·4)*	1·7% (0·9 to 2·6)*
Contact dermatitis	937 (618 to 1390)	1050 (692 to 1560)	1990 (1300 to 2950)	14·4% (12·3 to 16·4)*	14·4% (12·5 to 16·3)*	14·4% (12·6 to 16·2)*	24·0 (15·8 to 35·6)	26·6 (17·4 to 39·4)	25·3 (16·6 to 37·4)	−0·8% (−1·7 to 0·1)	−1·3% (−2·2 to −0·5)*	−1·1% (−1·7 to −0·5)*
Seborrhoeic dermatitis	69·0 (39·8 to 110)	66·6 (38·3 to 105)	136 (78·1 to 215)	8·9% (6·7 to 11·2)*	7·8% (5·6 to 10·1)*	8·4% (6·6 to 10·3)*	1·8 (1·0 to 2·8)	1·7 (1·0 to 2·7)	1·7 (1·0 to 2·7)	−6·7% (−8·3 to −5·1)*	−7·5% (−9·0 to −5·9)*	−7·1% (−8·3 to −5·8)*
Psoriasis	2710 (1920 to 3590)	2860 (2030 to 3770)	5570 (3960 to 7350)	21·2% (20·2 to 22·4)*	20·9% (19·8 to 22·0)*	21·1% (20·3 to 21·9)*	68·7 (48·7 to 90·9)	71·4 (50·7 to 94·2)	70·0 (49·7 to 92·5)	2·5% (1·7 to 3·4)*	2·1% (1·2 to 3·0)*	2·3% (1·6 to 2·9)*
Bacterial skin diseases	1160 (700 to 1590)	1110 (636 to 1540)	2270 (1560 to 2900)	24·2% (13·4 to 39·3)*	26·9% (17·9 to 42·0)*	25·5 (17·8 to 34·9)*	31·1 (18·7 to 42·9)	29·1 (16·6 to 40·7)	30·0 (20·6 to 38·2)	4·6% (−4·5 to 18·1)	7·2% (−1·0 to 20·4)	6·0% (−0·5 to 14·5)
Cellulitis	321 (178 to 465)	278 (155 to 393)	598 (377 to 760)	34·4% (22·9 to 48·1)*	30·1% (15·8 to 46·6)*	32·4% (26·4 to 41·1)*	8·4 (4·7 to 12·3)	7·0 (3·9 to 9·8)	7·7 (4·9 to 9·8)	12·4% (2·7 to 24·4)*	7·5% (−3·7 to 21·6)	10·0% (5·1 to 17·4)*
Pyoderma	843 (489 to 1170)	833 (441 to 1190)	1680 (1110 to 2200)	20·6% (9·4 to 37·5)*	25·9% (16·3 to 41·7)*	23·2% (14·5 to 34·3)*	22·7 (13·1 to 31·5)	22·1 (11·7 to 31·9)	22·3 (14·8 to 29·4)	2·0% (−7·5 to 17·2)	7·1% (−1·8 to 21·3)	4·7% (−2·7 to 14·7)
Scabies	2260 (1250 to 3700)	2270 (1260 to 3700)	4530 (2510 to 7410)	6·4% (5·0 to 7·9)*	6·7% (5·3 to 8·2)*	6·6% (5·3 to 8·0)*	59·4 (32·9 to 96·8)	60·6 (33·5 to 98·8)	60·0 (33·2 to 97·8)	−3·2% (−3·8 to −2·5)*	−3·1% (−3·7 to −2·5)*	−3·1% (−3·6 to −2·6)*
Fungal skin diseases	2100 (832 to 4370)	2050 (819 to 4260)	4150 (1650 to 8630)	9·8% (8·1 to 11·5)*	12·1% (10·4 to 13·7)*	10·9% (9·2 to 12·5)*	56·5 (22·4 to 117·4)	53·2 (21·2 to 110·5)	54·9 (21·8 to 114·2)	−4·6% (−5·6 to −3·6)*	−4·1% (−5·1 to −3·1)*	−4·4% (−5·4 to −3·4)*
Viral skin diseases	1940 (1250 to 2880)	2090 (1350 to 3110)	4030 (2600 to 6000)	7·1% (6·4 to 7·7)*	5·9% (5·3 to 6·4)*	6·4% (6·0 to 6·9)*	52·9 (33·9 to 78·5)	59·0 (38·0 to 87·7)	56·0 (36·0 to 83·2)	−1·0% (−1·5 to −0·4)*	−2·5% (−3·0 to −2·0)*	−1·8% (−2·2 to −1·4)*
Acne vulgaris	1150 (677 to 1830)	1400 (841 to 2230)	2550 (1520 to 4060)	17·3% (15·8 to 18·9)*	15·3% (14·1 to 16·6)*	16·2% (15·2 to 17·2)*	30·0 (17·7 to 47·9)	37·9 (22·7 to 60·5)	33·9 (20·2 to 54·1)	12·8% (11·3 to 14·6)*	10·2% (9·0 to 11·6)*	11·4% (10·3 to 12·5)*
Alopecia areata	241 (154 to 358)	282 (181 to 418)	523 (335 to 775)	12·8% (11·3 to 14·2)*	12·7% (11·4 to 14·0)*	12·7% (11·8 to 13·8)*	6·1 (3·9 to 9·1)	7·1 (4·6 to 10·6)	6·6 (4·2 to 9·8)	−0·6% (−1·9 to 0·7)	−2·2% (−3·3 to −1·2)*	−1·5% (−2·3 to −0·6)*
Pruritus	331 (156 to 632)	424 (200 to 801)	756 (356 to 1430)	19·3% (17·7 to 20·9)*	18·6% (17·1 to 20·2)*	18·9% (17·5 to 20·4)*	8·6 (4·0 to 16·1)	10·7 (5·0 to 20·2)	9·7 (4·5 to 18·2)	2·2% (1·6 to 2·8)*	1·2% (0·6 to 1·7)*	1·6% (1·2 to 2·0)*
Urticaria	2190 (1450 to 3090)	2830 (1880 to 3970)	5010 (3320 to 7050)	10·8% (9·6 to 12·1)*	10·8% (9·6 to 12·0)*	10·8% (9·8 to 11·9)*	58·4 (38·6 to 82·5)	77·2 (51·1 to 109·0)	67·7 (44·8 to 95·4)	0·6% (−0·2 to 1·5)	0·1% (−0·5 to 0·8)	0·3% (−0·2 to 0·8)
Decubitus ulcer	238 (169 to 357)	265 (184 to 371)	503 (379 to 661)	26·2% (18·8 to 41·0)*	28·1% (21·7 to 35·5)*	27·2% (21·5 to 37·7)*	6·7 (4·7 to 10·1)	6·3 (4·4 to 8·9)	6·5 (4·9 to 8·6)	−2·3% (−8·2 to 9·4)	−0·8% (−5·9 to 5·6)	−1·6% (−6·0 to 7·1)
Other skin and subcutaneous diseases	1470 (736 to 2680)	1640 (819 to 2970)	3110 (1560 to 5650)	25·8% (25·0 to 26·5)*	25·0% (24·2 to 26·0)*	25·4% (24·8 to 26·0)*	38·4 (19·3 to 69·9)	40·7 (20·4 to 73·9)	39·5 (19·8 to 71·9)	4·5% (3·9 to 5·0)*	3·9% (3·3 to 4·7)*	4·2% (3·8 to 4·7)*

** Sense organ diseases**	** 31 700** **(21 300 to 45 700)**	** 34 800** **(23 500 to 49 900)**	** 66 600** **(44 700 to 95 700)**	** 23·9%** (23·1 to 24·9)*	** 24·2%** (23·4 to 25·0)*	** 24·1%** (23·4 to 24·8)*	** 843·1** **(568·7 to 1205·8)**	** 841·0** **(567·7 to 1206·4)**	** 842·2** **(566·6 to 1206·5)**	** −0·9%** (−1·7 to −0·3)*	** −0·9%** (−1·6 to −0·2)*	** −0·9%** (−1·5 to −0·3)*

Blindness and vision impairment	13 500 (9060 to 20 000)	16 400 (11 100 to 24 100)	29 900 (20 300 to 44 000)	23·6% (22·4 to 24·8)*	22·6% (21·6 to 23·5)*	23·1% (22·0 to 24)*	356·2 (240·0 to 523·0)	395·7 (267·6 to 583·1)	376·6 (255·6 to 555)	−1·5% (−2·4 to −0·6)*	−2·1% (−3 to −1·2)*	−1·8% (−2·7 to −0·9)*
Glaucoma	342 (230 to 474)	344 (233 to 478)	686 (463 to 949)	28·8% (27·0 to 30·8)*	27·1% (25·1 to 28·9)*	27·9% (26·4 to 29·4)*	9·4 (6·3 to 12·9)	8·0 (5·4 to 11·2)	8·6 (5·8 to 12·0)	−4·7% (−6·1 to −3·4)*	−4·8% (−6·3 to −3·3)*	−4·7% (−5·8 to −3·6)*
Cataract	3350 (2340 to 4540)	4650 (3250 to 6270)	8010 (5580 to 10 800)	31·3% (28·5 to 34·1)*	28·4% (26·5 to 30·2)*	29·6% (27·4 to 31·7)*	93·2 (65·1 to 126·4)	109·0 (76 to 147)	101·8 (70·9 to 137·7)	−1·7% (−3·7 to 0·2)	−2·3% (−3·7 to −0·9)*	−2·0% (−3·6 to −0·5)*
Age-related macular degeneration	223 (153 to 307)	308 (210 to 422)	531 (364 to 729)	34·1% (31·3 to 37·1)*	28·3% (26·2 to 30·6)*	30·7% (28·6 to 32·9)*	6·3 (4·3 to 8·6)	7·2 (4·9 to 9·8)	6·8 (4·6 to 9·3)	−1·4% (−3·2 to 0·6)	−4·8% (−6·5 to −3·1)*	−3·7% (−5·3 to −2·1)*
Refraction disorders	3980 (2650 to 5780)	4000 (2700 to 5720)	7980 (5350 to 11 500)	17·0% (15·0 to 19·0)*	13·7% (12·0 to 15·2)*	15·3% (13·6 to 17·0)*	103·8 (68·9 to 150·7)	100·7 (67·6 to 144·2)	102·2 (68·2 to 147·3)	−1·5% (−2·9 to −0·1)*	−4·6% (−5·7 to −3·6)*	−3·1% (−4·2 to −1·9)*
Near vision loss	4260 (2030 to 7790)	5540 (2640 to 10 100)	9800 (4670 to 17 900)	23·7% (22·9 to 24·5)*	25·4% (24·6 to 26·1)*	24·6% (24 to 25·2)*	108·3 (51·7 to 198·2)	133·7 (63·6 to 244·5)	121·0 (57·7 to 221·3)	−0·7% (−1·2 to −0·2)*	1·4% (0·9 to 1·9)*	0·5% (0·1 to 0·9)*
Other vision loss	1350 (941 to 1810)	1540 (1070 to 2070)	2890 (2010 to 3880)	22·9% (20·9 to 24·7)*	19·5% (17·8 to 21·1)*	21·0% (19·4 to 22·6)*	35·3 (24·6 to 47·5)	37·1 (25·8 to 49·9)	36·3 (25·3 to 48·9)	−2·1% (−3·5 to −0·8)*	−5·0% (−6·1 to −3·9)*	−3·7% (−4·8 to −2·6)*
Age-related and other hearing loss	17 200 (11 500 to 24 500)	17 100 (11 600 to 24 200)	34 200 (23 200 to 48 700)	24·2% (22·8 to 25·6)*	25·7% (24·4 to 27·1)*	24·9% (23·9 to 26·0)*	458·7 (311·5 to 650·0)	411·6 (279·5 to 583·7)	434·6 (295·4 to 616·1)	−0·7% (−1·8 to 0·4)	0·2% (−0·9 to 1·2)	−0·2% (−1·0 to 0·5)
Other sense organ diseases	1070 (665 to 1570)	1380 (854 to 2010)	2450 (1520 to 3570)	24·9% (23·9 to 26·0)*	25·0% (24·1 to 26)*	25·0% (24·2 to 25·8)*	28·1 (17·5 to 41·2)	33·7 (20·9 to 49·0)	31·0 (19·2 to 45·2)	1·1% (0·4 to 1·8)*	0·8% (0·1 to 1·5)*	0·9% (0·4 to 1·4)*

** Musculoskeletal disorders**	** 60 800** **(44 700 to 80 400)**	** 77 900** **(57 500 to 103 000)**	** 139 000** **(102 000 to 183 000)**	** 19·5%** (18·3 to 20·7)*	** 20·2%** (19 to 21·6)*	** 19·9%** (18·8 to 21·2)*	** 1538·9** **(1134·0 to 2023·2)**	** 1894·7** **(1398·6 to 2491·4)**	** 1720·5** **(1264·4 to 2259·2)**	** −1%** (−1·7 to −0·4)*	** −1·2%** (−1·9 to −0·5)*	** −1·1%** (−1·7 to −0·5)*

Rheumatoid arthritis	977 (750 to 1220)	2510 (1910 to 3180)	3490 (2660 to 4410)	26·1% (22·9 to 28·9)*	30·6% (26·3 to 34·1)*	29·3% (26·0 to 32·1)*	25·4 (19·6 to 31·5)	60·1 (45·5 to 76·0)	43·3 (33·0 to 54·5)	−0·6% (−3·5 to 1·7)	3·4% (0·1 to 6·2)*	2·3% (−0·5 to 4·6)
Osteoarthritis	3840 (1930 to 7680)	5760 (2880 to 11 500)	9600 (4810 to 19 100)	32·0% (31·0 to 33·0)*	31·0% (30·3 to 31·8)*	31·4% (30·7 to 32·1)*	100·0 (50·3 to 199·1)	135·6 (67·7 to 269·7)	118·8 (59·5 to 236·2)	1·6% (0·9 to 2·3)*	0·8% (0·2 to 1·4)*	1·0% (0·5 to 1·6)*
Low back pain	29 500 (21 000 to 40 000)	35 500 (25 400 to 47 700)	64 900 (46 500 to 87 400)	17·8% (16·5 to 19·3)*	17·3% (15·8 to 18·8)*	17·5% (16·2 to 19·0)*	748·2 (537·6 to 1008·5)	869·1 (624·5 to 1164·5)	810·3 (582·4 to 1089·1)	−1·3% (−1·9 to −0·7)*	−2·7% (−3·3 to −2·2)*	−2·1% (−2·6 to −1·6)*
Neck pain	12 200 (8540 to 17 200)	16 400 (11 400 to 22 900)	28 600 (20 000 to 40 200)	22·3% (20·1 to 24·3)*	20·8% (18·4 to 23·7)*	21·4% (19·5 to 23·6)*	307·4 (215·7 to 430·8)	395·1 (275·9 to 551·8)	352·0 (245·6 to 493·3)	0·4% (−0·8 to 1·5)	−1·5% (−3·0 to 0·2)	−0·7% (−1·6 to 0·5)
Gout	953 (641 to 1300)	332 (226 to 451)	1280 (867 to 1770)	30·2% (28·2 to 32·2)*	32·9% (30·5 to 35·3)*	30·9% (29·2 to 32·6)*	24·6 (16·6 to 33·8)	7·8 (5·3 to 10·7)	15·9 (10·7 to 21·8)	2·4% (1·1 to 3·7)*	3·1% (1·3 to 4·9)*	2·7% (1·6 to 3·7)*

** Other musculoskeletal disorders**	** 13 300** **(9130 to 18 400)**	** 17 500** **(12 400 to 23 600)**	** 30 800** **(21 500 to 42 000)**	** 16·6%** (13·2 to 19·8)*	** 21·0%** (17·7 to 24·4)*	** 19·1%** (15·8 to 22·3)*	** 333·3** **(229·5 to 459·1)**	** 427·0** **(303·1 to 577·3)**	** 380·2** **(266·2 to 520·3)**	** −2·7%** (−5·2 to −0·4)*	** 0·9%** **(−1·6 to 3·2)**	** −0·7%** **(−3·0 to 1·4)**

Other non-communicable diseases	56 000 (48 900 to 64 900)	65 900 (54 700 to 79 500)	122 000 (104 000 to 144 000)	−3·8% (−10·8 to 0·6)	0·3% (−3·7 to 3·3)	−1·6% (−6·1 to 1·6)	1547·8 (1362·6 to 1778·5)	1812·4 (1524·5 to 2156·7)	1678·1 (1450·0 to 1961·2)	−12·8% (−18·8 to −9·3)*	−10·3% (−13·4 to −7·9)*	−11·5% (−15·4 to −8·9)*
Congenital anomalies	32 300 (29 800 to 35 300)	28 600 (26 400 to 30 700)	60 900 (56 400 to 65 400)	−12·1% (−21·3 to −7·0)*	−9·6% (−14·8 to −5·7)*	−10·9% (−17·3 to −7·1)*	917·1 (847·3 to 999·7)	859·6 (795·0 to 920·4)	889·0 (827·6 to 951·5)	−16·7% (−25·3 to −12)*	−14·8% (−19·6 to −11)*	−15·8% (−21·8 to −12·3)*
Neural tube defects	3140 (2380 to 4350)	3030 (2280 to 4150)	6170 (4840 to 8170)	−11·2% (−27·1 to 1·4)	−9·0% (−22·0 to 4·9)	−10·2% (−21·3 to 0·9)	90·1 (68·3 to 125·1)	92·7 (69·7 to 127·2)	91·3 (71·7 to 121·2)	−15·3% (−30·4 to −3·2)*	−13·6% (−26·0 to −0·2)*	−14·5% (−25·1 to −4·0)*
Congenital heart anomalies	11 500 (9480 to 14 000)	10 700 (8260 to 12 600)	22 200 (18 400 to 26 300)	−19·1% (−27·9 to −9·6)*	−17·6% (−23·4 to −10·1)*	−18·4% (−24·8 to −10·3)*	332·1 (272·0 to 404·0)	328·0 (253·0 to 388·2)	330·0 (272·3 to 390·8)	−22·5% (−30·9 to −13·4)*	−21·3% (−26·9 to −14·2)*	−21·9% (−28·1 to −14·3)*
Orofacial clefts	352 (194 to 657)	300 (187 to 599)	652 (411 to 1110)	21·9% (−37·7 to −4·3)*	−21·8% (−38·2 to −5·6)*	−21·9% (−35·2 to −8·1)*	9·8 (5·3 to 18·7)	8·7 (5·3 to 18·2)	9·3 (5·8 to 16·2)	−27·2% (−41·8 to −10·0)*	−28·3% (−42·6 to −13·9)*	−27·7% (−39·9 to −15·4)*
Down syndrome	1240 (963 to 1790)	989 (755 to 1520)	2230 (1760 to 3040)	−1·5% (−12·1 to 14·2)	2·6% (−9·0 to 16·7)	0·3% (−9·2 to 13·6)	34·8 (26·7 to 50·5)	29·0 (21·9 to 45·3)	32·0 (24·9 to 43·9)	−7·8% (−17·4 to 6·7)	−4·6% (−15·3 to 8·3)	−6·4% (−15·1 to 5·9)
Turner syndrome	··	10·1 (5·02 to 16·5)	10·1 (5·02 to 16·5)	··	9·1% (7·3 to 10·9)*	9·1% (7·3 to 10·9)*	··	0·3 (0·1 to 0·5)	0·1 (0·1 to 0·2)	··	1·0% (−0·6 to 2·6)	0·9% (−0·7 to 2·5)
Klinefelter syndrome	5·16 (2·43 to 9·74)	··	5·16 (2·43 to 9·74)	7·4% (4·3 to 10·7)*	··	7·4% (4·3 to 10·7)*	0·1 (0·1 to 0·3)	··	0·1 (0 to 0·1)	−1·9% (−4·7 to 1·0)	··	−1·7% (−4·5 to 1·1)
Other chromosomal abnormalities	928 (654 to 1380)	1170 (769 to 2010)	2100 (1510 to 2840)	10·2% (−2·8 to 22·0)	6·1% (−7·2 to 19·3)	7·9% (−1·9 to 18·2)	26·0 (18·3 to 38·8)	35·2 (22·6 to 61·2)	30·4 (21·8 to 41·3)	3·7% (−8·1 to 14·5)	0·2% (−11·8 to 12·6)	1·7% (−7·2 to 11·4)
Congenital musculoskeletal and limb anomalies	2890 (2030 to 3790)	2650 (1850 to 3510)	5530 (3880 to 7270)	8·6% (4·1 to 11·8)*	9·0% (4·2 to 12·8)*	8·8% (4·8 to 11·1)*	76·8 (54·5 to 100·5)	71·6 (50·4 to 94·3)	74·2 (52·6 to 97·2)	−2·3% (−5·9 to 0·3)	−2·7% (−6·7 to 0·4)	−2·5% (−5·6 to −0·5)*
Urogenital congenital anomalies	687 (459 to 879)	560 (411 to 698)	1250 (926 to 1500)	−4·9% (−15·5 to 8·5)	−3·9% (−13·3 to 7·3)	−4·5% (−13·2 to 6·1)	19·7 (13·0 to 25·3)	16·9 (12·3 to 21·0)	18·3 (13·6 to 22·1)	−9·1% (−19·1 to 3·5)	−9·1% (−18·1 to 1·8)	−9·1% (−17·2 to 0·8)
Digestive congenital anomalies	2970 (2190 to 3960)	2220 (1440 to 3300)	5190 (3990 to 7050)	−16·1% (−30·4 to −4·8)*	−9·5% (−17·8 to 1·3)	−13·4% (−24·5 to −4·0)*	85·3 (62·6 to 113·9)	67·5 (43·1 to 101·0)	76·7 (58·7 to 104·6)	−20·0% (−33·4 to −9·1)*	−14·1% (−21·9 to −4·1)*	−17·6% (−28·1 to −8·9)*
Other congenital anomalies	8570 (6620 to 10 500)	6980 (5170 to 8960)	15 500 (12 000 to 19 200)	−9·7% (−17·9 to 1·5)	−5·8% (−14·8 to 5·0)	−8·0% (−15·8 to 2·7)	242·4 (186·3 to 297·7)	209·8 (155·1 to 270·2)	226·5 (174·1 to 279·7)	−14·5% (−22·1 to −4·1)*	−11·0% (−19·4 to −1·0)*	−13·0% (−20·4 to −3·3)*
Urinary diseases and male infertility	6080 (5080 to 7320)	3310 (3140 to 3480)	9390 (8260 to 10 700)	21·8% (18·1 to 25·1)*	24·5% (18 to 28·2)*	22·8% (18·9 to 25·6)*	162·2 (136 to 194·8)	82·7 (78·4 to 87·1)	119·9 (105·9 to 136·8)	−2·7% (−5·3 to −0·2)*	2·3% (−3·3 to 5·4)	−0·8% (−3·9 to 1·6)
Urinary tract infections	2120 (1970 to 2410)	2570 (2410 to 2830)	4700 (4430 to 5190)	24·5% (17·7 to 31·7)*	36·4% (28·5 to 44·1)*	30·8% (24·2 to 37·8)*	57·8 (53·8 to 65·4)	63·8 (59·5 to 70·5)	60·7 (57·2 to 67·2)	2·3% (−3·0 to 7·9)	11·3% (4·8 to 17·6)*	7·0% (1·6 to 12·6)*
Urolithiasis	281 (227 to 346)	205 (165 to 252)	486 (397 to 591)	15·9% (9·5 to 25·0)*	23·7% (18·2 to 34·5)*	19·1% (13·6 to 28)*	7·3 (5·9 to 8·9)	4·9 (4·0 to 6·1)	6·0 (4·9 to 7·3)	−7·0% (−12·3 to 0·3)	−0·2% (−4·6 to 8·2)	−4·1% (−8·5 to 3·0)
Benign prostatic hyperplasia	2430 (1560 to 3460)	··	2430 (1560 to 3460)	32·0% (30·7 to 33·3)*	··	32·0% (30·7 to 33·3)*	63·7 (40·9 to 90·7)	··	29·9 (19·2 to 42·5)	−0·7% (−1·6 to 0·3)	··	−0·2% (−1·1 to 0·8)
Male infertility	181 (74·8 to 374)	··	181 (74·8 to 374)	17·0% (13·7 to 20·1)*	··	17·0% (13·7 to 20·1)*	4·6 (1·9 to 9·5)	··	2·3 (1·0 to 4·8)	6·7% (3·9 to 9·4)*	··	6·9% (4·1 to 9·7)*
Other urinary diseases	1080 (903 to 1240)	526 (346 to 654)	1600 (1290 to 1790)	1·7% (−6·3 to 11·6)	−12·4% (−23·3 to −3·3)*	−3·4% (−9·4 to 5·1)	28·9 (24·3 to 33·4)	13·9 (9·2 to 17·4)	21·0 (16·9 to 23·6)	−15·0% (−21·4 to −7·3)*	−24·8% (−33·4 to −17·3)*	−18·4% (−23·1 to −11·5)*
Gynaecological diseases	··	11 900 (8210 to 16 600)	11 900 (8210 to 16 600)	··	10·2% (9·0 to 11·6)*	10·2% (9·0 to 11·6)*	··	299·4 (206·4 to 415·8)	148·8 (102·6 to 206·6)	··	−2·4% (−3·3 to −1·4)*	−2·5% (−3·4 to −1·4)*
Uterine fibroids	··	1550 (947 to 2460)	1550 (947 to 2460)	··	8·3% (4·5 to 11·9)*	8·3% (4·5 to 11·9)*	··	37·7 (23·1 to 59·9)	18·9 (11·5 to 30·0)	··	−8·2% (−11·4 to −5·2)*	−7·9% (−11·1 to −5·0)*
Polycystic ovarian syndrome	··	460 (204 to 886)	460 (204 to 886)	··	13·1% (12·1 to 14·0)*	13·1% (12·1 to 14·0)*	··	11·7 (5·2 to 22·7)	5·8 (2·6 to 11·2)	··	2·0% (1·1 to 2·8)*	1·8% (1·0 to 2·7)*
Female infertility	··	343 (129 to 723)	343 (129 to 723)	··	27·5% (22·2 to 32·7)*	27·5% (22·2 to 32·7)*	··	8·8 (3·3 to 18·5)	4·4 (1·7 to 9·2)	··	16·7% (12·2 to 21·1)*	16·5% (12·0 to 20·8)*
Endometriosis	··	4130 (2760 to 5950)	4130 (2760 to 5950)	··	9·2% (7·9 to 10·5)*	9·2% (7·9 to 10·5)*	··	103·9 (69·5 to 149·1)	51·6 (34·5 to 74)	··	−2·9% (−3·8 to −1·9)*	−3·0% (−3·9 to −2·0)*
Genital prolapse	··	352 (173 to 652)	352 (173 to 652)	··	18·6% (16·0 to 20·4)*	18·6% (16·0 to 20·4)*	··	8·4 (4·1 to 15·6)	4·3 (2·1 to 8·0)	··	−4·5% (−6·7 to −3·4)*	−4·7% (−6·9 to −3·5)*
Premenstrual syndrome	··	3930 (2530 to 5900)	3930 (2530 to 5900)	··	8·8% (7·2 to 10·1)*	8·8% (7·2 to 10·1)*	··	100·0 (64·5 to 150·1)	49·5 (31·9 to 74·3)	··	−2·1% (−3·5 to −0·9)*	−2·2% (−3·6 to −1·0)*
Other gynaecological diseases	··	1150 (830 to 1580)	1150 (830 to 1580)	··	13·5% (10·1 to 16·6)*	13·5% (10·1 to 16·6)*	··	28·9 (20·8 to 39·6)	14·4 (10·3 to 19·7)	··	0·2% (−2·8 to 3·0)	0·2% (−2·8 to 3·0)
Haemoglobinopathies and haemolytic anaemias	4350 (3040 to 6130)	5800 (4510 to 7370)	10 100 (7740 to 13 300)	−1·0% (−9·7 to 7·3)	−5·6% (−10·3 to 0·2)	−3·7% (−9·3 to 2·3)	117·9 (82·4 to 165·8)	156·6 (122·3 to 198·9)	137·1 (104·6 to 179·3)	−10·5% (−18·6 to −2·8)*	−15·3% (−19·8 to −9·8)*	−13·2% (−18·5 to −7·6)*
Thalassaemias	308 (239 to 367)	274 (221 to 338)	582 (491 to 683)	−21·1% (−33·6 to −6·2)*	−28·0% (−36·5 to −13·4)*	−24·5% (−33·5 to −13·4)*	8·7 (6·8 to 10·4)	8·2 (6·6 to 10·1)	8·4 (7·1 to 9·9)	−25% (−37·2 to −10·7)*	−32·0% (−40·2 to −18·0)*	−28·5% (−37·2 to −17·8)*
Thalassaemias trait	477 (306 to 722)	1430 (968 to 2070)	1910 (1280 to 2780)	−13·8% (−20·0 to −7·0)*	−11·5% (−16 to −6·8)*	−12·1% (−16·1 to −7·7)*	12·8 (8·2 to 19·3)	37·2 (25·1 to 53·8)	25 (16·8 to 36·4)	−22·6% (−28·1 to −16·8)*	−21·4% (−25·4 to −17·3)*	−21·6% (−25·1 to −18·0)*
Sickle cell disorders	1580 (865 to 2640)	1470 (1050 to 2090)	3050 (2000 to 4200)	8·2% (−11·4 to 23·1)	−1·9% (−15·7 to 19·2)	3·1% (−11·6 to 16·9)	43·5 (23·8 to 72·8)	42·9 (30·6 to 61·2)	43·2 (28·3 to 59·4)	2·2% (−16·6 to 16·6)	−8·1% (−21·4 to 12·7)	−3·0% (−16·9 to 10·6)
Sickle cell trait	362 (237 to 534)	841 (567 to 1200)	1200 (812 to 1730)	−5·1% (−13·0 to 3·5)	0·4% (−3·4 to 4·4)	−1·3% (−5·1 to 2·9)	9·8 (6·4 to 14·6)	22·8 (15·4 to 32·5)	16·3 (11·0 to 23·4)	−13·4% (−20·4 to −5·4)*	−8·8% (−12·3 to −5·2)*	−10·3% (−13·9 to −6·4)*
G6PD deficiency	485 (330 to 655)	232 (187 to 289)	717 (546 to 920)	6·6% (−2·1 to 16·1)	−0·3% (−5·6 to 8·2)	4·3% (−2·2 to 11·9)	12·3 (8·4 to 16·6)	5·9 (4·8 to 7·4)	9·1 (7·0 to 11·7)	−7·8% (−14·3 to −0·5)*	−13·0% (−17·7 to −5·4)*	−9·6% (−14·7 to −3·5)*
G6PD trait	0 (0 to 0)	0·416 (0·281 to 0·580)	0·416 (0·281 to 0·580)	··	3·0% (−1·7 to 7·8)	3·0% (−1·7 to 7·8)	0 (0 to 0)	0 (0 to 0)	0 (0 to 0)	··	−8·1% (−12·1 to −3·9)*	−8·1% (−12·2 to −3·9)*
Other haemoglobinopathies and haemolytic anaemias	1140 (845 to 1550)	1550 (1180 to 2060)	2680 (2030 to 3580)	−1·2% (−5·5 to 3·8)	−1·6% (−4·1 to 1·0)	−1·4% (−3·8 to 1·4)	30·7 (22·9 to 41·6)	39·4 (29·9 to 52·8)	35·0 (26·4 to 46·7)	−15·3% (−19·2 to −11)*	−15·9% (−18·4 to −13·6)*	−15·6% (−18·1 to −12·9)*
Endocrine, metabolic, blood, and immune disorders	3310 (2750 to 3820)	4410 (3610 to 5350)	7720 (6500 to 9080)	10·3% (3·9 to 15·5)*	5·1% (1·6 to 8·5)*	7·3% (3·0 to 11·1)*	89·1 (73·8 to 102·7)	115·1 (94·0 to 140·0)	101·8 (85·9 to 120·0)	−4·7% (−9·8 to −0·5)*	−9·0% (−11·8 to −6·0)*	−7·2% (−10·7 to −4·1)*
Oral disorders	7860 (4620 to 12 300)	10 400 (6390 to 16 000)	18 300 (11 000 to 28 300)	20·5% (19·6 to 21·4)*	22·1% (21·2 to 23·0)*	21·4% (20·5 to 22·3)*	202·1 (119·1 to 315·7)	253·7 (154·8 to 388·5)	228·8 (137·5 to 353·7)	−1·8% (−2·6 to −1·1)*	−0·9% (−1·6 to −0·2)*	−1·3% (−2·0 to −0·6)*
Caries of deciduous teeth	72·4 (30·9 to 145)	66·5 (28·5 to 133)	139 (59·4 to 278)	4·6% (2·7 to 6·3)*	5·1% (3·0 to 6·9)*	4·9% (3·1 to 6·3)*	2·1 (0·9 to 4·1)	2·0 (0·9 to 4·0)	2·0 (0·9 to 4·1)	−2·2% (−3·9 to −0·6)*	−2·0% (−3·9 to −0·4)*	−2·1% (−3·7 to −0·8)*
Caries of permanent teeth	785 (338 to 1490)	834 (362 to 1590)	1620 (698 to 3090)	9·4% (8·5 to 10·4)*	9·5% (8·5 to 10·5)*	9·4% (8·6 to 10·3)*	20·0 (8·6 to 38·0)	21·3 (9·2 to 40·4)	20·7 (8·9 to 39·2)	−3·9% (−4·7 to −3·0)*	−4·1% (−5·0 to −3·3)*	−4·0% (−4·8 to −3·3)*
Periodontal diseases	2470 (967 to 5070)	2720 (1080 to 5580)	5190 (2040 to 10 700)	25·1% (23·5 to 26·6)*	27·9% (26·3 to 29·3)*	26·6% (25·1 to 27·9)*	61·6 (24·2 to 127·0)	65·4 (25·9 to 134·2)	63·5 (25·0 to 130·3)	1·8% (1·3 to 2·3)*	3·6% (3·0 to 4·3)*	2·8% (2·3 to 3·2)*
Edentulism and severe tooth loss	2770 (1850 to 3940)	4580 (3060 to 6480)	7350 (4890 to 10 400)	24·0% (23·1 to 24·8)*	25·1% (24·0 to 26·2)*	24·6% (23·7 to 25·5)*	73·5 (49·1 to 104·2)	108·0 (72·2 to 153·1)	91·7 (61·3 to 129·9)	−5·1% (−5·8 to −4·4)*	−3·3% (−3·9 to −2·5)*	−4·1% (−4·7 to −3·4)*
Other oral disorders	1770 (1100 to 2620)	2250 (1400 to 3310)	4020 (2510 to 5900)	15·5% (14·7 to 16·3)*	15·8% (15·0 to 16·6)*	15·7% (15 to 16·3)*	44·8 (28·0 to 66·7)	56·9 (35·6 to 83·8)	50·9 (31·8 to 74·7)	0·3% (−0·2 to 0·7)	0·2% (−0·3 to 0·6)	0·2% (−0·1 to 0·5)
Sudden infant death syndrome	2040 (816 to 3870)	1450 (572 to 3230)	3490 (1570 to 6730)	−18·1% (−32·9 to 2·1)	−16·1% (−30·7 to 6·4)	−17·3% (−28·6 to −1·4)*	59·4 (23·8 to 112·8)	45·4 (17·8 to 100·9)	52·7 (23·7 to 101·5)	−20·9% (−35·2 to −1·4)*	−19·2% (−33·2 to 2·5)	−20·2% (−31·2 to −4·9)*
**Injuries**	**171 000** **(161 000 to 182 000)**	**81 900** **(74 500 to 90 000)**	**252 000** **(236 000 to 271 000)**	**−1·8%** **(−3·6 to 0·2)**	0·**7%** **(−1·9 to 3·4)**	**−1·0%** **(−2·8 to 1·0)**	**4397·9** **(4149·6 to 4678·6)**	**2128·2** **(1943·4 to 2330·6)**	**3267·0** **(3058·2 to 3505·1)**	**−13·5%** (−15·1 to −11·8)*	**−12·7%** (−14·7 to −10·6)*	**−13·3%** (−14·7 to −11·7)*
**Transport injuries**	**54 900** **(52 000 to 57 800)**	**20 400** **(18 700 to 22 300)**	**75 300** **(71 000 to 79 800)**	**−6·0%** (−8·7 to −3·2)*	**−3·3%** (−6·1 to −0·3)*	**−5·3%** (−7·7 to −2·8)*	**1403·5** **(1328·7 to 1475·7)**	**528·6** **(484·5 to 575·5)**	**967·6** **(914·2 to 1023·9)**	**−16·8%** (−19·1 to −14·5)*	**−15·9%** (−18·3 to −13·4)*	**−16·6%** (−18·5 to −14·6)*

Road injuries	49 800 (47 300 to 52 100)	18 000 (16 600 to 19 400)	67 800 (64 300 to 71 500)	−6·5% (−9·3 to −3·6)*	−4·8% (−7·8 to −1·9)*	−6·1% (−8·5 to −3·5)*	1272·2 (1209·4 to 1331·3)	467·2 (431·8 to 501·5)	871·1 (827·9 to 917·3)	−17·3% (−19·6 to −14·8)*	−17·2% (−19·7 to −14·7)*	−17·2% (−19·2 to −15·1)*
Pedestrian road injuries	16 500 (15 200 to 18 500)	7090 (6430 to 7870)	23 600 (22 000 to 25 800)	−12·3% (−17·1 to −7·7)*	−11·2% (−15·0 to −6·8)*	−12·0% (−15·6 to −8·3)*	423·2 (390·0 to 474·3)	184·9 (167·7 to 204·9)	304·1 (283·9 to 332·1)	−23·0% (−27 to −19·1)*	−23·2% (−26·5 to −19·3)*	−23·1% (−26·1 to −20·0)*
Cyclist road injuries	3290 (2740 to 3790)	1270 (1050 to 1510)	4550 (3860 to 5270)	8·6% (2·2 to 14·9)*	12·3% (6·8 to 17·3)*	9·6% (4·2 to 14·9)*	83·5 (69·6 to 96·5)	31·5 (26·2 to 37·6)	57·5 (48·8 to 66·3)	−6·4% (−11·8 to −0·9)*	−5·6% (−9·9 to −1·4)*	−6·3% (−10·8 to −1·7)*
Motorcyclist road injuries	11 700 (10 300 to 12 600)	2310 (1960 to 2610)	14 000 (12 300 to 15 100)	−1·8% (−8·5 to 3·2)	−0·1% (−7·8 to 10·0)	−1·6% (−7·6 to 3·2)	295·8 (259·7 to 318·9)	59·0 (50·2 to 66·6)	177·9 (157·3 to 191·9)	−12·1% (−17·8 to −7·8)*	−12·8% (−19·4 to −4·4)*	−12·2% (−17·3 to −8·1)*
Motor vehicle road injuries	17 700 (16 500 to 19 000)	6850 (6230 to 7410)	24 600 (23 100 to 26 000)	−6·5% (−9·8 to −1·7)*	−3·7% (−7·1 to 1·9)	−5·7% (−8·4 to −1·3)*	453·4 (423·2 to 485·8)	179·5 (162·8 to 193·5)	317·2 (297·9 to 335·1)	−16·6% (−19·5 to −12·2)*	−15·0% (−18·1 to −9·9)*	−16·1% (−18·4 to −12·1)*
Other road injuries	640 (540 to 761)	491 (380 to 629)	1130 (932 to 1380)	1·7% (−5·2 to 17)	30·2% (22·0 to 40·2)*	12·4% (5·3 to 25·6)*	16·3 (13·8 to 19·4)	12·3 (9·6 to 15·6)	14·3 (11·9 to 17·4)	−11·8% (−17·3 to 1·4)	10·1% (3·7 to 18·4)*	−3·5% (−9·1 to 7·7)
Other transport injuries	5130 (4510 to 5920)	2400 (1940 to 2960)	7530 (6480 to 8760)	−0·2% (−4·6 to 4·8)	10·0% (5·6 to 14·3)*	2·9% (−1·0 to 6·7)	131·3 (115·3 to 151·4)	61·3 (49·7 to 75·3)	96·5 (83·0 to 112·1)	−12·1% (−15·9 to −7·9)*	−5·3% (−9·1 to −1·6)*	−10·0% (−13·3 to −6·7)*

**Unintentional injuries**	**65 300** **(58 800 to 72 800)**	**40 700** **(35 400 to 46 700)**	**106 000** **(94 200 to 119 000)**	**−5·0%** (−8·1 to −1·5)*	**2·3%** **(−1·6 to 6·3)**	**−2·3%** **(−5·4 to 1·3)**	**1715·6** **(1548·7 to 1908·9)**	**1058·9** **(924·2 to 1207·6)**	**1389·0** **(1241·3 to 1560·2)**	**−17%** (−19·5 to −14·3)*	**−13%** (−16·0 to −9·8)*	**−15·6%** (−18·0 to −12·7)*

Falls	21 000 (17 600 to 24 900)	15 000 (12 300 to 18 000)	35 900 (30 200 to 42 900)	15·3% (10·3 to 20·3)*	24·4% (20·7 to 27·5)*	18·9% (15·4 to 22·7)*	549·9 (462·2 to 652·6)	367·2 (304·1 to 442·2)	459·4 (387·1 to 547·5)	−3·8% (−7·7 to 0·4)	0·3% (−2·9 to 2·8)	−2·3% (−5·0 to 0·7)
Drowning	11 600 (11 100 to 12 100)	5110 (4680 to 5510)	16 700 (15 900 to 17 500)	−25·0% (−28·0 to −21·8)*	−27·8% (−32·5 to −20·3)*	−25·9% (−28·8 to −22·2)*	311·4 (298·2 to 325·9)	145·9 (133·3 to 158·3)	229·9 (219·0 to 241·2)	−31·6% (−34·3 to −28·6)*	−34·6% (−39·0 to −27·5)*	−32·6% (−35·4 to −29·2)*
Fire, heat, and hot substances	4060 (3380 to 4770)	4400 (3560 to 5210)	8460 (7030 to 9880)	−7·3% (−13·1 to 4·9)	−5·2% (−11·2 to 3·9)	−6·3% (−11·7 to 1·2)	106·3 (88·5 to 124·6)	116·3 (94·1 to 136·7)	111·1 (92·8 to 129·3)	−18·8% (−23·6 to −8·4)*	−16·6% (−21·6 to −8·4)*	−17·7% (−22·4 to −11·0)*
Poisonings	2320 (1740 to 2620)	1470 (1090 to 1640)	3790 (2930 to 4210)	−12·4% (−20·1 to −0·3)*	−8·2% (−14·8 to 2·6)	−10·8% (−17·8 to −0·5)*	60·6 (45·2 to 68·5)	39·5 (29·3 to 43·9)	50·1 (38·8 to 55·6)	−22·4% (−29 to −11·3)*	−18·3% (−24·2 to −8·5)*	−20·9% (−26·9 to −11·7)*
Poisoning by carbon monoxide	1040 (770 to 1150)	500 (329 to 558)	1540 (1150 to 1690)	−19·6% (−27·6 to −11·0)*	−12·8% (−20·8 to −5·4)*	−17·5% (−25·3 to −10·1)*	26·6 (19·7 to 29·4)	13·1 (8·6 to 14·7)	19·9 (14·8 to 21·9)	−29·5% (−36·4 to −21·9)*	−23·3% (−30·3 to −16·6)*	−27·6% (−34·5 to −21·0)*
Poisoning by other means	1290 (948 to 1500)	966 (737 to 1090)	2250 (1780 to 2560)	−5·6% (−15·0 to 12·3)	−5·6% (−13·5 to 6·8)	−5·6% (−13·8 to 7·5)	34·0 (25·1 to 39·7)	26·4 (20·1 to 29·8)	30·2 (23·9 to 34·3)	−15·7% (−24·1 to 0·8)	−15·6% (−22·8 to −4·2)*	−15·7% (−23·2 to −3·7)*
Exposure to mechanical forces	8110 (6970 to 9480)	3240 (2560 to 3980)	11 300 (9620 to 13 500)	−3·5% (−8·0 to 1·4)	13·6% (7·7 to 20·5)*	0·8% (−3·6 to 5·5)	208·5 (179·4 to 243·3)	83·8 (66·4 to 102·5)	146·1 (124·2 to 172·6)	−15·8% (−19·2 to −12·2)*	−2·4% (−7·1 to 2·8)	−12·5% (−15·8 to −8·9)*
Unintentional firearm injuries	1080 (972 to 1250)	334 (285 to 390)	1410 (1270 to 1620)	−4·6% (−9·4 to 2·8)	11·2% (3·5 to 21·7)*	−1·3% (−5·8 to 4·2)	28·0 (25·1 to 32·5)	8·7 (7·4 to 10·2)	18·4 (16·6 to 21·2)	−14·6% (−18·8 to −8·1)*	−2·9% (−9·7 to 6·5)	−12·1% (−16·2 to −7·3)*
Other exposure to mechanical forces	7030 (5970 to 8340)	2900 (2260 to 3600)	9930 (8310 to 11 900)	−3·4% (−8·2 to 1·8)	13·9% (8·0 to 21·4)*	1·1% (−3·6 to 6·1)	180·5 (153·2 to 213·8)	75·1 (58·4 to 93)	127·6 (107·2 to 152·3)	−16·0% (−19·7 to −12·1)*	−2·3% (−7·1 to 3·3)	−12·5% (−16·1 to −8·9)*
Adverse effects of medical treatment	2490 (2070 to 3100)	2230 (1760 to 2660)	4720 (3940 to 5600)	7·2% (1·0 to 14·4)*	5·5% (0 to 12·3)*	6·4% (1·2 to 13·2)*	66·6 (55·2 to 83·7)	58·7 (46·0 to 70·8)	62·6 (51·9 to 75·2)	−6·7% (−11·9 to −0·8)*	−9·3% (−13·9 to −3·5)*	−7·9% (−12·3 to −2·2)*
Animal contact	2650 (1630 to 3090)	2340 (1180 to 2870)	4990 (3180 to 5830)	−8·5% (−16·1 to 5·5)	0% (−7·8 to 9·9)	−4·7% (−10·7 to 3·6)	69·3 (42·6 to 81·1)	63·2 (31·6 to 77·4)	66·1 (42·1 to 77·4)	−19·1% (−25·8 to −6·5)*	−11·3% (−18·0 to −2·9)*	−15·6% (−21·2 to −8·0)*
Venomous animal contact	2160 (1250 to 2540)	1960 (898 to 2450)	4120 (2490 to 4910)	−10·3% (−18·2 to 4·6)	−0·4% (−8·8 to 9·3)	−5·8% (−11·7 to 2·8)	56·6 (32·7 to 66·6)	53·1 (23·8 to 66·6)	54·7 (32·8 to 65·3)	−20·5% (−27·6 to −7·0)*	−11·3% (−18·5 to −3·2)*	−16·4% (−21·9 to −8·5)*
Non-venomous animal contact	484 (343 to 612)	379 (247 to 648)	863 (640 to 1140)	0·3% (−8·5 to 9·4)	2·0% (−24·8 to 23·6)	1·1% (−16·0 to 10·7)	12·7 (9·0 to 16·1)	10·1 (6·5 to 17·8)	11·4 (8·4 to 15·1)	−12·3% (−19·5 to −4·7)*	−11·1% (−33·7 to 7·9)	−11·7% (−26·2 to −3·4)*
Foreign body	4140 (3830 to 4500)	2710 (2500 to 2910)	6860 (6340 to 7350)	−9·0% (−13·1 to −4·5)*	−8·6% (−15 to −3·2)*	−8·8% (−12·6 to −5·0)*	113·7 (104·7 to 123·4)	77·0 (70·8 to 82·6)	95·4 (88·2 to 102·2)	−17·7% (−21·3 to −13·8)*	−17·7% (−23·5 to −12·8)*	−17·7% (−21·2 to −14·3)*
Pulmonary aspiration and foreign body in airway	3460 (3250 to 3760)	2210 (2050 to 2350)	5680 (5350 to 6050)	−11·4% (−16 to −6·7)*	−11·7% (−18·8 to −5·9)*	−11·5% (−15·7 to −7·4)*	95·8 (89·8 to 104·1)	64·1 (59·1 to 68·3)	80·0 (75·2 to 85·5)	−19·3% (−23·4 to −15·0)*	−19·5% (−26·1 to −14·3)*	−19·4% (−23·3 to −15·6)*
Foreign body in eyes	149 (76·1 to 250)	52·8 (28·0 to 85·9)	202 (105 to 334)	21·6% (20·0 to 23·8)*	16·7% (15·4 to 18·3)*	20·3% (18·8 to 22·1)*	3·9 (2·0 to 6·5)	1·3 (0·7 to 2·2)	2·6 (1·3 to 4·3)	3·9% (2·6 to 4·7)*	−0·9% (−2·7 to 0·4)	2·7% (1·3 to 3·6)*
Foreign body in other body part	532 (420 to 657)	446 (357 to 547)	978 (797 to 1180)	2·1% (−7·5 to 9·6)	7·2% (−0·7 to 14·1)	4·4% (−1·9 to 10·2)	13·9 (11·0 to 17·3)	11·5 (9·2 to 14·1)	12·7 (10·4 to 15·4)	−11·0% (−18·7 to −5·1)*	−7·6% (−14·1 to −1·5)*	−9·6% (−14·7 to −4·7)*
Environmental heat and cold exposure	2240 (1690 to 2650)	1120 (817 to 1360)	3360 (2550 to 4000)	−9·3% (−13·9 to −5·0)*	2·0% (−3·3 to 6·6)	−5·8% (−10·6 to −1·7)*	57·5 (43·2 to 68·2)	28·4 (20·5 to 34·7)	42·8 (32·4 to 51·1)	−22·6% (−26·6 to −19)*	−13·7% (−18·4 to −9·7)*	−19·9% (−24·2 to −16·4)*
Exposure to forces of nature	749 (624 to 920)	454 (370 to 564)	1200 (1000 to 1470)	−18·7% (−25·1 to −11·5)*	−15·6% (−22·6 to −7·8)*	−17·6% (−23·3 to −10·9)*	19·2 (16·0 to 23·5)	11·9 (9·7 to 14·8)	15·5 (13·0 to 18·9)	−27·5% (−33·2 to −21·2)*	−25·8% (−32·0 to −18·8)*	−26·9% (−32·0 to −21·1)*
Other unintentional injuries	5950 (5220 to 6860)	2640 (2110 to 3340)	8580 (7380 to 10 200)	−7·7% (−11·4 to −3·6)*	5·4% (−0·3 to 10·8)	−4·0% (−8·2 to 0·4)	152·7 (134·1 to 175·9)	67·0 (54·1 to 84·1)	110·0 (94·9 to 130·3)	−19% (−21·9 to −15·8)*	−10·9% (−15·2 to −6·8)*	−16·8% (−19·9 to −13·6)*

**Self-harm and interpersonal violence**	**50 300** **(47 500 to 52 300)**	**20 800** **(19 600 to 21 900)**	**71 100** **(68 100 to 73 700)**	**8·1%** (4·7 to 10·9)*	**1·7%** **(−1·3 to 4·1)**	**6·2%** (3·8 to 8·2)*	**1278·8** **(1206·1 to 1328·3)**	**540·8** **(510·6 to 570·7)**	**910·5** **(871·7 to 944·2)**	**−4·0%** (−7·0 to −1·5)*	**−8·6%** (−11·2 to −6·3)*	**−5·3%** (−7·4 to −3·5)*

Self-harm	22 900 (20 900 to 24 000)	11 100 (10 600 to 11 500)	34 000 (31 800 to 35 100)	−0·8% (−6·2 to 3·6)	−8·0% (−12·6 to −5·1)*	−3·3% (−6·9 to −0·8)*	577·3 (525·5 to 603·9)	282·2 (268·3 to 293)	429·0 (401·6 to 443·5)	−13·5% (−18·2 to −9·7)*	−18·3% (−22·4 to −15·7)*	−15·1% (−18·3 to −12·9)*
Self-harm by firearm	2250 (1840 to 2880)	405 (334 to 476)	2660 (2250 to 3290)	2·3% (−3·7 to 7·8)	−6·2% (−14·1 to 3·1)	0·9% (−3·5 to 5·5)	57·0 (46·5 to 72·8)	10·3 (8·4 to 12·1)	33·5 (28·3 to 41·7)	−10·6% (−15·5 to −6·1)*	−16·6% (−23·1 to −8·5)*	−11·5% (−15·2 to −7·6)*
Self-harm by other specified means	20 700 (18 600 to 21 700)	10 700 (10 200 to 11 100)	31 400 (29 200 to 32 600)	−1·1% (−6·8 to 3·7)	−8·1% (−12·8 to −5·0)*	−3·6% (−7·4 to −1)*	520·3 (467·5 to 546·3)	271·9 (258·6 to 282·5)	395·4 (368·6 to 410·5)	−13·8% (−18·8 to −9·6)*	−18·4% (−22·5 to −15·7)*	−15·4% (−18·7 to −13·1)*
Interpersonal violence	19 800 (17 800 to 21 000)	6250 (5370 to 7230)	26 000 (23 700 to 28 000)	2·0% (−0·7 to 5·2)	−2·6% (−5·8 to 2·7)	0·9% (−1·4 to 3·6)	503·0 (451·5 to 534·9)	163·2 (139·9 to 188·5)	334·3 (304·7 to 360·5)	−8·3% (−10·6 to −5·5)*	−13·0% (−15·8 to −8·2)*	−9·4% (−11·5 to −6·9)*
Assault by firearm	8690 (7330 to 9500)	968 (804 to 1140)	9660 (8230 to 10 400)	6·8% (3·3 to 10·8)*	−4·5% (−8·6 to 1·3)	5·5% (2·4 to 9·2)*	221·5 (186·8 to 242·3)	25·4 (21·0 to 30·0)	124·4 (105·8 to 134·0)	−2·7% (−5·8 to 1·0)	−13·6% (−17·2 to −8·4)*	−3·7% (−6·6 to −0·4)*
Assault by sharp object	4150 (3240 to 5180)	959 (792 to 1100)	5110 (4170 to 6160)	−11·8% (−15·8 to −6·2)*	−12·6% (−15·7 to −7·4)*	−11·9% (−15·5 to −7·1)*	105·1 (81·7 to 131·1)	24·8 (20·4 to 28·3)	65·2 (53·2 to 78·5)	−21·0% (−24·6 to −16·0)*	−22·1% (−25 to −17·5)*	−21·2% (−24·3 to −16·9)*
Sexual violence	370 (248 to 540)	1770 (1190 to 2570)	2140 (1450 to 3110)	9·4% (7·7 to 11·1)*	13·0% (10·7 to 15·1)*	12·4% (10·2 to 14·3)*	9·4 (6·3 to 13·8)	45·4 (30·6 to 66·1)	27·3 (18·5 to 39·7)	−1·7% (−2·3 to−1·0)*	1·0% (−0·2 to 2·3)	0·6% (−0·6 to 1·6)
Assault by other means	6540 (5750 to 7730)	2550 (2140 to 2990)	9090 (8190 to 10 400)	5·9% (0·6 to 11·5)*	−6·9% (−11·3 to 1·5)	1·9% (−1·9 to 6·2)	167·0 (147·0 to 197·4)	67·6 (56·2 to 79·4)	117·4 (105·7 to 134·4)	−6·2% (−10·6 to−1·3)*	−17·0% (−20·9 to−9·2)*	−9·5% (−12·9 to−5·8)*
Conflict and terrorism	6840 (6400 to 7830)	3270 (2820 to 3870)	10 100 (8980 to 11 400)	81·9% (55·6 to 112·4)*	70·8% (48·8 to 99·3)*	78·1% (58·6 to 101·2)*	177·1 (155·8 to 203·0)	90·6 (78·2 to 107·0)	134·1 (119·3 to 151·9)	66·2% (42·8 to 94·5)*	58·4% (36·9 to 86·4)*	63·7% (45·4 to 85·1)*
Executions and police conflict	840 (800 to 894)	179 (170 to 190)	1020 (972 to 1080)	140·7% (123·6 to 160·4)*	280·6% (245·5 to 309·1)*	157·3% (139·7 to 178·0)*	21·4 (20·4 to 22·8)	4·8 (4·5 to 5·1)	13·1 (12·6 to 13·9)	117·3% (101·5 to 135·6)*	242·7% (210·3 to 269·5)*	133·1% (116·6 to 152·4)*

DALY counts are quoted to three significant figures and percentage changes and rates to one decimal place. DALY=disability-adjusted life-year. G6PD=glucose-6-phosphate dehydrogenase. *H influenzae*=*Haemophilus influenzae*. NASH=non-alcoholic steatohepatitis.

* Percentage changes that are statistically significant.

‡ Incidence estimates for stroke represent first-ever stroke only.

## Research in context

### Evidence before this study

The Global Burden of Diseases, Injuries, and Risk Factors Study 2016 (GBD 2016) estimated 333 causes of disability-adjusted life-years (DALYs) for 195 countries and territories from 1990 to 2016. GBD 2016 also provided estimates for life expectancy and healthy life expectancy (HALE) at birth and at age 65 years, by sex, for each location over time. GBD 2016 included analysis of the epidemiological transition as a function of the Socio-demographic Index. The WHO Global Health Estimates has also published estimates of HALE and DALYs, although these estimates largely relied on GBD 2016 results.

### Added value of this study

In GBD 2017, we expanded the scope of the study compared with previous iterations to include subnational estimates for five more countries (Ethiopia, Iran, New Zealand, Norway, and Russia) and 19 additional causes. The new causes estimated are invasive non-typhoidal salmonella disease; liver cancer due to non-alcoholic steatohepatitis; cirrhosis due to non-alcoholic steatohepatitis; myelodysplastic, myeloproliferative, and other haemopoietic neoplasms; benign and in-situ intestinal neoplasms; benign and in-situ cervical and uterine neoplasms; other benign and in-situ neoplasms; subarachnoid haemorrhage; non-rheumatic valvular heart disease; non-rheumatic calcific aortic valve disease; non-rheumatic degenerative mitral valve disease; other non-rheumatic valve diseases; gastro-oesophageal reflux disease; type 1 diabetes; type 2 diabetes; chronic kidney disease due to type 1 diabetes; chronic kidney disease due to type 2 diabetes; poisoning by carbon monoxide; and poisoning by other means. In addition to broadening our estimation by cause, location, and time, a substantial amount of new data were added for GBD 2017. For cause-specific non-fatal estimations, we added new data from epidemiological surveillance, disease registries, scientific literature sources, and survey sources. Similarly, for cause-specific fatal estimation, we added new data from verbal autopsy studies, vital registration, and cancer registries. For age-specific all-cause mortality estimations, we added vital registration data, complete birth history sources, summary birth history sources, and sibling history surveys. These improvements are reflected in the summary measures of population health, DALYs and HALE, reported in this paper. We also provided a more detailed assessment for HALE than in previous GBD papers by examining the following: distinguishing the years of life gained over the past 28 years into years spent in good health and in poor health, by sex, for each location; determining which extra years lived were spent in good health and in poor health for females compared with males for each location; and assessing the male–female difference in HALE and years lived in poor health for the period 1990–2017 across Socio-demographic Index (SDI) quintiles. With increasing longevity, such information has relevance for policy development, health systems planning, and resource allocation.

### Implications of all the available evidence

Over the past 28 years, the world has had tremendous gains in life expectancy; however, in many locations simply gaining years of life has not meant living those years in good health. In some locations, a large proportion of those years are spent in poor health. By distinguishing where, among whom, and how many of these additional years of life gained are spent in good health versus poor health, we have more insight to inform policy, planning, and resource prioritisation for improving health and reducing disparities. Our results showed large disparities in health and disease burden by SDI and sex, suggesting that much could be done to narrow these gaps, such as targeted approaches to reduce risk factors and scale up proven cost-effective interventions to decrease the burden of disease and make additional improvements to HALE more equitable. Our results not only provide the most up-to-date evidence, but also serve as a baseline for evaluating the effectiveness of interventions and programmes over time.

## GBD 2017 DALYs and HALE Collaborators

Hmwe Hmwe Kyu, Degu Abate, Kalkidan Hassen Abate, Solomon M Abay, Cristiana Abbafati, Nooshin Abbasi, Hedayat Abbastabar, Foad Abd-Allah, Jemal Abdela, Ahmed Abdelalim, Ibrahim Abdollahpour, Rizwan Suliankatchi Abdulkader, Molla Abebe, Zegeye Abebe, Olifan Zewdie Abil, Victor Aboyans, Aklilu Roba Abrham, Laith Jamal Abu-Raddad, Niveen M E Abu-Rmeileh, Manfred Mario Kokou Accrombessi, Dilaram Acharya, Pawan Acharya, Ilana N Ackerman, Abdu A Adamu, Oladimeji M Adebayo, Victor Adekanmbi, Zanfina Ademi, Olatunji O Adetokunboh, Mina G Adib, Jose C Adsuar, Kossivi Agbelenko Afanvi, Mohsen Afarideh, Ashkan Afshin, Gina Agarwal, Kareha M Agesa, Rakesh Aggarwal, Sargis Aghasi Aghayan, Anurag Agrawal, Alireza Ahmadi, Mehdi Ahmadi, Hamid Ahmadieh, Muktar Beshir Ahmed, Sayem Ahmed, Amani Nidhal Aichour, Ibtihel Aichour, Miloud Taki Eddine Aichour, Tomi Akinyemiju, Nadia Akseer, Ziyad Al-Aly, Ayman Al-Eyadhy, Hesham M Al-Mekhlafi, Rajaa M Al-Raddadi, Fares Alahdab, Khurshid Alam, Tahiya Alam, Alaa Alashi, Seyed Moayed Alavian, Kefyalew Addis Alene, Mehran Alijanzadeh, Reza Alizadeh-Navaei, Syed Mohamed Aljunid, Ala’a Alkerwi, François Alla, Peter Allebeck, Jordi Alonso, Ubai Alsharif, Khalid Altirkawi, Nelson Alvis-Guzman, Leopold N Aminde, Erfan Amini, Mohammadreza Amiresmaili, Walid Ammar, Yaw Ampem Amoako, Nahla Hamed Anber, Catalina Liliana Andrei, Sofia Androudi, Megbaru Debalkie Animut, Mina Anjomshoa, Mustafa Geleto Ansha, Carl Abelardo T Antonio, Palwasha Anwari, Jalal Arabloo, Olatunde Aremu, Johan Ärnlöv, Amit Arora, Megha Arora, Al Artaman, Krishna K Aryal, Hamid Asayesh, Zerihun Ataro, Marcel Ausloos, Leticia Avila-Burgos, Euripide F G A Avokpaho, Ashish Awasthi, Beatriz Paulina Ayala Quintanilla, Rakesh Ayer, Peter S Azzopardi, Arefeh Babazadeh, Hamid Badali, Kalpana Balakrishnan, Ayele Geleto Bali, Maciej Banach, Joseph Adel Mattar Banoub, Aleksandra Barac, Miguel A Barboza, Suzanne Lyn Barker-Collo, Till Winfried Bärnighausen, Simon Barquera, Lope H Barrero, Shahrzad Bazargan-Hejazi, Neeraj Bedi, Ettore Beghi, Masoud Behzadifar, Meysam Behzadifar, Bayu Begashaw Bekele, Eyasu Tamru Bekru, Abate Bekele Belachew, Yihalem Abebe Belay, Michelle L Bell, Aminu K Bello, Derrick A Bennett, Isabela M Bensenor, Adugnaw Berhane, Eduardo Bernabe, Robert S Bernstein, Mircea Beuran, Tina Beyranvand, Neeraj Bhala, Samir Bhatt, Soumyadeep Bhaumik, Zulfiqar A Bhutta, Belete Biadgo, Molly H Biehl, Ali Bijani, Boris Bikbov, Ver Bilano, Nigus Bililign, Muhammad Shahdaat Bin Sayeed, Donal Bisanzio, Tone Bjørge, Archie Bleyer, Eshetu Mulisa Bobasa, Ibrahim R Bou-Orm, Soufiane Boufous, Rupert Bourne, Oliver J Brady, Luisa C Brant, Carol Brayne, Alexandra Brazinova, Nicholas J K Breitborde, Hermann Brenner, Paul Svitil Briant, Andrey Nikolaevich Briko, Gabrielle Britton, Traolach Brugha, Rachelle Buchbinder, Reinhard Busse, Zahid A Butt, Lucero Cahuana-Hurtado, Julio Cesar Campuzano Rincon, Jorge Cano, Rosario Cárdenas, Juan J Carrero, Austin Carter, Félix Carvalho, Carlos A Castañeda-Orjuela, Jacqueline Castillo Rivas, Franz Castro, Ferrán Catalá-López, Kelly M Cercy, Ester Cerin, Yazan Chaiah, Jung-Chen Chang, Fiona J Charlson, Vijay Kumar Chattu, Peggy Pei-Chia Chiang, Abdulaal Chitheer, Jee-Young J Choi, Hanne Christensen, Devasahayam J Christopher, Sheng-Chia Chung, Flavia M Cicuttini, Massimo Cirillo, Daniel Collado-Mateo, Cyrus Cooper, Paolo Angelo Cortesi, Monica Cortinovis, Ewerton Cousin, Michael H Criqui, Elizabeth A Cromwell, Marita Cross, John A Crump, Alemneh Kabeta Daba, Berihun Assefa Dachew, Abel Fekadu Dadi, Lalit Dandona, Rakhi Dandona, Paul I Dargan, Ahmad Daryani, Rajat Das Gupta, José Das Neves, Tamirat Tesfaye Dasa, Dragos Virgil Davitoiu, Fernando Pio De La Hoz, Diego De Leo, Jan-Walter De Neve, Hans De Steur, Meaza Girma Degefa, Louisa Degenhardt, Selina Deiparine, Gebre Teklemariam Demoz, Edgar Denova-Gutiérrez, Kebede Deribe, Nikolaos Dervenis, Don C Des Jarlais, Subhojit Dey, Samath D Dharmaratne, Meghnath Dhimal, Mesfin Tadese Dinberu, M Ashworth Dirac, Shirin Djalalinia, Linh Doan, Klara Dokova, David Teye Doku, E Ray Dorsey, Kerrie E Doyle, Tim Robert Driscoll, Manisha Dubey, Eleonora Dubljanin, Eyasu Ejeta Duken, Bruce B Duncan, Andre R Duraes, Hedyeh Ebrahimi, Soheil Ebrahimpour, Michelle M Echko, Dumessa Edessa, David Edvardsson, Andem Effiong, Anne Elise Eggen, Joshua R Ehrlich, Charbel El Bcheraoui, Ziad El-Khatib, Iqbal R F Elyazar, Ahmadali Enayati, Melese Linger Endalifer, Aman Yesuf Endries, Benjamin Er, Holly E Erskine, Sharareh Eskandarieh, Alireza Esteghamati, Sadaf Esteghamati, Hamed Fakhim, Mahbobeh Faramarzi, Mohammad Fareed, Farzaneh Farhadi, Talha A Farid, Carla Sofia E sá Farinha, Andrea Farioli, Andre Faro, Farshad Farzadfar, Ali Akbar Fazaeli, Valery L Feigin, Netsanet Fentahun, Seyed-Mohammad Fereshtehnejad, Eduarda Fernandes, Joao C Fernandes, Alize J Ferrari, Manuela L Ferreira, Irina Filip, Florian Fischer, Christina Fitzmaurice, Nataliya A Foigt, Kyle J Foreman, Tahvi D Frank, Takeshi Fukumoto, Nancy Fullman, Thomas Fürst, João M Furtado, Emmanuela Gakidou, Seana Gall, Silvano Gallus, Morsaleh Ganji, Alberto L Garcia-Basteiro, William M Gardner, Abadi Kahsu Gebre, Amanuel Tesfay Gebremedhin, Teklu Gebrehiwo Gebremichael, Tilayie Feto Gelano, Johanna M Geleijnse, Ricard Genova-Maleras, Yilma Chisha Dea Geramo, Peter W Gething, Kebede Embaye Gezae, Mohammad Rasoul Ghadami, Keyghobad Ghadiri, Maryam Ghasemi-Kasman, Mamata Ghimire, Aloke Gopal Ghoshal, Paramjit Singh Gill, Tiffany K Gill, Ibrahim Abdelmageed Ginawi, Giorgia Giussani, Elena V Gnedovskaya, Ellen M Goldberg, Srinivas Goli, Hector Gómez-Dantés, Philimon N Gona, Sameer Vali Gopalani, Taren M Gorman, Alessandra C Goulart, Bárbara Niegia Garcia Goulart, Ayman Grada, Giuseppe Grosso, Harish Chander Gugnani, Francis Guillemin, Yuming Guo, Prakash C Gupta, Rahul Gupta, Rajeev Gupta, Tanush Gupta, Reyna Alma Gutiérrez, Bishal Gyawali, Juanita A Haagsma, Vladimir Hachinski, Nima Hafezi-Nejad, Hassan Haghparast Bidgoli, Tekleberhan B Hagos, Tewodros Tesfa Hailegiyorgis, Arvin Haj-Mirzaian, Arya Haj-Mirzaian, Randah R Hamadeh, Samer Hamidi, Alexis J Handal, Graeme J Hankey, Yuantao Hao, Hilda L Harb, Sivadasanpillai Harikrishnan, Hamidreza Haririan, Josep Maria Haro, Hadi Hassankhani, Hamid Yimam Hassen, Rasmus Havmoeller, Roderick J Hay, Simon I Hay, Akbar Hedayatizadeh-Omran, Behzad Heibati, Delia Hendrie, Andualem Henok, Ileana Heredia-Pi, Claudiu Herteliu, Fatemeh Heydarpour, Pouria Heydarpour, Desalegn Tsegaw Hibstu, Hans W Hoek, Howard J Hoffman, Michael K Hole, Enayatollah Homaie Rad, Praveen Hoogar, H Dean Hosgood, Seyed Mostafa Hosseini, Mehdi Hosseinzadeh, Mihaela Hostiuc, Sorin Hostiuc, Peter J Hotez, Damian G Hoy, Mohamed Hsairi, Aung Soe Htet, John J Huang, Kim Moesgaard Iburg, Chad Thomas Ikeda, Olayinka Stephen Ilesanmi, Seyed Sina Naghibi Irvani, Caleb Mackay Salpeter Irvine, Sheikh Mohammed Shariful Islam, Farhad Islami, Kathryn H Jacobsen, Leila Jahangiry, Nader Jahanmehr, Sudhir Kumar Jain, Mihajlo Jakovljevic, Spencer L James, Achala Upendra Jayatilleke, Panniyammakal Jeemon, Ravi Prakash Jha, Vivekanand Jha, John S Ji, Catherine O Johnson, Jost B Jonas, Jitendra Jonnagaddala, Zahra Jorjoran Shushtari, Ankur Joshi, Jacek Jerzy Jozwiak, Suresh Banayya Jungari, Mikk Jürisson, Zubair Kabir, Rajendra Kadel, Amaha Kahsay, Rizwan Kalani, Tanuj Kanchan, Chittaranjan Kar, Manoochehr Karami, Behzad Karami Matin, André Karch, Corine Karema, Narges Karimi, Seyed M Karimi, Amir Kasaeian, Dessalegn H Kassa, Getachew Mullu Kassa, Tesfaye Dessale Kassa, Nicholas J Kassebaum, Srinivasa Vittal Katikireddi, Anil Kaul, Norito Kawakami, Zhila Kazemi, Ali Kazemi Karyani, Masoud Masoud Keighobadi, Peter Njenga Keiyoro, Laura Kemmer, Grant Rodgers Kemp, Andre Pascal Kengne, Andre Keren, Yousef Saleh Khader, Behzad Khafaei, Morteza Abdullatif Khafaie, Alireza Khajavi, Nauman Khalid, Ibrahim A Khalil, Ejaz Ahmad Khan, Muhammad Shahzeb Khan, Muhammad Ali Khan, Young-Ho Khang, Mona M Khater, Mohammad Khazaei, Abdullah T Khoja, Ardeshir Khosravi, Mohammad Hossein Khosravi, Aliasghar A Kiadaliri, Zelalem Teklemariam Kidanemariam, Daniel N Kiirithio, Cho-Il Kim, Daniel Kim, Young-Eun Kim, Yun Jin Kim, Ruth W Kimokoti, Yohannes Kinfu, Adnan Kisa, Katarzyna Kissimova-Skarbek, Ann Kristin Skrindo Knudsen, Jonathan M Kocarnik, Sonali Kochhar, Yoshihiro Kokubo, Tufa Kolola, Jacek A Kopec, Soewarta Kosen, Georgios A Kotsakis, Parvaiz A Koul, Ai Koyanagi, Kewal Krishan, Sanjay Krishnaswami, Kristopher J Krohn, Barthelemy Kuate Defo, Burcu Kucuk Bicer, G Anil Kumar, Manasi Kumar, Igor Kuzin, Deepesh P Lad, Sheetal D Lad, Alessandra Lafranconi, Ratilal Lalloo, Tea Lallukka, Faris Hasan Lami, Justin J Lang, Sinéad M Langan, Van C Lansingh, Arman Latifi, Kathryn Mei-Ming Lau, Jeffrey V Lazarus, Janet L Leasher, Jorge R Ledesma, Paul H Lee, James Leigh, Mostafa Leili, Cheru Tesema Leshargie, Janni Leung, Miriam Levi, Sonia Lewycka, Shanshan Li, Yichong Li, Xiaofeng Liang, Yu Liao, Misgan Legesse Liben, Lee-Ling Lim, Stephen S Lim, Miteku Andualem Limenih, Shai Linn, Shiwei Liu, Katharine J Looker, Alan D Lopez, Stefan Lorkowski, Paulo A Lotufo, Rafael Lozano, Tim C D Lucas, Raimundas Lunevicius, Ronan A Lyons, Stefan Ma, Erlyn Rachelle King Macarayan, Mark T Mackay, Emilie R Maddison, Fabiana Madotto, Dhaval P Maghavani, Hue Thi Mai, Marek Majdan, Reza Majdzadeh, Azeem Majeed, Reza Malekzadeh, Deborah Carvalho Malta, Abdullah A Mamun, Ana-Laura Manda, Helena Manguerra, Mohammad Ali Mansournia, Ana Maria Mantilla Herrera, Lorenzo Giovanni Mantovani, Joemer C Maravilla, Wagner Marcenes, Ashley Marks, Francisco Rogerlândio Martins-Melo, Ira Martopullo, Winfried März, Melvin B Marzan, João Massano, Benjamin Ballard Massenburg, Manu Raj Mathur, Pallab K Maulik, Mohsen Mazidi, Colm McAlinden, John J McGrath, Martin McKee, Brian J McMahon, Suresh Mehata, Ravi Mehrotra, Kala M Mehta, Varshil Mehta, Fabiola Mejia-Rodriguez, Tesfa Mekonen, Addisu Melese, Mulugeta Melku, Peter T N Memiah, Ziad A Memish, Walter Mendoza, Getnet Mengistu, George A Mensah, Seid Tiku Mereta, Atte Meretoja, Tuomo J Meretoja, Tomislav Mestrovic, Bartosz Miazgowski, Tomasz Miazgowski, Anoushka I Millear, Ted R Miller, G K Mini, Mojde Mirarefin, Andreea Mirica, Erkin M Mirrakhimov, Awoke Temesgen Misganaw, Philip B Mitchell, Habtamu Mitiku, Babak Moazen, Bahram Mohajer, Karzan Abdulmuhsin Mohammad, Moslem Mohammadi, Noushin Mohammadifard, Mousa Mohammadnia-Afrouzi, Mohammed A Mohammed, Shafiu Mohammed, Farnam Mohebi, Ali H Mokdad, Mariam Molokhia, Lorenzo Monasta, Julio Cesar Montañez, Mahmood Moosazadeh, Ghobad Moradi, Mahmoudreza Moradi, Maziar Moradi-Lakeh, Mehdi Moradinazar, Paula Moraga, Lidia Morawska, Ilais Moreno Velásquez, Joana Morgado-Da-Costa, Shane Douglas Morrison, Marilita M Moschos, Seyyed Meysam Mousavi, Kalayu Brhane Mruts, Achenef Asmamaw Muche, Kindie Fentahun Muchie, Ulrich Otto Mueller, Oumer Sada Muhammed, Satinath Mukhopadhyay, Kate Muller, John Everett Mumford, G V S Murthy, Kamarul Imran Musa, Ghulam Mustafa, Ashraf F Nabhan, Chie Nagata, Gabriele Nagel, Mohsen Naghavi, Aliya Naheed, Azin Nahvijou, Gurudatta Naik, Farid Najafi, Hae Sung Nam, Vinay Nangia, Jobert Richie Nansseu, Nahid Neamati, Ionut Negoi, Ruxandra Irina Negoi, Subas Neupane, Charles Richard James Newton, Josephine W Ngunjiri, Anh Quynh Nguyen, Grant Nguyen, Ha Thu Nguyen, Huong Lan Thi Nguyen, Huong Thanh Nguyen, Long Hoang Nguyen, Minh Nguyen, Nam Ba Nguyen, Son Hoang Nguyen, Emma Nichols, Dina Nur Anggraini Ningrum, Molly R Nixon, Shuhei Nomura, Mehdi Noroozi, Bo Norrving, Jean Jacques Noubiap, Hamid Reza Nouri, Malihe Nourollahpour Shiadeh, Mohammad Reza Nowroozi, Elaine O Nsoesie, Peter S Nyasulu, Christopher M Odell, Richard Ofori-Asenso, Felix Akpojene Ogbo, In-Hwan Oh, Olanrewaju Oladimeji, Andrew T Olagunju, Tinuke O Olagunju, Pedro R Olivares, Helen Elizabeth Olsen, Bolajoko Olubukunola Olusanya, Jacob Olusegun Olusanya, Kanyin L Ong, Sok King Ong, Eyal Oren, Alberto Ortiz, Erika Ota, Stanislav S Otstavnov, Simon Øverland, Mayowa Ojo Owolabi, Mahesh P A, Rosana Pacella, Abhijit P Pakhare, Amir H Pakpour, Adrian Pana, Songhomitra Panda-Jonas, Eun-Kee Park, James Park, Charles D H Parry, Hadi Parsian, Yahya Pasdar, Shanti Patel, Snehal T Patil, Ajay Patle, George C Patton, Vishnupriya Rao Paturi, Deepak Paudel, Katherine R Paulson, Neil Pearce, Alexandre Pereira, David M Pereira, Norberto Perico, Konrad Pesudovs, Max Petzold, Hai Quang Pham, Michael R Phillips, David M Pigott, Julian David Pillay, Michael A Piradov, Meghdad Pirsaheb, Farhad Pishgar, Oleguer Plana-Ripoll, Suzanne Polinder, Svetlana Popova, Maarten J Postma, Akram Pourshams, Hossein Poustchi, Dorairaj Prabhakaran, Swayam Prakash, V Prakash, Narayan Prasad, Caroline A Purcell, Mostafa Qorbani, D Alex Quistberg, Amir Radfar, Anwar Rafay, Alireza Rafiei, Fakher Rahim, Kazem Rahimi, Zohreh Rahimi, Afarin Rahimi-Movaghar, Vafa Rahimi-Movaghar, Mahfuzar Rahman, Mohammad Hifz Ur Rahman, Muhammad Aziz Rahman, Sajjad Ur Rahman, Rajesh Kumar Rai, Fatemeh Rajati, Prabhat Ranjan, Puja C Rao, Davide Rasella, David Laith Rawaf, Salman Rawaf, K Srinath Reddy, Robert C Reiner, Marissa Bettay Reitsma, Giuseppe Remuzzi, Andre M N Renzaho, Serge Resnikoff, Satar Rezaei, Mohammad Sadegh Rezai, Antonio Luiz P Ribeiro, Nicholas L S Roberts, Stephen R Robinson, Leonardo Roever, Luca Ronfani, Gholamreza Roshandel, Ali Rostami, Gregory A Roth, Dietrich Rothenbacher, Enrico Rubagotti, Perminder S Sachdev, Nafis Sadat, Ehsan Sadeghi, Sahar Saeedi Moghaddam, Hosein Safari, Yahya Safari, Roya Safari-Faramani, Mahdi Safdarian, Sare Safi, Saeid Safiri, Rajesh Sagar, Amirhossein Sahebkar, Mohammad Ali Sahraian, Haniye Sadat Sajadi, Nasir Salam, Joseph S Salama, Payman Salamati, Zikria Saleem, Yahya Salimi, Hamideh Salimzadeh, Joshua A Salomon, Sundeep Santosh Salvi, Inbal Salz, Abdallah M Samy, Juan Sanabria, Maria Dolores Sanchez-Niño, Damian Francesco Santomauro, Itamar S Santos, João Vasco Santos, Milena M Santric Milicevic, Bruno Piassi Sao Jose, Mayank Sardana, Abdur Razzaque Sarker, Rodrigo Sarmiento-Suárez, Nizal Sarrafzadegan, Benn Sartorius, Shahabeddin Sarvi, Brijesh Sathian, Maheswar Satpathy, Arundhati R Sawant, Monika Sawhney, Sonia Saxena, Elke Schaeffner, Maria Inês Schmidt, Ione J C Schneider, Aletta Elisabeth Schutte, David C Schwebel, Falk Schwendicke, James G Scott, Mario Sekerija, Sadaf G Sepanlou, Edson Serván-Mori, Seyedmojtaba Seyedmousavi, Hosein Shabaninejad, Azadeh Shafieesabet, Mehdi Shahbazi, Amira A Shaheen, Masood Ali Shaikh, Mehran Shams-Beyranvand, Mohammadbagher Shamsi, Heidar Sharafi, Kiomars Sharafi, Mehdi Sharif, Mahdi Sharif-Alhoseini, Jayendra Sharma, Rajesh Sharma, Jun She, Aziz Sheikh, Peilin Shi, Kenji Shibuya, Mekonnen Sisay Shiferaw, Mika Shigematsu, Rahman Shiri, Reza Shirkoohi, Ivy Shiue, Yalda Shokoohinia, Farhad Shokraneh, Haitham Shoman, Mark G Shrime, Si Si, Soraya Siabani, Abla Mehio Sibai, Tariq J Siddiqi, Inga Dora Sigfusdottir, Rannveig Sigurvinsdottir, Diego Augusto Santos Silva, João Pedro Silva, Dayane Gabriele Alves Silveira, Narayana Sarma Venkata Singam, Jasvinder A Singh, Narinder Pal Singh, Virendra Singh, Dhirendra Narain Sinha, Eirini Skiadaresi, Vegard Skirbekk, Karen Sliwa, David L Smith, Mari Smith, Adauto Martins Soares Filho, Badr Hasan Sobaih, Soheila Sobhani, Moslem Soofi, Reed J D Sorensen, Ireneous N Soyiri, Luciano A Sposato, Chandrashekhar T Sreeramareddy, Vinay Srinivasan, Jeffrey D Stanaway, Vladimir I Starodubov, Dan J Stein, Caitlyn Steiner, Timothy J Steiner, Mark A Stokes, Lars Jacob Stovner, Michelle L Subart, Agus Sudaryanto, Mu’awiyyah Babale Sufiyan, Gerhard Sulo, Bruno F Sunguya, Patrick John Sur, Bryan L Sykes, P N Sylaja, Dillon O Sylte, Cassandra E I Szoeke, Rafael Tabarés-Seisdedos, Takahiro Tabuchi, Santosh Kumar Tadakamadla, Nikhil Tandon, Segen Gebremeskel Tassew, Mohammad Tavakkoli, Nuno Taveira, Hugh R Taylor, Arash Tehrani-Banihashemi, Tigist Gashaw Tekalign, Shishay Wahdey Tekelemedhin, Merhawi Gebremedhin Tekle, Mohamad-Hani Temsah, Omar Temsah,Abdullah Sulieman Terkawi, Belay Tessema, Mebrahtu Teweldemedhin, Kavumpurathu Raman Thankappan, Andrew Theis, Sathish Thirunavukkarasu, Nihal Thomas, Binyam Tilahun, Quyen G To, Marcello Tonelli, Roman Topor-Madry, Anna E Torre, Miguel Tortajada-Girbés, Mathilde Touvier, Marcos Roberto Tovani-Palone, Jeffrey A Towbin, Bach Xuan Tran, Khanh Bao Tran, Christopher E Troeger, Afewerki Gebremeskel Tsadik, Derrick Tsoi, Lorainne Tudor Car, Stefanos Tyrovolas, Kingsley Nnanna Ukwaja, Irfan Ullah, Eduardo A Undurraga, Rachel L Updike, Muhammad Shariq Usman, Olalekan A Uthman, Muthiah Vaduganathan, Afsane Vaezi, Pascual R Valdez, Elena Varavikova, Santosh Varughese, Tommi Juhani Vasankari, Narayanaswamy Venketasubramanian, Santos Villafaina, Francesco S Violante, Sergey Konstantinovitch Vladimirov, Vasily Vlassov, Stein Emil Vollset, Theo Vos, Kia Vosoughi, Isidora S Vujcic, Fasil Shiferaw Wagnew, Yasir Waheed, Yafeng Wang, Yuan-Pang Wang, Elisabete Weiderpass, Robert G Weintraub, Daniel J Weiss, Fitsum Weldegebreal, Kidu Gidey Weldegwergs, Andrea Werdecker, T Eoin West, Ronny Westerman, Harvey A Whiteford, Justyna Widecka, Tissa Wijeratne, Hywel C Williams, Lauren B Wilner, Shadrach Wilson, Andrea Sylvia Winkler, Alison B Wiyeh, Charles Shey Wiysonge, Charles D A Wolfe, Anthony D Woolf, Grant M A Wyper, Denis Xavier, Gelin Xu, Simon Yadgir, Seyed Hossein Yahyazadeh Jabbari, Tomohide Yamada, Lijing L Yan, Yuichiro Yano, Mehdi Yaseri, Yasin Jemal Yasin, Alex Yeshaneh, Ebrahim M Yimer, Paul Yip, Engida Yisma, Naohiro Yonemoto, Seok-Jun Yoon, Marcel Yotebieng, Mustafa Z Younis, Mahmoud Yousefifard, Chuanhua Yu, Vesna Zadnik, Zoubida Zaidi, Sojib Bin Zaman, Mohammad Zamani, Hamed Zandian, Heather J Zar, Zerihun Menlkalew Zenebe, Ben Zipkin, Maigeng Zhou, Sanjay Zodpey, Inbar Zucker, Liesl Joanna Zuhlke, Christopher J L Murray.

## Affiliations

Department of Health Metrics Sciences (H H Kyu PhD, A Afshin MD, E A Cromwell PhD, C El Bcheraoui PhD, Prof E Gakidou PhD, Prof S I Hay FMedSci, I A Khalil MD, Prof S S Lim PhD, Prof R Lozano MD, A T Misganaw PhD, Prof A H Mokdad PhD, Prof M Naghavi MD, D M Pigott DPhil, R C Reiner PhD, Prof D L Smith PhD, J D Stanaway PhD, Prof S E Vollset DrPH, Prof T Vos PhD, Prof C J L Murray DPhil), Institute for Health Metrics and Evaluation (H H Kyu PhD, A Afshin MD, K M Agesa BA, T Alam MPH, M Arora BSA, M H Biehl MPH, P S Briant BS, A Carter MPH, K M Cercy BS, E A Cromwell PhD, Prof L Dandona MD, Prof R Dandona PhD, Prof L Degenhardt PhD, S Deiparine BA, S D Dharmaratne MD, M A Dirac MD, M M Echko BSc, Prof V L Feigin PhD, C Fitzmaurice MD, K J Foreman PhD, T D Frank BS, N Fullman MPH, Prof E Gakidou PhD, W M Gardner AB, E M Goldberg MPH, T M Gorman BS, Prof S I Hay FMedSci, C T Ikeda BS, C M S Irvine BS, S L James MD, C O Johnson PhD, N J Kassebaum MD, L Kemmer PhD, G R Kemp BA, I A Khalil MD, J M Kocarnik PhD, K J Krohn MPH, K M Lau BS, J R Ledesma BA, Prof S S Lim PhD, Prof A D Lopez PhD, Prof R Lozano MD, E R Maddison BS, H Manguerra BS, A Marks MA, I Martopullo MPH, A I Millear MPH, A T Misganaw PhD, Prof A H Mokdad PhD, K Muller MPH, J E Mumford BA, Prof M Naghavi MD, G Nguyen MPH, M Nguyen BS, E Nichols BA, M R Nixon PhD, E O Nsoesie PhD, C M Odell MPP, H E Olsen MA, K L Ong PhD, K R Paulson BS, D M Pigott DPhil, C A Purcell BA, P C Rao MPH, R C Reiner PhD, M B Reitsma BS, N L S Roberts BS, N Sadat MA, J S Salama MSc, Prof D L Smith PhD, M Smith MPA, R J D Sorensen MPH, V Srinivasan BA, M L J D Stanaway PhD, C Steiner MPH, M Subart BA, P J Sur MPH, D O Sylte BA, A Theis BA, A E Torre BS, C E Troeger MPH, D Tsoi BS, R L Updike BA, Prof S E Vollset DrPH, Prof T Vos PhD, Prof H A Whiteford PhD, L B Wilner MPH, S Wilson BS, S Yadgir BS, B Zipkin BA, Prof C J L Murray DPhil), Department of Global Health (F J Charlson PhD, S Kochhar MD, R J D Sorensen MPH), Division of Hematology (C Fitzmaurice MD), Department of Neurology (R Kalani MD), Department of Periodontics (G A Kotsakis DDS), Division of Plastic Surgery (B B Massenburg MD), Department of Medicine (B J McMahon MD, T E West MD), Department of Surgery (S D Morrison MD), Department of Biomedical Informatics and Medical Education (E O Nsoesie PhD), Division of Cardiology, Department of Medicine (G A Roth MD), University of Washington, Seattle, WA, USA (I Kuzin MPH, Prof E Oren PhD); School of Pharmacy (J Abdela MSc, D Edessa MSc, G Mengistu MSc, M S Shiferaw MSc), Department of Pediatrics (A R Abrham MSc), Department of Medical Laboratory Science (Z Ataro MSc, F Weldegebreal MPH, H Mitiku MSc), School of Public Health (A G Bali MPH, M G Tekle MPH), School of Nursing and Midwifery (T T Dasa MSc), College of Health and Medical Sciences (Z T Kidanemariam MSc), Haramaya University, Harar, Ethiopia (D Abate MSc, T F Gelano MSc, T Hailegiyorgis MSc, H Mitiku MSc, T G Tekalign MS); Department of Population and Family Health (K H Abate PhD, A T Gebremedhin MPH), Department of Epidemiology (M B Ahmed MPH), School of Pharmacy (E M Bobasa MSc), Mycobacteriology Research Center (E Duken MSc), Department of Environmental Health Sciences and Technology (S Mereta PhD), Jimma University, Jimma, Ethiopia; Department of Pharmacology and Clinical Pharmacy (S M Abay PhD, O S Muhammed MSc), School of Public Health (A Berhane PhD, K Deribe PhD, Y J Yasin MPH), School of Allied Health Sciences (E Yisma MPH), Addis Ababa University, Addis Ababa, Ethiopia (G T Demoz MSc); Department of Law Philosophy and Economic Studies, La Sapienza University, Rome, Italy (C Abbafati PhD); Non-communicable Diseases Research Center (N Abbasi MD, F Farzadfar MD, S N Irvani MD, S Saeedi Moghaddam MSc, M Shams-Beyranvand MSc, H Ebrahimi MD, B Mohajer MD, F Mohebi MD, F Pishgar MD), Department of Health (H Abbastabar PhD), Endocrinology and Metabolism Research Center (M Afarideh MD, Prof A Esteghamati MD, M Ganji MD), Department of Urology (E Amini MD), Department of Health Management and Economics (M Anjomshoa PhD, S Mousavi PhD), Liver and Pancreaticobilliary Disease Research Center (H Ebrahimi MD), Multiple Sclerosis Research Center (S Eskandarieh PhD, P Heydarpour MD, B Mohajer MD, Prof M Sahraian MD), Endocrine Research Center (S Esteghamati MD), School of Medicine (N Hafezi-Nejad MD), Department of Pharmacology (A Haj-Mirzaian MD), Department of Epidemiology and Biostatistics (Prof S Hosseini PhD, M Mansournia PhD, M Yaseri PhD), Hematologic Malignancies Research Center (A Kasaeian PhD), Knowledge Utilization Research Center (Prof R Majdzadeh PhD), Digestive Diseases Research Institute (Prof R Malekzadeh MD, Prof A Pourshams MD, H Poustchi PhD, G Roshandel PhD, H Salimzadeh PhD, S G Sepanlou MD), Iran National Institute of Health Research (F Mohebi MD, H S Sajadi PhD), Cancer Research Center (A Nahvijou PhD, R Shirkoohi PhD), Uro-Oncology Research Center (M Nowroozi MD, F Pishgar MD), Iranian National Center for Addiction Studies (Prof A Rahimi-Movaghar MD), Sina Trauma and Surgery Research Center (Prof V Rahimi-Movaghar MD, M Safdarian MD, Prof P Salamati MD, M Sharif-Alhoseini PhD), Center of Expertise in Microbiology (Prof S Seyedmousavi PhD), Cancer Biology Research Center (R Shirkoohi PhD), Department of Anatomy (S Sobhani MD), Hematology-Oncology and Stem Cell Transplantation Research Center (A Kasaeian PhD), Community-Based Participatory-Research Center (Prof R Majdzadeh PhD), Tehran University of Medical Sciences, Tehran, Iran; Montreal Neuroimaging Center, McGill University, Montreal, QC, Canada (N Abbasi MD); Department of Neurology (Prof F Abd-Allah MD, Prof A Abdelalim MD), Department of Medical Parasitology (M M Khater MD), Cairo University, Cairo, Egypt; Department of Epidemiology, Arak University of Medical Sciences, Arak, Iran (I Abdollahpour PhD); Multiple Sclerosis Research Center, Tehran, Iran (I Abdollahpour PhD); Department of Statistics, Manonmaniam Sundaranar University, Tirunelveli, India (R S Abdulkader MD); Department of Clinical Chemistry (M Abebe MSc, B Biadgo MSc), Human Nutrition Department (Z Abebe MSc), Institute of Public Health (K A Alene MPH, B Bekele MPH, B A Dachew MPH, A F Dadi MPH, M A Limenih MSc, M Melku MSc, A A Muche MPH, K Muchie MSc, B Tilahun PhD), Department of Medical Microbiology (B Tessema PhD), University of Gondar, Gondar, Ethiopia; Department of Medical Laboratory Sciences (O Abil MSc), Department of Health Sciences (E Duken MSc), Wollega University, Nekemte, Ethiopia; School of Public Health, University of Medical Science, Ondo, Ondo, Nigeria (O Abil MSc); Department of Cardiology, Dupuytren University Hospital, Limoges, France (Prof V Aboyans MD); Institute of Epidemiology, University of Limoges, Limoges, France (Prof V Aboyans MD); Department of Healthcare Policy and Research, Weill Cornell Medical College in Qatar, Doha, Qatar (Prof L J Abu-Raddad PhD); Institute of Community and Public Health, Birzeit University, Birzeit, Palestine (N M E Abu-Rmeileh PhD); Bénin Clinical Research Institute (IRCB), Cotonou, Benin (M M K Accrombessi PhD, E F A Avokpaho MD); Department of Preventive Medicine, Dongguk University, Gyeongju, South Korea (D Acharya MPH); Department of Community Medicine, Kathmandu University, Devdaha, Nepal (D Acharya MPH); Department of Non-Communicable Diseases (B Gyawali MPH), Nepal Development Society, Pokhara, Nepal (P Acharya MPH); School of Public Health and Preventive Medicine (I N Ackerman PhD, Z Ademi PhD, Prof F M Cicuttini PhD, Prof Y Guo PhD, S Li PhD, S Si PhD), Department of Epidemiology and Preventive Medicine (Prof R Buchbinder PhD), Department of Medicine (S Gall PhD), Centre of Cardiovascular Research and Education in Therapeutics (R Ofori-Asenso MSc), Monash University, Melbourne, VIC, Australia; Department of Global Health (A A Adamu MSc, O O Adetokunboh MD, Prof C S Wiysonge MD), Faculty of Medicine & Health Sciences (Prof P S Nyasulu PhD), Department of Psychiatry (Prof C D H Parry PhD), Stellenbosch University, Cape Town, South Africa; Cochrane South Africa (A A Adamu MSc, O O Adetokunboh MD), Unit for Hypertension and Cardiovascular Disease (Prof A E Schutte PhD), South African Medical Research Council, Cape Town, South Africa (Prof D J Stein MD); Department of Medicine, University College Hospital, Ibadan, Ibadan, Nigeria (O M Adebayo MD); School of Medicine, Cardiff University, Cardiff, UK (V Adekanmbi PhD); Emergency Department, Saint Mark Hospital, Alexandria, Egypt (M G Adib MD); Sport Science Department (J C Adsuar PhD, S Villafaina MSc), University of Extremadura, Badajoz, Spain (D Collado-Mateo MSc); Department of Public Health, University of Lomé, Lomé, Togo (K A Afanvi MD); Prefectoral Direction of the Health of the Zio, Ministry of Health and Social Protection, Tsevie, Togo (K A Afanvi MD); Department of Family Medicine (G Agarwal MD), Department of Pathology and Molecular Medicine (T O Olagunju MD), McMaster University, Hamilton, ON, Canada; Department of Gastroenterology (Prof R Aggarwal MD), Department of Nephrology (S Prakash PhD, Prof N Prasad MD), Sanjay Gandhi Postgraduate Institute of Medical Sciences, Lucknow, India; Department of Zoology, Yerevan State University, Yerevan, Armenia (S A Aghayan PhD); Research Group of Molecular Parasitology, Scientific Center of Zoology and Hydroecology, Yerevan, Armenia (S A Aghayan PhD); Research Area for Informatics and Big Data, CSIR Institute of Genomics and Integrative Biology, Delhi, India (Prof A Agrawal PhD); Department of Internal Medicine (Prof A Agrawal PhD), National School of Tropical Medicine (Prof P J Hotez PhD), Baylor College of Medicine, Houston, TX, USA; Department of Anesthesiology (A Ahmadi PhD), Sleep Disorders Research Center (M Ghadami MD), Faculty of Nutrition and Food Sciences (F Heydarpour PhD), Faculty of Public Health (B Karami Matin PhD, A Kazemi Karyani PhD, R Safari-Faramani PhD), Department of Urology (Prof M Moradi MD), Research Center for Environmental Determinants of Health (M Moradinazar PhD), Department of Epidemiology & Biostatistics (Prof F Najafi PhD, Y Salimi PhD), Department of Nutritional Sciences (Y Pasdar PhD), Department of Clinical Biochemistry (Prof Z Rahimi PhD), Department of Health Education & Promotion (F Rajati PhD), Environmental Determinants of Health Research Center (S Rezaei PhD, M Soofi PhD), Department of Food Technology & Quality Control (E Sadeghi PhD), Sports Medicine & Rehabilitation (M Shamsi PhD), Pharmaceutical Sciences Research Center (Prof Y Shokoohinia PhD), Imam Ali Cardiovascular Research Center (S Siabani PhD), Kermanshah University of Medical Sciences, Kermanshah, Iran (K Ghadiri BEP, Prof M Pirsaheb PhD, Y Safari PhD, K Sharafi PhD); Environmental Technologies Research Center (M Ahmadi PhD), Department of Public Health (M A Khafaie PhD), Thalassemia and Hemoglobinopathy Research Center (F Rahim PhD), Department of Neurosurgery (H Safari MD), Ahvaz Jundishapur University of Medical Sciences, Ahvaz, Iran; Department of Ophthalmology (H Ahmadieh MD), Research Institute for Endocrine Sciences (A Haj-Mirzaian MD, S N Irvani MD), Safety Promotion and Injury Prevention Research Center (N Jahanmehr PhD), Department of Biostatistics (A Khajavi MSc), Ophthalmic Research Center (S Safi PhD, M Yaseri PhD, H Ahmadieh MD), School of Public Health (N Jahanmehr PhD), Ophthalmic Epidemiology Research Center (S Safi PhD), Shahid Beheshti University of Medical Sciences, Tehran, Iran; Health Systems and Population Studies Division (S Ahmed MSc), Initiative for Non Communicable Diseases (A Naheed PhD), Health Economics and Financing Research Group (A R Sarker MHE), Maternal and Child Health Division (S Zaman MPH), International Centre for Diarrhoeal Disease Research, Dhaka, Bangladesh; Department of Learning, Informatics, Management, and Ethics (S Ahmed MSc), Department of Public Health Sciences (Prof P Allebeck MD, Z El-Khatib PhD), Department of Neurobiology (Prof J Ärnlöv PhD), Department of Medical Epidemiology and Biostatistics (J J Carrero PhD, Prof E Weiderpass PhD), Department of Neurobiology, Care Sciences and Society (S Fereshtehnejad PhD), Karolinska Institutet, Stockholm, Sweden; University Ferhat Abbas of Setif, Algeria (A Aichour BMedSc, I Aichour BPharm); Higher National School of Veterinary Medicine, Algiers, Algeria (M Aichour MA); Department of Epidemiology, University of Kentucky, Lexington, KY, USA (T Akinyemiju PhD); The Hospital for Sick Children (N Akseer PhD), The Centre for Global Child Health, Hospital for Sick Children (Prof Z A Bhutta PhD), Dalla Lana School of Public Health (S Popova PhD), University of Toronto, Toronto, ON, Canada; Internal Medicine Department, Washington University in St Louis, St Louis, MO, USA (Z Al-Aly MD); Clinical Epidemiology Center, VA St Louis Health Care System, Department of Veterans Affairs, St Louis, MO, USA (Z Al-Aly MD); Pediatric Intensive Care Unit (A Al-Eyadhy MD), Department of Pediatrics (B H Sobaih MD, M Temsah MRCPCH), King Saud University, Riyadh, Saudi Arabia (K Altirkawi MD); Medical Research Center (H M Al-Mekhlafi PhD), Jazan University, Jazan, Saudi Arabia (Prof N Bedi MD); Department of Medical Parasitology, Sana’a University, Sana’a, Yemen (H M Al-Mekhlafi PhD); Department of Family and Community Medicine, King Abdulaziz University, Jeddah, Saudi Arabia (Prof R M Al-Raddadi PhD); Evidence Based Practice Center, Mayo Clinic Foundation for Medical Education and Research, Rochester, MN, USA (F Alahdab MD); School of Population and Global Health (K Alam PhD), School of Medicine (Prof G J Hankey MD), University of Western Australia, Perth, WA, Australia; Cardiovascular Medicine, Cleveland Clinic, Cleveland, OH, USA (A Alashi MD); Baqiyatallah Research Center for Gastroenterology and Liver Diseases (Prof S Alavian MD), Student Research Committee (M Khosravi MD), Baqiyatallah University of Medical Sciences, Tehran, Iran; Research School of Population Health (K A Alene MPH), National Centre for Epidemiology and Population Health (M Bin Sayeed MSPS), Australian National University, Canberra, ACT, Australia; Department of Public Health (Prof A H Pakpour PhD), Qazvin University of Medical Sciences, Qazvin, Iran (M Alijanzadeh PhD); Gastrointestinal Cancer Research Center (R Alizadeh-Navaei PhD), Department of Medical Mycology (H Badali PhD), Toxoplasmosis Research Center (Prof A Daryani PhD, S Sarvi PhD), School of Public Health (Prof A Enayati PhD), Department of Neurology (N Karimi MD), Department of Physiology and Pharmacology (M Mohammadi PhD), Health Sciences Research Center (M Moosazadeh PhD), Molecular and Cell Biology Research Center (Prof A Rafiei PhD), Department of Pediatrics (M Rezai MD), Invasive Fungi Research Center (Prof S Seyedmousavi PhD), Department of Medical Mycology and Parasitology (A Vaezi PhD), Immunogenetics Research Center (N Karimi MD), Pharmaceutical Sciences Research Center (M M Keighobadi PhD), Department of Immunology (Prof A Rafiei PhD), Mazandaran University of Medical Sciences, Sari, Iran (A Hedayatizadeh-Omran PhD, M M Keighobadi PhD, M Nourollahpour Shiadeh PhD); Department of Health Policy and Management, Kuwait University, Safat, Kuwait (Prof S M Aljunid PhD); International Centre for Casemix and Clinical Coding, National University of Malaysia, Bandar Tun Razak, Malaysia (Prof S M Aljunid PhD); Department of Population Health, Luxembourg Institute of Health, Strassen, Luxembourg (A Alkerwi PhD); University of Bordeaux, Bordeaux, France (Prof F Alla PhD); Swedish Research Council for Health, Working Life, and Welfare, Stockholm, Sweden (Prof P Allebeck MD); Research Program in Epidemiology & Public Health, Hospital Del Mar Medical Research Institute, Barcelona, Spain (Prof J Alonso MD); Department of Experimental and Health Sciences, Pompeu Fabra University, Barcelona, Spain (Prof J Alonso MD); Department of Oral and Maxillofacial Surgery, University Hospital Knappschaftskrankenhaus Bochum, Bochum, Germany (U Alsharif MD); Research Group on Health Economics, University of Cartagena, Cartagena, Colombia (Prof N Alvis-Guzman PhD); Research Group in Hospital Management and Health Policies, University of the Coast, Barranquilla, Colombia (Prof N Alvis-Guzman PhD); School of Public Health (L N Aminde MD, F J Charlson PhD, B A Dachew MPH, H E Erskine PhD, A J Ferrari PhD, A M Mantilla Herrera MHEcon, D F Santomauro PhD, J G Scott PhD), Queensland Alliance for Agriculture and Food Innovations (E M Bobasa MSc), School of Dentistry (R Lalloo PhD), Institute for Social Science Research (A A Mamun PhD, J C Maravilla PhD), Queensland Brain Institute (Prof J J McGrath MD), The University of Queensland, Brisbane, QLD, Australia (J Leung PhD, Prof H A Whiteford PhD); Department of Health Management, Policy and Economics, Kerman University of Medical Sciences, Kerman, Iran (M Amiresmaili PhD); Department of the Health Industrial Complex and Innovation in Health (Prof D A Silveira MD), Department of Diseases and Noncommunicable Diseases and Health Promotion (A M Soares Filho DSc), Federal Ministry of Health, Beirut, Lebanon (Prof W Ammar PhD); Faculty of Health Sciences (Prof W Ammar PhD), Department of Epidemiology and Population Health (Prof A M Sibai PhD), American University of Beirut, Beirut, Lebanon; Department of Internal Medicine, Komfo Anokye Teaching Hospital, Kumasi, Ghana (Y A Amoako MD); Faculty of Medicine (N H Anber PhD), Mansoura University, Mansoura, Egypt; Emergency Hospital of Bucharest (Prof M Beuran PhD, I Negoi PhD), Department of General Surgery (D V Davitoiu PhD, M Hostiuc PhD), Department of Legal Medicine and Bioethics (S Hostiuc PhD), Anatomy and Embryology Department (R I Negoi PhD), Carol Davila University of Medicine and Pharmacy, Bucharest, Romania (C Andrei PhD); Department of Medicine, University of Thessaly, Volos, Greece (S Androudi PhD); Department of Public Health (Y C D Geramo MSc), Arba Minch University, Arba Minch, Ethiopia (M D Animut MPH); Department of Public Health (M G Ansha MPH, T Kolola MPH, K B Mruts MPH), Department of Midwifery (M T Dinberu MA), Debre Berhan University, Debre Berhan, Ethiopia; Social Determinants of Health Research Center, Rafsanjan University of Medical Sciences, Rafsanjan, Iran (M Anjomshoa PhD); Department of Health Policy and Administration (C T Antonio MD), Development and Communication Studies (E K Macarayan PhD), University of the Philippines Manila, Manila, Philippines; Department of Applied Social Sciences (C T Antonio MD), School of Nursing (P H Lee PhD), Hong Kong Polytechnic University, Hong Kong, China; Independent Consultant, Kabul, Afghanistan (P Anwari MD); Health Management and Economics Research Center (J Arabloo PhD, M Behzadifar PhD), Air Pollution Research Center (B Heibati PhD), Preventive Medicine and Public Health Research Center (M Moradi-Lakeh MD, K Vosoughi MD, A Tehrani-Banihashemi PhD), Department of Neuroscience (M Safdarian MD), Department of Health Policy (H Shabaninejad PhD), Department of Community Medicine (A Tehrani-Banihashemi PhD), Physiology Research Center (M Yousefifard PhD), Iran University of Medical Sciences, Tehran, Iran (T Beyranvand PhD, F Farhadi MD, M Hosseinzadeh PhD); School of Health Sciences, Birmingham City University, Birmingham, UK (O Aremu PhD); School of Health and Social Studies, Dalarna University, Falun, Sweden (Prof J Ärnlöv PhD); School of Science and Health (A Arora PhD), School of Social Sciences and Psychology (Prof A M N Renzaho PhD), Western Sydney University, Sydney, NSW, Australia (F A Ogbo PhD); Oral Health Services, Sydney Local Health District, Sydney, NSW, Australia (A Arora PhD); Department of Community Health Sciences, University of Manitoba, Winnipeg, MB, Canada (A Artaman PhD); Monitoring Evaluation and Operational Research Project, Abt Associates Nepal, Lalitpur, Nepal (K K Aryal PhD); Qom University of Medical Sciences, Qom, Iran (H Asayesh MSc); School of Business (Prof M Ausloos PhD), Department of Health Sciences (Prof T Brugha MD), University of Leicester, Leicester, UK; Center for Health Systems Research (L Avila-Burgos PhD, L Cahuana-Hurtado PhD, H Gómez-Dantés MSc, Prof I Heredia-Pi DipSocSc, Prof E Serván-Mori DSc), Center for Nutrition and Health Research (E Denova-Gutiérrez DSc), Centro de Investigacion en Nutricion y Salud (F Mejia-Rodriguez MSc), National Institute of Public Health, Cuernavaca, Mexico (S Barquera PhD, J Campuzano Rincon PhD); Contrôle Des Maladies Infectieuses, Laboratory of Studies and Research-Action in Health, Porto Novo, Benin (E F A Avokpaho MD); Indian Institute of Public Health, Gandhinagar, India (A Awasthi PhD); Indian Institute of Public Health, Hyderabad (Prof G V S Murthy MD), Indian Institute of Public Health (Prof S Zodpey PhD), Public Health Foundation of India, Gurugram, India (A Awasthi PhD, Prof L Dandona MD, Prof R Dandona PhD, G Kumar PhD, M R Mathur PhD, Prof D Prabhakaran DM, K S Reddy DM); The Judith Lumley Centre (B Ayala Quintanilla PhD), School of Nursing and Midwifery (Prof D Edvardsson PhD), Austin Clinical School of Nursing (M Rahman PhD), Department of Psychology and Counselling (Prof T Wijeratne MD), La Trobe University, Melbourne, VIC, Australia; General Office for Research and Technological Transfer, Peruvian National Institute of Health, Lima, Peru (B Ayala Quintanilla PhD); Department of Community and Global Health (R Ayer MHSc), Department of Mental Health (Prof N Kawakami PhD), Department of Global Health Policy (S Nomura MSc, Prof K Shibuya MD), Department of Diabetes and Metabolic Diseases (T Yamada MD), University of Tokyo, Tokyo, Japan; Global Adolescent Health Group, Burnet Institute, Melbourne, VIC, Australia (P S Azzopardi PhD); Wardliparingga Aboriginal Research Unit, South Australian Health and Medical Research Institute, Adelaide, SA, Australia (P S Azzopardi PhD); Center for Infectious Diseases Research, Babol, Iran (A Babazadeh MD, S Ebrahimpour PhD); Department of Environmental Health Engineering, Sri Ramachandra Medical College and Research Institute, Chennai, India (Prof K Balakrishnan PhD); Department of Hypertension, Medical University of Lodz, Lodz, Poland (Prof M Banach PhD); Polish Mothers’ Memorial Hospital Research Institute, Lodz, Poland (Prof M Banach PhD); Faculty of Medicine, Alexandria University, Alexandria, Egypt (J A M Banoub MD); Department of Transplant Services, University Hospital Foundation Santa Fe de Bogotá, Bogotá, Colombia (J A M Banoub MD); Clinic for Infectious and Tropical Diseases, Clinical Center of Serbia, Belgrade, Serbia (A Barac PhD); Faculty of Medicine (A Barac PhD, E Dubljanin PhD), Centre School of Public Health and Health Management (Prof M M Santric Milicevic PhD), Faculty of Medicine Institute of Epidemiology (I S Vujcic PhD), University of Belgrade, Belgrade, Serbia; Department of Neurosciences (Prof M A Barboza MD), Area de Estadística, Dirección Actuarial (Prof J Castillo Rivas MSc), Costa Rican Department of Social Security, San Jose, Costa Rica; School of Medicine (Prof M A Barboza MD), School of Dentistry (Prof J Castillo Rivas MSc), University of Costa Rica, San Pedro, Costa Rica; School of Psychology (S L Barker-Collo PhD), Molecular Medicine and Pathology (K B Tran MD), University of Auckland, Auckland, New Zealand; Institute of Public Health (Prof T W Bärnighausen MD, B Moazen MSc, S Mohammed PhD), Institute of Global Health (Prof J De Neve MD), Department of Ophthalmology (Prof J B Jonas MD), Medical Clinic V (Prof W März MD), Augenpraxis Jonas (S Panda-Jonas MD), Heidelberg University, Heidelberg, Germany; Department of Global Health and Population (Prof T W Bärnighausen MD), T H Chan School of Public Health (P C Gupta DSc), Ariadne Labs (E K Macarayan PhD), Department of Biostatistics (J Park MS), Department of Genetics (A Pereira PhD), Division of General Internal Medicine and Primary Care (Prof A Sheikh MD), Heart and Vascular Center (M Vaduganathan MD), Harvard University, Boston, MA, USA (M G Shrime MD); Department of Industrial Engineering, Pontifical Javeriana University, Bogota, Colombia (Prof L H Barrero DSc); Department of Psychiatry, Charles R Drew University of Medicine and Science, Los Angeles, CA, USA (S Bazargan-Hejazi BEP); Department of Psychiatry and Biobehavioral Sciences, University of California Los Angeles, Los Angeles, CA, USA (S Bazargan-Hejazi BEP); Department of Community Medicine, Gandhi Medical College Bhopal, Bhopal, India (Prof N Bedi MD); Department of Neuroscience (E Beghi MD, G Giussani PhD), Department of Renal Medicine (B Bikbov MD, N Perico MD), Department of Oncology (M Cortinovis PhD), Department of Environmental Health Science (S Gallus DSc), Mario Negri Institute for Pharmacological Research, Milan, Italy (Prof G Remuzzi MD); Social Determinants of Health Research Center (M Behzadifar PhD), Lorestan University of Medical Sciences, Khorramabad, Iran (M Behzadifar MS); Public Health Department (B Bekele MPH, H Y Hassen MPH), Mizan-Tepi University, Teppi, Ethiopia (A Henok MPH); College of Health Science and Medicine, Wolaita Sodo University, Sodo, Ethiopia (E T Bekru MSc); School of Public Health (A B Belachew MSc), Department of Nutrition and Dietetics (M G Degefa BSc, A Kahsay MPH), School of Pharmacy (A K Gebre MSc, T G Gebremichael MSc, A G Tsadik MSc, E M Yimer MSc), Department of Biostatistics (K Gezae MSc), Anatomy Unit (T B Hagos MSc), Clinical Pharmacy Unit (T D Kassa MSc, K G Weldegwergs MSc), Department of Midwifery (Z M Zenebe MSc), Mekelle University, Mekelle, Ethiopia (S G Tassew MSc, S W Tekelemedhin MPH); Department of Public Health (Y A Belay MPH, C T Leshargie MPH), Department of Nursing (D H Kassa MSc, F S Wagnew MSc), College of Health Sciences (G M Kassa MSc), Debre Markos University, Debre Markos, Ethiopia; School of Forestry and Environmental Studies (Prof M L Bell PhD), Department of Ophthalmology and Visual Science (Prof J J Huang MD), Yale University, New Haven, CT, USA; Department of Medicine, University of Alberta, Edmonton, AB, Canada (A K Bello PhD); Nuffield Department of Population Health (D A Bennett PhD), Big Data Institute, Nuffield Department of Medicine (Prof P W Gething PhD, D J Weiss PhD), Centre for Tropical Medicine and Global Health, Nuffield Department of Medicine (S Lewycka PhD), The Big Data Institute (T C D Lucas PhD), Department of Psychiatry (Prof C R J Newton MD), Nuffield Department of Women’s and Reproductive Health (Prof K Rahimi MD), University of Oxford, Oxford, UK (Prof V Jha MD); Department of Internal Medicine (I M Bensenor PhD, Prof I S Santos PhD), Division of Ophthalmology (J M Furtado MD), University Hospital, Internal Medicine Department (A C Goulart PhD), Department of Medicine (Prof P A Lotufo DrPH), Laboratory of Genetics and Molecular Cardiology (A Pereira PhD), Department of Pathology and Legal Medicine (M R Tovani-Palone MSc), Department of Psychiatry (Y Wang PhD), Center for Clinical and Epidemiological Research (A C Goulart PhD), University of São Paulo, São Paulo, Brazil; Dental Institute (E Bernabe PhD), Faculty of Life Sciences and Medicine (Prof P I Dargan MB, M Molokhia PhD), St John’s Institute of Dermatology (Prof R J Hay MD), Division of Patient and Population (Prof W Marcenes PhD), School of Population Health & Environmental Sciences (Prof C D A Wolfe MD), King’s College London, London, UK; Hubert Department of Global Health, Emory University, Atlanta, GA, USA (R S Bernstein MD); Department of Global Health, University of South Florida, Tampa, FL, USA (R S Bernstein MD); Institutes of Applied Health Research and Translational Medicine, Queen Elizabeth Hospital Birmingham, Birmingham, UK (N Bhala DPhil); IAHR/ITM, University of Birmingham, Birmingham, UK (N Bhala DPhil); Department of Epidemiology and Biostatistics (V Bilano PhD), Department of Primary Care and Public Health (Prof A Majeed MD, Prof S Rawaf PhD), WHO Collaborating Centre for Public Health Education and Training (D L Rawaf MD, H Shoman MPH), School of Public Health (Prof S Saxena MD), Division of Brain Sciences (Prof T J Steiner PhD), Imperial College London, London, UK (S Bhatt PhD); The George Institute for Global Health, New Delhi, India (S Bhaumik MBBS, Prof V Jha MD, P K Maulik PhD); Center of Excellence in Women and Child Health, Aga Khan University, Karachi, Pakistan (Prof Z A Bhutta PhD); Social Determinants of HealthResearch Center (A Bijani PhD), Health Research Institute (M Ghasemi-Kasman PhD), Department of Immunology (M Mohammadnia-Afrouzi PhD), Department of Clinical Biochemistry (N Neamati MSc, H Parsian PhD), Cellular and Molecular Biology Research Center (H Nouri PhD), Infectious Diseases and Tropical Medicine Research Center (A Rostami PhD), Department of Microbiology and Immunology (M Shahbazi PhD), Student Research Committee (M Zamani MD), Babol University of Medical Sciences, Babol, Iran (M Faramarzi PhD); Woldia University, Woldia, Ethiopia (N Bililign BHlthSci, M L Endalifer MPH); Department of Clinical Pharmacy and Pharmacology, University of Dhaka, Ramna, Bangladesh (M Bin Sayeed MSPS); Global Health Division, Research Triangle Institute International, Research Triangle Park, NC, USA (D Bisanzio PhD); School of Medicine (D Bisanzio PhD, F Shokraneh MS), Centre of Evidence-based Dermatology (Prof H C Williams DSc), University of Nottingham, Nottingham, UK; Department of Global Public Health and Primary Care (Prof T Bjørge PhD), Department of Psychosocial Science (A S Knudsen PhD, Prof S Øverland PhD), University of Bergen, Bergen, Norway; Department of Research (Prof E Weiderpass PhD), Cancer Registry of Norway, Oslo, Norway (Prof T Bjørge PhD); Radiation Medicine (A Bleyer MD), Department of Surgery (S Krishnaswami MD), Oregon Health and Science University, Portland, OR, USA; Department of Pediatrics, University of Texas, Houston, TX, USA (A Bleyer MD); Department of Vital and Health Statistics (H L Harb MPH), Department of Disease, Epidemics, and Pandemics Control (J Nansseu MD), Ministry of Public Health, Beirut, Lebanon (I R Bou-Orm MD); Transport and Road Safety Research (S Boufous PhD), National Drug and Alcohol Research Centre (Prof L Degenhardt PhD), School of Public Health and Community Medicine (J Jonnagaddala PhD), School of Medicine (P K Maulik PhD), School of Psychiatry (Prof P B Mitchell MD, Prof P S Sachdev MD), University of New South Wales, Sydney, NSW, Australia; Vision & Eye Research Unit, Anglia Ruskin University, Cambridge, UK (Prof R Bourne MD); Department of Infectious Disease Epidemiology (O J Brady PhD), Department of Disease Control (J Cano PhD), Faculty of Epidemiology and Population Health (S M Langan PhD), Department of Health Services Research and Policy (Prof M McKee DSc), Department of Medical Statistics (Prof N Pearce PhD), Department of Non-Communicable Disease Epidemiology (Prof D Prabhakaran DM), London School of Hygiene & Tropical Medicine, London, UK; School of Medicine and Clinical Hospital (Prof L C Brant PhD), Department of Maternal and Child Nursing and Public Health (Prof D C Malta PhD), Hospital of the Federal University of Minas Gerais (Prof A P Ribeiro MD), Post-Graduate Program in Infectious Diseases and Tropical Medicine (B P Sao Jose PhD), Federal University of Minas Gerais, Belo Horizonte, Brazil; Department of Public Health and Primary Care, University of Cambridge, Cambridge, UK (Prof C Brayne MD); Institute of Epidemiology, Comenius University, Bratislava, Slovakia (A Brazinova MD); Department of Psychology (Prof N J K Breitborde PhD), College of Public Health (M Yotebieng PhD), Psychiatry and Behavioral Health Department (Prof N J K Breitborde PhD), The Ohio State University, Columbus, OH, USA; Division of Clinical Epidemiology and Aging Research, German Cancer Research Center, Heidelberg, Germany (Prof H Brenner MD); Committee for Comprehensive Assessment of Medical Devices and Information Technology, Health Technology Assessment Association, Moscow, Russia (A N Briko MSc); Neuroscience, Institute for Scientific Research and High Technology Services, City Of Knowledge, Panama (G Britton PhD); Department of Research and Health Technology Assessment (F Castro MD), Gorgas Memorial Institute for Health Studies, Panama City, Panama (G Britton PhD, I Moreno Velásquez PhD); Monash Department of Clinical Epidemiology, Cabrini Institute, Melbourne, VIC, Australia (Prof R Buchbinder PhD); Institute of Public Health (Prof R Busse PhD, Prof E Schaeffner MD), Department of Operative and Preventive Dentistry (Prof F Schwendicke MPH), Charité University Medical Center Berlin, Berlin, Germany; School of Population and Public Health (Z A Butt PhD, Prof N Sarrafzadegan MD), University of British Columbia, Vancouver, BC, Canada (J A Kopec PhD); Al Shifa School of Public Health, Al Shifa Trust Eye Hospital, Rawalpindi, Pakistan (Z A Butt PhD); School of Medicine, University of the Valley of Cuernavaca, Cuernavaca, Mexico (J Campuzano Rincon PhD); Department of Population and Health, Metropolitan Autonomous University, Mexico City, Mexico (Prof R Cárdenas DSc); Institute of Public Health (Prof F Carvalho PhD), Institute of Biomedical Engineering (J Das Neves PhD), REQUIMTE/LAQV (Prof E Fernandes PhD, Prof D M Pereira PhD), Department of Clinical Neurosciences and Mental Health, Faculty of Medicine (J Massano MD), Department of Community Medicine (J V Santos MD), UCIBIO (J P Silva PhD), Applied Molecular Biosciences Unit (Prof F Carvalho PhD), Institute for Research and Innovation in Health (J Das Neves PhD), University of Porto, Porto, Portugal; Colombian National Health Observatory (C A Castañeda-Orjuela MD), National Institute on Deafness and Other Communication Disorders (H J Hoffman MA), National Institute of Health, Bogota, Colombia; Epidemiology and Public Health Evaluation Group (C A Castañeda-Orjuela MD), Department of Public Health (Prof F P De La Hoz PhD), National University of Colombia, Bogota, Colombia; Department of Health Planning and Economics, Institute of Health Carlos III, Madrid, Spain (F Catalá-López PhD); Mary MacKillop Institute for Health Research (Prof E Cerin PhD), The Brain Institute (Prof C E I Szoeke PhD), Australian Catholic University, Melbourne, VIC, Australia; School of Public Health (Prof E Cerin PhD), Centre for Suicide Research and Prevention (Prof P Yip PhD), University of Hong Kong, Hong Kong, China (Prof P Yip PhD); College of Medicine, Alfaisal University, Riyadh, Saudi Arabia (Y Chaiah, Prof Z A Memish MD, M Temsah MRCPCH, O Temsah); College of Medicine, National Taiwan University, Taipei, Taiwan (J Chang PhD); Faculty of Medical Sciences, University of the West Indies, St Augustine, Trinidad and Tobago (V Chattu MD); Independent Consultant, Athens, Greece (V Chattu MD); Clinical Governance, Gold Coast Health, Gold Coast, QLD, Australia (P P Chiang PhD); Ministry of Health, Baghdad, Iraq (A Chitheer MD); Biochemistry, Biomedical Science, Seoul National University Hospital, Seoul, South Korea (J J Choi PhD); Institute of Clinical Medicine and Bispebjerg Hospital, University of Copenhagen, Copenhagen, Denmark (Prof H Christensen DMSci); Department of Pulmonary Medicine (Prof D J Christopher MD), Department of Endocrinology (Prof N Thomas PhD), Christian Medical College and Hospital, Vellore, India (Prof S Varughese MD); Department of Health Informatics (S Chung PhD), Institute for Global Health (H Haghparast Bidgoli PhD), Department of Psychology (M Kumar PhD), Department of Epidemiology and Public Health (M R Mathur PhD), University College London, London, UK; Health Data Research UK, London, UK (S Chung PhD); Scuola Medica Salernitana, University of Salerno, Baronissi, Italy (M Cirillo MD); Faculty of Education (D Collado-Mateo MSc), Institute of Physical Activity and Health (Prof P R Olivares PhD), Autonomous University of Chile, Talca, Chile; NIHR Oxford Biomedical Research Centre, University of Southampton, Southampton, UK (Prof C Cooper MEd); School of Medicine and Surgery, University of Milan Bicocca, Monza, Italy (P A Cortesi PhD, A Lafranconi MD, F Madotto PhD, Prof L G Mantovani DSc); Postgraduate Program in Epidemiology, Federal University of Rio Grande do Sul, Porto Alegre, Brazil (E Cousin MS, B B Duncan MD, Prof B N G Goulart DSc, Prof M I Schmidt PhD); Department of Family Medicine and Public Health, University of California San Diego, La Jolla, CA, USA (Prof M H Criqui MD); Institute of Bone and Joint Research (M Cross PhD, M L Ferreira PhD), Sydney School of Public Health (Prof T R Driscoll PhD), Sydney Medical School (S Islam PhD), Asbestos Diseases Research Institute (J Leigh MD), University of Sydney, Sydney, NSW, Australia (D G Hoy PhD, M A Mohammed PhD); Centre for International Health, University of Otago, Dunedin, New Zealand (Prof J A Crump MD); Division of Infectious Diseases and International Health (Prof J A Crump MD), Duke Global Health Institute (L L Yan PhD), Duke University, Durham, NC, USA; College of Medicine and Health Sciences (A K Daba MSc), Department of Reproductive Health (D T Hibstu MPH), Hawassa University, Hawassa, Ethiopia; Discipline of Public Health, Flinders University, Adelaide, SA, Australia (A F Dadi MPH); Clinical Toxicology Service (Prof P I Dargan MB), Biomedical Research Council (Prof C D A Wolfe MD), Guy’s and St Thomas’ NHS Foundation Trust, London, UK; James P Grant School of Public Health (R Das Gupta MPH), Research and Evaluation Division (M Rahman PhD), BRAC University, Dhaka, Bangladesh; Department of Surgery, Clinical Emergency Hospital Sf Pantelimon, Bucharest, Romania (D V Davitoiu PhD); Australian Institute for Suicide Research and Prevention (Prof D De Leo DSc), Menzies Health Institute Queensland (S K Tadakamadla PhD), Griffith University, Mount Gravatt, QLD, Australia; Department of Agricultural Economics, Ghent University, Ghent, Belgium (H De Steur PhD); Department of Clinical Pharmacy (G T Demoz MSc), Department of Medical Laboratory Sciences (M Teweldemedhin MSc), Aksum University, Aksum, Ethiopia; Department of Global Health and Infection, Brighton and Sussex Medical School, Brighton, UK (K Deribe PhD); Information Services Division (G M A Wyper MSc), National Health Service Scotland, Edinburgh, UK (N Dervenis MD); Aristotle University of Thessaloniki, Thessaloniki, Greece (N Dervenis MD); Department of Psychiatry, Icahn School of Medicine at Mount Sinai, New York, NY, USA (Prof D C Des Jarlais PhD); Disha Foundation, Gurgaon, India (S Dey PhD); Department of Community Medicine, University of Peradeniya, Peradeniya, Sri Lanka (S D Dharmaratne MD); Health Research Section, Nepal Health Research Council, Kathmandu, Nepal (M Dhimal PhD); Swedish Family Medicine – First Hill, Seattle, WA, USA (M A Dirac MD); Deputy of Research and Technology (S Djalalinia PhD), Ministry of Health and Medical Education, Tehran, Iran (Z Kazemi MSc, A Khosravi PhD); Center of Excellence in Health Service Management (L Doan BMedSc), Center for Excellence in Behavioral Health (L H Nguyen PhD), Center of Excellence in Behavioral Medicine (N B Nguyen MD, S H Nguyen BS), Nguyen Tat Thanh University, Ho Chi Minh City, Vietnam; Department of Social Medicine and Health Care Organisation, Medical University of Varna, Varna, Bulgaria (K Dokova PhD); Department of Population and Health, University of Cape Coast, Cape Coast, Ghana (D T Doku PhD); Faculty of Social Sciences, Health Sciences (D T Doku PhD), Faculty of Health Sciences, Health Sciences (S Neupane PhD), University of Tampere, Tampere, Finland; University of Rochester, Rochester, NY, USA (E Dorsey MD); School of Health and Biomedical Sciences (Prof K E Doyle PhD), Department of Psychology (Prof S R Robinson PhD), Royal Melbourne Institute of Technology University, Bundoora, VIC, Australia; United Nations World Food Programme, New Delhi, India (M Dubey PhD); School of Medicine (Prof A R Duraes PhD), Institute of Public Health (Prof D Rasella PhD), Federal University of Bahia, Salvador, Brazil; Diretoria Médica, Roberto Santos General Hospital, Salvador, Brazil (Prof A R Duraes PhD); Department of Nursing, Umeå University, Umeå, Sweden (Prof D Edvardsson PhD); Clinical Epidemiology and Biostatistics, University of Newcastle, Newcastle, NSW, Australia (A Effiong MB); Department of Community Medicine, University of Tromsø, Tromsø, Norway (Prof A E Eggen PhD); Institute for Health Care Policy and Innovation (J R Ehrlich MD), Department of Ophthalmology and Visual Sciences (J R Ehrlich MD), University of Michigan, Ann Arbor, MI, USA; Eijkman-OxfordClinical Research Unit, Eijkman Institute for Molecular Biology, Jakarta, Indonesia (I R F Elyazar PhD); Public Health Department, Saint Paul’s Hospital Millennium Medical College, Addis Ababa, Ethiopia (A Y Endries MPH); Epidemiology & Disease Control (S Ma PhD), Ministry of Health, Singapore, Singapore (B Er MSc); Psychiatric Epidemiology and Burden of Disease Research (A M Mantilla Herrera MHEcon), Policy and Epidemiology Group (D F Santomauro PhD), Child and Youth Mental Health (J G Scott PhD), Queensland Centre for Mental Health Research, Brisbane, QLD, Australia (H E Erskine PhD, A J Ferrari PhD); Department of Medical Parasitology and Mycology, Urmia University of Medical Science, Urmia, Iran (H Fakhim PhD); College of Medicine (M Fareed PhD), Department of Public Health (A T Khoja MD), Imam Muhammad Ibn Saud Islamic University, Riyadh, Saudi Arabia; Division of Cardiovascular Medicine, University of Louisville, Louisville, KY, USA (T A Farid MD, N V Singam MD); National Statistical Office, Lisbon, Portugal (C S E Farinha MSc); Department of Medical and Surgical Sciences, University of Bologna, Bologna, Italy (A Farioli PhD, Prof F S Violante MPH); Department of Psychology, Federal University of Sergipe, Sao Cristovao, Brazil (Prof A Faro PhD); Social Determinants of Health Research Center (A Fazaeli PhD), Department of Epidemiology (M Karami PhD), Department of Environmental Health Engineering (M Khazaei PhD, M Leili PhD), Hamadan University of Medical Sciences, Hamadan, Iran; National Institute for Stroke and Applied Neurosciences, Auckland University of Technology, Auckland, New Zealand (Prof V L Feigin PhD); Department of Public Health Nutrition (N Fentahun PhD), Department of Psychiatry (T Mekonen MSc), Bahir Dar University, Bahir Dar, Ethiopia; Division of Neurology, University of Ottawa, Ottawa, ON, Canada (S Fereshtehnejad PhD); Center for Biotechnology and Fine Chemistry, Catholic University of Portugal, Porto, Portugal (J C Fernandes PhD); Psychiatry, Kaiser Permanente, Fontana, CA, USA (I Filip MD); Department of Health Sciences (I Filip MD), A T Still University, Mesa, Arizona, USA (A Radfar MD); Department of Public Health Medicine, Bielefeld University, Bielefeld, Germany (F Fischer PhD); Institute of Gerontology, National Academy of Medical Sciences of Ukraine, Kyiv, Ukraine (N A Foigt PhD); Gene Expression & Regulation Program, Cancer Institute, Philadelphia, PA, USA (T Fukumoto PhD); Department of Dermatology, Kobe University, Kobe, Japan (T Fukumoto PhD); Epidemiology and Public Health (T Fürst PhD), Malaria Vaccines (C Karema MPH), Swiss Tropical and Public Health Institute, Basel, Switzerland; University of Basel, Basel, Switzerland (T Fürst PhD); Menzies Institute for Medical Research, University of Tasmania, Hobart, TAS, Australia (S Gall PhD); Tuberculosis, Manhiça Health Research Center, Manhiça, Mozambique (A L Garcia-Basteiro MD); Tuberculosis Department (A L Garcia-Basteiro MD), Barcelona Institute for Global Health, Barcelona, Spain (Prof J V Lazarus PhD); School of Public Health, Curtin University, Perth, WA, Australia (A T Gebremedhin MPH, D Hendrie PhD, T R Miller PhD); Division of Human Nutrition and Health, Wageningen University & Research, Wageningen, Netherlands (Prof J M Geleijnse PhD); Directorate General for Public Health, Regional Health Council, Madrid, Spain (R Genova-Maleras MSc); Department of Health Care Policy and Management, University of Tsukuba, Tsukuba, Japan (M Ghimire MA); Department of Respiratory Medicine, National Allergy, Asthma, and Bronchitis Institute, Kolkota, India (Prof A G Ghoshal MD); Department of Respiratory Medicine, Fortis Hospital, Kolkata, India (Prof A G Ghoshal MD); Unit of Academic Primary Care (Prof P S Gill DM), Division of Health Sciences (O A Uthman PhD), University of Warwick, Coventry, UK; Adelaide Medical School (T K Gill PhD), University of Adelaide, Adelaide, SA, Australia (A T Olagunju MD); Department of Family and Community Medicine, University of Hail, Hail, Saudi Arabia (Prof I A Ginawi MD); Research Center of Neurology, Moscow, Russia (E V Gnedovskaya PhD, Prof M A Piradov DSc); Center for the Study of Regional Development, Jawahar Lal Nehru University, New Delhi, India (S Goli PhD); Department of Population Studies (A Patle MPH), Department of Public Health & Mortality Studies (M H U Rahman MPhil), International Institute for Population Sciences, Mumbai, India (S Goli PhD); Nursing and Health Sciences Department, University of Massachusetts Boston, Boston, MA, USA (P N Gona PhD); Department of Biostatistics and Epidemiology, University of Oklahoma, Oklahoma City, OK, USA (S V Gopalani MPH); Department of Health and Social Affairs, Government of the Federated States of Micronesia, Palikir, Federated States of Micronesia (S V Gopalani MPH); School of Medicine, Boston University, Boston, MA, USA (A Grada MD); Registro Tumori Integrato, Vittorio Emanuele University Hospital Polyclinic, Catania, Italy (G Grosso PhD); Department of Epidemiology (Prof H C Gugnani PhD), Department of Microbiology, (Prof H C Gugnani PhD), Saint James School of Medicine, The Valley, Anguilla; School of Public Health, University of Lorraine, Vandoeuvre-les-Nancy, France (Prof F Guillemin PhD); Department of Epidemiology, Healis Sekhsaria Institute for Public Health, Mumbai, India (P C Gupta DSc, D N Sinha PhD); Commissioner of Public Health, West Virginia Bureau for Public Health, Charleston, WV, USA (Prof R Gupta MD); Department of Health Policy, Management & Leadership, West Virginia University School of Public Health, Morgantown, WV, USA (Prof R Gupta MD); Academics and Research, Rajasthan University of Health Sciences, Jaipur, India (Prof R Gupta MD); Department of Preventive Cardiology, Eternal Heart Care Centre & Research Institute, Jaipur, India (Prof R Gupta MD); Department of Cardiology, Montefiore Medical Center, Bronx, NY, USA (T Gupta MD); Department of Epidemiology and Population Health (H Hosgood PhD), Albert Einstein College of Medicine, Bronx, NY, USA (T Gupta MD); Department of Epidemiology and Psychosocial Research, Ramón de la Fuente Muñiz National Institute of Psychiatry, Mexico City, Mexico (R A Gutiérrez PhD); Department of Public Health (B Gyawali MPH, K M Iburg PhD), National Centre for Register-Based Research (Prof J J McGrath MD, O Plana-Ripoll PhD), Aarhus University, Aarhus, Denmark; Department of Public Health, Erasmus University Medical Center, Rotterdam, Netherlands (J A Haagsma PhD, S Kochhar MD, S Polinder MA); Department of Clinical Neurological Sciences (V Hachinski DSc), Clinical Neurological Sciences (L A Sposato MD), The University of Western Ontario, London, ON, Canada; Lawson Health Research Institute, London, ON, Canada (V Hachinski DSc); Department of Radiology (N Hafezi-Nejad MD, A Haj-Mirzaian MD), Department of Health Policy and Management (A T Khoja MD), Department of Gastroenterology and Hepatology (K Vosoughi MD), Johns Hopkins University, Baltimore, MD, USA; Department of Family and Community Medicine, Arabian Gulf University, Manama, Bahrain (Prof R R Hamadeh DPhil); School of Health and Environmental Studies, Hamdan Bin Mohammed Smart University, Dubai, United Arab Emirates (Prof S Hamidi DrPH); Population Health Department, University of New Mexico, Albuquerque, NM, USA (A J Handal PhD); Neurology Department, Sir Charles Gairdner Hospital, Perth, WA, Australia (Prof G J Hankey MD); Sun Yat-sen Global Health Institute (Prof Y Hao PhD), Department of Medical Statistics and Epidemiology (Y Liao PhD, Prof Y Hao PhD), Sun Yat-sen University, Guangzhou, China; Cardiology Department (Prof S Harikrishnan MD), Health Education and Health Promotion Department (L Jahangiry PhD), Achutha Menon Centre for Health Science Studies (P Jeemon PhD, G K Mini PhD, Prof K R Thankappan MD), Neurology Department (Prof P Sylaja MD), Sree Chitra Tirunal Institute for Medical Sciences and Technology, Trivandrum, India (Prof P Sylaja MD); Tabriz University of Medical Sciences, Tabriz, Iran (H Haririan PhD, H Hassankhani PhD); Research and Development Unit, San Juan de Dios Sanitary Park, Sant Boi De Llobregat, Spain (Prof J M Haro MD, A Koyanagi MD, S Tyrovolas PhD); Department of Medicine (Prof J M Haro MD), University of Barcelona, Barcelona, Spain (S Tyrovolas PhD); Independent Consultant, Tabriz, Iran (H Hassankhani PhD); Unit of Epidemiology and Social Medicine, University Hospital Antwerp, Wilrijk, Belgium (H Y Hassen MPH); Clinical Sciences, Karolinska University Hospital, Stockholm, Sweden (R Havmoeller PhD); International Foundation for Dermatology, London, UK (Prof R J Hay MD); Department of Statistics and Econometrics, Bucharest University of Economic Studies, Bucharest, Romania (Prof C Herteliu PhD, A Mirica PhD, A Pana MD); Department of Psychiatry (Prof H W Hoek MD), University Medical Center Groningen, Groningen, Netherlands (Prof M J Postma PhD); Department of Epidemiology (Prof H W Hoek MD), Department of Health and Behavior Studies (Prof I D Sigfusdottir PhD), Columbia University, New York, NY, USA; Division of Scientific Programs (H J Hoffman MA), Center for Translation Research and Implementation Science (G A Mensah MD), National Institutes of Health, Bethesda, MD, USA; University of Texas at Austin, Austin, TX, USA (M K Hole MD); School of Health (E Homaie Rad PhD), Guilan Road Trauma Research Center (E Homaie Rad PhD), Guilan University of Medical Sciences, Rasht, Iran; Transdisciplinary Centre for Qualitative Methods, Manipal University, Manipal, India (P Hoogar PhD); Department of Computer Science, University of Human Development, Sulaimaniyah, Iraq (M Hosseinzadeh PhD); Department of Internal Medicine, Bucharest Emergency Hospital, Bucharest, Romania (M Hostiuc PhD); Clinical Legal Medicine, National Institute of Legal Medicine Mina Minovici, Bucharest, Romania (S Hostiuc PhD); Faculty of Medicine Tunis, Medicine School of Tunis, Baab Saadoun, Tunisia (Prof M Hsairi MPH); International Relations Division, Ministry of Health and Sports, Myanmar (A Htet MD); Institute of Health and Society (A Htet MD, A S Winkler PhD), Department of Health Management and Health Economics (Prof A Kisa PhD), University of Oslo, Oslo, Norway; Department of Public Health and Community Medicine, University of Liberia, Monrovia, Liberia (O S Ilesanmi PhD); Institute for Physical Activity and Nutrition (S Islam PhD), School of Medicine (M Rahman PhD), Department of Psychology (M A Stokes PhD), Deakin University, Burwood, VIC, Australia; Surveillance and Health Services Research, American Cancer Society, Atlanta, GA, USA (F Islami PhD); Department of Global and Community Health, George Mason University, Fairfax, VA, USA (K H Jacobsen PhD); Department of Parasitic Diseases, National Centre for Disease Control Delhi, Delhi, India (S K Jain MD); Medical Sciences Department, University of Kragujevac, Kragujevac, Serbia (Prof M Jakovljevic PhD); Faculty of Graduate Studies (A U Jayatilleke PhD), Institute of Medicine (A U Jayatilleke PhD), University of Colombo, Colombo, Sri Lanka; Department of Community Medicine, Banaras Hindu University, Varanasi, India (R P Jha MSc); Environmental Research Center (J S Ji DSc), Global Health Research Center (L L Yan PhD), Duke Kunshan University, Kunshan, China; Beijing Institute of Ophthalmology, Beijing Tongren Hospital, Beijing, China (Prof J B Jonas MD); New South Wales Health, Sydney, NSW, Australia (J Jonnagaddala PhD); University of Social Welfare and Rehabilitation Sciences, Tehran, Iran (Z Jorjoran Shushtari MSc, M Noroozi PhD); Centre for Community Medicine (A Joshi MD, A P Pakhare MD), Department of Forensic Medicine and Toxicology (T Kanchan MD), Department of Psychiatry (Prof R Sagar MD), Department of Endocrinology, Metabolism, & Diabetes (Prof N Tandon PhD), All India Institute of Medical Sciences, New Delhi, India; Faculty of Medicine and Health Sciences (J J Jozwiak PhD), Department of Family Medicine and Public Health (J J Jozwiak PhD), University of Opole, Opole, Poland; School of Health Sciences, Savitribai Phule Pune University, Pune, India (S B Jungari MA); Institute of Family Medicine and Public Health, University of Tartu, Tartu, Estonia (M Jürisson PhD); School of Public Health, University College Cork, Cork, UK (Z Kabir PhD); Personal Social Services Research Unit, London School of Economics and Political Science, London, UK (R Kadel MPH); Department of Nephrology, Srirama Chandra Bhanja Medical College and Hospital, Cuttack, India (Prof C Kar MD); Department for Epidemiology, Helmholtz Centre for Infection Research, Braunschweig, Germany (A Karch MD); Quality and Equity Health Care, Kigali, Rwanda (C Karema MPH); School of Interdisciplinary Arts and Sciences, University of Washington Tacoma, Tacoma, WA, USA (S Karimi PhD); Department of Anesthesiology & Pain Medicine, Seattle Children’s Hospital, Seattle, WA, USA (N J Kassebaum MD); MRC/CSO Social and Public Health Sciences Unit, University of Glasgow, Glasgow, UK (S V Katikireddi PhD); School of Health Care Administration, Oklahoma State University, Tulsa, OK, USA (Prof A Kaul MD); Health Care Delivery Sciences, University of Tulsa, Tulsa, OK, USA (Prof A Kaul MD); ODeL Campus (Prof P N Keiyoro PhD), Department of Psychiatry (M Kumar PhD), University of Nairobi, Nairobi, Kenya; Department of Linguistics and Germanic, Slavic, Asian, and African Languages, Michigan State University, East Lansing, MI, USA (G R Kemp BA); Non-Communicable Diseases Research Unit (Prof A P Kengne PhD), Alcohol, Tobacco, & Other Drug Use Research Unit (Prof C D H Parry PhD), Cochrane South Africa (A B Wiyeh MD, Prof C S Wiysonge MD), Medical Research Council South Africa, Cape Town, South Africa; Department of Medicine (Prof A P Kengne PhD, G A Mensah MD, J Noubiap MD, Prof K Sliwa MD, L J Zuhlke PhD), Department of Psychiatry and Mental Health (Prof D J Stein MD), Department of Paediatrics and Child Health (Prof H J Zar PhD, L J Zuhlke PhD), University of Cape Town, Cape Town, South Africa; Institute of Cardiology, Assuta Hospital, Tel Aviv Yaffo, Israel (Prof A Keren MD); Heart Failure and Cardiomyopathies Center, Hadassah Hebrew University Hospital, Jerusalem, Israel (Prof A Keren MD); Department of Public Health and Community Medicine, Jordan University of Science and Technology, Ramtha, Jordan (Prof Y S Khader PhD); Department of Statistics, Azad University, Omidiyeh Branch, Iran (B Khafaei PhD); School of Food and Agricultural Sciences, University of Management and Technology, Lahore, Pakistan (N Khalid PhD); Epidemiology and Biostatistics Department, Health Services Academy, Islamabad, Pakistan (E A Khan MPH); Department of Internal Medicine, John H Stroger Jr Hospital of Cook County, Chicago, IL, USA (M S Khan MD); Department of Internal Medicine, Dow University of Health Sciences, Karachi, Pakistan (M S Khan MD, T J Siddiqi MB, M S Usman MB); Department of Epidemiology (G Naik MPH, J A Singh MD), Department of Medicine (P Ranjan PhD, J A Singh MD), Department of Psychology (D C Schwebel PhD), University of Alabama at Birmingham, Birmingham, AL, USA (M Khan MD, A R Sawant MD); Department of Pediatrics (Prof J A Towbin MD), University of Tennessee, Knoxville, TN, USA (M Khan MD); Institute of Health Policy and Management (Prof Y Khang MD), Department of Health Policy and Management (Prof Y Khang MD), Seoul National University, Seoul, South Korea; International Otorhinolaryngology Research Association, Tehran, Iran (M Khosravi MD); Clinical Epidemiology Unit (A A Kiadaliri PhD), Department of Clinical Sciences (Prof B Norrving PhD), Lund University, Lund, Sweden; Research Department, Kenya Revenue Authority, Nairobi, Kenya (D N Kiirithio MSc); Research and Data Solutions, Synotech Consultant, Nairobi, Kenya (D N Kiirithio MSc); Korea Health Industry Development Institute, Cheongju-si, South Korea (C Kim PhD); Department of Health Sciences, Northeastern University, Boston, MA, USA (Prof D Kim DrPH); Department of Preventive Medicine, Korea University, Seoul, South Korea (Y Kim PhD, Prof S Yoon PhD); School of Medicine, Xiamen University Malaysia, Sepang, Malaysia (Y Kim PhD); Department of Nutrition, Simmons College, Boston, MA, USA (R W Kimokoti MD); Faculty of Health, University of Canberra, Canberra, ACT, Australia (Y Kinfu PhD); Murdoch Childrens Research Institute (Y Kinfu PhD), Neurology Department (M T Mackay PhD), Cardiology Department (R G Weintraub MB), Royal Children’s Hospital, Melbourne, VIC, Australia; Department of Global Community Health and Behavioral Sciences, Tulane University, New Orleans, LA, USA (Prof A Kisa PhD); Department of Health Economics and Social Security (K Kissimova-Skarbek PhD), Institute of Public Health (R Topor-Madry PhD), Jagiellonian University Medical College, Krakow, Poland; Centre for Disease Burden (A S Knudsen PhD), Division of Mental and Physical Health (Prof S Øverland PhD), Norwegian Institute of Public Health, Bergen, Norway (Prof V Skirbekk PhD, G Sulo PhD); Public Health Sciences Division, Fred Hutchinson Cancer Research Center, Seattle, WA, USA (J M Kocarnik PhD); Department of Preventive Cardiology, National Cerebral and Cardiovascular Center, Suita, Japan (Prof Y Kokubo PhD); Arthritis Research Canada, Richmond, BC, Canada (J A Kopec PhD); Independent Consultant, Jakarta, Indonesia (S Kosen MD); Department of Internal and Pulmonary Medicine, Sheri Kashmir Institute of Medical Sciences, Srinagar, India (Prof P A Koul MD); Department of Anthropology, Panjab University, Chandigarh, India (K Krishan PhD); Department of Social and Preventive Medicine (Prof B Kuate Defo PhD), Department of Demography (Prof B Kuate Defo PhD), University of Montreal, Montreal, QC, Canada; Department of Public Health, Yuksek Ihtisas University, Ankara, Turkey (B Kucuk Bicer BEP); Department of Public Health, Hacettepe University, Ankara, Turkey (B Kucuk Bicer BEP); Department of Internal Medicine (D P Lad DM), Department of Pediatrics (S D Lad MD), Post Graduate Institute of Medical Education and Research, Chandigarh, India; Population and Work Ability Program (T Lallukka PhD), Finnish Institute of Occupational Health, Helsinki, Finland (R Shiri PhD); Department of Public Health (T Lallukka PhD), University of Helsinki, Helsinki, Finland (T J Meretoja MD); Department of Community and Family Medicine, Academy of Medical Science, Baghdad, Iraq (F H Lami PhD); Health Promotion and Chronic Disease Prevention Branch, Public Health Agency of Canada, Ottawa, ON, Canada (J J Lang PhD); HelpMeSee, New York, NY, USA (Prof V C Lansingh PhD); International Relations, Mexican Institute of Ophthalmology, Queretaro, Mexico (Prof V C Lansingh PhD); Department of Public Health (A Latifi PhD), Managerial Epidemiology Research Center (S Safiri PhD), Maragheh University of Medical Sciences, Maragheh, Iran; College of Optometry, Nova Southeastern University, Fort Lauderdale, FL, USA (J L Leasher OD); Regional Centre for the Analysis of Data on Occupational and Work-Related Injuries and Diseases, Local Health Unit Tuscany Centre, Florence, Italy (M Levi PhD); Department of Health Sciences, University of Florence, Florence, Italy (M Levi PhD); Oxford University Clinical Research Unit, Wellcome Trust Asia Programme, Hanoi, Vietnam (S Lewycka PhD); Department of Clinical Research and Epidemiology, Shenzhen Sun Yat-Sen Cardiovascular Hospital, Shenzhen, China (Y Li PhD); Noncommunicable Disease Control and Prevention Center (M Zhou PhD), Chinese Center for Disease Control and Prevention, Beijing, China (Prof X Liang MD); Alliance for Improving Health Outcomes, Inc, Quezon City, Philippines (Y Liao PhD); Department of Public Health, Samara University, Samara, Ethiopia (M L Liben MPH); Department of Medicine, University of Malaya, Kuala Lumpur, Malaysia (L Lim MD); Department of Medicine and Therapeutics, The Chinese University of Hong Kong, Shatin, China (L Lim MD); School of Public Health, University of Haifa, Haifa, Israel (Prof S Linn DrPH); Centre for Chronic Disease Control, Beijing, China (Prof S Liu PhD); Population Health Sciences, Bristol Medical School, University of Bristol, Bristol, UK (K J Looker PhD); Department of Paediatrics (M T Mackay PhD, Prof G C Patton MD), School of Health Sciences (A Meretoja MD, Prof C E I Szoeke PhD), School of Populationand Global Health (Prof H R Taylor MD), Department of Medicine (Prof T Wijeratne MD), University of Melbourne, Melbourne, QLD, Australia (Prof A D Lopez PhD); Institute of Nutrition, Friedrich Schiller University Jena, Jena, Germany (Prof S Lorkowski PhD); Competence Cluster for Nutrition and Cardiovascular Health (NUTRICARD), Jena, Germany (Prof S Lorkowski PhD); General Surgery Department, Aintree University Hospital National Health Service (NHS) Foundation Trust, Liverpool, UK (R Lunevicius PhD); Surgery Department, University of Liverpool, Liverpool, UK (R Lunevicius PhD); Health Data Research UK, Swansea University, Swansea, UK (Prof R A Lyons MD); Saw Swee Hock School of Public Health (S Ma PhD), Yong Loo Lin School of Medicine (Prof N Venketasubramanian MBBS), National University of Singapore, Singapore, Singapore; Department of Internal Medicine, Grant Medical College & Sir J J Group of Hospitals, Mumbai, India (D P Maghavani MBBS); Institute for Global Health Innovations, Duy Tan University, Hanoi, Vietnam (H T Mai MPH, H L T Nguyen MPH, H Q Pham MD); Department of Public Health, Trnava University, Trnava, Slovakia (M Majdan PhD); Non-Communicable Diseases Research Center, Shiraz University of Medical Sciences, Shiraz, Iran (Prof R Malekzadeh MD, S G Sepanlou MD); Surgery Department, Emergency University Hospital Bucharest, Bucharest, Romania (A Manda MD); Campus Caucaia, Federal Institute of Education, Science and Technology of Ceará, Caucaia, Brazil (F R Martins-Melo PhD); Clinical Institute of Medical and Chemical Laboratory Diagnostics, Medical University of Graz, Graz, Austria (Prof W März MD); Graduate School, University of the East Ramon Magsaysay Memorial Medical Center, Quezon City, Philippines (M B Marzan MSc); Department of Neurology, Hospital Center of St John, Porto, Portugal (J Massano MD); Department of Biology and Biological Engineering, Chalmers University of Technology, Gothenburg, Sweden (M Mazidi PhD); Department of Ophthalmology, Hywel Dda University Health Board, Carmarthen, UK (C McAlinden PhD, E Skiadaresi MD); Liver Disease and Hepatitis Program, Alaska Native Medical Center, Anchorage, AK, USA (B J McMahon MD); Research, Monitoring and Evaluation, Ipas Nepal, Kathmandu, Nepal (S Mehata PhD); Preventive Oncology, National Institute of Cancer Prevention and Research, Noida, India (Prof R Mehrotra PhD); Department of Epidemiology and Biostatistics, University of California San Francisco, San Francisco, CA, USA (K M Mehta DSc); Department of Internal Medicine, Sevenhills Hospital, Mumbai, India (V Mehta MD); College of Health Sciences, Debre Tabor University, Debre Tabor, Ethiopia (A Melese MSc); Department of Public Health, University of West Florida, Pensacola, FL, USA (P T N Memiah DrPH); Research Department Prince Mohammed Bin Abdulaziz Hospital, Ministry of Health, Riyadh, Saudi Arabia (Prof Z A Memish MD); Peru Country Office, United Nations Population Fund (UNFPA), Lima, Peru (W Mendoza MD); Department of Pharmacy, Wollo University, Dessie, Ethiopia (G Mengistu MSc); Neurocenter (A Meretoja MD), Breast Surgery Unit (T J Meretoja MD), Helsinki University Hospital, Helsinki, Finland; Clinical Microbiology and Parasitology Unit, Dr Zora ozic Polyclinic, Zagreb, Croatia (T Mestrovic PhD); University Centre Varazdin, University North, Varazdin, Croatia (T Mestrovic PhD); Department of Hypertension (Prof T Miazgowski MD), Zdroje Hospital (J Widecka PhD), Emergency Department (B Miazgowski MD), Pomeranian Medical University, Szczecin, Poland (B Miazgowski MD); Pacific Institute for Research & Evaluation, Calverton, MD, USA (T R Miller PhD); Nevada Division of Public and Behavioral Health, Carson City, NV, USA (M Mirarefin MPH); President’s Office, National Institute of Statistics, Bucharest, Romania (A Mirica PhD); Faculty of General Medicine, Kyrgyz State Medical Academy, Bishkek, Kyrgyzstan (Prof E M Mirrakhimov MD); Department of Atherosclerosis and Coronary Heart Disease, National Center of Cardiology and Internal Disease, Bishkek, Kyrgyzstan (Prof E M Mirrakhimov MD); Institute of Addiction Research (ISFF), Frankfurt University of Applied Sciences, Frankfurt, Germany (B Moazen MSc); Department of Biology, Salahaddin University, Erbil, Iraq (K A Mohammad PhD); Ishik University, Erbil, Iraq (K A Mohammad PhD); Cardiovascular Research Institute, Isfahan University of Medical Sciences, Isfahan, Iran (N Mohammadifard PhD, Prof N Sarrafzadegan MD); Department of Public Health, Jigjiga University, Jigjiga, Ethiopia (M A Mohammed PhD); Health Systems and Policy Research Unit (S Mohammed PhD), Department of Community Medicine (M B Sufiyan MD), Ahmadu Bello University, Zaria, Nigeria; Clinical Epidemiology and Public Health Research Unit, Burlo Garofolo Institute for Maternal and Child Health, Trieste, Italy (L Monasta DSc, L Ronfani PhD); Health Systems Research Center, National Health Research Institutes, Cuernavaca, Mexico (Prof J C Montañez MSc); Department of Epidemiology and Biostatistics (G Moradi PhD), Social Determinants of Health Research Center (G Moradi PhD), Kurdistan University of Medical Sciences, Sanandaj, Iran; Lancaster University, Lancaster, UK (P Moraga PhD); International Laboratory for Air Quality and Health (Prof L Morawska PhD), Australian Centre for Health Services Innovation (R Pacella PhD), School of Exercise and Nutrition Sciences (Q G To PhD), Queensland University of Technology, Brisbane, QLD, Australia; Hospital de Sto António, Hospital Center of Porto, Porto, Portugal (J Morgado-Da-Costa MSc); 1st Department of Ophthalmology, University of Athens, Athens, Greece (M M Moschos PhD); Biomedical Research Foundation, Academy of Athens, Athens, Greece (M M Moschos PhD); Demographic Change and Ageing Research Area (A Werdecker PhD), Competence Center Mortality-Follow-Up (R Westerman PhD), Federal Institute for Population Research, Wiesbaden, Germany (Prof U O Mueller MD); Center for Population and Health, Wiesbaden, Germany (Prof U O Mueller MD); Department of Endocrinology & Metabolism, Institute of Post Graduate Medical Education & Research, Kolkata, India (Prof S Mukhopadhyay MD); School of Medical Sciences, Science University of Malaysia, Kubang Kerian, Malaysia (K Musa PhD); Pediatrics Department, Nishtar Medical University, Multan, Pakistan (Prof G Mustafa MD); Pediatrics & Pediatric Pulmonology, Institute of Mother & Child Care, Multan, Pakistan (Prof G Mustafa MD); Department of Obstetrics and Gynecology (Prof A F Nabhan PhD), Department of Entomology (A M Samy PhD), Ain Shams University, Cairo, Egypt; National Center for Child Health and Development, Setagaya, Japan (C Nagata PhD); Institute of Epidemiology and Medical Biometry, Ulm University, Ulm, Germany (Prof G Nagel PhD, Prof D Rothenbacher MD); Department of Preventive Medicine and Public Health, Chungnam National University School of Medicine, Daejeon, South Korea (Prof H Nam PhD); Daejeon Regional Cancer Center, Chungnam National University Hospital, Daejeon, South Korea (Prof H Nam PhD); Suraj Eye Institute, Nagpur, India (V Nangia MD); Department of Public Heath, University of Yaoundé I, Yaoundé, Cameroon (J Nansseu MD); Department of Cardiology, Cardio-Aid, Bucharest, Romania (R I Negoi PhD); Neurosciences, Kenya Medical Research Institute/Wellcome Trust Research Programme, Kilifi, Kenya (Prof C R J Newton MD); Department off Biological Sciences, University of Embu, Embu, Kenya (J W Ngunjiri DrPH); Health Economics and Finance (A Q Nguyen PhD), Department of Health Economics (H T Nguyen MSc), Hanoi School of Public Health, Hanoi, Vietnam (H T Nguyen PhD); Public Health Science Department, State University of Semarang, Kota Semarang, Indonesia (D N A Ningrum MPH); Graduate Institute of Biomedical Informatics, Taipei Medical University, Taipei City, Taiwan (D N A Ningrum MPH); Institute for Global Health Policy Research, National Center for Global Health and Medicine, Shinjuku-ku, Japan (S Nomura MSc); Independent Consultant, Accra, Ghana (R Ofori-Asenso MSc); Department of Preventive Medicine, Kyung Hee University, Dongdaemun-gu, South Korea (I Oh PhD); Department of HIV/AIDS, STIs & TB, Human Sciences Research Council, Durban, South Africa (O Oladimeji MD); School of Public Health, University of Namibia, Oshakati Campus, Namibia (O Oladimeji MD); Department of Psychiatry, University of Lagos, Lagos, Nigeria (A T Olagunju MD); Centre for Healthy Start Initiative, Ikoyi, Nigeria (B O Olusanya PhD, J O Olusanya MBA); NCD Prevention & Control Unit, Ministry of Health, Bandar Seri Begawan, Brunei (S Ong FAMS); Institute of Health Science, University of Brunei Darussalam, Gadong, Brunei (S Ong FAMS); Graduate School of Public Health, San Diego State University, San Diego, CA, USA (Prof E Oren PhD); School of Medicine, Autonomous University of Madrid, Madrid, Spain (Prof A Ortiz MD); Department of Nephrology and Hypertension, The Institute for Health Research Foundation Jiménez Díaz University Hospital, Madrid, Spain (Prof A Ortiz MD); Department of Global Health Nursing, St Luke’s International University, Chuo-ku, Japan (Prof E Ota PhD); The Center for Healthcare Quality Assessment and Control, Ministry of Health of the Russian Federation, Moscow, Russia (S S Otstavnov PhD); Moscow Institute of Physics and Technology, Moscow State University, Dolgoprudny, Russia (S S Otstavnov PhD); Institute for Advanced Medical Research and Training, University of Ibadan, Ibadan, Nigeria (Prof M O Owolabi DrM); Department of TB & Respiratory Medicine, Jagadguru Sri Shivarathreeswara University, Mysore, India (Prof M P A DNB); University of Chichester, Chichester, UK (R Pacella PhD); Health Outcomes, Center for Health Outcomes & Evaluation, Bucharest, Romania (A Pana MD); Department of Medical Humanities and Social Medicine, Kosin University, Busan, South Korea (Prof E Park PhD); Department of Medicine, Maimonides Medical Center, Brooklyn, NY, USA (S Patel MD); Krishna Institute of Medical Sciences, Deemed University, Karad, India (S T Patil MPH); International Institute of Health Management Research, New Delhi, India (A Patle MPH); Population Health Group (Prof G C Patton MD), Murdoch Children’s Research Institute, Melbourne, VIC, Australia (R G Weintraub MB); Clinical Research Department, Diabetes Research Society, Hyderabad, India (Prof V R Paturi MD); Clinical Research Department, DiabetOmics, Portland, OR, USA (Prof V R Paturi MD); Health, Nutrition, and HIV/AIDS Program, Save the Children, Kathmandu, Nepal (D Paudel PhD); Center for International Health, Ludwig Maximilians University, Munich, Germany (D Paudel PhD); Cartagena University, Cartagena, Colombia (Prof D M Pereira PhD); Institute of Medicine, University of Gothenburg, Gothenburg, Sweden (Prof M Petzold PhD); School of Public Health, University of Witwatersrand, Johannesburg, South Africa (Prof M Petzold PhD); Shanghai Mental Health Center, Shanghai Jiao Tong University, Shanghai, China (Prof M R Phillips MD); Basic Medical Sciences Department, Durban University of Technology, Durban, South Africa (J D Pillay PhD); Institute for Mental Health Policy Research, Centre for Addiction and Mental Health, Toronto, ON, Canada (S Popova PhD); Ashok & Rita Patel Institute of Physiotherapy, Charotar University of Science and Technology, Anand, India (V Prakash PhD); Independent Consultant, Glenelg, SA, Australia (Prof K Pesudovs PhD); Non-communicable Diseases Research Center, Alborz University of Medical Sciences, Karaj, Iran (M Qorbani PhD); Department of Environmental & Occupational Health, Drexel University, Philadelphia, PA, USA (D Quistberg PhD); Medichem, Barcelona, Spain (A Radfar MD); Department of Epidemiology & Biostatistics, Contech School of Public Health, Lahore, Pakistan (A Rafay MS); Department of Clinical Pediatrics, Sweidi Hospital, Riyadh, Saudi Arabia (Prof S U Rahman MBBS); Department of Pediatrics (Prof S U Rahman), Hypertension in Africa Research Team (Prof A E Schutte PhD), North-West University, Peshawar, Pakistan; Society for Health and Demographic Surveillance, Suri, India (R Rai MPH); Department of Economics, University of Göttingen, Göttingen, Germany (R Rai); Gonçalo Moniz Institute, Oswaldo Cruz Foundation, Salvador, Brazil (Prof D Rasella PhD); University College London Hospitals, London, UK (D L Rawaf MD); Public Health England, London, UK (Prof S Rawaf PhD); Brien Holden Vision Institute, Sydney, NSW, Australia (Prof S Resnikoff MD); Organization for the Prevention of Blindness, Paris, France (Prof S Resnikoff MD); Department of Clinical Research, Federal University of Uberlândia, Uberlândia, Brazil (L Roever PhD); Golestan Research Center of Gastroenterology and Hepatology, Golestan University of Medical Sciences, Gorgan, Iran (G Roshandel PhD); Biotechnology, Ikiam Amazon Regional University, Ciudad De Tena, Ecuador (E Rubagotti PhD); Department of Ocean Science and Engineering, Southern University of Science and Technology, Shenzhen, China (E Rubagotti PhD); Neuropsychiatric Institute, Prince of Wales Hospital, Randwick, NSW, Australia (Prof P S Sachdev MD); Neurogenic Inflammation Research Center (A Sahebkar PhD), Biotechnology Research Center (A Sahebkar PhD), Mashhad University of Medical Sciences, Mashhad, Iran; College of Medicine, Al-Imam Mohammad Ibn Saud Islamic University, Riyadh, Saudi Arabia (N Salam PhD); School of Health and Policy Management, Faculty of Health, York University, Toronto, ON, Canada (Prof P Salamati MD); Punjab University College of Pharmacy, Anarkali, Pakistan (Z Saleem PharmD); Center for Health Policy & Center for Primary Care and Outcomes Research, Stanford University, Stanford, CA, USA (Prof J A Salomon PhD); Clinical Research Division, Chest Research Foundation, Pune, India (Prof S S Salvi MD); Health and Disability Intelligence Group, Ministry of Health, Wellington, New Zealand (I Salz MD); Department of Surgery, Marshall University, Huntington, WV, USA (Prof J Sanabria MD); Department of Nutrition and Preventive Medicine, Case Western Reserve University, Cleveland, OH, USA (Prof J Sanabria MD); Nephrology Group, Jimenez Diaz Foundation University Hospital Institute for Health Research, Madrid, Spain (M Sanchez-Niño PhD); Department of Public Health, Regional Health Administration Do Norte I P, Vila Nova De Gaia, Portugal (J V Santos MD); Department of Medicine, University of Massachusetts Medical School, Worcester, MA, USA (M Sardana MD); Department of Health and Society, Faculty of Medicine, University of Applied and Environmental Sciences, Bogotá, Colombia (Prof R Sarmiento-Suárez MPH); Department of Public Health Medicine, University of Kwazulu-Natal, Durban, South Africa (Prof B Sartorius PhD); Surgery Department, Hamad Medical Corporation, Doha, Qatar (B Sathian PhD); Faculty of Health & Social Sciences, Bournemouth University, Bournemouth, UK (B Sathian PhD); UGC Centre of Advanced Study in Psychology, Utkal University, Bhubaneswar, India (M Satpathy PhD); Udyam-Global Association for Sustainable Development, Bhubaneswar, India (M Satpathy PhD); Dr D Y Patil Vidyapeeth, Pune, India (A R Sawant MD); Department of Public Health Sciences, University of North Carolina at Charlotte, Charlotte, NC, USA (M Sawhney PhD); School of Health Sciences, Federal University of Santa Catarina, Ararangua, Brazil (Prof I J C Schneider PhD, Prof D A S Silva PhD); Department of Medical Statistics, Epidemiology and Medical Informatics, University of Zagreb, Zagreb, Croatia (M Sekerija PhD); Division of Epidemiology and Prevention of Chronic Noncommunicable Diseases, Croatian Institute of Public Health, Zagreb, Croatia (M Sekerija PhD); Langone Medical Center, New York University, New York, NY, USA (A Shafieesabet MD); Public Health Division, An-Najah National University, Nablus, Palestine (A A Shaheen PhD); Independent Consultant, Karachi, Pakistan (M A Shaikh MD); Department of Molecular Hepatology, Middle East Liver Disease Center, Tehran, Iran (H Sharafi PhD); Department of Laboratory Sciences (Prof M Sharif PhD), Department of Basic Sciences (Prof M Sharif PhD), Islamic Azad University, Sari, Iran; Policy and Planning Division, Ministry of Health, Thimphi, Bhutan (J Sharma MPH); University School of Management and Entrepreneurship, Delhi Technological University, New Delhi, India (R Sharma PhD); Department of Pulmonary Medicine, Fudan University, Shanghai, China (J She MD); Usher Institute of Population Health Sciences and Informatics, University of Edinburgh, Edinburgh, UK (Prof A Sheikh MD, I N Soyiri PhD); Friedman School of Nutrition Science and Policy, Tufts University, Boston, MA, USA (P Shi PhD); National Institute of Infectious Diseases, Tokyo, Japan (M Shigematsu PhD); Institute of Medical Epidemiology, Martin Luther University Halle-Wittenberg, Halle, Germany (I Shiue PhD); School of Health, University of Technology Sydney, Sydney, NSW, Australia (S Siabani PhD); Department of Psychology, Reykjavik University, Reykjavik, Iceland (Prof I D Sigfusdottir PhD, R Sigurvinsdottir PhD); Brasília University, Brasília, Brazil (Prof D A Silveira MD); Max Hospital, Ghaziabad, India (Prof N P Singh MD); Department of Pulmonary Medicine, Asthma Bhawan, Jaipur, India (Prof V Singh MD); Department of Epidemiology, School of Preventive Oncology, Patna, India (D N Sinha PhD); Pediatric Department, King Khalid University Hospital, Riyadh, Saudi Arabia (B H Sobaih MD); Division of Community Medicine, International Medical University, Kuala Lumpur, Malaysia (C T Sreeramareddy MD); Central Research Institute of Cytology and Genetics (E Varavikova PhD), Federal Research Institute for Health Organization and Informatics of the Ministry of Health (FRIHOI), Moscow, Russia (Prof V I Starodubov DSc, S K Vladimirov PhD); Department of Neuromedicine and Movement Science, Norwegian University of Science and Technology, Trondheim, Norway (Prof T J Steiner PhD, Prof L J Stovner PhD); Neuro Centre, St Olavs Hospital, Trondheim, Norway (Prof L J Stovner PhD); Department of Nursing, Muhammadiyah University of Surakarta, Kartasura, Indonesia (A Sudaryanto MPH); Department of Community Health (B F Sunguya PhD), Muhimbili University of Health and Allied Sciences, Dar Es Salaam, Tanzania (B F Sunguya PhD); School of Medicine, University of California Riverside, Riverside, CA, USA (P J Sur MPH); Department of Criminology, Law and Society, University of California Irvine, Irvine, CA, USA (Prof B L Sykes PhD); Department of Medicine (Prof R Tabarés-Seisdedos PhD), Department of Pediatrics, Obstetrics and Gynecology (Prof M Tortajada-Girbés PhD), University of Valencia, Valencia, Spain; Carlos III Health Institute, Biomedical Research Networking Center for Mental Health Network (CIBERSAM), Madrid, Spain (Prof R Tabarés-Seisdedos PhD); Cancer Control Center, Osaka International Cancer Institute, Osaka, Japan (T Tabuchi MD); Department of Psychiatry and Behavioral Sciences, New York Medical College, Valhalla, NY, USA (M Tavakkoli MD); University Institute “Egas Moniz”, Monte Da Caparica, Portugal (Prof N Taveira PhD); Research Institute for Medicines, Faculty of Pharmacy of Lisbon, University of Lisbon, Lisbon, Portugal (Prof N Taveira PhD); Anesthesiology Department, University of Virginia, Charlottesville, VA, USA (A S Terkawi MD); Syrian Expatriate Medical Association, Charlottesville, VA, USA (A S Terkawi MD); Lee Kong Chian School of Medicine (L Tudor Car PhD), Nanyang Technological University, Singapore, Singapore (S Thirunavukkarasu PhD); Department of Medicine, University of Calgary, Calgary, AB, Canada (Prof M Tonelli MD); Agency for Health Technology Assessment and Tariff System, Warszawa, Poland (R Topor-Madry PhD); Pediatric Department, University Hospital Doctor Peset, Valencia, Spain (Prof M Tortajada-Girbés PhD); Nutritional Epidemiology Research Team, National Institute of Health and Medical Research, Paris, France (M Touvier PhD); Department of Health Economics, Hanoi Medical University, Hanoi, Vietnam (B X Tran PhD); Clinical Hematology and Toxicology, Military Medical University, Hanoi, Vietnam (K B Tran MD); Department of Internal Medicine, Federal Teaching Hospital, Abakaliki, Nigeria (K N UkwajaMD); Gomal Center of Biochemistry and Biotechnology, Gomal University, Dera Ismail Khan, Pakistan (I Ullah PhD); TB Culture Laboratory, Mufti Mehmood Memorial Teaching Hospital, Dera Ismail Khan, Pakistan (I Ullah PhD); School of Government, Pontifical Catholic University of Chile, Santiago, Chile (E A Undurraga PhD); Schneider Institutes for Health Policy, Brandeis University, Waltham, MA, USA (E A Undurraga PhD); Argentine Society of Medicine, Ciudad De Buenos Aires, Argentina (Prof P R Valdez MEd); Velez Sarsfield Hospital, Buenos Aires, Argentina (Prof P R Valdez MEd); UKK Institute, Tampere, Finland (Prof T J Vasankari MD); Raffles Neuroscience Centre, Raffles Hospital, Singapore, Singapore (Prof N Venketasubramanian MBBS); Occupational Health Unit, Sant’Orsola Malpighi Hospital, Bologna, Italy (Prof F S Violante MPH); Department of Information and Internet Technologies, I M Sechenov First Moscow State Medical University, Moscow, Russia (S K Vladimirov PhD); Department of Health Care Administration and Economy, National Research University Higher School of Economics, Moscow, Russia (Prof V Vlassov MD); Foundation University Medical College, Foundation University, Rawalpindi, Pakistan (Y Waheed PhD); Department of Epidemiology and Biostatistics (Y Wang BSA, Prof C Yu PhD), Global Health Institute (Prof C Yu PhD), Wuhan University, Wuhan, China; Independent Consultant, Staufenberg, Germany (A Werdecker PhD); Department of Neurology, Technical University of Munich, Munich, Germany (A S Winkler PhD); Bone and Joint Research Group, Royal Cornwall Hospital, Truro, UK (Prof A D Woolf BSc); University of Strathclyde, Glasgow, UK (G M A Wyper MSc); Department of Pharmacology, St John’s National Academy of Health Sciences, Bangalore, India (Prof D Xavier MD); School of Medicine, Nanjing University, Nanjing, China (Prof G Xu MD); Clinical Cancer Research Center, Milad General Hospital, Tehran, Iran (S Yahyazadeh Jabbari MD); Department of Preventive Medicine, Northwestern University, Chicago, IL, USA (Y Yano MD); Department of Earth Science, King Fahd University of Petroleum and Minerals, Dhahran, Saudi Arabia (Y J Yasin MPH); Wolkite University, Wolkite, Ethiopia (A Yeshaneh BHlthSci); Department of Psychopharmacology, National Center of Neurology and Psychiatry, Tokyo, Japan (N Yonemoto MPH); School of Public Health, University of Kinshasa, Kinshasa, Democratic Republic of the Congo (M Yotebieng PhD); Department of Health Policy and Management, Jackson State University, Jackson, MS, USA (Prof M Z Younis DrPH); Tsinghua University, Tsinghua University, Beijing, China (Prof M Z Younis DrPH); Epidemiology and Cancer Registry Sector, Institute of Oncology Ljubljana, Ljubljana, Slovenia (Prof V Zadnik PhD); Department of Epidemiology, University Hospital of Setif, Setif, Algeria (Prof Z Zaidi PhD); Social Determinants of Health Research Center, Ardebil University of Medical Science, Ardabil, Iran (H Zandian PhD); Israeli Center for Disease Control, Ministry of Health, Ramat Gan, Israel (I Zucker MD); and School of Public Health, Tel Aviv University, Tel Aviv, Israel (I Zucker MD).
