# Department of Error

**DOI:** 10.1016/S0140-6736(19)31043-8

**Published:** 2019-06-22

*GBD 2017 DALYs and HALE Collaborators. Global, regional, and national disability-adjusted life-years (DALYs) for 359 diseases and injuries and healthy life expectancy (HALE) for 195 countries and territories, 1990–2017: a systematic analysis for the Global Burden of Disease Study 2017.* Lancet *2018; **392:** 1859–922—*In this Global Health Metrics paper, Joan B Soriano has been added to the collaborators list; affiliation details have been amended for Joseph Adel Mattar Banoub, Tanuj Kanchan, and Yasin Jemal Yasin; and the declaration of interests statement has been amended for Boris Bikbov. These corrections have been made to the online version as of June 20, 2019.

