# Supplementary material for: Global, regional, and national disability-adjusted life-years (DALYs) for 359 diseases and injuries and healthy life expectancy (HALE) for 195 countries and territories, 1990–2017: a systematic analysis for the Global Burden of Disease Study 2017
Source: Lancet. Author manuscript; Available in PMC 2018 Nov 24. (PMC6252083; doi:10.1016/S0140-6736(18)32335-3)
Supplement: Supplementary appendix 1 [file NIHMS80527-supplement-Supplementary_appendix_1.pdf]

THE LANCET

Supplementary appendix 1

This appendix formed part of the original submission and has been peer reviewed. We post it as supplied by the authors.

Supplement to: GBD 2017 DALYs and HALE Collaborators. Global, regional, and national disability-adjusted life-years (DALYs) for 359 diseases and injuries and healthy life expectancy (HALE) for 195 countries and territories, 1990–2017: a systematic analysis for the Global Burden of Disease Study 2017. *Lancet* 2018; **392:** 1859–1922.

Methods Appendix to Global, regional, and national disability-adjusted life-years (DALYs) for 359 diseases and injuries and healthy life expectancy (HALE) for 195 countries and territories, 1990–2017: a systematic analysis for the Global Burden of Disease Study 2017

This appendix provides methodological detail for disability-adjusted life years (DALYs) and healthy life expectancy (HALE). The appendix is organized into broad sections following the structure of the main paper.

Preamble

This study complies with the Guidelines for Accurate and Transparent Health Estimates Reporting (GATHER) recommendations. It includes detailed tables and information on data in an effort to maximize transparency in our estimation processes and provide a comprehensive description of analytical steps. We intend this to be a living document, to be updated with each annual iteration of the Global Burden of Disease.

Table of Contents

Preamble 2

List of Appendix Tables & Figures 4

Authors' Contributions........ i

Section 1. GBD Overview 5

- Section 1.1. Locations of the Analysis 5
- Section 1.2. Time Period of the Analysis 5
- Section 1.3. Statement of GATHER Compliance 5
- Section 1.4. GBD Glossary 6
- Section 1.5. GBD results overview 8
- Section 1.6. Data input sources overview 8
- Section 1.7. Funding Sources 8

Section 2. Estimation Process for DALYs/HALE 9

Section 2.1 Computing DALYs 9

Section 2.2 Computing HALE 10

Section 2.3 Socio-Demographic Index (SDI) Definitions & Method 10

- Overview 10
- Section 2.3.1 Development of revised SDI indicator 10
- Section 2.3 Socio-Demographic Index (SDI) analysis & Epidemiological Transition 12

Appendix Tables & Figures

Appendix Figure 1. Estimation Process for DALYs and HALE

Appendix Table 1. GATHER checklist of information that should be included in reports of global health estimates, with description of compliance and location of information

Appendix Table 2. GBD 2017 geography hierarchy with levels

Appendix Table 3. Socio-demographic Index values for all estimated GBD 2017 locations, 1990-2017

Appendix Table 4. Socio-demographic Index groupings by geography, based on 2017 values

Authors' Contributions

Managing the estimation process

Ashkan Afshin, Tahiya Alam, Molly Biehl, Elizabeth Cromwell, Lalit Dandona, Rakhi Dandona, Louisa Degenhardt, Samath Dharmaratne, Valery Feigin, Christina Fitzmaurice, Nancy Fullman, Emmanuela Gakidou, Simon Hay, Spencer James, Nicholas Kassebaum, Ibrahim Khalil, Hmwe Kyu, Stephen Lim, Alan Lopez, Rafael Lozano, Ashley Marks, Awoke Misganaw, Ali H Mokdad, Christopher Murray, Mohsen Naghavi, Elaine Nsoesie, Helen Olsen, David Pigott, Puja Rao, Robert Reiner, Joseph Salama, David Smith, Mari Smith, Jeffrey Stanaway, Caitlyn Steiner, Stein Emil Vollset, Theo Vos, and Harvey Whiteford

Writing the first draft of the manuscript

Megha Arora, Molly Biehl, Selina Deiparine, Chad Ikeda, Grant Rogers Kemp, Hmwe Kyu, Emilie Maddison, Minh Nguyen, and Benajmin Zipkin

Providing data or critical feedback on data sources

Aliasghar A Kiadaliri, Kalkidan Hassen Abate, Solomon Mequanente Abay, Cristiana Abbafati, Ibrahim Abdollahpour, Molla Abebe, Zegeye Abebe, Victor Aboyans, Aklilu Abrham Roba, Laith Abu-Raddad, Niveen Abu-Rmeileh, Manfred Accrombessi, Oladimeji Adebayo, Zanfina Ademi, Olatunji Adetokunboh, Mina Adib, Mohsen Afarideh, Ashkan Afshin, Rakesh Aggarwal, Sargis Aghayan, Alireza Ahmadi, Mehdi Ahmadi, Muktar Ahmed, Ibtihel Aichour, Tomi Akinyemiju, Nadia Akseer, Fares Alahdab, Khurshid Alam, Kefyalew Addis Alene, Syed Aljunid, François Alla, Peter Allebeck, Ubai Alsharif, Dayane Gabriele Alves Silveira, Nelson Alvis-Guzman, Erfan Amini, Walid Ammar, Yaw Ampem Amoako, Catalina Liliana Andrei, Mustafa Geleto Ansha, Carl Abelardo Antonio, Olatunde Aremu, Johan Ärnlöv, Amit Arora, Al Artaman, Krishna Kumar Aryal, Hamid Asayesh, Marcel Ausloos, Ashish Awasthi, Beatriz Paulina Ayala Quintanilla, Rakesh Ayer, Peter Azzopardi, Nam Ba Nguyen, Arefeh Babazadeh, Kalpana Balakrishnan, Maciej Banach, Joseph Banoub, Aleksandra Barac, Miguel A. Barboza, Till Bärnighausen, Simon Barquera, Lope Barrero, Neeraj Bedi, Masoud Behzadifar, Meysam Behzadifar, Bayu Begashaw Bekele, Yihalem Abebe Belay, Aminu Bello, Derrick Bennett, Isabela Bensenor, Eduardo Bernabe, Robert Bernstein, Mircea Beuran, Tina Beyranvand, Neeraj Bhala, Belete Biadgo, Boris Bikbov, Ver Bilano, Nigus Bililign, Muhammad Shahdaat Bin Sayeed, Ibrahim Bou-Orm, Rupert Bourne, Luisa Brant, Carol Brayne, Andrey Briko, Gabrielle Britton, Traolach Brugha, Julio Cesar Campuzano, Jorge Cano, Juan J Carrero, Deborah Carvalho Malta, Carlos Castañeda-Orjuela, Jacqueline Castillo Rivas, Franz Castro, Ferrán Catalá-López, Yazan Chaiah, Jung-Chen Chang, Peggy Chiang, Abdulaal Chitheer, Devasahayam Christopher, Sheng-Chia Chung, Paolo Angelo Cortesi, Monica Cortinovis, Michael Criqui, Marita Cross, John Crump, Alemneh Kabeta Daba, Lalit Dandona, Rakhi Dandona, Paul Dargan, Ahmad Daryani, José Das Neves, Fernando De La Hoz, Louisa Degenhardt, Kebede Deribe, Nikolaos Dervenis, Samath Dharmaratne, Meghnath Dhimal, M.Ashworth Dirac, Klara Dokova, David Teye Doku, Kerrie Doyle, Manisha Dubey, Eyasu Ejeta Duken, Andre Duraes, Hedyeh Ebrahimi, Soheil Ebrahimpour, Andem Effiong, Anne Elise Eggen, Joshua Ehrlich, Ziad El-Khatib, Iqbal Elyazar, Benjamin Er, Holly Erskine, Sharareh Eskandarieh, Alireza Esteghamati, Sadaf Esteghamati, Mohammad Fareed, Carla Farinha, Andre Faro, Farshad Farzadfar, Valery Feigin, Seyed-Mohammad Fereshtehnejad, Joao Fernandes, Irina Filip, Christina Fitzmaurice, Nataliya Foigt, Takeshi Fukumoto, Thomas Fürst, Seana Gall, Morsaleh Ganji, Alberto L. García-Basteiro, Tigist Gashaw, Amanuel Tesfay Gebremedhin, Merhawi Gebremedhin, Teklu Gebremichael, Tilayie Feto Gelano, Johanna Geleijnse, Ayele Geleto, Ricard Genova-Maleras, Mamata Ghimire, Kidu Gidey, Srinivas Goli, Hector Gomez-Dantes, Philimon Gona, Sameer Gopalani, Bárbara

Goulart, Ayman Grada, Giuseppe Grosso, Prakash Gupta, Rajat Das Gupta, Rajeev Gupta, Tanush Gupta, Juanita Haagsma, Nima Hafezi-Nejad, Tekleberhan Beyene Hagos, Arvin Haj-Mirzaian, Arya Haj-Mirzaian, Yuantao Hao, Hilda Harb, Sivadasanpillai Harikrishnan, Josep Maria Haro, Hadi Hassankhani, Hamid Y. Hassen, Rasmus Havmoeller, Roderick Hay, Delia Hendrie, Andualem Henok, Ileana Heredia-Pi, Claudiu Herteliu, Pouria Heydarpour, Long Hoang Nguyen, Son Hoang Nguyen, Hans Hoek, Howard J. Hoffman, Michael Hole, Enayatollah Homaie Rad, Praveen Hoogar, H Dean Hosgood, Mehdi Hosseinzadeh, Mihaela Hostiuc, Sorin Hostiuc, Damian Hoy, Aung Soe Htet, Kim Moesgaard Iburg, Farhad Islami, Kathryn H. Jacobsen, Nader Jahanmehr, Mihajlo Jakovljevic, Spencer James, Achala Jayatilleke, Panniyammakal Jeemon, Vivekanand Jha, Catherine Johnson, Jost B. Jonas, Zahra Jorjoran Shushtari, Jacek Jozwiak, Mikk Jürisson, Zubair Kabir, Rajendra Kadel, Amaha Kahsay, Tanuj Kanchan, André Karch, Corine Karema, Seyed M. Karimi, Amir Kasaeian, Getachew Mullu Kassa, Nicholas J Kassebaum, Srinivasa Vittal Katikireddi, Anil Kaul, Norito Kawakami, Peter Keiyoro, Andre Keren, Yousef Khader, Morteza Abdullatif Khafaie, Ibrahim Khalil, Muhammad Shahzeb Khan, Young-Ho Khang, Mona Khater, Abdullah Khoja, Ardeshir Khosravi, Mohammad Hossein Khosravi, Daniel Kiirithio, Cho-Il Kim, Daniel Kim, Young-Eun Kim, Yun Jin Kim, Adnan Kisa, Ann Kristin Knudsen, Sonali Kochhar, Soewarta Kosen, Georgios Kotsakis, Parvaiz Koul, Ai Koyanagi, Kewal Krishan, Barthelemy Kuate Defo, Burcu Kucuk Bicer, G Anil Kumar, Manasi Kumar, Ihor Kuzin, Deepesh P. Lad, Sheetal Lad, Huong Lan Nguyen, Justin Lang, Sinead Langan, Van Lansingh, Janet Leasher, Misgan Legesse , James Leigh, Mostafa Leili, Janni Leung, Yichong Li, Xiaofeng Liang, Yu Liao, Lee-Ling Lim, Miteku Limenih, Shiwei Liu, Alan Lopez, Stefan Lorkowski, Paulo Lotufo, Ronan Lyons, Stefan Ma, Erlyn Rachelle Macarayan, Dhaval Maghavani, P A Mahesh, Marek Majdan, Reza Majdzadeh, Azeem Majeed, Reza Malekzadeh, Mohammad Ali Mansournia, Lorenzo Mantovani, Joemer Maravilla, Wagner Marcenes, Francisco Rogerlândio Martins-Melo, Winfried März, Melvin Marzan, Benjamin Massenburg, Manu Mathur, Mohsen Mazidi, John Mcgrath, Abla Mehio Sibai, Varshil Mehta, Fabiola Mejia-Rodriguez, Tesfa Mekonen, Addisu Melese, Mulugeta Melku, Peter Memiah, Ziad Memish, Walter Mendoza, Getnet Mengistu, Zerihun Menlkalew , Atte Meretoja, Tuomo Meretoja, Tomislav Mestrovic, Tomasz Miazgowski, Ted R Miller, Erkin Mirrakhimov, Babak Moazen, Bahram Mohajer, Karzan Mohammad, Moslem Mohammadi, Noushin Mohammadifard, Shafiu Mohammed, Farnam Mohebi, Ali Mokdad, Lorenzo Monasta, Ghobad Moradi, Mahmoudreza Moradi, Maziar Moradi-Lakeh, Mehdi Moradinazar, Lidia Morawska, Ilais Moreno, Joana Morgado-Da-Costa, Marilita Moschos, Seyyed Meysam Mousavi, Achenef Muche, Ulrich Mueller, Kamarul Imran Musa, Ghulam Mustafa, Ashraf Nabhan, Gabriele Nagel, Mohsen Naghavi, Seyed Sina Naghibi Irvani, Azin Nahvijou, Farid Najafi, Hae-Sung Nam, Ionut Negoi, Ruxandra Irina Negoi, Charles Newton, Josephine Ngunjiri, Anh Nguyen, Ha Nguyen, Huong Nguyen, Shuhei Nomura, Mehdi Noroozi, Bo Norrving, Jean Jacques Noubiap, Hamid Reza Nouri, Malihe Nourollahpour, Mohammad Reza Nowroozi, Felix Ogbo, In-Hwan Oh, Olanrewaju Oladimeji, Andrew T. Olagunju, Tinuke O. Olagunju, Bolajoko Olusanya, Jacob Olusanya, Kanyin Ong, Sok King Ong, Eyal Oren, Alberto Ortiz, Stanislav Otstavnov, Simon Øverland, Mayowa Owolabi, Adrian Pana, Songhomitra Panda-Jonas, Hadi Parsian, Yahya Pasdar, Shanti Patel, Snehal Patil, Ajay Patle, Neil Pearce, Alexandre Pereira, David Pereira, Norberto Perico, Max Petzold, Michael R. Phillips, Linh Phuong Doan, David Pigott, Meghdad Pirsaheb, Farhad Pishgar, Suzanne Polinder, Hossein Poustchi, Swayam Prakash, Narayan Prasad, Mostafa Qorbani, Hai Quang Pham, D. Alex Quistberg, Amir Radfar, Anwar Rafay, Alireza Rafiei, Fakher Rahim, Zohreh Rahimi, Afarin Rahimi-Movaghar, Vafa Rahimi-Movaghar, Mohammad Hifz Ur Rahman, Sajjad Rahman, Rajesh Kumar Rai, Prabhat Ranjan, Paturi Rao, Puja Rao, David Laith Rawaf, Salman Rawaf, Kolli Srinath Reddy Reddy, Giuseppe Remuzzi, Andre Renzaho, Serge Resnikoff, Mohammad Sadegh Rezai, Antonio Luiz Ribeiro,

Leonardo Roever, Luca Ronfani, Gholamreza Roshandel, Ali Rostami, Enrico Rubagotti, Perminder Sachdev, Ehsan Sadeghi, Hosein Safari, Yahya Safari, Saeid Safiri, Mohammad Ali Sahraian, Nasir Salam, Joseph Salama, Payman Salamati, Yahya Salimi, Hamideh Salimzadeh, Abdallah M. Samy, Juan Sanabria, Milena Santric Milicevic, Bruno Sao Jose, Mayank Sardana, Rodrigo Sarmiento-Suárez, Nizal Sarrafzadegan, Shahabeddin Sarvi, Thirunavukkarasu Sathish, Maheswar Satpathy, Monika Sawhney, Sonia Saxena, Elke Schaeffner, David C Schwebel, Falk Schwendicke, James Scott, Mario Šekerija, Sadaf Sepanlou, Edson Serván-Mori, Seyedmojtaba Seyedmousavi, Amira Shaheen , Masood Ali Shaikh, Mehran Shams-Beyranvand, Kiomars Sharafi, Mehdi Sharif, Sheikh Mohammed Shariful Islam, Jayendra Sharma, Aziz Sheikh, Peilin Shi, Ivy Shiue, Yalda Shokoohinia, Farhad Shokraneh, Si Si, Soraya Siabani, Tariq Jamal Siddiqi, Diego Augusto Santos Silva, Jasvinder Singh, Narinder Pal Singh, Virendra Singh, Dhirendra Narain Sinha, Vegard Skirbekk, Adauto Martins Soares Filho, Soheila Sobhani, Reed Sorensen, Luciano Sposato, Chandrashekhar T Sreeramareddy, Timothy Steiner, Mark Stokes, Agus Sudaryanto, Muawiyyah Babale Sufiyan, Rizwan Suliankatchi Abdulkader, Patrick Sur, Bryan L. Sykes, Dillon Sylte, Cassandra Szoeke, Rafael Tabarés-Seisdedos, Mesfin Tadese, Eyasu Tamru, Nuno Taveira, Hugh Taylor, Gebre Teklemariam Demoz, Awoke Temesgen, Mohamad-Hani Temsah, Omar Temsah, Belay Tessema, Mebrahtu Teweldemedhin, Hue Thi Mai, Nihal Thomas, Marcello Tonelli, Miguel Tortajada-Girbés, Mathilde Touvier, Marcos Roberto Tovani-Palone, Bach Tran, Khanh Bao Tran, Derrick Tsoi, Stefanos Tyrovolas, Kingsley Nnanna Ukwaja, Irfan Ullah, Eduardo Undurraga, Muhammad Shariq Usman, Olalekan Uthman, Muthiah Vaduganathan, Afsane Vaezi, Pascual Valdez, Tommi Vasankari, Narayanaswamy Venketasubramanian, Sergey Vladimirov, Vasiliy Vlassov, Stein Emil Vollset, Theo Vos, Fasil Wagnew, Shishay Wahdey, Yasir Waheed, Yuan-Pang Wang, Elisabete Weiderpass, Robert Weintraub, Daniel Weiss, Inbal Weiss Salz, Andrea Werdecker, Ronny Westerman, Harvey Whiteford, Justyna Widecka, Tissa Wijeratne, Charles Shey Wiysonge, Charles Wolfe, Grant Wyper, Denis Xavier , Gelin Xu, Hossein Yahyazadeh, Lijing Yan, Yasin Jemal Yasin, Alex Yeshaneh, Ebrahim M. Yimer, Naohiro Yonemoto, Seok-Jun Yoon, Marcel Yotebieng, Mustafa Younis, Chuanhua Yu, Vesna Zadnik, Zoubida Zaidi, Sojib Bin Zaman, Mohammad Zamani, Hamed Zandian, Sanjay Zodpey, and Liesl Zuhlke.

Developing methods or computational machinery

Cristiana Abbafati, Mehran Alijanzadeh, Marcel Ausloos, Donal Bisanzio, Ahmad Daryani, Farshad Farzadfar, Christina Fitzmaurice, Kyle Foreman, Tahvi Frank, Ayele Geleto, Ellen M. Goldberg, Taren Gorman, Tanush Gupta, Claudiu Herteliu, Mehdi Hosseinzadeh, Spencer James, Catherine Johnson, Chittaranjan Kar, Nicholas J Kassebaum, Young-Eun Kim, Adnan Kisa, Kathryn Lau, Misgan Legesse , Tim Lucas, Zerihun Menlkalew , Mohsen Naghavi, Grant Nguyen, Kanyin Ong, Charles Parry, David Pigott, Robert Reiner, Gregory Roth, Joshua A Salomon, Abdallah M. Samy, Damian Santomauro, Maheswar Satpathy, David C Schwebel, Mehdi Sharif, David Smith, Vinay Srinivasan, Jeffrey Stanaway, Patrick Sur, Dillon Sylte, Eyasu Tamru, Derrick Tsoi, Rachel L. Updike, Theo Vos, Ronny Westerman, Lijing Yan, and Naohiro Yonemoto.

Applying analytical methods to produce estimates

Aklilu Abrham Roba, Kareha Agesa, Miloud Taki Eddine Aichour, Olatunde Aremu, Marcel Ausloos, Bayu Begashaw Bekele, Samir Bhatt, Donal Bisanzio, Kelly Cercy, Devasahayam Christopher, Cyrus Cooper, Elizabeth Cromwell, Ahmad Daryani, M.Ashworth Dirac, Michelle Echko, Aman Endries, Holly Erskine, Alize Ferrari, Christina Fitzmaurice, Tahvi Frank, William Gardner, Segen Gebremeskel, Ellen M. Goldberg, Hamid Y. Hassen, Claudiu Herteliu, Chad Ikeda, Spencer James, Catherine Johnson, Manoochehr Karami, Nicholas J Kassebaum, Yun Jin Kim, Adnan Kisa, Jonathan Kocarnik, Hmwe Kyu, Van

Lansingh, Kathryn Lau, Jorge Ledesma, Misgan Legesse , James Leigh, Janni Leung, Tim Lucas, Helena Manguerra, Ira Martopullo, Shafiu Mohammed, Ali Mokdad, Mohsen Naghavi, Grant Nguyen, Emma Nichols, Bolajoko Olusanya, Kanyin Ong, Katherine Paulson, David Pigott, Caroline Purcell, Alireza Rafiei, Fatemeh Rajati, Robert Reiner, Marissa Reitsma, Nicholas Roberts, Gregory Roth, Nafis Sadat, Abdallah M. Samy, Damian Santomauro, Shahabeddin Sarvi, Maheswar Satpathy, Seyedmojtaba Seyedmousavi, Masood Ali Shaikh, Mehdi Sharif, Reed Sorensen, Vinay Srinivasan, Jeffrey Stanaway, Patrick Sur, Bryan L. Sykes, Dillon Sylte, Eyasu Tamru, Andrew Theis, Christopher Troeger, Derrick Tsoi, Rachel L. Updike, Theo Vos, Inbal Weiss Salz, Ronny Westerman, Tissa Wijeratne, Lauren Wilner, and Simon Yadgir.

Providing critical feedback on methods or results

Aliasghar A Kiadaliri, Degu Abate, Kalkidan Hassen Abate, Solomon Mequanente Abay, Cristiana Abbafati, Nooshin Abbasi, Hedayat Abbastabar, Jemal Abdela, Ibrahim Abdollahpour, Molla Abebe, Zegeye Abebe, Aklilu Abrham Roba, Niveen Abu-Rmeileh, Pawan Acharya, Ilana Ackerman, Abdu Adamu, Oladimeji Adebayo, Victor Adekanmbi, Olatunji Adetokunboh, Mina Adib, José C. Adsuar, Kossivi Afanvi, Mohsen Afarideh, Gina Agarwal, Sargis Aghayan, Anurag Agrawal, Alireza Ahmadi, Mehdi Ahmadi, Hamid Ahmadieh, Muktar Ahmed, Sayem Ahmed, Amani Nidhal Aichour, Ibtihel Aichour, Miloud Taki Eddine Aichour, Tomi Akinyemiju, Nadia Akseer, Fares Alahdab, Ziyad Al-Aly, Khurshid Alam, Seyed Moayed Alavian, Kefyalew Addis Alene, Ayman Al-Eyadhy, Mehran Alijanzadeh, Reza Alizadeh-Navaei, Syed Aljunid, Ala'a Alkerwi, Peter Allebeck, Hesham Al-Mekhlafi, Jordi Alonso, Rajaa Al-Raddadi, Ubai Alsharif, Khalid Altirkawi, Dayane Gabriele Alves Silveira, Nelson Alvis-Guzman, Leopold N. Aminde, Erfan Amini, Mohammadreza Amiresmaili, Walid Ammar, Yaw Ampem Amoako, Mirica Andreea, Catalina Liliana Andrei, Sofia Androudi, Mina Anjomshoa, Mustafa Geleto Ansha, Carl Abelardo Antonio, Palwasha Anwari, Olatunde Aremu, Johan Ärnlöv, Amit Arora, Al Artaman, Krishna Kumar Aryal, Hamid Asayesh, Zerihun Ataro, Marcel Ausloos, Leticia Avila-Burgos, Euripide Avokpaho, Ashish Awasthi, Beatriz Paulina Ayala Quintanilla, Peter Azzopardi, Nam Ba Nguyen, Arefeh Babazadeh, Hamid Badali, Joseph Banoub, Aleksandra Barac, Miguel A. Barboza, Suzanne Barker-Collo, Till Bärnighausen, Lope Barrero, Shahrzad Bazargan-Hejazi, Neeraj Bedi, Ettore Beghi, Abate Bekele, Bayu Begashaw Bekele, Yihalem Abebe Belay, Michelle Bell, Aminu Bello, Derrick Bennett, Isabela Bensenor, Adugnaw Berhane, Eduardo Bernabe, Robert Bernstein, Mircea Beuran, Tina Beyranvand, Neeraj Bhala, Soumyadeep Bhaumik, Belete Biadgo, Molly Biehl, Ali Bijani, Boris Bikbov, Ver Bilano, Nigus Bililign, Muhammad Shahdaat Bin Sayeed, Donal Bisanzio, Tone Bjørge, Archie Bleyer, Eshetu Mulisa Bobasa, Soufiane Boufous, Rupert Bourne, Oliver Brady, Luisa Brant, Alexandra Brazinova, Nicholas Breitborde, Hermann Brenner, Andrey Briko, Gabrielle Britton, Traolach Brugha, Rachelle Buchbinder, Reinhard Busse, Zahid Butt, Alessandra C Goulart, Lucero Cahuana-Hurtado, Jorge Cano, Rosario Cárdenas, Juan J Carrero, Felix Carvalho, Deborah Carvalho Malta, Carlos Castañeda-Orjuela, Jacqueline Castillo Rivas, Franz Castro, Ferrán Catalá-López, Ester Cerin, Yazan Chaiah, Jung-Chen Chang, Vijay Kumar Chattu, Peggy Chiang, Yilma Chisha, Jee-Young Choi, Hanne Christensen, Devasahayam Christopher, Sheng-Chia Chung, Flavia Cicuttini, Massimo Cirillo, Daniel Collado-Mateo, Cyrus Cooper, Paolo Angelo Cortesi, Monica Cortinovis, Ewerton Cousin, Michael Criqui, John Crump, Alemneh Kabeta Daba, Berihun Dachew, Abel Dadi, Lalit Dandona, Rakhi Dandona, Paul Dargan, Ahmad Daryani, José Das Neves, Tamirat Tesfaye Dasa, Diego De Leo, Jan-Walter De Neve, Hans De Steur, Megbaru Debalkie, Louisa Degenhardt, Edgar Denova-Gutierrez, Kebede Deribe, Nikolaos Dervenis, Don Des Jarlais, Subhojit Dey, Samath Dharmaratne, Meghnath Dhimal, M.Ashworth Dirac, Shirin Djalalinia, Klara Dokova, David Teye Doku, E. Ray Dorsey, Kerrie Doyle, Tim Driscoll, Manisha Dubey, Eleonora Dubljanin, Eyasu Ejeta Duken, Bruce Duncan, Andre Duraes, Hedyeh Ebrahimi, Soheil Ebrahimpour, Michelle Echko, Dumessa Edessa, David Edvardsson,

Andem Effiong, Anne Elise Eggen, Joshua Ehrlich, Ziad El-Khatib, Iqbal Elyazar, Ahmadali Enayati, Aman Endries, Benjamin Er, Holly Erskine, Sharareh Eskandarieh, Alireza Esteghamati, Sadaf Esteghamati, Hamed Fakhim, Mahbobeh Faramarzi, Mohammad Fareed, Farzaneh Farhadi, Talha Farid, Carla Farinha, Andrea Farioli, Andre Faro, Farshad Farzadfar, Valery Feigin, Netsanet Fentahun, Seyed-Mohammad Fereshtehnejad, Eduarda Fernandes, Joao Fernandes, Manuela Ferreira, Irina Filip, Florian Fischer, Christina Fitzmaurice, Nataliya Foigt, Takeshi Fukumoto, Nancy Fullman, Thomas Fürst, João M. Furtado, Silvano Gallus, Morsaleh Ganji, Alberto L. García-Basteiro, Tigist Gashaw, Abadi Kahsu Gebre, Amanuel Tesfay Gebremedhin, Merhawi Gebremedhin, Afewerki Gebremeskel, Segen Gebremeskel, Teklu Gebremichael, Tilayie Feto Gelano, Johanna Geleijnse, Ayele Geleto, Ricard Genova-Maleras, Kebede Embaye Gezae, Maryam Ghasemi-Kasman, Mamata Ghimire, Kidu Gidey, Paramjit Gill, Tiffany Gill, Meaza Girma, Giorgia Giussani, Elena Gnedovskaya, Srinivas Goli, Philimon Gona, Aloke Gopal, Sameer Gopalani, Taren Gorman, Bárbara Goulart, Ayman Grada, Giuseppe Grosso, Harish Gugnani, Francis Guillemin, Yuming Guo, Rahul Gupta, Rajat Das Gupta, Rajeev Gupta, Tanush Gupta, Reyna Alma Gutiérrez, Bishal Gyawali, Juanita Haagsma, Vladimir Hachinski, Nima Hafezi-Nejad, Hassan Haghparast-Bidgoli, Tekleberhan Beyene Hagos, Dessalegn Haile, Arvin Haj-Mirzaian, Arya Haj-Mirzaian, Randah Hamadeh, Samer Hamidi, Alexis Jeannine Handal, Graeme Hankey, Yuantao Hao, Hilda Harb, Sivadasanpillai Harikrishnan, Hamidreza Haririan, Josep Maria Haro, Hadi Hassankhani, Hamid Y. Hassen, Rasmus Havmoeller, Roderick Hay, Akbar Hedayatizadeh-Omran, Behzad Heibati, Delia Hendrie, Ileana Heredia-Pi, Claudiu Herteliu, Fatemeh Heydarpour, Desalegn Hibstu, Long Hoang Nguyen, Son Hoang Nguyen, Michael Hole, Enayatollah Homaie Rad, Praveen Hoogar, H Dean Hosgood, Mostafa Hossini, Mihaela Hostiuc, Sorin Hostiuc, Peter Hotez, Damian Hoy, John Huang, Olayinka Ilesanmi, Farhad Islami, Kathryn H. Jacobsen, Nader Jahanmehr, Sudhir Kumar Jain, Mihajlo Jakovljevic, Spencer James, Achala Jayatilleke, Panniyammakal Jeemon, Ravi Prakash Jha, Vivekanand Jha, Catherine Johnson, Jost B. Jonas, Jitendra Jonnagaddala, Zahra Jorjoran Shushtari, Ankur Joshi, Jacek Jozwiak, Suresh Jungari, Mikk Jürisson, Rajendra Kadel, Amaha Kahsay, Rizwan Kalani, Tanuj Kanchan, Chittaranjan Kar, Manoochehr Karami, Behzad Karami Matin, André Karch, Seyed M. Karimi, Amir Kasaeian, Getachew Mullu Kassa, Tesfaye Kassa, Nicholas J Kassebaum, Srinivasa Vittal Katikireddi, Zhila Kazemi, Ali Kazemi Karyani, Peter Keiyoro, Andre P Kengne, Yousef Khader, Morteza Abdullatif Khafaie, Behzad Khafaie , Nauman Khalid, Ibrahim Khalil, Ejaz Khan, Muhammad Ali Khan, Muhammad Shahzeb Khan, Young-Ho Khang, Mona Khater, Abdullah Khoja, Ardeshir Khosravi, Mohammad Hossein Khosravi, Daniel Kiirithio, Daniel Kim, Yun Jin Kim, Ruth Kimokoti, Adnan Kisa, Katarzyna Kissimova-Skarbek, Ann Kristin Knudsen, Jonathan Kocarnik, Sonali Kochhar, Yoshihiro Kokubo, Tufa Kolola, Jacek Kopec, Soewarta Kosen, Georgios Kotsakis, Parvaiz Koul, Ai Koyanagi, Kewal Krishan, Sanjay Krishnaswami, Barthelemy Kuate Defo, G Anil Kumar, Manasi Kumar, Hmwe Kyu, Deepesh P. Lad, Sheetal Lad, Alessandra Lafranconi, Ratilal Lalloo, Tea Lallukka, Faris Lami, Huong Lan Nguyen, Justin Lang, Sinead Langan, Jeffrey Lazarus, Janet Leasher, Paul Lee, Misgan Legesse , James Leigh, Cheru T Leshargie, Janni Leung, Miriam Levi, Sonia Lewycka, Shanshan Li, Yu Liao, Lee-Ling Lim, Miteku Limenih, Shai Linn, Shiwei Liu, Katharine Looker, Alan Lopez, Stefan Lorkowski, Paulo Lotufo, Raimundas Lunevicius, Ronan Lyons, Kala M. Mehta, Stefan Ma, Erlyn Rachelle Macarayan, Mark Mackay, F Madotto, P A Mahesh, Marek Majdan, Reza Majdzadeh, Azeem Majeed, Reza Malekzadeh, Abdullah Mamun, Mohammad Ali Mansournia, Lorenzo Mantovani, Joemer Maravilla, Wagner Marcenes, Francisco Rogerlândio Martins-Melo, Melvin Marzan, João Massano, Benjamin Massenburg, Manu Mathur, Pallab K Maulik, Mohsen Mazidi, Colm Mcalinden, John Mcgrath, Martin Mckee, Brian Mcmahon, Suresh Mehata, Ravi Mehrotra, Varshil Mehta, Tesfa Mekonen, Addisu Melese, Mulugeta Melku, Peter Memiah, Ziad Memish, Walter Mendoza, Getnet Mengistu, Zerihun

Menlkalew , George Mensah, Seid Tiku Mereta, Atte Meretoja, Tuomo Meretoja, Tomislav Mestrovic, Bartosz Miazgowski, Tomasz Miazgowski, Ted R Miller, Gk Mini, Mojde Mirarefin, Erkin Mirrakhimov, Philip B. Mitchell, Habtamu Mitiku, Babak Moazen, Bahram Mohajer, Moslem Mohammadi, Mohammed Mohammed, Shafiu Mohammed, Farnam Mohebi, Ali Mokdad, Mariam Molokhia, Lorenzo Monasta, Julio Montañez, Mahmood Moosazadeh, Ghobad Moradi, Mahmoudreza Moradi, Maziar Moradi-Lakeh, Mehdi Moradinazar, Paula Moraga, Lidia Morawska, Ilais Moreno, Joana Morgado-Da-Costa, Shane Morrison, Marilita Moschos, Seyyed Meysam Mousavi, Kalayu Brhane Mruts, Achenef Muche, Kindie Fentahun Muchie, Ulrich Mueller, Satinath Mukhopadhyay, Kamarul Imran Musa, Ghulam Mustafa, Ashraf Nabhan, Chie Nagata, Gabriele Nagel, Mohsen Naghavi, Seyed Sina Naghibi Irvani, Aliya Naheed, Azin Nahvijou, Farid Najafi, Vinay Nangia, Jobert Richie Nansseu, Ionut Negoi, Ruxandra Irina Negoi, Subas Neupane, Charles Newton, Josephine Ngunjiri, Emma Nichols, Mehdi Noroozi, Bo Norrving, Jean Jacques Noubiap, Hamid Reza Nouri, Malihe Nourollahpour, Mohammad Reza Nowroozi, Dina Nur Anggraini Ningrum, Richard Ofori-Asenso, Felix Ogbo, In-Hwan Oh, Olanrewaju Oladimeji, Andrew T. Olagunju, Tinuke O. Olagunju, Pedro Olivares, Bolajoko Olusanya, Jacob Olusanya, Kanyin Ong, Eyal Oren, Alberto Ortiz, Erika Ota, Stanislav Otstavnov, Simon Øverland, Mayowa Owolabi, Rosana Pacella, Abhijit Pakhare, Amir Pakpour, Adrian Pana, Songhomitra Panda-Jonas, Eun-Kee Park, Hadi Parsian, Yahya Pasdar, Shanti Patel, Snehal Patil, George Patton, Deepak Paudel, Katherine Paulson, Neil Pearce, Alexandre Pereira, David Pereira, Norberto Perico, Michael R. Phillips, Linh Phuong Doan, David Pigott, Julian Pillay, Michael Piradov, Meghdad Pirsaheb, Farhad Pishgar, Oleguer Plana-Ripoll, Suzanne Polinder, Svetlana Popova, Maarten Postma, Akram Pourshams, Hossein Poustchi, Dorairaj Prabhakaran, Swayam Prakash, V Prakash, Mostafa Qorbani, Hai Quang Pham, D. Alex Quistberg, Amir Radfar, Anwar Rafay, Alireza Rafiei, Fakher Rahim, Kazem Rahimi, Afarin Rahimi-Movaghar, Vafa Rahimi-Movaghar, Mahfuzar Rahman, Muhammad Aziz Rahman, Sajjad Rahman, Rajesh Kumar Rai, Fatemeh Rajati, Prabhat Ranjan, Puja Rao, Davide Rasella, David Laith Rawaf, Salman Rawaf, Kolli Srinath Reddy Reddy, Robert Reiner, Giuseppe Remuzzi, Andre Renzaho, Serge Resnikoff, Mohammad Sadegh Rezai, Stephen Robinson, Leonardo Roever, Luca Ronfani, Gholamreza Roshandel, Ali Rostami, Dietrich Rothenbacher, Enrico Rubagotti, Perminder Sachdev, Hosein Safari, Yahya Safari, Roya Safari-Faramani, Mahdi Safdarian, Sare Safi, Saeid Safiri, Rajesh Sagar, Amirhossein Sahebkar, Mohammad Ali Sahraian, Haniye Sadat Sajadi, Nasir Salam, Yahya Salimi, Hamideh Salimzadeh, Joshua A Salomon, Sundeep Salvi, Abdallah M. Samy, Juan Sanabria, Maria Dolores Sanchez-Niño, Itamar Santos, João Vasco Santos, Milena Santric Milicevic, Bruno Sao Jose, Mayank Sardana, Abdur Razzaque Sarker, Rodrigo Sarmiento-Suárez, Benn Sartorius, Brijesh Sathian, Thirunavukkarasu Sathish, Maheswar Satpathy, Monika Sawhney, Sonia Saxena, Elke Schaeffner, Maria Inês Schmidt, Ione Schneider, Aletta Schutte, David C Schwebel, Falk Schwendicke, James Scott, Sadaf Sepanlou, Edson Serván-Mori, Seyedmojtaba Seyedmousavi, Hosein Shabaninejad, Azadeh Shafieesabet, Amira Shaheen , Masood Ali Shaikh, Mehran Shams-Beyranvand, Mohammadbagher Shamsi, Heidar Sharafi, Kiomars Sharafi, Mehdi Sharif, Mahdi Sharif-Alhoseini, Sheikh Mohammed Shariful Islam, Rajesh Sharma, Jun She, Aziz Sheikh, Mika Shigematsu, Rahman Shiri, Ivy Shiue, Yalda Shokoohinia, Mark Shrime, Si Si, Soraya Siabani, Tariq Jamal Siddiqi, Inga Dora Sigfusdottir, Rannveig Sigurvinsdottir, Diego Augusto Santos Silva, João Pedro Silva, Narayana Sarma Singam, Jasvinder Singh, Narinder Pal Singh, Virendra Singh, Dhirendra Narain Sinha, Mekonnen Sisay, Eirini Skiadaresi, Badr Sobaih, Soheila Sobhani, Moslem Soofi, Reed Sorensen, Ireneous Soyiri, Luciano Sposato, Chandrashekhar T Sreeramareddy, Dan J Stein, Timothy Steiner, Mark Stokes, Lars Jacob Stovner, Muawiyyah Babale Sufiyan, Rizwan Suliankatchi Abdulkader, Gerhard Sulo, Bruno Sunguya, Patrick Sur, Bryan L. Sykes, Pn Sylaja, Dillon Sylte, Cassandra Szoeke, Rafael Tabarés-

Seisdedos, Takahiro Tabuchi, Santosh Tadakamadla, Mesfin Tadese, Eyasu Tamru, Nuno Taveira, Hugh Taylor, Arash Tehrani-Banihashemi, Zelalem Teklemariam, Gebre Teklemariam Demoz, Awoke Temesgen, Mohamad-Hani Temsah, Omar Temsah, Abdullah Terkawi, Tewodros Tesfa, Belay Tessema, Mebrahtu Teweldemedhin, Kavumpurathu Thankappan, Hue Thi Mai, Nihal Thomas, Binyam Tilahun, Quyen G To, Marcello Tonelli, Roman Topor-Madry, Miguel Tortajada-Girbés, Mathilde Touvier, Marcos Roberto Tovani-Palone, Jeffrey Towbin, Bach Tran, Khanh Bao Tran, Christopher Troeger, Derrick Tsoi, Lorainne Tudor Car, Stefanos Tyrovolas, Kingsley Nnanna Ukwaja, Irfan Ullah, Eduardo Undurraga, Muhammad Shariq Usman, Olalekan Uthman, Muthiah Vaduganathan, Afsane Vaezi, Pascual Valdez, Santosh Varughese, Tommi Vasankari, Narayanaswamy Venketasubramanian, Santos Villafaina, Francesco S Violante, Sergey Vladimirov, Vasiliy Vlassov, Stein Emil Vollset, Theo Vos, Kia Vosoughi, Isidora Vujcic, Fasil Wagnew, Shishay Wahdey, Yasir Waheed, Yafeng Wang, Yuan-Pang Wang, Elisabete Weiderpass, Robert Weintraub, Inbal Weiss Salz, Fitsum Weldegebreal, Andrea Werdecker, T. Eoin West, Ronny Westerman, Harvey Whiteford, Tissa Wijeratne, Hywel Williams, Lauren Wilner, Andrea Winkler, Alison B. Wiyeh, Charles Shey Wiysonge, Charles Wolfe, Anthony Woolf, Grant Wyper, Denis Xavier , Gelin Xu, Tomohide Yamada, Lijing Yan, Yuichiro Yano, Mehdi Yaseri, Yasin Jemal Yasin, Alex Yeshaneh, Ebrahim M. Yimer, Engida Yisma, Naohiro Yonemoto, Seok-Jun Yoon, Marcel Yotebieng, Mustafa Younis, Mahmoud Yousefifard, Chuanhua Yu, Zoubida Zaidi, Sojib Bin Zaman, Mohammad Zamani, Hamed Zandian, Heather Zar, Olifan Zewdie, Inbar Zucker, and Liesl Zuhlke.

Drafting the work or revising is critically for important intellectual content

Nooshin Abbasi, Olatunji Adetokunboh, Mohsen Afarideh, Muktar Ahmed, Tomi Akinyemiju, Fares Alahdab, Khurshid Alam, Syed Aljunid, Yaw Ampem Amoako, Marcel Ausloos, Peter Azzopardi, Nam Ba Nguyen, Hamid Badali, Shahrzad Bazargan-Hejazi, Yihalem Abebe Belay, Neeraj Bhala, Molly Biehl, Oliver Brady, Alessandra C Goulart, Franz Castro, Devasahayam Christopher, Flavia Cicuttini, Cyrus Cooper, Ahmad Daryani, Dragos Davitoiu, Jan-Walter De Neve, Selina Deiparine, Samath Dharmaratne, David Edvardsson, Holly Erskine, Sadaf Esteghamati, Andre Faro, Seyed-Mohammad Fereshtehnejad, Joao Fernandes, Nataliya Foigt, Takeshi Fukumoto, Morsaleh Ganji, Segen Gebremeskel, Maryam Ghasemi-Kasman, Ibrahim Ginawi, Rajat Das Gupta, Alexis Jeannine Handal, Hadi Hassankhani, Rasmus Havmoeller, Claudiu Herteliu, Long Hoang Nguyen, Son Hoang Nguyen, Enayatollah Homaie Rad, Farhad Islami, Ravi Prakash Jha, Mikk Jürisson, Manoochehr Karami, Behzad Khafaie , Alireza Khajavi, Mona Khater, Yun Jin Kim, Hmwe Kyu, Huong Lan Nguyen, Mostafa Leili, Janni Leung, Alan Lopez, Ana Laura Manda, Mohammad Ali Mansournia, Joemer Maravilla, Francisco Rogerlândio Martins-Melo, Melvin Marzan, Benjamin Massenburg, Colm Mcalinden, Tuomo Meretoja, Bahram Mohajer, Karzan Mohammad, Ali Mokdad, Ghobad Moradi, Mehdi Moradinazar, Ghulam Mustafa, Nahid Neamati, Molly Nixon, Olanrewaju Oladimeji, Andrew T. Olagunju, Tinuke O. Olagunju, Bolajoko Olusanya, Jacob Olusanya, Simon Øverland, Konrad Pesudovs, Michael R. Phillips, Linh Phuong Doan, Hai Quang Pham, Anwar Rafay, Vafa Rahimi-Movaghar, Fatemeh Rajati, Leonardo Roever, Saeid Safiri, Juan Sanabria, Damian Santomauro, Milena Santric Milicevic, Bruno Sao Jose, Abdur Razzaque Sarker, Shahabeddin Sarvi, Maheswar Satpathy, Monika Sawhney, Seyedmojtaba Seyedmousavi, Mehdi Sharif, Mahdi Sharif-Alhoseini, Sheikh Mohammed Shariful Islam, Mika Shigematsu, Haitham Shoman, Inga Dora Sigfusdottir, Rannveig Sigurvinsdottir, Diego Augusto Santos Silva, Dhirendra Narain Sinha, Eirini Skiadaresi, Eyasu Tamru, Nuno Taveira, Hue Thi Mai, Nihal Thomas, Marcos Roberto Tovani-Palone, Bach Tran, Olalekan Uthman, Vasiliy Vlassov, Yuan-Pang Wang, and Robert Weintraub.

Extracting, cleaning, or cataloging data; designing or coding figures and tables

Hedayat Abbastabar, Ibrahim Abdollahpour, Olatunji Adetokunboh, Kareha Agesa, Rakesh Aggarwal, Muktar Ahmed, Khurshid Alam, Marcel Ausloos, Adugnaw Berhane, Donal Bisanzio, Rupert Bourne, Paul Briant, Julio Cesar Campuzano, John Crump, Ahmad Daryani, Selina Deiparine, M.Ashworth Dirac, Aman Endries, Benjamin Er, Holly Erskine, Hamed Fakhim, Andre Faro, Alize Ferrari, Takeshi Fukumoto, Nancy Fullman, William Gardner, Segen Gebremeskel, Johanna Geleijnse, Ayele Geleto, Ellen M. Goldberg, Taren Gorman, Yuming Guo, Roderick Hay, Behzad Heibati, Claudiu Herteliu, Chad Ikeda, Catherine Johnson, Chittaranjan Kar, Manoochehr Karami, André Karch, Tesfaye Kassa, Nicholas J Kassebaum, Laura Kemmer, Grant Rogers Kemp, Andre Keren, Mohammad Hossein Khosravi, Yun Jin Kim, Barthelemy Kuate Defo, Van Lansingh, Misgan Legesse , Mostafa Leili, Janni Leung, Shanshan Li, Stefan Ma, Emilie Maddison, Reza Malekzadeh, Helena Manguerra, Mohammad Ali Mansournia, Ana M. Mantilla-Herrera, Manu Mathur, Mohsen Mazidi, Maziar Moradi-Lakeh, Minh Nguyen, Emma Nichols, Kanyin Ong, Katherine Paulson, David Pigott, Hossein Poustchi, Caroline Purcell, Enrico Rubagotti, Hosein Safari, Saeid Safiri, Abdallah M. Samy, Damian Santomauro, Maheswar Satpathy, Sadaf Sepanlou, Seyedmojtaba Seyedmousavi, Mehdi Sharif, Farhad Shokraneh, Soraya Siabani, Chandrashekhar T Sreeramareddy, Jeffrey Stanaway, Lars Jacob Stovner, Michelle Subart, Patrick Sur, Dillon Sylte, Eyasu Tamru, Mohammad Tavakkoli, Awoke Temesgen, Anna Torre, Miguel Tortajada-Girbés, Bach Tran, Christopher Troeger, Derrick Tsoi, Irfan Ullah, Rachel L. Updike, Inbal Weiss Salz, Tissa Wijeratne, Simon Yadgir, Hossein Yahyazadeh, Mahmoud Yousefifard, and Zoubida Zaidi.

Managing the overall research enterprise

Ashkan Afshin, Tahiya Alam, Peter Allebeck, Megha Arora, Molly Biehl, Deborah Carvalho Malta, Elizabeth Cromwell, Lalit Dandona, Rakhi Dandona, Louisa Degenhardt, Samath Dharmaratne, Valery Feigin, Christina Fitzmaurice, Kyle Foreman, Nancy Fullman, Thomas Fürst, Emmanuela Gakidou, Simon Hay, Peter Hotez, Spencer James, Nicholas Kassebaum, Ibrahim Khalil, Kristopher Krohn, Hmwe Kyu, Xiaofeng Liang, Stephen Lim, Alan Lopez, Rafael Lozano, Ashley Marks, George Mensah, Awoke Misganaw, Ali Mokdad, Kate Muller, Christopher Murray, Mohsen Naghavi, Molly Nixon, Elaine Nsoesie, Helen Olsen, David Pigott, Puja Rao, Robert Reiner, Joseph Salama, Joshua Salomon, Benn Sartorius, David Smith, Mari Smith, Jeffrey Stanaway, Caitlyn Steiner, Roman Topor-Madry, Stein Emil Vollset, Theo Vos, and Harvey Whiteford.

Did not provide contribution information

Dilaram Acharya, Nahla Anber, Jalal Arabloo, Ali Akbar Fazaeli, Mohammad Rasoul Ghadami, Keyghobad Ghadiri, Mohamed Hsairi, Caleb Irvine, John Ji, Narges Karimi, Masoud Keighobadi, Mohammad Khazaei, Anoushka Millear, Mousa Mohammadnia-Afrouzi, Gvs Murthy, Peter Nyasulu, Satar Rezaei, Sahar Saeedi Moghaddam, Zikria Saleem, Mehdi Shahbazi, Reza Shirkoohi, Karen Sliwa, Vladimir Starodubov, Nikhil Tandon, Elena Varavikova, and Paul Yip.

Section 1. GBD Overview

Section 1.1. Locations of the Analysis

The locations included in GBD 2017 are organized into groups of seven super-regions which contain a total of 21 regions containing 195 countries and territories, as shown in Appendix Table 2. The locations for which GBD estimated global, regional, and national cause-specific mortality and years of life lost (YLLs) have not expanded following GBD 2015. Subnational estimation in GBD 2017 includes all countries with populations over 200 million (China, India, United States, Indonesia, and Brazil) as well as additional countries which have requested and undertaken subnational analyses collaboratively with the GBD Study (Japan, Ethiopia, Iran, Kenya, Mexico, Norway, Russia, South Africa, and Sweden at the administrative one level; New Zealand separately by Maori ethnicity; and the United Kingdom at the upper-tier local authority level). For this publication, we present all subnational results that have been already published elsewhere; given space constraints the results are presented in appendix tables and figures instead of the main text. For countries with populations over 200 million that have not yet been published elsewhere, we present results in maps.

Section 1.2. Time Period of the Analysis

We estimated a complete set of cause-specific mortality and years of life lost (YLL) numbers and rates for the years 1980-2017, and a set of non-fatal burden and years lived with disability (YLDs) numbers and rates for 1990-2017.

DALYs were calculated as the sum of YLLs and YLDs for each cause, location, age group, sex, and year.

The estimates of YLDs per capita for each location-age-sex-year from 1990 to 2017 are used to determine HALE by age group within abridged multiple-decrement life tables.

Section 1.3. Statement of GATHER Compliance

This study complies with the Guidelines for Accurate and Transparent Health Estimates Reporting (GATHER) recommendations. We have documented the steps involved in our analytical procedures and detailed the data sources used in compliance with the Guidelines for Accurate and Transparent Health Estimates Reporting (GATHER). See Appendix Table 1 for GATHER checklist.

The GATHER recommendations may be found here: http://gather-statement.org/

Section 1.4. GBD Glossary

Phrase	Acronym
Annualised Rate Of Change	ARC
Antenatal Care	ANC
Antiretroviral Therapy	ART
Basic Tabulation List	BTL
Body-mass index	BMI
Cancer Registry	CR
Case-Detection Rates	CDRs
Cause-Specific Mortality Rate	CSMR
Causes Of Death	COD
Causes-Of-Death Ensemble Modelling	CODEm
Centers for Disease Control & Prevention	CDC
Civil Registration System	CRS
Cluster Of Differentiation 4	CD4
Comparative Risk Assessment	CRA
Complete Birth Histories	CBH
Crude Death Rate	CDR
Data Representativeness Index	DRI
Demographic and Health Survey	DHS
Disability-Adjusted Life Year	DALY
Disease Model-Bayesian Meta-regression	DisMod-MR
Disease Surveillance Points	DSP
Emergency Obstetric Care	EmOC
Enzyme-Linked Immunosorbent Assay	ELISA
Epidemiology	Epi
Estimation and Projection Package	EPP
European Commission	EC
Fasting Plasma Glucose	FPG
Food Frequency Questionnaires	FFQs
Global Burden of Disease	GBD
Global Enteric Multicentre Study	GEMS
Guidelines for Accurate and Transparent Health Estimates Reporting	GATHER
Healthy Life Expectancy	HALE
Heart Outcomes Prevention Evaluation–3	HOPE-3
Human Development Index	HDI
Institute for Health Metrics and Evaluation	IHME
Integrated Exposure Response	IER
Inter-agency Group for Child Mortality Estimation	IGME
International Agency For Research On Cancer	IARC
International Classification of Diseases	ICD

International Diabetes Federation	IDF
Lagged Distributed Income	LDI
Low-Density Lipoprotein Cholesterol	LDL cholesterol
Low-Income And Middle-Income Countries	LMICs
Maternal Mortality	MM
Maternal Mortality Estimation Inter-Agency Group	MMEIG
Maternal Mortality Ratio	MMR
Maternal Mortality Surveillance	MMS
Medical Certification of Causes of Death	MCCD
Medical Expenditure Panel Surveys	MEPS
Millennium Development Goals	MDGs
Mortality/Incidence Ratio	MIR
Non-communicable Diseases	NCDs
Organisation for Economic Co-operation and Development	OECD
Polyunsaturated Fatty Acids	PUFAs
Pooled Resource Open-<br>Access ALS Clinical Trials	PROACT
Population-attributable Fraction	PAF
Prevention Of Mother-To-Child Transmission	PMTCT
Probability Of Death From Age 15 To 60 Years	45q15
Probability Of Death From Birth To Age 5 Years	5q0
Prospective Urban Rural Epidemiology	PURE
Quantitative Polymerase Chain Reaction Diagnostic	qPCR
Relative Risk	RR
Root Mean Square Error	RMSE
Sample Registration System	SRS
Short Form 12 questions	SF-12
Socio-demographic Index	SDI
Spatiotemporal Gaussian Process Regression	ST-GPR
Standardised Mortality Ratio	SMR
Stillbirth Epidemiology Investigator Group	SEIG
Summary Birth History	SBH
Summary Exposure Value	SEV
Super-Region Median Average Deviation	SR MAD
Surveillance, Epidemiology, and End Results Program	SEER
Survey of Causes of Death	SCD
Sustainable Development Goals	SDGs
Systolic Blood Pressure Intervention Trial	SPRINT
TaqMan Array Card	TAC
The Joint United Nations Programme on HIV and AIDS	UNAIDS
The United Nations Children's Fund	UNICEF
Theoretical Minimum Risk Level	TMREL
Uncertainty Intervals	UIs

Verbal Autopsy	VA
Vital Registration	VR
World Malaria Report	WMR
World Population Prospects	WPP
Years Lived with Disability	YLDs
Years of Life Lost	YLLs

Section 1.5. GBD results overview

Results from the Global Burden of Disease Study (GBD 2017) are now measured in terabytes. Results will be made available upon manuscript acceptance in an interactive data downloading tool on the Global Health Data exchange (GHDx).

The current version of the data download tool is available in the GHDx and will contains core summary results for the GBD 2017 upon manuscript acceptance: http://ghdx.healthdata.org/gbd-results-tool. The core summary results include deaths, YLLs, years lived with disability (YLDs), and disability-adjusted life-years (DALYs). The GHDx includes data for causes, risks, cause-risk attribution, aetiologies, and impairments.

In the GBD 2017 version, the GHDx tool also contains measures such as prevalence and incidence as well as rate of change data. Data above a certain size cannot be viewed online but can be downloaded. Depending on the size of the download, users may need to enter an email address; a download location will be sent to them when the files are prepared.

Section 1.6. Data input sources overview

GBD 2017 incorporated a large number and wide variety of input sources to estimate mortality, causes of death and illness, and risk factors for 195 countries and territories from 1990-2017. These input sources are accessible through an interactive citation tool available in IHME's GHDx.

Data and underlying code used for this analysis will be made publicly available pending acceptance.

Section 1.7. Funding Sources

Funding for this research was provided by the Bill & Melinda Gates Foundation.

Section 2. Estimation Process for DALYs/HALE

Section 2.1 Computing DALYs

To estimate DALYs for GBD 2017, we started by estimating cause-specific mortality and non-fatal health loss. For each year for which YLDs have been estimated (1990, 1995, 2000, 2007, 2010 and 2017), we compute DALYs by adding YLLs and YLDs for each age-sex-location. Uncertainty in YLLs was assumed to be independent of uncertainty in YLDs. We calculated 1,000 draws for DALYs by summing the first draw of the 1000 draws for YLLs and YLDs and then repeating for each subsequent draw. 95% uncertainty intervals (UI) were computed using the 25th and 975th ordered draw of the DALY uncertainty distribution. Please refer to the appendices of the GBD 2017 non-fatal capstone and cause of death capstone publications for information on how YLLs and YLDs were computed. We calculate DALYs as the sum of YLLs and YLDs for each cause, location, age group, sex, and year. For more information, please refer to Appendix Figure 1 below.

Appendix Figure 1. Estimation for DALYs & HALE for GBD 2017

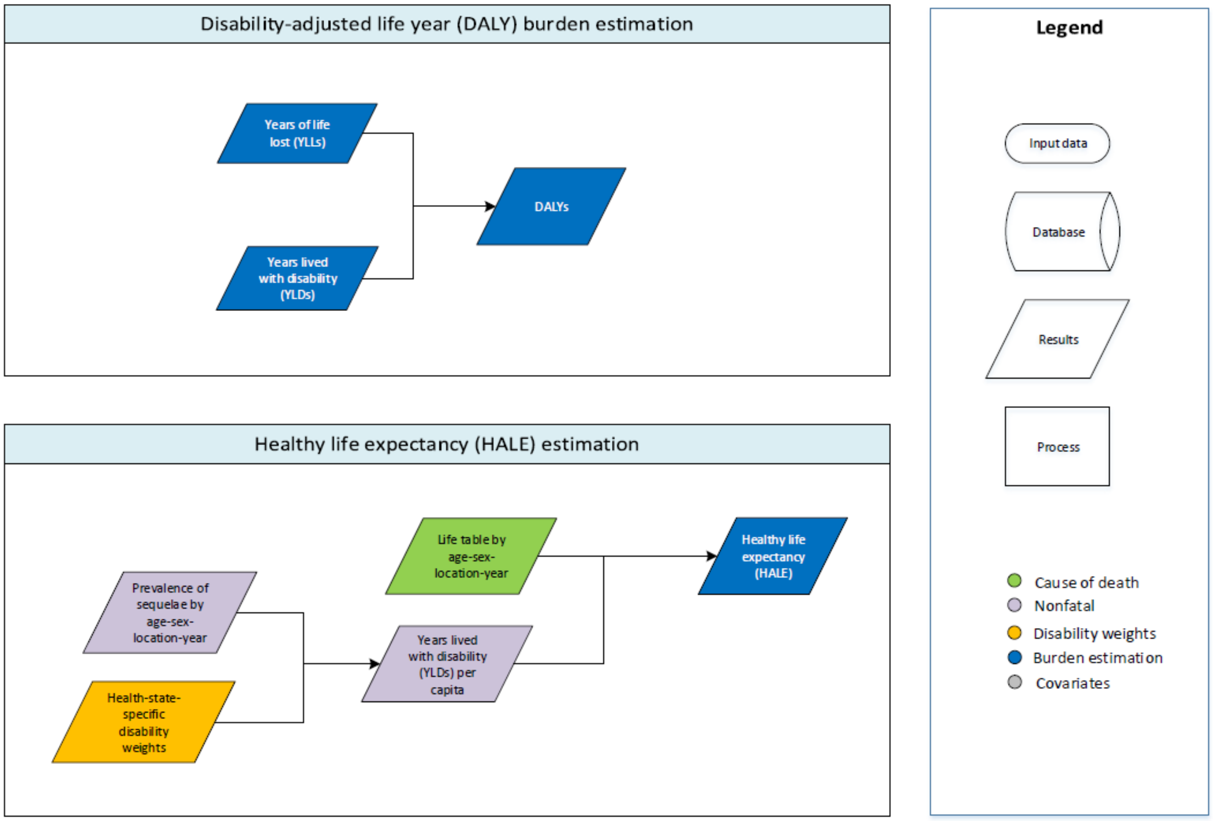

Section 2.2 Computing HALE

The first step to calculating healthy life expectancy for a population (defined by sex, country, and year) was to compute average health of individuals for every age group in that population. We combined information about prevalences for all sequelae and their associated disability weights, and accounted for comorbidity with a Monte Carlo simulation approach. We made the assumption that comorbidities were independent within each age group. We created simulations where individuals were exposed to each sequela with a probability equal to the estimated prevalence of that sequela in each age group. This created a simulated population where the frequencies of many possible multi-morbidities were consistent with the underlying estimates of prevalence. We define 1 minus the disability weight as the positive health associated with each sequela. The combined health for a simulated individual was the product of these positive health values for all relevant sequelae in the presence of multiple sequelae. Average health values are computed as 1 minute the YLD per person in a population, which are then used to compute health adjusted person years.

We incorporated average health values into the life table using Sullivan's method. First, we multiplied values in the nLx (average person-years lived within an age interval starting at age x) column of the life table by the corresponding average health value in that interval. We recalculated the rest of the life table using the adjusted nLx values. Sullivan's method began with an adjusted estimate of health adjusted life years within the terminal age interval (equal to nLx multiplied by the average health value for the terminal age group) and subsequent calculations we produced estimates by iterating through younger age intervals, summing the health-adjusted person-years with all age intervals above the current age interval to generate health adjusted person years lived above a certain age (adjusted Tx) for each age group. After calculating adjusted Tx for all age groups, HALE was calculated by dividing the adjusted Tx for each age group by the proportion of hypothetical birth cohort still alive at age x.

Section 2.3 Socio-Demographic Index (SDI) Definitions & Method

Overview

The Socio-demographic Index (SDI) is a composite indicator of development status strongly correlated with health outcomes. In short, it is the geometric mean of 0 to 1 indices of total fertility under 25 (TFU25), mean education for those aged 15 and older (EDU15+), and lag distributed income (LDI) per capita.

Section 2.3.1 Development of revised SDI indicator

SDI was originally constructed for GBD 2015 using the Human Development Index (HDI) methodology, wherein a 0 to 1 index value was determined for each of the original three covariate inputs (TFR in ages 15 to 49, EDU15+, and LDI per capita) using the observed minima and maxima over the estimation period to set the scales.[1]

In response to feedback from collaborators and the evolution of the GBD, we have refined the indicator with each GBD cycle. For GBD 2017, in conjunction with our expanded estimation of age-specific fertility, we replaced total fertility rate (TFR) with TFU25 as one of the three component indices. The TFU25 provides a better measure of women's status in society, as it focuses on ages where childbearing disrupts the pursuit of education and entrance into the workforce. The concordance correlation coefficient between SDI using the GBD 2016 method and the updated method for GBD 2017 was 0.981.

During GBD 2016 we moved from using relative index scales to absolute scales to enhance the stability of SDI's interpretation over time, as we noticed that the measure was highly sensitive to the addition of subnational units that tended to stretch the empirical minima and maxima.[2] We selected the minima and maxima of the scales by examining the relationships each of the inputs had with life expectancy at birth and under-5 mortality and identifying points of limiting returns at both high and low values, if they occurred prior to theoretical limits (e.g., a TFU25 of 0).

Thus, an index score of 0 represents the minimum level of each covariate input past which selected health outcomes can get no worse, while an index score of 1 represents the maximum level of each covariate input past which selected health outcomes cease to improve. As a composite, a location with an SDI of 0 would have a theoretical minimum level of development relevant to these health outcomes, while a location with an SDI of 1 would have a theoretical maximum level of development relevant to these health outcomes.

The final scales for GBD 2017 are summarized in table C below.

Table C. Final SDI scales

Input	Lower Bound	Upper Bound
TFU25	0	3
LDI per capita	250 USD (5.52 log USD)[a]	60,000 USD (11.00 log USD)
EDU15+	0 years	17 years

[a] The minimum for the LDI scale was originally set at the theoretical limit of 0 USD, as we did not observe an asymptotic relationship between log(LDI) and E_0 or 5q0 at lower values of log(LDI). Empirically, however, we also did not observe an LDI below 350 USD (5.86 log USD) for the estimation period 1970-2016. In log-space, this meant that approximately half of our scale was not being utilized, compressing the observed variation in LDI and diminishing its meaningful contribution to SDI. Accordingly, we set the lower limit on LDI to 250 USD (5.52 log USD) to ensure we were fully utilizing the range of the scale to capture its variation across space and time, as is the case with the other two inputs.

Using scales described above, we computed the index scores underlying SDI as follows:

$$I_{Cly} = \frac{(C_{ly} - C_{low})}{(C_{high} - C_{low})}$$

Where I_{cly} – the index for covariate *C*, location *l*, and year *y* – is equal to the difference between the value of that covariate in that location-year and the lower bound of the covariate divided by the difference between the upper and lower bounds for that covariate. If the values of input covariates fell outside the upper or lower bounds (e.g. LDI per capita greater than 60,000 USD), they were mapped to the respective upper or lower bounds. The index value for TFU25 was computed as $1 - I_{TFU25ly}$, as lower TFU25s correspond to higher levels of development, and thus higher index scores. For GBD 2017 we expanded the computation of SDI to 890 national and subnational locations spanning the time period 1950-2017.

The composite SCI was the geometric mean of these three indices for a given location-year. The cutoff values used to determine quintiles for analysis were then computed using country-level estimates of SDI for the year 2017, excluding countries with populations less than 1 million. SDI groupings by geography are provided in Appendix Table 7; SDI values by location are provided in Appendix Tables 8-10.

Example Calculation

Below we present the calculation of SDI for Mexico in the year 2010

$$TFU25 = 1.09;\ Mean\ educ\ yrs\ pc = 8.23;\ lnLDI = 9.60$$

$$I_{TFU25} = 1 - \frac{1.09 - 0}{3 - 0} = .637$$

$$I_{Educ} = \frac{8.23 - 0}{17 - 0} = .484$$

$$I_{lnLDI} = \frac{9.60 - 5.52}{11.00 - 5.52} = .744$$

$$SDI = \sqrt[3]{I_{TFU25} * I_{Educ} * I_{lnLDI}} = \sqrt[3]{.637 * .484 * .744} = .611$$

Section 2.3 Socio-Demographic Index (SDI) analysis & Epidemiological Transition

We derived expected DALYs on the basis of SDI using the separate YLL and YLD models described in the GBD 2016 cause of death and non-fatal papers.[1] Additionally, HALE was calculated using the same methods described in both the main text of the paper and earlier in the appendix, with expected YLD rates and expected life tables as inputs.

Section 3. Comparison of GBD 2017 to other global estimates

The GBD study is the only source of comprehensive quantification of population health summary measures, including YLLs, YLDs, DALYs and HALE. There are specific efforts to estimate burden within other organisations which are relevant to policy makers. In the following sections we explore how some of the major sources of DALYs and HALE compare with other global estimates. Since GBD 2015, most organisations we evaluated have not produced updated estimates for DALYs or HALE. The exception to this is WHO, which has released updated Global Health Estimates for DALYs and HALE for 183 countries from 2000 to 2015. These estimates draw heavily on the GBD 2015 results with revisions to the all-cause mortality envelope and revisions to selected cause-specific disability weights and severity distributions for YLDs.

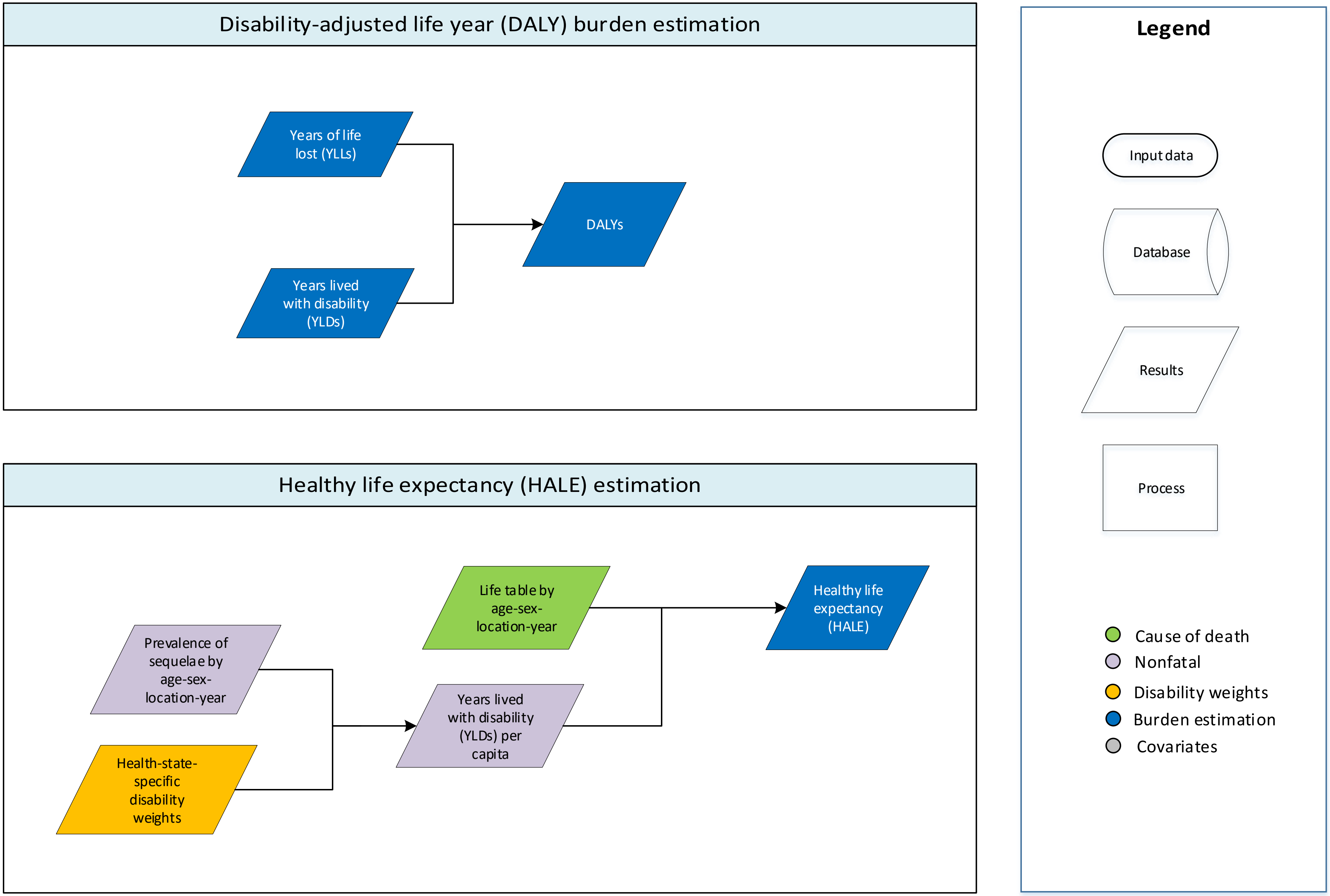
Disability-adjusted life year (DALY) burden estimation
Years of life lost (YLLs)
Years lived with disability (YLDs)
DALYs
Healthy life expectancy (HALE) estimation
Life table by age-sex-location-year
Healthy life expectancy (HALE)
Prevalence of sequelae by age-sex-location-year
Years lived with disability (YLDs) per capita
Health-state-specific disability weights
Legend
Input data
Database
Results
Process
Cause of death
Nonfatal
Disability weights
Burden estimation
Covariates

Methods Appendix Table 1. GATHER checklist of information that should be included in reports of global health estimates, with description of compliance and location of information for Global, regional, and national disability-adjusted life-years (DALYs) for 359 diseases and injuries and healthy life expectancy (HALE) for 195 countries and territories, 1990–2017: a systematic analysis for the Global Burden of Disease Study 2017

#	GATHER checklist item	Description of compliance	Reference
**Objectives and funding**			
1	Define the indicators, populations, and time periods for which estimates were made.	Narrative provided in paper and methods appendix describing indicators, definitions, and populations	Main text (Methods: Overview, Geographic units and time periods) and methods appendix
2	List the funding sources for the work.	Funding sources listed in paper	Summary (Funding)
**Data Inputs**			
For all data inputs from multiple sources that are synthesized as part of the study:			
3	Describe how the data were identified and how the data were accessed.	Narrative description of data seeking methods provided	Main text (Methods) and methods appendix
4	Specify the inclusion and exclusion criteria. Identify all ad-hoc exclusions.	Narrative about inclusion and exclusion criteria by data type provided	Main text (Methods) and methods appendix
5	Provide information on all included data sources and their main characteristics. For each data source used, report reference information or contact name/institution, population represented, data collection method, year(s) of data collection, sex and age range, diagnostic criteria or measurement method, and sample size, as relevant.	An interactive, online data source tool that provides metadata for data sources by component, geography, cause, risk, or impairment has been developed	Online data citation tools
6	Identify and describe any categories of input data that have potentially important biases (e.g., based on characteristics listed in item 5).	Summary of known biases by cause included in methods appendix	Methods appendix
For data inputs that contribute to the analysis but were not synthesized as part of the study:			
7	Describe and give sources for any other data inputs.	Included in online data source tool, http://ghdx.healthdata.org/gbd-2017	Online data citation tools
For all data inputs:			
8	Provide all data inputs in a file format from which data can be efficiently extracted (e.g., a spreadsheet as opposed to a PDF), including all relevant meta-data listed in item 5. For any	Downloads of input data available through	Online data visualization tools, data query tools, and

	data inputs that cannot be shared due to ethical or legal reasons, such as third-party ownership, provide a contact name or the name of the institution that retains the right to the data.	online tools, including data visualization tools and data query tools, http://ghdx.healthdata.org/gbd-2017; input data not available in tools will be made available upon request	the Global Health Data Exchange, http://ghdx.healthdata.org
**Data analysis**			
9	Provide a conceptual overview of the data analysis method. A diagram may be helpful.	Flow diagrams of the overall methodological processes, as well as cause-specific modelling processes, have been provided	Main text (Methods) and methods appendix
10	Provide a detailed description of all steps of the analysis, including mathematical formulae. This description should cover, as relevant, data cleaning, data pre-processing, data adjustments and weighting of data sources, and mathematical or statistical model(s).	Flow diagrams and corresponding methodological write-ups for each cause, as well as the demographics and causes of death databases and modelling processes, have been provided	Main text (Methods) and methods appendix
11	Describe how candidate models were evaluated and how the final model(s) were selected.	Provided in the methodological write-ups	Methods appendix
12	Provide the results of an evaluation of model performance, if done, as well as the results of any relevant sensitivity analysis.	Provided in the methodological write-ups	Methods appendix
13	Describe methods for calculating uncertainty of the estimates. State which sources of uncertainty were, and were not, accounted for in the uncertainty analysis.	Provided in the methodological write-ups	Methods appendix
14	State how analytic or statistical source code used to generate estimates can be accessed.	Access statement provided	Code is provided in an online repository
**Results and Discussion**			
15	Provide published estimates in a file format from which data can be efficiently extracted.	Results are available through online data visualization tools, the Global Health Data Exchange, and the online data query tool (http://ghdx.healthdata.org/gbd-2017)	Main text, methods appendix, and online data tools (data visualization tools, data query tools, and the Global Health Data Exchange, http://ghdx.healthdata.org/gbd-2017)
16	Report a quantitative measure of the uncertainty of the estimates (e.g. uncertainty intervals).	Uncertainty intervals are provided with all	Main text, methods appendix, and online

		results	data tools (data visualization tools, data query tools, and the Global Health Data Exchange, http://ghdx.healthdata.org/gbd-2017)
17	Interpret results in light of existing evidence. If updating a previous set of estimates, describe the reasons for changes in estimates.	Discussion of methodological changes between GBD rounds provided in the narrative of the Article and methods appendix	Main text (Methods and Discussion) and methods appendix
18	Discuss limitations of the estimates. Include a discussion of any modelling assumptions or data limitations that affect interpretation of the estimates.	Discussion of limitations provided in the narrative of the main paper, as well as in the methodological write-ups in the methods appendix	Main text (Limitations) and methods appendix

Methods Appendix Table 2. GBD 2017 location hierarchy with levels	
**Geography**	**Level**
Global	0
Low SDI	1
Low-middle SDI	1
Middle SDI	1
High-middle SDI	1
High SDI	1
Central Europe, Eastern Europe, and Central Asia	1
Central Asia	2
Armenia	3
Azerbaijan	3
Georgia	3
Kazakhstan	3
Kyrgyzstan	3
Mongolia	3
Tajikistan	3
Turkmenistan	3
Uzbekistan	3
Central Europe	2
Albania	3
Bosnia and Herzegovina	3
Bulgaria	3
Croatia	3
Czech Republic	3
Hungary	3
Macedonia	3
Montenegro	3
Poland	3
Romania	3
Serbia	3
Slovakia	3
Slovenia	3
Eastern Europe	2
Belarus	3
Estonia	3
Latvia	3
Lithuania	3
Moldova	3
Russian Federation	3
Ukraine	3
High-income	1
Australasia	2
Australia	3
New Zealand	3
High-income Asia-Pacific	2

Methods Appendix Table 2. GBD 2017 location hierarchy with levels	
**Geography**	**Level**
Brunei	3
Japan	3
Aichi	4
Akita	4
Aomori	4
Chiba	4
Ehime	4
Fukui	4
Fukuoka	4
Fukushima	4
Gifu	4
Gunma	4
Hiroshima	4
Hokkaidō	4
Hyōgo	4
Ibaraki	4
Ishikawa	4
Iwate	4
Kagawa	4
Kagoshima	4
Kanagawa	4
Kōchi	4
Kumamoto	4
Kyōto	4
Mie	4
Miyagi	4
Miyazaki	4
Nagano	4
Nagasaki	4
Nara	4
Niigata	4
Ōita	4
Okayama	4
Okinawa	4
Ōsaka	4
Saga	4
Saitama	4
Shiga	4
Shimane	4
Shizuoka	4
Tochigi	4
Tokushima	4
Tōkyō	4
Tottori	4
Toyama	4

Methods Appendix Table 2. GBD 2017 location hierarchy with levels

Geography	Level
Wakayama	4
Yamagata	4
Yamaguchi	4
Yamanashi	4
South Korea	3
Singapore	3
High-income North America	2
Canada	3
Greenland	3
USA	3
Alabama	4
Alaska	4
Arizona	4
Arkansas	4
California	4
Colorado	4
Connecticut	4
Delaware	4
Washington, DC	4
Florida	4
Georgia	4
Hawaii	4
Idaho	4
Illinois	4
Indiana	4
Iowa	4
Kansas	4
Kentucky	4
Louisiana	4
Maine	4
Maryland	4
Massachusetts	4
Michigan	4
Minnesota	4
Mississippi	4
Missouri	4
Montana	4
Nebraska	4
Nevada	4
New Hampshire	4
New Jersey	4
New Mexico	4
New York	4
North Carolina	4
North Dakota	4

Methods Appendix Table 2. GBD 2017 location hierarchy with levels	
**Geography**	**Level**
Ohio	4
Oklahoma	4
Oregon	4
Pennsylvania	4
Rhode Island	4
South Carolina	4
South Dakota	4
Tennessee	4
Texas	4
Utah	4
Vermont	4
Virginia	4
Washington	4
West Virginia	4
Wisconsin	4
Wyoming	4
Southern Latin America	2
Argentina	3
Chile	3
Uruguay	3
Western Europe	2
Andorra	3
Austria	3
Belgium	3
Cyprus	3
Denmark	3
Finland	3
France	3
Germany	3
Greece	3
Iceland	3
Ireland	3
Israel	3
Italy	3
Luxembourg	3
Malta	3
Netherlands	3
Norway	3
Portugal	3
Spain	3
Sweden	3
Stockholm	4
Sweden except Stockholm	4
Switzerland	3
United Kingdom	3

Methods Appendix Table 2. GBD 2017 location hierarchy with levels	
**Geography**	**Level**
England	4
East Midlands	5
Derby	6
Derbyshire	6
Leicester	6
Leicestershire	6
Lincolnshire	6
Northamptonshire	6
Nottingham	6
Nottinghamshire	6
Rutland	6
East of England	5
Bedford	6
Cambridgeshire	6
Central Bedfordshire	6
Essex	6
Hertfordshire	6
Luton	6
Norfolk	6
Peterborough	6
Southend-on-Sea	6
Suffolk	6
Thurrock	6
Greater London	5
Barking and Dagenham	6
Barnet	6
Bexley	6
Brent	6
Bromley	6
Camden	6
Croydon	6
Ealing	6
Enfield	6
Greenwich	6
Hackney	6
Hammersmith and Fulham	6
Haringey	6
Harrow	6
Havering	6
Hillingdon	6
Hounslow	6
Islington	6
Kensington and Chelsea	6
Kingston upon Thames	6
Lambeth	6

Methods Appendix Table 2. GBD 2017 location hierarchy with levels	
**Geography**	**Level**
Lewisham	6
Merton	6
Newham	6
Redbridge	6
Richmond upon Thames	6
Southwark	6
Sutton	6
Tower Hamlets	6
Waltham Forest	6
Wandsworth	6
Westminster	6
North East England	5
County Durham	6
Darlington	6
Gateshead	6
Hartlepool	6
Middlesbrough	6
Newcastle upon Tyne	6
North Tyneside	6
Northumberland	6
Redcar and Cleveland	6
South Tyneside	6
Stockton-on-Tees	6
Sunderland	6
North West England	5
Blackburn with Darwen	6
Blackpool	6
Bolton	6
Bury	6
Cheshire East	6
Cheshire West and Chester	6
Cumbria	6
Halton	6
Knowsley	6
Lancashire	6
Liverpool	6
Manchester	6
Oldham	6
Rochdale	6
Salford	6
Sefton	6
St Helens	6
Stockport	6
Tameside	6
Trafford	6

Methods Appendix Table 2. GBD 2017 location hierarchy with levels	
**Geography**	**Level**
Warrington	6
Wigan	6
Wirral	6
South East England	5
Bracknell Forest	6
Brighton and Hove	6
Buckinghamshire	6
East Sussex	6
Hampshire	6
Isle of Wight	6
Kent	6
Medway	6
Milton Keynes	6
Oxfordshire	6
Portsmouth	6
Reading	6
Slough	6
Southampton	6
Surrey	6
West Berkshire	6
West Sussex	6
Windsor and Maidenhead	6
Wokingham	6
South West England	5
Bath and North East Somerset	6
Bournemouth	6
Bristol, City of	6
Cornwall	6
Devon	6
Dorset	6
Gloucestershire	6
North Somerset	6
Plymouth	6
Poole	6
Somerset	6
South Gloucestershire	6
Swindon	6
Torbay	6
Wiltshire	6
West Midlands	5
Birmingham	6
Coventry	6
Dudley	6
Herefordshire, County of	6
Sandwell	6

Methods Appendix Table 2. GBD 2017 location hierarchy with levels	
**Geography**	**Level**
Shropshire	6
Solihull	6
Staffordshire	6
Stoke-on-Trent	6
Telford and Wrekin	6
Walsall	6
Warwickshire	6
Wolverhampton	6
Worcestershire	6
Yorkshire and the Humber	5
Barnsley	6
Bradford	6
Calderdale	6
Doncaster	6
East Riding of Yorkshire	6
Kingston upon Hull, City of	6
Kirklees	6
Leeds	6
North East Lincolnshire	6
North Lincolnshire	6
North Yorkshire	6
Rotherham	6
Sheffield	6
Wakefield	6
York	6
Northern Ireland	4
Scotland	4
Wales	4
Latin America and Caribbean	1
Andean Latin America	2
Bolivia	3
Ecuador	3
Peru	3
Caribbean	2
Antigua and Barbuda	3
The Bahamas	3
Barbados	3
Belize	3
Bermuda	3
Cuba	3
Dominica	3
Dominican Republic	3
Grenada	3
Guyana	3
Haiti	3

Methods Appendix Table 2. GBD 2017 location hierarchy with levels	
**Geography**	**Level**
Jamaica	3
Puerto Rico	3
Saint Lucia	3
Saint Vincent and the Grenadines	3
Suriname	3
Trinidad and Tobago	3
Virgin Islands	3
Central Latin America	2
Colombia	3
Costa Rica	3
El Salvador	3
Guatemala	3
Honduras	3
Mexico	3
Aguascalientes	4
Baja California	4
Baja California Sur	4
Campeche	4
Chiapas	4
Chihuahua	4
Coahuila	4
Colima	4
Mexico City	4
Durango	4
Guanajuato	4
Guerrero	4
Hidalgo	4
Jalisco	4
México	4
Michoacán de Ocampo	4
Morelos	4
Nayarit	4
Nuevo León	4
Oaxaca	4
Puebla	4
Querétaro	4
Quintana Roo	4
San Luis Potosí	4
Sinaloa	4
Sonora	4
Tabasco	4
Tamaulipas	4
Tlaxcala	4
Veracruz de Ignacio de la Llave	4
Yucatán	4

Methods Appendix Table 2. GBD 2017 location hierarchy with levels	
**Geography**	**Level**
Zacatecas	4
Nicaragua	3
Panama	3
Venezuela	3
Tropical Latin America	2
Brazil	3
Acre	4
Alagoas	4
Amapá	4
Amazonas	4
Bahia	4
Ceará	4
Distrito Federal	4
Espírito Santo	4
Goiás	4
Maranhão	4
Mato Grosso	4
Mato Grosso do Sul	4
Minas Gerais	4
Pará	4
Paraíba	4
Paraná	4
Pernambuco	4
Piauí	4
Rio de Janeiro	4
Rio Grande do Norte	4
Rio Grande do Sul	4
Rondônia	4
Roraima	4
Santa Catarina	4
São Paulo	4
Sergipe	4
Tocantins	4
Paraguay	3
North Africa and Middle East	1
North Africa and Middle East	2
Afghanistan	3
Algeria	3
Bahrain	3
Egypt	3
Iran	3
Iraq	3
Jordan	3
Kuwait	3
Lebanon	3

Methods Appendix Table 2. GBD 2017 location hierarchy with levels	
Geography	Level
Libya	3
Morocco	3
Palestine	3
Oman	3
Qatar	3
Saudi Arabia	3
Sudan	3
Syria	3
Tunisia	3
Turkey	3
United Arab Emirates	3
Yemen	3
South Asia	1
South Asia	2
Bangladesh	3
Bhutan	3
India	3
Andhra Pradesh	4
Arunachal Pradesh	4
Assam	4
Bihar	4
Chhattisgarh	4
Delhi	4
Goa	4
Gujarat	4
Haryana	4
Himachal Pradesh	4
Jammu and Kashmir	4
Jharkhand	4
Karnataka	4
Kerala	4
Madhya Pradesh	4
Maharashtra	4
Manipur	4
Meghalaya	4
Mizoram	4
Nagaland	4
Odisha	4
Punjab	4
Rajasthan	4
Sikkim	4
Tamil Nadu	4
Telangana	4
Tripura	4
Uttar Pradesh	4

Methods Appendix Table 2. GBD 2017 location hierarchy with levels	
**Geography**	**Level**
Uttarakhand	4
West Bengal	4
Union Territories other than Delhi	4
Nepal	3
Pakistan	3
Southeast Asia, East Asia, and Oceania	1
East Asia	2
China	3
North Korea	3
Taiwan (Province of China)	3
Oceania	2
American Samoa	3
Federated States of Micronesia	3
Fiji	3
Guam	3
Kiribati	3
Marshall Islands	3
Northern Mariana Islands	3
Papua New Guinea	3
Samoa	3
Solomon Islands	3
Tonga	3
Vanuatu	3
Southeast Asia	2
Cambodia	3
Indonesia	3
Laos	3
Malaysia	3
Maldives	3
Mauritius	3
Myanmar	3
Philippines	3
Sri Lanka	3
Seychelles	3
Thailand	3
Timor-Leste	3
Vietnam	3
Sub-Saharan Africa	1
Central sub-Saharan Africa	2
Angola	3
Central African Republic	3
Congo (Brazzaville)	3
DR Congo	3
Equatorial Guinea	3
Gabon	3

Methods Appendix Table 2. GBD 2017 location hierarchy with levels	
**Geography**	**Level**
Eastern sub-Saharan Africa	2
Burundi	3
Comoros	3
Djibouti	3
Eritrea	3
Ethiopia	3
Kenya	3
Baringo	4
Bomet	4
Bungoma	4
Busia	4
Elgeyo Marakwet	4
Embu	4
Garissa	4
Homa Bay	4
Isiolo	4
Kajiado	4
Kakamega	4
Kericho	4
Kiambu	4
Kilifi	4
Kirinyaga	4
Kisii	4
Kisumu	4
Kitui	4
Kwale	4
Laikipia	4
Lamu	4
Machakos	4
Makueni	4
Mandera	4
Marsabit	4
Meru	4
Migori	4
Mombasa	4
Murang'a	4
Nairobi	4
Nakuru	4
Nandi	4
Narok	4
Nyamira	4
Nyandarua	4
Nyeri	4
Samburu	4
Siaya	4

Methods Appendix Table 2. GBD 2017 location hierarchy with levels	
**Geography**	**Level**
Taita Taveta	4
Tana River	4
Tharaka Nithi	4
Trans Nzoia	4
Turkana	4
Uasin Gishu	4
Vihiga	4
Wajir	4
West Pokot	4
Madagascar	3
Malawi	3
Mozambique	3
Rwanda	3
Somalia	3
South Sudan	3
Tanzania	3
Uganda	3
Zambia	3
Southern sub-Saharan Africa	2
Botswana	3
Lesotho	3
Namibia	3
South Africa	3
Swaziland	3
Zimbabwe	3
Western sub-Saharan Africa	2
Benin	3
Burkina Faso	3
Cameroon	3
Cape Verde	3
Chad	3
Cote d'Ivoire	3
The Gambia	3
Ghana	3
Guinea	3
Guinea-Bissau	3
Liberia	3
Mali	3
Mauritania	3
Niger	3
Nigeria	3
Sao Tome and Principe	3
Senegal	3
Sierra Leone	3
Togo	3

Methods Appendix Table 3: Socio-Demographic Index values for all estimated GBD 2017 locations, 1990-2017																												
Location	1990	1991	1992	1993	1994	1995	1996	1997	1998	1999	2000	2001	2002	2003	2004	2005	2006	2007	2008	2009	2010	2011	2012	2013	2014	2015	2016	2017
Global	0.523	0.529	0.534	0.539	0.543	0.548	0.553	0.557	0.561	0.566	0.571	0.576	0.581	0.585	0.59	0.595	0.601	0.606	0.611	0.616	0.62	0.624	0.628	0.633	0.639	0.644	0.647	0.652
Central Europe, Eastern Europe, and Central Asia	0.656	0.662	0.67	0.674	0.677	0.682	0.686	0.689	0.691	0.694	0.698	0.701	0.705	0.709	0.715	0.72	0.725	0.73	0.735	0.739	0.743	0.747	0.75	0.753	0.757	0.76	0.763	0.766
Central Asia	0.563	0.567	0.57	0.573	0.575	0.577	0.578	0.579	0.58	0.582	0.585	0.588	0.593	0.598	0.603	0.609	0.615	0.621	0.627	0.633	0.639	0.644	0.649	0.654	0.659	0.664	0.669	0.673
Armenia	0.555	0.559	0.56	0.562	0.565	0.567	0.57	0.573	0.577	0.581	0.586	0.592	0.6	0.61	0.619	0.629	0.639	0.65	0.66	0.667	0.673	0.678	0.683	0.687	0.691	0.695	0.699	0.702
Azerbaijan	0.611	0.614	0.616	0.617	0.616	0.613	0.61	0.607	0.604	0.601	0.6	0.6	0.602	0.605	0.608	0.615	0.625	0.635	0.645	0.654	0.664	0.672	0.678	0.684	0.689	0.694	0.698	0.701
Georgia	0.654	0.66	0.661	0.658	0.653	0.645	0.639	0.634	0.63	0.625	0.621	0.62	0.622	0.624	0.628	0.633	0.638	0.644	0.649	0.654	0.659	0.665	0.671	0.676	0.682	0.688	0.695	0.7
Kazakhstan	0.613	0.615	0.619	0.625	0.632	0.638	0.643	0.645	0.646	0.647	0.651	0.656	0.661	0.666	0.671	0.677	0.683	0.689	0.696	0.702	0.705	0.707	0.708	0.711	0.716	0.723	0.73	0.735
Kyrgyzstan	0.565	0.571	0.576	0.578	0.577	0.572	0.569	0.567	0.564	0.562	0.56	0.559	0.56	0.562	0.565	0.566	0.567	0.569	0.572	0.575	0.576	0.581	0.584	0.589	0.594	0.598	0.603	0.607
Mongolia	0.537	0.545	0.55	0.555	0.559	0.564	0.569	0.573	0.577	0.581	0.585	0.589	0.594	0.598	0.603	0.608	0.614	0.619	0.624	0.628	0.632	0.636	0.641	0.646	0.65	0.654	0.658	0.662
Tajikistan	0.474	0.481	0.485	0.487	0.486	0.481	0.474	0.468	0.463	0.459	0.455	0.454	0.456	0.462	0.465	0.466	0.472	0.479	0.483	0.488	0.494	0.501	0.506	0.51	0.514	0.517	0.52	0.523
Turkmenistan	0.588	0.592	0.594	0.599	0.602	0.604	0.606	0.606	0.606	0.607	0.61	0.613	0.617	0.622	0.628	0.635	0.638	0.641	0.644	0.647	0.651	0.657	0.663	0.669	0.678	0.685	0.691	0.696
Uzbekistan	0.481	0.484	0.487	0.493	0.497	0.502	0.508	0.513	0.52	0.526	0.532	0.537	0.543	0.549	0.555	0.56	0.565	0.57	0.575	0.581	0.587	0.592	0.598	0.604	0.611	0.618	0.624	0.63
Central Europe	0.665	0.671	0.677	0.683	0.69	0.698	0.705	0.711	0.717	0.723	0.731	0.738	0.745	0.751	0.757	0.762	0.767	0.772	0.776	0.782	0.788	0.793	0.797	0.802	0.805	0.808	0.811	0.814
Albania	0.548	0.545	0.542	0.541	0.542	0.546	0.552	0.558	0.566	0.577	0.584	0.593	0.602	0.611	0.619	0.627	0.635	0.642	0.648	0.653	0.658	0.661	0.665	0.668	0.672	0.676	0.681	0.685
Bosnia and Herzegovina	0.497	0.499	0.5	0.5	0.501	0.507	0.525	0.549	0.571	0.592	0.607	0.619	0.63	0.639	0.647	0.654	0.66	0.667	0.673	0.679	0.685	0.69	0.694	0.699	0.703	0.706	0.71	0.713
Bulgaria	0.658	0.668	0.676	0.684	0.693	0.699	0.705	0.706	0.704	0.703	0.708	0.715	0.721	0.726	0.731	0.736	0.741	0.746	0.751	0.757	0.765	0.771	0.775	0.778	0.781	0.784	0.788	0.792
Croatia	0.725	0.73	0.732	0.732	0.731	0.731	0.732	0.737	0.743	0.749	0.755	0.762	0.768	0.773	0.778	0.782	0.787	0.792	0.797	0.801	0.805	0.809	0.813	0.816	0.818	0.821	0.823	0.825
Czech Republic	0.711	0.717	0.726	0.74	0.757	0.769	0.777	0.783	0.788	0.794	0.799	0.804	0.809	0.814	0.819	0.823	0.827	0.83	0.833	0.836	0.84	0.843	0.846	0.847	0.848	0.848	0.849	0.851
Hungary	0.678	0.683	0.691	0.699	0.707	0.716	0.724	0.732	0.739	0.745	0.751	0.758	0.764	0.77	0.776	0.781	0.786	0.791	0.795	0.799	0.803	0.806	0.807	0.808	0.809	0.811	0.814	0.817
Macedonia	0.626	0.629	0.63	0.631	0.632	0.635	0.64	0.647	0.654	0.661	0.665	0.67	0.677	0.685	0.693	0.699	0.704	0.709	0.715	0.719	0.724	0.729	0.734	0.739	0.744	0.748	0.751	0.754
Montenegro	0.705	0.706	0.705	0.701	0.698	0.696	0.696	0.698	0.7	0.703	0.706	0.711	0.716	0.721	0.726	0.731	0.737	0.743	0.75	0.756	0.761	0.767	0.771	0.775	0.779	0.782	0.785	0.788
Poland	0.662	0.668	0.678	0.686	0.697	0.707	0.714	0.724	0.733	0.741	0.75	0.759	0.767	0.773	0.779	0.784	0.789	0.792	0.797	0.804	0.811	0.818	0.823	0.829	0.833	0.837	0.841	0.844
Romania	0.652	0.66	0.663	0.666	0.671	0.678	0.682	0.685	0.689	0.694	0.7	0.707	0.713	0.718	0.724	0.73	0.734	0.739	0.745	0.751	0.758	0.763	0.768	0.772	0.774	0.777	0.78	0.784
Serbia	0.632	0.638	0.643	0.642	0.641	0.641	0.643	0.648	0.653	0.655	0.661	0.665	0.669	0.675	0.684	0.692	0.699	0.705	0.709	0.713	0.718	0.723	0.729	0.736	0.742	0.747	0.75	0.752
Slovakia	0.684	0.69	0.699	0.71	0.722	0.732	0.74	0.748	0.756	0.764	0.772	0.779	0.784	0.788	0.793	0.798	0.804	0.809	0.814	0.818	0.823	0.828	0.832	0.834	0.836	0.838	0.839	0.842
Slovenia	0.741	0.747	0.753	0.759	0.764	0.769	0.775	0.781	0.788	0.794	0.801	0.808	0.814	0.819	0.824	0.828	0.833	0.837	0.841	0.843	0.846	0.848	0.85	0.852	0.854	0.856	0.858	0.86
Eastern Europe	0.678	0.685	0.694	0.698	0.7	0.704	0.708	0.708	0.71	0.711	0.712	0.713	0.715	0.72	0.727	0.734	0.739	0.745	0.751	0.756	0.761	0.764	0.767	0.772	0.776	0.779	0.783	0.785
Belarus	0.625	0.631	0.636	0.641	0.645	0.647	0.65	0.654	0.657	0.661	0.665	0.67	0.676	0.682	0.689	0.696	0.704	0.712	0.72	0.727	0.733	0.74	0.747	0.753	0.759	0.764	0.769	0.773
Estonia	0.711	0.719	0.728	0.736	0.742	0.746	0.75	0.755	0.761	0.766	0.772	0.778	0.783	0.788	0.794	0.799	0.806	0.813	0.82	0.826	0.832	0.838	0.843	0.847	0.851	0.854	0.856	0.858
Latvia	0.696	0.703	0.712	0.721	0.727	0.731	0.733	0.734	0.735	0.738	0.741	0.745	0.75	0.757	0.763	0.769	0.776	0.783	0.792	0.8	0.806	0.81	0.814	0.816	0.817	0.819	0.822	0.825
Lithuania	0.707	0.71	0.717	0.725	0.728	0.731	0.733	0.736	0.74	0.746	0.753	0.76	0.765	0.772	0.779	0.785	0.79	0.796	0.802	0.808	0.815	0.822	0.828	0.833	0.836	0.838	0.839	0.841
Moldova	0.575	0.578	0.58	0.582	0.583	0.584	0.584	0.582	0.58	0.577	0.574	0.574	0.577	0.582	0.588	0.595	0.602	0.61	0.618	0.624	0.632	0.64	0.647	0.654	0.66	0.666	0.671	0.676
Russian Federation	0.683	0.692	0.704	0.708	0.708	0.714	0.718	0.719	0.72	0.722	0.722	0.722	0.724	0.728	0.734	0.742	0.747	0.752	0.757	0.763	0.768	0.77	0.772	0.777	0.781	0.785	0.789	0.792
Ukraine	0.664	0.667	0.67	0.673	0.675	0.676	0.676	0.675	0.673	0.672	0.672	0.673	0.675	0.68	0.687	0.694	0.7	0.707	0.714	0.717	0.721	0.725	0.729	0.732	0.735	0.736	0.738	0.74
High-income	0.769	0.774	0.779	0.783	0.787	0.792	0.796	0.798	0.801	0.804	0.807	0.811	0.814	0.817	0.82	0.822	0.823	0.826	0.829	0.832	0.836	0.839	0.842	0.845	0.848	0.851	0.853	0.854
Australasia	0.783	0.786	0.79	0.794	0.797	0.801	0.805	0.809	0.813	0.817	0.821	0.825	0.828	0.832	0.835	0.837	0.838	0.84	0.842	0.845	0.848	0.851	0.855	0.859	0.862	0.864	0.867	0.869
Australia	0.786	0.79	0.793	0.797	0.801	0.805	0.81	0.814	0.818	0.822	0.825	0.829	0.833	0.837	0.84	0.843	0.844	0.845	0.848	0.851	0.854	0.856	0.86	0.864	0.867	0.869	0.871	0.873
New Zealand	0.765	0.768	0.771	0.774	0.777	0.78	0.783	0.786	0.79	0.794	0.798	0.802	0.805	0.807	0.809	0.811	0.811	0.811	0.813	0.816	0.819	0.823	0.828	0.832	0.835	0.838	0.84	0.842
High-income Asia-Pacific	0.783	0.789	0.794	0.799	0.804	0.809	0.813	0.817	0.82	0.823	0.826	0.83	0.833	0.836	0.839	0.842	0.844	0.846	0.849	0.851	0.853	0.856	0.858	0.861	0.863	0.865	0.867	0.869
Brunei	0.728	0.733	0.739	0.745	0.751	0.757	0.763	0.769	0.774	0.779	0.784	0.789	0.795	0.802	0.808	0.814	0.819	0.824	0.828	0.831	0.835	0.838	0.842	0.845	0.848	0.851	0.854	0.856
Japan	0.803	0.807	0.812	0.816	0.82	0.823	0.826	0.829	0.831	0.833	0.834	0.836	0.838	0.84	0.842	0.844	0.846	0.847	0.849	0.851	0.853	0.855	0.857	0.859	0.861	0.862	0.863	0.865
Aichi	0.812	0.816	0.821	0.825	0.829	0.833	0.836	0.839	0.841	0.843	0.844	0.846	0.847	0.85	0.852	0.854	0.855	0.856	0.858	0.86	0.862	0.864	0.866	0.869	0.871	0.872	0.873	0.875
Akita	0.766	0.77	0.775	0.778	0.781	0.785	0.788	0.791	0.793	0.795	0.796	0.798	0.8	0.802	0.805	0.806	0.808	0.81	0.812	0.814	0.816	0.818	0.821	0.823	0.825	0.826	0.827	0.829
Aomori	0.761	0.765	0.77	0.773	0.777	0.78	0.783	0.786	0.788	0.79	0.791	0.793	0.795	0.798	0.801	0.803	0.805	0.807	0.809	0.81	0.812	0.815	0.817	0.819	0.821	0.822	0.823	0.825
Chiba	0.803	0.807	0.812	0.816	0.82	0.823	0.827	0.829	0.831	0.832	0.833	0.834	0.836	0.837	0.839	0.841	0.842	0.843	0.845	0.846	0.848	0.85	0.852	0.854	0.856	0.857	0.858	0.859
Ehime	0.776	0.78	0.785	0.788	0.792	0.796	0.799	0.802	0.804	0.806	0.808	0.809	0.811	0.814	0.816	0.818	0.819	0.82	0.822	0.824	0.825	0.828	0.83	0.832	0.834	0.835	0.836	0.838
Fukui	0.784	0.789	0.794	0.798	0.802	0.806	0.81	0.813	0.815	0.818	0.819	0.821	0.823	0.826	0.828	0.83	0.832	0.834	0.836	0.837	0.839	0.842	0.844	0.846	0.848	0.849	0.85	0.852
Fukuoka	0.797	0.801	0.806	0.809	0.813	0.816	0.819	0.822	0.824	0.826	0.828	0.83	0.832	0.834	0.836	0.837	0.838	0.839	0.841	0.842	0.843	0.845	0.847	0.849	0.851	0.853	0.854	0.855
Fukushima	0.769	0.773	0.778	0.781	0.784	0.788	0.791	0.793	0.794	0.795	0.797	0.798	0.8	0.803	0.807	0.809	0.811	0.813	0.814	0.816	0.819	0.821	0.823	0.825	0.827	0.828	0.829	0.831
Gifu	0.786	0.79	0.795	0.799	0.803	0.807	0.81	0.813	0.815	0.817	0.818	0.82	0.821	0.824	0.826	0.828	0.829	0.831	0.833	0.835	0.837	0.839	0.841	0.843	0.845	0.846	0.847	0.849
Gunma	0.787	0.791	0.796	0.8	0.803	0.807	0.81	0.813	0.815	0.816	0.818	0.819	0.821	0.824	0.827	0.829	0.831	0.833	0.835	0.837	0.839	0.841	0.843	0.845	0.847	0.848	0.849	0.851
Hiroshima	0.8	0.805	0.809	0.813	0.817	0.821	0.825	0.828	0.83	0.832	0.833	0.835	0.836	0.839	0.841	0.842	0.843	0.844	0.846	0.847	0.849	0.852	0.854	0.856	0.858	0.86	0.861	0.863
Hokkaidō	0.783	0.788	0.792	0.796	0.799	0.803	0.806	0.808	0.809	0.811	0.812	0.813	0.815	0.818	0.82	0.822	0.824	0.825	0.827	0.828	0.83	0.832	0.834	0.836	0.838	0.839	0.84	0.842
Hyōgo	0.798	0.803	0.807	0.811	0.815	0.819	0.822	0.825	0.827	0.829	0.831	0.833	0.835	0.837	0.839	0.841	0.842	0.843	0.845	0.846	0.848	0.85	0.852	0.854	0.856	0.857	0.858	0.86
Ibaraki	0.789	0.793	0.798	0.802	0.805	0.809	0.813	0.815	0.817	0.819	0.82	0.821	0.823	0.826	0.828	0.83	0.831	0.833	0.835	0.837	0.839	0.841	0.843	0.845	0.847	0.848	0.849	0.851
Ishikawa	0.789	0.795	0.8	0.804	0.808	0.812	0.815	0.818	0.82	0.822	0.824	0.826	0.828	0.831	0.833	0.835	0.837	0.839	0.84	0.842	0.844	0.846	0.848	0.85	0.852	0.853	0.854	0.856
Iwate	0.76	0.764	0.768	0.772	0.775	0.779	0.782	0.785	0.787	0.788	0.79	0.791	0.793	0.796	0.799	0.801	0.803	0.806	0.808	0.81	0.812	0.815	0.817	0.819	0.821	0.823	0.823	0.825
Kagawa	0.787	0.792	0.797	0.801	0.804	0.808	0.811	0.814	0.816	0.817	0.819	0.82	0.822	0.825	0.827	0.829	0.831	0.832	0.834	0.835	0.837	0.839	0.842	0.844	0.846	0.847	0.848	0.85
Kagoshima	0.768	0.772	0.777	0.781	0.784	0.788	0.791	0.793	0.795	0.797	0.798	0.799	0.801	0.804	0.806	0.808	0.809	0.81	0.812	0.814	0.816	0.818	0.82	0.823	0.825	0.827	0.828	0.83
Kanagawa	0.818	0.823	0.827	0.831	0.835	0.838	0.842	0.844	0.846	0.848	0.849	0.85	0.852	0.854	0.856	0.857	0.859	0.86	0.862	0.863	0.865	0.866	0.868	0.87	0.871	0.873	0.873	0.875
Kōchi	0.759	0.763	0.767	0.771	0.775	0.779	0.783	0.786	0.788	0.791	0.793	0.795	0.797	0.8	0.803	0.805	0.806	0.808	0.81	0.812	0.813	0.816	0.818	0.82	0.822	0.823	0.824	0.825
Kumamoto	0.77	0.774	0.778	0.782	0.785	0.789	0.792	0.794	0.796	0.798	0.799	0.801	0.803	0.805	0.808	0.81	0.811	0.812	0.814	0.815	0.817	0.82	0.822	0.825	0.827	0.829	0.83	0.832
Kyōto	0.813	0.817	0.822	0.826	0.83	0.833	0.837	0.839	0.841	0.843	0.845	0.847	0.849	0.851	0.853	0.854	0.856	0.857	0.859	0.86	0.862	0.863	0.865	0.867	0.869	0.87	0.871	0.873
Mie	0.787	0.792	0.797	0.801	0.805	0.81	0.813	0.815	0.818	0.819	0.821	0.823	0.825	0.827	0.83	0.832	0.833	0.835	0.836	0.838	0.84	0.842	0.845	0.847	0.849	0.851	0.852	0.854
Miyagi	0.788	0.793	0.798	0.801	0.805	0.809	0.812	0.814	0.816	0.817	0.818	0.819	0.821	0.824	0.827	0.829	0.831	0.833	0.835	0.836	0.838	0.841	0.843	0.845	0.847	0.848	0.849	0.85
Miyazaki	0.764	0.768	0.772	0.775	0.778	0.781	0.784	0.786	0.788	0.79	0.791	0.793	0.795	0.797	0.8	0.801	0.802	0.804	0.805	0.807	0.809	0.812	0.814	0.816	0.819	0.82	0.821	0.823
Nagano	0.792	0.797	0.801	0.805	0.808	0.811	0.814	0.817	0.818	0.82	0.821	0.823	0.824	0.827	0.829	0.831	0.832	0.834	0.836	0.838	0.839	0.842	0.844	0.845	0.847	0.849	0.849	0.851
Nagasaki	0.766	0.77	0.774	0.777	0.781	0.784	0.787	0.79	0.792	0.794	0.795	0.797	0.799	0.801	0.803	0.805	0.806	0.808	0.809	0.811	0.813	0.815	0.817	0.82	0.822	0.823	0.824	0.826
Nara	0.789	0.794	0.799	0.803	0.807	0.811	0.814	0.817	0.819	0.821	0.822	0.824	0.825	0.827	0.829	0.831	0.832	0.834	0.835	0.836	0.838	0.839	0.841	0.843	0.844	0.845	0.846	0.848
Niigata	0.776	0.78	0.784	0.788	0.792	0.795	0.799	0.801	0.804	0.806	0.808	0.81	0.813	0.815	0.818	0.82	0.822	0.824	0.826	0.828	0.83	0.832	0.835	0.837	0.839	0.84	0.842	0.843
Ōita	0.785	0.789	0.795	0.802	0.808	0.809	0.809	0.81	0.812	0.813	0.815	0.816	0.819	0.821	0.824	0.825	0.827	0.828	0.83	0.831	0.833	0.835	0.838	0.84	0.842	0.843	0.844	0.846
Okayama	0.79	0.794	0.799	0.803	0.807	0.811	0.814	0.817	0.819	0.821	0.823	0.824	0.827	0.829	0.832	0.834	0.836	0.837	0.839	0.841	0.843	0.846	0.848	0.85	0.852	0.853	0.854	0.856
Okinawa	0.753	0.757	0.764	0.772	0.778	0.779	0.777	0.777	0.778	0.78	0.781	0.783	0.786	0.789	0.793	0.795	0.797	0.799	0.8	0.802	0.804	0.807	0.809	0.812	0.814	0.815	0.816	0.818
Ōsaka	0.815	0.819	0.823	0.827	0.831	0.834	0.837	0.84	0.842	0.844	0.845	0.847	0.849	0.851	0.853	0.854	0.855	0.856	0.858	0.859	0.861	0.863	0.865	0.867	0.868	0.87	0.871	0.872
Saga	0.773	0.777	0.782	0.785	0.788	0.792	0.795	0.797	0.799	0.8	0.801	0.803	0.805	0.808	0.81	0.812	0.814	0.815	0.817	0.819	0.821	0.823	0.825	0.827	0.83	0.831	0.832	0.834
Saitama	0.793	0.798	0.802	0.806	0.81	0.814	0.818	0.82	0.822	0.824	0.825	0.826	0.827	0.829	0.831	0.833	0.835	0.836	0.838	0.839	0.841	0.843	0.845	0.847	0.848	0.849	0.85	0.852
Shiga	0.804	0.809	0.814	0.819	0.823	0.827	0.83	0.833	0.836	0.838	0.84	0.841	0.843	0.846	0.848	0.85	0.852	0.853	0.855	0.857	0.858	0.861	0.863	0.865	0.867	0.868	0.869	0.871
Shimane	0.762	0.766	0.771	0.775	0.779	0.783	0.787	0.79	0.793	0.795	0.797	0.798	0.801	0.803	0.806	0.808	0.81	0.812	0.814	0.816	0.818	0.82	0.823	0.825	0.827	0.828	0.829	0.831
Shizuoka	0.798	0.802	0.807	0.81	0.814	0.818	0.821	0.823	0.825	0.826	0.827	0.828	0.83	0.833	0.835	0.837	0.838	0.84	0.842	0.843	0.846	0.848	0.85	0.853	0.855	0.856	0.857	0.859
Tochigi	0.787	0.792	0.797	0.801	0.805	0.809	0.813	0.815	0.817	0.819	0.82	0.821	0.823	0.826	0.829	0.831	0.832	0.834	0.836	0.838	0.84	0.842	0.845	0.847	0.849	0.85	0.851	0.853
Tokushima	0.776	0.781	0.786	0.79	0.794	0.798	0.802	0.805	0.807	0.809	0.812	0.814	0.816	0.819	0.822	0.824	0.826	0.828	0.83	0.831	0.833	0.835	0.837	0.839	0.841	0.842	0.843	0.845
Tōkyō	0.87	0.875	0.879	0.883	0.887	0.892	0.896	0.899	0.901	0.903	0.905	0.906	0.908	0.91	0.911	0.913	0.914	0.915	0.916	0.917	0.918	0.919	0.92	0.921	0.922	0.923	0.923	0.924
Tottori	0.77	0.775	0.78	0.783	0.787	0.791	0.794	0.797	0.799	0.801	0.802	0.804	0.806	0.808	0.811	0.813	0.814	0.816	0.818	0.819	0.821	0.824	0.826	0.828	0.831	0.832	0.833	0.834
Toyama	0.79	0.795	0.8	0.805	0.808	0.812	0.816	0.818	0.821	0.823	0.825	0.827	0.829	0.832	0.835	0.837	0.839	0.841	0.843	0.845	0.847	0.849	0.852	0.854	0.856	0.857	0.858	0.86
Wakayama	0.775	0.78	0.785	0.789	0.792	0.796	0.8	0.802	0.805	0.806	0.808	0.81	0.812	0.815	0.817	0.819	0.821	0.822	0.824	0.825	0.827	0.829	0.831	0.833	0.836	0.837	0.838	0.84
Yamagata	0.766	0.77	0.775	0.778	0.782	0.785	0.788	0.791	0.792	0.794	0.795	0.796	0.799	0.802	0.805	0.807	0.809	0.812	0.814	0.816	0.819	0.821	0.824	0.826	0.828	0.829	0.83	0.832
Yamaguchi	0.79	0.794	0.799	0.802	0.806	0.809	0.812	0.815	0.817	0.819	0.82	0.822	0.824	0.826	0.828	0.83	0.831	0.832	0.834	0.835	0.837	0.839	0.841	0.843	0.846	0.847	0.848	0.849
Yamanashi	0.791	0.796	0.8	0.804	0.808	0.812	0.815	0.818	0.82	0.822	0.823	0.825	0.827	0.829	0.832	0.834	0.836	0.837	0.839	0.841	0.842	0.844	0.846	0.848	0.85	0.851	0.853	0.854
South Korea	0.713	0.724	0.733	0.742	0.751	0.76	0.768	0.777	0.783	0.79	0.799	0.806	0.814	0.82	0.825	0.83	0.834	0.839	0.843	0.846	0.85	0.853	0.857	0.86	0.864	0.867	0.869	0.872
Singapore	0.736	0.744	0.75	0.758	0.765	0.772	0.78	0.786	0.79	0.79	0.797	0.808	0.815	0.82	0.824	0.827	0.832	0.838	0.844	0.849	0.854	0.857	0.86	0.863	0.865	0.868	0.87	0.872
High-income North America	0.784	0.786	0.789	0.793	0.796	0.8	0.805	0.807	0.809	0.812	0.815	0.82	0.824	0.827	0.829	0.83	0.829	0.832	0.837	0.843	0.848	0.852	0.856	0.859	0.861	0.865	0.867	0.868
Canada	0.802	0.805	0.808	0.811	0.814	0.818	0.823	0.828	0.832	0.836	0.841	0.846	0.85	0.853	0.857	0.859	0.861	0.862	0.864	0.867	0.871	0.874	0.877	0.878	0.879	0.88	0.881	0.882
Greenland	0.671	0.67	0.67	0.671	0.672	0.675	0.679	0.683	0.687	0.691	0.695	0.699	0.703	0.708	0.713	0.717	0.722	0.726	0.731	0.737	0.743	0.747	0.751	0.754	0.756	0.757	0.759	0.76
USA	0.781	0.784	0.787	0.79	0.793	0.798	0.803	0.804	0.806	0.809	0.812	0.817	0.821	0.823	0.826	0.826	0.826	0.829	0.834	0.84	0.846	0.85	0.853	0.857	0.859	0.863	0.866	0.867
Alabama	0.745	0.749	0.753	0.757	0.76	0.765	0.769	0.769	0.771	0.773	0.777	0.783	0.787	0.791	0.794	0.794	0.793	0.796	0.801	0.809	0.816	0.82	0.824	0.827	0.83	0.834	0.837	0.837
Alaska	0.755	0.757	0.761	0.767	0.772	0.781	0.788	0.79	0.793	0.795	0.799	0.804	0.808	0.811	0.814	0.813	0.811	0.813	0.818	0.826	0.833	0.838	0.843	0.847	0.851	0.857	0.86	0.861
Arizona	0.751	0.753	0.755	0.758	0.76	0.764	0.769	0.77	0.771	0.772	0.775	0.781	0.784	0.787	0.79	0.791	0.791	0.797	0.806	0.815	0.823	0.828	0.832	0.836	0.839	0.842	0.845	0.845
Arkansas	0.723	0.727	0.733	0.738	0.741	0.746	0.751	0.751	0.753	0.754	0.757	0.763	0.767	0.77	0.773	0.772	0.77	0.774	0.78	0.789	0.797	0.802	0.807	0.811	0.816	0.821	0.825	0.826
California	0.771	0.771	0.773	0.776	0.78	0.787	0.794	0.798	0.803	0.807	0.812	0.819	0.823	0.826	0.829	0.83	0.831	0.835	0.84	0.846	0.852	0.856	0.859	0.863	0.865	0.869	0.871	0.872
Colorado	0.798	0.801	0.804	0.807	0.81	0.815	0.818	0.819	0.82	0.821	0.824	0.83	0.833	0.836	0.839	0.84	0.84	0.844	0.849	0.855	0.861	0.865	0.869	0.872	0.875	0.879	0.881	0.882
Connecticut	0.841	0.844	0.847	0.85	0.853	0.856	0.859	0.861	0.863	0.866	0.869	0.874	0.877	0.879	0.881	0.881	0.881	0.883	0.886	0.89	0.894	0.896	0.899	0.901	0.903	0.905	0.906	0.906
Delaware	0.801	0.804	0.808	0.811	0.813	0.816	0.819	0.819	0.82	0.822	0.825	0.83	0.833	0.836	0.838	0.839	0.838	0.841	0.846	0.852	0.858	0.861	0.864	0.866	0.868	0.871	0.873	0.874
Washington, DC	0.797	0.801	0.806	0.812	0.818	0.826	0.834	0.839	0.845	0.85	0.855	0.861	0.865	0.868	0.869	0.868	0.866	0.866	0.868	0.871	0.874	0.876	0.877	0.88	0.883	0.887	0.89	0.89
Florida	0.774	0.778	0.782	0.786	0.789	0.794	0.799	0.8	0.802	0.804	0.808	0.813	0.817	0.82	0.823	0.824	0.825	0.828	0.834	0.84	0.846	0.85	0.853	0.855	0.858	0.861	0.863	0.864
Georgia	0.755	0.759	0.764	0.769	0.772	0.778	0.782	0.783	0.784	0.785	0.788	0.793	0.796	0.798	0.801	0.801	0.8	0.804	0.81	0.818	0.825	0.83	0.834	0.837	0.841	0.845	0.848	0.848
Hawaii	0.781	0.784	0.788	0.792	0.797	0.805	0.811	0.813	0.815	0.817	0.82	0.824	0.828	0.83	0.833	0.833	0.832	0.834	0.839	0.845	0.851	0.854	0.858	0.861	0.864	0.868	0.871	0.872
Idaho	0.756	0.76	0.764	0.769	0.773	0.778	0.783	0.783	0.784	0.785	0.787	0.791	0.794	0.796	0.799	0.798	0.797	0.801	0.807	0.814	0.821	0.824	0.827	0.83	0.832	0.836	0.84	0.841
Illinois	0.787	0.789	0.792	0.796	0.799	0.805	0.81	0.812	0.816	0.818	0.823	0.828	0.833	0.837	0.84	0.841	0.841	0.845	0.85	0.855	0.861	0.864	0.867	0.87	0.873	0.876	0.879	0.879
Indiana	0.772	0.774	0.778	0.781	0.783	0.787	0.791	0.791	0.793	0.794	0.797	0.802	0.806	0.808	0.81	0.809	0.807	0.809	0.813	0.819	0.825	0.829	0.832	0.836	0.839	0.844	0.847	0.848
Iowa	0.794	0.797	0.8	0.803	0.806	0.809	0.813	0.814	0.816	0.818	0.821	0.825	0.828	0.829	0.832	0.831	0.83	0.832	0.837	0.843	0.848	0.852	0.856	0.86	0.863	0.867	0.87	0.87
Kansas	0.782	0.785	0.788	0.791	0.794	0.798	0.802	0.803	0.804	0.806	0.809	0.814	0.817	0.819	0.821	0.819	0.817	0.819	0.823	0.83	0.837	0.842	0.847	0.852	0.856	0.861	0.864	0.864
Kentucky	0.743	0.747	0.751	0.755	0.759	0.763	0.768	0.769	0.77	0.772	0.776	0.782	0.786	0.789	0.791	0.79	0.787	0.789	0.793	0.799	0.806	0.81	0.814	0.817	0.821	0.827	0.83	0.831
Louisiana	0.735	0.739	0.743	0.748	0.752	0.758	0.762	0.764	0.765	0.767	0.771	0.777	0.782	0.786	0.789	0.788	0.787	0.79	0.796	0.803	0.811	0.815	0.819	0.823	0.826	0.831	0.835	0.835
Maine	0.789	0.795	0.8	0.805	0.809	0.814	0.817	0.819	0.821	0.823	0.826	0.83	0.834	0.837	0.84	0.84	0.84	0.842	0.846	0.851	0.856	0.859	0.861	0.864	0.866	0.869	0.871	0.872
Maryland	0.813	0.817	0.821	0.825	0.828	0.833	0.836	0.838	0.839	0.841	0.845	0.85	0.854	0.857	0.86	0.861	0.861	0.864	0.868	0.874	0.879	0.883	0.886	0.889	0.891	0.894	0.896	0.896
Massachusetts	0.843	0.847	0.85	0.853	0.856	0.86	0.864	0.866	0.869	0.871	0.875	0.88	0.883	0.886	0.888	0.889	0.89	0.893	0.896	0.899	0.902	0.904	0.907	0.909	0.91	0.912	0.913	0.913
Michigan	0.788	0.791	0.795	0.799	0.803	0.808	0.813	0.815	0.817	0.82	0.823	0.829	0.832	0.835	0.838	0.838	0.837	0.839	0.841	0.845	0.849	0.852	0.854	0.857	0.86	0.864	0.867	0.868
Minnesota	0.818	0.821	0.825	0.828	0.83	0.834	0.837	0.839	0.841	0.843	0.845	0.849	0.852	0.855	0.857	0.858	0.858	0.86	0.865	0.87	0.874	0.877	0.881	0.884	0.886	0.89	0.892	0.893
Mississippi	0.719	0.722	0.727	0.732	0.736	0.741	0.746	0.747	0.748	0.75	0.753	0.758	0.762	0.764	0.765	0.764	0.76	0.763	0.77	0.779	0.789	0.794	0.8	0.804	0.809	0.814	0.818	0.819
Missouri	0.77	0.773	0.777	0.781	0.784	0.789	0.793	0.795	0.797	0.799	0.802	0.806	0.81	0.812	0.813	0.812	0.811	0.813	0.818	0.825	0.831	0.835	0.839	0.842	0.846	0.85	0.853	0.853
Montana	0.775	0.779	0.783	0.789	0.793	0.799	0.804	0.805	0.807	0.808	0.81	0.814	0.816	0.819	0.822	0.822	0.821	0.823	0.827	0.833	0.839	0.843	0.847	0.851	0.855	0.859	0.862	0.863
Nebraska	0.795	0.798	0.802	0.805	0.808	0.813	0.817	0.817	0.819	0.819	0.821	0.824	0.827	0.829	0.831	0.831	0.83	0.832	0.836	0.842	0.848	0.853	0.857	0.861	0.865	0.869	0.872	0.873
Nevada	0.76	0.761	0.762	0.765	0.767	0.773	0.779	0.781	0.784	0.787	0.791	0.798	0.802	0.805	0.808	0.809	0.81	0.814	0.82	0.827	0.833	0.836	0.838	0.84	0.842	0.845	0.847	0.847
New Hampshire	0.827	0.832	0.835	0.838	0.84	0.844	0.847	0.849	0.852	0.856	0.86	0.865	0.869	0.872	0.874	0.875	0.876	0.878	0.881	0.885	0.889	0.891	0.894	0.897	0.899	0.902	0.904	0.904
New Jersey	0.828	0.831	0.834	0.838	0.841	0.845	0.849	0.85	0.852	0.854	0.857	0.861	0.864	0.867	0.87	0.87	0.871	0.873	0.876	0.881	0.885	0.888	0.89	0.893	0.895	0.897	0.899	0.899
New Mexico	0.733	0.735	0.739	0.743	0.748	0.755	0.76	0.762	0.764	0.765	0.767	0.773	0.777	0.779	0.782	0.781	0.779	0.783	0.79	0.799	0.808	0.814	0.819	0.823	0.828	0.832	0.835	0.835
New York	0.816	0.819	0.822	0.825	0.828	0.832	0.836	0.838	0.841	0.843	0.847	0.852	0.855	0.858	0.86	0.861	0.861	0.864	0.868	0.872	0.877	0.88	0.883	0.886	0.889	0.892	0.893	0.893
North Carolina	0.763	0.766	0.77	0.774	0.777	0.781	0.785	0.785	0.786	0.788	0.791	0.796	0.8	0.802	0.805	0.805	0.805	0.809	0.815	0.822	0.829	0.833	0.836	0.84	0.843	0.846	0.849	0.85
North Dakota	0.79	0.792	0.796	0.799	0.802	0.807	0.813	0.816	0.82	0.823	0.827	0.83	0.833	0.834	0.835	0.835	0.833	0.835	0.839	0.844	0.85	0.854	0.859	0.864	0.87	0.875	0.879	0.88
Ohio	0.78	0.783	0.787	0.79	0.793	0.797	0.801	0.802	0.804	0.806	0.809	0.814	0.818	0.82	0.822	0.822	0.821	0.822	0.826	0.832	0.837	0.841	0.844	0.847	0.85	0.854	0.857	0.858

Methods Appendix Table 3: Socio-Demographic Index values for all estimated GBD 2017 locations, 1990-2017

Location	1990	1991	1992	1993	1994	1995	1996	1997	1998	1999	2000	2001	2002	2003	2004	2005	2006	2007	2008	2009	2010	2011	2012	2013	2014	2015	2016	2017
Oklahoma	0.749	0.751	0.755	0.758	0.76	0.764	0.768	0.768	0.768	0.769	0.772	0.777	0.781	0.784	0.786	0.785	0.784	0.786	0.792	0.8	0.808	0.813	0.818	0.824	0.829	0.835	0.838	0.838
Oregon	0.785	0.788	0.791	0.794	0.797	0.802	0.806	0.808	0.811	0.814	0.818	0.824	0.827	0.83	0.833	0.833	0.833	0.836	0.841	0.847	0.852	0.855	0.858	0.861	0.864	0.867	0.87	0.871
Pennsylvania	0.8	0.804	0.808	0.812	0.816	0.821	0.824	0.826	0.828	0.83	0.833	0.837	0.84	0.842	0.845	0.845	0.845	0.847	0.851	0.856	0.86	0.863	0.866	0.869	0.871	0.875	0.878	0.879
Rhode Island	0.815	0.818	0.822	0.825	0.827	0.831	0.834	0.835	0.838	0.84	0.843	0.848	0.851	0.854	0.857	0.858	0.859	0.862	0.866	0.87	0.875	0.877	0.88	0.882	0.885	0.887	0.889	0.89
South Carolina	0.752	0.757	0.762	0.768	0.772	0.777	0.781	0.782	0.783	0.785	0.787	0.793	0.796	0.799	0.801	0.8	0.799	0.802	0.808	0.815	0.822	0.826	0.83	0.834	0.838	0.842	0.845	0.846
South Dakota	0.769	0.772	0.777	0.783	0.788	0.794	0.799	0.801	0.804	0.805	0.808	0.811	0.813	0.814	0.816	0.814	0.812	0.814	0.819	0.826	0.833	0.838	0.842	0.847	0.851	0.856	0.859	0.86
Tennessee	0.749	0.752	0.757	0.761	0.765	0.77	0.774	0.775	0.777	0.779	0.781	0.786	0.788	0.789	0.79	0.789	0.786	0.789	0.795	0.803	0.81	0.815	0.819	0.823	0.827	0.832	0.836	0.837
Texas	0.743	0.745	0.747	0.75	0.752	0.757	0.761	0.763	0.764	0.766	0.769	0.775	0.779	0.782	0.784	0.783	0.782	0.785	0.792	0.801	0.809	0.815	0.82	0.824	0.829	0.834	0.837	0.838
Utah	0.761	0.765	0.769	0.773	0.776	0.781	0.786	0.787	0.79	0.792	0.795	0.8	0.803	0.806	0.808	0.808	0.807	0.811	0.817	0.825	0.832	0.837	0.841	0.845	0.848	0.852	0.855	0.856
Vermont	0.815	0.819	0.823	0.827	0.83	0.833	0.837	0.839	0.841	0.844	0.848	0.853	0.857	0.86	0.864	0.866	0.866	0.869	0.872	0.876	0.88	0.882	0.885	0.887	0.89	0.893	0.895	0.896
Virginia	0.8	0.803	0.807	0.81	0.814	0.818	0.822	0.823	0.824	0.826	0.829	0.834	0.838	0.841	0.845	0.846	0.847	0.851	0.856	0.862	0.867	0.871	0.874	0.877	0.88	0.883	0.885	0.885
Washington	0.797	0.8	0.804	0.807	0.811	0.816	0.821	0.823	0.827	0.83	0.834	0.84	0.844	0.846	0.849	0.848	0.848	0.85	0.855	0.86	0.865	0.868	0.871	0.874	0.877	0.881	0.883	0.884
West Virginia	0.749	0.752	0.756	0.761	0.764	0.769	0.773	0.774	0.775	0.776	0.778	0.783	0.786	0.787	0.789	0.787	0.784	0.784	0.787	0.793	0.799	0.802	0.806	0.81	0.814	0.82	0.824	0.825
Wisconsin	0.801	0.804	0.808	0.812	0.815	0.819	0.823	0.825	0.826	0.828	0.831	0.835	0.839	0.841	0.843	0.843	0.843	0.845	0.849	0.853	0.858	0.862	0.865	0.868	0.871	0.874	0.877	0.878
Wyoming	0.766	0.771	0.777	0.782	0.786	0.792	0.796	0.797	0.799	0.801	0.804	0.809	0.813	0.816	0.818	0.819	0.819	0.823	0.831	0.838	0.846	0.851	0.855	0.858	0.862	0.866	0.869	0.869
Southern Latin America	0.594	0.6	0.607	0.613	0.619	0.626	0.632	0.638	0.643	0.648	0.652	0.655	0.658	0.662	0.667	0.673	0.677	0.679	0.682	0.685	0.69	0.695	0.7	0.704	0.707	0.713	0.717	0.72
Argentina	0.59	0.595	0.604	0.61	0.617	0.624	0.63	0.635	0.64	0.644	0.647	0.649	0.65	0.653	0.658	0.665	0.669	0.672	0.675	0.677	0.681	0.686	0.691	0.693	0.696	0.702	0.707	0.71
Chile	0.6	0.608	0.615	0.62	0.626	0.633	0.64	0.647	0.654	0.661	0.667	0.674	0.681	0.687	0.692	0.696	0.698	0.701	0.704	0.708	0.714	0.721	0.727	0.732	0.738	0.742	0.746	0.748
Uruguay	0.592	0.597	0.6	0.602	0.606	0.609	0.613	0.618	0.625	0.632	0.637	0.64	0.643	0.647	0.652	0.656	0.659	0.661	0.663	0.666	0.671	0.675	0.68	0.685	0.691	0.697	0.702	0.707
Western Europe	0.764	0.77	0.776	0.782	0.787	0.791	0.795	0.798	0.801	0.805	0.809	0.813	0.817	0.82	0.822	0.825	0.828	0.83	0.833	0.836	0.838	0.842	0.845	0.848	0.851	0.853	0.855	0.857
Andorra	0.85	0.854	0.856	0.857	0.858	0.859	0.86	0.863	0.866	0.868	0.871	0.873	0.875	0.878	0.881	0.883	0.885	0.886	0.888	0.89	0.891	0.894	0.896	0.897	0.899	0.9	0.901	0.902
Austria	0.776	0.778	0.78	0.785	0.79	0.795	0.8	0.805	0.809	0.813	0.818	0.822	0.825	0.828	0.831	0.834	0.838	0.841	0.845	0.847	0.85	0.854	0.857	0.859	0.862	0.863	0.865	0.866
Belgium	0.803	0.808	0.813	0.818	0.822	0.826	0.829	0.832	0.835	0.837	0.84	0.843	0.847	0.849	0.852	0.854	0.856	0.858	0.861	0.863	0.867	0.871	0.875	0.879	0.882	0.884	0.885	0.886
Cyprus	0.724	0.73	0.74	0.75	0.758	0.765	0.771	0.778	0.784	0.789	0.795	0.803	0.81	0.817	0.824	0.83	0.837	0.842	0.847	0.851	0.854	0.857	0.859	0.861	0.862	0.863	0.864	0.865
Denmark	0.846	0.849	0.852	0.855	0.858	0.862	0.866	0.87	0.874	0.877	0.881	0.884	0.888	0.891	0.893	0.895	0.897	0.898	0.9	0.902	0.904	0.907	0.91	0.912	0.914	0.915	0.916	0.918
Finland	0.813	0.813	0.814	0.817	0.821	0.825	0.828	0.831	0.835	0.84	0.844	0.847	0.851	0.854	0.857	0.859	0.862	0.865	0.869	0.871	0.875	0.878	0.881	0.884	0.887	0.889	0.891	0.893
France	0.769	0.776	0.783	0.79	0.793	0.795	0.802	0.806	0.808	0.813	0.816	0.819	0.824	0.827	0.83	0.833	0.836	0.838	0.84	0.842	0.845	0.848	0.851	0.854	0.857	0.86	0.863	0.865
Germany	0.787	0.796	0.801	0.805	0.809	0.811	0.812	0.813	0.813	0.814	0.818	0.823	0.827	0.829	0.832	0.835	0.838	0.842	0.846	0.848	0.851	0.855	0.858	0.861	0.864	0.866	0.868	0.87
Greece	0.717	0.723	0.731	0.738	0.744	0.75	0.755	0.761	0.767	0.773	0.778	0.782	0.787	0.792	0.796	0.8	0.803	0.806	0.809	0.812	0.815	0.818	0.819	0.82	0.819	0.818	0.817	0.817
Iceland	0.814	0.818	0.821	0.825	0.828	0.83	0.833	0.835	0.839	0.843	0.848	0.854	0.859	0.862	0.865	0.869	0.872	0.876	0.88	0.883	0.886	0.889	0.892	0.895	0.899	0.902	0.905	0.907
Ireland	0.756	0.762	0.768	0.774	0.779	0.785	0.79	0.795	0.802	0.808	0.814	0.821	0.827	0.834	0.84	0.844	0.846	0.849	0.851	0.855	0.858	0.862	0.865	0.867	0.87	0.874	0.878	0.882
Israel	0.734	0.738	0.743	0.748	0.752	0.757	0.76	0.764	0.768	0.772	0.776	0.78	0.783	0.786	0.789	0.793	0.796	0.798	0.798	0.799	0.801	0.803	0.805	0.808	0.81	0.812	0.814	0.816
Italy	0.767	0.772	0.778	0.783	0.788	0.793	0.797	0.8	0.804	0.807	0.81	0.814	0.817	0.819	0.821	0.823	0.825	0.827	0.829	0.83	0.832	0.834	0.836	0.838	0.839	0.841	0.842	0.843
Luxembourg	0.845	0.849	0.851	0.854	0.858	0.862	0.866	0.869	0.873	0.876	0.878	0.88	0.881	0.883	0.885	0.888	0.891	0.894	0.896	0.899	0.901	0.904	0.906	0.909	0.912	0.913	0.915	0.916
Malta	0.729	0.733	0.737	0.743	0.748	0.752	0.756	0.761	0.766	0.773	0.779	0.784	0.788	0.792	0.796	0.799	0.802	0.805	0.808	0.811	0.814	0.817	0.82	0.823	0.826	0.829	0.833	0.836
Netherlands	0.827	0.832	0.837	0.841	0.845	0.849	0.852	0.855	0.858	0.862	0.866	0.87	0.873	0.876	0.879	0.882	0.885	0.887	0.89	0.892	0.895	0.898	0.901	0.904	0.906	0.908	0.91	0.912
Norway	0.811	0.816	0.821	0.827	0.831	0.835	0.84	0.846	0.85	0.855	0.86	0.866	0.87	0.873	0.876	0.878	0.88	0.882	0.885	0.888	0.892	0.896	0.9	0.903	0.906	0.909	0.91	0.911
Portugal	0.642	0.65	0.659	0.667	0.675	0.682	0.688	0.694	0.699	0.705	0.71	0.716	0.722	0.727	0.732	0.736	0.741	0.744	0.748	0.751	0.755	0.76	0.764	0.768	0.771	0.773	0.775	0.778
Spain	0.715	0.723	0.731	0.738	0.745	0.752	0.758	0.763	0.768	0.773	0.778	0.782	0.786	0.79	0.794	0.797	0.799	0.802	0.805	0.809	0.812	0.815	0.818	0.819	0.82	0.822	0.823	0.825
Sweden	0.784	0.789	0.795	0.802	0.808	0.815	0.82	0.825	0.831	0.835	0.838	0.841	0.844	0.847	0.85	0.853	0.855	0.857	0.86	0.862	0.865	0.868	0.871	0.874	0.876	0.879	0.881	0.883
Stockholm	0.825	0.83	0.835	0.84	0.845	0.85	0.854	0.859	0.864	0.867	0.871	0.873	0.876	0.879	0.882	0.885	0.888	0.891	0.893	0.896	0.899	0.902	0.904	0.907	0.909	0.911	0.913	0.914
Sweden except Stockholm	0.773	0.778	0.785	0.792	0.798	0.805	0.811	0.816	0.821	0.825	0.829	0.832	0.834	0.838	0.841	0.843	0.845	0.848	0.85	0.852	0.854	0.858	0.86	0.863	0.866	0.868	0.87	0.873
Switzerland	0.841	0.842	0.844	0.846	0.847	0.848	0.849	0.85	0.851	0.852	0.854	0.857	0.859	0.86	0.862	0.863	0.865	0.868	0.871	0.873	0.875	0.877	0.88	0.882	0.884	0.886	0.887	0.889
United Kingdom	0.723	0.729	0.736	0.743	0.749	0.754	0.758	0.762	0.767	0.774	0.78	0.786	0.791	0.794	0.797	0.801	0.804	0.807	0.81	0.813	0.817	0.821	0.826	0.832	0.836	0.839	0.841	0.843
England	0.732	0.738	0.745	0.752	0.758	0.763	0.766	0.77	0.776	0.782	0.788	0.794	0.798	0.801	0.804	0.807	0.81	0.813	0.816	0.819	0.823	0.827	0.832	0.838	0.842	0.845	0.847	0.849
East Midlands	0.71	0.716	0.723	0.73	0.737	0.741	0.745	0.75	0.755	0.762	0.768	0.773	0.778	0.781	0.785	0.788	0.791	0.794	0.797	0.8	0.804	0.808	0.813	0.819	0.823	0.826	0.828	0.83
Derby	0.715	0.722	0.73	0.738	0.745	0.75	0.754	0.758	0.764	0.771	0.778	0.785	0.79	0.793	0.797	0.801	0.803	0.806	0.809	0.811	0.815	0.82	0.826	0.832	0.837	0.841	0.844	0.846
Derbyshire	0.698	0.703	0.71	0.716	0.723	0.727	0.731	0.735	0.74	0.747	0.753	0.758	0.762	0.765	0.768	0.771	0.774	0.777	0.779	0.782	0.785	0.79	0.796	0.803	0.808	0.812	0.815	0.817
Leicester	0.708	0.715	0.723	0.73	0.737	0.743	0.747	0.751	0.756	0.763	0.769	0.775	0.78	0.785	0.79	0.794	0.798	0.802	0.805	0.809	0.813	0.817	0.822	0.828	0.832	0.835	0.837	0.839
Leicestershire	0.727	0.733	0.74	0.748	0.754	0.759	0.763	0.767	0.773	0.778	0.784	0.789	0.794	0.798	0.802	0.806	0.81	0.814	0.817	0.82	0.823	0.827	0.831	0.836	0.839	0.842	0.844	0.846
Lincolnshire	0.699	0.705	0.711	0.718	0.724	0.729	0.732	0.737	0.742	0.748	0.754	0.76	0.764	0.768	0.77	0.772	0.774	0.776	0.779	0.781	0.784	0.788	0.793	0.8	0.804	0.808	0.81	0.812
Northamptonshire	0.714	0.72	0.728	0.735	0.741	0.746	0.749	0.753	0.757	0.764	0.77	0.775	0.779	0.781	0.784	0.787	0.79	0.792	0.796	0.798	0.802	0.806	0.812	0.818	0.822	0.825	0.828	0.829
Nottingham	0.735	0.742	0.749	0.757	0.763	0.769	0.774	0.78	0.786	0.793	0.8	0.806	0.812	0.817	0.822	0.826	0.829	0.832	0.835	0.839	0.842	0.847	0.852	0.856	0.859	0.861	0.862	0.863
Nottinghamshire	0.696	0.702	0.708	0.715	0.722	0.726	0.73	0.734	0.74	0.747	0.754	0.76	0.765	0.768	0.771	0.775	0.778	0.781	0.783	0.785	0.788	0.792	0.797	0.803	0.807	0.81	0.812	0.814
Rutland	0.73	0.735	0.741	0.746	0.752	0.756	0.759	0.762	0.767	0.772	0.777	0.781	0.784	0.787	0.79	0.793	0.797	0.8	0.804	0.806	0.81	0.814	0.818	0.823	0.826	0.829	0.831	0.833
East of England	0.724	0.73	0.737	0.744	0.75	0.755	0.759	0.763	0.768	0.774	0.78	0.786	0.79	0.793	0.796	0.799	0.802	0.805	0.808	0.811	0.814	0.818	0.824	0.83	0.834	0.837	0.839	0.84
Bedford	0.73	0.736	0.743	0.749	0.756	0.76	0.764	0.768	0.774	0.78	0.785	0.79	0.794	0.797	0.8	0.803	0.805	0.807	0.81	0.812	0.816	0.82	0.825	0.83	0.833	0.836	0.837	0.838
Cambridgeshire	0.75	0.757	0.764	0.771	0.777	0.782	0.786	0.791	0.797	0.803	0.809	0.815	0.82	0.823	0.827	0.83	0.833	0.836	0.84	0.842	0.846	0.85	0.855	0.86	0.864	0.867	0.869	0.871
Central Bedfordshire	0.723	0.729	0.736	0.743	0.749	0.753	0.757	0.76	0.765	0.771	0.776	0.781	0.785	0.787	0.79	0.793	0.795	0.797	0.8	0.803	0.806	0.81	0.816	0.822	0.827	0.83	0.833	0.834
Essex	0.713	0.719	0.726	0.734	0.74	0.745	0.748	0.753	0.758	0.764	0.77	0.775	0.78	0.783	0.787	0.79	0.793	0.796	0.8	0.802	0.806	0.81	0.816	0.821	0.825	0.828	0.83	0.832
Hertfordshire	0.749	0.755	0.762	0.769	0.776	0.781	0.784	0.789	0.795	0.802	0.81	0.816	0.821	0.825	0.829	0.832	0.835	0.837	0.84	0.843	0.846	0.85	0.855	0.86	0.863	0.866	0.868	0.87
Luton	0.71	0.716	0.723	0.731	0.737	0.742	0.745	0.749	0.754	0.76	0.766	0.771	0.775	0.778	0.781	0.785	0.79	0.795	0.8	0.805	0.809	0.814	0.819	0.825	0.828	0.83	0.832	0.833
Norfolk	0.712	0.717	0.724	0.731	0.737	0.741	0.745	0.749	0.754	0.761	0.767	0.772	0.776	0.779	0.781	0.784	0.787	0.79	0.792	0.795	0.798	0.803	0.808	0.814	0.819	0.822	0.824	0.826
Peterborough	0.713	0.718	0.724	0.731	0.737	0.741	0.743	0.746	0.751	0.757	0.761	0.765	0.768	0.77	0.772	0.774	0.776	0.778	0.779	0.781	0.784	0.788	0.795	0.803	0.808	0.813	0.816	0.818
Southend-on-Sea	0.696	0.701	0.707	0.714	0.72	0.724	0.727	0.731	0.736	0.743	0.75	0.755	0.761	0.765	0.768	0.77	0.773	0.775	0.778	0.781	0.785	0.79	0.795	0.801	0.805	0.808	0.81	0.811
Suffolk	0.713	0.719	0.726	0.732	0.738	0.742	0.745	0.748	0.753	0.758	0.763	0.767	0.771	0.773	0.775	0.777	0.78	0.782	0.785	0.788	0.792	0.797	0.803	0.81	0.814	0.817	0.819	0.821
Thurrock	0.71	0.716	0.723	0.73	0.737	0.741	0.745	0.749	0.754	0.759	0.763	0.767	0.769	0.77	0.772	0.774	0.775	0.776	0.778	0.781	0.784	0.789	0.793	0.799	0.802	0.804	0.806	0.807
Greater London	0.78	0.786	0.793	0.8	0.806	0.81	0.814	0.818	0.823	0.83	0.836	0.842	0.846	0.849	0.852	0.855	0.858	0.861	0.863	0.866	0.87	0.875	0.88	0.885	0.889	0.891	0.893	0.894
Barking and Dagenham	0.704	0.709	0.716	0.723	0.73	0.734	0.737	0.741	0.746	0.752	0.756	0.76	0.762	0.762	0.763	0.763	0.763	0.763	0.764	0.765	0.768	0.773	0.78	0.787	0.793	0.797	0.8	0.802
Barnet	0.759	0.765	0.771	0.777	0.783	0.788	0.792	0.796	0.802	0.809	0.814	0.82	0.824	0.827	0.829	0.831	0.834	0.835	0.838	0.84	0.843	0.847	0.852	0.856	0.859	0.862	0.864	0.865
Bexley	0.721	0.727	0.734	0.74	0.746	0.75	0.753	0.757	0.761	0.767	0.772	0.777	0.781	0.784	0.787	0.789	0.791	0.793	0.796	0.798	0.801	0.804	0.809	0.815	0.819	0.822	0.824	0.826
Brent	0.753	0.758	0.764	0.771	0.777	0.781	0.785	0.789	0.795	0.801	0.806	0.811	0.814	0.816	0.818	0.819	0.821	0.821	0.822	0.823	0.825	0.828	0.833	0.839	0.842	0.845	0.847	0.849
Bromley	0.751	0.756	0.762	0.768	0.774	0.778	0.782	0.786	0.791	0.798	0.804	0.809	0.814	0.817	0.82	0.822	0.825	0.827	0.829	0.83	0.831	0.833	0.837	0.84	0.843	0.845	0.847	0.848
Camden	0.838	0.843	0.848	0.854	0.858	0.862	0.866	0.869	0.873	0.88	0.885	0.889	0.892	0.895	0.898	0.901	0.904	0.906	0.908	0.911	0.913	0.917	0.92	0.924	0.926	0.927	0.929	0.93
Croydon	0.741	0.747	0.754	0.761	0.767	0.771	0.774	0.777	0.782	0.788	0.794	0.799	0.803	0.805	0.808	0.81	0.812	0.813	0.815	0.816	0.818	0.82	0.824	0.827	0.83	0.831	0.832	0.833
Ealing	0.764	0.77	0.776	0.783	0.789	0.794	0.797	0.801	0.807	0.814	0.82	0.825	0.828	0.831	0.833	0.834	0.835	0.836	0.837	0.837	0.839	0.843	0.848	0.853	0.857	0.86	0.863	0.865
Enfield	0.737	0.742	0.749	0.755	0.761	0.765	0.768	0.772	0.777	0.783	0.788	0.793	0.797	0.799	0.801	0.802	0.804	0.806	0.809	0.811	0.814	0.818	0.824	0.829	0.833	0.836	0.838	0.839
Greenwich	0.725	0.732	0.74	0.747	0.754	0.758	0.762	0.766	0.771	0.777	0.783	0.788	0.791	0.793	0.795	0.797	0.799	0.801	0.803	0.805	0.809	0.813	0.819	0.824	0.828	0.83	0.832	0.833
Hackney	0.767	0.774	0.781	0.789	0.795	0.798	0.8	0.801	0.805	0.811	0.817	0.822	0.825	0.828	0.831	0.834	0.838	0.843	0.848	0.853	0.858	0.864	0.871	0.877	0.882	0.884	0.886	0.887
Hammersmith and Fulham	0.825	0.833	0.839	0.845	0.851	0.856	0.86	0.864	0.869	0.875	0.879	0.883	0.887	0.889	0.892	0.895	0.898	0.901	0.904	0.907	0.91	0.914	0.917	0.921	0.923	0.924	0.925	0.927
Haringey	0.755	0.761	0.766	0.773	0.778	0.782	0.785	0.788	0.792	0.798	0.804	0.809	0.813	0.815	0.818	0.821	0.823	0.825	0.828	0.829	0.832	0.835	0.84	0.845	0.848	0.851	0.852	0.854
Harrow	0.747	0.754	0.76	0.767	0.773	0.777	0.78	0.784	0.79	0.796	0.802	0.808	0.812	0.815	0.819	0.821	0.824	0.825	0.827	0.829	0.83	0.832	0.835	0.839	0.842	0.844	0.846	0.848
Havering	0.719	0.725	0.731	0.737	0.743	0.747	0.75	0.754	0.759	0.765	0.77	0.775	0.78	0.783	0.786	0.789	0.792	0.794	0.797	0.8	0.802	0.805	0.809	0.814	0.817	0.82	0.823	0.824
Hillingdon	0.781	0.787	0.794	0.801	0.807	0.811	0.815	0.818	0.822	0.828	0.832	0.836	0.84	0.842	0.845	0.846	0.848	0.85	0.852	0.854	0.857	0.862	0.867	0.872	0.876	0.879	0.881	0.882
Hounslow	0.769	0.775	0.781	0.788	0.793	0.797	0.801	0.805	0.811	0.818	0.824	0.83	0.834	0.837	0.84	0.842	0.844	0.845	0.848	0.849	0.852	0.856	0.862	0.868	0.872	0.875	0.877	0.879
Islington	0.812	0.818	0.825	0.832	0.838	0.843	0.847	0.851	0.856	0.862	0.867	0.872	0.876	0.879	0.882	0.885	0.888	0.891	0.895	0.898	0.902	0.906	0.91	0.914	0.917	0.919	0.921	0.922
Kensington and Chelsea	0.839	0.845	0.851	0.857	0.861	0.865	0.869	0.873	0.877	0.884	0.889	0.894	0.897	0.899	0.902	0.905	0.907	0.909	0.912	0.915	0.918	0.921	0.924	0.927	0.929	0.93	0.931	0.932
Kingston upon Thames	0.787	0.794	0.8	0.807	0.812	0.817	0.821	0.826	0.831	0.838	0.844	0.849	0.854	0.858	0.861	0.864	0.867	0.869	0.872	0.874	0.876	0.879	0.882	0.885	0.887	0.888	0.889	0.89
Lambeth	0.775	0.783	0.791	0.797	0.804	0.808	0.812	0.816	0.821	0.828	0.835	0.84	0.845	0.848	0.852	0.856	0.86	0.864	0.869	0.873	0.877	0.882	0.887	0.892	0.895	0.897	0.899	0.9
Lewisham	0.733	0.74	0.746	0.753	0.759	0.763	0.766	0.77	0.775	0.781	0.787	0.793	0.797	0.799	0.802	0.804	0.806	0.808	0.811	0.813	0.816	0.821	0.826	0.832	0.836	0.839	0.841	0.843
Merton	0.758	0.765	0.771	0.779	0.785	0.79	0.795	0.799	0.805	0.812	0.819	0.824	0.828	0.83	0.833	0.835	0.837	0.839	0.841	0.843	0.845	0.849	0.855	0.86	0.865	0.868	0.871	0.873
Newham	0.716	0.722	0.728	0.734	0.74	0.743	0.746	0.748	0.753	0.76	0.765	0.771	0.775	0.777	0.78	0.781	0.783	0.785	0.788	0.792	0.799	0.806	0.815	0.823	0.829	0.833	0.836	0.838
Redbridge	0.728	0.733	0.739	0.746	0.751	0.756	0.759	0.763	0.768	0.774	0.78	0.785	0.789	0.791	0.794	0.796	0.798	0.801	0.803	0.805	0.807	0.811	0.816	0.821	0.825	0.827	0.83	0.831
Richmond upon Thames	0.794	0.801	0.807	0.813	0.819	0.824	0.827	0.832	0.837	0.844	0.85	0.856	0.86	0.864	0.867	0.87	0.872	0.874	0.876	0.878	0.88	0.883	0.887	0.891	0.895	0.897	0.9	0.902
Southwark	0.793	0.8	0.807	0.815	0.821	0.827	0.83	0.835	0.84	0.847	0.852	0.857	0.86	0.863	0.866	0.869	0.872	0.875	0.879	0.883	0.887	0.893	0.898	0.904	0.907	0.909	0.911	0.912
Sutton	0.735	0.74	0.747	0.754	0.761	0.765	0.769	0.774	0.78	0.787	0.793	0.798	0.802	0.805	0.808	0.809	0.811	0.813	0.815	0.817	0.819	0.823	0.828	0.833	0.836	0.839	0.841	0.843
Tower Hamlets	0.766	0.773	0.78	0.788	0.795	0.801	0.805	0.81	0.817	0.825	0.832	0.839	0.844	0.848	0.852	0.857	0.861	0.866	0.871	0.876	0.881	0.886	0.891	0.896	0.9	0.902	0.904	0.905
Waltham Forest	0.712	0.718	0.724	0.731	0.738	0.742	0.745	0.749	0.754	0.76	0.765	0.77	0.773	0.775	0.777	0.778	0.78	0.781	0.783	0.784	0.787	0.792	0.798	0.805	0.81	0.814	0.817	0.819
Wandsworth	0.793	0.8	0.807	0.814	0.82	0.825	0.83	0.835	0.84	0.848	0.855	0.862	0.867	0.87	0.873	0.876	0.879	0.881	0.885	0.887	0.889	0.893	0.897	0.901	0.905	0.907	0.909	0.911
Westminster	0.831	0.837	0.843	0.849	0.854	0.858	0.862	0.865	0.87	0.875	0.881	0.885	0.889	0.891	0.895	0.898	0.901	0.903	0.905	0.907	0.91	0.913	0.916	0.92	0.922	0.924	0.925	0.927
North East England	0.697	0.703	0.711	0.718	0.725	0.73	0.733	0.738	0.744	0.75	0.757	0.763	0.767	0.771	0.774	0.778	0.781	0.784	0.787	0.79	0.794	0.798	0.804	0.81	0.814	0.817	0.819	0.821
County Durham	0.69	0.696	0.703	0.71	0.717	0.721	0.725	0.73	0.735	0.742	0.748	0.754	0.758	0.762	0.765	0.767	0.77	0.772	0.776	0.778	0.782	0.786	0.792	0.798	0.802	0.806	0.808	0.81
Darlington	0.703	0.709	0.716	0.723	0.73	0.734	0.737	0.74	0.745	0.752	0.758	0.764	0.769	0.773	0.777	0.78	0.783	0.785	0.788	0.791	0.795	0.8	0.806	0.812	0.817	0.821	0.823	0.825
Gateshead	0.7	0.706	0.713	0.72	0.727	0.732	0.735	0.739	0.745	0.751	0.758	0.764	0.769	0.773	0.777	0.781	0.784	0.787	0.791	0.794	0.798	0.803	0.808	0.814	0.818	0.821	0.824	0.826
Hartlepool	0.677	0.683	0.69	0.697	0.703	0.707	0.71	0.714	0.719	0.726	0.732	0.737	0.74	0.742	0.744	0.747	0.749	0.752	0.755	0.758	0.762	0.767	0.773	0.78	0.785	0.788	0.791	0.793
Middlesbrough	0.688	0.695	0.702	0.709	0.716	0.72	0.723	0.727	0.732	0.74	0.746	0.751	0.755	0.758	0.762	0.766	0.769	0.772	0.775	0.778	0.781	0.786	0.792	0.797	0.801	0.804	0.806	0.808
Newcastle upon Tyne	0.736	0.743	0.751	0.759	0.765	0.771	0.776	0.781	0.787	0.794	0.801	0.808	0.814	0.82	0.825	0.831	0.835	0.84	0.843	0.846	0.85	0.854	0.858	0.863	0.866	0.869	0.87	0.872
North Tyneside	0.696	0.703	0.71	0.718	0.724	0.729	0.733	0.737	0.743	0.749	0.756	0.762	0.768	0.772	0.776	0.78	0.784	0.787	0.79	0.793	0.797	0.802	0.807	0.813	0.817	0.821	0.823	0.825
Northumberland	0.697	0.703	0.709	0.716	0.722	0.727	0.73	0.735	0.74	0.746	0.752	0.757	0.761	0.764	0.767	0.77	0.773	0.776	0.779	0.781	0.784	0.787	0.793	0.798	0.802	0.805	0.807	0.808
Redcar and Cleveland	0.67	0.675	0.682	0.689	0.696	0.701	0.704	0.708	0.714	0.721	0.727	0.732	0.736	0.738	0.741	0.743	0.745	0.747	0.75	0.752	0.756	0.76	0.767	0.775	0.78	0.784	0.787	0.79
South Tyneside	0.664	0.671	0.678	0.685	0.692	0.697	0.7	0.705	0.711	0.718	0.725	0.731	0.737	0.74	0.744	0.748	0.751	0.754	0.757	0.759	0.762	0.767	0.773	0.78	0.785	0.789	0.792	0.794
Stockton-on-Tees	0.705	0.712	0.72	0.728	0.735	0.74	0.744	0.748	0.754	0.761	0.767	0.772	0.775	0.777	0.779	0.781	0.784	0.785	0.788	0.791	0.794	0.798	0.804	0.811	0.815	0.818	0.821	0.823
Sunderland	0.689	0.696	0.704	0.713	0.72	0.725	0.729	0.733	0.739	0.746	0.752	0.758	0.763	0.766	0.77	0.773	0.776	0.779	0.782	0.784	0.787	0.791	0.797	0.803	0.808	0.811	0.813	0.815
North West England	0.716	0.723	0.729	0.736	0.742	0.747	0.751	0.755	0.76	0.766	0.772	0.778	0.782	0.786	0.789	0.792	0.795	0.798	0.801	0.804	0.807	0.812	0.817	0.823	0.827	0.83	0.832	0.834
Blackburn with Darwen	0.691	0.695	0.701	0.707	0.713	0.717	0.719	0.723	0.728	0.734	0.74	0.745	0.748	0.75	0.753	0.756	0.758	0.761	0.764	0.766	0.769	0.774	0.78	0.788	0.793	0.797	0.8	0.802
Blackpool	0.68	0.685	0.691	0.698	0.703	0.707	0.71	0.713	0.718	0.724	0.729	0.733	0.737	0.738	0.741	0.743	0.745	0.747	0.748	0.75	0.752	0.756	0.762	0.768	0.773	0.776	0.779	0.781
Bolton	0.701	0.707	0.714	0.72	0.726	0.73	0.733	0.736	0.741	0.746	0.751	0.756	0.759	0.761	0.763	0.765	0.768	0.769	0.772	0.774	0.777	0.781	0.787	0.794	0.798	0.801	0.803	0.805
Bury	0.703	0.709	0.716	0.723	0.73	0.734	0.738	0.742	0.747	0.752	0.758	0.762	0.766	0.768	0.771	0.774	0.777	0.779	0.781	0.783	0.786	0.791	0.797	0.803	0.807	0.81	0.813	0.815
Cheshire East	0.748	0.754	0.76	0.767	0.773	0.778	0.782	0.787	0.792	0.798	0.803	0.809	0.813	0.817	0.82	0.823	0.826	0.829	0.832	0.834	0.836	0.84	0.845	0.851	0.856	0.859	0.861	0.864
Cheshire West and Chester	0.74	0.747	0.754	0.761	0.768	0.772	0.776	0.78	0.785	0.792	0.797	0.803	0.807	0.811	0.815	0.818	0.822	0.825	0.828	0.831	0.834	0.838	0.842	0.847	0.85	0.852	0.854	0.855
Cumbria	0.719	0.724	0.731	0.737	0.743	0.748	0.751	0.755	0.76	0.765	0.77	0.774	0.777	0.779	0.782	0.784	0.787	0.789	0.792	0.794	0.798	0.802	0.808	0.815	0.819	0.823	0.826	0.828
Halton	0.702	0.708	0.715	0.723	0.729	0.734	0.737	0.74	0.745	0.75	0.756	0.762	0.766	0.769	0.772	0.775	0.778	0.78	0.784	0.787	0.791	0.797	0.803	0.81	0.815	0.819	0.822	0.824
Knowsley	0.691	0.696	0.702	0.708	0.714	0.718	0.722	0.726	0.731	0.737	0.743	0.749	0.754	0.757	0.76	0.764	0.767	0.77	0.774	0.777	0.782	0.787	0.793	0.8	0.806	0.81	0.813	0.816
Lancashire	0.72	0.726	0.734	0.741	0.747	0.752	0.756	0.76	0.765	0.77	0.775	0.78	0.784	0.786	0.789	0.792	0.794	0.797	0.799	0.802	0.805	0.809	0.815	0.82	0.824	0.827	0.829	0.831
Liverpool	0.721	0.728	0.735	0.742	0.748	0.753	0.757	0.762	0.768	0.775	0.783	0.789	0.795	0.801	0.806	0.811	0.815	0.819	0.823	0.828	0.831	0.835	0.84	0.844	0.847	0.849	0.851	0.852
Manchester	0.746	0.753	0.76	0.768	0.775	0.78	0.785	0.79	0.796	0.804	0.812	0.819	0.825	0.83	0.835	0.84	0.844	0.849	0.853	0.856	0.86	0.864	0.869	0.874	0.878	0.88	0.883	0.885
Oldham	0.685	0.69	0.697	0.703	0.708	0.711	0.713	0.715	0.719	0.724	0.729	0.733	0.737	0.739	0.741	0.744	0.746	0.749	0.752	0.754	0.758	0.763	0.77	0.778	0.782	0.786	0.788	0.79
Rochdale	0.686	0.692	0.698	0.705	0.711	0.715	0.717	0.721	0.726	0.732	0.738	0.742	0.746	0.748	0.75	0.753	0.755	0.757	0.76	0.762	0.765	0.77	0.777	0.783	0.787	0.791	0.793	0.795
Salford	0.708	0.714	0.721	0.728	0.735	0.74	0.743	0.747	0.753	0.761	0.768	0.775	0.781	0.785	0.79	0.794	0.797	0.8	0.803	0.806	0.809	0.814	0.819	0.826	0.83	0.834	0.836	0.838
Sefton	0.706	0.713	0.72	0.727	0.733	0.738	0.741	0.746	0.751	0.757	0.763	0.768	0.773	0.776	0.78	0.783	0.786	0.788	0.79	0.792	0.794	0.796	0.8	0.803	0.806	0.809	0.81	0.812

Methods Appendix Table 3: Socio-Demographic Index values for all estimated GBD 2017 locations, 1990-2017

Location	1990	1991	1992	1993	1994	1995	1996	1997	1998	1999	2000	2001	2002	2003	2004	2005	2006	2007	2008	2009	2010	2011	2012	2013	2014	2015	2016	2017
St Helens	0.684	0.69	0.696	0.703	0.709	0.713	0.716	0.72	0.725	0.731	0.738	0.744	0.749	0.753	0.756	0.759	0.762	0.765	0.768	0.771	0.774	0.779	0.785	0.791	0.795	0.798	0.801	0.803
Stockport	0.727	0.734	0.741	0.748	0.755	0.759	0.763	0.767	0.772	0.779	0.785	0.791	0.796	0.799	0.803	0.806	0.809	0.812	0.814	0.817	0.82	0.823	0.828	0.833	0.837	0.839	0.841	0.843
Tameside	0.691	0.697	0.703	0.71	0.716	0.721	0.724	0.728	0.734	0.74	0.746	0.751	0.755	0.758	0.76	0.762	0.763	0.764	0.766	0.768	0.77	0.774	0.779	0.785	0.79	0.793	0.795	0.797
Trafford	0.751	0.757	0.764	0.771	0.778	0.782	0.786	0.79	0.795	0.802	0.809	0.815	0.82	0.824	0.829	0.832	0.836	0.839	0.842	0.844	0.848	0.852	0.856	0.862	0.865	0.868	0.871	0.873
Warrington	0.739	0.745	0.752	0.759	0.765	0.769	0.773	0.776	0.782	0.788	0.795	0.801	0.807	0.811	0.814	0.818	0.822	0.825	0.827	0.83	0.833	0.838	0.843	0.849	0.853	0.856	0.858	0.86
Wigan	0.691	0.697	0.703	0.71	0.716	0.72	0.723	0.727	0.731	0.737	0.742	0.747	0.75	0.753	0.755	0.757	0.76	0.762	0.764	0.766	0.769	0.774	0.78	0.786	0.79	0.793	0.796	0.798
Wirral	0.695	0.701	0.708	0.714	0.721	0.725	0.728	0.732	0.737	0.743	0.749	0.753	0.757	0.761	0.763	0.766	0.768	0.77	0.773	0.775	0.777	0.781	0.786	0.792	0.796	0.799	0.801	0.803
South East England	0.749	0.755	0.761	0.767	0.773	0.777	0.781	0.784	0.789	0.795	0.801	0.806	0.809	0.812	0.815	0.818	0.821	0.823	0.826	0.828	0.831	0.836	0.841	0.846	0.85	0.852	0.855	0.856
Bracknell Forest	0.759	0.764	0.77	0.776	0.781	0.785	0.788	0.791	0.797	0.804	0.811	0.817	0.822	0.825	0.829	0.832	0.835	0.838	0.841	0.843	0.845	0.849	0.854	0.859	0.862	0.865	0.867	0.869
Brighton and Hove	0.766	0.772	0.779	0.785	0.791	0.796	0.801	0.806	0.811	0.816	0.822	0.827	0.833	0.838	0.843	0.847	0.851	0.855	0.859	0.863	0.865	0.868	0.872	0.876	0.879	0.881	0.883	0.885
Buckinghamshire	0.764	0.769	0.775	0.782	0.788	0.792	0.795	0.799	0.804	0.81	0.815	0.82	0.824	0.826	0.829	0.832	0.834	0.836	0.838	0.84	0.842	0.846	0.851	0.855	0.859	0.861	0.863	0.865
East Sussex	0.712	0.718	0.724	0.73	0.736	0.74	0.742	0.745	0.749	0.754	0.759	0.763	0.766	0.769	0.771	0.773	0.776	0.778	0.781	0.783	0.787	0.791	0.797	0.803	0.807	0.81	0.812	0.814
Hampshire	0.744	0.75	0.756	0.762	0.768	0.772	0.775	0.778	0.782	0.788	0.793	0.798	0.802	0.804	0.807	0.81	0.812	0.815	0.818	0.82	0.824	0.828	0.834	0.839	0.843	0.846	0.848	0.85
Isle of Wight	0.704	0.709	0.715	0.722	0.727	0.732	0.735	0.739	0.744	0.749	0.756	0.761	0.765	0.767	0.768	0.771	0.773	0.776	0.779	0.781	0.784	0.788	0.794	0.801	0.806	0.809	0.812	0.814
Kent	0.723	0.728	0.734	0.74	0.746	0.75	0.752	0.756	0.76	0.765	0.77	0.774	0.777	0.779	0.782	0.785	0.787	0.79	0.793	0.796	0.8	0.805	0.811	0.817	0.822	0.824	0.826	0.828
Medway	0.703	0.709	0.715	0.722	0.728	0.731	0.734	0.737	0.742	0.747	0.752	0.756	0.76	0.762	0.765	0.768	0.771	0.773	0.776	0.778	0.781	0.785	0.791	0.797	0.802	0.805	0.807	0.809
Milton Keynes	0.754	0.76	0.767	0.774	0.78	0.784	0.786	0.789	0.793	0.798	0.802	0.806	0.81	0.812	0.815	0.817	0.819	0.821	0.823	0.825	0.829	0.834	0.84	0.847	0.852	0.856	0.859	0.86
Oxfordshire	0.769	0.775	0.781	0.788	0.794	0.798	0.801	0.805	0.81	0.816	0.822	0.827	0.831	0.835	0.838	0.841	0.844	0.847	0.849	0.852	0.855	0.859	0.864	0.869	0.872	0.875	0.878	0.879
Portsmouth	0.75	0.756	0.763	0.77	0.776	0.781	0.785	0.79	0.795	0.8	0.805	0.81	0.815	0.818	0.822	0.824	0.827	0.829	0.832	0.835	0.838	0.842	0.846	0.851	0.854	0.857	0.858	0.86
Reading	0.785	0.791	0.797	0.803	0.809	0.813	0.817	0.821	0.827	0.834	0.84	0.847	0.852	0.856	0.86	0.864	0.866	0.868	0.87	0.872	0.874	0.877	0.882	0.887	0.89	0.892	0.894	0.895
Slough	0.764	0.77	0.777	0.784	0.79	0.793	0.796	0.799	0.802	0.806	0.81	0.813	0.815	0.816	0.817	0.818	0.819	0.82	0.822	0.825	0.829	0.834	0.841	0.848	0.852	0.855	0.858	0.859
Southampton	0.752	0.758	0.765	0.772	0.779	0.784	0.789	0.794	0.8	0.805	0.81	0.815	0.819	0.823	0.826	0.829	0.831	0.834	0.836	0.837	0.839	0.842	0.845	0.849	0.852	0.855	0.856	0.858
Surrey	0.773	0.779	0.785	0.791	0.797	0.801	0.805	0.809	0.814	0.821	0.827	0.833	0.838	0.841	0.845	0.847	0.85	0.853	0.856	0.858	0.861	0.864	0.868	0.873	0.876	0.879	0.881	0.883
West Berkshire	0.774	0.78	0.786	0.793	0.799	0.803	0.805	0.808	0.813	0.819	0.824	0.829	0.832	0.835	0.836	0.836	0.837	0.838	0.84	0.842	0.846	0.851	0.857	0.863	0.867	0.869	0.871	0.872
West Sussex	0.74	0.745	0.751	0.757	0.763	0.767	0.77	0.773	0.777	0.783	0.788	0.793	0.796	0.799	0.802	0.804	0.807	0.809	0.812	0.814	0.818	0.822	0.827	0.833	0.837	0.84	0.842	0.843
Windsor and Maidenhead	0.778	0.783	0.789	0.795	0.8	0.805	0.808	0.811	0.816	0.823	0.829	0.835	0.839	0.843	0.847	0.851	0.854	0.857	0.86	0.863	0.866	0.87	0.874	0.88	0.883	0.885	0.887	0.889
Wokingham	0.778	0.784	0.79	0.797	0.802	0.806	0.81	0.814	0.82	0.826	0.832	0.837	0.842	0.845	0.849	0.853	0.856	0.858	0.861	0.863	0.865	0.868	0.871	0.876	0.879	0.882	0.883	0.885
South West England	0.729	0.735	0.741	0.748	0.754	0.758	0.762	0.766	0.771	0.777	0.783	0.788	0.792	0.796	0.799	0.802	0.805	0.807	0.81	0.813	0.816	0.82	0.825	0.831	0.835	0.838	0.84	0.841
Bath and North East Somerset	0.752	0.758	0.764	0.77	0.777	0.782	0.786	0.79	0.796	0.803	0.809	0.816	0.822	0.828	0.833	0.838	0.842	0.846	0.85	0.853	0.856	0.859	0.863	0.867	0.869	0.872	0.874	0.875
Bournemouth	0.736	0.743	0.75	0.757	0.763	0.768	0.773	0.778	0.784	0.79	0.797	0.803	0.808	0.812	0.816	0.821	0.824	0.828	0.831	0.834	0.836	0.839	0.843	0.848	0.851	0.853	0.856	0.858
Bristol, City of	0.763	0.77	0.777	0.784	0.791	0.796	0.8	0.805	0.81	0.817	0.823	0.828	0.833	0.836	0.839	0.843	0.846	0.849	0.853	0.856	0.859	0.863	0.868	0.873	0.877	0.88	0.882	0.884
Cornwall	0.7	0.706	0.713	0.721	0.727	0.731	0.734	0.738	0.743	0.749	0.755	0.76	0.764	0.768	0.771	0.774	0.777	0.78	0.783	0.786	0.789	0.793	0.799	0.806	0.81	0.813	0.815	0.817
Devon	0.72	0.726	0.733	0.74	0.746	0.75	0.753	0.757	0.762	0.769	0.775	0.78	0.785	0.789	0.793	0.796	0.8	0.803	0.806	0.808	0.811	0.816	0.821	0.826	0.83	0.833	0.835	0.837
Dorset	0.716	0.721	0.727	0.734	0.74	0.744	0.747	0.751	0.756	0.762	0.769	0.773	0.777	0.779	0.781	0.783	0.786	0.788	0.791	0.793	0.797	0.802	0.808	0.814	0.818	0.821	0.823	0.825
Gloucestershire	0.735	0.741	0.747	0.754	0.76	0.765	0.768	0.772	0.777	0.783	0.79	0.795	0.8	0.804	0.808	0.811	0.813	0.816	0.818	0.82	0.824	0.828	0.833	0.839	0.843	0.846	0.848	0.85
North Somerset	0.714	0.72	0.727	0.733	0.739	0.743	0.746	0.75	0.755	0.76	0.766	0.771	0.776	0.78	0.783	0.786	0.789	0.792	0.795	0.798	0.801	0.806	0.813	0.819	0.824	0.827	0.83	0.832
Plymouth	0.724	0.73	0.737	0.744	0.75	0.754	0.758	0.762	0.767	0.772	0.778	0.783	0.787	0.79	0.793	0.796	0.799	0.802	0.805	0.807	0.81	0.814	0.819	0.825	0.829	0.832	0.834	0.836
Poole	0.727	0.733	0.74	0.746	0.753	0.758	0.761	0.765	0.771	0.777	0.783	0.789	0.793	0.796	0.798	0.801	0.804	0.806	0.809	0.811	0.814	0.818	0.824	0.83	0.835	0.838	0.84	0.842
Somerset	0.713	0.718	0.724	0.731	0.737	0.741	0.744	0.748	0.752	0.757	0.763	0.767	0.77	0.772	0.775	0.777	0.78	0.782	0.785	0.787	0.789	0.794	0.799	0.805	0.809	0.812	0.814	0.816
South Gloucestershire	0.747	0.752	0.758	0.765	0.771	0.775	0.779	0.783	0.789	0.796	0.802	0.808	0.813	0.817	0.821	0.824	0.827	0.831	0.834	0.837	0.84	0.844	0.849	0.855	0.859	0.862	0.865	0.867
Swindon	0.747	0.753	0.76	0.767	0.773	0.776	0.778	0.781	0.786	0.792	0.797	0.801	0.805	0.806	0.807	0.809	0.811	0.813	0.815	0.818	0.82	0.825	0.831	0.837	0.841	0.844	0.846	0.847
Torbay	0.699	0.705	0.711	0.717	0.723	0.727	0.73	0.733	0.736	0.741	0.745	0.749	0.75	0.751	0.753	0.755	0.757	0.759	0.761	0.762	0.764	0.767	0.773	0.779	0.783	0.786	0.789	0.79
Wiltshire	0.726	0.731	0.737	0.743	0.749	0.753	0.756	0.759	0.763	0.769	0.775	0.78	0.784	0.786	0.789	0.791	0.793	0.795	0.797	0.799	0.803	0.808	0.813	0.819	0.823	0.826	0.828	0.829
West Midlands	0.707	0.713	0.72	0.727	0.734	0.739	0.743	0.747	0.752	0.759	0.765	0.771	0.776	0.779	0.783	0.786	0.789	0.792	0.795	0.798	0.802	0.806	0.812	0.818	0.822	0.825	0.828	0.829
Birmingham	0.707	0.714	0.721	0.728	0.735	0.739	0.743	0.747	0.753	0.76	0.766	0.773	0.778	0.782	0.787	0.791	0.794	0.798	0.803	0.807	0.811	0.817	0.823	0.829	0.834	0.837	0.839	0.84
Coventry	0.722	0.729	0.737	0.745	0.751	0.757	0.762	0.766	0.772	0.779	0.785	0.791	0.796	0.8	0.803	0.807	0.81	0.812	0.815	0.818	0.821	0.825	0.831	0.837	0.841	0.844	0.847	0.848
Dudley	0.692	0.698	0.704	0.71	0.716	0.721	0.724	0.728	0.733	0.739	0.746	0.751	0.756	0.759	0.762	0.765	0.767	0.77	0.772	0.774	0.776	0.779	0.784	0.789	0.793	0.795	0.797	0.799
Herefordshire, County of	0.706	0.712	0.719	0.726	0.733	0.738	0.742	0.747	0.752	0.759	0.766	0.771	0.776	0.779	0.783	0.786	0.789	0.792	0.795	0.798	0.801	0.806	0.812	0.818	0.822	0.825	0.827	0.828
Sandwell	0.682	0.688	0.695	0.702	0.708	0.712	0.715	0.719	0.724	0.729	0.735	0.74	0.744	0.747	0.75	0.752	0.754	0.756	0.759	0.761	0.765	0.769	0.776	0.783	0.788	0.792	0.795	0.797
Shropshire	0.708	0.714	0.721	0.728	0.734	0.739	0.743	0.747	0.752	0.759	0.766	0.771	0.776	0.781	0.785	0.789	0.793	0.795	0.799	0.802	0.806	0.811	0.816	0.821	0.825	0.828	0.83	0.832
Solihull	0.733	0.74	0.747	0.755	0.762	0.767	0.771	0.776	0.783	0.789	0.795	0.801	0.807	0.811	0.815	0.818	0.822	0.825	0.828	0.83	0.832	0.835	0.84	0.845	0.848	0.851	0.853	0.855
Staffordshire	0.708	0.714	0.722	0.729	0.736	0.74	0.744	0.749	0.755	0.761	0.767	0.772	0.777	0.78	0.783	0.787	0.79	0.793	0.796	0.799	0.802	0.806	0.811	0.816	0.819	0.822	0.824	0.826
Stoke-on-Trent	0.689	0.696	0.703	0.71	0.716	0.721	0.724	0.728	0.733	0.738	0.743	0.747	0.75	0.751	0.753	0.754	0.756	0.757	0.76	0.763	0.767	0.772	0.78	0.788	0.794	0.798	0.801	0.804
Telford and Wrekin	0.713	0.719	0.726	0.733	0.74	0.745	0.749	0.754	0.759	0.764	0.769	0.773	0.776	0.777	0.779	0.781	0.783	0.784	0.787	0.789	0.791	0.796	0.802	0.809	0.814	0.817	0.82	0.822
Walsall	0.681	0.686	0.692	0.698	0.704	0.707	0.709	0.712	0.717	0.723	0.729	0.734	0.738	0.74	0.743	0.746	0.749	0.752	0.755	0.757	0.761	0.766	0.772	0.779	0.783	0.787	0.789	0.791
Warwickshire	0.732	0.738	0.746	0.753	0.76	0.765	0.769	0.774	0.779	0.786	0.792	0.798	0.802	0.806	0.809	0.812	0.816	0.819	0.823	0.826	0.83	0.834	0.84	0.845	0.85	0.853	0.855	0.857
Wolverhampton	0.691	0.697	0.704	0.712	0.719	0.723	0.727	0.731	0.737	0.744	0.751	0.757	0.761	0.764	0.768	0.771	0.774	0.776	0.779	0.781	0.784	0.788	0.794	0.8	0.804	0.807	0.809	0.811
Worcestershire	0.707	0.713	0.72	0.727	0.733	0.738	0.742	0.747	0.754	0.762	0.771	0.778	0.782	0.786	0.791	0.794	0.798	0.8	0.803	0.805	0.808	0.811	0.816	0.822	0.826	0.829	0.831	0.833
Yorkshire and the Humber	0.711	0.717	0.724	0.731	0.737	0.742	0.745	0.75	0.755	0.762	0.768	0.773	0.778	0.781	0.785	0.788	0.791	0.794	0.797	0.8	0.803	0.808	0.813	0.819	0.823	0.826	0.828	0.83
Barnsley	0.675	0.68	0.686	0.692	0.698	0.702	0.705	0.709	0.715	0.721	0.726	0.731	0.735	0.738	0.741	0.743	0.746	0.748	0.75	0.752	0.755	0.759	0.765	0.772	0.778	0.781	0.785	0.787
Bradford	0.693	0.699	0.705	0.712	0.718	0.721	0.723	0.725	0.729	0.736	0.741	0.747	0.751	0.753	0.756	0.759	0.762	0.765	0.768	0.771	0.775	0.78	0.787	0.794	0.799	0.803	0.805	0.807
Calderdale	0.708	0.714	0.721	0.729	0.735	0.739	0.743	0.746	0.751	0.758	0.763	0.768	0.771	0.774	0.777	0.78	0.783	0.785	0.788	0.791	0.795	0.8	0.807	0.814	0.819	0.823	0.825	0.827
Doncaster	0.674	0.679	0.685	0.692	0.697	0.701	0.704	0.707	0.712	0.718	0.724	0.729	0.733	0.736	0.74	0.743	0.746	0.749	0.752	0.755	0.759	0.764	0.771	0.778	0.783	0.786	0.789	0.791
East Riding of Yorkshire	0.714	0.72	0.727	0.734	0.741	0.745	0.749	0.753	0.758	0.764	0.769	0.774	0.778	0.781	0.784	0.787	0.79	0.792	0.794	0.797	0.799	0.802	0.807	0.812	0.815	0.818	0.82	0.822
Kingston upon Hull, City of	0.689	0.695	0.702	0.71	0.717	0.722	0.726	0.73	0.736	0.742	0.749	0.755	0.76	0.763	0.767	0.77	0.773	0.776	0.779	0.782	0.785	0.789	0.795	0.801	0.806	0.809	0.811	0.813
Kirklees	0.703	0.709	0.716	0.723	0.73	0.734	0.737	0.741	0.746	0.752	0.758	0.763	0.767	0.77	0.773	0.776	0.779	0.781	0.783	0.785	0.788	0.793	0.798	0.805	0.809	0.812	0.814	0.816
Leeds	0.739	0.746	0.753	0.761	0.768	0.773	0.778	0.783	0.789	0.797	0.803	0.81	0.816	0.82	0.825	0.83	0.834	0.837	0.841	0.843	0.846	0.849	0.854	0.858	0.862	0.864	0.867	0.868
North East Lincolnshire	0.698	0.705	0.712	0.72	0.727	0.731	0.733	0.737	0.741	0.745	0.749	0.753	0.755	0.757	0.758	0.761	0.763	0.767	0.77	0.775	0.779	0.784	0.79	0.796	0.799	0.802	0.803	0.804
North Lincolnshire	0.702	0.707	0.714	0.72	0.727	0.731	0.734	0.737	0.742	0.748	0.752	0.757	0.76	0.762	0.764	0.766	0.768	0.77	0.773	0.776	0.78	0.785	0.792	0.799	0.803	0.807	0.809	0.811
North Yorkshire	0.724	0.73	0.737	0.743	0.75	0.754	0.758	0.762	0.767	0.774	0.78	0.785	0.79	0.794	0.798	0.801	0.803	0.806	0.808	0.81	0.813	0.817	0.822	0.827	0.831	0.834	0.836	0.839
Rotherham	0.681	0.687	0.693	0.7	0.707	0.711	0.714	0.717	0.722	0.728	0.734	0.739	0.744	0.747	0.75	0.753	0.756	0.759	0.761	0.763	0.766	0.77	0.776	0.782	0.787	0.791	0.794	0.796
Sheffield	0.727	0.733	0.74	0.747	0.753	0.758	0.762	0.767	0.773	0.78	0.786	0.792	0.798	0.803	0.807	0.811	0.815	0.819	0.823	0.826	0.83	0.834	0.839	0.843	0.847	0.849	0.852	0.853
Wakefield	0.692	0.698	0.705	0.712	0.718	0.722	0.726	0.729	0.734	0.741	0.747	0.752	0.755	0.758	0.76	0.763	0.765	0.766	0.768	0.769	0.772	0.777	0.784	0.791	0.797	0.801	0.804	0.806
York	0.762	0.769	0.776	0.783	0.789	0.795	0.799	0.804	0.81	0.816	0.822	0.829	0.834	0.839	0.844	0.848	0.851	0.854	0.857	0.859	0.862	0.865	0.868	0.872	0.874	0.876	0.878	0.879
Northern Ireland	0.71	0.718	0.726	0.735	0.742	0.747	0.751	0.755	0.761	0.767	0.774	0.78	0.786	0.79	0.795	0.798	0.801	0.805	0.808	0.812	0.815	0.818	0.822	0.825	0.828	0.831	0.833	0.835
Scotland	0.672	0.678	0.686	0.693	0.701	0.706	0.711	0.716	0.722	0.73	0.737	0.743	0.748	0.752	0.757	0.761	0.764	0.768	0.772	0.777	0.781	0.785	0.789	0.793	0.797	0.8	0.803	0.805
Wales	0.642	0.65	0.659	0.668	0.676	0.683	0.688	0.694	0.702	0.711	0.719	0.727	0.734	0.739	0.745	0.75	0.755	0.76	0.765	0.77	0.774	0.78	0.786	0.792	0.797	0.8	0.803	0.806
Latin America and Caribbean	0.497	0.502	0.508	0.514	0.521	0.527	0.534	0.54	0.546	0.552	0.557	0.563	0.567	0.571	0.576	0.581	0.587	0.592	0.598	0.603	0.608	0.613	0.618	0.624	0.628	0.633	0.637	0.64
Andean Latin America	0.483	0.486	0.49	0.495	0.501	0.507	0.512	0.518	0.526	0.533	0.539	0.546	0.552	0.558	0.563	0.567	0.572	0.577	0.581	0.585	0.59	0.595	0.601	0.606	0.612	0.618	0.624	0.628
Bolivia	0.403	0.408	0.416	0.424	0.431	0.438	0.447	0.456	0.466	0.475	0.485	0.494	0.504	0.513	0.52	0.526	0.532	0.536	0.539	0.543	0.547	0.551	0.555	0.559	0.565	0.573	0.581	0.587
Ecuador	0.506	0.51	0.515	0.52	0.524	0.524	0.522	0.521	0.526	0.532	0.539	0.548	0.557	0.564	0.57	0.575	0.581	0.585	0.588	0.59	0.594	0.603	0.611	0.618	0.623	0.628	0.632	0.636
Peru	0.493	0.495	0.497	0.501	0.509	0.517	0.526	0.534	0.542	0.549	0.555	0.56	0.564	0.568	0.571	0.575	0.578	0.584	0.59	0.594	0.6	0.604	0.608	0.614	0.62	0.626	0.632	0.636
Caribbean	0.531	0.536	0.539	0.543	0.546	0.549	0.552	0.555	0.559	0.563	0.568	0.573	0.578	0.583	0.589	0.596	0.602	0.607	0.61	0.613	0.617	0.621	0.624	0.626	0.628	0.631	0.635	0.638
Antigua and Barbuda	0.61	0.616	0.622	0.627	0.631	0.633	0.636	0.64	0.644	0.649	0.653	0.657	0.661	0.666	0.67	0.675	0.68	0.685	0.69	0.694	0.696	0.698	0.7	0.703	0.706	0.709	0.712	0.715
The Bahamas	0.674	0.669	0.665	0.667	0.671	0.676	0.681	0.687	0.695	0.703	0.71	0.715	0.718	0.721	0.723	0.727	0.732	0.737	0.742	0.746	0.751	0.753	0.755	0.755	0.755	0.755	0.755	0.756
Barbados	0.65	0.655	0.661	0.667	0.672	0.675	0.678	0.68	0.682	0.683	0.686	0.689	0.693	0.698	0.703	0.707	0.71	0.713	0.717	0.72	0.723	0.726	0.729	0.732	0.734	0.736	0.738	0.739
Belize	0.402	0.414	0.429	0.445	0.458	0.471	0.482	0.49	0.496	0.501	0.506	0.511	0.518	0.525	0.533	0.541	0.549	0.557	0.563	0.569	0.576	0.581	0.586	0.59	0.594	0.598	0.6	0.602
Bermuda	0.73	0.732	0.734	0.737	0.738	0.74	0.741	0.743	0.746	0.749	0.752	0.754	0.757	0.761	0.765	0.769	0.773	0.777	0.782	0.786	0.79	0.794	0.797	0.8	0.803	0.804	0.805	0.805
Cuba	0.592	0.6	0.604	0.605	0.604	0.603	0.601	0.6	0.601	0.604	0.607	0.611	0.617	0.623	0.629	0.636	0.644	0.646	0.647	0.649	0.652	0.656	0.663	0.668	0.671	0.676	0.683	0.688
Dominica	0.534	0.535	0.539	0.546	0.555	0.563	0.57	0.577	0.584	0.592	0.599	0.606	0.614	0.621	0.63	0.636	0.641	0.645	0.65	0.656	0.662	0.668	0.673	0.677	0.68	0.682	0.684	0.687
Dominican Republic	0.442	0.443	0.445	0.449	0.454	0.459	0.464	0.469	0.475	0.48	0.484	0.49	0.495	0.499	0.511	0.525	0.536	0.547	0.555	0.562	0.568	0.572	0.575	0.576	0.578	0.583	0.589	0.593
Grenada	0.46	0.472	0.485	0.497	0.508	0.519	0.529	0.54	0.55	0.558	0.566	0.573	0.578	0.584	0.589	0.594	0.599	0.604	0.608	0.612	0.615	0.618	0.62	0.624	0.628	0.632	0.637	0.64
Guyana	0.442	0.446	0.45	0.454	0.458	0.465	0.472	0.481	0.489	0.497	0.503	0.51	0.516	0.521	0.526	0.53	0.533	0.537	0.542	0.547	0.552	0.557	0.563	0.568	0.573	0.576	0.58	0.584
Haiti	0.328	0.332	0.336	0.339	0.342	0.345	0.35	0.355	0.361	0.367	0.373	0.379	0.384	0.389	0.393	0.397	0.401	0.405	0.409	0.413	0.416	0.42	0.423	0.427	0.431	0.435	0.439	0.442
Jamaica	0.547	0.553	0.56	0.565	0.571	0.578	0.585	0.591	0.596	0.6	0.607	0.615	0.621	0.626	0.63	0.635	0.639	0.644	0.649	0.653	0.658	0.663	0.667	0.671	0.673	0.676	0.677	0.679
Puerto Rico	0.684	0.689	0.693	0.698	0.703	0.708	0.714	0.719	0.724	0.729	0.736	0.745	0.752	0.756	0.759	0.762	0.766	0.77	0.774	0.778	0.782	0.786	0.791	0.797	0.805	0.811	0.813	0.813
Saint Lucia	0.509	0.516	0.524	0.532	0.54	0.549	0.558	0.566	0.573	0.58	0.585	0.589	0.593	0.598	0.603	0.606	0.609	0.613	0.618	0.623	0.628	0.633	0.636	0.64	0.643	0.646	0.65	0.653
Saint Vincent and the Grenadines	0.465	0.472	0.479	0.486	0.492	0.499	0.506	0.512	0.518	0.524	0.53	0.537	0.543	0.549	0.553	0.557	0.561	0.566	0.571	0.575	0.579	0.584	0.588	0.593	0.597	0.601	0.605	0.608
Suriname	0.529	0.534	0.538	0.541	0.543	0.543	0.544	0.547	0.551	0.555	0.56	0.568	0.576	0.585	0.592	0.597	0.601	0.604	0.608	0.612	0.616	0.62	0.625	0.628	0.632	0.636	0.639	0.641
Trinidad and Tobago	0.601	0.604	0.608	0.611	0.615	0.619	0.624	0.629	0.634	0.64	0.646	0.652	0.657	0.663	0.669	0.675	0.68	0.685	0.689	0.691	0.693	0.694	0.695	0.696	0.697	0.698	0.698	0.698
Virgin Islands	0.673	0.683	0.691	0.699	0.706	0.713	0.719	0.725	0.73	0.734	0.737	0.74	0.748	0.755	0.762	0.768	0.774	0.78	0.786	0.79	0.795	0.799	0.802	0.804	0.805	0.806	0.806	0.807
Central Latin America	0.492	0.496	0.501	0.507	0.515	0.521	0.528	0.534	0.54	0.545	0.551	0.557	0.561	0.564	0.567	0.571	0.577	0.583	0.588	0.593	0.597	0.601	0.606	0.61	0.614	0.617	0.621	0.623
Colombia	0.482	0.483	0.485	0.49	0.496	0.503	0.511	0.518	0.524	0.528	0.533	0.537	0.541	0.544	0.548	0.553	0.558	0.565	0.573	0.581	0.588	0.596	0.604	0.611	0.617	0.624	0.629	0.634
Costa Rica	0.524	0.53	0.536	0.54	0.545	0.55	0.555	0.56	0.566	0.573	0.581	0.589	0.594	0.598	0.603	0.607	0.609	0.612	0.616	0.623	0.629	0.634	0.64	0.645	0.649	0.654	0.658	0.662
El Salvador	0.406	0.409	0.413	0.418	0.424	0.431	0.44	0.449	0.459	0.47	0.481	0.491	0.5	0.508	0.515	0.523	0.531	0.539	0.545	0.55	0.556	0.561	0.567	0.573	0.578	0.584	0.589	0.593
Guatemala	0.313	0.318	0.326	0.338	0.353	0.363	0.371	0.381	0.397	0.411	0.42	0.427	0.433	0.44	0.449	0.458	0.465	0.47	0.476	0.482	0.489	0.496	0.501	0.507	0.513	0.517	0.521	0.524
Honduras	0.34	0.344	0.347	0.352	0.358	0.365	0.372	0.381	0.39	0.4	0.41	0.419	0.428	0.437	0.445	0.452	0.459	0.465	0.47	0.475	0.481	0.486	0.49	0.495	0.499	0.504	0.508	0.512
Mexico	0.513	0.517	0.521	0.529	0.537	0.543	0.549	0.555	0.561	0.566	0.572	0.577	0.581	0.586	0.59	0.595	0.598	0.602	0.606	0.608	0.611	0.613	0.616	0.619	0.622	0.624	0.626	0.628
Aguascalientes	0.558	0.561	0.565	0.571	0.577	0.583	0.591	0.597	0.603	0.606	0.609	0.612	0.615	0.621	0.626	0.632	0.636	0.64	0.643	0.644	0.645	0.647	0.649	0.651	0.653	0.655	0.657	0.659
Baja California	0.57	0.572	0.575	0.579	0.582	0.585	0.589	0.594	0.599	0.604	0.608	0.611	0.615	0.618	0.622	0.626	0.63	0.634	0.639	0.642	0.644	0.646	0.649	0.651	0.653	0.654	0.656	0.657
Baja California Sur	0.569	0.573	0.577	0.582	0.586	0.589	0.593	0.598	0.602	0.605	0.609	0.612	0.614	0.617	0.621	0.624	0.628	0.633	0.637	0.641	0.644	0.647	0.649	0.652	0.654	0.656	0.657	0.659
Campeche	0.485	0.49	0.496	0.504	0.511	0.517	0.525	0.534	0.543	0.551	0.558	0.564	0.569	0.573	0.577	0.581	0.585	0.589	0.593	0.595	0.597	0.599	0.602	0.605	0.608	0.611	0.614	0.616
Chiapas	0.403	0.407	0.413	0.422	0.43	0.436	0.444	0.452	0.459	0.465	0.47	0.475	0.48	0.484	0.489	0.494	0.498	0.503	0.508	0.512	0.514	0.517	0.52	0.522	0.525	0.528	0.531	0.533
Chihuahua	0.546	0.549	0.552	0.555	0.558	0.559	0.562	0.566	0.57	0.574	0.577	0.581	0.585	0.589	0.594	0.599	0.604	0.61	0.615	0.619	0.622	0.625	0.628	0.63	0.633	0.635	0.637	0.639
Coahuila	0.549	0.555	0.561	0.567	0.573	0.576	0.581	0.587	0.592	0.597	0.601	0.604	0.607	0.611	0.616	0.62	0.624	0.628	0.631	0.633	0.634	0.635	0.637	0.638	0.64	0.642	0.644	0.645
Colima	0.538	0.543	0.549	0.556	0.561	0.565	0.571	0.577	0.583	0.588	0.594	0.598	0.603	0.607	0.611	0.616	0.621	0.626	0.631	0.634	0.637	0.64	0.643	0.645	0.648	0.65	0.652	0.654
Mexico City	0.602	0.606	0.61	0.617	0.629	0.64	0.65	0.656	0.661	0.666	0.671	0.676	0.681	0.685	0.689	0.693	0.696	0.698	0.7	0.701	0.702	0.704	0.706	0.708	0.71	0.712	0.714	0.716
Durango	0.506	0.508	0.511	0.516	0.52	0.523	0.528	0.535	0.542	0.55	0.556	0.562	0.568	0.574	0.581	0.586	0.592	0.597	0.602	0.605	0.607	0.609	0.612	0.614	0.617	0.619	0.622	0.624
Guanajuato	0.494	0.499	0.504	0.511	0.519	0.527	0.534	0.541	0.546	0.551	0.558	0.565	0.571	0.575	0.579	0.583	0.587	0.591	0.595	0.598	0.601	0.604	0.607	0.611	0.614	0.616	0.619	0.621
Guerrero	0.426	0.429	0.433	0.44	0.445	0.45	0.456	0.465	0.473	0.48	0.487	0.492	0.498	0.505	0.511	0.517	0.523	0.529	0.535	0.539	0.542	0.545	0.548	0.552	0.555	0.558	0.56	0.562
Hidalgo	0.417	0.424	0.433	0.444	0.454	0.462	0.471	0.481	0.491	0.501	0.511	0.52	0.527	0.531	0.535	0.538	0.543	0.548	0.554	0.558	0.562	0.567	0.572	0.576	0.58	0.583	0.585	0.587
Jalisco	0.532	0.538	0.545	0.552	0.558	0.563	0.569	0.576	0.582	0.588	0.593	0.598	0.602	0.606	0.611	0.615	0.619	0.624	0.628	0.63	0.632	0.635	0.637	0.64	0.642	0.645	0.647	0.649
México	0.529	0.524	0.525	0.539	0.558	0.568	0.574	0.577	0.58	0.585	0.59	0.596	0.6	0.603	0.607	0.611	0.613	0.614	0.614	0.614	0.615	0.617	0.621	0.625	0.628	0.631	0.633	0.635
Michoacán de Ocampo	0.464	0.47	0.476	0.483	0.489	0.494	0.501	0.508	0.515	0.522	0.527	0.532	0.537	0.542	0.546	0.551	0.555	0.559	0.563	0.566	0.568	0.571	0.573	0.576	0.579	0.581	0.584	0.586
Morelos	0.525	0.532	0.539	0.547	0.553	0.558	0.564	0.571	0.577	0.582	0.587	0.591	0.595	0.598	0.601	0.605	0.608	0.612	0.615	0.617	0.619	0.622	0.624	0.627	0.629	0.631	0.634	0.635
Nayarit	0.495	0.5	0.506	0.512	0.518	0.523	0.529	0.537	0.545	0.553	0.56	0.566	0.571	0.576	0.581	0.585	0.59	0.594	0.598	0.6	0.602	0.604	0.607	0.61	0.613	0.615	0.618	0.62
Nuevo León	0.598	0.602	0.606	0.611	0.615	0.617	0.62	0.625	0.63	0.635	0.64	0.644	0.647	0.65	0.653	0.656	0.659	0.662	0.665	0.666	0.667	0.669	0.67	0.672	0.673	0.675	0.676	0.677
Oaxaca	0.431	0.435	0.441	0.447	0.452	0.455	0.459	0.466	0.473	0.479	0.485	0.49	0.496	0.501	0.507	0.512	0.517	0.523	0.529	0.533	0.537	0.541	0.545	0.549	0.552	0.555	0.558	0.561
Puebla	0.456	0.463	0.47	0.478	0.487	0.493	0.496	0.497	0.498	0.499	0.504	0.509	0.516	0.524	0.532	0.538	0.544	0.549	0.554	0.558	0.563	0.567	0.571	0.575	0.577	0.58	0.582	0.584
Querétaro	0.497	0.502	0.508	0.518	0.53	0.543	0.556	0.566	0.575	0.583	0.591	0.597	0.602	0.606	0.609	0.612	0.616	0.619	0.623	0.624	0.626	0.629	0.631	0.633	0.635	0.636	0.638	0.639

Methods Appendix Table 3: Socio-Demographic Index values for all estimated GBD 2017 locations, 1990-2017

Location	1990	1991	1992	1993	1994	1995	1996	1997	1998	1999	2000	2001	2002	2003	2004	2005	2006	2007	2008	2009	2010	2011	2012	2013	2014	2015	2016	2017
Quintana Roo	0.52	0.526	0.533	0.541	0.548	0.555	0.562	0.57	0.577	0.582	0.587	0.591	0.594	0.596	0.599	0.601	0.605	0.608	0.612	0.614	0.616	0.618	0.62	0.621	0.623	0.624	0.625	0.626
San Luis Potosí	0.482	0.486	0.492	0.5	0.508	0.514	0.522	0.53	0.538	0.545	0.551	0.556	0.561	0.566	0.571	0.575	0.58	0.586	0.591	0.595	0.599	0.602	0.606	0.61	0.613	0.616	0.619	0.621
Sinaloa	0.523	0.528	0.533	0.539	0.544	0.549	0.555	0.562	0.57	0.577	0.583	0.589	0.594	0.599	0.604	0.609	0.614	0.619	0.623	0.627	0.63	0.633	0.636	0.639	0.642	0.644	0.646	0.649
Sonora	0.553	0.557	0.562	0.566	0.57	0.573	0.578	0.583	0.588	0.593	0.597	0.601	0.605	0.608	0.612	0.616	0.621	0.625	0.629	0.632	0.635	0.637	0.64	0.643	0.645	0.647	0.649	0.65
Tabasco	0.474	0.479	0.486	0.493	0.5	0.507	0.515	0.524	0.533	0.541	0.548	0.553	0.558	0.563	0.568	0.573	0.578	0.583	0.588	0.591	0.594	0.596	0.599	0.602	0.604	0.607	0.609	0.611
Tamaulipas	0.548	0.553	0.558	0.564	0.568	0.571	0.574	0.579	0.586	0.592	0.598	0.602	0.606	0.609	0.613	0.616	0.62	0.624	0.628	0.63	0.633	0.635	0.637	0.64	0.642	0.643	0.645	0.647
Tlaxcala	0.478	0.482	0.487	0.495	0.506	0.515	0.524	0.531	0.536	0.541	0.545	0.55	0.556	0.561	0.567	0.573	0.578	0.583	0.587	0.59	0.591	0.594	0.596	0.598	0.6	0.601	0.603	0.604
Veracruz de Ignacio de la Llave	0.461	0.463	0.467	0.472	0.477	0.48	0.485	0.492	0.5	0.509	0.517	0.523	0.529	0.534	0.54	0.546	0.551	0.557	0.563	0.567	0.571	0.574	0.578	0.581	0.584	0.587	0.59	0.592
Yucatán	0.497	0.502	0.508	0.516	0.524	0.531	0.54	0.548	0.556	0.562	0.569	0.575	0.581	0.587	0.592	0.596	0.599	0.602	0.605	0.608	0.61	0.613	0.617	0.62	0.623	0.626	0.628	0.63
Zacatecas	0.483	0.489	0.495	0.502	0.509	0.514	0.52	0.527	0.534	0.54	0.546	0.551	0.555	0.56	0.564	0.568	0.572	0.577	0.581	0.584	0.586	0.589	0.592	0.595	0.598	0.602	0.605	0.608
Nicaragua	0.357	0.363	0.368	0.374	0.381	0.389	0.397	0.406	0.415	0.424	0.432	0.439	0.446	0.453	0.46	0.466	0.47	0.475	0.481	0.486	0.492	0.499	0.504	0.509	0.514	0.52	0.525	0.53
Panama	0.542	0.546	0.55	0.555	0.56	0.565	0.569	0.573	0.578	0.583	0.589	0.595	0.6	0.604	0.608	0.611	0.614	0.618	0.622	0.626	0.63	0.635	0.641	0.648	0.656	0.664	0.671	0.677
Venezuela	0.528	0.536	0.553	0.559	0.566	0.578	0.587	0.592	0.591	0.587	0.591	0.602	0.602	0.588	0.576	0.579	0.594	0.61	0.621	0.625	0.629	0.633	0.64	0.646	0.648	0.651	0.654	0.655
Tropical Latin America	0.494	0.5	0.507	0.514	0.521	0.529	0.537	0.544	0.55	0.556	0.561	0.566	0.571	0.577	0.582	0.588	0.594	0.601	0.608	0.614	0.621	0.628	0.635	0.642	0.648	0.654	0.659	0.662
Brazil	0.494	0.501	0.508	0.515	0.522	0.53	0.537	0.545	0.551	0.556	0.562	0.567	0.572	0.577	0.583	0.589	0.595	0.602	0.608	0.615	0.622	0.629	0.636	0.643	0.649	0.655	0.66	0.663
Acre	0.376	0.386	0.395	0.405	0.415	0.424	0.435	0.445	0.453	0.46	0.466	0.472	0.479	0.485	0.492	0.5	0.508	0.517	0.527	0.536	0.546	0.556	0.565	0.575	0.583	0.591	0.597	0.602
Alagoas	0.355	0.363	0.371	0.379	0.387	0.395	0.404	0.412	0.419	0.425	0.431	0.436	0.442	0.448	0.455	0.462	0.47	0.478	0.487	0.496	0.505	0.514	0.523	0.531	0.539	0.546	0.552	0.556
Amapá	0.467	0.475	0.483	0.491	0.5	0.508	0.517	0.526	0.534	0.54	0.546	0.552	0.558	0.564	0.57	0.576	0.583	0.591	0.598	0.605	0.613	0.621	0.629	0.636	0.643	0.65	0.655	0.659
Amazonas	0.438	0.447	0.457	0.466	0.475	0.483	0.492	0.499	0.505	0.51	0.514	0.519	0.523	0.528	0.533	0.539	0.546	0.553	0.561	0.568	0.577	0.585	0.594	0.602	0.611	0.618	0.625	0.629
Bahia	0.402	0.41	0.419	0.427	0.435	0.443	0.451	0.459	0.465	0.47	0.475	0.48	0.485	0.491	0.496	0.503	0.51	0.518	0.526	0.534	0.542	0.551	0.559	0.567	0.575	0.582	0.587	0.591
Ceará	0.411	0.419	0.426	0.433	0.44	0.448	0.455	0.463	0.469	0.475	0.48	0.486	0.492	0.498	0.505	0.512	0.52	0.528	0.536	0.544	0.553	0.561	0.569	0.577	0.584	0.591	0.596	0.6
Distrito Federal	0.63	0.636	0.642	0.649	0.656	0.663	0.671	0.679	0.685	0.691	0.696	0.702	0.707	0.713	0.719	0.725	0.731	0.738	0.744	0.75	0.756	0.763	0.769	0.775	0.78	0.785	0.789	0.792
Espírito Santo	0.499	0.507	0.515	0.524	0.532	0.54	0.549	0.557	0.564	0.57	0.576	0.582	0.588	0.593	0.599	0.606	0.612	0.618	0.625	0.631	0.638	0.644	0.651	0.657	0.663	0.669	0.673	0.677
Goiás	0.46	0.468	0.476	0.484	0.493	0.501	0.51	0.518	0.526	0.532	0.538	0.545	0.551	0.558	0.564	0.571	0.579	0.586	0.594	0.601	0.608	0.616	0.623	0.63	0.636	0.642	0.647	0.65
Maranhão	0.313	0.322	0.33	0.339	0.347	0.355	0.364	0.371	0.377	0.38	0.383	0.386	0.389	0.392	0.396	0.402	0.409	0.418	0.427	0.436	0.446	0.456	0.467	0.477	0.486	0.495	0.502	0.507
Mato Grosso	0.475	0.484	0.492	0.501	0.509	0.518	0.527	0.535	0.543	0.548	0.554	0.559	0.564	0.57	0.576	0.582	0.589	0.596	0.604	0.611	0.618	0.626	0.633	0.641	0.648	0.654	0.659	0.662
Mato Grosso do Sul	0.465	0.473	0.481	0.489	0.497	0.506	0.515	0.523	0.531	0.537	0.543	0.549	0.555	0.56	0.566	0.573	0.58	0.587	0.594	0.6	0.607	0.614	0.622	0.629	0.636	0.642	0.647	0.65
Minas Gerais	0.491	0.498	0.506	0.513	0.521	0.53	0.538	0.545	0.551	0.557	0.562	0.567	0.573	0.579	0.585	0.591	0.598	0.604	0.611	0.618	0.624	0.631	0.637	0.643	0.649	0.654	0.658	0.661
Pará	0.41	0.418	0.425	0.432	0.44	0.447	0.454	0.461	0.466	0.47	0.473	0.476	0.48	0.483	0.488	0.493	0.499	0.506	0.514	0.521	0.529	0.538	0.546	0.554	0.562	0.569	0.575	0.579
Paraíba	0.399	0.406	0.413	0.42	0.427	0.434	0.441	0.447	0.453	0.457	0.461	0.465	0.469	0.474	0.48	0.486	0.493	0.501	0.509	0.517	0.526	0.535	0.543	0.551	0.559	0.565	0.571	0.574
Paraná	0.513	0.519	0.525	0.532	0.539	0.548	0.556	0.564	0.572	0.578	0.585	0.591	0.597	0.603	0.609	0.615	0.622	0.628	0.634	0.64	0.646	0.652	0.658	0.664	0.67	0.675	0.679	0.682
Pernambuco	0.416	0.423	0.43	0.437	0.444	0.451	0.458	0.466	0.472	0.477	0.481	0.486	0.492	0.497	0.503	0.51	0.517	0.525	0.533	0.54	0.548	0.556	0.564	0.572	0.579	0.585	0.59	0.594
Piauí	0.365	0.372	0.379	0.386	0.393	0.4	0.408	0.415	0.42	0.425	0.429	0.434	0.439	0.444	0.45	0.457	0.465	0.473	0.482	0.491	0.5	0.51	0.518	0.527	0.535	0.542	0.548	0.552
Rio de Janeiro	0.576	0.581	0.585	0.59	0.595	0.601	0.608	0.614	0.619	0.624	0.628	0.632	0.637	0.641	0.645	0.65	0.655	0.66	0.665	0.67	0.675	0.681	0.686	0.692	0.697	0.702	0.706	0.709
Rio Grande do Norte	0.415	0.422	0.429	0.436	0.444	0.451	0.46	0.467	0.474	0.48	0.485	0.491	0.497	0.503	0.509	0.516	0.524	0.532	0.541	0.549	0.558	0.567	0.575	0.583	0.59	0.597	0.602	0.605
Rio Grande do Sul	0.543	0.549	0.555	0.561	0.567	0.574	0.581	0.587	0.593	0.598	0.603	0.608	0.614	0.619	0.624	0.63	0.635	0.641	0.647	0.653	0.659	0.665	0.67	0.676	0.681	0.686	0.69	0.693
Rondônia	0.423	0.433	0.441	0.45	0.458	0.467	0.475	0.484	0.491	0.497	0.502	0.508	0.515	0.521	0.528	0.535	0.543	0.551	0.559	0.567	0.575	0.584	0.592	0.599	0.606	0.613	0.618	0.622
Roraima	0.428	0.438	0.447	0.456	0.465	0.474	0.483	0.492	0.499	0.504	0.509	0.514	0.521	0.527	0.534	0.543	0.552	0.562	0.572	0.581	0.591	0.601	0.611	0.62	0.628	0.636	0.642	0.646
Santa Catarina	0.541	0.548	0.554	0.56	0.567	0.574	0.582	0.589	0.595	0.601	0.606	0.612	0.618	0.623	0.629	0.635	0.641	0.647	0.653	0.659	0.665	0.672	0.678	0.684	0.69	0.695	0.699	0.702
São Paulo	0.558	0.565	0.572	0.579	0.587	0.595	0.603	0.611	0.618	0.624	0.63	0.636	0.641	0.646	0.652	0.657	0.663	0.669	0.674	0.68	0.685	0.691	0.697	0.703	0.708	0.713	0.717	0.72
Sergipe	0.425	0.433	0.441	0.448	0.456	0.464	0.473	0.481	0.488	0.494	0.5	0.506	0.512	0.518	0.524	0.531	0.538	0.546	0.554	0.562	0.57	0.578	0.586	0.594	0.601	0.607	0.612	0.616
Tocantins	0.396	0.404	0.412	0.42	0.428	0.436	0.445	0.453	0.46	0.466	0.471	0.477	0.484	0.491	0.498	0.507	0.517	0.527	0.537	0.547	0.558	0.568	0.577	0.586	0.594	0.601	0.607	0.611
Paraguay	0.467	0.471	0.475	0.48	0.485	0.491	0.497	0.504	0.512	0.519	0.525	0.532	0.538	0.544	0.548	0.553	0.558	0.562	0.566	0.569	0.573	0.577	0.582	0.589	0.598	0.606	0.613	0.619
North Africa and Middle East	0.456	0.464	0.472	0.478	0.485	0.492	0.499	0.506	0.513	0.521	0.531	0.539	0.547	0.553	0.561	0.568	0.574	0.582	0.589	0.596	0.602	0.607	0.612	0.617	0.622	0.628	0.635	0.639
North Africa and Middle East	0.456	0.464	0.472	0.478	0.485	0.492	0.499	0.506	0.513	0.521	0.531	0.539	0.547	0.553	0.561	0.568	0.574	0.582	0.589	0.596	0.602	0.607	0.612	0.617	0.622	0.628	0.635	0.639
Afghanistan	0.149	0.15	0.151	0.152	0.151	0.151	0.152	0.152	0.154	0.155	0.157	0.159	0.164	0.171	0.177	0.184	0.191	0.199	0.208	0.218	0.228	0.238	0.248	0.258	0.267	0.276	0.284	0.29
Algeria	0.495	0.504	0.513	0.522	0.531	0.54	0.549	0.558	0.567	0.574	0.583	0.591	0.599	0.608	0.617	0.625	0.633	0.641	0.648	0.653	0.659	0.665	0.67	0.675	0.68	0.685	0.691	0.696
Bahrain	0.612	0.618	0.624	0.63	0.635	0.639	0.644	0.648	0.653	0.657	0.662	0.668	0.673	0.677	0.68	0.684	0.687	0.69	0.693	0.695	0.696	0.697	0.698	0.7	0.702	0.706	0.709	0.712
Egypt	0.441	0.451	0.461	0.47	0.477	0.485	0.493	0.5	0.507	0.514	0.52	0.526	0.532	0.537	0.542	0.547	0.55	0.553	0.556	0.559	0.563	0.565	0.564	0.566	0.572	0.584	0.596	0.604
Iran	0.503	0.513	0.522	0.532	0.541	0.548	0.553	0.556	0.56	0.572	0.589	0.604	0.616	0.626	0.634	0.641	0.649	0.656	0.662	0.667	0.672	0.677	0.68	0.683	0.687	0.691	0.696	0.7
Iraq	0.433	0.433	0.437	0.441	0.444	0.446	0.45	0.455	0.463	0.473	0.481	0.488	0.494	0.496	0.502	0.507	0.512	0.517	0.522	0.528	0.535	0.542	0.551	0.559	0.566	0.572	0.58	0.585
Jordan	0.552	0.556	0.561	0.566	0.57	0.574	0.578	0.584	0.589	0.594	0.597	0.6	0.603	0.607	0.612	0.616	0.621	0.626	0.633	0.643	0.653	0.662	0.671	0.679	0.685	0.69	0.694	0.697
Kuwait	0.656	0.651	0.642	0.637	0.633	0.632	0.638	0.651	0.665	0.674	0.681	0.683	0.685	0.687	0.693	0.701	0.711	0.721	0.73	0.737	0.743	0.748	0.754	0.762	0.769	0.776	0.781	0.786
Lebanon	0.519	0.528	0.536	0.544	0.552	0.56	0.569	0.578	0.588	0.597	0.604	0.611	0.618	0.626	0.635	0.643	0.65	0.658	0.667	0.676	0.686	0.694	0.702	0.711	0.717	0.722	0.726	0.73
Libya	0.645	0.657	0.669	0.679	0.688	0.696	0.705	0.713	0.72	0.725	0.729	0.732	0.736	0.742	0.747	0.753	0.759	0.765	0.771	0.777	0.783	0.781	0.784	0.783	0.779	0.773	0.766	0.761
Morocco	0.389	0.397	0.404	0.411	0.419	0.426	0.434	0.442	0.449	0.456	0.463	0.47	0.477	0.484	0.49	0.496	0.503	0.51	0.517	0.525	0.532	0.54	0.547	0.554	0.561	0.567	0.574	0.579
Palestine	0.362	0.366	0.371	0.377	0.384	0.39	0.397	0.405	0.413	0.421	0.427	0.431	0.433	0.435	0.439	0.444	0.448	0.453	0.46	0.468	0.479	0.491	0.503	0.513	0.521	0.528	0.536	0.541
Oman	0.473	0.485	0.497	0.509	0.521	0.533	0.546	0.558	0.57	0.582	0.594	0.606	0.618	0.629	0.639	0.649	0.659	0.668	0.678	0.686	0.695	0.703	0.711	0.72	0.727	0.734	0.739	0.744
Qatar	0.603	0.615	0.626	0.637	0.647	0.656	0.665	0.674	0.681	0.688	0.695	0.701	0.706	0.712	0.717	0.721	0.725	0.728	0.731	0.735	0.738	0.743	0.747	0.751	0.755	0.759	0.762	0.766
Saudi Arabia	0.436	0.457	0.477	0.496	0.514	0.53	0.545	0.56	0.573	0.586	0.598	0.61	0.621	0.633	0.645	0.658	0.67	0.682	0.695	0.706	0.716	0.728	0.738	0.748	0.757	0.765	0.773	0.779
Sudan	0.221	0.228	0.236	0.244	0.252	0.26	0.267	0.275	0.283	0.291	0.3	0.308	0.317	0.326	0.336	0.346	0.357	0.367	0.378	0.389	0.401	0.414	0.425	0.437	0.448	0.46	0.47	0.478
Syria	0.389	0.394	0.401	0.409	0.419	0.429	0.439	0.448	0.458	0.466	0.474	0.481	0.489	0.496	0.506	0.518	0.527	0.536	0.545	0.556	0.566	0.576	0.584	0.589	0.595	0.601	0.606	0.611
Tunisia	0.453	0.463	0.473	0.482	0.491	0.501	0.511	0.521	0.532	0.543	0.553	0.563	0.572	0.582	0.592	0.6	0.609	0.617	0.625	0.632	0.639	0.645	0.65	0.656	0.661	0.666	0.671	0.675
Turkey	0.507	0.516	0.524	0.533	0.54	0.548	0.556	0.564	0.572	0.579	0.587	0.594	0.601	0.608	0.616	0.625	0.633	0.643	0.652	0.66	0.669	0.678	0.688	0.697	0.706	0.715	0.723	0.729
United Arab Emirates	0.621	0.637	0.651	0.663	0.675	0.686	0.696	0.707	0.716	0.726	0.734	0.742	0.749	0.755	0.76	0.765	0.77	0.776	0.781	0.784	0.788	0.79	0.791	0.793	0.791	0.794	0.794	0.795
Yemen	0.203	0.208	0.214	0.22	0.228	0.238	0.247	0.256	0.266	0.275	0.284	0.294	0.304	0.313	0.324	0.336	0.349	0.362	0.373	0.382	0.393	0.401	0.408	0.415	0.422	0.426	0.429	0.43
South Asia	0.312	0.317	0.324	0.331	0.338	0.345	0.352	0.358	0.363	0.371	0.38	0.388	0.395	0.403	0.412	0.421	0.43	0.438	0.446	0.455	0.462	0.468	0.476	0.489	0.504	0.517	0.526	0.534
South Asia	0.312	0.317	0.324	0.331	0.338	0.345	0.352	0.358	0.363	0.371	0.38	0.388	0.395	0.403	0.412	0.421	0.43	0.438	0.446	0.455	0.462	0.468	0.476	0.489	0.504	0.517	0.526	0.534
Bangladesh	0.256	0.264	0.272	0.281	0.289	0.298	0.306	0.314	0.322	0.329	0.335	0.34	0.345	0.35	0.356	0.362	0.369	0.377	0.385	0.393	0.401	0.41	0.418	0.427	0.435	0.443	0.451	0.458
Bhutan	0.324	0.333	0.343	0.352	0.362	0.372	0.382	0.392	0.402	0.412	0.422	0.432	0.442	0.452	0.461	0.47	0.479	0.489	0.498	0.507	0.516	0.525	0.534	0.542	0.549	0.557	0.564	0.57
India	0.32	0.324	0.331	0.339	0.346	0.353	0.36	0.365	0.37	0.378	0.387	0.395	0.403	0.412	0.422	0.431	0.44	0.448	0.457	0.465	0.472	0.477	0.485	0.5	0.517	0.532	0.542	0.55
Andhra Pradesh	0.28	0.286	0.294	0.303	0.312	0.322	0.331	0.337	0.344	0.352	0.363	0.374	0.384	0.395	0.406	0.415	0.424	0.434	0.442	0.451	0.459	0.466	0.473	0.487	0.503	0.517	0.528	0.536
Arunachal Pradesh	0.311	0.317	0.323	0.33	0.336	0.345	0.354	0.36	0.366	0.374	0.381	0.388	0.395	0.405	0.416	0.424	0.432	0.44	0.447	0.458	0.467	0.473	0.479	0.498	0.52	0.536	0.548	0.556
Assam	0.327	0.332	0.339	0.345	0.351	0.358	0.363	0.367	0.371	0.379	0.389	0.397	0.406	0.415	0.424	0.431	0.439	0.446	0.453	0.459	0.464	0.467	0.472	0.485	0.5	0.513	0.523	0.53
Bihar	0.29	0.293	0.297	0.298	0.298	0.298	0.298	0.297	0.292	0.291	0.292	0.294	0.296	0.299	0.303	0.308	0.316	0.324	0.334	0.342	0.349	0.355	0.365	0.382	0.4	0.414	0.425	0.433
Chhattisgarh	0.292	0.294	0.298	0.303	0.306	0.311	0.316	0.319	0.322	0.328	0.336	0.344	0.352	0.362	0.372	0.382	0.392	0.403	0.413	0.421	0.427	0.431	0.438	0.455	0.474	0.49	0.503	0.512
Delhi	0.486	0.493	0.504	0.516	0.522	0.526	0.531	0.535	0.537	0.545	0.555	0.565	0.575	0.584	0.593	0.602	0.612	0.62	0.628	0.637	0.644	0.649	0.656	0.67	0.686	0.7	0.709	0.715
Goa	0.499	0.505	0.512	0.519	0.526	0.534	0.542	0.55	0.56	0.571	0.58	0.588	0.597	0.606	0.616	0.626	0.634	0.643	0.655	0.665	0.674	0.683	0.691	0.702	0.714	0.724	0.733	0.74
Gujarat	0.35	0.353	0.361	0.37	0.379	0.388	0.396	0.402	0.409	0.418	0.426	0.433	0.438	0.447	0.457	0.469	0.478	0.487	0.496	0.506	0.515	0.521	0.528	0.541	0.556	0.569	0.577	0.584
Haryana	0.342	0.348	0.356	0.364	0.372	0.38	0.388	0.394	0.398	0.405	0.414	0.423	0.431	0.44	0.451	0.462	0.472	0.481	0.491	0.5	0.508	0.512	0.521	0.54	0.564	0.582	0.593	0.6
Himachal Pradesh	0.34	0.347	0.356	0.366	0.376	0.386	0.397	0.408	0.418	0.431	0.445	0.457	0.468	0.479	0.491	0.502	0.513	0.524	0.534	0.544	0.553	0.562	0.571	0.585	0.601	0.614	0.625	0.633
Jammu and Kashmir	0.334	0.337	0.341	0.349	0.356	0.363	0.37	0.38	0.389	0.402	0.417	0.43	0.442	0.454	0.466	0.476	0.485	0.493	0.503	0.511	0.519	0.527	0.535	0.547	0.562	0.574	0.582	0.59
Jharkhand	0.281	0.283	0.286	0.29	0.293	0.297	0.3	0.304	0.307	0.312	0.319	0.325	0.333	0.342	0.353	0.362	0.371	0.38	0.388	0.397	0.405	0.411	0.419	0.434	0.451	0.466	0.478	0.487
Karnataka	0.323	0.331	0.339	0.349	0.357	0.366	0.375	0.38	0.386	0.396	0.406	0.415	0.424	0.434	0.444	0.454	0.463	0.472	0.481	0.49	0.498	0.504	0.512	0.526	0.542	0.556	0.566	0.574
Kerala	0.404	0.408	0.418	0.429	0.438	0.447	0.456	0.463	0.472	0.483	0.496	0.506	0.515	0.525	0.536	0.548	0.557	0.565	0.574	0.581	0.587	0.592	0.599	0.612	0.629	0.642	0.652	0.659
Madhya Pradesh	0.284	0.288	0.293	0.299	0.303	0.309	0.315	0.317	0.319	0.326	0.334	0.342	0.348	0.355	0.363	0.369	0.373	0.378	0.384	0.39	0.394	0.398	0.406	0.425	0.447	0.465	0.477	0.487
Maharashtra	0.363	0.369	0.379	0.39	0.399	0.41	0.419	0.426	0.431	0.44	0.451	0.459	0.468	0.478	0.489	0.5	0.51	0.52	0.529	0.538	0.546	0.552	0.559	0.572	0.588	0.602	0.611	0.618
Manipur	0.381	0.387	0.393	0.4	0.405	0.411	0.417	0.421	0.427	0.436	0.445	0.454	0.461	0.469	0.479	0.49	0.498	0.505	0.512	0.52	0.526	0.532	0.539	0.549	0.561	0.572	0.582	0.59
Meghalaya	0.334	0.339	0.345	0.352	0.357	0.364	0.37	0.376	0.382	0.39	0.401	0.411	0.42	0.43	0.441	0.45	0.46	0.469	0.478	0.487	0.495	0.501	0.507	0.52	0.534	0.547	0.557	0.565
Mizoram	0.392	0.399	0.408	0.417	0.424	0.433	0.443	0.449	0.456	0.465	0.475	0.483	0.492	0.502	0.511	0.52	0.526	0.531	0.537	0.545	0.55	0.552	0.557	0.57	0.587	0.6	0.609	0.616
Nagaland	0.395	0.399	0.406	0.414	0.421	0.429	0.435	0.441	0.445	0.452	0.462	0.472	0.482	0.492	0.503	0.513	0.522	0.532	0.543	0.552	0.56	0.567	0.574	0.587	0.602	0.614	0.625	0.633
Odisha	0.283	0.287	0.292	0.299	0.305	0.314	0.32	0.325	0.331	0.34	0.349	0.357	0.364	0.373	0.384	0.393	0.404	0.415	0.425	0.434	0.441	0.447	0.456	0.472	0.49	0.504	0.515	0.524
Punjab	0.383	0.388	0.396	0.404	0.413	0.422	0.429	0.435	0.441	0.449	0.459	0.468	0.475	0.483	0.492	0.501	0.511	0.52	0.53	0.539	0.547	0.554	0.563	0.576	0.592	0.605	0.614	0.622
Rajasthan	0.263	0.268	0.274	0.28	0.287	0.295	0.303	0.309	0.314	0.322	0.332	0.342	0.349	0.359	0.369	0.377	0.385	0.391	0.398	0.404	0.41	0.415	0.421	0.438	0.458	0.473	0.484	0.492
Sikkim	0.343	0.349	0.355	0.362	0.368	0.375	0.382	0.388	0.395	0.403	0.413	0.421	0.431	0.442	0.453	0.463	0.473	0.482	0.492	0.512	0.528	0.541	0.555	0.574	0.593	0.608	0.619	0.628
Tamil Nadu	0.35	0.354	0.363	0.373	0.383	0.391	0.399	0.406	0.414	0.424	0.436	0.446	0.455	0.465	0.475	0.486	0.497	0.506	0.514	0.522	0.529	0.535	0.543	0.559	0.579	0.595	0.607	0.615
Telangana	0.288	0.294	0.3	0.307	0.315	0.323	0.331	0.337	0.344	0.352	0.363	0.373	0.383	0.395	0.407	0.418	0.429	0.441	0.454	0.465	0.478	0.488	0.5	0.517	0.536	0.552	0.565	0.575
Tripura	0.333	0.336	0.34	0.346	0.35	0.356	0.363	0.368	0.373	0.385	0.398	0.409	0.419	0.43	0.44	0.449	0.457	0.462	0.469	0.476	0.48	0.483	0.488	0.5	0.515	0.527	0.536	0.543
Uttar Pradesh	0.272	0.276	0.281	0.286	0.291	0.297	0.303	0.307	0.31	0.317	0.325	0.333	0.34	0.348	0.358	0.366	0.374	0.382	0.39	0.398	0.404	0.41	0.419	0.436	0.454	0.469	0.48	0.488
Uttarakhand	0.304	0.306	0.31	0.315	0.321	0.329	0.336	0.341	0.347	0.355	0.367	0.378	0.391	0.405	0.42	0.434	0.449	0.463	0.478	0.494	0.509	0.522	0.535	0.552	0.57	0.586	0.598	0.607
West Bengal	0.321	0.322	0.329	0.338	0.345	0.353	0.36	0.366	0.371	0.38	0.389	0.398	0.406	0.416	0.425	0.434	0.441	0.448	0.455	0.462	0.467	0.471	0.476	0.49	0.506	0.519	0.53	0.538
Union Territories other than Delhi	0.406	0.408	0.412	0.417	0.423	0.43	0.439	0.446	0.453	0.463	0.474	0.486	0.498	0.511	0.524	0.537	0.548	0.559	0.569	0.579	0.586	0.591	0.597	0.61	0.624	0.636	0.646	0.653
Nepal	0.216	0.222	0.228	0.235	0.242	0.249	0.257	0.265	0.273	0.281	0.29	0.298	0.307	0.315	0.323	0.331	0.339	0.347	0.355	0.364	0.373	0.382	0.39	0.399	0.407	0.415	0.422	0.429
Pakistan	0.305	0.31	0.314	0.319	0.324	0.33	0.337	0.343	0.35	0.359	0.367	0.375	0.382	0.389	0.396	0.404	0.412	0.42	0.428	0.436	0.443	0.449	0.458	0.466	0.474	0.48	0.486	0.492
Southeast Asia, East Asia, and Oceania	0.468	0.479	0.489	0.498	0.506	0.517	0.528	0.538	0.546	0.553	0.561	0.568	0.574	0.58	0.588	0.596	0.606	0.615	0.624	0.632	0.641	0.649	0.656	0.662	0.67	0.673	0.675	0.685
East Asia	0.463	0.476	0.486	0.495	0.504	0.516	0.528	0.54	0.55	0.559	0.568	0.577	0.584	0.591	0.599	0.61	0.621	0.632	0.642	0.652	0.663	0.672	0.679	0.685	0.694	0.696	0.696	0.709
China	0.456	0.469	0.479	0.489	0.498	0.51	0.523	0.535	0.545	0.554	0.563	0.573	0.58	0.587	0.595	0.606	0.618	0.63	0.64	0.65	0.661	0.671	0.678	0.683	0.693	0.695	0.694	0.707
North Korea	0.512	0.517	0.52	0.524	0.524	0.522	0.519	0.515	0.51	0.506	0.503	0.5	0.498	0.498	0.499	0.501	0.503	0.506	0.508	0.511	0.514	0.517	0.52	0.524	0.527	0.531	0.535	0.538
Taiwan (Province of China)	0.691	0.7	0.708	0.715	0.723	0.731	0.739	0.75	0.759	0.764	0.77	0.778	0.786	0.793	0.8	0.807	0.813	0.821	0.827	0.833	0.838	0.842	0.846	0.85	0.854	0.858	0.861	0.864
Oceania	0.406	0.408	0.41	0.412	0.415	0.418	0.421	0.423	0.426	0.428	0.43	0.431	0.432	0.433	0.435	0.436	0.438	0.44	0.442	0.444	0.446	0.449	0.452	0.455	0.459	0.463	0.467	0.471
American Samoa	0.609	0.612	0.615	0.62	0.624	0.629	0.633	0.636	0.639	0.643	0.647	0.651	0.655	0.659	0.662	0.666	0.668	0.671	0.673	0.675	0.678	0.682	0.687	0.691	0.694	0.697	0.7	0.702
Federated States of Micronesia	0.462	0.469	0.476	0.483	0.489	0.496	0.501	0.505	0.509	0.514	0.518	0.523	0.527	0.532	0.536	0.54	0.543	0.547	0.549	0.552	0.555	0.559	0.562	0.565	0.567	0.57	0.573	0.575
Fiji	0.533	0.537	0.542	0.547	0.552	0.558	0.563	0.568	0.574	0.58	0.585	0.589	0.594	0.598	0.602	0.604	0.607	0.61	0.611	0.613	0.615	0.617	0.619	0.623	0.627	0.632	0.637	0.641
Guam	0.698	0.695	0.693	0.694	0.698	0.703	0.709	0.717	0.727	0.739	0.75	0.759	0.765	0.768	0.77	0.77	0.769	0.77	0.771	0.774	0.776	0.78	0.784	0.788	0.792	0.794	0.794	0.794
Kiribati	0.355	0.357	0.359	0.361	0.363	0.365	0.368	0.37	0.373	0.376	0.379	0.382	0.385	0.388	0.39	0.393	0.395	0.397	0.398	0.399	0.401	0.403	0.406	0.41	0.414	0.418	0.423	0.427
Marshall Islands	0.413	0.421	0.429	0.436	0.444	0.451	0.457	0.462	0.465	0.469	0.473	0.477	0.482	0.485	0.489	0.493	0.498	0.503	0.507	0.512	0.518	0.523	0.528	0.533	0.538	0.542	0.547	0.55
Northern Mariana Islands	0.738	0.744	0.748	0.75	0.752	0.754	0.756	0.757	0.758	0.759	0.763	0.766	0.767	0.767	0.767	0.766	0.765	0.764	0.763	0.761	0.759	0.758	0.757	0.756	0.756	0.756	0.757	0.758
Papua New Guinea	0.318	0.321	0.324	0.33	0.335	0.34	0.344	0.348	0.351	0.354	0.356	0.358	0.36	0.361	0.363	0.365	0.368	0.371	0.375	0.379	0.383	0.388	0.393	0.398	0.404	0.409	0.415	0.419
Samoa	0.538	0.539	0.538	0.536	0.535	0.536	0.536	0.537	0.538	0.54	0.542	0.544	0.546	0.548	0.55	0.552	0.554	0.556	0.558	0.559	0.561	0.563	0.56	0.558	0.562	0.567	0.572	0.576
Solomon Islands	0.316	0.32	0.326	0.331	0.337	0.344	0.35	0.355	0.36	0.364	0.365	0.366	0.366	0.366	0.367	0.369	0.371	0.375	0.38	0.384	0.388	0.394	0.4	0.406	0.411	0.416	0.421	0.425
Tonga	0.522	0.528	0.533	0.538	0.544	0.549	0.553	0.557	0.56	0.564	0.566	0.569	0.573	0.577	0.581	0.584	0.587	0.589	0.592	0.595	0.599	0.603	0.607	0.61	0.614	0.617	0.621	0.625
Vanuatu	0.38	0.384	0.388	0.391	0.395	0.398	0.402	0.406	0.409	0.413	0.416	0.419	0.421	0.423	0.425	0.428	0.431	0.435	0.44	0.445	0.449	0.453	0.458	0.462	0.465	0.469	0.472	0.475
Southeast Asia	0.467	0.477	0.486	0.495	0.504	0.513	0.521	0.529	0.534	0.538	0.543	0.548	0.552	0.557	0.562	0.567	0.572	0.578	0.584	0.589	0.595	0.602	0.609	0.616	0.622	0.629	0.635	0.641
Cambodia	0.259	0.266	0.273	0.279	0.286	0.294	0.302	0.31	0.317	0.326	0.335	0.344	0.353	0.362	0.372	0.382	0.392	0.401	0.411	0.42	0.427	0.435	0.443	0.451	0.459	0.467	0.475	0.482
Indonesia	0.465	0.476	0.487	0.499	0.51	0.52	0.53	0.537	0.542	0.546	0.55	0.554	0.558	0.562	0.566	0.571	0.576	0.581	0.587	0.594	0.6	0.608	0.615	0.622	0.629	0.636	0.642	0.648
Laos	0.307	0.313	0.32	0.326	0.332	0.339	0.345	0.352	0.358	0.364	0.371	0.378	0.385	0.392	0.4	0.407	0.416	0.425	0.434	0.444	0.453	0.463	0.473	0.483	0.493	0.502	0.511	0.519
Malaysia	0.57	0.575	0.581	0.589	0.598	0.606	0.616	0.626	0.638	0.654	0.661	0.664	0.671	0.679	0.684	0.689	0.697	0.704	0.709	0.714	0.721	0.727	0.733	0.739	0.744	0.749	0.754	0.759
Maldives	0.386	0.399	0.412	0.424	0.435	0.446	0.46	0.477	0.493	0.507	0.518	0.528	0.537	0.545	0.554	0.56	0.567	0.575	0.584	0.593	0.602	0.611	0.62	0.629	0.636	0.643	0.65	0.655
Mauritius	0.554	0.561	0.568	0.576	0.585	0.595	0.603	0.609	0.613	0.616	0.621	0.627	0.633	0.639	0.646	0.651	0.657	0.663	0.669	0.675	0.68	0.687	0.694	0.701	0.708	0.713	0.717	0.72
Myanmar	0.33	0.333	0.337	0.341	0.347	0.353	0.36	0.367	0.375	0.383	0.392	0.402	0.412	0.423	0.434	0.446	0.458	0.47	0.481	0.492	0.501	0.51	0.518	0.527	0.535	0.542	0.549	0.556

Methods Appendix Table 3: Socio-Demographic Index values for all estimated GBD 2017 locations, 1990-2017

Location	1990	1991	1992	1993	1994	1995	1996	1997	1998	1999	2000	2001	2002	2003	2004	2005	2006	2007	2008	2009	2010	2011	2012	2013	2014	2015	2016	2017
Philippines	0.511	0.516	0.521	0.525	0.53	0.534	0.539	0.542	0.545	0.547	0.55	0.553	0.555	0.557	0.559	0.561	0.563	0.566	0.568	0.569	0.572	0.579	0.586	0.593	0.599	0.605	0.612	0.617
Sri Lanka	0.49	0.495	0.501	0.508	0.516	0.524	0.532	0.54	0.547	0.553	0.559	0.565	0.571	0.578	0.584	0.59	0.597	0.604	0.611	0.618	0.626	0.634	0.642	0.65	0.658	0.666	0.673	0.68
Seychelles	0.549	0.557	0.565	0.573	0.582	0.589	0.597	0.605	0.613	0.62	0.626	0.631	0.636	0.64	0.643	0.646	0.65	0.653	0.656	0.658	0.66	0.663	0.667	0.671	0.675	0.68	0.686	0.692
Thailand	0.502	0.514	0.525	0.534	0.542	0.552	0.561	0.567	0.569	0.572	0.579	0.587	0.594	0.6	0.605	0.61	0.616	0.623	0.629	0.635	0.641	0.647	0.654	0.66	0.667	0.673	0.679	0.684
Timor-Leste	0.276	0.283	0.29	0.296	0.302	0.307	0.314	0.321	0.325	0.321	0.32	0.325	0.332	0.345	0.362	0.379	0.4	0.419	0.437	0.449	0.46	0.471	0.481	0.49	0.495	0.5	0.504	0.505
Vietnam	0.406	0.413	0.42	0.427	0.435	0.444	0.452	0.461	0.469	0.477	0.483	0.49	0.497	0.504	0.511	0.518	0.525	0.532	0.54	0.547	0.554	0.562	0.57	0.578	0.585	0.593	0.6	0.607
Sub-Saharan Africa	0.304	0.307	0.311	0.314	0.317	0.32	0.324	0.328	0.332	0.335	0.339	0.343	0.348	0.353	0.359	0.365	0.371	0.379	0.386	0.393	0.4	0.407	0.414	0.421	0.428	0.435	0.441	0.446
Central sub-Saharan Africa	0.298	0.303	0.307	0.309	0.311	0.313	0.316	0.318	0.32	0.323	0.325	0.328	0.332	0.336	0.341	0.348	0.355	0.364	0.373	0.382	0.391	0.402	0.413	0.423	0.433	0.443	0.452	0.457
Angola	0.235	0.24	0.245	0.249	0.253	0.258	0.263	0.269	0.276	0.282	0.288	0.293	0.299	0.305	0.312	0.32	0.329	0.34	0.351	0.363	0.375	0.389	0.401	0.414	0.428	0.441	0.453	0.461
Central African Republic	0.22	0.225	0.228	0.232	0.236	0.24	0.242	0.245	0.249	0.254	0.257	0.261	0.265	0.268	0.271	0.275	0.28	0.285	0.29	0.296	0.304	0.313	0.323	0.325	0.328	0.33	0.333	0.334
Congo (Brazzaville)	0.382	0.39	0.398	0.405	0.41	0.416	0.421	0.426	0.43	0.434	0.439	0.444	0.449	0.455	0.46	0.467	0.475	0.482	0.49	0.499	0.509	0.52	0.531	0.542	0.552	0.561	0.569	0.574
DR Congo	0.293	0.296	0.298	0.298	0.296	0.294	0.291	0.288	0.283	0.279	0.274	0.269	0.265	0.263	0.262	0.264	0.264	0.265	0.267	0.27	0.278	0.288	0.3	0.315	0.33	0.344	0.356	0.364
Equatorial Guinea	0.2	0.204	0.212	0.22	0.229	0.241	0.26	0.292	0.316	0.339	0.363	0.388	0.41	0.429	0.449	0.467	0.483	0.499	0.516	0.53	0.544	0.559	0.573	0.587	0.599	0.61	0.62	0.625
Gabon	0.433	0.443	0.453	0.462	0.472	0.481	0.49	0.498	0.506	0.514	0.522	0.529	0.535	0.542	0.549	0.556	0.562	0.569	0.576	0.582	0.589	0.598	0.607	0.616	0.625	0.634	0.644	0.651
Eastern sub-Saharan Africa	0.23	0.233	0.236	0.239	0.241	0.245	0.249	0.254	0.259	0.262	0.266	0.271	0.276	0.282	0.288	0.294	0.301	0.308	0.316	0.324	0.332	0.34	0.348	0.356	0.365	0.373	0.381	0.387
Burundi	0.247	0.252	0.257	0.258	0.263	0.265	0.265	0.266	0.268	0.268	0.268	0.267	0.268	0.269	0.271	0.272	0.274	0.276	0.278	0.282	0.286	0.29	0.295	0.299	0.303	0.306	0.308	0.31
Comoros	0.272	0.279	0.286	0.293	0.298	0.303	0.306	0.31	0.314	0.319	0.325	0.331	0.338	0.344	0.351	0.358	0.365	0.372	0.378	0.384	0.39	0.396	0.403	0.41	0.417	0.423	0.429	0.434
Djibouti	0.313	0.317	0.32	0.322	0.325	0.329	0.333	0.337	0.339	0.341	0.342	0.347	0.359	0.374	0.388	0.4	0.407	0.412	0.419	0.425	0.432	0.439	0.446	0.454	0.462	0.47	0.478	0.485
Eritrea	0.202	0.214	0.223	0.234	0.247	0.26	0.272	0.285	0.296	0.306	0.315	0.323	0.331	0.337	0.343	0.348	0.353	0.357	0.36	0.364	0.368	0.372	0.378	0.383	0.39	0.396	0.403	0.409
Ethiopia	0.138	0.141	0.143	0.146	0.148	0.15	0.155	0.161	0.166	0.169	0.172	0.177	0.183	0.189	0.195	0.202	0.21	0.221	0.233	0.245	0.257	0.268	0.28	0.292	0.303	0.314	0.325	0.334
Kenya	0.341	0.349	0.357	0.364	0.372	0.377	0.382	0.387	0.392	0.398	0.401	0.403	0.406	0.411	0.416	0.42	0.425	0.432	0.438	0.445	0.452	0.459	0.465	0.473	0.481	0.488	0.494	0.499
Baringo	0.254	0.266	0.278	0.289	0.299	0.307	0.313	0.319	0.326	0.333	0.338	0.341	0.345	0.352	0.358	0.362	0.368	0.376	0.384	0.393	0.401	0.408	0.414	0.421	0.428	0.434	0.439	0.444
Bomet	0.306	0.315	0.325	0.333	0.341	0.347	0.351	0.355	0.361	0.367	0.371	0.373	0.378	0.385	0.392	0.398	0.406	0.414	0.423	0.433	0.442	0.449	0.456	0.465	0.475	0.483	0.49	0.496
Bungoma	0.316	0.325	0.333	0.341	0.348	0.353	0.357	0.36	0.365	0.37	0.373	0.373	0.376	0.38	0.384	0.387	0.391	0.397	0.403	0.41	0.417	0.423	0.429	0.436	0.445	0.451	0.458	0.463
Busia	0.297	0.304	0.312	0.32	0.327	0.332	0.336	0.339	0.344	0.349	0.352	0.353	0.356	0.361	0.367	0.37	0.375	0.381	0.386	0.393	0.4	0.404	0.409	0.415	0.423	0.428	0.434	0.438
Elgeyo Marakwet	0.292	0.302	0.312	0.321	0.329	0.336	0.342	0.348	0.355	0.362	0.368	0.372	0.378	0.386	0.394	0.4	0.408	0.417	0.425	0.435	0.443	0.451	0.458	0.467	0.475	0.483	0.49	0.496
Embu	0.375	0.384	0.393	0.4	0.407	0.413	0.417	0.422	0.427	0.431	0.434	0.437	0.44	0.444	0.449	0.452	0.458	0.464	0.47	0.478	0.486	0.493	0.499	0.507	0.514	0.521	0.527	0.533
Garissa	0.153	0.16	0.168	0.177	0.184	0.19	0.195	0.201	0.207	0.213	0.217	0.219	0.223	0.228	0.233	0.237	0.242	0.249	0.255	0.263	0.272	0.28	0.288	0.298	0.309	0.318	0.326	0.334
Homa Bay	0.214	0.222	0.232	0.243	0.253	0.26	0.265	0.271	0.279	0.288	0.292	0.293	0.297	0.305	0.313	0.319	0.328	0.338	0.346	0.356	0.366	0.374	0.382	0.392	0.403	0.411	0.419	0.425
Isiolo	0.264	0.27	0.276	0.282	0.288	0.292	0.295	0.298	0.301	0.305	0.307	0.308	0.31	0.314	0.318	0.321	0.326	0.331	0.337	0.343	0.35	0.355	0.36	0.365	0.372	0.377	0.381	0.385
Kajiado	0.384	0.392	0.4	0.407	0.414	0.42	0.425	0.429	0.434	0.438	0.442	0.445	0.448	0.452	0.456	0.46	0.464	0.47	0.475	0.481	0.486	0.492	0.498	0.506	0.514	0.521	0.528	0.534
Kakamega	0.295	0.303	0.311	0.319	0.326	0.332	0.337	0.342	0.348	0.356	0.36	0.361	0.365	0.37	0.375	0.378	0.383	0.389	0.394	0.4	0.407	0.412	0.417	0.425	0.433	0.439	0.445	0.45
Kericho	0.266	0.277	0.288	0.299	0.309	0.317	0.324	0.331	0.339	0.348	0.353	0.356	0.362	0.37	0.378	0.385	0.394	0.404	0.414	0.425	0.436	0.445	0.454	0.464	0.475	0.485	0.493	0.5
Kiambu	0.435	0.443	0.45	0.457	0.464	0.469	0.473	0.476	0.48	0.484	0.487	0.489	0.492	0.496	0.5	0.504	0.509	0.516	0.521	0.528	0.535	0.541	0.548	0.555	0.562	0.569	0.575	0.58
Kilifi	0.292	0.3	0.307	0.314	0.321	0.327	0.331	0.336	0.34	0.346	0.348	0.349	0.352	0.357	0.361	0.365	0.371	0.378	0.385	0.392	0.4	0.408	0.415	0.424	0.434	0.442	0.45	0.456
Kirinyaga	0.389	0.396	0.402	0.407	0.411	0.415	0.418	0.422	0.425	0.429	0.432	0.434	0.437	0.442	0.447	0.451	0.457	0.464	0.471	0.479	0.486	0.493	0.5	0.507	0.514	0.521	0.527	0.533
Kisii	0.34	0.35	0.36	0.368	0.377	0.383	0.389	0.395	0.401	0.407	0.411	0.414	0.418	0.424	0.429	0.434	0.44	0.448	0.454	0.463	0.471	0.479	0.486	0.495	0.503	0.51	0.517	0.522
Kisumu	0.315	0.325	0.334	0.342	0.349	0.355	0.36	0.364	0.37	0.376	0.381	0.384	0.388	0.395	0.402	0.407	0.415	0.424	0.432	0.442	0.451	0.459	0.466	0.475	0.484	0.491	0.497	0.503
Kitui	0.28	0.288	0.297	0.304	0.311	0.317	0.322	0.327	0.332	0.338	0.343	0.346	0.351	0.357	0.363	0.369	0.376	0.383	0.391	0.399	0.408	0.416	0.423	0.432	0.44	0.448	0.455	0.461
Kwale	0.294	0.301	0.308	0.314	0.321	0.326	0.33	0.334	0.338	0.342	0.344	0.346	0.348	0.352	0.357	0.36	0.366	0.374	0.381	0.39	0.399	0.407	0.414	0.424	0.433	0.442	0.45	0.457
Laikipia	0.346	0.354	0.361	0.368	0.375	0.38	0.385	0.389	0.395	0.401	0.405	0.409	0.415	0.423	0.433	0.442	0.451	0.462	0.472	0.483	0.494	0.502	0.511	0.521	0.531	0.54	0.549	0.556
Lamu	0.295	0.303	0.312	0.321	0.328	0.334	0.338	0.343	0.347	0.352	0.355	0.356	0.359	0.364	0.369	0.373	0.378	0.384	0.39	0.397	0.405	0.411	0.417	0.425	0.434	0.441	0.448	0.453
Machakos	0.352	0.362	0.372	0.38	0.389	0.396	0.403	0.411	0.418	0.426	0.431	0.435	0.44	0.444	0.449	0.452	0.456	0.461	0.465	0.471	0.476	0.482	0.487	0.493	0.5	0.507	0.512	0.518
Makueni	0.3	0.307	0.314	0.322	0.328	0.333	0.336	0.339	0.343	0.348	0.35	0.351	0.354	0.36	0.367	0.373	0.381	0.391	0.401	0.413	0.423	0.432	0.439	0.447	0.454	0.46	0.465	0.469
Mandera	0.102	0.109	0.118	0.126	0.135	0.142	0.149	0.156	0.164	0.172	0.178	0.181	0.187	0.194	0.201	0.206	0.212	0.218	0.224	0.231	0.238	0.244	0.251	0.261	0.271	0.28	0.288	0.295
Marsabit	0.22	0.223	0.227	0.231	0.236	0.239	0.241	0.244	0.247	0.252	0.255	0.256	0.259	0.264	0.268	0.271	0.276	0.281	0.286	0.293	0.299	0.304	0.309	0.315	0.323	0.329	0.335	0.34
Meru	0.338	0.346	0.354	0.362	0.369	0.376	0.381	0.387	0.393	0.399	0.403	0.407	0.411	0.417	0.423	0.428	0.434	0.441	0.447	0.454	0.462	0.469	0.475	0.482	0.49	0.496	0.503	0.508
Migori	0.229	0.237	0.246	0.255	0.262	0.267	0.27	0.274	0.279	0.285	0.287	0.286	0.289	0.296	0.303	0.307	0.315	0.325	0.333	0.344	0.355	0.364	0.372	0.383	0.395	0.404	0.412	0.419
Mombasa	0.396	0.405	0.414	0.422	0.43	0.437	0.443	0.449	0.455	0.461	0.465	0.468	0.471	0.475	0.478	0.481	0.486	0.491	0.496	0.502	0.51	0.517	0.525	0.534	0.543	0.552	0.561	0.568
Murang'a	0.381	0.389	0.396	0.402	0.408	0.413	0.418	0.424	0.429	0.436	0.441	0.444	0.449	0.454	0.46	0.465	0.47	0.476	0.482	0.488	0.494	0.499	0.504	0.51	0.515	0.52	0.524	0.528
Nairobi	0.499	0.506	0.513	0.519	0.525	0.53	0.534	0.539	0.544	0.549	0.553	0.556	0.56	0.566	0.572	0.577	0.585	0.593	0.602	0.611	0.619	0.627	0.634	0.643	0.652	0.66	0.667	0.674
Nakuru	0.337	0.347	0.357	0.367	0.375	0.383	0.389	0.394	0.399	0.405	0.409	0.413	0.417	0.423	0.43	0.436	0.445	0.454	0.464	0.474	0.484	0.493	0.502	0.511	0.521	0.53	0.538	0.545
Nandi	0.323	0.333	0.344	0.354	0.362	0.369	0.375	0.381	0.388	0.394	0.399	0.402	0.406	0.412	0.418	0.422	0.428	0.435	0.441	0.449	0.456	0.462	0.468	0.475	0.483	0.49	0.496	0.501
Narok	0.217	0.225	0.234	0.242	0.25	0.255	0.259	0.264	0.272	0.28	0.284	0.285	0.289	0.297	0.305	0.31	0.318	0.326	0.333	0.342	0.35	0.356	0.362	0.371	0.381	0.389	0.396	0.402
Nyamira	0.361	0.37	0.379	0.387	0.395	0.401	0.406	0.41	0.414	0.419	0.421	0.423	0.426	0.431	0.437	0.443	0.451	0.46	0.468	0.478	0.488	0.496	0.504	0.513	0.523	0.531	0.538	0.544
Nyandarua	0.353	0.361	0.369	0.376	0.382	0.388	0.392	0.397	0.402	0.409	0.413	0.416	0.421	0.428	0.436	0.442	0.449	0.458	0.466	0.475	0.484	0.491	0.498	0.506	0.514	0.521	0.528	0.534
Nyeri	0.407	0.414	0.421	0.427	0.434	0.439	0.444	0.449	0.454	0.459	0.463	0.466	0.47	0.475	0.479	0.484	0.489	0.496	0.502	0.509	0.516	0.522	0.527	0.533	0.539	0.544	0.549	0.554
Samburu	0.205	0.21	0.215	0.221	0.225	0.228	0.23	0.232	0.235	0.239	0.24	0.239	0.24	0.243	0.247	0.249	0.253	0.258	0.262	0.267	0.273	0.277	0.28	0.287	0.294	0.299	0.304	0.308
Siaya	0.227	0.236	0.246	0.255	0.265	0.272	0.279	0.287	0.294	0.303	0.309	0.313	0.318	0.326	0.334	0.34	0.349	0.359	0.368	0.379	0.39	0.4	0.409	0.421	0.432	0.443	0.452	0.46
Taita Taveta	0.352	0.362	0.371	0.38	0.389	0.396	0.403	0.409	0.416	0.422	0.427	0.43	0.434	0.438	0.443	0.447	0.452	0.458	0.464	0.472	0.48	0.487	0.494	0.502	0.509	0.516	0.523	0.529
Tana River	0.231	0.237	0.244	0.25	0.255	0.26	0.263	0.267	0.27	0.274	0.276	0.276	0.278	0.281	0.285	0.288	0.292	0.298	0.304	0.311	0.319	0.326	0.333	0.342	0.353	0.362	0.371	0.379
Tharaka Nithi	0.342	0.35	0.357	0.364	0.371	0.377	0.382	0.387	0.392	0.397	0.402	0.405	0.41	0.417	0.423	0.429	0.437	0.445	0.453	0.463	0.472	0.48	0.488	0.497	0.506	0.514	0.522	0.528
Trans Nzoia	0.318	0.328	0.337	0.346	0.353	0.359	0.363	0.368	0.373	0.38	0.384	0.388	0.392	0.399	0.406	0.411	0.418	0.426	0.433	0.441	0.448	0.454	0.46	0.468	0.477	0.484	0.491	0.496
Turkana	0.211	0.216	0.221	0.226	0.231	0.234	0.237	0.239	0.242	0.245	0.247	0.247	0.248	0.25	0.252	0.252	0.253	0.255	0.257	0.26	0.264	0.266	0.269	0.274	0.281	0.286	0.291	0.295
Uasin Gishu	0.363	0.373	0.382	0.391	0.399	0.405	0.41	0.416	0.421	0.428	0.433	0.436	0.441	0.447	0.453	0.458	0.465	0.472	0.48	0.488	0.496	0.503	0.51	0.517	0.526	0.533	0.539	0.545
Vihiga	0.328	0.337	0.346	0.355	0.362	0.367	0.371	0.374	0.376	0.378	0.378	0.378	0.379	0.382	0.387	0.392	0.399	0.408	0.417	0.427	0.437	0.444	0.45	0.457	0.464	0.469	0.474	0.477
Wajir	0.104	0.111	0.12	0.128	0.136	0.142	0.146	0.151	0.156	0.162	0.165	0.166	0.168	0.172	0.176	0.178	0.181	0.185	0.189	0.195	0.201	0.206	0.21	0.218	0.226	0.233	0.238	0.243
West Pokot	0.213	0.219	0.226	0.233	0.24	0.245	0.249	0.253	0.258	0.265	0.268	0.27	0.274	0.28	0.286	0.291	0.298	0.305	0.312	0.319	0.327	0.333	0.34	0.349	0.359	0.368	0.376	0.382
Madagascar	0.262	0.264	0.265	0.265	0.265	0.265	0.266	0.268	0.27	0.272	0.274	0.278	0.282	0.286	0.291	0.294	0.295	0.295	0.298	0.3	0.302	0.303	0.306	0.31	0.315	0.321	0.326	0.331
Malawi	0.199	0.202	0.204	0.206	0.208	0.212	0.216	0.221	0.225	0.23	0.234	0.238	0.244	0.249	0.256	0.262	0.27	0.279	0.288	0.296	0.305	0.313	0.32	0.327	0.333	0.339	0.345	0.349
Mozambique	0.133	0.135	0.136	0.138	0.14	0.142	0.147	0.154	0.162	0.169	0.175	0.182	0.189	0.197	0.206	0.215	0.224	0.235	0.246	0.257	0.268	0.279	0.29	0.301	0.311	0.322	0.332	0.34
Rwanda	0.267	0.269	0.272	0.274	0.268	0.267	0.269	0.272	0.276	0.28	0.284	0.29	0.297	0.304	0.311	0.319	0.327	0.334	0.342	0.349	0.356	0.363	0.371	0.378	0.386	0.393	0.401	0.407
Somalia	0.153	0.156	0.157	0.159	0.16	0.161	0.162	0.163	0.163	0.164	0.165	0.167	0.169	0.172	0.176	0.179	0.183	0.188	0.193	0.198	0.203	0.208	0.212	0.217	0.222	0.227	0.231	0.235
South Sudan	0.179	0.18	0.182	0.183	0.184	0.186	0.187	0.189	0.191	0.194	0.197	0.2	0.204	0.207	0.211	0.215	0.22	0.225	0.229	0.234	0.239	0.244	0.249	0.254	0.259	0.265	0.27	0.275
Tanzania	0.275	0.28	0.285	0.289	0.291	0.294	0.297	0.3	0.304	0.307	0.311	0.315	0.32	0.325	0.33	0.334	0.339	0.344	0.35	0.355	0.36	0.366	0.373	0.381	0.388	0.397	0.405	0.412
Uganda	0.163	0.164	0.166	0.169	0.173	0.178	0.185	0.193	0.202	0.212	0.222	0.232	0.243	0.254	0.265	0.276	0.287	0.298	0.31	0.32	0.33	0.339	0.348	0.357	0.366	0.374	0.382	0.388
Zambia	0.312	0.314	0.316	0.319	0.321	0.324	0.326	0.329	0.332	0.335	0.338	0.343	0.348	0.353	0.358	0.365	0.373	0.382	0.392	0.402	0.412	0.421	0.431	0.44	0.449	0.458	0.466	0.472
Southern sub-Saharan Africa	0.521	0.525	0.53	0.535	0.539	0.543	0.546	0.55	0.555	0.56	0.565	0.569	0.574	0.578	0.582	0.585	0.59	0.596	0.603	0.607	0.612	0.616	0.619	0.622	0.627	0.633	0.637	0.64
Botswana	0.463	0.473	0.483	0.492	0.501	0.509	0.517	0.524	0.532	0.539	0.546	0.553	0.56	0.567	0.574	0.581	0.588	0.597	0.605	0.612	0.619	0.626	0.633	0.639	0.646	0.652	0.658	0.663
Lesotho	0.333	0.34	0.347	0.354	0.362	0.369	0.376	0.383	0.389	0.396	0.402	0.407	0.413	0.417	0.422	0.426	0.431	0.436	0.441	0.446	0.452	0.458	0.464	0.47	0.476	0.482	0.488	0.493
Namibia	0.454	0.459	0.465	0.471	0.477	0.483	0.489	0.495	0.501	0.507	0.512	0.516	0.521	0.525	0.53	0.534	0.54	0.546	0.552	0.558	0.565	0.572	0.579	0.587	0.595	0.603	0.61	0.616
South Africa	0.557	0.56	0.565	0.57	0.574	0.576	0.579	0.583	0.587	0.593	0.598	0.602	0.607	0.611	0.615	0.619	0.624	0.631	0.639	0.644	0.649	0.653	0.655	0.658	0.664	0.669	0.674	0.677
Swaziland	0.426	0.435	0.444	0.452	0.459	0.466	0.473	0.478	0.483	0.489	0.494	0.499	0.503	0.508	0.513	0.518	0.523	0.528	0.533	0.538	0.543	0.547	0.552	0.557	0.563	0.569	0.574	0.578
Zimbabwe	0.408	0.417	0.424	0.43	0.436	0.44	0.446	0.45	0.453	0.456	0.457	0.459	0.458	0.456	0.453	0.449	0.443	0.438	0.43	0.426	0.424	0.425	0.43	0.436	0.444	0.451	0.458	0.463
Western sub-Saharan Africa	0.293	0.296	0.299	0.303	0.306	0.309	0.313	0.316	0.32	0.324	0.328	0.333	0.337	0.343	0.35	0.357	0.365	0.373	0.381	0.389	0.396	0.403	0.411	0.417	0.424	0.43	0.437	0.441
Benin	0.218	0.223	0.228	0.233	0.238	0.243	0.248	0.253	0.258	0.263	0.268	0.273	0.278	0.284	0.29	0.296	0.301	0.307	0.313	0.32	0.326	0.332	0.338	0.345	0.353	0.36	0.367	0.373
Burkina Faso	0.139	0.143	0.147	0.151	0.154	0.158	0.163	0.168	0.173	0.179	0.184	0.19	0.195	0.201	0.208	0.214	0.22	0.226	0.232	0.237	0.242	0.248	0.253	0.259	0.266	0.272	0.279	0.284
Cameroon	0.332	0.338	0.343	0.347	0.35	0.352	0.355	0.357	0.359	0.36	0.361	0.361	0.361	0.362	0.366	0.373	0.38	0.388	0.396	0.405	0.414	0.423	0.433	0.443	0.453	0.464	0.474	0.482
Cape Verde	0.307	0.315	0.322	0.329	0.337	0.347	0.357	0.367	0.377	0.387	0.399	0.41	0.42	0.431	0.441	0.451	0.461	0.472	0.482	0.491	0.5	0.509	0.517	0.524	0.531	0.538	0.544	0.549
Chad	0.119	0.123	0.128	0.131	0.135	0.138	0.141	0.144	0.146	0.148	0.15	0.152	0.154	0.156	0.161	0.166	0.171	0.178	0.185	0.192	0.2	0.207	0.216	0.224	0.232	0.24	0.248	0.253
Cote d'Ivoire	0.273	0.28	0.287	0.292	0.297	0.301	0.306	0.312	0.317	0.322	0.326	0.33	0.334	0.338	0.342	0.346	0.349	0.353	0.357	0.361	0.367	0.371	0.377	0.384	0.391	0.398	0.406	0.412
The Gambia	0.245	0.251	0.257	0.263	0.269	0.275	0.282	0.288	0.294	0.3	0.307	0.314	0.319	0.325	0.332	0.338	0.343	0.349	0.355	0.361	0.367	0.372	0.378	0.384	0.389	0.395	0.401	0.405
Ghana	0.38	0.386	0.392	0.396	0.4	0.403	0.408	0.413	0.416	0.418	0.422	0.425	0.43	0.435	0.439	0.443	0.45	0.459	0.467	0.474	0.482	0.49	0.498	0.505	0.513	0.521	0.53	0.537
Guinea	0.164	0.168	0.171	0.175	0.178	0.183	0.188	0.194	0.2	0.206	0.211	0.217	0.222	0.228	0.234	0.24	0.246	0.252	0.26	0.266	0.273	0.281	0.288	0.295	0.303	0.31	0.318	0.325
Guinea-Bissau	0.183	0.189	0.194	0.199	0.205	0.211	0.217	0.224	0.229	0.235	0.241	0.246	0.252	0.257	0.263	0.268	0.274	0.28	0.286	0.293	0.3	0.308	0.315	0.322	0.329	0.336	0.343	0.349
Liberia	0.207	0.209	0.21	0.206	0.199	0.19	0.178	0.174	0.175	0.183	0.198	0.214	0.228	0.234	0.239	0.245	0.251	0.257	0.264	0.27	0.276	0.284	0.292	0.301	0.31	0.318	0.324	0.328
Mali	0.13	0.134	0.137	0.141	0.145	0.148	0.152	0.157	0.161	0.166	0.171	0.176	0.181	0.187	0.192	0.198	0.203	0.209	0.215	0.22	0.226	0.232	0.238	0.243	0.249	0.255	0.262	0.267
Mauritania	0.299	0.307	0.314	0.321	0.328	0.335	0.341	0.346	0.351	0.356	0.359	0.363	0.367	0.371	0.377	0.382	0.39	0.398	0.406	0.412	0.42	0.427	0.435	0.442	0.45	0.458	0.465	0.471
Niger	0.092	0.097	0.101	0.105	0.108	0.111	0.113	0.115	0.118	0.121	0.123	0.125	0.127	0.13	0.134	0.137	0.14	0.144	0.147	0.151	0.156	0.16	0.165	0.17	0.176	0.181	0.186	0.191
Nigeria	0.344	0.346	0.347	0.349	0.352	0.355	0.358	0.362	0.365	0.369	0.374	0.379	0.385	0.391	0.4	0.41	0.419	0.429	0.437	0.445	0.452	0.459	0.466	0.473	0.478	0.484	0.49	0.493
Sao Tome and Principe	0.287	0.292	0.296	0.3	0.305	0.309	0.314	0.32	0.326	0.332	0.338	0.345	0.353	0.362	0.371	0.381	0.39	0.399	0.408	0.416	0.426	0.435	0.444	0.453	0.463	0.472	0.481	0.488
Senegal	0.244	0.25	0.257	0.262	0.267	0.272	0.277	0.281	0.286	0.291	0.295	0.299	0.303	0.307	0.311	0.316	0.319	0.324	0.328	0.333	0.338	0.342	0.347	0.352	0.357	0.362	0.368	0.373
Sierra Leone	0.202	0.205	0.207	0.211	0.216	0.219	0.222	0.225	0.226	0.228	0.23	0.233	0.238	0.244	0.249	0.256	0.262	0.27	0.278	0.286	0.294	0.302	0.312	0.325	0.336	0.344	0.351	0.357
Togo	0.263	0.27	0.276	0.281	0.286	0.292	0.297	0.303	0.307	0.311	0.315	0.318	0.321	0.325	0.329	0.333	0.337	0.343	0.348	0.353	0.36	0.366	0.373	0.381	0.39	0.398	0.406	0.413

Methods Appendix Table 4. Socio-Demographic Index groupings by geography, based on 2017 values		
**Geography**	**2017 SDI**	**SDI Quintile**
Global	0.652	
Central Europe, Eastern Europe, and Central Asia	0.766	
Central Asia	0.673	
Armenia	0.702	High-middle SDI
Azerbaijan	0.701	High-middle SDI
Georgia	0.7	High-middle SDI
Kazakhstan	0.735	High-middle SDI
Kyrgyzstan	0.607	Low-middle SDI
Mongolia	0.662	Middle SDI
Tajikistan	0.523	Low-middle SDI
Turkmenistan	0.696	Middle SDI
Uzbekistan	0.63	Middle SDI
Central Europe	0.814	
Albania	0.685	Middle SDI
Bosnia and Herzegovina	0.713	High-middle SDI
Bulgaria	0.792	High-middle SDI
Croatia	0.825	High SDI
Czech Republic	0.851	High SDI
Hungary	0.817	High-middle SDI
Macedonia	0.754	High-middle SDI
Montenegro	0.788	High-middle SDI
Poland	0.844	High SDI
Romania	0.784	High-middle SDI
Serbia	0.752	High-middle SDI
Slovakia	0.842	High SDI
Slovenia	0.86	High SDI
Eastern Europe	0.785	
Belarus	0.773	High-middle SDI
Estonia	0.858	High SDI
Latvia	0.825	High SDI
Lithuania	0.841	High SDI
Moldova	0.676	Middle SDI
Russian Federation	0.792	High-middle SDI
Ukraine	0.74	High-middle SDI
High-income	0.854	
Australasia	0.869	
Australia	0.873	High SDI
New Zealand	0.842	High SDI
High-income Asia-Pacific	0.869	
Brunei	0.856	High SDI
Japan	0.865	High SDI
Aichi	0.875	High SDI
Akita	0.829	High SDI
Aomori	0.825	High SDI

Methods Appendix Table 4. Socio-Demographic Index groupings by geography, based on 2017 values		
**Geography**	**2017 SDI**	**SDI Quintile**
Chiba	0.859	High SDI
Ehime	0.838	High SDI
Fukui	0.852	High SDI
Fukuoka	0.855	High SDI
Fukushima	0.831	High SDI
Gifu	0.849	High SDI
Gunma	0.851	High SDI
Hiroshima	0.863	High SDI
Hokkaidō	0.842	High SDI
Hyōgo	0.86	High SDI
Ibaraki	0.851	High SDI
Ishikawa	0.856	High SDI
Iwate	0.825	High SDI
Kagawa	0.85	High SDI
Kagoshima	0.83	High SDI
Kanagawa	0.875	High SDI
Kōchi	0.825	High SDI
Kumamoto	0.832	High SDI
Kyōto	0.873	High SDI
Mie	0.854	High SDI
Miyagi	0.85	High SDI
Miyazaki	0.823	High SDI
Nagano	0.851	High SDI
Nagasaki	0.826	High SDI
Nara	0.848	High SDI
Niigata	0.843	High SDI
Ōita	0.846	High SDI
Okayama	0.856	High SDI
Okinawa	0.818	High SDI
Ōsaka	0.872	High SDI
Saga	0.834	High SDI
Saitama	0.852	High SDI
Shiga	0.871	High SDI
Shimane	0.831	High SDI
Shizuoka	0.859	High SDI
Tochigi	0.853	High SDI
Tokushima	0.845	High SDI
Tōkyō	0.924	High SDI
Tottori	0.834	High SDI
Toyama	0.86	High SDI
Wakayama	0.84	High SDI
Yamagata	0.832	High SDI
Yamaguchi	0.849	High SDI
Yamanashi	0.854	High SDI
South Korea	0.872	High SDI

Methods Appendix Table 4. Socio-Demographic Index groupings by geography, based on 2017 values		
**Geography**	**2017 SDI**	**SDI Quintile**
Singapore	0.872	High SDI
High-income North America	0.868	
Canada	0.882	High SDI
Greenland	0.76	High-middle SDI
USA	0.867	High SDI
Alabama	0.837	High SDI
Alaska	0.861	High SDI
Arizona	0.845	High SDI
Arkansas	0.826	High SDI
California	0.872	High SDI
Colorado	0.882	High SDI
Connecticut	0.906	High SDI
Delaware	0.874	High SDI
Washington, DC	0.89	High SDI
Florida	0.864	High SDI
Georgia	0.848	High SDI
Hawaii	0.872	High SDI
Idaho	0.841	High SDI
Illinois	0.879	High SDI
Indiana	0.848	High SDI
Iowa	0.87	High SDI
Kansas	0.864	High SDI
Kentucky	0.831	High SDI
Louisiana	0.835	High SDI
Maine	0.872	High SDI
Maryland	0.896	High SDI
Massachusetts	0.913	High SDI
Michigan	0.868	High SDI
Minnesota	0.893	High SDI
Mississippi	0.819	High SDI
Missouri	0.853	High SDI
Montana	0.863	High SDI
Nebraska	0.873	High SDI
Nevada	0.847	High SDI
New Hampshire	0.904	High SDI
New Jersey	0.899	High SDI
New Mexico	0.835	High SDI
New York	0.893	High SDI
North Carolina	0.85	High SDI
North Dakota	0.88	High SDI
Ohio	0.858	High SDI
Oklahoma	0.838	High SDI
Oregon	0.871	High SDI
Pennsylvania	0.879	High SDI
Rhode Island	0.89	High SDI

Methods Appendix Table 4. Socio-Demographic Index groupings by geography, based on 2017 values		
**Geography**	**2017 SDI**	**SDI Quintile**
South Carolina	0.846	High SDI
South Dakota	0.86	High SDI
Tennessee	0.837	High SDI
Texas	0.838	High SDI
Utah	0.856	High SDI
Vermont	0.896	High SDI
Virginia	0.885	High SDI
Washington	0.884	High SDI
West Virginia	0.825	High SDI
Wisconsin	0.878	High SDI
Wyoming	0.869	High SDI
Southern Latin America	0.72	
Argentina	0.71	High-middle SDI
Chile	0.748	High-middle SDI
Uruguay	0.707	High-middle SDI
Western Europe	0.857	
Andorra	0.902	High SDI
Austria	0.866	High SDI
Belgium	0.886	High SDI
Cyprus	0.865	High SDI
Denmark	0.918	High SDI
Finland	0.893	High SDI
France	0.865	High SDI
Germany	0.87	High SDI
Greece	0.817	High SDI
Iceland	0.907	High SDI
Ireland	0.882	High SDI
Israel	0.816	High-middle SDI
Italy	0.843	High SDI
Luxembourg	0.916	High SDI
Malta	0.836	High SDI
Netherlands	0.912	High SDI
Norway	0.911	High SDI
Portugal	0.778	High-middle SDI
Spain	0.825	High SDI
Sweden	0.883	High SDI
Stockholm	0.914	High SDI
Sweden except Stockholm	0.873	High SDI
Switzerland	0.889	High SDI
United Kingdom	0.843	High SDI
England	0.849	High SDI
East Midlands	0.83	High SDI
Derby	0.846	High SDI
Derbyshire	0.817	High SDI
Leicester	0.839	High SDI

Methods Appendix Table 4. Socio-Demographic Index groupings by geography, based on 2017 values		
**Geography**	**2017 SDI**	**SDI Quintile**
Leicestershire	0.846	High SDI
Lincolnshire	0.812	High SDI
Northamptonshire	0.829	High SDI
Nottingham	0.863	High SDI
Nottinghamshire	0.814	High SDI
Rutland	0.833	High SDI
East of England	0.84	High SDI
Bedford	0.838	High SDI
Cambridgeshire	0.871	High SDI
Central Bedfordshire	0.834	High SDI
Essex	0.832	High SDI
Hertfordshire	0.87	High SDI
Luton	0.833	High SDI
Norfolk	0.826	High SDI
Peterborough	0.818	High SDI
Southend-on-Sea	0.811	High SDI
Suffolk	0.821	High SDI
Thurrock	0.807	High SDI
Greater London	0.894	High SDI
Barking and Dagenham	0.802	High SDI
Barnet	0.865	High SDI
Bexley	0.826	High SDI
Brent	0.849	High SDI
Bromley	0.848	High SDI
Camden	0.93	High SDI
Croydon	0.833	High SDI
Ealing	0.865	High SDI
Enfield	0.839	High SDI
Greenwich	0.833	High SDI
Hackney	0.887	High SDI
Hammersmith and Fulham	0.927	High SDI
Haringey	0.854	High SDI
Harrow	0.848	High SDI
Havering	0.824	High SDI
Hillingdon	0.882	High SDI
Hounslow	0.879	High SDI
Islington	0.922	High SDI
Kensington and Chelsea	0.932	High SDI
Kingston upon Thames	0.89	High SDI
Lambeth	0.9	High SDI
Lewisham	0.843	High SDI
Merton	0.873	High SDI
Newham	0.838	High SDI
Redbridge	0.831	High SDI
Richmond upon Thames	0.902	High SDI

Methods Appendix Table 4. Socio-Demographic Index groupings by geography, based on 2017 values		
**Geography**	**2017 SDI**	**SDI Quintile**
Southwark	0.912	High SDI
Sutton	0.843	High SDI
Tower Hamlets	0.905	High SDI
Waltham Forest	0.819	High SDI
Wandsworth	0.911	High SDI
Westminster	0.927	High SDI
North East England	0.821	High SDI
County Durham	0.81	High SDI
Darlington	0.825	High SDI
Gateshead	0.826	High SDI
Hartlepool	0.793	High SDI
Middlesbrough	0.808	High SDI
Newcastle upon Tyne	0.872	High SDI
North Tyneside	0.825	High SDI
Northumberland	0.808	High SDI
Redcar and Cleveland	0.79	High SDI
South Tyneside	0.794	High SDI
Stockton-on-Tees	0.823	High SDI
Sunderland	0.815	High SDI
North West England	0.834	High SDI
Blackburn with Darwen	0.802	High SDI
Blackpool	0.781	High SDI
Bolton	0.805	High SDI
Bury	0.815	High SDI
Cheshire East	0.864	High SDI
Cheshire West and Chester	0.855	High SDI
Cumbria	0.828	High SDI
Halton	0.824	High SDI
Knowsley	0.816	High SDI
Lancashire	0.831	High SDI
Liverpool	0.852	High SDI
Manchester	0.885	High SDI
Oldham	0.79	High SDI
Rochdale	0.795	High SDI
Salford	0.838	High SDI
Sefton	0.812	High SDI
St Helens	0.803	High SDI
Stockport	0.843	High SDI
Tameside	0.797	High SDI
Trafford	0.873	High SDI
Warrington	0.86	High SDI
Wigan	0.798	High SDI
Wirral	0.803	High SDI
South East England	0.856	High SDI
Bracknell Forest	0.869	High SDI

Methods Appendix Table 4. Socio-Demographic Index groupings by geography, based on 2017 values		
**Geography**	**2017 SDI**	**SDI Quintile**
Brighton and Hove	0.885	High SDI
Buckinghamshire	0.865	High SDI
East Sussex	0.814	High SDI
Hampshire	0.85	High SDI
Isle of Wight	0.814	High SDI
Kent	0.828	High SDI
Medway	0.809	High SDI
Milton Keynes	0.86	High SDI
Oxfordshire	0.879	High SDI
Portsmouth	0.86	High SDI
Reading	0.895	High SDI
Slough	0.859	High SDI
Southampton	0.858	High SDI
Surrey	0.883	High SDI
West Berkshire	0.872	High SDI
West Sussex	0.843	High SDI
Windsor and Maidenhead	0.889	High SDI
Wokingham	0.885	High SDI
South West England	0.841	High SDI
Bath and North East Somerset	0.875	High SDI
Bournemouth	0.858	High SDI
Bristol, City of	0.884	High SDI
Cornwall	0.817	High SDI
Devon	0.837	High SDI
Dorset	0.825	High SDI
Gloucestershire	0.85	High SDI
North Somerset	0.832	High SDI
Plymouth	0.836	High SDI
Poole	0.842	High SDI
Somerset	0.816	High SDI
South Gloucestershire	0.867	High SDI
Swindon	0.847	High SDI
Torbay	0.79	High SDI
Wiltshire	0.829	High SDI
West Midlands	0.829	High SDI
Birmingham	0.84	High SDI
Coventry	0.848	High SDI
Dudley	0.799	High SDI
Herefordshire, County of	0.828	High SDI
Sandwell	0.797	High SDI
Shropshire	0.832	High SDI
Solihull	0.855	High SDI
Staffordshire	0.826	High SDI
Stoke-on-Trent	0.804	High SDI
Telford and Wrekin	0.822	High SDI

Methods Appendix Table 4. Socio-Demographic Index groupings by geography, based on 2017 values		
**Geography**	**2017 SDI**	**SDI Quintile**
Walsall	0.791	High SDI
Warwickshire	0.857	High SDI
Wolverhampton	0.811	High SDI
Worcestershire	0.833	High SDI
Yorkshire and the Humber	0.83	High SDI
Barnsley	0.787	High SDI
Bradford	0.807	High SDI
Calderdale	0.827	High SDI
Doncaster	0.791	High SDI
East Riding of Yorkshire	0.822	High SDI
Kingston upon Hull, City of	0.813	High SDI
Kirklees	0.816	High SDI
Leeds	0.868	High SDI
North East Lincolnshire	0.804	High SDI
North Lincolnshire	0.811	High SDI
North Yorkshire	0.839	High SDI
Rotherham	0.796	High SDI
Sheffield	0.853	High SDI
Wakefield	0.806	High SDI
York	0.879	High SDI
Northern Ireland	0.835	High SDI
Scotland	0.805	High SDI
Wales	0.806	High SDI
Latin America and Caribbean	0.64	
Andean Latin America	0.628	
Bolivia	0.587	Low-middle SDI
Ecuador	0.636	Middle SDI
Peru	0.636	Middle SDI
Caribbean	0.638	
Antigua and Barbuda	0.715	High-middle SDI
The Bahamas	0.756	High-middle SDI
Barbados	0.739	High-middle SDI
Belize	0.602	Low-middle SDI
Bermuda	0.805	High-middle SDI
Cuba	0.688	Middle SDI
Dominica	0.687	Middle SDI
Dominican Republic	0.593	Low-middle SDI
Grenada	0.64	Middle SDI
Guyana	0.584	Low-middle SDI
Haiti	0.442	Low SDI
Jamaica	0.679	Middle SDI
Puerto Rico	0.813	High-middle SDI
Saint Lucia	0.653	Middle SDI
Saint Vincent and the Grenadines	0.608	Middle SDI
Suriname	0.641	Middle SDI

Methods Appendix Table 4. Socio-Demographic Index groupings by geography, based on 2017 values		
**Geography**	**2017 SDI**	**SDI Quintile**
Trinidad and Tobago	0.698	Middle SDI
Virgin Islands	0.807	High-middle SDI
Central Latin America	0.623	
Colombia	0.634	Middle SDI
Costa Rica	0.662	Middle SDI
El Salvador	0.593	Low-middle SDI
Guatemala	0.524	Low-middle SDI
Honduras	0.512	Low-middle SDI
Mexico	0.628	Middle SDI
Aguascalientes	0.659	Middle SDI
Baja California	0.657	Middle SDI
Baja California Sur	0.659	Middle SDI
Campeche	0.616	Middle SDI
Chiapas	0.533	Middle SDI
Chihuahua	0.639	Middle SDI
Coahuila	0.645	Middle SDI
Colima	0.654	Middle SDI
Mexico City	0.716	Middle SDI
Durango	0.624	Middle SDI
Guanajuato	0.621	Middle SDI
Guerrero	0.562	Middle SDI
Hidalgo	0.587	Middle SDI
Jalisco	0.649	Middle SDI
México	0.635	Middle SDI
Michoacán de Ocampo	0.586	Middle SDI
Morelos	0.635	Middle SDI
Nayarit	0.62	Middle SDI
Nuevo León	0.677	Middle SDI
Oaxaca	0.561	Middle SDI
Puebla	0.584	Middle SDI
Querétaro	0.639	Middle SDI
Quintana Roo	0.626	Middle SDI
San Luis Potosí	0.621	Middle SDI
Sinaloa	0.649	Middle SDI
Sonora	0.65	Middle SDI
Tabasco	0.611	Middle SDI
Tamaulipas	0.647	Middle SDI
Tlaxcala	0.604	Middle SDI
Veracruz de Ignacio de la Llave	0.592	Middle SDI
Yucatán	0.63	Middle SDI
Zacatecas	0.608	Middle SDI
Nicaragua	0.53	Low-middle SDI
Panama	0.677	Middle SDI
Venezuela	0.655	Middle SDI
Tropical Latin America	0.662	

Methods Appendix Table 4. Socio-Demographic Index groupings by geography, based on 2017 values		
**Geography**	**2017 SDI**	**SDI Quintile**
Brazil	0.663	Middle SDI
Acre	0.602	Low-middle SDI
Alagoas	0.556	Low-middle SDI
Amapá	0.659	Middle SDI
Amazonas	0.629	Middle SDI
Bahia	0.591	Low-middle SDI
Ceará	0.6	Low-middle SDI
Distrito Federal	0.792	High-middle SDI
Espírito Santo	0.677	Middle SDI
Goiás	0.65	Middle SDI
Maranhão	0.507	Low-middle SDI
Mato Grosso	0.662	Middle SDI
Mato Grosso do Sul	0.65	Middle SDI
Minas Gerais	0.661	Middle SDI
Pará	0.579	Low-middle SDI
Paraíba	0.574	Low-middle SDI
Paraná	0.682	Middle SDI
Pernambuco	0.594	Low-middle SDI
Piauí	0.552	Low-middle SDI
Rio de Janeiro	0.709	High-middle SDI
Rio Grande do Norte	0.605	Low-middle SDI
Rio Grande do Sul	0.693	Middle SDI
Rondônia	0.622	Middle SDI
Roraima	0.646	Middle SDI
Santa Catarina	0.702	High-middle SDI
São Paulo	0.72	High-middle SDI
Sergipe	0.616	Middle SDI
Tocantins	0.611	Middle SDI
Paraguay	0.619	Middle SDI
North Africa and Middle East	0.639	
North Africa and Middle East	0.639	
Afghanistan	0.29	Low SDI
Algeria	0.696	Middle SDI
Bahrain	0.712	High-middle SDI
Egypt	0.604	Low-middle SDI
Iran	0.7	High-middle SDI
Iraq	0.585	Low-middle SDI
Jordan	0.697	Middle SDI
Kuwait	0.786	High-middle SDI
Lebanon	0.73	High-middle SDI
Libya	0.761	High-middle SDI
Morocco	0.579	Low-middle SDI
Palestine	0.541	Low-middle SDI
Oman	0.744	High-middle SDI
Qatar	0.766	High-middle SDI

Methods Appendix Table 4. Socio-Demographic Index groupings by geography, based on 2017 values		
**Geography**	**2017 SDI**	**SDI Quintile**
Saudi Arabia	0.779	High-middle SDI
Sudan	0.478	Low-middle SDI
Syria	0.611	Middle SDI
Tunisia	0.675	Middle SDI
Turkey	0.729	High-middle SDI
United Arab Emirates	0.795	High-middle SDI
Yemen	0.43	Low SDI
South Asia	0.534	
South Asia	0.534	
Bangladesh	0.458	Low SDI
Bhutan	0.57	Low-middle SDI
India	0.55	Low-middle SDI
Andhra Pradesh	0.536	Low-middle SDI
Arunachal Pradesh	0.556	Low-middle SDI
Assam	0.53	Low-middle SDI
Bihar	0.433	Low SDI
Chhattisgarh	0.512	Low-middle SDI
Delhi	0.715	High-middle SDI
Goa	0.74	High-middle SDI
Gujarat	0.584	Low-middle SDI
Haryana	0.6	Low-middle SDI
Himachal Pradesh	0.633	Middle SDI
Jammu and Kashmir	0.59	Low-middle SDI
Jharkhand	0.487	Low-middle SDI
Karnataka	0.574	Low-middle SDI
Kerala	0.659	Middle SDI
Madhya Pradesh	0.487	Low-middle SDI
Maharashtra	0.618	Middle SDI
Manipur	0.59	Low-middle SDI
Meghalaya	0.565	Low-middle SDI
Mizoram	0.616	Middle SDI
Nagaland	0.633	Middle SDI
Odisha	0.524	Low-middle SDI
Punjab	0.622	Middle SDI
Rajasthan	0.492	Low-middle SDI
Sikkim	0.628	Middle SDI
Tamil Nadu	0.615	Middle SDI
Telangana	0.575	Low-middle SDI
Tripura	0.543	Low-middle SDI
Uttar Pradesh	0.488	Low-middle SDI
Uttarakhand	0.607	Middle SDI
West Bengal	0.538	Low-middle SDI
Union Territories other than Delhi	0.653	Middle SDI
Nepal	0.429	Low SDI
Pakistan	0.492	Low-middle SDI

Methods Appendix Table 4. Socio-Demographic Index groupings by geography, based on 2017 values		
**Geography**	**2017 SDI**	**SDI Quintile**
Southeast Asia, East Asia, and Oceania	0.685	
East Asia	0.709	
China	0.707	High-middle SDI
North Korea	0.538	Low-middle SDI
Taiwan (Province of China)	0.864	High SDI
Oceania	0.471	
American Samoa	0.702	High-middle SDI
Federated States of Micronesia	0.575	Low-middle SDI
Fiji	0.641	Middle SDI
Guam	0.794	High-middle SDI
Kiribati	0.427	Low SDI
Marshall Islands	0.55	Low-middle SDI
Northern Mariana Islands	0.758	High-middle SDI
Papua New Guinea	0.419	Low SDI
Samoa	0.576	Low-middle SDI
Solomon Islands	0.425	Low SDI
Tonga	0.625	Middle SDI
Vanuatu	0.475	Low-middle SDI
Southeast Asia	0.641	
Cambodia	0.482	Low-middle SDI
Indonesia	0.648	Middle SDI
Laos	0.519	Low-middle SDI
Malaysia	0.759	High-middle SDI
Maldives	0.655	Middle SDI
Mauritius	0.72	High-middle SDI
Myanmar	0.556	Low-middle SDI
Philippines	0.617	Middle SDI
Sri Lanka	0.68	Middle SDI
Seychelles	0.692	Middle SDI
Thailand	0.684	Middle SDI
Timor-Leste	0.505	Low-middle SDI
Vietnam	0.607	Middle SDI
Sub-Saharan Africa	0.446	
Central sub-Saharan Africa	0.457	
Angola	0.461	Low-middle SDI
Central African Republic	0.334	Low SDI
Congo (Brazzaville)	0.574	Low-middle SDI
DR Congo	0.364	Low SDI
Equatorial Guinea	0.625	Middle SDI
Gabon	0.651	Middle SDI
Eastern sub-Saharan Africa	0.387	
Burundi	0.31	Low SDI
Comoros	0.434	Low SDI
Djibouti	0.485	Low-middle SDI
Eritrea	0.409	Low SDI

Methods Appendix Table 4. Socio-Demographic Index groupings by geography, based on 2017 values		
**Geography**	**2017 SDI**	**SDI Quintile**
Ethiopia	0.334	Low SDI
Kenya	0.499	Low-middle SDI
Baringo	0.444	Low-middle SDI
Bomet	0.496	Low-middle SDI
Bungoma	0.463	Low-middle SDI
Busia	0.438	Low-middle SDI
Elgeyo Marakwet	0.496	Low-middle SDI
Embu	0.533	Low-middle SDI
Garissa	0.334	Low-middle SDI
Homa Bay	0.425	Low-middle SDI
Isiolo	0.385	Low-middle SDI
Kajiado	0.534	Low-middle SDI
Kakamega	0.45	Low-middle SDI
Kericho	0.5	Low-middle SDI
Kiambu	0.58	Low-middle SDI
Kilifi	0.456	Low-middle SDI
Kirinyaga	0.533	Low-middle SDI
Kisii	0.522	Low-middle SDI
Kisumu	0.503	Low-middle SDI
Kitui	0.461	Low-middle SDI
Kwale	0.457	Low-middle SDI
Laikipia	0.556	Low-middle SDI
Lamu	0.453	Low-middle SDI
Machakos	0.518	Low-middle SDI
Makueni	0.469	Low-middle SDI
Mandera	0.295	Low-middle SDI
Marsabit	0.34	Low-middle SDI
Meru	0.508	Low-middle SDI
Migori	0.419	Low-middle SDI
Mombasa	0.568	Low-middle SDI
Murang’a	0.528	Low-middle SDI
Nairobi	0.674	Low-middle SDI
Nakuru	0.545	Low-middle SDI
Nandi	0.501	Low-middle SDI
Narok	0.402	Low-middle SDI
Nyamira	0.544	Low-middle SDI
Nyandarua	0.534	Low-middle SDI
Nyeri	0.554	Low-middle SDI
Samburu	0.308	Low-middle SDI
Siaya	0.46	Low-middle SDI
Taita Taveta	0.529	Low-middle SDI
Tana River	0.379	Low-middle SDI
Tharaka Nithi	0.528	Low-middle SDI
Trans Nzoia	0.496	Low-middle SDI
Turkana	0.295	Low-middle SDI

Methods Appendix Table 4. Socio-Demographic Index groupings by geography, based on 2017 values		
**Geography**	**2017 SDI**	**SDI Quintile**
Uasin Gishu	0.545	Low-middle SDI
Vihiga	0.477	Low-middle SDI
Wajir	0.243	Low-middle SDI
West Pokot	0.382	Low-middle SDI
Madagascar	0.331	Low SDI
Malawi	0.349	Low SDI
Mozambique	0.34	Low SDI
Rwanda	0.407	Low SDI
Somalia	0.235	Low SDI
South Sudan	0.275	Low SDI
Tanzania	0.412	Low SDI
Uganda	0.388	Low SDI
Zambia	0.472	Low-middle SDI
Southern sub-Saharan Africa	0.64	
Botswana	0.663	Middle SDI
Lesotho	0.493	Low-middle SDI
Namibia	0.616	Middle SDI
South Africa	0.677	Middle SDI
Swaziland	0.578	Low-middle SDI
Zimbabwe	0.463	Low-middle SDI
Western sub-Saharan Africa	0.441	
Benin	0.373	Low SDI
Burkina Faso	0.284	Low SDI
Cameroon	0.482	Low-middle SDI
Cape Verde	0.549	Low-middle SDI
Chad	0.253	Low SDI
Cote d'Ivoire	0.412	Low SDI
The Gambia	0.405	Low SDI
Ghana	0.537	Low-middle SDI
Guinea	0.325	Low SDI
Guinea-Bissau	0.349	Low SDI
Liberia	0.328	Low SDI
Mali	0.267	Low SDI
Mauritania	0.471	Low-middle SDI
Niger	0.191	Low SDI
Nigeria	0.493	Low-middle SDI
Sao Tome and Principe	0.488	Low-middle SDI
Senegal	0.373	Low SDI
Sierra Leone	0.357	Low SDI
Togo	0.413	Low SDI
