# Supplementary material for: Global, regional, and national disability-adjusted life-years (DALYs) for 359 diseases and injuries and healthy life expectancy (HALE) for 195 countries and territories, 1990–2017: a systematic analysis for the Global Burden of Disease Study 2017
Source: Lancet. Author manuscript; Available in PMC 2018 Nov 24. (PMC6252083; doi:10.1016/S0140-6736(18)32335-3)
Supplement: Supplementary appendix 2 [file NIHMS80527-supplement-Supplementary_appendix_2.pdf]

THE LANCET

Supplementary appendix 2

This appendix formed part of the original submission and has been peer reviewed. We post it as supplied by the authors.

Supplement to: GBD 2017 DALYs and HALE Collaborators. Global, regional, and national disability-adjusted life-years (DALYs) for 359 diseases and injuries and healthy life expectancy (HALE) for 195 countries and territories, 1990–2017: a systematic analysis for the Global Burden of Disease Study 2017. *Lancet* 2018; **392:** 1859–1922.

Supplementary Results Appendix to Global, regional, and national disability-adjusted life-years (DALYs) for 359 diseases and injuries and healthy life expectancy (HALE) for 195 countries and territories, 1990–2017: a systematic analysis for the Global Burden of Disease Study 2017

This appendix provides supplementary results for disability-adjusted life years (DALYs) and healthy life expectancy (HALE).

Preamble

This study complies with the Guidelines for Accurate and Transparent Health Estimates Reporting (GATHER) recommendations. It includes detailed tables and information on data in an effort to maximize transparency in our estimation processes and provide a comprehensive description of analytical steps. We intend this to be a living document, to be updated with each annual iteration of the Global Burden of Disease.

Table of Contents

Preamble 1

List of Supplemental Results Figures and Tables 2

- SR Appendix Figure 1........ 4
- SR Appendix Figure 2........ 6
- SR Appendix Figure 3........ 7
- SR Appendix Figure 4........ 9
- SR Appendix Figure 5........ 10
- SR Appendix Figure 6........ 12
- SR Appendix Figure 7........ 16
- SR Appendix Figure 8........ 17
- SR Appendix Figure 9........ 21
- SR Appendix Figure 10........ 23
- SR Appendix Figure 11........ 33
- SR Appendix Figure 12........ 34
- SR Appendix Figure 13........ 35
- SR Appendix Table 1........ 36
- SR Appendix Table 2........ 41
- SR Appendix Tables 3........ 5653
- SR Appendix Tables 4........ 16969
- SR Appendix Table 5........ 18195

List of Supplemental Results Figures and Tables

SR Appendix Figure 1a-b. Life expectancy compared to HALE at birth (A) and age 65 (B) for 21 GBD regions and 195 countries and territories, by sex in 2017

SR Appendix Figure 2. Life expectancy at birth for females compared to males for 21 GBD regions and 195 countries and territories in 2017

SR Appendix Figure 3. Years of life gained at birth by sex and functional health status for 195 countries and territories from 1990 to 2017

SR Appendix Figure 4. Years of life gained at age 65 by sex and functional health status for five SDI quintiles, 21 GBD regions, and 28 countries with the largest and smallest percentages of years spent in poor health from 1990 to 2017

SR Appendix Figure 5. Years of life gained at age 65 by sex and functional health status for 195 countries and territories from 1990 to 2017

SR Appendix Figure 6. Extra years of life expected at birth for females compared to males by functional health status for 195 countries and territories in 2017

SR Appendix Figure 7. Extra years of life expected at age 65 for females compared to males by functional health status for five SDI quintiles, 21 GBD regions, and 28 countries with the largest and smallest percentages of years spent in poor health in 2017

SR Appendix Figure 8. Extra years of life expected at age 65 for females compared to males by functional health status for 195 countries and territories in 2017

SR Appendix Figure 9a-d. Leading level 3 causes of global all-age number of DALYs by location for Females in 1990 (A) Males in 1990 (B) Females in 2017 (C) and Males in 2017 (D)

SR Appendix Figure 10. Leading 30 level 3 causes of global DALYs for 1990, 2007, and 2017 with percent change in number of DALYs, and age-standardised DALY rates for low SDI males (A) low SDI females (B) low-middle SDI males (C) low-middle SDI females (D) middle SDI males (E) middle SDI females (F) high-middle SDI males (G) high-middle SDI females (H) high SDI males (I) high SDI females (J)

SR Appendix Figure 11a-b. Difference in years between observed and expected HALE at age 65 on the basis of SDI for females (A) and males (B), 2017

SR Appendix Figure 12a-b. Ratio of observed to expected age−standardized DALY rates per 100,000 on the basis of SDI for females (A) and males (B), 2017

SR Appendix Figure 13. Difference in HALE at birth between females and males globally and for 21 GBD regions, from 1990 to 2017

SR Appendix Table 1. Life expectancy and healthy life expectancy at birth and at age 65 by location for 1990, 2007, and 2017, both sexes combined

SR Appendix Table 2. All-age DALYs counts and age standardized DALYs rates per 100,000 individuals for all causes and level 1 cause groups by location for 1990, 2007, and 2017, both sexes combined

SR Appendix Tables 3a-c. Global DALYS by cause and age group for Males (A) Females (B) both sexes combined (C) with 95% uncertainty intervals, by location for 2017

SR Appendix Tables 4a-g. Percentage change in DALYs between 1990 and 2007, and 1990 and 2017 for global (A) low SDI quintile (B) low-middle SDI quintile (C) middle SDI quintile (D) high-middle SDI quintile (E) high SDI quintile (F) and 195 countries and territories (G), for all causes, both sexes

SR Appendix Table 5. Total years of life gained between 1990 and 2017, and number of years gained spent in good health and poor health, all ages, both sexes

SR Appendix Figure 1a-b. Life expectancy compared to HALE at birth (A) and age 65 (B) for 21 GBD regions and 195 countries and territories, by sex in 2017

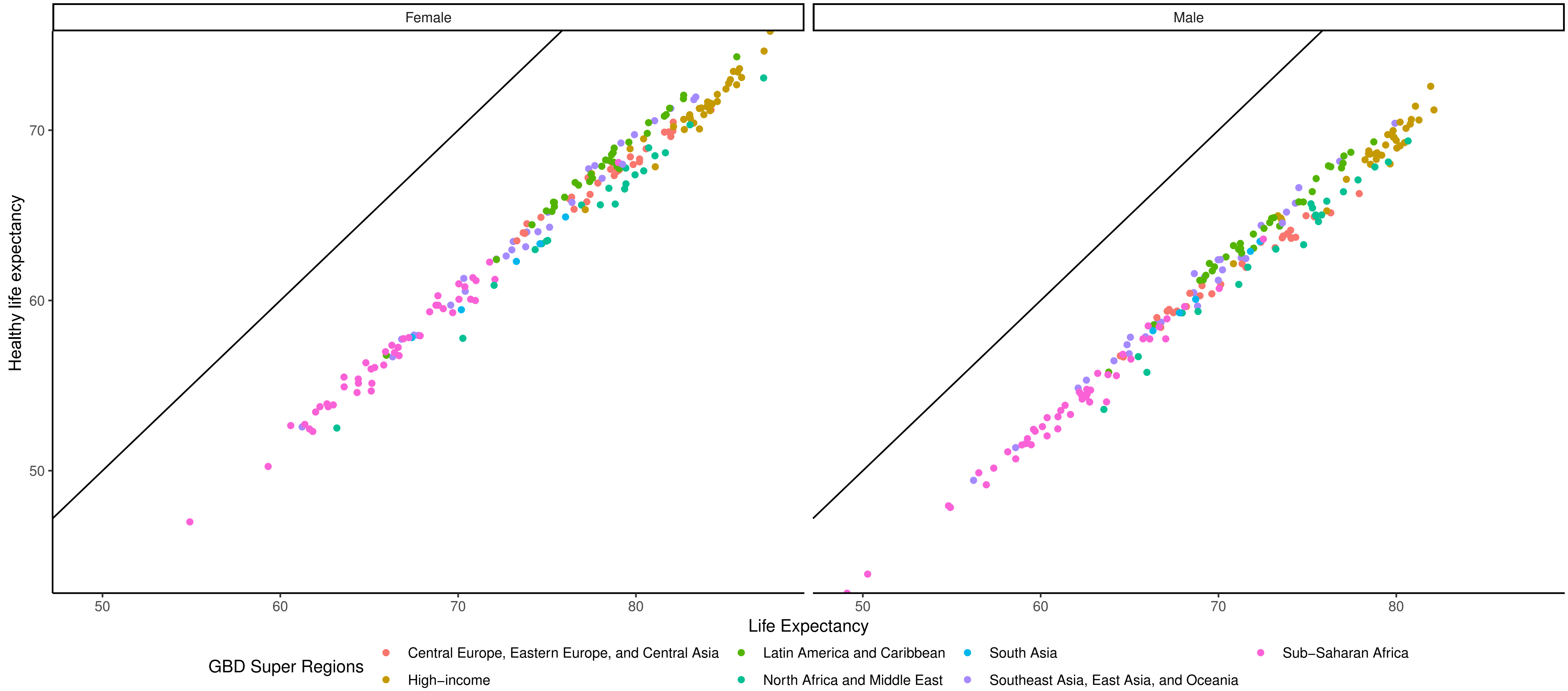

(A) At birth

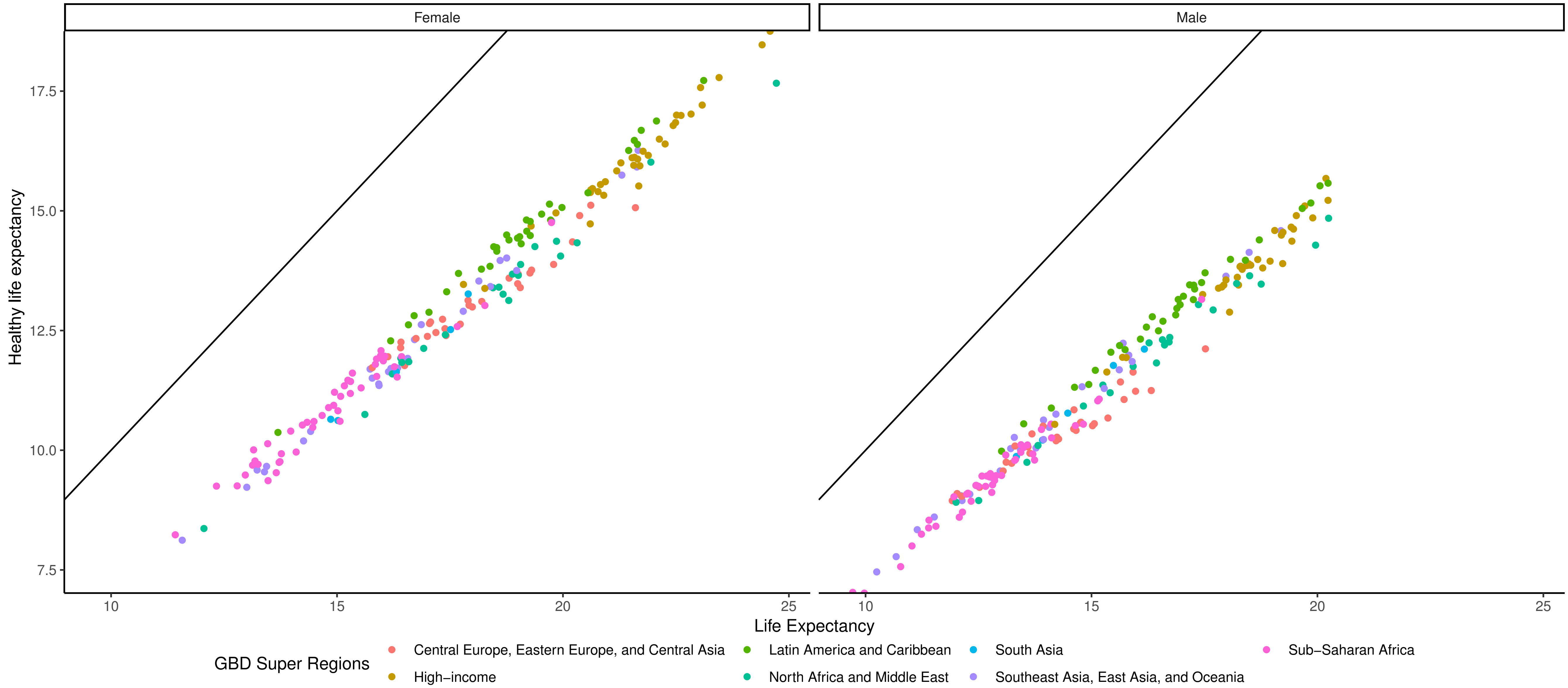

Female
Male
Healthy life expectancy
17.5
15.0
12.5
10.0
7.5
10
15
20
25
Life Expectancy
GBD Super Regions
Central Europe, Eastern Europe, and Central Asia
High−income
Latin America and Caribbean
North Africa and Middle East
South Asia
Southeast Asia, East Asia, and Oceania
Sub−Saharan Africa

(B) Age 65

SR Appendix Figure 2. Life expectancy at birth for females compared to males for 21 GBD regions and 195 countries and territories in 2017

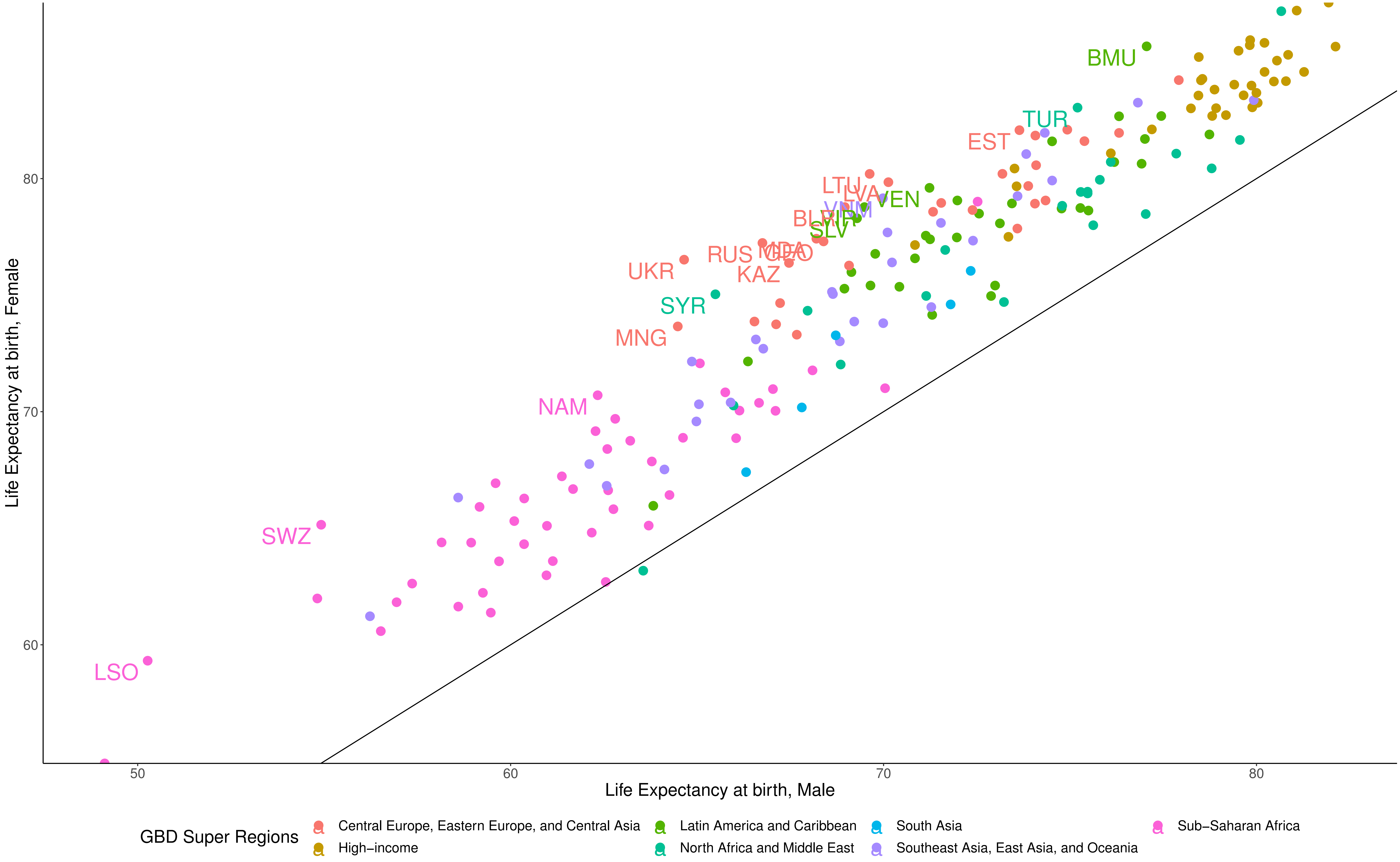

Locations where there is no statistically significant difference between male and female life expectancy at birth: Haiti, Bolivia, Honduras, Morocco, Qatar, Afghanistan, Pakistan, Congo (Brazzaville), Madagascar, Liberia, Mali, Mauritania, Niger, Sierra Leone, Sudan

SR Appendix Figure 3. Years of life gained at birth by sex and functional health status for 195 countries and territories from 1990 to 2017

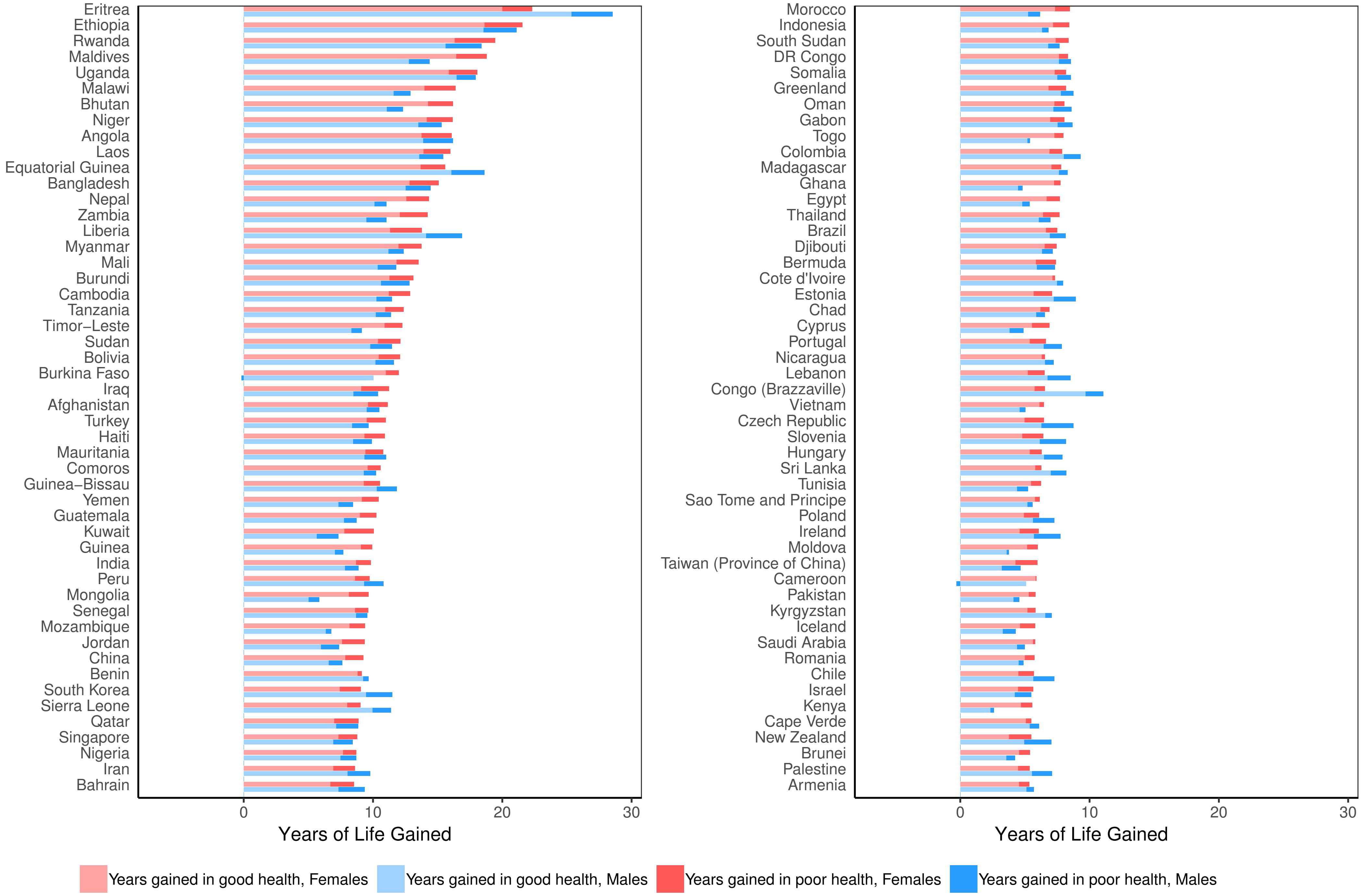

Locations where years gained by females or males were not statistically significant:
American Samoa, Andorra, Antigua and Barbuda, Botswana, Dominica, Dominican Republic, Fiji, Guam, Jamaica, Libya, Marshall Islands, North Korea, Northern Mariana Islands, Paraguay, Philippines, Saint Vincent and the Grenadines, Samoa, South Africa, Swaziland, Tonga, United Arab Emirates, Uzbekistan, Vanuatu, Venezuela, Zimbabwe

SR Appendix Figure 3. Years of life gained at birth by sex and functional health status for 195 countries and territories from 1990 to 2017

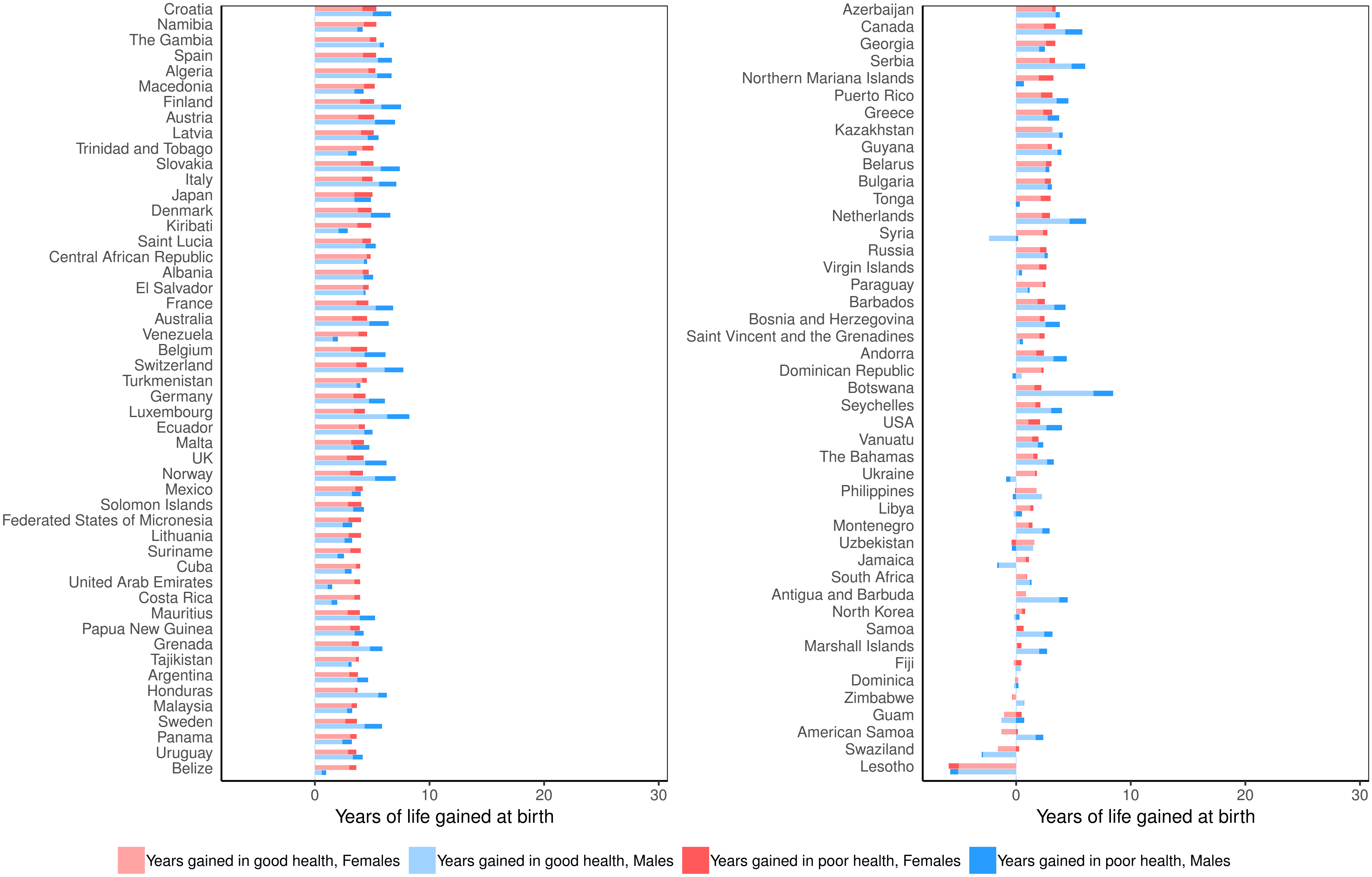

Locations where years gained by females or males were not statistically significant:
American Samoa, Andorra, Antigua and Barbuda, Botswana, Dominica, Dominican Republic, Fiji, Guam, Jamaica, Libya, Marshall Islands, North Korea, Northern Mariana Islands, Paraguay, Philippines, Saint Vincent and the Grenadines, Samoa, South Africa, Swaziland, Tonga, United Arab Emirates, Uzbekistan, Vanuatu, Venezuela, Zimbabwe

SR Appendix Figure 4. Years of life gained at age 65 by sex and functional health status for five SDI quintiles, 21 GBD regions, and 28 countries with the largest and smallest percentages of years spent in poor health from 1990 to 2017

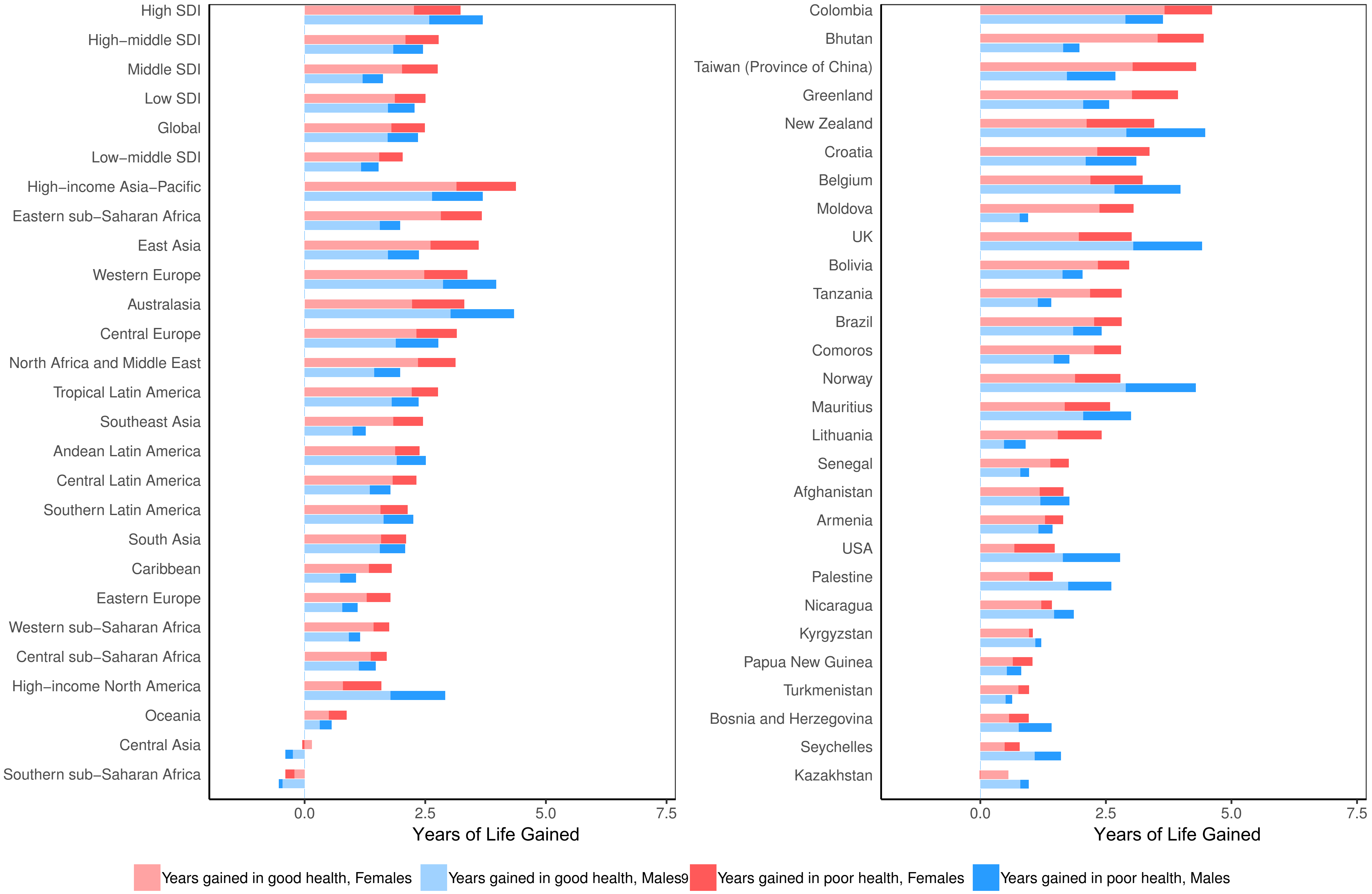

SR Appendix Figure 5. Years of life gained at age 65 by sex and functional health status for 195 countries and territories from 1990 to 2017

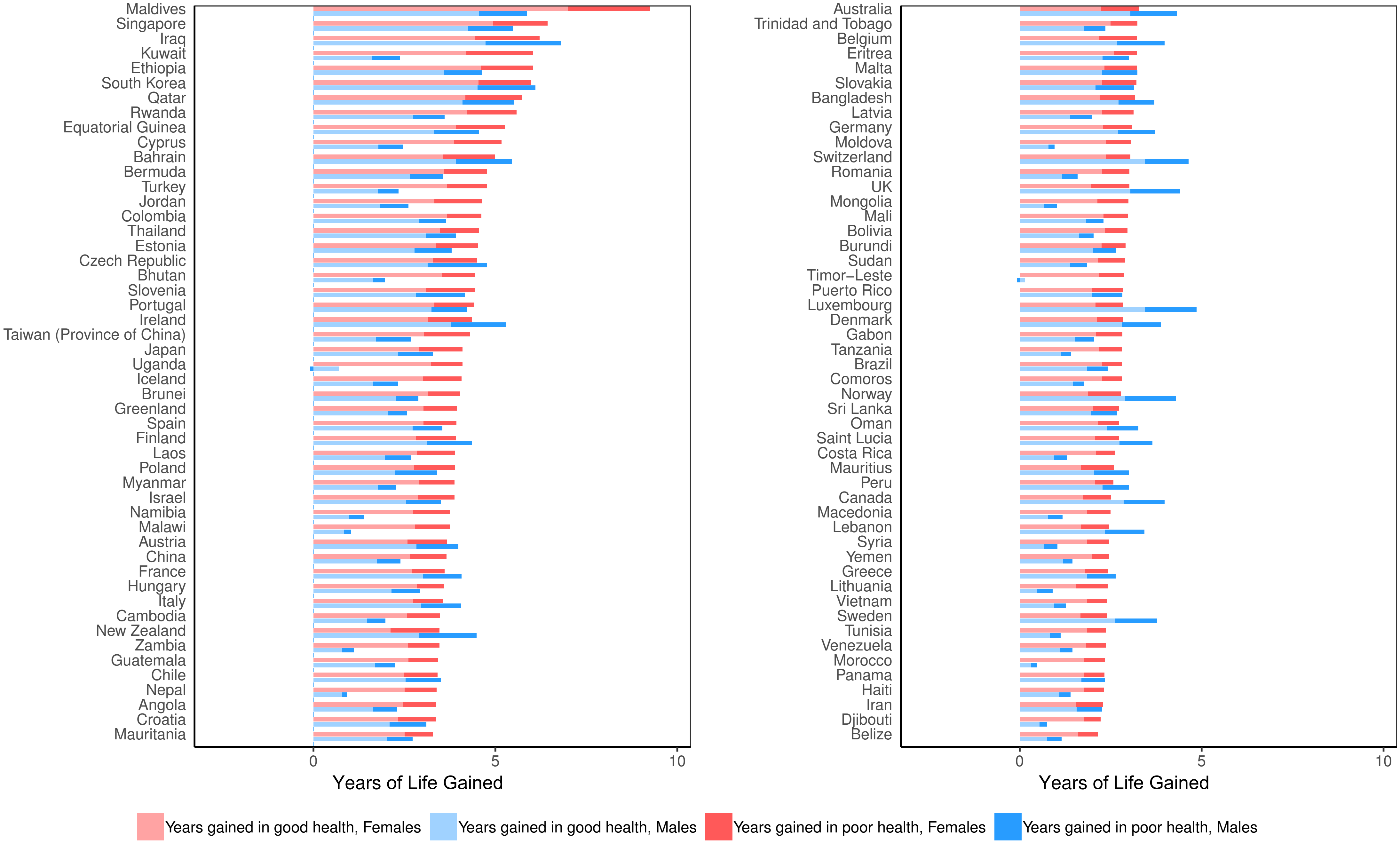

Locations where years gained by females or males were not statistically significant:
Albania, American Samoa, Andorra, Antigua and Barbuda, Azerbaijan, Belarus, Benin, Botswana, Burkina Faso, Cameroon, Central African Republic, Chad, Cote d'Ivoire, Djibouti, Dominican Republic, Egypt, El Salvador, Federated States of Micronesia, Fiji, Georgia, Ghana, Guam, Guinea, Honduras, Indonesia, Jamaica, Kiribati, Lesotho, Liberia, Libya, Madagascar, Marshall Islands, Montenegro, Morocco, Mozambique, Nepal, Nigeria, North Korea, Northern Mariana Islands, Pakistan, Paraguay, Philippines, Saint Vincent and the Grenadines, Samoa, Sao Tome and Principe, Saudi Arabia, Sierra Leone, Solomon Islands, Somalia, South Sudan, Suriname, Swaziland, Syria, Tajikistan, The Bahamas, The Gambia, Timor−Leste, Togo, Tonga, Tunisia, Uganda, Ukraine, United Arab Emirates, Vanuatu, Yemen, Zimbabwe

SR Appendix Figure 5. Years of life gained at age 65 by sex and functional health status for 195 countries and territories from 1990 to 2017

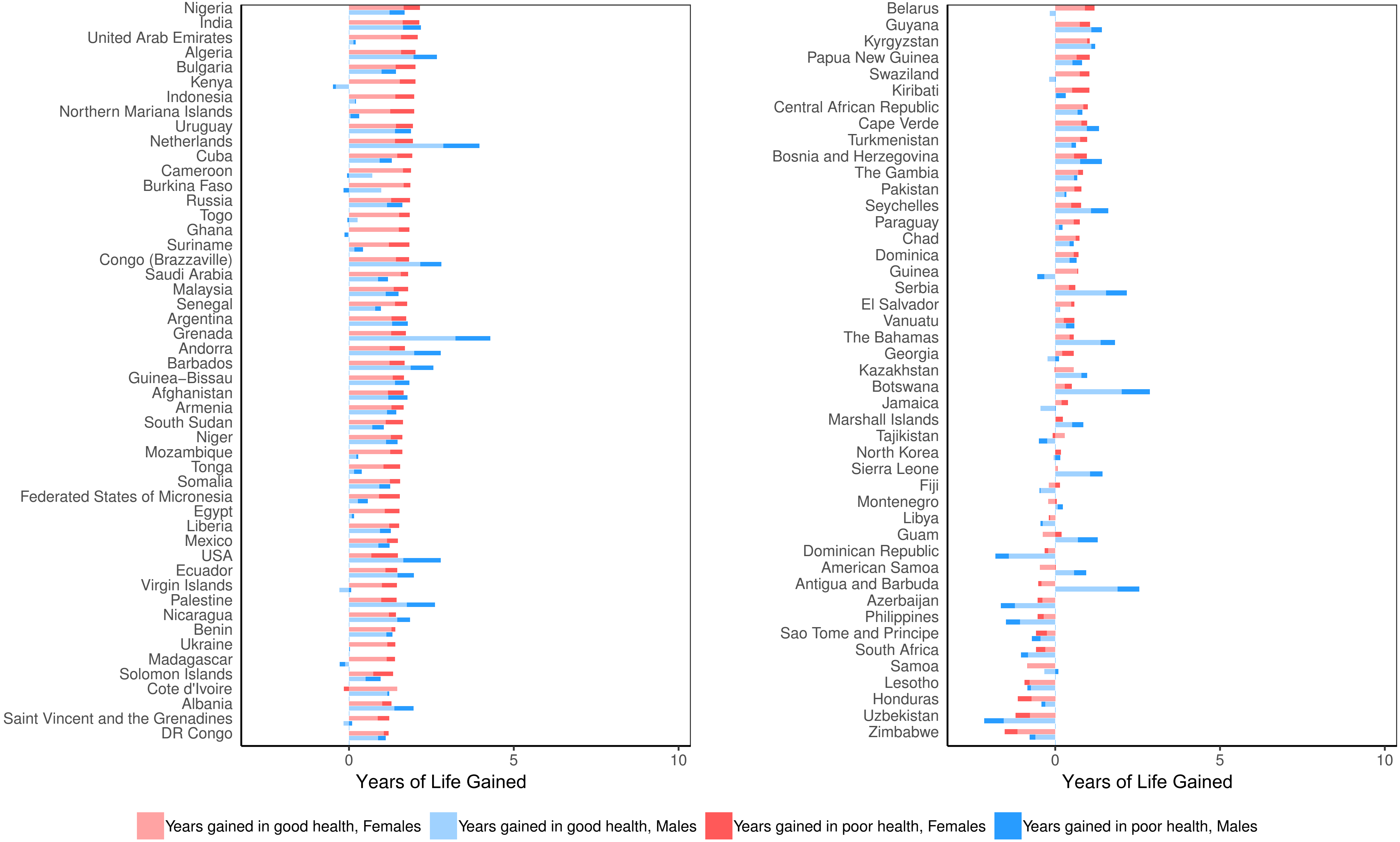

Locations where years gained by females or males were not statistically significant:

Albania, American Samoa, Andorra, Antigua and Barbuda, Azerbaijan, Belarus, Benin, Botswana, Burkina Faso, Cameroon, Central African Republic, Chad, Cote d'Ivoire, Djibouti, Dominican Republic, Egypt, El Salvador, Federated States of Micronesia, Fiji, Georgia, Ghana, Guam, Guinea, Honduras, Indonesia, Jamaica, Kiribati, Lesotho, Liberia, Libya, Madagascar, Marshall Islands, Montenegro, Morocco, Mozambique, Nepal, Nigeria, North Korea, Northern Mariana Islands, Pakistan, Paraguay, Philippines, Saint Vincent and the Grenadines, Samoa, Sao Tome and Principe, Saudi Arabia, Sierra Leone, Solomon Islands, Somalia, South Sudan, Suriname, Swaziland, Syria, Tajikistan, The Bahamas, The Gambia, Timor−Leste, Togo, Tonga, Tunisia, Uganda, Ukraine, United Arab Emirates, Vanuatu, Yemen, Zimbabwe

SR Appendix Figure 6. Extra years of life expected at birth for females compared to males by functional health status for 195 countries and territories in 2017

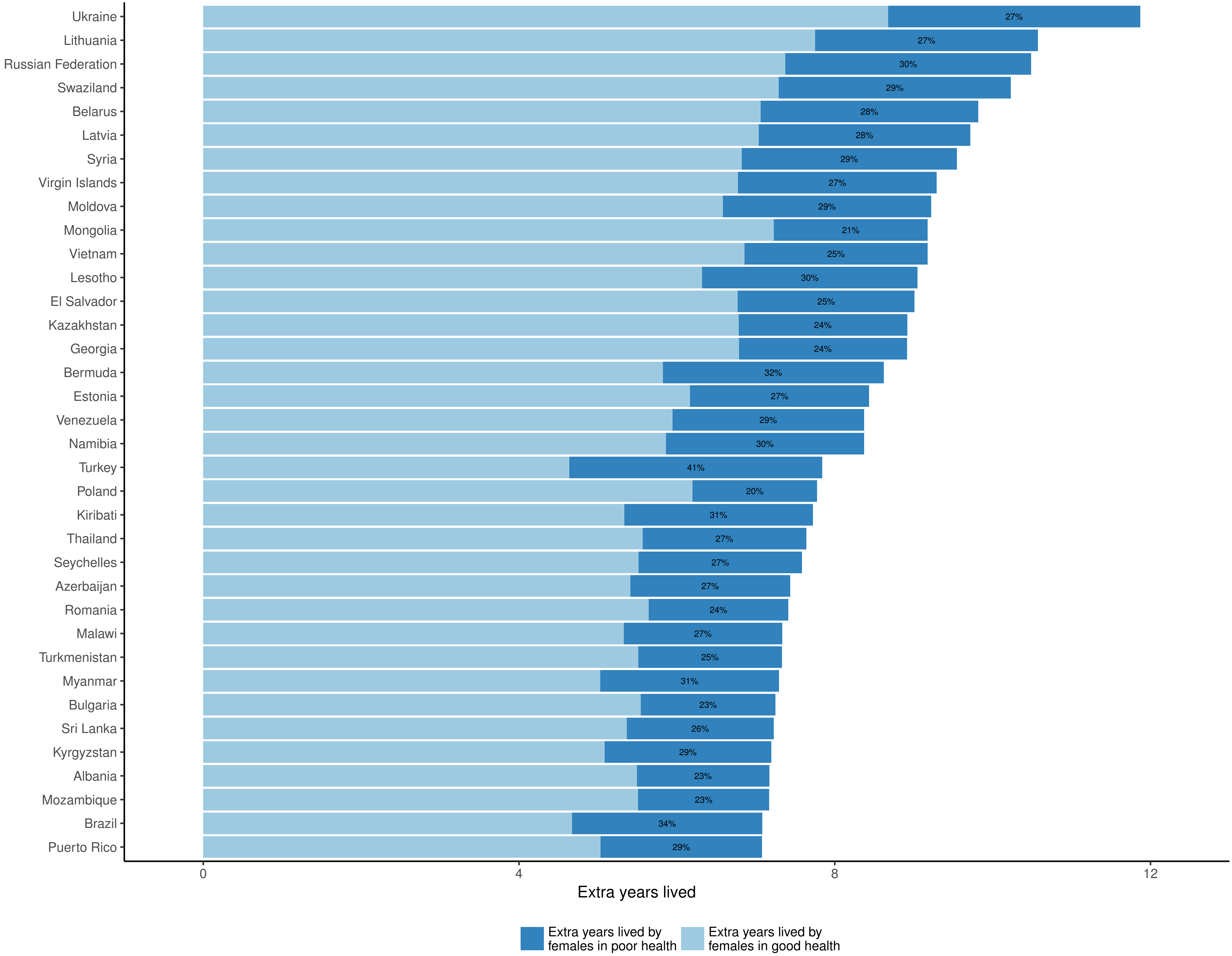

Locations where extra years lived by females were not statistically significant:
Haiti, Bolivia, Honduras, Morocco, Qatar, Afghanistan, Pakistan, Congo (Brazzaville), Madagascar, Liberia, Mali, Mauritania, Niger, Sierra Leone, Sudan

SR Appendix Figure 6. Extra years of life expected at birth for females compared to males by functional health status for 195 countries and territories in 2017

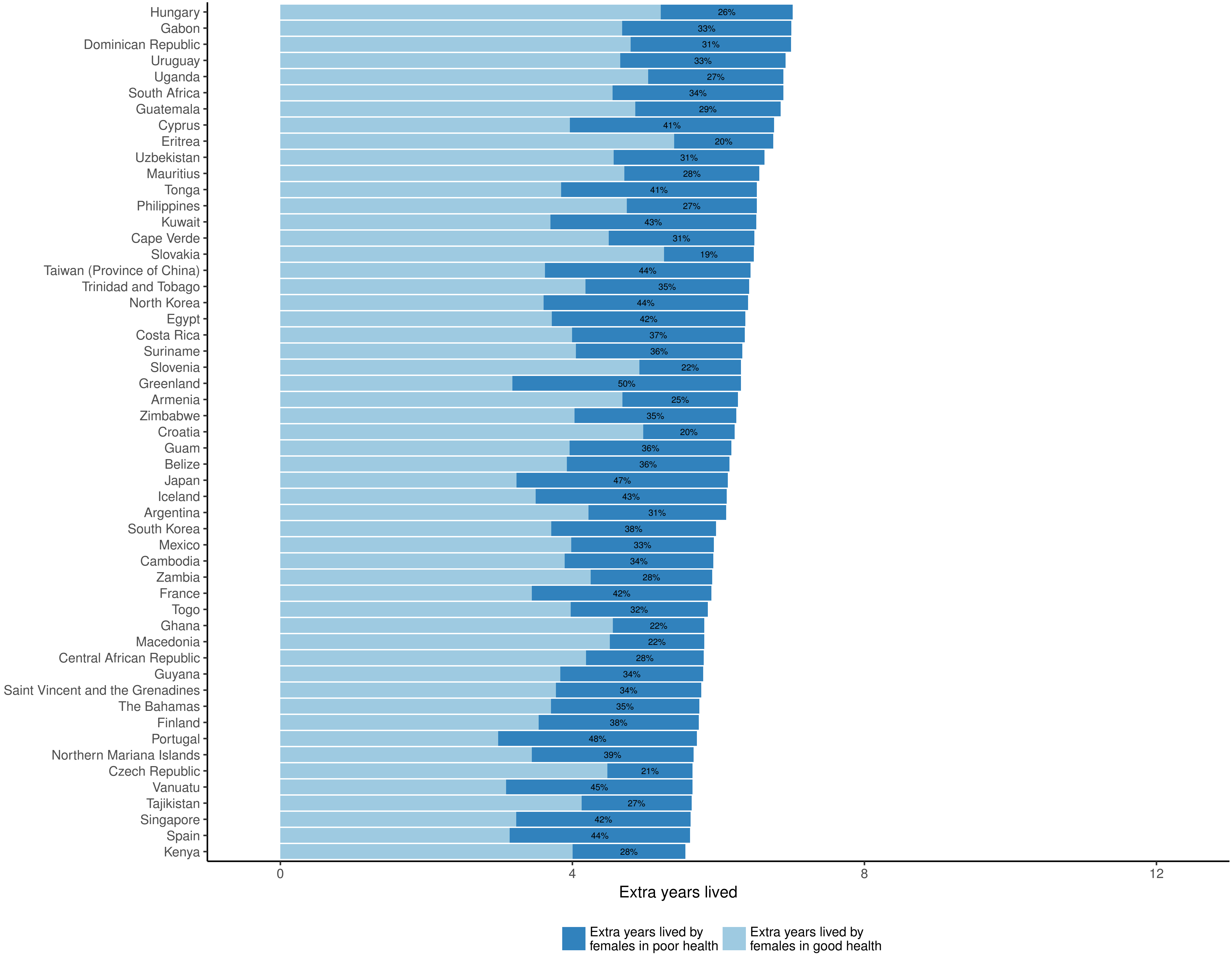

Locations where extra years lived by females were not statistically significant:
Haiti, Bolivia, Honduras, Morocco, Qatar, Afghanistan, Pakistan, Congo (Brazzaville), Madagascar, Liberia, Mali, Mauritania, Niger, Sierra Leone, Sudan

SR Appendix Figure 6. Extra years of life expected at birth for females compared to males by functional health status for 195 countries and territories in 2017

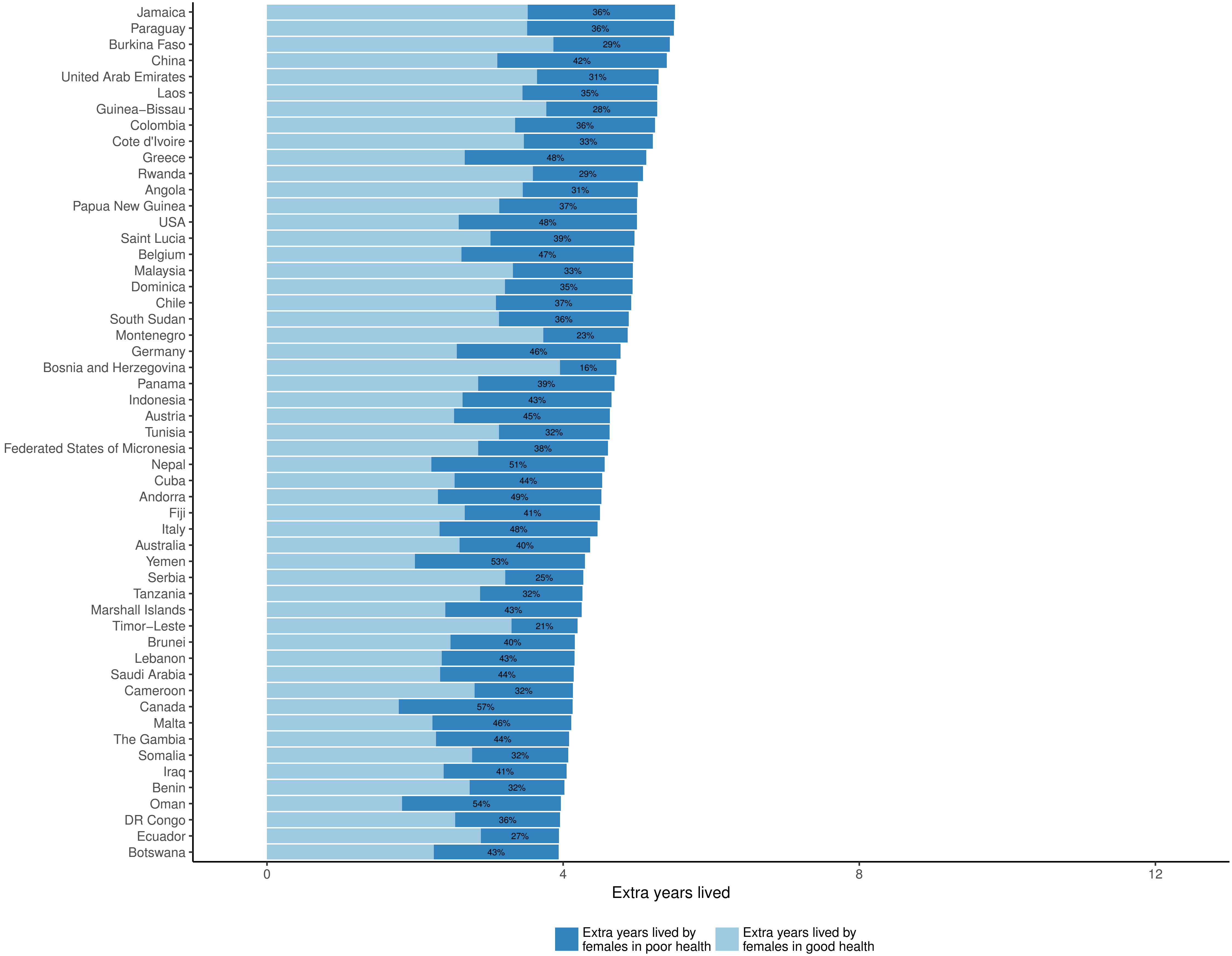

Locations where extra years lived by females were not statistically significant:
Haiti, Bolivia, Honduras, Morocco, Qatar, Afghanistan, Pakistan, Congo (Brazzaville), Madagascar, Liberia, Mali, Mauritania, Niger, Sierra Leone, Sudan

SR Appendix Figure 6. Extra years of life expected at birth for females compared to males by functional health status for 195 countries and territories in 2017

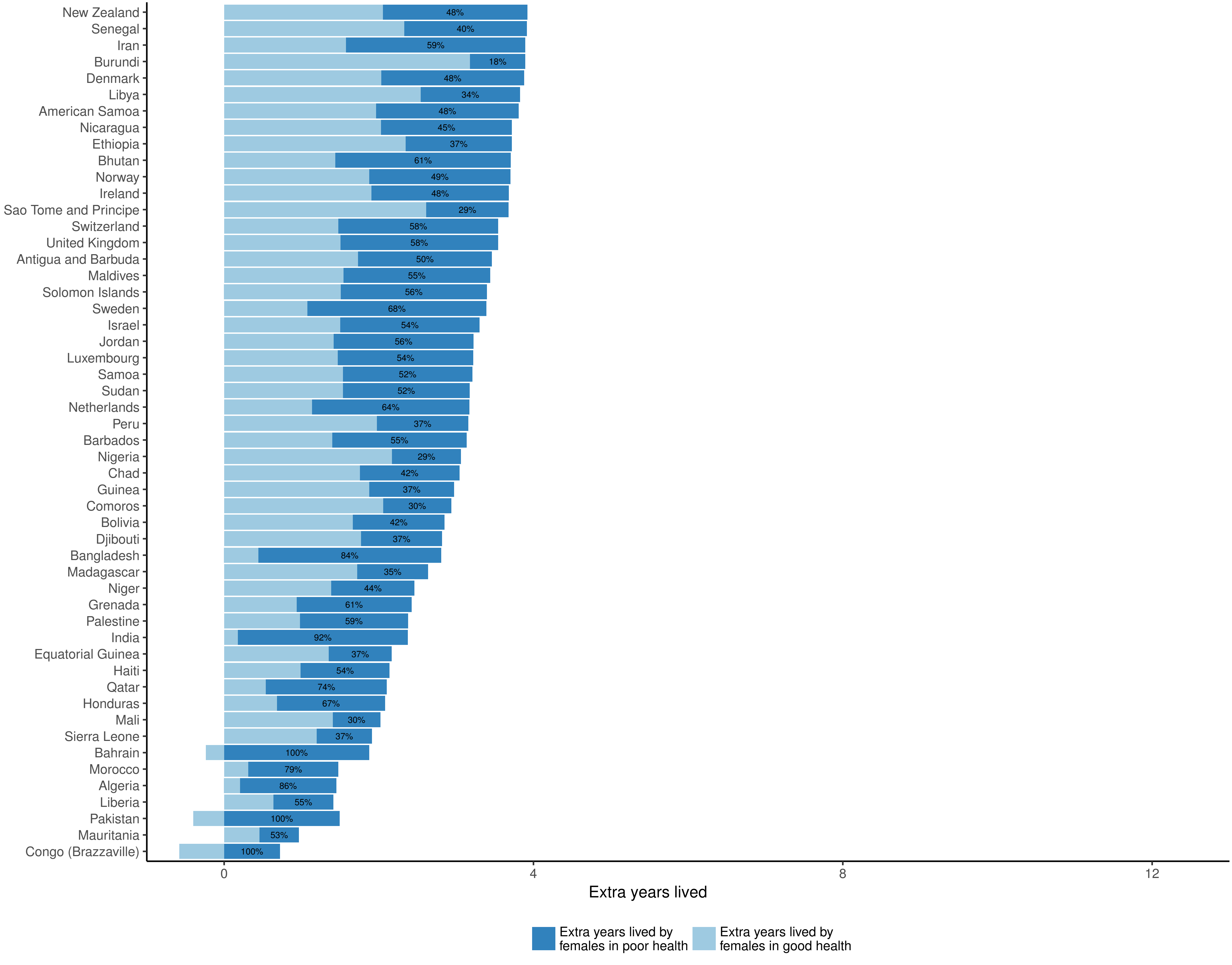

Locations where extra years lived by females were not statistically significant:
Haiti, Bolivia, Honduras, Morocco, Qatar, Afghanistan, Pakistan, Congo (Brazzaville), Madagascar, Liberia, Mali, Mauritania, Niger, Sierra Leone, Sudan

SR Appendix Figure 7. Extra years of life expected at age 65 for females compared to males by functional health status for five SDI quintiles, 21 GBD regions, and 28 countries with the largest and smallest percentages of years spent in poor health in 2017

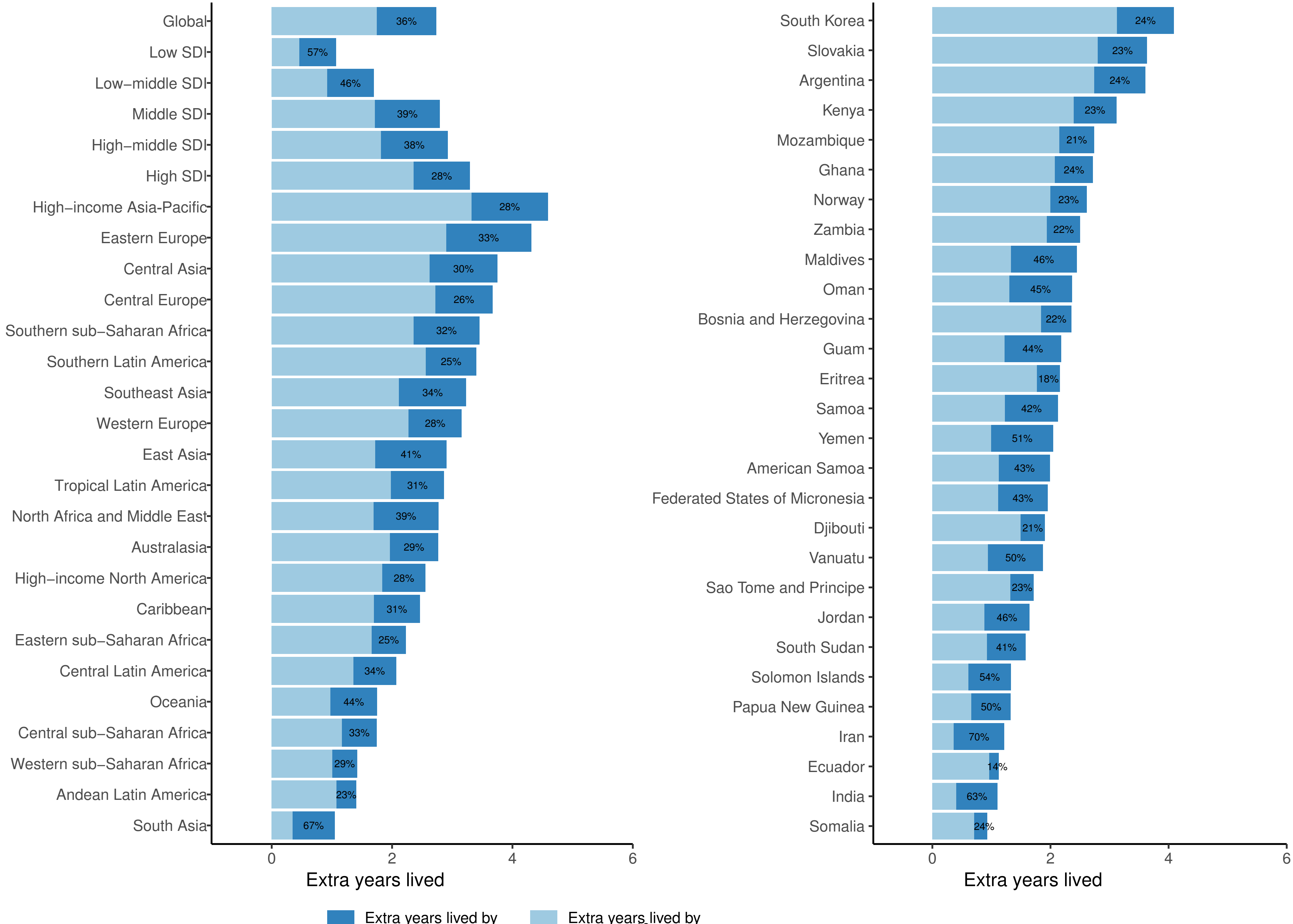

SDI = Socio−demographic Index. GBD = Global Burden of Disease.

SR Appendix Figure 8. Extra years of life expected at age 65 for females compared to males by functional health status for 195 countries and territories in 2017

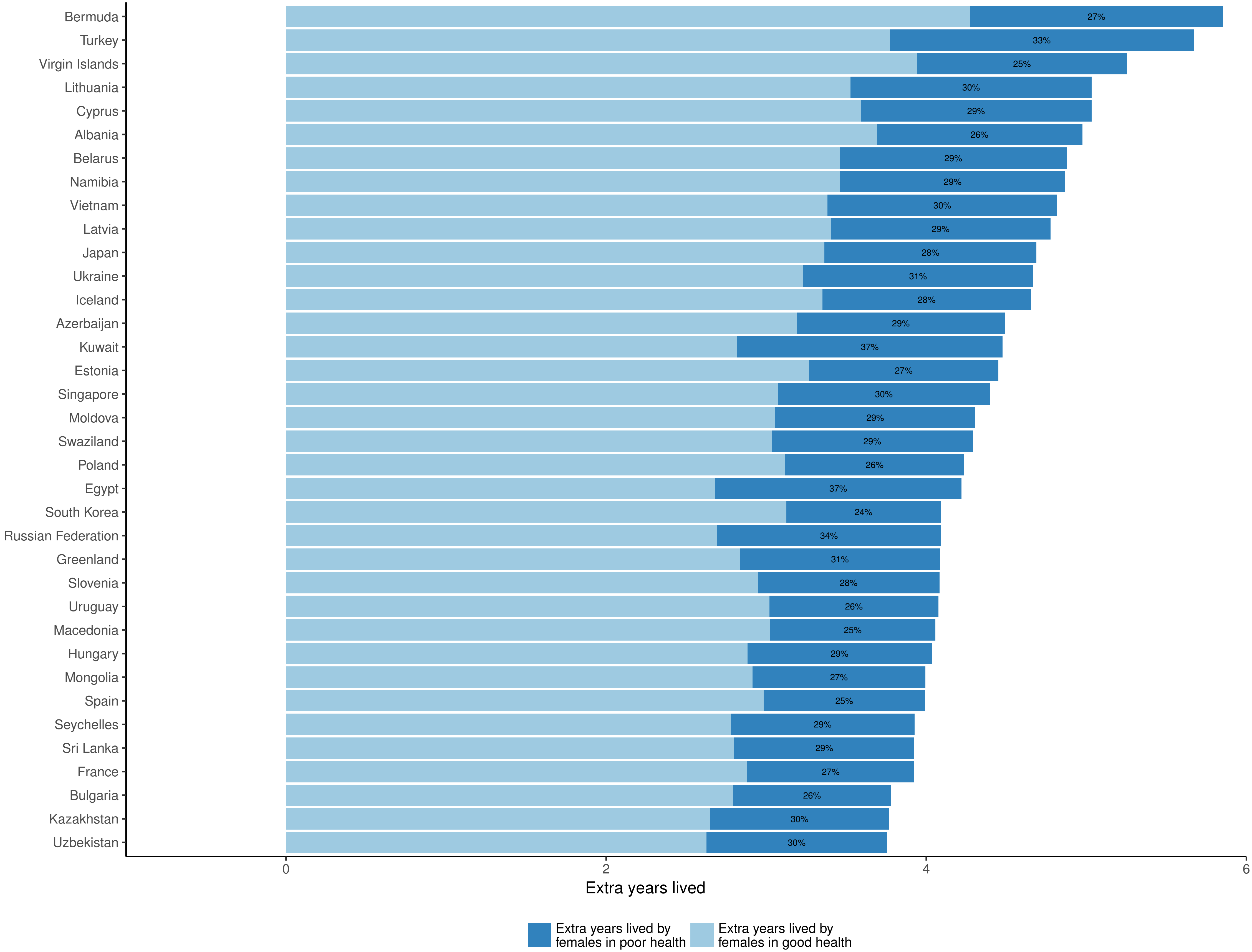

Locations where extra years lived by females were not statistically significant:
Grenada, Haiti, Bolivia, Peru, Honduras, Nicaragua, Algeria, Bahrain, Iraq, Libya, Morocco, Qatar, Afghanistan, Pakistan, Congo (Brazzaville), DR Congo, Burundi, Madagascar, Benin, Chad, Guinea, Liberia, Mali, Mauritania, Niger, Sierra Leone, Sudan

SR Appendix Figure 8. Extra years of life expected at age 65 for females compared to males by functional health status for 195 countries and territories in 2017

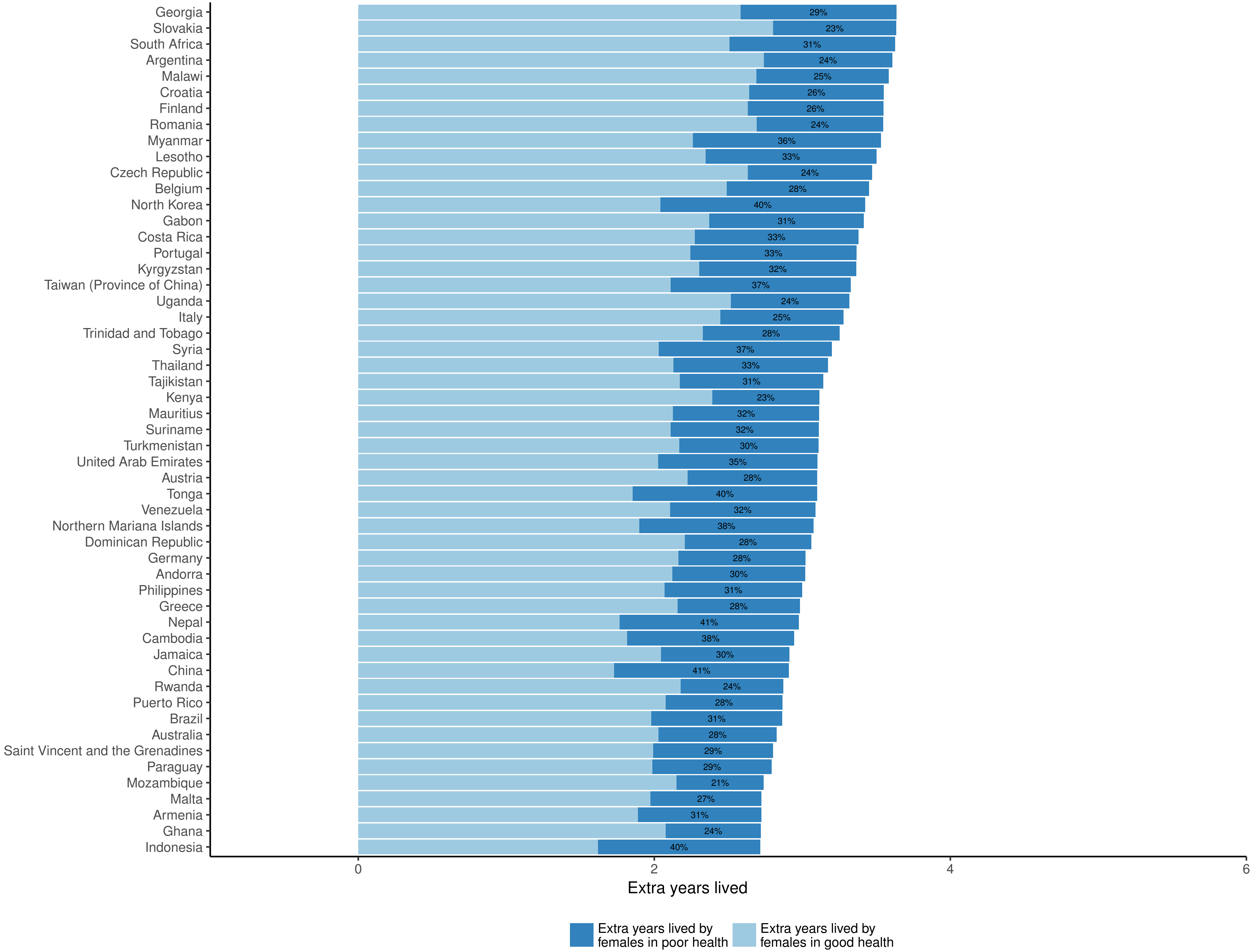

Locations where extra years lived by females were not statistically significant:
Grenada, Haiti, Bolivia, Peru, Honduras, Nicaragua, Algeria, Bahrain, Iraq, Libya, Morocco, Qatar, Afghanistan, Pakistan, Congo (Brazzaville), DR Congo, Burundi, Madagascar, Benin, Chad, Guinea, Liberia, Mali, Mauritania, Niger, Sierra Leone, Sudan

SR Appendix Figure 8. Extra years of life expected at age 65 for females compared to males by functional health status for 195 countries and territories in 2017

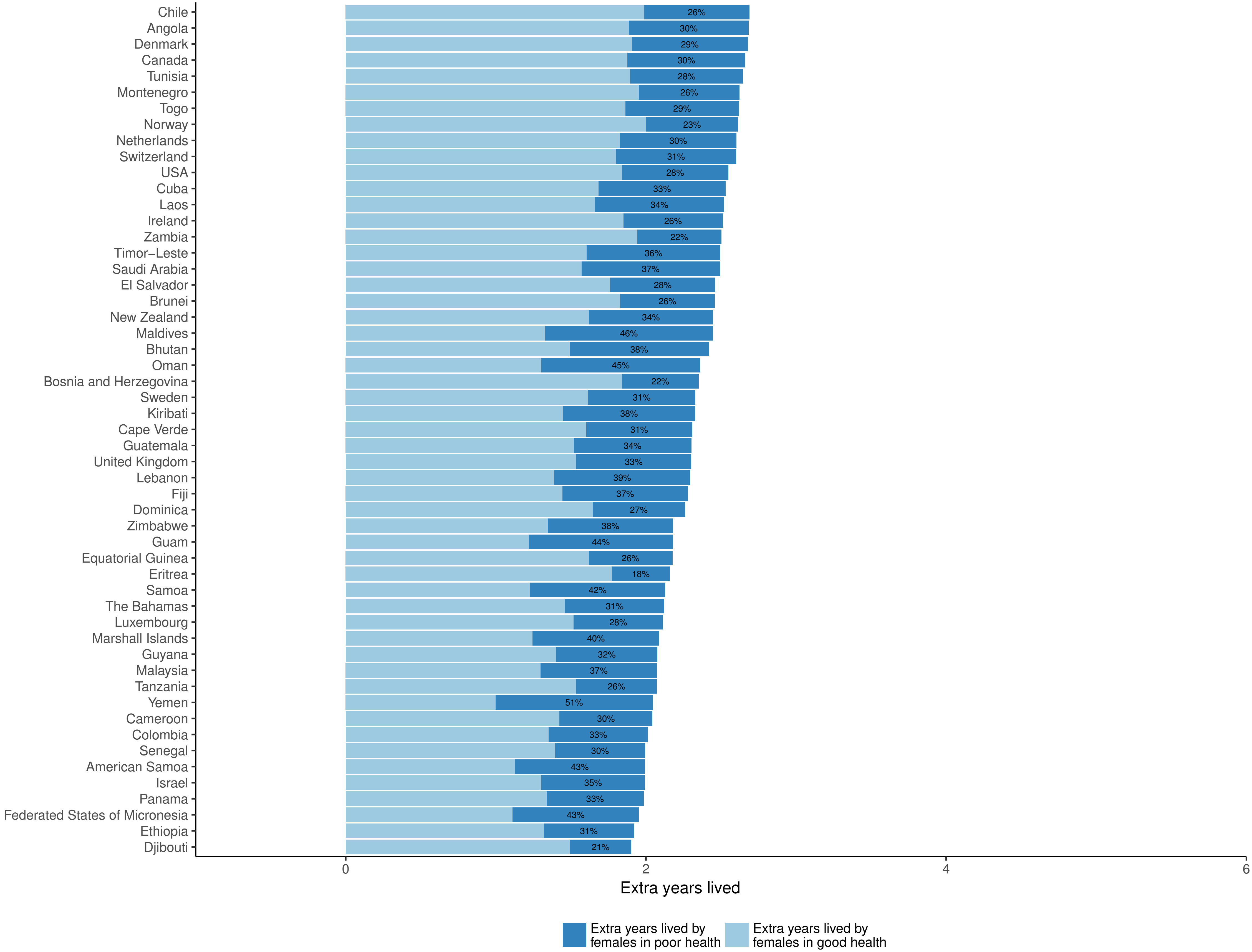

Locations where extra years lived by females were not statistically significant:
Grenada, Haiti, Bolivia, Peru, Honduras, Nicaragua, Algeria, Bahrain, Iraq, Libya, Morocco, Qatar, Afghanistan, Pakistan, Congo (Brazzaville), DR Congo, Burundi, Madagascar, Benin, Chad, Guinea, Liberia, Mali, Mauritania, Niger, Sierra Leone, Sudan

SR Appendix Figure 8. Extra years of life expected at age 65 for females compared to males by functional health status for 195 countries and territories in 2017

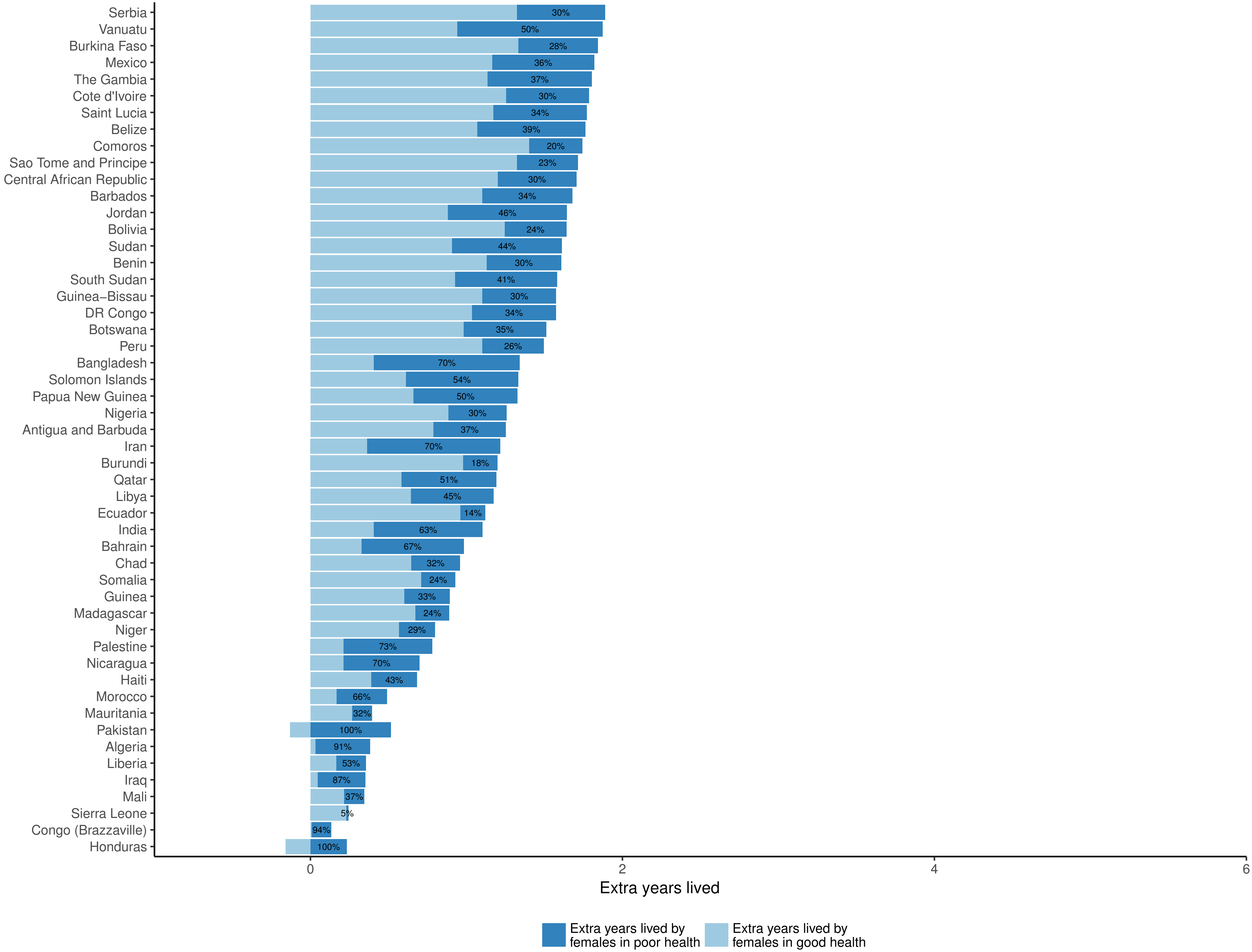

Locations where extra years lived by females were not statistically significant:
Grenada, Haiti, Bolivia, Peru, Honduras, Nicaragua, Algeria, Bahrain, Iraq, Libya, Morocco, Qatar, Afghanistan, Pakistan, Congo (Brazzaville), DR Congo, Burundi, Madagascar, Benin, Chad, Guinea, Liberia, Mali, Mauritania, Niger, Sierra Leone, Sudan

SR Appendix Figure 9a–d. Leading level 3 causes of global all-age number of DALYs by location for Females in 1990 (A) Males in 1990 (B) Females in 2017 (C) and Males in 2017 (D)

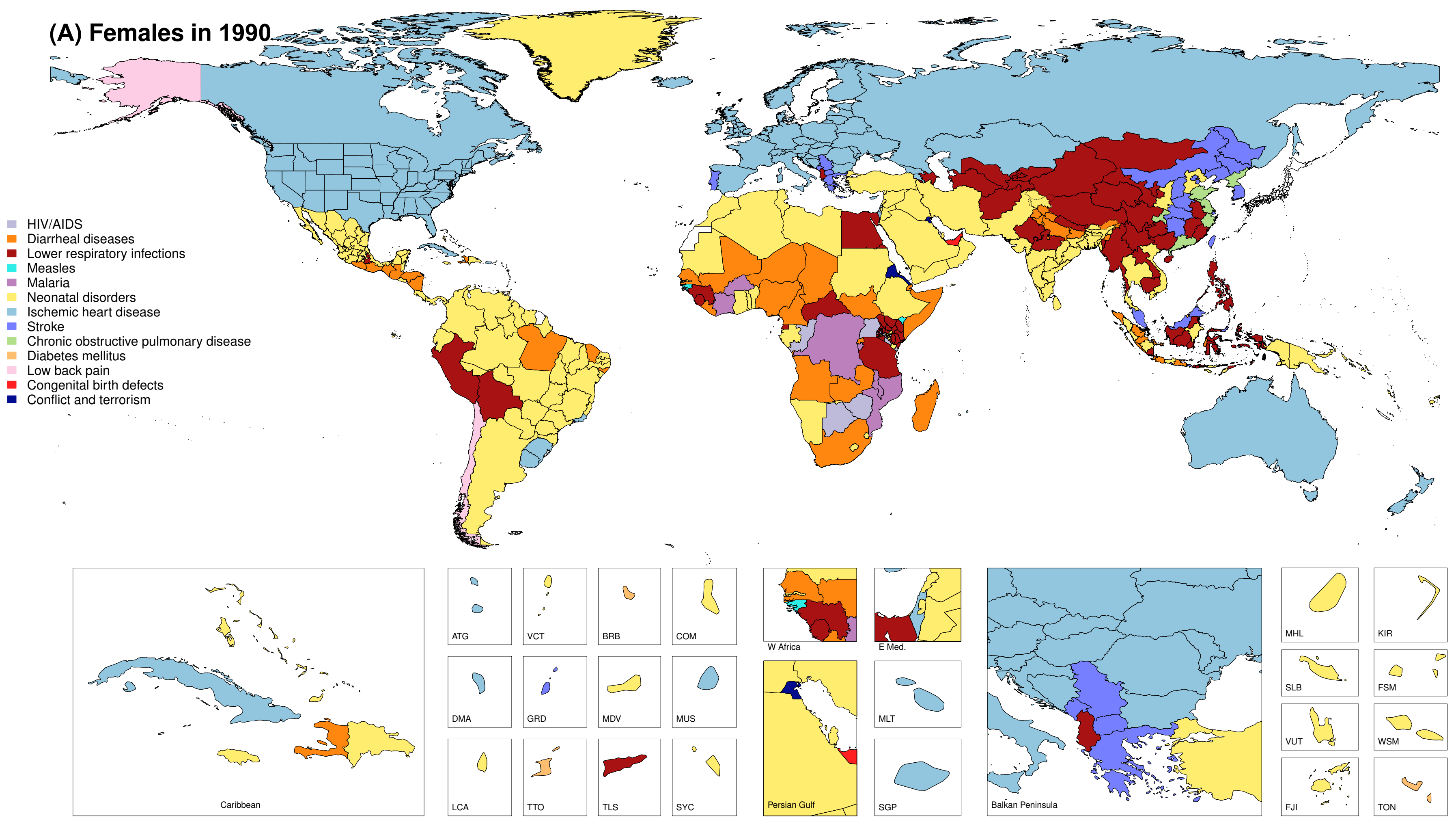

(B) Males in 1990

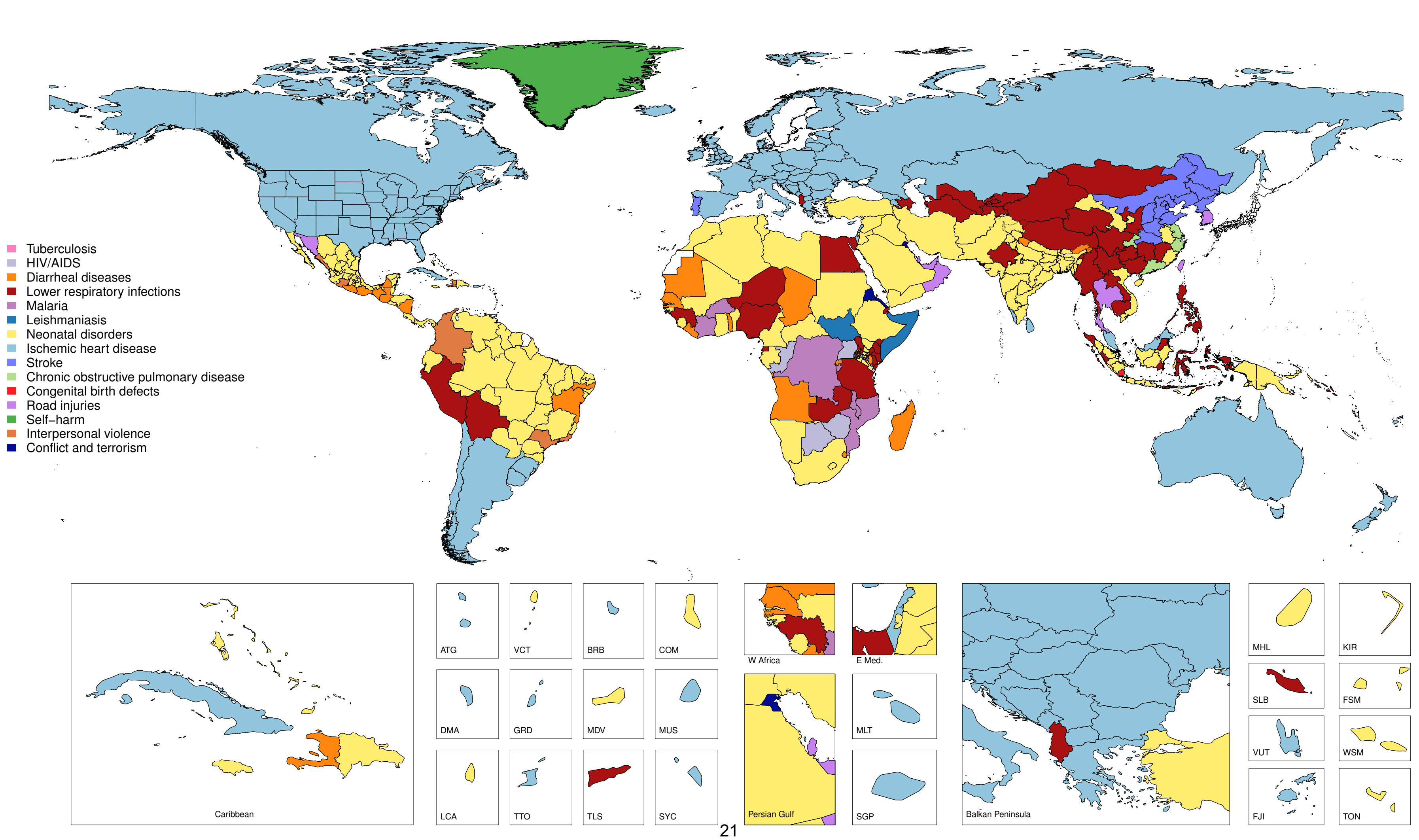

(C) Females in 2017

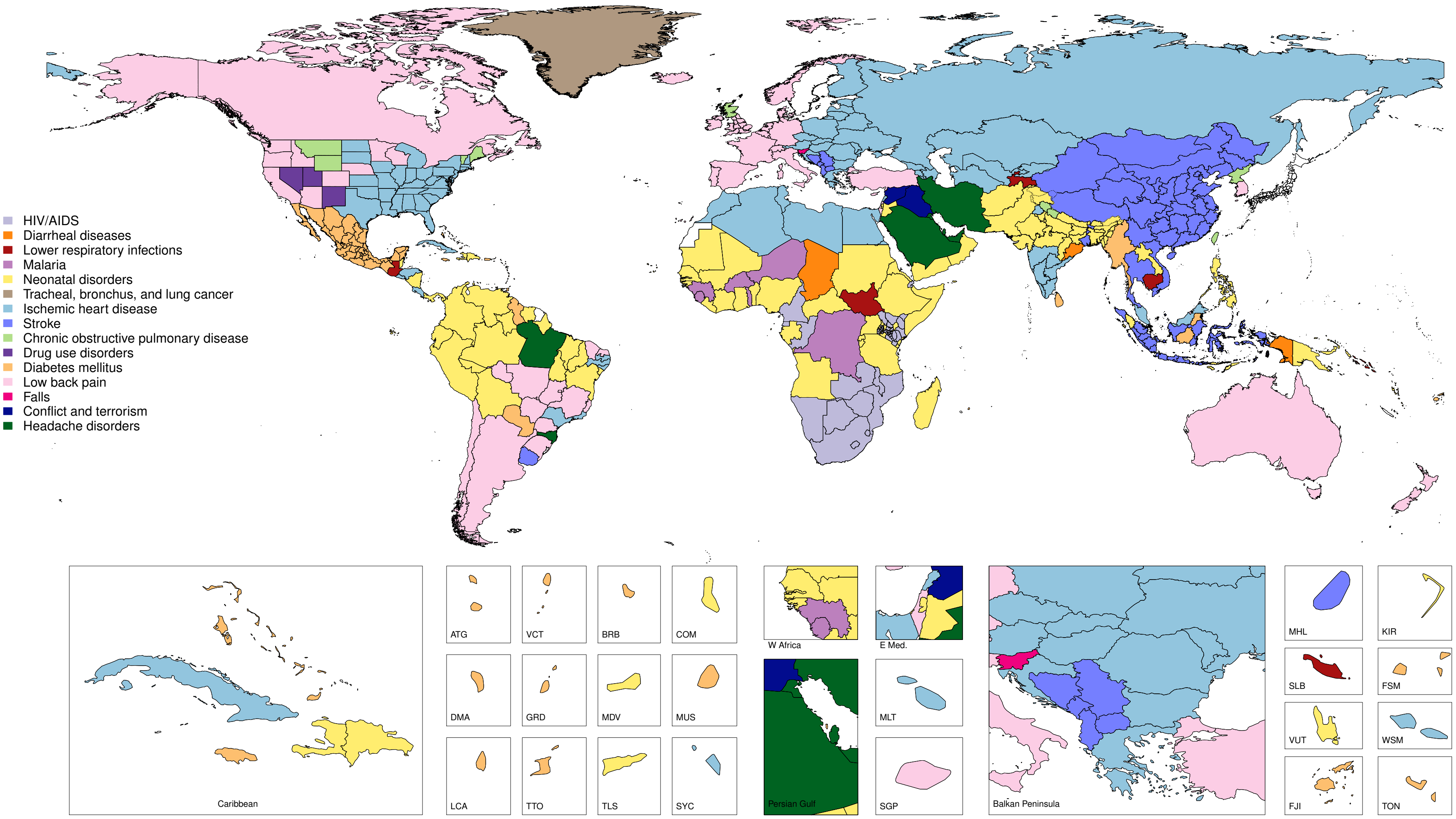

(D) Males in 2017

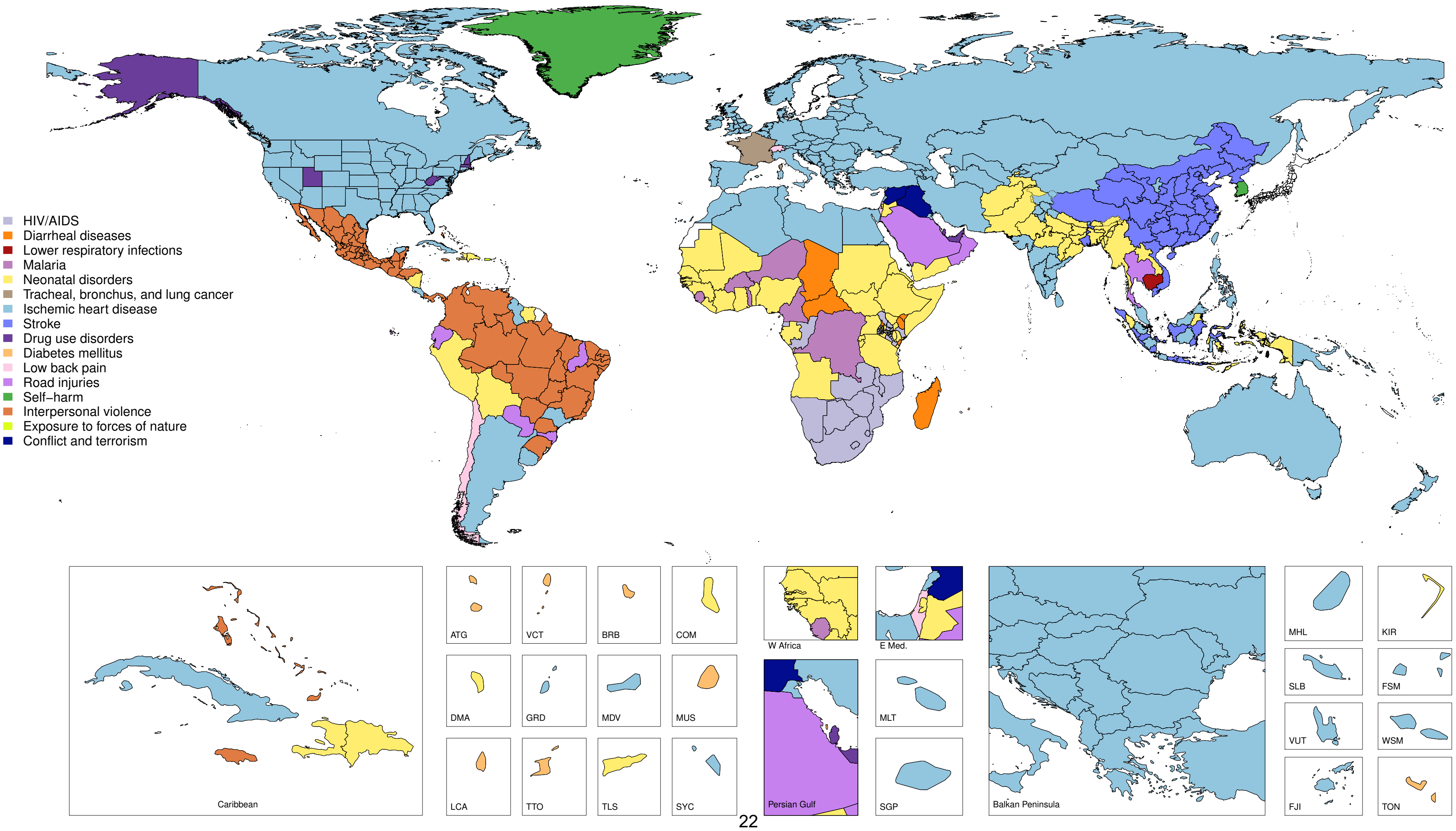

SR Appendix Figure 10a-j. Leading 30 level 3 causes of global DALYs for 1990, 2007, and 2017 with percent change in number of DALYs, and age-standardised DALY rates for low SDI males (A) low SDI females (B) low-middle SDI males (C) low-middle SDI females (D) middle SDI males (E) middle SDI females (F) high-middle SDI males (G) high-middle SDI females (H) high SDI males (I) high SDI females (J)

(A) Low SDI Males

Leading causes 1990	Leading causes 2007	Mean % change number of DALYs 1990-2007	Mean % change age-standardised DALY rate 1990-2007	Leading causes 2017	Mean % change number of DALYs 2007-2017	Mean % change age-standardised DALY rate 2007-2017
1 Neonatal disorders	1 Neonatal disorders	-2.9	**-22.2**	1 Neonatal disorders	**-16.8**	**-20.4**
2 Lower respiratory infect	2 Lower respiratory infect	**-27.8**	**-41.4**	2 Lower respiratory infect	**-29.1**	**-31.6**
3 Diarrhoeal diseases	3 Diarrhoeal diseases	**-26.2**	**-41.2**	3 Diarrhoeal diseases	**-20.2**	**-27.9**
4 Malaria	4 Malaria	15.6	-13.3	4 Malaria	**-31.9**	**-38.3**
5 Measles	5 HIV/AIDS	**137.4**	**54.9**	5 Ischaemic heart disease	**31.3**	-1.4
6 Tuberculosis	6 Tuberculosis	-5.2	**-33.5**	6 Tuberculosis	**-12.5**	**-29.7**
7 Protein-energy malnutrition	7 Congenital defects	18.1	-9.4	7 Congenital defects	3.8	-3.8
8 Leishmaniasis	8 Ischaemic heart disease	**53.4**	1.5	8 Road injuries	4.7	**-14.3**
9 Congenital defects	9 Road injuries	**34.3**	-4.8	9 HIV/AIDS	**-53.3**	**-64.1**
10 Tetanus	10 Protein-energy malnutrition	**-41.3**	**-56.0**	10 COPD	**30.3**	-3.7
11 HIV/AIDS	11 Meningitis	-14.1	**-34.8**	11 Stroke	**17.6**	**-12.0**
12 Meningitis	12 Stroke	**35.1**	**-8.3**	12 Meningitis	**-23.9**	**-30.5**
13 Road injuries	13 COPD	**13.4**	**-23.4**	13 Diabetes	**31.7**	-0.7
14 Ischaemic heart disease	14 Measles	**-66.0**	**-74.8**	14 Protein-energy malnutrition	**-38.7**	**-43.5**
15 Drowning	15 Drowning	**-25.0**	**-44.0**	15 Cirrhosis	8.6	**-15.3**
16 COPD	16 Dietary iron deficiency	**32.9**	**-10.9**	16 Dietary iron deficiency	-1.3	**-17.1**
17 Stroke	17 Cirrhosis	**35.4**	-5.5	17 Low back pain	**30.5**	-0.4
18 Typhoid & paratyph	18 Typhoid & paratyph	**-19.0**	**-43.4**	18 Headache disorders	**28.7**	-0.2
19 Asthma	19 STIs	-3.6	**-25.5**	19 Asthma	-1.3	**-25.4**
20 STIs	20 Diabetes	**69.5**	**13.8**	20 STIs	-9.2	**-13.9**
21 Conflict & terror	21 Asthma	**-21.8**	**-44.6**	21 Self-harm	**20.4**	-4.8
22 Dietary iron deficiency	22 Whooping cough	6.8	-19.8	22 Drowning	**-30.6**	**-36.8**
23 Whooping cough	23 Low back pain	**49.5**	**-2.1**	23 Falls	**18.7**	-4.1
24 Cirrhosis	24 Self-harm	**32.0**	-12.4	24 Interpersonal violence	**7.5**	**-14.6**
25 Self-harm	25 Interpersonal violence	**36.0**	-8.9	25 Typhoid & paratyph	**-24.3**	**-35.3**
26 Upper digest diseases	26 Headache disorders	**52.3**	-0.3	26 Chronic kidney disease	**14.4**	**-8.5**
27 Diabetes	27 Chronic kidney disease	**24.8**	**-11.8**	27 Depressive disorders	**26.0**	**-3.6**
28 Vitamin A deficiency	28 Falls	**29.9**	-7.5	28 Blindness and vision impairment	**31.9**	0.4
29 Low back pain	29 Tetanus	**-73.4**	**-78.0**	29 Whooping cough	-24.6	-30.2
30 Interpersonal violence	30 Depressive disorders	**53.3**	1.5	30 Conflict & terror	**76.9**	**36.3**
31 Chronic kidney disease	31 Vitamin A deficiency			33 Measles		
32 Falls	33 Upper digest diseases			34 Upper digest diseases		
33 Headache disorders	35 Blindness and vision impairment			37 Vitamin A deficiency		
38 Depressive disorders	43 Conflict & terror			67 Tetanus		
41 Blindness and vision impairment	62 Leishmaniasis			101 Leishmaniasis		

Causes are connected by lines between time periods - solid lines are increases and dashed lines are decreases. For the time period of 1990 to 2007 and for 2007 to 2017, two measures of change are shown: percentage change in the number of DALYs; and percentage change in the age-standardised DALY rate.
DALYs=disability adjusted life years. Communicable, maternal, neonatal and nutritional disorders are shown in red, non-communicable causes in blue and injuries in green.
COPD=chronic obstructive pulmonary disease. Lower respiratory infect=lower respiratory infections. Bolded values are stastically significant.

(B) Low SDI Females

Leading causes 1990
1 Diarrhoeal diseases
2 Lower respiratory infect
3 Neonatal disorders
4 Malaria
5 Measles
6 Tuberculosis
7 Protein-energy malnutrition
8 Maternal disorders
9 Congenital defects
10 Meningitis
11 Leishmaniasis
12 Tetanus
13 HIV/AIDS
14 Dietary iron deficiency
15 Stroke
16 COPD
17 Ischaemic heart disease
18 Drowning
19 Typhoid & paratyph
20 Asthma
21 Whooping cough
22 Road injuries
23 Headache disorders
24 STIs
25 Vitamin A deficiency
26 Depressive disorders
27 Low back pain
28 Self-harm
29 Conflict & terror
30 Chronic kidney disease
32 Diabetes
36 Blindness and vision impairment
42 Other musculoskeletal
43 Anxiety disorders

Leading causes 2007	Mean % change number of DALYs 1990-2007	Mean % change age-standardised DALY rate 1990-2007
1 Neonatal disorders	-6.4	**-24.6**
2 Lower respiratory infect	**-28.9**	**-43.9**
3 Diarrhoeal diseases	**-39.6**	**-51.9**
4 HIV/AIDS	**313.6**	**209.2**
5 Malaria	**18.3**	-12.6
6 Congenital defects	**21.6**	-7.1
7 Tuberculosis	**-17.8**	**-42.3**
8 Maternal disorders	**-12.9**	**-44.1**
9 Protein-energy malnutrition	**-34.2**	**-50.6**
10 Dietary iron deficiency	**29.2**	**-14.7**
11 Meningitis	-11.9	**-34.9**
12 Stroke	**26.3**	**-18.7**
13 Ischaemic heart disease	**36.1**	**-14.3**
14 COPD	**16.7**	**-25.2**
15 Measles	**-65.6**	**-74.5**
16 Headache disorders	**55.8**	0.4
17 Whooping cough	7.1	-19.6
18 Road injuries	11.3	**-21.6**
19 Low back pain	**60.2**	2.3
20 Depressive disorders	**55.0**	0.4
21 Asthma	-15.6	**-42.0**
22 Typhoid & paratyph	**-18.7**	**-43.6**
23 Diabetes	**71.6**	**9.9**
24 Drowning	**-27.0**	**-46.3**
25 Vitamin A deficiency	**15.3**	**-17.4**
26 STIs	-2.9	**-26.2**
27 Blindness and vision impairment	**50.6**	**-4.7**
28 Self-harm	7.6	**-28.7**
29 Other musculoskeletal	**56.1**	0.6
30 Chronic kidney disease	**10.1**	**-24.6**
31 Anxiety disorders		
34 Tetanus		
59 Conflict & terror		
77 Leishmaniasis		

Leading causes 2017	Mean % change number of DALYs 2007-2017	Mean % change age-standardised DALY rate 2007-2017
1 Neonatal disorders	**-16.3**	**-20.0**
2 Lower respiratory infect	**-29.2**	**-32.1**
3 Diarrhoeal diseases	**-27.1**	**-31.5**
4 Malaria	**-32.9**	**-39.3**
5 Congenital defects	6.0	-2.1
6 HIV/AIDS	**-60.7**	**-70.8**
7 Tuberculosis	**-15.3**	**-30.5**
8 Ischaemic heart disease	**35.2**	-2.7
9 COPD	**42.5**	2.1
10 Stroke	**24.6**	**-9.8**
11 Dietary iron deficiency	1.5	**-18.6**
12 Maternal disorders	**-23.0**	**-39.4**
13 Headache disorders	**30.8**	0.1
14 Protein-energy malnutrition	**-29.3**	**-34.8**
15 Meningitis	**-26.2**	**-33.2**
16 Low back pain	**30.0**	**-2.9**
17 Diabetes	**41.9**	**4.9**
18 Depressive disorders	**25.1**	**-6.1**
19 Asthma	**16.2**	-11.1
20 Road injuries	3.6	**-12.1**
21 Blindness and vision impairment	**33.2**	**-2.3**
22 Whooping cough	-22.3	-28.2
23 Other musculoskeletal	**27.6**	-2.3
24 Anxiety disorders	**30.2**	0.4
25 Chronic kidney disease	**20.8**	**-4.3**
26 Typhoid & paratyph	**-20.5**	**-32.5**
27 Vitamin A deficiency	**-11.0**	**-21.9**
28 STIs	-5.5	**-12.0**
29 Measles	**-55.8**	**-59.7**
30 Self-harm	4.5	**-16.7**
35 Drowning		
45 Conflict & terror		
74 Tetanus		
107 Leishmaniasis		

Causes are connected by lines between time periods - solid lines are increases and dashed lines are decreases. For the time period of 1990 to 2007 and for 2007 to 2017, two measures of change are shown: percentage change in the number of DALYs; and percentage change in the age-standardised DALY rate. DALYs=disability adjusted life years. Communicable, maternal, neonatal and nutritional disorders are shown in red, non-communicable causes in blue and injuries in green. COPD=chronic obstructive pulmonary disease. Lower respiratory infect=lower respiratory infections. Bolded values are stastically significant.

(C) Low-Middle SDI Males

Leading causes 1990	Leading causes 2007	Mean % change number of DALYs 1990-2007	Mean % change age-standardised DALY rate 1990-2007	Leading causes 2017	Mean % change number of DALYs 2007-2017	Mean % change age-standardised DALY rate 2007-2017
1 Neonatal disorders	1 Neonatal disorders	**-9.6**	**-19.4**	1 Neonatal disorders	**-19.3**	**-22.5**
2 Lower respiratory infect	2 Lower respiratory infect	**-29.0**	**-37.1**	2 Ischaemic heart disease	**27.3**	-1.9
3 Diarrhoeal diseases	3 Diarrhoeal diseases	**-30.3**	**-40.8**	3 Lower respiratory infect	**-24.9**	**-28.8**
4 Tuberculosis	4 HIV/AIDS	**612.9**	**422.9**	4 Diarrhoeal diseases	**-37.2**	**-41.4**
5 Ischaemic heart disease	5 Ischaemic heart disease	**51.3**	0.1	5 Stroke	**18.2**	**-8.7**
6 Measles	6 Malaria	**61.7**	**32.9**	6 Road injuries	0.7	**-14.8**
7 Congenital defects	7 Road injuries	**23.4**	**-8.1**	7 HIV/AIDS	**-46.1**	**-55.0**
8 Road injuries	8 Tuberculosis	**-8.7**	**-35.6**	8 Congenital defects	**-9.8**	**-15.4**
9 Malaria	9 Stroke	**29.8**	**-12.1**	9 Malaria	**-34.9**	**-40.6**
10 Stroke	10 Congenital defects	-0.4	**-14.3**	10 Tuberculosis	**-20.7**	**-35.3**
11 COPD	11 COPD	**10.7**	**-25.6**	11 COPD	**18.9**	**-9.3**
12 Meningitis	12 Cirrhosis	**34.5**	-8.7	12 Cirrhosis	**15.9**	-7.9
13 Protein-energy malnutrition	13 Meningitis	**-17.9**	**-31.4**	13 Diabetes	**36.5**	**6.1**
14 Cirrhosis	14 Diabetes	**83.7**	**24.0**	14 Low back pain	**24.8**	0.4
15 Drowning	15 Self-harm	**30.2**	-9.7	15 Self-harm	5.1	**-12.3**
16 Asthma	16 Dietary iron deficiency	**14.0**	**-14.4**	16 Headache disorders	**22.1**	0.7
17 Dietary iron deficiency	17 Interpersonal violence	**50.8**	5.6	17 Meningitis	-18.4	**-25.0**
18 Self-harm	18 Low back pain	**45.7**	**-1.9**	18 Chronic kidney disease	**17.8**	**-5.3**
19 Tetanus	19 Protein-energy malnutrition	**-39.5**	**-47.9**	19 Interpersonal violence	5.7	**-11.0**
20 Typhoid & paratyph	20 Chronic kidney disease	**41.3**	0.4	20 Dietary iron deficiency	-2.6	**-15.0**
21 Low back pain	21 Drowning	**-23.4**	**-37.8**	21 Falls	**16.3**	-4.1
22 Interpersonal violence	22 Headache disorders	**44.8**	0.1	22 Depressive disorders	**17.3**	**-5.2**
23 Diabetes	23 Measles	**-66.9**	**-71.8**	23 Blindness and vision impairment	**22.3**	**-4.6**
24 Chronic kidney disease	24 Falls	**29.1**	-5.2	24 Drowning	**-20.9**	**-29.2**
25 Falls	25 Asthma	**-18.5**	**-42.9**	25 Age-related hearing loss	**25.4**	-0.9
26 Headache disorders	26 Depressive disorders	**48.8**	**2.4**	26 Drug use disorders	**31.4**	**8.0**
27 HIV/AIDS	27 Blindness and vision impairment	**34.8**	**-9.3**	27 Asthma	-7.1	**-26.7**
28 Upper digest diseases	28 Age-related hearing loss	**45.7**	**-1.2**	28 Other musculoskeletal	**16.7**	**-6.9**
29 STIs	29 Other musculoskeletal	**46.9**	-0.0	29 Protein-energy malnutrition	**-36.8**	**-41.2**
30 Depressive disorders	30 Upper digest diseases	5.3	**-28.7**	30 Alcohol use disorders	**16.8**	**-5.5**
32 Blindness and vision impairment	31 STIs			31 Upper digest diseases		
36 Age-related hearing loss	32 Drug use disorders			36 STIs		
37 Other musculoskeletal	33 Typhoid & paratyph			39 Typhoid & paratyph		
39 Drug use disorders	34 Alcohol use disorders			40 Measles		
43 Alcohol use disorders	68 Tetanus			92 Tetanus		

Causes are connected by lines between time periods - solid lines are increases and dashed lines are decreases. For the time period of 1990 to 2007 and for 2007 to 2017, two measures of change are shown: percentage change in the number of DALYs; and percentage change in the age-standardised DALY rate. DALYs=disability adjusted life years. Communicable, maternal, neonatal and nutritional disorders are shown in red, non-communicable causes in blue and injuries in green. COPD=chronic obstructive pulmonary disease. Lower respiratory infect=lower respiratory infections. Bolded values are stastically significant.

(D) Low-Middle SDI Females

Leading causes 1990
1 Neonatal disorders
2 Lower respiratory infect
3 Diarrhoeal diseases
4 Measles
5 Congenital defects
6 Tuberculosis
7 Malaria
8 Ischaemic heart disease
9 Stroke
10 Maternal disorders
11 Dietary iron deficiency
12 Meningitis
13 COPD
14 Protein-energy malnutrition
15 Road injuries
16 Headache disorders
17 Self-harm
18 Asthma
19 Low back pain
20 Depressive disorders
21 Tetanus
22 Diabetes
23 Chronic kidney disease
24 Drowning
25 Typhoid & paratyph
26 Cirrhosis
27 Whooping cough
28 Blindness and vision impairment
29 Vitamin A deficiency
30 HIV/AIDS
31 Other musculoskeletal
33 Anxiety disorders
34 Upper digest diseases
38 Falls
39 Age-related hearing loss
41 Breast cancer

Leading causes 2007	Mean % change number of DALYs 1990-2007	Mean % change age-standardised DALY rate 1990-2007
1 Neonatal disorders	**-8.3**	**-17.8**
2 Lower respiratory infect	**-31.3**	**-39.9**
3 Diarrhoeal diseases	**-35.5**	**-44.3**
4 HIV/AIDS	**941.1**	**706.3**
5 Malaria	**51.4**	**23.4**
6 Ischaemic heart disease	**31.5**	**-16.1**
7 Congenital defects	-0.7	**-14.5**
8 Stroke	**23.9**	**-19.9**
9 Tuberculosis	**-21.3**	**-44.0**
10 Dietary iron deficiency	**6.8**	**-23.2**
11 COPD	**12.7**	**-27.8**
12 Headache disorders	**48.1**	0.7
13 Maternal disorders	**-15.8**	**-42.3**
14 Diabetes	**83.4**	**19.4**
15 Meningitis	**-16.9**	**-31.0**
16 Low back pain	**53.3**	0.6
17 Depressive disorders	**49.2**	0.2
18 Road injuries	8.4	**-17.1**
19 Measles	**-65.5**	**-70.3**
20 Protein-energy malnutrition	**-35.4**	**-44.3**
21 Self-harm	-4.3	**-31.8**
22 Chronic kidney disease	**26.6**	**-12.6**
23 Blindness and vision impairment	**43.5**	**-7.1**
24 Cirrhosis	**24.7**	**-17.6**
25 Other musculoskeletal	**54.7**	2.0
26 Asthma	-10.4	**-38.8**
27 Anxiety disorders	**47.2**	1.6
28 Breast cancer	**77.4**	13.1
29 Age-related hearing loss	**51.5**	-0.9
30 Upper digest diseases	**24.6**	**-17.5**
31 Falls		
33 Vitamin A deficiency		
35 Drowning		
37 Typhoid & paratyph		
39 Whooping cough		
63 Tetanus		

Leading causes 2017	Mean % change number of DALYs 2007-2017	Mean % change age-standardised DALY rate 2007-2017
1 Neonatal disorders	**-18.7**	**-22.2**
2 Lower respiratory infect	**-27.2**	**-31.7**
3 Diarrhoeal diseases	**-31.2**	**-37.0**
4 Ischaemic heart disease	**29.8**	**-4.4**
5 Stroke	**20.6**	**-10.4**
6 HIV/AIDS	**-48.1**	**-57.2**
7 COPD	**26.6**	**-7.1**
8 Congenital defects	**-8.0**	**-14.3**
9 Malaria	**-39.2**	**-44.7**
10 Headache disorders	**24.0**	0.6
11 Diabetes	**37.0**	2.9
12 Dietary iron deficiency	**-5.5**	**-20.8**
13 Low back pain	**25.2**	**-1.8**
14 Tuberculosis	**-22.1**	**-36.0**
15 Depressive disorders	**16.7**	**-7.5**
16 Maternal disorders	**-22.6**	**-35.3**
17 Road injuries	-0.1	**-15.2**
18 Chronic kidney disease	**20.9**	**-4.9**
19 Meningitis	**-22.5**	**-29.5**
20 Blindness and vision impairment	**24.0**	**-6.3**
21 Other musculoskeletal	**24.3**	-2.0
22 Cirrhosis	**17.7**	-8.1
23 Breast cancer	**40.9**	6.5
24 Anxiety disorders	**22.2**	-0.4
25 Self-harm	**-6.1**	**-20.9**
26 Asthma	**10.1**	**-14.8**
27 Age-related hearing loss	**30.3**	-0.7
28 Falls	**27.3**	-0.2
29 Protein-energy malnutrition	**-28.7**	**-34.0**
30 Upper digest diseases	**14.4**	**-11.1**
39 Vitamin A deficiency		
40 Measles		
43 Drowning		
44 Typhoid & paratyph		
46 Whooping cough		
96 Tetanus		

Causes are connected by lines between time periods - solid lines are increases and dashed lines are decreases. For the time period of 1990 to 2007 and for 2007 to 2017, two measures of change are shown: percentage change in the number of DALYs; and percentage change in the age-standardised DALY rate. DALYs=disability adjusted life years. Communicable, maternal, neonatal and nutritional disorders are shown in red, non-communicable causes in blue and injuries in green. COPD=chronic obstructive pulmonary disease. Lower respiratory infect=lower respiratory infections. Bolded values are stastically significant.

(E) Middle SDI Males

Leading causes 1990
1 Neonatal disorders
2 Lower respiratory infect
3 Road injuries
4 Stroke
5 Ischaemic heart disease
6 Congenital defects
7 Diarrhoeal diseases
8 COPD
9 Tuberculosis
10 Drowning
11 Interpersonal violence
12 Cirrhosis
13 Self-harm
14 Diabetes
15 Low back pain
16 Measles
17 Drug use disorders
18 Liver cancer
19 Lung cancer
20 Headache disorders
21 Falls
22 Chronic kidney disease
23 Meningitis
24 Stomach cancer
25 Asthma
26 Protein-energy malnutrition
27 Dietary iron deficiency
28 Age-related hearing loss
29 Depressive disorders
30 Other musculoskeletal

31 Blindness and vision impairment
37 Neck pain
38 Alcohol use disorders
58 Alzheimer's disease
80 HIV/AIDS

Leading causes 2007	Mean % change number of DALYs 1990-2007	Mean % change age-standardised DALY rate 1990-2007
1 Ischaemic heart disease	**54.9**	**-4.8**
2 Neonatal disorders	**-37.6**	**-28.6**
3 Stroke	**36.6**	**-13.4**
4 Road injuries	**7.2**	**-13.6**
5 HIV/AIDS	**2048.0**	**1587.4**
6 COPD	**8.1**	**-33.2**
7 Lower respiratory infect	**-61.3**	**-58.0**
8 Congenital defects	**-31.0**	**-24.2**
9 Diabetes	**89.1**	**19.9**
10 Cirrhosis	**23.0**	**-18.6**
11 Tuberculosis	**-24.9**	**-46.8**
12 Interpersonal violence	**11.5**	**-11.5**
13 Lung cancer	**64.2**	4.7
14 Low back pain	**35.9**	**-5.9**
15 Diarrhoeal diseases	**-56.2**	**-55.7**
16 Self-harm	-7.2	**-29.5**
17 Chronic kidney disease	**45.1**	0.8
18 Headache disorders	**37.9**	**3.6**
19 Liver cancer	**36.2**	**-11.0**
20 Drowning	**-52.8**	**-54.3**
21 Falls	21.6	-7.7
22 Age-related hearing loss	**48.0**	**-1.1**
23 Depressive disorders	**37.8**	-0.4
24 Drug use disorders	2.6	**-20.3**
25 Stomach cancer	**10.9**	**-29.8**
26 Other musculoskeletal	**41.3**	**-3.6**
27 Blindness and vision impairment	**42.1**	**-7.4**
28 Alcohol use disorders	**58.6**	11.5
29 Neck pain	**49.0**	-1.9
30 Asthma	**-11.2**	**-35.8**

35 Dietary iron deficiency
36 Alzheimer's disease
44 Meningitis
53 Protein-energy malnutrition
74 Measles

Leading causes 2017	Mean % change number of DALYs 2007-2017	Mean % change age-standardised DALY rate 2007-2017
1 Ischaemic heart disease	**34.5**	0.6
2 Stroke	**27.5**	**-6.3**
3 Neonatal disorders	**-28.5**	**-30.9**
4 Road injuries	**-6.1**	**-15.1**
5 COPD	**8.8**	**-20.9**
6 Diabetes	**38.7**	**6.6**
7 Lower respiratory infect	**-27.1**	**-35.8**
8 Lung cancer	**43.1**	5.4
9 Cirrhosis	**14.8**	**-8.4**
10 Interpersonal violence	**12.9**	**6.0**
11 HIV/AIDS	**-39.0**	**-45.9**
12 Low back pain	**20.1**	**1.5**
13 Congenital defects	**-23.1**	**-26.3**
14 Chronic kidney disease	**30.1**	2.4
15 Liver cancer	**26.4**	-3.0
16 Tuberculosis	**-21.6**	**-35.8**
17 Headache disorders	**13.4**	**2.5**
18 Self-harm	3.4	**-7.8**
19 Age-related hearing loss	**24.1**	**-1.4**
20 Falls	**19.9**	0.6
21 Depressive disorders	**14.3**	**-2.3**
22 Blindness and vision impairment	**25.0**	**-3.1**
23 Drug use disorders	**14.5**	**5.1**
24 Other musculoskeletal	**15.3**	**-3.7**
25 Diarrhoeal diseases	**-24.5**	**-33.3**
26 Stomach cancer	**10.4**	**-18.4**
27 Neck pain	**21.7**	-0.6
28 Drowning	**-22.6**	**-27.0**
29 Alcohol use disorders	**12.7**	-2.2
30 Alzheimer's disease	**50.5**	2.4

32 Asthma
44 Dietary iron deficiency
61 Meningitis
69 Protein-energy malnutrition
105 Measles

Causes are connected by lines between time periods - solid lines are increases and dashed lines are decreases. For the time period of 1990 to 2007 and for 2007 to 2017, two measures of change are shown: percentage change in the number of DALYs; and percentage change in the age-standardised DALY rate. DALYs=disability adjusted life years. Communicable, maternal, neonatal and nutritional disorders are shown in red, non-communicable causes in blue and injuries in green. COPD=chronic obstructive pulmonary disease. Lower respiratory infect=lower respiratory infections. Bolded values are stastically significant.

(F) Middle SDI Females

Leading causes 1990
1 Neonatal disorders
2 Lower respiratory infect
3 Stroke
4 Diarrhoeal diseases
5 COPD
6 Congenital defects
7 Ischaemic heart disease
8 Tuberculosis
9 Road injuries
10 Headache disorders
11 Self-harm
12 Dietary iron deficiency
13 Low back pain
14 Diabetes
15 Drowning
16 Depressive disorders
17 Maternal disorders
18 Chronic kidney disease
19 Measles
20 Anxiety disorders
21 Other musculoskeletal
22 Protein-energy malnutrition
23 Blindness and vision impairment
24 Cirrhosis
25 Age-related hearing loss
26 Asthma
27 Rheumatic heart disease
28 Neck pain
29 Meningitis
30 Gynecological diseases
31 Hypertensive heart disease
34 Breast cancer
36 Falls
38 Alzheimer's disease
46 Oral disorders
48 Lung cancer
93 HIV/AIDS

Leading causes 2007	Mean % change number of DALYs 1990-2007	Mean % change age-standardised DALY rate 1990-2007
1 Neonatal disorders	**-37.8**	**-29.5**
2 Stroke	**15.5**	**-29.1**
3 HIV/AIDS	**4017.2**	**3221.1**
4 Ischaemic heart disease	**41.0**	**-15.3**
5 Lower respiratory infect	**-64.5**	**-61.6**
6 COPD	-10.7	**-46.2**
7 Diabetes	**76.8**	**10.0**
8 Headache disorders	**40.4**	**3.2**
9 Congenital defects	**-31.5**	**-24.0**
10 Low back pain	**38.1**	**-6.9**
11 Depressive disorders	**32.3**	**-6.1**
12 Diarrhoeal diseases	**-57.1**	**-56.5**
13 Road injuries	**-6.2**	**-23.5**
14 Other musculoskeletal	**60.5**	**8.0**
15 Chronic kidney disease	**31.0**	**-11.2**
16 Dietary iron deficiency	**-13.1**	**-30.8**
17 Blindness and vision impairment	**49.9**	**-4.2**
18 Tuberculosis	**-38.4**	**-54.6**
19 Age-related hearing loss	**52.7**	**-1.3**
20 Anxiety disorders	**36.9**	**2.3**
21 Neck pain	**57.8**	0.4
22 Breast cancer	**64.5**	0.7
23 Gynecological diseases	**37.7**	**-4.0**
24 Alzheimer's disease	**72.1**	**-8.9**
25 Self-harm	**-42.8**	**-55.7**
26 Cirrhosis	5.3	**-30.4**
27 Oral disorders	**58.2**	**4.4**
28 Asthma	-6.5	**-30.9**
29 Falls	**22.4**	**-10.7**
30 Lung cancer	**53.9**	**-4.8**
35 Hypertensive heart disease		
36 Maternal disorders		
39 Drowning		
42 Rheumatic heart disease		
47 Meningitis		
53 Protein-energy malnutrition		
69 Measles		

Leading causes 2017	Mean % change number of DALYs 2007-2017	Mean % change age-standardised DALY rate 2007-2017
1 Stroke	**22.7**	**-11.2**
2 Ischaemic heart disease	**34.5**	-2.8
3 Neonatal disorders	**-22.7**	**-27.1**
4 Diabetes	**36.6**	2.3
5 COPD	**16.7**	**-15.4**
6 Headache disorders	**14.4**	**2.1**
7 Low back pain	**21.1**	-0.3
8 Depressive disorders	**15.0**	**-3.6**
9 Lower respiratory infect	**-29.4**	**-39.0**
10 HIV/AIDS	**-56.5**	**-61.3**
11 Chronic kidney disease	**25.4**	**-3.2**
12 Other musculoskeletal	**22.7**	1.5
13 Congenital defects	**-21.6**	**-25.5**
14 Age-related hearing loss	**28.0**	-0.9
15 Blindness and vision impairment	**23.3**	**-6.0**
16 Road injuries	**-5.5**	**-17.4**
17 Neck pain	**21.7**	**-2.9**
18 Alzheimer's disease	**48.2**	0.0
19 Breast cancer	**36.7**	5.2
20 Anxiety disorders	**9.9**	**-2.4**
21 Diarrhoeal diseases	**-24.5**	**-36.4**
22 Dietary iron deficiency	**-8.0**	**-16.6**
23 Gynecological diseases	**7.6**	**-3.0**
24 Lung cancer	**44.6**	6.0
25 Oral disorders	**27.7**	1.0
26 Cirrhosis	**8.8**	**-15.6**
27 Falls	**31.1**	2.6
28 Tuberculosis	**-28.1**	**-40.2**
29 Hypertensive heart disease	**47.2**	6.0
30 Self-harm	**-10.8**	**-19.8**
31 Asthma		
44 Rheumatic heart disease		
49 Drowning		
50 Maternal disorders		
67 Protein-energy malnutrition		
69 Meningitis		
99 Measles		

Causes are connected by lines between time periods - solid lines are increases and dashed lines are decreases. For the time period of 1990 to 2007 and for 2007 to 2017, two measures of change are shown: percentage change in the number of DALYs; and percentage change in the age-standardised DALY rate. DALYs=disability adjusted life years. Communicable, maternal, neonatal and nutritional disorders are shown in red, non-communicable causes in blue and injuries in green. COPD=chronic obstructive pulmonary disease. Lower respiratory infect=lower respiratory infections. Bolded values are stastically significant.

(G) High-Middle SDI Males

Leading causes 1990	Leading causes 2007	Mean % change number of DALYs 1990-2007	Mean % change age-standardised DALY rate 1990-2007	Leading causes 2017	Mean % change number of DALYs 2007-2017	Mean % change age-standardised DALY rate 2007-2017
1 Ischaemic heart disease	1 Ischaemic heart disease	**36.0**	**-10.7**	1 Ischaemic heart disease	**3.3**	**-20.2**
2 Neonatal disorders	2 Stroke	**19.1**	**-21.3**	2 Stroke	**11.0**	**-15.2**
3 Stroke	3 Road injuries	3.9	**-14.6**	3 Road injuries	**-17.1**	**-24.2**
4 Road injuries	4 Neonatal disorders	**-43.4**	**-33.1**	4 Lung cancer	**23.8**	**-5.2**
5 Lower respiratory infect	5 Lung cancer	**19.8**	**-16.4**	5 COPD	0.9	**-24.0**
6 Congenital defects	6 COPD	**-13.7**	**-43.4**	6 Diabetes	**19.8**	**-4.6**
7 COPD	7 Self-harm	**8.4**	**-17.5**	7 Neonatal disorders	**-27.0**	**-34.4**
8 Lung cancer	8 Diabetes	**67.2**	**14.9**	8 Low back pain	**14.9**	-1.2
9 Self-harm	9 Low back pain	**20.0**	**-12.5**	9 Drug use disorders	**12.1**	3.8
10 Drowning	10 Congenital defects	**-38.3**	**-26.0**	10 Cirrhosis	0.7	**-17.8**
11 Low back pain	11 Lower respiratory infect	**-47.0**	**-49.5**	11 Self-harm	**-17.0**	**-24.2**
12 Stomach cancer	12 Cirrhosis	**43.6**	-1.0	12 Falls	**8.5**	**-7.6**
13 Interpersonal violence	13 Falls	**11.8**	**-14.7**	13 Liver cancer	**23.7**	-2.3
14 Falls	14 Alcohol use disorders	**48.0**	**8.3**	14 Lower respiratory infect	**-13.9**	**-31.9**
15 Cirrhosis	15 Drug use disorders	**49.5**	**19.5**	15 Stomach cancer	3.1	**-20.5**
16 Diabetes	16 Interpersonal violence	-0.4	**-19.9**	16 Headache disorders	**10.9**	**1.7**
17 Liver cancer	17 Stomach cancer	**-6.1**	**-35.7**	17 Alcohol use disorders	**-15.0**	**-25.9**
18 Alcohol use disorders	18 Liver cancer	**24.6**	**-13.9**	18 Age-related hearing loss	**22.1**	**-1.9**
19 Drug use disorders	19 Cardiomyopathy	**171.4**	**85.4**	19 Congenital defects	**-30.7**	**-37.8**
20 Tuberculosis	20 Headache disorders	**28.2**	0.1	20 Depressive disorders	**11.6**	**-1.6**
21 Headache disorders	21 Depressive disorders	**31.3**	-0.6	21 Neck pain	**20.8**	0.7
22 Diarrhoeal diseases	22 Age-related hearing loss	**40.3**	-1.1	22 Interpersonal violence	**-23.4**	**-27.0**
23 Depressive disorders	23 Neck pain	**45.0**	2.0	23 Colorectal cancer	**29.9**	-0.4
24 Age-related hearing loss	24 Drowning	**-46.0**	**-48.3**	24 Cardiomyopathy	-22.6	**-34.3**
25 Chronic kidney disease	25 Tuberculosis	**-17.0**	**-41.0**	25 Alzheimer's disease	**44.9**	1.7
26 Mechanical forces	26 Chronic kidney disease	**13.8**	**-16.4**	26 Chronic kidney disease	**19.5**	**-5.4**
27 Upper digest diseases	27 Blindness and vision impairment	**33.2**	**-5.0**	27 Blindness and vision impairment	**20.1**	**-3.6**
28 Neck pain	28 Colorectal cancer	**46.2**	0.1	28 Other musculoskeletal	**21.8**	3.1
29 Esophageal cancer	29 Esophageal cancer	**19.1**	**-20.0**	29 Esophageal cancer	**7.7**	**-17.7**
30 Blindness and vision impairment	30 Other musculoskeletal	**38.8**	0.6	30 Upper digest diseases	**7.0**	**-10.2**
33 Colorectal cancer	31 Mechanical forces			33 Mechanical forces		
35 Other musculoskeletal	32 Upper digest diseases			34 Drowning		
39 Cardiomyopathy	33 Alzheimer's disease			43 Tuberculosis		
43 Alzheimer's disease	53 Diarrhoeal diseases			54 Diarrhoeal diseases		

Causes are connected by lines between time periods - solid lines are increases and dashed lines are decreases. For the time period of 1990 to 2007 and for 2007 to 2017, two measures of change are shown: percentage change in the number of DALYs; and percentage change in the age-standardised DALY rate.
DALYs=disability adjusted life years. Communicable, maternal, neonatal and nutritional disorders are shown in red, non-communicable causes in blue and injuries in green.
COPD=chronic obstructive pulmonary disease. Lower respiratory infect=lower respiratory infections. Bolded values are stastically significant.

(H) High-Middle SDI Females

Leading causes 1990
1 Stroke
2 Ischaemic heart disease
3 Neonatal disorders
4 COPD
5 Lower respiratory infect
6 Congenital defects
7 Low back pain
8 Headache disorders
9 Depressive disorders
10 Road injuries
11 Diabetes
12 Self-harm
13 Diarrhoeal diseases
14 Dietary iron deficiency
15 Anxiety disorders
16 Neck pain
17 Stomach cancer
18 Falls
19 Alzheimer's disease
20 Breast cancer
21 Age-related hearing loss
22 Blindness and vision impairment
23 Gynecological diseases
24 Chronic kidney disease
25 Lung cancer
26 Oral disorders
27 Cirrhosis
28 Upper digest diseases
29 Other musculoskeletal
30 Interpersonal violence
31 Colorectal cancer
33 Hypertensive heart disease
38 Liver cancer
39 Drug use disorders
40 Alcohol use disorders

Leading causes 2007	Mean % change number of DALYs 1990-2007	Mean % change age-standardised DALY rate 1990-2007
1 Ischaemic heart disease	**24.9**	**-15.0**
2 Stroke	**4.1**	**-27.9**
3 Neonatal disorders	**-38.2**	**-28.8**
4 Low back pain	**16.0**	**-13.9**
5 Headache disorders	**26.7**	-1.4
6 COPD	**-24.1**	**-46.7**
7 Diabetes	**50.4**	**7.1**
8 Depressive disorders	**26.9**	**-3.6**
9 Congenital defects	**-39.0**	**-25.5**
10 Road injuries	1.5	**-13.9**
11 Neck pain	**51.8**	**7.6**
12 Alzheimer's disease	**48.0**	**-4.9**
13 Anxiety disorders	**32.2**	**6.2**
14 Breast cancer	**37.7**	-4.7
15 Age-related hearing loss	**38.3**	-0.5
16 Lower respiratory infect	**-60.8**	**-60.7**
17 Blindness and vision impairment	**34.4**	**-2.8**
18 Gynecological diseases	**31.4**	**-2.1**
19 Lung cancer	**46.5**	3.1
20 Falls	**8.3**	**-17.5**
21 Other musculoskeletal	**57.7**	**16.8**
22 Cirrhosis	**43.4**	3.6
23 Oral disorders	**36.3**	-0.5
24 Chronic kidney disease	**6.9**	**-21.4**
25 Stomach cancer	**-16.9**	**-41.4**
26 Self-harm	**-40.3**	**-53.4**
27 Colorectal cancer	**24.7**	**-12.2**
28 Upper digest diseases	**13.6**	**-14.0**
29 Dietary iron deficiency	**-35.3**	**-44.7**
30 Alcohol use disorders	**28.8**	**-4.7**
31 Drug use disorders		
32 Interpersonal violence		
34 Hypertensive heart disease		
35 Liver cancer		
43 Diarrhoeal diseases		

Leading causes 2017	Mean % change number of DALYs 2007-2017	Mean % change age-standardised DALY rate 2007-2017
1 Ischaemic heart disease	1.3	**-21.8**
2 Stroke	2.2	**-20.5**
3 Low back pain	**13.9**	-1.1
4 Headache disorders	**8.2**	**0.9**
5 COPD	**6.4**	**-15.9**
6 Diabetes	**15.9**	**-7.0**
7 Neonatal disorders	**-20.4**	**-30.1**
8 Depressive disorders	**9.7**	**-2.4**
9 Alzheimer's disease	**36.5**	-0.5
10 Neck pain	**16.5**	-1.3
11 Age-related hearing loss	**21.6**	**-1.3**
12 Lung cancer	**39.3**	**9.0**
13 Breast cancer	**14.9**	**-7.3**
14 Road injuries	**-14.7**	**-24.6**
15 Anxiety disorders	**5.2**	-1.5
16 Blindness and vision impairment	**15.8**	**-5.7**
17 Falls	**19.6**	-0.6
18 Other musculoskeletal	**21.0**	**4.4**
19 Congenital defects	**-30.3**	**-38.1**
20 Gynecological diseases	2.1	**-2.7**
21 Lower respiratory infect	**-13.4**	**-34.9**
22 Oral disorders	**16.8**	**-3.2**
23 Chronic kidney disease	**18.3**	**-5.1**
24 Cirrhosis	-1.3	**-18.8**
25 Colorectal cancer	**18.0**	**-6.9**
26 Upper digest diseases	**9.4**	**-5.5**
27 Stomach cancer	-5.0	**-24.6**
28 Hypertensive heart disease	**28.5**	-1.3
29 Drug use disorders	**8.4**	**3.8**
30 Liver cancer	**16.8**	-8.0
32 Self-harm		
33 Alcohol use disorders		
34 Dietary iron deficiency		
37 Interpersonal violence		
49 Diarrhoeal diseases		

Causes are connected by lines between time periods - solid lines are increases and dashed lines are decreases. For the time period of 1990 to 2007 and for 2007 to 2017, two measures of change are shown: percentage change in the number of DALYs; and percentage change in the age-standardised DALY rate. DALYs=disability adjusted life years. Communicable, maternal, neonatal and nutritional disorders are shown in red, non-communicable causes in blue and injuries in green. COPD=chronic obstructive pulmonary disease. Lower respiratory infect=lower respiratory infections. Bolded values are stastically significant.

(I) High SDI Males

Leading causes 1990	Leading causes 2007	Mean % change number of DALYs 1990-2007	Mean % change age-standardised DALY rate 1990-2007	Leading causes 2017	Mean % change number of DALYs 2007-2017	Mean % change age-standardised DALY rate 2007-2017
1 Ischaemic heart disease	1 Ischaemic heart disease	**-27.2**	**-48.3**	1 Ischaemic heart disease	**-2.5**	**-21.6**
2 Stroke	2 Lung cancer	**1.2**	**-27.1**	2 Lung cancer	1.9	**-17.7**
3 Lung cancer	3 Low back pain	**19.8**	**-2.3**	3 Low back pain	**8.9**	**-1.9**
4 Road injuries	4 Stroke	**-13.0**	**-38.8**	4 Stroke	**3.2**	**-17.7**
5 Low back pain	5 COPD	**14.7**	**-19.5**	5 COPD	**12.8**	**-10.6**
6 COPD	6 Road injuries	**-31.5**	**-39.5**	6 Diabetes	**20.9**	1.6
7 Self-harm	7 Diabetes	**51.3**	**12.1**	7 Drug use disorders	**49.0**	**44.2**
8 Neonatal disorders	8 Self-harm	**11.0**	**-3.9**	8 Self-harm	**-3.4**	**-7.7**
9 Falls	9 Falls	**9.8**	**-13.8**	9 Falls	**14.7**	-0.6
10 Cirrhosis	10 Cirrhosis	0.3	**-24.7**	10 Road injuries	**-19.4**	**-25.0**
11 Diabetes	11 Drug use disorders	**61.6**	**50.5**	11 Alzheimer's disease	**35.7**	-1.6
12 Headache disorders	12 Colorectal cancer	**22.0**	**-12.8**	12 Colorectal cancer	**10.2**	**-10.4**
13 Colorectal cancer	13 Headache disorders	**12.9**	-0.4	13 Cirrhosis	1.7	**-11.9**
14 Congenital defects	14 Neonatal disorders	**-19.7**	**-22.8**	14 Age-related hearing loss	**21.4**	-0.5
15 Stomach cancer	15 Alzheimer's disease	**57.1**	**-2.5**	15 Headache disorders	**4.8**	0.1
16 Lower respiratory infect	16 Age-related hearing loss	**37.4**	0.3	16 Lower respiratory infect	**14.9**	**-10.4**
17 Depressive disorders	17 Depressive disorders	**14.4**	-1.0	17 Neonatal disorders	**-7.4**	**-9.5**
18 Drug use disorders	18 Lower respiratory infect	**3.6**	**-28.8**	18 Depressive disorders	**7.6**	**1.3**
19 Age-related hearing loss	19 Neck pain	**29.6**	1.3	19 Neck pain	**14.7**	**1.1**
20 Alzheimer's disease	20 Alcohol use disorders	**22.2**	**3.2**	20 Prostate cancer	**15.5**	**-11.0**
21 HIV/AIDS	21 Prostate cancer	**21.8**	**-17.4**	21 Chronic kidney disease	**21.0**	-2.5
22 Neck pain	22 Stomach cancer	**-19.4**	**-42.2**	22 Alcohol use disorders	**4.4**	-1.7
23 Interpersonal violence	23 Chronic kidney disease	**36.2**	**-2.7**	23 Liver cancer	**7.7**	**-11.0**
24 Alcohol use disorders	24 Liver cancer	**30.5**	**-4.8**	24 Other musculoskeletal	**11.3**	1.5
25 Prostate cancer	25 Congenital defects	**-26.0**	**-30.7**	25 Stomach cancer	**-6.0**	**-23.8**
26 Anxiety disorders	26 Other musculoskeletal	**27.9**	**6.3**	26 Anxiety disorders	**4.2**	-0.3
27 Liver cancer	27 Anxiety disorders	**14.5**	1.5	27 Pancreatic cancer	**20.0**	-2.4
28 Chronic kidney disease	28 Pancreatic cancer	**38.6**	-0.2	28 Congenital defects	**-8.6**	**-12.4**
29 Other musculoskeletal	29 Interpersonal violence	**-16.2**	**-21.1**	29 Oral disorders	**10.8**	**-3.1**
30 Asthma	30 Oral disorders	**10.3**	**-14.1**	30 Interpersonal violence	**-3.8**	**-6.9**
34 Oral disorders	38 Asthma			41 Asthma		
37 Pancreatic cancer	59 HIV/AIDS			65 HIV/AIDS		

Causes are connected by lines between time periods - solid lines are increases and dashed lines are decreases. For the time period of 1990 to 2007 and for 2007 to 2017, two measures of change are shown: percentage change in the number of DALYs; and percentage change in the age-standardised DALY rate.
DALYs=disability adjusted life years. Communicable, maternal, neonatal and nutritional disorders are shown in red, non-communicable causes in blue and injuries in green.
COPD=chronic obstructive pulmonary disease. Lower respiratory infect=lower respiratory infections. Bolded values are stastically significant.

(J) High SDI Females

Leading causes 1990	Leading causes 2007	Mean % change number of DALYs 1990-2007	Mean % change age-standardised DALY rate 1990-2007	Leading causes 2017	Mean % change number of DALYs 2007-2017	Mean % change age-standardised DALY rate 2007-2017
1 Ischaemic heart disease	1 Low back pain	**17.0**	**-2.5**	1 Low back pain	**8.3**	**-1.5**
2 Stroke	2 Ischaemic heart disease	**-30.8**	**-49.9**	2 Ischaemic heart disease	**-5.1**	**-22.1**
3 Low back pain	3 Stroke	**-18.4**	**-40.0**	3 Stroke	0.8	**-16.6**
4 Headache disorders	4 Headache disorders	**12.8**	-0.1	4 Alzheimer's disease	**24.7**	**-2.6**
5 Depressive disorders	5 Alzheimer's disease	**43.5**	**-5.4**	5 Headache disorders	**4.3**	0.1
6 Breast cancer	6 COPD	**29.6**	-2.4	6 COPD	**18.1**	-0.4
7 Alzheimer's disease	7 Depressive disorders	**13.0**	-0.0	7 Diabetes	**15.7**	0.3
8 COPD	8 Diabetes	**23.6**	-2.9	8 Depressive disorders	**7.7**	**2.1**
9 Diabetes	9 Breast cancer	**3.2**	**-20.7**	9 Lung cancer	**14.4**	**-4.0**
10 Neonatal disorders	10 Lung cancer	**41.1**	**7.7**	10 Falls	**19.3**	**2.6**
11 Falls	11 Falls	**13.1**	**-13.6**	11 Breast cancer	2.1	**-11.6**
12 Anxiety disorders	12 Anxiety disorders	**13.1**	0.7	12 Neck pain	**12.7**	**1.1**
13 Road injuries	13 Neck pain	**25.0**	1.1	13 Anxiety disorders	**4.2**	-0.4
14 Neck pain	14 Age-related hearing loss	**33.0**	0.3	14 Age-related hearing loss	**19.1**	0.4
15 Lung cancer	15 Other musculoskeletal	**22.6**	2.9	15 Other musculoskeletal	**10.2**	1.4
16 Colorectal cancer	16 Neonatal disorders	**-16.3**	**-20.8**	16 Drug use disorders	**51.0**	**43.8**
17 Congenital defects	17 Colorectal cancer	**5.8**	**-19.5**	17 Colorectal cancer	**4.6**	**-11.5**
18 Age-related hearing loss	18 Road injuries	**-23.3**	**-34.9**	18 Neonatal disorders	-5.2	**-8.4**
19 Other musculoskeletal	19 Chronic kidney disease	**29.7**	**-3.9**	19 Chronic kidney disease	**18.0**	-0.5
20 Lower respiratory infect	20 Gynecological diseases	**10.9**	0.3	20 Road injuries	**-7.1**	**-15.8**
21 Oral disorders	21 Drug use disorders	**60.7**	**51.1**	21 Oral disorders	**11.7**	**-1.0**
22 Gynecological diseases	22 Oral disorders	**5.2**	**-14.1**	22 Lower respiratory infect	**10.8**	**-10.4**
23 Self-harm	23 Lower respiratory infect	0.9	**-30.6**	23 Gynecological diseases	**2.2**	**1.7**
24 Cirrhosis	24 Self-harm	**5.9**	**-6.2**	24 Osteoarthritis	**19.7**	0.7
25 Asthma	25 Congenital defects	**-25.9**	**-30.6**	25 Cirrhosis	**7.2**	**-6.3**
26 Stomach cancer	26 Cirrhosis	**3.2**	**-19.8**	26 Self-harm	**-4.3**	**-8.8**
27 Chronic kidney disease	27 Osteoarthritis	**48.1**	**12.6**	27 Pancreatic cancer	**22.2**	2.1
28 Other cardiovascular	28 Asthma	**-9.8**	**-18.4**	28 Congenital defects	**-8.6**	**-12.6**
29 Drug use disorders	29 Pancreatic cancer	**34.2**	**2.8**	29 Blindness and vision impairment	**14.6**	**-2.0**
30 Dermatitis	30 Blindness and vision impairment	**20.7**	**-6.1**	30 Asthma	2.6	**-3.9**
33 Blindness and vision impairment	31 Other cardiovascular			31 Dermatitis		
34 Osteoarthritis	32 Dermatitis			32 Other cardiovascular		
35 Pancreatic cancer	35 Stomach cancer			39 Stomach cancer		

Causes are connected by lines between time periods - solid lines are increases and dashed lines are decreases. For the time period of 1990 to 2007 and for 2007 to 2017, two measures of change are shown: percentage change in the number of DALYs; and percentage change in the age-standardised DALY rate.
DALYs=disability adjusted life years. Communicable, maternal, neonatal and nutritional disorders are shown in red, non-communicable causes in blue and injuries in green.
COPD=chronic obstructive pulmonary disease. Lower respiratory infect=lower respiratory infections. Bolded values are stastically significant.

SR Appendix Figure 11a–b. Difference in years between observed and expected HALE at age 65 on the basis of SDI for females (A) and males (B), 2017

(A) Females

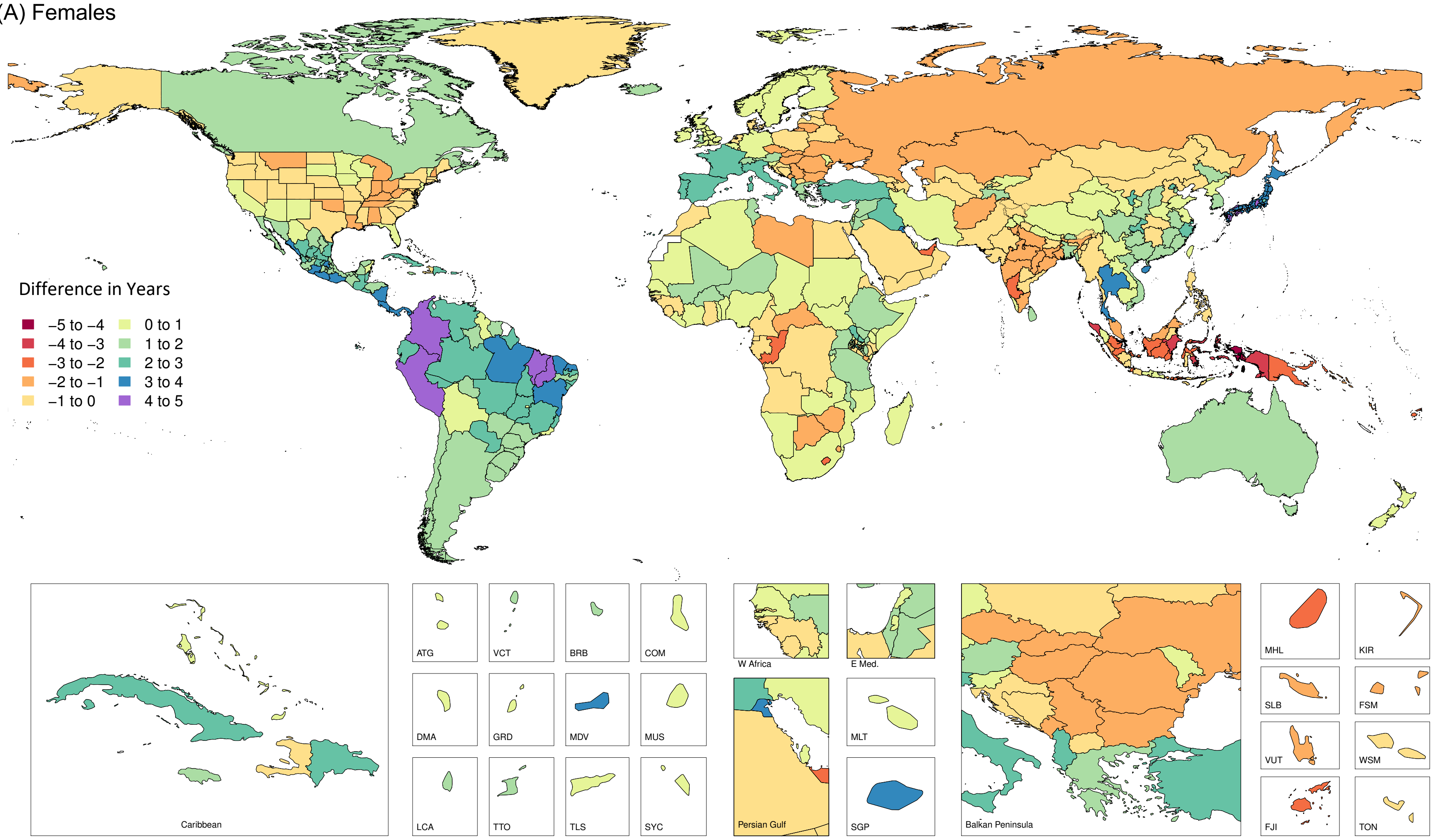

(B) Males

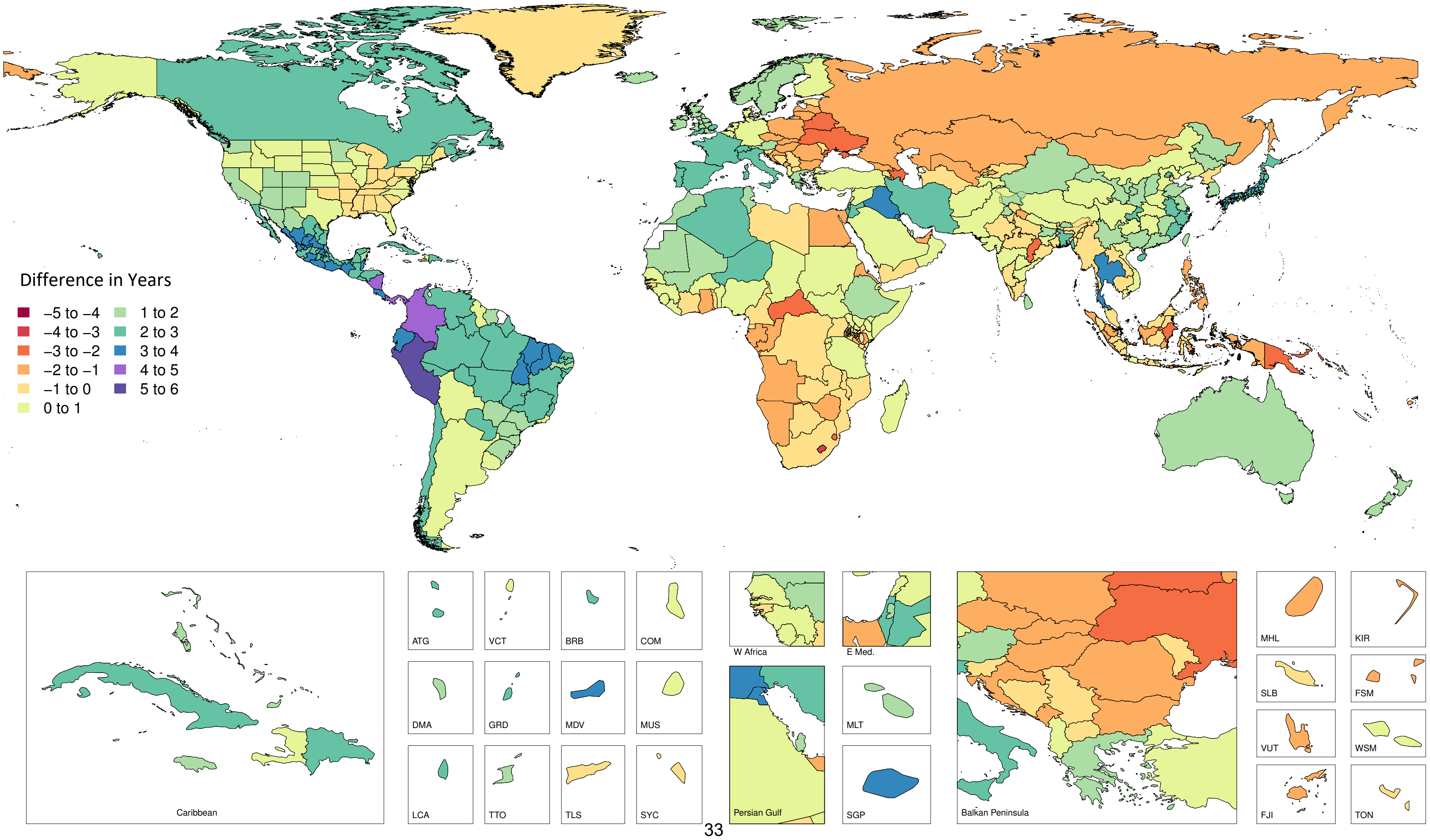

SR Appendix Figure 12a–b. Ratio of observed to expected age−standardized DALY rates per 100,000 on the basis of SDI for females (A) and males (B), 2017

(A) Females

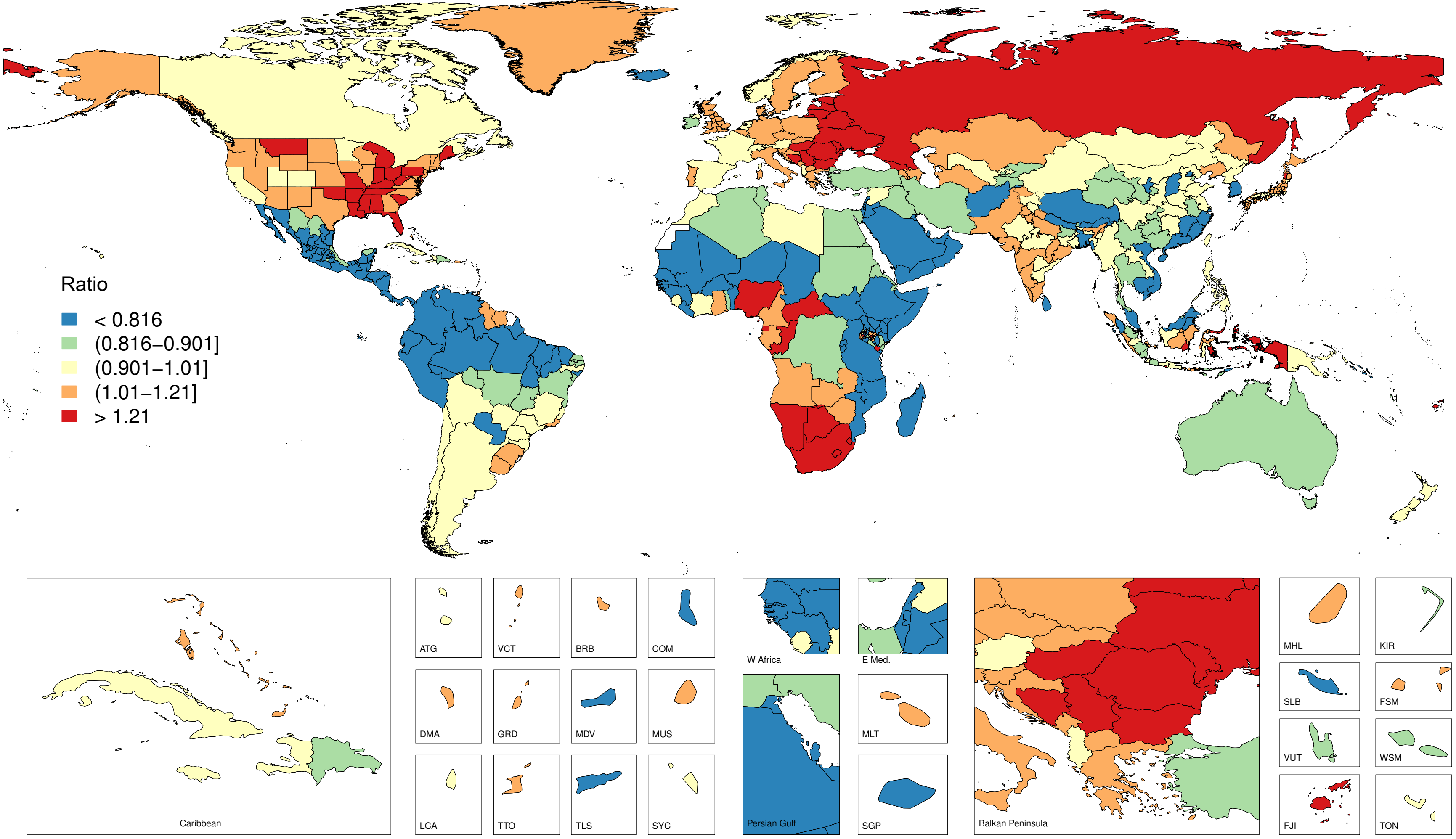

(B) Males

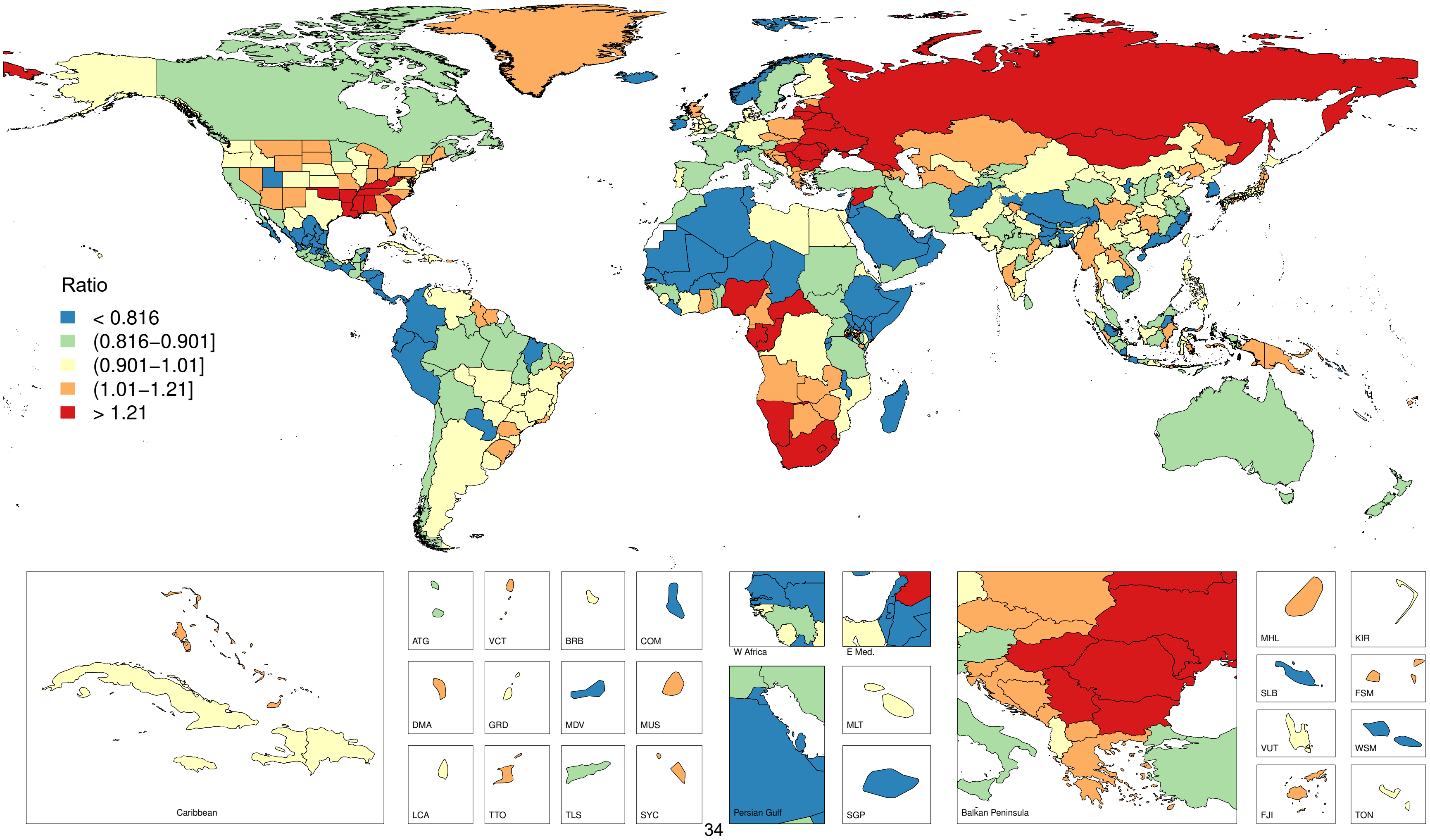

SR Appendix Figure 13. Difference in HALE at birth between females and males globally and for 21 GBD regions, from 1990 to 2017

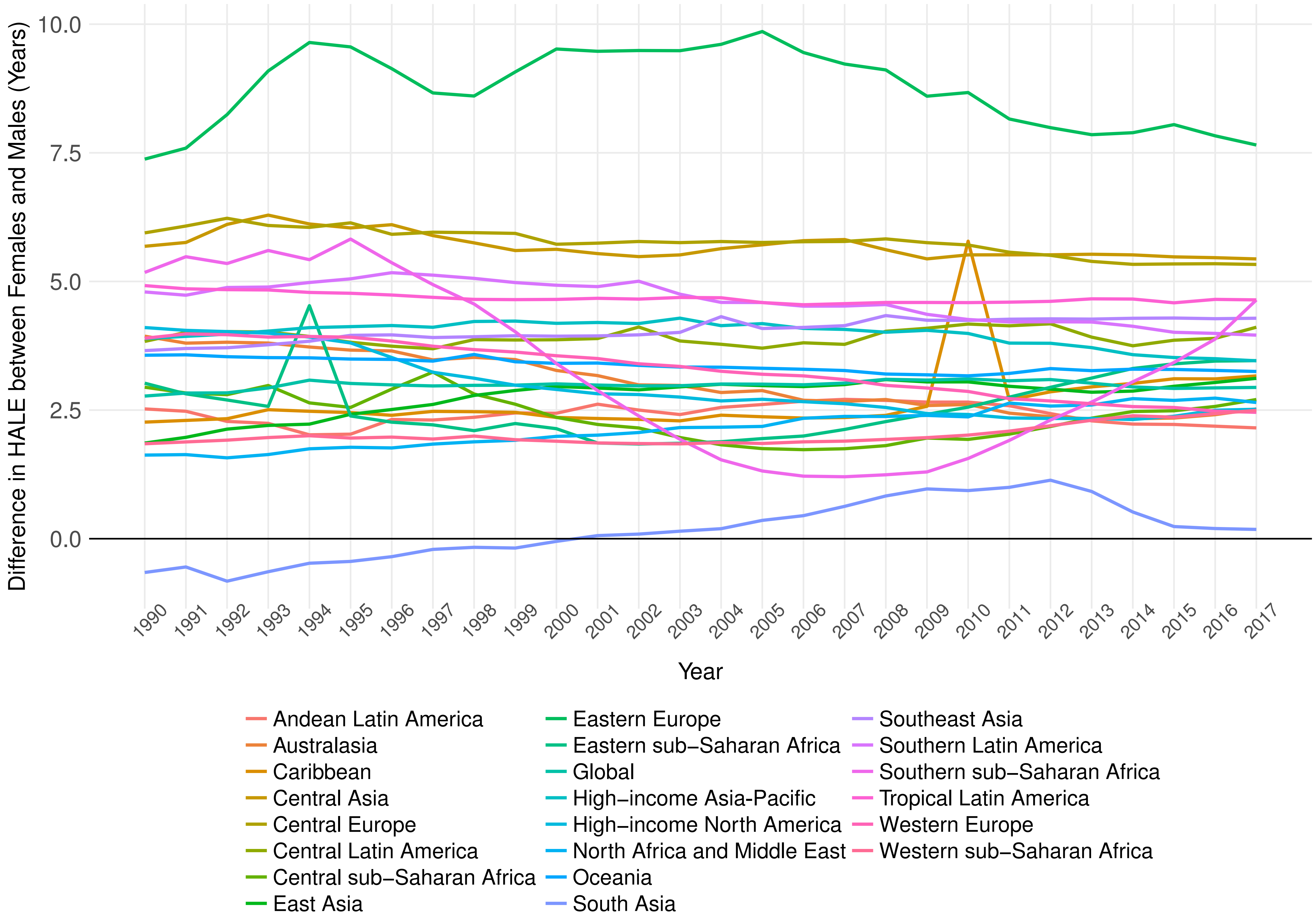

[35] GBD = Global Burden of Disease Study   HALE = Healthy life expectancy

SR Appendix Table 1. Life expectancy and healthy life expectancy (HALE) for both sexes combined, at birth and at age 65, 2007 and 2017, with 95% uncertainty intervals

Location	Life expectancy				HALE (Healthy life expectancy)			
	1990		2017		1990		2017	
	At birth	At age 65 years	At birth	At age 65 years	At birth	At age 65 years	At birth	At age 65 years
Global	65.6 (65.3 to 65.8)	15 (14.9 to 15)	73 (72.7 to 73.3)	17.4 (17.3 to 17.4)	57 (54.6 to 59.1)	11.2 (10.1 to 12)	63.3 (60.5 to 65.7)	12.9 (11.7 to 14)
Low SDI	53.8 (53.4 to 54.3)	11.8 (11.6 to 12)	65.9 (65.3 to 66.4)	14.2 (14 to 14.4)	46.2 (44.1 to 48.2)	8.5 (7.6 to 9.2)	56.7 (54.2 to 59.1)	10.3 (9.3 to 11.2)
Low-middle SDI	60.4 (60 to 60.8)	13.2 (13 to 13.4)	68.1 (67.6 to 68.6)	15 (14.8 to 15.2)	52 (49.7 to 54.2)	9.6 (8.7 to 10.4)	58.9 (56.3 to 61.2)	11 (9.9 to 11.9)
Middle SDI	67.8 (67.4 to 68.1)	14.6 (14.5 to 14.7)	74.5 (74.2 to 74.7)	16.8 (16.6 to 17)	59.5 (57.2 to 61.5)	11.1 (10.1 to 11.9)	65.2 (62.6 to 67.5)	12.7 (11.6 to 13.7)
High-middle SDI	69.9 (69.7 to 70.1)	14.6 (14.5 to 14.7)	76.3 (76.1 to 76.5)	17.1 (17 to 17.3)	61 (58.6 to 63.2)	10.9 (9.9 to 11.7)	66.5 (63.8 to 68.9)	12.8 (11.6 to 13.8)
High SDI	76 (75.9 to 76)	17 (17 to 17)	81.1 (81 to 81.2)	20.4 (20.3 to 20.4)	65.9 (63 to 68.4)	12.8 (11.6 to 13.8)	69.7 (66.5 to 72.5)	15.1 (13.7 to 16.3)
Central Europe, Eastern Europe, and Central Asia	69.5 (69.4 to 69.6)	14.6 (14.6 to 14.6)	73.1 (72.9 to 73.2)	16.3 (16.3 to 16.4)	60 (57.3 to 62.3)	10.5 (9.4 to 11.5)	63.1 (60.2 to 65.5)	11.7 (10.5 to 12.8)
Central Asia	68 (67.7 to 68.3)	15.2 (15.1 to 15.2)	71.1 (70.6 to 71.5)	14.8 (14.6 to 15)	59.2 (56.6 to 61.4)	11.2 (10.1 to 12.1)	62.1 (59.5 to 64.4)	11 (9.9 to 11.9)
Armenia	70.1 (69.6 to 70.5)	14.7 (14.5 to 14.9)	75.6 (75.3 to 75.9)	16.1 (15.9 to 16.3)	61 (58.4 to 63.3)	10.7 (9.7 to 11.7)	65.8 (62.9 to 68.2)	11.9 (10.7 to 12.9)
Azerbaijan	67.4 (66.7 to 68.1)	15.6 (15.4 to 15.9)	70.8 (70 to 71.5)	14.2 (13.7 to 14.5)	58.9 (56.4 to 61.2)	11.6 (10.5 to 12.6)	62 (59.5 to 64.3)	10.5 (9.5 to 11.5)
Georgia	70 (69.5 to 70.5)	15.1 (14.8 to 15.3)	72.8 (72.5 to 73.2)	15.2 (15 to 15.3)	61.6 (59.1 to 63.8)	11.3 (10.3 to 12.3)	63.8 (61.2 to 66)	11.2 (10.1 to 12.2)
Kazakhstan	68.5 (68.2 to 68.7)	15 (14.9 to 15)	72 (71.5 to 72.5)	15.4 (15.2 to 15.7)	59.3 (56.7 to 61.6)	10.8 (9.7 to 11.8)	62.7 (60.1 to 64.9)	11.3 (10.2 to 12.2)
Kyrgyzstan	66.3 (65.7 to 66.9)	14.7 (14.4 to 14.9)	72.7 (72.4 to 73)	15.6 (15.4 to 15.7)	57.6 (55 to 59.7)	10.8 (9.7 to 11.7)	63.4 (60.8 to 65.7)	11.6 (10.5 to 12.6)
Mongolia	61.2 (60.6 to 61.8)	12.2 (12 to 12.4)	68.9 (67.9 to 69.9)	14.3 (13.8 to 14.8)	53.7 (51.5 to 55.7)	9.1 (8.3 to 9.9)	60.3 (57.7 to 62.6)	10.6 (9.6 to 11.6)
Tajikistan	67 (66.4 to 67.5)	15.8 (15.7 to 15.9)	70.3 (69.3 to 71.3)	15.4 (14.9 to 15.9)	58.2 (55.6 to 60.4)	11.7 (10.5 to 12.7)	61.3 (58.6 to 63.8)	11.5 (10.4 to 12.6)
Turkmenistan	66 (65.5 to 66.5)	14.4 (14.3 to 14.4)	70.1 (69.3 to 70.9)	15 (14.5 to 15.4)	57.9 (55.5 to 60)	10.8 (9.7 to 11.6)	61.7 (59.1 to 64)	11.3 (10.2 to 12.3)
Uzbekistan	69.4 (69 to 69.7)	15.9 (15.9 to 15.9)	70.3 (69.2 to 71.5)	14 (13.3 to 14.7)	60.2 (57.4 to 62.5)	11.8 (10.7 to 12.8)	61.6 (58.9 to 64.2)	10.4 (9.4 to 11.5)
Central Europe	71 (70.9 to 71)	14.4 (14.4 to 14.4)	77 (76.8 to 77.2)	17.4 (17.2 to 17.5)	61.2 (58.4 to 63.6)	10.2 (9.1 to 11.2)	66.1 (63.1 to 68.9)	12.3 (11 to 13.5)
Albania	73.3 (73 to 73.7)	16.5 (16.4 to 16.5)	78.2 (76.7 to 79.7)	17.9 (16.9 to 19)	63.3 (60.4 to 65.9)	12.1 (10.8 to 13.1)	67.5 (64.2 to 70.4)	13.1 (11.7 to 14.7)
Bosnia and Herzegovina	73.6 (73.5 to 73.7)	15.3 (15.2 to 15.3)	76.7 (76.2 to 77.2)	16.3 (16 to 16.7)	63.4 (60.5 to 65.9)	11 (9.8 to 12)	65.7 (62.7 to 68.5)	11.6 (10.3 to 12.7)
Bulgaria	71.7 (71.6 to 71.8)	14.4 (14.4 to 14.4)	74.9 (74.4 to 75.4)	16.3 (16 to 16.6)	62.2 (59.4 to 64.6)	10.4 (9.3 to 11.4)	64.9 (62 to 67.4)	11.7 (10.5 to 12.9)
Croatia	72.5 (72.4 to 72.6)	14.6 (14.5 to 14.6)	78.5 (78 to 79)	17.7 (17.3 to 18)	62.8 (60.1 to 65.3)	10.4 (9.3 to 11.4)	67.4 (64.3 to 70.1)	12.5 (11.1 to 13.7)
Czech Republic	71.5 (71.4 to 71.5)	13.6 (13.6 to 13.7)	79.2 (78.7 to 79.7)	18.2 (17.9 to 18.6)	61.7 (58.9 to 64.2)	9.5 (8.4 to 10.5)	67.4 (64 to 70.3)	12.7 (11.2 to 14)
Hungary	69.5 (69.4 to 69.6)	14 (13.9 to 14)	76.8 (76.3 to 77.3)	17.3 (17 to 17.7)	59.7 (56.9 to 62.1)	9.6 (8.5 to 10.6)	65.8 (62.8 to 68.5)	12.2 (10.8 to 13.4)
Macedonia	71.9 (71.8 to 72.1)	14.9 (14.8 to 15)	76.5 (76.1 to 77)	16.7 (16.4 to 17)	62.2 (59.5 to 64.6)	10.7 (9.6 to 11.7)	66 (63 to 68.6)	12 (10.7 to 13.1)
Montenegro	74.3 (74.1 to 74.6)	16.2 (16.1 to 16.3)	76.5 (75.8 to 77.2)	16.2 (15.7 to 16.6)	64.3 (61.3 to 66.8)	11.8 (10.5 to 12.9)	66 (62.9 to 68.6)	11.6 (10.4 to 12.7)
Poland	71.2 (71.2 to 71.2)	14.7 (14.7 to 14.7)	78 (77.5 to 78.5)	18.3 (18 to 18.7)	61.5 (58.7 to 63.9)	10.5 (9.4 to 11.5)	66.8 (63.7 to 69.6)	13 (11.6 to 14.3)
Romania	69.9 (69.8 to 69.9)	14.2 (14.2 to 14.3)	75.2 (74.7 to 75.7)	16.6 (16.3 to 16.9)	59.9 (57.1 to 62.4)	10.1 (9.1 to 11.1)	64.7 (61.6 to 67.4)	11.9 (10.6 to 13)
Serbia	70.9 (70.8 to 71)	14.2 (14.1 to 14.2)	75.7 (75.3 to 76.2)	15.6 (15.3 to 15.9)	61.3 (58.6 to 63.7)	10.1 (9.1 to 11.1)	65.3 (62.3 to 67.9)	11.2 (10 to 12.2)
Slovakia	71 (70.8 to 71.1)	14.2 (14.1 to 14.2)	77.4 (76.9 to 77.9)	17.4 (17.1 to 17.8)	61.3 (58.5 to 63.7)	10 (8.9 to 11)	66.3 (63.2 to 69)	12.3 (10.9 to 13.5)
Slovenia	73.9 (73.7 to 74)	15.6 (15.5 to 15.7)	81.1 (80.5 to 81.7)	19.7 (19.3 to 20.2)	63.3 (60.4 to 66)	10.9 (9.7 to 12)	68.7 (65.3 to 71.8)	13.7 (12.1 to 15.2)
Eastern Europe	69.8 (69.7 to 69.8)	14.7 (14.7 to 14.7)	71.9 (71.8 to 72.1)	16 (15.9 to 16)	60.2 (57.5 to 62.5)	10.6 (9.5 to 11.6)	62.1 (59.3 to 64.5)	11.5 (10.3 to 12.6)
Belarus	71.1 (70.9 to 71.3)	15.5 (15.4 to 15.5)	74 (73.5 to 74.5)	16 (15.6 to 16.3)	61.4 (58.6 to 63.8)	11.3 (10.2 to 12.3)	63.9 (61 to 66.5)	11.6 (10.5 to 12.8)
Estonia	70 (69.8 to 70.1)	14.5 (14.4 to 14.6)	78.1 (76.9 to 79.3)	18.6 (17.9 to 19.4)	60.5 (57.8 to 62.9)	10.6 (9.5 to 11.5)	67 (63.8 to 70.1)	13.6 (12.2 to 15)
Latvia	69.8 (69.7 to 69.9)	14.6 (14.5 to 14.6)	75.2 (74 to 76.3)	17.1 (16.5 to 17.8)	60.2 (57.5 to 62.7)	10.5 (9.4 to 11.5)	64.6 (61.6 to 67.4)	12.3 (11.1 to 13.5)
Lithuania	71.4 (71.3 to 71.5)	15.5 (15.4 to 15.6)	75 (74.4 to 75.6)	17.3 (16.9 to 17.6)	61.6 (58.8 to 64)	11.3 (10.1 to 12.3)	64.3 (61.3 to 67)	12.3 (11 to 13.5)
Moldova	68 (67.8 to 68.3)	14 (13.9 to 14)	72.9 (72.6 to 73.2)	16.1 (15.9 to 16.3)	58.6 (56 to 61)	10.2 (9.1 to 11.1)	63 (60.2 to 65.4)	11.9 (10.7 to 12.9)
Russian Federation	69.5 (69.5 to 69.5)	14.6 (14.6 to 14.7)	72.1 (72 to 72.2)	16.1 (16.1 to 16.2)	59.9 (57.2 to 62.3)	10.5 (9.4 to 11.5)	62.2 (59.4 to 64.6)	11.6 (10.4 to 12.7)
Ukraine	70.3 (70.1 to 70.5)	14.7 (14.6 to 14.7)	70.5 (70 to 71.1)	15.3 (15 to 15.6)	60.6 (57.9 to 63)	10.6 (9.5 to 11.5)	61 (58.3 to 63.4)	11.1 (10 to 12.2)
High-income	76.1 (76.1 to 76.2)	17.1 (17.1 to 17.1)	81 (80.9 to 81.2)	20.4 (20.3 to 20.5)	66 (63.1 to 68.5)	12.9 (11.7 to 13.9)	69.6 (66.3 to 72.4)	15.1 (13.7 to 16.4)
Australasia	76.6 (76.5 to 76.7)	17.1 (17 to 17.1)	82.3 (81.5 to 83)	20.8 (20.3 to 21.3)	66.1 (63 to 68.7)	12.7 (11.5 to 13.8)	70.2 (66.6 to 73.1)	15.3 (13.7 to 16.7)
Australia	76.9 (76.8 to 76.9)	17.2 (17.1 to 17.2)	82.4 (81.5 to 83.2)	20.9 (20.3 to 21.5)	66.4 (63.3 to 68.9)	12.8 (11.6 to 13.9)	70.4 (66.9 to 73.3)	15.4 (13.9 to 16.8)
New Zealand	75.4 (75.2 to 75.5)	16.6 (16.5 to 16.7)	81.6 (81.2 to 82.1)	20.5 (20.1 to 20.8)	64.7 (61.6 to 67.3)	12.3 (11.1 to 13.3)	69.1 (65.4 to 72.2)	14.7 (13.1 to 16.1)
High-income Asia-Pacific	77.8 (77.8 to 77.8)	18 (18 to 18)	83.9 (83.7 to 84.1)	22 (21.8 to 22.1)	68.3 (65.5 to 70.6)	13.8 (12.7 to 14.9)	72.8 (69.7 to 75.6)	16.7 (15.2 to 17.9)
Brunei	70.5 (70 to 70.9)	13.1 (12.8 to 13.3)	75.3 (74.6 to 76.1)	16.6 (16 to 17.1)	62.1 (59.7 to 64.2)	9.8 (8.9 to 10.6)	66.2 (63.4 to 68.6)	12.6 (11.4 to 13.6)
Japan	79.3 (79.3 to 79.3)	18.6 (18.6 to 18.6)	84.2 (84 to 84.4)	22.2 (22.1 to 22.3)	69.7 (66.9 to 72)	14.3 (13.1 to 15.4)	73.1 (69.9 to 75.9)	16.9 (15.4 to 18.1)
Singapore	76.1 (76 to 76.2)	16.5 (16.4 to 16.6)	84.8 (84.3 to 85.3)	22.5 (22.1 to 22.8)	67.1 (64.4 to 69.3)	12.7 (11.6 to 13.6)	74.2 (71.2 to 76.8)	17.3 (15.8 to 18.6)
South Korea	72.2 (72.1 to 72.3)	14.8 (14.8 to 14.9)	82.6 (82.1 to 83.1)	20.8 (20.4 to 21.1)	63.1 (60.5 to 65.4)	11.1 (10.1 to 12)	71.7 (68.5 to 74.3)	15.6 (14.2 to 16.8)
High-income North America	75.7 (75.7 to 75.8)	17.4 (17.4 to 17.4)	78.9 (78.7 to 79.1)	19.6 (19.4 to 19.7)	64.9 (61.9 to 67.6)	12.8 (11.6 to 13.9)	67 (63.7 to 69.8)	14 (12.6 to 15.3)
Canada	77.3 (77.3 to 77.4)	17.7 (17.7 to 17.7)	82 (81.5 to 82.4)	20.9 (20.5 to 21.2)	67.1 (64.2 to 69.7)	13.4 (12.1 to 14.4)	70.5 (67.2 to 73.3)	15.6 (14.1 to 16.9)
Greenland	65.2 (64.7 to 65.6)	13.1 (12.9 to 13.2)	73.5 (73 to 74.1)	16 (15.7 to 16.6)	56.3 (53.8 to 58.4)	9.5 (8.5 to 10.3)	63.5 (60.7 to 65.9)	11.8 (10.7 to 12.9)
USA	75.6 (75.6 to 75.6)	17.4 (17.4 to 17.4)	78.6 (78.4 to 78.8)	19.4 (19.3 to 19.5)	64.7 (61.6 to 67.4)	12.8 (11.6 to 13.9)	66.6 (63.3 to 69.5)	13.9 (12.5 to 15.2)
Southern Latin America	72.6 (72.6 to 72.7)	15.9 (15.8 to 15.9)	77.5 (76.7 to 78.2)	18.1 (17.6 to 18.5)	63.5 (60.8 to 65.8)	12.1 (11 to 13)	67.3 (64.3 to 69.9)	13.7 (12.4 to 14.8)
Argentina	72.4 (72.3 to 72.4)	15.8 (15.8 to 15.9)	76.6 (75.5 to 77.7)	17.6 (16.9 to 18.3)	63.4 (60.7 to 65.6)	12.1 (11 to 13)	66.8 (63.8 to 69.5)	13.4 (12.1 to 14.6)

SR Appendix Table 1. Life expectancy and healthy life expectancy (HALE) for both sexes combined, at birth and at age 65, 2007 and 2017, with 95% uncertainty intervals

Location	Life expectancy				HALE (Healthy life expectancy)			
	1990		2017		1990		2017	
	At birth	At age 65 years	At birth	At age 65 years	At birth	At age 65 years	At birth	At age 65 years
Chile	73.2 (73.1 to 73.2)	15.9 (15.9 to 16)	79.7 (78.7 to 80.7)	19.4 (18.7 to 20)	63.6 (60.8 to 66)	12 (10.9 to 13)	68.7 (65.5 to 71.4)	14.5 (13.1 to 15.7)
Uruguay	73 (72.9 to 73.2)	16 (15.9 to 16.1)	77 (76 to 78.1)	18 (17.3 to 18.7)	64 (61.4 to 66.3)	12.1 (11 to 13.1)	67.2 (64.3 to 69.8)	13.6 (12.3 to 14.8)
Western Europe	76.3 (76.3 to 76.3)	16.8 (16.8 to 16.8)	81.9 (81.7 to 82.1)	20.4 (20.2 to 20.5)	66.3 (63.4 to 68.8)	12.7 (11.6 to 13.7)	70.6 (67.4 to 73.4)	15.3 (13.9 to 16.5)
Andorra	79 (77.8 to 80)	18.5 (17.7 to 19.2)	82.7 (81.8 to 83.6)	20.9 (20.3 to 21.6)	68.5 (65.3 to 71.3)	14 (12.6 to 15.3)	71.2 (67.9 to 74.1)	15.7 (14.2 to 17)
Austria	75.8 (75.7 to 75.9)	16.6 (16.5 to 16.6)	81.8 (81.3 to 82.2)	20.2 (19.8 to 20.5)	66 (63.2 to 68.5)	12.6 (11.4 to 13.6)	70.4 (67.2 to 73.3)	15.1 (13.7 to 16.4)
Belgium	76 (76 to 76.1)	16.6 (16.5 to 16.6)	81.4 (80.9 to 81.8)	20.1 (19.7 to 20.4)	65.9 (63 to 68.4)	12.4 (11.3 to 13.4)	69.6 (66.2 to 72.5)	14.8 (13.3 to 16.1)
Cyprus	75.9 (75.7 to 76.1)	16.2 (16.1 to 16.3)	81.7 (81 to 82.4)	19.9 (19.4 to 20.4)	66.1 (63.2 to 68.5)	12.2 (11.2 to 13.2)	70.7 (67.5 to 73.4)	15 (13.6 to 16.3)
Denmark	75 (74.9 to 75.1)	16.1 (16 to 16.1)	80.8 (80.2 to 81.3)	19.4 (19 to 19.7)	65.3 (62.4 to 67.7)	12.1 (11 to 13.1)	69.6 (66.4 to 72.4)	14.5 (13.2 to 15.7)
Finland	75.1 (75 to 75.2)	16.2 (16.2 to 16.3)	81.4 (80.9 to 81.9)	20.1 (19.7 to 20.4)	64.9 (62 to 67.5)	12.2 (11.1 to 13.2)	69.8 (66.4 to 72.7)	15 (13.6 to 16.3)
France	77 (77 to 77.1)	17.9 (17.9 to 17.9)	82.8 (82.4 to 83.3)	21.6 (21.3 to 21.9)	67.2 (64.4 to 69.7)	13.6 (12.5 to 14.7)	71.7 (68.6 to 74.5)	16.4 (15 to 17.7)
Germany	75.5 (75.5 to 75.6)	16.3 (16.3 to 16.3)	80.6 (79.7 to 81.5)	19.4 (18.8 to 20)	65.6 (62.7 to 68.1)	12.3 (11.2 to 13.2)	69.5 (66.3 to 72.5)	14.5 (13.1 to 15.8)
Greece	77.5 (77.4 to 77.6)	17.3 (17.3 to 17.4)	81 (80.5 to 81.5)	19.8 (19.5 to 20.2)	67.3 (64.4 to 69.9)	13.1 (12 to 14.1)	69.9 (66.9 to 72.6)	15 (13.7 to 16.2)
Iceland	77.8 (77.6 to 78.1)	17.6 (17.4 to 17.8)	82.7 (82.4 to 83.1)	20.7 (20.4 to 20.9)	67.4 (64.4 to 69.9)	13.2 (12.1 to 14.3)	71.3 (67.9 to 74.1)	15.5 (14 to 16.7)
Ireland	74.9 (74.7 to 75)	15.2 (15.1 to 15.2)	81.8 (81.4 to 82.3)	20 (19.6 to 20.3)	65.2 (62.4 to 67.6)	11.5 (10.5 to 12.4)	70.4 (67.1 to 73.1)	14.9 (13.5 to 16.2)
Israel	77.4 (77.3 to 77.5)	17.3 (17.2 to 17.3)	83 (82.5 to 83.4)	21 (20.6 to 21.3)	67 (64.1 to 69.6)	12.8 (11.6 to 13.9)	71.4 (68 to 74.4)	15.5 (14 to 17)
Italy	77.1 (77 to 77.1)	17.3 (17.2 to 17.3)	83.2 (82.7 to 83.6)	21 (20.7 to 21.3)	67 (64.1 to 69.5)	13.1 (11.9 to 14.1)	71.9 (68.7 to 74.7)	15.9 (14.4 to 17.2)
Luxembourg	75.4 (75.2 to 75.6)	16.3 (16.2 to 16.4)	81.7 (81 to 82.4)	19.9 (19.4 to 20.4)	64.9 (61.9 to 67.5)	12 (10.8 to 13)	69.7 (66.3 to 72.6)	14.6 (13.2 to 15.9)
Malta	76.5 (76.3 to 76.7)	16.1 (16 to 16.2)	81 (80.6 to 81.4)	19.3 (19.1 to 19.6)	66.6 (63.7 to 69.1)	12.2 (11 to 13.2)	69.8 (66.5 to 72.6)	14.5 (13.1 to 15.7)
Netherlands	77 (76.9 to 77.1)	16.9 (16.8 to 16.9)	81.5 (81 to 82)	19.7 (19.4 to 20)	66.7 (63.8 to 69.3)	12.7 (11.6 to 13.7)	70.2 (66.9 to 73)	14.7 (13.4 to 16)
Norway	76.6 (76.6 to 76.7)	16.8 (16.8 to 16.9)	82.3 (82.2 to 82.5)	20.3 (20.2 to 20.4)	66 (63 to 68.7)	12.6 (11.5 to 13.7)	70.2 (66.8 to 73.2)	15 (13.5 to 16.3)
Portugal	74.1 (74.1 to 74.2)	15.8 (15.7 to 15.8)	81.4 (81 to 81.9)	20.1 (19.8 to 20.4)	64.2 (61.2 to 66.6)	11.8 (10.7 to 12.7)	70.1 (66.9 to 73)	15 (13.6 to 16.3)
Spain	77 (77 to 77.1)	17.5 (17.5 to 17.5)	83.1 (82.7 to 83.5)	21.2 (20.9 to 21.5)	67.2 (64.4 to 69.6)	13.3 (12.2 to 14.3)	72.1 (68.9 to 74.8)	16.2 (14.8 to 17.4)
Sweden	77.7 (77.6 to 77.8)	17.4 (17.3 to 17.4)	82.5 (82.1 to 82.9)	20.4 (20.1 to 20.7)	67.4 (64.4 to 69.9)	13.2 (12.1 to 14.2)	70.9 (67.6 to 73.9)	15.3 (13.9 to 16.6)
Switzerland	77.8 (77.7 to 77.9)	17.9 (17.8 to 18)	84 (83.5 to 84.4)	21.6 (21.3 to 22)	67.1 (64.1 to 69.7)	13.4 (12.1 to 14.5)	72 (68.6 to 75)	16.2 (14.7 to 17.6)
United Kingdom	75.8 (75.7 to 75.8)	16.1 (16.1 to 16.1)	81 (80.9 to 81.1)	19.7 (19.6 to 19.7)	65.7 (62.9 to 68.2)	12.3 (11.2 to 13.2)	69.3 (66 to 72.1)	14.7 (13.3 to 15.9)
England	76 (76 to 76)	16.2 (16.2 to 16.2)	81.2 (81.2 to 81.3)	19.8 (19.8 to 19.9)	65.9 (63.1 to 68.4)	12.4 (11.3 to 13.3)	69.4 (66.1 to 72.2)	14.7 (13.3 to 16)
Northern Ireland	74.4 (74.3 to 74.6)	15.3 (15.2 to 15.4)	80.7 (79.9 to 81.4)	19.6 (19.1 to 20.1)	64.8 (62 to 67.2)	11.6 (10.6 to 12.5)	69.4 (66.3 to 72.2)	14.8 (13.4 to 16)
Scotland	74.1 (74 to 74.2)	15.1 (15.1 to 15.2)	79.1 (78.4 to 79.8)	18.7 (18.2 to 19.1)	64.2 (61.4 to 66.6)	11.4 (10.5 to 12.3)	68 (64.9 to 70.8)	14.1 (12.8 to 15.2)
Wales	75.8 (75.7 to 75.9)	16 (16 to 16.1)	80.4 (79.8 to 80.9)	19.4 (19 to 19.8)	65.7 (62.8 to 68.2)	12.1 (11.1 to 13.1)	69.2 (66 to 72.1)	14.7 (13.3 to 15.9)
Latin America and Caribbean	69.2 (69 to 69.4)	16.3 (16.3 to 16.3)	75.9 (75.6 to 76.1)	18.6 (18.5 to 18.7)	60.5 (58 to 62.7)	12.4 (11.3 to 13.3)	66.2 (63.4 to 68.6)	14.1 (12.9 to 15.2)
Andean Latin America	68.6 (68.1 to 69.1)	17 (16.8 to 17.2)	77.8 (77 to 78.7)	19.5 (18.9 to 20)	60.1 (57.6 to 62.3)	13 (11.9 to 14)	68.1 (65.3 to 70.7)	14.9 (13.6 to 16.2)
Bolivia	60.9 (60 to 61.8)	13.3 (12.9 to 13.7)	72.7 (71 to 74.6)	15.8 (14.7 to 17.1)	53.3 (50.9 to 55.4)	10 (9.1 to 10.9)	63.6 (60.7 to 66.4)	12 (10.7 to 13.4)
Ecuador	72 (71.8 to 72.2)	17.2 (17.2 to 17.3)	76.7 (75.7 to 77.6)	19 (18.4 to 19.5)	63.1 (60.5 to 65.3)	13.2 (12 to 14.2)	67.2 (64.4 to 69.6)	14.5 (13.2 to 15.6)
Peru	70 (69.2 to 70.8)	18.2 (17.8 to 18.6)	80.3 (78.9 to 81.6)	21 (20.1 to 21.9)	61.3 (58.7 to 63.6)	14 (12.7 to 15.1)	70.3 (67.3 to 73.3)	16.1 (14.7 to 17.6)
Caribbean	68.3 (68 to 68.7)	16.2 (16.1 to 16.3)	72.8 (72.1 to 73.7)	17.7 (17.3 to 18)	60 (57.6 to 62.1)	12.6 (11.5 to 13.5)	63.7 (61.1 to 66)	13.6 (12.5 to 14.6)
Antigua and Barbuda	74.3 (73.8 to 74.8)	17 (16.7 to 17.2)	77 (76.5 to 77.6)	17.9 (17.6 to 18.4)	64.9 (62.1 to 67.4)	13.1 (11.9 to 14.1)	67.3 (64.3 to 69.8)	13.8 (12.5 to 14.9)
The Bahamas	71.1 (70.8 to 71.5)	16.4 (16.2 to 16.5)	73.7 (72.9 to 74.6)	17.5 (17 to 18)	63 (60.6 to 65)	12.7 (11.7 to 13.6)	65.1 (62.5 to 67.6)	13.6 (12.4 to 14.7)
Barbados	73.8 (73.5 to 74)	16.4 (16.2 to 16.5)	77.1 (76.4 to 77.9)	18.4 (18 to 18.9)	65.3 (62.8 to 67.5)	12.8 (11.8 to 13.7)	67.9 (65.1 to 70.3)	14.3 (13.1 to 15.4)
Belize	72 (71.4 to 72.5)	16.7 (16.4 to 17)	74.2 (73.7 to 74.6)	18.2 (18 to 18.5)	63.2 (60.6 to 65.4)	12.9 (11.8 to 13.9)	64.9 (62.3 to 67.3)	14 (12.8 to 15.1)
Bermuda	73.9 (73.6 to 74.2)	16.1 (15.9 to 16.3)	81.3 (80.8 to 81.9)	20.3 (19.8 to 20.7)	65.4 (63 to 67.6)	12.5 (11.5 to 13.5)	71.4 (68.5 to 73.9)	15.7 (14.4 to 16.8)
Cuba	74.8 (74.7 to 74.9)	16.8 (16.8 to 16.8)	78.4 (77.4 to 79.4)	18.5 (17.8 to 19.1)	66 (63.4 to 68.2)	13.1 (12 to 14)	69.1 (66.4 to 71.5)	14.3 (13.1 to 15.4)
Dominica	72.9 (72.6 to 73.3)	16 (15.8 to 16.2)	72.8 (72 to 73.6)	16.6 (16.2 to 17)	64.3 (61.7 to 66.5)	12.4 (11.4 to 13.3)	64.1 (61.6 to 66.4)	12.9 (11.8 to 13.9)
Dominican Republic	72 (71.1 to 72.7)	18.8 (18.4 to 19.3)	73.1 (71.7 to 74.6)	17.7 (17 to 18.6)	63 (60.4 to 65.3)	14.5 (13.3 to 15.6)	64.2 (61.4 to 66.7)	13.6 (12.4 to 14.8)
Grenada	69.4 (69 to 69.8)	14 (13.8 to 14.1)	74.1 (73.5 to 74.6)	16.8 (16.4 to 17.1)	61.3 (58.9 to 63.4)	10.8 (9.9 to 11.6)	65.2 (62.6 to 67.5)	13 (11.8 to 14)
Guyana	65.5 (65.2 to 65.9)	13.9 (13.8 to 14)	69.2 (67.9 to 70.5)	15.2 (14.5 to 15.9)	57.2 (54.7 to 59.3)	10.6 (9.7 to 11.5)	60.4 (57.6 to 62.8)	11.6 (10.5 to 12.6)
Haiti	54.5 (53.2 to 55.6)	11.5 (11 to 12)	64.9 (63.1 to 66.9)	13.4 (12.5 to 14.4)	47.4 (44.9 to 49.4)	8.7 (7.9 to 9.5)	56.3 (53.4 to 58.9)	10.2 (9.1 to 11.3)
Jamaica	75 (74.5 to 75.5)	17.1 (16.8 to 17.4)	74.6 (73.2 to 76.1)	17.1 (16.2 to 17.9)	66 (63.3 to 68.2)	13.4 (12.2 to 14.3)	65.6 (62.7 to 68)	13.2 (11.9 to 14.3)
Puerto Rico	74.1 (74 to 74.3)	17.3 (17.3 to 17.4)	78.1 (77.5 to 78.6)	20.2 (19.9 to 20.6)	65.4 (62.9 to 67.6)	13.5 (12.4 to 14.4)	68.3 (65.5 to 70.8)	15.5 (14.2 to 16.7)
Saint Lucia	70.5 (70.2 to 70.8)	14.8 (14.7 to 14.9)	75.5 (74.9 to 76.1)	17.9 (17.6 to 18.3)	62.1 (59.7 to 64.3)	11.4 (10.5 to 12.3)	66.3 (63.7 to 68.6)	13.8 (12.7 to 14.9)
Saint Vincent and the Grenadines	71 (70.6 to 71.4)	15.5 (15.3 to 15.7)	72.3 (71.7 to 72.9)	15.9 (15.6 to 16.2)	62.4 (59.9 to 64.7)	12 (10.9 to 12.9)	63.5 (60.9 to 65.8)	12.2 (11.2 to 13.2)
Suriname	68.8 (68 to 69.5)	15.5 (15.2 to 15.9)	72 (70.9 to 73.2)	16.7 (16.1 to 17.4)	60.6 (58.2 to 62.8)	12 (11 to 13)	63.1 (60.5 to 65.5)	12.8 (11.6 to 13.9)
Trinidad and Tobago	69.9 (69.6 to 70.2)	14.6 (14.5 to 14.7)	74.2 (72.2 to 76.2)	17.4 (16.2 to 18.6)	61.5 (59.1 to 63.6)	11.1 (10.2 to 12)	65 (61.9 to 67.8)	13.3 (11.8 to 14.7)
Virgin Islands	72.5 (71.8 to 73.1)	15.6 (15.1 to 15.9)	73.9 (72.8 to 75.4)	16.1 (15.4 to 17.2)	64.3 (61.9 to 66.4)	12.2 (11.2 to 13.1)	65.4 (62.7 to 67.9)	12.5 (11.3 to 13.7)
Central Latin America	71 (70.8 to 71.2)	16.8 (16.8 to 16.8)	76.4 (76 to 76.7)	18.9 (18.7 to 19.1)	62.4 (60.1 to 64.5)	12.7 (11.6 to 13.7)	67 (64.3 to 69.3)	14.4 (13.1 to 15.5)

SR Appendix Table 1. Life expectancy and healthy life expectancy (HALE) for both sexes combined, at birth and at age 65, 2007 and 2017, with 95% uncertainty intervals								
	Life expectancy				HALE (Healthy life expectancy)			
	1990		2017		1990		2017	
Location	At birth	At age 65 years	At birth	At age 65 years	At birth	At age 65 years	At birth	At age 65 years
Colombia	71.4 (71.2 to 71.6)	16.9 (16.9 to 17)	80.1 (79 to 81.1)	21.1 (20.4 to 21.8)	62.9 (60.5 to 65)	12.9 (11.8 to 13.9)	70.4 (67.5 to 72.9)	16.2 (14.8 to 17.6)
Costa Rica	76.5 (76.4 to 76.6)	17.8 (17.7 to 17.9)	79.5 (78.9 to 80)	19.8 (19.4 to 20.2)	67.4 (64.8 to 69.6)	13.6 (12.4 to 14.7)	69.8 (67 to 72.2)	15.1 (13.9 to 16.3)
El Salvador	69.2 (68.8 to 69.5)	17.5 (17.4 to 17.5)	74 (72.2 to 75.8)	17.9 (16.9 to 18.9)	60.6 (58.2 to 62.8)	13.3 (12.1 to 14.3)	65 (62.1 to 67.7)	13.6 (12.3 to 14.9)
Guatemala	63 (62.6 to 63.4)	14.4 (14.3 to 14.4)	72.6 (71.4 to 73.7)	17.3 (16.7 to 17.8)	55.2 (52.9 to 57.3)	10.9 (10 to 11.8)	63.7 (61 to 66.2)	13.1 (11.9 to 14.2)
Honduras	68.9 (67.7 to 70.1)	17.8 (17.1 to 18.4)	74 (72.1 to 75.9)	17 (16 to 18.2)	60.4 (57.7 to 62.8)	13.5 (12.2 to 14.6)	64.9 (61.8 to 67.6)	12.9 (11.6 to 14.3)
Mexico	71.4 (71.1 to 71.7)	16.8 (16.8 to 16.8)	75.5 (75.3 to 75.8)	18.2 (18.1 to 18.3)	62.8 (60.5 to 64.9)	12.7 (11.6 to 13.7)	66.2 (63.6 to 68.5)	13.8 (12.5 to 14.8)
Nicaragua	71.9 (71.1 to 72.6)	18.6 (18.2 to 19)	78.8 (77.7 to 79.8)	20.2 (19.5 to 20.8)	62.4 (59.7 to 64.8)	13.9 (12.7 to 15.1)	68.8 (66 to 71.4)	15.3 (13.9 to 16.6)
Panama	75.8 (75.5 to 76.1)	18.3 (18.2 to 18.4)	79.3 (78.7 to 79.9)	20.6 (20.3 to 21)	66.7 (64 to 69)	14 (12.7 to 15)	69.4 (66.7 to 71.9)	15.7 (14.4 to 17)
Venezuela	72.1 (72 to 72.2)	16.6 (16.5 to 16.6)	75.3 (73.6 to 76.8)	18.5 (17.6 to 19.4)	63.6 (61.2 to 65.7)	12.6 (11.5 to 13.6)	66.2 (63.3 to 68.8)	14 (12.7 to 15.3)
Tropical Latin America	67.7 (67.4 to 68)	15.8 (15.7 to 15.8)	75.5 (75.3 to 75.8)	18.4 (18.3 to 18.4)	58.7 (56.1 to 60.9)	11.8 (10.7 to 12.8)	65.4 (62.5 to 67.8)	13.9 (12.6 to 15)
Brazil	67.6 (67.2 to 67.9)	15.7 (15.7 to 15.7)	75.5 (75.3 to 75.7)	18.4 (18.4 to 18.4)	58.5 (55.9 to 60.8)	11.8 (10.7 to 12.7)	65.4 (62.5 to 67.8)	13.9 (12.6 to 15)
Paraguay	74.3 (73.7 to 74.9)	17.4 (17.1 to 17.8)	76.1 (74.4 to 77.7)	17.9 (16.8 to 18.9)	64.4 (61.5 to 67)	13.2 (11.9 to 14.3)	66 (62.9 to 68.9)	13.5 (12.1 to 14.8)
North Africa and Middle East	66.3 (66 to 66.6)	14.6 (14.5 to 14.7)	74.2 (73.9 to 74.6)	17.1 (16.9 to 17.3)	56.6 (54 to 59.1)	10.6 (9.6 to 11.6)	63.4 (60.3 to 66)	12.5 (11.2 to 13.6)
Afghanistan	52.6 (51 to 54.1)	10.6 (10 to 11.3)	63.4 (61.6 to 65.1)	12.3 (11.8 to 12.9)	43.5 (40.8 to 46.3)	7.5 (6.6 to 8.4)	53 (49.9 to 55.9)	8.6 (7.6 to 9.6)
Algeria	71.7 (71.1 to 72.4)	16.3 (16 to 16.7)	77.7 (77.3 to 78.2)	18.7 (18.4 to 19)	61.4 (58.5 to 64.1)	11.9 (10.6 to 13)	66.5 (63.3 to 69.3)	13.7 (12.2 to 14.9)
Bahrain	70.5 (70.1 to 70.9)	12.9 (12.7 to 13.1)	79.5 (78.8 to 80.2)	18.2 (17.6 to 18.7)	60.7 (58 to 63.1)	9.3 (8.3 to 10.2)	67.8 (64.5 to 70.8)	13.1 (11.7 to 14.4)
Egypt	64.4 (63.9 to 64.8)	13.1 (13 to 13.1)	70.6 (69.6 to 71.7)	13.7 (13.2 to 14.3)	55.2 (52.6 to 57.6)	9.5 (8.6 to 10.4)	60.8 (58 to 63.4)	10 (9 to 11)
Iran	68.1 (67.5 to 68.6)	15.7 (15.5 to 15.9)	77.4 (77.3 to 77.4)	18 (17.9 to 18)	58.2 (55.4 to 60.7)	11.6 (10.5 to 12.6)	65.7 (62.5 to 68.6)	13.2 (11.9 to 14.4)
Iraq	65.9 (64.6 to 67.2)	13.6 (13 to 14.3)	76.7 (76.1 to 77.3)	20.2 (19.7 to 20.6)	55.6 (52.8 to 58.4)	9.7 (8.6 to 10.8)	64.4 (61.1 to 67.5)	14.3 (12.7 to 15.8)
Jordan	71 (70 to 72.1)	15.4 (14.8 to 16)	79.3 (78.3 to 80.3)	19 (18.2 to 19.7)	61 (58.2 to 63.6)	11.3 (10.2 to 12.4)	67.7 (64.4 to 70.6)	13.9 (12.4 to 15.2)
Kuwait	74.9 (74.8 to 75.1)	18.3 (18.2 to 18.4)	83.1 (82.6 to 83.6)	21.9 (21.5 to 22.2)	64.4 (61.4 to 67.1)	13.4 (12 to 14.6)	70.7 (67.2 to 73.9)	15.9 (14.2 to 17.4)
Lebanon	70.3 (69.4 to 71.4)	15 (14.6 to 15.7)	78 (77.4 to 78.5)	18 (17.6 to 18.4)	60.2 (57.4 to 62.9)	11 (9.8 to 12.1)	66.3 (63 to 69.1)	13 (11.6 to 14.3)
Libya	72 (70.7 to 73.4)	16.3 (15.6 to 17.1)	72.9 (71.7 to 74.2)	16 (15.2 to 16.8)	61.7 (58.6 to 64.4)	11.8 (10.5 to 13.1)	62.1 (59.1 to 65.1)	11.5 (10.2 to 12.8)
Morocco	66.6 (65.9 to 67.3)	14.7 (14.3 to 15.1)	73.9 (72.4 to 75.6)	16.2 (15.2 to 17.2)	56.8 (54.1 to 59.2)	10.8 (9.7 to 11.8)	63.1 (59.9 to 66.1)	11.8 (10.5 to 13.2)
Palestine	70.6 (69.3 to 72)	15 (14.1 to 16)	76.8 (76.2 to 77.4)	17.1 (16.6 to 17.6)	60.1 (57 to 63.2)	11 (9.7 to 12.3)	65.1 (61.9 to 68)	12.3 (11 to 13.6)
Oman	68.8 (66.7 to 70.7)	14.5 (13.5 to 15.6)	77.1 (75.6 to 78.6)	17.5 (16.7 to 18.5)	58.6 (55.4 to 61.6)	10.1 (8.8 to 11.5)	65.8 (62.8 to 68.9)	12.4 (11 to 13.9)
Qatar	71.5 (70.3 to 72.7)	13.7 (13 to 14.4)	80.2 (78.7 to 81.6)	19.2 (18.2 to 20.2)	61.3 (58.4 to 64)	9.6 (8.4 to 10.7)	68.4 (65 to 71.6)	13.7 (12 to 15.3)
Saudi Arabia	71.7 (70 to 73.2)	16.2 (15.2 to 17.1)	76.8 (75.8 to 77.7)	17.5 (16.8 to 17.9)	61.5 (58.4 to 64.5)	11.8 (10.4 to 13.2)	66.3 (63.4 to 69.1)	12.9 (11.6 to 14.1)
Sudan	58.6 (57.2 to 59.9)	13.2 (12.6 to 13.9)	70.3 (68.4 to 72.4)	15.5 (14.4 to 16.8)	50 (47.3 to 52.5)	9.6 (8.5 to 10.7)	60 (56.9 to 63.1)	11.3 (9.9 to 12.7)
Syria	69.8 (68.9 to 70.7)	15 (14.5 to 15.5)	69.8 (68.6 to 70.9)	16.6 (15.8 to 17.4)	60 (57.4 to 62.6)	11.1 (10 to 12.1)	59.8 (57 to 62.4)	12.2 (10.9 to 13.5)
Tunisia	72.5 (72.1 to 73)	16.2 (16 to 16.5)	78.3 (76.5 to 80.1)	18 (16.8 to 19.2)	62.4 (59.5 to 64.9)	11.9 (10.8 to 13)	67.3 (64.1 to 70.3)	13.3 (11.7 to 14.8)
Turkey	68.6 (68 to 69.3)	15.6 (15.3 to 15.9)	78.9 (78.1 to 79.7)	19.1 (18.5 to 19.6)	59 (56.2 to 61.4)	11.4 (10.3 to 12.4)	67.9 (64.8 to 70.7)	14.1 (12.8 to 15.4)
United Arab Emirates	71.2 (69.8 to 72.9)	14.1 (13.2 to 15.1)	73.3 (71.4 to 75.2)	15 (13.9 to 16.1)	61.4 (58.6 to 64.1)	10.2 (9 to 11.4)	63.1 (60.1 to 65.9)	10.8 (9.5 to 12.1)
Yemen	58.6 (56.1 to 61.3)	12.7 (11.3 to 14.1)	68 (65.5 to 70.4)	14.5 (13.2 to 16)	48.5 (45.1 to 52)	8.7 (7.4 to 10.1)	56.7 (53.3 to 60.3)	10.2 (8.9 to 11.7)
South Asia	59.6 (59.1 to 60.1)	12.6 (12.4 to 12.7)	69 (68.6 to 69.5)	14.7 (14.6 to 14.8)	51.2 (48.8 to 53.3)	9 (8.1 to 9.8)	59.5 (56.9 to 61.8)	10.6 (9.5 to 11.5)
Bangladesh	58.2 (57.4 to 59)	13.2 (12.9 to 13.6)	73.1 (71.9 to 74.2)	16.8 (16.1 to 17.4)	50.4 (48 to 52.4)	9.8 (8.9 to 10.6)	63.1 (60.2 to 65.6)	12.3 (11.1 to 13.5)
Bhutan	59.9 (58.2 to 61.6)	13.5 (12.7 to 14.2)	74 (72.4 to 75.8)	16.6 (15.5 to 17.8)	51.5 (48.8 to 54.1)	9.9 (8.8 to 10.9)	64.1 (61.1 to 66.9)	12.5 (11.2 to 13.8)
India	59.6 (59 to 60.2)	12.3 (12.1 to 12.5)	69 (68.4 to 69.5)	14.5 (14.3 to 14.6)	51.1 (48.7 to 53.3)	8.8 (7.9 to 9.6)	59.4 (56.7 to 61.7)	10.4 (9.4 to 11.3)
Nepal	58.3 (57.1 to 59.6)	12.7 (12.1 to 13.3)	70.9 (69.8 to 72.1)	14.8 (14.2 to 15.5)	49.8 (47.3 to 52.2)	9.1 (8.1 to 10.1)	61.1 (58.4 to 63.5)	10.7 (9.7 to 11.8)
Pakistan	61.7 (61 to 62.3)	14.1 (13.8 to 14.4)	66.8 (64.9 to 68.7)	14.7 (13.7 to 15.7)	53.4 (51 to 55.6)	10.3 (9.3 to 11.2)	58 (55.2 to 60.6)	10.7 (9.5 to 11.9)
Southeast Asia, East Asia, and Oceania	67.8 (67.4 to 68.1)	14.2 (14.1 to 14.3)	75.6 (75.3 to 75.9)	16.9 (16.7 to 17.1)	59.9 (57.7 to 61.8)	10.8 (10 to 11.6)	66.6 (64.1 to 68.8)	12.8 (11.7 to 13.8)
East Asia	68.8 (68.3 to 69.3)	14.2 (14.1 to 14.3)	77 (76.7 to 77.4)	17.1 (16.9 to 17.4)	61 (58.9 to 62.9)	10.9 (10.1 to 11.7)	68 (65.6 to 70.2)	13.1 (12 to 14)
China	68.7 (68.1 to 69.2)	14.2 (14 to 14.3)	77.1 (76.7 to 77.4)	17.1 (16.9 to 17.3)	60.9 (58.8 to 62.8)	10.9 (10.1 to 11.7)	68.1 (65.6 to 70.3)	13.1 (12 to 14)
North Korea	71.8 (70.2 to 73.4)	15.3 (14.6 to 16.3)	72 (70.6 to 73.4)	15.4 (14.6 to 16.2)	63.4 (60.8 to 65.8)	11.6 (10.6 to 12.6)	63.5 (61.1 to 65.9)	11.5 (10.5 to 12.6)
Taiwan (Province of China)	74.5 (74.4 to 74.5)	16.1 (16 to 16.1)	79.9 (79.5 to 80.4)	19.7 (19.3 to 20)	66.1 (63.7 to 68.1)	12.3 (11.3 to 13.2)	69.9 (67.2 to 72.3)	14.7 (13.5 to 15.8)
Oceania	57.8 (56.5 to 59.2)	11.1 (10.8 to 11.4)	60.5 (58.8 to 62.2)	11.8 (11.4 to 12.2)	50.5 (48.1 to 52.6)	8.1 (7.2 to 8.8)	52.6 (49.9 to 54.9)	8.5 (7.6 to 9.3)
American Samoa	70.8 (70.3 to 71.4)	14.5 (14.3 to 14.9)	71.8 (70.9 to 72.8)	14.9 (14.3 to 15.6)	61.7 (59.1 to 64)	10.6 (9.6 to 11.6)	62.1 (59.3 to 64.7)	10.8 (9.6 to 11.9)
Federated States of Micronesia	63.5 (61.9 to 65.1)	12.2 (11.7 to 12.8)	67.2 (65.6 to 68.8)	13.3 (12.7 to 13.8)	55.5 (52.8 to 58)	9 (8.1 to 9.9)	58.2 (55.2 to 60.7)	9.7 (8.6 to 10.6)
Fiji	67.7 (66.6 to 68.8)	13.5 (12.9 to 14.1)	68.1 (66.7 to 69.5)	13.3 (12.6 to 14.1)	59 (56.3 to 61.5)	10 (8.9 to 10.9)	59.1 (56.4 to 61.6)	9.7 (8.6 to 10.8)
Guam	73.6 (73.3 to 74)	16.2 (15.9 to 16.4)	73.1 (72.3 to 73.9)	16.7 (16.3 to 17.1)	64.8 (62.3 to 67.1)	12.1 (11 to 13.1)	63.6 (60.9 to 66)	12.3 (11.1 to 13.4)
Kiribati	58.5 (57.4 to 59.5)	11.3 (10.9 to 11.6)	62.4 (60.6 to 64.1)	12.1 (11.5 to 12.8)	51.1 (48.8 to 53.2)	8.3 (7.4 to 9)	54 (51.3 to 56.5)	8.6 (7.7 to 9.7)
Marshall Islands	62.8 (62.2 to 63.3)	11.6 (11.4 to 11.8)	64.5 (63 to 66)	12.1 (11.5 to 12.7)	55.2 (53 to 57.2)	8.7 (7.8 to 9.4)	56.4 (53.9 to 58.8)	8.9 (8 to 9.8)
Northern Mariana Islands	74.2 (72.7 to 75.5)	16.2 (15.3 to 17)	76.2 (75.3 to 77.2)	17.3 (16.5 to 18)	65.2 (62.4 to 67.6)	12.1 (10.9 to 13.2)	66.1 (63 to 68.6)	12.7 (11.4 to 13.9)
Papua New Guinea	54.4 (52.8 to 56.2)	9.9 (9.6 to 10.3)	58.5 (56.4 to 60.5)	10.8 (10.3 to 11.4)	47.5 (45.1 to 49.7)	7.2 (6.4 to 7.9)	50.8 (48.1 to 53.2)	7.7 (6.9 to 8.6)

SR Appendix Table 1. Life expectancy and healthy life expectancy (HALE) for both sexes combined, at birth and at age 65, 2007 and 2017, with 95% uncertainty intervals

Location	Life expectancy				HALE (Healthy life expectancy)			
	1990		2017		1990		2017	
	At birth	At age 65 years	At birth	At age 65 years	At birth	At age 65 years	At birth	At age 65 years
Samoa	70.7 (69.2 to 72.3)	15.6 (14.8 to 16.4)	72.9 (71.8 to 74)	15.2 (14.6 to 15.9)	61.8 (59.1 to 64.5)	11.6 (10.4 to 12.8)	63.3 (60.4 to 65.7)	11.1 (10 to 12.3)
Solomon Islands	61.5 (59.6 to 63.2)	11.5 (11 to 12.2)	65.7 (64.2 to 67.2)	12.7 (12.3 to 13.2)	54 (51.3 to 56.5)	8.7 (7.8 to 9.6)	57.2 (54.5 to 59.7)	9.3 (8.4 to 10.2)
Tonga	70.1 (69.5 to 70.8)	14 (13.7 to 14.3)	71.8 (70.4 to 73)	15.1 (14.4 to 15.8)	61.3 (58.6 to 63.5)	10.4 (9.3 to 11.3)	62.3 (59.5 to 64.8)	11 (9.8 to 12.1)
Vanuatu	62.4 (59.7 to 65.1)	11.7 (11 to 12.6)	64.7 (61.8 to 67.4)	12.3 (11.5 to 13.3)	54.5 (51.1 to 57.6)	8.7 (7.7 to 9.7)	56.3 (53.2 to 59.2)	9 (8 to 10)
Southeast Asia	65.1 (64.8 to 65.5)	14.1 (13.9 to 14.3)	72.5 (72.1 to 73)	16 (15.8 to 16.2)	57 (54.7 to 59.1)	10.4 (9.4 to 11.3)	63.6 (61 to 65.8)	11.9 (10.7 to 12.9)
Cambodia	57.6 (56.7 to 58.6)	11.8 (11.4 to 12.3)	69.9 (68.6 to 71.1)	14.7 (14 to 15.1)	49.9 (47.6 to 52)	8.5 (7.6 to 9.3)	60.7 (58 to 63.2)	10.6 (9.5 to 11.6)
Indonesia	63.9 (63.4 to 64.4)	13.7 (13.5 to 13.9)	71.5 (70.8 to 72.1)	14.8 (14.5 to 15.2)	55.9 (53.6 to 58)	10 (9.1 to 10.9)	62.7 (60 to 64.9)	10.9 (9.8 to 11.8)
Laos	51.9 (50.3 to 53.6)	11.2 (10.6 to 11.9)	67.6 (66 to 69.1)	14.5 (13.7 to 15.3)	45.8 (43.4 to 47.9)	8.5 (7.6 to 9.3)	59.5 (56.9 to 62)	10.9 (9.8 to 11.9)
Malaysia	71.3 (71.3 to 71.4)	14.2 (14.2 to 14.2)	74.7 (73.9 to 75.5)	15.8 (15.3 to 16.3)	63 (60.6 to 65.1)	10.7 (9.8 to 11.6)	66 (63.6 to 68.3)	12 (10.9 to 13)
Maldives	65.1 (64.6 to 65.6)	13 (12.9 to 13.1)	81.4 (80.9 to 82)	20.3 (19.9 to 20.7)	56.7 (54.3 to 58.8)	9.6 (8.7 to 10.4)	71.1 (68.2 to 73.7)	15.2 (13.8 to 16.5)
Mauritius	70.1 (69.8 to 70.3)	14.1 (14 to 14.3)	74.8 (74.1 to 75.4)	17 (16.6 to 17.4)	61.3 (58.7 to 63.5)	10.6 (9.6 to 11.4)	64.8 (61.9 to 67.3)	12.4 (11.2 to 13.6)
Myanmar	55.3 (53.5 to 57.2)	11.5 (10.9 to 12.3)	68.5 (67.2 to 69.9)	14.7 (14.1 to 15.4)	48.2 (45.7 to 50.7)	8.3 (7.3 to 9.3)	59.9 (57.3 to 62.3)	10.7 (9.6 to 11.7)
Philippines	67.9 (67.2 to 68.5)	15.3 (15 to 15.7)	69.7 (68.4 to 71.2)	14.4 (13.7 to 15.2)	58.9 (56.4 to 61.2)	11.2 (10.1 to 12.2)	61 (58.4 to 63.3)	10.5 (9.5 to 11.6)
Sri Lanka	69.7 (69.5 to 69.9)	14.8 (14.7 to 15)	77.4 (76 to 79)	17.9 (17.1 to 18.9)	61.1 (58.6 to 63.3)	11.2 (10.2 to 12.1)	67.9 (64.8 to 70.6)	13.5 (12.1 to 14.8)
Seychelles	70.5 (70.2 to 70.8)	15.1 (14.9 to 15.2)	73.6 (73.1 to 74.1)	16.3 (16 to 16.6)	62.6 (60.3 to 64.6)	11.4 (10.4 to 12.3)	65 (62.5 to 67.2)	12.2 (11.1 to 13.2)
Thailand	70.7 (70.3 to 71.2)	15.9 (15.7 to 16.1)	78.1 (77.1 to 79.2)	20.2 (19.6 to 20.8)	62.2 (59.7 to 64.3)	12 (10.9 to 12.9)	68.5 (65.7 to 70.9)	15.3 (13.9 to 16.5)
Timor-Leste	60.2 (58.8 to 61.6)	13.5 (12.9 to 14.3)	70.8 (69.6 to 72.1)	15 (14.3 to 15.7)	51.7 (49 to 54.2)	9.7 (8.6 to 10.8)	61.2 (58.4 to 63.8)	10.8 (9.7 to 12)
Vietnam	68.9 (67.9 to 70)	14.7 (14.3 to 15.3)	74.5 (73.3 to 75.6)	16.6 (15.9 to 17.2)	60.5 (57.8 to 62.9)	11.1 (10 to 12.1)	65.8 (63.2 to 68.1)	12.5 (11.3 to 13.6)
Sub-Saharan Africa	53.6 (53 to 54.1)	12.7 (12.3 to 13)	63.9 (63.1 to 64.6)	14.5 (14.1 to 14.7)	46.1 (43.9 to 48.1)	9.2 (8.2 to 10)	55.2 (52.7 to 57.6)	10.6 (9.6 to 11.6)
Central sub-Saharan Africa	52.3 (51.3 to 53.4)	11.4 (11 to 11.8)	62.4 (61.1 to 63.6)	13.1 (12.5 to 13.7)	44.5 (42.1 to 46.6)	8.1 (7.1 to 8.9)	53.4 (50.6 to 56)	9.4 (8.3 to 10.4)
Angola	47.8 (46.1 to 49.6)	10.8 (10.1 to 11.6)	64.2 (62.5 to 65.8)	13.7 (13 to 14.6)	41.1 (38.7 to 43.2)	7.8 (6.9 to 8.7)	55 (52.2 to 57.8)	9.9 (8.7 to 11.1)
Central African Republic	47.2 (45.7 to 48.7)	9.6 (9.3 to 10)	51.8 (49.8 to 54.1)	10.6 (9.9 to 11.6)	40.4 (38 to 42.5)	6.8 (6.1 to 7.6)	44.8 (42.2 to 47.3)	7.7 (6.7 to 8.7)
Congo (Brazzaville)	53.8 (52.1 to 55.4)	10.5 (10.1 to 11)	62.6 (60.8 to 64.6)	12.7 (12.1 to 13.6)	46.3 (43.8 to 48.7)	7.5 (6.7 to 8.3)	54 (51.2 to 56.7)	9.2 (8.2 to 10.3)
DR Congo	53.9 (52.5 to 55.4)	11.8 (11.1 to 12.4)	62.3 (60.7 to 64.1)	13 (12.3 to 13.9)	45.7 (42.9 to 48.1)	8.2 (7.2 to 9.2)	53.3 (50.4 to 56.1)	9.3 (8.2 to 10.4)
Equatorial Guinea	48.2 (45.7 to 50.8)	9.9 (9.1 to 10.9)	65.4 (62 to 68.8)	14.9 (12.9 to 17.2)	41.3 (38.6 to 44)	7.1 (6.3 to 8.1)	56.3 (52.7 to 59.9)	10.8 (9.1 to 12.6)
Gabon	60 (58.8 to 61.2)	12.2 (11.7 to 12.7)	68.4 (66.9 to 69.7)	14.6 (13.9 to 15.3)	51.5 (48.9 to 53.9)	8.8 (7.8 to 9.7)	58.7 (55.8 to 61.3)	10.6 (9.4 to 11.6)
Eastern sub-Saharan Africa	50.7 (50.2 to 51.3)	11.3 (11.2 to 11.5)	64.9 (64.2 to 65.5)	14.2 (14 to 14.4)	44.1 (42.1 to 45.9)	8.4 (7.6 to 9.1)	56.5 (54.1 to 58.7)	10.6 (9.6 to 11.4)
Burundi	48.7 (47.3 to 50.2)	9.8 (9.4 to 10.3)	61.5 (59.7 to 63.4)	12.5 (11.8 to 13.5)	43 (40.9 to 44.9)	7.4 (6.6 to 8.1)	53.8 (51.3 to 56.3)	9.5 (8.5 to 10.5)
Comoros	58.1 (56.6 to 59.7)	12.1 (11.4 to 12.8)	68.6 (67.1 to 70.2)	14.4 (13.7 to 15.3)	50.4 (47.9 to 52.8)	8.9 (7.9 to 9.8)	60 (57.2 to 62.5)	10.8 (9.7 to 11.9)
Djibouti	60.1 (57.4 to 62.8)	12.9 (11.5 to 14.3)	67.3 (64.1 to 70.2)	14.3 (12.7 to 16)	52.9 (49.8 to 55.7)	9.7 (8.4 to 11)	59.3 (56.1 to 62.5)	10.8 (9.3 to 12.4)
Eritrea	36 (35.2 to 36.7)	9.2 (8.8 to 9.5)	62.6 (60.6 to 64.8)	12.4 (11.7 to 13.5)	30.7 (28.9 to 32.2)	6.6 (5.8 to 7.3)	54.4 (51.5 to 57.1)	9.2 (8.2 to 10.2)
Ethiopia	47.1 (46.2 to 48.1)	9.7 (9.5 to 10)	68.4 (67.5 to 69.3)	15 (14.5 to 15.4)	41 (38.9 to 42.8)	7.1 (6.4 to 7.7)	59.5 (56.9 to 61.9)	11.1 (10 to 12.1)
Kenya	61.9 (61.3 to 62.5)	13.5 (13.3 to 13.8)	65.9 (65.2 to 66.5)	14.3 (14.1 to 14.6)	54.2 (51.8 to 56.3)	10.1 (9.2 to 11)	57.6 (55.1 to 59.7)	10.7 (9.7 to 11.6)
Madagascar	55.4 (54.5 to 56.4)	12.5 (12.1 to 12.9)	63.5 (61.6 to 65.4)	13 (12.2 to 14.1)	48.1 (45.9 to 50.1)	9.3 (8.4 to 10.1)	55.4 (52.7 to 58.1)	9.8 (8.7 to 10.9)
Malawi	48.6 (46.9 to 50)	11.7 (11.3 to 12.1)	63.2 (61.9 to 64.6)	14.2 (13.5 to 14.8)	42.3 (40 to 44.3)	8.6 (7.7 to 9.5)	55 (52.4 to 57.5)	10.5 (9.5 to 11.6)
Mozambique	50.3 (49.1 to 51.5)	11.6 (11.3 to 12.1)	58.3 (56.5 to 60.1)	12.7 (11.9 to 13.5)	43.4 (41.2 to 45.4)	8.6 (7.7 to 9.4)	50.6 (47.9 to 53)	9.4 (8.3 to 10.4)
Rwanda	49.4 (48.4 to 50.5)	10 (9.6 to 10.3)	68.4 (67.1 to 69.9)	14.8 (14 to 15.5)	43.6 (41.6 to 45.3)	7.5 (6.8 to 8.2)	59.6 (57 to 62.2)	11.1 (10 to 12.2)
Somalia	50 (47.6 to 52.6)	10.5 (9.4 to 11.6)	58.5 (55.6 to 61.2)	11.9 (10.6 to 13.3)	43.8 (41.1 to 46.5)	7.8 (6.8 to 8.9)	51.2 (48 to 54.2)	8.9 (7.8 to 10.2)
South Sudan	51.1 (48.5 to 53.9)	11.4 (10.3 to 12.7)	59.2 (56.1 to 62.4)	12.7 (11.3 to 14.2)	43.5 (40.5 to 46.3)	8.1 (6.9 to 9.3)	50.6 (47.1 to 53.9)	9 (7.7 to 10.4)
Tanzania	54.9 (53.2 to 56.3)	12.8 (12.3 to 13.3)	66.7 (65.5 to 68)	14.9 (14.3 to 15.5)	47.7 (45.2 to 49.8)	9.5 (8.6 to 10.4)	58.2 (55.5 to 60.7)	11.2 (10.2 to 12.3)
Uganda	47.5 (44.4 to 50.7)	12 (11.4 to 12.8)	65.7 (64.2 to 67.2)	14.5 (13.8 to 15.3)	40.7 (37.6 to 43.9)	8.7 (7.7 to 9.7)	57 (54.5 to 59.6)	10.8 (9.7 to 11.9)
Zambia	50.7 (48.5 to 52.4)	11.4 (11 to 11.8)	63.2 (61.7 to 64.6)	13.7 (13.1 to 14.4)	44.4 (41.9 to 46.8)	8.5 (7.7 to 9.3)	55.1 (52.5 to 57.5)	10.2 (9.2 to 11.3)
Southern sub-Saharan Africa	64.1 (63.2 to 64.7)	16.3 (16.2 to 16.5)	65 (64.3 to 65.7)	16 (15.8 to 16.1)	55.1 (52.7 to 57.3)	11.7 (10.5 to 12.7)	56 (53.4 to 58.2)	11.4 (10.2 to 12.4)
Botswana	63.5 (60.7 to 65.6)	14 (13.3 to 14.5)	69.1 (67.4 to 70.4)	15.7 (14.9 to 16.1)	54.6 (51.6 to 57.4)	9.9 (8.9 to 10.9)	58.9 (55.8 to 61.6)	11.1 (9.8 to 12.2)
Lesotho	60.3 (59 to 61.7)	12.8 (12.2 to 13.3)	54.6 (52.7 to 56.7)	12.1 (11.1 to 13.3)	51.9 (49.4 to 54.3)	9 (8 to 10)	47 (44.3 to 49.5)	8.4 (7.3 to 9.6)
Namibia	61.6 (60.1 to 62.8)	12.7 (12.3 to 13.1)	66.5 (64.6 to 68.2)	15.4 (14.5 to 16.1)	53 (50.3 to 55.5)	9 (8.1 to 9.9)	57.1 (54.1 to 59.9)	11 (9.8 to 12.2)
South Africa	65.1 (64.6 to 65.6)	17.4 (17.3 to 17.6)	66.3 (65.5 to 67)	16.7 (16.6 to 16.8)	56 (53.5 to 58.2)	12.4 (11.2 to 13.5)	57.1 (54.5 to 59.4)	12 (10.8 to 13)
Swaziland	62.1 (60.8 to 63.4)	12.6 (12 to 13.3)	59.8 (57.9 to 61.8)	13.3 (12.2 to 14.5)	53.7 (51.2 to 55.8)	8.9 (8 to 9.9)	51.3 (48.5 to 53.9)	9.3 (8.1 to 10.5)
Zimbabwe	60.9 (56.9 to 63.5)	13.8 (13.2 to 14.4)	61.3 (59.8 to 62.9)	12.8 (12 to 13.6)	52.8 (49.2 to 55.9)	9.9 (8.9 to 11)	53.2 (50.6 to 55.4)	9.2 (8.1 to 10.2)
Western sub-Saharan Africa	54.7 (53.4 to 55.8)	13.2 (12.5 to 13.9)	63.5 (61.9 to 64.8)	14.6 (13.8 to 15.3)	46.8 (44.3 to 49)	9.5 (8.5 to 10.5)	54.7 (52 to 57.3)	10.7 (9.5 to 11.8)
Benin	55.2 (54 to 56.3)	12.7 (12.2 to 13.2)	64.6 (62.7 to 66.5)	14.2 (13.2 to 15.1)	46.8 (44.1 to 49.3)	9.1 (8.1 to 10.1)	55.8 (53 to 58.7)	10.4 (9.2 to 11.6)
Burkina Faso	50.7 (49.5 to 52.1)	12.1 (11.7 to 12.5)	61.7 (59.9 to 63.2)	13.4 (12.8 to 14.1)	42.9 (40.4 to 45.3)	8.6 (7.6 to 9.5)	53.4 (50.7 to 55.9)	9.9 (9 to 11)
Cameroon	57.7 (56.7 to 58.6)	12.5 (12.1 to 13)	62.9 (61.1 to 64.9)	13.8 (12.8 to 14.9)	49.1 (46.5 to 51.4)	9 (8.1 to 9.9)	54.5 (51.6 to 57.2)	10.2 (9 to 11.4)
Cape Verde	70.3 (69.9 to 70.6)	17.6 (17.4 to 17.8)	75.8 (74.9 to 76.6)	18.8 (18.4 to 19.2)	60.8 (58.1 to 63.2)	13.2 (12 to 14.2)	65.9 (63.1 to 68.5)	14.1 (12.7 to 15.3)

SR Appendix Table 1. Life expectancy and healthy life expectancy (HALE) for both sexes combined, at birth and at age 65, 2007 and 2017, with 95% uncertainty intervals

Location	Life expectancy				HALE (Healthy life expectancy)			
	1990		2017		1990		2017	
	At birth	At age 65 years	At birth	At age 65 years	At birth	At age 65 years	At birth	At age 65 years
Chad	53.4 (52.1 to 54.6)	12.6 (12.1 to 13.1)	60 (58.2 to 61.8)	13.2 (12.4 to 14)	45.5 (43 to 47.8)	9.1 (8.1 to 10)	51.5 (48.7 to 54.1)	9.5 (8.5 to 10.6)
Cote d'Ivoire	54.8 (51.8 to 56.6)	12.4 (11.8 to 13)	62.5 (60.6 to 64.3)	13.7 (12.9 to 14.5)	46.8 (43.5 to 49.4)	8.7 (7.6 to 9.7)	54.2 (51.3 to 56.7)	10 (8.9 to 11.1)
The Gambia	59.9 (58.2 to 61.7)	12.8 (12 to 13.6)	65.7 (64.2 to 67.3)	13.6 (12.8 to 14.3)	51.4 (48.7 to 54.2)	9.3 (8.3 to 10.4)	56.7 (53.9 to 59.3)	10 (9 to 11.1)
Ghana	59.2 (57.7 to 60.6)	13 (12.2 to 13.7)	65.5 (64.3 to 66.8)	13.9 (13.3 to 14.5)	51.2 (48.6 to 53.5)	9.6 (8.5 to 10.6)	57 (54.4 to 59.5)	10.4 (9.4 to 11.3)
Guinea	51.9 (50.7 to 53.1)	12.6 (12.2 to 13)	60.7 (59.1 to 62.3)	12.7 (12 to 13.4)	44.7 (42.4 to 46.9)	9.2 (8.3 to 10.1)	52.8 (50 to 55.4)	9.4 (8.4 to 10.4)
Guinea-Bissau	48.6 (46.9 to 50.4)	10.3 (9.8 to 11)	60 (58.3 to 61.6)	12.2 (11.5 to 13)	42.1 (39.8 to 44.3)	7.5 (6.6 to 8.3)	52 (49.3 to 54.3)	9 (8 to 9.9)
Liberia	49 (47.7 to 50.4)	12.5 (11.9 to 13.1)	64.4 (62.8 to 66.1)	13.9 (13.1 to 14.7)	41.6 (39.1 to 43.8)	8.8 (7.7 to 9.8)	54.3 (51.2 to 57.3)	9.9 (8.7 to 11)
Mali	49.3 (48.3 to 50.4)	11.6 (11.3 to 12)	61.9 (60.2 to 63.5)	14.3 (13.6 to 15.1)	42 (39.7 to 44.1)	8.3 (7.4 to 9.1)	53.1 (50.3 to 55.7)	10.4 (9.2 to 11.5)
Mauritania	59.6 (58.6 to 60.7)	12.3 (11.8 to 12.8)	70.5 (69 to 72)	15.3 (14.4 to 16.3)	51.5 (49.1 to 53.7)	8.9 (7.9 to 9.8)	60.9 (58 to 63.6)	11.2 (9.9 to 12.4)
Niger	46.6 (45.2 to 47.9)	12.3 (11.8 to 12.7)	62.3 (60.5 to 64.1)	13.8 (13.1 to 14.6)	40.3 (38.2 to 42.4)	9 (8.1 to 9.9)	54.2 (51.6 to 56.8)	10.2 (9.2 to 11.3)
Nigeria	55.5 (53 to 57.9)	13.9 (12.5 to 15.4)	64.3 (61 to 67.1)	15.8 (13.9 to 17.5)	47.5 (44.4 to 50.4)	10.1 (8.7 to 11.5)	55.1 (51.6 to 58.3)	11.5 (9.8 to 13.1)
Sao Tome and Principe	64.1 (63.1 to 65)	15.1 (14.7 to 15.6)	69.9 (68.7 to 71.2)	14.5 (13.9 to 15.2)	55.5 (52.9 to 57.7)	11.2 (10.1 to 12.1)	60.9 (58.3 to 63.5)	10.8 (9.7 to 11.9)
Senegal	58.4 (57.5 to 59.4)	12.9 (12.4 to 13.3)	68 (66.7 to 69.3)	14.3 (13.6 to 15)	50.2 (47.7 to 52.6)	9.4 (8.4 to 10.3)	58.8 (56 to 61.5)	10.5 (9.3 to 11.5)
Sierra Leone	50.1 (48.6 to 51.6)	12.3 (11.7 to 12.9)	60.4 (58.7 to 62.2)	13.1 (12.4 to 13.9)	43 (40.8 to 45.2)	9 (8 to 9.8)	52.1 (49.4 to 54.7)	9.6 (8.5 to 10.6)
Togo	57.6 (56.3 to 58.9)	12.7 (12.2 to 13.3)	64.3 (62.6 to 66.2)	14 (13.1 to 14.9)	49.6 (47 to 51.8)	9.3 (8.3 to 10.2)	55.8 (53 to 58.6)	10.3 (9.2 to 11.4)

SR Appendix Table 2. All-age DALYs counts and age standardized DALYs rates per 100,000 individuals for all causes and level 1 cause groups by location for 1990, 2007, and 2017, both sexes combined

Location	Cause	All-age DALYs			Age-standardized rate of DALYs per 100,000		
		1990	2007	2017	1990	2000	2007
Global	All causes	2560000 (2420000 to 2720000)	2540000 (2350000 to 2740000)	2500000 (2290000 to 2740000)	48595.2 (45720.1 to 51786.7)	39144.9 (36363.5 to 42263.6)	32796.9 (30041.9 to 35849.3)
Global	Communicable, maternal, neonatal, and nutritional disorders	1190000 (1140000 to 1230000)	946000 (910000 to 987000)	696000 (660000 to 740000)	19631.4 (18899.3 to 20409.3)	14525.7 (13978.2 to 15143.9)	9853.3 (9349.7 to 10459.3)
Global	HIV/AIDS and sexually transmitted infections	33700 (26100 to 44000)	121000 (112000 to 132000)	65900 (58700 to 74700)	583.8 (462 to 746.1)	1767.3 (1633.8 to 1935.5)	874.1 (767.5 to 1006.2)
Global	HIV/AIDS	20900 (18800 to 23100)	108000 (103000 to 113000)	54400 (51200 to 57700)	380.2 (342.5 to 422.1)	1572.7 (1500.8 to 1648.4)	704.8 (662.6 to 747.9)
Global	HIV/AIDS - Drug-susceptible Tuberculosis	6270 (4800 to 7510)	24500 (17100 to 31600)	11100 (7940 to 14200)	112.3 (85.8 to 135.4)	361.2 (252.3 to 464)	145.1 (104.4 to 185.3)
Global	HIV/AIDS - Multidrug-resistant Tuberculosis without extensive drug resistance	48 (26.8 to 85.4)	2600 (1520 to 3930)	1260 (755 to 1920)	0.9 (0.5 to 1.6)	38.3 (22.4 to 57.6)	16.6 (9.9 to 25.3)
Global	HIV/AIDS - Extensively drug-resistant Tuberculosis	0 (0 to 0)	70.5 (43.5 to 103)	63.3 (38.8 to 93.3)	0 (0 to 0)	1 (0.6 to 1.5)	0.8 (0.5 to 1.2)
Global	HIV/AIDS resulting in other diseases	14600 (12400 to 17000)	80700 (71500 to 90000)	42100 (37800 to 46700)	267.1 (226.9 to 313.7)	1172.2 (1038.1 to 1306.9)	542.3 (487 to 603.1)
Global	Sexually transmitted infections excluding HIV	12800 (5630 to 22600)	12600 (5690 to 22400)	11500 (5320 to 20200)	203.6 (91.7 to 357.2)	194.6 (86.9 to 347.2)	169.3 (76.7 to 300.4)
Global	Syphilis	11600 (4520 to 21500)	11200 (4370 to 20900)	9910 (3930 to 18700)	181.3 (71 to 335.5)	174.4 (68.1 to 325.7)	149.5 (59.1 to 283.2)
Global	Chlamydial infection	272 (176 to 443)	329 (205 to 555)	355 (219 to 606)	5.1 (3.3 to 8.2)	4.6 (2.9 to 7.8)	4.5 (2.8 to 7.7)
Global	Gonococcal infection	286 (198 to 406)	290 (206 to 437)	303 (216 to 468)	5.3 (3.7 to 7.4)	4.1 (2.9 to 6.2)	3.9 (2.7 to 6)
Global	Trichomoniasis	152 (61.5 to 332)	209 (84.4 to 454)	243 (97.6 to 524)	2.9 (1.2 to 6.3)	3 (1.2 to 6.5)	3.1 (1.2 to 6.6)
Global	Genital herpes	146 (48 to 345)	207 (67.4 to 492)	247 (79.8 to 594)	3 (1 to 7.1)	3 (1 to 7.2)	3.1 (1 to 7.4)
Global	Other sexually transmitted infections	325 (224 to 500)	385 (257 to 604)	416 (276 to 659)	6.1 (4.2 to 9.2)	5.4 (3.6 to 8.5)	5.3 (3.5 to 8.4)
Global	Respiratory infections and tuberculosis	309000 (287000 to 329000)	207000 (198000 to 217000)	160000 (152000 to 168000)	5315.9 (4952.2 to 5621.5)	3219.1 (3067.9 to 3363.3)	2209.6 (2095.9 to 2327.9)
Global	Tuberculosis	68200 (63600 to 72600)	56000 (53700 to 58500)	45000 (42800 to 47300)	1344.1 (1258.3 to 1429.7)	842.3 (807.1 to 879.8)	572.8 (544 to 602.4)
Global	Latent tuberculosis infection	0 (0 to 0)	0 (0 to 0)	0 (0 to 0)	0 (0 to 0)	0 (0 to 0)	0 (0 to 0)
Global	Drug-susceptible tuberculosis	67500 (63100 to 71800)	50000 (47300 to 52400)	39900 (36400 to 43100)	1330.2 (1246.1 to 1414.9)	752 (712.3 to 788.4)	508.2 (463.4 to 548.7)
Global	Multidrug-resistant tuberculosis without extensive drug resistance	700 (357 to 1310)	5600 (4520 to 6950)	4650 (2660 to 7220)	13.9 (7.1 to 26.2)	84.1 (67.8 to 104.2)	59 (34 to 91.4)
Global	Extensively drug-resistant tuberculosis	0 (0 to 0)	422 (340 to 513)	447 (311 to 626)	0 (0 to 0)	6.3 (5.1 to 7.6)	5.6 (3.9 to 7.8)
Global	Lower respiratory infections	233000 (213000 to 250000)	143000 (135000 to 151000)	106000 (100000 to 112000)	3830.9 (3517.2 to 4100.8)	2257.7 (2126 to 2380.8)	1523.9 (1433 to 1610.4)
Global	Upper respiratory infections	5990 (3910 to 8790)	5980 (3700 to 9130)	6340 (3870 to 9820)	106.7 (69.8 to 156.8)	88.8 (55.4 to 134.9)	84.6 (51.7 to 130.7)
Global	Otitis media	1950 (1250 to 2990)	2040 (1260 to 3210)	2080 (1280 to 3290)	34.2 (21.8 to 52.3)	30.3 (18.7 to 47.6)	28.2 (17.3 to 44.7)
Global	Enteric infections	199000 (171000 to 226000)	130000 (116000 to 150000)	95200 (83900 to 112000)	3315.2 (2843.3 to 3790.9)	2034.3 (1804 to 2337.5)	1350.2 (1195.2 to 1579.1)
Global	Diarrhoeal diseases	179000 (154000 to 204000)	112000 (98800 to 130000)	81000 (70100 to 97200)	2972.5 (2542.7 to 3412.9)	1758.3 (1548 to 2037.9)	1149 (1005.2 to 1359.9)
Global	Typhoid and paratyphoid	17200 (9930 to 27800)	12900 (7400 to 20500)	9800 (5580 to 15800)	286.5 (165.2 to 462.1)	193.4 (111.1 to 311)	137.9 (78.4 to 223.3)
Global	Typhoid fever	15100 (8490 to 24700)	11300 (6350 to 18200)	8440 (4730 to 13500)	251.6 (141.1 to 409.9)	169.9 (95.4 to 274.9)	118.8 (66.9 to 190.4)
Global	Paratyphoid fever	2090 (925 to 4180)	1570 (716 to 3030)	1360 (633 to 2630)	34.9 (15.5 to 70.2)	23.5 (10.7 to 45.6)	19.1 (8.9 to 36.7)
Global	Invasive non-typhoidal salmonella	3000 (1630 to 5220)	5150 (2940 to 8440)	4260 (2380 to 7380)	50.1 (27.3 to 86.8)	79.7 (45.5 to 130.1)	61.7 (34.7 to 107.6)
Global	Other intestinal infectious diseases	384 (258 to 604)	187 (123 to 305)	105 (68.7 to 172)	6.1 (4.2 to 9.6)	2.9 (1.9 to 4.7)	1.5 (1 to 2.5)
Global	Neglected tropical diseases and malaria	92900 (61900 to 147000)	88600 (70700 to 112000)	62300 (48600 to 79900)	1573.7 (1056.9 to 2481.3)	1363.8 (1086 to 1725.5)	877.6 (679.5 to 1129.2)
Global	Malaria	52800 (36300 to 73400)	68400 (51000 to 89800)	45000 (31700 to 61000)	861.7 (597.5 to 1190)	1063.4 (790 to 1398.1)	649.8 (457.1 to 878.7)
Global	Chagas disease	314 (294 to 336)	239 (220 to 262)	232 (210 to 261)	7 (6.6 to 7.5)	3.8 (3.5 to 4.1)	2.9 (2.6 to 3.2)
Global	Leishmaniasis	19000 (206 to 75200)	1610 (165 to 7690)	774 (199 to 2720)	325.9 (3.8 to 1272.5)	24.2 (2.4 to 116.1)	10.6 (2.6 to 38.2)
Global	Visceral leishmaniasis	18900 (90.8 to 75100)	1410 (6.48 to 7500)	511 (1.02 to 2440)	323.5 (1.6 to 1269.9)	21.3 (0.1 to 112.7)	7.2 (0 to 34.7)
Global	Cutaneous and mucocutaneous leishmaniasis	121 (63.5 to 211)	200 (126 to 308)	264 (172 to 389)	2.4 (1.3 to 4.2)	2.9 (1.8 to 4.5)	3.4 (2.2 to 5)
Global	African trypanosomiasis	951 (478 to 1590)	410 (207 to 687)	79 (15.4 to 287)	17.1 (8.5 to 28.5)	5.9 (3 to 10)	1.1 (0.2 to 3.9)
Global	Schistosomiasis	1470 (996 to 2260)	1780 (1090 to 3000)	1430 (876 to 2440)	28.5 (19.5 to 43.7)	25.9 (15.9 to 43.4)	18.3 (11.2 to 30.9)

SR Appendix Table 2. All-age DALYs counts and age standardized DALYs rates per 100,000 individuals for all causes and level 1 cause groups by location for 1990, 2007, and 2017, both sexes combined

		All-age DALYs			Age-standardized rate of DALYs per 100,000		
Global	Cysticercosis	1350 (951 to 1790)	1500 (1010 to 2040)	1610 (1050 to 2230)	27.5 (19.4 to 36.4)	22.3 (15.1 to 30.3)	20.1 (13.2 to 27.8)
Global	Cystic echinococcosis	195 (154 to 243)	126 (94.8 to 160)	100 (72.8 to 139)	3.5 (2.8 to 4.4)	1.9 (1.4 to 2.4)	1.3 (1 to 1.8)
Global	Lymphatic filariasis	1730 (889 to 2900)	2170 (1150 to 3590)	1360 (752 to 2160)	33.6 (17.4 to 56.4)	31.5 (16.7 to 52)	17.4 (9.6 to 27.5)
Global	Onchocerciasis	1450 (799 to 2300)	1290 (722 to 2040)	1340 (639 to 2370)	27.9 (15.8 to 44.4)	18.8 (10.6 to 29.7)	17.3 (8.2 to 30.5)
Global	Trachoma	368 (249 to 498)	321 (219 to 435)	303 (202 to 425)	9.1 (6.2 to 12.3)	5.3 (3.6 to 7.2)	3.8 (2.6 to 5.4)
Global	Dengue	1410 (624 to 2510)	2070 (1100 to 2760)	2920 (1630 to 3970)	23.7 (10.9 to 41.5)	31.4 (16.5 to 41.5)	39.6 (22 to 53.3)
Global	Yellow fever	759 (160 to 2240)	374 (78.6 to 1110)	314 (67.2 to 900)	13 (2.8 to 38.5)	5.5 (1.2 to 16.3)	4.3 (0.9 to 12.4)
Global	Rabies	2360 (1810 to 3010)	1310 (1100 to 1870)	634 (504 to 836)	41.2 (32 to 52.3)	19.6 (16.4 to 28)	8.6 (6.8 to 11.5)
Global	Intestinal nematode infections	4620 (3060 to 6820)	2840 (1830 to 4280)	1920 (1200 to 2980)	80.8 (53.1 to 120.1)	42 (27.2 to 63.2)	25.9 (16.4 to 40.1)
Global	Ascariasis	2410 (1700 to 3440)	1380 (955 to 2030)	861 (569 to 1290)	41 (28.7 to 59.2)	20.8 (14.5 to 30.5)	12 (8 to 17.8)
Global	Trichuriasis	485 (270 to 806)	276 (157 to 460)	213 (120 to 354)	8.5 (4.7 to 14.1)	4 (2.2 to 6.6)	2.8 (1.6 to 4.7)
Global	Hookworm disease	1730 (1060 to 2660)	1180 (708 to 1840)	845 (510 to 1340)	31.3 (19.1 to 48.1)	17.2 (10.4 to 26.9)	11.2 (6.7 to 17.8)
Global	Food-borne trematodiases	1580 (788 to 2890)	1720 (979 to 2920)	1870 (1070 to 3150)	29.9 (15 to 54.4)	24.9 (14.2 to 42.2)	23.4 (13.4 to 39.3)
Global	Leprosy	23.6 (16 to 33.5)	31.9 (21.7 to 44.9)	31.5 (21.5 to 44.6)	0.5 (0.4 to 0.7)	0.5 (0.3 to 0.7)	0.4 (0.3 to 0.6)
Global	Ebola virus disease	0 (0 to 0)	26.4 (26.3 to 26.4)	0.503 (0.502 to 0.505)	0 (0 to 0)	0.4 (0.4 to 0.4)	0 (0 to 0)
Global	Zika virus disease	0 (0 to 0)	0.0000687 (0 to 0.000406)	2.24 (1.27 to 4.66)	0 (0 to 0)	0 (0 to 0)	0 (0 to 0.1)
Global	Guinea worm disease	26.5 (16.8 to 39)	0.103 (0.0638 to 0.156)	0.00055 (0.000294 to 0.000883)	0.5 (0.3 to 0.8)	0 (0 to 0)	0 (0 to 0)
Global	Other neglected tropical diseases	2480 (1680 to 4490)	2400 (1620 to 4350)	2340 (1560 to 4190)	42.2 (28.8 to 74.8)	36.5 (24.5 to 66.7)	32.8 (21.8 to 60.1)
Global	Other infectious diseases	160000 (123000 to 218000)	83200 (69100 to 103000)	57100 (48800 to 67300)	2597.3 (2020.1 to 3514.7)	1288.8 (1069.2 to 1598.6)	816.8 (693.8 to 970)
Global	Meningitis	35800 (30600 to 39700)	26900 (23800 to 29700)	20400 (17800 to 23400)	584.6 (501.5 to 648)	416 (367.3 to 458.5)	293 (254.3 to 337.3)
Global	Pneumococcal meningitis	4720 (3940 to 5490)	3810 (3260 to 4380)	3080 (2640 to 3600)	78.1 (65.6 to 90.4)	58.6 (50.1 to 67.3)	43.9 (37.5 to 51.4)
Global	H influenzae type B meningitis	12200 (10300 to 14000)	8400 (7270 to 9630)	4990 (4310 to 5890)	199.2 (167.7 to 227.9)	129.9 (112.3 to 149)	71.6 (61.6 to 85)
Global	Meningococcal infection	4370 (3670 to 5070)	3450 (3000 to 3940)	2280 (1920 to 2720)	70.5 (59.3 to 81.7)	53.6 (46.6 to 61.3)	33.2 (27.8 to 39.7)
Global	Other meningitis	14500 (12200 to 16600)	11300 (9900 to 12600)	10000 (8600 to 11500)	236.8 (200.1 to 270.1)	173.9 (152.7 to 195)	144.2 (123.6 to 166.2)
Global	Encephalitis	6400 (4840 to 9170)	5710 (5240 to 6390)	5110 (4540 to 5760)	110 (84.6 to 155)	87.4 (80.2 to 97.9)	70.8 (62.8 to 79.3)
Global	Diphtheria	1440 (1050 to 1930)	393 (285 to 543)	299 (182 to 510)	23 (16.9 to 30.9)	6.2 (4.5 to 8.5)	4.4 (2.7 to 7.6)
Global	Whooping cough	14800 (6660 to 27800)	10400 (4840 to 18800)	7980 (4020 to 14100)	233.7 (105.2 to 439)	163.4 (76.1 to 295.5)	119.3 (60.1 to 210.7)
Global	Tetanus	24400 (21300 to 27500)	6020 (5060 to 7170)	2450 (1740 to 3200)	392.8 (340.3 to 442.2)	92.8 (78.1 to 110.5)	35.2 (25 to 46.4)
Global	Measles	59800 (22000 to 123000)	18900 (7010 to 38900)	8160 (2950 to 17600)	949.3 (349.9 to 1958.3)	297.5 (110.4 to 612.7)	121.5 (44 to 261.7)
Global	Varicella and herpes zoster	1600 (1400 to 1790)	1330 (1180 to 1510)	1140 (985 to 1320)	27.7 (24.4 to 30.9)	20.7 (18.4 to 23.4)	16 (13.9 to 18.4)
Global	Acute hepatitis	8510 (6390 to 9660)	7470 (5430 to 8410)	5990 (4580 to 6900)	152.3 (114.5 to 172.1)	112 (81.3 to 126.3)	78.9 (60.1 to 91.2)
Global	Acute hepatitis A	2690 (2000 to 3260)	2210 (1560 to 2660)	1500 (1130 to 1870)	44.4 (33.1 to 53.8)	33.2 (23.4 to 40)	20.9 (15.7 to 26.2)
Global	Acute hepatitis B	4300 (3130 to 5070)	3960 (2830 to 4630)	3530 (2620 to 4120)	82.1 (60 to 95.8)	59.7 (42.6 to 69.8)	45.1 (33.3 to 53)
Global	Acute hepatitis C	521 (283 to 877)	326 (172 to 550)	228 (124 to 383)	8.4 (4.6 to 14.2)	5.1 (2.7 to 8.6)	3.3 (1.8 to 5.6)
Global	Acute hepatitis E	996 (710 to 1260)	979 (670 to 1250)	739 (517 to 935)	17.4 (12.4 to 22)	14 (9.6 to 17.8)	9.7 (6.8 to 12.2)
Global	Other unspecified infectious diseases	7230 (5300 to 8300)	6070 (4600 to 6980)	5570 (4300 to 6430)	123.8 (92.8 to 141.9)	92.8 (70.2 to 106.4)	77.6 (59.3 to 89.5)
Global	Maternal and neonatal disorders	298000 (280000 to 317000)	246000 (236000 to 256000)	198000 (187000 to 209000)	4684.6 (4418.1 to 4979.1)	3784.1 (3639.8 to 3946)	2916.4 (2761 to 3077.1)
Global	Maternal disorders	19900 (18300 to 21200)	15600 (14700 to 16400)	11800 (11000 to 12800)	353 (326.3 to 377.2)	217.5 (206 to 229)	151.2 (140.7 to 163.6)
Global	Maternal haemorrhage	7520 (6300 to 8840)	4690 (4020 to 5440)	2230 (1910 to 2610)	134 (113.2 to 156.9)	65.7 (56.4 to 76)	28.6 (24.5 to 33.4)
Global	Maternal sepsis and other pregnancy related infections	2310 (1800 to 3030)	1740 (1440 to 2190)	1250 (1080 to 1480)	40.9 (32.2 to 52.9)	24.2 (20 to 30.4)	16.1 (13.8 to 19)

SR Appendix Table 2. All-age DALYs counts and age standardized DALYs rates per 100,000 individuals for all causes and level 1 cause groups by location for 1990, 2007, and 2017, both sexes combined

		All-age DALYs			Age-standardized rate of DALYs per 100,000		
Global	Maternal hypertensive disorders	2320 (1870 to 2920)	2000 (1710 to 2410)	1870 (1620 to 2190)	40.4 (32.8 to 50.6)	27.9 (23.9 to 33.5)	24.2 (20.9 to 28.2)
Global	Maternal obstructed labour and uterine rupture	1680 (1280 to 2260)	1330 (1080 to 1690)	1120 (901 to 1380)	30.6 (23.5 to 40.7)	18.7 (15.1 to 23.7)	14.3 (11.5 to 17.6)
Global	Maternal abortive outcome	1510 (1210 to 1920)	1080 (884 to 1330)	982 (827 to 1180)	27.2 (22 to 34)	15 (12.4 to 18.6)	12.5 (10.5 to 15.1)
Global	Ectopic pregnancy	804 (482 to 1390)	694 (457 to 1120)	606 (424 to 896)	14.2 (8.6 to 23.9)	9.7 (6.4 to 15.6)	7.8 (5.4 to 11.6)
Global	Indirect maternal deaths	2020 (1550 to 2720)	2060 (1750 to 2510)	1930 (1690 to 2220)	35.7 (27.6 to 47.6)	28.8 (24.5 to 35.1)	24.8 (21.7 to 28.5)
Global	Late maternal deaths	203 (163 to 256)	199 (160 to 250)	195 (152 to 251)	3.6 (2.9 to 4.5)	2.8 (2.2 to 3.5)	2.5 (2 to 3.2)
Global	Maternal deaths aggravated by HIV/AIDS	40.5 (23.6 to 56.9)	115 (74.5 to 150)	84.4 (53 to 114)	0.7 (0.4 to 1)	1.6 (1.1 to 2.1)	1.1 (0.7 to 1.4)
Global	Other maternal disorders	1450 (1080 to 2030)	1650 (1320 to 2120)	1520 (1280 to 1810)	25.8 (19.4 to 35.6)	23 (18.4 to 29.5)	19.4 (16.4 to 23.1)
Global	Neonatal disorders	278000 (260000 to 297000)	230000 (221000 to 240000)	186000 (175000 to 197000)	4331.6 (4060.3 to 4625.8)	3566.6 (3426.8 to 3721.8)	2765.2 (2611.8 to 2922.7)
Global	Neonatal preterm birth	120000 (110000 to 131000)	87700 (82100 to 97300)	70200 (64400 to 77200)	1870.9 (1719.7 to 2048.9)	1357.2 (1271.8 to 1508.7)	1041.2 (958.9 to 1138.5)
Global	Neonatal encephalopathy due to birth asphyxia and trauma	76100 (68400 to 84200)	69900 (62500 to 75500)	56500 (50200 to 63100)	1187.9 (1066.8 to 1315.4)	1082.3 (965.8 to 1166.5)	838.9 (746.3 to 930.9)
Global	Neonatal sepsis and other neonatal infections	22700 (18300 to 31700)	23700 (20600 to 30200)	22000 (18900 to 28000)	358.4 (287.9 to 496.4)	367 (318.4 to 467.6)	325.4 (281.2 to 417.5)
Global	Hemolytic disease and other neonatal jaundice	12100 (10100 to 14200)	7630 (6750 to 8580)	5190 (4610 to 5790)	188.4 (158.2 to 222)	118.3 (104.7 to 133)	77.1 (68.4 to 86)
Global	Other neonatal disorders	46900 (39900 to 55800)	41100 (35800 to 45000)	31800 (27100 to 34800)	726.1 (617.8 to 863.7)	641.7 (557.9 to 701.3)	482.6 (410.4 to 527.5)
Global	Nutritional deficiencies	92400 (74700 to 115000)	69900 (55200 to 89400)	58000 (44300 to 76900)	1560.9 (1258.7 to 1956.2)	1068.3 (845.3 to 1358.1)	808.7 (619.2 to 1065.8)
Global	Protein-energy malnutrition	41500 (35200 to 47200)	24100 (21800 to 26400)	16200 (14500 to 18000)	670.2 (569.2 to 759.7)	378.8 (342.7 to 415.5)	238 (212.7 to 264.8)
Global	Iodine deficiency	3330 (2030 to 5320)	2210 (1360 to 3470)	2060 (1250 to 3260)	60.5 (36.9 to 97)	32.4 (20 to 50.8)	26.7 (16.2 to 42.3)
Global	Dietary iron deficiency	31700 (21600 to 45500)	31700 (21400 to 45200)	30000 (20300 to 43600)	569.3 (387.8 to 815.6)	470.4 (318.5 to 671.1)	403 (272.4 to 586.6)
Global	Other nutritional deficiencies	3300 (2590 to 3920)	1960 (1750 to 2190)	1450 (1250 to 1640)	57.9 (45.8 to 68)	30.4 (27.1 to 34)	19.5 (16.8 to 22.3)
Global	Non-communicable diseases	1110000 (1000000 to 1230000)	1340000 (1190000 to 1510000)	1550000 (1380000 to 1750000)	24017.4 (21861.4 to 26509.6)	20852.2 (18710.2 to 23388.5)	19676.5 (17509.8 to 22177.9)
Global	Neoplasms	160000 (155000 to 165000)	194000 (191000 to 197000)	234000 (229000 to 238000)	3583.2 (3499.2 to 3692.3)	3057.9 (3010.6 to 3103.3)	2900.9 (2842.8 to 2957)
Global	Lip and oral cavity cancer	2810 (2700 to 2940)	4020 (3910 to 4110)	5250 (4980 to 5500)	63.9 (61.4 to 66.8)	62.2 (60.6 to 63.6)	64.2 (60.9 to 67.2)
Global	Nasopharynx cancer	1770 (1660 to 1860)	1760 (1710 to 1800)	2090 (2000 to 2170)	38.2 (35.9 to 40.2)	26.8 (26 to 27.4)	25.4 (24.4 to 26.5)
Global	Other pharynx cancer	1660 (1450 to 1790)	2400 (2050 to 2500)	3260 (2820 to 3460)	37.6 (32.8 to 40.5)	37.1 (31.8 to 38.7)	39.6 (34.3 to 42)
Global	Oesophageal cancer	7680 (7420 to 7970)	8970 (8810 to 9140)	9780 (9530 to 10000)	179.9 (174.1 to 186.8)	143 (140.6 to 145.8)	119.9 (116.9 to 123)
Global	Stomach cancer	19100 (18700 to 19800)	18200 (17900 to 18500)	19100 (18700 to 19600)	445.9 (435 to 461.2)	289 (285.1 to 294.4)	235.9 (231.1 to 241.3)
Global	Colon and rectum cancer	11500 (11100 to 12200)	15200 (15000 to 15600)	19000 (18500 to 19500)	275.6 (265.9 to 291)	245.7 (241.1 to 251.5)	235.7 (229.7 to 242)
Global	Liver cancer	13300 (12400 to 14400)	17100 (16600 to 18000)	20800 (19900 to 21800)	297.4 (278.6 to 323.3)	265.6 (257.9 to 280)	253.6 (243.2 to 266.2)
Global	Liver cancer due to hepatitis B	6860 (6270 to 7530)	8310 (7860 to 8940)	9550 (8920 to 10300)	149.5 (136.6 to 163.9)	126.2 (119.3 to 135.8)	115.8 (108.3 to 124.4)
Global	Liver cancer due to hepatitis C	2810 (2580 to 3110)	3900 (3640 to 4210)	4960 (4600 to 5330)	66.3 (61.2 to 73.2)	62.9 (58.8 to 67.9)	61.1 (56.9 to 65.6)
Global	Liver cancer due to alcohol use	1700 (1460 to 2000)	2400 (2100 to 2790)	3080 (2680 to 3590)	39.5 (34 to 46.5)	37.8 (33.2 to 43.8)	37.6 (32.9 to 43.8)
Global	Liver cancer due to NASH	712 (625 to 817)	1060 (946 to 1190)	1460 (1300 to 1620)	16.5 (14.5 to 18.9)	16.9 (15.1 to 18.9)	18 (16.1 to 20)
Global	Liver cancer due to other causes	1170 (1020 to 1310)	1420 (1270 to 1580)	1720 (1540 to 1920)	25.5 (22.3 to 28.8)	21.8 (19.5 to 24.2)	21.1 (19 to 23.5)
Global	Gallbladder and biliary tract cancer	2290 (2170 to 2600)	2860 (2530 to 3060)	3480 (3040 to 3710)	55.2 (52.5 to 62.5)	46.3 (41.2 to 49.6)	43.2 (37.7 to 46.1)
Global	Pancreatic cancer	4380 (4310 to 4480)	6680 (6620 to 6760)	9080 (8890 to 9260)	104.9 (103.2 to 107)	107.9 (106.9 to 109.2)	112.2 (110 to 114.4)
Global	Larynx cancer	2620 (2530 to 2700)	2780 (2710 to 2860)	3280 (3190 to 3380)	60.3 (58.2 to 62.1)	43.7 (42.5 to 44.9)	39.9 (38.8 to 41)
Global	Tracheal, bronchus, and lung cancer	25400 (24900 to 26200)	32700 (32400 to 33100)	40900 (40000 to 41900)	594 (582.8 to 613.2)	524 (518.1 to 530.1)	503.1 (491.9 to 514.3)
Global	Malignant skin melanoma	1020 (829 to 1280)	1410 (1130 to 1650)	1650 (1330 to 1940)	22.9 (18.6 to 28.6)	21.8 (17.4 to 25.5)	20.5 (16.5 to 24)
Global	Non-melanoma skin cancer	693 (660 to 734)	1020 (973 to 1070)	1330 (1270 to 1400)	16.5 (15.7 to 17.5)	16.6 (15.8 to 17.4)	16.6 (15.9 to 17.5)
Global	Non-melanoma skin cancer (squamous-cell carcinoma)	691 (659 to 732)	1020 (972 to 1070)	1330 (1270 to 1390)	16.5 (15.7 to 17.5)	16.6 (15.7 to 17.4)	16.6 (15.9 to 17.5)

SR Appendix Table 2. All-age DALYs counts and age standardized DALYs rates per 100,000 individuals for all causes and level 1 cause groups by location for 1990, 2007, and 2017, both sexes combined

		All-age DALYs			Age-standardized rate of DALYs per 100,000		
Global	Non-melanoma skin cancer (basal-cell carcinoma)	1.31 (0.472 to 2.85)	1.89 (0.745 to 3.93)	2.47 (0.945 to 5.2)	0 (0 to 0.1)	0 (0 to 0.1)	0 (0 to 0.1)
Global	Breast cancer	10400 (9760 to 11600)	14200 (13700 to 14900)	17700 (16900 to 18700)	238.3 (223.6 to 263)	219 (211.5 to 229.8)	216.3 (206.4 to 228.1)
Global	Cervical cancer	6120 (5580 to 6810)	7000 (6500 to 7340)	8060 (7530 to 8400)	135.5 (123.9 to 150.7)	105.6 (98.1 to 110.8)	98.2 (91.7 to 102.4)
Global	Uterine cancer	1530 (1440 to 1600)	1840 (1770 to 1900)	2140 (2060 to 2230)	35.6 (33.8 to 37.3)	29.1 (28.1 to 30.2)	26.3 (25.2 to 27.3)
Global	Ovarian cancer	2630 (2490 to 2830)	3620 (3530 to 3780)	4670 (4530 to 4830)	60.2 (57.3 to 64.6)	56.4 (55.1 to 58.8)	57.1 (55.4 to 59)
Global	Prostate cancer	3690 (2930 to 4200)	5440 (4490 to 6250)	7060 (6050 to 8350)	96 (75.9 to 108.4)	92.4 (76.2 to 106.2)	90 (77.2 to 106.6)
Global	Testicular cancer	348 (328 to 371)	365 (349 to 382)	375 (356 to 398)	6.6 (6.2 to 7)	5.2 (5 to 5.5)	4.7 (4.5 to 5)
Global	Kidney cancer	1920 (1740 to 2040)	2670 (2490 to 2750)	3280 (3090 to 3390)	42.7 (38.9 to 44.9)	42.5 (39.6 to 43.9)	41.1 (38.7 to 42.5)
Global	Bladder cancer	2320 (2170 to 2410)	2920 (2830 to 3010)	3600 (3480 to 3770)	57.6 (54.1 to 59.8)	48.3 (46.9 to 49.9)	45.3 (43.7 to 47.4)
Global	Thyroid cancer	648 (596 to 713)	916 (866 to 970)	1130 (1070 to 1230)	14.4 (13.4 to 15.8)	14.2 (13.5 to 15)	14.1 (13.3 to 15.3)
Global	Mesothelioma	432 (352 to 527)	554 (527 to 598)	671 (648 to 693)	10 (8.2 to 12.1)	8.8 (8.4 to 9.4)	8.3 (8 to 8.6)
Global	Hodgkin lymphoma	1660 (1230 to 1840)	1440 (1210 to 1710)	1380 (1160 to 1620)	31.5 (23.6 to 34.7)	21.3 (17.9 to 25.2)	17.8 (14.9 to 20.9)
Global	Non-Hodgkin's lymphoma	4570 (4240 to 5020)	5730 (5570 to 5930)	7020 (6790 to 7230)	94.6 (88.9 to 102.5)	88.9 (86.4 to 91.9)	89.3 (86.1 to 92.2)
Global	Multiple myeloma	1220 (1130 to 1390)	1780 (1660 to 2000)	2330 (2180 to 2610)	28.8 (26.7 to 33.3)	28.6 (26.6 to 32.2)	28.8 (26.9 to 32.2)
Global	Leukaemia	12000 (9870 to 14000)	11700 (10500 to 12500)	12000 (10700 to 12800)	225.4 (190.2 to 259.3)	177.7 (159.5 to 190.7)	156.8 (140.8 to 168.1)
Global	Acute lymphoid leukaemia	2280 (1870 to 3220)	2560 (2300 to 2960)	2700 (2380 to 2990)	39.7 (32.9 to 55.1)	38.4 (34.5 to 44.5)	36.7 (32.2 to 40.7)
Global	Chronic lymphoid leukaemia	452 (420 to 476)	588 (555 to 613)	709 (667 to 755)	10.9 (10.3 to 11.4)	9.7 (9.1 to 10.1)	8.9 (8.4 to 9.5)
Global	Acute myeloid leukaemia	2060 (1710 to 2910)	2770 (2520 to 3150)	3220 (2890 to 3440)	40.4 (34.4 to 54.7)	42.3 (38.5 to 48)	41.7 (37.3 to 44.5)
Global	Chronic myeloid leukaemia	735 (666 to 806)	666 (604 to 719)	655 (595 to 713)	16 (14.6 to 17.4)	10.2 (9.3 to 11)	8.2 (7.4 to 8.9)
Global	Other leukaemia	6440 (4680 to 7950)	5080 (4280 to 5630)	4690 (4040 to 5120)	118.4 (88.7 to 143.3)	77.2 (65.2 to 85.6)	61.4 (53 to 67)
Global	Other malignant cancers	8280 (7400 to 9160)	9720 (9180 to 10200)	11900 (11000 to 12400)	165.6 (150.9 to 179.2)	149.1 (140.7 to 155.8)	153.2 (142 to 160.8)
Global	Other neoplasms	1210 (950 to 1400)	1870 (1490 to 2170)	2480 (2080 to 2980)	25.7 (20 to 30)	29.4 (23.4 to 34.2)	31.8 (26.6 to 38.2)
Global	Myelodysplastic, myeloproliferative, and other hematopoietic neoplasms	1080 (853 to 1270)	1680 (1340 to 1970)	2240 (1880 to 2720)	23.3 (18.4 to 27.8)	26.7 (21.1 to 31.3)	28.6 (23.9 to 34.6)
Global	Benign and in situ intestinal neoplasms	0 (0 to 0)	0 (0 to 0)	0 (0 to 0)	0 (0 to 0)	0 (0 to 0)	0 (0 to 0)
Global	Benign and in situ cervical and uterine neoplasms	0 (0 to 0)	0 (0 to 0)	0 (0 to 0)	0 (0 to 0)	0 (0 to 0)	0 (0 to 0)
Global	Other benign and in situ neoplasms	135 (90.3 to 177)	189 (141 to 222)	237 (186 to 278)	2.4 (1.6 to 3.1)	2.8 (2.1 to 3.3)	3.2 (2.5 to 3.7)
Global	Cardiovascular diseases	267000 (260000 to 274000)	314000 (306000 to 322000)	366000 (355000 to 377000)	6446.5 (6295.2 to 6604.5)	5105.3 (4978.1 to 5235.5)	4597.9 (4463.7 to 4734.2)
Global	Rheumatic heart disease	12400 (11500 to 13500)	9980 (9200 to 10800)	9390 (8580 to 10300)	255.4 (238.8 to 278.8)	151.2 (139.7 to 163.9)	118.7 (108.5 to 130.7)
Global	Ischaemic heart disease	119000 (117000 to 123000)	145000 (143000 to 148000)	170000 (167000 to 174000)	2948.6 (2889.4 to 3022)	2359.9 (2321.1 to 2413.4)	2132.1 (2093.7 to 2179.8)
Global	Stroke	98900 (95600 to 103000)	114000 (110000 to 118000)	132000 (126000 to 137000)	2392.7 (2316.5 to 2478.9)	1861.9 (1801.6 to 1919.1)	1657.2 (1587.4 to 1723.8)
Global	Ischaemic stroke	36600 (34300 to 39000)	44200 (41300 to 47100)	55100 (50900 to 59400)	948.8 (891.4 to 1008.2)	746.5 (698.7 to 794.1)	702.8 (649.5 to 756.5)
Global	Intracerebral hemorrhage	49300 (46600 to 52800)	58900 (56800 to 60800)	64500 (62300 to 66600)	1158.4 (1095.2 to 1242.3)	943.5 (909.5 to 973.1)	800.3 (773.3 to 826.1)
Global	Subarachnoid hemorrhage	12900 (10700 to 14500)	11100 (10200 to 12300)	12400 (11600 to 13700)	285.5 (235.2 to 321.9)	171.9 (159 to 191.4)	154.1 (143.7 to 170.1)
Global	Hypertensive heart disease	11100 (8800 to 12000)	12200 (10100 to 13200)	16500 (12700 to 17900)	275.6 (218.1 to 298.2)	202 (168.4 to 218.1)	209.4 (160.5 to 226.3)
Global	Non-rheumatic valvular heart disease	1490 (1350 to 1680)	2050 (1870 to 2240)	2530 (2290 to 2770)	37.5 (34.1 to 41.9)	34.6 (31.4 to 38)	32.7 (29.5 to 36)
Global	Non-rheumatic calcific aortic valve disease	751 (660 to 868)	1150 (1020 to 1270)	1510 (1340 to 1650)	19.7 (17.5 to 22.8)	20.1 (17.7 to 22.1)	19.8 (17.5 to 21.5)
Global	Non-rheumatic degenerative mitral valve disease	649 (554 to 753)	763 (656 to 876)	874 (748 to 1010)	15.8 (13.5 to 18.3)	12.5 (10.7 to 14.4)	11.2 (9.5 to 12.9)
Global	Other non-rheumatic valve diseases	88 (62.8 to 109)	131 (91.2 to 158)	142 (109 to 190)	1.9 (1.4 to 2.4)	2.1 (1.4 to 2.4)	1.8 (1.4 to 2.4)
Global	Cardiomyopathy and myocarditis	7360 (6680 to 8120)	10700 (9090 to 11400)	10200 (9500 to 10900)	158.4 (143.7 to 174.1)	164.1 (140.7 to 175.5)	130.3 (121 to 138.8)
Global	Myocarditis	1240 (1010 to 1470)	1380 (1160 to 1550)	1390 (1220 to 1560)	23.5 (19.3 to 27.7)	21.3 (17.9 to 23.9)	18.3 (16.1 to 20.4)

SR Appendix Table 2. All-age DALYs counts and age standardized DALYs rates per 100,000 individuals for all causes and level 1 cause groups by location for 1990, 2007, and 2017, both sexes combined							
		All-age DALYs			Age-standardized rate of DALYs per 100,000		
Global	Alcoholic cardiomyopathy	1790 (1580 to 2030)	4230 (3190 to 4450)	2990 (2720 to 3210)	39.8 (35.4 to 45.7)	63 (47.7 to 66.3)	36.4 (33.2 to 39.1)
Global	Other cardiomyopathy	4340 (3740 to 4830)	5050 (4570 to 5490)	5870 (5290 to 6390)	95 (80.6 to 105.6)	79.8 (72.6 to 86.6)	75.6 (68.3 to 82.1)
Global	Atrial fibrillation and flutter	2870 (2400 to 3450)	4400 (3700 to 5250)	5980 (5040 to 7090)	80 (67.6 to 95.3)	77.6 (65.7 to 92.1)	78 (66.1 to 92)
Global	Aortic aneurysm	2090 (2000 to 2240)	2550 (2470 to 2690)	3040 (2880 to 3190)	51.1 (49 to 54.5)	41.7 (40.4 to 44)	38.2 (36.2 to 40)
Global	Peripheral vascular disease	587 (378 to 852)	1020 (685 to 1490)	1430 (970 to 2130)	15.6 (10 to 22.7)	17.7 (11.8 to 26.1)	18.4 (12.5 to 27.6)
Global	Endocarditis	1600 (1390 to 1940)	1900 (1740 to 2130)	2230 (2080 to 2420)	31.5 (27.6 to 37.4)	29.7 (27.1 to 33.3)	29 (27.1 to 31.7)
Global	Other cardiovascular and circulatory diseases	9010 (7950 to 10300)	10500 (9200 to 12000)	12200 (10600 to 14000)	200.2 (176.7 to 228.1)	164.9 (144.9 to 188.7)	153.8 (134.7 to 176.5)
Global	Chronic respiratory diseases	99100 (91700 to 105000)	98100 (91700 to 104000)	112000 (105000 to 120000)	2304.4 (2138.5 to 2422.4)	1585 (1486.6 to 1675.3)	1422.9 (1326.6 to 1517.2)
Global	Chronic obstructive pulmonary disease	68200 (61500 to 74700)	69800 (65000 to 74700)	81600 (76000 to 86800)	1663.1 (1505 to 1814)	1147.6 (1072 to 1225.1)	1028.8 (960.4 to 1092.5)
Global	Pneumoconiosis	519 (436 to 604)	459 (424 to 501)	507 (472 to 548)	12.2 (10.3 to 14.2)	7.4 (6.8 to 8.1)	6.3 (5.9 to 6.8)
Global	Silicosis	288 (227 to 358)	230 (205 to 266)	261 (233 to 286)	6.7 (5.3 to 8.4)	3.7 (3.3 to 4.3)	3.2 (2.9 to 3.5)
Global	Asbestosis	38.1 (26.7 to 50.4)	58 (41.3 to 70.4)	69.1 (52.2 to 81.4)	0.9 (0.6 to 1.2)	1 (0.7 to 1.2)	0.9 (0.7 to 1)
Global	Coal workers pneumoconiosis	113 (78.9 to 162)	80.8 (65.3 to 99)	80.8 (69 to 100)	2.7 (1.9 to 3.9)	1.3 (1.1 to 1.6)	1 (0.9 to 1.3)
Global	Other pneumoconiosis	79.9 (55.7 to 107)	90.1 (74.3 to 110)	96.9 (81.8 to 117)	1.8 (1.3 to 2.5)	1.4 (1.2 to 1.7)	1.2 (1 to 1.5)
Global	Asthma	26100 (19900 to 31300)	22000 (17400 to 27200)	22800 (18100 to 28300)	536.8 (401.7 to 646.2)	339.9 (268.6 to 419.6)	293.3 (232.4 to 365.2)
Global	Interstitial lung disease and pulmonary sarcoidosis	1590 (1210 to 2130)	2400 (1940 to 2950)	3360 (2770 to 4080)	37.7 (28.9 to 49.7)	39.1 (31.5 to 48)	42.3 (34.8 to 51.4)
Global	Other chronic respiratory diseases	2760 (2260 to 3330)	3460 (3030 to 3910)	4080 (3600 to 4560)	54.5 (45.1 to 65.1)	51.1 (44.8 to 57.7)	52.1 (46.1 to 58.2)
Global	Digestive diseases	67400 (62200 to 73600)	77400 (71500 to 85000)	85300 (78000 to 94500)	1422.6 (1317.6 to 1544.5)	1181.7 (1093 to 1294.2)	1070.8 (979.3 to 1187.9)
Global	Cirrhosis and other chronic liver diseases	30500 (28600 to 32200)	37700 (36100 to 40000)	41400 (39600 to 45100)	656.4 (612.8 to 689.2)	571 (546.2 to 605.8)	510.7 (487.6 to 557.1)
Global	Cirrhosis and other chronic liver diseases due to hepatitis B	9790 (8720 to 10700)	11700 (10700 to 12900)	12200 (11100 to 13900)	210.7 (187.7 to 231.1)	176 (161.1 to 193.8)	150.1 (136.2 to 170.3)
Global	Cirrhosis and other chronic liver diseases due to hepatitis C	7240 (6500 to 7980)	9210 (8350 to 10100)	10400 (9470 to 11600)	160 (143.7 to 176.6)	139.7 (127 to 153.3)	127.2 (115.9 to 141.8)
Global	Cirrhosis and other chronic liver diseases due to alcohol use	6810 (6170 to 7400)	9000 (8290 to 9790)	10200 (9310 to 11300)	152.3 (137.8 to 165.7)	136.6 (125.8 to 148.6)	123.9 (113.2 to 138.1)
Global	Cirrhosis due to NASH	1880 (1690 to 2060)	2780 (2560 to 3020)	3430 (3150 to 3740)	42.4 (38.1 to 46.5)	42.7 (39.2 to 46.3)	41.8 (38.5 to 45.5)
Global	Cirrhosis and other chronic liver diseases due to other causes	4810 (4080 to 6000)	5000 (4560 to 5700)	5160 (4650 to 5760)	91 (78.4 to 110.9)	76.1 (69.3 to 86.9)	67.7 (61.1 to 75.4)
Global	Upper digestive system diseases	16900 (13900 to 21300)	18000 (14000 to 23700)	19800 (15100 to 26600)	360.8 (299.4 to 447.9)	273.6 (213.4 to 355.9)	248.7 (189.1 to 333.3)
Global	Peptic ulcer disease	8130 (7580 to 8860)	6620 (6190 to 7120)	6350 (5900 to 6870)	181.3 (169.3 to 196.1)	103.2 (96.6 to 110.9)	79.6 (73.9 to 86.2)
Global	Gastritis and duodenitis	5190 (3700 to 7050)	6440 (4570 to 8780)	7470 (5240 to 10200)	105.1 (75.5 to 140.9)	96.6 (68.6 to 130.9)	94.3 (66.4 to 129.1)
Global	GERD	3600 (1930 to 6120)	4980 (2670 to 8460)	6010 (3220 to 10200)	74.3 (39.8 to 126)	73.8 (39.5 to 125.1)	74.8 (40.1 to 126.9)
Global	Appendicitis	2060 (1760 to 2550)	1990 (1820 to 2230)	1860 (1680 to 2020)	38.4 (33.4 to 46.7)	29.8 (27.2 to 33.3)	24.4 (22 to 26.5)
Global	Paralytic ileus and intestinal obstruction	6790 (6170 to 7880)	6840 (5970 to 7300)	7290 (5910 to 8030)	126.6 (116.2 to 145.1)	106.8 (93.6 to 113.8)	97.6 (79.4 to 107.4)
Global	Inguinal, femoral, and abdominal hernia	2710 (2100 to 3410)	3060 (2290 to 3900)	3480 (2610 to 4480)	55.9 (43.8 to 70)	47.2 (35.6 to 60)	44.8 (33.6 to 57.5)
Global	Inflammatory bowel disease	1250 (965 to 1610)	1590 (1290 to 1900)	1850 (1510 to 2230)	26.5 (21 to 33)	24.6 (20.1 to 29.3)	23.2 (19.1 to 27.8)
Global	Vascular intestinal disorders	1020 (922 to 1090)	1360 (1270 to 1420)	1600 (1460 to 1700)	25.4 (23.2 to 26.8)	22.6 (21.1 to 23.6)	20.4 (18.7 to 21.6)
Global	Gallbladder and biliary diseases	1690 (1490 to 1790)	1690 (1600 to 1780)	2010 (1890 to 2130)	39.6 (35 to 41.6)	27.6 (26.1 to 29)	25.7 (24.1 to 27.2)
Global	Pancreatitis	1960 (1780 to 2210)	2830 (2560 to 3080)	3250 (2890 to 3610)	42.7 (38.8 to 47.9)	42.8 (38.6 to 46.6)	40.3 (35.9 to 44.7)
Global	Other digestive diseases	2460 (2160 to 2840)	2250 (2000 to 2540)	2720 (2380 to 3110)	50.3 (44.9 to 57)	35.7 (31.7 to 40.1)	35 (30.7 to 40)
Global	Neurological disorders	68900 (54300 to 86700)	92200 (73000 to 115000)	111000 (88500 to 139000)	1497.7 (1213.3 to 1846.4)	1447.6 (1164.1 to 1786.8)	1444.4 (1154 to 1792.7)
Global	Alzheimer's disease and other dementias	14200 (13300 to 15200)	22100 (20700 to 23600)	30500 (28500 to 32600)	439.7 (412.4 to 469.2)	415.1 (389.5 to 443)	412.6 (385.7 to 440.3)
Global	Parkinson's disease	2620 (2400 to 2920)	4170 (3840 to 4540)	5580 (5150 to 6080)	71.2 (65.4 to 79.5)	72.2 (66.6 to 78.5)	72.5 (66.9 to 79)
Global	Epilepsy	13000 (10300 to 15900)	14000 (11300 to 17300)	14800 (11400 to 19000)	233.5 (186.4 to 285.9)	206.6 (166.3 to 257.3)	195.8 (151.4 to 251.8)

SR Appendix Table 2. All-age DALYs counts and age standardized DALYs rates per 100,000 individuals for all causes and level 1 cause groups by location for 1990, 2007, and 2017, both sexes combined

		All-age DALYs			Age-standardized rate of DALYs per 100,000		
Global	Multiple sclerosis	683 (587 to 811)	922 (800 to 1060)	1080 (943 to 1240)	14.8 (12.8 to 17.5)	13.9 (12 to 15.8)	13.3 (11.5 to 15.2)
Global	Motor neuron disease	484 (451 to 601)	691 (668 to 744)	879 (841 to 966)	10.4 (9.8 to 12.5)	10.9 (10.6 to 11.7)	11 (10.5 to 12)
Global	Headache disorders	35100 (22800 to 49700)	47100 (30700 to 66700)	54300 (35600 to 76800)	677 (441.9 to 953.4)	679.7 (443.5 to 960.6)	686.5 (448.7 to 970.5)
Global	Migraine	30500 (19300 to 44400)	41000 (25900 to 59500)	47200 (30000 to 68700)	586.6 (370.4 to 850.1)	590.2 (373.4 to 857.6)	596.8 (378.2 to 866)
Global	Tension-type headache	4630 (2620 to 7350)	6140 (3480 to 9750)	7100 (4040 to 11200)	90.4 (51.5 to 142.9)	89.5 (50.9 to 141.7)	89.7 (51 to 142.2)
Global	Other neurological disorders	2820 (2110 to 3480)	3290 (2720 to 3980)	3970 (3270 to 4850)	51.1 (39.4 to 62.2)	49.2 (40.8 to 59.3)	52.7 (43.3 to 64.7)
Global	Mental disorders	82200 (61300 to 106000)	108000 (80900 to 139000)	123000 (91600 to 158000)	1586.7 (1184 to 2044.1)	1577.9 (1179.2 to 2030.6)	1560.4 (1165.1 to 2010.5)
Global	Schizophrenia	7790 (5830 to 9610)	10800 (8040 to 13300)	12700 (9480 to 15600)	157 (117.5 to 193.5)	157 (116.8 to 193.1)	156.4 (117.1 to 192.3)
Global	Depressive disorders	28300 (20100 to 38700)	37700 (26700 to 51400)	43100 (30500 to 58900)	565.8 (402.9 to 771.6)	555 (392.9 to 755.3)	540.5 (382.4 to 737.8)
Global	Major depressive disorder	22100 (15400 to 30200)	29200 (20400 to 39600)	32800 (23100 to 44300)	438.9 (308.3 to 594)	428.5 (299.5 to 579.9)	413 (289.8 to 558.9)
Global	Dysthymia	6160 (4130 to 8960)	8510 (5680 to 12500)	10300 (6880 to 15000)	126.8 (84.9 to 185.2)	126.4 (84.2 to 184.3)	127.4 (85.4 to 185.6)
Global	Bipolar disorder	6020 (3800 to 8890)	8060 (5090 to 11900)	9290 (5870 to 13700)	115.6 (72.8 to 171)	116.1 (73.5 to 171.5)	117.5 (74.1 to 173.5)
Global	Anxiety disorders	18200 (12900 to 24300)	24000 (17000 to 32000)	27100 (19200 to 36100)	348.5 (246 to 462.6)	349.9 (247.6 to 463.7)	345.7 (245.6 to 459.7)
Global	Eating disorders	2060 (1320 to 2990)	2830 (1820 to 4120)	3370 (2170 to 4890)	36.7 (23.6 to 53.1)	39.6 (25.5 to 57.5)	43.4 (27.9 to 62.9)
Global	Anorexia nervosa	489 (302 to 720)	642 (398 to 950)	728 (454 to 1080)	8.5 (5.3 to 12.6)	9 (5.6 to 13.3)	9.5 (5.9 to 14.1)
Global	Bulimia nervosa	1570 (990 to 2340)	2190 (1390 to 3250)	2640 (1660 to 3940)	28.3 (17.9 to 42.2)	30.7 (19.4 to 45.5)	33.8 (21.3 to 50.5)
Global	Autism spectrum disorders	3460 (2350 to 4760)	4250 (2900 to 5850)	4730 (3240 to 6520)	63.1 (42.9 to 86.7)	62.6 (42.7 to 86.2)	62.4 (42.7 to 86)
Global	Attention-deficit/hyperactivity disorder	692 (418 to 1100)	831 (504 to 1320)	889 (543 to 1410)	12 (7.3 to 19)	12 (7.3 to 18.9)	11.9 (7.3 to 18.9)
Global	Conduct disorder	5300 (3200 to 8460)	6150 (3700 to 9810)	6450 (3870 to 10300)	86.6 (52.4 to 138.2)	87.9 (52.9 to 140.4)	90 (54 to 143.9)
Global	Idiopathic developmental intellectual disability	3580 (1810 to 5940)	4040 (1990 to 6810)	4050 (1940 to 6860)	63.1 (31.8 to 104.6)	59.4 (29.3 to 100)	54.2 (26.1 to 91.9)
Global	Other mental disorders	6860 (4530 to 9490)	9450 (6250 to 13100)	11100 (7360 to 15300)	138.4 (91.6 to 191)	138.4 (91.6 to 191.1)	138.4 (91.6 to 191)
Global	Substance use disorders	27500 (21800 to 33400)	38100 (30500 to 46300)	44700 (35900 to 54100)	527.6 (420.8 to 638.7)	546.9 (439.1 to 664.3)	559.2 (448.2 to 677.9)
Global	Alcohol use disorders	11600 (9300 to 14400)	16600 (13600 to 20400)	17500 (14100 to 21600)	231.7 (187.2 to 285.1)	241.1 (197.2 to 295.4)	216.4 (174.1 to 268.5)
Global	Drug use disorders	15900 (12300 to 19800)	21400 (16600 to 26600)	27200 (21100 to 33600)	295.9 (228.6 to 366.4)	305.8 (237 to 379.4)	342.8 (266.4 to 424.1)
Global	Opioid use disorders	11600 (8590 to 14800)	16400 (12400 to 20800)	21500 (16300 to 27100)	216.6 (160.7 to 276.9)	234.7 (177.3 to 297.2)	270.5 (204.4 to 342.2)
Global	Cocaine use disorders	588 (411 to 804)	833 (620 to 1090)	992 (747 to 1290)	11 (7.8 to 15.1)	11.9 (8.9 to 15.6)	12.5 (9.4 to 16.3)
Global	Amphetamine use disorders	1070 (633 to 1600)	1120 (708 to 1670)	1180 (757 to 1740)	18.5 (11 to 27.5)	15.7 (9.9 to 23.2)	15.3 (9.7 to 22.6)
Global	Cannabis use disorders	400 (252 to 601)	496 (314 to 727)	518 (329 to 766)	7.1 (4.5 to 10.5)	6.9 (4.4 to 10.1)	6.7 (4.2 to 9.9)
Global	Other drug use disorders	2270 (1790 to 2900)	2560 (2130 to 3090)	3010 (2550 to 3540)	42.6 (34.1 to 53.9)	36.6 (30.5 to 44.1)	37.9 (32.1 to 44.7)
Global	Diabetes and kidney diseases	54800 (48400 to 62600)	82000 (71300 to 94900)	104000 (90100 to 121000)	1223.1 (1079 to 1396.4)	1284.6 (1120.3 to 1482.5)	1294.2 (1122 to 1496.7)
Global	Diabetes mellitus	31300 (26100 to 37800)	52200 (42900 to 63400)	67900 (55400 to 82600)	717.7 (599.2 to 863.6)	818.8 (675.2 to 992.5)	839 (685.1 to 1020)
Global	Diabetes mellitus type 1	7510 (6980 to 8260)	9370 (8720 to 10100)	10400 (9790 to 11100)	164 (151.8 to 180.7)	143.4 (133.4 to 155.1)	129.4 (121.3 to 137.6)
Global	Diabetes mellitus type 2	23800 (18500 to 30100)	42900 (33400 to 53700)	57400 (45000 to 71900)	553.6 (435.1 to 696.5)	675.4 (531 to 843.6)	709.6 (557.2 to 888.3)
Global	Chronic kidney disease	22900 (21500 to 24500)	29400 (27700 to 31200)	35800 (33700 to 38000)	493.5 (463.3 to 528)	460.8 (435 to 488.3)	451.3 (424.9 to 478.3)
Global	Chronic kidney disease due to diabetes mellitus type 1	1830 (1500 to 2200)	2440 (2010 to 2950)	2890 (2370 to 3500)	38.9 (31.7 to 47)	36.1 (29.7 to 43.8)	35.2 (28.9 to 42.6)
Global	Chronic kidney disease due to diabetes mellitus type 2	3820 (3300 to 4390)	6050 (5290 to 6850)	8120 (7120 to 9250)	92.1 (79.9 to 105.3)	98.6 (86.4 to 111.6)	100.9 (88.4 to 114.8)
Global	Chronic kidney disease due to hypertension	3780 (3320 to 4270)	5550 (4900 to 6230)	7350 (6450 to 8220)	91.7 (80.3 to 103.3)	90.8 (80.1 to 101.8)	92.7 (81.7 to 103.7)
Global	Chronic kidney disease due to glomerulonephritis	4910 (4360 to 5490)	5800 (5180 to 6450)	6600 (5860 to 7420)	98.1 (87.8 to 109.7)	87.5 (78.4 to 97.3)	83.7 (74.4 to 93.7)
Global	Chronic kidney disease due to other and unspecified causes	8560 (7650 to 9490)	9580 (8570 to 10700)	10900 (9660 to 12200)	172.8 (153.8 to 193.4)	147.8 (132.4 to 164.6)	138.8 (123.7 to 155.9)
Global	Acute glomerulonephritis	617 (538 to 686)	329 (302 to 350)	311 (285 to 339)	11.9 (10.5 to 13.2)	5 (4.6 to 5.3)	4 (3.6 to 4.3)

SR Appendix Table 2. All-age DALYs counts and age standardized DALYs rates per 100,000 individuals for all causes and level 1 cause groups by location for 1990, 2007, and 2017, both sexes combined

		All-age DALYs			Age-standardized rate of DALYs per 100,000		
Global	Skin and subcutaneous diseases	31200<br>(21000 to 45500)	38800<br>(26200 to 56600)	44100<br>(29900 to 64200)	574.9<br>(388.9 to 835.7)	580.4<br>(392 to 842.6)	585<br>(396.2 to 851.8)
Global	Dermatitis	8320<br>(4830 to 13300)	9930<br>(5780 to 15800)	11100<br>(6480 to 17700)	148.8<br>(86.9 to 235.6)	148.7<br>(86.3 to 237.2)	150.3<br>(87 to 240.3)
Global	Atopic dermatitis	6890<br>(3720 to 11500)	8070<br>(4380 to 13400)	9000<br>(4890 to 15000)	120.8<br>(65.4 to 201)	121.3<br>(65.7 to 202.2)	123.3<br>(66.8 to 205.2)
Global	Contact dermatitis	1330<br>(868 to 1970)	1740<br>(1140 to 2580)	1990<br>(1300 to 2950)	26<br>(17.1 to 38.4)	25.6<br>(16.8 to 37.9)	25.3<br>(16.6 to 37.4)
Global	Seborrhoeic dermatitis	104<br>(59.5 to 163)	125<br>(71.6 to 198)	136<br>(78.1 to 215)	2<br>(1.1 to 3.2)	1.9<br>(1.1 to 2.9)	1.7<br>(1 to 2.7)
Global	Psoriasis	3210<br>(2280 to 4250)	4600<br>(3250 to 6090)	5570<br>(3960 to 7350)	65.2<br>(46.2 to 86)	68.5<br>(48.4 to 90.6)	70<br>(49.7 to 92.5)
Global	Bacterial skin diseases	1420<br>(846 to 1710)	1810<br>(1200 to 2210)	2270<br>(1560 to 2900)	27.2<br>(17 to 32.4)	28.3<br>(18.8 to 34.3)	30<br>(20.6 to 38.2)
Global	Cellulitis	317<br>(200 to 420)	452<br>(286 to 560)	598<br>(377 to 760)	6.2<br>(4.1 to 8)	7<br>(4.4 to 8.7)	7.7<br>(4.9 to 9.8)
Global	Pyoderma	1100<br>(620 to 1350)	1360<br>(866 to 1680)	1680<br>(1110 to 2200)	21<br>(12.3 to 25.4)	21.3<br>(13.5 to 26.3)	22.3<br>(14.8 to 29.4)
Global	Scabies	3660<br>(2030 to 6000)	4250<br>(2350 to 6960)	4530<br>(2510 to 7410)	64.7<br>(36 to 105.9)	61.9<br>(34.2 to 101)	60<br>(33.2 to 97.8)
Global	Fungal skin diseases	3080<br>(1220 to 6400)	3750<br>(1480 to 7790)	4150<br>(1650 to 8630)	59.2<br>(23.5 to 123.1)	57.4<br>(22.6 to 119)	54.9<br>(21.8 to 114.2)
Global	Viral skin diseases	3430<br>(2200 to 5100)	3790<br>(2430 to 5630)	4030<br>(2600 to 6000)	58.6<br>(37.6 to 87.1)	57<br>(36.5 to 84.7)	56<br>(36 to 83.2)
Global	Acne vulgaris	1500<br>(891 to 2400)	2190<br>(1300 to 3500)	2550<br>(1520 to 4060)	25.4<br>(15.2 to 40.6)	30.5<br>(18.2 to 48.6)	33.9<br>(20.2 to 54.1)
Global	Alopecia areata	360<br>(230 to 535)	464<br>(296 to 691)	523<br>(335 to 775)	6.9<br>(4.4 to 10.2)	6.7<br>(4.3 to 10)	6.6<br>(4.2 to 9.8)
Global	Pruritus	466<br>(218 to 884)	635<br>(301 to 1200)	756<br>(356 to 1430)	9.2<br>(4.3 to 17.4)	9.5<br>(4.5 to 17.8)	9.7<br>(4.5 to 18.2)
Global	Urticaria	3790<br>(2500 to 5360)	4530<br>(3000 to 6400)	5010<br>(3320 to 7050)	67<br>(44.2 to 94.6)	67.4<br>(44.5 to 95.1)	67.7<br>(44.8 to 95.4)
Global	Decubitus ulcer	276<br>(200 to 356)	395<br>(295 to 513)	503<br>(379 to 661)	6.9<br>(5 to 8.8)	6.6<br>(4.9 to 8.6)	6.5<br>(4.9 to 8.6)
Global	Other skin and subcutaneous diseases	1720<br>(873 to 3120)	2480<br>(1240 to 4510)	3110<br>(1560 to 5650)	35.7<br>(18.1 to 64.7)	37.9<br>(19 to 68.9)	39.5<br>(19.8 to 71.9)
Global	Sense organ diseases	37800<br>(25500 to 54300)	53700<br>(36200 to 77100)	66600<br>(44700 to 95700)	857.9<br>(580.9 to 1220.3)	849.6<br>(575 to 1211.6)	842.2<br>(566.6 to 1206.5)
Global	Blindness and vision impairment	17400<br>(11800 to 25800)	24300<br>(16400 to 35900)	29900<br>(20300 to 44000)	394<br>(267.5 to 572.6)	383.4<br>(261.4 to 562.4)	376.6<br>(255.6 to 555)
Global	Glaucoma	379<br>(255 to 531)	536<br>(363 to 744)	686<br>(463 to 949)	9.6<br>(6.5 to 13.4)	9.1<br>(6.2 to 12.6)	8.6<br>(5.8 to 12)
Global	Cataract	4130<br>(2890 to 5570)	6180<br>(4300 to 8310)	8010<br>(5580 to 10800)	106.8<br>(75 to 143.7)	103.8<br>(72.4 to 139.8)	101.8<br>(70.9 to 137.7)
Global	Age-related macular degeneration	255<br>(172 to 353)	406<br>(277 to 559)	531<br>(364 to 729)	6.8<br>(4.6 to 9.4)	7<br>(4.8 to 9.6)	6.8<br>(4.6 to 9.3)
Global	Refraction disorders	5560<br>(3660 to 8050)	6920<br>(4620 to 9940)	7980<br>(5350 to 11500)	112.3<br>(74.5 to 162.8)	105.4<br>(70.8 to 150.7)	102.2<br>(68.2 to 147.3)
Global	Near vision loss	5370<br>(2570 to 9820)	7870<br>(3750 to 14400)	9800<br>(4670 to 17900)	118.9<br>(56.8 to 217.6)	120.4<br>(57.4 to 220.8)	121<br>(57.7 to 221.3)
Global	Other vision loss	1740<br>(1210 to 2350)	2380<br>(1660 to 3210)	2890<br>(2010 to 3880)	39.5<br>(27.6 to 53.1)	37.7<br>(26.4 to 50.7)	36.3<br>(25.3 to 48.9)
Global	Age-related and other hearing loss	19000<br>(12900 to 27000)	27400<br>(18600 to 39000)	34200<br>(23200 to 48700)	433.8<br>(298 to 611.2)	435.5<br>(297.6 to 615.4)	434.6<br>(295.4 to 616.1)
Global	Other sense organ diseases	1380<br>(858 to 2010)	1960<br>(1220 to 2860)	2450<br>(1520 to 3570)	30.2<br>(18.8 to 44)	30.7<br>(19 to 44.7)	31<br>(19.2 to 45.2)
Global	Musculoskeletal disorders	83700<br>(61700 to 110000)	116000<br>(85600 to 152000)	139000<br>(102000 to 183000)	1782.6<br>(1316.8 to 2342.9)	1740.3<br>(1284.1 to 2288.4)	1720.5<br>(1264.4 to 2259.2)
Global	Rheumatoid arthritis	1970<br>(1540 to 2430)	2700<br>(2080 to 3370)	3490<br>(2660 to 4410)	44.9<br>(35.2 to 55.1)	42.3<br>(32.8 to 52.8)	43.3<br>(33 to 54.5)
Global	Osteoarthritis	4480<br>(2250 to 8900)	7310<br>(3660 to 14600)	9600<br>(4810 to 19100)	108.3<br>(54.4 to 214.9)	117.6<br>(59 to 234.6)	118.8<br>(59.5 to 236.2)
Global	Low back pain	42500<br>(30200 to 57200)	55300<br>(39400 to 74500)	64900<br>(46500 to 87400)	892.3<br>(636.6 to 1195.5)	827.7<br>(595.9 to 1112.8)	810.3<br>(582.4 to 1089.1)
Global	Neck pain	16300<br>(11300 to 22900)	23600<br>(16500 to 33200)	28600<br>(20000 to 40200)	354.4<br>(246.9 to 497.1)	354.4<br>(247.6 to 495.8)	352<br>(245.6 to 493.3)
Global	Gout	635<br>(426 to 872)	982<br>(663 to 1350)	1280<br>(867 to 1770)	14.8<br>(9.9 to 20.4)	15.5<br>(10.5 to 21.2)	15.9<br>(10.7 to 21.8)
Global	Other musculoskeletal disorders	17800<br>(12600 to 24300)	25800<br>(18200 to 35400)	30800<br>(21500 to 42000)	367.9<br>(260.6 to 503.9)	382.8<br>(270.7 to 523.8)	380.2<br>(266.2 to 520.3)
Global	Other non-communicable diseases	128000<br>(113000 to 148000)	124000<br>(108000 to 144000)	122000<br>(104000 to 144000)	2210.2<br>(1927.1 to 2578.4)	1895.1<br>(1654.7 to 2194.1)	1678.1<br>(1450 to 1961.2)
Global	Congenital anomalies	79300<br>(71500 to 94600)	68400<br>(63400 to 75500)	60900<br>(56400 to 65400)	1268.6<br>(1143.4 to 1510.2)	1056.2<br>(979.8 to 1166.8)	889<br>(827.6 to 951.5)
Global	Neural tube defects	8920<br>(5820 to 12600)	6870<br>(4950 to 9410)	6170<br>(4840 to 8170)	141<br>(92.4 to 198.9)	106.8<br>(76.8 to 146.6)	91.3<br>(71.7 to 121.2)
Global	Congenital heart anomalies	34300<br>(23100 to 43500)	27200<br>(20400 to 32400)	22200<br>(18400 to 26300)	541.1<br>(366.2 to 685.9)	422.6<br>(316.2 to 503.1)	330<br>(272.3 to 390.8)
Global	Orofacial clefts	1540<br>(756 to 3080)	835<br>(502 to 1530)	652<br>(411 to 1110)	24.7<br>(12.3 to 48.7)	12.8<br>(7.6 to 23.6)	9.3<br>(5.8 to 16.2)

SR Appendix Table 2. All-age DALYs counts and age standardized DALYs rates per 100,000 individuals for all causes and level 1 cause groups by location for 1990, 2007, and 2017, both sexes combined							
		All-age DALYs			Age-standardized rate of DALYs per 100,000		
Global	Down's syndrome	2510 (1270 to 3880)	2230 (1620 to 3150)	2230 (1760 to 3040)	40.6 (21.2 to 62.1)	34.2 (24.7 to 48.6)	32 (24.9 to 43.9)
Global	Turner syndrome	7.62 (3.75 to 12.6)	9.26 (4.59 to 15.3)	10.1 (5.02 to 16.5)	0.1 (0.1 to 0.2)	0.1 (0.1 to 0.2)	0.1 (0.1 to 0.2)
Global	Klinefelter syndrome	3.74 (1.77 to 7.07)	4.81 (2.24 to 9.03)	5.16 (2.43 to 9.74)	0.1 (0 to 0.1)	0.1 (0 to 0.1)	0.1 (0 to 0.1)
Global	Other chromosomal abnormalities	1610 (976 to 2740)	1950 (1310 to 2770)	2100 (1510 to 2840)	26.1 (16 to 43.8)	29.9 (20 to 42.9)	30.4 (21.8 to 41.3)
Global	Congenital musculoskeletal and limb anomalies	4380 (3200 to 5670)	5090 (3610 to 6670)	5530 (3880 to 7270)	78.3 (56.5 to 102.4)	76.1 (54.1 to 99.4)	74.2 (52.6 to 97.2)
Global	Urogenital congenital anomalies	1250 (846 to 1700)	1310 (931 to 1660)	1250 (926 to 1500)	20 (13.6 to 27.1)	20.2 (14.3 to 25.7)	18.3 (13.6 to 22.1)
Global	Digestive congenital anomalies	6990 (4590 to 12400)	6000 (4410 to 8930)	5190 (3990 to 7050)	111.1 (73.1 to 196.5)	93.1 (68.4 to 138.8)	76.7 (58.7 to 104.6)
Global	Other congenital anomalies	17800 (10700 to 24700)	16900 (12000 to 21500)	15500 (12000 to 19200)	285.4 (173.9 to 393.9)	260.3 (184.8 to 333.1)	226.5 (174.1 to 279.7)
Global	Urinary diseases and male infertility	6410 (5690 to 7290)	7650 (6790 to 8690)	9390 (8260 to 10700)	134.8 (119.2 to 154.1)	120.8 (107.2 to 137.3)	119.9 (105.9 to 136.8)
Global	Urinary tract infections	2770 (2350 to 3150)	3590 (3330 to 4010)	4700 (4430 to 5190)	57.3 (49.7 to 64.5)	56.7 (52.7 to 63.1)	60.7 (57.2 to 67.2)
Global	Urolithiasis	426 (342 to 504)	408 (338 to 488)	486 (397 to 591)	9.4 (7.6 to 11)	6.3 (5.2 to 7.5)	6 (4.9 to 7.3)
Global	Benign prostatic hyperplasia	1350 (860 to 1930)	1840 (1180 to 2620)	2430 (1560 to 3460)	32.5 (20.8 to 46.4)	29.9 (19.2 to 42.7)	29.9 (19.2 to 42.5)
Global	Male infertility	116 (48.5 to 241)	155 (64.6 to 320)	181 (74.8 to 374)	2.1 (0.9 to 4.4)	2.2 (0.9 to 4.5)	2.3 (1 to 4.8)
Global	Other urinary diseases	1750 (1300 to 2110)	1660 (1270 to 1920)	1600 (1290 to 1790)	33.4 (25.4 to 39.8)	25.7 (19.7 to 29.8)	21 (16.9 to 23.6)
Global	Gynecological diseases	8130 (5700 to 11200)	10800 (7510 to 15000)	11900 (8210 to 16600)	156.6 (109.3 to 215.6)	152.6 (106 to 211.2)	148.8 (102.6 to 206.6)
Global	Uterine fibroids	1010 (639 to 1560)	1430 (889 to 2260)	1550 (947 to 2460)	21.3 (13.5 to 33.1)	20.5 (12.7 to 32.5)	18.9 (11.5 to 30)
Global	Polycystic ovarian syndrome	306 (135 to 592)	407 (180 to 791)	460 (204 to 886)	5.7 (2.5 to 10.9)	5.7 (2.5 to 11.1)	5.8 (2.6 to 11.2)
Global	Female infertility	203 (77.3 to 429)	269 (102 to 565)	343 (129 to 723)	3.7 (1.4 to 7.9)	3.7 (1.4 to 7.9)	4.4 (1.7 to 9.2)
Global	Endometriosis	2880 (1920 to 4140)	3780 (2530 to 5390)	4130 (2760 to 5950)	54.9 (36.4 to 79.1)	53.1 (35.5 to 75.9)	51.6 (34.5 to 74)
Global	Genital prolapse	241 (122 to 439)	296 (148 to 546)	352 (173 to 652)	5.4 (2.7 to 9.8)	4.5 (2.3 to 8.4)	4.3 (2.1 to 8)
Global	Premenstrual syndrome	2730 (1750 to 4120)	3610 (2310 to 5430)	3930 (2530 to 5900)	50.9 (32.7 to 76.7)	50.6 (32.4 to 76)	49.5 (31.9 to 74.3)
Global	Other gynecological diseases	756 (562 to 999)	1010 (741 to 1380)	1150 (830 to 1580)	14.7 (11 to 19.4)	14.3 (10.5 to 19.5)	14.4 (10.3 to 19.7)
Global	Hemoglobinopathies and hemolytic anaemias	11000 (8610 to 13600)	10500 (8160 to 13400)	10100 (7740 to 13300)	197.4 (154.3 to 244.8)	158 (122.8 to 201.2)	137.1 (104.6 to 179.3)
Global	Thalassemias	1320 (961 to 1770)	771 (654 to 870)	582 (491 to 683)	21.2 (15.5 to 28.4)	11.8 (10 to 13.4)	8.4 (7.1 to 9.9)
Global	Thalassaemias trait	2370 (1610 to 3420)	2170 (1470 to 3170)	1910 (1280 to 2780)	42.9 (29.2 to 61.9)	31.9 (21.5 to 46.5)	25 (16.8 to 36.4)
Global	Sickle cell disorders	2910 (1780 to 3790)	2960 (1890 to 3900)	3050 (2000 to 4200)	47.5 (29.3 to 61.9)	44.6 (28.2 to 58.6)	43.2 (28.3 to 59.4)
Global	Sickle cell trait	1010 (690 to 1440)	1220 (825 to 1740)	1200 (812 to 1730)	17.9 (12.2 to 25.6)	18.1 (12.3 to 26)	16.3 (11 to 23.4)
Global	G6PD deficiency	558 (449 to 666)	688 (540 to 858)	717 (546 to 920)	10.5 (8.4 to 12.7)	10.1 (7.9 to 12.6)	9.1 (7 to 11.7)
Global	G6PD trait	0.314 (0.215 to 0.443)	0.403 (0.274 to 0.564)	0.416 (0.281 to 0.58)	0 (0 to 0)	0 (0 to 0)	0 (0 to 0)
Global	Other hemoglobinopathies and hemolytic anaemias	2880 (2200 to 3730)	2720 (2060 to 3610)	2680 (2030 to 3580)	57.4 (44.4 to 73.4)	41.5 (31.6 to 54.7)	35 (26.4 to 46.7)
Global	Endocrine, metabolic, blood, and immune disorders	6760 (5410 to 8220)	7200 (6040 to 8550)	7720 (6500 to 9080)	125.9 (102.3 to 152.7)	109.7 (92.2 to 129.8)	101.8 (85.9 to 120)
Global	Oral disorders	10900 (6570 to 16800)	15100 (9020 to 23300)	18300 (11000 to 28300)	238.3 (145.5 to 366)	231.8 (140.4 to 357.5)	228.8 (137.5 to 353.7)
Global	Caries of deciduous teeth	139 (59.6 to 273)	132 (56.9 to 263)	139 (59.4 to 278)	2.3 (1 to 4.4)	2.1 (0.9 to 4.2)	2 (0.9 to 4.1)
Global	Caries of permanent teeth	1230 (531 to 2300)	1480 (638 to 2820)	1620 (698 to 3090)	23.4 (10.2 to 44.6)	21.5 (9.3 to 41)	20.7 (8.9 to 39.2)
Global	Periodontal disease	2730 (1070 to 5600)	4100 (1610 to 8420)	5190 (2040 to 10700)	59.9 (23.6 to 123.3)	61.8 (24.3 to 127)	63.5 (25 to 130.3)
Global	Edentulism and severe tooth loss	4190 (2790 to 5910)	5890 (3930 to 8340)	7350 (4890 to 10400)	102 (68.2 to 143.5)	95.6 (64 to 134.7)	91.7 (61.3 to 129.9)
Global	Other oral disorders	2620 (1640 to 3860)	3470 (2170 to 5110)	4020 (2510 to 5900)	50.8 (31.7 to 74.5)	50.8 (31.8 to 74.6)	50.9 (31.8 to 74.7)
Global	Sudden infant death syndrome	5650 (2300 to 12000)	4220 (1700 to 8460)	3490 (1570 to 6730)	88.5 (35.9 to 188.2)	66 (26.6 to 132.2)	52.7 (23.7 to 101.5)
Global	Injuries	270000 (254000 to 284000)	255000 (240000 to 271000)	252000 (236000 to 271000)	4946.5 (4654.6 to 5233.3)	3767 (3543.5 to 4002)	3267 (3058.2 to 3505.1)
Global	Transport injuries	75500 (70700 to 79900)	79500 (75900 to 83200)	75300 (71000 to 79800)	1384.6 (1297.4 to 1463.2)	1159.6 (1104.5 to 1214.8)	967.6 (914.2 to 1023.9)

SR Appendix Table 2. All-age DALYs counts and age standardized DALYs rates per 100,000 individuals for all causes and level 1 cause groups by location for 1990, 2007, and 2017, both sexes combined

		All-age DALYs			Age-standardized rate of DALYs per 100,000		
Global	Road injuries	68900 (64500 to 72900)	72200 (69000 to 75200)	67800 (64300 to 71500)	1259.3 (1181.9 to 1330.1)	1052.4 (1004.7 to 1098.1)	871.1 (827.9 to 917.3)
Global	Pedestrian road injuries	28900 (26000 to 31500)	26800 (25300 to 29200)	23600 (22000 to 25800)	525.9 (475.8 to 572)	395.6 (374.3 to 431.5)	304.1 (283.9 to 332.1)
Global	Cyclist road injuries	2880 (2440 to 3400)	4160 (3580 to 4700)	4550 (3860 to 5270)	55.9 (47.4 to 66.2)	61.3 (52.8 to 69.5)	57.5 (48.8 to 66.3)
Global	Motorcyclist road injuries	11000 (9920 to 12900)	14200 (12800 to 15200)	14000 (12300 to 15100)	201.1 (180.4 to 235.4)	202.6 (182 to 217.4)	177.9 (157.3 to 191.9)
Global	Motor vehicle road injuries	25300 (22200 to 27500)	26100 (24100 to 27600)	24600 (23100 to 26000)	460.7 (408.5 to 500.3)	378 (349.5 to 400.5)	317.2 (297.9 to 335.1)
Global	Other road injuries	825 (599 to 1000)	1010 (847 to 1180)	1130 (932 to 1380)	15.8 (11.7 to 18.9)	14.9 (12.5 to 17.5)	14.3 (11.9 to 17.4)
Global	Other transport injuries	6670 (5900 to 7620)	7320 (6490 to 8340)	7530 (6480 to 8760)	125.2 (110.3 to 143.1)	107.2 (94.9 to 122)	96.5 (83 to 112.1)
Global	Unintentional injuries	126000 (115000 to 137000)	108000 (98200 to 120000)	106000 (94200 to 119000)	2310.4 (2106.7 to 2513.1)	1645.8 (1490.5 to 1816.8)	1389 (1241.3 to 1560.2)
Global	Falls	25400 (21300 to 30000)	30200 (25700 to 35700)	35900 (30200 to 42900)	533.5 (446.5 to 632.8)	470.2 (399.7 to 554.6)	459.4 (387.1 to 547.5)
Global	Drowning	38100 (34100 to 41400)	22500 (21300 to 23700)	16700 (15900 to 17500)	634.9 (571 to 688.7)	341.4 (321.4 to 359.3)	229.9 (219 to 241.2)
Global	Fire, heat, and hot substances	10800 (8420 to 12500)	9030 (7520 to 10400)	8460 (7030 to 9880)	197.2 (156.9 to 227.8)	134.9 (112.8 to 154.5)	111.1 (92.8 to 129.3)
Global	Poisonings	4810 (3870 to 5850)	4250 (3480 to 4600)	3790 (2930 to 4210)	85.8 (70.2 to 104.2)	63.3 (51.8 to 68.6)	50.1 (38.8 to 55.6)
Global	Poisoning by carbon monoxide	2020 (1750 to 2500)	1860 (1510 to 1980)	1540 (1150 to 1690)	37.3 (32.6 to 45.9)	27.4 (22.2 to 29.1)	19.9 (14.8 to 21.9)
Global	Poisoning by other means	2790 (2120 to 3490)	2380 (1930 to 2660)	2250 (1780 to 2560)	48.5 (37.5 to 60.1)	35.8 (28.9 to 40.1)	30.2 (23.9 to 34.3)
Global	Exposure to mechanical forces	11200 (10100 to 12600)	11300 (9870 to 12900)	11300 (9620 to 13500)	211 (187.8 to 240)	166.9 (146.3 to 192.1)	146.1 (124.2 to 172.6)
Global	Unintentional firearm injuries	1780 (1520 to 1980)	1430 (1300 to 1650)	1410 (1270 to 1620)	32.4 (27.7 to 36.1)	21 (19 to 24.3)	18.4 (16.6 to 21.2)
Global	Other exposure to mechanical forces	9460 (8330 to 10800)	9820 (8470 to 11400)	9930 (8310 to 11900)	178.6 (156.9 to 206.8)	145.9 (125.5 to 170.3)	127.6 (107.2 to 152.3)
Global	Adverse effects of medical treatment	4570 (3220 to 5660)	4440 (3570 to 5240)	4720 (3940 to 5600)	84.7 (62.3 to 101.9)	67.9 (54.6 to 80.6)	62.6 (51.9 to 75.2)
Global	Animal contact	5660 (3140 to 7350)	5230 (3180 to 6090)	4990 (3180 to 5830)	102.3 (58 to 130.8)	78.4 (47.7 to 91.4)	66.1 (42.1 to 77.4)
Global	Venomous animal contact	4650 (2450 to 5990)	4380 (2490 to 5120)	4120 (2490 to 4910)	83.8 (45.1 to 106.7)	65.5 (37.2 to 76.6)	54.7 (32.8 to 65.3)
Global	Non-venomous animal contact	1000 (621 to 1770)	854 (619 to 1290)	863 (640 to 1140)	18.4 (11.7 to 30.9)	12.9 (9.3 to 19.6)	11.4 (8.4 to 15.1)
Global	Foreign body	10100 (8820 to 11200)	7520 (6900 to 8070)	6860 (6340 to 7350)	169.8 (150 to 187.6)	115.9 (106.2 to 124.4)	95.4 (88.2 to 102.2)
Global	Pulmonary aspiration and foreign body in airway	8520 (7420 to 9610)	6420 (5870 to 6880)	5680 (5350 to 6050)	142 (125 to 159.4)	99.3 (90.8 to 106.4)	80 (75.2 to 85.5)
Global	Foreign body in eyes	139 (70.8 to 233)	168 (86.6 to 278)	202 (105 to 334)	2.8 (1.5 to 4.6)	2.5 (1.3 to 4.2)	2.6 (1.3 to 4.3)
Global	Foreign body in other body part	1410 (926 to 1710)	937 (758 to 1130)	978 (797 to 1180)	25 (17 to 29.8)	14.1 (11.4 to 17)	12.7 (10.4 to 15.4)
Global	Environmental heat and cold exposure	3600 (2750 to 4140)	3560 (2820 to 4110)	3360 (2550 to 4000)	71 (54.9 to 81.3)	53.4 (42.2 to 61.6)	42.8 (32.4 to 51.1)
Global	Exposure to forces of nature	3240 (3190 to 3310)	1460 (1290 to 1680)	1200 (1000 to 1450)	56.7 (55.7 to 57.9)	21.3 (18.9 to 24.4)	15.5 (13 to 18.7)
Global	Other unintentional injuries	8690 (7740 to 9790)	8940 (7930 to 10200)	8580 (7380 to 10200)	163.6 (143.7 to 186.2)	132.2 (116.7 to 151.5)	110 (94.9 to 130.3)
Global	Self-harm and interpersonal violence	67800 (63300 to 70700)	67000 (63700 to 69100)	71100 (68100 to 73700)	1251.5 (1166.3 to 1305.3)	961.6 (913.8 to 992.6)	910.5 (871.7 to 944.2)
Global	Self-harm	36300 (32800 to 38300)	35200 (33200 to 36000)	34000 (31800 to 35100)	686.9 (620.5 to 723.2)	505.3 (476 to 516.6)	429 (401.6 to 443.5)
Global	Self-harm by firearm	2700 (2300 to 3350)	2630 (2220 to 3290)	2660 (2250 to 3290)	51.5 (43.9 to 63.9)	37.9 (31.9 to 47.1)	33.5 (28.3 to 41.7)
Global	Self-harm by other specified means	33600 (30000 to 35600)	32500 (30500 to 33500)	31400 (29200 to 32600)	635.4 (567 to 672.4)	467.4 (438 to 480.5)	395.4 (368.6 to 410.5)
Global	Interpersonal violence	23100 (20800 to 24800)	25800 (23100 to 27500)	26000 (23700 to 28000)	420.2 (378.8 to 450.7)	368.8 (330.8 to 392.9)	334.3 (304.7 to 360.5)
Global	Assault by firearm	6850 (5870 to 7480)	9150 (7700 to 9930)	9660 (8230 to 10400)	123.1 (105.5 to 134.4)	129.1 (108.5 to 140)	124.4 (105.8 to 134)
Global	Assault by sharp object	5380 (4310 to 6280)	5800 (4720 to 6690)	5110 (4170 to 6160)	98.7 (79.8 to 115)	82.7 (67.2 to 95.2)	65.2 (53.2 to 78.5)
Global	Sexual violence	1510 (1020 to 2210)	1910 (1300 to 2770)	2140 (1450 to 3110)	27.7 (18.6 to 40.4)	27.2 (18.5 to 39.5)	27.3 (18.5 to 39.7)
Global	Assault by other means	9380 (8050 to 10500)	8920 (7820 to 10100)	9090 (8190 to 10400)	170.7 (148.2 to 191.8)	129.8 (113.8 to 147)	117.4 (105.7 to 134.4)
Global	Conflict and terrorism	7930 (7430 to 8650)	5670 (5010 to 6650)	10100 (9400 to 11200)	136.4 (127.5 to 149)	81.9 (72.5 to 95.8)	134.1 (125.3 to 147.4)
Global	Executions and police conflict	439 (243 to 492)	396 (357 to 447)	1020 (972 to 1080)	8 (4.4 to 8.9)	5.6 (5.1 to 6.4)	13.1 (12.6 to 13.9)
Low SDI	All causes	654000 (632000 to 679000)	617000 (589000 to 651000)	553000 (517000 to 593000)	81773.9 (78126.2 to 85816.2)	59851.2 (56683.3 to 63486.6)	47422.5 (44312.4 to 50971.8)

SR Appendix Table 2. All-age DALYs counts and age standardized DALYs rates per 100,000 individuals for all causes and level 1 cause groups by location for 1990, 2007, and 2017, both sexes combined

		All-age DALYs			Age-standardized rate of DALYs per 100,000		
Low SDI	Communicable, maternal, neonatal, and nutritional disorders	472000 (454000 to 493000)	394000 (380000 to 409000)	288000 (275000 to 303000)	47533 (45250.9 to 50139.6)	30799.4 (29548.6 to 32117.1)	19941.6 (18968.4 to 21088.2)
Low SDI	HIV/AIDS and sexually transmitted infections	18300 (14100 to 23400)	44200 (40000 to 49600)	21700 (18200 to 26000)	2325.9 (1902.8 to 2827.9)	4325.8 (3990.3 to 4726.2)	1583.9 (1377.3 to 1834)
Low SDI	HIV/AIDS	12400 (10500 to 14500)	38600 (36100 to 41400)	16400 (15100 to 18000)	1850 (1527.4 to 2202.1)	3972.3 (3722.5 to 4245)	1276.7 (1181.2 to 1393.2)
Low SDI	HIV/AIDS - Drug-susceptible Tuberculosis	4040 (3000 to 4950)	10400 (7290 to 13000)	4810 (3630 to 5800)	570.8 (417.9 to 710.9)	1021.2 (703.5 to 1304.9)	366.5 (277 to 441.7)
Low SDI	HIV/AIDS - Multidrug-resistant Tuberculosis without extensive drug resistance	21.7 (9.27 to 45.8)	834 (508 to 1200)	528 (305 to 829)	3.1 (1.3 to 6.8)	81.5 (48.6 to 119.4)	39.6 (23 to 61.8)
Low SDI	HIV/AIDS - Extensively drug-resistant Tuberculosis	0 (0 to 0)	9.58 (5.99 to 13.8)	10.6 (6.15 to 16.5)	0 (0 to 0)	0.9 (0.6 to 1.4)	0.8 (0.5 to 1.3)
Low SDI	HIV/AIDS resulting in other diseases	8360 (6560 to 10400)	27400 (23700 to 31300)	11100 (9680 to 13000)	1276.1 (964.2 to 1620)	2868.7 (2490.5 to 3262.3)	869.8 (760 to 1014.5)
Low SDI	Sexually transmitted infections excluding HIV	5860 (2380 to 10700)	5670 (2380 to 10300)	5230 (2250 to 9420)	475.9 (205.2 to 836)	353.5 (156 to 631.3)	307.2 (136.1 to 546.8)
Low SDI	Syphilis	5620 (2190 to 10400)	5400 (2110 to 10100)	4910 (1940 to 9050)	432.7 (170.6 to 793.8)	323.8 (127.8 to 601.6)	279.9 (111.2 to 514.2)
Low SDI	Chlamydial infection	45.1 (30.3 to 62.3)	50.6 (35.2 to 76.6)	60.6 (40.9 to 94.4)	7.9 (5.3 to 10.8)	5.6 (4 to 8.3)	5.1 (3.5 to 7.9)
Low SDI	Gonococcal infection	84.9 (52.6 to 109)	75 (54.9 to 105)	82.8 (60 to 120)	15.5 (9.4 to 19.2)	8.4 (6.3 to 11.2)	7.1 (5.4 to 9.9)
Low SDI	Trichomoniasis	24.4 (9.85 to 51.8)	38.3 (15.5 to 81.9)	49.9 (20 to 108)	4.2 (1.7 to 9)	4.2 (1.7 to 9.1)	4.2 (1.7 to 9.1)
Low SDI	Genital herpes	17.5 (5.86 to 41.2)	27.6 (9.3 to 64.6)	37 (12.3 to 87.5)	3.4 (1.1 to 8.1)	3.4 (1.1 to 8.2)	3.5 (1.1 to 8.4)
Low SDI	Other sexually transmitted infections	68.9 (46.3 to 91.2)	73.5 (52.4 to 108)	87 (60.7 to 132)	12.2 (8.1 to 15.8)	8.1 (5.9 to 11.6)	7.4 (5.3 to 10.9)
Low SDI	Respiratory infections and tuberculosis	108000 (95200 to 118000)	82200 (77200 to 87100)	62300 (58700 to 65800)	11994.7 (10817.8 to 12932.1)	7154.2 (6779.3 to 7495.1)	4952.2 (4688 to 5194)
Low SDI	Tuberculosis	24500 (21700 to 26800)	21900 (20500 to 23300)	18900 (17600 to 20200)	4398.4 (3912.8 to 4789.9)	2750.3 (2582.3 to 2923.5)	1919.4 (1791.4 to 2051.7)
Low SDI	Latent tuberculosis infection	0 (0 to 0)	0 (0 to 0)	0 (0 to 0)	0 (0 to 0)	0 (0 to 0)	0 (0 to 0)
Low SDI	Drug-susceptible tuberculosis	24400 (21600 to 26700)	20000 (18400 to 21500)	17000 (15100 to 18500)	4383 (3898.4 to 4773.7)	2516.8 (2323.3 to 2705)	1723.6 (1521.2 to 1887.4)
Low SDI	Multidrug-resistant tuberculosis without extensive drug resistance	88.7 (45.7 to 183)	1790 (1190 to 2570)	1830 (967 to 3320)	15.4 (7.9 to 32.2)	227.3 (147.9 to 331.9)	187.6 (95.9 to 348.1)
Low SDI	Extensively drug-resistant tuberculosis	0 (0 to 0)	48.3 (28.1 to 76.2)	78.6 (30.5 to 174)	0 (0 to 0)	6.2 (3.5 to 9.9)	8.3 (3.1 to 18.6)
Low SDI	Lower respiratory infections	81800 (71200 to 90700)	58600 (54300 to 62900)	41500 (38700 to 44500)	7438.8 (6553.3 to 8173.3)	4266.2 (3937.5 to 4543.1)	2905.9 (2706.5 to 3082.7)
Low SDI	Upper respiratory infections	948 (464 to 1500)	1130 (638 to 1650)	1250 (756 to 1890)	107.7 (58.8 to 161.6)	94 (55.8 to 138.8)	88.2 (53.8 to 133.6)
Low SDI	Otitis media	404 (261 to 606)	514 (326 to 798)	555 (343 to 871)	49.7 (32.5 to 74.6)	43.7 (27.4 to 67.3)	38.7 (24.2 to 60.4)
Low SDI	Enteric infections	85200 (71400 to 97900)	59600 (52900 to 68100)	45600 (39900 to 53600)	8961.3 (7341 to 10645.5)	4903.4 (4197.7 to 6068.4)	3422.9 (2896 to 4377.2)
Low SDI	Diarrhoeal diseases	75500 (63500 to 86900)	50200 (44300 to 58100)	38300 (33400 to 46000)	7950.8 (6473.3 to 9551.6)	4215.4 (3531.8 to 5396.2)	2968.2 (2443.8 to 3911.1)
Low SDI	Typhoid and paratyphoid	7840 (4380 to 12800)	6360 (3630 to 10300)	4920 (2830 to 7930)	822.1 (462.1 to 1325.2)	464.4 (266.3 to 743.8)	306.2 (174.8 to 489.1)
Low SDI	Typhoid fever	6960 (3820 to 11500)	5640 (3130 to 9160)	4310 (2430 to 6840)	727 (401.2 to 1195.9)	410.3 (228.5 to 662.6)	268.2 (150.4 to 424)
Low SDI	Paratyphoid fever	884 (385 to 1760)	723 (330 to 1410)	607 (277 to 1160)	95.1 (42.7 to 187.8)	54.1 (24.7 to 105.5)	38 (17.6 to 72.7)
Low SDI	Invasive non-typhoidal salmonella	1590 (838 to 2820)	2930 (1650 to 4830)	2300 (1260 to 3920)	168.2 (91.3 to 293.3)	215.6 (125 to 350.8)	144.6 (79.9 to 241.9)
Low SDI	Other intestinal infectious diseases	234 (124 to 452)	122 (67.9 to 231)	65.4 (37.2 to 129)	20.1 (11.2 to 37.4)	7.9 (4.6 to 14.6)	3.9 (2.3 to 7.5)
Low SDI	Neglected tropical diseases and malaria	56400 (34000 to 96400)	46900 (36000 to 60000)	32300 (24300 to 42200)	6212.6 (3738.2 to 10838.1)	3588.5 (2794 to 4572.3)	2202.7 (1678.6 to 2822.9)
Low SDI	Malaria	33000 (22200 to 45900)	38500 (27900 to 50900)	26100 (18300 to 35400)	3161.4 (2177.1 to 4356.8)	2751.5 (1989.3 to 3653.8)	1684.1 (1189.9 to 2277.8)
Low SDI	Chagas disease	0 (0 to 0)	0 (0 to 0)	0 (0 to 0)	0 (0 to 0)	0 (0 to 0)	0 (0 to 0)
Low SDI	Leishmaniasis	15900 (150 to 59100)	1260 (112 to 5970)	580 (139 to 2030)	1915.1 (23.6 to 6822.5)	107.5 (12.8 to 469)	42.4 (12 to 134.4)
Low SDI	Visceral leishmaniasis	15800 (74.9 to 59100)	1110 (2.43 to 5810)	394 (0.718 to 1820)	1898.8 (9.1 to 6807.4)	90.6 (0.2 to 449.3)	25.7 (0 to 114.5)
Low SDI	Cutaneous and mucocutaneous leishmaniasis	80.3 (31.8 to 157)	143 (84.1 to 229)	186 (118 to 279)	16.3 (6.5 to 31.7)	17 (9.6 to 27.9)	16.7 (10.2 to 25.9)
Low SDI	African trypanosomiasis	753 (371 to 1270)	369 (184 to 627)	75.3 (13.4 to 283)	113.9 (56.5 to 192)	36.3 (17.8 to 61.9)	5.8 (1 to 20.3)
Low SDI	Schistosomiasis	763 (533 to 1150)	952 (587 to 1590)	787 (494 to 1340)	136.8 (95.3 to 206.8)	111 (69.5 to 182.9)	71.3 (45.4 to 120.4)
Low SDI	Cysticercosis	272 (183 to 381)	316 (211 to 448)	366 (240 to 524)	54 (35.8 to 75)	41.4 (27.4 to 58.1)	37.1 (24.1 to 52.7)
Low SDI	Cystic echinococcosis	91 (63.5 to 123)	50 (37.2 to 62.7)	31.7 (23.4 to 41.5)	11.3 (8.2 to 14.7)	4.6 (3.5 to 5.8)	2.6 (2 to 3.3)

SR Appendix Table 2. All-age DALYs counts and age standardized DALYs rates per 100,000 individuals for all causes and level 1 cause groups by location for 1990, 2007, and 2017, both sexes combined

		All-age DALYs			Age-standardized rate of DALYs per 100,000		
Low SDI	Lymphatic filariasis	580 (301 to 975)	866 (445 to 1480)	445 (237 to 728)	105.2 (55.2 to 176.4)	102.4 (52.7 to 173.5)	40.4 (21.7 to 65.5)
Low SDI	Onchocerciasis	967 (527 to 1540)	918 (526 to 1440)	998 (480 to 1760)	176.9 (101.6 to 276)	106.4 (62.7 to 165.3)	88.8 (45.1 to 155.8)
Low SDI	Trachoma	137 (93.3 to 187)	129 (87.7 to 174)	108 (71.7 to 150)	44.2 (30.1 to 59.8)	27 (18.7 to 36.7)	16.7 (11 to 23.1)
Low SDI	Dengue	203 (66.3 to 342)	364 (161 to 518)	621 (268 to 913)	27 (9 to 44.1)	35.6 (15.6 to 50.8)	52.6 (22.1 to 76.8)
Low SDI	Yellow fever	252 (54.5 to 736)	188 (40.8 to 556)	130 (26.2 to 388)	32.5 (7.1 to 95.9)	16.1 (3.5 to 47.5)	8.9 (1.8 to 26.7)
Low SDI	Rabies	1140 (789 to 1560)	615 (477 to 1070)	271 (214 to 380)	137.7 (99.9 to 183.4)	54 (42.2 to 93.1)	20.6 (16.6 to 27.5)
Low SDI	Intestinal nematode infections	1580 (1100 to 2240)	1310 (860 to 1940)	914 (578 to 1400)	201.8 (135 to 295.9)	116.8 (74.5 to 175.6)	67.2 (41.8 to 103.6)
Low SDI	Ascariasis	769 (576 to 1030)	578 (412 to 828)	355 (240 to 528)	81.7 (59.2 to 113)	45.4 (31 to 67.8)	24.2 (16.2 to 36.8)
Low SDI	Trichuriasis	73 (39.3 to 123)	96 (53.8 to 161)	69.9 (38.4 to 120)	10 (5.4 to 16.9)	8.8 (4.9 to 14.7)	5.1 (2.8 to 8.8)
Low SDI	Hookworm disease	742 (459 to 1140)	637 (379 to 976)	489 (291 to 765)	110.1 (67.7 to 169.7)	62.6 (37.2 to 96.3)	37.9 (22.6 to 59.3)
Low SDI	Food-borne trematodiases	0 (0 to 0)	0 (0 to 0)	0 (0 to 0)	0 (0 to 0)	0 (0 to 0)	0 (0 to 0)
Low SDI	Leprosy	7.55 (5.17 to 10.6)	10.7 (7.31 to 15)	10.4 (7.06 to 14.7)	1.9 (1.3 to 2.7)	1.8 (1.2 to 2.5)	1.3 (0.9 to 1.8)
Low SDI	Ebola virus disease	0 (0 to 0)	26.3 (26.3 to 26.4)	0.503 (0.502 to 0.505)	0 (0 to 0)	2.5 (2.5 to 2.5)	0 (0 to 0)
Low SDI	Zika virus disease	0 (0 to 0)	0 (0 to 0)	0.00824 (0.00188 to 0.0329)	0 (0 to 0)	0 (0 to 0)	0 (0 to 0)
Low SDI	Guinea worm disease	18 (11.4 to 26.6)	0.0675 (0.0416 to 0.102)	0.00055 (0.000293 to 0.000882)	3.5 (2.2 to 5.1)	0 (0 to 0)	0 (0 to 0)
Low SDI	Other neglected tropical diseases	822 (504 to 1860)	953 (624 to 1810)	951 (622 to 1770)	89.5 (57.5 to 178.9)	73.7 (49.3 to 131)	62.9 (41.6 to 112.7)
Low SDI	Other infectious diseases	70100 (51700 to 98700)	39000 (31300 to 49800)	25600 (21100 to 31300)	6317.3 (4738.8 to 8768.2)	2727.9 (2244.5 to 3405)	1652 (1396.3 to 1976.2)
Low SDI	Meningitis	13800 (11500 to 16200)	12000 (10800 to 13600)	8990 (7960 to 10600)	1322.6 (1108.5 to 1582.1)	861.3 (777.5 to 990.1)	587.1 (524 to 698.8)
Low SDI	Pneumococcal meningitis	2100 (1700 to 2520)	1790 (1560 to 2070)	1440 (1240 to 1700)	211.3 (174 to 250.7)	136.5 (120.2 to 157.7)	97.2 (85.1 to 114.9)
Low SDI	H influenzae type B meningitis	4020 (3300 to 4890)	3260 (2790 to 3810)	1880 (1590 to 2300)	380.3 (313.8 to 468.1)	229.2 (197.9 to 270.1)	122 (103.9 to 150.3)
Low SDI	Meningococcal infection	1860 (1520 to 2230)	1760 (1550 to 2000)	1080 (927 to 1300)	168.8 (139.6 to 201.4)	120 (106.8 to 137.1)	68 (59 to 82.3)
Low SDI	Other meningitis	5800 (4710 to 6920)	5180 (4550 to 5980)	4590 (4030 to 5400)	562.2 (460.2 to 681.2)	375.5 (332.8 to 431.7)	299.9 (264.2 to 352)
Low SDI	Encephalitis	1620 (1100 to 2900)	1780 (1520 to 2130)	1580 (1410 to 1800)	184 (136.4 to 303)	146.1 (129.8 to 168.6)	114.6 (103.5 to 131.5)
Low SDI	Diphtheria	969 (650 to 1350)	237 (175 to 320)	108 (77.7 to 150)	85.4 (58.2 to 117.8)	15.4 (11.5 to 20.6)	6.4 (4.6 to 8.8)
Low SDI	Whooping cough	5960 (2330 to 12400)	6370 (2530 to 12300)	4880 (2200 to 8990)	490.5 (191.9 to 1026.8)	393.7 (156.5 to 759.9)	279.2 (125.4 to 513.6)
Low SDI	Tetanus	14000 (11400 to 16300)	3690 (2950 to 4610)	1290 (906 to 1780)	1197.3 (965.2 to 1385.3)	255.2 (204.7 to 320.9)	84.3 (59.5 to 115.2)
Low SDI	Measles	27800 (9780 to 59300)	9510 (3400 to 20400)	4160 (1490 to 9160)	2350.7 (827 to 5028)	596.3 (213.2 to 1276.5)	238 (85 to 523.6)
Low SDI	Varicella and herpes zoster	609 (488 to 729)	555 (480 to 633)	473 (411 to 545)	67.6 (56.3 to 78.3)	44.1 (39.1 to 49.3)	32.8 (28.9 to 37.1)
Low SDI	Acute hepatitis	2870 (2110 to 3710)	2560 (1960 to 3140)	1960 (1540 to 2380)	371 (275.4 to 475.2)	237 (185.2 to 292.4)	163.2 (129.3 to 194.2)
Low SDI	Acute hepatitis A	1020 (725 to 1320)	845 (617 to 1070)	527 (400 to 699)	99.3 (69.1 to 128.5)	59.4 (43.1 to 75.2)	32.8 (24.5 to 43.4)
Low SDI	Acute hepatitis B	1350 (972 to 1860)	1270 (964 to 1630)	1110 (872 to 1360)	210.9 (154.4 to 284.8)	139.5 (108 to 176.8)	106 (85.2 to 128.2)
Low SDI	Acute hepatitis C	157 (74.3 to 293)	119 (59.6 to 218)	80.4 (39.2 to 144)	13.9 (6.6 to 25.4)	8.1 (4.1 to 14.7)	5.1 (2.6 to 9.2)
Low SDI	Acute hepatitis E	348 (241 to 454)	326 (240 to 416)	246 (182 to 312)	46.9 (33.4 to 60.8)	30 (21.9 to 38.3)	19.3 (14.4 to 24.3)
Low SDI	Other unspecified infectious diseases	2400 (1710 to 2880)	2350 (1600 to 2720)	2140 (1460 to 2480)	248.2 (192.7 to 286)	178.8 (128.4 to 204.9)	146.5 (101.4 to 169.7)
Low SDI	Maternal and neonatal disorders	97200 (89800 to 107000)	92200 (88400 to 96200)	76400 (72300 to 81000)	7949.4 (7375.8 to 8653.5)	5830.5 (5590.6 to 6079)	4509.9 (4265.5 to 4778.2)
Low SDI	Maternal disorders	8370 (7220 to 9210)	7290 (6850 to 7690)	5610 (5210 to 6040)	1321.7 (1146.4 to 1452.1)	743.2 (699.3 to 785.2)	451.3 (419.3 to 484.9)
Low SDI	Maternal haemorrhage	3080 (2350 to 3910)	2410 (1960 to 2880)	1030 (870 to 1240)	491.6 (378.8 to 612.7)	246.8 (204.6 to 291.3)	83.5 (70.9 to 99.5)
Low SDI	Maternal sepsis and other pregnancy related infections	1020 (734 to 1420)	924 (733 to 1220)	735 (611 to 900)	159.3 (117.9 to 214.2)	94.2 (76.6 to 122)	59 (49.1 to 71.6)
Low SDI	Maternal hypertensive disorders	867 (628 to 1210)	734 (585 to 966)	694 (575 to 860)	130.9 (95.8 to 179.4)	72 (58.1 to 93.9)	54 (44.9 to 66)
Low SDI	Maternal obstructed labour and uterine rupture	816 (593 to 1130)	586 (456 to 797)	533 (427 to 666)	134.6 (99.2 to 186.1)	62.6 (49.2 to 83.1)	44 (35.5 to 54.2)

SR Appendix Table 2. All-age DALYs counts and age standardized DALYs rates per 100,000 individuals for all causes and level 1 cause groups by location for 1990, 2007, and 2017, both sexes combined

		All-age DALYs			Age-standardized rate of DALYs per 100,000		
Low SDI	Maternal abortive outcome	628 (461 to 870)	433 (333 to 575)	421 (340 to 526)	100.3 (75.5 to 134.2)	45.2 (35.4 to 58.4)	34.7 (28 to 43.2)
Low SDI	Ectopic pregnancy	340 (177 to 670)	305 (183 to 548)	277 (187 to 445)	52.1 (28 to 99.4)	30.3 (18.7 to 52.5)	21.8 (14.9 to 34.6)
Low SDI	Indirect maternal deaths	941 (668 to 1340)	1110 (922 to 1400)	1150 (1000 to 1340)	146.4 (106.6 to 201.6)	112.4 (94.7 to 140.4)	92.3 (80.5 to 106.6)
Low SDI	Late maternal deaths	65.9 (53 to 80.6)	68.5 (56.4 to 84.9)	59.3 (46.8 to 76.9)	10.3 (8.3 to 12.4)	6.8 (5.6 to 8.4)	4.7 (3.7 to 6)
Low SDI	Maternal deaths aggravated by HIV/AIDS	29.8 (17 to 42.4)	53.8 (33.6 to 71.6)	40.8 (25.4 to 54.8)	4.8 (2.8 to 6.8)	5.9 (3.7 to 7.8)	3.5 (2.2 to 4.7)
Low SDI	Other maternal disorders	579 (402 to 890)	664 (479 to 927)	672 (535 to 850)	91.3 (65.1 to 133.9)	67.1 (49.6 to 91.8)	53.8 (43.5 to 67)
Low SDI	Neonatal disorders	88800 (81200 to 98500)	84900 (81200 to 88900)	70800 (66600 to 75400)	6627.7 (6065.1 to 7353)	5087.2 (4854.5 to 5326.8)	4058.6 (3811.5 to 4319.6)
Low SDI	Neonatal preterm birth	31900 (27500 to 39500)	28600 (26100 to 35000)	24400 (21800 to 28800)	2385.7 (2059.3 to 2954)	1722.2 (1574.5 to 2101.5)	1401.4 (1256.3 to 1647.2)
Low SDI	Neonatal encephalopathy due to birth asphyxia and trauma	23500 (20200 to 26900)	25000 (22300 to 27200)	20600 (18500 to 22700)	1745.3 (1507.5 to 2003.4)	1500 (1336.9 to 1639.3)	1183 (1057.6 to 1314.3)
Low SDI	Neonatal sepsis and other neonatal infections	8570 (6720 to 11900)	9040 (7700 to 11500)	8620 (7160 to 10600)	642.9 (502.1 to 890.3)	544.6 (464.1 to 689.1)	495.7 (413.2 to 605.9)
Low SDI	Hemolytic disease and other neonatal jaundice	4810 (3670 to 6050)	2980 (2510 to 3480)	1940 (1630 to 2280)	362.7 (278.4 to 456.2)	180.6 (152.9 to 210.4)	113.1 (95.4 to 132)
Low SDI	Other neonatal disorders	20100 (15900 to 24100)	19300 (15200 to 21600)	15300 (11900 to 17400)	1491.1 (1175.9 to 1788.5)	1139.8 (897 to 1276.1)	865.4 (669.7 to 983.7)
Low SDI	Nutritional deficiencies	36800 (30500 to 43700)	29800 (24600 to 36500)	24200 (19000 to 30900)	3771.9 (3103.5 to 4561.4)	2269.1 (1841.2 to 2837.9)	1617.9 (1265.2 to 2071.9)
Low SDI	Protein-energy malnutrition	21900 (17900 to 25700)	13600 (12100 to 15000)	8990 (7890 to 10300)	1954.6 (1609.7 to 2269.1)	909.5 (816.3 to 1000.7)	554 (487.2 to 629.9)
Low SDI	Iodine deficiency	1830 (1070 to 3040)	1050 (656 to 1600)	786 (493 to 1190)	261.6 (151.2 to 435.4)	102.9 (64.2 to 157.1)	62.8 (39.6 to 94.5)
Low SDI	Dietary iron deficiency	7690 (5220 to 10900)	10000 (6830 to 14300)	10100 (6820 to 14400)	1030.8 (700.9 to 1457.1)	896 (606.3 to 1268.2)	735.9 (497.1 to 1051.7)
Low SDI	Other nutritional deficiencies	1390 (996 to 1750)	838 (694 to 999)	567 (447 to 684)	165.1 (125.5 to 200.5)	78.8 (68.2 to 89.8)	49.9 (40.4 to 58.8)
Low SDI	Non-communicable diseases	137000 (123000 to 153000)	177000 (159000 to 198000)	217000 (194000 to 244000)	28047.5 (25524.8 to 30875.1)	24469.8 (22129.7 to 26958.7)	23494.4 (21207.7 to 26022.1)
Low SDI	Neoplasms	12800 (10800 to 14900)	16700 (15800 to 17600)	21800 (20700 to 22700)	2865.1 (2547.3 to 3184.4)	2496.5 (2351.8 to 2628)	2519.5 (2386.8 to 2628.1)
Low SDI	Lip and oral cavity cancer	398 (349 to 459)	592 (552 to 627)	777 (723 to 834)	102.2 (90.2 to 117.6)	100.5 (93.6 to 106.5)	99.6 (92.5 to 106.9)
Low SDI	Nasopharynx cancer	148 (121 to 172)	177 (160 to 196)	227 (209 to 244)	33.4 (27.9 to 38.6)	26.8 (24.3 to 29.5)	26.3 (24.4 to 28.3)
Low SDI	Other pharynx cancer	336 (260 to 389)	467 (347 to 518)	690 (520 to 773)	85.1 (65.6 to 98.6)	79 (58.2 to 87.7)	87.2 (65.7 to 97.4)
Low SDI	Oesophageal cancer	500 (451 to 544)	589 (554 to 623)	798 (752 to 849)	134.4 (122.8 to 145.7)	104.6 (98.3 to 110.9)	104.5 (98.7 to 111.2)
Low SDI	Stomach cancer	1290 (1150 to 1420)	1420 (1330 to 1510)	1750 (1650 to 1860)	334.1 (301 to 369.1)	243.2 (227.2 to 258)	222 (209 to 234.7)
Low SDI	Colon and rectum cancer	673 (539 to 881)	938 (862 to 1070)	1330 (1260 to 1410)	181.4 (148.2 to 234.1)	165.5 (152 to 187.6)	175.4 (166.1 to 186)
Low SDI	Liver cancer	730 (546 to 1310)	978 (809 to 1460)	1200 (1080 to 1450)	183.6 (138.3 to 327.6)	161.6 (133.2 to 239.1)	148.3 (133.3 to 178.8)
Low SDI	Liver cancer due to hepatitis B	314 (222 to 618)	412 (326 to 674)	482 (419 to 619)	71.6 (50.7 to 141.3)	61.7 (48.4 to 100.6)	54.4 (47 to 70.4)
Low SDI	Liver cancer due to hepatitis C	149 (109 to 244)	202 (156 to 284)	258 (213 to 318)	42.9 (31.6 to 70.2)	38.1 (29.5 to 53.2)	35.5 (29.5 to 43.5)
Low SDI	Liver cancer due to alcohol use	137 (97.1 to 266)	186 (144 to 288)	238 (206 to 293)	37 (26.1 to 72.2)	32.5 (25.4 to 50)	30.7 (26.5 to 37.6)
Low SDI	Liver cancer due to NASH	58.1 (43 to 97.1)	83.7 (66.6 to 119)	111 (95.2 to 132)	16.1 (11.9 to 26.9)	15.2 (12.1 to 21.4)	14.8 (12.7 to 17.6)
Low SDI	Liver cancer due to other causes	71.9 (53.1 to 116)	95 (77.2 to 132)	115 (98.2 to 135)	15.9 (11.9 to 25.8)	14 (11.3 to 19.5)	12.9 (11 to 15.3)
Low SDI	Gallbladder and biliary tract cancer	158 (123 to 235)	261 (213 to 304)	368 (290 to 426)	45.1 (35.4 to 67.3)	48.6 (39.6 to 57.2)	50.4 (39.8 to 58.3)
Low SDI	Pancreatic cancer	151 (127 to 188)	257 (230 to 295)	416 (388 to 446)	42.2 (36.4 to 52.2)	47.4 (42.1 to 54.5)	56.4 (52.4 to 60.6)
Low SDI	Larynx cancer	382 (328 to 427)	419 (372 to 458)	533 (485 to 577)	100 (85.9 to 111.5)	73.1 (64.9 to 80.1)	68.6 (62.6 to 74.2)
Low SDI	Tracheal, bronchus, and lung cancer	885 (796 to 996)	1200 (1110 to 1310)	1740 (1640 to 1860)	245.4 (221.2 to 275.6)	218.2 (201.1 to 237.2)	235.4 (221 to 250.3)
Low SDI	Malignant skin melanoma	46.3 (33.3 to 67.1)	67.8 (53.8 to 87)	91 (74.3 to 115)	10.7 (7.8 to 15.1)	10.1 (8.1 to 12.9)	10.2 (8.4 to 12.9)
Low SDI	Non-melanoma skin cancer	56.6 (48.6 to 68.4)	86.3 (70.6 to 100)	118 (101 to 133)	16.4 (13.8 to 19.9)	16.1 (12.9 to 18.9)	16.2 (13.6 to 18.4)
Low SDI	Non-melanoma skin cancer (squamous-cell carcinoma)	56.5 (48.6 to 68.4)	86.3 (70.6 to 100)	118 (101 to 133)	16.4 (13.8 to 19.9)	16.1 (12.9 to 18.9)	16.2 (13.6 to 18.4)
Low SDI	Non-melanoma skin cancer (basal-cell carcinoma)	0.0185 (0.00698 to 0.0414)	0.0292 (0.0111 to 0.0661)	0.0397 (0.015 to 0.0908)	0 (0 to 0)	0 (0 to 0)	0 (0 to 0)
Low SDI	Breast cancer	731 (567 to 1030)	1130 (1020 to 1340)	1820 (1680 to 1980)	174.9 (138.2 to 244.7)	178.2 (161.9 to 211.2)	214.7 (198.4 to 233.7)

SR Appendix Table 2. All-age DALYs counts and age standardized DALYs rates per 100,000 individuals for all causes and level 1 cause groups by location for 1990, 2007, and 2017, both sexes combined

		All-age DALYs			Age-standardized rate of DALYs per 100,000		
Low SDI	Cervical cancer	1290 (1060 to 1460)	1400 (1250 to 1530)	1710 (1540 to 1880)	300.3 (249.9 to 340.6)	214.9 (192.6 to 235)	196.5 (177.1 to 216.7)
Low SDI	Uterine cancer	106 (82.3 to 125)	122 (102 to 141)	170 (149 to 192)	28.4 (22.6 to 33.5)	21.7 (18.2 to 24.9)	22.2 (19.5 to 25.1)
Low SDI	Ovarian cancer	151 (114 to 224)	235 (205 to 286)	384 (340 to 445)	36.9 (28.3 to 53.8)	38 (33.1 to 46)	46.3 (41.1 to 53.7)
Low SDI	Prostate cancer	259 (205 to 301)	428 (321 to 481)	614 (466 to 686)	93.3 (74.6 to 109.2)	96.3 (72.6 to 108.2)	98.2 (74.3 to 110)
Low SDI	Testicular cancer	28 (21 to 34.3)	27.4 (22.5 to 32.2)	30.6 (25.8 to 34.8)	4.9 (3.7 to 6)	3.1 (2.5 to 3.6)	2.7 (2.3 to 3.1)
Low SDI	Kidney cancer	104 (66.2 to 155)	143 (115 to 178)	189 (161 to 214)	17.6 (13.1 to 24.3)	17.5 (13.8 to 21)	19.2 (16.3 to 21.5)
Low SDI	Bladder cancer	129 (109 to 149)	174 (156 to 198)	238 (218 to 276)	39.8 (34.3 to 46)	34.8 (31.3 to 39.6)	34.5 (31.6 to 40.1)
Low SDI	Thyroid cancer	89.8 (63.6 to 114)	123 (106 to 140)	153 (137 to 170)	19.4 (14.4 to 24.4)	17.6 (15.2 to 19.9)	17.1 (15.3 to 18.9)
Low SDI	Mesothelioma	22.1 (12.6 to 39.7)	28.4 (19.4 to 45.2)	40.3 (30.3 to 58.5)	5.4 (3.1 to 9.5)	4.5 (3.1 to 7.1)	4.8 (3.7 to 7)
Low SDI	Hodgkin lymphoma	319 (223 to 394)	300 (254 to 402)	286 (241 to 391)	49.2 (35.9 to 58.8)	31.4 (26.6 to 42.7)	24.2 (20.5 to 32.9)
Low SDI	Non-Hodgkin's lymphoma	611 (436 to 849)	818 (708 to 944)	1010 (884 to 1100)	102.1 (80.3 to 133.4)	95 (80.8 to 107.2)	95 (84.1 to 102.6)
Low SDI	Multiple myeloma	79.9 (64.9 to 110)	122 (109 to 148)	179 (164 to 207)	21.8 (18 to 29.4)	22 (19.5 to 26.4)	23.6 (21.6 to 27)
Low SDI	Leukaemia	1220 (802 to 2040)	1550 (1290 to 1950)	1690 (1450 to 1920)	167.2 (122.4 to 246.7)	151.7 (126.3 to 178.8)	139 (118.4 to 156.2)
Low SDI	Acute lymphoid leukaemia	315 (169 to 697)	424 (325 to 644)	445 (381 to 555)	34.3 (19.8 to 70.5)	32.8 (25.9 to 47.9)	29.4 (25.3 to 36.5)
Low SDI	Chronic lymphoid leukaemia	15.4 (11.4 to 18.9)	25.2 (17.6 to 29.7)	35.9 (27 to 41)	4.5 (3.4 to 5.6)	4.8 (3.3 to 5.7)	5 (3.7 to 5.7)
Low SDI	Acute myeloid leukaemia	250 (133 to 575)	382 (292 to 564)	482 (385 to 561)	37.2 (23.5 to 72.5)	39.8 (30.9 to 52.9)	41.2 (33.1 to 47)
Low SDI	Chronic myeloid leukaemia	106 (82.8 to 138)	133 (109 to 155)	152 (127 to 172)	23.1 (18.5 to 29.2)	19 (15.7 to 22.1)	16.7 (14 to 18.8)
Low SDI	Other leukaemia	529 (290 to 1030)	591 (427 to 852)	579 (461 to 705)	68.1 (43.2 to 117)	55.4 (41.6 to 74.1)	46.7 (37.7 to 56)
Low SDI	Other malignant cancers	1160 (874 to 1590)	1600 (1460 to 1740)	1990 (1790 to 2170)	178.5 (146.7 to 219.2)	174 (158.8 to 187.5)	177.2 (159.9 to 191.5)
Low SDI	Other neoplasms	59.8 (38.1 to 86.2)	94.4 (58.6 to 137)	145 (94.4 to 200)	10.2 (5.5 to 16.8)	11.3 (6.2 to 17.8)	14.3 (8.8 to 20.5)
Low SDI	Myelodysplastic, myeloproliferative, and other hematopoietic neoplasms	48.9 (33.2 to 64.4)	74.4 (48.3 to 102)	115 (78.1 to 153)	8.9 (4.9 to 14)	9.6 (5.3 to 14.8)	12.2 (7.7 to 17.2)
Low SDI	Benign and in situ intestinal neoplasms	0 (0 to 0)	0 (0 to 0)	0 (0 to 0)	0 (0 to 0)	0 (0 to 0)	0 (0 to 0)
Low SDI	Benign and in situ cervical and uterine neoplasms	0 (0 to 0)	0 (0 to 0)	0 (0 to 0)	0 (0 to 0)	0 (0 to 0)	0 (0 to 0)
Low SDI	Other benign and in situ neoplasms	11 (4.25 to 22.7)	20 (9.88 to 36.4)	29.5 (16.5 to 47.7)	1.3 (0.5 to 2.9)	1.7 (0.8 to 3.1)	2 (1.1 to 3.3)
Low SDI	Cardiovascular diseases	25100 (22800 to 26800)	33400 (31900 to 34700)	41900 (40000 to 43700)	6719.6 (6215.1 to 7171.9)	5957.4 (5697.1 to 6172.4)	5586.4 (5325 to 5818.8)
Low SDI	Rheumatic heart disease	2260 (1870 to 2700)	2420 (2090 to 2740)	2500 (2170 to 2840)	418.5 (352.2 to 508.4)	309.9 (266.4 to 354.6)	258.3 (224.2 to 294.5)
Low SDI	Ischaemic heart disease	9830 (8840 to 10700)	14400 (13700 to 15100)	19100 (18300 to 20000)	2823.6 (2573.3 to 3078.4)	2666.2 (2541.8 to 2804.8)	2604 (2487 to 2736.1)
Low SDI	Stroke	8850 (8130 to 9550)	11600 (11000 to 12100)	14000 (13300 to 14700)	2493 (2307.3 to 2670.1)	2155.8 (2055.5 to 2248.8)	1919.7 (1829 to 2018.6)
Low SDI	Ischaemic stroke	2640 (2220 to 3150)	3730 (3160 to 4460)	4710 (3990 to 5610)	872.1 (735.6 to 1033.1)	793.4 (679.3 to 941.8)	722.5 (614.9 to 853.4)
Low SDI	Intracerebral hemorrhage	5260 (4510 to 5990)	6680 (5860 to 7460)	7920 (7030 to 8700)	1419.6 (1221 to 1603.1)	1191.1 (1033.8 to 1335.7)	1041.5 (915.4 to 1152.2)
Low SDI	Subarachnoid hemorrhage	952 (693 to 1190)	1170 (916 to 1460)	1370 (1140 to 1750)	201.2 (144 to 265)	171.3 (134.4 to 218.1)	155.6 (127.8 to 199.2)
Low SDI	Hypertensive heart disease	1320 (739 to 1670)	1630 (1070 to 2140)	2090 (1460 to 2620)	404.6 (233.4 to 518.3)	326 (217.5 to 433)	305.5 (216.4 to 386.9)
Low SDI	Non-rheumatic valvular heart disease	119 (93 to 165)	164 (138 to 206)	224 (190 to 270)	31.5 (25.8 to 41.7)	28.1 (24 to 34.2)	29 (24.6 to 34.1)
Low SDI	Non-rheumatic calcific aortic valve disease	29.9 (16.8 to 56.6)	46.2 (31.3 to 72.6)	72.1 (52.1 to 102)	8.6 (5.1 to 15)	8.6 (5.9 to 12.8)	10 (7.4 to 13.6)
Low SDI	Non-rheumatic degenerative mitral valve disease	81.4 (57.8 to 105)	106 (79.5 to 127)	136 (103 to 165)	21.3 (15.5 to 26.2)	18 (13.5 to 21.6)	17.4 (13.2 to 20.9)
Low SDI	Other non-rheumatic valve diseases	7.68 (4.29 to 11.5)	12.1 (7.54 to 16.3)	16.4 (10.6 to 22)	1.6 (0.9 to 2.3)	1.6 (1 to 2.1)	1.6 (1 to 2.1)
Low SDI	Cardiomyopathy and myocarditis	871 (644 to 1160)	957 (731 to 1240)	1120 (895 to 1410)	139.4 (100.3 to 190.7)	113.4 (85.5 to 150.1)	113.1 (89.2 to 144)
Low SDI	Myocarditis	160 (109 to 235)	162 (124 to 221)	175 (130 to 223)	22 (15.6 to 32.7)	16.9 (12.3 to 23.3)	15.8 (11.3 to 20.4)
Low SDI	Alcoholic cardiomyopathy	78.6 (50 to 117)	85.6 (53.9 to 131)	110 (74.3 to 160)	18.6 (12.2 to 27.8)	13.4 (8.4 to 20.7)	13.1 (8.8 to 18.9)
Low SDI	Other cardiomyopathy	633 (455 to 847)	710 (528 to 931)	837 (655 to 1070)	98.8 (70.2 to 137.8)	83.1 (61.3 to 112.5)	84.2 (65.7 to 110.3)

SR Appendix Table 2. All-age DALYs counts and age standardized DALYs rates per 100,000 individuals for all causes and level 1 cause groups by location for 1990, 2007, and 2017, both sexes combined

		All-age DALYs			Age-standardized rate of DALYs per 100,000		
Low SDI	Atrial fibrillation and flutter	148 (120 to 183)	237 (190 to 292)	355 (288 to 433)	59.4 (48.2 to 72.8)	59.1 (47.5 to 72.6)	63.1 (51.5 to 76.4)
Low SDI	Aortic aneurysm	143 (103 to 201)	179 (144 to 234)	251 (209 to 313)	41.1 (31 to 56.7)	33.3 (27.1 to 43.7)	34.5 (29 to 42.7)
Low SDI	Peripheral vascular disease	24.4 (13 to 40.6)	38.1 (20.6 to 61.5)	55.9 (31.3 to 88.5)	8.9 (4.7 to 14.8)	8.8 (4.8 to 14.1)	9.2 (5.2 to 14.5)
Low SDI	Endocarditis	292 (189 to 482)	307 (221 to 455)	365 (289 to 479)	40.7 (25.9 to 65.7)	31.6 (22.8 to 47)	32.6 (25.8 to 43.1)
Low SDI	Other cardiovascular and circulatory diseases	1210 (937 to 1620)	1520 (1210 to 1900)	1870 (1530 to 2310)	259 (205.9 to 341.8)	225 (179.3 to 291.2)	217.4 (178.1 to 271.6)
Low SDI	Chronic respiratory diseases	18000 (15900 to 19400)	18200 (16800 to 19300)	23000 (21200 to 24500)	4763.2 (4221 to 5108.1)	3301.1 (3022 to 3481.2)	3109.2 (2844.3 to 3298.1)
Low SDI	Chronic obstructive pulmonary disease	9580 (7250 to 12700)	11000 (8970 to 13300)	15000 (12500 to 16900)	2939.4 (2247.4 to 3783.6)	2221.1 (1805.5 to 2666)	2192.6 (1827.5 to 2476.4)
Low SDI	Pneumoconiosis	37.7 (23.6 to 55.5)	40.2 (28.7 to 61.2)	48.8 (37.5 to 68)	11.2 (6.9 to 16.5)	7.8 (5.5 to 12)	6.8 (5.3 to 9.6)
Low SDI	Silicosis	16.8 (7.78 to 30.8)	16.8 (8.55 to 31.1)	19.7 (11.8 to 31.9)	5 (2.3 to 9.3)	3.3 (1.7 to 6.2)	2.8 (1.7 to 4.5)
Low SDI	Asbestosis	4.42 (1.12 to 9.75)	5.45 (1.74 to 11.6)	7.34 (2.83 to 13.7)	1.3 (0.3 to 3)	1 (0.3 to 2.2)	1 (0.4 to 2)
Low SDI	Coal workers pneumoconiosis	6.36 (1.53 to 13.3)	5.95 (1.8 to 12)	6.38 (2.58 to 11.5)	1.9 (0.4 to 4)	1.2 (0.3 to 2.4)	0.9 (0.4 to 1.6)
Low SDI	Other pneumoconiosis	10.1 (4.83 to 18.6)	12 (6.53 to 21.1)	15.4 (9.31 to 24.4)	3 (1.4 to 5.4)	2.3 (1.2 to 4)	2.1 (1.3 to 3.4)
Low SDI	Asthma	7560 (4450 to 9460)	6120 (4010 to 8030)	6550 (4550 to 8640)	1650.1 (826.5 to 2197.4)	929.3 (535.5 to 1270.7)	753.9 (472 to 1026.3)
Low SDI	Interstitial lung disease and pulmonary sarcoidosis	225 (77.9 to 510)	301 (128 to 572)	481 (238 to 851)	61.5 (22.4 to 132.7)	56.2 (24.1 to 108.3)	66.9 (33.2 to 120.3)
Low SDI	Other chronic respiratory diseases	569 (364 to 842)	724 (564 to 901)	943 (790 to 1100)	101 (65.7 to 144.2)	86.7 (66.8 to 108.5)	88.9 (73.7 to 104.9)
Low SDI	Digestive diseases	11300 (9550 to 13500)	13100 (12100 to 14400)	14700 (13500 to 16200)	2260.4 (1983.8 to 2597.7)	1774.2 (1643.8 to 1931)	1548.8 (1430.1 to 1721.6)
Low SDI	Cirrhosis and other chronic liver diseases	4330 (3490 to 5490)	5450 (4930 to 6480)	5950 (5190 to 7440)	894.9 (747.9 to 1087)	778.3 (708 to 918.2)	657.9 (574.3 to 828.1)
Low SDI	Cirrhosis and other chronic liver diseases due to hepatitis B	1320 (1080 to 1600)	1790 (1580 to 2150)	1910 (1610 to 2430)	295.8 (246.1 to 358.6)	264.9 (233.3 to 318.4)	214.9 (180.3 to 277.3)
Low SDI	Cirrhosis and other chronic liver diseases due to hepatitis C	829 (657 to 1020)	1100 (943 to 1310)	1260 (1060 to 1530)	196.3 (157.8 to 239.8)	172.4 (147.2 to 204.2)	148.1 (124.6 to 181)
Low SDI	Cirrhosis and other chronic liver diseases due to alcohol use	805 (652 to 985)	1140 (986 to 1360)	1350 (1130 to 1770)	194.7 (159.2 to 236.2)	181.3 (157.6 to 219.3)	161.3 (135.8 to 212)
Low SDI	Cirrhosis due to NASH	174 (143 to 210)	244 (214 to 286)	300 (250 to 372)	43 (35.5 to 51.5)	39.6 (34.6 to 46.3)	36.4 (30.3 to 45.5)
Low SDI	Cirrhosis and other chronic liver diseases due to other causes	1200 (851 to 1800)	1170 (1010 to 1500)	1130 (966 to 1390)	165 (124.6 to 229)	120.1 (103.9 to 149.4)	97.1 (81.9 to 122)
Low SDI	Upper digestive system diseases	3330 (2840 to 3970)	3160 (2580 to 3900)	3640 (2900 to 4620)	747.1 (648.5 to 874.3)	442.6 (369.7 to 536.7)	382.5 (311.4 to 475.4)
Low SDI	Peptic ulcer disease	2120 (1830 to 2550)	1480 (1330 to 1720)	1460 (1290 to 1670)	508.8 (448.9 to 600.9)	230 (208.3 to 267.2)	171.1 (152.9 to 198.2)
Low SDI	Gastritis and duodenitis	857 (637 to 1110)	1130 (826 to 1500)	1440 (1040 to 1950)	164.3 (123.4 to 210.4)	139 (103.3 to 180.5)	136.8 (100.5 to 182.3)
Low SDI	GERD	357 (192 to 604)	551 (295 to 932)	741 (397 to 1250)	73.9 (39.9 to 125.3)	73.6 (39.6 to 123.5)	74.6 (40 to 125.8)
Low SDI	Appendicitis	668 (471 to 968)	760 (658 to 933)	708 (620 to 803)	103.3 (79.7 to 140.2)	81.3 (71.4 to 96.4)	62.7 (55.4 to 71.7)
Low SDI	Paralytic ileus and intestinal obstruction	1820 (1510 to 2220)	2280 (1710 to 2530)	2510 (1750 to 2900)	279.9 (209.4 to 331.3)	260.3 (182.2 to 290.9)	238.3 (162.1 to 276.7)
Low SDI	Inguinal, femoral, and abdominal hernia	338 (270 to 416)	437 (349 to 537)	510 (405 to 622)	70.5 (57.5 to 84.4)	59.2 (47.8 to 71.6)	53.3 (43.2 to 65.2)
Low SDI	Inflammatory bowel disease	94.5 (44.4 to 206)	119 (77 to 196)	148 (111 to 199)	16.7 (10.1 to 29.6)	15.3 (11.3 to 21.9)	15.4 (12.2 to 19.6)
Low SDI	Vascular intestinal disorders	93.3 (55.6 to 124)	129 (94.3 to 152)	170 (132 to 202)	23.4 (16.3 to 29.4)	22.9 (17.5 to 26.8)	22.8 (17.7 to 27)
Low SDI	Gallbladder and biliary diseases	185 (144 to 228)	220 (185 to 253)	279 (233 to 327)	41.9 (33.3 to 50.3)	33.8 (28.8 to 39.2)	33 (27.7 to 38.9)
Low SDI	Pancreatitis	160 (113 to 248)	280 (230 to 346)	382 (322 to 468)	35.8 (26.4 to 54.5)	41.5 (34.2 to 50.5)	43 (36.4 to 52.7)
Low SDI	Other digestive diseases	266 (187 to 410)	311 (252 to 417)	405 (321 to 506)	46.9 (35.4 to 69.8)	39 (31.9 to 56.5)	40.1 (31 to 51)
Low SDI	Neurological disorders	8660 (6630 to 10900)	11900 (9200 to 15200)	15000 (11400 to 19200)	1668.7 (1342.6 to 2046.6)	1538.8 (1231.8 to 1902.7)	1519.2 (1198.1 to 1880.5)
Low SDI	Alzheimer's disease and other dementias	796 (709 to 927)	1200 (1100 to 1340)	1740 (1610 to 1900)	409.4 (368.6 to 464.3)	373.6 (340.9 to 412.5)	376.1 (346.5 to 406.7)
Low SDI	Parkinson's disease	183 (150 to 229)	283 (234 to 348)	409 (352 to 478)	74.1 (60 to 91.9)	70.8 (58 to 86.8)	72 (61.8 to 84.1)
Low SDI	Epilepsy	3010 (2080 to 3900)	3400 (2660 to 4280)	3720 (2870 to 4800)	402.5 (289 to 517.3)	315 (249.9 to 396.4)	285.4 (222.7 to 365)
Low SDI	Multiple sclerosis	33.6 (23 to 65.4)	51.3 (38.5 to 82.8)	71.1 (56.4 to 106)	7.4 (5.1 to 14.3)	7.3 (5.4 to 11.7)	7.7 (6.1 to 11.4)
Low SDI	Motor neuron disease	8.89 (6.71 to 29.5)	12.5 (9.46 to 29.9)	18.4 (14.6 to 34.1)	1.7 (1.2 to 5.3)	1.7 (1.2 to 4.1)	2 (1.6 to 3.8)

SR Appendix Table 2. All-age DALYs counts and age standardized DALYs rates per 100,000 individuals for all causes and level 1 cause groups by location for 1990, 2007, and 2017, both sexes combined

		All-age DALYs			Age-standardized rate of DALYs per 100,000		
Low SDI	Headache disorders	4150 (2690 to 5870)	6410 (4180 to 9060)	8330 (5400 to 11900)	718 (467.1 to 1019.4)	720.9 (471.5 to 1024.3)	722.4 (469.9 to 1024.6)
Low SDI	Migraine	3610 (2270 to 5280)	5580 (3520 to 8170)	7260 (4590 to 10600)	624.1 (393.7 to 912.6)	627.5 (396.6 to 915.9)	628.7 (400.3 to 915.4)
Low SDI	Tension-type headache	540 (305 to 860)	824 (462 to 1320)	1070 (600 to 1700)	94 (53.4 to 148.5)	93.4 (52.9 to 147.6)	93.7 (53.2 to 147.9)
Low SDI	Other neurological disorders	472 (257 to 748)	585 (410 to 825)	754 (553 to 1020)	55.4 (34.9 to 85.1)	49.5 (37.1 to 69.3)	53.5 (40.3 to 71.3)
Low SDI	Mental disorders	9620 (7080 to 12400)	14700 (10900 to 18900)	18600 (13700 to 24000)	1627.5 (1215.9 to 2088.7)	1633 (1219.3 to 2093)	1595.6 (1189.3 to 2049.1)
Low SDI	Schizophrenia	689 (512 to 858)	1050 (782 to 1300)	1360 (1020 to 1700)	134.3 (100 to 166.1)	132.6 (99.3 to 163.6)	131.1 (98.2 to 162.5)
Low SDI	Depressive disorders	3230 (2290 to 4410)	4980 (3520 to 6820)	6250 (4430 to 8510)	620.6 (441.7 to 838.9)	627.8 (444.2 to 849.5)	597.3 (423.1 to 810.8)
Low SDI	Major depressive disorder	2660 (1860 to 3630)	4100 (2850 to 5620)	5070 (3550 to 6940)	508 (359.4 to 683.3)	514 (359.9 to 694.7)	482.4 (339 to 651.8)
Low SDI	Dysthymia	571 (383 to 834)	888 (596 to 1300)	1180 (790 to 1730)	112.5 (75.5 to 163.8)	113.8 (76.5 to 164.8)	114.9 (76.8 to 167.2)
Low SDI	Bipolar disorder	674 (423 to 984)	1050 (660 to 1550)	1380 (863 to 2030)	116.4 (73.1 to 169.9)	117.6 (74.1 to 171.8)	118.3 (73.7 to 173.1)
Low SDI	Anxiety disorders	1980 (1400 to 2640)	3050 (2150 to 4060)	3940 (2790 to 5280)	326 (231.7 to 433.4)	328.4 (233.2 to 436.3)	329.8 (235.6 to 438.4)
Low SDI	Eating disorders	139 (87.5 to 203)	227 (144 to 331)	337 (212 to 491)	20.9 (13.2 to 30.5)	22 (14 to 32)	25.5 (16.2 to 37.3)
Low SDI	Anorexia nervosa	32.8 (19.5 to 49.4)	52.4 (31.5 to 78.2)	75.6 (45.9 to 114)	4.5 (2.7 to 6.7)	4.6 (2.8 to 6.9)	5.3 (3.3 to 7.9)
Low SDI	Bulimia nervosa	106 (66.4 to 159)	174 (108 to 262)	261 (160 to 391)	16.4 (10.2 to 24.5)	17.3 (10.8 to 25.7)	20.2 (12.4 to 30)
Low SDI	Autism spectrum disorders	422 (287 to 580)	632 (429 to 870)	783 (534 to 1080)	56.9 (38.8 to 77.9)	57.2 (39 to 78.7)	57.6 (39.5 to 79.1)
Low SDI	Attention-deficit/hyperactivity disorder	78.9 (48 to 127)	123 (74.4 to 198)	159 (95.8 to 254)	9.9 (6 to 15.8)	10.2 (6.2 to 16.5)	10.6 (6.4 to 17)
Low SDI	Conduct disorder	820 (494 to 1310)	1240 (750 to 1980)	1550 (933 to 2470)	88.4 (53.2 to 141.6)	88.7 (53.7 to 141.1)	90.4 (54.5 to 144.1)
Low SDI	Idiopathic developmental intellectual disability	897 (462 to 1480)	1270 (663 to 2100)	1370 (715 to 2260)	118.4 (61.4 to 195.9)	112.2 (58.2 to 185.2)	98.7 (51.1 to 162.9)
Low SDI	Other mental disorders	695 (460 to 959)	1080 (718 to 1490)	1420 (941 to 1970)	135.7 (90 to 186.3)	136.1 (90.4 to 187)	136.3 (90.3 to 187.3)
Low SDI	Substance use disorders	2030 (1560 to 2540)	3270 (2500 to 4090)	4370 (3390 to 5430)	373 (289.7 to 460.9)	384.9 (299.3 to 477)	394.9 (309.8 to 485.5)
Low SDI	Alcohol use disorders	989 (731 to 1300)	1510 (1140 to 1970)	1910 (1460 to 2460)	188.5 (143 to 245.3)	185 (142.4 to 237.5)	179.3 (140.3 to 226.9)
Low SDI	Drug use disorders	1040 (780 to 1340)	1760 (1320 to 2250)	2460 (1850 to 3170)	184.5 (139.5 to 234.1)	199.9 (152.1 to 253.4)	215.6 (162.9 to 274.1)
Low SDI	Opioid use disorders	879 (628 to 1170)	1500 (1080 to 1980)	2120 (1530 to 2810)	153.4 (109.4 to 201.1)	167.9 (121.8 to 219.4)	183 (133.7 to 239.6)
Low SDI	Cocaine use disorders	10.4 (5.58 to 22.5)	18.7 (11.2 to 34.4)	26.8 (17.1 to 42.8)	2 (1.1 to 4.5)	2.3 (1.4 to 4.4)	2.6 (1.6 to 4.2)
Low SDI	Amphetamine use disorders	23.2 (10.7 to 41.3)	35.4 (19.4 to 53.1)	45.2 (27.7 to 64.3)	4 (1.7 to 7.3)	3.9 (2 to 6)	3.8 (2.3 to 5.5)
Low SDI	Cannabis use disorders	29.4 (18.5 to 44.1)	46.7 (29.4 to 69)	60.6 (38.2 to 89.7)	4.5 (2.8 to 6.6)	4.6 (2.9 to 6.7)	4.6 (2.9 to 6.8)
Low SDI	Other drug use disorders	99 (68.5 to 157)	159 (117 to 244)	211 (163 to 307)	20.6 (14.6 to 31.6)	21.2 (15.9 to 31.9)	21.6 (16.9 to 31.1)
Low SDI	Diabetes and kidney diseases	7170 (6330 to 8080)	10300 (9170 to 11600)	13200 (11600 to 15000)	1663.9 (1481.7 to 1872.7)	1635.1 (1458.4 to 1844.9)	1611.1 (1426.3 to 1821.8)
Low SDI	Diabetes mellitus	3530 (3020 to 4130)	6010 (5110 to 7100)	8200 (6880 to 9760)	923.5 (800.6 to 1073.1)	1032.3 (887.2 to 1209.6)	1050.3 (893.3 to 1230.9)
Low SDI	Diabetes mellitus type 1	1140 (950 to 1370)	1610 (1400 to 1900)	1970 (1720 to 2240)	271.2 (222.1 to 335.7)	251.9 (213.4 to 303.9)	232.6 (200.2 to 270.4)
Low SDI	Diabetes mellitus type 2	2390 (1870 to 2980)	4400 (3470 to 5470)	6230 (4920 to 7740)	652.2 (523.6 to 805.3)	780.4 (624.6 to 952.3)	817.7 (661 to 998.4)
Low SDI	Chronic kidney disease	3570 (3140 to 3970)	4200 (3930 to 4630)	4930 (4590 to 5430)	729.2 (653.4 to 828.4)	597 (556.2 to 674.4)	556.7 (518 to 619)
Low SDI	Chronic kidney disease due to diabetes mellitus type 1	209 (161 to 265)	275 (220 to 346)	346 (276 to 432)	45.9 (35.5 to 59.8)	39.8 (31.6 to 50.8)	37.9 (30 to 48.2)
Low SDI	Chronic kidney disease due to diabetes mellitus type 2	433 (359 to 532)	589 (500 to 718)	786 (670 to 931)	127.5 (106.1 to 156.3)	114 (97.6 to 140.3)	111.4 (95.4 to 132)
Low SDI	Chronic kidney disease due to hypertension	520 (443 to 613)	666 (587 to 771)	867 (760 to 995)	150.9 (129.7 to 178)	124.4 (109.4 to 144.6)	119.4 (104.9 to 136.6)
Low SDI	Chronic kidney disease due to glomerulonephritis	935 (774 to 1090)	1070 (941 to 1200)	1200 (1060 to 1350)	158.8 (135 to 183.6)	125.2 (111.2 to 142.9)	113.9 (100.8 to 128.9)
Low SDI	Chronic kidney disease due to other and unspecified causes	1470 (1250 to 1690)	1610 (1440 to 1780)	1740 (1550 to 1950)	246.1 (211 to 282.8)	193.6 (171.3 to 219)	174.2 (152.7 to 197.3)
Low SDI	Acute glomerulonephritis	75.3 (37.7 to 106)	53.4 (34 to 66.8)	44.6 (30.1 to 58.2)	11.3 (7 to 15)	5.7 (4.2 to 7.3)	4 (3 to 5.3)
Low SDI	Skin and subcutaneous diseases	4240 (2850 to 6240)	6060 (4070 to 8850)	7450 (5000 to 10900)	562.7 (383.3 to 829.7)	552.4 (375.1 to 810.1)	559.1 (381.6 to 811.8)
Low SDI	Dermatitis	1150 (641 to 1860)	1700 (941 to 2730)	2090 (1170 to 3370)	139.7 (81 to 222)	140.4 (80.7 to 222.6)	143.1 (82.2 to 227.2)

SR Appendix Table 2. All-age DALYs counts and age standardized DALYs rates per 100,000 individuals for all causes and level 1 cause groups by location for 1990, 2007, and 2017, both sexes combined

		All-age DALYs			Age-standardized rate of DALYs per 100,000		
Low SDI	Atopic dermatitis	1030 (547 to 1710)	1500 (804 to 2500)	1840 (976 to 3060)	118 (63.5 to 194.6)	118.6 (64 to 197.3)	121.2 (64.7 to 201.3)
Low SDI	Contact dermatitis	125 (80.5 to 188)	193 (124 to 288)	250 (162 to 374)	21.5 (14.1 to 31.7)	21.6 (14.1 to 32)	21.7 (14.2 to 32.2)
Low SDI	Seborrhoeic dermatitis	1.33 (0.762 to 2.13)	2.1 (1.18 to 3.35)	2.67 (1.51 to 4.27)	0.2 (0.1 to 0.3)	0.2 (0.1 to 0.3)	0.2 (0.1 to 0.3)
Low SDI	Psoriasis	249 (178 to 328)	381 (269 to 504)	508 (358 to 671)	44.3 (31.7 to 58.2)	44.6 (31.9 to 59)	46.1 (32.5 to 60.6)
Low SDI	Bacterial skin diseases	385 (189 to 530)	497 (279 to 619)	601 (365 to 785)	56.9 (30.9 to 71.5)	54.2 (30.5 to 67)	56.5 (33.9 to 73)
Low SDI	Cellulitis	58.6 (19.5 to 106)	67.5 (30.7 to 97.4)	84.9 (42.4 to 117)	7.9 (3.1 to 12.6)	7 (3.3 to 9.6)	7.8 (3.8 to 10.8)
Low SDI	Pyoderma	327 (165 to 436)	429 (239 to 543)	516 (314 to 694)	49 (26.5 to 62.6)	47.2 (26.5 to 58.7)	48.7 (28.9 to 63.7)
Low SDI	Scabies	407 (224 to 665)	588 (324 to 965)	723 (399 to 1190)	53.1 (29.5 to 86.5)	51.6 (28.9 to 83.8)	51.3 (28.4 to 83.5)
Low SDI	Fungal skin diseases	664 (261 to 1390)	818 (320 to 1720)	901 (353 to 1880)	88.9 (35.6 to 186.7)	78.1 (31 to 162.5)	72.1 (28.5 to 149.1)
Low SDI	Viral skin diseases	447 (286 to 658)	651 (418 to 964)	772 (498 to 1140)	47.2 (30.3 to 69.7)	47.5 (30.6 to 70.3)	47.8 (30.9 to 70.8)
Low SDI	Acne vulgaris	107 (62.3 to 175)	205 (119 to 338)	321 (185 to 521)	14 (8.2 to 22.4)	17.2 (10 to 28.1)	21 (12.2 to 33.8)
Low SDI	Alopecia areata	33.6 (21.6 to 49.7)	51.8 (33.3 to 78)	66.8 (42.4 to 99.1)	5.6 (3.6 to 8.2)	5.6 (3.6 to 8.4)	5.6 (3.6 to 8.3)
Low SDI	Pruritus	54 (25.2 to 103)	81.6 (38.3 to 156)	106 (49.7 to 203)	9.3 (4.4 to 17.6)	9.3 (4.4 to 17.4)	9.5 (4.5 to 17.9)
Low SDI	Urticaria	565 (372 to 805)	826 (539 to 1170)	994 (654 to 1420)	69.3 (45.9 to 97.3)	69.5 (45.9 to 97.8)	69.5 (46.1 to 97.8)
Low SDI	Decubitus ulcer	23.2 (15.4 to 34.8)	33.6 (23.2 to 47.1)	44.8 (32.1 to 62.6)	6.3 (3.9 to 8.9)	5.9 (3.9 to 8)	6 (4.1 to 8.1)
Low SDI	Other skin and subcutaneous diseases	153 (77.4 to 276)	233 (117 to 420)	316 (159 to 571)	28.3 (14.3 to 50.6)	28.7 (14.4 to 51.6)	30.4 (15.3 to 54.9)
Low SDI	Sense organ diseases	4670 (3170 to 6710)	6980 (4720 to 10000)	9270 (6250 to 13300)	1173.2 (805.4 to 1647.6)	1134.7 (781.6 to 1597)	1127.2 (774.1 to 1586.8)
Low SDI	Blindness and vision impairment	2560 (1730 to 3780)	3750 (2510 to 5540)	4970 (3380 to 7310)	662.2 (454.3 to 942.4)	626.4 (427.7 to 894.5)	620.5 (423.2 to 889.6)
Low SDI	Glaucoma	41.2 (28 to 57.6)	65.2 (44.3 to 91.4)	89.7 (60.6 to 126)	13.9 (9.4 to 19.2)	14 (9.5 to 19.2)	13.9 (9.3 to 19.2)
Low SDI	Cataract	683 (482 to 924)	1020 (720 to 1380)	1480 (1030 to 1990)	241.4 (171.4 to 324.4)	226.7 (159.3 to 303.4)	232.9 (163.3 to 312.5)
Low SDI	Age-related macular degeneration	24.7 (16.8 to 34)	39.1 (26.5 to 53.9)	58.1 (39.5 to 80.3)	8.4 (5.7 to 11.5)	8.6 (5.9 to 11.8)	9.1 (6.2 to 12.6)
Low SDI	Refraction disorders	790 (529 to 1140)	1080 (723 to 1550)	1360 (909 to 1940)	158.9 (107.9 to 226.8)	142 (97.3 to 200.4)	137.1 (93.1 to 193.8)
Low SDI	Near vision loss	835 (399 to 1530)	1260 (600 to 2300)	1610 (765 to 2930)	194.7 (93.2 to 356.2)	191 (91.1 to 350.1)	182.6 (87.3 to 333.2)
Low SDI	Other vision loss	187 (131 to 253)	278 (194 to 378)	375 (262 to 505)	45 (31.8 to 61)	44.1 (31.2 to 59.6)	44.9 (31.6 to 60.5)
Low SDI	Age-related and other hearing loss	1980 (1360 to 2810)	3030 (2090 to 4300)	4030 (2760 to 5700)	482.4 (334.7 to 674)	479.3 (331.2 to 668.7)	477.2 (330 to 667.2)
Low SDI	Other sense organ diseases	132 (82.5 to 192)	204 (127 to 300)	271 (169 to 398)	28.5 (17.8 to 41.1)	29 (18.1 to 42.2)	29.5 (18.3 to 43.1)
Low SDI	Musculoskeletal disorders	7810 (5810 to 10300)	12000 (8850 to 15700)	15600 (11600 to 20600)	1716 (1280.3 to 2244.8)	1709.2 (1274.2 to 2238.2)	1688.3 (1252.6 to 2212.3)
Low SDI	Rheumatoid arthritis	254 (199 to 319)	346 (273 to 423)	478 (376 to 583)	68.8 (54.7 to 85)	61.3 (48.6 to 74.2)	62.7 (49.7 to 75.3)
Low SDI	Osteoarthritis	333 (166 to 662)	536 (268 to 1070)	770 (384 to 1540)	96.1 (48.1 to 189.9)	100.5 (50.4 to 199.4)	105 (52.7 to 209.4)
Low SDI	Low back pain	3760 (2700 to 5090)	5830 (4180 to 7890)	7600 (5420 to 10300)	796.2 (573.1 to 1059.8)	798.4 (572 to 1071.7)	785.7 (562.9 to 1055.4)
Low SDI	Neck pain	1160 (806 to 1620)	1750 (1210 to 2450)	2420 (1690 to 3410)	262.8 (183.5 to 367.6)	256.8 (179.8 to 360)	266.3 (185.6 to 373.1)
Low SDI	Gout	48.4 (32.3 to 66.1)	75.6 (50.2 to 104)	105 (70.5 to 144)	13.4 (9 to 18.2)	13.5 (9.1 to 18.5)	13.7 (9.4 to 18.9)
Low SDI	Other musculoskeletal disorders	2250 (1590 to 3050)	3440 (2410 to 4700)	4260 (2980 to 5780)	478.7 (339 to 650.6)	478.7 (337.7 to 650.7)	454.9 (321.6 to 616.6)
Low SDI	Other non-communicable diseases	25600 (21300 to 33100)	30400 (26600 to 34900)	32400 (28600 to 36600)	2654.3 (2229 to 3290)	2352.5 (2037.6 to 2747.4)	2235.1 (1942.8 to 2574.2)
Low SDI	Congenital anomalies	16300 (13800 to 23400)	19500 (17600 to 23300)	20500 (18600 to 22400)	1367.4 (1159.5 to 1938.6)	1253.5 (1130.3 to 1484.8)	1216.7 (1105.8 to 1338.1)
Low SDI	Neural tube defects	2780 (1520 to 4790)	3170 (2060 to 4930)	3070 (2190 to 4470)	223.4 (123.8 to 387)	196.2 (128.1 to 303.4)	177.9 (127.9 to 257.2)
Low SDI	Congenital heart anomalies	5880 (2250 to 9420)	7040 (3920 to 9860)	7280 (4920 to 9570)	473.8 (183.9 to 757.3)	435.3 (245.4 to 607.4)	420.6 (286.5 to 551.2)
Low SDI	Orofacial clefts	500 (130 to 1320)	360 (128 to 873)	284 (120 to 688)	40.6 (12.2 to 102.4)	23.3 (9.4 to 54.2)	17.3 (7.9 to 40.4)
Low SDI	Down's syndrome	502 (122 to 1100)	588 (233 to 1140)	702 (358 to 1270)	42.7 (12.1 to 91.6)	38.2 (16.3 to 72.3)	42.1 (22.4 to 74.8)
Low SDI	Turner syndrome	1.24 (0.6 to 2.08)	1.9 (0.916 to 3.15)	2.4 (1.18 to 4)	0.1 (0.1 to 0.2)	0.2 (0.1 to 0.3)	0.2 (0.1 to 0.3)

SR Appendix Table 2. All-age DALYs counts and age standardized DALYs rates per 100,000 individuals for all causes and level 1 cause groups by location for 1990, 2007, and 2017, both sexes combined							
		All-age DALYs			Age-standardized rate of DALYs per 100,000		
Low SDI	Klinefelter syndrome	0.407 (0.188 to 0.743)	0.621 (0.286 to 1.13)	0.775 (0.361 to 1.44)	0.1 (0 to 0.1)	0.1 (0 to 0.1)	0.1 (0 to 0.1)
Low SDI	Other chromosomal abnormalities	340 (104 to 849)	440 (157 to 899)	542 (237 to 963)	30.7 (11.4 to 70.8)	29.6 (11.9 to 57.4)	33 (15.2 to 56.8)
Low SDI	Congenital musculoskeletal and limb anomalies	661 (465 to 932)	951 (695 to 1230)	1130 (834 to 1460)	79.2 (56.2 to 105.5)	80.3 (57.5 to 104.3)	81.1 (58.6 to 105.2)
Low SDI	Urogenital congenital anomalies	255 (93 to 414)	382 (187 to 547)	431 (245 to 569)	20.6 (7.8 to 32.8)	23.9 (12.1 to 33.7)	25.3 (14.6 to 33)
Low SDI	Digestive congenital anomalies	1620 (787 to 3970)	1760 (1040 to 3190)	1780 (1150 to 2820)	128.1 (63.1 to 311.1)	108.3 (64.5 to 194.4)	103.2 (66.7 to 162.4)
Low SDI	Other congenital anomalies	3740 (1440 to 6300)	4800 (2540 to 6960)	5220 (3290 to 7110)	328.2 (143.4 to 529.9)	318.2 (178.9 to 452.2)	316.2 (204.9 to 424.3)
Low SDI	Urinary diseases and male infertility	1580 (1370 to 1800)	1680 (1540 to 1840)	1970 (1800 to 2160)	278.6 (247.7 to 316)	224.1 (202.7 to 248.9)	218.4 (197.5 to 243.3)
Low SDI	Urinary tract infections	566 (363 to 808)	705 (540 to 932)	943 (792 to 1190)	100.7 (73.4 to 138.8)	91.7 (75.1 to 118.5)	102.5 (89.1 to 127.2)
Low SDI	Urolithiasis	31.3 (19.9 to 42.1)	40.1 (28 to 54.4)	52.6 (38.1 to 69.5)	7.2 (4.5 to 9.9)	6.1 (4.2 to 8.3)	6 (4.3 to 8.1)
Low SDI	Benign prostatic hyperplasia	153 (96.4 to 220)	221 (141 to 318)	302 (191 to 437)	47.5 (30.3 to 68.3)	44.9 (28.8 to 64.7)	44.2 (28.1 to 63.7)
Low SDI	Male infertility	14.2 (6 to 29.3)	21.1 (8.8 to 43.5)	31.1 (13 to 64.2)	2.3 (1 to 4.8)	2.2 (0.9 to 4.6)	2.5 (1 to 5.1)
Low SDI	Other urinary diseases	814 (488 to 1060)	690 (434 to 880)	638 (424 to 789)	120.9 (73.7 to 152.5)	79.3 (50.4 to 97.6)	63.1 (42.6 to 75.8)
Low SDI	Gynecological diseases	823 (585 to 1140)	1240 (869 to 1710)	1610 (1120 to 2230)	146.7 (104.1 to 202.9)	141.5 (98.7 to 196.1)	140.1 (98.3 to 195.7)
Low SDI	Uterine fibroids	101 (65.3 to 153)	148 (94.3 to 227)	189 (120 to 294)	21.5 (14 to 32.7)	20.4 (13.1 to 31.5)	19.9 (12.9 to 30.6)
Low SDI	Polycystic ovarian syndrome	29.2 (12.9 to 56.8)	46.4 (20.5 to 89.4)	61.7 (27.4 to 118)	4.8 (2.1 to 9.2)	4.9 (2.2 to 9.4)	5 (2.2 to 9.6)
Low SDI	Female infertility	27.3 (10.9 to 57.3)	37.5 (15.2 to 76.5)	59.6 (22.6 to 125)	4.5 (1.8 to 9.4)	3.9 (1.6 to 8.1)	4.8 (1.8 to 10.1)
Low SDI	Endometriosis	218 (146 to 313)	340 (225 to 489)	442 (290 to 640)	36.9 (24.4 to 53.1)	36.9 (24.6 to 53.5)	37 (24.3 to 53.6)
Low SDI	Genital prolapse	41.7 (21.5 to 74.7)	54.5 (27.1 to 99.1)	63.9 (31.6 to 117)	10 (5.2 to 17.5)	8.5 (4.3 to 15.5)	7.5 (3.8 to 13.7)
Low SDI	Premenstrual syndrome	313 (202 to 471)	487 (314 to 737)	622 (397 to 935)	52.1 (33.7 to 78)	52.1 (33.6 to 78.5)	51 (32.8 to 76.7)
Low SDI	Other gynecological diseases	92.6 (69.1 to 127)	127 (96.9 to 168)	168 (131 to 220)	16.9 (12.8 to 22.7)	14.8 (11.4 to 19.4)	14.9 (11.7 to 19.4)
Low SDI	Hemoglobinopathies and hemolytic anaemias	2960 (1850 to 3980)	3360 (2150 to 4830)	3500 (2120 to 5280)	372.4 (241.7 to 521.8)	298.5 (185.4 to 439.3)	258 (155.1 to 394)
Low SDI	Thalassemias	160 (78.7 to 287)	123 (76.1 to 173)	88.6 (57.7 to 127)	13.7 (6.7 to 24.8)	7.8 (4.9 to 11)	5.1 (3.3 to 7.3)
Low SDI	Thalassaemias trait	257 (175 to 365)	299 (200 to 425)	302 (202 to 430)	33.4 (22.8 to 47.5)	27.2 (18.2 to 38.7)	22.6 (15.3 to 32.3)
Low SDI	Sickle cell disorders	1520 (665 to 2290)	1630 (729 to 2680)	1730 (773 to 3020)	155.5 (70.1 to 245)	120.4 (53.1 to 204.2)	109.8 (48.2 to 194.2)
Low SDI	Sickle cell trait	332 (227 to 470)	448 (301 to 630)	463 (310 to 661)	43.5 (29.6 to 61.9)	40.3 (27 to 57.1)	34.1 (23 to 48.8)
Low SDI	G6PD deficiency	128 (60.4 to 210)	173 (82.4 to 298)	198 (86.9 to 359)	23.1 (10.5 to 41.2)	22.3 (9.7 to 41.5)	20.3 (8.1 to 38.5)
Low SDI	G6PD trait	0.0475 (0.0321 to 0.0667)	0.0695 (0.0471 to 0.0972)	0.0848 (0.0578 to 0.119)	0 (0 to 0)	0 (0 to 0)	0 (0 to 0)
Low SDI	Other hemoglobinopathies and hemolytic anaemias	569 (384 to 795)	688 (470 to 968)	712 (481 to 1010)	103.1 (68.3 to 149.5)	80.6 (53.7 to 116.7)	66 (44 to 95.4)
Low SDI	Endocrine, metabolic, blood, and immune disorders	1100 (789 to 1420)	1290 (1000 to 1610)	1370 (1080 to 1700)	149.8 (114.3 to 187.3)	124.5 (98.9 to 155.1)	110.9 (89 to 137.3)
Low SDI	Oral disorders	909 (517 to 1460)	1460 (834 to 2350)	1930 (1090 to 3130)	190.8 (109 to 307.3)	201.6 (116.1 to 321.9)	201.5 (115 to 324.1)
Low SDI	Caries of deciduous teeth	21.3 (9.01 to 41.8)	29.1 (12.4 to 57.2)	31.8 (13.4 to 63.3)	1.9 (0.8 to 3.8)	1.9 (0.8 to 3.7)	1.8 (0.8 to 3.6)
Low SDI	Caries of permanent teeth	125 (53.3 to 236)	191 (81.5 to 361)	234 (99.3 to 439)	20.5 (8.9 to 38)	20.3 (8.7 to 37.8)	19.3 (8.4 to 36.1)
Low SDI	Periodontal disease	297 (116 to 606)	483 (188 to 989)	679 (265 to 1390)	68.7 (27.1 to 141)	72.2 (28.4 to 148.2)	75.6 (29.8 to 154.7)
Low SDI	Edentulism and severe tooth loss	171 (113 to 244)	307 (203 to 435)	406 (269 to 578)	49.5 (32.9 to 69.9)	56.8 (37.6 to 79.5)	54.2 (35.9 to 76.7)
Low SDI	Other oral disorders	294 (185 to 433)	452 (283 to 665)	584 (366 to 862)	50.2 (31.5 to 73.5)	50.4 (31.7 to 73.8)	50.6 (31.7 to 74.2)
Low SDI	Sudden infant death syndrome	1930 (213 to 5250)	1810 (310 to 4140)	1570 (357 to 3360)	148.8 (16.4 to 405.8)	108.7 (18.6 to 248)	89.4 (20.3 to 191.8)
Low SDI	Injuries	45800 (38700 to 51000)	46300 (43000 to 49200)	47900 (44400 to 51300)	6193.4 (5399.5 to 6780.9)	4582 (4246.7 to 4865.1)	3986.5 (3693.2 to 4276.7)
Low SDI	Transport injuries	9990 (8130 to 11300)	12800 (12000 to 13700)	13400 (12600 to 14400)	1441.7 (1214.1 to 1601)	1305.4 (1221.3 to 1393.2)	1124.6 (1055.4 to 1200.7)
Low SDI	Road injuries	8850 (7070 to 10100)	11200 (10500 to 12000)	11700 (11000 to 12600)	1265.5 (1054.9 to 1415.9)	1135.4 (1065.1 to 1211.6)	976.4 (916.9 to 1041.4)
Low SDI	Pedestrian road injuries	4160 (3300 to 4960)	4640 (4150 to 5220)	4400 (4010 to 4930)	566.6 (464.1 to 658)	458.6 (416.2 to 517.1)	367.4 (336.5 to 411.4)

SR Appendix Table 2. All-age DALYs counts and age standardized DALYs rates per 100,000 individuals for all causes and level 1 cause groups by location for 1990, 2007, and 2017, both sexes combined

		All-age DALYs			Age-standardized rate of DALYs per 100,000		
Low SDI	Cyclist road injuries	370 (283 to 474)	586 (495 to 697)	680 (564 to 850)	59.7 (47.1 to 74.3)	65 (54 to 77.8)	61.2 (50.5 to 75.2)
Low SDI	Motorcyclist road injuries	1190 (918 to 1570)	1880 (1670 to 2220)	2110 (1890 to 2390)	185.9 (146.6 to 242.3)	198.6 (176.8 to 233.1)	179 (160.1 to 200.2)
Low SDI	Motor vehicle road injuries	3000 (2170 to 3570)	3980 (3380 to 4400)	4370 (3890 to 4810)	434.6 (328.4 to 503.9)	396.2 (339.4 to 437.3)	353.1 (315 to 386.5)
Low SDI	Other road injuries	121 (58.8 to 153)	157 (116 to 185)	175 (145 to 209)	18.8 (10.9 to 22.8)	17 (13.2 to 20.5)	15.7 (13.1 to 18.9)
Low SDI	Other transport injuries	1150 (964 to 1350)	1590 (1410 to 1860)	1690 (1470 to 2000)	176.2 (149.6 to 205.9)	170.1 (150.1 to 199.8)	148.2 (127.7 to 175.7)
Low SDI	Unintentional injuries	24300 (19600 to 28300)	23400 (20800 to 25500)	22100 (19600 to 24300)	3070.9 (2574.8 to 3486.8)	2232.1 (1960.8 to 2442.7)	1847 (1638.9 to 2040.3)
Low SDI	Falls	2940 (2320 to 3680)	3850 (3380 to 4320)	4740 (4180 to 5350)	556.7 (465.6 to 656.3)	512.9 (450.2 to 576.3)	504.5 (447.7 to 567.1)
Low SDI	Drowning	9140 (7110 to 10800)	6780 (5940 to 7520)	4590 (4160 to 5080)	896.4 (705.8 to 1046.1)	493 (438.5 to 542.4)	304.7 (278 to 334.4)
Low SDI	Fire, heat, and hot substances	2590 (1700 to 3200)	2430 (1910 to 2780)	2440 (2060 to 2830)	342.6 (251.8 to 406.2)	229.7 (188.7 to 261.5)	195.7 (164.8 to 225.6)
Low SDI	Poisonings	716 (406 to 1030)	736 (565 to 860)	710 (563 to 826)	78.4 (49 to 108.8)	59.7 (47.3 to 67.8)	50.3 (39.9 to 58.3)
Low SDI	Poisoning by carbon monoxide	81.1 (51 to 111)	117 (85.9 to 139)	133 (98.1 to 166)	11.2 (7.3 to 14.4)	11.3 (8.3 to 13.5)	10.6 (7.8 to 13.1)
Low SDI	Poisoning by other means	635 (354 to 935)	620 (480 to 733)	576 (459 to 667)	67.2 (41.3 to 94.5)	48.4 (38.4 to 56.1)	39.7 (31.8 to 45.6)
Low SDI	Exposure to mechanical forces	1880 (1570 to 2180)	2010 (1730 to 2250)	2120 (1830 to 2390)	259.7 (221.7 to 298.7)	198.7 (172.2 to 225.5)	175.1 (151 to 199)
Low SDI	Unintentional firearm injuries	279 (211 to 385)	290 (237 to 365)	312 (262 to 386)	41 (31.4 to 52.5)	29.3 (24.1 to 35.7)	25.4 (21.4 to 30.8)
Low SDI	Other exposure to mechanical forces	1600 (1290 to 1870)	1720 (1440 to 1950)	1810 (1520 to 2080)	218.7 (183.3 to 254.8)	169.4 (142.5 to 193.5)	149.7 (125.8 to 172.8)
Low SDI	Adverse effects of medical treatment	1070 (578 to 1590)	1170 (794 to 1500)	1240 (937 to 1560)	147.7 (96.4 to 193.6)	119.4 (86.9 to 138.3)	108 (82.8 to 127)
Low SDI	Animal contact	2160 (913 to 3160)	2200 (1080 to 2650)	2080 (1120 to 2530)	296.5 (138.8 to 410.1)	211.8 (108 to 252.1)	169.5 (92.1 to 203.5)
Low SDI	Venomous animal contact	1930 (781 to 2720)	1950 (908 to 2370)	1820 (920 to 2260)	266.1 (121.1 to 356.9)	188 (92.1 to 224.8)	148.5 (77.6 to 179.7)
Low SDI	Non-venomous animal contact	232 (102 to 669)	249 (143 to 490)	262 (162 to 386)	30.5 (16.4 to 74.5)	23.8 (14.6 to 41.8)	21 (13.4 to 29.5)
Low SDI	Foreign body	1250 (891 to 1540)	1160 (968 to 1390)	1190 (1050 to 1360)	118.6 (90 to 145.6)	86.4 (74 to 102)	80.6 (71.6 to 90.6)
Low SDI	Pulmonary aspiration and foreign body in airway	1090 (775 to 1410)	996 (837 to 1230)	1000 (878 to 1160)	98.7 (74.1 to 125.8)	70.3 (60.3 to 84.8)	65.1 (58 to 74.6)
Low SDI	Foreign body in eyes	17.6 (7.73 to 31.6)	27.1 (11.9 to 48.6)	34.7 (15.4 to 61.9)	3 (1.4 to 5.1)	3 (1.5 to 5.2)	3 (1.5 to 5.2)
Low SDI	Foreign body in other body part	139 (80.2 to 212)	141 (99.4 to 188)	154 (118 to 195)	16.9 (11.3 to 23.1)	13.1 (10 to 16.4)	12.4 (9.7 to 15.1)
Low SDI	Environmental heat and cold exposure	616 (327 to 864)	727 (400 to 901)	777 (442 to 971)	100.5 (58 to 130.4)	83.4 (46.9 to 104.2)	72.9 (41 to 91.5)
Low SDI	Exposure to forces of nature	148 (135 to 168)	581 (524 to 656)	418 (334 to 522)	20.5 (18.7 to 23.1)	57.5 (51.3 to 65.4)	35.2 (28 to 44.2)
Low SDI	Other unintentional injuries	1780 (1450 to 2050)	1790 (1610 to 1950)	1740 (1540 to 1960)	253.2 (210.8 to 288.5)	179.8 (160 to 200.1)	150.5 (130.7 to 172.8)
Low SDI	Self-harm and interpersonal violence	11500 (10500 to 12500)	10100 (9360 to 10800)	12400 (11600 to 13200)	1680.8 (1529.9 to 1831)	1044.5 (971.2 to 1126.4)	1014.9 (946.5 to 1086.8)
Low SDI	Self-harm	3790 (3220 to 4340)	4560 (4260 to 5080)	5170 (4790 to 5650)	621.5 (530.5 to 712.3)	496.4 (464.1 to 553.6)	447 (416.2 to 484.8)
Low SDI	Self-harm by firearm	290 (177 to 481)	348 (238 to 554)	380 (270 to 571)	48.1 (30.2 to 78.9)	38.1 (26.7 to 59.4)	32.9 (23.4 to 49.6)
Low SDI	Self-harm by other specified means	3500 (2940 to 4050)	4210 (3880 to 4700)	4790 (4430 to 5230)	573.4 (484.1 to 661.9)	458.3 (423.8 to 510.6)	414 (384.8 to 451.5)
Low SDI	Interpersonal violence	2730 (2160 to 3340)	3530 (2790 to 3980)	3800 (2990 to 4310)	409.4 (326 to 492)	356.2 (278.5 to 398.5)	306.4 (239.4 to 346.7)
Low SDI	Assault by firearm	596 (329 to 790)	858 (462 to 1080)	956 (532 to 1240)	93.8 (51 to 120.8)	88.3 (47 to 109.6)	77.3 (42.7 to 98.7)
Low SDI	Assault by sharp object	438 (262 to 619)	625 (365 to 805)	704 (426 to 907)	71.1 (43.1 to 98.5)	66.1 (38.9 to 84.8)	58.4 (35.6 to 74.2)
Low SDI	Sexual violence	157 (105 to 229)	249 (169 to 360)	318 (215 to 460)	24.6 (16.6 to 36.1)	25.2 (17.2 to 36.5)	25.2 (17.2 to 36.9)
Low SDI	Assault by other means	1540 (1170 to 2070)	1800 (1460 to 2300)	1820 (1480 to 2300)	219.8 (172.2 to 295.2)	176.6 (144.7 to 224)	145.5 (119.1 to 184.6)
Low SDI	Conflict and terrorism	4960 (4740 to 5280)	1930 (1580 to 2420)	3280 (2970 to 3740)	647.9 (615.1 to 692.6)	188.4 (151.9 to 241.9)	250.3 (221.7 to 290)
Low SDI	Executions and police conflict	12.5 (9.54 to 16.3)	34.9 (31.9 to 39.1)	147 (142 to 154)	1.9 (1.5 to 2.5)	3.6 (3.2 to 4.1)	11.3 (10.8 to 12)
Low-middle SDI	All causes	662000 (630000 to 694000)	684000 (642000 to 732000)	641000 (594000 to 698000)	62139.2 (58780.2 to 65625.9)	51205.4 (48013.6 to 54757.3)	41768.9 (38715.5 to 45331.7)
Low-middle SDI	Communicable, maternal, neonatal, and nutritional disorders	401000 (386000 to 417000)	354000 (340000 to 370000)	258000 (241000 to 277000)	29950.4 (28686.6 to 31314.8)	22437.6 (21460.4 to 23512.1)	14739 (13794.5 to 15883.3)
Low-middle SDI	HIV/AIDS and sexually transmitted infections	8200 (5910 to 11200)	44100 (40000 to 48700)	24800 (21700 to 28600)	678 (520.6 to 882.5)	3086.5 (2826.1 to 3374.6)	1450.3 (1277.1 to 1657.4)

SR Appendix Table 2. All-age DALYs counts and age standardized DALYs rates per 100,000 individuals for all causes and level 1 cause groups by location for 1990, 2007, and 2017, both sexes combined

		All-age DALYs			Age-standardized rate of DALYs per 100,000		
Low-middle SDI	HIV/AIDS	4690 (3900 to 5700)	40100 (36800 to 43500)	21200 (19000 to 23600)	446.4 (368.2 to 551.9)	2860.3 (2631.6 to 3096.3)	1253.3 (1129.9 to 1390.1)
Low-middle SDI	HIV/AIDS - Drug-susceptible Tuberculosis	1620 (1300 to 1990)	8750 (6080 to 11700)	3700 (2590 to 4980)	152.3 (120.1 to 188.8)	617.1 (429.3 to 823.4)	216.8 (151.8 to 292.3)
Low-middle SDI	HIV/AIDS - Multidrug-resistant Tuberculosis without extensive drug resistance	11.6 (4.05 to 32.1)	1040 (560 to 1790)	471 (243 to 829)	1.1 (0.4 to 3)	73.6 (39.4 to 127.1)	27.6 (14.2 to 48.6)
Low-middle SDI	HIV/AIDS - Extensively drug-resistant Tuberculosis	0 (0 to 0)	23 (13.7 to 35.5)	16.3 (9.3 to 25.8)	0 (0 to 0)	1.7 (1 to 2.6)	1 (0.6 to 1.5)
Low-middle SDI	HIV/AIDS resulting in other diseases	3050 (2450 to 3960)	30300 (25800 to 34500)	17000 (14400 to 19800)	293 (233 to 388.3)	2167.9 (1857 to 2470.2)	1008 (858.8 to 1167.8)
Low-middle SDI	Sexually transmitted infections excluding HIV	3510 (1490 to 6400)	3970 (1670 to 7340)	3660 (1550 to 6710)	231.5 (104.6 to 410.3)	226.2 (98.3 to 412.1)	197 (85.1 to 359.4)
Low-middle SDI	Syphilis	3260 (1260 to 6180)	3640 (1360 to 6950)	3270 (1230 to 6310)	204.2 (79.7 to 386.5)	202.2 (76.2 to 385.1)	174.1 (65.8 to 335.6)
Low-middle SDI	Chlamydial infection	52.5 (35.9 to 79.6)	68.1 (45.4 to 109)	79.4 (51.6 to 130)	5.5 (3.8 to 8.2)	4.9 (3.3 to 7.6)	4.6 (3 to 7.5)
Low-middle SDI	Gonococcal infection	74.7 (49 to 104)	84.2 (61.2 to 122)	91.8 (66.6 to 138)	7.9 (5.2 to 10.5)	6 (4.5 to 8.5)	5.4 (4 to 7.9)
Low-middle SDI	Trichomoniasis	30.4 (12.3 to 65.8)	46.9 (18.9 to 101)	57.9 (23.4 to 125)	3.3 (1.3 to 7)	3.4 (1.4 to 7.3)	3.4 (1.4 to 7.4)
Low-middle SDI	Genital herpes	24.7 (8.25 to 57.9)	38.3 (12.7 to 90.4)	49.3 (16.3 to 117)	3 (1 to 7.1)	3.1 (1 to 7.4)	3.2 (1 to 7.5)
Low-middle SDI	Other sexually transmitted infections	72.3 (50.4 to 105)	92.5 (64 to 141)	107 (73 to 166)	7.6 (5.4 to 10.9)	6.6 (4.6 to 9.9)	6.2 (4.3 to 9.6)
Low-middle SDI	Respiratory infections and tuberculosis	98600 (90700 to 105000)	73200 (68800 to 78200)	55800 (51300 to 60600)	8073.8 (7549.7 to 8534.2)	4976.5 (4709.6 to 5272.7)	3406.6 (3156.1 to 3673.1)
Low-middle SDI	Tuberculosis	22900 (21400 to 24500)	19700 (18700 to 20900)	15500 (14500 to 16500)	2689.8 (2526.2 to 2896.9)	1633.1 (1550.6 to 1735.9)	1047.6 (979.3 to 1119.1)
Low-middle SDI	Latent tuberculosis infection	0 (0 to 0)	0 (0 to 0)	0 (0 to 0)	0 (0 to 0)	0 (0 to 0)	0 (0 to 0)
Low-middle SDI	Drug-susceptible tuberculosis	22700 (21300 to 24400)	17500 (16400 to 18800)	13600 (12100 to 15000)	2675.7 (2513 to 2879.9)	1455.8 (1359.3 to 1564.3)	918.9 (812.8 to 1015.9)
Low-middle SDI	Multidrug-resistant tuberculosis without extensive drug resistance	123 (50.4 to 271)	2040 (1450 to 2770)	1780 (938 to 2970)	14.1 (5.7 to 30.8)	169.9 (120.1 to 230.2)	120.7 (61.9 to 205.4)
Low-middle SDI	Extensively drug-resistant tuberculosis	0 (0 to 0)	87.1 (64.3 to 117)	116 (63 to 187)	0 (0 to 0)	7.4 (5.4 to 9.9)	7.9 (4.3 to 13)
Low-middle SDI	Lower respiratory infections	74300 (67300 to 80400)	51900 (47900 to 56100)	38400 (34700 to 42600)	5256.6 (4792.1 to 5656.7)	3233.1 (2992.6 to 3468.9)	2253.8 (2046.6 to 2480.1)
Low-middle SDI	Upper respiratory infections	1010 (635 to 1480)	1130 (702 to 1730)	1290 (785 to 2010)	87.5 (55.6 to 129.9)	75.5 (46.9 to 115.2)	73.5 (44.9 to 113.4)
Low-middle SDI	Otitis media	469 (297 to 731)	541 (335 to 852)	573 (350 to 910)	39.9 (25.3 to 61.9)	34.8 (21.8 to 54.9)	31.7 (19.5 to 50.3)
Low-middle SDI	Enteric infections	79200 (65800 to 91800)	54700 (47000 to 63800)	36800 (31800 to 43700)	6130.1 (5124.4 to 7225.2)	3588.3 (3066.4 to 4286.9)	2221.4 (1867.1 to 2698.8)
Low-middle SDI	Diarrhoeal diseases	72200 (60000 to 84500)	48400 (41500 to 56800)	31800 (26800 to 37900)	5595.6 (4625.9 to 6720.7)	3218.4 (2699.2 to 3886.7)	1957.3 (1594.8 to 2413.2)
Low-middle SDI	Typhoid and paratyphoid	5840 (3310 to 9420)	4160 (2370 to 6820)	3180 (1790 to 5230)	441.2 (251.5 to 714.9)	246.1 (140.1 to 401.3)	167.2 (93.9 to 273.7)
Low-middle SDI	Typhoid fever	5000 (2820 to 8190)	3560 (1990 to 5790)	2660 (1480 to 4370)	376.5 (211.1 to 620.1)	209.9 (117.7 to 340.7)	139.6 (77.6 to 228.5)
Low-middle SDI	Paratyphoid fever	843 (370 to 1710)	606 (271 to 1170)	525 (237 to 1030)	64.7 (28.7 to 129.8)	36.2 (16.1 to 70.4)	27.6 (12.5 to 54.1)
Low-middle SDI	Invasive non-typhoidal salmonella	1120 (618 to 1910)	2010 (1140 to 3330)	1780 (977 to 3170)	87.4 (48.1 to 144.2)	121.1 (69.7 to 198.1)	95.5 (52.6 to 168.2)
Low-middle SDI	Other intestinal infectious diseases	86.1 (54.2 to 155)	44.6 (26 to 89.6)	27.8 (15.7 to 56.2)	5.9 (3.8 to 10.4)	2.6 (1.6 to 5.1)	1.5 (0.9 to 3)
Low-middle SDI	Neglected tropical diseases and malaria	27400 (19500 to 40300)	34600 (26400 to 44800)	23300 (17600 to 30600)	2187.6 (1593.1 to 3205.9)	2140.8 (1660.5 to 2757.3)	1299.7 (993.7 to 1705.5)
Low-middle SDI	Malaria	18300 (12500 to 26000)	28700 (21100 to 38300)	18100 (12300 to 25300)	1342.3 (932.6 to 1881.4)	1719.4 (1268.5 to 2300)	987.2 (674.8 to 1377.5)
Low-middle SDI	Chagas disease	53.5 (48.1 to 58)	55.1 (50.6 to 60.3)	55.6 (50.1 to 63.6)	8.1 (7.3 to 8.9)	5.7 (5.2 to 6.2)	4.4 (4 to 5.1)
Low-middle SDI	Leishmaniasis	2700 (40.8 to 14200)	270 (34.6 to 1410)	140 (40.4 to 595)	224.8 (4.6 to 1139.2)	18.1 (2.8 to 88.1)	8.3 (2.6 to 32.8)
Low-middle SDI	Visceral leishmaniasis	2670 (11.3 to 14100)	226 (0.69 to 1370)	87.1 (0.231 to 527)	220.6 (0.9 to 1135.5)	14.4 (0 to 84.4)	4.8 (0 to 28.3)
Low-middle SDI	Cutaneous and mucocutaneous leishmaniasis	33.2 (19.2 to 52.2)	43.8 (28 to 63.8)	53.2 (35.6 to 76.2)	4.2 (2.4 to 6.5)	3.7 (2.3 to 5.4)	3.5 (2.3 to 5.1)
Low-middle SDI	African trypanosomiasis	183 (88.1 to 311)	34.5 (18.5 to 58.2)	1.46 (0.266 to 5.45)	17.6 (8.2 to 30.1)	2.3 (1.3 to 3.8)	0.1 (0 to 0.3)
Low-middle SDI	Schistosomiasis	514 (327 to 842)	641 (378 to 1110)	461 (279 to 776)	58.9 (37.6 to 95.3)	49 (29.3 to 83.9)	28.4 (17.4 to 47.2)
Low-middle SDI	Cysticercosis	327 (227 to 443)	363 (244 to 502)	380 (248 to 534)	40.4 (27.7 to 54.5)	30.8 (20.6 to 42.1)	25.9 (17 to 36.1)
Low-middle SDI	Cystic echinococcosis	53.5 (42.4 to 66.3)	33.3 (25.3 to 42.3)	26.7 (19.4 to 37.4)	5.1 (4.2 to 6.2)	2.5 (1.9 to 3.1)	1.7 (1.2 to 2.3)
Low-middle SDI	Lymphatic filariasis	610 (307 to 1050)	823 (433 to 1330)	727 (402 to 1140)	69.4 (35 to 119.5)	63 (33.3 to 101.9)	44.6 (24.7 to 70)
Low-middle SDI	Onchocerciasis	468 (263 to 766)	370 (198 to 593)	342 (153 to 637)	52 (29.7 to 84)	28 (15.4 to 45)	21 (9.7 to 38.3)

SR Appendix Table 2. All-age DALYs counts and age standardized DALYs rates per 100,000 individuals for all causes and level 1 cause groups by location for 1990, 2007, and 2017, both sexes combined

		All-age DALYs			Age-standardized rate of DALYs per 100,000		
Low-middle SDI	Trachoma	148 (100 to 202)	123 (84.7 to 169)	121 (80.9 to 171)	29.9 (20.3 to 40.3)	15.9 (11 to 21.6)	11.4 (7.7 to 16.1)
Low-middle SDI	Dengue	360 (141 to 611)	541 (283 to 774)	810 (397 to 1130)	30.6 (12.8 to 50.3)	37.5 (19.8 to 53.4)	49.8 (24.8 to 69.2)
Low-middle SDI	Yellow fever	476 (101 to 1390)	171 (33.4 to 516)	176 (36.9 to 507)	40 (8.4 to 116.3)	10.6 (2.1 to 32.1)	9.4 (2 to 27.2)
Low-middle SDI	Rabies	848 (653 to 1100)	459 (369 to 594)	258 (181 to 380)	74 (58 to 95.7)	30.7 (24.9 to 39.5)	14.7 (10.4 to 21.2)
Low-middle SDI	Intestinal nematode infections	1300 (868 to 1860)	915 (598 to 1370)	574 (360 to 869)	111.9 (73 to 164.8)	59.2 (38.4 to 88.9)	31.9 (19.9 to 48.8)
Low-middle SDI	Ascariasis	711 (494 to 975)	502 (345 to 734)	342 (224 to 512)	55.8 (38.3 to 77.8)	31.1 (21.3 to 45.7)	18.6 (12.1 to 27.9)
Low-middle SDI	Trichuriasis	114 (63.1 to 188)	74.1 (41 to 122)	44.1 (23.6 to 76.3)	10.3 (5.7 to 17)	4.8 (2.7 to 8)	2.5 (1.3 to 4.3)
Low-middle SDI	Hookworm disease	474 (289 to 736)	339 (202 to 528)	188 (111 to 298)	45.9 (27.9 to 71.3)	23.3 (13.9 to 36.2)	10.9 (6.4 to 17.3)
Low-middle SDI	Food-borne trematodiases	86.6 (34.4 to 170)	85 (37.4 to 156)	84.4 (37.4 to 156)	9 (3.6 to 17.5)	6.5 (2.9 to 11.9)	5.3 (2.3 to 9.7)
Low-middle SDI	Leprosy	7.75 (5.22 to 10.9)	10 (6.78 to 14)	9.43 (6.37 to 13.3)	1.2 (0.8 to 1.6)	1 (0.7 to 1.4)	0.7 (0.5 to 1)
Low-middle SDI	Ebola virus disease	0 (0 to 0)	0 (0 to 0)	0 (0 to 0)	0 (0 to 0)	0 (0 to 0)	0 (0 to 0)
Low-middle SDI	Zika virus disease	0 (0 to 0)	0.0000645 (0 to 0.000381)	0.407 (0.19 to 0.936)	0 (0 to 0)	0 (0 to 0)	0 (0 to 0.1)
Low-middle SDI	Guinea worm disease	8.49 (5.36 to 12.5)	0.0355 (0.0222 to 0.0524)	0 (0 to 0)	1 (0.6 to 1.5)	0 (0 to 0)	0 (0 to 0)
Low-middle SDI	Other neglected tropical diseases	904 (584 to 1860)	979 (628 to 1980)	997 (635 to 2010)	71.3 (47.4 to 136.8)	60.9 (39.7 to 119.7)	54.7 (35 to 108.7)
Low-middle SDI	Other infectious diseases	56100 (42100 to 77700)	31400 (25600 to 39600)	22100 (18200 to 26600)	3977.9 (3038.8 to 5417.5)	1906.7 (1571 to 2375.8)	1231.6 (1024.6 to 1464.7)
Low-middle SDI	Meningitis	13600 (11000 to 15400)	11200 (9280 to 12700)	8920 (7260 to 10600)	973.6 (800 to 1099.7)	670 (560.1 to 757.8)	487.9 (400.3 to 582.4)
Low-middle SDI	Pneumococcal meningitis	1740 (1350 to 2120)	1590 (1250 to 1910)	1340 (1040 to 1660)	128.8 (102.8 to 154.2)	96.6 (77.7 to 115.3)	73.8 (58.2 to 91.1)
Low-middle SDI	H influenzae type B meningitis	4880 (3930 to 5720)	3790 (3150 to 4490)	2240 (1830 to 2730)	350 (286.8 to 407.2)	226.1 (189.9 to 267.5)	123.2 (102.2 to 150.1)
Low-middle SDI	Meningococcal infection	1720 (1380 to 2030)	1390 (1110 to 1660)	1010 (775 to 1270)	119.6 (96.6 to 140)	81.3 (65.6 to 96.5)	54.7 (42.5 to 68.6)
Low-middle SDI	Other meningitis	5220 (4190 to 6110)	4440 (3640 to 5190)	4330 (3390 to 5280)	375.3 (305.7 to 435)	266 (220.2 to 309.4)	236.2 (186.1 to 286.4)
Low-middle SDI	Encephalitis	2150 (1650 to 3150)	1900 (1720 to 2180)	1750 (1550 to 1990)	186.5 (147 to 260.1)	130.1 (118.5 to 146.9)	105.7 (94.1 to 120.4)
Low-middle SDI	Diphtheria	369 (236 to 612)	140 (70.9 to 269)	181 (71.5 to 394)	25.1 (16.2 to 41.3)	8 (4.1 to 15.4)	9.5 (3.8 to 20.6)
Low-middle SDI	Whooping cough	4210 (1730 to 8780)	2890 (1250 to 5480)	2500 (1100 to 5050)	275.3 (113.1 to 574)	163.1 (70.3 to 309.5)	131.5 (57.6 to 265.6)
Low-middle SDI	Tetanus	6420 (5280 to 7780)	1730 (1420 to 2150)	906 (629 to 1240)	448.7 (368.3 to 546.4)	105 (85.2 to 131.5)	50.9 (35.1 to 69.5)
Low-middle SDI	Measles	23600 (8810 to 48100)	7990 (3030 to 16200)	3190 (1140 to 6880)	1568 (585 to 3198.8)	453.3 (171.9 to 916.9)	167.2 (59.9 to 360.3)
Low-middle SDI	Varicella and herpes zoster	464 (402 to 534)	408 (356 to 472)	327 (277 to 385)	38.8 (34.4 to 43.7)	27.4 (24.2 to 31)	19.4 (16.6 to 22.7)
Low-middle SDI	Acute hepatitis	2880 (2010 to 3290)	3030 (1850 to 3440)	2400 (1540 to 2860)	256.1 (182.8 to 287.7)	212.6 (131.7 to 241.5)	148.2 (94.8 to 175.1)
Low-middle SDI	Acute hepatitis A	943 (618 to 1180)	872 (514 to 1070)	590 (387 to 761)	68.2 (45.3 to 85.2)	51.6 (30.5 to 63.8)	31.3 (20.5 to 40.5)
Low-middle SDI	Acute hepatitis B	1340 (909 to 1600)	1550 (928 to 1830)	1380 (863 to 1660)	139 (98.2 to 162.2)	122 (74.5 to 142.9)	91.9 (58 to 109.9)
Low-middle SDI	Acute hepatitis C	226 (123 to 368)	159 (85 to 263)	115 (63.6 to 191)	15.4 (8.5 to 25.2)	9.5 (5.1 to 15.8)	6.4 (3.5 to 10.5)
Low-middle SDI	Acute hepatitis E	376 (261 to 480)	449 (271 to 584)	321 (208 to 417)	33.5 (23.4 to 42.6)	29.6 (18 to 38.3)	18.6 (12 to 24.1)
Low-middle SDI	Other unspecified infectious diseases	2470 (1780 to 2930)	2100 (1610 to 2440)	1930 (1550 to 2250)	205.7 (151.8 to 243.8)	137.1 (107.4 to 158.3)	111.2 (89.7 to 128.5)
Low-middle SDI	Maternal and neonatal disorders	101000 (95100 to 108000)	91800 (87100 to 96600)	74100 (68600 to 79700)	6554.1 (6172.7 to 6959.2)	5173.6 (4910.8 to 5446)	3963.5 (3670.1 to 4259.6)
Low-middle SDI	Maternal disorders	7000 (6530 to 7480)	5890 (5400 to 6490)	4560 (4010 to 5200)	679.4 (633.7 to 725.9)	395.2 (361.8 to 434.9)	256.3 (225 to 291.7)
Low-middle SDI	Maternal haemorrhage	2480 (2000 to 3050)	1720 (1410 to 2040)	931 (726 to 1210)	242.9 (198.5 to 294.2)	115.5 (95.7 to 137.6)	52.6 (41.1 to 67.4)
Low-middle SDI	Maternal sepsis and other pregnancy related infections	857 (637 to 1150)	617 (495 to 778)	391 (329 to 471)	81.3 (61.4 to 106.5)	41 (33.2 to 51.1)	21.9 (18.5 to 26.3)
Low-middle SDI	Maternal hypertensive disorders	862 (673 to 1130)	849 (687 to 1040)	849 (685 to 1040)	80.9 (63.9 to 104.2)	55.5 (45.1 to 67.8)	46.7 (37.7 to 57)
Low-middle SDI	Maternal obstructed labour and uterine rupture	661 (485 to 920)	628 (475 to 822)	496 (375 to 658)	67.4 (51 to 89.9)	43.7 (33.2 to 56.7)	28.5 (21.7 to 37.7)
Low-middle SDI	Maternal abortive outcome	511 (380 to 694)	432 (330 to 563)	407 (316 to 526)	49.7 (37.6 to 65.8)	29.1 (22.5 to 37.3)	23 (18 to 29.7)
Low-middle SDI	Ectopic pregnancy	320 (175 to 560)	286 (170 to 454)	256 (162 to 387)	30.7 (17.2 to 52.2)	18.8 (11.4 to 29.6)	14.2 (9.1 to 21.4)

SR Appendix Table 2. All-age DALYs counts and age standardized DALYs rates per 100,000 individuals for all causes and level 1 cause groups by location for 1990, 2007, and 2017, both sexes combined

		All-age DALYs			Age-standardized rate of DALYs per 100,000		
Low-middle SDI	Indirect maternal deaths	714 (518 to 1010)	677 (545 to 838)	606 (501 to 728)	68.7 (50.5 to 95.4)	45.3 (36.8 to 56)	34.1 (28.2 to 40.9)
Low-middle SDI	Late maternal deaths	71.1 (54.7 to 92.1)	72.2 (54.5 to 94.6)	73.2 (54.3 to 97.8)	6.8 (5.3 to 8.8)	4.8 (3.6 to 6.3)	4.1 (3 to 5.4)
Low-middle SDI	Maternal deaths aggravated by HIV/AIDS	9.92 (5.79 to 14.2)	44.4 (26.1 to 61.4)	37.4 (22.1 to 52.9)	1 (0.6 to 1.4)	3.1 (1.8 to 4.3)	2.2 (1.3 to 3.1)
Low-middle SDI	Other maternal disorders	518 (362 to 748)	570 (443 to 747)	516 (420 to 630)	50 (35.8 to 70.1)	38.4 (30.2 to 49.4)	29.1 (23.7 to 35.2)
Low-middle SDI	Neonatal disorders	94400 (88000 to 101000)	85900 (81300 to 90700)	69600 (64300 to 74900)	5874.7 (5473 to 6275.7)	4778.4 (4521.8 to 5047.4)	3707.2 (3424.2 to 3989.9)
Low-middle SDI	Neonatal preterm birth	38600 (34800 to 42700)	32000 (29300 to 34700)	25200 (22900 to 28000)	2409.8 (2168.9 to 2659.2)	1782.4 (1634.9 to 1937)	1344.7 (1220.5 to 1496.3)
Low-middle SDI	Neonatal encephalopathy due to birth asphyxia and trauma	27700 (23900 to 31500)	28100 (24200 to 31200)	23300 (19800 to 26700)	1721.6 (1483.2 to 1956.1)	1563.7 (1351.5 to 1737.8)	1239.4 (1055.6 to 1423.3)
Low-middle SDI	Neonatal sepsis and other neonatal infections	7060 (5550 to 10800)	7900 (6510 to 11000)	7470 (6200 to 10400)	443 (348.7 to 673.4)	441.8 (363.5 to 616)	398.8 (331.4 to 554)
Low-middle SDI	Hemolytic disease and other neonatal jaundice	4740 (3930 to 5680)	3490 (2920 to 4090)	2420 (1970 to 2890)	296.8 (246.7 to 355.2)	194.9 (163.7 to 227.8)	129.2 (105.5 to 154.3)
Low-middle SDI	Other neonatal disorders	16300 (13500 to 20300)	14400 (12400 to 17400)	11200 (9550 to 13300)	1003.5 (835.2 to 1249.3)	795.6 (685.5 to 961.4)	595 (506.9 to 706.7)
Low-middle SDI	Nutritional deficiencies	29800 (23900 to 37200)	24600 (19100 to 31600)	20700 (15500 to 27500)	2348.9 (1868.4 to 2967)	1565.2 (1213.1 to 2012.4)	1165.9 (874.1 to 1542.3)
Low-middle SDI	Protein-energy malnutrition	12700 (10500 to 14600)	7910 (6880 to 9110)	5300 (4610 to 6120)	873.1 (726.7 to 1003.7)	468.8 (409.9 to 537)	291.6 (254.8 to 334.1)
Low-middle SDI	Iodine deficiency	848 (536 to 1280)	587 (368 to 879)	582 (358 to 884)	83.8 (53 to 127.1)	42.4 (26.6 to 63.4)	35 (21.4 to 53.2)
Low-middle SDI	Dietary iron deficiency	10900 (7420 to 15500)	11900 (8050 to 17000)	11400 (7590 to 16500)	1000.7 (683.5 to 1423.4)	800.7 (542.9 to 1142.7)	652.5 (435.5 to 951)
Low-middle SDI	Other nutritional deficiencies	1110 (841 to 1360)	694 (622 to 772)	518 (457 to 581)	98.8 (75.2 to 115.2)	51.4 (46.9 to 56.3)	34.3 (30.5 to 38.2)
Low-middle SDI	Non-communicable diseases	205000 (185000 to 227000)	268000 (241000 to 298000)	321000 (286000 to 360000)	26949.9 (24568.1 to 29549.3)	24358.7 (22081.6 to 26857.1)	23224.4 (21024.7 to 25820.8)
Low-middle SDI	Neoplasms	19500 (18100 to 21700)	27800 (26700 to 29600)	35700 (34000 to 37600)	2695.4 (2533 to 2939.2)	2625.2 (2524.4 to 2780.4)	2627.3 (2508.2 to 2765.9)
Low-middle SDI	Lip and oral cavity cancer	750 (693 to 814)	1210 (1140 to 1280)	1700 (1540 to 1870)	112.4 (104.1 to 122.2)	117.9 (111.5 to 124.4)	125.9 (114.2 to 138.8)
Low-middle SDI	Nasopharynx cancer	237 (212 to 257)	293 (276 to 311)	355 (335 to 376)	32.2 (29.1 to 35)	27.1 (25.5 to 28.6)	25.5 (24.1 to 26.9)
Low-middle SDI	Other pharynx cancer	451 (367 to 508)	720 (570 to 768)	1030 (842 to 1140)	67.7 (55.1 to 76.6)	71.4 (56.7 to 76.2)	77.7 (63.7 to 86)
Low-middle SDI	Oesophageal cancer	816 (766 to 865)	1120 (1060 to 1180)	1380 (1310 to 1460)	129.8 (121.8 to 137.7)	116.8 (110.8 to 123.5)	108.5 (103.1 to 114.4)
Low-middle SDI	Stomach cancer	2060 (1940 to 2220)	2330 (2240 to 2450)	2610 (2500 to 2740)	321.5 (303.5 to 346.9)	241.7 (232.2 to 254.3)	206.3 (197.7 to 215.9)
Low-middle SDI	Colon and rectum cancer	973 (849 to 1180)	1520 (1410 to 1660)	2110 (1990 to 2230)	155.4 (136.1 to 188.1)	160.1 (149.3 to 175)	168.9 (159 to 178.2)
Low-middle SDI	Liver cancer	1210 (1050 to 1500)	1840 (1680 to 2170)	2350 (2180 to 2680)	178.9 (155.9 to 222.6)	183.2 (167.6 to 215.6)	178.9 (166.1 to 203.7)
Low-middle SDI	Liver cancer due to hepatitis B	571 (483 to 739)	774 (681 to 962)	904 (801 to 1110)	78.4 (66.2 to 102.1)	71.9 (63.1 to 90)	64.9 (57.6 to 79.9)
Low-middle SDI	Liver cancer due to hepatitis C	292 (251 to 363)	506 (450 to 576)	684 (614 to 764)	48.3 (41.5 to 59.9)	54.5 (48.7 to 62.2)	55.2 (49.7 to 61.6)
Low-middle SDI	Liver cancer due to alcohol use	156 (128 to 198)	262 (223 to 315)	360 (311 to 429)	24.6 (20.2 to 31.2)	27 (23.1 to 32.4)	28.2 (24.4 to 33.3)
Low-middle SDI	Liver cancer due to NASH	76.6 (64.4 to 94.7)	137 (119 to 160)	196 (172 to 223)	12.4 (10.4 to 15.2)	14.5 (12.6 to 16.9)	15.8 (13.8 to 18)
Low-middle SDI	Liver cancer due to other causes	112 (94.7 to 136)	164 (143 to 190)	205 (180 to 233)	15.3 (12.8 to 18.7)	15.2 (13.2 to 17.8)	14.8 (13 to 16.9)
Low-middle SDI	Gallbladder and biliary tract cancer	284 (252 to 386)	453 (405 to 553)	614 (539 to 738)	47.4 (42.1 to 64.8)	48.9 (43.9 to 60.5)	49.8 (43.8 to 60.4)
Low-middle SDI	Pancreatic cancer	298 (275 to 322)	577 (542 to 616)	913 (863 to 981)	49.1 (45.5 to 53.1)	62.2 (58.7 to 66.3)	74 (70.1 to 79.5)
Low-middle SDI	Larynx cancer	502 (454 to 536)	641 (605 to 680)	786 (735 to 844)	77.9 (70.5 to 83.1)	65.4 (61.6 to 69.2)	60.5 (56.6 to 64.8)
Low-middle SDI	Tracheal, bronchus, and lung cancer	1580 (1470 to 1720)	2350 (2250 to 2480)	3090 (2900 to 3300)	254.4 (236.7 to 276.6)	249.8 (238.3 to 262.5)	247.2 (232.5 to 263.9)
Low-middle SDI	Malignant skin melanoma	64.4 (47 to 85.9)	101 (78.3 to 122)	136 (107 to 161)	8.9 (6.7 to 11.8)	9.2 (7.2 to 11.1)	9.7 (7.6 to 11.5)
Low-middle SDI	Non-melanoma skin cancer	91.6 (84.1 to 104)	146 (137 to 161)	198 (187 to 213)	15.1 (13.7 to 17.1)	15.9 (14.9 to 17.5)	16.3 (15.5 to 17.5)
Low-middle SDI	Non-melanoma skin cancer (squamous-cell carcinoma)	91.6 (84.1 to 104)	146 (137 to 161)	198 (187 to 213)	15.1 (13.6 to 17.1)	15.9 (14.9 to 17.4)	16.3 (15.5 to 17.5)
Low-middle SDI	Non-melanoma skin cancer (basal-cell carcinoma)	0.0394 (0.0152 to 0.0862)	0.0565 (0.0218 to 0.124)	0.0942 (0.0341 to 0.207)	0 (0 to 0)	0 (0 to 0)	0 (0 to 0)
Low-middle SDI	Breast cancer	1430 (1180 to 1900)	2530 (2300 to 3040)	3570 (3180 to 4460)	206.4 (171.1 to 272.3)	237.8 (216.3 to 287.5)	257.2 (229.1 to 322.6)
Low-middle SDI	Cervical cancer	1280 (1080 to 1520)	1590 (1420 to 1830)	1890 (1710 to 2140)	180.9 (152 to 216.5)	147.3 (132.2 to 171)	135.2 (122.6 to 152.9)
Low-middle SDI	Uterine cancer	191 (163 to 214)	271 (249 to 293)	351 (322 to 386)	30 (25.8 to 33.7)	27.9 (25.6 to 30.1)	27.3 (25 to 30)

SR Appendix Table 2. All-age DALYs counts and age standardized DALYs rates per 100,000 individuals for all causes and level 1 cause groups by location for 1990, 2007, and 2017, both sexes combined

		All-age DALYs			Age-standardized rate of DALYs per 100,000		
Low-middle SDI	Ovarian cancer	286 (248 to 360)	553 (504 to 606)	865 (778 to 1000)	41.5 (36.2 to 51.9)	52.6 (48.1 to 57.7)	63 (56.9 to 72.5)
Low-middle SDI	Prostate cancer	465 (373 to 540)	784 (610 to 897)	1090 (853 to 1250)	95.3 (76.9 to 110.1)	102 (79.3 to 116.5)	103.9 (81 to 118.5)
Low-middle SDI	Testicular cancer	66.4 (58.7 to 74.6)	79.8 (70.8 to 89.8)	82.3 (70.6 to 97.1)	7 (6.3 to 7.9)	5.7 (5.1 to 6.4)	4.9 (4.2 to 5.7)
Low-middle SDI	Kidney cancer	155 (128 to 188)	240 (213 to 265)	333 (306 to 358)	18.4 (15.9 to 21.5)	21.2 (19.3 to 23.1)	23.7 (21.9 to 25.4)
Low-middle SDI	Bladder cancer	255 (221 to 275)	383 (352 to 408)	520 (480 to 569)	44.3 (39.1 to 47.9)	43 (39.9 to 46)	43.5 (40.4 to 47.6)
Low-middle SDI	Thyroid cancer	115 (100 to 135)	186 (170 to 204)	237 (215 to 270)	15.7 (13.9 to 18.5)	16.8 (15.5 to 18.5)	16.9 (15.6 to 19.2)
Low-middle SDI	Mesothelioma	40.2 (26.3 to 63.6)	59 (46.5 to 78.1)	76.8 (66.8 to 90.3)	5.7 (3.9 to 8.8)	5.6 (4.5 to 7.4)	5.7 (5 to 6.7)
Low-middle SDI	Hodgkin lymphoma	456 (330 to 534)	509 (401 to 608)	509 (405 to 611)	45.7 (34.5 to 53.2)	36.2 (29.1 to 43.7)	30.2 (24.3 to 36.3)
Low-middle SDI	Non-Hodgkin's lymphoma	773 (665 to 909)	1160 (1070 to 1240)	1450 (1320 to 1570)	85.7 (76.8 to 97.4)	93.1 (87.2 to 99.6)	95.7 (87.7 to 102.6)
Low-middle SDI	Multiple myeloma	129 (115 to 157)	214 (196 to 249)	310 (284 to 355)	20.6 (18.5 to 24.9)	22.3 (20.4 to 25.8)	24.4 (22.3 to 27.8)
Low-middle SDI	Leukaemia	1890 (1530 to 2600)	2270 (1970 to 2640)	2440 (2200 to 2790)	177.4 (149.7 to 232.5)	162.8 (143 to 189.3)	149.9 (134.9 to 171.4)
Low-middle SDI	Acute lymphoid leukaemia	413 (303 to 643)	554 (466 to 701)	586 (510 to 705)	32.9 (24.9 to 49.6)	34.8 (29.5 to 43.9)	32.5 (28.3 to 38.8)
Low-middle SDI	Chronic lymphoid leukaemia	23.7 (18.6 to 27.3)	36.7 (30.8 to 40.5)	52 (44.2 to 58.9)	3.7 (2.9 to 4.2)	3.9 (3.2 to 4.3)	4.2 (3.6 to 4.7)
Low-middle SDI	Acute myeloid leukaemia	330 (231 to 545)	512 (428 to 644)	651 (568 to 755)	32.9 (24.6 to 50)	37.7 (32.2 to 46.3)	40.4 (35.6 to 47)
Low-middle SDI	Chronic myeloid leukaemia	101 (86 to 129)	134 (117 to 156)	147 (128 to 172)	13.2 (11.5 to 16.8)	11.8 (10.4 to 13.7)	10.3 (8.9 to 12.1)
Low-middle SDI	Other leukaemia	1020 (723 to 1440)	1030 (838 to 1270)	1010 (870 to 1180)	94.6 (71.7 to 125.8)	74.6 (61.6 to 91)	62.5 (54.5 to 74.2)
Low-middle SDI	Other malignant cancers	1590 (1360 to 1940)	2210 (1980 to 2460)	2770 (2500 to 3030)	165.5 (148.1 to 191.3)	169 (155.5 to 185.2)	176.7 (161.5 to 190.6)
Low-middle SDI	Other neoplasms	116 (86.8 to 142)	202 (158 to 237)	309 (244 to 360)	13 (9.3 to 16.9)	16.7 (12.9 to 19.8)	21.2 (16.8 to 24.8)
Low-middle SDI	Myelodysplastic, myeloproliferative, and other hematopoietic neoplasms	95.6 (73.9 to 113)	164 (132 to 198)	255 (205 to 304)	11.3 (8.1 to 14.3)	14.3 (11.3 to 17.5)	18.2 (14.7 to 21.7)
Low-middle SDI	Benign and in situ intestinal neoplasms	0 (0 to 0)	0 (0 to 0)	0 (0 to 0)	0 (0 to 0)	0 (0 to 0)	0 (0 to 0)
Low-middle SDI	Benign and in situ cervical and uterine neoplasms	0 (0 to 0)	0 (0 to 0)	0 (0 to 0)	0 (0 to 0)	0 (0 to 0)	0 (0 to 0)
Low-middle SDI	Other benign and in situ neoplasms	20.8 (12.4 to 32.3)	37.5 (25.4 to 49.3)	53.9 (37.8 to 66)	1.8 (1 to 2.8)	2.4 (1.6 to 3.2)	3 (2.1 to 3.6)
Low-middle SDI	Cardiovascular diseases	45900 (44000 to 47600)	60900 (58700 to 63100)	74900 (72000 to 77800)	7424.9 (7126.4 to 7719.3)	6533.1 (6301.3 to 6777.7)	6126 (5899.3 to 6359.3)
Low-middle SDI	Rheumatic heart disease	3150 (2840 to 3580)	3010 (2720 to 3330)	2850 (2540 to 3190)	379.1 (340.3 to 437.5)	256.6 (231.3 to 284.5)	197.1 (175.7 to 219.9)
Low-middle SDI	Ischaemic heart disease	20600 (19700 to 21700)	29600 (28500 to 30800)	37900 (36400 to 39500)	3465.3 (3302.8 to 3644.7)	3215.9 (3100 to 3357.5)	3108.6 (2983.8 to 3236.4)
Low-middle SDI	Stroke	16400 (15600 to 17100)	20800 (20000 to 21600)	24800 (23700 to 26000)	2721.9 (2596.1 to 2855.2)	2286.1 (2199.6 to 2381.9)	2065.2 (1972.6 to 2163.7)
Low-middle SDI	Ischaemic stroke	5050 (4590 to 5630)	7010 (6400 to 7770)	8920 (8090 to 9940)	962.7 (877.9 to 1069.6)	852.3 (780.6 to 941.9)	806 (734.4 to 893.7)
Low-middle SDI	Intracerebral hemorrhage	9090 (8190 to 9900)	11600 (10700 to 12300)	13300 (12400 to 14100)	1463 (1311.7 to 1603)	1228 (1134.8 to 1309)	1070.3 (992.2 to 1137.2)
Low-middle SDI	Subarachnoid hemorrhage	2210 (1830 to 2610)	2210 (1870 to 2580)	2570 (2260 to 2990)	296.2 (242.5 to 360)	205.8 (174.4 to 241.6)	188.9 (167.2 to 219.8)
Low-middle SDI	Hypertensive heart disease	1810 (1270 to 2060)	2350 (1770 to 2730)	3020 (2350 to 3470)	328 (234.2 to 371.8)	276.4 (211.2 to 317.1)	266 (208.6 to 302.7)
Low-middle SDI	Non-rheumatic valvular heart disease	184 (154 to 231)	274 (239 to 326)	360 (318 to 412)	29 (24.7 to 35.4)	28.3 (25 to 33.1)	28.8 (25.6 to 32.6)
Low-middle SDI	Non-rheumatic calcific aortic valve disease	58.7 (41.6 to 89.8)	100 (76.6 to 134)	149 (122 to 183)	9.8 (7.1 to 14.3)	10.8 (8.4 to 14.1)	12.3 (10.1 to 14.9)
Low-middle SDI	Non-rheumatic degenerative mitral valve disease	108 (81.3 to 134)	148 (117 to 178)	180 (144 to 215)	16.9 (13 to 20.3)	15.2 (12 to 18)	14.3 (11.5 to 16.8)
Low-middle SDI	Other non-rheumatic valve diseases	16.8 (10.8 to 21.6)	25.6 (17.2 to 33.5)	31.3 (22.6 to 42.2)	2.3 (1.5 to 3)	2.4 (1.5 to 3)	2.3 (1.6 to 3)
Low-middle SDI	Cardiomyopathy and myocarditis	1110 (927 to 1350)	1400 (1230 to 1580)	1600 (1440 to 1790)	123.8 (101.3 to 153.2)	116.8 (101.8 to 133.8)	111.9 (100.2 to 125.6)
Low-middle SDI	Myocarditis	264 (192 to 341)	295 (235 to 345)	303 (250 to 364)	24.8 (18.2 to 32.4)	22.1 (17.5 to 26.2)	19.7 (16 to 24)
Low-middle SDI	Alcoholic cardiomyopathy	109 (77.4 to 144)	139 (111 to 178)	170 (142 to 210)	15.5 (11 to 20.6)	13 (10.4 to 16.6)	12.3 (10.3 to 15.3)
Low-middle SDI	Other cardiomyopathy	738 (614 to 892)	961 (841 to 1100)	1120 (1010 to 1270)	83.5 (68 to 104)	81.6 (70.9 to 94.6)	79.9 (71.4 to 90.5)
Low-middle SDI	Atrial fibrillation and flutter	278 (229 to 336)	460 (383 to 553)	673 (564 to 804)	62.7 (52.5 to 74.7)	64.6 (54.5 to 76.6)	68.4 (58.2 to 81)
Low-middle SDI	Aortic aneurysm	204 (170 to 252)	303 (266 to 357)	421 (379 to 476)	34.6 (29.4 to 42.6)	33.2 (29.5 to 39)	34.9 (31.5 to 39.3)

SR Appendix Table 2. All-age DALYs counts and age standardized DALYs rates per 100,000 individuals for all causes and level 1 cause groups by location for 1990, 2007, and 2017, both sexes combined

		All-age DALYs			Age-standardized rate of DALYs per 100,000		
Low-middle SDI	Peripheral vascular disease	42.6 (22.9 to 70)	72.9 (42.1 to 114)	109 (66.9 to 163)	8.9 (4.8 to 14.6)	9.6 (5.6 to 15)	10.5 (6.4 to 15.6)
Low-middle SDI	Endocarditis	364 (298 to 476)	415 (370 to 482)	478 (435 to 532)	34.6 (28.4 to 45.4)	31.1 (28.2 to 36.1)	31.2 (28.5 to 34.5)
Low-middle SDI	Other cardiovascular and circulatory diseases	1770 (1540 to 2080)	2290 (2010 to 2680)	2720 (2360 to 3160)	237 (205.6 to 286.5)	214.5 (186.9 to 256)	203.5 (176.1 to 238.5)
Low-middle SDI	Chronic respiratory diseases	21800 (20000 to 23100)	22600 (21100 to 23900)	26500 (24600 to 28200)	3518.1 (3222.9 to 3716.1)	2457.9 (2313.6 to 2581.7)	2196 (2053.5 to 2320.1)
Low-middle SDI	Chronic obstructive pulmonary disease	13500 (11700 to 15800)	15000 (13700 to 16600)	18400 (17000 to 19900)	2408.5 (2100.6 to 2798.1)	1763.6 (1601.8 to 1933.4)	1615.3 (1490.3 to 1744.3)
Low-middle SDI	Pneumoconiosis	55.6 (41.4 to 69.7)	56.3 (47.4 to 66.2)	65.3 (57.5 to 74.6)	9.1 (6.8 to 11.4)	6.1 (5.2 to 7.2)	5.4 (4.8 to 6.1)
Low-middle SDI	Silicosis	27.3 (17 to 39.4)	26.2 (19.6 to 34.3)	29.4 (24.2 to 36)	4.5 (2.8 to 6.5)	2.8 (2.1 to 3.8)	2.4 (2 to 3)
Low-middle SDI	Asbestosis	4.22 (1.92 to 7.24)	5.55 (2.9 to 8.41)	7.56 (4.46 to 10.6)	0.7 (0.3 to 1.2)	0.6 (0.3 to 0.9)	0.6 (0.4 to 0.9)
Low-middle SDI	Coal workers pneumoconiosis	10.6 (4.85 to 17.2)	8.9 (5.63 to 12.4)	9.17 (6.72 to 11.9)	1.8 (0.8 to 2.9)	1 (0.6 to 1.4)	0.8 (0.6 to 1)
Low-middle SDI	Other pneumoconiosis	13.5 (8.37 to 19.8)	15.7 (11.1 to 20.7)	19.1 (14.6 to 24)	2.2 (1.4 to 3.3)	1.7 (1.2 to 2.3)	1.6 (1.2 to 2)
Low-middle SDI	Asthma	7320 (4930 to 8740)	6240 (4610 to 7790)	6300 (5000 to 7760)	982.1 (589.1 to 1225)	578.3 (392.6 to 730.1)	457 (356.6 to 564.7)
Low-middle SDI	Interstitial lung disease and pulmonary sarcoidosis	244 (137 to 412)	357 (239 to 513)	567 (424 to 752)	40.4 (22.8 to 66.7)	39.3 (26.6 to 56.8)	47.5 (35.6 to 63)
Low-middle SDI	Other chronic respiratory diseases	681 (519 to 869)	906 (771 to 1070)	1120 (981 to 1260)	77.9 (59.6 to 98.6)	70.6 (59.9 to 83.1)	70.9 (62.3 to 80.3)
Low-middle SDI	Digestive diseases	16100 (14900 to 17600)	19800 (18000 to 21800)	22700 (20200 to 25300)	2063.7 (1880.5 to 2234.6)	1742.1 (1576.4 to 1909.4)	1585.8 (1422.4 to 1759.3)
Low-middle SDI	Cirrhosis and other chronic liver diseases	7880 (7130 to 8620)	10400 (9340 to 11500)	12100 (10900 to 13400)	1059.4 (911.9 to 1174.6)	929.6 (830.2 to 1035.9)	850.3 (768.8 to 945.2)
Low-middle SDI	Cirrhosis and other chronic liver diseases due to hepatitis B	2900 (2410 to 3310)	3810 (3270 to 4440)	4190 (3630 to 4870)	405.2 (328.8 to 466.9)	346.6 (295.4 to 401.9)	296.9 (257.4 to 344.5)
Low-middle SDI	Cirrhosis and other chronic liver diseases due to hepatitis C	1660 (1380 to 1930)	2260 (1980 to 2560)	2800 (2440 to 3180)	239.8 (194.5 to 278.6)	211.4 (184.7 to 240)	201.6 (175.6 to 228.5)
Low-middle SDI	Cirrhosis and other chronic liver diseases due to alcohol use	1400 (1180 to 1630)	1950 (1650 to 2260)	2410 (2090 to 2750)	203.2 (168.5 to 237)	184 (155.1 to 212.5)	174.3 (151.9 to 199.4)
Low-middle SDI	Cirrhosis due to NASH	441 (366 to 503)	663 (580 to 742)	886 (778 to 996)	66.4 (53.8 to 75.9)	64.9 (56.3 to 72.7)	66.2 (58.1 to 74.3)
Low-middle SDI	Cirrhosis and other chronic liver diseases due to other causes	1470 (1220 to 1950)	1660 (1450 to 1930)	1780 (1490 to 2060)	144.8 (126 to 173.3)	122.7 (105.8 to 142.2)	111.2 (93.3 to 128.6)
Low-middle SDI	Upper digestive system diseases	3980 (3360 to 4800)	4520 (3690 to 5640)	4960 (3910 to 6430)	530.4 (453.1 to 630.7)	402.6 (333.8 to 492.2)	346.5 (278.2 to 442.3)
Low-middle SDI	Peptic ulcer disease	2230 (2040 to 2450)	2110 (1900 to 2280)	1970 (1740 to 2170)	318.8 (290.5 to 348)	204.7 (183.3 to 220.1)	149.7 (132.8 to 164.5)
Low-middle SDI	Gastritis and duodenitis	1150 (830 to 1540)	1500 (1060 to 2040)	1840 (1310 to 2520)	136.9 (100.4 to 179)	122.8 (88.6 to 162.5)	121.1 (86.8 to 164)
Low-middle SDI	GERD	596 (318 to 1010)	900 (481 to 1530)	1150 (613 to 1940)	74.8 (40.3 to 126.6)	75 (40 to 126.7)	75.7 (40.5 to 128)
Low-middle SDI	Appendicitis	620 (535 to 762)	658 (592 to 727)	623 (532 to 713)	63.7 (56.4 to 74.5)	49.3 (44.3 to 54.7)	39.3 (34 to 45)
Low-middle SDI	Paralytic ileus and intestinal obstruction	1830 (1620 to 2280)	2030 (1780 to 2280)	2230 (1760 to 2560)	177.1 (158 to 207.4)	154.3 (131.1 to 174.8)	146.1 (113 to 167.4)
Low-middle SDI	Inguinal, femoral, and abdominal hernia	529 (418 to 652)	660 (506 to 820)	743 (578 to 929)	65.4 (52.7 to 79.7)	56.7 (44.7 to 69.9)	51.7 (41.1 to 64.2)
Low-middle SDI	Inflammatory bowel disease	135 (92.3 to 208)	167 (130 to 211)	211 (174 to 243)	16.7 (12.3 to 22.5)	14.8 (11.8 to 17.6)	15 (12.5 to 17.1)
Low-middle SDI	Vascular intestinal disorders	116 (83.3 to 136)	167 (132 to 195)	218 (172 to 253)	18.6 (14.1 to 22)	18.3 (14.4 to 21.2)	18.3 (14.5 to 21.1)
Low-middle SDI	Gallbladder and biliary diseases	275 (241 to 305)	327 (281 to 369)	406 (333 to 460)	39.7 (34.3 to 43.9)	32.1 (27.8 to 35.8)	31.1 (25.9 to 34.9)
Low-middle SDI	Pancreatitis	303 (250 to 368)	501 (407 to 600)	662 (525 to 791)	41.1 (33.8 to 49.3)	45.2 (36.3 to 54)	46.9 (37.2 to 55.7)
Low-middle SDI	Other digestive diseases	460 (391 to 554)	455 (378 to 574)	586 (478 to 723)	51.5 (43.6 to 61.5)	39.2 (32.3 to 50)	40.6 (33.3 to 50.2)
Low-middle SDI	Neurological disorders	12800 (9870 to 16300)	18000 (13800 to 22900)	22200 (17000 to 28500)	1601 (1281.9 to 1967.6)	1554.7 (1239.4 to 1930.9)	1554 (1233.9 to 1938.1)
Low-middle SDI	Alzheimer's disease and other dementias	1520 (1400 to 1670)	2400 (2220 to 2610)	3440 (3200 to 3680)	417.2 (385.1 to 452.9)	398.8 (370.5 to 430.1)	403.8 (376.8 to 431)
Low-middle SDI	Parkinson's disease	332 (294 to 387)	545 (491 to 618)	769 (708 to 854)	73.9 (65.3 to 85.5)	75.8 (68.3 to 85.5)	77.5 (71.3 to 85.9)
Low-middle SDI	Epilepsy	3440 (2690 to 4250)	4060 (3250 to 5040)	4420 (3350 to 5800)	314.2 (245.6 to 391.1)	277.2 (221 to 346.8)	258.3 (196.3 to 336.6)
Low-middle SDI	Multiple sclerosis	54.6 (41.7 to 88)	87.3 (71.5 to 120)	120 (101 to 146)	7.2 (5.6 to 11.6)	7.6 (6.3 to 10.3)	8.1 (6.9 to 9.9)
Low-middle SDI	Motor neuron disease	21 (18 to 42.2)	30.9 (27.4 to 48.5)	46.2 (41.5 to 62.4)	2.5 (2.1 to 4.9)	2.7 (2.4 to 4.3)	3.3 (3 to 4.5)
Low-middle SDI	Headache disorders	6880 (4470 to 9710)	10100 (6570 to 14300)	12400 (8100 to 17700)	738 (481.5 to 1040.7)	743.1 (483.5 to 1051.3)	749 (489.1 to 1062.1)
Low-middle SDI	Migraine	6030 (3810 to 8770)	8870 (5600 to 12800)	10900 (6890 to 15800)	645.6 (407.1 to 937)	650.8 (412.2 to 941.8)	655.9 (414.3 to 948.8)

SR Appendix Table 2. All-age DALYs counts and age standardized DALYs rates per 100,000 individuals for all causes and level 1 cause groups by location for 1990, 2007, and 2017, both sexes combined

		All-age DALYs			Age-standardized rate of DALYs per 100,000		
Low-middle SDI	Tension-type headache	849<br>(473 to 1370)	1230<br>(694 to 1970)	1520<br>(860 to 2420)	92.4<br>(52.3 to 146.4)	92.3<br>(52.4 to 146.6)	93<br>(52.6 to 147.7)
Low-middle SDI	Other neurological disorders	563<br>(365 to 752)	749<br>(549 to 948)	950<br>(719 to 1250)	48<br>(33.5 to 63.8)	49.4<br>(37.5 to 61.9)	54<br>(41.6 to 70.3)
Low-middle SDI	Mental disorders	15300<br>(11300 to 19800)	22000<br>(16300 to 28300)	26100<br>(19400 to 33800)	1647.4<br>(1228.9 to 2119)	1645<br>(1227.5 to 2118.4)	1597.9<br>(1189.3 to 2062.8)
Low-middle SDI	Schizophrenia	1210<br>(905 to 1510)	1810<br>(1350 to 2250)	2240<br>(1660 to 2780)	144.6<br>(108.3 to 178.9)	143.7<br>(107.2 to 177)	142.1<br>(105.3 to 175.7)
Low-middle SDI	Depressive disorders	5250<br>(3720 to 7170)	7830<br>(5550 to 10600)	9160<br>(6470 to 12400)	625.2<br>(443.4 to 844.9)	633.8<br>(447.1 to 858.5)	593.1<br>(419 to 802.7)
Low-middle SDI	Major depressive disorder	4310<br>(3000 to 5920)	6420<br>(4460 to 8690)	7370<br>(5140 to 9980)	509.3<br>(358 to 686.7)	516.9<br>(361.2 to 693.4)	475.3<br>(332.6 to 638.4)
Low-middle SDI	Dysthymia	943<br>(632 to 1380)	1410<br>(952 to 2060)	1790<br>(1200 to 2620)	115.9<br>(77.8 to 168.9)	116.9<br>(78.3 to 169.9)	117.8<br>(79 to 170.9)
Low-middle SDI	Bipolar disorder	1100<br>(697 to 1610)	1640<br>(1030 to 2400)	2020<br>(1270 to 2970)	118.2<br>(74.3 to 173.8)	120.1<br>(75.9 to 176.7)	121.3<br>(76.1 to 178)
Low-middle SDI	Anxiety disorders	3170<br>(2240 to 4250)	4610<br>(3240 to 6150)	5610<br>(3980 to 7500)	332.9<br>(235.1 to 442.2)	337.2<br>(238.9 to 446.7)	337.7<br>(240.6 to 449.7)
Low-middle SDI	Eating disorders	260<br>(164 to 380)	433<br>(276 to 635)	602<br>(379 to 875)	24.7<br>(15.8 to 36.2)	28.6<br>(18.3 to 41.8)	33.3<br>(21.1 to 48.5)
Low-middle SDI	Anorexia nervosa	59.1<br>(35.6 to 88)	93.2<br>(56.1 to 139)	125<br>(75.8 to 187)	5.2<br>(3.1 to 7.7)	5.8<br>(3.5 to 8.7)	6.7<br>(4.1 to 10)
Low-middle SDI	Bulimia nervosa	201<br>(125 to 299)	340<br>(214 to 505)	477<br>(299 to 711)	19.5<br>(12.3 to 29.2)	22.7<br>(14.3 to 33.8)	26.7<br>(16.8 to 39.4)
Low-middle SDI	Autism spectrum disorders	604<br>(413 to 830)	828<br>(564 to 1140)	978<br>(669 to 1350)	55.1<br>(37.8 to 75.9)	55.6<br>(37.9 to 76.4)	55.8<br>(38.2 to 76.7)
Low-middle SDI	Attention-deficit/hyperactivity disorder	115<br>(69.1 to 183)	161<br>(97.2 to 255)	191<br>(117 to 304)	9.7<br>(5.9 to 15.5)	10<br>(6.1 to 15.8)	10.2<br>(6.3 to 16.3)
Low-middle SDI	Conduct disorder	1180<br>(713 to 1890)	1560<br>(939 to 2490)	1780<br>(1070 to 2850)	86.9<br>(52.5 to 139.4)	87.7<br>(52.6 to 139.8)	89.5<br>(53.6 to 142.9)
Low-middle SDI	Idiopathic developmental intellectual disability	1290<br>(689 to 2100)	1400<br>(729 to 2310)	1390<br>(698 to 2320)	114.1<br>(60.4 to 185.9)	92.2<br>(47.9 to 152.1)	78.4<br>(39.1 to 130.6)
Low-middle SDI	Other mental disorders	1140<br>(749 to 1560)	1700<br>(1130 to 2360)	2140<br>(1410 to 2940)	136<br>(90.2 to 186.3)	136.2<br>(90.1 to 187.3)	136.4<br>(90.4 to 186.8)
Low-middle SDI	Substance use disorders	4110<br>(3190 to 5070)	6130<br>(4800 to 7590)	7680<br>(5990 to 9510)	456.1<br>(356 to 558.4)	462.8<br>(366.7 to 568.1)	469.3<br>(370.1 to 578.1)
Low-middle SDI	Alcohol use disorders	1770<br>(1370 to 2250)	2740<br>(2160 to 3430)	3280<br>(2600 to 4050)	208.5<br>(162.4 to 262.2)	216.8<br>(171.5 to 268.9)	207.7<br>(165.5 to 255.7)
Low-middle SDI	Drug use disorders	2330<br>(1730 to 2980)	3390<br>(2530 to 4330)	4400<br>(3230 to 5700)	247.6<br>(186.8 to 313.7)	246.1<br>(184.9 to 312.3)	261.5<br>(192.8 to 335.4)
Low-middle SDI	Opioid use disorders	1930<br>(1370 to 2540)	2910<br>(2110 to 3780)	3830<br>(2730 to 5020)	205.5<br>(147.7 to 268.9)	211.7<br>(153.7 to 273.5)	227<br>(161.9 to 295.9)
Low-middle SDI	Cocaine use disorders	34.2<br>(22.5 to 50.6)	52.4<br>(36.2 to 72.7)	71.3<br>(51.6 to 96.2)	3.6<br>(2.4 to 5.5)	3.9<br>(2.7 to 5.4)	4.3<br>(3.2 to 5.8)
Low-middle SDI	Amphetamine use disorders	93.2<br>(52.9 to 142)	101<br>(60.6 to 151)	114<br>(70.4 to 171)	8.6<br>(4.9 to 13)	6.6<br>(4 to 9.8)	6.3<br>(3.9 to 9.4)
Low-middle SDI	Cannabis use disorders	49.9<br>(31.4 to 74.9)	72.4<br>(46 to 107)	85.5<br>(53.9 to 127)	4.8<br>(3.1 to 7)	4.8<br>(3.1 to 7.1)	4.8<br>(3 to 7.1)
Low-middle SDI	Other drug use disorders	227<br>(172 to 314)	247<br>(195 to 322)	303<br>(248 to 374)	25.1<br>(19.2 to 34.3)	19<br>(15.3 to 24.7)	19.1<br>(16 to 23.4)
Low-middle SDI	Diabetes and kidney diseases	11600<br>(10400 to 13000)	18300<br>(16400 to 20700)	23700<br>(21000 to 27000)	1656.4<br>(1485.7 to 1855.3)	1796.5<br>(1611.8 to 2022.7)	1806<br>(1610.4 to 2041.9)
Low-middle SDI	Diabetes mellitus	5800<br>(4920 to 6870)	10700<br>(8990 to 12700)	14600<br>(12200 to 17300)	891.5<br>(763 to 1044.4)	1084.8<br>(926.9 to 1276.4)	1132.8<br>(957.8 to 1335.6)
Low-middle SDI	Diabetes mellitus type 1	1700<br>(1520 to 1940)	2520<br>(2300 to 2790)	2960<br>(2710 to 3220)	239.7<br>(213 to 277)	238.1<br>(214.7 to 266.9)	216.8<br>(197.4 to 238.1)
Low-middle SDI	Diabetes mellitus type 2	4110<br>(3220 to 5180)	8130<br>(6480 to 10100)	11600<br>(9310 to 14400)	651.9<br>(520.5 to 810.5)	846.7<br>(686.6 to 1033.2)	916<br>(745.1 to 1118.7)
Low-middle SDI	Chronic kidney disease	5690<br>(5280 to 6180)	7620<br>(7150 to 8080)	9090<br>(8520 to 9650)	753.6<br>(698.4 to 818.5)	706.9<br>(661.3 to 749.6)	669.8<br>(627.5 to 710.8)
Low-middle SDI	Chronic kidney disease due to diabetes mellitus type 1	429<br>(340 to 529)	639<br>(516 to 786)	773<br>(621 to 946)	56.7<br>(45.1 to 70.1)	56.1<br>(45.1 to 69.2)	52.9<br>(42.2 to 64.7)
Low-middle SDI	Chronic kidney disease due to diabetes mellitus type 2	866<br>(733 to 1020)	1370<br>(1180 to 1570)	1800<br>(1540 to 2070)	147.6<br>(126.1 to 173)	153<br>(132.5 to 174.4)	150.1<br>(129.3 to 171.8)
Low-middle SDI	Chronic kidney disease due to hypertension	881<br>(762 to 1000)	1270<br>(1110 to 1430)	1630<br>(1410 to 1830)	148.4<br>(128.6 to 169.8)	138.9<br>(121.6 to 156.7)	134.3<br>(117.4 to 151.5)
Low-middle SDI	Chronic kidney disease due to glomerulonephritis	1250<br>(1090 to 1420)	1600<br>(1420 to 1810)	1840<br>(1610 to 2080)	141.2<br>(125 to 159.7)	129.6<br>(115.2 to 146.4)	121.9<br>(107.4 to 138.4)
Low-middle SDI	Chronic kidney disease due to other and unspecified causes	2260<br>(2010 to 2530)	2740<br>(2450 to 3040)	3060<br>(2710 to 3430)	259.8<br>(228.9 to 293.7)	229.3<br>(204.1 to 256.4)	210.6<br>(186.1 to 236.4)
Low-middle SDI	Acute glomerulonephritis	104<br>(86.4 to 121)	58<br>(51.3 to 63.6)	47.4<br>(42.5 to 52.9)	11.2<br>(9.5 to 13)	4.9<br>(4.4 to 5.3)	3.4<br>(3.1 to 3.7)
Low-middle SDI	Skin and subcutaneous diseases	5850<br>(3920 to 8590)	7900<br>(5290 to 11500)	9380<br>(6360 to 13600)	537.3<br>(364.4 to 793.4)	542.8<br>(368.8 to 796.5)	549.7<br>(376.7 to 798)
Low-middle SDI	Dermatitis	1530<br>(875 to 2460)	2050<br>(1170 to 3300)	2440<br>(1400 to 3930)	130.6<br>(76.2 to 207.8)	132.4<br>(76.6 to 211.4)	135.9<br>(78.5 to 217.4)
Low-middle SDI	Atopic dermatitis	1320<br>(725 to 2210)	1750<br>(941 to 2940)	2070<br>(1130 to 3460)	108.3<br>(59 to 180.2)	110.1<br>(59.5 to 183.9)	113.6<br>(61.7 to 189.1)
Low-middle SDI	Contact dermatitis	198<br>(129 to 298)	288<br>(187 to 431)	353<br>(231 to 527)	21.6<br>(14.2 to 32.1)	21.7<br>(14.1 to 32.1)	21.7<br>(14.3 to 32.2)

SR Appendix Table 2. All-age DALYs counts and age standardized DALYs rates per 100,000 individuals for all causes and level 1 cause groups by location for 1990, 2007, and 2017, both sexes combined

		All-age DALYs			Age-standardized rate of DALYs per 100,000		
Low-middle SDI	Seborrhoeic dermatitis	6.65 (3.79 to 10.4)	8.59 (4.9 to 13.5)	9.66 (5.58 to 15.3)	0.7 (0.4 to 1.1)	0.6 (0.4 to 1)	0.6 (0.3 to 0.9)
Low-middle SDI	Psoriasis	411 (290 to 545)	641 (455 to 842)	834 (591 to 1110)	46.3 (32.8 to 61.1)	49.9 (35.5 to 65.7)	52.6 (37.3 to 69.7)
Low-middle SDI	Bacterial skin diseases	385 (191 to 491)	512 (279 to 630)	632 (378 to 846)	40.5 (21.4 to 50)	41.5 (23.2 to 50.4)	43.3 (25.8 to 57.4)
Low-middle SDI	Cellulitis	58 (26.5 to 83)	83.2 (41.8 to 113)	114 (59.3 to 159)	5.8 (2.8 to 7.9)	6.5 (3.3 to 8.7)	7.7 (3.9 to 10.6)
Low-middle SDI	Pyoderma	327 (159 to 412)	429 (234 to 536)	518 (307 to 700)	34.7 (17.8 to 43.5)	35 (19.3 to 42.5)	35.6 (20.8 to 47.6)
Low-middle SDI	Scabies	717 (396 to 1180)	906 (503 to 1480)	1020 (570 to 1680)	63.7 (35.5 to 103.9)	59.6 (33.3 to 97.5)	57.6 (32 to 93.7)
Low-middle SDI	Fungal skin diseases	722 (285 to 1510)	916 (363 to 1910)	992 (392 to 2050)	70.1 (27.9 to 146.5)	66.8 (26.5 to 138.9)	61.8 (24.6 to 128.6)
Low-middle SDI	Viral skin diseases	639 (410 to 947)	803 (518 to 1190)	899 (578 to 1330)	48.9 (31.4 to 72.6)	48.4 (31.2 to 71.8)	47.9 (30.8 to 70.8)
Low-middle SDI	Acne vulgaris	196 (114 to 318)	347 (204 to 562)	475 (280 to 765)	16.4 (9.6 to 26.4)	20.9 (12.3 to 33.7)	24.7 (14.6 to 39.7)
Low-middle SDI	Alopecia areata	54.5 (35.1 to 80.7)	79.1 (50.9 to 118)	96.5 (61.6 to 144)	5.7 (3.7 to 8.4)	5.7 (3.7 to 8.6)	5.7 (3.7 to 8.5)
Low-middle SDI	Pruritus	85.9 (40.3 to 162)	124 (58.5 to 236)	155 (72.5 to 293)	9.5 (4.5 to 17.9)	9.7 (4.6 to 18.2)	9.8 (4.6 to 18.4)
Low-middle SDI	Urticaria	823 (539 to 1180)	1080 (718 to 1540)	1260 (829 to 1780)	70.3 (46.4 to 99.2)	70.3 (46.5 to 99.5)	70.4 (46.6 to 99.4)
Low-middle SDI	Decubitus ulcer	30.5 (22.6 to 40.8)	51 (37.2 to 66.9)	68.9 (50.9 to 91.1)	4.9 (3.7 to 6.5)	5.6 (4 to 7.2)	5.9 (4.3 to 7.7)
Low-middle SDI	Other skin and subcutaneous diseases	257 (129 to 464)	390 (195 to 708)	512 (256 to 931)	30.4 (15.3 to 54.9)	32.1 (16.1 to 58.3)	34.1 (17.1 to 61.8)
Low-middle SDI	Sense organ diseases	7600 (5150 to 10900)	10900 (7360 to 15600)	13700 (9220 to 19600)	1149 (786.9 to 1614.2)	1092.1 (748.6 to 1538.2)	1056.5 (723 to 1491.2)
Low-middle SDI	Blindness and vision impairment	4190 (2830 to 6090)	5840 (3980 to 8530)	7190 (4870 to 10500)	648.4 (443.2 to 920)	596.4 (406.6 to 849.4)	564.3 (385.5 to 807.7)
Low-middle SDI	Glaucoma	68.9 (46.6 to 96)	102 (69.2 to 141)	133 (89.3 to 183)	13.5 (9.1 to 18.6)	12.7 (8.6 to 17.4)	12.2 (8.2 to 16.7)
Low-middle SDI	Cataract	1170 (820 to 1580)	1680 (1170 to 2260)	2170 (1520 to 2930)	235.3 (167.1 to 316.1)	212.6 (149.6 to 283.9)	202.5 (141.9 to 272.1)
Low-middle SDI	Age-related macular degeneration	41.6 (28.4 to 57.1)	65.6 (45.5 to 89.9)	91.4 (63.2 to 126)	8.1 (5.6 to 11.1)	8.2 (5.7 to 11.2)	8.4 (5.8 to 11.5)
Low-middle SDI	Refraction disorders	1370 (912 to 1980)	1760 (1180 to 2530)	2020 (1360 to 2900)	169.9 (115.6 to 243)	151.1 (103.8 to 213.1)	137.6 (92.8 to 197.2)
Low-middle SDI	Near vision loss	1230 (585 to 2240)	1780 (850 to 3270)	2210 (1050 to 4060)	175.6 (84.1 to 322.2)	168.4 (80.4 to 309.6)	161.2 (76.7 to 296.2)
Low-middle SDI	Other vision loss	316 (222 to 427)	444 (310 to 602)	560 (393 to 756)	46.1 (32.1 to 62.1)	43.4 (30.6 to 58.3)	42.4 (29.6 to 57)
Low-middle SDI	Age-related and other hearing loss	3200 (2180 to 4550)	4750 (3220 to 6780)	6060 (4110 to 8630)	471.3 (325.5 to 662.4)	465.9 (320 to 653.9)	461.9 (316.3 to 651.7)
Low-middle SDI	Other sense organ diseases	217 (136 to 317)	324 (202 to 474)	418 (260 to 610)	29.3 (18.3 to 42.7)	29.9 (18.6 to 43.4)	30.3 (18.9 to 44.1)
Low-middle SDI	Musculoskeletal disorders	13100 (9660 to 17300)	19800 (14700 to 26100)	25000 (18400 to 33100)	1729 (1285.1 to 2263.8)	1734.1 (1288.7 to 2276.8)	1725 (1278.6 to 2263.2)
Low-middle SDI	Rheumatoid arthritis	337 (262 to 420)	497 (389 to 611)	678 (525 to 837)	52.5 (41.7 to 65)	51.2 (40.2 to 61.9)	53.4 (41.7 to 65.6)
Low-middle SDI	Osteoarthritis	608 (304 to 1210)	1020 (510 to 2050)	1440 (720 to 2860)	102.3 (51.4 to 201.9)	110.7 (55.5 to 219)	116.6 (58.4 to 230.2)
Low-middle SDI	Low back pain	6290 (4480 to 8530)	9420 (6730 to 12700)	11800 (8390 to 16100)	796 (570.6 to 1067.7)	792.7 (567 to 1065.8)	787.3 (561.6 to 1062.2)
Low-middle SDI	Neck pain	2210 (1540 to 3090)	3310 (2310 to 4650)	4360 (3050 to 6140)	300.7 (210.1 to 421.3)	296.5 (207.7 to 415.6)	303.6 (211.7 to 426)
Low-middle SDI	Gout	80.7 (54.3 to 111)	129 (87.4 to 177)	175 (116 to 240)	13 (8.8 to 17.9)	13.4 (9.1 to 18.4)	13.7 (9.2 to 18.7)
Low-middle SDI	Other musculoskeletal disorders	3620 (2550 to 4920)	5470 (3850 to 7440)	6610 (4630 to 8950)	464.5 (327.2 to 634.1)	469.6 (332 to 641.4)	450.5 (317.6 to 613.6)
Low-middle SDI	Other non-communicable diseases	31700 (27900 to 37000)	33700 (29900 to 38600)	33200 (28900 to 38600)	2471.7 (2132.6 to 2901.4)	2172.4 (1895.3 to 2522.5)	1930.8 (1670.3 to 2259.6)
Low-middle SDI	Congenital anomalies	20800 (18500 to 25500)	20700 (19100 to 23300)	18900 (17400 to 20500)	1393.7 (1236.2 to 1692.7)	1193 (1097 to 1336.9)	1015.6 (937.7 to 1106.1)
Low-middle SDI	Neural tube defects	2220 (1280 to 3480)	2110 (1410 to 3150)	1800 (1330 to 2530)	144.7 (84 to 225.1)	119.8 (80.3 to 177.5)	96.3 (71.2 to 134.9)
Low-middle SDI	Congenital heart anomalies	8890 (5220 to 12200)	8220 (5610 to 10500)	7040 (5470 to 8920)	578.7 (341.7 to 788)	463.6 (317 to 592.8)	374.2 (291.1 to 473.4)
Low-middle SDI	Orofacial clefts	363 (156 to 771)	198 (119 to 342)	153 (102 to 227)	24.4 (11.6 to 49.9)	11.9 (7.3 to 20.1)	8.5 (5.7 to 12.5)
Low-middle SDI	Down's syndrome	551 (233 to 999)	568 (340 to 901)	592 (416 to 884)	37.6 (17.1 to 67)	33.1 (20.4 to 51.9)	32 (22.6 to 47.5)
Low-middle SDI	Turner syndrome	1.55 (0.752 to 2.57)	2.15 (1.05 to 3.56)	2.53 (1.26 to 4.17)	0.1 (0.1 to 0.2)	0.1 (0.1 to 0.2)	0.1 (0.1 to 0.2)
Low-middle SDI	Klinefelter syndrome	0.677 (0.317 to 1.23)	0.94 (0.436 to 1.72)	1.11 (0.522 to 2.08)	0.1 (0 to 0.1)	0.1 (0 to 0.1)	0.1 (0 to 0.1)
Low-middle SDI	Other chromosomal abnormalities	386 (179 to 745)	504 (287 to 811)	581 (371 to 850)	27 (13.4 to 49.9)	29.5 (17.1 to 46.6)	31.4 (20.2 to 45.7)

SR Appendix Table 2. All-age DALYs counts and age standardized DALYs rates per 100,000 individuals for all causes and level 1 cause groups by location for 1990, 2007, and 2017, both sexes combined

		All-age DALYs			Age-standardized rate of DALYs per 100,000		
Low-middle SDI	Congenital musculoskeletal and limb anomalies	965 (691 to 1270)	1220 (881 to 1580)	1370 (989 to 1770)	82.4 (58.8 to 108.1)	80.9 (57.7 to 104.6)	79.1 (56.8 to 102.4)
Low-middle SDI	Urogenital congenital anomalies	349 (202 to 502)	408 (264 to 548)	391 (270 to 502)	22.8 (13.5 to 32.7)	23.3 (15.2 to 31.1)	21 (14.6 to 26.9)
Low-middle SDI	Digestive congenital anomalies	1860 (1090 to 3690)	1830 (1190 to 3080)	1590 (1110 to 2420)	119.6 (70.4 to 236.2)	102.9 (66.9 to 172.6)	85.1 (59.8 to 128.8)
Low-middle SDI	Other congenital anomalies	5230 (2750 to 7680)	5650 (3620 to 7560)	5330 (3840 to 6830)	356.1 (196.8 to 513.8)	327.9 (213.5 to 435.1)	287.8 (208.5 to 367.7)
Low-middle SDI	Urinary diseases and male infertility	1460 (1310 to 1620)	1920 (1740 to 2130)	2340 (2090 to 2620)	179.6 (159.5 to 203.3)	173.7 (155.3 to 194.9)	172.8 (154 to 194.4)
Low-middle SDI	Urinary tract infections	688 (558 to 808)	995 (900 to 1150)	1310 (1200 to 1470)	79.1 (66.2 to 91.3)	85.9 (77.6 to 98.4)	94.4 (86.6 to 105.7)
Low-middle SDI	Urolithiasis	55.5 (37.5 to 69.4)	65.9 (50.1 to 83.5)	81.9 (63.3 to 103)	7.7 (5.2 to 9.7)	6.2 (4.7 to 7.9)	6 (4.6 to 7.5)
Low-middle SDI	Benign prostatic hyperplasia	226 (143 to 326)	328 (211 to 471)	439 (278 to 630)	40.3 (25.9 to 57.8)	37.9 (24.4 to 54.4)	37.3 (23.7 to 53.5)
Low-middle SDI	Male infertility	24.1 (10.1 to 49.8)	33 (13.9 to 68.3)	46.9 (19.5 to 96.5)	2.4 (1 to 5)	2.3 (0.9 to 4.7)	2.7 (1.1 to 5.4)
Low-middle SDI	Other urinary diseases	464 (337 to 577)	498 (387 to 597)	464 (386 to 544)	50 (37.2 to 60.5)	41.4 (32.6 to 49.9)	32.5 (27.3 to 38.3)
Low-middle SDI	Gynecological diseases	1340 (938 to 1850)	1950 (1350 to 2710)	2350 (1640 to 3300)	147.1 (102.7 to 204.4)	143.2 (99.4 to 199.8)	140.4 (97.2 to 196.9)
Low-middle SDI	Uterine fibroids	171 (110 to 259)	246 (155 to 381)	290 (177 to 456)	22 (14.2 to 33.3)	20.5 (13 to 31.9)	19 (11.7 to 30)
Low-middle SDI	Polycystic ovarian syndrome	54.4 (24.1 to 104)	81.8 (36.2 to 158)	101 (44.7 to 196)	5.6 (2.5 to 10.7)	5.7 (2.5 to 11)	5.8 (2.6 to 11.2)
Low-middle SDI	Female infertility	42.8 (17.2 to 88.6)	56.3 (22.6 to 118)	85.8 (33.4 to 180)	4.4 (1.7 to 9.1)	3.9 (1.5 to 8.1)	4.9 (1.9 to 10.2)
Low-middle SDI	Endometriosis	365 (243 to 526)	547 (363 to 791)	669 (444 to 966)	39 (25.8 to 56.8)	39.2 (26 to 56.5)	39.1 (26.1 to 57)
Low-middle SDI	Genital prolapse	59.6 (30.3 to 107)	76.1 (37.9 to 140)	89.3 (44.7 to 166)	8.5 (4.4 to 15.2)	7.1 (3.6 to 12.9)	6.4 (3.2 to 11.9)
Low-middle SDI	Premenstrual syndrome	503 (325 to 756)	732 (471 to 1100)	866 (560 to 1300)	51.7 (33.4 to 77.6)	51.2 (32.9 to 76.8)	49.8 (32.3 to 74.8)
Low-middle SDI	Other gynecological diseases	143 (111 to 187)	211 (162 to 284)	253 (187 to 352)	16 (12.5 to 20.7)	15.7 (12.1 to 21)	15.3 (11.4 to 21.1)
Low-middle SDI	Hemoglobinopathies and hemolytic anaemias	3320 (2510 to 4140)	3500 (2750 to 4330)	3470 (2780 to 4300)	293.3 (228.5 to 361.5)	234.9 (186.1 to 291.5)	199.9 (160 to 246.6)
Low-middle SDI	Thalassemias	315 (203 to 487)	274 (227 to 337)	237 (187 to 304)	21.5 (14 to 32.9)	15.6 (13 to 19.1)	12.4 (9.7 to 15.8)
Low-middle SDI	Thalassaemias trait	511 (344 to 737)	519 (347 to 753)	495 (330 to 721)	46.9 (31.6 to 67.5)	35.9 (24 to 52.1)	29.1 (19.5 to 42.2)
Low-middle SDI	Sickle cell disorders	1110 (705 to 1590)	1110 (782 to 1530)	1140 (839 to 1530)	81.5 (53.6 to 114.2)	65.7 (46.4 to 89.9)	60.2 (44.3 to 80.7)
Low-middle SDI	Sickle cell trait	412 (283 to 583)	499 (338 to 712)	498 (332 to 730)	36.8 (25.2 to 51.9)	33.2 (22.5 to 47.5)	28.4 (19 to 41.3)
Low-middle SDI	G6PD deficiency	142 (117 to 163)	203 (158 to 242)	221 (166 to 279)	14.7 (11.8 to 16.9)	15.1 (11.7 to 17.8)	13.5 (10.3 to 16.8)
Low-middle SDI	G6PD trait	0.0674 (0.0462 to 0.0936)	0.0912 (0.0627 to 0.127)	0.104 (0.0706 to 0.145)	0 (0 to 0)	0 (0 to 0)	0 (0 to 0)
Low-middle SDI	Other hemoglobinopathies and hemolytic anaemias	829 (621 to 1110)	892 (656 to 1210)	876 (639 to 1210)	91.9 (70.5 to 120.8)	69.4 (52.4 to 93.1)	56.3 (41.7 to 77.1)
Low-middle SDI	Endocrine, metabolic, blood, and immune disorders	1600 (1180 to 2040)	1790 (1400 to 2230)	1870 (1490 to 2320)	147.4 (111.2 to 185.7)	125.9 (99.8 to 157.1)	113.2 (91.4 to 139.2)
Low-middle SDI	Oral disorders	1610 (929 to 2550)	2530 (1470 to 3990)	3230 (1890 to 5100)	215.4 (126.5 to 337.6)	229 (134.8 to 358.5)	228.3 (134.7 to 358)
Low-middle SDI	Caries of deciduous teeth	28.7 (12.3 to 57.1)	31 (13.2 to 61.7)	33.6 (14.3 to 66.7)	2 (0.8 to 3.9)	1.8 (0.8 to 3.5)	1.7 (0.7 to 3.5)
Low-middle SDI	Caries of permanent teeth	181 (77.5 to 342)	244 (106 to 456)	280 (121 to 525)	18.7 (8.1 to 34.9)	17.6 (7.7 to 33.2)	16.7 (7.3 to 31.6)
Low-middle SDI	Periodontal disease	518 (204 to 1050)	828 (325 to 1680)	1100 (431 to 2230)	71.7 (28.5 to 146.3)	74.7 (29.6 to 151.8)	76.7 (30.3 to 155.5)
Low-middle SDI	Edentulism and severe tooth loss	415 (273 to 586)	752 (497 to 1060)	989 (651 to 1400)	72.7 (48.3 to 101.8)	84.4 (56.1 to 118)	82.4 (54.8 to 115.9)
Low-middle SDI	Other oral disorders	467 (293 to 688)	676 (424 to 999)	829 (519 to 1230)	50.4 (31.6 to 74)	50.6 (31.7 to 74.7)	50.7 (31.7 to 74.4)
Low-middle SDI	Sudden infant death syndrome	1510 (322 to 3590)	1310 (373 to 2980)	1140 (418 to 2590)	95.2 (20.3 to 226.8)	72.6 (20.7 to 165.4)	60.7 (22.3 to 138)
Low-middle SDI	Injuries	55300 (50600 to 59000)	61700 (58300 to 64800)	62600 (59000 to 66200)	5239 (4863 to 5564.8)	4409.1 (4141 to 4652.1)	3805.4 (3564.1 to 4030.6)
Low-middle SDI	Transport injuries	15700 (14000 to 17100)	18800 (17600 to 20000)	19000 (17800 to 20300)	1504.5 (1376.3 to 1621.6)	1339.1 (1254.6 to 1413.6)	1142.5 (1066.5 to 1217.3)
Low-middle SDI	Road injuries	14300 (12600 to 15600)	17000 (15800 to 18100)	17100 (15900 to 18200)	1356.9 (1241.4 to 1464.9)	1206.3 (1126.2 to 1275.7)	1023.2 (950.1 to 1086)
Low-middle SDI	Pedestrian road injuries	6260 (5270 to 7020)	6430 (5800 to 7350)	5880 (5220 to 6970)	580.9 (502.9 to 647.2)	459.4 (417.7 to 522.8)	358 (319 to 419)
Low-middle SDI	Cyclist road injuries	449 (369 to 543)	777 (654 to 924)	905 (740 to 1110)	47 (38.6 to 55.9)	58.2 (49 to 68.7)	56.4 (46 to 68.1)
Low-middle SDI	Motorcyclist road injuries	2270 (1920 to 2770)	3540 (3080 to 3900)	3850 (3210 to 4300)	225 (191.5 to 271.7)	249.6 (216.8 to 274.9)	227 (189.4 to 253)

SR Appendix Table 2. All-age DALYs counts and age standardized DALYs rates per 100,000 individuals for all causes and level 1 cause groups by location for 1990, 2007, and 2017, both sexes combined

		All-age DALYs			Age-standardized rate of DALYs per 100,000		
Low-middle SDI	Motor vehicle road injuries	5150 (3970 to 5900)	6060 (5320 to 6660)	6230 (5510 to 6770)	486.6 (396.5 to 549.8)	423.7 (373.2 to 464.5)	367.5 (323.9 to 399.3)
Low-middle SDI	Other road injuries	168 (108 to 236)	203 (163 to 251)	225 (191 to 270)	17.3 (11.7 to 23.2)	15.4 (12.6 to 18.6)	14.3 (12.1 to 17)
Low-middle SDI	Other transport injuries	1450 (1270 to 1680)	1810 (1570 to 2100)	1950 (1640 to 2350)	147.6 (129.1 to 170.2)	132.8 (115.4 to 153.5)	119.3 (100.5 to 143.7)
Low-middle SDI	Unintentional injuries	26400 (23300 to 29300)	25700 (23500 to 27800)	25200 (23000 to 27500)	2437.4 (2175 to 2668.1)	1875.7 (1704.5 to 2042.6)	1590.9 (1449 to 1742.1)
Low-middle SDI	Falls	4330 (3610 to 5030)	5640 (4930 to 6330)	6790 (6040 to 7660)	533.9 (454.3 to 607.5)	509 (447 to 569.5)	495.4 (443.9 to 556)
Low-middle SDI	Drowning	8000 (6680 to 9060)	5900 (5370 to 6400)	4640 (4290 to 5020)	607.6 (514.8 to 682.8)	365.9 (335.6 to 395.2)	257.2 (238.9 to 277.8)
Low-middle SDI	Fire, heat, and hot substances	2710 (1900 to 3280)	2480 (1920 to 2880)	2350 (1920 to 2730)	247.8 (187.1 to 293.5)	172.3 (136.4 to 198.8)	140.2 (115.3 to 162.4)
Low-middle SDI	Poisonings	879 (593 to 1130)	838 (622 to 970)	777 (582 to 861)	73.6 (51.7 to 91.8)	55.3 (41.2 to 63.3)	44.7 (33.5 to 49.5)
Low-middle SDI	Poisoning by carbon monoxide	220 (146 to 270)	226 (153 to 258)	218 (146 to 249)	20.1 (13.7 to 24)	15.6 (10.7 to 17.8)	12.9 (8.6 to 14.6)
Low-middle SDI	Poisoning by other means	659 (437 to 873)	613 (459 to 720)	558 (434 to 628)	53.4 (37.3 to 68.6)	39.7 (30 to 46)	31.9 (24.6 to 35.8)
Low-middle SDI	Exposure to mechanical forces	2250 (2030 to 2470)	2460 (2230 to 2680)	2500 (2230 to 2820)	216.3 (195.4 to 238.4)	176.8 (159.3 to 194)	152.7 (134.8 to 172.8)
Low-middle SDI	Unintentional firearm injuries	390 (325 to 502)	406 (357 to 514)	417 (368 to 510)	37 (31.3 to 45.1)	28.4 (25 to 35.4)	24.6 (21.8 to 30)
Low-middle SDI	Other exposure to mechanical forces	1860 (1630 to 2080)	2050 (1790 to 2270)	2090 (1820 to 2380)	179.3 (158.8 to 200.3)	148.4 (129.7 to 165.4)	128.1 (110.7 to 146.7)
Low-middle SDI	Adverse effects of medical treatment	1210 (795 to 1590)	1380 (1030 to 1690)	1490 (1160 to 1820)	117 (83.5 to 142.6)	103.1 (79.5 to 122)	94.6 (74.9 to 114.2)
Low-middle SDI	Animal contact	2020 (1080 to 2590)	1960 (1120 to 2310)	1840 (1110 to 2170)	193.8 (104.8 to 238.5)	140.8 (81.2 to 165.3)	112.8 (67.8 to 133.2)
Low-middle SDI	Venomous animal contact	1800 (930 to 2260)	1740 (946 to 2050)	1620 (929 to 1930)	173 (90.2 to 212)	125 (68.4 to 146.7)	99.3 (56.8 to 118.3)
Low-middle SDI	Non-venomous animal contact	220 (127 to 475)	219 (146 to 373)	222 (153 to 313)	20.8 (12.9 to 39.9)	15.8 (10.8 to 25.7)	13.6 (9.4 to 19)
Low-middle SDI	Foreign body	2040 (1570 to 2360)	1800 (1460 to 1990)	1710 (1490 to 1950)	149.7 (120.1 to 171.8)	111.7 (92.9 to 123.5)	97.1 (84.9 to 110.2)
Low-middle SDI	Pulmonary aspiration and foreign body in airway	1770 (1370 to 2100)	1550 (1250 to 1740)	1450 (1240 to 1680)	126 (100.9 to 147.5)	94.4 (77.9 to 105.4)	81.4 (70.3 to 93.6)
Low-middle SDI	Foreign body in eyes	26.2 (12 to 45.6)	37.2 (17.1 to 64.1)	45.2 (20.9 to 78)	3 (1.5 to 5)	3 (1.5 to 5)	2.9 (1.4 to 4.9)
Low-middle SDI	Foreign body in other body part	243 (166 to 326)	205 (156 to 262)	212 (169 to 264)	20.7 (15.2 to 26.4)	14.3 (11.2 to 17.9)	12.8 (10.3 to 15.7)
Low-middle SDI	Environmental heat and cold exposure	588 (407 to 715)	668 (459 to 804)	738 (505 to 910)	64.7 (46.2 to 76.9)	53.3 (36.8 to 64.4)	48.6 (33.2 to 59.7)
Low-middle SDI	Exposure to forces of nature	74 (69.6 to 79.9)	315 (271 to 375)	237 (183 to 309)	6.8 (6.4 to 7.3)	21.3 (18.5 to 25.3)	14 (10.9 to 18.1)
Low-middle SDI	Other unintentional injuries	2310 (2070 to 2530)	2280 (2080 to 2510)	2140 (1890 to 2460)	226.1 (201.6 to 250.1)	166.1 (148.5 to 185.7)	133.5 (117.1 to 155.2)
Low-middle SDI	Self-harm and interpersonal violence	13100 (11700 to 14100)	17200 (15400 to 17900)	18400 (16700 to 19200)	1297.1 (1145 to 1400.8)	1194.3 (1066.8 to 1249.3)	1072 (971.4 to 1122.4)
Low-middle SDI	Self-harm	7490 (6220 to 8360)	8440 (7260 to 8900)	8460 (7150 to 9020)	760.9 (626.1 to 850.7)	603.9 (513.7 to 638.1)	506.9 (424.6 to 540.4)
Low-middle SDI	Self-harm by firearm	416 (286 to 565)	493 (344 to 683)	468 (319 to 653)	42.4 (29.3 to 56.9)	34.9 (23.9 to 48)	27.7 (18.7 to 38)
Low-middle SDI	Self-harm by other specified means	7070 (5810 to 7940)	7950 (6760 to 8440)	8000 (6740 to 8520)	718.5 (586.3 to 808.2)	569.1 (480.6 to 604.6)	479.2 (401 to 511.8)
Low-middle SDI	Interpersonal violence	4100 (3540 to 4630)	5820 (4790 to 6240)	6090 (5160 to 6590)	396 (342.7 to 444.7)	397.4 (329.3 to 427.2)	349.9 (296.8 to 378.7)
Low-middle SDI	Assault by firearm	1100 (912 to 1390)	2220 (1690 to 2480)	2560 (2010 to 2800)	107.8 (89 to 136.5)	149.2 (113.2 to 166.6)	144.5 (113.4 to 159.4)
Low-middle SDI	Assault by sharp object	865 (613 to 1050)	1130 (796 to 1360)	1170 (832 to 1430)	87.1 (61.5 to 104.8)	78.9 (55.9 to 95.5)	68.5 (48.6 to 83.4)
Low-middle SDI	Sexual violence	226 (151 to 332)	310 (210 to 449)	377 (252 to 554)	22.6 (15.2 to 33.3)	21.7 (14.8 to 31.4)	21.8 (14.7 to 32)
Low-middle SDI	Assault by other means	1900 (1510 to 2210)	2160 (1720 to 2500)	1980 (1630 to 2370)	178.5 (142.7 to 207)	147.6 (117.8 to 171.7)	115 (94.9 to 138.2)
Low-middle SDI	Conflict and terrorism	1500 (1330 to 1750)	2820 (2630 to 3110)	3260 (3040 to 3590)	134.3 (118.5 to 157.2)	186.2 (171.7 to 207.6)	183.6 (170.1 to 203.7)
Low-middle SDI	Executions and police conflict	56.3 (41.8 to 70.4)	92.4 (79.8 to 108)	552 (536 to 573)	5.9 (4.4 to 7.4)	6.7 (5.7 to 7.9)	31.6 (30.7 to 32.9)
Middle SDI	All causes	578000 (542000 to 620000)	553000 (507000 to 608000)	585000 (529000 to 647000)	41646 (39040 to 44658.9)	31721.6 (29297 to 34664.2)	27609.1 (25073.7 to 30443.9)
Middle SDI	Communicable, maternal, neonatal, and nutritional disorders	219000 (210000 to 230000)	140000 (133000 to 149000)	100000 (93000 to 109000)	13051.3 (12483.3 to 13686.2)	8173.2 (7770.5 to 8646.3)	5334.8 (4968.5 to 5746.5)
Middle SDI	HIV/AIDS and sexually transmitted infections	3380 (2020 to 5390)	28300 (26000 to 30900)	15500 (14100 to 17100)	197.3 (122.9 to 308.3)	1444.7 (1320.8 to 1588.8)	714.8 (642.8 to 802.6)
Middle SDI	HIV/AIDS	925 (854 to 1010)	26200 (24100 to 28600)	13700 (12700 to 14800)	58.6 (53.8 to 64.5)	1315.3 (1212.3 to 1429.6)	607.3 (565.6 to 658.1)
Middle SDI	HIV/AIDS - Drug-susceptible Tuberculosis	303 (249 to 352)	5140 (3360 to 6850)	2330 (1570 to 3220)	19.2 (15.8 to 22.4)	264.2 (173.6 to 349.7)	104 (70 to 143.3)

SR Appendix Table 2. All-age DALYs counts and age standardized DALYs rates per 100,000 individuals for all causes and level 1 cause groups by location for 1990, 2007, and 2017, both sexes combined

		All-age DALYs			Age-standardized rate of DALYs per 100,000		
Middle SDI	HIV/AIDS - Multidrug-resistant Tuberculosis without extensive drug resistance	6.79 (2.83 to 14.6)	653 (281 to 1310)	222 (112 to 382)	0.4 (0.2 to 0.9)	33.7 (14.4 to 68.5)	9.9 (5 to 17.1)
Middle SDI	HIV/AIDS - Extensively drug-resistant Tuberculosis	0 (0 to 0)	17.7 (10 to 28.4)	15.8 (8.6 to 26.4)	0 (0 to 0)	0.9 (0.5 to 1.4)	0.7 (0.4 to 1.2)
Middle SDI	HIV/AIDS resulting in other diseases	616 (550 to 700)	20400 (17600 to 23300)	11100 (9800 to 12400)	39 (34.6 to 44.5)	1016.6 (884.8 to 1159.3)	492.8 (435.4 to 551.5)
Middle SDI	Sexually transmitted infections excluding HIV	2460 (1100 to 4460)	2100 (1010 to 3670)	1820 (910 to 3040)	138.7 (64 to 248.3)	129.4 (59.6 to 231.8)	107.5 (50.5 to 184.5)
Middle SDI	Syphilis	2140 (823 to 4150)	1700 (638 to 3270)	1390 (529 to 2580)	117.9 (45.9 to 227.7)	110.1 (41.1 to 212.6)	88.6 (33.4 to 165.3)
Middle SDI	Chlamydial infection	84.6 (52.5 to 143)	105 (63.8 to 182)	109 (66.2 to 188)	5.3 (3.3 to 8.9)	5 (3 to 8.6)	4.8 (2.9 to 8.3)
Middle SDI	Gonococcal infection	64.6 (45.7 to 97.9)	68.6 (47.3 to 108)	69.5 (47.7 to 110)	4.1 (3 to 6)	3.3 (2.3 to 5.2)	3.1 (2.1 to 5)
Middle SDI	Trichomoniasis	41.5 (16.7 to 90.1)	57.5 (23.1 to 124)	65.2 (26.1 to 142)	2.8 (1.1 to 6)	2.8 (1.1 to 6.1)	2.8 (1.1 to 6.1)
Middle SDI	Genital herpes	40.4 (13.5 to 95.4)	59.7 (19.4 to 142)	70.9 (22.8 to 170)	3 (1 to 7.2)	3 (1 to 7.3)	3 (1 to 7.3)
Middle SDI	Other sexually transmitted infections	87.7 (58.1 to 140)	109 (70.7 to 176)	113 (73.8 to 183)	5.6 (3.8 to 8.8)	5.2 (3.3 to 8.3)	5 (3.2 to 8.1)
Middle SDI	Respiratory infections and tuberculosis	74200 (70300 to 78100)	34000 (32800 to 35500)	25500 (24200 to 27100)	4632.9 (4401.6 to 4853.2)	2037.1 (1966.4 to 2123.8)	1311.5 (1240.9 to 1391.5)
Middle SDI	Tuberculosis	16000 (15100 to 16900)	11100 (10700 to 11500)	8450 (7980 to 8940)	1211 (1142 to 1285.8)	606.2 (584.9 to 629.3)	379.1 (357.8 to 400.3)
Middle SDI	Latent tuberculosis infection	0 (0 to 0)	0 (0 to 0)	0 (0 to 0)	0 (0 to 0)	0 (0 to 0)	0 (0 to 0)
Middle SDI	Drug-susceptible tuberculosis	15600 (14700 to 16600)	9930 (9380 to 10500)	7620 (6960 to 8210)	1186.5 (1113.9 to 1261.6)	542.4 (512.5 to 571.8)	341.9 (312.6 to 368.3)
Middle SDI	Multidrug-resistant tuberculosis without extensive drug resistance	326 (126 to 691)	1070 (775 to 1440)	726 (375 to 1190)	24.5 (9.5 to 51.3)	58.3 (42.3 to 78.3)	32.4 (16.8 to 52.8)
Middle SDI	Extensively drug-resistant tuberculosis	0 (0 to 0)	100 (74.6 to 134)	108 (71.5 to 158)	0 (0 to 0)	5.5 (4 to 7.4)	4.8 (3.2 to 7.1)
Middle SDI	Lower respiratory infections	55800 (52400 to 59500)	20800 (20000 to 21600)	14900 (14300 to 15900)	3272.5 (3076.6 to 3471.9)	1317 (1271.2 to 1370.6)	824.7 (788.3 to 879.5)
Middle SDI	Upper respiratory infections	1850 (1260 to 2630)	1600 (988 to 2480)	1650 (994 to 2590)	115.6 (79.2 to 163.5)	85.8 (53.8 to 131.8)	81.7 (49.1 to 127.5)
Middle SDI	Otitis media	560 (352 to 864)	524 (321 to 821)	507 (311 to 802)	33.8 (21.4 to 51.9)	28.1 (17.3 to 44)	26 (15.8 to 41.1)
Middle SDI	Enteric infections	28500 (25200 to 31900)	13100 (11300 to 15600)	9800 (8170 to 12200)	1722.2 (1514.7 to 1951.2)	794.9 (691 to 938.6)	526.4 (443.9 to 643.3)
Middle SDI	Diarrhoeal diseases	25100 (22300 to 28200)	10900 (9370 to 13200)	8230 (6700 to 10600)	1534.5 (1332 to 1759)	675 (581.3 to 812.9)	440.5 (366.3 to 552.5)
Middle SDI	Typhoid and paratyphoid	3100 (1850 to 4840)	2040 (1190 to 3230)	1460 (850 to 2340)	174.9 (105.1 to 272.3)	111.4 (65.7 to 175.6)	79.4 (46.4 to 127.1)
Middle SDI	Typhoid fever	2790 (1650 to 4340)	1820 (1060 to 2860)	1260 (713 to 2010)	157.2 (93.3 to 244.4)	99.8 (58.4 to 156.1)	68.4 (39.1 to 110.5)
Middle SDI	Paratyphoid fever	313 (144 to 616)	212 (98.6 to 412)	201 (92.1 to 386)	17.7 (8.2 to 34.8)	11.6 (5.4 to 22.6)	11 (5.1 to 21.2)
Middle SDI	Invasive non-typhoidal salmonella	174 (98.3 to 295)	135 (75.7 to 231)	110 (59.7 to 191)	10.2 (5.8 to 17.2)	7.7 (4.4 to 13.1)	6 (3.3 to 10.5)
Middle SDI	Other intestinal infectious diseases	45.6 (35.6 to 57)	14.1 (9.26 to 20.1)	8.15 (5.2 to 12.1)	2.6 (2 to 3.2)	0.9 (0.5 to 1.2)	0.5 (0.3 to 0.7)
Middle SDI	Neglected tropical diseases and malaria	6820 (5150 to 8950)	5260 (4270 to 6440)	4730 (3810 to 5800)	439.3 (334 to 570.2)	287.3 (235.6 to 347.7)	232.7 (188.9 to 283.9)
Middle SDI	Malaria	1400 (906 to 1870)	1080 (868 to 1500)	816 (634 to 1150)	83.6 (54.5 to 110.6)	61.7 (49.3 to 84)	43.5 (33.4 to 60.7)
Middle SDI	Chagas disease	158 (150 to 168)	109 (101 to 119)	109 (99.2 to 123)	14.4 (13.6 to 15.3)	6.5 (6 to 7.1)	4.9 (4.5 to 5.5)
Middle SDI	Leishmaniasis	371 (5.84 to 1870)	70.8 (11.5 to 318)	46.4 (16.1 to 134)	22.5 (0.4 to 109.9)	3.9 (0.6 to 17.3)	2.4 (0.8 to 7.1)
Middle SDI	Visceral leishmaniasis	366 (1.31 to 1860)	58.5 (2.04 to 306)	23 (0.0519 to 111)	22.2 (0.1 to 109.4)	3.2 (0.1 to 16.7)	1.2 (0 to 5.9)
Middle SDI	Cutaneous and mucocutaneous leishmaniasis	5.6 (3.09 to 9.47)	12.3 (8.19 to 17.4)	23.5 (14.7 to 34.8)	0.4 (0.2 to 0.6)	0.7 (0.4 to 0.9)	1.2 (0.7 to 1.8)
Middle SDI	African trypanosomiasis	14.1 (6.61 to 25.5)	7.04 (2.96 to 13.7)	2.25 (0.0771 to 11.4)	0.9 (0.4 to 1.6)	0.4 (0.2 to 0.7)	0.1 (0 to 0.6)
Middle SDI	Schistosomiasis	146 (108 to 210)	136 (84.1 to 226)	127 (75.9 to 214)	11.5 (8.7 to 16.2)	7.1 (4.5 to 11.8)	5.6 (3.4 to 9.4)
Middle SDI	Cysticercosis	448 (311 to 598)	502 (333 to 697)	549 (356 to 771)	34.2 (23.8 to 45.5)	26.6 (17.6 to 36.8)	24.2 (15.5 to 33.8)
Middle SDI	Cystic echinococcosis	20.4 (13.7 to 29.8)	18.9 (10.6 to 32.8)	19.6 (10.3 to 35.5)	1.4 (1 to 2)	1 (0.6 to 1.6)	0.9 (0.5 to 1.6)
Middle SDI	Lymphatic filariasis	502 (263 to 835)	437 (233 to 725)	173 (94.1 to 281)	35.4 (18.7 to 58.8)	22 (11.7 to 36.6)	7.6 (4.1 to 12.4)
Middle SDI	Onchocerciasis	11.6 (7.85 to 16.1)	4.25 (2.73 to 6.31)	2.83 (1.5 to 4.89)	0.8 (0.6 to 1.1)	0.2 (0.1 to 0.3)	0.1 (0.1 to 0.2)
Middle SDI	Trachoma	65.5 (43.6 to 89.2)	52.8 (35.5 to 73.6)	58.1 (38 to 82.9)	7.9 (5.3 to 10.8)	3.7 (2.5 to 5.1)	2.9 (1.9 to 4.1)
Middle SDI	Dengue	766 (333 to 1430)	1040 (553 to 1330)	1310 (823 to 1700)	43.9 (19.8 to 80.9)	60.4 (32.1 to 77.2)	69.8 (43.3 to 89.9)

SR Appendix Table 2. All-age DALYs counts and age standardized DALYs rates per 100,000 individuals for all causes and level 1 cause groups by location for 1990, 2007, and 2017, both sexes combined

		All-age DALYs			Age-standardized rate of DALYs per 100,000		
Middle SDI	Yellow fever	21.3 (4.55 to 61.5)	9.71 (2.11 to 28.9)	5.84 (1.27 to 17)	1.2 (0.3 to 3.6)	0.5 (0.1 to 1.5)	0.3 (0.1 to 0.9)
Middle SDI	Rabies	344 (279 to 431)	196 (158 to 212)	91.1 (70.8 to 102)	21.4 (17.5 to 26.6)	10.6 (8.4 to 11.5)	4.4 (3.5 to 5)
Middle SDI	Intestinal nematode infections	1360 (829 to 2130)	533 (319 to 846)	354 (204 to 585)	83.6 (50.9 to 131.6)	27.8 (16.8 to 44)	17.2 (10 to 28.4)
Middle SDI	Ascariasis	742 (467 to 1160)	282 (169 to 460)	153 (87.1 to 256)	44.9 (28 to 70.8)	15 (9.1 to 24.3)	7.7 (4.5 to 12.8)
Middle SDI	Trichuriasis	229 (128 to 383)	94.9 (53.3 to 159)	82.2 (45.9 to 136)	13.6 (7.6 to 22.8)	4.7 (2.7 to 7.9)	3.9 (2.2 to 6.5)
Middle SDI	Hookworm disease	388 (226 to 624)	156 (91 to 252)	119 (69.6 to 199)	25.1 (14.7 to 40.4)	8 (4.7 to 12.9)	5.6 (3.3 to 9.3)
Middle SDI	Food-borne trematodiases	675 (320 to 1270)	728 (399 to 1260)	781 (427 to 1350)	44.9 (21.7 to 82.7)	36.3 (19.9 to 62.7)	34.1 (18.8 to 58.5)
Middle SDI	Leprosy	6.41 (4.23 to 9.04)	8.67 (5.81 to 12.4)	9 (6.04 to 12.7)	0.6 (0.4 to 0.8)	0.5 (0.3 to 0.7)	0.4 (0.3 to 0.6)
Middle SDI	Ebola virus disease	0 (0 to 0)	0 (0 to 0)	0 (0 to 0)	0 (0 to 0)	0 (0 to 0)	0 (0 to 0)
Middle SDI	Zika virus disease	0 (0 to 0)	0 (0 to 0)	1.15 (0.659 to 2.22)	0 (0 to 0)	0 (0 to 0)	0.1 (0 to 0.1)
Middle SDI	Guinea worm disease	0 (0 to 0)	0 (0 to 0)	0 (0 to 0)	0 (0 to 0)	0 (0 to 0)	0 (0 to 0)
Middle SDI	Other neglected tropical diseases	510 (361 to 682)	328 (239 to 450)	279 (203 to 383)	31 (22 to 41.5)	18.1 (13.2 to 24.6)	14.2 (10.3 to 19.4)
Middle SDI	Other infectious diseases	25300 (20600 to 32100)	9410 (8300 to 10800)	6610 (5880 to 7440)	1461.7 (1197.6 to 1832.8)	555.9 (488.4 to 645)	356.2 (313.7 to 405.5)
Middle SDI	Meningitis	5720 (5080 to 6150)	2750 (2480 to 2940)	1730 (1550 to 1930)	326.5 (290.5 to 351.2)	162.7 (147 to 173.5)	95 (85.4 to 104.6)
Middle SDI	Pneumococcal meningitis	533 (473 to 601)	275 (244 to 308)	185 (163 to 211)	31.1 (27.7 to 34.9)	15.9 (14.1 to 17.7)	9.7 (8.6 to 11)
Middle SDI	H influenzae type B meningitis	2140 (1840 to 2360)	958 (858 to 1050)	604 (545 to 694)	122.5 (105.2 to 134.7)	57.2 (51.6 to 62.8)	33.4 (30 to 37.9)
Middle SDI	Meningococcal infection	492 (428 to 552)	220 (198 to 244)	127 (114 to 142)	27.9 (24.4 to 31.2)	13.2 (11.8 to 14.7)	7.1 (6.3 to 8)
Middle SDI	Other meningitis	2550 (2240 to 2810)	1290 (1160 to 1400)	816 (719 to 896)	145 (127.8 to 159.4)	76.4 (68.5 to 82.7)	44.7 (39.6 to 49.3)
Middle SDI	Encephalitis	1890 (1460 to 2400)	1470 (1390 to 1540)	1270 (1080 to 1540)	113.9 (88.8 to 141.9)	85.8 (81 to 90.2)	68 (57.5 to 82.3)
Middle SDI	Diphtheria	81 (68.3 to 97)	13.4 (11.7 to 15.2)	8.08 (6.83 to 9.91)	4.5 (3.8 to 5.4)	0.8 (0.7 to 0.9)	0.5 (0.4 to 0.6)
Middle SDI	Whooping cough	3530 (1240 to 7590)	932 (384 to 1860)	511 (215 to 1040)	193 (67.8 to 415.2)	59.5 (24.5 to 118.7)	32.1 (13.5 to 65.2)
Middle SDI	Tetanus	3490 (2640 to 4270)	539 (414 to 610)	229 (144 to 316)	207.6 (155.6 to 253.1)	31.8 (25.3 to 36.1)	11.9 (7.9 to 17.1)
Middle SDI	Measles	6870 (3030 to 13900)	1260 (491 to 2690)	700 (267 to 1450)	375 (165.5 to 760.7)	79.9 (31.1 to 169.7)	43.8 (16.7 to 90.5)
Middle SDI	Varicella and herpes zoster	331 (293 to 379)	215 (180 to 262)	184 (144 to 239)	21.5 (19.1 to 24.4)	12.8 (10.9 to 15.4)	9.4 (7.5 to 12.1)
Middle SDI	Acute hepatitis	1930 (1380 to 2180)	1290 (979 to 1410)	1130 (867 to 1260)	127.8 (91.8 to 143.5)	69.2 (52.7 to 75.6)	52.5 (40.8 to 59)
Middle SDI	Acute hepatitis A	504 (353 to 605)	318 (238 to 384)	243 (185 to 302)	28.5 (20.1 to 34.5)	17 (12.7 to 20.3)	12.5 (9.6 to 15.4)
Middle SDI	Acute hepatitis B	1110 (784 to 1300)	775 (581 to 874)	731 (560 to 833)	80.7 (57.3 to 93.1)	42.2 (31.8 to 47.3)	32.4 (24.8 to 36.9)
Middle SDI	Acute hepatitis C	106 (57.1 to 176)	34.3 (19.5 to 57.1)	22.3 (12.5 to 38)	6 (3.3 to 10)	2.2 (1.2 to 3.6)	1.3 (0.7 to 2.2)
Middle SDI	Acute hepatitis E	207 (139 to 269)	158 (109 to 202)	135 (91.3 to 172)	12.6 (8.7 to 16.4)	7.9 (5.5 to 9.9)	6.3 (4.3 to 8)
Middle SDI	Other unspecified infectious diseases	1480 (1030 to 1720)	947 (774 to 1130)	843 (710 to 1000)	91.8 (65.3 to 106.8)	53.4 (43.5 to 63.1)	42.9 (36 to 50.9)
Middle SDI	Maternal and neonatal disorders	63600 (60400 to 67300)	39300 (37300 to 41800)	29000 (26700 to 31700)	3517.4 (3342.6 to 3719.8)	2441.7 (2320.4 to 2574.2)	1721.5 (1601.7 to 1863.2)
Middle SDI	Maternal disorders	3430 (3260 to 3630)	1830 (1760 to 1910)	1200 (1140 to 1270)	203.1 (193.1 to 214.6)	86.5 (83.3 to 90)	54.4 (51.6 to 57.6)
Middle SDI	Maternal haemorrhage	1580 (1420 to 1750)	454 (387 to 523)	209 (175 to 251)	94.1 (84.8 to 104.5)	21.4 (18.3 to 24.8)	9.5 (7.9 to 11.4)
Middle SDI	Maternal sepsis and other pregnancy related infections	307 (245 to 383)	147 (117 to 186)	88.1 (71.3 to 110)	17.9 (14.4 to 22.2)	7 (5.5 to 8.8)	4 (3.2 to 5)
Middle SDI	Maternal hypertensive disorders	430 (351 to 529)	324 (278 to 382)	256 (222 to 300)	24.9 (20.5 to 30.5)	15.3 (13.1 to 18.1)	11.8 (10.2 to 13.8)
Middle SDI	Maternal obstructed labour and uterine rupture	164 (114 to 240)	95.7 (70.8 to 135)	69.7 (52.6 to 97.2)	9.8 (6.8 to 14.3)	4.5 (3.3 to 6.4)	3.1 (2.4 to 4.4)
Middle SDI	Maternal abortive outcome	241 (194 to 298)	152 (125 to 186)	111 (90.4 to 137)	14.8 (11.9 to 18.3)	7.2 (6 to 8.8)	5 (4.1 to 6.1)
Middle SDI	Ectopic pregnancy	116 (70.6 to 188)	85.5 (54.7 to 132)	60.9 (39 to 96.3)	6.9 (4.3 to 10.9)	4 (2.6 to 6.2)	2.8 (1.8 to 4.4)
Middle SDI	Indirect maternal deaths	286 (223 to 368)	221 (178 to 271)	133 (109 to 165)	16.6 (13 to 21.1)	10.5 (8.4 to 12.9)	6.1 (5 to 7.5)
Middle SDI	Late maternal deaths	42.2 (33.6 to 53.7)	34 (27.9 to 42.1)	36.1 (29.2 to 45.3)	2.5 (2 to 3.1)	1.6 (1.3 to 2)	1.7 (1.3 to 2.1)

SR Appendix Table 2. All-age DALYs counts and age standardized DALYs rates per 100,000 individuals for all causes and level 1 cause groups by location for 1990, 2007, and 2017, both sexes combined

		All-age DALYs			Age-standardized rate of DALYs per 100,000		
Middle SDI	Maternal deaths aggravated by HIV/AIDS	0.583<br>(0.316 to 0.881)	16.3<br>(12.3 to 19.7)	5.67<br>(3.89 to 7.16)	0<br>(0 to 0.1)	0.8<br>(0.6 to 0.9)	0.3<br>(0.2 to 0.3)
Middle SDI	Other maternal disorders	265<br>(206 to 338)	302<br>(256 to 358)	227<br>(191 to 268)	15.7<br>(12.3 to 19.9)	14.1<br>(11.9 to 16.8)	10.2<br>(8.6 to 12.1)
Middle SDI	Neonatal disorders	60200<br>(57000 to 63900)	37500<br>(35500 to 39900)	27800<br>(25500 to 30500)	3314.3<br>(3138.6 to 3519.1)	2355.2<br>(2235.4 to 2488.7)	1667.1<br>(1547.8 to 1810.1)
Middle SDI	Neonatal preterm birth	30500<br>(28100 to 32500)	16800<br>(15700 to 18100)	12600<br>(11300 to 14200)	1676.9<br>(1548.9 to 1791.2)	1053.9<br>(986.2 to 1131.7)	754.9<br>(682.4 to 834.9)
Middle SDI	Neonatal encephalopathy due to birth asphyxia and trauma	16500<br>(15000 to 18900)	10900<br>(9620 to 12300)	7700<br>(6440 to 9370)	913<br>(828.9 to 1041.6)	673.3<br>(602.3 to 747)	448.6<br>(385.5 to 530.6)
Middle SDI	Neonatal sepsis and other neonatal infections	4290<br>(3550 to 5970)	4130<br>(3590 to 5120)	3410<br>(2870 to 4300)	237.6<br>(197 to 329.5)	258.3<br>(227.7 to 316.4)	205.1<br>(176.2 to 256.5)
Middle SDI	Hemolytic disease and other neonatal jaundice	1900<br>(1700 to 2120)	863<br>(790 to 931)	590<br>(517 to 665)	104.9<br>(93.9 to 116.8)	53.6<br>(49.2 to 57.7)	34.4<br>(30.4 to 38.4)
Middle SDI	Other neonatal disorders	6980<br>(6280 to 7980)	4850<br>(4460 to 5300)	3470<br>(3180 to 3710)	381.8<br>(343.2 to 436.5)	316.1<br>(290.6 to 345.9)	224.1<br>(205.9 to 239.6)
Middle SDI	Nutritional deficiencies	17700<br>(13700 to 23000)	10800<br>(7990 to 14700)	9160<br>(6590 to 12600)	1080.3<br>(839.9 to 1405.4)	611.6<br>(455.9 to 824.5)	471.5<br>(342.2 to 647.5)
Middle SDI	Protein-energy malnutrition	5690<br>(5200 to 6160)	2110<br>(1940 to 2300)	1530<br>(1380 to 1710)	335.9<br>(307.9 to 362.8)	133.6<br>(122.9 to 144.9)	88.1<br>(78.9 to 98.5)
Middle SDI	Iodine deficiency	366<br>(216 to 562)	311<br>(183 to 515)	396<br>(233 to 648)	24.3<br>(14.3 to 37.4)	16<br>(9.4 to 26.5)	18.3<br>(10.8 to 29.9)
Middle SDI	Dietary iron deficiency	7940<br>(5360 to 11400)	6400<br>(4310 to 9240)	5710<br>(3860 to 8350)	508.4<br>(343.3 to 731.8)	340.2<br>(229.7 to 489.9)	276.6<br>(187.3 to 404.7)
Middle SDI	Other nutritional deficiencies	650<br>(530 to 765)	320<br>(289 to 358)	261<br>(228 to 297)	43.7<br>(35.3 to 53)	19.3<br>(17.6 to 21.7)	13.1<br>(11.6 to 14.9)
Middle SDI	Non-communicable diseases	282000<br>(255000 to 313000)	347000<br>(310000 to 392000)	418000<br>(374000 to 471000)	23763.7<br>(21727.1 to 26164.9)	20155.5<br>(18147.4 to 22534.3)	19196.8<br>(17165.6 to 21576.4)
Middle SDI	Neoplasms	38700<br>(37300 to 40600)	49600<br>(48700 to 50500)	63600<br>(61500 to 65600)	3360.2<br>(3263 to 3516.5)	2860.8<br>(2809.5 to 2911.7)	2801.1<br>(2710.7 to 2887.5)
Middle SDI	Lip and oral cavity cancer	600<br>(575 to 626)	928<br>(905 to 950)	1330<br>(1250 to 1400)	54.7<br>(52.4 to 57)	52.6<br>(51.3 to 53.8)	57.1<br>(53.3 to 60)
Middle SDI	Nasopharynx cancer	669<br>(625 to 715)	637<br>(609 to 668)	781<br>(732 to 835)	55<br>(51.5 to 58.8)	34.6<br>(33.1 to 36.3)	33<br>(30.9 to 35.2)
Middle SDI	Other pharynx cancer	295<br>(255 to 319)	460<br>(408 to 503)	692<br>(617 to 783)	26.7<br>(23.2 to 28.9)	25.8<br>(23 to 28.3)	29.2<br>(25.9 to 33)
Middle SDI	Oesophageal cancer	2740<br>(2610 to 2880)	3060<br>(2970 to 3150)	3180<br>(3020 to 3350)	268.7<br>(256.8 to 282.8)	185.3<br>(180.4 to 190.8)	140.1<br>(133.1 to 147.5)
Middle SDI	Stomach cancer	5250<br>(5060 to 5540)	5450<br>(5330 to 5600)	5970<br>(5750 to 6200)	500<br>(482.1 to 527.6)	324.9<br>(317.6 to 333.6)	262.8<br>(253.1 to 272.9)
Middle SDI	Colon and rectum cancer	1930<br>(1820 to 2110)	3070<br>(3010 to 3150)	4410<br>(4190 to 4610)	181.6<br>(171.5 to 197.7)	182.7<br>(178.9 to 187.3)	194.9<br>(185.3 to 203.4)
Middle SDI	Liver cancer	5010<br>(4660 to 5320)	6500<br>(6250 to 6710)	8100<br>(7610 to 8560)	438.8<br>(408.7 to 465.6)	364.9<br>(350.7 to 376.3)	344.7<br>(323.9 to 363.6)
Middle SDI	Liver cancer due to hepatitis B	2980<br>(2720 to 3200)	3650<br>(3440 to 3830)	4260<br>(3910 to 4600)	249<br>(226.7 to 267.3)	197.6<br>(186.2 to 207.4)	177.8<br>(163.5 to 192.4)
Middle SDI	Liver cancer due to hepatitis C	789<br>(717 to 874)	1120<br>(1020 to 1210)	1510<br>(1380 to 1650)	77.3<br>(70.5 to 85.6)	67.7<br>(62.3 to 73)	66.6<br>(60.9 to 72.5)
Middle SDI	Liver cancer due to alcohol use	525<br>(429 to 647)	807<br>(673 to 983)	1100<br>(913 to 1350)	48.8<br>(40.2 to 59.8)	46.4<br>(38.8 to 55.9)	47.1<br>(39.3 to 57.3)
Middle SDI	Liver cancer due to NASH	249<br>(218 to 282)	377<br>(334 to 419)	537<br>(470 to 603)	23.7<br>(20.8 to 26.8)	22.6<br>(20 to 25)	23.7<br>(20.9 to 26.4)
Middle SDI	Liver cancer due to other causes	469<br>(408 to 527)	553<br>(491 to 613)	691<br>(611 to 776)	40<br>(35 to 44.8)	30.7<br>(27.3 to 33.9)	29.4<br>(26.1 to 32.9)
Middle SDI	Gallbladder and biliary tract cancer	466<br>(428 to 555)	651<br>(558 to 713)	880<br>(721 to 948)	46.3<br>(42.8 to 55.7)	39.5<br>(33.9 to 43.3)	38.9<br>(32 to 42)
Middle SDI	Pancreatic cancer	620<br>(602 to 640)	1170<br>(1140 to 1190)	1850<br>(1760 to 1920)	59.7<br>(58 to 61.5)	69.8<br>(68.2 to 71.3)	81.1<br>(77.2 to 84.3)
Middle SDI	Larynx cancer	498<br>(478 to 524)	624<br>(604 to 660)	827<br>(791 to 892)	46.8<br>(45 to 49.2)	36.3<br>(35.2 to 38.4)	35.5<br>(34 to 38.2)
Middle SDI	Tracheal, bronchus, and lung cancer	4980<br>(4780 to 5300)	8030<br>(7840 to 8270)	11500<br>(10900 to 12100)	472.3<br>(453.8 to 501.5)	478.6<br>(467.8 to 492.3)	503.9<br>(478.9 to 530.3)
Middle SDI	Malignant skin melanoma	114<br>(92.5 to 148)	166<br>(141 to 200)	220<br>(180 to 257)	9.6<br>(7.9 to 12.4)	9.1<br>(7.7 to 10.9)	9.6<br>(7.8 to 11.2)
Middle SDI	Non-melanoma skin cancer	188<br>(176 to 201)	294<br>(285 to 304)	408<br>(394 to 422)	18.2<br>(17 to 19.4)	17.9<br>(17.4 to 18.5)	18.5<br>(17.9 to 19.1)
Middle SDI	Non-melanoma skin cancer (squamous-cell carcinoma)	188<br>(176 to 201)	294<br>(285 to 304)	408<br>(394 to 422)	18.2<br>(17 to 19.4)	17.9<br>(17.4 to 18.5)	18.5<br>(17.9 to 19.1)
Middle SDI	Non-melanoma skin cancer (basal-cell carcinoma)	0.107<br>(0.0417 to 0.23)	0.189<br>(0.0748 to 0.4)	0.289<br>(0.11 to 0.607)	0<br>(0 to 0)	0<br>(0 to 0)	0<br>(0 to 0)
Middle SDI	Breast cancer	1960<br>(1790 to 2220)	3230<br>(3050 to 3340)	4430<br>(4000 to 4720)	170.4<br>(156.8 to 192.3)	174.5<br>(165 to 180.5)	185.9<br>(167.2 to 197.9)
Middle SDI	Cervical cancer	1610<br>(1510 to 1870)	2080<br>(1900 to 2150)	2460<br>(2010 to 2580)	138.9<br>(130.4 to 160.5)	111.4<br>(101.6 to 115.5)	102.9<br>(84.4 to 108.1)
Middle SDI	Uterine cancer	375<br>(350 to 398)	469<br>(452 to 487)	529<br>(502 to 560)	34.2<br>(32.1 to 36.3)	26.7<br>(25.8 to 27.8)	22.6<br>(21.5 to 23.9)
Middle SDI	Ovarian cancer	398<br>(372 to 448)	744<br>(718 to 786)	1140<br>(1090 to 1190)	34.2<br>(32 to 38.4)	41<br>(39.6 to 43.1)	48<br>(46 to 50.1)
Middle SDI	Prostate cancer	589<br>(505 to 697)	1060<br>(901 to 1240)	1530<br>(1330 to 1840)	70.3<br>(60.5 to 83.6)	72.9<br>(61.9 to 85.8)	74.4<br>(64.6 to 89.1)

SR Appendix Table 2. All-age DALYs counts and age standardized DALYs rates per 100,000 individuals for all causes and level 1 cause groups by location for 1990, 2007, and 2017, both sexes combined

		All-age DALYs			Age-standardized rate of DALYs per 100,000		
Middle SDI	Testicular cancer	68.2 (63.3 to 76)	89 (86.2 to 91.9)	104 (98.8 to 110)	4.4 (4.1 to 4.9)	4.4 (4.2 to 4.5)	4.7 (4.5 to 5)
Middle SDI	Kidney cancer	294 (274 to 320)	440 (408 to 453)	620 (576 to 661)	23.5 (22.2 to 25.5)	25.6 (23.8 to 26.4)	27.8 (26.1 to 29.6)
Middle SDI	Bladder cancer	338 (298 to 358)	479 (452 to 512)	670 (632 to 735)	35.5 (31.3 to 37.5)	30.5 (28.7 to 32.5)	30.8 (29 to 33.9)
Middle SDI	Thyroid cancer	149 (140 to 172)	228 (218 to 251)	315 (295 to 347)	13.2 (12.4 to 15.4)	13.1 (12.5 to 14.3)	13.8 (13 to 15.3)
Middle SDI	Mesothelioma	61.2 (52.1 to 75.4)	92.1 (88.4 to 97.6)	122 (116 to 128)	5.2 (4.5 to 6.4)	5.1 (4.9 to 5.3)	5.2 (5 to 5.5)
Middle SDI	Hodgkin lymphoma	351 (246 to 402)	252 (209 to 289)	241 (198 to 276)	24.2 (17.1 to 27.6)	13.3 (11 to 15.2)	10.9 (9 to 12.6)
Middle SDI	Non-Hodgkin's lymphoma	1020 (962 to 1070)	1230 (1210 to 1260)	1650 (1580 to 1720)	75.4 (72.1 to 78.7)	68.6 (67.1 to 70)	73.8 (70.7 to 76.8)
Middle SDI	Multiple myeloma	206 (181 to 224)	323 (295 to 349)	475 (425 to 506)	18.9 (16.8 to 20.4)	18.8 (17.2 to 20.3)	20.7 (18.5 to 22)
Middle SDI	Leukaemia	3840 (2980 to 4500)	3320 (2890 to 3460)	3400 (2970 to 3580)	244.5 (194.9 to 281.7)	182 (159.1 to 190.2)	165 (145.2 to 174.4)
Middle SDI	Acute lymphoid leukaemia	687 (579 to 924)	824 (736 to 898)	916 (771 to 988)	41 (34.9 to 54.5)	45.2 (40.5 to 49.3)	47.1 (39.7 to 50.9)
Middle SDI	Chronic lymphoid leukaemia	60 (48.8 to 70)	84.4 (73.2 to 92.7)	130 (110 to 143)	5 (4.2 to 5.7)	4.8 (4.2 to 5.3)	5.8 (4.9 to 6.4)
Middle SDI	Acute myeloid leukaemia	420 (339 to 590)	581 (512 to 652)	705 (616 to 765)	28.1 (23.3 to 38.2)	31.5 (27.8 to 35.2)	33.3 (29.2 to 36.2)
Middle SDI	Chronic myeloid leukaemia	111 (97 to 129)	128 (113 to 141)	138 (120 to 148)	8.7 (7.7 to 10.1)	6.9 (6.1 to 7.5)	6 (5.3 to 6.5)
Middle SDI	Other leukaemia	2560 (1790 to 2990)	1700 (1380 to 1800)	1510 (1260 to 1640)	161.7 (118.4 to 185.8)	93.6 (76.6 to 99.1)	72.7 (61.4 to 79.5)
Middle SDI	Other malignant cancers	2060 (1820 to 2180)	2160 (2080 to 2310)	2720 (2560 to 2840)	151.8 (138.4 to 158.5)	120.9 (116.5 to 129.7)	125.3 (119 to 131.5)
Middle SDI	Other neoplasms	294 (235 to 331)	480 (390 to 539)	682 (576 to 799)	21.2 (17.1 to 24.2)	26.9 (21.8 to 30.3)	31.8 (27 to 37.2)
Middle SDI	Myelodysplastic, myeloproliferative, and other hematopoietic neoplasms	252 (203 to 288)	416 (338 to 473)	603 (510 to 709)	18.7 (15.2 to 21.3)	23.6 (19.2 to 26.8)	27.8 (23.6 to 32.7)
Middle SDI	Benign and in situ intestinal neoplasms	0 (0 to 0)	0 (0 to 0)	0 (0 to 0)	0 (0 to 0)	0 (0 to 0)	0 (0 to 0)
Middle SDI	Benign and in situ cervical and uterine neoplasms	0 (0 to 0)	0 (0 to 0)	0 (0 to 0)	0 (0 to 0)	0 (0 to 0)	0 (0 to 0)
Middle SDI	Other benign and in situ neoplasms	41.9 (29.6 to 53.7)	63.9 (48.4 to 72.6)	79.5 (62.1 to 92.5)	2.5 (1.8 to 3.2)	3.4 (2.5 to 3.8)	4 (3.1 to 4.7)
Middle SDI	Cardiovascular diseases	62800 (61100 to 64800)	81500 (79300 to 83800)	105000 (102000 to 109000)	6280.6 (6112.1 to 6496.1)	5090.5 (4956.8 to 5229.8)	4865.9 (4709 to 5028.7)
Middle SDI	Rheumatic heart disease	4030 (3810 to 4340)	2730 (2530 to 2980)	2480 (2250 to 2760)	324.4 (306.6 to 353.8)	151.2 (141.1 to 163.7)	112.6 (102.6 to 125)
Middle SDI	Ischaemic heart disease	23100 (22400 to 24200)	34500 (33800 to 35300)	46400 (45200 to 47700)	2369.6 (2301.4 to 2483.5)	2150.8 (2109.4 to 2200.1)	2132.1 (2078.5 to 2190.5)
Middle SDI	Stroke	27300 (26300 to 28900)	34500 (33400 to 35700)	43300 (41400 to 45200)	2767 (2667.4 to 2950.6)	2179.5 (2109.2 to 2249.5)	1994.4 (1906.4 to 2080.7)
Middle SDI	Ischaemic stroke	7630 (7050 to 8360)	10700 (9970 to 11400)	15400 (14200 to 16600)	867.1 (804.1 to 952.1)	721 (675.5 to 766.2)	739.5 (683.8 to 793.3)
Middle SDI	Intracerebral hemorrhage	15000 (14200 to 17100)	20600 (20100 to 21300)	24000 (23000 to 25000)	1478.3 (1390.8 to 1690.4)	1270.7 (1237.5 to 1311)	1079.3 (1034.7 to 1126.8)
Middle SDI	Subarachnoid hemorrhage	4640 (3360 to 5340)	3270 (3020 to 3680)	3970 (3620 to 4340)	421.5 (289.7 to 490.2)	187.8 (172.5 to 211)	175.6 (159.8 to 191.6)
Middle SDI	Hypertensive heart disease	3870 (2670 to 4280)	3670 (3220 to 3920)	5570 (4120 to 6080)	428.9 (298.4 to 476.3)	240.6 (211.8 to 256.6)	265.6 (195.4 to 289.9)
Middle SDI	Non-rheumatic valvular heart disease	238 (215 to 270)	348 (313 to 376)	435 (393 to 481)	23 (20.9 to 25.8)	21.6 (19.2 to 23.5)	20.4 (18.3 to 22.5)
Middle SDI	Non-rheumatic calcific aortic valve disease	91.5 (75.1 to 116)	151 (132 to 170)	216 (191 to 237)	8.9 (7.4 to 11)	9.3 (8.1 to 10.4)	10 (8.8 to 11)
Middle SDI	Non-rheumatic degenerative mitral valve disease	115 (96.6 to 135)	147 (124 to 167)	169 (140 to 199)	11.1 (9.3 to 13)	9.1 (7.7 to 10.5)	7.9 (6.6 to 9.4)
Middle SDI	Other non-rheumatic valve diseases	31.7 (22.1 to 39.1)	50 (32.8 to 58.3)	50.7 (37 to 66.5)	3 (2 to 3.7)	3.2 (2 to 3.7)	2.4 (1.8 to 3.2)
Middle SDI	Cardiomyopathy and myocarditis	1220 (1120 to 1350)	1570 (1470 to 1680)	1790 (1590 to 1930)	89.8 (81.9 to 99.1)	92 (85.9 to 97.9)	84.9 (75.7 to 90.9)
Middle SDI	Myocarditis	405 (324 to 471)	430 (360 to 485)	442 (378 to 518)	26.7 (21.4 to 30.9)	25 (20.8 to 28.1)	21.9 (18.8 to 25.4)
Middle SDI	Alcoholic cardiomyopathy	102 (87.7 to 119)	171 (151 to 193)	229 (188 to 279)	8.7 (7.4 to 10)	9.3 (8.2 to 10.4)	9.7 (8 to 11.7)
Middle SDI	Other cardiomyopathy	716 (655 to 779)	973 (902 to 1040)	1120 (987 to 1210)	54.4 (49.9 to 59.6)	57.8 (53.7 to 61.4)	53.3 (47.2 to 57.6)
Middle SDI	Atrial fibrillation and flutter	511 (424 to 624)	920 (766 to 1110)	1390 (1160 to 1670)	66.4 (55.9 to 79.4)	66.7 (56.3 to 79.3)	70.4 (59.7 to 83.5)
Middle SDI	Aortic aneurysm	299 (274 to 345)	444 (419 to 480)	608 (549 to 660)	29.7 (27.1 to 33.7)	27.2 (25.6 to 29.3)	27.7 (25 to 30.1)
Middle SDI	Peripheral vascular disease	91.2 (52.6 to 146)	163 (101 to 253)	246 (158 to 368)	11 (6.3 to 17.8)	11.3 (6.9 to 17.4)	12 (7.6 to 17.8)
Middle SDI	Endocarditis	414 (385 to 449)	463 (435 to 502)	547 (500 to 600)	28.8 (26.8 to 31.4)	26.2 (24.6 to 28.3)	25.6 (23.5 to 28.2)

SR Appendix Table 2. All-age DALYs counts and age standardized DALYs rates per 100,000 individuals for all causes and level 1 cause groups by location for 1990, 2007, and 2017, both sexes combined							
		All-age DALYs			Age-standardized rate of DALYs per 100,000		
Middle SDI	Other cardiovascular and circulatory diseases	1680 (1490 to 1930)	2100 (1830 to 2410)	2620 (2280 to 3020)	142.1 (125.3 to 163.5)	123.4 (108.2 to 141)	120.3 (105 to 138)
Middle SDI	Chronic respiratory diseases	27600 (25400 to 29200)	27300 (25600 to 29000)	30300 (28200 to 32500)	2773.5 (2561.7 to 2911.9)	1734.7 (1631.6 to 1829.8)	1443.8 (1343.9 to 1547.5)
Middle SDI	Chronic obstructive pulmonary disease	20900 (19000 to 22200)	20700 (19500 to 21700)	23200 (21700 to 24600)	2247 (2039.8 to 2376.4)	1355 (1283.2 to 1416.6)	1102.9 (1036.4 to 1164.5)
Middle SDI	Pneumoconiosis	169 (134 to 216)	157 (147 to 170)	189 (173 to 207)	15.7 (12.5 to 20.6)	9.3 (8.7 to 10)	8.3 (7.6 to 9.1)
Middle SDI	Silicosis	103 (75.3 to 137)	89.3 (81.9 to 103)	117 (98.8 to 130)	9.4 (6.9 to 12.7)	5.2 (4.8 to 6)	5 (4.3 to 5.6)
Middle SDI	Asbestosis	7.88 (5.28 to 11.3)	11.1 (8.02 to 13.8)	12.8 (9.92 to 17.5)	0.7 (0.5 to 1.1)	0.7 (0.5 to 0.8)	0.6 (0.4 to 0.8)
Middle SDI	Coal workers pneumoconiosis	33.6 (16.7 to 61)	28.9 (22.2 to 34.9)	28.9 (23.8 to 38.5)	3.3 (1.7 to 6)	1.8 (1.4 to 2.2)	1.3 (1.1 to 1.7)
Middle SDI	Other pneumoconiosis	24.8 (16.1 to 34)	27.9 (24.1 to 32.8)	30.6 (26.6 to 39.4)	2.2 (1.5 to 3.1)	1.6 (1.4 to 1.9)	1.4 (1.2 to 1.7)
Middle SDI	Asthma	5580 (4510 to 6720)	5070 (4130 to 6150)	5170 (4150 to 6410)	438.1 (346.7 to 522.8)	291.6 (238 to 351.4)	251.3 (199.1 to 314.9)
Middle SDI	Interstitial lung disease and pulmonary sarcoidosis	246 (196 to 315)	471 (402 to 543)	714 (625 to 811)	23.8 (19.1 to 30.2)	28.9 (24.7 to 33.3)	32.9 (28.9 to 37.2)
Middle SDI	Other chronic respiratory diseases	711 (615 to 811)	965 (854 to 1070)	1060 (933 to 1190)	48.8 (42.5 to 55.4)	50 (44.5 to 55.4)	48.5 (42.8 to 54)
Middle SDI	Digestive diseases	17900 (16500 to 19400)	19300 (17800 to 21100)	21400 (19600 to 23800)	1445.1 (1324.6 to 1555.1)	1075.8 (999.9 to 1173.2)	955.2 (874.6 to 1059.7)
Middle SDI	Cirrhosis and other chronic liver diseases	8410 (7520 to 8890)	9880 (9520 to 10400)	11200 (10700 to 12400)	697 (618.6 to 738.4)	538 (516.6 to 566.1)	480.3 (457.8 to 533)
Middle SDI	Cirrhosis and other chronic liver diseases due to hepatitis B	3030 (2560 to 3310)	3310 (3070 to 3610)	3490 (3180 to 4020)	250.6 (210.9 to 275.3)	178.7 (165.7 to 194.4)	149.5 (136.1 to 172.1)
Middle SDI	Cirrhosis and other chronic liver diseases due to hepatitis C	2210 (1920 to 2420)	2710 (2490 to 2940)	3090 (2820 to 3460)	187.8 (163.3 to 206.1)	146.8 (135.1 to 158.4)	130.9 (119.8 to 145.6)
Middle SDI	Cirrhosis and other chronic liver diseases due to alcohol use	1590 (1420 to 1730)	2080 (1910 to 2280)	2570 (2340 to 2910)	138.2 (123.7 to 151)	113.2 (104.1 to 123.9)	108.3 (98.7 to 122.5)
Middle SDI	Cirrhosis due to NASH	576 (512 to 627)	829 (769 to 892)	1060 (976 to 1160)	51.3 (45.5 to 55.8)	46.2 (42.9 to 49.5)	44.9 (41.6 to 49)
Middle SDI	Cirrhosis and other chronic liver diseases due to other causes	1010 (895 to 1190)	949 (877 to 1040)	965 (879 to 1070)	69.1 (61.7 to 78.7)	53.1 (49.2 to 58)	46.7 (42.5 to 51.5)
Middle SDI	Upper digestive system diseases	4030 (3270 to 5120)	4500 (3420 to 5990)	4940 (3700 to 6710)	335.6 (276.8 to 419)	249.6 (193.1 to 326.9)	219.5 (165.9 to 296.4)
Middle SDI	Peptic ulcer disease	1760 (1620 to 1880)	1420 (1340 to 1510)	1370 (1280 to 1500)	160.6 (146.8 to 171.2)	84.6 (79.6 to 89.8)	62.4 (58.4 to 68.2)
Middle SDI	Gastritis and duodenitis	1340 (975 to 1800)	1730 (1240 to 2330)	1930 (1380 to 2600)	105.1 (77.3 to 138.8)	95.1 (69.2 to 126.8)	86 (61.8 to 115.8)
Middle SDI	GERD	932 (497 to 1590)	1360 (728 to 2320)	1640 (873 to 2770)	69.8 (37.3 to 118.4)	69.9 (37.4 to 118.1)	71.1 (38.1 to 120.6)
Middle SDI	Appendicitis	506 (465 to 562)	386 (362 to 418)	353 (326 to 384)	34.2 (31.5 to 37.3)	21.2 (19.9 to 22.8)	16.9 (15.6 to 18.4)
Middle SDI	Paralytic ileus and intestinal obstruction	1940 (1730 to 2330)	1430 (1350 to 1640)	1390 (1250 to 1550)	129.6 (116.5 to 155.2)	88.1 (83.5 to 100.7)	72 (64.9 to 78.9)
Middle SDI	Inguinal, femoral, and abdominal hernia	756 (580 to 954)	818 (612 to 1050)	935 (704 to 1200)	57.7 (44.9 to 71.5)	46.9 (35.7 to 59.9)	44.8 (33.8 to 57.6)
Middle SDI	Inflammatory bowel disease	306 (220 to 392)	333 (273 to 395)	386 (313 to 468)	24.4 (18.1 to 29.4)	19.1 (15.5 to 22.3)	17.3 (14.2 to 20.7)
Middle SDI	Vascular intestinal disorders	154 (133 to 167)	225 (208 to 247)	289 (272 to 328)	16 (13.9 to 17.3)	14.6 (13.4 to 16)	13.8 (12.9 to 15.6)
Middle SDI	Gallbladder and biliary diseases	481 (407 to 511)	479 (447 to 506)	558 (535 to 603)	45.8 (38.6 to 48.5)	29.6 (27.5 to 31.3)	26 (24.9 to 28.1)
Middle SDI	Pancreatitis	491 (405 to 550)	640 (545 to 706)	779 (664 to 868)	40.1 (33 to 44.7)	34.8 (29.7 to 38.4)	34.2 (29.1 to 38.2)
Middle SDI	Other digestive diseases	862 (766 to 979)	569 (508 to 634)	645 (569 to 732)	64.8 (58.4 to 72)	33.9 (30.4 to 37.6)	30.6 (26.9 to 34.5)
Middle SDI	Neurological disorders	16300 (12500 to 20900)	22400 (17300 to 28500)	27400 (21500 to 34400)	1355 (1093.3 to 1659.7)	1308.8 (1045.1 to 1618.9)	1324.7 (1052.3 to 1645.7)
Middle SDI	Alzheimer's disease and other dementias	2830 (2640 to 3030)	4940 (4600 to 5330)	7370 (6850 to 7910)	437.1 (407.6 to 466.8)	406 (377.3 to 436.7)	409.4 (380.6 to 440)
Middle SDI	Parkinson's disease	586 (531 to 651)	1010 (919 to 1110)	1470 (1330 to 1620)	74.5 (67.7 to 82.5)	72.3 (65.9 to 79.3)	73.8 (67.4 to 81.1)
Middle SDI	Epilepsy	3320 (2630 to 4140)	3340 (2570 to 4310)	3480 (2600 to 4570)	207.3 (163 to 258.2)	175.8 (134.8 to 226.6)	167.8 (125.4 to 220.9)
Middle SDI	Multiple sclerosis	69.6 (59.8 to 88.8)	107 (96.3 to 120)	142 (125 to 158)	5.6 (4.8 to 7.1)	5.5 (5 to 6.2)	5.9 (5.2 to 6.6)
Middle SDI	Motor neuron disease	54.9 (47.2 to 78.4)	74.2 (69.5 to 89.4)	111 (104 to 132)	4 (3.5 to 5.5)	4.2 (3.9 to 5)	5 (4.6 to 5.9)
Middle SDI	Headache disorders	8750 (5680 to 12500)	12200 (7940 to 17400)	13900 (9050 to 19900)	584.2 (380.2 to 834.1)	604.9 (394.2 to 863.5)	619 (402.2 to 883.6)
Middle SDI	Migraine	7580 (4760 to 11100)	10600 (6650 to 15600)	12100 (7620 to 17700)	504.8 (316.2 to 741.9)	524.5 (328.3 to 768.2)	537.8 (337.5 to 785.3)
Middle SDI	Tension-type headache	1160 (648 to 1880)	1590 (898 to 2560)	1810 (1030 to 2900)	79.4 (44.9 to 127.3)	80.4 (45.4 to 128.9)	81.2 (45.9 to 130.2)
Middle SDI	Other neurological disorders	681 (521 to 842)	748 (599 to 927)	890 (705 to 1130)	42.4 (33.3 to 51.9)	40.2 (32.4 to 49.7)	43.8 (34.4 to 55.6)

SR Appendix Table 2. All-age DALYs counts and age standardized DALYs rates per 100,000 individuals for all causes and level 1 cause groups by location for 1990, 2007, and 2017, both sexes combined

		All-age DALYs			Age-standardized rate of DALYs per 100,000		
Middle SDI	Mental disorders	21200 (15800 to 27600)	28300 (21100 to 36600)	31700 (23600 to 40900)	1450.1 (1082.4 to 1872.2)	1440.9 (1073.7 to 1864.7)	1426.6 (1063.9 to 1843.3)
Middle SDI	Schizophrenia	2240 (1670 to 2760)	3240 (2410 to 4010)	3780 (2800 to 4650)	161.5 (120.4 to 198.7)	160.8 (120.2 to 198.3)	160.6 (119.3 to 197.6)
Middle SDI	Depressive disorders	7090 (5010 to 9720)	9530 (6740 to 13000)	10900 (7730 to 14900)	515.4 (365.6 to 703.9)	496.3 (351.7 to 677.7)	481.4 (341.2 to 658.6)
Middle SDI	Major depressive disorder	5380 (3720 to 7410)	7000 (4890 to 9500)	7880 (5530 to 10600)	382.5 (268.3 to 519.9)	363.3 (254.2 to 492.7)	349.4 (245.3 to 473.6)
Middle SDI	Dysthymia	1710 (1140 to 2490)	2530 (1690 to 3720)	3050 (2040 to 4480)	132.9 (89.2 to 195.2)	133 (89.2 to 195.5)	132.1 (89 to 193.6)
Middle SDI	Bipolar disorder	1510 (950 to 2220)	2120 (1330 to 3130)	2420 (1530 to 3580)	100.7 (63.5 to 149.2)	105 (66 to 155.7)	107.9 (67.9 to 158.8)
Middle SDI	Anxiety disorders	4550 (3210 to 6140)	6130 (4300 to 8180)	6770 (4810 to 9030)	306.4 (216.1 to 410.9)	311.4 (219.1 to 416.3)	307 (217.1 to 409.8)
Middle SDI	Eating disorders	442 (281 to 651)	676 (431 to 988)	824 (524 to 1210)	26 (16.6 to 37.9)	31.9 (20.4 to 46.6)	37.8 (24.2 to 55)
Middle SDI	Anorexia nervosa	103 (62 to 155)	146 (88.2 to 219)	169 (104 to 252)	5.8 (3.5 to 8.6)	7 (4.2 to 10.4)	8.1 (4.9 to 12)
Middle SDI	Bulimia nervosa	339 (213 to 506)	530 (332 to 787)	655 (414 to 971)	20.3 (12.6 to 30.1)	24.9 (15.7 to 37)	29.7 (18.8 to 43.9)
Middle SDI	Autism spectrum disorders	871 (591 to 1210)	1050 (719 to 1460)	1130 (773 to 1570)	54.4 (37 to 75.3)	55.1 (37.7 to 76.4)	55.3 (37.7 to 76.6)
Middle SDI	Attention-deficit/hyperactivity disorder	212 (129 to 339)	234 (142 to 370)	230 (141 to 365)	12.3 (7.4 to 19.5)	11.8 (7.1 to 18.7)	11.4 (7 to 18.2)
Middle SDI	Conduct disorder	1560 (939 to 2480)	1680 (1010 to 2710)	1600 (957 to 2560)	83 (50.2 to 132.9)	85.8 (51.5 to 137.2)	88.1 (52.9 to 141)
Middle SDI	Idiopathic developmental intellectual disability	893 (441 to 1510)	891 (429 to 1530)	826 (382 to 1430)	54.2 (26.7 to 91.9)	46.4 (22.4 to 79.5)	40.7 (18.9 to 70.6)
Middle SDI	Other mental disorders	1880 (1230 to 2600)	2700 (1780 to 3720)	3150 (2080 to 4340)	136.2 (89.8 to 187.8)	136.4 (90.1 to 188)	136.4 (90.2 to 188.2)
Middle SDI	Substance use disorders	7620 (6000 to 9340)	8950 (6960 to 11100)	10200 (7960 to 12600)	499.3 (397 to 607.3)	439 (342.2 to 543.7)	447.4 (347.7 to 553.7)
Middle SDI	Alcohol use disorders	2410 (1890 to 3060)	3690 (2870 to 4670)	4200 (3300 to 5290)	170.4 (133.9 to 212.9)	182.7 (142.6 to 229.9)	180.3 (140.9 to 226.7)
Middle SDI	Drug use disorders	5210 (4040 to 6470)	5260 (3940 to 6720)	6010 (4480 to 7640)	329 (257.1 to 406)	256.3 (192.4 to 326.7)	267.1 (199 to 339.8)
Middle SDI	Opioid use disorders	3730 (2780 to 4760)	4050 (2950 to 5230)	4730 (3420 to 6130)	239.1 (180 to 303.7)	198.1 (144.5 to 255.7)	208.4 (150.4 to 271.2)
Middle SDI	Cocaine use disorders	99.4 (69 to 137)	119 (83.6 to 164)	143 (102 to 195)	6.2 (4.4 to 8.7)	5.8 (4.1 to 7.9)	6.4 (4.6 to 8.8)
Middle SDI	Amphetamine use disorders	469 (273 to 711)	400 (231 to 620)	395 (229 to 618)	25.8 (15.2 to 38.9)	18.9 (11 to 29.3)	18.7 (10.7 to 29.5)
Middle SDI	Cannabis use disorders	103 (64.4 to 156)	125 (78.8 to 185)	128 (81.6 to 190)	6 (3.8 to 9)	5.9 (3.8 to 8.7)	5.9 (3.8 to 8.8)
Middle SDI	Other drug use disorders	809 (621 to 1080)	564 (427 to 735)	608 (471 to 771)	51.7 (40.5 to 68.4)	27.5 (21.1 to 35.6)	27.6 (21.2 to 35.2)
Middle SDI	Diabetes and kidney diseases	15800 (14000 to 17900)	25400 (22300 to 29100)	34000 (29900 to 39000)	1387.1 (1241.7 to 1567.3)	1463.2 (1290.7 to 1668.6)	1501.2 (1321.5 to 1717.3)
Middle SDI	Diabetes mellitus	8710 (7290 to 10500)	15900 (13200 to 19300)	21900 (18200 to 26300)	798.2 (676.1 to 953.3)	916.3 (764.3 to 1101.3)	957.2 (795.4 to 1143.1)
Middle SDI	Diabetes mellitus type 1	2160 (2020 to 2380)	2740 (2610 to 2920)	3140 (2960 to 3350)	185.7 (172.6 to 206.9)	152.1 (144.5 to 162.7)	136.4 (128.5 to 145.1)
Middle SDI	Diabetes mellitus type 2	6550 (5100 to 8250)	13200 (10400 to 16400)	18800 (15000 to 23100)	612.6 (486.4 to 761.7)	764.2 (611.7 to 943.9)	820.8 (658.8 to 1007.9)
Middle SDI	Chronic kidney disease	6770 (6410 to 7160)	9350 (8890 to 9810)	12000 (11300 to 12600)	569 (537.1 to 603.2)	539.2 (512.6 to 565.4)	537.6 (507.6 to 566.5)
Middle SDI	Chronic kidney disease due to diabetes mellitus type 1	661 (540 to 796)	951 (778 to 1150)	1150 (931 to 1390)	51.6 (42.2 to 62.4)	48.3 (39.7 to 58.2)	47.3 (38.7 to 56.9)
Middle SDI	Chronic kidney disease due to diabetes mellitus type 2	1160 (996 to 1320)	2050 (1800 to 2320)	2930 (2550 to 3330)	118.4 (102.6 to 134.7)	127.1 (112 to 143.3)	131.7 (114.7 to 149.3)
Middle SDI	Chronic kidney disease due to hypertension	1080 (933 to 1220)	1770 (1540 to 2000)	2480 (2140 to 2820)	110.1 (96.1 to 124.3)	111 (96.8 to 124.9)	115 (99.6 to 130.2)
Middle SDI	Chronic kidney disease due to glomerulonephritis	1360 (1190 to 1550)	1730 (1520 to 1950)	2050 (1780 to 2340)	100.4 (88.6 to 113.4)	93.6 (82.7 to 105.8)	91 (79.9 to 102.9)
Middle SDI	Chronic kidney disease due to other and unspecified causes	2510 (2240 to 2800)	2840 (2530 to 3210)	3350 (2950 to 3810)	188.4 (167.8 to 212.1)	159.2 (141.4 to 178.8)	152.6 (134.5 to 172.7)
Middle SDI	Acute glomerulonephritis	277 (253 to 304)	139 (133 to 145)	140 (130 to 151)	19.9 (18.2 to 21.8)	7.7 (7.4 to 8.1)	6.4 (6 to 6.9)
Middle SDI	Skin and subcutaneous diseases	8730 (5780 to 12900)	10400 (6930 to 15300)	11500 (7700 to 16900)	557.3 (373 to 821.1)	559.3 (373.3 to 823.2)	566.7 (379.4 to 830.1)
Middle SDI	Dermatitis	2130 (1240 to 3400)	2430 (1420 to 3840)	2610 (1540 to 4130)	130.5 (76.1 to 206.9)	132 (76.9 to 208.9)	133.7 (77.5 to 213)
Middle SDI	Atopic dermatitis	1770 (951 to 2970)	1950 (1060 to 3270)	2070 (1130 to 3470)	105.7 (57 to 177)	107.5 (58.3 to 180.4)	109.5 (59.2 to 183.5)
Middle SDI	Contact dermatitis	329 (214 to 494)	441 (286 to 656)	497 (325 to 741)	22.6 (14.7 to 33.6)	22.5 (14.7 to 33.7)	22.5 (14.7 to 33.5)
Middle SDI	Seborrhoeic dermatitis	33.3 (19.2 to 52.4)	37.9 (21.6 to 59.4)	39.2 (22.6 to 61.8)	2.2 (1.3 to 3.5)	2 (1.1 to 3.1)	1.8 (1 to 2.8)
Middle SDI	Psoriasis	635 (448 to 840)	1000 (706 to 1330)	1240 (881 to 1650)	45.7 (32.4 to 60.2)	51.9 (36.7 to 69)	55.2 (39.1 to 73.2)

SR Appendix Table 2. All-age DALYs counts and age standardized DALYs rates per 100,000 individuals for all causes and level 1 cause groups by location for 1990, 2007, and 2017, both sexes combined

		All-age DALYs			Age-standardized rate of DALYs per 100,000		
Middle SDI	Bacterial skin diseases	339 (202 to 406)	383 (281 to 510)	504 (370 to 690)	24.5 (15.1 to 29)	22.5 (16.5 to 30.1)	24.3 (17.8 to 33.3)
Middle SDI	Cellulitis	69.1 (39.4 to 91)	94.5 (64.3 to 132)	138 (93 to 207)	4.8 (2.9 to 6.2)	5.4 (3.6 to 7.5)	6.5 (4.4 to 9.7)
Middle SDI	Pyoderma	270 (150 to 329)	288 (199 to 381)	366 (257 to 502)	19.6 (11.4 to 23.7)	17.1 (11.9 to 22.6)	17.8 (12.6 to 24.5)
Middle SDI	Scabies	1560 (860 to 2550)	1680 (928 to 2740)	1700 (941 to 2780)	94.9 (52.7 to 154.4)	87 (48 to 141.6)	82.9 (45.9 to 135.2)
Middle SDI	Fungal skin diseases	823 (324 to 1700)	974 (388 to 2030)	1090 (434 to 2260)	58.5 (23.2 to 122)	54.8 (21.9 to 114.1)	52.6 (21.1 to 109.8)
Middle SDI	Viral skin diseases	1040 (666 to 1550)	1050 (677 to 1570)	1050 (677 to 1570)	60.3 (38.5 to 89.8)	58.3 (37.3 to 87)	57.3 (36.6 to 85.3)
Middle SDI	Acne vulgaris	353 (208 to 569)	533 (315 to 855)	610 (364 to 975)	19.3 (11.4 to 30.8)	25.5 (15 to 41)	30.6 (18.2 to 48.9)
Middle SDI	Alopecia areata	89.8 (57.4 to 134)	119 (75.7 to 177)	131 (84.2 to 196)	5.9 (3.8 to 8.8)	5.9 (3.8 to 8.8)	5.9 (3.8 to 8.8)
Middle SDI	Pruritus	134 (62.3 to 255)	185 (87.8 to 351)	219 (104 to 414)	9.6 (4.5 to 18.1)	9.9 (4.6 to 18.7)	10.1 (4.7 to 19)
Middle SDI	Urticaria	1130 (743 to 1610)	1280 (849 to 1810)	1360 (903 to 1920)	68.4 (45.1 to 96.6)	69 (45.6 to 97.5)	69.2 (45.7 to 97.5)
Middle SDI	Decubitus ulcer	72.8 (52.5 to 99.6)	114 (86.7 to 160)	153 (117 to 217)	7.6 (5.5 to 10.3)	7.4 (5.7 to 10.4)	7.5 (5.8 to 10.6)
Middle SDI	Other skin and subcutaneous diseases	425 (217 to 768)	638 (320 to 1160)	817 (411 to 1480)	32.2 (16.3 to 58)	35.1 (17.7 to 63.6)	37.5 (18.8 to 67.9)
Middle SDI	Sense organ diseases	10800 (7220 to 15700)	16000 (10700 to 23200)	20000 (13400 to 29100)	981.4 (663.9 to 1399.8)	949.7 (640.6 to 1359.8)	923.5 (620.1 to 1329.2)
Middle SDI	Blindness and vision impairment	5130 (3430 to 7650)	7500 (5010 to 11100)	9310 (6270 to 13800)	475.2 (322.7 to 691.4)	448.5 (305.3 to 656.2)	427.5 (290 to 628.7)
Middle SDI	Glaucoma	97.1 (65.3 to 137)	149 (102 to 210)	198 (134 to 279)	11.1 (7.5 to 15.6)	10.1 (6.8 to 14)	9.3 (6.4 to 13)
Middle SDI	Cataract	1140 (789 to 1540)	1870 (1290 to 2520)	2440 (1700 to 3300)	137.3 (95.8 to 185.1)	128.5 (89.5 to 172.9)	118.9 (82.3 to 161)
Middle SDI	Age-related macular degeneration	50.2 (34.3 to 68.9)	97.2 (67.2 to 134)	128 (87.6 to 178)	5.8 (4 to 8)	6.6 (4.6 to 9.1)	6.1 (4.2 to 8.4)
Middle SDI	Refraction disorders	1590 (1040 to 2300)	1990 (1340 to 2830)	2320 (1550 to 3320)	120.7 (80.6 to 173.1)	110.9 (75.6 to 157.3)	107.4 (71.8 to 153.3)
Middle SDI	Near vision loss	1720 (818 to 3130)	2640 (1260 to 4830)	3320 (1580 to 6070)	151.3 (72.2 to 277.3)	147.2 (70.2 to 270.6)	144.1 (68.8 to 264.4)
Middle SDI	Other vision loss	538 (375 to 732)	760 (532 to 1030)	914 (639 to 1230)	48.9 (34.2 to 66.1)	45.2 (31.6 to 61.1)	41.7 (29.1 to 56.2)
Middle SDI	Age-related and other hearing loss	5310 (3590 to 7620)	7980 (5380 to 11500)	10100 (6740 to 14400)	476 (325.6 to 672.7)	470.3 (318.6 to 666.9)	464.7 (315.3 to 661.1)
Middle SDI	Other sense organ diseases	357 (222 to 524)	534 (331 to 781)	682 (420 to 996)	30.2 (18.8 to 44.3)	30.9 (19.2 to 45.1)	31.3 (19.3 to 45.7)
Middle SDI	Musculoskeletal disorders	20100 (14800 to 26500)	29600 (21800 to 39200)	36100 (26500 to 47700)	1592.3 (1174.1 to 2095.5)	1564.5 (1147.9 to 2065.6)	1561.7 (1150.2 to 2059.7)
Middle SDI	Rheumatoid arthritis	437 (346 to 535)	651 (504 to 805)	870 (663 to 1100)	39.4 (31.4 to 47.9)	37.4 (29.4 to 45.7)	38.4 (29.3 to 48)
Middle SDI	Osteoarthritis	974 (484 to 1940)	1790 (892 to 3580)	2460 (1230 to 4910)	96.7 (48.2 to 191.3)	106.1 (52.9 to 210.5)	107.5 (53.7 to 213.4)
Middle SDI	Low back pain	8920 (6320 to 12100)	12200 (8740 to 16600)	14800 (10500 to 19900)	689.4 (490.8 to 930.3)	645 (463.4 to 867.9)	648.8 (466.2 to 871.9)
Middle SDI	Neck pain	4310 (3020 to 6060)	6640 (4610 to 9390)	8080 (5610 to 11400)	351.8 (244.7 to 494.5)	350 (243.1 to 492.6)	343.7 (238.6 to 483.5)
Middle SDI	Gout	130 (86.9 to 180)	219 (146 to 302)	297 (199 to 407)	12.1 (8.1 to 16.7)	12.6 (8.4 to 17.2)	13 (8.7 to 17.8)
Middle SDI	Other musculoskeletal disorders	5310 (3750 to 7230)	8030 (5680 to 11000)	9590 (6720 to 13100)	402.9 (286.4 to 549.7)	413.4 (292.5 to 564.9)	410.5 (288 to 559.1)
Middle SDI	Other non-communicable diseases	34100 (30500 to 38700)	28700 (24500 to 34000)	26800 (22000 to 32900)	2081.7 (1840.8 to 2398.5)	1668.3 (1446.7 to 1948.9)	1379 (1160.2 to 1649.3)
Middle SDI	Congenital anomalies	22400 (20600 to 24800)	15400 (14200 to 16600)	12000 (10800 to 13100)	1250.8 (1148.7 to 1380.1)	949.7 (875.9 to 1016)	702.8 (640.3 to 759.7)
Middle SDI	Neural tube defects	2200 (1560 to 2780)	871 (756 to 995)	706 (614 to 804)	121.7 (87 to 153.7)	53.8 (46.7 to 61.5)	41.4 (36.3 to 46.7)
Middle SDI	Congenital heart anomalies	11000 (8500 to 13200)	7130 (6160 to 7740)	4900 (4300 to 5310)	603.1 (467.5 to 726.4)	448.7 (386.4 to 488.3)	301.6 (264 to 327.4)
Middle SDI	Orofacial clefts	412 (230 to 719)	143 (112 to 181)	111 (80.9 to 152)	23.2 (13.1 to 40)	8.4 (6.7 to 10.5)	5.8 (4.3 to 7.8)
Middle SDI	Down's syndrome	688 (399 to 908)	489 (440 to 555)	412 (364 to 494)	38.5 (22.7 to 50.4)	29.4 (26.5 to 33.7)	23.4 (20.8 to 28.2)
Middle SDI	Turner syndrome	1.68 (0.825 to 2.78)	2.01 (1 to 3.31)	2 (1 to 3.28)	0.1 (0 to 0.2)	0.1 (0.1 to 0.2)	0.1 (0 to 0.2)
Middle SDI	Klinefelter syndrome	1.18 (0.556 to 2.24)	1.52 (0.707 to 2.86)	1.54 (0.715 to 2.91)	0.1 (0 to 0.1)	0.1 (0 to 0.1)	0.1 (0 to 0.1)
Middle SDI	Other chromosomal abnormalities	334 (227 to 528)	389 (315 to 491)	396 (326 to 499)	19.1 (13 to 29.8)	23.7 (19.3 to 30.4)	23 (19.2 to 29.2)
Middle SDI	Congenital musculoskeletal and limb anomalies	1110 (808 to 1410)	1220 (855 to 1620)	1290 (885 to 1730)	68.8 (49.5 to 88.6)	66.6 (47 to 87.3)	63.8 (44.4 to 85.2)
Middle SDI	Urogenital congenital anomalies	305 (235 to 405)	244 (204 to 303)	206 (181 to 252)	17.1 (13.2 to 22.6)	14.9 (12.5 to 18.7)	12 (10.5 to 14.7)

SR Appendix Table 2. All-age DALYs counts and age standardized DALYs rates per 100,000 individuals for all causes and level 1 cause groups by location for 1990, 2007, and 2017, both sexes combined

		All-age DALYs			Age-standardized rate of DALYs per 100,000		
Middle SDI	Digestive congenital anomalies	1820 (1280 to 2700)	1230 (1060 to 1460)	909 (814 to 1050)	100.9 (71.3 to 148.9)	78 (66.8 to 92.3)	55.5 (50 to 63.5)
Middle SDI	Other congenital anomalies	4590 (3330 to 5990)	3710 (3140 to 4330)	3040 (2640 to 3490)	258.3 (188.2 to 335.5)	226.2 (191.8 to 263.8)	176 (154.6 to 200.1)
Middle SDI	Urinary diseases and male infertility	1340 (1200 to 1510)	1680 (1500 to 1910)	2170 (1910 to 2470)	111.5 (98.9 to 126.8)	98.7 (88 to 112.1)	99.7 (88.2 to 113.4)
Middle SDI	Urinary tract infections	655 (563 to 714)	849 (801 to 894)	1140 (1090 to 1210)	50.3 (43.7 to 54.6)	49.4 (46.8 to 52.2)	53.6 (51 to 56.8)
Middle SDI	Urolithiasis	132 (85.3 to 159)	125 (105 to 148)	147 (120 to 178)	11.3 (7.4 to 13.4)	7 (6 to 8.3)	6.5 (5.3 to 7.8)
Middle SDI	Benign prostatic hyperplasia	249 (158 to 361)	376 (242 to 543)	535 (342 to 769)	26 (16.6 to 37.7)	23.5 (15.1 to 33.9)	23.8 (15.3 to 34.2)
Middle SDI	Male infertility	32.8 (13.4 to 68.5)	45.2 (18.4 to 94.1)	48.1 (19.6 to 99.2)	2 (0.8 to 4.3)	2.1 (0.9 to 4.3)	2.1 (0.9 to 4.4)
Middle SDI	Other urinary diseases	272 (229 to 340)	284 (254 to 324)	294 (253 to 332)	21.8 (18.7 to 27.1)	16.7 (15 to 19)	13.8 (11.9 to 15.5)
Middle SDI	Gynecological diseases	2240 (1560 to 3110)	3090 (2120 to 4340)	3320 (2280 to 4700)	150.1 (104 to 207.9)	146 (100.3 to 204.9)	142 (97.4 to 201.1)
Middle SDI	Uterine fibroids	299 (189 to 461)	469 (289 to 742)	505 (306 to 811)	23.2 (14.6 to 35.8)	22.6 (13.9 to 35.6)	20.7 (12.6 to 33.1)
Middle SDI	Polycystic ovarian syndrome	94.5 (41.9 to 184)	129 (57.6 to 249)	144 (64.1 to 277)	5.9 (2.7 to 11.5)	6.1 (2.7 to 11.8)	6.3 (2.8 to 12.2)
Middle SDI	Female infertility	67.3 (25 to 143)	93.3 (34.9 to 195)	108 (40.1 to 227)	4.3 (1.6 to 8.9)	4.3 (1.6 to 9)	4.8 (1.8 to 10.1)
Middle SDI	Endometriosis	718 (474 to 1040)	982 (650 to 1430)	1060 (702 to 1540)	47.9 (31.8 to 69.6)	45.9 (30.3 to 66.5)	44.7 (29.7 to 64.7)
Middle SDI	Genital prolapse	52.4 (27.1 to 95.5)	67.5 (34 to 124)	83.1 (41.3 to 156)	4.6 (2.4 to 8.4)	3.7 (1.9 to 6.9)	3.6 (1.8 to 6.7)
Middle SDI	Premenstrual syndrome	824 (529 to 1240)	1090 (697 to 1640)	1160 (744 to 1730)	51.8 (33.2 to 77.9)	51.5 (32.8 to 77.5)	50.5 (32.5 to 75.7)
Middle SDI	Other gynecological diseases	184 (138 to 245)	251 (182 to 348)	270 (194 to 379)	12.4 (9.3 to 16.5)	11.9 (8.6 to 16.4)	11.5 (8.3 to 16.2)
Middle SDI	Hemoglobinopathies and hemolytic anaemias	2800 (2150 to 3550)	2140 (1640 to 2800)	1850 (1430 to 2450)	181.2 (140.2 to 228.6)	115 (89 to 149.8)	89.1 (69.1 to 117.3)
Middle SDI	Thalassemias	584 (439 to 765)	250 (180 to 284)	171 (125 to 201)	32 (24 to 41.8)	14.6 (10.6 to 16.6)	9.9 (7.2 to 11.6)
Middle SDI	Thalassaemias trait	1000 (667 to 1450)	800 (533 to 1170)	657 (432 to 976)	64.6 (43.2 to 93.8)	41.3 (27.4 to 60.1)	30.5 (20.1 to 45)
Middle SDI	Sickle cell disorders	139 (115 to 169)	114 (102 to 128)	94.7 (83 to 107)	7.7 (6.4 to 9.4)	6.3 (5.6 to 7)	5.1 (4.4 to 5.7)
Middle SDI	Sickle cell trait	159 (108 to 230)	179 (121 to 258)	166 (112 to 242)	9.9 (6.7 to 14.2)	9.5 (6.4 to 13.7)	8.2 (5.5 to 11.9)
Middle SDI	G6PD deficiency	163 (133 to 184)	181 (153 to 200)	177 (151 to 196)	10.8 (8.7 to 12.1)	9.2 (7.7 to 10.1)	7.9 (6.7 to 8.8)
Middle SDI	G6PD trait	0.0916 (0.0632 to 0.128)	0.117 (0.0786 to 0.163)	0.114 (0.0766 to 0.158)	0 (0 to 0)	0 (0 to 0)	0 (0 to 0)
Middle SDI	Other hemoglobinopathies and hemolytic anaemias	755 (579 to 982)	613 (476 to 803)	588 (457 to 766)	56.2 (43.7 to 71.9)	34.2 (26.8 to 44.4)	27.6 (21.5 to 36)
Middle SDI	Endocrine, metabolic, blood, and immune disorders	1830 (1430 to 2190)	1670 (1400 to 1960)	1770 (1480 to 2100)	120.6 (96.5 to 143.6)	92.8 (78.3 to 108.5)	85.7 (72 to 101.3)
Middle SDI	Oral disorders	2730 (1610 to 4280)	4240 (2500 to 6620)	5330 (3160 to 8330)	227.2 (135.1 to 353.3)	236.3 (140.9 to 366.9)	237.2 (141.3 to 369)
Middle SDI	Caries of deciduous teeth	43.6 (18.6 to 86.1)	39.5 (16.9 to 77.8)	38.4 (16.5 to 76.2)	2.4 (1 to 4.8)	2.4 (1 to 4.8)	2.4 (1 to 4.7)
Middle SDI	Caries of permanent teeth	327 (141 to 612)	412 (178 to 776)	444 (192 to 839)	21.5 (9.2 to 39.9)	20.8 (9 to 39.2)	20.3 (8.8 to 38)
Middle SDI	Periodontal disease	773 (304 to 1580)	1270 (499 to 2590)	1650 (651 to 3360)	65.9 (26 to 134.3)	67.9 (26.8 to 138.2)	69.6 (27.5 to 142)
Middle SDI	Edentulism and severe tooth loss	839 (556 to 1180)	1520 (1010 to 2150)	2060 (1370 to 2920)	86.5 (57.9 to 122.4)	93.9 (62.7 to 133)	93.8 (62.5 to 133)
Middle SDI	Other oral disorders	744 (464 to 1100)	1000 (626 to 1480)	1140 (710 to 1680)	51 (31.8 to 75.2)	51.1 (31.9 to 75.4)	51.1 (31.9 to 75.5)
Middle SDI	Sudden infant death syndrome	736 (362 to 1430)	462 (323 to 697)	352 (259 to 442)	40.3 (19.8 to 78.4)	29.9 (20.9 to 45.1)	22.4 (16.5 to 28.1)
Middle SDI	Injuries	76700 (73700 to 79600)	65000 (62200 to 67900)	66000 (62400 to 70100)	4831 (4627.4 to 5027.4)	3392.9 (3240.9 to 3547)	3077.5 (2917.8 to 3257.1)
Middle SDI	Transport injuries	22000 (20900 to 23400)	22800 (21900 to 23700)	21500 (20300 to 22900)	1398 (1323.9 to 1485)	1158.4 (1110.6 to 1208.6)	979.4 (923.4 to 1039.4)
Middle SDI	Road injuries	20100 (19100 to 21300)	20800 (20100 to 21500)	19600 (18500 to 20600)	1266.9 (1207 to 1344.3)	1056.2 (1018.8 to 1095.3)	888.8 (843.7 to 936.5)
Middle SDI	Pedestrian road injuries	9470 (8630 to 10400)	8300 (7880 to 9000)	7360 (6890 to 7940)	603.4 (548 to 663.2)	434.6 (412.7 to 470.8)	337.2 (316.4 to 362.2)
Middle SDI	Cyclist road injuries	688 (568 to 835)	1120 (978 to 1240)	1270 (1100 to 1440)	46.1 (38.5 to 55.6)	57.3 (50.2 to 63.5)	56.5 (49 to 63.9)
Middle SDI	Motorcyclist road injuries	3720 (3290 to 4610)	4750 (4300 to 5060)	4740 (4210 to 5100)	230.9 (203.5 to 284.7)	232.3 (210 to 248)	213.4 (189.5 to 229.1)
Middle SDI	Motor vehicle road injuries	5930 (5040 to 6590)	6300 (5860 to 6870)	5800 (5410 to 6230)	368.8 (315.3 to 409.1)	314.9 (293.5 to 342.9)	265.1 (248.9 to 284.3)
Middle SDI	Other road injuries	263 (195 to 331)	330 (279 to 383)	370 (307 to 450)	17.7 (13.4 to 22.1)	17.1 (14.4 to 19.8)	16.6 (13.8 to 20.1)

SR Appendix Table 2. All-age DALYs counts and age standardized DALYs rates per 100,000 individuals for all causes and level 1 cause groups by location for 1990, 2007, and 2017, both sexes combined

		All-age DALYs			Age-standardized rate of DALYs per 100,000		
Middle SDI	Other transport injuries	1980 (1730 to 2270)	1980 (1740 to 2270)	1990 (1710 to 2330)	131.1 (114 to 151.2)	102.2 (89.2 to 117.3)	90.6 (77.6 to 105.8)
Middle SDI	Unintentional injuries	34500 (32400 to 36200)	24500 (22400 to 26300)	23600 (21300 to 26100)	2152 (2017.9 to 2275.5)	1356.8 (1241.2 to 1455.2)	1143.2 (1036.5 to 1254.4)
Middle SDI	Falls	5280 (4640 to 5970)	6430 (5480 to 7340)	7970 (6750 to 9220)	404.4 (355.8 to 452.9)	367.5 (313.9 to 416.8)	371.7 (317.4 to 427.9)
Middle SDI	Drowning	13700 (12700 to 14700)	6010 (5790 to 6230)	4600 (4410 to 4800)	780.1 (722.1 to 832.2)	335.3 (321.9 to 347.7)	240.4 (230.3 to 251.7)
Middle SDI	Fire, heat, and hot substances	2360 (1810 to 2710)	1610 (1300 to 1880)	1480 (1160 to 1820)	150.1 (119 to 173.2)	86.5 (70.2 to 101)	70.4 (55.3 to 85.8)
Middle SDI	Poisonings	1300 (1030 to 1740)	1020 (724 to 1110)	951 (618 to 1090)	79.2 (63.4 to 105.3)	54.3 (38.8 to 59.1)	45.2 (29.7 to 52.2)
Middle SDI	Poisoning by carbon monoxide	550 (440 to 801)	425 (283 to 471)	394 (237 to 459)	33.6 (27 to 49.2)	22.5 (14.9 to 25)	18.7 (11.3 to 21.8)
Middle SDI	Poisoning by other means	755 (594 to 971)	593 (439 to 643)	557 (377 to 640)	45.6 (36.2 to 58.4)	31.8 (23.7 to 34.5)	26.6 (18.2 to 30.6)
Middle SDI	Exposure to mechanical forces	2820 (2600 to 3120)	2670 (2110 to 2950)	2480 (2100 to 2840)	181 (166.7 to 201.1)	138.4 (110.1 to 153.5)	113.2 (95.8 to 129.5)
Middle SDI	Unintentional firearm injuries	557 (443 to 616)	338 (296 to 370)	318 (283 to 353)	34.7 (27.9 to 38.3)	17.1 (15.1 to 18.8)	14.7 (13.2 to 16.3)
Middle SDI	Other exposure to mechanical forces	2260 (2070 to 2610)	2330 (1800 to 2590)	2160 (1810 to 2500)	146.3 (133.7 to 169.1)	121.3 (94.3 to 135.4)	98.5 (82.1 to 113.6)
Middle SDI	Adverse effects of medical treatment	1190 (858 to 1370)	885 (747 to 957)	901 (784 to 1000)	78 (58.8 to 87.5)	50.1 (42.4 to 54.3)	43.8 (38.3 to 48.8)
Middle SDI	Animal contact	1000 (702 to 1140)	703 (597 to 807)	686 (588 to 813)	64.6 (45.5 to 73.3)	38 (32.1 to 43.5)	32.7 (28.1 to 38.6)
Middle SDI	Venomous animal contact	664 (493 to 780)	492 (417 to 557)	484 (409 to 560)	42.6 (31.8 to 49.9)	26.5 (22.4 to 29.9)	23.2 (19.6 to 26.9)
Middle SDI	Non-venomous animal contact	337 (199 to 418)	212 (170 to 260)	202 (162 to 264)	22 (13.3 to 27.4)	11.5 (9.2 to 14.2)	9.5 (7.7 to 12.4)
Middle SDI	Foreign body	3870 (3480 to 4230)	2160 (2000 to 2280)	1830 (1690 to 1960)	223.1 (201.7 to 242.6)	130.9 (120.9 to 137.9)	101.1 (93.4 to 108.2)
Middle SDI	Pulmonary aspiration and foreign body in airway	3260 (2940 to 3650)	1860 (1690 to 1940)	1500 (1380 to 1580)	185.4 (167.8 to 207.1)	114.2 (103.5 to 119.2)	85.8 (78.7 to 90.8)
Middle SDI	Foreign body in eyes	27.6 (14.1 to 45.8)	37.5 (19.8 to 61.8)	47.2 (25.4 to 77.7)	2.1 (1.1 to 3.4)	2.1 (1.1 to 3.4)	2.2 (1.2 to 3.6)
Middle SDI	Foreign body in other body part	585 (334 to 716)	267 (224 to 329)	278 (226 to 343)	35.5 (21.4 to 43)	14.6 (12.2 to 18.1)	13.1 (10.7 to 16.2)
Middle SDI	Environmental heat and cold exposure	753 (517 to 898)	522 (409 to 633)	579 (441 to 724)	52.9 (37.4 to 63.1)	28.3 (22.2 to 34.3)	26.4 (20.1 to 32.9)
Middle SDI	Exposure to forces of nature	406 (387 to 431)	398 (351 to 456)	287 (242 to 343)	24.8 (23.6 to 26.2)	20.5 (18.2 to 23.5)	13.3 (11.3 to 15.7)
Middle SDI	Other unintentional injuries	1770 (1640 to 1930)	2110 (1960 to 2290)	1880 (1690 to 2110)	113.9 (105 to 125.4)	107 (99 to 116.6)	85.1 (76.8 to 95.2)
Middle SDI	Self-harm and interpersonal violence	20200 (18100 to 21100)	17700 (16900 to 18300)	20800 (20100 to 21600)	1280.9 (1143.9 to 1346.2)	877.7 (833 to 903.2)	955 (922.2 to 988.8)
Middle SDI	Self-harm	10700 (9210 to 11300)	8070 (7510 to 8340)	7930 (7420 to 8340)	695.4 (598.6 to 739.3)	403.1 (372.9 to 415.3)	353.8 (331 to 371.8)
Middle SDI	Self-harm by firearm	382 (299 to 503)	400 (326 to 535)	380 (302 to 495)	24.2 (18.9 to 31.7)	19.4 (15.8 to 25.9)	17.1 (13.6 to 22.3)
Middle SDI	Self-harm by other specified means	10300 (8890 to 10900)	7670 (7100 to 7950)	7550 (7020 to 7930)	671.3 (576.4 to 713)	383.7 (352.6 to 395.8)	336.7 (313.5 to 353.6)
Middle SDI	Interpersonal violence	8400 (7390 to 8930)	8990 (8330 to 9570)	9880 (9320 to 10500)	518.6 (457.5 to 552.3)	440.8 (408 to 469.1)	452.6 (427.5 to 482.1)
Middle SDI	Assault by firearm	2980 (2560 to 3170)	3980 (3640 to 4240)	4230 (3830 to 4520)	181.5 (155.6 to 193.6)	191.8 (175.3 to 204.1)	193.7 (175.5 to 207)
Middle SDI	Assault by sharp object	2220 (1760 to 2630)	2330 (1930 to 2760)	2010 (1750 to 2480)	137.6 (109.2 to 162.1)	113.3 (93.7 to 133.9)	90.9 (79 to 111.5)
Middle SDI	Sexual violence	546 (366 to 799)	647 (436 to 935)	704 (473 to 1020)	34.4 (23.2 to 50.1)	31.6 (21.4 to 45.8)	31.6 (21.3 to 46)
Middle SDI	Assault by other means	2650 (2180 to 3010)	2030 (1770 to 2380)	2940 (2740 to 3320)	165 (137.5 to 187)	104.1 (90.9 to 121.5)	136.4 (127.5 to 153.7)
Middle SDI	Conflict and terrorism	938 (902 to 991)	554 (501 to 636)	2860 (2760 to 3020)	55.3 (53.1 to 58.3)	28 (25.5 to 32.1)	143 (138.4 to 150.3)
Middle SDI	Executions and police conflict	190 (80.7 to 223)	117 (99.7 to 136)	127 (111 to 147)	11.6 (5 to 13.6)	5.7 (4.8 to 6.6)	5.7 (5 to 6.6)
High-middle SDI	All causes	374000 (346000 to 406000)	381000 (346000 to 421000)	394000 (354000 to 441000)	36624 (33950.3 to 39605.4)	28975.9 (26467.8 to 31901.3)	24713 (22215.2 to 27639.6)
High-middle SDI	Communicable, maternal, neonatal, and nutritional disorders	71300 (66700 to 76100)	40000 (36600 to 43800)	33000 (29600 to 36700)	6737.5 (6330 to 7174.9)	3863.9 (3584.4 to 4180.1)	2743.7 (2485.8 to 3027.8)
High-middle SDI	HIV/AIDS and sexually transmitted infections	1440 (1120 to 1920)	2650 (2360 to 3020)	2980 (2700 to 3300)	131.1 (99.7 to 177.3)	203.9 (172.9 to 247.5)	202.4 (177.5 to 233.9)
High-middle SDI	HIV/AIDS	739 (720 to 764)	2020 (1960 to 2110)	2440 (2340 to 2580)	65 (63.4 to 67.3)	142.3 (137.8 to 148.5)	155.4 (147.7 to 164.3)
High-middle SDI	HIV/AIDS - Drug-susceptible Tuberculosis	158 (113 to 211)	236 (169 to 323)	196 (144 to 260)	13.9 (9.9 to 18.5)	16.7 (12 to 22.8)	12.8 (9.5 to 17)
High-middle SDI	HIV/AIDS - Multidrug-resistant Tuberculosis without extensive drug resistance	2.63 (1.22 to 5.03)	70.6 (42.1 to 114)	40.8 (23.7 to 64.9)	0.2 (0.1 to 0.4)	5 (3 to 8)	2.6 (1.5 to 4.1)
High-middle SDI	HIV/AIDS - Extensively drug-resistant Tuberculosis	0 (0 to 0)	19.5 (11.3 to 32.1)	19.8 (11.4 to 31.6)	0 (0 to 0)	1.4 (0.8 to 2.2)	1.2 (0.7 to 2)

SR Appendix Table 2. All-age DALYs counts and age standardized DALYs rates per 100,000 individuals for all causes and level 1 cause groups by location for 1990, 2007, and 2017, both sexes combined

		All-age DALYs			Age-standardized rate of DALYs per 100,000		
High-middle SDI	HIV/AIDS resulting in other diseases	579 (524 to 631)	1700 (1570 to 1820)	2190 (2060 to 2340)	50.9 (46.1 to 55.5)	119.3 (109.7 to 128.1)	138.7 (129.8 to 148.7)
High-middle SDI	Sexually transmitted infections excluding HIV	705 (381 to 1190)	621 (354 to 992)	534 (319 to 834)	66 (35 to 111.4)	61.7 (32 to 103.9)	47 (26.3 to 76.8)
High-middle SDI	Syphilis	493 (201 to 971)	363 (146 to 685)	268 (116 to 488)	47.7 (19.4 to 93.9)	44.3 (16.8 to 85.2)	29.9 (11.5 to 56.2)
High-middle SDI	Chlamydial infection	54.3 (32.7 to 95.3)	66.9 (39.3 to 117)	68.6 (40.1 to 120)	4.6 (2.8 to 8)	4.5 (2.6 to 7.9)	4.5 (2.6 to 7.8)
High-middle SDI	Gonococcal infection	38.9 (25.5 to 64.4)	41.5 (26.3 to 70.4)	39 (25.1 to 66)	3.3 (2.2 to 5.4)	2.9 (1.8 to 4.9)	2.7 (1.7 to 4.7)
High-middle SDI	Trichomoniasis	28.8 (11.6 to 62.8)	36.3 (14.6 to 78.5)	38.6 (15.5 to 83.4)	2.5 (1 to 5.5)	2.4 (1 to 5.3)	2.4 (1 to 5.2)
High-middle SDI	Genital herpes	31.6 (10.3 to 75.1)	42.7 (13.9 to 102)	49.2 (15.8 to 117)	2.9 (0.9 to 6.9)	2.9 (0.9 to 6.9)	2.9 (0.9 to 6.9)
High-middle SDI	Other sexually transmitted infections	58.4 (37.3 to 96.5)	70.6 (44.4 to 116)	71.2 (45.3 to 116)	5 (3.2 to 8.2)	4.7 (3 to 7.8)	4.6 (2.9 to 7.7)
High-middle SDI	Respiratory infections and tuberculosis	21500 (20400 to 22800)	11800 (11200 to 12700)	9690 (9070 to 10600)	2059.7 (1955.1 to 2179.5)	1020.6 (971.4 to 1088.5)	686 (636.8 to 752.8)
High-middle SDI	Tuberculosis	3890 (3710 to 4060)	2950 (2830 to 3070)	1840 (1710 to 1970)	365.9 (349.4 to 381.6)	205.4 (197.1 to 214.2)	110.4 (102.9 to 118.5)
High-middle SDI	Latent tuberculosis infection	0 (0 to 0)	0 (0 to 0)	0 (0 to 0)	0 (0 to 0)	0 (0 to 0)	0 (0 to 0)
High-middle SDI	Drug-susceptible tuberculosis	3750 (3530 to 3960)	2100 (1870 to 2340)	1390 (1220 to 1550)	353 (332.2 to 372.3)	147.7 (132.1 to 163.8)	84.5 (74.5 to 94.4)
High-middle SDI	Multidrug-resistant tuberculosis without extensive drug resistance	136 (47.5 to 310)	671 (517 to 833)	302 (223 to 396)	12.9 (4.5 to 29.4)	45.6 (35.3 to 56.5)	17.8 (13 to 23.4)
High-middle SDI	Extensively drug-resistant tuberculosis	0 (0 to 0)	181 (134 to 226)	140 (107 to 174)	0 (0 to 0)	12.1 (9 to 15.2)	8.1 (6.3 to 10.1)
High-middle SDI	Lower respiratory infections	16100 (15200 to 17100)	7550 (7390 to 7830)	6520 (6270 to 6770)	1559.7 (1472.9 to 1653.7)	705.7 (688.3 to 731.4)	471.8 (455.9 to 491.1)
High-middle SDI	Upper respiratory infections	1160 (766 to 1690)	1050 (639 to 1640)	1080 (649 to 1700)	105.5 (70.7 to 153.4)	85.6 (52.3 to 133.2)	82 (48.9 to 128.9)
High-middle SDI	Otitis media	319 (196 to 491)	278 (168 to 438)	261 (159 to 410)	28.5 (17.6 to 43.6)	23.8 (14.5 to 37.6)	21.8 (13.2 to 34.6)
High-middle SDI	Enteric infections	5530 (4930 to 6210)	2040 (1720 to 2440)	1880 (1510 to 2330)	518 (461.8 to 580.6)	204.6 (174.4 to 241.6)	165.5 (133 to 204.4)
High-middle SDI	Diarrhoeal diseases	5010 (4460 to 5630)	1680 (1390 to 2050)	1600 (1260 to 2050)	472.1 (419.9 to 529.2)	169 (142.9 to 202)	138.5 (109.5 to 174.3)
High-middle SDI	Typhoid and paratyphoid	432 (247 to 711)	303 (174 to 490)	231 (131 to 381)	38.2 (21.9 to 62.7)	29.7 (17.3 to 47.8)	22.8 (13 to 37.4)
High-middle SDI	Typhoid fever	390 (218 to 640)	273 (155 to 447)	201 (114 to 331)	34.4 (19.3 to 56.6)	26.9 (15.3 to 43.3)	19.9 (11.2 to 32.8)
High-middle SDI	Paratyphoid fever	42.9 (19.9 to 84.1)	29.8 (13.4 to 58.3)	29.7 (13.4 to 59.4)	3.8 (1.8 to 7.4)	2.9 (1.3 to 5.6)	2.9 (1.3 to 5.8)
High-middle SDI	Invasive non-typhoidal salmonella	70.7 (37.7 to 122)	55.6 (29.4 to 96.7)	43.4 (22.4 to 78)	6.4 (3.4 to 10.9)	5.4 (2.9 to 9.5)	3.9 (2 to 7.2)
High-middle SDI	Other intestinal infectious diseases	14.6 (10.2 to 19.8)	4.22 (2.67 to 6.13)	2.57 (1.62 to 3.65)	1.4 (0.9 to 1.8)	0.4 (0.2 to 0.7)	0.2 (0.1 to 0.3)
High-middle SDI	Neglected tropical diseases and malaria	1950 (1360 to 2710)	1620 (1150 to 2250)	1660 (1160 to 2320)	175.7 (123.3 to 241.4)	120.7 (87.4 to 164.5)	110.8 (78.2 to 152.4)
High-middle SDI	Malaria	69.6 (46.9 to 106)	53.3 (40.9 to 98.5)	40 (29 to 79.2)	6.2 (4.2 to 9.6)	4.9 (3.6 to 9.1)	3.5 (2.4 to 7)
High-middle SDI	Chagas disease	100 (92.4 to 108)	70.2 (63.6 to 79.1)	64.5 (57.8 to 73.2)	9.8 (9.1 to 10.6)	5 (4.5 to 5.6)	3.6 (3.2 to 4.2)
High-middle SDI	Leishmaniasis	71 (1.56 to 600)	10.8 (1.18 to 65.3)	6.02 (0.728 to 29.8)	6.3 (0.1 to 53.2)	1 (0.1 to 6.3)	0.5 (0.1 to 2.8)
High-middle SDI	Visceral leishmaniasis	69.2 (0.233 to 599)	9.51 (0.144 to 63)	5.02 (0.0122 to 28.7)	6.1 (0 to 53.1)	0.9 (0 to 6.1)	0.4 (0 to 2.7)
High-middle SDI	Cutaneous and mucocutaneous leishmaniasis	1.8 (0.966 to 3.11)	1.32 (0.816 to 1.99)	1.01 (0.541 to 1.71)	0.2 (0.1 to 0.3)	0.1 (0.1 to 0.2)	0.1 (0 to 0.1)
High-middle SDI	African trypanosomiasis	0 (0 to 0)	0 (0 to 0)	0 (0 to 0)	0 (0 to 0)	0 (0 to 0)	0 (0 to 0)
High-middle SDI	Schistosomiasis	41.3 (26.1 to 67.6)	49 (27.2 to 87.6)	55.7 (29.7 to 100)	3.9 (2.5 to 6.3)	3.3 (1.9 to 6)	3.3 (1.8 to 5.9)
High-middle SDI	Cysticercosis	182 (124 to 250)	187 (121 to 273)	176 (110 to 264)	17 (11.7 to 23.3)	12.9 (8.4 to 18.8)	10.3 (6.5 to 15.4)
High-middle SDI	Cystic echinococcosis	27.3 (20.3 to 35.6)	21.1 (14.7 to 29.1)	20.2 (14 to 29.2)	2.5 (1.9 to 3.3)	1.5 (1.1 to 2.1)	1.3 (0.9 to 1.8)
High-middle SDI	Lymphatic filariasis	23.5 (12.5 to 37.9)	31 (16.6 to 49.6)	15.8 (8.63 to 25.4)	2.1 (1.1 to 3.4)	2.2 (1.2 to 3.5)	1 (0.6 to 1.7)
High-middle SDI	Onchocerciasis	0 (0 to 0)	0 (0 to 0)	0 (0 to 0)	0 (0 to 0)	0 (0 to 0)	0 (0 to 0)
High-middle SDI	Trachoma	15.9 (10.7 to 22.1)	15.1 (10.1 to 21)	15.1 (9.91 to 21.3)	1.8 (1.3 to 2.6)	1.2 (0.8 to 1.7)	0.9 (0.6 to 1.3)
High-middle SDI	Dengue	75.8 (38.7 to 128)	123 (76.8 to 174)	175 (113 to 241)	6.8 (3.5 to 11.5)	11.8 (7.2 to 16.5)	15.1 (9.9 to 20.3)
High-middle SDI	Yellow fever	9.55 (1.59 to 31.7)	4.72 (0.792 to 15.3)	2.7 (0.456 to 9.03)	0.8 (0.1 to 2.8)	0.4 (0.1 to 1.4)	0.2 (0 to 0.8)
High-middle SDI	Rabies	29.5 (23.6 to 53)	32.9 (24.3 to 36.4)	12.3 (9.92 to 14.2)	2.7 (2.1 to 4.8)	2.9 (2.1 to 3.3)	1 (0.8 to 1.1)

SR Appendix Table 2. All-age DALYs counts and age standardized DALYs rates per 100,000 individuals for all causes and level 1 cause groups by location for 1990, 2007, and 2017, both sexes combined

		All-age DALYs			Age-standardized rate of DALYs per 100,000		
High-middle SDI	Intestinal nematode infections	344 (201 to 565)	69.9 (40.5 to 115)	69.4 (39.1 to 116)	30.2 (17.7 to 49.5)	5.4 (3.2 to 8.8)	5 (2.9 to 8.5)
High-middle SDI	Ascariasis	167 (93.8 to 290)	14.6 (8.59 to 24.1)	10.1 (5.81 to 16.4)	14.7 (8.3 to 25.5)	1.3 (0.8 to 2.2)	0.9 (0.5 to 1.4)
High-middle SDI	Trichuriasis	63.5 (32.8 to 109)	11 (5.47 to 19.2)	15.9 (8.19 to 28.6)	5.4 (2.8 to 9.3)	0.8 (0.4 to 1.5)	1.2 (0.6 to 2.2)
High-middle SDI	Hookworm disease	114 (62 to 195)	44.3 (24.5 to 75.4)	43.4 (23.4 to 75.9)	10.1 (5.5 to 17.3)	3.2 (1.8 to 5.5)	3 (1.6 to 5.2)
High-middle SDI	Food-borne trematodiases	745 (383 to 1330)	829 (476 to 1390)	910 (528 to 1510)	65.6 (33.9 to 116.7)	57.3 (32.9 to 96.4)	57.5 (33.2 to 95)
High-middle SDI	Leprosy	1.81 (1.21 to 2.57)	2.37 (1.59 to 3.36)	2.53 (1.71 to 3.53)	0.2 (0.1 to 0.3)	0.2 (0.1 to 0.2)	0.1 (0.1 to 0.2)
High-middle SDI	Ebola virus disease	0 (0 to 0)	0 (0 to 0)	0 (0 to 0)	0 (0 to 0)	0 (0 to 0)	0 (0 to 0)
High-middle SDI	Zika virus disease	0 (0 to 0)	0 (0 to 0)	0.639 (0.312 to 1.41)	0 (0 to 0)	0 (0 to 0)	0.1 (0 to 0.1)
High-middle SDI	Guinea worm disease	0 (0 to 0)	0 (0 to 0)	0 (0 to 0)	0 (0 to 0)	0 (0 to 0)	0 (0 to 0)
High-middle SDI	Other neglected tropical diseases	219 (158 to 299)	120 (86.2 to 166)	91.4 (65.6 to 125)	19.6 (14.2 to 26.7)	10.7 (7.8 to 14.6)	7.4 (5.4 to 10.2)
High-middle SDI	Other infectious diseases	6950 (5500 to 8960)	2410 (2170 to 2690)	1870 (1700 to 2070)	641.7 (508.5 to 825.7)	233.7 (209.2 to 264.6)	156.1 (141.3 to 174)
High-middle SDI	Meningitis	2170 (1560 to 2450)	751 (673 to 797)	527 (460 to 567)	200.7 (143.6 to 226.8)	75.2 (67 to 79.7)	45.7 (40.3 to 49)
High-middle SDI	Pneumococcal meningitis	225 (167 to 263)	92.6 (81 to 105)	68.8 (59.8 to 78.6)	20.9 (15.5 to 24.5)	8.6 (7.5 to 9.6)	5.6 (4.8 to 6.3)
High-middle SDI	H influenzae type B meningitis	988 (688 to 1140)	326 (286 to 344)	218 (187 to 234)	91.5 (63.5 to 105.1)	33.5 (29.4 to 35.7)	19.5 (16.8 to 21.1)
High-middle SDI	Meningococcal infection	233 (163 to 278)	65.5 (58.6 to 72.4)	44 (38.5 to 49)	21.6 (15.1 to 25.9)	6.8 (6 to 7.6)	3.9 (3.4 to 4.4)
High-middle SDI	Other meningitis	722 (530 to 829)	268 (237 to 289)	196 (171 to 214)	66.7 (48.9 to 76.7)	26.4 (23.4 to 28.6)	16.7 (14.6 to 18.2)
High-middle SDI	Encephalitis	593 (502 to 695)	439 (413 to 469)	383 (342 to 429)	54.2 (45.8 to 63.5)	42 (39.7 to 44.5)	32.1 (28.2 to 37.6)
High-middle SDI	Diphtheria	17.1 (14.4 to 20.1)	2.54 (2.18 to 2.99)	1.2 (1.04 to 1.42)	1.6 (1.3 to 1.8)	0.3 (0.2 to 0.3)	0.1 (0.1 to 0.1)
High-middle SDI	Whooping cough	1020 (263 to 2600)	171 (68.8 to 385)	67.9 (33 to 127)	93.9 (24.3 to 241.5)	21.4 (8.6 to 48.3)	7.9 (3.8 to 14.9)
High-middle SDI	Tetanus	445 (344 to 560)	57.9 (38.1 to 68.3)	26.8 (16.1 to 34.4)	42.8 (33.2 to 54.1)	5.6 (4.2 to 6.5)	2.1 (1.4 to 2.8)
High-middle SDI	Measles	1420 (545 to 3010)	122 (46.6 to 265)	91 (33 to 199)	129 (49.6 to 274.1)	15.3 (5.8 to 33.1)	10.6 (3.8 to 23.2)
High-middle SDI	Varicella and herpes zoster	130 (110 to 154)	86.5 (63.7 to 115)	87.5 (60 to 122)	12.3 (10.4 to 14.6)	7.5 (5.8 to 9.6)	6.1 (4.4 to 8.2)
High-middle SDI	Acute hepatitis	604 (469 to 673)	415 (344 to 473)	350 (307 to 413)	55.8 (43.2 to 62.2)	31.5 (26.1 to 35.9)	23.2 (20.2 to 27.3)
High-middle SDI	Acute hepatitis A	146 (116 to 175)	103 (82.4 to 126)	84.6 (66.4 to 106)	12.8 (10.2 to 15.3)	8.6 (6.9 to 10.4)	6.9 (5.5 to 8.6)
High-middle SDI	Acute hepatitis B	387 (295 to 444)	272 (220 to 311)	233 (201 to 285)	36.7 (28 to 42)	19.6 (15.8 to 22.4)	13.9 (12 to 17)
High-middle SDI	Acute hepatitis C	25.2 (13.8 to 41.4)	9.39 (5.45 to 15.2)	6.48 (3.72 to 10.8)	2.4 (1.3 to 3.9)	1 (0.6 to 1.7)	0.6 (0.3 to 1)
High-middle SDI	Acute hepatitis E	45.3 (32.9 to 58.9)	30.9 (23.2 to 39.1)	25.5 (19.8 to 31.9)	4 (2.9 to 5.1)	2.3 (1.7 to 2.8)	1.8 (1.4 to 2.2)
High-middle SDI	Other unspecified infectious diseases	558 (464 to 664)	369 (314 to 440)	337 (284 to 402)	51.5 (43.1 to 61.2)	35 (30.2 to 41)	28.2 (23.7 to 33.5)
High-middle SDI	Maternal and neonatal disorders	27600 (25700 to 29800)	16100 (14800 to 17500)	12200 (10800 to 14000)	2638.1 (2457.6 to 2851)	1794.5 (1678.9 to 1919.7)	1213.3 (1098.2 to 1347.5)
High-middle SDI	Maternal disorders	872 (824 to 926)	394 (374 to 417)	289 (269 to 311)	71.5 (67.6 to 75.8)	26.9 (25.5 to 28.5)	19.8 (18.4 to 21.3)
High-middle SDI	Maternal haemorrhage	325 (289 to 364)	85.8 (74.1 to 98.7)	48.3 (41.1 to 56.9)	26.5 (23.6 to 29.6)	5.9 (5 to 6.7)	3.3 (2.8 to 3.8)
High-middle SDI	Maternal sepsis and other pregnancy related infections	108 (91.6 to 129)	35.7 (30 to 42.3)	28.6 (23.1 to 35.9)	8.9 (7.6 to 10.6)	2.4 (2 to 2.9)	2 (1.6 to 2.5)
High-middle SDI	Maternal hypertensive disorders	125 (105 to 150)	65.8 (55.3 to 76.8)	50.7 (43.1 to 59.7)	10.2 (8.6 to 12.2)	4.5 (3.8 to 5.3)	3.6 (3 to 4.2)
High-middle SDI	Maternal obstructed labour and uterine rupture	32 (24.8 to 41.2)	17.3 (13.3 to 22.7)	13.8 (10.3 to 19.5)	2.6 (2 to 3.4)	1.2 (0.9 to 1.6)	0.9 (0.7 to 1.3)
High-middle SDI	Maternal abortive outcome	108 (95.4 to 123)	44.9 (39.1 to 50.9)	32 (28.1 to 36.7)	9 (8 to 10.3)	3 (2.6 to 3.4)	2.1 (1.9 to 2.5)
High-middle SDI	Ectopic pregnancy	23.2 (17.8 to 30.2)	14.3 (11.2 to 19.6)	9.41 (7.25 to 13.2)	1.9 (1.5 to 2.5)	1 (0.8 to 1.3)	0.7 (0.5 to 1)
High-middle SDI	Indirect maternal deaths	69.9 (59.5 to 82.8)	42.2 (36.3 to 49.1)	29.5 (24.9 to 35)	5.7 (4.9 to 6.7)	2.9 (2.5 to 3.4)	2 (1.7 to 2.4)
High-middle SDI	Late maternal deaths	16.1 (11.3 to 22.6)	12.5 (9.61 to 16.5)	13.8 (10.7 to 18.1)	1.3 (0.9 to 1.8)	0.9 (0.7 to 1.1)	1 (0.8 to 1.2)
High-middle SDI	Maternal deaths aggravated by HIV/AIDS	0.0819 (0.0418 to 0.13)	0.252 (0.135 to 0.39)	0.317 (0.169 to 0.494)	0 (0 to 0)	0 (0 to 0)	0 (0 to 0)
High-middle SDI	Other maternal disorders	64.8 (56.4 to 74.9)	74.9 (66.8 to 83.4)	62.6 (55.9 to 70.1)	5.3 (4.6 to 6.2)	5.1 (4.5 to 5.6)	4.2 (3.7 to 4.7)

SR Appendix Table 2. All-age DALYs counts and age standardized DALYs rates per 100,000 individuals for all causes and level 1 cause groups by location for 1990, 2007, and 2017, both sexes combined

		All-age DALYs			Age-standardized rate of DALYs per 100,000		
High-middle SDI	Neonatal disorders	26700 (24800 to 28900)	15700 (14400 to 17100)	12000 (10500 to 13700)	2566.6 (2385.1 to 2779.9)	1767.7 (1652.7 to 1893.4)	1193.4 (1078.6 to 1326.4)
High-middle SDI	Neonatal preterm birth	14900 (13800 to 16100)	7360 (6810 to 7990)	5310 (4630 to 6140)	1438 (1330.6 to 1556.9)	850.5 (797.7 to 911.9)	547.4 (491.1 to 615.6)
High-middle SDI	Neonatal encephalopathy due to birth asphyxia and trauma	6530 (5690 to 7830)	4430 (3650 to 5470)	3590 (2740 to 4760)	623.7 (543.1 to 748.2)	467.1 (403.2 to 550.3)	329.6 (265.5 to 414.8)
High-middle SDI	Neonatal sepsis and other neonatal infections	1870 (1460 to 2430)	1700 (1280 to 2320)	1560 (1100 to 2200)	175.1 (137.9 to 227.2)	172.1 (135.6 to 220.9)	142.1 (106.5 to 190.2)
High-middle SDI	Hemolytic disease and other neonatal jaundice	562 (497 to 636)	255 (228 to 283)	192 (162 to 224)	54 (47.7 to 61.1)	27.7 (25.2 to 30.4)	18.1 (15.7 to 20.8)
High-middle SDI	Other neonatal disorders	2840 (2440 to 3590)	1970 (1790 to 2100)	1300 (1180 to 1410)	275.8 (236.6 to 348)	250.3 (227.5 to 266.3)	156.2 (141.7 to 168.7)
High-middle SDI	Nutritional deficiencies	6330 (4600 to 8660)	3290 (2260 to 4750)	2660 (1810 to 3880)	573.3 (417.9 to 781.8)	285.7 (198.4 to 406.3)	209.7 (143.2 to 302.2)
High-middle SDI	Protein-energy malnutrition	1130 (1030 to 1240)	314 (285 to 345)	248 (225 to 274)	107.4 (98 to 116.9)	32.8 (29.8 to 36.3)	21 (18.8 to 23.5)
High-middle SDI	Iodine deficiency	176 (97.5 to 289)	167 (89 to 298)	186 (97.6 to 334)	15.8 (8.8 to 25.9)	12.1 (6.5 to 21.6)	12.3 (6.5 to 22.2)
High-middle SDI	Dietary iron deficiency	3850 (2550 to 5660)	2340 (1540 to 3510)	1860 (1200 to 2790)	343.3 (228.1 to 504.8)	186.6 (122.8 to 278.1)	137.4 (88.6 to 206.1)
High-middle SDI	Other nutritional deficiencies	112 (97.1 to 130)	76.3 (66.1 to 92.9)	68.9 (59 to 80.6)	10.6 (9.2 to 12.4)	6.8 (6 to 8.1)	4.9 (4.3 to 5.6)
High-middle SDI	Non-communicable diseases	247000 (225000 to 274000)	291000 (261000 to 326000)	317000 (283000 to 356000)	24855.3 (22782.3 to 27336.2)	21431.4 (19340.6 to 23947.8)	19050.6 (16961 to 21525.8)
High-middle SDI	Neoplasms	41200 (40100 to 42000)	48000 (46900 to 48900)	56300 (54400 to 58200)	4064.1 (3967.7 to 4147.2)	3411 (3327.6 to 3472.5)	3173.3 (3061.4 to 3275.4)
High-middle SDI	Lip and oral cavity cancer	447 (433 to 491)	604 (594 to 613)	715 (690 to 739)	44.4 (43.1 to 48.8)	41.5 (40.8 to 42.1)	38.6 (37.3 to 39.9)
High-middle SDI	Nasopharynx cancer	481 (436 to 526)	432 (414 to 451)	502 (461 to 550)	46 (41.8 to 50.4)	29.2 (28 to 30.5)	27.6 (25.4 to 30.1)
High-middle SDI	Other pharynx cancer	233 (217 to 252)	319 (310 to 331)	372 (352 to 394)	22.9 (21.4 to 24.8)	21.7 (21.1 to 22.5)	19.8 (18.7 to 21)
High-middle SDI	Oesophageal cancer	2360 (2250 to 2470)	2680 (2610 to 2760)	2790 (2630 to 2940)	240.6 (229.5 to 252.1)	189.3 (184.2 to 194.3)	151.4 (143.3 to 160.2)
High-middle SDI	Stomach cancer	6290 (6130 to 6480)	5660 (5570 to 5780)	5680 (5430 to 5940)	634.4 (617.9 to 653.1)	397.6 (390.7 to 405.4)	311.4 (297.8 to 325.2)
High-middle SDI	Colon and rectum cancer	2850 (2750 to 2980)	3860 (3800 to 3920)	4810 (4640 to 4970)	289.7 (279.6 to 302.7)	273.9 (269.4 to 278.1)	265.6 (256 to 274.5)
High-middle SDI	Liver cancer	4120 (3800 to 4350)	4940 (4750 to 5090)	6030 (5630 to 6460)	403.3 (372.4 to 426.9)	337.3 (324.3 to 348)	326.2 (304.8 to 349.8)
High-middle SDI	Liver cancer due to hepatitis B	2320 (2110 to 2500)	2680 (2520 to 2820)	3070 (2820 to 3350)	224.1 (203.2 to 241.2)	180.2 (169.5 to 189.5)	165.9 (152.5 to 180.7)
High-middle SDI	Liver cancer due to hepatitis C	781 (719 to 840)	1020 (953 to 1080)	1370 (1260 to 1500)	79.2 (73.3 to 85)	71.8 (67 to 76.2)	74.2 (68.2 to 80.8)
High-middle SDI	Liver cancer due to alcohol use	422 (357 to 495)	529 (457 to 621)	681 (577 to 804)	42.2 (35.8 to 49.4)	36.5 (31.6 to 42.5)	36.7 (31.4 to 43.2)
High-middle SDI	Liver cancer due to NASH	206 (182 to 230)	274 (244 to 303)	386 (340 to 432)	20.8 (18.3 to 23.3)	19.3 (17.2 to 21.3)	21.1 (18.7 to 23.6)
High-middle SDI	Liver cancer due to other causes	382 (332 to 424)	435 (387 to 482)	517 (456 to 581)	37.1 (32 to 41.1)	29.7 (26.4 to 32.9)	28.2 (25 to 31.6)
High-middle SDI	Gallbladder and biliary tract cancer	475 (450 to 526)	591 (506 to 611)	681 (568 to 722)	49.2 (46.7 to 54.6)	42.2 (36.2 to 43.6)	37.5 (31.3 to 39.7)
High-middle SDI	Pancreatic cancer	1180 (1140 to 1210)	1760 (1740 to 1780)	2360 (2290 to 2430)	118.9 (115.2 to 122.6)	124.1 (122.5 to 125.6)	128.6 (124.6 to 132.5)
High-middle SDI	Larynx cancer	650 (636 to 674)	638 (629 to 648)	678 (659 to 698)	64.3 (62.9 to 66.7)	44.1 (43.4 to 44.8)	36.3 (35.3 to 37.4)
High-middle SDI	Tracheal, bronchus, and lung cancer	7540 (7310 to 7840)	9490 (9320 to 9650)	12100 (11600 to 12600)	749.7 (727 to 778.9)	663.9 (652.2 to 675.6)	656.8 (628.8 to 682.8)
High-middle SDI	Malignant skin melanoma	239 (204 to 310)	342 (281 to 411)	377 (315 to 454)	23.3 (19.8 to 30.2)	23.4 (19.3 to 28.2)	21.2 (17.7 to 25.5)
High-middle SDI	Non-melanoma skin cancer	155 (149 to 160)	227 (224 to 231)	286 (275 to 296)	16.1 (15.5 to 16.6)	16.5 (16.2 to 16.7)	16.1 (15.5 to 16.7)
High-middle SDI	Non-melanoma skin cancer (squamous-cell carcinoma)	155 (149 to 160)	227 (223 to 231)	285 (275 to 296)	16.1 (15.5 to 16.6)	16.5 (16.2 to 16.7)	16.1 (15.5 to 16.7)
High-middle SDI	Non-melanoma skin cancer (basal-cell carcinoma)	0.129 (0.0486 to 0.277)	0.182 (0.0705 to 0.391)	0.253 (0.0964 to 0.544)	0 (0 to 0)	0 (0 to 0)	0 (0 to 0)
High-middle SDI	Breast cancer	2220 (2110 to 2380)	3070 (2950 to 3170)	3540 (3260 to 3720)	218.9 (208.5 to 234.7)	208.8 (200.8 to 215.5)	192.3 (177 to 202.4)
High-middle SDI	Cervical cancer	1060 (1010 to 1230)	1150 (1120 to 1190)	1230 (1070 to 1290)	103.4 (98.8 to 120.3)	77.6 (75.5 to 80.1)	67.2 (58.9 to 70.6)
High-middle SDI	Uterine cancer	430 (411 to 446)	471 (455 to 488)	480 (457 to 504)	43.2 (41.3 to 44.8)	32.9 (31.9 to 34)	26.1 (24.9 to 27.4)
High-middle SDI	Ovarian cancer	619 (585 to 650)	837 (821 to 857)	987 (952 to 1020)	60.7 (57.4 to 63.7)	57.3 (56.1 to 58.6)	53.6 (51.7 to 55.4)
High-middle SDI	Prostate cancer	612 (530 to 758)	1020 (825 to 1160)	1320 (1110 to 1540)	67.8 (58.7 to 83.8)	76.1 (61.4 to 86.8)	75.6 (63.4 to 87.7)
High-middle SDI	Testicular cancer	86.7 (80.5 to 93)	89 (86 to 92.5)	82.9 (78.2 to 88.2)	7.5 (7 to 8)	6.1 (5.9 to 6.3)	5.3 (5 to 5.7)
High-middle SDI	Kidney cancer	529 (470 to 569)	711 (671 to 728)	843 (799 to 879)	52 (46.3 to 55.9)	51.8 (49 to 53)	48.2 (45.8 to 50.2)

SR Appendix Table 2. All-age DALYs counts and age standardized DALYs rates per 100,000 individuals for all causes and level 1 cause groups by location for 1990, 2007, and 2017, both sexes combined

		All-age DALYs			Age-standardized rate of DALYs per 100,000		
High-middle SDI	Bladder cancer	561 (496 to 595)	705 (679 to 726)	832 (800 to 876)	59.2 (52.4 to 62.8)	51.2 (49.3 to 52.7)	46.5 (44.7 to 48.9)
High-middle SDI	Thyroid cancer	129 (120 to 136)	167 (159 to 177)	204 (191 to 221)	12.8 (12 to 13.5)	11.7 (11.1 to 12.4)	11.5 (10.7 to 12.4)
High-middle SDI	Mesothelioma	71.6 (60.1 to 89.5)	97.6 (93.3 to 102)	118 (111 to 123)	7 (5.9 to 8.7)	6.7 (6.4 to 7)	6.5 (6.1 to 6.8)
High-middle SDI	Hodgkin lymphoma	292 (203 to 340)	212 (172 to 227)	191 (152 to 208)	26.5 (18.4 to 30.8)	15.2 (12.2 to 16.2)	12.2 (9.7 to 13.3)
High-middle SDI	Non-Hodgkin's lymphoma	789 (759 to 830)	986 (970 to 1000)	1250 (1200 to 1290)	74.5 (71.8 to 78.2)	72.1 (71 to 73.3)	75 (72.3 to 77.5)
High-middle SDI	Multiple myeloma	228 (214 to 264)	346 (317 to 377)	462 (421 to 499)	22.7 (21.3 to 26.6)	24.3 (22.3 to 26.5)	25.4 (23 to 27.3)
High-middle SDI	Leukaemia	2810 (2310 to 3120)	2360 (2080 to 2450)	2320 (2030 to 2450)	256.4 (211.9 to 283.4)	191.8 (168.2 to 199.6)	165.2 (143.7 to 174.6)
High-middle SDI	Acute lymphoid leukaemia	518 (454 to 628)	480 (429 to 526)	508 (419 to 549)	46.4 (40.8 to 56.2)	42.7 (38 to 46.9)	42.1 (34.3 to 46)
High-middle SDI	Chronic lymphoid leukaemia	109 (99.7 to 116)	145 (136 to 154)	182 (167 to 198)	10.8 (10 to 11.5)	10.3 (9.7 to 11)	10.3 (9.4 to 11.2)
High-middle SDI	Acute myeloid leukaemia	447 (391 to 561)	483 (434 to 510)	515 (458 to 545)	40.8 (35.8 to 51)	37.9 (34.1 to 40.1)	35 (31.1 to 37.2)
High-middle SDI	Chronic myeloid leukaemia	129 (112 to 145)	109 (100 to 114)	91.5 (85 to 100)	12.2 (10.7 to 13.6)	7.5 (6.9 to 7.9)	5.3 (4.9 to 5.8)
High-middle SDI	Other leukaemia	1610 (1180 to 1840)	1150 (925 to 1210)	1030 (850 to 1100)	146.2 (108.2 to 167.1)	93.4 (74.8 to 98.8)	72.5 (59.2 to 78.1)
High-middle SDI	Other malignant cancers	1840 (1640 to 1940)	2140 (1840 to 2230)	2530 (2190 to 2730)	175.4 (156.8 to 184)	159.8 (139 to 166.6)	161.7 (140.4 to 176.5)
High-middle SDI	Other neoplasms	263 (201 to 294)	387 (331 to 460)	511 (447 to 656)	25.2 (19.3 to 28)	29.6 (25.4 to 35.2)	32.3 (28.2 to 41.7)
High-middle SDI	Myelodysplastic, myeloproliferative, and other hematopoietic neoplasms	231 (179 to 261)	348 (296 to 416)	466 (410 to 596)	22.4 (17.4 to 25.1)	26.4 (22.5 to 31.4)	28.7 (25.2 to 36.7)
High-middle SDI	Benign and in situ intestinal neoplasms	0 (0 to 0)	0 (0 to 0)	0 (0 to 0)	0 (0 to 0)	0 (0 to 0)	0 (0 to 0)
High-middle SDI	Benign and in situ cervical and uterine neoplasms	0 (0 to 0)	0 (0 to 0)	0 (0 to 0)	0 (0 to 0)	0 (0 to 0)	0 (0 to 0)
High-middle SDI	Other benign and in situ neoplasms	31.7 (22 to 38.9)	38.6 (32.5 to 47.9)	44.5 (37.1 to 60.6)	2.8 (1.9 to 3.4)	3.2 (2.7 to 4)	3.6 (3 to 4.9)
High-middle SDI	Cardiovascular diseases	71200 (69700 to 72700)	88100 (86200 to 90100)	92000 (88900 to 95100)	7721 (7562 to 7877.4)	6474.6 (6338.3 to 6620.9)	5254.9 (5081.4 to 5432.6)
High-middle SDI	Rheumatic heart disease	2010 (1920 to 2110)	1250 (1160 to 1360)	1020 (929 to 1140)	194.6 (186.3 to 204.1)	90 (84.1 to 97.6)	61.2 (55.6 to 68.9)
High-middle SDI	Ischaemic heart disease	32000 (31500 to 32600)	42100 (41500 to 42800)	43100 (42100 to 44100)	3509.8 (3454.7 to 3571.7)	3089.3 (3053.3 to 3141.2)	2449 (2393.1 to 2502.5)
High-middle SDI	Stroke	29500 (28500 to 30400)	32900 (31700 to 34000)	35100 (33400 to 37000)	3195.7 (3097.4 to 3294)	2423.2 (2340.4 to 2502.9)	1998.4 (1900 to 2103.5)
High-middle SDI	Ischaemic stroke	13000 (12300 to 13600)	15500 (14700 to 16300)	18200 (16900 to 19600)	1480.2 (1409.8 to 1549.2)	1178.5 (1118.1 to 1234.2)	1048.1 (971.5 to 1125.1)
High-middle SDI	Intracerebral hemorrhage	13500 (13000 to 14100)	14800 (14500 to 15200)	14400 (13800 to 14900)	1413 (1360.7 to 1475.7)	1067.2 (1045.4 to 1088.7)	803.6 (773.9 to 835.2)
High-middle SDI	Subarachnoid hemorrhage	3010 (2520 to 3290)	2480 (2360 to 2710)	2570 (2380 to 2810)	302.4 (250.8 to 330.8)	177.5 (169 to 193.7)	146.7 (135.8 to 160)
High-middle SDI	Hypertensive heart disease	2460 (2180 to 2670)	2720 (2440 to 2890)	3620 (2880 to 3930)	274.2 (243.1 to 298)	205.5 (183.5 to 218.4)	209.7 (165.2 to 227.3)
High-middle SDI	Non-rheumatic valvular heart disease	245 (223 to 280)	348 (322 to 378)	400 (368 to 444)	26.3 (23.9 to 29.7)	26 (24 to 28.3)	23.5 (21.6 to 26)
High-middle SDI	Non-rheumatic calcific aortic valve disease	113 (100 to 134)	180 (162 to 198)	230 (206 to 250)	12.3 (10.9 to 14.6)	13.6 (12.2 to 14.9)	13.4 (12.1 to 14.7)
High-middle SDI	Non-rheumatic degenerative mitral valve disease	111 (98.2 to 128)	134 (117 to 152)	138 (120 to 160)	11.7 (10.4 to 13.6)	9.9 (8.7 to 11.2)	8.1 (7 to 9.3)
High-middle SDI	Other non-rheumatic valve diseases	21.5 (16.9 to 28.4)	33.6 (24.7 to 41.3)	33 (27.1 to 48)	2.2 (1.7 to 2.9)	2.5 (1.8 to 3.1)	2 (1.7 to 2.9)
High-middle SDI	Cardiomyopathy and myocarditis	1970 (1780 to 2240)	4790 (3690 to 4980)	3750 (3380 to 3890)	195.1 (176.5 to 220.9)	333.4 (261.7 to 345.9)	220.9 (201.6 to 228.6)
High-middle SDI	Myocarditis	233 (197 to 261)	265 (238 to 286)	267 (245 to 302)	22.8 (19.1 to 25.5)	22.5 (20.3 to 24.2)	18.9 (17.5 to 21.4)
High-middle SDI	Alcoholic cardiomyopathy	979 (820 to 1210)	3370 (2400 to 3570)	2010 (1870 to 2220)	94 (79.2 to 116.2)	221.5 (158.9 to 234.3)	110.7 (103.2 to 122.9)
High-middle SDI	Other cardiomyopathy	758 (699 to 834)	1150 (995 to 1210)	1480 (1180 to 1580)	78.3 (72.6 to 84.9)	89.5 (78.5 to 93.1)	91.2 (75.1 to 97.2)
High-middle SDI	Atrial fibrillation and flutter	621 (515 to 749)	931 (775 to 1120)	1280 (1080 to 1530)	75.7 (64.1 to 90.1)	73.9 (62.3 to 88)	75.7 (64.2 to 89.7)
High-middle SDI	Aortic aneurysm	430 (412 to 459)	591 (573 to 627)	710 (678 to 742)	44.2 (42.4 to 47.1)	42 (40.8 to 44.5)	39.6 (37.8 to 41.5)
High-middle SDI	Peripheral vascular disease	175 (109 to 253)	296 (199 to 427)	407 (278 to 604)	19.9 (12.4 to 29.1)	22.4 (15 to 32.7)	23.2 (15.8 to 34.6)
High-middle SDI	Endocarditis	219 (199 to 246)	304 (285 to 347)	352 (326 to 396)	21.2 (19.2 to 23.8)	23.6 (22.2 to 26.8)	22.3 (20.6 to 25.3)
High-middle SDI	Other cardiovascular and circulatory diseases	1610 (1460 to 1790)	1950 (1760 to 2190)	2210 (1970 to 2500)	164.3 (149.3 to 182.6)	145.3 (131.9 to 162.6)	131.3 (117.4 to 148.6)
High-middle SDI	Chronic respiratory diseases	19000 (17600 to 20100)	15500 (14300 to 16600)	16200 (14800 to 17800)	2034 (1895.8 to 2146)	1166.9 (1075.7 to 1253.7)	975.8 (884.8 to 1074.8)

SR Appendix Table 2. All-age DALYs counts and age standardized DALYs rates per 100,000 individuals for all causes and level 1 cause groups by location for 1990, 2007, and 2017, both sexes combined							
		All-age DALYs			Age-standardized rate of DALYs per 100,000		
High-middle SDI	Chronic obstructive pulmonary disease	15400 (14400 to 16100)	12500 (11700 to 13300)	12900 (12000 to 14000)	1690.7 (1581.9 to 1769.7)	934.6 (879.2 to 989.2)	747.2 (692.2 to 810)
High-middle SDI	Pneumoconiosis	117 (99.4 to 137)	98.8 (92 to 106)	102 (93.4 to 114)	11.9 (10.1 to 13.9)	7 (6.5 to 7.5)	5.7 (5.2 to 6.3)
High-middle SDI	Silicosis	74.7 (60.1 to 92)	58.8 (54.1 to 63.4)	63.4 (54.5 to 70.4)	7.6 (6.1 to 9.3)	4.1 (3.8 to 4.4)	3.5 (3 to 3.9)
High-middle SDI	Asbestosis	6.53 (4.83 to 8.04)	6.83 (5.52 to 8.42)	7.64 (6.27 to 10.1)	0.7 (0.5 to 0.8)	0.5 (0.4 to 0.6)	0.4 (0.4 to 0.6)
High-middle SDI	Coal workers pneumoconiosis	17.9 (13 to 26)	14.6 (12.5 to 18.2)	13.2 (10.6 to 17.2)	1.9 (1.4 to 2.7)	1 (0.9 to 1.3)	0.7 (0.6 to 1)
High-middle SDI	Other pneumoconiosis	18.1 (13.7 to 22.8)	18.5 (15.8 to 21.3)	18.2 (15.7 to 22.6)	1.8 (1.4 to 2.3)	1.3 (1.1 to 1.5)	1 (0.9 to 1.3)
High-middle SDI	Asthma	2570 (2040 to 3250)	2030 (1550 to 2620)	2170 (1600 to 2870)	246.1 (195.4 to 308.5)	162.9 (123 to 214.3)	159.6 (114 to 217)
High-middle SDI	Interstitial lung disease and pulmonary sarcoidosis	362 (257 to 449)	341 (292 to 405)	447 (385 to 525)	37.9 (26.3 to 47)	24.7 (21.2 to 29.2)	25.5 (22.1 to 30)
High-middle SDI	Other chronic respiratory diseases	511 (459 to 559)	519 (456 to 580)	575 (497 to 651)	47.5 (42.9 to 51.8)	37.7 (33.2 to 42.1)	37.8 (32.7 to 42.9)
High-middle SDI	Digestive diseases	11500 (10300 to 12900)	14000 (12700 to 15600)	14600 (13100 to 16600)	1115.8 (1007.4 to 1240.4)	993.7 (900.8 to 1112.3)	863.1 (768 to 979.8)
High-middle SDI	Cirrhosis and other chronic liver diseases	4750 (4520 to 4980)	6820 (6630 to 7330)	6830 (6500 to 7530)	459.8 (437.5 to 482.1)	464 (450.9 to 498.2)	381.3 (363.1 to 420)
High-middle SDI	Cirrhosis and other chronic liver diseases due to hepatitis B	1660 (1530 to 1810)	2010 (1870 to 2280)	1880 (1700 to 2250)	159.4 (146.4 to 174.4)	135.9 (126.4 to 154.3)	104.3 (94.5 to 124.6)
High-middle SDI	Cirrhosis and other chronic liver diseases due to hepatitis C	1070 (977 to 1180)	1620 (1480 to 1770)	1680 (1530 to 1880)	104.5 (95.1 to 114.5)	108.6 (99.5 to 118.7)	92.5 (84.7 to 103.2)
High-middle SDI	Cirrhosis and other chronic liver diseases due to alcohol use	1210 (1130 to 1290)	2000 (1880 to 2140)	2010 (1880 to 2170)	118.4 (110.7 to 126.5)	133.7 (125.6 to 142.9)	109.8 (102.7 to 118.1)
High-middle SDI	Cirrhosis due to NASH	348 (322 to 375)	615 (570 to 662)	708 (653 to 766)	34.2 (31.6 to 36.8)	41.4 (38.4 to 44.4)	38.4 (35.6 to 41.5)
High-middle SDI	Cirrhosis and other chronic liver diseases due to other causes	460 (408 to 515)	576 (526 to 631)	548 (494 to 603)	43.4 (38.7 to 48.4)	44.5 (40.8 to 48.5)	36.2 (33 to 39.5)
High-middle SDI	Upper digestive system diseases	3220 (2460 to 4300)	3490 (2570 to 4840)	3770 (2730 to 5270)	307.8 (237.1 to 406.8)	247.6 (182.9 to 341.3)	228.3 (164.1 to 319.2)
High-middle SDI	Peptic ulcer disease	1130 (1050 to 1210)	969 (904 to 1050)	937 (858 to 1040)	113.2 (105.9 to 120.9)	68.8 (64.3 to 74.3)	53.6 (48.9 to 59.2)
High-middle SDI	Gastritis and duodenitis	1240 (832 to 1750)	1400 (944 to 1970)	1520 (1020 to 2160)	115.1 (77.7 to 162.3)	102.6 (68.6 to 145.1)	97.1 (64.2 to 138.6)
High-middle SDI	GERD	862 (465 to 1460)	1120 (601 to 1900)	1310 (701 to 2220)	79.5 (42.7 to 134.8)	76.2 (40.9 to 129.3)	77.6 (41.8 to 131.8)
High-middle SDI	Appendicitis	177 (163 to 195)	119 (109 to 131)	108 (97.1 to 120)	16.5 (15.2 to 18.2)	9.4 (8.5 to 10.3)	7.6 (6.7 to 8.5)
High-middle SDI	Paralytic ileus and intestinal obstruction	739 (664 to 887)	546 (507 to 606)	539 (480 to 596)	73.2 (65.9 to 87.7)	50.3 (47.3 to 54.8)	39.2 (35 to 42.5)
High-middle SDI	Inguinal, femoral, and abdominal hernia	626 (450 to 830)	673 (475 to 900)	763 (538 to 1030)	59.8 (43.3 to 79)	54.1 (38.2 to 72)	53.1 (37.1 to 71)
High-middle SDI	Inflammatory bowel disease	288 (233 to 351)	334 (264 to 412)	397 (309 to 499)	27.8 (22.5 to 33.7)	23.8 (19.1 to 29.2)	23 (18 to 28.8)
High-middle SDI	Vascular intestinal disorders	278 (258 to 296)	362 (348 to 383)	417 (386 to 437)	30.1 (28 to 32.1)	26.7 (25.5 to 28.3)	23.9 (22.2 to 25.1)
High-middle SDI	Gallbladder and biliary diseases	413 (372 to 433)	338 (328 to 373)	370 (351 to 424)	43.6 (39.4 to 45.8)	25.2 (24.5 to 27.7)	21.6 (20.5 to 24.6)
High-middle SDI	Pancreatitis	555 (517 to 613)	896 (840 to 958)	918 (849 to 1000)	53.2 (49.6 to 58.7)	60.7 (56.8 to 64.9)	52.2 (48.2 to 57)
High-middle SDI	Other digestive diseases	459 (389 to 533)	413 (360 to 472)	516 (449 to 590)	44 (37.5 to 51)	31.9 (27.8 to 36.5)	32.9 (28.3 to 38)
High-middle SDI	Neurological disorders	13900 (11000 to 17500)	17800 (14200 to 22200)	21100 (16900 to 26000)	1433.7 (1167.6 to 1755.6)	1360.5 (1094.9 to 1669.7)	1351.5 (1078 to 1671.3)
High-middle SDI	Alzheimer's disease and other dementias	3320 (3110 to 3550)	5060 (4730 to 5440)	7060 (6590 to 7610)	465.7 (435.9 to 496.9)	439.6 (411.2 to 471.4)	439.3 (410.2 to 472.5)
High-middle SDI	Parkinson's disease	648 (596 to 716)	976 (895 to 1090)	1270 (1160 to 1420)	77.5 (71.5 to 85.7)	74.8 (68.8 to 83)	74.3 (67.7 to 82.6)
High-middle SDI	Epilepsy	1960 (1540 to 2490)	1780 (1330 to 2340)	1720 (1230 to 2360)	175.2 (137.6 to 223.3)	141.8 (107.5 to 186.7)	127.2 (90.9 to 176.8)
High-middle SDI	Multiple sclerosis	153 (128 to 174)	179 (157 to 203)	200 (168 to 238)	14.3 (12 to 16.3)	11.9 (10.4 to 13.5)	11.3 (9.5 to 13.5)
High-middle SDI	Motor neuron disease	86.6 (68.3 to 127)	113 (106 to 130)	152 (142 to 185)	8.2 (6.5 to 12.2)	9.1 (8.2 to 10.2)	9.6 (8.9 to 10.9)
High-middle SDI	Headache disorders	7240 (4710 to 10200)	9220 (6020 to 13000)	10100 (6620 to 14200)	646.3 (422.9 to 912.7)	639.8 (417.3 to 904.7)	644.5 (420.6 to 913.1)
High-middle SDI	Migraine	6180 (3910 to 9020)	7890 (5030 to 11500)	8610 (5520 to 12500)	549.7 (349.5 to 799.9)	546.6 (346.9 to 794.2)	552.3 (350.7 to 803.6)
High-middle SDI	Tension-type headache	1070 (607 to 1680)	1330 (755 to 2080)	1450 (827 to 2270)	96.6 (55 to 151.4)	93.2 (52.9 to 146.4)	92.2 (52.3 to 144.9)
High-middle SDI	Other neurological disorders	510 (401 to 615)	526 (443 to 638)	595 (494 to 725)	46.4 (36.6 to 55.7)	43.5 (36.5 to 52.5)	45.4 (37.2 to 56.6)
High-middle SDI	Mental disorders	16900 (12600 to 22000)	21600 (16100 to 28000)	23600 (17600 to 30500)	1524.6 (1135.2 to 1972.7)	1536.6 (1142.9 to 1989.5)	1535.6 (1138.3 to 1989.6)
High-middle SDI	Schizophrenia	1760 (1320 to 2150)	2440 (1830 to 2990)	2850 (2140 to 3490)	157.3 (118.3 to 192.7)	162.5 (122.1 to 198.9)	166.9 (125.2 to 204.5)

SR Appendix Table 2. All-age DALYs counts and age standardized DALYs rates per 100,000 individuals for all causes and level 1 cause groups by location for 1990, 2007, and 2017, both sexes combined

		All-age DALYs			Age-standardized rate of DALYs per 100,000		
High-middle SDI	Depressive disorders	6260 (4450 to 8560)	8060 (5700 to 10900)	8890 (6290 to 12000)	574.7 (407.2 to 785.5)	559.1 (396.1 to 760.8)	545.5 (387.9 to 746.5)
High-middle SDI	Major depressive disorder	4850 (3400 to 6630)	6100 (4270 to 8200)	6600 (4620 to 8910)	443.1 (313.3 to 598.3)	425 (297.3 to 574.3)	411.1 (287.1 to 555)
High-middle SDI	Dysthymia	1410 (942 to 2050)	1960 (1300 to 2860)	2300 (1540 to 3350)	131.6 (88.1 to 191)	134.1 (89.5 to 195.4)	134.4 (90.3 to 195.6)
High-middle SDI	Bipolar disorder	1230 (780 to 1820)	1580 (998 to 2340)	1730 (1090 to 2550)	109.3 (68.7 to 161.2)	109.4 (69.3 to 161.4)	112 (70.6 to 166)
High-middle SDI	Anxiety disorders	3670 (2580 to 4890)	4790 (3400 to 6380)	5120 (3620 to 6780)	329.3 (231.6 to 437.5)	345.3 (243.5 to 460.7)	342.1 (240.6 to 458.1)
High-middle SDI	Eating disorders	383 (244 to 557)	539 (343 to 793)	644 (412 to 954)	31.9 (20.4 to 46.6)	37.5 (23.9 to 54.7)	45.4 (29.1 to 66.5)
High-middle SDI	Anorexia nervosa	83.1 (50.2 to 125)	113 (68.9 to 170)	125 (76.4 to 189)	6.9 (4.2 to 10.4)	8.2 (5 to 12.2)	9.7 (5.8 to 14.5)
High-middle SDI	Bulimia nervosa	299 (187 to 446)	426 (269 to 638)	519 (325 to 780)	25 (15.7 to 37.2)	29.3 (18.5 to 43.7)	35.7 (22.4 to 53.1)
High-middle SDI	Autism spectrum disorders	685 (466 to 942)	772 (526 to 1060)	821 (561 to 1130)	61 (41.5 to 83.8)	61.4 (41.9 to 85.1)	61.7 (42.1 to 85)
High-middle SDI	Attention-deficit/hyperactivity disorder	177 (107 to 280)	197 (120 to 308)	194 (119 to 306)	15.4 (9.3 to 24.3)	15.9 (9.6 to 25)	16 (9.8 to 25.2)
High-middle SDI	Conduct disorder	971 (587 to 1550)	931 (556 to 1490)	814 (486 to 1290)	85.1 (51.2 to 135.7)	86.3 (51.5 to 138.7)	88.4 (52.7 to 140.8)
High-middle SDI	Idiopathic developmental intellectual disability	286 (112 to 520)	283 (106 to 518)	265 (94.7 to 491)	25.2 (9.8 to 45.9)	23 (8.7 to 42)	20.7 (7.6 to 38.4)
High-middle SDI	Other mental disorders	1500 (988 to 2060)	2010 (1330 to 2770)	2290 (1510 to 3150)	135.5 (89.6 to 186.3)	136.1 (89.9 to 187.7)	136.8 (90.4 to 189.3)
High-middle SDI	Substance use disorders	7930 (6370 to 9570)	11300 (9260 to 13500)	11100 (8900 to 13600)	695.8 (561.7 to 837.3)	760.2 (621.9 to 906.8)	689.2 (546.1 to 841)
High-middle SDI	Alcohol use disorders	3980 (3280 to 4810)	5670 (4820 to 6700)	4890 (4010 to 6000)	359.8 (298.3 to 433.7)	376.3 (319 to 445.6)	287.3 (233.3 to 353.7)
High-middle SDI	Drug use disorders	3950 (3040 to 4930)	5630 (4370 to 6980)	6260 (4670 to 7910)	335.9 (259.5 to 418.1)	383.9 (297.9 to 475.5)	401.9 (300 to 507.8)
High-middle SDI	Opioid use disorders	2970 (2190 to 3830)	4510 (3400 to 5700)	5230 (3800 to 6710)	252.7 (186.5 to 323.7)	306.6 (231.4 to 388.5)	330.9 (239.3 to 426.9)
High-middle SDI	Cocaine use disorders	112 (83.2 to 149)	173 (134 to 219)	166 (126 to 218)	9.7 (7.2 to 12.8)	11.8 (9.1 to 15)	11 (8.3 to 14.6)
High-middle SDI	Amphetamine use disorders	269 (162 to 400)	290 (181 to 440)	258 (158 to 393)	22 (13.4 to 32.7)	20.2 (12.5 to 30.8)	19.1 (11.4 to 29.5)
High-middle SDI	Cannabis use disorders	76.4 (47.7 to 116)	91 (57.1 to 134)	87.2 (54.8 to 129)	6.4 (4 to 9.7)	6.3 (4 to 9.4)	6.3 (4 to 9.5)
High-middle SDI	Other drug use disorders	524 (423 to 652)	569 (471 to 693)	521 (425 to 639)	45.1 (36.7 to 56)	39.1 (32.3 to 47.6)	34.5 (27.7 to 42.9)
High-middle SDI	Diabetes and kidney diseases	10300 (8890 to 12200)	14400 (12000 to 17300)	17000 (14100 to 20400)	1031 (886.3 to 1210)	1022.9 (857.9 to 1224.5)	966.7 (801 to 1162)
High-middle SDI	Diabetes mellitus	6430 (5180 to 8000)	10200 (7970 to 12800)	12000 (9390 to 15100)	643.4 (519.8 to 797.9)	713.8 (561.2 to 893.3)	673.9 (527.1 to 848.1)
High-middle SDI	Diabetes mellitus type 1	1250 (1170 to 1360)	1270 (1160 to 1340)	1240 (1100 to 1330)	121.1 (113.3 to 132.1)	89.6 (82.3 to 94.6)	72 (64.1 to 77.4)
High-middle SDI	Diabetes mellitus type 2	5180 (3930 to 6730)	8930 (6740 to 11500)	10800 (8210 to 13800)	522.3 (397.5 to 675.9)	624.2 (474.4 to 803.3)	601.9 (460 to 770)
High-middle SDI	Chronic kidney disease	3770 (3520 to 4050)	4160 (3870 to 4480)	4940 (4580 to 5360)	374.1 (349.5 to 402)	303.9 (283 to 326.7)	288.7 (267.2 to 312.5)
High-middle SDI	Chronic kidney disease due to diabetes mellitus type 1	321 (264 to 386)	340 (280 to 411)	366 (297 to 443)	30.1 (24.8 to 36.2)	22.4 (18.5 to 27)	20.3 (16.7 to 24.4)
High-middle SDI	Chronic kidney disease due to diabetes mellitus type 2	630 (545 to 726)	864 (752 to 987)	1150 (995 to 1320)	66.4 (57.5 to 76.4)	62.9 (54.9 to 71.6)	63.7 (55.4 to 72.9)
High-middle SDI	Chronic kidney disease due to hypertension	609 (528 to 690)	825 (720 to 932)	1090 (951 to 1240)	65.6 (57 to 73.9)	61.5 (54.1 to 69.2)	63 (54.9 to 71.2)
High-middle SDI	Chronic kidney disease due to glomerulonephritis	829 (746 to 918)	809 (721 to 905)	857 (751 to 972)	78.1 (70.3 to 86.5)	57.9 (51.8 to 64.6)	51.2 (45.1 to 57.6)
High-middle SDI	Chronic kidney disease due to other and unspecified causes	1380 (1220 to 1560)	1320 (1150 to 1500)	1480 (1280 to 1690)	133.9 (119.1 to 151.5)	99.2 (87.6 to 112)	90.4 (78.8 to 102.9)
High-middle SDI	Acute glomerulonephritis	146 (137 to 155)	70.7 (68.1 to 73.3)	68 (63.6 to 74.2)	13.6 (12.7 to 14.4)	5.2 (5 to 5.4)	4.1 (3.9 to 4.5)
High-middle SDI	Skin and subcutaneous diseases	5850 (3910 to 8600)	6700 (4490 to 9850)	7350 (4950 to 10700)	529.4 (354.5 to 777.5)	540.5 (361.5 to 792.8)	549.9 (368.7 to 805.4)
High-middle SDI	Dermatitis	1420 (835 to 2250)	1510 (892 to 2360)	1620 (967 to 2530)	127.9 (75.1 to 201.5)	129.2 (75.8 to 204.3)	131.3 (76.7 to 208.8)
High-middle SDI	Atopic dermatitis	1130 (611 to 1890)	1140 (620 to 1910)	1220 (664 to 2040)	101.6 (54.9 to 169.6)	102.8 (55.7 to 171.7)	104.9 (56.7 to 175.9)
High-middle SDI	Contact dermatitis	263 (172 to 393)	335 (219 to 499)	366 (239 to 543)	23.8 (15.6 to 35.3)	23.8 (15.6 to 35.4)	23.8 (15.6 to 35.3)
High-middle SDI	Seborrhoeic dermatitis	28.2 (16.1 to 43.9)	35.6 (20.5 to 56.1)	38.5 (22.1 to 60.5)	2.5 (1.4 to 3.9)	2.6 (1.5 to 4.1)	2.6 (1.5 to 4)
High-middle SDI	Psoriasis	594 (421 to 783)	830 (588 to 1100)	991 (703 to 1310)	54.7 (38.9 to 72)	58.8 (41.8 to 77.8)	61.8 (43.7 to 81.6)
High-middle SDI	Bacterial skin diseases	172 (107 to 205)	202 (138 to 265)	258 (170 to 331)	16.6 (10.3 to 19.7)	15.5 (11 to 20.6)	16.2 (11.3 to 21.5)
High-middle SDI	Cellulitis	65.2 (45.7 to 89.7)	93.3 (62.8 to 125)	117 (74.2 to 157)	6.1 (4.3 to 8.5)	6.8 (4.7 to 9.2)	7.2 (4.6 to 9.7)

SR Appendix Table 2. All-age DALYs counts and age standardized DALYs rates per 100,000 individuals for all causes and level 1 cause groups by location for 1990, 2007, and 2017, both sexes combined

		All-age DALYs			Age-standardized rate of DALYs per 100,000		
High-middle SDI	Pyoderma	107 (58.5 to 132)	109 (73.6 to 150)	141 (92.2 to 193)	10.4 (5.7 to 12.8)	8.7 (6.1 to 12.3)	9.1 (6.2 to 12.8)
High-middle SDI	Scabies	830 (460 to 1360)	901 (493 to 1450)	897 (489 to 1470)	72.7 (40.3 to 118.3)	70.7 (39 to 115)	68.1 (37.5 to 111.4)
High-middle SDI	Fungal skin diseases	492 (197 to 1030)	580 (234 to 1200)	658 (264 to 1370)	47.4 (19 to 98.8)	44.7 (18.1 to 93.1)	43.5 (17.6 to 90.1)
High-middle SDI	Viral skin diseases	686 (439 to 1020)	646 (414 to 955)	653 (421 to 970)	61 (38.9 to 90.4)	61 (38.8 to 90.3)	60.4 (38.7 to 89.6)
High-middle SDI	Acne vulgaris	272 (162 to 435)	405 (242 to 644)	410 (243 to 655)	22.9 (13.6 to 36.6)	30.6 (18.2 to 48.7)	35.7 (21 to 57.4)
High-middle SDI	Alopecia areata	71.7 (45.8 to 106)	89.4 (57.2 to 133)	97.4 (62.2 to 145)	6.3 (4 to 9.3)	6.3 (4 to 9.4)	6.3 (4 to 9.4)
High-middle SDI	Pruritus	104 (48.9 to 198)	135 (64.3 to 256)	157 (74.5 to 295)	9.7 (4.5 to 18.2)	10 (4.7 to 18.7)	10.1 (4.8 to 19.2)
High-middle SDI	Urticaria	781 (514 to 1110)	810 (536 to 1140)	855 (568 to 1200)	69.6 (45.8 to 98.5)	69.4 (45.7 to 98.1)	69.4 (45.7 to 98.1)
High-middle SDI	Decubitus ulcer	50.3 (35.6 to 62.3)	80.7 (54.8 to 96.7)	103 (73.3 to 130)	5.6 (4 to 6.9)	6.4 (4.3 to 7.7)	6.3 (4.5 to 8)
High-middle SDI	Other skin and subcutaneous diseases	369 (184 to 670)	514 (256 to 939)	646 (322 to 1180)	35.1 (17.6 to 63.8)	38 (19 to 69.2)	40.8 (20.3 to 74.4)
High-middle SDI	Sense organ diseases	8070 (5400 to 11700)	11000 (7400 to 16000)	13300 (8880 to 19200)	828.8 (559.5 to 1190.5)	812.4 (547.8 to 1167.6)	788.8 (528.6 to 1136.5)
High-middle SDI	Blindness and vision impairment	3600 (2410 to 5320)	4810 (3220 to 7130)	5670 (3800 to 8390)	369.2 (249.6 to 544.6)	354.9 (239.6 to 524.9)	337.6 (226.3 to 500.1)
High-middle SDI	Glaucoma	98.6 (66.9 to 137)	132 (89.8 to 183)	160 (109 to 220)	10.9 (7.5 to 15.2)	10 (6.8 to 13.8)	9.1 (6.2 to 12.4)
High-middle SDI	Cataract	760 (532 to 1020)	1100 (778 to 1490)	1310 (918 to 1760)	89.6 (62.9 to 120.2)	85.1 (59.8 to 114.5)	76.5 (53.5 to 102.6)
High-middle SDI	Age-related macular degeneration	46.4 (31.5 to 63.5)	83.7 (57.5 to 114)	107 (73 to 146)	5.3 (3.6 to 7.2)	6.4 (4.4 to 8.8)	6.1 (4.2 to 8.3)
High-middle SDI	Refraction disorders	1100 (718 to 1600)	1300 (876 to 1860)	1440 (964 to 2050)	103.7 (68 to 151)	98.6 (66 to 141.9)	95.4 (62.9 to 138)
High-middle SDI	Near vision loss	1180 (561 to 2150)	1630 (774 to 2990)	2010 (954 to 3660)	116.9 (55.6 to 214.3)	114 (54.2 to 209.3)	112.8 (53.8 to 206.3)
High-middle SDI	Other vision loss	415 (287 to 559)	557 (387 to 749)	642 (446 to 862)	42.9 (29.8 to 57.8)	40.8 (28.4 to 54.9)	37.7 (26.2 to 50.8)
High-middle SDI	Age-related and other hearing loss	4160 (2820 to 5940)	5800 (3900 to 8290)	7060 (4750 to 10100)	428.9 (293.4 to 606.2)	426.1 (289.8 to 604.8)	419.5 (285 to 597.5)
High-middle SDI	Other sense organ diseases	310 (193 to 454)	425 (263 to 621)	526 (328 to 767)	30.7 (19.1 to 44.8)	31.4 (19.5 to 45.9)	31.7 (19.7 to 46.2)
High-middle SDI	Musculoskeletal disorders	17800 (13100 to 23600)	23600 (17400 to 31500)	27800 (20500 to 37100)	1688.8 (1239.1 to 2246.8)	1622.3 (1195.7 to 2157.4)	1622.5 (1197.2 to 2147)
High-middle SDI	Rheumatoid arthritis	339 (257 to 426)	464 (345 to 594)	611 (446 to 791)	33.5 (25.5 to 41.9)	32.4 (24.1 to 41.2)	34.4 (25.2 to 44.7)
High-middle SDI	Osteoarthritis	948 (472 to 1890)	1550 (774 to 3090)	1990 (990 to 3950)	99 (49.6 to 196.1)	109.7 (54.8 to 218.5)	109 (54.3 to 217.2)
High-middle SDI	Low back pain	9490 (6740 to 12800)	11200 (7970 to 15000)	12800 (9030 to 17200)	898.8 (639.6 to 1205.7)	778.3 (558.6 to 1049.4)	768.2 (547 to 1029.6)
High-middle SDI	Neck pain	4000 (2780 to 5630)	5950 (4130 to 8380)	7040 (4880 to 9940)	382.3 (266.1 to 537.9)	401.9 (280.4 to 564.3)	399.3 (278.4 to 559.3)
High-middle SDI	Gout	132 (88.5 to 183)	202 (135 to 278)	263 (177 to 362)	13.3 (9 to 18.4)	14.1 (9.5 to 19.4)	14.6 (9.8 to 20.1)
High-middle SDI	Other musculoskeletal disorders	2850 (2000 to 3920)	4240 (2930 to 5880)	5140 (3560 to 7110)	261.9 (184.4 to 360.1)	286 (198.3 to 393.7)	297 (205.7 to 408.9)
High-middle SDI	Other non-communicable diseases	23200 (20500 to 26500)	18500 (15500 to 22300)	16400 (13200 to 20600)	2188.1 (1939.4 to 2497.4)	1729.7 (1508.8 to 2002.7)	1279.2 (1073.4 to 1542.1)
High-middle SDI	Congenital anomalies	14600 (13100 to 16100)	8960 (8320 to 9620)	6230 (5600 to 6880)	1378.6 (1236.7 to 1518.5)	1024 (958.1 to 1084)	635.9 (582.4 to 689.5)
High-middle SDI	Neural tube defects	1410 (1110 to 1800)	533 (480 to 589)	392 (339 to 453)	133.7 (105.3 to 171)	61 (55.7 to 67.2)	40.1 (35.3 to 45.9)
High-middle SDI	Congenital heart anomalies	6600 (5300 to 7650)	3830 (3510 to 4050)	2260 (2070 to 2460)	623.6 (500.1 to 723.9)	464.3 (424.9 to 492)	253.7 (232.5 to 276.3)
High-middle SDI	Orofacial clefts	221 (97.8 to 490)	93 (75 to 116)	64.9 (46.8 to 88.1)	20.9 (9.2 to 46.8)	9.6 (8 to 11.6)	5.5 (4.1 to 7.3)
High-middle SDI	Down's syndrome	580 (336 to 747)	374 (337 to 423)	294 (247 to 377)	54.1 (31.1 to 69.8)	40.6 (36.7 to 46.4)	28.4 (23.9 to 37.1)
High-middle SDI	Turner syndrome	1.11 (0.558 to 1.83)	1.16 (0.586 to 1.88)	1.14 (0.576 to 1.85)	0.1 (0 to 0.2)	0.1 (0 to 0.2)	0.1 (0 to 0.1)
High-middle SDI	Klinefelter syndrome	0.663 (0.306 to 1.24)	0.867 (0.399 to 1.66)	0.873 (0.404 to 1.66)	0.1 (0 to 0.1)	0.1 (0 to 0.1)	0.1 (0 to 0.1)
High-middle SDI	Other chromosomal abnormalities	242 (179 to 347)	267 (228 to 316)	269 (225 to 321)	22.8 (16.8 to 32.9)	30 (25.8 to 36)	27.7 (23.3 to 32.9)
High-middle SDI	Congenital musculoskeletal and limb anomalies	908 (653 to 1180)	941 (660 to 1250)	958 (654 to 1290)	83.1 (60.3 to 107.4)	78.7 (56.8 to 103)	72.6 (50.6 to 96.7)
High-middle SDI	Urogenital congenital anomalies	216 (161 to 275)	155 (135 to 184)	118 (100 to 143)	20.4 (15.3 to 26.1)	18 (15.8 to 21.3)	12.7 (10.8 to 15.3)
High-middle SDI	Digestive congenital anomalies	1190 (863 to 1660)	762 (675 to 858)	526 (456 to 609)	113.9 (82.4 to 159)	88.6 (79.3 to 99.6)	55.1 (48.7 to 63.1)
High-middle SDI	Other congenital anomalies	3230 (2180 to 3990)	2010 (1820 to 2190)	1350 (1180 to 1540)	305.9 (205.6 to 378.8)	233 (212.1 to 252)	140.1 (124.3 to 160.8)

SR Appendix Table 2. All-age DALYs counts and age standardized DALYs rates per 100,000 individuals for all causes and level 1 cause groups by location for 1990, 2007, and 2017, both sexes combined

		All-age DALYs			Age-standardized rate of DALYs per 100,000		
High-middle SDI	Urinary diseases and male infertility	1080 (925 to 1270)	1170 (976 to 1410)	1460 (1220 to 1760)	107.5 (91.9 to 126)	86 (72.2 to 102.9)	85 (71.2 to 101.7)
High-middle SDI	Urinary tract infections	488 (445 to 520)	506 (489 to 530)	641 (613 to 675)	48 (43.9 to 51.1)	38.4 (37.1 to 40.2)	39 (37.2 to 41.1)
High-middle SDI	Urolithiasis	134 (115 to 159)	104 (88.1 to 123)	117 (97.5 to 140)	13.3 (11.4 to 15.8)	7.3 (6.2 to 8.5)	6.6 (5.5 to 8)
High-middle SDI	Benign prostatic hyperplasia	322 (205 to 461)	418 (268 to 596)	545 (349 to 783)	33.3 (21.3 to 47.6)	29.9 (19.3 to 42.7)	29.6 (18.9 to 42.4)
High-middle SDI	Male infertility	28.8 (11.7 to 60.4)	37.6 (15.3 to 78.1)	37.3 (15.1 to 77.7)	2.4 (1 to 5)	2.5 (1 to 5.2)	2.4 (1 to 5.1)
High-middle SDI	Other urinary diseases	108 (89 to 132)	105 (89.9 to 118)	120 (95.4 to 139)	10.4 (8.6 to 12.8)	7.9 (6.8 to 8.9)	7.4 (5.9 to 8.5)
High-middle SDI	Gynecological diseases	1920 (1340 to 2660)	2520 (1750 to 3510)	2570 (1780 to 3570)	168.8 (117.5 to 233.1)	165.2 (114.8 to 229.9)	158.1 (109.3 to 219)
High-middle SDI	Uterine fibroids	239 (153 to 373)	340 (211 to 542)	336 (203 to 550)	22.6 (14.4 to 35.3)	21.7 (13.5 to 34.6)	18.8 (11.3 to 30.6)
High-middle SDI	Polycystic ovarian syndrome	62.7 (27.8 to 121)	81.6 (36.5 to 157)	85.6 (38.4 to 165)	5.4 (2.4 to 10.4)	5.5 (2.4 to 10.5)	5.6 (2.5 to 10.7)
High-middle SDI	Female infertility	49.8 (18.2 to 107)	63.7 (23.1 to 134)	68.4 (25 to 144)	4.1 (1.5 to 8.9)	4.2 (1.5 to 8.9)	4.4 (1.6 to 9.2)
High-middle SDI	Endometriosis	753 (499 to 1090)	994 (651 to 1430)	1030 (677 to 1500)	65.3 (43.3 to 94.4)	64.8 (42.7 to 93.2)	62.6 (41.4 to 90.6)
High-middle SDI	Genital prolapse	42.6 (20.8 to 78.8)	47.4 (23.4 to 88.3)	59.6 (29 to 113)	4.2 (2.1 to 7.8)	3.2 (1.6 to 6)	3.3 (1.6 to 6.2)
High-middle SDI	Premenstrual syndrome	595 (382 to 891)	761 (489 to 1140)	751 (482 to 1120)	51.4 (33.1 to 77)	50.7 (32.5 to 76)	48.7 (31.2 to 72.9)
High-middle SDI	Other gynecological diseases	177 (126 to 241)	234 (162 to 324)	244 (169 to 346)	15.7 (11.2 to 21.4)	15.2 (10.6 to 21.1)	14.8 (10.2 to 21)
High-middle SDI	Hemoglobinopathies and hemolytic anaemias	1390 (1080 to 1770)	1080 (835 to 1430)	896 (686 to 1190)	126.9 (98.1 to 160.9)	87.2 (68.4 to 114.4)	65.4 (50.5 to 85.8)
High-middle SDI	Thalassemias	221 (170 to 281)	104 (83.3 to 113)	67.6 (56.2 to 75)	20.1 (15.4 to 25.6)	12 (9.7 to 13.2)	7.5 (6.2 to 8.3)
High-middle SDI	Thalassaemias trait	497 (336 to 735)	470 (309 to 701)	379 (247 to 569)	44.6 (30.2 to 65.7)	35.7 (23.6 to 53.5)	26.9 (17.3 to 40.4)
High-middle SDI	Sickle cell disorders	108 (79.8 to 154)	71.4 (63.3 to 84.9)	58.4 (51.7 to 70.7)	9.6 (7.1 to 13.9)	6.4 (5.7 to 7.9)	5 (4.4 to 6.1)
High-middle SDI	Sickle cell trait	63.6 (42 to 93.4)	55.5 (36.1 to 83.3)	47.4 (30.5 to 71.4)	5.7 (3.8 to 8.4)	4.5 (2.9 to 6.7)	3.6 (2.3 to 5.4)
High-middle SDI	G6PD deficiency	85.2 (70.3 to 98.8)	86.6 (69.8 to 95.7)	80.5 (68.8 to 89.1)	7.5 (6.2 to 8.6)	6.4 (5.2 to 7)	5.5 (4.8 to 6.1)
High-middle SDI	G6PD trait	0.0591 (0.0403 to 0.0836)	0.0743 (0.0513 to 0.106)	0.0697 (0.0472 to 0.0993)	0 (0 to 0)	0 (0 to 0)	0 (0 to 0)
High-middle SDI	Other hemoglobinopathies and hemolytic anaemias	419 (327 to 537)	293 (232 to 379)	263 (210 to 337)	39.4 (31 to 50.1)	22.1 (17.5 to 28.6)	16.9 (13.4 to 21.7)
High-middle SDI	Endocrine, metabolic, blood, and immune disorders	1110 (910 to 1360)	979 (837 to 1130)	1040 (873 to 1210)	104.1 (85.2 to 126.6)	84.6 (71.8 to 97)	76.6 (63.5 to 88.7)
High-middle SDI	Oral disorders	2540 (1570 to 3850)	3490 (2150 to 5310)	4070 (2510 to 6240)	250.3 (155.2 to 379.2)	248.2 (153.4 to 378.3)	238.4 (146.8 to 364.1)
High-middle SDI	Caries of deciduous teeth	28.3 (12.2 to 55.9)	19.4 (8.32 to 38.2)	20.9 (8.98 to 41.6)	2.6 (1.1 to 5)	2.4 (1 to 4.8)	2.5 (1.1 to 4.9)
High-middle SDI	Caries of permanent teeth	268 (117 to 506)	301 (130 to 581)	311 (137 to 608)	23.9 (10.5 to 45.2)	21.6 (9.4 to 41.3)	20.9 (9.1 to 39.9)
High-middle SDI	Periodontal disease	555 (220 to 1150)	855 (336 to 1750)	1030 (408 to 2110)	54.1 (21.4 to 111)	57.8 (22.7 to 118.1)	56.9 (22.4 to 116.5)
High-middle SDI	Edentulism and severe tooth loss	1120 (749 to 1590)	1590 (1070 to 2250)	1910 (1280 to 2700)	118.7 (79.5 to 167.9)	115.3 (77.3 to 161.8)	107.1 (71.6 to 151.6)
High-middle SDI	Other oral disorders	567 (353 to 832)	718 (446 to 1060)	793 (494 to 1170)	51 (31.8 to 75)	51.1 (31.8 to 75.3)	51 (31.8 to 75.3)
High-middle SDI	Sudden infant death syndrome	541 (336 to 956)	273 (225 to 334)	170 (142 to 200)	51.9 (32.3 to 91.8)	34.5 (28.4 to 42.2)	19.9 (16.6 to 23.4)
High-middle SDI	Injuries	56100 (53300 to 59200)	50000 (46900 to 53700)	44000 (40200 to 48700)	5031.2 (4764.8 to 5320.6)	3680.7 (3466.8 to 3944.6)	2918.8 (2699.1 to 3194)
High-middle SDI	Transport injuries	16100 (15200 to 17100)	16600 (15800 to 17500)	14000 (13000 to 15100)	1418.6 (1344.7 to 1510.8)	1212.2 (1157.5 to 1273.4)	934.6 (876.2 to 1001.4)
High-middle SDI	Road injuries	14900 (14200 to 15900)	15400 (14700 to 16200)	12900 (12000 to 13900)	1316.7 (1250.3 to 1403.9)	1126.9 (1080.8 to 1179.4)	859.1 (806.4 to 916.9)
High-middle SDI	Pedestrian road injuries	6520 (5870 to 7310)	5800 (5520 to 6350)	4540 (4240 to 4890)	583.9 (526.7 to 653.5)	436.5 (416.2 to 475.8)	302.9 (284.4 to 325.6)
High-middle SDI	Cyclist road injuries	645 (525 to 786)	1010 (838 to 1170)	1020 (855 to 1200)	58.7 (47.8 to 71.5)	71.9 (59.6 to 82.4)	63.2 (53.3 to 73.7)
High-middle SDI	Motorcyclist road injuries	2330 (2050 to 2760)	2790 (2250 to 3030)	2180 (1910 to 2430)	200.4 (177 to 238)	193.7 (157.2 to 210.4)	144.9 (128.2 to 159.5)
High-middle SDI	Motor vehicle road injuries	5250 (4860 to 5930)	5600 (5340 to 5910)	4880 (4550 to 5220)	458.4 (423.9 to 519.1)	410.2 (391.8 to 432.8)	333.2 (312.8 to 354)
High-middle SDI	Other road injuries	168 (133 to 201)	205 (167 to 255)	244 (189 to 321)	15.4 (12.2 to 18.4)	14.6 (12 to 18)	15 (11.7 to 19.5)
High-middle SDI	Other transport injuries	1140 (993 to 1310)	1180 (1060 to 1340)	1150 (995 to 1340)	101.9 (88.9 to 117.1)	85.2 (76.1 to 96.4)	75.5 (65.6 to 87.4)
High-middle SDI	Unintentional injuries	25900 (23900 to 28300)	20400 (18200 to 23100)	19000 (16400 to 22300)	2378.4 (2186.3 to 2598.9)	1569.4 (1414.9 to 1760.6)	1269.7 (1111.9 to 1468.9)

SR Appendix Table 2. All-age DALYs counts and age standardized DALYs rates per 100,000 individuals for all causes and level 1 cause groups by location for 1990, 2007, and 2017, both sexes combined							
		All-age DALYs			Age-standardized rate of DALYs per 100,000		
High-middle SDI	Falls	6110 (4960 to 7430)	6750 (5510 to 8320)	7600 (6060 to 9610)	585.8 (476.7 to 714.1)	493.9 (405.7 to 606.3)	470.1 (378.5 to 588.7)
High-middle SDI	Drowning	5740 (5460 to 6060)	2990 (2920 to 3050)	2130 (2060 to 2200)	510.3 (485.5 to 538.7)	260.6 (253.8 to 267.5)	173.3 (167.4 to 180.1)
High-middle SDI	Fire, heat, and hot substances	2090 (1790 to 2380)	1700 (1490 to 1960)	1350 (1140 to 1650)	190.2 (163.8 to 217.3)	128.8 (113.1 to 147.3)	89.6 (76.3 to 108.2)
High-middle SDI	Poisonings	1420 (1280 to 1670)	1300 (1150 to 1370)	1000 (821 to 1120)	128.5 (116.3 to 150.8)	98.9 (86.6 to 104.5)	69.1 (56.3 to 77.8)
High-middle SDI	Poisoning by carbon monoxide	941 (857 to 1110)	944 (835 to 981)	657 (532 to 722)	85.1 (77.9 to 99.8)	69.9 (61.2 to 72.9)	44.6 (35.6 to 49.5)
High-middle SDI	Poisoning by other means	481 (416 to 579)	357 (307 to 403)	345 (281 to 407)	43.4 (37.5 to 52.1)	29 (25 to 32.5)	24.5 (20.2 to 29.3)
High-middle SDI	Exposure to mechanical forces	2390 (2080 to 2800)	2430 (2060 to 2900)	2370 (1920 to 2940)	217.1 (187.8 to 254.6)	176.8 (150.7 to 209.7)	150.9 (123.2 to 185)
High-middle SDI	Unintentional firearm injuries	342 (288 to 391)	268 (240 to 292)	245 (208 to 278)	30.4 (25.5 to 34.7)	19.7 (17.7 to 21.4)	16.9 (14.3 to 19)
High-middle SDI	Other exposure to mechanical forces	2050 (1760 to 2460)	2160 (1810 to 2610)	2120 (1700 to 2670)	186.6 (159.5 to 225.3)	157.1 (132.4 to 188.8)	134 (107.6 to 167.1)
High-middle SDI	Adverse effects of medical treatment	653 (537 to 717)	545 (496 to 602)	551 (498 to 643)	61.1 (50.4 to 67)	44.7 (40.9 to 49.7)	38.1 (34.7 to 44.6)
High-middle SDI	Animal contact	300 (243 to 354)	230 (189 to 278)	220 (175 to 272)	27.5 (22.3 to 32.4)	18.2 (15.1 to 21.7)	15.3 (12.3 to 18.9)
High-middle SDI	Venomous animal contact	173 (138 to 208)	136 (110 to 167)	128 (101 to 160)	15.8 (12.6 to 19)	10.9 (8.8 to 13.3)	9.2 (7.2 to 11.4)
High-middle SDI	Non-venomous animal contact	127 (101 to 157)	93.8 (73 to 125)	91.7 (68.3 to 127)	11.7 (9.3 to 14.4)	7.3 (5.7 to 9.5)	6.2 (4.7 to 8.5)
High-middle SDI	Foreign body	1930 (1810 to 2110)	1510 (1440 to 1580)	1270 (1190 to 1340)	180.1 (169.5 to 197.6)	140.8 (133.8 to 147.6)	102.7 (96.5 to 108.6)
High-middle SDI	Pulmonary aspiration and foreign body in airway	1590 (1480 to 1860)	1290 (1250 to 1340)	1030 (986 to 1080)	149 (138.8 to 174.2)	124.5 (118.9 to 129.7)	87.3 (82.5 to 92.3)
High-middle SDI	Foreign body in eyes	21.3 (10.4 to 36)	24.7 (12.5 to 41.8)	28.9 (15 to 48.1)	2 (1 to 3.3)	1.8 (0.9 to 3.1)	1.9 (0.9 to 3.2)
High-middle SDI	Foreign body in other body part	315 (193 to 384)	189 (156 to 225)	202 (164 to 247)	29.1 (17.7 to 35.5)	14.6 (12.2 to 17.2)	13.5 (11 to 16.3)
High-middle SDI	Environmental heat and cold exposure	1350 (1180 to 1510)	1340 (1230 to 1460)	964 (846 to 1100)	124.7 (109.5 to 139.8)	91.4 (83.8 to 100)	57 (49.9 to 65.3)
High-middle SDI	Exposure to forces of nature	2560 (2550 to 2580)	127 (107 to 154)	229 (211 to 253)	225.7 (224 to 227.5)	9.5 (8.1 to 11.4)	16.5 (15.4 to 18)
High-middle SDI	Other unintentional injuries	1410 (1250 to 1600)	1460 (1290 to 1680)	1350 (1130 to 1630)	127.5 (112.7 to 145.8)	105.8 (93.7 to 121.5)	87.1 (74.1 to 104.6)
High-middle SDI	Self-harm and interpersonal violence	14100 (13400 to 14600)	13100 (12700 to 13500)	11000 (10500 to 11500)	1234.2 (1172.9 to 1282)	899.2 (874.6 to 927.5)	714.6 (686.8 to 746.2)
High-middle SDI	Self-harm	8150 (7570 to 8510)	7380 (7240 to 7630)	5990 (5740 to 6310)	719.3 (668.5 to 751.1)	501.9 (492.4 to 518.3)	377.4 (361.9 to 397.3)
High-middle SDI	Self-harm by firearm	375 (301 to 505)	340 (261 to 458)	274 (207 to 356)	33.1 (26.5 to 44.5)	23.2 (17.9 to 31.3)	18 (13.8 to 23.3)
High-middle SDI	Self-harm by other specified means	7780 (7190 to 8150)	7040 (6870 to 7290)	5710 (5480 to 6010)	686.2 (633.5 to 719.8)	478.7 (467.3 to 495.3)	359.4 (344.5 to 377.9)
High-middle SDI	Interpersonal violence	5300 (5000 to 5610)	5270 (4990 to 5610)	4150 (3850 to 4500)	459.6 (433.9 to 486.8)	368.6 (349.3 to 392.4)	277.7 (258.7 to 300.7)
High-middle SDI	Assault by firearm	1060 (976 to 1180)	1180 (1100 to 1240)	1030 (945 to 1100)	88.9 (81.9 to 98.8)	82.5 (77 to 86.5)	75.9 (70.1 to 80.8)
High-middle SDI	Assault by sharp object	1360 (1170 to 1530)	1370 (1280 to 1490)	916 (846 to 1040)	117.6 (100.7 to 132.1)	94.3 (87.5 to 102.3)	59.2 (54.7 to 67.6)
High-middle SDI	Sexual violence	413 (278 to 601)	521 (352 to 756)	553 (375 to 799)	35.5 (24 to 51.9)	36.3 (24.6 to 52.6)	36.7 (24.9 to 53.2)
High-middle SDI	Assault by other means	2460 (2240 to 2700)	2200 (2030 to 2430)	1650 (1490 to 1880)	217.6 (197.9 to 238.9)	155.6 (144 to 171.7)	105.9 (96 to 119.9)
High-middle SDI	Conflict and terrorism	509 (433 to 632)	295 (227 to 414)	673 (606 to 793)	43.5 (37.1 to 54.1)	20.9 (16.3 to 28.9)	49.5 (45.5 to 56.6)
High-middle SDI	Executions and police conflict	141 (71.2 to 164)	113 (98.4 to 127)	144 (129 to 161)	11.8 (6 to 13.7)	7.8 (6.8 to 8.7)	10 (9 to 11.2)
High SDI	All causes	286000 (257000 to 320000)	296000 (262000 to 336000)	319000 (280000 to 364000)	25660.7 (22991.3 to 28823.4)	21149.5 (18524.3 to 24224.8)	19982.1 (17281.9 to 23098.6)
High SDI	Communicable, maternal, neonatal, and nutritional disorders	19500 (17700 to 21600)	16200 (14600 to 18200)	16100 (14300 to 18200)	2241.8 (2048.7 to 2474.1)	1616.7 (1445.8 to 1815.9)	1459.1 (1287.9 to 1657.4)
High SDI	HIV/AIDS and sexually transmitted infections	2300 (2190 to 2450)	1170 (1030 to 1350)	893 (732 to 1110)	222.9 (211.8 to 238.3)	96.7 (84.9 to 112.9)	70.4 (57.7 to 87.5)
High SDI	HIV/AIDS	2090 (2020 to 2170)	956 (871 to 1070)	679 (576 to 812)	199.7 (193.5 to 207.5)	75.5 (69 to 83.8)	49.8 (42.9 to 58.8)
High SDI	HIV/AIDS - Drug-susceptible Tuberculosis	141 (90.7 to 212)	42.1 (27.6 to 62.3)	29 (19.2 to 41.9)	13.6 (8.8 to 20.5)	3.4 (2.3 to 5.1)	2.2 (1.5 to 3.2)
High SDI	HIV/AIDS - Multidrug-resistant Tuberculosis without extensive drug resistance	5.15 (2.93 to 8.44)	3.03 (1.95 to 4.54)	1.78 (1.09 to 2.73)	0.5 (0.3 to 0.8)	0.3 (0.2 to 0.4)	0.1 (0.1 to 0.2)
High SDI	HIV/AIDS - Extensively drug-resistant Tuberculosis	0 (0 to 0)	0.642 (0.409 to 0.965)	0.64 (0.398 to 0.982)	0 (0 to 0)	0.1 (0 to 0.1)	0.1 (0 to 0.1)
High SDI	HIV/AIDS resulting in other diseases	1940 (1850 to 2030)	910 (828 to 1020)	648 (549 to 780)	185.5 (176.7 to 194.4)	71.8 (65.5 to 80.1)	47.4 (40.6 to 56.4)
High SDI	Sexually transmitted infections excluding HIV	214 (133 to 350)	213 (127 to 355)	214 (124 to 359)	23.3 (14.1 to 36.9)	21.2 (12.5 to 34.3)	20.6 (11.9 to 34.2)

SR Appendix Table 2. All-age DALYs counts and age standardized DALYs rates per 100,000 individuals for all causes and level 1 cause groups by location for 1990, 2007, and 2017, both sexes combined

		All-age DALYs			Age-standardized rate of DALYs per 100,000		
High SDI	Syphilis	65.2 (36.1 to 108)	52.4 (29.3 to 88)	51.7 (29.3 to 88.7)	9 (4.4 to 15.8)	7.2 (3.3 to 13.4)	6.9 (3 to 13.6)
High SDI	Chlamydial infection	34.2 (18.8 to 64.5)	36.6 (19.9 to 69.2)	35.7 (19.3 to 67.5)	3.3 (1.8 to 6.3)	3.4 (1.8 to 6.4)	3.3 (1.8 to 6.3)
High SDI	Gonococcal infection	21.5 (13.9 to 36.3)	19.9 (12.6 to 33.9)	19.3 (12.3 to 33)	2.1 (1.3 to 3.7)	1.8 (1.1 to 3.3)	1.8 (1.1 to 3.2)
High SDI	Trichomoniasis	26.3 (10.6 to 56.2)	29.5 (11.9 to 63.3)	30.5 (12.3 to 65.1)	2.5 (1 to 5.3)	2.5 (1 to 5.4)	2.5 (1 to 5.4)
High SDI	Genital herpes	30.9 (9.94 to 74)	37.4 (12 to 89.9)	40.2 (12.8 to 96.1)	2.8 (0.9 to 6.7)	2.8 (0.9 to 6.8)	2.8 (0.9 to 6.7)
High SDI	Other sexually transmitted infections	36 (22.1 to 61.5)	37.7 (23.1 to 64.4)	36.9 (22.7 to 63.3)	3.5 (2.1 to 6)	3.5 (2.1 to 6)	3.4 (2 to 5.9)
High SDI	Respiratory infections and tuberculosis	6270 (5790 to 6950)	5920 (5410 to 6640)	6430 (5920 to 7150)	589.4 (536.8 to 664)	422.3 (369.7 to 495.3)	384 (333.9 to 456.2)
High SDI	Tuberculosis	820 (796 to 848)	343 (328 to 360)	260 (244 to 278)	71.3 (69 to 73.9)	22.3 (21.1 to 23.6)	14.4 (13.3 to 15.6)
High SDI	Latent tuberculosis infection	0 (0 to 0)	0 (0 to 0)	0 (0 to 0)	0 (0 to 0)	0 (0 to 0)	0 (0 to 0)
High SDI	Drug-susceptible tuberculosis	801 (774 to 828)	315 (299 to 332)	245 (227 to 262)	69.6 (67.2 to 72.1)	20.4 (19.2 to 21.7)	13.5 (12.4 to 14.7)
High SDI	Multidrug-resistant tuberculosis without extensive drug resistance	19.7 (12.8 to 31.7)	22.9 (17.2 to 30.6)	11.6 (7.67 to 18.1)	1.7 (1.1 to 2.8)	1.5 (1.2 to 2)	0.7 (0.4 to 1)
High SDI	Extensively drug-resistant tuberculosis	0 (0 to 0)	4.76 (3.76 to 6)	4.03 (2.9 to 5.88)	0 (0 to 0)	0.3 (0.3 to 0.4)	0.2 (0.2 to 0.3)
High SDI	Lower respiratory infections	4260 (4220 to 4310)	4370 (4320 to 4410)	4940 (4830 to 5070)	387.2 (382.3 to 394.4)	275.1 (271.8 to 278.6)	248.1 (242.6 to 253.9)
High SDI	Upper respiratory infections	993 (596 to 1560)	1030 (613 to 1640)	1050 (623 to 1660)	109 (65.2 to 170.5)	105.8 (61.9 to 167.5)	102.7 (60 to 162.6)
High SDI	Otitis media	190 (117 to 292)	177 (107 to 279)	179 (109 to 280)	21.9 (13.7 to 33.7)	19.2 (11.5 to 30.5)	18.8 (11.3 to 30.1)
High SDI	Enteric infections	633 (488 to 798)	940 (758 to 1160)	1030 (836 to 1280)	72.5 (56.3 to 91.9)	89.1 (70.6 to 111.9)	87.4 (68 to 110.8)
High SDI	Diarrhoeal diseases	594 (451 to 764)	911 (732 to 1130)	1010 (806 to 1250)	67.8 (51.8 to 87.5)	85.8 (67 to 108.5)	84.6 (65 to 108)
High SDI	Typhoid and paratyphoid	5.07 (3.19 to 7.3)	2.05 (1.26 to 3.35)	1.81 (1.09 to 2.93)	0.6 (0.4 to 0.9)	0.2 (0.1 to 0.4)	0.2 (0.1 to 0.3)
High SDI	Typhoid fever	3.39 (2.23 to 5.25)	1.59 (0.938 to 2.6)	1.36 (0.788 to 2.19)	0.4 (0.2 to 0.6)	0.2 (0.1 to 0.3)	0.2 (0.1 to 0.3)
High SDI	Paratyphoid fever	1.68 (0.659 to 2.39)	0.464 (0.269 to 0.847)	0.459 (0.271 to 0.8)	0.2 (0.1 to 0.3)	0.1 (0 to 0.1)	0.1 (0 to 0.1)
High SDI	Invasive non-typhoidal salmonella	31.4 (16.4 to 54.7)	25.4 (13 to 44.6)	23.4 (11.8 to 41.6)	3.7 (2 to 6.7)	2.9 (1.4 to 5.4)	2.5 (1.2 to 4.8)
High SDI	Other intestinal infectious diseases	2.64 (1.32 to 4.39)	1.57 (0.867 to 2.36)	1.27 (0.734 to 1.9)	0.3 (0.1 to 0.6)	0.2 (0.1 to 0.3)	0.1 (0.1 to 0.2)
High SDI	Neglected tropical diseases and malaria	227 (158 to 305)	224 (152 to 306)	239 (160 to 330)	21.5 (15.1 to 28.8)	17.6 (12 to 23.9)	17.1 (11.5 to 23.3)
High SDI	Malaria	2.48 (1.81 to 3.69)	0.627 (0.467 to 0.862)	0.25 (0.187 to 0.342)	0.3 (0.2 to 0.4)	0.1 (0 to 0.1)	0 (0 to 0)
High SDI	Chagas disease	2.32 (1.71 to 3.09)	3.94 (3.04 to 4.96)	2.58 (1.68 to 3.75)	0.2 (0.1 to 0.3)	0.2 (0.2 to 0.3)	0.2 (0.1 to 0.2)
High SDI	Leishmaniasis	5.36 (0.634 to 40.4)	3.35 (0.754 to 22.8)	1.06 (0.00551 to 9.18)	0.6 (0.1 to 4.9)	0.3 (0 to 2.7)	0.1 (0 to 1.1)
High SDI	Visceral leishmaniasis	5.35 (0.633 to 40.4)	3.35 (0.751 to 22.8)	1.05 (0.00361 to 9.18)	0.6 (0.1 to 4.9)	0.3 (0 to 2.7)	0.1 (0 to 1.1)
High SDI	Cutaneous and mucocutaneous leishmaniasis	0.00158 (0.000722 to 0.00282)	0.00242 (0.00142 to 0.00393)	0.00225 (0.00122 to 0.00389)	0 (0 to 0)	0 (0 to 0)	0 (0 to 0)
High SDI	African trypanosomiasis	0 (0 to 0)	0.0496 (0.00273 to 0.161)	0 (0 to 0)	0 (0 to 0)	0 (0 to 0)	0 (0 to 0)
High SDI	Schistosomiasis	0.0139 (0.00822 to 0.0211)	0.00765 (0.0044 to 0.0117)	0.00702 (0.00407 to 0.011)	0 (0 to 0)	0 (0 to 0)	0 (0 to 0)
High SDI	Cysticercosis	115 (71.5 to 169)	124 (74 to 186)	133 (76.5 to 203)	10.1 (6.2 to 14.8)	8.9 (5.3 to 13.4)	8.4 (4.8 to 12.8)
High SDI	Cystic echinococcosis	2.69 (1.98 to 3.48)	2.32 (1.66 to 3.06)	2.02 (1.46 to 2.76)	0.2 (0.2 to 0.3)	0.2 (0.1 to 0.2)	0.1 (0.1 to 0.2)
High SDI	Lymphatic filariasis	7.9 (1.5 to 22.9)	5.79 (1.26 to 14.1)	0.0139 (0.00102 to 0.0466)	0.8 (0.1 to 2.3)	0.5 (0.1 to 1.2)	0 (0 to 0)
High SDI	Onchocerciasis	0 (0 to 0)	0 (0 to 0)	0 (0 to 0)	0 (0 to 0)	0 (0 to 0)	0 (0 to 0)
High SDI	Trachoma	0.274 (0.142 to 0.474)	0.278 (0.15 to 0.463)	0.308 (0.137 to 0.611)	0 (0 to 0)	0 (0 to 0)	0 (0 to 0)
High SDI	Dengue	1.64 (0.479 to 3.24)	3.41 (1.79 to 6.02)	8.21 (4.23 to 14.2)	0.2 (0.1 to 0.4)	0.3 (0.2 to 0.6)	0.7 (0.4 to 1.3)
High SDI	Yellow fever	0 (0 to 0)	0 (0 to 0)	0 (0 to 0)	0 (0 to 0)	0 (0 to 0)	0 (0 to 0)
High SDI	Rabies	0.637 (0.558 to 0.875)	0.797 (0.709 to 0.859)	0.625 (0.554 to 0.707)	0.1 (0.1 to 0.1)	0.1 (0.1 to 0.1)	0.1 (0.1 to 0.1)
High SDI	Intestinal nematode infections	17.8 (9.49 to 29.9)	2.69 (1.54 to 4.46)	3.82 (2.13 to 6.53)	1.9 (1 to 3.2)	0.3 (0.1 to 0.4)	0.3 (0.2 to 0.6)
High SDI	Ascariasis	12.4 (6.4 to 21)	0.205 (0.11 to 0.358)	0.178 (0.0956 to 0.318)	1.4 (0.7 to 2.3)	0 (0 to 0)	0 (0 to 0)

SR Appendix Table 2. All-age DALYs counts and age standardized DALYs rates per 100,000 individuals for all causes and level 1 cause groups by location for 1990, 2007, and 2017, both sexes combined							
		All-age DALYs			Age-standardized rate of DALYs per 100,000		
High SDI	Trichuriasis	2.88 (1.45 to 5.11)	0.176 (0.082 to 0.345)	0.277 (0.124 to 0.542)	0.3 (0.2 to 0.5)	0 (0 to 0)	0 (0 to 0)
High SDI	Hookworm disease	2.48 (1.39 to 4.11)	2.31 (1.31 to 3.81)	3.37 (1.84 to 5.84)	0.3 (0.1 to 0.4)	0.2 (0.1 to 0.3)	0.3 (0.2 to 0.5)
High SDI	Food-borne trematodiases	49.2 (29.4 to 75.6)	62.2 (38 to 94.2)	72.7 (45.2 to 110)	4.8 (2.8 to 7.4)	5.2 (3.2 to 7.9)	5.7 (3.5 to 8.6)
High SDI	Leprosy	0.0917 (0.0557 to 0.144)	0.101 (0.0642 to 0.155)	0.0974 (0.0617 to 0.147)	0 (0 to 0)	0 (0 to 0)	0 (0 to 0)
High SDI	Ebola virus disease	0 (0 to 0)	0 (0 to 0)	0 (0 to 0)	0 (0 to 0)	0 (0 to 0)	0 (0 to 0)
High SDI	Zika virus disease	0 (0 to 0)	0 (0 to 0)	0.0178 (0.0082 to 0.0408)	0 (0 to 0)	0 (0 to 0)	0 (0 to 0)
High SDI	Guinea worm disease	0 (0 to 0)	0 (0 to 0)	0 (0 to 0)	0 (0 to 0)	0 (0 to 0)	0 (0 to 0)
High SDI	Other neglected tropical diseases	21.4 (15.6 to 29.8)	15 (9.42 to 19.6)	14.9 (8.91 to 19.7)	2.4 (1.7 to 3.4)	1.5 (1 to 2)	1.4 (0.9 to 1.9)
High SDI	Other infectious diseases	1240 (1150 to 1350)	905 (824 to 1010)	832 (752 to 939)	140.4 (130.7 to 152.3)	84.3 (76.6 to 93.5)	70.4 (63.4 to 79.4)
High SDI	Meningitis	494 (455 to 526)	238 (220 to 265)	188 (173 to 217)	61.8 (57.7 to 66.1)	25.9 (24 to 29)	18.7 (17.2 to 22.3)
High SDI	Pneumococcal meningitis	111 (98.7 to 122)	62.8 (54.9 to 72)	47.8 (42.3 to 55.2)	12.9 (11.6 to 14.2)	6.1 (5.4 to 7)	4.2 (3.7 to 5)
High SDI	H influenzae type B meningitis	153 (141 to 163)	65.7 (61.9 to 73.7)	47.7 (44.8 to 57.5)	20.2 (18.7 to 21.8)	7.8 (7.3 to 9)	5.4 (5 to 6.7)
High SDI	Meningococcal infection	57.9 (53.1 to 63.1)	23.2 (21.2 to 25.8)	16.2 (14.6 to 18.8)	7.7 (7.1 to 8.4)	2.7 (2.5 to 3.1)	1.7 (1.6 to 2.1)
High SDI	Other meningitis	173 (157 to 185)	86.8 (79.7 to 97)	76.4 (68.8 to 88.7)	21.1 (19.4 to 22.7)	9.2 (8.4 to 10.4)	7.3 (6.6 to 8.7)
High SDI	Encephalitis	128 (115 to 138)	119 (109 to 131)	119 (107 to 133)	14.3 (12.8 to 15.3)	11.4 (10.5 to 12.4)	10.4 (9.4 to 11.7)
High SDI	Diphtheria	0.528 (0.438 to 0.65)	0.196 (0.184 to 0.21)	0.183 (0.17 to 0.198)	0.1 (0.1 to 0.1)	0 (0 to 0)	0 (0 to 0)
High SDI	Whooping cough	19.7 (9.61 to 44)	9.55 (5.18 to 15)	8.12 (4.44 to 12.2)	3.1 (1.5 to 6.9)	1.6 (0.9 to 2.5)	1.4 (0.8 to 2.1)
High SDI	Tetanus	8.48 (7 to 9.58)	1.81 (1.65 to 2.09)	1.38 (1.21 to 1.65)	0.9 (0.8 to 1)	0.2 (0.1 to 0.2)	0.1 (0.1 to 0.1)
High SDI	Measles	20.6 (15.6 to 27.8)	1.69 (1.15 to 2.64)	1.7 (0.971 to 2.97)	3.1 (2.3 to 4.2)	0.3 (0.2 to 0.4)	0.3 (0.2 to 0.5)
High SDI	Varicella and herpes zoster	59.6 (41.9 to 84.3)	64.5 (43.5 to 92.9)	70.6 (47.4 to 101)	6 (4.2 to 8.7)	5.1 (3.4 to 7.4)	4.9 (3.2 to 7.1)
High SDI	Acute hepatitis	202 (186 to 223)	178 (162 to 200)	135 (118 to 161)	18.9 (17.2 to 21)	13.5 (12 to 15.5)	9.8 (8.3 to 11.9)
High SDI	Acute hepatitis A	74.9 (63 to 88.3)	68.4 (57.3 to 81.7)	51.6 (41.3 to 65.1)	7.5 (6.3 to 8.9)	5.9 (4.8 to 7.2)	4.5 (3.5 to 5.8)
High SDI	Acute hepatitis B	103 (91 to 116)	90.5 (79.8 to 102)	69.9 (60.2 to 83.4)	9.2 (8.1 to 10.3)	6.3 (5.5 to 7.1)	4.4 (3.7 to 5.2)
High SDI	Acute hepatitis C	5.76 (3.34 to 9.9)	4.64 (2.7 to 7.86)	3.22 (1.85 to 5.67)	0.6 (0.4 to 1)	0.4 (0.2 to 0.6)	0.2 (0.1 to 0.4)
High SDI	Acute hepatitis E	18.3 (14.8 to 22.4)	14.7 (12 to 18)	10.7 (8.58 to 13.6)	1.6 (1.3 to 2)	1 (0.9 to 1.3)	0.7 (0.6 to 0.9)
High SDI	Other unspecified infectious diseases	308 (253 to 358)	292 (245 to 338)	308 (256 to 358)	32.3 (26.9 to 37.7)	26.3 (22.8 to 31)	24.9 (21.2 to 30)
High SDI	Maternal and neonatal disorders	7120 (6410 to 7930)	5830 (5090 to 6680)	5460 (4590 to 6500)	1010.8 (929 to 1103.8)	789.9 (711.7 to 880.1)	719.2 (631.5 to 822.9)
High SDI	Maternal disorders	151 (136 to 170)	130 (119 to 144)	124 (111 to 139)	14.9 (13.4 to 16.8)	12.5 (11.4 to 13.9)	11.8 (10.6 to 13.3)
High SDI	Maternal haemorrhage	28.6 (23.9 to 34.1)	17.2 (14 to 21.3)	14 (11 to 17.9)	2.8 (2.3 to 3.3)	1.7 (1.3 to 2.1)	1.4 (1.1 to 1.7)
High SDI	Maternal sepsis and other pregnancy related infections	20.2 (16.1 to 26.1)	11.7 (8.53 to 16)	10.8 (7.4 to 15.5)	2 (1.6 to 2.6)	1.1 (0.8 to 1.5)	1 (0.7 to 1.5)
High SDI	Maternal hypertensive disorders	27.9 (20.8 to 37.5)	22.1 (16.4 to 29)	20.9 (15 to 29)	2.8 (2.1 to 3.7)	2.2 (1.6 to 2.9)	2 (1.5 to 2.8)
High SDI	Maternal obstructed labour and uterine rupture	6.73 (4.86 to 9.51)	5.36 (3.92 to 7.21)	4.77 (3.23 to 7.04)	0.7 (0.5 to 0.9)	0.5 (0.4 to 0.7)	0.5 (0.3 to 0.7)
High SDI	Maternal abortive outcome	21 (18.4 to 24.2)	12.2 (9.81 to 15.2)	9.48 (7.31 to 12.3)	2 (1.8 to 2.4)	1.1 (0.9 to 1.4)	0.8 (0.6 to 1.1)
High SDI	Ectopic pregnancy	4.84 (3.77 to 6.26)	2.96 (2.17 to 4.07)	2.08 (1.49 to 2.99)	0.5 (0.4 to 0.6)	0.3 (0.2 to 0.4)	0.2 (0.1 to 0.3)
High SDI	Indirect maternal deaths	9.91 (7.93 to 12.3)	9.75 (6.75 to 14.4)	11.9 (7.3 to 18.9)	1 (0.8 to 1.2)	1 (0.6 to 1.4)	1.1 (0.7 to 1.8)
High SDI	Late maternal deaths	7.56 (5.44 to 10.5)	11.3 (8.83 to 14.8)	12.1 (8.52 to 17.3)	0.7 (0.5 to 1)	1.1 (0.8 to 1.4)	1.2 (0.8 to 1.7)
High SDI	Maternal deaths aggravated by HIV/AIDS	0.0559 (0.0263 to 0.0976)	0.115 (0.0494 to 0.207)	0.132 (0.059 to 0.226)	0 (0 to 0)	0 (0 to 0)	0 (0 to 0)
High SDI	Other maternal disorders	24 (21 to 27.4)	37.5 (32 to 43)	37.5 (31.1 to 43.5)	2.4 (2.1 to 2.7)	3.6 (3.1 to 4.1)	3.6 (2.9 to 4.2)
High SDI	Neonatal disorders	6970 (6260 to 7780)	5700 (4960 to 6550)	5340 (4470 to 6380)	995.9 (914.2 to 1088.5)	777.4 (699.9 to 867.3)	707.4 (619.9 to 811.1)
High SDI	Neonatal preterm birth	3770 (3450 to 4150)	2790 (2460 to 3140)	2560 (2120 to 3090)	554.1 (515.9 to 600.7)	394.8 (359.9 to 432.6)	352.9 (306 to 407.8)

SR Appendix Table 2. All-age DALYs counts and age standardized DALYs rates per 100,000 individuals for all causes and level 1 cause groups by location for 1990, 2007, and 2017, both sexes combined							
		All-age DALYs			Age-standardized rate of DALYs per 100,000		
High SDI	Neonatal encephalopathy due to birth asphyxia and trauma	1630 (1330 to 2040)	1350 (1020 to 1810)	1320 (974 to 1810)	224.5 (191.7 to 269.9)	170.4 (137.9 to 216.3)	161.1 (125.9 to 207.9)
High SDI	Neonatal sepsis and other neonatal infections	912 (585 to 1390)	933 (580 to 1450)	924 (554 to 1470)	111.5 (75.9 to 163.5)	104.8 (70 to 154.9)	99 (64.1 to 148.8)
High SDI	Hemolytic disease and other neonatal jaundice	51.2 (44.3 to 58.8)	38.8 (31.5 to 46.9)	37.9 (30.3 to 46.4)	6.8 (6 to 7.7)	4.5 (3.8 to 5.3)	4.3 (3.5 to 5.1)
High SDI	Other neonatal disorders	608 (577 to 653)	593 (551 to 620)	496 (446 to 533)	99.1 (94 to 106.4)	102.9 (95.7 to 107.7)	90.2 (81.1 to 96.8)
High SDI	Nutritional deficiencies	1710 (1130 to 2570)	1240 (834 to 1880)	1250 (838 to 1890)	184.2 (122.2 to 279.1)	116.7 (77.4 to 179)	110.5 (72.4 to 168)
High SDI	Protein-energy malnutrition	74.8 (58.3 to 86.7)	95.6 (71.8 to 106)	115 (85.1 to 125)	7.1 (5.7 to 8.1)	6.2 (4.8 to 6.8)	5.9 (4.6 to 6.4)
High SDI	Iodine deficiency	104 (51.7 to 188)	98 (47.4 to 183)	105 (50.9 to 195)	9.9 (4.9 to 18)	7.9 (3.9 to 14.8)	7.8 (3.8 to 14.7)
High SDI	Dietary iron deficiency	1300 (843 to 2010)	936 (602 to 1440)	937 (590 to 1440)	135.5 (88 to 208.6)	87.8 (56.5 to 134.6)	84.5 (53.3 to 128.4)
High SDI	Other nutritional deficiencies	35.6 (24.5 to 52.6)	28.7 (18.7 to 52.7)	30.2 (19.6 to 58.7)	3.2 (2.3 to 4.6)	2 (1.4 to 3.3)	1.7 (1.2 to 3)
High SDI	Non-communicable diseases	232000 (207000 to 260000)	248000 (219000 to 282000)	271000 (238000 to 309000)	20035.5 (17788.8 to 22691.9)	16994 (14720.5 to 19632.9)	16236.2 (13938.6 to 18865.6)
High SDI	Neoplasms	46600 (45900 to 47100)	51200 (50300 to 52000)	55000 (53700 to 56300)	3812.5 (3758.3 to 3858.3)	3140.2 (3089.7 to 3197)	2820.5 (2756.1 to 2890)
High SDI	Lip and oral cavity cancer	608 (596 to 621)	673 (661 to 685)	718 (696 to 743)	50.6 (49.6 to 51.6)	42.2 (41.4 to 43)	38 (36.8 to 39.3)
High SDI	Nasopharynx cancer	220 (216 to 224)	210 (207 to 213)	206 (198 to 215)	18.9 (18.5 to 19.3)	13.8 (13.6 to 14)	11.7 (11.3 to 12.3)
High SDI	Other pharynx cancer	343 (337 to 350)	429 (422 to 436)	479 (459 to 499)	28.9 (28.4 to 29.4)	27 (26.5 to 27.4)	25.5 (24.4 to 26.5)
High SDI	Oesophageal cancer	1200 (1180 to 1210)	1440 (1430 to 1450)	1550 (1510 to 1590)	96.8 (95.8 to 98)	86.8 (86 to 87.6)	77.8 (75.8 to 79.6)
High SDI	Stomach cancer	4120 (4080 to 4160)	3190 (3160 to 3230)	2970 (2890 to 3060)	330.1 (326.8 to 333.5)	188.9 (187 to 190.9)	145.1 (141.2 to 149.3)
High SDI	Colon and rectum cancer	5060 (4990 to 5150)	5790 (5690 to 5910)	6240 (6050 to 6440)	399.9 (394.1 to 406.4)	340 (333.9 to 346.6)	304.2 (295 to 313.8)
High SDI	Liver cancer	2050 (2010 to 2090)	2680 (2650 to 2710)	2890 (2800 to 3010)	167.6 (164.3 to 171)	161.8 (160.2 to 164.2)	145.9 (141.5 to 151.9)
High SDI	Liver cancer due to hepatitis B	586 (546 to 626)	693 (655 to 739)	719 (668 to 788)	50.5 (47.1 to 53.9)	45.2 (42.8 to 48)	40.1 (37.3 to 44)
High SDI	Liver cancer due to hepatitis C	776 (738 to 814)	1030 (989 to 1080)	1100 (1030 to 1160)	61.5 (58.5 to 64.5)	59.7 (57 to 62.5)	51.8 (48.5 to 55.1)
High SDI	Liver cancer due to alcohol use	455 (419 to 496)	606 (563 to 657)	678 (622 to 743)	36.4 (33.5 to 39.7)	36.3 (33.5 to 39.3)	33.7 (30.9 to 37.1)
High SDI	Liver cancer due to NASH	116 (103 to 129)	182 (163 to 203)	219 (195 to 246)	9.2 (8.2 to 10.2)	10.6 (9.5 to 11.8)	10.6 (9.4 to 11.7)
High SDI	Liver cancer due to other causes	118 (105 to 131)	158 (142 to 176)	178 (159 to 200)	9.9 (8.9 to 11.1)	10.1 (9 to 11.2)	9.6 (8.6 to 10.8)
High SDI	Gallbladder and biliary tract cancer	896 (813 to 913)	890 (820 to 906)	928 (872 to 970)	69.9 (63.3 to 71.2)	50.6 (46.7 to 51.5)	43 (40.6 to 44.9)
High SDI	Pancreatic cancer	2120 (2100 to 2140)	2900 (2880 to 2920)	3510 (3430 to 3590)	167.4 (166 to 168.8)	170.2 (169 to 171.5)	169.6 (165.9 to 173.6)
High SDI	Larynx cancer	579 (568 to 592)	454 (445 to 464)	446 (431 to 462)	47.4 (46.5 to 48.5)	27.7 (27.1 to 28.3)	22.6 (21.8 to 23.4)
High SDI	Tracheal, bronchus, and lung cancer	10300 (10200 to 10300)	11500 (11400 to 11600)	12200 (11900 to 12400)	817.7 (811.1 to 823.9)	678.9 (673.9 to 684.2)	593.1 (582 to 604.1)
High SDI	Malignant skin melanoma	557 (423 to 700)	730 (526 to 892)	827 (591 to 1010)	47.4 (35.9 to 59.2)	48 (35.2 to 59.3)	47 (34.5 to 59.2)
High SDI	Non-melanoma skin cancer	199 (189 to 216)	263 (235 to 303)	313 (275 to 368)	15.9 (15.2 to 17.3)	15.2 (13.6 to 17.4)	14.7 (12.9 to 17.2)
High SDI	Non-melanoma skin cancer (squamous-cell carcinoma)	198 (188 to 215)	262 (233 to 301)	311 (274 to 366)	15.9 (15.1 to 17.2)	15.1 (13.6 to 17.3)	14.6 (12.9 to 17.1)
High SDI	Non-melanoma skin cancer (basal-cell carcinoma)	1.01 (0.354 to 2.23)	1.43 (0.559 to 3)	1.79 (0.675 to 3.76)	0.1 (0 to 0.2)	0.1 (0 to 0.2)	0.1 (0 to 0.2)
High SDI	Breast cancer	4060 (3960 to 4190)	4200 (4060 to 4390)	4290 (4080 to 4510)	338.3 (329.7 to 348.5)	263.8 (254.7 to 275.5)	231.2 (219.9 to 243.1)
High SDI	Cervical cancer	871 (856 to 889)	756 (742 to 771)	743 (719 to 768)	73.8 (72.5 to 75.4)	49.8 (48.8 to 50.9)	43.5 (42 to 45)
High SDI	Uterine cancer	418 (404 to 434)	493 (471 to 519)	604 (571 to 640)	32.9 (31.8 to 34.2)	29.2 (27.9 to 30.8)	29.8 (28.2 to 31.7)
High SDI	Ovarian cancer	1170 (1150 to 1190)	1240 (1220 to 1260)	1290 (1240 to 1330)	95.6 (94 to 97.2)	76.7 (75.5 to 78)	67.5 (65.3 to 69.8)
High SDI	Prostate cancer	1760 (1230 to 1940)	2140 (1690 to 2710)	2470 (2090 to 3330)	130.4 (91.4 to 144.4)	115.5 (91.3 to 146.5)	106.9 (90.6 to 144.8)
High SDI	Testicular cancer	97.6 (93 to 103)	79.3 (73.4 to 86.1)	74.5 (67.9 to 82.1)	9.3 (8.9 to 9.9)	7 (6.4 to 7.6)	6.3 (5.8 to 7)
High SDI	Kidney cancer	829 (750 to 852)	1130 (1040 to 1150)	1290 (1210 to 1340)	68.3 (61.8 to 70.2)	69.3 (64.2 to 71)	66.1 (62.7 to 68.4)
High SDI	Bladder cancer	1030 (1010 to 1060)	1170 (1140 to 1200)	1320 (1280 to 1380)	79 (77.1 to 81.1)	65.1 (63.4 to 67)	59.7 (57.4 to 62.1)
High SDI	Thyroid cancer	163 (155 to 172)	209 (196 to 225)	220 (205 to 240)	13.2 (12.6 to 14)	13.1 (12.2 to 14.1)	11.6 (10.7 to 12.7)

SR Appendix Table 2. All-age DALYs counts and age standardized DALYs rates per 100,000 individuals for all causes and level 1 cause groups by location for 1990, 2007, and 2017, both sexes combined

		All-age DALYs			Age-standardized rate of DALYs per 100,000		
High SDI	Mesothelioma	236 (197 to 265)	275 (271 to 279)	311 (300 to 324)	18.5 (15.5 to 20.7)	16.1 (15.9 to 16.4)	14.9 (14.3 to 15.5)
High SDI	Hodgkin lymphoma	235 (180 to 252)	168 (146 to 210)	149 (135 to 195)	21.5 (16.4 to 23.1)	13.1 (11.6 to 16.6)	10.6 (9.5 to 14.2)
High SDI	Non-Hodgkin's lymphoma	1370 (1350 to 1380)	1520 (1500 to 1550)	1640 (1600 to 1690)	115.9 (114.5 to 117.4)	96.8 (95.2 to 98.6)	87.5 (85 to 90.3)
High SDI	Multiple myeloma	569 (505 to 680)	766 (681 to 902)	891 (815 to 1080)	44.6 (39.4 to 53.3)	44.4 (39.6 to 52.6)	42.5 (39.1 to 51.9)
High SDI	Leukaemia	2140 (2110 to 2160)	2110 (2070 to 2140)	2070 (2010 to 2130)	199.3 (196.3 to 201.9)	152.6 (149.7 to 154.9)	127.6 (123.2 to 131.5)
High SDI	Acute lymphoid leukaemia	340 (331 to 350)	269 (265 to 276)	239 (231 to 249)	38.7 (37.6 to 39.9)	27.3 (26.7 to 28)	22.7 (21.7 to 23.6)
High SDI	Chronic lymphoid leukaemia	243 (234 to 251)	296 (285 to 307)	306 (292 to 323)	18.7 (18 to 19.3)	16.6 (15.9 to 17.2)	13.9 (13.3 to 14.7)
High SDI	Acute myeloid leukaemia	610 (594 to 628)	807 (776 to 817)	861 (826 to 889)	56.1 (54.2 to 57.9)	57.2 (54.9 to 57.9)	52 (49.7 to 53.9)
High SDI	Chronic myeloid leukaemia	286 (278 to 295)	161 (158 to 164)	126 (121 to 131)	24.2 (23.5 to 25)	10.3 (10.1 to 10.5)	6.8 (6.6 to 7.1)
High SDI	Other leukaemia	657 (633 to 674)	578 (563 to 589)	539 (521 to 559)	61.6 (58.7 to 63.3)	41.3 (39.9 to 42.2)	32.3 (30.9 to 33.7)
High SDI	Other malignant cancers	1600 (1390 to 1650)	1590 (1490 to 1710)	1810 (1650 to 1950)	146.2 (128 to 151)	115.8 (110 to 126.5)	116.4 (107.2 to 127.2)
High SDI	Other neoplasms	476 (348 to 632)	697 (491 to 877)	825 (580 to 1100)	42.2 (30.5 to 56)	46.5 (33.2 to 59.6)	46.3 (33.1 to 63.5)
High SDI	Myelodysplastic, myeloproliferative, and other hematopoietic neoplasms	447 (329 to 592)	668 (466 to 838)	797 (559 to 1060)	38.8 (28.2 to 51.3)	43.2 (30.5 to 55.1)	43 (30.6 to 58.7)
High SDI	Benign and in situ intestinal neoplasms	0 (0 to 0)	0 (0 to 0)	0 (0 to 0)	0 (0 to 0)	0 (0 to 0)	0 (0 to 0)
High SDI	Benign and in situ cervical and uterine neoplasms	0 (0 to 0)	0 (0 to 0)	0 (0 to 0)	0 (0 to 0)	0 (0 to 0)	0 (0 to 0)
High SDI	Other benign and in situ neoplasms	28.6 (18.2 to 39.4)	28.8 (20.3 to 39.7)	28.5 (20.8 to 42.5)	3.4 (2.1 to 4.7)	3.4 (2.4 to 4.6)	3.3 (2.4 to 4.9)
High SDI	Cardiovascular diseases	60700 (59200 to 62500)	49100 (47300 to 51100)	50000 (47700 to 52300)	4777.6 (4652.6 to 4918)	2816 (2705.6 to 2937.1)	2338.6 (2226.9 to 2454.5)
High SDI	Rheumatic heart disease	848 (837 to 863)	524 (517 to 533)	514 (499 to 535)	70 (69 to 71.2)	31.6 (31.1 to 32)	25.4 (24.7 to 26.4)
High SDI	Ischaemic heart disease	33600 (33200 to 34100)	24000 (23600 to 24500)	23100 (22600 to 23900)	2614.6 (2583.7 to 2657.1)	1354.5 (1333.7 to 1382.2)	1065 (1039.9 to 1096.9)
High SDI	Stroke	16300 (15600 to 17000)	13700 (12900 to 14600)	14000 (12900 to 15100)	1286.1 (1227.8 to 1341)	784.1 (733 to 834.7)	650.3 (597.1 to 703)
High SDI	Ischaemic stroke	8210 (7680 to 8730)	7050 (6380 to 7680)	7590 (6720 to 8430)	627.8 (585.2 to 669.2)	380.3 (341.5 to 418.3)	326 (285.5 to 364.6)
High SDI	Intracerebral hemorrhage	6110 (5970 to 6240)	4830 (4720 to 4940)	4570 (4420 to 4720)	487 (475.7 to 497.9)	282.6 (275.8 to 289.7)	221.3 (213.4 to 229.5)
High SDI	Subarachnoid hemorrhage	2010 (1910 to 2100)	1870 (1760 to 1990)	1850 (1720 to 2010)	171.3 (162.9 to 179.4)	121.3 (114.3 to 128.8)	102.9 (95.2 to 111.2)
High SDI	Hypertensive heart disease	1550 (1250 to 1740)	1770 (1400 to 1960)	2150 (1570 to 2350)	121.4 (99 to 136.8)	100.7 (79.3 to 111.1)	99.6 (72.6 to 108.8)
High SDI	Non-rheumatic valvular heart disease	698 (616 to 777)	907 (784 to 1010)	1100 (950 to 1230)	54.2 (47.8 to 60.2)	48.9 (42.7 to 54.3)	46.7 (40.9 to 51.9)
High SDI	Non-rheumatic calcific aortic valve disease	457 (402 to 517)	672 (567 to 736)	844 (701 to 929)	35.3 (31.2 to 39.8)	35.7 (30.4 to 39)	35.1 (30 to 38.3)
High SDI	Non-rheumatic degenerative mitral valve disease	232 (186 to 270)	227 (186 to 274)	250 (203 to 307)	18.1 (14.5 to 20.9)	12.5 (10.3 to 15)	11 (9 to 13.4)
High SDI	Other non-rheumatic valve diseases	9.69 (6.97 to 12.7)	8.67 (7.02 to 12.6)	9.68 (7.32 to 13.3)	0.9 (0.6 to 1.1)	0.6 (0.5 to 0.9)	0.6 (0.4 to 0.8)
High SDI	Cardiomyopathy and myocarditis	2170 (1870 to 2280)	1920 (1820 to 2030)	1970 (1860 to 2120)	187.4 (164 to 196.3)	129.8 (124.1 to 137.2)	112.9 (107.3 to 123)
High SDI	Myocarditis	171 (149 to 217)	216 (163 to 244)	197 (168 to 216)	17 (15 to 21.5)	17.5 (13.5 to 19.6)	14.4 (12.2 to 15.7)
High SDI	Alcoholic cardiomyopathy	515 (466 to 627)	462 (384 to 498)	468 (389 to 514)	43.4 (39.2 to 52.2)	29.8 (25.2 to 32.2)	26.2 (22 to 28.8)
High SDI	Other cardiomyopathy	1490 (1180 to 1590)	1240 (1170 to 1370)	1300 (1220 to 1470)	127 (103.2 to 134.8)	82.5 (78.4 to 92.2)	72.3 (68 to 82.8)
High SDI	Atrial fibrillation and flutter	1300 (1090 to 1570)	1830 (1550 to 2170)	2260 (1920 to 2670)	98.8 (82.6 to 119.2)	95.9 (80.6 to 114)	93.5 (78.6 to 111.2)
High SDI	Aortic aneurysm	1010 (994 to 1030)	1030 (1020 to 1040)	1040 (1010 to 1070)	77.7 (76.6 to 79.3)	59 (58.2 to 59.7)	49.1 (47.7 to 50.4)
High SDI	Peripheral vascular disease	252 (156 to 373)	449 (293 to 710)	611 (394 to 1020)	19.1 (11.8 to 28.2)	24.1 (15.6 to 37.8)	26.1 (16.9 to 43.2)
High SDI	Endocarditis	307 (248 to 371)	409 (336 to 491)	481 (403 to 583)	26 (21.2 to 31.3)	25.7 (21.3 to 30.6)	25.2 (21.6 to 30.4)
High SDI	Other cardiovascular and circulatory diseases	2710 (2340 to 3180)	2580 (2200 to 3050)	2710 (2270 to 3250)	222.4 (191.6 to 260.4)	161.8 (138.1 to 191.7)	144.9 (121.3 to 174.4)
High SDI	Chronic respiratory diseases	12200 (11100 to 13400)	14000 (12700 to 15300)	15800 (14300 to 17300)	1016.5 (908.6 to 1132.2)	891.5 (790.8 to 998.2)	848 (748.6 to 954.8)
High SDI	Chronic obstructive pulmonary disease	8370 (7790 to 8820)	10100 (9420 to 10700)	11700 (10800 to 12500)	650.2 (603.7 to 686.5)	578.6 (534.7 to 613.7)	548 (501.7 to 589.1)
High SDI	Pneumoconiosis	135 (130 to 141)	103 (97.4 to 108)	97.5 (90.7 to 105)	10.3 (9.9 to 10.8)	5.6 (5.3 to 5.9)	4.3 (4 to 4.6)

SR Appendix Table 2. All-age DALYs counts and age standardized DALYs rates per 100,000 individuals for all causes and level 1 cause groups by location for 1990, 2007, and 2017, both sexes combined							
		All-age DALYs			Age-standardized rate of DALYs per 100,000		
High SDI	Silicosis	63.6 (60.1 to 69)	36.6 (33.7 to 46.1)	28.5 (25.3 to 37)	4.8 (4.6 to 5.3)	2 (1.8 to 2.4)	1.2 (1.1 to 1.6)
High SDI	Asbestosis	14.9 (10.3 to 17.8)	28.8 (17.8 to 32.3)	33.5 (21.3 to 38.2)	1.1 (0.8 to 1.3)	1.5 (1 to 1.7)	1.4 (0.9 to 1.6)
High SDI	Coal workers pneumoconiosis	43.6 (40.6 to 48.2)	21.7 (19.7 to 26)	22.5 (20 to 26.1)	3.3 (3.1 to 3.7)	1.2 (1.1 to 1.4)	1 (0.9 to 1.2)
High SDI	Other pneumoconiosis	12.8 (8.83 to 15.1)	15.5 (12.4 to 17.9)	13 (11 to 17.3)	1 (0.7 to 1.2)	0.9 (0.7 to 1)	0.6 (0.5 to 0.8)
High SDI	Asthma	2950 (2240 to 3840)	2510 (1810 to 3400)	2510 (1790 to 3410)	289.4 (216.1 to 380.7)	225.8 (159.1 to 308)	212.7 (147.3 to 294.2)
High SDI	Interstitial lung disease and pulmonary sarcoidosis	510 (444 to 631)	927 (725 to 1050)	1150 (868 to 1320)	40.8 (35.6 to 50.4)	54.1 (42.8 to 61.3)	54.7 (42.3 to 62.5)
High SDI	Other chronic respiratory diseases	274 (251 to 296)	331 (295 to 364)	366 (322 to 407)	25.8 (23.5 to 28)	27.4 (24.3 to 30.3)	28.3 (24.6 to 31.8)
High SDI	Digestive diseases	10300 (9480 to 11200)	10900 (9920 to 12100)	11600 (10500 to 13000)	881.1 (808.7 to 969.8)	724.3 (651.5 to 813)	668.8 (594.9 to 760.3)
High SDI	Cirrhosis and other chronic liver diseases	5040 (4930 to 5160)	5100 (4980 to 5260)	5280 (5070 to 5500)	431.3 (421.5 to 442.3)	333.8 (325 to 344.8)	300.1 (288.5 to 313.3)
High SDI	Cirrhosis and other chronic liver diseases due to hepatitis B	826 (753 to 900)	724 (654 to 795)	694 (624 to 765)	71.3 (65 to 77.7)	48.1 (43.5 to 52.8)	40.4 (36.5 to 44.5)
High SDI	Cirrhosis and other chronic liver diseases due to hepatitis C	1440 (1320 to 1570)	1490 (1380 to 1630)	1560 (1440 to 1690)	122.4 (112.4 to 133.8)	97 (89.4 to 105.8)	87.9 (81.1 to 95.8)
High SDI	Cirrhosis and other chronic liver diseases due to alcohol use	1790 (1640 to 1930)	1810 (1670 to 1950)	1820 (1680 to 1970)	152.5 (139.5 to 164)	118.2 (108.8 to 127.1)	103.7 (95.3 to 111.9)
High SDI	Cirrhosis due to NASH	338 (286 to 394)	426 (369 to 487)	472 (416 to 535)	28.5 (24.2 to 33.3)	27.4 (23.7 to 31.4)	26.3 (23.2 to 29.9)
High SDI	Cirrhosis and other chronic liver diseases due to other causes	651 (562 to 756)	644 (559 to 743)	730 (639 to 836)	56.6 (49.3 to 65.6)	43.2 (37.8 to 49.7)	41.9 (36.8 to 47.7)
High SDI	Upper digestive system diseases	2290 (1730 to 3090)	2290 (1630 to 3250)	2450 (1690 to 3510)	198.6 (147.7 to 272)	162.7 (113 to 234.5)	155.3 (104.8 to 226.4)
High SDI	Peptic ulcer disease	869 (825 to 921)	616 (570 to 673)	596 (541 to 661)	70 (66.3 to 74.5)	37.3 (34.2 to 41)	30.4 (27.2 to 34.3)
High SDI	Gastritis and duodenitis	582 (392 to 825)	641 (429 to 907)	697 (465 to 990)	54.1 (36.3 to 77.1)	49 (32.4 to 70.1)	47.8 (31.4 to 68.5)
High SDI	GERD	839 (449 to 1430)	1030 (551 to 1760)	1150 (616 to 1950)	74.5 (40 to 126.8)	76.5 (41.2 to 130.2)	77.2 (41.4 to 132)
High SDI	Appendicitis	84.8 (79.3 to 90.8)	65.4 (59.8 to 71.6)	62.7 (57 to 69)	8 (7.4 to 8.6)	5.1 (4.6 to 5.7)	4.4 (3.8 to 5)
High SDI	Paralytic ileus and intestinal obstruction	427 (368 to 504)	541 (456 to 626)	614 (522 to 717)	38.1 (33.5 to 44.4)	34.7 (29.9 to 39.7)	31.9 (27.5 to 36.8)
High SDI	Inguinal, femoral, and abdominal hernia	449 (338 to 576)	457 (338 to 595)	516 (381 to 679)	40.4 (30.3 to 52.4)	33.4 (24.1 to 44.3)	32.9 (23.6 to 44)
High SDI	Inflammatory bowel disease	417 (330 to 515)	629 (490 to 782)	698 (540 to 867)	37.1 (29.2 to 46)	45.6 (35.1 to 57.3)	45.1 (34.1 to 56.9)
High SDI	Vascular intestinal disorders	381 (356 to 399)	472 (447 to 492)	500 (467 to 523)	29.4 (27.6 to 30.7)	26.3 (25 to 27.3)	22.8 (21.2 to 23.8)
High SDI	Gallbladder and biliary diseases	330 (289 to 340)	325 (316 to 344)	390 (367 to 410)	25.9 (22.7 to 26.7)	18.1 (17.6 to 19.2)	17.3 (16.4 to 18.1)
High SDI	Pancreatitis	449 (405 to 504)	505 (453 to 576)	506 (445 to 583)	38.4 (34.7 to 43.3)	33.7 (30.1 to 38.6)	29.6 (25.9 to 34.5)
High SDI	Other digestive diseases	398 (366 to 434)	497 (460 to 536)	558 (514 to 609)	33.9 (30.8 to 37.5)	30.9 (28.2 to 33.9)	29.3 (26.4 to 32.7)
High SDI	Neurological disorders	17000 (13800 to 20700)	21800 (17900 to 26100)	25200 (21100 to 29800)	1509.3 (1200.9 to 1864.4)	1491 (1177.8 to 1850.8)	1477.6 (1168.3 to 1843)
High SDI	Alzheimer's disease and other dementias	5670 (5320 to 6040)	8380 (7850 to 8940)	10800 (10100 to 11500)	429.7 (403.3 to 457.5)	409.6 (383.9 to 436.5)	399.3 (372.9 to 426.3)
High SDI	Parkinson's disease	859 (794 to 1010)	1330 (1220 to 1440)	1640 (1460 to 1780)	63.2 (58.4 to 74.1)	67.9 (62.2 to 73.8)	66.9 (59.5 to 72.9)
High SDI	Epilepsy	1230 (868 to 1680)	1370 (927 to 1940)	1430 (928 to 2080)	125.2 (88.4 to 171.3)	122.5 (82.4 to 172.7)	118.3 (76.8 to 173)
High SDI	Multiple sclerosis	371 (319 to 429)	496 (416 to 570)	551 (458 to 636)	32.5 (27.9 to 37.8)	34.7 (29 to 40)	34.4 (28.7 to 40.3)
High SDI	Motor neuron disease	311 (302 to 319)	459 (449 to 469)	548 (527 to 564)	28.2 (27.2 to 29.2)	31.2 (30.4 to 31.9)	31.2 (30 to 32.2)
High SDI	Headache disorders	8010 (5300 to 11300)	9040 (5990 to 12700)	9450 (6280 to 13200)	769.7 (507.8 to 1089.9)	766.8 (504.1 to 1087.6)	766.8 (505.9 to 1084.2)
High SDI	Migraine	7030 (4510 to 10100)	7900 (5090 to 11300)	8230 (5320 to 11800)	676.4 (433.3 to 975)	674 (432.9 to 971.1)	673.9 (430.9 to 969.6)
High SDI	Tension-type headache	987 (562 to 1570)	1140 (651 to 1790)	1220 (695 to 1910)	93.3 (53 to 147.1)	92.9 (52.7 to 147.4)	93 (52.7 to 147.8)
High SDI	Other neurological disorders	585 (525 to 661)	671 (592 to 759)	766 (675 to 882)	60.8 (54 to 69.6)	58.4 (50.5 to 67.7)	60.6 (51.6 to 72.2)
High SDI	Mental disorders	18700 (13900 to 24200)	21200 (15800 to 27300)	22400 (16700 to 28700)	1818.5 (1353.1 to 2349.4)	1827.8 (1357.9 to 2365.2)	1839.1 (1368.7 to 2378.6)
High SDI	Schizophrenia	1840 (1390 to 2260)	2200 (1670 to 2690)	2360 (1790 to 2890)	168.1 (126.8 to 206.2)	170.3 (128.9 to 208.7)	171.2 (128.4 to 210.8)
High SDI	Depressive disorders	6290 (4450 to 8520)	7140 (5060 to 9630)	7690 (5460 to 10400)	590.6 (417.9 to 801.2)	586.6 (415.9 to 787.2)	596.5 (423.3 to 805.4)
High SDI	Major depressive disorder	4800 (3360 to 6490)	5460 (3820 to 7380)	5810 (4080 to 7830)	453.8 (317.2 to 619.7)	456.1 (319.3 to 621.3)	461.4 (321.6 to 629.5)

SR Appendix Table 2. All-age DALYs counts and age standardized DALYs rates per 100,000 individuals for all causes and level 1 cause groups by location for 1990, 2007, and 2017, both sexes combined

		All-age DALYs			Age-standardized rate of DALYs per 100,000		
High SDI	Dysthymia	1490 (1000 to 2180)	1680 (1120 to 2460)	1880 (1260 to 2750)	136.8 (91.8 to 200.3)	130.5 (87.3 to 190.8)	135.1 (90.6 to 198.6)
High SDI	Bipolar disorder	1490 (942 to 2200)	1650 (1050 to 2450)	1720 (1100 to 2560)	142.5 (90.4 to 211.3)	140.3 (88.9 to 207.5)	139.6 (88.3 to 206.3)
High SDI	Anxiety disorders	4730 (3380 to 6280)	5370 (3840 to 7110)	5590 (4030 to 7380)	462.6 (329.6 to 616.3)	465.9 (331.6 to 615.7)	463.7 (329.3 to 617)
High SDI	Eating disorders	828 (538 to 1200)	950 (621 to 1370)	953 (623 to 1380)	83.6 (54.5 to 120.3)	94 (61.9 to 135)	96.1 (62.9 to 139)
High SDI	Anorexia nervosa	209 (131 to 306)	235 (149 to 344)	231 (146 to 340)	21.7 (13.5 to 31.8)	24.3 (15.3 to 35.7)	24.5 (15.4 to 36.1)
High SDI	Bulimia nervosa	619 (393 to 927)	715 (459 to 1070)	722 (463 to 1080)	61.9 (39.3 to 92.6)	69.8 (44.7 to 104.1)	71.6 (45.8 to 107.5)
High SDI	Autism spectrum disorders	862 (589 to 1180)	948 (649 to 1300)	1000 (686 to 1370)	92 (62.8 to 126.6)	93 (63.5 to 127.8)	93.8 (64.1 to 128.8)
High SDI	Attention-deficit/hyperactivity disorder	105 (63.4 to 164)	112 (68.3 to 175)	112 (67 to 175)	12.1 (7.3 to 19)	12.8 (7.8 to 20.3)	13 (7.8 to 20.5)
High SDI	Conduct disorder	750 (451 to 1190)	714 (430 to 1130)	694 (420 to 1100)	95.9 (57.9 to 152.4)	96.4 (57.8 to 153.4)	98.1 (59.1 to 154.8)
High SDI	Idiopathic developmental intellectual disability	212 (77.7 to 377)	195 (68.4 to 356)	185 (62.3 to 341)	22.9 (8.4 to 40.8)	19.9 (7 to 36.1)	18.4 (6.3 to 33.6)
High SDI	Other mental disorders	1620 (1070 to 2230)	1910 (1270 to 2620)	2060 (1360 to 2840)	148.2 (97.8 to 204)	148.7 (98.1 to 203.9)	148.8 (98.4 to 204.8)
High SDI	Substance use disorders	5700 (4510 to 6980)	8260 (6800 to 9830)	11100 (9370 to 12900)	547.3 (432.1 to 672)	722 (589.8 to 867.9)	936.3 (782.5 to 1101.2)
High SDI	Alcohol use disorders	2410 (1940 to 2980)	2960 (2450 to 3600)	3120 (2570 to 3800)	225.5 (180.9 to 280.9)	236.5 (191.7 to 292.1)	236.3 (189.3 to 292.4)
High SDI	Drug use disorders	3290 (2550 to 4070)	5310 (4340 to 6330)	7950 (6720 to 9200)	321.8 (249 to 398.5)	485.5 (392.2 to 582.8)	699.9 (585.8 to 821.2)
High SDI	Opioid use disorders	2020 (1550 to 2500)	3380 (2710 to 4050)	5490 (4600 to 6360)	196.2 (150.9 to 243.2)	310.5 (247.6 to 373.3)	481.8 (401.6 to 563.8)
High SDI	Cocaine use disorders	330 (228 to 458)	469 (351 to 613)	584 (443 to 758)	32.4 (22.3 to 45)	41.7 (30.8 to 54.9)	50.7 (37.8 to 66.5)
High SDI	Amphetamine use disorders	210 (127 to 320)	289 (201 to 400)	366 (250 to 500)	21.4 (12.9 to 32.9)	28.2 (19.3 to 39.7)	35.7 (24.1 to 50.2)
High SDI	Cannabis use disorders	140 (87.5 to 208)	159 (102 to 232)	154 (97.3 to 226)	14.3 (8.9 to 21.4)	16.1 (10.3 to 23.6)	15.9 (9.9 to 23.5)
High SDI	Other drug use disorders	590 (473 to 737)	1010 (872 to 1170)	1350 (1160 to 1570)	57.5 (45.9 to 72.2)	89 (76.2 to 104.5)	115.8 (98.5 to 135.3)
High SDI	Diabetes and kidney diseases	9750 (8330 to 11500)	13200 (11100 to 15700)	15700 (13000 to 18900)	792.9 (673.8 to 931.5)	812.9 (675.3 to 971.5)	817.3 (666.2 to 991.7)
High SDI	Diabetes mellitus	6740 (5520 to 8180)	9240 (7370 to 11400)	10900 (8500 to 13800)	546.1 (444.8 to 666)	573.4 (453.9 to 713.7)	580.5 (445.8 to 739.7)
High SDI	Diabetes mellitus type 1	1240 (1070 to 1360)	1200 (1050 to 1340)	1100 (971 to 1240)	106.2 (92.7 to 117.1)	84.4 (73.5 to 95.7)	71.5 (62.3 to 82.2)
High SDI	Diabetes mellitus type 2	5500 (4350 to 6880)	8040 (6280 to 10100)	9850 (7500 to 12600)	439.9 (346.1 to 552.5)	489 (377.3 to 621.1)	509 (380.7 to 657.8)
High SDI	Chronic kidney disease	3010 (2750 to 3270)	4000 (3660 to 4340)	4780 (4370 to 5190)	246.1 (225.1 to 268.1)	239.2 (216.4 to 261.5)	236.4 (214 to 259.6)
High SDI	Chronic kidney disease due to diabetes mellitus type 1	197 (164 to 236)	229 (189 to 276)	247 (201 to 299)	17.2 (14.3 to 20.5)	15.3 (12.7 to 18.3)	14.7 (12.2 to 17.7)
High SDI	Chronic kidney disease due to diabetes mellitus type 2	719 (622 to 827)	1150 (1000 to 1300)	1430 (1250 to 1610)	55.5 (48.2 to 63.8)	64.1 (56.4 to 72.5)	64.8 (57 to 73.4)
High SDI	Chronic kidney disease due to hypertension	684 (594 to 775)	995 (870 to 1120)	1250 (1090 to 1410)	53.6 (46.5 to 60.5)	55.3 (48.1 to 62.1)	56.3 (49 to 63.3)
High SDI	Chronic kidney disease due to glomerulonephritis	513 (445 to 585)	578 (500 to 662)	647 (552 to 744)	44.2 (38.4 to 50)	38.3 (33.3 to 43.6)	37.1 (31.7 to 42.3)
High SDI	Chronic kidney disease due to other and unspecified causes	896 (771 to 1030)	1050 (895 to 1210)	1210 (1020 to 1400)	75.7 (65.4 to 86.8)	66.1 (56.7 to 76.1)	63.5 (54.3 to 73.4)
High SDI	Acute glomerulonephritis	7.96 (7.64 to 8.28)	4.83 (4.63 to 5.07)	7.8 (7.49 to 8.16)	0.7 (0.7 to 0.7)	0.3 (0.3 to 0.3)	0.4 (0.4 to 0.5)
High SDI	Skin and subcutaneous diseases	6450 (4440 to 9080)	7620 (5280 to 10800)	8320 (5820 to 11700)	676.8 (458.9 to 956.9)	719.3 (490.7 to 1014.5)	735.8 (503.6 to 1036.4)
High SDI	Dermatitis	2060 (1220 to 3240)	2220 (1320 to 3470)	2330 (1390 to 3640)	234.1 (136.3 to 371.1)	239.1 (138.3 to 378.3)	240.3 (139.6 to 379.8)
High SDI	Atopic dermatitis	1620 (879 to 2710)	1710 (929 to 2840)	1780 (965 to 2960)	191.8 (103.6 to 322.5)	195.7 (105.6 to 327.3)	196.3 (105.6 to 329.6)
High SDI	Contact dermatitis	406 (269 to 597)	475 (315 to 699)	515 (339 to 753)	39.1 (25.7 to 57.9)	40.1 (26.4 to 59.2)	40.5 (26.5 to 59.9)
High SDI	Seborrhoeic dermatitis	32.9 (18.9 to 52.2)	39.6 (22.6 to 62.9)	44.3 (25.4 to 70.2)	3.2 (1.8 to 5)	3.3 (1.9 to 5.3)	3.4 (2 to 5.4)
High SDI	Psoriasis	1320 (935 to 1740)	1730 (1230 to 2290)	1980 (1410 to 2610)	123 (87.2 to 162.8)	138.3 (97.6 to 182.8)	145.6 (103.4 to 192)
High SDI	Bacterial skin diseases	129 (96.2 to 203)	216 (135 to 309)	276 (160 to 394)	11.7 (8.9 to 17.7)	14.7 (9.5 to 20.5)	15.7 (9.8 to 22)
High SDI	Cellulitis	65.5 (48.3 to 96.4)	113 (71.4 to 153)	144 (86.1 to 194)	6.1 (4.5 to 8.7)	8 (5.4 to 10.7)	8.7 (5.6 to 11.5)
High SDI	Pyoderma	63.7 (39.8 to 111)	103 (53.1 to 160)	132 (63.3 to 201)	5.6 (3.5 to 9.6)	6.6 (3.5 to 10.5)	7 (3.6 to 10.9)
High SDI	Scabies	124 (68.1 to 201)	147 (80.8 to 238)	159 (88.3 to 259)	13.6 (7.5 to 22.2)	15.2 (8.3 to 24.8)	15.8 (8.8 to 26)

SR Appendix Table 2. All-age DALYs counts and age standardized DALYs rates per 100,000 individuals for all causes and level 1 cause groups by location for 1990, 2007, and 2017, both sexes combined

		All-age DALYs			Age-standardized rate of DALYs per 100,000		
High SDI	Fungal skin diseases	370 (149 to 775)	445 (179 to 927)	505 (203 to 1050)	34.2 (13.8 to 71.4)	32.7 (13.1 to 68.1)	32.3 (13 to 67.4)
High SDI	Viral skin diseases	598 (387 to 886)	618 (400 to 916)	637 (412 to 941)	71.9 (46.4 to 106.5)	72.9 (47 to 108.1)	73.2 (47.1 to 108.2)
High SDI	Acne vulgaris	568 (342 to 908)	696 (422 to 1110)	725 (438 to 1170)	62.4 (37.6 to 100.2)	77.3 (46.6 to 123.5)	83.3 (50.6 to 133.6)
High SDI	Alopecia areata	109 (69.9 to 163)	123 (79.6 to 182)	129 (82.8 to 191)	10.5 (6.7 to 15.6)	10.5 (6.8 to 15.6)	10.5 (6.7 to 15.6)
High SDI	Pruritus	85.8 (40.7 to 161)	106 (50.6 to 198)	116 (55.7 to 215)	8.1 (3.8 to 15.2)	8.4 (4 to 15.8)	8.4 (4 to 15.8)
High SDI	Urticaria	479 (320 to 671)	509 (341 to 712)	529 (354 to 737)	53.2 (35.2 to 74.7)	52.6 (34.9 to 74.1)	52.4 (34.8 to 73.8)
High SDI	Decubitus ulcer	98.1 (66 to 132)	114 (83.5 to 168)	131 (96.5 to 197)	7.9 (5.4 to 10.7)	6.8 (4.9 to 9.8)	6.3 (4.7 to 9.3)
High SDI	Other skin and subcutaneous diseases	511 (257 to 928)	693 (346 to 1260)	803 (402 to 1460)	46.2 (23.1 to 83.7)	50.9 (25.3 to 92.7)	52 (25.9 to 94.9)
High SDI	Sense organ diseases	6490 (4420 to 9090)	8480 (5780 to 11900)	10100 (6870 to 14100)	543.2 (367.4 to 766.1)	535.3 (361.2 to 758.9)	532.4 (359.1 to 755.4)
High SDI	Blindness and vision impairment	1900 (1280 to 2720)	2300 (1550 to 3300)	2650 (1790 to 3780)	166.5 (110.9 to 238.7)	156 (103.5 to 225.2)	152.7 (101.4 to 221.2)
High SDI	Glaucoma	71.8 (47.6 to 101)	85.9 (57.5 to 120)	103 (69.4 to 142)	5.4 (3.6 to 7.6)	4.6 (3.1 to 6.4)	4.4 (2.9 to 6.1)
High SDI	Cataract	373 (258 to 506)	482 (334 to 658)	588 (407 to 804)	29 (20.1 to 39.6)	25.8 (17.9 to 35.2)	24.9 (17.2 to 34.1)
High SDI	Age-related macular degeneration	91 (60.2 to 128)	119 (78.7 to 165)	144 (96.2 to 198)	6.9 (4.6 to 9.7)	6.1 (4.1 to 8.5)	5.8 (3.9 to 8)
High SDI	Refraction disorders	688 (439 to 1020)	765 (493 to 1130)	825 (537 to 1220)	67.9 (42.6 to 100.9)	64.9 (41 to 96.1)	63.2 (40.1 to 94)
High SDI	Near vision loss	398 (190 to 723)	516 (246 to 947)	608 (289 to 1110)	33.5 (16 to 61)	33.2 (15.9 to 61)	34 (16.2 to 62.1)
High SDI	Other vision loss	280 (195 to 379)	334 (233 to 453)	382 (267 to 518)	23.7 (16.5 to 32.3)	21.4 (14.7 to 29)	20.6 (14.2 to 27.9)
High SDI	Age-related and other hearing loss	4230 (2910 to 5910)	5710 (3930 to 7970)	6870 (4760 to 9570)	346 (238.3 to 484)	348.1 (238.1 to 489.5)	348.3 (238.8 to 490.6)
High SDI	Other sense organ diseases	358 (221 to 520)	464 (287 to 674)	543 (338 to 786)	30.8 (19.1 to 44.7)	31.2 (19.3 to 45.4)	31.3 (19.5 to 45.4)
High SDI	Musculoskeletal disorders	24600 (18100 to 32600)	30300 (22300 to 40100)	33700 (24800 to 44600)	2168.3 (1598.8 to 2879.4)	2166.7 (1589.5 to 2865.4)	2157.2 (1586.3 to 2855.3)
High SDI	Rheumatoid arthritis	598 (455 to 746)	734 (544 to 925)	841 (618 to 1070)	49.4 (37.4 to 62.1)	47.4 (34.7 to 60.1)	47.1 (34.1 to 60.3)
High SDI	Osteoarthritis	1600 (801 to 3160)	2380 (1200 to 4710)	2910 (1470 to 5760)	127.3 (63.8 to 251.3)	140.1 (70.4 to 276.7)	142.1 (71.6 to 281.8)
High SDI	Low back pain	13900 (9890 to 18800)	16500 (11800 to 22100)	17900 (12900 to 24000)	1252.7 (892.6 to 1692.1)	1220.2 (866.8 to 1635.2)	1199.1 (854.5 to 1612.1)
High SDI	Neck pain	4560 (3180 to 6360)	5790 (4070 to 8020)	6570 (4620 to 9070)	396.2 (276.7 to 551.7)	400.2 (281.7 to 554.5)	404.3 (284 to 559.5)
High SDI	Gout	241 (162 to 333)	352 (240 to 484)	441 (300 to 604)	19.8 (13.3 to 27.3)	22 (14.9 to 30.2)	23.4 (15.9 to 32.2)
High SDI	Other musculoskeletal disorders	3640 (2560 to 5000)	4540 (3210 to 6190)	5020 (3530 to 6900)	322.9 (225.3 to 444)	336.9 (239 to 458.1)	341.2 (239.5 to 468)
High SDI	Other non-communicable diseases	13200 (10800 to 16100)	12300 (9730 to 15400)	12700 (9980 to 16000)	1491.3 (1261.6 to 1763.2)	1146.8 (951.1 to 1397.2)	1064.5 (874.9 to 1311.1)
High SDI	Congenital anomalies	4840 (4200 to 5400)	3580 (3080 to 4100)	3280 (2780 to 3820)	673 (588.5 to 739.5)	466.9 (415.8 to 523.1)	408.7 (359.4 to 461.9)
High SDI	Neural tube defects	280 (242 to 339)	170 (142 to 198)	191 (157 to 224)	39.8 (34.8 to 48.8)	22.2 (19.1 to 25.1)	26.2 (21.6 to 30.2)
High SDI	Congenital heart anomalies	1760 (1400 to 1900)	905 (808 to 1030)	683 (614 to 796)	264.6 (208.3 to 286.5)	137 (122.1 to 155.9)	104 (93.6 to 120.1)
High SDI	Orofacial clefts	40 (27.9 to 56.4)	37.2 (25.8 to 52.7)	37 (25.1 to 53)	4.7 (3.3 to 6.7)	3.9 (2.7 to 5.4)	3.6 (2.5 to 5.1)
High SDI	Down's syndrome	175 (147 to 222)	199 (166 to 232)	225 (182 to 262)	22.1 (18.5 to 28.3)	21.7 (18.4 to 26.2)	23.2 (19.2 to 29)
High SDI	Turner syndrome	2.01 (1.02 to 3.28)	2.02 (1.02 to 3.29)	2 (1.01 to 3.28)	0.2 (0.1 to 0.4)	0.2 (0.1 to 0.3)	0.2 (0.1 to 0.3)
High SDI	Klinefelter syndrome	0.795 (0.372 to 1.53)	0.84 (0.394 to 1.59)	0.842 (0.392 to 1.57)	0.1 (0 to 0.2)	0.1 (0 to 0.2)	0.1 (0 to 0.2)
High SDI	Other chromosomal abnormalities	300 (237 to 417)	343 (257 to 398)	310 (236 to 370)	43.5 (34 to 61.8)	50.6 (37.2 to 58.9)	45.9 (33.7 to 55.5)
High SDI	Congenital musculoskeletal and limb anomalies	724 (502 to 964)	737 (509 to 992)	768 (526 to 1030)	78.3 (55.6 to 103.3)	71.4 (50.4 to 95.3)	69.7 (48.7 to 92.3)
High SDI	Urogenital congenital anomalies	126 (89.7 to 170)	114 (89.9 to 138)	99.1 (77 to 124)	18.1 (12.5 to 24.8)	16.1 (12.4 to 18.9)	13.7 (10.5 to 16.9)
High SDI	Digestive congenital anomalies	467 (378 to 578)	398 (308 to 499)	372 (282 to 483)	61.6 (51.2 to 75.2)	48.4 (39.1 to 58.3)	42.6 (33.8 to 52.6)
High SDI	Other congenital anomalies	965 (805 to 1100)	680 (590 to 792)	588 (492 to 707)	140 (116.6 to 157.2)	95.3 (85 to 110.7)	79.5 (68.7 to 96.1)
High SDI	Urinary diseases and male infertility	933 (761 to 1150)	1190 (981 to 1440)	1440 (1200 to 1740)	77.2 (63 to 94.9)	72.2 (59.1 to 87.8)	71.9 (58.8 to 87.9)
High SDI	Urinary tract infections	363 (348 to 386)	529 (505 to 547)	655 (628 to 677)	29.8 (28.4 to 31.8)	31.1 (29.3 to 32.6)	31 (29.5 to 32.5)

SR Appendix Table 2. All-age DALYs counts and age standardized DALYs rates per 100,000 individuals for all causes and level 1 cause groups by location for 1990, 2007, and 2017, both sexes combined

		All-age DALYs			Age-standardized rate of DALYs per 100,000		
High SDI	Urolithiasis	69.9 (57 to 84.8)	71.3 (55.7 to 89.2)	86.3 (67.1 to 109)	5.9 (4.8 to 7.2)	4.8 (3.7 to 6)	5 (3.8 to 6.3)
High SDI	Benign prostatic hyperplasia	392 (253 to 565)	491 (319 to 700)	599 (393 to 853)	31.3 (20 to 45.1)	28.9 (18.8 to 41.2)	29.2 (19.1 to 41.5)
High SDI	Male infertility	15.6 (6.54 to 32.3)	17 (6.99 to 34.9)	16.7 (6.92 to 34.3)	1.5 (0.6 to 3.1)	1.5 (0.6 to 3.2)	1.5 (0.6 to 3.2)
High SDI	Other urinary diseases	92.6 (73.3 to 107)	78.2 (66.7 to 95.5)	82.5 (70.9 to 102)	8.6 (7.1 to 10)	5.8 (4.9 to 7.1)	5.2 (4.4 to 6.2)
High SDI	Gynecological diseases	1770 (1230 to 2440)	1960 (1390 to 2700)	2000 (1410 to 2750)	169.6 (118.2 to 234.1)	169.8 (119.8 to 231.8)	172.5 (121.6 to 235)
High SDI	Uterine fibroids	194 (113 to 325)	219 (124 to 375)	221 (126 to 375)	17.7 (10.3 to 29.5)	16.3 (9.3 to 27.7)	15.9 (9.1 to 27)
High SDI	Polycystic ovarian syndrome	63.7 (28.3 to 121)	66.2 (29.8 to 126)	66.1 (29.8 to 125)	6.2 (2.8 to 11.9)	6.1 (2.7 to 11.6)	6.1 (2.8 to 11.6)
High SDI	Female infertility	14.7 (5.53 to 32.3)	15.9 (5.76 to 35.1)	19 (6.86 to 42.2)	1.4 (0.5 to 3)	1.4 (0.5 to 3.1)	1.7 (0.6 to 3.7)
High SDI	Endometriosis	813 (543 to 1170)	897 (605 to 1250)	912 (618 to 1280)	78.3 (52.2 to 112.6)	79.6 (53.8 to 111.1)	80.9 (55 to 112.4)
High SDI	Genital prolapse	43.6 (21.7 to 80.4)	50.1 (24.5 to 94.1)	54.8 (26.7 to 103)	3.6 (1.8 to 6.6)	3.1 (1.5 to 5.8)	3 (1.4 to 5.5)
High SDI	Premenstrual syndrome	484 (312 to 719)	526 (339 to 782)	520 (333 to 777)	47.4 (30.6 to 70.4)	47.1 (30.4 to 69.8)	46.8 (30.1 to 69.7)
High SDI	Other gynecological diseases	156 (108 to 216)	188 (130 to 260)	213 (146 to 295)	14.9 (10.3 to 20.8)	16.1 (11.2 to 22.5)	18.2 (12.5 to 25.2)
High SDI	Hemoglobinopathies and hemolytic anaemias	518 (423 to 645)	417 (345 to 517)	405 (336 to 498)	50 (40.8 to 63.1)	33.3 (27.1 to 42.3)	28.7 (23.2 to 36.5)
High SDI	Thalassemias	29.6 (24.5 to 33.2)	17.3 (15.5 to 21)	15.7 (13.9 to 19.3)	3.9 (3.2 to 4.4)	2.3 (2.1 to 2.8)	2.1 (1.9 to 2.6)
High SDI	Thalassaemias trait	79.2 (49.9 to 122)	66.9 (42.9 to 102)	62.4 (39 to 94.1)	8.2 (5.2 to 12.6)	6.1 (3.9 to 9.4)	5.3 (3.4 to 8)
High SDI	Sickle cell disorders	31.5 (26.4 to 37.8)	21.4 (18.6 to 25.3)	19.1 (16.7 to 23)	3.9 (3.3 to 4.6)	2.5 (2.2 to 3)	2.1 (1.8 to 2.6)
High SDI	Sickle cell trait	44.6 (27.9 to 69.8)	37 (23.3 to 56.8)	28.2 (17.4 to 44.2)	4.8 (3 to 7.4)	3.5 (2.2 to 5.4)	2.6 (1.6 to 3.9)
High SDI	G6PD deficiency	37.2 (32.4 to 41.7)	42.5 (36.3 to 47.9)	39.4 (33.8 to 44.9)	3.7 (3.2 to 4.2)	3.6 (3.2 to 4.1)	3.2 (2.8 to 3.7)
High SDI	G6PD trait	0.0476 (0.0317 to 0.0681)	0.0503 (0.0335 to 0.0713)	0.0419 (0.0272 to 0.0624)	0 (0 to 0)	0 (0 to 0)	0 (0 to 0)
High SDI	Other hemoglobinopathies and hemolytic anaemias	296 (239 to 353)	232 (198 to 272)	240 (205 to 283)	25.6 (20.7 to 31.1)	15.2 (12.7 to 18.5)	13.4 (11 to 16.6)
High SDI	Endocrine, metabolic, blood, and immune disorders	1100 (949 to 1330)	1460 (1160 to 1650)	1660 (1280 to 1880)	109.2 (94.5 to 132.5)	117.8 (96.2 to 133.5)	116.5 (94.6 to 132.3)
High SDI	Oral disorders	3070 (1930 to 4630)	3290 (2060 to 4950)	3660 (2300 to 5510)	261.7 (164.3 to 395.2)	223.9 (139.2 to 338.7)	219.4 (136 to 332.8)
High SDI	Caries of deciduous teeth	16.2 (6.92 to 33.3)	13.1 (5.63 to 26.8)	13.8 (5.96 to 27.8)	2.4 (1 to 5)	2.2 (0.9 to 4.4)	2.2 (1 to 4.5)
High SDI	Caries of permanent teeth	321 (138 to 615)	327 (142 to 628)	346 (151 to 660)	31 (13.4 to 59.5)	27.5 (11.9 to 53.2)	27.6 (12 to 53.6)
High SDI	Periodontal disease	573 (227 to 1180)	639 (252 to 1320)	702 (276 to 1450)	49.6 (19.6 to 102.3)	43.2 (17.1 to 88.9)	42.1 (16.6 to 86.7)
High SDI	Edentulism and severe tooth loss	1630 (1090 to 2310)	1710 (1150 to 2410)	1950 (1300 to 2760)	127.8 (85.2 to 181.7)	100.7 (67.2 to 142.7)	96.7 (64.3 to 138.1)
High SDI	Other oral disorders	531 (332 to 786)	606 (376 to 896)	653 (406 to 963)	50.8 (31.8 to 75.2)	50.3 (31.4 to 74.7)	50.8 (31.7 to 75.3)
High SDI	Sudden infant death syndrome	935 (608 to 1040)	367 (338 to 480)	261 (226 to 379)	150.6 (98 to 166.9)	62.9 (58.1 to 82.5)	46.8 (40.5 to 67.9)
High SDI	Injuries	34500 (31400 to 38600)	31000 (27600 to 35500)	31100 (27000 to 36200)	3383.4 (3095.3 to 3747.9)	2538.8 (2285.2 to 2859.5)	2286.9 (2027.1 to 2617.9)
High SDI	Transport injuries	11400 (10700 to 12200)	8150 (7420 to 8990)	6990 (6210 to 7910)	1149.1 (1087.5 to 1224.3)	715.8 (661.8 to 777.6)	564 (512.5 to 625.4)
High SDI	Road injuries	10500 (9900 to 11100)	7410 (6780 to 8170)	6260 (5600 to 7050)	1059.7 (1010.1 to 1121.3)	655.1 (608.5 to 709.4)	509.4 (465 to 561.5)
High SDI	Pedestrian road injuries	2290 (2170 to 2440)	1440 (1320 to 1570)	1240 (1110 to 1400)	236.6 (225.3 to 251)	123.3 (114.8 to 133.3)	96.2 (87.4 to 106.5)
High SDI	Cyclist road injuries	714 (604 to 847)	639 (516 to 788)	646 (511 to 808)	69.5 (59.5 to 81.7)	50 (40.8 to 60.9)	45 (36.1 to 55.8)
High SDI	Motorcyclist road injuries	1470 (1360 to 1610)	1170 (1050 to 1320)	1040 (901 to 1200)	147.8 (137.7 to 160.8)	102.6 (93.5 to 113.7)	84.4 (75.1 to 95.1)
High SDI	Motor vehicle road injuries	5890 (5630 to 6190)	4060 (3790 to 4380)	3220 (2950 to 3570)	596.1 (572.2 to 623.5)	370.7 (350.7 to 394.9)	276 (258 to 302.3)
High SDI	Other road injuries	102 (83.9 to 126)	106 (84.7 to 136)	110 (85.1 to 144)	9.8 (8.2 to 11.9)	8.5 (6.9 to 10.7)	7.8 (6.2 to 10.1)
High SDI	Other transport injuries	920 (797 to 1060)	732 (635 to 852)	726 (607 to 874)	89.4 (77.8 to 103)	60.7 (53.4 to 69.5)	54.6 (46.8 to 64.2)
High SDI	Unintentional injuries	14600 (12200 to 17700)	14100 (11400 to 17500)	15600 (12500 to 19700)	1403.3 (1182.6 to 1686.5)	1066 (873.3 to 1314.2)	1028.8 (827 to 1289.7)
High SDI	Falls	6700 (5240 to 8560)	7460 (5810 to 9570)	8720 (6780 to 11200)	602.8 (470.6 to 769.2)	518.6 (400.3 to 669.9)	522.4 (400.2 to 677.7)
High SDI	Drowning	1230 (1210 to 1250)	779 (770 to 790)	661 (647 to 674)	142.6 (140 to 145.6)	77.4 (76.1 to 78.6)	60.5 (59.1 to 61.9)

SR Appendix Table 2. All-age DALYs counts and age standardized DALYs rates per 100,000 individuals for all causes and level 1 cause groups by location for 1990, 2007, and 2017, both sexes combined

		All-age DALYs			Age-standardized rate of DALYs per 100,000		
High SDI	Fire, heat, and hot substances	1070 (910 to 1300)	801 (641 to 1030)	813 (621 to 1090)	110.5 (95.6 to 131.6)	66.1 (54 to 83)	59.2 (46.2 to 77.7)
High SDI	Poisonings	458 (409 to 517)	335 (286 to 396)	330 (272 to 401)	46.6 (41.7 to 52.5)	30.2 (25.8 to 35.8)	28.1 (23.3 to 34.2)
High SDI	Poisoning by carbon monoxide	216 (207 to 228)	143 (136 to 152)	126 (118 to 138)	22.1 (21.2 to 23.4)	12.6 (12 to 13.4)	10.6 (9.9 to 11.6)
High SDI	Poisoning by other means	242 (199 to 297)	192 (149 to 248)	204 (154 to 268)	24.5 (20.2 to 30.1)	17.7 (13.8 to 22.6)	17.6 (13.2 to 23)
High SDI	Exposure to mechanical forces	1860 (1470 to 2410)	1640 (1240 to 2180)	1830 (1340 to 2480)	177.2 (141.7 to 226.6)	127.8 (98.2 to 168)	128.1 (95.6 to 171.6)
High SDI	Unintentional firearm injuries	209 (191 to 234)	128 (112 to 147)	120 (102 to 142)	21.7 (20 to 24)	11.3 (10.1 to 12.7)	9.8 (8.4 to 11.3)
High SDI	Other exposure to mechanical forces	1650 (1270 to 2170)	1510 (1130 to 2040)	1710 (1240 to 2350)	155.5 (121.1 to 202.4)	116.5 (87.7 to 155.4)	118.3 (86.7 to 160.5)
High SDI	Adverse effects of medical treatment	429 (352 to 475)	449 (395 to 533)	528 (451 to 642)	39.8 (33.1 to 44.2)	32.9 (29 to 39.2)	32.9 (28.4 to 40.2)
High SDI	Animal contact	155 (114 to 207)	142 (104 to 190)	150 (109 to 204)	15.1 (11.2 to 20)	11.5 (8.6 to 15.2)	11.1 (8.2 to 14.9)
High SDI	Venomous animal contact	73.7 (53 to 98.9)	64.1 (46.3 to 85.5)	67.6 (48.4 to 91.2)	7.2 (5.2 to 9.7)	5.4 (3.9 to 7.3)	5.3 (3.8 to 7.2)
High SDI	Non-venomous animal contact	81.7 (55.6 to 122)	77.6 (53.2 to 116)	82.9 (55.7 to 125)	7.9 (5.5 to 11.5)	6.1 (4.3 to 8.9)	5.8 (4 to 8.6)
High SDI	Foreign body	928 (875 to 997)	862 (811 to 924)	839 (783 to 903)	107.1 (101.4 to 114.7)	82.6 (78 to 88.2)	72.2 (67.5 to 77.3)
High SDI	Pulmonary aspiration and foreign body in airway	764 (744 to 806)	688 (672 to 700)	666 (650 to 682)	91.1 (88.1 to 97.7)	68.8 (66.2 to 70.3)	59.6 (57.1 to 61.5)
High SDI	Foreign body in eyes	46.3 (25.4 to 74.5)	40.7 (22.6 to 65.3)	45.1 (25.5 to 72)	4.4 (2.3 to 7.2)	3.3 (1.8 to 5.4)	3.4 (1.8 to 5.5)
High SDI	Foreign body in other body part	118 (90.5 to 152)	133 (100 to 169)	128 (96.7 to 163)	11.6 (8.8 to 14.7)	10.5 (8 to 13.5)	9.2 (7 to 11.7)
High SDI	Environmental heat and cold exposure	280 (249 to 319)	298 (262 to 343)	289 (248 to 342)	25.7 (22.9 to 29.2)	21.6 (19.1 to 24.8)	18.4 (15.8 to 21.7)
High SDI	Exposure to forces of nature	47.2 (46.1 to 48.7)	34.9 (32.7 to 37.9)	23.1 (20 to 27.4)	5.1 (5 to 5.2)	3.3 (3.1 to 3.5)	1.9 (1.7 to 2.2)
High SDI	Other unintentional injuries	1410 (1080 to 1840)	1280 (915 to 1770)	1450 (1010 to 2030)	130.7 (101.6 to 170.2)	93.9 (68.1 to 129)	93.9 (66.5 to 131.1)
High SDI	Self-harm and interpersonal violence	8580 (8420 to 8770)	8810 (8620 to 9020)	8440 (8180 to 8710)	831.1 (815.8 to 848.1)	757 (741.7 to 774.4)	694.1 (674.8 to 714.6)
High SDI	Self-harm	6030 (5960 to 6090)	6610 (6530 to 6690)	6370 (6190 to 6510)	567.9 (561.7 to 573.7)	544 (537.3 to 550.5)	501.4 (488.8 to 511.7)
High SDI	Self-harm by firearm	1240 (988 to 1480)	1050 (843 to 1260)	1150 (979 to 1410)	118.4 (94.2 to 141)	87.4 (70 to 105.5)	92.6 (77.9 to 113.9)
High SDI	Self-harm by other specified means	4790 (4530 to 5040)	5560 (5320 to 5770)	5210 (4930 to 5430)	449.5 (425.2 to 474.5)	456.6 (436.6 to 474.1)	408.7 (385.4 to 426.8)
High SDI	Interpersonal violence	2510 (2390 to 2640)	2090 (1960 to 2260)	2020 (1860 to 2200)	258.3 (246.7 to 270.8)	202.2 (191.2 to 215.6)	187.7 (175.9 to 201.7)
High SDI	Assault by firearm	1110 (969 to 1140)	903 (804 to 928)	876 (779 to 922)	113.1 (98.9 to 115.6)	90.7 (80.8 to 93.1)	87.8 (77.8 to 92.2)
High SDI	Assault by sharp object	464 (438 to 564)	324 (300 to 374)	289 (263 to 336)	46 (43.5 to 56)	29.1 (27.2 to 33.3)	24.8 (22.8 to 28.7)
High SDI	Sexual violence	155 (104 to 227)	162 (109 to 237)	172 (117 to 249)	15.8 (10.6 to 23.1)	15.8 (10.6 to 23)	16.3 (10.9 to 23.9)
High SDI	Assault by other means	782 (697 to 899)	706 (612 to 842)	680 (571 to 841)	83.4 (75.5 to 94.7)	66.6 (59.2 to 77.3)	58.7 (51.1 to 70)
High SDI	Conflict and terrorism	13.9 (13 to 15.3)	70.5 (67.4 to 76)	10.2 (7.12 to 16.1)	1.5 (1.4 to 1.6)	7.4 (7.1 to 7.8)	0.8 (0.5 to 1.2)
High SDI	Executions and police conflict	34.1 (31.5 to 37)	37 (34.1 to 41)	47.8 (42.7 to 54.2)	3.4 (3.2 to 3.7)	3.4 (3.2 to 3.8)	4.3 (3.9 to 4.9)
Central Europe, Eastern Europe, and Central Asia	All causes	165000 (152000 to 180000)	177000 (163000 to 192000)	158000 (144000 to 173000)	37776.6 (34830.9 to 41049.9)	37055.8 (34248.9 to 40308.3)	30575.6 (27769.1 to 33745.7)
Central Europe, Eastern Europe, and Central Asia	Communicable, maternal, neonatal, and nutritional disorders	21900 (20600 to 23400)	16400 (15300 to 17600)	13500 (12300 to 14700)	6133.8 (5789.4 to 6524.9)	4918.3 (4592.6 to 5272.6)	3811.1 (3502.8 to 4142.1)
Central Europe, Eastern Europe, and Central Asia	HIV/AIDS and sexually transmitted infections	425 (381 to 497)	1260 (1200 to 1340)	1570 (1490 to 1670)	101.5 (90.8 to 118.4)	296.8 (283 to 316.6)	350.6 (334.2 to 372.8)
Central Europe, Eastern Europe, and Central Asia	HIV/AIDS	303 (295 to 314)	1150 (1110 to 1200)	1470 (1420 to 1530)	72 (70.2 to 74.4)	270.3 (263.2 to 282.1)	326.5 (316.3 to 340.6)
Central Europe, Eastern Europe, and Central Asia	HIV/AIDS - Drug-susceptible Tuberculosis	45.8 (29.2 to 69.1)	80.7 (45 to 128)	40.5 (22.3 to 67.9)	10.9 (6.9 to 16.4)	19.1 (10.6 to 30.3)	9.1 (5 to 15.3)
Central Europe, Eastern Europe, and Central Asia	HIV/AIDS - Multidrug-resistant Tuberculosis without extensive drug resistance	1.55 (0.607 to 3.59)	60.3 (35 to 100)	34.3 (20 to 55.3)	0.4 (0.1 to 0.9)	14.3 (8.3 to 23.6)	7.7 (4.5 to 12.4)
Central Europe, Eastern Europe, and Central Asia	HIV/AIDS - Extensively drug-resistant Tuberculosis	0 (0 to 0)	19.1 (11 to 31.9)	19.6 (11.3 to 31.4)	0 (0 to 0)	4.5 (2.6 to 7.5)	4.4 (2.5 to 7)
Central Europe, Eastern Europe, and Central Asia	HIV/AIDS resulting in other diseases	256 (231 to 276)	985 (900 to 1060)	1370 (1310 to 1440)	60.7 (54.9 to 65.4)	232.4 (212.2 to 250.1)	305.3 (291.1 to 321.2)
Central Europe, Eastern Europe, and Central Asia	Sexually transmitted infections excluding HIV	122 (79.5 to 195)	112 (69.3 to 185)	102 (61.4 to 171)	29.5 (19.1 to 47.3)	26.5 (16.5 to 43.6)	24.1 (14.5 to 40.2)
Central Europe, Eastern Europe, and Central Asia	Syphilis	34.2 (22.7 to 50.8)	20.9 (13.8 to 31)	16.7 (11.6 to 24.6)	9.1 (5.5 to 14.1)	6.4 (3.6 to 10.2)	4.7 (2.8 to 7.7)
Central Europe, Eastern Europe, and Central Asia	Chlamydial infection	18.1 (10.5 to 32.4)	19 (11 to 34.3)	18 (10.3 to 32.8)	4.2 (2.5 to 7.7)	4.3 (2.5 to 7.8)	4.2 (2.4 to 7.8)

SR Appendix Table 2. All-age DALYs counts and age standardized DALYs rates per 100,000 individuals for all causes and level 1 cause groups by location for 1990, 2007, and 2017, both sexes combined

		All-age DALYs			Age-standardized rate of DALYs per 100,000		
Central Europe, Eastern Europe, and Central Asia	Gonococcal infection	19.3 (12.4 to 32.8)	19.1 (12.1 to 32.7)	15.6 (9.88 to 27.1)	4.6 (2.9 to 8)	4.3 (2.7 to 7.6)	3.9 (2.4 to 7.1)
Central Europe, Eastern Europe, and Central Asia	Trichomoniasis	13.2 (5.32 to 28.4)	14.3 (5.72 to 30.7)	14 (5.66 to 29.6)	3 (1.2 to 6.5)	3.1 (1.2 to 6.6)	3 (1.2 to 6.5)
Central Europe, Eastern Europe, and Central Asia	Genital herpes	13.1 (4.21 to 31.2)	14 (4.5 to 33.6)	14.5 (4.64 to 34.5)	2.9 (0.9 to 6.8)	2.9 (0.9 to 6.8)	2.9 (0.9 to 6.8)
Central Europe, Eastern Europe, and Central Asia	Other sexually transmitted infections	24.3 (15.5 to 40.5)	24.6 (15.6 to 40.7)	22.7 (14.4 to 37.8)	5.7 (3.6 to 9.5)	5.6 (3.5 to 9.3)	5.4 (3.3 to 9.1)
Central Europe, Eastern Europe, and Central Asia	Respiratory infections and tuberculosis	8610 (8320 to 8940)	7150 (6870 to 7460)	5050 (4790 to 5320)	2368.7 (2286.9 to 2460.3)	1967.1 (1871.5 to 2071.6)	1293.1 (1214 to 1374.8)
Central Europe, Eastern Europe, and Central Asia	Tuberculosis	1120 (1090 to 1160)	1910 (1880 to 1950)	967 (919 to 1010)	255.4 (248.2 to 263.8)	410 (402.7 to 418.4)	201.3 (191.7 to 209.2)
Central Europe, Eastern Europe, and Central Asia	Latent tuberculosis infection	0 (0 to 0)	0 (0 to 0)	0 (0 to 0)	0 (0 to 0)	0 (0 to 0)	0 (0 to 0)
Central Europe, Eastern Europe, and Central Asia	Drug-susceptible tuberculosis	1100 (1060 to 1140)	1090 (895 to 1280)	502 (417 to 601)	250.3 (241.1 to 259.2)	232.9 (193 to 274.2)	104.7 (87.3 to 125)
Central Europe, Eastern Europe, and Central Asia	Multidrug-resistant tuberculosis without extensive drug resistance	23.2 (11.4 to 51.2)	627 (481 to 774)	296 (238 to 356)	5.1 (2.5 to 11.3)	134.5 (103.7 to 165.5)	61.6 (49.6 to 73.9)
Central Europe, Eastern Europe, and Central Asia	Extensively drug-resistant tuberculosis	0 (0 to 0)	198 (151 to 245)	169 (134 to 202)	0 (0 to 0)	42.5 (32.4 to 52.4)	35 (27.9 to 41.9)
Central Europe, Eastern Europe, and Central Asia	Lower respiratory infections	6970 (6740 to 7190)	4820 (4640 to 5020)	3690 (3530 to 3870)	1982.5 (1914.9 to 2046.6)	1445.2 (1373.1 to 1522.5)	989.8 (930.9 to 1057)
Central Europe, Eastern Europe, and Central Asia	Upper respiratory infections	411 (287 to 584)	324 (210 to 484)	302 (190 to 464)	104.1 (74.2 to 146.9)	86.4 (57.5 to 127.3)	78 (49.8 to 118)
Central Europe, Eastern Europe, and Central Asia	Otitis media	106 (66.2 to 163)	93.1 (56.2 to 147)	86.6 (52.5 to 136)	26.6 (16.6 to 40.9)	25.5 (15.5 to 40.6)	24 (14.4 to 38.2)
Central Europe, Eastern Europe, and Central Asia	Enteric infections	1680 (1550 to 1840)	573 (485 to 675)	558 (456 to 683)	485.1 (446.5 to 528.7)	198.2 (169.3 to 230.9)	175.9 (144.1 to 215.7)
Central Europe, Eastern Europe, and Central Asia	Diarrhoeal diseases	1650 (1520 to 1810)	554 (469 to 657)	539 (438 to 665)	477.8 (439.4 to 521.6)	192.5 (164.6 to 225.8)	170.6 (138.7 to 209.2)
Central Europe, Eastern Europe, and Central Asia	Typhoid and paratyphoid	1.7 (0.565 to 2.75)	0.357 (0.193 to 0.528)	0.196 (0.117 to 0.279)	0.4 (0.1 to 0.6)	0.1 (0 to 0.1)	0 (0 to 0.1)
Central Europe, Eastern Europe, and Central Asia	Typhoid fever	0.601 (0.445 to 0.713)	0.171 (0.133 to 0.22)	0.103 (0.0758 to 0.144)	0.1 (0.1 to 0.2)	0 (0 to 0.1)	0 (0 to 0)
Central Europe, Eastern Europe, and Central Asia	Paratyphoid fever	1.1 (0.0843 to 2.1)	0.186 (0.0491 to 0.342)	0.0925 (0.0325 to 0.153)	0.2 (0 to 0.5)	0 (0 to 0.1)	0 (0 to 0)
Central Europe, Eastern Europe, and Central Asia	Invasive non-typhoidal salmonella	24.3 (13.4 to 41.6)	17 (9.33 to 28.8)	17 (9.17 to 29.4)	6.2 (3.4 to 10.7)	5 (2.7 to 8.8)	4.9 (2.5 to 8.9)
Central Europe, Eastern Europe, and Central Asia	Other intestinal infectious diseases	2.68 (1.3 to 4.5)	1.58 (0.798 to 2.46)	1.06 (0.554 to 1.64)	0.7 (0.3 to 1.3)	0.5 (0.2 to 0.8)	0.3 (0.1 to 0.5)
Central Europe, Eastern Europe, and Central Asia	Neglected tropical diseases and malaria	335 (239 to 442)	317 (224 to 424)	306 (213 to 412)	76.9 (55 to 101.7)	70.5 (50.2 to 93.3)	65.7 (45.8 to 89)
Central Europe, Eastern Europe, and Central Asia	Malaria	5.81 (2.21 to 13.7)	2.22 (1.47 to 3.81)	0.107 (0.0769 to 0.145)	1.6 (0.6 to 3.9)	0.8 (0.5 to 1.4)	0 (0 to 0)
Central Europe, Eastern Europe, and Central Asia	Chagas disease	0 (0 to 0)	0 (0 to 0)	0 (0 to 0)	0 (0 to 0)	0 (0 to 0)	0 (0 to 0)
Central Europe, Eastern Europe, and Central Asia	Leishmaniasis	6.15 (0.471 to 56.7)	3.62 (0.362 to 30.9)	2.32 (0.151 to 18.1)	1.5 (0.1 to 14.7)	1.1 (0.1 to 9.8)	0.7 (0 to 5.7)
Central Europe, Eastern Europe, and Central Asia	Visceral leishmaniasis	5.75 (0.149 to 56.1)	3.44 (0.204 to 30.8)	2.09 (0.00594 to 17.9)	1.4 (0 to 14.5)	1 (0 to 9.7)	0.6 (0 to 5.7)
Central Europe, Eastern Europe, and Central Asia	Cutaneous and mucocutaneous leishmaniasis	0.409 (0.217 to 0.713)	0.178 (0.101 to 0.292)	0.229 (0.12 to 0.398)	0.1 (0.1 to 0.2)	0 (0 to 0.1)	0.1 (0 to 0.1)
Central Europe, Eastern Europe, and Central Asia	African trypanosomiasis	0 (0 to 0)	0 (0 to 0)	0 (0 to 0)	0 (0 to 0)	0 (0 to 0)	0 (0 to 0)
Central Europe, Eastern Europe, and Central Asia	Schistosomiasis	0 (0 to 0)	0 (0 to 0)	0 (0 to 0)	0 (0 to 0)	0 (0 to 0)	0 (0 to 0)
Central Europe, Eastern Europe, and Central Asia	Cysticercosis	89.9 (58 to 125)	80.8 (51.1 to 116)	76.7 (46.4 to 113)	19.6 (12.7 to 27.1)	15.9 (10 to 22.8)	14.1 (8.5 to 20.7)
Central Europe, Eastern Europe, and Central Asia	Cystic echinococcosis	27.3 (17.3 to 42.4)	29 (17.4 to 48.1)	31.3 (17.7 to 54.7)	6.2 (3.9 to 9.8)	6.4 (3.7 to 10.8)	7 (3.8 to 12.5)
Central Europe, Eastern Europe, and Central Asia	Lymphatic filariasis	0 (0 to 0)	0 (0 to 0)	0 (0 to 0)	0 (0 to 0)	0 (0 to 0)	0 (0 to 0)
Central Europe, Eastern Europe, and Central Asia	Onchocerciasis	0 (0 to 0)	0 (0 to 0)	0 (0 to 0)	0 (0 to 0)	0 (0 to 0)	0 (0 to 0)
Central Europe, Eastern Europe, and Central Asia	Trachoma	0 (0 to 0)	0 (0 to 0)	0 (0 to 0)	0 (0 to 0)	0 (0 to 0)	0 (0 to 0)
Central Europe, Eastern Europe, and Central Asia	Dengue	0.601 (0.58 to 0.675)	0.632 (0.567 to 0.769)	0.692 (0.612 to 0.847)	0.1 (0.1 to 0.2)	0.2 (0.1 to 0.2)	0.2 (0.2 to 0.2)
Central Europe, Eastern Europe, and Central Asia	Yellow fever	0 (0 to 0)	0 (0 to 0)	0 (0 to 0)	0 (0 to 0)	0 (0 to 0)	0 (0 to 0)
Central Europe, Eastern Europe, and Central Asia	Rabies	3.26 (2.76 to 3.99)	2.14 (1.98 to 2.32)	1.49 (1.35 to 1.66)	0.8 (0.7 to 1)	0.5 (0.5 to 0.6)	0.3 (0.3 to 0.4)
Central Europe, Eastern Europe, and Central Asia	Intestinal nematode infections	5.1 (3.27 to 7.77)	2.6 (1.63 to 4.02)	2.06 (1.27 to 3.28)	1.3 (0.8 to 1.9)	0.7 (0.5 to 1.1)	0.6 (0.4 to 0.9)
Central Europe, Eastern Europe, and Central Asia	Ascariasis	0.913 (0.598 to 1.41)	0.755 (0.434 to 1.25)	0.611 (0.341 to 1.04)	0.2 (0.2 to 0.4)	0.2 (0.1 to 0.4)	0.2 (0.1 to 0.3)
Central Europe, Eastern Europe, and Central Asia	Trichuriasis	0.0000299 (0.000014 to 0.0000582)	0.0000156 (0.00000687 to 0.0000307)	0.0000187 (0.00000797 to 0.0000381)	0 (0 to 0)	0 (0 to 0)	0 (0 to 0)
Central Europe, Eastern Europe, and Central Asia	Hookworm disease	4.18 (2.64 to 6.4)	1.85 (1.15 to 2.92)	1.45 (0.908 to 2.28)	1 (0.7 to 1.6)	0.5 (0.3 to 0.8)	0.4 (0.2 to 0.6)

SR Appendix Table 2. All-age DALYs counts and age standardized DALYs rates per 100,000 individuals for all causes and level 1 cause groups by location for 1990, 2007, and 2017, both sexes combined

		All-age DALYs			Age-standardized rate of DALYs per 100,000		
Central Europe, Eastern Europe, and Central Asia	Food-borne trematodiases	155 (101 to 219)	162 (106 to 232)	161 (104 to 228)	35 (22.9 to 49.6)	34.6 (22.7 to 49.4)	33.7 (22.1 to 47.8)
Central Europe, Eastern Europe, and Central Asia	Leprosy	0.00016 (0.000107 to 0.000229)	0.00015 (0.0000995 to 0.000216)	0.000126 (0.0000831 to 0.000184)	0 (0 to 0)	0 (0 to 0)	0 (0 to 0)
Central Europe, Eastern Europe, and Central Asia	Ebola virus disease	0 (0 to 0)	0 (0 to 0)	0 (0 to 0)	0 (0 to 0)	0 (0 to 0)	0 (0 to 0)
Central Europe, Eastern Europe, and Central Asia	Zika virus disease	0 (0 to 0)	0 (0 to 0)	0.000283 (0.0000303 to 0.000835)	0 (0 to 0)	0 (0 to 0)	0 (0 to 0)
Central Europe, Eastern Europe, and Central Asia	Guinea worm disease	0 (0 to 0)	0 (0 to 0)	0 (0 to 0)	0 (0 to 0)	0 (0 to 0)	0 (0 to 0)
Central Europe, Eastern Europe, and Central Asia	Other neglected tropical diseases	41.3 (30 to 55.1)	33.8 (24.5 to 47)	30.4 (21.9 to 43)	10.8 (7.8 to 14.4)	10.4 (7.5 to 14.5)	9 (6.4 to 12.7)
Central Europe, Eastern Europe, and Central Asia	Other infectious diseases	1400 (1300 to 1490)	834 (779 to 908)	687 (629 to 759)	378.4 (353.3 to 403)	256.7 (238.5 to 281.7)	198 (179.9 to 219.6)
Central Europe, Eastern Europe, and Central Asia	Meningitis	597 (527 to 632)	319 (300 to 345)	203 (187 to 234)	163.1 (143.9 to 172.2)	97.5 (91 to 106.6)	57.5 (52.4 to 67.2)
Central Europe, Eastern Europe, and Central Asia	Pneumococcal meningitis	52.4 (44.9 to 61.3)	34.6 (29.2 to 41)	20.4 (17.2 to 24.1)	13.3 (11.4 to 15.3)	8.9 (7.5 to 10.4)	5 (4.2 to 5.9)
Central Europe, Eastern Europe, and Central Asia	H influenzae type B meningitis	298 (262 to 317)	150 (141 to 163)	91.3 (84.2 to 107)	83.2 (72.7 to 88.4)	49.1 (45.6 to 53.9)	27.6 (25.2 to 32.9)
Central Europe, Eastern Europe, and Central Asia	Meningococcal infection	70.1 (61.6 to 79)	30.6 (26.9 to 34.9)	19.4 (17 to 23.2)	19.3 (16.9 to 21.9)	9.5 (8.3 to 11.1)	5.6 (4.8 to 6.7)
Central Europe, Eastern Europe, and Central Asia	Other meningitis	177 (154 to 191)	104 (96.4 to 112)	72.2 (65.4 to 82.1)	47.3 (41.1 to 51.3)	30 (27.5 to 33.1)	19.3 (17.3 to 22.4)
Central Europe, Eastern Europe, and Central Asia	Encephalitis	269 (238 to 291)	217 (199 to 233)	220 (191 to 240)	70.1 (61.6 to 76.4)	64.9 (59.1 to 69.9)	61.7 (53 to 69)
Central Europe, Eastern Europe, and Central Asia	Diphtheria	9.14 (7.32 to 11.7)	1.16 (0.91 to 1.5)	0.395 (0.299 to 0.527)	2.4 (1.9 to 3.1)	0.4 (0.3 to 0.5)	0.1 (0.1 to 0.1)
Central Europe, Eastern Europe, and Central Asia	Whooping cough	59 (30.8 to 103)	31.2 (11.3 to 71.6)	23 (8.7 to 50.9)	17.1 (8.9 to 29.8)	12.9 (4.6 to 29.7)	8.5 (3.2 to 18.9)
Central Europe, Eastern Europe, and Central Asia	Tetanus	5.3 (3.97 to 5.83)	1.28 (1.15 to 1.51)	1.13 (0.901 to 1.77)	1.2 (0.9 to 1.3)	0.3 (0.2 to 0.3)	0.2 (0.2 to 0.4)
Central Europe, Eastern Europe, and Central Asia	Measles	33 (25.9 to 41.7)	0.763 (0.644 to 0.842)	0.889 (0.707 to 1.07)	9.3 (7.3 to 11.8)	0.3 (0.2 to 0.3)	0.3 (0.2 to 0.4)
Central Europe, Eastern Europe, and Central Asia	Varicella and herpes zoster	20.5 (15.3 to 28)	18 (12.9 to 25)	18 (12.5 to 24.9)	5 (3.8 to 6.8)	4.6 (3.5 to 6.2)	4.2 (3.1 to 5.6)
Central Europe, Eastern Europe, and Central Asia	Acute hepatitis	202 (166 to 222)	93.3 (84.7 to 105)	78 (68.7 to 90.4)	53.4 (43.8 to 58.8)	25.9 (23.4 to 29.4)	20.8 (18.3 to 24.2)
Central Europe, Eastern Europe, and Central Asia	Acute hepatitis A	74.4 (58.9 to 88.5)	33.4 (28.4 to 39.8)	26.9 (22.3 to 32.6)	19.8 (15.7 to 23.6)	9.9 (8.5 to 11.7)	8.1 (6.7 to 9.8)
Central Europe, Eastern Europe, and Central Asia	Acute hepatitis B	74.2 (60 to 86.7)	44 (39.3 to 50.5)	39.1 (34.3 to 45.9)	18.5 (14.7 to 21.9)	10.6 (9.4 to 12.4)	9 (7.9 to 10.6)
Central Europe, Eastern Europe, and Central Asia	Acute hepatitis C	42.6 (28.1 to 57.5)	9.02 (6.1 to 12.9)	6.33 (4.03 to 9.71)	12.5 (8.3 to 16.8)	3.6 (2.4 to 5.2)	2.3 (1.4 to 3.5)
Central Europe, Eastern Europe, and Central Asia	Acute hepatitis E	10.9 (8.72 to 12.8)	6.94 (5.77 to 8.43)	5.55 (4.6 to 6.74)	2.7 (2.2 to 3.1)	1.7 (1.4 to 2.1)	1.4 (1.2 to 1.7)
Central Europe, Eastern Europe, and Central Asia	Other unspecified infectious diseases	205 (175 to 253)	152 (131 to 185)	142 (116 to 177)	56.9 (49 to 71.8)	50 (43.7 to 60.8)	44.7 (36.3 to 57.3)
Central Europe, Eastern Europe, and Central Asia	Maternal and neonatal disorders	7640 (7130 to 8250)	5000 (4540 to 5520)	4190 (3690 to 4750)	2276.9 (2142.5 to 2430.9)	1780.9 (1644.2 to 1918.1)	1431.7 (1287.5 to 1599.5)
Central Europe, Eastern Europe, and Central Asia	Maternal disorders	212 (202 to 224)	109 (102 to 117)	74.1 (67.2 to 81.9)	50.7 (48.2 to 53.7)	25.4 (23.9 to 27.4)	18.5 (16.8 to 20.6)
Central Europe, Eastern Europe, and Central Asia	Maternal haemorrhage	48.5 (41.5 to 56)	22.1 (18.7 to 26)	13.6 (11.3 to 16.4)	11.5 (9.9 to 13.4)	5.2 (4.4 to 6.1)	3.4 (2.8 to 4.1)
Central Europe, Eastern Europe, and Central Asia	Maternal sepsis and other pregnancy related infections	26.4 (22.5 to 30.7)	10.5 (8.77 to 12.9)	7.52 (5.89 to 10)	6.4 (5.4 to 7.5)	2.4 (2 to 3)	1.9 (1.5 to 2.6)
Central Europe, Eastern Europe, and Central Asia	Maternal hypertensive disorders	22.8 (18.5 to 28.1)	13.6 (11.2 to 16.7)	10 (8.01 to 12.4)	5.5 (4.5 to 6.8)	3.2 (2.6 to 3.9)	2.6 (2.1 to 3.2)
Central Europe, Eastern Europe, and Central Asia	Maternal obstructed labour and uterine rupture	7.7 (5.89 to 10.2)	5.3 (3.99 to 6.92)	4.26 (2.94 to 6.2)	1.9 (1.4 to 2.5)	1.2 (0.9 to 1.6)	1.1 (0.7 to 1.6)
Central Europe, Eastern Europe, and Central Asia	Maternal abortive outcome	46.5 (40.2 to 53.4)	15.1 (12.9 to 17.6)	8.29 (7.01 to 9.75)	11.1 (9.6 to 12.7)	3.5 (2.9 to 4.1)	2 (1.7 to 2.4)
Central Europe, Eastern Europe, and Central Asia	Ectopic pregnancy	10.3 (6.65 to 14.8)	3.28 (2.21 to 4.74)	1.79 (1.2 to 2.62)	2.4 (1.6 to 3.5)	0.8 (0.5 to 1.1)	0.4 (0.3 to 0.6)
Central Europe, Eastern Europe, and Central Asia	Indirect maternal deaths	13.7 (10.9 to 17.3)	9.86 (8.01 to 12)	5.86 (4.74 to 7.17)	3.3 (2.6 to 4.2)	2.3 (1.9 to 2.8)	1.5 (1.2 to 1.8)
Central Europe, Eastern Europe, and Central Asia	Late maternal deaths	7.22 (4.16 to 11.4)	4.75 (3.02 to 7.27)	3.08 (2.09 to 4.96)	1.7 (1 to 2.7)	1.1 (0.7 to 1.7)	0.8 (0.5 to 1.2)
Central Europe, Eastern Europe, and Central Asia	Maternal deaths aggravated by HIV/AIDS	0.0111 (0.00541 to 0.0197)	0.0748 (0.0371 to 0.126)	0.119 (0.0631 to 0.19)	0 (0 to 0)	0 (0 to 0)	0 (0 to 0)
Central Europe, Eastern Europe, and Central Asia	Other maternal disorders	28.4 (24.7 to 32.6)	24.3 (21.4 to 27.5)	19.6 (17.4 to 22)	6.8 (5.9 to 7.8)	5.7 (5 to 6.4)	4.9 (4.3 to 5.5)
Central Europe, Eastern Europe, and Central Asia	Neonatal disorders	7430 (6920 to 8050)	4890 (4430 to 5400)	4120 (3620 to 4680)	2226.2 (2091 to 2378.5)	1755.5 (1618.7 to 1893.9)	1413.2 (1268.3 to 1579.4)
Central Europe, Eastern Europe, and Central Asia	Neonatal preterm birth	2960 (2700 to 3210)	1720 (1590 to 1870)	1410 (1240 to 1590)	902.8 (822.9 to 977.7)	644.3 (600.8 to 690)	513.9 (457.4 to 572.1)
Central Europe, Eastern Europe, and Central Asia	Neonatal encephalopathy due to birth asphyxia and trauma	2650 (2350 to 3000)	1760 (1500 to 2070)	1450 (1180 to 1810)	796.9 (712.5 to 888.4)	624.1 (549.7 to 711.1)	483.8 (410.7 to 584)
Central Europe, Eastern Europe, and Central Asia	Neonatal sepsis and other neonatal infections	938 (661 to 1320)	820 (575 to 1160)	825 (572 to 1170)	254.2 (185.6 to 348.5)	252.6 (189.6 to 336.2)	248.5 (183.1 to 336.9)

SR Appendix Table 2. All-age DALYs counts and age standardized DALYs rates per 100,000 individuals for all causes and level 1 cause groups by location for 1990, 2007, and 2017, both sexes combined							
		All-age DALYs			Age-standardized rate of DALYs per 100,000		
Central Europe, Eastern Europe, and Central Asia	Hemolytic disease and other neonatal jaundice	134<br>(115 to 151)	83.2<br>(71.1 to 95.9)	68.8<br>(56.8 to 81.2)	38.9<br>(33.6 to 43.8)	27.3<br>(23.7 to 31)	21.1<br>(17.7 to 24.8)
Central Europe, Eastern Europe, and Central Asia	Other neonatal disorders	744<br>(675 to 849)	507<br>(473 to 540)	368<br>(325 to 399)	233.4<br>(211.4 to 266.5)	207.1<br>(193.1 to 220.7)	145.8<br>(128.5 to 158.7)
Central Europe, Eastern Europe, and Central Asia	Nutritional deficiencies	1780<br>(1210 to 2560)	1250<br>(833 to 1840)	1110<br>(739 to 1630)	446.3<br>(304 to 639.7)	348<br>(233 to 508.6)	296.1<br>(195.6 to 432.3)
Central Europe, Eastern Europe, and Central Asia	Protein-energy malnutrition	86.9<br>(73.5 to 103)	44.2<br>(34.8 to 54.2)	34.6<br>(26.7 to 44.4)	23.9<br>(20.4 to 28.3)	14<br>(11.2 to 17.3)	9.6<br>(7.5 to 12.4)
Central Europe, Eastern Europe, and Central Asia	Iodine deficiency	55.2<br>(29.4 to 93.1)	52<br>(27.2 to 89.2)	51.8<br>(26.5 to 90.3)	12.8<br>(6.8 to 21.6)	11.9<br>(6.3 to 20.5)	11.8<br>(6 to 20.5)
Central Europe, Eastern Europe, and Central Asia	Dietary iron deficiency	1280<br>(848 to 1880)	984<br>(642 to 1480)	878<br>(560 to 1320)	312.8<br>(206.2 to 459)	259.5<br>(169.7 to 387.8)	227.5<br>(145.9 to 339.5)
Central Europe, Eastern Europe, and Central Asia	Other nutritional deficiencies	52.6<br>(45.8 to 59.4)	37.5<br>(30.9 to 45.3)	31.9<br>(25.4 to 38.2)	13.2<br>(11.7 to 14.7)	9.6<br>(8.2 to 11.2)	7<br>(5.7 to 8.2)
Central Europe, Eastern Europe, and Central Asia	Non-communicable diseases	118000<br>(108000 to 129000)	135000<br>(124000 to 147000)	124000<br>(113000 to 137000)	25621.8<br>(23402.6 to 28250.3)	26395.5<br>(24224.3 to 29017)	22439.8<br>(20262.6 to 24981)
Central Europe, Eastern Europe, and Central Asia	Neoplasms	19500<br>(19200 to 19800)	20600<br>(20200 to 20900)	19900<br>(19400 to 20300)	4047.8<br>(3981.9 to 4107.9)	3869.1<br>(3791.7 to 3912.2)	3386.4<br>(3303.4 to 3453.4)
Central Europe, Eastern Europe, and Central Asia	Lip and oral cavity cancer	344<br>(330 to 388)	418<br>(411 to 424)	409<br>(397 to 423)	70.9<br>(68.1 to 80.1)	77.4<br>(76.2 to 78.5)	69<br>(66.9 to 71.3)
Central Europe, Eastern Europe, and Central Asia	Nasopharynx cancer	52.2<br>(49.6 to 55.7)	65.5<br>(64.2 to 66.8)	61.5<br>(59.1 to 64)	11.2<br>(10.6 to 11.9)	12.8<br>(12.5 to 13)	11.4<br>(10.9 to 11.9)
Central Europe, Eastern Europe, and Central Asia	Other pharynx cancer	184<br>(169 to 199)	259<br>(253 to 264)	277<br>(267 to 286)	37.7<br>(34.7 to 40.7)	47.8<br>(46.8 to 48.7)	46.2<br>(44.7 to 47.9)
Central Europe, Eastern Europe, and Central Asia	Oesophageal cancer	556<br>(546 to 566)	500<br>(495 to 506)	493<br>(481 to 504)	112.1<br>(110.1 to 114.2)	91.1<br>(90 to 92.2)	80.5<br>(78.6 to 82.4)
Central Europe, Eastern Europe, and Central Asia	Stomach cancer	2800<br>(2740 to 2840)	2060<br>(2040 to 2080)	1670<br>(1630 to 1710)	568.6<br>(557 to 577.7)	374.2<br>(371.2 to 377.7)	275.3<br>(269.3 to 282.3)
Central Europe, Eastern Europe, and Central Asia	Colon and rectum cancer	1860<br>(1830 to 1910)	2270<br>(2240 to 2290)	2270<br>(2210 to 2340)	378.8<br>(372.3 to 389.2)	407.6<br>(402.8 to 412.3)	367.9<br>(358.2 to 379)
Central Europe, Eastern Europe, and Central Asia	Liver cancer	551<br>(521 to 586)	646<br>(634 to 657)	729<br>(705 to 757)	112.5<br>(106.4 to 119.8)	118.8<br>(116.6 to 120.8)	121.9<br>(117.8 to 126.6)
Central Europe, Eastern Europe, and Central Asia	Liver cancer due to hepatitis B	135<br>(117 to 155)	154<br>(138 to 171)	160<br>(141 to 182)	28.3<br>(24.7 to 32.4)	29.6<br>(26.5 to 32.8)	28.7<br>(25.6 to 32.4)
Central Europe, Eastern Europe, and Central Asia	Liver cancer due to hepatitis C	169<br>(149 to 192)	197<br>(177 to 217)	230<br>(205 to 256)	33.9<br>(29.8 to 38.3)	35.4<br>(31.8 to 39.1)	36.8<br>(32.9 to 41)
Central Europe, Eastern Europe, and Central Asia	Liver cancer due to alcohol use	173<br>(155 to 192)	208<br>(194 to 224)	238<br>(220 to 259)	35<br>(31.5 to 38.8)	37.6<br>(34.9 to 40.5)	39.3<br>(36.2 to 42.6)
Central Europe, Eastern Europe, and Central Asia	Liver cancer due to NASH	38.4<br>(33.6 to 43.9)	47.6<br>(42.5 to 52.9)	57.2<br>(51 to 64.1)	7.8<br>(6.8 to 8.8)	8.6<br>(7.7 to 9.6)	9.3<br>(8.4 to 10.4)
Central Europe, Eastern Europe, and Central Asia	Liver cancer due to other causes	35.9<br>(31.3 to 40.7)	39.7<br>(35.5 to 44.3)	43.7<br>(39 to 48.9)	7.5<br>(6.6 to 8.5)	7.6<br>(6.8 to 8.4)	7.7<br>(6.9 to 8.6)
Central Europe, Eastern Europe, and Central Asia	Gallbladder and biliary tract cancer	254<br>(243 to 271)	259<br>(247 to 266)	239<br>(230 to 248)	51.7<br>(49.5 to 55)	46.4<br>(44 to 47.6)	38.3<br>(36.8 to 39.8)
Central Europe, Eastern Europe, and Central Asia	Pancreatic cancer	789<br>(753 to 829)	969<br>(959 to 981)	1060<br>(1040 to 1080)	160.1<br>(152.8 to 168.3)	175.6<br>(173.7 to 177.8)	172.1<br>(168.8 to 176.3)
Central Europe, Eastern Europe, and Central Asia	Larynx cancer	464<br>(454 to 478)	406<br>(400 to 412)	350<br>(341 to 360)	94<br>(92 to 97)	74.2<br>(73.1 to 75.3)	57.4<br>(55.9 to 59)
Central Europe, Eastern Europe, and Central Asia	Tracheal, bronchus, and lung cancer	4250<br>(4190 to 4300)	3930<br>(3890 to 3960)	3760<br>(3690 to 3830)	847.7<br>(835.7 to 857.9)	710.7<br>(705 to 717.3)	605.1<br>(593.4 to 616.5)
Central Europe, Eastern Europe, and Central Asia	Malignant skin melanoma	193<br>(152 to 256)	270<br>(203 to 335)	276<br>(207 to 335)	41.2<br>(32.3 to 54.3)	51.8<br>(39.1 to 64.3)	49<br>(37.2 to 60)
Central Europe, Eastern Europe, and Central Asia	Non-melanoma skin cancer	86.7<br>(82.3 to 91.4)	116<br>(114 to 118)	117<br>(113 to 122)	18.4<br>(17.5 to 19.3)	21.6<br>(21.2 to 22)	19.3<br>(18.6 to 20.2)
Central Europe, Eastern Europe, and Central Asia	Non-melanoma skin cancer (squamous-cell carcinoma)	86.6<br>(82.2 to 91.3)	116<br>(114 to 118)	117<br>(112 to 122)	18.4<br>(17.5 to 19.3)	21.5<br>(21.2 to 21.9)	19.2<br>(18.5 to 20.1)
Central Europe, Eastern Europe, and Central Asia	Non-melanoma skin cancer (basal-cell carcinoma)	0.102<br>(0.0364 to 0.23)	0.131<br>(0.0464 to 0.294)	0.163<br>(0.0552 to 0.375)	0<br>(0 to 0)	0<br>(0 to 0.1)	0<br>(0 to 0.1)
Central Europe, Eastern Europe, and Central Asia	Breast cancer	1350<br>(1320 to 1380)	1660<br>(1620 to 1690)	1560<br>(1520 to 1620)	283.3<br>(277.2 to 290.4)	308.1<br>(301.7 to 315.1)	265.9<br>(257.5 to 275.1)
Central Europe, Eastern Europe, and Central Asia	Cervical cancer	631<br>(617 to 646)	651<br>(640 to 664)	522<br>(505 to 541)	132.8<br>(129.9 to 136.1)	125.1<br>(122.9 to 127.7)	93.4<br>(90.3 to 96.8)
Central Europe, Eastern Europe, and Central Asia	Uterine cancer	275<br>(267 to 284)	295<br>(288 to 304)	295<br>(283 to 307)	55.8<br>(54.1 to 57.6)	53.5<br>(52.2 to 55.1)	48<br>(46 to 50.1)
Central Europe, Eastern Europe, and Central Asia	Ovarian cancer	485<br>(459 to 524)	574<br>(565 to 584)	534<br>(516 to 553)	100.1<br>(94.7 to 108.2)	106.1<br>(104.3 to 108)	90.6<br>(87.4 to 93.8)
Central Europe, Eastern Europe, and Central Asia	Prostate cancer	352<br>(305 to 470)	560<br>(397 to 611)	629<br>(442 to 681)	71.6<br>(62.2 to 95.3)	97.4<br>(68.5 to 105.3)	97.6<br>(68.6 to 105.8)
Central Europe, Eastern Europe, and Central Asia	Testicular cancer	52.8<br>(48.5 to 56.8)	57.1<br>(55.1 to 59.3)	41.9<br>(39.9 to 44.1)	12.1<br>(11.1 to 13)	12.6<br>(12.2 to 13.1)	9.2<br>(8.7 to 9.7)
Central Europe, Eastern Europe, and Central Asia	Kidney cancer	386<br>(342 to 421)	530<br>(493 to 542)	546<br>(515 to 564)	79.9<br>(71 to 87)	99.4<br>(92.6 to 101.7)	92<br>(87 to 95.1)
Central Europe, Eastern Europe, and Central Asia	Bladder cancer	351<br>(326 to 373)	437<br>(429 to 446)	435<br>(420 to 449)	70.8<br>(65.8 to 75.1)	77.3<br>(75.8 to 78.9)	68.7<br>(66.4 to 71)
Central Europe, Eastern Europe, and Central Asia	Thyroid cancer	70.3<br>(67 to 75.7)	78.1<br>(74.6 to 82.4)	74.6<br>(70 to 80.2)	14.6<br>(13.9 to 15.7)	14.5<br>(13.9 to 15.4)	12.7<br>(11.9 to 13.7)
Central Europe, Eastern Europe, and Central Asia	Mesothelioma	28.6<br>(25.8 to 32.8)	42.4<br>(40.4 to 43.8)	42.3<br>(40.5 to 44.4)	5.9<br>(5.3 to 6.8)	8<br>(7.6 to 8.2)	7.2<br>(6.9 to 7.6)
Central Europe, Eastern Europe, and Central Asia	Hodgkin lymphoma	160<br>(114 to 182)	132<br>(108 to 152)	101<br>(86.3 to 127)	36.2<br>(25.6 to 41.1)	28.4<br>(23.4 to 32.7)	21.6<br>(18.4 to 27.3)

SR Appendix Table 2. All-age DALYs counts and age standardized DALYs rates per 100,000 individuals for all causes and level 1 cause groups by location for 1990, 2007, and 2017, both sexes combined

		All-age DALYs			Age-standardized rate of DALYs per 100,000		
Central Europe, Eastern Europe, and Central Asia	Non-Hodgkin's lymphoma	334 (318 to 345)	404 (398 to 411)	421 (411 to 433)	74.2 (70.6 to 76.4)	82.3 (80.9 to 83.9)	79.6 (77.4 to 82)
Central Europe, Eastern Europe, and Central Asia	Multiple myeloma	121 (110 to 144)	174 (148 to 189)	203 (167 to 216)	24.4 (22.2 to 29.1)	31.4 (26.7 to 34.2)	33.1 (27.2 to 35.3)
Central Europe, Eastern Europe, and Central Asia	Leukaemia	887 (856 to 914)	821 (806 to 836)	748 (716 to 777)	206.5 (198.8 to 213)	182.6 (179.1 to 186.4)	155.3 (147.5 to 162.8)
Central Europe, Eastern Europe, and Central Asia	Acute lymphoid leukaemia	231 (213 to 247)	165 (159 to 173)	146 (138 to 155)	58 (53.3 to 62.2)	44.3 (42.4 to 46.7)	37.9 (35.3 to 40.6)
Central Europe, Eastern Europe, and Central Asia	Chronic lymphoid leukaemia	84.6 (80.1 to 88.9)	112 (108 to 117)	124 (118 to 130)	17.2 (16.3 to 18.1)	20.1 (19.3 to 20.9)	19.8 (18.9 to 20.8)
Central Europe, Eastern Europe, and Central Asia	Acute myeloid leukaemia	225 (207 to 241)	215 (205 to 225)	224 (211 to 235)	51.9 (47.9 to 56.1)	47.1 (44.6 to 49.8)	46.3 (43.4 to 48.9)
Central Europe, Eastern Europe, and Central Asia	Chronic myeloid leukaemia	79.3 (74.2 to 87.2)	68 (65.9 to 70)	52.7 (50.5 to 54.9)	16.8 (15.8 to 18.5)	13 (12.6 to 13.4)	9.4 (9 to 9.8)
Central Europe, Eastern Europe, and Central Asia	Other leukaemia	267 (250 to 289)	261 (249 to 269)	201 (192 to 212)	62.7 (58.3 to 68.2)	58 (54.8 to 60.4)	41.8 (39.3 to 44.7)
Central Europe, Eastern Europe, and Central Asia	Other malignant cancers	950 (860 to 994)	1200 (933 to 1260)	1110 (917 to 1170)	209.5 (190 to 218.9)	242.9 (192.6 to 255.7)	213.3 (178.9 to 225)
Central Europe, Eastern Europe, and Central Asia	Other neoplasms	100 (72.2 to 136)	141 (104 to 177)	163 (117 to 208)	22 (15.8 to 30.1)	28.5 (21.1 to 36.1)	30.4 (21.9 to 39.6)
Central Europe, Eastern Europe, and Central Asia	Myelodysplastic, myeloproliferative, and other hematopoietic neoplasms	87.5 (63.1 to 118)	127 (93.5 to 158)	149 (106 to 189)	18.8 (13.5 to 25.5)	24.9 (18.3 to 31)	26.6 (18.9 to 34.2)
Central Europe, Eastern Europe, and Central Asia	Benign and in situ intestinal neoplasms	0 (0 to 0)	0 (0 to 0)	0 (0 to 0)	0 (0 to 0)	0 (0 to 0)	0 (0 to 0)
Central Europe, Eastern Europe, and Central Asia	Benign and in situ cervical and uterine neoplasms	0 (0 to 0)	0 (0 to 0)	0 (0 to 0)	0 (0 to 0)	0 (0 to 0)	0 (0 to 0)
Central Europe, Eastern Europe, and Central Asia	Other benign and in situ neoplasms	12.9 (9.01 to 18.4)	13.5 (9.93 to 18)	14.2 (10.7 to 20.4)	3.2 (2.2 to 4.6)	3.6 (2.7 to 4.8)	3.8 (2.9 to 5.5)
Central Europe, Eastern Europe, and Central Asia	Cardiovascular diseases	43800 (43000 to 44600)	54700 (53800 to 55500)	46000 (44900 to 47200)	9278.8 (9104.2 to 9447.2)	10000.3 (9851.6 to 10156)	7491.8 (7306.1 to 7676.4)
Central Europe, Eastern Europe, and Central Asia	Rheumatic heart disease	861 (827 to 893)	511 (496 to 531)	340 (322 to 359)	183.5 (176.1 to 190.1)	101.8 (98.6 to 106.2)	64.4 (60.6 to 68.8)
Central Europe, Eastern Europe, and Central Asia	Ischaemic heart disease	24100 (23700 to 24500)	30000 (29700 to 30900)	25200 (24700 to 25900)	5107.1 (5021.1 to 5186.7)	5435 (5375.5 to 5607.1)	4040.9 (3957.2 to 4152.6)
Central Europe, Eastern Europe, and Central Asia	Stroke	14500 (14000 to 15000)	16100 (15600 to 16600)	13400 (12800 to 14000)	3046.9 (2942 to 3150.1)	2903.8 (2817.1 to 3000.4)	2151.8 (2052.8 to 2242.2)
Central Europe, Eastern Europe, and Central Asia	Ischaemic stroke	8260 (7880 to 8600)	8990 (8620 to 9370)	7870 (7400 to 8320)	1759.3 (1679.8 to 1831.3)	1605.1 (1537.9 to 1674.4)	1232.3 (1157.7 to 1303.9)
Central Europe, Eastern Europe, and Central Asia	Intracerebral hemorrhage	5110 (4990 to 5260)	5770 (5670 to 5990)	4440 (4310 to 4590)	1055.3 (1029.1 to 1084.5)	1046 (1025.9 to 1087.4)	730.4 (707.9 to 755.5)
Central Europe, Eastern Europe, and Central Asia	Subarachnoid hemorrhage	1100 (1050 to 1150)	1340 (1280 to 1420)	1100 (1050 to 1200)	232.4 (221.4 to 243.2)	252.7 (241.8 to 267.1)	189.1 (180.7 to 205.9)
Central Europe, Eastern Europe, and Central Asia	Hypertensive heart disease	727 (675 to 976)	1210 (874 to 1280)	1150 (856 to 1250)	153 (142.1 to 205.1)	218.1 (157.6 to 230.3)	184.2 (137.8 to 198.8)
Central Europe, Eastern Europe, and Central Asia	Non-rheumatic valvular heart disease	115 (103 to 133)	163 (142 to 182)	188 (151 to 210)	24.6 (22 to 28.4)	30.3 (26.3 to 33.8)	31 (25.1 to 34.7)
Central Europe, Eastern Europe, and Central Asia	Non-rheumatic calcific aortic valve disease	53.8 (47.7 to 64.5)	92.2 (77.7 to 103)	116 (86.8 to 130)	11.4 (10.1 to 13.8)	17 (14.3 to 19)	18.9 (14.2 to 21.2)
Central Europe, Eastern Europe, and Central Asia	Non-rheumatic degenerative mitral valve disease	54 (46.2 to 62.6)	62.7 (52.7 to 73)	63.5 (51.8 to 75.7)	11.5 (9.8 to 13.4)	11.6 (9.9 to 13.5)	10.5 (8.6 to 12.5)
Central Europe, Eastern Europe, and Central Asia	Other non-rheumatic valve diseases	7.65 (4.96 to 8.88)	7.83 (6.05 to 10.9)	8.25 (6.19 to 11)	1.7 (1.1 to 1.9)	1.6 (1.2 to 2.2)	1.6 (1.2 to 2.1)
Central Europe, Eastern Europe, and Central Asia	Cardiomyopathy and myocarditis	1610 (1420 to 1940)	4400 (3300 to 4580)	3280 (2890 to 3430)	354.8 (314.1 to 425.7)	885.2 (665.8 to 921.6)	614.7 (540.8 to 642.2)
Central Europe, Eastern Europe, and Central Asia	Myocarditis	104 (77.6 to 121)	128 (102 to 140)	130 (113 to 149)	24.3 (18.4 to 28.1)	27.9 (22.5 to 30.4)	25.6 (22.6 to 29)
Central Europe, Eastern Europe, and Central Asia	Alcoholic cardiomyopathy	966 (808 to 1220)	3420 (2440 to 3630)	2060 (1900 to 2280)	207.1 (173.2 to 261.9)	683 (487.6 to 725.2)	386.8 (357.6 to 428.6)
Central Europe, Eastern Europe, and Central Asia	Other cardiomyopathy	542 (491 to 624)	843 (681 to 890)	1090 (802 to 1190)	123.4 (112.1 to 140.8)	174.3 (140.4 to 184)	202.2 (148.4 to 222.5)
Central Europe, Eastern Europe, and Central Asia	Atrial fibrillation and flutter	393 (327 to 475)	498 (418 to 598)	589 (497 to 701)	86.1 (72.4 to 103.2)	90.7 (76.4 to 108)	92 (77.4 to 109.6)
Central Europe, Eastern Europe, and Central Asia	Aortic aneurysm	259 (247 to 271)	355 (342 to 377)	377 (367 to 388)	53.4 (51 to 55.8)	65.1 (62.6 to 69.4)	62.3 (60.5 to 64.1)
Central Europe, Eastern Europe, and Central Asia	Peripheral vascular disease	136 (77.9 to 205)	230 (144 to 353)	297 (186 to 484)	28.5 (16.2 to 42.8)	41 (25.7 to 63.2)	46.1 (28.9 to 75.4)
Central Europe, Eastern Europe, and Central Asia	Endocarditis	66.2 (56.4 to 82.3)	127 (113 to 155)	126 (111 to 145)	14.9 (12.8 to 18.3)	27 (24.2 to 33.1)	25.4 (22.3 to 29)
Central Europe, Eastern Europe, and Central Asia	Other cardiovascular and circulatory diseases	1070 (982 to 1160)	1060 (970 to 1170)	1040 (932 to 1150)	225.9 (208.3 to 247.1)	202.4 (184.7 to 223.4)	179 (161 to 198.5)
Central Europe, Eastern Europe, and Central Asia	Chronic respiratory diseases	5920 (5490 to 6330)	4670 (4260 to 5040)	4320 (3910 to 4720)	1250.5 (1157.2 to 1339.4)	888.4 (803.9 to 964.7)	757.1 (676.9 to 836.6)
Central Europe, Eastern Europe, and Central Asia	Chronic obstructive pulmonary disease	4250 (3970 to 4520)	3570 (3320 to 3790)	3290 (3020 to 3540)	885.2 (827.5 to 940.9)	652 (605.5 to 692.6)	540 (496.5 to 582.8)
Central Europe, Eastern Europe, and Central Asia	Pneumoconiosis	33.2 (30.1 to 37.1)	22 (20.3 to 24.1)	15.8 (14.1 to 17.7)	6.7 (6.1 to 7.5)	3.9 (3.6 to 4.3)	2.5 (2.3 to 2.9)
Central Europe, Eastern Europe, and Central Asia	Silicosis	10.6 (9.03 to 13.1)	5.16 (4.55 to 6.01)	3.4 (2.91 to 3.99)	2.1 (1.8 to 2.6)	0.9 (0.8 to 1.1)	0.5 (0.5 to 0.6)
Central Europe, Eastern Europe, and Central Asia	Asbestosis	3.2 (1.94 to 3.93)	2.43 (1.89 to 2.99)	2.33 (1.86 to 2.87)	0.7 (0.4 to 0.8)	0.4 (0.4 to 0.5)	0.4 (0.3 to 0.5)

SR Appendix Table 2. All-age DALYs counts and age standardized DALYs rates per 100,000 individuals for all causes and level 1 cause groups by location for 1990, 2007, and 2017, both sexes combined

		All-age DALYs			Age-standardized rate of DALYs per 100,000		
Central Europe, Eastern Europe, and Central Asia	Coal workers pneumoconiosis	10.9 (9.19 to 14.2)	6.62 (5.85 to 7.96)	3.89 (3.36 to 4.56)	2.2 (1.8 to 2.8)	1.2 (1 to 1.4)	0.6 (0.5 to 0.7)
Central Europe, Eastern Europe, and Central Asia	Other pneumoconiosis	8.46 (5.74 to 10.4)	7.79 (5.27 to 9.04)	6.18 (4.63 to 7.22)	1.7 (1.2 to 2.1)	1.4 (0.9 to 1.6)	1 (0.7 to 1.2)
Central Europe, Eastern Europe, and Central Asia	Asthma	1090 (876 to 1340)	738 (560 to 952)	683 (497 to 913)	240.2 (191.8 to 300.2)	165.1 (122.8 to 218.6)	150 (106.9 to 202.8)
Central Europe, Eastern Europe, and Central Asia	Interstitial lung disease and pulmonary sarcoidosis	311 (200 to 399)	186 (148 to 231)	197 (158 to 245)	65.3 (42.2 to 83.7)	35.2 (28.2 to 43.6)	34.1 (27.6 to 42.2)
Central Europe, Eastern Europe, and Central Asia	Other chronic respiratory diseases	243 (228 to 259)	148 (132 to 164)	142 (124 to 160)	53.1 (49.6 to 56.7)	32.1 (28.4 to 35.6)	30.4 (26.6 to 34.6)
Central Europe, Eastern Europe, and Central Asia	Digestive diseases	5880 (5300 to 6590)	8830 (8250 to 9530)	8440 (7760 to 9180)	1289.1 (1155.3 to 1451.5)	1795.2 (1659.7 to 1946.3)	1607.1 (1465.9 to 1768.1)
Central Europe, Eastern Europe, and Central Asia	Cirrhosis and other chronic liver diseases	2340 (2260 to 2390)	5030 (4930 to 5160)	4690 (4500 to 4860)	494.8 (479.5 to 507.6)	1001 (980.7 to 1025.7)	872.7 (834.6 to 906.6)
Central Europe, Eastern Europe, and Central Asia	Cirrhosis and other chronic liver diseases due to hepatitis B	544 (494 to 600)	1140 (1040 to 1240)	952 (863 to 1050)	115.1 (104.5 to 126.7)	228.5 (208.4 to 248.5)	178.6 (162.6 to 196.6)
Central Europe, Eastern Europe, and Central Asia	Cirrhosis and other chronic liver diseases due to hepatitis C	507 (458 to 562)	1150 (1040 to 1270)	1100 (995 to 1210)	106.4 (96.3 to 117.8)	226.7 (204.8 to 249.2)	203.2 (183.1 to 224)
Central Europe, Eastern Europe, and Central Asia	Cirrhosis and other chronic liver diseases due to alcohol use	873 (817 to 931)	1870 (1750 to 1980)	1790 (1660 to 1910)	182.2 (170.9 to 193.6)	363.9 (341.9 to 385.7)	324.8 (301.5 to 348)
Central Europe, Eastern Europe, and Central Asia	Cirrhosis due to NASH	171 (157 to 186)	425 (392 to 459)	438 (401 to 473)	35.5 (32.5 to 38.6)	82.4 (75.9 to 88.6)	78.9 (72 to 85.5)
Central Europe, Eastern Europe, and Central Asia	Cirrhosis and other chronic liver diseases due to other causes	241 (219 to 266)	448 (406 to 493)	408 (365 to 454)	55.8 (51.1 to 61)	99.5 (90.8 to 109)	87.2 (77.9 to 96.3)
Central Europe, Eastern Europe, and Central Asia	Upper digestive system diseases	1610 (1230 to 2160)	1630 (1250 to 2180)	1580 (1190 to 2130)	360.4 (272.8 to 485.6)	344.6 (260.7 to 463.4)	319.2 (236.1 to 435.7)
Central Europe, Eastern Europe, and Central Asia	Peptic ulcer disease	582 (546 to 619)	617 (585 to 652)	576 (539 to 617)	123.6 (115.8 to 131.7)	118.7 (112.2 to 125.7)	101.4 (94.3 to 109)
Central Europe, Eastern Europe, and Central Asia	Gastritis and duodenitis	590 (386 to 851)	539 (355 to 764)	512 (335 to 733)	140.7 (91.5 to 203.9)	130.6 (85.1 to 188.2)	122 (78.7 to 175.3)
Central Europe, Eastern Europe, and Central Asia	GERD	438 (236 to 743)	471 (251 to 790)	492 (263 to 830)	96.1 (51.4 to 162.4)	95.3 (51.1 to 160.5)	95.8 (51.4 to 162.2)
Central Europe, Eastern Europe, and Central Asia	Appendicitis	91.5 (85.8 to 97.5)	49.5 (45.2 to 54.5)	42.1 (37.9 to 47)	21.4 (20 to 22.8)	11.6 (10.4 to 12.9)	9.7 (8.5 to 11.1)
Central Europe, Eastern Europe, and Central Asia	Paralytic ileus and intestinal obstruction	270 (235 to 296)	215 (190 to 246)	216 (184 to 246)	67.7 (59.5 to 73.7)	51.6 (46.8 to 58)	47.2 (39.8 to 53)
Central Europe, Eastern Europe, and Central Asia	Inguinal, femoral, and abdominal hernia	365 (270 to 480)	343 (247 to 455)	355 (254 to 475)	82.4 (60.5 to 107.8)	77.7 (54.9 to 104.4)	74.1 (52.1 to 99.9)
Central Europe, Eastern Europe, and Central Asia	Inflammatory bowel disease	166 (134 to 202)	154 (126 to 186)	150 (120 to 182)	36.5 (29.4 to 44.4)	31.1 (25.4 to 37.7)	28.4 (22.5 to 34.7)
Central Europe, Eastern Europe, and Central Asia	Vascular intestinal disorders	226 (211 to 240)	290 (266 to 304)	311 (271 to 326)	47.3 (44.1 to 50.1)	51.7 (47.8 to 54.3)	49.5 (42.9 to 51.9)
Central Europe, Eastern Europe, and Central Asia	Gallbladder and biliary diseases	191 (150 to 198)	134 (130 to 149)	132 (126 to 145)	39.9 (31.5 to 41.5)	24.6 (23.9 to 27.3)	21.6 (20.7 to 23.7)
Central Europe, Eastern Europe, and Central Asia	Pancreatitis	465 (430 to 503)	779 (717 to 829)	726 (643 to 791)	101 (93.3 to 109.2)	156.8 (144.8 to 166.7)	137.6 (121.8 to 149.7)
Central Europe, Eastern Europe, and Central Asia	Other digestive diseases	159 (127 to 197)	205 (181 to 231)	235 (206 to 266)	37.7 (29.9 to 47)	44.5 (38.7 to 51.1)	47 (40.4 to 54.4)
Central Europe, Eastern Europe, and Central Asia	Neurological disorders	6860 (5390 to 8570)	7630 (6120 to 9510)	8090 (6490 to 9950)	1595.5 (1261.2 to 1988.8)	1608.3 (1280.4 to 2015.3)	1572.5 (1236.1 to 1959.5)
Central Europe, Eastern Europe, and Central Asia	Alzheimer's disease and other dementias	1830 (1710 to 1970)	2290 (2150 to 2450)	2790 (2600 to 2980)	447.9 (415.8 to 480.1)	446.5 (418.9 to 476.5)	432.3 (403.8 to 462.1)
Central Europe, Eastern Europe, and Central Asia	Parkinson's disease	324 (297 to 358)	428 (391 to 474)	460 (421 to 507)	69.9 (63.9 to 77.1)	73.5 (67.3 to 81.3)	70.5 (64.5 to 77.8)
Central Europe, Eastern Europe, and Central Asia	Epilepsy	740 (569 to 973)	764 (599 to 988)	710 (528 to 955)	177 (136.5 to 232.2)	185.6 (145 to 240.2)	173.3 (128.4 to 231.4)
Central Europe, Eastern Europe, and Central Asia	Multiple sclerosis	134 (104 to 152)	132 (117 to 150)	122 (102 to 156)	29.4 (22.8 to 33.5)	26.3 (23.4 to 30.1)	23.3 (19.4 to 30.1)
Central Europe, Eastern Europe, and Central Asia	Motor neuron disease	26.8 (24.3 to 30)	38.1 (35.9 to 39.1)	46.2 (44 to 47.8)	5.9 (5.3 to 6.6)	7.7 (7.2 to 7.9)	8.4 (7.9 to 8.7)
Central Europe, Eastern Europe, and Central Asia	Headache disorders	3600 (2360 to 5130)	3770 (2470 to 5360)	3750 (2460 to 5360)	816.7 (530.7 to 1162.9)	815.2 (534.9 to 1158.4)	809.9 (532.4 to 1151.2)
Central Europe, Eastern Europe, and Central Asia	Migraine	3030 (1920 to 4460)	3180 (2020 to 4660)	3150 (2010 to 4660)	689.5 (435.7 to 1010.6)	688.5 (435.4 to 1007.5)	684.6 (433.4 to 1001.5)
Central Europe, Eastern Europe, and Central Asia	Tension-type headache	565 (325 to 874)	591 (341 to 916)	592 (341 to 915)	127.2 (72.6 to 197)	126.7 (72.4 to 196.9)	125.3 (71.6 to 194.3)
Central Europe, Eastern Europe, and Central Asia	Other neurological disorders	197 (166 to 238)	213 (181 to 251)	219 (182 to 262)	48.7 (40.8 to 58.8)	53.4 (45.2 to 63.6)	54.9 (44.8 to 66.6)
Central Europe, Eastern Europe, and Central Asia	Mental disorders	6210 (4610 to 8020)	6390 (4770 to 8250)	6320 (4730 to 8200)	1419.6 (1052.9 to 1841.8)	1404.8 (1042.5 to 1821.2)	1389 (1031.6 to 1803.8)
Central Europe, Eastern Europe, and Central Asia	Schizophrenia	525 (395 to 653)	558 (415 to 689)	590 (440 to 729)	115.9 (87 to 144.6)	116.1 (85.9 to 143.6)	118.5 (88.4 to 147.6)
Central Europe, Eastern Europe, and Central Asia	Depressive disorders	2390 (1700 to 3250)	2500 (1780 to 3380)	2470 (1750 to 3310)	531.1 (377.5 to 723.7)	516.1 (367.6 to 700.5)	498.6 (355.6 to 675.9)
Central Europe, Eastern Europe, and Central Asia	Major depressive disorder	1860 (1310 to 2510)	1940 (1360 to 2610)	1890 (1320 to 2530)	415 (291.8 to 560.4)	400.4 (281.9 to 541.7)	383.7 (269.2 to 519.7)
Central Europe, Eastern Europe, and Central Asia	Dysthymia	530 (357 to 769)	569 (381 to 824)	585 (392 to 847)	116 (78.2 to 167.6)	115.7 (77.7 to 166.7)	114.9 (77 to 165.5)
Central Europe, Eastern Europe, and Central Asia	Bipolar disorder	576 (363 to 849)	610 (385 to 904)	595 (376 to 875)	132.1 (83 to 194.7)	132.3 (83.2 to 195.9)	132.2 (83 to 194.5)

SR Appendix Table 2. All-age DALYs counts and age standardized DALYs rates per 100,000 individuals for all causes and level 1 cause groups by location for 1990, 2007, and 2017, both sexes combined							
		All-age DALYs			Age-standardized rate of DALYs per 100,000		
Central Europe, Eastern Europe, and Central Asia	Anxiety disorders	1180 (841 to 1560)	1210 (863 to 1610)	1200 (862 to 1590)	271.8 (193.1 to 361)	270.8 (191.8 to 360.6)	268.3 (190.5 to 356.6)
Central Europe, Eastern Europe, and Central Asia	Eating disorders	159 (102 to 236)	156 (99.1 to 227)	164 (105 to 242)	38.1 (24.3 to 55.6)	36.9 (23.6 to 53.8)	41.5 (26.4 to 60.7)
Central Europe, Eastern Europe, and Central Asia	Anorexia nervosa	30.8 (19 to 45.8)	30.4 (18.5 to 45.4)	30.1 (19 to 45.2)	7.6 (4.7 to 11.2)	7.4 (4.5 to 11.1)	8.3 (5.1 to 12.4)
Central Europe, Eastern Europe, and Central Asia	Bulimia nervosa	128 (81 to 191)	125 (78.4 to 186)	134 (84.7 to 201)	30.5 (19.1 to 45.2)	29.5 (18.5 to 43.6)	33.2 (21.1 to 49.5)
Central Europe, Eastern Europe, and Central Asia	Autism spectrum disorders	266 (182 to 366)	254 (174 to 347)	255 (175 to 350)	64.3 (44 to 88.5)	64.6 (44.2 to 88.8)	64.8 (44.2 to 89.1)
Central Europe, Eastern Europe, and Central Asia	Attention-deficit/hyperactivity disorder	47 (28.4 to 75.4)	43.5 (26.5 to 68.7)	40.9 (25 to 65)	11.7 (7.1 to 18.7)	11.7 (7.1 to 18.7)	11.8 (7.2 to 18.8)
Central Europe, Eastern Europe, and Central Asia	Conduct disorder	380 (228 to 600)	321 (192 to 506)	276 (165 to 436)	99.9 (59.9 to 157.7)	99.9 (59.5 to 158)	100.2 (60.1 to 158)
Central Europe, Eastern Europe, and Central Asia	Idiopathic developmental intellectual disability	82.2 (24.3 to 159)	80.8 (26 to 155)	66.3 (17.2 to 129)	20.1 (6 to 38.9)	21.3 (6.9 to 40.7)	17.6 (4.8 to 34.5)
Central Europe, Eastern Europe, and Central Asia	Other mental disorders	604 (401 to 826)	648 (430 to 892)	667 (443 to 908)	134.6 (89.3 to 184.3)	135.1 (89.6 to 186)	135.6 (89.9 to 186.4)
Central Europe, Eastern Europe, and Central Asia	Substance use disorders	4250 (3600 to 4990)	6170 (5450 to 6980)	4820 (4180 to 5610)	954.6 (808.6 to 1122.8)	1304.5 (1148.1 to 1481.2)	1000.6 (861.2 to 1170.3)
Central Europe, Eastern Europe, and Central Asia	Alcohol use disorders	3280 (2800 to 3870)	4530 (4010 to 5160)	3520 (3030 to 4140)	732.2 (622 to 864.8)	937.9 (827.1 to 1072)	707.3 (603.3 to 835.1)
Central Europe, Eastern Europe, and Central Asia	Drug use disorders	963 (784 to 1140)	1640 (1430 to 1860)	1300 (1110 to 1500)	222.4 (180.4 to 263.3)	366.6 (320.2 to 417.7)	293.3 (248.9 to 338.5)
Central Europe, Eastern Europe, and Central Asia	Opioid use disorders	639 (519 to 760)	1120 (976 to 1280)	910 (769 to 1060)	146.2 (118.8 to 174.7)	250.6 (217.9 to 286.1)	201 (169.4 to 234.6)
Central Europe, Eastern Europe, and Central Asia	Cocaine use disorders	57.2 (44.1 to 72.9)	96.5 (80.8 to 115)	74 (60.7 to 90.1)	13.2 (10.1 to 17)	21.6 (18.1 to 25.9)	16.8 (13.6 to 20.7)
Central Europe, Eastern Europe, and Central Asia	Amphetamine use disorders	75 (49.2 to 110)	105 (74.8 to 144)	80.7 (55.8 to 115)	18 (11.7 to 26.7)	24.2 (17.2 to 33.3)	20.4 (13.8 to 29.2)
Central Europe, Eastern Europe, and Central Asia	Cannabis use disorders	28.9 (18.1 to 43.1)	31 (19.3 to 46)	25.7 (16.1 to 37.7)	7 (4.4 to 10.5)	7.2 (4.5 to 10.7)	6.7 (4.2 to 10)
Central Europe, Eastern Europe, and Central Asia	Other drug use disorders	163 (138 to 195)	280 (248 to 320)	213 (185 to 244)	37.9 (31.7 to 45.6)	63 (55.7 to 72.2)	48.5 (41.8 to 56.4)
Central Europe, Eastern Europe, and Central Asia	Diabetes and kidney diseases	4100 (3400 to 4930)	4860 (4000 to 5880)	5300 (4320 to 6440)	870.6 (722.6 to 1046.6)	927.9 (766.5 to 1120.5)	920.4 (747.8 to 1118.5)
Central Europe, Eastern Europe, and Central Asia	Diabetes mellitus	2800 (2200 to 3530)	3470 (2710 to 4380)	3960 (3080 to 5010)	587.6 (460.4 to 741.9)	652.5 (508.2 to 824.3)	676.1 (521.3 to 856.4)
Central Europe, Eastern Europe, and Central Asia	Diabetes mellitus type 1	476 (449 to 505)	490 (455 to 519)	433 (393 to 466)	101 (95.2 to 107.2)	97.2 (90.5 to 103.3)	81.4 (74.1 to 88)
Central Europe, Eastern Europe, and Central Asia	Diabetes mellitus type 2	2330 (1730 to 3040)	2980 (2240 to 3870)	3530 (2670 to 4540)	486.6 (361.4 to 637.1)	555.3 (415.6 to 723.8)	594.8 (445.2 to 768.9)
Central Europe, Eastern Europe, and Central Asia	Chronic kidney disease	1250 (1150 to 1350)	1360 (1270 to 1470)	1320 (1210 to 1440)	272.4 (251.7 to 295.5)	270.9 (252.1 to 291.3)	241.5 (221.8 to 261.1)
Central Europe, Eastern Europe, and Central Asia	Chronic kidney disease due to diabetes mellitus type 1	86.2 (69.8 to 105)	97.2 (79.4 to 117)	90 (74.1 to 108)	18.6 (15.1 to 22.6)	19.2 (15.8 to 23)	16.9 (13.9 to 20.1)
Central Europe, Eastern Europe, and Central Asia	Chronic kidney disease due to diabetes mellitus type 2	160 (134 to 190)	197 (167 to 228)	208 (177 to 243)	32.9 (27.7 to 38.9)	35.4 (30.3 to 41)	33.4 (28.6 to 38.7)
Central Europe, Eastern Europe, and Central Asia	Chronic kidney disease due to hypertension	140 (120 to 161)	165 (141 to 188)	174 (149 to 199)	30.3 (25.8 to 34.8)	31.2 (26.6 to 35.5)	29.4 (25 to 33.4)
Central Europe, Eastern Europe, and Central Asia	Chronic kidney disease due to glomerulonephritis	393 (357 to 428)	415 (378 to 453)	376 (340 to 415)	86.6 (79.1 to 93.8)	85 (78 to 92.3)	72.4 (65.7 to 79.5)
Central Europe, Eastern Europe, and Central Asia	Chronic kidney disease due to other and unspecified causes	467 (406 to 541)	488 (427 to 561)	475 (414 to 548)	104 (90.7 to 119.5)	100 (88.2 to 113.6)	89.4 (78.6 to 101.8)
Central Europe, Eastern Europe, and Central Asia	Acute glomerulonephritis	46.5 (43.6 to 49.6)	20.1 (19.2 to 21.1)	12.7 (12 to 13.7)	10.6 (9.9 to 11.3)	4.5 (4.3 to 4.8)	2.8 (2.6 to 3)
Central Europe, Eastern Europe, and Central Asia	Skin and subcutaneous diseases	2090 (1420 to 3020)	2050 (1410 to 2980)	2100 (1440 to 3060)	500.6 (337.8 to 726.7)	508.4 (346.4 to 733.7)	512.4 (348.2 to 740.2)
Central Europe, Eastern Europe, and Central Asia	Dermatitis	533 (312 to 846)	479 (284 to 750)	489 (289 to 766)	130.8 (76 to 208.4)	128.9 (75.7 to 205.4)	128.8 (75.3 to 205.1)
Central Europe, Eastern Europe, and Central Asia	Atopic dermatitis	430 (235 to 719)	371 (204 to 618)	381 (211 to 635)	107.3 (58.5 to 179.1)	105.3 (57.4 to 175.7)	105.2 (57.8 to 175.1)
Central Europe, Eastern Europe, and Central Asia	Contact dermatitis	103 (67.2 to 153)	108 (70.7 to 160)	108 (70.9 to 159)	23.4 (15.5 to 34.9)	23.5 (15.4 to 34.9)	23.4 (15.2 to 34.6)
Central Europe, Eastern Europe, and Central Asia	Seborrhoeic dermatitis	0.461 (0.259 to 0.745)	0.453 (0.252 to 0.732)	0.465 (0.258 to 0.75)	0.1 (0.1 to 0.2)	0.1 (0.1 to 0.2)	0.1 (0.1 to 0.2)
Central Europe, Eastern Europe, and Central Asia	Psoriasis	317 (226 to 417)	340 (242 to 447)	353 (251 to 462)	70.9 (50.2 to 93)	72.5 (51.6 to 95.3)	72.8 (51.7 to 95)
Central Europe, Eastern Europe, and Central Asia	Bacterial skin diseases	65.7 (47.3 to 104)	94.3 (55.8 to 131)	102 (55.3 to 131)	14.7 (10.5 to 22.7)	19.5 (11.8 to 26.8)	19.6 (10.9 to 25.2)
Central Europe, Eastern Europe, and Central Asia	Cellulitis	39.8 (27.9 to 66.5)	60 (34.7 to 81.4)	66.8 (34.7 to 83.5)	8.7 (6.2 to 14.5)	12.3 (7.2 to 16.7)	12.8 (6.8 to 15.9)
Central Europe, Eastern Europe, and Central Asia	Pyoderma	25.9 (15.2 to 42.7)	34.3 (17.4 to 51.5)	35 (16.5 to 50.1)	6 (3.4 to 9.6)	7.2 (3.7 to 10.8)	6.8 (3.3 to 9.9)
Central Europe, Eastern Europe, and Central Asia	Scabies	118 (65.4 to 191)	111 (61 to 179)	106 (58.5 to 171)	29.3 (16.3 to 47.5)	28.9 (16.2 to 47.1)	28.7 (16 to 46.7)
Central Europe, Eastern Europe, and Central Asia	Fungal skin diseases	207 (83 to 430)	212 (85 to 438)	222 (89.2 to 457)	48.1 (19.3 to 100.2)	47.1 (18.9 to 97.7)	46 (18.4 to 95.5)
Central Europe, Eastern Europe, and Central Asia	Viral skin diseases	175 (112 to 260)	138 (88.5 to 206)	144 (92.4 to 214)	45.6 (29.1 to 67.6)	45.7 (29.1 to 67.7)	45.7 (29.3 to 68)

SR Appendix Table 2. All-age DALYs counts and age standardized DALYs rates per 100,000 individuals for all causes and level 1 cause groups by location for 1990, 2007, and 2017, both sexes combined

		All-age DALYs			Age-standardized rate of DALYs per 100,000		
Central Europe, Eastern Europe, and Central Asia	Acne vulgaris	114 (68.3 to 185)	135 (80.1 to 220)	119 (70.9 to 191)	28.8 (17.2 to 46.7)	33.6 (19.9 to 54.9)	36.4 (21.6 to 59.2)
Central Europe, Eastern Europe, and Central Asia	Alopecia areata	25.7 (16.6 to 38.6)	26.2 (16.8 to 38.9)	26.3 (17 to 39.3)	5.9 (3.8 to 8.9)	5.9 (3.8 to 8.8)	5.9 (3.8 to 8.8)
Central Europe, Eastern Europe, and Central Asia	Pruritus	43.6 (20.7 to 81.8)	45.1 (21.4 to 84.7)	47.5 (22.9 to 88.8)	9.9 (4.6 to 18.6)	9.9 (4.6 to 18.6)	10 (4.7 to 18.9)
Central Europe, Eastern Europe, and Central Asia	Urticaria	309 (203 to 438)	276 (184 to 388)	283 (188 to 396)	76.6 (50.4 to 108)	76.5 (50.5 to 108)	76.5 (50.6 to 108.1)
Central Europe, Eastern Europe, and Central Asia	Decubitus ulcer	13.5 (9.88 to 18.2)	16.5 (12 to 21.6)	19.8 (14.3 to 25.9)	3.1 (2.2 to 4.1)	3.3 (2.4 to 4.4)	3.6 (2.6 to 4.7)
Central Europe, Eastern Europe, and Central Asia	Other skin and subcutaneous diseases	165 (82 to 302)	174 (86.6 to 317)	193 (96.3 to 350)	36.8 (18.3 to 67.4)	36.6 (18.2 to 66.8)	38.4 (19.1 to 70.2)
Central Europe, Eastern Europe, and Central Asia	Sense organ diseases	3810 (2560 to 5450)	4250 (2860 to 6070)	4580 (3090 to 6500)	832.7 (560.7 to 1190)	828.6 (558.2 to 1187.8)	812.5 (545.9 to 1165.3)
Central Europe, Eastern Europe, and Central Asia	Blindness and vision impairment	1780 (1200 to 2590)	1970 (1340 to 2860)	2090 (1420 to 3010)	388.1 (261.1 to 565.3)	385 (259.1 to 560.5)	372 (250.5 to 542.7)
Central Europe, Eastern Europe, and Central Asia	Glaucoma	49.9 (33.6 to 68.9)	59.1 (40.3 to 81.3)	65 (44.1 to 88.9)	10.2 (6.9 to 14)	10.3 (7 to 14.2)	9.9 (6.7 to 13.6)
Central Europe, Eastern Europe, and Central Asia	Cataract	534 (373 to 722)	635 (444 to 859)	695 (483 to 938)	115.2 (80.4 to 155.5)	114.3 (79.6 to 154.8)	109.5 (76.2 to 147.8)
Central Europe, Eastern Europe, and Central Asia	Age-related macular degeneration	14.3 (9.56 to 19.9)	17.8 (11.9 to 24.8)	20.2 (13.6 to 28.2)	3 (2 to 4.2)	3.1 (2.1 to 4.4)	3.1 (2.1 to 4.3)
Central Europe, Eastern Europe, and Central Asia	Refraction disorders	500 (321 to 751)	511 (326 to 766)	507 (325 to 761)	114.1 (72.5 to 172.3)	114.5 (72.7 to 172.8)	110 (70 to 166.4)
Central Europe, Eastern Europe, and Central Asia	Near vision loss	471 (225 to 853)	510 (242 to 929)	545 (259 to 991)	100.1 (47.7 to 181.7)	96.9 (46.1 to 176.6)	95.5 (45.4 to 173.8)
Central Europe, Eastern Europe, and Central Asia	Other vision loss	212 (147 to 284)	241 (168 to 323)	253 (176 to 339)	45.4 (31.6 to 60.7)	45.8 (31.9 to 61.2)	43.9 (30.5 to 58.7)
Central Europe, Eastern Europe, and Central Asia	Age-related and other hearing loss	1890 (1280 to 2670)	2120 (1440 to 3000)	2320 (1580 to 3270)	413.5 (283.2 to 582.9)	412.2 (282 to 582.9)	409 (279.1 to 579.2)
Central Europe, Eastern Europe, and Central Asia	Other sense organ diseases	143 (89.4 to 207)	158 (98.2 to 229)	173 (109 to 252)	31.1 (19.4 to 45.3)	31.4 (19.5 to 45.7)	31.6 (19.8 to 46.1)
Central Europe, Eastern Europe, and Central Asia	Musculoskeletal disorders	7810 (5690 to 10500)	8290 (6070 to 11200)	8740 (6340 to 11700)	1690.6 (1232.5 to 2270.2)	1635.9 (1192.3 to 2197.6)	1625.4 (1183.1 to 2193.5)
Central Europe, Eastern Europe, and Central Asia	Rheumatoid arthritis	171 (132 to 214)	183 (138 to 230)	197 (147 to 252)	35.9 (27.5 to 44.9)	34.7 (26.1 to 43.8)	34.5 (25.5 to 44.1)
Central Europe, Eastern Europe, and Central Asia	Osteoarthritis	454 (228 to 892)	543 (274 to 1070)	627 (314 to 1230)	94.2 (47.5 to 184.9)	98.7 (49.7 to 193.7)	101.9 (51.3 to 200.6)
Central Europe, Eastern Europe, and Central Asia	Low back pain	5590 (4040 to 7510)	5760 (4150 to 7720)	6040 (4370 to 8120)	1216.2 (869.2 to 1636)	1151.8 (821.5 to 1542.2)	1143.5 (817.4 to 1535.8)
Central Europe, Eastern Europe, and Central Asia	Neck pain	1410 (982 to 1980)	1570 (1100 to 2200)	1650 (1160 to 2350)	304 (213.4 to 425)	304.3 (213.3 to 426.1)	303.7 (213 to 425.2)
Central Europe, Eastern Europe, and Central Asia	Gout	57.6 (39 to 79.1)	69.3 (47.1 to 94.7)	78.2 (53.3 to 108)	12 (8.1 to 16.6)	12.8 (8.7 to 17.6)	13.1 (8.9 to 18.2)
Central Europe, Eastern Europe, and Central Asia	Other musculoskeletal disorders	127 (80 to 188)	161 (105 to 235)	143 (90.5 to 218)	28.4 (17.8 to 41.8)	33.7 (22.1 to 48.9)	28.7 (17.9 to 43.1)
Central Europe, Eastern Europe, and Central Asia	Other non-communicable diseases	7320 (6190 to 8740)	6080 (4900 to 7530)	5600 (4420 to 7080)	1891.5 (1627.7 to 2210.3)	1623.9 (1367 to 1940.2)	1364.5 (1119.2 to 1662.8)
Central Europe, Eastern Europe, and Central Asia	Congenital anomalies	3650 (3270 to 4020)	2400 (2110 to 2670)	1900 (1640 to 2200)	1056.6 (950.5 to 1165)	835.7 (742.9 to 916.3)	618 (543.7 to 712.4)
Central Europe, Eastern Europe, and Central Asia	Neural tube defects	277 (232 to 359)	136 (118 to 154)	112 (90.7 to 138)	82.4 (68.8 to 107.5)	50 (43.8 to 56.5)	38.6 (31.1 to 48)
Central Europe, Eastern Europe, and Central Asia	Congenital heart anomalies	1380 (1150 to 1520)	850 (715 to 917)	576 (500 to 658)	413.9 (345.5 to 456.2)	325 (272.2 to 350.9)	211.6 (182.8 to 242.2)
Central Europe, Eastern Europe, and Central Asia	Orofacial clefts	23.5 (17.8 to 30)	14.4 (10.7 to 19.3)	12.2 (8.39 to 17.2)	6.5 (4.9 to 8.2)	4.2 (3.2 to 5.5)	3.3 (2.3 to 4.5)
Central Europe, Eastern Europe, and Central Asia	Down's syndrome	103 (74.9 to 140)	68.1 (57 to 80.6)	61.2 (50.1 to 76)	28.6 (20.6 to 39.7)	21.6 (18.6 to 25.2)	18.4 (15.2 to 23.1)
Central Europe, Eastern Europe, and Central Asia	Turner syndrome	0.397 (0.201 to 0.656)	0.372 (0.188 to 0.606)	0.349 (0.175 to 0.572)	0.1 (0 to 0.2)	0.1 (0 to 0.2)	0.1 (0 to 0.2)
Central Europe, Eastern Europe, and Central Asia	Klinefelter syndrome	0.112 (0.0524 to 0.211)	0.108 (0.0498 to 0.208)	0.103 (0.048 to 0.199)	0 (0 to 0.1)	0 (0 to 0.1)	0 (0 to 0.1)
Central Europe, Eastern Europe, and Central Asia	Other chromosomal abnormalities	91.2 (68.6 to 141)	72.8 (59.4 to 95.1)	69.8 (55.5 to 93.6)	26.4 (19.9 to 41.7)	25.7 (20.8 to 34)	23.3 (18.8 to 31.5)
Central Europe, Eastern Europe, and Central Asia	Congenital musculoskeletal and limb anomalies	440 (302 to 589)	422 (287 to 571)	420 (285 to 568)	107.3 (74.3 to 142.8)	105.1 (72.6 to 141.8)	102.7 (70.5 to 138.1)
Central Europe, Eastern Europe, and Central Asia	Urogenital congenital anomalies	45.2 (34.1 to 71.9)	31.6 (26 to 41)	27.2 (21.5 to 36.1)	13.1 (9.8 to 21.2)	11.3 (9.3 to 14.7)	9.3 (7.4 to 12.5)
Central Europe, Eastern Europe, and Central Asia	Digestive congenital anomalies	379 (301 to 505)	263 (219 to 312)	204 (163 to 249)	109.5 (86.9 to 149)	90.1 (76.8 to 103.6)	65.3 (53.3 to 78.2)
Central Europe, Eastern Europe, and Central Asia	Other congenital anomalies	909 (717 to 1090)	540 (479 to 611)	415 (363 to 503)	268.7 (210.5 to 322.7)	202.6 (179.5 to 228)	145.4 (127.7 to 179.4)
Central Europe, Eastern Europe, and Central Asia	Urinary diseases and male infertility	719 (626 to 834)	675 (571 to 800)	744 (628 to 882)	151.4 (132.3 to 175.4)	130.9 (111.8 to 154.7)	131.9 (112.6 to 155)
Central Europe, Eastern Europe, and Central Asia	Urinary tract infections	355 (323 to 375)	318 (309 to 330)	348 (335 to 361)	76.7 (70.1 to 80.9)	64.7 (62.7 to 67.4)	66 (63.5 to 68.8)
Central Europe, Eastern Europe, and Central Asia	Urolithiasis	89.8 (71.1 to 114)	62.1 (53.1 to 72)	69.5 (58.5 to 82.2)	18.6 (14.7 to 23.5)	11.7 (10 to 13.6)	11.9 (10 to 14.2)
Central Europe, Eastern Europe, and Central Asia	Benign prostatic hyperplasia	208 (134 to 298)	240 (155 to 342)	277 (178 to 396)	41.4 (26.6 to 59.1)	42.7 (27.6 to 60.9)	43.9 (28.1 to 62.7)

SR Appendix Table 2. All-age DALYs counts and age standardized DALYs rates per 100,000 individuals for all causes and level 1 cause groups by location for 1990, 2007, and 2017, both sexes combined

		All-age DALYs			Age-standardized rate of DALYs per 100,000		
Central Europe, Eastern Europe, and Central Asia	Male infertility	11.6 (4.81 to 23.9)	11.9 (4.91 to 24.8)	10.9 (4.52 to 22.7)	2.7 (1.1 to 5.6)	2.8 (1.1 to 5.8)	2.6 (1.1 to 5.4)
Central Europe, Eastern Europe, and Central Asia	Other urinary diseases	54.2 (45.5 to 68)	43.2 (35.7 to 49.7)	38.6 (30.8 to 44.9)	12 (10.1 to 14.9)	9 (7.4 to 10.4)	7.4 (6 to 8.7)
Central Europe, Eastern Europe, and Central Asia	Gynecological diseases	805 (563 to 1110)	848 (591 to 1170)	799 (556 to 1120)	187.6 (131.2 to 258.9)	184.5 (128 to 255)	179.7 (124.5 to 249.6)
Central Europe, Eastern Europe, and Central Asia	Uterine fibroids	121 (74.9 to 196)	125 (74.8 to 206)	112 (64.1 to 186)	27.2 (16.8 to 43.8)	24.9 (14.9 to 41)	21.8 (12.6 to 36.2)
Central Europe, Eastern Europe, and Central Asia	Polycystic ovarian syndrome	22 (9.75 to 42)	23.4 (10.4 to 45.1)	22.5 (10 to 43.1)	5.2 (2.3 to 9.9)	5.2 (2.3 to 10.1)	5.3 (2.4 to 10.3)
Central Europe, Eastern Europe, and Central Asia	Female infertility	11.7 (4.41 to 24.9)	11 (4.03 to 23.9)	12.4 (4.61 to 26.6)	2.7 (1 to 5.8)	2.6 (1 to 5.6)	2.9 (1.1 to 6.2)
Central Europe, Eastern Europe, and Central Asia	Endometriosis	308 (201 to 444)	320 (211 to 453)	303 (198 to 436)	72.2 (47.4 to 103.6)	71.9 (47 to 102.5)	70.1 (45.9 to 100.3)
Central Europe, Eastern Europe, and Central Asia	Genital prolapse	21.7 (10.8 to 40.4)	18.6 (9.19 to 34.6)	21.4 (10.4 to 40.5)	4.5 (2.2 to 8.4)	3.5 (1.7 to 6.4)	3.6 (1.8 to 6.9)
Central Europe, Eastern Europe, and Central Asia	Premenstrual syndrome	215 (139 to 321)	231 (149 to 345)	210 (135 to 314)	51.3 (33.1 to 77)	51.1 (32.9 to 76.5)	49.8 (32 to 74.5)
Central Europe, Eastern Europe, and Central Asia	Other gynecological diseases	106 (74.2 to 145)	120 (81.9 to 169)	119 (80.2 to 172)	24.4 (17.2 to 33.7)	25.4 (17.4 to 35.9)	26.1 (17.6 to 37.5)
Central Europe, Eastern Europe, and Central Asia	Hemoglobinopathies and hemolytic anaemias	275 (214 to 361)	226 (175 to 299)	199 (155 to 262)	66.3 (51.7 to 86.8)	56.6 (43.8 to 74.7)	48.4 (37.4 to 63.3)
Central Europe, Eastern Europe, and Central Asia	Thalassemias	24.5 (20.3 to 29.6)	17.3 (12.2 to 20.1)	15.3 (11.3 to 18.2)	7 (5.8 to 8.4)	7 (4.9 to 8.1)	5.6 (4.1 to 6.6)
Central Europe, Eastern Europe, and Central Asia	Thalassaemias trait	74 (49.8 to 108)	61.9 (41.2 to 91)	53.9 (35.6 to 79)	17.8 (12 to 26.1)	15.4 (10.3 to 22.8)	13.2 (8.7 to 19.6)
Central Europe, Eastern Europe, and Central Asia	Sickle cell disorders	8.49 (7.15 to 10.3)	5.87 (4.88 to 6.93)	5 (4.18 to 5.9)	2.3 (1.9 to 2.8)	1.9 (1.6 to 2.3)	1.6 (1.3 to 1.9)
Central Europe, Eastern Europe, and Central Asia	Sickle cell trait	13.9 (9 to 21)	10.1 (6.39 to 15.7)	8.14 (5.19 to 12.4)	3.4 (2.2 to 5)	2.5 (1.6 to 3.9)	2 (1.3 to 3)
Central Europe, Eastern Europe, and Central Asia	G6PD deficiency	14.8 (12.8 to 17.4)	19.2 (15.8 to 21.3)	17.4 (15.2 to 19.4)	3.6 (3.1 to 4.2)	4.5 (3.7 to 4.9)	4.2 (3.7 to 4.7)
Central Europe, Eastern Europe, and Central Asia	G6PD trait	0.0178 (0.0121 to 0.0249)	0.0155 (0.0106 to 0.0218)	0.0125 (0.00844 to 0.0178)	0 (0 to 0)	0 (0 to 0)	0 (0 to 0)
Central Europe, Eastern Europe, and Central Asia	Other hemoglobinopathies and hemolytic anaemias	139 (108 to 183)	111 (85.9 to 147)	99.6 (76.7 to 133)	32.2 (24.9 to 42.6)	25.4 (19.4 to 34.3)	21.9 (16.4 to 30)
Central Europe, Eastern Europe, and Central Asia	Endocrine, metabolic, blood, and immune disorders	353 (292 to 424)	314 (261 to 377)	310 (255 to 374)	85.8 (71.1 to 102.5)	79.4 (66.8 to 94.8)	74 (61.1 to 89.1)
Central Europe, Eastern Europe, and Central Asia	Oral disorders	1360 (860 to 2040)	1530 (965 to 2290)	1580 (1000 to 2370)	293.2 (186 to 440.5)	298.5 (189.2 to 448.3)	288 (182.5 to 432.7)
Central Europe, Eastern Europe, and Central Asia	Caries of deciduous teeth	12 (5.17 to 23.8)	7.81 (3.36 to 15.6)	9.09 (3.91 to 18.2)	3.3 (1.4 to 6.5)	3.3 (1.4 to 6.6)	3.2 (1.4 to 6.5)
Central Europe, Eastern Europe, and Central Asia	Caries of permanent teeth	154 (67.9 to 297)	159 (70 to 302)	151 (66.9 to 291)	36.1 (15.8 to 68.8)	36.4 (15.9 to 69.1)	35 (15.2 to 66.5)
Central Europe, Eastern Europe, and Central Asia	Periodontal disease	217 (85.9 to 449)	249 (98.9 to 515)	254 (101 to 526)	46.2 (18.3 to 95.8)	47.9 (19 to 99.9)	45.4 (18 to 94.7)
Central Europe, Eastern Europe, and Central Asia	Edentulism and severe tooth loss	753 (502 to 1060)	880 (591 to 1240)	937 (627 to 1320)	156.7 (104.8 to 221.4)	159.9 (106.5 to 224.8)	153.4 (102.4 to 216.3)
Central Europe, Eastern Europe, and Central Asia	Other oral disorders	224 (140 to 328)	232 (144 to 340)	234 (146 to 343)	51 (31.8 to 74.7)	51 (31.9 to 74.8)	50.9 (32 to 75)
Central Europe, Eastern Europe, and Central Asia	Sudden infant death syndrome	165 (128 to 200)	93.4 (70.7 to 115)	63 (54.1 to 78.6)	50.6 (39.4 to 61.5)	38.3 (29 to 47.2)	24.6 (21.1 to 30.7)
Central Europe, Eastern Europe, and Central Asia	Injuries	25900 (24000 to 28400)	25900 (24000 to 28400)	20100 (18000 to 22800)	6021 (5588.3 to 6565.2)	5742.1 (5334.3 to 6237.5)	4324.8 (3912.7 to 4836.5)
Central Europe, Eastern Europe, and Central Asia	Transport injuries	6060 (5730 to 6430)	5260 (4950 to 5630)	3740 (3440 to 4100)	1420.1 (1349.3 to 1500.8)	1189 (1126.4 to 1263)	845.7 (787.9 to 913.7)
Central Europe, Eastern Europe, and Central Asia	Road injuries	5680 (5350 to 6040)	4850 (4560 to 5190)	3430 (3150 to 3760)	1333.1 (1259.5 to 1410.1)	1097.2 (1038.5 to 1166.4)	775.4 (721.7 to 839.9)
Central Europe, Eastern Europe, and Central Asia	Pedestrian road injuries	2290 (2050 to 2440)	1550 (1470 to 1650)	942 (872 to 1030)	535.8 (481.4 to 569.2)	350.9 (334.6 to 372)	209 (194.5 to 225.9)
Central Europe, Eastern Europe, and Central Asia	Cyclist road injuries	297 (244 to 356)	295 (243 to 358)	269 (212 to 337)	67.1 (55.4 to 80)	62.8 (52.2 to 75.6)	54.3 (43.3 to 67.4)
Central Europe, Eastern Europe, and Central Asia	Motorcyclist road injuries	466 (401 to 515)	430 (395 to 469)	320 (284 to 360)	110.8 (95.7 to 121.8)	97.9 (90.9 to 106.2)	75.2 (68 to 83)
Central Europe, Eastern Europe, and Central Asia	Motor vehicle road injuries	2550 (2380 to 2850)	2510 (2370 to 2660)	1840 (1710 to 2000)	601.4 (562.9 to 669.1)	571.3 (543.8 to 601.9)	425.4 (399.4 to 455.8)
Central Europe, Eastern Europe, and Central Asia	Other road injuries	78.9 (66.3 to 94.4)	66.5 (55.1 to 82.4)	56.5 (44.9 to 72.3)	18 (15.2 to 21.3)	14.2 (11.9 to 17.5)	11.5 (9.3 to 14.7)
Central Europe, Eastern Europe, and Central Asia	Other transport injuries	371 (348 to 440)	410 (388 to 434)	311 (288 to 335)	87 (81.8 to 103)	91.8 (87.1 to 96.9)	70.3 (65.7 to 75.4)
Central Europe, Eastern Europe, and Central Asia	Unintentional injuries	13300 (11700 to 15400)	12700 (11100 to 14600)	10700 (8930 to 12900)	3104.9 (2744.6 to 3554.4)	2799.6 (2478.2 to 3205.3)	2227.6 (1888.2 to 2653.2)
Central Europe, Eastern Europe, and Central Asia	Falls	4990 (3890 to 6370)	4890 (3820 to 6270)	4990 (3810 to 6530)	1118 (875.3 to 1425.9)	1010.5 (792.9 to 1286)	968.9 (741.8 to 1257.1)
Central Europe, Eastern Europe, and Central Asia	Drowning	2070 (2020 to 2120)	1500 (1480 to 1530)	936 (909 to 966)	513.3 (501.2 to 526.8)	384.6 (376.7 to 392.8)	238.8 (231.4 to 247.2)
Central Europe, Eastern Europe, and Central Asia	Fire, heat, and hot substances	1150 (1030 to 1280)	1140 (1040 to 1270)	790 (689 to 924)	273.2 (248 to 304.4)	259.4 (239.3 to 286.9)	170.6 (150.1 to 197.2)
Central Europe, Eastern Europe, and Central Asia	Poisonings	855 (813 to 904)	826 (793 to 864)	527 (487 to 576)	201.4 (191.7 to 213.1)	188 (180.6 to 197)	117.8 (108.5 to 129.2)

SR Appendix Table 2. All-age DALYs counts and age standardized DALYs rates per 100,000 individuals for all causes and level 1 cause groups by location for 1990, 2007, and 2017, both sexes combined

		All-age DALYs			Age-standardized rate of DALYs per 100,000		
Central Europe, Eastern Europe, and Central Asia	Poisoning by carbon monoxide	610 (595 to 632)	665 (654 to 677)	380 (368 to 396)	141.7 (138.3 to 146.7)	148.3 (145.9 to 151.1)	83.2 (80.5 to 86.8)
Central Europe, Eastern Europe, and Central Asia	Poisoning by other means	245 (210 to 288)	161 (134 to 198)	147 (113 to 192)	59.7 (51.5 to 69.7)	39.6 (33.3 to 47.9)	34.5 (26.6 to 45.1)
Central Europe, Eastern Europe, and Central Asia	Exposure to mechanical forces	1270 (1040 to 1570)	1160 (949 to 1450)	1070 (826 to 1400)	290.6 (240 to 356.3)	249.2 (205.9 to 306.7)	218.7 (170.7 to 282)
Central Europe, Eastern Europe, and Central Asia	Unintentional firearm injuries	144 (128 to 170)	127 (119 to 139)	92.9 (84.5 to 103)	34.3 (30.6 to 40.4)	28.9 (27.1 to 31.3)	20.8 (19.1 to 22.9)
Central Europe, Eastern Europe, and Central Asia	Other exposure to mechanical forces	1130 (902 to 1420)	1030 (824 to 1310)	980 (739 to 1300)	256.3 (206.6 to 319.9)	220.3 (177.9 to 277.5)	198 (151.1 to 258.4)
Central Europe, Eastern Europe, and Central Asia	Adverse effects of medical treatment	171 (152 to 197)	203 (167 to 218)	185 (166 to 218)	41 (36.4 to 46.8)	46.8 (38.7 to 50.3)	40.2 (36.2 to 48.2)
Central Europe, Eastern Europe, and Central Asia	Animal contact	116 (91.8 to 143)	109 (87.3 to 135)	96.8 (75.4 to 122)	27.1 (21.6 to 33.5)	25 (20.1 to 30.9)	21.6 (16.9 to 27.2)
Central Europe, Eastern Europe, and Central Asia	Venomous animal contact	71.3 (53.3 to 93.9)	68.8 (52.3 to 89.4)	62.6 (46.4 to 83)	16.6 (12.5 to 21.9)	15.7 (11.8 to 20.5)	14.1 (10.3 to 18.8)
Central Europe, Eastern Europe, and Central Asia	Non-venomous animal contact	44.3 (35.8 to 55.9)	40.2 (31.8 to 51.6)	34.2 (26.8 to 45)	10.5 (8.6 to 13.1)	9.3 (7.5 to 11.8)	7.5 (6 to 9.8)
Central Europe, Eastern Europe, and Central Asia	Foreign body	795 (765 to 827)	869 (844 to 897)	636 (604 to 667)	203.5 (196 to 211.8)	223.3 (216.8 to 230.4)	153.5 (145.5 to 161.4)
Central Europe, Eastern Europe, and Central Asia	Pulmonary aspiration and foreign body in airway	685 (667 to 707)	755 (742 to 771)	530 (512 to 548)	177.7 (172.6 to 184.1)	197.2 (192.5 to 202.3)	129.7 (124.5 to 135.3)
Central Europe, Eastern Europe, and Central Asia	Foreign body in eyes	9.35 (4.42 to 16.2)	9.71 (4.73 to 16.7)	10.4 (5.11 to 18)	2.2 (1 to 3.8)	2.2 (1 to 3.9)	2.3 (1.1 to 4)
Central Europe, Eastern Europe, and Central Asia	Foreign body in other body part	101 (85.4 to 118)	104 (89.3 to 122)	95.9 (80.1 to 114)	23.7 (20.1 to 27.5)	24 (20.7 to 28)	21.5 (18.1 to 25.6)
Central Europe, Eastern Europe, and Central Asia	Environmental heat and cold exposure	1080 (1010 to 1200)	1240 (1170 to 1320)	806 (738 to 878)	241.9 (224.9 to 266.8)	253.7 (239.4 to 269.5)	158.3 (145 to 172.7)
Central Europe, Eastern Europe, and Central Asia	Exposure to forces of nature	14.7 (12.7 to 17.4)	12.4 (11 to 14.2)	8.03 (7 to 9.32)	3.6 (3.1 to 4.2)	3 (2.7 to 3.4)	1.9 (1.7 to 2.2)
Central Europe, Eastern Europe, and Central Asia	Other unintentional injuries	821 (719 to 956)	697 (605 to 819)	642 (522 to 802)	191.3 (168.7 to 220.8)	156 (137.3 to 181.1)	137.2 (113.8 to 168.4)
Central Europe, Eastern Europe, and Central Asia	Self-harm and interpersonal violence	6530 (6410 to 6660)	8030 (7890 to 8170)	5680 (5500 to 5870)	1495.9 (1469.2 to 1525)	1753.5 (1724 to 1783.3)	1251.5 (1212.8 to 1291.2)
Central Europe, Eastern Europe, and Central Asia	Self-harm	4010 (3960 to 4110)	5150 (5060 to 5220)	3790 (3650 to 3950)	910.6 (899 to 934.3)	1118.2 (1097.6 to 1132.6)	835.8 (804.6 to 871)
Central Europe, Eastern Europe, and Central Asia	Self-harm by firearm	208 (151 to 310)	205 (148 to 305)	139 (94.3 to 191)	47.3 (34.2 to 70.1)	44 (31.7 to 65.6)	29.7 (20.2 to 40.9)
Central Europe, Eastern Europe, and Central Asia	Self-harm by other specified means	3800 (3690 to 3920)	4950 (4840 to 5030)	3650 (3510 to 3810)	863.3 (837.7 to 890.7)	1074.3 (1050.5 to 1092)	806.1 (776.4 to 840.9)
Central Europe, Eastern Europe, and Central Asia	Interpersonal violence	2480 (2370 to 2600)	2780 (2690 to 2890)	1800 (1710 to 1910)	575.2 (550 to 600.6)	613.5 (594.4 to 636.6)	394.1 (374.3 to 416.7)
Central Europe, Eastern Europe, and Central Asia	Assault by firearm	231 (179 to 254)	216 (201 to 227)	131 (118 to 141)	54.6 (42.2 to 60)	49.2 (45.7 to 51.6)	30.5 (27.2 to 33.1)
Central Europe, Eastern Europe, and Central Asia	Assault by sharp object	844 (705 to 924)	978 (943 to 1030)	569 (536 to 601)	196.5 (164.7 to 215)	216.5 (208.7 to 228.3)	126.2 (118.5 to 133.5)
Central Europe, Eastern Europe, and Central Asia	Sexual violence	46.7 (31.7 to 67.7)	49.1 (33.3 to 71.8)	48.1 (32.6 to 69)	10.9 (7.4 to 16.1)	11.2 (7.6 to 16.4)	11.3 (7.6 to 16.6)
Central Europe, Eastern Europe, and Central Asia	Assault by other means	1360 (1260 to 1480)	1540 (1440 to 1650)	1060 (974 to 1160)	313.1 (291.3 to 340.5)	336.6 (317.1 to 359.1)	226.1 (209.8 to 246.5)
Central Europe, Eastern Europe, and Central Asia	Conflict and terrorism	25.3 (25 to 25.7)	79.9 (60.7 to 112)	82.9 (66.1 to 112)	6.2 (6.1 to 6.3)	18.5 (14.1 to 26.2)	18.9 (15.3 to 25.1)
Central Europe, Eastern Europe, and Central Asia	Executions and police conflict	16.5 (14.9 to 18.3)	13.9 (12.6 to 15.7)	11.4 (10 to 13.3)	4 (3.6 to 4.4)	3.2 (2.9 to 3.6)	2.8 (2.4 to 3.2)
Central Asia	All causes	27300 (25500 to 29400)	27800 (25800 to 30200)	27900 (25400 to 30600)	41472.1 (38597.8 to 44683.1)	40071.9 (37282.2 to 43341.9)	33621.9 (30774.6 to 36767.6)
Central Asia	Communicable, maternal, neonatal, and nutritional disorders	10300 (9770 to 10800)	6430 (5970 to 6950)	5190 (4720 to 5690)	11673.1 (11062.9 to 12373.7)	8214.4 (7636.2 to 8860.7)	5721.7 (5212.5 to 6263.9)
Central Asia	HIV/AIDS and sexually transmitted infections	44.7 (34.3 to 60.6)	89.5 (78.5 to 107)	97.9 (86 to 118)	63 (49.4 to 84.4)	109.8 (96.6 to 129.8)	101.8 (89.4 to 122.9)
Central Asia	HIV/AIDS	16.4 (15.7 to 17.1)	62.8 (60.6 to 66.4)	71 (68.1 to 74)	24.3 (23.1 to 25.3)	77.7 (74.9 to 82)	73.3 (70.4 to 76.5)
Central Asia	HIV/AIDS - Drug-susceptible Tuberculosis	4.76 (3.08 to 6.68)	6.85 (4.23 to 10.6)	4.03 (2.32 to 6.6)	7.1 (4.5 to 9.9)	8.5 (5.2 to 13)	4.2 (2.4 to 6.8)
Central Asia	HIV/AIDS - Multidrug-resistant Tuberculosis without extensive drug resistance	0.0191 (0.00615 to 0.0484)	5.66 (3.41 to 8.63)	3.07 (1.84 to 4.78)	0 (0 to 0.1)	7 (4.2 to 10.7)	3.2 (1.9 to 4.9)
Central Asia	HIV/AIDS - Extensively drug-resistant Tuberculosis	0 (0 to 0)	1.79 (1.08 to 2.74)	1.75 (1.04 to 2.72)	0 (0 to 0)	2.2 (1.3 to 3.4)	1.8 (1.1 to 2.8)
Central Asia	HIV/AIDS resulting in other diseases	11.7 (9.68 to 13.4)	48.5 (41.4 to 54.5)	62.1 (57.1 to 66.7)	17.2 (14.3 to 19.8)	60 (51.2 to 67.3)	64.2 (59 to 68.9)
Central Asia	Sexually transmitted infections excluding HIV	28.3 (18 to 43.9)	26.6 (16.2 to 43.6)	27 (16.1 to 45.2)	38.7 (25.3 to 60.8)	32.1 (19.6 to 52.2)	28.4 (17 to 47.4)
Central Asia	Syphilis	11.7 (5.55 to 21.6)	7.22 (3.3 to 13.3)	5.52 (2.51 to 10.6)	13.8 (7.1 to 24.2)	9.2 (4.2 to 16.9)	6.1 (2.8 to 11.8)
Central Asia	Chlamydial infection	3.82 (2.24 to 6.77)	4.65 (2.64 to 8.54)	5.24 (2.92 to 9.65)	5.7 (3.3 to 9.9)	5.4 (3.1 to 9.8)	5.3 (3 to 9.8)
Central Asia	Gonococcal infection	3.98 (2.58 to 6.69)	4.07 (2.41 to 7.3)	4.13 (2.44 to 7.37)	5.8 (3.9 to 9.4)	4.6 (2.8 to 8.1)	4.3 (2.5 to 7.7)
Central Asia	Trichomoniasis	2.63 (1.05 to 5.67)	3.36 (1.34 to 7.22)	3.85 (1.55 to 8.2)	4.1 (1.7 to 8.8)	4.1 (1.6 to 8.7)	4 (1.6 to 8.6)

SR Appendix Table 2. All-age DALYs counts and age standardized DALYs rates per 100,000 individuals for all causes and level 1 cause groups by location for 1990, 2007, and 2017, both sexes combined

		All-age DALYs			Age-standardized rate of DALYs per 100,000		
Central Asia	Genital herpes	1.55<br>(0.503 to 3.67)	1.98<br>(0.65 to 4.7)	2.4<br>(0.774 to 5.68)	2.6<br>(0.8 to 6.2)	2.6<br>(0.8 to 6.3)	2.6<br>(0.8 to 6.2)
Central Asia	Other sexually transmitted infections	4.58<br>(2.9 to 7.68)	5.37<br>(3.27 to 9.22)	5.83<br>(3.6 to 9.95)	6.8<br>(4.4 to 11.2)	6.2<br>(3.8 to 10.5)	6<br>(3.7 to 10.3)
Central Asia	Respiratory infections and tuberculosis	4820<br>(4610 to 5030)	2770<br>(2580 to 2970)	2000<br>(1840 to 2200)	5445.5<br>(5223.4 to 5675.5)	3601.9<br>(3359.4 to 3856.2)	2215.2<br>(2036.6 to 2429.7)
Central Asia	Tuberculosis	340<br>(324 to 355)	503<br>(489 to 518)	295<br>(277 to 317)	504.2<br>(486.3 to 522.1)	644.6<br>(626.4 to 663.4)	314.6<br>(296 to 338.4)
Central Asia	Latent tuberculosis infection	0<br>(0 to 0)	0<br>(0 to 0)	0<br>(0 to 0)	0<br>(0 to 0)	0<br>(0 to 0)	0<br>(0 to 0)
Central Asia	Drug-susceptible tuberculosis	338<br>(323 to 354)	277<br>(235 to 317)	160<br>(129 to 196)	502.4<br>(483.5 to 520.6)	354.6<br>(300.6 to 407)	170.8<br>(137.8 to 209.2)
Central Asia	Multidrug-resistant tuberculosis without extensive drug resistance	1.22<br>(0.523 to 2.7)	172<br>(140 to 204)	85.9<br>(65.1 to 105)	1.9<br>(0.8 to 4.2)	220.3<br>(178.8 to 261.7)	91.6<br>(69.3 to 112.2)
Central Asia	Extensively drug-resistant tuberculosis	0<br>(0 to 0)	54.4<br>(43.9 to 64.8)	48.9<br>(36.6 to 60.4)	0<br>(0 to 0)	69.7<br>(56.2 to 83)	52.2<br>(39 to 64.3)
Central Asia	Lower respiratory infections	4360<br>(4160 to 4560)	2180<br>(2000 to 2360)	1620<br>(1470 to 1800)	4796.8<br>(4582.1 to 5014.4)	2844.1<br>(2612.1 to 3080.2)	1806.4<br>(1637.8 to 2007.3)
Central Asia	Upper respiratory infections	98.5<br>(68.1 to 128)	67.6<br>(49.5 to 91.1)	63.2<br>(44.4 to 88.2)	117.8<br>(83.6 to 154.7)	85.3<br>(63 to 114.1)	68.8<br>(48.5 to 95.7)
Central Asia	Otitis media	20<br>(11.9 to 31.9)	22.5<br>(13.6 to 36)	23.2<br>(14 to 36.6)	26.6<br>(15.9 to 42)	27.9<br>(16.9 to 44.4)	25.4<br>(15.3 to 40.3)
Central Asia	Enteric infections	1180<br>(1090 to 1260)	295<br>(259 to 336)	282<br>(229 to 343)	1284.6<br>(1194.6 to 1380.6)	380.3<br>(333.2 to 431.1)	308.1<br>(250.8 to 375.6)
Central Asia	Diarrhoeal diseases	1170<br>(1090 to 1260)	289<br>(253 to 331)	276<br>(224 to 338)	1274.6<br>(1183.4 to 1367.9)	372.5<br>(326.1 to 425.8)	301.5<br>(245.6 to 369.3)
Central Asia	Typhoid and paratyphoid	0.441<br>(0.143 to 1)	0.192<br>(0.0631 to 0.346)	0.079<br>(0.0333 to 0.128)	0.7<br>(0.2 to 1.8)	0.3<br>(0.1 to 0.6)	0.1<br>(0 to 0.1)
Central Asia	Typhoid fever	0.173<br>(0.115 to 0.244)	0.08<br>(0.0516 to 0.116)	0.0414<br>(0.0255 to 0.0662)	0.3<br>(0.2 to 0.4)	0.1<br>(0.1 to 0.1)	0<br>(0 to 0.1)
Central Asia	Paratyphoid fever	0.268<br>(0.0137 to 0.827)	0.112<br>(0.00574 to 0.253)	0.0376<br>(0.00417 to 0.0784)	0.5<br>(0 to 1.5)	0.2<br>(0 to 0.5)	0<br>(0 to 0.1)
Central Asia	Invasive non-typhoidal salmonella	6.11<br>(3.4 to 10.5)	5.08<br>(2.71 to 8.74)	5.36<br>(2.83 to 9.39)	7.7<br>(4.3 to 13.2)	6.6<br>(3.6 to 11.1)	6<br>(3.2 to 10.4)
Central Asia	Other intestinal infectious diseases	1.3<br>(0.546 to 2.2)	0.717<br>(0.282 to 1.23)	0.506<br>(0.2 to 0.893)	1.5<br>(0.7 to 2.4)	0.9<br>(0.4 to 1.6)	0.6<br>(0.2 to 1)
Central Asia	Neglected tropical diseases and malaria	65.5<br>(44.9 to 95.1)	57.8<br>(37.9 to 88.2)	57.3<br>(35.8 to 88)	91.1<br>(62.5 to 130.7)	73<br>(47.9 to 110.4)	62<br>(39.1 to 95.3)
Central Asia	Malaria	5.81<br>(2.21 to 13.7)	2.22<br>(1.47 to 3.81)	0.107<br>(0.0769 to 0.145)	6.9<br>(2.9 to 15.9)	2.8<br>(1.9 to 4.9)	0.1<br>(0.1 to 0.2)
Central Asia	Chagas disease	0<br>(0 to 0)	0<br>(0 to 0)	0<br>(0 to 0)	0<br>(0 to 0)	0<br>(0 to 0)	0<br>(0 to 0)
Central Asia	Leishmaniasis	1.69<br>(0.255 to 13.3)	2.31<br>(0.129 to 19.5)	2.14<br>(0.147 to 16.3)	2.2<br>(0.4 to 16.7)	3<br>(0.2 to 24.7)	2.3<br>(0.2 to 17.7)
Central Asia	Visceral leishmaniasis	1.28<br>(0.00399 to 12.8)	2.14<br>(0.00698 to 19.3)	1.91<br>(0.00522 to 16.1)	1.7<br>(0 to 16.2)	2.8<br>(0 to 24.5)	2.1<br>(0 to 17.4)
Central Asia	Cutaneous and mucocutaneous leishmaniasis	0.407<br>(0.215 to 0.711)	0.177<br>(0.101 to 0.29)	0.227<br>(0.12 to 0.396)	0.6<br>(0.3 to 1)	0.2<br>(0.1 to 0.4)	0.3<br>(0.1 to 0.4)
Central Asia	African trypanosomiasis	0<br>(0 to 0)	0<br>(0 to 0)	0<br>(0 to 0)	0<br>(0 to 0)	0<br>(0 to 0)	0<br>(0 to 0)
Central Asia	Schistosomiasis	0<br>(0 to 0)	0<br>(0 to 0)	0<br>(0 to 0)	0<br>(0 to 0)	0<br>(0 to 0)	0<br>(0 to 0)
Central Asia	Cysticercosis	0.713<br>(0.408 to 1.1)	1.07<br>(0.586 to 1.69)	1.33<br>(0.731 to 2.14)	1.2<br>(0.7 to 1.8)	1.4<br>(0.8 to 2.2)	1.4<br>(0.8 to 2.3)
Central Asia	Cystic echinococcosis	18.1<br>(10 to 31.2)	21.2<br>(11 to 39.6)	24.9<br>(12.6 to 46.8)	28.6<br>(16.2 to 47.9)	26.8<br>(14.4 to 47.5)	26.9<br>(13.8 to 49.1)
Central Asia	Lymphatic filariasis	0<br>(0 to 0)	0<br>(0 to 0)	0<br>(0 to 0)	0<br>(0 to 0)	0<br>(0 to 0)	0<br>(0 to 0)
Central Asia	Onchocerciasis	0<br>(0 to 0)	0<br>(0 to 0)	0<br>(0 to 0)	0<br>(0 to 0)	0<br>(0 to 0)	0<br>(0 to 0)
Central Asia	Trachoma	0<br>(0 to 0)	0<br>(0 to 0)	0<br>(0 to 0)	0<br>(0 to 0)	0<br>(0 to 0)	0<br>(0 to 0)
Central Asia	Dengue	0<br>(0 to 0)	0<br>(0 to 0)	0<br>(0 to 0)	0<br>(0 to 0)	0<br>(0 to 0)	0<br>(0 to 0)
Central Asia	Yellow fever	0<br>(0 to 0)	0<br>(0 to 0)	0<br>(0 to 0)	0<br>(0 to 0)	0<br>(0 to 0)	0<br>(0 to 0)
Central Asia	Rabies	2.13<br>(1.67 to 2.87)	1.13<br>(1 to 1.28)	0.802<br>(0.693 to 0.954)	3<br>(2.5 to 3.9)	1.5<br>(1.4 to 1.7)	0.9<br>(0.8 to 1.1)
Central Asia	Intestinal nematode infections	4.99<br>(3.18 to 7.67)	2.57<br>(1.6 to 3.98)	2.03<br>(1.24 to 3.25)	7.1<br>(4.5 to 10.9)	3.2<br>(2 to 4.9)	2.2<br>(1.3 to 3.5)
Central Asia	Ascariasis	0.807<br>(0.494 to 1.3)	0.721<br>(0.402 to 1.22)	0.586<br>(0.315 to 1.01)	1<br>(0.6 to 1.6)	0.9<br>(0.5 to 1.5)	0.6<br>(0.3 to 1.1)
Central Asia	Trichuriasis	0.0000299<br>(0.000014 to 0.0000582)	0.0000156<br>(0.00000687 to 0.0000307)	0.0000187<br>(0.00000797 to 0.0000381)	0<br>(0 to 0)	0<br>(0 to 0)	0<br>(0 to 0)
Central Asia	Hookworm disease	4.18<br>(2.64 to 6.4)	1.85<br>(1.15 to 2.92)	1.45<br>(0.908 to 2.28)	6.1<br>(3.8 to 9.3)	2.3<br>(1.4 to 3.6)	1.6<br>(1 to 2.5)
Central Asia	Food-borne trematodiases	0.75<br>(0.336 to 1.34)	0.754<br>(0.338 to 1.38)	0.886<br>(0.386 to 1.63)	1.3<br>(0.6 to 2.4)	1<br>(0.5 to 1.9)	1<br>(0.4 to 1.9)
Central Asia	Leprosy	0<br>(0 to 0)	0.0000204<br>(0.0000109 to 0.0000335)	0.0000206<br>(0.0000114 to 0.0000341)	0<br>(0 to 0)	0<br>(0 to 0)	0<br>(0 to 0)

SR Appendix Table 2. All-age DALYs counts and age standardized DALYs rates per 100,000 individuals for all causes and level 1 cause groups by location for 1990, 2007, and 2017, both sexes combined							
		All-age DALYs			Age-standardized rate of DALYs per 100,000		
Central Asia	Ebola virus disease	0 (0 to 0)	0 (0 to 0)	0 (0 to 0)	0 (0 to 0)	0 (0 to 0)	0 (0 to 0)
Central Asia	Zika virus disease	0 (0 to 0)	0 (0 to 0)	0 (0 to 0)	0 (0 to 0)	0 (0 to 0)	0 (0 to 0)
Central Asia	Guinea worm disease	0 (0 to 0)	0 (0 to 0)	0 (0 to 0)	0 (0 to 0)	0 (0 to 0)	0 (0 to 0)
Central Asia	Other neglected tropical diseases	31.4 (21.2 to 44.3)	26.6 (17.8 to 39.2)	25.1 (16.7 to 37.3)	40.7 (27.6 to 57.6)	33.2 (22.1 to 48.6)	27.1 (18 to 40.2)
Central Asia	Other infectious diseases	570 (517 to 621)	351 (316 to 393)	295 (265 to 333)	673.3 (612.4 to 731.3)	445.4 (401 to 501.6)	321.5 (288.2 to 362)
Central Asia	Meningitis	236 (204 to 254)	126 (116 to 141)	80.1 (70.7 to 101)	277.2 (239.5 to 296.6)	159.2 (146.1 to 178.5)	87.2 (77 to 110.1)
Central Asia	Pneumococcal meningitis	10.9 (8.9 to 13.1)	8.79 (6.93 to 11)	5.24 (4.08 to 6.5)	14.3 (11.7 to 17.3)	10.9 (8.6 to 13.5)	5.7 (4.4 to 7)
Central Asia	H influenzae type B meningitis	105 (90.8 to 115)	56.3 (51.2 to 63.3)	34.5 (29.8 to 44.5)	121.5 (105.2 to 132.3)	71.4 (64.8 to 80.4)	37.6 (32.5 to 48.4)
Central Asia	Meningococcal infection	37.1 (31.1 to 44.4)	15.9 (13.6 to 19.2)	10.3 (8.54 to 12.8)	43.1 (36.4 to 50.8)	20.2 (17.2 to 24.4)	11.2 (9.3 to 14)
Central Asia	Other meningitis	83.2 (71 to 92.1)	45.1 (40.6 to 51.2)	30.2 (26.1 to 38.5)	98.3 (83.1 to 107.8)	56.8 (51.1 to 64.7)	32.8 (28.4 to 41.9)
Central Asia	Encephalitis	94.5 (82.5 to 114)	93.3 (83.3 to 102)	103 (87.3 to 117)	112.7 (99.2 to 134.6)	119.1 (106.5 to 130.8)	112.4 (94.9 to 127.7)
Central Asia	Diphtheria	3.98 (2.34 to 6.39)	0.707 (0.469 to 1.05)	0.247 (0.181 to 0.338)	4.9 (3.1 to 7.5)	0.9 (0.6 to 1.4)	0.3 (0.2 to 0.4)
Central Asia	Whooping cough	34.5 (11.6 to 75)	27.2 (7.63 to 67.4)	18.8 (5.46 to 45.5)	37.1 (12.5 to 80.4)	36 (10.1 to 89.1)	20.3 (5.9 to 49)
Central Asia	Tetanus	0.795 (0.475 to 0.887)	0.411 (0.277 to 0.505)	0.399 (0.259 to 0.578)	1.2 (0.7 to 1.3)	0.6 (0.4 to 0.7)	0.4 (0.3 to 0.6)
Central Asia	Measles	6.41 (4.83 to 7.9)	0.234 (0.164 to 0.351)	0.449 (0.331 to 0.597)	6.9 (5.2 to 8.5)	0.3 (0.2 to 0.5)	0.5 (0.4 to 0.6)
Central Asia	Varicella and herpes zoster	4.58 (3.49 to 5.78)	3.95 (3.02 to 5.14)	4.13 (3.05 to 5.45)	6.3 (4.8 to 7.9)	5.5 (4.1 to 7.1)	4.8 (3.5 to 6.3)
Central Asia	Acute hepatitis	134 (106 to 151)	50.6 (46.3 to 59.4)	41.5 (36.7 to 51.7)	159 (125.9 to 177)	63 (57.6 to 74.2)	44.8 (39.6 to 55.8)
Central Asia	Acute hepatitis A	48.5 (36.4 to 60.7)	16 (13.8 to 19.3)	12.8 (10.7 to 16.4)	55.1 (41.6 to 68)	19.4 (16.7 to 23.6)	13.9 (11.6 to 17.8)
Central Asia	Acute hepatitis B	42.5 (32.4 to 51.3)	23.8 (21.2 to 27.6)	21 (18.5 to 25.3)	56.9 (44.1 to 66.9)	30 (26.9 to 34.6)	22.5 (19.8 to 27.1)
Central Asia	Acute hepatitis C	37.5 (25.1 to 50.1)	7.52 (5.15 to 10.9)	5.24 (3.26 to 8.5)	40 (26.8 to 53.5)	9.8 (6.7 to 14.3)	5.7 (3.6 to 9.3)
Central Asia	Acute hepatitis E	5.48 (4.24 to 6.6)	3.23 (2.64 to 4)	2.43 (1.95 to 3.16)	7 (5.5 to 8.4)	3.8 (3.1 to 4.6)	2.6 (2.2 to 3.4)
Central Asia	Other unspecified infectious diseases	54.6 (42.9 to 67.9)	48.2 (37.7 to 60.3)	46.5 (36.9 to 58.9)	68.1 (53.5 to 85.2)	60.9 (47.7 to 75.6)	50.8 (40.4 to 64.3)
Central Asia	Maternal and neonatal disorders	2840 (2690 to 3020)	2260 (2100 to 2440)	1880 (1720 to 2090)	3114.4 (2944.4 to 3307.7)	2836.5 (2639.4 to 3057.8)	2088.2 (1901.2 to 2312.4)
Central Asia	Maternal disorders	66.1 (63.4 to 68.9)	43.4 (41.1 to 46)	31 (28.3 to 33.8)	94.1 (90.4 to 97.9)	49.8 (47.2 to 52.8)	31 (28.4 to 33.9)
Central Asia	Maternal haemorrhage	17.1 (14.7 to 19.8)	8.62 (7.25 to 10.1)	5.06 (4.18 to 6.06)	24.6 (21.2 to 28.3)	10.1 (8.5 to 11.8)	5.1 (4.2 to 6)
Central Asia	Maternal sepsis and other pregnancy related infections	11.3 (9.98 to 12.9)	5.44 (4.71 to 6.32)	3.99 (3.33 to 4.94)	16.2 (14.4 to 18.2)	6.1 (5.3 to 7.1)	4 (3.4 to 5)
Central Asia	Maternal hypertensive disorders	6.74 (5.54 to 8.11)	5.81 (4.72 to 7.26)	4.49 (3.54 to 5.64)	9.3 (7.7 to 11.1)	6.6 (5.4 to 8.1)	4.5 (3.5 to 5.6)
Central Asia	Maternal obstructed labour and uterine rupture	2.71 (2.13 to 3.45)	1.81 (1.37 to 2.36)	1.58 (1.11 to 2.2)	3.9 (3.1 to 4.9)	2 (1.5 to 2.6)	1.6 (1.1 to 2.2)
Central Asia	Maternal abortive outcome	10.6 (8.77 to 12.4)	5.44 (4.57 to 6.47)	3.16 (2.7 to 3.65)	15.5 (13 to 18)	6.3 (5.3 to 7.5)	3.2 (2.8 to 3.7)
Central Asia	Ectopic pregnancy	1.47 (0.912 to 2.37)	0.989 (0.572 to 1.77)	0.719 (0.438 to 1.2)	2.1 (1.3 to 3.3)	1.1 (0.7 to 2)	0.7 (0.4 to 1.2)
Central Asia	Indirect maternal deaths	3.07 (2.33 to 4.22)	3.69 (2.79 to 4.93)	2.49 (1.91 to 3.41)	4.3 (3.3 to 5.9)	4.2 (3.2 to 5.6)	2.5 (1.9 to 3.3)
Central Asia	Late maternal deaths	1.93 (1.43 to 2.54)	1.68 (1.39 to 1.99)	1.45 (1.18 to 1.74)	2.7 (2 to 3.5)	1.9 (1.6 to 2.3)	1.4 (1.2 to 1.7)
Central Asia	Maternal deaths aggravated by HIV/AIDS	0.00251 (0.00124 to 0.0042)	0.00673 (0.00327 to 0.0119)	0.00925 (0.00487 to 0.0153)	0 (0 to 0)	0 (0 to 0)	0 (0 to 0)
Central Asia	Other maternal disorders	11.2 (9.38 to 13.1)	9.91 (8.43 to 11.7)	8.04 (6.73 to 9.45)	15.6 (13.2 to 18.2)	11.3 (9.6 to 13.3)	8 (6.7 to 9.4)
Central Asia	Neonatal disorders	2780 (2620 to 2950)	2220 (2060 to 2390)	1850 (1690 to 2060)	3020.3 (2849.7 to 3212.1)	2786.8 (2588.5 to 3007.3)	2057.2 (1869.6 to 2283.8)
Central Asia	Neonatal preterm birth	1210 (1030 to 1360)	921 (842 to 1000)	792 (691 to 892)	1312.4 (1117.9 to 1482.6)	1156.8 (1057 to 1256.1)	880 (768.1 to 992.3)
Central Asia	Neonatal encephalopathy due to birth asphyxia and trauma	1120 (978 to 1290)	890 (786 to 1000)	695 (595 to 819)	1214.7 (1059.8 to 1397.5)	1118.9 (988.1 to 1262.2)	770.7 (660.2 to 907.3)
Central Asia	Neonatal sepsis and other neonatal infections	170 (133 to 227)	171 (144 to 213)	186 (152 to 226)	188 (146.1 to 251.1)	215.3 (181.4 to 267.9)	206.5 (168.3 to 250.3)
Central Asia	Hemolytic disease and other neonatal jaundice	47.7 (40.4 to 55.5)	38.2 (31.7 to 44)	32 (26.6 to 38)	55.3 (46.9 to 64.3)	47.5 (39.4 to 54.7)	34.9 (29.1 to 41.5)
Central Asia	Other neonatal disorders	234 (198 to 284)	196 (174 to 223)	149 (129 to 172)	249.8 (211.4 to 304.1)	248.4 (220.3 to 283.2)	165 (143.7 to 190.7)

SR Appendix Table 2. All-age DALYs counts and age standardized DALYs rates per 100,000 individuals for all causes and level 1 cause groups by location for 1990, 2007, and 2017, both sexes combined

		All-age DALYs			Age-standardized rate of DALYs per 100,000		
Central Asia	Nutritional deficiencies	754<br>(517 to 1060)	612<br>(409 to 884)	571<br>(381 to 842)	1001.2<br>(691.8 to 1412.8)	767.5<br>(517.1 to 1106.9)	624.9<br>(417.4 to 919.8)
Central Asia	Protein-energy malnutrition	43.3<br>(36.1 to 52)	18.5<br>(15 to 22.8)	13.9<br>(10.5 to 18.4)	49.2<br>(41.2 to 58.6)	24.2<br>(19.5 to 30)	15.1<br>(11.5 to 20)
Central Asia	Iodine deficiency	15.8<br>(8.64 to 26.6)	17.9<br>(9.9 to 30.1)	17.9<br>(9.79 to 30.7)	23.7<br>(12.9 to 39.9)	22.5<br>(12.5 to 37.8)	19.7<br>(10.8 to 33.9)
Central Asia	Dietary iron deficiency	521<br>(349 to 750)	481<br>(319 to 707)	457<br>(299 to 675)	728.1<br>(488.2 to 1047.1)	597.7<br>(397.5 to 878.1)	498.1<br>(325.4 to 739.6)
Central Asia	Other nutritional deficiencies	25.6<br>(23.3 to 28)	13.2<br>(11.5 to 15.1)	7.72<br>(6.66 to 8.97)	32.2<br>(29.6 to 34.8)	17.4<br>(15.1 to 20)	8.6<br>(7.4 to 10)
Central Asia	Non-communicable diseases	13600<br>(12300 to 15100)	18000<br>(16500 to 19900)	19600<br>(17700 to 21800)	24739.1<br>(22605.7 to 27212.1)	27640.7<br>(25498.2 to 30149.9)	24547.5<br>(22327.8 to 27073.5)
Central Asia	Neoplasms	1880<br>(1830 to 1920)	1990<br>(1960 to 2020)	2290<br>(2210 to 2370)	3476.7<br>(3391 to 3551.7)	3040.1<br>(2990.1 to 3089.1)	2780.6<br>(2682.1 to 2874)
Central Asia	Lip and oral cavity cancer	25.9<br>(24.3 to 29.6)	31.7<br>(30.8 to 32.7)	37.7<br>(35.6 to 39.8)	49.5<br>(46.5 to 56.7)	48.8<br>(47.4 to 50.3)	45.1<br>(42.7 to 47.6)
Central Asia	Nasopharynx cancer	7.05<br>(6.67 to 7.53)	9.22<br>(8.85 to 9.59)	10.7<br>(9.95 to 11.4)	12.1<br>(11.4 to 12.9)	12.6<br>(12.1 to 13.1)	12<br>(11.3 to 12.9)
Central Asia	Other pharynx cancer	12.2<br>(10.7 to 13.7)	14.8<br>(14.1 to 15.4)	18.4<br>(17 to 19.7)	23.1<br>(20.3 to 25.9)	22.8<br>(21.9 to 23.7)	21.6<br>(20.1 to 23.2)
Central Asia	Oesophageal cancer	153<br>(150 to 157)	102<br>(99.2 to 105)	106<br>(100 to 112)	306.6<br>(299.2 to 313.7)	168.5<br>(164.2 to 173.3)	135.2<br>(128.7 to 142)
Central Asia	Stomach cancer	303<br>(297 to 310)	261<br>(255 to 267)	273<br>(261 to 286)	593.4<br>(580.6 to 605.9)	415.6<br>(407 to 425.3)	340<br>(325 to 354.6)
Central Asia	Colon and rectum cancer	112<br>(110 to 114)	125<br>(122 to 128)	150<br>(143 to 158)	217<br>(213.1 to 221.4)	201.5<br>(196.6 to 206.3)	193.5<br>(184.4 to 203.1)
Central Asia	Liver cancer	107<br>(96.7 to 119)	138<br>(134 to 143)	179<br>(170 to 190)	207<br>(186.4 to 230.6)	219.8<br>(212.3 to 226.7)	222.7<br>(211.1 to 234.5)
Central Asia	Liver cancer due to hepatitis B	30.7<br>(24.4 to 37.7)	39.5<br>(33.7 to 45.5)	48.6<br>(40.4 to 57.1)	56.1<br>(44.5 to 69.2)	58.2<br>(49.1 to 67.8)	56.1<br>(47 to 65.5)
Central Asia	Liver cancer due to hepatitis C	36.9<br>(30.3 to 44)	47<br>(41.4 to 52.9)	62.7<br>(53.8 to 71)	73.9<br>(60.9 to 87.5)	79.7<br>(69.8 to 89.5)	81.2<br>(70.8 to 91.3)
Central Asia	Liver cancer due to alcohol use	26.7<br>(22.5 to 31.8)	34.6<br>(31.1 to 38.4)	45.6<br>(40.6 to 51.4)	53<br>(44.6 to 62.5)	55.1<br>(49.7 to 61.4)	57.3<br>(51.4 to 64.5)
Central Asia	Liver cancer due to NASH	6.06<br>(5.03 to 7.26)	8.25<br>(7.29 to 9.33)	11.6<br>(10.1 to 13.3)	12<br>(10 to 14.3)	13.6<br>(12 to 15.5)	15<br>(13 to 17.2)
Central Asia	Liver cancer due to other causes	6.67<br>(5.59 to 7.84)	8.72<br>(7.68 to 9.82)	11.1<br>(9.61 to 12.7)	12.2<br>(10.2 to 14.4)	13.1<br>(11.6 to 14.9)	13.1<br>(11.5 to 15)
Central Asia	Gallbladder and biliary tract cancer	13.3<br>(11.6 to 16.4)	17.1<br>(15.3 to 17.8)	18.6<br>(16.8 to 19.8)	26.9<br>(23.6 to 33.6)	28.3<br>(25.6 to 29.4)	24.2<br>(22 to 25.7)
Central Asia	Pancreatic cancer	29.8<br>(27.8 to 31.7)	63.6<br>(62.1 to 65.1)	84.1<br>(80.5 to 88.3)	59.8<br>(55.3 to 63.7)	104.3<br>(101.7 to 106.7)	108.2<br>(103.6 to 113.4)
Central Asia	Larynx cancer	36.5<br>(35.4 to 37.7)	33.8<br>(32.8 to 34.9)	34.8<br>(32.8 to 36.9)	69.4<br>(67.4 to 71.5)	53.8<br>(52.2 to 55.4)	42.3<br>(40 to 44.6)
Central Asia	Tracheal, bronchus, and lung cancer	327<br>(320 to 333)	266<br>(261 to 272)	314<br>(299 to 328)	625.7<br>(613.2 to 637.9)	430.6<br>(421.2 to 439.6)	388.1<br>(370.8 to 405.1)
Central Asia	Malignant skin melanoma	10.4<br>(7.41 to 12.2)	11.5<br>(9.94 to 16.1)	13<br>(11.4 to 19.2)	19.6<br>(13.9 to 22.9)	17.3<br>(14.9 to 24.1)	15.7<br>(13.7 to 23)
Central Asia	Non-melanoma skin cancer	5.5<br>(5.03 to 5.96)	9.97<br>(9.64 to 10.3)	12.5<br>(11.8 to 13.3)	10.8<br>(9.8 to 11.8)	16.1<br>(15.6 to 16.7)	16.4<br>(15.5 to 17.3)
Central Asia	Non-melanoma skin cancer (squamous-cell carcinoma)	5.48<br>(5.01 to 5.95)	9.95<br>(9.61 to 10.3)	12.5<br>(11.7 to 13.2)	10.8<br>(9.8 to 11.8)	16.1<br>(15.6 to 16.6)	16.3<br>(15.5 to 17.3)
Central Asia	Non-melanoma skin cancer (basal-cell carcinoma)	0.0151<br>(0.0053 to 0.0349)	0.0189<br>(0.00624 to 0.0445)	0.0241<br>(0.00827 to 0.0534)	0<br>(0 to 0.1)	0<br>(0 to 0.1)	0<br>(0 to 0.1)
Central Asia	Breast cancer	117<br>(114 to 121)	166<br>(160 to 172)	198<br>(185 to 213)	223.7<br>(217.8 to 230.4)	244.4<br>(236.6 to 252.4)	229.3<br>(214.8 to 245.2)
Central Asia	Cervical cancer	67.7<br>(65.7 to 70.6)	97.7<br>(94 to 102)	97.1<br>(90.1 to 105)	129.6<br>(125.8 to 134.9)	138.8<br>(133.8 to 144.5)	109.5<br>(101.8 to 118)
Central Asia	Uterine cancer	19.9<br>(19.1 to 20.7)	25.8<br>(24.7 to 27)	32.8<br>(30.4 to 35.1)	39.1<br>(37.6 to 40.7)	40.1<br>(38.4 to 42)	39.4<br>(36.7 to 42.1)
Central Asia	Ovarian cancer	26.9<br>(23.5 to 31.8)	42.2<br>(40.5 to 43.8)	51.4<br>(48.4 to 54.7)	51.1<br>(44.6 to 60.6)	62.5<br>(60.1 to 64.8)	60<br>(56.6 to 63.8)
Central Asia	Prostate cancer	22.2<br>(18.5 to 28.3)	33.8<br>(24.9 to 37.3)	44.2<br>(31.6 to 48.7)	48.4<br>(40.2 to 61.2)	61<br>(45 to 67.6)	67.1<br>(48 to 73.7)
Central Asia	Testicular cancer	5.72<br>(3.81 to 7.44)	6.55<br>(5.95 to 7.2)	5.26<br>(4.82 to 5.76)	9.1<br>(6.1 to 11.9)	8.6<br>(7.8 to 9.4)	5.8<br>(5.3 to 6.3)
Central Asia	Kidney cancer	30.1<br>(25.6 to 34.9)	39.2<br>(37.5 to 40.8)	52.3<br>(49.3 to 55.1)	53.4<br>(44.6 to 63.2)	60.2<br>(57.7 to 62.8)	62.4<br>(58.9 to 65.6)
Central Asia	Bladder cancer	24.2<br>(19.7 to 28.2)	29.3<br>(28.3 to 30.4)	33.2<br>(31.4 to 35.1)	49.1<br>(40.1 to 57.2)	49.4<br>(47.8 to 51.5)	44.6<br>(42.1 to 47.1)
Central Asia	Thyroid cancer	6.63<br>(6.17 to 7.5)	6.03<br>(5.73 to 6.33)	7.63<br>(7.09 to 8.23)	12.6<br>(11.7 to 14.3)	9.3<br>(8.8 to 9.7)	9.5<br>(8.8 to 10.2)
Central Asia	Mesothelioma	2.63<br>(2.39 to 3.03)	4.19<br>(3.77 to 4.47)	4.38<br>(4.16 to 4.58)	4.8<br>(4.4 to 5.6)	6.2<br>(5.6 to 6.6)	5.2<br>(5 to 5.5)
Central Asia	Hodgkin lymphoma	16.9<br>(13.1 to 19.7)	16.9<br>(14.3 to 20.1)	15.8<br>(13.2 to 18.3)	26.3<br>(20.4 to 30.7)	21.9<br>(18.6 to 26.1)	17.3<br>(14.5 to 20)
Central Asia	Non-Hodgkin's lymphoma	44<br>(41.3 to 46.4)	48.9<br>(47 to 50.7)	56.6<br>(53.4 to 59.9)	68.9<br>(64.1 to 72.6)	66.2<br>(63.7 to 68.7)	64.8<br>(61.3 to 68.4)
Central Asia	Multiple myeloma	8.24<br>(6.8 to 8.98)	9.72<br>(9.13 to 11.9)	13.2<br>(12.1 to 15.6)	16<br>(13.2 to 17.4)	15.3<br>(14.4 to 18.8)	16.2<br>(14.7 to 19.2)

SR Appendix Table 2. All-age DALYs counts and age standardized DALYs rates per 100,000 individuals for all causes and level 1 cause groups by location for 1990, 2007, and 2017, both sexes combined

		All-age DALYs			Age-standardized rate of DALYs per 100,000		
Central Asia	Leukaemia	139 (132 to 145)	138 (132 to 143)	134 (123 to 144)	192.6 (184.9 to 200.1)	179.2 (171.4 to 186.1)	151.5 (140.1 to 162.4)
Central Asia	Acute lymphoid leukaemia	35.8 (32.1 to 40.6)	34.3 (31 to 38.6)	35.3 (31.5 to 39.3)	45.5 (41 to 51)	42.7 (38.5 to 48.2)	39.4 (35.2 to 43.8)
Central Asia	Chronic lymphoid leukaemia	4.34 (3.91 to 4.68)	5.2 (4.65 to 5.7)	5.9 (5.44 to 6.51)	8.1 (7.3 to 8.8)	7.9 (7.1 to 8.8)	7.4 (6.8 to 8.1)
Central Asia	Acute myeloid leukaemia	30.9 (26.3 to 37.1)	35.5 (31.1 to 40.7)	40.4 (36.8 to 44.3)	44.1 (38.4 to 52.2)	45.3 (39.7 to 51.8)	44.9 (41 to 49.1)
Central Asia	Chronic myeloid leukaemia	7.39 (6.31 to 8.47)	7.6 (6.74 to 8.33)	6.69 (5.93 to 7.44)	12.8 (10.9 to 14.6)	10.6 (9.4 to 11.6)	7.6 (6.8 to 8.4)
Central Asia	Other leukaemia	60.1 (53.8 to 66.5)	55.4 (49.2 to 60.6)	45.4 (40.4 to 50.5)	82.2 (74.9 to 90)	72.6 (64.9 to 79.2)	52.2 (46.7 to 57.9)
Central Asia	Other malignant cancers	130 (104 to 143)	126 (121 to 136)	141 (133 to 153)	219 (170.9 to 241.8)	178.3 (171.5 to 192.3)	164.4 (156 to 178.4)
Central Asia	Other neoplasms	8.62 (5.64 to 12.4)	11.8 (8.34 to 14.9)	15.5 (11.6 to 18.5)	14.2 (9.6 to 19.7)	17.1 (12.1 to 21.4)	18.6 (13.8 to 22.1)
Central Asia	Myelodysplastic, myeloproliferative, and other hematopoietic neoplasms	6.76 (4.48 to 9.44)	9.39 (6.6 to 11.8)	12.5 (9.13 to 14.9)	11.7 (8 to 15.8)	14.1 (10 to 17.6)	15.3 (11.2 to 18.1)
Central Asia	Benign and in situ intestinal neoplasms	0 (0 to 0)	0 (0 to 0)	0 (0 to 0)	0 (0 to 0)	0 (0 to 0)	0 (0 to 0)
Central Asia	Benign and in situ cervical and uterine neoplasms	0 (0 to 0)	0 (0 to 0)	0 (0 to 0)	0 (0 to 0)	0 (0 to 0)	0 (0 to 0)
Central Asia	Other benign and in situ neoplasms	1.86 (1.16 to 3.11)	2.43 (1.72 to 3.27)	3.05 (2.33 to 3.79)	2.5 (1.6 to 4)	3.1 (2.2 to 4)	3.3 (2.5 to 4.1)
Central Asia	Cardiovascular diseases	4430 (4330 to 4530)	6780 (6650 to 6930)	6900 (6620 to 7180)	9220.7 (9015.3 to 9416.8)	11606.5 (11381.9 to 11840)	9580.2 (9211.4 to 9959.8)
Central Asia	Rheumatic heart disease	174 (166 to 183)	175 (165 to 187)	143 (130 to 158)	289.5 (277.1 to 303.6)	233.3 (220.8 to 248.7)	161 (147 to 177.6)
Central Asia	Ischaemic heart disease	2490 (2440 to 2540)	3900 (3830 to 3990)	4060 (3880 to 4260)	5289 (5180.9 to 5392.3)	6830 (6706.6 to 6975)	5762.4 (5516.4 to 6021)
Central Asia	Stroke	1400 (1350 to 1450)	1990 (1930 to 2050)	1930 (1830 to 2020)	2923.4 (2822.1 to 3026.9)	3435 (3329.8 to 3537)	2664.7 (2539.4 to 2789.3)
Central Asia	Ischaemic stroke	506 (472 to 540)	694 (655 to 731)	666 (619 to 713)	1104.1 (1031.2 to 1174.3)	1260.8 (1194.9 to 1325.6)	997.5 (930.6 to 1064.1)
Central Asia	Intracerebral hemorrhage	770 (749 to 790)	1140 (1110 to 1170)	1110 (1060 to 1150)	1578.5 (1535.4 to 1620.5)	1934.3 (1887.1 to 1977.4)	1472.8 (1409.4 to 1535.4)
Central Asia	Subarachnoid hemorrhage	125 (117 to 136)	155 (147 to 174)	158 (147 to 180)	240.8 (224.8 to 261.8)	240 (228 to 267)	194.5 (181.5 to 221.2)
Central Asia	Hypertensive heart disease	119 (108 to 152)	192 (159 to 210)	224 (185 to 249)	251.3 (227.2 to 321.9)	331.4 (275.7 to 366.3)	321.6 (262 to 353.5)
Central Asia	Non-rheumatic valvular heart disease	7.33 (6.33 to 8.47)	11.4 (10.1 to 12.7)	15.7 (12.7 to 17.7)	14.6 (12.5 to 17.2)	18.4 (16.1 to 20.9)	21.2 (17 to 24.2)
Central Asia	Non-rheumatic calcific aortic valve disease	2.84 (2.42 to 3.39)	5.06 (4.41 to 5.9)	8.16 (6.26 to 9.29)	5.8 (4.9 to 7.1)	8.4 (7.3 to 9.9)	11.2 (8.6 to 12.9)
Central Asia	Non-rheumatic degenerative mitral valve disease	3.22 (2.71 to 3.82)	4.39 (3.71 to 5.17)	5.26 (4.12 to 6.24)	6.5 (5.4 to 7.8)	7.1 (5.9 to 8.5)	7.1 (5.6 to 8.5)
Central Asia	Other non-rheumatic valve diseases	1.26 (0.849 to 1.5)	1.97 (1.35 to 2.3)	2.32 (1.62 to 2.88)	2.3 (1.5 to 2.8)	2.9 (2 to 3.4)	2.9 (2 to 3.6)
Central Asia	Cardiomyopathy and myocarditis	81.6 (75 to 101)	281 (184 to 299)	273 (202 to 304)	140.1 (127.8 to 175.1)	390.4 (259 to 414.7)	309.7 (230.5 to 342.9)
Central Asia	Myocarditis	14.1 (12.3 to 16.5)	28.8 (25.4 to 33.5)	31.2 (28.1 to 36.9)	22.2 (19.3 to 26.1)	38 (33.4 to 43.7)	34.7 (31.5 to 40.5)
Central Asia	Alcoholic cardiomyopathy	22.7 (18.7 to 31)	103 (49.2 to 126)	96.4 (48.4 to 120)	41.8 (34.4 to 57.1)	143.4 (69 to 174.2)	105.4 (52.9 to 130.1)
Central Asia	Other cardiomyopathy	44.8 (41 to 56.1)	148 (104 to 161)	145 (118 to 161)	76 (68.9 to 96.4)	209 (149.6 to 226.3)	169.6 (139.7 to 186.8)
Central Asia	Atrial fibrillation and flutter	33.1 (27.4 to 40.3)	42.1 (34.9 to 50.9)	57.7 (48.8 to 68.7)	77.3 (64.2 to 93.1)	84.2 (71.2 to 100.4)	93.7 (80.4 to 109.7)
Central Asia	Aortic aneurysm	15.1 (13.9 to 16.8)	25.9 (23.7 to 28.5)	31.8 (30.4 to 33.4)	30.1 (27.5 to 33.9)	42.2 (39 to 46)	42.2 (40.4 to 44.1)
Central Asia	Peripheral vascular disease	3.23 (1.78 to 5.3)	4.08 (2.33 to 6.46)	5.3 (3.16 to 8.15)	7.3 (4 to 11.9)	7.8 (4.5 to 12.4)	8.2 (4.8 to 12.7)
Central Asia	Endocarditis	7.6 (6.47 to 8.9)	11.9 (10.7 to 14.7)	13.7 (12.4 to 16.6)	11.6 (9.7 to 13.7)	16.2 (14.7 to 20)	15.9 (14.4 to 19.3)
Central Asia	Other cardiovascular and circulatory diseases	102 (90.8 to 115)	146 (134 to 162)	146 (132 to 162)	186.4 (162.7 to 210.7)	217.6 (200.5 to 239.4)	179.5 (162.8 to 199)
Central Asia	Chronic respiratory diseases	765 (711 to 822)	723 (667 to 784)	728 (658 to 803)	1483.4 (1388.1 to 1577.8)	1157 (1075.4 to 1242)	950.9 (865.7 to 1036.3)
Central Asia	Chronic obstructive pulmonary disease	502 (475 to 529)	487 (459 to 512)	470 (433 to 505)	1017.5 (962.4 to 1070.7)	812.5 (768.4 to 854.3)	641.6 (595.9 to 686.8)
Central Asia	Pneumoconiosis	2.11 (1.75 to 2.5)	1.85 (1.62 to 2.15)	1.81 (1.58 to 2.07)	4.2 (3.5 to 4.9)	3.1 (2.7 to 3.6)	2.4 (2.1 to 2.7)
Central Asia	Silicosis	0.126 (0.0869 to 0.233)	0.0857 (0.0646 to 0.121)	0.0742 (0.0566 to 0.0993)	0.2 (0.2 to 0.4)	0.1 (0.1 to 0.2)	0.1 (0.1 to 0.1)
Central Asia	Asbestosis	0.459 (0.217 to 0.66)	0.375 (0.199 to 0.501)	0.381 (0.231 to 0.486)	0.9 (0.4 to 1.2)	0.6 (0.3 to 0.8)	0.5 (0.3 to 0.6)
Central Asia	Coal workers pneumoconiosis	0.249 (0.143 to 0.52)	0.257 (0.166 to 0.507)	0.195 (0.136 to 0.34)	0.5 (0.3 to 0.9)	0.4 (0.2 to 0.8)	0.2 (0.2 to 0.4)
Central Asia	Other pneumoconiosis	1.28 (1.01 to 1.56)	1.14 (0.933 to 1.36)	1.16 (0.978 to 1.35)	2.6 (2.1 to 3.2)	1.9 (1.6 to 2.3)	1.6 (1.3 to 1.8)

SR Appendix Table 2. All-age DALYs counts and age standardized DALYs rates per 100,000 individuals for all causes and level 1 cause groups by location for 1990, 2007, and 2017, both sexes combined

		All-age DALYs			Age-standardized rate of DALYs per 100,000		
Central Asia	Asthma	184 (150 to 224)	155 (123 to 195)	165 (125 to 215)	322.2 (267.6 to 385.4)	226.4 (183 to 279.9)	197.7 (151.4 to 253.5)
Central Asia	Interstitial lung disease and pulmonary sarcoidosis	42.8 (33.9 to 50.5)	41.7 (35.4 to 49)	51.4 (43.8 to 59.9)	82.6 (66.2 to 96.8)	64.4 (55 to 74.9)	64.5 (55.6 to 74.4)
Central Asia	Other chronic respiratory diseases	34.6 (31.5 to 37.7)	37.5 (33.8 to 41.3)	39.8 (35.4 to 44.1)	57 (52.2 to 61.5)	50.6 (46.1 to 55.4)	44.8 (40.2 to 49.3)
Central Asia	Digestive diseases	854 (800 to 921)	1350 (1270 to 1430)	1530 (1390 to 1660)	1445.5 (1360.3 to 1556.7)	1905.5 (1802.7 to 2020.2)	1748.9 (1593 to 1897.9)
Central Asia	Cirrhosis and other chronic liver diseases	432 (421 to 449)	908 (881 to 943)	1050 (967 to 1120)	776.3 (757.6 to 807.3)	1291.4 (1253.2 to 1341.5)	1197.1 (1107.7 to 1275.3)
Central Asia	Cirrhosis and other chronic liver diseases due to hepatitis B	102 (90.5 to 115)	226 (199 to 253)	234 (203 to 269)	186.4 (165.2 to 210.7)	317.2 (281.1 to 354.6)	264.5 (230 to 301.1)
Central Asia	Cirrhosis and other chronic liver diseases due to hepatitis C	92.1 (80.9 to 105)	204 (178 to 234)	247 (212 to 285)	173.1 (152.6 to 198.5)	294.2 (256.3 to 334.2)	281.5 (242.7 to 323.3)
Central Asia	Cirrhosis and other chronic liver diseases due to alcohol use	136 (123 to 150)	302 (271 to 331)	370 (324 to 412)	258.7 (234.8 to 284.9)	440.6 (399.3 to 482.9)	423.4 (371.2 to 469.8)
Central Asia	Cirrhosis due to NASH	26.3 (23.3 to 29.2)	59.6 (53.1 to 66.5)	80.4 (69.3 to 91.7)	49.9 (44.3 to 55.4)	88.2 (78.9 to 97.8)	92.5 (80.5 to 104.7)
Central Asia	Cirrhosis and other chronic liver diseases due to other causes	75.6 (69 to 82.8)	116 (104 to 130)	117 (102 to 134)	108.2 (98.2 to 119.2)	151.2 (134.6 to 169.7)	135.2 (118.2 to 154.5)
Central Asia	Upper digestive system diseases	147 (115 to 194)	181 (139 to 242)	199 (150 to 271)	253.4 (200.8 to 331.1)	251.2 (196.4 to 332.1)	226.8 (173 to 308.4)
Central Asia	Peptic ulcer disease	70.6 (67.2 to 75.1)	86.6 (82 to 91.6)	85.1 (78.1 to 93)	128 (121.7 to 135.8)	126.1 (119.5 to 133.3)	100.9 (93 to 109.6)
Central Asia	Gastritis and duodenitis	22 (14.9 to 31)	25.4 (17 to 36.6)	30.2 (20.3 to 43.5)	33.6 (22.9 to 47.1)	33.1 (22.3 to 47.2)	33.8 (22.9 to 48.4)
Central Asia	GERD	54 (28.8 to 92.9)	68.7 (36.4 to 117)	83.3 (44.7 to 142)	91.9 (48.9 to 156.7)	92 (49.1 to 156.5)	92 (49.1 to 157.2)
Central Asia	Appendicitis	22.6 (20 to 24.7)	12.6 (11 to 14.4)	12 (10.2 to 13.9)	31.2 (28 to 33.7)	15.7 (13.8 to 17.7)	13.3 (11.4 to 15.4)
Central Asia	Paralytic ileus and intestinal obstruction	82.7 (68.7 to 90.2)	49.8 (45.7 to 55.5)	53.5 (42.5 to 61.5)	100.9 (85.3 to 109.5)	67 (61.4 to 74.8)	61.4 (48.6 to 70.4)
Central Asia	Inguinal, femoral, and abdominal hernia	65.9 (47.1 to 87.6)	73.4 (51.2 to 99)	81.5 (56.7 to 111)	101 (73.1 to 133.7)	101.6 (71.2 to 136.5)	92.9 (64.6 to 126.7)
Central Asia	Inflammatory bowel disease	12.7 (10.4 to 16.7)	11.4 (9.64 to 13.2)	13.5 (10.8 to 15.9)	19.7 (16.3 to 24.3)	15.7 (13.4 to 18.2)	15.2 (12.1 to 17.8)
Central Asia	Vascular intestinal disorders	9.64 (8.62 to 10.3)	11.6 (10.4 to 13)	12.9 (10.1 to 14)	19.8 (17.7 to 21.1)	19.7 (17.7 to 22)	18 (14.2 to 19.4)
Central Asia	Gallbladder and biliary diseases	16.9 (14.5 to 17.6)	16.6 (15.7 to 17.9)	16.4 (15.1 to 17.8)	32.6 (27.9 to 34)	26.4 (25.1 to 28.4)	21.2 (19.6 to 22.9)
Central Asia	Pancreatitis	38 (34.4 to 42.5)	57.3 (49.9 to 63.2)	56.9 (47 to 64.8)	69.6 (62.8 to 77.7)	80.2 (70.8 to 88.4)	64.1 (53.1 to 73)
Central Asia	Other digestive diseases	27.5 (23.2 to 31.8)	25.5 (22.5 to 28.7)	33.3 (29 to 38.3)	40.9 (35.4 to 46.7)	36.6 (32.5 to 41.1)	39.1 (34.3 to 44.7)
Central Asia	Neurological disorders	888 (679 to 1140)	1140 (887 to 1440)	1290 (987 to 1650)	1531.8 (1190.8 to 1931.8)	1634.1 (1313.1 to 2017.2)	1610 (1264.4 to 2005.9)
Central Asia	Alzheimer's disease and other dementias	159 (135 to 177)	182 (170 to 194)	231 (216 to 247)	411.8 (350.6 to 459.6)	430.8 (403.6 to 459.3)	438.8 (410.1 to 468.7)
Central Asia	Parkinson's disease	27.7 (24 to 32.9)	38.9 (35.5 to 43.5)	45.4 (41.2 to 50.7)	66.2 (57.3 to 78.9)	76.3 (69.8 to 84.7)	75.9 (69.3 to 84.4)
Central Asia	Epilepsy	197 (148 to 263)	280 (222 to 354)	280 (209 to 369)	274.3 (205.4 to 365.9)	339.5 (268.9 to 429.5)	303.6 (224.7 to 401.7)
Central Asia	Multiple sclerosis	8.78 (7.04 to 10.6)	10.6 (8.43 to 13.2)	12.9 (10.1 to 15.8)	16 (12.9 to 19.2)	14.6 (11.7 to 18)	14.3 (11.3 to 17.5)
Central Asia	Motor neuron disease	1.13 (0.978 to 1.3)	1.62 (1.21 to 1.85)	1.89 (1.56 to 2.12)	2 (1.7 to 2.3)	2.4 (1.8 to 2.7)	2.1 (1.8 to 2.4)
Central Asia	Headache disorders	462 (300 to 661)	582 (378 to 833)	668 (434 to 959)	715.9 (466.5 to 1027.3)	716.5 (468.8 to 1027)	715.8 (466.4 to 1026)
Central Asia	Migraine	401 (251 to 597)	506 (315 to 752)	581 (363 to 864)	621.3 (391.8 to 922.1)	621.8 (389.9 to 921.2)	621.1 (387.7 to 922.5)
Central Asia	Tension-type headache	60.6 (33.9 to 97.3)	75.3 (42.2 to 121)	86.8 (49.3 to 138)	94.7 (53.3 to 151.9)	94.7 (53.6 to 151.3)	94.7 (53.7 to 151)
Central Asia	Other neurological disorders	33 (24.2 to 44.5)	43.2 (32.3 to 56.3)	52.8 (38.5 to 71)	45.6 (34.2 to 60.7)	54 (41.1 to 69.4)	59.6 (44 to 79.5)
Central Asia	Mental disorders	857 (633 to 1110)	1050 (778 to 1360)	1200 (890 to 1550)	1327.7 (983.8 to 1715.5)	1321.7 (979.5 to 1701.8)	1317.4 (981.6 to 1706.1)
Central Asia	Schizophrenia	69.7 (51.9 to 86.8)	88.9 (66 to 111)	111 (82.5 to 139)	115.6 (86.3 to 143.3)	114.7 (85.6 to 142.7)	117.4 (87.4 to 147.1)
Central Asia	Depressive disorders	287 (205 to 391)	358 (253 to 486)	411 (290 to 561)	474.6 (336.7 to 646.5)	469.6 (333.8 to 636.2)	462.1 (326.5 to 628.5)
Central Asia	Major depressive disorder	223 (156 to 305)	277 (193 to 375)	314 (219 to 426)	366.8 (256.8 to 499.5)	361.9 (253 to 490.4)	354.6 (247.6 to 481.2)
Central Asia	Dysthymia	64.2 (42.6 to 93.3)	80.7 (53.4 to 118)	96.8 (64 to 143)	107.8 (71.7 to 156.3)	107.6 (71.5 to 156.1)	107.5 (71.7 to 156.2)
Central Asia	Bipolar disorder	85.1 (53.4 to 126)	108 (67.7 to 161)	122 (77.1 to 180)	131.6 (82.8 to 195.4)	131.7 (82.9 to 196.8)	131.6 (82.7 to 194.3)
Central Asia	Anxiety disorders	154 (109 to 207)	189 (133 to 253)	214 (151 to 285)	235.7 (168.5 to 315.3)	234.8 (167 to 312.9)	234.9 (165.9 to 313.4)
Central Asia	Eating disorders	23 (14.6 to 33.7)	26.6 (16.9 to 39.2)	34.4 (21.9 to 50.9)	32.3 (20.9 to 47.4)	30.1 (19.2 to 44.4)	35.4 (22.6 to 52.4)

SR Appendix Table 2. All-age DALYs counts and age standardized DALYs rates per 100,000 individuals for all causes and level 1 cause groups by location for 1990, 2007, and 2017, both sexes combined							
		All-age DALYs			Age-standardized rate of DALYs per 100,000		
Central Asia	Anorexia nervosa	4.82 (2.96 to 7.16)	5.62 (3.33 to 8.48)	6.74 (4.1 to 10.2)	6.5 (4 to 9.6)	6.1 (3.7 to 9.2)	7.1 (4.3 to 10.8)
Central Asia	Bulimia nervosa	18.1 (11.4 to 27)	21 (13.1 to 31.2)	27.6 (17.4 to 41.2)	25.8 (16.4 to 38.3)	23.9 (14.9 to 35.5)	28.3 (17.8 to 42.1)
Central Asia	Autism spectrum disorders	46 (31.4 to 63.6)	51.6 (35.2 to 71.1)	59 (40.2 to 81.6)	63.6 (43.3 to 87.8)	64 (43.6 to 88.1)	64.3 (43.8 to 88.8)
Central Asia	Attention-deficit/hyperactivity disorder	8.81 (5.36 to 14.1)	10.1 (6.13 to 16.1)	10.8 (6.53 to 17.4)	11.7 (7.1 to 18.7)	11.7 (7.1 to 18.6)	11.7 (7.1 to 18.9)
Central Asia	Conduct disorder	78.8 (47.3 to 125)	89.7 (53.6 to 141)	82.9 (50.3 to 132)	97.2 (58.3 to 153.9)	97.6 (58.1 to 156)	98 (59.4 to 156.3)
Central Asia	Idiopathic developmental intellectual disability	19.9 (6.94 to 37.3)	23.5 (8.63 to 44)	21.1 (6.89 to 40)	26.8 (9 to 50.4)	28.6 (10.5 to 53.7)	22.8 (7.4 to 43.2)
Central Asia	Other mental disorders	84.5 (56 to 117)	107 (70.5 to 150)	129 (85.8 to 180)	138.5 (92.1 to 190.3)	139 (91.3 to 193.1)	139.2 (92.8 to 193.5)
Central Asia	Substance use disorders	319 (254 to 391)	554 (461 to 656)	555 (457 to 670)	500.7 (405.1 to 609.4)	690.5 (577 to 814.6)	580.4 (478.7 to 699.8)
Central Asia	Alcohol use disorders	235 (190 to 290)	378 (312 to 454)	370 (300 to 454)	375.2 (305.7 to 461.4)	477 (396.6 to 570.5)	388.4 (316.2 to 476.1)
Central Asia	Drug use disorders	83.8 (63.2 to 105)	176 (147 to 208)	185 (150 to 221)	125.5 (95.5 to 156.8)	213.5 (179.3 to 250.5)	192 (156 to 228.8)
Central Asia	Opioid use disorders	57.3 (42.5 to 73.5)	127 (105 to 151)	137 (111 to 166)	87.3 (64.9 to 111)	155.5 (128.7 to 184.1)	141.9 (114.8 to 171.6)
Central Asia	Cocaine use disorders	3.06 (2.23 to 4.15)	7.06 (5.55 to 9.42)	7.07 (5.66 to 8.83)	4.6 (3.4 to 6.2)	8.6 (6.8 to 11.5)	7.4 (5.9 to 9.3)
Central Asia	Amphetamine use disorders	8.65 (5.21 to 13.7)	12.9 (8.39 to 19.2)	12.9 (8.22 to 19.6)	11.9 (7.3 to 18.5)	14.7 (9.7 to 21.5)	13.2 (8.4 to 20.1)
Central Asia	Cannabis use disorders	3.31 (2.05 to 4.87)	4.19 (2.62 to 6.21)	4.4 (2.76 to 6.52)	4.6 (2.9 to 6.8)	4.6 (2.9 to 6.8)	4.6 (2.9 to 6.9)
Central Asia	Other drug use disorders	11.5 (8.46 to 15.2)	24.9 (20.2 to 31)	23.6 (19.1 to 29.3)	17 (12.8 to 22.1)	30.1 (24.5 to 37.1)	24.8 (20.3 to 30.5)
Central Asia	Diabetes and kidney diseases	623 (522 to 747)	1070 (921 to 1250)	1310 (1100 to 1560)	1147.6 (951.1 to 1382.1)	1620.7 (1399.3 to 1893.9)	1583.1 (1334.3 to 1872.6)
Central Asia	Diabetes mellitus	405 (316 to 515)	694 (561 to 860)	933 (742 to 1160)	775.5 (606 to 984.8)	1081.8 (879.4 to 1328.2)	1130.8 (903.8 to 1398.1)
Central Asia	Diabetes mellitus type 1	81.8 (76 to 86.7)	131 (120 to 140)	141 (126 to 154)	148.2 (137.3 to 157.5)	193.7 (176 to 207.2)	164.9 (146.5 to 179.7)
Central Asia	Diabetes mellitus type 2	323 (236 to 432)	563 (435 to 724)	791 (608 to 1020)	627.4 (461.3 to 833.3)	888.1 (696.1 to 1132.1)	965.9 (750.5 to 1230.9)
Central Asia	Chronic kidney disease	206 (192 to 222)	366 (346 to 387)	372 (346 to 400)	355.2 (328.1 to 385)	527.3 (498.8 to 558)	446.1 (415.2 to 479.2)
Central Asia	Chronic kidney disease due to diabetes mellitus type 1	15.8 (12.7 to 19.1)	34.3 (27.7 to 41.9)	34.7 (27.9 to 42.4)	28 (22.3 to 34)	45.7 (36.9 to 55.3)	37.4 (30.2 to 45.7)
Central Asia	Chronic kidney disease due to diabetes mellitus type 2	26.1 (22.1 to 30.6)	52.4 (44.7 to 60.5)	59.8 (51.1 to 69.2)	52.6 (44.9 to 61)	88.4 (75.5 to 102)	79.2 (68.1 to 91.1)
Central Asia	Chronic kidney disease due to hypertension	18 (15.1 to 20.6)	33.1 (28.3 to 38)	35.7 (30.4 to 41.3)	35.1 (29.5 to 40.2)	53.4 (45.5 to 61.4)	47.3 (40.3 to 54.7)
Central Asia	Chronic kidney disease due to glomerulonephritis	59.1 (52.5 to 66)	108 (96.2 to 121)	105 (92.7 to 120)	94.6 (84.4 to 105.4)	144.9 (128.8 to 162.3)	118 (104.5 to 133.9)
Central Asia	Chronic kidney disease due to other and unspecified causes	87.5 (77.1 to 98.8)	137 (122 to 154)	137 (121 to 155)	145 (127 to 166.3)	194.9 (172.6 to 219.8)	164.2 (145.5 to 186.3)
Central Asia	Acute glomerulonephritis	11.4 (9.52 to 13.6)	9 (8.26 to 9.78)	5.6 (4.94 to 6.46)	16.9 (14.1 to 20)	11.6 (10.6 to 12.6)	6.1 (5.4 to 7.1)
Central Asia	Skin and subcutaneous diseases	319 (211 to 471)	354 (237 to 518)	409 (273 to 602)	448.5 (299.4 to 662)	450.7 (301.6 to 662.2)	456.7 (304.4 to 673.1)
Central Asia	Dermatitis	79.2 (46.5 to 128)	84.3 (50 to 133)	96.9 (57.2 to 154)	106.6 (63.2 to 169.3)	106.8 (63.1 to 169.2)	107.3 (63.5 to 169.9)
Central Asia	Atopic dermatitis	64.4 (34.7 to 109)	66 (35.3 to 110)	76.1 (40.8 to 128)	83.5 (45.1 to 140.7)	83.6 (45.2 to 139.6)	84.2 (45.1 to 141.5)
Central Asia	Contact dermatitis	14.8 (9.45 to 22.1)	18.2 (11.8 to 27.1)	20.7 (13.4 to 30.6)	23 (15 to 34.2)	23.1 (15.2 to 34)	23.1 (15 to 34.1)
Central Asia	Seborrhoeic dermatitis	0.0734 (0.0411 to 0.118)	0.0829 (0.0461 to 0.134)	0.0964 (0.0538 to 0.154)	0.1 (0.1 to 0.2)	0.1 (0.1 to 0.2)	0.1 (0.1 to 0.2)
Central Asia	Psoriasis	38.6 (27.5 to 51)	47 (33.2 to 62.2)	56.9 (40.1 to 74.6)	62.5 (44.5 to 82.5)	61.7 (44.1 to 81.6)	62.9 (44.3 to 82.1)
Central Asia	Bacterial skin diseases	2.41 (1.86 to 3.12)	3.7 (2.24 to 4.66)	4.93 (2.74 to 6.34)	3.4 (2.7 to 4.6)	5.1 (3.1 to 6.4)	5.7 (3.2 to 7.3)
Central Asia	Cellulitis	1.01 (0.732 to 1.42)	1.63 (1 to 2.12)	2.3 (1.27 to 3.09)	1.5 (1.1 to 2.2)	2.2 (1.3 to 2.8)	2.6 (1.4 to 3.5)
Central Asia	Pyoderma	1.4 (0.935 to 1.85)	2.06 (1.12 to 2.67)	2.63 (1.37 to 3.46)	1.9 (1.4 to 2.6)	2.9 (1.6 to 3.7)	3.1 (1.6 to 4.1)
Central Asia	Scabies	22.4 (12.4 to 36.7)	24.6 (13.7 to 40.5)	26.4 (14.5 to 42.9)	30.2 (16.8 to 49.3)	29.5 (16.5 to 48.1)	28.9 (16 to 47.1)
Central Asia	Fungal skin diseases	31.6 (12.6 to 65.6)	34.7 (14 to 72.2)	39.3 (15.7 to 81.7)	49.9 (20 to 104.3)	48.6 (19.5 to 101.3)	47 (18.8 to 97.3)
Central Asia	Viral skin diseases	37.6 (23.9 to 56.2)	36.4 (23.3 to 54.5)	41.7 (26.5 to 62.5)	45.7 (29 to 68.5)	45.8 (29.4 to 68.4)	45.8 (29.1 to 68.8)
Central Asia	Acne vulgaris	18.8 (11.1 to 31)	27.2 (15.7 to 45.3)	28.5 (16.8 to 47.1)	24.9 (14.6 to 40.5)	28.7 (16.6 to 47.6)	31.5 (18.5 to 52)
Central Asia	Alopecia areata	3.93 (2.51 to 5.86)	4.78 (3.03 to 7.15)	5.61 (3.59 to 8.43)	5.9 (3.8 to 8.8)	5.9 (3.7 to 8.8)	5.9 (3.8 to 8.9)

SR Appendix Table 2. All-age DALYs counts and age standardized DALYs rates per 100,000 individuals for all causes and level 1 cause groups by location for 1990, 2007, and 2017, both sexes combined

		All-age DALYs			Age-standardized rate of DALYs per 100,000		
Central Asia	Pruritus	5.91 (2.77 to 11.2)	6.94 (3.34 to 13.3)	8.39 (3.99 to 16.2)	9.4 (4.4 to 17.8)	9.3 (4.4 to 17.5)	9.5 (4.5 to 18)
Central Asia	Urticaria	56.7 (37.6 to 80.7)	59.3 (39.3 to 84.3)	68.4 (45.1 to 96.9)	74.6 (49.4 to 106.2)	74.6 (49.6 to 106.1)	74.6 (49.6 to 106.4)
Central Asia	Decubitus ulcer	1.23 (0.881 to 1.64)	1.56 (1.11 to 2.03)	1.97 (1.4 to 2.59)	2.3 (1.6 to 3.1)	2.4 (1.7 to 3.2)	2.6 (1.9 to 3.4)
Central Asia	Other skin and subcutaneous diseases	20.1 (9.71 to 37.1)	23.2 (11.2 to 42.5)	29.9 (14.4 to 54.7)	33.2 (16.1 to 61.4)	32.3 (15.6 to 59.3)	34.9 (16.8 to 63.8)
Central Asia	Sense organ diseases	458 (305 to 669)	546 (363 to 804)	658 (435 to 971)	863.2 (578.8 to 1254.5)	856.7 (572.5 to 1246.9)	841.2 (562.7 to 1224.1)
Central Asia	Blindness and vision impairment	218 (139 to 330)	257 (164 to 391)	305 (196 to 461)	407.6 (262.3 to 610.8)	400.6 (258 to 601.7)	388.4 (250.6 to 582.8)
Central Asia	Glaucoma	1.7 (1.12 to 2.36)	1.87 (1.24 to 2.62)	2.18 (1.43 to 3.1)	3.7 (2.5 to 5.2)	3.6 (2.4 to 5)	3.3 (2.2 to 4.6)
Central Asia	Cataract	66.6 (44.9 to 92.2)	79.2 (53.6 to 110)	96.9 (65.2 to 135)	148.4 (99.4 to 203.3)	146.8 (99.5 to 201.4)	143.1 (96.5 to 197.2)
Central Asia	Age-related macular degeneration	0.519 (0.335 to 0.768)	0.608 (0.386 to 0.909)	0.784 (0.504 to 1.19)	1.1 (0.7 to 1.7)	1.2 (0.7 to 1.7)	1.2 (0.8 to 1.7)
Central Asia	Refraction disorders	75.5 (47.6 to 116)	86.8 (54.4 to 133)	98.9 (62.5 to 152)	115.2 (73.7 to 175.7)	114.6 (72.7 to 175.9)	111.8 (71 to 171.4)
Central Asia	Near vision loss	61.6 (29.3 to 112)	74.3 (35.2 to 136)	89.8 (43 to 163)	117.6 (56 to 214.3)	113.2 (53.8 to 207.8)	108.4 (51.9 to 197.3)
Central Asia	Other vision loss	11.7 (7.76 to 16.4)	13.9 (9.03 to 19.8)	16.8 (11.1 to 23.7)	21.5 (14.3 to 30)	21.2 (14 to 29.9)	20.7 (13.7 to 29.2)
Central Asia	Age-related and other hearing loss	223 (153 to 315)	269 (183 to 383)	327 (221 to 467)	424.9 (292.7 to 595.4)	425.1 (292.6 to 596.5)	421.6 (286.7 to 594.9)
Central Asia	Other sense organ diseases	17.1 (10.7 to 25)	20.4 (12.6 to 29.7)	25.2 (15.7 to 37)	30.7 (19.2 to 44.7)	30.9 (19.2 to 45)	31.1 (19.6 to 45.5)
Central Asia	Musculoskeletal disorders	808 (589 to 1090)	1030 (753 to 1400)	1260 (912 to 1710)	1454.5 (1059.8 to 1950.3)	1463.7 (1067 to 1967.1)	1452.1 (1058.5 to 1951.1)
Central Asia	Rheumatoid arthritis	19.9 (15.3 to 25.1)	25.7 (19.8 to 32.1)	29.7 (22 to 38.3)	36.7 (28.3 to 46.2)	38 (29.4 to 47.1)	35.6 (26.4 to 45.4)
Central Asia	Osteoarthritis	37.4 (18.8 to 74.1)	47.6 (24 to 95.4)	64.4 (32.5 to 128)	77.5 (39.1 to 153.3)	80.1 (40.5 to 158)	84.2 (42.4 to 165.9)
Central Asia	Low back pain	560 (404 to 752)	703 (503 to 950)	850 (607 to 1150)	992.6 (711.9 to 1329.7)	985 (708.1 to 1322)	979.4 (704 to 1314)
Central Asia	Neck pain	161 (113 to 227)	212 (148 to 298)	261 (182 to 370)	298.1 (209.2 to 418.7)	298.2 (207.6 to 418.9)	298.1 (208.5 to 417.3)
Central Asia	Gout	6.32 (4.18 to 8.76)	8.37 (5.67 to 11.7)	11 (7.43 to 15.2)	12.6 (8.3 to 17.4)	13.3 (9 to 18.4)	13.8 (9.4 to 18.9)
Central Asia	Other musculoskeletal disorders	22.8 (13.9 to 34.9)	38.2 (24.4 to 57.6)	39.1 (23.1 to 61.2)	37.1 (22.6 to 56.2)	49.2 (31.4 to 72.6)	41.1 (24.5 to 63.2)
Central Asia	Other non-communicable diseases	1390 (1200 to 1610)	1440 (1220 to 1710)	1460 (1210 to 1770)	1838.9 (1556.9 to 2197.8)	1893.4 (1592.3 to 2253.7)	1645.9 (1360.3 to 1998.3)
Central Asia	Congenital anomalies	817 (745 to 920)	751 (647 to 831)	685 (587 to 791)	924.5 (839.5 to 1041)	953.1 (819.9 to 1056)	755.1 (646.4 to 872.1)
Central Asia	Neural tube defects	82.4 (69.8 to 97.7)	55.4 (46.3 to 64.9)	43.1 (35.1 to 53.5)	90.6 (77 to 107.3)	70.2 (58.8 to 82.3)	47.7 (38.8 to 59.2)
Central Asia	Congenital heart anomalies	335 (279 to 381)	313 (246 to 362)	271 (215 to 328)	364.5 (303.9 to 414.4)	396.8 (310.3 to 458.4)	298.4 (236.9 to 361.3)
Central Asia	Orofacial clefts	6.69 (4.22 to 10.1)	3.7 (2.75 to 4.95)	3.37 (2.31 to 4.66)	8 (5.2 to 11.8)	4.7 (3.5 to 6.2)	3.7 (2.5 to 5.1)
Central Asia	Down's syndrome	17.8 (11.6 to 26.3)	15.6 (12.2 to 18.2)	15.7 (12.7 to 18.8)	20.7 (14.1 to 29.9)	19.7 (15.4 to 23.1)	17.1 (13.8 to 20.4)
Central Asia	Turner syndrome	0.0779 (0.0382 to 0.13)	0.087 (0.0436 to 0.142)	0.0943 (0.0467 to 0.154)	0.1 (0.1 to 0.2)	0.1 (0.1 to 0.2)	0.1 (0 to 0.2)
Central Asia	Klinefelter syndrome	0.0191 (0.00906 to 0.0355)	0.0222 (0.0103 to 0.0415)	0.0251 (0.0116 to 0.0481)	0 (0 to 0)	0 (0 to 0)	0 (0 to 0)
Central Asia	Other chromosomal abnormalities	15.9 (11.1 to 28.7)	17.4 (13 to 27.8)	18.1 (13.6 to 27)	18.6 (13.3 to 32.4)	22 (16.4 to 34.7)	19.8 (14.9 to 29.5)
Central Asia	Congenital musculoskeletal and limb anomalies	81.2 (58 to 107)	87.6 (61.7 to 117)	97.5 (67.7 to 131)	112 (78.4 to 147.8)	111.1 (78.5 to 147.8)	108.4 (75.3 to 145.5)
Central Asia	Urogenital congenital anomalies	8.99 (6.58 to 15.1)	8.91 (6.94 to 13.1)	9.44 (7.1 to 13.1)	10.3 (7.6 to 16.9)	11.2 (8.7 to 16.4)	10.4 (7.8 to 14.5)
Central Asia	Digestive congenital anomalies	82.5 (61.9 to 112)	77.7 (65.8 to 93.5)	70.5 (57.4 to 85.6)	93.8 (71 to 124.8)	98.6 (83.4 to 118.6)	77.9 (63.4 to 94.6)
Central Asia	Other congenital anomalies	186 (150 to 242)	171 (140 to 205)	156 (133 to 188)	205.9 (166.4 to 265.8)	218.7 (178.6 to 262.5)	171.6 (145.7 to 206.6)
Central Asia	Urinary diseases and male infertility	92.6 (82.8 to 104)	134 (121 to 148)	157 (141 to 176)	159 (140.9 to 180.9)	190.8 (171.7 to 213.5)	185.4 (166.4 to 208.1)
Central Asia	Urinary tract infections	57.7 (55 to 59.9)	94.2 (91.1 to 97.4)	108 (102 to 114)	93.2 (88.5 to 96.9)	127.2 (123 to 131.3)	123 (116.8 to 129.6)
Central Asia	Urolithiasis	5.36 (3.59 to 7.38)	5.25 (4.2 to 6.48)	6.45 (5.02 to 8.24)	10.2 (6.6 to 14.4)	8 (6.4 to 10)	8.3 (6.4 to 10.9)
Central Asia	Benign prostatic hyperplasia	20 (12.8 to 28.5)	24.2 (15.4 to 34.8)	33.3 (21.3 to 47.6)	39.9 (25.5 to 56.6)	41.5 (26.3 to 59.5)	42.1 (27.2 to 60.4)
Central Asia	Male infertility	1.55 (0.632 to 3.26)	1.92 (0.775 to 4.04)	2.18 (0.89 to 4.57)	2.2 (0.9 to 4.7)	2.2 (0.9 to 4.7)	2.2 (0.9 to 4.6)
Central Asia	Other urinary diseases	8 (6.86 to 9.94)	8.06 (6.58 to 9.54)	7.73 (6.37 to 9.19)	13.5 (11.6 to 16.9)	11.9 (9.6 to 13.9)	9.8 (7.9 to 11.6)

SR Appendix Table 2. All-age DALYs counts and age standardized DALYs rates per 100,000 individuals for all causes and level 1 cause groups by location for 1990, 2007, and 2017, both sexes combined							
		All-age DALYs			Age-standardized rate of DALYs per 100,000		
Central Asia	Gynecological diseases	104 (73.3 to 144)	139 (97 to 193)	154 (106 to 218)	165.8 (115.6 to 229.6)	164.3 (113.9 to 229.3)	159.4 (110 to 225.6)
Central Asia	Uterine fibroids	17 (11.2 to 26)	23.9 (15 to 37.7)	24.6 (14.9 to 39.7)	31.4 (20.4 to 48.7)	30.3 (19.1 to 47.8)	26.3 (16 to 42.7)
Central Asia	Polycystic ovarian syndrome	3.86 (1.7 to 7.45)	5.03 (2.23 to 9.73)	5.68 (2.54 to 11)	5.8 (2.6 to 11.2)	5.8 (2.6 to 11.2)	5.8 (2.6 to 11.3)
Central Asia	Female infertility	1.56 (0.563 to 3.41)	1.69 (0.598 to 3.67)	2.29 (0.822 to 4.96)	2.2 (0.8 to 4.7)	2 (0.7 to 4.3)	2.3 (0.8 to 4.8)
Central Asia	Endometriosis	33.8 (22.4 to 49.1)	43.7 (28.9 to 62.8)	50 (33.4 to 72.5)	51 (34 to 73.5)	51 (33.7 to 73.6)	50.5 (33.7 to 72.9)
Central Asia	Genital prolapse	2.03 (1.02 to 3.73)	2.03 (1.03 to 3.74)	2.54 (1.25 to 4.78)	4 (2 to 7.3)	3.1 (1.6 to 5.7)	3 (1.5 to 5.7)
Central Asia	Premenstrual syndrome	33.6 (21.7 to 50.5)	45.3 (29 to 68.3)	48.4 (31.1 to 73)	51.5 (33.2 to 77.1)	51.7 (33 to 77.8)	50 (32.1 to 75.3)
Central Asia	Other gynecological diseases	12.4 (8.75 to 17.6)	17.3 (11.8 to 25.5)	20.8 (14 to 31.6)	20 (14 to 28.9)	20.5 (14 to 30.3)	21.5 (14.3 to 32.8)
Central Asia	Hemoglobinopathies and hemolytic anaemias	96.3 (74.1 to 124)	91.2 (70.4 to 121)	86.1 (66.7 to 113)	134.4 (102.9 to 173.5)	116.9 (90.2 to 154.4)	96 (74.4 to 126.8)
Central Asia	Thalassemias	16.5 (13 to 21.1)	13.5 (8.67 to 16.2)	11.9 (8.29 to 14.8)	18.1 (14.2 to 22.9)	17.6 (11.3 to 21.2)	13 (9.1 to 16.1)
Central Asia	Thalassaemias trait	28.2 (19.1 to 40.5)	26.7 (17.8 to 39.4)	26 (17.1 to 38.2)	39.2 (26.7 to 56.1)	33.8 (22.7 to 49.8)	28.8 (19 to 42.3)
Central Asia	Sickle cell disorders	1.57 (1.14 to 2.14)	1.32 (0.916 to 1.79)	1.2 (0.858 to 1.62)	1.7 (1.3 to 2.3)	1.7 (1.2 to 2.3)	1.3 (0.9 to 1.8)
Central Asia	Sickle cell trait	2.23 (1.43 to 3.27)	1.94 (1.23 to 2.88)	1.86 (1.2 to 2.81)	3 (2 to 4.5)	2.4 (1.5 to 3.6)	2 (1.3 to 3.1)
Central Asia	G6PD deficiency	3.09 (2.55 to 3.61)	5.21 (4.32 to 5.91)	5.62 (4.86 to 6.38)	4.4 (3.6 to 5.1)	6.1 (5.1 to 7)	6 (5.1 to 6.8)
Central Asia	G6PD trait	0.00268 (0.00183 to 0.00376)	0.00285 (0.00192 to 0.004)	0.0029 (0.002 to 0.00405)	0 (0 to 0)	0 (0 to 0)	0 (0 to 0)
Central Asia	Other hemoglobinopathies and hemolytic anaemias	44.7 (33.5 to 59.5)	42.6 (31.8 to 57.5)	39.4 (29.6 to 52.8)	68 (51.5 to 90)	55.2 (42 to 73.7)	44.8 (33.7 to 60.3)
Central Asia	Endocrine, metabolic, blood, and immune disorders	70.3 (55.2 to 89.2)	80.7 (62.4 to 102)	86.1 (66.1 to 109)	97.5 (76.8 to 124.8)	103.2 (80.1 to 129.5)	95.4 (72.9 to 120.2)
Central Asia	Oral disorders	178 (112 to 268)	223 (141 to 336)	270 (171 to 407)	326.8 (207.7 to 489)	334.2 (212.8 to 502.5)	329.6 (209.7 to 493.5)
Central Asia	Caries of deciduous teeth	3.11 (1.35 to 6.05)	2.52 (1.09 to 4.98)	3.14 (1.35 to 6.32)	3.5 (1.5 to 6.8)	3.4 (1.5 to 6.8)	3.4 (1.5 to 6.8)
Central Asia	Caries of permanent teeth	26.1 (11.3 to 49.1)	32.3 (14.2 to 61.1)	34.5 (15.1 to 65.2)	39.2 (17.1 to 73.9)	39.5 (17.4 to 74.7)	37.5 (16.4 to 70.9)
Central Asia	Periodontal disease	23.1 (9.14 to 47.9)	30.6 (12 to 65.5)	38.6 (15.2 to 82.3)	43 (16.9 to 89.3)	43.8 (17.2 to 92.1)	43.6 (17.2 to 92)
Central Asia	Edentulism and severe tooth loss	92.8 (61.7 to 132)	118 (78.2 to 167)	147 (97 to 209)	190.2 (127.4 to 269.4)	196.5 (130.8 to 277.5)	194.2 (129.5 to 274.7)
Central Asia	Other oral disorders	32.7 (20.5 to 48.7)	40.1 (25.1 to 59.6)	46.7 (29.5 to 69.4)	50.9 (32 to 75.3)	50.9 (32 to 75.5)	50.9 (32.1 to 75.3)
Central Asia	Sudden infant death syndrome	28.8 (17.4 to 52.3)	24.2 (17.1 to 38)	22.8 (17.5 to 31.5)	30.8 (18.5 to 55.8)	30.9 (21.8 to 48.5)	25.1 (19.2 to 34.6)
Central Asia	Injuries	3490 (3280 to 3720)	3350 (3140 to 3590)	3090 (2810 to 3410)	5059.9 (4726.4 to 5446)	4216.8 (3927.7 to 4544.2)	3352.7 (3035.7 to 3713.1)
Central Asia	Transport injuries	810 (777 to 844)	767 (729 to 806)	692 (640 to 748)	1192.3 (1139.8 to 1249.2)	951.7 (902.1 to 1003.4)	740.3 (683.8 to 801.3)
Central Asia	Road injuries	769 (739 to 800)	713 (679 to 749)	654 (606 to 706)	1133 (1084.3 to 1184.3)	886.3 (841.2 to 934.2)	699.8 (647.1 to 756.4)
Central Asia	Pedestrian road injuries	283 (269 to 299)	233 (219 to 255)	200 (183 to 218)	410.3 (385.6 to 434.3)	295.4 (277.1 to 321.7)	216.8 (199.3 to 236.9)
Central Asia	Cyclist road injuries	25.2 (21.1 to 29.6)	24.8 (20.5 to 29.9)	28.8 (23.1 to 35.6)	40.2 (33.3 to 47.6)	32.3 (26.6 to 39.1)	31.9 (25.5 to 39.6)
Central Asia	Motorcyclist road injuries	72.1 (65.8 to 78.1)	66.1 (61 to 73.5)	59.8 (54.3 to 65.9)	106.4 (96.9 to 115.8)	79.6 (73.1 to 88.6)	62.8 (56.8 to 69.3)
Central Asia	Motor vehicle road injuries	376 (358 to 393)	377 (347 to 399)	354 (329 to 380)	556 (528 to 585)	463.7 (427.8 to 490.9)	375.6 (349.1 to 404.2)
Central Asia	Other road injuries	12.9 (11.1 to 14.8)	11.9 (10.3 to 13.9)	11.4 (9.49 to 14)	20.1 (17.1 to 23.3)	15.3 (13.1 to 18)	12.6 (10.5 to 15.4)
Central Asia	Other transport injuries	40.5 (37.2 to 45.2)	53.2 (48.7 to 57.8)	37.8 (33.7 to 42.3)	59.3 (54.3 to 66.5)	65.4 (59.7 to 71.2)	40.5 (36 to 45.5)
Central Asia	Unintentional injuries	1980 (1820 to 2170)	1660 (1500 to 1860)	1620 (1400 to 1870)	2813 (2547.4 to 3131.6)	2154.1 (1930.2 to 2420.1)	1789.6 (1540.9 to 2078.4)
Central Asia	Falls	497 (406 to 609)	442 (351 to 557)	541 (418 to 695)	795.6 (638.9 to 990.8)	597 (473.7 to 754.5)	615.3 (474.5 to 792.3)
Central Asia	Drowning	524 (500 to 554)	329 (312 to 347)	255 (239 to 273)	656.3 (629.4 to 688.8)	411.7 (389.6 to 435.6)	274.7 (257.2 to 294)
Central Asia	Fire, heat, and hot substances	297 (268 to 326)	216 (192 to 242)	190 (162 to 222)	410.7 (370 to 454.1)	277.1 (247.1 to 311.9)	206 (176 to 241.1)
Central Asia	Poisonings	94.4 (86.1 to 100)	80.9 (76.2 to 88.6)	71 (63.4 to 84.2)	137 (125 to 145.2)	102.1 (96.1 to 111.9)	76.2 (68.2 to 90.1)
Central Asia	Poisoning by carbon monoxide	56.4 (52.4 to 59.1)	58.7 (56 to 63.8)	47.4 (43.2 to 56.4)	85.9 (79.8 to 89.7)	73.8 (70.5 to 80.1)	50.5 (46.1 to 59.8)
Central Asia	Poisoning by other means	38 (33.1 to 42.4)	22.2 (19.2 to 25.9)	23.6 (18.8 to 29.4)	51.2 (44.2 to 57.4)	28.3 (24.6 to 32.9)	25.7 (20.5 to 32)

SR Appendix Table 2. All-age DALYs counts and age standardized DALYs rates per 100,000 individuals for all causes and level 1 cause groups by location for 1990, 2007, and 2017, both sexes combined

		All-age DALYs			Age-standardized rate of DALYs per 100,000		
Central Asia	Exposure to mechanical forces	136 (115 to 161)	144 (121 to 172)	151 (122 to 190)	210.7 (176.3 to 254.2)	186.8 (156.5 to 225.1)	166.7 (133.2 to 210.3)
Central Asia	Unintentional firearm injuries	35.6 (32.6 to 39.2)	38.6 (34.8 to 41)	30.4 (27.7 to 33.2)	52.6 (48.2 to 58.1)	48.5 (43.6 to 51.5)	32.8 (30 to 35.9)
Central Asia	Other exposure to mechanical forces	100 (80.3 to 123)	105 (84.2 to 131)	121 (92.6 to 158)	158.1 (124.7 to 197.8)	138.3 (110.1 to 174.8)	133.9 (101.9 to 176)
Central Asia	Adverse effects of medical treatment	35.6 (29.9 to 40)	36.6 (30 to 39.8)	38.2 (33.2 to 42.9)	49.4 (41.7 to 54.1)	48 (39.4 to 52.2)	42.5 (37 to 47.7)
Central Asia	Animal contact	14 (11.3 to 17)	14.5 (11.6 to 18)	14.8 (11.7 to 18.5)	19.9 (15.9 to 24.3)	18.6 (14.8 to 23)	16.3 (12.9 to 20.4)
Central Asia	Venomous animal contact	7.89 (5.95 to 10.2)	8.32 (6.19 to 11)	8.84 (6.43 to 11.8)	11.3 (8.5 to 14.6)	10.5 (7.9 to 13.8)	9.7 (7.1 to 12.9)
Central Asia	Non-venomous animal contact	6.11 (5.01 to 7.38)	6.14 (5.01 to 7.52)	5.98 (4.89 to 7.39)	8.6 (7 to 10.4)	8 (6.5 to 9.8)	6.6 (5.4 to 8.3)
Central Asia	Foreign body	185 (172 to 199)	170 (160 to 180)	142 (131 to 154)	226.7 (212.7 to 243.3)	217.5 (205.8 to 230.8)	154.8 (142.6 to 168.5)
Central Asia	Pulmonary aspiration and foreign body in airway	152 (141 to 167)	133 (125 to 142)	106 (97 to 117)	179.6 (167.5 to 195.4)	170.5 (160.3 to 182.1)	114.6 (105.3 to 126.6)
Central Asia	Foreign body in eyes	1.3 (0.588 to 2.31)	1.69 (0.764 to 3)	2.05 (0.933 to 3.6)	2 (0.9 to 3.5)	2.2 (1 to 3.8)	2.3 (1.1 to 4)
Central Asia	Foreign body in other body part	31 (27.1 to 35.6)	35.2 (31.5 to 39.6)	34.4 (30.2 to 39.1)	45.1 (39.9 to 51.3)	44.8 (40.1 to 50.5)	37.9 (33.2 to 43.1)
Central Asia	Environmental heat and cold exposure	89.8 (78.9 to 104)	115 (103 to 126)	103 (90.5 to 117)	145.8 (128 to 169.2)	150.8 (136.1 to 166.4)	112.8 (98.3 to 127.5)
Central Asia	Exposure to forces of nature	9.18 (7.25 to 11.9)	7.71 (6.48 to 9.38)	6.38 (5.47 to 7.53)	13.4 (10.5 to 17.4)	9.9 (8.3 to 12.1)	7.1 (6.1 to 8.4)
Central Asia	Other unintentional injuries	102 (91.4 to 116)	107 (95.4 to 122)	107 (89.9 to 130)	147.5 (130 to 170.3)	134.8 (118.9 to 154.7)	117.2 (98.2 to 142.4)
Central Asia	Self-harm and interpersonal violence	692 (670 to 710)	922 (894 to 953)	779 (736 to 825)	1054.7 (1023.1 to 1082.9)	1111.1 (1076.6 to 1148.7)	822.8 (778.3 to 869.8)
Central Asia	Self-harm	338 (330 to 346)	595 (573 to 612)	509 (478 to 541)	521.9 (511 to 533.9)	712.1 (685.9 to 732.9)	538.3 (505.4 to 571.9)
Central Asia	Self-harm by firearm	10.8 (8.54 to 16)	13.7 (9.93 to 19.9)	11.4 (8.65 to 15.6)	17.4 (13.9 to 25.5)	17.2 (12.5 to 24.8)	12.1 (9.2 to 16.5)
Central Asia	Self-harm by other specified means	327 (319 to 335)	581 (560 to 598)	498 (467 to 530)	504.5 (492.7 to 516.5)	695 (670.4 to 715.6)	526.2 (494.4 to 560)
Central Asia	Interpersonal violence	326 (304 to 341)	294 (280 to 319)	244 (225 to 274)	492.7 (460 to 516.5)	359.2 (341.1 to 389.1)	256.5 (236.4 to 287.8)
Central Asia	Assault by firearm	48.3 (31.5 to 56.5)	35 (29.4 to 39.4)	26.7 (19.6 to 31.2)	69.6 (46.6 to 80.5)	40.7 (34.6 to 45.7)	27.7 (20.5 to 32.2)
Central Asia	Assault by sharp object	134 (122 to 147)	132 (121 to 150)	103 (93.9 to 122)	202.8 (185.5 to 221)	159 (146.7 to 181.4)	107.2 (97.9 to 127.3)
Central Asia	Sexual violence	5.49 (3.69 to 8.01)	6.85 (4.64 to 9.96)	7.96 (5.38 to 11.6)	8.1 (5.4 to 11.8)	8.1 (5.5 to 11.9)	8.4 (5.7 to 12.3)
Central Asia	Assault by other means	138 (128 to 151)	121 (105 to 138)	106 (93.1 to 124)	212.3 (195.6 to 232.2)	151.3 (132.7 to 172.8)	113.1 (99.3 to 132.3)
Central Asia	Conflict and terrorism	24.7 (24.5 to 25)	28.7 (20.2 to 42.2)	22.6 (15.4 to 34.1)	34.5 (34.2 to 35)	34.5 (24.3 to 50.5)	23.7 (16.2 to 35.7)
Central Asia	Executions and police conflict	3.71 (2.69 to 5.39)	4.42 (3.39 to 6.03)	4.15 (3 to 5.88)	5.5 (4 to 7.9)	5.2 (4.1 to 7.1)	4.3 (3.1 to 6.1)
Armenia	All causes	1160 (1070 to 1270)	954 (865 to 1060)	911 (820 to 1020)	36927.8 (33933.8 to 40260.9)	27813.1 (25213 to 30932.5)	25136.1 (22552.8 to 28218.3)
Armenia	Communicable, maternal, neonatal, and nutritional disorders	284 (255 to 316)	80.4 (70.8 to 91.8)	63.9 (54.8 to 74.8)	7902.2 (7085.2 to 8786.3)	3161.4 (2810.2 to 3566.6)	2553.8 (2191.4 to 2966.9)
Armenia	HIV/AIDS and sexually transmitted infections	1.57 (0.99 to 2.47)	1.36 (0.968 to 2.03)	2.16 (1.75 to 2.83)	45.6 (29.3 to 71.8)	43.8 (30.8 to 64.1)	69.1 (56 to 90.4)
Armenia	HIV/AIDS	0.0621 (0.0591 to 0.0653)	0.449 (0.42 to 0.485)	1.37 (1.27 to 1.5)	1.8 (1.7 to 1.9)	14.7 (13.7 to 15.8)	43.1 (40 to 47.2)
Armenia	HIV/AIDS - Drug-susceptible Tuberculosis	0.0297 (0.0279 to 0.0315)	0.104 (0.0886 to 0.118)	0.21 (0.0883 to 0.382)	0.9 (0.8 to 0.9)	3.4 (2.9 to 3.9)	6.7 (2.8 to 12.1)
Armenia	HIV/AIDS - Multidrug-resistant Tuberculosis without extensive drug resistance	0.0000781 (0.0000113 to 0.000294)	0.0652 (0.0539 to 0.0764)	0.1 (0.0322 to 0.201)	0 (0 to 0)	2.1 (1.8 to 2.5)	3.2 (1 to 6.4)
Armenia	HIV/AIDS - Extensively drug-resistant Tuberculosis	0 (0 to 0)	0.0205 (0.0169 to 0.0244)	0.0573 (0.0181 to 0.116)	0 (0 to 0)	0.7 (0.6 to 0.8)	1.8 (0.6 to 3.7)
Armenia	HIV/AIDS resulting in other diseases	0.0324 (0.0309 to 0.034)	0.26 (0.237 to 0.291)	1 (0.807 to 1.19)	0.9 (0.9 to 1)	8.5 (7.7 to 9.4)	31.4 (25.4 to 37.1)
Armenia	Sexually transmitted infections excluding HIV	1.51 (0.929 to 2.41)	0.915 (0.523 to 1.59)	0.788 (0.435 to 1.42)	43.8 (27.5 to 70)	29.1 (16.2 to 50)	26 (14.3 to 46.3)
Armenia	Syphilis	0.623 (0.317 to 1.31)	0.155 (0.0578 to 0.371)	0.0866 (0.0308 to 0.232)	18.1 (9.5 to 37.1)	7.3 (2.4 to 18.2)	4.3 (1.3 to 12.2)
Armenia	Chlamydial infection	0.198 (0.118 to 0.359)	0.179 (0.0986 to 0.338)	0.167 (0.0936 to 0.306)	5.7 (3.4 to 10.2)	5.2 (2.9 to 9.8)	5.2 (2.9 to 9.7)
Armenia	Gonococcal infection	0.212 (0.139 to 0.356)	0.14 (0.0747 to 0.26)	0.119 (0.0709 to 0.209)	6.2 (4.1 to 10.2)	4 (2.1 to 7.4)	4 (2.3 to 7.2)
Armenia	Trichomoniasis	0.141 (0.0565 to 0.305)	0.147 (0.0592 to 0.314)	0.137 (0.0554 to 0.292)	4.1 (1.7 to 8.8)	4.2 (1.7 to 8.9)	4.1 (1.6 to 8.7)
Armenia	Genital herpes	0.0858 (0.0279 to 0.205)	0.0935 (0.0302 to 0.223)	0.0946 (0.0304 to 0.226)	2.6 (0.8 to 6.3)	2.6 (0.8 to 6.2)	2.6 (0.8 to 6.2)
Armenia	Other sexually transmitted infections	0.245 (0.157 to 0.401)	0.202 (0.119 to 0.347)	0.183 (0.112 to 0.308)	7.1 (4.6 to 11.5)	5.9 (3.5 to 10.1)	5.8 (3.5 to 10)

SR Appendix Table 2. All-age DALYs counts and age standardized DALYs rates per 100,000 individuals for all causes and level 1 cause groups by location for 1990, 2007, and 2017, both sexes combined							
		All-age DALYs			Age-standardized rate of DALYs per 100,000		
Armenia	Respiratory infections and tuberculosis	99.8<br>(90.8 to 109)	25.9<br>(23.9 to 28.3)	20.3<br>(18.6 to 22.3)	2764.4<br>(2520.3 to 3014)	954.2<br>(872.5 to 1056.9)	743.6<br>(670.4 to 821.8)
Armenia	Tuberculosis	5.48<br>(4.75 to 6.19)	5.85<br>(5.47 to 6.23)	2.77<br>(2.53 to 3.04)	162.7<br>(142.2 to 182.2)	167.3<br>(156.3 to 178.2)	76.9<br>(70.1 to 84.1)
Armenia	Latent tuberculosis infection	0<br>(0 to 0)	0<br>(0 to 0)	0<br>(0 to 0)	0<br>(0 to 0)	0<br>(0 to 0)	0<br>(0 to 0)
Armenia	Drug-susceptible tuberculosis	5.46<br>(4.73 to 6.18)	3.55<br>(3.14 to 3.96)	1.75<br>(0.974 to 2.49)	162.4<br>(142 to 181.5)	101.5<br>(89.7 to 113.2)	48.5<br>(27.2 to 69.5)
Armenia	Multidrug-resistant tuberculosis without extensive drug resistance	0.0124<br>(0.00184 to 0.0473)	1.75<br>(1.48 to 2.06)	0.657<br>(0.219 to 1.18)	0.4<br>(0.1 to 1.4)	50.1<br>(42.3 to 58.9)	18.2<br>(6 to 32.5)
Armenia	Extensively drug-resistant tuberculosis	0<br>(0 to 0)	0.552<br>(0.463 to 0.654)	0.369<br>(0.123 to 0.662)	0<br>(0 to 0)	15.8<br>(13.2 to 18.6)	10.2<br>(3.4 to 18.3)
Armenia	Lower respiratory infections	89.4<br>(80.6 to 97.9)	16.8<br>(15.4 to 18.5)	14.8<br>(13.6 to 16)	2463.6<br>(2219.5 to 2696.2)	681.2<br>(613.1 to 765.9)	569.9<br>(514.2 to 631.1)
Armenia	Upper respiratory infections	3.89<br>(2.45 to 5.7)	2.36<br>(1.46 to 3.64)	2.08<br>(1.24 to 3.33)	109.9<br>(69.5 to 160.8)	77.9<br>(49.2 to 118.4)	71.3<br>(42.8 to 113.7)
Armenia	Otitis media	0.985<br>(0.583 to 1.56)	0.827<br>(0.494 to 1.32)	0.698<br>(0.429 to 1.09)	28.1<br>(16.8 to 44.5)	27.8<br>(16.6 to 44.7)	25.5<br>(15.5 to 40.3)
Armenia	Enteric infections	34.9<br>(30.3 to 40)	4.72<br>(3.66 to 6.01)	3.91<br>(2.92 to 5.09)	956.9<br>(831.5 to 1094.6)	183.9<br>(144.2 to 231.3)	149.3<br>(111.9 to 193.7)
Armenia	Diarrhoeal diseases	34.7<br>(30 to 39.7)	4.58<br>(3.51 to 5.89)	3.79<br>(2.8 to 4.99)	949.1<br>(820.8 to 1084)	178.7<br>(140 to 225.6)	144.8<br>(107.8 to 189.1)
Armenia	Typhoid and paratyphoid	0.00214<br>(0.00113 to 0.00369)	0.00159<br>(0.000845 to 0.00277)	0.00131<br>(0.000691 to 0.00225)	0.1<br>(0 to 0.1)	0<br>(0 to 0.1)	0<br>(0 to 0.1)
Armenia	Typhoid fever	0.0021<br>(0.00111 to 0.0036)	0.00145<br>(0.000776 to 0.00253)	0.00117<br>(0.00061 to 0.002)	0.1<br>(0 to 0.1)	0<br>(0 to 0.1)	0<br>(0 to 0.1)
Armenia	Paratyphoid fever	0.0000408<br>(0.0000161 to 0.0000834)	0.000141<br>(0.0000581 to 0.00029)	0.000147<br>(0.000058 to 0.000303)	0<br>(0 to 0)	0<br>(0 to 0)	0<br>(0 to 0)
Armenia	Invasive non-typhoidal salmonella	0.237<br>(0.128 to 0.395)	0.133<br>(0.0704 to 0.229)	0.111<br>(0.058 to 0.194)	6.7<br>(3.7 to 11.2)	4.9<br>(2.6 to 8.6)	4.3<br>(2.2 to 7.9)
Armenia	Other intestinal infectious diseases	0.0374<br>(0.00634 to 0.0866)	0.0075<br>(0.00325 to 0.0135)	0.0049<br>(0.00247 to 0.008)	1<br>(0.2 to 2.4)	0.3<br>(0.1 to 0.6)	0.2<br>(0.1 to 0.3)
Armenia	Neglected tropical diseases and malaria	1.8<br>(1.15 to 2.72)	1.41<br>(0.83 to 2.22)	1.24<br>(0.705 to 2.23)	52.3<br>(33.7 to 77.6)	49.6<br>(29.5 to 80)	44.1<br>(24.8 to 81.6)
Armenia	Malaria	0.0599<br>(0.0437 to 0.0784)	0.00351<br>(0.00217 to 0.00536)	0<br>(0 to 0)	1.7<br>(1.3 to 2.3)	0.1<br>(0.1 to 0.2)	0<br>(0 to 0)
Armenia	Chagas disease	0<br>(0 to 0)	0<br>(0 to 0)	0<br>(0 to 0)	0<br>(0 to 0)	0<br>(0 to 0)	0<br>(0 to 0)
Armenia	Leishmaniasis	0.0565<br>(0.000149 to 0.512)	0.0563<br>(0.000129 to 0.557)	0.0906<br>(0.000332 to 0.888)	1.6<br>(0 to 14.1)	2.1<br>(0 to 21.4)	3.5<br>(0 to 36.5)
Armenia	Visceral leishmaniasis	0.0565<br>(0.000148 to 0.511)	0.0563<br>(0.000129 to 0.557)	0.0905<br>(0.000332 to 0.888)	1.6<br>(0 to 14.1)	2.1<br>(0 to 21.4)	3.5<br>(0 to 36.5)
Armenia	Cutaneous and mucocutaneous leishmaniasis	0.0000236<br>(0.000000000000000000000<br>000000000000562 to	0.0000121<br>(0 to 0.0000838)	0.0000036<br>(0 to 0.000000000148)	0<br>(0 to 0)	0<br>(0 to 0)	0<br>(0 to 0)
Armenia	African trypanosomiasis	0<br>(0 to 0)	0<br>(0 to 0)	0<br>(0 to 0)	0<br>(0 to 0)	0<br>(0 to 0)	0<br>(0 to 0)
Armenia	Schistosomiasis	0<br>(0 to 0)	0<br>(0 to 0)	0<br>(0 to 0)	0<br>(0 to 0)	0<br>(0 to 0)	0<br>(0 to 0)
Armenia	Cysticercosis	0.00241<br>(0.00134 to 0.00373)	0.000835<br>(0.000426 to 0.00131)	0.000545<br>(0.00028 to 0.000847)	0.1<br>(0 to 0.1)	0<br>(0 to 0)	0<br>(0 to 0)
Armenia	Cystic echinococcosis	0.542<br>(0.227 to 1.09)	0.55<br>(0.226 to 1.08)	0.534<br>(0.224 to 1.04)	16.1<br>(6.9 to 31.6)	15.7<br>(6.4 to 31)	15.5<br>(6.4 to 30.8)
Armenia	Lymphatic filariasis	0<br>(0 to 0)	0<br>(0 to 0)	0<br>(0 to 0)	0<br>(0 to 0)	0<br>(0 to 0)	0<br>(0 to 0)
Armenia	Onchocerciasis	0<br>(0 to 0)	0<br>(0 to 0)	0<br>(0 to 0)	0<br>(0 to 0)	0<br>(0 to 0)	0<br>(0 to 0)
Armenia	Trachoma	0<br>(0 to 0)	0<br>(0 to 0)	0<br>(0 to 0)	0<br>(0 to 0)	0<br>(0 to 0)	0<br>(0 to 0)
Armenia	Dengue	0<br>(0 to 0)	0<br>(0 to 0)	0<br>(0 to 0)	0<br>(0 to 0)	0<br>(0 to 0)	0<br>(0 to 0)
Armenia	Yellow fever	0<br>(0 to 0)	0<br>(0 to 0)	0<br>(0 to 0)	0<br>(0 to 0)	0<br>(0 to 0)	0<br>(0 to 0)
Armenia	Rabies	0.086<br>(0.0549 to 0.133)	0.0445<br>(0.03 to 0.0638)	0.00863<br>(0.00526 to 0.0139)	3.3<br>(2.1 to 5.1)	1.4<br>(0.9 to 2.1)	0.2<br>(0.1 to 0.4)
Armenia	Intestinal nematode infections	0.0705<br>(0.0358 to 0.125)	0.0605<br>(0.0325 to 0.102)	0.039<br>(0.0204 to 0.0663)	2.1<br>(1 to 3.6)	1.9<br>(1 to 3.2)	1.3<br>(0.7 to 2.2)
Armenia	Ascariasis	0.00322<br>(0.000669 to 0.00777)	0.000608<br>(0.000238 to 0.0013)	0.000474<br>(0.000176 to 0.00104)	0.1<br>(0 to 0.2)	0<br>(0 to 0.1)	0<br>(0 to 0)
Armenia	Trichuriasis	0.000000001<br>(0.000000000435 to<br>0.00000000207)	0.000000000329<br>(0.000000000146 to<br>0.000000000638)	0.000000000292<br>(0.000000000126 to<br>0.000000000594)	0<br>(0 to 0)	0<br>(0 to 0)	0<br>(0 to 0)
Armenia	Hookworm disease	0.0672<br>(0.033 to 0.121)	0.0599<br>(0.0317 to 0.101)	0.0385<br>(0.0197 to 0.0658)	2<br>(1 to 3.5)	1.9<br>(1 to 3.1)	1.3<br>(0.6 to 2.2)
Armenia	Food-borne trematodiases	0<br>(0 to 0)	0<br>(0 to 0)	0<br>(0 to 0)	0<br>(0 to 0)	0<br>(0 to 0)	0<br>(0 to 0)
Armenia	Leprosy	0<br>(0 to 0)	0<br>(0 to 0)	0<br>(0 to 0)	0<br>(0 to 0)	0<br>(0 to 0)	0<br>(0 to 0)
Armenia	Ebola virus disease	0<br>(0 to 0)	0<br>(0 to 0)	0<br>(0 to 0)	0<br>(0 to 0)	0<br>(0 to 0)	0<br>(0 to 0)
Armenia	Zika virus disease	0<br>(0 to 0)	0<br>(0 to 0)	0<br>(0 to 0)	0<br>(0 to 0)	0<br>(0 to 0)	0<br>(0 to 0)

SR Appendix Table 2. All-age DALYs counts and age standardized DALYs rates per 100,000 individuals for all causes and level 1 cause groups by location for 1990, 2007, and 2017, both sexes combined

		All-age DALYs			Age-standardized rate of DALYs per 100,000		
Armenia	Guinea worm disease	0 (0 to 0)	0 (0 to 0)	0 (0 to 0)	0 (0 to 0)	0 (0 to 0)	0 (0 to 0)
Armenia	Other neglected tropical diseases	0.984 (0.614 to 1.48)	0.693 (0.427 to 1.06)	0.568 (0.344 to 0.888)	27.5 (17.4 to 41.1)	28.5 (17.4 to 44)	23.6 (14 to 37.2)
Armenia	Other infectious diseases	5.21 (4.41 to 6.17)	3.58 (3.06 to 4.21)	2.89 (2.45 to 3.42)	147.9 (125 to 174.5)	123.8 (105 to 147.5)	105 (88.5 to 125.5)
Armenia	Meningitis	2.22 (1.98 to 2.5)	1.2 (1.02 to 1.42)	0.746 (0.615 to 0.898)	62.9 (56 to 70.6)	40 (34 to 47.4)	25.7 (21.4 to 30.9)
Armenia	Pneumococcal meningitis	0.283 (0.204 to 0.371)	0.261 (0.182 to 0.356)	0.0992 (0.0709 to 0.133)	8.2 (5.9 to 10.7)	8 (5.6 to 10.9)	3.2 (2.3 to 4.3)
Armenia	H influenzae type B meningitis	0.858 (0.756 to 0.964)	0.377 (0.332 to 0.436)	0.184 (0.164 to 0.231)	24.1 (21.3 to 27)	13.1 (11.4 to 15.3)	6.7 (5.9 to 8.2)
Armenia	Meningococcal infection	0.292 (0.243 to 0.35)	0.171 (0.137 to 0.211)	0.113 (0.0887 to 0.143)	8.3 (6.9 to 9.9)	5.6 (4.5 to 6.8)	3.8 (3 to 4.7)
Armenia	Other meningitis	0.791 (0.702 to 0.893)	0.395 (0.345 to 0.453)	0.35 (0.282 to 0.432)	22.3 (19.9 to 25.1)	13.3 (11.6 to 15.4)	12 (9.7 to 14.9)
Armenia	Encephalitis	0.881 (0.692 to 1.27)	0.719 (0.634 to 0.923)	0.766 (0.676 to 0.887)	25 (19.9 to 35.4)	25.5 (22 to 35.2)	28 (24.3 to 34.3)
Armenia	Diphtheria	0.00087 (0.000449 to 0.00168)	0.000272 (0.000194 to 0.000459)	0.000384 (0.000254 to 0.000626)	0 (0 to 0)	0 (0 to 0)	0 (0 to 0)
Armenia	Whooping cough	0.0824 (0.047 to 0.147)	0.0224 (0.0126 to 0.0425)	0.0248 (0.0138 to 0.0407)	2.2 (1.3 to 3.9)	1.1 (0.6 to 2.1)	1.2 (0.7 to 2)
Armenia	Tetanus	0.0326 (0.0238 to 0.0434)	0.0116 (0.00363 to 0.0165)	0.00277 (0.00201 to 0.00359)	0.9 (0.7 to 1.2)	0.3 (0.1 to 0.5)	0.1 (0.1 to 0.1)
Armenia	Measles	0.00699 (0.00457 to 0.00838)	0.00461 (0.00301 to 0.00629)	0.0017 (0.000952 to 0.0024)	0.2 (0.1 to 0.2)	0.2 (0.1 to 0.3)	0.1 (0 to 0.1)
Armenia	Varicella and herpes zoster	0.107 (0.0697 to 0.156)	0.101 (0.0628 to 0.148)	0.101 (0.0627 to 0.152)	3.3 (2.1 to 4.8)	3.1 (1.9 to 4.5)	3 (1.8 to 4.5)
Armenia	Acute hepatitis	0.733 (0.626 to 0.878)	0.689 (0.576 to 0.806)	0.459 (0.372 to 0.565)	21 (17.9 to 25.1)	21.5 (18 to 25)	15.2 (12.5 to 18.8)
Armenia	Acute hepatitis A	0.246 (0.194 to 0.31)	0.204 (0.158 to 0.256)	0.142 (0.107 to 0.188)	6.8 (5.4 to 8.6)	6.8 (5.3 to 8.6)	5.5 (4.2 to 7.3)
Armenia	Acute hepatitis B	0.343 (0.279 to 0.423)	0.408 (0.333 to 0.486)	0.272 (0.219 to 0.339)	10.2 (8.4 to 12.5)	11.7 (9.6 to 13.9)	8 (6.4 to 10)
Armenia	Acute hepatitis C	0.102 (0.063 to 0.152)	0.0365 (0.0224 to 0.055)	0.0208 (0.0128 to 0.0335)	2.8 (1.7 to 4.1)	1.7 (1 to 2.5)	0.9 (0.6 to 1.5)
Armenia	Acute hepatitis E	0.0415 (0.0324 to 0.0526)	0.0408 (0.031 to 0.0515)	0.0247 (0.019 to 0.0316)	1.2 (0.9 to 1.5)	1.2 (1 to 1.6)	0.9 (0.7 to 1.1)
Armenia	Other unspecified infectious diseases	1.15 (0.75 to 1.69)	0.832 (0.583 to 1.18)	0.787 (0.564 to 1.08)	32.4 (21.2 to 47.5)	32.1 (22.5 to 45.5)	31.7 (22.8 to 43.8)
Armenia	Maternal and neonatal disorders	121 (100 to 143)	29.8 (26.4 to 33.9)	22.4 (19 to 26.4)	3351.2 (2783.9 to 3980.9)	1318.6 (1178.9 to 1473.6)	1037.7 (892.1 to 1204.3)
Armenia	Maternal disorders	2.15 (1.96 to 2.34)	0.7 (0.642 to 0.772)	0.582 (0.525 to 0.638)	58.5 (53.4 to 63.7)	20.7 (19 to 22.8)	18.7 (16.9 to 20.6)
Armenia	Maternal haemorrhage	0.461 (0.265 to 0.735)	0.173 (0.116 to 0.249)	0.118 (0.0714 to 0.178)	12.7 (7.5 to 19.6)	5.1 (3.4 to 7.4)	3.7 (2.3 to 5.6)
Armenia	Maternal sepsis and other pregnancy related infections	0.105 (0.0695 to 0.152)	0.038 (0.0244 to 0.0569)	0.0327 (0.0213 to 0.0511)	2.8 (1.9 to 4.2)	1.1 (0.7 to 1.6)	1.1 (0.7 to 1.7)
Armenia	Maternal hypertensive disorders	0.302 (0.136 to 0.562)	0.036 (0.0177 to 0.0683)	0.02 (0.0113 to 0.0346)	8.1 (3.7 to 15)	1.1 (0.5 to 2)	0.6 (0.4 to 1.2)
Armenia	Maternal obstructed labour and uterine rupture	0.164 (0.0932 to 0.266)	0.0437 (0.025 to 0.0713)	0.036 (0.0206 to 0.057)	4.4 (2.5 to 7.2)	1.2 (0.7 to 2)	1.2 (0.7 to 2)
Armenia	Maternal abortive outcome	0.406 (0.233 to 0.649)	0.107 (0.0638 to 0.166)	0.0682 (0.0407 to 0.106)	11.3 (6.7 to 17.7)	3.2 (1.8 to 4.9)	2.2 (1.3 to 3.4)
Armenia	Ectopic pregnancy	0.0582 (0.0167 to 0.15)	0.0172 (0.00496 to 0.0468)	0.0157 (0.00477 to 0.0417)	1.6 (0.5 to 4)	0.5 (0.1 to 1.4)	0.5 (0.2 to 1.3)
Armenia	Indirect maternal deaths	0.0502 (0.0216 to 0.105)	0.0485 (0.0224 to 0.0896)	0.0479 (0.0218 to 0.0878)	1.3 (0.6 to 2.8)	1.5 (0.7 to 2.7)	1.5 (0.7 to 2.8)
Armenia	Late maternal deaths	0.0776 (0.0566 to 0.105)	0.0346 (0.0258 to 0.0455)	0.0425 (0.0309 to 0.0574)	2.1 (1.5 to 2.8)	1 (0.8 to 1.4)	1.4 (1 to 1.8)
Armenia	Maternal deaths aggravated by HIV/AIDS	0.00000216 (0 to 0.00000466)	0.0000353 (0.0000155 to 0.00007)	0.000166 (0.0000792 to 0.000312)	0 (0 to 0)	0 (0 to 0)	0 (0 to 0)
Armenia	Other maternal disorders	0.522 (0.343 to 0.732)	0.202 (0.146 to 0.271)	0.201 (0.148 to 0.259)	14.2 (9.4 to 19.6)	6 (4.3 to 8.2)	6.5 (4.8 to 8.4)
Armenia	Neonatal disorders	118 (98.1 to 141)	29.1 (25.7 to 33.2)	21.8 (18.4 to 25.9)	3292.7 (2726.2 to 3922.4)	1297.9 (1157.6 to 1453.8)	1019 (874 to 1185.6)
Armenia	Neonatal preterm birth	66.7 (51.8 to 82.8)	13.7 (11.8 to 15.8)	9.59 (8.12 to 11.4)	1856 (1441.4 to 2304.5)	625.5 (535.5 to 715.8)	466.3 (396.7 to 548.9)
Armenia	Neonatal encephalopathy due to birth asphyxia and trauma	11.2 (7.45 to 16.4)	5.34 (3.55 to 7.87)	4.69 (3.02 to 7.36)	313.2 (208.4 to 456.2)	198.5 (137.5 to 281.7)	177.4 (117.7 to 270.9)
Armenia	Neonatal sepsis and other neonatal infections	21.1 (12 to 28.4)	5.96 (4.71 to 7.37)	4.64 (3.61 to 5.92)	585 (330.8 to 787.7)	277.9 (219.3 to 340.4)	229.4 (179.7 to 286.2)
Armenia	Hemolytic disease and other neonatal jaundice	3.54 (1.9 to 5.28)	0.719 (0.563 to 0.893)	0.602 (0.454 to 0.768)	98.3 (53.1 to 146.1)	26.9 (21.6 to 33.2)	23.3 (18 to 29.2)
Armenia	Other neonatal disorders	15.9 (10.6 to 22.8)	3.35 (2.67 to 4.07)	2.26 (1.8 to 2.75)	440.2 (292.6 to 633.2)	169.2 (134.9 to 205.6)	122.6 (97.7 to 149.8)
Armenia	Nutritional deficiencies	20.3 (13.8 to 28.9)	13.6 (8.66 to 20.5)	11 (6.76 to 16.8)	583.9 (398.6 to 830.1)	487.4 (308.5 to 729.4)	404.9 (250.9 to 620.2)
Armenia	Protein-energy malnutrition	1.3 (1.2 to 1.44)	0.14 (0.114 to 0.188)	0.0928 (0.0516 to 0.164)	37.4 (34.4 to 41.9)	4.6 (3.4 to 6.8)	3.5 (1.6 to 6.7)

SR Appendix Table 2. All-age DALYs counts and age standardized DALYs rates per 100,000 individuals for all causes and level 1 cause groups by location for 1990, 2007, and 2017, both sexes combined

		All-age DALYs			Age-standardized rate of DALYs per 100,000		
Armenia	Iodine deficiency	1.32 (0.713 to 2.23)	0.917 (0.441 to 1.7)	0.792 (0.387 to 1.45)	38.8 (20.9 to 65.6)	27.5 (13.2 to 51.1)	24.6 (12 to 45.2)
Armenia	Dietary iron deficiency	15.5 (9.73 to 23.2)	12.3 (7.77 to 18.7)	9.9 (6.12 to 15.1)	443.7 (279.8 to 662.2)	444.1 (277 to 662.7)	369.5 (227.4 to 568.6)
Armenia	Other nutritional deficiencies	1.69 (1.51 to 1.82)	0.162 (0.145 to 0.174)	0.078 (0.0678 to 0.0886)	49.7 (44.4 to 53.7)	4.7 (4.3 to 5.1)	2.3 (2 to 2.6)
Armenia	Non-communicable diseases	712 (641 to 788)	788 (715 to 878)	769 (694 to 856)	24102.8 (21830.8 to 26454.3)	22134.6 (20071.7 to 24700.2)	20244.4 (18171.2 to 22720.1)
Armenia	Neoplasms	120 (114 to 125)	135 (129 to 138)	135 (130 to 140)	3898.5 (3687.8 to 4062.8)	3631.6 (3474.9 to 3717.2)	3359.3 (3238.3 to 3471.9)
Armenia	Lip and oral cavity cancer	1.04 (0.881 to 1.45)	1.18 (1.09 to 1.26)	1.2 (1.1 to 1.31)	33.6 (28.5 to 47)	32.2 (29.8 to 34.5)	29.1 (26.9 to 31.6)
Armenia	Nasopharynx cancer	0.284 (0.251 to 0.362)	0.261 (0.241 to 0.282)	0.268 (0.245 to 0.292)	8.6 (7.6 to 10.8)	7.2 (6.7 to 7.8)	7.2 (6.7 to 7.9)
Armenia	Other pharynx cancer	0.466 (0.365 to 0.572)	0.494 (0.448 to 0.544)	0.426 (0.376 to 0.479)	14.7 (11.6 to 17.8)	13.5 (12.2 to 14.9)	10.1 (9 to 11.3)
Armenia	Oesophageal cancer	1.57 (1.45 to 1.69)	1.21 (1.07 to 1.32)	1.49 (1.37 to 1.63)	53.5 (49.8 to 57.4)	31.8 (28.2 to 34.7)	35.7 (32.9 to 38.7)
Armenia	Stomach cancer	15.4 (14.6 to 16.3)	12 (11.5 to 12.5)	11.5 (10.9 to 12.2)	513 (488.2 to 539.1)	314.2 (301.5 to 326.5)	278.8 (264 to 294.3)
Armenia	Colon and rectum cancer	8.22 (7.8 to 8.65)	9.78 (9.23 to 10.3)	11.1 (10.4 to 11.7)	276.3 (262 to 290.4)	259.6 (244.5 to 272.8)	268.3 (252.7 to 282.9)
Armenia	Liver cancer	5.85 (5.04 to 7.04)	8.03 (7.62 to 8.46)	8.56 (8.05 to 9.13)	205.2 (175.6 to 247.7)	211 (200.6 to 222.1)	208.1 (195.6 to 221)
Armenia	Liver cancer due to hepatitis B	1.45 (1.12 to 1.88)	1.78 (1.46 to 2.13)	1.76 (1.42 to 2.15)	47.8 (37.2 to 61.7)	47.4 (38.8 to 56.6)	44.3 (36.2 to 53.6)
Armenia	Liver cancer due to hepatitis C	2.35 (1.91 to 2.92)	3.35 (2.98 to 3.73)	3.67 (3.21 to 4.11)	84.5 (69.1 to 104.6)	88.3 (78.1 to 98.3)	87.1 (76.7 to 97.6)
Armenia	Liver cancer due to alcohol use	1.35 (1.09 to 1.72)	1.93 (1.72 to 2.16)	2.08 (1.84 to 2.36)	48.2 (38.9 to 61.5)	49.8 (44.4 to 55.7)	50.8 (45.1 to 57.4)
Armenia	Liver cancer due to NASH	0.357 (0.291 to 0.451)	0.537 (0.465 to 0.615)	0.61 (0.531 to 0.706)	13 (10.6 to 16.4)	14.2 (12.3 to 16.3)	14.7 (12.9 to 16.8)
Armenia	Liver cancer due to other causes	0.345 (0.288 to 0.425)	0.428 (0.373 to 0.488)	0.452 (0.392 to 0.524)	11.6 (9.8 to 14.4)	11.5 (10 to 13.1)	11.2 (9.8 to 12.9)
Armenia	Gallbladder and biliary tract cancer	0.497 (0.4 to 0.773)	0.651 (0.601 to 0.792)	1.26 (0.943 to 1.37)	17.6 (14.1 to 26.7)	17.2 (15.9 to 20.9)	30.2 (22.6 to 32.6)
Armenia	Pancreatic cancer	4.67 (4.24 to 5.08)	7.12 (6.77 to 7.48)	8.03 (7.59 to 8.49)	160.5 (146.8 to 174)	187.9 (179 to 196.8)	191.5 (180.7 to 202.2)
Armenia	Larynx cancer	3.19 (2.95 to 3.47)	2.76 (2.57 to 2.95)	2.53 (2.3 to 2.78)	101.4 (93.7 to 109.9)	73.4 (68.2 to 78.4)	59.8 (54.6 to 65.6)
Armenia	Tracheal, bronchus, and lung cancer	24.6 (23.3 to 26.2)	28.8 (27.7 to 29.9)	28.7 (27 to 30.3)	781.2 (741.4 to 826.7)	764.6 (736.3 to 793.7)	679.6 (642.2 to 715.9)
Armenia	Malignant skin melanoma	0.353 (0.254 to 0.48)	0.459 (0.362 to 0.6)	0.535 (0.395 to 0.676)	11.2 (8.3 to 15.7)	12.6 (9.8 to 16.3)	13.8 (10 to 17)
Armenia	Non-melanoma skin cancer	0.149 (0.135 to 0.165)	0.264 (0.248 to 0.282)	0.67 (0.627 to 0.716)	5.3 (4.8 to 5.8)	7.2 (6.8 to 7.7)	16.5 (15.5 to 17.6)
Armenia	Non-melanoma skin cancer (squamous-cell carcinoma)	0.148 (0.134 to 0.164)	0.263 (0.247 to 0.281)	0.669 (0.626 to 0.714)	5.3 (4.8 to 5.8)	7.2 (6.8 to 7.6)	16.5 (15.5 to 17.5)
Armenia	Non-melanoma skin cancer (basal-cell carcinoma)	0.000941 (0.000305 to 0.00218)	0.00118 (0.000365 to 0.00302)	0.00127 (0.000389 to 0.00306)	0 (0 to 0.1)	0 (0 to 0.1)	0 (0 to 0.1)
Armenia	Breast cancer	11.5 (10.8 to 12.3)	14.9 (14.3 to 15.6)	14 (13 to 15.1)	375.9 (352.6 to 400.5)	399.7 (381.8 to 419.9)	349.5 (326 to 376)
Armenia	Cervical cancer	4.82 (4.43 to 5.21)	5.22 (4.85 to 5.56)	4.4 (4 to 4.79)	155.3 (143.5 to 167.7)	140.8 (130.7 to 150.5)	113.5 (103.1 to 123.6)
Armenia	Uterine cancer	1.15 (1.05 to 1.26)	1.47 (1.31 to 1.63)	2.07 (1.86 to 2.29)	38.5 (35 to 42.3)	39.2 (34.8 to 43.7)	50.5 (45.4 to 55.7)
Armenia	Ovarian cancer	2.23 (1.91 to 2.79)	3.08 (2.85 to 3.31)	2.92 (2.66 to 3.18)	74.7 (64.3 to 92.4)	81.1 (75.3 to 87.5)	72.6 (66.2 to 79.2)
Armenia	Prostate cancer	1.17 (0.991 to 1.63)	3.36 (1.76 to 3.84)	4.33 (1.87 to 5.16)	45.4 (37.9 to 62.1)	86.2 (45.7 to 98)	103.1 (44.3 to 122.8)
Armenia	Testicular cancer	0.456 (0.271 to 0.898)	0.301 (0.258 to 0.352)	0.254 (0.219 to 0.293)	13.2 (7.9 to 25.8)	8.5 (7.3 to 9.9)	7.7 (6.6 to 8.9)
Armenia	Kidney cancer	0.459 (0.39 to 0.688)	0.824 (0.745 to 1.03)	2.38 (2.03 to 2.61)	14.7 (12.5 to 22.2)	22.6 (20.5 to 28.6)	59.1 (50.4 to 64.5)
Armenia	Bladder cancer	3.31 (2.46 to 4.5)	3.96 (3.7 to 4.23)	3.81 (3.53 to 4.1)	118.2 (88.6 to 161.2)	103.2 (96.5 to 110.1)	90.7 (84.1 to 97.9)
Armenia	Thyroid cancer	0.153 (0.133 to 0.177)	0.232 (0.215 to 0.253)	0.553 (0.502 to 0.609)	5.3 (4.6 to 6.1)	6.2 (5.8 to 6.8)	13.5 (12.3 to 14.8)
Armenia	Mesothelioma	0.424 (0.347 to 0.551)	0.591 (0.547 to 0.639)	0.525 (0.487 to 0.567)	14.3 (11.8 to 18.5)	15.9 (14.7 to 17.2)	13.2 (12.3 to 14.2)
Armenia	Hodgkin lymphoma	0.18 (0.134 to 0.326)	0.172 (0.147 to 0.253)	0.275 (0.151 to 0.336)	5.5 (4.1 to 9.7)	4.9 (4.2 to 7.2)	7.7 (4.3 to 9.4)
Armenia	Non-Hodgkin's lymphoma	1.45 (1.28 to 1.64)	1.64 (1.54 to 1.74)	1.94 (1.81 to 2.08)	45.5 (40.6 to 51.1)	45.9 (43.1 to 48.6)	51.9 (48.5 to 55.4)
Armenia	Multiple myeloma	0.379 (0.303 to 0.446)	0.534 (0.427 to 0.587)	0.7 (0.5 to 0.791)	12.6 (10.2 to 14.7)	14.2 (11.4 to 15.7)	16.9 (12.3 to 19)
Armenia	Leukaemia	7.4 (6.89 to 7.89)	5.4 (5.1 to 5.71)	4.94 (4.61 to 5.26)	217.4 (203.4 to 231.1)	163.2 (153 to 174.1)	148.4 (138.2 to 159.5)
Armenia	Acute lymphoid leukaemia	1.88 (1.46 to 2.29)	1.11 (0.984 to 1.26)	0.997 (0.89 to 1.11)	53 (41.6 to 64.5)	38.7 (33.9 to 44.4)	36.7 (32.2 to 41.7)

SR Appendix Table 2. All-age DALYs counts and age standardized DALYs rates per 100,000 individuals for all causes and level 1 cause groups by location for 1990, 2007, and 2017, both sexes combined

		All-age DALYs			Age-standardized rate of DALYs per 100,000		
Armenia	Chronic lymphoid leukaemia	0.404<br>(0.346 to 0.471)	0.485<br>(0.432 to 0.551)	0.501<br>(0.447 to 0.577)	12.9<br>(11.1 to 14.9)	13<br>(11.6 to 14.7)	12.6<br>(11.3 to 14.4)
Armenia	Acute myeloid leukaemia	1.6<br>(1.25 to 1.93)	1.1<br>(0.975 to 1.23)	1.01<br>(0.912 to 1.12)	45.3<br>(35.7 to 54.8)	35.2<br>(30.7 to 40.6)	33.1<br>(29.3 to 37.6)
Armenia	Chronic myeloid leukaemia	0.628<br>(0.543 to 0.734)	0.482<br>(0.426 to 0.542)	0.433<br>(0.376 to 0.493)	19.2<br>(16.6 to 22)	13.6<br>(12 to 15.3)	10.8<br>(9.4 to 12.2)
Armenia	Other leukaemia	2.9<br>(2.5 to 3.27)	2.23<br>(2.05 to 2.39)	1.99<br>(1.79 to 2.18)	87<br>(75.5 to 97.5)	62.8<br>(57.9 to 67.3)	55.2<br>(50.3 to 60.6)
Armenia	Other malignant cancers	12.4<br>(8.34 to 14.5)	13.6<br>(8.83 to 15.3)	8.67<br>(8.05 to 9.58)	398.4<br>(261.1 to 464.7)	375.5<br>(253.9 to 422)	231.8<br>(215.9 to 263.5)
Armenia	Other neoplasms	0.274<br>(0.169 to 0.47)	0.389<br>(0.273 to 0.514)	0.466<br>(0.319 to 0.568)	8.8<br>(5.4 to 14.7)	11.1<br>(7.9 to 14.8)	12.9<br>(8.9 to 15.9)
Armenia	Myelodysplastic, myeloproliferative, and other hematopoietic neoplasms	0.216<br>(0.133 to 0.358)	0.332<br>(0.23 to 0.427)	0.403<br>(0.273 to 0.484)	7.2<br>(4.4 to 11.6)	9.2<br>(6.4 to 12)	10.6<br>(7.2 to 12.8)
Armenia	Benign and in situ intestinal neoplasms	0<br>(0 to 0)	0<br>(0 to 0)	0<br>(0 to 0)	0<br>(0 to 0)	0<br>(0 to 0)	0<br>(0 to 0)
Armenia	Benign and in situ cervical and uterine neoplasms	0<br>(0 to 0)	0<br>(0 to 0)	0<br>(0 to 0)	0<br>(0 to 0)	0<br>(0 to 0)	0<br>(0 to 0)
Armenia	Other benign and in situ neoplasms	0.0578<br>(0.0319 to 0.112)	0.0565<br>(0.0426 to 0.0835)	0.0628<br>(0.0467 to 0.0864)	1.6<br>(0.9 to 3.2)	2<br>(1.5 to 2.9)	2.3<br>(1.7 to 3.2)
Armenia	Cardiovascular diseases	221<br>(214 to 229)	259<br>(253 to 267)	235<br>(227 to 244)	8385.7<br>(8109.4 to 8681.6)	7055.5<br>(6882.8 to 7242.9)	5735.4<br>(5513.6 to 5945.4)
Armenia	Rheumatic heart disease	8.79<br>(8.14 to 9.56)	4.96<br>(4.41 to 5.69)	4.39<br>(3.86 to 5.02)	271.8<br>(252 to 294.5)	139.2<br>(123.7 to 160.1)	119.7<br>(104.2 to 138.4)
Armenia	Ischaemic heart disease	135<br>(130 to 141)	157<br>(154 to 162)	153<br>(148 to 159)	5165.3<br>(4982.4 to 5403.1)	4291<br>(4195.9 to 4432.5)	3723.3<br>(3587.8 to 3860.5)
Armenia	Stroke	58.1<br>(55 to 61.4)	70.8<br>(67.5 to 74.2)	50.8<br>(47.5 to 53.9)	2215.2<br>(2101.4 to 2337.1)	1894.7<br>(1806.9 to 1986.2)	1230.9<br>(1150.2 to 1305.3)
Armenia	Ischaemic stroke	32.7<br>(30 to 35.4)	39.7<br>(37 to 42.5)	29.5<br>(27.1 to 31.7)	1299.3<br>(1198.3 to 1408)	1067.6<br>(995.9 to 1136.6)	712<br>(656.9 to 767.4)
Armenia	Intracerebral hemorrhage	20.6<br>(19.3 to 22)	25.1<br>(23.8 to 26.6)	16.7<br>(15.8 to 17.7)	753.3<br>(704.1 to 805.7)	668<br>(634.5 to 705.4)	404.3<br>(382.9 to 427.6)
Armenia	Subarachnoid hemorrhage	4.83<br>(4.26 to 5.44)	5.98<br>(5.42 to 6.47)	4.63<br>(4.16 to 5.04)	162.7<br>(143 to 184.6)	159.1<br>(144.7 to 171.8)	114.6<br>(103 to 124.4)
Armenia	Hypertensive heart disease	5.29<br>(2.8 to 6.42)	7.74<br>(3.88 to 8.94)	7.91<br>(4.03 to 9.03)	228.7<br>(107.3 to 283)	214.7<br>(102.2 to 247.1)	191.7<br>(97.2 to 218.5)
Armenia	Non-rheumatic valvular heart disease	0.266<br>(0.223 to 0.329)	0.381<br>(0.319 to 0.472)	0.53<br>(0.411 to 0.633)	9.9<br>(8.2 to 12.3)	10.7<br>(9 to 13.2)	13.2<br>(10.4 to 15.7)
Armenia	Non-rheumatic calcific aortic valve disease	0.167<br>(0.142 to 0.206)	0.256<br>(0.222 to 0.313)	0.377<br>(0.299 to 0.437)	6.2<br>(5.2 to 7.5)	7.2<br>(6.3 to 8.7)	9.4<br>(7.5 to 10.9)
Armenia	Non-rheumatic degenerative mitral valve disease	0.0916<br>(0.068 to 0.119)	0.115<br>(0.0839 to 0.155)	0.139<br>(0.0961 to 0.188)	3.5<br>(2.6 to 4.6)	3.2<br>(2.3 to 4.3)	3.4<br>(2.4 to 4.6)
Armenia	Other non-rheumatic valve diseases	0.00798<br>(0.0049 to 0.0117)	0.00995<br>(0.00541 to 0.0137)	0.0142<br>(0.0057 to 0.0223)	0.3<br>(0.2 to 0.4)	0.3<br>(0.2 to 0.4)	0.3<br>(0.2 to 0.5)
Armenia	Cardiomyopathy and myocarditis	4.19<br>(3.64 to 4.7)	5.04<br>(4.43 to 5.42)	3.52<br>(3.19 to 4.38)	145<br>(124.7 to 162.3)	143.6<br>(127.7 to 154.4)	91.3<br>(82.6 to 115.4)
Armenia	Myocarditis	0.182<br>(0.114 to 0.231)	0.176<br>(0.142 to 0.214)	0.164<br>(0.119 to 0.283)	5.7<br>(3.4 to 7.3)	5.6<br>(4.3 to 6.7)	4.9<br>(3.7 to 8)
Armenia	Alcoholic cardiomyopathy	2.93<br>(2.36 to 3.39)	3.22<br>(2.45 to 3.54)	0.808<br>(0.614 to 1.57)	101.6<br>(83.3 to 117.4)	88.1<br>(70.3 to 96.2)	20.5<br>(15.7 to 41.1)
Armenia	Other cardiomyopathy	1.07<br>(0.897 to 1.39)	1.65<br>(1.44 to 1.94)	2.55<br>(2.24 to 2.83)	37.7<br>(31.1 to 45.8)	49.9<br>(42.7 to 55.7)	65.8<br>(58.5 to 74)
Armenia	Atrial fibrillation and flutter	1.86<br>(1.5 to 2.27)	2.89<br>(2.38 to 3.5)	3.51<br>(2.93 to 4.19)	77.4<br>(63.3 to 94)	81.7<br>(68.2 to 97.9)	85.1<br>(71 to 101.7)
Armenia	Aortic aneurysm	3.44<br>(3.01 to 3.99)	5.34<br>(4.87 to 5.85)	6.25<br>(5.83 to 6.71)	123.9<br>(108.6 to 143.8)	141.9<br>(130.3 to 154.5)	150.3<br>(140.2 to 161.2)
Armenia	Peripheral vascular disease	0.187<br>(0.1 to 0.314)	0.291<br>(0.158 to 0.474)	0.357<br>(0.197 to 0.586)	7.5<br>(4 to 12.6)	7.9<br>(4.3 to 12.8)	8.5<br>(4.7 to 14)
Armenia	Endocarditis	0.177<br>(0.125 to 0.231)	0.22<br>(0.183 to 0.257)	0.236<br>(0.201 to 0.278)	5.6<br>(3.9 to 7.1)	6.3<br>(5.2 to 7.4)	6.4<br>(5.6 to 7.7)
Armenia	Other cardiovascular and circulatory diseases	3.88<br>(3.36 to 4.51)	4.55<br>(3.92 to 5.36)	4.51<br>(3.89 to 5.27)	135.4<br>(117.4 to 157.6)	123.8<br>(107.3 to 144.8)	114.9<br>(98.5 to 134.6)
Armenia	Chronic respiratory diseases	37.3<br>(34.3 to 40.2)	34.7<br>(31.9 to 37.6)	33.7<br>(30.5 to 36.9)	1342.2<br>(1243.8 to 1435.9)	959.4<br>(879.7 to 1043.1)	862<br>(774.8 to 952.5)
Armenia	Chronic obstructive pulmonary disease	28.6<br>(26.8 to 30.5)	26.9<br>(25 to 28.6)	25.7<br>(23.6 to 27.7)	1058.8<br>(993.5 to 1125.5)	731<br>(681.5 to 778.4)	631.8<br>(580.9 to 683.1)
Armenia	Pneumoconiosis	0.0381<br>(0.0307 to 0.0485)	0.0359<br>(0.0291 to 0.0444)	0.0388<br>(0.0318 to 0.0477)	1.3<br>(1.1 to 1.7)	1<br>(0.8 to 1.2)	0.9<br>(0.8 to 1.1)
Armenia	Silicosis	0.0186<br>(0.0131 to 0.0277)	0.0125<br>(0.01 to 0.0168)	0.0154<br>(0.0126 to 0.0186)	0.6<br>(0.4 to 0.9)	0.4<br>(0.3 to 0.5)	0.4<br>(0.3 to 0.4)
Armenia	Asbestosis	0.00303<br>(0.00192 to 0.00455)	0.0037<br>(0.00243 to 0.00541)	0.00418<br>(0.00277 to 0.00621)	0.1<br>(0.1 to 0.2)	0.1<br>(0.1 to 0.1)	0.1<br>(0.1 to 0.2)
Armenia	Coal workers pneumoconiosis	0.00346<br>(0.00225 to 0.00512)	0.00591<br>(0.00401 to 0.00843)	0.00736<br>(0.0052 to 0.0101)	0.1<br>(0.1 to 0.2)	0.1<br>(0.1 to 0.2)	0.2<br>(0.1 to 0.2)
Armenia	Other pneumoconiosis	0.013<br>(0.00887 to 0.0173)	0.0138<br>(0.01 to 0.0181)	0.0119<br>(0.00816 to 0.0164)	0.5<br>(0.3 to 0.6)	0.3<br>(0.3 to 0.5)	0.3<br>(0.2 to 0.4)
Armenia	Asthma	5.15<br>(3.8 to 6.81)	4.26<br>(3.05 to 5.77)	4.48<br>(3.1 to 6.18)	162.5<br>(122.2 to 210.8)	130.6<br>(91.6 to 176.4)	137.2<br>(93.5 to 190.4)
Armenia	Interstitial lung disease and pulmonary sarcoidosis	2.73<br>(2.27 to 3.3)	2.8<br>(2.21 to 3.38)	2.73<br>(2.11 to 3.27)	98.4<br>(78.4 to 119.3)	75.2<br>(58.7 to 90.2)	67.9<br>(52.7 to 81.7)

SR Appendix Table 2. All-age DALYs counts and age standardized DALYs rates per 100,000 individuals for all causes and level 1 cause groups by location for 1990, 2007, and 2017, both sexes combined							
		All-age DALYs			Age-standardized rate of DALYs per 100,000		
Armenia	Other chronic respiratory diseases	0.713 (0.597 to 0.844)	0.727 (0.601 to 0.864)	0.785 (0.646 to 0.94)	21.3 (17.9 to 24.9)	21.7 (17.9 to 25.9)	24.2 (19.7 to 28.9)
Armenia	Digestive diseases	34.9 (31.9 to 38.8)	42.1 (39.2 to 45.8)	44.6 (41 to 48.8)	1109.5 (1016.2 to 1224.3)	1179.2 (1097.4 to 1285.5)	1175.5 (1077.4 to 1293.4)
Armenia	Cirrhosis and other chronic liver diseases	12.5 (11.8 to 13.3)	22.1 (21.2 to 23.2)	24.5 (23 to 26.1)	409.8 (388.6 to 434.4)	598.8 (574.6 to 627)	623 (586.9 to 661.7)
Armenia	Cirrhosis and other chronic liver diseases due to hepatitis B	3.11 (2.72 to 3.6)	5.46 (4.78 to 6.15)	5.31 (4.6 to 6.08)	101.7 (88.9 to 117.5)	146.7 (129.8 to 164.9)	134.7 (116.9 to 153.5)
Armenia	Cirrhosis and other chronic liver diseases due to hepatitis C	2.91 (2.53 to 3.38)	5.34 (4.68 to 6.09)	6.11 (5.27 to 6.98)	96.2 (83.9 to 111)	143.2 (126.4 to 161.8)	153.6 (133.3 to 175.6)
Armenia	Cirrhosis and other chronic liver diseases due to alcohol use	4.26 (3.79 to 4.82)	7.65 (6.89 to 8.48)	8.79 (7.87 to 9.74)	140.3 (125.3 to 158.1)	204.2 (185.2 to 226.2)	219.5 (196.6 to 243.3)
Armenia	Cirrhosis due to NASH	0.827 (0.73 to 0.933)	1.61 (1.43 to 1.8)	2.01 (1.78 to 2.25)	27.3 (24.3 to 30.9)	43.1 (38.6 to 47.9)	49.9 (43.9 to 55.9)
Armenia	Cirrhosis and other chronic liver diseases due to other causes	1.41 (1.23 to 1.63)	2.07 (1.79 to 2.4)	2.29 (1.97 to 2.69)	44.3 (38.7 to 51)	61.5 (53.7 to 70.3)	65.3 (57.3 to 74.6)
Armenia	Upper digestive system diseases	8.42 (6.67 to 10.9)	9.06 (7.21 to 11.8)	9.03 (7.09 to 11.8)	266 (212.6 to 341.3)	250.5 (198.7 to 328.4)	238.3 (185.2 to 314.5)
Armenia	Peptic ulcer disease	4.28 (3.95 to 4.62)	4.7 (4.32 to 5.11)	4.6 (4.23 to 4.98)	139.6 (128.9 to 151.4)	127 (116.9 to 137.5)	113.9 (105.1 to 123.4)
Armenia	Gastritis and duodenitis	1.14 (0.785 to 1.6)	1.06 (0.745 to 1.48)	1.08 (0.758 to 1.5)	34.5 (23.8 to 48)	31.5 (21.8 to 44.3)	32.4 (22.2 to 45.5)
Armenia	GERD	2.99 (1.6 to 5.11)	3.3 (1.75 to 5.57)	3.35 (1.79 to 5.69)	91.9 (48.9 to 156.3)	92 (48.6 to 156.1)	92.1 (49.3 to 157.1)
Armenia	Appendicitis	0.777 (0.683 to 0.891)	0.355 (0.297 to 0.424)	0.342 (0.294 to 0.401)	23.1 (20.4 to 26.2)	10.8 (9 to 13)	11.4 (9.6 to 13.7)
Armenia	Paralytic ileus and intestinal obstruction	4.74 (4.08 to 5.51)	1.46 (1.25 to 1.67)	1.3 (0.985 to 1.52)	132.9 (114.8 to 154.2)	58.1 (50 to 67.6)	50.1 (38.2 to 59.6)
Armenia	Inguinal, femoral, and abdominal hernia	3.66 (2.6 to 4.93)	3.61 (2.58 to 4.81)	3.37 (2.44 to 4.55)	115.4 (82.7 to 154.2)	108.4 (76.9 to 143.1)	99.2 (71.3 to 134.6)
Armenia	Inflammatory bowel disease	0.451 (0.366 to 0.55)	0.474 (0.384 to 0.573)	0.454 (0.354 to 0.547)	14 (11.4 to 16.9)	13.2 (10.9 to 15.9)	12.4 (9.7 to 15)
Armenia	Vascular intestinal disorders	1.45 (1.23 to 1.75)	2.01 (1.73 to 2.25)	2.09 (1.76 to 2.28)	54 (46.2 to 64.7)	54.6 (45.5 to 60.7)	50.7 (42.9 to 55.2)
Armenia	Gallbladder and biliary diseases	1.25 (1.04 to 1.36)	1.34 (1.15 to 1.46)	1.73 (1.29 to 1.9)	41.5 (35.7 to 45.2)	36.3 (31 to 39.3)	42.7 (32 to 46.7)
Armenia	Pancreatitis	0.835 (0.714 to 1.01)	0.929 (0.804 to 1.09)	0.966 (0.82 to 1.17)	27 (23 to 32.7)	25.9 (22.4 to 30.5)	25.7 (21.7 to 31.3)
Armenia	Other digestive diseases	0.851 (0.616 to 1.07)	0.734 (0.614 to 0.867)	0.771 (0.65 to 0.911)	25.7 (18.7 to 32)	22.5 (18.7 to 26.9)	22 (18.2 to 26.4)
Armenia	Neurological disorders	43 (32 to 55.9)	48.1 (37.3 to 62)	50.6 (39.5 to 62.9)	1449.9 (1116.2 to 1838.4)	1444.8 (1127 to 1851.2)	1442.1 (1099.1 to 1816.3)
Armenia	Alzheimer's disease and other dementias	8.39 (7.73 to 9.04)	13.2 (12.3 to 14.2)	16.9 (15.7 to 18.1)	411.4 (380.7 to 443.3)	427.4 (398 to 459.1)	419.5 (389.9 to 449.8)
Armenia	Parkinson's disease	1.51 (1.22 to 1.72)	2.71 (2.24 to 3.01)	2.91 (2.39 to 3.24)	66.5 (53.8 to 75.5)	70.3 (58 to 77.9)	69.1 (56.8 to 76.7)
Armenia	Epilepsy	6.58 (3.41 to 11)	5.08 (2.78 to 8.57)	4.75 (1.66 to 9.21)	190.1 (97.3 to 320)	160 (88 to 270.1)	159 (54.9 to 308.7)
Armenia	Multiple sclerosis	0.41 (0.339 to 0.492)	0.568 (0.451 to 0.678)	0.609 (0.471 to 0.743)	12.8 (10.6 to 15.3)	15.9 (12.5 to 19)	16.5 (12.6 to 20.2)
Armenia	Motor neuron disease	0.0333 (0.028 to 0.0397)	0.0438 (0.039 to 0.0494)	0.0591 (0.0529 to 0.0659)	1 (0.9 to 1.2)	1.2 (1.1 to 1.4)	1.5 (1.3 to 1.7)
Armenia	Headache disorders	24.3 (15.8 to 35.2)	25 (16.3 to 36.1)	23.8 (15.4 to 34.3)	717.1 (467.7 to 1032.6)	721.8 (467.5 to 1039.7)	719.6 (465.7 to 1037.2)
Armenia	Migraine	21.2 (13.3 to 31.5)	21.7 (13.6 to 32.1)	20.6 (12.9 to 30.7)	622.4 (391.3 to 925.3)	626.7 (392.5 to 924.1)	624.7 (393.5 to 926.8)
Armenia	Tension-type headache	3.18 (1.78 to 5.11)	3.29 (1.85 to 5.2)	3.16 (1.79 to 5)	94.7 (52.9 to 151.1)	95.2 (54 to 151.3)	94.9 (53.7 to 150.9)
Armenia	Other neurological disorders	1.78 (1.04 to 2.67)	1.51 (1.11 to 2.13)	1.57 (1.02 to 2.35)	51 (30.4 to 75.8)	48.2 (35 to 68.8)	56.8 (35.6 to 86.9)
Armenia	Mental disorders	42 (31.2 to 54.3)	43.7 (32.3 to 56.4)	42 (31.3 to 54.5)	1256.9 (934.4 to 1627.8)	1274.2 (945.5 to 1649.3)	1283.9 (957 to 1673.6)
Armenia	Schizophrenia	3.96 (2.93 to 5.01)	4.15 (3.08 to 5.19)	4.28 (3.18 to 5.38)	117.6 (87.3 to 148.1)	117.9 (87.1 to 148)	119.9 (89.1 to 151.9)
Armenia	Depressive disorders	12.8 (9.09 to 17.6)	14.5 (10.3 to 19.6)	14.6 (10.4 to 19.8)	396.7 (281.7 to 540.8)	407.6 (288.7 to 553)	415.9 (296.1 to 568.8)
Armenia	Major depressive disorder	9.3 (6.47 to 12.7)	10.7 (7.35 to 14.5)	10.7 (7.47 to 14.5)	288.9 (202.4 to 390.7)	299.4 (206.6 to 404.2)	308 (216 to 419.5)
Armenia	Dysthymia	3.52 (2.32 to 5.19)	3.86 (2.56 to 5.65)	3.88 (2.58 to 5.68)	107.7 (71.1 to 157.2)	108.2 (71.8 to 158.2)	107.9 (71.8 to 157.9)
Armenia	Bipolar disorder	4.73 (2.92 to 7.02)	4.94 (3.11 to 7.38)	4.59 (2.9 to 6.88)	139.3 (86.6 to 206.9)	139.9 (88 to 208.6)	139.8 (87.7 to 209.9)
Armenia	Anxiety disorders	7.91 (5.62 to 10.6)	8.08 (5.76 to 10.8)	7.62 (5.39 to 10.2)	235.6 (167.4 to 315)	236.3 (168.2 to 316.4)	235.6 (166.5 to 315.3)
Armenia	Eating disorders	1.05 (0.652 to 1.56)	0.988 (0.62 to 1.47)	1.03 (0.653 to 1.54)	29.1 (18.2 to 43)	29.5 (18.4 to 43.6)	34.1 (21.7 to 51.1)
Armenia	Anorexia nervosa	0.211 (0.125 to 0.325)	0.205 (0.121 to 0.313)	0.192 (0.112 to 0.295)	5.9 (3.6 to 9.1)	6.1 (3.6 to 9.2)	6.9 (4.1 to 10.5)
Armenia	Bulimia nervosa	0.838 (0.512 to 1.26)	0.782 (0.48 to 1.18)	0.838 (0.525 to 1.27)	23.2 (14.1 to 34.9)	23.4 (14.5 to 35.3)	27.2 (17 to 41.4)

SR Appendix Table 2. All-age DALYs counts and age standardized DALYs rates per 100,000 individuals for all causes and level 1 cause groups by location for 1990, 2007, and 2017, both sexes combined

		All-age DALYs			Age-standardized rate of DALYs per 100,000		
Armenia	Autism spectrum disorders	2.26 (1.54 to 3.12)	2 (1.36 to 2.74)	1.9 (1.3 to 2.63)	64.9 (44.5 to 89.4)	65 (43.7 to 89.3)	65.5 (44.7 to 91)
Armenia	Attention-deficit/hyperactivity disorder	0.412 (0.248 to 0.655)	0.357 (0.213 to 0.561)	0.311 (0.19 to 0.496)	11.7 (7 to 18.5)	11.7 (7 to 18.4)	11.8 (7.1 to 18.9)
Armenia	Conduct disorder	3.38 (2.04 to 5.45)	2.85 (1.69 to 4.49)	2.06 (1.25 to 3.25)	97.7 (59.2 to 157)	97.9 (58.1 to 154.6)	98.7 (60.1 to 156.1)
Armenia	Idiopathic developmental intellectual disability	0.905 (0.288 to 1.7)	0.914 (0.359 to 1.65)	0.68 (0.227 to 1.27)	25.8 (8.2 to 48.5)	29.8 (11.7 to 54)	23.8 (8.1 to 44.4)
Armenia	Other mental disorders	4.63 (3.05 to 6.44)	4.89 (3.25 to 6.84)	4.91 (3.25 to 6.82)	138.6 (91.7 to 192.9)	138.5 (92.4 to 193.1)	138.8 (91.6 to 193.3)
Armenia	Substance use disorders	14.4 (11.2 to 18.1)	13.5 (10.4 to 16.9)	13.4 (10.3 to 16.8)	419.2 (327.7 to 522.4)	385.7 (293.6 to 483)	395.2 (300.2 to 495.8)
Armenia	Alcohol use disorders	10.6 (8.3 to 13.4)	9.16 (7.08 to 11.7)	8.18 (6.12 to 10.7)	311.9 (247.2 to 393.9)	260.2 (200.7 to 333.6)	239.2 (177.9 to 312.9)
Armenia	Drug use disorders	3.82 (2.74 to 5.12)	4.36 (3.27 to 5.57)	5.24 (4.08 to 6.55)	107.3 (76.7 to 142.8)	125.5 (93.7 to 160.8)	156 (120.8 to 195.8)
Armenia	Opioid use disorders	2.74 (1.94 to 3.74)	3.28 (2.49 to 4.22)	4.17 (3.24 to 5.19)	77.1 (54.4 to 104.8)	94.8 (71.9 to 121.9)	121.8 (94.8 to 151.6)
Armenia	Cocaine use disorders	0.111 (0.0619 to 0.174)	0.108 (0.0636 to 0.168)	0.121 (0.075 to 0.179)	3.2 (1.8 to 4.9)	3.1 (1.8 to 4.8)	3.8 (2.3 to 5.7)
Armenia	Amphetamine use disorders	0.388 (0.212 to 0.647)	0.373 (0.202 to 0.597)	0.344 (0.195 to 0.548)	10.6 (5.8 to 17.7)	10.7 (5.7 to 17.1)	11.4 (6.4 to 18.5)
Armenia	Cannabis use disorders	0.16 (0.0982 to 0.24)	0.16 (0.0968 to 0.241)	0.132 (0.0821 to 0.2)	4.5 (2.8 to 6.8)	4.5 (2.8 to 6.8)	4.6 (2.8 to 6.9)
Armenia	Other drug use disorders	0.423 (0.265 to 0.634)	0.437 (0.284 to 0.635)	0.466 (0.325 to 0.654)	11.9 (7.6 to 17.7)	12.4 (8.1 to 18.2)	14.4 (9.8 to 20.7)
Armenia	Diabetes and kidney diseases	38.8 (32.2 to 47.1)	65.7 (55.8 to 77.3)	63.8 (53.8 to 76.3)	1294.6 (1075.9 to 1563.9)	1759.7 (1494.2 to 2078.5)	1580.9 (1328.9 to 1896)
Armenia	Diabetes mellitus	30.6 (24.6 to 38)	56.6 (47.5 to 67.9)	52 (42.7 to 64)	1028.2 (826 to 1272.9)	1508.6 (1262.2 to 1809.7)	1279.9 (1052 to 1576.9)
Armenia	Diabetes mellitus type 1	6.82 (5.07 to 8.05)	7.2 (5.53 to 8.04)	5.01 (3.8 to 5.61)	221 (162.8 to 264.3)	196.4 (152.6 to 218.8)	128.4 (99.2 to 143)
Armenia	Diabetes mellitus type 2	23.8 (17.7 to 31.3)	49.4 (40.5 to 60.6)	47 (38.1 to 58.4)	807.2 (601.8 to 1057.7)	1312.2 (1071.7 to 1611.4)	1151.6 (927.9 to 1434.7)
Armenia	Chronic kidney disease	8.16 (7.37 to 9.15)	9.03 (8.1 to 10)	11.8 (10.7 to 12.9)	266.1 (239.5 to 297.2)	250.9 (224.9 to 277.8)	300.8 (273.4 to 329.1)
Armenia	Chronic kidney disease due to diabetes mellitus type 1	0.623 (0.497 to 0.773)	0.678 (0.549 to 0.82)	0.782 (0.632 to 0.967)	19.5 (15.6 to 24.1)	18.3 (14.9 to 22)	20.7 (16.7 to 25)
Armenia	Chronic kidney disease due to diabetes mellitus type 2	1.37 (1.15 to 1.63)	1.9 (1.61 to 2.19)	2.81 (2.39 to 3.24)	47.4 (40 to 55.7)	50.8 (43.4 to 58.5)	66.7 (56.8 to 76.5)
Armenia	Chronic kidney disease due to hypertension	0.854 (0.704 to 1.01)	1.14 (0.949 to 1.34)	1.58 (1.31 to 1.85)	30.1 (25 to 35.5)	31.9 (26.4 to 37.5)	39.4 (32.7 to 46.2)
Armenia	Chronic kidney disease due to glomerulonephritis	2.02 (1.77 to 2.33)	1.97 (1.73 to 2.26)	2.49 (2.15 to 2.88)	62.2 (54.8 to 71.1)	55.3 (48.5 to 63)	66.8 (58.1 to 76.1)
Armenia	Chronic kidney disease due to other and unspecified causes	3.29 (2.78 to 3.92)	3.34 (2.79 to 3.97)	4.09 (3.49 to 4.79)	106.8 (90.2 to 127.6)	94.6 (79.3 to 111.9)	107.3 (92.4 to 125.4)
Armenia	Acute glomerulonephritis	0.0116 (0.0093 to 0.0145)	0.00586 (0.00478 to 0.00714)	0.00474 (0.00385 to 0.00585)	0.4 (0.3 to 0.4)	0.2 (0.1 to 0.2)	0.1 (0.1 to 0.2)
Armenia	Skin and subcutaneous diseases	15.2 (10.1 to 22.4)	14 (9.38 to 20.5)	13.6 (9.14 to 20)	445.2 (295.5 to 658)	453.7 (302.8 to 667.1)	461.2 (307.8 to 678.4)
Armenia	Dermatitis	3.76 (2.18 to 6.04)	3.16 (1.88 to 4.94)	3.02 (1.77 to 4.73)	108.3 (64.1 to 173.1)	108.3 (63.7 to 172.5)	108.3 (62.3 to 172.6)
Armenia	Atopic dermatitis	2.99 (1.6 to 5.05)	2.36 (1.25 to 3.95)	2.25 (1.2 to 3.75)	85.1 (45.7 to 143.2)	85.1 (45.4 to 144.3)	85.1 (45.8 to 142.9)
Armenia	Contact dermatitis	0.766 (0.492 to 1.14)	0.798 (0.52 to 1.18)	0.763 (0.499 to 1.13)	23.1 (14.9 to 34.2)	23.1 (15 to 33.8)	23.1 (14.9 to 34.2)
Armenia	Seborrhoeic dermatitis	0.00362 (0.00203 to 0.0058)	0.00339 (0.00188 to 0.00544)	0.00331 (0.00184 to 0.0053)	0.1 (0.1 to 0.2)	0.1 (0.1 to 0.2)	0.1 (0.1 to 0.2)
Armenia	Psoriasis	2.02 (1.42 to 2.68)	2.23 (1.58 to 2.96)	2.27 (1.63 to 3.01)	61.1 (43 to 81.4)	64.6 (45.7 to 85.5)	66.1 (47 to 87.9)
Armenia	Bacterial skin diseases	0.0741 (0.0526 to 0.106)	0.0897 (0.0625 to 0.118)	0.172 (0.0654 to 0.245)	2.2 (1.6 to 3.2)	2.8 (2 to 3.7)	4.9 (2 to 6.8)
Armenia	Cellulitis	0.0403 (0.0283 to 0.056)	0.0439 (0.0302 to 0.0597)	0.0703 (0.0322 to 0.101)	1.2 (0.8 to 1.7)	1.3 (0.9 to 1.8)	2 (1 to 2.9)
Armenia	Pyoderma	0.0339 (0.0213 to 0.0572)	0.0458 (0.0262 to 0.0661)	0.102 (0.0276 to 0.151)	1 (0.7 to 1.8)	1.4 (0.9 to 2.1)	2.8 (0.9 to 4.1)
Armenia	Scabies	1.04 (0.571 to 1.69)	0.888 (0.491 to 1.43)	0.778 (0.43 to 1.26)	29.8 (16.3 to 48.6)	28.8 (16.1 to 46.7)	28.2 (15.7 to 45.8)
Armenia	Fungal skin diseases	1.51 (0.597 to 3.14)	1.53 (0.616 to 3.14)	1.53 (0.61 to 3.16)	47.9 (19.1 to 100.4)	46.6 (18.6 to 96)	45.5 (18.1 to 94.9)
Armenia	Viral skin diseases	1.65 (1.05 to 2.45)	1.14 (0.726 to 1.69)	1.09 (0.694 to 1.61)	45.8 (29 to 68.1)	45.8 (28.9 to 67.8)	45.9 (29.2 to 68.2)
Armenia	Acne vulgaris	0.923 (0.535 to 1.53)	1.13 (0.66 to 1.87)	0.893 (0.521 to 1.46)	26.3 (15.3 to 43.5)	32.6 (18.9 to 54.1)	35.6 (20.4 to 58.8)
Armenia	Alopecia areata	0.206 (0.13 to 0.306)	0.198 (0.126 to 0.295)	0.194 (0.124 to 0.293)	5.9 (3.8 to 8.8)	5.9 (3.7 to 8.8)	5.9 (3.8 to 8.9)
Armenia	Pruritus	0.302 (0.141 to 0.567)	0.313 (0.146 to 0.586)	0.318 (0.152 to 0.598)	9.2 (4.3 to 17.3)	9.3 (4.3 to 17.5)	9.4 (4.4 to 17.8)
Armenia	Urticaria	2.64 (1.75 to 3.73)	2.13 (1.43 to 3.01)	2.04 (1.36 to 2.87)	74.7 (49.6 to 106)	74.7 (49.8 to 106.4)	74.6 (49 to 106.5)

SR Appendix Table 2. All-age DALYs counts and age standardized DALYs rates per 100,000 individuals for all causes and level 1 cause groups by location for 1990, 2007, and 2017, both sexes combined							
		All-age DALYs			Age-standardized rate of DALYs per 100,000		
Armenia	Decubitus ulcer	0.0659 (0.0473 to 0.088)	0.081 (0.0559 to 0.109)	0.0944 (0.066 to 0.128)	2.3 (1.6 to 3.1)	2.4 (1.7 to 3.1)	2.5 (1.8 to 3.4)
Armenia	Other skin and subcutaneous diseases	1.01 (0.485 to 1.87)	1.1 (0.532 to 2.02)	1.2 (0.576 to 2.18)	31.6 (15.3 to 58.4)	31.9 (15.4 to 58.9)	34.3 (16.5 to 62.5)
Armenia	Sense organ diseases	25.1 (16.6 to 36.8)	29.9 (20.1 to 43.7)	31.8 (21.3 to 46)	864.5 (575.1 to 1256.4)	846.7 (569 to 1230.9)	833.6 (557.6 to 1210.8)
Armenia	Blindness and vision impairment	11.9 (7.57 to 18.1)	13.9 (8.82 to 20.9)	14.5 (9.35 to 21.6)	407.7 (264.3 to 614.3)	392.3 (250.1 to 588.5)	381.5 (245.5 to 574.2)
Armenia	Glaucoma	0.0889 (0.0575 to 0.128)	0.116 (0.0772 to 0.165)	0.124 (0.0798 to 0.177)	3.4 (2.2 to 4.8)	3.2 (2.1 to 4.4)	2.9 (1.9 to 4.2)
Armenia	Cataract	3.72 (2.5 to 5.14)	5.25 (3.54 to 7.22)	5.79 (3.89 to 8.05)	146.3 (98.6 to 200.5)	142.7 (96.2 to 196.2)	139.8 (94.1 to 194.3)
Armenia	Age-related macular degeneration	0.0284 (0.0171 to 0.0427)	0.0411 (0.0258 to 0.0624)	0.0483 (0.03 to 0.0742)	1.1 (0.7 to 1.6)	1.1 (0.7 to 1.7)	1.1 (0.7 to 1.8)
Armenia	Refraction disorders	3.86 (2.44 to 5.96)	3.7 (2.35 to 5.6)	3.57 (2.28 to 5.48)	116.1 (73.5 to 178.8)	113.4 (71.7 to 174.1)	110.8 (70.7 to 171.5)
Armenia	Near vision loss	3.6 (1.72 to 6.56)	4.07 (1.93 to 7.49)	4.18 (2 to 7.64)	120.1 (57.2 to 219)	111.6 (53 to 205.1)	106.8 (51 to 194.5)
Armenia	Other vision loss	0.624 (0.413 to 0.882)	0.716 (0.464 to 1.02)	0.749 (0.495 to 1.06)	20.7 (13.6 to 29.2)	20.4 (13.4 to 29)	20 (13.2 to 28.3)
Armenia	Age-related and other hearing loss	12.2 (8.32 to 17.3)	15 (10.2 to 21.2)	16.1 (11 to 22.7)	426.2 (293.2 to 597.7)	423.2 (290.7 to 595.3)	420.8 (286.7 to 590.4)
Armenia	Other sense organ diseases	0.923 (0.571 to 1.34)	1.09 (0.668 to 1.6)	1.17 (0.734 to 1.71)	30.6 (18.9 to 44.9)	31.1 (19.2 to 45.6)	31.3 (19.5 to 45.6)
Armenia	Musculoskeletal disorders	45.1 (32.7 to 61.3)	53.5 (38.9 to 72.6)	56.3 (40.6 to 75.8)	1447 (1050.7 to 1957.6)	1476.7 (1076.7 to 1999.9)	1502.2 (1094.1 to 2026.9)
Armenia	Rheumatoid arthritis	0.79 (0.563 to 1.05)	1 (0.722 to 1.31)	1.26 (0.922 to 1.66)	25.9 (18.4 to 34.3)	27.4 (19.8 to 35.7)	32.2 (23.5 to 42.6)
Armenia	Osteoarthritis	2.17 (1.09 to 4.34)	2.99 (1.51 to 5.9)	3.48 (1.76 to 6.94)	77.2 (38.8 to 154)	80.8 (40.7 to 159.3)	84.7 (42.7 to 169.2)
Armenia	Low back pain	31.9 (22.8 to 43.3)	36.7 (26.4 to 49.5)	38.4 (27.4 to 52.2)	1013.2 (728.6 to 1383.6)	1021.2 (730.4 to 1383)	1035.7 (738.4 to 1408.5)
Armenia	Neck pain	9.16 (6.41 to 12.9)	11 (7.63 to 15.4)	11.2 (7.8 to 15.7)	297.6 (207.9 to 419.8)	298.6 (209 to 417.2)	298.6 (207.4 to 417.3)
Armenia	Gout	0.367 (0.241 to 0.509)	0.49 (0.331 to 0.677)	0.551 (0.374 to 0.767)	12.6 (8.5 to 17.3)	13.2 (8.8 to 18.2)	13.7 (9.2 to 18.9)
Armenia	Other musculoskeletal disorders	0.695 (0.248 to 1.29)	1.25 (0.656 to 2.06)	1.34 (0.723 to 2.17)	20.5 (7.4 to 38)	35.6 (19 to 59.1)	37.4 (20.2 to 59.4)
Armenia	Other non-communicable diseases	75.5 (62.2 to 94.4)	48.7 (39.4 to 60.5)	49 (39.4 to 60.6)	2189.7 (1806.2 to 2733)	1667.5 (1368.3 to 2030.6)	1613.3 (1322.1 to 1972.2)
Armenia	Congenital anomalies	49.2 (40.3 to 65.4)	18.7 (15.6 to 21.3)	15.3 (12.5 to 18.1)	1365.9 (1117.9 to 1811.9)	810.6 (669.1 to 928)	688.9 (561 to 814.3)
Armenia	Neural tube defects	5.56 (3.51 to 7.77)	1.77 (1.34 to 2.46)	1.14 (0.844 to 1.51)	153.8 (97.4 to 214.6)	83.4 (61.7 to 118.1)	54.9 (39.9 to 75.1)
Armenia	Congenital heart anomalies	16.8 (10.7 to 23)	5.91 (4.1 to 7.16)	4.35 (3.23 to 5.24)	464.1 (296 to 634.3)	278.8 (189.3 to 340.7)	216.8 (159.3 to 264.1)
Armenia	Orofacial clefts	0.33 (0.149 to 1.28)	0.117 (0.0831 to 0.163)	0.0968 (0.0646 to 0.14)	9.2 (4.2 to 35.5)	4.3 (3.1 to 6.1)	3.6 (2.4 to 5.1)
Armenia	Down's syndrome	0.533 (0.371 to 0.996)	0.395 (0.308 to 0.478)	0.388 (0.295 to 0.464)	15.2 (10.7 to 28)	13 (10.6 to 16.6)	13.6 (10.8 to 16.9)
Armenia	Turner syndrome	0.00309 (0.00153 to 0.00511)	0.00271 (0.00137 to 0.00447)	0.0024 (0.00119 to 0.00391)	0.1 (0 to 0.1)	0.1 (0 to 0.1)	0.1 (0 to 0.1)
Armenia	Klinefelter syndrome	0.000881 (0.000414 to 0.00165)	0.000757 (0.000354 to 0.00145)	0.00071 (0.000329 to 0.00136)	0 (0 to 0)	0 (0 to 0)	0 (0 to 0)
Armenia	Other chromosomal abnormalities	0.643 (0.335 to 1.42)	0.296 (0.215 to 0.391)	0.327 (0.236 to 0.425)	17.8 (9.4 to 38.9)	11.8 (8.6 to 15.2)	13.5 (9.7 to 17.4)
Armenia	Congenital musculoskeletal and limb anomalies	5.76 (3.81 to 8.95)	3.78 (2.7 to 4.93)	3.5 (2.51 to 4.61)	165.1 (110.3 to 253.7)	129.9 (95.1 to 168.2)	125.8 (92.3 to 162.5)
Armenia	Urogenital congenital anomalies	0.33 (0.14 to 0.632)	0.159 (0.0919 to 0.232)	0.159 (0.0852 to 0.239)	9.1 (3.9 to 17.5)	6.6 (3.6 to 9.8)	6.9 (3.5 to 10.6)
Armenia	Digestive congenital anomalies	8.91 (4.01 to 19)	2.43 (1.54 to 3.13)	2.02 (1.29 to 2.59)	247 (111.8 to 522.7)	108.2 (63.1 to 142)	94.2 (55.5 to 123.2)
Armenia	Other congenital anomalies	10.3 (6.14 to 22.9)	3.83 (3.17 to 4.86)	3.36 (2.75 to 4.2)	284.5 (169.1 to 627.4)	174.6 (141.8 to 226.7)	159.6 (130.3 to 207)
Armenia	Urinary diseases and male infertility	4.61 (4 to 5.34)	6.95 (6.25 to 7.75)	11.4 (10.5 to 12.5)	150.5 (131.1 to 174.1)	189.2 (170.2 to 210.8)	284 (262.3 to 309.8)
Armenia	Urinary tract infections	1.92 (1.71 to 2.16)	3.62 (3.17 to 3.93)	6.85 (5.46 to 7.79)	60 (53.2 to 68)	99 (86.8 to 106.8)	173 (139.6 to 195.6)
Armenia	Urolithiasis	0.611 (0.384 to 0.81)	0.731 (0.543 to 1.06)	1.36 (0.818 to 2.3)	22 (13.6 to 29.1)	20 (14.9 to 29)	33.2 (20.2 to 55.4)
Armenia	Benign prostatic hyperplasia	1.26 (0.806 to 1.82)	1.56 (0.991 to 2.24)	1.81 (1.16 to 2.6)	41.9 (26.9 to 60.9)	41.5 (26.5 to 59.8)	42.4 (27.3 to 60.6)
Armenia	Male infertility	0.0752 (0.0301 to 0.159)	0.056 (0.0228 to 0.117)	0.0643 (0.0259 to 0.134)	2.1 (0.8 to 4.3)	1.7 (0.7 to 3.6)	2 (0.8 to 4.2)
Armenia	Other urinary diseases	0.748 (0.574 to 0.896)	0.983 (0.778 to 1.24)	1.34 (1.07 to 2.01)	24.6 (18.7 to 30.1)	27 (21.3 to 33.9)	33.5 (26.8 to 49.1)
Armenia	Gynecological diseases	5.62 (3.96 to 7.75)	5.91 (4.1 to 8.33)	5.23 (3.61 to 7.44)	166.2 (117 to 230.4)	165.4 (115.2 to 232.5)	160.4 (110.4 to 227.7)
Armenia	Uterine fibroids	0.983 (0.659 to 1.52)	1.12 (0.688 to 1.77)	0.885 (0.524 to 1.45)	31 (20.3 to 47.8)	29.5 (18.2 to 47.2)	24.7 (14.4 to 40.6)

SR Appendix Table 2. All-age DALYs counts and age standardized DALYs rates per 100,000 individuals for all causes and level 1 cause groups by location for 1990, 2007, and 2017, both sexes combined

		All-age DALYs			Age-standardized rate of DALYs per 100,000		
Armenia	Polycystic ovarian syndrome	0.201 (0.0908 to 0.389)	0.207 (0.0917 to 0.402)	0.185 (0.0812 to 0.366)	5.8 (2.6 to 11.2)	5.9 (2.6 to 11.5)	5.9 (2.6 to 11.6)
Armenia	Female infertility	0.0797 (0.0268 to 0.181)	0.0379 (0.0133 to 0.0844)	0.0742 (0.0249 to 0.165)	2.1 (0.7 to 4.7)	1.2 (0.4 to 2.6)	2.2 (0.8 to 5)
Armenia	Endometriosis	1.81 (1.18 to 2.64)	1.78 (1.16 to 2.56)	1.66 (1.08 to 2.43)	51.3 (33.5 to 74.8)	51.9 (34 to 74.6)	51.2 (33.3 to 75.6)
Armenia	Genital prolapse	0.104 (0.0534 to 0.191)	0.0946 (0.0492 to 0.175)	0.0996 (0.0487 to 0.191)	3.5 (1.8 to 6.4)	2.5 (1.3 to 4.7)	2.5 (1.2 to 4.8)
Armenia	Premenstrual syndrome	1.83 (1.18 to 2.75)	1.94 (1.26 to 2.9)	1.63 (1.05 to 2.48)	53.8 (34.8 to 80.8)	54.2 (35.1 to 81)	52.3 (33.8 to 80)
Armenia	Other gynecological diseases	0.617 (0.409 to 0.901)	0.731 (0.471 to 1.12)	0.704 (0.456 to 1.09)	18.6 (12.3 to 27.5)	20.3 (13.2 to 31.1)	21.6 (13.9 to 33.5)
Armenia	Hemoglobinopathies and hemolytic anaemias	2.69 (1.84 to 3.83)	2.03 (1.41 to 2.86)	1.69 (1.14 to 2.43)	78.3 (54.1 to 111.1)	66.4 (46.4 to 93.9)	56.9 (38.5 to 83.2)
Armenia	Thalassemias	0.133 (0.0924 to 0.212)	0.0639 (0.0508 to 0.0832)	0.0656 (0.05 to 0.0852)	3.6 (2.5 to 5.7)	3 (2.4 to 4)	3.1 (2.4 to 4.1)
Armenia	Thalassaemias trait	1.23 (0.782 to 1.86)	0.86 (0.57 to 1.27)	0.689 (0.428 to 1.06)	35.1 (22.2 to 52.8)	27.5 (18.3 to 40.1)	22.3 (13.8 to 33.9)
Armenia	Sickle cell disorders	0.0159 (0.00901 to 0.0283)	0.00914 (0.00597 to 0.0137)	0.00864 (0.00544 to 0.0132)	0.4 (0.2 to 0.8)	0.4 (0.2 to 0.6)	0.4 (0.2 to 0.6)
Armenia	Sickle cell trait	0.0942 (0.0565 to 0.148)	0.0601 (0.036 to 0.094)	0.0446 (0.026 to 0.0718)	2.7 (1.6 to 4.2)	2 (1.2 to 3.1)	1.5 (0.9 to 2.5)
Armenia	G6PD deficiency	0.0543 (0.0443 to 0.072)	0.0599 (0.0496 to 0.0772)	0.0622 (0.0535 to 0.0728)	1.6 (1.3 to 2.1)	1.8 (1.5 to 2.3)	2.2 (1.8 to 2.5)
Armenia	G6PD trait	0.000154 (0.0000999 to 0.000227)	0.000125 (0.000082 to 0.000179)	0.000091 (0.0000574 to 0.000135)	0 (0 to 0)	0 (0 to 0)	0 (0 to 0)
Armenia	Other hemoglobinopathies and hemolytic anaemias	1.17 (0.806 to 1.66)	0.977 (0.674 to 1.4)	0.82 (0.542 to 1.2)	35 (24.4 to 49.3)	31.7 (21.5 to 45.4)	27.4 (17.8 to 40.9)
Armenia	Endocrine, metabolic, blood, and immune disorders	1.94 (1.49 to 2.54)	1.79 (1.4 to 2.28)	1.98 (1.45 to 2.52)	57 (44.1 to 73.6)	58.9 (46.3 to 75)	65.5 (48.2 to 83.9)
Armenia	Oral disorders	10.5 (6.61 to 15.9)	13 (8.31 to 19.5)	13.1 (8.44 to 19.5)	347.4 (220 to 521.5)	360.9 (230.2 to 541)	344.5 (219.2 to 518.6)
Armenia	Caries of deciduous teeth	0.143 (0.0613 to 0.288)	0.0737 (0.0312 to 0.146)	0.0801 (0.0348 to 0.161)	3.8 (1.6 to 7.8)	3.8 (1.6 to 7.5)	3.8 (1.6 to 7.6)
Armenia	Caries of permanent teeth	1.48 (0.626 to 2.81)	1.46 (0.635 to 2.74)	1.3 (0.569 to 2.44)	43.6 (18.6 to 82.7)	43.1 (18.6 to 81.2)	40.9 (17.6 to 77)
Armenia	Periodontal disease	1.51 (0.601 to 3.18)	1.82 (0.719 to 3.83)	1.8 (0.724 to 3.78)	48.4 (19.2 to 101.6)	49.9 (19.8 to 104.9)	47 (18.7 to 99.4)
Armenia	Edentulism and severe tooth loss	5.64 (3.74 to 7.94)	7.9 (5.31 to 11.2)	8.23 (5.49 to 11.6)	200.6 (133.7 to 284.4)	213 (143 to 303.4)	201.7 (134.4 to 284.5)
Armenia	Other oral disorders	1.71 (1.07 to 2.54)	1.75 (1.09 to 2.56)	1.7 (1.06 to 2.5)	50.9 (31.7 to 75.2)	51.1 (32.1 to 75.3)	51 (31.7 to 75.1)
Armenia	Sudden infant death syndrome	0.9 (0.517 to 1.79)	0.319 (0.183 to 0.5)	0.243 (0.107 to 0.395)	24.5 (14.1 to 48.5)	16 (9.2 to 25.1)	13 (5.7 to 21.1)
Armenia	Injuries	168 (155 to 184)	85.9 (76.9 to 96.2)	78.5 (68.1 to 91.1)	4922.8 (4507.1 to 5398.2)	2517.1 (2265.7 to 2811.6)	2337.9 (2049.3 to 2691.7)
Armenia	Transport injuries	33.5 (31.4 to 35.6)	16 (14.8 to 17.4)	15.2 (13.8 to 16.8)	973.8 (912.5 to 1037.9)	466.9 (432.4 to 503.1)	461.4 (422.7 to 507.1)
Armenia	Road injuries	32 (30 to 34)	14.9 (13.8 to 16.2)	14.2 (13 to 15.7)	930.1 (872.4 to 991.5)	435.5 (403.8 to 469.1)	430.6 (394.8 to 472.7)
Armenia	Pedestrian road injuries	11.2 (10.3 to 12.7)	4.52 (4.15 to 4.94)	4.15 (3.75 to 4.61)	330.2 (301.9 to 370.1)	132.5 (121.8 to 143.9)	123.6 (112.3 to 137.1)
Armenia	Cyclist road injuries	0.776 (0.595 to 0.985)	0.638 (0.483 to 0.825)	0.849 (0.672 to 1.07)	23.7 (18.2 to 30)	18.2 (13.8 to 23.4)	24.4 (19.6 to 30.5)
Armenia	Motorcyclist road injuries	4.67 (4.04 to 5.36)	2.2 (1.97 to 2.46)	2.05 (1.8 to 2.33)	131.4 (114 to 150.2)	64.2 (57.6 to 71.5)	64.4 (56.7 to 73.2)
Armenia	Motor vehicle road injuries	14.3 (12.6 to 15.4)	7.16 (6.63 to 7.69)	6.75 (6.13 to 7.41)	415.3 (369.3 to 450.2)	208.5 (193.4 to 224)	206.8 (188.5 to 226.5)
Armenia	Other road injuries	1.02 (0.841 to 1.23)	0.414 (0.35 to 0.494)	0.385 (0.322 to 0.469)	29.6 (24.5 to 35.4)	12.1 (10.3 to 14.3)	11.3 (9.5 to 13.6)
Armenia	Other transport injuries	1.51 (1.33 to 1.77)	1.07 (0.911 to 1.23)	0.993 (0.85 to 1.15)	43.6 (38.4 to 51)	31.4 (26.9 to 36.2)	30.9 (26.5 to 35.7)
Armenia	Unintentional injuries	108 (97.2 to 121)	51.9 (44.4 to 61.1)	45.4 (36.8 to 56.2)	3193.3 (2849.9 to 3601.5)	1535.4 (1321.8 to 1794.3)	1343.3 (1100.9 to 1632.1)
Armenia	Falls	29.2 (23.6 to 36.1)	16.3 (12.3 to 21.3)	18.6 (13.8 to 25)	900.7 (726.9 to 1120.9)	465.3 (354.3 to 607.8)	522.9 (390 to 698.6)
Armenia	Drowning	16 (14.9 to 17.3)	3.35 (3.1 to 3.62)	2.42 (2.22 to 2.63)	447.9 (415.3 to 482.8)	107.1 (98.9 to 116.1)	88.3 (80.1 to 97.3)
Armenia	Fire, heat, and hot substances	14.1 (12.5 to 15.9)	4.04 (3.28 to 4.98)	3.61 (2.76 to 4.73)	406.4 (360.3 to 455.1)	123.9 (101.8 to 151.1)	108.3 (84.5 to 139.7)
Armenia	Poisonings	3.54 (3.27 to 3.81)	1.28 (1.14 to 1.44)	1.29 (1.11 to 1.5)	101.5 (93.9 to 109.4)	38.4 (34.4 to 43.1)	40.1 (34.3 to 46.9)
Armenia	Poisoning by carbon monoxide	2.55 (2.34 to 2.76)	0.643 (0.587 to 0.701)	0.564 (0.514 to 0.624)	72.6 (67 to 78.5)	19.4 (17.7 to 21)	17.5 (15.9 to 19.3)
Armenia	Poisoning by other means	0.988 (0.854 to 1.15)	0.638 (0.524 to 0.779)	0.723 (0.573 to 0.91)	28.9 (25.1 to 33.8)	19 (15.7 to 23.3)	22.7 (17.9 to 28.8)
Armenia	Exposure to mechanical forces	9.65 (8.16 to 11.5)	6.23 (5.12 to 7.74)	5.32 (4.11 to 6.9)	286.6 (241.9 to 343.2)	181.4 (150.3 to 223.5)	155.4 (121.6 to 199.2)
Armenia	Unintentional firearm injuries	2.82 (2.4 to 3.36)	1.73 (1.56 to 1.93)	1.13 (1.02 to 1.25)	83 (71.2 to 98.2)	50.4 (45.5 to 56.4)	34.7 (31.2 to 38.8)

SR Appendix Table 2. All-age DALYs counts and age standardized DALYs rates per 100,000 individuals for all causes and level 1 cause groups by location for 1990, 2007, and 2017, both sexes combined

		All-age DALYs			Age-standardized rate of DALYs per 100,000		
Armenia	Other exposure to mechanical forces	6.84 (5.41 to 8.59)	4.5 (3.45 to 5.9)	4.19 (3.08 to 5.68)	203.6 (160.5 to 257.4)	131 (101.3 to 170.3)	120.7 (89.4 to 162.1)
Armenia	Adverse effects of medical treatment	1.09 (0.841 to 1.4)	0.885 (0.785 to 1.14)	0.995 (0.843 to 1.14)	31.9 (25.2 to 40.7)	28.3 (25.1 to 37.5)	32.1 (27.3 to 37.7)
Armenia	Animal contact	0.69 (0.551 to 0.849)	0.553 (0.429 to 0.694)	0.505 (0.395 to 0.638)	20.4 (16.2 to 25.1)	17 (13.2 to 21.2)	16 (12.6 to 20.2)
Armenia	Venomous animal contact	0.422 (0.314 to 0.551)	0.336 (0.246 to 0.445)	0.305 (0.222 to 0.411)	12.4 (9.2 to 16.1)	10.4 (7.6 to 13.8)	9.8 (7.2 to 13.1)
Armenia	Non-venomous animal contact	0.268 (0.213 to 0.349)	0.217 (0.171 to 0.278)	0.2 (0.16 to 0.259)	8 (6.3 to 10.3)	6.5 (5.2 to 8.3)	6.2 (5 to 8)
Armenia	Foreign body	10.7 (9.41 to 12.2)	4.71 (4.38 to 5.08)	3.58 (3.28 to 3.88)	301 (267 to 342.7)	157.3 (144.9 to 172.1)	118.5 (108.3 to 129)
Armenia	Pulmonary aspiration and foreign body in airway	8.57 (7.4 to 10)	3 (2.75 to 3.3)	2.04 (1.86 to 2.27)	238.2 (206.6 to 277.7)	106.6 (96.3 to 119.5)	72.9 (65.6 to 81.3)
Armenia	Foreign body in eyes	0.0675 (0.0307 to 0.119)	0.0755 (0.0355 to 0.131)	0.0784 (0.038 to 0.137)	2 (0.9 to 3.5)	2.3 (1.1 to 4)	2.4 (1.1 to 4.2)
Armenia	Foreign body in other body part	2.02 (1.71 to 2.45)	1.64 (1.45 to 1.82)	1.46 (1.24 to 1.66)	60.8 (52.1 to 72.4)	48.4 (43.3 to 53.9)	43.2 (37.2 to 49)
Armenia	Environmental heat and cold exposure	9.25 (8.34 to 10.2)	6.45 (5.9 to 7.1)	2.64 (2.25 to 3.09)	283 (255.1 to 314.6)	180.8 (165.1 to 199.2)	74.6 (63.4 to 87.4)
Armenia	Exposure to forces of nature	6.81 (4.92 to 9.54)	3.91 (2.84 to 5.43)	2.72 (1.97 to 3.71)	199.4 (144.4 to 279)	110.6 (80 to 153.5)	72.3 (52.6 to 99.8)
Armenia	Other unintentional injuries	7.3 (6.3 to 8.41)	4.17 (3.53 to 4.88)	3.71 (3.02 to 4.62)	214.4 (184.6 to 246.7)	125.2 (107.4 to 145.5)	114.8 (94.8 to 140.7)
Armenia	Self-harm and interpersonal violence	26.5 (25.6 to 27.4)	18 (17.1 to 18.9)	17.9 (16.8 to 19)	755.7 (729.4 to 782.9)	514.8 (490.7 to 540.2)	533.1 (499.7 to 565)
Armenia	Self-harm	4.8 (4.48 to 5.1)	9.78 (9.26 to 10.3)	10.8 (10.1 to 11.4)	142 (132.8 to 150.6)	277 (262.4 to 293.3)	316.9 (297.6 to 338.2)
Armenia	Self-harm by firearm	0.311 (0.212 to 0.423)	0.614 (0.382 to 0.794)	0.67 (0.419 to 0.83)	9.2 (6.3 to 12.4)	17.5 (10.9 to 22.6)	20 (12.1 to 25.4)
Armenia	Self-harm by other specified means	4.49 (4.19 to 4.79)	9.17 (8.64 to 9.77)	10.1 (9.41 to 10.8)	132.8 (124 to 141.3)	259.5 (244.5 to 277)	296.8 (276.5 to 318.6)
Armenia	Interpersonal violence	9.28 (8.55 to 10)	7.23 (6.7 to 7.81)	6.55 (5.96 to 7.25)	265.2 (243.8 to 286.4)	210.2 (195 to 227)	198.2 (180.2 to 218.8)
Armenia	Assault by firearm	1.69 (1.23 to 2.07)	1.32 (1.03 to 1.58)	1.11 (0.861 to 1.31)	47.2 (34.4 to 57.4)	38.6 (30.2 to 46.4)	35.9 (28 to 42.4)
Armenia	Assault by sharp object	2.22 (1.9 to 2.67)	1.63 (1.44 to 1.85)	1.48 (1.31 to 1.67)	61.7 (53.3 to 73.5)	47.5 (41.7 to 54.3)	43.4 (38.3 to 49.2)
Armenia	Sexual violence	0.265 (0.179 to 0.384)	0.255 (0.171 to 0.367)	0.24 (0.162 to 0.345)	7.6 (5.2 to 11)	7.5 (5 to 10.9)	7.7 (5.2 to 11)
Armenia	Assault by other means	5.11 (4.48 to 5.78)	4.03 (3.63 to 4.53)	3.72 (3.26 to 4.33)	148.6 (130.1 to 168.6)	116.6 (105.2 to 131.5)	111.2 (97.8 to 128.4)
Armenia	Conflict and terrorism	12.3 (12.2 to 12.5)	0.889 (0.678 to 1.23)	0.599 (0.417 to 0.904)	346.3 (342.6 to 350.7)	25.6 (19.7 to 35.2)	17.1 (11.7 to 26.1)
Armenia	Executions and police conflict	0.0806 (0.0526 to 0.123)	0.0665 (0.0509 to 0.0878)	0.0265 (0.0228 to 0.0326)	2.3 (1.5 to 3.4)	2 (1.6 to 2.6)	0.9 (0.8 to 1.1)
Azerbaijan	All causes	3040 (2800 to 3320)	2990 (2730 to 3270)	3230 (2910 to 3610)	43509.8 (40205.9 to 47413)	39840.4 (36673.5 to 43156.9)	34532.2 (31176.7 to 38289.5)
Azerbaijan	Communicable, maternal, neonatal, and nutritional disorders	1280 (1170 to 1430)	755 (672 to 845)	595 (489 to 722)	15010.6 (13608.4 to 16682.9)	10161.8 (9019.4 to 11389.6)	7041.7 (5742.3 to 8625.2)
Azerbaijan	HIV/AIDS and sexually transmitted infections	4.7 (3.16 to 6.93)	6.35 (4.97 to 8.49)	5.04 (3.59 to 7.28)	61.3 (41.9 to 88.7)	68.4 (52.9 to 91.3)	46.5 (33.1 to 67.7)
Azerbaijan	HIV/AIDS	0.86 (0.309 to 1.27)	3.1 (2.64 to 3.47)	1.94 (1.48 to 2.29)	11.6 (3.8 to 17.3)	31.9 (27.3 to 35.7)	17 (12.8 to 20)
Azerbaijan	HIV/AIDS - Drug-susceptible Tuberculosis	0.378 (0.135 to 0.557)	0.739 (0.376 to 1.13)	0.327 (0.144 to 0.538)	5.1 (1.7 to 7.6)	7.6 (3.9 to 11.6)	2.9 (1.3 to 4.7)
Azerbaijan	HIV/AIDS - Multidrug-resistant Tuberculosis without extensive drug resistance	0.000704 (0.0000702 to 0.00278)	0.355 (0.126 to 0.637)	0.203 (0.103 to 0.334)	0 (0 to 0)	3.7 (1.3 to 6.5)	1.8 (0.9 to 2.9)
Azerbaijan	HIV/AIDS - Extensively drug-resistant Tuberculosis	0 (0 to 0)	0.112 (0.0395 to 0.204)	0.116 (0.059 to 0.189)	0 (0 to 0)	1.2 (0.4 to 2.1)	1 (0.5 to 1.7)
Azerbaijan	HIV/AIDS resulting in other diseases	0.482 (0.174 to 0.716)	1.89 (1.48 to 2.41)	1.3 (0.945 to 1.71)	6.5 (2.1 to 9.7)	19.5 (15.4 to 24.7)	11.3 (8.2 to 14.9)
Azerbaijan	Sexually transmitted infections excluding HIV	3.84 (2.33 to 5.99)	3.25 (1.94 to 5.35)	3.1 (1.75 to 5.26)	49.7 (30.8 to 76.1)	36.5 (21.6 to 59.6)	29.6 (17.2 to 50.5)
Azerbaijan	Syphilis	1.95 (0.882 to 3.64)	1.03 (0.436 to 2.06)	0.628 (0.26 to 1.28)	23.7 (11.3 to 43)	14.4 (6 to 29)	7.8 (3.1 to 16)
Azerbaijan	Chlamydial infection	0.428 (0.254 to 0.771)	0.536 (0.297 to 0.99)	0.605 (0.331 to 1.12)	5.8 (3.5 to 10.4)	5.3 (2.9 to 9.7)	5.2 (2.9 to 9.8)
Azerbaijan	Gonococcal infection	0.469 (0.3 to 0.785)	0.426 (0.226 to 0.798)	0.441 (0.24 to 0.819)	6.2 (4.2 to 10)	4.1 (2.2 to 7.8)	4 (2.2 to 7.5)
Azerbaijan	Trichomoniasis	0.301 (0.122 to 0.664)	0.405 (0.161 to 0.879)	0.463 (0.186 to 0.994)	4.2 (1.7 to 9.2)	4.1 (1.6 to 8.9)	4 (1.6 to 8.6)
Azerbaijan	Genital herpes	0.17 (0.056 to 0.399)	0.237 (0.0785 to 0.558)	0.295 (0.092 to 0.686)	2.6 (0.9 to 6.2)	2.6 (0.9 to 6.2)	2.6 (0.8 to 6.1)
Azerbaijan	Other sexually transmitted infections	0.521 (0.333 to 0.86)	0.615 (0.359 to 1.08)	0.664 (0.396 to 1.13)	7.1 (4.6 to 11.5)	6 (3.5 to 10.4)	5.9 (3.5 to 10.1)
Azerbaijan	Respiratory infections and tuberculosis	719 (644 to 809)	324 (286 to 368)	247 (203 to 299)	8382.9 (7536.2 to 9405.9)	4429.7 (3881.9 to 5053.5)	2924.4 (2377.2 to 3555)
Azerbaijan	Tuberculosis	42 (37.5 to 46.9)	45.8 (42.4 to 49.5)	32.3 (28.2 to 38)	581.3 (529.7 to 640.6)	518.3 (479.1 to 561.3)	299 (260.8 to 353.4)

SR Appendix Table 2. All-age DALYs counts and age standardized DALYs rates per 100,000 individuals for all causes and level 1 cause groups by location for 1990, 2007, and 2017, both sexes combined							
		All-age DALYs			Age-standardized rate of DALYs per 100,000		
Azerbaijan	Latent tuberculosis infection	0 (0 to 0)	0 (0 to 0)	0 (0 to 0)	0 (0 to 0)	0 (0 to 0)	0 (0 to 0)
Azerbaijan	Drug-susceptible tuberculosis	41.9 (37.4 to 46.9)	30.5 (18.6 to 41.2)	18.4 (11 to 25.9)	580.4 (527.9 to 639.6)	345.4 (211.6 to 462.5)	170.3 (102.4 to 238.7)
Azerbaijan	Multidrug-resistant tuberculosis without extensive drug resistance	0.0635 (0.00878 to 0.224)	11.6 (4.21 to 20.2)	8.85 (4.72 to 13.8)	0.9 (0.1 to 3.1)	131.4 (47.9 to 228)	82.1 (44 to 125.8)
Azerbaijan	Extensively drug-resistant tuberculosis	0 (0 to 0)	3.66 (1.34 to 6.4)	5.03 (2.73 to 7.75)	0 (0 to 0)	41.5 (15.1 to 72.3)	46.6 (25.4 to 71.3)
Azerbaijan	Lower respiratory infections	659 (586 to 741)	269 (232 to 311)	205 (164 to 254)	7587.2 (6757 to 8527.4)	3793.1 (3275.1 to 4393.4)	2524.5 (2009.2 to 3127.4)
Azerbaijan	Upper respiratory infections	15.9 (6.44 to 26.4)	7.45 (5.21 to 10.8)	7.36 (5.03 to 10.7)	189.1 (82 to 308.4)	92 (64.6 to 137.2)	78.2 (53.4 to 115.2)
Azerbaijan	Otitis media	1.98 (1.18 to 3.13)	2.31 (1.38 to 3.68)	2.21 (1.31 to 3.44)	25.4 (15.4 to 40)	26.4 (15.7 to 42)	22.7 (13.6 to 35.7)
Azerbaijan	Enteric infections	134 (109 to 162)	32.5 (25.3 to 40.4)	26 (19.8 to 34.2)	1546.8 (1263 to 1868)	437.1 (337.9 to 544.8)	299.6 (223.7 to 397.4)
Azerbaijan	Diarrhoeal diseases	133 (109 to 161)	31.9 (24.8 to 39.8)	25.5 (19.2 to 33.7)	1538.3 (1257 to 1857.9)	429.6 (330.7 to 538.5)	293.9 (219.6 to 391.7)
Azerbaijan	Typhoid and paratyphoid	0.00307 (0.00148 to 0.00577)	0.00337 (0.00159 to 0.00624)	0.00331 (0.00153 to 0.0061)	0 (0 to 0.1)	0 (0 to 0.1)	0 (0 to 0.1)
Azerbaijan	Typhoid fever	0.00302 (0.00146 to 0.00569)	0.00308 (0.00146 to 0.00573)	0.00295 (0.00138 to 0.00546)	0 (0 to 0.1)	0 (0 to 0.1)	0 (0 to 0.1)
Azerbaijan	Paratyphoid fever	0.0000507 (0.0000202 to 0.00011)	0.000285 (0.000111 to 0.0006)	0.000362 (0.000141 to 0.000815)	0 (0 to 0)	0 (0 to 0)	0 (0 to 0)
Azerbaijan	Invasive non-typhoidal salmonella	0.541 (0.285 to 0.943)	0.527 (0.271 to 0.929)	0.469 (0.24 to 0.843)	6.9 (3.7 to 12)	6.5 (3.4 to 11.1)	5.1 (2.6 to 9.3)
Azerbaijan	Other intestinal infectious diseases	0.138 (0.0304 to 0.29)	0.0716 (0.0178 to 0.147)	0.0496 (0.0146 to 0.103)	1.6 (0.4 to 3.3)	1 (0.2 to 2.1)	0.6 (0.2 to 1.3)
Azerbaijan	Neglected tropical diseases and malaria	5.15 (3.48 to 7.45)	4.54 (2.82 to 7.38)	4.16 (2.39 to 7.24)	68.8 (46.9 to 99)	51.1 (31.7 to 82.3)	39.6 (23.1 to 70.2)
Azerbaijan	Malaria	0.267 (0.225 to 0.317)	0.0859 (0.0701 to 0.104)	0 (0 to 0)	3.6 (3.1 to 4.3)	1 (0.8 to 1.2)	0 (0 to 0)
Azerbaijan	Chagas disease	0 (0 to 0)	0 (0 to 0)	0 (0 to 0)	0 (0 to 0)	0 (0 to 0)	0 (0 to 0)
Azerbaijan	Leishmaniasis	0.204 (0.0116 to 1.46)	0.297 (0.0435 to 2.26)	0.343 (0.0491 to 2.74)	2.7 (0.2 to 17.8)	3.5 (0.5 to 27.4)	3.5 (0.5 to 29.2)
Azerbaijan	Visceral leishmaniasis	0.146 (0.000457 to 1.42)	0.214 (0.000631 to 2.21)	0.257 (0.000864 to 2.65)	1.8 (0 to 17.2)	2.6 (0 to 26.5)	2.7 (0 to 28.5)
Azerbaijan	Cutaneous and mucocutaneous leishmaniasis	0.058 (0.00661 to 0.162)	0.0836 (0.0385 to 0.162)	0.0856 (0.0399 to 0.163)	0.9 (0.1 to 2.5)	1 (0.4 to 1.9)	0.8 (0.4 to 1.5)
Azerbaijan	African trypanosomiasis	0 (0 to 0)	0 (0 to 0)	0 (0 to 0)	0 (0 to 0)	0 (0 to 0)	0 (0 to 0)
Azerbaijan	Schistosomiasis	0 (0 to 0)	0 (0 to 0)	0 (0 to 0)	0 (0 to 0)	0 (0 to 0)	0 (0 to 0)
Azerbaijan	Cysticercosis	0.000309 (0.000162 to 0.000486)	0.000287 (0.000153 to 0.000439)	0.000144 (0.000072 to 0.000218)	0 (0 to 0)	0 (0 to 0)	0 (0 to 0)
Azerbaijan	Cystic echinococcosis	1.13 (0.459 to 2.32)	1.46 (0.577 to 3)	1.73 (0.686 to 3.52)	16.3 (7.1 to 32.3)	15.8 (6.6 to 30.9)	15.5 (6.2 to 31.3)
Azerbaijan	Lymphatic filariasis	0 (0 to 0)	0 (0 to 0)	0 (0 to 0)	0 (0 to 0)	0 (0 to 0)	0 (0 to 0)
Azerbaijan	Onchocerciasis	0 (0 to 0)	0 (0 to 0)	0 (0 to 0)	0 (0 to 0)	0 (0 to 0)	0 (0 to 0)
Azerbaijan	Trachoma	0 (0 to 0)	0 (0 to 0)	0 (0 to 0)	0 (0 to 0)	0 (0 to 0)	0 (0 to 0)
Azerbaijan	Dengue	0 (0 to 0)	0 (0 to 0)	0 (0 to 0)	0 (0 to 0)	0 (0 to 0)	0 (0 to 0)
Azerbaijan	Yellow fever	0 (0 to 0)	0 (0 to 0)	0 (0 to 0)	0 (0 to 0)	0 (0 to 0)	0 (0 to 0)
Azerbaijan	Rabies	0.922 (0.517 to 1.67)	0.282 (0.19 to 0.401)	0.201 (0.126 to 0.317)	12 (7 to 20.7)	3.2 (2.1 to 4.5)	2 (1.2 to 3.3)
Azerbaijan	Intestinal nematode infections	0.563 (0.313 to 0.928)	0.288 (0.157 to 0.489)	0.15 (0.0756 to 0.269)	7.8 (4.3 to 12.6)	3.1 (1.7 to 5.3)	1.4 (0.7 to 2.5)
Azerbaijan	Ascariasis	0.0281 (0.00648 to 0.0662)	0.0154 (0.00693 to 0.0279)	0.00991 (0.00472 to 0.0178)	0.3 (0.1 to 0.8)	0.2 (0.1 to 0.4)	0.1 (0 to 0.2)
Azerbaijan	Trichuriasis	0.000000000956 (0.000000000416 to 0.00000000182)	0.000000000241 (0.000000000107 to 0.000000000488)	0.000000000251 (0.000000000103 to 0.000000000502)	0 (0 to 0)	0 (0 to 0)	0 (0 to 0)
Azerbaijan	Hookworm disease	0.535 (0.286 to 0.897)	0.273 (0.145 to 0.472)	0.14 (0.0695 to 0.256)	7.4 (4 to 12.3)	2.9 (1.6 to 5.1)	1.3 (0.7 to 2.4)
Azerbaijan	Food-borne trematodiases	0 (0 to 0)	0 (0 to 0)	0 (0 to 0)	0 (0 to 0)	0 (0 to 0)	0 (0 to 0)
Azerbaijan	Leprosy	0 (0 to 0)	0 (0 to 0)	0 (0 to 0)	0 (0 to 0)	0 (0 to 0)	0 (0 to 0)
Azerbaijan	Ebola virus disease	0 (0 to 0)	0 (0 to 0)	0 (0 to 0)	0 (0 to 0)	0 (0 to 0)	0 (0 to 0)
Azerbaijan	Zika virus disease	0 (0 to 0)	0 (0 to 0)	0 (0 to 0)	0 (0 to 0)	0 (0 to 0)	0 (0 to 0)
Azerbaijan	Guinea worm disease	0 (0 to 0)	0 (0 to 0)	0 (0 to 0)	0 (0 to 0)	0 (0 to 0)	0 (0 to 0)
Azerbaijan	Other neglected tropical diseases	2.06 (1.32 to 3.08)	2.13 (1.36 to 3.22)	1.74 (1.11 to 2.65)	26.4 (16.9 to 39.2)	24.5 (15.8 to 37)	17.1 (10.9 to 25.9)

SR Appendix Table 2. All-age DALYs counts and age standardized DALYs rates per 100,000 individuals for all causes and level 1 cause groups by location for 1990, 2007, and 2017, both sexes combined

		All-age DALYs			Age-standardized rate of DALYs per 100,000		
Azerbaijan	Other infectious diseases	57.6 (41.7 to 82.1)	45.8 (33.7 to 65.4)	33.5 (25.3 to 43.8)	680.2 (499.5 to 959.3)	609.7 (435.7 to 895.5)	380.5 (279.1 to 506.9)
Azerbaijan	Meningitis	11.9 (9.9 to 16.3)	10.6 (9.04 to 12.4)	7.42 (6.14 to 8.97)	143.2 (119.1 to 195.1)	134.1 (114 to 157.1)	81.2 (66.6 to 99.4)
Azerbaijan	Pneumococcal meningitis	0.741 (0.562 to 0.939)	0.89 (0.675 to 1.15)	0.501 (0.387 to 0.63)	9.7 (7.3 to 12.2)	10 (7.8 to 12.7)	5 (3.9 to 6.2)
Azerbaijan	H influenzae type B meningitis	6.92 (5.66 to 9.61)	5.95 (5 to 7.04)	3.97 (3.17 to 4.88)	82.1 (67.3 to 114.3)	76.8 (64.5 to 92.1)	44.6 (35.1 to 56)
Azerbaijan	Meningococcal infection	1.79 (1.4 to 2.5)	1.45 (1.18 to 1.79)	1.03 (0.809 to 1.28)	21.5 (16.9 to 29.9)	18.5 (14.8 to 23.2)	11.2 (8.8 to 14.1)
Azerbaijan	Other meningitis	2.48 (1.99 to 3.55)	2.31 (1.9 to 2.77)	1.93 (1.59 to 2.36)	29.9 (24.2 to 42.8)	28.9 (23.6 to 35)	20.5 (16.6 to 25.3)
Azerbaijan	Encephalitis	15.9 (7.9 to 24.7)	13.9 (8.14 to 17.4)	13 (8.16 to 18.1)	186.8 (96.9 to 287.1)	186.5 (104.1 to 237.4)	152 (91.3 to 214.1)
Azerbaijan	Diphtheria	2.62 (1.08 to 4.97)	0.402 (0.202 to 0.728)	0.0661 (0.0346 to 0.116)	30.6 (13.3 to 57.1)	5.4 (2.6 to 10.1)	0.7 (0.4 to 1.3)
Azerbaijan	Whooping cough	13.9 (1.45 to 38.2)	10.3 (1.12 to 30)	4.14 (0.415 to 12.3)	157.4 (16.4 to 434.5)	149.6 (16.3 to 433.1)	50.3 (5 to 149.1)
Azerbaijan	Tetanus	0.158 (0.0682 to 0.2)	0.0981 (0.0662 to 0.116)	0.0791 (0.06 to 0.11)	2 (0.9 to 2.5)	1.1 (0.8 to 1.3)	0.8 (0.6 to 1.1)
Azerbaijan	Measles	0.331 (0.236 to 0.452)	0.00248 (0.00145 to 0.00425)	0.000314 (0 to 0.00153)	3.8 (2.7 to 5.1)	0 (0 to 0.1)	0 (0 to 0)
Azerbaijan	Varicella and herpes zoster	0.891 (0.594 to 1.32)	0.709 (0.493 to 0.983)	0.561 (0.397 to 0.755)	12 (8.4 to 17)	9.8 (6.7 to 13.7)	6.2 (4.4 to 8.5)
Azerbaijan	Acute hepatitis	5.41 (4.44 to 6.73)	4.09 (3.35 to 4.88)	3.31 (2.73 to 4.11)	66.8 (55.2 to 82.2)	49.4 (40.5 to 59.5)	34 (27.8 to 42.6)
Azerbaijan	Acute hepatitis A	1.54 (1.2 to 2.09)	1.2 (0.971 to 1.5)	0.952 (0.736 to 1.23)	18.1 (14.1 to 24.5)	14 (11.3 to 17.7)	10.2 (7.9 to 13.5)
Azerbaijan	Acute hepatitis B	1.9 (1.47 to 2.54)	2.04 (1.54 to 2.42)	1.77 (1.43 to 2.21)	26 (20.8 to 34.1)	24 (18 to 28.7)	16.9 (13.6 to 21.1)
Azerbaijan	Acute hepatitis C	1.76 (1.08 to 2.55)	0.627 (0.396 to 0.991)	0.421 (0.234 to 0.723)	20.1 (12.4 to 29.1)	9 (5.6 to 14.3)	5.2 (2.9 to 9)
Azerbaijan	Acute hepatitis E	0.209 (0.161 to 0.295)	0.226 (0.175 to 0.293)	0.166 (0.127 to 0.215)	2.6 (2 to 3.6)	2.4 (1.9 to 3.1)	1.7 (1.3 to 2.2)
Azerbaijan	Other unspecified infectious diseases	6.46 (3.48 to 8.83)	5.69 (3.28 to 7.47)	4.87 (2.9 to 6.63)	77.7 (43.4 to 105.6)	73.8 (40.2 to 96.4)	55.3 (30.6 to 75.8)
Azerbaijan	Maternal and neonatal disorders	304 (262 to 348)	290 (251 to 333)	234 (181 to 302)	3490.8 (3016.9 to 4000.5)	3965.1 (3432.8 to 4554)	2910.1 (2237 to 3780)
Azerbaijan	Maternal disorders	4.92 (4.28 to 5.61)	3.4 (2.9 to 3.98)	2.85 (2.32 to 3.49)	63 (55 to 71.4)	33.4 (28.3 to 39.2)	24.7 (20 to 30.2)
Azerbaijan	Maternal haemorrhage	1.26 (0.939 to 1.64)	0.675 (0.486 to 0.895)	0.387 (0.257 to 0.543)	16.3 (12.2 to 21)	6.7 (4.8 to 8.9)	3.3 (2.2 to 4.6)
Azerbaijan	Maternal sepsis and other pregnancy related infections	0.938 (0.719 to 1.2)	0.453 (0.347 to 0.593)	0.308 (0.217 to 0.427)	12.3 (9.6 to 15.6)	4.3 (3.3 to 5.8)	2.7 (1.9 to 3.8)
Azerbaijan	Maternal hypertensive disorders	0.363 (0.271 to 0.48)	0.162 (0.119 to 0.211)	0.166 (0.116 to 0.227)	4.4 (3.3 to 5.7)	1.6 (1.2 to 2.1)	1.4 (1 to 2)
Azerbaijan	Maternal obstructed labour and uterine rupture	0.283 (0.173 to 0.424)	0.229 (0.141 to 0.339)	0.207 (0.129 to 0.317)	3.4 (2.1 to 5.1)	2.2 (1.4 to 3.2)	1.8 (1.2 to 2.8)
Azerbaijan	Maternal abortive outcome	0.497 (0.388 to 0.634)	0.47 (0.364 to 0.612)	0.415 (0.303 to 0.553)	6.6 (5.2 to 8.3)	4.5 (3.5 to 5.9)	3.6 (2.7 to 4.8)
Azerbaijan	Ectopic pregnancy	0.093 (0.0285 to 0.268)	0.0636 (0.0201 to 0.163)	0.0546 (0.0174 to 0.142)	1.2 (0.4 to 3.3)	0.6 (0.2 to 1.6)	0.5 (0.2 to 1.2)
Azerbaijan	Indirect maternal deaths	0.243 (0.102 to 0.466)	0.247 (0.128 to 0.417)	0.166 (0.0736 to 0.316)	3.3 (1.5 to 6.3)	2.5 (1.3 to 4.4)	1.4 (0.6 to 2.7)
Azerbaijan	Late maternal deaths	0.131 (0.093 to 0.18)	0.112 (0.0799 to 0.152)	0.163 (0.11 to 0.234)	1.6 (1.2 to 2.2)	1.1 (0.8 to 1.5)	1.4 (0.9 to 2)
Azerbaijan	Maternal deaths aggravated by HIV/AIDS	0.0000944 (0.0000432 to 0.00017)	0.000179 (0.0000834 to 0.000316)	0.000484 (0.000233 to 0.000825)	0 (0 to 0)	0 (0 to 0)	0 (0 to 0)
Azerbaijan	Other maternal disorders	1.11 (0.787 to 1.52)	0.983 (0.712 to 1.3)	0.986 (0.698 to 1.33)	13.9 (10.1 to 18.8)	9.7 (7.1 to 13)	8.4 (6 to 11.3)
Azerbaijan	Neonatal disorders	299 (257 to 343)	287 (248 to 330)	231 (178 to 299)	3427.8 (2952.4 to 3934.2)	3931.7 (3398 to 4523.4)	2885.5 (2209.7 to 3753.8)
Azerbaijan	Neonatal preterm birth	127 (94.7 to 160)	115 (86.1 to 147)	87.6 (58.3 to 125)	1461.8 (1091.1 to 1837.7)	1569.5 (1168.8 to 2017.4)	1094.2 (721.2 to 1576)
Azerbaijan	Neonatal encephalopathy due to birth asphyxia and trauma	144 (113 to 179)	143 (110 to 181)	116 (81.8 to 164)	1643.9 (1299.9 to 2044.3)	1974.2 (1513 to 2486.7)	1456.7 (1023.2 to 2056.2)
Azerbaijan	Neonatal sepsis and other neonatal infections	17.4 (11.1 to 26.2)	18.1 (12.2 to 26.4)	18.2 (11.6 to 26.4)	200.9 (129 to 302.5)	246.7 (165.9 to 362.5)	223.6 (142.3 to 329.9)
Azerbaijan	Hemolytic disease and other neonatal jaundice	4 (2.99 to 5.48)	4.76 (3.38 to 6.62)	3.84 (2.69 to 5.31)	48.3 (36.3 to 64.9)	60.6 (42.1 to 84.9)	42.7 (29.4 to 61.2)
Azerbaijan	Other neonatal disorders	6.4 (4.31 to 10.6)	5.71 (4.06 to 8.81)	5.39 (3.49 to 8.36)	72.8 (49.1 to 120.1)	80.8 (57.6 to 124.2)	68.3 (44 to 106.5)
Azerbaijan	Nutritional deficiencies	60.6 (42.3 to 84.1)	52 (34.6 to 75.8)	44.5 (29.4 to 66.1)	779.8 (541.5 to 1082.7)	600.6 (402 to 881)	440.8 (293.2 to 652.4)
Azerbaijan	Protein-energy malnutrition	10.4 (8.42 to 12.8)	2.63 (2.14 to 3.24)	2.08 (1.55 to 2.77)	120.3 (98.3 to 147.8)	36.1 (29.2 to 44.9)	23.8 (17.5 to 32.1)
Azerbaijan	Iodine deficiency	1.85 (0.983 to 3.14)	1.95 (1.03 to 3.22)	1.59 (0.812 to 2.7)	25.8 (13.8 to 43.8)	21.1 (11.3 to 35)	15 (7.7 to 25.5)
Azerbaijan	Dietary iron deficiency	36.9 (23.6 to 54.3)	41.7 (26.8 to 62.1)	36.6 (23.4 to 54.7)	498.2 (323.1 to 732.6)	466.2 (301.5 to 702.7)	352.5 (226.7 to 532.5)

SR Appendix Table 2. All-age DALYs counts and age standardized DALYs rates per 100,000 individuals for all causes and level 1 cause groups by location for 1990, 2007, and 2017, both sexes combined

		All-age DALYs			Age-standardized rate of DALYs per 100,000		
Azerbaijan	Other nutritional deficiencies	1.57 (1.15 to 2.21)	0.524 (0.433 to 0.644)	0.479 (0.38 to 0.595)	19.3 (14.3 to 26.6)	6.6 (5.4 to 8.3)	5.1 (4 to 6.4)
Azerbaijan	Non-communicable diseases	1450 (1310 to 1610)	1990 (1810 to 2210)	2370 (2130 to 2640)	24305 (22154.1 to 26794.5)	26978.1 (24826.7 to 29589.5)	24970.6 (22615.5 to 27680)
Azerbaijan	Neoplasms	188 (179 to 197)	250 (236 to 262)	324 (299 to 350)	3170 (3025.8 to 3315.4)	3214.8 (3058.5 to 3353.2)	3165.5 (2937.9 to 3414.6)
Azerbaijan	Lip and oral cavity cancer	1.28 (1.15 to 1.44)	2.69 (2.47 to 2.94)	3.26 (2.85 to 3.7)	22.8 (20.5 to 25.6)	34.6 (31.7 to 37.8)	31.2 (27.5 to 35.2)
Azerbaijan	Nasopharynx cancer	0.398 (0.331 to 0.477)	0.845 (0.763 to 0.94)	0.799 (0.688 to 0.947)	6 (5 to 7.1)	9.6 (8.7 to 10.6)	7.4 (6.4 to 8.8)
Azerbaijan	Other pharynx cancer	0.548 (0.439 to 0.763)	1.16 (0.851 to 1.33)	1.54 (1.12 to 1.99)	9.3 (7.5 to 12.9)	14.6 (11.2 to 16.7)	14.1 (10.3 to 18.1)
Azerbaijan	Oesophageal cancer	11.3 (10.7 to 12)	13.4 (12.2 to 14.8)	18.9 (16.4 to 22.3)	205.6 (194.4 to 217.9)	184.8 (168.8 to 204)	191.6 (166.5 to 225.1)
Azerbaijan	Stomach cancer	32.6 (30.8 to 34.5)	36.6 (34 to 39)	44.2 (38.7 to 49.7)	585.5 (555.1 to 616.2)	503.4 (468.7 to 537.5)	446.8 (392.7 to 499.4)
Azerbaijan	Colon and rectum cancer	11.7 (11 to 12.5)	15.1 (14 to 16.2)	21 (17.8 to 24.2)	205.2 (193.4 to 218)	200.1 (186.3 to 216.2)	207.8 (177.1 to 237.2)
Azerbaijan	Liver cancer	9.96 (8.35 to 11.9)	13.2 (12.2 to 14.5)	18.9 (15.1 to 23.4)	179.8 (148.7 to 215.7)	178.7 (163.7 to 196.4)	191 (152.5 to 234.4)
Azerbaijan	Liver cancer due to hepatitis B	2.85 (2.26 to 3.67)	3.64 (2.99 to 4.38)	4.83 (3.5 to 6.27)	47.2 (37.1 to 60.2)	44.9 (36.5 to 54.3)	45.4 (33.2 to 59.4)
Azerbaijan	Liver cancer due to hepatitis C	3.58 (2.79 to 4.46)	4.74 (4.06 to 5.5)	7.05 (5.4 to 9.03)	67.8 (53 to 84.9)	68.5 (58.7 to 78.3)	73.7 (56.5 to 93.5)
Azerbaijan	Liver cancer due to alcohol use	2.29 (1.82 to 2.9)	3.22 (2.78 to 3.76)	4.61 (3.53 to 6.01)	42.8 (34 to 53.6)	43.2 (37.3 to 50.8)	47.5 (36.3 to 60.4)
Azerbaijan	Liver cancer due to NASH	0.586 (0.466 to 0.731)	0.81 (0.693 to 0.949)	1.28 (0.989 to 1.66)	10.9 (8.6 to 14)	11.5 (9.8 to 13.5)	13.6 (10.4 to 17.6)
Azerbaijan	Liver cancer due to other causes	0.658 (0.54 to 0.803)	0.83 (0.714 to 0.957)	1.12 (0.867 to 1.42)	11.1 (9 to 13.5)	10.6 (9.2 to 12.4)	10.8 (8.4 to 13.6)
Azerbaijan	Gallbladder and biliary tract cancer	1.67 (1.4 to 2.17)	2.7 (2.08 to 3.04)	3.3 (2.65 to 3.92)	31.6 (25.9 to 42)	37.1 (29.4 to 42)	33.1 (27.5 to 39)
Azerbaijan	Pancreatic cancer	4.58 (4.04 to 5.3)	8.23 (7.67 to 8.83)	12.8 (11.3 to 14.3)	85.4 (73.9 to 99.8)	112.7 (104.9 to 120.7)	129.9 (115.4 to 144.9)
Azerbaijan	Larynx cancer	4.09 (3.77 to 4.46)	4.67 (4.22 to 5.15)	6.25 (5.33 to 7.33)	70.2 (64.7 to 76.3)	60.8 (55 to 66.6)	57.7 (49.4 to 67.4)
Azerbaijan	Tracheal, bronchus, and lung cancer	30.1 (28.2 to 32.1)	34.9 (32 to 38)	54 (43.5 to 63.1)	520.4 (488.1 to 553.9)	463.8 (426.2 to 504.6)	507.5 (413.2 to 589.4)
Azerbaijan	Malignant skin melanoma	0.759 (0.525 to 0.907)	1.01 (0.747 to 1.2)	1.32 (0.932 to 1.6)	12.9 (8.9 to 15.4)	12.8 (9.4 to 15.1)	12.5 (9 to 15.2)
Azerbaijan	Non-melanoma skin cancer	0.889 (0.81 to 0.984)	1.63 (1.5 to 1.76)	2.07 (1.81 to 2.34)	15.6 (14.1 to 17.3)	21.9 (20.1 to 23.7)	20.6 (18.3 to 23.1)
Azerbaijan	Non-melanoma skin cancer (squamous-cell carcinoma)	0.887 (0.808 to 0.983)	1.63 (1.5 to 1.76)	2.07 (1.81 to 2.34)	15.6 (14.1 to 17.3)	21.8 (20.1 to 23.6)	20.6 (18.2 to 23)
Azerbaijan	Non-melanoma skin cancer (basal-cell carcinoma)	0.00171 (0.00056 to 0.00412)	0.00223 (0.000751 to 0.00521)	0.00305 (0.00104 to 0.00691)	0 (0 to 0.1)	0 (0 to 0.1)	0 (0 to 0.1)
Azerbaijan	Breast cancer	11.3 (10.2 to 12.4)	19.6 (17.4 to 21.5)	24.6 (20.6 to 28.8)	200.7 (179.6 to 218.3)	227.8 (203.6 to 248.4)	216.8 (184.2 to 252.1)
Azerbaijan	Cervical cancer	4.46 (3.97 to 5.71)	9.21 (7.95 to 10.4)	9.19 (7.52 to 11.1)	77.5 (69.3 to 100.3)	107.4 (94 to 120)	81.5 (67.2 to 97.3)
Azerbaijan	Uterine cancer	1.75 (1.55 to 1.95)	2.78 (2.45 to 3.18)	3.52 (2.86 to 4.27)	31.6 (28.1 to 35.1)	36.3 (32.1 to 41.5)	32.9 (26.8 to 39.7)
Azerbaijan	Ovarian cancer	2.55 (2.14 to 2.97)	4.97 (4.41 to 5.59)	6.24 (5.18 to 7.33)	43.9 (37.1 to 50.5)	58.3 (51.8 to 65.7)	55.1 (46 to 64.8)
Azerbaijan	Prostate cancer	2.79 (2.43 to 3.91)	5.22 (3.73 to 5.94)	7.22 (5.58 to 8.7)	56.9 (49.2 to 81.3)	82.5 (60.5 to 95.3)	89.6 (71.2 to 108.4)
Azerbaijan	Testicular cancer	0.228 (0.153 to 0.317)	0.227 (0.169 to 0.299)	0.232 (0.163 to 0.327)	2.6 (1.8 to 3.6)	2.3 (1.7 to 3.1)	2 (1.4 to 2.7)
Azerbaijan	Kidney cancer	5 (3.69 to 5.86)	5.43 (4.88 to 6.11)	8.95 (7.52 to 10.6)	82 (59.7 to 97)	70.8 (63.7 to 79.8)	84.1 (71.1 to 99.2)
Azerbaijan	Bladder cancer	2.82 (2.01 to 3.45)	3.58 (3.25 to 3.98)	5.01 (4.35 to 5.75)	52.3 (37.8 to 63.7)	51 (46.1 to 58.3)	52.1 (45.8 to 59.8)
Azerbaijan	Thyroid cancer	0.639 (0.559 to 0.752)	0.858 (0.752 to 1.02)	1.15 (0.961 to 1.41)	11.1 (9.6 to 13.4)	10.9 (9.6 to 13.5)	11 (9.3 to 13.9)
Azerbaijan	Mesothelioma	0.143 (0.126 to 0.158)	0.275 (0.25 to 0.3)	0.291 (0.248 to 0.334)	2.5 (2.2 to 2.7)	3.5 (3.2 to 3.8)	2.8 (2.4 to 3.2)
Azerbaijan	Hodgkin lymphoma	2.55 (1.64 to 3.18)	2.98 (1.86 to 3.69)	2.59 (1.68 to 3.35)	35 (23.2 to 43.1)	32.6 (21 to 39.8)	23.8 (15.4 to 30.4)
Azerbaijan	Non-Hodgkin's lymphoma	3.27 (2.91 to 3.74)	5.46 (4.98 to 5.95)	6.35 (5.18 to 7.47)	48.9 (43.8 to 54.6)	65.7 (60 to 71.7)	63 (51.2 to 73.9)
Azerbaijan	Multiple myeloma	0.752 (0.648 to 0.917)	1.23 (1.11 to 1.46)	1.8 (1.47 to 2.24)	13.5 (11.6 to 16.3)	16.2 (14.6 to 19.2)	17.4 (14.2 to 21.6)
Azerbaijan	Leukaemia	15.2 (13.4 to 18.2)	21.4 (15.8 to 24.9)	21.4 (16.1 to 26.1)	201.1 (178.1 to 241.8)	254.2 (187.8 to 297.8)	222.4 (165.2 to 275.2)
Azerbaijan	Acute lymphoid leukaemia	2.45 (1.87 to 3.25)	3.61 (2.45 to 4.57)	3.72 (2.43 to 5)	30.7 (23.7 to 40.5)	43.1 (28.5 to 55.5)	40.6 (26.5 to 55.4)
Azerbaijan	Chronic lymphoid leukaemia	0.254 (0.2 to 0.375)	0.42 (0.339 to 0.522)	0.499 (0.398 to 0.647)	4.6 (3.6 to 6.9)	5.3 (4.3 to 6.7)	5.1 (4 to 6.4)
Azerbaijan	Acute myeloid leukaemia	2.24 (1.75 to 3.28)	3.89 (2.9 to 4.64)	4.44 (3.43 to 5.4)	30.5 (24.3 to 43.9)	44.2 (32.7 to 52.7)	44.3 (33.7 to 54.4)

SR Appendix Table 2. All-age DALYs counts and age standardized DALYs rates per 100,000 individuals for all causes and level 1 cause groups by location for 1990, 2007, and 2017, both sexes combined

		All-age DALYs			Age-standardized rate of DALYs per 100,000		
Azerbaijan	Chronic myeloid leukaemia	0.494 (0.377 to 0.676)	0.694 (0.53 to 0.853)	0.723 (0.553 to 0.909)	7.8 (6.1 to 10.7)	8.1 (6.3 to 9.9)	6.7 (5.1 to 8.3)
Azerbaijan	Other leukaemia	9.81 (8.3 to 11.5)	12.8 (8.78 to 15.5)	12 (8.65 to 15.1)	127.6 (109.9 to 149.4)	153.5 (105.8 to 187.9)	125.6 (89.9 to 161.1)
Azerbaijan	Other malignant cancers	13.6 (11.5 to 15.4)	16 (14.4 to 17.6)	19.2 (16.6 to 22.3)	200.3 (172.8 to 224.8)	191 (173.5 to 212.5)	189.5 (164.5 to 219)
Azerbaijan	Other neoplasms	0.58 (0.468 to 0.84)	0.841 (0.705 to 1.11)	1.28 (1.05 to 1.66)	9.4 (7.6 to 12.5)	11 (9.2 to 14.3)	13.6 (10.9 to 17.1)
Azerbaijan	Myelodysplastic, myeloproliferative, and other hematopoietic neoplasms	0.45 (0.365 to 0.605)	0.673 (0.55 to 0.85)	1.06 (0.847 to 1.32)	7.7 (6.2 to 9.8)	9.2 (7.3 to 11.2)	11.3 (8.7 to 13.8)
Azerbaijan	Benign and in situ intestinal neoplasms	0 (0 to 0)	0 (0 to 0)	0 (0 to 0)	0 (0 to 0)	0 (0 to 0)	0 (0 to 0)
Azerbaijan	Benign and in situ cervical and uterine neoplasms	0 (0 to 0)	0 (0 to 0)	0 (0 to 0)	0 (0 to 0)	0 (0 to 0)	0 (0 to 0)
Azerbaijan	Other benign and in situ neoplasms	0.13 (0.0906 to 0.224)	0.168 (0.126 to 0.266)	0.222 (0.154 to 0.351)	1.7 (1.2 to 2.8)	1.9 (1.4 to 3.1)	2.2 (1.5 to 3.6)
Azerbaijan	Cardiovascular diseases	473 (455 to 493)	734 (706 to 763)	845 (794 to 903)	9103.2 (8763.4 to 9471.2)	11090.1 (10689.5 to 11512.7)	9660.4 (9110.8 to 10289.9)
Azerbaijan	Rheumatic heart disease	10 (9.01 to 11.4)	13.9 (12.1 to 15.7)	11.4 (9.63 to 13.5)	152.3 (137.6 to 171.3)	164.4 (145.3 to 184.3)	109.2 (93.2 to 127.5)
Azerbaijan	Ischaemic heart disease	297 (285 to 310)	445 (424 to 464)	538 (500 to 579)	5818.3 (5575 to 6072.6)	6798.4 (6502.3 to 7068.4)	6201.9 (5793.3 to 6660.9)
Azerbaijan	Stroke	113 (107 to 118)	204 (193 to 214)	209 (194 to 226)	2194.6 (2083.9 to 2311.8)	3149.3 (2993 to 3298.8)	2436.2 (2252.5 to 2625.3)
Azerbaijan	Ischaemic stroke	26.3 (23.7 to 29)	49.3 (44.7 to 53.9)	54.6 (48.7 to 60.4)	544.3 (490.8 to 600.5)	812.6 (741.9 to 882.1)	683.8 (615.2 to 754.2)
Azerbaijan	Intracerebral hemorrhage	80.1 (75.7 to 84.2)	145 (136 to 153)	144 (132 to 157)	1545.1 (1460.7 to 1623.6)	2205.9 (2082.8 to 2322.4)	1650.9 (1511.9 to 1794.9)
Azerbaijan	Subarachnoid hemorrhage	6.11 (5.12 to 8.65)	9.94 (8.37 to 14.6)	10.5 (8.57 to 15.7)	105.1 (86.9 to 153.5)	130.8 (110.2 to 196.2)	101.6 (82.8 to 154.3)
Azerbaijan	Hypertensive heart disease	8.47 (7.12 to 11.2)	13.2 (11.8 to 18)	14.8 (12.6 to 20.1)	167.5 (137.5 to 227)	199.6 (176.8 to 276.9)	169.8 (144.3 to 233.6)
Azerbaijan	Non-rheumatic valvular heart disease	0.63 (0.535 to 0.749)	1.15 (0.973 to 1.31)	1.32 (1.12 to 1.55)	11.6 (9.5 to 14)	16 (13.8 to 18.5)	14.9 (12.4 to 17.8)
Azerbaijan	Non-rheumatic calcific aortic valve disease	0.27 (0.217 to 0.33)	0.532 (0.418 to 0.625)	0.67 (0.55 to 0.8)	5 (4 to 6.1)	7.4 (6.1 to 8.8)	7.6 (6.2 to 9.1)
Azerbaijan	Non-rheumatic degenerative mitral valve disease	0.255 (0.205 to 0.322)	0.407 (0.33 to 0.498)	0.424 (0.33 to 0.543)	4.8 (3.7 to 6.2)	5.7 (4.6 to 7.1)	4.9 (3.8 to 6.4)
Azerbaijan	Other non-rheumatic valve diseases	0.105 (0.0701 to 0.14)	0.216 (0.144 to 0.287)	0.225 (0.149 to 0.294)	1.9 (1.2 to 2.6)	2.9 (1.9 to 3.7)	2.4 (1.6 to 3.1)
Azerbaijan	Cardiomyopathy and myocarditis	22.9 (20.9 to 25.8)	24.1 (21.6 to 37.5)	30.8 (26.5 to 45.3)	366.9 (332.1 to 416.2)	304.6 (276.1 to 456.2)	303 (261.7 to 427.8)
Azerbaijan	Myocarditis	3.37 (2.65 to 4.73)	4.08 (3.24 to 5.67)	4.45 (3.38 to 6.61)	50.3 (39.2 to 72.2)	48.1 (38.6 to 64.3)	42.4 (32.8 to 60.7)
Azerbaijan	Alcoholic cardiomyopathy	3.68 (2.16 to 5.34)	4.14 (2.2 to 6.47)	5.02 (2.85 to 8.22)	61.8 (36 to 88.4)	48.6 (27.3 to 75.3)	44.7 (26.3 to 73)
Azerbaijan	Other cardiomyopathy	15.9 (13.9 to 18.8)	15.8 (13.1 to 27.9)	21.4 (17.6 to 33.9)	254.8 (222.6 to 304.2)	207.9 (175 to 342.7)	215.9 (181.8 to 320.8)
Azerbaijan	Atrial fibrillation and flutter	3.44 (2.63 to 4.25)	4.57 (3.75 to 5.56)	6.64 (5.5 to 8.04)	75.4 (56.9 to 92.8)	86.1 (72.8 to 102.1)	90.6 (76.2 to 107.1)
Azerbaijan	Aortic aneurysm	1.4 (1.15 to 1.74)	2.49 (2.22 to 2.79)	3.36 (2.82 to 3.87)	25.9 (20.8 to 33.5)	35.5 (31.5 to 39.9)	36.8 (30.4 to 42.4)
Azerbaijan	Peripheral vascular disease	0.32 (0.168 to 0.54)	0.427 (0.228 to 0.719)	0.573 (0.324 to 0.931)	6.9 (3.6 to 11.5)	7.4 (4 to 12.3)	7.4 (4.2 to 11.9)
Azerbaijan	Endocarditis	1.27 (0.984 to 1.57)	1.84 (1.68 to 2.02)	2.06 (1.79 to 2.34)	17.8 (13.6 to 22.2)	22.6 (20.7 to 24.7)	21 (18.3 to 23.7)
Azerbaijan	Other cardiovascular and circulatory diseases	15.1 (13.1 to 17.7)	23.2 (20.9 to 25.9)	26.5 (22.7 to 30.6)	266.1 (226.7 to 317.1)	306.3 (276.5 to 342.7)	269.6 (232.3 to 309.4)
Azerbaijan	Chronic respiratory diseases	70.4 (64.6 to 77)	72.5 (65.1 to 83.2)	88.2 (77.6 to 99.9)	1257.6 (1156.6 to 1369.4)	1019.6 (920.5 to 1183.5)	961.1 (848.7 to 1093.3)
Azerbaijan	Chronic obstructive pulmonary disease	48.2 (44.7 to 52.3)	50.1 (45.6 to 58.4)	60.5 (53.4 to 69.5)	901.9 (835.7 to 982.8)	737.4 (671.6 to 872.7)	679.6 (598.3 to 788.1)
Azerbaijan	Pneumoconiosis	0.0519 (0.0424 to 0.0633)	0.0621 (0.0515 to 0.075)	0.0604 (0.0471 to 0.0757)	0.9 (0.7 to 1)	0.9 (0.7 to 1.1)	0.6 (0.5 to 0.8)
Azerbaijan	Silicosis	0.0176 (0.00799 to 0.0243)	0.0184 (0.0102 to 0.0242)	0.0153 (0.00838 to 0.022)	0.2 (0.1 to 0.3)	0.2 (0.1 to 0.3)	0.1 (0.1 to 0.2)
Azerbaijan	Asbestosis	0.00866 (0.00593 to 0.0124)	0.0106 (0.00725 to 0.0146)	0.0124 (0.00853 to 0.0173)	0.1 (0.1 to 0.2)	0.1 (0.1 to 0.2)	0.1 (0.1 to 0.2)
Azerbaijan	Coal workers pneumoconiosis	0.00402 (0.00248 to 0.00783)	0.00439 (0.00278 to 0.00863)	0.00303 (0.00173 to 0.00554)	0.1 (0 to 0.1)	0.1 (0 to 0.1)	0 (0 to 0.1)
Azerbaijan	Other pneumoconiosis	0.0216 (0.0164 to 0.029)	0.0287 (0.0217 to 0.0387)	0.0296 (0.0215 to 0.0399)	0.4 (0.3 to 0.5)	0.4 (0.3 to 0.6)	0.3 (0.2 to 0.5)
Azerbaijan	Asthma	13.5 (10.3 to 17.4)	13.5 (10.1 to 17.6)	16.3 (11.8 to 21.8)	215.8 (168.9 to 271.9)	171.3 (131.1 to 221.5)	167.7 (121.4 to 222.9)
Azerbaijan	Interstitial lung disease and pulmonary sarcoidosis	3.38 (2.77 to 4.14)	3.7 (3.03 to 4.57)	4.98 (4.01 to 6.15)	58.5 (47.3 to 73.1)	47.2 (38.8 to 58.5)	50.3 (40.8 to 61.9)
Azerbaijan	Other chronic respiratory diseases	5.26 (4.58 to 6.02)	5.09 (4.48 to 5.77)	6.31 (5.34 to 7.49)	80.6 (70.1 to 93.7)	62.8 (55.4 to 70.6)	62.7 (53.6 to 74)
Azerbaijan	Digestive diseases	84 (77 to 95.4)	123 (110 to 135)	138 (122 to 160)	1357.7 (1254.7 to 1514.5)	1581.4 (1410.2 to 1739.9)	1368.1 (1200.7 to 1574.6)

SR Appendix Table 2. All-age DALYs counts and age standardized DALYs rates per 100,000 individuals for all causes and level 1 cause groups by location for 1990, 2007, and 2017, both sexes combined							
		All-age DALYs			Age-standardized rate of DALYs per 100,000		
Azerbaijan	Cirrhosis and other chronic liver diseases	42.1 (39 to 46.7)	78.5 (69.7 to 85.8)	89.2 (75.8 to 104)	720.2 (669.7 to 790.4)	1031.8 (896.4 to 1140.7)	881.6 (751 to 1027.7)
Azerbaijan	Cirrhosis and other chronic liver diseases due to hepatitis B	10.4 (9.01 to 12.2)	19.4 (16.6 to 22.4)	20.1 (16.2 to 24.6)	176.9 (155.4 to 206.1)	250.6 (211.9 to 290.5)	197.1 (159.5 to 238.1)
Azerbaijan	Cirrhosis and other chronic liver diseases due to hepatitis C	9.49 (8.19 to 11)	18.1 (15.1 to 21.1)	21.3 (17 to 26.2)	166.2 (143.6 to 192.1)	238.8 (199.2 to 279.6)	208.4 (166.8 to 256.7)
Azerbaijan	Cirrhosis and other chronic liver diseases due to alcohol use	13.7 (12.1 to 15.5)	26.6 (23.1 to 30.5)	31.2 (25.8 to 37.3)	241.8 (216.4 to 272.8)	352.3 (301.1 to 406.7)	304.2 (249.6 to 362.9)
Azerbaijan	Cirrhosis due to NASH	2.74 (2.39 to 3.11)	5.64 (4.81 to 6.48)	7.65 (6.27 to 9.24)	48.4 (42.4 to 54.4)	76.1 (62.9 to 87.8)	74.8 (61.3 to 90.4)
Azerbaijan	Cirrhosis and other chronic liver diseases due to other causes	5.86 (5.07 to 6.89)	8.82 (7.58 to 10.3)	8.9 (7.34 to 10.7)	86.9 (76.1 to 99.7)	114 (97 to 133.2)	97.1 (79.7 to 116.7)
Azerbaijan	Upper digestive system diseases	16.6 (13.1 to 22)	18 (13 to 24.9)	20.6 (14.8 to 29.7)	264.2 (209.6 to 343.8)	211.5 (154.3 to 288.7)	191.6 (138.1 to 273.6)
Azerbaijan	Peptic ulcer disease	8.56 (7.78 to 9.65)	7.1 (6.21 to 8.07)	7.19 (6.08 to 8.76)	141.1 (129.4 to 157.5)	88.4 (77.4 to 100.1)	69 (58.8 to 83.2)
Azerbaijan	Gastritis and duodenitis	2.15 (1.44 to 3.07)	2.72 (1.77 to 3.99)	3.17 (2.07 to 4.68)	31 (21 to 44.3)	31 (20.3 to 45.1)	30.5 (20 to 44.9)
Azerbaijan	GERD	5.94 (3.17 to 10.2)	8.21 (4.33 to 14)	10.2 (5.45 to 17.3)	92.1 (48.8 to 157.1)	92.1 (49.1 to 156.1)	92.1 (49.2 to 156.3)
Azerbaijan	Appendicitis	2.3 (1.92 to 2.71)	1.25 (1.03 to 1.59)	1.13 (0.909 to 1.45)	30.2 (25.6 to 35.3)	13.9 (11.4 to 17.7)	11.4 (9.1 to 14.7)
Azerbaijan	Paralytic ileus and intestinal obstruction	6.21 (4.96 to 9.71)	5.41 (4.37 to 6.85)	5.04 (3.7 to 6.72)	77.7 (63 to 119.5)	74.8 (60.5 to 93.4)	59.9 (43.4 to 80.4)
Azerbaijan	Inguinal, femoral, and abdominal hernia	5.58 (3.84 to 7.64)	6.89 (4.68 to 9.34)	7.39 (5.14 to 10.1)	82.3 (56.9 to 111.6)	85.1 (58.5 to 115.8)	73.6 (51.2 to 101.2)
Azerbaijan	Inflammatory bowel disease	0.6 (0.474 to 0.748)	0.771 (0.622 to 0.945)	0.979 (0.771 to 1.22)	9.4 (7.5 to 11.7)	9.1 (7.4 to 11)	9.1 (7.2 to 11.3)
Azerbaijan	Vascular intestinal disorders	0.523 (0.408 to 0.611)	0.735 (0.572 to 0.855)	0.906 (0.693 to 1.13)	9.9 (7.7 to 11.8)	10.7 (8.3 to 12.3)	9.8 (7.6 to 12.3)
Azerbaijan	Gallbladder and biliary diseases	1.41 (1.22 to 1.55)	1.17 (0.977 to 1.57)	1.39 (1.12 to 1.82)	25.2 (21.4 to 27.7)	16.2 (13.5 to 21.6)	14.6 (11.7 to 19.1)
Azerbaijan	Pancreatitis	1.33 (1.11 to 1.7)	1.93 (1.58 to 2.42)	2.45 (1.94 to 3.18)	22.3 (18.5 to 28.2)	22.9 (18.8 to 28.6)	23 (18.3 to 29.6)
Azerbaijan	Other digestive diseases	7.28 (5.86 to 9.17)	7.93 (6.97 to 8.91)	9.34 (7.6 to 11.3)	116.3 (94.5 to 143.5)	105.5 (92.4 to 118.3)	93.6 (76 to 111.5)
Azerbaijan	Neurological disorders	96.8 (71 to 127)	125 (94.5 to 164)	149 (112 to 193)	1544.9 (1169.9 to 1977.3)	1651.5 (1309.8 to 2073.4)	1635 (1281.1 to 2042.7)
Azerbaijan	Alzheimer's disease and other dementias	18.5 (14.5 to 21.4)	19.7 (18 to 21.6)	30.1 (27.3 to 33.5)	434.7 (337.8 to 502.4)	490.4 (452.8 to 533)	500 (455.5 to 551.8)
Azerbaijan	Parkinson's disease	2.82 (2.27 to 3.86)	4.31 (3.84 to 4.77)	5.75 (5.02 to 6.48)	65.5 (52.2 to 90.8)	80.1 (70.8 to 87.9)	81.4 (70.5 to 91.6)
Azerbaijan	Epilepsy	20.7 (12.4 to 32.7)	26.7 (17.7 to 39.2)	26.8 (14.8 to 42.8)	269 (157.2 to 427.8)	299.2 (196.4 to 440.8)	267.8 (148.1 to 428.2)
Azerbaijan	Multiple sclerosis	0.627 (0.495 to 0.765)	0.962 (0.755 to 1.18)	1.25 (0.962 to 1.54)	10.3 (8.1 to 12.5)	10.8 (8.5 to 13.2)	11 (8.5 to 13.6)
Azerbaijan	Motor neuron disease	0.0817 (0.0661 to 0.104)	0.128 (0.11 to 0.148)	0.192 (0.161 to 0.229)	1.3 (1.1 to 1.7)	1.6 (1.4 to 1.8)	1.7 (1.5 to 2)
Azerbaijan	Headache disorders	50.6 (32.9 to 72.9)	68.7 (44.4 to 98.9)	79.5 (51.6 to 115)	720.1 (468.7 to 1037.1)	717.7 (466.8 to 1032.7)	716.2 (464.5 to 1032.7)
Azerbaijan	Migraine	44 (27.5 to 65.7)	59.8 (37.4 to 89.6)	69.2 (43 to 103)	625 (391.1 to 925.2)	622.9 (390.4 to 929.3)	621.5 (387.2 to 926.3)
Azerbaijan	Tension-type headache	6.6 (3.68 to 10.6)	8.84 (4.89 to 14.1)	10.3 (5.82 to 16.4)	95.1 (53.3 to 151.9)	94.9 (52.6 to 151.2)	94.8 (53.5 to 150.4)
Azerbaijan	Other neurological disorders	3.39 (1.93 to 5.37)	4.52 (3.02 to 6.55)	5.52 (3.3 to 8.61)	44 (25.5 to 69.1)	51.7 (35.1 to 74)	56.9 (33.7 to 88.7)
Azerbaijan	Mental disorders	87.2 (64.2 to 113)	115 (85.1 to 149)	133 (99.8 to 172)	1251.3 (926 to 1620.1)	1237 (916.8 to 1599.1)	1249.3 (933.4 to 1620.9)
Azerbaijan	Schizophrenia	7.86 (5.81 to 9.99)	11 (8.21 to 13.9)	14.1 (10.4 to 17.9)	118.5 (88 to 149.2)	117.4 (87.7 to 147.2)	121.5 (89.5 to 153.6)
Azerbaijan	Depressive disorders	25.6 (18.3 to 35.5)	33.8 (24.1 to 46.1)	40.8 (28.9 to 56.2)	391.4 (278.1 to 538.3)	380.1 (270.7 to 516.6)	384.3 (273.5 to 527.8)
Azerbaijan	Major depressive disorder	18.6 (12.9 to 25.6)	24.2 (16.9 to 33)	29 (20.3 to 39.5)	283.1 (198.7 to 387.8)	272.5 (190.1 to 373)	276.8 (192.6 to 375.2)
Azerbaijan	Dysthymia	7.05 (4.69 to 10.3)	9.58 (6.34 to 14)	11.8 (7.88 to 17.3)	108.3 (72 to 157.3)	107.6 (71.9 to 156.9)	107.5 (71.3 to 156)
Azerbaijan	Bipolar disorder	9.51 (5.98 to 14.1)	13 (8.21 to 19.3)	14.8 (9.35 to 22.2)	134.3 (84.4 to 199.8)	134.4 (84.7 to 199.7)	134.2 (84.4 to 201.4)
Azerbaijan	Anxiety disorders	16.7 (11.7 to 22.4)	21.9 (15.5 to 29)	25 (17.7 to 33.7)	237.1 (168 to 318)	234.9 (167.7 to 310.6)	235 (166 to 316)
Azerbaijan	Eating disorders	2.69 (1.68 to 3.93)	3.32 (2.1 to 4.89)	4.65 (2.94 to 6.93)	34.5 (21.7 to 50.5)	32.4 (20.5 to 47.4)	41.7 (26.3 to 61.7)
Azerbaijan	Anorexia nervosa	0.562 (0.332 to 0.864)	0.684 (0.41 to 1.05)	0.879 (0.511 to 1.34)	7 (4.2 to 10.7)	6.6 (4 to 10.1)	8.3 (4.9 to 12.7)
Azerbaijan	Bulimia nervosa	2.13 (1.3 to 3.23)	2.64 (1.63 to 3.92)	3.78 (2.31 to 5.64)	27.5 (17.1 to 41)	25.8 (15.9 to 38.2)	33.4 (20.3 to 50.2)
Azerbaijan	Autism spectrum disorders	4.82 (3.29 to 6.65)	5.78 (3.88 to 7.98)	6.59 (4.39 to 9.09)	63.8 (43.6 to 87.9)	64.5 (43.2 to 89)	65 (43 to 89.5)
Azerbaijan	Attention-deficit/hyperactivity disorder	0.925 (0.559 to 1.48)	1.13 (0.675 to 1.79)	1.18 (0.71 to 1.87)	11.7 (7 to 18.6)	11.7 (7 to 18.7)	11.9 (7.1 to 18.9)

SR Appendix Table 2. All-age DALYs counts and age standardized DALYs rates per 100,000 individuals for all causes and level 1 cause groups by location for 1990, 2007, and 2017, both sexes combined							
		All-age DALYs			Age-standardized rate of DALYs per 100,000		
Azerbaijan	Conduct disorder	8.04 (4.89 to 12.8)	9.72 (5.8 to 15.5)	8.27 (5 to 13.1)	97.9 (59.6 to 155.5)	98.2 (58.8 to 156.2)	99 (59.6 to 157.6)
Azerbaijan	Idiopathic developmental intellectual disability	1.79 (0.529 to 3.38)	2.19 (0.685 to 4.15)	1.74 (0.451 to 3.44)	23.2 (6.8 to 43.8)	24.1 (7.6 to 45.7)	17.1 (4.5 to 33.8)
Azerbaijan	Other mental disorders	9.31 (6.17 to 13)	12.8 (8.49 to 17.9)	15.8 (10.4 to 21.7)	138.7 (92.3 to 191.7)	139.4 (92.3 to 193.3)	139.5 (92.4 to 191.9)
Azerbaijan	Substance use disorders	28.7 (21.8 to 36.1)	46.1 (36.7 to 56.4)	54 (42.2 to 67.5)	399.4 (306.2 to 501.2)	485.3 (391.4 to 592.5)	475.8 (374.8 to 592.2)
Azerbaijan	Alcohol use disorders	20.7 (15.8 to 26.6)	33.1 (26.4 to 41.1)	38.5 (30.1 to 49.2)	293.9 (225.5 to 382.7)	352.5 (284 to 435.1)	340.3 (266 to 430.6)
Azerbaijan	Drug use disorders	7.96 (5.65 to 10.4)	13 (10.2 to 16.1)	15.5 (11.8 to 19.5)	105.5 (76.1 to 136.5)	132.8 (104.5 to 164.4)	135.5 (103.8 to 170.1)
Azerbaijan	Opioid use disorders	5.44 (3.83 to 7.18)	9.29 (7.15 to 11.6)	11.3 (8.46 to 14.5)	73.4 (52.1 to 96)	95.7 (73.8 to 120.2)	98.1 (73.8 to 124.7)
Azerbaijan	Cocaine use disorders	0.266 (0.164 to 0.395)	0.44 (0.295 to 0.662)	0.528 (0.35 to 0.784)	3.6 (2.3 to 5.2)	4.6 (3.1 to 6.7)	4.7 (3.2 to 7)
Azerbaijan	Amphetamine use disorders	0.888 (0.49 to 1.46)	1.17 (0.674 to 1.88)	1.32 (0.752 to 2.13)	10.8 (6.1 to 17.5)	11.5 (6.7 to 18.1)	11.8 (6.6 to 19.3)
Azerbaijan	Cannabis use disorders	0.368 (0.223 to 0.556)	0.478 (0.291 to 0.737)	0.497 (0.299 to 0.74)	4.6 (2.9 to 6.9)	4.6 (2.8 to 7.1)	4.6 (2.8 to 7)
Azerbaijan	Other drug use disorders	0.999 (0.652 to 1.48)	1.58 (1.11 to 2.25)	1.79 (1.23 to 2.53)	13.1 (8.9 to 18.8)	16.4 (11.8 to 22.9)	16.3 (11.4 to 22.5)
Azerbaijan	Diabetes and kidney diseases	71.3 (59.6 to 85.7)	129 (110 to 154)	165 (137 to 200)	1209 (1004.6 to 1453.2)	1690.2 (1445.1 to 1996.9)	1612.3 (1358.4 to 1941.1)
Azerbaijan	Diabetes mellitus	47.7 (37.6 to 60.9)	90.7 (73.2 to 113)	121 (96.6 to 154)	840.6 (662 to 1066.8)	1188.9 (969.3 to 1464.7)	1170.4 (937.9 to 1474.9)
Azerbaijan	Diabetes mellitus type 1	10.8 (9.43 to 12.7)	19.9 (18.1 to 21.7)	21.7 (18.9 to 25)	182.2 (155.9 to 216.1)	253.3 (230.2 to 278.3)	208.2 (182.1 to 237.9)
Azerbaijan	Diabetes mellitus type 2	36.9 (26.5 to 49.8)	70.8 (54.3 to 92.3)	99.6 (74.9 to 131)	658.4 (472.7 to 885.5)	935.6 (726.6 to 1203.6)	962.2 (735.6 to 1252.6)
Azerbaijan	Chronic kidney disease	21.3 (19.3 to 23.6)	37.6 (33.5 to 40.6)	42.6 (38.2 to 46.8)	339 (306.4 to 377.3)	489.6 (430.5 to 526.5)	434.6 (388.5 to 477.2)
Azerbaijan	Chronic kidney disease due to diabetes mellitus type 1	1.52 (1.22 to 1.88)	3.37 (2.67 to 4.1)	3.87 (3.01 to 4.77)	24.7 (19.8 to 30.5)	37.4 (29.8 to 45.7)	33.5 (26.4 to 41)
Azerbaijan	Chronic kidney disease due to diabetes mellitus type 2	2.78 (2.34 to 3.27)	6.13 (5.06 to 7.13)	7.67 (6.35 to 9.01)	52.1 (43.8 to 61.4)	91.2 (74.6 to 106)	82.8 (69 to 96.8)
Azerbaijan	Chronic kidney disease due to hypertension	1.85 (1.52 to 2.2)	3.65 (3.02 to 4.3)	4.32 (3.54 to 5.12)	33.5 (27.8 to 39.5)	53.9 (44 to 63)	48.6 (39.3 to 58.1)
Azerbaijan	Chronic kidney disease due to glomerulonephritis	5.88 (5.12 to 6.8)	10.3 (8.96 to 11.8)	11.4 (9.83 to 13.2)	86.3 (75.8 to 99.1)	123.6 (107.6 to 140.5)	109.1 (94.9 to 126)
Azerbaijan	Chronic kidney disease due to other and unspecified causes	9.23 (8 to 10.6)	14.2 (12.2 to 16)	15.4 (13.2 to 17.7)	142.5 (122.5 to 165.7)	183.5 (156.1 to 206.9)	160.7 (138.7 to 183.5)
Azerbaijan	Acute glomerulonephritis	2.31 (1.56 to 3.26)	0.977 (0.788 to 1.22)	0.705 (0.553 to 0.936)	29.4 (20.3 to 40.5)	11.7 (9.4 to 14.9)	7.3 (5.6 to 9.6)
Azerbaijan	Skin and subcutaneous diseases	33.7 (22.4 to 49.9)	39.4 (26.4 to 58.3)	45.7 (30.5 to 67.1)	456 (303.8 to 674.9)	454.4 (303.1 to 674.1)	462.2 (308.2 to 676)
Azerbaijan	Dermatitis	8.29 (4.7 to 13.3)	9.3 (5.47 to 14.6)	10.5 (6.18 to 16.7)	108.5 (63.3 to 171.6)	108.4 (63.6 to 171.8)	108.4 (63.4 to 173.3)
Azerbaijan	Atopic dermatitis	6.69 (3.56 to 11.4)	7.16 (3.86 to 12.1)	8.08 (4.32 to 13.7)	85.3 (45.4 to 144.9)	85.2 (46.1 to 144)	85.2 (45.8 to 144.9)
Azerbaijan	Contact dermatitis	1.6 (1.02 to 2.38)	2.12 (1.37 to 3.13)	2.45 (1.58 to 3.66)	23.1 (14.9 to 34.3)	23.1 (15 to 33.9)	23.1 (15 to 34.2)
Azerbaijan	Seborrhoeic dermatitis	0.00771 (0.00431 to 0.0124)	0.00932 (0.0052 to 0.0151)	0.0109 (0.00606 to 0.0175)	0.1 (0.1 to 0.2)	0.1 (0.1 to 0.2)	0.1 (0.1 to 0.2)
Azerbaijan	Psoriasis	4.43 (3.13 to 5.88)	5.67 (3.98 to 7.55)	7.16 (5.12 to 9.48)	66.5 (47.2 to 88.2)	63.3 (44.5 to 84.1)	66.1 (47.2 to 87.8)
Azerbaijan	Bacterial skin diseases	0.152 (0.107 to 0.213)	0.246 (0.154 to 0.329)	0.3 (0.19 to 0.399)	2.2 (1.6 to 3.1)	3.1 (1.9 to 4.1)	3.1 (1.9 to 4.1)
Azerbaijan	Cellulitis	0.0833 (0.0563 to 0.117)	0.115 (0.0828 to 0.156)	0.145 (0.103 to 0.199)	1.2 (0.8 to 1.6)	1.3 (1 to 1.8)	1.4 (1 to 1.9)
Azerbaijan	Pyoderma	0.0683 (0.0458 to 0.111)	0.131 (0.0642 to 0.188)	0.155 (0.0762 to 0.224)	1 (0.7 to 1.6)	1.8 (0.8 to 2.5)	1.6 (0.8 to 2.4)
Azerbaijan	Scabies	2.38 (1.32 to 3.94)	2.78 (1.55 to 4.57)	2.88 (1.59 to 4.72)	30.6 (16.9 to 50.5)	30.3 (16.5 to 49.6)	29.1 (16.2 to 47.2)
Azerbaijan	Fungal skin diseases	3.4 (1.35 to 7.08)	3.95 (1.59 to 8.25)	4.46 (1.76 to 9.34)	50.9 (20.3 to 106.7)	49.2 (19.9 to 103)	46.5 (18.5 to 96.4)
Azerbaijan	Viral skin diseases	3.77 (2.41 to 5.71)	3.76 (2.4 to 5.71)	4.18 (2.66 to 6.29)	45.8 (29.2 to 69.3)	45.8 (29.1 to 69.3)	45.9 (29.3 to 69.1)
Azerbaijan	Acne vulgaris	2.04 (1.19 to 3.42)	3.04 (1.73 to 5.09)	3.21 (1.84 to 5.23)	25 (14.5 to 41.9)	28.6 (16.4 to 47.6)	33.1 (18.8 to 54.3)
Azerbaijan	Alopecia areata	0.428 (0.27 to 0.647)	0.559 (0.351 to 0.842)	0.661 (0.422 to 0.988)	5.9 (3.7 to 9)	5.9 (3.8 to 8.9)	5.9 (3.8 to 8.8)
Azerbaijan	Pruritus	0.639 (0.298 to 1.19)	0.811 (0.389 to 1.56)	1.01 (0.478 to 1.91)	9.5 (4.4 to 17.7)	9.4 (4.5 to 17.9)	9.7 (4.6 to 18.3)
Azerbaijan	Urticaria	5.83 (3.84 to 8.28)	6.4 (4.25 to 9.11)	7.28 (4.76 to 10.3)	74.8 (49.5 to 105.4)	74.6 (49.5 to 106.4)	74.5 (48.7 to 105.9)
Azerbaijan	Decubitus ulcer	0.13 (0.0933 to 0.175)	0.171 (0.123 to 0.228)	0.221 (0.159 to 0.296)	2.3 (1.6 to 3.1)	2.4 (1.7 to 3.2)	2.4 (1.7 to 3.3)
Azerbaijan	Other skin and subcutaneous diseases	2.2 (1.05 to 4.01)	2.76 (1.33 to 5.09)	3.8 (1.84 to 6.93)	34.1 (16.5 to 62.4)	33.3 (16.1 to 61.3)	37.3 (18.1 to 68.2)

SR Appendix Table 2. All-age DALYs counts and age standardized DALYs rates per 100,000 individuals for all causes and level 1 cause groups by location for 1990, 2007, and 2017, both sexes combined

		All-age DALYs			Age-standardized rate of DALYs per 100,000		
Azerbaijan	Sense organ diseases	48.8 (32.4 to 71.2)	62.7 (41.5 to 92.5)	78.9 (52 to 116)	857.9 (572.8 to 1243.3)	851.6 (569.5 to 1241.1)	830.3 (552.5 to 1207)
Azerbaijan	Blindness and vision impairment	23.1 (14.7 to 35.2)	29.3 (18.8 to 44.7)	36.3 (23.3 to 54.6)	403.7 (257.7 to 609.7)	395.4 (255.8 to 594.5)	380.2 (245.4 to 568.1)
Azerbaijan	Glaucoma	0.174 (0.114 to 0.25)	0.199 (0.128 to 0.285)	0.249 (0.161 to 0.357)	3.6 (2.4 to 5.1)	3.4 (2.2 to 4.8)	3 (1.9 to 4.4)
Azerbaijan	Cataract	7.04 (4.73 to 9.78)	8.86 (5.95 to 12.4)	11.7 (7.94 to 16.3)	147.2 (99.6 to 202.8)	143.6 (96.5 to 196.6)	139.5 (93.5 to 190.6)
Azerbaijan	Age-related macular degeneration	0.0559 (0.0355 to 0.0858)	0.0667 (0.0413 to 0.103)	0.0955 (0.0587 to 0.146)	1.2 (0.7 to 1.7)	1.1 (0.7 to 1.7)	1.1 (0.7 to 1.7)
Azerbaijan	Refraction disorders	7.99 (5.01 to 12.3)	9.75 (6.12 to 15)	11.1 (7.05 to 17.1)	114.8 (72.6 to 176.6)	113.5 (72 to 173.3)	109.6 (68.9 to 168)
Azerbaijan	Near vision loss	6.56 (3.12 to 12.1)	8.84 (4.19 to 16.1)	11.1 (5.27 to 20.2)	115.5 (54.7 to 211.3)	113.2 (53.9 to 207.1)	106.9 (50.9 to 194.2)
Azerbaijan	Other vision loss	1.26 (0.836 to 1.8)	1.57 (1.03 to 2.27)	1.99 (1.29 to 2.81)	21.4 (14.2 to 30.4)	20.6 (13.5 to 29.3)	20 (13.2 to 28.2)
Azerbaijan	Age-related and other hearing loss	23.9 (16.2 to 33.9)	31.1 (21 to 44.4)	39.6 (26.6 to 56.8)	423.1 (290.3 to 594.7)	425.2 (292.9 to 598.3)	418.7 (285.6 to 591.6)
Azerbaijan	Other sense organ diseases	1.84 (1.15 to 2.69)	2.35 (1.44 to 3.42)	3.05 (1.9 to 4.46)	31.1 (19.5 to 44.9)	31 (19.1 to 44.5)	31.3 (19.6 to 45.8)
Azerbaijan	Musculoskeletal disorders	86.3 (62.5 to 117)	122 (87.9 to 165)	155 (112 to 210)	1429.4 (1035.7 to 1934.2)	1439.7 (1043 to 1933.2)	1447.8 (1053.9 to 1940.3)
Azerbaijan	Rheumatoid arthritis	1.46 (1.03 to 1.95)	2.07 (1.42 to 2.74)	3.05 (2.1 to 4.12)	25 (17.7 to 33.1)	25.6 (17.6 to 33.8)	29.1 (20.2 to 38.9)
Azerbaijan	Osteoarthritis	3.98 (2.01 to 7.83)	5.62 (2.8 to 11.2)	8.33 (4.22 to 16.3)	76.6 (38.6 to 151.7)	80 (40.1 to 158.4)	86.1 (43.5 to 169.3)
Azerbaijan	Low back pain	60.3 (43.2 to 81.5)	83.3 (59.4 to 113)	106 (75.4 to 144)	980.5 (698.2 to 1329.3)	978.4 (695.3 to 1316.6)	986.5 (703.4 to 1326.9)
Azerbaijan	Neck pain	17.6 (12.2 to 24.6)	25.8 (17.8 to 36.4)	32.7 (22.7 to 46.4)	299.2 (208.4 to 421.5)	298.4 (208.4 to 418.4)	298.2 (208.4 to 419.7)
Azerbaijan	Gout	0.68 (0.449 to 0.954)	1.01 (0.67 to 1.39)	1.41 (0.938 to 1.96)	12.5 (8.3 to 17.4)	13.5 (9.1 to 18.6)	14 (9.4 to 19.5)
Azerbaijan	Other musculoskeletal disorders	2.34 (1.26 to 3.79)	4.03 (2.46 to 6.24)	4.02 (2.1 to 6.4)	35.6 (19.1 to 56.9)	43.7 (26.9 to 65.7)	34 (18.2 to 54.5)
Azerbaijan	Other non-communicable diseases	178 (154 to 206)	176 (148 to 211)	191 (153 to 237)	2268.6 (1936.2 to 2666.3)	2262.5 (1912.3 to 2673.6)	2102.8 (1670.3 to 2626)
Azerbaijan	Congenital anomalies	112 (98.9 to 127)	88.8 (72.9 to 106)	93.9 (68.5 to 123)	1303.3 (1153 to 1480.9)	1199.8 (981.9 to 1436.7)	1136.3 (815.3 to 1497.4)
Azerbaijan	Neural tube defects	14.2 (10.1 to 20.3)	4.9 (3.05 to 7.86)	5.25 (2.92 to 8.75)	162.9 (116.9 to 233.1)	66.9 (40.8 to 108.5)	64.8 (34.9 to 110.1)
Azerbaijan	Congenital heart anomalies	62.4 (42.7 to 80.1)	49.6 (35.8 to 63.9)	50.2 (34 to 72.2)	713.2 (489 to 913.7)	691.4 (497.1 to 892)	631.7 (423.6 to 912.2)
Azerbaijan	Orofacial clefts	0.521 (0.275 to 1.11)	0.34 (0.215 to 0.504)	0.344 (0.224 to 0.517)	6.5 (3.5 to 13.3)	4.1 (2.6 to 6.1)	3.6 (2.3 to 5.6)
Azerbaijan	Down's syndrome	1.68 (0.997 to 2.92)	1.42 (0.881 to 2.23)	1.44 (0.944 to 2.19)	20 (12.1 to 34)	18.2 (10.9 to 29.7)	16.1 (10.2 to 25.2)
Azerbaijan	Turner syndrome	0.00805 (0.00386 to 0.0134)	0.00946 (0.0046 to 0.0157)	0.00927 (0.00452 to 0.0153)	0.1 (0 to 0.2)	0.1 (0 to 0.2)	0.1 (0 to 0.1)
Azerbaijan	Klinefelter syndrome	0.00194 (0.000895 to 0.00371)	0.00242 (0.00113 to 0.0047)	0.00273 (0.00128 to 0.0053)	0 (0 to 0)	0 (0 to 0)	0 (0 to 0)
Azerbaijan	Other chromosomal abnormalities	1.59 (0.986 to 2.45)	1.57 (1.02 to 2.28)	2 (1.16 to 3.27)	18.8 (11.8 to 29)	20.5 (13.2 to 30.2)	23.5 (13.3 to 39.4)
Azerbaijan	Congenital musculoskeletal and limb anomalies	9.26 (6.5 to 12.2)	10.8 (7.61 to 14.6)	12.2 (8.6 to 16.4)	122.2 (85.5 to 160.6)	128.7 (90.8 to 173)	128.2 (89.2 to 172.4)
Azerbaijan	Urogenital congenital anomalies	1.61 (0.938 to 4.07)	1.65 (0.936 to 3.83)	1.93 (0.96 to 4.4)	19.1 (11.3 to 46.9)	21.6 (11.9 to 52.4)	22.7 (10.7 to 54.4)
Azerbaijan	Digestive congenital anomalies	4.55 (2.87 to 8.23)	4.84 (3.2 to 8.2)	5.47 (3.48 to 9.75)	55.1 (35.4 to 97.7)	62.8 (40.7 to 110.8)	63.2 (38.8 to 117.1)
Azerbaijan	Other congenital anomalies	15.9 (9.32 to 27.6)	13.6 (8.41 to 21.7)	15 (8.92 to 23.3)	185.3 (110.1 to 318.5)	185.5 (113.5 to 301.2)	182.4 (105.6 to 287.3)
Azerbaijan	Urinary diseases and male infertility	6.77 (5.63 to 8.1)	13.4 (11.8 to 15.1)	16.8 (14.3 to 19.5)	106.2 (87.4 to 128.4)	174 (152.6 to 197.5)	168.4 (145.2 to 194.9)
Azerbaijan	Urinary tract infections	3.61 (3.33 to 3.97)	9.21 (8.59 to 9.83)	11 (9.89 to 12.1)	52.3 (48.3 to 57.1)	115 (107.4 to 122.6)	111.1 (100.1 to 122.3)
Azerbaijan	Urolithiasis	0.251 (0.178 to 0.344)	0.378 (0.273 to 0.508)	0.501 (0.367 to 0.664)	4 (2.9 to 5.4)	4.9 (3.6 to 6.4)	5 (3.7 to 6.5)
Azerbaijan	Benign prostatic hyperplasia	2.25 (1.42 to 3.23)	2.95 (1.84 to 4.27)	4.35 (2.73 to 6.34)	40.5 (25.6 to 57.8)	43.4 (27.1 to 62.8)	42.5 (26.5 to 61.6)
Azerbaijan	Male infertility	0.204 (0.0822 to 0.432)	0.241 (0.102 to 0.507)	0.316 (0.129 to 0.664)	2.6 (1.1 to 5.5)	2.4 (1 to 4.9)	2.7 (1.1 to 5.7)
Azerbaijan	Other urinary diseases	0.454 (0.338 to 0.566)	0.628 (0.506 to 0.793)	0.635 (0.501 to 0.801)	6.7 (4.9 to 8.3)	8.3 (6.8 to 10.8)	7.1 (5.6 to 8.9)
Azerbaijan	Gynecological diseases	11.7 (8.2 to 16.3)	16.8 (11.6 to 23.3)	18.5 (12.7 to 26.3)	169.4 (117.1 to 235.9)	163.4 (112.7 to 228.4)	159.8 (109.7 to 226.7)
Azerbaijan	Uterine fibroids	1.64 (1 to 2.63)	2.71 (1.6 to 4.39)	2.75 (1.56 to 4.54)	28.6 (17.5 to 45.7)	27.1 (16 to 43.7)	23.5 (13.3 to 38.7)
Azerbaijan	Polycystic ovarian syndrome	0.436 (0.195 to 0.847)	0.611 (0.269 to 1.18)	0.673 (0.301 to 1.29)	6 (2.7 to 11.5)	5.9 (2.6 to 11.5)	5.9 (2.6 to 11.3)
Azerbaijan	Female infertility	0.239 (0.0893 to 0.529)	0.199 (0.075 to 0.431)	0.363 (0.133 to 0.783)	3 (1.1 to 6.6)	2 (0.7 to 4.2)	3.1 (1.1 to 6.5)

SR Appendix Table 2. All-age DALYs counts and age standardized DALYs rates per 100,000 individuals for all causes and level 1 cause groups by location for 1990, 2007, and 2017, both sexes combined

		All-age DALYs			Age-standardized rate of DALYs per 100,000		
Azerbaijan	Endometriosis	3.83 (2.54 to 5.63)	5.28 (3.5 to 7.73)	5.92 (3.94 to 8.71)	52.2 (34.3 to 75.9)	51.5 (34.1 to 76)	50.4 (33.4 to 74)
Azerbaijan	Genital prolapse	0.208 (0.105 to 0.385)	0.221 (0.109 to 0.407)	0.293 (0.145 to 0.551)	3.8 (1.9 to 6.9)	2.8 (1.4 to 5.2)	2.8 (1.4 to 5.2)
Azerbaijan	Premenstrual syndrome	3.8 (2.42 to 5.78)	5.51 (3.49 to 8.2)	5.78 (3.7 to 8.7)	52.9 (33.9 to 80.3)	52.6 (33.4 to 78.3)	50.6 (32.4 to 76.2)
Azerbaijan	Other gynecological diseases	1.56 (1.14 to 2.16)	2.23 (1.52 to 3.29)	2.75 (1.83 to 4.2)	22.9 (16.4 to 32.2)	21.5 (14.7 to 31.7)	23.5 (15.8 to 36.1)
Azerbaijan	Hemoglobinopathies and hemolytic anaemias	14.7 (11.4 to 18.6)	16.5 (10.9 to 20.9)	13.6 (9.92 to 17.8)	189.5 (149 to 237.4)	206.4 (130.2 to 262.1)	143.8 (101.8 to 188.7)
Azerbaijan	Thalassemias	5.88 (3.12 to 8.64)	6 (2.23 to 8.35)	4.34 (1.84 to 6.78)	67.3 (35.7 to 98.4)	84.6 (30.6 to 118.3)	52.3 (22 to 81.7)
Azerbaijan	Thalassaemias trait	3.67 (2.34 to 5.48)	3.5 (2.31 to 5.2)	3.24 (2.06 to 4.89)	50.1 (32.3 to 74.3)	39.5 (26.3 to 59)	31.5 (20 to 47.1)
Azerbaijan	Sickle cell disorders	0.387 (0.216 to 0.594)	0.398 (0.165 to 0.633)	0.298 (0.136 to 0.484)	4.4 (2.5 to 6.8)	5.5 (2.2 to 8.8)	3.6 (1.6 to 5.9)
Azerbaijan	Sickle cell trait	0.214 (0.131 to 0.323)	0.199 (0.124 to 0.306)	0.173 (0.107 to 0.273)	2.9 (1.8 to 4.3)	2.2 (1.4 to 3.4)	1.7 (1.1 to 2.6)
Azerbaijan	G6PD deficiency	0.5 (0.391 to 0.711)	1.13 (0.896 to 1.32)	1.07 (0.841 to 1.34)	6.4 (5.1 to 9.2)	11.7 (9.1 to 13.8)	10.3 (8.1 to 13)
Azerbaijan	G6PD trait	0.000278 (0.000182 to 0.000402)	0.000301 (0.000203 to 0.000429)	0.000291 (0.000192 to 0.000417)	0 (0 to 0)	0 (0 to 0)	0 (0 to 0)
Azerbaijan	Other hemoglobinopathies and hemolytic anaemias	4.01 (3.1 to 5.18)	5.32 (4.24 to 6.75)	4.44 (3.51 to 5.65)	58.3 (45.3 to 74.8)	62.8 (50 to 78.9)	44.4 (35.1 to 56.4)
Azerbaijan	Endocrine, metabolic, blood, and immune disorders	11.7 (6.93 to 14.9)	13.4 (8.34 to 17.1)	14.4 (8.93 to 19.1)	146.9 (93.2 to 185.2)	167.8 (99.5 to 215.4)	156.8 (91.3 to 211.1)
Azerbaijan	Oral disorders	18.5 (11.7 to 27.7)	24.6 (15.6 to 37.1)	31.1 (19.8 to 46.2)	321.3 (205.2 to 474)	316.3 (202.4 to 468.6)	310.6 (200.4 to 456.7)
Azerbaijan	Caries of deciduous teeth	0.199 (0.0828 to 0.396)	0.159 (0.0667 to 0.311)	0.192 (0.081 to 0.379)	2.3 (1 to 4.6)	2.3 (1 to 4.5)	2.2 (0.9 to 4.4)
Azerbaijan	Caries of permanent teeth	1.82 (0.78 to 3.45)	2.44 (1.05 to 4.72)	2.54 (1.1 to 4.92)	25.3 (11 to 48.3)	25.8 (11.1 to 49.2)	23.7 (10.3 to 45.7)
Azerbaijan	Periodontal disease	2.66 (1.05 to 5.61)	3.69 (1.44 to 7.92)	4.75 (1.85 to 9.97)	45.3 (17.8 to 94.5)	43.4 (17.1 to 91.1)	42.6 (16.7 to 88.9)
Azerbaijan	Edentulism and severe tooth loss	10.3 (6.83 to 14.7)	13.6 (9.06 to 19.5)	18.1 (11.9 to 25.8)	197.3 (132.6 to 280.6)	193.8 (129.1 to 276.5)	191.2 (127 to 270.7)
Azerbaijan	Other oral disorders	3.55 (2.21 to 5.28)	4.69 (2.92 to 7.01)	5.54 (3.49 to 8.16)	51.1 (32 to 75.5)	51 (31.8 to 75.7)	50.9 (32.1 to 74.7)
Azerbaijan	Sudden infant death syndrome	2.8 (1.24 to 7.51)	2.45 (1.31 to 4.92)	2.14 (1.14 to 4.25)	32 (14.1 to 86)	34.9 (18.7 to 70.1)	27.2 (14.4 to 54)
Azerbaijan	Injuries	308 (284 to 336)	241 (213 to 271)	265 (229 to 308)	4194.2 (3820.1 to 4599.4)	2700.5 (2379.2 to 3038.2)	2520 (2176.7 to 2923.4)
Azerbaijan	Transport injuries	68.8 (64 to 74.3)	40.6 (36.8 to 44.8)	45.3 (39.5 to 51.6)	960.8 (893.4 to 1037.3)	444.4 (402.3 to 490)	421 (366.8 to 479.6)
Azerbaijan	Road injuries	66.2 (61.7 to 71.4)	37.9 (34.5 to 41.5)	42.4 (36.8 to 48.3)	926 (862.3 to 998.9)	414.4 (376.9 to 454.1)	393.6 (341.9 to 447.3)
Azerbaijan	Pedestrian road injuries	18.6 (16.2 to 21.1)	9.89 (8.37 to 11.2)	10.7 (8.66 to 12.5)	260.8 (225.1 to 294.5)	115.8 (99 to 130.3)	102.3 (84.9 to 120.2)
Azerbaijan	Cyclist road injuries	4.88 (3.69 to 5.9)	3.37 (2.59 to 4.05)	4.22 (3.18 to 5.32)	73 (54.3 to 88.1)	37 (29 to 44.7)	38.6 (29.5 to 48.1)
Azerbaijan	Motorcyclist road injuries	15.9 (13.4 to 19.2)	8.56 (6.55 to 10.4)	8.95 (6.75 to 11.2)	218.6 (181.4 to 261.8)	88.7 (68.7 to 106.6)	80.6 (61.5 to 100.3)
Azerbaijan	Motor vehicle road injuries	25.1 (21.8 to 30.8)	14.8 (13.1 to 18.2)	17.2 (14.5 to 20.9)	346.4 (303.1 to 429.9)	159.1 (141 to 194.2)	159.5 (135.9 to 192.2)
Azerbaijan	Other road injuries	1.84 (1.41 to 2.3)	1.26 (0.992 to 1.57)	1.38 (1.05 to 1.9)	27.3 (20.5 to 34.4)	13.9 (11.1 to 17.1)	12.6 (9.9 to 16.9)
Azerbaijan	Other transport injuries	2.57 (2.2 to 2.95)	2.71 (2.3 to 3.21)	2.92 (2.42 to 3.49)	34.8 (29.9 to 40.1)	30.1 (25.6 to 35.6)	27.4 (22.8 to 32.8)
Azerbaijan	Unintentional injuries	178 (159 to 201)	150 (128 to 172)	169 (141 to 203)	2421.3 (2135.9 to 2747.9)	1734.9 (1479.9 to 1990.9)	1636.8 (1374.7 to 1957.3)
Azerbaijan	Falls	46.8 (37.2 to 58.4)	43.7 (33.3 to 55.3)	60.7 (45.5 to 79.5)	701.7 (554.7 to 883.1)	511.4 (389.6 to 645.1)	577.8 (435.1 to 757.1)
Azerbaijan	Drowning	38.1 (34.5 to 42.9)	17.5 (15.7 to 20.4)	17.8 (15.2 to 21)	470.2 (426.9 to 526.2)	199.6 (177.9 to 236.9)	181.7 (153.4 to 217.5)
Azerbaijan	Fire, heat, and hot substances	40.8 (33.4 to 47)	30.2 (24.4 to 38.9)	29.8 (22.9 to 41.5)	536.4 (446.8 to 614.8)	351.4 (282.1 to 452.3)	291.5 (221.9 to 406.4)
Azerbaijan	Poisonings	6.54 (5.07 to 7.43)	5.25 (4.4 to 7.87)	5.97 (4.76 to 8.69)	87.8 (68 to 99.4)	58.5 (49.3 to 87.1)	56.3 (44.7 to 81.1)
Azerbaijan	Poisoning by carbon monoxide	3.48 (2.48 to 4.04)	2.44 (1.98 to 4.01)	2.67 (2.05 to 4.15)	48.1 (34.4 to 55.4)	26.1 (21.2 to 42.2)	23.9 (18.6 to 36.7)
Azerbaijan	Poisoning by other means	3.06 (2.46 to 3.65)	2.81 (2.31 to 3.97)	3.3 (2.52 to 4.68)	39.6 (32.2 to 47.2)	32.4 (26.7 to 45.2)	32.3 (25 to 45.5)
Azerbaijan	Exposure to mechanical forces	12 (9.9 to 14.6)	15.8 (13 to 18.9)	17 (13.2 to 21.6)	171.4 (139.4 to 210.4)	177.7 (146.4 to 214.4)	159.6 (124.3 to 203.2)
Azerbaijan	Unintentional firearm injuries	4.19 (3.59 to 4.92)	4.82 (4.13 to 5.4)	4.4 (3.72 to 5.12)	55.3 (47.7 to 64.9)	53.7 (46.1 to 60.2)	42.3 (35.5 to 49.1)
Azerbaijan	Other exposure to mechanical forces	7.81 (5.92 to 10.1)	11 (8.54 to 13.9)	12.6 (9.21 to 17.1)	116.1 (87.2 to 152.3)	124 (96.5 to 158.1)	117.3 (85.6 to 159.6)
Azerbaijan	Adverse effects of medical treatment	2.86 (2.28 to 3.75)	3.71 (2.39 to 4.64)	3.33 (2.36 to 4.18)	37 (30.5 to 47.5)	45.2 (28.8 to 56.8)	34.7 (23.6 to 44.3)

SR Appendix Table 2. All-age DALYs counts and age standardized DALYs rates per 100,000 individuals for all causes and level 1 cause groups by location for 1990, 2007, and 2017, both sexes combined

		All-age DALYs			Age-standardized rate of DALYs per 100,000		
Azerbaijan	Animal contact	1.24 (0.934 to 1.57)	1.61 (1.23 to 2.05)	1.54 (1.17 to 1.99)	16.8 (12.7 to 21.3)	18.8 (14.2 to 23.8)	15.3 (11.6 to 19.8)
Azerbaijan	Venomous animal contact	0.677 (0.463 to 0.927)	0.885 (0.621 to 1.22)	0.892 (0.627 to 1.23)	9.3 (6.4 to 12.7)	9.9 (7 to 13.7)	8.6 (6 to 11.8)
Azerbaijan	Non-venomous animal contact	0.562 (0.441 to 0.728)	0.73 (0.534 to 0.933)	0.651 (0.488 to 0.843)	7.5 (6 to 9.7)	8.9 (6.4 to 11.4)	6.7 (5 to 8.6)
Azerbaijan	Foreign body	13.1 (10.8 to 17.3)	13 (11.5 to 14.8)	12.3 (10.6 to 14.4)	162.8 (136.6 to 211.2)	158.7 (139.4 to 187.3)	128.5 (109.6 to 153.5)
Azerbaijan	Pulmonary aspiration and foreign body in airway	9.13 (6.97 to 13.8)	8.1 (6.76 to 10.2)	7.52 (6.11 to 9.76)	109.6 (85.1 to 163.5)	103.7 (85.7 to 134.3)	82.7 (66.5 to 109.5)
Azerbaijan	Foreign body in eyes	0.161 (0.0725 to 0.286)	0.211 (0.0948 to 0.375)	0.303 (0.141 to 0.541)	2.3 (1.1 to 4)	2.4 (1.1 to 4.2)	2.9 (1.3 to 5.1)
Azerbaijan	Foreign body in other body part	3.76 (2.67 to 4.63)	4.64 (3.53 to 5.48)	4.48 (3.46 to 5.4)	50.9 (36.9 to 62.3)	52.6 (40 to 62.5)	42.9 (33.2 to 51.8)
Azerbaijan	Environmental heat and cold exposure	8.02 (6.06 to 10)	8.58 (6.79 to 10.8)	8.56 (6.44 to 11.1)	118.7 (89.6 to 150)	94.3 (75 to 118.7)	78.2 (58.8 to 101.2)
Azerbaijan	Exposure to forces of nature	0 (0 to 0)	0.0166 (0.0123 to 0.022)	0.014 (0.0105 to 0.0183)	0 (0 to 0)	0.2 (0.1 to 0.2)	0.1 (0.1 to 0.2)
Azerbaijan	Other unintentional injuries	8.72 (7.42 to 10.2)	10.7 (9.06 to 12.5)	11.7 (9.41 to 14.6)	118.5 (100.1 to 139.5)	119.1 (100.4 to 139.8)	113 (91 to 139.6)
Azerbaijan	Self-harm and interpersonal violence	61.5 (48.8 to 67.2)	50.1 (44.5 to 63)	51.4 (44.2 to 62.2)	812.1 (651.1 to 886.3)	521.1 (462.6 to 656.4)	462.2 (398.2 to 560.9)
Azerbaijan	Self-harm	10.3 (9.17 to 13.8)	21.5 (14.5 to 24.3)	19.8 (14.9 to 23.6)	144.6 (130.2 to 188.8)	223.3 (151.1 to 252.1)	178.4 (135.1 to 210.3)
Azerbaijan	Self-harm by firearm	0.912 (0.68 to 1.34)	1.72 (0.992 to 2.13)	1.51 (0.921 to 1.91)	13.1 (9.7 to 18.7)	18.3 (10.6 to 22.6)	13.4 (8.3 to 17.1)
Azerbaijan	Self-harm by other specified means	9.36 (8.31 to 12.8)	19.7 (13.2 to 22.3)	18.3 (13.7 to 21.8)	131.6 (117.9 to 174.4)	205 (136.8 to 231.3)	165 (124.6 to 195.2)
Azerbaijan	Interpersonal violence	38.7 (23.7 to 44.5)	20.4 (16.4 to 36.7)	25.4 (19.7 to 39.5)	512.1 (321.6 to 583.4)	213 (171.4 to 384.6)	230.5 (177.7 to 353.3)
Azerbaijan	Assault by firearm	11.6 (1.75 to 17.3)	4.48 (1.6 to 6.01)	5.94 (1.78 to 9.01)	142.5 (23.3 to 210.1)	44.7 (16.3 to 59.5)	53.6 (16.5 to 81.1)
Azerbaijan	Assault by sharp object	18 (11.9 to 26.6)	9.16 (6.47 to 21)	10.9 (7.29 to 22.3)	242.5 (157.5 to 345.4)	94.5 (67.2 to 216.3)	97.5 (64.6 to 199.6)
Azerbaijan	Sexual violence	0.708 (0.47 to 1.04)	0.943 (0.637 to 1.37)	1.06 (0.712 to 1.54)	9.6 (6.4 to 14)	9.6 (6.6 to 14)	9.7 (6.5 to 14.1)
Azerbaijan	Assault by other means	8.4 (6.46 to 14.1)	5.83 (3.96 to 11.2)	7.55 (5.18 to 12.5)	117.4 (90.7 to 190.9)	64.2 (44.4 to 120.9)	69.7 (48.9 to 114.4)
Azerbaijan	Conflict and terrorism	12.3 (12.2 to 12.5)	8.13 (5.74 to 11.8)	6.08 (4.16 to 9.02)	154.1 (152.5 to 155.9)	83.9 (59.3 to 121.9)	52.4 (35.7 to 77.6)
Azerbaijan	Executions and police conflict	0.102 (0.0638 to 0.185)	0.0789 (0.0703 to 0.0918)	0.0876 (0.0762 to 0.104)	1.3 (0.8 to 2.2)	0.9 (0.8 to 1)	0.9 (0.7 to 1)
Georgia	All causes	2020 (1860 to 2190)	1440 (1320 to 1570)	1440 (1320 to 1570)	36292.2 (33423.3 to 39287.1)	29153.1 (26581.2 to 32032.6)	29519.9 (26869.7 to 32599.7)
Georgia	Communicable, maternal, neonatal, and nutritional disorders	379 (343 to 418)	141 (123 to 159)	94.7 (82.5 to 109)	8685.3 (7828.9 to 9572.6)	4531.1 (3953.4 to 5131.6)	3039.1 (2610.2 to 3506)
Georgia	HIV/AIDS and sexually transmitted infections	2.08 (1.27 to 3.37)	1.73 (1.19 to 2.6)	3.19 (2.58 to 4.21)	41.2 (25.1 to 67.3)	45.6 (31.1 to 67.6)	87 (70.8 to 114.6)
Georgia	HIV/AIDS	0.103 (0.0974 to 0.108)	0.433 (0.365 to 0.607)	2.19 (1.91 to 3)	1.9 (1.8 to 1.9)	10.6 (9 to 14.8)	57.5 (50.7 to 77.9)
Georgia	HIV/AIDS - Drug-susceptible Tuberculosis	0.0482 (0.0455 to 0.051)	0.091 (0.0739 to 0.106)	0.245 (0.148 to 0.386)	0.9 (0.8 to 0.9)	2.3 (1.8 to 2.6)	6.6 (3.9 to 10.4)
Georgia	HIV/AIDS - Multidrug-resistant Tuberculosis without extensive drug resistance	0.000242 (0.0000424 to 0.000831)	0.0471 (0.0359 to 0.0587)	0.147 (0.0883 to 0.228)	0 (0 to 0)	1.2 (0.9 to 1.5)	4 (2.4 to 6.2)
Georgia	HIV/AIDS - Extensively drug-resistant Tuberculosis	0 (0 to 0)	0.0148 (0.0112 to 0.0185)	0.0835 (0.049 to 0.129)	0 (0 to 0)	0.4 (0.3 to 0.5)	2.3 (1.3 to 3.5)
Georgia	HIV/AIDS resulting in other diseases	0.0542 (0.0515 to 0.0573)	0.28 (0.215 to 0.455)	1.71 (1.33 to 2.53)	1 (0.9 to 1)	6.8 (5.3 to 11)	44.6 (34.7 to 65.3)
Georgia	Sexually transmitted infections excluding HIV	1.97 (1.17 to 3.26)	1.3 (0.77 to 2.18)	1 (0.581 to 1.68)	39.4 (23.2 to 65.5)	35 (20.8 to 58.1)	29.6 (17.4 to 49.3)
Georgia	Syphilis	0.699 (0.286 to 1.46)	0.357 (0.137 to 0.719)	0.201 (0.0846 to 0.419)	16.7 (6.4 to 35.7)	13.4 (4.8 to 27.4)	8.1 (3.1 to 17.3)
Georgia	Chlamydial infection	0.295 (0.161 to 0.526)	0.22 (0.123 to 0.405)	0.186 (0.105 to 0.346)	5.3 (2.9 to 9.4)	5.2 (2.9 to 9.6)	5.2 (2.9 to 9.7)
Georgia	Gonococcal infection	0.246 (0.145 to 0.454)	0.161 (0.0929 to 0.293)	0.131 (0.077 to 0.228)	4.4 (2.6 to 8.3)	3.9 (2.2 to 7.2)	3.9 (2.2 to 7)
Georgia	Trichomoniasis	0.239 (0.0947 to 0.504)	0.185 (0.0749 to 0.39)	0.16 (0.065 to 0.341)	4.2 (1.7 to 8.8)	4.1 (1.7 to 8.7)	4 (1.6 to 8.6)
Georgia	Genital herpes	0.156 (0.0506 to 0.374)	0.128 (0.0403 to 0.302)	0.117 (0.0377 to 0.276)	2.6 (0.9 to 6.3)	2.6 (0.8 to 6.2)	2.6 (0.9 to 6.1)
Georgia	Other sexually transmitted infections	0.34 (0.204 to 0.572)	0.247 (0.149 to 0.42)	0.204 (0.124 to 0.352)	6.1 (3.7 to 10.3)	5.8 (3.5 to 10)	5.8 (3.4 to 10)
Georgia	Respiratory infections and tuberculosis	155 (139 to 174)	30.9 (28.3 to 33.9)	27.2 (25.1 to 29.8)	3455 (3064.2 to 3909.7)	837.6 (748.2 to 939.7)	666.7 (604.6 to 745.6)
Georgia	Tuberculosis	19.5 (17.9 to 21.2)	8.33 (7.82 to 8.87)	6.26 (5.68 to 6.85)	352 (320.7 to 387.2)	186.1 (174.3 to 198.4)	147 (133.1 to 161.8)
Georgia	Latent tuberculosis infection	0 (0 to 0)	0 (0 to 0)	0 (0 to 0)	0 (0 to 0)	0 (0 to 0)	0 (0 to 0)
Georgia	Drug-susceptible tuberculosis	19.4 (17.8 to 21.1)	5.43 (4.69 to 6.15)	3.64 (2.98 to 4.31)	350.5 (318.6 to 385.4)	121.3 (104.5 to 137.4)	85.7 (70.2 to 100.9)

SR Appendix Table 2. All-age DALYs counts and age standardized DALYs rates per 100,000 individuals for all causes and level 1 cause groups by location for 1990, 2007, and 2017, both sexes combined

		All-age DALYs			Age-standardized rate of DALYs per 100,000		
Georgia	Multidrug-resistant tuberculosis without extensive drug resistance	0.0848 (0.0152 to 0.295)	2.21 (1.71 to 2.74)	1.67 (1.31 to 2.08)	1.5 (0.3 to 5.3)	49.4 (38.3 to 61.2)	39.2 (30.8 to 48.6)
Georgia	Extensively drug-resistant tuberculosis	0 (0 to 0)	0.692 (0.541 to 0.865)	0.943 (0.727 to 1.18)	0 (0 to 0)	15.5 (12.1 to 19.3)	22.1 (17 to 27.5)
Georgia	Lower respiratory infections	132 (116 to 150)	19.5 (17.5 to 21.9)	18.4 (16.8 to 20.4)	3025.7 (2642.9 to 3470.9)	574.8 (498.3 to 664.2)	446.3 (397.8 to 513.5)
Georgia	Upper respiratory infections	2.8 (1.72 to 4.35)	1.95 (1.15 to 3.1)	1.7 (1.01 to 2.73)	52.1 (32.2 to 80.3)	48.6 (28.4 to 77)	48.3 (28.4 to 77.7)
Georgia	Otitis media	1.34 (0.788 to 2.11)	1.06 (0.642 to 1.69)	0.828 (0.504 to 1.3)	25.1 (14.9 to 39.8)	28.2 (16.9 to 44.7)	25.1 (15.2 to 39.9)
Georgia	Enteric infections	17.3 (14.4 to 20.6)	4.4 (3.39 to 5.56)	3.82 (2.89 to 4.87)	386.7 (320.3 to 466.7)	132.6 (102.5 to 164.2)	124.4 (94.2 to 158.7)
Georgia	Diarrhoeal diseases	16.9 (14 to 20.2)	4.16 (3.17 to 5.28)	3.63 (2.71 to 4.68)	379.7 (312.5 to 457.4)	125.6 (96.7 to 157.3)	118.3 (88.1 to 153.1)
Georgia	Typhoid and paratyphoid	0.00367 (0.0019 to 0.00638)	0.00269 (0.00143 to 0.00459)	0.00207 (0.00108 to 0.00358)	0.1 (0 to 0.1)	0.1 (0 to 0.1)	0.1 (0 to 0.1)
Georgia	Typhoid fever	0.00361 (0.00187 to 0.00625)	0.00247 (0.0013 to 0.00423)	0.00185 (0.000968 to 0.00322)	0.1 (0 to 0.1)	0.1 (0 to 0.1)	0 (0 to 0.1)
Georgia	Paratyphoid fever	0.0000614 (0.0000251 to 0.000128)	0.000224 (0.0000889 to 0.000452)	0.000222 (0.0000902 to 0.000459)	0 (0 to 0)	0 (0 to 0)	0 (0 to 0)
Georgia	Invasive non-typhoidal salmonella	0.32 (0.168 to 0.544)	0.232 (0.123 to 0.389)	0.183 (0.0962 to 0.312)	6.2 (3.3 to 10.6)	6.6 (3.5 to 11.4)	5.8 (2.9 to 10.2)
Georgia	Other intestinal infectious diseases	0.033 (0.00855 to 0.0711)	0.012 (0.00504 to 0.0216)	0.00874 (0.00425 to 0.0148)	0.7 (0.2 to 1.7)	0.4 (0.1 to 0.7)	0.3 (0.1 to 0.5)
Georgia	Neglected tropical diseases and malaria	1.8 (1.08 to 4.22)	2.11 (0.712 to 11.8)	0.972 (0.485 to 3.55)	32.7 (19.3 to 80)	57.1 (17.4 to 349.3)	27.9 (13 to 114.5)
Georgia	Malaria	0.0878 (0.0658 to 0.114)	0.0396 (0.031 to 0.0527)	0 (0 to 0)	1.6 (1.2 to 2.1)	0.9 (0.7 to 1.3)	0 (0 to 0)
Georgia	Chagas disease	0 (0 to 0)	0 (0 to 0)	0 (0 to 0)	0 (0 to 0)	0 (0 to 0)	0 (0 to 0)
Georgia	Leishmaniasis	0.319 (0.00133 to 2.61)	1.16 (0.00429 to 10.8)	0.308 (0.00115 to 2.8)	6.1 (0 to 51.5)	33.3 (0.1 to 323.8)	9.8 (0 to 93.8)
Georgia	Visceral leishmaniasis	0.319 (0.001 to 2.61)	1.16 (0.00394 to 10.8)	0.308 (0.00107 to 2.8)	6.1 (0 to 51.5)	33.3 (0.1 to 323.7)	9.8 (0 to 93.8)
Georgia	Cutaneous and mucocutaneous leishmaniasis	0.000462 (0.0000248 to 0.00147)	0.00046 (0.00015 to 0.00101)	0.000138 (0.0000011 to 0.000571)	0 (0 to 0)	0 (0 to 0)	0 (0 to 0)
Georgia	African trypanosomiasis	0 (0 to 0)	0 (0 to 0)	0 (0 to 0)	0 (0 to 0)	0 (0 to 0)	0 (0 to 0)
Georgia	Schistosomiasis	0 (0 to 0)	0 (0 to 0)	0 (0 to 0)	0 (0 to 0)	0 (0 to 0)	0 (0 to 0)
Georgia	Cysticercosis	0.00314 (0.00171 to 0.00486)	0.0011 (0.000534 to 0.00174)	0.000828 (0.000399 to 0.0013)	0.1 (0 to 0.1)	0 (0 to 0)	0 (0 to 0)
Georgia	Cystic echinococcosis	0.167 (0.112 to 0.231)	0.1 (0.0665 to 0.142)	0.0868 (0.058 to 0.124)	2.8 (1.9 to 3.9)	2.1 (1.4 to 3.1)	2 (1.3 to 2.9)
Georgia	Lymphatic filariasis	0 (0 to 0)	0 (0 to 0)	0 (0 to 0)	0 (0 to 0)	0 (0 to 0)	0 (0 to 0)
Georgia	Onchocerciasis	0 (0 to 0)	0 (0 to 0)	0 (0 to 0)	0 (0 to 0)	0 (0 to 0)	0 (0 to 0)
Georgia	Trachoma	0 (0 to 0)	0 (0 to 0)	0 (0 to 0)	0 (0 to 0)	0 (0 to 0)	0 (0 to 0)
Georgia	Dengue	0 (0 to 0)	0 (0 to 0)	0 (0 to 0)	0 (0 to 0)	0 (0 to 0)	0 (0 to 0)
Georgia	Yellow fever	0 (0 to 0)	0 (0 to 0)	0 (0 to 0)	0 (0 to 0)	0 (0 to 0)	0 (0 to 0)
Georgia	Rabies	0.282 (0.203 to 0.382)	0.124 (0.101 to 0.153)	0.0864 (0.0613 to 0.118)	4.8 (3.5 to 6.5)	2.9 (2.3 to 3.6)	2 (1.4 to 2.8)
Georgia	Intestinal nematode infections	0.0628 (0.0303 to 0.113)	0.0526 (0.0266 to 0.0907)	0.0331 (0.0148 to 0.0596)	1.1 (0.5 to 2.1)	1.3 (0.7 to 2.2)	0.9 (0.4 to 1.7)
Georgia	Ascariasis	0.0000494 (0.0000222 to 0.0000952)	0.0000212 (0.00000905 to 0.0000411)	0.0000108 (0.00000435 to 0.0000219)	0 (0 to 0)	0 (0 to 0)	0 (0 to 0)
Georgia	Trichuriasis	0.000000000396 (0.000000000168 to 0.000000000804)	0.000000000173 (0.0000000000784 to 0.000000000359)	0.000000000142 (0.000000000062 to 0.00000000029)	0 (0 to 0)	0 (0 to 0)	0 (0 to 0)
Georgia	Hookworm disease	0.0628 (0.0303 to 0.113)	0.0526 (0.0266 to 0.0907)	0.0331 (0.0148 to 0.0596)	1.1 (0.5 to 2.1)	1.3 (0.7 to 2.2)	0.9 (0.4 to 1.7)
Georgia	Food-borne trematodiases	0 (0 to 0)	0 (0 to 0)	0 (0 to 0)	0 (0 to 0)	0 (0 to 0)	0 (0 to 0)
Georgia	Leprosy	0 (0 to 0)	0 (0 to 0)	0 (0 to 0)	0 (0 to 0)	0 (0 to 0)	0 (0 to 0)
Georgia	Ebola virus disease	0 (0 to 0)	0 (0 to 0)	0 (0 to 0)	0 (0 to 0)	0 (0 to 0)	0 (0 to 0)
Georgia	Zika virus disease	0 (0 to 0)	0 (0 to 0)	0 (0 to 0)	0 (0 to 0)	0 (0 to 0)	0 (0 to 0)
Georgia	Guinea worm disease	0 (0 to 0)	0 (0 to 0)	0 (0 to 0)	0 (0 to 0)	0 (0 to 0)	0 (0 to 0)
Georgia	Other neglected tropical diseases	0.874 (0.531 to 1.36)	0.634 (0.4 to 0.93)	0.457 (0.288 to 0.688)	16.1 (9.8 to 24.9)	16.6 (10 to 24.5)	13.2 (8.3 to 19.8)
Georgia	Other infectious diseases	11.9 (10.5 to 13.8)	6.95 (6.15 to 8.12)	5.94 (5.17 to 7.36)	240.8 (211 to 284.4)	191 (165 to 232.2)	170.9 (145.7 to 228.7)
Georgia	Meningitis	6.04 (5.14 to 6.82)	2.34 (2.04 to 2.76)	1.72 (1.53 to 1.95)	124.3 (104.9 to 141.8)	66.9 (57.6 to 79.2)	51.7 (45.5 to 60.3)

SR Appendix Table 2. All-age DALYs counts and age standardized DALYs rates per 100,000 individuals for all causes and level 1 cause groups by location for 1990, 2007, and 2017, both sexes combined							
		All-age DALYs			Age-standardized rate of DALYs per 100,000		
Georgia	Pneumococcal meningitis	0.456 (0.347 to 0.58)	0.296 (0.217 to 0.387)	0.143 (0.11 to 0.181)	8.5 (6.5 to 10.8)	7.3 (5.3 to 9.5)	4 (3 to 5)
Georgia	H influenzae type B meningitis	2.06 (1.73 to 2.37)	0.717 (0.629 to 0.864)	0.509 (0.449 to 0.591)	43 (35.7 to 50.2)	21.3 (18.2 to 25.9)	15.9 (13.6 to 18.9)
Georgia	Meningococcal infection	0.951 (0.801 to 1.1)	0.37 (0.317 to 0.44)	0.271 (0.236 to 0.315)	19.7 (16.5 to 23.1)	10.5 (8.9 to 12.6)	8.1 (6.9 to 9.6)
Georgia	Other meningitis	2.58 (2.18 to 2.93)	0.958 (0.848 to 1.15)	0.795 (0.707 to 0.903)	53.1 (44.4 to 61.2)	27.7 (24 to 33.5)	23.8 (20.9 to 27.8)
Georgia	Encephalitis	1.57 (1.26 to 2.22)	1.03 (0.871 to 1.51)	1.11 (0.973 to 1.39)	31.3 (24.8 to 44.9)	28.5 (23.4 to 46)	32.4 (27.2 to 46.5)
Georgia	Diphtheria	0.13 (0.0711 to 0.233)	0.00425 (0.00285 to 0.00632)	0.00168 (0.00105 to 0.00272)	2.1 (1.2 to 3.7)	0.1 (0.1 to 0.2)	0 (0 to 0.1)
Georgia	Whooping cough	0.169 (0.1 to 0.277)	0.11 (0.0416 to 0.179)	0.0853 (0.0391 to 0.141)	3.8 (2.2 to 6.3)	4.1 (1.5 to 6.8)	3.4 (1.5 to 5.7)
Georgia	Tetanus	0.0458 (0.0353 to 0.0579)	0.00447 (0.00307 to 0.00799)	0.00614 (0.00467 to 0.00839)	0.8 (0.6 to 1)	0.1 (0.1 to 0.2)	0.1 (0.1 to 0.2)
Georgia	Measles	0.00778 (0.00367 to 0.0125)	0.007 (0.00485 to 0.00868)	0.00386 (0.00197 to 0.00535)	0.2 (0.1 to 0.2)	0.2 (0.2 to 0.3)	0.1 (0.1 to 0.2)
Georgia	Varicella and herpes zoster	0.181 (0.115 to 0.266)	0.146 (0.0922 to 0.214)	0.14 (0.0889 to 0.204)	3.2 (2 to 4.7)	3.2 (2 to 4.6)	3.3 (2.1 to 4.7)
Georgia	Acute hepatitis	1.03 (0.857 to 1.23)	1.16 (0.979 to 1.35)	1.54 (1.3 to 1.8)	19.9 (16.6 to 23.9)	29.1 (24.5 to 34.2)	40.9 (33.9 to 47.8)
Georgia	Acute hepatitis A	0.303 (0.228 to 0.394)	0.256 (0.2 to 0.319)	0.289 (0.225 to 0.364)	6 (4.6 to 7.8)	7.7 (6 to 9.6)	9.9 (7.7 to 12.5)
Georgia	Acute hepatitis B	0.531 (0.437 to 0.644)	0.754 (0.638 to 0.889)	1.07 (0.891 to 1.29)	9.7 (7.9 to 11.8)	16.5 (14 to 19.5)	24.8 (20.5 to 29.7)
Georgia	Acute hepatitis C	0.141 (0.0834 to 0.215)	0.0958 (0.0518 to 0.146)	0.105 (0.0565 to 0.167)	3.3 (2 to 5.1)	3.6 (1.9 to 5.5)	4.1 (2.2 to 6.6)
Georgia	Acute hepatitis E	0.0498 (0.0392 to 0.0651)	0.0572 (0.0452 to 0.0705)	0.0763 (0.0567 to 0.0985)	0.9 (0.7 to 1.2)	1.4 (1.1 to 1.7)	2.1 (1.6 to 2.6)
Georgia	Other unspecified infectious diseases	2.71 (2.1 to 4.11)	2.15 (1.8 to 2.76)	1.33 (0.979 to 2.41)	55.3 (42.2 to 88)	58.7 (48 to 83.8)	38.9 (26.3 to 82.6)
Georgia	Maternal and neonatal disorders	165 (146 to 186)	77.7 (66.8 to 89.7)	41.1 (34.2 to 49.7)	4055.7 (3558.2 to 4555.7)	2843 (2440 to 3275.3)	1623.8 (1346.4 to 1978.3)
Georgia	Maternal disorders	2.11 (1.93 to 2.31)	1.03 (0.952 to 1.12)	1.15 (1.05 to 1.26)	38 (34.8 to 41.6)	25.4 (23.4 to 27.5)	33.5 (30.4 to 37)
Georgia	Maternal haemorrhage	0.56 (0.352 to 0.808)	0.151 (0.0945 to 0.223)	0.184 (0.112 to 0.275)	10 (6.3 to 14.4)	3.7 (2.3 to 5.5)	5.2 (3.2 to 7.8)
Georgia	Maternal sepsis and other pregnancy related infections	0.268 (0.208 to 0.343)	0.18 (0.144 to 0.222)	0.179 (0.142 to 0.22)	4.8 (3.8 to 6.2)	4.4 (3.6 to 5.5)	5.3 (4.2 to 6.6)
Georgia	Maternal hypertensive disorders	0.153 (0.0789 to 0.279)	0.0815 (0.0452 to 0.147)	0.117 (0.0633 to 0.199)	2.8 (1.4 to 5)	2 (1.1 to 3.6)	3.4 (1.9 to 5.9)
Georgia	Maternal obstructed labour and uterine rupture	0.0476 (0.0257 to 0.0795)	0.0312 (0.0173 to 0.0525)	0.0282 (0.0159 to 0.0467)	0.9 (0.5 to 1.4)	0.8 (0.4 to 1.3)	0.9 (0.5 to 1.4)
Georgia	Maternal abortive outcome	0.498 (0.338 to 0.688)	0.199 (0.14 to 0.271)	0.188 (0.128 to 0.256)	9.1 (6.1 to 12.4)	4.7 (3.3 to 6.5)	5.2 (3.5 to 7.1)
Georgia	Ectopic pregnancy	0.0751 (0.0219 to 0.204)	0.0316 (0.00993 to 0.0773)	0.0302 (0.0102 to 0.0771)	1.4 (0.4 to 3.7)	0.8 (0.2 to 1.9)	0.9 (0.3 to 2.3)
Georgia	Indirect maternal deaths	0.0237 (0.015 to 0.0363)	0.0556 (0.0366 to 0.0824)	0.0792 (0.053 to 0.113)	0.4 (0.3 to 0.6)	1.4 (0.9 to 2.1)	2.3 (1.6 to 3.4)
Georgia	Late maternal deaths	0.0948 (0.0691 to 0.129)	0.0474 (0.0347 to 0.0631)	0.067 (0.0493 to 0.0872)	1.7 (1.2 to 2.3)	1.2 (0.9 to 1.6)	2 (1.4 to 2.5)
Georgia	Maternal deaths aggravated by HIV/AIDS	0.00000628 (0.00000228 to 0.0000155)	0.0000821 (0.0000367 to 0.000152)	0.000368 (0.000166 to 0.000692)	0 (0 to 0)	0 (0 to 0)	0 (0 to 0)
Georgia	Other maternal disorders	0.392 (0.286 to 0.528)	0.255 (0.199 to 0.32)	0.277 (0.211 to 0.349)	7 (5.2 to 9.4)	6.4 (5 to 8)	8.3 (6.3 to 10.4)
Georgia	Neonatal disorders	163 (144 to 184)	76.7 (65.8 to 88.6)	40 (33 to 48.5)	4017.7 (3519.8 to 4521.5)	2817.6 (2414.9 to 3248.4)	1590.3 (1314 to 1945.2)
Georgia	Neonatal preterm birth	56.4 (44.1 to 72)	22.9 (19.4 to 26.6)	12.1 (9.66 to 14.9)	1374.3 (1061.8 to 1766.5)	829 (697 to 967.3)	480.8 (379 to 594.8)
Georgia	Neonatal encephalopathy due to birth asphyxia and trauma	78 (62.3 to 95.5)	32.2 (27 to 38)	13.3 (10.3 to 16.9)	1934.5 (1537.5 to 2372)	1181.6 (991.1 to 1387.4)	509.7 (400.1 to 648.2)
Georgia	Neonatal sepsis and other neonatal infections	7.59 (5.4 to 10.8)	8.06 (5.93 to 10.2)	5.6 (4.11 to 7.48)	179.6 (127.7 to 257)	292.1 (212.4 to 371.9)	220.2 (162.3 to 293.6)
Georgia	Hemolytic disease and other neonatal jaundice	2.17 (1.68 to 2.69)	1.11 (0.885 to 1.37)	0.727 (0.547 to 0.926)	47.8 (36.5 to 59.9)	33.3 (26.6 to 40.9)	22.5 (16.9 to 28.6)
Georgia	Other neonatal disorders	19.2 (14 to 26.1)	12.4 (10.1 to 15.1)	8.3 (6.35 to 10.6)	481.5 (350 to 655.4)	481.6 (392.3 to 585.4)	357 (273 to 454.8)
Georgia	Nutritional deficiencies	25.7 (16.2 to 38.9)	17 (11 to 25.6)	12.5 (7.78 to 19.1)	473.3 (299 to 719.7)	424.2 (269.7 to 639)	338.3 (210.4 to 506.9)
Georgia	Protein-energy malnutrition	0.201 (0.162 to 0.271)	0.0408 (0.0299 to 0.0661)	0.109 (0.0816 to 0.133)	4 (3.2 to 5.6)	1.2 (0.8 to 2.2)	3 (2.4 to 3.6)
Georgia	Iodine deficiency	1.69 (0.809 to 3.12)	0.985 (0.483 to 1.82)	0.798 (0.39 to 1.5)	30 (14.4 to 55.3)	22.3 (10.8 to 41.1)	20.1 (9.8 to 37.7)
Georgia	Dietary iron deficiency	19 (11.9 to 29.3)	14.2 (9.03 to 21.2)	10.1 (6.24 to 15.5)	338.7 (209.7 to 516.3)	334.1 (212.3 to 498.6)	262.6 (162.5 to 395.5)
Georgia	Other nutritional deficiencies	0.447 (0.395 to 0.506)	0.0708 (0.064 to 0.0769)	0.212 (0.185 to 0.247)	8.6 (7.5 to 9.9)	1.6 (1.4 to 1.8)	4.9 (4.3 to 5.6)
Georgia	Non-communicable diseases	1410 (1290 to 1540)	1180 (1080 to 1290)	1200 (1110 to 1320)	23567.7 (21468.1 to 25822.1)	21917 (19895.2 to 24260.1)	23146.5 (21022.5 to 25610)

SR Appendix Table 2. All-age DALYs counts and age standardized DALYs rates per 100,000 individuals for all causes and level 1 cause groups by location for 1990, 2007, and 2017, both sexes combined

		All-age DALYs			Age-standardized rate of DALYs per 100,000		
Georgia	Neoplasms	184 (175 to 194)	148 (145 to 150)	197 (189 to 205)	2929.1 (2783.2 to 3081.9)	2721.5 (2653.6 to 2775.6)	3710.4 (3545.8 to 3856.2)
Georgia	Lip and oral cavity cancer	3.05 (2.51 to 3.51)	2.98 (2.8 to 3.19)	3.95 (3.59 to 4.29)	47.7 (39.4 to 54.6)	54 (50.6 to 57.8)	73 (66.4 to 79)
Georgia	Nasopharynx cancer	0.872 (0.702 to 1.03)	0.526 (0.486 to 0.567)	0.52 (0.471 to 0.572)	14.3 (11.5 to 16.9)	10.5 (9.7 to 11.2)	11 (10 to 12.1)
Georgia	Other pharynx cancer	1.31 (0.918 to 1.66)	1.07 (0.983 to 1.17)	1.85 (1.58 to 2.1)	20.3 (14.4 to 25.5)	19.6 (18 to 21.4)	34.3 (29.3 to 38.7)
Georgia	Oesophageal cancer	2.72 (2.51 to 2.94)	3.55 (3.35 to 3.76)	3.63 (3.3 to 3.93)	42 (38.9 to 45.2)	62 (58.6 to 65.8)	65.9 (60.2 to 71.3)
Georgia	Stomach cancer	23.9 (22.6 to 25.2)	17.2 (16.4 to 18)	18.8 (17.8 to 20)	372.5 (353.5 to 392.5)	298 (285.1 to 311.8)	337.2 (317.8 to 357.3)
Georgia	Colon and rectum cancer	13.2 (12.5 to 13.9)	10.1 (9.62 to 10.5)	13.9 (12.9 to 14.9)	207.1 (196.3 to 219.1)	176.5 (168.8 to 185.2)	249.1 (230.7 to 265.8)
Georgia	Liver cancer	8.47 (7.33 to 9.67)	6.08 (5.77 to 6.42)	10.3 (9.3 to 11.3)	131.9 (114.3 to 149.4)	107.5 (102 to 113.3)	187.2 (169.6 to 206.7)
Georgia	Liver cancer due to hepatitis B	2.14 (1.67 to 2.71)	1.44 (1.18 to 1.7)	2.28 (1.81 to 2.8)	33.9 (26.9 to 42.6)	27.2 (22.6 to 31.8)	45 (36.1 to 54.6)
Georgia	Liver cancer due to hepatitis C	3.21 (2.64 to 3.84)	2.36 (2.08 to 2.65)	4.05 (3.44 to 4.71)	49 (40.7 to 58.2)	40.3 (35.2 to 45.1)	70 (59.6 to 81.7)
Georgia	Liver cancer due to alcohol use	2.15 (1.8 to 2.57)	1.58 (1.41 to 1.76)	2.74 (2.34 to 3.19)	33.6 (28.4 to 40)	27.4 (24.4 to 30.6)	49.9 (42.5 to 57.9)
Georgia	Liver cancer due to NASH	0.502 (0.409 to 0.617)	0.395 (0.345 to 0.453)	0.701 (0.595 to 0.824)	7.8 (6.4 to 9.5)	6.8 (6 to 7.8)	12.4 (10.5 to 14.4)
Georgia	Liver cancer due to other causes	0.472 (0.385 to 0.568)	0.315 (0.274 to 0.36)	0.519 (0.443 to 0.607)	7.5 (6.1 to 8.9)	5.9 (5.1 to 6.7)	9.9 (8.4 to 11.5)
Georgia	Gallbladder and biliary tract cancer	1.78 (1.34 to 2.74)	3.02 (2.72 to 3.26)	2.87 (2.58 to 3.13)	27.9 (21.2 to 43.3)	52.1 (46.5 to 56.4)	50.3 (45.2 to 54.5)
Georgia	Pancreatic cancer	4.75 (4.19 to 5.65)	5.45 (5.15 to 5.74)	7.27 (6.74 to 7.81)	73.6 (65.4 to 87.2)	94 (88.8 to 99.1)	129.1 (120.1 to 138.9)
Georgia	Larynx cancer	6.01 (5.57 to 6.45)	3.95 (3.69 to 4.23)	4.83 (4.36 to 5.33)	91.2 (84.7 to 98.2)	70.1 (65.7 to 75)	87 (78.5 to 96)
Georgia	Tracheal, bronchus, and lung cancer	35.5 (33.4 to 37.7)	24.1 (23 to 25.2)	37.5 (34.9 to 40.4)	538.9 (506.3 to 570.9)	426.8 (408.8 to 447.4)	668.4 (623.8 to 715.8)
Georgia	Malignant skin melanoma	2.19 (1.65 to 2.84)	1.4 (1.2 to 2.29)	1.99 (1.49 to 2.72)	36.1 (26.9 to 46.6)	26.5 (22.8 to 42.6)	39.4 (28.9 to 52.8)
Georgia	Non-melanoma skin cancer	0.648 (0.561 to 0.728)	1.71 (1.6 to 1.82)	2.02 (1.86 to 2.19)	10.3 (9 to 11.5)	29.8 (28 to 31.8)	36.1 (33.3 to 39)
Georgia	Non-melanoma skin cancer (squamous-cell carcinoma)	0.646 (0.559 to 0.726)	1.7 (1.6 to 1.82)	2.02 (1.86 to 2.19)	10.3 (9 to 11.5)	29.8 (28 to 31.7)	36 (33.3 to 39)
Georgia	Non-melanoma skin cancer (basal-cell carcinoma)	0.00202 (0.000649 to 0.00506)	0.00182 (0.000541 to 0.00462)	0.00178 (0.000527 to 0.00427)	0 (0 to 0.1)	0 (0 to 0.1)	0 (0 to 0.1)
Georgia	Breast cancer	23.7 (22.2 to 25.3)	14.2 (13.4 to 15)	20.8 (19.1 to 22.6)	383.9 (357.6 to 409)	263.5 (249.4 to 278.1)	393.8 (361.6 to 427.8)
Georgia	Cervical cancer	9.44 (8.71 to 10.2)	5.91 (5.5 to 6.35)	7.02 (6.37 to 7.7)	153.6 (141.7 to 166.1)	111.6 (103.9 to 119.7)	138.6 (125.2 to 152.1)
Georgia	Uterine cancer	2.77 (2.51 to 3.05)	4.79 (4.41 to 5.22)	4.55 (3.98 to 5.12)	43.4 (39.4 to 47.6)	86.9 (80 to 95)	83.1 (72.9 to 92.9)
Georgia	Ovarian cancer	3.18 (2.63 to 3.77)	3.54 (3.25 to 3.85)	5.12 (4.61 to 5.67)	50.1 (41.5 to 59.4)	65.1 (59.8 to 70.8)	95.7 (86.2 to 105.5)
Georgia	Prostate cancer	2.95 (2.33 to 3.85)	4.14 (2.73 to 4.65)	5.78 (2.8 to 7.05)	47.6 (37.5 to 61.9)	65.7 (42.7 to 73.6)	96 (45.4 to 117.3)
Georgia	Testicular cancer	0.547 (0.356 to 0.793)	0.675 (0.595 to 0.764)	1.02 (0.886 to 1.16)	9.3 (6.1 to 13.6)	14.1 (12.4 to 16.1)	23.5 (20.3 to 27.1)
Georgia	Kidney cancer	3.1 (2.68 to 3.65)	2.37 (2.18 to 2.57)	3.78 (3.34 to 4.24)	49.1 (42.5 to 57.5)	44.4 (40.8 to 48.2)	71.2 (63.2 to 79.4)
Georgia	Bladder cancer	3.37 (2.97 to 3.85)	3.31 (3.08 to 3.54)	4.7 (4.31 to 5.14)	52.1 (46.3 to 59)	54.7 (51.1 to 58.7)	80 (73.3 to 87.6)
Georgia	Thyroid cancer	0.523 (0.444 to 0.586)	0.392 (0.361 to 0.429)	0.785 (0.698 to 0.87)	8.3 (7.1 to 9.2)	7 (6.5 to 7.7)	14.6 (13 to 16.2)
Georgia	Mesothelioma	0.143 (0.113 to 0.165)	0.294 (0.273 to 0.318)	0.569 (0.524 to 0.613)	2.3 (1.8 to 2.7)	5.2 (4.8 to 5.6)	11 (10.1 to 11.9)
Georgia	Hodgkin lymphoma	3.08 (2.11 to 3.6)	1.61 (1.36 to 2.11)	1.5 (1.26 to 1.98)	51.8 (35.6 to 60.8)	33.2 (28.7 to 44.5)	33.5 (28.1 to 45.6)
Georgia	Non-Hodgkin's lymphoma	2.51 (2.18 to 2.96)	2.81 (2.65 to 2.98)	4.24 (3.91 to 4.64)	41.4 (36 to 48.8)	56 (52.4 to 59.5)	87.6 (80.9 to 95.3)
Georgia	Multiple myeloma	1.01 (0.863 to 1.28)	0.948 (0.827 to 1.11)	1.2 (0.952 to 1.37)	15.8 (13.5 to 19.8)	17 (14.6 to 19.8)	22.5 (17.4 to 25.7)
Georgia	Leukaemia	10.7 (10 to 11.5)	9.46 (8.52 to 10.1)	8.5 (7.84 to 9.18)	196.9 (182.6 to 212.4)	218.2 (192.4 to 236.4)	206.5 (189.3 to 225.5)
Georgia	Acute lymphoid leukaemia	2.12 (1.68 to 2.53)	1.03 (0.91 to 1.18)	1.14 (0.991 to 1.32)	40.8 (32.4 to 49.1)	27.8 (23.6 to 32.6)	33.3 (28.4 to 39.6)
Georgia	Chronic lymphoid leukaemia	0.218 (0.176 to 0.251)	0.19 (0.166 to 0.22)	0.445 (0.387 to 0.514)	3.5 (2.8 to 4)	3.3 (2.9 to 3.8)	7.9 (6.9 to 9.1)
Georgia	Acute myeloid leukaemia	1.88 (1.43 to 2.36)	1.02 (0.912 to 1.36)	2.09 (1.87 to 2.32)	33.9 (25.2 to 42.7)	23.2 (20.4 to 31.3)	50.7 (45.2 to 56.6)
Georgia	Chronic myeloid leukaemia	0.417 (0.32 to 0.508)	0.219 (0.194 to 0.248)	0.416 (0.364 to 0.477)	6.9 (5.3 to 8.4)	4.3 (3.8 to 4.9)	9 (7.8 to 10.4)
Georgia	Other leukaemia	6.09 (5.43 to 6.85)	6.99 (5.77 to 7.49)	4.41 (4.01 to 4.85)	111.7 (98.7 to 126.1)	159.5 (129.8 to 174.6)	105.7 (95.4 to 116.9)

SR Appendix Table 2. All-age DALYs counts and age standardized DALYs rates per 100,000 individuals for all causes and level 1 cause groups by location for 1990, 2007, and 2017, both sexes combined

		All-age DALYs			Age-standardized rate of DALYs per 100,000		
Georgia	Other malignant cancers	6.38 (5.18 to 8.94)	7 (6.39 to 7.47)	9.62 (6.26 to 11.1)	107.6 (87.9 to 149.5)	140.7 (127.1 to 151.8)	200.9 (134 to 229.9)
Georgia	Other neoplasms	0.582 (0.435 to 0.879)	0.564 (0.418 to 0.758)	0.867 (0.461 to 1.22)	9.8 (7.4 to 15.1)	11 (8.3 to 15.3)	18.2 (9.7 to 25.9)
Georgia	Myelodysplastic, myeloproliferative, and other hematopoietic neoplasms	0.49 (0.377 to 0.726)	0.496 (0.359 to 0.649)	0.778 (0.405 to 1.11)	8.1 (6.2 to 12.1)	9.1 (6.8 to 12.3)	15.5 (8 to 22.6)
Georgia	Benign and in situ intestinal neoplasms	0 (0 to 0)	0 (0 to 0)	0 (0 to 0)	0 (0 to 0)	0 (0 to 0)	0 (0 to 0)
Georgia	Benign and in situ cervical and uterine neoplasms	0 (0 to 0)	0 (0 to 0)	0 (0 to 0)	0 (0 to 0)	0 (0 to 0)	0 (0 to 0)
Georgia	Other benign and in situ neoplasms	0.0923 (0.0564 to 0.155)	0.0687 (0.0492 to 0.11)	0.0885 (0.053 to 0.116)	1.8 (1.1 to 3)	1.9 (1.3 to 3)	2.7 (1.6 to 3.6)
Georgia	Cardiovascular diseases	635 (612 to 660)	519 (508 to 529)	495 (478 to 511)	10434.5 (10059.4 to 10834.7)	8908.3 (8714.5 to 9096.4)	8505.7 (8205.3 to 8803.3)
Georgia	Rheumatic heart disease	9.08 (8.2 to 10.1)	9.49 (8.74 to 10.4)	10.6 (9.75 to 11.6)	155.8 (140.2 to 174.7)	198.7 (182.1 to 218.6)	217.2 (199.4 to 239)
Georgia	Ischaemic heart disease	375 (357 to 392)	271 (265 to 277)	239 (228 to 252)	6176.7 (5890.2 to 6438.6)	4640.7 (4544.2 to 4748)	4098.2 (3920.4 to 4310.4)
Georgia	Stroke	214 (203 to 225)	191 (185 to 197)	173 (163 to 184)	3485.3 (3301.9 to 3657.1)	3233.2 (3129.3 to 3334.9)	2926.6 (2765.2 to 3113.4)
Georgia	Ischaemic stroke	52.3 (47.9 to 57.2)	43.4 (40 to 46.5)	62.2 (57.1 to 67.9)	886.3 (811.9 to 970.2)	718 (662.7 to 771.3)	1021.4 (938.3 to 1116.8)
Georgia	Intracerebral hemorrhage	141 (132 to 150)	131 (127 to 135)	91.3 (86 to 97.6)	2254.8 (2114.3 to 2404.6)	2211.9 (2145.2 to 2276.8)	1556 (1466.8 to 1656.6)
Georgia	Subarachnoid hemorrhage	21.3 (18.2 to 23.7)	16.9 (15.9 to 18.2)	19.4 (17.6 to 21.4)	344.2 (296.8 to 382.6)	303.3 (285.5 to 329.6)	349.2 (319 to 382.4)
Georgia	Hypertensive heart disease	12.6 (9.88 to 24.3)	19.2 (16.9 to 25.6)	38.9 (19.9 to 44.1)	208.8 (165.9 to 392.5)	318.1 (281.1 to 425.4)	637.6 (333.5 to 719.2)
Georgia	Non-rheumatic valvular heart disease	1.11 (0.91 to 1.57)	1.46 (1.27 to 1.66)	2.23 (1.42 to 2.54)	18.8 (15.5 to 26.3)	27 (23.2 to 30.5)	41.1 (25.8 to 46.5)
Georgia	Non-rheumatic calcific aortic valve disease	0.429 (0.338 to 0.602)	0.554 (0.467 to 0.676)	1.04 (0.637 to 1.23)	7.1 (5.6 to 9.9)	9.8 (8.3 to 12)	18.3 (11.2 to 21.7)
Georgia	Non-rheumatic degenerative mitral valve disease	0.372 (0.273 to 0.633)	0.505 (0.42 to 0.616)	0.88 (0.466 to 1.23)	6.3 (4.6 to 10.6)	9.1 (7.7 to 11.1)	16.3 (8.4 to 22.6)
Georgia	Other non-rheumatic valve diseases	0.307 (0.231 to 0.416)	0.402 (0.268 to 0.49)	0.311 (0.213 to 0.484)	5.3 (4 to 7.1)	8.1 (5.2 to 9.8)	6.6 (4.5 to 9.7)
Georgia	Cardiomyopathy and myocarditis	6.38 (5.42 to 8.39)	5.76 (5.33 to 6.83)	7.32 (6.52 to 8.39)	110.4 (94.1 to 142.8)	119.2 (110.2 to 139.7)	151.1 (135.1 to 174.5)
Georgia	Myocarditis	2.32 (1.57 to 2.94)	1.78 (0.905 to 2.4)	2.21 (0.818 to 3.13)	40.1 (27.6 to 50.8)	37.8 (20 to 50.5)	46.5 (18.9 to 64.8)
Georgia	Alcoholic cardiomyopathy	0.437 (0.325 to 0.802)	0.455 (0.315 to 0.836)	0.669 (0.414 to 1.3)	7.4 (5.5 to 13.1)	9.2 (6.4 to 16.5)	14.4 (8.9 to 27.6)
Georgia	Other cardiomyopathy	3.62 (2.73 to 5.82)	3.53 (2.94 to 4.73)	4.44 (3.45 to 6.2)	62.9 (48.1 to 97.6)	72.2 (60.2 to 95.9)	90.2 (70.6 to 125.1)
Georgia	Atrial fibrillation and flutter	4.56 (3.69 to 5.63)	5.46 (4.62 to 6.49)	7.66 (6.7 to 8.71)	77.9 (63.6 to 95.8)	90.2 (76.8 to 106.7)	124.4 (108.6 to 141.9)
Georgia	Aortic aneurysm	1.31 (1.15 to 1.5)	2.69 (2.51 to 2.89)	3.65 (3.33 to 4.01)	21 (18.5 to 23.9)	47.5 (44.4 to 50.9)	66.6 (61.1 to 73.1)
Georgia	Peripheral vascular disease	0.436 (0.239 to 0.725)	0.484 (0.271 to 0.774)	0.559 (0.31 to 0.918)	7.2 (3.9 to 11.8)	7.8 (4.4 to 12.4)	9.1 (5.1 to 14.9)
Georgia	Endocarditis	0.809 (0.68 to 1.04)	0.991 (0.81 to 1.1)	0.886 (0.775 to 1.06)	14.4 (12.1 to 18.6)	20.4 (16.8 to 22.8)	18.7 (16.4 to 22)
Georgia	Other cardiovascular and circulatory diseases	9.48 (8.08 to 10.9)	10.9 (9.9 to 12.1)	11 (9.78 to 12.3)	158.3 (135.5 to 181.8)	205.5 (187.4 to 228.5)	215 (190.6 to 240.8)
Georgia	Chronic respiratory diseases	49.6 (45 to 54.1)	60.4 (56.1 to 64.7)	50.5 (46 to 55)	821.3 (744.9 to 900.5)	1095.5 (1014 to 1178.5)	953.8 (861.1 to 1045.9)
Georgia	Chronic obstructive pulmonary disease	30.2 (27.4 to 32.8)	46.1 (42.9 to 49.1)	37.2 (34.3 to 40.2)	494.1 (448.9 to 535.2)	799.8 (746.3 to 850.6)	662.9 (610.3 to 712.9)
Georgia	Pneumoconiosis	0.0546 (0.0432 to 0.067)	0.0531 (0.0434 to 0.0643)	0.0864 (0.0703 to 0.101)	0.9 (0.7 to 1)	0.9 (0.7 to 1.1)	1.5 (1.2 to 1.7)
Georgia	Silicosis	0.00527 (0.00312 to 0.00767)	0.0054 (0.00344 to 0.00785)	0.00785 (0.005 to 0.0118)	0.1 (0.1 to 0.1)	0.1 (0.1 to 0.1)	0.1 (0.1 to 0.2)
Georgia	Asbestosis	0.00717 (0.0049 to 0.0105)	0.0073 (0.00519 to 0.00992)	0.0133 (0.00836 to 0.0179)	0.1 (0.1 to 0.2)	0.1 (0.1 to 0.2)	0.2 (0.2 to 0.3)
Georgia	Coal workers pneumoconiosis	0.00781 (0.00519 to 0.0112)	0.00755 (0.0053 to 0.0103)	0.0127 (0.00898 to 0.0173)	0.1 (0.1 to 0.2)	0.1 (0.1 to 0.2)	0.2 (0.2 to 0.3)
Georgia	Other pneumoconiosis	0.0344 (0.0255 to 0.0424)	0.0329 (0.0263 to 0.0401)	0.0526 (0.0415 to 0.0628)	0.5 (0.4 to 0.6)	0.5 (0.4 to 0.6)	0.9 (0.7 to 1)
Georgia	Asthma	13.3 (10.7 to 16.4)	6.75 (5.22 to 8.65)	6.77 (5.13 to 8.83)	226.4 (182.2 to 281.7)	151.6 (115.5 to 195)	161.3 (120.4 to 212.8)
Georgia	Interstitial lung disease and pulmonary sarcoidosis	3.79 (3.13 to 4.56)	3.76 (3.05 to 4.44)	3.69 (3.01 to 4.37)	61.5 (50.9 to 73.8)	67.2 (54.4 to 79.1)	68.4 (56 to 81.6)
Georgia	Other chronic respiratory diseases	2.29 (2 to 2.62)	3.81 (3.48 to 4.2)	2.67 (2.36 to 2.98)	38.4 (33.8 to 43.8)	76.1 (69.9 to 83.6)	59.7 (52.9 to 66.6)
Georgia	Digestive diseases	68.7 (63.5 to 74.3)	50.3 (46.6 to 55)	60.6 (55.1 to 66.3)	1129.9 (1043 to 1226.5)	982.2 (905.7 to 1077.8)	1237.7 (1127.4 to 1351.7)
Georgia	Cirrhosis and other chronic liver diseases	43.5 (41.2 to 45.8)	32.7 (31.2 to 34.3)	40.4 (36.8 to 44.7)	691 (654.2 to 729.6)	618.5 (590.4 to 651)	810.7 (737.8 to 892.5)
Georgia	Cirrhosis and other chronic liver diseases due to hepatitis B	10.6 (9.21 to 12.1)	8.08 (7.07 to 9.11)	9 (7.55 to 10.6)	168.4 (147.1 to 191.9)	154.4 (135.2 to 175)	183 (153.2 to 214)

SR Appendix Table 2. All-age DALYs counts and age standardized DALYs rates per 100,000 individuals for all causes and level 1 cause groups by location for 1990, 2007, and 2017, both sexes combined

		All-age DALYs			Age-standardized rate of DALYs per 100,000		
Georgia	Cirrhosis and other chronic liver diseases due to hepatitis C	10.1 (8.7 to 11.7)	7.77 (6.7 to 8.9)	9.78 (8.18 to 11.6)	159.8 (137.8 to 184.8)	145.9 (125.5 to 168.2)	195.4 (162.9 to 231.3)
Georgia	Cirrhosis and other chronic liver diseases due to alcohol use	16.1 (14.4 to 17.9)	12.1 (11 to 13.3)	15.7 (13.7 to 18)	253.2 (227.8 to 280.4)	226.7 (205.6 to 247.8)	312.4 (272.2 to 354.5)
Georgia	Cirrhosis due to NASH	2.98 (2.63 to 3.37)	2.35 (2.09 to 2.67)	3.24 (2.75 to 3.78)	46.4 (41.1 to 52.2)	43.5 (38.5 to 49.1)	63.1 (53.6 to 73.7)
Georgia	Cirrhosis and other chronic liver diseases due to other causes	3.68 (3.16 to 4.26)	2.33 (2.02 to 2.7)	2.62 (2.17 to 3.15)	63.1 (54.9 to 71.8)	47.9 (41.8 to 54.7)	56.8 (48.3 to 66.4)
Georgia	Upper digestive system diseases	14.4 (11.4 to 18.9)	9.49 (6.97 to 13.1)	9.64 (7.3 to 13)	238.5 (187.2 to 315.3)	186.6 (135.1 to 260.6)	199.4 (147.9 to 273.3)
Georgia	Peptic ulcer disease	7.51 (6.95 to 8.15)	3.95 (3.65 to 4.28)	4.61 (4.14 to 5.14)	121.1 (112.3 to 130.8)	72.5 (66.7 to 78.8)	87.5 (78.7 to 97.1)
Georgia	Gastritis and duodenitis	1.43 (1.01 to 1.95)	0.981 (0.68 to 1.39)	0.831 (0.596 to 1.13)	25.2 (17.7 to 34.5)	21.9 (15.1 to 30.9)	19.8 (13.9 to 27.2)
Georgia	GERD	5.5 (2.92 to 9.39)	4.56 (2.4 to 7.7)	4.2 (2.23 to 7.15)	92.2 (49.1 to 157.4)	92.3 (48.5 to 155.7)	92.1 (49.1 to 157.3)
Georgia	Appendicitis	0.586 (0.492 to 0.696)	0.41 (0.34 to 0.492)	0.487 (0.422 to 0.563)	10.7 (8.9 to 12.8)	9.9 (8 to 12.1)	12.5 (10.6 to 14.8)
Georgia	Paralytic ileus and intestinal obstruction	3.45 (2.99 to 4.03)	1.84 (1.58 to 2.16)	2.49 (1.48 to 3.22)	72.7 (62.5 to 85.5)	46 (39.5 to 54.2)	58.9 (38 to 74.6)
Georgia	Inguinal, femoral, and abdominal hernia	3.29 (2.34 to 4.4)	2.77 (1.95 to 3.77)	2.69 (2 to 3.5)	58 (41.5 to 77.2)	61.7 (42.9 to 84.5)	61 (44.7 to 80.5)
Georgia	Inflammatory bowel disease	0.717 (0.548 to 0.87)	0.409 (0.323 to 0.513)	0.56 (0.416 to 0.683)	12 (9.3 to 14.6)	8.1 (6.3 to 10.3)	11.5 (8.9 to 14.2)
Georgia	Vascular intestinal disorders	0.426 (0.378 to 0.484)	0.461 (0.408 to 0.507)	0.84 (0.58 to 0.996)	7.1 (6.3 to 8)	7.7 (6.8 to 8.4)	14.1 (9.9 to 16.5)
Georgia	Gallbladder and biliary diseases	0.677 (0.62 to 0.792)	0.727 (0.672 to 0.807)	0.884 (0.791 to 1.03)	11.2 (10.3 to 13)	13.6 (12.6 to 14.9)	16.6 (15 to 19.2)
Georgia	Pancreatitis	0.852 (0.679 to 1.09)	0.777 (0.633 to 0.976)	1.22 (1.03 to 1.47)	14 (11.1 to 18.1)	14.7 (11.9 to 18.6)	24.4 (20.4 to 29.3)
Georgia	Other digestive diseases	0.831 (0.668 to 1.01)	0.735 (0.627 to 0.859)	1.42 (1.23 to 1.63)	14.8 (11.8 to 18.2)	15.4 (12.9 to 18.3)	28.7 (25 to 32.5)
Georgia	Neurological disorders	82.9 (63.9 to 105)	70.6 (56.1 to 87.6)	71.1 (57.3 to 88.2)	1494 (1157.3 to 1886.4)	1436.8 (1113.5 to 1804.6)	1511.2 (1170 to 1943)
Georgia	Alzheimer's disease and other dementias	21.4 (19.5 to 23.6)	24.9 (22.9 to 27)	27.8 (25.5 to 30.3)	408.3 (372.5 to 449.6)	434.4 (397.8 to 470.5)	448.8 (411 to 488.6)
Georgia	Parkinson's disease	4.31 (3.71 to 5.06)	4.73 (4.29 to 5.31)	5.1 (4.55 to 6.06)	74.4 (64.2 to 87)	74.2 (67.4 to 82.6)	80.3 (71.5 to 94.6)
Georgia	Epilepsy	12.7 (6.94 to 20.6)	6.35 (3.17 to 10.8)	6.95 (2.88 to 12.9)	236.9 (130.7 to 379.8)	155.2 (77.5 to 263.5)	191.5 (80.2 to 355.5)
Georgia	Multiple sclerosis	0.634 (0.504 to 0.775)	0.565 (0.445 to 0.704)	0.658 (0.487 to 0.821)	10.5 (8.4 to 12.8)	11.2 (8.8 to 14)	13.9 (10.3 to 17.3)
Georgia	Motor neuron disease	0.174 (0.142 to 0.226)	0.112 (0.103 to 0.122)	0.168 (0.145 to 0.188)	2.7 (2.2 to 3.5)	2.2 (2.1 to 2.4)	3.4 (3 to 3.8)
Georgia	Headache disorders	41.6 (27 to 60)	32.4 (21.2 to 46.6)	28.4 (18.6 to 40.9)	723.4 (468.7 to 1042)	721.9 (472.5 to 1038.2)	716.8 (467.8 to 1034)
Georgia	Migraine	36.1 (22.6 to 53.6)	28.1 (17.7 to 41.7)	24.6 (15.4 to 36.3)	628.2 (392.6 to 931.3)	626.9 (392.5 to 927.9)	622.1 (391.4 to 921)
Georgia	Tension-type headache	5.5 (3.07 to 8.79)	4.32 (2.45 to 6.83)	3.84 (2.16 to 6.09)	95.2 (53.3 to 152)	95 (53.6 to 150.9)	94.7 (53.1 to 151.3)
Georgia	Other neurological disorders	2.03 (1.14 to 3.22)	1.51 (0.989 to 2.22)	2 (1.3 to 3.08)	37.8 (20.8 to 60.8)	37.6 (23.9 to 56.3)	56.4 (34 to 90.1)
Georgia	Mental disorders	73.8 (54.4 to 95.7)	58.4 (43.4 to 75.2)	51.6 (38.5 to 66.8)	1290.6 (952.6 to 1673.5)	1286.3 (955.6 to 1666.8)	1286.6 (956.2 to 1673.4)
Georgia	Schizophrenia	7.07 (5.27 to 8.81)	5.5 (4.08 to 6.87)	5.15 (3.8 to 6.45)	119.5 (88.7 to 149.6)	116.5 (86.4 to 146.3)	119 (87.3 to 149.3)
Georgia	Depressive disorders	24.9 (17.6 to 33.9)	20.8 (14.7 to 28.1)	18.7 (13.3 to 25.1)	422.1 (298.8 to 577.2)	421.9 (298.3 to 571.6)	421.4 (300.6 to 568.3)
Georgia	Major depressive disorder	18.4 (12.7 to 25)	15.5 (10.7 to 20.8)	13.8 (9.67 to 18.5)	313.4 (215.7 to 429.4)	313.4 (218.5 to 424.1)	313.5 (220.1 to 423.9)
Georgia	Dysthymia	6.49 (4.27 to 9.43)	5.31 (3.52 to 7.83)	4.87 (3.24 to 7.1)	108.7 (71.8 to 158.3)	108.5 (71.6 to 159.1)	107.9 (72.1 to 156.3)
Georgia	Bipolar disorder	7.94 (5 to 11.7)	6.26 (3.91 to 9.4)	5.42 (3.4 to 8.04)	138.1 (87 to 203.8)	138 (86.4 to 206.6)	137.4 (86.1 to 205)
Georgia	Anxiety disorders	13.6 (9.66 to 18.1)	10.6 (7.52 to 14.1)	9.29 (6.56 to 12.3)	238.2 (169.3 to 318.4)	237.1 (168.4 to 316.9)	235.4 (165.6 to 313.8)
Georgia	Eating disorders	1.93 (1.22 to 2.82)	1.18 (0.752 to 1.75)	1.17 (0.723 to 1.74)	35.2 (22.2 to 51.4)	29.2 (18.5 to 43.4)	35 (21.8 to 52.7)
Georgia	Anorexia nervosa	0.381 (0.223 to 0.591)	0.237 (0.144 to 0.36)	0.218 (0.129 to 0.333)	7.1 (4.2 to 11)	6 (3.6 to 9.2)	7 (4.1 to 10.7)
Georgia	Bulimia nervosa	1.55 (0.966 to 2.32)	0.945 (0.587 to 1.44)	0.951 (0.583 to 1.46)	28.1 (17.5 to 42.2)	23.2 (14.4 to 35)	28 (17.2 to 42.5)
Georgia	Autism spectrum disorders	3.49 (2.35 to 4.85)	2.56 (1.72 to 3.58)	2.28 (1.54 to 3.15)	64.5 (43.5 to 89.5)	65 (43.4 to 91.3)	65.5 (44.1 to 91.4)
Georgia	Attention-deficit/hyperactivity disorder	0.616 (0.368 to 0.982)	0.433 (0.258 to 0.693)	0.36 (0.215 to 0.571)	11.7 (7 to 18.7)	11.7 (6.9 to 18.7)	11.9 (7 to 18.9)
Georgia	Conduct disorder	4.87 (2.89 to 7.71)	3.31 (2 to 5.2)	2.42 (1.46 to 3.81)	98 (58.2 to 155.4)	98.3 (59.8 to 154.9)	99.1 (59.8 to 156.4)
Georgia	Idiopathic developmental intellectual disability	1.31 (0.462 to 2.39)	1.14 (0.424 to 2.07)	0.763 (0.254 to 1.45)	24.4 (8.6 to 44.5)	29.4 (11.1 to 53.5)	22.6 (7.6 to 42.5)

SR Appendix Table 2. All-age DALYs counts and age standardized DALYs rates per 100,000 individuals for all causes and level 1 cause groups by location for 1990, 2007, and 2017, both sexes combined

		All-age DALYs			Age-standardized rate of DALYs per 100,000		
Georgia	Other mental disorders	8.17 (5.41 to 11.3)	6.61 (4.38 to 9.08)	6.02 (4 to 8.3)	138.9 (92 to 193.5)	139.2 (91.8 to 192.5)	139.3 (92.4 to 193.6)
Georgia	Substance use disorders	23.9 (18.8 to 29.7)	16.3 (12.4 to 20.4)	17.2 (13.4 to 21.3)	412.8 (324 to 513)	362.4 (275.3 to 455.9)	428.9 (330.1 to 534.8)
Georgia	Alcohol use disorders	17.5 (13.9 to 22.1)	10.2 (7.51 to 13.5)	10.3 (7.64 to 13.3)	301.1 (238.3 to 379.9)	226.5 (165.2 to 299.4)	254.6 (189.2 to 333.3)
Georgia	Drug use disorders	6.41 (4.68 to 8.21)	6 (4.67 to 7.47)	6.92 (5.6 to 8.29)	111.7 (81 to 144)	135.9 (104.9 to 170.9)	174.3 (138.8 to 211.3)
Georgia	Opioid use disorders	4.68 (3.37 to 6.1)	4.39 (3.33 to 5.44)	5.17 (4.12 to 6.26)	80.7 (58.1 to 105.2)	98.6 (74.3 to 123)	128.1 (101.4 to 157.4)
Georgia	Cocaine use disorders	0.205 (0.129 to 0.307)	0.219 (0.163 to 0.287)	0.275 (0.212 to 0.351)	3.6 (2.2 to 5.4)	4.8 (3.6 to 6.4)	6.8 (5 to 8.8)
Georgia	Amphetamine use disorders	0.595 (0.331 to 0.982)	0.477 (0.291 to 0.749)	0.44 (0.272 to 0.667)	10.7 (5.9 to 17.7)	11.6 (7 to 18.4)	12.9 (7.6 to 20.2)
Georgia	Cannabis use disorders	0.228 (0.141 to 0.339)	0.17 (0.108 to 0.255)	0.139 (0.0864 to 0.206)	4.1 (2.6 to 6.2)	4.1 (2.6 to 6.2)	4.2 (2.6 to 6.3)
Georgia	Other drug use disorders	0.711 (0.468 to 1.01)	0.746 (0.569 to 0.988)	0.899 (0.723 to 1.11)	12.6 (8.2 to 18.1)	16.7 (12.5 to 22.6)	22.4 (17.5 to 28.7)
Georgia	Diabetes and kidney diseases	63.5 (51.5 to 78)	68.1 (55.5 to 83.7)	87.9 (73.3 to 106)	1021.2 (828.5 to 1253.4)	1237.8 (1007.8 to 1518.5)	1629.1 (1352.4 to 1977.3)
Georgia	Diabetes mellitus	47.4 (36.8 to 60.8)	49 (37.4 to 63.4)	66.5 (53.1 to 82.6)	753.8 (580.6 to 969.3)	882.4 (670.4 to 1141.7)	1217.4 (963 to 1526.9)
Georgia	Diabetes mellitus type 1	11.4 (10.1 to 12.2)	8.2 (7.61 to 8.74)	11.4 (8.91 to 12.7)	177.8 (157.9 to 190.4)	146.8 (136.7 to 156.2)	210.3 (168.7 to 231.7)
Georgia	Diabetes mellitus type 2	36 (25.7 to 49.2)	40.8 (29.4 to 55)	55.1 (42 to 71)	575.9 (409.5 to 786.3)	735.6 (526.5 to 988.6)	1007.1 (762.4 to 1313.6)
Georgia	Chronic kidney disease	15.4 (14 to 17.2)	19.1 (17.6 to 20.7)	21.4 (19.6 to 23.4)	256.2 (231.8 to 285.8)	354.6 (326.5 to 383.7)	411.3 (378.5 to 447.3)
Georgia	Chronic kidney disease due to diabetes mellitus type 1	1.15 (0.913 to 1.44)	1.42 (1.15 to 1.74)	1.61 (1.29 to 1.98)	19 (15.1 to 23.4)	28 (22.7 to 34.5)	33.9 (27.6 to 41.4)
Georgia	Chronic kidney disease due to diabetes mellitus type 2	2.79 (2.33 to 3.32)	4.06 (3.5 to 4.68)	5.18 (4.47 to 5.92)	43.5 (36.7 to 51.4)	67.5 (58.6 to 77.3)	88.1 (76.6 to 100.4)
Georgia	Chronic kidney disease due to hypertension	1.73 (1.43 to 2.04)	2.36 (1.99 to 2.74)	2.65 (2.21 to 3.09)	28.6 (23.7 to 33.8)	41.2 (34.7 to 47.7)	47.1 (39.5 to 54.6)
Georgia	Chronic kidney disease due to glomerulonephritis	3.75 (3.28 to 4.31)	4.38 (3.84 to 4.96)	4.7 (4.06 to 5.43)	62.9 (55.2 to 71.3)	86.9 (76.6 to 98.3)	98 (85.7 to 111.3)
Georgia	Chronic kidney disease due to other and unspecified causes	6.02 (5.12 to 7.04)	6.85 (5.85 to 8)	7.28 (6.23 to 8.55)	102.2 (87.5 to 119.4)	130.9 (113.2 to 152.1)	144.3 (124.7 to 167.5)
Georgia	Acute glomerulonephritis	0.669 (0.526 to 0.845)	0.0391 (0.0342 to 0.0439)	0.0171 (0.0146 to 0.0197)	11.3 (8.8 to 14.2)	0.8 (0.7 to 0.9)	0.4 (0.3 to 0.4)
Georgia	Skin and subcutaneous diseases	24.9 (16.7 to 36.5)	18.2 (12.4 to 27)	16.7 (11.4 to 24.7)	455.8 (306.4 to 667.4)	448.8 (302.2 to 659.2)	458.9 (308.3 to 674.2)
Georgia	Dermatitis	5.41 (3.23 to 8.44)	3.82 (2.3 to 5.9)	3.47 (2.08 to 5.33)	101.1 (59.8 to 158.6)	99.8 (58.7 to 157.4)	101.5 (59.4 to 158.4)
Georgia	Atopic dermatitis	4.08 (2.18 to 6.84)	2.75 (1.49 to 4.61)	2.53 (1.36 to 4.19)	77.8 (41.3 to 131.1)	76.5 (41.6 to 128.4)	78.3 (42.5 to 130.8)
Georgia	Contact dermatitis	1.33 (0.866 to 1.97)	1.06 (0.705 to 1.56)	0.942 (0.613 to 1.39)	23.2 (15.1 to 34.1)	23.2 (15.1 to 34.6)	23.1 (15 to 34.4)
Georgia	Seborrhoeic dermatitis	0.00593 (0.00331 to 0.0095)	0.00452 (0.00251 to 0.00725)	0.00409 (0.00227 to 0.00657)	0.1 (0.1 to 0.2)	0.1 (0.1 to 0.2)	0.1 (0.1 to 0.2)
Georgia	Psoriasis	4.18 (2.95 to 5.53)	3.04 (2.14 to 4.01)	2.84 (2 to 3.75)	71.3 (50.3 to 94)	64.7 (45.2 to 86.5)	66.3 (46.6 to 87.8)
Georgia	Bacterial skin diseases	0.137 (0.0912 to 0.209)	0.193 (0.0967 to 0.26)	0.338 (0.0951 to 0.497)	2.4 (1.6 to 3.7)	4.1 (2.2 to 5.5)	7.2 (2.3 to 10.5)
Georgia	Cellulitis	0.0766 (0.0529 to 0.107)	0.0722 (0.047 to 0.099)	0.136 (0.0469 to 0.207)	1.4 (0.9 to 1.9)	1.6 (1.1 to 2.2)	2.9 (1.2 to 4.3)
Georgia	Pyoderma	0.0607 (0.0355 to 0.118)	0.121 (0.0425 to 0.168)	0.201 (0.0397 to 0.312)	1.1 (0.6 to 2.1)	2.5 (1 to 3.5)	4.3 (1 to 6.7)
Georgia	Scabies	1.64 (0.918 to 2.66)	1.19 (0.666 to 1.93)	0.982 (0.546 to 1.59)	30.8 (17.2 to 50.5)	31 (17.3 to 50.8)	30 (16.7 to 49.2)
Georgia	Fungal skin diseases	2.64 (1.05 to 5.45)	2.19 (0.868 to 4.55)	2.02 (0.812 to 4.16)	47.1 (18.8 to 97.5)	46.6 (18.5 to 96.6)	46.1 (18.3 to 95.2)
Georgia	Viral skin diseases	2.3 (1.47 to 3.46)	1.44 (0.919 to 2.15)	1.3 (0.826 to 1.94)	45.9 (29.2 to 69.1)	46 (29.4 to 68.6)	46 (29.3 to 69)
Georgia	Acne vulgaris	1.59 (0.95 to 2.65)	1.28 (0.743 to 2.13)	0.976 (0.569 to 1.61)	30 (17.9 to 50.2)	32.5 (18.8 to 54.2)	34 (19.6 to 56.5)
Georgia	Alopecia areata	0.339 (0.215 to 0.512)	0.258 (0.164 to 0.385)	0.23 (0.145 to 0.339)	5.9 (3.7 to 9)	5.9 (3.8 to 8.9)	5.9 (3.8 to 8.8)
Georgia	Pruritus	0.548 (0.258 to 1.02)	0.43 (0.206 to 0.808)	0.404 (0.194 to 0.757)	9.5 (4.4 to 17.8)	9.3 (4.4 to 17.6)	9.5 (4.5 to 18)
Georgia	Urticaria	3.96 (2.6 to 5.61)	2.74 (1.83 to 3.89)	2.46 (1.63 to 3.49)	75 (49.2 to 107.2)	74.8 (49.3 to 106.9)	74.7 (49.4 to 106)
Georgia	Decubitus ulcer	0.131 (0.0906 to 0.176)	0.126 (0.0857 to 0.17)	0.157 (0.0955 to 0.214)	2.3 (1.6 to 3.1)	2.4 (1.7 to 3.2)	3 (1.9 to 4.1)
Georgia	Other skin and subcutaneous diseases	2.01 (0.977 to 3.68)	1.55 (0.741 to 2.85)	1.57 (0.755 to 2.87)	34.3 (16.7 to 62.7)	31.7 (15.2 to 58.2)	34.7 (16.6 to 63.8)
Georgia	Sense organ diseases	51.1 (34 to 74.7)	46.9 (31.4 to 68)	44.2 (29.7 to 64)	860 (572.1 to 1256.9)	868.8 (578.9 to 1271.9)	847.8 (567 to 1238.7)
Georgia	Blindness and vision impairment	24.3 (15.6 to 36.7)	22.2 (14.3 to 33.6)	20.5 (13.2 to 30.6)	407.2 (260.5 to 611.9)	411.9 (263.9 to 625.7)	395.7 (253.3 to 596)

SR Appendix Table 2. All-age DALYs counts and age standardized DALYs rates per 100,000 individuals for all causes and level 1 cause groups by location for 1990, 2007, and 2017, both sexes combined

		All-age DALYs			Age-standardized rate of DALYs per 100,000		
Georgia	Glaucoma	0.206 (0.13 to 0.297)	0.198 (0.127 to 0.282)	0.184 (0.121 to 0.265)	3.3 (2.1 to 4.7)	3.2 (2.1 to 4.5)	3 (2 to 4.3)
Georgia	Cataract	8.71 (5.81 to 12.1)	8.81 (5.92 to 12.1)	8.42 (5.67 to 11.6)	144.4 (96.1 to 199.3)	145.1 (97.5 to 198.6)	139.8 (94.1 to 193)
Georgia	Age-related macular degeneration	0.0722 (0.0452 to 0.107)	0.0699 (0.0437 to 0.106)	0.0699 (0.0431 to 0.104)	1.2 (0.7 to 1.7)	1.1 (0.7 to 1.7)	1.1 (0.7 to 1.7)
Georgia	Refraction disorders	6.35 (4.01 to 9.75)	5.07 (3.2 to 7.71)	4.47 (2.81 to 6.84)	111.5 (70.7 to 169.6)	114.2 (72.5 to 174.8)	110.6 (69.6 to 169.3)
Georgia	Near vision loss	7.76 (3.68 to 14.3)	6.95 (3.29 to 12.8)	6.35 (3.03 to 11.6)	126.1 (59.7 to 232.9)	127.8 (60.8 to 234.2)	120.9 (57.4 to 219.4)
Georgia	Other vision loss	1.24 (0.821 to 1.77)	1.08 (0.701 to 1.52)	1.01 (0.667 to 1.44)	20.8 (13.6 to 29.2)	20.6 (13.4 to 29.3)	20.2 (13.4 to 28.4)
Georgia	Age-related and other hearing loss	24.9 (17 to 35.3)	23.1 (15.9 to 32.4)	22.1 (15.1 to 30.9)	421.3 (289.8 to 591.3)	425.5 (293.4 to 597.7)	420.7 (288.3 to 591.7)
Georgia	Other sense organ diseases	1.88 (1.17 to 2.75)	1.65 (1.03 to 2.43)	1.59 (0.988 to 2.31)	31.5 (19.4 to 46.2)	31.4 (19.6 to 46.1)	31.4 (19.4 to 45.8)
Georgia	Musculoskeletal disorders	79.2 (57.4 to 106)	70 (51.2 to 93.7)	65.8 (48 to 88)	1310.3 (956 to 1763.3)	1350 (991.2 to 1805)	1345.3 (984.3 to 1807.1)
Georgia	Rheumatoid arthritis	1.62 (1.12 to 2.19)	1.37 (0.947 to 1.81)	1.52 (1.08 to 2.03)	26.4 (18.3 to 35.8)	25.6 (17.7 to 34.1)	29.7 (20.8 to 39.4)
Georgia	Osteoarthritis	4.99 (2.52 to 9.82)	4.83 (2.44 to 9.62)	4.87 (2.45 to 9.57)	79.9 (40.4 to 157.5)	83 (41.9 to 164.8)	85.1 (43.1 to 168)
Georgia	Low back pain	51.7 (36.8 to 70.3)	45.1 (32.1 to 61.4)	42.2 (30.1 to 57.5)	858.1 (609.6 to 1165)	881.5 (622.4 to 1196.4)	875.3 (624.2 to 1192.1)
Georgia	Neck pain	18.1 (12.5 to 25.6)	15.7 (10.9 to 21.9)	14.6 (10.2 to 20.5)	300.2 (208.3 to 421.9)	300 (209.3 to 421.5)	298.6 (208.6 to 418.6)
Georgia	Gout	0.779 (0.509 to 1.1)	0.758 (0.508 to 1.03)	0.749 (0.507 to 1.03)	12.5 (8.2 to 17.6)	13.3 (8.8 to 18.3)	13.6 (9.2 to 18.9)
Georgia	Other musculoskeletal disorders	1.94 (1.07 to 3.04)	2.22 (1.32 to 3.43)	1.87 (1.15 to 2.85)	33.2 (18.4 to 52.5)	46.5 (27.5 to 71.9)	43 (26.2 to 64.4)
Georgia	Other non-communicable diseases	73 (56.9 to 93.2)	50.9 (38.5 to 66.1)	47.6 (35.3 to 62.1)	1408 (1108.5 to 1791.9)	1218.6 (948.1 to 1534.7)	1231 (922.8 to 1571.7)
Georgia	Congenital anomalies	31.1 (25.5 to 41.9)	17 (14 to 20.3)	15.2 (11 to 19)	694.3 (565.2 to 962.3)	535.2 (443.6 to 645)	526.5 (370.5 to 667.5)
Georgia	Neural tube defects	2.78 (2.12 to 4.14)	0.872 (0.681 to 1.11)	0.968 (0.706 to 1.3)	63.8 (48.3 to 97.9)	27.9 (21.9 to 36.2)	34.9 (24.9 to 48.4)
Georgia	Congenital heart anomalies	11.8 (9.24 to 17.1)	5.42 (4.46 to 6.73)	4.91 (2.58 to 6.71)	282 (219.3 to 411.5)	190.6 (156.7 to 248.7)	192.6 (103.7 to 265.8)
Georgia	Orofacial clefts	0.361 (0.235 to 0.553)	0.152 (0.1 to 0.221)	0.121 (0.0792 to 0.176)	7.7 (4.7 to 12.1)	4 (2.6 to 5.7)	3.5 (2.3 to 5)
Georgia	Down's syndrome	0.535 (0.402 to 0.793)	0.441 (0.332 to 0.548)	0.501 (0.303 to 0.658)	10.9 (8.1 to 17)	13.5 (10 to 16.8)	17.2 (9.7 to 23.2)
Georgia	Turner syndrome	0.00483 (0.00235 to 0.00789)	0.00332 (0.00168 to 0.00544)	0.0028 (0.00142 to 0.00462)	0.1 (0 to 0.1)	0.1 (0 to 0.1)	0.1 (0 to 0.1)
Georgia	Klinefelter syndrome	0.00133 (0.000612 to 0.00256)	0.000976 (0.000451 to 0.00187)	0.000836 (0.000382 to 0.00158)	0 (0 to 0)	0 (0 to 0)	0 (0 to 0)
Georgia	Other chromosomal abnormalities	1.48 (0.582 to 2.49)	0.736 (0.366 to 1.02)	0.479 (0.309 to 0.646)	34.2 (12.3 to 59.1)	24.7 (11.2 to 35.4)	16.4 (9.9 to 22.1)
Georgia	Congenital musculoskeletal and limb anomalies	5.62 (3.75 to 7.67)	4.24 (2.82 to 5.78)	3.78 (2.54 to 5.18)	103.4 (68.9 to 140.6)	102.1 (67.8 to 139.7)	102.1 (69.4 to 139.7)
Georgia	Urogenital congenital anomalies	0.383 (0.241 to 0.711)	0.301 (0.184 to 0.409)	0.337 (0.13 to 0.512)	8.5 (5.3 to 16.5)	10.3 (6.3 to 14.1)	12.6 (4.7 to 19.4)
Georgia	Digestive congenital anomalies	2.14 (1.52 to 3.36)	1.77 (1.37 to 2.26)	1.6 (1.07 to 2.09)	45.8 (32 to 74.6)	56.8 (43.7 to 72.8)	56.2 (35.8 to 74.7)
Georgia	Other congenital anomalies	6.03 (4.32 to 10.6)	3.09 (2.4 to 4.24)	2.49 (1.9 to 3.16)	138 (97.9 to 251.9)	105.1 (81.8 to 149.7)	91.1 (67.9 to 115.6)
Georgia	Urinary diseases and male infertility	5.49 (4.41 to 6.83)	3.49 (2.61 to 4.55)	4.12 (3.19 to 5.2)	89.3 (72.3 to 110)	64.1 (48.3 to 83.7)	78.5 (61.8 to 97.5)
Georgia	Urinary tract infections	2.36 (2.18 to 2.56)	0.875 (0.792 to 0.968)	1.44 (1.32 to 1.6)	40.8 (37.7 to 44)	17.7 (16 to 19.7)	30.4 (27.9 to 33.7)
Georgia	Urolithiasis	0.218 (0.151 to 0.298)	0.175 (0.124 to 0.241)	0.207 (0.155 to 0.273)	3.6 (2.5 to 4.9)	3.4 (2.4 to 4.7)	4.1 (3.1 to 5.4)
Georgia	Benign prostatic hyperplasia	2.43 (1.54 to 3.54)	2.11 (1.35 to 3.02)	2.13 (1.35 to 3.09)	36.3 (23.2 to 52.7)	36 (23.1 to 51.3)	36.2 (23 to 52.5)
Georgia	Male infertility	0.128 (0.0506 to 0.27)	0.0993 (0.0396 to 0.208)	0.0804 (0.0326 to 0.168)	2.3 (0.9 to 4.8)	2.4 (1 to 5.1)	2.3 (0.9 to 4.8)
Georgia	Other urinary diseases	0.358 (0.275 to 0.466)	0.221 (0.156 to 0.283)	0.259 (0.179 to 0.318)	6.3 (4.9 to 8.3)	4.6 (3.3 to 6)	5.5 (3.9 to 6.7)
Georgia	Gynecological diseases	9.35 (6.51 to 13)	7.36 (5.07 to 10.6)	6.09 (4.17 to 8.65)	167.7 (116.5 to 231.6)	163.4 (112 to 234.1)	158.5 (108.2 to 224.5)
Georgia	Uterine fibroids	1.86 (1.18 to 2.88)	1.51 (0.886 to 2.46)	1.26 (0.737 to 2.07)	32.5 (20.6 to 50.4)	30.4 (18 to 49.3)	28.2 (16.5 to 46.3)
Georgia	Polycystic ovarian syndrome	0.326 (0.145 to 0.631)	0.269 (0.119 to 0.521)	0.223 (0.0987 to 0.427)	5.9 (2.6 to 11.5)	6.2 (2.7 to 12.1)	6.2 (2.7 to 11.9)
Georgia	Female infertility	0.137 (0.0465 to 0.305)	0.091 (0.0305 to 0.204)	0.0874 (0.0295 to 0.197)	2.4 (0.8 to 5.4)	2.3 (0.8 to 5.1)	2.5 (0.8 to 5.5)
Georgia	Endometriosis	2.88 (1.88 to 4.28)	2.13 (1.4 to 3.06)	1.77 (1.16 to 2.58)	51.8 (34 to 76.7)	49.2 (32.1 to 70.9)	48.1 (31.6 to 69.7)
Georgia	Genital prolapse	0.184 (0.0898 to 0.349)	0.139 (0.0652 to 0.266)	0.139 (0.065 to 0.267)	2.9 (1.4 to 5.6)	2.5 (1.2 to 4.8)	2.6 (1.2 to 5)

SR Appendix Table 2. All-age DALYs counts and age standardized DALYs rates per 100,000 individuals for all causes and level 1 cause groups by location for 1990, 2007, and 2017, both sexes combined							
		All-age DALYs			Age-standardized rate of DALYs per 100,000		
Georgia	Premenstrual syndrome	2.91 (1.84 to 4.34)	2.34 (1.48 to 3.51)	1.85 (1.17 to 2.77)	53.2 (33.7 to 79.2)	53.5 (33.6 to 80.4)	51.2 (32.6 to 77.2)
Georgia	Other gynecological diseases	1.05 (0.693 to 1.59)	0.885 (0.563 to 1.38)	0.766 (0.49 to 1.21)	18.9 (12.4 to 28.8)	19.3 (12.4 to 30.1)	19.8 (12.7 to 31.2)
Georgia	Hemoglobinopathies and hemolytic anaemias	3.68 (2.78 to 4.9)	3.04 (2.34 to 3.93)	2.65 (1.98 to 3.39)	65.1 (48.9 to 87.3)	66.8 (51.8 to 86.7)	62.7 (49.1 to 81.8)
Georgia	Thalassemias	0.121 (0.0627 to 0.3)	0.0774 (0.044 to 0.184)	0.0659 (0.0343 to 0.176)	2.7 (1.4 to 6.6)	2.9 (1.6 to 7)	2.6 (1.3 to 7)
Georgia	Thalassaemias trait	0.741 (0.458 to 1.12)	0.622 (0.394 to 0.92)	0.509 (0.317 to 0.781)	13.5 (8.3 to 20.4)	14.5 (9.1 to 21.5)	13.3 (8.1 to 20.2)
Georgia	Sickle cell disorders	0.0524 (0.0268 to 0.115)	0.0366 (0.0199 to 0.0758)	0.0323 (0.0171 to 0.0714)	1.2 (0.6 to 2.5)	1.3 (0.7 to 2.8)	1.2 (0.6 to 2.8)
Georgia	Sickle cell trait	0.156 (0.0928 to 0.247)	0.0922 (0.0564 to 0.143)	0.0679 (0.038 to 0.111)	2.9 (1.7 to 4.5)	2.2 (1.4 to 3.4)	1.9 (1.1 to 3.2)
Georgia	G6PD deficiency	0.187 (0.143 to 0.284)	0.24 (0.197 to 0.282)	0.291 (0.202 to 0.346)	3.4 (2.5 to 5.3)	5.5 (4.6 to 6.6)	7.4 (5.9 to 8.8)
Georgia	G6PD trait	0.000254 (0.000149 to 0.000403)	0.000166 (0.000106 to 0.000238)	0.000152 (0.0000922 to 0.000227)	0 (0 to 0)	0 (0 to 0)	0 (0 to 0)
Georgia	Other hemoglobinopathies and hemolytic anaemias	2.42 (1.88 to 3.2)	1.97 (1.45 to 2.52)	1.68 (1.13 to 2.15)	41.5 (32.1 to 54.6)	40.4 (30.4 to 51.7)	36.2 (25.5 to 46.9)
Georgia	Endocrine, metabolic, blood, and immune disorders	2.82 (2.1 to 3.82)	2.62 (2.02 to 3.35)	3.43 (1.78 to 4.39)	50.6 (37.5 to 68.2)	59.6 (46.4 to 75.5)	82.2 (46.1 to 103.8)
Georgia	Oral disorders	20.3 (12.9 to 30.6)	17.2 (11 to 25.8)	16.1 (10.3 to 23.7)	335.4 (212 to 505.7)	323.5 (205.7 to 487.2)	317.5 (201.9 to 477.4)
Georgia	Caries of deciduous teeth	0.179 (0.078 to 0.358)	0.0976 (0.0426 to 0.197)	0.1 (0.0425 to 0.202)	3.8 (1.6 to 7.6)	3.9 (1.7 to 7.8)	3.9 (1.7 to 7.9)
Georgia	Caries of permanent teeth	2.41 (1.05 to 4.59)	1.91 (0.832 to 3.6)	1.6 (0.701 to 3.07)	42.7 (18.5 to 81)	43.8 (18.9 to 82.7)	42.4 (18.5 to 81.1)
Georgia	Periodontal disease	2.8 (1.12 to 5.89)	2.23 (0.885 to 4.66)	2.07 (0.823 to 4.25)	45.6 (18.2 to 95.1)	42.5 (16.8 to 88.7)	41.4 (16.4 to 85.4)
Georgia	Edentulism and severe tooth loss	12 (7.93 to 16.9)	10.7 (7.21 to 15.2)	10.2 (6.78 to 14.5)	192.1 (127.4 to 270.9)	182.1 (121.9 to 257.8)	178.9 (119 to 254.5)
Georgia	Other oral disorders	2.95 (1.85 to 4.33)	2.31 (1.45 to 3.41)	2.07 (1.3 to 3.05)	51.3 (32.1 to 75.3)	51.2 (32 to 75.7)	51 (32 to 75.4)
Georgia	Sudden infant death syndrome	0.229 (0.136 to 0.433)	0.153 (0.0999 to 0.218)	0.122 (0.0658 to 0.191)	5.6 (3.3 to 10.6)	5.9 (3.9 to 8.4)	5.1 (2.8 to 8)
Georgia	Injuries	227 (207 to 253)	120 (108 to 135)	135 (121 to 152)	4039.2 (3681.3 to 4466.8)	2705 (2447.2 to 3000.4)	3334.4 (3023.8 to 3705.3)
Georgia	Transport injuries	67.8 (63.1 to 72.9)	31.7 (29.5 to 34.1)	41.4 (37.9 to 44.9)	1194.1 (1112 to 1282.5)	724.3 (675.6 to 777.2)	1070.9 (986.6 to 1153.5)
Georgia	Road injuries	65.2 (60.7 to 70)	29.1 (27.1 to 31.3)	38.8 (35.5 to 42.1)	1149.3 (1071.4 to 1231.8)	664.7 (620.3 to 712.4)	1003.8 (923.8 to 1080.5)
Georgia	Pedestrian road injuries	17 (14.9 to 20.5)	6.38 (5.8 to 7.01)	9.79 (8.89 to 11)	298.8 (262.7 to 360.5)	142 (129.2 to 155.5)	239.2 (217.3 to 268)
Georgia	Cyclist road injuries	2.66 (2.22 to 3.23)	1.49 (1.23 to 1.79)	1.71 (1.4 to 2.08)	46 (38.4 to 56)	31.9 (26.7 to 38)	40.4 (33.4 to 48.3)
Georgia	Motorcyclist road injuries	6.07 (5.19 to 7.51)	4.68 (4.19 to 5.21)	3.75 (3.28 to 4.33)	108 (92.8 to 134.2)	110.6 (99.2 to 123.3)	102 (88.8 to 117.7)
Georgia	Motor vehicle road injuries	38.4 (34.2 to 41.9)	15.9 (14.7 to 17.1)	23 (20.8 to 25)	678.4 (603.3 to 741.9)	366.9 (340.6 to 393.7)	609.7 (549.9 to 656.9)
Georgia	Other road injuries	1.04 (0.871 to 1.27)	0.618 (0.525 to 0.726)	0.528 (0.438 to 0.645)	18.1 (15.3 to 22.2)	13.3 (11.3 to 15.6)	12.6 (10.5 to 15.1)
Georgia	Other transport injuries	2.58 (2.25 to 2.93)	2.61 (2.34 to 2.91)	2.63 (2.3 to 2.97)	44.9 (39.2 to 50.7)	59.7 (53.5 to 66.4)	67.1 (58.6 to 76.2)
Georgia	Unintentional injuries	132 (115 to 151)	67.5 (57.9 to 79.4)	71.6 (60.8 to 84.9)	2353 (2075.7 to 2687.1)	1496.9 (1297.1 to 1735.6)	1700.9 (1464.1 to 1985)
Georgia	Falls	48.2 (38.7 to 60.3)	23.1 (17.7 to 29.7)	28 (21.9 to 35.7)	831 (668.7 to 1034.7)	468.2 (361.8 to 600.4)	605.8 (475.4 to 765.2)
Georgia	Drowning	24.1 (22.4 to 26)	6.09 (5.64 to 6.5)	6.28 (5.78 to 6.86)	450 (417.3 to 485.9)	152.3 (139.7 to 164.4)	179.1 (162.8 to 196.9)
Georgia	Fire, heat, and hot substances	22 (19.7 to 24.7)	10.7 (9.38 to 12.3)	9.21 (7.89 to 10.8)	403 (360.6 to 452.5)	245.8 (216.1 to 279.3)	223.9 (192.6 to 261.5)
Georgia	Poisonings	5.26 (4.84 to 5.69)	2.07 (1.87 to 2.31)	2.06 (1.81 to 2.35)	95.5 (87.7 to 103.4)	49.4 (44.4 to 55.1)	54.2 (47.6 to 61.6)
Georgia	Poisoning by carbon monoxide	3.47 (3.2 to 3.79)	0.932 (0.848 to 1.01)	0.881 (0.795 to 0.974)	62.1 (57.1 to 67.7)	21.5 (19.6 to 23.5)	22.7 (20.5 to 25.2)
Georgia	Poisoning by other means	1.78 (1.55 to 2.05)	1.14 (0.987 to 1.33)	1.18 (0.98 to 1.42)	33.4 (29.1 to 38.3)	27.9 (24.1 to 32.4)	31.5 (26.3 to 37.9)
Georgia	Exposure to mechanical forces	13 (10.7 to 15.8)	9.7 (8 to 11.9)	10.7 (8.78 to 13.2)	226 (188.8 to 273.7)	210.4 (175.8 to 255.3)	252.9 (211.4 to 307)
Georgia	Unintentional firearm injuries	3.19 (2.49 to 3.67)	1.6 (1.43 to 1.86)	1.95 (1.72 to 2.39)	56.9 (44.2 to 65.4)	37.1 (33 to 43.1)	51.2 (45.2 to 62.7)
Georgia	Other exposure to mechanical forces	9.81 (7.63 to 12.8)	8.1 (6.46 to 10.2)	8.73 (6.92 to 11)	169.1 (132.5 to 219.3)	173.3 (139.8 to 216.2)	201.6 (162.4 to 252.9)
Georgia	Adverse effects of medical treatment	2.08 (1.72 to 3.5)	2.62 (2.16 to 3.07)	2.99 (2.24 to 3.28)	38.4 (31.6 to 64.6)	60.2 (51.7 to 74.6)	71.9 (57.6 to 81.9)
Georgia	Animal contact	1.03 (0.813 to 1.27)	0.746 (0.577 to 0.939)	0.708 (0.574 to 0.861)	18.9 (15 to 23.2)	17.2 (13.5 to 21.6)	18 (14.6 to 21.8)
Georgia	Venomous animal contact	0.561 (0.406 to 0.743)	0.388 (0.271 to 0.524)	0.326 (0.234 to 0.437)	10.2 (7.5 to 13.5)	9 (6.3 to 12.3)	8.4 (5.9 to 11.3)

SR Appendix Table 2. All-age DALYs counts and age standardized DALYs rates per 100,000 individuals for all causes and level 1 cause groups by location for 1990, 2007, and 2017, both sexes combined

		All-age DALYs			Age-standardized rate of DALYs per 100,000		
Georgia	Non-venomous animal contact	0.473 (0.367 to 0.591)	0.358 (0.29 to 0.449)	0.382 (0.323 to 0.452)	8.7 (6.7 to 10.7)	8.2 (6.8 to 10.2)	9.6 (8.2 to 11.3)
Georgia	Foreign body	5.45 (4.91 to 6.06)	5.12 (4.69 to 5.54)	4.23 (3.85 to 4.62)	105.1 (94 to 117.7)	132.4 (119.9 to 144.3)	118.3 (107.8 to 129.7)
Georgia	Pulmonary aspiration and foreign body in airway	2.55 (2.19 to 2.97)	2.86 (2.59 to 3.11)	2.46 (2.25 to 2.67)	51.4 (43.5 to 60.5)	76.2 (67.7 to 84.5)	70.9 (63.8 to 78.7)
Georgia	Foreign body in eyes	0.112 (0.0534 to 0.194)	0.1 (0.0492 to 0.172)	0.0921 (0.0459 to 0.156)	2 (0.9 to 3.4)	2.3 (1.1 to 4)	2.3 (1.1 to 4)
Georgia	Foreign body in other body part	2.79 (2.38 to 3.21)	2.16 (1.89 to 2.44)	1.67 (1.45 to 1.96)	51.8 (43.4 to 60.2)	53.9 (46.8 to 60.5)	45.1 (39.6 to 52.2)
Georgia	Environmental heat and cold exposure	3.77 (3.12 to 4.52)	3.56 (3.08 to 4.11)	3.31 (2.82 to 3.87)	66 (54.8 to 78.8)	78 (67.7 to 89.7)	78.3 (67.3 to 90.4)
Georgia	Exposure to forces of nature	0.00406 (0.0031 to 0.00532)	0.0928 (0.0817 to 0.109)	0.0323 (0.024 to 0.0439)	0.1 (0.1 to 0.1)	2.1 (1.9 to 2.5)	0.7 (0.5 to 0.9)
Georgia	Other unintentional injuries	6.79 (5.64 to 8.08)	3.71 (3.04 to 4.54)	4.08 (3.27 to 5.16)	119.2 (99.6 to 141.3)	80.9 (66.8 to 97.9)	97.7 (79.5 to 121.6)
Georgia	Self-harm and interpersonal violence	28 (26.4 to 29.8)	21.3 (20.2 to 22.5)	22.4 (20.9 to 24)	492.1 (463 to 522.8)	483.7 (457.7 to 511)	562.6 (525.4 to 603.2)
Georgia	Self-harm	11.8 (11 to 12.6)	9.42 (8.88 to 9.97)	12.8 (11.8 to 13.9)	203.9 (189.8 to 217.4)	206.7 (193.9 to 219.3)	315.3 (289.9 to 342)
Georgia	Self-harm by firearm	0.951 (0.68 to 1.4)	0.597 (0.361 to 0.775)	0.936 (0.652 to 1.36)	16.5 (11.8 to 24.3)	13.6 (8.2 to 17.9)	24 (16.4 to 34.8)
Georgia	Self-harm by other specified means	10.9 (10 to 11.6)	8.83 (8.26 to 9.39)	11.9 (11 to 12.9)	187.3 (172.6 to 200.6)	193.1 (180.3 to 206.8)	291.3 (267 to 317.2)
Georgia	Interpersonal violence	16.2 (15 to 17.4)	10.1 (9.47 to 10.8)	8.33 (7.5 to 9.27)	287.3 (267.1 to 309.9)	234.8 (219.9 to 250.2)	217 (195.6 to 240.5)
Georgia	Assault by firearm	9.12 (7.71 to 10.1)	5.46 (5.02 to 5.92)	2.64 (2.25 to 3.7)	162.1 (136.6 to 179.2)	129.1 (118.4 to 140.3)	72.3 (61.5 to 101.7)
Georgia	Assault by sharp object	2.82 (2.36 to 3.66)	1.29 (1.07 to 1.48)	2.13 (1.7 to 2.45)	50.1 (41.8 to 65)	29.5 (24.4 to 34.1)	55.5 (43 to 64.6)
Georgia	Sexual violence	0.329 (0.219 to 0.477)	0.231 (0.155 to 0.334)	0.223 (0.149 to 0.321)	5.9 (3.9 to 8.6)	5.4 (3.6 to 7.9)	6.1 (4.1 to 8.9)
Georgia	Assault by other means	3.88 (3.32 to 4.62)	3.13 (2.7 to 3.68)	3.34 (2.69 to 3.96)	69.3 (59.3 to 81.8)	70.8 (61 to 82.5)	83.1 (66.3 to 97.9)
Georgia	Conflict and terrorism	0.0125 (0.00827 to 0.0203)	1.75 (1.32 to 2.53)	1.22 (0.832 to 1.95)	0.2 (0.2 to 0.4)	41.5 (31.4 to 60)	29.5 (19.8 to 48.1)
Georgia	Executions and police conflict	0.0335 (0.0314 to 0.0358)	0.0273 (0.0246 to 0.0301)	0.0255 (0.0231 to 0.0282)	0.6 (0.6 to 0.7)	0.7 (0.6 to 0.8)	0.8 (0.7 to 0.9)
Kazakhstan	All causes	6170 (5710 to 6690)	6620 (6180 to 7120)	5610 (5120 to 6190)	40093 (37233.6 to 43401.1)	43778 (41012.3 to 47048.9)	31859.5 (29106.3 to 35108)
Kazakhstan	Communicable, maternal, neonatal, and nutritional disorders	1420 (1300 to 1550)	944 (861 to 1040)	635 (545 to 735)	8036.2 (7378.8 to 8797.3)	6219.3 (5656.3 to 6872.8)	3594 (3070.5 to 4175)
Kazakhstan	HIV/AIDS and sexually transmitted infections	9.03 (7.07 to 12.3)	18.9 (16.9 to 22.3)	18.8 (16.5 to 22.5)	53.1 (41.7 to 72)	113.5 (101.7 to 132.8)	97.4 (85.3 to 117.6)
Kazakhstan	HIV/AIDS	4.05 (3.86 to 4.27)	15 (14.3 to 15.8)	14.6 (13.8 to 15.5)	23.7 (22.6 to 25)	90.6 (86.3 to 95.6)	75.2 (71 to 79.8)
Kazakhstan	HIV/AIDS - Drug-susceptible Tuberculosis	1.27 (0.791 to 1.75)	1.14 (0.586 to 1.96)	0.491 (0.291 to 0.8)	7.5 (4.6 to 10.2)	6.9 (3.5 to 11.8)	2.5 (1.5 to 4.1)
Kazakhstan	HIV/AIDS - Multidrug-resistant Tuberculosis without extensive drug resistance	0.0116 (0.00198 to 0.0367)	1.95 (1.17 to 2.98)	0.367 (0.218 to 0.564)	0.1 (0 to 0.2)	11.8 (7.1 to 18)	1.9 (1.1 to 2.9)
Kazakhstan	HIV/AIDS - Extensively drug-resistant Tuberculosis	0 (0 to 0)	0.618 (0.371 to 0.948)	0.209 (0.126 to 0.322)	0 (0 to 0)	3.7 (2.2 to 5.7)	1.1 (0.6 to 1.7)
Kazakhstan	HIV/AIDS resulting in other diseases	2.76 (2.24 to 3.3)	11.3 (9.24 to 12.9)	13.6 (12.6 to 14.6)	16.2 (13.1 to 19.3)	68.2 (55.7 to 77.7)	69.7 (64.9 to 74.9)
Kazakhstan	Sexually transmitted infections excluding HIV	4.98 (3.07 to 8.23)	3.92 (2.11 to 7.16)	4.14 (2.19 to 7.53)	29.4 (18.2 to 48.3)	22.9 (12.4 to 41.5)	22.2 (11.8 to 40.4)
Kazakhstan	Syphilis	1.01 (0.549 to 1.72)	0.208 (0.0968 to 0.412)	0.119 (0.0821 to 0.188)	5.9 (3.3 to 10)	1.4 (0.6 to 2.8)	0.7 (0.5 to 1.1)
Kazakhstan	Chlamydial infection	0.938 (0.532 to 1.67)	0.896 (0.501 to 1.69)	0.981 (0.526 to 1.8)	5.4 (3.1 to 9.6)	5.2 (2.9 to 9.7)	5.2 (2.8 to 9.5)
Kazakhstan	Gonococcal infection	0.856 (0.526 to 1.5)	0.679 (0.364 to 1.3)	0.691 (0.375 to 1.29)	5 (3.1 to 8.5)	3.8 (2.1 to 7.3)	3.9 (2.1 to 7.2)
Kazakhstan	Trichomoniasis	0.677 (0.273 to 1.47)	0.701 (0.282 to 1.48)	0.771 (0.308 to 1.65)	4.1 (1.6 to 8.8)	4.1 (1.7 to 8.7)	4.1 (1.6 to 8.7)
Kazakhstan	Genital herpes	0.412 (0.135 to 0.973)	0.427 (0.14 to 1.04)	0.494 (0.161 to 1.17)	2.6 (0.9 to 6.2)	2.6 (0.9 to 6.4)	2.6 (0.9 to 6.2)
Kazakhstan	Other sexually transmitted infections	1.09 (0.679 to 1.83)	1.01 (0.587 to 1.76)	1.08 (0.636 to 1.85)	6.4 (4 to 10.7)	5.8 (3.4 to 10.1)	5.8 (3.4 to 10)
Kazakhstan	Respiratory infections and tuberculosis	558 (511 to 606)	367 (344 to 392)	203 (182 to 228)	3165.6 (2915 to 3431.3)	2365.2 (2209.3 to 2543.6)	1122.2 (1006.2 to 1265.1)
Kazakhstan	Tuberculosis	90.3 (85.1 to 95.7)	140 (135 to 147)	39.8 (36.1 to 43.7)	552.1 (521.7 to 582.3)	842.6 (810.1 to 877.9)	207 (187.8 to 227)
Kazakhstan	Latent tuberculosis infection	0 (0 to 0)	0 (0 to 0)	0 (0 to 0)	0 (0 to 0)	0 (0 to 0)	0 (0 to 0)
Kazakhstan	Drug-susceptible tuberculosis	89.7 (84.4 to 95.2)	53.6 (38 to 69.6)	21.1 (17.6 to 24.7)	548 (518.5 to 578.6)	321.5 (227.8 to 417.9)	109.9 (91.6 to 128.8)
Kazakhstan	Multidrug-resistant tuberculosis without extensive drug resistance	0.668 (0.121 to 2.16)	66 (53.4 to 78.4)	11.9 (9.71 to 14.2)	4.1 (0.7 to 13.3)	395.9 (320.8 to 470.4)	62 (50.4 to 73.8)
Kazakhstan	Extensively drug-resistant tuberculosis	0 (0 to 0)	20.9 (16.7 to 25)	6.77 (5.46 to 8.15)	0 (0 to 0)	125.2 (100.4 to 149.8)	35.1 (28.3 to 42.3)

SR Appendix Table 2. All-age DALYs counts and age standardized DALYs rates per 100,000 individuals for all causes and level 1 cause groups by location for 1990, 2007, and 2017, both sexes combined

		All-age DALYs			Age-standardized rate of DALYs per 100,000		
Kazakhstan	Lower respiratory infections	450 (407 to 495)	212 (192 to 235)	147 (130 to 168)	2512.7 (2280.6 to 2759.8)	1429.4 (1292 to 1591.3)	825 (728 to 944)
Kazakhstan	Upper respiratory infections	13.3 (8.92 to 19.3)	10.9 (6.86 to 16.5)	12.3 (7.75 to 18.8)	77 (51.1 to 111.4)	69.7 (44.2 to 105.6)	68.9 (43.4 to 104.3)
Kazakhstan	Otitis media	4.15 (2.48 to 6.53)	3.55 (2.14 to 5.59)	3.76 (2.26 to 5.96)	23.7 (14.2 to 37.5)	23.5 (14.1 to 37.1)	21.4 (12.8 to 33.8)
Kazakhstan	Enteric infections	136 (117 to 156)	22.8 (19 to 27.3)	19.9 (15.1 to 25)	748.8 (647.4 to 859.4)	156.1 (130.5 to 186.9)	112.6 (85.4 to 141.5)
Kazakhstan	Diarrhoeal diseases	134 (115 to 155)	21.8 (18.1 to 26.4)	18.9 (14.2 to 24)	739 (636.4 to 850)	149.4 (123.9 to 180)	107.2 (80.5 to 136.1)
Kazakhstan	Typhoid and paratyphoid	0.412 (0.118 to 0.974)	0.163 (0.0381 to 0.312)	0.0493 (0.0113 to 0.0939)	2.7 (0.7 to 6.6)	1.2 (0.2 to 2.3)	0.3 (0.1 to 0.5)
Kazakhstan	Typhoid fever	0.145 (0.0896 to 0.211)	0.0538 (0.0279 to 0.083)	0.0148 (0.00771 to 0.033)	0.9 (0.5 to 1.3)	0.3 (0.2 to 0.5)	0.1 (0 to 0.2)
Kazakhstan	Paratyphoid fever	0.268 (0.0131 to 0.827)	0.109 (0.00378 to 0.251)	0.0345 (0.0018 to 0.075)	1.9 (0.1 to 5.8)	0.8 (0 to 1.9)	0.2 (0 to 0.4)
Kazakhstan	Invasive non-typhoidal salmonella	1.1 (0.59 to 1.89)	0.698 (0.365 to 1.2)	0.837 (0.431 to 1.5)	6.3 (3.4 to 10.6)	4.9 (2.6 to 8.5)	4.8 (2.4 to 8.5)
Kazakhstan	Other intestinal infectious diseases	0.152 (0.0533 to 0.281)	0.0932 (0.0385 to 0.167)	0.056 (0.0223 to 0.103)	0.9 (0.3 to 1.5)	0.6 (0.2 to 1.1)	0.3 (0.1 to 0.6)
Kazakhstan	Neglected tropical diseases and malaria	15.1 (10.3 to 21.1)	10.5 (7.28 to 14.3)	10.7 (7.3 to 14.8)	90.5 (62.2 to 126.2)	65.5 (45.6 to 89.1)	58.6 (39.8 to 80.7)
Kazakhstan	Malaria	0 (0 to 0)	0 (0 to 0)	0 (0 to 0)	0 (0 to 0)	0 (0 to 0)	0 (0 to 0)
Kazakhstan	Chagas disease	0 (0 to 0)	0 (0 to 0)	0 (0 to 0)	0 (0 to 0)	0 (0 to 0)	0 (0 to 0)
Kazakhstan	Leishmaniasis	0.0918 (0.00277 to 0.746)	0.0141 (0.000547 to 0.111)	0.026 (0.000298 to 0.231)	0.5 (0 to 4.2)	0.1 (0 to 0.8)	0.1 (0 to 1.3)
Kazakhstan	Visceral leishmaniasis	0.0815 (0.000159 to 0.741)	0.0131 (0.000044 to 0.109)	0.0251 (0.0000468 to 0.23)	0.5 (0 to 4.1)	0.1 (0 to 0.7)	0.1 (0 to 1.3)
Kazakhstan	Cutaneous and mucocutaneous leishmaniasis	0.0104 (0.00131 to 0.0268)	0.00107 (0.000351 to 0.00228)	0.000869 (0.0000893 to 0.00263)	0.1 (0 to 0.2)	0 (0 to 0)	0 (0 to 0)
Kazakhstan	African trypanosomiasis	0 (0 to 0)	0 (0 to 0)	0 (0 to 0)	0 (0 to 0)	0 (0 to 0)	0 (0 to 0)
Kazakhstan	Schistosomiasis	0 (0 to 0)	0 (0 to 0)	0 (0 to 0)	0 (0 to 0)	0 (0 to 0)	0 (0 to 0)
Kazakhstan	Cysticercosis	0.00225 (0.00123 to 0.00355)	0.00138 (0.000639 to 0.00227)	0.000639 (0.000302 to 0.001)	0 (0 to 0)	0 (0 to 0)	0 (0 to 0)
Kazakhstan	Cystic echinococcosis	6.5 (4.11 to 9.74)	5.16 (3.28 to 7.56)	5.73 (3.64 to 8.47)	40.1 (25.7 to 59.6)	31.5 (20.2 to 45.8)	31 (19.6 to 45.8)
Kazakhstan	Lymphatic filariasis	0 (0 to 0)	0 (0 to 0)	0 (0 to 0)	0 (0 to 0)	0 (0 to 0)	0 (0 to 0)
Kazakhstan	Onchocerciasis	0 (0 to 0)	0 (0 to 0)	0 (0 to 0)	0 (0 to 0)	0 (0 to 0)	0 (0 to 0)
Kazakhstan	Trachoma	0 (0 to 0)	0 (0 to 0)	0 (0 to 0)	0 (0 to 0)	0 (0 to 0)	0 (0 to 0)
Kazakhstan	Dengue	0 (0 to 0)	0 (0 to 0)	0 (0 to 0)	0 (0 to 0)	0 (0 to 0)	0 (0 to 0)
Kazakhstan	Yellow fever	0 (0 to 0)	0 (0 to 0)	0 (0 to 0)	0 (0 to 0)	0 (0 to 0)	0 (0 to 0)
Kazakhstan	Rabies	0.296 (0.224 to 0.408)	0.296 (0.248 to 0.348)	0.27 (0.205 to 0.355)	1.7 (1.3 to 2.3)	2 (1.6 to 2.3)	1.5 (1.2 to 2)
Kazakhstan	Intestinal nematode infections	1.45 (0.839 to 2.42)	0.375 (0.201 to 0.663)	0.292 (0.152 to 0.525)	8.6 (5 to 14.2)	2.4 (1.3 to 4.3)	1.6 (0.9 to 3)
Kazakhstan	Ascariasis	0.165 (0.0763 to 0.306)	0.169 (0.0765 to 0.321)	0.128 (0.0573 to 0.243)	0.9 (0.4 to 1.7)	1.1 (0.5 to 2.2)	0.7 (0.3 to 1.4)
Kazakhstan	Trichuriasis	0.00000000208 (0.000000000921 to 0.00000000417)	0.00000000114 (0.000000000494 to 0.00000000232)	0.00000000116 (0.00000000049 to 0.00000000238)	0 (0 to 0)	0 (0 to 0)	0 (0 to 0)
Kazakhstan	Hookworm disease	1.29 (0.73 to 2.19)	0.206 (0.102 to 0.366)	0.164 (0.0771 to 0.3)	7.7 (4.4 to 13)	1.3 (0.6 to 2.3)	0.9 (0.4 to 1.7)
Kazakhstan	Food-borne trematodiases	0.75 (0.336 to 1.34)	0.754 (0.338 to 1.38)	0.886 (0.386 to 1.63)	4.9 (2.1 to 8.9)	4.7 (2.1 to 8.6)	4.8 (2.1 to 8.9)
Kazakhstan	Leprosy	0 (0 to 0)	0.00000124 (0.000000726 to 0.00000198)	0.000000912 (0.000000521 to 0.00000148)	0 (0 to 0)	0 (0 to 0)	0 (0 to 0)
Kazakhstan	Ebola virus disease	0 (0 to 0)	0 (0 to 0)	0 (0 to 0)	0 (0 to 0)	0 (0 to 0)	0 (0 to 0)
Kazakhstan	Zika virus disease	0 (0 to 0)	0 (0 to 0)	0 (0 to 0)	0 (0 to 0)	0 (0 to 0)	0 (0 to 0)
Kazakhstan	Guinea worm disease	0 (0 to 0)	0 (0 to 0)	0 (0 to 0)	0 (0 to 0)	0 (0 to 0)	0 (0 to 0)
Kazakhstan	Other neglected tropical diseases	5.98 (4.04 to 8.57)	3.87 (2.69 to 5.52)	3.5 (2.31 to 5.1)	34.6 (23.3 to 49.6)	24.7 (17.2 to 35.4)	19.5 (12.9 to 28.3)
Kazakhstan	Other infectious diseases	74.2 (66.7 to 82.3)	47.6 (42.6 to 53.3)	46.1 (38.3 to 54.6)	416.3 (375.7 to 460.8)	320.1 (285.4 to 360)	257.3 (213.4 to 305.5)
Kazakhstan	Meningitis	33 (29.2 to 37.1)	19.8 (17.2 to 23)	14.4 (11.9 to 17.5)	184.6 (164 to 206.8)	132.7 (115.1 to 155.2)	79.9 (66.1 to 97.3)
Kazakhstan	Pneumococcal meningitis	1.81 (1.44 to 2.26)	1.29 (1.02 to 1.6)	0.899 (0.702 to 1.13)	10.5 (8.3 to 13.1)	8.2 (6.5 to 10.1)	5 (3.9 to 6.2)
Kazakhstan	H influenzae type B meningitis	16.9 (14.8 to 19.2)	9.67 (8.29 to 11.4)	6.67 (5.37 to 8.38)	93.9 (82.3 to 106.3)	65.6 (55.8 to 77.4)	37.1 (29.8 to 46.8)

SR Appendix Table 2. All-age DALYs counts and age standardized DALYs rates per 100,000 individuals for all causes and level 1 cause groups by location for 1990, 2007, and 2017, both sexes combined

		All-age DALYs			Age-standardized rate of DALYs per 100,000		
Kazakhstan	Meningococcal infection	3.74 (3.04 to 4.7)	2.2 (1.79 to 2.72)	1.69 (1.34 to 2.19)	21 (17.1 to 26.1)	14.8 (11.9 to 18.5)	9.4 (7.4 to 12.1)
Kazakhstan	Other meningitis	10.6 (9.28 to 11.9)	6.6 (5.74 to 7.71)	5.14 (4.28 to 6.14)	59.3 (52.5 to 66.6)	44 (38.1 to 51.7)	28.4 (23.7 to 34.1)
Kazakhstan	Encephalitis	18.9 (16 to 21.9)	13.4 (11.6 to 16.1)	16.8 (13.3 to 21.6)	104.8 (89.2 to 121)	93 (79.5 to 111.8)	94 (74.3 to 120.1)
Kazakhstan	Diphtheria	0.401 (0.296 to 0.544)	0.0609 (0.0437 to 0.0863)	0.0251 (0.016 to 0.0391)	2.3 (1.7 to 3.1)	0.4 (0.3 to 0.5)	0.1 (0.1 to 0.2)
Kazakhstan	Whooping cough	0.571 (0.318 to 0.985)	0.168 (0.101 to 0.257)	0.467 (0.288 to 0.721)	3.1 (1.7 to 5.3)	1.2 (0.7 to 1.9)	2.6 (1.6 to 4)
Kazakhstan	Tetanus	0.199 (0.132 to 0.233)	0.0599 (0.0374 to 0.0837)	0.0381 (0.0146 to 0.0983)	1.3 (0.9 to 1.5)	0.4 (0.2 to 0.5)	0.2 (0.1 to 0.5)
Kazakhstan	Measles	0.124 (0.0852 to 0.171)	0.0442 (0.0313 to 0.059)	0.0176 (0.00904 to 0.0285)	0.7 (0.5 to 0.9)	0.3 (0.2 to 0.4)	0.1 (0.1 to 0.2)
Kazakhstan	Varicella and herpes zoster	0.533 (0.342 to 0.778)	0.484 (0.307 to 0.723)	0.558 (0.354 to 0.825)	3.4 (2.2 to 4.9)	3.3 (2.1 to 4.8)	3.1 (2 to 4.7)
Kazakhstan	Acute hepatitis	10.8 (9.29 to 12.3)	5.26 (4.65 to 6.1)	3.55 (2.93 to 4.43)	60.5 (52.4 to 68.4)	33.8 (29.9 to 39.1)	19.6 (16.2 to 24.6)
Kazakhstan	Acute hepatitis A	3.73 (2.89 to 4.58)	1.74 (1.45 to 2.06)	1.24 (0.977 to 1.59)	20.2 (15.9 to 24.7)	11.3 (9.4 to 13.6)	7.1 (5.6 to 9.1)
Kazakhstan	Acute hepatitis B	4.47 (3.69 to 5.39)	2.6 (2.24 to 3.09)	1.74 (1.42 to 2.21)	26 (21.8 to 31.1)	16.2 (13.9 to 19.3)	9.3 (7.6 to 11.7)
Kazakhstan	Acute hepatitis C	2.07 (1.2 to 3.02)	0.575 (0.369 to 0.856)	0.369 (0.206 to 0.578)	11.2 (6.5 to 16.4)	4.1 (2.6 to 6.2)	2.1 (1.2 to 3.3)
Kazakhstan	Acute hepatitis E	0.545 (0.438 to 0.668)	0.353 (0.278 to 0.454)	0.198 (0.154 to 0.263)	3.1 (2.5 to 3.8)	2.2 (1.7 to 2.8)	1.1 (0.9 to 1.5)
Kazakhstan	Other unspecified infectious diseases	9.71 (7.66 to 12.5)	8.34 (6.41 to 10.3)	10.2 (6.33 to 13.3)	55.7 (43.9 to 71.7)	55 (42.7 to 67.6)	57.6 (35.9 to 75.5)
Kazakhstan	Maternal and neonatal disorders	460 (412 to 505)	381 (335 to 432)	244 (197 to 302)	2596.9 (2325.7 to 2849.3)	2565.4 (2252.9 to 2908)	1428.3 (1150 to 1778.1)
Kazakhstan	Maternal disorders	13.7 (13 to 14.5)	8.39 (7.88 to 8.95)	3.61 (3.18 to 4.04)	77.3 (73.1 to 81.7)	48.8 (45.9 to 52.2)	19 (16.7 to 21.3)
Kazakhstan	Maternal haemorrhage	3.01 (1.76 to 4.54)	1.58 (0.939 to 2.35)	0.585 (0.357 to 0.891)	16.8 (10 to 25.3)	9.3 (5.5 to 14)	3 (1.9 to 4.7)
Kazakhstan	Maternal sepsis and other pregnancy related infections	1.68 (1.25 to 2.23)	0.652 (0.488 to 0.867)	0.366 (0.256 to 0.527)	9.5 (7.1 to 12.5)	3.7 (2.8 to 5)	2 (1.4 to 2.8)
Kazakhstan	Maternal hypertensive disorders	1.84 (1.15 to 2.8)	0.928 (0.593 to 1.39)	0.328 (0.22 to 0.473)	10.2 (6.4 to 15.5)	5.4 (3.5 to 8.2)	1.7 (1.2 to 2.5)
Kazakhstan	Maternal obstructed labour and uterine rupture	0.442 (0.309 to 0.606)	0.245 (0.162 to 0.361)	0.194 (0.107 to 0.332)	2.5 (1.8 to 3.4)	1.4 (0.9 to 2.1)	1 (0.6 to 1.7)
Kazakhstan	Maternal abortive outcome	4.36 (2.92 to 6.02)	2.4 (1.66 to 3.31)	0.702 (0.475 to 0.983)	24.8 (16.9 to 33.6)	13.9 (9.5 to 19.1)	3.7 (2.5 to 5.1)
Kazakhstan	Ectopic pregnancy	0.25 (0.0732 to 0.726)	0.184 (0.0544 to 0.548)	0.0911 (0.0307 to 0.246)	1.4 (0.4 to 3.9)	1.1 (0.3 to 3)	0.5 (0.2 to 1.2)
Kazakhstan	Indirect maternal deaths	0.374 (0.165 to 0.761)	0.516 (0.229 to 0.969)	0.196 (0.0948 to 0.353)	2.1 (0.9 to 4.2)	3 (1.3 to 5.8)	1 (0.5 to 1.8)
Kazakhstan	Late maternal deaths	0.497 (0.36 to 0.656)	0.346 (0.26 to 0.449)	0.23 (0.163 to 0.318)	2.8 (2 to 3.6)	2 (1.5 to 2.7)	1.2 (0.9 to 1.6)
Kazakhstan	Maternal deaths aggravated by HIV/AIDS	0.000691 (0.00034 to 0.0012)	0.00146 (0.000723 to 0.00257)	0.00165 (0.000841 to 0.00262)	0 (0 to 0)	0 (0 to 0)	0 (0 to 0)
Kazakhstan	Other maternal disorders	1.29 (0.818 to 1.94)	1.53 (1.01 to 2.15)	0.913 (0.661 to 1.21)	7.2 (4.7 to 10.7)	8.9 (5.9 to 12.5)	4.8 (3.5 to 6.3)
Kazakhstan	Neonatal disorders	447 (398 to 491)	373 (326 to 424)	240 (193 to 298)	2519.5 (2246.9 to 2768.8)	2516.5 (2204.2 to 2861)	1409.4 (1130 to 1757.9)
Kazakhstan	Neonatal preterm birth	246 (207 to 286)	185 (155 to 217)	107 (82.8 to 137)	1387.6 (1168.3 to 1615.4)	1246.8 (1046.1 to 1466.9)	633 (488.8 to 813.6)
Kazakhstan	Neonatal encephalopathy due to birth asphyxia and trauma	97.8 (72.5 to 131)	86.2 (68.1 to 107)	59.9 (44.3 to 78.9)	552.5 (409.5 to 739.2)	576.4 (455.4 to 716.9)	346.5 (257.5 to 455.8)
Kazakhstan	Neonatal sepsis and other neonatal infections	30.7 (21.1 to 41.7)	30.6 (20.6 to 40)	26.4 (18.8 to 35.7)	173.4 (118.8 to 235.9)	206.3 (138.4 to 268.3)	154.4 (110 to 208.5)
Kazakhstan	Hemolytic disease and other neonatal jaundice	8.19 (6.34 to 10.4)	7.45 (5.57 to 9.56)	4.66 (3.62 to 5.78)	46.5 (36 to 58.8)	49.3 (36.8 to 63.5)	26.5 (20.6 to 32.8)
Kazakhstan	Other neonatal disorders	64 (48.5 to 82.6)	64.1 (50.8 to 78.9)	42.1 (31.7 to 54.9)	359.5 (272.3 to 464.4)	437.9 (346.9 to 538.3)	249 (187.1 to 326.4)
Kazakhstan	Nutritional deficiencies	166 (111 to 238)	96.7 (65.3 to 142)	92.5 (59.7 to 139)	965 (646.7 to 1371.2)	633.6 (422.5 to 923.8)	517.6 (334.8 to 771.1)
Kazakhstan	Protein-energy malnutrition	5.18 (4.26 to 6.14)	3.36 (2.66 to 3.97)	2.59 (2.06 to 3.15)	29.5 (24.3 to 34.6)	22.1 (17.8 to 26.2)	14.2 (11.3 to 17.3)
Kazakhstan	Iodine deficiency	3.35 (1.75 to 5.74)	2.96 (1.43 to 5.5)	3.22 (1.56 to 6.08)	20.2 (10.6 to 34.8)	18.5 (8.9 to 34.2)	17.9 (8.7 to 33.8)
Kazakhstan	Dietary iron deficiency	111 (73.3 to 161)	71.7 (46.7 to 106)	66.3 (42.5 to 99.8)	660.3 (440.3 to 964)	447 (295.3 to 664.3)	367.3 (234.7 to 550.3)
Kazakhstan	Other nutritional deficiencies	1.04 (0.632 to 1.68)	1.16 (0.692 to 1.88)	0.77 (0.41 to 1.26)	6.8 (4.1 to 10.8)	7.5 (4.5 to 11.9)	4.4 (2.3 to 7)
Kazakhstan	Non-communicable diseases	3680 (3350 to 4060)	4500 (4170 to 4880)	4130 (3760 to 4580)	25711.6 (23572.3 to 28162.5)	30396.4 (28302.6 to 32853.9)	23691.3 (21633.8 to 26243.1)
Kazakhstan	Neoplasms	646 (618 to 668)	559 (550 to 571)	508 (482 to 535)	4482.3 (4289.9 to 4630.9)	3738.4 (3679.8 to 3818.3)	2806.7 (2671.4 to 2954)
Kazakhstan	Lip and oral cavity cancer	9.95 (9.11 to 11.9)	9.76 (9.2 to 10.2)	9.92 (9.13 to 10.9)	69.5 (63.8 to 83.4)	64.6 (61.1 to 67.6)	53.8 (49.6 to 58.6)

SR Appendix Table 2. All-age DALYs counts and age standardized DALYs rates per 100,000 individuals for all causes and level 1 cause groups by location for 1990, 2007, and 2017, both sexes combined

		All-age DALYs			Age-standardized rate of DALYs per 100,000		
Kazakhstan	Nasopharynx cancer	2.02 (1.8 to 2.29)	1.84 (1.7 to 1.98)	2.16 (1.92 to 2.44)	13.3 (11.9 to 15)	11.7 (10.8 to 12.6)	11.7 (10.5 to 13.2)
Kazakhstan	Other pharynx cancer	5.57 (4.87 to 6.66)	5.16 (4.81 to 5.5)	4.32 (3.84 to 4.92)	38.4 (33.6 to 46)	34 (31.7 to 36.1)	23 (20.6 to 26.1)
Kazakhstan	Oesophageal cancer	57.6 (55.5 to 59.6)	31.3 (30.2 to 32.5)	25.8 (23.7 to 28)	424.6 (410.5 to 439.2)	220.5 (212.7 to 228.3)	147.2 (136.3 to 159.5)
Kazakhstan	Stomach cancer	104 (101 to 108)	71.1 (69 to 73.2)	55.6 (51.6 to 59.9)	743.1 (718.8 to 767.4)	478.5 (464.4 to 492.6)	308.7 (287.4 to 331.7)
Kazakhstan	Colon and rectum cancer	39.9 (38.5 to 41.3)	43.8 (42.1 to 45.2)	42.8 (39.7 to 46.7)	287.6 (277.6 to 297.9)	302.2 (291.1 to 312.1)	244.8 (227.7 to 265.8)
Kazakhstan	Liver cancer	36.4 (32.4 to 41.1)	27.9 (26.8 to 29)	25 (22.4 to 28)	255.3 (228 to 287.2)	189 (181.3 to 196.1)	138.7 (124.7 to 154.9)
Kazakhstan	Liver cancer due to hepatitis B	9.73 (7.5 to 12.2)	7 (5.73 to 8.29)	5.77 (4.5 to 7.27)	65.4 (50.2 to 82)	45.5 (37.2 to 54.5)	30.9 (24.2 to 38.8)
Kazakhstan	Liver cancer due to hepatitis C	12.6 (10.4 to 15.3)	10.1 (8.74 to 11.3)	9.25 (7.66 to 10.9)	90.8 (74.9 to 109.1)	70.3 (60.9 to 78.8)	52.3 (43.8 to 61.2)
Kazakhstan	Liver cancer due to alcohol use	9.98 (8.32 to 12)	7.61 (6.78 to 8.47)	6.96 (5.87 to 8.18)	70.8 (59.3 to 85.2)	51.3 (45.7 to 57.1)	38.8 (32.9 to 45.3)
Kazakhstan	Liver cancer due to NASH	2.01 (1.66 to 2.47)	1.65 (1.45 to 1.88)	1.59 (1.32 to 1.9)	14.4 (12 to 17.6)	11.4 (10 to 13)	9 (7.6 to 10.7)
Kazakhstan	Liver cancer due to other causes	2.05 (1.69 to 2.49)	1.59 (1.38 to 1.82)	1.41 (1.18 to 1.68)	13.9 (11.5 to 16.9)	10.6 (9.2 to 12.1)	7.7 (6.5 to 9.1)
Kazakhstan	Gallbladder and biliary tract cancer	4.45 (3.52 to 6.65)	5.25 (4.5 to 5.57)	4.33 (3.83 to 4.8)	33 (26.2 to 49.4)	36.3 (31.3 to 38.6)	24.6 (21.7 to 27.2)
Kazakhstan	Pancreatic cancer	3.98 (3.46 to 4.46)	22.4 (21.6 to 23.2)	23.6 (21.7 to 25.9)	29.7 (25.8 to 33.1)	151.6 (146.4 to 157.4)	131.3 (121.2 to 143.4)
Kazakhstan	Larynx cancer	12.7 (12 to 13.4)	8.11 (7.7 to 8.56)	6.73 (6.12 to 7.47)	87.6 (83 to 92.4)	54.8 (51.9 to 57.6)	36.3 (33.2 to 40.1)
Kazakhstan	Tracheal, bronchus, and lung cancer	140 (135 to 145)	95.3 (91.9 to 98.8)	83.7 (77.6 to 90.6)	973.5 (938.7 to 1008.2)	646 (624.4 to 669.3)	457.7 (425.3 to 493.3)
Kazakhstan	Malignant skin melanoma	4.26 (2.95 to 5.43)	4.83 (4.07 to 7.54)	4.44 (3.52 to 7.84)	29.8 (20.2 to 37.6)	31.6 (26.7 to 48.5)	24.5 (19.5 to 42.7)
Kazakhstan	Non-melanoma skin cancer	1.09 (0.944 to 1.28)	2.1 (1.97 to 2.22)	2.29 (2.01 to 2.61)	7.8 (6.8 to 9.1)	14.4 (13.6 to 15.2)	13.2 (11.8 to 15.1)
Kazakhstan	Non-melanoma skin cancer (squamous-cell carcinoma)	1.09 (0.941 to 1.28)	2.09 (1.97 to 2.22)	2.28 (2.01 to 2.61)	7.8 (6.8 to 9.1)	14.4 (13.6 to 15.2)	13.2 (11.7 to 15)
Kazakhstan	Non-melanoma skin cancer (basal-cell carcinoma)	0.00411 (0.00136 to 0.00946)	0.00437 (0.00145 to 0.0103)	0.00524 (0.00167 to 0.0119)	0 (0 to 0.1)	0 (0 to 0.1)	0 (0 to 0.1)
Kazakhstan	Breast cancer	32.9 (31.4 to 34.5)	45.4 (43.5 to 47.3)	41.2 (37 to 45.5)	226.3 (216.1 to 236.8)	293.8 (281.4 to 305.5)	221.3 (198.9 to 243.7)
Kazakhstan	Cervical cancer	22.6 (21.3 to 24)	29.4 (27.9 to 30.9)	22.3 (20 to 24.7)	157.6 (149 to 167.4)	185.3 (176.5 to 194.7)	117.5 (105.5 to 130)
Kazakhstan	Uterine cancer	8.36 (7.82 to 8.96)	5.99 (5.44 to 6.5)	7.52 (6.7 to 8.39)	60.3 (56.4 to 64.5)	40.6 (37.1 to 43.9)	41.7 (37.1 to 46.3)
Kazakhstan	Ovarian cancer	10.5 (7.97 to 14.2)	15.1 (14.3 to 16)	15.8 (14 to 17.7)	73.3 (55.5 to 98.2)	97.1 (91.8 to 102.6)	84.4 (74.9 to 94.4)
Kazakhstan	Prostate cancer	6.65 (5.67 to 10.2)	9.3 (6.77 to 11)	9.94 (7.28 to 12)	52 (44.4 to 79.8)	67.7 (50.1 to 80.4)	61.3 (45.3 to 74.4)
Kazakhstan	Testicular cancer	2.63 (1.22 to 4.13)	2.63 (2.06 to 3.33)	1.27 (1.1 to 1.46)	16.4 (7.7 to 25.6)	16.3 (12.8 to 20.6)	6.9 (6 to 7.9)
Kazakhstan	Kidney cancer	11.9 (8.54 to 15.7)	14.2 (13.3 to 15.1)	14 (12.7 to 15.4)	81.7 (58.9 to 107.6)	95.8 (89.6 to 101.6)	76.3 (69.7 to 83.7)
Kazakhstan	Bladder cancer	8.23 (5.68 to 10.7)	8.92 (8.44 to 9.4)	7.56 (6.78 to 8.43)	60.9 (42.4 to 78.9)	62.2 (59 to 65.5)	43.7 (39.4 to 48.5)
Kazakhstan	Thyroid cancer	3.79 (3.43 to 4.54)	2.46 (2.27 to 2.67)	2.49 (2.22 to 2.85)	26 (23.6 to 31.1)	16.1 (14.9 to 17.4)	13.6 (12.2 to 15.5)
Kazakhstan	Mesothelioma	0.907 (0.802 to 1.08)	1.43 (1.11 to 1.61)	1.15 (1.06 to 1.25)	6.3 (5.6 to 7.5)	9.5 (7.5 to 10.8)	6.3 (5.8 to 6.8)
Kazakhstan	Hodgkin lymphoma	4.24 (3.27 to 5.63)	4.11 (3.23 to 5.5)	3.2 (2.68 to 3.91)	25.8 (19.9 to 34.3)	25 (19.8 to 33.4)	17.2 (14.4 to 21)
Kazakhstan	Non-Hodgkin's lymphoma	15.5 (13.7 to 17.2)	8.52 (8.02 to 9.07)	8.38 (7.58 to 9.29)	95.7 (84.2 to 105.5)	55.1 (51.7 to 58.6)	46.4 (41.9 to 51.4)
Kazakhstan	Multiple myeloma	3.4 (2.21 to 3.96)	2.62 (2.32 to 3.86)	3 (2.47 to 4.3)	23.8 (15.8 to 27.7)	17.4 (15.5 to 25.5)	16.4 (13.5 to 23.5)
Kazakhstan	Leukaemia	33 (30.6 to 35.7)	26.2 (24.5 to 28.1)	23.5 (20.8 to 26.6)	195.7 (182.4 to 209.8)	171.8 (160 to 185.3)	131.8 (116.6 to 149.2)
Kazakhstan	Acute lymphoid leukaemia	11.8 (9.84 to 13.7)	8.21 (7.06 to 9.88)	7.78 (6.5 to 9.37)	66 (55.5 to 76.7)	54.8 (47.1 to 65.8)	44.6 (37.4 to 53.6)
Kazakhstan	Chronic lymphoid leukaemia	1.72 (1.5 to 1.95)	1.91 (1.59 to 2.22)	1.77 (1.52 to 2.03)	12 (10.4 to 13.5)	12.5 (10.5 to 14.4)	9.9 (8.5 to 11.3)
Kazakhstan	Acute myeloid leukaemia	10.2 (8.38 to 12.3)	9.05 (7.1 to 11.2)	8.73 (7.44 to 9.89)	59.9 (50 to 72.2)	57.7 (45.5 to 71.4)	48.1 (40.9 to 54.6)
Kazakhstan	Chronic myeloid leukaemia	2.89 (2.32 to 3.79)	2.61 (2 to 3.4)	1.74 (1.33 to 2.33)	18.8 (15.1 to 24.6)	16.3 (12.6 to 21.1)	9.3 (7.1 to 12.3)
Kazakhstan	Other leukaemia	6.5 (5.35 to 8.07)	4.39 (3.76 to 5.18)	3.45 (2.88 to 4.12)	39.1 (32.6 to 47.8)	30.4 (26.2 to 35.9)	19.9 (16.7 to 23.6)
Kazakhstan	Other malignant cancers	49.6 (32.1 to 57.5)	30.8 (27.9 to 42.3)	31 (26.5 to 42.3)	325.3 (206.4 to 376.3)	201.2 (182.9 to 271.8)	171.4 (146.6 to 233)
Kazakhstan	Other neoplasms	3.46 (1.93 to 5.25)	4.67 (2.76 to 6.57)	5.61 (3.64 to 6.95)	22.5 (12.8 to 33.1)	30.8 (18.4 to 42.8)	30.9 (20.3 to 37.9)

SR Appendix Table 2. All-age DALYs counts and age standardized DALYs rates per 100,000 individuals for all causes and level 1 cause groups by location for 1990, 2007, and 2017, both sexes combined

		All-age DALYs			Age-standardized rate of DALYs per 100,000		
Kazakhstan	Myelodysplastic, myeloproliferative, and other hematopoietic neoplasms	2.93 (1.64 to 4.43)	3.99 (2.36 to 5.64)	4.75 (3.05 to 5.87)	19.5 (11.2 to 28.7)	26.4 (15.9 to 36.7)	26.1 (17 to 32.2)
Kazakhstan	Benign and in situ intestinal neoplasms	0 (0 to 0)	0 (0 to 0)	0 (0 to 0)	0 (0 to 0)	0 (0 to 0)	0 (0 to 0)
Kazakhstan	Benign and in situ cervical and uterine neoplasms	0 (0 to 0)	0 (0 to 0)	0 (0 to 0)	0 (0 to 0)	0 (0 to 0)	0 (0 to 0)
Kazakhstan	Other benign and in situ neoplasms	0.531 (0.277 to 1.07)	0.681 (0.35 to 1.1)	0.859 (0.51 to 1.14)	3 (1.6 to 6)	4.4 (2.3 to 7.1)	4.8 (2.9 to 6.2)
Kazakhstan	Cardiovascular diseases	1190 (1150 to 1220)	1820 (1790 to 1850)	1430 (1370 to 1500)	9073.4 (8830.1 to 9306)	12842 (12622.6 to 13070.9)	8627.8 (8257.9 to 9021.3)
Kazakhstan	Rheumatic heart disease	40.9 (38.8 to 43.3)	28.2 (26.5 to 30.6)	15 (13.1 to 17.2)	264.2 (250.9 to 279.5)	177.2 (167.5 to 192.6)	80.9 (71.2 to 92.3)
Kazakhstan	Ischaemic heart disease	640 (623 to 657)	945 (920 to 1000)	714 (673 to 775)	4951.6 (4826 to 5087.6)	6845.1 (6681.5 to 7179.7)	4418.4 (4184.1 to 4735)
Kazakhstan	Stroke	404 (384 to 424)	529 (507 to 558)	436 (404 to 471)	3123.2 (2976.5 to 3273.7)	3757.7 (3602.1 to 3933.8)	2642.1 (2464.5 to 2831.9)
Kazakhstan	Ischaemic stroke	196 (181 to 209)	278 (262 to 294)	236 (218 to 254)	1576.9 (1463.6 to 1684.1)	2054.4 (1938.8 to 2170.4)	1505.9 (1400 to 1617.4)
Kazakhstan	Intracerebral hemorrhage	170 (160 to 181)	205 (195 to 216)	165 (151 to 179)	1273.4 (1197.5 to 1358.5)	1402.6 (1336.2 to 1473.8)	943.3 (868.6 to 1019.3)
Kazakhstan	Subarachnoid hemorrhage	38.9 (35.2 to 47.1)	46.5 (41.6 to 64.5)	35.3 (30.6 to 56.4)	272.9 (246.9 to 332.8)	300.7 (268.3 to 418.8)	192.9 (168.1 to 307.6)
Kazakhstan	Hypertensive heart disease	32.7 (28.4 to 46.7)	56 (41.8 to 62.5)	38.2 (33 to 61.8)	245.1 (212.3 to 364.8)	387.6 (296.9 to 444.7)	223.9 (193.4 to 366.2)
Kazakhstan	Non-rheumatic valvular heart disease	1.77 (1.36 to 2.18)	2.68 (2.02 to 3.04)	2.26 (1.95 to 2.72)	13.2 (10.2 to 16.3)	18.3 (14.1 to 21)	13.6 (11.6 to 16.4)
Kazakhstan	Non-rheumatic calcific aortic valve disease	0.715 (0.558 to 0.886)	1.21 (0.943 to 1.46)	1.13 (0.957 to 1.42)	5.4 (4.2 to 6.8)	8.3 (6.6 to 10)	6.8 (5.8 to 8.5)
Kazakhstan	Non-rheumatic degenerative mitral valve disease	0.867 (0.648 to 1.08)	1.2 (0.84 to 1.49)	0.932 (0.742 to 1.13)	6.5 (4.8 to 8.1)	8.3 (5.9 to 10.3)	5.6 (4.5 to 6.9)
Kazakhstan	Other non-rheumatic valve diseases	0.193 (0.0956 to 0.268)	0.273 (0.122 to 0.385)	0.202 (0.115 to 0.271)	1.3 (0.6 to 1.7)	1.7 (0.8 to 2.3)	1.1 (0.7 to 1.5)
Kazakhstan	Cardiomyopathy and myocarditis	13.7 (10.2 to 30.8)	179 (89.1 to 197)	163 (88.1 to 195)	89.8 (67.5 to 201.9)	1119.1 (565.9 to 1226.7)	865.8 (475.1 to 1030.2)
Kazakhstan	Myocarditis	1.01 (0.714 to 2.07)	9.89 (7.91 to 14.6)	8.92 (7.05 to 12.7)	6.3 (4.5 to 12.8)	62.4 (49.6 to 89.2)	49 (39.2 to 67.1)
Kazakhstan	Alcoholic cardiomyopathy	5.73 (3.88 to 15.5)	74.2 (27.2 to 95.9)	68.8 (25.6 to 90.2)	38 (25.8 to 103)	451.2 (164.5 to 582)	351.9 (131 to 460.2)
Kazakhstan	Other cardiomyopathy	6.92 (5.18 to 13.6)	95.4 (50.7 to 109)	85.5 (52 to 104)	45.5 (34.3 to 87.6)	605.5 (330.7 to 686.9)	464.9 (289.1 to 561.4)
Kazakhstan	Atrial fibrillation and flutter	9.58 (7.94 to 11.9)	10.4 (8.45 to 12.9)	13 (10.7 to 16.1)	83.3 (69.5 to 101.9)	85.2 (70.9 to 105.5)	88.8 (74.4 to 108.8)
Kazakhstan	Aortic aneurysm	5.41 (4.73 to 6.45)	8.5 (6.78 to 10.7)	8.44 (7.65 to 9.56)	38.4 (33.4 to 45.9)	56 (45.2 to 69.6)	46.7 (42.5 to 52.3)
Kazakhstan	Peripheral vascular disease	0.85 (0.458 to 1.43)	0.978 (0.56 to 1.57)	1.17 (0.684 to 1.84)	7.1 (3.8 to 11.9)	7.7 (4.4 to 12.2)	7.6 (4.4 to 11.9)
Kazakhstan	Endocarditis	2.19 (1.49 to 2.67)	3.39 (2.63 to 4.72)	3.23 (2.8 to 4.06)	13.5 (9.1 to 16.5)	21.3 (16.7 to 29.4)	17.6 (15.2 to 22.2)
Kazakhstan	Other cardiovascular and circulatory diseases	35.5 (31.6 to 40.3)	56.6 (50.6 to 66.3)	40.1 (35.4 to 45.8)	243.8 (216.8 to 276.7)	366.9 (328 to 425.4)	222.5 (197.7 to 252.7)
Kazakhstan	Chronic respiratory diseases	225 (211 to 240)	193 (181 to 206)	190 (174 to 208)	1648.8 (1550.5 to 1747.3)	1341.6 (1256.3 to 1423.3)	1124.3 (1030.4 to 1227.3)
Kazakhstan	Chronic obstructive pulmonary disease	166 (156 to 175)	148 (139 to 155)	143 (132 to 156)	1240 (1169.2 to 1305.4)	1032.7 (973.3 to 1084.4)	856.4 (790.5 to 930.8)
Kazakhstan	Pneumoconiosis	1.6 (1.29 to 1.98)	1.33 (1.13 to 1.6)	1.14 (0.963 to 1.34)	11.5 (9.3 to 14)	9.2 (7.8 to 11.1)	6.6 (5.5 to 7.7)
Kazakhstan	Silicosis	0.0484 (0.0199 to 0.159)	0.0204 (0.0104 to 0.0559)	0.0125 (0.00685 to 0.0284)	0.3 (0.1 to 1)	0.2 (0.1 to 0.5)	0.1 (0 to 0.1)
Kazakhstan	Asbestosis	0.401 (0.171 to 0.596)	0.313 (0.148 to 0.433)	0.303 (0.163 to 0.402)	2.8 (1.2 to 4.1)	2.1 (1 to 2.9)	1.7 (0.9 to 2.2)
Kazakhstan	Coal workers pneumoconiosis	0.178 (0.0788 to 0.443)	0.178 (0.0937 to 0.423)	0.0967 (0.0538 to 0.241)	1.2 (0.5 to 2.8)	1.1 (0.6 to 2.6)	0.5 (0.3 to 1.2)
Kazakhstan	Other pneumoconiosis	0.973 (0.731 to 1.23)	0.816 (0.639 to 1.01)	0.728 (0.579 to 0.882)	7.2 (5.5 to 9)	5.9 (4.6 to 7.2)	4.3 (3.5 to 5.2)
Kazakhstan	Asthma	44.4 (36.3 to 53.8)	32.4 (26.2 to 40)	31.2 (24.2 to 39.9)	306.2 (251.1 to 369.7)	220.8 (179.3 to 271.1)	180.2 (140.2 to 229.9)
Kazakhstan	Interstitial lung disease and pulmonary sarcoidosis	5.63 (4.47 to 6.97)	5.16 (4.06 to 6.38)	6.58 (5.27 to 8.13)	39.6 (31.6 to 48.7)	34.1 (27.2 to 42.1)	36.8 (29.7 to 45.4)
Kazakhstan	Other chronic respiratory diseases	7.65 (6.65 to 8.82)	6.97 (5.89 to 8.59)	8 (6.96 to 9.14)	51.5 (45.1 to 58.7)	44.8 (38 to 55.1)	44.4 (38.9 to 50.5)
Kazakhstan	Digestive diseases	177 (164 to 195)	322 (305 to 341)	340 (277 to 385)	1180 (1096.8 to 1297)	2036.5 (1927.9 to 2151.5)	1820.6 (1491.4 to 2061.2)
Kazakhstan	Cirrhosis and other chronic liver diseases	66.7 (63.4 to 69.7)	202 (193 to 213)	225 (185 to 255)	459.6 (438.2 to 479.8)	1263.2 (1205.7 to 1325.6)	1196.7 (984.6 to 1351.3)
Kazakhstan	Cirrhosis and other chronic liver diseases due to hepatitis B	15.4 (13.5 to 17.8)	48.9 (42.3 to 55.5)	47.5 (36.9 to 57.3)	106 (92.7 to 121.7)	302.9 (264.3 to 345)	251.4 (196.6 to 300.3)
Kazakhstan	Cirrhosis and other chronic liver diseases due to hepatitis C	14.7 (12.8 to 17)	46.6 (39.7 to 54.1)	53.3 (40.5 to 64.7)	102.9 (89.5 to 118.7)	290 (248.5 to 334.1)	282 (216.9 to 342.3)
Kazakhstan	Cirrhosis and other chronic liver diseases due to alcohol use	23.8 (21.4 to 26.4)	75.8 (67.8 to 83.7)	87.8 (69.3 to 103)	166.9 (149.5 to 184.8)	473.1 (424.3 to 519.7)	464 (367.3 to 542)

SR Appendix Table 2. All-age DALYs counts and age standardized DALYs rates per 100,000 individuals for all causes and level 1 cause groups by location for 1990, 2007, and 2017, both sexes combined

		All-age DALYs			Age-standardized rate of DALYs per 100,000		
Kazakhstan	Cirrhosis due to NASH	4.53 (4.01 to 5.15)	13.8 (12.1 to 15.6)	17.4 (13.4 to 20.8)	31.8 (28.3 to 35.9)	86.9 (76.8 to 97.7)	92.4 (71.6 to 109.4)
Kazakhstan	Cirrhosis and other chronic liver diseases due to other causes	8.17 (7.22 to 9.28)	17.3 (14.9 to 20)	19.1 (14.6 to 23.4)	52 (45.9 to 59.3)	110.3 (95.6 to 127.3)	106.9 (82.4 to 129.6)
Kazakhstan	Upper digestive system diseases	33.5 (25.2 to 45.3)	38.7 (30 to 51)	39.7 (28.9 to 55.2)	218.4 (165.9 to 293.1)	244.3 (190.8 to 321.7)	214.6 (156.2 to 297.5)
Kazakhstan	Peptic ulcer disease	13.7 (12.4 to 14.9)	18.9 (17.5 to 20.5)	16.7 (13.4 to 19.1)	92.7 (83.5 to 100.2)	120.2 (111.7 to 130.1)	90.3 (73.5 to 103.4)
Kazakhstan	Gastritis and duodenitis	5.49 (3.68 to 7.74)	5 (3.4 to 7.11)	5.85 (4.01 to 8.32)	34 (22.9 to 48)	32.2 (21.9 to 45.4)	32.3 (22.1 to 46.1)
Kazakhstan	GERD	14.3 (7.66 to 24.3)	14.8 (7.96 to 25.2)	17.2 (9.17 to 29.2)	91.7 (48.8 to 155.9)	91.8 (49.5 to 156.1)	92 (49 to 156.8)
Kazakhstan	Appendicitis	5.47 (4.89 to 6.14)	2.51 (2.19 to 2.91)	2.05 (1.58 to 2.51)	32.3 (29 to 35.9)	16.1 (14 to 18.7)	11.7 (9 to 14.4)
Kazakhstan	Paralytic ileus and intestinal obstruction	13.8 (11.1 to 15.7)	10 (8.76 to 11.7)	10.6 (7.14 to 13.6)	82.9 (67.1 to 93.8)	68.2 (59.6 to 80.4)	59.7 (40.7 to 77.1)
Kazakhstan	Inguinal, femoral, and abdominal hernia	15.6 (11.6 to 20.4)	13.7 (10 to 17.9)	13.1 (9.1 to 17.7)	100.7 (75.2 to 131.1)	92 (67.7 to 121.2)	72.1 (50.4 to 97.1)
Kazakhstan	Inflammatory bowel disease	3.66 (3 to 4.43)	3.61 (2.91 to 4.57)	3.81 (2.74 to 4.6)	24 (19.8 to 28.7)	23.2 (18.8 to 29)	20.5 (14.7 to 24.7)
Kazakhstan	Vascular intestinal disorders	4.24 (3.59 to 4.67)	4.92 (3.96 to 5.97)	4.42 (2.84 to 5.14)	33.2 (27.2 to 36.1)	34.8 (27.7 to 42)	26.4 (17 to 30.6)
Kazakhstan	Gallbladder and biliary diseases	5.68 (4.86 to 6.11)	5.9 (5.37 to 6.49)	3.98 (3.27 to 4.6)	42 (35.3 to 45.2)	40 (36.3 to 44)	22.9 (18.8 to 26.2)
Kazakhstan	Pancreatitis	25.1 (22.3 to 28)	37 (30.7 to 41.5)	31.1 (23.8 to 36.7)	168.2 (149.5 to 188.4)	231.8 (194.3 to 259.5)	164 (125.7 to 193.5)
Kazakhstan	Other digestive diseases	3.09 (2.41 to 3.89)	3.42 (2.87 to 4.08)	5.86 (4.56 to 7.08)	18.8 (14.8 to 23.5)	22.9 (19.2 to 27.3)	32.1 (25.1 to 38.6)
Kazakhstan	Neurological disorders	224 (168 to 295)	237 (184 to 304)	255 (193 to 333)	1546.5 (1201.3 to 1990.9)	1634 (1300.4 to 2040.1)	1550.4 (1203.8 to 1987.5)
Kazakhstan	Alzheimer's disease and other dementias	44.9 (38.7 to 49.9)	46.2 (43.1 to 49.3)	55.4 (51.1 to 60.2)	447.6 (385.9 to 497.3)	454.5 (424.5 to 486.1)	442.8 (409.9 to 478.9)
Kazakhstan	Parkinson's disease	7.85 (6.59 to 11.6)	10.1 (8.54 to 16.3)	10.8 (9.07 to 16.7)	68.6 (57.6 to 101.4)	81.4 (68.2 to 131.4)	74.3 (62.6 to 114.5)
Kazakhstan	Epilepsy	41.3 (22.3 to 66.6)	47.3 (31.2 to 68.1)	41.7 (19.7 to 70.7)	242.2 (129.5 to 391.1)	293.6 (191.6 to 424.7)	232.9 (108.7 to 399.6)
Kazakhstan	Multiple sclerosis	3.72 (2.89 to 4.72)	3.86 (2.91 to 5.46)	4.14 (2.99 to 5.61)	24.8 (19.3 to 31.3)	24.1 (18.2 to 33.6)	21.9 (16 to 29.5)
Kazakhstan	Motor neuron disease	0.447 (0.326 to 0.57)	0.705 (0.344 to 0.9)	0.663 (0.415 to 0.82)	3 (2.2 to 3.8)	4.5 (2.2 to 5.7)	3.4 (2.2 to 4.2)
Kazakhstan	Headache disorders	118 (76.9 to 169)	120 (77.7 to 171)	133 (86.2 to 190)	715.4 (466.1 to 1026.4)	718.6 (466.6 to 1027.9)	718.7 (467.7 to 1029.3)
Kazakhstan	Migraine	103 (64.4 to 152)	104 (64.9 to 154)	115 (71.9 to 171)	620.8 (390.3 to 917.5)	623.7 (387.4 to 920.4)	623.7 (388.2 to 926.4)
Kazakhstan	Tension-type headache	15.5 (8.67 to 24.7)	15.5 (8.8 to 24.7)	17.3 (9.76 to 27.7)	94.6 (53.5 to 150.9)	94.9 (53.8 to 151.1)	94.9 (53.6 to 151.6)
Kazakhstan	Other neurological disorders	7.63 (4.37 to 11.9)	9.1 (5.55 to 13.2)	9.9 (5.57 to 15.5)	45 (26.3 to 69.5)	57.4 (35.2 to 83.1)	56.4 (31.8 to 89.1)
Kazakhstan	Mental disorders	228 (168 to 296)	233 (171 to 302)	257 (190 to 333)	1400.7 (1035.4 to 1810.3)	1426.4 (1047.7 to 1846)	1424.7 (1052.1 to 1852.6)
Kazakhstan	Schizophrenia	18.8 (14 to 23.7)	19.4 (14.4 to 24.2)	23.3 (17.2 to 29.4)	116.7 (87 to 146.5)	117.2 (87.2 to 146.5)	120.8 (89.4 to 152.2)
Kazakhstan	Depressive disorders	86.4 (61.7 to 118)	92.8 (65 to 126)	102 (71.2 to 138)	552.2 (394.5 to 747.5)	573.6 (404.4 to 776.3)	564.2 (394.8 to 761.5)
Kazakhstan	Major depressive disorder	69.5 (48.4 to 94.3)	75.4 (52.7 to 103)	82.1 (56.7 to 111)	444.3 (310.1 to 603.4)	465.6 (326.3 to 634.9)	456.2 (314 to 618.1)
Kazakhstan	Dysthymia	16.9 (11.2 to 24.4)	17.4 (11.5 to 25.2)	20 (13.2 to 29.2)	107.9 (71.6 to 156.1)	108.1 (71.6 to 155.7)	108 (71.7 to 157.6)
Kazakhstan	Bipolar disorder	21.2 (13.1 to 31.7)	21.8 (13.6 to 32.8)	23.7 (14.8 to 35.4)	128.5 (79.7 to 191.5)	129.1 (80.9 to 194.7)	129.4 (81.1 to 193.2)
Kazakhstan	Anxiety disorders	38.7 (27.3 to 51.5)	38.5 (27.1 to 51.6)	42.5 (30.1 to 56.8)	235.9 (167.4 to 313.3)	236 (166.9 to 316.4)	236 (167.4 to 315.5)
Kazakhstan	Eating disorders	6.81 (4.36 to 10)	6.83 (4.36 to 10.1)	8.49 (5.27 to 12.7)	38.6 (24.7 to 56.7)	39.4 (25.2 to 57.9)	46.2 (28.6 to 69)
Kazakhstan	Anorexia nervosa	1.36 (0.811 to 2.09)	1.38 (0.812 to 2.14)	1.62 (0.972 to 2.45)	7.7 (4.6 to 11.7)	7.9 (4.7 to 12.2)	9.3 (5.5 to 14.1)
Kazakhstan	Bulimia nervosa	5.45 (3.37 to 8.17)	5.45 (3.38 to 8.34)	6.87 (4.24 to 10.3)	30.9 (19.2 to 46.2)	31.4 (19.6 to 47.9)	36.9 (22.6 to 55.5)
Kazakhstan	Autism spectrum disorders	10.9 (7.41 to 15)	9.89 (6.79 to 13.6)	11.4 (7.72 to 15.6)	63.1 (43 to 86.9)	63.3 (43.4 to 87.7)	63.6 (43.3 to 87.6)
Kazakhstan	Attention-deficit/hyperactivity disorder	2.07 (1.23 to 3.28)	1.87 (1.12 to 3.05)	2 (1.22 to 3.18)	11.7 (6.9 to 18.5)	11.7 (7 to 19)	11.7 (7.1 to 18.6)
Kazakhstan	Conduct disorder	17.6 (10.4 to 27.9)	15.5 (9.24 to 24.3)	14.7 (8.85 to 23.2)	97.3 (57.4 to 154)	97.5 (58.5 to 153.5)	97.9 (58.9 to 154.4)
Kazakhstan	Idiopathic developmental intellectual disability	3.32 (0.85 to 6.49)	3.26 (1.07 to 6.15)	2.89 (0.807 to 5.49)	19 (4.8 to 37.3)	20.7 (6.8 to 39)	16.2 (4.6 to 30.8)
Kazakhstan	Other mental disorders	22.1 (14.7 to 30.8)	22.6 (14.9 to 31.3)	26.1 (17.4 to 36.1)	137.8 (92.2 to 191.4)	137.9 (90.7 to 190.4)	138.7 (92.2 to 191.3)
Kazakhstan	Substance use disorders	97.4 (78.8 to 118)	216 (191 to 246)	191 (163 to 224)	589.2 (478.8 to 712.6)	1288.6 (1135.2 to 1466.4)	988.6 (844.3 to 1159.1)

SR Appendix Table 2. All-age DALYs counts and age standardized DALYs rates per 100,000 individuals for all causes and level 1 cause groups by location for 1990, 2007, and 2017, both sexes combined

		All-age DALYs			Age-standardized rate of DALYs per 100,000		
Kazakhstan	Alcohol use disorders	69.5 (56.2 to 85.5)	139 (120 to 161)	126 (106 to 152)	424.5 (344.5 to 521.5)	833.2 (717.3 to 966)	652 (547.4 to 785)
Kazakhstan	Drug use disorders	27.9 (22 to 34.2)	77 (67.2 to 87.3)	64.3 (54.6 to 74.5)	164.7 (129.8 to 200)	455.4 (397.6 to 515.9)	336.6 (285.8 to 389.5)
Kazakhstan	Opioid use disorders	19.1 (14.5 to 24)	55.5 (46.1 to 64.5)	48.3 (40.6 to 56.3)	113.3 (87.1 to 141)	328.1 (272.3 to 381)	251.3 (211.9 to 292.5)
Kazakhstan	Cocaine use disorders	1.12 (0.806 to 1.57)	3.65 (2.75 to 5.77)	2.84 (2.28 to 3.51)	6.7 (4.9 to 9.3)	21.8 (16.3 to 34.4)	14.8 (11.9 to 18.3)
Kazakhstan	Amphetamine use disorders	2.56 (1.66 to 3.8)	4.45 (3.12 to 6.4)	3.25 (2.3 to 4.66)	14.6 (9.6 to 21.6)	26 (18.4 to 37)	17.4 (12.2 to 25.1)
Kazakhstan	Cannabis use disorders	0.805 (0.489 to 1.23)	0.817 (0.507 to 1.21)	0.81 (0.501 to 1.19)	4.6 (2.8 to 7)	4.6 (2.9 to 6.9)	4.6 (2.8 to 6.8)
Kazakhstan	Other drug use disorders	4.29 (3.37 to 5.38)	12.6 (9.83 to 16.6)	9.12 (7.58 to 10.9)	25.5 (20.3 to 31.9)	74.9 (58.6 to 99)	48.4 (40.2 to 57.5)
Kazakhstan	Diabetes and kidney diseases	145 (118 to 178)	175 (147 to 211)	182 (146 to 224)	1004.9 (815.5 to 1230.9)	1158.9 (969.9 to 1397)	1012.4 (818.5 to 1240.7)
Kazakhstan	Diabetes mellitus	98.8 (75.3 to 127)	114 (88.2 to 146)	126 (93.8 to 166)	693 (528.2 to 889.7)	757.1 (590.3 to 968.4)	699.3 (519.6 to 913.4)
Kazakhstan	Diabetes mellitus type 1	15.4 (13.1 to 17.5)	13.3 (10.9 to 15.4)	9.37 (7.48 to 11.6)	102.1 (85.9 to 116.3)	84.5 (69.3 to 98.5)	50.4 (40.3 to 61.9)
Kazakhstan	Diabetes mellitus type 2	83.3 (60.7 to 111)	101 (75.6 to 132)	117 (85.7 to 155)	590.9 (432 to 787.4)	672.6 (508.7 to 880)	648.9 (474.8 to 855.3)
Kazakhstan	Chronic kidney disease	46 (41.8 to 50.6)	61.1 (57.1 to 65.6)	55 (50.3 to 60.1)	308.9 (279.5 to 341.8)	398.7 (372.1 to 428.1)	311.7 (285.2 to 340.3)
Kazakhstan	Chronic kidney disease due to diabetes mellitus type 1	3.81 (3.05 to 4.67)	5.83 (4.66 to 7.16)	4.59 (3.67 to 5.66)	24.7 (19.7 to 30.3)	35.1 (28.2 to 42.7)	23.8 (19.1 to 29.2)
Kazakhstan	Chronic kidney disease due to diabetes mellitus type 2	6.46 (5.4 to 7.61)	9.49 (7.98 to 11)	10.2 (8.58 to 12)	47.6 (40 to 55.8)	66.2 (56.2 to 76.7)	59.2 (50.3 to 69.4)
Kazakhstan	Chronic kidney disease due to hypertension	4.4 (3.65 to 5.17)	6.01 (5.08 to 6.98)	6.17 (5.08 to 7.23)	32 (26.6 to 37.4)	41.8 (35.4 to 48.5)	37.1 (30.7 to 43.2)
Kazakhstan	Chronic kidney disease due to glomerulonephritis	12.6 (11.1 to 14.3)	17.3 (15.3 to 19.6)	13.9 (12 to 16.1)	79.4 (70 to 89.7)	107.9 (95.5 to 122)	75.8 (65.8 to 87.2)
Kazakhstan	Chronic kidney disease due to other and unspecified causes	18.7 (16.1 to 21.8)	22.4 (19.6 to 25.7)	20.2 (17.3 to 23.6)	125.2 (107 to 147.1)	147.6 (128.8 to 169.3)	115.8 (99.5 to 135.4)
Kazakhstan	Acute glomerulonephritis	0.468 (0.259 to 0.791)	0.511 (0.447 to 0.584)	0.258 (0.212 to 0.32)	3.1 (1.7 to 5.2)	3.1 (2.8 to 3.6)	1.4 (1.1 to 1.7)
Kazakhstan	Skin and subcutaneous diseases	76.4 (51.2 to 112)	70.3 (47.3 to 103)	82.5 (55.7 to 121)	453.2 (304.9 to 665.8)	459.7 (309 to 673.7)	468.2 (314.7 to 684.2)
Kazakhstan	Dermatitis	17.9 (10.5 to 28.7)	15.3 (9.15 to 24.1)	18.4 (10.8 to 29.2)	103.7 (61.2 to 165.1)	102.3 (60.1 to 161.5)	104.1 (61.3 to 165.4)
Kazakhstan	Atopic dermatitis	14.2 (7.64 to 23.9)	11.6 (6.29 to 19.2)	14.2 (7.68 to 24.1)	80.6 (43.8 to 135.9)	79.1 (42.8 to 131.9)	80.9 (43.6 to 136.3)
Kazakhstan	Contact dermatitis	3.73 (2.41 to 5.59)	3.74 (2.39 to 5.58)	4.13 (2.69 to 6.14)	23 (14.9 to 34.4)	23.1 (14.8 to 34.3)	23.1 (14.9 to 34.1)
Kazakhstan	Seborrhoeic dermatitis	0.0178 (0.00996 to 0.0286)	0.0165 (0.00918 to 0.0266)	0.0192 (0.0107 to 0.0308)	0.1 (0.1 to 0.2)	0.1 (0.1 to 0.2)	0.1 (0.1 to 0.2)
Kazakhstan	Psoriasis	10.7 (7.62 to 14.2)	11.1 (7.9 to 14.7)	13 (9.03 to 17.1)	67.3 (47.7 to 88.4)	69.4 (49.4 to 91.6)	70.5 (49.1 to 92.6)
Kazakhstan	Bacterial skin diseases	0.425 (0.309 to 0.632)	0.622 (0.38 to 0.809)	0.861 (0.461 to 1.16)	2.6 (1.9 to 4)	4.1 (2.5 to 5.3)	4.8 (2.6 to 6.5)
Kazakhstan	Cellulitis	0.264 (0.187 to 0.382)	0.388 (0.242 to 0.505)	0.549 (0.294 to 0.736)	1.6 (1.2 to 2.4)	2.5 (1.6 to 3.2)	3 (1.6 to 4)
Kazakhstan	Pyoderma	0.162 (0.102 to 0.276)	0.234 (0.111 to 0.347)	0.312 (0.138 to 0.461)	1 (0.6 to 1.7)	1.6 (0.8 to 2.4)	1.8 (0.8 to 2.6)
Kazakhstan	Scabies	5.21 (2.88 to 8.5)	4.65 (2.59 to 7.62)	5.03 (2.77 to 8.12)	30 (16.6 to 48.7)	29.5 (16.4 to 47.6)	28.9 (16.1 to 46.8)
Kazakhstan	Fungal skin diseases	7.48 (3 to 15.7)	7.07 (2.85 to 14.7)	7.85 (3.14 to 16.4)	48.3 (19.5 to 100.8)	48.1 (19.4 to 100.1)	45.8 (18.4 to 94.5)
Kazakhstan	Viral skin diseases	8.31 (5.34 to 12.5)	6.41 (4.08 to 9.52)	7.97 (5.15 to 11.9)	45.7 (29.4 to 68.6)	45.8 (29.3 to 68.2)	45.9 (29.7 to 69)
Kazakhstan	Acne vulgaris	4.79 (2.78 to 7.79)	5.56 (3.25 to 9.16)	5.77 (3.39 to 9.35)	27 (15.7 to 43.8)	31.2 (18.4 to 51.6)	35.8 (20.7 to 58.9)
Kazakhstan	Alopecia areata	0.997 (0.626 to 1.48)	0.973 (0.615 to 1.48)	1.11 (0.692 to 1.66)	5.9 (3.7 to 8.7)	5.9 (3.7 to 8.9)	5.9 (3.7 to 8.9)
Kazakhstan	Pruritus	1.53 (0.719 to 2.88)	1.5 (0.716 to 2.85)	1.78 (0.84 to 3.38)	9.6 (4.5 to 18.1)	9.6 (4.6 to 18.2)	9.9 (4.7 to 18.8)
Kazakhstan	Urticaria	13.1 (8.71 to 18.7)	11.2 (7.42 to 15.9)	13.4 (8.78 to 19)	74.7 (49.7 to 106.1)	74.8 (49.8 to 106.6)	74.9 (49.8 to 106.5)
Kazakhstan	Decubitus ulcer	0.328 (0.232 to 0.436)	0.349 (0.248 to 0.461)	0.41 (0.292 to 0.544)	2.4 (1.7 to 3.2)	2.5 (1.8 to 3.3)	2.5 (1.8 to 3.3)
Kazakhstan	Other skin and subcutaneous diseases	5.55 (2.68 to 10.3)	5.56 (2.69 to 10.2)	6.98 (3.39 to 12.9)	35.9 (17.4 to 66)	36.4 (17.6 to 66.8)	39.1 (19 to 72.2)
Kazakhstan	Sense organ diseases	116 (77.6 to 171)	117 (78.4 to 172)	138 (91.8 to 202)	833.7 (557.6 to 1208.2)	822.6 (550.8 to 1193.8)	811 (541.3 to 1178)
Kazakhstan	Blindness and vision impairment	54.1 (34.7 to 80.6)	53.7 (34.6 to 80.5)	62.7 (40.5 to 93.6)	385.2 (248.1 to 570.5)	374.9 (241.4 to 555.9)	367.6 (237.6 to 546)
Kazakhstan	Glaucoma	0.419 (0.271 to 0.596)	0.424 (0.276 to 0.607)	0.472 (0.304 to 0.681)	3.4 (2.2 to 4.8)	3.3 (2.2 to 4.7)	3 (1.9 to 4.2)
Kazakhstan	Cataract	17.7 (11.9 to 24.5)	18.7 (12.6 to 25.8)	22.3 (14.9 to 31.1)	145.4 (97.7 to 199)	143.2 (96.3 to 197.1)	141 (94.4 to 193.8)

SR Appendix Table 2. All-age DALYs counts and age standardized DALYs rates per 100,000 individuals for all causes and level 1 cause groups by location for 1990, 2007, and 2017, both sexes combined

		All-age DALYs			Age-standardized rate of DALYs per 100,000		
Kazakhstan	Age-related macular degeneration	0.14 (0.0857 to 0.211)	0.151 (0.0937 to 0.226)	0.188 (0.121 to 0.287)	1.1 (0.7 to 1.7)	1.2 (0.7 to 1.8)	1.2 (0.7 to 1.8)
Kazakhstan	Refraction disorders	18.4 (11.7 to 28.1)	17.1 (10.8 to 26.2)	19.6 (12.5 to 30.2)	113.1 (72 to 172.3)	111.4 (70.6 to 170.2)	110.1 (70.3 to 168.4)
Kazakhstan	Near vision loss	14.4 (6.86 to 26.1)	14.2 (6.84 to 26)	16.5 (7.83 to 29.8)	101.3 (48.4 to 185.2)	95.1 (45.7 to 173.9)	91.9 (43.5 to 166.8)
Kazakhstan	Other vision loss	3 (2.01 to 4.22)	3.05 (1.97 to 4.31)	3.59 (2.34 to 5.11)	20.8 (13.9 to 29.2)	20.8 (13.5 to 29.5)	20.5 (13.4 to 29)
Kazakhstan	Age-related and other hearing loss	57.8 (39.6 to 82.5)	59.2 (40 to 84.2)	69.5 (46.7 to 99.3)	417.6 (288.3 to 587.7)	416.3 (285 to 588.1)	411.9 (280.2 to 588.2)
Kazakhstan	Other sense organ diseases	4.47 (2.77 to 6.55)	4.57 (2.83 to 6.64)	5.5 (3.43 to 8)	30.9 (19.1 to 45.1)	31.3 (19.4 to 45.3)	31.6 (19.6 to 45.9)
Kazakhstan	Musculoskeletal disorders	219 (158 to 294)	228 (165 to 306)	266 (191 to 360)	1478.1 (1073 to 1979.5)	1462.9 (1068.3 to 1969.1)	1460.7 (1053.4 to 1971)
Kazakhstan	Rheumatoid arthritis	3.81 (2.69 to 5.11)	4.15 (2.95 to 5.56)	5.35 (3.67 to 7.22)	26.4 (18.7 to 35.3)	27.4 (19.4 to 36.4)	29.6 (20.5 to 39.8)
Kazakhstan	Osteoarthritis	10.6 (5.35 to 21.2)	11.6 (5.83 to 23.2)	14.9 (7.51 to 29.4)	80.3 (40.3 to 159.4)	82 (41.3 to 162.8)	86.1 (43.3 to 171.1)
Kazakhstan	Low back pain	155 (111 to 207)	159 (115 to 212)	183 (132 to 244)	1038.1 (742.3 to 1379.5)	1017 (730.6 to 1357.9)	1005.9 (715.7 to 1338.2)
Kazakhstan	Neck pain	44 (30.7 to 62)	47.3 (33 to 66.8)	55.6 (38.8 to 78.3)	297.6 (208.1 to 416.8)	298.1 (208.7 to 417.5)	298.6 (209.7 to 415.2)
Kazakhstan	Gout	1.73 (1.15 to 2.4)	1.93 (1.29 to 2.68)	2.43 (1.6 to 3.42)	12.5 (8.3 to 17.4)	13.1 (8.7 to 18.2)	13.7 (9.1 to 19.2)
Kazakhstan	Other musculoskeletal disorders	3.82 (1.78 to 6.45)	4.21 (2.09 to 6.93)	5.28 (2.31 to 9.4)	23.2 (10.7 to 39.3)	25.3 (12.6 to 41.6)	26.9 (12.1 to 47.5)
Kazakhstan	Other non-communicable diseases	339 (285 to 400)	327 (268 to 389)	283 (224 to 353)	2020.9 (1695.4 to 2397.9)	2184.9 (1791 to 2578.8)	1595.8 (1266.4 to 2000.4)
Kazakhstan	Congenital anomalies	192 (164 to 220)	188 (145 to 222)	137 (105 to 180)	1077.1 (918.2 to 1229)	1291.4 (987.6 to 1528.4)	788 (607.1 to 1032.2)
Kazakhstan	Neural tube defects	15.9 (10.8 to 20.6)	5.2 (3.9 to 6.7)	3.41 (2.37 to 4.65)	88.7 (60.3 to 114.6)	35.5 (26.5 to 45.9)	19.5 (13.5 to 26.7)
Kazakhstan	Congenital heart anomalies	66.4 (39.4 to 84.3)	78.5 (40 to 102)	45.2 (26 to 63.2)	369.1 (219.1 to 468.6)	539.1 (269 to 701.2)	262.2 (150.2 to 366.5)
Kazakhstan	Orofacial clefts	1.86 (0.688 to 3.74)	1.01 (0.654 to 1.45)	0.708 (0.457 to 0.987)	10.5 (4 to 21.1)	6.8 (4.4 to 9.8)	4 (2.6 to 5.6)
Kazakhstan	Down's syndrome	2.48 (1.77 to 3.19)	2.57 (1.94 to 3.15)	2.46 (1.72 to 3.19)	14 (10.1 to 18)	17.1 (12.8 to 21.1)	13.7 (9.5 to 17.9)
Kazakhstan	Turner syndrome	0.0167 (0.00809 to 0.0283)	0.0156 (0.00777 to 0.0252)	0.0165 (0.00825 to 0.0277)	0.1 (0 to 0.2)	0.1 (0 to 0.2)	0.1 (0 to 0.2)
Kazakhstan	Klinefelter syndrome	0.00484 (0.00225 to 0.00897)	0.00443 (0.00198 to 0.00829)	0.00493 (0.00224 to 0.00889)	0 (0 to 0.1)	0 (0 to 0.1)	0 (0 to 0)
Kazakhstan	Other chromosomal abnormalities	5.19 (2.24 to 16.1)	6.33 (3.04 to 17.3)	5.94 (3.02 to 15.9)	29.2 (12.8 to 89.6)	42.3 (20 to 118.1)	33.9 (16.8 to 92.8)
Kazakhstan	Congenital musculoskeletal and limb anomalies	20.2 (14.3 to 26.7)	18.3 (13.1 to 24)	19.4 (13.5 to 26.3)	119.1 (84.4 to 158.5)	118.5 (85.2 to 154.4)	110.1 (76.9 to 148.8)
Kazakhstan	Urogenital congenital anomalies	1.48 (0.899 to 2.21)	1.32 (0.805 to 1.85)	1.18 (0.726 to 1.7)	8.2 (5 to 12.3)	9 (5.5 to 12.7)	6.9 (4.2 to 9.9)
Kazakhstan	Digestive congenital anomalies	23.6 (16.2 to 42.6)	21.9 (16 to 39.8)	15.9 (9.75 to 29.3)	132.9 (91.8 to 240.2)	148.5 (108.3 to 272.5)	91.8 (56.1 to 171.6)
Kazakhstan	Other congenital anomalies	55.1 (42 to 73.2)	53.3 (40.6 to 71.6)	42.9 (29.3 to 60.9)	305.2 (232.6 to 404.2)	374.6 (284.1 to 505.8)	245.9 (168.8 to 349.2)
Kazakhstan	Urinary diseases and male infertility	26.1 (23.6 to 29.3)	21.1 (18.5 to 24)	18.8 (15.6 to 22.7)	176.9 (159.1 to 198.5)	138.9 (121.7 to 159)	104.8 (87.4 to 126.6)
Kazakhstan	Urinary tract infections	15.2 (12.9 to 16.8)	11.2 (10.6 to 12.2)	7.79 (6.86 to 9.14)	98.7 (82.8 to 109.2)	70.9 (66.7 to 77.6)	42.7 (37.6 to 50.3)
Kazakhstan	Urolithiasis	2.64 (1.35 to 4.37)	1.78 (1.46 to 2.15)	1.75 (1.45 to 2.09)	19.6 (9.8 to 33)	12.2 (10 to 15)	10.2 (8.4 to 12.3)
Kazakhstan	Benign prostatic hyperplasia	5.15 (3.28 to 7.44)	5.48 (3.45 to 7.87)	7.16 (4.57 to 10.3)	37.3 (23.8 to 53.6)	38.4 (24.4 to 55.1)	39.8 (25.5 to 57.1)
Kazakhstan	Male infertility	0.395 (0.164 to 0.828)	0.389 (0.158 to 0.826)	0.415 (0.169 to 0.866)	2.2 (0.9 to 4.7)	2.3 (0.9 to 4.8)	2.2 (0.9 to 4.5)
Kazakhstan	Other urinary diseases	2.72 (2.11 to 3.94)	2.21 (1.35 to 2.77)	1.71 (0.939 to 2.24)	19.1 (15.2 to 27.8)	15.2 (8.8 to 19.1)	10 (5.3 to 13.2)
Kazakhstan	Gynecological diseases	27.6 (19.1 to 38.1)	29.5 (20.6 to 41)	31.3 (21.4 to 44.1)	167.1 (116 to 232.6)	169.5 (118.4 to 235.1)	163.7 (112.3 to 230.2)
Kazakhstan	Uterine fibroids	4.87 (3.14 to 7.46)	5.71 (3.72 to 9.1)	5.5 (3.34 to 9)	32.4 (20.8 to 49.9)	33.2 (21.7 to 52.8)	28.3 (17.2 to 46.3)
Kazakhstan	Polycystic ovarian syndrome	0.988 (0.429 to 1.92)	1.02 (0.453 to 1.97)	1.12 (0.505 to 2.13)	5.8 (2.5 to 11.2)	5.8 (2.6 to 11.2)	5.9 (2.7 to 11.3)
Kazakhstan	Female infertility	0.35 (0.135 to 0.751)	0.34 (0.116 to 0.771)	0.425 (0.15 to 0.945)	1.9 (0.7 to 4.1)	2 (0.7 to 4.5)	2.2 (0.8 to 4.8)
Kazakhstan	Endometriosis	8.68 (5.72 to 12.8)	8.9 (5.84 to 13)	9.86 (6.48 to 14.5)	50.6 (33.2 to 74)	51.4 (33.8 to 75.2)	51.1 (33.6 to 74.9)
Kazakhstan	Genital prolapse	0.516 (0.257 to 0.959)	0.482 (0.24 to 0.898)	0.568 (0.274 to 1.06)	3.7 (1.9 to 6.8)	3.2 (1.6 to 6)	3.1 (1.5 to 5.8)
Kazakhstan	Premenstrual syndrome	8.64 (5.54 to 12.9)	9.14 (5.8 to 13.7)	9.48 (6.09 to 14.1)	51.5 (33 to 76.7)	51.6 (32.7 to 77.1)	50.6 (32.5 to 75.4)
Kazakhstan	Other gynecological diseases	3.52 (2.41 to 5.09)	3.91 (2.69 to 5.68)	4.34 (2.83 to 6.63)	21.3 (14.3 to 30.9)	22.4 (15.4 to 32.4)	22.4 (14.6 to 34.3)

SR Appendix Table 2. All-age DALYs counts and age standardized DALYs rates per 100,000 individuals for all causes and level 1 cause groups by location for 1990, 2007, and 2017, both sexes combined							
		All-age DALYs			Age-standardized rate of DALYs per 100,000		
Kazakhstan	Hemoglobinopathies and hemolytic anaemias	20.5 (15.1 to 27.5)	14.7 (11.1 to 19.6)	13.5 (9.99 to 18.4)	123.4 (91.2 to 165)	94.1 (71.8 to 125.6)	75.5 (55.6 to 102.8)
Kazakhstan	Thalassemias	1.58 (1.23 to 2.02)	1.1 (0.865 to 1.4)	1.13 (0.811 to 1.65)	8.5 (6.6 to 10.9)	8.1 (6.4 to 10.4)	6.5 (4.7 to 9.4)
Kazakhstan	Thalassaemias trait	6.92 (4.47 to 9.92)	4.93 (3.22 to 7.34)	4.86 (3.14 to 7.31)	40.6 (26.5 to 58.5)	31.9 (20.9 to 47.4)	26.9 (17.3 to 40)
Kazakhstan	Sickle cell disorders	0.206 (0.13 to 0.313)	0.166 (0.106 to 0.245)	0.176 (0.106 to 0.28)	1.1 (0.7 to 1.7)	1.2 (0.7 to 1.7)	1 (0.6 to 1.6)
Kazakhstan	Sickle cell trait	0.557 (0.346 to 0.838)	0.362 (0.229 to 0.552)	0.33 (0.185 to 0.518)	3.2 (2 to 4.9)	2.4 (1.5 to 3.6)	1.8 (1 to 2.9)
Kazakhstan	G6PD deficiency	0.678 (0.486 to 0.839)	0.922 (0.714 to 1.08)	0.843 (0.715 to 1.01)	3.9 (2.8 to 4.9)	5.5 (4.2 to 6.4)	4.6 (3.9 to 5.5)
Kazakhstan	G6PD trait	0.000648 (0.000425 to 0.000931)	0.000587 (0.000382 to 0.000843)	0.00057 (0.000368 to 0.00085)	0 (0 to 0)	0 (0 to 0)	0 (0 to 0)
Kazakhstan	Other hemoglobinopathies and hemolytic anaemias	10.6 (7.74 to 14.1)	7.18 (5.46 to 9.65)	6.19 (4.5 to 8.36)	66 (48.8 to 88.6)	45.1 (34.4 to 59.7)	34.7 (25.3 to 47)
Kazakhstan	Endocrine, metabolic, blood, and immune disorders	14.2 (10.6 to 18.8)	15.4 (12 to 19.1)	16.5 (12 to 20.6)	85.8 (63.8 to 114.1)	98.8 (77 to 121.4)	91.1 (66.4 to 114)
Kazakhstan	Oral disorders	49.3 (31.2 to 74.3)	51.1 (32.3 to 77.1)	58.7 (37.4 to 87.7)	341.6 (218.6 to 510.1)	342.2 (217.7 to 514.6)	332.7 (213.3 to 494.8)
Kazakhstan	Caries of deciduous teeth	0.696 (0.299 to 1.38)	0.447 (0.194 to 0.898)	0.651 (0.276 to 1.35)	3.7 (1.6 to 7.4)	3.7 (1.6 to 7.4)	3.6 (1.5 to 7.5)
Kazakhstan	Caries of permanent teeth	6.75 (2.95 to 12.7)	6.64 (2.88 to 12.7)	6.95 (3.06 to 13.3)	40.4 (17.7 to 76.3)	40.3 (17.5 to 76.8)	38.7 (17.1 to 74.8)
Kazakhstan	Periodontal disease	6.51 (2.57 to 13.6)	6.75 (2.68 to 14.2)	7.83 (3.08 to 16.7)	44 (17.3 to 91.1)	42.7 (17.1 to 89.6)	41.6 (16.3 to 88)
Kazakhstan	Edentulism and severe tooth loss	27 (17.9 to 38.2)	29 (19.3 to 41.2)	33.9 (22.5 to 48.1)	202.6 (135.2 to 286)	204.4 (135.8 to 290.1)	197.8 (132.3 to 280.3)
Kazakhstan	Other oral disorders	8.32 (5.23 to 12.4)	8.26 (5.12 to 12.3)	9.37 (5.89 to 13.8)	50.9 (31.9 to 75.7)	51 (31.7 to 75.8)	51.1 (32.1 to 75.1)
Kazakhstan	Sudden infant death syndrome	8.86 (4.59 to 16.9)	7.23 (4.53 to 11.5)	6.85 (4.63 to 10.3)	49 (25.4 to 93.5)	50.1 (31.4 to 80.1)	40 (27 to 60.1)
Kazakhstan	Injuries	1070 (1010 to 1140)	1170 (1120 to 1240)	848 (777 to 930)	6345.2 (5982.3 to 6783.2)	7162.3 (6792.1 to 7583.7)	4574.2 (4191.6 to 5016.7)
Kazakhstan	Transport injuries	252 (240 to 265)	272 (258 to 286)	179 (164 to 195)	1478.4 (1406.6 to 1558.9)	1651.4 (1565.4 to 1739.5)	965.6 (883.9 to 1050.4)
Kazakhstan	Road injuries	235 (223 to 248)	245 (233 to 258)	169 (155 to 183)	1377.8 (1304.8 to 1455.4)	1490.1 (1414 to 1575.1)	911.1 (835.1 to 990.1)
Kazakhstan	Pedestrian road injuries	75.1 (68.7 to 81.7)	72.4 (64.7 to 86)	46.3 (41.8 to 51.2)	444.3 (406.7 to 483.3)	453.2 (405.8 to 535.8)	251.5 (227.3 to 278.4)
Kazakhstan	Cyclist road injuries	6.03 (4.83 to 7.34)	6.82 (5.53 to 8.42)	6.82 (5.37 to 8.48)	37.4 (29.7 to 45.6)	42.6 (34.5 to 52.4)	37.3 (29.5 to 46.4)
Kazakhstan	Motorcyclist road injuries	15.2 (13 to 17.4)	17.3 (14.7 to 22.4)	11.5 (9.98 to 13.1)	89 (76 to 101.7)	102.3 (86.8 to 131.5)	61.4 (53.5 to 69.9)
Kazakhstan	Motor vehicle road injuries	136 (127 to 144)	145 (125 to 157)	102 (93.1 to 111)	788.2 (736.2 to 838.5)	874.6 (752.8 to 945.2)	548 (501.2 to 595.7)
Kazakhstan	Other road injuries	3.06 (2.55 to 3.59)	2.85 (2.35 to 3.53)	2.38 (1.9 to 2.98)	18.8 (15.6 to 22.2)	17.5 (14.5 to 21.7)	12.9 (10.3 to 16.1)
Kazakhstan	Other transport injuries	17.2 (15.3 to 21.2)	26.9 (23.7 to 29.8)	10.1 (8.95 to 11.4)	100.7 (89.8 to 123.2)	161.3 (141.4 to 178.4)	54.4 (48.3 to 61.3)
Kazakhstan	Unintentional injuries	524 (479 to 579)	465 (425 to 516)	383 (333 to 443)	3117.5 (2840.5 to 3460.7)	2939.4 (2678.4 to 3258.3)	2082.2 (1811.1 to 2414.3)
Kazakhstan	Falls	127 (103 to 157)	114 (91.9 to 142)	123 (95.8 to 158)	803.3 (648.6 to 999.2)	723 (583.5 to 900.7)	677 (525.9 to 865.8)
Kazakhstan	Drowning	131 (121 to 141)	84.1 (79.2 to 89.6)	52.7 (46.8 to 59.5)	735.2 (679.9 to 785.8)	537.7 (502.2 to 577.3)	291.2 (258.9 to 329.1)
Kazakhstan	Fire, heat, and hot substances	64.9 (58 to 72.7)	41 (36.7 to 46.7)	31.9 (27 to 38)	381.7 (341.2 to 428)	261.7 (233.6 to 295.5)	171.7 (145.5 to 205.2)
Kazakhstan	Poisonings	34.6 (32.3 to 37.2)	32.7 (30.9 to 34.5)	23.7 (21.5 to 26.2)	204.2 (191.2 to 218.6)	204 (192.6 to 216.5)	126.8 (115.4 to 139.8)
Kazakhstan	Poisoning by carbon monoxide	21.2 (19.7 to 22.6)	26 (24.7 to 27.4)	18.4 (16.8 to 20.1)	128 (119.2 to 136.2)	159.5 (150.7 to 168.1)	97.7 (89.4 to 106.7)
Kazakhstan	Poisoning by other means	13.3 (11.8 to 15.2)	6.66 (5.76 to 7.74)	5.3 (4.31 to 6.52)	76.2 (67.7 to 86.3)	44.5 (38.5 to 52)	29.2 (23.6 to 35.9)
Kazakhstan	Exposure to mechanical forces	46.8 (39.7 to 55.8)	49.3 (42.8 to 57.6)	38.7 (32 to 47.7)	286.8 (241.1 to 344)	303.9 (263.3 to 356.8)	207.9 (171.5 to 256.2)
Kazakhstan	Unintentional firearm injuries	16.9 (15.2 to 18.9)	21.6 (18.6 to 23.2)	13.8 (12 to 15.3)	99.7 (90.3 to 111.9)	130.8 (113.3 to 140.8)	73.2 (64.1 to 81.3)
Kazakhstan	Other exposure to mechanical forces	29.9 (23.6 to 38.1)	27.7 (21.6 to 35.9)	25 (18.6 to 33.6)	187.1 (146.7 to 239.6)	173.1 (135 to 224.3)	134.7 (100.6 to 180.5)
Kazakhstan	Adverse effects of medical treatment	9.59 (7.78 to 10.9)	8.68 (7.37 to 10.3)	8.8 (7.74 to 12)	57.8 (46.7 to 65.3)	55.4 (47.8 to 66.2)	48.2 (42.4 to 65.9)
Kazakhstan	Animal contact	3.59 (3 to 4.29)	3.25 (2.73 to 3.87)	2.99 (2.42 to 3.63)	21.3 (17.7 to 25.5)	20.7 (17.4 to 24.6)	16.4 (13.3 to 19.9)
Kazakhstan	Venomous animal contact	1.7 (1.27 to 2.22)	1.71 (1.31 to 2.19)	1.66 (1.25 to 2.16)	10.2 (7.6 to 13.3)	10.7 (8.2 to 13.7)	9.1 (6.8 to 11.9)
Kazakhstan	Non-venomous animal contact	1.89 (1.56 to 2.27)	1.54 (1.27 to 1.84)	1.33 (1.1 to 1.62)	11.1 (9.2 to 13.2)	10 (8.3 to 12)	7.3 (6.1 to 8.9)
Kazakhstan	Foreign body	50.8 (45.9 to 56)	58 (53.7 to 63.7)	42.3 (36.2 to 50.3)	290 (263.4 to 318.7)	379.8 (348.9 to 420.3)	232.8 (198.7 to 277.8)

SR Appendix Table 2. All-age DALYs counts and age standardized DALYs rates per 100,000 individuals for all causes and level 1 cause groups by location for 1990, 2007, and 2017, both sexes combined

		All-age DALYs			Age-standardized rate of DALYs per 100,000		
Kazakhstan	Pulmonary aspiration and foreign body in airway	44.6 (39.8 to 49.9)	51.2 (46.8 to 56.6)	36 (30.5 to 43.7)	251.9 (226 to 280.6)	336.6 (306.1 to 375.9)	198.4 (167.5 to 242.4)
Kazakhstan	Foreign body in eyes	0.303 (0.137 to 0.535)	0.364 (0.165 to 0.64)	0.384 (0.177 to 0.673)	1.8 (0.8 to 3.2)	2.3 (1.1 to 4.1)	2.1 (1 to 3.7)
Kazakhstan	Foreign body in other body part	5.9 (5.07 to 7.5)	6.51 (5.43 to 8.32)	5.92 (5.05 to 7.21)	36.3 (31.2 to 45.3)	40.8 (34.1 to 52.2)	32.3 (27.6 to 39.2)
Kazakhstan	Environmental heat and cold exposure	36.6 (32.4 to 44.3)	48.5 (45.2 to 51.7)	38.2 (34.2 to 42.5)	227.1 (200.5 to 273.3)	295.7 (275.3 to 316.4)	200.2 (179.4 to 223.3)
Kazakhstan	Exposure to forces of nature	0.0552 (0.055 to 0.0555)	0.0182 (0.0138 to 0.0237)	0.0189 (0.0145 to 0.0241)	0.3 (0.3 to 0.3)	0.1 (0.1 to 0.1)	0.1 (0.1 to 0.1)
Kazakhstan	Other unintentional injuries	18.3 (15.8 to 21.4)	25.4 (22.7 to 28.9)	20.1 (16.7 to 24.9)	109.8 (94.1 to 130)	157.3 (140.2 to 179.2)	110 (91.5 to 136.1)
Kazakhstan	Self-harm and interpersonal violence	294 (286 to 302)	436 (422 to 449)	287 (266 to 307)	1749.3 (1701 to 1796.4)	2571.5 (2487.6 to 2651)	1526.4 (1418.8 to 1631.3)
Kazakhstan	Self-harm	159 (154 to 164)	294 (279 to 306)	200 (184 to 219)	955.4 (926.1 to 986.3)	1727.2 (1641.2 to 1797.1)	1066.7 (983.8 to 1170.3)
Kazakhstan	Self-harm by firearm	3.59 (2.5 to 5.95)	4.99 (3.07 to 8.32)	3.55 (2.51 to 5.36)	22.2 (15.8 to 36.4)	29.9 (18.6 to 49.9)	18.6 (13.2 to 28)
Kazakhstan	Self-harm by other specified means	155 (150 to 161)	289 (274 to 301)	196 (180 to 215)	933.2 (903.9 to 964.9)	1697.3 (1612.6 to 1764.3)	1048.1 (968.3 to 1148.3)
Kazakhstan	Interpersonal violence	132 (126 to 138)	138 (130 to 145)	83.3 (75.9 to 90.8)	777.5 (743.1 to 813.2)	818.1 (774.7 to 863.7)	440.7 (400.9 to 479.2)
Kazakhstan	Assault by firearm	9.66 (7.19 to 12.2)	11.2 (7.7 to 14.2)	6.36 (5.23 to 7.7)	55.5 (41.6 to 69.4)	64.9 (45 to 81.8)	34.4 (28.2 to 41.3)
Kazakhstan	Assault by sharp object	50.6 (45.5 to 55.6)	64.7 (57.2 to 79.3)	35.7 (31.9 to 40)	295.8 (265.4 to 324.8)	380.1 (335.4 to 465.1)	187.7 (167.7 to 210)
Kazakhstan	Sexual violence	1.29 (0.868 to 1.88)	1.21 (0.808 to 1.75)	1.4 (0.937 to 2.05)	7.6 (5.1 to 11)	7.2 (4.8 to 10.3)	7.6 (5.1 to 11.1)
Kazakhstan	Assault by other means	70.6 (65.1 to 76.4)	60.5 (50.4 to 67.6)	39.9 (35.4 to 44.4)	418.7 (387.9 to 453.2)	366 (306.9 to 407.6)	211 (187.8 to 234.8)
Kazakhstan	Conflict and terrorism	0 (0 to 0)	0.924 (0.889 to 0.982)	0.223 (0.15 to 0.355)	0 (0 to 0)	5.4 (5.2 to 5.8)	1.2 (0.8 to 1.9)
Kazakhstan	Executions and police conflict	2.84 (1.82 to 4.45)	3.55 (2.54 to 5.08)	3.35 (2.22 to 5.1)	16.3 (10.5 to 25.4)	20.7 (14.9 to 29.6)	17.8 (11.8 to 27.1)
Kyrgyzstan	All causes	1940 (1800 to 2090)	1900 (1770 to 2050)	1700 (1540 to 1880)	45881.3 (42612 to 49458.3)	41231.4 (38524.7 to 44266.5)	30701.8 (28012.8 to 33883.7)
Kyrgyzstan	Communicable, maternal, neonatal, and nutritional disorders	733 (671 to 803)	490 (460 to 522)	367 (330 to 411)	12748.9 (11682.1 to 13950.4)	8752.5 (8222.7 to 9341.9)	5331.3 (4791.5 to 5967.4)
Kyrgyzstan	HIV/AIDS and sexually transmitted infections	2.35 (1.89 to 3.15)	9.39 (8.62 to 10.6)	11.9 (10.9 to 13.2)	54.8 (43.8 to 73.8)	174.3 (160.4 to 195.4)	185.1 (170.4 to 205.8)
Kyrgyzstan	HIV/AIDS	1.27 (1.21 to 1.33)	7.83 (7.45 to 8.26)	10.3 (9.8 to 10.8)	29.4 (28.1 to 30.7)	146.6 (139.2 to 154.9)	161.3 (153.1 to 169.1)
Kyrgyzstan	HIV/AIDS - Drug-susceptible Tuberculosis	0.408 (0.253 to 0.557)	0.797 (0.382 to 1.39)	0.404 (0.141 to 0.783)	9.5 (5.9 to 12.9)	14.9 (7.1 to 26)	6.3 (2.2 to 12.2)
Kyrgyzstan	HIV/AIDS - Multidrug-resistant Tuberculosis without extensive drug resistance	0.000987 (0.000133 to 0.00358)	0.628 (0.318 to 1.09)	0.406 (0.188 to 0.682)	0 (0 to 0.1)	11.7 (5.9 to 20.4)	6.3 (2.9 to 10.6)
Kyrgyzstan	HIV/AIDS - Extensively drug-resistant Tuberculosis	0 (0 to 0)	0.199 (0.0993 to 0.346)	0.232 (0.107 to 0.396)	0 (0 to 0)	3.7 (1.9 to 6.5)	3.6 (1.7 to 6.2)
Kyrgyzstan	HIV/AIDS resulting in other diseases	0.857 (0.695 to 1.02)	6.21 (5.33 to 6.96)	9.28 (8.63 to 9.89)	19.9 (16.1 to 23.8)	116.3 (100 to 130.3)	145 (134.8 to 154.9)
Kyrgyzstan	Sexually transmitted infections excluding HIV	1.08 (0.628 to 1.9)	1.55 (0.916 to 2.64)	1.57 (0.883 to 2.81)	25.4 (14.6 to 44.6)	27.6 (16.5 to 46.4)	23.8 (13.4 to 42.8)
Kyrgyzstan	Syphilis	0.151 (0.0798 to 0.289)	0.217 (0.0994 to 0.455)	0.133 (0.0566 to 0.315)	3 (1.8 to 5.3)	4 (2 to 8.1)	2 (0.9 to 4.5)
Kyrgyzstan	Chlamydial infection	0.223 (0.125 to 0.403)	0.317 (0.181 to 0.577)	0.355 (0.198 to 0.656)	5.3 (3 to 9.6)	5.5 (3.2 to 9.9)	5.3 (2.9 to 9.7)
Kyrgyzstan	Gonococcal infection	0.197 (0.107 to 0.358)	0.301 (0.183 to 0.523)	0.278 (0.158 to 0.507)	4.4 (2.5 to 7.8)	5 (3.2 to 8.5)	4.1 (2.4 to 7.3)
Kyrgyzstan	Trichomoniasis	0.162 (0.065 to 0.346)	0.219 (0.0877 to 0.461)	0.257 (0.104 to 0.553)	4.1 (1.6 to 8.6)	4.1 (1.6 to 8.5)	4 (1.6 to 8.5)
Kyrgyzstan	Genital herpes	0.0961 (0.031 to 0.23)	0.126 (0.0407 to 0.301)	0.155 (0.0511 to 0.37)	2.6 (0.8 to 6.2)	2.6 (0.8 to 6.2)	2.6 (0.9 to 6.3)
Kyrgyzstan	Other sexually transmitted infections	0.256 (0.153 to 0.443)	0.373 (0.229 to 0.639)	0.391 (0.232 to 0.683)	6 (3.7 to 10.4)	6.4 (4 to 10.8)	5.9 (3.5 to 10.2)
Kyrgyzstan	Respiratory infections and tuberculosis	334 (303 to 368)	161 (153 to 169)	84.4 (76.2 to 93.2)	5779.9 (5266.8 to 6330.1)	3000.3 (2857.1 to 3143.2)	1261 (1143.9 to 1382.3)
Kyrgyzstan	Tuberculosis	22.4 (20.1 to 24.9)	37.7 (36.2 to 39.5)	20.7 (18.6 to 23)	523.3 (480.1 to 567.6)	754.7 (725.5 to 788.1)	340 (307.9 to 376)
Kyrgyzstan	Latent tuberculosis infection	0 (0 to 0)	0 (0 to 0)	0 (0 to 0)	0 (0 to 0)	0 (0 to 0)	0 (0 to 0)
Kyrgyzstan	Drug-susceptible tuberculosis	22.3 (20 to 24.9)	21 (13.3 to 28.4)	9.51 (4.87 to 14.9)	522.3 (478.2 to 567)	419 (266.2 to 566.8)	156.3 (80.1 to 244.9)
Kyrgyzstan	Multidrug-resistant tuberculosis without extensive drug resistance	0.0439 (0.00632 to 0.162)	12.7 (7.37 to 18.6)	7.14 (3.76 to 10.3)	1 (0.1 to 3.7)	255 (147.6 to 372.2)	117.2 (61.5 to 168.9)
Kyrgyzstan	Extensively drug-resistant tuberculosis	0 (0 to 0)	4.03 (2.3 to 5.9)	4.05 (2.19 to 5.87)	0 (0 to 0)	80.7 (46 to 117.9)	66.5 (35.9 to 96.1)
Kyrgyzstan	Lower respiratory infections	305 (276 to 337)	117 (110 to 124)	57.2 (50.2 to 64.9)	5127.9 (4648 to 5655.5)	2124.2 (1998.9 to 2255.3)	824.8 (729.6 to 928.3)
Kyrgyzstan	Upper respiratory infections	5.51 (3.34 to 7.98)	5.01 (3.46 to 6.67)	4.53 (3.13 to 6.14)	101 (63.7 to 143.2)	90 (62.4 to 119.9)	66.4 (45.9 to 91.2)

SR Appendix Table 2. All-age DALYs counts and age standardized DALYs rates per 100,000 individuals for all causes and level 1 cause groups by location for 1990, 2007, and 2017, both sexes combined							
		All-age DALYs			Age-standardized rate of DALYs per 100,000		
Kyrgyzstan	Otitis media	1.34 (0.794 to 2.16)	1.76 (1.06 to 2.83)	1.98 (1.19 to 3.17)	27.8 (16.6 to 44.1)	31.5 (18.8 to 50.4)	29.8 (18.1 to 47.7)
Kyrgyzstan	Enteric infections	63.9 (54.8 to 74.4)	26.5 (23.5 to 29.8)	16.2 (13.4 to 19.3)	1066.9 (917.1 to 1241.9)	472.5 (418.1 to 530.6)	228.8 (190.4 to 271.8)
Kyrgyzstan	Diarrhoeal diseases	63.3 (54.3 to 73.9)	25.9 (22.8 to 29.2)	15.6 (12.9 to 18.7)	1055.5 (907.8 to 1231.9)	461.5 (406.4 to 518.5)	220.7 (182.7 to 263.9)
Kyrgyzstan	Typhoid and paratyphoid	0.00334 (0.00173 to 0.00592)	0.00315 (0.00161 to 0.00557)	0.00318 (0.00166 to 0.00566)	0.1 (0 to 0.1)	0.1 (0 to 0.1)	0.1 (0 to 0.1)
Kyrgyzstan	Typhoid fever	0.00328 (0.00169 to 0.00581)	0.00289 (0.00149 to 0.00511)	0.00286 (0.00147 to 0.00503)	0.1 (0 to 0.1)	0.1 (0 to 0.1)	0 (0 to 0.1)
Kyrgyzstan	Paratyphoid fever	0.0000564 (0.0000233 to 0.000114)	0.000257 (0.000104 to 0.000522)	0.000322 (0.000127 to 0.000666)	0 (0 to 0)	0 (0 to 0)	0 (0 to 0)
Kyrgyzstan	Invasive non-typhoidal salmonella	0.507 (0.278 to 0.846)	0.548 (0.303 to 0.947)	0.51 (0.271 to 0.88)	9.9 (5.5 to 16.4)	10 (5.6 to 16.7)	7.6 (4 to 12.9)
Kyrgyzstan	Other intestinal infectious diseases	0.0796 (0.016 to 0.16)	0.0512 (0.0156 to 0.102)	0.0285 (0.00992 to 0.0534)	1.4 (0.4 to 2.7)	0.9 (0.3 to 1.8)	0.4 (0.2 to 0.8)
Kyrgyzstan	Neglected tropical diseases and malaria	4.28 (2.87 to 6.51)	4.51 (2.89 to 7.76)	5.04 (2.99 to 11)	89.3 (60.7 to 136.1)	82.7 (53.9 to 138.9)	76 (45.6 to 161.8)
Kyrgyzstan	Malaria	0.0673 (0.0489 to 0.0884)	0.067 (0.0531 to 0.0887)	0 (0 to 0)	1.5 (1.1 to 2)	1.3 (1 to 1.6)	0 (0 to 0)
Kyrgyzstan	Chagas disease	0 (0 to 0)	0 (0 to 0)	0 (0 to 0)	0 (0 to 0)	0 (0 to 0)	0 (0 to 0)
Kyrgyzstan	Leishmaniasis	0.216 (0.000262 to 2.07)	0.384 (0.000373 to 3.26)	0.755 (0.000726 to 6.97)	4.3 (0 to 40.5)	6.9 (0 to 58.4)	11 (0 to 99.8)
Kyrgyzstan	Visceral leishmaniasis	0.216 (0.000202 to 2.07)	0.384 (0.000362 to 3.26)	0.755 (0.000725 to 6.97)	4.3 (0 to 40.5)	6.9 (0 to 58.4)	10.9 (0 to 99.8)
Kyrgyzstan	Cutaneous and mucocutaneous leishmaniasis	0.000114 (0.000000168 to 0.000604)	0.0000223 (0.000000000000000129 to 0.000216)	0.0000159 (0.000000000000000000000000000000000319 to 0.000208)	0 (0 to 0)	0 (0 to 0)	0 (0 to 0)
Kyrgyzstan	African trypanosomiasis	0 (0 to 0)	0 (0 to 0)	0 (0 to 0)	0 (0 to 0)	0 (0 to 0)	0 (0 to 0)
Kyrgyzstan	Schistosomiasis	0 (0 to 0)	0 (0 to 0)	0 (0 to 0)	0 (0 to 0)	0 (0 to 0)	0 (0 to 0)
Kyrgyzstan	Cysticercosis	0.000316 (0.000169 to 0.0005)	0.000165 (0.0000838 to 0.00026)	0.0000942 (0.0000465 to 0.000143)	0 (0 to 0)	0 (0 to 0)	0 (0 to 0)
Kyrgyzstan	Cystic echinococcosis	0.814 (0.549 to 1.14)	1.35 (0.872 to 1.99)	1.55 (0.993 to 2.29)	20.5 (13.9 to 28.5)	25.8 (16.9 to 37)	24.7 (16 to 35.9)
Kyrgyzstan	Lymphatic filariasis	0 (0 to 0)	0 (0 to 0)	0 (0 to 0)	0 (0 to 0)	0 (0 to 0)	0 (0 to 0)
Kyrgyzstan	Onchocerciasis	0 (0 to 0)	0 (0 to 0)	0 (0 to 0)	0 (0 to 0)	0 (0 to 0)	0 (0 to 0)
Kyrgyzstan	Trachoma	0 (0 to 0)	0 (0 to 0)	0 (0 to 0)	0 (0 to 0)	0 (0 to 0)	0 (0 to 0)
Kyrgyzstan	Dengue	0 (0 to 0)	0 (0 to 0)	0 (0 to 0)	0 (0 to 0)	0 (0 to 0)	0 (0 to 0)
Kyrgyzstan	Yellow fever	0 (0 to 0)	0 (0 to 0)	0 (0 to 0)	0 (0 to 0)	0 (0 to 0)	0 (0 to 0)
Kyrgyzstan	Rabies	0.21 (0.126 to 0.357)	0.176 (0.136 to 0.233)	0.0628 (0.0451 to 0.0878)	4.3 (2.8 to 6.8)	3.4 (2.7 to 4.3)	1.1 (0.8 to 1.5)
Kyrgyzstan	Intestinal nematode infections	0.475 (0.267 to 0.805)	0.381 (0.208 to 0.669)	0.326 (0.167 to 0.591)	10 (5.6 to 17.1)	6.8 (3.7 to 12)	4.9 (2.5 to 8.9)
Kyrgyzstan	Ascariasis	0.274 (0.131 to 0.516)	0.255 (0.121 to 0.486)	0.23 (0.107 to 0.456)	5.4 (2.6 to 10.2)	4.6 (2.2 to 8.7)	3.4 (1.6 to 6.8)
Kyrgyzstan	Trichuriasis	0.000000000886 (0.000000000374 to 0.00000000176)	0.000000000433 (0.000000000187 to 0.000000000834)	0.000000000499 (0.000000000216 to 0.000000000969)	0 (0 to 0)	0 (0 to 0)	0 (0 to 0)
Kyrgyzstan	Hookworm disease	0.201 (0.108 to 0.343)	0.126 (0.0672 to 0.221)	0.0959 (0.043 to 0.18)	4.6 (2.5 to 7.9)	2.3 (1.2 to 4)	1.5 (0.7 to 2.8)
Kyrgyzstan	Food-borne trematodiases	0 (0 to 0)	0 (0 to 0)	0 (0 to 0)	0 (0 to 0)	0 (0 to 0)	0 (0 to 0)
Kyrgyzstan	Leprosy	0 (0 to 0)	0 (0 to 0)	0 (0 to 0)	0 (0 to 0)	0 (0 to 0)	0 (0 to 0)
Kyrgyzstan	Ebola virus disease	0 (0 to 0)	0 (0 to 0)	0 (0 to 0)	0 (0 to 0)	0 (0 to 0)	0 (0 to 0)
Kyrgyzstan	Zika virus disease	0 (0 to 0)	0 (0 to 0)	0 (0 to 0)	0 (0 to 0)	0 (0 to 0)	0 (0 to 0)
Kyrgyzstan	Guinea worm disease	0 (0 to 0)	0 (0 to 0)	0 (0 to 0)	0 (0 to 0)	0 (0 to 0)	0 (0 to 0)
Kyrgyzstan	Other neglected tropical diseases	2.5 (1.63 to 3.54)	2.14 (1.42 to 3.16)	2.35 (1.56 to 3.46)	48.6 (32 to 69.2)	38.6 (25.2 to 56.3)	34.4 (23.1 to 50.7)
Kyrgyzstan	Other infectious diseases	40.7 (27.7 to 46.8)	22.4 (18.8 to 24.9)	15.3 (13.4 to 17.8)	741.8 (517.8 to 844.8)	401.1 (337.8 to 445.1)	225.7 (196.2 to 260.9)
Kyrgyzstan	Meningitis	19.4 (13.7 to 22.3)	8.05 (7.17 to 9.7)	4.57 (3.87 to 6.13)	348.4 (246.4 to 395.6)	144.1 (128.8 to 173.3)	67.2 (57.3 to 89.9)
Kyrgyzstan	Pneumococcal meningitis	0.921 (0.721 to 1.12)	0.677 (0.522 to 0.845)	0.365 (0.278 to 0.466)	18.6 (14.7 to 22.6)	12.2 (9.5 to 15.2)	5.6 (4.3 to 7.1)
Kyrgyzstan	H influenzae type B meningitis	12.3 (8.41 to 14.2)	4.7 (4.13 to 5.73)	2.4 (2.02 to 3.47)	216.5 (148.9 to 249.1)	83.9 (73.9 to 102.4)	34.7 (29.5 to 49.3)
Kyrgyzstan	Meningococcal infection	1.96 (1.39 to 2.31)	0.853 (0.743 to 1.01)	0.524 (0.429 to 0.687)	35.3 (25.3 to 41.2)	15.3 (13.4 to 18.1)	7.8 (6.4 to 10.1)
Kyrgyzstan	Other meningitis	4.29 (3.1 to 4.96)	1.82 (1.61 to 2.22)	1.28 (1.05 to 1.65)	78 (56.3 to 89.4)	32.7 (29.1 to 39.9)	19 (15.8 to 24.4)

SR Appendix Table 2. All-age DALYs counts and age standardized DALYs rates per 100,000 individuals for all causes and level 1 cause groups by location for 1990, 2007, and 2017, both sexes combined							
		All-age DALYs			Age-standardized rate of DALYs per 100,000		
Kyrgyzstan	Encephalitis	6.04 (3.92 to 7.79)	5.69 (4.23 to 6.4)	4.67 (3.89 to 5.57)	112.7 (73.5 to 141.1)	102.4 (76.4 to 115.2)	68.3 (57.4 to 80.4)
Kyrgyzstan	Diphtheria	0.00884 (0.00361 to 0.0182)	0.000699 (0.000562 to 0.000898)	0.000623 (0.000465 to 0.000875)	0.3 (0.1 to 0.6)	0 (0 to 0)	0 (0 to 0)
Kyrgyzstan	Whooping cough	0.251 (0.112 to 0.42)	0.098 (0.0521 to 0.167)	0.146 (0.0825 to 0.249)	4.2 (1.8 to 6.9)	1.8 (0.9 to 3)	2 (1.1 to 3.4)
Kyrgyzstan	Tetanus	0.0208 (0.00277 to 0.0329)	0.00309 (0.00114 to 0.00498)	0.0111 (0.00129 to 0.0187)	0.5 (0.1 to 0.7)	0.1 (0 to 0.1)	0.2 (0 to 0.3)
Kyrgyzstan	Measles	0.08 (0.0521 to 0.111)	0.0054 (0.00321 to 0.00682)	0.00827 (0.00427 to 0.0116)	1.3 (0.9 to 1.8)	0.1 (0.1 to 0.1)	0.1 (0.1 to 0.2)
Kyrgyzstan	Varicella and herpes zoster	0.122 (0.0755 to 0.184)	0.146 (0.0929 to 0.224)	0.169 (0.104 to 0.254)	3 (1.9 to 4.6)	3.1 (1.9 to 4.6)	3 (1.8 to 4.4)
Kyrgyzstan	Acute hepatitis	8.56 (6.23 to 9.87)	3.56 (3.2 to 4)	2.65 (2.25 to 3.13)	161.5 (118.5 to 183.9)	63.5 (57.2 to 71.7)	39.3 (33.7 to 46.2)
Kyrgyzstan	Acute hepatitis A	2.94 (2.11 to 3.76)	1.22 (1.02 to 1.44)	0.931 (0.735 to 1.16)	51.5 (36.8 to 65)	20.6 (17 to 24.5)	13.2 (10.6 to 16.4)
Kyrgyzstan	Acute hepatitis B	3.13 (2.27 to 3.77)	1.57 (1.37 to 1.81)	1.22 (1.02 to 1.46)	67.8 (51.1 to 78.7)	29.4 (25.8 to 34.2)	19.1 (16 to 22.9)
Kyrgyzstan	Acute hepatitis C	2.09 (1.31 to 2.92)	0.551 (0.383 to 0.758)	0.346 (0.211 to 0.532)	33.9 (21.4 to 47.6)	9.9 (6.9 to 13.6)	4.7 (2.9 to 7.3)
Kyrgyzstan	Acute hepatitis E	0.412 (0.276 to 0.532)	0.221 (0.176 to 0.274)	0.151 (0.121 to 0.186)	8.3 (5.7 to 10.7)	3.7 (3 to 4.5)	2.3 (1.8 to 2.8)
Kyrgyzstan	Other unspecified infectious diseases	6.15 (2.26 to 9.28)	4.89 (2.08 to 6.51)	3.12 (2.18 to 4.26)	110 (44.6 to 162.6)	86.1 (37.2 to 114.3)	45.6 (32.1 to 62.1)
Kyrgyzstan	Maternal and neonatal disorders	239 (209 to 271)	222 (207 to 237)	189 (167 to 210)	4019.7 (3522 to 4546)	3823.6 (3578 to 4080.8)	2655 (2353.3 to 2961.9)
Kyrgyzstan	Maternal disorders	6.2 (5.71 to 6.77)	4.82 (4.52 to 5.11)	3.67 (3.29 to 4.05)	140.6 (129.4 to 153.3)	82.5 (77.4 to 87.2)	54 (48.5 to 59.3)
Kyrgyzstan	Maternal haemorrhage	1.52 (1.2 to 1.88)	0.971 (0.782 to 1.2)	0.696 (0.553 to 0.854)	35.4 (28.3 to 43.4)	17.1 (13.8 to 21.1)	10.5 (8.3 to 12.8)
Kyrgyzstan	Maternal sepsis and other pregnancy related infections	0.735 (0.592 to 0.907)	0.525 (0.423 to 0.641)	0.413 (0.331 to 0.515)	16.4 (13.3 to 20)	8.7 (7 to 10.5)	6 (4.9 to 7.5)
Kyrgyzstan	Maternal hypertensive disorders	0.851 (0.651 to 1.09)	1.09 (0.872 to 1.3)	0.851 (0.688 to 1.05)	18.8 (14.5 to 23.7)	18.4 (14.8 to 22.2)	12.4 (10.1 to 15.2)
Kyrgyzstan	Maternal obstructed labour and uterine rupture	0.121 (0.0764 to 0.185)	0.0971 (0.0625 to 0.144)	0.093 (0.0531 to 0.15)	2.7 (1.7 to 4.1)	1.7 (1.1 to 2.4)	1.3 (0.8 to 2.1)
Kyrgyzstan	Maternal abortive outcome	0.967 (0.659 to 1.31)	0.341 (0.245 to 0.464)	0.24 (0.171 to 0.336)	22.6 (15.9 to 30.4)	5.9 (4.2 to 8)	3.6 (2.6 to 5)
Kyrgyzstan	Ectopic pregnancy	0.101 (0.0316 to 0.276)	0.0679 (0.0223 to 0.161)	0.0524 (0.0194 to 0.134)	2.3 (0.7 to 6)	1.2 (0.4 to 2.8)	0.8 (0.3 to 2)
Kyrgyzstan	Indirect maternal deaths	0.837 (0.679 to 1)	0.917 (0.755 to 1.08)	0.651 (0.528 to 0.789)	18.4 (14.9 to 22)	15.5 (12.7 to 18.1)	9.4 (7.7 to 11.4)
Kyrgyzstan	Late maternal deaths	0.154 (0.111 to 0.208)	0.141 (0.108 to 0.182)	0.136 (0.0996 to 0.181)	3.4 (2.5 to 4.6)	2.4 (1.8 to 3.1)	2 (1.5 to 2.6)
Kyrgyzstan	Maternal deaths aggravated by HIV/AIDS	0.000211 (0.000102 to 0.000377)	0.000747 (0.000343 to 0.0014)	0.00187 (0.000966 to 0.00308)	0 (0 to 0)	0 (0 to 0)	0 (0 to 0)
Kyrgyzstan	Other maternal disorders	0.914 (0.749 to 1.1)	0.671 (0.572 to 0.784)	0.539 (0.445 to 0.64)	20.7 (16.8 to 24.9)	11.6 (9.8 to 13.5)	8 (6.6 to 9.4)
Kyrgyzstan	Neonatal disorders	233 (202 to 265)	217 (203 to 232)	185 (163 to 207)	3879.1 (3378 to 4404)	3741.1 (3495.8 to 3996.1)	2601 (2302.9 to 2907.5)
Kyrgyzstan	Neonatal preterm birth	95.1 (72.1 to 119)	97.7 (89.1 to 106)	84.5 (72.8 to 96.5)	1589.9 (1208.2 to 1980.2)	1683.2 (1535.5 to 1833.3)	1186.6 (1024.1 to 1355.9)
Kyrgyzstan	Neonatal encephalopathy due to birth asphyxia and trauma	101 (78.1 to 129)	89.4 (79.6 to 99.3)	72.8 (61.3 to 85.7)	1681.6 (1301.3 to 2134.7)	1542.9 (1375.3 to 1712.1)	1027.2 (863.9 to 1209.6)
Kyrgyzstan	Neonatal sepsis and other neonatal infections	8.75 (5.3 to 11.5)	7.14 (5.58 to 9.05)	8.39 (6.35 to 11.3)	149.3 (91.1 to 194.7)	123.8 (96.6 to 156.8)	119.4 (90.1 to 160.4)
Kyrgyzstan	Hemolytic disease and other neonatal jaundice	5.04 (3.71 to 6.5)	3.45 (2.95 to 4.05)	2.35 (1.88 to 2.89)	88.3 (66 to 112.7)	60.1 (51.5 to 70.5)	34.4 (27.4 to 42.4)
Kyrgyzstan	Other neonatal disorders	22.5 (17.2 to 29)	19.2 (16.6 to 21.8)	16.8 (12.8 to 21.4)	370 (282.1 to 477)	331.1 (287.2 to 377.4)	233.5 (178 to 298.1)
Kyrgyzstan	Nutritional deficiencies	48.4 (31.9 to 69.5)	43.5 (28.5 to 64)	45.9 (29.5 to 68.8)	996.5 (661 to 1429.6)	797.9 (523.7 to 1162.9)	699.7 (455.8 to 1033)
Kyrgyzstan	Protein-energy malnutrition	1.1 (0.811 to 1.33)	0.595 (0.361 to 0.723)	0.368 (0.223 to 0.465)	23.7 (16.2 to 28.8)	12.1 (6.9 to 14.7)	5.9 (3.5 to 7.5)
Kyrgyzstan	Iodine deficiency	0.991 (0.537 to 1.64)	1.52 (0.835 to 2.53)	1.61 (0.857 to 2.71)	23.3 (12.9 to 38.7)	28.3 (15.8 to 47)	25.7 (13.8 to 43.2)
Kyrgyzstan	Dietary iron deficiency	37.1 (24.2 to 53.7)	34.9 (22.6 to 51.5)	37.2 (23.9 to 55.5)	789.2 (512.6 to 1147.6)	640.4 (415.9 to 937.2)	574 (372.7 to 858.4)
Kyrgyzstan	Other nutritional deficiencies	0.699 (0.538 to 0.968)	0.566 (0.46 to 0.817)	0.401 (0.315 to 0.543)	15.2 (11.4 to 22.5)	11.1 (8.9 to 16.6)	6.5 (5.1 to 8.9)
Kyrgyzstan	Non-communicable diseases	939 (850 to 1030)	1180 (1080 to 1300)	1130 (1010 to 1270)	26910.2 (24560.6 to 29446)	28026.9 (25859.4 to 30501.7)	22193.4 (20101.8 to 24608.8)
Kyrgyzstan	Neoplasms	117 (112 to 123)	105 (103 to 107)	110 (105 to 115)	3486.8 (3337.6 to 3645.5)	2605.7 (2558 to 2655.1)	2185 (2082 to 2285.7)
Kyrgyzstan	Lip and oral cavity cancer	2.36 (2.11 to 2.75)	1.61 (1.5 to 1.72)	1.8 (1.63 to 2.08)	72.5 (64.8 to 84.5)	42.7 (39.8 to 45.6)	36.2 (32.9 to 41.6)
Kyrgyzstan	Nasopharynx cancer	0.394 (0.359 to 0.433)	0.555 (0.513 to 0.598)	0.515 (0.452 to 0.595)	11.2 (10.2 to 12.4)	12 (11.1 to 12.9)	9.3 (8.2 to 10.6)
Kyrgyzstan	Other pharynx cancer	0.631 (0.535 to 0.737)	0.866 (0.792 to 0.939)	0.866 (0.77 to 1.01)	19.3 (16.4 to 22.6)	22.6 (20.6 to 24.7)	17.3 (15.5 to 20.1)

SR Appendix Table 2. All-age DALYs counts and age standardized DALYs rates per 100,000 individuals for all causes and level 1 cause groups by location for 1990, 2007, and 2017, both sexes combined

		All-age DALYs			Age-standardized rate of DALYs per 100,000		
Kyrgyzstan	Oesophageal cancer	5.95 (5.56 to 6.4)	3.73 (3.52 to 3.97)	3.43 (3.18 to 3.7)	186.6 (175.1 to 199.7)	104.6 (98.8 to 111.1)	77.4 (71.8 to 83.3)
Kyrgyzstan	Stomach cancer	24.4 (23 to 25.8)	17.9 (17.3 to 18.7)	16.9 (15.9 to 18)	757.8 (718.7 to 799)	468.3 (451.1 to 485.8)	350.1 (329.4 to 372.5)
Kyrgyzstan	Colon and rectum cancer	7.95 (7.49 to 8.44)	6.39 (6.09 to 6.7)	6.71 (6.18 to 7.64)	245.8 (231.9 to 260.7)	168.5 (160.7 to 176.5)	144 (132.9 to 161.8)
Kyrgyzstan	Liver cancer	5.89 (5.3 to 6.52)	6.94 (6.49 to 7.35)	8.44 (7.56 to 9.45)	180.3 (162.1 to 200.4)	182.8 (171.6 to 193.6)	176.4 (158.5 to 196.5)
Kyrgyzstan	Liver cancer due to hepatitis B	1.69 (1.37 to 2.05)	2.02 (1.69 to 2.37)	2.22 (1.74 to 2.76)	49.2 (39.5 to 59.9)	48.5 (39.6 to 58.1)	42 (33.1 to 52.6)
Kyrgyzstan	Liver cancer due to hepatitis C	2.09 (1.71 to 2.49)	2.41 (2.1 to 2.74)	3.15 (2.63 to 3.71)	65.9 (54.4 to 77.8)	68.3 (59.3 to 77.8)	69.6 (58.8 to 80.8)
Kyrgyzstan	Liver cancer due to alcohol use	1.39 (1.18 to 1.64)	1.67 (1.46 to 1.88)	2 (1.69 to 2.39)	43.8 (37.4 to 51.9)	44.4 (39.1 to 50.4)	42.8 (36.3 to 50.5)
Kyrgyzstan	Liver cancer due to NASH	0.327 (0.275 to 0.39)	0.394 (0.343 to 0.449)	0.522 (0.439 to 0.629)	10.2 (8.5 to 12.1)	10.7 (9.2 to 12.4)	11.4 (9.6 to 13.6)
Kyrgyzstan	Liver cancer due to other causes	0.389 (0.333 to 0.455)	0.444 (0.387 to 0.506)	0.545 (0.458 to 0.65)	11.2 (9.6 to 13.2)	10.8 (9.3 to 12.4)	10.6 (9 to 12.5)
Kyrgyzstan	Gallbladder and biliary tract cancer	0.907 (0.805 to 1.08)	0.919 (0.838 to 0.987)	0.959 (0.857 to 1.09)	29.1 (25.9 to 34.8)	25.4 (23 to 27.3)	21.3 (19 to 23.8)
Kyrgyzstan	Pancreatic cancer	3.11 (2.87 to 3.38)	3.39 (3.19 to 3.57)	4.46 (4.07 to 4.97)	97.8 (90.5 to 106.1)	92 (86.7 to 97.1)	94.4 (86.8 to 104.8)
Kyrgyzstan	Larynx cancer	1.89 (1.72 to 2.07)	1.03 (0.95 to 1.12)	0.935 (0.839 to 1.05)	57.5 (52.3 to 63.2)	28.5 (26.2 to 31.1)	19.1 (17.3 to 21.5)
Kyrgyzstan	Tracheal, bronchus, and lung cancer	20.2 (18.9 to 21.5)	12.2 (11.7 to 12.8)	13 (12.1 to 13.9)	621.4 (582.3 to 661.6)	336.5 (322.4 to 350.8)	272.9 (254.8 to 290.6)
Kyrgyzstan	Malignant skin melanoma	0.721 (0.431 to 0.881)	0.683 (0.544 to 0.951)	0.718 (0.571 to 0.983)	21.4 (12.9 to 26.1)	16.3 (12.8 to 22.3)	13.8 (11 to 18.9)
Kyrgyzstan	Non-melanoma skin cancer	0.671 (0.604 to 0.74)	0.763 (0.718 to 0.812)	0.78 (0.694 to 0.881)	21.6 (19.3 to 23.9)	20.5 (19.3 to 21.8)	17.3 (15.5 to 19.4)
Kyrgyzstan	Non-melanoma skin cancer (squamous-cell carcinoma)	0.671 (0.603 to 0.739)	0.762 (0.717 to 0.812)	0.778 (0.693 to 0.879)	21.6 (19.3 to 23.9)	20.5 (19.3 to 21.8)	17.3 (15.5 to 19.3)
Kyrgyzstan	Non-melanoma skin cancer (basal-cell carcinoma)	0.000499 (0.00021 to 0.00106)	0.00114 (0.000381 to 0.00271)	0.00146 (0.00048 to 0.00332)	0 (0 to 0)	0 (0 to 0.1)	0 (0 to 0.1)
Kyrgyzstan	Breast cancer	7.28 (6.74 to 7.89)	8.11 (7.72 to 8.54)	8.18 (7.46 to 8.96)	226.5 (209.8 to 246)	194.9 (185.6 to 204.6)	157.5 (144.1 to 172.4)
Kyrgyzstan	Cervical cancer	5.73 (5.27 to 6.27)	7.49 (7.01 to 7.94)	7.24 (6.55 to 7.97)	176.4 (162.6 to 192.7)	169.8 (160.4 to 179.3)	133.7 (121.8 to 146.5)
Kyrgyzstan	Uterine cancer	1.1 (0.988 to 1.23)	1.61 (1.47 to 1.75)	1.63 (1.44 to 1.86)	34.4 (30.9 to 38.2)	42 (38.4 to 45.8)	33.1 (29.6 to 37.5)
Kyrgyzstan	Ovarian cancer	2.1 (1.83 to 2.45)	2.76 (2.55 to 2.99)	3.26 (2.89 to 3.7)	64.4 (55.7 to 74.8)	67.2 (62.3 to 72.5)	61.9 (55.1 to 70.2)
Kyrgyzstan	Prostate cancer	1.35 (0.855 to 1.54)	1.5 (1.03 to 1.71)	1.67 (1.13 to 1.92)	45.2 (28.7 to 51.6)	45 (30.4 to 50.6)	43 (28.8 to 49.5)
Kyrgyzstan	Testicular cancer	0.379 (0.268 to 0.49)	0.333 (0.29 to 0.376)	0.281 (0.236 to 0.329)	9.4 (6.6 to 12.1)	6.5 (5.7 to 7.3)	4.7 (4 to 5.4)
Kyrgyzstan	Kidney cancer	0.97 (0.865 to 1.21)	1.98 (1.78 to 2.13)	2.3 (2 to 2.65)	26.9 (24 to 32.9)	49.2 (44.5 to 52.9)	44.2 (38.8 to 50.4)
Kyrgyzstan	Bladder cancer	1.23 (1.07 to 1.42)	1.28 (1.18 to 1.38)	1.32 (1.13 to 1.5)	39.8 (34.6 to 45.8)	36.5 (33.7 to 39.7)	29.6 (25.7 to 33.4)
Kyrgyzstan	Thyroid cancer	0.512 (0.458 to 0.565)	0.452 (0.415 to 0.489)	0.47 (0.422 to 0.535)	15.2 (13.6 to 16.8)	11.3 (10.4 to 12.3)	9.5 (8.6 to 10.7)
Kyrgyzstan	Mesothelioma	0.0719 (0.0633 to 0.0812)	0.124 (0.116 to 0.132)	0.148 (0.131 to 0.175)	2.1 (1.8 to 2.4)	3 (2.8 to 3.2)	2.8 (2.5 to 3.3)
Kyrgyzstan	Hodgkin lymphoma	0.865 (0.526 to 1.04)	0.642 (0.558 to 0.837)	0.535 (0.448 to 0.736)	21.4 (13.2 to 25.5)	13 (11.3 to 17)	9 (7.6 to 12.3)
Kyrgyzstan	Non-Hodgkin's lymphoma	1.98 (1.8 to 2.18)	1.94 (1.83 to 2.06)	2.03 (1.82 to 2.25)	51.8 (47.6 to 56.6)	42.7 (40.4 to 45.4)	37.3 (33.7 to 41)
Kyrgyzstan	Multiple myeloma	0.368 (0.286 to 0.412)	0.434 (0.35 to 0.482)	0.531 (0.424 to 0.604)	11.7 (8.9 to 13.1)	11.8 (9.1 to 13)	11.2 (8.7 to 12.7)
Kyrgyzstan	Leukaemia	8.28 (7.62 to 8.95)	6.6 (6.17 to 7.06)	6.5 (5.68 to 7.47)	180.1 (167.1 to 192.5)	126.7 (119.5 to 135.1)	106.5 (94.1 to 121)
Kyrgyzstan	Acute lymphoid leukaemia	1.77 (1.48 to 2.14)	1.73 (1.54 to 1.95)	1.84 (1.58 to 2.17)	34.2 (29 to 40.8)	30 (26.9 to 33.7)	28 (24.3 to 32.9)
Kyrgyzstan	Chronic lymphoid leukaemia	0.321 (0.284 to 0.356)	0.241 (0.216 to 0.268)	0.211 (0.178 to 0.255)	9.4 (8.3 to 10.5)	5.8 (5.2 to 6.5)	4.3 (3.7 to 5.1)
Kyrgyzstan	Acute myeloid leukaemia	1.48 (1.16 to 1.88)	1.62 (1.48 to 1.78)	2.07 (1.68 to 2.49)	32.7 (26.5 to 40.7)	30.9 (28.3 to 33.7)	33.6 (27.3 to 39.9)
Kyrgyzstan	Chronic myeloid leukaemia	0.38 (0.328 to 0.438)	0.428 (0.383 to 0.475)	0.263 (0.226 to 0.308)	10.7 (9.2 to 12.4)	9.7 (8.7 to 10.7)	4.8 (4.2 to 5.6)
Kyrgyzstan	Other leukaemia	4.34 (3.71 to 4.88)	2.58 (2.37 to 2.84)	2.11 (1.82 to 2.51)	93.1 (81 to 103.4)	50.3 (46.6 to 54.8)	35.7 (31.3 to 41.8)
Kyrgyzstan	Other malignant cancers	6.48 (4.64 to 7.41)	6.69 (6.08 to 7.14)	7.36 (6.09 to 8.17)	170.5 (124.8 to 193)	149.1 (131.5 to 159.3)	134.1 (109.7 to 148.7)
Kyrgyzstan	Other neoplasms	1.17 (0.703 to 1.55)	1.41 (0.913 to 1.72)	1.56 (1.06 to 2.03)	29.1 (18.7 to 37)	29.8 (20.2 to 36.2)	27.4 (19 to 36.7)
Kyrgyzstan	Myelodysplastic, myeloproliferative, and other hematopoietic neoplasms	0.821 (0.531 to 1.05)	1.01 (0.669 to 1.22)	1.15 (0.797 to 1.54)	22.2 (15 to 28.2)	22.7 (15.8 to 28.5)	21.3 (15.2 to 29.4)
Kyrgyzstan	Benign and in situ intestinal neoplasms	0 (0 to 0)	0 (0 to 0)	0 (0 to 0)	0 (0 to 0)	0 (0 to 0)	0 (0 to 0)

SR Appendix Table 2. All-age DALYs counts and age standardized DALYs rates per 100,000 individuals for all causes and level 1 cause groups by location for 1990, 2007, and 2017, both sexes combined

		All-age DALYs			Age-standardized rate of DALYs per 100,000		
Kyrgyzstan	Benign and in situ cervical and uterine neoplasms	0 (0 to 0)	0 (0 to 0)	0 (0 to 0)	0 (0 to 0)	0 (0 to 0)	0 (0 to 0)
Kyrgyzstan	Other benign and in situ neoplasms	0.346 (0.169 to 0.534)	0.407 (0.235 to 0.535)	0.413 (0.258 to 0.534)	7 (3.5 to 10.6)	7.1 (4.2 to 9.3)	6.1 (3.8 to 7.8)
Kyrgyzstan	Cardiovascular diseases	292 (280 to 304)	396 (389 to 403)	348 (335 to 361)	9464.1 (9082.2 to 9840.5)	10869.1 (10691.3 to 11049.7)	8066.4 (7807 to 8347.2)
Kyrgyzstan	Rheumatic heart disease	15.2 (14.3 to 16.4)	13 (12.1 to 14)	9.01 (7.85 to 10.3)	407.6 (381.8 to 437.1)	265.3 (249 to 283.9)	151.1 (132.6 to 171)
Kyrgyzstan	Ischaemic heart disease	136 (130 to 142)	206 (202 to 211)	193 (185 to 203)	4524.3 (4328.8 to 4719.7)	5827.1 (5732.6 to 5948.1)	4718.9 (4546 to 4922.2)
Kyrgyzstan	Stroke	117 (111 to 123)	143 (139 to 148)	111 (105 to 117)	3799.9 (3619.4 to 3990.9)	3953.3 (3842.5 to 4070.3)	2490.2 (2362.9 to 2623.1)
Kyrgyzstan	Ischaemic stroke	42.5 (39 to 45.9)	45.7 (43.3 to 48.4)	37.4 (34.5 to 40.3)	1445.6 (1324.9 to 1559.7)	1333.2 (1262.6 to 1407.8)	928 (862.6 to 997.8)
Kyrgyzstan	Intracerebral hemorrhage	61.9 (58 to 66.2)	80.3 (77.4 to 84.7)	58.7 (54.7 to 64.8)	1978 (1855.4 to 2115.4)	2187.2 (2110.3 to 2301.6)	1269.7 (1189.4 to 1384.1)
Kyrgyzstan	Subarachnoid hemorrhage	12.2 (9.3 to 13.6)	17.2 (13.2 to 18.5)	14.7 (12.3 to 16.1)	376.3 (285.9 to 420.4)	432.9 (324.8 to 465.6)	292.5 (242.3 to 319.3)
Kyrgyzstan	Hypertensive heart disease	7.52 (5.93 to 9.04)	8.75 (7.41 to 10.8)	9.35 (7.55 to 11.5)	246.2 (198.1 to 299.8)	242.6 (206.7 to 299.8)	212.9 (173.1 to 263.5)
Kyrgyzstan	Non-rheumatic valvular heart disease	0.545 (0.38 to 0.631)	0.557 (0.483 to 0.644)	0.52 (0.431 to 0.651)	16.3 (11.9 to 19)	13.5 (11.7 to 15.9)	11.2 (9 to 14.2)
Kyrgyzstan	Non-rheumatic calcific aortic valve disease	0.208 (0.16 to 0.244)	0.261 (0.22 to 0.309)	0.272 (0.224 to 0.333)	6.5 (5.1 to 7.7)	6.7 (5.7 to 8.1)	6 (4.8 to 7.4)
Kyrgyzstan	Non-rheumatic degenerative mitral valve disease	0.291 (0.177 to 0.354)	0.241 (0.198 to 0.287)	0.201 (0.155 to 0.271)	8.5 (5.6 to 10.3)	5.7 (4.6 to 6.9)	4.4 (3.3 to 5.8)
Kyrgyzstan	Other non-rheumatic valve diseases	0.0472 (0.0259 to 0.063)	0.0543 (0.0384 to 0.0736)	0.047 (0.035 to 0.0715)	1.3 (0.7 to 1.7)	1.1 (0.8 to 1.5)	0.9 (0.6 to 1.3)
Kyrgyzstan	Cardiomyopathy and myocarditis	6.87 (5.49 to 7.92)	13.8 (8.3 to 15.3)	14.2 (8.37 to 16.8)	198.2 (154 to 231.1)	301 (176 to 332.6)	245.1 (144 to 287.1)
Kyrgyzstan	Myocarditis	0.751 (0.478 to 0.979)	1.05 (0.668 to 1.31)	0.977 (0.647 to 1.25)	18.4 (11.1 to 25.1)	19.8 (12.3 to 24.8)	15.7 (10.2 to 20.2)
Kyrgyzstan	Alcoholic cardiomyopathy	5.57 (4.26 to 6.46)	11.9 (6.85 to 13.4)	12.3 (6.94 to 14.5)	166.1 (124.6 to 194.2)	264.5 (149.6 to 298.1)	214.3 (120.8 to 251.1)
Kyrgyzstan	Other cardiomyopathy	0.549 (0.462 to 0.669)	0.865 (0.672 to 0.974)	0.91 (0.653 to 1.09)	13.6 (11.5 to 16.3)	16.7 (13.1 to 18.8)	15.2 (11.1 to 18)
Kyrgyzstan	Atrial fibrillation and flutter	2.15 (1.78 to 2.58)	2.46 (2.03 to 2.99)	2.77 (2.26 to 3.41)	77.4 (64.5 to 92.3)	75.3 (62.6 to 90.7)	72.4 (59.6 to 88.3)
Kyrgyzstan	Aortic aneurysm	0.592 (0.541 to 0.687)	0.763 (0.709 to 0.816)	0.766 (0.687 to 0.876)	18.5 (16.8 to 21.4)	19.5 (18.1 to 20.8)	16.2 (14.6 to 18.5)
Kyrgyzstan	Peripheral vascular disease	0.353 (0.204 to 0.572)	0.418 (0.258 to 0.614)	0.52 (0.343 to 0.769)	12.1 (7.1 to 19.2)	12.6 (7.9 to 18.4)	12.9 (8.4 to 19.1)
Kyrgyzstan	Endocarditis	0.422 (0.328 to 0.502)	0.557 (0.481 to 0.642)	0.482 (0.402 to 0.561)	10.5 (8.1 to 12.5)	11.4 (9.9 to 13.1)	8.4 (7 to 9.8)
Kyrgyzstan	Other cardiovascular and circulatory diseases	5.35 (4.83 to 5.98)	6.38 (5.81 to 7.01)	6.74 (5.87 to 7.71)	153.1 (137.6 to 171.2)	147.3 (134.2 to 161.8)	127 (111 to 144.6)
Kyrgyzstan	Chronic respiratory diseases	78.8 (74 to 83.6)	67 (63 to 71.4)	48.8 (44.1 to 54.2)	2466.2 (2324.6 to 2601.1)	1763.4 (1671.6 to 1857.5)	1050.2 (966.6 to 1145.8)
Kyrgyzstan	Chronic obstructive pulmonary disease	64.9 (61.4 to 68.1)	55.1 (52.8 to 57.2)	35.4 (33.1 to 37.8)	2094.5 (1984 to 2195.6)	1509.1 (1448.8 to 1565.9)	818.9 (768.1 to 872.6)
Kyrgyzstan	Pneumoconiosis	0.0203 (0.0158 to 0.0255)	0.0214 (0.0162 to 0.0276)	0.0254 (0.0186 to 0.0335)	0.6 (0.5 to 0.8)	0.6 (0.4 to 0.7)	0.6 (0.4 to 0.7)
Kyrgyzstan	Silicosis	0.00277 (0.00168 to 0.00412)	0.00224 (0.00161 to 0.00304)	0.00228 (0.00141 to 0.00335)	0.1 (0.1 to 0.1)	0.1 (0 to 0.1)	0 (0 to 0.1)
Kyrgyzstan	Asbestosis	0.00637 (0.00487 to 0.00827)	0.00664 (0.00513 to 0.0084)	0.00752 (0.00566 to 0.00981)	0.2 (0.1 to 0.2)	0.2 (0.1 to 0.2)	0.1 (0.1 to 0.2)
Kyrgyzstan	Coal workers pneumoconiosis	0.00306 (0.00188 to 0.00472)	0.0038 (0.00231 to 0.0058)	0.00513 (0.00297 to 0.00811)	0.1 (0.1 to 0.1)	0.1 (0.1 to 0.1)	0.1 (0.1 to 0.2)
Kyrgyzstan	Other pneumoconiosis	0.00807 (0.00571 to 0.0111)	0.00868 (0.006 to 0.0122)	0.0105 (0.00706 to 0.0146)	0.3 (0.2 to 0.4)	0.2 (0.2 to 0.3)	0.3 (0.2 to 0.4)
Kyrgyzstan	Asthma	10.8 (8.34 to 13.8)	9.04 (6.83 to 11.8)	10.4 (7.32 to 14.4)	284.3 (222.8 to 353.9)	192.2 (149.1 to 246.2)	178.8 (128.2 to 242)
Kyrgyzstan	Interstitial lung disease and pulmonary sarcoidosis	1.45 (0.943 to 1.9)	1.1 (0.855 to 1.4)	1.23 (0.896 to 1.63)	45 (29.2 to 58.8)	27.2 (21.2 to 34.5)	24.3 (17.8 to 32.1)
Kyrgyzstan	Other chronic respiratory diseases	1.62 (1.43 to 1.83)	1.8 (1.55 to 2.03)	1.76 (1.46 to 2.07)	41.8 (36.9 to 46.6)	34.4 (29.9 to 38.6)	27.7 (23.1 to 32.5)
Kyrgyzstan	Digestive diseases	62.7 (57.5 to 68.8)	111 (104 to 119)	111 (97.3 to 124)	1731.4 (1598.5 to 1886.7)	2467.2 (2312.8 to 2626.3)	1976.6 (1743.1 to 2179.6)
Kyrgyzstan	Cirrhosis and other chronic liver diseases	34.7 (32.9 to 36.6)	80 (77.1 to 83.7)	76.8 (67.6 to 83.8)	1018.1 (964.5 to 1075.1)	1810.1 (1746.3 to 1888.9)	1386.6 (1232.1 to 1506)
Kyrgyzstan	Cirrhosis and other chronic liver diseases due to hepatitis B	8.68 (7.65 to 9.97)	21.1 (18.3 to 24.1)	17.9 (15 to 21.1)	255.5 (224.5 to 292.6)	471.2 (409.6 to 535.5)	320.2 (268.6 to 374)
Kyrgyzstan	Cirrhosis and other chronic liver diseases due to hepatitis C	7.82 (6.85 to 9.04)	18.9 (16.1 to 21.9)	19 (15.6 to 22.5)	236.7 (206 to 272.6)	432.6 (371.2 to 498.4)	344.8 (284.9 to 404.4)
Kyrgyzstan	Cirrhosis and other chronic liver diseases due to alcohol use	11.3 (10.1 to 12.5)	26.8 (23.7 to 29.9)	26.9 (22.5 to 30.7)	345.6 (308.3 to 383.2)	623.9 (553.8 to 694.7)	491.6 (415.2 to 557.1)
Kyrgyzstan	Cirrhosis due to NASH	2.17 (1.91 to 2.44)	5.03 (4.39 to 5.66)	5.6 (4.64 to 6.5)	66.1 (58.2 to 74.8)	119.3 (104.5 to 133.8)	103 (85.9 to 118.9)
Kyrgyzstan	Cirrhosis and other chronic liver diseases due to other causes	4.76 (4.22 to 5.39)	8.14 (7.2 to 9.23)	7.33 (6.11 to 8.57)	114.3 (100.6 to 130.2)	163 (142.3 to 186.1)	126.9 (106.8 to 148.6)

SR Appendix Table 2. All-age DALYs counts and age standardized DALYs rates per 100,000 individuals for all causes and level 1 cause groups by location for 1990, 2007, and 2017, both sexes combined							
		All-age DALYs			Age-standardized rate of DALYs per 100,000		
Kyrgyzstan	Upper digestive system diseases	11 (8.54 to 14.4)	12.2 (9.06 to 16.8)	13.2 (9.21 to 18.8)	300.4 (237.4 to 387.8)	258.4 (194.4 to 348.6)	226.9 (160.1 to 321.2)
Kyrgyzstan	Peptic ulcer disease	4.86 (4.47 to 5.35)	4.25 (3.9 to 4.63)	3.37 (2.88 to 3.86)	143.5 (131.9 to 157.2)	97.8 (90 to 106)	62.9 (54.6 to 71.7)
Kyrgyzstan	Gastritis and duodenitis	2.75 (1.85 to 3.93)	3.58 (2.34 to 5.19)	4.45 (2.86 to 6.42)	65.1 (43.8 to 92.2)	68.5 (44.7 to 98.7)	71.8 (46.7 to 103)
Kyrgyzstan	GERD	3.36 (1.8 to 5.79)	4.41 (2.33 to 7.51)	5.38 (2.84 to 9.15)	91.8 (48.9 to 156.4)	92.1 (48.7 to 155.3)	92.1 (49.1 to 157.3)
Kyrgyzstan	Appendicitis	1.21 (1.07 to 1.35)	0.671 (0.567 to 0.799)	0.654 (0.532 to 0.808)	27.4 (24.6 to 30.3)	12.4 (10.6 to 14.4)	10.5 (8.6 to 12.9)
Kyrgyzstan	Paralytic ileus and intestinal obstruction	3.1 (2.33 to 3.75)	2.54 (2.13 to 2.87)	2.34 (1.7 to 2.81)	66.6 (47.9 to 80.4)	50.4 (41.5 to 56.8)	38.3 (27.9 to 45.5)
Kyrgyzstan	Inguinal, femoral, and abdominal hernia	6.31 (4.42 to 8.64)	8.79 (6.02 to 12)	10.6 (7.21 to 14.5)	146.6 (102.9 to 200.7)	177.8 (122.5 to 241.3)	173.2 (117.5 to 235.9)
Kyrgyzstan	Inflammatory bowel disease	1.08 (0.568 to 1.41)	0.748 (0.583 to 0.857)	0.809 (0.618 to 0.982)	25.7 (15.4 to 32.1)	15.5 (12.6 to 17.6)	13.5 (10.7 to 16.2)
Kyrgyzstan	Vascular intestinal disorders	0.431 (0.391 to 0.478)	0.407 (0.373 to 0.449)	0.424 (0.339 to 0.506)	13.6 (12.4 to 15)	10.5 (9.6 to 11.7)	9.2 (7.5 to 10.8)
Kyrgyzstan	Gallbladder and biliary diseases	1.33 (1.21 to 1.46)	1.26 (1.17 to 1.39)	1.07 (0.94 to 1.24)	40.5 (36.4 to 44.4)	31.3 (29 to 35.4)	22.3 (19.7 to 25.9)
Kyrgyzstan	Pancreatitis	1.83 (1.59 to 2.1)	3.06 (2.77 to 3.37)	3.39 (2.59 to 4)	52.9 (45.9 to 61)	65.2 (59.1 to 71.8)	59.4 (45.6 to 69.5)
Kyrgyzstan	Other digestive diseases	1.77 (1.35 to 2.25)	1.72 (1.39 to 2.1)	2.2 (1.73 to 2.76)	39.5 (30.4 to 48.7)	35.7 (29.1 to 43.2)	36.7 (29.2 to 45.6)
Kyrgyzstan	Neurological disorders	60.3 (45.7 to 77.3)	78.6 (60.9 to 99.9)	81.1 (59.5 to 107)	1627.1 (1275.8 to 2041.8)	1630.4 (1290.5 to 2039.8)	1465.6 (1113.5 to 1877.6)
Kyrgyzstan	Alzheimer's disease and other dementias	11.2 (10 to 12.3)	11.7 (10.9 to 12.7)	11.7 (10.6 to 12.8)	438.3 (389.8 to 480.4)	379.9 (351.9 to 410.5)	339.8 (308.1 to 371.8)
Kyrgyzstan	Parkinson's disease	1.9 (1.57 to 2.21)	2.31 (1.94 to 2.56)	2.2 (1.89 to 2.53)	68.6 (56.7 to 79.8)	68.5 (57.7 to 76)	61.7 (52.6 to 70.4)
Kyrgyzstan	Epilepsy	15.7 (9.77 to 22.9)	22.6 (16.7 to 30.5)	18.4 (11.1 to 28.8)	347.1 (215.5 to 507.8)	401.4 (294 to 543.5)	285.4 (171.3 to 448.5)
Kyrgyzstan	Multiple sclerosis	0.419 (0.307 to 0.518)	0.517 (0.408 to 0.636)	0.617 (0.475 to 0.777)	12.2 (9 to 15)	11.2 (8.9 to 13.8)	10.7 (8.2 to 13.4)
Kyrgyzstan	Motor neuron disease	0.0954 (0.0826 to 0.111)	0.141 (0.129 to 0.151)	0.156 (0.14 to 0.178)	2.7 (2.4 to 3.2)	3.2 (2.9 to 3.4)	2.6 (2.4 to 3)
Kyrgyzstan	Headache disorders	28.9 (18.7 to 41.5)	38.5 (25 to 55.3)	44.8 (29.3 to 64.8)	714.7 (464.7 to 1025.7)	716.3 (466.7 to 1029.7)	716.9 (469 to 1029.5)
Kyrgyzstan	Migraine	25.1 (15.7 to 37.3)	33.5 (20.8 to 50)	39 (24.3 to 58.1)	620.1 (389.7 to 921)	621.5 (388.4 to 921.5)	622.1 (388.3 to 925)
Kyrgyzstan	Tension-type headache	3.8 (2.13 to 6.14)	4.98 (2.76 to 7.92)	5.81 (3.28 to 9.3)	94.6 (53.7 to 152.1)	94.8 (53.2 to 150.2)	94.8 (53.6 to 150.9)
Kyrgyzstan	Other neurological disorders	2.03 (1.02 to 3.27)	2.82 (1.76 to 4.26)	3.12 (1.62 to 5.32)	43.4 (22.8 to 69)	49.9 (31.8 to 74.3)	48.4 (25.8 to 81.2)
Kyrgyzstan	Mental disorders	55.2 (40.6 to 71.5)	70.9 (52.2 to 91.7)	81.1 (59.9 to 105)	1358.5 (1003.7 to 1755.1)	1339.6 (991.9 to 1726.8)	1328 (984.8 to 1716.6)
Kyrgyzstan	Schizophrenia	4.22 (3.13 to 5.29)	5.48 (4.03 to 6.88)	6.86 (5.08 to 8.7)	113.2 (84.1 to 141)	110.4 (81.9 to 137.3)	111.8 (83.3 to 140.2)
Kyrgyzstan	Depressive disorders	19.4 (13.7 to 26.4)	24.5 (17.1 to 33.2)	28.1 (19.9 to 38.4)	511.6 (359.1 to 694.4)	493.8 (346.3 to 659.4)	482 (342.2 to 654.1)
Kyrgyzstan	Major depressive disorder	15.4 (10.6 to 21.1)	19.3 (13.4 to 26.1)	21.8 (15.2 to 29.9)	403.9 (281.3 to 548.2)	386.1 (267.7 to 520.5)	374.3 (261.8 to 513)
Kyrgyzstan	Dysthymia	4.01 (2.67 to 5.83)	5.19 (3.44 to 7.58)	6.29 (4.17 to 9.22)	107.7 (71.2 to 157.9)	107.7 (71.4 to 156.7)	107.7 (72 to 156.5)
Kyrgyzstan	Bipolar disorder	5.27 (3.27 to 7.76)	7.12 (4.44 to 10.6)	8.17 (5.07 to 12.2)	130.1 (81.4 to 192.8)	130.6 (81.6 to 193.7)	130.6 (80.7 to 195.4)
Kyrgyzstan	Anxiety disorders	9.69 (6.86 to 13.1)	12.6 (8.96 to 16.8)	14.5 (10.2 to 19.4)	235.3 (167 to 315.7)	235 (168 to 311.9)	235.1 (166.8 to 312.9)
Kyrgyzstan	Eating disorders	1.35 (0.856 to 2)	1.49 (0.947 to 2.18)	1.8 (1.11 to 2.67)	30.2 (19.3 to 44.2)	24.6 (15.8 to 35.9)	26.7 (16.6 to 39.3)
Kyrgyzstan	Anorexia nervosa	0.298 (0.18 to 0.453)	0.343 (0.208 to 0.528)	0.386 (0.232 to 0.587)	6.4 (4 to 9.7)	5.4 (3.3 to 8.2)	5.7 (3.4 to 8.7)
Kyrgyzstan	Bulimia nervosa	1.05 (0.644 to 1.58)	1.14 (0.709 to 1.73)	1.42 (0.867 to 2.16)	23.8 (14.7 to 35.6)	19.2 (12 to 29.2)	21 (12.8 to 31.7)
Kyrgyzstan	Autism spectrum disorders	2.94 (1.99 to 4.05)	3.55 (2.39 to 4.93)	4.18 (2.82 to 5.76)	63.3 (42.9 to 87.2)	64 (43 to 88.5)	64.1 (43.2 to 88)
Kyrgyzstan	Attention-deficit/hyperactivity disorder	0.566 (0.335 to 0.919)	0.711 (0.428 to 1.13)	0.782 (0.468 to 1.27)	11.6 (6.9 to 18.7)	11.7 (7 to 18.5)	11.7 (7 to 18.9)
Kyrgyzstan	Conduct disorder	5.18 (3.14 to 8.22)	6.57 (3.92 to 10.4)	6.33 (3.81 to 10.1)	97.1 (58.7 to 153.8)	97.4 (58.1 to 154.6)	97.5 (58.8 to 155.1)
Kyrgyzstan	Idiopathic developmental intellectual disability	1.33 (0.448 to 2.51)	1.87 (0.657 to 3.46)	1.94 (0.651 to 3.65)	27.9 (9.3 to 52.8)	33 (11.5 to 61)	29.2 (9.7 to 55.1)
Kyrgyzstan	Other mental disorders	5.24 (3.47 to 7.23)	6.96 (4.58 to 9.74)	8.45 (5.56 to 11.7)	138.2 (91.8 to 191)	139.1 (92.3 to 193.3)	139.3 (91.9 to 192)
Kyrgyzstan	Substance use disorders	31.2 (26.2 to 37.1)	49.5 (42.5 to 57.3)	44.8 (37.4 to 53.7)	826.8 (699.3 to 975.4)	994.9 (866.6 to 1145.7)	727.6 (611.3 to 867.8)
Kyrgyzstan	Alcohol use disorders	24.7 (20.9 to 29.7)	35 (30.1 to 41)	31.7 (26.3 to 38.6)	665.8 (563.3 to 789.3)	718.1 (622.3 to 833.3)	519.4 (433.5 to 628.6)
Kyrgyzstan	Drug use disorders	6.49 (5.15 to 7.96)	14.5 (12.3 to 16.8)	13.1 (10.8 to 15.8)	161 (129.1 to 195.1)	276.8 (236.1 to 320)	208.2 (172 to 247.1)

SR Appendix Table 2. All-age DALYs counts and age standardized DALYs rates per 100,000 individuals for all causes and level 1 cause groups by location for 1990, 2007, and 2017, both sexes combined

		All-age DALYs			Age-standardized rate of DALYs per 100,000		
Kyrgyzstan	Opioid use disorders	4.6 (3.61 to 5.7)	10.9 (9.17 to 12.7)	9.87 (7.99 to 11.9)	116.6 (92.1 to 142.4)	212.2 (179.9 to 245)	158.2 (130.4 to 188.3)
Kyrgyzstan	Cocaine use disorders	0.267 (0.193 to 0.359)	0.562 (0.454 to 0.685)	0.47 (0.356 to 0.62)	6.7 (4.9 to 8.9)	10.8 (8.9 to 13)	7.4 (5.6 to 9.7)
Kyrgyzstan	Amphetamine use disorders	0.559 (0.327 to 0.85)	0.875 (0.567 to 1.33)	0.906 (0.547 to 1.44)	12.4 (7.4 to 18.7)	14.9 (9.8 to 22.2)	13.2 (8 to 20.7)
Kyrgyzstan	Cannabis use disorders	0.209 (0.128 to 0.314)	0.288 (0.178 to 0.435)	0.311 (0.19 to 0.473)	4.6 (2.9 to 6.9)	4.6 (2.9 to 6.9)	4.6 (2.9 to 7.1)
Kyrgyzstan	Other drug use disorders	0.853 (0.639 to 1.13)	1.84 (1.52 to 2.26)	1.58 (1.24 to 2.02)	20.7 (16.1 to 26.9)	34.3 (28.8 to 41.1)	24.7 (19.5 to 31)
Kyrgyzstan	Diabetes and kidney diseases	36.8 (30.8 to 44.3)	56.6 (49 to 66.2)	54.5 (44.8 to 66.2)	1065.8 (879.7 to 1294.2)	1306.1 (1121.1 to 1537.4)	1036.5 (853.3 to 1257.2)
Kyrgyzstan	Diabetes mellitus	22.2 (16.9 to 28.5)	28.4 (21.7 to 36.9)	33.3 (24.8 to 43.7)	677.2 (517.4 to 872.7)	704.3 (544.7 to 911.4)	651.7 (486.2 to 853.4)
Kyrgyzstan	Diabetes mellitus type 1	3.94 (3.46 to 4.4)	4.14 (3.82 to 4.66)	3.82 (3.42 to 4.33)	111.6 (96.9 to 125.6)	96 (88.1 to 110.4)	70.5 (63.3 to 80.2)
Kyrgyzstan	Diabetes mellitus type 2	18.2 (13.2 to 24.5)	24.2 (17.9 to 32.4)	29.5 (21.2 to 39.6)	565.6 (411.8 to 758.5)	608.4 (453.1 to 809.8)	581.2 (419.3 to 776.4)
Kyrgyzstan	Chronic kidney disease	13.9 (12.8 to 15.1)	27.8 (26.4 to 29.3)	20.9 (19.3 to 22.7)	371.8 (338.7 to 405.6)	593.5 (562.3 to 626.7)	380.2 (351.5 to 414.8)
Kyrgyzstan	Chronic kidney disease due to diabetes mellitus type 1	1.04 (0.828 to 1.29)	2.72 (2.12 to 3.35)	1.86 (1.46 to 2.3)	29.8 (23.3 to 36.6)	55.6 (43.7 to 67.8)	31.5 (24.9 to 38.9)
Kyrgyzstan	Chronic kidney disease due to diabetes mellitus type 2	1.53 (1.28 to 1.8)	2.89 (2.41 to 3.45)	2.69 (2.23 to 3.24)	48.3 (40.8 to 56.6)	77.6 (65 to 92)	56.9 (47.7 to 67.1)
Kyrgyzstan	Chronic kidney disease due to hypertension	1.14 (0.947 to 1.32)	2.19 (1.85 to 2.52)	1.86 (1.57 to 2.2)	34.4 (28.8 to 39.9)	52.9 (44.7 to 61.1)	38 (32.1 to 44.7)
Kyrgyzstan	Chronic kidney disease due to glomerulonephritis	4.18 (3.66 to 4.72)	9.13 (7.93 to 10.3)	6.31 (5.5 to 7.2)	104.7 (91.7 to 118.6)	181.1 (158.9 to 204.1)	105.8 (92.5 to 121)
Kyrgyzstan	Chronic kidney disease due to other and unspecified causes	6.03 (5.24 to 6.93)	10.9 (9.65 to 12.2)	8.19 (7.16 to 9.41)	154.6 (134.1 to 179.4)	226.3 (200.2 to 257.2)	148 (128.9 to 170.8)
Kyrgyzstan	Acute glomerulonephritis	0.733 (0.558 to 0.935)	0.436 (0.388 to 0.496)	0.297 (0.254 to 0.347)	16.8 (12.9 to 21.2)	8.3 (7.4 to 9.4)	4.7 (4.1 to 5.5)
Kyrgyzstan	Skin and subcutaneous diseases	20.5 (13.7 to 30.2)	24.3 (16.2 to 35.3)	28.8 (19.2 to 42.2)	448.5 (299.6 to 657.2)	450.6 (301.1 to 649.7)	452.1 (302.2 to 663.5)
Kyrgyzstan	Dermatitis	5.15 (2.94 to 8.25)	5.9 (3.41 to 9.4)	7.14 (4.18 to 11.5)	107.3 (62.6 to 168.5)	107.5 (62.3 to 170.2)	108.9 (63.9 to 173.8)
Kyrgyzstan	Atopic dermatitis	4.22 (2.27 to 7.06)	4.68 (2.51 to 7.83)	5.74 (3.06 to 9.7)	84.2 (45.5 to 140.3)	84.4 (45.2 to 141.9)	85.8 (45.9 to 144.8)
Kyrgyzstan	Contact dermatitis	0.93 (0.607 to 1.38)	1.21 (0.778 to 1.78)	1.39 (0.901 to 2.07)	23 (15.1 to 33.8)	23.1 (14.9 to 33.8)	23.1 (15 to 33.9)
Kyrgyzstan	Seborrhoeic dermatitis	0.0047 (0.00264 to 0.00756)	0.00562 (0.00313 to 0.00906)	0.00671 (0.00376 to 0.0107)	0.1 (0.1 to 0.2)	0.1 (0.1 to 0.2)	0.1 (0.1 to 0.2)
Kyrgyzstan	Psoriasis	2.41 (1.71 to 3.18)	2.88 (2.01 to 3.82)	3.46 (2.42 to 4.61)	62.1 (43.8 to 81.3)	57.9 (40.8 to 76.8)	57.9 (40.5 to 77)
Kyrgyzstan	Bacterial skin diseases	0.149 (0.0915 to 0.3)	0.535 (0.162 to 0.757)	0.567 (0.191 to 0.806)	3.5 (2.1 to 7.4)	10.8 (3.4 to 15)	9.4 (3.3 to 13.2)
Kyrgyzstan	Cellulitis	0.0836 (0.0535 to 0.167)	0.266 (0.0856 to 0.383)	0.297 (0.0992 to 0.43)	2 (1.3 to 4.1)	5.4 (1.8 to 7.7)	5 (1.7 to 7.1)
Kyrgyzstan	Pyoderma	0.0652 (0.0351 to 0.149)	0.269 (0.0667 to 0.399)	0.27 (0.0777 to 0.394)	1.5 (0.8 to 3.6)	5.4 (1.4 to 7.9)	4.4 (1.3 to 6.4)
Kyrgyzstan	Scabies	1.54 (0.848 to 2.5)	1.82 (1 to 3)	2 (1.12 to 3.28)	32.1 (17.9 to 52.2)	31.2 (17.5 to 51.4)	30.2 (17 to 49.5)
Kyrgyzstan	Fungal skin diseases	2.03 (0.798 to 4.22)	2.33 (0.935 to 4.85)	2.69 (1.07 to 5.6)	49.7 (20.1 to 103.9)	48.5 (19.4 to 100.5)	47.3 (19 to 97.8)
Kyrgyzstan	Viral skin diseases	2.47 (1.59 to 3.68)	2.63 (1.67 to 3.93)	3.19 (2.04 to 4.82)	45.6 (29.3 to 67.8)	45.7 (29.1 to 68.2)	45.8 (29.3 to 69)
Kyrgyzstan	Acne vulgaris	1.2 (0.697 to 1.99)	1.86 (1.08 to 3.12)	1.97 (1.13 to 3.27)	24.7 (14.5 to 40.9)	27.7 (16.2 to 46.7)	29.6 (16.9 to 49.1)
Kyrgyzstan	Alopecia areata	0.247 (0.156 to 0.373)	0.317 (0.202 to 0.477)	0.379 (0.239 to 0.565)	5.9 (3.7 to 8.9)	5.9 (3.8 to 8.9)	5.9 (3.8 to 8.8)
Kyrgyzstan	Pruritus	0.37 (0.174 to 0.698)	0.445 (0.213 to 0.846)	0.539 (0.257 to 1.01)	9.3 (4.3 to 17.4)	9 (4.2 to 17)	9.1 (4.3 to 17)
Kyrgyzstan	Urticaria	3.67 (2.4 to 5.23)	4.14 (2.76 to 5.92)	5 (3.31 to 7.2)	74.4 (49.2 to 105.5)	74.6 (49.6 to 105.9)	74.7 (49.3 to 107.2)
Kyrgyzstan	Decubitus ulcer	0.0746 (0.0526 to 0.101)	0.11 (0.074 to 0.146)	0.131 (0.0896 to 0.174)	2.2 (1.5 to 3)	2.5 (1.7 to 3.4)	2.6 (1.8 to 3.5)
Kyrgyzstan	Other skin and subcutaneous diseases	1.22 (0.587 to 2.27)	1.38 (0.668 to 2.51)	1.73 (0.828 to 3.15)	31.7 (15.3 to 59.1)	29.3 (14.2 to 53.1)	30.5 (14.7 to 55.6)
Kyrgyzstan	Sense organ diseases	29.2 (19.4 to 42.5)	35.6 (23.8 to 52.1)	42.8 (28.5 to 62.8)	864.2 (580.3 to 1257.8)	871.6 (581.4 to 1265.2)	864.2 (578.7 to 1253.2)
Kyrgyzstan	Blindness and vision impairment	13.9 (8.87 to 20.9)	16.9 (10.8 to 25.9)	20.1 (12.8 to 30.6)	408.6 (260.5 to 610.3)	412.2 (265.7 to 624.3)	404.7 (259.3 to 607.2)
Kyrgyzstan	Glaucoma	0.106 (0.0694 to 0.152)	0.11 (0.0717 to 0.156)	0.127 (0.0817 to 0.182)	3.6 (2.4 to 5.1)	3.4 (2.2 to 4.9)	3.2 (2.1 to 4.5)
Kyrgyzstan	Cataract	4.35 (2.95 to 6)	5.1 (3.45 to 7.04)	6.04 (4.05 to 8.42)	149.5 (101.6 to 205.2)	150.7 (101.4 to 206.9)	148.7 (99.9 to 206.1)
Kyrgyzstan	Age-related macular degeneration	0.0335 (0.0209 to 0.051)	0.0362 (0.0223 to 0.0547)	0.0451 (0.0277 to 0.0685)	1.1 (0.7 to 1.7)	1.1 (0.7 to 1.7)	1.1 (0.7 to 1.7)
Kyrgyzstan	Refraction disorders	4.76 (3.01 to 7.32)	5.91 (3.73 to 9.13)	6.95 (4.36 to 10.7)	114.2 (73.2 to 175.2)	117.1 (74.9 to 179.7)	115.6 (73.4 to 177.4)

SR Appendix Table 2. All-age DALYs counts and age standardized DALYs rates per 100,000 individuals for all causes and level 1 cause groups by location for 1990, 2007, and 2017, both sexes combined

		All-age DALYs			Age-standardized rate of DALYs per 100,000		
Kyrgyzstan	Near vision loss	3.92 (1.89 to 7.17)	4.87 (2.32 to 8.93)	5.93 (2.83 to 10.8)	119.3 (57.6 to 217.5)	119.1 (56.7 to 216.9)	115.6 (55 to 210.3)
Kyrgyzstan	Other vision loss	0.717 (0.464 to 1.02)	0.862 (0.56 to 1.23)	1.05 (0.672 to 1.51)	20.9 (13.7 to 29.5)	20.7 (13.5 to 29.2)	20.4 (13.2 to 29)
Kyrgyzstan	Age-related and other hearing loss	14.2 (9.82 to 20)	17.4 (12 to 24.6)	21 (14.3 to 29.8)	425 (293.1 to 594.1)	428.8 (296.9 to 600.1)	428.7 (294.5 to 603.2)
Kyrgyzstan	Other sense organ diseases	1.09 (0.668 to 1.6)	1.29 (0.804 to 1.91)	1.59 (0.981 to 2.37)	30.6 (18.9 to 44.8)	30.6 (19.1 to 45)	30.8 (19.1 to 45.7)
Kyrgyzstan	Musculoskeletal disorders	52.1 (38 to 69.1)	68.7 (50.4 to 92.4)	81.3 (59.2 to 110)	1495.7 (1095.2 to 1995.7)	1530.7 (1131.2 to 2048.2)	1486.5 (1088.4 to 2000.7)
Kyrgyzstan	Rheumatoid arthritis	1.49 (1.15 to 1.87)	2.44 (2.01 to 2.94)	2.64 (2.07 to 3.26)	45.5 (35.6 to 57.1)	61.2 (50.7 to 73.4)	55.1 (43.6 to 67.8)
Kyrgyzstan	Osteoarthritis	2.31 (1.17 to 4.59)	2.81 (1.41 to 5.57)	3.69 (1.85 to 7.39)	75.6 (38.3 to 149.5)	77.3 (39 to 152.9)	80.4 (40.2 to 159.7)
Kyrgyzstan	Low back pain	35.8 (25.7 to 48)	45.4 (32.3 to 61.8)	54.3 (38.9 to 73.7)	1010.8 (728.9 to 1360.7)	995.8 (714.7 to 1337.6)	980.6 (701.8 to 1313.4)
Kyrgyzstan	Neck pain	9.99 (6.97 to 14)	13.3 (9.26 to 18.7)	16.4 (11.4 to 23)	297.7 (208 to 414.6)	298.5 (206.2 to 418.3)	298.6 (208.5 to 417.6)
Kyrgyzstan	Gout	0.388 (0.259 to 0.542)	0.5 (0.326 to 0.696)	0.648 (0.421 to 0.911)	12.2 (8.2 to 17)	13 (8.5 to 18)	13.4 (8.8 to 18.5)
Kyrgyzstan	Other musculoskeletal disorders	2.06 (1.39 to 2.91)	4.3 (3.08 to 5.95)	3.6 (2.32 to 5.44)	53.9 (35.4 to 76.5)	84.9 (60.8 to 118.5)	58.4 (37.7 to 88)
Kyrgyzstan	Other non-communicable diseases	103 (84.8 to 132)	116 (99.8 to 134)	97.6 (77.9 to 121)	2075.2 (1707.4 to 2597.3)	2197.7 (1875.3 to 2567.4)	1554.6 (1234.5 to 1933.8)
Kyrgyzstan	Congenital anomalies	65.3 (53.7 to 93.5)	66 (59.1 to 78.7)	46.8 (37.6 to 64)	1129.4 (930.5 to 1588.2)	1158.3 (1035.4 to 1376.3)	669.8 (538.6 to 912.2)
Kyrgyzstan	Neural tube defects	6.67 (4.96 to 9.32)	5.24 (4.25 to 6.81)	3.35 (2.4 to 5.37)	112 (83.8 to 154.8)	91.4 (74.2 to 118.5)	47 (33.9 to 74.7)
Kyrgyzstan	Congenital heart anomalies	26.2 (18.9 to 43.2)	26.4 (22.7 to 36.5)	16.7 (12.2 to 27.4)	440.3 (320.5 to 719.1)	459.3 (395.9 to 632.5)	233.4 (170.9 to 382.9)
Kyrgyzstan	Orofacial clefts	0.497 (0.257 to 0.866)	0.295 (0.223 to 0.385)	0.207 (0.137 to 0.309)	9 (5 to 15.2)	5.3 (4 to 6.9)	3.2 (2.1 to 4.7)
Kyrgyzstan	Down's syndrome	1.35 (0.813 to 2.57)	1.13 (0.88 to 1.38)	0.925 (0.696 to 1.23)	24.2 (15 to 44.2)	20.1 (15.8 to 24.6)	13.4 (10.1 to 17.7)
Kyrgyzstan	Turner syndrome	0.00508 (0.00241 to 0.00858)	0.00627 (0.00313 to 0.0104)	0.00744 (0.00355 to 0.0124)	0.1 (0.1 to 0.2)	0.1 (0.1 to 0.2)	0.1 (0.1 to 0.2)
Kyrgyzstan	Klinefelter syndrome	0.00126 (0.000585 to 0.00242)	0.00161 (0.000728 to 0.00305)	0.00187 (0.000848 to 0.00373)	0 (0 to 0.1)	0 (0 to 0.1)	0 (0 to 0.1)
Kyrgyzstan	Other chromosomal abnormalities	0.708 (0.492 to 1.2)	0.831 (0.632 to 1.17)	0.857 (0.619 to 1.22)	13.3 (9.2 to 21.3)	14.7 (11.2 to 20.6)	12.5 (9 to 17.7)
Kyrgyzstan	Congenital musculoskeletal and limb anomalies	4.97 (3.47 to 6.61)	5.8 (4.01 to 7.8)	6.6 (4.44 to 8.91)	107.9 (74.4 to 144.2)	107.4 (74.1 to 144.1)	104 (69.6 to 140.9)
Kyrgyzstan	Urogenital congenital anomalies	0.661 (0.437 to 1.08)	0.665 (0.515 to 0.883)	0.554 (0.385 to 0.879)	11.5 (7.7 to 18.5)	11.5 (8.9 to 15.3)	7.9 (5.5 to 12.4)
Kyrgyzstan	Digestive congenital anomalies	9.99 (6.25 to 18)	9.6 (8.04 to 13.8)	6.58 (4.78 to 11)	170.7 (108.5 to 302.9)	167.8 (140.6 to 239.9)	93.7 (68.4 to 156.2)
Kyrgyzstan	Other congenital anomalies	14.2 (10.3 to 19.4)	16.1 (10.2 to 19.4)	11 (8.14 to 16.7)	240.4 (175.5 to 326.6)	280.6 (179 to 339.2)	154.7 (114.4 to 232.6)
Kyrgyzstan	Urinary diseases and male infertility	7.5 (6.74 to 8.42)	14 (13.1 to 15.1)	10.7 (9.57 to 12)	206.2 (184.5 to 231.9)	310.8 (287.3 to 337.6)	198.4 (176.2 to 224.8)
Kyrgyzstan	Urinary tract infections	5.1 (4.75 to 5.47)	11.3 (10.6 to 11.9)	7.56 (7.03 to 8.12)	135.9 (126.3 to 145.7)	241 (227.5 to 254.2)	135.3 (126.4 to 144.9)
Kyrgyzstan	Urolithiasis	0.561 (0.39 to 0.744)	0.599 (0.439 to 0.868)	0.46 (0.353 to 0.609)	15.5 (10.8 to 20.7)	13 (9.7 to 18.7)	8.2 (6.4 to 10.7)
Kyrgyzstan	Benign prostatic hyperplasia	1.36 (0.848 to 1.97)	1.57 (0.995 to 2.27)	2.17 (1.4 to 3.15)	42.8 (26.7 to 62.2)	45.4 (29.1 to 65.4)	46.4 (29.8 to 67)
Kyrgyzstan	Male infertility	0.125 (0.0499 to 0.263)	0.163 (0.067 to 0.344)	0.184 (0.0759 to 0.381)	2.9 (1.2 to 6.1)	2.8 (1.2 to 6)	2.7 (1.1 to 5.5)
Kyrgyzstan	Other urinary diseases	0.354 (0.277 to 0.434)	0.4 (0.307 to 0.548)	0.325 (0.265 to 0.407)	9.1 (7.2 to 11)	8.6 (6.5 to 11.9)	5.8 (4.8 to 7.2)
Kyrgyzstan	Gynecological diseases	6.5 (4.51 to 9.05)	9.14 (6.35 to 12.7)	10.5 (7.3 to 14.5)	167.3 (116.1 to 232.8)	164.9 (113.9 to 230.5)	162 (112.1 to 224.9)
Kyrgyzstan	Uterine fibroids	1.07 (0.706 to 1.66)	1.38 (0.851 to 2.22)	1.48 (0.874 to 2.38)	32 (21 to 50.4)	28.1 (17.3 to 45.4)	25.1 (14.8 to 40.4)
Kyrgyzstan	Polycystic ovarian syndrome	0.236 (0.106 to 0.455)	0.329 (0.146 to 0.648)	0.383 (0.167 to 0.737)	5.7 (2.6 to 11)	5.7 (2.5 to 11.2)	5.8 (2.5 to 11.1)
Kyrgyzstan	Female infertility	0.147 (0.0537 to 0.325)	0.188 (0.0707 to 0.401)	0.233 (0.0913 to 0.506)	3.4 (1.2 to 7.3)	3.3 (1.3 to 7.1)	3.3 (1.3 to 7.2)
Kyrgyzstan	Endometriosis	2.09 (1.34 to 3.11)	2.87 (1.87 to 4.16)	3.39 (2.2 to 4.97)	50.8 (33 to 74.9)	50.6 (32.9 to 73.2)	50.5 (32.6 to 74.2)
Kyrgyzstan	Genital prolapse	0.127 (0.06 to 0.241)	0.127 (0.0612 to 0.241)	0.164 (0.0768 to 0.322)	4 (1.9 to 7.4)	3.2 (1.5 to 6.1)	3.2 (1.5 to 6.3)
Kyrgyzstan	Premenstrual syndrome	2.04 (1.3 to 3.07)	3.01 (1.92 to 4.56)	3.33 (2.13 to 5.03)	50.6 (32.4 to 76.1)	51.6 (33 to 78.2)	50.6 (32.4 to 76.5)
Kyrgyzstan	Other gynecological diseases	0.797 (0.552 to 1.13)	1.24 (0.84 to 1.79)	1.51 (1.02 to 2.21)	20.9 (14.4 to 29.6)	22.4 (15.2 to 32.4)	23.4 (15.8 to 34.1)
Kyrgyzstan	Hemoglobinopathies and hemolytic anaemias	5.5 (4 to 7.48)	5.31 (3.91 to 7.34)	5.43 (3.88 to 7.45)	121.2 (88.5 to 164.5)	99.8 (73.8 to 137.4)	86.1 (61.3 to 118.6)
Kyrgyzstan	Thalassemias	0.523 (0.324 to 0.677)	0.457 (0.328 to 0.573)	0.432 (0.331 to 0.542)	8.7 (5.4 to 11.2)	8 (5.7 to 10.1)	5.9 (4.6 to 7.4)

SR Appendix Table 2. All-age DALYs counts and age standardized DALYs rates per 100,000 individuals for all causes and level 1 cause groups by location for 1990, 2007, and 2017, both sexes combined

		All-age DALYs			Age-standardized rate of DALYs per 100,000		
Kyrgyzstan	Thalassaemias trait	1.85 (1.21 to 2.68)	1.82 (1.2 to 2.7)	1.92 (1.22 to 2.89)	40.4 (26.8 to 58.8)	34.4 (22.8 to 50.9)	30.5 (19.4 to 45.5)
Kyrgyzstan	Sickle cell disorders	0.0641 (0.0358 to 0.103)	0.0579 (0.0353 to 0.0887)	0.0546 (0.0348 to 0.0815)	1.1 (0.6 to 1.7)	1 (0.6 to 1.6)	0.8 (0.5 to 1.1)
Kyrgyzstan	Sickle cell trait	0.141 (0.0883 to 0.211)	0.128 (0.076 to 0.201)	0.136 (0.079 to 0.218)	3 (1.9 to 4.6)	2.4 (1.4 to 3.7)	2.1 (1.2 to 3.3)
Kyrgyzstan	G6PD deficiency	0.147 (0.115 to 0.177)	0.242 (0.188 to 0.288)	0.263 (0.21 to 0.31)	3.3 (2.6 to 3.9)	4.1 (3.2 to 4.9)	4 (3.2 to 4.7)
Kyrgyzstan	G6PD trait	0.000171 (0.000111 to 0.000244)	0.00018 (0.000121 to 0.000258)	0.000199 (0.000127 to 0.000291)	0 (0 to 0)	0 (0 to 0)	0 (0 to 0)
Kyrgyzstan	Other hemoglobinopathies and hemolytic anaemias	2.78 (1.96 to 3.85)	2.6 (1.88 to 3.64)	2.62 (1.82 to 3.71)	64.6 (46.5 to 88.3)	49.9 (36.3 to 68.8)	42.9 (30 to 60.3)
Kyrgyzstan	Endocrine, metabolic, blood, and immune disorders	4.17 (3.18 to 5.37)	5.26 (3.98 to 6.79)	5.55 (4.23 to 7.3)	92.5 (70.5 to 120.4)	99.6 (76.1 to 127.8)	87.9 (66.8 to 115.4)
Kyrgyzstan	Oral disorders	10.8 (6.76 to 16.5)	14.1 (8.82 to 21.4)	17 (10.7 to 25.7)	311.5 (197.4 to 470.5)	330.1 (209.8 to 496.8)	327.3 (208.8 to 493.8)
Kyrgyzstan	Caries of deciduous teeth	0.23 (0.101 to 0.459)	0.212 (0.0926 to 0.423)	0.29 (0.126 to 0.578)	3.8 (1.7 to 7.6)	3.9 (1.7 to 7.8)	3.9 (1.7 to 7.8)
Kyrgyzstan	Caries of permanent teeth	1.82 (0.784 to 3.43)	2.5 (1.07 to 4.75)	2.76 (1.21 to 5.24)	43.3 (18.9 to 81.2)	45.7 (19.8 to 86.8)	44.1 (19.3 to 83.2)
Kyrgyzstan	Periodontal disease	1.29 (0.515 to 2.72)	1.83 (0.713 to 3.89)	2.33 (0.929 to 5.01)	38.9 (15.5 to 81.4)	42.2 (16.8 to 88.5)	42.5 (17 to 90.4)
Kyrgyzstan	Edentulism and severe tooth loss	5.42 (3.62 to 7.71)	6.88 (4.55 to 9.76)	8.48 (5.6 to 12)	174.7 (116.9 to 247.4)	187.4 (124.6 to 265.5)	185.8 (124 to 264.1)
Kyrgyzstan	Other oral disorders	2.06 (1.28 to 3.05)	2.65 (1.66 to 3.94)	3.14 (1.97 to 4.61)	50.9 (31.8 to 75.4)	50.9 (31.9 to 75.7)	51 (31.8 to 74.3)
Kyrgyzstan	Sudden infant death syndrome	2.88 (1.6 to 4.58)	1.95 (1.49 to 2.66)	1.68 (1.11 to 2.47)	47.2 (26.2 to 75.2)	34.1 (26.1 to 46.5)	23.2 (15.3 to 33.9)
Kyrgyzstan	Injuries	270 (252 to 289)	235 (222 to 250)	198 (181 to 218)	6222.2 (5787.3 to 6695.3)	4452 (4190.2 to 4758.1)	3177.1 (2894.6 to 3529.7)
Kyrgyzstan	Transport injuries	66.7 (62.5 to 70.9)	65 (61.9 to 68.1)	54.4 (50.6 to 58.4)	1558.6 (1459.9 to 1660.6)	1218 (1159 to 1281.2)	861.6 (800.1 to 929.6)
Kyrgyzstan	Road injuries	64.6 (60.6 to 68.6)	61.9 (59 to 64.8)	51.9 (48.4 to 55.7)	1508.9 (1411.7 to 1606.7)	1160.9 (1104.7 to 1218.5)	821.4 (762.8 to 884.6)
Kyrgyzstan	Pedestrian road injuries	21.6 (19.8 to 23.7)	18.5 (17.3 to 19.7)	16.6 (15.2 to 18.1)	497.6 (456.4 to 541.9)	354.7 (332.7 to 377.2)	266.1 (244.1 to 289)
Kyrgyzstan	Cyclist road injuries	1.91 (1.6 to 2.23)	1.86 (1.58 to 2.18)	1.9 (1.54 to 2.32)	47.8 (39.7 to 55.9)	37.2 (31.3 to 43.9)	32.2 (25.8 to 39.4)
Kyrgyzstan	Motorcyclist road injuries	8.46 (7.34 to 9.6)	7.61 (6.93 to 8.43)	5.32 (4.68 to 6)	196.2 (170.2 to 222)	137.2 (125.1 to 151.3)	81.9 (72.2 to 92.1)
Kyrgyzstan	Motor vehicle road injuries	30.7 (28.2 to 33.1)	31.7 (29.7 to 33.5)	26.5 (24 to 30.1)	723.8 (667.7 to 782.4)	589.9 (554.5 to 624.2)	417 (378.8 to 470.6)
Kyrgyzstan	Other road injuries	1.89 (1.61 to 2.19)	2.23 (1.98 to 2.49)	1.49 (1.3 to 1.7)	43.6 (37.3 to 50.1)	42 (37.5 to 47)	24.2 (21.1 to 27.6)
Kyrgyzstan	Other transport injuries	2.07 (1.84 to 2.33)	3.04 (2.74 to 3.34)	2.52 (2.19 to 2.93)	49.6 (44.2 to 55.9)	57 (51.5 to 62.9)	40.2 (35 to 46.7)
Kyrgyzstan	Unintentional injuries	145 (133 to 159)	107 (97.5 to 119)	97.4 (84 to 114)	3225.1 (2922.7 to 3569.4)	2087.4 (1885.9 to 2329)	1585.2 (1354.8 to 1858.4)
Kyrgyzstan	Falls	38 (31.5 to 45.6)	27.2 (21.8 to 34)	31.3 (24.1 to 40.5)	946.9 (768.2 to 1160.1)	561.7 (446.7 to 703.1)	541 (413.7 to 704.3)
Kyrgyzstan	Drowning	42.8 (39 to 47.5)	23.2 (21.7 to 24.7)	17.3 (15.4 to 19.5)	847.2 (781.1 to 927.8)	423.2 (396.3 to 450.7)	256.8 (230.3 to 287.2)
Kyrgyzstan	Fire, heat, and hot substances	15.5 (13.9 to 17.3)	12 (10.6 to 13.5)	9.96 (8.32 to 11.9)	338.9 (302.6 to 378.6)	232.9 (207.5 to 263.9)	160.2 (133.8 to 192)
Kyrgyzstan	Poisonings	7.27 (6.69 to 7.89)	5.92 (5.54 to 6.3)	4.43 (3.98 to 5)	174.3 (161.2 to 187.6)	116.8 (109.7 to 124.3)	71.7 (64.5 to 80.8)
Kyrgyzstan	Poisoning by carbon monoxide	5.16 (4.74 to 5.6)	4.29 (4.02 to 4.56)	2.81 (2.56 to 3.12)	131.5 (121 to 142.8)	86.4 (81.3 to 91.8)	46.6 (42.6 to 51.6)
Kyrgyzstan	Poisoning by other means	2.11 (1.84 to 2.42)	1.63 (1.45 to 1.85)	1.62 (1.32 to 2.02)	42.8 (37.6 to 48.8)	30.4 (27 to 34.6)	25.1 (20.4 to 31.3)
Kyrgyzstan	Exposure to mechanical forces	7.5 (6.35 to 8.8)	7.75 (6.55 to 9.26)	8.5 (6.74 to 10.6)	184.6 (153.3 to 219.8)	153.2 (128.5 to 185.4)	142.1 (112.3 to 179.5)
Kyrgyzstan	Unintentional firearm injuries	1.37 (1.18 to 1.62)	1.48 (1.34 to 1.7)	1.36 (1.19 to 1.57)	32.7 (28.3 to 38.4)	28 (25.3 to 32.1)	22.1 (19.4 to 25.5)
Kyrgyzstan	Other exposure to mechanical forces	6.13 (5.04 to 7.41)	6.26 (5.16 to 7.74)	7.14 (5.51 to 9.21)	151.9 (123 to 188.2)	125.1 (102 to 156.1)	120 (91.5 to 155.6)
Kyrgyzstan	Adverse effects of medical treatment	2.23 (1.63 to 2.55)	2.33 (1.74 to 2.56)	2.28 (1.85 to 2.62)	50.9 (35.9 to 57.7)	45.7 (33.9 to 50.2)	36.8 (29.5 to 41.5)
Kyrgyzstan	Animal contact	1.21 (0.927 to 1.47)	1.14 (0.929 to 1.39)	1.16 (0.898 to 1.44)	26 (20.4 to 31.7)	21.6 (17.6 to 26.4)	18.5 (14.2 to 23.4)
Kyrgyzstan	Venomous animal contact	0.777 (0.584 to 0.968)	0.704 (0.55 to 0.897)	0.746 (0.548 to 0.976)	16.8 (12.8 to 20.8)	13.3 (10.4 to 16.9)	11.9 (8.8 to 15.6)
Kyrgyzstan	Non-venomous animal contact	0.433 (0.32 to 0.56)	0.433 (0.352 to 0.533)	0.411 (0.317 to 0.535)	9.2 (6.9 to 11.9)	8.3 (6.7 to 10.3)	6.6 (5.1 to 8.7)
Kyrgyzstan	Foreign body	16.6 (14.4 to 19.5)	12.5 (11.5 to 13.5)	8.84 (7.85 to 9.97)	304.9 (268.5 to 353.5)	228.4 (210.7 to 246.9)	131.7 (117.7 to 147.4)
Kyrgyzstan	Pulmonary aspiration and foreign body in airway	14.4 (12.2 to 17.2)	10.1 (9.17 to 11.1)	6.55 (5.72 to 7.57)	253.9 (218 to 300.7)	182.8 (166.7 to 200)	94.8 (83.4 to 108.4)
Kyrgyzstan	Foreign body in eyes	0.0784 (0.035 to 0.141)	0.0996 (0.0441 to 0.176)	0.125 (0.0554 to 0.222)	1.9 (0.9 to 3.3)	1.9 (0.9 to 3.4)	2 (0.9 to 3.6)

SR Appendix Table 2. All-age DALYs counts and age standardized DALYs rates per 100,000 individuals for all causes and level 1 cause groups by location for 1990, 2007, and 2017, both sexes combined

		All-age DALYs			Age-standardized rate of DALYs per 100,000		
Kyrgyzstan	Foreign body in other body part	2.15 (1.78 to 2.62)	2.31 (2.04 to 2.58)	2.16 (1.84 to 2.51)	49.1 (41.9 to 57.6)	43.6 (38.6 to 48.9)	34.9 (29.7 to 40.4)
Kyrgyzstan	Environmental heat and cold exposure	5.67 (5.06 to 6.43)	8.45 (7.8 to 9.17)	6.37 (5.62 to 7.21)	155 (137.6 to 177.3)	177.5 (164 to 192.6)	108.1 (95.4 to 122.1)
Kyrgyzstan	Exposure to forces of nature	0 (0 to 0)	0.255 (0.229 to 0.288)	1.47 (1.44 to 1.52)	0 (0 to 0)	4.3 (3.8 to 4.9)	22.5 (21.9 to 23.2)
Kyrgyzstan	Other unintentional injuries	8.64 (7.67 to 9.82)	6.43 (5.7 to 7.32)	5.82 (4.8 to 7.18)	196.4 (172.3 to 224.4)	122.1 (106.9 to 140.8)	95.8 (77.8 to 119.4)
Kyrgyzstan	Self-harm and interpersonal violence	57.5 (54.6 to 60.7)	63 (60.8 to 65)	46.1 (43.2 to 49.3)	1438.5 (1363.9 to 1518.6)	1146.6 (1107.4 to 1184.6)	730.3 (684.9 to 781.3)
Kyrgyzstan	Self-harm	30.3 (28.5 to 32.3)	38.7 (36.8 to 40.4)	29.5 (27.2 to 32.7)	763.1 (716.9 to 813)	701.4 (667.4 to 729.2)	466.4 (430.6 to 516.2)
Kyrgyzstan	Self-harm by firearm	0.935 (0.714 to 1.45)	0.781 (0.555 to 1.19)	0.591 (0.377 to 0.859)	24.5 (18.6 to 38)	15.1 (10.7 to 22.5)	9.5 (6.1 to 13.9)
Kyrgyzstan	Self-harm by other specified means	29.3 (27.6 to 31.3)	37.9 (36.1 to 39.6)	28.9 (26.6 to 32.1)	738.6 (694.5 to 786.9)	686.3 (652.9 to 715)	456.9 (421.7 to 506.1)
Kyrgyzstan	Interpersonal violence	27.2 (25.5 to 29)	24 (23 to 25.1)	16.2 (15 to 17.7)	674.8 (631.4 to 721.3)	441.2 (422.4 to 461.9)	257.4 (237.8 to 280.9)
Kyrgyzstan	Assault by firearm	3.97 (3.06 to 4.82)	2.9 (2.42 to 3.32)	1.75 (1.44 to 2.11)	94.3 (74 to 112.8)	50.7 (42.7 to 57.7)	26.9 (22.2 to 32.2)
Kyrgyzstan	Assault by sharp object	12.1 (10.8 to 13.5)	11.5 (10.7 to 12.3)	6.39 (5.51 to 7.23)	304 (274.4 to 337.8)	211.3 (197.9 to 225.2)	101.3 (87.8 to 114)
Kyrgyzstan	Sexual violence	0.496 (0.333 to 0.731)	0.614 (0.414 to 0.901)	0.685 (0.468 to 0.993)	11.6 (7.9 to 17.1)	10.9 (7.4 to 15.8)	10.6 (7.3 to 15.3)
Kyrgyzstan	Assault by other means	10.7 (9.64 to 11.9)	9.01 (8.22 to 9.9)	7.38 (6.54 to 8.35)	264.9 (238.5 to 295.3)	168.4 (153.6 to 185.8)	118.6 (105.4 to 134.7)
Kyrgyzstan	Conflict and terrorism	0 (0 to 0)	0.18 (0.131 to 0.266)	0.363 (0.25 to 0.555)	0 (0 to 0)	3.1 (2.3 to 4.6)	5.7 (4 to 8.7)
Kyrgyzstan	Executions and police conflict	0.0298 (0.028 to 0.0319)	0.0456 (0.0428 to 0.0492)	0.0494 (0.0448 to 0.0545)	0.6 (0.6 to 0.7)	0.8 (0.7 to 0.9)	0.7 (0.7 to 0.8)
Mongolia	All causes	1150 (1080 to 1240)	1020 (948 to 1110)	1090 (982 to 1200)	57137.2 (53822.7 to 60996.3)	47554.9 (44395.7 to 51029)	37723.7 (34215.9 to 41381.7)
Mongolia	Communicable, maternal, neonatal, and nutritional disorders	576 (529 to 629)	254 (233 to 278)	205 (181 to 234)	18759.5 (17230.3 to 20453.2)	9313.1 (8556.9 to 10153)	5835.4 (5135.2 to 6638.4)
Mongolia	HIV/AIDS and sexually transmitted infections	3.7 (1.45 to 8.22)	3.7 (1.85 to 6.77)	3.91 (1.87 to 7.31)	123.3 (54.1 to 259.2)	131.7 (66.2 to 241.7)	110.5 (53.2 to 203)
Mongolia	HIV/AIDS	0.00076 (0.00000281 to 0.0000186)	0.333 (0.19 to 0.539)	0.926 (0.164 to 1.98)	0 (0 to 0)	13.1 (7.3 to 21.2)	27.7 (4.9 to 59.9)
Mongolia	HIV/AIDS - Drug-susceptible Tuberculosis	0.0000218 (0.0000028 to 0.0000186)	0.0319 (0.0189 to 0.0507)	0.0347 (0.016 to 0.0602)	0 (0 to 0)	1.3 (0.7 to 2)	1 (0.5 to 1.8)
Mongolia	HIV/AIDS - Multidrug-resistant Tuberculosis without extensive drug resistance	0.0000000775 (0.00000000163 to 0.0000000729)	0.00406 (0.00242 to 0.00654)	0.00843 (0.00206 to 0.0185)	0 (0 to 0)	0.2 (0.1 to 0.3)	0.3 (0.1 to 0.6)
Mongolia	HIV/AIDS - Extensively drug-resistant Tuberculosis	0 (0 to 0)	0.00129 (0.000762 to 0.00206)	0.00482 (0.00119 to 0.0109)	0 (0 to 0)	0.1 (0 to 0.1)	0.1 (0 to 0.3)
Mongolia	HIV/AIDS resulting in other diseases	0.000738 (0 to 0)	0.295 (0.155 to 0.505)	0.878 (0.132 to 1.93)	0 (0 to 0)	11.6 (6.1 to 19.8)	26.2 (3.8 to 58.3)
Mongolia	Sexually transmitted infections excluding HIV	3.7 (1.45 to 8.22)	3.37 (1.55 to 6.51)	2.98 (1.32 to 6.32)	123.2 (54.1 to 259.2)	118.6 (54.4 to 231.8)	82.8 (36.6 to 174.4)
Mongolia	Syphilis	3.21 (0.979 to 7.81)	2.61 (0.905 to 5.73)	2.15 (0.593 to 5.45)	97.5 (30.1 to 236.2)	93.6 (32.6 to 205.6)	59.3 (16.5 to 150.2)
Mongolia	Chlamydial infection	0.116 (0.069 to 0.209)	0.18 (0.106 to 0.322)	0.204 (0.118 to 0.37)	6 (3.6 to 10.5)	5.8 (3.4 to 10.2)	5.6 (3.2 to 10.1)
Mongolia	Gonococcal infection	0.122 (0.0761 to 0.21)	0.178 (0.107 to 0.31)	0.17 (0.105 to 0.282)	6.2 (3.8 to 9.9)	5.7 (3.5 to 9.5)	4.9 (3 to 8.3)
Mongolia	Trichomoniasis	0.0742 (0.0298 to 0.162)	0.122 (0.0487 to 0.264)	0.142 (0.0562 to 0.302)	4 (1.6 to 8.6)	4.2 (1.7 to 9)	4 (1.6 to 8.5)
Mongolia	Genital herpes	0.0408 (0.0137 to 0.0956)	0.0649 (0.0216 to 0.152)	0.086 (0.028 to 0.207)	2.6 (0.9 to 6.1)	2.6 (0.8 to 6.2)	2.6 (0.8 to 6.3)
Mongolia	Other sexually transmitted infections	0.136 (0.0847 to 0.231)	0.212 (0.131 to 0.355)	0.23 (0.148 to 0.386)	7 (4.5 to 11.5)	6.8 (4.3 to 11.1)	6.4 (4.1 to 10.7)
Mongolia	Respiratory infections and tuberculosis	323 (295 to 356)	105 (92.2 to 118)	68 (57.5 to 81.6)	10492.8 (9648.9 to 11484.3)	3945.6 (3493.8 to 4467.4)	1943.8 (1660.7 to 2315.3)
Mongolia	Tuberculosis	25.9 (23.1 to 28.8)	21.7 (19.9 to 23.7)	17.4 (15.3 to 19.8)	1197.1 (1091.8 to 1322.4)	831.9 (764.6 to 907.4)	506.2 (448.9 to 572.8)
Mongolia	Latent tuberculosis infection	0 (0 to 0)	0 (0 to 0)	0 (0 to 0)	0 (0 to 0)	0 (0 to 0)	0 (0 to 0)
Mongolia	Drug-susceptible tuberculosis	25.7 (23 to 28.7)	19.1 (17.3 to 21.1)	13.3 (8.72 to 17.2)	1191.5 (1086.1 to 1316.3)	731.9 (665.3 to 805.9)	386.7 (254.9 to 499)
Mongolia	Multidrug-resistant tuberculosis without extensive drug resistance	0.122 (0.0243 to 0.4)	1.98 (1.44 to 2.63)	2.62 (0.749 to 5.41)	5.6 (1.1 to 18.3)	76 (55.5 to 101.4)	76.1 (21.6 to 156.9)
Mongolia	Extensively drug-resistant tuberculosis	0 (0 to 0)	0.627 (0.463 to 0.831)	1.5 (0.429 to 3.24)	0 (0 to 0)	24.1 (17.7 to 31.9)	43.4 (12.4 to 94)
Mongolia	Lower respiratory infections	295 (268 to 328)	80.4 (69.6 to 93.2)	48 (38.5 to 60.8)	9192.2 (8376.2 to 10165.5)	3023.8 (2612.4 to 3508.3)	1358.6 (1099.9 to 1713.6)
Mongolia	Upper respiratory infections	1.97 (1 to 3.23)	1.74 (1.06 to 2.54)	1.84 (1.13 to 2.8)	75 (40.8 to 115.6)	62.5 (38.6 to 91.7)	54.6 (33.7 to 83.2)
Mongolia	Otitis media	0.688 (0.409 to 1.1)	0.745 (0.445 to 1.19)	0.795 (0.477 to 1.27)	28.5 (17.2 to 45.1)	27.3 (16.5 to 43.3)	24.3 (14.6 to 38.4)
Mongolia	Enteric infections	13.6 (9.94 to 18.1)	5.99 (4.74 to 7.43)	6.22 (4.74 to 7.96)	437.2 (327.9 to 573.3)	220.5 (175.2 to 273.7)	180.4 (137.9 to 230.9)

SR Appendix Table 2. All-age DALYs counts and age standardized DALYs rates per 100,000 individuals for all causes and level 1 cause groups by location for 1990, 2007, and 2017, both sexes combined

		All-age DALYs			Age-standardized rate of DALYs per 100,000		
Mongolia	Diarrhoeal diseases	13.3 (9.61 to 17.7)	5.72 (4.43 to 7.18)	5.9 (4.45 to 7.56)	425 (315.5 to 562.9)	210 (163.6 to 263.2)	170.7 (129.3 to 218.7)
Mongolia	Typhoid and paratyphoid	0.00135 (0.000685 to 0.00242)	0.00184 (0.000954 to 0.00332)	0.0023 (0.00117 to 0.00404)	0.1 (0 to 0.1)	0.1 (0 to 0.1)	0.1 (0 to 0.1)
Mongolia	Typhoid fever	0.00133 (0.000673 to 0.00237)	0.00168 (0.000875 to 0.00307)	0.00205 (0.00105 to 0.00366)	0.1 (0 to 0.1)	0.1 (0 to 0.1)	0.1 (0 to 0.1)
Mongolia	Paratyphoid fever	0.0000231 (0.00000901 to 0.0000499)	0.000153 (0.0000589 to 0.000318)	0.000246 (0.0000985 to 0.000508)	0 (0 to 0)	0 (0 to 0)	0 (0 to 0)
Mongolia	Invasive non-typhoidal salmonella	0.246 (0.136 to 0.423)	0.239 (0.126 to 0.407)	0.297 (0.157 to 0.529)	9.3 (5.2 to 15.6)	9.3 (5 to 15.5)	9.1 (4.9 to 15.9)
Mongolia	Other intestinal infectious diseases	0.0865 (0.0123 to 0.186)	0.032 (0.00743 to 0.0686)	0.0193 (0.00603 to 0.0385)	2.8 (0.5 to 5.7)	1.2 (0.3 to 2.5)	0.6 (0.2 to 1.1)
Mongolia	Neglected tropical diseases and malaria	2.64 (1.81 to 3.58)	2.4 (1.58 to 3.4)	2.67 (1.77 to 3.84)	130.6 (89.5 to 176.2)	93.9 (62.8 to 132)	82.1 (54.5 to 117.8)
Mongolia	Malaria	0 (0 to 0)	0 (0 to 0)	0 (0 to 0)	0 (0 to 0)	0 (0 to 0)	0 (0 to 0)
Mongolia	Chagas disease	0 (0 to 0)	0 (0 to 0)	0 (0 to 0)	0 (0 to 0)	0 (0 to 0)	0 (0 to 0)
Mongolia	Leishmaniasis	0 (0 to 0)	0 (0 to 0)	0 (0 to 0)	0 (0 to 0)	0 (0 to 0)	0 (0 to 0)
Mongolia	Visceral leishmaniasis	0 (0 to 0)	0 (0 to 0)	0 (0 to 0)	0 (0 to 0)	0 (0 to 0)	0 (0 to 0)
Mongolia	Cutaneous and mucocutaneous leishmaniasis	0 (0 to 0)	0 (0 to 0)	0 (0 to 0)	0 (0 to 0)	0 (0 to 0)	0 (0 to 0)
Mongolia	African trypanosomiasis	0 (0 to 0)	0 (0 to 0)	0 (0 to 0)	0 (0 to 0)	0 (0 to 0)	0 (0 to 0)
Mongolia	Schistosomiasis	0 (0 to 0)	0 (0 to 0)	0 (0 to 0)	0 (0 to 0)	0 (0 to 0)	0 (0 to 0)
Mongolia	Cysticercosis	0.703 (0.398 to 1.09)	1.07 (0.582 to 1.68)	1.32 (0.727 to 2.14)	45.5 (26 to 69.5)	44.5 (24.2 to 69.5)	41.6 (23.1 to 66)
Mongolia	Cystic echinococcosis	0.308 (0.133 to 0.625)	0.433 (0.167 to 0.899)	0.515 (0.202 to 1.11)	17.4 (8.1 to 32.8)	16.4 (7 to 31.6)	15.7 (6.4 to 31.8)
Mongolia	Lymphatic filariasis	0 (0 to 0)	0 (0 to 0)	0 (0 to 0)	0 (0 to 0)	0 (0 to 0)	0 (0 to 0)
Mongolia	Onchocerciasis	0 (0 to 0)	0 (0 to 0)	0 (0 to 0)	0 (0 to 0)	0 (0 to 0)	0 (0 to 0)
Mongolia	Trachoma	0 (0 to 0)	0 (0 to 0)	0 (0 to 0)	0 (0 to 0)	0 (0 to 0)	0 (0 to 0)
Mongolia	Dengue	0 (0 to 0)	0 (0 to 0)	0 (0 to 0)	0 (0 to 0)	0 (0 to 0)	0 (0 to 0)
Mongolia	Yellow fever	0 (0 to 0)	0 (0 to 0)	0 (0 to 0)	0 (0 to 0)	0 (0 to 0)	0 (0 to 0)
Mongolia	Rabies	0.0293 (0.0101 to 0.0679)	0.00311 (0.00168 to 0.00528)	0.00323 (0.0016 to 0.00575)	0.9 (0.3 to 2)	0.2 (0.1 to 0.3)	0.1 (0.1 to 0.2)
Mongolia	Intestinal nematode infections	0.476 (0.276 to 0.765)	0.19 (0.108 to 0.312)	0.175 (0.0976 to 0.302)	22.9 (13.2 to 36.8)	6.8 (3.8 to 11.2)	5.3 (3 to 9.2)
Mongolia	Ascariasis	0.0000000177 (0.00000000779 to 0.0000000362)	0.0000000592 (0.0000000256 to 0.000000119)	0.0000000618 (0.0000000258 to 0.000000131)	0 (0 to 0)	0 (0 to 0)	0 (0 to 0)
Mongolia	Trichuriasis	0.000000000613 (0.000000000269 to 0.00000000122)	0.000000000186 (0.0000000000786 to 0.000000000383)	0.000000000202 (0.0000000000876 to 0.000000000408)	0 (0 to 0)	0 (0 to 0)	0 (0 to 0)
Mongolia	Hookworm disease	0.476 (0.276 to 0.765)	0.19 (0.108 to 0.312)	0.175 (0.0976 to 0.302)	22.9 (13.2 to 36.8)	6.8 (3.8 to 11.2)	5.3 (3 to 9.2)
Mongolia	Food-borne trematodiases	0 (0 to 0)	0 (0 to 0)	0 (0 to 0)	0 (0 to 0)	0 (0 to 0)	0 (0 to 0)
Mongolia	Leprosy	0 (0 to 0)	0 (0 to 0)	0.00000494 (0.00000296 to 0.00000785)	0 (0 to 0)	0 (0 to 0)	0 (0 to 0)
Mongolia	Ebola virus disease	0 (0 to 0)	0 (0 to 0)	0 (0 to 0)	0 (0 to 0)	0 (0 to 0)	0 (0 to 0)
Mongolia	Zika virus disease	0 (0 to 0)	0 (0 to 0)	0 (0 to 0)	0 (0 to 0)	0 (0 to 0)	0 (0 to 0)
Mongolia	Guinea worm disease	0 (0 to 0)	0 (0 to 0)	0 (0 to 0)	0 (0 to 0)	0 (0 to 0)	0 (0 to 0)
Mongolia	Other neglected tropical diseases	1.12 (0.73 to 1.62)	0.708 (0.468 to 1.04)	0.656 (0.426 to 0.969)	43.9 (29 to 63)	26.1 (17.2 to 38.8)	19.3 (12.5 to 28.6)
Mongolia	Other infectious diseases	49.2 (23.8 to 65.3)	12.1 (9.61 to 16.1)	10.3 (7.79 to 14.3)	1630 (806.2 to 2130.8)	449.3 (354.7 to 599.5)	295.7 (227.2 to 404)
Mongolia	Meningitis	27.3 (8.96 to 36.6)	3.37 (2.72 to 4.34)	2.35 (1.85 to 3.5)	895 (303.1 to 1184.9)	124.6 (101.3 to 159.8)	68.7 (54.3 to 101.3)
Mongolia	Pneumococcal meningitis	0.782 (0.383 to 1.05)	0.256 (0.192 to 0.334)	0.141 (0.107 to 0.186)	29.8 (15.8 to 39)	9.2 (6.9 to 11.8)	4.2 (3.2 to 5.5)
Mongolia	H influenzae type B meningitis	14.2 (4.48 to 19.5)	1.55 (1.24 to 2.03)	1.03 (0.763 to 1.58)	460.7 (149.9 to 623.1)	57.8 (46.2 to 75)	29.7 (22.3 to 45.6)
Mongolia	Meningococcal infection	3.04 (0.968 to 4.57)	0.377 (0.295 to 0.492)	0.273 (0.209 to 0.401)	99.7 (33.5 to 146.4)	14 (11 to 18.2)	8 (6.2 to 11.6)
Mongolia	Other meningitis	9.21 (2.86 to 12.9)	1.18 (0.951 to 1.57)	0.913 (0.71 to 1.36)	304.8 (97.8 to 419.4)	43.7 (35 to 57.7)	26.8 (20.9 to 39.9)
Mongolia	Encephalitis	3.86 (2.84 to 5.45)	3.27 (2.12 to 4.45)	3.44 (1.58 to 5.19)	127.4 (97.4 to 174.5)	121.7 (79.3 to 165.5)	97.3 (45.8 to 144.9)
Mongolia	Diphtheria	0.15 (0.0527 to 0.314)	0.0167 (0.0083 to 0.0294)	0.0117 (0.00601 to 0.0207)	4.7 (1.8 to 9.7)	0.6 (0.3 to 1.1)	0.3 (0.2 to 0.6)

SR Appendix Table 2. All-age DALYs counts and age standardized DALYs rates per 100,000 individuals for all causes and level 1 cause groups by location for 1990, 2007, and 2017, both sexes combined

		All-age DALYs			Age-standardized rate of DALYs per 100,000		
Mongolia	Whooping cough	6.23 (0.534 to 18.6)	1.8 (0.165 to 5.75)	1.39 (0.131 to 4.75)	188.6 (16.1 to 561.6)	68.7 (6.3 to 217.7)	37.5 (3.5 to 128)
Mongolia	Tetanus	0.0348 (0.0164 to 0.062)	0.0262 (0.0182 to 0.0462)	0.0273 (0.0191 to 0.0474)	2 (0.7 to 4.2)	0.9 (0.6 to 1.7)	0.8 (0.6 to 1.4)
Mongolia	Measles	0.0904 (0.0626 to 0.125)	0.00264 (0.00111 to 0.00471)	0.000167 (0 to 0.000919)	2.7 (1.9 to 3.8)	0.1 (0 to 0.2)	0 (0 to 0)
Mongolia	Varicella and herpes zoster	0.69 (0.401 to 1.11)	0.348 (0.221 to 0.546)	0.336 (0.209 to 0.52)	26.7 (17.4 to 39.6)	14.6 (9.7 to 22.1)	10.6 (7 to 15.7)
Mongolia	Acute hepatitis	8.74 (2.01 to 11.9)	2.24 (1.15 to 2.77)	1.7 (1.05 to 2.15)	298.4 (75.1 to 399.2)	78.4 (42.8 to 97)	50.3 (31 to 62.8)
Mongolia	Acute hepatitis A	3.09 (0.635 to 4.61)	0.689 (0.285 to 0.919)	0.514 (0.261 to 0.695)	99.5 (20.5 to 147.8)	24.2 (10.2 to 32.9)	15.7 (7.8 to 21.2)
Mongolia	Acute hepatitis B	2.69 (0.679 to 3.81)	1.05 (0.614 to 1.32)	0.863 (0.626 to 1.07)	107 (32.2 to 144.8)	36.2 (23.2 to 44.9)	25.4 (18.6 to 31.2)
Mongolia	Acute hepatitis C	2.6 (0.59 to 4.15)	0.367 (0.173 to 0.565)	0.232 (0.11 to 0.409)	78.5 (18 to 125)	13.8 (6.5 to 21.3)	6.4 (3.1 to 11.2)
Mongolia	Acute hepatitis E	0.367 (0.0666 to 0.561)	0.132 (0.0497 to 0.194)	0.087 (0.0399 to 0.122)	13.4 (2.5 to 20.3)	4.2 (1.6 to 5.9)	2.7 (1.2 to 3.8)
Mongolia	Other unspecified infectious diseases	2.18 (1.69 to 2.76)	1.07 (0.769 to 1.58)	1.03 (0.742 to 1.51)	84.5 (66.4 to 104.8)	39.6 (28.8 to 57.9)	30.3 (22 to 43.6)
Mongolia	Maternal and neonatal disorders	155 (128 to 186)	106 (96.9 to 117)	95.6 (80.4 to 113)	4802.4 (3987 to 5738.1)	3756.9 (3422.6 to 4144.2)	2658.1 (2237 to 3128.6)
Mongolia	Maternal disorders	5.53 (4.75 to 6.37)	2.44 (2.17 to 2.75)	1.96 (1.59 to 2.42)	265.7 (228.9 to 304.6)	75 (66.7 to 84.6)	52.8 (42.8 to 65.3)
Mongolia	Maternal haemorrhage	1.84 (0.924 to 2.96)	0.546 (0.264 to 0.911)	0.388 (0.18 to 0.682)	83.9 (44.3 to 133.6)	16.6 (8.1 to 27.7)	10.5 (5 to 18.1)
Mongolia	Maternal sepsis and other pregnancy related infections	1.7 (1.12 to 2.39)	0.495 (0.352 to 0.667)	0.352 (0.245 to 0.488)	84.3 (56.8 to 116.4)	15.3 (10.9 to 20.4)	9.5 (6.7 to 13.2)
Mongolia	Maternal hypertensive disorders	0.16 (0.0837 to 0.303)	0.112 (0.0611 to 0.184)	0.101 (0.0554 to 0.173)	7.3 (3.9 to 13.2)	3.3 (1.8 to 5.4)	2.8 (1.5 to 4.7)
Mongolia	Maternal obstructed labour and uterine rupture	0.106 (0.0654 to 0.168)	0.0785 (0.0497 to 0.117)	0.0742 (0.0453 to 0.114)	5.3 (3.3 to 8.7)	2.4 (1.6 to 3.7)	2 (1.2 to 3)
Mongolia	Maternal abortive outcome	1.28 (0.718 to 1.97)	0.339 (0.189 to 0.538)	0.24 (0.14 to 0.384)	62.8 (36.6 to 93.9)	10.5 (6 to 16.7)	6.5 (3.8 to 10.1)
Mongolia	Ectopic pregnancy	0.111 (0.0314 to 0.302)	0.0717 (0.022 to 0.178)	0.0581 (0.0167 to 0.156)	5.5 (1.6 to 15)	2.2 (0.7 to 5.8)	1.6 (0.4 to 4.2)
Mongolia	Indirect maternal deaths	0.129 (0.0554 to 0.255)	0.414 (0.216 to 0.689)	0.337 (0.172 to 0.572)	7 (2.9 to 13.8)	12.8 (6.8 to 21)	9 (4.6 to 15.2)
Mongolia	Late maternal deaths	0.081 (0.0574 to 0.111)	0.0669 (0.0488 to 0.0903)	0.0644 (0.0431 to 0.0909)	3.8 (2.7 to 5.3)	2.1 (1.5 to 2.8)	1.7 (1.2 to 2.5)
Mongolia	Maternal deaths aggravated by HIV/AIDS	0.000000175 (0 to 0)	0.0000248 (0.000000543 to 0.000128)	0.000199 (0.00000533 to 0.0011)	0 (0 to 0)	0 (0 to 0)	0 (0 to 0)
Mongolia	Other maternal disorders	0.118 (0.0693 to 0.193)	0.315 (0.195 to 0.458)	0.345 (0.212 to 0.521)	5.7 (3.5 to 9.1)	9.7 (6 to 14.2)	9.2 (5.7 to 13.7)
Mongolia	Neonatal disorders	149 (122 to 180)	104 (94.4 to 115)	93.6 (78.5 to 111)	4536.7 (3732.6 to 5469.6)	3681.9 (3344.4 to 4069.4)	2605.3 (2188.3 to 3090.2)
Mongolia	Neonatal preterm birth	56.9 (41.5 to 75.4)	35.6 (29.2 to 43.2)	32.2 (25.3 to 40.3)	1733 (1264.6 to 2294.6)	1259.2 (1032.6 to 1527)	895.3 (703.1 to 1121.6)
Mongolia	Neonatal encephalopathy due to birth asphyxia and trauma	76.4 (56.5 to 95.9)	55.4 (46.3 to 64.3)	48.3 (37.3 to 60)	2311.7 (1710.4 to 2904.2)	1963.1 (1640.6 to 2279)	1345.1 (1039.7 to 1673.6)
Mongolia	Neonatal sepsis and other neonatal infections	6.37 (3.58 to 13)	5.77 (4.14 to 10.2)	6.21 (4.34 to 9.66)	197.2 (112.5 to 398.4)	204.1 (145.8 to 360)	173.8 (121.5 to 268.2)
Mongolia	Hemolytic disease and other neonatal jaundice	2.74 (1.76 to 4.37)	1.8 (1.35 to 2.39)	1.52 (1.16 to 1.97)	89 (59.5 to 136.8)	63.8 (47.7 to 84.9)	43.4 (33.1 to 55.9)
Mongolia	Other neonatal disorders	6.84 (4.42 to 10.7)	5.35 (4 to 7.36)	5.37 (3.92 to 7.23)	205.9 (133 to 322)	191.7 (143.5 to 262.9)	147.7 (107.9 to 199.1)
Mongolia	Nutritional deficiencies	28.6 (19.9 to 40.1)	18.9 (12.6 to 27.2)	18.5 (12.3 to 27)	1143.1 (800.4 to 1603.9)	715.2 (480.5 to 1016.9)	564.7 (376 to 821.9)
Mongolia	Protein-energy malnutrition	0.614 (0.405 to 0.862)	0.158 (0.126 to 0.201)	0.129 (0.0984 to 0.165)	20.6 (14.4 to 28.4)	5.5 (4.4 to 7.1)	3.7 (2.8 to 4.6)
Mongolia	Iodine deficiency	0.626 (0.33 to 1.03)	0.778 (0.42 to 1.32)	0.804 (0.412 to 1.4)	30.8 (16.2 to 50.9)	28.8 (15.4 to 48.8)	25 (12.8 to 43.6)
Mongolia	Dietary iron deficiency	16.9 (11.2 to 24.4)	13.2 (8.7 to 19.3)	13 (8.56 to 19)	747.4 (503.2 to 1075.2)	494.9 (328.5 to 714.7)	394.5 (261.4 to 577.3)
Mongolia	Other nutritional deficiencies	2.63 (1.93 to 3.58)	0.658 (0.551 to 0.803)	0.432 (0.367 to 0.508)	93 (71.6 to 120.7)	26.4 (22.5 to 31.9)	13 (11.2 to 15.2)
Mongolia	Non-communicable diseases	454 (418 to 496)	607 (556 to 664)	717 (640 to 798)	32485 (30209.8 to 34990.7)	32340.9 (30084.1 to 34809.7)	26985.1 (24404.1 to 29759.3)
Mongolia	Neoplasms	73.6 (69.9 to 78.2)	102 (96.6 to 107)	134 (122 to 146)	6202.3 (5901.7 to 6534.4)	6161.8 (5878.4 to 6440.2)	5405.5 (4934.5 to 5873.6)
Mongolia	Lip and oral cavity cancer	1.25 (1.14 to 1.38)	0.953 (0.854 to 1.06)	1.29 (1.12 to 1.5)	108.2 (98 to 119.2)	56 (50.2 to 62.2)	49.8 (43.6 to 57.5)
Mongolia	Nasopharynx cancer	0.1 (0.0889 to 0.114)	0.197 (0.176 to 0.223)	0.247 (0.204 to 0.296)	7.8 (6.8 to 8.9)	9.6 (8.5 to 10.8)	8 (6.6 to 9.5)
Mongolia	Other pharynx cancer	0.16 (0.136 to 0.181)	0.193 (0.169 to 0.225)	0.27 (0.226 to 0.331)	15.1 (12.6 to 17)	12.4 (10.5 to 14.2)	11.4 (9.3 to 13.8)
Mongolia	Oesophageal cancer	5.61 (4.73 to 6.59)	6.48 (5.97 to 7.05)	8.06 (7.11 to 9.04)	536.6 (451.9 to 629.2)	468.8 (431.1 to 512.3)	386.1 (342.6 to 434)
Mongolia	Stomach cancer	15.9 (14.6 to 17.4)	18.1 (16.7 to 19.3)	21 (18.7 to 23.5)	1427.5 (1315.4 to 1552.8)	1096.2 (1022 to 1166.5)	842.9 (754.7 to 941)

SR Appendix Table 2. All-age DALYs counts and age standardized DALYs rates per 100,000 individuals for all causes and level 1 cause groups by location for 1990, 2007, and 2017, both sexes combined

		All-age DALYs			Age-standardized rate of DALYs per 100,000		
Mongolia	Colon and rectum cancer	2.09 (1.9 to 2.32)	2.86 (2.63 to 3.13)	3.83 (3.29 to 4.32)	180.9 (164.3 to 201.5)	168.2 (156.3 to 182.1)	152.4 (131.4 to 171.8)
Mongolia	Liver cancer	17.2 (15.2 to 19.5)	36.8 (33.2 to 40.1)	52.9 (45.7 to 59.7)	1517.5 (1345.6 to 1712.4)	2354.4 (2100.5 to 2544.4)	2186.4 (1904.6 to 2451.6)
Mongolia	Liver cancer due to hepatitis B	5.55 (4.5 to 6.74)	11.5 (9.51 to 13.5)	15.9 (12.7 to 19.5)	470.1 (379.7 to 570.4)	648.2 (528.1 to 767.6)	582.3 (462.4 to 705.8)
Mongolia	Liver cancer due to hepatitis C	5.34 (4.33 to 6.48)	11.6 (9.64 to 13.5)	16.8 (13.7 to 20.4)	489.1 (397.6 to 590.4)	823.2 (695.8 to 953.7)	762.3 (630.4 to 902.1)
Mongolia	Liver cancer due to alcohol use	4.37 (3.64 to 5.18)	9.48 (8.06 to 10.9)	13.9 (11.5 to 16.5)	390.4 (326 to 461.4)	600.2 (514.2 to 685.8)	575.4 (478 to 673.7)
Mongolia	Liver cancer due to NASH	0.958 (0.792 to 1.15)	2.17 (1.83 to 2.53)	3.26 (2.67 to 3.94)	87.2 (71.8 to 104.8)	151.9 (127.1 to 177.9)	148.2 (122 to 177.4)
Mongolia	Liver cancer due to other causes	0.941 (0.789 to 1.14)	2.14 (1.81 to 2.5)	2.98 (2.47 to 3.59)	80.7 (67.5 to 97.6)	131 (110.8 to 152.6)	118.2 (98.7 to 142.3)
Mongolia	Gallbladder and biliary tract cancer	0.766 (0.266 to 1.1)	0.859 (0.329 to 1.16)	1.21 (0.466 to 1.7)	69.1 (23.8 to 100)	54.9 (20.6 to 75.2)	50.3 (19 to 71.4)
Mongolia	Pancreatic cancer	0.757 (0.692 to 0.833)	1.69 (1.55 to 1.84)	2.75 (2.41 to 3.14)	68 (62.4 to 74.6)	104.4 (96.1 to 113.1)	108.1 (95 to 122.5)
Mongolia	Larynx cancer	0.218 (0.196 to 0.242)	0.415 (0.37 to 0.462)	0.539 (0.46 to 0.649)	20.4 (18.4 to 22.6)	30.5 (27.3 to 33.9)	26 (22.3 to 30.7)
Mongolia	Tracheal, bronchus, and lung cancer	9.09 (8.32 to 10)	9.3 (8.54 to 10.1)	12.7 (11.1 to 14.5)	831.7 (762.9 to 914.7)	630.7 (580.8 to 685)	558.4 (487.7 to 629.1)
Mongolia	Malignant skin melanoma	0.0502 (0.0425 to 0.0683)	0.0728 (0.0617 to 0.103)	0.0945 (0.0707 to 0.141)	3.9 (3.3 to 5.3)	3.6 (3.1 to 5.1)	3.4 (2.6 to 4.9)
Mongolia	Non-melanoma skin cancer	0.0772 (0.0706 to 0.0844)	0.116 (0.107 to 0.126)	0.159 (0.14 to 0.179)	7.2 (6.6 to 7.9)	7.8 (7.2 to 8.4)	7.2 (6.3 to 8.1)
Mongolia	Non-melanoma skin cancer (squamous-cell carcinoma)	0.0768 (0.0702 to 0.0839)	0.116 (0.106 to 0.126)	0.158 (0.139 to 0.179)	7.2 (6.6 to 7.9)	7.7 (7.1 to 8.4)	7.2 (6.3 to 8)
Mongolia	Non-melanoma skin cancer (basal-cell carcinoma)	0.000345 (0.000116 to 0.000787)	0.00049 (0.000168 to 0.00111)	0.000734 (0.000261 to 0.00171)	0 (0 to 0.1)	0 (0 to 0.1)	0 (0 to 0.1)
Mongolia	Breast cancer	1.56 (1.3 to 2.06)	3.02 (2.62 to 3.62)	3.95 (3.14 to 5.16)	128.1 (107.4 to 167.8)	155.8 (137.3 to 180.1)	136.1 (110.5 to 169.2)
Mongolia	Cervical cancer	3.14 (2.71 to 3.83)	4.05 (3.51 to 4.82)	4.66 (3.87 to 5.69)	261.4 (226.7 to 311.7)	203.8 (178.3 to 233.4)	159.4 (132.2 to 191.8)
Mongolia	Uterine cancer	0.458 (0.386 to 0.551)	0.717 (0.615 to 0.817)	0.842 (0.674 to 1.04)	33.7 (28.8 to 40.4)	35.7 (31.4 to 40.6)	29.8 (24.2 to 36.7)
Mongolia	Ovarian cancer	0.427 (0.352 to 0.495)	0.995 (0.876 to 1.14)	1.5 (1.23 to 1.81)	35.1 (28.9 to 40.5)	52.5 (46.1 to 59.7)	52.4 (43.5 to 62.8)
Mongolia	Prostate cancer	0.449 (0.382 to 0.627)	0.597 (0.504 to 0.757)	0.825 (0.671 to 1.03)	45.2 (38.7 to 61.5)	47.1 (39.7 to 59.9)	44.3 (36.2 to 56.5)
Mongolia	Testicular cancer	0.00338 (0.00289 to 0.00418)	0.0115 (0.00856 to 0.0158)	0.0109 (0.00821 to 0.0148)	0.2 (0.2 to 0.2)	0.4 (0.3 to 0.5)	0.4 (0.3 to 0.5)
Mongolia	Kidney cancer	0.415 (0.335 to 0.655)	0.847 (0.756 to 0.942)	1.19 (1.04 to 1.38)	30 (24.6 to 44.9)	49.1 (43.3 to 54.4)	46.8 (40.3 to 53.7)
Mongolia	Bladder cancer	0.519 (0.469 to 0.577)	0.37 (0.338 to 0.415)	0.501 (0.429 to 0.574)	46 (41.6 to 51)	24.7 (22.5 to 27.1)	22.2 (18.7 to 25.6)
Mongolia	Thyroid cancer	0.137 (0.105 to 0.157)	0.23 (0.167 to 0.265)	0.31 (0.221 to 0.375)	11.8 (8.7 to 13.6)	14.3 (9.3 to 16.7)	12.6 (8 to 15.5)
Mongolia	Mesothelioma	0.0987 (0.0695 to 0.129)	0.0975 (0.0864 to 0.109)	0.0986 (0.083 to 0.118)	8.4 (6 to 10.9)	5.3 (4.7 to 5.9)	3.5 (3 to 4.2)
Mongolia	Hodgkin lymphoma	0.146 (0.109 to 0.225)	0.184 (0.155 to 0.24)	0.187 (0.154 to 0.242)	9.7 (7.9 to 13.2)	7.7 (6.1 to 9.3)	6 (4.8 to 7.5)
Mongolia	Non-Hodgkin's lymphoma	1.27 (1.15 to 1.42)	1.36 (1.24 to 1.5)	1.69 (1.45 to 1.94)	83.2 (75.9 to 91.5)	66.7 (61.1 to 73.4)	59.5 (51.4 to 67.4)
Mongolia	Multiple myeloma	0.146 (0.122 to 0.162)	0.213 (0.167 to 0.241)	0.322 (0.245 to 0.382)	12.9 (10.3 to 14.3)	12.6 (9.7 to 14.4)	12.6 (9.4 to 15.1)
Mongolia	Leukaemia	2.6 (2.02 to 4.03)	3.08 (2.63 to 3.86)	3.35 (2.7 to 4.23)	121.7 (99.5 to 171.5)	125.2 (109.4 to 151.1)	108.8 (88.8 to 134.8)
Mongolia	Acute lymphoid leukaemia	0.889 (0.609 to 1.5)	0.805 (0.605 to 1.13)	0.829 (0.594 to 1.2)	31.4 (22 to 50.7)	30.3 (22.7 to 41.8)	25.5 (18.7 to 36)
Mongolia	Chronic lymphoid leukaemia	0.0229 (0.0152 to 0.0393)	0.0367 (0.0277 to 0.0561)	0.0448 (0.0337 to 0.0642)	1.5 (1.1 to 2.2)	1.7 (1.3 to 2.3)	1.6 (1.2 to 2.3)
Mongolia	Acute myeloid leukaemia	1.15 (0.886 to 1.76)	1.67 (1.35 to 1.98)	1.89 (1.47 to 2.33)	57.4 (46.1 to 81.5)	65.1 (52.5 to 76.4)	59.7 (46.7 to 73.1)
Mongolia	Chronic myeloid leukaemia	0.146 (0.115 to 0.193)	0.191 (0.151 to 0.299)	0.21 (0.162 to 0.308)	10.7 (8.5 to 13.7)	9.1 (7.3 to 13.3)	7.1 (5.6 to 9.8)
Mongolia	Other leukaemia	0.39 (0.221 to 1.06)	0.378 (0.283 to 0.754)	0.384 (0.28 to 0.626)	20.7 (14.2 to 42.3)	19 (15.1 to 30.5)	14.8 (11.5 to 20.8)
Mongolia	Other malignant cancers	7.44 (4.64 to 9.05)	4.82 (3.93 to 6.72)	5.76 (4.51 to 8.29)	500.9 (288.8 to 627.8)	232.9 (196.2 to 306.1)	200.9 (160.5 to 279.2)
Mongolia	Other neoplasms	0.223 (0.162 to 0.382)	0.347 (0.284 to 0.493)	0.473 (0.381 to 0.659)	13 (10.2 to 18.9)	16.5 (13.7 to 22.6)	16.7 (13.6 to 22.6)
Mongolia	Myelodysplastic, myeloproliferative, and other hematopoietic neoplasms	0.162 (0.121 to 0.27)	0.252 (0.208 to 0.342)	0.359 (0.292 to 0.482)	10.5 (8.3 to 14.9)	13.2 (11 to 17.2)	13.3 (11 to 17.4)
Mongolia	Benign and in situ intestinal neoplasms	0 (0 to 0)	0 (0 to 0)	0 (0 to 0)	0 (0 to 0)	0 (0 to 0)	0 (0 to 0)
Mongolia	Benign and in situ cervical and uterine neoplasms	0 (0 to 0)	0 (0 to 0)	0 (0 to 0)	0 (0 to 0)	0 (0 to 0)	0 (0 to 0)
Mongolia	Other benign and in situ neoplasms	0.0612 (0.0393 to 0.112)	0.0944 (0.0699 to 0.152)	0.114 (0.0826 to 0.181)	2.5 (1.7 to 4.3)	3.3 (2.4 to 5.5)	3.3 (2.4 to 5.2)

SR Appendix Table 2. All-age DALYs counts and age standardized DALYs rates per 100,000 individuals for all causes and level 1 cause groups by location for 1990, 2007, and 2017, both sexes combined

		All-age DALYs			Age-standardized rate of DALYs per 100,000		
Mongolia	Cardiovascular diseases	127 (120 to 134)	187 (180 to 195)	205 (188 to 224)	11438.8 (10905.7 to 12049.5)	12018.9 (11601.7 to 12429.5)	8890.9 (8225 to 9629.7)
Mongolia	Rheumatic heart disease	5.34 (4.53 to 6.37)	4.93 (4.39 to 5.59)	4.69 (4.04 to 5.4)	390.7 (327.7 to 465.8)	237.3 (216.3 to 262.8)	162.6 (142.7 to 183.5)
Mongolia	Ischaemic heart disease	64.6 (60.3 to 71)	85.1 (81.1 to 89.4)	91.6 (83.3 to 101)	6257.7 (5882.6 to 6856.4)	5783.4 (5545.7 to 6056.6)	4225.2 (3866.4 to 4607.7)
Mongolia	Stroke	42.5 (39.4 to 46.2)	83.4 (79.4 to 87.6)	92.1 (84.2 to 101)	3608.6 (3361.9 to 3921.6)	5191.6 (4964.5 to 5433.3)	3808 (3489.5 to 4146)
Mongolia	Ischaemic stroke	5.78 (5.18 to 6.42)	7.51 (6.75 to 8.25)	9.3 (8.16 to 10.5)	555.9 (501.7 to 615)	521.6 (472.7 to 571.2)	445.5 (391.2 to 499.8)
Mongolia	Intracerebral hemorrhage	27.4 (24.9 to 30.7)	68.3 (63.4 to 72.3)	74.6 (67.8 to 81.8)	2334.7 (2139.1 to 2594.9)	4238.5 (3991.2 to 4459)	3044.1 (2774.9 to 3318.7)
Mongolia	Subarachnoid hemorrhage	9.32 (8.21 to 10.3)	7.61 (6.45 to 10.8)	8.25 (6.72 to 12.4)	718 (637.6 to 809.3)	431.5 (373.7 to 592.3)	318.4 (265.8 to 453.3)
Mongolia	Hypertensive heart disease	4.05 (2.47 to 4.64)	3.41 (2.83 to 3.82)	4.02 (3.39 to 4.62)	380.9 (233.6 to 434.7)	227.1 (194.8 to 253)	179.7 (153.7 to 207)
Mongolia	Non-rheumatic valvular heart disease	0.447 (0.384 to 0.504)	0.62 (0.494 to 0.691)	0.808 (0.617 to 0.938)	39.4 (30.9 to 44.5)	38.9 (29.2 to 43.4)	35.3 (26.6 to 41.1)
Mongolia	Non-rheumatic calcific aortic valve disease	0.0962 (0.0725 to 0.139)	0.155 (0.125 to 0.193)	0.225 (0.179 to 0.274)	8.7 (6.5 to 12.7)	9.9 (8 to 12.7)	10 (8.2 to 12.2)
Mongolia	Non-rheumatic degenerative mitral valve disease	0.305 (0.227 to 0.356)	0.399 (0.271 to 0.455)	0.493 (0.329 to 0.585)	26.8 (17.7 to 31.3)	24.7 (15.5 to 29)	21 (13.3 to 24.7)
Mongolia	Other non-rheumatic valve diseases	0.0455 (0.0298 to 0.0625)	0.065 (0.0443 to 0.0898)	0.0893 (0.0552 to 0.126)	4 (2.1 to 5.8)	4.4 (2.3 to 6)	4.3 (2.1 to 6.5)
Mongolia	Cardiomyopathy and myocarditis	3.65 (3.09 to 4.69)	5.53 (4.93 to 7.28)	6.85 (5.9 to 9.02)	243.2 (206.3 to 300.6)	261.4 (235.9 to 327)	231.4 (202 to 290.6)
Mongolia	Myocarditis	1.12 (0.832 to 1.43)	1.45 (1.07 to 1.8)	1.76 (1.28 to 2.24)	68.9 (47.5 to 93)	65.9 (46.1 to 79.5)	57.5 (41 to 72.4)
Mongolia	Alcoholic cardiomyopathy	0.246 (0.18 to 0.407)	0.457 (0.322 to 0.715)	0.708 (0.502 to 1.06)	20.3 (14.7 to 33.5)	21.8 (15.6 to 33.9)	21.8 (15.5 to 33.1)
Mongolia	Other cardiomyopathy	2.29 (1.94 to 3.1)	3.63 (3.2 to 4.79)	4.37 (3.72 to 5.86)	154.1 (134.1 to 201.4)	173.7 (155.3 to 220.6)	152 (130.1 to 193.2)
Mongolia	Atrial fibrillation and flutter	0.759 (0.633 to 0.913)	0.914 (0.748 to 1.11)	1.36 (1.11 to 1.66)	83 (70.2 to 98.2)	82.2 (68.9 to 97.5)	81.7 (68 to 97.6)
Mongolia	Aortic aneurysm	0.231 (0.196 to 0.303)	0.367 (0.324 to 0.458)	0.467 (0.398 to 0.569)	20.5 (17.5 to 26.7)	21.8 (19.5 to 26.5)	19.1 (16.4 to 22.7)
Mongolia	Peripheral vascular disease	0.0741 (0.0384 to 0.126)	0.0861 (0.0479 to 0.143)	0.126 (0.0691 to 0.208)	7.7 (4.1 to 13.1)	7.6 (4.1 to 12.5)	7.5 (4.1 to 12.3)
Mongolia	Endocarditis	0.162 (0.119 to 0.21)	0.225 (0.201 to 0.25)	0.273 (0.229 to 0.318)	10.6 (7.8 to 13.9)	12 (10.9 to 13.3)	10.6 (9 to 12.2)
Mongolia	Other cardiovascular and circulatory diseases	5.16 (2.55 to 6.35)	2.64 (2.16 to 3.64)	3.11 (2.48 to 4.32)	396.2 (172.1 to 493.7)	155.6 (130.6 to 193.9)	129.9 (107 to 164.1)
Mongolia	Chronic respiratory diseases	19.5 (17.6 to 21.7)	17 (14.9 to 19.1)	20 (17.3 to 22.8)	1457.4 (1331.9 to 1576.6)	991.2 (890 to 1103.4)	808.4 (713 to 912.4)
Mongolia	Chronic obstructive pulmonary disease	8.93 (7.81 to 10.1)	9.14 (8.16 to 10.3)	10.7 (9.34 to 12.2)	775 (677.8 to 875.8)	581.4 (523.3 to 658.1)	465.8 (410.4 to 534)
Mongolia	Pneumoconiosis	0.0457 (0.0345 to 0.0606)	0.0439 (0.036 to 0.0542)	0.0487 (0.0388 to 0.0594)	4.4 (3.3 to 5.9)	3.2 (2.6 to 4)	2.3 (1.9 to 2.8)
Mongolia	Silicosis	0.00244 (0.0012 to 0.00914)	0.00208 (0.00123 to 0.00752)	0.00223 (0.00129 to 0.00709)	0.2 (0.1 to 1)	0.1 (0.1 to 0.6)	0.1 (0.1 to 0.4)
Mongolia	Asbestosis	0.00185 (0.00122 to 0.00303)	0.00228 (0.00152 to 0.00385)	0.00297 (0.00177 to 0.00501)	0.1 (0.1 to 0.3)	0.1 (0.1 to 0.3)	0.1 (0.1 to 0.2)
Mongolia	Coal workers pneumoconiosis	0.0309 (0.0209 to 0.0428)	0.0304 (0.0216 to 0.0395)	0.0328 (0.0225 to 0.0429)	2.9 (1.9 to 4)	2.2 (1.5 to 2.8)	1.5 (1 to 2)
Mongolia	Other pneumoconiosis	0.0105 (0.00751 to 0.014)	0.00916 (0.00701 to 0.0121)	0.0107 (0.008 to 0.0143)	1.2 (0.8 to 1.6)	0.8 (0.6 to 1.1)	0.6 (0.5 to 0.8)
Mongolia	Asthma	6.42 (5.04 to 7.95)	4.9 (3.76 to 6.23)	5.8 (4.33 to 7.58)	473.2 (366 to 568.8)	265.7 (213.8 to 326.6)	218.4 (167.7 to 276.8)
Mongolia	Interstitial lung disease and pulmonary sarcoidosis	2.67 (1.41 to 3.68)	1.94 (1.65 to 2.32)	2.33 (1.96 to 2.78)	149.8 (101.6 to 187.1)	105.7 (89.5 to 125.2)	90.1 (76.2 to 105.7)
Mongolia	Other chronic respiratory diseases	1.43 (0.748 to 2.3)	0.94 (0.764 to 1.16)	1.08 (0.89 to 1.29)	55 (34.4 to 81.7)	35.1 (28.8 to 43.5)	31.8 (26.5 to 37.7)
Mongolia	Digestive diseases	53.3 (46.6 to 59.1)	55.4 (49.7 to 66.5)	68.3 (59.6 to 78.1)	3272.6 (2982.7 to 3582.8)	2786.3 (2545.5 to 3124.9)	2410.4 (2126.2 to 2694.4)
Mongolia	Cirrhosis and other chronic liver diseases	24.5 (21.8 to 27.6)	34.7 (30.8 to 44)	44.4 (38 to 52.2)	1764 (1574.2 to 2051.7)	1808 (1638.3 to 2112.6)	1583.6 (1379.9 to 1782.4)
Mongolia	Cirrhosis and other chronic liver diseases due to hepatitis B	5.64 (4.67 to 7.05)	8.17 (6.77 to 11)	9.96 (8.22 to 12.3)	435.2 (364.3 to 543.4)	412.1 (349.7 to 516.1)	347.5 (293.1 to 412.4)
Mongolia	Cirrhosis and other chronic liver diseases due to hepatitis C	4.97 (4.11 to 6.18)	8.34 (6.83 to 10.9)	10.9 (8.81 to 13.4)	402.8 (335.3 to 498.7)	439.5 (373.8 to 528.7)	387.9 (322.9 to 462.2)
Mongolia	Cirrhosis and other chronic liver diseases due to alcohol use	6.71 (5.57 to 8.25)	12 (10.2 to 15.6)	16 (13.4 to 19.2)	559.8 (470.8 to 678.4)	641.2 (556.4 to 768.8)	569.3 (483.1 to 657.7)
Mongolia	Cirrhosis due to NASH	1.3 (1.07 to 1.63)	2.29 (1.95 to 2.88)	3.25 (2.7 to 3.89)	107.9 (89.9 to 133.9)	126.6 (109.6 to 149.2)	118.8 (100.2 to 138.6)
Mongolia	Cirrhosis and other chronic liver diseases due to other causes	5.84 (3.97 to 7.41)	3.89 (3.3 to 4.74)	4.28 (3.51 to 5.21)	258.3 (200.2 to 313.1)	188.5 (160.3 to 220.4)	160.1 (131.1 to 191.8)
Mongolia	Upper digestive system diseases	6.5 (5.46 to 7.93)	7.98 (6.41 to 10.2)	9.58 (7.55 to 12.4)	446.8 (380.5 to 534.3)	401.6 (333.8 to 491)	344.7 (280 to 432.3)
Mongolia	Peptic ulcer disease	4.27 (3.66 to 4.88)	4.64 (4.04 to 5.6)	5.18 (4.43 to 6.18)	309 (270.3 to 352.5)	261.3 (230.1 to 304.9)	203.3 (177.3 to 237.9)

SR Appendix Table 2. All-age DALYs counts and age standardized DALYs rates per 100,000 individuals for all causes and level 1 cause groups by location for 1990, 2007, and 2017, both sexes combined

		All-age DALYs			Age-standardized rate of DALYs per 100,000		
Mongolia	Gastritis and duodenitis	0.802 (0.555 to 1.1)	1.1 (0.762 to 1.55)	1.46 (1 to 2.06)	45.8 (32.8 to 62.1)	48.3 (34 to 66)	49.4 (33.6 to 68.5)
Mongolia	GERD	1.43 (0.765 to 2.45)	2.24 (1.18 to 3.86)	2.94 (1.57 to 4.99)	92 (48.9 to 157.5)	92 (49.1 to 157.9)	92 (48.3 to 156.1)
Mongolia	Appendicitis	3.91 (1.39 to 5.05)	1.83 (0.98 to 2.28)	1.6 (0.848 to 2.11)	153 (61.6 to 190.8)	70.2 (37.8 to 87.7)	49.1 (27 to 62.9)
Mongolia	Paralytic ileus and intestinal obstruction	7.35 (5.55 to 9.43)	3.4 (2.8 to 4.31)	3.56 (2.75 to 4.54)	292.8 (215.6 to 363.5)	142.4 (120 to 173.7)	113.5 (88.8 to 141)
Mongolia	Inguinal, femoral, and abdominal hernia	3.6 (2.25 to 4.74)	2.17 (1.47 to 2.99)	2.5 (1.71 to 3.43)	181.6 (120.7 to 233.9)	94.9 (63.9 to 128.6)	80.5 (55.5 to 108.8)
Mongolia	Inflammatory bowel disease	0.585 (0.3 to 1.06)	0.453 (0.311 to 0.606)	0.593 (0.418 to 0.833)	31.1 (17.7 to 46.3)	19.8 (13.6 to 26.2)	18.8 (13.4 to 26.2)
Mongolia	Vascular intestinal disorders	0.197 (0.14 to 0.275)	0.171 (0.146 to 0.212)	0.221 (0.184 to 0.275)	16.8 (12.1 to 23.5)	11.9 (10 to 14.3)	10.3 (8.4 to 12.4)
Mongolia	Gallbladder and biliary diseases	1.38 (1.05 to 1.6)	0.82 (0.72 to 0.985)	0.928 (0.785 to 1.11)	105.1 (82.9 to 119.4)	50.5 (44.3 to 58.2)	40 (34.2 to 46.4)
Mongolia	Pancreatitis	1.59 (1.13 to 1.99)	2.24 (1.69 to 2.81)	3.11 (2.14 to 3.8)	125.3 (84.3 to 156.6)	111.4 (80.3 to 136.6)	106.1 (69.5 to 130.1)
Mongolia	Other digestive diseases	3.76 (2.41 to 5.16)	1.6 (1.36 to 1.89)	1.84 (1.5 to 2.3)	156 (111.1 to 196.4)	75.4 (65.3 to 88.6)	63.9 (53.7 to 77.6)
Mongolia	Neurological disorders	24.2 (17.9 to 31.6)	33.9 (25.2 to 45.3)	40.7 (29.6 to 54.3)	1551.3 (1215.4 to 1937.4)	1535.8 (1203.9 to 1957.5)	1480.4 (1129.5 to 1895.2)
Mongolia	Alzheimer's disease and other dementias	3.5 (3.22 to 3.87)	3.29 (3.05 to 3.55)	4.6 (4.06 to 5.14)	453.1 (413.5 to 505.6)	387.7 (357 to 419.9)	364.5 (325.1 to 405.1)
Mongolia	Parkinson's disease	0.729 (0.649 to 0.809)	0.756 (0.684 to 0.854)	0.976 (0.846 to 1.14)	78.2 (69.8 to 86.3)	69.1 (62.7 to 77.3)	60.5 (52.6 to 70.6)
Mongolia	Epilepsy	5.29 (2.97 to 8.65)	8.1 (5.45 to 11.5)	8.67 (4.78 to 13.7)	238.6 (133.4 to 387.9)	287.7 (192.4 to 409.9)	263 (140.6 to 418.9)
Mongolia	Multiple sclerosis	0.228 (0.179 to 0.292)	0.4 (0.321 to 0.493)	0.538 (0.437 to 0.654)	16 (12.9 to 20.5)	16.8 (13.6 to 20.6)	16.2 (13.3 to 19.6)
Mongolia	Motor neuron disease	0.0196 (0.0146 to 0.0248)	0.0308 (0.0244 to 0.037)	0.0456 (0.0369 to 0.0536)	1.4 (1 to 1.8)	1.7 (1.2 to 2)	1.6 (1.2 to 1.9)
Mongolia	Headache disorders	13.3 (8.65 to 19.1)	19.9 (12.9 to 28.6)	24 (15.5 to 34.5)	711.6 (463.1 to 1021.8)	714.8 (464.5 to 1030.5)	714.9 (463.9 to 1021.9)
Mongolia	Migraine	11.5 (7.17 to 17.1)	17.3 (10.8 to 25.8)	20.9 (13 to 31)	617.2 (387.8 to 911.8)	620.1 (388.6 to 918)	620.2 (386.2 to 915.6)
Mongolia	Tension-type headache	1.74 (0.965 to 2.83)	2.53 (1.4 to 4.06)	3.08 (1.72 to 4.89)	94.4 (52.6 to 149.9)	94.7 (53.5 to 151.3)	94.7 (52.9 to 150.8)
Mongolia	Other neurological disorders	1.14 (0.671 to 1.76)	1.51 (1.02 to 2.1)	1.92 (1.13 to 2.96)	52.4 (34.1 to 76.3)	58.1 (41.2 to 77.9)	59.7 (35.5 to 91.7)
Mongolia	Mental disorders	26.3 (19.2 to 34.2)	38.3 (28 to 49.5)	45.6 (33.5 to 59.1)	1390.8 (1025.2 to 1798.7)	1421.9 (1042.6 to 1841.9)	1419 (1043 to 1843.5)
Mongolia	Schizophrenia	1.79 (1.29 to 2.28)	2.96 (2.17 to 3.73)	4.03 (2.97 to 5.04)	111.8 (81.5 to 140.4)	112.9 (83.9 to 141)	115.8 (85.5 to 144.3)
Mongolia	Depressive disorders	9.36 (6.59 to 12.8)	15 (10.5 to 20.5)	18 (12.6 to 24.4)	546.5 (386.9 to 740.7)	584.5 (410.4 to 796.1)	573 (403.2 to 771.8)
Mongolia	Major depressive disorder	7.64 (5.27 to 10.8)	12.4 (8.52 to 16.8)	14.6 (10 to 19.8)	439.7 (306.1 to 599.6)	477 (331 to 645.8)	465.6 (321.6 to 630.2)
Mongolia	Dysthymia	1.71 (1.14 to 2.49)	2.62 (1.73 to 3.83)	3.39 (2.23 to 4.96)	106.9 (71.2 to 154.8)	107.4 (71.7 to 158.2)	107.5 (71.2 to 155.7)
Mongolia	Bipolar disorder	2.4 (1.49 to 3.57)	3.62 (2.24 to 5.34)	4.24 (2.62 to 6.33)	127.5 (80.4 to 189.7)	127.7 (79.3 to 188.9)	127.6 (79.7 to 189.7)
Mongolia	Anxiety disorders	4.5 (3.17 to 6.07)	6.22 (4.39 to 8.27)	7.54 (5.32 to 10.2)	233.6 (166.3 to 311.3)	230.1 (163.7 to 305.3)	234.9 (165.7 to 316.2)
Mongolia	Eating disorders	0.624 (0.394 to 0.938)	0.992 (0.621 to 1.47)	1.32 (0.83 to 1.98)	28.5 (18.3 to 42)	30.3 (19 to 44.9)	36.9 (23 to 54.9)
Mongolia	Anorexia nervosa	0.139 (0.0793 to 0.213)	0.205 (0.119 to 0.313)	0.251 (0.154 to 0.393)	5.8 (3.4 to 8.8)	6.1 (3.6 to 9.4)	7.4 (4.6 to 11.5)
Mongolia	Bulimia nervosa	0.485 (0.298 to 0.736)	0.788 (0.479 to 1.19)	1.07 (0.656 to 1.6)	22.7 (14 to 34.2)	24.2 (14.7 to 36.6)	29.4 (18 to 44.5)
Mongolia	Autism spectrum disorders	1.45 (0.986 to 2.02)	1.75 (1.18 to 2.41)	2.1 (1.43 to 2.9)	63.7 (43.4 to 88.5)	63.5 (43.1 to 87.4)	63.6 (43.3 to 87.5)
Mongolia	Attention-deficit/hyperactivity disorder	0.289 (0.172 to 0.463)	0.354 (0.211 to 0.564)	0.381 (0.232 to 0.605)	11.6 (7 to 18.4)	11.7 (6.9 to 18.5)	11.7 (7.1 to 18.8)
Mongolia	Conduct disorder	2.82 (1.66 to 4.46)	3.07 (1.86 to 4.93)	2.76 (1.66 to 4.3)	97.1 (57.3 to 152.8)	97.4 (58.8 to 154.3)	97.7 (58.9 to 152.9)
Mongolia	Idiopathic developmental intellectual disability	0.736 (0.274 to 1.35)	0.703 (0.232 to 1.33)	0.632 (0.16 to 1.25)	30.9 (11.1 to 56.8)	24.8 (8 to 47)	18.9 (4.8 to 37.4)
Mongolia	Other mental disorders	2.3 (1.51 to 3.19)	3.62 (2.38 to 5.05)	4.65 (3.07 to 6.48)	139.5 (92.6 to 192.8)	139 (92.6 to 193.9)	138.9 (91.9 to 193.2)
Mongolia	Substance use disorders	7.49 (5.74 to 9.5)	30.9 (17.8 to 37.1)	39 (22 to 47.6)	426.3 (330.9 to 534.3)	1132.5 (634.5 to 1355.4)	1085.5 (622.1 to 1324.8)
Mongolia	Alcohol use disorders	5.08 (3.81 to 6.78)	26.1 (13 to 31.7)	33.2 (16.3 to 41.2)	301.4 (228.7 to 400.2)	966.6 (477.1 to 1174.5)	919.9 (458.5 to 1141.2)
Mongolia	Drug use disorders	2.41 (1.82 to 3.05)	4.81 (3.85 to 5.94)	5.8 (4.51 to 7.1)	125 (94.4 to 156.1)	165.9 (134.1 to 201.7)	165.6 (129.6 to 200.3)
Mongolia	Opioid use disorders	1.6 (1.19 to 2.07)	3.5 (2.79 to 4.31)	4.33 (3.35 to 5.34)	86.7 (65.3 to 108.9)	123.7 (99.4 to 150)	122.8 (95.6 to 150.5)
Mongolia	Cocaine use disorders	0.0884 (0.0572 to 0.13)	0.158 (0.106 to 0.219)	0.196 (0.133 to 0.27)	4.6 (3.1 to 6.5)	5.4 (3.7 to 7.3)	5.7 (3.9 to 7.8)

SR Appendix Table 2. All-age DALYs counts and age standardized DALYs rates per 100,000 individuals for all causes and level 1 cause groups by location for 1990, 2007, and 2017, both sexes combined

		All-age DALYs			Age-standardized rate of DALYs per 100,000		
Mongolia	Amphetamine use disorders	0.261 (0.149 to 0.428)	0.399 (0.235 to 0.646)	0.432 (0.261 to 0.678)	11.5 (6.8 to 18.3)	12.1 (7.4 to 19.4)	12.4 (7.5 to 19.3)
Mongolia	Cannabis use disorders	0.121 (0.0739 to 0.187)	0.178 (0.11 to 0.265)	0.185 (0.114 to 0.283)	5.3 (3.3 to 8.1)	5.4 (3.4 to 8)	5.6 (3.4 to 8.4)
Mongolia	Other drug use disorders	0.338 (0.242 to 0.474)	0.578 (0.431 to 0.776)	0.659 (0.486 to 0.876)	16.9 (12.6 to 22.9)	19.2 (14.6 to 25.3)	19.1 (14.3 to 25.2)
Mongolia	Diabetes and kidney diseases	22.5 (19.8 to 25.3)	24.4 (21 to 28.7)	29.6 (24.6 to 35.9)	1649 (1437.1 to 1865.7)	1221.7 (1049.9 to 1437.6)	1056.3 (872.3 to 1275.7)
Mongolia	Diabetes mellitus	6.36 (4.7 to 8.45)	11.4 (8.65 to 15)	16.1 (11.9 to 21.4)	511.3 (376.6 to 678)	586.4 (445.3 to 764.3)	569.8 (423.1 to 750)
Mongolia	Diabetes mellitus type 1	1.28 (1.14 to 1.45)	2.33 (2.12 to 2.58)	2.62 (2.3 to 2.97)	93.8 (83.8 to 105)	111.7 (101.9 to 123)	90.1 (79.6 to 101.7)
Mongolia	Diabetes mellitus type 2	5.08 (3.47 to 7.08)	9.04 (6.45 to 12.4)	13.4 (9.37 to 18.6)	417.6 (289.2 to 580.3)	474.7 (335.8 to 645.7)	479.8 (336.4 to 654.1)
Mongolia	Chronic kidney disease	16.1 (14.2 to 17.6)	13 (12 to 14.8)	13.6 (12.1 to 16.3)	1136.9 (977.1 to 1237.5)	635 (584.3 to 704.2)	486 (436 to 569.8)
Mongolia	Chronic kidney disease due to diabetes mellitus type 1	1.21 (0.949 to 1.53)	1.34 (1.02 to 1.71)	1.4 (1.04 to 1.85)	89.4 (69.2 to 114.2)	55 (42.5 to 70.3)	40.9 (31.2 to 53.5)
Mongolia	Chronic kidney disease due to diabetes mellitus type 2	2.02 (1.65 to 2.42)	1.54 (1.27 to 1.85)	1.86 (1.53 to 2.28)	187.6 (153.1 to 224.6)	100.8 (84.7 to 120.4)	80.6 (67.1 to 97)
Mongolia	Chronic kidney disease due to hypertension	1.36 (1.08 to 1.62)	1.13 (0.942 to 1.33)	1.26 (1.03 to 1.52)	123 (96.4 to 145.7)	69 (57.9 to 79.9)	54.3 (45.3 to 64)
Mongolia	Chronic kidney disease due to glomerulonephritis	4.92 (4.21 to 5.74)	4.14 (3.53 to 4.9)	4.11 (3.45 to 5.1)	319.6 (271.3 to 372.6)	176.7 (152.5 to 208.2)	131.7 (111.7 to 161.1)
Mongolia	Chronic kidney disease due to other and unspecified causes	6.6 (5.7 to 7.5)	4.91 (4.2 to 5.78)	4.93 (4.18 to 6.01)	417.4 (353.3 to 477.9)	233.4 (202.4 to 269.3)	178.6 (152.4 to 211.5)
Mongolia	Acute glomerulonephritis	0.0116 (0.00606 to 0.0193)	0.00988 (0.00752 to 0.0132)	0.00992 (0.00753 to 0.0132)	0.7 (0.4 to 1.3)	0.4 (0.3 to 0.6)	0.4 (0.3 to 0.5)
Mongolia	Skin and subcutaneous diseases	10.7 (7.21 to 15.8)	12.4 (8.44 to 17.9)	15.2 (10.3 to 22.1)	475.7 (323.8 to 694.9)	476 (325.7 to 689.4)	478.5 (325.3 to 691.8)
Mongolia	Dermatitis	2.6 (1.5 to 4.17)	2.87 (1.69 to 4.52)	3.51 (2.06 to 5.6)	107.9 (64 to 170.9)	108.2 (63.9 to 170.2)	108.5 (64 to 172.4)
Mongolia	Atopic dermatitis	2.17 (1.15 to 3.66)	2.26 (1.22 to 3.77)	2.78 (1.48 to 4.71)	84.8 (45.6 to 142.5)	85.1 (45.6 to 140.7)	85.3 (45.6 to 143.8)
Mongolia	Contact dermatitis	0.429 (0.272 to 0.644)	0.607 (0.39 to 0.927)	0.723 (0.461 to 1.07)	23 (15 to 34)	23 (15 to 34.5)	23 (14.9 to 34)
Mongolia	Seborrhoeic dermatitis	0.00224 (0.00125 to 0.00362)	0.00277 (0.00154 to 0.00446)	0.00344 (0.00192 to 0.00553)	0.1 (0.1 to 0.2)	0.1 (0.1 to 0.2)	0.1 (0.1 to 0.2)
Mongolia	Psoriasis	1.04 (0.73 to 1.39)	1.56 (1.08 to 2.05)	2.02 (1.41 to 2.7)	60.4 (42.5 to 80.1)	62.6 (43.9 to 82.3)	63.1 (44.3 to 83.8)
Mongolia	Bacterial skin diseases	0.664 (0.277 to 1)	0.555 (0.324 to 0.74)	0.638 (0.42 to 0.928)	28 (15.4 to 38.1)	23.9 (15.1 to 33.3)	20.7 (14.2 to 31.2)
Mongolia	Cellulitis	0.0597 (0.0323 to 0.0847)	0.0669 (0.0421 to 0.0877)	0.0827 (0.0533 to 0.109)	2.7 (1.6 to 3.8)	2.7 (1.6 to 3.6)	2.7 (1.7 to 3.6)
Mongolia	Pyoderma	0.604 (0.24 to 0.925)	0.489 (0.279 to 0.672)	0.556 (0.353 to 0.825)	25.3 (13.5 to 34.7)	21.2 (13 to 30.1)	18.1 (12.1 to 28)
Mongolia	Scabies	0.744 (0.408 to 1.21)	0.903 (0.5 to 1.46)	1.04 (0.579 to 1.69)	30.8 (17.2 to 49.9)	31.4 (17.6 to 51.3)	32.2 (17.9 to 52.4)
Mongolia	Fungal skin diseases	1.16 (0.454 to 2.41)	1.16 (0.461 to 2.43)	1.45 (0.572 to 3.05)	58.2 (23.3 to 121.8)	51.3 (20.5 to 107.1)	50.2 (20 to 105.8)
Mongolia	Viral skin diseases	1.29 (0.821 to 1.93)	1.23 (0.79 to 1.84)	1.51 (0.971 to 2.24)	45.6 (29.1 to 67.9)	45.8 (29.2 to 67.9)	45.8 (29.5 to 68.1)
Mongolia	Acne vulgaris	0.519 (0.297 to 0.876)	0.911 (0.529 to 1.55)	0.881 (0.515 to 1.52)	20.5 (11.9 to 34.2)	26.8 (15.7 to 45)	28.5 (16.7 to 49.1)
Mongolia	Alopecia areata	0.114 (0.0715 to 0.174)	0.167 (0.106 to 0.255)	0.205 (0.13 to 0.307)	5.9 (3.8 to 9)	5.9 (3.8 to 8.9)	5.9 (3.8 to 8.9)
Mongolia	Pruritus	0.166 (0.077 to 0.318)	0.226 (0.105 to 0.432)	0.296 (0.14 to 0.56)	9.2 (4.4 to 17.3)	9.3 (4.3 to 17.6)	9.6 (4.5 to 18.1)
Mongolia	Urticaria	1.84 (1.2 to 2.63)	2.02 (1.36 to 2.9)	2.5 (1.64 to 3.55)	74.4 (49.1 to 104.7)	74.7 (49.6 to 106.6)	74.8 (49 to 107)
Mongolia	Decubitus ulcer	0.0434 (0.0322 to 0.0605)	0.0569 (0.0435 to 0.0759)	0.0736 (0.0555 to 0.0978)	2.8 (2.1 to 3.8)	2.9 (2.2 to 3.9)	2.9 (2.2 to 3.9)
Mongolia	Other skin and subcutaneous diseases	0.549 (0.27 to 0.99)	0.75 (0.367 to 1.36)	1.06 (0.523 to 1.94)	32 (15.6 to 58.3)	33.2 (16.2 to 60.1)	36.1 (17.8 to 65.7)
Mongolia	Sense organ diseases	11.6 (7.69 to 16.9)	15.3 (10.1 to 22.8)	21 (13.7 to 31)	868.1 (581.7 to 1263.8)	847.1 (568.6 to 1237.1)	837.7 (558.3 to 1218.4)
Mongolia	Blindness and vision impairment	5.53 (3.51 to 8.54)	7.09 (4.5 to 11)	9.69 (6.11 to 14.9)	408.9 (260.2 to 615.9)	390.5 (248.9 to 592.8)	384.2 (243.7 to 578.5)
Mongolia	Glaucoma	0.0213 (0.0139 to 0.0305)	0.023 (0.0147 to 0.0338)	0.0344 (0.0216 to 0.0499)	2.1 (1.4 to 3)	1.9 (1.2 to 2.8)	1.9 (1.2 to 2.7)
Mongolia	Cataract	1.47 (0.981 to 2.05)	1.81 (1.22 to 2.5)	2.73 (1.82 to 3.83)	148.8 (99.3 to 205.4)	143.6 (96.6 to 197)	142.5 (95.6 to 196.3)
Mongolia	Age-related macular degeneration	0.00994 (0.00607 to 0.015)	0.0121 (0.00738 to 0.0186)	0.0191 (0.0113 to 0.0301)	1 (0.6 to 1.5)	1 (0.6 to 1.5)	1 (0.6 to 1.6)
Mongolia	Refraction disorders	2.24 (1.4 to 3.49)	2.75 (1.71 to 4.24)	3.4 (2.13 to 5.25)	116.8 (73.4 to 179)	111.9 (70.9 to 171.4)	110.7 (68.7 to 171)
Mongolia	Near vision loss	1.54 (0.734 to 2.8)	2.16 (1.03 to 3.93)	3.02 (1.44 to 5.52)	122.2 (58 to 220.5)	114.3 (54.7 to 208.1)	110.3 (52.5 to 200.8)
Mongolia	Other vision loss	0.253 (0.162 to 0.367)	0.347 (0.223 to 0.508)	0.485 (0.304 to 0.706)	18 (11.4 to 26.1)	17.8 (11.3 to 25.6)	17.9 (11.4 to 25.7)

SR Appendix Table 2. All-age DALYs counts and age standardized DALYs rates per 100,000 individuals for all causes and level 1 cause groups by location for 1990, 2007, and 2017, both sexes combined

		All-age DALYs			Age-standardized rate of DALYs per 100,000		
Mongolia	Age-related and other hearing loss	5.6 (3.82 to 7.89)	7.62 (5.12 to 10.9)	10.5 (6.97 to 15)	428.7 (294.9 to 601.1)	425.7 (291.4 to 598.8)	422.5 (288.7 to 596.3)
Mongolia	Other sense organ diseases	0.445 (0.275 to 0.65)	0.594 (0.362 to 0.878)	0.823 (0.512 to 1.21)	30.5 (18.9 to 43.9)	30.9 (19 to 45.2)	31.1 (19.2 to 45.7)
Mongolia	Musculoskeletal disorders	21.3 (15.5 to 28.7)	32.2 (23.4 to 43.6)	43 (31.1 to 58.1)	1491.1 (1087 to 2003.1)	1469.1 (1079.8 to 1983.6)	1439.6 (1049.8 to 1926.4)
Mongolia	Rheumatoid arthritis	0.482 (0.375 to 0.605)	0.659 (0.487 to 0.844)	0.932 (0.671 to 1.23)	36 (27.8 to 45.4)	33.9 (25.2 to 43.5)	33.9 (24.5 to 44.3)
Mongolia	Osteoarthritis	0.803 (0.398 to 1.6)	1.17 (0.586 to 2.36)	1.88 (0.953 to 3.74)	74.1 (36.8 to 146.5)	76.2 (38.3 to 151.7)	79.6 (40.6 to 157.7)
Mongolia	Low back pain	14.9 (10.7 to 20.4)	22.1 (15.7 to 30.2)	29.1 (20.8 to 39.6)	1017.7 (730 to 1376.8)	992.3 (712.4 to 1343.3)	972.2 (694.2 to 1300.4)
Mongolia	Neck pain	4 (2.78 to 5.62)	6.46 (4.49 to 9.01)	9.13 (6.3 to 12.9)	296.8 (207.1 to 413.6)	297.5 (208 to 416)	297.7 (207.8 to 416)
Mongolia	Gout	0.156 (0.104 to 0.214)	0.231 (0.155 to 0.322)	0.344 (0.228 to 0.476)	13.4 (9 to 18.4)	13.5 (9 to 18.7)	13.4 (9 to 18.7)
Mongolia	Other musculoskeletal disorders	0.895 (0.602 to 1.26)	1.54 (1.07 to 2.12)	1.55 (0.967 to 2.25)	52.9 (35.3 to 75.9)	55.7 (38.6 to 76.8)	42.7 (27.1 to 61.2)
Mongolia	Other non-communicable diseases	56.8 (49.6 to 64.9)	58.7 (50.5 to 68.8)	54.9 (45.2 to 65.5)	2261.6 (1956.7 to 2621.5)	2278.6 (1946.8 to 2668.9)	1672.7 (1370.8 to 2008.5)
Mongolia	Congenital anomalies	37.6 (31.8 to 43.7)	37.2 (32.8 to 42)	29.7 (23.9 to 35.9)	1207.1 (1031 to 1395.3)	1350.9 (1190 to 1524.6)	841.3 (680.7 to 1016.2)
Mongolia	Neural tube defects	1.28 (0.814 to 1.98)	0.942 (0.679 to 1.28)	0.711 (0.508 to 1.05)	41.5 (27.1 to 62.4)	34.1 (24.6 to 46.1)	20.4 (14.7 to 29.6)
Mongolia	Congenital heart anomalies	14.9 (9.36 to 23.9)	15.3 (12 to 21.2)	11.7 (7.92 to 18.1)	463.5 (295.7 to 735.1)	556.1 (433.7 to 762.8)	328 (224.2 to 502.7)
Mongolia	Orofacial clefts	0.278 (0.129 to 0.727)	0.108 (0.0689 to 0.185)	0.108 (0.0724 to 0.158)	9.4 (4.7 to 22.9)	4 (2.5 to 6.8)	3.2 (2.2 to 4.7)
Mongolia	Down's syndrome	1.63 (0.474 to 3.01)	1.66 (0.635 to 2.28)	1.45 (0.738 to 2.05)	52.3 (17 to 94.2)	60 (23 to 82.7)	40.2 (20.8 to 56.6)
Mongolia	Turner syndrome	0.00301 (0.00145 to 0.00515)	0.00369 (0.0018 to 0.00612)	0.00405 (0.00195 to 0.00673)	0.1 (0.1 to 0.2)	0.1 (0.1 to 0.2)	0.1 (0.1 to 0.2)
Mongolia	Klinefelter syndrome	0.000638 (0.000291 to 0.00123)	0.000854 (0.000386 to 0.00157)	0.000979 (0.00044 to 0.00193)	0 (0 to 0.1)	0 (0 to 0.1)	0 (0 to 0.1)
Mongolia	Other chromosomal abnormalities	0.841 (0.247 to 1.44)	1.16 (0.373 to 1.81)	1.12 (0.458 to 1.69)	27.8 (9.4 to 45.8)	41.7 (13.4 to 64.8)	31.3 (13.2 to 47.2)
Mongolia	Congenital musculoskeletal and limb anomalies	2.98 (2.01 to 3.99)	3.44 (2.43 to 4.55)	3.72 (2.61 to 4.95)	124.1 (87.3 to 164.7)	128.1 (90.7 to 169.2)	114.3 (80.1 to 151.7)
Mongolia	Urogenital congenital anomalies	0.318 (0.205 to 0.469)	0.347 (0.254 to 0.512)	0.325 (0.222 to 0.55)	10.5 (6.9 to 15.1)	12.5 (9.2 to 18.6)	9.4 (6.5 to 15.6)
Mongolia	Digestive congenital anomalies	5.9 (1.28 to 9.67)	5.68 (1.55 to 7.67)	3.95 (1.48 to 5.48)	185.7 (46.3 to 299.3)	204.2 (56.3 to 275.5)	110.8 (42.7 to 154)
Mongolia	Other congenital anomalies	9.4 (3.72 to 14.6)	8.5 (5.68 to 10.8)	6.58 (4.72 to 8.9)	292.2 (119.9 to 448.2)	310.2 (207.2 to 392.7)	183.5 (133.3 to 246.5)
Mongolia	Urinary diseases and male infertility	3.64 (3.29 to 4.06)	2.7 (2.34 to 3.12)	3.24 (2.73 to 3.87)	252.9 (227.4 to 282.2)	143.8 (123.6 to 168.6)	121.7 (102 to 145.1)
Mongolia	Urinary tract infections	2.52 (2.27 to 2.82)	1.65 (1.47 to 1.82)	1.77 (1.56 to 2.03)	164.9 (145.9 to 184.4)	79.6 (68.4 to 89.6)	62.4 (54.7 to 72.7)
Mongolia	Urolithiasis	0.135 (0.0759 to 0.213)	0.11 (0.0806 to 0.144)	0.137 (0.0985 to 0.185)	9.8 (5.3 to 16.7)	4.9 (3.7 to 6.5)	4.6 (3.3 to 6.1)
Mongolia	Benign prostatic hyperplasia	0.485 (0.308 to 0.698)	0.613 (0.39 to 0.878)	0.997 (0.631 to 1.43)	45.1 (28.8 to 64.7)	42.7 (27.1 to 61.3)	42 (26.9 to 60.1)
Mongolia	Male infertility	0.0462 (0.0184 to 0.1)	0.0743 (0.0302 to 0.155)	0.0864 (0.0349 to 0.181)	2.3 (0.9 to 4.7)	2.3 (0.9 to 4.7)	2.3 (0.9 to 4.8)
Mongolia	Other urinary diseases	0.459 (0.312 to 0.668)	0.252 (0.153 to 0.356)	0.255 (0.159 to 0.373)	30.8 (20 to 49.9)	14.2 (7.6 to 22.6)	10.4 (5.8 to 15.8)
Mongolia	Gynecological diseases	2.99 (2.12 to 4.1)	4.97 (3.42 to 7)	5.82 (4.03 to 8.2)	168.1 (118.3 to 231.7)	167.2 (114.7 to 236.5)	158.9 (110.1 to 223.8)
Mongolia	Uterine fibroids	0.411 (0.254 to 0.645)	0.87 (0.529 to 1.38)	0.971 (0.58 to 1.6)	30.8 (18.9 to 48)	33.9 (20.5 to 53.7)	27.4 (16.5 to 45.1)
Mongolia	Polycystic ovarian syndrome	0.121 (0.0543 to 0.233)	0.19 (0.0849 to 0.364)	0.219 (0.0969 to 0.423)	6.2 (2.8 to 11.8)	6.1 (2.7 to 11.6)	6 (2.7 to 11.6)
Mongolia	Female infertility	0.051 (0.018 to 0.113)	0.0773 (0.0269 to 0.172)	0.0961 (0.0324 to 0.216)	2.5 (0.9 to 5.5)	2.4 (0.8 to 5.3)	2.5 (0.8 to 5.7)
Mongolia	Endometriosis	1.01 (0.677 to 1.47)	1.62 (1.05 to 2.35)	1.94 (1.28 to 2.84)	53 (35.6 to 76.4)	51.8 (33.9 to 74.8)	51.5 (34.1 to 75.2)
Mongolia	Genital prolapse	0.0583 (0.0312 to 0.101)	0.0579 (0.029 to 0.106)	0.0918 (0.0452 to 0.171)	4.6 (2.4 to 8)	3.1 (1.6 to 5.7)	3.3 (1.7 to 6.2)
Mongolia	Premenstrual syndrome	0.952 (0.605 to 1.43)	1.58 (1.01 to 2.41)	1.76 (1.13 to 2.65)	49.1 (31.1 to 74.2)	50.5 (32.5 to 76.5)	48.4 (31 to 72.8)
Mongolia	Other gynecological diseases	0.381 (0.268 to 0.524)	0.573 (0.392 to 0.851)	0.741 (0.496 to 1.11)	21.9 (15.4 to 30.3)	19.4 (13.3 to 28.9)	19.8 (13.2 to 29.7)
Mongolia	Hemoglobinopathies and hemolytic anaemias	2.79 (1.95 to 3.76)	2.55 (1.81 to 3.49)	2.51 (1.78 to 3.46)	126.5 (89.5 to 171)	97.9 (70 to 133.5)	79.1 (56.5 to 107.4)
Mongolia	Thalassemias	0.347 (0.138 to 0.642)	0.17 (0.115 to 0.243)	0.143 (0.104 to 0.195)	10.7 (4.4 to 19.5)	6.4 (4.3 to 9.2)	4.2 (3 to 5.6)
Mongolia	Thalassaemias trait	1.07 (0.709 to 1.54)	1.16 (0.76 to 1.69)	1.18 (0.78 to 1.74)	48.4 (32.6 to 69.3)	43.8 (28.9 to 63.4)	36.8 (24.1 to 54)
Mongolia	Sickle cell disorders	0.0468 (0.0153 to 0.0986)	0.0254 (0.0145 to 0.0426)	0.0213 (0.013 to 0.0339)	1.5 (0.5 to 3)	0.9 (0.5 to 1.6)	0.6 (0.4 to 1)

SR Appendix Table 2. All-age DALYs counts and age standardized DALYs rates per 100,000 individuals for all causes and level 1 cause groups by location for 1990, 2007, and 2017, both sexes combined

		All-age DALYs			Age-standardized rate of DALYs per 100,000		
Mongolia	Sickle cell trait	0.0785 (0.0496 to 0.116)	0.0791 (0.0482 to 0.123)	0.0854 (0.052 to 0.128)	3.4 (2.1 to 5)	2.9 (1.8 to 4.5)	2.6 (1.6 to 3.9)
Mongolia	G6PD deficiency	0.0556 (0.0385 to 0.0714)	0.0822 (0.0664 to 0.0996)	0.0844 (0.0702 to 0.103)	2.4 (1.7 to 3.1)	2.7 (2.2 to 3.4)	2.5 (2.1 to 3.1)
Mongolia	G6PD trait	0.0000774 (0.0000509 to 0.00011)	0.000109 (0.0000721 to 0.000154)	0.000117 (0.000077 to 0.000172)	0 (0 to 0)	0 (0 to 0)	0 (0 to 0)
Mongolia	Other hemoglobinopathies and hemolytic anaemias	1.2 (0.84 to 1.67)	1.04 (0.731 to 1.44)	0.993 (0.702 to 1.39)	60.1 (42.8 to 82.4)	41.2 (29.6 to 55.9)	32.3 (23.1 to 44.5)
Mongolia	Endocrine, metabolic, blood, and immune disorders	4.99 (3.5 to 6.02)	3.91 (3.26 to 4.62)	4.11 (3.31 to 5.19)	208.6 (154 to 248.9)	151 (127.1 to 179.2)	124.2 (100.6 to 154.3)
Mongolia	Oral disorders	3.65 (2.28 to 5.49)	6.42 (4 to 9.63)	8.67 (5.45 to 13)	263.7 (168.7 to 391.4)	334.5 (214.3 to 493)	324.9 (208.7 to 481.2)
Mongolia	Caries of deciduous teeth	0.0739 (0.0315 to 0.143)	0.0566 (0.0237 to 0.112)	0.0799 (0.0331 to 0.158)	2.3 (1 to 4.5)	2.3 (1 to 4.6)	2.3 (1 to 4.5)
Mongolia	Caries of permanent teeth	0.517 (0.22 to 0.97)	0.73 (0.312 to 1.39)	0.806 (0.345 to 1.54)	26 (11.2 to 49.2)	26.2 (11.3 to 49.6)	24.6 (10.6 to 46.8)
Mongolia	Periodontal disease	0.424 (0.167 to 0.883)	1.02 (0.402 to 2.16)	1.42 (0.558 to 3.05)	33 (13.1 to 68.7)	47.6 (18.8 to 97.6)	45.8 (18.2 to 96.2)
Mongolia	Edentulism and severe tooth loss	1.7 (1.13 to 2.43)	3.26 (2.16 to 4.63)	4.7 (3.08 to 6.71)	151.7 (100.5 to 215.7)	207.6 (139 to 293.1)	201.3 (133.8 to 286.9)
Mongolia	Other oral disorders	0.94 (0.591 to 1.4)	1.35 (0.845 to 1.98)	1.66 (1.04 to 2.46)	50.7 (32 to 75.1)	50.9 (32 to 75.1)	50.9 (31.7 to 74.8)
Mongolia	Sudden infant death syndrome	1.15 (0.453 to 3.16)	0.924 (0.483 to 1.93)	0.837 (0.503 to 1.33)	34.7 (13.7 to 95.3)	33.2 (17.3 to 69.5)	22.8 (13.7 to 36.3)
Mongolia	Injuries	120 (111 to 130)	161 (147 to 176)	166 (147 to 186)	5892.7 (5374 to 6463.2)	5900.8 (5326.7 to 6477.3)	4903.2 (4355 to 5519.7)
Mongolia	Transport injuries	22.4 (19.4 to 26.7)	34.8 (31.7 to 38.1)	35.3 (31.2 to 39.6)	1043 (908.1 to 1225.2)	1262.9 (1153.2 to 1380.1)	1034.9 (919.4 to 1155.9)
Mongolia	Road injuries	19.4 (16.5 to 23.9)	32.1 (29.1 to 35.2)	33.3 (29.6 to 37.4)	906.1 (775.5 to 1088.7)	1166.7 (1065.2 to 1274.8)	978.3 (871.4 to 1092.8)
Mongolia	Pedestrian road injuries	8.46 (5.96 to 13.2)	8.81 (7.56 to 11)	8.49 (7.02 to 11.1)	370.5 (270.1 to 575)	341.1 (294.7 to 421.2)	258.6 (216.3 to 333.9)
Mongolia	Cyclist road injuries	0.459 (0.331 to 0.618)	0.656 (0.491 to 0.896)	0.955 (0.702 to 1.26)	26.4 (19.1 to 34.1)	26.3 (19.7 to 35.3)	30.2 (22.4 to 39.5)
Mongolia	Motorcyclist road injuries	2.41 (1.66 to 3.15)	5.85 (4.61 to 7.33)	6.1 (4.71 to 7.55)	126.3 (88.9 to 164.7)	201.8 (156.6 to 253.2)	170.4 (133.3 to 209.3)
Mongolia	Motor vehicle road injuries	7.33 (4.91 to 9.12)	15.5 (13.2 to 17.5)	16.5 (14.3 to 18.8)	346 (244.2 to 420.5)	550.3 (466.2 to 617.2)	480.3 (419.2 to 544.2)
Mongolia	Other road injuries	0.733 (0.479 to 1.17)	1.26 (0.736 to 1.61)	1.29 (0.779 to 1.75)	37 (24.7 to 61.7)	47.2 (28.3 to 60)	38.6 (24.1 to 51.6)
Mongolia	Other transport injuries	3 (2.61 to 3.56)	2.69 (2.41 to 3)	1.95 (1.62 to 2.36)	136.9 (122.3 to 158)	96.2 (86.2 to 107.8)	56.7 (47.2 to 67.9)
Mongolia	Unintentional injuries	70.1 (62.3 to 79.3)	79.9 (68.7 to 90.7)	86.8 (73.9 to 102)	3499.6 (3034.2 to 4049.7)	3083 (2628.8 to 3523.7)	2619.7 (2215.3 to 3065.7)
Mongolia	Falls	15.1 (12 to 18.7)	22 (17.5 to 27.1)	28.9 (22.4 to 36.2)	929.6 (742 to 1163)	915.2 (723.6 to 1130.6)	922.5 (712.1 to 1157.8)
Mongolia	Drowning	10.7 (8.6 to 13.2)	9.63 (8.48 to 10.9)	8.44 (7.13 to 10)	398.1 (329.1 to 482.6)	344 (302.9 to 392.2)	249.3 (211.6 to 295.3)
Mongolia	Fire, heat, and hot substances	8.74 (6.27 to 11.2)	9.87 (5.83 to 14)	10.1 (6.25 to 14.6)	355.8 (281.9 to 429.1)	372.5 (217.6 to 534.5)	292.3 (183.3 to 421.3)
Mongolia	Poisonings	10.5 (5.86 to 12.7)	5.03 (3.94 to 8.52)	4.85 (3.69 to 8.61)	525.7 (276.6 to 642.4)	185.7 (147.3 to 313.6)	141.6 (108.7 to 245.7)
Mongolia	Poisoning by carbon monoxide	3.13 (1.49 to 4.33)	3.94 (3.11 to 6.66)	3.78 (2.87 to 6.74)	171.4 (81.8 to 235.1)	143.5 (114.6 to 236.6)	109.6 (84 to 191)
Mongolia	Poisoning by other means	7.38 (3.72 to 9.38)	1.08 (0.752 to 2.09)	1.07 (0.709 to 2.02)	354.3 (139.3 to 445.4)	42.2 (29.7 to 80.8)	32 (21.6 to 59.9)
Mongolia	Exposure to mechanical forces	5.56 (4.81 to 6.73)	7.73 (5.83 to 9.09)	8.33 (6.54 to 10.1)	287.3 (244.7 to 345.1)	290.1 (225.4 to 342.8)	249.5 (197.8 to 305.1)
Mongolia	Unintentional firearm injuries	1.08 (0.73 to 1.56)	1.13 (0.797 to 1.48)	0.955 (0.741 to 1.31)	53.4 (35.8 to 77.1)	38.9 (28.2 to 50.8)	27.9 (22 to 37.8)
Mongolia	Other exposure to mechanical forces	4.48 (3.73 to 5.63)	6.6 (5.05 to 7.84)	7.37 (5.73 to 9.05)	233.8 (191.3 to 288)	251.2 (194.8 to 300.9)	221.5 (174.5 to 273.9)
Mongolia	Adverse effects of medical treatment	1.11 (0.884 to 1.67)	1.2 (0.894 to 1.44)	1.24 (0.941 to 1.56)	46.6 (39.1 to 65.2)	47.3 (34.7 to 56.9)	37.6 (28.8 to 46.6)
Mongolia	Animal contact	0.578 (0.341 to 0.892)	0.615 (0.362 to 0.819)	0.639 (0.388 to 0.854)	23.7 (15.5 to 33.8)	23.9 (13.8 to 32)	19.6 (12.1 to 26.1)
Mongolia	Venomous animal contact	0.296 (0.159 to 0.554)	0.268 (0.178 to 0.389)	0.295 (0.199 to 0.408)	12.6 (7.4 to 20.8)	10.2 (6.7 to 14.8)	9 (6.1 to 12.4)
Mongolia	Non-venomous animal contact	0.282 (0.166 to 0.487)	0.347 (0.157 to 0.475)	0.345 (0.155 to 0.506)	11.1 (7.3 to 17.2)	13.8 (6.3 to 19)	10.6 (4.8 to 15.1)
Mongolia	Foreign body	4.05 (3.14 to 7.79)	9.13 (6.39 to 11.2)	7.51 (5.45 to 9.35)	148.3 (119.9 to 272.3)	338 (238.9 to 412.7)	212.5 (156.2 to 262.3)
Mongolia	Pulmonary aspiration and foreign body in airway	3.58 (2.71 to 7.3)	8.32 (5.73 to 10.2)	6.56 (4.65 to 8.34)	123.9 (96.7 to 245.2)	307.1 (213.4 to 377.9)	183.4 (131.8 to 232.1)
Mongolia	Foreign body in eyes	0.0389 (0.0168 to 0.0705)	0.0516 (0.0225 to 0.0914)	0.0654 (0.0287 to 0.116)	2 (1 to 3.5)	2 (0.9 to 3.5)	2 (0.9 to 3.6)
Mongolia	Foreign body in other body part	0.431 (0.327 to 0.649)	0.762 (0.611 to 0.968)	0.883 (0.704 to 1.06)	22.4 (17.2 to 30.1)	29 (23.2 to 36.8)	27 (21.5 to 32.5)
Mongolia	Environmental heat and cold exposure	9.71 (5.87 to 16.1)	10.7 (7.04 to 14.4)	11.3 (7.51 to 15.6)	601.1 (343.6 to 990)	423.6 (277.4 to 558.6)	330.9 (222.5 to 455.5)

SR Appendix Table 2. All-age DALYs counts and age standardized DALYs rates per 100,000 individuals for all causes and level 1 cause groups by location for 1990, 2007, and 2017, both sexes combined							
		All-age DALYs			Age-standardized rate of DALYs per 100,000		
Mongolia	Exposure to forces of nature	2.2 (2.2 to 2.21)	0.0271 (0.0198 to 0.0369)	0.991 (0.976 to 1.01)	95.1 (94.9 to 95.3)	1 (0.7 to 1.3)	29.9 (29.5 to 30.5)
Mongolia	Other unintentional injuries	1.86 (1.58 to 2.21)	3.91 (3.37 to 4.5)	4.48 (3.65 to 5.34)	88.2 (73.1 to 105.8)	141.5 (120.8 to 164.1)	134 (108.1 to 161.3)
Mongolia	Self-harm and interpersonal violence	27.2 (23.4 to 30.2)	46.8 (37.7 to 50.9)	43.9 (37.2 to 50.1)	1350.2 (1189.4 to 1511.3)	1554.9 (1290.9 to 1681.5)	1248.6 (1064.4 to 1419.4)
Mongolia	Self-harm	16.3 (13.3 to 18.5)	29.9 (22.6 to 33.4)	27.7 (21.9 to 32.2)	792.4 (685.1 to 895)	976.4 (773 to 1083.9)	793.7 (629.6 to 918.8)
Mongolia	Self-harm by firearm	0.658 (0.319 to 1.07)	1.1 (0.443 to 1.44)	1 (0.394 to 1.34)	36.4 (17.9 to 58.8)	37.9 (16.2 to 49)	28.2 (11.4 to 37.1)
Mongolia	Self-harm by other specified means	15.6 (12.9 to 17.7)	28.8 (21.8 to 32.2)	26.7 (21.2 to 31)	756 (660.1 to 850.9)	938.6 (743.6 to 1040.8)	765.6 (607.8 to 885.1)
Mongolia	Interpersonal violence	10.9 (8.72 to 13.3)	16.9 (12.9 to 18.9)	16.1 (13 to 19.7)	557.1 (448.3 to 679)	576.7 (442 to 643.3)	453.2 (368.4 to 549)
Mongolia	Assault by firearm	0.3 (0.156 to 0.541)	0.601 (0.299 to 1.03)	0.501 (0.275 to 0.984)	13.2 (7.2 to 25)	18.4 (9.7 to 32.2)	14.2 (7.9 to 27.1)
Mongolia	Assault by sharp object	3.23 (1.86 to 4.8)	5.38 (3 to 8.22)	5.02 (3.06 to 7.41)	164.7 (94.9 to 246.8)	176.8 (101.2 to 272.2)	138.2 (85.8 to 202.3)
Mongolia	Sexual violence	0.16 (0.105 to 0.236)	0.245 (0.165 to 0.36)	0.3 (0.2 to 0.435)	7.8 (5.2 to 11.4)	8.3 (5.6 to 12.1)	8.7 (5.9 to 12.7)
Mongolia	Assault by other means	7.25 (5.54 to 9.47)	10.7 (7.7 to 13)	10.3 (7.66 to 12.9)	371.4 (276 to 491.2)	373.3 (271.8 to 445.2)	292 (219.6 to 362.7)
Mongolia	Conflict and terrorism	0 (0 to 0)	0.0284 (0.0247 to 0.0344)	0.0296 (0.0199 to 0.0454)	0 (0 to 0)	0.9 (0.8 to 1.1)	0.8 (0.6 to 1.3)
Mongolia	Executions and police conflict	0.0165 (0.0145 to 0.0196)	0.0231 (0.021 to 0.0258)	0.0266 (0.0231 to 0.0311)	0.7 (0.6 to 0.8)	0.8 (0.7 to 0.9)	0.8 (0.7 to 0.9)
Tajikistan	All causes	2580 (2390 to 2790)	2350 (2150 to 2580)	2910 (2600 to 3270)	44165.5 (40895 to 47744.4)	37196.5 (34069.9 to 40584.7)	35255.3 (31770.3 to 39066.8)
Tajikistan	Communicable, maternal, neonatal, and nutritional disorders	1490 (1360 to 1630)	972 (876 to 1070)	1070 (899 to 1270)	16998.1 (15531.5 to 18528.2)	10729.3 (9716.8 to 11761.8)	9070.9 (7764 to 10690.7)
Tajikistan	HIV/AIDS and sexually transmitted infections	3.14 (2.15 to 4.55)	9.17 (7.9 to 11.1)	6.44 (4.93 to 8.77)	57 (41.8 to 80.5)	138.9 (122.4 to 163.4)	68.2 (52.9 to 92.5)
Tajikistan	HIV/AIDS	0.403 (0.221 to 0.601)	5.74 (5.29 to 6.23)	2.58 (2.02 to 3.37)	9.4 (5 to 14.4)	94.3 (86.6 to 102.7)	29 (22.8 to 38.2)
Tajikistan	HIV/AIDS - Drug-susceptible Tuberculosis	0.119 (0.0698 to 0.181)	0.511 (0.269 to 0.866)	0.226 (0.125 to 0.372)	2.7 (1.6 to 4.2)	8.4 (4.4 to 14.2)	2.5 (1.4 to 4.2)
Tajikistan	HIV/AIDS - Multidrug-resistant Tuberculosis without extensive drug resistance	0.000211 (0.000028 to 0.000762)	0.205 (0.0744 to 0.401)	0.106 (0.0569 to 0.177)	0 (0 to 0)	3.4 (1.2 to 6.6)	1.2 (0.6 to 2)
Tajikistan	HIV/AIDS - Extensively drug-resistant Tuberculosis	0 (0 to 0)	0.065 (0.0239 to 0.129)	0.0605 (0.0324 to 0.101)	0 (0 to 0)	1.1 (0.4 to 2.1)	0.7 (0.4 to 1.1)
Tajikistan	HIV/AIDS resulting in other diseases	0.284 (0.139 to 0.476)	4.96 (4.32 to 5.54)	2.18 (1.58 to 2.98)	6.6 (3 to 11.5)	81.5 (71.1 to 90.8)	24.7 (17.8 to 33.8)
Tajikistan	Sexually transmitted infections excluding HIV	2.73 (1.77 to 4.13)	3.43 (2.22 to 5.29)	3.86 (2.5 to 6.02)	47.6 (33.5 to 70.7)	44.5 (29.9 to 68.3)	39.1 (25.6 to 62.1)
Tajikistan	Syphilis	1.4 (0.688 to 2.58)	1.54 (0.77 to 2.73)	1.45 (0.739 to 2.65)	18.8 (11.3 to 31)	17.9 (10.1 to 30.7)	13 (7.1 to 22.8)
Tajikistan	Chlamydial infection	0.294 (0.181 to 0.513)	0.427 (0.248 to 0.767)	0.558 (0.327 to 0.999)	6.2 (3.9 to 10.8)	5.9 (3.5 to 10.4)	5.9 (3.5 to 10.3)
Tajikistan	Gonococcal infection	0.379 (0.26 to 0.606)	0.501 (0.321 to 0.844)	0.612 (0.4 to 1.02)	7.9 (5.7 to 11.9)	6.7 (4.6 to 10.7)	6.5 (4.4 to 10.3)
Tajikistan	Trichomoniasis	0.181 (0.0722 to 0.387)	0.273 (0.11 to 0.575)	0.361 (0.144 to 0.766)	4.1 (1.6 to 8.7)	4 (1.6 to 8.6)	4 (1.6 to 8.7)
Tajikistan	Genital herpes	0.1 (0.0331 to 0.236)	0.149 (0.0498 to 0.348)	0.207 (0.0681 to 0.486)	2.6 (0.8 to 6.2)	2.6 (0.8 to 6.2)	2.6 (0.9 to 6.1)
Tajikistan	Other sexually transmitted infections	0.375 (0.251 to 0.604)	0.539 (0.34 to 0.892)	0.676 (0.435 to 1.1)	8 (5.5 to 12.5)	7.4 (4.8 to 11.9)	7.2 (4.7 to 11.5)
Tajikistan	Respiratory infections and tuberculosis	660 (588 to 740)	384 (333 to 437)	417 (348 to 503)	7591.2 (6823.5 to 8413.2)	4443.8 (3917.4 to 5009.3)	3675.6 (3124.7 to 4351.6)
Tajikistan	Tuberculosis	24.2 (20 to 28.4)	43.7 (39.9 to 47.7)	40.4 (35.9 to 45)	412.5 (365.3 to 459.3)	651.9 (598.5 to 706.4)	447.4 (401.7 to 496.4)
Tajikistan	Latent tuberculosis infection	0 (0 to 0)	0 (0 to 0)	0 (0 to 0)	0 (0 to 0)	0 (0 to 0)	0 (0 to 0)
Tajikistan	Drug-susceptible tuberculosis	24.1 (19.9 to 28.3)	30.7 (21 to 39.3)	25.5 (19.2 to 31.6)	411.9 (364.5 to 458.4)	457.7 (313.1 to 582.7)	283.1 (215.3 to 345)
Tajikistan	Multidrug-resistant tuberculosis without extensive drug resistance	0.0352 (0.00495 to 0.125)	9.9 (3.68 to 17.2)	9.44 (5.97 to 13.1)	0.6 (0.1 to 2.1)	147.5 (54.4 to 258)	104.5 (65.7 to 144.6)
Tajikistan	Extensively drug-resistant tuberculosis	0 (0 to 0)	3.13 (1.17 to 5.48)	5.4 (3.39 to 7.46)	0 (0 to 0)	46.7 (17.4 to 81.8)	59.7 (37.9 to 83.4)
Tajikistan	Lower respiratory infections	628 (558 to 704)	331 (283 to 383)	367 (301 to 448)	7068.7 (6328.2 to 7852.7)	3688.3 (3202.8 to 4232)	3134.7 (2612.3 to 3772)
Tajikistan	Upper respiratory infections	6.25 (2.48 to 10.4)	6.02 (3.07 to 9.37)	6.76 (3.81 to 10.2)	81.8 (38.1 to 128.5)	70.1 (37.8 to 106.1)	62.5 (37 to 90.9)
Tajikistan	Otitis media	1.73 (1.02 to 2.71)	2.63 (1.59 to 4.16)	3.06 (1.82 to 4.83)	28.3 (16.9 to 44.6)	33.5 (20.4 to 52.7)	31.1 (18.6 to 49.1)
Tajikistan	Enteric infections	347 (293 to 405)	135 (106 to 167)	161 (118 to 218)	3751.4 (3181.5 to 4352.7)	1429.1 (1133.1 to 1753.4)	1322.8 (977 to 1762.2)
Tajikistan	Diarrhoeal diseases	346 (292 to 404)	134 (105 to 166)	160 (117 to 216)	3737.7 (3169 to 4338)	1417.6 (1124.2 to 1742.1)	1311.3 (963.1 to 1743.3)
Tajikistan	Typhoid and paratyphoid	0.00356 (0.00185 to 0.00635)	0.00385 (0.00199 to 0.00687)	0.00472 (0.00244 to 0.00833)	0.1 (0 to 0.1)	0.1 (0 to 0.1)	0.1 (0 to 0.1)

SR Appendix Table 2. All-age DALYs counts and age standardized DALYs rates per 100,000 individuals for all causes and level 1 cause groups by location for 1990, 2007, and 2017, both sexes combined

		All-age DALYs			Age-standardized rate of DALYs per 100,000		
Tajikistan	Typhoid fever	0.0035 (0.00181 to 0.00627)	0.00354 (0.00182 to 0.00624)	0.00424 (0.00219 to 0.00756)	0.1 (0 to 0.1)	0.1 (0 to 0.1)	0 (0 to 0.1)
Tajikistan	Paratyphoid fever	0.0000603 (0.0000243 to 0.000125)	0.000315 (0.000126 to 0.00064)	0.000482 (0.000196 to 0.000999)	0 (0 to 0)	0 (0 to 0)	0 (0 to 0)
Tajikistan	Invasive non-typhoidal salmonella	0.745 (0.404 to 1.25)	0.798 (0.435 to 1.37)	1.05 (0.542 to 1.78)	11 (6 to 18)	9.7 (5.3 to 16.3)	10.2 (5.4 to 17.1)
Tajikistan	Other intestinal infectious diseases	0.241 (0.0713 to 0.506)	0.157 (0.0411 to 0.316)	0.153 (0.0394 to 0.327)	2.7 (0.9 to 5.5)	1.7 (0.5 to 3.3)	1.3 (0.4 to 2.6)
Tajikistan	Neglected tropical diseases and malaria	9.44 (5.02 to 17.2)	6.94 (4.48 to 10.6)	5.99 (3.43 to 9.77)	142.4 (79.7 to 255.4)	92.5 (59.3 to 138.1)	65.3 (38.4 to 104.1)
Tajikistan	Malaria	4.63 (1.07 to 12.5)	1.81 (1.08 to 3.37)	0.107 (0.0769 to 0.145)	56 (13.1 to 153.7)	21.1 (13.2 to 37)	1.1 (0.8 to 1.5)
Tajikistan	Chagas disease	0 (0 to 0)	0 (0 to 0)	0 (0 to 0)	0 (0 to 0)	0 (0 to 0)	0 (0 to 0)
Tajikistan	Leishmaniasis	0.271 (0.00223 to 2.53)	0.174 (0.00691 to 1.76)	0.207 (0.022 to 1.77)	4.1 (0 to 38.2)	2.2 (0.1 to 20.9)	2 (0.2 to 16.7)
Tajikistan	Visceral leishmaniasis	0.267 (0.000775 to 2.52)	0.164 (0.000629 to 1.75)	0.173 (0.000466 to 1.73)	4.1 (0 to 38.1)	2 (0 to 20.8)	1.7 (0 to 16.2)
Tajikistan	Cutaneous and mucocutaneous leishmaniasis	0.00349 (0.000504 to 0.0097)	0.0103 (0.00502 to 0.0186)	0.0342 (0.0193 to 0.0551)	0.1 (0 to 0.2)	0.1 (0.1 to 0.3)	0.4 (0.2 to 0.6)
Tajikistan	African trypanosomiasis	0 (0 to 0)	0 (0 to 0)	0 (0 to 0)	0 (0 to 0)	0 (0 to 0)	0 (0 to 0)
Tajikistan	Schistosomiasis	0 (0 to 0)	0 (0 to 0)	0 (0 to 0)	0 (0 to 0)	0 (0 to 0)	0 (0 to 0)
Tajikistan	Cysticercosis	0.000285 (0.00012 to 0.000522)	0.000194 (0.0000946 to 0.000299)	0.00017 (0.0000845 to 0.000262)	0 (0 to 0)	0 (0 to 0)	0 (0 to 0)
Tajikistan	Cystic echinococcosis	1.67 (0.766 to 3.23)	2.25 (0.932 to 4.71)	2.92 (1.2 to 6.05)	37.8 (18.3 to 70.3)	35.7 (16 to 69.2)	34.5 (14.9 to 65.2)
Tajikistan	Lymphatic filariasis	0 (0 to 0)	0 (0 to 0)	0 (0 to 0)	0 (0 to 0)	0 (0 to 0)	0 (0 to 0)
Tajikistan	Onchocerciasis	0 (0 to 0)	0 (0 to 0)	0 (0 to 0)	0 (0 to 0)	0 (0 to 0)	0 (0 to 0)
Tajikistan	Trachoma	0 (0 to 0)	0 (0 to 0)	0 (0 to 0)	0 (0 to 0)	0 (0 to 0)	0 (0 to 0)
Tajikistan	Dengue	0 (0 to 0)	0 (0 to 0)	0 (0 to 0)	0 (0 to 0)	0 (0 to 0)	0 (0 to 0)
Tajikistan	Yellow fever	0 (0 to 0)	0 (0 to 0)	0 (0 to 0)	0 (0 to 0)	0 (0 to 0)	0 (0 to 0)
Tajikistan	Rabies	0.0115 (0.00541 to 0.0215)	0.00632 (0.00372 to 0.0102)	0.00633 (0.00358 to 0.0108)	0.3 (0.1 to 0.5)	0.1 (0.1 to 0.2)	0.1 (0.1 to 0.2)
Tajikistan	Intestinal nematode infections	0.563 (0.308 to 0.903)	0.434 (0.241 to 0.713)	0.364 (0.2 to 0.613)	9.5 (5.4 to 15.6)	5.5 (3.1 to 9.1)	3.7 (2 to 6.2)
Tajikistan	Ascariasis	0.272 (0.129 to 0.474)	0.259 (0.128 to 0.457)	0.203 (0.0964 to 0.358)	3.9 (1.9 to 6.8)	3.2 (1.6 to 5.6)	2 (0.9 to 3.5)
Tajikistan	Trichuriasis	0.0000299 (0.000014 to 0.0000582)	0.0000155 (0.00000687 to 0.0000307)	0.0000187 (0.00000796 to 0.0000381)	0 (0 to 0)	0 (0 to 0)	0 (0 to 0)
Tajikistan	Hookworm disease	0.291 (0.149 to 0.511)	0.174 (0.0942 to 0.303)	0.161 (0.0806 to 0.294)	5.6 (2.9 to 9.6)	2.3 (1.3 to 4.1)	1.7 (0.9 to 3.1)
Tajikistan	Food-borne trematodiases	0 (0 to 0)	0 (0 to 0)	0 (0 to 0)	0 (0 to 0)	0 (0 to 0)	0 (0 to 0)
Tajikistan	Leprosy	0 (0 to 0)	0 (0 to 0)	0 (0 to 0)	0 (0 to 0)	0 (0 to 0)	0 (0 to 0)
Tajikistan	Ebola virus disease	0 (0 to 0)	0 (0 to 0)	0 (0 to 0)	0 (0 to 0)	0 (0 to 0)	0 (0 to 0)
Tajikistan	Zika virus disease	0 (0 to 0)	0 (0 to 0)	0 (0 to 0)	0 (0 to 0)	0 (0 to 0)	0 (0 to 0)
Tajikistan	Guinea worm disease	0 (0 to 0)	0 (0 to 0)	0 (0 to 0)	0 (0 to 0)	0 (0 to 0)	0 (0 to 0)
Tajikistan	Other neglected tropical diseases	2.3 (1.46 to 3.35)	2.26 (1.42 to 3.31)	2.39 (1.43 to 3.65)	34.7 (22.5 to 50.3)	27.9 (17.7 to 41)	23.8 (14.3 to 35.9)
Tajikistan	Other infectious diseases	81.2 (65.2 to 111)	60.6 (44.3 to 92.6)	49.6 (33.7 to 75.8)	1026.7 (852.1 to 1350.6)	696 (522.2 to 1029.6)	448.5 (316.2 to 667.2)
Tajikistan	Meningitis	44.5 (39.2 to 55)	33.6 (28.2 to 39.7)	22.6 (16.7 to 33.9)	555.4 (496.1 to 677.9)	381.9 (323.8 to 447.8)	200.9 (149.5 to 309.8)
Tajikistan	Pneumococcal meningitis	1.3 (1.04 to 1.61)	1.5 (1.18 to 1.85)	1.3 (0.945 to 1.75)	20.3 (16.4 to 24.8)	19.2 (15.1 to 23.6)	13.3 (9.6 to 18.1)
Tajikistan	H influenzae type B meningitis	20.2 (17 to 26)	17.5 (14.4 to 20.9)	11.5 (8.2 to 17.7)	248.2 (211.9 to 314.4)	195.5 (162.1 to 232.7)	99.6 (71.6 to 157.7)
Tajikistan	Meningococcal infection	11.2 (10.1 to 12.8)	3.95 (3.12 to 5.07)	2.67 (1.89 to 4.05)	136.4 (124.4 to 154)	45.1 (36.2 to 57)	23.9 (17.2 to 36.8)
Tajikistan	Other meningitis	11.8 (9.5 to 15.6)	10.6 (8.6 to 13.1)	7.13 (5.04 to 11.1)	150.6 (125 to 196.3)	122.2 (99.9 to 147.9)	64 (46.2 to 103.1)
Tajikistan	Encephalitis	0.766 (0.574 to 1.14)	1.07 (0.853 to 1.49)	1.33 (1.08 to 1.94)	13.5 (10.7 to 17.9)	14.7 (11.9 to 18.8)	14.2 (11.6 to 18.5)
Tajikistan	Diphtheria	0.214 (0.0762 to 0.446)	0.0629 (0.0261 to 0.122)	0.062 (0.0274 to 0.118)	2.4 (0.9 to 4.9)	0.7 (0.3 to 1.3)	0.5 (0.2 to 1)
Tajikistan	Whooping cough	12.3 (1.36 to 42.7)	14 (1.28 to 46.6)	11.9 (1.19 to 36.5)	132.6 (14.6 to 458.9)	148.7 (13.6 to 497.2)	96.2 (9.6 to 295.2)
Tajikistan	Tetanus	0.0963 (0.0477 to 0.141)	0.071 (0.0529 to 0.0806)	0.0837 (0.068 to 0.0919)	1.5 (0.8 to 2)	0.9 (0.7 to 1.1)	0.8 (0.7 to 0.9)

SR Appendix Table 2. All-age DALYs counts and age standardized DALYs rates per 100,000 individuals for all causes and level 1 cause groups by location for 1990, 2007, and 2017, both sexes combined

		All-age DALYs			Age-standardized rate of DALYs per 100,000		
Tajikistan	Measles	2.77 (1.96 to 3.78)	0.00258 (0.00146 to 0.00413)	0.246 (0.165 to 0.35)	30.7 (21.7 to 41.7)	0 (0 to 0)	2 (1.4 to 2.9)
Tajikistan	Varicella and herpes zoster	1.3 (0.782 to 2.08)	1.13 (0.716 to 1.77)	1.21 (0.779 to 1.86)	19.3 (13.3 to 28.3)	14.8 (10.1 to 21.9)	12.3 (8.7 to 17.7)
Tajikistan	Acute hepatitis	16.4 (12.9 to 22.3)	7.67 (5.85 to 13.5)	8.98 (6.71 to 15)	226.4 (188.2 to 288.9)	96.1 (75.8 to 162.7)	88.6 (68.8 to 139.5)
Tajikistan	Acute hepatitis A	5.89 (4.18 to 8.39)	2.55 (1.85 to 4.49)	2.87 (2.01 to 4.88)	73.2 (54 to 101.4)	28.5 (20.7 to 49.9)	26.2 (18.7 to 43.6)
Tajikistan	Acute hepatitis B	4.66 (3.68 to 6.14)	2.85 (2.21 to 4.49)	3.59 (2.8 to 5.24)	87.2 (71.5 to 104.4)	42.8 (35 to 63)	41 (33.1 to 56.6)
Tajikistan	Acute hepatitis C	5.12 (3.18 to 8.33)	1.77 (0.902 to 4.1)	1.97 (0.957 to 4.44)	54.2 (33.9 to 87.9)	18.4 (9.4 to 42.1)	15.7 (7.7 to 35.1)
Tajikistan	Acute hepatitis E	0.717 (0.551 to 0.906)	0.51 (0.378 to 0.803)	0.544 (0.392 to 0.855)	11.8 (9.1 to 14.9)	6.4 (4.8 to 9.8)	5.6 (4.2 to 8.6)
Tajikistan	Other unspecified infectious diseases	2.86 (1.84 to 6.21)	2.99 (1.98 to 5.61)	3.23 (2.14 to 6.01)	44.9 (30.7 to 79.5)	38.2 (26.6 to 63.2)	32.9 (22.7 to 54.3)
Tajikistan	Maternal and neonatal disorders	330 (266 to 406)	309 (273 to 348)	360 (296 to 444)	3508.3 (2855.1 to 4255.9)	3092 (2738.7 to 3482.8)	2839.1 (2348.6 to 3479.8)
Tajikistan	Maternal disorders	7.89 (7.12 to 8.64)	5.44 (4.63 to 6.32)	4.58 (3.76 to 5.55)	162 (147.4 to 177.9)	73 (62.2 to 84.6)	46.5 (38.4 to 56.2)
Tajikistan	Maternal haemorrhage	2.3 (1.57 to 3.21)	1.22 (0.816 to 1.72)	0.693 (0.443 to 1.05)	48.4 (33.5 to 66.1)	17 (11.3 to 24.5)	7.2 (4.6 to 10.8)
Tajikistan	Maternal sepsis and other pregnancy related infections	1.82 (1.39 to 2.29)	1.08 (0.818 to 1.39)	1.03 (0.77 to 1.33)	38.4 (29.8 to 47.7)	14.4 (11 to 18.5)	10.6 (8 to 13.7)
Tajikistan	Maternal hypertensive disorders	1.06 (0.81 to 1.34)	0.764 (0.581 to 0.98)	0.558 (0.411 to 0.726)	20.8 (16.2 to 26.1)	10.1 (7.6 to 12.9)	5.6 (4.2 to 7.3)
Tajikistan	Maternal obstructed labour and uterine rupture	0.38 (0.254 to 0.554)	0.355 (0.231 to 0.516)	0.342 (0.212 to 0.513)	7.9 (5.3 to 11.4)	4.7 (3.1 to 7)	3.5 (2.2 to 5.2)
Tajikistan	Maternal abortive outcome	0.587 (0.352 to 0.936)	0.374 (0.237 to 0.578)	0.361 (0.229 to 0.559)	12.3 (7.7 to 19.6)	5.1 (3.2 to 7.9)	3.8 (2.4 to 5.8)
Tajikistan	Ectopic pregnancy	0.217 (0.0692 to 0.546)	0.173 (0.0592 to 0.429)	0.152 (0.0514 to 0.382)	4.4 (1.4 to 11.3)	2.3 (0.8 to 6.1)	1.6 (0.5 to 3.8)
Tajikistan	Indirect maternal deaths	0.0405 (0.0177 to 0.0778)	0.0497 (0.0225 to 0.0965)	0.0432 (0.0203 to 0.0832)	0.8 (0.4 to 1.5)	0.6 (0.3 to 1.2)	0.4 (0.2 to 0.8)
Tajikistan	Late maternal deaths	0.142 (0.101 to 0.194)	0.122 (0.0872 to 0.169)	0.13 (0.0891 to 0.185)	2.9 (2.1 to 3.9)	1.6 (1.2 to 2.3)	1.3 (0.9 to 1.9)
Tajikistan	Maternal deaths aggravated by HIV/AIDS	0.000273 (0.000138 to 0.000471)	0.000564 (0.000269 to 0.00103)	0.00105 (0.000525 to 0.00175)	0 (0 to 0)	0 (0 to 0)	0 (0 to 0)
Tajikistan	Other maternal disorders	1.34 (0.897 to 1.94)	1.31 (0.889 to 1.8)	1.26 (0.848 to 1.75)	26.1 (17.4 to 37.3)	17.1 (11.8 to 23.4)	12.5 (8.5 to 17.2)
Tajikistan	Neonatal disorders	323 (258 to 398)	303 (268 to 343)	356 (292 to 440)	3346.3 (2700.3 to 4094.4)	3019 (2665.8 to 3410.6)	2792.6 (2302.2 to 3435.4)
Tajikistan	Neonatal preterm birth	252 (163 to 334)	222 (157 to 264)	258 (177 to 329)	2595.3 (1679.6 to 3423.2)	2185.8 (1548.9 to 2598.7)	1995.7 (1378.6 to 2535.8)
Tajikistan	Neonatal encephalopathy due to birth asphyxia and trauma	24.1 (8.12 to 95.8)	31.5 (16.6 to 91)	35.9 (18 to 110)	266.2 (98.4 to 987.6)	335.8 (180 to 916.5)	309 (157.8 to 873.5)
Tajikistan	Neonatal sepsis and other neonatal infections	29.6 (19.2 to 45.4)	34.5 (23 to 48.5)	44.1 (28 to 63.7)	307.6 (201.8 to 469.3)	344.2 (230.5 to 482.5)	345.7 (221.5 to 495.1)
Tajikistan	Hemolytic disease and other neonatal jaundice	8.96 (5.67 to 13.7)	7.21 (5.04 to 10)	7.98 (5.33 to 11.3)	100.2 (66.7 to 149.2)	75.6 (53.5 to 103.9)	66.4 (45.1 to 92.7)
Tajikistan	Other neonatal disorders	7.53 (4.37 to 14.3)	7.8 (5.08 to 13.4)	9.79 (5.87 to 16.6)	77 (45 to 146)	77.6 (51.1 to 132.2)	75.7 (46.1 to 127.6)
Tajikistan	Nutritional deficiencies	59.6 (39.7 to 83.5)	67.4 (46.8 to 93.2)	64.9 (41.6 to 95.8)	921.1 (614.4 to 1288.3)	837.1 (583.4 to 1156.7)	651.4 (418.5 to 961)
Tajikistan	Protein-energy malnutrition	2.88 (1.97 to 4.09)	6.32 (4.79 to 8.08)	5.79 (3.83 to 8.49)	32.9 (22.6 to 46.5)	65.8 (49.8 to 84)	46.2 (30.6 to 67.8)
Tajikistan	Iodine deficiency	1.29 (0.704 to 2.13)	2.35 (1.3 to 3.77)	2.28 (1.26 to 3.71)	26 (14.4 to 43.3)	33.2 (18.7 to 53.8)	25.5 (14.1 to 41.5)
Tajikistan	Dietary iron deficiency	35.8 (22.8 to 51.9)	38.7 (24.8 to 56.7)	40.9 (25 to 61.8)	621.1 (399.7 to 885.9)	514 (334.7 to 743.4)	437.5 (273.6 to 657.3)
Tajikistan	Other nutritional deficiencies	4.39 (3.61 to 5.21)	6.47 (5.06 to 8.17)	2.82 (2.09 to 3.88)	56.6 (48.2 to 65.6)	78.7 (63.6 to 96.5)	24.9 (19.2 to 33.2)
Tajikistan	Non-communicable diseases	860 (773 to 961)	1150 (1030 to 1300)	1560 (1380 to 1760)	22831.6 (20707.7 to 25270.9)	23189.8 (21032.5 to 25644.9)	23050.7 (20615.5 to 25642.6)
Tajikistan	Neoplasms	101 (95.4 to 109)	113 (106 to 121)	161 (148 to 175)	2806.9 (2689.1 to 2921.9)	2306.7 (2180.7 to 2459.3)	2360.5 (2169.5 to 2551)
Tajikistan	Lip and oral cavity cancer	0.672 (0.59 to 0.762)	0.915 (0.816 to 1.02)	1.31 (1.16 to 1.48)	21.6 (18.9 to 24.6)	20.7 (18.4 to 23.1)	21 (18.6 to 23.5)
Tajikistan	Nasopharynx cancer	0.549 (0.468 to 0.64)	0.733 (0.653 to 0.818)	1.03 (0.901 to 1.16)	16.3 (13.9 to 19.1)	15.3 (13.7 to 17)	14.9 (13.1 to 16.9)
Tajikistan	Other pharynx cancer	0.462 (0.372 to 0.56)	0.56 (0.482 to 0.658)	0.845 (0.707 to 0.975)	14.9 (12 to 18.1)	13 (11.1 to 15.5)	13.4 (11.2 to 15.5)
Tajikistan	Oesophageal cancer	7.4 (7.01 to 7.84)	8.27 (7.52 to 9.15)	7.33 (6.45 to 8.32)	246.4 (232.7 to 260.6)	214.9 (194.3 to 237.2)	125.5 (110.4 to 142.3)
Tajikistan	Stomach cancer	18.4 (17.6 to 19.2)	17.6 (16.3 to 19.1)	26.3 (23.5 to 29.1)	600.2 (575.2 to 625.5)	435.3 (400.9 to 470)	438.4 (392.7 to 485.5)
Tajikistan	Colon and rectum cancer	5.38 (5.03 to 5.75)	5.94 (5.47 to 6.49)	9.15 (8.11 to 10.3)	165 (155.5 to 175)	134 (123.7 to 149.6)	150.8 (134.2 to 168.8)
Tajikistan	Liver cancer	5.32 (4.21 to 7.39)	7.52 (6.55 to 8.77)	10.7 (9.45 to 12)	152.7 (120.8 to 207.6)	149.1 (130.9 to 174.5)	154.7 (137.8 to 176.2)

SR Appendix Table 2. All-age DALYs counts and age standardized DALYs rates per 100,000 individuals for all causes and level 1 cause groups by location for 1990, 2007, and 2017, both sexes combined

		All-age DALYs			Age-standardized rate of DALYs per 100,000		
Tajikistan	Liver cancer due to hepatitis B	1.64 (1.26 to 2.38)	2.43 (2 to 2.95)	3.16 (2.67 to 3.7)	39.6 (30.3 to 57.3)	39.1 (32.7 to 47.2)	38 (32.3 to 44.4)
Tajikistan	Liver cancer due to hepatitis C	1.78 (1.37 to 2.39)	2.38 (2.01 to 2.86)	3.62 (3.06 to 4.26)	57.9 (44.8 to 78.1)	56.3 (48.1 to 67.8)	60 (51.4 to 71)
Tajikistan	Liver cancer due to alcohol use	1.11 (0.86 to 1.62)	1.55 (1.3 to 1.86)	2.24 (1.9 to 2.67)	34.8 (26.8 to 50.1)	33 (28.1 to 40.5)	34.6 (29.6 to 41.4)
Tajikistan	Liver cancer due to NASH	0.298 (0.23 to 0.407)	0.434 (0.362 to 0.518)	0.653 (0.554 to 0.777)	8.6 (6.6 to 11.6)	8.7 (7.4 to 10.4)	9.7 (8.2 to 11.4)
Tajikistan	Liver cancer due to other causes	0.492 (0.378 to 0.651)	0.732 (0.611 to 0.89)	1.01 (0.828 to 1.21)	12 (9.4 to 15.8)	12 (10.1 to 14.3)	12.4 (10.5 to 14.7)
Tajikistan	Gallbladder and biliary tract cancer	0.433 (0.338 to 0.561)	0.468 (0.405 to 0.603)	0.711 (0.613 to 0.899)	14.7 (11.5 to 19.1)	11.8 (10.1 to 15.7)	12.5 (10.8 to 16.1)
Tajikistan	Pancreatic cancer	2.1 (1.85 to 2.46)	2.77 (2.51 to 3.04)	4.61 (4.13 to 5.12)	71.9 (63 to 83.8)	71.3 (64.2 to 78.1)	82.8 (74.6 to 91.9)
Tajikistan	Larynx cancer	0.994 (0.91 to 1.1)	1.03 (0.922 to 1.15)	1.15 (0.989 to 1.3)	31.6 (28.9 to 34.8)	24.1 (21.4 to 27)	18.2 (15.9 to 20.6)
Tajikistan	Tracheal, bronchus, and lung cancer	10.5 (9.87 to 11.1)	7.68 (6.97 to 8.39)	11 (9.51 to 12.5)	335.7 (316.8 to 355)	186.6 (169.8 to 204.7)	185.6 (159.9 to 210.3)
Tajikistan	Malignant skin melanoma	0.317 (0.253 to 0.419)	0.459 (0.361 to 0.606)	0.671 (0.533 to 0.899)	9.2 (7.5 to 12.3)	9.4 (7.5 to 12.6)	10.2 (7.8 to 13.1)
Tajikistan	Non-melanoma skin cancer	0.291 (0.25 to 0.327)	0.394 (0.353 to 0.442)	0.581 (0.517 to 0.655)	9.8 (8.4 to 11)	9.5 (8.5 to 10.6)	10.3 (9.2 to 11.6)
Tajikistan	Non-melanoma skin cancer (squamous-cell carcinoma)	0.29 (0.249 to 0.325)	0.393 (0.352 to 0.44)	0.58 (0.515 to 0.654)	9.8 (8.4 to 11)	9.4 (8.5 to 10.6)	10.3 (9.2 to 11.6)
Tajikistan	Non-melanoma skin cancer (basal-cell carcinoma)	0.000956 (0.000316 to 0.00228)	0.00124 (0.000413 to 0.00281)	0.00182 (0.000635 to 0.00411)	0 (0 to 0.1)	0 (0 to 0.1)	0 (0 to 0.1)
Tajikistan	Breast cancer	4.23 (3.67 to 4.63)	7.02 (6.11 to 8)	11.5 (9.53 to 13.4)	135.1 (116.1 to 147.1)	143.5 (126.6 to 163.1)	164.7 (136.7 to 192.3)
Tajikistan	Cervical cancer	2.53 (2.09 to 2.8)	2.14 (1.81 to 3.18)	3.43 (2.83 to 4.32)	81.1 (65.1 to 89.7)	44.7 (38.5 to 63.4)	49.8 (41.3 to 60.9)
Tajikistan	Uterine cancer	0.647 (0.578 to 0.727)	1.2 (1.03 to 1.37)	2.76 (2.23 to 3.26)	20.9 (18.7 to 23.5)	25.7 (22.2 to 29.1)	40.2 (32.6 to 47.1)
Tajikistan	Ovarian cancer	1.33 (1.03 to 1.78)	1.97 (1.72 to 2.25)	3.26 (2.74 to 3.8)	42.1 (32.9 to 55.9)	42.5 (37.4 to 48.6)	48.5 (41.7 to 56.4)
Tajikistan	Prostate cancer	1.37 (1.09 to 1.64)	1.55 (1.28 to 1.94)	2.43 (2.03 to 3.14)	50.4 (39.9 to 59.7)	47 (38.3 to 58.2)	57.6 (47.7 to 72.3)
Tajikistan	Testicular cancer	0.00986 (0.0092 to 0.0108)	0.0151 (0.0139 to 0.0166)	0.0207 (0.019 to 0.023)	0.2 (0.2 to 0.2)	0.2 (0.2 to 0.2)	0.2 (0.2 to 0.2)
Tajikistan	Kidney cancer	1.86 (1.56 to 2.3)	2.45 (2.14 to 2.81)	3.99 (3.44 to 4.56)	48.1 (40.3 to 58.4)	49 (42.8 to 57.3)	56.1 (48.8 to 64.4)
Tajikistan	Bladder cancer	0.785 (0.588 to 0.99)	0.962 (0.82 to 1.38)	1.4 (1.18 to 2.05)	26.9 (20 to 34.2)	24.3 (20.2 to 38.4)	26 (21.6 to 39.7)
Tajikistan	Thyroid cancer	0.127 (0.111 to 0.148)	0.166 (0.148 to 0.186)	0.251 (0.219 to 0.286)	4.1 (3.4 to 4.9)	3.9 (3.4 to 4.4)	4.2 (3.7 to 4.8)
Tajikistan	Mesothelioma	0.29 (0.218 to 0.358)	0.532 (0.453 to 0.613)	0.652 (0.58 to 0.738)	6.4 (4.9 to 7.7)	7.7 (6.7 to 8.8)	7.3 (6.6 to 8.2)
Tajikistan	Hodgkin lymphoma	0.37 (0.299 to 0.509)	0.471 (0.389 to 0.67)	0.596 (0.491 to 0.804)	9.2 (7.3 to 11.7)	8.3 (6.9 to 10.4)	7.7 (6.1 to 9.4)
Tajikistan	Non-Hodgkin's lymphoma	3.74 (3.26 to 4.24)	4.96 (4.41 to 5.6)	7.08 (6.23 to 7.99)	82.2 (72.5 to 91.3)	76.4 (68.9 to 85.6)	83.9 (74.7 to 93.4)
Tajikistan	Multiple myeloma	0.414 (0.362 to 0.48)	0.547 (0.482 to 0.657)	0.855 (0.739 to 1.02)	13.6 (11.8 to 15.5)	13.8 (12.2 to 16.1)	14.5 (12.6 to 16.9)
Tajikistan	Leukaemia	11.4 (8.42 to 13.2)	10.4 (8.68 to 13.8)	13 (10.6 to 17.4)	193.5 (151.2 to 218.3)	140.3 (120.7 to 182.8)	141.8 (119 to 186.3)
Tajikistan	Acute lymphoid leukaemia	2.87 (2.02 to 3.92)	2.86 (2.12 to 4.23)	3.54 (2.63 to 5.27)	41.7 (29.7 to 54)	33.5 (25.4 to 48.8)	34.7 (26.1 to 50.6)
Tajikistan	Chronic lymphoid leukaemia	0.185 (0.123 to 0.24)	0.194 (0.155 to 0.26)	0.282 (0.221 to 0.39)	5.5 (3.8 to 7.2)	4.3 (3.4 to 5.6)	4.5 (3.5 to 6.1)
Tajikistan	Acute myeloid leukaemia	2.26 (1.67 to 2.91)	2.66 (2.13 to 3.51)	3.71 (2.97 to 4.8)	42 (31.5 to 51.1)	36.8 (30 to 47.6)	40.8 (33 to 51.8)
Tajikistan	Chronic myeloid leukaemia	0.495 (0.364 to 0.633)	0.401 (0.295 to 0.583)	0.478 (0.36 to 0.699)	13.4 (9.7 to 17.4)	7.4 (5.5 to 10.5)	6.4 (4.8 to 9.1)
Tajikistan	Other leukaemia	5.57 (3.61 to 6.83)	4.25 (3.45 to 5.74)	4.98 (3.91 to 7)	90.9 (64.1 to 107.6)	58.4 (48.8 to 77.7)	55.5 (45.2 to 75.9)
Tajikistan	Other malignant cancers	8.01 (6.98 to 9.75)	9.68 (8.62 to 10.8)	13.9 (12.4 to 15.4)	184 (157.7 to 206.9)	163 (147.3 to 180.4)	176.4 (158.1 to 194.1)
Tajikistan	Other neoplasms	0.564 (0.392 to 0.96)	0.647 (0.535 to 0.915)	1.06 (0.855 to 1.47)	11.9 (9.3 to 16.3)	11.7 (9.2 to 14.6)	14.4 (11.3 to 17.6)
Tajikistan	Myelodysplastic, myeloproliferative, and other hematopoietic neoplasms	0.426 (0.283 to 0.75)	0.457 (0.368 to 0.617)	0.761 (0.601 to 0.974)	9.6 (7.4 to 12.8)	9.3 (7.1 to 11.1)	11.4 (8.7 to 13.6)
Tajikistan	Benign and in situ intestinal neoplasms	0 (0 to 0)	0 (0 to 0)	0 (0 to 0)	0 (0 to 0)	0 (0 to 0)	0 (0 to 0)
Tajikistan	Benign and in situ cervical and uterine neoplasms	0 (0 to 0)	0 (0 to 0)	0 (0 to 0)	0 (0 to 0)	0 (0 to 0)	0 (0 to 0)
Tajikistan	Other benign and in situ neoplasms	0.138 (0.0993 to 0.228)	0.19 (0.143 to 0.3)	0.296 (0.226 to 0.482)	2.2 (1.7 to 3.4)	2.4 (1.9 to 3.7)	3 (2.3 to 4.7)
Tajikistan	Cardiovascular diseases	220 (213 to 227)	312 (297 to 328)	424 (390 to 460)	7623.7 (7387.4 to 7843.1)	8483 (8078 to 8915)	8270.2 (7625.2 to 8934.5)
Tajikistan	Rheumatic heart disease	12.6 (11.7 to 13.5)	13.9 (12.6 to 15.6)	11.7 (10.1 to 13.5)	313 (291.7 to 336)	235 (213.5 to 259.4)	142.3 (124.3 to 163.4)

SR Appendix Table 2. All-age DALYs counts and age standardized DALYs rates per 100,000 individuals for all causes and level 1 cause groups by location for 1990, 2007, and 2017, both sexes combined

		All-age DALYs			Age-standardized rate of DALYs per 100,000		
Tajikistan	Ischaemic heart disease	114 (110 to 119)	170 (160 to 179)	244 (223 to 266)	4062.2 (3897.8 to 4231.3)	4711 (4456.4 to 4975.4)	4849.9 (4459.3 to 5268.2)
Tajikistan	Stroke	67.7 (64.5 to 70.9)	95.9 (90 to 102)	121 (111 to 132)	2365 (2254.7 to 2479.2)	2672.5 (2515.3 to 2843.4)	2380.1 (2180.8 to 2597.4)
Tajikistan	Ischaemic stroke	20.4 (18.3 to 22.7)	28.8 (25.6 to 32.2)	35.6 (31.1 to 40.4)	748.8 (668.1 to 833.3)	849.2 (758.9 to 948.9)	787.9 (692.3 to 886.9)
Tajikistan	Intracerebral hemorrhage	40.7 (37.6 to 43.5)	56.6 (51.4 to 61.7)	70.9 (63.1 to 79.1)	1408.2 (1302.7 to 1506.8)	1565.6 (1427.5 to 1708.5)	1350.8 (1211.6 to 1507.7)
Tajikistan	Subarachnoid hemorrhage	6.54 (5.08 to 8.93)	10.6 (8.18 to 14.1)	14.7 (11.1 to 20.5)	208.1 (161.3 to 285.5)	257.6 (197.7 to 346.8)	241.4 (181.3 to 340.1)
Tajikistan	Hypertensive heart disease	16.9 (13.8 to 19.2)	21.4 (17.6 to 24.3)	30.5 (24.3 to 34.3)	601.7 (490.4 to 685.9)	585.9 (478 to 666.2)	596.9 (482 to 666.8)
Tajikistan	Non-rheumatic valvular heart disease	0.528 (0.461 to 0.605)	0.674 (0.584 to 0.778)	1.06 (0.903 to 1.21)	17.3 (14.6 to 20.2)	16.4 (13.8 to 19.1)	18.7 (15.4 to 21.6)
Tajikistan	Non-rheumatic calcific aortic valve disease	0.214 (0.172 to 0.266)	0.299 (0.245 to 0.367)	0.483 (0.394 to 0.569)	6.9 (5.6 to 8.7)	7.2 (5.8 to 9.1)	8.4 (6.9 to 10.2)
Tajikistan	Non-rheumatic degenerative mitral valve disease	0.193 (0.149 to 0.248)	0.229 (0.181 to 0.303)	0.333 (0.26 to 0.442)	6.5 (4.9 to 8.4)	5.9 (4.4 to 7.9)	6.2 (4.7 to 8.2)
Tajikistan	Other non-rheumatic valve diseases	0.12 (0.0639 to 0.164)	0.146 (0.0918 to 0.191)	0.243 (0.142 to 0.327)	3.8 (1.8 to 5.5)	3.3 (1.9 to 4.5)	4 (2.1 to 5.6)
Tajikistan	Cardiomyopathy and myocarditis	1.3 (1.18 to 1.46)	1.9 (1.69 to 2.12)	2.81 (2.46 to 3.21)	30.6 (27.5 to 34.4)	31.5 (28.3 to 35.1)	34.3 (30.6 to 38.9)
Tajikistan	Myocarditis	0.448 (0.319 to 0.551)	0.665 (0.439 to 0.832)	0.958 (0.623 to 1.23)	10.2 (7.2 to 12.6)	10.3 (6.9 to 12.8)	10.9 (7.3 to 13.9)
Tajikistan	Alcoholic cardiomyopathy	0.107 (0.0688 to 0.184)	0.141 (0.0972 to 0.234)	0.192 (0.138 to 0.314)	3 (1.9 to 5.3)	2.5 (1.8 to 4.2)	2.4 (1.8 to 3.9)
Tajikistan	Other cardiomyopathy	0.749 (0.656 to 0.883)	1.1 (0.963 to 1.28)	1.66 (1.45 to 1.91)	17.5 (15.3 to 20.8)	18.7 (16.5 to 22.1)	20.9 (18.4 to 24)
Tajikistan	Atrial fibrillation and flutter	1.88 (1.54 to 2.29)	2.22 (1.78 to 2.71)	3.24 (2.64 to 3.9)	72.1 (59 to 87.1)	69.8 (56.8 to 84.7)	77.2 (64 to 91.9)
Tajikistan	Aortic aneurysm	0.873 (0.751 to 1.03)	1.19 (1.06 to 1.34)	1.86 (1.63 to 2.09)	30.3 (25.9 to 35.8)	31.4 (27.8 to 35.3)	35.5 (30.1 to 39.6)
Tajikistan	Peripheral vascular disease	0.185 (0.0972 to 0.314)	0.214 (0.113 to 0.356)	0.281 (0.155 to 0.468)	7 (3.7 to 12)	6.9 (3.6 to 11.5)	6.7 (3.7 to 11.2)
Tajikistan	Endocarditis	0.373 (0.306 to 0.462)	0.454 (0.403 to 0.513)	0.698 (0.604 to 0.794)	8.4 (6.5 to 10.4)	8.9 (8 to 9.9)	10.1 (9 to 11.4)
Tajikistan	Other cardiovascular and circulatory diseases	3.69 (2.82 to 4.61)	4.66 (3.64 to 5.48)	6.66 (5.04 to 7.97)	116.1 (75.9 to 152.3)	113.7 (77.7 to 138.3)	118.6 (77.7 to 146.3)
Tajikistan	Chronic respiratory diseases	54.4 (49.7 to 58.9)	54.2 (49.1 to 59.5)	64.8 (57.4 to 72.7)	1685 (1552.6 to 1814.2)	1207.9 (1109.7 to 1308.6)	1037.8 (929.9 to 1156.6)
Tajikistan	Chronic obstructive pulmonary disease	32.8 (30 to 35.6)	31.8 (29.1 to 34.3)	36.3 (32.7 to 40.2)	1053.8 (963 to 1147.4)	753.7 (688.6 to 810.1)	616.5 (553.6 to 687.6)
Tajikistan	Pneumoconiosis	0.0159 (0.0118 to 0.021)	0.0204 (0.0148 to 0.0271)	0.0283 (0.0203 to 0.0389)	0.5 (0.4 to 0.7)	0.5 (0.4 to 0.7)	0.5 (0.4 to 0.7)
Tajikistan	Silicosis	0.00199 (0.00123 to 0.00276)	0.00247 (0.00169 to 0.00339)	0.00318 (0.0021 to 0.0046)	0.1 (0 to 0.1)	0.1 (0 to 0.1)	0.1 (0 to 0.1)
Tajikistan	Asbestosis	0.00307 (0.00199 to 0.00442)	0.00382 (0.0025 to 0.00562)	0.00563 (0.00355 to 0.00854)	0.1 (0.1 to 0.1)	0.1 (0.1 to 0.1)	0.1 (0.1 to 0.1)
Tajikistan	Coal workers pneumoconiosis	0.00347 (0.00229 to 0.0051)	0.00481 (0.00309 to 0.00698)	0.0071 (0.00434 to 0.011)	0.1 (0.1 to 0.2)	0.1 (0.1 to 0.2)	0.1 (0.1 to 0.2)
Tajikistan	Other pneumoconiosis	0.00737 (0.00517 to 0.0102)	0.0093 (0.00643 to 0.0129)	0.0124 (0.0083 to 0.0176)	0.3 (0.2 to 0.4)	0.3 (0.2 to 0.4)	0.3 (0.2 to 0.4)
Tajikistan	Asthma	11.4 (8.68 to 14.5)	10.9 (8.19 to 14.3)	13.6 (9.94 to 18)	319.7 (248.6 to 397.5)	213.9 (171.8 to 265.2)	194.2 (150.2 to 247.5)
Tajikistan	Interstitial lung disease and pulmonary sarcoidosis	6.23 (5.18 to 7.49)	6.57 (5.54 to 7.87)	9.09 (7.81 to 10.6)	209.3 (173.1 to 255)	158.1 (133.6 to 192.1)	156 (134.7 to 188.1)
Tajikistan	Other chronic respiratory diseases	3.93 (3.24 to 4.53)	4.84 (4.18 to 5.56)	5.8 (5.03 to 6.64)	101.5 (80.8 to 119.4)	81.6 (70.2 to 92.7)	70.5 (61 to 80.5)
Tajikistan	Digestive diseases	78.3 (65.7 to 86.2)	85 (77.2 to 93.7)	111 (98.5 to 125)	1608.7 (1487.9 to 1755)	1528.1 (1403.8 to 1679.7)	1424 (1276.6 to 1589.4)
Tajikistan	Cirrhosis and other chronic liver diseases	32 (29 to 35.9)	47 (42.2 to 51)	60.4 (53.7 to 67.2)	798.9 (732.5 to 958.3)	908.6 (834.7 to 988.8)	816.8 (729.3 to 911.1)
Tajikistan	Cirrhosis and other chronic liver diseases due to hepatitis B	6.32 (5.45 to 7.88)	11.2 (9.64 to 12.9)	12.9 (10.9 to 15.3)	177.7 (152.4 to 226.7)	219.8 (190.3 to 253)	175.5 (148.7 to 207.9)
Tajikistan	Cirrhosis and other chronic liver diseases due to hepatitis C	5.52 (4.69 to 6.98)	9.69 (8.19 to 11.2)	13.2 (11.1 to 15.5)	165.3 (139 to 213.6)	202.2 (171.6 to 234.2)	186.6 (158.5 to 219.8)
Tajikistan	Cirrhosis and other chronic liver diseases due to alcohol use	7.94 (6.89 to 10.3)	13.5 (12 to 15.3)	18.3 (15.7 to 21)	248.5 (215.9 to 321.5)	295 (261.9 to 337.1)	267.1 (230 to 312.5)
Tajikistan	Cirrhosis due to NASH	1.45 (1.25 to 1.85)	2.56 (2.23 to 2.92)	3.76 (3.19 to 4.39)	44.8 (38.5 to 57.7)	56 (49 to 63.8)	54.9 (46.8 to 65)
Tajikistan	Cirrhosis and other chronic liver diseases due to other causes	10.8 (7.98 to 13.2)	10 (7.8 to 11.8)	12.3 (9.44 to 14.7)	162.6 (133.7 to 191.6)	135.7 (110.3 to 156.6)	132.7 (107.1 to 155.8)
Tajikistan	Upper digestive system diseases	11.2 (8.87 to 14.4)	14.6 (11.4 to 19.4)	18.1 (13.7 to 24.6)	287.8 (229.7 to 370.8)	270.1 (214.1 to 352.8)	238 (183.1 to 319.5)
Tajikistan	Peptic ulcer disease	5.82 (5.29 to 6.97)	6.92 (6.17 to 7.9)	7.63 (6.7 to 8.71)	158.5 (142.9 to 194)	141.3 (126.2 to 162.6)	108 (95 to 124.6)
Tajikistan	Gastritis and duodenitis	1.83 (1.28 to 2.57)	2.47 (1.69 to 3.52)	3.25 (2.21 to 4.58)	37.3 (26.2 to 51.8)	36.5 (25.4 to 51.3)	37.9 (26.3 to 53)
Tajikistan	GERD	3.53 (1.88 to 6.07)	5.2 (2.73 to 8.98)	7.2 (3.81 to 12.3)	92 (48.9 to 157.1)	92.2 (49.3 to 157.3)	92.1 (49 to 157.9)

SR Appendix Table 2. All-age DALYs counts and age standardized DALYs rates per 100,000 individuals for all causes and level 1 cause groups by location for 1990, 2007, and 2017, both sexes combined

		All-age DALYs			Age-standardized rate of DALYs per 100,000		
Tajikistan	Appendicitis	2 (1.58 to 2.43)	1.52 (1.28 to 1.78)	1.78 (1.48 to 2.11)	33 (28.2 to 38.5)	20.5 (17.6 to 23.6)	19 (16.1 to 22.3)
Tajikistan	Paralytic ileus and intestinal obstruction	21.8 (9.7 to 27.3)	8.57 (6.85 to 10.5)	11.7 (8.52 to 15.4)	246.1 (122.3 to 301.3)	97.9 (80.5 to 119.3)	105.6 (79.4 to 135.4)
Tajikistan	Inguinal, femoral, and abdominal hernia	4.78 (3.26 to 6.52)	7.43 (5.12 to 10.3)	9.83 (6.65 to 13.7)	94.4 (64.3 to 129.4)	116.4 (80 to 161.1)	117.2 (79.7 to 161.5)
Tajikistan	Inflammatory bowel disease	1.46 (0.736 to 3.08)	0.844 (0.653 to 1.12)	1.38 (1.11 to 1.69)	23.6 (15.6 to 40.5)	13.7 (11.2 to 16.8)	16.6 (13.6 to 20.1)
Tajikistan	Vascular intestinal disorders	1.05 (0.738 to 1.35)	1.1 (0.716 to 1.3)	1.7 (1.02 to 2.04)	29.1 (22.6 to 36)	23.2 (17.8 to 27.1)	26.5 (19.4 to 31.7)
Tajikistan	Gallbladder and biliary diseases	1.1 (0.91 to 1.3)	0.852 (0.757 to 0.999)	1.19 (1.02 to 1.39)	27.2 (24 to 30.9)	19.1 (17 to 22.8)	18.9 (16.3 to 22.6)
Tajikistan	Pancreatitis	0.746 (0.59 to 0.979)	0.93 (0.754 to 1.2)	1.51 (1.2 to 1.94)	21.1 (17.3 to 27)	19 (15.6 to 24)	22.3 (18 to 28.1)
Tajikistan	Other digestive diseases	2.19 (1.76 to 2.66)	2.21 (1.84 to 2.62)	3.21 (2.67 to 3.83)	47.5 (39.7 to 56.4)	39.6 (33.5 to 47)	43.1 (36.4 to 50.8)
Tajikistan	Neurological disorders	67.5 (50.9 to 88.8)	103 (79.2 to 132)	128 (96.2 to 168)	1621.4 (1267.5 to 2036.8)	1708.1 (1355.1 to 2138.4)	1700.5 (1337 to 2135.8)
Tajikistan	Alzheimer's disease and other dementias	10.1 (9.08 to 11.1)	10.7 (9.64 to 11.9)	14.4 (13.3 to 15.7)	415.9 (372.3 to 455.5)	379.3 (344.5 to 419.1)	404.5 (371.7 to 438.6)
Tajikistan	Parkinson's disease	1.91 (1.29 to 2.28)	2.48 (1.74 to 2.84)	3.23 (2.21 to 3.66)	75.3 (50.5 to 89.8)	78.8 (54.2 to 90.2)	84.7 (57.2 to 95.9)
Tajikistan	Epilepsy	20.7 (12.7 to 31.6)	37.2 (26.2 to 49.6)	42.6 (29.1 to 60.3)	368.8 (223.4 to 562.3)	484.2 (336.7 to 643.8)	441.4 (298.2 to 627)
Tajikistan	Multiple sclerosis	0.336 (0.268 to 0.414)	0.533 (0.427 to 0.657)	0.819 (0.648 to 1.02)	9.7 (7.8 to 12)	10.1 (8.1 to 12.4)	10.8 (8.5 to 13.4)
Tajikistan	Motor neuron disease	0.0333 (0.0272 to 0.0403)	0.0466 (0.039 to 0.0546)	0.0767 (0.0629 to 0.0889)	0.9 (0.7 to 1.1)	1 (0.8 to 1.1)	1.1 (0.9 to 1.2)
Tajikistan	Headache disorders	32.2 (20.9 to 45.8)	48.2 (31.5 to 69.2)	62.8 (41 to 90.3)	713 (462.5 to 1024)	711.8 (461.5 to 1022.2)	712 (467 to 1021.9)
Tajikistan	Migraine	27.9 (17.3 to 41.1)	42 (26.3 to 62.3)	54.7 (34.1 to 81.4)	618.5 (384.2 to 915.9)	617.4 (385.2 to 913.5)	617.6 (387.5 to 919.7)
Tajikistan	Tension-type headache	4.24 (2.36 to 6.8)	6.25 (3.45 to 10.1)	8.11 (4.53 to 13)	94.5 (53 to 152)	94.5 (52.9 to 150.6)	94.3 (53.1 to 150.6)
Tajikistan	Other neurological disorders	2.22 (0.938 to 4)	3.38 (1.72 to 5.53)	4.46 (1.94 to 8.12)	37.7 (16.7 to 66.4)	42.8 (22.9 to 68.3)	46.1 (21.2 to 82.7)
Tajikistan	Mental disorders	58.6 (42.9 to 76.3)	84.5 (61.8 to 111)	107 (79.6 to 140)	1269.9 (941.4 to 1642.2)	1245 (922.5 to 1610.1)	1239.4 (922.8 to 1606)
Tajikistan	Schizophrenia	4.47 (3.29 to 5.64)	6.56 (4.83 to 8.34)	9.35 (6.83 to 11.9)	114.3 (84.7 to 143.4)	110.9 (81.9 to 139.9)	112.2 (83.1 to 141.3)
Tajikistan	Depressive disorders	17.1 (12 to 23.4)	23.5 (16.6 to 32.2)	30.9 (21.9 to 42.1)	416.8 (298.4 to 566.3)	389.4 (275.8 to 531.6)	388.2 (274.6 to 529)
Tajikistan	Major depressive disorder	12.9 (8.97 to 17.7)	17.3 (12 to 23.7)	22.4 (15.6 to 30.8)	309.7 (217.4 to 419.2)	282.8 (197.2 to 384.8)	281.6 (196.1 to 382.1)
Tajikistan	Dysthymia	4.23 (2.82 to 6.16)	6.17 (4.11 to 8.97)	8.43 (5.63 to 12.4)	107.2 (71.2 to 156.3)	106.6 (71.3 to 156)	106.7 (72.3 to 156.1)
Tajikistan	Bipolar disorder	5.95 (3.71 to 8.98)	9 (5.6 to 13.6)	11.6 (7.2 to 17.3)	131.7 (81.9 to 197.3)	131.7 (81.8 to 197.2)	131.6 (81.7 to 196.2)
Tajikistan	Anxiety disorders	11 (7.7 to 14.8)	16 (11.2 to 21.4)	20.4 (14.3 to 27.2)	234.4 (166.1 to 313.5)	232.6 (163.4 to 309.9)	232.7 (165.9 to 309.1)
Tajikistan	Eating disorders	1.49 (0.94 to 2.21)	1.73 (1.08 to 2.56)	2.45 (1.53 to 3.59)	28.7 (18 to 42.3)	21.8 (13.8 to 32)	24.6 (15.4 to 36)
Tajikistan	Anorexia nervosa	0.333 (0.194 to 0.517)	0.399 (0.23 to 0.632)	0.521 (0.301 to 0.83)	5.9 (3.5 to 9)	4.6 (2.7 to 7.2)	5.1 (3 to 8)
Tajikistan	Bulimia nervosa	1.16 (0.706 to 1.75)	1.33 (0.799 to 2.04)	1.93 (1.18 to 2.93)	22.8 (13.9 to 34.2)	17.2 (10.4 to 26.1)	19.5 (11.9 to 29.3)
Tajikistan	Autism spectrum disorders	3.64 (2.47 to 5.02)	4.91 (3.33 to 6.81)	6.18 (4.22 to 8.58)	64 (43.6 to 88.2)	64.4 (43.6 to 89.6)	64.5 (44 to 89.2)
Tajikistan	Attention-deficit/hyperactivity disorder	0.704 (0.415 to 1.13)	1.01 (0.602 to 1.61)	1.19 (0.714 to 1.91)	11.7 (7 to 18.5)	11.7 (7 to 18.7)	11.8 (7 to 18.9)
Tajikistan	Conduct disorder	6.73 (3.99 to 10.6)	9.79 (5.92 to 15.5)	10.2 (6.09 to 16.2)	97.1 (57.8 to 153.7)	97.6 (59.1 to 154.8)	97.9 (58.5 to 155.9)
Tajikistan	Idiopathic developmental intellectual disability	1.89 (0.654 to 3.51)	3.54 (1.44 to 6.42)	3.55 (1.29 to 6.46)	32 (11 to 59.8)	44.7 (17.9 to 81.1)	35.9 (13 to 65.5)
Tajikistan	Other mental disorders	5.63 (3.71 to 7.8)	8.41 (5.51 to 11.7)	11.6 (7.59 to 16.2)	139.4 (92.5 to 192.2)	140.2 (92.7 to 193.4)	139.9 (92.5 to 195.4)
Tajikistan	Substance use disorders	19.3 (15 to 24.1)	32.2 (25.4 to 39.4)	38.9 (29.7 to 48.8)	444.3 (346.1 to 550.7)	484 (385.6 to 590.9)	431.8 (332 to 539.7)
Tajikistan	Alcohol use disorders	14.2 (11.1 to 17.8)	24.4 (19.2 to 29.9)	27.4 (20.6 to 35.4)	336.6 (265.2 to 420)	372.2 (295.6 to 452.1)	308.9 (233.2 to 403.1)
Tajikistan	Drug use disorders	5.08 (3.7 to 6.6)	7.79 (5.62 to 10.1)	11.5 (8.37 to 14.8)	107.6 (79 to 137.4)	111.9 (82.3 to 142.3)	123 (90.6 to 157)
Tajikistan	Opioid use disorders	3.32 (2.35 to 4.49)	5.22 (3.78 to 6.91)	7.96 (5.69 to 10.8)	73.5 (52.7 to 97.5)	78.4 (57.6 to 102.6)	87.1 (62.8 to 115.5)
Tajikistan	Cocaine use disorders	0.169 (0.0993 to 0.257)	0.241 (0.142 to 0.371)	0.359 (0.209 to 0.55)	3.6 (2.2 to 5.3)	3.4 (2.1 to 5)	3.9 (2.4 to 5.9)
Tajikistan	Amphetamine use disorders	0.592 (0.314 to 0.992)	0.884 (0.474 to 1.44)	1.17 (0.632 to 1.94)	10.7 (5.9 to 17.8)	10.8 (5.9 to 17.6)	11.4 (6.2 to 18.9)
Tajikistan	Cannabis use disorders	0.249 (0.149 to 0.383)	0.38 (0.228 to 0.579)	0.459 (0.274 to 0.69)	4.6 (2.8 to 7)	4.6 (2.8 to 6.9)	4.6 (2.8 to 6.9)

SR Appendix Table 2. All-age DALYs counts and age standardized DALYs rates per 100,000 individuals for all causes and level 1 cause groups by location for 1990, 2007, and 2017, both sexes combined

		All-age DALYs			Age-standardized rate of DALYs per 100,000		
Tajikistan	Other drug use disorders	0.746 (0.514 to 1.06)	1.07 (0.725 to 1.52)	1.52 (1.03 to 2.13)	15.3 (10.9 to 20.9)	14.6 (10.3 to 20)	16 (11.2 to 22)
Tajikistan	Diabetes and kidney diseases	45.2 (38.6 to 53.3)	72.4 (61.7 to 85.4)	117 (99.1 to 139)	1294 (1090 to 1547.8)	1548.6 (1322.7 to 1838.9)	1787.9 (1519.7 to 2112.2)
Tajikistan	Diabetes mellitus	27.7 (21.9 to 35.2)	46.7 (37.5 to 58.1)	86.2 (70.1 to 106)	869.3 (688.6 to 1100)	1085.1 (882.3 to 1336.1)	1368.2 (1118.7 to 1664.2)
Tajikistan	Diabetes mellitus type 1	5.7 (4.3 to 6.53)	10.1 (7.39 to 11.3)	18.2 (11.9 to 20.9)	159.2 (117.1 to 185.2)	202.8 (141.4 to 231.7)	254 (161.2 to 293.3)
Tajikistan	Diabetes mellitus type 2	22 (16.3 to 29)	36.6 (27.8 to 47.7)	68 (51.9 to 87.1)	710.2 (530.9 to 933.1)	882.3 (688.2 to 1136.3)	1114.1 (869.1 to 1402.2)
Tajikistan	Chronic kidney disease	16.7 (15.4 to 18.2)	25.2 (22.8 to 27.6)	30.1 (26.9 to 33.3)	406.6 (372.3 to 444.8)	456.8 (414.9 to 500.2)	414.8 (369.6 to 458.8)
Tajikistan	Chronic kidney disease due to diabetes mellitus type 1	1.15 (0.911 to 1.41)	2.24 (1.77 to 2.79)	2.66 (2.08 to 3.33)	32.3 (25.7 to 39.9)	39.6 (31.4 to 48.9)	33.8 (26.3 to 41.8)
Tajikistan	Chronic kidney disease due to diabetes mellitus type 2	1.73 (1.46 to 2.04)	2.53 (2.11 to 2.99)	3.44 (2.85 to 4.09)	58.2 (49 to 68.4)	66.5 (55.5 to 78.4)	63.9 (53.5 to 75.6)
Tajikistan	Chronic kidney disease due to hypertension	1.25 (1.04 to 1.47)	1.95 (1.62 to 2.3)	2.48 (2.04 to 2.92)	38.7 (32.3 to 45)	44.5 (36.8 to 51.9)	42.5 (35.1 to 50)
Tajikistan	Chronic kidney disease due to glomerulonephritis	4.93 (4.29 to 5.58)	8.02 (6.9 to 9.24)	9.3 (7.98 to 10.7)	110.2 (97.5 to 125.5)	128.9 (111.3 to 147.5)	113.1 (97.6 to 129.7)
Tajikistan	Chronic kidney disease due to other and unspecified causes	7.59 (6.65 to 8.6)	10.5 (9.04 to 12)	12.3 (10.5 to 14.2)	167.2 (145.3 to 193.3)	177.3 (153.7 to 204.1)	161.5 (137.8 to 187.4)
Tajikistan	Acute glomerulonephritis	0.878 (0.575 to 1.36)	0.456 (0.292 to 0.759)	0.433 (0.293 to 0.669)	18.1 (11.6 to 29.4)	6.8 (4.3 to 11.5)	4.9 (3.3 to 7.7)
Tajikistan	Skin and subcutaneous diseases	24.9 (16.3 to 36.8)	32.2 (21.2 to 47.7)	40.6 (26.8 to 60.1)	439.9 (289.1 to 650.5)	431.6 (284.9 to 640.4)	434.3 (287.1 to 648.4)
Tajikistan	Dermatitis	6.56 (3.73 to 10.6)	8.45 (4.88 to 13.6)	10.5 (6.17 to 16.8)	108 (63.7 to 171.1)	108.2 (63.4 to 171.5)	108.3 (64.7 to 171.7)
Tajikistan	Atopic dermatitis	5.51 (2.93 to 9.39)	6.91 (3.66 to 11.7)	8.55 (4.64 to 14.4)	84.9 (45.4 to 144.4)	85.1 (45 to 142.9)	85.1 (46.1 to 143.2)
Tajikistan	Contact dermatitis	1.04 (0.672 to 1.58)	1.53 (0.977 to 2.32)	1.96 (1.26 to 2.96)	23 (15 to 34.3)	23 (15 to 34.1)	23 (15 to 34.3)
Tajikistan	Seborrhoeic dermatitis	0.00561 (0.00313 to 0.00904)	0.0075 (0.00418 to 0.0121)	0.00963 (0.00539 to 0.0155)	0.1 (0.1 to 0.2)	0.1 (0.1 to 0.2)	0.1 (0.1 to 0.2)
Tajikistan	Psoriasis	2.32 (1.62 to 3.15)	3.12 (2.2 to 4.15)	4.2 (2.94 to 5.57)	54.4 (38.7 to 73.2)	50.5 (35.6 to 67)	51 (36.1 to 67.6)
Tajikistan	Bacterial skin diseases	0.118 (0.0843 to 0.173)	0.154 (0.115 to 0.208)	0.227 (0.171 to 0.3)	2.3 (1.7 to 3.2)	2.4 (1.8 to 3.2)	2.9 (2 to 3.8)
Tajikistan	Cellulitis	0.0602 (0.0414 to 0.0844)	0.0802 (0.0566 to 0.11)	0.111 (0.08 to 0.151)	1.2 (0.8 to 1.6)	1.2 (0.8 to 1.6)	1.3 (0.9 to 1.7)
Tajikistan	Pyoderma	0.0579 (0.0381 to 0.0968)	0.0736 (0.0515 to 0.112)	0.117 (0.0792 to 0.167)	1.1 (0.8 to 1.8)	1.3 (0.9 to 1.8)	1.6 (0.9 to 2.2)
Tajikistan	Scabies	1.87 (1.02 to 3.04)	2.47 (1.36 to 4.08)	2.96 (1.64 to 4.84)	31.3 (17.4 to 51.4)	30.4 (17.1 to 49.6)	29.9 (16.7 to 48.9)
Tajikistan	Fungal skin diseases	2.64 (1.05 to 5.49)	3.23 (1.28 to 6.71)	3.97 (1.57 to 8.29)	53.7 (21.4 to 111.6)	51.3 (20.4 to 107)	49.7 (19.8 to 103.9)
Tajikistan	Viral skin diseases	3.3 (2.12 to 4.89)	4.02 (2.58 to 5.98)	4.9 (3.13 to 7.32)	45.7 (29.5 to 67.6)	45.8 (29.2 to 67.9)	45.8 (29.3 to 68.3)
Tajikistan	Acne vulgaris	1.27 (0.728 to 2.12)	2.18 (1.25 to 3.6)	2.61 (1.53 to 4.35)	21.2 (12.3 to 35.4)	23.6 (13.6 to 38.5)	25.4 (14.8 to 42.3)
Tajikistan	Alopecia areata	0.278 (0.175 to 0.42)	0.403 (0.252 to 0.608)	0.538 (0.341 to 0.817)	5.9 (3.8 to 8.8)	5.9 (3.7 to 8.9)	5.9 (3.7 to 9)
Tajikistan	Pruritus	0.416 (0.195 to 0.791)	0.554 (0.26 to 1.05)	0.739 (0.35 to 1.4)	9.2 (4.4 to 17.5)	8.8 (4.2 to 16.6)	9 (4.3 to 16.7)
Tajikistan	Urticaria	4.7 (3.06 to 6.73)	5.94 (3.91 to 8.57)	7.47 (4.87 to 10.7)	74.5 (49 to 105.6)	74.5 (49.3 to 106.2)	74.4 (49 to 105.3)
Tajikistan	Decubitus ulcer	0.0832 (0.0606 to 0.111)	0.112 (0.0823 to 0.149)	0.155 (0.112 to 0.206)	2.3 (1.6 to 3.1)	2.3 (1.6 to 3.1)	2.4 (1.7 to 3.3)
Tajikistan	Other skin and subcutaneous diseases	1.34 (0.644 to 2.48)	1.61 (0.777 to 2.97)	2.27 (1.09 to 4.14)	31.5 (15.1 to 58.2)	27.8 (13.5 to 50.8)	29.5 (14.2 to 53.6)
Tajikistan	Sense organ diseases	30.6 (20.3 to 44.5)	41.2 (27.2 to 60.3)	55.2 (36.5 to 81.8)	885.1 (594.8 to 1288.3)	891.2 (595.8 to 1302.5)	879.9 (588.5 to 1283.9)
Tajikistan	Blindness and vision impairment	14.7 (9.39 to 22.5)	19.8 (12.5 to 30.6)	26.3 (16.7 to 40.8)	421.7 (274.4 to 632.7)	424.5 (272 to 643.2)	414.6 (267 to 629.6)
Tajikistan	Glaucoma	0.103 (0.0671 to 0.147)	0.112 (0.0734 to 0.161)	0.146 (0.0946 to 0.211)	3.8 (2.5 to 5.4)	3.6 (2.4 to 5.2)	3.4 (2.2 to 4.8)
Tajikistan	Cataract	4.05 (2.75 to 5.59)	5.07 (3.42 to 7.01)	6.82 (4.62 to 9.51)	150.2 (102.1 to 205.2)	151.2 (101.6 to 208.9)	148.6 (100.7 to 205.8)
Tajikistan	Age-related macular degeneration	0.0291 (0.0181 to 0.0445)	0.0333 (0.0205 to 0.0511)	0.0474 (0.0297 to 0.0735)	1.1 (0.7 to 1.6)	1.1 (0.7 to 1.6)	1.1 (0.7 to 1.7)
Tajikistan	Refraction disorders	5.61 (3.52 to 8.65)	7.82 (4.9 to 12.1)	9.97 (6.26 to 15.5)	117 (74 to 178.6)	119.8 (75.6 to 184.6)	118.1 (74.9 to 182.1)
Tajikistan	Near vision loss	4.17 (1.99 to 7.56)	5.76 (2.73 to 10.6)	7.93 (3.77 to 14.5)	128.6 (61.6 to 232.4)	128.4 (61.1 to 235.7)	123.3 (58.3 to 223.9)
Tajikistan	Other vision loss	0.745 (0.488 to 1.05)	0.976 (0.651 to 1.4)	1.34 (0.869 to 1.91)	21.1 (14 to 29.7)	20.5 (13.7 to 29.1)	20.2 (13.2 to 28.3)
Tajikistan	Age-related and other hearing loss	14.8 (10.2 to 20.8)	19.9 (13.7 to 28)	26.9 (18.2 to 38)	433.2 (299.4 to 606.6)	436.6 (302.5 to 609.8)	435 (298.3 to 610)
Tajikistan	Other sense organ diseases	1.13 (0.705 to 1.64)	1.49 (0.919 to 2.18)	2.04 (1.27 to 3.02)	30.2 (18.7 to 43.5)	30.1 (18.7 to 44)	30.3 (18.8 to 44.2)

SR Appendix Table 2. All-age DALYs counts and age standardized DALYs rates per 100,000 individuals for all causes and level 1 cause groups by location for 1990, 2007, and 2017, both sexes combined

		All-age DALYs			Age-standardized rate of DALYs per 100,000		
Tajikistan	Musculoskeletal disorders	51.1 (36.9 to 68.8)	72.9 (52.7 to 98.5)	101 (73.5 to 136)	1439.4 (1041.4 to 1939.1)	1420.7 (1039.9 to 1916)	1412.9 (1030.6 to 1883.6)
Tajikistan	Rheumatoid arthritis	1.16 (0.881 to 1.49)	1.56 (1.2 to 2)	2.38 (1.79 to 3.02)	34.2 (25.8 to 44)	33 (25.4 to 41.8)	35.3 (26.9 to 44.3)
Tajikistan	Osteoarthritis	2.07 (1.04 to 4.11)	2.78 (1.4 to 5.55)	4.21 (2.11 to 8.46)	71.9 (36.1 to 142.8)	74.2 (37.6 to 147.6)	77.2 (38.7 to 153.8)
Tajikistan	Low back pain	35.9 (25.6 to 48.7)	50.9 (36.7 to 69.3)	69.6 (49.7 to 94.4)	982.8 (698.2 to 1323.6)	962.6 (701.2 to 1294.7)	954.5 (678.2 to 1290.4)
Tajikistan	Neck pain	9.98 (6.98 to 13.9)	14.9 (10.4 to 20.9)	21 (14.8 to 29.6)	297.1 (207.6 to 414.9)	297.2 (207.1 to 414.6)	296.9 (208.2 to 418.1)
Tajikistan	Gout	0.38 (0.252 to 0.525)	0.543 (0.356 to 0.76)	0.802 (0.527 to 1.13)	12.6 (8.5 to 17.4)	13.4 (8.9 to 18.7)	13.8 (9.2 to 19.3)
Tajikistan	Other musculoskeletal disorders	1.59 (0.973 to 2.45)	2.23 (1.27 to 3.52)	2.85 (1.42 to 4.67)	40.7 (24.3 to 61.8)	40.2 (23 to 62.7)	35.2 (18 to 57.2)
Tajikistan	Other non-communicable diseases	110 (94.1 to 128)	150 (129 to 176)	210 (176 to 250)	1713.4 (1425.4 to 2054.2)	1934.8 (1642.5 to 2311)	2071.3 (1734 to 2493.6)
Tajikistan	Congenital anomalies	64.8 (56.1 to 75.3)	90.1 (77.6 to 105)	129 (102 to 160)	757.4 (657.6 to 869.6)	953.8 (823.6 to 1107.1)	1062.9 (850.5 to 1314.3)
Tajikistan	Neural tube defects	4.69 (3.25 to 6.46)	5.67 (4.26 to 7.51)	7.01 (4.78 to 10.2)	52 (37 to 70.1)	58.6 (44.4 to 76.7)	56.7 (39.3 to 80.8)
Tajikistan	Congenital heart anomalies	24.3 (17.5 to 34.6)	38 (29.2 to 48.6)	56 (41.9 to 76.1)	261 (190.2 to 367.5)	386.6 (298.4 to 491.9)	448.4 (338.9 to 607.3)
Tajikistan	Orofacial clefts	0.816 (0.467 to 1.39)	0.487 (0.295 to 0.821)	0.61 (0.363 to 1.05)	9.9 (6.2 to 15.6)	5.6 (3.5 to 9.1)	5.5 (3.4 to 9.1)
Tajikistan	Down's syndrome	2.54 (0.841 to 3.68)	3.2 (1.53 to 4.39)	4.28 (2.32 to 5.93)	29.8 (11.8 to 42.1)	34.3 (17.5 to 46.6)	35.9 (20.6 to 49.4)
Tajikistan	Turner syndrome	0.00714 (0.00344 to 0.012)	0.00927 (0.0046 to 0.0154)	0.0113 (0.00546 to 0.0188)	0.1 (0.1 to 0.2)	0.1 (0.1 to 0.2)	0.1 (0.1 to 0.2)
Tajikistan	Klinefelter syndrome	0.00141 (0.000668 to 0.00268)	0.00202 (0.000945 to 0.0038)	0.00257 (0.00121 to 0.00482)	0 (0 to 0)	0 (0 to 0)	0 (0 to 0)
Tajikistan	Other chromosomal abnormalities	1.15 (0.655 to 1.79)	1.53 (0.917 to 2.37)	2.29 (1.45 to 3.45)	14.8 (9.1 to 22)	16.9 (10.4 to 25.6)	19.5 (12.7 to 28.7)
Tajikistan	Congenital musculoskeletal and limb anomalies	6.22 (4.35 to 8.34)	8.37 (5.9 to 11.1)	10.9 (7.74 to 14.6)	107.5 (73.8 to 145.6)	110.4 (76.7 to 147.8)	113.1 (78.9 to 151)
Tajikistan	Urogenital congenital anomalies	0.807 (0.55 to 1.12)	1.03 (0.75 to 1.42)	1.45 (0.984 to 2.08)	9.4 (6.6 to 13)	10.9 (8 to 14.8)	12 (8.3 to 17.1)
Tajikistan	Digestive congenital anomalies	8.1 (5.04 to 11.9)	11.2 (7.06 to 15.3)	16.3 (10.3 to 22.9)	92.5 (59.5 to 132.7)	116.1 (75.6 to 157.4)	131.6 (85 to 182)
Tajikistan	Other congenital anomalies	16.3 (9.63 to 22.8)	20.6 (14.1 to 29.1)	29.8 (19.5 to 43.7)	180.3 (110.8 to 248.6)	214.3 (147.6 to 301.4)	240.1 (158.7 to 347.5)
Tajikistan	Urinary diseases and male infertility	8.7 (7.9 to 9.66)	14.2 (12.9 to 15.7)	24.2 (21.8 to 26.8)	203.7 (182 to 228.9)	256.9 (231.6 to 285.5)	316.3 (284.8 to 352.1)
Tajikistan	Urinary tract infections	6.78 (6.3 to 7.31)	11.7 (10.8 to 12.7)	20.5 (18.4 to 22.7)	145.2 (136.3 to 155)	194.2 (179.3 to 209.3)	253.9 (230.6 to 280)
Tajikistan	Urolithiasis	0.274 (0.189 to 0.359)	0.415 (0.301 to 0.529)	0.627 (0.453 to 0.826)	7.3 (5.1 to 9.5)	8.2 (6.1 to 10.3)	8.8 (6.8 to 11.1)
Tajikistan	Benign prostatic hyperplasia	1.28 (0.817 to 1.86)	1.63 (1.04 to 2.35)	2.52 (1.6 to 3.66)	43.7 (27.9 to 63)	47.4 (30.5 to 68.2)	47.1 (30.3 to 68.2)
Tajikistan	Male infertility	0.111 (0.0447 to 0.233)	0.166 (0.0687 to 0.347)	0.214 (0.0896 to 0.453)	2.3 (0.9 to 4.7)	2.2 (0.9 to 4.6)	2.1 (0.9 to 4.5)
Tajikistan	Other urinary diseases	0.254 (0.189 to 0.331)	0.295 (0.225 to 0.383)	0.35 (0.272 to 0.444)	5.3 (4 to 6.9)	4.8 (3.7 to 6.1)	4.3 (3.5 to 5.4)
Tajikistan	Gynecological diseases	6.87 (4.81 to 9.53)	11.1 (7.82 to 15.5)	14 (9.77 to 19.5)	160.7 (111.6 to 223.5)	162.6 (113.9 to 226.7)	154.3 (107.1 to 217.5)
Tajikistan	Uterine fibroids	0.925 (0.557 to 1.46)	1.68 (1.05 to 2.64)	1.95 (1.16 to 3.17)	28.4 (17.1 to 45)	29.7 (18.4 to 46.7)	25.3 (15 to 41)
Tajikistan	Polycystic ovarian syndrome	0.277 (0.124 to 0.535)	0.434 (0.196 to 0.838)	0.56 (0.255 to 1.08)	5.9 (2.7 to 11.4)	5.9 (2.7 to 11.2)	5.9 (2.7 to 11.5)
Tajikistan	Female infertility	0.116 (0.0408 to 0.266)	0.159 (0.0587 to 0.339)	0.209 (0.081 to 0.438)	2.3 (0.8 to 5.2)	2.2 (0.8 to 4.6)	2 (0.8 to 4.3)
Tajikistan	Endometriosis	2.34 (1.53 to 3.45)	3.64 (2.39 to 5.26)	4.82 (3.17 to 7.08)	50.8 (33.9 to 75)	51 (33.5 to 74.3)	50.5 (33 to 74.1)
Tajikistan	Genital prolapse	0.182 (0.104 to 0.303)	0.202 (0.119 to 0.337)	0.249 (0.128 to 0.446)	5.5 (3.1 to 9.5)	4.2 (2.4 to 7.3)	3.8 (1.9 to 7)
Tajikistan	Premenstrual syndrome	2.22 (1.4 to 3.39)	3.7 (2.39 to 5.64)	4.52 (2.86 to 6.77)	48.7 (31.1 to 74.1)	50.3 (32.3 to 76.2)	48.4 (30.8 to 72.6)
Tajikistan	Other gynecological diseases	0.802 (0.586 to 1.09)	1.31 (0.926 to 1.83)	1.65 (1.13 to 2.4)	19 (13.5 to 26.3)	19.3 (13.5 to 27.1)	18.5 (12.5 to 26.9)
Tajikistan	Hemoglobinopathies and hemolytic anaemias	11.1 (7.93 to 14.5)	11.3 (8.6 to 14.9)	12.6 (9.42 to 16.5)	178.5 (131.4 to 230.1)	149.8 (114.6 to 196.2)	132.6 (100.2 to 175.6)
Tajikistan	Thalassemias	3.67 (1.91 to 5.86)	2.66 (1.85 to 3.57)	3.12 (2.05 to 4.15)	40.8 (21.6 to 64.7)	28.3 (19.7 to 38.3)	26.1 (17.2 to 34.6)
Tajikistan	Thalassaemias trait	3.83 (2.42 to 5.61)	4.54 (2.94 to 6.6)	4.84 (3.02 to 7.29)	66.8 (43 to 95.9)	60.9 (39.7 to 87.9)	52.6 (33.7 to 78.3)
Tajikistan	Sickle cell disorders	0.161 (0.0765 to 0.266)	0.124 (0.0804 to 0.177)	0.145 (0.0921 to 0.209)	1.8 (0.9 to 3)	1.3 (0.9 to 1.9)	1.2 (0.8 to 1.8)
Tajikistan	Sickle cell trait	0.185 (0.114 to 0.279)	0.206 (0.127 to 0.318)	0.216 (0.12 to 0.354)	3.2 (2 to 4.8)	2.7 (1.7 to 4.2)	2.3 (1.3 to 3.7)
Tajikistan	G6PD deficiency	0.246 (0.191 to 0.298)	0.468 (0.386 to 0.558)	0.663 (0.549 to 0.786)	4.7 (3.7 to 5.6)	6.3 (5.2 to 7.4)	7 (5.8 to 8.2)

SR Appendix Table 2. All-age DALYs counts and age standardized DALYs rates per 100,000 individuals for all causes and level 1 cause groups by location for 1990, 2007, and 2017, both sexes combined							
		All-age DALYs			Age-standardized rate of DALYs per 100,000		
Tajikistan	G6PD trait	0.000201 (0.000128 to 0.000293)	0.000254 (0.000172 to 0.00035)	0.000287 (0.000184 to 0.000433)	0 (0 to 0)	0 (0 to 0)	0 (0 to 0)
Tajikistan	Other hemoglobinopathies and hemolytic anaemias	2.97 (2.18 to 3.95)	3.33 (2.45 to 4.5)	3.57 (2.61 to 4.78)	61.3 (45.4 to 80.3)	50.2 (38 to 65.9)	43.3 (32.6 to 57.4)
Tajikistan	Endocrine, metabolic, blood, and immune disorders	4.48 (3.35 to 6.09)	5.69 (4.37 to 7.25)	7.65 (5.54 to 9.9)	79 (58.7 to 105.1)	77.4 (60.2 to 98.9)	81.6 (59.2 to 106.1)
Tajikistan	Oral disorders	10.4 (6.57 to 15.7)	14.3 (8.88 to 21.6)	19.2 (12 to 28.8)	300.6 (192 to 446.4)	299.8 (191.5 to 444.6)	291.3 (186.2 to 433.8)
Tajikistan	Caries of deciduous teeth	0.197 (0.0825 to 0.389)	0.216 (0.0926 to 0.422)	0.277 (0.114 to 0.544)	2.3 (1 to 4.6)	2.4 (1 to 4.7)	2.4 (1 to 4.7)
Tajikistan	Caries of permanent teeth	1.26 (0.542 to 2.38)	1.97 (0.85 to 3.75)	2.38 (1.03 to 4.54)	26.3 (11.3 to 49.7)	27.7 (12 to 52.8)	26.6 (11.5 to 50.7)
Tajikistan	Periodontal disease	1.34 (0.534 to 2.82)	1.92 (0.763 to 4.04)	2.72 (1.06 to 5.69)	41.1 (16.4 to 85.6)	40.2 (16 to 82.6)	39.2 (15.6 to 80.8)
Tajikistan	Edentulism and severe tooth loss	5.29 (3.52 to 7.59)	6.82 (4.49 to 9.73)	9.39 (6.13 to 13.4)	180 (120.6 to 257.4)	178.7 (119.2 to 253.2)	172.3 (113.7 to 244.1)
Tajikistan	Other oral disorders	2.3 (1.45 to 3.4)	3.34 (2.09 to 4.95)	4.38 (2.74 to 6.5)	50.8 (31.9 to 74.9)	50.8 (31.8 to 75.1)	50.8 (31.7 to 74.6)
Tajikistan	Sudden infant death syndrome	3.28 (1.57 to 7.91)	3.49 (1.52 to 7.61)	4.21 (1.87 to 9.26)	33.6 (16 to 80.9)	34.6 (15 to 75.4)	32.3 (14.3 to 71)
Tajikistan	Injuries	230 (211 to 253)	231 (207 to 259)	283 (251 to 318)	4335.8 (3932.4 to 4756)	3277.5 (2926.5 to 3700.6)	3133.7 (2764.2 to 3541.8)
Tajikistan	Transport injuries	44.6 (41.7 to 47.8)	30.3 (26.8 to 35)	47.6 (42.7 to 53.1)	897.5 (841 to 959.7)	437.9 (387.2 to 506)	524.7 (470.9 to 584.5)
Tajikistan	Road injuries	40.9 (38.1 to 43.6)	26.7 (23.6 to 31)	42.3 (37.9 to 47.2)	825 (770.4 to 883.4)	386.9 (343.1 to 448.7)	467.5 (418 to 521.5)
Tajikistan	Pedestrian road injuries	12.2 (9.53 to 15.6)	5.32 (4.29 to 6.56)	8.53 (7.23 to 9.8)	241.3 (192.8 to 311.3)	80.4 (65.6 to 97.6)	97.3 (83.2 to 112.1)
Tajikistan	Cyclist road injuries	1.29 (0.983 to 1.66)	0.986 (0.777 to 1.25)	1.59 (1.24 to 2)	26.8 (20.7 to 33.8)	16.3 (12.6 to 20.7)	19.5 (15 to 24.6)
Tajikistan	Motorcyclist road injuries	3.8 (2.74 to 4.54)	2.82 (2.11 to 3.55)	4.48 (3.46 to 5.39)	74.7 (57.6 to 88.7)	38.7 (30.1 to 48.1)	47.5 (37.8 to 56.3)
Tajikistan	Motor vehicle road injuries	22.7 (19.5 to 25.5)	16.9 (14.8 to 19.7)	26.7 (23.8 to 29.9)	463.8 (395.1 to 518)	240.9 (211.9 to 281.1)	291.2 (259.8 to 326)
Tajikistan	Other road injuries	0.901 (0.619 to 1.2)	0.687 (0.504 to 0.87)	1.03 (0.753 to 1.29)	18.5 (13.6 to 23.4)	10.6 (8.1 to 13.2)	12.1 (9.1 to 15.1)
Tajikistan	Other transport injuries	3.79 (3.08 to 4.36)	3.6 (2.83 to 4.59)	5.33 (4.47 to 6.39)	72.5 (60.7 to 83.6)	51 (40.3 to 64.7)	57.2 (48.5 to 68.2)
Tajikistan	Unintentional injuries	158 (140 to 180)	153 (134 to 175)	178 (153 to 206)	2809.8 (2470.8 to 3165)	2157.1 (1873.1 to 2491.2)	1958.7 (1670.4 to 2296.7)
Tajikistan	Falls	41.3 (33.3 to 49.7)	40.2 (32.6 to 49)	56.8 (44.7 to 70.7)	928 (741.4 to 1147.3)	664 (529.8 to 821.9)	703.6 (547.6 to 887.5)
Tajikistan	Drowning	51.7 (45.9 to 59.5)	48.2 (41 to 56.8)	47.8 (41.4 to 56.2)	738.2 (669.5 to 827.4)	583.7 (503.6 to 682.9)	455.5 (398.9 to 527.2)
Tajikistan	Fire, heat, and hot substances	30.1 (22.8 to 41.9)	25.2 (19.8 to 30.1)	24.5 (19 to 32.4)	512.3 (416.7 to 635.1)	349.1 (276.1 to 417.2)	260 (203.8 to 335.9)
Tajikistan	Poisonings	5.55 (3.89 to 6.33)	5.63 (4.75 to 6.91)	4.42 (3.03 to 8.82)	108 (73.1 to 123.2)	81.5 (66 to 97.6)	47.4 (32.9 to 93.7)
Tajikistan	Poisoning by carbon monoxide	4.73 (3.2 to 5.42)	4.77 (3.88 to 5.93)	2.77 (1.82 to 6.34)	93.4 (59.7 to 107.5)	69.7 (53.8 to 85)	29.6 (19.6 to 67.9)
Tajikistan	Poisoning by other means	0.828 (0.585 to 1.23)	0.861 (0.643 to 1.14)	1.65 (1.09 to 2.66)	14.5 (10.7 to 20.1)	11.9 (8.8 to 15.7)	17.9 (12 to 27.8)
Tajikistan	Exposure to mechanical forces	5.96 (4.72 to 7.42)	7.05 (5.6 to 8.75)	11.4 (8.78 to 14.7)	137.1 (107.2 to 171.8)	115.2 (90.6 to 145.3)	141 (107.3 to 183.1)
Tajikistan	Unintentional firearm injuries	1.37 (0.979 to 1.68)	1.57 (1.14 to 1.87)	2 (1.45 to 2.38)	28.1 (21.2 to 34.1)	22.8 (16.8 to 26.8)	22.4 (16.2 to 26.6)
Tajikistan	Other exposure to mechanical forces	4.59 (3.41 to 5.93)	5.48 (4.09 to 7.11)	9.41 (6.87 to 12.5)	108.9 (80.6 to 143)	92.4 (68.7 to 121.6)	118.6 (85.8 to 159.6)
Tajikistan	Adverse effects of medical treatment	3.62 (1.69 to 6.13)	3.36 (1.95 to 4.59)	4.23 (2.5 to 5.76)	50.9 (30.7 to 76.1)	42 (27 to 54.6)	41 (26.9 to 53.2)
Tajikistan	Animal contact	0.839 (0.581 to 1.11)	1.13 (0.832 to 1.5)	1.45 (1.07 to 1.91)	15.7 (11.3 to 20.5)	16.5 (12.1 to 21.7)	16.3 (11.9 to 21.7)
Tajikistan	Venomous animal contact	0.502 (0.337 to 0.698)	0.704 (0.475 to 0.997)	0.93 (0.628 to 1.3)	9.5 (6.5 to 13)	10.1 (6.9 to 14.1)	10.4 (7.1 to 14.4)
Tajikistan	Non-venomous animal contact	0.337 (0.226 to 0.475)	0.429 (0.322 to 0.572)	0.521 (0.387 to 0.701)	6.1 (4.4 to 8.3)	6.4 (4.8 to 8.6)	6 (4.4 to 8)
Tajikistan	Foreign body	10.8 (7.61 to 16.4)	9.65 (8.08 to 13.3)	12 (10 to 16)	150.1 (116.6 to 207.3)	118.8 (101.6 to 157.9)	116 (99.1 to 148.6)
Tajikistan	Pulmonary aspiration and foreign body in airway	9.27 (6.28 to 15.2)	7.93 (6.43 to 11.9)	9.58 (7.85 to 13.7)	120.8 (90 to 183.2)	93.4 (77.4 to 134.8)	88.5 (74.4 to 121.1)
Tajikistan	Foreign body in eyes	0.0978 (0.0416 to 0.174)	0.151 (0.065 to 0.27)	0.205 (0.0908 to 0.367)	2.1 (1 to 3.6)	2.3 (1.1 to 4)	2.4 (1.1 to 4.2)
Tajikistan	Foreign body in other body part	1.45 (0.915 to 1.99)	1.56 (1.11 to 1.93)	2.19 (1.61 to 2.71)	27.3 (19.2 to 35.1)	23.1 (17.2 to 28.2)	25.1 (19.1 to 30.7)
Tajikistan	Environmental heat and cold exposure	2.31 (1.73 to 2.89)	2.98 (2.21 to 3.77)	4.4 (3.27 to 5.68)	53.4 (40.2 to 67.2)	48.7 (36.6 to 62.2)	55 (41 to 70.7)
Tajikistan	Exposure to forces of nature	0.107 (0.0766 to 0.148)	3.33 (3.22 to 3.48)	1.12 (1.02 to 1.24)	2.1 (1.5 to 2.9)	43.9 (42.3 to 46.1)	12.4 (11.2 to 13.8)
Tajikistan	Other unintentional injuries	5.46 (4.6 to 6.46)	6.46 (5.47 to 7.63)	9.67 (8.03 to 11.7)	114.2 (95.1 to 135.8)	93.6 (78.1 to 112.3)	110.5 (90 to 135.5)

SR Appendix Table 2. All-age DALYs counts and age standardized DALYs rates per 100,000 individuals for all causes and level 1 cause groups by location for 1990, 2007, and 2017, both sexes combined

		All-age DALYs			Age-standardized rate of DALYs per 100,000		
Tajikistan	Self-harm and interpersonal violence	28<br>(23.7 to 30.3)	47.5<br>(40.4 to 58.9)	58<br>(50.9 to 68.6)	628.4<br>(502.9 to 680.4)	682.5<br>(582 to 836.8)	650.3<br>(570 to 765.7)
Tajikistan	Self-harm	12.2<br>(11 to 13.2)	17<br>(15.1 to 19.6)	25.6<br>(21.6 to 29)	275.4<br>(237.4 to 296.2)	242.1<br>(217.5 to 275.2)	278.7<br>(233.5 to 313.4)
Tajikistan	Self-harm by firearm	0.126<br>(0.0923 to 0.188)	0.166<br>(0.123 to 0.274)	0.239<br>(0.162 to 0.375)	3.3<br>(2.4 to 4.6)	2.7<br>(2 to 4.3)	2.8<br>(1.9 to 4.3)
Tajikistan	Self-harm by other specified means	12.1<br>(10.8 to 13.1)	16.9<br>(14.9 to 19.4)	25.4<br>(21.4 to 28.7)	272.1<br>(233.8 to 292.8)	239.4<br>(215 to 272.5)	275.9<br>(231.2 to 310.2)
Tajikistan	Interpersonal violence	15.7<br>(11.7 to 17.6)	14.4<br>(12.3 to 21.8)	18.8<br>(15.7 to 28)	352.4<br>(243.5 to 394.1)	209.1<br>(178 to 308.7)	206.9<br>(173 to 301.3)
Tajikistan	Assault by firearm	2.45<br>(0.601 to 3.77)	2.23<br>(0.743 to 3.13)	2.85<br>(0.947 to 4.05)	48.8<br>(12.5 to 74)	28.5<br>(10.1 to 39.8)	28.6<br>(10.1 to 39.9)
Tajikistan	Assault by sharp object	5.83<br>(3.19 to 7.83)	4.89<br>(3.63 to 7.87)	6.26<br>(4.69 to 10.3)	135.9<br>(69.8 to 178.2)	69.9<br>(51.9 to 111.6)	68.2<br>(51.5 to 108.2)
Tajikistan	Sexual violence	0.542<br>(0.361 to 0.796)	0.734<br>(0.497 to 1.08)	0.909<br>(0.615 to 1.32)	11<br>(7.4 to 16.2)	10<br>(6.8 to 14.5)	9.8<br>(6.6 to 14.2)
Tajikistan	Assault by other means	6.86<br>(5.58 to 9.79)	6.59<br>(5.03 to 11.9)	8.81<br>(6.67 to 15.1)	156.6<br>(128 to 211.9)	100.6<br>(77.8 to 169.9)	100.3<br>(76.8 to 164.7)
Tajikistan	Conflict and terrorism	0<br>(0 to 0)	16<br>(10.5 to 24.8)	13.5<br>(9.03 to 20.7)	0<br>(0 to 0)	230.5<br>(155 to 352.4)	163.7<br>(111 to 248.5)
Tajikistan	Executions and police conflict	0.0412<br>(0.0385 to 0.0442)	0.0645<br>(0.0587 to 0.0716)	0.09<br>(0.0805 to 0.101)	0.7<br>(0.6 to 0.7)	0.8<br>(0.7 to 0.9)	0.9<br>(0.8 to 1)
Turkmenistan	All causes	1600<br>(1470 to 1730)	1640<br>(1510 to 1780)	1560<br>(1390 to 1720)	45390.1<br>(42200.8 to 48928)	44232.8<br>(41063.5 to 47591.2)	34468.1<br>(31033.2 to 37939.8)
Turkmenistan	Communicable, maternal, neonatal, and nutritional disorders	735<br>(661 to 811)	432<br>(378 to 493)	280<br>(227 to 344)	13632.2<br>(12345.9 to 14985.7)	9891.7<br>(8644 to 11267.9)	5432.3<br>(4410.5 to 6653)
Turkmenistan	HIV/AIDS and sexually transmitted infections	4.23<br>(3.56 to 5.39)	7.08<br>(6.42 to 8.1)	6.61<br>(5.9 to 7.64)	120.6<br>(105.1 to 145.7)	152.3<br>(138.1 to 173.4)	122<br>(108.9 to 141.7)
Turkmenistan	HIV/AIDS	2.65<br>(2.52 to 2.79)	5.84<br>(5.53 to 6.24)	5.4<br>(5.07 to 5.83)	80.3<br>(76.5 to 84.3)	125.9<br>(119.3 to 134.7)	99.1<br>(93 to 107)
Turkmenistan	HIV/AIDS - Drug-susceptible Tuberculosis	0.856<br>(0.542 to 1.17)	1.1<br>(0.585 to 1.72)	0.614<br>(0.309 to 1.06)	25.9<br>(16.4 to 35.5)	23.8<br>(12.6 to 37.1)	11.3<br>(5.7 to 19.5)
Turkmenistan	HIV/AIDS - Multidrug-resistant Tuberculosis without extensive drug resistance	0.00161<br>(0.00022 to 0.00596)	0.414<br>(0.151 to 0.805)	0.336<br>(0.157 to 0.579)	0<br>(0 to 0.2)	8.9<br>(3.3 to 17.4)	6.2<br>(2.9 to 10.6)
Turkmenistan	HIV/AIDS - Extensively drug-resistant Tuberculosis	0<br>(0 to 0)	0.131<br>(0.0478 to 0.257)	0.193<br>(0.0893 to 0.329)	0<br>(0 to 0)	2.8<br>(1 to 5.6)	3.5<br>(1.6 to 6)
Turkmenistan	HIV/AIDS resulting in other diseases	1.79<br>(1.46 to 2.15)	4.19<br>(3.33 to 4.93)	4.26<br>(3.57 to 4.86)	54.3<br>(44.2 to 64.9)	90.4<br>(71.8 to 106.4)	78.1<br>(65.5 to 89.3)
Turkmenistan	Sexually transmitted infections excluding HIV	1.58<br>(0.942 to 2.76)	1.25<br>(0.688 to 2.24)	1.21<br>(0.66 to 2.18)	40.3<br>(25.6 to 65.1)	26.4<br>(14.7 to 47.5)	23<br>(12.5 to 41.4)
Turkmenistan	Syphilis	0.681<br>(0.253 to 1.73)	0.221<br>(0.0789 to 0.589)	0.118<br>(0.0424 to 0.276)	13.1<br>(5.6 to 30.9)	5.1<br>(1.8 to 13.5)	2.3<br>(0.8 to 5.4)
Turkmenistan	Chlamydial infection	0.207<br>(0.124 to 0.363)	0.257<br>(0.143 to 0.471)	0.28<br>(0.147 to 0.529)	6.1<br>(3.8 to 10.3)	5.2<br>(2.9 to 9.5)	5.2<br>(2.7 to 9.7)
Turkmenistan	Gonococcal infection	0.238<br>(0.155 to 0.393)	0.195<br>(0.101 to 0.37)	0.185<br>(0.0998 to 0.357)	7<br>(4.6 to 10.8)	3.8<br>(2 to 7.1)	3.5<br>(1.9 to 6.7)
Turkmenistan	Trichomoniasis	0.132<br>(0.0535 to 0.284)	0.182<br>(0.0735 to 0.392)	0.201<br>(0.0814 to 0.428)	4.1<br>(1.7 to 8.9)	4<br>(1.6 to 8.5)	3.8<br>(1.6 to 8.1)
Turkmenistan	Genital herpes	0.0732<br>(0.0247 to 0.17)	0.107<br>(0.0359 to 0.254)	0.129<br>(0.0422 to 0.303)	2.6<br>(0.8 to 6.2)	2.6<br>(0.9 to 6.2)	2.6<br>(0.8 to 6.1)
Turkmenistan	Other sexually transmitted infections	0.251<br>(0.161 to 0.415)	0.285<br>(0.167 to 0.509)	0.295<br>(0.176 to 0.508)	7.4<br>(4.8 to 11.9)	5.7<br>(3.4 to 10.2)	5.5<br>(3.3 to 9.5)
Turkmenistan	Respiratory infections and tuberculosis	367<br>(323 to 414)	195<br>(170 to 223)	114<br>(92.8 to 142)	6848.9<br>(6088.4 to 7668.7)	4542.9<br>(3954.4 to 5172.5)	2206.7<br>(1801.4 to 2738.6)
Turkmenistan	Tuberculosis	23.7<br>(21.4 to 26.1)	33.5<br>(32.1 to 35)	24.6<br>(21.9 to 29.1)	666.9<br>(624 to 711.7)	774<br>(741.5 to 807.9)	472.2<br>(420.9 to 558.6)
Turkmenistan	Latent tuberculosis infection	0<br>(0 to 0)	0<br>(0 to 0)	0<br>(0 to 0)	0<br>(0 to 0)	0<br>(0 to 0)	0<br>(0 to 0)
Turkmenistan	Drug-susceptible tuberculosis	23.6<br>(21.4 to 26)	24<br>(16.3 to 30.3)	14.7<br>(9.91 to 19.8)	665.8<br>(623.1 to 711)	554.5<br>(375.8 to 700)	281.8<br>(189.7 to 378.2)
Turkmenistan	Multidrug-resistant tuberculosis without extensive drug resistance	0.037<br>(0.0052 to 0.136)	7.2<br>(2.75 to 13.2)	6.31<br>(3.51 to 9.58)	1<br>(0.1 to 3.7)	166.6<br>(63.6 to 304.3)	121<br>(67.7 to 183.6)
Turkmenistan	Extensively drug-resistant tuberculosis	0<br>(0 to 0)	2.28<br>(0.892 to 4.09)	3.61<br>(2.01 to 5.55)	0<br>(0 to 0)	52.8<br>(20.6 to 95)	69.3<br>(38.6 to 106.1)
Turkmenistan	Lower respiratory infections	339<br>(297 to 385)	158<br>(133 to 183)	85.4<br>(65.9 to 112)	6084.2<br>(5361.1 to 6866.3)	3670.2<br>(3100.5 to 4261.8)	1652.8<br>(1282.7 to 2154.5)
Turkmenistan	Upper respiratory infections	3.21<br>(2.01 to 4.49)	3.25<br>(2.18 to 4.59)	3.01<br>(1.99 to 4.45)	72.3<br>(46.5 to 102.8)	72.7<br>(48.8 to 101.4)	59<br>(39.2 to 87.2)
Turkmenistan	Otitis media	1.07<br>(0.629 to 1.72)	1.18<br>(0.702 to 1.91)	1.15<br>(0.676 to 1.82)	25.5<br>(15.1 to 40.6)	26.1<br>(15.6 to 41.9)	22.7<br>(13.4 to 36)
Turkmenistan	Enteric infections	91.6<br>(78.8 to 106)	24.1<br>(19.8 to 28.7)	9.01<br>(6.92 to 11.4)	1631.1<br>(1409.7 to 1875.3)	554.3<br>(456.8 to 660.8)	174.8<br>(135.1 to 220.1)
Turkmenistan	Diarrhoeal diseases	90.9<br>(78.3 to 105)	23.6<br>(19.4 to 28.2)	8.66<br>(6.6 to 11)	1617.1<br>(1397.5 to 1861.6)	544.3<br>(446.8 to 651.2)	167.7<br>(128.7 to 213.6)
Turkmenistan	Typhoid and paratyphoid	0.00241<br>(0.00115 to 0.00442)	0.00193<br>(0.000892 to 0.00355)	0.00182<br>(0.000853 to 0.00334)	0.1<br>(0 to 0.1)	0<br>(0 to 0.1)	0<br>(0 to 0.1)
Turkmenistan	Typhoid fever	0.00237<br>(0.00112 to 0.00435)	0.00177<br>(0.000825 to 0.00325)	0.00163<br>(0.000759 to 0.00298)	0.1<br>(0 to 0.1)	0<br>(0 to 0.1)	0<br>(0 to 0.1)
Turkmenistan	Paratyphoid fever	0.0000408<br>(0.0000157 to 0.0000894)	0.000159<br>(0.0000601 to 0.000332)	0.00019<br>(0.0000755 to 0.000408)	0<br>(0 to 0)	0<br>(0 to 0)	0<br>(0 to 0)

SR Appendix Table 2. All-age DALYs counts and age standardized DALYs rates per 100,000 individuals for all causes and level 1 cause groups by location for 1990, 2007, and 2017, both sexes combined							
		All-age DALYs			Age-standardized rate of DALYs per 100,000		
Turkmenistan	Invasive non-typhoidal salmonella	0.567 (0.311 to 0.955)	0.381 (0.196 to 0.649)	0.327 (0.171 to 0.566)	12.4 (6.9 to 20.8)	8.9 (4.6 to 15)	6.6 (3.4 to 11.4)
Turkmenistan	Other intestinal infectious diseases	0.0795 (0.0122 to 0.17)	0.0468 (0.0111 to 0.099)	0.0269 (0.00776 to 0.0529)	1.5 (0.3 to 3)	1.1 (0.3 to 2.3)	0.5 (0.2 to 1)
Turkmenistan	Neglected tropical diseases and malaria	2.84 (1.85 to 4.22)	2.08 (1.27 to 3.31)	1.89 (1.07 to 3.31)	76.5 (50.5 to 112.3)	47.7 (29.5 to 74.7)	37.5 (21.2 to 65.3)
Turkmenistan	Malaria	0.0672 (0.0491 to 0.0875)	0 (0 to 0)	0 (0 to 0)	1.8 (1.3 to 2.3)	0 (0 to 0)	0 (0 to 0)
Turkmenistan	Chagas disease	0 (0 to 0)	0 (0 to 0)	0 (0 to 0)	0 (0 to 0)	0 (0 to 0)	0 (0 to 0)
Turkmenistan	Leishmaniasis	0.348 (0.131 to 1.08)	0.0925 (0.00995 to 0.635)	0.128 (0.0058 to 0.941)	9.3 (3.7 to 24.9)	2.1 (0.2 to 14.3)	2.5 (0.1 to 18)
Turkmenistan	Visceral leishmaniasis	0.0911 (0.000147 to 0.764)	0.0696 (0.000127 to 0.614)	0.104 (0.000157 to 0.925)	2 (0 to 17.1)	1.6 (0 to 13.8)	2 (0 to 17.8)
Turkmenistan	Cutaneous and mucocutaneous leishmaniasis	0.257 (0.118 to 0.456)	0.0228 (0.00779 to 0.0509)	0.0244 (0.00301 to 0.0678)	7.3 (3.3 to 12.7)	0.5 (0.2 to 1.1)	0.5 (0.1 to 1.4)
Turkmenistan	African trypanosomiasis	0 (0 to 0)	0 (0 to 0)	0 (0 to 0)	0 (0 to 0)	0 (0 to 0)	0 (0 to 0)
Turkmenistan	Schistosomiasis	0 (0 to 0)	0 (0 to 0)	0 (0 to 0)	0 (0 to 0)	0 (0 to 0)	0 (0 to 0)
Turkmenistan	Cysticercosis	0.000273 (0.000132 to 0.00046)	0.000152 (0.0000801 to 0.000233)	0.000108 (0.0000529 to 0.00017)	0 (0 to 0)	0 (0 to 0)	0 (0 to 0)
Turkmenistan	Cystic echinococcosis	0.527 (0.221 to 1.09)	0.693 (0.269 to 1.47)	0.785 (0.304 to 1.63)	16.8 (7.4 to 32.7)	16 (6.8 to 31.3)	15.7 (6.3 to 30.8)
Turkmenistan	Lymphatic filariasis	0 (0 to 0)	0 (0 to 0)	0 (0 to 0)	0 (0 to 0)	0 (0 to 0)	0 (0 to 0)
Turkmenistan	Onchocerciasis	0 (0 to 0)	0 (0 to 0)	0 (0 to 0)	0 (0 to 0)	0 (0 to 0)	0 (0 to 0)
Turkmenistan	Trachoma	0 (0 to 0)	0 (0 to 0)	0 (0 to 0)	0 (0 to 0)	0 (0 to 0)	0 (0 to 0)
Turkmenistan	Dengue	0 (0 to 0)	0 (0 to 0)	0 (0 to 0)	0 (0 to 0)	0 (0 to 0)	0 (0 to 0)
Turkmenistan	Yellow fever	0 (0 to 0)	0 (0 to 0)	0 (0 to 0)	0 (0 to 0)	0 (0 to 0)	0 (0 to 0)
Turkmenistan	Rabies	0.217 (0.145 to 0.329)	0.092 (0.0673 to 0.127)	0.0764 (0.0534 to 0.107)	6.8 (4.9 to 9.6)	3 (2.2 to 4.2)	1.7 (1.2 to 2.3)
Turkmenistan	Intestinal nematode infections	0.563 (0.312 to 0.935)	0.28 (0.153 to 0.471)	0.136 (0.073 to 0.239)	16 (8.9 to 26.6)	6.2 (3.4 to 10.3)	2.7 (1.5 to 4.8)
Turkmenistan	Ascariasis	0.0188 (0.00207 to 0.0568)	0.00555 (0.00148 to 0.0136)	0.00392 (0.00111 to 0.0096)	0.3 (0.1 to 1)	0.1 (0 to 0.3)	0.1 (0 to 0.2)
Turkmenistan	Trichuriasis	0.000000000528 (0.000000000242 to 0.00000000103)	0.000000000344 (0.000000000154 to 0.000000000721)	0.000000000363 (0.00000000016 to 0.000000000755)	0 (0 to 0)	0 (0 to 0)	0 (0 to 0)
Turkmenistan	Hookworm disease	0.544 (0.297 to 0.919)	0.275 (0.148 to 0.464)	0.132 (0.0689 to 0.234)	15.7 (8.7 to 26.4)	6.1 (3.3 to 10.1)	2.7 (1.4 to 4.7)
Turkmenistan	Food-borne trematodiases	0 (0 to 0)	0 (0 to 0)	0 (0 to 0)	0 (0 to 0)	0 (0 to 0)	0 (0 to 0)
Turkmenistan	Leprosy	0 (0 to 0)	0.00000163 (0.000000947 to 0.0000026)	0.00000125 (0.000000702 to 0.00000206)	0 (0 to 0)	0 (0 to 0)	0 (0 to 0)
Turkmenistan	Ebola virus disease	0 (0 to 0)	0 (0 to 0)	0 (0 to 0)	0 (0 to 0)	0 (0 to 0)	0 (0 to 0)
Turkmenistan	Zika virus disease	0 (0 to 0)	0 (0 to 0)	0 (0 to 0)	0 (0 to 0)	0 (0 to 0)	0 (0 to 0)
Turkmenistan	Guinea worm disease	0 (0 to 0)	0 (0 to 0)	0 (0 to 0)	0 (0 to 0)	0 (0 to 0)	0 (0 to 0)
Turkmenistan	Other neglected tropical diseases	1.12 (0.688 to 1.68)	0.927 (0.57 to 1.47)	0.764 (0.413 to 1.26)	25.7 (16.1 to 38.1)	20.4 (12.5 to 32.4)	14.9 (8.1 to 24.9)
Turkmenistan	Other infectious diseases	32.7 (29 to 37)	21.7 (18.5 to 24.6)	16.1 (13.7 to 18.9)	662.8 (595.1 to 739.1)	486.9 (416.8 to 554.6)	311.4 (264.9 to 364.3)
Turkmenistan	Meningitis	13.5 (11.8 to 15.5)	8.93 (7.59 to 10.4)	4.89 (4.12 to 6.02)	265.7 (235.3 to 300.2)	199.8 (169 to 234.3)	94.7 (79.8 to 116.3)
Turkmenistan	Pneumococcal meningitis	0.533 (0.429 to 0.651)	0.476 (0.374 to 0.588)	0.416 (0.311 to 0.535)	12.6 (10.1 to 15.4)	10.5 (8.2 to 12.9)	8.2 (6.1 to 10.5)
Turkmenistan	H influenzae type B meningitis	7.25 (6.21 to 8.49)	4.65 (3.9 to 5.55)	2.37 (1.93 to 2.97)	139.7 (122.1 to 160.8)	104.4 (87.3 to 124.7)	45.7 (37.4 to 57.2)
Turkmenistan	Meningococcal infection	1.58 (1.25 to 2.07)	1.03 (0.82 to 1.31)	0.563 (0.45 to 0.729)	31.1 (25.2 to 39.3)	23.1 (18.3 to 29.8)	10.9 (8.7 to 14.1)
Turkmenistan	Other meningitis	4.13 (3.43 to 4.98)	2.77 (2.28 to 3.36)	1.55 (1.26 to 1.92)	82.2 (70.1 to 96.6)	61.8 (50.6 to 74.9)	29.9 (24.5 to 37.1)
Turkmenistan	Encephalitis	2.14 (1.71 to 3.33)	5.26 (2.64 to 6.5)	4.32 (2.52 to 5.59)	42.4 (34.2 to 68.2)	118.8 (60.3 to 147.4)	83 (48.6 to 107)
Turkmenistan	Diphtheria	0.122 (0.0774 to 0.198)	0.00389 (0.00194 to 0.00764)	0.000913 (0.000504 to 0.00171)	2.4 (1.6 to 3.8)	0.1 (0 to 0.2)	0 (0 to 0)
Turkmenistan	Whooping cough	0.167 (0.0773 to 0.615)	0.269 (0.0622 to 0.454)	0.22 (0.0636 to 0.421)	3 (1.4 to 10.8)	6.3 (1.5 to 10.6)	4.2 (1.2 to 7.9)
Turkmenistan	Tetanus	0.0475 (0.0372 to 0.0604)	0.0674 (0.0351 to 0.0853)	0.0784 (0.0283 to 0.118)	1.1 (0.9 to 1.4)	1.9 (1 to 2.4)	1.6 (0.6 to 2.4)
Turkmenistan	Measles	0.564 (0.33 to 0.794)	0.00426 (0.00197 to 0.00628)	0.00166 (0.000523 to 0.00349)	9.9 (5.8 to 14)	0.1 (0 to 0.1)	0 (0 to 0.1)
Turkmenistan	Varicella and herpes zoster	0.126 (0.0843 to 0.18)	0.139 (0.0911 to 0.201)	0.152 (0.0965 to 0.221)	3.6 (2.4 to 5.2)	3.6 (2.3 to 5.1)	3.3 (2.1 to 4.8)

SR Appendix Table 2. All-age DALYs counts and age standardized DALYs rates per 100,000 individuals for all causes and level 1 cause groups by location for 1990, 2007, and 2017, both sexes combined

		All-age DALYs			Age-standardized rate of DALYs per 100,000		
Turkmenistan	Acute hepatitis	12.7 (10 to 15.2)	4.13 (3.68 to 5.02)	3.88 (3.28 to 4.63)	268.1 (211.6 to 312.1)	91.4 (81 to 111.1)	75.3 (63.7 to 89.6)
Turkmenistan	Acute hepatitis A	4.08 (2.99 to 5.37)	1.13 (0.921 to 1.45)	0.967 (0.771 to 1.21)	78.3 (58.6 to 101)	23.9 (19.3 to 31)	18.8 (14.9 to 23.3)
Turkmenistan	Acute hepatitis B	4.27 (3.22 to 5.3)	2.08 (1.84 to 2.47)	2.38 (1.97 to 2.83)	110.7 (85 to 129.6)	46.7 (41.3 to 55.4)	46.1 (38.4 to 54.7)
Turkmenistan	Acute hepatitis C	3.93 (2.49 to 5.53)	0.684 (0.437 to 0.984)	0.319 (0.179 to 0.551)	68.1 (43.3 to 95.7)	15.8 (10.1 to 22.8)	6.1 (3.4 to 10.4)
Turkmenistan	Acute hepatitis E	0.46 (0.363 to 0.57)	0.238 (0.188 to 0.303)	0.221 (0.167 to 0.288)	11 (8.6 to 13.4)	5 (4 to 6.2)	4.4 (3.4 to 5.6)
Turkmenistan	Other unspecified infectious diseases	3.28 (2.45 to 4)	2.87 (2.17 to 3.57)	2.54 (1.88 to 3.25)	66.7 (50.7 to 80.8)	65 (49.4 to 80.5)	49.3 (36.6 to 63.1)
Turkmenistan	Maternal and neonatal disorders	207 (185 to 230)	158 (134 to 186)	114 (87.6 to 146)	3604.8 (3226.2 to 3992.6)	3587.3 (3030.5 to 4225.8)	2206.5 (1697.6 to 2828.2)
Turkmenistan	Maternal disorders	4.34 (4.09 to 4.63)	1.31 (1.2 to 1.42)	0.773 (0.664 to 0.897)	120.5 (113.6 to 127.8)	26.4 (24.3 to 28.8)	14.2 (12.2 to 16.4)
Turkmenistan	Maternal haemorrhage	1.16 (0.947 to 1.38)	0.32 (0.262 to 0.388)	0.179 (0.14 to 0.221)	32.4 (26.6 to 38.5)	6.6 (5.3 to 8)	3.3 (2.6 to 4)
Turkmenistan	Maternal sepsis and other pregnancy related infections	1.2 (0.968 to 1.43)	0.214 (0.171 to 0.264)	0.11 (0.0762 to 0.156)	33.4 (27.4 to 39.6)	4.3 (3.4 to 5.3)	2 (1.4 to 2.9)
Turkmenistan	Maternal hypertensive disorders	0.383 (0.316 to 0.469)	0.103 (0.0842 to 0.126)	0.0658 (0.0508 to 0.084)	10 (8.4 to 12.1)	2.1 (1.7 to 2.5)	1.2 (0.9 to 1.5)
Turkmenistan	Maternal obstructed labour and uterine rupture	0.128 (0.0849 to 0.189)	0.0604 (0.0341 to 0.0946)	0.0551 (0.0272 to 0.0987)	3.5 (2.4 to 5.2)	1.2 (0.7 to 1.9)	1 (0.5 to 1.8)
Turkmenistan	Maternal abortive outcome	0.517 (0.383 to 0.68)	0.187 (0.141 to 0.239)	0.112 (0.0829 to 0.148)	14.6 (11 to 18.9)	3.8 (2.9 to 4.9)	2.1 (1.5 to 2.7)
Turkmenistan	Ectopic pregnancy	0.108 (0.0318 to 0.28)	0.0303 (0.0106 to 0.0731)	0.0167 (0.00581 to 0.0415)	3 (0.9 to 7.7)	0.6 (0.2 to 1.4)	0.3 (0.1 to 0.8)
Turkmenistan	Indirect maternal deaths	0.202 (0.0992 to 0.384)	0.107 (0.0568 to 0.191)	0.0431 (0.0224 to 0.0767)	5.6 (2.8 to 10.4)	2.2 (1.1 to 3.8)	0.8 (0.4 to 1.4)
Turkmenistan	Late maternal deaths	0.0958 (0.0687 to 0.13)	0.0374 (0.0276 to 0.0499)	0.0302 (0.0216 to 0.042)	2.6 (1.9 to 3.5)	0.8 (0.6 to 1)	0.6 (0.4 to 0.8)
Turkmenistan	Maternal deaths aggravated by HIV/AIDS	0.00062 (0.000314 to 0.00105)	0.000281 (0.000135 to 0.000537)	0.000129 (0.0000579 to 0.000274)	0 (0 to 0)	0 (0 to 0)	0 (0 to 0)
Turkmenistan	Other maternal disorders	0.553 (0.366 to 0.808)	0.248 (0.178 to 0.327)	0.161 (0.118 to 0.212)	15.3 (10.4 to 21.8)	5 (3.6 to 6.6)	3 (2.2 to 3.9)
Turkmenistan	Neonatal disorders	203 (181 to 226)	157 (132 to 185)	113 (86.7 to 145)	3484.3 (3108 to 3873.2)	3560.9 (3005.5 to 4199.6)	2192.4 (1681.4 to 2815.9)
Turkmenistan	Neonatal preterm birth	89.4 (63.5 to 116)	68.2 (53.4 to 84.7)	49.1 (35.6 to 64.1)	1541 (1100.7 to 1999.9)	1544.8 (1211.4 to 1918.4)	952.9 (691.5 to 1244.5)
Turkmenistan	Neonatal encephalopathy due to birth asphyxia and trauma	82.8 (59.7 to 111)	52.9 (39.3 to 67.1)	34.7 (24.6 to 47.7)	1411.9 (1018.9 to 1896)	1199.7 (890.6 to 1518.9)	673.1 (476 to 926)
Turkmenistan	Neonatal sepsis and other neonatal infections	10.6 (6.94 to 14.8)	13.8 (9 to 18.2)	12.1 (8.02 to 16.7)	184.4 (121.3 to 256.7)	312.5 (203.3 to 414.4)	234.2 (155.7 to 322.7)
Turkmenistan	Hemolytic disease and other neonatal jaundice	2.74 (2.02 to 3.87)	2.81 (2.07 to 3.73)	2.14 (1.62 to 2.82)	54.3 (41 to 72.8)	62.7 (46 to 83.4)	41.7 (31.6 to 54.8)
Turkmenistan	Other neonatal disorders	17.3 (11.8 to 25.1)	19.3 (14 to 27)	15 (10.3 to 21)	292.7 (199.1 to 424)	441.2 (319.1 to 614.6)	290.6 (198.5 to 407.1)
Turkmenistan	Nutritional deficiencies	29.1 (19.2 to 42.7)	23.4 (15 to 35.5)	18.9 (11.7 to 30)	687.4 (455.9 to 990.7)	520.4 (336.8 to 774.2)	373.2 (232.2 to 595.8)
Turkmenistan	Protein-energy malnutrition	3.06 (2.47 to 3.69)	1.02 (0.775 to 1.29)	0.224 (0.158 to 0.315)	59 (46.6 to 71.4)	23.7 (18 to 30.1)	4.3 (3.1 to 6.1)
Turkmenistan	Iodine deficiency	0.828 (0.399 to 1.55)	1.11 (0.587 to 1.87)	0.83 (0.399 to 1.57)	24.3 (11.8 to 45.6)	25.1 (13.3 to 42.2)	16.9 (8.1 to 31.9)
Turkmenistan	Dietary iron deficiency	18.5 (11.5 to 27.3)	17.5 (10.8 to 26.6)	14.7 (8.83 to 24)	470.1 (297.1 to 681.9)	382.5 (238.9 to 575.8)	291.6 (174.7 to 476.7)
Turkmenistan	Other nutritional deficiencies	0.653 (0.48 to 0.863)	0.547 (0.466 to 0.612)	0.29 (0.232 to 0.341)	18.3 (13.3 to 25.2)	13.3 (11.2 to 14.9)	5.8 (4.6 to 6.8)
Turkmenistan	Non-communicable diseases	682 (621 to 750)	1040 (953 to 1140)	1130 (1020 to 1260)	27002.6 (24918 to 29415.2)	30629.6 (28447.2 to 33200.2)	26206.4 (23635.5 to 28958.3)
Turkmenistan	Neoplasms	76.9 (74.9 to 79.3)	89.8 (87.1 to 92.3)	112 (104 to 120)	3214.3 (3147.5 to 3281.7)	2610.6 (2555.1 to 2674.5)	2503.8 (2336.9 to 2682.6)
Turkmenistan	Lip and oral cavity cancer	1.34 (1.24 to 1.44)	1.94 (1.78 to 2.07)	2.56 (2.32 to 2.81)	60.2 (56 to 64.9)	58.7 (54.1 to 62.4)	57.6 (52.6 to 63.3)
Turkmenistan	Nasopharynx cancer	0.33 (0.304 to 0.362)	0.522 (0.481 to 0.565)	0.527 (0.472 to 0.584)	10.9 (10.1 to 11.7)	12.6 (11.7 to 13.6)	11.2 (10 to 12.4)
Turkmenistan	Other pharynx cancer	0.721 (0.643 to 0.808)	0.898 (0.817 to 0.968)	1.3 (1.12 to 1.5)	32.3 (28.8 to 36.2)	27.5 (24.8 to 29.8)	28.8 (24.8 to 32.9)
Turkmenistan	Oesophageal cancer	16.1 (15.5 to 16.6)	7.6 (7.25 to 8.01)	8.98 (8.12 to 9.88)	772.3 (745 to 798.5)	255.5 (244.3 to 268.8)	216.7 (195.6 to 237.9)
Turkmenistan	Stomach cancer	10.4 (10 to 10.8)	9.04 (8.66 to 9.45)	10.1 (9.29 to 11)	486 (467.9 to 504.5)	294.8 (282.3 to 307.1)	238.9 (221.1 to 260)
Turkmenistan	Colon and rectum cancer	3.56 (3.39 to 3.75)	4.64 (4.32 to 4.89)	6.29 (5.77 to 6.84)	157.7 (149.6 to 165.8)	153.2 (142.5 to 161.4)	156.4 (143.8 to 169.6)
Turkmenistan	Liver cancer	2.32 (2.05 to 2.63)	4.86 (4.43 to 5.27)	6.75 (5.97 to 7.53)	101.9 (90.3 to 116.2)	147.6 (134.6 to 159.7)	152.9 (135.6 to 170.1)
Turkmenistan	Liver cancer due to hepatitis B	0.73 (0.587 to 0.893)	1.53 (1.27 to 1.79)	1.92 (1.54 to 2.31)	28.6 (22.7 to 35.3)	41.2 (33.6 to 49)	40.2 (32.4 to 48.5)
Turkmenistan	Liver cancer due to hepatitis C	0.779 (0.637 to 0.938)	1.57 (1.31 to 1.85)	2.29 (1.86 to 2.73)	37.1 (30.6 to 44.4)	52.4 (44.2 to 61.3)	54.6 (45 to 64.3)

SR Appendix Table 2. All-age DALYs counts and age standardized DALYs rates per 100,000 individuals for all causes and level 1 cause groups by location for 1990, 2007, and 2017, both sexes combined

		All-age DALYs			Age-standardized rate of DALYs per 100,000		
Turkmenistan	Liver cancer due to alcohol use	0.505 (0.421 to 0.614)	1.15 (0.983 to 1.32)	1.67 (1.4 to 1.95)	23.5 (19.6 to 28.5)	35.5 (30.4 to 40.9)	38.3 (32.2 to 44.9)
Turkmenistan	Liver cancer due to NASH	0.126 (0.105 to 0.155)	0.282 (0.24 to 0.329)	0.431 (0.359 to 0.512)	5.8 (4.8 to 7.1)	9 (7.7 to 10.5)	10.2 (8.5 to 12.1)
Turkmenistan	Liver cancer due to other causes	0.178 (0.149 to 0.212)	0.341 (0.294 to 0.394)	0.449 (0.377 to 0.527)	6.9 (5.8 to 8.2)	9.5 (8.1 to 10.9)	9.7 (8.2 to 11.3)
Turkmenistan	Gallbladder and biliary tract cancer	1.23 (0.702 to 1.38)	0.725 (0.66 to 0.853)	1.03 (0.929 to 1.14)	59.3 (34.2 to 66.3)	24.3 (22.1 to 28.7)	24.8 (22.5 to 27.4)
Turkmenistan	Pancreatic cancer	0.641 (0.599 to 0.685)	2.02 (1.9 to 2.15)	3.25 (2.97 to 3.54)	31.8 (29.7 to 34)	68.1 (63.7 to 72.3)	80.7 (74 to 87.8)
Turkmenistan	Larynx cancer	1.36 (1.26 to 1.46)	1.23 (1.15 to 1.31)	1.06 (0.931 to 1.17)	61.8 (57.4 to 66.3)	39.4 (36.9 to 42)	24.4 (21.7 to 26.9)
Turkmenistan	Tracheal, bronchus, and lung cancer	8.29 (7.93 to 8.69)	7.88 (7.53 to 8.24)	10.8 (9.75 to 11.9)	371.4 (355.2 to 388.5)	250.5 (239.5 to 261.9)	248.8 (225.4 to 273.4)
Turkmenistan	Malignant skin melanoma	0.497 (0.218 to 0.607)	0.503 (0.393 to 0.682)	0.593 (0.453 to 0.932)	21.1 (9.2 to 25.6)	14.3 (11.4 to 19.4)	13.1 (10.1 to 20.5)
Turkmenistan	Non-melanoma skin cancer	0.281 (0.264 to 0.299)	0.513 (0.478 to 0.547)	0.583 (0.526 to 0.642)	12 (11.2 to 12.8)	14.8 (13.8 to 15.9)	13.4 (12.2 to 14.7)
Turkmenistan	Non-melanoma skin cancer (squamous-cell carcinoma)	0.281 (0.263 to 0.298)	0.512 (0.476 to 0.547)	0.582 (0.525 to 0.641)	11.9 (11.2 to 12.7)	14.8 (13.8 to 15.9)	13.4 (12.1 to 14.7)
Turkmenistan	Non-melanoma skin cancer (basal-cell carcinoma)	0.000652 (0.000221 to 0.00153)	0.000925 (0.000312 to 0.0022)	0.00124 (0.000421 to 0.0029)	0 (0 to 0.1)	0 (0 to 0.1)	0 (0 to 0.1)
Turkmenistan	Breast cancer	3.71 (3.47 to 3.97)	7.88 (7.47 to 8.32)	11.2 (9.81 to 12.8)	160.5 (150.1 to 171.7)	215.9 (205.3 to 227.2)	236.2 (206.5 to 268.6)
Turkmenistan	Cervical cancer	2.54 (2.35 to 2.72)	5.44 (5.08 to 5.85)	6.52 (5.62 to 7.47)	111.5 (104 to 119.8)	143.2 (134.5 to 152.9)	134.1 (116 to 153.3)
Turkmenistan	Uterine cancer	0.604 (0.555 to 0.66)	0.451 (0.405 to 0.498)	0.562 (0.484 to 0.651)	28.2 (25.9 to 30.8)	13.9 (12.5 to 15.3)	12.8 (11.1 to 14.8)
Turkmenistan	Ovarian cancer	1.36 (1.21 to 1.51)	2.08 (1.87 to 2.26)	2.7 (2.33 to 3.1)	55.8 (49.2 to 62.5)	55.5 (50 to 60.4)	57.2 (49.6 to 65.4)
Turkmenistan	Prostate cancer	0.927 (0.807 to 1.48)	1.78 (1.55 to 2.54)	2.25 (1.85 to 3.14)	49.5 (42.5 to 81.3)	64.1 (55.7 to 100.6)	63.2 (52.5 to 94.7)
Turkmenistan	Testicular cancer	0.358 (0.308 to 0.411)	0.524 (0.335 to 0.794)	0.48 (0.352 to 0.676)	12.6 (10.9 to 14.4)	12.2 (7.8 to 18.3)	9.5 (7 to 13.3)
Turkmenistan	Kidney cancer	1.62 (1.41 to 1.84)	2.73 (2.5 to 2.98)	3.74 (3.29 to 4.18)	61.6 (53.4 to 70.4)	81.5 (74.7 to 88.8)	82.1 (72.3 to 91.7)
Turkmenistan	Bladder cancer	0.445 (0.407 to 0.486)	0.78 (0.72 to 0.851)	1.22 (1.08 to 1.35)	22.5 (20.6 to 24.5)	27.1 (25 to 29.5)	30.2 (26.9 to 33.5)
Turkmenistan	Thyroid cancer	0.287 (0.263 to 0.314)	0.357 (0.329 to 0.385)	0.415 (0.368 to 0.463)	12.4 (11.4 to 13.7)	10.5 (9.7 to 11.4)	9.4 (8.4 to 10.4)
Turkmenistan	Mesothelioma	0.0673 (0.0564 to 0.0788)	0.152 (0.127 to 0.17)	0.168 (0.143 to 0.189)	2.9 (2.4 to 3.3)	4.3 (3.7 to 4.8)	3.7 (3.2 to 4.1)
Turkmenistan	Hodgkin lymphoma	0.891 (0.599 to 1.06)	1.29 (0.772 to 1.54)	1.05 (0.671 to 1.26)	26.1 (18.4 to 30.4)	28.6 (18 to 33.6)	20.6 (13.5 to 24.4)
Turkmenistan	Non-Hodgkin's lymphoma	0.941 (0.86 to 1.03)	1.59 (1.5 to 1.72)	2.07 (1.86 to 2.3)	34.7 (32 to 37.6)	44.1 (41.5 to 47.3)	45.6 (41.2 to 50.7)
Turkmenistan	Multiple myeloma	0.443 (0.362 to 0.609)	0.83 (0.734 to 1.06)	1.42 (1.12 to 1.63)	18.3 (14.5 to 27.6)	23.1 (20.6 to 33.1)	30.5 (25.1 to 34.7)
Turkmenistan	Leukaemia	7.49 (6.74 to 8.32)	6.33 (5.83 to 6.89)	7.05 (6.27 to 7.99)	182.5 (168.2 to 199.2)	147.6 (136.2 to 160.3)	146.4 (130.5 to 165.1)
Turkmenistan	Acute lymphoid leukaemia	2 (1.61 to 2.55)	1.74 (1.46 to 2.1)	2.05 (1.65 to 2.5)	43 (35 to 53.4)	37.8 (31.9 to 45.5)	41.5 (33.5 to 50.5)
Turkmenistan	Chronic lymphoid leukaemia	0.137 (0.118 to 0.157)	0.197 (0.168 to 0.228)	0.242 (0.205 to 0.285)	5.9 (5.1 to 6.7)	6.1 (5.1 to 7)	5.8 (4.9 to 6.8)
Turkmenistan	Acute myeloid leukaemia	1.41 (1.06 to 1.82)	1.56 (1.25 to 1.92)	2 (1.59 to 2.42)	35.8 (28.7 to 45)	35.7 (29.1 to 43.4)	40.7 (32.7 to 49.2)
Turkmenistan	Chronic myeloid leukaemia	0.269 (0.194 to 0.349)	0.307 (0.23 to 0.384)	0.301 (0.218 to 0.39)	10 (7.2 to 12.8)	8 (6.1 to 10)	6.4 (4.7 to 8.2)
Turkmenistan	Other leukaemia	3.68 (2.97 to 4.39)	2.53 (2.21 to 2.86)	2.46 (2.14 to 2.88)	87.8 (74.3 to 102)	60 (53 to 67.3)	52 (45.4 to 60.3)
Turkmenistan	Other malignant cancers	6.16 (4.88 to 6.85)	7.75 (6.98 to 8.46)	8.65 (7.66 to 9.6)	200 (167.4 to 218.6)	194.1 (180.2 to 214)	182.7 (164.1 to 201.9)
Turkmenistan	Other neoplasms	0.509 (0.299 to 0.845)	0.781 (0.526 to 1.01)	1.02 (0.689 to 1.27)	15.8 (10.2 to 24.2)	21.3 (14.5 to 27.1)	22.5 (15.3 to 28.2)
Turkmenistan	Myelodysplastic, myeloproliferative, and other hematopoietic neoplasms	0.352 (0.212 to 0.597)	0.585 (0.399 to 0.755)	0.795 (0.538 to 1)	12.4 (8.1 to 18.8)	17 (11.7 to 21.6)	18.2 (12.3 to 23)
Turkmenistan	Benign and in situ intestinal neoplasms	0 (0 to 0)	0 (0 to 0)	0 (0 to 0)	0 (0 to 0)	0 (0 to 0)	0 (0 to 0)
Turkmenistan	Benign and in situ cervical and uterine neoplasms	0 (0 to 0)	0 (0 to 0)	0 (0 to 0)	0 (0 to 0)	0 (0 to 0)	0 (0 to 0)
Turkmenistan	Other benign and in situ neoplasms	0.157 (0.0821 to 0.282)	0.197 (0.127 to 0.265)	0.221 (0.147 to 0.287)	3.5 (1.9 to 5.9)	4.3 (2.8 to 5.8)	4.3 (2.8 to 5.6)
Turkmenistan	Cardiovascular diseases	230 (226 to 233)	417 (411 to 423)	423 (396 to 450)	11213.6 (11027 to 11392.3)	14204.9 (14015.6 to 14412.6)	10860.5 (10204.9 to 11533.8)
Turkmenistan	Rheumatic heart disease	8.35 (7.8 to 9.02)	9.24 (8.46 to 10.1)	7.21 (6.32 to 8.25)	273.3 (256.7 to 294.8)	215.2 (198.1 to 234.6)	143.9 (126.3 to 164)
Turkmenistan	Ischaemic heart disease	130 (127 to 133)	229 (225 to 233)	227 (212 to 243)	6767.1 (6639.1 to 6901.6)	8272.9 (8135.1 to 8399.9)	6091.7 (5716.3 to 6499.5)
Turkmenistan	Stroke	58.6 (56.6 to 60.7)	119 (116 to 122)	135 (125 to 144)	2878.7 (2779.7 to 2978.3)	4002.4 (3900 to 4110.4)	3379 (3154.9 to 3603.1)

SR Appendix Table 2. All-age DALYs counts and age standardized DALYs rates per 100,000 individuals for all causes and level 1 cause groups by location for 1990, 2007, and 2017, both sexes combined

		All-age DALYs			Age-standardized rate of DALYs per 100,000		
Turkmenistan	Ischaemic stroke	5.6 (4.94 to 6.24)	9.16 (8.17 to 10.2)	11.5 (9.83 to 13)	328.8 (294.6 to 362.3)	383.6 (345.9 to 422.1)	356.7 (308.5 to 401.7)
Turkmenistan	Intracerebral hemorrhage	50.7 (49 to 52.3)	105 (103 to 108)	118 (109 to 126)	2452.7 (2370.6 to 2532.7)	3487.3 (3397.6 to 3569.9)	2902.8 (2690.2 to 3109.1)
Turkmenistan	Subarachnoid hemorrhage	2.38 (2.1 to 3.61)	4.75 (4.16 to 7.46)	5.53 (4.54 to 9.02)	97.2 (85.1 to 162.3)	131.6 (114.1 to 224.7)	119.5 (97.9 to 205.5)
Turkmenistan	Hypertensive heart disease	6.57 (4.91 to 7.43)	13.5 (9.44 to 15.3)	15 (10.5 to 18.4)	332.3 (259.4 to 389.6)	456.1 (336.6 to 524.1)	378.5 (281.7 to 468.3)
Turkmenistan	Non-rheumatic valvular heart disease	0.249 (0.195 to 0.29)	0.445 (0.378 to 0.514)	0.486 (0.414 to 0.586)	11.5 (9.3 to 13.8)	14 (11.9 to 16.6)	12.7 (10.6 to 15.6)
Turkmenistan	Non-rheumatic calcific aortic valve disease	0.0934 (0.0755 to 0.117)	0.211 (0.175 to 0.261)	0.266 (0.222 to 0.339)	4.7 (3.7 to 5.9)	7 (5.8 to 8.6)	7 (5.8 to 8.6)
Turkmenistan	Non-rheumatic degenerative mitral valve disease	0.119 (0.0907 to 0.146)	0.18 (0.14 to 0.223)	0.176 (0.137 to 0.222)	5.5 (4.3 to 6.8)	5.7 (4.5 to 7)	4.7 (3.6 to 6)
Turkmenistan	Other non-rheumatic valve diseases	0.0367 (0.0161 to 0.0537)	0.0538 (0.0277 to 0.0733)	0.044 (0.0238 to 0.06)	1.3 (0.6 to 1.9)	1.3 (0.7 to 1.8)	1 (0.5 to 1.3)
Turkmenistan	Cardiomyopathy and myocarditis	16.1 (13.5 to 17.8)	30.6 (26.9 to 32.4)	21.8 (19 to 28.6)	572.8 (488.3 to 632.6)	784.8 (682.1 to 830.2)	444.3 (388.3 to 580.7)
Turkmenistan	Myocarditis	1.35 (0.981 to 1.67)	2.22 (1.85 to 3.01)	1.65 (1.21 to 2.6)	39.3 (33.4 to 50)	53.2 (45 to 71.1)	33.6 (25.2 to 51.4)
Turkmenistan	Alcoholic cardiomyopathy	3.75 (3.13 to 4.51)	8.35 (6.72 to 9.39)	6.86 (5.31 to 8.17)	154.9 (129.1 to 186)	222 (175.4 to 250)	138.9 (108.3 to 165.5)
Turkmenistan	Other cardiomyopathy	11 (8.41 to 12.3)	20.1 (16.9 to 21.7)	13.2 (11.3 to 18.9)	378.6 (293.5 to 423.7)	509.5 (434.8 to 550.1)	271.8 (232.3 to 386.3)
Turkmenistan	Atrial fibrillation and flutter	1.31 (1.08 to 1.59)	2.29 (1.96 to 2.71)	3.06 (2.56 to 3.64)	80.3 (67.6 to 96.2)	101.7 (86.8 to 117.9)	99.5 (82.2 to 117.4)
Turkmenistan	Aortic aneurysm	0.501 (0.455 to 0.567)	1.49 (1.35 to 1.68)	2.02 (1.71 to 2.41)	24 (21.7 to 27.6)	48.6 (43.8 to 55.5)	49.5 (41.4 to 60.1)
Turkmenistan	Peripheral vascular disease	0.135 (0.073 to 0.22)	0.224 (0.131 to 0.356)	0.324 (0.194 to 0.508)	7.8 (4.2 to 12.6)	9.4 (5.4 to 14.9)	9.9 (5.9 to 15.5)
Turkmenistan	Endocarditis	0.378 (0.232 to 0.576)	0.789 (0.545 to 1.18)	0.882 (0.614 to 1.22)	11.6 (7 to 16.2)	19.7 (13.9 to 28.5)	18.6 (13.2 to 25.2)
Turkmenistan	Other cardiovascular and circulatory diseases	7.25 (6.69 to 7.88)	9.99 (9.09 to 11)	10.2 (8.57 to 12.1)	254.2 (236.1 to 274.7)	280.2 (254.9 to 307.1)	233 (196 to 276.3)
Turkmenistan	Chronic respiratory diseases	37 (33.9 to 40.3)	26.4 (23.5 to 29.4)	26.2 (22.7 to 30)	1571.1 (1468.2 to 1688.5)	788.2 (711.6 to 865.3)	621.8 (547.1 to 704)
Turkmenistan	Chronic obstructive pulmonary disease	22.8 (21.1 to 24.4)	17.2 (15.8 to 18.4)	16.3 (14.6 to 18)	1086 (1005.7 to 1153.9)	554.4 (512 to 593.3)	412.4 (370.3 to 450.7)
Turkmenistan	Pneumoconiosis	0.0193 (0.015 to 0.0242)	0.0214 (0.0172 to 0.0259)	0.0228 (0.0171 to 0.0291)	0.9 (0.7 to 1.2)	0.7 (0.6 to 0.9)	0.6 (0.4 to 0.7)
Turkmenistan	Silicosis	0.00211 (0.00127 to 0.00362)	0.00204 (0.0013 to 0.0029)	0.00213 (0.00125 to 0.00322)	0.1 (0.1 to 0.2)	0.1 (0 to 0.1)	0.1 (0 to 0.1)
Turkmenistan	Asbestosis	0.00706 (0.00381 to 0.00991)	0.0056 (0.00369 to 0.00754)	0.00628 (0.00394 to 0.00899)	0.3 (0.2 to 0.4)	0.2 (0.1 to 0.2)	0.1 (0.1 to 0.2)
Turkmenistan	Coal workers pneumoconiosis	0.0008 (0.000437 to 0.00133)	0.00114 (0.000737 to 0.00173)	0.000714 (0.000403 to 0.00119)	0 (0 to 0.1)	0 (0 to 0)	0 (0 to 0)
Turkmenistan	Other pneumoconiosis	0.00934 (0.0067 to 0.0139)	0.0126 (0.00973 to 0.016)	0.0137 (0.0101 to 0.0181)	0.5 (0.3 to 0.7)	0.4 (0.3 to 0.6)	0.4 (0.3 to 0.5)
Turkmenistan	Asthma	10.6 (8.11 to 13.7)	6.5 (4.76 to 8.65)	7.03 (4.96 to 9.63)	354.2 (270.5 to 451.5)	163.5 (122.1 to 216.2)	149.5 (106.1 to 203.2)
Turkmenistan	Interstitial lung disease and pulmonary sarcoidosis	1.5 (1.14 to 1.82)	1.22 (0.95 to 1.54)	1.38 (1.05 to 1.77)	66.3 (51.5 to 79.9)	35.8 (28.2 to 44.8)	31.9 (24.5 to 40.9)
Turkmenistan	Other chronic respiratory diseases	2.04 (1.68 to 2.61)	1.49 (1.27 to 1.72)	1.4 (1.19 to 1.64)	63.7 (54 to 76.1)	33.8 (29.1 to 38.7)	27.4 (23.3 to 31.9)
Turkmenistan	Digestive diseases	50.6 (47.3 to 54.2)	91 (85.9 to 97)	116 (106 to 128)	1797.1 (1693.8 to 1915.8)	2354.7 (2226.1 to 2507.3)	2380.3 (2160.5 to 2606.6)
Turkmenistan	Cirrhosis and other chronic liver diseases	30.1 (28.8 to 31.3)	66.9 (64.3 to 70.7)	90.7 (82.6 to 99)	1183.7 (1132.6 to 1226.4)	1739 (1670.9 to 1837.5)	1842.5 (1681.7 to 2006.2)
Turkmenistan	Cirrhosis and other chronic liver diseases due to hepatitis B	7.27 (6.47 to 8.2)	17.4 (15.2 to 19.8)	20.9 (17.3 to 24.3)	293 (260 to 329.7)	443.7 (389.1 to 501.8)	418.1 (349.6 to 483.6)
Turkmenistan	Cirrhosis and other chronic liver diseases due to hepatitis C	6.3 (5.5 to 7.18)	14.9 (12.7 to 17.1)	21.4 (17.8 to 25)	267.8 (234 to 304.2)	393.1 (340.6 to 449.2)	435.2 (367.3 to 503.4)
Turkmenistan	Cirrhosis and other chronic liver diseases due to alcohol use	8.81 (7.88 to 9.73)	21.4 (19.3 to 23.8)	31.1 (27.3 to 35.7)	385.7 (346 to 426)	578.4 (520.8 to 643)	634.2 (559.3 to 723.1)
Turkmenistan	Cirrhosis due to NASH	1.7 (1.5 to 1.91)	4.32 (3.8 to 4.86)	6.85 (5.82 to 7.88)	74.1 (65.8 to 83)	118.6 (105.2 to 132.4)	140.9 (120.6 to 160.7)
Turkmenistan	Cirrhosis and other chronic liver diseases due to other causes	6.05 (5.34 to 6.79)	8.97 (7.84 to 10.3)	10.5 (8.87 to 12)	163 (144.8 to 182.6)	205.3 (181.1 to 234.4)	214.1 (183.6 to 244.9)
Turkmenistan	Upper digestive system diseases	6.94 (5.31 to 9.24)	10.2 (7.98 to 13.5)	11.3 (8.55 to 15.3)	250.9 (194.1 to 329.7)	260.2 (204.9 to 340.9)	234.1 (177.9 to 315.5)
Turkmenistan	Peptic ulcer disease	3.04 (2.82 to 3.28)	4.98 (4.57 to 5.42)	5.12 (4.5 to 5.79)	119.3 (110.7 to 128.4)	131.9 (121.4 to 142.9)	107.1 (94.4 to 120.3)
Turkmenistan	Gastritis and duodenitis	1.35 (0.873 to 1.96)	1.52 (1.01 to 2.22)	1.7 (1.13 to 2.46)	39.5 (26.2 to 56.3)	36 (24.1 to 52)	34.7 (23.3 to 49.9)
Turkmenistan	GERD	2.55 (1.36 to 4.4)	3.73 (1.98 to 6.42)	4.5 (2.42 to 7.68)	92.1 (48.6 to 156.8)	92.3 (48.9 to 157.6)	92.3 (49.2 to 158.1)
Turkmenistan	Appendicitis	1.22 (1.08 to 1.38)	0.727 (0.621 to 0.845)	0.718 (0.602 to 0.843)	32 (28.8 to 35.4)	15.9 (13.8 to 18.3)	14.4 (12.1 to 16.9)
Turkmenistan	Paralytic ileus and intestinal obstruction	5.78 (4.88 to 6.73)	4.85 (4.03 to 5.84)	4.47 (3.3 to 5.94)	111.3 (97 to 127.3)	115.8 (96.5 to 139.8)	89 (67 to 116.7)

SR Appendix Table 2. All-age DALYs counts and age standardized DALYs rates per 100,000 individuals for all causes and level 1 cause groups by location for 1990, 2007, and 2017, both sexes combined

		All-age DALYs			Age-standardized rate of DALYs per 100,000		
Turkmenistan	Inguinal, femoral, and abdominal hernia	3.27 (2.33 to 4.39)	3.7 (2.61 to 4.95)	3.86 (2.7 to 5.21)	101.9 (74.1 to 135.8)	97.5 (69.8 to 129.3)	82.1 (58.1 to 110.1)
Turkmenistan	Inflammatory bowel disease	0.619 (0.401 to 0.937)	0.578 (0.458 to 0.755)	0.723 (0.575 to 0.882)	18.9 (13.6 to 25.2)	14.9 (11.9 to 19.1)	14.9 (12.1 to 17.9)
Turkmenistan	Vascular intestinal disorders	0.281 (0.246 to 0.355)	0.488 (0.438 to 0.582)	0.594 (0.501 to 0.682)	13.8 (12.4 to 17.2)	17.3 (15.7 to 20.2)	16 (13.1 to 18.5)
Turkmenistan	Gallbladder and biliary diseases	0.701 (0.641 to 0.757)	0.879 (0.785 to 1.1)	0.965 (0.839 to 1.15)	29.7 (27.2 to 32.1)	27 (24 to 34.4)	22.5 (19.5 to 27.2)
Turkmenistan	Pancreatitis	0.783 (0.665 to 0.942)	1.79 (1.53 to 2.07)	2.18 (1.76 to 2.57)	31.5 (27 to 37.8)	47.4 (41 to 54.5)	45.7 (37.4 to 53.7)
Turkmenistan	Other digestive diseases	0.909 (0.711 to 1.14)	0.773 (0.624 to 0.955)	0.898 (0.708 to 1.14)	23.4 (18.7 to 28.5)	19.8 (15.9 to 24.4)	19 (15.1 to 24)
Turkmenistan	Neurological disorders	42.1 (31.2 to 55.9)	60.4 (45.1 to 78.3)	67.6 (51 to 88.1)	1515.8 (1183.1 to 1933.5)	1675.9 (1326.1 to 2078.8)	1598.8 (1258 to 2006.6)
Turkmenistan	Alzheimer's disease and other dementias	5.64 (5.16 to 6.1)	9.44 (8.81 to 10.1)	12.3 (11.4 to 13.3)	420.4 (381.7 to 454.4)	519.7 (486.7 to 554.9)	477 (443.2 to 515)
Turkmenistan	Parkinson's disease	0.977 (0.867 to 1.11)	1.68 (1.51 to 1.87)	1.97 (1.7 to 2.25)	60.3 (53.8 to 68.5)	71.6 (64.5 to 79.6)	65.5 (56.4 to 74.7)
Turkmenistan	Epilepsy	10 (5.8 to 15.5)	13.6 (9.15 to 19.2)	13.3 (7.57 to 21.1)	254.7 (142.4 to 397)	289.5 (191.8 to 412.5)	263.7 (147.4 to 422.3)
Turkmenistan	Multiple sclerosis	0.403 (0.316 to 0.497)	0.698 (0.55 to 0.856)	0.814 (0.629 to 1.02)	16.7 (13.4 to 20.2)	18.7 (15.2 to 22.3)	17.2 (13.5 to 21)
Turkmenistan	Motor neuron disease	0.0743 (0.0568 to 0.0873)	0.116 (0.0878 to 0.131)	0.155 (0.113 to 0.186)	2.8 (2.2 to 3.3)	3.3 (2.5 to 3.7)	3.2 (2.4 to 3.9)
Turkmenistan	Headache disorders	23.2 (15.1 to 33.2)	32.3 (20.8 to 46.5)	36.1 (23.4 to 52.2)	717.2 (468.5 to 1026.7)	716 (464.2 to 1028.2)	712.2 (461.4 to 1026.4)
Turkmenistan	Migraine	20.2 (12.6 to 30)	28.2 (17.5 to 41.8)	31.4 (19.7 to 46.9)	622.4 (389.7 to 920.4)	621.2 (388.3 to 919.2)	617.7 (387.4 to 921.2)
Turkmenistan	Tension-type headache	3.04 (1.71 to 4.91)	4.17 (2.34 to 6.65)	4.69 (2.64 to 7.5)	94.8 (54.1 to 151.6)	94.9 (53.8 to 149.5)	94.5 (53.4 to 150.6)
Turkmenistan	Other neurological disorders	1.77 (0.96 to 2.79)	2.58 (1.69 to 3.71)	2.96 (1.71 to 4.66)	43.6 (25.2 to 67.7)	57.2 (38.2 to 80.4)	59.9 (35.2 to 94)
Turkmenistan	Mental disorders	43 (31.9 to 55.8)	57.2 (42.2 to 74.2)	63.5 (47.2 to 82.3)	1313.1 (981.2 to 1694.3)	1295.3 (961.3 to 1675)	1291.4 (961.6 to 1673.9)
Turkmenistan	Schizophrenia	3.33 (2.48 to 4.23)	4.93 (3.64 to 6.19)	6.17 (4.59 to 7.84)	116 (86.9 to 145.9)	115.6 (85.8 to 143.6)	119.9 (89.1 to 151.3)
Turkmenistan	Depressive disorders	13.5 (9.54 to 18.4)	18.4 (13 to 25)	20.5 (14.4 to 28.1)	456.5 (326.5 to 621.4)	443 (315.3 to 601.6)	431.1 (304.5 to 586)
Turkmenistan	Major depressive disorder	10.4 (7.18 to 14.3)	14 (9.7 to 19.1)	15.3 (10.6 to 20.8)	348.6 (244.2 to 476)	335.3 (232.6 to 456)	324 (227.5 to 438.8)
Turkmenistan	Dysthymia	3.06 (2.05 to 4.49)	4.37 (2.89 to 6.38)	5.19 (3.41 to 7.6)	108 (71.8 to 157.8)	107.7 (72.2 to 156.5)	107.1 (70.5 to 155)
Turkmenistan	Bipolar disorder	4.24 (2.67 to 6.26)	5.97 (3.69 to 8.68)	6.58 (4.09 to 9.71)	130.2 (82 to 192.8)	130.4 (81.1 to 192.3)	130.3 (81.1 to 191.4)
Turkmenistan	Anxiety disorders	7.84 (5.54 to 10.6)	10.4 (7.34 to 13.9)	11.5 (8.1 to 15.3)	236.1 (169.2 to 316.1)	234.7 (165.7 to 311.1)	233.3 (164.2 to 310.2)
Turkmenistan	Eating disorders	1.29 (0.809 to 1.89)	1.66 (1.04 to 2.46)	2.18 (1.4 to 3.22)	34.2 (21.5 to 50.3)	32.6 (20.5 to 48.1)	41 (26.1 to 60.3)
Turkmenistan	Anorexia nervosa	0.278 (0.162 to 0.422)	0.342 (0.207 to 0.53)	0.419 (0.253 to 0.653)	6.9 (4.2 to 10.4)	6.5 (4 to 10.1)	8.1 (4.9 to 12.6)
Turkmenistan	Bulimia nervosa	1.01 (0.608 to 1.54)	1.32 (0.807 to 1.96)	1.77 (1.09 to 2.66)	27.3 (16.6 to 41.4)	26.1 (16.3 to 38.7)	32.9 (20.5 to 49.5)
Turkmenistan	Autism spectrum disorders	2.48 (1.68 to 3.42)	2.93 (1.97 to 4.1)	3.28 (2.21 to 4.48)	63.5 (42.7 to 86.9)	64.5 (43.5 to 90.7)	65 (43.8 to 89.1)
Turkmenistan	Attention-deficit/hyperactivity disorder	0.489 (0.292 to 0.784)	0.587 (0.357 to 0.93)	0.601 (0.362 to 0.96)	11.7 (7 to 18.6)	11.8 (7.2 to 18.8)	11.9 (7.1 to 19.1)
Turkmenistan	Conduct disorder	4.6 (2.76 to 7.27)	5.22 (3.17 to 8.29)	4.57 (2.76 to 7.16)	97.4 (58.4 to 154.1)	98.6 (60.4 to 158.8)	98.6 (59.7 to 154.8)
Turkmenistan	Idiopathic developmental intellectual disability	1.15 (0.455 to 2.08)	1.11 (0.358 to 2.13)	0.994 (0.32 to 1.89)	28.5 (11.3 to 51.8)	23.9 (7.7 to 45.9)	19.5 (6.3 to 37.1)
Turkmenistan	Other mental disorders	4.08 (2.68 to 5.67)	5.96 (3.92 to 8.25)	7.08 (4.64 to 9.78)	139 (91.4 to 192)	140.2 (92.8 to 193.7)	140.8 (92.4 to 193.6)
Turkmenistan	Substance use disorders	15.1 (11.9 to 18.6)	34.1 (28.6 to 40.2)	28.7 (22.9 to 35.2)	475.9 (380.5 to 582)	745.9 (626.2 to 877.4)	544.4 (434.6 to 666.2)
Turkmenistan	Alcohol use disorders	11.3 (9 to 14.1)	25.1 (21 to 29.6)	18.9 (14.7 to 23.8)	368.3 (296.4 to 456.9)	558.6 (468.3 to 659.4)	359.9 (280.4 to 451.1)
Turkmenistan	Drug use disorders	3.75 (2.71 to 4.89)	9.03 (7.34 to 10.9)	9.84 (7.82 to 11.9)	107.6 (78.4 to 138.7)	187.3 (152.8 to 225.2)	184.5 (147 to 222.8)
Turkmenistan	Opioid use disorders	2.55 (1.81 to 3.36)	6.8 (5.54 to 8.15)	7.52 (6.05 to 9.19)	75.7 (54.1 to 98.7)	142.6 (115.9 to 170.9)	140.9 (113 to 171.7)
Turkmenistan	Cocaine use disorders	0.128 (0.0759 to 0.193)	0.292 (0.202 to 0.398)	0.338 (0.222 to 0.472)	3.7 (2.3 to 5.4)	6.1 (4.2 to 8.2)	6.4 (4.2 to 8.9)
Turkmenistan	Amphetamine use disorders	0.428 (0.237 to 0.712)	0.645 (0.371 to 1.04)	0.666 (0.377 to 1.03)	10.8 (6 to 17.5)	12.5 (7.3 to 19.8)	12.5 (7.1 to 19.2)
Turkmenistan	Cannabis use disorders	0.177 (0.108 to 0.27)	0.243 (0.144 to 0.366)	0.243 (0.15 to 0.366)	4.6 (2.9 to 6.9)	4.7 (2.8 to 7)	4.7 (2.9 to 7.1)
Turkmenistan	Other drug use disorders	0.473 (0.31 to 0.7)	1.05 (0.762 to 1.37)	1.07 (0.773 to 1.43)	12.9 (8.6 to 18.2)	21.4 (15.7 to 27.8)	20.1 (14.5 to 26.7)
Turkmenistan	Diabetes and kidney diseases	30.7 (25.8 to 36.4)	61 (52.8 to 71.2)	77.5 (65.8 to 91.4)	1255.7 (1044.2 to 1506.2)	1772.8 (1543.9 to 2067.5)	1729.9 (1469.9 to 2034.3)

SR Appendix Table 2. All-age DALYs counts and age standardized DALYs rates per 100,000 individuals for all causes and level 1 cause groups by location for 1990, 2007, and 2017, both sexes combined

		All-age DALYs			Age-standardized rate of DALYs per 100,000		
Turkmenistan	Diabetes mellitus	19.1 (15 to 24.3)	37.2 (29.9 to 46.2)	52.1 (41.5 to 65)	844.6 (660.5 to 1069.9)	1124.3 (917.2 to 1377.8)	1169.1 (935.9 to 1458.9)
Turkmenistan	Diabetes mellitus type 1	5 (4.62 to 5.38)	8.84 (8.18 to 9.49)	9.85 (8.53 to 11.1)	204.3 (187.8 to 221.2)	253.7 (231.7 to 273.9)	210.4 (182.1 to 237.1)
Turkmenistan	Diabetes mellitus type 2	14.1 (10.1 to 19.1)	28.4 (21.3 to 37.3)	42.2 (32 to 55)	640.3 (461.6 to 861)	870.6 (666.5 to 1127.2)	958.7 (733.5 to 1241.5)
Turkmenistan	Chronic kidney disease	10.4 (9.68 to 11.3)	22.8 (21.5 to 24.1)	25.2 (23.3 to 27.3)	382.4 (352.3 to 419.1)	626.9 (593 to 664)	556.4 (515.2 to 602.3)
Turkmenistan	Chronic kidney disease due to diabetes mellitus type 1	0.81 (0.644 to 0.988)	2.37 (1.87 to 2.87)	2.66 (2.12 to 3.28)	31.3 (25 to 38.5)	58.4 (46.6 to 71.1)	52.6 (42 to 64.7)
Turkmenistan	Chronic kidney disease due to diabetes mellitus type 2	1.19 (0.996 to 1.39)	3.19 (2.71 to 3.74)	3.89 (3.24 to 4.57)	57.8 (48.7 to 67.3)	109.8 (93.7 to 128.3)	97.3 (82.5 to 113.2)
Turkmenistan	Chronic kidney disease due to hypertension	0.83 (0.696 to 0.98)	1.98 (1.67 to 2.3)	2.28 (1.91 to 2.68)	37.5 (31.6 to 43.9)	63.8 (53.8 to 74.2)	56.2 (47.5 to 65.7)
Turkmenistan	Chronic kidney disease due to glomerulonephritis	3.11 (2.72 to 3.51)	6.93 (6.12 to 7.83)	7.51 (6.5 to 8.73)	102.1 (90.3 to 115.2)	173 (153.1 to 195.7)	154.4 (134.3 to 178.6)
Turkmenistan	Chronic kidney disease due to other and unspecified causes	4.47 (3.91 to 5.13)	8.28 (7.29 to 9.34)	8.88 (7.73 to 10.2)	153.7 (133.7 to 178.4)	222 (195.6 to 249.4)	195.9 (170.8 to 223.9)
Turkmenistan	Acute glomerulonephritis	1.2 (0.854 to 1.64)	0.998 (0.878 to 1.11)	0.225 (0.185 to 0.28)	28.8 (21.5 to 37.7)	21.6 (19.1 to 24)	4.4 (3.6 to 5.4)
Turkmenistan	Skin and subcutaneous diseases	17.5 (11.5 to 25.8)	20.1 (13.4 to 29.5)	22.8 (15.2 to 33.6)	457.9 (303 to 679.5)	460.8 (306.6 to 673.2)	465.4 (311 to 687.4)
Turkmenistan	Dermatitis	4.44 (2.51 to 7.18)	4.81 (2.83 to 7.64)	5.4 (3.18 to 8.49)	108.4 (63.4 to 172.7)	108.5 (64 to 172.9)	108.5 (63.7 to 170.4)
Turkmenistan	Atopic dermatitis	3.7 (1.96 to 6.24)	3.79 (2.03 to 6.42)	4.28 (2.31 to 7.18)	85.3 (45.8 to 142.7)	85.3 (45.8 to 143.4)	85.4 (46.1 to 143)
Turkmenistan	Contact dermatitis	0.743 (0.481 to 1.11)	1.01 (0.655 to 1.5)	1.12 (0.726 to 1.65)	23.1 (15.1 to 34.2)	23.1 (15.1 to 34.2)	23 (15.1 to 33.9)
Turkmenistan	Seborrhoeic dermatitis	0.00386 (0.00216 to 0.00623)	0.0046 (0.00256 to 0.00742)	0.00526 (0.00294 to 0.00842)	0.1 (0.1 to 0.2)	0.1 (0.1 to 0.2)	0.1 (0.1 to 0.2)
Turkmenistan	Psoriasis	1.94 (1.36 to 2.58)	2.66 (1.86 to 3.54)	3.22 (2.27 to 4.27)	64.1 (45.6 to 84.4)	64 (45 to 85)	65.7 (46.4 to 86.9)
Turkmenistan	Bacterial skin diseases	0.117 (0.0858 to 0.185)	0.236 (0.124 to 0.302)	0.249 (0.148 to 0.32)	3.4 (2.5 to 5.4)	6.2 (3.2 to 7.9)	5.4 (3.1 to 7)
Turkmenistan	Cellulitis	0.055 (0.039 to 0.0826)	0.104 (0.0589 to 0.139)	0.126 (0.0755 to 0.17)	1.7 (1.2 to 2.5)	2.7 (1.5 to 3.6)	2.7 (1.6 to 3.7)
Turkmenistan	Pyoderma	0.0618 (0.0414 to 0.111)	0.132 (0.0585 to 0.177)	0.122 (0.0626 to 0.181)	1.7 (1.1 to 3.1)	3.5 (1.6 to 4.7)	2.7 (1.3 to 3.9)
Turkmenistan	Scabies	1.37 (0.757 to 2.27)	1.48 (0.816 to 2.44)	1.5 (0.828 to 2.45)	33.5 (18.9 to 55.2)	31.3 (17.5 to 51.2)	29.9 (16.6 to 49)
Turkmenistan	Fungal skin diseases	1.74 (0.683 to 3.64)	1.94 (0.774 to 4.04)	2.14 (0.853 to 4.48)	52.8 (20.9 to 111.1)	50.1 (19.9 to 105)	47.6 (18.9 to 98.8)
Turkmenistan	Viral skin diseases	2.19 (1.39 to 3.27)	2.08 (1.35 to 3.1)	2.34 (1.48 to 3.5)	45.8 (29.2 to 68.6)	45.9 (29.6 to 68.1)	46 (29.1 to 68.9)
Turkmenistan	Acne vulgaris	0.987 (0.574 to 1.61)	1.54 (0.873 to 2.57)	1.57 (0.92 to 2.6)	23.4 (13.6 to 38.3)	28.2 (16 to 47.5)	31.9 (18.5 to 53.2)
Turkmenistan	Alopecia areata	0.2 (0.127 to 0.302)	0.269 (0.17 to 0.406)	0.306 (0.193 to 0.46)	5.9 (3.8 to 8.9)	5.9 (3.8 to 8.9)	5.9 (3.8 to 8.9)
Turkmenistan	Pruritus	0.299 (0.14 to 0.559)	0.383 (0.181 to 0.728)	0.464 (0.218 to 0.889)	9.5 (4.5 to 17.7)	9.4 (4.5 to 17.9)	9.7 (4.6 to 18.4)
Turkmenistan	Urticaria	3.17 (2.08 to 4.51)	3.34 (2.2 to 4.75)	3.79 (2.52 to 5.4)	74.8 (49.3 to 105.4)	74.7 (48.9 to 106.5)	74.7 (49.8 to 106.5)
Turkmenistan	Decubitus ulcer	0.057 (0.0408 to 0.0767)	0.0857 (0.0613 to 0.112)	0.106 (0.0757 to 0.141)	2.3 (1.6 to 3.1)	2.6 (1.9 to 3.4)	2.6 (1.9 to 3.5)
Turkmenistan	Other skin and subcutaneous diseases	1.01 (0.486 to 1.86)	1.3 (0.622 to 2.39)	1.73 (0.829 to 3.16)	34 (16.5 to 62.9)	33.8 (16.4 to 62.2)	37.5 (18.1 to 68.4)
Turkmenistan	Sense organ diseases	21.3 (14.2 to 31.2)	28.6 (19 to 42.1)	34.9 (23.3 to 51.2)	902.7 (606.5 to 1303.5)	883.8 (597.6 to 1271.6)	852.3 (571.6 to 1233.7)
Turkmenistan	Blindness and vision impairment	10.6 (6.88 to 15.9)	13.8 (8.95 to 20.8)	16.4 (10.7 to 24.4)	446.6 (299.1 to 655)	427.1 (283.8 to 632.5)	400.5 (261.3 to 589.9)
Turkmenistan	Glaucoma	0.178 (0.118 to 0.249)	0.217 (0.147 to 0.305)	0.248 (0.164 to 0.343)	10 (6.7 to 14)	9.1 (6.2 to 12.7)	7.5 (5 to 10.6)
Turkmenistan	Cataract	2.88 (1.94 to 3.94)	3.98 (2.73 to 5.41)	5.01 (3.37 to 6.93)	162.5 (110.3 to 220.4)	157.9 (107.8 to 213.1)	146.5 (99.1 to 199.9)
Turkmenistan	Age-related macular degeneration	0.0316 (0.0204 to 0.0446)	0.0419 (0.0276 to 0.0599)	0.0541 (0.0348 to 0.078)	1.8 (1.2 to 2.5)	1.8 (1.2 to 2.5)	1.6 (1.1 to 2.3)
Turkmenistan	Refraction disorders	3.89 (2.42 to 5.95)	4.59 (2.89 to 7.03)	5.08 (3.2 to 7.81)	116.7 (73.4 to 179.1)	111 (70.4 to 168.2)	106.4 (67.2 to 163.3)
Turkmenistan	Near vision loss	2.69 (1.28 to 4.95)	3.79 (1.79 to 6.93)	4.71 (2.24 to 8.63)	117.6 (56.6 to 216)	112.7 (53.4 to 204.8)	107.7 (51 to 198)
Turkmenistan	Other vision loss	0.886 (0.604 to 1.21)	1.13 (0.767 to 1.55)	1.29 (0.872 to 1.76)	37.8 (26 to 51.6)	34.7 (23.8 to 47.6)	30.8 (21.1 to 42.1)
Turkmenistan	Age-related and other hearing loss	9.92 (6.76 to 14.1)	13.7 (9.31 to 19.6)	17.1 (11.5 to 24.5)	425.2 (291.6 to 596.7)	425.6 (293.6 to 598.5)	420.5 (286.7 to 593.6)
Turkmenistan	Other sense organ diseases	0.799 (0.495 to 1.18)	1.06 (0.657 to 1.53)	1.34 (0.818 to 1.96)	30.9 (19.3 to 45.1)	31.2 (19.4 to 44.7)	31.4 (19.2 to 45.5)
Turkmenistan	Musculoskeletal disorders	36.8 (26.6 to 49.9)	54.3 (39.3 to 73.4)	66.4 (48.2 to 90)	1446.6 (1045.3 to 1957.8)	1452.2 (1057.4 to 1964)	1436.6 (1041.9 to 1920.8)
Turkmenistan	Rheumatoid arthritis	0.618 (0.442 to 0.817)	0.929 (0.648 to 1.23)	1.32 (0.92 to 1.79)	25.7 (18.4 to 33.8)	27 (19.3 to 35.3)	30.2 (21.2 to 40.2)

SR Appendix Table 2. All-age DALYs counts and age standardized DALYs rates per 100,000 individuals for all causes and level 1 cause groups by location for 1990, 2007, and 2017, both sexes combined

		All-age DALYs			Age-standardized rate of DALYs per 100,000		
Turkmenistan	Osteoarthritis	1.5 (0.754 to 3.02)	2.4 (1.2 to 4.82)	3.42 (1.72 to 6.73)	75.8 (38 to 151.3)	82.4 (41.2 to 163.6)	86.2 (43.1 to 171.9)
Turkmenistan	Low back pain	26 (18.6 to 35.5)	37.3 (26.4 to 50.9)	45.3 (32.2 to 61.1)	995.8 (711.7 to 1350.5)	982.3 (702 to 1326.4)	972.8 (695.4 to 1308.1)
Turkmenistan	Neck pain	7.23 (4.99 to 10.2)	11.2 (7.82 to 15.8)	14 (9.69 to 19.9)	298.7 (206.2 to 418.6)	299 (208.1 to 420.4)	298.4 (207.4 to 420.3)
Turkmenistan	Gout	0.269 (0.179 to 0.37)	0.439 (0.292 to 0.618)	0.612 (0.408 to 0.844)	12.7 (8.4 to 17.5)	13.9 (9.3 to 19.3)	14.7 (9.7 to 19.9)
Turkmenistan	Other musculoskeletal disorders	1.11 (0.642 to 1.79)	1.97 (1.07 to 3.22)	1.78 (0.893 to 3)	38 (21.5 to 62)	47.5 (25.9 to 74.8)	34.4 (17.5 to 56.7)
Turkmenistan	Other non-communicable diseases	81.2 (69.2 to 97)	101 (70.2 to 125)	95.4 (65.3 to 123)	1838.7 (1533.4 to 2221.7)	2384.4 (1665.8 to 2936.5)	1921.1 (1327.6 to 2469.7)
Turkmenistan	Congenital anomalies	55.2 (47.4 to 68.1)	61.6 (36.9 to 78.5)	53.3 (28 to 76.5)	1012.5 (870.2 to 1238.8)	1402.7 (840 to 1791)	1030.7 (547.5 to 1475.5)
Turkmenistan	Neural tube defects	3.81 (2.83 to 4.78)	2.95 (1.29 to 4.44)	2.4 (0.973 to 4.17)	67.9 (51 to 84.8)	67.2 (29.4 to 101.5)	46.4 (19 to 80.2)
Turkmenistan	Congenital heart anomalies	23.2 (18.9 to 30.5)	19.8 (12.9 to 26.8)	16.5 (9.18 to 25.3)	403 (328.9 to 530.2)	450 (292.7 to 610.5)	317 (177.1 to 486.2)
Turkmenistan	Orofacial clefts	0.369 (0.224 to 0.631)	0.195 (0.122 to 0.306)	0.178 (0.114 to 0.26)	7.5 (4.8 to 12.1)	4.4 (2.7 to 7)	3.5 (2.3 to 5.1)
Turkmenistan	Down's syndrome	1.21 (0.786 to 2.25)	1.25 (0.664 to 1.7)	1.15 (0.565 to 1.66)	23.1 (15.7 to 41.4)	28.4 (15 to 38.7)	21.9 (10.9 to 31.8)
Turkmenistan	Turner syndrome	0.00485 (0.00231 to 0.00819)	0.00516 (0.00252 to 0.00871)	0.00519 (0.00247 to 0.00869)	0.1 (0.1 to 0.2)	0.1 (0.1 to 0.2)	0.1 (0 to 0.2)
Turkmenistan	Klinefelter syndrome	0.00105 (0.000488 to 0.00197)	0.00133 (0.000613 to 0.00256)	0.00145 (0.000677 to 0.00274)	0 (0 to 0)	0 (0 to 0.1)	0 (0 to 0.1)
Turkmenistan	Other chromosomal abnormalities	1.14 (0.766 to 1.98)	1.85 (0.743 to 2.9)	1.77 (0.717 to 2.88)	22.1 (15 to 36.5)	41.9 (16.7 to 66.1)	34 (13.9 to 55.4)
Turkmenistan	Congenital musculoskeletal and limb anomalies	4.37 (3.11 to 5.81)	5.34 (3.72 to 7.11)	5.69 (3.92 to 7.74)	110.8 (77 to 149.2)	122 (84.9 to 162.3)	114.9 (78.9 to 156.3)
Turkmenistan	Urogenital congenital anomalies	0.575 (0.393 to 0.826)	0.809 (0.366 to 1.24)	0.758 (0.303 to 1.17)	10.5 (7.3 to 15)	18.2 (8.1 to 27.9)	14.7 (5.9 to 22.7)
Turkmenistan	Digestive congenital anomalies	6.44 (4.73 to 8.38)	10.7 (4.47 to 15.2)	8.78 (3.4 to 13.5)	117.8 (88.5 to 150.8)	243.1 (101.9 to 347.4)	169.8 (66.5 to 259.7)
Turkmenistan	Other congenital anomalies	14.1 (10.7 to 21.1)	18.7 (8.22 to 25.9)	16.1 (6.55 to 24.3)	249.6 (190.5 to 370.5)	427.3 (187.6 to 593.2)	308.3 (126.5 to 465.2)
Turkmenistan	Urinary diseases and male infertility	3.32 (2.86 to 3.85)	9.39 (8.74 to 10.2)	10.6 (9.54 to 11.9)	116.1 (97.2 to 138.5)	245.1 (224.9 to 270.7)	227.5 (203.1 to 256)
Turkmenistan	Urinary tract infections	1.98 (1.85 to 2.11)	7.41 (7 to 7.8)	8.05 (7.37 to 8.76)	59.3 (55.6 to 63.1)	181.9 (171.8 to 191.4)	166 (152.3 to 180.5)
Turkmenistan	Urolithiasis	0.123 (0.0876 to 0.162)	0.235 (0.182 to 0.297)	0.308 (0.239 to 0.389)	4.7 (3.3 to 6.2)	6.6 (5.2 to 8.2)	7 (5.5 to 8.7)
Turkmenistan	Benign prostatic hyperplasia	0.834 (0.519 to 1.21)	1.18 (0.739 to 1.7)	1.76 (1.11 to 2.57)	41 (25.6 to 58.9)	42.3 (26.4 to 60.7)	43.6 (27.6 to 63.3)
Turkmenistan	Male infertility	0.0825 (0.033 to 0.176)	0.124 (0.0498 to 0.259)	0.133 (0.0533 to 0.279)	2.3 (0.9 to 4.8)	2.5 (1 to 5.2)	2.4 (1 to 5)
Turkmenistan	Other urinary diseases	0.294 (0.23 to 0.377)	0.436 (0.298 to 0.679)	0.382 (0.283 to 0.564)	8.8 (6.9 to 11.1)	11.8 (8.2 to 18.2)	8.4 (6.3 to 12.6)
Turkmenistan	Gynecological diseases	5.11 (3.61 to 7.05)	7.5 (5.19 to 10.4)	8.01 (5.5 to 11.3)	163.2 (114 to 228.3)	158 (109 to 220.2)	150.6 (103.3 to 212.4)
Turkmenistan	Uterine fibroids	0.756 (0.483 to 1.16)	1.18 (0.718 to 1.89)	1.22 (0.711 to 2.03)	30.6 (19.1 to 47.7)	27.6 (16.7 to 44.2)	23.9 (13.9 to 39.6)
Turkmenistan	Polycystic ovarian syndrome	0.197 (0.0886 to 0.387)	0.274 (0.121 to 0.527)	0.297 (0.13 to 0.577)	5.8 (2.6 to 11.3)	5.6 (2.5 to 10.6)	5.6 (2.4 to 10.7)
Turkmenistan	Female infertility	0.0878 (0.0304 to 0.198)	0.108 (0.0365 to 0.239)	0.13 (0.0456 to 0.288)	2.4 (0.8 to 5.3)	2.2 (0.8 to 4.9)	2.3 (0.8 to 5.1)
Turkmenistan	Endometriosis	1.73 (1.14 to 2.52)	2.42 (1.61 to 3.56)	2.63 (1.7 to 3.9)	51.4 (33.6 to 73.9)	49.7 (33 to 73.2)	48.2 (31.3 to 71.3)
Turkmenistan	Genital prolapse	0.0978 (0.0502 to 0.179)	0.107 (0.0534 to 0.2)	0.143 (0.0691 to 0.265)	4.5 (2.3 to 8.2)	3.3 (1.7 to 6.1)	3.3 (1.6 to 6)
Turkmenistan	Premenstrual syndrome	1.67 (1.07 to 2.5)	2.44 (1.55 to 3.71)	2.47 (1.57 to 3.71)	49.9 (32.2 to 74.7)	49.2 (31.3 to 74.7)	46.6 (29.6 to 70)
Turkmenistan	Other gynecological diseases	0.572 (0.409 to 0.794)	0.967 (0.663 to 1.43)	1.12 (0.741 to 1.68)	18.7 (13.2 to 26.1)	20.4 (14 to 30.1)	20.8 (13.8 to 31.4)
Turkmenistan	Hemoglobinopathies and hemolytic anaemias	4.86 (3.61 to 6.26)	4.48 (3.36 to 5.81)	3.77 (2.84 to 5.02)	121.9 (92.5 to 156.7)	102 (78 to 131.5)	75.8 (57 to 100.5)
Turkmenistan	Thalassemias	1.23 (0.628 to 1.68)	0.824 (0.483 to 1.1)	0.578 (0.405 to 0.778)	21.9 (11.5 to 30)	18.8 (11 to 25.3)	11.1 (7.8 to 14.9)
Turkmenistan	Thalassaemias trait	1.32 (0.824 to 1.96)	1.34 (0.847 to 2.01)	1.3 (0.798 to 1.96)	36.1 (23.3 to 52.6)	30.7 (19.6 to 45.2)	26 (16.3 to 39)
Turkmenistan	Sickle cell disorders	0.162 (0.0696 to 0.283)	0.123 (0.0613 to 0.208)	0.091 (0.0518 to 0.144)	2.9 (1.3 to 5)	2.8 (1.4 to 4.8)	1.7 (1 to 2.7)
Turkmenistan	Sickle cell trait	0.108 (0.0638 to 0.166)	0.0959 (0.0574 to 0.154)	0.092 (0.0527 to 0.15)	2.8 (1.6 to 4.2)	2.1 (1.3 to 3.4)	1.8 (1.1 to 3)
Turkmenistan	G6PD deficiency	0.212 (0.161 to 0.26)	0.339 (0.259 to 0.403)	0.295 (0.245 to 0.358)	5.5 (4.3 to 6.5)	6.9 (5.4 to 8.2)	5.7 (4.7 to 7)
Turkmenistan	G6PD trait	0.00014 (0.0000883 to 0.000202)	0.000162 (0.000101 to 0.000236)	0.000161 (0.0001 to 0.000244)	0 (0 to 0)	0 (0 to 0)	0 (0 to 0)
Turkmenistan	Other hemoglobinopathies and hemolytic anaemias	1.83 (1.39 to 2.38)	1.76 (1.33 to 2.35)	1.42 (1.03 to 1.98)	52.7 (40.6 to 66.5)	40.7 (31.2 to 53.1)	29.4 (21.6 to 40.6)

SR Appendix Table 2. All-age DALYs counts and age standardized DALYs rates per 100,000 individuals for all causes and level 1 cause groups by location for 1990, 2007, and 2017, both sexes combined							
		All-age DALYs			Age-standardized rate of DALYs per 100,000		
Turkmenistan	Endocrine, metabolic, blood, and immune disorders	3.14 (2.5 to 3.95)	5.21 (3.27 to 6.38)	4.52 (3.26 to 5.54)	77.6 (60.8 to 99)	119.7 (76.1 to 146.3)	90.5 (65.9 to 110.9)
Turkmenistan	Oral disorders	7.95 (4.92 to 12.1)	11.2 (6.94 to 17)	14.1 (8.85 to 21.3)	319.2 (202.4 to 480.8)	321.2 (202.3 to 485.7)	324.3 (207.8 to 487)
Turkmenistan	Caries of deciduous teeth	0.203 (0.0881 to 0.407)	0.158 (0.0667 to 0.315)	0.195 (0.0828 to 0.389)	3.8 (1.6 to 7.5)	3.8 (1.6 to 7.5)	3.7 (1.6 to 7.4)
Turkmenistan	Caries of permanent teeth	1.45 (0.617 to 2.75)	2 (0.874 to 3.81)	2.05 (0.895 to 3.88)	42.3 (18.2 to 80)	43.9 (19.2 to 83.5)	40.9 (17.9 to 77.4)
Turkmenistan	Periodontal disease	0.952 (0.37 to 1.99)	1.44 (0.563 to 3.07)	1.94 (0.762 to 4.08)	40.4 (15.9 to 84.5)	39.4 (15.7 to 83.4)	41.1 (16.3 to 85.8)
Turkmenistan	Edentulism and severe tooth loss	3.7 (2.45 to 5.27)	5.36 (3.56 to 7.64)	7.35 (4.87 to 10.4)	181.8 (120.9 to 258.3)	183.2 (122.1 to 259.5)	187.7 (125.2 to 264.8)
Turkmenistan	Other oral disorders	1.64 (1.02 to 2.45)	2.22 (1.38 to 3.28)	2.53 (1.58 to 3.78)	51 (31.8 to 75.6)	50.9 (31.6 to 75.3)	50.8 (31.7 to 75.5)
Turkmenistan	Sudden infant death syndrome	1.65 (0.905 to 2.82)	1.56 (0.856 to 2.97)	1.14 (0.66 to 1.86)	28.2 (15.4 to 48.2)	35.8 (19.6 to 67.9)	21.7 (12.6 to 35.5)
Turkmenistan	Injuries	182 (166 to 199)	167 (155 to 180)	143 (126 to 162)	4755.4 (4356 to 5199)	3711.5 (3418 to 4031.3)	2829.5 (2497.3 to 3228.6)
Turkmenistan	Transport injuries	41.6 (39 to 44.5)	31.7 (29.7 to 33.9)	23.9 (20.9 to 27)	1152.1 (1084.5 to 1228.4)	699.9 (652.4 to 749.5)	467.4 (410 to 530.4)
Turkmenistan	Road injuries	40.5 (38.1 to 43.2)	30.3 (28.4 to 32.2)	22.7 (19.9 to 25.6)	1120.5 (1057.5 to 1192.8)	668.8 (626 to 715.2)	443.4 (390.2 to 502)
Turkmenistan	Pedestrian road injuries	17 (15.3 to 18.7)	10.6 (9.56 to 11.9)	7.17 (6.17 to 8.41)	454.5 (406.6 to 496.8)	238.8 (217.5 to 265.5)	141.3 (122 to 165.1)
Turkmenistan	Cyclist road injuries	1.16 (0.951 to 1.4)	1.18 (0.943 to 1.44)	1.28 (0.969 to 1.63)	36.7 (29.6 to 44.4)	28.1 (22.4 to 34.5)	26.2 (19.9 to 33.6)
Turkmenistan	Motorcyclist road injuries	3.99 (3.18 to 4.57)	3.41 (2.98 to 3.99)	2.69 (2.18 to 3.3)	111.3 (87.5 to 127.4)	72 (63.2 to 83.9)	52 (42.2 to 63.5)
Turkmenistan	Motor vehicle road injuries	17.7 (16 to 19.7)	14.6 (13.1 to 16)	11.1 (9.68 to 12.7)	499.2 (450.8 to 569.1)	318.1 (285.9 to 347.8)	215.2 (188 to 245.6)
Turkmenistan	Other road injuries	0.638 (0.528 to 0.739)	0.501 (0.422 to 0.598)	0.419 (0.332 to 0.536)	18.7 (15.4 to 21.8)	11.8 (9.9 to 14.2)	8.6 (6.8 to 11)
Turkmenistan	Other transport injuries	1.1 (0.944 to 1.29)	1.41 (1.17 to 1.64)	1.21 (0.969 to 1.47)	31.6 (26.9 to 37)	31.1 (26 to 36.3)	24 (19.2 to 29.4)
Turkmenistan	Unintentional injuries	113 (100 to 127)	91.4 (82 to 102)	82.4 (69.7 to 97.3)	2775.7 (2443.6 to 3143.3)	2103.1 (1870.5 to 2367.8)	1661.8 (1402.4 to 1967)
Turkmenistan	Falls	21.4 (17.3 to 26.2)	19.2 (15 to 24.6)	26.7 (20.1 to 34.6)	691.5 (547.5 to 863.8)	479.2 (370.9 to 613.2)	563.6 (423.7 to 735.9)
Turkmenistan	Drowning	38.8 (33 to 45.4)	18.3 (15.7 to 21.1)	9.64 (8.3 to 11.5)	786.2 (682.1 to 902.4)	411.7 (349.9 to 478.3)	185.3 (159.9 to 221.8)
Turkmenistan	Fire, heat, and hot substances	21.1 (18.7 to 23.7)	17.8 (15.9 to 19.7)	10.7 (8.95 to 12.6)	540.9 (478.5 to 608.7)	389.4 (346.3 to 436.7)	209.6 (175.8 to 247.4)
Turkmenistan	Poisonings	3.69 (3.34 to 4.06)	3.41 (3.14 to 3.72)	2.44 (2.09 to 2.83)	93.3 (85.8 to 101)	74.3 (68.5 to 81)	47.7 (40.8 to 55.3)
Turkmenistan	Poisoning by carbon monoxide	1.38 (1.26 to 1.51)	1.93 (1.78 to 2.09)	0.983 (0.865 to 1.11)	39.1 (35.8 to 42.5)	41.1 (38.1 to 44.5)	19 (16.8 to 21.5)
Turkmenistan	Poisoning by other means	2.31 (2.01 to 2.64)	1.48 (1.29 to 1.71)	1.46 (1.19 to 1.77)	54.2 (48.1 to 61.1)	33.2 (29 to 38.3)	28.6 (23.2 to 34.8)
Turkmenistan	Exposure to mechanical forces	5.74 (4.83 to 6.83)	7.63 (6.4 to 9.09)	9.46 (7.64 to 11.8)	165.6 (136 to 202.6)	175.9 (146.1 to 211.7)	190.1 (152.7 to 238.6)
Turkmenistan	Unintentional firearm injuries	0.38 (0.317 to 0.453)	0.424 (0.352 to 0.504)	0.491 (0.393 to 0.596)	10.1 (8.3 to 12)	9.8 (8.1 to 11.6)	10 (8 to 12.1)
Turkmenistan	Other exposure to mechanical forces	5.36 (4.48 to 6.4)	7.21 (6.03 to 8.62)	8.97 (7.23 to 11.3)	155.5 (127.1 to 190.4)	166.2 (137.6 to 201.5)	180.1 (144.2 to 227.7)
Turkmenistan	Adverse effects of medical treatment	1.52 (1.07 to 1.8)	1.67 (1.36 to 1.93)	1.77 (1.43 to 2.11)	34.6 (26.3 to 39.7)	38.5 (31.8 to 44.4)	35.2 (28.6 to 41.7)
Turkmenistan	Animal contact	0.59 (0.463 to 0.734)	0.717 (0.567 to 0.901)	0.696 (0.533 to 0.883)	15.9 (12.3 to 20)	16.6 (13.1 to 20.8)	14.1 (10.7 to 17.9)
Turkmenistan	Venomous animal contact	0.36 (0.262 to 0.476)	0.428 (0.315 to 0.578)	0.428 (0.303 to 0.572)	9.8 (7 to 13)	9.8 (7.2 to 13.1)	8.6 (6.1 to 11.5)
Turkmenistan	Non-venomous animal contact	0.231 (0.168 to 0.3)	0.289 (0.232 to 0.363)	0.269 (0.212 to 0.338)	6.1 (4.5 to 7.9)	6.8 (5.4 to 8.6)	5.5 (4.3 to 6.9)
Turkmenistan	Foreign body	14.2 (12.2 to 16.4)	10.5 (9.24 to 11.9)	8.7 (7.39 to 10.1)	284.5 (251.3 to 324.7)	239.4 (209.7 to 269.6)	169.2 (144.2 to 195.4)
Turkmenistan	Pulmonary aspiration and foreign body in airway	12 (10.3 to 14.2)	8.64 (7.45 to 9.93)	6.8 (5.64 to 8.11)	230.1 (199.4 to 267.1)	195.9 (169.1 to 225.3)	131 (109 to 156)
Turkmenistan	Foreign body in eyes	0.0659 (0.0289 to 0.119)	0.0894 (0.0398 to 0.159)	0.111 (0.0503 to 0.195)	2 (0.9 to 3.4)	2.1 (1 to 3.7)	2.3 (1.1 to 3.9)
Turkmenistan	Foreign body in other body part	2.08 (1.57 to 2.75)	1.82 (1.51 to 2.2)	1.79 (1.48 to 2.17)	52.5 (42.1 to 65.3)	41.4 (34.5 to 50.3)	35.9 (29.8 to 43.3)
Turkmenistan	Environmental heat and cold exposure	1.69 (1.32 to 2.09)	6.78 (6.19 to 7.41)	6.44 (5.49 to 7.36)	55.6 (43.2 to 69)	158.1 (143.6 to 173.1)	128.2 (109.4 to 146.3)
Turkmenistan	Exposure to forces of nature	0 (0 to 0)	0.0462 (0.0458 to 0.0467)	0.00202 (0.0015 to 0.00267)	0 (0 to 0)	1 (1 to 1)	0 (0 to 0.1)
Turkmenistan	Other unintentional injuries	3.98 (3.42 to 4.64)	5.35 (4.71 to 6.17)	5.87 (4.8 to 7.19)	107.7 (91 to 128)	119 (103.9 to 138.2)	118.8 (96.9 to 146.8)
Turkmenistan	Self-harm and interpersonal violence	28 (26.9 to 29.1)	43.8 (42 to 45.6)	36.5 (33.3 to 39.9)	827.6 (795.5 to 860.1)	908.5 (873.1 to 945.2)	700.3 (640.8 to 767.3)
Turkmenistan	Self-harm	14.7 (14 to 15.5)	30.1 (28.6 to 31.7)	23.1 (20.9 to 25.7)	436.7 (417.6 to 456.7)	618 (588.4 to 648.8)	443.4 (401.8 to 493.3)

SR Appendix Table 2. All-age DALYs counts and age standardized DALYs rates per 100,000 individuals for all causes and level 1 cause groups by location for 1990, 2007, and 2017, both sexes combined

		All-age DALYs			Age-standardized rate of DALYs per 100,000		
Turkmenistan	Self-harm by firearm	0.722 (0.523 to 1.11)	1.09 (0.756 to 1.74)	0.819 (0.557 to 1.32)	22 (15.8 to 33.8)	22.9 (16 to 36.8)	15.5 (10.7 to 24.7)
Turkmenistan	Self-harm by other specified means	14 (13.2 to 14.7)	29.1 (27.5 to 30.6)	22.3 (20.1 to 24.8)	414.6 (392.7 to 434.9)	595.1 (564 to 625.5)	427.9 (386.5 to 475)
Turkmenistan	Interpersonal violence	13.2 (12.4 to 14.1)	13.2 (12.4 to 14.1)	13.1 (11.7 to 14.6)	389.2 (365.2 to 413.6)	282.4 (264.6 to 301.6)	252.5 (224.6 to 281.4)
Turkmenistan	Assault by firearm	2.96 (1.93 to 3.55)	2.28 (1.79 to 2.76)	2.03 (1.59 to 2.57)	83.6 (54.7 to 99.2)	45.8 (36.2 to 55.3)	38.4 (30.1 to 48.1)
Turkmenistan	Assault by sharp object	4.57 (4.03 to 5.48)	5.11 (4.5 to 5.9)	4.91 (4.16 to 5.84)	136.9 (121.7 to 161.2)	108 (96.1 to 123.5)	93.5 (79.6 to 110.4)
Turkmenistan	Sexual violence	0.257 (0.171 to 0.37)	0.368 (0.246 to 0.537)	0.392 (0.263 to 0.575)	7.3 (4.9 to 10.5)	7.8 (5.2 to 11.4)	7.6 (5.1 to 11.1)
Turkmenistan	Assault by other means	5.39 (4.79 to 6.09)	5.49 (4.83 to 6.23)	5.8 (4.82 to 6.75)	161.4 (143.3 to 182)	120.9 (106.2 to 137)	113 (94.1 to 131.7)
Turkmenistan	Conflict and terrorism	0 (0 to 0)	0.293 (0.278 to 0.316)	0.0752 (0.0495 to 0.119)	0 (0 to 0)	5.7 (5.4 to 6.1)	1.4 (0.9 to 2.3)
Turkmenistan	Executions and police conflict	0.0674 (0.0468 to 0.1)	0.12 (0.0942 to 0.155)	0.155 (0.112 to 0.224)	1.6 (1.2 to 2.4)	2.4 (1.9 to 3)	3 (2.1 to 4.4)
Uzbekistan	All causes	7690 (7080 to 8400)	8900 (8060 to 9830)	9430 (8350 to 10500)	38919.8 (35816.6 to 42496.7)	40794.9 (37478.8 to 44492.4)	35452.5 (31734.8 to 39320.5)
Uzbekistan	Communicable, maternal, neonatal, and nutritional disorders	3370 (3090 to 3680)	2370 (2040 to 2740)	1880 (1610 to 2200)	11169.3 (10215.2 to 12224.8)	8295.7 (7163.9 to 9578.8)	5843.5 (5008.2 to 6810.9)
Uzbekistan	HIV/AIDS and sexually transmitted infections	13.9 (11.5 to 17.8)	31.8 (28.1 to 37.7)	39.9 (35.8 to 47.2)	69.8 (58.5 to 89.7)	113.6 (101.2 to 133.8)	117.1 (105 to 138.1)
Uzbekistan	HIV/AIDS	7.04 (6.75 to 7.34)	24.1 (22.8 to 25.7)	31.6 (30.2 to 33.1)	36 (34.6 to 37.4)	87.1 (82.4 to 92.8)	92.8 (88.7 to 97.2)
Uzbekistan	HIV/AIDS - Drug-susceptible Tuberculosis	1.65 (1.01 to 2.56)	2.33 (1.2 to 4.01)	1.48 (0.527 to 2.81)	8.4 (5.2 to 13.1)	8.4 (4.3 to 14.5)	4.3 (1.5 to 8.3)
Uzbekistan	HIV/AIDS - Multidrug-resistant Tuberculosis without extensive drug resistance	0.0037 (0.000472 to 0.0134)	1.99 (1.03 to 3.35)	1.4 (0.643 to 2.33)	0 (0 to 0.1)	7.2 (3.7 to 12.1)	4.1 (1.9 to 6.9)
Uzbekistan	HIV/AIDS - Extensively drug-resistant Tuberculosis	0 (0 to 0)	0.632 (0.337 to 1.07)	0.799 (0.364 to 1.33)	0 (0 to 0)	2.3 (1.2 to 3.9)	2.3 (1.1 to 3.9)
Uzbekistan	HIV/AIDS resulting in other diseases	5.39 (4.42 to 6.09)	19.1 (16.3 to 21.6)	27.9 (25.7 to 30)	27.5 (22.8 to 31)	69.2 (58.8 to 78)	82 (75.3 to 87.9)
Uzbekistan	Sexually transmitted infections excluding HIV	6.84 (4.55 to 10.9)	7.66 (4.54 to 13.2)	8.33 (4.75 to 14.6)	33.8 (22.7 to 53.6)	26.5 (15.8 to 45.4)	24.3 (13.9 to 42.2)
Uzbekistan	Syphilis	1.96 (1.11 to 3.32)	0.885 (0.41 to 1.72)	0.636 (0.303 to 1.17)	8 (5 to 12.1)	3.2 (1.5 to 6)	2 (1 to 3.7)
Uzbekistan	Chlamydial infection	1.12 (0.661 to 1.99)	1.64 (0.927 to 2.98)	1.91 (1.07 to 3.5)	5.8 (3.4 to 10.2)	5.5 (3.1 to 9.9)	5.4 (3 to 9.8)
Uzbekistan	Gonococcal infection	1.26 (0.811 to 2.09)	1.48 (0.903 to 2.59)	1.51 (0.888 to 2.71)	6.3 (4.3 to 9.9)	4.9 (3.1 to 8.3)	4.3 (2.6 to 7.7)
Uzbekistan	Trichomoniasis	0.724 (0.291 to 1.53)	1.12 (0.447 to 2.41)	1.36 (0.543 to 2.92)	4 (1.7 to 8.5)	4 (1.6 to 8.6)	4 (1.6 to 8.5)
Uzbekistan	Genital herpes	0.413 (0.134 to 0.975)	0.643 (0.213 to 1.51)	0.819 (0.271 to 1.93)	2.6 (0.8 to 6.3)	2.6 (0.9 to 6.1)	2.6 (0.8 to 6.1)
Uzbekistan	Other sexually transmitted infections	1.36 (0.874 to 2.31)	1.88 (1.15 to 3.25)	2.11 (1.29 to 3.56)	7.1 (4.6 to 11.6)	6.3 (3.9 to 10.6)	6 (3.7 to 10.1)
Uzbekistan	Respiratory infections and tuberculosis	1600 (1460 to 1750)	1180 (1010 to 1360)	821 (702 to 967)	5184.9 (4746.6 to 5641.8)	4216.5 (3642.2 to 4856.6)	2577.5 (2211.5 to 3031.7)
Uzbekistan	Tuberculosis	86.3 (75.3 to 98.1)	166 (155 to 179)	111 (97.4 to 127)	457.8 (422.6 to 494.8)	655.1 (610.9 to 703.1)	348.9 (308 to 400.4)
Uzbekistan	Latent tuberculosis infection	0 (0 to 0)	0 (0 to 0)	0 (0 to 0)	0 (0 to 0)	0 (0 to 0)	0 (0 to 0)
Uzbekistan	Drug-susceptible tuberculosis	86.1 (75.2 to 97.9)	89.2 (62 to 117)	52.2 (26.8 to 82.1)	457 (421.1 to 493.6)	351.2 (244.3 to 462.3)	164.7 (84.1 to 258.3)
Uzbekistan	Multidrug-resistant tuberculosis without extensive drug resistance	0.155 (0.0223 to 0.535)	58.6 (36.4 to 81.7)	37.3 (19.3 to 53.6)	0.8 (0.1 to 2.9)	230.8 (143.4 to 320.9)	117.3 (60.5 to 167.6)
Uzbekistan	Extensively drug-resistant tuberculosis	0 (0 to 0)	18.5 (11.5 to 25.5)	21.3 (11 to 30.5)	0 (0 to 0)	73 (45.6 to 100.2)	66.9 (34.5 to 96)
Uzbekistan	Lower respiratory infections	1460 (1320 to 1610)	972 (816 to 1150)	678 (567 to 817)	4546.1 (4131.3 to 4986.8)	3432 (2901.4 to 4042.7)	2130.2 (1789.8 to 2559.4)
Uzbekistan	Upper respiratory infections	45.6 (27.4 to 60.9)	28.9 (20.3 to 37.8)	23.6 (16.5 to 32)	152 (92.9 to 200.1)	100.2 (71.3 to 131.3)	71.9 (50.2 to 97.2)
Uzbekistan	Otitis media	6.75 (4.01 to 10.8)	8.46 (5.09 to 13.5)	8.75 (5.29 to 13.9)	29 (17.3 to 46.1)	29.2 (17.6 to 46)	26.6 (16.1 to 42.5)
Uzbekistan	Enteric infections	339 (292 to 387)	39.4 (32.1 to 47.1)	35.5 (28.1 to 44)	1047 (906 to 1190.9)	137.5 (112.4 to 163.7)	109.2 (86.6 to 135.4)
Uzbekistan	Diarrhoeal diseases	336 (290 to 384)	37.6 (30.6 to 45.1)	33.8 (26.4 to 42.4)	1038.2 (896.6 to 1179.3)	131.1 (106.9 to 156.5)	103.7 (81.3 to 129.7)
Uzbekistan	Typhoid and paratyphoid	0.00923 (0.00481 to 0.0164)	0.0101 (0.00522 to 0.0182)	0.011 (0.00559 to 0.0196)	0 (0 to 0.1)	0 (0 to 0.1)	0 (0 to 0.1)
Uzbekistan	Typhoid fever	0.00907 (0.00473 to 0.0161)	0.00924 (0.00479 to 0.0165)	0.00986 (0.00499 to 0.0176)	0 (0 to 0.1)	0 (0 to 0.1)	0 (0 to 0.1)
Uzbekistan	Paratyphoid fever	0.000158 (0.0000625 to 0.000335)	0.000829 (0.000322 to 0.00177)	0.00116 (0.000467 to 0.00238)	0 (0 to 0)	0 (0 to 0)	0 (0 to 0)
Uzbekistan	Invasive non-typhoidal salmonella	1.85 (1.03 to 3.2)	1.52 (0.795 to 2.62)	1.57 (0.844 to 2.77)	7.3 (4 to 12.5)	5.4 (2.9 to 9.1)	5 (2.6 to 8.7)
Uzbekistan	Other intestinal infectious diseases	0.457 (0.185 to 0.876)	0.246 (0.0977 to 0.447)	0.16 (0.0689 to 0.274)	1.5 (0.7 to 2.7)	0.9 (0.4 to 1.6)	0.5 (0.2 to 0.9)

SR Appendix Table 2. All-age DALYs counts and age standardized DALYs rates per 100,000 individuals for all causes and level 1 cause groups by location for 1990, 2007, and 2017, both sexes combined

		All-age DALYs			Age-standardized rate of DALYs per 100,000		
Uzbekistan	Neglected tropical diseases and malaria	22.5 (14.8 to 33.3)	23.4 (14.6 to 36)	24.6 (14.7 to 39.3)	103.2 (68.4 to 152.9)	83.8 (53.3 to 127)	75 (45.3 to 115.9)
Uzbekistan	Malaria	0.638 (0.535 to 0.767)	0.208 (0.176 to 0.247)	0 (0 to 0)	3.1 (2.6 to 3.7)	0.8 (0.7 to 0.9)	0 (0 to 0)
Uzbekistan	Chagas disease	0 (0 to 0)	0 (0 to 0)	0 (0 to 0)	0 (0 to 0)	0 (0 to 0)	0 (0 to 0)
Uzbekistan	Leishmaniasis	0.18 (0.0177 to 1.02)	0.135 (0.0329 to 0.833)	0.279 (0.0259 to 1.97)	0.8 (0.1 to 4.1)	0.5 (0.1 to 2.8)	0.8 (0.1 to 5.9)
Uzbekistan	Visceral leishmaniasis	0.102 (0.000241 to 0.952)	0.0761 (0.000319 to 0.758)	0.197 (0.000678 to 1.92)	0.4 (0 to 3.7)	0.3 (0 to 2.6)	0.6 (0 to 5.7)
Uzbekistan	Cutaneous and mucocutaneous leishmaniasis	0.0783 (0.01 to 0.229)	0.0588 (0.0297 to 0.102)	0.0822 (0.0158 to 0.202)	0.4 (0 to 1.1)	0.2 (0.1 to 0.4)	0.3 (0 to 0.6)
Uzbekistan	African trypanosomiasis	0 (0 to 0)	0 (0 to 0)	0 (0 to 0)	0 (0 to 0)	0 (0 to 0)	0 (0 to 0)
Uzbekistan	Schistosomiasis	0 (0 to 0)	0 (0 to 0)	0 (0 to 0)	0 (0 to 0)	0 (0 to 0)	0 (0 to 0)
Uzbekistan	Cysticercosis	0.000819 (0.000422 to 0.00135)	0.000447 (0.000237 to 0.000677)	0.000384 (0.000192 to 0.000592)	0 (0 to 0)	0 (0 to 0)	0 (0 to 0)
Uzbekistan	Cystic echinococcosis	6.45 (2.78 to 13)	9.16 (3.78 to 19)	11.1 (4.38 to 22.6)	36.8 (17 to 70.4)	35.2 (15.5 to 67)	34.4 (14.3 to 68.3)
Uzbekistan	Lymphatic filariasis	0 (0 to 0)	0 (0 to 0)	0 (0 to 0)	0 (0 to 0)	0 (0 to 0)	0 (0 to 0)
Uzbekistan	Onchocerciasis	0 (0 to 0)	0 (0 to 0)	0 (0 to 0)	0 (0 to 0)	0 (0 to 0)	0 (0 to 0)
Uzbekistan	Trachoma	0 (0 to 0)	0 (0 to 0)	0 (0 to 0)	0 (0 to 0)	0 (0 to 0)	0 (0 to 0)
Uzbekistan	Dengue	0 (0 to 0)	0 (0 to 0)	0 (0 to 0)	0 (0 to 0)	0 (0 to 0)	0 (0 to 0)
Uzbekistan	Yellow fever	0 (0 to 0)	0 (0 to 0)	0 (0 to 0)	0 (0 to 0)	0 (0 to 0)	0 (0 to 0)
Uzbekistan	Rabies	0.0737 (0.0458 to 0.116)	0.11 (0.0812 to 0.145)	0.0876 (0.06 to 0.127)	0.4 (0.3 to 0.7)	0.4 (0.3 to 0.6)	0.3 (0.2 to 0.4)
Uzbekistan	Intestinal nematode infections	0.766 (0.389 to 1.39)	0.508 (0.285 to 0.863)	0.519 (0.284 to 0.871)	3.5 (1.8 to 6.4)	1.8 (1 to 3)	1.5 (0.8 to 2.6)
Uzbekistan	Ascariasis	0.0461 (0.0066 to 0.115)	0.0151 (0.00271 to 0.0375)	0.0106 (0.00203 to 0.0267)	0.1 (0 to 0.4)	0.1 (0 to 0.1)	0 (0 to 0.1)
Uzbekistan	Trichuriasis	0.00000000518 (0.00000000237 to 0.0000000106)	0.00000000175 (0.000000000765 to 0.00000000343)	0.00000000196 (0.000000000859 to 0.00000000378)	0 (0 to 0)	0 (0 to 0)	0 (0 to 0)
Uzbekistan	Hookworm disease	0.72 (0.36 to 1.32)	0.493 (0.273 to 0.85)	0.508 (0.277 to 0.857)	3.4 (1.7 to 6.2)	1.7 (0.9 to 2.9)	1.5 (0.8 to 2.6)
Uzbekistan	Food-borne trematodiases	0 (0 to 0)	0 (0 to 0)	0 (0 to 0)	0 (0 to 0)	0 (0 to 0)	0 (0 to 0)
Uzbekistan	Leprosy	0 (0 to 0)	0.0000175 (0.0000091 to 0.0000291)	0.0000135 (0.00000669 to 0.0000232)	0 (0 to 0)	0 (0 to 0)	0 (0 to 0)
Uzbekistan	Ebola virus disease	0 (0 to 0)	0 (0 to 0)	0 (0 to 0)	0 (0 to 0)	0 (0 to 0)	0 (0 to 0)
Uzbekistan	Zika virus disease	0 (0 to 0)	0 (0 to 0)	0 (0 to 0)	0 (0 to 0)	0 (0 to 0)	0 (0 to 0)
Uzbekistan	Guinea worm disease	0 (0 to 0)	0 (0 to 0)	0 (0 to 0)	0 (0 to 0)	0 (0 to 0)	0 (0 to 0)
Uzbekistan	Other neglected tropical diseases	14.4 (9.55 to 20.9)	13.2 (8.64 to 19.9)	12.7 (8.2 to 19.3)	58.5 (38.8 to 84.1)	45.2 (29.5 to 67.5)	37.9 (24.5 to 57.5)
Uzbekistan	Other infectious diseases	217 (193 to 243)	130 (115 to 152)	116 (101 to 134)	790.3 (709.9 to 868.5)	452.8 (402 to 528.2)	352.9 (307.2 to 410.1)
Uzbekistan	Meningitis	78.6 (63.1 to 89.7)	38.2 (32.9 to 46.7)	21.5 (18.1 to 28.8)	283.6 (224.4 to 318)	132.1 (113.7 to 162.4)	65.1 (54.9 to 87.6)
Uzbekistan	Pneumococcal meningitis	4.03 (3.21 to 4.94)	3.13 (2.42 to 3.98)	1.37 (1.08 to 1.72)	17.2 (13.7 to 21.4)	11 (8.5 to 13.8)	4.2 (3.3 to 5.2)
Uzbekistan	H influenzae type B meningitis	24.6 (19.4 to 28.8)	11.2 (9.42 to 14)	5.89 (4.78 to 8.2)	86.6 (67.3 to 99.6)	38.5 (32.5 to 48.4)	17.9 (14.5 to 25)
Uzbekistan	Meningococcal infection	12.5 (9.68 to 15.6)	5.51 (4.49 to 7.06)	3.13 (2.53 to 4.12)	44.2 (34.9 to 53.9)	19.2 (15.6 to 24.7)	9.5 (7.7 to 12.5)
Uzbekistan	Other meningitis	37.4 (30 to 43)	18.4 (15.9 to 22.6)	11.1 (9.31 to 14.9)	135.5 (106.4 to 153.2)	63.5 (54.9 to 78.7)	33.5 (28.2 to 45.2)
Uzbekistan	Encephalitis	44.4 (35.7 to 61.7)	49 (42.4 to 58.1)	57.7 (47.2 to 69.1)	164.1 (136 to 226.3)	171.9 (149.3 to 203.6)	176.5 (144.8 to 211.9)
Uzbekistan	Diphtheria	0.327 (0.0898 to 0.701)	0.155 (0.0852 to 0.265)	0.0785 (0.0434 to 0.136)	2.6 (0.7 to 5.5)	0.6 (0.4 to 1.1)	0.2 (0.1 to 0.4)
Uzbekistan	Whooping cough	0.841 (0.525 to 1.73)	0.415 (0.242 to 0.754)	0.427 (0.259 to 0.714)	2.8 (1.7 to 5.3)	1.5 (0.9 to 2.6)	1.3 (0.8 to 2.2)
Uzbekistan	Tetanus	0.16 (0.0829 to 0.192)	0.0696 (0.0358 to 0.111)	0.072 (0.0296 to 0.138)	0.8 (0.4 to 1)	0.3 (0.2 to 0.5)	0.2 (0.1 to 0.5)
Uzbekistan	Measles	2.43 (1.38 to 3.25)	0.161 (0.102 to 0.272)	0.169 (0.103 to 0.28)	7.4 (4.2 to 9.9)	0.6 (0.4 to 0.9)	0.5 (0.3 to 0.8)
Uzbekistan	Varicella and herpes zoster	0.627 (0.417 to 0.927)	0.749 (0.48 to 1.09)	0.904 (0.573 to 1.33)	3.3 (2.1 to 4.8)	3.1 (2 to 4.7)	3.1 (2 to 4.5)
Uzbekistan	Acute hepatitis	69.5 (50.7 to 83.3)	21.8 (19.4 to 25.5)	15.4 (13 to 19.7)	246.7 (181.9 to 289.2)	75.6 (67.2 to 88.6)	46.6 (39.4 to 59.3)
Uzbekistan	Acute hepatitis A	26.7 (18.7 to 35.7)	7.05 (5.84 to 8.51)	4.91 (3.94 to 6.26)	88.4 (62.3 to 116.5)	22.7 (18.7 to 27.5)	14.5 (11.5 to 18.4)

SR Appendix Table 2. All-age DALYs counts and age standardized DALYs rates per 100,000 individuals for all causes and level 1 cause groups by location for 1990, 2007, and 2017, both sexes combined

		All-age DALYs			Age-standardized rate of DALYs per 100,000		
Uzbekistan	Acute hepatitis B	20.5 (15.3 to 25.5)	10.5 (9.19 to 12.2)	8.11 (6.75 to 10.5)	88.7 (67.8 to 105.4)	38.4 (34 to 45)	24.8 (20.7 to 32.3)
Uzbekistan	Acute hepatitis C	19.7 (12.1 to 27.8)	2.81 (1.8 to 4.13)	1.46 (0.841 to 2.36)	58.9 (36.2 to 83.4)	9.7 (6.2 to 14.3)	4.5 (2.6 to 7.2)
Uzbekistan	Acute hepatitis E	2.68 (1.96 to 3.36)	1.45 (1.15 to 1.84)	0.964 (0.731 to 1.32)	10.8 (8 to 13.3)	4.8 (3.8 to 6)	2.9 (2.2 to 4)
Uzbekistan	Other unspecified infectious diseases	20.1 (15.3 to 26)	19.4 (14.7 to 25.5)	19.4 (14.6 to 25.6)	79 (60 to 102.8)	67.1 (51.2 to 88.1)	59.3 (45.1 to 77.8)
Uzbekistan	Maternal and neonatal disorders	862 (776 to 973)	686 (568 to 830)	585 (485 to 710)	2635 (2376.8 to 2957.5)	2320.5 (1919.8 to 2804.4)	1810.8 (1499.1 to 2202.2)
Uzbekistan	Maternal disorders	19.2 (18.1 to 20.4)	15.9 (14.3 to 17.5)	11.8 (9.87 to 13.9)	96.6 (91.3 to 102.5)	51.3 (46.1 to 56.6)	32 (26.8 to 37.7)
Uzbekistan	Maternal haemorrhage	5.01 (3.61 to 6.6)	2.99 (2.06 to 4.17)	1.83 (1.21 to 2.67)	26.4 (19.3 to 34.3)	9.9 (6.9 to 13.8)	5 (3.3 to 7.2)
Uzbekistan	Maternal sepsis and other pregnancy related infections	2.86 (2.17 to 3.64)	1.81 (1.34 to 2.41)	1.19 (0.843 to 1.67)	14.3 (11.1 to 18)	5.8 (4.3 to 7.6)	3.2 (2.3 to 4.5)
Uzbekistan	Maternal hypertensive disorders	1.64 (1.01 to 2.52)	2.53 (1.57 to 3.97)	2.28 (1.38 to 3.44)	7.6 (4.7 to 11.4)	8 (5 to 12.4)	6.1 (3.8 to 9.1)
Uzbekistan	Maternal obstructed labour and uterine rupture	1.04 (0.686 to 1.51)	0.671 (0.439 to 1.01)	0.546 (0.315 to 0.863)	5.3 (3.5 to 7.7)	2.2 (1.4 to 3.2)	1.5 (0.9 to 2.3)
Uzbekistan	Maternal abortive outcome	1.46 (1.15 to 1.83)	1.03 (0.785 to 1.29)	0.831 (0.609 to 1.09)	7.7 (6.1 to 9.5)	3.4 (2.6 to 4.2)	2.3 (1.7 to 3.1)
Uzbekistan	Ectopic pregnancy	0.455 (0.133 to 1.16)	0.35 (0.106 to 0.968)	0.248 (0.0779 to 0.681)	2.3 (0.7 to 5.8)	1.1 (0.4 to 3.1)	0.7 (0.2 to 1.8)
Uzbekistan	Indirect maternal deaths	1.17 (0.563 to 2.17)	1.34 (0.613 to 2.52)	0.923 (0.442 to 1.77)	5.7 (2.9 to 10.1)	4.3 (2.1 to 8)	2.5 (1.2 to 4.7)
Uzbekistan	Late maternal deaths	0.655 (0.486 to 0.869)	0.77 (0.586 to 0.984)	0.583 (0.433 to 0.78)	3.2 (2.4 to 4.3)	2.5 (1.9 to 3.2)	1.6 (1.2 to 2.1)
Uzbekistan	Maternal deaths aggravated by HIV/AIDS	0.000607 (0.000254 to 0.00107)	0.00335 (0.00164 to 0.00576)	0.00332 (0.00148 to 0.00631)	0 (0 to 0)	0 (0 to 0)	0 (0 to 0)
Uzbekistan	Other maternal disorders	4.93 (3.47 to 6.55)	4.4 (3.03 to 5.9)	3.35 (2.25 to 4.56)	24.1 (17 to 31.8)	14.2 (9.8 to 18.9)	9 (6.1 to 12.3)
Uzbekistan	Neonatal disorders	843 (757 to 953)	671 (552 to 813)	573 (474 to 699)	2538.4 (2280.4 to 2863.9)	2269.2 (1867.5 to 2750.7)	1778.8 (1470.9 to 2170.9)
Uzbekistan	Neonatal preterm birth	215 (154 to 285)	162 (130 to 198)	152 (122 to 187)	665.2 (484.2 to 864.1)	545.8 (440.2 to 669.4)	468.1 (375 to 578.3)
Uzbekistan	Neonatal encephalopathy due to birth asphyxia and trauma	505 (416 to 612)	394 (319 to 488)	309 (246 to 390)	1499.1 (1235.8 to 1812.7)	1333.6 (1078.5 to 1650.1)	961.7 (766.7 to 1213.3)
Uzbekistan	Neonatal sepsis and other neonatal infections	38.4 (24.1 to 75.8)	47.4 (34.2 to 79.3)	60.8 (44.6 to 85.7)	121.2 (77.5 to 231.6)	160.3 (115.8 to 268.3)	188.5 (138.4 to 266.6)
Uzbekistan	Hemolytic disease and other neonatal jaundice	10.3 (7.96 to 12.8)	8.86 (6.98 to 11)	8.16 (6.36 to 10.3)	36.4 (28.1 to 45.2)	30.2 (23.9 to 37.2)	24.6 (19.3 to 31.3)
Uzbekistan	Other neonatal disorders	73.9 (53.7 to 97.3)	58.7 (45.8 to 74.9)	43.6 (32.5 to 56.1)	216.5 (157.2 to 284.6)	199.4 (155.5 to 254.1)	135.8 (101 to 175)
Uzbekistan	Nutritional deficiencies	316 (214 to 450)	279 (184 to 411)	263 (171 to 396)	1339.1 (902 to 1902)	971 (636.4 to 1436.6)	801 (521.3 to 1207.5)
Uzbekistan	Protein-energy malnutrition	18.6 (13.2 to 25.7)	4.21 (2.2 to 7.05)	2.47 (0.861 to 5.15)	57.5 (40.5 to 79.2)	14.9 (7.8 to 25.1)	7.5 (2.7 to 15.5)
Uzbekistan	Iodine deficiency	3.9 (2.12 to 6.27)	5.36 (2.96 to 8.58)	5.95 (3.18 to 9.83)	20.1 (11.1 to 32.7)	19.6 (10.8 to 31.4)	18.5 (10 to 30.6)
Uzbekistan	Dietary iron deficiency	231 (149 to 335)	237 (155 to 354)	228 (147 to 344)	1049.9 (682.7 to 1519.3)	825.9 (544.6 to 1224)	694.7 (448.3 to 1050.5)
Uzbekistan	Other nutritional deficiencies	12.5 (10.9 to 14.3)	3.03 (2.7 to 3.42)	2.25 (1.93 to 2.59)	48.1 (43 to 53.5)	11.3 (10.1 to 12.7)	7 (6.1 to 8.1)
Uzbekistan	Non-communicable diseases	3400 (3060 to 3820)	5600 (5050 to 6180)	6570 (5790 to 7420)	23385.7 (21327.8 to 25863.7)	29059.5 (26702.6 to 31558.7)	26607.1 (23793.9 to 29703.5)
Uzbekistan	Neoplasms	371 (356 to 386)	493 (468 to 516)	609 (549 to 672)	2636.7 (2527 to 2737.2)	2395.1 (2275.5 to 2503.3)	2270.4 (2051.2 to 2499.5)
Uzbekistan	Lip and oral cavity cancer	4.99 (4.21 to 6.5)	9.65 (8.94 to 10.4)	12.4 (10.8 to 14)	37.1 (31 to 49.1)	48.3 (44.9 to 52.1)	45.5 (40.2 to 51.2)
Uzbekistan	Nasopharynx cancer	2.1 (1.9 to 2.38)	3.73 (3.43 to 4.07)	4.59 (4.05 to 5.2)	13.1 (11.8 to 14.8)	15.6 (14.4 to 16.9)	15.3 (13.5 to 17.3)
Uzbekistan	Other pharynx cancer	2.34 (1.83 to 2.99)	4.35 (3.98 to 4.72)	6.94 (5.98 to 7.93)	17.6 (13.7 to 22.8)	22.4 (20.5 to 24.3)	25.9 (22.3 to 29.6)
Uzbekistan	Oesophageal cancer	45.1 (43.4 to 46.7)	26.3 (24.6 to 28.1)	28.2 (24.8 to 31.9)	369.2 (355.8 to 382.9)	149 (139 to 159)	115.2 (101.8 to 129.2)
Uzbekistan	Stomach cancer	57.9 (55.9 to 60.1)	61.5 (57.9 to 65.2)	68.8 (61.3 to 77.8)	463.1 (447.5 to 479.4)	332.6 (313.9 to 352.2)	268.4 (239.2 to 302.3)
Uzbekistan	Colon and rectum cancer	19.8 (19.1 to 20.6)	26.2 (24.6 to 27.8)	35.1 (31 to 39.4)	150.9 (145.5 to 156.4)	144.6 (135.5 to 153.1)	149.8 (133 to 167.3)
Uzbekistan	Liver cancer	15.7 (11.6 to 20.4)	26.7 (25 to 28.5)	38 (33.4 to 42.7)	120.9 (88.4 to 158.2)	141 (132 to 150.3)	148.8 (131.6 to 167)
Uzbekistan	Liver cancer due to hepatitis B	4.88 (3.46 to 6.73)	8.24 (6.94 to 9.63)	10.7 (8.64 to 13.2)	34.2 (23.5 to 47.9)	38.2 (31.4 to 45.2)	37.4 (30.1 to 45.5)
Uzbekistan	Liver cancer due to hepatitis C	5.18 (3.43 to 7.19)	8.58 (7.33 to 9.96)	12.8 (10.4 to 15.3)	42.7 (28.1 to 59.7)	50.3 (43.5 to 58.1)	53.9 (44.5 to 63.5)
Uzbekistan	Liver cancer due to alcohol use	3.59 (2.5 to 4.94)	6.4 (5.62 to 7.26)	9.41 (7.78 to 11.1)	29 (20.1 to 40.1)	34.7 (30.3 to 39.6)	37.9 (31.7 to 44.5)
Uzbekistan	Liver cancer due to NASH	0.894 (0.615 to 1.23)	1.59 (1.37 to 1.81)	2.5 (2.07 to 2.95)	7.1 (4.8 to 9.8)	8.8 (7.6 to 10.3)	10.4 (8.6 to 12.3)

SR Appendix Table 2. All-age DALYs counts and age standardized DALYs rates per 100,000 individuals for all causes and level 1 cause groups by location for 1990, 2007, and 2017, both sexes combined

		All-age DALYs			Age-standardized rate of DALYs per 100,000		
Uzbekistan	Liver cancer due to other causes	1.15 (0.835 to 1.51)	1.89 (1.66 to 2.16)	2.56 (2.13 to 3.04)	8 (5.6 to 10.6)	9 (7.8 to 10.2)	9.2 (7.7 to 10.9)
Uzbekistan	Gallbladder and biliary tract cancer	1.52 (1.24 to 2.27)	2.46 (2.23 to 2.75)	2.97 (2.61 to 3.45)	12.6 (10.2 to 19.2)	14.5 (13.2 to 16.4)	13 (11.5 to 14.9)
Uzbekistan	Pancreatic cancer	5.24 (3.91 to 6.09)	10.6 (9.9 to 11.3)	17.4 (15.5 to 19.5)	43.1 (31.6 to 50.5)	61.8 (57.7 to 65.7)	75.8 (67.7 to 84.7)
Uzbekistan	Larynx cancer	6.1 (5.79 to 6.42)	10.6 (9.91 to 11.3)	10.8 (9.54 to 12.3)	47.6 (45.2 to 50.1)	56.6 (52.9 to 60.3)	41.6 (36.7 to 47.1)
Uzbekistan	Tracheal, bronchus, and lung cancer	49 (47.3 to 50.8)	46.1 (43.1 to 49)	62.2 (54.8 to 70.7)	384.2 (371.6 to 397.7)	250.6 (234.6 to 266.6)	246.5 (217.9 to 278.5)
Uzbekistan	Malignant skin melanoma	1.23 (0.684 to 1.66)	2.06 (1.53 to 2.6)	2.65 (2.08 to 3.68)	9 (5 to 12.2)	10.2 (7.6 to 12.9)	10 (7.9 to 13.9)
Uzbekistan	Non-melanoma skin cancer	1.4 (1.13 to 1.66)	2.49 (2.3 to 2.67)	3.33 (2.95 to 3.75)	11.1 (8.8 to 13.4)	13.9 (12.8 to 15)	14.1 (12.5 to 15.9)
Uzbekistan	Non-melanoma skin cancer (squamous-cell carcinoma)	1.4 (1.12 to 1.66)	2.48 (2.29 to 2.67)	3.32 (2.95 to 3.74)	11.1 (8.8 to 13.4)	13.9 (12.8 to 14.9)	14.1 (12.5 to 15.8)
Uzbekistan	Non-melanoma skin cancer (basal-cell carcinoma)	0.00389 (0.00129 to 0.00902)	0.00546 (0.00185 to 0.0126)	0.00748 (0.00258 to 0.017)	0 (0 to 0.1)	0 (0 to 0.1)	0 (0 to 0.1)
Uzbekistan	Breast cancer	20.7 (19.6 to 21.8)	45.8 (41.9 to 49.8)	62.8 (53.1 to 75.3)	158.9 (151.2 to 167)	215.9 (197.8 to 234.3)	217 (184.6 to 258.8)
Uzbekistan	Cervical cancer	12.5 (11.7 to 13.3)	28.9 (26.2 to 32)	32.3 (26.8 to 39.1)	96.5 (90.8 to 102.3)	130.9 (119.5 to 143.7)	108.8 (90.8 to 131.4)
Uzbekistan	Uterine cancer	3.08 (2.86 to 3.3)	6.83 (6.16 to 7.57)	9.31 (7.7 to 11.2)	24.5 (22.8 to 26.3)	33.8 (30.5 to 37.2)	32.7 (27.1 to 39)
Uzbekistan	Ovarian cancer	3.18 (2.25 to 4.43)	7.69 (6.89 to 8.56)	10.7 (8.82 to 12.8)	24 (16.9 to 33.9)	37.1 (33.5 to 41.1)	38.5 (32 to 45.6)
Uzbekistan	Prostate cancer	4.59 (2.76 to 5.06)	6.4 (4.43 to 7.63)	9.75 (6.73 to 12)	42.4 (25 to 47)	46.6 (31.1 to 54.4)	58.7 (38.9 to 71.4)
Uzbekistan	Testicular cancer	1.11 (0.735 to 1.51)	1.83 (1.55 to 2.15)	1.69 (1.39 to 2.07)	6.4 (4.3 to 8.7)	7.2 (6.1 to 8.4)	5.2 (4.3 to 6.3)
Uzbekistan	Kidney cancer	4.81 (3.75 to 5.83)	8.3 (7.65 to 9.02)	12 (10.6 to 13.6)	29.2 (21.6 to 37.4)	40.1 (37 to 44)	43.4 (38.3 to 49)
Uzbekistan	Bladder cancer	3.45 (2.73 to 4.22)	6.13 (5.65 to 6.64)	7.62 (6.65 to 8.72)	28.6 (22.5 to 35.4)	36 (33.3 to 38.9)	33.2 (29.3 to 37.6)
Uzbekistan	Thyroid cancer	0.456 (0.401 to 0.608)	0.88 (0.797 to 0.964)	1.21 (1.04 to 1.38)	3.5 (3.1 to 4.9)	4.8 (4.4 to 5.2)	4.9 (4.3 to 5.6)
Uzbekistan	Mesothelioma	0.482 (0.393 to 0.621)	0.701 (0.647 to 0.754)	0.773 (0.675 to 0.876)	3.2 (2.6 to 4.2)	3.1 (2.9 to 3.3)	2.7 (2.4 to 3.1)
Uzbekistan	Hodgkin lymphoma	4.54 (3.65 to 5.95)	5.47 (4.7 to 7.48)	5.85 (4.9 to 7.47)	23.8 (18.8 to 34.1)	21 (17.9 to 29.7)	18.6 (15.6 to 23.8)
Uzbekistan	Non-Hodgkin's lymphoma	13.4 (11.9 to 15.2)	20.6 (19 to 22.3)	22.8 (20.3 to 25.5)	69.2 (60.2 to 77.9)	81.4 (75.5 to 87.5)	75.7 (67.5 to 84.4)
Uzbekistan	Multiple myeloma	1.32 (0.944 to 1.63)	2.37 (2.1 to 2.89)	3.43 (2.9 to 4.25)	10.6 (7.5 to 13.1)	13.2 (11.6 to 15.7)	13.9 (11.7 to 16.6)
Uzbekistan	Leukaemia	42.4 (39.4 to 45.8)	49.2 (45.6 to 53.5)	45.5 (40.3 to 51.6)	192.7 (181 to 204.4)	178.1 (165.8 to 192.4)	146.1 (129.9 to 164.9)
Uzbekistan	Acute lymphoid leukaemia	10.1 (8.34 to 12.4)	13.2 (11 to 16)	13.4 (11.2 to 16.4)	40.5 (34 to 48.3)	44.7 (37.5 to 53.9)	41.2 (34.5 to 50.2)
Uzbekistan	Chronic lymphoid leukaemia	1.08 (0.904 to 1.22)	1.53 (1.25 to 1.79)	1.91 (1.57 to 2.28)	8.1 (6.7 to 9.1)	7.8 (6.4 to 9.2)	7.6 (6.3 to 9.1)
Uzbekistan	Acute myeloid leukaemia	8.78 (7 to 11.3)	12.9 (10.6 to 15.6)	14.5 (12.2 to 17)	42.4 (35.6 to 52.4)	47.2 (39.2 to 56.6)	45.6 (38.4 to 53.2)
Uzbekistan	Chronic myeloid leukaemia	1.67 (1.27 to 2.04)	2.26 (1.79 to 2.77)	2.13 (1.68 to 2.61)	10.8 (8.2 to 13.3)	9.9 (7.9 to 12)	7.3 (5.8 to 8.9)
Uzbekistan	Other leukaemia	20.8 (17.6 to 24.4)	19.3 (16.5 to 22.5)	13.6 (11.2 to 16.4)	91 (79.6 to 104.2)	68.4 (59.3 to 79.1)	44.3 (36.8 to 53.2)
Uzbekistan	Other malignant cancers	20.3 (16.6 to 23.5)	30.1 (26.8 to 32.6)	36.6 (31.7 to 41.3)	124.1 (95.7 to 144.8)	131.8 (118.9 to 142.1)	129.4 (112.9 to 145.4)
Uzbekistan	Other neoplasms	1.26 (0.819 to 2.14)	2.17 (1.61 to 3.04)	3.22 (2.29 to 4.53)	7.5 (5.1 to 11.3)	10.3 (7.4 to 13.7)	12.4 (8.4 to 16.6)
Uzbekistan	Myelodysplastic, myeloproliferative, and other hematopoietic neoplasms	0.916 (0.609 to 1.47)	1.59 (1.18 to 2.22)	2.45 (1.75 to 3.42)	5.9 (4.1 to 8.8)	8.1 (5.8 to 10.9)	9.9 (6.8 to 13.2)
Uzbekistan	Benign and in situ intestinal neoplasms	0 (0 to 0)	0 (0 to 0)	0 (0 to 0)	0 (0 to 0)	0 (0 to 0)	0 (0 to 0)
Uzbekistan	Benign and in situ cervical and uterine neoplasms	0 (0 to 0)	0 (0 to 0)	0 (0 to 0)	0 (0 to 0)	0 (0 to 0)	0 (0 to 0)
Uzbekistan	Other benign and in situ neoplasms	0.344 (0.2 to 0.664)	0.571 (0.404 to 0.8)	0.769 (0.543 to 1.12)	1.6 (0.9 to 2.5)	2.2 (1.4 to 3)	2.5 (1.6 to 3.4)
Uzbekistan	Cardiovascular diseases	1050 (1020 to 1080)	2140 (2040 to 2230)	2490 (2260 to 2730)	8837.5 (8599.4 to 9078.2)	13656.9 (13079.2 to 14246.4)	12113.3 (11057.8 to 13174.8)
Uzbekistan	Rheumatic heart disease	64 (60.6 to 67.9)	77.1 (71.1 to 83.3)	68.6 (60.5 to 77.6)	383.7 (364.2 to 405.9)	307.4 (285.4 to 331.4)	221 (195.6 to 248.8)
Uzbekistan	Ischaemic heart disease	596 (577 to 615)	1400 (1330 to 1460)	1660 (1500 to 1830)	5221.4 (5049.1 to 5391)	9131.3 (8737.5 to 9556.4)	8346.1 (7591.9 to 9115.7)
Uzbekistan	Stroke	327 (312 to 341)	553 (525 to 582)	602 (543 to 661)	2758.3 (2634.8 to 2880)	3577.5 (3404.8 to 3752.2)	2828.4 (2559.8 to 3083.7)
Uzbekistan	Ischaemic stroke	125 (114 to 136)	192 (178 to 206)	190 (169 to 210)	1081 (987.8 to 1172.4)	1309.3 (1221.4 to 1395.9)	973.9 (872.1 to 1075.4)
Uzbekistan	Intracerebral hemorrhage	178 (168 to 187)	325 (308 to 343)	367 (329 to 406)	1493.3 (1411.3 to 1570.9)	2075.4 (1969.3 to 2185.8)	1679 (1517.8 to 1850.2)

SR Appendix Table 2. All-age DALYs counts and age standardized DALYs rates per 100,000 individuals for all causes and level 1 cause groups by location for 1990, 2007, and 2017, both sexes combined

		All-age DALYs			Age-standardized rate of DALYs per 100,000		
Uzbekistan	Subarachnoid hemorrhage	23.8 (17.4 to 29.5)	35.6 (26.4 to 39)	45.4 (34.1 to 52)	184 (128.4 to 232.5)	192.8 (140.3 to 211.1)	175.5 (132.4 to 199.8)
Uzbekistan	Hypertensive heart disease	24.6 (19.7 to 34.3)	48.4 (36.3 to 59.2)	65.2 (50.6 to 80.4)	213.7 (170.5 to 296.1)	314.3 (236.5 to 385.8)	330.1 (252.7 to 402.2)
Uzbekistan	Non-rheumatic valvular heart disease	1.78 (1.35 to 2.27)	3.45 (3.09 to 4.08)	6.53 (4.8 to 7.61)	14 (10.4 to 17.6)	19.6 (17.3 to 22.7)	29.2 (20.6 to 34)
Uzbekistan	Non-rheumatic calcific aortic valve disease	0.652 (0.492 to 0.853)	1.58 (1.37 to 1.92)	3.7 (2.44 to 4.37)	5.5 (4.1 to 7.1)	9.6 (8.2 to 11.6)	17.3 (11.2 to 20.4)
Uzbekistan	Non-rheumatic degenerative mitral valve disease	0.729 (0.537 to 0.96)	1.12 (0.934 to 1.45)	1.69 (1.3 to 2.05)	5.8 (4.1 to 7.6)	6.3 (5.2 to 7.9)	7.3 (5.7 to 9)
Uzbekistan	Other non-rheumatic valve diseases	0.396 (0.234 to 0.558)	0.752 (0.506 to 0.97)	1.15 (0.745 to 1.51)	2.7 (1.6 to 3.9)	3.6 (2.4 to 4.7)	4.5 (2.8 to 6)
Uzbekistan	Cardiomyopathy and myocarditis	6.43 (5.08 to 8.47)	14.4 (13.1 to 17.8)	22.5 (19.6 to 27.3)	36.1 (27.5 to 52.3)	56.2 (50.8 to 73.6)	71.3 (61.6 to 88.7)
Uzbekistan	Myocarditis	3.56 (2.69 to 4.27)	7.47 (6.6 to 8.82)	10.1 (8.38 to 13.1)	19.1 (14.1 to 24.5)	28.4 (25 to 35.1)	31.5 (26.1 to 41.7)
Uzbekistan	Alcoholic cardiomyopathy	0.214 (0.125 to 0.374)	0.546 (0.436 to 0.899)	1.05 (0.779 to 1.7)	1.4 (0.8 to 2.7)	2.2 (1.8 to 3.8)	3.3 (2.5 to 5.4)
Uzbekistan	Other cardiomyopathy	2.66 (1.91 to 4.07)	6.41 (5.6 to 8.6)	11.4 (9.51 to 13.6)	15.6 (10.3 to 26)	25.7 (22.3 to 37)	36.5 (30.9 to 44.4)
Uzbekistan	Atrial fibrillation and flutter	7.59 (6.07 to 9.38)	11 (9.11 to 13.3)	16.5 (13.8 to 19.7)	71.8 (57.3 to 88.3)	84.8 (71.9 to 100.4)	101.3 (87.3 to 117.8)
Uzbekistan	Aortic aneurysm	1.34 (1.06 to 1.61)	3.02 (2.76 to 3.28)	4.96 (4.35 to 5.64)	11 (8.6 to 13.5)	18.2 (16.7 to 19.8)	22.8 (20.1 to 26)
Uzbekistan	Peripheral vascular disease	0.693 (0.367 to 1.15)	0.96 (0.54 to 1.53)	1.38 (0.812 to 2.16)	6.5 (3.4 to 10.8)	7.1 (3.9 to 11.4)	7.8 (4.6 to 12)
Uzbekistan	Endocarditis	1.82 (1.44 to 2.37)	3.41 (2.9 to 4.66)	4.96 (4.15 to 6.54)	8.7 (6.2 to 12.6)	13.8 (11.7 to 19.3)	17.1 (14.2 to 22.7)
Uzbekistan	Other cardiovascular and circulatory diseases	17 (14 to 20.2)	27 (24 to 30.4)	37.2 (32.5 to 42.2)	112.2 (90.7 to 135.5)	126.6 (113.4 to 141.9)	138.3 (121.4 to 156.2)
Uzbekistan	Chronic respiratory diseases	193 (176 to 210)	198 (178 to 220)	206 (180 to 234)	1458.1 (1349.7 to 1568.1)	1038.4 (950.1 to 1136.5)	835.7 (743.6 to 933.4)
Uzbekistan	Chronic obstructive pulmonary disease	99.1 (90.8 to 107)	103 (95 to 111)	104 (92.8 to 115)	812.2 (745.3 to 876.5)	594.6 (550.1 to 635.6)	464.6 (417.8 to 511.5)
Uzbekistan	Pneumoconiosis	0.263 (0.216 to 0.31)	0.269 (0.225 to 0.307)	0.363 (0.282 to 0.438)	2.1 (1.7 to 2.5)	1.5 (1.2 to 1.7)	1.5 (1.2 to 1.8)
Uzbekistan	Silicosis	0.0267 (0.0132 to 0.0462)	0.0201 (0.0116 to 0.0284)	0.0134 (0.00863 to 0.0189)	0.2 (0.1 to 0.4)	0.1 (0.1 to 0.2)	0.1 (0 to 0.1)
Uzbekistan	Asbestosis	0.0204 (0.0127 to 0.0286)	0.0218 (0.0133 to 0.0307)	0.0259 (0.0167 to 0.0383)	0.2 (0.1 to 0.2)	0.1 (0.1 to 0.2)	0.1 (0.1 to 0.1)
Uzbekistan	Coal workers pneumoconiosis	0.0165 (0.0107 to 0.024)	0.021 (0.0139 to 0.031)	0.0291 (0.0174 to 0.0448)	0.1 (0.1 to 0.2)	0.1 (0.1 to 0.2)	0.1 (0.1 to 0.2)
Uzbekistan	Other pneumoconiosis	0.199 (0.163 to 0.246)	0.206 (0.174 to 0.239)	0.294 (0.225 to 0.358)	1.6 (1.3 to 2)	1.1 (1 to 1.3)	1.2 (0.9 to 1.5)
Uzbekistan	Asthma	68.1 (55.7 to 82.4)	67.2 (53.7 to 83.2)	69.6 (53.4 to 89.4)	463.1 (392.8 to 543.8)	316 (263.4 to 379.7)	252.5 (198.5 to 317.7)
Uzbekistan	Interstitial lung disease and pulmonary sarcoidosis	15.4 (10.6 to 19.4)	15.5 (12.2 to 18.4)	19.4 (15.5 to 23)	121.1 (85.6 to 151.9)	79.5 (65.6 to 93.2)	78.7 (64.6 to 92.8)
Uzbekistan	Other chronic respiratory diseases	9.63 (8.66 to 10.9)	11.8 (10.5 to 13.1)	12 (10.4 to 13.7)	59.4 (53.2 to 67.8)	46.9 (41.8 to 51.8)	38.3 (33.3 to 43.4)
Uzbekistan	Digestive diseases	245 (228 to 265)	466 (435 to 500)	537 (476 to 600)	1565.2 (1471.3 to 1682.8)	2093.9 (1959.7 to 2232.2)	1841.1 (1636.5 to 2048.1)
Uzbekistan	Cirrhosis and other chronic liver diseases	146 (141 to 150)	343 (324 to 364)	397 (352 to 445)	1010.8 (979.2 to 1043.8)	1566.2 (1480.4 to 1656.8)	1362.3 (1209.8 to 1520.6)
Uzbekistan	Cirrhosis and other chronic liver diseases due to hepatitis B	34.6 (30.5 to 38.8)	85.9 (75.4 to 96.8)	90.8 (76.3 to 107)	247.6 (219 to 279.5)	386.6 (340.3 to 438)	305.6 (258 to 358.5)
Uzbekistan	Cirrhosis and other chronic liver diseases due to hepatitis C	30.2 (26.5 to 34.5)	74.7 (64.5 to 86.1)	91.9 (76.5 to 109)	226.4 (197.4 to 259.4)	351.9 (307.2 to 402.2)	317 (265.5 to 376.4)
Uzbekistan	Cirrhosis and other chronic liver diseases due to alcohol use	43.1 (38.6 to 47.9)	106 (94.1 to 117)	134 (115 to 156)	332.1 (297.7 to 368.8)	514.6 (459 to 571)	468.4 (403 to 539.5)
Uzbekistan	Cirrhosis due to NASH	8.59 (7.6 to 9.54)	22 (19.4 to 24.8)	30.6 (25.6 to 36)	65.6 (58.1 to 73)	107.9 (95.6 to 121)	107.5 (91.3 to 125.5)
Uzbekistan	Cirrhosis and other chronic liver diseases due to other causes	29.1 (26.3 to 32.1)	54.9 (48.5 to 61.7)	49.8 (42 to 58.3)	139.1 (125.2 to 155.1)	205.1 (180 to 231.7)	163.8 (139.4 to 191)
Uzbekistan	Upper digestive system diseases	38.1 (29.7 to 50.6)	60.3 (46.7 to 79.9)	67.5 (50.9 to 92.4)	249.6 (197 to 326.8)	265.3 (209.5 to 345.1)	229.9 (174.9 to 310.1)
Uzbekistan	Peptic ulcer disease	18.6 (17.4 to 19.8)	31.2 (28.6 to 33.7)	30.8 (26.5 to 35.6)	131.7 (123.7 to 140.6)	146.9 (135.5 to 158.1)	110.8 (96.1 to 128.1)
Uzbekistan	Gastritis and duodenitis	5.07 (3.29 to 7.38)	6.93 (4.47 to 10.1)	8.45 (5.46 to 12.4)	26.1 (17.1 to 37.5)	26.4 (17.2 to 38.4)	27.1 (17.6 to 39.8)
Uzbekistan	GERD	14.4 (7.7 to 24.9)	22.2 (11.7 to 37.7)	28.3 (15.1 to 48.4)	91.9 (48.9 to 156.5)	92 (48.8 to 156.3)	92 (49.1 to 157.6)
Uzbekistan	Appendicitis	5.15 (4.61 to 5.8)	3.33 (2.71 to 4.01)	3.22 (2.61 to 4.01)	24.8 (22.4 to 27.5)	11.7 (9.7 to 13.9)	10 (8.1 to 12.4)
Uzbekistan	Paralytic ileus and intestinal obstruction	16.4 (14.7 to 18.4)	11.7 (9.83 to 14.2)	12 (9.6 to 15.1)	60.1 (54.5 to 67.8)	45.2 (38.4 to 54.2)	41.4 (33 to 50.9)
Uzbekistan	Inguinal, femoral, and abdominal hernia	19.8 (13.6 to 27.1)	24.4 (16.7 to 33)	28.3 (19.6 to 38.9)	101.1 (70.8 to 136.9)	98.4 (68.2 to 132.8)	93.7 (65 to 129)
Uzbekistan	Inflammatory bowel disease	3.55 (2.05 to 4.7)	3.47 (2.65 to 4.11)	4.19 (3.18 to 5.13)	17.5 (12.6 to 21.3)	14 (11.4 to 16.3)	13.6 (10.7 to 16.4)

SR Appendix Table 2. All-age DALYs counts and age standardized DALYs rates per 100,000 individuals for all causes and level 1 cause groups by location for 1990, 2007, and 2017, both sexes combined

		All-age DALYs			Age-standardized rate of DALYs per 100,000		
Uzbekistan	Vascular intestinal disorders	1.04 (0.871 to 1.21)	1.35 (1.22 to 1.68)	1.75 (1.45 to 2.06)	8.2 (6.4 to 10)	8.1 (7.3 to 10.5)	8.4 (6.9 to 10.1)
Uzbekistan	Gallbladder and biliary diseases	3.39 (2.62 to 3.66)	3.64 (3.34 to 4.12)	4.25 (3.66 to 5.03)	26.3 (20.2 to 28.4)	20.3 (18.6 to 23.1)	18 (15.5 to 21.1)
Uzbekistan	Pancreatitis	5 (4.24 to 6.02)	8.57 (7.55 to 9.72)	10.9 (9.22 to 12.8)	34.4 (28.7 to 41.4)	38 (33.6 to 43.4)	37 (31.4 to 43.3)
Uzbekistan	Other digestive diseases	6.79 (5.54 to 8.24)	6.33 (5.38 to 7.35)	7.75 (6.44 to 9.19)	32.6 (27.6 to 38.4)	26.6 (22.8 to 30.6)	26.8 (22.7 to 31.6)
Uzbekistan	Neurological disorders	247 (179 to 332)	382 (292 to 491)	448 (332 to 583)	1489.8 (1108.3 to 1959)	1663.9 (1324.6 to 2070.6)	1684.8 (1309.7 to 2113)
Uzbekistan	Alzheimer's disease and other dementias	35.1 (25 to 42.1)	42.5 (39.5 to 45.6)	57.8 (52.2 to 63.8)	357.1 (254 to 429)	409.3 (379.7 to 439.5)	453.6 (409 to 500.2)
Uzbekistan	Parkinson's disease	5.7 (4.26 to 8.19)	9.81 (7.78 to 11)	12.5 (9.97 to 14.3)	55.7 (41.5 to 80.4)	76.5 (61.4 to 85.2)	80.6 (64.2 to 92.3)
Uzbekistan	Epilepsy	63.6 (39.3 to 96.3)	113 (81.7 to 154)	117 (75.5 to 173)	298 (182.9 to 453.4)	390.5 (280.8 to 534.5)	351.6 (225.1 to 529.2)
Uzbekistan	Multiple sclerosis	2.01 (1.5 to 2.5)	2.51 (1.94 to 3.16)	3.43 (2.63 to 4.4)	14.4 (10.7 to 17.8)	10.8 (8.4 to 13.6)	11.2 (8.7 to 14.3)
Uzbekistan	Motor neuron disease	0.168 (0.138 to 0.221)	0.295 (0.263 to 0.33)	0.377 (0.328 to 0.432)	1.1 (0.9 to 1.6)	1.4 (1.3 to 1.6)	1.3 (1.1 to 1.5)
Uzbekistan	Headache disorders	130 (84.6 to 186)	197 (129 to 282)	236 (152 to 341)	712.7 (464.2 to 1024.4)	714.1 (467.1 to 1025.2)	714.5 (462.4 to 1029.3)
Uzbekistan	Migraine	113 (70.4 to 168)	172 (108 to 255)	205 (128 to 305)	618.2 (390.8 to 918.7)	619.6 (391.3 to 921.8)	619.9 (388.1 to 920.7)
Uzbekistan	Tension-type headache	17 (9.48 to 27.4)	25.4 (13.9 to 40.8)	30.4 (17.1 to 48.4)	94.5 (52.7 to 150.4)	94.5 (52.8 to 150)	94.6 (53.4 to 150.8)
Uzbekistan	Other neurological disorders	11 (6.81 to 16.8)	16.3 (10.8 to 23.9)	21.3 (12.9 to 33.2)	50.9 (32.3 to 76.1)	61.2 (43.1 to 86.6)	71.9 (46.7 to 107.4)
Uzbekistan	Mental disorders	243 (179 to 316)	352 (259 to 458)	414 (307 to 538)	1316.3 (975.2 to 1702.1)	1302.1 (966.2 to 1681.1)	1297.3 (965.9 to 1684.4)
Uzbekistan	Schizophrenia	18.2 (13.4 to 23)	28.9 (21.6 to 36.3)	37.9 (28.1 to 47.6)	112.6 (82.7 to 140.8)	113 (84.6 to 140.2)	115.4 (86.1 to 144.4)
Uzbekistan	Depressive disorders	78 (55.1 to 107)	115 (80.8 to 157)	137 (96.2 to 189)	467.6 (329 to 637.3)	454.9 (323.3 to 622.5)	447.9 (315.2 to 613.2)
Uzbekistan	Major depressive disorder	60.7 (42.2 to 83.5)	88.3 (60.9 to 120)	104 (71.8 to 141)	360.2 (251.3 to 492)	347.6 (242.7 to 470)	340.7 (236.9 to 461.1)
Uzbekistan	Dysthymia	17.3 (11.4 to 25.3)	26.2 (17.3 to 38.5)	33 (21.8 to 48.4)	107.4 (71.5 to 154.2)	107.3 (71.1 to 157.1)	107.2 (71.2 to 156.6)
Uzbekistan	Bipolar disorder	23.9 (14.9 to 35.9)	36.5 (22.6 to 54.8)	43.3 (27 to 65)	130.9 (82.1 to 195.9)	131.1 (81.3 to 196.8)	131.3 (82.1 to 195.2)
Uzbekistan	Anxiety disorders	43.9 (30.5 to 59.2)	64.2 (45.1 to 85.9)	75.3 (53 to 101)	234.6 (166.5 to 316.1)	234.2 (165.3 to 312.3)	234.5 (164.7 to 312.9)
Uzbekistan	Eating disorders	5.72 (3.64 to 8.54)	8.42 (5.34 to 12.6)	11.3 (7.22 to 16.6)	27.1 (17.5 to 39.9)	27 (17 to 39.9)	31.6 (20.2 to 46.2)
Uzbekistan	Anorexia nervosa	1.25 (0.718 to 1.89)	1.82 (1.05 to 2.78)	2.26 (1.31 to 3.48)	5.6 (3.2 to 8.4)	5.6 (3.2 to 8.5)	6.4 (3.7 to 9.9)
Uzbekistan	Bulimia nervosa	4.47 (2.77 to 6.72)	6.6 (4.08 to 9.97)	9.01 (5.66 to 13.5)	21.6 (13.5 to 32.1)	21.4 (13.3 to 32.5)	25.2 (15.9 to 37.6)
Uzbekistan	Autism spectrum disorders	14 (9.55 to 19.5)	18.3 (12.4 to 25.5)	21.1 (14.4 to 29.4)	63.6 (43 to 88.3)	64 (43.5 to 89.2)	64.2 (43.8 to 89.5)
Uzbekistan	Attention-deficit/hyperactivity disorder	2.73 (1.65 to 4.42)	3.68 (2.23 to 5.81)	3.98 (2.36 to 6.34)	11.6 (7.1 to 18.7)	11.7 (7.1 to 18.4)	11.7 (6.9 to 18.6)
Uzbekistan	Conduct disorder	25.6 (15.2 to 40.7)	33.6 (19.9 to 53.5)	31.7 (19.2 to 50.5)	96.7 (57.5 to 153.4)	97.2 (57.7 to 155.5)	97.6 (59.4 to 155.6)
Uzbekistan	Idiopathic developmental intellectual disability	7.51 (2.74 to 13.8)	8.75 (3.2 to 16.5)	7.89 (2.44 to 14.8)	32.8 (11.8 to 60.8)	29.8 (10.7 to 56.3)	23.6 (7.2 to 44.4)
Uzbekistan	Other mental disorders	23 (15.2 to 32.1)	35.5 (22.8 to 49.8)	44.7 (29.6 to 62.4)	138.8 (92.4 to 191.8)	139.1 (90.3 to 195.5)	139.3 (92.8 to 193.4)
Uzbekistan	Substance use disorders	81.4 (64.2 to 102)	115 (88.7 to 144)	128 (97.1 to 161)	452.4 (360.1 to 559)	419.1 (325.5 to 521.9)	376.2 (287.1 to 473.6)
Uzbekistan	Alcohol use disorders	61.5 (49.1 to 77.4)	75.3 (56.2 to 97.3)	75.2 (54.7 to 98.8)	349.1 (281.3 to 435.5)	276.3 (207.9 to 354.5)	221.8 (162.2 to 290.7)
Uzbekistan	Drug use disorders	19.9 (14.4 to 25.9)	40 (31.3 to 49.5)	52.8 (41.1 to 64.8)	103.3 (75.6 to 133.2)	142.8 (112.9 to 174.3)	154.4 (121.1 to 188.5)
Uzbekistan	Opioid use disorders	13.2 (9.46 to 17.6)	28.4 (22.1 to 35.3)	38.4 (29.3 to 48.2)	71.2 (51.8 to 93.4)	104.2 (81.4 to 128.3)	112.9 (86.8 to 139.9)
Uzbekistan	Cocaine use disorders	0.707 (0.438 to 1.07)	1.39 (0.975 to 1.93)	1.94 (1.41 to 2.64)	3.7 (2.4 to 5.4)	4.9 (3.6 to 6.7)	5.7 (4.2 to 7.7)
Uzbekistan	Amphetamine use disorders	2.38 (1.3 to 3.96)	3.66 (2.16 to 5.66)	4.4 (2.53 to 6.91)	10.8 (6.1 to 17.6)	11.6 (7 to 18)	12.2 (7.1 to 19.1)
Uzbekistan	Cannabis use disorders	0.99 (0.598 to 1.48)	1.47 (0.885 to 2.25)	1.62 (1 to 2.45)	4.6 (2.8 to 6.9)	4.6 (2.8 to 7)	4.6 (2.9 to 7)
Uzbekistan	Other drug use disorders	2.63 (1.68 to 3.83)	5.06 (3.79 to 6.79)	6.49 (4.77 to 8.58)	13 (8.7 to 18.6)	17.5 (13.4 to 23.1)	18.9 (14.1 to 24.7)
Uzbekistan	Diabetes and kidney diseases	169 (142 to 202)	416 (363 to 475)	534 (452 to 631)	1199.6 (997.9 to 1443.3)	2102.3 (1842.7 to 2389.8)	2008.6 (1706.5 to 2362.2)
Uzbekistan	Diabetes mellitus	105 (81.5 to 133)	260 (216 to 314)	379 (307 to 459)	806.1 (624.2 to 1022.8)	1382.6 (1160.6 to 1646.3)	1451.7 (1186.1 to 1759.1)
Uzbekistan	Diabetes mellitus type 1	21.4 (19.5 to 23.2)	56.9 (48.8 to 63.1)	59.4 (47.5 to 70.2)	151.7 (138.4 to 165.7)	281.3 (234.4 to 317.4)	213.3 (167.5 to 253.9)

SR Appendix Table 2. All-age DALYs counts and age standardized DALYs rates per 100,000 individuals for all causes and level 1 cause groups by location for 1990, 2007, and 2017, both sexes combined

		All-age DALYs			Age-standardized rate of DALYs per 100,000		
Uzbekistan	Diabetes mellitus type 2	83.7 (61.1 to 112)	204 (159 to 257)	319 (251 to 400)	654.4 (481.9 to 863.3)	1101.2 (879.6 to 1372.6)	1238.4 (979.6 to 1547.9)
Uzbekistan	Chronic kidney disease	58.5 (54 to 63.6)	150 (140 to 160)	152 (136 to 168)	363.9 (334.2 to 397.9)	697.3 (652.3 to 741.7)	545.1 (488.4 to 603.5)
Uzbekistan	Chronic kidney disease due to diabetes mellitus type 1	4.47 (3.57 to 5.48)	14.4 (11.5 to 17.7)	15.2 (12 to 19.1)	30.5 (24.1 to 37.4)	60.5 (48.9 to 74.4)	48.1 (38 to 59.6)
Uzbekistan	Chronic kidney disease due to diabetes mellitus type 2	6.24 (5.25 to 7.38)	20.7 (17.5 to 24.2)	22.1 (18.3 to 26.4)	50.8 (43.1 to 59.9)	124.7 (105.5 to 145.4)	96.1 (80.5 to 113.9)
Uzbekistan	Chronic kidney disease due to hypertension	4.56 (3.81 to 5.36)	12.7 (10.7 to 14.8)	13.1 (10.8 to 15.7)	34 (28.4 to 39.8)	70.7 (59.5 to 82.3)	55.4 (46.2 to 65.6)
Uzbekistan	Chronic kidney disease due to glomerulonephritis	17.6 (15.5 to 20)	46.1 (40.3 to 52.1)	45.5 (38.4 to 53.8)	99.8 (88.4 to 112.4)	191.5 (167.9 to 216)	147.7 (126.4 to 173)
Uzbekistan	Chronic kidney disease due to other and unspecified causes	25.6 (22.4 to 28.8)	56.1 (49.4 to 63.5)	55.6 (47.8 to 64.6)	148.8 (129 to 171.2)	249.9 (220.9 to 282.8)	197.9 (171.4 to 229.4)
Uzbekistan	Acute glomerulonephritis	5.16 (3.94 to 6.47)	5.57 (4.91 to 6.27)	3.65 (3.04 to 4.39)	29.6 (22.3 to 37.2)	22.4 (19.8 to 25.1)	11.7 (9.8 to 14.1)
Uzbekistan	Skin and subcutaneous diseases	94.8 (61.9 to 140)	122 (80.8 to 179)	143 (94.9 to 210)	435.3 (287.3 to 645.6)	443.3 (295.4 to 649.9)	450.2 (298.9 to 664.6)
Uzbekistan	Dermatitis	25.1 (14.5 to 40.7)	30.6 (18.1 to 49)	35 (20.2 to 55.7)	108 (63.6 to 171.6)	108.1 (64.5 to 172.9)	108.3 (62.8 to 172.2)
Uzbekistan	Atopic dermatitis	20.9 (11.2 to 35.6)	24.4 (13 to 41.4)	27.7 (14.7 to 47.1)	84.9 (45.4 to 143.5)	85 (45.9 to 144)	85.1 (45.2 to 144.5)
Uzbekistan	Contact dermatitis	4.18 (2.67 to 6.28)	6.14 (3.98 to 9.25)	7.25 (4.67 to 10.8)	23 (14.8 to 33.8)	23 (15.2 to 34.1)	23 (15 to 33.9)
Uzbekistan	Seborrhoeic dermatitis	0.0219 (0.0123 to 0.0353)	0.0287 (0.016 to 0.0463)	0.0339 (0.0189 to 0.0543)	0.1 (0.1 to 0.2)	0.1 (0.1 to 0.2)	0.1 (0.1 to 0.2)
Uzbekistan	Psoriasis	9.48 (6.7 to 12.6)	14.7 (10.3 to 19.7)	18.7 (13.2 to 24.7)	55.1 (39.1 to 72.7)	58 (41 to 77)	59.8 (42.2 to 78.6)
Uzbekistan	Bacterial skin diseases	0.57 (0.425 to 0.845)	1.07 (0.641 to 1.4)	1.58 (0.827 to 2.08)	2.8 (2 to 4.4)	4.4 (2.7 to 5.8)	5.5 (2.9 to 7.2)
Uzbekistan	Cellulitis	0.285 (0.202 to 0.411)	0.498 (0.326 to 0.649)	0.782 (0.427 to 1.06)	1.5 (1 to 2.2)	2 (1.3 to 2.6)	2.6 (1.4 to 3.5)
Uzbekistan	Pyoderma	0.286 (0.193 to 0.489)	0.569 (0.276 to 0.79)	0.796 (0.349 to 1.09)	1.4 (0.9 to 2.5)	2.5 (1.2 to 3.3)	2.9 (1.3 to 3.9)
Uzbekistan	Scabies	6.65 (3.65 to 11)	8.43 (4.67 to 13.8)	9.21 (5.08 to 15)	28.8 (15.8 to 47)	28.2 (15.4 to 45.7)	27.8 (15.4 to 45.1)
Uzbekistan	Fungal skin diseases	9.05 (3.58 to 18.8)	11.3 (4.51 to 23.7)	13.2 (5.2 to 27.4)	49.2 (19.7 to 102.7)	47.9 (19.2 to 99.2)	46.7 (18.7 to 97)
Uzbekistan	Viral skin diseases	12.4 (7.92 to 18.5)	13.7 (8.82 to 20.5)	15.2 (9.64 to 22.8)	45.6 (29.2 to 68.3)	45.7 (29.3 to 68.1)	45.7 (29 to 68.6)
Uzbekistan	Acne vulgaris	5.52 (3.17 to 9.1)	9.69 (5.47 to 16.6)	10.6 (6.18 to 17.4)	23.5 (13.7 to 38.4)	28.3 (16.1 to 48.3)	31 (17.9 to 51)
Uzbekistan	Alopecia areata	1.12 (0.713 to 1.69)	1.64 (1.05 to 2.48)	1.99 (1.24 to 3.01)	5.9 (3.7 to 8.8)	5.9 (3.8 to 8.9)	5.9 (3.7 to 8.9)
Uzbekistan	Pruritus	1.64 (0.791 to 3.18)	2.28 (1.08 to 4.36)	2.84 (1.36 to 5.48)	9.2 (4.3 to 17.4)	9.1 (4.3 to 17.2)	9.3 (4.5 to 17.8)
Uzbekistan	Urticaria	17.8 (11.6 to 25.5)	21.4 (14.1 to 30.4)	24.5 (16.1 to 34.7)	74.3 (49.2 to 106.1)	74.4 (49.2 to 105.3)	74.5 (48.9 to 105.1)
Uzbekistan	Decubitus ulcer	0.319 (0.228 to 0.43)	0.465 (0.332 to 0.616)	0.619 (0.434 to 0.82)	2.2 (1.6 to 3)	2.4 (1.7 to 3.2)	2.6 (1.8 to 3.4)
Uzbekistan	Other skin and subcutaneous diseases	5.2 (2.5 to 9.55)	7.19 (3.48 to 13.2)	9.59 (4.61 to 17.5)	30.7 (14.9 to 56.4)	30.7 (14.9 to 55.9)	33.1 (16 to 60.4)
Uzbekistan	Sense organ diseases	124 (82.3 to 181)	168 (112 to 248)	211 (140 to 314)	887.1 (596.4 to 1289.1)	870.8 (583.5 to 1268.1)	853.5 (570.4 to 1247.8)
Uzbekistan	Blindness and vision impairment	59.6 (37.9 to 92.3)	80.2 (51.3 to 123)	99 (63.5 to 151)	424.5 (272.9 to 639.2)	411.2 (264.1 to 618.7)	396.3 (256.9 to 599.6)
Uzbekistan	Glaucoma	0.398 (0.266 to 0.569)	0.471 (0.308 to 0.67)	0.597 (0.385 to 0.874)	3.6 (2.4 to 5.2)	3.4 (2.2 to 4.8)	3.2 (2.1 to 4.6)
Uzbekistan	Cataract	16.6 (11.2 to 23)	21.7 (14.6 to 30.1)	28.1 (18.9 to 39.4)	152.1 (102.9 to 209.7)	149.9 (100.7 to 206.9)	145.4 (98.4 to 200.8)
Uzbekistan	Age-related macular degeneration	0.119 (0.074 to 0.177)	0.155 (0.0948 to 0.236)	0.216 (0.135 to 0.327)	1.1 (0.7 to 1.6)	1.1 (0.7 to 1.7)	1.1 (0.7 to 1.7)
Uzbekistan	Refraction disorders	22.5 (14 to 34.6)	30.1 (19 to 46.2)	34.7 (21.8 to 53.1)	118.9 (76.4 to 182.3)	117.4 (73.9 to 180.3)	113.2 (70.1 to 173.1)
Uzbekistan	Near vision loss	16.9 (8.09 to 30.6)	23.6 (11.3 to 43.3)	30.1 (14.3 to 54.5)	127.8 (61.3 to 231.2)	118.7 (56.7 to 218.5)	113.1 (53.7 to 205)
Uzbekistan	Other vision loss	2.98 (1.94 to 4.23)	4.18 (2.69 to 5.95)	5.32 (3.48 to 7.54)	20.9 (13.6 to 29.3)	20.7 (13.5 to 29.4)	20.2 (13.3 to 28.5)
Uzbekistan	Age-related and other hearing loss	59.5 (41.2 to 83.7)	81.9 (55.7 to 117)	104 (70.2 to 150)	432.4 (297.6 to 608.6)	429 (296.7 to 599.3)	426.3 (290.3 to 599.3)
Uzbekistan	Other sense organ diseases	4.53 (2.85 to 6.58)	6.26 (3.87 to 9.22)	8.07 (5 to 11.8)	30.2 (19.1 to 43.6)	30.6 (19 to 44.8)	30.9 (19.1 to 45.2)
Uzbekistan	Musculoskeletal disorders	217 (158 to 292)	333 (244 to 447)	421 (308 to 567)	1492.4 (1088.4 to 2000.8)	1490.4 (1095.2 to 1985.4)	1461.6 (1072 to 1962.2)
Uzbekistan	Rheumatoid arthritis	8.44 (6.7 to 10.3)	11.5 (9.31 to 13.9)	11.3 (8.65 to 14.2)	60 (47.8 to 72.8)	54.3 (44 to 65.2)	41.1 (31.8 to 51.2)
Uzbekistan	Osteoarthritis	8.92 (4.47 to 17.6)	13.4 (6.74 to 26.5)	19.6 (9.83 to 39)	76 (38.1 to 150.4)	79.2 (39.8 to 157.7)	84.1 (42.3 to 166.4)
Uzbekistan	Low back pain	149 (106 to 200)	223 (158 to 304)	283 (201 to 383)	996.6 (709.2 to 1345)	982.2 (703.7 to 1320.3)	973.7 (700.4 to 1309.8)

SR Appendix Table 2. All-age DALYs counts and age standardized DALYs rates per 100,000 individuals for all causes and level 1 cause groups by location for 1990, 2007, and 2017, both sexes combined

		All-age DALYs			Age-standardized rate of DALYs per 100,000		
Uzbekistan	Neck pain	41.1 (28.6 to 57.7)	66.2 (46.2 to 93.3)	86.5 (60.3 to 122)	297.6 (207.8 to 417.1)	297.6 (207.4 to 416.1)	297.8 (207.5 to 416.1)
Uzbekistan	Gout	1.57 (1.04 to 2.21)	2.47 (1.63 to 3.47)	3.48 (2.3 to 4.85)	12.7 (8.4 to 17.7)	13.4 (8.9 to 18.8)	13.9 (9.4 to 19.3)
Uzbekistan	Other musculoskeletal disorders	8.37 (5.38 to 12.6)	16.4 (10.8 to 24.4)	16.8 (10.4 to 25.7)	49.6 (31.2 to 75.4)	63.6 (41.9 to 93.9)	51 (31.6 to 76.7)
Uzbekistan	Other non-communicable diseases	371 (309 to 443)	414 (332 to 511)	433 (345 to 543)	1615.2 (1298 to 1978.7)	1583.5 (1262.4 to 1967.2)	1414.6 (1122.8 to 1780.3)
Uzbekistan	Congenital anomalies	210 (175 to 244)	183 (144 to 222)	165 (129 to 205)	691.1 (580.6 to 800.9)	630.5 (498.5 to 763.6)	509.6 (400.2 to 633.5)
Uzbekistan	Neural tube defects	27.5 (21.8 to 36.5)	27.8 (20.9 to 36.4)	18.9 (13.8 to 26.2)	84.2 (67.3 to 110.9)	94.8 (71.4 to 123.9)	58.6 (42.8 to 81.3)
Uzbekistan	Congenital heart anomalies	88.9 (69.1 to 104)	74.3 (49.6 to 94.8)	65.1 (41.7 to 87.6)	270.9 (210.8 to 315)	250.9 (167.8 to 320.4)	200.1 (128.5 to 269.4)
Uzbekistan	Orofacial clefts	1.66 (1.15 to 2.33)	0.995 (0.672 to 1.43)	0.996 (0.64 to 1.47)	6.1 (4.3 to 8.4)	3.5 (2.4 to 5)	3.1 (2 to 4.5)
Uzbekistan	Down's syndrome	5.82 (3.26 to 9.27)	3.51 (2.7 to 4.36)	3.11 (2.34 to 4.16)	19.9 (11.9 to 30.3)	12.2 (9.4 to 15.2)	9.4 (7.1 to 12.6)
Uzbekistan	Turner syndrome	0.0252 (0.0121 to 0.0422)	0.0315 (0.0151 to 0.0512)	0.0353 (0.0171 to 0.0583)	0.1 (0.1 to 0.2)	0.1 (0 to 0.2)	0.1 (0 to 0.2)
Uzbekistan	Klinefelter syndrome	0.00577 (0.00268 to 0.0109)	0.00779 (0.00369 to 0.0144)	0.00899 (0.00416 to 0.0171)	0 (0 to 0)	0 (0 to 0)	0 (0 to 0)
Uzbekistan	Other chromosomal abnormalities	3.15 (2.02 to 4.68)	3.14 (2.14 to 4.22)	3.32 (2.34 to 4.54)	11.4 (7.6 to 16.7)	10.8 (7.4 to 14.6)	10.1 (7.1 to 13.8)
Uzbekistan	Congenital musculoskeletal and limb anomalies	21.9 (15.1 to 29.7)	27.5 (18.3 to 37.8)	31.6 (21.3 to 44.1)	101.7 (69 to 138.8)	100.3 (67.2 to 137.6)	99.5 (67.1 to 139.3)
Uzbekistan	Urogenital congenital anomalies	2.82 (1.62 to 6.38)	2.63 (1.61 to 4.92)	2.75 (1.73 to 4.56)	9.4 (5.7 to 20)	8.9 (5.5 to 16.6)	8.4 (5.3 to 14)
Uzbekistan	Digestive congenital anomalies	12.9 (9.27 to 16.6)	9.73 (7.41 to 12.4)	10 (7.39 to 13)	45.1 (33.5 to 57.5)	34 (25.8 to 43.2)	30.9 (22.9 to 40.1)
Uzbekistan	Other congenital anomalies	44.9 (35.1 to 56.7)	33.5 (26 to 41.8)	29 (22.5 to 38.7)	142.2 (111.9 to 178.7)	114.9 (89.6 to 143.5)	89.4 (69.5 to 119.2)
Uzbekistan	Urinary diseases and male infertility	26.4 (23.7 to 29.7)	48.4 (44.1 to 53.1)	57.3 (50.1 to 64.7)	165.2 (146.1 to 188.6)	211.1 (188.9 to 235.7)	198.1 (172.9 to 225.9)
Uzbekistan	Urinary tract infections	18.2 (17.2 to 19.1)	37.2 (34.9 to 39.6)	42.6 (37.8 to 47.6)	105.3 (100.4 to 110.3)	150.9 (141.7 to 160.2)	140.5 (124.9 to 156.5)
Uzbekistan	Urolithiasis	0.542 (0.365 to 0.759)	0.828 (0.553 to 1.16)	1.1 (0.744 to 1.55)	3.6 (2.4 to 5.1)	3.6 (2.5 to 5.1)	3.8 (2.6 to 5.3)
Uzbekistan	Benign prostatic hyperplasia	5 (3.23 to 7.2)	7.06 (4.46 to 10.2)	10.4 (6.6 to 15)	41.7 (26.8 to 59.7)	43.3 (27.5 to 61.9)	43.1 (27.4 to 61.9)
Uzbekistan	Male infertility	0.386 (0.156 to 0.818)	0.607 (0.243 to 1.28)	0.691 (0.276 to 1.46)	1.9 (0.8 to 4)	2 (0.8 to 4.3)	1.9 (0.8 to 4)
Uzbekistan	Other urinary diseases	2.36 (1.87 to 2.9)	2.63 (2.19 to 3.28)	2.47 (2.06 to 3.15)	12.7 (10.5 to 15.5)	11.3 (9.4 to 14.5)	8.8 (7.3 to 11.3)
Uzbekistan	Gynecological diseases	28.6 (20.2 to 39.5)	46.6 (32.3 to 64.4)	54.8 (38 to 78.2)	163.6 (114.5 to 228.2)	162.4 (112 to 225.1)	159.1 (110.2 to 225.8)
Uzbekistan	Uterine fibroids	4.48 (3.02 to 6.76)	7.69 (4.81 to 11.9)	8.56 (5.08 to 13.8)	32 (21 to 49.3)	30.2 (18.9 to 46.9)	26.8 (15.8 to 43.2)
Uzbekistan	Polycystic ovarian syndrome	1.08 (0.479 to 2.09)	1.7 (0.748 to 3.26)	2.03 (0.907 to 3.95)	5.7 (2.5 to 10.9)	5.7 (2.5 to 10.9)	5.8 (2.6 to 11.2)
Uzbekistan	Female infertility	0.353 (0.116 to 0.785)	0.494 (0.154 to 1.1)	0.676 (0.219 to 1.53)	1.7 (0.5 to 3.8)	1.7 (0.5 to 3.7)	1.8 (0.6 to 4.2)
Uzbekistan	Endometriosis	9.44 (6.21 to 13.7)	15 (9.8 to 21.5)	18 (12 to 26.2)	50.2 (32.9 to 72)	50.8 (32.8 to 73.3)	50.4 (33.6 to 73.6)
Uzbekistan	Genital prolapse	0.553 (0.277 to 1.02)	0.599 (0.292 to 1.12)	0.793 (0.384 to 1.51)	4.4 (2.2 to 8)	3.1 (1.5 to 5.7)	3 (1.4 to 5.7)
Uzbekistan	Premenstrual syndrome	9.58 (6.13 to 14.5)	15.6 (10 to 23.3)	17.5 (11.2 to 26.3)	51.4 (33 to 77.4)	51.7 (33.2 to 77.1)	50.2 (32.2 to 75.3)
Uzbekistan	Other gynecological diseases	3.09 (2.12 to 4.44)	5.46 (3.62 to 8.25)	7.25 (4.75 to 11.1)	18.2 (12.5 to 26.5)	19.2 (12.7 to 28.9)	21.1 (13.8 to 32.2)
Uzbekistan	Hemoglobinopathies and hemolytic anaemias	30.5 (22.5 to 40.7)	31.3 (22.9 to 43.3)	30.4 (22.3 to 42)	145 (107.3 to 194.3)	114.7 (84.7 to 156.3)	96.6 (70.6 to 132.9)
Uzbekistan	Thalassemias	3.07 (2.32 to 3.95)	2.14 (1.69 to 2.69)	2.07 (1.58 to 2.65)	9.7 (7.3 to 12.4)	7.3 (5.7 to 9.2)	6.2 (4.8 to 8)
Uzbekistan	Thalassaemias trait	7.57 (4.98 to 11.1)	7.91 (5.16 to 11.9)	7.48 (4.84 to 11.3)	34.5 (23.1 to 50.3)	28.9 (18.9 to 43.3)	23.7 (15.4 to 35.7)
Uzbekistan	Sickle cell disorders	0.471 (0.339 to 0.667)	0.382 (0.28 to 0.531)	0.375 (0.269 to 0.525)	1.5 (1.1 to 2.2)	1.3 (0.9 to 1.8)	1.1 (0.8 to 1.6)
Uzbekistan	Sickle cell trait	0.691 (0.417 to 1.07)	0.715 (0.437 to 1.11)	0.712 (0.438 to 1.12)	3 (1.9 to 4.6)	2.5 (1.6 to 3.9)	2.2 (1.4 to 3.4)
Uzbekistan	G6PD deficiency	1.01 (0.724 to 1.25)	1.73 (1.35 to 2.05)	2.05 (1.58 to 2.46)	4.8 (3.4 to 5.9)	5.8 (4.6 to 6.9)	6.1 (4.6 to 7.2)
Uzbekistan	G6PD trait	0.000762 (0.000491 to 0.00112)	0.000964 (0.000631 to 0.00142)	0.00103 (0.00066 to 0.00151)	0 (0 to 0)	0 (0 to 0)	0 (0 to 0)
Uzbekistan	Other hemoglobinopathies and hemolytic anaemias	17.7 (12.7 to 24.3)	18.4 (13.1 to 25.9)	17.7 (12.6 to 24.9)	91.5 (66.5 to 124.4)	68.9 (50.1 to 96)	57.3 (41 to 79.8)
Uzbekistan	Endocrine, metabolic, blood, and immune disorders	22.9 (16.8 to 30.7)	27.4 (20.5 to 37.1)	27.9 (21 to 37.8)	107.2 (78.1 to 144.3)	99 (74.2 to 132.6)	87.2 (65.9 to 118.1)
Uzbekistan	Oral disorders	46.4 (28.9 to 70.8)	71.3 (44.5 to 108)	92.3 (57.8 to 139)	322.2 (204.8 to 481.8)	344.9 (218.5 to 517.2)	346.9 (220.7 to 522.4)

SR Appendix Table 2. All-age DALYs counts and age standardized DALYs rates per 100,000 individuals for all causes and level 1 cause groups by location for 1990, 2007, and 2017, both sexes combined							
		All-age DALYs			Age-standardized rate of DALYs per 100,000		
Uzbekistan	Caries of deciduous teeth	1.19<br>(0.512 to 2.39)	1.1<br>(0.475 to 2.18)	1.28<br>(0.545 to 2.61)	3.9<br>(1.7 to 7.7)	3.8<br>(1.7 to 7.6)	3.8<br>(1.6 to 7.8)
Uzbekistan	Caries of permanent teeth	8.55<br>(3.67 to 16)	12.7<br>(5.43 to 23.8)	14.1<br>(6.13 to 26.8)	44.5<br>(19.3 to 83.5)	45.1<br>(19.9 to 85.3)	43.1<br>(18.8 to 81.8)
Uzbekistan	Periodontal disease	5.58<br>(2.22 to 11.6)	9.9<br>(3.89 to 21.3)	13.7<br>(5.38 to 29.3)	41.3<br>(16.3 to 85.3)	45.4<br>(17.8 to 95)	46.7<br>(18.5 to 98.6)
Uzbekistan	Edentulism and severe tooth loss	21.8<br>(14.4 to 31)	34.1<br>(22.4 to 48.4)	46.9<br>(31.1 to 66.6)	181.7<br>(121.2 to 257.4)	199.8<br>(133.2 to 281.6)	202.5<br>(134.7 to 288.3)
Uzbekistan	Other oral disorders	9.24<br>(5.75 to 13.8)	13.5<br>(8.49 to 20)	16.4<br>(10.3 to 24.3)	50.8<br>(31.9 to 75.3)	50.8<br>(31.9 to 74.3)	50.8<br>(32.2 to 75.4)
Uzbekistan	Sudden infant death syndrome	7.1<br>(3.06 to 13.7)	6.12<br>(3.16 to 11)	5.54<br>(3.37 to 8.5)	20.8<br>(9 to 40.3)	20.9<br>(10.7 to 37.3)	17.1<br>(10.4 to 26.2)
Uzbekistan	Injuries	910<br>(843 to 984)	936<br>(860 to 1020)	973<br>(858 to 1100)	4364.7<br>(4037.9 to 4732.7)	3439.7<br>(3137.4 to 3770.5)	3001.8<br>(2640.6 to 3406)
Uzbekistan	Transport injuries	212<br>(202 to 224)	245<br>(228 to 262)	250<br>(220 to 282)	1075.4<br>(1019.7 to 1134)	898.4<br>(835.2 to 963.3)	758.8<br>(668.9 to 853.6)
Uzbekistan	Road injuries	206<br>(196 to 217)	236<br>(220 to 252)	239<br>(211 to 271)	1040.9<br>(989.8 to 1096.9)	864.9<br>(804.4 to 927)	727.9<br>(642.7 to 819.5)
Uzbekistan	Pedestrian road injuries	102<br>(91 to 111)	97<br>(89.6 to 105)	88.3<br>(76.9 to 101)	495<br>(434.2 to 536.9)	353.6<br>(327.7 to 380.6)	268.9<br>(235 to 307.4)
Uzbekistan	Cyclist road injuries	6.03<br>(4.96 to 7.07)	7.77<br>(6.42 to 9.33)	9.51<br>(7.51 to 11.8)	35.3<br>(28.7 to 41.8)	30.7<br>(25.2 to 37.1)	30.5<br>(24.1 to 38)
Uzbekistan	Motorcyclist road injuries	11.6<br>(9.27 to 13.4)	13.6<br>(12.1 to 15.4)	15<br>(12.6 to 17.7)	60.3<br>(48.5 to 69.6)	48.8<br>(43.1 to 54.9)	44.7<br>(37.5 to 52.5)
Uzbekistan	Motor vehicle road injuries	84.1<br>(76.3 to 96.7)	115<br>(106 to 124)	124<br>(109 to 141)	439.4<br>(398.6 to 506.8)	423.4<br>(390.9 to 455.7)	375.4<br>(329.4 to 425)
Uzbekistan	Other road injuries	1.83<br>(1.47 to 2.23)	2.06<br>(1.64 to 2.62)	2.53<br>(1.93 to 3.35)	10.9<br>(8.6 to 13.4)	8.5<br>(6.7 to 10.8)	8.3<br>(6.3 to 11)
Uzbekistan	Other transport injuries	6.62<br>(5.79 to 7.69)	9.19<br>(8.1 to 10.4)	10.1<br>(8.46 to 11.9)	34.5<br>(30 to 40.5)	33.4<br>(29.3 to 37.8)	31<br>(25.9 to 36.5)
Uzbekistan	Unintentional injuries	557<br>(501 to 617)	496<br>(440 to 562)	507<br>(434 to 593)	2507.1<br>(2241.7 to 2815.6)	1860.7<br>(1634.7 to 2130.7)	1606<br>(1366.9 to 1887.9)
Uzbekistan	Falls	130<br>(107 to 157)	137<br>(109 to 171)	167<br>(128 to 218)	721.8<br>(576.2 to 899.2)	559.1<br>(440.9 to 712.1)	557.8<br>(426.9 to 723.8)
Uzbekistan	Drowning	171<br>(152 to 192)	119<br>(106 to 132)	92.8<br>(81.3 to 106)	634.7<br>(574.4 to 703)	410.9<br>(365.9 to 457.2)	277.7<br>(243.1 to 317.5)
Uzbekistan	Fire, heat, and hot substances	80<br>(70.9 to 89.4)	64.7<br>(57.1 to 73.2)	59.9<br>(50.5 to 69.8)	359.2<br>(318.4 to 405)	241.2<br>(211.6 to 275.3)	185.6<br>(156.2 to 217)
Uzbekistan	Poisonings	17.5<br>(16.2 to 19)	19.6<br>(18 to 21.4)	21.8<br>(19.2 to 24.9)	83.1<br>(77.5 to 89.8)	71.2<br>(65.4 to 77.3)	66<br>(58.1 to 75.1)
Uzbekistan	Poisoning by carbon monoxide	11.3<br>(10.5 to 12.2)	13.8<br>(12.7 to 14.9)	14.6<br>(12.8 to 16.6)	57<br>(53.7 to 60.7)	49.9<br>(46.2 to 53.9)	43.6<br>(38.7 to 49.6)
Uzbekistan	Poisoning by other means	6.19<br>(5.29 to 7.33)	5.86<br>(4.95 to 7.03)	7.28<br>(5.67 to 9.26)	26.1<br>(22.2 to 30.9)	21.2<br>(18 to 25.4)	22.4<br>(17.4 to 28.5)
Uzbekistan	Exposure to mechanical forces	29.4<br>(24.5 to 35)	32.5<br>(26.5 to 39.9)	41.9<br>(32.6 to 54.1)	160.6<br>(131.6 to 194.5)	128.4<br>(103.9 to 159.3)	134.7<br>(104.4 to 174.9)
Uzbekistan	Unintentional firearm injuries	4.28<br>(3.62 to 5.42)	4.26<br>(3.79 to 4.87)	4.36<br>(3.72 to 5.12)	22<br>(18.7 to 27.6)	16.6<br>(14.8 to 18.9)	13.9<br>(11.8 to 16.4)
Uzbekistan	Other exposure to mechanical forces	25.1<br>(20.4 to 30.4)	28.3<br>(22.5 to 35.4)	37.6<br>(28.6 to 49.3)	138.6<br>(110.4 to 170.3)	111.8<br>(88.3 to 141.2)	120.8<br>(91.7 to 159.2)
Uzbekistan	Adverse effects of medical treatment	11.5<br>(7.94 to 13.2)	12.1<br>(9.25 to 13.7)	12.6<br>(10.6 to 14.8)	53.7<br>(36.3 to 60.7)	46<br>(35.5 to 51.7)	39.7<br>(33.8 to 47)
Uzbekistan	Animal contact	4.24<br>(3.41 to 5.18)	4.7<br>(3.64 to 5.89)	5.13<br>(3.94 to 6.52)	20<br>(15.9 to 24.8)	17.4<br>(13.5 to 21.9)	16.1<br>(12.3 to 20.5)
Uzbekistan	Venomous animal contact	2.6<br>(1.97 to 3.33)	2.9<br>(2.12 to 3.86)	3.26<br>(2.35 to 4.36)	12.2<br>(9.2 to 15.9)	10.6<br>(7.7 to 14.1)	10.1<br>(7.3 to 13.5)
Uzbekistan	Non-venomous animal contact	1.64<br>(1.29 to 2.1)	1.79<br>(1.42 to 2.29)	1.88<br>(1.47 to 2.43)	7.8<br>(6.1 to 10)	6.8<br>(5.3 to 8.7)	6<br>(4.6 to 7.7)
Uzbekistan	Foreign body	59.1<br>(50.5 to 68.9)	47<br>(41 to 53.6)	42.6<br>(37.4 to 48.7)	209.1<br>(183.6 to 239.1)	165.4<br>(144.9 to 188.3)	130.1<br>(114.1 to 148.7)
Uzbekistan	Pulmonary aspiration and foreign body in airway	48.3<br>(40.5 to 57.5)	32.6<br>(27.4 to 38.6)	28<br>(23.9 to 33.5)	156.8<br>(133 to 184.5)	113.3<br>(95.4 to 133.4)	85<br>(72.4 to 101.7)
Uzbekistan	Foreign body in eyes	0.375<br>(0.166 to 0.678)	0.549<br>(0.239 to 0.975)	0.686<br>(0.305 to 1.22)	2<br>(0.9 to 3.5)	2.1<br>(1 to 3.7)	2.2<br>(1 to 3.8)
Uzbekistan	Foreign body in other body part	10.4<br>(9.07 to 12.5)	13.8<br>(12.3 to 15.6)	13.9<br>(12 to 16.1)	50.3<br>(44.7 to 57.8)	50<br>(44.7 to 56.7)	42.9<br>(36.9 to 49.7)
Uzbekistan	Environmental heat and cold exposure	12.8<br>(10.9 to 15.4)	18.6<br>(15.9 to 21.4)	22.3<br>(18.2 to 26.6)	72.4<br>(61.2 to 87.7)	72.9<br>(62.1 to 84)	70.6<br>(57.4 to 84.6)
Uzbekistan	Exposure to forces of nature	0<br>(0 to 0)	0.0108<br>(0.00802 to 0.0145)	0.0143<br>(0.0107 to 0.0189)	0<br>(0 to 0)	0<br>(0 to 0.1)	0<br>(0 to 0.1)
Uzbekistan	Other unintentional injuries	41.3<br>(36.5 to 46.7)	41.3<br>(36.7 to 47.1)	41.3<br>(34.7 to 49.6)	192.4<br>(168.3 to 219.8)	148.1<br>(130.5 to 170.3)	127.8<br>(106.9 to 155.2)
Uzbekistan	Self-harm and interpersonal violence	141<br>(136 to 147)	196<br>(183 to 208)	216<br>(192 to 243)	782.2<br>(755.2 to 810.3)	680.7<br>(636 to 723.4)	637<br>(567.6 to 716.1)
Uzbekistan	Self-harm	78.1<br>(74.8 to 81.7)	144<br>(134 to 155)	160<br>(140 to 182)	429.7<br>(412.8 to 447.9)	496.9<br>(461.4 to 532.9)	468.9<br>(412.3 to 530.6)
Uzbekistan	Self-harm by firearm	2.58<br>(1.84 to 4.55)	2.63<br>(1.98 to 4.1)	2.11<br>(1.51 to 3.38)	16<br>(11.5 to 27.7)	10.1<br>(7.6 to 15.4)	6.5<br>(4.7 to 10.2)
Uzbekistan	Self-harm by other specified means	75.5<br>(72.1 to 78.7)	142<br>(131 to 152)	158<br>(139 to 179)	413.7<br>(396.6 to 429.7)	486.8<br>(452 to 521.2)	462.4<br>(406.8 to 524)

SR Appendix Table 2. All-age DALYs counts and age standardized DALYs rates per 100,000 individuals for all causes and level 1 cause groups by location for 1990, 2007, and 2017, both sexes combined

		All-age DALYs			Age-standardized rate of DALYs per 100,000		
Uzbekistan	Interpersonal violence	62.7 (59.3 to 66.3)	50.4 (46.4 to 54.4)	55.6 (49.2 to 62.9)	349.4 (329.8 to 368.9)	180.3 (166.2 to 195.1)	165.5 (146.5 to 186.8)
Uzbekistan	Assault by firearm	6.6 (3.79 to 8.16)	4.51 (3.03 to 5.33)	3.53 (2.72 to 4.39)	34.5 (19.9 to 42.2)	15 (10.3 to 17.7)	10.2 (7.9 to 12.5)
Uzbekistan	Assault by sharp object	34.4 (30.6 to 38.9)	28.1 (25.9 to 30.6)	30.2 (25.9 to 34.7)	194.8 (174.6 to 216.1)	100.2 (92.4 to 108.6)	88.6 (76.3 to 101.3)
Uzbekistan	Sexual violence	1.45 (0.964 to 2.1)	2.26 (1.51 to 3.3)	2.75 (1.84 to 3.95)	7.3 (4.9 to 10.7)	7.7 (5.2 to 11.4)	8.1 (5.4 to 11.7)
Uzbekistan	Assault by other means	20.3 (17.6 to 23.7)	15.4 (13.3 to 18)	19.2 (15.9 to 23.2)	112.8 (97.6 to 132.3)	57.3 (49 to 67.2)	58.7 (48.6 to 71.5)
Uzbekistan	Conflict and terrorism	0 (0 to 0)	0.546 (0.419 to 0.757)	0.502 (0.335 to 0.821)	0 (0 to 0)	1.8 (1.4 to 2.5)	1.5 (1 to 2.4)
Uzbekistan	Executions and police conflict	0.494 (0.361 to 0.71)	0.447 (0.369 to 0.557)	0.334 (0.286 to 0.396)	3.2 (2.2 to 4.8)	1.7 (1.4 to 2.1)	1.1 (0.9 to 1.3)
Central Europe	All causes	46100 (42100 to 50600)	41900 (37900 to 46500)	40000 (35700 to 44700)	34406.9 (31475.8 to 37689.5)	27337.8 (24526.2 to 30537.6)	24009 (21159.3 to 27223.2)
Central Europe	Communicable, maternal, neonatal, and nutritional disorders	4300 (3970 to 4670)	2270 (2020 to 2560)	1920 (1690 to 2180)	4315.3 (4024.4 to 4640.2)	2380.1 (2144.9 to 2655.3)	1929.3 (1696.6 to 2191.1)
Central Europe	HIV/AIDS and sexually transmitted infections	61.8 (49.8 to 81.1)	56 (44.7 to 75.1)	50.5 (39.7 to 67.6)	54.9 (44.5 to 71.3)	48.4 (38.9 to 64.5)	44.7 (35.3 to 60.1)
Central Europe	HIV/AIDS	31.3 (28.9 to 33.1)	30 (28.7 to 32.4)	27.6 (25.1 to 31.4)	28.7 (26.6 to 30.3)	25.7 (24.6 to 27.5)	23.8 (21.9 to 26.8)
Central Europe	HIV/AIDS - Drug-susceptible Tuberculosis	6.48 (4.29 to 8.85)	5.36 (3.5 to 6.98)	3.43 (2.14 to 5.19)	5.8 (3.8 to 8)	4.9 (3.2 to 6.3)	3.1 (1.9 to 4.7)
Central Europe	HIV/AIDS - Multidrug-resistant Tuberculosis without extensive drug resistance	0.106 (0.0486 to 0.209)	0.408 (0.182 to 0.912)	0.211 (0.114 to 0.354)	0.1 (0 to 0.2)	0.4 (0.2 to 0.9)	0.2 (0.1 to 0.3)
Central Europe	HIV/AIDS - Extensively drug-resistant Tuberculosis	0 (0 to 0)	0.129 (0.0562 to 0.286)	0.121 (0.0656 to 0.2)	0 (0 to 0)	0.1 (0.1 to 0.3)	0.1 (0.1 to 0.2)
Central Europe	HIV/AIDS resulting in other diseases	24.7 (22.1 to 27.3)	24.1 (22.1 to 27.1)	23.9 (20.8 to 28)	22.8 (20.4 to 25.1)	20.3 (18.5 to 22.8)	20.5 (18 to 23.8)
Central Europe	Sexually transmitted infections excluding HIV	30.5 (18.7 to 50.1)	25.9 (15 to 44.1)	22.9 (13.2 to 39.5)	26.3 (16.1 to 43.1)	22.7 (13.6 to 38.6)	20.8 (12.1 to 35.1)
Central Europe	Syphilis	8.26 (4.47 to 14.2)	4.81 (2.96 to 7.48)	3.76 (2.48 to 5.75)	8.8 (4.2 to 16.1)	6.1 (3.1 to 10.6)	4.7 (2.4 to 8.4)
Central Europe	Chlamydial infection	4.84 (2.65 to 9.04)	4.62 (2.48 to 8.66)	4.17 (2.23 to 7.93)	3.9 (2.1 to 7.3)	3.7 (2 to 7.1)	3.7 (1.9 to 7.1)
Central Europe	Gonococcal infection	4.35 (2.51 to 7.89)	3.66 (1.96 to 6.97)	2.92 (1.62 to 5.54)	3.6 (2 to 6.6)	3.1 (1.6 to 6)	2.9 (1.5 to 5.7)
Central Europe	Trichomoniasis	3.9 (1.57 to 8.26)	4.02 (1.62 to 8.49)	3.77 (1.53 to 7.91)	3 (1.2 to 6.4)	3 (1.2 to 6.4)	3 (1.2 to 6.2)
Central Europe	Genital herpes	2.88 (0.927 to 6.87)	3.09 (0.986 to 7.32)	3.12 (1 to 7.48)	2.1 (0.7 to 4.9)	2.1 (0.7 to 4.9)	2.1 (0.7 to 5)
Central Europe	Other sexually transmitted infections	6.26 (3.8 to 10.7)	5.75 (3.46 to 9.94)	5.12 (3.04 to 8.73)	4.9 (3 to 8.5)	4.7 (2.8 to 8.2)	4.6 (2.6 to 7.9)
Central Europe	Respiratory infections and tuberculosis	1500 (1440 to 1580)	824 (779 to 888)	719 (674 to 776)	1397.6 (1338.1 to 1466.1)	694.7 (654.3 to 754.1)	527.3 (484.1 to 585)
Central Europe	Tuberculosis	204 (197 to 211)	121 (117 to 125)	69 (64.7 to 73.4)	147.5 (141.6 to 153.3)	81.3 (78.5 to 84)	43.9 (41.1 to 46.8)
Central Europe	Latent tuberculosis infection	0 (0 to 0)	0 (0 to 0)	0 (0 to 0)	0 (0 to 0)	0 (0 to 0)	0 (0 to 0)
Central Europe	Drug-susceptible tuberculosis	201 (194 to 208)	112 (100 to 119)	63.8 (59.4 to 68.2)	145.5 (139.8 to 151.2)	74.9 (66.6 to 79.4)	40.5 (37.6 to 43.5)
Central Europe	Multidrug-resistant tuberculosis without extensive drug resistance	2.63 (1.34 to 4.74)	7.12 (3.56 to 16.1)	3.27 (2.1 to 4.65)	2 (1 to 3.6)	4.9 (2.4 to 11.3)	2.2 (1.4 to 3.1)
Central Europe	Extensively drug-resistant tuberculosis	0 (0 to 0)	2.23 (1.11 to 5.09)	1.85 (1.19 to 2.66)	0 (0 to 0)	1.5 (0.7 to 3.6)	1.2 (0.8 to 1.8)
Central Europe	Lower respiratory infections	1170 (1140 to 1210)	598 (587 to 613)	553 (535 to 572)	1144.2 (1101.7 to 1184.5)	518.3 (504.3 to 533.4)	390.7 (375.3 to 407.9)
Central Europe	Upper respiratory infections	90.7 (55.7 to 141)	80.4 (48.1 to 126)	75.7 (45 to 119)	75.6 (47 to 117.4)	71.8 (42.8 to 114.3)	70.9 (41.8 to 112.7)
Central Europe	Otitis media	33.7 (22.2 to 49.9)	23.8 (14.3 to 37.6)	20.8 (12.7 to 32.8)	30.2 (20.2 to 44.2)	23.2 (14 to 36.8)	21.8 (12.9 to 34.7)
Central Europe	Enteric infections	178 (143 to 223)	110 (80.8 to 144)	116 (87.2 to 150)	178.3 (145.6 to 220.1)	125.1 (92.5 to 165.6)	123 (89.4 to 164.2)
Central Europe	Diarrhoeal diseases	172 (137 to 216)	107 (77.9 to 142)	113 (85 to 148)	172.9 (140.2 to 214.9)	122.4 (90.1 to 163.8)	120.8 (87.2 to 161.7)
Central Europe	Typhoid and paratyphoid	1.08 (0.254 to 1.92)	0.0946 (0.066 to 0.134)	0.0777 (0.0514 to 0.114)	0.8 (0.2 to 1.4)	0.1 (0 to 0.1)	0.1 (0 to 0.1)
Central Europe	Typhoid fever	0.292 (0.196 to 0.35)	0.0492 (0.0356 to 0.072)	0.042 (0.0289 to 0.0637)	0.2 (0.2 to 0.3)	0 (0 to 0)	0 (0 to 0)
Central Europe	Paratyphoid fever	0.789 (0.0441 to 1.62)	0.0454 (0.0231 to 0.0773)	0.0356 (0.0184 to 0.0644)	0.6 (0 to 1.2)	0 (0 to 0.1)	0 (0 to 0)
Central Europe	Invasive non-typhoidal salmonella	4.55 (2.52 to 7.66)	2.41 (1.29 to 4.12)	2.1 (1.08 to 3.67)	4 (2.2 to 6.9)	2.4 (1.2 to 4.2)	2.1 (1 to 3.9)
Central Europe	Other intestinal infectious diseases	0.568 (0.238 to 1)	0.204 (0.113 to 0.313)	0.133 (0.0736 to 0.196)	0.6 (0.2 to 1.1)	0.2 (0.1 to 0.4)	0.1 (0.1 to 0.2)
Central Europe	Neglected tropical diseases and malaria	47 (29 to 84.6)	39 (24.1 to 57.8)	36.4 (21.7 to 55.5)	34.9 (21.2 to 69.8)	26.1 (16 to 40)	23.3 (13.8 to 35.7)
Central Europe	Malaria	0 (0 to 0)	0 (0 to 0)	0 (0 to 0)	0 (0 to 0)	0 (0 to 0)	0 (0 to 0)

SR Appendix Table 2. All-age DALYs counts and age standardized DALYs rates per 100,000 individuals for all causes and level 1 cause groups by location for 1990, 2007, and 2017, both sexes combined

		All-age DALYs			Age-standardized rate of DALYs per 100,000		
Central Europe	Chagas disease	0<br>(0 to 0)	0<br>(0 to 0)	0<br>(0 to 0)	0<br>(0 to 0)	0<br>(0 to 0)	0<br>(0 to 0)
Central Europe	Leishmaniasis	4.47<br>(0.143 to 42.2)	1.3<br>(0.182 to 10.1)	0.181<br>(0.00135 to 1.83)	4<br>(0.1 to 39.1)	1.3<br>(0.1 to 11.7)	0.2<br>(0 to 2.3)
Central Europe	Visceral leishmaniasis	4.47<br>(0.142 to 42.2)	1.3<br>(0.18 to 10.1)	0.179<br>(0.000662 to 1.83)	4<br>(0.1 to 39.1)	1.3<br>(0.1 to 11.7)	0.2<br>(0 to 2.3)
Central Europe	Cutaneous and mucocutaneous leishmaniasis	0.00187<br>(0.000439 to 0.00486)	0.00115<br>(0.000462 to 0.00218)	0.00129<br>(0.000312 to 0.003)	0<br>(0 to 0)	0<br>(0 to 0)	0<br>(0 to 0)
Central Europe	African trypanosomiasis	0<br>(0 to 0)	0<br>(0 to 0)	0<br>(0 to 0)	0<br>(0 to 0)	0<br>(0 to 0)	0<br>(0 to 0)
Central Europe	Schistosomiasis	0<br>(0 to 0)	0<br>(0 to 0)	0<br>(0 to 0)	0<br>(0 to 0)	0<br>(0 to 0)	0<br>(0 to 0)
Central Europe	Cysticercosis	38.8<br>(24.6 to 56.9)	35.5<br>(21.2 to 53.8)	34.4<br>(19.7 to 53.5)	28<br>(17.7 to 41.1)	23.2<br>(13.8 to 35.6)	21.8<br>(12.3 to 34.1)
Central Europe	Cystic echinococcosis	2.18<br>(1.57 to 2.8)	1.37<br>(0.986 to 1.79)	1.07<br>(0.763 to 1.43)	1.6<br>(1.1 to 2.1)	1<br>(0.7 to 1.2)	0.7<br>(0.5 to 1)
Central Europe	Lymphatic filariasis	0<br>(0 to 0)	0<br>(0 to 0)	0<br>(0 to 0)	0<br>(0 to 0)	0<br>(0 to 0)	0<br>(0 to 0)
Central Europe	Onchocerciasis	0<br>(0 to 0)	0<br>(0 to 0)	0<br>(0 to 0)	0<br>(0 to 0)	0<br>(0 to 0)	0<br>(0 to 0)
Central Europe	Trachoma	0<br>(0 to 0)	0<br>(0 to 0)	0<br>(0 to 0)	0<br>(0 to 0)	0<br>(0 to 0)	0<br>(0 to 0)
Central Europe	Dengue	0<br>(0 to 0)	0<br>(0 to 0)	0<br>(0 to 0)	0<br>(0 to 0)	0<br>(0 to 0)	0<br>(0 to 0)
Central Europe	Yellow fever	0<br>(0 to 0)	0<br>(0 to 0)	0<br>(0 to 0)	0<br>(0 to 0)	0<br>(0 to 0)	0<br>(0 to 0)
Central Europe	Rabies	0.203<br>(0.173 to 0.236)	0.0853<br>(0.0687 to 0.106)	0.0648<br>(0.0514 to 0.0825)	0.2<br>(0.1 to 0.2)	0.1<br>(0 to 0.1)	0<br>(0 to 0)
Central Europe	Intestinal nematode infections	0.0407<br>(0.0216 to 0.0676)	0.00917<br>(0.0062 to 0.0128)	0.00534<br>(0.00378 to 0.00719)	0<br>(0 to 0.1)	0<br>(0 to 0)	0<br>(0 to 0)
Central Europe	Ascariasis	0.0407<br>(0.0216 to 0.0676)	0.00917<br>(0.0062 to 0.0128)	0.00534<br>(0.00378 to 0.00719)	0<br>(0 to 0.1)	0<br>(0 to 0)	0<br>(0 to 0)
Central Europe	Trichuriasis	0<br>(0 to 0)	0<br>(0 to 0)	0<br>(0 to 0)	0<br>(0 to 0)	0<br>(0 to 0)	0<br>(0 to 0)
Central Europe	Hookworm disease	0<br>(0 to 0)	0<br>(0 to 0)	0<br>(0 to 0)	0<br>(0 to 0)	0<br>(0 to 0)	0<br>(0 to 0)
Central Europe	Food-borne trematodiases	0<br>(0 to 0)	0<br>(0 to 0)	0<br>(0 to 0)	0<br>(0 to 0)	0<br>(0 to 0)	0<br>(0 to 0)
Central Europe	Leprosy	0<br>(0 to 0)	0.00000122<br>(0.000000789 to 0.00000181)	0.000000906<br>(0.000000583 to 0.00000135)	0<br>(0 to 0)	0<br>(0 to 0)	0<br>(0 to 0)
Central Europe	Ebola virus disease	0<br>(0 to 0)	0<br>(0 to 0)	0<br>(0 to 0)	0<br>(0 to 0)	0<br>(0 to 0)	0<br>(0 to 0)
Central Europe	Zika virus disease	0<br>(0 to 0)	0<br>(0 to 0)	0.000283<br>(0.0000303 to 0.000835)	0<br>(0 to 0)	0<br>(0 to 0)	0<br>(0 to 0)
Central Europe	Guinea worm disease	0<br>(0 to 0)	0<br>(0 to 0)	0<br>(0 to 0)	0<br>(0 to 0)	0<br>(0 to 0)	0<br>(0 to 0)
Central Europe	Other neglected tropical diseases	1.31<br>(0.647 to 1.55)	0.788<br>(0.429 to 0.915)	0.649<br>(0.35 to 0.771)	1.2<br>(0.6 to 1.4)	0.7<br>(0.4 to 0.9)	0.5<br>(0.3 to 0.7)
Central Europe	Other infectious diseases	292<br>(244 to 319)	105<br>(96.3 to 117)	84.3<br>(75.7 to 95.8)	286.3<br>(237.9 to 314.7)	105.8<br>(97.4 to 116.5)	83<br>(74.5 to 94)
Central Europe	Meningitis	114<br>(99 to 121)	36.5<br>(34.3 to 40)	23.2<br>(21.6 to 25.7)	115.5<br>(101.3 to 122.9)	39.7<br>(37.2 to 44.2)	24<br>(22.1 to 26.8)
Central Europe	Pneumococcal meningitis	16.8<br>(14.5 to 18.7)	6.97<br>(6.24 to 7.91)	4.55<br>(4.06 to 5.21)	15.4<br>(13.4 to 17.3)	6.4<br>(5.7 to 7.3)	4<br>(3.5 to 4.6)
Central Europe	H influenzae type B meningitis	44.3<br>(37.4 to 47.6)	11.7<br>(11 to 13)	6.8<br>(6.38 to 7.61)	46.8<br>(39.7 to 50.5)	14.4<br>(13.2 to 16.2)	8<br>(7.4 to 9.1)
Central Europe	Meningococcal infection	17.2<br>(15.3 to 19.2)	5.44<br>(4.98 to 6.03)	3.38<br>(3.08 to 3.78)	17.7<br>(15.6 to 19.8)	6<br>(5.4 to 6.8)	3.5<br>(3.2 to 4.1)
Central Europe	Other meningitis	35.5<br>(31.2 to 38.1)	12.3<br>(11.2 to 13.6)	8.48<br>(7.64 to 9.52)	35.7<br>(31.4 to 38.5)	12.9<br>(11.8 to 14.4)	8.4<br>(7.6 to 9.6)
Central Europe	Encephalitis	50.8<br>(32.1 to 55.2)	26<br>(23.5 to 27.6)	19.7<br>(17.8 to 22.2)	47.8<br>(28.5 to 52.5)	26<br>(22.8 to 27.6)	19.3<br>(17.2 to 22.1)
Central Europe	Diphtheria	0.106<br>(0.0814 to 0.138)	0.0274<br>(0.0225 to 0.0327)	0.022<br>(0.0184 to 0.0264)	0.1<br>(0.1 to 0.1)	0<br>(0 to 0)	0<br>(0 to 0)
Central Europe	Whooping cough	19<br>(7.79 to 33.6)	2.84<br>(1.32 to 5.35)	2.74<br>(1.17 to 5.24)	21.6<br>(8.9 to 38.2)	4.8<br>(2.2 to 9.1)	4.9<br>(2.1 to 9.3)
Central Europe	Tetanus	1.9<br>(1.49 to 2.1)	0.519<br>(0.458 to 0.57)	0.331<br>(0.293 to 0.406)	1.4<br>(1.1 to 1.5)	0.4<br>(0.3 to 0.4)	0.2<br>(0.2 to 0.3)
Central Europe	Measles	25.3<br>(19.3 to 32.3)	0.157<br>(0.123 to 0.18)	0.129<br>(0.0996 to 0.158)	28<br>(21.4 to 35.9)	0.2<br>(0.2 to 0.3)	0.2<br>(0.2 to 0.3)
Central Europe	Varicella and herpes zoster	5.47<br>(3.94 to 7.27)	4.56<br>(3.08 to 6.55)	4.48<br>(2.9 to 6.49)	4.5<br>(3.4 to 5.9)	3.5<br>(2.5 to 4.9)	3.2<br>(2.1 to 4.4)
Central Europe	Acute hepatitis	33.4<br>(26 to 37.2)	12.8<br>(10.8 to 15.5)	10.5<br>(8.63 to 13)	28.2<br>(21.7 to 31.5)	10.9<br>(9 to 13.2)	9.2<br>(7.4 to 11.5)
Central Europe	Acute hepatitis A	11.5<br>(8.83 to 13.6)	5.14<br>(3.99 to 6.53)	4.06<br>(3.03 to 5.34)	10.3<br>(7.8 to 12.2)	5.1<br>(3.9 to 6.5)	4.4<br>(3.3 to 5.9)
Central Europe	Acute hepatitis B	16.2<br>(13.1 to 18.2)	6.21<br>(5.33 to 7.37)	5.34<br>(4.5 to 6.64)	12.5<br>(9.9 to 14.3)	4.4<br>(3.8 to 5.3)	3.7<br>(3.1 to 4.6)
Central Europe	Acute hepatitis C	2.63<br>(1.32 to 3.97)	0.335<br>(0.2 to 0.533)	0.222<br>(0.131 to 0.359)	3<br>(1.5 to 4.5)	0.5<br>(0.3 to 0.7)	0.3<br>(0.2 to 0.5)

SR Appendix Table 2. All-age DALYs counts and age standardized DALYs rates per 100,000 individuals for all causes and level 1 cause groups by location for 1990, 2007, and 2017, both sexes combined

		All-age DALYs			Age-standardized rate of DALYs per 100,000		
Central Europe	Acute hepatitis E	3 (2.31 to 3.67)	1.16 (0.941 to 1.45)	0.916 (0.73 to 1.15)	2.3 (1.8 to 2.8)	0.9 (0.7 to 1.1)	0.7 (0.6 to 0.9)
Central Europe	Other unspecified infectious diseases	42.2 (27.7 to 51.2)	21.7 (17.9 to 27.4)	23.2 (19.6 to 28.1)	39.1 (24.2 to 47.1)	20.3 (16.9 to 25.3)	22 (18.7 to 26.9)
Central Europe	Maternal and neonatal disorders	1790 (1630 to 1970)	899 (767 to 1060)	723 (593 to 891)	1987.8 (1845.6 to 2157.2)	1148.3 (1025.1 to 1299.8)	935.6 (797.6 to 1098.6)
Central Europe	Maternal disorders	44.5 (42.5 to 46.8)	11.7 (10.7 to 13)	8.1 (7.16 to 9.2)	36.4 (34.7 to 38.4)	9.8 (8.9 to 10.9)	7.7 (6.7 to 8.8)
Central Europe	Maternal haemorrhage	10.9 (7.87 to 14.6)	3.04 (2.38 to 3.88)	2.02 (1.52 to 2.6)	8.9 (6.4 to 11.9)	2.6 (2 to 3.3)	2 (1.5 to 2.5)
Central Europe	Maternal sepsis and other pregnancy related infections	7.5 (5.08 to 10.1)	0.989 (0.729 to 1.32)	0.64 (0.434 to 0.946)	6.2 (4.2 to 8.4)	0.8 (0.6 to 1.1)	0.6 (0.4 to 1)
Central Europe	Maternal hypertensive disorders	3.18 (2.32 to 4.38)	0.981 (0.78 to 1.22)	0.73 (0.568 to 0.919)	2.7 (2 to 3.6)	0.8 (0.7 to 1)	0.7 (0.6 to 0.9)
Central Europe	Maternal obstructed labour and uterine rupture	1.22 (0.888 to 1.7)	0.638 (0.458 to 0.857)	0.485 (0.323 to 0.706)	1 (0.7 to 1.4)	0.5 (0.4 to 0.7)	0.5 (0.3 to 0.7)
Central Europe	Maternal abortive outcome	15.4 (11.7 to 19.3)	2.14 (1.73 to 2.63)	1.25 (0.996 to 1.58)	12.5 (9.3 to 15.8)	1.7 (1.4 to 2.1)	1.1 (0.9 to 1.4)
Central Europe	Ectopic pregnancy	0.735 (0.537 to 0.986)	0.154 (0.12 to 0.204)	0.0828 (0.0636 to 0.109)	0.6 (0.4 to 0.8)	0.1 (0.1 to 0.2)	0.1 (0.1 to 0.1)
Central Europe	Indirect maternal deaths	1.37 (0.956 to 1.95)	0.62 (0.468 to 0.807)	0.426 (0.317 to 0.557)	1.1 (0.8 to 1.6)	0.5 (0.4 to 0.7)	0.4 (0.3 to 0.5)
Central Europe	Late maternal deaths	0.888 (0.675 to 1.15)	0.351 (0.287 to 0.424)	0.283 (0.223 to 0.352)	0.7 (0.5 to 0.9)	0.3 (0.2 to 0.4)	0.3 (0.2 to 0.3)
Central Europe	Maternal deaths aggravated by HIV/AIDS	0.00105 (0.000452 to 0.00201)	0.00168 (0.000821 to 0.0029)	0.00165 (0.000849 to 0.00283)	0 (0 to 0)	0 (0 to 0)	0 (0 to 0)
Central Europe	Other maternal disorders	3.27 (2.79 to 4.08)	2.82 (2.36 to 3.42)	2.17 (1.81 to 2.61)	2.7 (2.3 to 3.4)	2.4 (2 to 2.9)	2 (1.7 to 2.4)
Central Europe	Neonatal disorders	1740 (1590 to 1930)	888 (756 to 1050)	715 (585 to 883)	1951.4 (1809.3 to 2120.8)	1138.5 (1015 to 1290.1)	928 (790.5 to 1090.6)
Central Europe	Neonatal preterm birth	831 (766 to 905)	334 (301 to 370)	250 (211 to 295)	968.2 (899.3 to 1055.5)	475.4 (442.8 to 512.4)	379.3 (331.7 to 432.9)
Central Europe	Neonatal encephalopathy due to birth asphyxia and trauma	421 (353 to 514)	227 (166 to 311)	197 (136 to 283)	458.7 (395.5 to 539.7)	260.4 (204.8 to 339.9)	224.5 (165.8 to 305.9)
Central Europe	Neonatal sepsis and other neonatal infections	245 (158 to 376)	213 (132 to 322)	204 (122 to 315)	225.6 (151.1 to 329.8)	211.3 (137.9 to 309.6)	204.8 (126.2 to 311)
Central Europe	Hemolytic disease and other neonatal jaundice	20.9 (17.8 to 24.2)	9.71 (7.92 to 11.7)	7.59 (6 to 9.37)	22.5 (19.3 to 26.2)	11 (9.3 to 13)	8.6 (7 to 10.3)
Central Europe	Other neonatal disorders	223 (198 to 259)	104 (96.2 to 111)	57.1 (50.9 to 63.9)	276.4 (245 to 321.3)	180.5 (167.1 to 192.6)	110.8 (98.6 to 124)
Central Europe	Nutritional deficiencies	431 (283 to 639)	237 (154 to 360)	193 (125 to 297)	375.5 (246.8 to 554.2)	231.7 (149.6 to 351.2)	192.3 (124.9 to 288.8)
Central Europe	Protein-energy malnutrition	9.4 (7.57 to 11.9)	6.35 (4.81 to 7.49)	6.39 (4.56 to 7.49)	10.1 (8 to 12.8)	6.5 (5.1 to 7.9)	5.4 (4.1 to 6.7)
Central Europe	Iodine deficiency	15.1 (8 to 26)	9.19 (4.4 to 17.6)	12 (6.11 to 21.2)	11.7 (6.2 to 19.9)	6.8 (3.3 to 13)	9.3 (4.7 to 16.7)
Central Europe	Dietary iron deficiency	268 (174 to 402)	175 (111 to 270)	142 (90.2 to 222)	214.4 (139.9 to 321.4)	147.7 (93.8 to 228.4)	125.8 (79 to 193.6)
Central Europe	Other nutritional deficiencies	3.76 (3.15 to 4.3)	3.63 (2.99 to 5.09)	2.88 (2.3 to 4.71)	3.2 (2.7 to 3.6)	3.2 (2.8 to 4.1)	2.4 (2 to 3.5)
Central Europe	Non-communicable diseases	35500 (32600 to 38900)	34600 (31600 to 38300)	33500 (30300 to 37200)	25193.7 (23074.5 to 27767.3)	21316.8 (19206.9 to 23877.2)	18909 (16772 to 21430.4)
Central Europe	Neoplasms	6290 (6220 to 6350)	7030 (6940 to 7100)	6990 (6820 to 7170)	4224 (4173.4 to 4265.1)	4041.3 (3989.9 to 4083.3)	3627.4 (3537.3 to 3723.5)
Central Europe	Lip and oral cavity cancer	116 (113 to 120)	147 (144 to 149)	138 (132 to 144)	78.2 (76.2 to 80.6)	85 (83.5 to 86.5)	74.2 (71 to 77.4)
Central Europe	Nasopharynx cancer	16.2 (15.8 to 16.6)	24.3 (23.8 to 24.9)	20.5 (19.6 to 21.5)	11.2 (10.9 to 11.5)	14.9 (14.6 to 15.2)	12.2 (11.6 to 12.8)
Central Europe	Other pharynx cancer	66.9 (64.5 to 71.1)	107 (102 to 110)	106 (100 to 112)	45 (43.4 to 47.6)	62.1 (59 to 63.7)	58 (54.5 to 61)
Central Europe	Oesophageal cancer	117 (114 to 119)	136 (134 to 138)	133 (128 to 139)	76.8 (75.3 to 78.4)	76.5 (75.2 to 77.8)	68.7 (66 to 71.6)
Central Europe	Stomach cancer	578 (565 to 590)	444 (438 to 449)	379 (368 to 391)	379.4 (370.9 to 386.5)	247 (244 to 250.1)	189.9 (184 to 196)
Central Europe	Colon and rectum cancer	688 (677 to 699)	865 (853 to 875)	891 (863 to 919)	451.7 (444.6 to 458.2)	472.7 (466.3 to 478.3)	432.6 (418.9 to 446.3)
Central Europe	Liver cancer	175 (170 to 180)	220 (217 to 224)	236 (227 to 245)	114.4 (111.4 to 117.9)	122.8 (120.9 to 124.8)	118.4 (113.8 to 123)
Central Europe	Liver cancer due to hepatitis B	38.4 (31.5 to 45.9)	45.3 (36.8 to 54.4)	44.5 (35.6 to 54.3)	25.8 (21.4 to 30.7)	26.9 (22.2 to 32)	24.5 (19.9 to 29.4)
Central Europe	Liver cancer due to hepatitis C	46.3 (36.2 to 58.4)	60.1 (47.9 to 74.2)	65.7 (51.7 to 82.1)	29.8 (23.5 to 37.3)	32.4 (25.8 to 40.1)	31.3 (24.7 to 39.3)
Central Europe	Liver cancer due to alcohol use	66.8 (59.2 to 74.4)	84.6 (75.4 to 93.8)	93.3 (83.5 to 104)	43.4 (38.6 to 48.2)	46.5 (41.5 to 51.6)	46 (41.3 to 51.1)
Central Europe	Liver cancer due to NASH	12.5 (10.8 to 14.3)	17.3 (15 to 20)	19.7 (16.9 to 22.8)	8.1 (7 to 9.3)	9.4 (8.2 to 10.8)	9.5 (8.2 to 11)
Central Europe	Liver cancer due to other causes	10.9 (9.46 to 12.5)	12.9 (11.3 to 14.9)	13.2 (11.4 to 15.3)	7.3 (6.4 to 8.4)	7.6 (6.7 to 8.7)	7.1 (6.2 to 8.1)
Central Europe	Gallbladder and biliary tract cancer	143 (137 to 148)	134 (128 to 140)	124 (118 to 131)	92.7 (89.4 to 96)	72.2 (69 to 75.4)	59.1 (56.4 to 62.8)

SR Appendix Table 2. All-age DALYs counts and age standardized DALYs rates per 100,000 individuals for all causes and level 1 cause groups by location for 1990, 2007, and 2017, both sexes combined

		All-age DALYs			Age-standardized rate of DALYs per 100,000		
Central Europe	Pancreatic cancer	288 (283 to 293)	358 (354 to 363)	400 (389 to 413)	187.9 (184.5 to 191.1)	197.5 (195.2 to 200.1)	197.2 (191.3 to 203.5)
Central Europe	Larynx cancer	151 (148 to 155)	145 (143 to 148)	127 (122 to 133)	99.2 (97 to 101.9)	82.1 (80.5 to 83.8)	65.8 (63 to 68.7)
Central Europe	Tracheal, bronchus, and lung cancer	1440 (1420 to 1460)	1660 (1640 to 1680)	1660 (1610 to 1710)	933.3 (920.5 to 946.4)	920.5 (910.2 to 930.9)	822.5 (796.4 to 847.5)
Central Europe	Malignant skin melanoma	71 (58.4 to 91.9)	91.4 (69.8 to 108)	96 (71.4 to 111)	49.2 (40.4 to 63.2)	55.9 (42.7 to 66)	54.6 (41.1 to 63.7)
Central Europe	Non-melanoma skin cancer	35.7 (34.9 to 36.4)	41.2 (40.3 to 42.4)	41.2 (39.7 to 43.3)	24.6 (24.1 to 25)	23.2 (22.7 to 23.8)	20 (19.2 to 21)
Central Europe	Non-melanoma skin cancer (squamous-cell carcinoma)	35.7 (34.9 to 36.4)	41.2 (40.3 to 42.4)	41.2 (39.6 to 43.2)	24.5 (24.1 to 25)	23.2 (22.7 to 23.8)	20 (19.2 to 20.9)
Central Europe	Non-melanoma skin cancer (basal-cell carcinoma)	0.0254 (0.0101 to 0.0562)	0.044 (0.0169 to 0.103)	0.0612 (0.0216 to 0.148)	0 (0 to 0)	0 (0 to 0.1)	0 (0 to 0.1)
Central Europe	Breast cancer	449 (437 to 461)	503 (490 to 516)	488 (466 to 513)	302.9 (295.1 to 311.1)	291.4 (283.8 to 299.3)	259.5 (247.4 to 272.9)
Central Europe	Cervical cancer	242 (235 to 250)	224 (219 to 230)	182 (173 to 192)	165.7 (161.1 to 171.4)	137 (133.4 to 140.4)	104 (99 to 109.5)
Central Europe	Uterine cancer	78.7 (76.3 to 81)	79.4 (76.4 to 82.5)	88.5 (83.2 to 94)	51.2 (49.7 to 52.7)	43.9 (42.2 to 45.6)	43.5 (40.9 to 46.2)
Central Europe	Ovarian cancer	161 (156 to 165)	189 (186 to 193)	187 (178 to 196)	107.7 (104.8 to 110.4)	109 (106.9 to 111.2)	99.6 (95 to 104.5)
Central Europe	Prostate cancer	156 (133 to 198)	233 (181 to 269)	264 (199 to 296)	101.8 (87.2 to 129.5)	119.8 (92.3 to 137.4)	117.1 (88.1 to 131.2)
Central Europe	Testicular cancer	25.8 (24.7 to 27)	21.5 (20.4 to 22.6)	17.3 (16.1 to 18.7)	20.4 (19.5 to 21.3)	16.6 (15.8 to 17.6)	14.3 (13.2 to 15.5)
Central Europe	Kidney cancer	111 (104 to 114)	168 (147 to 173)	175 (156 to 184)	74.8 (69.8 to 77)	96.5 (84.8 to 99.1)	90.3 (80.1 to 94.7)
Central Europe	Bladder cancer	133 (128 to 136)	171 (166 to 175)	188 (180 to 196)	85.8 (83 to 88)	91 (88.6 to 93.1)	87.5 (83.8 to 91.3)
Central Europe	Thyroid cancer	27.9 (26.8 to 29.1)	23.1 (22 to 24.3)	22.2 (20.8 to 23.9)	18.5 (17.8 to 19.3)	13.3 (12.6 to 14.1)	11.7 (10.9 to 12.6)
Central Europe	Mesothelioma	8.43 (7.61 to 9.7)	13.2 (12.8 to 13.5)	15.2 (14.3 to 16.3)	5.7 (5.1 to 6.5)	7.7 (7.5 to 7.9)	8.1 (7.6 to 8.6)
Central Europe	Hodgkin lymphoma	53.3 (40.1 to 57.5)	34.1 (27.8 to 37.9)	25.3 (22 to 30.1)	39.5 (29.9 to 42.5)	23.8 (19.5 to 26.5)	17.7 (15.4 to 20.9)
Central Europe	Non-Hodgkin's lymphoma	112 (109 to 114)	136 (134 to 138)	137 (133 to 142)	80.1 (78.5 to 81.9)	85.7 (84.4 to 87.2)	79.8 (77 to 82.9)
Central Europe	Multiple myeloma	43.3 (40.1 to 53)	67.1 (55.5 to 70.4)	73.5 (61.7 to 78.9)	28.1 (26 to 34.4)	36.8 (30.5 to 38.8)	36.1 (30.4 to 38.9)
Central Europe	Leukaemia	254 (248 to 262)	245 (232 to 250)	225 (214 to 234)	193.1 (188.2 to 200)	167.5 (159.3 to 171.3)	141.4 (133.2 to 147.8)
Central Europe	Acute lymphoid leukaemia	55.3 (51.2 to 59.8)	37.5 (35.8 to 38.9)	29.6 (27.7 to 31.3)	47.7 (44.2 to 51.8)	35.1 (33.4 to 36.6)	27.7 (25.4 to 29.7)
Central Europe	Chronic lymphoid leukaemia	29 (27 to 30.9)	40.7 (39.2 to 42.4)	47.6 (44.7 to 50.2)	18.9 (17.7 to 20.2)	22 (21.2 to 22.9)	22.5 (21.1 to 23.8)
Central Europe	Acute myeloid leukaemia	68.3 (63.1 to 74.5)	76.2 (71.6 to 78.3)	83.3 (77 to 87.5)	51.3 (46.8 to 56.4)	51.5 (48.3 to 53)	52.3 (48.2 to 55.4)
Central Europe	Chronic myeloid leukaemia	27.5 (26.1 to 29)	18.7 (18 to 19.4)	13.5 (12.8 to 14.3)	19.1 (18.1 to 20.2)	11.3 (10.8 to 11.7)	7.5 (7.1 to 8)
Central Europe	Other leukaemia	73.6 (68.6 to 78.7)	71.5 (64.4 to 73.7)	51.4 (48.8 to 54)	56 (52.1 to 60.2)	47.6 (43.7 to 49.2)	31.4 (29.7 to 33.1)
Central Europe	Other malignant cancers	275 (230 to 288)	238 (224 to 250)	230 (213 to 243)	200.3 (166.7 to 209.6)	155.4 (147.1 to 164)	141 (131.2 to 150.5)
Central Europe	Other neoplasms	30.5 (20.8 to 43.4)	48.5 (35.2 to 56.5)	56.5 (40 to 67.9)	21.6 (14.7 to 30.9)	29.4 (21.4 to 34.5)	30.7 (21.8 to 37.8)
Central Europe	Myelodysplastic, myeloproliferative, and other hematopoietic neoplasms	28.3 (19.3 to 40)	46.3 (33.6 to 53.9)	54.5 (38.4 to 65.5)	19.6 (13.4 to 27.9)	27.2 (19.8 to 31.8)	28.5 (20.1 to 35)
Central Europe	Benign and in situ intestinal neoplasms	0 (0 to 0)	0 (0 to 0)	0 (0 to 0)	0 (0 to 0)	0 (0 to 0)	0 (0 to 0)
Central Europe	Benign and in situ cervical and uterine neoplasms	0 (0 to 0)	0 (0 to 0)	0 (0 to 0)	0 (0 to 0)	0 (0 to 0)	0 (0 to 0)
Central Europe	Other benign and in situ neoplasms	2.23 (1.44 to 3.4)	2.19 (1.58 to 2.66)	2.01 (1.47 to 2.59)	2 (1.2 to 3)	2.2 (1.6 to 2.7)	2.2 (1.6 to 2.8)
Central Europe	Cardiovascular diseases	13300 (13000 to 13600)	11600 (11300 to 11900)	10600 (10200 to 10900)	9033.5 (8853 to 9225.4)	6409.8 (6248.7 to 6579.1)	5013.9 (4820.3 to 5199.6)
Central Europe	Rheumatic heart disease	235 (230 to 240)	80.9 (79.2 to 82.9)	61.6 (58.9 to 64.9)	160.7 (157.3 to 164.5)	48 (47 to 49.2)	33.1 (31.7 to 34.8)
Central Europe	Ischaemic heart disease	7120 (7030 to 7230)	5800 (5720 to 5930)	5140 (5000 to 5310)	4833.9 (4770 to 4907.2)	3192.2 (3146 to 3260.6)	2418.9 (2353.5 to 2505.3)
Central Europe	Stroke	4060 (3910 to 4200)	3780 (3640 to 3930)	3310 (3130 to 3480)	2744.4 (2647.4 to 2840.9)	2058.6 (1978.7 to 2138.2)	1556.7 (1468.3 to 1635.6)
Central Europe	Ischaemic stroke	2090 (1970 to 2200)	2080 (1960 to 2190)	1960 (1810 to 2100)	1424.3 (1348.2 to 1497.5)	1104.8 (1042.8 to 1167.8)	882 (813.4 to 945.1)
Central Europe	Intracerebral hemorrhage	1630 (1590 to 1670)	1390 (1360 to 1430)	1090 (1050 to 1130)	1088 (1062.4 to 1111.9)	764.2 (747.3 to 787.9)	529.3 (508.8 to 552.3)
Central Europe	Subarachnoid hemorrhage	338 (314 to 354)	320 (298 to 334)	268 (253 to 288)	232.2 (215.9 to 242.8)	189.6 (177.2 to 198.3)	145.3 (137.3 to 156.2)
Central Europe	Hypertensive heart disease	387 (351 to 495)	512 (386 to 549)	504 (390 to 562)	260.2 (236.8 to 334)	274.8 (206.5 to 294.2)	231.1 (178.9 to 256.9)

SR Appendix Table 2. All-age DALYs counts and age standardized DALYs rates per 100,000 individuals for all causes and level 1 cause groups by location for 1990, 2007, and 2017, both sexes combined

		All-age DALYs			Age-standardized rate of DALYs per 100,000		
Central Europe	Non-rheumatic valvular heart disease	72.5 (65.8 to 81)	94 (80.6 to 104)	108 (87.3 to 120)	49.1 (44.7 to 55.2)	52 (44.7 to 57.2)	51.6 (41.8 to 57)
Central Europe	Non-rheumatic calcific aortic valve disease	32.1 (28 to 38.9)	52.4 (42.7 to 58.5)	66.8 (48.8 to 74.9)	21.7 (18.9 to 26.4)	28.8 (23.4 to 32)	31.4 (23.2 to 35.1)
Central Europe	Non-rheumatic degenerative mitral valve disease	36.3 (31.4 to 40.4)	38.7 (32.7 to 44.5)	38.4 (32.3 to 46)	24.5 (21.3 to 27.4)	21.4 (18.2 to 24.6)	18.4 (15.6 to 21.9)
Central Europe	Other non-rheumatic valve diseases	4.08 (2.3 to 5.1)	2.95 (2.37 to 4.2)	3.23 (2.45 to 4.48)	2.9 (1.6 to 3.5)	1.8 (1.5 to 2.6)	1.8 (1.4 to 2.5)
Central Europe	Cardiomyopathy and myocarditis	485 (439 to 528)	507 (463 to 530)	539 (488 to 571)	349.6 (318.5 to 379.1)	305.4 (279.4 to 318.9)	283.6 (256.3 to 300.4)
Central Europe	Myocarditis	64.4 (43.4 to 81.3)	64.4 (48.7 to 72.6)	60.4 (50.5 to 67.2)	49.1 (33.8 to 60.5)	42.5 (33.2 to 47.4)	35.4 (30 to 39)
Central Europe	Alcoholic cardiomyopathy	120 (101 to 147)	144 (112 to 161)	144 (102 to 163)	81.6 (69 to 99.2)	85.9 (66.2 to 95.1)	79.8 (55.8 to 89.6)
Central Europe	Other cardiomyopathy	300 (258 to 324)	298 (276 to 318)	334 (306 to 357)	219 (188.6 to 235.3)	177 (164.4 to 188.6)	168.4 (154.2 to 179.8)
Central Europe	Atrial fibrillation and flutter	136 (114 to 164)	178 (150 to 213)	211 (177 to 250)	94.5 (79.6 to 113.3)	95.8 (80.7 to 113.9)	94.3 (79 to 111.9)
Central Europe	Aortic aneurysm	83 (77.4 to 89.7)	112 (109 to 114)	111 (106 to 116)	55.3 (51.6 to 59.8)	63.1 (61.1 to 64.4)	56.1 (53.4 to 58.4)
Central Europe	Peripheral vascular disease	35.9 (20.2 to 59.5)	59.7 (38.7 to 90.8)	79.9 (51.3 to 129)	24 (13.4 to 39.8)	32 (20.6 to 48.6)	36 (23.1 to 58.1)
Central Europe	Endocarditis	24.6 (20.4 to 27.7)	29.8 (26.4 to 34.5)	30.2 (27.2 to 36.2)	18 (15 to 20.2)	19.1 (17 to 21.9)	17.9 (16.2 to 21.4)
Central Europe	Other cardiovascular and circulatory diseases	657 (605 to 717)	471 (416 to 534)	459 (393 to 532)	443.7 (409.1 to 483.5)	269 (237.9 to 304.9)	234.7 (201.4 to 272.2)
Central Europe	Chronic respiratory diseases	1710 (1560 to 1870)	1520 (1370 to 1660)	1510 (1350 to 1660)	1169.9 (1061.9 to 1278.3)	886.9 (790.2 to 980.3)	796.8 (703.2 to 893.8)
Central Europe	Chronic obstructive pulmonary disease	1270 (1180 to 1370)	1180 (1090 to 1270)	1190 (1080 to 1310)	851.1 (786.7 to 912)	648.8 (593.9 to 696.4)	572.9 (518.4 to 628.8)
Central Europe	Pneumoconiosis	16.6 (15.2 to 18.1)	8.31 (7.57 to 9.12)	6.54 (5.76 to 7.45)	10.7 (9.8 to 11.6)	4.5 (4.1 to 4.9)	3.1 (2.7 to 3.6)
Central Europe	Silicosis	7.62 (6.91 to 8.38)	3.1 (2.77 to 3.51)	2.26 (1.93 to 2.64)	4.9 (4.4 to 5.4)	1.7 (1.5 to 1.9)	1.1 (0.9 to 1.3)
Central Europe	Asbestosis	0.537 (0.417 to 0.708)	0.665 (0.486 to 0.782)	0.819 (0.567 to 0.963)	0.4 (0.3 to 0.5)	0.4 (0.3 to 0.4)	0.4 (0.3 to 0.5)
Central Europe	Coal workers pneumoconiosis	5.93 (5.28 to 6.64)	2.55 (2.3 to 2.86)	1.93 (1.67 to 2.22)	3.8 (3.4 to 4.2)	1.3 (1.2 to 1.5)	0.9 (0.8 to 1)
Central Europe	Other pneumoconiosis	2.47 (1.75 to 3.02)	2 (1.51 to 2.35)	1.53 (1.23 to 1.89)	1.7 (1.2 to 2)	1.1 (0.8 to 1.3)	0.7 (0.6 to 0.9)
Central Europe	Asthma	324 (255 to 410)	229 (168 to 305)	214 (153 to 291)	239.2 (185.5 to 307.3)	173.4 (124.9 to 235.3)	162.1 (112.4 to 223.5)
Central Europe	Interstitial lung disease and pulmonary sarcoidosis	63.8 (52.6 to 76.4)	59.9 (49.1 to 72.6)	64.7 (53.3 to 78.6)	43.6 (36 to 52.2)	35.4 (29.1 to 42.8)	34.8 (28.4 to 42.4)
Central Europe	Other chronic respiratory diseases	33.9 (30 to 37.9)	33.8 (29.3 to 38)	31.5 (26.8 to 36.6)	25.4 (22.4 to 28.4)	24.8 (21.4 to 28)	23.9 (20.1 to 27.9)
Central Europe	Digestive diseases	2010 (1850 to 2210)	2080 (1920 to 2280)	1860 (1690 to 2060)	1425 (1300.2 to 1578.7)	1326.1 (1207.2 to 1475.2)	1120.8 (1001.6 to 1265.2)
Central Europe	Cirrhosis and other chronic liver diseases	944 (920 to 974)	1080 (1050 to 1110)	910 (872 to 951)	642.1 (625.6 to 662.9)	655.6 (638.9 to 674.2)	520.1 (496.6 to 544.2)
Central Europe	Cirrhosis and other chronic liver diseases due to hepatitis B	242 (219 to 270)	259 (231 to 288)	205 (183 to 230)	165 (149.2 to 183.4)	158.6 (141.9 to 176.2)	118.5 (106 to 131.6)
Central Europe	Cirrhosis and other chronic liver diseases due to hepatitis C	184 (162 to 209)	223 (197 to 253)	186 (164 to 212)	124.7 (110 to 141.9)	134.4 (118.6 to 152.5)	106.5 (94 to 121.5)
Central Europe	Cirrhosis and other chronic liver diseases due to alcohol use	400 (376 to 426)	464 (434 to 495)	399 (370 to 428)	268.4 (252.9 to 285.9)	277.7 (260.1 to 295.6)	223.8 (207.4 to 239.9)
Central Europe	Cirrhosis due to NASH	55.5 (50.2 to 61.6)	72.4 (65.1 to 80.4)	66.2 (59.4 to 73.6)	37.1 (33.6 to 41.2)	43 (38.7 to 47.5)	36.9 (33.1 to 41.2)
Central Europe	Cirrhosis and other chronic liver diseases due to other causes	62.1 (54.1 to 70.8)	60.9 (51.8 to 71.1)	52.6 (44.3 to 61.9)	46.9 (41.3 to 53)	41.9 (36.2 to 47.9)	34.4 (29.7 to 39.6)
Central Europe	Upper digestive system diseases	474 (364 to 626)	445 (334 to 599)	416 (309 to 561)	349.2 (264.6 to 465.4)	309.9 (226.8 to 426)	280.5 (201.6 to 390.2)
Central Europe	Peptic ulcer disease	171 (163 to 180)	143 (135 to 152)	131 (122 to 141)	116.4 (110.9 to 122.8)	83.2 (78.3 to 88.7)	68.5 (63.3 to 74.4)
Central Europe	Gastritis and duodenitis	194 (126 to 279)	184 (122 to 262)	164 (109 to 233)	153.8 (100.1 to 222.5)	148.1 (96.1 to 211.3)	132.8 (86.6 to 190.3)
Central Europe	GERD	109 (58.5 to 183)	117 (62.5 to 199)	121 (64.3 to 201)	79 (42 to 132.8)	78.5 (42.4 to 132.6)	79.2 (42.2 to 133.7)
Central Europe	Appendicitis	20.5 (19.4 to 21.9)	10.9 (10.1 to 11.8)	8.88 (8.07 to 9.77)	15.6 (14.7 to 16.8)	7.8 (7 to 8.7)	6.2 (5.4 to 7.1)
Central Europe	Paralytic ileus and intestinal obstruction	74.4 (67.4 to 84.4)	68 (58.1 to 76.5)	67.7 (57.2 to 77.3)	60.7 (55.7 to 67.7)	44.3 (38.5 to 49.2)	38.6 (32.7 to 43.3)
Central Europe	Inguinal, femoral, and abdominal hernia	123 (93.6 to 160)	100 (73 to 133)	102 (73.3 to 135)	92.2 (69.4 to 118.7)	75.3 (53.6 to 100.7)	72.2 (51.1 to 96.9)
Central Europe	Inflammatory bowel disease	49.8 (39.6 to 61)	57.6 (45.6 to 71.4)	54 (42 to 67.2)	37.2 (30 to 45.2)	38.5 (30.4 to 47.9)	34.4 (26.3 to 43.3)
Central Europe	Vascular intestinal disorders	59.2 (55.9 to 65.4)	68.9 (65.3 to 71.7)	66.8 (61 to 71)	39.6 (37.4 to 43.9)	37.2 (35.4 to 38.7)	31.4 (28.8 to 33.4)
Central Europe	Gallbladder and biliary diseases	60.5 (50.5 to 63.3)	35.2 (34 to 40.9)	38.4 (36.5 to 41.4)	40.7 (34.1 to 42.6)	19.4 (18.8 to 22.5)	18.1 (17.2 to 19.6)

SR Appendix Table 2. All-age DALYs counts and age standardized DALYs rates per 100,000 individuals for all causes and level 1 cause groups by location for 1990, 2007, and 2017, both sexes combined

		All-age DALYs			Age-standardized rate of DALYs per 100,000		
Central Europe	Pancreatitis	154 (140 to 169)	157 (145 to 173)	137 (122 to 153)	108 (98.1 to 118.9)	98.8 (91.1 to 108.6)	81 (72.6 to 91)
Central Europe	Other digestive diseases	49.3 (39.1 to 60.6)	56.5 (48.6 to 65)	59.5 (51.4 to 68.7)	39.7 (31.2 to 49.7)	39.3 (32.9 to 46.3)	38.5 (31.9 to 46)
Central Europe	Neurological disorders	2040 (1630 to 2550)	2260 (1830 to 2800)	2400 (1970 to 2910)	1563.2 (1247.1 to 1952.4)	1526.2 (1208 to 1931.2)	1482.3 (1153.6 to 1867.3)
Central Europe	Alzheimer's disease and other dementias	588 (551 to 626)	784 (735 to 838)	970 (903 to 1040)	454.4 (426.8 to 483.7)	440.1 (412.8 to 469.7)	418.8 (389.8 to 450.3)
Central Europe	Parkinson's disease	113 (104 to 128)	150 (139 to 170)	168 (154 to 192)	77 (71.3 to 87.8)	76.3 (70.5 to 86.4)	72.9 (66.6 to 83)
Central Europe	Epilepsy	239 (174 to 326)	219 (157 to 299)	199 (132 to 287)	192.7 (140.2 to 263.6)	176.8 (125.7 to 244)	163.2 (107.4 to 239.6)
Central Europe	Multiple sclerosis	55 (42.5 to 62.4)	50.4 (44.6 to 56.4)	47.6 (41 to 55.3)	38.9 (30 to 44.2)	32.7 (28.8 to 36.8)	30.1 (25.7 to 35.3)
Central Europe	Motor neuron disease	14.4 (13.3 to 15.8)	16.5 (15.9 to 17)	19.2 (18.1 to 20.2)	10.7 (9.9 to 11.8)	11 (10.6 to 11.3)	11.6 (10.9 to 12.2)
Central Europe	Headache disorders	963 (619 to 1420)	976 (632 to 1430)	938 (605 to 1390)	730.5 (469.7 to 1073.7)	732.2 (470.9 to 1077.3)	730.5 (468.6 to 1070.9)
Central Europe	Migraine	824 (510 to 1240)	834 (516 to 1260)	799 (495 to 1210)	626.2 (387.2 to 944.5)	627.7 (389.3 to 947.3)	626.2 (386 to 941.9)
Central Europe	Tension-type headache	139 (79.1 to 218)	142 (81.2 to 221)	139 (79.9 to 217)	104.3 (59.1 to 163.2)	104.5 (59.2 to 163)	104.3 (59.5 to 162.8)
Central Europe	Other neurological disorders	69.6 (57.8 to 85.6)	65.8 (55.8 to 78.4)	61.8 (50.3 to 76.7)	59 (48.6 to 72.6)	57.2 (47.8 to 68.7)	55.1 (43.4 to 70.1)
Central Europe	Mental disorders	1760 (1310 to 2270)	1750 (1310 to 2240)	1690 (1260 to 2170)	1340.6 (995.3 to 1730.6)	1327.2 (984.1 to 1711.3)	1317.6 (981.1 to 1697.3)
Central Europe	Schizophrenia	163 (122 to 202)	173 (130 to 215)	174 (130 to 216)	119.6 (89.5 to 148.6)	121.6 (91 to 151.4)	123 (92 to 152.1)
Central Europe	Depressive disorders	552 (390 to 746)	558 (396 to 750)	546 (387 to 733)	406 (287.3 to 551.6)	384.6 (271.7 to 521.3)	374.5 (265.7 to 509.7)
Central Europe	Major depressive disorder	436 (304 to 587)	434 (303 to 586)	421 (293 to 567)	321.7 (223.7 to 434.6)	300.7 (209.6 to 406.9)	290.1 (202.4 to 393.7)
Central Europe	Dysthymia	116 (78.2 to 169)	124 (83.5 to 178)	126 (85.7 to 183)	84.3 (57 to 122.9)	83.9 (56.5 to 121.3)	84.3 (57.1 to 123.2)
Central Europe	Bipolar disorder	180 (113 to 268)	183 (115 to 272)	174 (110 to 259)	137.1 (86.2 to 203.4)	137.4 (86.4 to 204.4)	137.3 (86.2 to 204.2)
Central Europe	Anxiety disorders	401 (285 to 532)	403 (286 to 534)	388 (277 to 511)	307.1 (218.5 to 409.4)	308.8 (218.1 to 410.7)	307 (218.1 to 409.8)
Central Europe	Eating disorders	43.3 (27.7 to 64.1)	45.5 (29.2 to 66.8)	45.4 (28.8 to 66.7)	35.3 (22.5 to 51.6)	39.3 (25 to 57.6)	43.9 (27.9 to 64.7)
Central Europe	Anorexia nervosa	8.46 (5.19 to 12.5)	8.76 (5.42 to 13.1)	8.34 (5.23 to 12.4)	7.1 (4.4 to 10.5)	8 (4.9 to 11.7)	8.9 (5.4 to 13.3)
Central Europe	Bulimia nervosa	34.8 (21.8 to 52.1)	36.8 (22.8 to 54.9)	37.1 (23.1 to 55.5)	28.2 (17.5 to 42)	31.3 (19.5 to 46.8)	35.1 (22.2 to 52.3)
Central Europe	Autism spectrum disorders	80.7 (55.2 to 111)	74.7 (51.4 to 102)	71.5 (49.2 to 97.4)	66.5 (45.4 to 91.6)	66.9 (45.8 to 91.7)	67.2 (45.9 to 92)
Central Europe	Attention-deficit/hyperactivity disorder	13.8 (8.34 to 22.2)	11.9 (7.13 to 18.9)	10.5 (6.47 to 16.6)	11.7 (7 to 18.8)	11.7 (7 to 18.8)	11.8 (7.2 to 18.8)
Central Europe	Conduct disorder	111 (67 to 174)	81.1 (48.6 to 127)	66.2 (39.9 to 104)	97.6 (59.2 to 153.5)	97.9 (59.1 to 154.4)	98 (58.9 to 154.7)
Central Europe	Idiopathic developmental intellectual disability	25.4 (8.1 to 48)	21.6 (7.34 to 40.9)	16 (4.05 to 31.4)	21.2 (6.8 to 40.1)	19.8 (6.8 to 37.4)	15.7 (4.1 to 30.8)
Central Europe	Other mental disorders	187 (124 to 258)	198 (132 to 273)	198 (131 to 272)	138.6 (91.9 to 191.3)	139.2 (92.1 to 193)	139.3 (92.1 to 192.9)
Central Europe	Substance use disorders	675 (556 to 806)	735 (616 to 868)	693 (576 to 817)	506.5 (414.7 to 607.1)	527.8 (436 to 629.5)	514.4 (420.4 to 615.6)
Central Europe	Alcohol use disorders	509 (431 to 604)	535 (456 to 634)	503 (427 to 599)	374.4 (315.1 to 447.3)	370.7 (312.3 to 444.1)	353.9 (293.9 to 427.6)
Central Europe	Drug use disorders	167 (127 to 209)	200 (157 to 247)	189 (148 to 232)	132.1 (100.3 to 166.2)	157.1 (122.2 to 194.7)	160.5 (123.6 to 198.1)
Central Europe	Opioid use disorders	97.7 (72.3 to 126)	123 (95.2 to 152)	120 (91.8 to 150)	76 (56.5 to 98)	93.5 (72.3 to 115.8)	96.4 (73.2 to 121.3)
Central Europe	Cocaine use disorders	9.82 (6.39 to 14.3)	10.5 (7.02 to 14.8)	9.63 (6.41 to 13.7)	7.8 (5 to 11.4)	8.3 (5.5 to 11.9)	8.3 (5.5 to 11.9)
Central Europe	Amphetamine use disorders	17.9 (10.6 to 27.4)	20.2 (12.9 to 29.5)	17.6 (11.3 to 25.8)	15.1 (8.9 to 23.2)	17.5 (11.2 to 25.7)	18.2 (11.5 to 27.2)
Central Europe	Cannabis use disorders	9.41 (5.91 to 13.8)	9.82 (6.34 to 14.3)	7.69 (4.9 to 11.2)	7.8 (4.9 to 11.4)	8.5 (5.5 to 12.5)	7.7 (4.8 to 11.1)
Central Europe	Other drug use disorders	31.9 (24.7 to 40.9)	37.2 (30 to 45.7)	34.6 (28.1 to 42.1)	25.5 (19.5 to 32.9)	29.2 (23.1 to 36.5)	29.9 (23.4 to 37.5)
Central Europe	Diabetes and kidney diseases	1420 (1210 to 1670)	1600 (1340 to 1920)	1710 (1400 to 2080)	961.6 (820.3 to 1134.5)	925.3 (770.2 to 1111.6)	900.2 (730 to 1104.1)
Central Europe	Diabetes mellitus	993 (810 to 1220)	1180 (947 to 1480)	1320 (1030 to 1660)	665.3 (541.1 to 819.4)	680.9 (539.4 to 855.2)	694.8 (534.8 to 883.5)
Central Europe	Diabetes mellitus type 1	198 (188 to 211)	178 (154 to 188)	148 (126 to 159)	133.1 (126.5 to 142)	104.9 (91.9 to 111.5)	81.7 (71 to 88.5)
Central Europe	Diabetes mellitus type 2	795 (612 to 1020)	1010 (771 to 1290)	1170 (886 to 1500)	532.1 (409 to 683.9)	576 (436.6 to 744.2)	613.1 (456.9 to 799)
Central Europe	Chronic kidney disease	423 (397 to 451)	418 (390 to 446)	393 (362 to 423)	294 (276 to 313.2)	243.8 (227.4 to 260.9)	205 (187.8 to 221.6)

SR Appendix Table 2. All-age DALYs counts and age standardized DALYs rates per 100,000 individuals for all causes and level 1 cause groups by location for 1990, 2007, and 2017, both sexes combined

		All-age DALYs			Age-standardized rate of DALYs per 100,000		
Central Europe	Chronic kidney disease due to diabetes mellitus type 1	29.5 (23.6 to 36.8)	26.3 (21.3 to 32.3)	21.8 (17.5 to 26.8)	20.5 (16.3 to 25.3)	16.2 (13.3 to 19.7)	12.9 (10.4 to 15.7)
Central Europe	Chronic kidney disease due to diabetes mellitus type 2	70.8 (59.4 to 84.8)	80.9 (69.2 to 93.7)	77.8 (66.4 to 90.6)	46.3 (39 to 55)	43.3 (37.3 to 50)	36.4 (31.3 to 42.2)
Central Europe	Chronic kidney disease due to hypertension	60.9 (52 to 70.5)	70.6 (61 to 80.9)	71.8 (62.5 to 81.6)	41.6 (35.6 to 48.1)	39.2 (33.8 to 44.7)	34.5 (29.9 to 39.1)
Central Europe	Chronic kidney disease due to glomerulonephritis	101 (88.7 to 114)	89.7 (78.8 to 102)	79 (68.1 to 90.5)	71.6 (63.3 to 80.7)	54.9 (49 to 61.7)	44.8 (39.3 to 50.5)
Central Europe	Chronic kidney disease due to other and unspecified causes	160 (140 to 183)	150 (132 to 170)	142 (124 to 162)	113.9 (100.5 to 128.6)	90.1 (79.9 to 101.1)	76.3 (67.5 to 86.2)
Central Europe	Acute glomerulonephritis	3.03 (2.84 to 3.26)	0.919 (0.868 to 0.973)	0.754 (0.697 to 0.817)	2.3 (2.2 to 2.5)	0.6 (0.6 to 0.7)	0.5 (0.4 to 0.5)
Central Europe	Skin and subcutaneous diseases	607 (413 to 880)	587 (397 to 853)	581 (395 to 842)	496.8 (335.4 to 721.5)	511 (344.5 to 737.6)	515.8 (348.8 to 746.1)
Central Europe	Dermatitis	163 (95.4 to 259)	145 (86.2 to 227)	139 (82.3 to 216)	138 (79.8 to 221.1)	140.4 (81.1 to 223.8)	139.7 (81.1 to 221.6)
Central Europe	Atopic dermatitis	132 (72.1 to 219)	114 (62.5 to 190)	109 (59.6 to 180)	114.9 (62.4 to 191.4)	117.3 (64 to 195.2)	116.6 (63.3 to 194.2)
Central Europe	Contact dermatitis	30.3 (20.1 to 44.6)	31 (20.4 to 45.6)	30.4 (20.1 to 44.4)	22.9 (15.1 to 33.9)	23 (15.2 to 34)	23 (15 to 33.7)
Central Europe	Seborrhoeic dermatitis	0.135 (0.0755 to 0.216)	0.131 (0.0728 to 0.21)	0.129 (0.0717 to 0.207)	0.1 (0.1 to 0.2)	0.1 (0.1 to 0.2)	0.1 (0.1 to 0.2)
Central Europe	Psoriasis	96.2 (68 to 125)	107 (76.1 to 140)	109 (76.7 to 142)	71 (50.1 to 92.7)	76.7 (54.1 to 100.3)	77.2 (54.6 to 101.4)
Central Europe	Bacterial skin diseases	12.6 (8.07 to 15.6)	11.7 (8.45 to 17.3)	14.2 (8.83 to 18.8)	10.1 (6.2 to 12.4)	8.2 (6 to 12)	9.2 (6 to 12.3)
Central Europe	Cellulitis	4.8 (3.38 to 6.16)	4.38 (3.35 to 6.24)	5.07 (3.68 to 7.13)	3.7 (2.6 to 4.8)	3.2 (2.4 to 4.5)	3.5 (2.6 to 4.9)
Central Europe	Pyoderma	7.78 (4.41 to 9.97)	7.27 (4.61 to 11.4)	9.15 (4.67 to 12.5)	6.4 (3.4 to 8)	5 (3.2 to 8)	5.7 (3 to 8)
Central Europe	Scabies	32.6 (18.2 to 52.9)	28.7 (15.8 to 46.2)	26.1 (14.3 to 42)	27.6 (15.3 to 44.7)	26.9 (15 to 43.7)	26.6 (14.8 to 43.5)
Central Europe	Fungal skin diseases	60.6 (24.2 to 125)	62.5 (25 to 129)	65.7 (26.3 to 135)	46.7 (18.7 to 96.9)	44.9 (17.8 to 92.7)	44.3 (17.6 to 91.1)
Central Europe	Viral skin diseases	49.4 (31.4 to 73.3)	37 (23.7 to 54.8)	34.7 (22.1 to 51.3)	45.7 (29 to 67.8)	45.8 (29.1 to 67.8)	45.8 (29.4 to 67.6)
Central Europe	Acne vulgaris	33.4 (19.8 to 55)	38.6 (22.9 to 63.5)	33.5 (20.1 to 53.9)	28.4 (16.7 to 47)	36.4 (21.5 to 59.9)	39.5 (23.4 to 64)
Central Europe	Alopecia areata	7.61 (4.97 to 11.4)	7.57 (4.87 to 11.2)	7.26 (4.68 to 10.8)	5.9 (3.8 to 8.9)	5.9 (3.8 to 8.8)	5.9 (3.8 to 8.8)
Central Europe	Pruritus	12.6 (5.97 to 23.6)	13.2 (6.3 to 24.5)	13.6 (6.54 to 25.2)	9.5 (4.4 to 17.9)	9.6 (4.5 to 18.2)	9.8 (4.6 to 18.4)
Central Europe	Urticaria	86.4 (57.5 to 122)	75.1 (49.7 to 106)	71.7 (47.8 to 101)	74.6 (49.2 to 105.2)	74.7 (49.1 to 106.2)	74.7 (49.8 to 105.7)
Central Europe	Decubitus ulcer	4.44 (3.29 to 6)	5.82 (4.36 to 7.5)	7.25 (5.25 to 9.28)	3.3 (2.4 to 4.4)	3.6 (2.7 to 4.6)	3.9 (2.8 to 5)
Central Europe	Other skin and subcutaneous diseases	48.9 (24.4 to 88.9)	54.8 (27.2 to 99.6)	59.4 (29.5 to 108)	36 (18 to 65.8)	37.8 (18.6 to 69)	39.4 (19.4 to 72.2)
Central Europe	Sense organ diseases	1060 (709 to 1530)	1190 (797 to 1710)	1290 (865 to 1840)	752.2 (505.1 to 1086.9)	731.3 (488.4 to 1061.5)	717.4 (479.1 to 1039.8)
Central Europe	Blindness and vision impairment	436 (281 to 648)	471 (307 to 694)	501 (330 to 736)	309.6 (198.6 to 460.1)	292.5 (188 to 434)	283 (181.8 to 419.2)
Central Europe	Glaucoma	5.81 (3.83 to 8.17)	6.51 (4.3 to 9.16)	7.15 (4.78 to 10)	3.8 (2.5 to 5.3)	3.4 (2.3 to 4.8)	3.2 (2.1 to 4.4)
Central Europe	Cataract	144 (97.3 to 198)	175 (118 to 238)	200 (134 to 272)	98.5 (66.8 to 134.3)	94.3 (63.3 to 128.4)	91.4 (61.7 to 124.6)
Central Europe	Age-related macular degeneration	1.33 (0.87 to 1.94)	1.69 (1.1 to 2.46)	2 (1.29 to 2.92)	0.9 (0.6 to 1.3)	0.9 (0.6 to 1.3)	0.9 (0.6 to 1.3)
Central Europe	Refraction disorders	141 (89.8 to 216)	136 (86.4 to 206)	134 (85.1 to 201)	107.1 (67.8 to 163.4)	101.8 (65 to 155.2)	98.8 (62.9 to 151.1)
Central Europe	Near vision loss	109 (52.1 to 198)	115 (55.4 to 207)	119 (57.4 to 216)	75.2 (36.1 to 136.7)	69.1 (33.2 to 125.1)	66.5 (32 to 119.7)
Central Europe	Other vision loss	34.5 (23.1 to 47.5)	37.8 (25.5 to 52.2)	40.3 (27.1 to 56)	24.1 (16.3 to 32.9)	23 (15.5 to 31.7)	22.3 (15 to 30.7)
Central Europe	Age-related and other hearing loss	579 (394 to 817)	668 (455 to 944)	733 (500 to 1030)	411.7 (282.3 to 578.5)	407.3 (277.9 to 576.5)	402.8 (273.7 to 569.9)
Central Europe	Other sense organ diseases	43.6 (27.4 to 62.9)	49.9 (31.3 to 72.3)	54.7 (34.1 to 79.2)	30.9 (19.3 to 44.8)	31.5 (19.7 to 45.9)	31.6 (19.7 to 46.1)
Central Europe	Musculoskeletal disorders	2520 (1850 to 3390)	2790 (2020 to 3770)	2910 (2110 to 3890)	1786.2 (1299.3 to 2408.2)	1790.2 (1294 to 2420.4)	1789.9 (1293.5 to 2419.1)
Central Europe	Rheumatoid arthritis	54.4 (41 to 68.3)	58.4 (43.2 to 74.4)	62.4 (45.5 to 80.2)	36.8 (27.8 to 46.2)	34.7 (25.6 to 44.6)	34.5 (25 to 44.6)
Central Europe	Osteoarthritis	138 (69.2 to 270)	173 (86.7 to 340)	199 (99.6 to 390)	91.5 (46.1 to 179.4)	96 (48.4 to 188.4)	98.7 (49.6 to 194.1)
Central Europe	Low back pain	1860 (1320 to 2500)	2030 (1460 to 2710)	2100 (1530 to 2810)	1328.6 (949.1 to 1787.2)	1325.7 (951.7 to 1777.6)	1327.6 (949.3 to 1772.4)
Central Europe	Neck pain	422 (295 to 593)	471 (329 to 660)	491 (343 to 686)	295.5 (206.9 to 413.2)	296.6 (207.6 to 415.6)	296 (206.9 to 413.9)
Central Europe	Gout	17.1 (11.5 to 23.4)	20.9 (14.1 to 28.7)	23.5 (16.1 to 32.2)	11.5 (7.7 to 15.8)	12 (8.1 to 16.5)	12.3 (8.3 to 16.8)

SR Appendix Table 2. All-age DALYs counts and age standardized DALYs rates per 100,000 individuals for all causes and level 1 cause groups by location for 1990, 2007, and 2017, both sexes combined

		All-age DALYs			Age-standardized rate of DALYs per 100,000		
Central Europe	Other musculoskeletal disorders	30.8 (20.5 to 46.3)	37.7 (24.7 to 55.5)	32.4 (21.3 to 49.4)	22.3 (14.8 to 33.6)	25.1 (15.8 to 37.3)	20.8 (12.9 to 32.9)
Central Europe	Other non-communicable diseases	2090 (1750 to 2500)	1450 (1130 to 1850)	1310 (998 to 1710)	1934.3 (1655.9 to 2250.3)	1313.5 (1075.3 to 1602)	1112.4 (886.2 to 1401.7)
Central Europe	Congenital anomalies	1080 (933 to 1190)	529 (453 to 608)	404 (330 to 482)	1175 (1009.2 to 1279.6)	672.9 (600.8 to 744.7)	509.5 (437.6 to 587.2)
Central Europe	Neural tube defects	73.5 (58 to 102)	23 (19.6 to 26.8)	18.5 (15.3 to 22)	84.6 (65.5 to 119.8)	31.5 (27.8 to 36.8)	26.3 (22.2 to 30.9)
Central Europe	Congenital heart anomalies	422 (329 to 474)	152 (138 to 167)	91.4 (79.8 to 103)	491.1 (381.3 to 553.6)	236.9 (216.1 to 260)	153.7 (134.4 to 174.1)
Central Europe	Orofacial clefts	4.86 (3.52 to 6.55)	3.28 (2.18 to 4.67)	2.88 (1.89 to 4.13)	4.5 (3.3 to 5.9)	3.1 (2.1 to 4.4)	2.8 (1.8 to 4)
Central Europe	Down's syndrome	30.7 (20.7 to 43.2)	16.9 (14.2 to 19.9)	15.2 (12.5 to 18.5)	31.6 (20.2 to 46)	19.3 (16.6 to 22.5)	17.9 (15.1 to 22.3)
Central Europe	Turner syndrome	0.117 (0.0581 to 0.191)	0.107 (0.0532 to 0.174)	0.0923 (0.0464 to 0.152)	0.1 (0 to 0.2)	0.1 (0 to 0.2)	0.1 (0 to 0.2)
Central Europe	Klinefelter syndrome	0.021 (0.00982 to 0.0405)	0.0193 (0.00901 to 0.0376)	0.0176 (0.00814 to 0.0342)	0 (0 to 0)	0 (0 to 0)	0 (0 to 0)
Central Europe	Other chromosomal abnormalities	31.1 (21.1 to 54.5)	20.2 (15.7 to 24.1)	17.5 (13.5 to 21.6)	35.5 (23.6 to 63.2)	30.1 (22.9 to 35.9)	27.6 (20.9 to 34.6)
Central Europe	Congenital musculoskeletal and limb anomalies	164 (112 to 220)	152 (102 to 206)	147 (98.6 to 198)	136.3 (94.7 to 181.2)	129.4 (87.4 to 174.9)	127 (86.2 to 171.4)
Central Europe	Urogenital congenital anomalies	16.2 (11.6 to 26.2)	8.91 (7.09 to 11)	6.55 (4.91 to 8.28)	18.6 (13 to 30.6)	13.3 (10.5 to 16.1)	10.3 (7.6 to 13.1)
Central Europe	Digestive congenital anomalies	113 (88.2 to 146)	59.1 (47.1 to 73.2)	44.2 (33.5 to 57.3)	121.7 (94.7 to 162.3)	72.4 (60.8 to 86.1)	53.2 (42.6 to 65.8)
Central Europe	Other congenital anomalies	221 (155 to 275)	93.9 (80.9 to 106)	61.4 (51.7 to 72.7)	250.9 (171.7 to 314.2)	136.7 (116.5 to 150)	90.5 (77.2 to 105.6)
Central Europe	Urinary diseases and male infertility	184 (160 to 215)	132 (103 to 168)	153 (121 to 193)	125.6 (108.8 to 145.9)	76.9 (60.4 to 97.7)	80.2 (63.3 to 100.8)
Central Europe	Urinary tract infections	86.4 (79.9 to 89.9)	38.8 (37 to 41.3)	52.6 (50.3 to 55.3)	60 (55.5 to 62.5)	23.4 (22.1 to 25.1)	27.6 (26.2 to 29.3)
Central Europe	Urolithiasis	18.3 (15.1 to 22.7)	9.29 (7.09 to 11.8)	9.65 (7.33 to 12.2)	12.4 (10.2 to 15.3)	5.9 (4.4 to 7.5)	5.8 (4.3 to 7.4)
Central Europe	Benign prostatic hyperplasia	59 (37.9 to 84.5)	71.5 (46.4 to 103)	80.9 (52.5 to 115)	38 (24.4 to 54.3)	38.7 (25.1 to 55.9)	39.7 (25.7 to 56.6)
Central Europe	Male infertility	2.7 (1.09 to 5.6)	2.82 (1.13 to 5.96)	2.33 (0.944 to 4.92)	2.1 (0.9 to 4.5)	2.3 (0.9 to 5)	2.1 (0.8 to 4.4)
Central Europe	Other urinary diseases	17.8 (15.6 to 20.9)	9.39 (7.33 to 11.1)	7.96 (6.62 to 9.22)	13.1 (11.4 to 15.4)	6.5 (5.2 to 7.8)	5.2 (4.3 to 6.1)
Central Europe	Gynecological diseases	194 (132 to 274)	190 (129 to 267)	175 (119 to 251)	151 (102.8 to 210.6)	146 (99.3 to 205)	143.7 (98 to 201.6)
Central Europe	Uterine fibroids	32.8 (19.5 to 53.2)	30.3 (17 to 50.2)	27.1 (14.9 to 45.7)	23.6 (14.1 to 38.3)	20.6 (11.6 to 34.1)	18.1 (10 to 30.5)
Central Europe	Polycystic ovarian syndrome	6.28 (2.76 to 12)	6.19 (2.74 to 12)	5.69 (2.52 to 11)	5 (2.2 to 9.6)	5 (2.2 to 9.6)	5 (2.2 to 9.7)
Central Europe	Female infertility	2.42 (0.866 to 5.35)	2.38 (0.8 to 5.28)	2.66 (0.9 to 5.94)	1.9 (0.7 to 4.1)	2 (0.7 to 4.4)	2.3 (0.8 to 5.2)
Central Europe	Endometriosis	52.6 (34.8 to 76.6)	50.8 (33.9 to 72.1)	46.7 (31.1 to 66)	42.1 (27.6 to 61.3)	41.3 (27.6 to 58.4)	41.4 (27.5 to 58.6)
Central Europe	Genital prolapse	7.06 (3.51 to 13.1)	6.06 (2.96 to 11.4)	6.77 (3.26 to 12.7)	4.7 (2.3 to 8.7)	3.5 (1.7 to 6.6)	3.6 (1.7 to 6.7)
Central Europe	Premenstrual syndrome	64 (41 to 95.2)	62.4 (39.8 to 93.2)	55.4 (35.5 to 83)	51.2 (32.8 to 76.2)	50 (32 to 74.8)	48.8 (31.3 to 73)
Central Europe	Other gynecological diseases	29.2 (20.2 to 41)	31.9 (21.5 to 45.8)	30.9 (20.4 to 45.2)	22.5 (15.5 to 31.6)	23.8 (15.9 to 34.2)	24.4 (16.1 to 35.6)
Central Europe	Hemoglobinopathies and hemolytic anaemias	69.2 (54.8 to 89.1)	50.8 (40.4 to 65.5)	43.2 (34.3 to 55.6)	54.6 (43.1 to 70.4)	38.7 (30.6 to 50.4)	31.6 (24.8 to 41.4)
Central Europe	Thalassemias	2.03 (1.58 to 2.64)	0.816 (0.646 to 1.01)	0.564 (0.431 to 0.71)	2.2 (1.7 to 2.9)	1.3 (1 to 1.6)	1 (0.7 to 1.2)
Central Europe	Thalassaemias trait	12.8 (8.23 to 18.7)	8.54 (5.48 to 12.7)	6.65 (4.32 to 9.78)	10.2 (6.6 to 15.1)	7.1 (4.5 to 10.6)	5.5 (3.6 to 8.2)
Central Europe	Sickle cell disorders	4.75 (4.06 to 5.88)	3.12 (2.61 to 3.61)	2.48 (2.07 to 2.87)	4.5 (3.9 to 5.6)	3.5 (2.9 to 4)	2.9 (2.5 to 3.4)
Central Europe	Sickle cell trait	6.63 (4.25 to 10.2)	4.4 (2.77 to 6.82)	3.29 (2.04 to 5.06)	5.5 (3.5 to 8.4)	3.8 (2.4 to 5.9)	2.9 (1.8 to 4.5)
Central Europe	G6PD deficiency	5.23 (4.41 to 6.35)	5.99 (4.64 to 6.83)	4.91 (3.56 to 5.69)	4.1 (3.5 to 5)	4.4 (3.6 to 5)	3.7 (2.8 to 4.2)
Central Europe	G6PD trait	0.00504 (0.0034 to 0.00701)	0.00429 (0.00296 to 0.00616)	0.00322 (0.00221 to 0.00456)	0 (0 to 0)	0 (0 to 0)	0 (0 to 0)
Central Europe	Other hemoglobinopathies and hemolytic anaemias	37.8 (30.2 to 48.3)	27.9 (22.4 to 35.5)	25.3 (20.2 to 32.4)	28.1 (22.2 to 36.2)	18.6 (14.5 to 24.2)	15.7 (12.1 to 21)
Central Europe	Endocrine, metabolic, blood, and immune disorders	118 (98.5 to 139)	90.3 (75.4 to 109)	86.1 (72.3 to 103)	98.9 (83.5 to 116.5)	74.2 (62.5 to 89.6)	68.3 (57.2 to 82.3)
Central Europe	Oral disorders	416 (263 to 629)	447 (283 to 669)	443 (279 to 663)	292.6 (183.7 to 441.2)	281.5 (176.2 to 422.2)	262.6 (164.1 to 396.8)
Central Europe	Caries of deciduous teeth	3.47 (1.51 to 6.89)	2.11 (0.916 to 4.18)	2.01 (0.875 to 3.98)	3.6 (1.6 to 7.1)	3.5 (1.5 to 6.9)	3.3 (1.5 to 6.6)
Central Europe	Caries of permanent teeth	51.5 (22.7 to 98.2)	49.5 (22 to 94)	44.7 (19.8 to 84.8)	40.3 (17.7 to 76.6)	39.6 (17.4 to 75.2)	37.7 (16.5 to 71.4)

SR Appendix Table 2. All-age DALYs counts and age standardized DALYs rates per 100,000 individuals for all causes and level 1 cause groups by location for 1990, 2007, and 2017, both sexes combined

		All-age DALYs			Age-standardized rate of DALYs per 100,000		
Central Europe	Periodontal disease	76.8 (30.4 to 158)	79.7 (31.6 to 165)	80.2 (31.8 to 164)	53.2 (21 to 110.2)	49.7 (19.7 to 103.1)	47.1 (18.7 to 98)
Central Europe	Edentulism and severe tooth loss	218 (145 to 307)	248 (167 to 348)	250 (168 to 349)	144.8 (96.5 to 203.9)	137.9 (92.6 to 193.1)	123.8 (83.2 to 174.3)
Central Europe	Other oral disorders	66.8 (41.8 to 98.4)	67.8 (42.4 to 99.8)	66.3 (41.6 to 97.4)	50.7 (31.8 to 75.1)	50.8 (31.8 to 74.9)	50.7 (31.8 to 74.8)
Central Europe	Sudden infant death syndrome	30.3 (22.6 to 45.8)	13.4 (10.4 to 15.5)	8.58 (6.65 to 10.1)	36.6 (27.3 to 55.4)	23.2 (18 to 26.9)	16.4 (12.7 to 19.4)
Central Europe	Injuries	6340 (5610 to 7230)	5030 (4360 to 5890)	4530 (3790 to 5490)	4897.9 (4366 to 5547.9)	3641 (3186.7 to 4218.3)	3170.7 (2691.1 to 3781.6)
Central Europe	Transport injuries	1620 (1510 to 1740)	1160 (1060 to 1290)	847 (744 to 971)	1281.7 (1201.8 to 1370)	898.1 (828.2 to 981.6)	658.9 (590.6 to 740.9)
Central Europe	Road injuries	1470 (1370 to 1590)	1070 (971 to 1190)	775 (677 to 894)	1165.7 (1092.1 to 1250.2)	825.3 (759.1 to 905.2)	601.6 (536.6 to 678.7)
Central Europe	Pedestrian road injuries	486 (455 to 521)	274 (251 to 301)	185 (162 to 213)	383.8 (360.2 to 411.1)	209.7 (194.3 to 228.8)	137 (121.5 to 155.9)
Central Europe	Cyclist road injuries	143 (119 to 172)	134 (109 to 164)	118 (92.8 to 149)	106.8 (88.6 to 127.7)	95.4 (78.4 to 115.9)	81.1 (64.5 to 101.3)
Central Europe	Motorcyclist road injuries	136 (122 to 152)	114 (102 to 128)	88.8 (76.4 to 103)	108.9 (98.2 to 121)	90.8 (82.4 to 100.5)	74.4 (65.9 to 84.4)
Central Europe	Motor vehicle road injuries	685 (639 to 735)	529 (487 to 579)	369 (328 to 420)	547.3 (512.4 to 585.2)	416.2 (387.4 to 449.9)	298.6 (270.7 to 333.1)
Central Europe	Other road injuries	24.8 (21 to 29.4)	18.5 (15.3 to 23.2)	15.2 (12.2 to 19.7)	18.8 (16 to 22.2)	13.2 (11 to 16.3)	10.6 (8.5 to 13.6)
Central Europe	Other transport injuries	147 (140 to 154)	94.4 (89.2 to 99.7)	71.9 (66.2 to 78)	116 (110.9 to 121.6)	72.8 (69.2 to 76.7)	57.3 (53.2 to 62)
Central Europe	Unintentional injuries	3550 (2960 to 4300)	2830 (2270 to 3550)	2860 (2230 to 3680)	2723.3 (2296 to 3266.5)	1980.6 (1608.5 to 2464.6)	1888.2 (1479.1 to 2409.9)
Central Europe	Falls	1970 (1520 to 2540)	1730 (1310 to 2290)	1890 (1410 to 2510)	1440.1 (1111.8 to 1850.8)	1141.5 (858.9 to 1501.8)	1174.5 (874.6 to 1558.5)
Central Europe	Drowning	293 (286 to 301)	155 (152 to 159)	103 (98.3 to 108)	250.7 (243.9 to 257.7)	136.3 (133.3 to 139.4)	91.7 (87.7 to 96.4)
Central Europe	Fire, heat, and hot substances	179 (155 to 208)	119 (99.8 to 143)	103 (82.7 to 132)	143.1 (125.2 to 165)	88.1 (75.3 to 105.8)	73 (59.1 to 91.8)
Central Europe	Poisonings	159 (144 to 175)	81.7 (71.2 to 95.7)	63.7 (51.5 to 79.6)	132.9 (120.9 to 146.8)	67.2 (58.8 to 78.8)	52.7 (42.6 to 65.8)
Central Europe	Poisoning by carbon monoxide	78.8 (75 to 83.4)	44 (42.5 to 45.8)	27.6 (25.9 to 29.5)	63.9 (60.6 to 67.4)	35 (33.7 to 36.4)	21.8 (20.4 to 23.3)
Central Europe	Poisoning by other means	80.5 (67.1 to 95.3)	37.7 (28.2 to 50.8)	36.2 (25 to 51.4)	69 (58.2 to 81.1)	32.2 (24.4 to 43)	30.9 (21.7 to 43.6)
Central Europe	Exposure to mechanical forces	375 (297 to 476)	327 (250 to 427)	339 (246 to 459)	282.1 (225.6 to 355)	229.9 (177.6 to 299.4)	231.3 (170.8 to 311.3)
Central Europe	Unintentional firearm injuries	30.8 (27.7 to 33.2)	19.2 (17.7 to 21.8)	14.4 (12.7 to 16.4)	24.7 (22.3 to 26.7)	14.5 (13.4 to 16.5)	11 (9.8 to 12.6)
Central Europe	Other exposure to mechanical forces	344 (267 to 444)	308 (231 to 407)	324 (233 to 443)	257.4 (201.8 to 329.7)	215.4 (163.6 to 283)	220.3 (160.7 to 299.1)
Central Europe	Adverse effects of medical treatment	51.3 (43.6 to 55.2)	49.5 (43.6 to 54.4)	47.1 (42.6 to 53.9)	40.9 (34.1 to 44.1)	35.2 (30.9 to 38.6)	31 (28.1 to 35.8)
Central Europe	Animal contact	26.2 (20.8 to 33)	22.9 (18.1 to 28.9)	19.8 (15.4 to 25.1)	20.4 (16.2 to 25.6)	17.4 (13.7 to 22.1)	15 (11.6 to 19.3)
Central Europe	Venomous animal contact	15.7 (11.8 to 20.7)	13.7 (10.3 to 18.1)	11.9 (8.73 to 15.9)	12.2 (9.1 to 16.1)	10.7 (7.9 to 14.2)	9.4 (6.8 to 12.7)
Central Europe	Non-venomous animal contact	10.5 (8.39 to 13.4)	9.12 (7.26 to 11.8)	7.86 (6.14 to 10.3)	8.2 (6.7 to 10.4)	6.8 (5.4 to 8.7)	5.6 (4.4 to 7.3)
Central Europe	Foreign body	188 (179 to 197)	125 (120 to 132)	96 (89.9 to 103)	176.8 (168.2 to 185.3)	109.4 (104.3 to 114.7)	80.2 (75.2 to 85.9)
Central Europe	Pulmonary aspiration and foreign body in airway	166 (160 to 173)	106 (104 to 109)	78.4 (75.3 to 81.7)	160 (153 to 167.6)	95 (92.1 to 98.1)	67.1 (64.1 to 70.3)
Central Europe	Foreign body in eyes	2.55 (1.22 to 4.36)	2.74 (1.36 to 4.73)	2.93 (1.49 to 4.99)	2 (0.9 to 3.4)	2.1 (1 to 3.7)	2.3 (1.1 to 4)
Central Europe	Foreign body in other body part	19 (15.5 to 22.8)	16.4 (13.2 to 19.9)	14.7 (11.6 to 18.3)	14.8 (12.2 to 17.8)	12.2 (9.9 to 14.9)	10.9 (8.6 to 13.6)
Central Europe	Environmental heat and cold exposure	77.6 (68.6 to 87.6)	79 (70.7 to 88.2)	66.7 (57.5 to 76.5)	57.9 (51.2 to 65.5)	54 (48.1 to 60.3)	44.5 (38.1 to 51.3)
Central Europe	Exposure to forces of nature	0.752 (0.743 to 0.762)	1.56 (1.53 to 1.59)	0.529 (0.498 to 0.568)	0.6 (0.6 to 0.6)	1.3 (1.3 to 1.3)	0.5 (0.5 to 0.5)
Central Europe	Other unintentional injuries	229 (202 to 263)	135 (112 to 168)	132 (100 to 175)	177.8 (158.4 to 203.4)	100.4 (84.2 to 123.2)	94 (73 to 123)
Central Europe	Self-harm and interpersonal violence	1170 (1140 to 1200)	1040 (1010 to 1070)	824 (785 to 866)	892.9 (871.7 to 914.1)	762.2 (741.6 to 785.2)	623.6 (594.2 to 652.4)
Central Europe	Self-harm	888 (871 to 906)	825 (808 to 844)	657 (630 to 685)	670 (657.2 to 684.2)	598 (585.6 to 610.9)	494.2 (472.9 to 516.2)
Central Europe	Self-harm by firearm	47.3 (37.4 to 61.5)	39.7 (30.9 to 51.1)	30.2 (22.7 to 39.1)	36.9 (29.1 to 48)	29.3 (22.9 to 37.6)	23 (17.5 to 29.6)
Central Europe	Self-harm by other specified means	841 (821 to 861)	785 (765 to 803)	627 (598 to 657)	633.1 (617.5 to 648.4)	568.6 (554.2 to 581.6)	471.2 (449.3 to 493.7)
Central Europe	Interpersonal violence	280 (259 to 301)	189 (173 to 207)	150 (133 to 171)	221.8 (205.8 to 237.5)	145.7 (133.8 to 158.7)	117.2 (105.1 to 132.3)
Central Europe	Assault by firearm	42.2 (30.3 to 47.1)	26.3 (20.3 to 29.7)	17.2 (14 to 20.5)	34.1 (24.5 to 38.3)	21.3 (16.5 to 24)	15 (12.2 to 18)

SR Appendix Table 2. All-age DALYs counts and age standardized DALYs rates per 100,000 individuals for all causes and level 1 cause groups by location for 1990, 2007, and 2017, both sexes combined							
		All-age DALYs			Age-standardized rate of DALYs per 100,000		
Central Europe	Assault by sharp object	78.9 (73.1 to 86.2)	45.8 (42.8 to 51.1)	33.3 (30.7 to 37.3)	62.1 (57.4 to 67.5)	35.1 (32.9 to 39.1)	26.3 (24.2 to 29.4)
Central Europe	Sexual violence	15.4 (10.5 to 22.4)	13.2 (8.94 to 19)	12.1 (8.23 to 17.4)	12.1 (8.2 to 17.7)	10.7 (7.2 to 15.6)	10.5 (7.1 to 15.3)
Central Europe	Assault by other means	144 (128 to 161)	104 (90.3 to 119)	87.5 (72.8 to 105)	113.5 (101.8 to 126.5)	78.6 (69.3 to 89.4)	65.4 (55.3 to 77.5)
Central Europe	Conflict and terrorism	0.372 (0.276 to 0.55)	22.5 (15.5 to 35.2)	16.4 (11.1 to 25.6)	0.3 (0.2 to 0.5)	17.7 (12.1 to 28.2)	11.5 (7.7 to 18.6)
Central Europe	Executions and police conflict	1.02 (0.965 to 1.09)	0.823 (0.772 to 0.899)	0.871 (0.794 to 0.967)	0.8 (0.8 to 0.9)	0.8 (0.7 to 0.8)	0.8 (0.7 to 0.9)
Albania	All causes	946 (856 to 1040)	743 (654 to 838)	770 (653 to 885)	31497.5 (28521.4 to 34763.8)	25418.6 (22518.7 to 28560.6)	23013.7 (19678.7 to 26569.4)
Albania	Communicable, maternal, neonatal, and nutritional disorders	308 (281 to 334)	79.4 (69.2 to 91.4)	55.5 (45.6 to 67.1)	8051.5 (7345 to 8751.2)	3673.9 (3207 to 4182.9)	2579.2 (2081.1 to 3151.6)
Albania	HIV/AIDS and sexually transmitted infections	1.23 (0.712 to 2.03)	0.73 (0.458 to 1.23)	0.634 (0.386 to 1.06)	34.7 (20.7 to 56.5)	26.2 (16.5 to 43.3)	23.4 (14.5 to 39.9)
Albania	HIV/AIDS	0.063 (0.0558 to 0.0689)	0.0962 (0.0898 to 0.103)	0.0673 (0.0597 to 0.0775)	2 (1.7 to 2.2)	3.5 (3.2 to 3.8)	2.6 (2.3 to 3)
Albania	HIV/AIDS - Drug-susceptible Tuberculosis	0.0289 (0.0255 to 0.0317)	0.0201 (0.0119 to 0.0319)	0.0172 (0.0101 to 0.0267)	0.9 (0.8 to 1)	0.7 (0.4 to 1.1)	0.7 (0.4 to 1)
Albania	HIV/AIDS - Multidrug-resistant Tuberculosis without extensive drug resistance	0.000173 (0.000027 to 0.000655)	0.000848 (0.000247 to 0.00205)	0.000369 (0.0000877 to 0.00105)	0 (0 to 0)	0 (0 to 0.1)	0 (0 to 0)
Albania	HIV/AIDS - Extensively drug-resistant Tuberculosis	0 (0 to 0)	0.000263 (0.0000752 to 0.000649)	0.000206 (0.0000477 to 0.000592)	0 (0 to 0)	0 (0 to 0)	0 (0 to 0)
Albania	HIV/AIDS resulting in other diseases	0.0339 (0.03 to 0.0372)	0.075 (0.0604 to 0.0861)	0.0495 (0.0372 to 0.0618)	1.1 (0.9 to 1.2)	2.7 (2.2 to 3.1)	1.9 (1.4 to 2.4)
Albania	Sexually transmitted infections excluding HIV	1.16 (0.648 to 1.98)	0.634 (0.362 to 1.13)	0.567 (0.318 to 0.998)	32.7 (18.7 to 54.4)	22.7 (13 to 39.8)	20.8 (11.8 to 37.3)
Albania	Syphilis	0.617 (0.251 to 1.24)	0.135 (0.0731 to 0.263)	0.106 (0.0611 to 0.201)	16 (6.9 to 31.5)	6.3 (2.9 to 13.2)	4.7 (2.2 to 9.9)
Albania	Chlamydial infection	0.124 (0.0646 to 0.236)	0.109 (0.0551 to 0.212)	0.101 (0.0512 to 0.196)	3.7 (2 to 7)	3.6 (1.8 to 7.1)	3.7 (1.9 to 7.2)
Albania	Gonococcal infection	0.115 (0.0575 to 0.231)	0.091 (0.0438 to 0.183)	0.0791 (0.0382 to 0.157)	3.3 (1.7 to 6.4)	3 (1.5 to 6.1)	2.9 (1.4 to 5.7)
Albania	Trichomoniasis	0.0931 (0.0368 to 0.201)	0.0942 (0.0379 to 0.199)	0.0895 (0.0361 to 0.189)	3 (1.2 to 6.4)	3.1 (1.2 to 6.5)	3 (1.2 to 6.2)
Albania	Genital herpes	0.0582 (0.0193 to 0.137)	0.0659 (0.0212 to 0.159)	0.0685 (0.0221 to 0.163)	2 (0.7 to 4.8)	2.1 (0.7 to 5)	2.1 (0.7 to 4.9)
Albania	Other sexually transmitted infections	0.156 (0.0884 to 0.279)	0.139 (0.0788 to 0.248)	0.123 (0.0702 to 0.217)	4.7 (2.7 to 8.3)	4.6 (2.6 to 8.2)	4.5 (2.6 to 8.1)
Albania	Respiratory infections and tuberculosis	141 (124 to 157)	19.5 (16.8 to 22.6)	14.3 (11.6 to 17.9)	3717.5 (3256.8 to 4138)	894.8 (760.7 to 1041.3)	611.3 (480 to 801.2)
Albania	Tuberculosis	1.63 (1.15 to 2.42)	0.399 (0.348 to 0.462)	0.349 (0.284 to 0.424)	57 (44.5 to 76.9)	13 (11.1 to 16)	10 (8 to 12.7)
Albania	Latent tuberculosis infection	0 (0 to 0)	0 (0 to 0)	0 (0 to 0)	0 (0 to 0)	0 (0 to 0)	0 (0 to 0)
Albania	Drug-susceptible tuberculosis	1.62 (1.14 to 2.41)	0.382 (0.333 to 0.447)	0.341 (0.276 to 0.413)	56.8 (44.2 to 76.6)	12.5 (10.6 to 15.3)	9.8 (7.8 to 12.5)
Albania	Multidrug-resistant tuberculosis without extensive drug resistance	0.00803 (0.0011 to 0.0305)	0.0125 (0.00397 to 0.0269)	0.00557 (0.0014 to 0.0149)	0.3 (0 to 1.1)	0.4 (0.1 to 0.9)	0.2 (0 to 0.4)
Albania	Extensively drug-resistant tuberculosis	0 (0 to 0)	0.00372 (0.00115 to 0.00802)	0.00294 (0.000752 to 0.00792)	0 (0 to 0)	0.1 (0 to 0.3)	0.1 (0 to 0.2)
Albania	Lower respiratory infections	134 (118 to 151)	15.8 (13.5 to 18.7)	11 (8.76 to 14.5)	3531.1 (3035.6 to 3950.5)	759.7 (641.2 to 900.2)	487.3 (365 to 658.4)
Albania	Upper respiratory infections	3.55 (2.31 to 5.9)	2.58 (1.69 to 3.72)	2.25 (1.48 to 3.39)	100.6 (65.3 to 161.1)	95.3 (64.1 to 135.7)	89.4 (58.9 to 133.9)
Albania	Otitis media	1 (0.598 to 1.6)	0.768 (0.463 to 1.21)	0.617 (0.373 to 0.973)	28.7 (17.2 to 45.5)	26.8 (16.2 to 42.3)	24.5 (14.8 to 38.8)
Albania	Enteric infections	9.12 (7.41 to 11.2)	2.4 (1.8 to 3.11)	2.23 (1.63 to 2.97)	236.6 (192.5 to 288.2)	95.7 (72.5 to 123.8)	99 (71.7 to 134.8)
Albania	Diarrhoeal diseases	8.85 (7.14 to 10.9)	2.24 (1.65 to 2.98)	2.11 (1.52 to 2.86)	228.6 (185.1 to 280)	89.9 (67.4 to 119.1)	94.3 (67.4 to 129.7)
Albania	Typhoid and paratyphoid	0.008 (0.00355 to 0.0159)	0.00587 (0.00256 to 0.0114)	0.00512 (0.00228 to 0.0101)	0.3 (0.1 to 0.5)	0.2 (0.1 to 0.4)	0.2 (0.1 to 0.4)
Albania	Typhoid fever	0.00154 (0.000719 to 0.00293)	0.00112 (0.000514 to 0.00207)	0.00102 (0.000457 to 0.00187)	0 (0 to 0.1)	0 (0 to 0.1)	0 (0 to 0.1)
Albania	Paratyphoid fever	0.00646 (0.00259 to 0.0134)	0.00475 (0.00193 to 0.00959)	0.0041 (0.00167 to 0.00857)	0.2 (0.1 to 0.4)	0.2 (0.1 to 0.3)	0.1 (0.1 to 0.3)
Albania	Invasive non-typhoidal salmonella	0.223 (0.122 to 0.369)	0.138 (0.0727 to 0.236)	0.105 (0.0543 to 0.179)	6.5 (3.6 to 10.6)	5.1 (2.8 to 8.8)	4.2 (2.2 to 7.4)
Albania	Other intestinal infectious diseases	0.0479 (0.00804 to 0.106)	0.0105 (0.00373 to 0.0206)	0.00668 (0.00262 to 0.0131)	1.2 (0.2 to 2.7)	0.5 (0.1 to 1)	0.3 (0.1 to 0.7)
Albania	Neglected tropical diseases and malaria	1.7 (0.144 to 14.8)	0.751 (0.0833 to 6.86)	0.154 (0.0537 to 0.954)	48.6 (4.3 to 421.1)	28.2 (3 to 257.5)	6.1 (1.8 to 41.6)
Albania	Malaria	0 (0 to 0)	0 (0 to 0)	0 (0 to 0)	0 (0 to 0)	0 (0 to 0)	0 (0 to 0)
Albania	Chagas disease	0 (0 to 0)	0 (0 to 0)	0 (0 to 0)	0 (0 to 0)	0 (0 to 0)	0 (0 to 0)
Albania	Leishmaniasis	1.51 (0.00683 to 14.6)	0.653 (0.00273 to 6.76)	0.0874 (0.000507 to 0.877)	42.7 (0.2 to 414)	24.6 (0.1 to 254)	3.7 (0 to 39.5)

SR Appendix Table 2. All-age DALYs counts and age standardized DALYs rates per 100,000 individuals for all causes and level 1 cause groups by location for 1990, 2007, and 2017, both sexes combined

		All-age DALYs			Age-standardized rate of DALYs per 100,000		
Albania	Visceral leishmaniasis	1.51 (0.0057 to 14.6)	0.653 (0.00223 to 6.76)	0.0868 (0.000293 to 0.876)	42.7 (0.2 to 414)	24.6 (0.1 to 254)	3.7 (0 to 39.5)
Albania	Cutaneous and mucocutaneous leishmaniasis	0.0013 (0.000157 to 0.00398)	0.00057 (0.000164 to 0.00129)	0.00057 (0.0000379 to 0.00172)	0 (0 to 0.1)	0 (0 to 0)	0 (0 to 0.1)
Albania	African trypanosomiasis	0 (0 to 0)	0 (0 to 0)	0 (0 to 0)	0 (0 to 0)	0 (0 to 0)	0 (0 to 0)
Albania	Schistosomiasis	0 (0 to 0)	0 (0 to 0)	0 (0 to 0)	0 (0 to 0)	0 (0 to 0)	0 (0 to 0)
Albania	Cysticercosis	0.000124 (0.0000643 to 0.0002)	0.0000397 (0.0000203 to 0.0000603)	0.0000245 (0.0000123 to 0.0000378)	0 (0 to 0)	0 (0 to 0)	0 (0 to 0)
Albania	Cystic echinococcosis	0.0392 (0.0233 to 0.0587)	0.0236 (0.0159 to 0.0319)	0.0191 (0.0134 to 0.0261)	1.3 (0.8 to 1.8)	0.8 (0.5 to 1.1)	0.6 (0.4 to 0.8)
Albania	Lymphatic filariasis	0 (0 to 0)	0 (0 to 0)	0 (0 to 0)	0 (0 to 0)	0 (0 to 0)	0 (0 to 0)
Albania	Onchocerciasis	0 (0 to 0)	0 (0 to 0)	0 (0 to 0)	0 (0 to 0)	0 (0 to 0)	0 (0 to 0)
Albania	Trachoma	0 (0 to 0)	0 (0 to 0)	0 (0 to 0)	0 (0 to 0)	0 (0 to 0)	0 (0 to 0)
Albania	Dengue	0 (0 to 0)	0 (0 to 0)	0 (0 to 0)	0 (0 to 0)	0 (0 to 0)	0 (0 to 0)
Albania	Yellow fever	0 (0 to 0)	0 (0 to 0)	0 (0 to 0)	0 (0 to 0)	0 (0 to 0)	0 (0 to 0)
Albania	Rabies	0.000515 (0.000372 to 0.000769)	0.000173 (0.00013 to 0.000223)	0.000178 (0.000123 to 0.000267)	0 (0 to 0)	0 (0 to 0)	0 (0 to 0)
Albania	Intestinal nematode infections	0.00477 (0.000531 to 0.0125)	0.00106 (0.000227 to 0.00265)	0.00054 (0.000156 to 0.00137)	0.1 (0 to 0.3)	0 (0 to 0.1)	0 (0 to 0.1)
Albania	Ascariasis	0.00477 (0.000531 to 0.0125)	0.00106 (0.000227 to 0.00265)	0.00054 (0.000156 to 0.00137)	0.1 (0 to 0.3)	0 (0 to 0.1)	0 (0 to 0.1)
Albania	Trichuriasis	0 (0 to 0)	0 (0 to 0)	0 (0 to 0)	0 (0 to 0)	0 (0 to 0)	0 (0 to 0)
Albania	Hookworm disease	0 (0 to 0)	0 (0 to 0)	0 (0 to 0)	0 (0 to 0)	0 (0 to 0)	0 (0 to 0)
Albania	Food-borne trematodiases	0 (0 to 0)	0 (0 to 0)	0 (0 to 0)	0 (0 to 0)	0 (0 to 0)	0 (0 to 0)
Albania	Leprosy	0 (0 to 0)	0.00000122 (0.000000789 to 0.00000181)	0.000000906 (0.000000583 to 0.00000135)	0 (0 to 0)	0 (0 to 0)	0 (0 to 0)
Albania	Ebola virus disease	0 (0 to 0)	0 (0 to 0)	0 (0 to 0)	0 (0 to 0)	0 (0 to 0)	0 (0 to 0)
Albania	Zika virus disease	0 (0 to 0)	0 (0 to 0)	0 (0 to 0)	0 (0 to 0)	0 (0 to 0)	0 (0 to 0)
Albania	Guinea worm disease	0 (0 to 0)	0 (0 to 0)	0 (0 to 0)	0 (0 to 0)	0 (0 to 0)	0 (0 to 0)
Albania	Other neglected tropical diseases	0.144 (0.0644 to 0.249)	0.0726 (0.0405 to 0.104)	0.0471 (0.0314 to 0.0824)	4.5 (1.9 to 6.6)	2.8 (1.6 to 4.2)	1.7 (1.1 to 3.4)
Albania	Other infectious diseases	43.3 (35.9 to 52.8)	5.8 (4.88 to 7.3)	3.57 (2.91 to 4.78)	1116.5 (930 to 1352.7)	243.8 (198.9 to 313.8)	151.6 (120.4 to 211)
Albania	Meningitis	8.19 (7.04 to 9.64)	1.92 (1.54 to 2.86)	1.03 (0.772 to 1.64)	213.5 (184.5 to 249.2)	84.1 (66.2 to 128)	46.7 (34 to 75.5)
Albania	Pneumococcal meningitis	1.19 (0.929 to 1.5)	0.368 (0.287 to 0.531)	0.175 (0.127 to 0.286)	32.1 (25.4 to 40.1)	14.7 (11.1 to 21.9)	7.4 (5.2 to 12.4)
Albania	H influenzae type B meningitis	0.822 (0.663 to 1.02)	0.197 (0.152 to 0.298)	0.0928 (0.0643 to 0.152)	21.2 (17.1 to 26.1)	8.8 (6.6 to 13.6)	4.6 (3.1 to 7.6)
Albania	Meningococcal infection	1.62 (1.27 to 2.04)	0.352 (0.264 to 0.538)	0.183 (0.127 to 0.302)	42 (33.2 to 52.4)	15.7 (11.4 to 24.6)	8.5 (5.7 to 14.3)
Albania	Other meningitis	4.56 (3.87 to 5.47)	1 (0.796 to 1.53)	0.576 (0.435 to 0.915)	118.1 (100.6 to 140.9)	44.8 (34.8 to 68.4)	26.2 (19.1 to 42.1)
Albania	Encephalitis	4.45 (2.33 to 5.37)	1.61 (1.38 to 1.89)	0.99 (0.737 to 1.48)	117.2 (66.8 to 140.3)	67.5 (56.9 to 80.8)	44.2 (31.6 to 63.3)
Albania	Diphtheria	0.0224 (0.00988 to 0.0438)	0.00506 (0.00255 to 0.00905)	0.0028 (0.00148 to 0.00488)	0.6 (0.3 to 1.1)	0.2 (0.1 to 0.4)	0.1 (0.1 to 0.3)
Albania	Whooping cough	3.08 (0.279 to 9.17)	0.471 (0.0546 to 1.54)	0.106 (0.0104 to 0.345)	77 (7 to 229.2)	24.7 (2.9 to 80.8)	6.2 (0.6 to 20)
Albania	Tetanus	0.0352 (0.0284 to 0.044)	0.02 (0.018 to 0.0215)	0.0172 (0.0147 to 0.0199)	1 (0.8 to 1.2)	0.7 (0.6 to 0.8)	0.7 (0.6 to 0.8)
Albania	Measles	22.8 (17.1 to 29.5)	0.00393 (0.002 to 0.00661)	0.00155 (0.000565 to 0.00288)	570.9 (428.9 to 739.5)	0.2 (0.1 to 0.3)	0.1 (0 to 0.2)
Albania	Varicella and herpes zoster	0.281 (0.19 to 0.406)	0.144 (0.103 to 0.196)	0.127 (0.0904 to 0.174)	8.4 (5.8 to 11.4)	5.4 (3.8 to 7.4)	4.5 (3.1 to 6.2)
Albania	Acute hepatitis	1.8 (0.682 to 2.28)	0.285 (0.213 to 0.409)	0.24 (0.177 to 0.347)	49.6 (19.6 to 62.4)	9.9 (7.4 to 14.4)	8.9 (6.5 to 12.8)
Albania	Acute hepatitis A	0.815 (0.331 to 1.05)	0.15 (0.106 to 0.209)	0.114 (0.0785 to 0.16)	21.2 (8.9 to 27.3)	5.3 (3.7 to 7.4)	4.8 (3.3 to 6.8)
Albania	Acute hepatitis B	0.627 (0.229 to 0.894)	0.105 (0.078 to 0.161)	0.102 (0.0742 to 0.155)	19 (7.5 to 26)	3.5 (2.6 to 5.4)	3.1 (2.3 to 4.8)
Albania	Acute hepatitis C	0.235 (0.0515 to 0.446)	0.00858 (0.00429 to 0.0173)	0.0059 (0.00285 to 0.0123)	5.9 (1.3 to 11.2)	0.4 (0.2 to 0.9)	0.3 (0.1 to 0.6)
Albania	Acute hepatitis E	0.12 (0.0547 to 0.16)	0.022 (0.0162 to 0.0349)	0.0185 (0.013 to 0.0296)	3.4 (1.6 to 4.5)	0.7 (0.5 to 1.2)	0.7 (0.5 to 1.1)
Albania	Other unspecified infectious diseases	2.7 (2.22 to 4.51)	1.34 (1.14 to 1.85)	1.06 (0.86 to 1.42)	78.3 (65.8 to 121.6)	51 (42.8 to 76.7)	40.2 (32.1 to 59.1)

SR Appendix Table 2. All-age DALYs counts and age standardized DALYs rates per 100,000 individuals for all causes and level 1 cause groups by location for 1990, 2007, and 2017, both sexes combined

		All-age DALYs			Age-standardized rate of DALYs per 100,000		
Albania	Maternal and neonatal disorders	88.9 (75 to 103)	40 (33.8 to 47.2)	27.2 (20.8 to 34.9)	2265.1 (1912 to 2632.7)	2014.9 (1694.7 to 2371.2)	1384.7 (1045.7 to 1791.3)
Albania	Maternal disorders	1.64 (1.4 to 1.88)	0.371 (0.312 to 0.441)	0.271 (0.205 to 0.347)	47.3 (40.5 to 54)	13.2 (11.1 to 15.8)	10.2 (7.7 to 13.1)
Albania	Maternal haemorrhage	0.426 (0.262 to 0.662)	0.0876 (0.0584 to 0.124)	0.0624 (0.0399 to 0.094)	12.2 (7.7 to 18.9)	3.1 (2 to 4.5)	2.4 (1.5 to 3.6)
Albania	Maternal sepsis and other pregnancy related infections	0.0462 (0.0273 to 0.0762)	0.0315 (0.0205 to 0.0448)	0.0206 (0.012 to 0.0331)	1.3 (0.8 to 2.1)	1.1 (0.7 to 1.6)	0.8 (0.4 to 1.2)
Albania	Maternal hypertensive disorders	0.069 (0.0394 to 0.113)	0.0205 (0.0134 to 0.0304)	0.0171 (0.0109 to 0.0255)	1.9 (1.1 to 3.2)	0.7 (0.5 to 1.1)	0.6 (0.4 to 0.9)
Albania	Maternal obstructed labour and uterine rupture	0.0264 (0.0137 to 0.0475)	0.0227 (0.014 to 0.0351)	0.0188 (0.0108 to 0.0299)	0.7 (0.4 to 1.3)	0.8 (0.5 to 1.3)	0.7 (0.4 to 1.1)
Albania	Maternal abortive outcome	0.596 (0.396 to 0.818)	0.106 (0.0745 to 0.146)	0.076 (0.0485 to 0.114)	17.6 (12.1 to 23.8)	3.7 (2.5 to 5.2)	2.8 (1.8 to 4.3)
Albania	Ectopic pregnancy	0.0551 (0.0289 to 0.0975)	0.0132 (0.00702 to 0.0225)	0.00888 (0.00447 to 0.0159)	1.6 (0.9 to 2.8)	0.5 (0.2 to 0.8)	0.3 (0.2 to 0.6)
Albania	Indirect maternal deaths	0.00986 (0.00595 to 0.0154)	0.0104 (0.00658 to 0.0165)	0.00605 (0.00342 to 0.00952)	0.3 (0.2 to 0.4)	0.4 (0.2 to 0.6)	0.2 (0.1 to 0.4)
Albania	Late maternal deaths	0.0249 (0.0172 to 0.0354)	0.00944 (0.00649 to 0.013)	0.00861 (0.00534 to 0.013)	0.7 (0.5 to 1)	0.3 (0.2 to 0.5)	0.3 (0.2 to 0.5)
Albania	Maternal deaths aggravated by HIV/AIDS	0.00000361 (0.00000174 to 0.00000644)	0.00000158 (0.000000687 to 0.00000325)	0.00000113 (0.000000412 to 0.00000268)	0 (0 to 0)	0 (0 to 0)	0 (0 to 0)
Albania	Other maternal disorders	0.387 (0.24 to 0.563)	0.0696 (0.0465 to 0.1)	0.0529 (0.0344 to 0.0762)	10.9 (6.9 to 15.9)	2.5 (1.6 to 3.7)	2 (1.3 to 2.9)
Albania	Neonatal disorders	87.2 (73.4 to 102)	39.7 (33.4 to 46.8)	26.9 (20.5 to 34.6)	2217.8 (1868.3 to 2584.5)	2001.7 (1678.7 to 2357.7)	1374.5 (1036.9 to 1781.2)
Albania	Neonatal preterm birth	37.1 (29.3 to 45)	11.1 (8.64 to 15.1)	7.69 (5.67 to 10.1)	942.1 (747.8 to 1136.3)	543.8 (414.8 to 759.6)	390.3 (283.6 to 526.7)
Albania	Neonatal encephalopathy due to birth asphyxia and trauma	15.7 (10.5 to 20.7)	6.47 (4.49 to 9.23)	5.2 (3.51 to 7.55)	406.7 (273.7 to 532.9)	279.5 (197.5 to 386.1)	232.7 (157.5 to 330)
Albania	Neonatal sepsis and other neonatal infections	3.24 (1.81 to 5.59)	3.49 (1.82 to 6.26)	3.16 (1.69 to 5.69)	91.8 (50.8 to 159.2)	124.1 (67.1 to 219.8)	123.9 (66.6 to 220.9)
Albania	Hemolytic disease and other neonatal jaundice	3.47 (2.56 to 5.21)	0.868 (0.668 to 1.09)	0.627 (0.469 to 0.804)	89.6 (66.9 to 132.7)	35.9 (27.4 to 45.8)	25.8 (19.3 to 33.4)
Albania	Other neonatal disorders	27.6 (21.4 to 37.1)	17.8 (11.9 to 22.7)	10.2 (6.69 to 14.8)	687.7 (532.4 to 922.6)	1018.5 (678.5 to 1299.1)	601.8 (392.9 to 870.7)
Albania	Nutritional deficiencies	22.9 (15.7 to 32.6)	10.1 (6.64 to 15.3)	7.43 (4.85 to 11.1)	632.6 (432.3 to 905.4)	370.3 (241.8 to 551.2)	303.1 (197.6 to 448.5)
Albania	Protein-energy malnutrition	4.22 (3.3 to 5.38)	0.803 (0.576 to 1.1)	0.543 (0.348 to 0.803)	106.8 (83.5 to 136.1)	39.5 (28.1 to 53.4)	28.1 (17.9 to 41.8)
Albania	Iodine deficiency	0.6 (0.3 to 1.02)	0.276 (0.129 to 0.532)	0.409 (0.205 to 0.708)	18.9 (9.5 to 31.9)	8.8 (4.1 to 17.1)	13.7 (6.9 to 23.8)
Albania	Dietary iron deficiency	10.2 (6.49 to 15.2)	6.62 (4.28 to 10)	5.08 (3.09 to 7.84)	308 (196.2 to 459.8)	220.1 (141.2 to 335.9)	181 (112.5 to 278.8)
Albania	Other nutritional deficiencies	0.202 (0.14 to 0.285)	0.119 (0.0882 to 0.16)	0.089 (0.0624 to 0.121)	5.8 (4.1 to 8)	4.6 (3.4 to 6.1)	3.4 (2.3 to 4.6)
Albania	Non-communicable diseases	519 (457 to 589)	577 (508 to 654)	628 (533 to 721)	19705.7 (17553.1 to 22071.1)	18891.2 (16664.9 to 21344.3)	17652 (15010.4 to 20406.3)
Albania	Neoplasms	72.8 (69.1 to 80.5)	89.6 (84.3 to 95.1)	102 (85.7 to 121)	2853 (2715.2 to 3114.7)	2747.1 (2587.8 to 2915.4)	2678.9 (2260.7 to 3158.3)
Albania	Lip and oral cavity cancer	1.04 (0.958 to 1.14)	0.986 (0.88 to 1.1)	1.12 (0.906 to 1.36)	43.2 (39.7 to 47)	30.2 (27 to 33.7)	28.6 (23.1 to 34.8)
Albania	Nasopharynx cancer	0.405 (0.367 to 0.447)	0.342 (0.301 to 0.388)	0.341 (0.279 to 0.417)	15.7 (14.2 to 17.1)	10.5 (9.3 to 11.9)	9.3 (7.7 to 11.4)
Albania	Other pharynx cancer	0.566 (0.501 to 0.723)	0.552 (0.466 to 0.913)	0.669 (0.497 to 1.1)	23.7 (20.8 to 30)	16.3 (13.9 to 26.6)	16.7 (12.3 to 27.6)
Albania	Oesophageal cancer	1.12 (1.03 to 1.21)	1.1 (1 to 1.21)	1.29 (1.04 to 1.61)	47.9 (43.9 to 51.8)	32.9 (30 to 36.1)	31.8 (25.7 to 39.8)
Albania	Stomach cancer	8.03 (7.59 to 8.51)	9.27 (8.6 to 9.97)	9.78 (8.03 to 11.8)	339.9 (320.8 to 360.4)	276.8 (257.2 to 297.2)	242.6 (200.3 to 292.9)
Albania	Colon and rectum cancer	3.65 (3.4 to 4.09)	5.1 (4.64 to 5.59)	6.34 (5.16 to 7.7)	152.6 (141.9 to 177.3)	154.1 (140.4 to 169.1)	159 (130.2 to 193.2)
Albania	Liver cancer	5.5 (5.14 to 5.86)	5.45 (4.8 to 6.17)	6.7 (5.37 to 8.65)	234.9 (218.7 to 251.1)	162 (142.7 to 183.1)	163.3 (131.7 to 208.5)
Albania	Liver cancer due to hepatitis B	1.6 (1.32 to 1.92)	1.35 (1.06 to 1.73)	1.52 (1.11 to 2.05)	63.4 (51.6 to 76.6)	40.5 (32.3 to 51.2)	39.9 (29.7 to 52.8)
Albania	Liver cancer due to hepatitis C	1.46 (1.13 to 1.88)	1.5 (1.12 to 1.98)	1.9 (1.35 to 2.6)	66.3 (51.2 to 84.4)	44.5 (33.4 to 58.6)	44.3 (32 to 60.6)
Albania	Liver cancer due to alcohol use	1.59 (1.38 to 1.84)	1.8 (1.5 to 2.2)	2.3 (1.73 to 3.07)	70.1 (60.6 to 81.4)	53 (44.2 to 64.7)	55 (41.9 to 72.7)
Albania	Liver cancer due to NASH	0.403 (0.337 to 0.47)	0.433 (0.352 to 0.542)	0.573 (0.433 to 0.766)	17.9 (15 to 21.2)	13 (10.7 to 16.1)	13.6 (10.4 to 18)
Albania	Liver cancer due to other causes	0.437 (0.374 to 0.508)	0.362 (0.306 to 0.439)	0.405 (0.309 to 0.521)	17.3 (14.7 to 20.2)	11 (9.3 to 13.3)	10.5 (8.1 to 13.3)
Albania	Gallbladder and biliary tract cancer	0.538 (0.475 to 0.684)	0.775 (0.63 to 1.08)	0.987 (0.767 to 1.35)	23.5 (20.7 to 31)	23.2 (18.9 to 32.1)	23.8 (18.4 to 32)
Albania	Pancreatic cancer	1.64 (1.51 to 1.79)	3.94 (3.54 to 4.35)	5.06 (4.14 to 6.12)	71.1 (65.4 to 77.5)	116.2 (104.6 to 128.6)	122.4 (100.6 to 148.2)
Albania	Larynx cancer	1.75 (1.61 to 1.9)	1.75 (1.57 to 1.99)	2.03 (1.59 to 2.61)	75.2 (69.3 to 81.6)	51.5 (46.2 to 58.2)	49.3 (38.6 to 63.1)

SR Appendix Table 2. All-age DALYs counts and age standardized DALYs rates per 100,000 individuals for all causes and level 1 cause groups by location for 1990, 2007, and 2017, both sexes combined

		All-age DALYs			Age-standardized rate of DALYs per 100,000		
Albania	Tracheal, bronchus, and lung cancer	13.9 (13.1 to 14.6)	17.8 (16.4 to 19.1)	22.6 (18.4 to 27.9)	599.3 (565 to 630.6)	520.8 (479.4 to 560.9)	540.6 (439.9 to 668.4)
Albania	Malignant skin melanoma	0.535 (0.451 to 0.733)	0.567 (0.394 to 1.03)	0.653 (0.436 to 1.15)	20.9 (17.4 to 28.3)	17.5 (12.3 to 31.2)	18 (12 to 31.6)
Albania	Non-melanoma skin cancer	0.484 (0.449 to 0.527)	0.654 (0.569 to 0.722)	0.765 (0.631 to 0.917)	21.4 (19.8 to 23.4)	20.6 (18.1 to 22.7)	19.1 (15.8 to 22.9)
Albania	Non-melanoma skin cancer (squamous-cell carcinoma)	0.483 (0.448 to 0.526)	0.653 (0.568 to 0.721)	0.763 (0.629 to 0.916)	21.3 (19.7 to 23.4)	20.6 (18 to 22.7)	19 (15.7 to 22.9)
Albania	Non-melanoma skin cancer (basal-cell carcinoma)	0.000793 (0.000251 to 0.00194)	0.00114 (0.000339 to 0.00277)	0.0014 (0.000407 to 0.00362)	0 (0 to 0.1)	0 (0 to 0.1)	0 (0 to 0.1)
Albania	Breast cancer	3.24 (2.89 to 3.59)	6.42 (5.12 to 7.2)	6.71 (4.81 to 8.83)	128 (115.1 to 142.1)	190.5 (151.9 to 213.5)	182.3 (129.4 to 239.2)
Albania	Cervical cancer	1.49 (1.31 to 1.75)	1.8 (1.54 to 2.1)	1.67 (1.18 to 2.22)	56.1 (49.7 to 67.1)	54.5 (46.6 to 63.3)	47 (33.2 to 62.6)
Albania	Uterine cancer	0.695 (0.621 to 0.785)	0.902 (0.775 to 1.05)	0.991 (0.721 to 1.32)	27.8 (24.8 to 31.4)	26.7 (23.1 to 31.1)	26 (18.9 to 34.3)
Albania	Ovarian cancer	0.725 (0.639 to 0.826)	1.26 (1.1 to 1.44)	1.43 (1.05 to 1.9)	27.8 (24.5 to 31.8)	37.5 (32.6 to 42.8)	38 (27.9 to 50.5)
Albania	Prostate cancer	2.2 (1.78 to 3.91)	4.25 (3.17 to 8.36)	5.46 (3.61 to 10.7)	105.7 (84.2 to 192.1)	131.2 (98.4 to 257.7)	126.2 (83.8 to 242.8)
Albania	Testicular cancer	0.423 (0.351 to 0.507)	0.321 (0.267 to 0.389)	0.365 (0.266 to 0.488)	12.9 (10.9 to 15.4)	11.3 (9.3 to 14)	12.9 (9.2 to 17.4)
Albania	Kidney cancer	0.947 (0.831 to 1.22)	1.7 (1.45 to 2.03)	1.91 (1.55 to 2.4)	36.2 (32 to 47.2)	53.1 (45.3 to 61.8)	50.1 (41 to 61.5)
Albania	Bladder cancer	0.428 (0.383 to 0.556)	0.651 (0.551 to 0.757)	0.803 (0.632 to 1.05)	19 (16.9 to 25.6)	19.9 (17 to 22.9)	19.1 (15.1 to 24.8)
Albania	Thyroid cancer	0.348 (0.299 to 0.383)	0.383 (0.336 to 0.436)	0.444 (0.354 to 0.544)	13.9 (12 to 15.4)	11.6 (10.2 to 13.2)	11.7 (9.3 to 14.4)
Albania	Mesothelioma	0.0687 (0.0446 to 0.104)	0.114 (0.071 to 0.195)	0.133 (0.0801 to 0.238)	2.8 (1.8 to 4.2)	3.4 (2.1 to 5.8)	3.4 (2.1 to 6)
Albania	Hodgkin lymphoma	0.935 (0.75 to 1.5)	0.68 (0.547 to 1.08)	0.572 (0.407 to 0.956)	30.4 (24.7 to 50.2)	22 (17.7 to 35.2)	18.3 (13.1 to 29.8)
Albania	Non-Hodgkin's lymphoma	1.63 (1.48 to 1.78)	1.5 (1.34 to 1.66)	1.66 (1.36 to 1.99)	54.5 (49.9 to 59)	47.6 (42.5 to 52.6)	48.1 (39.4 to 57.3)
Albania	Multiple myeloma	0.317 (0.275 to 0.352)	0.565 (0.446 to 0.638)	0.723 (0.549 to 0.894)	13.5 (12 to 15.1)	16.7 (13.2 to 18.8)	18 (13.3 to 22.5)
Albania	Leukaemia	5.82 (4.67 to 7.32)	5.14 (4.46 to 6.31)	4.77 (3.83 to 6.22)	178.1 (156.6 to 217.8)	175.6 (152.4 to 204.9)	163.7 (132.2 to 200.8)
Albania	Acute lymphoid leukaemia	1.85 (1.17 to 2.53)	1.43 (1.09 to 1.79)	1.13 (0.846 to 1.44)	49.9 (33.6 to 66.6)	53.2 (38.5 to 68.6)	49.7 (35.8 to 65.5)
Albania	Chronic lymphoid leukaemia	0.294 (0.217 to 0.486)	0.484 (0.329 to 0.881)	0.598 (0.385 to 1.09)	13.1 (9.6 to 21.9)	14.5 (9.9 to 26.5)	14.2 (9.2 to 25.7)
Albania	Acute myeloid leukaemia	1.47 (1.06 to 1.9)	1.76 (1.28 to 2.09)	1.75 (1.25 to 2.26)	47.1 (35.6 to 58.3)	57.8 (42.5 to 68.9)	56.8 (41.3 to 72.6)
Albania	Chronic myeloid leukaemia	0.333 (0.24 to 0.514)	0.29 (0.2 to 0.505)	0.28 (0.177 to 0.482)	11.8 (8.4 to 18.8)	9.2 (6.5 to 15.5)	8.1 (5.3 to 13.2)
Albania	Other leukaemia	1.87 (1.46 to 2.7)	1.18 (0.959 to 1.72)	1 (0.745 to 1.59)	56.2 (46.6 to 76.9)	40.9 (33.2 to 56.1)	34.9 (26.5 to 48.8)
Albania	Other malignant cancers	8.49 (7.49 to 9.42)	8.04 (6.94 to 10.4)	8.33 (6.66 to 11.2)	283.5 (258.8 to 311.2)	262.4 (229.8 to 323.4)	250 (203.3 to 321.1)
Albania	Other neoplasms	0.256 (0.21 to 0.359)	0.463 (0.343 to 0.576)	0.587 (0.432 to 0.736)	9.2 (7.7 to 12.5)	15.1 (11.5 to 18.4)	16.8 (12.4 to 20.8)
Albania	Myelodysplastic, myeloproliferative, and other hematopoietic neoplasms	0.209 (0.169 to 0.286)	0.398 (0.286 to 0.514)	0.527 (0.385 to 0.67)	7.9 (6.5 to 10.9)	12.7 (9.2 to 15.8)	14.2 (10.5 to 17.7)
Albania	Benign and in situ intestinal neoplasms	0 (0 to 0)	0 (0 to 0)	0 (0 to 0)	0 (0 to 0)	0 (0 to 0)	0 (0 to 0)
Albania	Benign and in situ cervical and uterine neoplasms	0 (0 to 0)	0 (0 to 0)	0 (0 to 0)	0 (0 to 0)	0 (0 to 0)	0 (0 to 0)
Albania	Other benign and in situ neoplasms	0.047 (0.0343 to 0.0719)	0.0647 (0.0445 to 0.0823)	0.0602 (0.0415 to 0.0794)	1.3 (1 to 1.9)	2.4 (1.6 to 3.1)	2.6 (1.7 to 3.5)
Albania	Cardiovascular diseases	137 (132 to 142)	179 (169 to 190)	210 (181 to 242)	6122.5 (5907.9 to 6338.5)	5801.6 (5482.8 to 6133.1)	5227.7 (4539.3 to 6010.5)
Albania	Rheumatic heart disease	4.46 (3.97 to 5.06)	2.66 (2.24 to 3.18)	2.24 (1.79 to 2.74)	158 (142.1 to 176.8)	88.7 (74.2 to 105.7)	71.9 (57.3 to 88.7)
Albania	Ischaemic heart disease	56.4 (53.9 to 58.5)	81.1 (76 to 86.4)	96.4 (81.3 to 114)	2608.5 (2493.3 to 2706.1)	2565.3 (2408.8 to 2726.6)	2359.6 (1990.7 to 2791.4)
Albania	Stroke	50.6 (48.3 to 52.9)	65.5 (61.5 to 69.4)	77.7 (67.2 to 89.8)	2310.6 (2202.4 to 2415.4)	2161.2 (2034.3 to 2281.6)	1918.1 (1661.7 to 2208.2)
Albania	Ischaemic stroke	10.2 (9.19 to 11.2)	13.2 (11.7 to 14.5)	17.3 (14.5 to 20.1)	472.4 (427 to 517.3)	424.4 (377.8 to 467)	418.7 (353.9 to 485.4)
Albania	Intracerebral hemorrhage	37.2 (35.5 to 38.9)	47.9 (45.2 to 50.6)	56.1 (47.8 to 65.3)	1713.4 (1632.9 to 1791)	1590.9 (1511 to 1674.7)	1382.2 (1183.3 to 1601.8)
Albania	Subarachnoid hemorrhage	3.16 (2.77 to 4.11)	4.47 (3.49 to 5.13)	4.38 (3.46 to 5.31)	124.8 (110 to 161.9)	145.9 (111.8 to 166)	117.2 (94.4 to 139.7)
Albania	Hypertensive heart disease	2.11 (1.48 to 2.34)	1.87 (1.65 to 2.3)	2.11 (1.72 to 2.89)	102.4 (71.9 to 114.3)	60.8 (54.1 to 73.8)	50.4 (41.6 to 69)
Albania	Non-rheumatic valvular heart disease	0.998 (0.791 to 1.12)	0.945 (0.786 to 1.23)	1.23 (0.98 to 1.6)	46.8 (35.6 to 52.7)	31 (26 to 39.1)	30.3 (24.2 to 39.7)
Albania	Non-rheumatic calcific aortic valve disease	0.441 (0.335 to 0.528)	0.5 (0.402 to 0.677)	0.695 (0.533 to 0.904)	21 (15.7 to 25.2)	16.3 (13.2 to 21.5)	17 (13.1 to 22.1)

SR Appendix Table 2. All-age DALYs counts and age standardized DALYs rates per 100,000 individuals for all causes and level 1 cause groups by location for 1990, 2007, and 2017, both sexes combined							
		All-age DALYs			Age-standardized rate of DALYs per 100,000		
Albania	Non-rheumatic degenerative mitral valve disease	0.471 (0.355 to 0.575)	0.345 (0.248 to 0.484)	0.422 (0.305 to 0.58)	22.4 (15.6 to 27.8)	11.5 (8.5 to 15.4)	10.3 (7.5 to 14.3)
Albania	Other non-rheumatic valve diseases	0.0862 (0.0466 to 0.114)	0.0995 (0.0473 to 0.133)	0.112 (0.0522 to 0.163)	3.4 (1.8 to 4.5)	3.2 (1.5 to 4.2)	3 (1.4 to 4.3)
Albania	Cardiomyopathy and myocarditis	8.59 (7.96 to 9.2)	6.84 (6.15 to 7.63)	7.12 (6.04 to 8.59)	333.9 (308.8 to 356.6)	239.7 (215.3 to 269)	206.9 (175.2 to 248)
Albania	Myocarditis	4.53 (3.67 to 5.07)	3.25 (2.83 to 3.69)	3.26 (2.65 to 4.02)	175 (136.4 to 197.8)	115 (100.5 to 131.6)	96.5 (78.8 to 120.3)
Albania	Alcoholic cardiomyopathy	0.617 (0.529 to 0.846)	0.482 (0.277 to 0.652)	0.535 (0.341 to 0.746)	26.3 (22.2 to 37)	15.1 (8.7 to 20.2)	13.9 (8.7 to 19.6)
Albania	Other cardiomyopathy	3.44 (3.05 to 4.09)	3.11 (2.71 to 3.62)	3.32 (2.73 to 4.22)	132.7 (116.3 to 160.5)	109.5 (95.5 to 128.2)	96.5 (80.1 to 118.7)
Albania	Atrial fibrillation and flutter	1.5 (1.19 to 1.84)	2.62 (2.13 to 3.18)	3.59 (2.91 to 4.42)	74.3 (59.5 to 90.7)	85.5 (70.1 to 102.6)	85.3 (69.4 to 104.9)
Albania	Aortic aneurysm	0.523 (0.472 to 0.604)	0.73 (0.615 to 0.852)	0.846 (0.67 to 1.05)	21.8 (19.6 to 25.6)	22.6 (19.1 to 26.3)	21.5 (17 to 26.5)
Albania	Peripheral vascular disease	0.142 (0.0769 to 0.235)	0.214 (0.12 to 0.349)	0.29 (0.168 to 0.46)	7 (3.8 to 11.6)	6.7 (3.8 to 10.9)	6.7 (3.9 to 10.6)
Albania	Endocarditis	0.694 (0.614 to 0.781)	0.612 (0.558 to 0.67)	0.643 (0.542 to 0.761)	22 (19.9 to 24.4)	22.8 (20.5 to 25.3)	21 (17.6 to 24.7)
Albania	Other cardiovascular and circulatory diseases	10.7 (9 to 13.5)	16 (13.9 to 18.5)	17.3 (14.2 to 20.7)	437.2 (363.6 to 557)	517.4 (450 to 590)	456.1 (381.4 to 543)
Albania	Chronic respiratory diseases	26.6 (23.9 to 29.2)	21.8 (19.2 to 24.3)	25.2 (21.9 to 28.6)	1155 (1040.4 to 1258.8)	692 (609.9 to 772.6)	651.9 (564.6 to 746.5)
Albania	Chronic obstructive pulmonary disease	16.9 (15.4 to 18.4)	13.9 (12.3 to 15.4)	17.2 (15 to 19.5)	782.7 (712.4 to 850.6)	438.5 (391.3 to 485.7)	419.7 (365.8 to 475.6)
Albania	Pneumoconiosis	0.276 (0.237 to 0.326)	0.216 (0.179 to 0.252)	0.256 (0.193 to 0.338)	11.8 (10.2 to 13.9)	6.4 (5.4 to 7.5)	6.2 (4.7 to 8.1)
Albania	Silicosis	0.227 (0.154 to 0.278)	0.107 (0.0816 to 0.133)	0.123 (0.0839 to 0.176)	9.8 (6.8 to 11.9)	3.1 (2.4 to 3.9)	2.9 (2 to 4.1)
Albania	Asbestosis	0.00315 (0.00205 to 0.00454)	0.00387 (0.00262 to 0.00553)	0.00506 (0.0034 to 0.0072)	0.1 (0.1 to 0.2)	0.1 (0.1 to 0.2)	0.1 (0.1 to 0.2)
Albania	Coal workers pneumoconiosis	0.014 (0.00982 to 0.0324)	0.0288 (0.0213 to 0.0381)	0.0362 (0.0241 to 0.0528)	0.6 (0.4 to 1.4)	0.9 (0.6 to 1.1)	0.9 (0.6 to 1.3)
Albania	Other pneumoconiosis	0.0313 (0.0226 to 0.0813)	0.077 (0.0555 to 0.096)	0.0927 (0.0584 to 0.131)	1.3 (0.9 to 3.4)	2.4 (1.7 to 2.9)	2.3 (1.5 to 3.3)
Albania	Asthma	6.49 (5.18 to 7.94)	5.24 (4.11 to 6.64)	5.03 (3.85 to 6.42)	248.4 (205.1 to 297.4)	170.1 (132.9 to 215.5)	151.1 (111.8 to 197.7)
Albania	Interstitial lung disease and pulmonary sarcoidosis	1.31 (0.916 to 1.61)	1.28 (1.02 to 1.61)	1.52 (1.2 to 1.93)	57.4 (36.5 to 71.2)	39.6 (31.9 to 49.8)	38.9 (30.5 to 49.5)
Albania	Other chronic respiratory diseases	1.62 (1.41 to 1.82)	1.11 (0.964 to 1.28)	1.11 (0.943 to 1.31)	54.7 (48.3 to 60.9)	37.2 (32 to 42.7)	36 (30.6 to 42)
Albania	Digestive diseases	30.2 (24.7 to 35.6)	22.4 (18.4 to 27.2)	23 (18.5 to 28.8)	1025.6 (863.6 to 1196.3)	727.1 (599.5 to 880.7)	692.8 (550.9 to 862.7)
Albania	Cirrhosis and other chronic liver diseases	7.47 (6.87 to 8.17)	7.78 (6.56 to 10.2)	8.66 (6.97 to 11.7)	298.9 (273.9 to 324.2)	238.3 (201.9 to 309.9)	232.3 (186.4 to 315.7)
Albania	Cirrhosis and other chronic liver diseases due to hepatitis B	2.15 (1.91 to 2.45)	1.97 (1.61 to 2.68)	2.17 (1.69 to 2.94)	87.4 (77.7 to 98.7)	60 (49 to 81.2)	57.8 (45.1 to 80.2)
Albania	Cirrhosis and other chronic liver diseases due to hepatitis C	1.52 (1.33 to 1.75)	1.7 (1.38 to 2.29)	1.89 (1.47 to 2.58)	63 (55 to 72.5)	51 (41.5 to 68.6)	49.3 (38.1 to 67.9)
Albania	Cirrhosis and other chronic liver diseases due to alcohol use	2.23 (2.02 to 2.49)	2.7 (2.23 to 3.63)	3.13 (2.47 to 4.31)	94.5 (84.5 to 105)	80.9 (67 to 108.4)	80.1 (63.2 to 112.5)
Albania	Cirrhosis due to NASH	0.407 (0.357 to 0.457)	0.511 (0.414 to 0.68)	0.636 (0.498 to 0.862)	17.1 (15 to 19.2)	15.2 (12.4 to 20.2)	16.2 (12.7 to 22.3)
Albania	Cirrhosis and other chronic liver diseases due to other causes	1.16 (0.864 to 1.36)	0.899 (0.769 to 1.06)	0.837 (0.673 to 1.03)	36.9 (27.8 to 43.1)	31.2 (26.7 to 36.6)	28.9 (23.6 to 35.1)
Albania	Upper digestive system diseases	8.99 (6.23 to 12.8)	7.38 (4.98 to 10.7)	7.01 (4.71 to 10.1)	300.9 (213.3 to 422.2)	236.6 (161.1 to 344.4)	223.2 (150 to 327.6)
Albania	Peptic ulcer disease	1.54 (1.37 to 1.73)	0.991 (0.823 to 1.18)	1.03 (0.836 to 1.28)	63.5 (56.2 to 71.1)	31.3 (26.1 to 37.1)	27.3 (22.2 to 34)
Albania	Gastritis and duodenitis	5.23 (3.37 to 7.68)	3.91 (2.52 to 5.65)	3.38 (2.18 to 4.87)	159.1 (102.6 to 232.8)	127.3 (81.2 to 185)	118.3 (75.6 to 171.6)
Albania	GERD	2.21 (1.18 to 3.77)	2.48 (1.32 to 4.2)	2.61 (1.4 to 4.37)	78.4 (41.8 to 134.7)	78.1 (41.9 to 132.3)	77.6 (41.8 to 132)
Albania	Appendicitis	0.311 (0.25 to 0.366)	0.198 (0.166 to 0.238)	0.144 (0.117 to 0.176)	9.6 (8 to 11.1)	6.7 (5.7 to 8)	5.2 (4.2 to 6.5)
Albania	Paralytic ileus and intestinal obstruction	3.03 (1.62 to 3.95)	0.889 (0.735 to 1.15)	0.824 (0.632 to 1.09)	82.1 (47.4 to 104.5)	38.5 (31.5 to 47.5)	32.5 (23.3 to 44.7)
Albania	Inguinal, femoral, and abdominal hernia	3.23 (2.27 to 4.36)	2.65 (1.85 to 3.58)	2.67 (1.87 to 3.6)	107.3 (76.7 to 144.6)	91.2 (63.8 to 123)	87.6 (60.5 to 118.1)
Albania	Inflammatory bowel disease	3.63 (1.18 to 4.68)	1.13 (0.84 to 1.48)	1.15 (0.821 to 1.55)	103.7 (42.1 to 131.2)	38.8 (29.5 to 49.6)	36.3 (26.1 to 48.1)
Albania	Vascular intestinal disorders	0.181 (0.15 to 0.205)	0.262 (0.219 to 0.347)	0.319 (0.252 to 0.432)	7.8 (6.6 to 8.7)	8.4 (7.1 to 10.9)	7.9 (6.3 to 10.6)
Albania	Gallbladder and biliary diseases	0.267 (0.231 to 0.299)	0.257 (0.22 to 0.315)	0.313 (0.252 to 0.395)	11.2 (9.7 to 12.5)	8.3 (7.1 to 10.1)	8 (6.5 to 10.1)
Albania	Pancreatitis	0.652 (0.497 to 0.972)	0.92 (0.707 to 1.41)	0.994 (0.703 to 1.55)	25.3 (19.6 to 36.6)	28.4 (21.8 to 44.1)	27.4 (18.9 to 45.6)
Albania	Other digestive diseases	2.47 (1.98 to 2.96)	0.918 (0.73 to 1.13)	0.977 (0.771 to 1.2)	78.7 (65.3 to 91.7)	31.8 (25.3 to 39.3)	32.3 (25.1 to 40.4)

SR Appendix Table 2. All-age DALYs counts and age standardized DALYs rates per 100,000 individuals for all causes and level 1 cause groups by location for 1990, 2007, and 2017, both sexes combined

		All-age DALYs			Age-standardized rate of DALYs per 100,000		
Albania	Neurological disorders	44<br>(33.1 to 58)	49.1<br>(38.2 to 62.4)	52.1<br>(41.1 to 65.2)	1558.3<br>(1208.1 to 1992.5)	1668.5<br>(1315.6 to 2101.6)	1579<br>(1223.9 to 2014)
Albania	Alzheimer's disease and other dementias	6.57<br>(5.91 to 7.35)	11.7<br>(10.6 to 12.7)	16.8<br>(14.5 to 19.2)	358.2<br>(320.8 to 402.5)	438<br>(400.5 to 476.4)	417.7<br>(361.7 to 475.8)
Albania	Parkinson's disease	1.54<br>(1.3 to 2.48)	2.68<br>(2.31 to 3.92)	3.71<br>(3.03 to 5.65)	79.7<br>(66.7 to 130.4)	87.5<br>(75.8 to 128.9)	85.9<br>(70.3 to 130.1)
Albania	Epilepsy	8.61<br>(4.87 to 14.2)	7.57<br>(4.73 to 11.6)	5.79<br>(2.94 to 9.97)	250<br>(138.3 to 416)	266.3<br>(169.8 to 401.9)	216.1<br>(114.9 to 365.2)
Albania	Multiple sclerosis	1.18<br>(0.942 to 1.63)	1.27<br>(0.893 to 2.16)	1.15<br>(0.77 to 2.15)	46.3<br>(38.1 to 60.8)	39.3<br>(28 to 65.6)	33.1<br>(21.7 to 62.7)
Albania	Motor neuron disease	0.119<br>(0.0784 to 0.133)	0.0979<br>(0.0781 to 0.132)	0.127<br>(0.0977 to 0.163)	4.4<br>(2.8 to 4.9)	2.9<br>(2.3 to 4.1)	3.3<br>(2.6 to 4.4)
Albania	Headache disorders	22.6<br>(14.6 to 33.1)	23.1<br>(14.9 to 33.9)	22.1<br>(14.3 to 32.5)	724.5<br>(466.9 to 1063)	734.5<br>(473.3 to 1082.8)	732.3<br>(467.5 to 1076.9)
Albania	Migraine	19.5<br>(12 to 29.5)	19.8<br>(12.2 to 29.9)	18.9<br>(11.8 to 28.5)	620.7<br>(381.9 to 942.1)	629.7<br>(387.8 to 953.2)	627.9<br>(392.1 to 945.1)
Albania	Tension-type headache	3.19<br>(1.83 to 4.98)	3.31<br>(1.87 to 5.16)	3.24<br>(1.84 to 5.02)	103.8<br>(58.9 to 162.3)	104.8<br>(59.3 to 163.9)	104.4<br>(59 to 164.6)
Albania	Other neurological disorders	3.34<br>(2.54 to 4.9)	2.76<br>(2.16 to 3.59)	2.41<br>(1.79 to 3.33)	95.1<br>(73.4 to 135.8)	99.9<br>(77.6 to 130.4)	90.5<br>(65 to 125.3)
Albania	Mental disorders	39.9<br>(29.4 to 51.8)	40.2<br>(30 to 51.9)	38.3<br>(28.7 to 49.5)	1264.7<br>(942.1 to 1639.3)	1283.4<br>(958.6 to 1654.3)	1275.9<br>(949.5 to 1656.6)
Albania	Schizophrenia	3.49<br>(2.55 to 4.35)	3.79<br>(2.82 to 4.75)	3.85<br>(2.84 to 4.78)	118.8<br>(87.5 to 147.5)	121<br>(90.2 to 151.6)	122.2<br>(88.9 to 152.8)
Albania	Depressive disorders	9.66<br>(6.89 to 13.3)	10.8<br>(7.75 to 14.7)	11<br>(7.83 to 15)	327.4<br>(234.9 to 446.7)	340.3<br>(243.1 to 463.4)	335.8<br>(240.4 to 457.6)
Albania	Major depressive disorder	7.23<br>(5.04 to 9.97)	8.09<br>(5.7 to 11)	8.13<br>(5.72 to 11)	243<br>(170.2 to 331.5)	255.1<br>(179.5 to 346.5)	250.8<br>(174.8 to 342.6)
Albania	Dysthymia	2.43<br>(1.63 to 3.56)	2.72<br>(1.82 to 3.96)	2.84<br>(1.89 to 4.15)	84.4<br>(56.6 to 123.1)	85.2<br>(57.3 to 124.5)	85.1<br>(56.7 to 123.7)
Albania	Bipolar disorder	4.29<br>(2.66 to 6.44)	4.35<br>(2.72 to 6.51)	4.2<br>(2.64 to 6.25)	136.9<br>(85.3 to 204.5)	137.4<br>(85.9 to 203.9)	137.2<br>(86.5 to 204.3)
Albania	Anxiety disorders	9.71<br>(6.8 to 13)	9.74<br>(6.91 to 12.8)	9.23<br>(6.56 to 12.2)	306.5<br>(215.8 to 409.2)	310.5<br>(220 to 409.8)	308.4<br>(217.7 to 410.1)
Albania	Eating disorders	0.946<br>(0.59 to 1.42)	0.919<br>(0.574 to 1.36)	0.923<br>(0.58 to 1.37)	27<br>(17 to 40.5)	31.1<br>(19.5 to 45.9)	35.5<br>(22.1 to 52.4)
Albania	Anorexia nervosa	0.2<br>(0.113 to 0.311)	0.191<br>(0.113 to 0.295)	0.181<br>(0.107 to 0.281)	5.5<br>(3.1 to 8.6)	6.3<br>(3.7 to 9.8)	7<br>(4.2 to 10.8)
Albania	Bulimia nervosa	0.746<br>(0.454 to 1.14)	0.728<br>(0.446 to 1.1)	0.742<br>(0.459 to 1.14)	21.5<br>(13.1 to 32.7)	24.8<br>(15.2 to 37.4)	28.4<br>(17.5 to 43.7)
Albania	Autism spectrum disorders	2.27<br>(1.54 to 3.14)	1.93<br>(1.32 to 2.66)	1.76<br>(1.2 to 2.4)	66.7<br>(45.2 to 92.2)	65.9<br>(44.9 to 91.5)	66.5<br>(45.1 to 91.8)
Albania	Attention-deficit/hyperactivity disorder	0.431<br>(0.256 to 0.686)	0.352<br>(0.211 to 0.55)	0.286<br>(0.169 to 0.456)	11.8<br>(7.1 to 18.8)	11.7<br>(7 to 18.3)	11.9<br>(6.9 to 18.9)
Albania	Conduct disorder	3.87<br>(2.31 to 6.1)	3.11<br>(1.85 to 4.89)	2.1<br>(1.24 to 3.31)	97.8<br>(58.6 to 153.9)	97.7<br>(58.1 to 153.9)	98.4<br>(58.4 to 156)
Albania	Idiopathic developmental intellectual disability	1.09<br>(0.378 to 2)	0.815<br>(0.316 to 1.46)	0.509<br>(0.148 to 0.967)	31.3<br>(10.6 to 57.6)	27.9<br>(10.9 to 50)	19.8<br>(6 to 37.4)
Albania	Other mental disorders	4.14<br>(2.75 to 5.82)	4.37<br>(2.88 to 6.01)	4.48<br>(2.99 to 6.23)	140.5<br>(93.4 to 196)	139.9<br>(91.8 to 192.7)	140.3<br>(93 to 195)
Albania	Substance use disorders	8.9<br>(6.31 to 11.7)	9.47<br>(7.06 to 12.2)	9.63<br>(7.09 to 12.3)	276.6<br>(197.1 to 362.9)	311.3<br>(231.9 to 402.4)	324<br>(238.3 to 415.9)
Albania	Alcohol use disorders	5.55<br>(3.83 to 7.69)	5.65<br>(3.98 to 7.7)	5.73<br>(4.1 to 7.76)	175.3<br>(121.1 to 240.3)	184.5<br>(129.5 to 251.8)	190.9<br>(135.8 to 258.7)
Albania	Drug use disorders	3.34<br>(2.42 to 4.37)	3.82<br>(2.92 to 4.7)	3.9<br>(2.95 to 4.88)	101.3<br>(73.8 to 131.5)	126.8<br>(97.1 to 155.9)	133.1<br>(100.1 to 168.3)
Albania	Opioid use disorders	2.1<br>(1.47 to 2.86)	2.31<br>(1.71 to 2.95)	2.5<br>(1.84 to 3.21)	65.2<br>(45.9 to 88)	77.5<br>(57.2 to 99.5)	85.2<br>(62.4 to 109.8)
Albania	Cocaine use disorders	0.181<br>(0.102 to 0.28)	0.176<br>(0.109 to 0.266)	0.173<br>(0.102 to 0.255)	5.5<br>(3.2 to 8.3)	5.7<br>(3.5 to 8.5)	5.9<br>(3.4 to 8.7)
Albania	Amphetamine use disorders	0.353<br>(0.192 to 0.587)	0.325<br>(0.182 to 0.524)	0.304<br>(0.177 to 0.486)	9.8<br>(5.4 to 16.1)	10.8<br>(6.2 to 17.3)	11<br>(6.4 to 17.6)
Albania	Cannabis use disorders	0.216<br>(0.132 to 0.326)	0.185<br>(0.115 to 0.277)	0.162<br>(0.0993 to 0.245)	6.1<br>(3.8 to 9.1)	6<br>(3.7 to 8.9)	5.8<br>(3.5 to 8.9)
Albania	Other drug use disorders	0.497<br>(0.332 to 0.707)	0.821<br>(0.626 to 1.02)	0.753<br>(0.567 to 0.965)	14.7<br>(10 to 20.5)	26.8<br>(20.6 to 33.4)	25.2<br>(18.8 to 32.6)
Albania	Diabetes and kidney diseases	19.2<br>(16.5 to 22.4)	22.2<br>(18.2 to 26.9)	25.9<br>(20.7 to 32)	747.2<br>(637.1 to 875.8)	691.2<br>(570.3 to 833.7)	690.8<br>(551.3 to 848.1)
Albania	Diabetes mellitus	8.63<br>(6.41 to 11.3)	12.8<br>(9.25 to 16.8)	15.9<br>(11.4 to 21)	355<br>(264 to 461.7)	388.8<br>(281.6 to 511)	412.5<br>(296.2 to 547)
Albania	Diabetes mellitus type 1	1.43<br>(1.27 to 1.64)	1.22<br>(1.04 to 1.49)	1.14<br>(0.905 to 1.52)	51.9<br>(46.1 to 60.4)	38.6<br>(32.9 to 46.7)	34.1<br>(27.3 to 43.3)
Albania	Diabetes mellitus type 2	7.2<br>(5.11 to 9.76)	11.6<br>(8.2 to 15.5)	14.7<br>(10.4 to 19.8)	303.1<br>(217.3 to 406.8)	350.2<br>(248.2 to 470.1)	378.4<br>(265.8 to 509)
Albania	Chronic kidney disease	9.97<br>(9.25 to 10.8)	9.37<br>(8.56 to 10.2)	10<br>(8.61 to 11.8)	373.3<br>(346.7 to 403.5)	300<br>(274.4 to 326.2)	277.3<br>(238.3 to 322.7)
Albania	Chronic kidney disease due to diabetes mellitus type 1	0.477<br>(0.37 to 0.596)	0.466<br>(0.362 to 0.592)	0.431<br>(0.32 to 0.571)	17.6<br>(13.7 to 22.1)	14.3<br>(11.2 to 17.9)	12.8<br>(9.5 to 16.8)
Albania	Chronic kidney disease due to diabetes mellitus type 2	0.877<br>(0.726 to 1.07)	1.09<br>(0.897 to 1.34)	1.32<br>(1.04 to 1.65)	39.7<br>(32.9 to 48.8)	33<br>(27.1 to 40.1)	31.3<br>(24.9 to 39.2)

SR Appendix Table 2. All-age DALYs counts and age standardized DALYs rates per 100,000 individuals for all causes and level 1 cause groups by location for 1990, 2007, and 2017, both sexes combined

		All-age DALYs			Age-standardized rate of DALYs per 100,000		
Albania	Chronic kidney disease due to hypertension	1.13 (0.951 to 1.31)	1.29 (1.08 to 1.52)	1.54 (1.23 to 1.89)	49.4 (41.6 to 57.7)	41.1 (34.6 to 47.8)	38.6 (31.1 to 47.5)
Albania	Chronic kidney disease due to glomerulonephritis	2.6 (2.26 to 2.94)	2.26 (1.95 to 2.6)	2.24 (1.8 to 2.75)	91 (78.9 to 103.2)	72.4 (62.8 to 83.1)	65.8 (53.3 to 80)
Albania	Chronic kidney disease due to other and unspecified causes	4.89 (4.4 to 5.46)	4.26 (3.8 to 4.78)	4.52 (3.77 to 5.38)	175.6 (156.8 to 196.5)	139.3 (124.7 to 155.7)	128.8 (108.6 to 150.3)
Albania	Acute glomerulonephritis	0.597 (0.513 to 0.696)	0.0697 (0.0581 to 0.0828)	0.0316 (0.0242 to 0.0397)	18.9 (16.5 to 21.6)	2.4 (2 to 2.9)	1 (0.8 to 1.3)
Albania	Skin and subcutaneous diseases	15 (10.1 to 21.7)	13.1 (8.86 to 19.3)	12.3 (8.33 to 18.2)	446.5 (304 to 648.5)	447.1 (302.6 to 655.7)	452 (306.4 to 662.6)
Albania	Dermatitis	3.43 (2.02 to 5.47)	2.83 (1.72 to 4.41)	2.56 (1.56 to 3.98)	99.2 (58.6 to 156)	99 (59.4 to 155.4)	100.6 (59.6 to 157.9)
Albania	Atopic dermatitis	2.72 (1.47 to 4.58)	2.1 (1.14 to 3.51)	1.85 (1 to 3.06)	76.1 (41 to 127.9)	75.8 (41.2 to 126.3)	77.5 (41.8 to 128.5)
Albania	Contact dermatitis	0.712 (0.456 to 1.06)	0.728 (0.479 to 1.07)	0.712 (0.461 to 1.05)	23 (14.8 to 34)	23.1 (15.1 to 33.9)	23 (14.9 to 34.6)
Albania	Seborrhoeic dermatitis	0.0035 (0.00197 to 0.0057)	0.00318 (0.00178 to 0.00514)	0.00305 (0.0017 to 0.00491)	0.1 (0.1 to 0.2)	0.1 (0.1 to 0.2)	0.1 (0.1 to 0.2)
Albania	Psoriasis	1.78 (1.25 to 2.35)	1.99 (1.38 to 2.62)	2.02 (1.44 to 2.66)	59.8 (42.3 to 78.4)	62.9 (43.5 to 83.1)	63.5 (45.1 to 83.6)
Albania	Bacterial skin diseases	0.452 (0.149 to 0.713)	0.179 (0.13 to 0.282)	0.191 (0.137 to 0.313)	12.8 (5.1 to 19.3)	6.4 (4.7 to 10.1)	6.3 (4.6 to 9.9)
Albania	Cellulitis	0.0971 (0.0602 to 0.17)	0.0718 (0.0505 to 0.101)	0.0765 (0.0541 to 0.111)	3 (1.9 to 4.9)	2.4 (1.7 to 3.4)	2.5 (1.8 to 3.6)
Albania	Pyoderma	0.355 (0.0824 to 0.604)	0.107 (0.0743 to 0.193)	0.114 (0.0763 to 0.213)	9.8 (2.9 to 15.9)	4 (2.8 to 7.1)	3.8 (2.6 to 6.7)
Albania	Scabies	1.08 (0.594 to 1.75)	0.851 (0.476 to 1.38)	0.724 (0.397 to 1.18)	30.6 (17 to 49.7)	29 (16.2 to 46.8)	28.2 (15.6 to 46)
Albania	Fungal skin diseases	1.44 (0.575 to 3)	1.37 (0.55 to 2.86)	1.44 (0.577 to 2.96)	48.6 (19.4 to 101.7)	45.7 (18.2 to 94.8)	44.9 (17.9 to 93.1)
Albania	Viral skin diseases	1.74 (1.11 to 2.58)	1.21 (0.765 to 1.81)	0.941 (0.597 to 1.4)	45.7 (29 to 67.8)	45.8 (29.1 to 68.1)	45.8 (29.1 to 68.1)
Albania	Acne vulgaris	0.91 (0.527 to 1.51)	1 (0.581 to 1.67)	0.871 (0.514 to 1.42)	24.6 (14.3 to 40.7)	31.3 (18.2 to 51.8)	33.5 (19.7 to 54.7)
Albania	Alopecia areata	0.19 (0.12 to 0.287)	0.179 (0.113 to 0.268)	0.172 (0.109 to 0.256)	5.9 (3.7 to 8.9)	5.9 (3.7 to 8.9)	5.9 (3.7 to 8.8)
Albania	Pruritus	0.275 (0.13 to 0.524)	0.287 (0.136 to 0.529)	0.298 (0.143 to 0.55)	9.2 (4.4 to 17.3)	9.3 (4.4 to 17.4)	9.5 (4.5 to 17.7)
Albania	Urticaria	2.64 (1.74 to 3.77)	2.07 (1.37 to 2.95)	1.81 (1.21 to 2.57)	74.3 (49.2 to 105.5)	74.8 (49.4 to 106.3)	74.6 (49.5 to 105)
Albania	Decubitus ulcer	0.0792 (0.0591 to 0.102)	0.0878 (0.0651 to 0.116)	0.11 (0.0792 to 0.145)	3.1 (2.3 to 4)	2.9 (2.2 to 3.9)	3.1 (2.3 to 4)
Albania	Other skin and subcutaneous diseases	0.945 (0.462 to 1.73)	1.07 (0.523 to 1.97)	1.2 (0.585 to 2.2)	32.7 (16.1 to 59.8)	34.1 (16.7 to 62.9)	36 (17.6 to 66.2)
Albania	Sense organ diseases	19.8 (13.2 to 28.8)	24.2 (16.1 to 35.3)	28 (18.7 to 40.4)	791.3 (531 to 1143.1)	769.2 (513 to 1113.5)	754.1 (502.1 to 1096.7)
Albania	Blindness and vision impairment	8.43 (5.41 to 12.8)	9.95 (6.44 to 14.8)	11.2 (7.26 to 16.6)	333.1 (214.6 to 496.7)	314.3 (203.4 to 466.4)	304 (195.6 to 452.6)
Albania	Glaucoma	0.0871 (0.058 to 0.124)	0.121 (0.078 to 0.17)	0.152 (0.0991 to 0.214)	4.3 (2.8 to 6)	3.7 (2.4 to 5.3)	3.5 (2.3 to 4.9)
Albania	Cataract	2.18 (1.47 to 2.99)	3.18 (2.13 to 4.37)	4.1 (2.74 to 5.65)	106.2 (72 to 144.9)	100.7 (68 to 137.5)	97.3 (65 to 133.3)
Albania	Age-related macular degeneration	0.018 (0.0113 to 0.0265)	0.0275 (0.0171 to 0.0406)	0.0371 (0.0225 to 0.0541)	0.9 (0.6 to 1.3)	0.9 (0.5 to 1.3)	0.9 (0.5 to 1.2)
Albania	Refraction disorders	3.51 (2.21 to 5.39)	3.4 (2.13 to 5.17)	3.32 (2.12 to 5.01)	115.4 (73.1 to 176)	110.2 (69.5 to 167.8)	106.9 (67.9 to 162.6)
Albania	Near vision loss	2.01 (0.953 to 3.69)	2.46 (1.18 to 4.51)	2.72 (1.31 to 4.96)	80.7 (38.2 to 148.9)	75 (35.8 to 137.3)	72.3 (34.9 to 132.4)
Albania	Other vision loss	0.634 (0.424 to 0.876)	0.762 (0.514 to 1.06)	0.872 (0.574 to 1.21)	25.7 (17.2 to 35.4)	23.9 (16.1 to 33.1)	23.2 (15.3 to 32)
Albania	Age-related and other hearing loss	10.6 (7.21 to 14.8)	13.3 (9.03 to 18.9)	15.7 (10.7 to 22.1)	427.8 (294.3 to 596.7)	424 (289.8 to 598.8)	419.1 (286.5 to 591.3)
Albania	Other sense organ diseases	0.791 (0.488 to 1.17)	0.977 (0.605 to 1.42)	1.13 (0.701 to 1.63)	30.4 (18.9 to 44.4)	30.9 (19.2 to 45)	31 (19.1 to 45.4)
Albania	Musculoskeletal disorders	49.8 (35.9 to 68)	60 (43.6 to 81.3)	64.6 (46.6 to 87.2)	1838.5 (1329.1 to 2495)	1857.7 (1346.1 to 2518.1)	1848.1 (1333.3 to 2486.7)
Albania	Rheumatoid arthritis	0.609 (0.417 to 0.828)	0.98 (0.703 to 1.28)	1.16 (0.817 to 1.52)	23.7 (16.4 to 32.1)	29.8 (21.4 to 39)	30.8 (21.7 to 40.8)
Albania	Osteoarthritis	1.88 (0.945 to 3.74)	2.97 (1.47 to 5.86)	3.8 (1.91 to 7.42)	83.9 (42.3 to 167.7)	89.4 (44.6 to 176.7)	92.6 (46.5 to 181.1)
Albania	Low back pain	38.8 (27.7 to 52.6)	45.2 (32 to 60.8)	47.9 (34.3 to 64.8)	1405.3 (996 to 1901.8)	1405.4 (994 to 1912)	1393.9 (987.3 to 1887.8)
Albania	Neck pain	7.71 (5.42 to 10.8)	9.78 (6.82 to 13.7)	10.5 (7.3 to 14.8)	295.7 (207.6 to 411.8)	297.4 (207.7 to 418.2)	297.1 (206.2 to 414)
Albania	Gout	0.266 (0.174 to 0.374)	0.391 (0.259 to 0.549)	0.475 (0.315 to 0.665)	11.3 (7.5 to 15.8)	11.8 (7.8 to 16.5)	12 (8 to 16.7)
Albania	Other musculoskeletal disorders	0.524 (0.212 to 0.921)	0.742 (0.369 to 1.21)	0.701 (0.36 to 1.17)	18.5 (7.9 to 32.6)	23.8 (12.3 to 38.6)	21.7 (10.5 to 37.1)
Albania	Other non-communicable diseases	56 (48 to 66.2)	45.2 (37.6 to 54.4)	37 (29 to 46.6)	1626.5 (1383 to 1929.3)	1895.1 (1605.7 to 2221.6)	1476.6 (1157.3 to 1846.1)

SR Appendix Table 2. All-age DALYs counts and age standardized DALYs rates per 100,000 individuals for all causes and level 1 cause groups by location for 1990, 2007, and 2017, both sexes combined							
		All-age DALYs			Age-standardized rate of DALYs per 100,000		
Albania	Congenital anomalies	30.2<br>(26.4 to 34.3)	24.4<br>(20.9 to 28.4)	16.6<br>(12.9 to 20.8)	788.9<br>(688.1 to 896.9)	1195.5<br>(1018.6 to 1404.3)	827.1<br>(632.3 to 1069)
Albania	Neural tube defects	3.98<br>(2.61 to 5.4)	2.07<br>(1.46 to 3.01)	1.06<br>(0.63 to 1.7)	100<br>(65.7 to 135.2)	112.4<br>(78.3 to 165.1)	58.8<br>(33.6 to 95.3)
Albania	Congenital heart anomalies	14.1<br>(9.87 to 17.7)	12.6<br>(9.28 to 15.8)	7.62<br>(5.63 to 10.5)	352.7<br>(247.6 to 442.9)	666.2<br>(485 to 843.2)	429.2<br>(313 to 594.3)
Albania	Orofacial clefts	0.121<br>(0.0802 to 0.175)	0.088<br>(0.0583 to 0.128)	0.0722<br>(0.048 to 0.107)	3.5<br>(2.3 to 5.1)	3.2<br>(2.1 to 4.6)	2.8<br>(1.8 to 4.2)
Albania	Down's syndrome	1.26<br>(0.536 to 2.69)	0.692<br>(0.405 to 1.23)	0.486<br>(0.321 to 0.764)	33.1<br>(15.2 to 68.3)	31.5<br>(17 to 59.8)	23.2<br>(14.2 to 39)
Albania	Turner syndrome	0.00337<br>(0.00163 to 0.00556)	0.00294<br>(0.00146 to 0.00494)	0.0025<br>(0.0012 to 0.00414)	0.1<br>(0 to 0.2)	0.1<br>(0.1 to 0.2)	0.1<br>(0 to 0.2)
Albania	Klinefelter syndrome	0.000678<br>(0.000312 to 0.00128)	0.000516<br>(0.000241 to 0.000973)	0.000472<br>(0.000219 to 0.000905)	0<br>(0 to 0)	0<br>(0 to 0)	0<br>(0 to 0)
Albania	Other chromosomal abnormalities	0.73<br>(0.395 to 1.03)	0.763<br>(0.356 to 1.19)	0.556<br>(0.287 to 0.9)	19.1<br>(10.6 to 26.8)	40<br>(16.9 to 64.1)	30.1<br>(14.7 to 49.7)
Albania	Congenital musculoskeletal and limb anomalies	4.39<br>(3.01 to 6.01)	3.86<br>(2.61 to 5.25)	3.61<br>(2.43 to 4.92)	132.5<br>(91.2 to 181.8)	134.6<br>(91.8 to 181.6)	130.6<br>(88.7 to 178.8)
Albania	Urogenital congenital anomalies	0.616<br>(0.308 to 1.23)	0.388<br>(0.233 to 0.834)	0.267<br>(0.16 to 0.492)	15.6<br>(7.9 to 30.9)	19.7<br>(11.3 to 44.8)	14.3<br>(8.2 to 27.4)
Albania	Digestive congenital anomalies	1.67<br>(1.26 to 2.22)	1.52<br>(1.13 to 2.09)	1.16<br>(0.812 to 1.61)	45.6<br>(34.2 to 60.1)	68.5<br>(51.1 to 97.2)	51.6<br>(35.5 to 75)
Albania	Other congenital anomalies	3.33<br>(2.26 to 4.8)	2.45<br>(1.75 to 3.72)	1.72<br>(1.19 to 2.64)	86.7<br>(60.4 to 123.9)	119.4<br>(83.8 to 189.7)	86.4<br>(57.3 to 137.8)
Albania	Urinary diseases and male infertility	2.67<br>(2.2 to 3.24)	2.25<br>(1.64 to 3.01)	2.62<br>(1.9 to 3.53)	99.9<br>(81 to 122.9)	69.8<br>(51.7 to 92.5)	67.2<br>(49.5 to 89.8)
Albania	Urinary tract infections	0.697<br>(0.516 to 0.874)	0.328<br>(0.274 to 0.403)	0.36<br>(0.284 to 0.466)	23.2<br>(18.6 to 29)	11.4<br>(9.6 to 13.8)	11.1<br>(8.9 to 14)
Albania	Urolithiasis	0.17<br>(0.118 to 0.224)	0.152<br>(0.104 to 0.212)	0.166<br>(0.113 to 0.231)	6.3<br>(4.4 to 8.2)	4.7<br>(3.2 to 6.5)	4.8<br>(3.3 to 6.6)
Albania	Benign prostatic hyperplasia	0.926<br>(0.584 to 1.36)	1.42<br>(0.915 to 2.08)	1.77<br>(1.13 to 2.59)	41.1<br>(25.9 to 60.3)	41.6<br>(26.8 to 61)	40.8<br>(26 to 59.9)
Albania	Male infertility	0.0618<br>(0.0255 to 0.128)	0.0461<br>(0.0188 to 0.0945)	0.045<br>(0.0187 to 0.0964)	1.8<br>(0.8 to 4)	1.6<br>(0.7 to 3.3)	1.7<br>(0.7 to 3.7)
Albania	Other urinary diseases	0.816<br>(0.672 to 1.02)	0.296<br>(0.223 to 0.352)	0.28<br>(0.189 to 0.358)	27.4<br>(22.8 to 32.6)	10.5<br>(8.1 to 12.4)	8.8<br>(6.3 to 11.1)
Albania	Gynecological diseases	4.58<br>(3.15 to 6.35)	4.67<br>(3.2 to 6.53)	4.19<br>(2.85 to 5.88)	145.2<br>(99.4 to 202)	151.5<br>(104.5 to 211.9)	146.9<br>(100.2 to 206)
Albania	Uterine fibroids	0.608<br>(0.359 to 1)	0.788<br>(0.485 to 1.26)	0.689<br>(0.419 to 1.13)	22.7<br>(13.4 to 37.4)	23.5<br>(14.5 to 37.5)	21.6<br>(13 to 35)
Albania	Polycystic ovarian syndrome	0.163<br>(0.0723 to 0.314)	0.157<br>(0.0699 to 0.303)	0.141<br>(0.0629 to 0.271)	4.9<br>(2.2 to 9.5)	5.2<br>(2.3 to 10)	5.1<br>(2.3 to 9.9)
Albania	Female infertility	0.0438<br>(0.013 to 0.1)	0.0239<br>(0.00847 to 0.0509)	0.0359<br>(0.0112 to 0.0844)	1.3<br>(0.4 to 2.9)	0.9<br>(0.3 to 1.9)	1.4<br>(0.4 to 3.3)
Albania	Endometriosis	1.43<br>(0.933 to 2.15)	1.29<br>(0.836 to 1.92)	1.15<br>(0.752 to 1.67)	42.6<br>(27.7 to 63.6)	44.6<br>(28.8 to 66.1)	42.7<br>(27.9 to 62.3)
Albania	Genital prolapse	0.126<br>(0.0621 to 0.237)	0.125<br>(0.0611 to 0.232)	0.149<br>(0.0732 to 0.283)	5.3<br>(2.6 to 9.9)	3.7<br>(1.8 to 6.9)	3.8<br>(1.9 to 7.1)
Albania	Premenstrual syndrome	1.63<br>(1.05 to 2.45)	1.61<br>(1.04 to 2.42)	1.39<br>(0.876 to 2.08)	49.8<br>(32.1 to 75)	52.2<br>(33.8 to 78.6)	50<br>(31.7 to 74.9)
Albania	Other gynecological diseases	0.571<br>(0.387 to 0.793)	0.673<br>(0.449 to 0.959)	0.641<br>(0.435 to 0.914)	18.7<br>(12.7 to 25.9)	21.3<br>(14.4 to 30.4)	22.2<br>(15.1 to 31.9)
Albania	Hemoglobinopathies and hemolytic anaemias	4.71<br>(3.8 to 5.77)	2.66<br>(2.13 to 3.38)	2.14<br>(1.64 to 2.75)	138.8<br>(113.8 to 170.5)	93.7<br>(75.4 to 117.7)	78<br>(60.4 to 99.5)
Albania	Thalassemias	0.897<br>(0.587 to 1.17)	0.281<br>(0.167 to 0.375)	0.176<br>(0.1 to 0.273)	22.5<br>(14.7 to 29.3)	13.4<br>(7.5 to 18.1)	10.2<br>(5.8 to 15.9)
Albania	Thalassaemias trait	1.08<br>(0.689 to 1.6)	0.675<br>(0.44 to 1.01)	0.536<br>(0.331 to 0.823)	32.4<br>(21 to 48.5)	22.8<br>(14.7 to 34.5)	19.6<br>(12.2 to 29.7)
Albania	Sickle cell disorders	0.824<br>(0.591 to 1.03)	0.356<br>(0.28 to 0.441)	0.25<br>(0.188 to 0.338)	21.3<br>(15.5 to 26.5)	13.6<br>(10.4 to 17.1)	11.4<br>(8.4 to 15.8)
Albania	Sickle cell trait	0.281<br>(0.175 to 0.431)	0.167<br>(0.104 to 0.262)	0.13<br>(0.0804 to 0.199)	8.4<br>(5.2 to 12.8)	5.7<br>(3.5 to 8.9)	4.8<br>(3 to 7.4)
Albania	G6PD deficiency	0.348<br>(0.264 to 0.43)	0.307<br>(0.239 to 0.39)	0.24<br>(0.173 to 0.331)	10.2<br>(7.9 to 12.4)	10.1<br>(7.8 to 12.6)	8.5<br>(6.1 to 11.7)
Albania	G6PD trait	0.000114<br>(0.0000736 to 0.000165)	0.0000889<br>(0.0000591 to 0.000125)	0.0000713<br>(0.0000476 to 0.0001)	0<br>(0 to 0)	0<br>(0 to 0)	0<br>(0 to 0)
Albania	Other hemoglobinopathies and hemolytic anaemias	1.28<br>(1.01 to 1.64)	0.875<br>(0.681 to 1.15)	0.809<br>(0.603 to 1.1)	44<br>(35.1 to 55.8)	28.1<br>(21.8 to 37.1)	23.5<br>(17.4 to 31.8)
Albania	Endocrine, metabolic, blood, and immune disorders	5.47<br>(4.11 to 9.13)	3.38<br>(2.66 to 5.02)	3.1<br>(2.44 to 4.45)	168<br>(126.1 to 251.1)	120.6<br>(92.8 to 199.7)	108.2<br>(80.4 to 173.1)
Albania	Oral disorders	5.93<br>(3.61 to 8.96)	7.09<br>(4.42 to 10.7)	7.77<br>(4.91 to 11.6)	225.8<br>(141.3 to 339.4)	220.3<br>(137.3 to 331.9)	215.3<br>(134.6 to 323.3)
Albania	Caries of deciduous teeth	0.0971<br>(0.041 to 0.188)	0.054<br>(0.0229 to 0.106)	0.0398<br>(0.017 to 0.0772)	2.4<br>(1 to 4.7)	2.4<br>(1 to 4.8)	2.4<br>(1 to 4.7)
Albania	Caries of permanent teeth	0.895<br>(0.386 to 1.72)	0.837<br>(0.362 to 1.6)	0.732<br>(0.316 to 1.39)	27.5<br>(11.9 to 52.9)	26.9<br>(11.6 to 51.2)	25.4<br>(10.9 to 48)
Albania	Periodontal disease	0.928<br>(0.365 to 1.91)	1.15<br>(0.458 to 2.37)	1.28<br>(0.505 to 2.6)	36.6<br>(14.4 to 75.1)	35<br>(13.8 to 71.5)	34.8<br>(13.8 to 72.7)
Albania	Edentulism and severe tooth loss	2.45<br>(1.63 to 3.47)	3.46<br>(2.29 to 4.92)	4.18<br>(2.79 to 5.94)	108.8<br>(72.6 to 154.2)	105.1<br>(69.7 to 148.7)	101.9<br>(68.1 to 143.9)

SR Appendix Table 2. All-age DALYs counts and age standardized DALYs rates per 100,000 individuals for all causes and level 1 cause groups by location for 1990, 2007, and 2017, both sexes combined

		All-age DALYs			Age-standardized rate of DALYs per 100,000		
Albania	Other oral disorders	1.57 (0.979 to 2.33)	1.58 (0.991 to 2.34)	1.54 (0.966 to 2.27)	50.5 (31.8 to 74.9)	50.9 (31.8 to 75.4)	50.7 (31.6 to 75.4)
Albania	Sudden infant death syndrome	2.42 (0.764 to 7.06)	0.776 (0.343 to 1.69)	0.587 (0.249 to 1.15)	59.9 (18.9 to 174.8)	43.6 (19.3 to 94.9)	33.9 (14.3 to 66.2)
Albania	Injuries	119 (106 to 134)	87 (73.6 to 104)	86.4 (69.9 to 106)	3740.3 (3282.6 to 4276.6)	2853.6 (2428.8 to 3390.5)	2782.6 (2272 to 3379.6)
Albania	Transport injuries	28.7 (26.3 to 31.1)	20.9 (18.9 to 23.3)	19.4 (16 to 23.2)	885.7 (812.2 to 966.9)	691.9 (623 to 767)	645.8 (533.1 to 768.6)
Albania	Road injuries	26.6 (24.5 to 29)	18.9 (17 to 21.1)	17.7 (14.6 to 21.3)	824.7 (755.3 to 895.5)	623.1 (560.1 to 695.1)	587.9 (485 to 703.9)
Albania	Pedestrian road injuries	12 (10.5 to 13.3)	7.25 (6.31 to 8.11)	6.04 (4.8 to 7.26)	369.8 (331.6 to 405.7)	241.7 (207.8 to 270.7)	203.8 (162.2 to 244.7)
Albania	Cyclist road injuries	1.82 (1.44 to 2.27)	2.03 (1.57 to 2.57)	2.22 (1.65 to 2.88)	62.4 (49.3 to 77.8)	63.9 (49.5 to 80.8)	67.4 (49.5 to 87.7)
Albania	Motorcyclist road injuries	2.36 (1.95 to 3.62)	2.24 (1.92 to 2.65)	2.22 (1.7 to 2.82)	71.4 (59 to 106.3)	74.3 (63.8 to 87.7)	76.7 (58.8 to 96.9)
Albania	Motor vehicle road injuries	9.76 (8.8 to 11.3)	7.04 (6.18 to 8.13)	6.91 (5.55 to 8.41)	302.2 (274.2 to 345.4)	231 (203 to 265.6)	228.5 (183.3 to 279.9)
Albania	Other road injuries	0.629 (0.449 to 0.763)	0.367 (0.29 to 0.46)	0.339 (0.252 to 0.453)	18.8 (14.1 to 22.6)	12.2 (9.7 to 15.2)	11.5 (8.2 to 15.6)
Albania	Other transport injuries	2.04 (1.79 to 2.31)	2 (1.77 to 2.24)	1.65 (1.36 to 2.01)	61 (54 to 68.6)	68.9 (61.1 to 77.2)	57.9 (47.7 to 70.5)
Albania	Unintentional injuries	73.2 (61.8 to 86.2)	51.3 (40.5 to 65.3)	54.2 (41.2 to 70.6)	2324.7 (1935.1 to 2776.2)	1675.7 (1337.7 to 2115.9)	1694.6 (1304.9 to 2180.4)
Albania	Falls	26 (19.4 to 34.5)	26.8 (19.5 to 36.1)	32.7 (23.5 to 44.6)	910.7 (676.3 to 1205.3)	847.7 (619.8 to 1139.2)	964.6 (694.3 to 1316.4)
Albania	Drowning	10.2 (8.92 to 11.4)	2.79 (2.45 to 3.13)	2.07 (1.69 to 2.51)	269.9 (237.4 to 300.6)	101.2 (87.7 to 114.9)	87 (70.7 to 105.3)
Albania	Fire, heat, and hot substances	3.8 (2.89 to 4.51)	1.86 (1.41 to 2.41)	1.79 (1.29 to 2.46)	117.1 (92.5 to 139)	62.4 (47.6 to 80.2)	58.4 (42.8 to 78.5)
Albania	Poisonings	1.42 (1.07 to 2.04)	1.33 (1.05 to 1.67)	1.32 (1 to 1.74)	42.5 (31.9 to 59.4)	45.1 (36 to 56.3)	47.7 (36.1 to 62.8)
Albania	Poisoning by carbon monoxide	0.277 (0.215 to 0.507)	0.185 (0.147 to 0.229)	0.192 (0.148 to 0.252)	8.4 (6.5 to 14.6)	6.1 (4.8 to 7.4)	6.6 (5 to 8.5)
Albania	Poisoning by other means	1.15 (0.835 to 1.64)	1.14 (0.9 to 1.46)	1.13 (0.847 to 1.52)	34.1 (24.9 to 47.3)	39 (30.7 to 49.5)	41.2 (31 to 55.2)
Albania	Exposure to mechanical forces	15.2 (12.5 to 18.3)	9.98 (7.79 to 12.9)	8.52 (6.41 to 11.4)	495.4 (402.7 to 602)	320.1 (252.8 to 411.1)	266.6 (205.6 to 352.5)
Albania	Unintentional firearm injuries	1.89 (1.1 to 2.3)	0.462 (0.379 to 0.671)	0.369 (0.284 to 0.523)	54.9 (32 to 66.4)	15.4 (12.6 to 22.5)	12.9 (9.8 to 18.5)
Albania	Other exposure to mechanical forces	13.3 (10.7 to 16.3)	9.52 (7.4 to 12.4)	8.15 (6.08 to 11)	440.5 (351.7 to 545.3)	304.7 (238.4 to 393.4)	253.7 (193.5 to 337.8)
Albania	Adverse effects of medical treatment	1.41 (1.16 to 2.07)	1.27 (0.947 to 1.48)	1.09 (0.866 to 1.31)	41.9 (34.4 to 60.2)	47.5 (33.8 to 58.1)	39.6 (29.1 to 49.5)
Albania	Animal contact	0.576 (0.42 to 0.764)	0.461 (0.332 to 0.623)	0.403 (0.29 to 0.547)	18.4 (13.5 to 24.5)	15 (10.8 to 20.3)	13.5 (9.7 to 18.2)
Albania	Venomous animal contact	0.38 (0.263 to 0.522)	0.3 (0.206 to 0.424)	0.263 (0.179 to 0.368)	11.9 (8.3 to 16.2)	9.8 (6.7 to 13.8)	9 (6.2 to 12.8)
Albania	Non-venomous animal contact	0.196 (0.14 to 0.27)	0.16 (0.114 to 0.228)	0.14 (0.0965 to 0.204)	6.5 (4.6 to 8.9)	5.2 (3.7 to 7.4)	4.4 (3.1 to 6.4)
Albania	Foreign body	4.86 (3.91 to 5.81)	1.68 (1.45 to 1.95)	1.47 (1.2 to 1.8)	128.4 (104.4 to 152.1)	68.6 (58.4 to 80.6)	60.1 (47.6 to 77.5)
Albania	Pulmonary aspiration and foreign body in airway	4.47 (3.55 to 5.4)	1.26 (1.08 to 1.51)	1.07 (0.856 to 1.37)	115.6 (92.9 to 138.9)	54.6 (45.5 to 67)	46.8 (35.9 to 62.4)
Albania	Foreign body in eyes	0.0674 (0.0306 to 0.119)	0.0745 (0.0353 to 0.129)	0.0785 (0.0388 to 0.135)	2.2 (1 to 3.8)	2.5 (1.1 to 4.3)	2.6 (1.2 to 4.6)
Albania	Foreign body in other body part	0.324 (0.237 to 0.423)	0.344 (0.244 to 0.449)	0.322 (0.228 to 0.421)	10.6 (7.8 to 13.7)	11.6 (7.9 to 15.1)	10.8 (7.4 to 14.1)
Albania	Environmental heat and cold exposure	0.844 (0.644 to 1.07)	0.757 (0.561 to 0.969)	1 (0.787 to 1.25)	28.2 (21.4 to 35.9)	24.3 (17.9 to 31.1)	31.1 (24.2 to 38.8)
Albania	Exposure to forces of nature	0.0164 (0.0117 to 0.0228)	0.0116 (0.00855 to 0.0157)	0.0529 (0.0505 to 0.0561)	0.5 (0.4 to 0.7)	0.4 (0.3 to 0.5)	1.9 (1.9 to 2)
Albania	Other unintentional injuries	8.84 (7.87 to 9.87)	4.39 (3.59 to 5.42)	3.77 (2.96 to 4.82)	271.8 (240.8 to 306.8)	143.5 (118.3 to 175.3)	124 (98.9 to 156.6)
Albania	Self-harm and interpersonal violence	17.4 (14.5 to 18.9)	14.8 (12.7 to 16.3)	12.9 (10.6 to 15.2)	530 (439.2 to 577.2)	485.9 (425.2 to 534.5)	442.1 (365.8 to 520.7)
Albania	Self-harm	6.3 (5.73 to 7.24)	7.98 (6.21 to 8.84)	7.21 (5.37 to 8.97)	192.8 (176.5 to 221.7)	258.5 (202.5 to 287.1)	246 (181.6 to 306.7)
Albania	Self-harm by firearm	1.5 (1.21 to 2.27)	1.97 (1.26 to 2.38)	1.58 (0.979 to 2.14)	44.7 (36.5 to 68.5)	63.8 (40.6 to 77.3)	54.9 (33.8 to 74.2)
Albania	Self-harm by other specified means	4.8 (4.09 to 5.48)	6.01 (4.62 to 6.74)	5.63 (4.14 to 7.01)	148.1 (126.3 to 168.9)	194.8 (150.2 to 218.5)	191.2 (138.9 to 237.5)
Albania	Interpersonal violence	11 (7.75 to 12.5)	5.86 (4.95 to 6.96)	4.99 (4 to 6.29)	335.3 (231.6 to 381)	197 (167.7 to 239.6)	173.3 (139.2 to 225.1)
Albania	Assault by firearm	5.29 (3.8 to 7.51)	3.2 (2.55 to 4.11)	2.54 (1.87 to 3.74)	157 (109.3 to 220.7)	109.6 (86.4 to 142.3)	92.3 (67.7 to 136.5)
Albania	Assault by sharp object	2.32 (0.566 to 3.25)	0.651 (0.348 to 0.866)	0.589 (0.328 to 0.832)	69.2 (17.8 to 95.6)	21.7 (11.5 to 28.8)	20.2 (11 to 28.8)
Albania	Sexual violence	0.228 (0.153 to 0.335)	0.236 (0.159 to 0.342)	0.216 (0.145 to 0.315)	6.9 (4.6 to 10)	7.7 (5.2 to 11.3)	7.7 (5.2 to 11.2)

SR Appendix Table 2. All-age DALYs counts and age standardized DALYs rates per 100,000 individuals for all causes and level 1 cause groups by location for 1990, 2007, and 2017, both sexes combined

		All-age DALYs			Age-standardized rate of DALYs per 100,000		
Albania	Assault by other means	3.19 (2.35 to 3.95)	1.77 (1.35 to 2.34)	1.65 (1.24 to 2.15)	102.2 (77.6 to 126.1)	58 (44.1 to 80.3)	53 (39.9 to 69.9)
Albania	Conflict and terrorism	0.00857 (0.0056 to 0.0136)	0.89 (0.594 to 1.47)	0.668 (0.447 to 1.09)	0.3 (0.2 to 0.4)	29.3 (19.4 to 48.3)	21.8 (14.4 to 35.9)
Albania	Executions and police conflict	0.0539 (0.0271 to 0.0823)	0.0304 (0.0252 to 0.036)	0.0269 (0.0226 to 0.032)	1.5 (0.8 to 2.3)	1 (0.9 to 1.2)	1 (0.9 to 1.2)
Bosnia and Herzegovina	All causes	1250 (1120 to 1400)	1240 (1110 to 1380)	1160 (1040 to 1310)	29460.2 (26591.5 to 32719.7)	25560.3 (22785.5 to 28758.9)	24302.2 (21374 to 27583.3)
Bosnia and Herzegovina	Communicable, maternal, neonatal, and nutritional disorders	158 (140 to 179)	65.6 (56 to 77.5)	50.2 (42.1 to 59.8)	4190.6 (3752 to 4677.9)	2116.4 (1846.4 to 2451.2)	1898.9 (1596.7 to 2237.3)
Bosnia and Herzegovina	HIV/AIDS and sexually transmitted infections	1.32 (0.845 to 2.17)	0.902 (0.544 to 1.53)	0.717 (0.427 to 1.22)	29.9 (18.6 to 48.3)	21.6 (12.9 to 36.7)	21.5 (12.9 to 36.6)
Bosnia and Herzegovina	HIV/AIDS	0.171 (0.131 to 0.203)	0.116 (0.106 to 0.127)	0.0698 (0.0627 to 0.0794)	3.5 (2.7 to 4.1)	2.6 (2.4 to 2.8)	1.9 (1.7 to 2.2)
Bosnia and Herzegovina	HIV/AIDS - Drug-susceptible Tuberculosis	0.0767 (0.0585 to 0.0912)	0.0505 (0.0452 to 0.0546)	0.0296 (0.0261 to 0.0335)	1.6 (1.2 to 1.9)	1.1 (1 to 1.2)	0.8 (0.7 to 0.9)
Bosnia and Herzegovina	HIV/AIDS - Multidrug-resistant Tuberculosis without extensive drug resistance	0.000575 (0.000126 to 0.00159)	0.000877 (0.000564 to 0.00132)	0.000384 (0.000134 to 0.000887)	0 (0 to 0)	0 (0 to 0)	0 (0 to 0)
Bosnia and Herzegovina	HIV/AIDS - Extensively drug-resistant Tuberculosis	0 (0 to 0)	0.000276 (0.000177 to 0.00042)	0.000219 (0.0000755 to 0.000503)	0 (0 to 0)	0 (0 to 0)	0 (0 to 0)
Bosnia and Herzegovina	HIV/AIDS resulting in other diseases	0.094 (0.0715 to 0.112)	0.0643 (0.058 to 0.0726)	0.0397 (0.0349 to 0.0466)	1.9 (1.5 to 2.3)	1.4 (1.3 to 1.6)	1.1 (0.9 to 1.3)
Bosnia and Herzegovina	Sexually transmitted infections excluding HIV	1.15 (0.675 to 1.98)	0.786 (0.431 to 1.41)	0.647 (0.36 to 1.15)	26.4 (15.4 to 44.3)	19 (10.4 to 34.1)	19.6 (11 to 34.7)
Bosnia and Herzegovina	Syphilis	0.35 (0.156 to 0.784)	0.0923 (0.0618 to 0.151)	0.0946 (0.0658 to 0.137)	10 (4.1 to 23.4)	2.9 (1.6 to 6)	3.7 (2 to 6.7)
Bosnia and Herzegovina	Chlamydial infection	0.182 (0.0957 to 0.348)	0.154 (0.0792 to 0.293)	0.12 (0.0621 to 0.227)	3.7 (2 to 7.1)	3.6 (1.9 to 6.9)	3.6 (1.9 to 6.9)
Bosnia and Herzegovina	Gonococcal infection	0.15 (0.0764 to 0.298)	0.115 (0.057 to 0.237)	0.0825 (0.0405 to 0.165)	3.1 (1.6 to 6.1)	2.9 (1.4 to 6)	2.8 (1.3 to 5.7)
Bosnia and Herzegovina	Trichomoniasis	0.146 (0.0592 to 0.314)	0.136 (0.055 to 0.289)	0.113 (0.0453 to 0.243)	3 (1.2 to 6.4)	3 (1.2 to 6.4)	3 (1.2 to 6.3)
Bosnia and Herzegovina	Genital herpes	0.099 (0.032 to 0.234)	0.1 (0.0316 to 0.241)	0.0913 (0.0294 to 0.215)	2.1 (0.7 to 4.9)	2.1 (0.7 to 4.9)	2.1 (0.7 to 4.8)
Bosnia and Herzegovina	Other sexually transmitted infections	0.226 (0.131 to 0.405)	0.189 (0.109 to 0.333)	0.146 (0.0846 to 0.256)	4.6 (2.7 to 8.3)	4.5 (2.5 to 8)	4.4 (2.5 to 7.8)
Bosnia and Herzegovina	Respiratory infections and tuberculosis	26.7 (24.1 to 29.9)	15.7 (13.8 to 18.4)	12.4 (10.8 to 14.9)	650.6 (588.6 to 728.6)	357.3 (311.3 to 420.9)	306.1 (261.6 to 370.2)
Bosnia and Herzegovina	Tuberculosis	9.31 (8.73 to 9.99)	4.81 (4.32 to 5.31)	3.02 (2.72 to 3.35)	212.7 (199.8 to 227.1)	89.8 (81.1 to 98.7)	55.8 (50.3 to 61.9)
Bosnia and Herzegovina	Latent tuberculosis infection	0 (0 to 0)	0 (0 to 0)	0 (0 to 0)	0 (0 to 0)	0 (0 to 0)	0 (0 to 0)
Bosnia and Herzegovina	Drug-susceptible tuberculosis	9.25 (8.66 to 9.91)	4.72 (4.25 to 5.23)	2.97 (2.68 to 3.3)	211.4 (198.1 to 225.8)	88.2 (79.9 to 96.9)	54.9 (49.4 to 61.2)
Bosnia and Herzegovina	Multidrug-resistant tuberculosis without extensive drug resistance	0.0569 (0.0129 to 0.157)	0.0664 (0.043 to 0.0973)	0.031 (0.0111 to 0.0679)	1.3 (0.3 to 3.6)	1.2 (0.8 to 1.8)	0.6 (0.2 to 1.2)
Bosnia and Herzegovina	Extensively drug-resistant tuberculosis	0 (0 to 0)	0.0208 (0.0137 to 0.0307)	0.0175 (0.00613 to 0.038)	0 (0 to 0)	0.4 (0.3 to 0.6)	0.3 (0.1 to 0.7)
Bosnia and Herzegovina	Lower respiratory infections	12.6 (11.1 to 14.2)	7.28 (6.44 to 9.52)	6.41 (5.59 to 8.77)	332 (291 to 377.1)	171.2 (153.9 to 214.9)	155.7 (136.9 to 194.8)
Bosnia and Herzegovina	Upper respiratory infections	3.17 (1.85 to 5.1)	2.69 (1.57 to 4.31)	2.23 (1.32 to 3.55)	70.1 (40.9 to 112.7)	70.2 (41 to 113.1)	70 (40.7 to 111.8)
Bosnia and Herzegovina	Otitis media	1.58 (0.986 to 2.5)	0.927 (0.563 to 1.46)	0.719 (0.433 to 1.13)	35.7 (22.4 to 55.5)	26 (15.7 to 40.9)	24.6 (14.8 to 38.9)
Bosnia and Herzegovina	Enteric infections	5.19 (3.95 to 6.66)	2.64 (1.93 to 3.46)	2.44 (1.79 to 3.2)	137.2 (104 to 174.9)	87.3 (64 to 114.7)	97.4 (71.4 to 127.4)
Bosnia and Herzegovina	Diarrhoeal diseases	4.95 (3.79 to 6.39)	2.52 (1.82 to 3.36)	2.35 (1.7 to 3.13)	131.4 (99.2 to 169.5)	83.8 (60.9 to 111.5)	94.3 (68.1 to 124.7)
Bosnia and Herzegovina	Typhoid and paratyphoid	0.00774 (0.00342 to 0.0155)	0.0056 (0.00247 to 0.011)	0.00461 (0.00209 to 0.00917)	0.2 (0.1 to 0.3)	0.1 (0.1 to 0.3)	0.1 (0.1 to 0.2)
Bosnia and Herzegovina	Typhoid fever	0.00147 (0.00069 to 0.00278)	0.00098 (0.00046 to 0.00186)	0.000837 (0.00039 to 0.00159)	0 (0 to 0.1)	0 (0 to 0)	0 (0 to 0.1)
Bosnia and Herzegovina	Paratyphoid fever	0.00627 (0.00246 to 0.013)	0.00462 (0.00189 to 0.00949)	0.00377 (0.00157 to 0.00783)	0.1 (0.1 to 0.3)	0.1 (0 to 0.2)	0.1 (0 to 0.2)
Bosnia and Herzegovina	Invasive non-typhoidal salmonella	0.217 (0.113 to 0.369)	0.108 (0.0554 to 0.185)	0.0837 (0.0424 to 0.142)	5.2 (2.7 to 8.9)	3.2 (1.6 to 5.6)	2.9 (1.4 to 5.3)
Bosnia and Herzegovina	Other intestinal infectious diseases	0.0137 (0.00674 to 0.023)	0.00582 (0.00344 to 0.00837)	0.00405 (0.00237 to 0.006)	0.3 (0.2 to 0.6)	0.2 (0.1 to 0.2)	0.1 (0.1 to 0.2)
Bosnia and Herzegovina	Neglected tropical diseases and malaria	2.32 (0.95 to 9.67)	1.12 (0.608 to 1.89)	0.954 (0.506 to 1.66)	51 (20.6 to 224.2)	22.7 (12.3 to 38)	20.6 (10.8 to 36)
Bosnia and Herzegovina	Malaria	0 (0 to 0)	0 (0 to 0)	0 (0 to 0)	0 (0 to 0)	0 (0 to 0)	0 (0 to 0)
Bosnia and Herzegovina	Chagas disease	0 (0 to 0)	0 (0 to 0)	0 (0 to 0)	0 (0 to 0)	0 (0 to 0)	0 (0 to 0)
Bosnia and Herzegovina	Leishmaniasis	0.831 (0.00229 to 8.26)	0.00942 (0.0000316 to 0.0911)	0.00458 (0.0000151 to 0.0399)	19.3 (0.1 to 189.7)	0.3 (0 to 3)	0.2 (0 to 1.6)
Bosnia and Herzegovina	Visceral leishmaniasis	0.831 (0.00229 to 8.26)	0.00939 (0.0000294 to 0.0911)	0.00458 (0.0000151 to 0.0399)	19.3 (0.1 to 189.7)	0.3 (0 to 3)	0.2 (0 to 1.6)
Bosnia and Herzegovina	Cutaneous and mucocutaneous leishmaniasis	0.00000628 (0 to 0.00000582)	0.0000334 (0.00000000000951 to 0.000253)	0.00000192 (0 to 0.0000000000315)	0 (0 to 0)	0 (0 to 0)	0 (0 to 0)

SR Appendix Table 2. All-age DALYs counts and age standardized DALYs rates per 100,000 individuals for all causes and level 1 cause groups by location for 1990, 2007, and 2017, both sexes combined

		All-age DALYs			Age-standardized rate of DALYs per 100,000		
Bosnia and Herzegovina	African trypanosomiasis	0 (0 to 0)	0 (0 to 0)	0 (0 to 0)	0 (0 to 0)	0 (0 to 0)	0 (0 to 0)
Bosnia and Herzegovina	Schistosomiasis	0 (0 to 0)	0 (0 to 0)	0 (0 to 0)	0 (0 to 0)	0 (0 to 0)	0 (0 to 0)
Bosnia and Herzegovina	Cysticercosis	1.25 (0.656 to 2.02)	0.988 (0.492 to 1.71)	0.864 (0.424 to 1.56)	26.5 (13.9 to 42.9)	20 (9.8 to 34.7)	18.6 (9.1 to 33.9)
Bosnia and Herzegovina	Cystic echinococcosis	0.172 (0.116 to 0.23)	0.0977 (0.0673 to 0.132)	0.068 (0.0464 to 0.092)	3.8 (2.6 to 5.1)	2 (1.4 to 2.7)	1.5 (1.1 to 2.1)
Bosnia and Herzegovina	Lymphatic filariasis	0 (0 to 0)	0 (0 to 0)	0 (0 to 0)	0 (0 to 0)	0 (0 to 0)	0 (0 to 0)
Bosnia and Herzegovina	Onchocerciasis	0 (0 to 0)	0 (0 to 0)	0 (0 to 0)	0 (0 to 0)	0 (0 to 0)	0 (0 to 0)
Bosnia and Herzegovina	Trachoma	0 (0 to 0)	0 (0 to 0)	0 (0 to 0)	0 (0 to 0)	0 (0 to 0)	0 (0 to 0)
Bosnia and Herzegovina	Dengue	0 (0 to 0)	0 (0 to 0)	0 (0 to 0)	0 (0 to 0)	0 (0 to 0)	0 (0 to 0)
Bosnia and Herzegovina	Yellow fever	0 (0 to 0)	0 (0 to 0)	0 (0 to 0)	0 (0 to 0)	0 (0 to 0)	0 (0 to 0)
Bosnia and Herzegovina	Rabies	0.0203 (0.0068 to 0.0322)	0.0131 (0.00554 to 0.0255)	0.00697 (0.00276 to 0.0141)	0.6 (0.2 to 0.9)	0.2 (0.1 to 0.4)	0.1 (0 to 0.2)
Bosnia and Herzegovina	Intestinal nematode infections	0 (0 to 0)	0 (0 to 0)	0 (0 to 0)	0 (0 to 0)	0 (0 to 0)	0 (0 to 0)
Bosnia and Herzegovina	Ascariasis	0 (0 to 0)	0 (0 to 0)	0 (0 to 0)	0 (0 to 0)	0 (0 to 0)	0 (0 to 0)
Bosnia and Herzegovina	Trichuriasis	0 (0 to 0)	0 (0 to 0)	0 (0 to 0)	0 (0 to 0)	0 (0 to 0)	0 (0 to 0)
Bosnia and Herzegovina	Hookworm disease	0 (0 to 0)	0 (0 to 0)	0 (0 to 0)	0 (0 to 0)	0 (0 to 0)	0 (0 to 0)
Bosnia and Herzegovina	Food-borne trematodiases	0 (0 to 0)	0 (0 to 0)	0 (0 to 0)	0 (0 to 0)	0 (0 to 0)	0 (0 to 0)
Bosnia and Herzegovina	Leprosy	0 (0 to 0)	0 (0 to 0)	0 (0 to 0)	0 (0 to 0)	0 (0 to 0)	0 (0 to 0)
Bosnia and Herzegovina	Ebola virus disease	0 (0 to 0)	0 (0 to 0)	0 (0 to 0)	0 (0 to 0)	0 (0 to 0)	0 (0 to 0)
Bosnia and Herzegovina	Zika virus disease	0 (0 to 0)	0 (0 to 0)	0 (0 to 0)	0 (0 to 0)	0 (0 to 0)	0 (0 to 0)
Bosnia and Herzegovina	Guinea worm disease	0 (0 to 0)	0 (0 to 0)	0 (0 to 0)	0 (0 to 0)	0 (0 to 0)	0 (0 to 0)
Bosnia and Herzegovina	Other neglected tropical diseases	0.0403 (0.00752 to 0.0604)	0.00876 (0.00475 to 0.0124)	0.0109 (0.00379 to 0.0154)	0.9 (0.2 to 1.3)	0.2 (0.1 to 0.3)	0.2 (0.1 to 0.3)
Bosnia and Herzegovina	Other infectious diseases	13.4 (7.4 to 21.6)	3.72 (2.99 to 4.83)	3.19 (2.47 to 4.18)	355.4 (183.6 to 589.9)	115.2 (83.7 to 167.5)	122.5 (83 to 186.9)
Bosnia and Herzegovina	Meningitis	2.39 (1.94 to 2.85)	0.821 (0.707 to 0.976)	0.733 (0.595 to 0.87)	60.7 (48.3 to 73.3)	26 (22.2 to 30.7)	30.2 (23 to 37.5)
Bosnia and Herzegovina	Pneumococcal meningitis	0.425 (0.333 to 0.527)	0.18 (0.144 to 0.225)	0.157 (0.122 to 0.197)	9.9 (7.7 to 12.3)	4.8 (3.8 to 6)	5.1 (4 to 6.4)
Bosnia and Herzegovina	H influenzae type B meningitis	0.804 (0.631 to 0.977)	0.224 (0.194 to 0.271)	0.206 (0.159 to 0.249)	21.3 (16.5 to 26.3)	8.1 (6.7 to 9.8)	10 (7.4 to 13)
Bosnia and Herzegovina	Meningococcal infection	0.355 (0.276 to 0.443)	0.115 (0.0955 to 0.139)	0.102 (0.0816 to 0.126)	9 (6.9 to 11.5)	3.7 (3 to 4.5)	4.3 (3.1 to 5.6)
Bosnia and Herzegovina	Other meningitis	0.806 (0.652 to 0.962)	0.302 (0.254 to 0.357)	0.267 (0.216 to 0.32)	20.5 (16.3 to 24.7)	9.4 (7.9 to 11.1)	10.9 (8.2 to 13.5)
Bosnia and Herzegovina	Encephalitis	1.37 (1.2 to 1.54)	0.7 (0.621 to 0.812)	0.555 (0.482 to 0.668)	30.9 (26.8 to 35.1)	16.7 (14.6 to 22.9)	14.6 (12.4 to 22.3)
Bosnia and Herzegovina	Diphtheria	0.0178 (0.0106 to 0.0277)	0.00304 (0.00189 to 0.00453)	0.00442 (0.0027 to 0.00673)	0.4 (0.2 to 0.7)	0.1 (0.1 to 0.1)	0.2 (0.1 to 0.2)
Bosnia and Herzegovina	Whooping cough	7.31 (0.818 to 16)	0.659 (0.0806 to 1.8)	0.687 (0.108 to 1.73)	209.4 (23.5 to 456.6)	34.1 (4.2 to 92.9)	44.4 (7.1 to 111.5)
Bosnia and Herzegovina	Tetanus	0.0351 (0.0256 to 0.0346)	0.0226 (0.021 to 0.0239)	0.0185 (0.0164 to 0.021)	0.8 (0.6 to 0.8)	0.6 (0.6 to 0.7)	0.7 (0.6 to 0.7)
Bosnia and Herzegovina	Measles	0.137 (0.081 to 0.208)	0.0191 (0.0124 to 0.0282)	0.00114 (0.000411 to 0.00202)	3.8 (2.2 to 5.8)	1 (0.6 to 1.4)	0.1 (0 to 0.1)
Bosnia and Herzegovina	Varicella and herpes zoster	0.366 (0.26 to 0.503)	0.193 (0.138 to 0.259)	0.16 (0.113 to 0.22)	9.4 (6.5 to 13.1)	5.2 (3.7 to 6.9)	4.4 (3.1 to 5.9)
Bosnia and Herzegovina	Acute hepatitis	0.516 (0.419 to 0.644)	0.497 (0.338 to 0.615)	0.36 (0.26 to 0.451)	11.3 (9.1 to 14)	11.9 (8.1 to 14.8)	10.1 (7.3 to 12.9)
Bosnia and Herzegovina	Acute hepatitis A	0.234 (0.176 to 0.311)	0.193 (0.134 to 0.255)	0.133 (0.0945 to 0.177)	5.2 (3.9 to 7)	5.5 (3.8 to 7.3)	5 (3.5 to 6.7)
Bosnia and Herzegovina	Acute hepatitis B	0.232 (0.191 to 0.288)	0.255 (0.164 to 0.314)	0.192 (0.129 to 0.24)	4.9 (4 to 6)	5.1 (3.3 to 6.4)	4.1 (2.9 to 5.2)
Bosnia and Herzegovina	Acute hepatitis C	0.00933 (0.00505 to 0.0166)	0.007 (0.00351 to 0.0128)	0.00512 (0.00258 to 0.00964)	0.2 (0.1 to 0.4)	0.2 (0.1 to 0.4)	0.2 (0.1 to 0.3)
Bosnia and Herzegovina	Acute hepatitis E	0.0411 (0.0322 to 0.0517)	0.0427 (0.0262 to 0.0567)	0.0302 (0.0199 to 0.0395)	0.9 (0.7 to 1.1)	1 (0.6 to 1.3)	0.8 (0.6 to 1.1)
Bosnia and Herzegovina	Other unspecified infectious diseases	1.28 (0.983 to 1.67)	0.809 (0.624 to 1.05)	0.667 (0.496 to 0.84)	28.6 (22.2 to 37.4)	19.7 (15.5 to 25.9)	17.9 (14.2 to 23)
Bosnia and Herzegovina	Maternal and neonatal disorders	82.4 (74.2 to 90.6)	30.5 (25.4 to 37)	22.7 (18.1 to 28.3)	2345.9 (2136 to 2547.8)	1198.5 (1037.8 to 1382.1)	1068.4 (873.5 to 1302.7)
Bosnia and Herzegovina	Maternal disorders	1.06 (0.905 to 1.24)	0.315 (0.266 to 0.371)	0.172 (0.14 to 0.208)	21.4 (18.3 to 24.9)	7.7 (6.5 to 9)	5.5 (4.4 to 6.7)

SR Appendix Table 2. All-age DALYs counts and age standardized DALYs rates per 100,000 individuals for all causes and level 1 cause groups by location for 1990, 2007, and 2017, both sexes combined

		All-age DALYs			Age-standardized rate of DALYs per 100,000		
Bosnia and Herzegovina	Maternal haemorrhage	0.307 (0.192 to 0.461)	0.0606 (0.0396 to 0.0918)	0.0396 (0.0257 to 0.0592)	6.2 (3.9 to 9.3)	1.5 (1 to 2.3)	1.3 (0.8 to 1.9)
Bosnia and Herzegovina	Maternal sepsis and other pregnancy related infections	0.151 (0.08 to 0.259)	0.0161 (0.00924 to 0.0261)	0.0102 (0.00523 to 0.0179)	3.1 (1.6 to 5.2)	0.4 (0.2 to 0.6)	0.3 (0.2 to 0.6)
Bosnia and Herzegovina	Maternal hypertensive disorders	0.0465 (0.028 to 0.077)	0.026 (0.0166 to 0.0392)	0.0135 (0.00853 to 0.0198)	0.9 (0.6 to 1.5)	0.6 (0.4 to 1)	0.4 (0.3 to 0.7)
Bosnia and Herzegovina	Maternal obstructed labour and uterine rupture	0.0325 (0.0193 to 0.0561)	0.0155 (0.00958 to 0.0236)	0.00986 (0.00581 to 0.0154)	0.7 (0.4 to 1.1)	0.4 (0.2 to 0.6)	0.3 (0.2 to 0.5)
Bosnia and Herzegovina	Maternal abortive outcome	0.348 (0.217 to 0.514)	0.0792 (0.0489 to 0.119)	0.0339 (0.0208 to 0.0527)	7.1 (4.4 to 10.4)	1.9 (1.1 to 2.8)	1 (0.6 to 1.6)
Bosnia and Herzegovina	Ectopic pregnancy	0.0148 (0.00628 to 0.0307)	0.00333 (0.00146 to 0.00693)	0.00131 (0.000614 to 0.00271)	0.3 (0.1 to 0.6)	0.1 (0 to 0.2)	0 (0 to 0.1)
Bosnia and Herzegovina	Indirect maternal deaths	0.0632 (0.0241 to 0.14)	0.0269 (0.0114 to 0.0531)	0.0128 (0.00547 to 0.0253)	1.3 (0.5 to 2.8)	0.7 (0.3 to 1.3)	0.4 (0.2 to 0.8)
Bosnia and Herzegovina	Late maternal deaths	0.0127 (0.00901 to 0.0179)	0.00668 (0.00474 to 0.00895)	0.00396 (0.00267 to 0.00554)	0.3 (0.2 to 0.4)	0.2 (0.1 to 0.2)	0.1 (0.1 to 0.2)
Bosnia and Herzegovina	Maternal deaths aggravated by HIV/AIDS	0.00000241 (0.00000119 to 0.00000421)	0.000000622 (0.000000273 to 0.00000126)	0.000000372 (0.000000152 to 0.000000808)	0 (0 to 0)	0 (0 to 0)	0 (0 to 0)
Bosnia and Herzegovina	Other maternal disorders	0.0842 (0.0513 to 0.13)	0.0808 (0.0527 to 0.113)	0.0472 (0.0328 to 0.0638)	1.7 (1.1 to 2.6)	2 (1.3 to 2.8)	1.5 (1.1 to 2.1)
Bosnia and Herzegovina	Neonatal disorders	81.4 (73.2 to 89.5)	30.1 (25.1 to 36.7)	22.5 (18 to 28.1)	2324.5 (2114.7 to 2527)	1190.8 (1030.9 to 1373.6)	1062.9 (867.4 to 1297.3)
Bosnia and Herzegovina	Neonatal preterm birth	46 (40.2 to 51.3)	13.2 (11.4 to 15)	9.31 (7.45 to 11.5)	1351.5 (1177.1 to 1513.8)	594.4 (518.8 to 668.2)	519.7 (419.9 to 636)
Bosnia and Herzegovina	Neonatal encephalopathy due to birth asphyxia and trauma	19.1 (13.8 to 25.1)	8.1 (5.88 to 11.2)	6.01 (4.04 to 8.75)	540.6 (387.3 to 709.8)	287.3 (219.8 to 380.7)	242.3 (172.7 to 339.3)
Bosnia and Herzegovina	Neonatal sepsis and other neonatal infections	8.7 (5.45 to 13.6)	6.47 (3.72 to 10.7)	5.41 (3.05 to 9.09)	208 (133.2 to 317.7)	189.2 (115.7 to 301.7)	190 (112.5 to 305.5)
Bosnia and Herzegovina	Hemolytic disease and other neonatal jaundice	1.13 (0.794 to 1.58)	0.556 (0.415 to 0.705)	0.441 (0.33 to 0.562)	29.2 (19.5 to 42)	16 (12.2 to 20.4)	15.3 (11.5 to 19.3)
Bosnia and Herzegovina	Other neonatal disorders	6.36 (4.46 to 8.61)	1.85 (1.47 to 2.31)	1.32 (0.981 to 1.84)	195.2 (136.5 to 264.9)	103.9 (82.4 to 129.7)	95.6 (70.7 to 134.2)
Bosnia and Herzegovina	Nutritional deficiencies	26.6 (17.2 to 39)	11 (6.91 to 17.1)	7.86 (4.88 to 12)	620.6 (401.5 to 915.1)	313.9 (198.5 to 478)	262.3 (160.5 to 405.4)
Bosnia and Herzegovina	Protein-energy malnutrition	0.335 (0.149 to 0.637)	0.0761 (0.0546 to 0.116)	0.0471 (0.0401 to 0.0579)	9.1 (4 to 17.4)	2.2 (1.4 to 4)	1.1 (0.9 to 1.7)
Bosnia and Herzegovina	Iodine deficiency	1.6 (0.881 to 2.62)	0.759 (0.365 to 1.46)	0.867 (0.462 to 1.51)	34.4 (19 to 56.2)	17.4 (8.4 to 33.3)	23.6 (12.4 to 41)
Bosnia and Herzegovina	Dietary iron deficiency	15.8 (10.1 to 23.2)	8.08 (5 to 12.7)	5.55 (3.38 to 8.57)	340.8 (217.2 to 499.6)	200.9 (126.3 to 308.6)	159.5 (99 to 242.9)
Bosnia and Herzegovina	Other nutritional deficiencies	0.0662 (0.0436 to 0.0983)	0.0712 (0.0631 to 0.081)	0.0691 (0.0604 to 0.0791)	1.6 (1.1 to 2.4)	1.5 (1.3 to 1.7)	1.3 (1.2 to 1.5)
Bosnia and Herzegovina	Non-communicable diseases	946 (849 to 1060)	1040 (939 to 1160)	998 (892 to 1110)	22122.8 (19985.4 to 24611.1)	20610.5 (18468 to 23157)	19597.4 (17267.7 to 22113.4)
Bosnia and Herzegovina	Neoplasms	152 (148 to 157)	205 (198 to 212)	202 (190 to 214)	3329.9 (3235.5 to 3427.8)	3748.9 (3618.2 to 3866.8)	3590.3 (3369.8 to 3810.7)
Bosnia and Herzegovina	Lip and oral cavity cancer	2.05 (1.87 to 2.23)	2.39 (2.11 to 2.68)	2.24 (1.96 to 2.51)	44.9 (41.2 to 48.7)	43.3 (38.5 to 48.4)	39.5 (34.9 to 44.3)
Bosnia and Herzegovina	Nasopharynx cancer	0.169 (0.156 to 0.184)	0.368 (0.33 to 0.411)	0.323 (0.289 to 0.364)	3.5 (3.2 to 3.8)	7 (6.4 to 7.8)	6.2 (5.6 to 7)
Bosnia and Herzegovina	Other pharynx cancer	1.65 (1.45 to 2.29)	1.92 (1.59 to 2.98)	1.74 (1.43 to 2.89)	34.1 (30.3 to 46.8)	34.6 (28.6 to 53.3)	31.1 (25.7 to 51)
Bosnia and Herzegovina	Oesophageal cancer	2.76 (2.56 to 2.97)	3.24 (2.9 to 3.6)	2.98 (2.63 to 3.35)	60.3 (56.3 to 64.6)	57.4 (51.6 to 63.6)	50.1 (44.4 to 56.3)
Bosnia and Herzegovina	Stomach cancer	13.5 (12.8 to 14.3)	14.2 (13.2 to 15.2)	12.3 (11.2 to 13.3)	299.8 (285.4 to 315.5)	251.6 (234 to 269.8)	211.8 (193.3 to 228.9)
Bosnia and Herzegovina	Colon and rectum cancer	13.4 (12.5 to 15.1)	23.7 (22.1 to 25.7)	25 (22.9 to 27.3)	303 (283.7 to 346.1)	425.5 (396.6 to 459.6)	428.9 (392.6 to 467.5)
Bosnia and Herzegovina	Liver cancer	5.88 (5.34 to 6.38)	10.2 (9.11 to 11.2)	9.95 (8.83 to 11)	128.9 (117.5 to 139.2)	178.5 (161.6 to 195.3)	166.8 (148.9 to 184.1)
Bosnia and Herzegovina	Liver cancer due to hepatitis B	1.57 (1.23 to 1.94)	2.34 (1.84 to 2.93)	2.06 (1.58 to 2.58)	32.7 (26.2 to 40)	42.2 (33.5 to 52.4)	36.5 (28.8 to 44.9)
Bosnia and Herzegovina	Liver cancer due to hepatitis C	1.64 (1.21 to 2.16)	3.16 (2.42 to 3.99)	3.25 (2.47 to 4.17)	36.8 (27.8 to 47.6)	55.1 (42.5 to 69.4)	53 (40.9 to 67.4)
Bosnia and Herzegovina	Liver cancer due to alcohol use	1.86 (1.55 to 2.19)	3.04 (2.55 to 3.6)	3.01 (2.49 to 3.57)	41.3 (35 to 48.1)	52.5 (44.3 to 61.6)	49.8 (41.3 to 59.2)
Bosnia and Herzegovina	Liver cancer due to NASH	0.425 (0.349 to 0.513)	0.925 (0.764 to 1.13)	0.971 (0.791 to 1.18)	9.6 (8 to 11.6)	16.3 (13.6 to 19.7)	16 (13.2 to 19.3)
Bosnia and Herzegovina	Liver cancer due to other causes	0.391 (0.324 to 0.469)	0.685 (0.567 to 0.821)	0.662 (0.548 to 0.798)	8.4 (7 to 10)	12.4 (10.3 to 14.8)	11.6 (9.7 to 13.8)
Bosnia and Herzegovina	Gallbladder and biliary tract cancer	5.35 (4.01 to 5.92)	4.26 (3.52 to 6.84)	4.05 (3.24 to 6.87)	123.2 (93.9 to 135.7)	75.7 (62.9 to 120.5)	67.1 (53.9 to 113.5)
Bosnia and Herzegovina	Pancreatic cancer	6.93 (6.56 to 7.35)	10.6 (9.75 to 11.4)	11.3 (10.3 to 12.3)	155.7 (147.2 to 164.8)	186.8 (172.7 to 200.7)	189.1 (173.1 to 206.5)
Bosnia and Herzegovina	Larynx cancer	4.67 (4.33 to 5.02)	3.69 (3.32 to 4.15)	3.42 (3.02 to 3.86)	99.2 (92.4 to 106.3)	64.4 (57.8 to 72.4)	56.4 (49.9 to 63.5)
Bosnia and Herzegovina	Tracheal, bronchus, and lung cancer	40.5 (38.5 to 42.6)	52.9 (48.4 to 56.9)	51.7 (47.1 to 56.8)	856.5 (817.2 to 899)	922.4 (846.9 to 994.4)	863.3 (786.2 to 946.4)
Bosnia and Herzegovina	Malignant skin melanoma	1.65 (1.39 to 2.33)	2.28 (1.66 to 2.82)	2.16 (1.51 to 2.73)	35.7 (30.3 to 50.1)	44.2 (31.7 to 54.3)	42.6 (28.8 to 53.5)

SR Appendix Table 2. All-age DALYs counts and age standardized DALYs rates per 100,000 individuals for all causes and level 1 cause groups by location for 1990, 2007, and 2017, both sexes combined

		All-age DALYs			Age-standardized rate of DALYs per 100,000		
Bosnia and Herzegovina	Non-melanoma skin cancer	0.326<br>(0.301 to 0.356)	0.817<br>(0.749 to 0.891)	0.987<br>(0.888 to 1.1)	7.3<br>(6.8 to 8)	15.1<br>(13.9 to 16.4)	17.2<br>(15.5 to 19)
Bosnia and Herzegovina	Non-melanoma skin cancer (squamous-cell carcinoma)	0.325<br>(0.299 to 0.355)	0.815<br>(0.747 to 0.89)	0.986<br>(0.885 to 1.1)	7.3<br>(6.7 to 8)	15.1<br>(13.9 to 16.4)	17.2<br>(15.5 to 19)
Bosnia and Herzegovina	Non-melanoma skin cancer (basal-cell carcinoma)	0.0014<br>(0.000421 to 0.00332)	0.00176<br>(0.000512 to 0.00443)	0.00187<br>(0.00056 to 0.00484)	0<br>(0 to 0.1)	0<br>(0 to 0.1)	0<br>(0 to 0.1)
Bosnia and Herzegovina	Breast cancer	9.28<br>(8.34 to 10.1)	14.2<br>(11 to 16)	14.7<br>(10.7 to 16.7)	196.1<br>(177.3 to 212.6)	261.7<br>(201.9 to 293.1)	266<br>(193.9 to 303.2)
Bosnia and Herzegovina	Cervical cancer	4.65<br>(4.15 to 5.4)	5.27<br>(4.39 to 5.92)	4.46<br>(3.76 to 5.11)	98.6<br>(88.6 to 114.9)	99.2<br>(83.2 to 111.1)	86.8<br>(72.8 to 100)
Bosnia and Herzegovina	Uterine cancer	1.31<br>(1.17 to 1.49)	2.4<br>(2.09 to 2.73)	2.23<br>(1.91 to 2.61)	28.7<br>(25.7 to 32.3)	43.2<br>(37.9 to 49.3)	38.6<br>(33.1 to 45)
Bosnia and Herzegovina	Ovarian cancer	3.1<br>(2.81 to 3.42)	4.95<br>(4.36 to 5.62)	5.57<br>(4.88 to 6.34)	64.9<br>(59 to 70.9)	90.1<br>(79.6 to 101.9)	100.1<br>(88.4 to 113.2)
Bosnia and Herzegovina	Prostate cancer	2.95<br>(2.52 to 4.71)	6.3<br>(5.08 to 7.51)	7.28<br>(5.44 to 8.38)	78.8<br>(67.2 to 124.1)	111.2<br>(88.7 to 131.7)	117.1<br>(87.5 to 134.4)
Bosnia and Herzegovina	Testicular cancer	0.282<br>(0.234 to 0.357)	0.473<br>(0.389 to 0.57)	0.372<br>(0.311 to 0.448)	5.7<br>(4.7 to 7.2)	10.7<br>(8.7 to 12.9)	10.4<br>(8.6 to 12.7)
Bosnia and Herzegovina	Kidney cancer	2.21<br>(1.98 to 2.82)	4.19<br>(3.69 to 4.74)	4.29<br>(3.74 to 4.87)	49.1<br>(44.2 to 62.2)	77.3<br>(67.9 to 86.6)	76.5<br>(66.8 to 86.8)
Bosnia and Herzegovina	Bladder cancer	2.43<br>(2 to 2.66)	4.06<br>(3.39 to 4.52)	4.48<br>(3.62 to 5.06)	57.6<br>(48.7 to 62.7)	71.5<br>(59.3 to 79.2)	72.8<br>(58.7 to 81.9)
Bosnia and Herzegovina	Thyroid cancer	0.55<br>(0.454 to 0.621)	0.669<br>(0.517 to 0.77)	0.647<br>(0.513 to 0.739)	12.2<br>(10.2 to 13.6)	12.2<br>(9.4 to 13.9)	11.4<br>(9.1 to 13.1)
Bosnia and Herzegovina	Mesothelioma	0.222<br>(0.176 to 0.303)	0.275<br>(0.243 to 0.316)	0.205<br>(0.183 to 0.231)	4.7<br>(3.7 to 6.4)	5.1<br>(4.5 to 5.8)	3.8<br>(3.4 to 4.3)
Bosnia and Herzegovina	Hodgkin lymphoma	1.01<br>(0.758 to 1.17)	0.903<br>(0.521 to 1.11)	0.65<br>(0.384 to 0.806)	21.3<br>(15.8 to 24.5)	18.5<br>(10.9 to 22.8)	14.8<br>(9 to 18.3)
Bosnia and Herzegovina	Non-Hodgkin's lymphoma	2.33<br>(2.12 to 2.54)	3.55<br>(3.26 to 3.86)	3.24<br>(2.97 to 3.56)	51.2<br>(46.6 to 56.2)	74.2<br>(68 to 80.3)	71.7<br>(65.6 to 78.6)
Bosnia and Herzegovina	Multiple myeloma	0.598<br>(0.54 to 0.682)	1.25<br>(0.875 to 1.43)	1.3<br>(0.87 to 1.51)	13.1<br>(11.9 to 15.4)	22.2<br>(15.3 to 25.2)	22<br>(14.7 to 25.6)
Bosnia and Herzegovina	Leukaemia	6.23<br>(5.39 to 6.98)	6.54<br>(5.54 to 7.23)	6.08<br>(4.97 to 6.84)	139.8<br>(121.7 to 158.7)	142.2<br>(117.8 to 157)	140.3<br>(111.4 to 157.7)
Bosnia and Herzegovina	Acute lymphoid leukaemia	1.73<br>(1.34 to 2.05)	1.35<br>(1.06 to 1.59)	1.13<br>(0.849 to 1.36)	40.2<br>(31 to 48.1)	37.7<br>(28.8 to 45.2)	38.1<br>(27.3 to 47)
Bosnia and Herzegovina	Chronic lymphoid leukaemia	0.743<br>(0.598 to 0.983)	1.25<br>(1.06 to 1.54)	1.34<br>(1.1 to 1.61)	17.1<br>(13.9 to 23.1)	21.9<br>(18.7 to 26.8)	22.1<br>(18.1 to 26.4)
Bosnia and Herzegovina	Acute myeloid leukaemia	2.08<br>(1.37 to 2.5)	2.48<br>(1.88 to 2.86)	2.38<br>(1.69 to 2.8)	45.2<br>(29.9 to 54.2)	52.4<br>(38.7 to 61.1)	53.3<br>(37.5 to 63.8)
Bosnia and Herzegovina	Chronic myeloid leukaemia	0.723<br>(0.539 to 1.04)	0.628<br>(0.481 to 0.826)	0.533<br>(0.414 to 0.686)	15.3<br>(11.3 to 22)	12<br>(9.2 to 15.8)	10.2<br>(7.8 to 13.2)
Bosnia and Herzegovina	Other leukaemia	0.951<br>(0.647 to 1.36)	0.818<br>(0.637 to 1.16)	0.701<br>(0.56 to 0.99)	22<br>(15.1 to 31.2)	18.2<br>(14 to 24.4)	16.6<br>(13.2 to 21.9)
Bosnia and Herzegovina	Other malignant cancers	7.63<br>(6.67 to 8.32)	8.01<br>(7.2 to 9.31)	7.25<br>(6.4 to 8.46)	168.1<br>(147.9 to 182.8)	159.9<br>(144 to 188.8)	147.2<br>(130 to 174)
Bosnia and Herzegovina	Other neoplasms	0.339<br>(0.237 to 0.469)	0.757<br>(0.583 to 0.899)	0.879<br>(0.656 to 1.07)	7.9<br>(5.5 to 10.9)	14.8<br>(11.4 to 17.3)	17.1<br>(12.7 to 20.3)
Bosnia and Herzegovina	Myelodysplastic, myeloproliferative, and other hematopoietic neoplasms	0.296<br>(0.206 to 0.413)	0.688<br>(0.533 to 0.82)	0.816<br>(0.609 to 0.996)	6.9<br>(4.8 to 9.7)	12.9<br>(10 to 15.3)	14.9<br>(11.1 to 17.8)
Bosnia and Herzegovina	Benign and in situ intestinal neoplasms	0<br>(0 to 0)	0<br>(0 to 0)	0<br>(0 to 0)	0<br>(0 to 0)	0<br>(0 to 0)	0<br>(0 to 0)
Bosnia and Herzegovina	Benign and in situ cervical and uterine neoplasms	0<br>(0 to 0)	0<br>(0 to 0)	0<br>(0 to 0)	0<br>(0 to 0)	0<br>(0 to 0)	0<br>(0 to 0)
Bosnia and Herzegovina	Other benign and in situ neoplasms	0.0429<br>(0.0277 to 0.0631)	0.0694<br>(0.0469 to 0.0858)	0.0633<br>(0.0426 to 0.0786)	0.9<br>(0.6 to 1.4)	1.9<br>(1.3 to 2.3)	2.2<br>(1.5 to 2.7)
Bosnia and Herzegovina	Cardiovascular diseases	302<br>(292 to 312)	330<br>(315 to 345)	313<br>(294 to 333)	7602.7<br>(7372 to 7823.4)	6162<br>(5900.6 to 6428.4)	5411.8<br>(5091.2 to 5745.1)
Bosnia and Herzegovina	Rheumatic heart disease	1.47<br>(1.34 to 1.63)	1.58<br>(1.42 to 1.75)	1.17<br>(1.06 to 1.31)	34.8<br>(31.9 to 38.4)	30<br>(27.1 to 33)	22<br>(20 to 24.3)
Bosnia and Herzegovina	Ischaemic heart disease	160<br>(153 to 169)	153<br>(143 to 160)	143<br>(132 to 153)	4044.5<br>(3875.8 to 4241.9)	2861.3<br>(2697.6 to 2993.3)	2472.3<br>(2292.6 to 2633.8)
Bosnia and Herzegovina	Stroke	95.7<br>(90.2 to 102)	126<br>(117 to 133)	119<br>(110 to 128)	2426<br>(2296.3 to 2582.8)	2337.7<br>(2188.1 to 2465.5)	2041.5<br>(1881.1 to 2195.7)
Bosnia and Herzegovina	Ischaemic stroke	38.3<br>(35.3 to 41.2)	88.5<br>(78.4 to 95.5)	89.1<br>(79.5 to 96.9)	1067.7<br>(991.5 to 1145.2)	1649<br>(1475 to 1768.9)	1495.8<br>(1334.9 to 1628.1)
Bosnia and Herzegovina	Intracerebral hemorrhage	42.6<br>(40.1 to 45.4)	24.6<br>(21.1 to 27.1)	20.1<br>(17.3 to 22.3)	1026.5<br>(969.5 to 1095.6)	448<br>(387.1 to 491.2)	349.2<br>(299.5 to 387.3)
Bosnia and Herzegovina	Subarachnoid hemorrhage	14.9<br>(13.2 to 19.3)	12.6<br>(10.5 to 19.1)	10.1<br>(8.26 to 16.1)	331.8<br>(295.8 to 432.7)	240.7<br>(203.6 to 358.2)	196.5<br>(163.4 to 299.1)
Bosnia and Herzegovina	Hypertensive heart disease	4.48<br>(4.04 to 5.83)	5.3<br>(4.66 to 7.28)	4.79<br>(4.09 to 7.29)	106.3<br>(94.2 to 153.7)	99.6<br>(88.4 to 137.2)	83.1<br>(71.5 to 124)
Bosnia and Herzegovina	Non-rheumatic valvular heart disease	1.9<br>(1.61 to 2.27)	2.88<br>(2.54 to 3.25)	2.95<br>(2.58 to 3.32)	46.8<br>(40.1 to 55.8)	54.5<br>(48.1 to 61)	51.6<br>(45.1 to 57.6)
Bosnia and Herzegovina	Non-rheumatic calcific aortic valve disease	0.516<br>(0.378 to 0.724)	1.08<br>(0.867 to 1.31)	1.25<br>(1.01 to 1.47)	13.8<br>(10.1 to 18.9)	20.7<br>(16.8 to 24.8)	21.5<br>(17.5 to 25.3)
Bosnia and Herzegovina	Non-rheumatic degenerative mitral valve disease	1.33<br>(1.12 to 1.56)	1.73<br>(1.37 to 2.01)	1.63<br>(1.33 to 1.89)	31.9<br>(27 to 36.8)	32.3<br>(26.1 to 37.4)	28.7<br>(23.4 to 33.3)
Bosnia and Herzegovina	Other non-rheumatic valve diseases	0.0504<br>(0.0271 to 0.0708)	0.0698<br>(0.0374 to 0.0967)	0.0614<br>(0.0355 to 0.0835)	1.2<br>(0.6 to 1.7)	1.5<br>(0.8 to 2.2)	1.4<br>(0.7 to 1.9)

SR Appendix Table 2. All-age DALYs counts and age standardized DALYs rates per 100,000 individuals for all causes and level 1 cause groups by location for 1990, 2007, and 2017, both sexes combined

		All-age DALYs			Age-standardized rate of DALYs per 100,000		
Bosnia and Herzegovina	Cardiomyopathy and myocarditis	7.57 (5.29 to 9.97)	12.1 (8.74 to 15.4)	12.6 (9.32 to 15.9)	183 (128.1 to 241)	238 (172.8 to 301.3)	237.3 (175.6 to 298.9)
Bosnia and Herzegovina	Myocarditis	1.36 (0.841 to 1.91)	2.05 (1.17 to 2.84)	2.08 (1.21 to 2.87)	33.5 (20.6 to 47.1)	43.7 (25.2 to 59.9)	42.7 (25 to 58)
Bosnia and Herzegovina	Alcoholic cardiomyopathy	2.06 (0.942 to 3.2)	3.11 (1.67 to 4.71)	2.96 (1.61 to 4.48)	45.3 (20.7 to 70)	56.7 (30.2 to 86.1)	53.5 (28.5 to 82.7)
Bosnia and Herzegovina	Other cardiomyopathy	4.15 (2.85 to 5.67)	6.9 (5.25 to 8.92)	7.56 (5.93 to 9.47)	104.1 (71.7 to 140.5)	137.6 (104.7 to 177.3)	141.1 (111.3 to 176.8)
Bosnia and Herzegovina	Atrial fibrillation and flutter	3.18 (2.61 to 3.87)	5.27 (4.44 to 6.24)	6.05 (5.12 to 7.13)	88 (72.5 to 106)	102.6 (87.4 to 119.7)	103.7 (88.1 to 121.9)
Bosnia and Herzegovina	Aortic aneurysm	2.39 (1.96 to 2.91)	3.51 (2.87 to 4.03)	3.37 (2.69 to 3.82)	54.5 (45.5 to 66.1)	64.2 (52.6 to 72.8)	58.8 (47.5 to 66.4)
Bosnia and Herzegovina	Peripheral vascular disease	0.268 (0.143 to 0.457)	0.438 (0.259 to 0.7)	0.493 (0.306 to 0.769)	7.2 (3.8 to 11.9)	8.1 (4.9 to 12.8)	8.1 (5.1 to 12.5)
Bosnia and Herzegovina	Endocarditis	0.266 (0.227 to 0.35)	0.479 (0.428 to 0.534)	0.503 (0.45 to 0.561)	6.1 (5.2 to 7.9)	9.5 (8.5 to 10.6)	9.9 (8.9 to 11)
Bosnia and Herzegovina	Other cardiovascular and circulatory diseases	24.8 (18 to 28.6)	19.7 (15.7 to 26.4)	18.8 (14.5 to 26)	605.6 (424.4 to 697.6)	356.4 (285.6 to 481.1)	323.5 (249.9 to 449.6)
Bosnia and Herzegovina	Chronic respiratory diseases	46.1 (40.9 to 51)	47 (41.9 to 52.1)	45.1 (40.1 to 50.1)	1125.3 (1007.5 to 1239.5)	899.8 (798.5 to 1006.9)	837.6 (735.6 to 946.1)
Bosnia and Herzegovina	Chronic obstructive pulmonary disease	35.1 (31.7 to 38.8)	36.7 (33 to 40.3)	35.4 (31.8 to 39.1)	874.4 (794.7 to 954.9)	671.3 (607.5 to 737.3)	606.9 (545.3 to 668.9)
Bosnia and Herzegovina	Pneumoconiosis	0.0508 (0.0378 to 0.068)	0.0726 (0.0562 to 0.0921)	0.0975 (0.0768 to 0.122)	1.2 (0.9 to 1.6)	1.3 (1 to 1.6)	1.6 (1.3 to 2)
Bosnia and Herzegovina	Silicosis	0.0139 (0.0087 to 0.0214)	0.02 (0.0142 to 0.0278)	0.0312 (0.0225 to 0.0415)	0.3 (0.2 to 0.5)	0.3 (0.2 to 0.5)	0.5 (0.4 to 0.7)
Bosnia and Herzegovina	Asbestosis	0.00911 (0.00625 to 0.0135)	0.0135 (0.00986 to 0.0178)	0.0154 (0.0115 to 0.0214)	0.2 (0.2 to 0.3)	0.2 (0.2 to 0.3)	0.3 (0.2 to 0.4)
Bosnia and Herzegovina	Coal workers pneumoconiosis	0.0126 (0.00782 to 0.0182)	0.0166 (0.0113 to 0.0243)	0.021 (0.014 to 0.0308)	0.3 (0.2 to 0.4)	0.3 (0.2 to 0.4)	0.3 (0.2 to 0.5)
Bosnia and Herzegovina	Other pneumoconiosis	0.0152 (0.0104 to 0.0212)	0.0225 (0.0162 to 0.0306)	0.0299 (0.0216 to 0.0393)	0.4 (0.3 to 0.5)	0.4 (0.3 to 0.6)	0.5 (0.4 to 0.7)
Bosnia and Herzegovina	Asthma	8.5 (6.25 to 11.2)	7.38 (5.34 to 9.91)	6.85 (4.9 to 9.14)	195.8 (145.6 to 255.8)	170.6 (120.4 to 232.1)	173.8 (121.5 to 237)
Bosnia and Herzegovina	Interstitial lung disease and pulmonary sarcoidosis	1.47 (1.08 to 1.95)	1.88 (1.49 to 2.31)	1.79 (1.44 to 2.2)	34.5 (25 to 45.8)	35.4 (28 to 43.4)	32.7 (26.4 to 40.4)
Bosnia and Herzegovina	Other chronic respiratory diseases	0.913 (0.764 to 1.07)	0.943 (0.788 to 1.11)	0.871 (0.718 to 1.03)	19.4 (16.2 to 22.6)	21.2 (17.5 to 24.9)	22.6 (18.5 to 27)
Bosnia and Herzegovina	Digestive diseases	54.7 (48.4 to 62.4)	42.6 (36.2 to 52.1)	39.3 (33.9 to 46.3)	1208.2 (1069.9 to 1375.4)	855.8 (723.5 to 1034.2)	805.9 (681.6 to 953.7)
Bosnia and Herzegovina	Cirrhosis and other chronic liver diseases	24.1 (22.4 to 27.9)	18.4 (15.8 to 26.2)	16.6 (14.4 to 22.6)	505.7 (472.8 to 576.6)	341.8 (293.7 to 482.5)	307 (266.9 to 416.3)
Bosnia and Herzegovina	Cirrhosis and other chronic liver diseases due to hepatitis B	7.11 (6.2 to 8.63)	5.24 (4.3 to 7.57)	4.27 (3.56 to 5.99)	148.5 (130 to 177.7)	96.9 (80.2 to 139.5)	79.2 (66.3 to 110.5)
Bosnia and Herzegovina	Cirrhosis and other chronic liver diseases due to hepatitis C	5.36 (4.63 to 6.4)	4.18 (3.38 to 6.09)	3.81 (3.14 to 5.3)	111.4 (97.1 to 131.6)	76.5 (61.9 to 111.5)	69.6 (57.5 to 97)
Bosnia and Herzegovina	Cirrhosis and other chronic liver diseases due to alcohol use	8.32 (7.47 to 9.92)	6.38 (5.32 to 9.31)	6.02 (5.08 to 8.38)	174 (157.2 to 202.8)	116.1 (96.9 to 168.9)	108.2 (91.4 to 149.9)
Bosnia and Herzegovina	Cirrhosis due to NASH	1.46 (1.27 to 1.73)	1.38 (1.12 to 2.03)	1.36 (1.13 to 1.91)	30.4 (26.6 to 35.4)	25 (20.4 to 36.6)	24.4 (20.3 to 34)
Bosnia and Herzegovina	Cirrhosis and other chronic liver diseases due to other causes	1.82 (1.53 to 2.14)	1.26 (1.01 to 1.75)	1.15 (0.92 to 1.56)	41.5 (34.5 to 48.2)	27.3 (22.3 to 35.9)	25.7 (21.2 to 32.6)
Bosnia and Herzegovina	Upper digestive system diseases	15.4 (11.6 to 20.6)	11.1 (7.99 to 15.5)	9.79 (7.04 to 13.7)	344.7 (261.6 to 457.4)	239.4 (171.3 to 338.4)	227.4 (160.1 to 323.6)
Bosnia and Herzegovina	Peptic ulcer disease	5.68 (5.17 to 6.34)	3.06 (2.6 to 3.8)	2.61 (2.26 to 3.19)	131.3 (120 to 145.4)	57.1 (49 to 69.8)	46.9 (40.8 to 56.8)
Bosnia and Herzegovina	Gastritis and duodenitis	5.98 (3.88 to 8.72)	4.21 (2.71 to 6.07)	3.68 (2.37 to 5.32)	135.1 (87.2 to 196.7)	104 (66.9 to 151.6)	102.3 (65.3 to 150.5)
Bosnia and Herzegovina	GERD	3.72 (1.98 to 6.32)	3.82 (2.04 to 6.51)	3.5 (1.87 to 5.87)	78.3 (41.8 to 133.1)	78.2 (41.8 to 132.7)	78.1 (42.1 to 131.8)
Bosnia and Herzegovina	Appendicitis	0.881 (0.689 to 1.01)	0.365 (0.309 to 0.464)	0.314 (0.269 to 0.394)	20.6 (15.9 to 23.9)	8.4 (7.1 to 10.2)	7.6 (6.4 to 9)
Bosnia and Herzegovina	Paralytic ileus and intestinal obstruction	1.82 (1.52 to 2.16)	2.08 (1.54 to 2.41)	2.17 (1.5 to 2.54)	45.2 (38 to 53.9)	43 (32.2 to 49.1)	42.1 (29.8 to 48.8)
Bosnia and Herzegovina	Inguinal, femoral, and abdominal hernia	6.21 (4.52 to 8.22)	3.78 (2.69 to 5.08)	3.51 (2.49 to 4.7)	144.4 (105.8 to 190.6)	89 (61.9 to 119.7)	87.5 (61.8 to 118.3)
Bosnia and Herzegovina	Inflammatory bowel disease	1.06 (0.794 to 1.35)	1.12 (0.848 to 1.41)	1.1 (0.82 to 1.39)	22.8 (17.1 to 28.8)	22.3 (17.3 to 28.2)	23.1 (17.4 to 29.5)
Bosnia and Herzegovina	Vascular intestinal disorders	0.662 (0.482 to 0.781)	0.983 (0.806 to 1.18)	1.06 (0.825 to 1.25)	17 (12.1 to 20)	18.1 (14.6 to 21.3)	17.7 (13.9 to 20.9)
Bosnia and Herzegovina	Gallbladder and biliary diseases	1.15 (0.934 to 1.29)	1.12 (0.948 to 1.43)	1.12 (0.965 to 1.4)	27.5 (23 to 30.6)	20.7 (17.7 to 26.2)	19.2 (16.7 to 23.9)
Bosnia and Herzegovina	Pancreatitis	1.9 (1.27 to 2.37)	2.37 (1.61 to 2.96)	2.33 (1.45 to 2.92)	42.5 (27.3 to 53.7)	44 (30.7 to 54.4)	42.9 (28.4 to 53.2)
Bosnia and Herzegovina	Other digestive diseases	1.56 (1.16 to 2.01)	1.27 (1.03 to 1.53)	1.33 (1.09 to 1.6)	37.7 (28.1 to 49.1)	29.3 (23.5 to 36)	31.5 (25.2 to 38.6)
Bosnia and Herzegovina	Neurological disorders	63.3 (47.5 to 83.2)	68.4 (54.4 to 86.6)	67.7 (54.3 to 83.3)	1527 (1185.6 to 1944.6)	1543.7 (1224.1 to 1959.2)	1534.2 (1189.8 to 1931.4)
Bosnia and Herzegovina	Alzheimer's disease and other dementias	11.8 (11 to 12.7)	20.1 (18.7 to 21.6)	25.5 (23.5 to 27.6)	413.4 (383.2 to 446)	463.7 (424.1 to 500.8)	466.9 (431.2 to 502.8)

SR Appendix Table 2. All-age DALYs counts and age standardized DALYs rates per 100,000 individuals for all causes and level 1 cause groups by location for 1990, 2007, and 2017, both sexes combined

		All-age DALYs			Age-standardized rate of DALYs per 100,000		
Bosnia and Herzegovina	Parkinson's disease	3.34 (3.08 to 3.86)	4.74 (4.24 to 6.51)	5.16 (4.54 to 7.38)	96.6 (88.6 to 117)	88.2 (79.3 to 121.6)	84.3 (74.4 to 120.5)
Bosnia and Herzegovina	Epilepsy	9.96 (5.56 to 16)	7.59 (4.68 to 11.6)	6.13 (3.08 to 10.5)	220.7 (123.1 to 353.4)	188.9 (115.9 to 286.5)	177.7 (90.8 to 305.8)
Bosnia and Herzegovina	Multiple sclerosis	1.24 (1.09 to 1.43)	1.29 (1.09 to 1.67)	1.14 (0.924 to 1.57)	25.7 (22.4 to 29.5)	25.8 (21.8 to 32.8)	24.7 (20.1 to 32.7)
Bosnia and Herzegovina	Motor neuron disease	0.0443 (0.0256 to 0.0583)	0.0654 (0.0326 to 0.0756)	0.0673 (0.0317 to 0.0781)	0.9 (0.6 to 1.2)	1.3 (0.7 to 1.5)	1.3 (0.7 to 1.5)
Bosnia and Herzegovina	Headache disorders	35.2 (22.8 to 51.5)	32.8 (21.1 to 48.3)	28 (18.2 to 41.3)	729.8 (473.8 to 1067.6)	729.4 (466.1 to 1069.3)	728.4 (468.7 to 1065.1)
Bosnia and Herzegovina	Migraine	30.2 (18.6 to 45.4)	28 (17.3 to 42.7)	23.9 (14.8 to 36.3)	625.6 (386.4 to 941.4)	625.3 (386.7 to 949.4)	624.4 (384.1 to 946.9)
Bosnia and Herzegovina	Tension-type headache	4.97 (2.82 to 7.8)	4.73 (2.7 to 7.36)	4.14 (2.4 to 6.42)	104.2 (58.9 to 163.7)	104.1 (59 to 163.5)	104 (59.4 to 162.7)
Bosnia and Herzegovina	Other neurological disorders	1.78 (1.07 to 2.7)	1.86 (1.39 to 2.47)	1.69 (1.19 to 2.43)	39.8 (23.9 to 60.5)	46.5 (33.5 to 63.3)	51 (33.8 to 77.2)
Bosnia and Herzegovina	Mental disorders	64.4 (47.6 to 83.1)	57.8 (43 to 74.3)	49.1 (36.7 to 63.3)	1369.8 (1012 to 1769.8)	1315.4 (974.6 to 1697.5)	1291.2 (957.5 to 1671.5)
Bosnia and Herzegovina	Schizophrenia	5.7 (4.19 to 7.15)	5.72 (4.23 to 7.09)	5.07 (3.78 to 6.34)	116.1 (85.5 to 145.9)	120.1 (89.5 to 148.6)	120.9 (89.8 to 152.8)
Bosnia and Herzegovina	Depressive disorders	20.3 (14.3 to 27.4)	17.6 (12.6 to 23.7)	15.1 (10.8 to 20.3)	430.6 (303.6 to 583.4)	374.2 (267.5 to 505.3)	353.7 (252.5 to 477.2)
Bosnia and Herzegovina	Major depressive disorder	16.2 (11.3 to 22)	13.5 (9.49 to 18.4)	11.4 (7.91 to 15.4)	345.6 (241 to 466.9)	289.4 (203.8 to 394.2)	269 (188.1 to 364.3)
Bosnia and Herzegovina	Dysthymia	4.04 (2.71 to 5.97)	4.09 (2.76 to 5.93)	3.74 (2.52 to 5.44)	85 (57.1 to 124.7)	84.8 (57.3 to 122.5)	84.7 (56.8 to 123.7)
Bosnia and Herzegovina	Bipolar disorder	6.73 (4.21 to 9.98)	6.25 (3.86 to 9.2)	5.28 (3.32 to 7.83)	139.1 (87.1 to 206.3)	139.1 (86.4 to 204.6)	139 (87.5 to 203.8)
Bosnia and Herzegovina	Anxiety disorders	14.5 (10.3 to 19.3)	13.5 (9.61 to 17.8)	11.6 (8.25 to 15.4)	308.4 (219.3 to 411)	308.9 (219.7 to 406.4)	307.5 (215.6 to 409.4)
Bosnia and Herzegovina	Eating disorders	1.01 (0.639 to 1.47)	1.26 (0.799 to 1.86)	1.06 (0.675 to 1.55)	20.8 (13.1 to 30.1)	31.5 (20.1 to 46.8)	35 (22.2 to 51.1)
Bosnia and Herzegovina	Anorexia nervosa	0.207 (0.12 to 0.326)	0.241 (0.138 to 0.379)	0.196 (0.12 to 0.299)	4.4 (2.5 to 6.8)	6.3 (3.6 to 9.9)	7 (4.3 to 10.8)
Bosnia and Herzegovina	Bulimia nervosa	0.804 (0.488 to 1.19)	1.02 (0.635 to 1.51)	0.862 (0.541 to 1.29)	16.4 (10 to 24.5)	25.2 (15.6 to 37.3)	28 (17.7 to 41.8)
Bosnia and Herzegovina	Autism spectrum disorders	3.05 (2.08 to 4.18)	2.58 (1.77 to 3.51)	2.16 (1.46 to 2.94)	67.8 (46 to 92.9)	67.9 (46.2 to 93.1)	68 (46 to 93.2)
Bosnia and Herzegovina	Attention-deficit/hyperactivity disorder	0.535 (0.323 to 0.854)	0.418 (0.25 to 0.661)	0.318 (0.191 to 0.5)	11.8 (7.1 to 18.8)	11.8 (7.1 to 18.8)	11.8 (7 to 18.6)
Bosnia and Herzegovina	Conduct disorder	4.19 (2.45 to 6.71)	2.98 (1.77 to 4.7)	2.09 (1.24 to 3.28)	97.5 (57.4 to 155.2)	97.9 (58.6 to 155.1)	97.9 (58.3 to 152.9)
Bosnia and Herzegovina	Idiopathic developmental intellectual disability	1.74 (0.633 to 3.18)	0.935 (0.346 to 1.71)	0.571 (0.143 to 1.1)	38.7 (14.1 to 71.1)	24.9 (9.3 to 45.6)	18.7 (4.7 to 35.8)
Bosnia and Herzegovina	Other mental disorders	6.7 (4.45 to 9.32)	6.56 (4.39 to 9.07)	5.82 (3.91 to 7.98)	139 (92.2 to 193.5)	138.9 (93.1 to 192.7)	138.9 (92.7 to 192)
Bosnia and Herzegovina	Substance use disorders	27.3 (22.1 to 33.6)	22.6 (17.4 to 29)	18.1 (13.8 to 23.5)	556.5 (450.9 to 683.4)	487.5 (371.1 to 626.8)	463.1 (344.9 to 597.5)
Bosnia and Herzegovina	Alcohol use disorders	23.3 (18.8 to 28.8)	17 (12.8 to 22.6)	13.5 (10 to 18.7)	476.5 (385 to 587.7)	357.2 (265.5 to 478.3)	332.2 (241.2 to 452.5)
Bosnia and Herzegovina	Drug use disorders	3.94 (2.78 to 5.2)	5.59 (4.39 to 6.79)	4.55 (3.55 to 5.57)	80 (56.6 to 105.4)	130.3 (102.3 to 158.7)	130.9 (102.3 to 161)
Bosnia and Herzegovina	Opioid use disorders	3.04 (2.1 to 4.13)	4.64 (3.63 to 5.69)	3.74 (2.87 to 4.61)	61.5 (42.8 to 83.6)	107.8 (84.3 to 132.9)	106.8 (82.2 to 133.7)
Bosnia and Herzegovina	Cocaine use disorders	0.0842 (0.0522 to 0.126)	0.0834 (0.0546 to 0.119)	0.0731 (0.0471 to 0.103)	1.7 (1.1 to 2.6)	1.9 (1.2 to 2.8)	2 (1.3 to 3)
Bosnia and Herzegovina	Amphetamine use disorders	0.22 (0.118 to 0.365)	0.192 (0.106 to 0.309)	0.149 (0.0825 to 0.236)	4.5 (2.4 to 7.4)	4.8 (2.6 to 7.7)	5 (2.7 to 8.1)
Bosnia and Herzegovina	Cannabis use disorders	0.26 (0.16 to 0.39)	0.211 (0.133 to 0.317)	0.155 (0.099 to 0.227)	5.4 (3.3 to 8.1)	5.2 (3.3 to 7.9)	5 (3.1 to 7.4)
Bosnia and Herzegovina	Other drug use disorders	0.34 (0.221 to 0.48)	0.464 (0.358 to 0.591)	0.434 (0.344 to 0.539)	6.9 (4.5 to 9.7)	10.6 (8.1 to 13.6)	12.1 (9.4 to 15.3)
Bosnia and Herzegovina	Diabetes and kidney diseases	45.2 (38.4 to 53.4)	83.6 (72.9 to 96.9)	89.2 (76.5 to 105)	1030.2 (879.2 to 1211.9)	1523.5 (1324.6 to 1779.1)	1565.4 (1336.9 to 1853.4)
Bosnia and Herzegovina	Diabetes mellitus	31.2 (25.3 to 38.5)	67.4 (57.3 to 80)	74.3 (62.4 to 89)	709.4 (581.2 to 868.7)	1220.1 (1032.3 to 1452.4)	1293.2 (1080 to 1559.8)
Bosnia and Herzegovina	Diabetes mellitus type 1	8.6 (5.22 to 9.68)	16.4 (7.28 to 19.2)	12.5 (5.6 to 14.8)	200.2 (114.9 to 227.3)	295.3 (136.3 to 343.3)	221.4 (109.1 to 259.1)
Bosnia and Herzegovina	Diabetes mellitus type 2	22.6 (16.5 to 30.1)	51.1 (40.6 to 64.5)	61.8 (49.7 to 76.6)	509.2 (375.1 to 679.9)	924.8 (732.4 to 1166.2)	1071.8 (854 to 1339.7)
Bosnia and Herzegovina	Chronic kidney disease	14 (12.9 to 15.2)	16.2 (14.6 to 17.8)	14.9 (13.4 to 16.3)	320.6 (295.6 to 348.3)	303.3 (274.3 to 331.3)	272.1 (244.5 to 297.4)
Bosnia and Herzegovina	Chronic kidney disease due to diabetes mellitus type 1	1.05 (0.818 to 1.34)	1.12 (0.882 to 1.38)	1.04 (0.792 to 1.31)	21.7 (17.1 to 27.8)	20.9 (16.7 to 25.4)	19.7 (15.4 to 24.4)
Bosnia and Herzegovina	Chronic kidney disease due to diabetes mellitus type 2	1.86 (1.5 to 2.3)	2.61 (2.15 to 3.12)	2.57 (2.08 to 3.09)	44.3 (36 to 54.1)	46.1 (38.6 to 54.8)	42.1 (34.4 to 50.3)
Bosnia and Herzegovina	Chronic kidney disease due to hypertension	1.8 (1.51 to 2.13)	2.32 (1.92 to 2.74)	2.42 (2.02 to 2.84)	44.2 (37.3 to 51.6)	43.4 (36.1 to 50.7)	42.3 (35.5 to 49.6)
Bosnia and Herzegovina	Chronic kidney disease due to glomerulonephritis	3.33 (2.84 to 3.91)	3.51 (3 to 4.08)	3 (2.5 to 3.56)	72.5 (62.1 to 84.3)	67.2 (58.1 to 76.8)	58 (49.4 to 67.8)

SR Appendix Table 2. All-age DALYs counts and age standardized DALYs rates per 100,000 individuals for all causes and level 1 cause groups by location for 1990, 2007, and 2017, both sexes combined							
		All-age DALYs			Age-standardized rate of DALYs per 100,000		
Bosnia and Herzegovina	Chronic kidney disease due to other and unspecified causes	5.95 (5.18 to 6.79)	6.61 (5.71 to 7.56)	5.91 (5.1 to 6.79)	137.9 (120.7 to 157.5)	125.7 (110.1 to 142.2)	110 (95.5 to 125)
Bosnia and Herzegovina	Acute glomerulonephritis	0.00574 (0.00407 to 0.00837)	0.00542 (0.00383 to 0.00806)	0.00586 (0.00387 to 0.00871)	0.2 (0.1 to 0.2)	0.1 (0.1 to 0.2)	0.1 (0.1 to 0.2)
Bosnia and Herzegovina	Skin and subcutaneous diseases	19.7 (13.1 to 28.8)	18 (12.2 to 26.4)	15.8 (10.6 to 23.2)	449.4 (299.3 to 660.5)	470.8 (316.3 to 694.5)	476.5 (320.3 to 698.3)
Bosnia and Herzegovina	Dermatitis	5.28 (3.04 to 8.43)	4.32 (2.55 to 6.77)	3.66 (2.18 to 5.66)	122.1 (70.5 to 195.5)	122.3 (71.4 to 194.3)	123.3 (71.7 to 195.8)
Bosnia and Herzegovina	Atopic dermatitis	4.2 (2.26 to 7.03)	3.29 (1.79 to 5.49)	2.76 (1.5 to 4.61)	99 (53 to 165.9)	99.3 (53.7 to 168.7)	100.3 (55.3 to 170)
Bosnia and Herzegovina	Contact dermatitis	1.07 (0.694 to 1.61)	1.02 (0.676 to 1.51)	0.899 (0.589 to 1.31)	23 (14.9 to 34.3)	22.9 (15.2 to 33.8)	22.9 (14.8 to 33.8)
Bosnia and Herzegovina	Seborrhoeic dermatitis	0.00482 (0.0027 to 0.0078)	0.00435 (0.00243 to 0.00702)	0.0038 (0.00211 to 0.00608)	0.1 (0.1 to 0.2)	0.1 (0.1 to 0.2)	0.1 (0.1 to 0.2)
Bosnia and Herzegovina	Psoriasis	2.63 (1.85 to 3.48)	3.03 (2.12 to 4.01)	2.75 (1.94 to 3.6)	55.8 (39.3 to 73.6)	65.3 (46 to 87.2)	66 (45.9 to 86.7)
Bosnia and Herzegovina	Bacterial skin diseases	0.169 (0.124 to 0.227)	0.236 (0.132 to 0.322)	0.267 (0.119 to 0.378)	3.8 (2.8 to 5.2)	5.1 (3 to 7)	5.8 (3 to 8.2)
Bosnia and Herzegovina	Cellulitis	0.0909 (0.0648 to 0.127)	0.111 (0.0676 to 0.153)	0.122 (0.063 to 0.174)	2 (1.4 to 2.8)	2.5 (1.6 to 3.4)	2.8 (1.7 to 4)
Bosnia and Herzegovina	Pyoderma	0.0781 (0.0518 to 0.117)	0.125 (0.0576 to 0.18)	0.145 (0.0517 to 0.214)	1.8 (1.2 to 2.7)	2.7 (1.3 to 3.8)	3 (1.2 to 4.5)
Bosnia and Herzegovina	Scabies	1.27 (0.695 to 2.07)	0.981 (0.544 to 1.59)	0.783 (0.433 to 1.27)	28.4 (15.6 to 46.1)	26.9 (15.1 to 43.7)	26.7 (14.7 to 43.6)
Bosnia and Herzegovina	Fungal skin diseases	2.07 (0.834 to 4.31)	1.99 (0.795 to 4.11)	1.87 (0.754 to 3.82)	48.9 (19.7 to 101.2)	45.9 (18.3 to 95)	45.2 (17.9 to 93.9)
Bosnia and Herzegovina	Viral skin diseases	1.87 (1.19 to 2.78)	1.3 (0.829 to 1.91)	1.02 (0.656 to 1.53)	45.6 (29.1 to 67.7)	45.8 (29.2 to 67.5)	45.8 (29.1 to 68.4)
Bosnia and Herzegovina	Acne vulgaris	1.17 (0.673 to 1.98)	1.2 (0.699 to 1.98)	0.925 (0.547 to 1.55)	24.7 (14.2 to 42.1)	32 (18.6 to 52.7)	34.4 (20.1 to 57.6)
Bosnia and Herzegovina	Alopecia areata	0.284 (0.18 to 0.432)	0.256 (0.162 to 0.378)	0.214 (0.136 to 0.32)	5.9 (3.8 to 9)	5.9 (3.7 to 8.7)	5.9 (3.7 to 8.8)
Bosnia and Herzegovina	Pruritus	0.401 (0.188 to 0.763)	0.417 (0.2 to 0.783)	0.384 (0.183 to 0.707)	8.8 (4.1 to 16.7)	9.3 (4.4 to 17.7)	9.4 (4.5 to 17.7)
Bosnia and Herzegovina	Urticaria	3.22 (2.12 to 4.56)	2.55 (1.71 to 3.61)	2.11 (1.42 to 2.99)	74.4 (48.8 to 105.1)	74.7 (49.4 to 106)	74.6 (49.6 to 106.1)
Bosnia and Herzegovina	Decubitus ulcer	0.11 (0.081 to 0.145)	0.164 (0.117 to 0.214)	0.208 (0.134 to 0.275)	2.7 (2 to 3.6)	3.4 (2.4 to 4.4)	4 (2.7 to 5.2)
Bosnia and Herzegovina	Other skin and subcutaneous diseases	1.26 (0.606 to 2.32)	1.59 (0.773 to 2.92)	1.56 (0.759 to 2.87)	28.1 (13.5 to 51.7)	34.1 (16.4 to 62.5)	35.4 (17.1 to 65.6)
Bosnia and Herzegovina	Sense organ diseases	37.9 (25 to 56.3)	41.8 (27.7 to 61.6)	41.7 (27.7 to 60.8)	892.5 (597 to 1312.2)	838.4 (558.8 to 1233.9)	820.7 (545.6 to 1207.2)
Bosnia and Herzegovina	Blindness and vision impairment	18.7 (11.6 to 29.7)	19.6 (12.3 to 30.5)	19.1 (12 to 29.3)	434.8 (274.9 to 681.1)	390.9 (244.9 to 603.8)	376.9 (236.5 to 583.8)
Bosnia and Herzegovina	Glaucoma	0.167 (0.109 to 0.236)	0.205 (0.134 to 0.293)	0.215 (0.141 to 0.304)	4.3 (2.8 to 6)	3.7 (2.4 to 5.3)	3.5 (2.3 to 4.9)
Bosnia and Herzegovina	Cataract	4.05 (2.74 to 5.62)	5.23 (3.51 to 7.19)	5.62 (3.77 to 7.68)	108.5 (74 to 148.6)	98.4 (66.6 to 134.7)	94.9 (64.1 to 129.7)
Bosnia and Herzegovina	Age-related macular degeneration	0.0328 (0.0201 to 0.0484)	0.0474 (0.0303 to 0.0713)	0.0533 (0.0333 to 0.0801)	0.9 (0.5 to 1.3)	0.9 (0.6 to 1.3)	0.9 (0.6 to 1.3)
Bosnia and Herzegovina	Refraction disorders	5.37 (3.37 to 8.14)	4.72 (3.03 to 7.12)	4.18 (2.66 to 6.28)	119.4 (75.4 to 181.9)	108.1 (68.3 to 165.2)	104.9 (66.7 to 161.4)
Bosnia and Herzegovina	Near vision loss	7.99 (3.84 to 14.6)	8.2 (3.96 to 14.8)	7.85 (3.78 to 13.9)	175.9 (84.8 to 320)	156.1 (75.3 to 282.3)	149.7 (72.5 to 265.2)
Bosnia and Herzegovina	Other vision loss	1.11 (0.75 to 1.54)	1.2 (0.801 to 1.65)	1.19 (0.8 to 1.66)	25.8 (17.4 to 35.4)	23.7 (16 to 32.5)	23 (15.4 to 32.1)
Bosnia and Herzegovina	Age-related and other hearing loss	17.8 (12.1 to 25.4)	20.7 (14 to 29.1)	21 (14.3 to 29.5)	427.7 (296.8 to 597)	416.7 (285.6 to 585.4)	412.9 (282.4 to 578.3)
Bosnia and Herzegovina	Other sense organ diseases	1.3 (0.815 to 1.89)	1.52 (0.947 to 2.19)	1.54 (0.956 to 2.22)	30.1 (18.8 to 43.5)	30.8 (19.1 to 44.7)	30.9 (19.2 to 45.2)
Bosnia and Herzegovina	Musculoskeletal disorders	73.4 (53.7 to 97.6)	79.5 (58.3 to 107)	76.6 (56.1 to 101)	1576.6 (1155.3 to 2094)	1581.3 (1156 to 2115.1)	1593.8 (1162.2 to 2117.5)
Bosnia and Herzegovina	Rheumatoid arthritis	1.37 (0.989 to 1.81)	1.93 (1.41 to 2.49)	1.88 (1.34 to 2.46)	30.7 (22.6 to 39.8)	36.6 (26.9 to 47.4)	35.5 (25.3 to 46.7)
Bosnia and Herzegovina	Osteoarthritis	3.65 (1.86 to 7.17)	5.13 (2.58 to 10.1)	5.48 (2.76 to 10.7)	85 (43.6 to 166.8)	93.5 (47.2 to 184)	94.6 (47.9 to 185)
Bosnia and Herzegovina	Low back pain	52.9 (38 to 71.4)	55.7 (39.9 to 74.8)	53.5 (38.4 to 71.2)	1130.8 (817.6 to 1522.5)	1126.1 (810.1 to 1508.4)	1142.8 (823.7 to 1525.2)
Bosnia and Herzegovina	Neck pain	13.8 (9.59 to 19.6)	15.3 (10.6 to 21.5)	14.4 (10.1 to 20.2)	296.2 (207.5 to 414.8)	295.7 (207.5 to 415.2)	294.7 (207.6 to 412.2)
Bosnia and Herzegovina	Gout	0.485 (0.317 to 0.678)	0.634 (0.421 to 0.863)	0.659 (0.438 to 0.907)	11 (7.3 to 15.4)	11.7 (7.8 to 16)	11.9 (7.9 to 16.5)
Bosnia and Herzegovina	Other musculoskeletal disorders	1.1 (0.464 to 1.91)	0.876 (0.506 to 1.56)	0.688 (0.465 to 1.22)	22.9 (10.1 to 39.4)	17.6 (10.3 to 31)	14.5 (9.7 to 25.1)
Bosnia and Herzegovina	Other non-communicable diseases	59.5 (47.4 to 74.4)	45.1 (34.1 to 58.4)	39.8 (29.9 to 52)	1454.6 (1178.1 to 1795.1)	1183.3 (945.9 to 1460.2)	1206.7 (956.9 to 1518.3)
Bosnia and Herzegovina	Congenital anomalies	25.6 (21.7 to 30)	14.8 (12.3 to 17.6)	12.8 (10.2 to 15.6)	695.4 (590.7 to 811.3)	546.6 (473.5 to 628.7)	602.6 (472.3 to 730.8)
Bosnia and Herzegovina	Neural tube defects	1.98 (1.39 to 2.82)	0.719 (0.557 to 0.94)	0.682 (0.479 to 0.991)	58.1 (40.3 to 83.8)	32.5 (24.4 to 44.2)	41.2 (26.8 to 64.4)

SR Appendix Table 2. All-age DALYs counts and age standardized DALYs rates per 100,000 individuals for all causes and level 1 cause groups by location for 1990, 2007, and 2017, both sexes combined

		All-age DALYs			Age-standardized rate of DALYs per 100,000		
Bosnia and Herzegovina	Congenital heart anomalies	8.06 (6.3 to 10.7)	3.67 (3.11 to 4.33)	3.27 (2.39 to 4.2)	235.9 (182.1 to 315)	175 (144 to 208.7)	212.7 (148.9 to 280.4)
Bosnia and Herzegovina	Orofacial clefts	0.253 (0.146 to 0.431)	0.136 (0.0882 to 0.204)	0.0939 (0.0616 to 0.134)	6.6 (3.7 to 11.7)	4.3 (2.6 to 6.7)	3.3 (2.1 to 5)
Bosnia and Herzegovina	Down's syndrome	0.72 (0.522 to 0.993)	0.587 (0.457 to 0.728)	0.598 (0.407 to 0.774)	17.9 (12.7 to 25.3)	19.5 (15.2 to 24.5)	28.8 (16.6 to 40.5)
Bosnia and Herzegovina	Turner syndrome	0.00447 (0.00217 to 0.00742)	0.00381 (0.00186 to 0.00634)	0.00296 (0.00147 to 0.00487)	0.1 (0 to 0.2)	0.1 (0 to 0.2)	0.1 (0 to 0.2)
Bosnia and Herzegovina	Klinefelter syndrome	0.000966 (0.000447 to 0.00184)	0.000763 (0.000355 to 0.00147)	0.000593 (0.000268 to 0.00113)	0 (0 to 0)	0 (0 to 0)	0 (0 to 0)
Bosnia and Herzegovina	Other chromosomal abnormalities	0.524 (0.225 to 0.941)	0.359 (0.216 to 0.497)	0.364 (0.207 to 0.53)	14.5 (5.6 to 27.2)	14.8 (8 to 21.1)	19.6 (9.4 to 30)
Bosnia and Herzegovina	Congenital musculoskeletal and limb anomalies	6.01 (4.06 to 8.07)	5.13 (3.43 to 7)	4.41 (3 to 6.01)	138.4 (95.3 to 184.6)	132.2 (90.3 to 178.8)	131.1 (90.1 to 177.9)
Bosnia and Herzegovina	Urogenital congenital anomalies	0.269 (0.177 to 0.484)	0.173 (0.13 to 0.247)	0.136 (0.104 to 0.183)	7.2 (4.5 to 13.6)	6.5 (4.6 to 10.3)	6.2 (4.5 to 9.4)
Bosnia and Herzegovina	Digestive congenital anomalies	2.66 (1.42 to 3.86)	1.5 (0.992 to 2.01)	1.34 (0.847 to 1.83)	71.4 (35.8 to 106.6)	52.3 (31.8 to 70)	58.3 (31.7 to 86.2)
Bosnia and Herzegovina	Other congenital anomalies	5.15 (4 to 6.36)	2.54 (1.91 to 3.22)	1.89 (1.47 to 2.41)	145.4 (111.6 to 179.8)	109.3 (79.3 to 140.9)	101.2 (75.5 to 134)
Bosnia and Herzegovina	Urinary diseases and male infertility	4.76 (3.98 to 5.73)	3.34 (2.42 to 4.46)	3.39 (2.45 to 4.57)	109.1 (92 to 130.4)	62.3 (45.2 to 82.6)	61.1 (44.5 to 82.2)
Bosnia and Herzegovina	Urinary tract infections	1.88 (1.62 to 2.23)	0.607 (0.535 to 0.687)	0.565 (0.491 to 0.65)	44.9 (38.8 to 52.9)	12.2 (10.8 to 13.9)	11.4 (9.8 to 13.2)
Bosnia and Herzegovina	Urolithiasis	0.283 (0.211 to 0.369)	0.237 (0.164 to 0.319)	0.228 (0.161 to 0.308)	6.2 (4.6 to 8)	4.7 (3.3 to 6.4)	4.8 (3.4 to 6.5)
Bosnia and Herzegovina	Benign prostatic hyperplasia	1.66 (1.06 to 2.44)	2.15 (1.37 to 3.12)	2.33 (1.5 to 3.41)	36.3 (23.1 to 53.3)	37.6 (24 to 54.4)	38.4 (24.8 to 56.2)
Bosnia and Herzegovina	Male infertility	0.0994 (0.0397 to 0.214)	0.0883 (0.0349 to 0.189)	0.0588 (0.0235 to 0.123)	2 (0.8 to 4.3)	2.1 (0.8 to 4.6)	1.8 (0.7 to 3.8)
Bosnia and Herzegovina	Other urinary diseases	0.828 (0.585 to 1.04)	0.26 (0.188 to 0.321)	0.205 (0.144 to 0.254)	19.7 (13.6 to 24.6)	5.6 (4.3 to 7)	4.7 (3.6 to 5.8)
Bosnia and Herzegovina	Gynecological diseases	7.34 (5.01 to 10.2)	6.56 (4.43 to 9.17)	5.28 (3.59 to 7.56)	151.1 (103.2 to 209.2)	146 (99.1 to 202)	146.8 (100.3 to 205.7)
Bosnia and Herzegovina	Uterine fibroids	1.13 (0.666 to 1.81)	1.02 (0.563 to 1.73)	0.883 (0.497 to 1.47)	23.6 (14 to 38)	19.9 (11 to 34.1)	19.9 (11.1 to 33.2)
Bosnia and Herzegovina	Polycystic ovarian syndrome	0.243 (0.106 to 0.472)	0.216 (0.0967 to 0.417)	0.17 (0.0746 to 0.332)	5 (2.2 to 9.7)	5 (2.2 to 9.7)	5 (2.2 to 9.9)
Bosnia and Herzegovina	Female infertility	0.0847 (0.0271 to 0.19)	0.0645 (0.0196 to 0.149)	0.0616 (0.0192 to 0.142)	1.7 (0.6 to 3.8)	1.6 (0.5 to 3.6)	1.9 (0.6 to 4.4)
Bosnia and Herzegovina	Endometriosis	2.1 (1.37 to 3.06)	1.82 (1.21 to 2.64)	1.41 (0.92 to 2.07)	42.5 (27.9 to 61.7)	43.1 (28.5 to 62.6)	43.1 (28.1 to 64)
Bosnia and Herzegovina	Genital prolapse	0.215 (0.113 to 0.381)	0.179 (0.0939 to 0.326)	0.178 (0.0869 to 0.333)	4.7 (2.5 to 8.3)	3.3 (1.7 to 5.9)	3.2 (1.6 to 6)
Bosnia and Herzegovina	Premenstrual syndrome	2.58 (1.65 to 3.88)	2.23 (1.44 to 3.38)	1.71 (1.1 to 2.56)	53.2 (34 to 80)	51.3 (32.9 to 77.7)	50.8 (32.4 to 75.6)
Bosnia and Herzegovina	Other gynecological diseases	0.991 (0.69 to 1.36)	1.03 (0.691 to 1.47)	0.861 (0.572 to 1.25)	20.3 (14.2 to 28.1)	22 (14.7 to 31.5)	22.8 (15.2 to 33.2)
Bosnia and Herzegovina	Hemoglobinopathies and hemolytic anaemias	2.72 (1.98 to 3.66)	1.7 (1.3 to 2.31)	1.35 (1.04 to 1.77)	61.8 (45.2 to 82.8)	38.9 (29.2 to 53.1)	33.2 (24.8 to 44.7)
Bosnia and Herzegovina	Thalassemias	0.029 (0.017 to 0.0489)	0.0114 (0.00761 to 0.017)	0.00809 (0.00544 to 0.0118)	0.8 (0.5 to 1.3)	0.5 (0.3 to 0.7)	0.5 (0.3 to 0.7)
Bosnia and Herzegovina	Thalassaemias trait	0.535 (0.342 to 0.814)	0.315 (0.199 to 0.482)	0.252 (0.158 to 0.388)	12.3 (7.9 to 18.4)	7.7 (4.8 to 11.7)	7 (4.4 to 10.7)
Bosnia and Herzegovina	Sickle cell disorders	0.131 (0.1 to 0.185)	0.0846 (0.0685 to 0.105)	0.0635 (0.0516 to 0.0777)	3.1 (2.3 to 4.3)	2.4 (2 to 3.1)	2.3 (1.9 to 2.9)
Bosnia and Herzegovina	Sickle cell trait	0.291 (0.183 to 0.444)	0.14 (0.0864 to 0.218)	0.117 (0.0715 to 0.187)	6.7 (4.2 to 10.2)	3.6 (2.2 to 5.6)	3.5 (2.1 to 5.6)
Bosnia and Herzegovina	G6PD deficiency	0.128 (0.0953 to 0.156)	0.128 (0.0983 to 0.153)	0.0939 (0.072 to 0.115)	2.7 (2 to 3.3)	2.8 (2.2 to 3.3)	2.3 (1.8 to 2.7)
Bosnia and Herzegovina	G6PD trait	0.000165 (0.000109 to 0.000234)	0.000119 (0.0000771 to 0.000173)	0.000103 (0.0000665 to 0.000152)	0 (0 to 0)	0 (0 to 0)	0 (0 to 0)
Bosnia and Herzegovina	Other hemoglobinopathies and hemolytic anaemias	1.6 (1.16 to 2.15)	1.02 (0.776 to 1.4)	0.82 (0.637 to 1.06)	36.3 (26.5 to 48.4)	21.9 (16.4 to 30.3)	17.7 (13.3 to 23.6)
Bosnia and Herzegovina	Endocrine, metabolic, blood, and immune disorders	3.77 (3.04 to 4.72)	2.89 (2.35 to 3.58)	2.41 (1.96 to 2.92)	84.8 (68.7 to 105.4)	68.3 (55.1 to 85.9)	62.8 (50.8 to 78.5)
Bosnia and Herzegovina	Oral disorders	14 (8.81 to 20.8)	15.4 (9.84 to 22.8)	14.3 (9.26 to 21.1)	313.5 (198.5 to 467.8)	300.2 (189.8 to 445.7)	283.4 (179.9 to 423.2)
Bosnia and Herzegovina	Caries of deciduous teeth	0.0949 (0.0397 to 0.184)	0.0525 (0.022 to 0.103)	0.0425 (0.0181 to 0.0836)	2.5 (1.1 to 5)	2.5 (1 to 4.9)	2.5 (1.1 to 4.9)
Bosnia and Herzegovina	Caries of permanent teeth	1.33 (0.574 to 2.53)	1.12 (0.484 to 2.13)	0.903 (0.393 to 1.72)	28.3 (12.2 to 53.4)	26.4 (11.4 to 50)	25.4 (11 to 48.4)
Bosnia and Herzegovina	Periodontal disease	2.53 (0.995 to 5.32)	2.5 (0.992 to 5.16)	2.37 (0.933 to 4.94)	53.3 (21 to 111.2)	48.2 (19.1 to 99.6)	47.3 (18.8 to 98.4)
Bosnia and Herzegovina	Edentulism and severe tooth loss	7.59 (5.02 to 10.6)	9.44 (6.33 to 13.2)	9.07 (6.02 to 12.8)	178.7 (119.2 to 251.5)	172.3 (114.9 to 240.9)	157.7 (104.7 to 221.8)
Bosnia and Herzegovina	Other oral disorders	2.4 (1.5 to 3.55)	2.26 (1.41 to 3.34)	1.97 (1.23 to 2.9)	50.7 (31.7 to 74.5)	50.7 (31.7 to 74.5)	50.6 (31.6 to 75.1)
Bosnia and Herzegovina	Sudden infant death syndrome	1.29 (0.411 to 2.73)	0.378 (0.138 to 0.649)	0.23 (0.121 to 0.367)	39 (12.4 to 82)	21 (7.7 to 36.1)	16.8 (8.9 to 26.8)

SR Appendix Table 2. All-age DALYs counts and age standardized DALYs rates per 100,000 individuals for all causes and level 1 cause groups by location for 1990, 2007, and 2017, both sexes combined

		All-age DALYs			Age-standardized rate of DALYs per 100,000		
Bosnia and Herzegovina	Injuries	147 (124 to 172)	129 (107 to 155)	117 (95.3 to 143)	3146.8 (2653.4 to 3694.7)	2833.4 (2367.2 to 3377.2)	2805.9 (2312.5 to 3417)
Bosnia and Herzegovina	Transport injuries	20.7 (18.2 to 23.8)	21.1 (18.6 to 23.8)	20.3 (17.3 to 23.6)	444.4 (391.9 to 509.7)	471.2 (419 to 528.1)	523.2 (452.4 to 600.5)
Bosnia and Herzegovina	Road injuries	18.3 (16.1 to 21)	19.6 (17.3 to 22.2)	18.9 (16 to 22)	393.6 (345.6 to 450.8)	438.2 (389.1 to 491.2)	485.5 (419 to 558.6)
Bosnia and Herzegovina	Pedestrian road injuries	4.26 (3.6 to 5.11)	3.99 (3.41 to 4.66)	4.13 (3.4 to 4.94)	95.3 (80.8 to 113.7)	88.1 (75.6 to 102.5)	100.4 (82.9 to 119.4)
Bosnia and Herzegovina	Cyclist road injuries	2.28 (1.74 to 2.92)	2.64 (2 to 3.38)	2.78 (2.06 to 3.62)	48.5 (36.9 to 61.7)	55.9 (42.2 to 70.9)	64.9 (48.1 to 83.7)
Bosnia and Herzegovina	Motorcyclist road injuries	3.12 (2.49 to 3.83)	3.17 (2.44 to 3.69)	2.69 (2.11 to 3.19)	64.8 (52.1 to 79.3)	73 (56.2 to 85.1)	76.3 (58.9 to 91.3)
Bosnia and Herzegovina	Motor vehicle road injuries	8.08 (6.81 to 10.4)	9.28 (8.19 to 10.4)	8.82 (7.57 to 10.1)	172 (145.6 to 219.6)	209.5 (184.4 to 236.3)	232 (200.6 to 262.7)
Bosnia and Herzegovina	Other road injuries	0.602 (0.409 to 0.771)	0.538 (0.392 to 0.687)	0.498 (0.369 to 0.642)	13 (8.8 to 16.6)	11.6 (8.6 to 14.8)	12 (8.9 to 15.4)
Bosnia and Herzegovina	Other transport injuries	2.36 (2.08 to 2.76)	1.46 (1.26 to 1.71)	1.42 (1.21 to 1.64)	50.8 (44.7 to 59.1)	33 (28.5 to 38.5)	37.6 (32.1 to 43.5)
Bosnia and Herzegovina	Unintentional injuries	93.6 (72.1 to 117)	74.6 (58.5 to 95)	71.3 (53.7 to 92.9)	2029.7 (1565 to 2529.5)	1606.8 (1266.1 to 2033.8)	1646.6 (1256.7 to 2133.9)
Bosnia and Herzegovina	Falls	42 (31 to 56.3)	42.1 (30.7 to 56.7)	43.5 (31.4 to 59.2)	914.9 (676.8 to 1223.3)	888.3 (647.6 to 1200.2)	968.1 (695.3 to 1318.9)
Bosnia and Herzegovina	Drowning	2.65 (2.15 to 3.26)	1.95 (1.74 to 2.15)	1.45 (1.3 to 1.62)	59 (47.9 to 73.6)	50.6 (45 to 56.2)	46 (40.4 to 52.2)
Bosnia and Herzegovina	Fire, heat, and hot substances	5.45 (4.11 to 6.9)	3.66 (2.9 to 4.53)	2.86 (2.16 to 3.75)	117.4 (88.8 to 148)	80.6 (63.8 to 99.7)	68.5 (51.8 to 88.5)
Bosnia and Herzegovina	Poisonings	12.2 (3.59 to 16)	2.11 (1.64 to 2.89)	1.7 (1.22 to 2.45)	258.9 (77.7 to 338.7)	50.1 (38.6 to 69)	47.2 (33.5 to 67.9)
Bosnia and Herzegovina	Poisoning by carbon monoxide	0.796 (0.304 to 1.09)	0.496 (0.268 to 0.725)	0.388 (0.254 to 0.641)	17.3 (6.7 to 23.6)	11.7 (6.3 to 17.3)	10.7 (6.9 to 18.1)
Bosnia and Herzegovina	Poisoning by other means	11.4 (3.25 to 14.9)	1.62 (1.19 to 2.31)	1.31 (0.886 to 1.94)	241.6 (70.4 to 316.1)	38.4 (28.2 to 55.1)	36.4 (24.6 to 53.9)
Bosnia and Herzegovina	Exposure to mechanical forces	14.4 (10.9 to 18)	13 (9.67 to 16.7)	11.9 (8.7 to 16)	302.3 (230.2 to 377.9)	270.7 (203.3 to 347.2)	271 (197.8 to 362.8)
Bosnia and Herzegovina	Unintentional firearm injuries	0.268 (0.189 to 0.37)	0.218 (0.16 to 0.291)	0.198 (0.147 to 0.262)	5.7 (4.1 to 7.9)	4.9 (3.6 to 6.5)	4.9 (3.6 to 6.7)
Bosnia and Herzegovina	Other exposure to mechanical forces	14.1 (10.7 to 17.7)	12.8 (9.52 to 16.5)	11.7 (8.53 to 15.8)	296.6 (225.6 to 371)	265.8 (199.6 to 341.3)	266.1 (193.6 to 356.8)
Bosnia and Herzegovina	Adverse effects of medical treatment	6.13 (4.22 to 7.54)	3.3 (2.44 to 5.39)	2.33 (1.37 to 4.64)	140.1 (95.9 to 174.7)	78.4 (61.5 to 116.6)	60.9 (39.6 to 106.8)
Bosnia and Herzegovina	Animal contact	1.63 (1.14 to 2.06)	0.958 (0.724 to 1.21)	0.645 (0.494 to 0.83)	35.3 (25 to 44.6)	22 (16.5 to 27.9)	16.8 (12.8 to 21.6)
Bosnia and Herzegovina	Venomous animal contact	1.16 (0.791 to 1.49)	0.671 (0.499 to 0.864)	0.43 (0.319 to 0.561)	24.9 (17.1 to 31.8)	15.4 (11.4 to 19.8)	11.4 (8.4 to 15.1)
Bosnia and Herzegovina	Non-venomous animal contact	0.467 (0.315 to 0.633)	0.287 (0.21 to 0.389)	0.215 (0.156 to 0.301)	10.5 (7 to 14.2)	6.6 (4.8 to 8.9)	5.4 (4 to 7.4)
Bosnia and Herzegovina	Foreign body	3.8 (3.24 to 4.34)	2.87 (2.39 to 3.2)	2.02 (1.76 to 2.29)	83.6 (71.3 to 95.4)	67.8 (57.2 to 76)	55.4 (47.9 to 62.8)
Bosnia and Herzegovina	Pulmonary aspiration and foreign body in airway	1.34 (0.984 to 2.13)	1.22 (1.04 to 1.59)	1.07 (0.921 to 1.21)	31 (23 to 48.3)	31.3 (27 to 39)	31.3 (26.4 to 35.5)
Bosnia and Herzegovina	Foreign body in eyes	0.0914 (0.0419 to 0.161)	0.0933 (0.046 to 0.161)	0.0874 (0.0445 to 0.15)	2 (0.9 to 3.5)	2.2 (1 to 3.8)	2.3 (1.1 to 4)
Bosnia and Herzegovina	Foreign body in other body part	2.37 (1.57 to 2.84)	1.55 (1.03 to 1.85)	0.864 (0.697 to 1.02)	50.5 (33.8 to 60)	34.3 (22.8 to 40.9)	21.8 (17.6 to 25.8)
Bosnia and Herzegovina	Environmental heat and cold exposure	1.16 (0.842 to 1.5)	0.963 (0.691 to 1.25)	0.964 (0.696 to 1.28)	24.9 (18.1 to 32.3)	20.9 (15 to 27.2)	22.8 (16.4 to 30.2)
Bosnia and Herzegovina	Exposure to forces of nature	0 (0 to 0)	0.00177 (0.00135 to 0.00228)	0.00461 (0.00355 to 0.00597)	0 (0 to 0)	0 (0 to 0.1)	0.1 (0.1 to 0.2)
Bosnia and Herzegovina	Other unintentional injuries	4.32 (3.56 to 5.27)	3.6 (2.88 to 4.6)	3.86 (2.88 to 5.13)	93.3 (76.9 to 113.8)	77.4 (62.1 to 98.9)	89.9 (67.4 to 119.5)
Bosnia and Herzegovina	Self-harm and interpersonal violence	32.5 (29.1 to 35.2)	33.7 (29 to 42.1)	25.5 (21.7 to 31.1)	672.7 (601.9 to 729)	755.5 (647.6 to 944.9)	636.1 (540.8 to 768.3)
Bosnia and Herzegovina	Self-harm	22 (20 to 24.7)	13.5 (12.1 to 17.6)	11.2 (9.94 to 13.9)	453.3 (411.6 to 507.8)	295.2 (265.7 to 380.4)	283.8 (254 to 348.9)
Bosnia and Herzegovina	Self-harm by firearm	0.456 (0.256 to 1.24)	0.471 (0.33 to 0.902)	0.75 (0.262 to 0.969)	9.4 (5.3 to 25.3)	10.5 (7.3 to 19.3)	19.7 (7 to 25.6)
Bosnia and Herzegovina	Self-harm by other specified means	21.6 (19.4 to 24)	13.1 (11.7 to 16.8)	10.4 (9.28 to 13.3)	443.9 (399.9 to 492.9)	284.7 (257 to 364.1)	264.2 (234 to 335.8)
Bosnia and Herzegovina	Interpersonal violence	10.4 (7.66 to 12.7)	7.63 (5.59 to 8.81)	5.39 (4.35 to 6.26)	218.8 (162 to 264.9)	173.7 (126 to 200.8)	145.3 (115 to 169.1)
Bosnia and Herzegovina	Assault by firearm	1.78 (0.754 to 2.71)	1.36 (0.54 to 1.83)	0.893 (0.389 to 1.16)	36.4 (15.6 to 55.1)	32.4 (12.8 to 43.6)	27.2 (11.3 to 36.4)
Bosnia and Herzegovina	Assault by sharp object	2.66 (1.8 to 4.17)	1.86 (1.33 to 2.62)	1.2 (0.933 to 1.69)	55.1 (37.3 to 86.1)	42.3 (29.9 to 60.4)	33.2 (25.1 to 48.4)
Bosnia and Herzegovina	Sexual violence	0.525 (0.352 to 0.767)	0.428 (0.288 to 0.621)	0.348 (0.234 to 0.498)	11 (7.4 to 16.1)	10.2 (6.9 to 14.9)	10.2 (6.8 to 14.6)
Bosnia and Herzegovina	Assault by other means	5.47 (3.9 to 7.2)	3.98 (2.87 to 4.77)	2.95 (2.33 to 3.55)	116.3 (83.5 to 150.9)	88.9 (64.5 to 106.5)	74.7 (58.6 to 89.8)
Bosnia and Herzegovina	Conflict and terrorism	0 (0 to 0)	12.6 (8.45 to 19.8)	8.85 (5.99 to 13.8)	0 (0 to 0)	285.8 (190 to 460.7)	206.2 (136.8 to 335.3)

SR Appendix Table 2. All-age DALYs counts and age standardized DALYs rates per 100,000 individuals for all causes and level 1 cause groups by location for 1990, 2007, and 2017, both sexes combined

		All-age DALYs			Age-standardized rate of DALYs per 100,000		
Bosnia and Herzegovina	Executions and police conflict	0.0265 (0.0236 to 0.0315)	0.0268 (0.025 to 0.0292)	0.0224 (0.0205 to 0.0251)	0.6 (0.5 to 0.7)	0.7 (0.7 to 0.8)	0.8 (0.7 to 0.8)
Bulgaria	All causes	3440 (3150 to 3770)	3210 (2950 to 3510)	2940 (2660 to 3240)	32562.4 (29720 to 35721)	29461.8 (26767.4 to 32520.5)	26541.8 (23671.2 to 29673.5)
Bulgaria	Communicable, maternal, neonatal, and nutritional disorders	255 (234 to 278)	150 (135 to 167)	113 (98.1 to 129)	3641.6 (3356.5 to 3962.3)	2585.5 (2333.9 to 2851.9)	2031.6 (1707.6 to 2356.3)
Bulgaria	HIV/AIDS and sexually transmitted infections	3.97 (2.94 to 5.6)	6.46 (5.62 to 7.89)	4.14 (3.44 to 5.3)	48.9 (34.5 to 71.8)	84.1 (70.6 to 106.7)	61.7 (49.8 to 81.3)
Bulgaria	HIV/AIDS	1.64 (1.56 to 1.72)	4.53 (4.33 to 4.78)	2.6 (2.47 to 2.73)	18.3 (17.5 to 19.2)	55.1 (52.8 to 57.8)	36 (34.4 to 37.9)
Bulgaria	HIV/AIDS - Drug-susceptible Tuberculosis	0.0361 (0.022 to 0.056)	0.0466 (0.029 to 0.0716)	0.0224 (0.0138 to 0.0345)	0.4 (0.2 to 0.6)	0.6 (0.4 to 0.9)	0.3 (0.2 to 0.5)
Bulgaria	HIV/AIDS - Multidrug-resistant Tuberculosis without extensive drug resistance	0.000232 (0.0000404 to 0.000843)	0.00414 (0.00187 to 0.00758)	0.00168 (0.00054 to 0.00377)	0 (0 to 0)	0.1 (0 to 0.1)	0 (0 to 0.1)
Bulgaria	HIV/AIDS - Extensively drug-resistant Tuberculosis	0 (0 to 0)	0.00131 (0.00059 to 0.0024)	0.000957 (0.00031 to 0.00216)	0 (0 to 0)	0 (0 to 0)	0 (0 to 0)
Bulgaria	HIV/AIDS resulting in other diseases	1.6 (1.52 to 1.69)	4.48 (4.28 to 4.73)	2.57 (2.45 to 2.71)	17.9 (17 to 18.8)	54.4 (52.1 to 57.2)	35.7 (34 to 37.5)
Bulgaria	Sexually transmitted infections excluding HIV	2.33 (1.3 to 3.97)	1.92 (1.13 to 3.31)	1.54 (0.889 to 2.68)	30.6 (16.2 to 53.5)	29.1 (15.9 to 51.7)	25.7 (14 to 45.2)
Bulgaria	Syphilis	0.756 (0.308 to 1.56)	0.536 (0.241 to 1.09)	0.369 (0.187 to 0.728)	13.2 (4.3 to 29.6)	12 (3.8 to 27)	9 (3 to 21.6)
Bulgaria	Chlamydial infection	0.348 (0.181 to 0.664)	0.305 (0.158 to 0.58)	0.256 (0.134 to 0.5)	4 (2.1 to 7.6)	3.9 (2 to 7.5)	3.9 (2 to 7.6)
Bulgaria	Gonococcal infection	0.261 (0.143 to 0.497)	0.214 (0.112 to 0.415)	0.156 (0.0833 to 0.298)	3.2 (1.7 to 6.2)	3 (1.5 to 5.9)	2.8 (1.4 to 5.5)
Bulgaria	Trichomoniasis	0.285 (0.115 to 0.609)	0.261 (0.105 to 0.565)	0.228 (0.0921 to 0.488)	3 (1.2 to 6.5)	3 (1.2 to 6.5)	2.9 (1.2 to 6.4)
Bulgaria	Genital herpes	0.263 (0.0839 to 0.626)	0.247 (0.0794 to 0.592)	0.235 (0.0746 to 0.559)	2.5 (0.8 to 5.8)	2.5 (0.8 to 5.9)	2.5 (0.8 to 5.8)
Bulgaria	Other sexually transmitted infections	0.421 (0.249 to 0.729)	0.36 (0.212 to 0.629)	0.298 (0.176 to 0.514)	4.8 (2.8 to 8.4)	4.7 (2.7 to 8.3)	4.6 (2.6 to 8)
Bulgaria	Respiratory infections and tuberculosis	125 (119 to 131)	59.7 (56.5 to 63.9)	44.4 (40.5 to 48.8)	1597.9 (1508.5 to 1704.2)	850.3 (793.1 to 916.8)	575.5 (508.6 to 652.2)
Bulgaria	Tuberculosis	6.59 (6.08 to 7.17)	5.29 (4.97 to 5.61)	3.18 (2.83 to 3.6)	58.3 (51.7 to 66.6)	48 (44.2 to 52)	28.4 (25.1 to 32.3)
Bulgaria	Latent tuberculosis infection	0 (0 to 0)	0 (0 to 0)	0 (0 to 0)	0 (0 to 0)	0 (0 to 0)	0 (0 to 0)
Bulgaria	Drug-susceptible tuberculosis	6.55 (6.03 to 7.14)	4.84 (4.45 to 5.25)	2.91 (2.49 to 3.31)	58 (51.2 to 66.3)	44 (39.9 to 48.2)	26 (22.3 to 29.7)
Bulgaria	Multidrug-resistant tuberculosis without extensive drug resistance	0.0342 (0.00573 to 0.124)	0.342 (0.177 to 0.578)	0.173 (0.062 to 0.365)	0.3 (0.1 to 1.1)	3.1 (1.6 to 5.2)	1.5 (0.5 to 3.2)
Bulgaria	Extensively drug-resistant tuberculosis	0 (0 to 0)	0.106 (0.0555 to 0.18)	0.0968 (0.0342 to 0.199)	0 (0 to 0)	1 (0.5 to 1.6)	0.8 (0.3 to 1.8)
Bulgaria	Lower respiratory infections	110 (106 to 115)	47.7 (45.9 to 49.5)	35.3 (32.5 to 38.4)	1439.1 (1365.7 to 1533.6)	705.4 (667.6 to 745.4)	453.6 (400.5 to 512.9)
Bulgaria	Upper respiratory infections	6.4 (3.93 to 9.96)	5.17 (3.1 to 8.19)	4.6 (2.73 to 7.31)	75.9 (46.8 to 117.4)	72.7 (43.4 to 115.2)	71.1 (41.7 to 113.4)
Bulgaria	Otitis media	1.98 (1.21 to 3.12)	1.55 (0.915 to 2.42)	1.28 (0.78 to 2.02)	24.6 (14.9 to 39.1)	24.1 (14.3 to 38)	22.4 (13.5 to 35.8)
Bulgaria	Enteric infections	9.76 (7.49 to 12.4)	7.01 (5.36 to 9.02)	6.35 (4.74 to 8.37)	145.4 (112.8 to 182.9)	137.4 (105.4 to 176.9)	126.1 (93.5 to 166.3)
Bulgaria	Diarrhoeal diseases	9.42 (7.2 to 12.1)	6.82 (5.19 to 8.85)	6.19 (4.58 to 8.25)	141.1 (108.9 to 179.8)	134.4 (103.1 to 174.4)	123.6 (91.2 to 164)
Bulgaria	Typhoid and paratyphoid	0.0234 (0.0117 to 0.0308)	0.00385 (0.00272 to 0.00759)	0.00203 (0.0012 to 0.00474)	0.3 (0.1 to 0.4)	0 (0 to 0.1)	0 (0 to 0.1)
Bulgaria	Typhoid fever	0.00978 (0.00678 to 0.012)	0.00129 (0.000818 to 0.00311)	0.000741 (0.000411 to 0.00214)	0.1 (0.1 to 0.1)	0 (0 to 0)	0 (0 to 0)
Bulgaria	Paratyphoid fever	0.0136 (0.00376 to 0.0202)	0.00256 (0.00164 to 0.00526)	0.00129 (0.00071 to 0.00318)	0.2 (0 to 0.3)	0 (0 to 0.1)	0 (0 to 0)
Bulgaria	Invasive non-typhoidal salmonella	0.278 (0.152 to 0.487)	0.171 (0.0887 to 0.291)	0.14 (0.0715 to 0.24)	3.5 (1.9 to 6.5)	2.7 (1.4 to 4.7)	2.3 (1.1 to 4.3)
Bulgaria	Other intestinal infectious diseases	0.0349 (0.015 to 0.0635)	0.018 (0.00904 to 0.0292)	0.0112 (0.00617 to 0.0171)	0.5 (0.2 to 1)	0.3 (0.1 to 0.6)	0.2 (0.1 to 0.3)
Bulgaria	Neglected tropical diseases and malaria	0.502 (0.296 to 1.53)	0.449 (0.232 to 0.789)	0.324 (0.176 to 0.447)	5.5 (2.9 to 20.2)	5.1 (2.8 to 11.6)	3.7 (2.2 to 6)
Bulgaria	Malaria	0 (0 to 0)	0 (0 to 0)	0 (0 to 0)	0 (0 to 0)	0 (0 to 0)	0 (0 to 0)
Bulgaria	Chagas disease	0 (0 to 0)	0 (0 to 0)	0 (0 to 0)	0 (0 to 0)	0 (0 to 0)	0 (0 to 0)
Bulgaria	Leishmaniasis	0.115 (0.000388 to 1.12)	0.038 (0.000167 to 0.376)	0.012 (0.0000455 to 0.116)	1.5 (0 to 15.2)	0.7 (0 to 7.1)	0.2 (0 to 2.2)
Bulgaria	Visceral leishmaniasis	0.115 (0.000388 to 1.12)	0.038 (0.000167 to 0.376)	0.012 (0.0000455 to 0.116)	1.5 (0 to 15.2)	0.7 (0 to 7.1)	0.2 (0 to 2.2)
Bulgaria	Cutaneous and mucocutaneous leishmaniasis	0 (0 to 0)	0 (0 to 0)	0 (0 to 0)	0 (0 to 0)	0 (0 to 0)	0 (0 to 0)
Bulgaria	African trypanosomiasis	0 (0 to 0)	0 (0 to 0)	0 (0 to 0)	0 (0 to 0)	0 (0 to 0)	0 (0 to 0)
Bulgaria	Schistosomiasis	0 (0 to 0)	0 (0 to 0)	0 (0 to 0)	0 (0 to 0)	0 (0 to 0)	0 (0 to 0)

SR Appendix Table 2. All-age DALYs counts and age standardized DALYs rates per 100,000 individuals for all causes and level 1 cause groups by location for 1990, 2007, and 2017, both sexes combined

		All-age DALYs			Age-standardized rate of DALYs per 100,000		
Bulgaria	Cysticercosis	0.00509 (0.00277 to 0.00787)	0.00202 (0.00105 to 0.00311)	0.00122 (0.000603 to 0.00187)	0.1 (0 to 0.1)	0 (0 to 0)	0 (0 to 0)
Bulgaria	Cystic echinococcosis	0.264 (0.185 to 0.346)	0.163 (0.114 to 0.216)	0.123 (0.0844 to 0.166)	2.5 (1.8 to 3.4)	1.7 (1.2 to 2.2)	1.4 (0.9 to 1.8)
Bulgaria	Lymphatic filariasis	0 (0 to 0)	0 (0 to 0)	0 (0 to 0)	0 (0 to 0)	0 (0 to 0)	0 (0 to 0)
Bulgaria	Onchocerciasis	0 (0 to 0)	0 (0 to 0)	0 (0 to 0)	0 (0 to 0)	0 (0 to 0)	0 (0 to 0)
Bulgaria	Trachoma	0 (0 to 0)	0 (0 to 0)	0 (0 to 0)	0 (0 to 0)	0 (0 to 0)	0 (0 to 0)
Bulgaria	Dengue	0 (0 to 0)	0 (0 to 0)	0 (0 to 0)	0 (0 to 0)	0 (0 to 0)	0 (0 to 0)
Bulgaria	Yellow fever	0 (0 to 0)	0 (0 to 0)	0 (0 to 0)	0 (0 to 0)	0 (0 to 0)	0 (0 to 0)
Bulgaria	Rabies	0.000273 (0.000229 to 0.000327)	0.000767 (0.000627 to 0.000967)	0.000635 (0.000486 to 0.00083)	0 (0 to 0)	0 (0 to 0)	0 (0 to 0)
Bulgaria	Intestinal nematode infections	0 (0 to 0)	0 (0 to 0)	0 (0 to 0)	0 (0 to 0)	0 (0 to 0)	0 (0 to 0)
Bulgaria	Ascariasis	0 (0 to 0)	0 (0 to 0)	0 (0 to 0)	0 (0 to 0)	0 (0 to 0)	0 (0 to 0)
Bulgaria	Trichuriasis	0 (0 to 0)	0 (0 to 0)	0 (0 to 0)	0 (0 to 0)	0 (0 to 0)	0 (0 to 0)
Bulgaria	Hookworm disease	0 (0 to 0)	0 (0 to 0)	0 (0 to 0)	0 (0 to 0)	0 (0 to 0)	0 (0 to 0)
Bulgaria	Food-borne trematodiases	0 (0 to 0)	0 (0 to 0)	0 (0 to 0)	0 (0 to 0)	0 (0 to 0)	0 (0 to 0)
Bulgaria	Leprosy	0 (0 to 0)	0 (0 to 0)	0 (0 to 0)	0 (0 to 0)	0 (0 to 0)	0 (0 to 0)
Bulgaria	Ebola virus disease	0 (0 to 0)	0 (0 to 0)	0 (0 to 0)	0 (0 to 0)	0 (0 to 0)	0 (0 to 0)
Bulgaria	Zika virus disease	0 (0 to 0)	0 (0 to 0)	0 (0 to 0)	0 (0 to 0)	0 (0 to 0)	0 (0 to 0)
Bulgaria	Guinea worm disease	0 (0 to 0)	0 (0 to 0)	0 (0 to 0)	0 (0 to 0)	0 (0 to 0)	0 (0 to 0)
Bulgaria	Other neglected tropical diseases	0.118 (0.0869 to 0.261)	0.245 (0.071 to 0.324)	0.187 (0.0588 to 0.262)	1.3 (0.9 to 3.4)	2.7 (1 to 3.4)	2.1 (0.9 to 2.8)
Bulgaria	Other infectious diseases	15.4 (14.1 to 16.6)	10.2 (9.29 to 11.1)	7.07 (6.34 to 7.88)	218.7 (198.9 to 238.2)	175.2 (153.8 to 191.3)	123.5 (106.5 to 141.5)
Bulgaria	Meningitis	6.97 (6.37 to 7.68)	3.87 (3.55 to 4.32)	2.22 (1.98 to 2.51)	106.4 (95.9 to 118.6)	72.9 (65.3 to 82.5)	41.6 (35.5 to 49.2)
Bulgaria	Pneumococcal meningitis	1 (0.846 to 1.19)	0.617 (0.523 to 0.729)	0.31 (0.262 to 0.36)	13.1 (10.8 to 15.8)	9.4 (7.7 to 11.3)	4.8 (3.9 to 5.7)
Bulgaria	H influenzae type B meningitis	2.48 (2.21 to 2.79)	1.25 (1.12 to 1.41)	0.673 (0.584 to 0.783)	40.6 (35.6 to 46.3)	26.6 (23.2 to 31)	14.6 (11.9 to 17.9)
Bulgaria	Meningococcal infection	1.2 (1.01 to 1.44)	0.653 (0.562 to 0.771)	0.369 (0.315 to 0.43)	18.7 (15.2 to 23.2)	12.6 (10.3 to 15.7)	7 (5.6 to 8.7)
Bulgaria	Other meningitis	2.28 (2.06 to 2.54)	1.35 (1.23 to 1.5)	0.87 (0.769 to 0.988)	34 (30.2 to 38.5)	24.2 (21.5 to 27.7)	15.2 (13 to 18.2)
Bulgaria	Encephalitis	2.95 (2.45 to 3.27)	2.86 (2 to 3.16)	2.25 (1.68 to 2.57)	42.6 (32.4 to 48.6)	50.5 (31.8 to 57.6)	41.3 (28.5 to 49.7)
Bulgaria	Diphtheria	0.000453 (0.000442 to 0.000468)	0.00052 (0.000419 to 0.000678)	0.00036 (0.000299 to 0.000458)	0 (0 to 0)	0 (0 to 0)	0 (0 to 0)
Bulgaria	Whooping cough	0.0405 (0.0219 to 0.0807)	0.0491 (0.0165 to 0.0762)	0.0643 (0.0249 to 0.107)	0.7 (0.4 to 1.5)	1.4 (0.5 to 2.1)	2 (0.8 to 3.5)
Bulgaria	Tetanus	0.0693 (0.0552 to 0.0808)	0.0199 (0.0159 to 0.0251)	0.0132 (0.0093 to 0.0192)	0.6 (0.5 to 0.7)	0.2 (0.1 to 0.2)	0.1 (0.1 to 0.1)
Bulgaria	Measles	0.00877 (0.00617 to 0.0111)	0.0424 (0.0294 to 0.055)	0.0137 (0.00623 to 0.0213)	0.1 (0.1 to 0.2)	1.2 (0.8 to 1.5)	0.4 (0.2 to 0.7)
Bulgaria	Varicella and herpes zoster	0.317 (0.206 to 0.47)	0.28 (0.176 to 0.413)	0.271 (0.169 to 0.404)	3.3 (2.2 to 4.7)	3 (2 to 4.5)	2.9 (1.9 to 4.2)
Bulgaria	Acute hepatitis	2.73 (2.38 to 3.03)	1.64 (1.47 to 1.84)	1.16 (1.01 to 1.36)	33.8 (28.3 to 38)	23.3 (20.4 to 26.3)	17.6 (15.1 to 20.8)
Bulgaria	Acute hepatitis A	0.931 (0.747 to 1.11)	0.545 (0.444 to 0.646)	0.363 (0.295 to 0.451)	13 (10.4 to 15.4)	9.8 (8.1 to 11.6)	7.6 (6.1 to 9.5)
Bulgaria	Acute hepatitis B	1.48 (1.31 to 1.65)	0.903 (0.799 to 1.04)	0.679 (0.576 to 0.809)	16.5 (14 to 18.9)	10.5 (9.1 to 12)	8 (6.8 to 9.4)
Bulgaria	Acute hepatitis C	0.1 (0.0446 to 0.177)	0.0452 (0.0218 to 0.0782)	0.0231 (0.0115 to 0.0412)	1.8 (0.8 to 3.2)	1.1 (0.5 to 2)	0.6 (0.3 to 1.2)
Bulgaria	Acute hepatitis E	0.219 (0.178 to 0.269)	0.147 (0.118 to 0.181)	0.0967 (0.0762 to 0.123)	2.6 (2.1 to 3.1)	1.9 (1.5 to 2.3)	1.4 (1.1 to 1.7)
Bulgaria	Other unspecified infectious diseases	2.28 (1.82 to 2.77)	1.45 (1.21 to 1.83)	1.07 (0.837 to 1.57)	31.1 (22.9 to 37.1)	22.8 (19.3 to 27.8)	17.5 (13.6 to 25.2)
Bulgaria	Maternal and neonatal disorders	78.4 (67.1 to 91.8)	53.5 (44.7 to 64.6)	41.5 (32.2 to 52.9)	1329.8 (1155.3 to 1514.7)	1132 (991.6 to 1299.9)	984.9 (781.9 to 1210.3)
Bulgaria	Maternal disorders	2.48 (2.31 to 2.67)	1.09 (0.998 to 1.19)	0.719 (0.636 to 0.806)	29.1 (26.9 to 31.4)	14.3 (13 to 15.8)	11.3 (9.9 to 12.8)
Bulgaria	Maternal haemorrhage	0.64 (0.421 to 0.902)	0.243 (0.167 to 0.338)	0.138 (0.0899 to 0.197)	7.7 (5.1 to 10.8)	3.3 (2.3 to 4.5)	2.3 (1.5 to 3.3)
Bulgaria	Maternal sepsis and other pregnancy related infections	0.281 (0.138 to 0.515)	0.095 (0.0529 to 0.16)	0.0639 (0.0323 to 0.117)	3.3 (1.6 to 6.2)	1.2 (0.7 to 2.1)	1 (0.5 to 1.7)

SR Appendix Table 2. All-age DALYs counts and age standardized DALYs rates per 100,000 individuals for all causes and level 1 cause groups by location for 1990, 2007, and 2017, both sexes combined

		All-age DALYs			Age-standardized rate of DALYs per 100,000		
Bulgaria	Maternal hypertensive disorders	0.163 (0.0938 to 0.256)	0.0984 (0.063 to 0.143)	0.0745 (0.0502 to 0.105)	2.1 (1.2 to 3.3)	1.4 (0.9 to 2)	1.3 (0.9 to 1.9)
Bulgaria	Maternal obstructed labour and uterine rupture	0.105 (0.0553 to 0.194)	0.0498 (0.0295 to 0.0856)	0.0354 (0.0181 to 0.068)	1.2 (0.6 to 2.2)	0.6 (0.4 to 1)	0.5 (0.3 to 0.9)
Bulgaria	Maternal abortive outcome	0.979 (0.642 to 1.34)	0.288 (0.173 to 0.429)	0.189 (0.108 to 0.28)	11.1 (7.1 to 15.3)	3.5 (2.2 to 5.3)	2.6 (1.5 to 3.9)
Bulgaria	Ectopic pregnancy	0.0727 (0.0345 to 0.136)	0.0182 (0.00876 to 0.0342)	0.00899 (0.00444 to 0.0173)	0.9 (0.4 to 1.7)	0.2 (0.1 to 0.5)	0.1 (0.1 to 0.3)
Bulgaria	Indirect maternal deaths	0.0415 (0.0133 to 0.109)	0.0304 (0.0117 to 0.0638)	0.0188 (0.00716 to 0.0403)	0.5 (0.2 to 1.4)	0.4 (0.2 to 0.9)	0.3 (0.1 to 0.7)
Bulgaria	Late maternal deaths	0.0467 (0.0355 to 0.0613)	0.0253 (0.0189 to 0.0328)	0.0202 (0.0151 to 0.0267)	0.6 (0.4 to 0.7)	0.3 (0.3 to 0.4)	0.3 (0.2 to 0.4)
Bulgaria	Maternal deaths aggravated by HIV/AIDS	0.0000352 (0 to 0.000068)	0.0000846 (0.0000437 to 0.000138)	0.0000726 (0.0000366 to 0.00012)	0 (0 to 0)	0 (0 to 0)	0 (0 to 0)
Bulgaria	Other maternal disorders	0.153 (0.0735 to 0.273)	0.238 (0.139 to 0.351)	0.169 (0.102 to 0.251)	1.8 (0.9 to 3.3)	3.1 (1.9 to 4.7)	2.7 (1.7 to 3.9)
Bulgaria	Neonatal disorders	75.9 (64.7 to 89.4)	52.4 (43.6 to 63.4)	40.7 (31.5 to 52.2)	1300.7 (1126.8 to 1487.3)	1117.8 (977.5 to 1285.2)	973.6 (769.7 to 1198.7)
Bulgaria	Neonatal preterm birth	28.6 (24.9 to 32.6)	20 (17.7 to 22.5)	14.8 (11.7 to 18.4)	513.3 (451.6 to 584.5)	474.5 (427.7 to 523.6)	417.2 (331 to 517.9)
Bulgaria	Neonatal encephalopathy due to birth asphyxia and trauma	17.4 (12.9 to 24.1)	15.9 (11.6 to 21.8)	12.4 (8.49 to 18.1)	269.1 (209.4 to 351.9)	319.9 (249.6 to 405.9)	272.7 (195.5 to 367)
Bulgaria	Neonatal sepsis and other neonatal infections	11.8 (6.45 to 19.8)	11.3 (6.3 to 18.5)	10.6 (5.87 to 17.6)	149.2 (85.5 to 244.8)	179.8 (106.6 to 282.9)	186.4 (109.7 to 302.7)
Bulgaria	Hemolytic disease and other neonatal jaundice	0.832 (0.664 to 1.03)	0.48 (0.368 to 0.603)	0.389 (0.291 to 0.497)	12.8 (10.1 to 16)	8.1 (6.4 to 10)	7.1 (5.5 to 9)
Bulgaria	Other neonatal disorders	17.3 (14.9 to 20.2)	4.77 (4.17 to 5.46)	2.58 (1.94 to 3.38)	356.3 (304.8 to 415.3)	135.5 (118.4 to 155)	90.2 (67.9 to 118.6)
Bulgaria	Nutritional deficiencies	22.2 (14 to 33.8)	12.7 (8.02 to 19.6)	9.5 (5.86 to 14.8)	295.4 (185.8 to 447.9)	201.3 (126.5 to 310.9)	156.1 (93.6 to 251.6)
Bulgaria	Protein-energy malnutrition	0.2 (0.0656 to 0.478)	0.245 (0.153 to 0.415)	0.143 (0.0868 to 0.301)	3.3 (1 to 7.9)	4.9 (3.2 to 8.7)	2.7 (1.7 to 6.2)
Bulgaria	Iodine deficiency	0.688 (0.331 to 1.3)	0.602 (0.288 to 1.13)	0.547 (0.263 to 1.02)	7 (3.4 to 13.3)	6.7 (3.2 to 12.7)	6.6 (3.2 to 12.4)
Bulgaria	Dietary iron deficiency	13.5 (8.4 to 20.5)	9.19 (5.61 to 14.2)	6.91 (4.13 to 10.9)	153.6 (95.9 to 234.2)	115 (70.4 to 179.6)	93.6 (56.9 to 148.6)
Bulgaria	Other nutritional deficiencies	0.116 (0.0931 to 0.155)	0.218 (0.181 to 0.279)	0.158 (0.128 to 0.206)	1.6 (1.3 to 2.2)	3.1 (2.5 to 3.9)	2.1 (1.7 to 2.8)
Bulgaria	Non-communicable diseases	2760 (2540 to 3030)	2750 (2540 to 3000)	2560 (2340 to 2800)	24463.5 (22331.8 to 26943)	23395.6 (21272.9 to 25823)	21449.3 (19184.5 to 23919.7)
Bulgaria	Neoplasms	411 (404 to 418)	444 (436 to 451)	432 (408 to 455)	3389 (3328.3 to 3441.8)	3639.6 (3567.4 to 3702.2)	3508.3 (3308.6 to 3698.1)
Bulgaria	Lip and oral cavity cancer	5.36 (5.06 to 5.71)	7.34 (7.01 to 7.69)	6.83 (6.19 to 7.53)	44.1 (41.7 to 46.9)	60.7 (57.8 to 63.6)	57.8 (52.2 to 63.6)
Bulgaria	Nasopharynx cancer	0.768 (0.718 to 0.82)	1.2 (1.13 to 1.28)	1.16 (1.06 to 1.29)	6.7 (6.3 to 7.2)	10.7 (10.1 to 11.5)	10.8 (9.8 to 12)
Bulgaria	Other pharynx cancer	2.88 (2.67 to 3.12)	3.99 (3.76 to 4.22)	4.32 (3.81 to 4.85)	23.8 (22 to 25.7)	33.8 (31.8 to 35.7)	37.5 (33.3 to 42.1)
Bulgaria	Oesophageal cancer	7.35 (7.02 to 7.69)	5.85 (5.58 to 6.16)	6.02 (5.42 to 6.62)	57.9 (55.2 to 60.5)	46.3 (44.1 to 48.8)	48 (43.1 to 52.9)
Bulgaria	Stomach cancer	51.2 (49.1 to 53.3)	34.4 (33.5 to 35.3)	27.9 (25.9 to 30)	397.7 (382.2 to 413.6)	261.3 (254 to 268.3)	210.3 (194.6 to 226.1)
Bulgaria	Colon and rectum cancer	50.3 (48.6 to 51.9)	59.8 (58.1 to 61.4)	59.2 (54.8 to 63.3)	391.1 (378.4 to 403.1)	446.5 (434.1 to 457.7)	430.6 (398.9 to 461.7)
Bulgaria	Liver cancer	19.8 (17.8 to 22.5)	18.3 (17.7 to 19)	17.5 (16.1 to 19)	153.9 (138.1 to 175.5)	142.2 (137.5 to 147.2)	135.1 (124.2 to 146.4)
Bulgaria	Liver cancer due to hepatitis B	4.38 (3.4 to 5.54)	3.73 (3.03 to 4.49)	3.44 (2.71 to 4.24)	36.1 (28.5 to 45.2)	32 (26.4 to 38)	30 (24.1 to 36.9)
Bulgaria	Liver cancer due to hepatitis C	4.91 (3.62 to 6.52)	4.87 (3.84 to 6.11)	4.62 (3.53 to 5.84)	36.7 (27.5 to 48.4)	35.6 (28 to 44.9)	33.1 (25.3 to 42.3)
Bulgaria	Liver cancer due to alcohol use	7.96 (6.67 to 9.51)	7.23 (6.41 to 8.1)	7.09 (6.12 to 8.1)	60.8 (51.2 to 72.3)	55.2 (49.2 to 61.5)	53.6 (46.6 to 61.2)
Bulgaria	Liver cancer due to NASH	1.42 (1.17 to 1.74)	1.41 (1.21 to 1.64)	1.39 (1.16 to 1.65)	10.9 (9.1 to 13.2)	10.5 (9 to 12.1)	10.1 (8.5 to 11.9)
Bulgaria	Liver cancer due to other causes	1.16 (0.956 to 1.4)	1.07 (0.918 to 1.24)	0.976 (0.832 to 1.15)	9.5 (8 to 11.5)	9 (7.8 to 10.3)	8.2 (7.1 to 9.6)
Bulgaria	Gallbladder and biliary tract cancer	4.51 (4.26 to 5.22)	5.54 (5.25 to 5.83)	4.89 (4.47 to 5.36)	34.6 (32.8 to 39.8)	40.6 (38.4 to 42.6)	35.3 (32.2 to 38.6)
Bulgaria	Pancreatic cancer	18.5 (17.8 to 19.2)	24.8 (24 to 25.6)	26.9 (24.9 to 28.7)	143.5 (138.3 to 149)	188.9 (182.9 to 195)	200.9 (186.3 to 216.4)
Bulgaria	Larynx cancer	9.07 (8.6 to 9.56)	10.8 (10.3 to 11.3)	9.7 (8.74 to 10.7)	71.7 (68 to 75.8)	86.8 (82.7 to 91.1)	78.1 (70.1 to 86.6)
Bulgaria	Tracheal, bronchus, and lung cancer	83.9 (81.1 to 86.6)	91.7 (89 to 94.5)	92.1 (84.5 to 99.7)	651.3 (630.9 to 671.6)	721.3 (700.5 to 743.3)	718 (656.4 to 778.6)
Bulgaria	Malignant skin melanoma	3.08 (2.65 to 4.65)	4.16 (3.05 to 5.32)	4.57 (3.15 to 5.77)	27.1 (23.1 to 40.1)	36.4 (26.7 to 46.5)	40.4 (27.7 to 50.6)
Bulgaria	Non-melanoma skin cancer	1.7 (1.61 to 1.79)	2.67 (2.54 to 2.79)	2.64 (2.45 to 2.86)	14.7 (14 to 15.5)	20.9 (19.9 to 21.9)	19.7 (18.2 to 21.3)
Bulgaria	Non-melanoma skin cancer (squamous-cell carcinoma)	1.69 (1.6 to 1.79)	2.66 (2.54 to 2.78)	2.64 (2.45 to 2.85)	14.7 (13.9 to 15.5)	20.8 (19.9 to 21.8)	19.7 (18.2 to 21.3)

SR Appendix Table 2. All-age DALYs counts and age standardized DALYs rates per 100,000 individuals for all causes and level 1 cause groups by location for 1990, 2007, and 2017, both sexes combined

		All-age DALYs			Age-standardized rate of DALYs per 100,000		
Bulgaria	Non-melanoma skin cancer (basal-cell carcinoma)	0.00507<br>(0.00143 to 0.0134)	0.00424<br>(0.00178 to 0.00932)	0.0046<br>(0.00198 to 0.00968)	0<br>(0 to 0.1)	0<br>(0 to 0.1)	0<br>(0 to 0.1)
Bulgaria	Breast cancer	33<br>(31.6 to 34.4)	35.7<br>(34.2 to 37.2)	35<br>(31.8 to 38.4)	276.6<br>(264.9 to 287.8)	295.2<br>(282.7 to 306.8)	290.4<br>(262.7 to 319)
Bulgaria	Cervical cancer	13.7<br>(13 to 14.5)	16.1<br>(15.3 to 16.8)	13.5<br>(12.1 to 15)	121.3<br>(114.8 to 127.9)	147<br>(140.2 to 154.3)	127.2<br>(113 to 140.9)
Bulgaria	Uterine cancer	7.23<br>(6.77 to 7.71)	7.6<br>(7.17 to 8.08)	7.81<br>(6.88 to 8.8)	57.2<br>(53.8 to 60.9)	60<br>(56.4 to 64.1)	61.2<br>(53.8 to 69.2)
Bulgaria	Ovarian cancer	9.43<br>(8.93 to 9.95)	12.9<br>(12.3 to 13.5)	12.1<br>(10.7 to 13.5)	77.8<br>(73.7 to 82.1)	106.7<br>(101.6 to 111.9)	102.2<br>(90.1 to 115.3)
Bulgaria	Prostate cancer	11.5<br>(9.53 to 15.7)	16<br>(12.1 to 19.8)	17.2<br>(12.7 to 20.3)	87.2<br>(74.1 to 121.5)	106.7<br>(80.3 to 129.9)	108<br>(78.4 to 125.7)
Bulgaria	Testicular cancer	2.47<br>(2.23 to 2.73)	2.01<br>(1.82 to 2.22)	1.32<br>(1.12 to 1.54)	27.9<br>(25.1 to 31)	24.4<br>(21.9 to 27.1)	18.1<br>(15.3 to 21.3)
Bulgaria	Kidney cancer	3.55<br>(3.3 to 3.95)	7.04<br>(6.28 to 7.42)	7.74<br>(6.86 to 8.49)	29.7<br>(27.8 to 32.9)	58.5<br>(52.3 to 61.7)	63.8<br>(56.4 to 70)
Bulgaria	Bladder cancer	8.75<br>(8.35 to 9.17)	11.1<br>(10.6 to 11.5)	11.5<br>(10.5 to 12.6)	66.3<br>(63.3 to 69.3)	79.7<br>(76.5 to 82.9)	80.4<br>(73.4 to 87.6)
Bulgaria	Thyroid cancer	1.7<br>(1.56 to 1.83)	1.52<br>(1.42 to 1.63)	1.38<br>(1.24 to 1.54)	13.9<br>(12.9 to 15)	12.5<br>(11.7 to 13.5)	11.5<br>(10.4 to 12.9)
Bulgaria	Mesothelioma	0.264<br>(0.223 to 0.309)	0.3<br>(0.282 to 0.319)	0.285<br>(0.261 to 0.313)	2.2<br>(1.9 to 2.6)	2.6<br>(2.4 to 2.8)	2.5<br>(2.3 to 2.8)
Bulgaria	Hodgkin lymphoma	3.58<br>(3.01 to 4.65)	2.68<br>(2.23 to 3.29)	2.12<br>(1.77 to 2.61)	36.3<br>(29.6 to 44.9)	29.1<br>(23.8 to 34.5)	24.7<br>(20.1 to 29.4)
Bulgaria	Non-Hodgkin's lymphoma	6.02<br>(5.75 to 6.31)	6.97<br>(6.7 to 7.25)	7.5<br>(6.89 to 8.09)	57.4<br>(54.6 to 60.4)	66.8<br>(64 to 69.9)	72.3<br>(66.7 to 78.1)
Bulgaria	Multiple myeloma	1.92<br>(1.73 to 2.64)	2.48<br>(2.12 to 3.06)	2.73<br>(2.28 to 3.25)	14.9<br>(13.4 to 20)	19.2<br>(16 to 22.5)	21.2<br>(17.1 to 24.1)
Bulgaria	Leukaemia	16<br>(15.4 to 16.6)	15.1<br>(14.4 to 15.6)	13.7<br>(12.6 to 14.8)	171.9<br>(163.2 to 180.5)	166.5<br>(157.2 to 174.5)	148<br>(134.2 to 163.3)
Bulgaria	Acute lymphoid leukaemia	4.41<br>(3.85 to 5.1)	3.14<br>(2.91 to 3.39)	2.18<br>(1.95 to 2.45)	55.9<br>(48.6 to 65.2)	46.8<br>(42.5 to 51.6)	32.6<br>(28.4 to 37.5)
Bulgaria	Chronic lymphoid leukaemia	0.916<br>(0.752 to 1.07)	1.35<br>(1.19 to 1.51)	1.65<br>(1.43 to 1.85)	6.9<br>(5.7 to 8.1)	9.7<br>(8.5 to 10.7)	11.4<br>(9.9 to 12.8)
Bulgaria	Acute myeloid leukaemia	4.38<br>(3.73 to 5.09)	4.75<br>(4.26 to 5.1)	4.74<br>(4.26 to 5.21)	43<br>(35.9 to 51.2)	47.7<br>(42.9 to 51.6)	48.1<br>(43 to 53.2)
Bulgaria	Chronic myeloid leukaemia	1.69<br>(1.49 to 1.95)	1.41<br>(1.28 to 1.56)	0.913<br>(0.804 to 1.03)	14.5<br>(12.7 to 16.7)	11.7<br>(10.6 to 12.9)	7.3<br>(6.4 to 8.3)
Bulgaria	Other leukaemia	4.6<br>(4.07 to 5.2)	4.4<br>(4.12 to 4.72)	4.23<br>(3.84 to 4.71)	51.5<br>(44.6 to 59.3)	50.6<br>(45.9 to 54.7)	48.7<br>(42.8 to 55.2)
Bulgaria	Other malignant cancers	15.4<br>(14.6 to 17.3)	15.7<br>(14.2 to 16.5)	13.9<br>(12.2 to 15.4)	146.4<br>(137.7 to 158.6)	152.4<br>(135 to 160.9)	137.1<br>(117.6 to 151.4)
Bulgaria	Other neoplasms	1.16<br>(0.87 to 1.56)	1.71<br>(1.14 to 2.05)	1.86<br>(1.27 to 2.43)	10.6<br>(7.6 to 13.9)	15.5<br>(9.7 to 19.2)	16.2<br>(10.3 to 20.4)
Bulgaria	Myelodysplastic, myeloproliferative, and other hematopoietic neoplasms	1.03<br>(0.789 to 1.38)	1.56<br>(1.05 to 1.87)	1.73<br>(1.19 to 2.29)	8.8<br>(6.6 to 11.7)	13<br>(8.4 to 15.8)	13.9<br>(9 to 17.6)
Bulgaria	Benign and in situ intestinal neoplasms	0<br>(0 to 0)	0<br>(0 to 0)	0<br>(0 to 0)	0<br>(0 to 0)	0<br>(0 to 0)	0<br>(0 to 0)
Bulgaria	Benign and in situ cervical and uterine neoplasms	0<br>(0 to 0)	0<br>(0 to 0)	0<br>(0 to 0)	0<br>(0 to 0)	0<br>(0 to 0)	0<br>(0 to 0)
Bulgaria	Other benign and in situ neoplasms	0.137<br>(0.0761 to 0.183)	0.157<br>(0.0811 to 0.221)	0.134<br>(0.074 to 0.186)	1.7<br>(0.9 to 2.4)	2.4<br>(1.2 to 3.5)	2.3<br>(1.2 to 3.3)
Bulgaria	Cardiovascular diseases	1210<br>(1190 to 1240)	1240<br>(1210 to 1270)	1100<br>(1040 to 1150)	9957.2<br>(9733.3 to 10202.2)	9172.8<br>(8967.6 to 9402.4)	7732.1<br>(7293.1 to 8114.4)
Bulgaria	Rheumatic heart disease	22.3<br>(21.3 to 23.3)	8.65<br>(8.27 to 9.17)	6.62<br>(6.07 to 7.3)	191.7<br>(183.8 to 199.5)	75.9<br>(72.4 to 80.7)	58.5<br>(53.5 to 64.6)
Bulgaria	Ischaemic heart disease	588<br>(567 to 602)	601<br>(588 to 624)	528<br>(500 to 556)	4887.4<br>(4709.1 to 4995.1)	4446.9<br>(4355.1 to 4609)	3718.6<br>(3503.5 to 3924.9)
Bulgaria	Stroke	448<br>(426 to 465)	410<br>(394 to 427)	365<br>(341 to 388)	3584.1<br>(3408.9 to 3717.9)	2947.1<br>(2831.4 to 3076.1)	2472<br>(2304.8 to 2625.2)
Bulgaria	Ischaemic stroke	178<br>(166 to 190)	214<br>(202 to 226)	210<br>(194 to 226)	1447<br>(1351.3 to 1540.3)	1479.4<br>(1393.6 to 1565.5)	1347.4<br>(1245.3 to 1452)
Bulgaria	Intracerebral hemorrhage	243<br>(231 to 251)	174<br>(168 to 182)	134<br>(125 to 144)	1916.3<br>(1827.1 to 1979.6)	1289.9<br>(1246.4 to 1345.3)	957.8<br>(894 to 1025.5)
Bulgaria	Subarachnoid hemorrhage	26.8<br>(22.6 to 28.6)	21.7<br>(20.3 to 23.7)	20.6<br>(18.8 to 22.6)	220.7<br>(191.2 to 234.1)	177.9<br>(166.8 to 199.4)	166.8<br>(151.9 to 184.2)
Bulgaria	Hypertensive heart disease	49.6<br>(44.7 to 76.4)	99.2<br>(59.5 to 107)	89.5<br>(57.8 to 100)	393.7<br>(353.6 to 621.9)	706.5<br>(420.6 to 763.1)	603.1<br>(385.5 to 670.6)
Bulgaria	Non-rheumatic valvular heart disease	3.59<br>(3.12 to 4.32)	3.49<br>(2.87 to 4.86)	3.94<br>(3.28 to 5.29)	31.5<br>(27.3 to 36.8)	28.3<br>(24.1 to 37)	30.6<br>(26.4 to 37.4)
Bulgaria	Non-rheumatic calcific aortic valve disease	1.23<br>(0.895 to 1.85)	1.44<br>(1.09 to 2.09)	1.92<br>(1.46 to 2.47)	10.5<br>(7.8 to 15.3)	11.3<br>(8.8 to 14.9)	14.5<br>(10.9 to 17.9)
Bulgaria	Non-rheumatic degenerative mitral valve disease	1.14<br>(0.831 to 1.61)	1.48<br>(1.16 to 1.88)	1.51<br>(1.17 to 1.89)	9.8<br>(7.3 to 13.5)	11.9<br>(9.5 to 14.4)	11.6<br>(9.2 to 14)
Bulgaria	Other non-rheumatic valve diseases	1.23<br>(0.621 to 1.6)	0.571<br>(0.372 to 1.09)	0.51<br>(0.255 to 1.12)	11.2<br>(5.6 to 14.5)	5.1<br>(3.4 to 9.6)	4.5<br>(2.3 to 9.7)
Bulgaria	Cardiomyopathy and myocarditis	7.07<br>(5.9 to 14)	14.8<br>(14 to 15.7)	13.3<br>(12.2 to 15.8)	70.3<br>(60.1 to 128.1)	141.6<br>(129.3 to 149.3)	124.4<br>(114 to 139.1)
Bulgaria	Myocarditis	3.49<br>(2.65 to 5.09)	5.7<br>(4.83 to 6.42)	5.16<br>(4.53 to 6.14)	35.9<br>(28.6 to 50.8)	55.7<br>(47.5 to 64.8)	49.2<br>(43.4 to 59.7)

SR Appendix Table 2. All-age DALYs counts and age standardized DALYs rates per 100,000 individuals for all causes and level 1 cause groups by location for 1990, 2007, and 2017, both sexes combined

		All-age DALYs			Age-standardized rate of DALYs per 100,000		
Bulgaria	Alcoholic cardiomyopathy	0.727 (0.409 to 1.82)	1.03 (0.87 to 1.41)	0.909 (0.734 to 1.25)	6.4 (3.7 to 15.4)	9.1 (7.7 to 12.5)	8.4 (6.7 to 11.4)
Bulgaria	Other cardiomyopathy	2.85 (1.9 to 7.38)	8.1 (7.26 to 9.05)	7.28 (6.35 to 8.89)	27.9 (19.8 to 64.5)	76.8 (62.4 to 86)	66.8 (57.2 to 75)
Bulgaria	Atrial fibrillation and flutter	11.3 (9.15 to 14.1)	13.9 (11.7 to 16.9)	15.8 (13.4 to 18.7)	94.4 (77.4 to 117.6)	97.1 (82 to 117.2)	100.7 (85.8 to 119)
Bulgaria	Aortic aneurysm	4.97 (4.58 to 5.31)	7.19 (6.84 to 7.61)	6.79 (6.23 to 7.38)	42.1 (39 to 44.8)	61.2 (58.3 to 64.8)	57.9 (53.1 to 63.1)
Bulgaria	Peripheral vascular disease	0.977 (0.552 to 1.58)	1.16 (0.674 to 1.81)	1.38 (0.793 to 2.21)	7.6 (4.3 to 12.1)	7.9 (4.6 to 12.4)	8.7 (5 to 13.9)
Bulgaria	Endocarditis	2.62 (2.22 to 3.81)	2.99 (2.5 to 4.69)	3.06 (2.37 to 4.92)	26 (22.1 to 35.3)	29.7 (25.8 to 42.9)	30.1 (24.4 to 44.4)
Bulgaria	Other cardiovascular and circulatory diseases	75.8 (68 to 85.4)	78.2 (70.6 to 86.8)	66.7 (58.6 to 75.7)	628.5 (564.8 to 705)	630.7 (569.5 to 701)	527.5 (461.4 to 602)
Bulgaria	Chronic respiratory diseases	120 (109 to 131)	113 (103 to 123)	107 (96 to 119)	992.7 (896.2 to 1081.6)	893 (803.6 to 980.3)	827.3 (732.4 to 924.2)
Bulgaria	Chronic obstructive pulmonary disease	96.6 (87.8 to 105)	95.7 (87.6 to 103)	90.7 (81.9 to 100)	766.4 (697.8 to 828.5)	702.7 (646.2 to 757.7)	636.8 (575.7 to 701)
Bulgaria	Pneumoconiosis	1.99 (1.72 to 2.27)	0.971 (0.811 to 1.09)	0.671 (0.562 to 0.785)	14.3 (12.5 to 16.2)	6.9 (5.7 to 7.7)	4.6 (3.9 to 5.4)
Bulgaria	Silicosis	1.72 (1.49 to 1.98)	0.356 (0.278 to 0.514)	0.204 (0.149 to 0.298)	12.2 (10.6 to 13.9)	2.5 (1.9 to 3.6)	1.4 (1 to 2)
Bulgaria	Asbestosis	0.0374 (0.0291 to 0.0573)	0.113 (0.0576 to 0.144)	0.111 (0.0591 to 0.147)	0.3 (0.2 to 0.5)	0.9 (0.4 to 1.1)	0.8 (0.4 to 1.1)
Bulgaria	Coal workers pneumoconiosis	0.136 (0.108 to 0.174)	0.0747 (0.0556 to 0.104)	0.0629 (0.0441 to 0.085)	1 (0.8 to 1.3)	0.6 (0.4 to 0.8)	0.4 (0.3 to 0.6)
Bulgaria	Other pneumoconiosis	0.0953 (0.0698 to 0.137)	0.428 (0.176 to 0.52)	0.293 (0.167 to 0.37)	0.8 (0.6 to 1.1)	3 (1.3 to 3.6)	2 (1.2 to 2.5)
Bulgaria	Asthma	16.4 (12.4 to 21.2)	11.2 (8.05 to 15.1)	10.9 (7.62 to 15.1)	161.8 (120.2 to 213.5)	128.4 (89.1 to 176.2)	132.7 (90.2 to 183.4)
Bulgaria	Interstitial lung disease and pulmonary sarcoidosis	2.94 (2.18 to 3.79)	2.5 (1.86 to 3.26)	2.55 (1.88 to 3.32)	24.5 (18.1 to 31.6)	20.8 (15.3 to 27.3)	21 (15.4 to 27.5)
Bulgaria	Other chronic respiratory diseases	2.47 (2.2 to 2.73)	2.98 (2.65 to 3.28)	2.62 (2.28 to 2.99)	25.6 (22.7 to 28.2)	34.2 (30.2 to 37.9)	32.2 (27.9 to 36.7)
Bulgaria	Digestive diseases	120 (109 to 135)	115 (106 to 127)	116 (105 to 130)	1076.4 (965.2 to 1220.9)	1071.9 (968.3 to 1200.5)	1091 (973.8 to 1235.7)
Bulgaria	Cirrhosis and other chronic liver diseases	56.3 (54 to 58.6)	58.5 (56.3 to 61)	60.3 (55.4 to 65.4)	472.2 (451.6 to 491.5)	517.1 (497.3 to 540.7)	545.7 (499 to 592.8)
Bulgaria	Cirrhosis and other chronic liver diseases due to hepatitis B	14.7 (13.1 to 16.7)	14 (12.4 to 15.7)	14 (12.3 to 16)	123.4 (110.9 to 138.9)	124.2 (110.3 to 138.9)	128.3 (112.9 to 146.5)
Bulgaria	Cirrhosis and other chronic liver diseases due to hepatitis C	10.8 (9.49 to 12.2)	12.3 (10.9 to 13.9)	12.5 (10.7 to 14.6)	89.2 (78.6 to 101.2)	108 (94.9 to 122.5)	112.8 (96.5 to 132.1)
Bulgaria	Cirrhosis and other chronic liver diseases due to alcohol use	23.7 (21.9 to 25.5)	25 (23.2 to 27)	26.3 (23.6 to 28.9)	192.1 (178.4 to 206.5)	214.8 (198.8 to 231.6)	231.4 (206.9 to 255)
Bulgaria	Cirrhosis due to NASH	3.4 (3 to 3.84)	3.78 (3.38 to 4.21)	4.16 (3.64 to 4.72)	27.4 (24.3 to 30.9)	32.3 (28.9 to 36)	36.5 (31.9 to 41.7)
Bulgaria	Cirrhosis and other chronic liver diseases due to other causes	3.79 (3.28 to 4.37)	3.43 (2.92 to 4.01)	3.37 (2.83 to 4)	40 (35.3 to 45.6)	37.9 (32.7 to 43.8)	36.8 (31.4 to 42.4)
Bulgaria	Upper digestive system diseases	30.9 (23.6 to 41)	25.5 (19.1 to 34.4)	23.9 (17.9 to 32.5)	291.1 (218.8 to 396.7)	255.4 (185.9 to 352.8)	245.1 (177.1 to 342)
Bulgaria	Peptic ulcer disease	11.8 (11 to 12.6)	8.46 (7.84 to 9.11)	7.47 (6.69 to 8.28)	97.3 (91 to 104.1)	67.5 (62.2 to 73.1)	58 (51.8 to 64.7)
Bulgaria	Gastritis and duodenitis	10.7 (7.01 to 15.4)	9.03 (6.09 to 12.8)	8.83 (6.1 to 12.5)	115.4 (74.8 to 166.9)	109.3 (72.3 to 158)	108.6 (72.2 to 155.9)
Bulgaria	GERD	8.39 (4.49 to 14)	8.03 (4.27 to 13.5)	7.64 (4.03 to 12.9)	78.3 (42 to 132.3)	78.5 (41.9 to 133.8)	78.5 (41.9 to 132.8)
Bulgaria	Appendicitis	1.24 (1.14 to 1.36)	1.17 (1.08 to 1.27)	1.02 (0.915 to 1.13)	12.8 (11.6 to 14.2)	12 (10.9 to 13.4)	10.5 (9.2 to 11.9)
Bulgaria	Paralytic ileus and intestinal obstruction	4.66 (4 to 6.24)	5.12 (4.32 to 6.01)	5.6 (4.16 to 6.51)	50 (43.5 to 64.2)	50.7 (43.6 to 57.6)	51.4 (38.1 to 60.2)
Bulgaria	Inguinal, femoral, and abdominal hernia	10.9 (8.28 to 14)	8.15 (5.88 to 10.8)	7.4 (5.42 to 9.8)	101.9 (76.1 to 133.5)	85.7 (60.5 to 115.1)	79.6 (56.8 to 106.1)
Bulgaria	Inflammatory bowel disease	2.96 (2.19 to 3.95)	2.47 (1.84 to 3.23)	3 (2.19 to 4.01)	28.8 (21.4 to 38.2)	25.7 (18.8 to 33.6)	31.1 (21.9 to 41.8)
Bulgaria	Vascular intestinal disorders	2.56 (2.37 to 2.86)	3.23 (3 to 3.45)	3.66 (3.08 to 4.1)	20.5 (19.1 to 22.9)	23.4 (21.6 to 24.8)	25.2 (21 to 28.2)
Bulgaria	Gallbladder and biliary diseases	2.41 (2.03 to 2.6)	1.45 (1.34 to 1.72)	1.63 (1.47 to 1.82)	19.2 (16.4 to 20.6)	10.7 (9.9 to 12.5)	11.4 (10.3 to 12.6)
Bulgaria	Pancreatitis	5.33 (4.59 to 6.36)	6.82 (6.19 to 7.64)	6.4 (5.59 to 7.38)	47.1 (40.8 to 55.3)	59.6 (54 to 66.8)	56.8 (49.4 to 65.9)
Bulgaria	Other digestive diseases	2.96 (2.35 to 3.66)	2.92 (2.48 to 3.4)	3.2 (2.7 to 3.76)	33 (25.7 to 41.7)	31.6 (26.2 to 37.5)	34.2 (28.3 to 41.3)
Bulgaria	Neurological disorders	150 (117 to 189)	160 (130 to 196)	162 (134 to 195)	1530.5 (1189.8 to 1949.2)	1533.2 (1205.8 to 1921.6)	1502.2 (1174.9 to 1917.8)
Bulgaria	Alzheimer's disease and other dementias	45.8 (42.8 to 49.2)	63.4 (59.3 to 68)	72.6 (66.7 to 78.9)	463.2 (433.4 to 495.8)	455.1 (426.9 to 485.7)	441.9 (406.3 to 479.9)
Bulgaria	Parkinson's disease	9.47 (8.63 to 11.4)	12.3 (11.3 to 14.9)	12.6 (11.2 to 15.2)	78.7 (72.2 to 93.8)	79.7 (73.1 to 96.2)	75.3 (67.2 to 90.4)
Bulgaria	Epilepsy	16.3 (8.13 to 28.1)	14.2 (8.5 to 21.6)	12.8 (6.22 to 22.9)	184.8 (95.2 to 312.6)	180.7 (109.8 to 274.4)	173 (84.9 to 306.9)

SR Appendix Table 2. All-age DALYs counts and age standardized DALYs rates per 100,000 individuals for all causes and level 1 cause groups by location for 1990, 2007, and 2017, both sexes combined

		All-age DALYs			Age-standardized rate of DALYs per 100,000		
Bulgaria	Multiple sclerosis	2.74 (2.1 to 4.27)	2.78 (2.2 to 4.17)	2.51 (1.91 to 4.08)	25 (19.3 to 38.2)	26.7 (21.2 to 39.6)	25 (19 to 40)
Bulgaria	Motor neuron disease	0.594 (0.521 to 0.681)	0.207 (0.191 to 0.225)	0.233 (0.208 to 0.257)	6.1 (5.4 to 7)	1.9 (1.8 to 2.1)	2.3 (2 to 2.5)
Bulgaria	Headache disorders	71.1 (45.3 to 105)	63.7 (41 to 94.7)	57.5 (37.4 to 84.8)	731.9 (465.1 to 1076.5)	734.4 (471.9 to 1077.7)	730.9 (473 to 1075.3)
Bulgaria	Migraine	60.8 (37.5 to 92.2)	54.3 (33.8 to 82.3)	48.9 (30.4 to 74.7)	627.6 (385.9 to 947.3)	629.6 (389.6 to 953.3)	626.4 (384.3 to 949.6)
Bulgaria	Tension-type headache	10.4 (5.93 to 16.2)	9.39 (5.43 to 14.6)	8.64 (4.98 to 13.5)	104.3 (59.2 to 163.1)	104.8 (59.4 to 163.8)	104.5 (59.1 to 165)
Bulgaria	Other neurological disorders	3.46 (2.31 to 5.02)	3.8 (2.97 to 4.88)	3.46 (2.44 to 4.88)	40.9 (26.7 to 60.3)	54.6 (42.1 to 71.2)	54 (36 to 79.4)
Bulgaria	Mental disorders	134 (99.8 to 173)	117 (87.6 to 150)	107 (80 to 137)	1378.1 (1017.9 to 1779.2)	1344.8 (994.9 to 1738.9)	1335 (987.8 to 1728.5)
Bulgaria	Schizophrenia	12.3 (9.23 to 15.4)	11.4 (8.48 to 14.3)	10.8 (8.02 to 13.6)	120.3 (89.4 to 151.9)	120.8 (89.8 to 152.9)	122.4 (90.4 to 155.7)
Bulgaria	Depressive disorders	45.8 (31.9 to 61.3)	40.1 (28.2 to 54.1)	37 (26 to 49.6)	437 (305.7 to 589)	401.3 (280.7 to 544.2)	388.7 (274.6 to 525.9)
Bulgaria	Major depressive disorder	35.9 (24.8 to 48.4)	30.8 (21.1 to 41.6)	28.1 (19.7 to 37.7)	343.9 (236.9 to 465.1)	309.1 (215.4 to 420.4)	295.5 (206.7 to 397.5)
Bulgaria	Dysthymia	9.9 (6.64 to 14.4)	9.27 (6.28 to 13.2)	8.88 (5.98 to 12.9)	93 (62.4 to 135.9)	92.3 (62.1 to 133)	93.2 (62.6 to 135.6)
Bulgaria	Bipolar disorder	11.8 (7.4 to 17.7)	10.6 (6.61 to 15.6)	9.51 (6.03 to 14.1)	122.1 (76.1 to 181.6)	122.5 (76.3 to 180.3)	122.4 (77.6 to 182.3)
Bulgaria	Anxiety disorders	31.2 (22.1 to 41.6)	27.9 (20 to 36.8)	25.5 (17.8 to 33.7)	325.4 (229.7 to 434.9)	326.3 (232.6 to 432.6)	325.4 (227.5 to 433.8)
Bulgaria	Eating disorders	2.99 (1.86 to 4.41)	2.61 (1.65 to 3.87)	2.43 (1.53 to 3.54)	35.9 (22.4 to 53.2)	36.4 (23.2 to 53.4)	41.2 (25.7 to 60.4)
Bulgaria	Anorexia nervosa	0.575 (0.339 to 0.879)	0.48 (0.284 to 0.744)	0.428 (0.254 to 0.645)	7.2 (4.2 to 11.1)	7.3 (4.3 to 11.1)	8.1 (4.8 to 12.4)
Bulgaria	Bulimia nervosa	2.41 (1.48 to 3.65)	2.13 (1.31 to 3.19)	2 (1.24 to 2.99)	28.8 (17.6 to 43.2)	29.1 (18.3 to 43.8)	33 (20.6 to 49.3)
Bulgaria	Autism spectrum disorders	5.67 (3.89 to 7.75)	4.7 (3.22 to 6.48)	4.29 (2.96 to 5.96)	66.4 (45.6 to 91.5)	66.5 (45.1 to 92.6)	66.8 (46.2 to 92.7)
Bulgaria	Attention-deficit/hyperactivity disorder	0.925 (0.563 to 1.46)	0.707 (0.424 to 1.12)	0.606 (0.363 to 0.958)	11.7 (7.1 to 18.5)	11.8 (7.1 to 18.6)	11.8 (7 to 18.7)
Bulgaria	Conduct disorder	7.09 (4.3 to 11.1)	4.39 (2.65 to 6.93)	3.62 (2.15 to 5.73)	97.8 (59.2 to 153.6)	98 (59.6 to 153.5)	98.2 (58.4 to 155.6)
Bulgaria	Idiopathic developmental intellectual disability	1.87 (0.68 to 3.39)	1.46 (0.5 to 2.71)	1.1 (0.319 to 2.06)	22.4 (8.3 to 40.4)	21.5 (7.5 to 39.6)	18.2 (5.7 to 34)
Bulgaria	Other mental disorders	14.2 (9.45 to 19.6)	13.3 (8.84 to 18.4)	12.4 (8.24 to 17.2)	139.2 (92.4 to 192.8)	139.6 (91.9 to 194)	139.9 (91.7 to 194.3)
Bulgaria	Substance use disorders	38.4 (30.1 to 47.6)	31.2 (23.7 to 38.9)	28.5 (22 to 35.9)	400.1 (310.7 to 501)	367 (276.5 to 463.8)	383.4 (291.4 to 483.7)
Bulgaria	Alcohol use disorders	26.9 (21.4 to 33.8)	18.7 (14 to 24.4)	16.7 (12.7 to 21.6)	272.3 (213.3 to 344.8)	211.3 (157 to 276.8)	213.2 (156.2 to 279.5)
Bulgaria	Drug use disorders	11.4 (8.42 to 14.7)	12.5 (9.59 to 15.5)	11.7 (9.17 to 14.5)	127.8 (93.7 to 165.4)	155.7 (119.2 to 193.2)	170.2 (131.5 to 211.9)
Bulgaria	Opioid use disorders	6.28 (4.43 to 8.33)	7.25 (5.42 to 9.12)	7.22 (5.45 to 9.13)	67.9 (47.3 to 90.9)	86.9 (64.9 to 110.6)	98.7 (74.6 to 125.3)
Bulgaria	Cocaine use disorders	1.02 (0.628 to 1.53)	0.972 (0.63 to 1.42)	0.813 (0.532 to 1.21)	11.4 (6.9 to 17.1)	12.3 (7.8 to 18)	12.1 (7.9 to 18.2)
Bulgaria	Amphetamine use disorders	1.1 (0.632 to 1.79)	1.15 (0.732 to 1.7)	0.9 (0.558 to 1.37)	13.7 (7.7 to 22.3)	16.4 (10.4 to 24.5)	16.9 (10.2 to 26.4)
Bulgaria	Cannabis use disorders	0.643 (0.399 to 0.964)	0.546 (0.345 to 0.82)	0.447 (0.285 to 0.642)	7.7 (4.8 to 11.6)	7.7 (4.9 to 11.6)	8 (5 to 11.6)
Bulgaria	Other drug use disorders	2.38 (1.81 to 3.1)	2.57 (2.05 to 3.21)	2.36 (1.92 to 2.88)	27 (20.1 to 35.8)	32.4 (25.5 to 41.1)	34.5 (27.3 to 43.9)
Bulgaria	Diabetes and kidney diseases	111 (94.5 to 132)	125 (108 to 145)	120 (100 to 141)	914.4 (775.6 to 1084.8)	1004.5 (864.7 to 1177.5)	953.2 (799 to 1140.4)
Bulgaria	Diabetes mellitus	83.4 (68.4 to 102)	87.5 (71.7 to 106)	83 (65.6 to 103)	670.5 (547.4 to 819.3)	683.9 (554.7 to 838)	649.5 (507.3 to 813.9)
Bulgaria	Diabetes mellitus type 1	20.2 (18.1 to 22.1)	22.6 (20.7 to 23.9)	16.6 (14.9 to 18.1)	165.2 (149.4 to 178.7)	179.1 (164 to 188.8)	134.7 (121.9 to 146.8)
Bulgaria	Diabetes mellitus type 2	63.3 (48.4 to 80.9)	64.9 (49.6 to 83.4)	66.4 (49.5 to 86.3)	505.3 (383.2 to 649.1)	504.8 (379.8 to 653.2)	514.8 (378.5 to 676.8)
Bulgaria	Chronic kidney disease	27.8 (25.5 to 30.4)	37.7 (35.5 to 40.2)	36.8 (33.7 to 39.9)	241.6 (222.1 to 263.6)	319.8 (300.9 to 338.7)	303.2 (278.6 to 328.6)
Bulgaria	Chronic kidney disease due to diabetes mellitus type 1	1.86 (1.46 to 2.31)	2.58 (2.04 to 3.22)	2.4 (1.88 to 3.03)	16.6 (13.2 to 20.4)	24.3 (19.3 to 30.3)	23.2 (18.4 to 28.9)
Bulgaria	Chronic kidney disease due to diabetes mellitus type 2	4.78 (3.94 to 5.75)	6.77 (5.62 to 8.17)	6.86 (5.63 to 8.41)	36.5 (30.5 to 43.7)	49.1 (41 to 58.7)	47.8 (39.6 to 58)
Bulgaria	Chronic kidney disease due to hypertension	4.02 (3.4 to 4.73)	6.06 (5.13 to 7.09)	6.21 (5.23 to 7.32)	33.6 (28.6 to 39)	46.1 (39.3 to 53.3)	45 (37.9 to 52.4)
Bulgaria	Chronic kidney disease due to glomerulonephritis	5.99 (5.24 to 6.81)	8.44 (7.28 to 9.68)	7.97 (6.77 to 9.26)	55 (48.6 to 62)	77.8 (67.9 to 87.9)	73.7 (63.7 to 85.5)
Bulgaria	Chronic kidney disease due to other and unspecified causes	11.1 (9.49 to 12.9)	13.9 (12 to 15.8)	13.3 (11.4 to 15.3)	99.9 (86.8 to 114.8)	122.6 (108.1 to 137.6)	113.4 (98.6 to 128.9)
Bulgaria	Acute glomerulonephritis	0.221 (0.194 to 0.253)	0.0742 (0.0658 to 0.0847)	0.054 (0.0455 to 0.064)	2.3 (2 to 2.6)	0.8 (0.7 to 0.9)	0.6 (0.5 to 0.7)

SR Appendix Table 2. All-age DALYs counts and age standardized DALYs rates per 100,000 individuals for all causes and level 1 cause groups by location for 1990, 2007, and 2017, both sexes combined

		All-age DALYs			Age-standardized rate of DALYs per 100,000		
Bulgaria	Skin and subcutaneous diseases	41.3 (28 to 60.3)	35.9 (24.4 to 52.4)	34.1 (23.2 to 49.7)	471 (319.1 to 691.1)	479.6 (323.8 to 699.3)	486.4 (330 to 708.5)
Bulgaria	Dermatitis	9.39 (5.58 to 14.7)	7.5 (4.51 to 11.6)	7.01 (4.26 to 10.8)	114.5 (66.9 to 182.6)	113.3 (66.8 to 178.2)	114.7 (67.3 to 180.8)
Bulgaria	Atopic dermatitis	7.11 (3.89 to 11.9)	5.43 (2.99 to 8.99)	5.09 (2.77 to 8.56)	91.4 (49.7 to 154.8)	90.2 (49.4 to 151.4)	91.5 (49.1 to 153.8)
Bulgaria	Contact dermatitis	2.26 (1.48 to 3.35)	2.06 (1.35 to 3.04)	1.91 (1.25 to 2.79)	23 (15 to 34.2)	23 (15 to 34)	23.1 (14.8 to 33.9)
Bulgaria	Seborrhoeic dermatitis	0.00981 (0.00547 to 0.0158)	0.0086 (0.00476 to 0.0137)	0.00807 (0.00446 to 0.0129)	0.1 (0.1 to 0.2)	0.1 (0.1 to 0.2)	0.1 (0.1 to 0.2)
Bulgaria	Psoriasis	7.24 (5.13 to 9.55)	6.91 (4.86 to 9.07)	6.6 (4.7 to 8.71)	70.3 (49.8 to 92.9)	73.6 (51.5 to 97.5)	74.2 (52.3 to 98.8)
Bulgaria	Bacterial skin diseases	0.815 (0.61 to 1.37)	0.921 (0.605 to 1.49)	1.03 (0.617 to 1.64)	8.5 (6.1 to 13.4)	10.1 (6.4 to 14.9)	11.2 (6.6 to 16.1)
Bulgaria	Cellulitis	0.276 (0.182 to 0.366)	0.221 (0.156 to 0.317)	0.224 (0.158 to 0.352)	2.8 (1.9 to 3.7)	2.6 (1.9 to 3.7)	2.7 (2 to 4)
Bulgaria	Pyoderma	0.539 (0.373 to 1.11)	0.7 (0.411 to 1.16)	0.807 (0.416 to 1.28)	5.6 (3.9 to 10.4)	7.4 (4.2 to 11.3)	8.5 (4.2 to 12)
Bulgaria	Scabies	2.19 (1.2 to 3.52)	1.77 (0.972 to 2.84)	1.54 (0.85 to 2.49)	26.9 (14.7 to 43.2)	26.8 (14.9 to 43.6)	26.5 (14.7 to 43.3)
Bulgaria	Fungal skin diseases	4.55 (1.83 to 9.35)	4.38 (1.75 to 9.05)	4.35 (1.74 to 8.95)	46.3 (18.5 to 95.1)	45.6 (18.2 to 94.9)	44.8 (17.9 to 92.3)
Bulgaria	Viral skin diseases	3.18 (2.03 to 4.71)	2.17 (1.38 to 3.23)	2.01 (1.27 to 2.98)	45.7 (29.2 to 68.1)	45.8 (29.2 to 68.3)	45.9 (29.3 to 68.5)
Bulgaria	Acne vulgaris	2.27 (1.35 to 3.63)	2.1 (1.24 to 3.38)	1.72 (1.01 to 2.76)	28.5 (17 to 46.2)	33.4 (19.4 to 54)	36.5 (21 to 59.3)
Bulgaria	Alopecia areata	0.552 (0.353 to 0.825)	0.491 (0.313 to 0.725)	0.441 (0.278 to 0.663)	5.9 (3.8 to 8.8)	5.9 (3.8 to 8.8)	5.9 (3.7 to 9)
Bulgaria	Pruritus	0.952 (0.454 to 1.78)	0.883 (0.422 to 1.64)	0.861 (0.412 to 1.59)	9.5 (4.5 to 17.9)	9.6 (4.5 to 17.9)	9.7 (4.6 to 18.3)
Bulgaria	Urticaria	5.93 (3.95 to 8.4)	4.7 (3.13 to 6.66)	4.3 (2.85 to 6.07)	74.6 (49.3 to 106.2)	74.9 (49.8 to 106.4)	74.8 (49.5 to 106.2)
Bulgaria	Decubitus ulcer	0.312 (0.225 to 0.42)	0.349 (0.248 to 0.462)	0.375 (0.27 to 0.502)	3 (2.2 to 3.9)	3.1 (2.3 to 4.1)	3.2 (2.3 to 4.2)
Bulgaria	Other skin and subcutaneous diseases	3.94 (1.98 to 7.07)	3.77 (1.92 to 6.8)	3.83 (1.92 to 6.97)	37.4 (18.6 to 67.1)	37.5 (19 to 67.8)	38.9 (19.5 to 71.3)
Bulgaria	Sense organ diseases	85.3 (57 to 123)	86.9 (58.2 to 124)	87.9 (59.3 to 125)	750.9 (501.9 to 1084.5)	737.2 (492 to 1066.2)	722.8 (483.1 to 1046.7)
Bulgaria	Blindness and vision impairment	34.8 (22.6 to 51.4)	34.2 (22.4 to 50.2)	34.1 (22.6 to 50)	306.2 (198.1 to 452.8)	294.3 (190.9 to 436.1)	284.8 (183.9 to 423.7)
Bulgaria	Glaucoma	0.5 (0.327 to 0.714)	0.529 (0.348 to 0.736)	0.532 (0.345 to 0.763)	3.8 (2.5 to 5.3)	3.6 (2.4 to 5.1)	3.3 (2.2 to 4.7)
Bulgaria	Cataract	12.1 (8.09 to 16.8)	13.5 (9.04 to 18.5)	14.2 (9.58 to 19.4)	97.7 (65.4 to 133.3)	95.1 (63.8 to 130.3)	92.4 (62.6 to 126.3)
Bulgaria	Age-related macular degeneration	0.113 (0.0722 to 0.165)	0.131 (0.0815 to 0.192)	0.142 (0.0889 to 0.207)	0.9 (0.6 to 1.3)	0.9 (0.6 to 1.3)	0.9 (0.6 to 1.3)
Bulgaria	Refraction disorders	10.6 (6.74 to 16.2)	9.23 (5.82 to 14)	8.56 (5.4 to 12.9)	106.2 (67.5 to 163.2)	102.5 (64.6 to 157.7)	99.1 (62.8 to 151.5)
Bulgaria	Near vision loss	8.73 (4.21 to 16)	8.09 (3.85 to 14.8)	7.86 (3.73 to 14.3)	73.7 (35.6 to 135.2)	68.7 (32.8 to 125.4)	66.3 (31.3 to 120.3)
Bulgaria	Other vision loss	2.8 (1.88 to 3.89)	2.79 (1.89 to 3.85)	2.77 (1.86 to 3.83)	24 (16.2 to 32.8)	23.5 (16 to 32.6)	22.7 (15.2 to 31.8)
Bulgaria	Age-related and other hearing loss	46.9 (32 to 66.4)	49 (33.5 to 69.2)	50.1 (34.3 to 70.5)	413.8 (283.9 to 583.4)	411.5 (281.1 to 583.9)	406.4 (276.6 to 575.5)
Bulgaria	Other sense organ diseases	3.53 (2.2 to 5.09)	3.59 (2.23 to 5.19)	3.67 (2.29 to 5.3)	30.9 (19.1 to 44.9)	31.4 (19.4 to 45.3)	31.6 (19.5 to 46)
Bulgaria	Musculoskeletal disorders	188 (135 to 252)	178 (128 to 238)	175 (127 to 234)	1680.3 (1214.1 to 2262.6)	1643.1 (1182.6 to 2223.1)	1655.2 (1192.5 to 2237.6)
Bulgaria	Rheumatoid arthritis	3.2 (2.24 to 4.33)	3.19 (2.25 to 4.25)	3.48 (2.42 to 4.66)	27.1 (19 to 36.7)	27.6 (19.3 to 36.6)	29.9 (20.7 to 40.7)
Bulgaria	Osteoarthritis	12.3 (6.13 to 24.2)	12.5 (6.3 to 24.7)	13.4 (6.74 to 26.6)	96.2 (48 to 189.6)	94.2 (47.4 to 186.1)	97.3 (49 to 192.6)
Bulgaria	Low back pain	136 (96.7 to 185)	126 (91 to 169)	123 (88.8 to 164)	1235.4 (883 to 1677.6)	1194.8 (854.4 to 1607.9)	1201.7 (856.2 to 1627.5)
Bulgaria	Neck pain	33.7 (23.5 to 47.4)	32.8 (23 to 46.1)	32.2 (22.4 to 45.1)	295.9 (206.9 to 413.3)	297.5 (206.5 to 415.2)	297 (207.3 to 415.7)
Bulgaria	Gout	1.51 (0.996 to 2.1)	1.5 (1.01 to 2.09)	1.57 (1.06 to 2.18)	12.1 (8.1 to 17)	11.8 (7.9 to 16.6)	12.1 (8.1 to 16.6)
Bulgaria	Other musculoskeletal disorders	1.35 (0.595 to 2.64)	1.61 (0.828 to 2.86)	1.57 (0.825 to 2.77)	13.6 (5.9 to 26.5)	17.2 (8.5 to 29.5)	17.4 (8.5 to 31.9)
Bulgaria	Other non-communicable diseases	151 (126 to 182)	104 (82.2 to 132)	88.1 (67.9 to 113)	1923.1 (1617.2 to 2244.1)	1509 (1267.7 to 1803.9)	1252.4 (1012.5 to 1559.5)
Bulgaria	Congenital anomalies	67.1 (54.2 to 76.4)	40.1 (34.3 to 46.1)	28.4 (23 to 34.7)	1130.7 (901.4 to 1270.5)	850.7 (746.9 to 957.2)	633.1 (516.7 to 775.3)
Bulgaria	Neural tube defects	4.19 (3.31 to 5.88)	1.61 (1.26 to 2.06)	1.09 (0.821 to 1.4)	76 (58.7 to 108)	35.2 (27.7 to 47)	24.6 (18.1 to 33.8)
Bulgaria	Congenital heart anomalies	25.3 (19.6 to 29)	15.3 (13.4 to 17.4)	8.78 (7.08 to 10.9)	470.5 (363 to 541.9)	392.5 (346.3 to 451.7)	262 (207.7 to 334.7)
Bulgaria	Orofacial clefts	0.231 (0.161 to 0.323)	0.156 (0.106 to 0.222)	0.129 (0.0855 to 0.187)	3.1 (2.2 to 4.4)	2.5 (1.8 to 3.4)	2.1 (1.4 to 3)

SR Appendix Table 2. All-age DALYs counts and age standardized DALYs rates per 100,000 individuals for all causes and level 1 cause groups by location for 1990, 2007, and 2017, both sexes combined							
		**All-age DALYs**			**Age-standardized rate of DALYs per 100,000**		
Bulgaria	Down's syndrome	1.12 (0.797 to 1.85)	0.871 (0.678 to 1.13)	0.722 (0.565 to 0.927)	16.3 (11.3 to 29.8)	16.8 (12.5 to 23.4)	14.7 (11.3 to 19.5)
Bulgaria	Turner syndrome	0.00891 (0.00441 to 0.0145)	0.00784 (0.0038 to 0.0129)	0.00643 (0.00313 to 0.0107)	0.1 (0.1 to 0.2)	0.1 (0.1 to 0.2)	0.1 (0.1 to 0.2)
Bulgaria	Klinefelter syndrome	0.00162 (0.000754 to 0.00304)	0.00139 (0.000644 to 0.00271)	0.0012 (0.00055 to 0.00233)	0 (0 to 0)	0 (0 to 0)	0 (0 to 0)
Bulgaria	Other chromosomal abnormalities	1.29 (0.722 to 1.7)	0.908 (0.663 to 1.26)	0.741 (0.538 to 1.03)	22.6 (12.3 to 30.1)	21.3 (15.1 to 30.8)	19 (13.6 to 27.5)
Bulgaria	Congenital musculoskeletal and limb anomalies	11.8 (8.09 to 15.8)	10.2 (6.91 to 13.7)	9.18 (6.2 to 12.6)	136.1 (94.6 to 181.3)	135.5 (95.1 to 182.2)	128.2 (86.8 to 175.6)
Bulgaria	Urogenital congenital anomalies	0.936 (0.589 to 2.08)	0.558 (0.393 to 1.02)	0.385 (0.252 to 0.672)	17.1 (10.2 to 39.8)	14.1 (9.7 to 26.8)	11.1 (6.6 to 20.6)
Bulgaria	Digestive congenital anomalies	8.21 (5.41 to 10.4)	4.15 (3.11 to 5.43)	3.26 (2.33 to 4.42)	131.2 (77.9 to 167.9)	73.9 (58.5 to 93.8)	59.5 (44.1 to 79.3)
Bulgaria	Other congenital anomalies	14.1 (6.75 to 17.6)	6.45 (4.65 to 7.69)	4.16 (3.18 to 5.34)	257.6 (112.6 to 323.9)	158.7 (108.9 to 190.1)	111.8 (82 to 148.8)
Bulgaria	Urinary diseases and male infertility	24.1 (21.7 to 26.9)	9.33 (7.28 to 11.9)	9.18 (7.02 to 11.8)	199.7 (181.4 to 222.2)	76.2 (59.4 to 97.2)	73.7 (56.8 to 94)
Bulgaria	Urinary tract infections	13.9 (11.4 to 15.6)	2.82 (2.63 to 3.05)	2.69 (2.44 to 2.96)	118.4 (98.2 to 131.8)	24.6 (22.9 to 26.6)	23.1 (20.9 to 25.4)
Bulgaria	Urolithiasis	2.76 (1.95 to 4.06)	0.583 (0.415 to 0.776)	0.586 (0.42 to 0.784)	22.1 (15.8 to 32.2)	5.3 (3.8 to 7.1)	5.4 (3.9 to 7.3)
Bulgaria	Benign prostatic hyperplasia	5.22 (3.32 to 7.6)	5.12 (3.29 to 7.48)	5.29 (3.41 to 7.68)	39 (24.9 to 56.6)	37.6 (24.2 to 55.2)	38.2 (24.5 to 55.2)
Bulgaria	Male infertility	0.173 (0.0708 to 0.365)	0.159 (0.0637 to 0.338)	0.122 (0.0485 to 0.257)	2 (0.8 to 4.3)	2.1 (0.8 to 4.5)	1.9 (0.7 to 3.8)
Bulgaria	Other urinary diseases	1.98 (1.3 to 3.34)	0.649 (0.502 to 0.774)	0.497 (0.373 to 0.615)	18.2 (12.2 to 29.7)	6.6 (5.2 to 7.9)	5.1 (4 to 6.3)
Bulgaria	Gynecological diseases	14.7 (10.1 to 20.8)	12.2 (8.41 to 17.2)	10.5 (7.03 to 14.9)	158.8 (109.9 to 222.4)	147.2 (101.6 to 207.4)	143.5 (98 to 201.3)
Bulgaria	Uterine fibroids	3.08 (1.72 to 5.16)	2.1 (1.2 to 3.51)	1.76 (0.959 to 2.94)	28.9 (16.1 to 48.8)	21.6 (12.2 to 37)	18.8 (10.2 to 31.7)
Bulgaria	Polycystic ovarian syndrome	0.442 (0.194 to 0.854)	0.385 (0.169 to 0.738)	0.33 (0.15 to 0.639)	5 (2.2 to 9.7)	4.9 (2.2 to 9.5)	5 (2.2 to 9.6)
Bulgaria	Female infertility	0.146 (0.0463 to 0.331)	0.125 (0.0387 to 0.28)	0.124 (0.0385 to 0.286)	1.7 (0.5 to 3.9)	1.6 (0.5 to 3.7)	1.9 (0.6 to 4.4)
Bulgaria	Endometriosis	3.82 (2.46 to 5.59)	3.35 (2.16 to 4.96)	2.8 (1.8 to 4.14)	44.2 (28.4 to 64.8)	43.3 (27.9 to 64)	42.7 (27.4 to 62.7)
Bulgaria	Genital prolapse	0.494 (0.235 to 0.934)	0.414 (0.199 to 0.777)	0.429 (0.204 to 0.804)	3.9 (1.9 to 7.4)	3.3 (1.6 to 6.2)	3.4 (1.6 to 6.3)
Bulgaria	Premenstrual syndrome	4.57 (2.95 to 6.84)	3.94 (2.54 to 5.92)	3.27 (2.1 to 4.87)	51.8 (33.4 to 77.6)	50.4 (32.4 to 75.6)	48.9 (31.2 to 73.2)
Bulgaria	Other gynecological diseases	2.18 (1.44 to 3.14)	1.88 (1.27 to 2.65)	1.75 (1.15 to 2.56)	23.2 (15.5 to 33.1)	22.1 (15 to 31.3)	22.8 (15 to 33)
Bulgaria	Hemoglobinopathies and hemolytic anaemias	6.97 (4.87 to 10.4)	7.13 (5.62 to 8.37)	6.11 (4.65 to 7.14)	74.5 (53.1 to 108.5)	79.7 (61.6 to 94.6)	68.8 (51.5 to 82.4)
Bulgaria	Thalassemias	0.196 (0.136 to 0.311)	0.177 (0.116 to 0.232)	0.129 (0.0841 to 0.178)	3.5 (2.4 to 5.5)	4.9 (3.2 to 6.5)	4 (2.6 to 5.6)
Bulgaria	Thalassaemias trait	1.65 (1.01 to 2.49)	0.822 (0.489 to 1.26)	0.633 (0.38 to 0.96)	17.9 (11.1 to 27.4)	10.5 (6.3 to 16)	8.5 (4.9 to 13.4)
Bulgaria	Sickle cell disorders	0.464 (0.313 to 0.739)	0.529 (0.369 to 0.677)	0.425 (0.287 to 0.562)	6.8 (4.7 to 10.7)	10.4 (7.2 to 13.5)	9.4 (6.3 to 12.5)
Bulgaria	Sickle cell trait	0.584 (0.338 to 0.925)	0.284 (0.165 to 0.443)	0.211 (0.121 to 0.344)	6.6 (3.8 to 10.5)	3.8 (2.2 to 5.9)	3 (1.7 to 5)
Bulgaria	G6PD deficiency	0.868 (0.668 to 1.66)	1.65 (1.09 to 1.88)	1.4 (0.883 to 1.61)	9.5 (7.3 to 17.7)	19.2 (12.8 to 21.9)	17.5 (11 to 20.3)
Bulgaria	G6PD trait	0.000687 (0.000422 to 0.00102)	0.000388 (0.000248 to 0.000569)	0.000298 (0.000187 to 0.000448)	0 (0 to 0)	0 (0 to 0)	0 (0 to 0)
Bulgaria	Other hemoglobinopathies and hemolytic anaemias	3.21 (2.12 to 5.25)	3.67 (3.04 to 4.21)	3.31 (2.65 to 3.81)	30.2 (20.3 to 47.7)	30.8 (24.8 to 36.7)	26.5 (20.3 to 31.4)
Bulgaria	Endocrine, metabolic, blood, and immune disorders	6.39 (5.31 to 8.04)	5.99 (4.81 to 7.13)	5.39 (4.33 to 6.46)	74.2 (62 to 94.7)	76.1 (61 to 90.3)	68.6 (56.2 to 82.3)
Bulgaria	Oral disorders	30.4 (19.2 to 45.7)	29.1 (18.5 to 43.8)	28.3 (18 to 42.1)	269 (168.2 to 407.4)	262 (163 to 399.8)	255.1 (158 to 387.6)
Bulgaria	Caries of deciduous teeth	0.227 (0.097 to 0.452)	0.132 (0.0575 to 0.261)	0.131 (0.0574 to 0.257)	3.9 (1.7 to 7.8)	3.9 (1.7 to 7.7)	3.8 (1.7 to 7.5)
Bulgaria	Caries of permanent teeth	4.27 (1.88 to 8.06)	3.72 (1.65 to 7.03)	3.2 (1.41 to 6.03)	46.2 (20.3 to 87.3)	46.1 (20.5 to 86.6)	43.8 (19.2 to 82.5)
Bulgaria	Periodontal disease	4.83 (1.89 to 9.84)	4.47 (1.75 to 9.17)	4.36 (1.71 to 8.89)	41.5 (16.5 to 85.1)	39.4 (15.5 to 80.9)	38.6 (15.3 to 79.2)
Bulgaria	Edentulism and severe tooth loss	16.1 (10.7 to 23)	16.3 (10.8 to 23.1)	16.5 (11 to 23.3)	126.7 (84 to 180.7)	121.8 (81.5 to 172.9)	118 (78.7 to 167.4)
Bulgaria	Other oral disorders	4.96 (3.1 to 7.32)	4.47 (2.78 to 6.61)	4.13 (2.58 to 6.04)	50.7 (31.8 to 75)	50.9 (31.5 to 75.1)	50.8 (31.5 to 74.7)
Bulgaria	Sudden infant death syndrome	0.825 (0.595 to 1.2)	0.602 (0.388 to 0.769)	0.277 (0.177 to 0.396)	16.2 (11.7 to 23.4)	17 (11 to 21.7)	9.5 (6.1 to 13.6)
Bulgaria	Injuries	423 (370 to 488)	308 (264 to 364)	266 (220 to 324)	4457.3 (3959.3 to 5088)	3480.8 (3039.7 to 4033.9)	3060.9 (2594.6 to 3640.4)
Bulgaria	Transport injuries	97.5 (89.8 to 106)	72.7 (66.1 to 80.2)	53.6 (46.6 to 61.3)	1068.1 (991.3 to 1148.8)	892.4 (826.1 to 968.9)	697.3 (619.3 to 784)

SR Appendix Table 2. All-age DALYs counts and age standardized DALYs rates per 100,000 individuals for all causes and level 1 cause groups by location for 1990, 2007, and 2017, both sexes combined

		All-age DALYs			Age-standardized rate of DALYs per 100,000		
Bulgaria	Road injuries	87.9 (80.7 to 96.1)	67.2 (60.8 to 74.5)	49.5 (42.9 to 57)	964.2 (893 to 1041.2)	826 (762.3 to 898.2)	645.5 (572.3 to 729.8)
Bulgaria	Pedestrian road injuries	34 (31.5 to 36.7)	14 (12.6 to 15.7)	9.91 (8.53 to 11.6)	372.7 (343.9 to 399.7)	167.5 (152.3 to 184.7)	117.6 (102.3 to 135.4)
Bulgaria	Cyclist road injuries	7.47 (5.85 to 9.3)	7.04 (5.55 to 8.8)	6.62 (5.05 to 8.43)	72.8 (57.3 to 89.9)	75.2 (60.4 to 92.7)	71.7 (55.7 to 91)
Bulgaria	Motorcyclist road injuries	10.5 (9.16 to 11.8)	8.16 (7.18 to 9.23)	5.67 (4.74 to 6.65)	118.5 (104.9 to 132.4)	105.7 (94.2 to 118)	81.8 (69.6 to 95)
Bulgaria	Motor vehicle road injuries	34.6 (31.7 to 38.1)	36.2 (33.1 to 39.5)	26.2 (23.1 to 29.7)	384.3 (353.9 to 418.4)	452.3 (420.9 to 485.8)	358.4 (320.2 to 400.1)
Bulgaria	Other road injuries	1.38 (1.15 to 1.68)	1.79 (1.55 to 2.1)	1.1 (0.919 to 1.33)	15.9 (13.5 to 18.9)	25.2 (22.2 to 28.5)	16 (13.7 to 18.7)
Bulgaria	Other transport injuries	9.6 (8.97 to 10.3)	5.51 (5.09 to 5.98)	4.02 (3.58 to 4.54)	103.9 (97 to 111.3)	66.4 (61.1 to 72.1)	51.8 (45.7 to 58.5)
Bulgaria	Unintentional injuries	248 (205 to 304)	182 (146 to 229)	170 (131 to 221)	2583.9 (2174.9 to 3112.2)	1994.9 (1634.9 to 2474.4)	1846.8 (1459.3 to 2339.7)
Bulgaria	Falls	134 (102 to 176)	105 (78.6 to 140)	107 (79.2 to 144)	1287.7 (983.8 to 1682.2)	1046.5 (781.7 to 1381.2)	1073.7 (796.4 to 1426.4)
Bulgaria	Drowning	17.7 (16.7 to 18.6)	8.86 (8.42 to 9.31)	5.89 (5.38 to 6.45)	226 (212.8 to 239.5)	138.5 (129.9 to 147.1)	95.7 (86.1 to 106.2)
Bulgaria	Fire, heat, and hot substances	12.3 (10.5 to 14.5)	7.92 (6.64 to 9.62)	6.34 (5.05 to 8.06)	140.2 (121.1 to 161.5)	97.2 (83.4 to 115.6)	74.9 (60.7 to 93.4)
Bulgaria	Poisonings	7.87 (6.95 to 8.91)	4.13 (3.51 to 4.97)	3.44 (2.71 to 4.4)	92.6 (81.9 to 104.3)	54 (46 to 64.5)	46.7 (36.6 to 60.1)
Bulgaria	Poisoning by carbon monoxide	2.94 (2.71 to 3.17)	1.61 (1.48 to 1.74)	1.14 (1.03 to 1.26)	34.3 (31.5 to 37.4)	20.7 (19 to 22.5)	15.2 (13.6 to 17.1)
Bulgaria	Poisoning by other means	4.93 (4.12 to 5.93)	2.53 (1.95 to 3.31)	2.29 (1.61 to 3.19)	58.3 (49.3 to 69.1)	33.2 (25.9 to 42.9)	31.5 (22.2 to 43.9)
Bulgaria	Exposure to mechanical forces	26.2 (20.4 to 33.6)	25.7 (20 to 33)	23.7 (17.6 to 31.8)	261.9 (207.9 to 334.1)	277.4 (221 to 349.7)	261.1 (198.9 to 342)
Bulgaria	Unintentional firearm injuries	1.95 (1.68 to 2.16)	1.82 (1.63 to 2.39)	1.43 (1.21 to 1.87)	22.5 (19.3 to 25.1)	23.2 (20.6 to 30.9)	19.6 (16.6 to 26.1)
Bulgaria	Other exposure to mechanical forces	24.3 (18.6 to 31.6)	23.9 (18.3 to 31)	22.2 (16.3 to 29.9)	239.4 (185.7 to 310.2)	254.1 (198.7 to 324.6)	241.5 (180.2 to 319.4)
Bulgaria	Adverse effects of medical treatment	2.9 (2.54 to 3.45)	2.84 (2.49 to 3.36)	2.71 (2.38 to 3.23)	32.7 (27 to 36.2)	33.8 (27.5 to 37.5)	32.7 (27 to 36.9)
Bulgaria	Animal contact	1.68 (1.31 to 2.15)	1.52 (1.19 to 1.94)	1.27 (0.98 to 1.62)	17.8 (13.9 to 22.7)	17.7 (13.9 to 22.6)	15.4 (11.9 to 19.9)
Bulgaria	Venomous animal contact	1.03 (0.757 to 1.37)	0.939 (0.696 to 1.23)	0.775 (0.575 to 1.02)	11 (8 to 14.7)	11.2 (8.1 to 14.9)	9.9 (7.2 to 13.2)
Bulgaria	Non-venomous animal contact	0.654 (0.512 to 0.862)	0.585 (0.453 to 0.776)	0.491 (0.375 to 0.652)	6.8 (5.4 to 8.9)	6.6 (5.2 to 8.5)	5.5 (4.3 to 7.3)
Bulgaria	Foreign body	13.8 (12.9 to 14.8)	7.1 (6.59 to 7.66)	5.04 (4.56 to 5.58)	196.6 (181.4 to 214.1)	116 (106.1 to 126.4)	80.1 (70.1 to 91.3)
Bulgaria	Pulmonary aspiration and foreign body in airway	12.5 (11.7 to 13.5)	5.93 (5.57 to 6.32)	3.91 (3.55 to 4.31)	182.9 (168.4 to 200.2)	102 (93.2 to 111.7)	65.9 (56.9 to 76.7)
Bulgaria	Foreign body in eyes	0.195 (0.0972 to 0.332)	0.181 (0.0915 to 0.31)	0.177 (0.0914 to 0.301)	2 (1 to 3.5)	2.1 (1 to 3.7)	2.2 (1 to 3.8)
Bulgaria	Foreign body in other body part	1.1 (0.856 to 1.41)	0.989 (0.788 to 1.23)	0.96 (0.748 to 1.19)	11.7 (9.3 to 15)	11.9 (9.6 to 14.4)	12 (9.5 to 14.7)
Bulgaria	Environmental heat and cold exposure	5.74 (5.04 to 6.52)	5.81 (5.2 to 6.53)	4.95 (4.31 to 5.66)	57.4 (50.7 to 65.3)	60.9 (54.5 to 68.3)	53.4 (46.6 to 61.6)
Bulgaria	Exposure to forces of nature	0.0571 (0.0566 to 0.0576)	0.538 (0.535 to 0.541)	0.00952 (0.00737 to 0.0121)	0.7 (0.7 to 0.7)	7.6 (7.6 to 7.7)	0.1 (0.1 to 0.1)
Bulgaria	Other unintentional injuries	25.2 (22.2 to 29.3)	12.8 (10.6 to 15.6)	9.61 (7.54 to 12.3)	270.3 (241.2 to 307.7)	145.4 (124.3 to 172.2)	112.9 (90.9 to 142.5)
Bulgaria	Self-harm and interpersonal violence	77.5 (75 to 80.1)	53.1 (51.2 to 55.1)	42.5 (39.4 to 45.3)	805.3 (779.4 to 833.4)	593.5 (570.8 to 615.3)	516.8 (478.9 to 553.9)
Bulgaria	Self-harm	55.4 (53.7 to 57)	39.3 (37.9 to 40.7)	31.8 (29.3 to 34.3)	560 (541.6 to 577.5)	425.9 (409.7 to 442.1)	376.6 (346.1 to 407.4)
Bulgaria	Self-harm by firearm	3.05 (2.05 to 4.34)	3.19 (2 to 4.05)	2.41 (1.41 to 3.15)	33.7 (22.8 to 48)	37.2 (23.3 to 47.5)	31.2 (18.1 to 40.9)
Bulgaria	Self-harm by other specified means	52.4 (50.5 to 54.1)	36.1 (34.5 to 37.9)	29.3 (26.8 to 32)	526.4 (506.8 to 546.7)	388.6 (369.7 to 410.7)	345.4 (314.9 to 379.1)
Bulgaria	Interpersonal violence	22 (20.4 to 23.8)	13.8 (12.6 to 15)	10.6 (9.42 to 11.9)	244.5 (227.2 to 263.3)	166.8 (153.1 to 180.7)	138.6 (124 to 153.9)
Bulgaria	Assault by firearm	3.38 (2.97 to 3.86)	2.86 (2.55 to 3.16)	1.87 (1.58 to 2.16)	39.5 (34.4 to 45.4)	36.8 (32.5 to 41.1)	27.6 (23.1 to 32.2)
Bulgaria	Assault by sharp object	7.39 (6.74 to 8.05)	3.12 (2.82 to 3.42)	2.18 (1.88 to 2.47)	84 (76.6 to 91.7)	38.3 (34.3 to 42.3)	29.6 (25.2 to 33.7)
Bulgaria	Sexual violence	1.28 (0.846 to 1.85)	0.919 (0.608 to 1.34)	0.812 (0.541 to 1.19)	14.2 (9.4 to 20.5)	11.6 (7.7 to 17)	11.7 (7.7 to 16.9)
Bulgaria	Assault by other means	9.95 (8.69 to 11.4)	6.87 (5.98 to 7.99)	5.77 (4.89 to 6.88)	106.8 (93.7 to 121)	80.2 (70.7 to 92.1)	69.7 (59.4 to 81.8)
Bulgaria	Conflict and terrorism	0.000983 (0.000659 to 0.00157)	0.00822 (0.0055 to 0.0133)	0.0101 (0.0068 to 0.0162)	0 (0 to 0)	0.1 (0.1 to 0.2)	0.1 (0.1 to 0.2)
Bulgaria	Executions and police conflict	0.0677 (0.0609 to 0.0759)	0.0496 (0.0463 to 0.0546)	0.144 (0.107 to 0.2)	0.8 (0.7 to 0.8)	0.8 (0.7 to 0.8)	1.5 (1.2 to 1.9)
Croatia	All causes	1750 (1590 to 1920)	1570 (1420 to 1740)	1440 (1290 to 1620)	30519.4 (27780.5 to 33690.3)	25194.2 (22516 to 28255.5)	21599.1 (18978.5 to 24737.4)

SR Appendix Table 2. All-age DALYs counts and age standardized DALYs rates per 100,000 individuals for all causes and level 1 cause groups by location for 1990, 2007, and 2017, both sexes combined

		All-age DALYs			Age-standardized rate of DALYs per 100,000		
Croatia	Communicable, maternal, neonatal, and nutritional disorders	100 (89.3 to 112)	65.5 (57.1 to 74.1)	51.3 (42.6 to 60.3)	2569.5 (2316.4 to 2847.4)	1726 (1509.2 to 1956.3)	1430.1 (1199.6 to 1689.5)
Croatia	HIV/AIDS and sexually transmitted infections	1.28 (0.865 to 2.04)	1.01 (0.645 to 1.67)	1.14 (0.793 to 1.77)	25.5 (17.2 to 40.4)	22.5 (14.4 to 37)	26.3 (18.2 to 40.8)
Croatia	HIV/AIDS	0.372 (0.355 to 0.393)	0.238 (0.215 to 0.265)	0.432 (0.404 to 0.471)	7.4 (7.1 to 7.8)	5.4 (4.9 to 5.9)	9.7 (9.1 to 10.5)
Croatia	HIV/AIDS - Drug-susceptible Tuberculosis	0.163 (0.156 to 0.17)	0.0604 (0.0355 to 0.0885)	0.0456 (0.0274 to 0.072)	3.3 (3.1 to 3.4)	1.4 (0.8 to 2)	1 (0.6 to 1.6)
Croatia	HIV/AIDS - Multidrug-resistant Tuberculosis without extensive drug resistance	0.00134 (0.000293 to 0.00402)	0.00133 (0.000596 to 0.00247)	0.000485 (0.000132 to 0.00132)	0 (0 to 0.1)	0 (0 to 0.1)	0 (0 to 0)
Croatia	HIV/AIDS - Extensively drug-resistant Tuberculosis	0 (0 to 0)	0.000419 (0.000188 to 0.000769)	0.000277 (0.0000735 to 0.000753)	0 (0 to 0)	0 (0 to 0)	0 (0 to 0)
Croatia	HIV/AIDS resulting in other diseases	0.207 (0.195 to 0.226)	0.176 (0.139 to 0.215)	0.386 (0.348 to 0.43)	4.1 (3.9 to 4.4)	3.9 (3.1 to 4.8)	8.6 (7.8 to 9.6)
Croatia	Sexually transmitted infections excluding HIV	0.906 (0.495 to 1.66)	0.77 (0.412 to 1.41)	0.705 (0.368 to 1.31)	18 (9.7 to 33.1)	17.1 (9.1 to 31.4)	16.6 (8.6 to 31.1)
Croatia	Syphilis	0.0889 (0.0586 to 0.151)	0.0578 (0.0417 to 0.0827)	0.0545 (0.0392 to 0.0762)	2.2 (1.2 to 4.5)	1.4 (0.9 to 2.5)	1.3 (0.8 to 2.3)
Croatia	Chlamydial infection	0.185 (0.0952 to 0.362)	0.161 (0.0824 to 0.313)	0.143 (0.0727 to 0.282)	3.7 (1.9 to 7.2)	3.7 (1.9 to 7.3)	3.6 (1.8 to 7.1)
Croatia	Gonococcal infection	0.137 (0.0698 to 0.275)	0.11 (0.0541 to 0.212)	0.0937 (0.0478 to 0.182)	2.9 (1.4 to 5.9)	2.8 (1.3 to 5.4)	2.6 (1.3 to 5.2)
Croatia	Trichomoniasis	0.16 (0.0642 to 0.343)	0.146 (0.0586 to 0.313)	0.137 (0.0545 to 0.292)	3 (1.2 to 6.4)	3 (1.2 to 6.4)	2.9 (1.2 to 6.4)
Croatia	Genital herpes	0.117 (0.0375 to 0.274)	0.112 (0.0351 to 0.266)	0.113 (0.0363 to 0.273)	2 (0.6 to 4.7)	2 (0.6 to 4.7)	2 (0.7 to 4.8)
Croatia	Other sexually transmitted infections	0.218 (0.125 to 0.39)	0.183 (0.104 to 0.321)	0.163 (0.0913 to 0.293)	4.3 (2.4 to 7.8)	4.3 (2.4 to 7.6)	4.1 (2.3 to 7.5)
Croatia	Respiratory infections and tuberculosis	31.7 (29.6 to 34.3)	22.1 (20.4 to 24.4)	13.1 (11.6 to 15.2)	595.5 (549.1 to 651.9)	376.1 (338 to 431.4)	226.7 (188.1 to 280.5)
Croatia	Tuberculosis	10.4 (9.93 to 11)	2.68 (2.53 to 2.85)	1.24 (1.11 to 1.38)	166.2 (157.9 to 175.2)	39.6 (37.4 to 42.3)	17.5 (15.6 to 19.5)
Croatia	Latent tuberculosis infection	0 (0 to 0)	0 (0 to 0)	0 (0 to 0)	0 (0 to 0)	0 (0 to 0)	0 (0 to 0)
Croatia	Drug-susceptible tuberculosis	10.4 (9.86 to 10.9)	2.62 (2.46 to 2.79)	1.23 (1.1 to 1.36)	165.1 (156.8 to 174.1)	38.7 (36.4 to 41.3)	17.3 (15.4 to 19.2)
Croatia	Multidrug-resistant tuberculosis without extensive drug resistance	0.0695 (0.0158 to 0.204)	0.0464 (0.0248 to 0.0795)	0.0104 (0.00304 to 0.0261)	1.1 (0.2 to 3.2)	0.7 (0.4 to 1.2)	0.1 (0 to 0.4)
Croatia	Extensively drug-resistant tuberculosis	0 (0 to 0)	0.0145 (0.00773 to 0.025)	0.0058 (0.0017 to 0.0148)	0 (0 to 0)	0.2 (0.1 to 0.4)	0.1 (0 to 0.2)
Croatia	Lower respiratory infections	16.9 (16.1 to 17.6)	15.6 (15 to 16.3)	8.35 (7.77 to 8.98)	336.1 (318.3 to 355.2)	243.7 (234.1 to 253.9)	117.4 (109.7 to 125.7)
Croatia	Upper respiratory infections	3.34 (1.99 to 5.37)	2.91 (1.72 to 4.66)	2.77 (1.63 to 4.43)	70.4 (41.6 to 112.8)	70.4 (41.1 to 112.7)	70.2 (40.9 to 112.2)
Croatia	Otitis media	1.01 (0.606 to 1.61)	0.84 (0.511 to 1.33)	0.763 (0.461 to 1.19)	22.8 (13.6 to 36.4)	22.4 (13.4 to 35.3)	21.6 (12.9 to 34.8)
Croatia	Enteric infections	4.79 (3.44 to 6.43)	4 (2.9 to 5.35)	3.7 (2.74 to 4.87)	122.2 (87.6 to 164.2)	113.2 (81.7 to 153.7)	104.3 (75.8 to 140.7)
Croatia	Diarrhoeal diseases	4.65 (3.32 to 6.27)	3.91 (2.82 to 5.24)	3.63 (2.68 to 4.79)	118.9 (84.4 to 161.3)	110.9 (79.5 to 151.4)	102.4 (73.6 to 138.3)
Croatia	Typhoid and paratyphoid	0.00291 (0.00103 to 0.00437)	0.000461 (0.000319 to 0.000788)	0.000404 (0.000266 to 0.000687)	0.1 (0 to 0.1)	0 (0 to 0)	0 (0 to 0)
Croatia	Typhoid fever	0.000462 (0.000358 to 0.000613)	0.000119 (0.0000857 to 0.000175)	0.000117 (0.0000801 to 0.000174)	0 (0 to 0)	0 (0 to 0)	0 (0 to 0)
Croatia	Paratyphoid fever	0.00245 (0.000585 to 0.00388)	0.000342 (0.000219 to 0.000664)	0.000287 (0.000168 to 0.000577)	0.1 (0 to 0.1)	0 (0 to 0)	0 (0 to 0)
Croatia	Invasive non-typhoidal salmonella	0.124 (0.065 to 0.22)	0.0866 (0.0441 to 0.152)	0.07 (0.0342 to 0.124)	2.9 (1.5 to 5.4)	2.2 (1.1 to 4.1)	1.8 (0.9 to 3.4)
Croatia	Other intestinal infectious diseases	0.0111 (0.00565 to 0.0181)	0.00554 (0.00331 to 0.00807)	0.00363 (0.00207 to 0.00541)	0.3 (0.1 to 0.5)	0.1 (0.1 to 0.2)	0.1 (0 to 0.1)
Croatia	Neglected tropical diseases and malaria	2.18 (1.03 to 3.75)	1.62 (0.746 to 2.9)	1.54 (0.652 to 2.83)	37.4 (17.7 to 63.9)	28 (12.9 to 49.8)	25.7 (10.8 to 47.5)
Croatia	Malaria	0 (0 to 0)	0 (0 to 0)	0 (0 to 0)	0 (0 to 0)	0 (0 to 0)	0 (0 to 0)
Croatia	Chagas disease	0 (0 to 0)	0 (0 to 0)	0 (0 to 0)	0 (0 to 0)	0 (0 to 0)	0 (0 to 0)
Croatia	Leishmaniasis	0.0323 (0.000144 to 0.308)	0.0248 (0.000169 to 0.235)	0.00703 (0.0000934 to 0.0602)	0.8 (0 to 7.5)	0.7 (0 to 7.3)	0.2 (0 to 2.1)
Croatia	Visceral leishmaniasis	0.0322 (0.0000891 to 0.308)	0.0246 (0.0000882 to 0.235)	0.00673 (0.0000262 to 0.06)	0.8 (0 to 7.5)	0.7 (0 to 7.3)	0.2 (0 to 2)
Croatia	Cutaneous and mucocutaneous leishmaniasis	0.000124 (0.000000241 to 0.000596)	0.00017 (0.0000157 to 0.000535)	0.000292 (0.0000165 to 0.000969)	0 (0 to 0)	0 (0 to 0)	0 (0 to 0)
Croatia	African trypanosomiasis	0 (0 to 0)	0 (0 to 0)	0 (0 to 0)	0 (0 to 0)	0 (0 to 0)	0 (0 to 0)
Croatia	Schistosomiasis	0 (0 to 0)	0 (0 to 0)	0 (0 to 0)	0 (0 to 0)	0 (0 to 0)	0 (0 to 0)
Croatia	Cysticercosis	2.08 (0.931 to 3.62)	1.55 (0.687 to 2.79)	1.49 (0.6 to 2.79)	35.3 (15.8 to 61.9)	26.4 (11.5 to 46.9)	24.8 (10 to 46.7)
Croatia	Cystic echinococcosis	0.0626 (0.0438 to 0.0841)	0.0428 (0.0298 to 0.0586)	0.0355 (0.0245 to 0.0498)	1.1 (0.8 to 1.5)	0.8 (0.5 to 1.1)	0.7 (0.5 to 0.9)

SR Appendix Table 2. All-age DALYs counts and age standardized DALYs rates per 100,000 individuals for all causes and level 1 cause groups by location for 1990, 2007, and 2017, both sexes combined							
		All-age DALYs			Age-standardized rate of DALYs per 100,000		
Croatia	Lymphatic filariasis	0<br>(0 to 0)	0<br>(0 to 0)	0<br>(0 to 0)	0<br>(0 to 0)	0<br>(0 to 0)	0<br>(0 to 0)
Croatia	Onchocerciasis	0<br>(0 to 0)	0<br>(0 to 0)	0<br>(0 to 0)	0<br>(0 to 0)	0<br>(0 to 0)	0<br>(0 to 0)
Croatia	Trachoma	0<br>(0 to 0)	0<br>(0 to 0)	0<br>(0 to 0)	0<br>(0 to 0)	0<br>(0 to 0)	0<br>(0 to 0)
Croatia	Dengue	0<br>(0 to 0)	0<br>(0 to 0)	0<br>(0 to 0)	0<br>(0 to 0)	0<br>(0 to 0)	0<br>(0 to 0)
Croatia	Yellow fever	0<br>(0 to 0)	0<br>(0 to 0)	0<br>(0 to 0)	0<br>(0 to 0)	0<br>(0 to 0)	0<br>(0 to 0)
Croatia	Rabies	0.000299<br>(0.000252 to 0.000354)	0.000233<br>(0.000178 to 0.000305)	0.000255<br>(0.000182 to 0.000366)	0<br>(0 to 0)	0<br>(0 to 0)	0<br>(0 to 0)
Croatia	Intestinal nematode infections	0<br>(0 to 0)	0<br>(0 to 0)	0<br>(0 to 0)	0<br>(0 to 0)	0<br>(0 to 0)	0<br>(0 to 0)
Croatia	Ascariasis	0<br>(0 to 0)	0<br>(0 to 0)	0<br>(0 to 0)	0<br>(0 to 0)	0<br>(0 to 0)	0<br>(0 to 0)
Croatia	Trichuriasis	0<br>(0 to 0)	0<br>(0 to 0)	0<br>(0 to 0)	0<br>(0 to 0)	0<br>(0 to 0)	0<br>(0 to 0)
Croatia	Hookworm disease	0<br>(0 to 0)	0<br>(0 to 0)	0<br>(0 to 0)	0<br>(0 to 0)	0<br>(0 to 0)	0<br>(0 to 0)
Croatia	Food-borne trematodiases	0<br>(0 to 0)	0<br>(0 to 0)	0<br>(0 to 0)	0<br>(0 to 0)	0<br>(0 to 0)	0<br>(0 to 0)
Croatia	Leprosy	0<br>(0 to 0)	0<br>(0 to 0)	0<br>(0 to 0)	0<br>(0 to 0)	0<br>(0 to 0)	0<br>(0 to 0)
Croatia	Ebola virus disease	0<br>(0 to 0)	0<br>(0 to 0)	0<br>(0 to 0)	0<br>(0 to 0)	0<br>(0 to 0)	0<br>(0 to 0)
Croatia	Zika virus disease	0<br>(0 to 0)	0<br>(0 to 0)	0<br>(0 to 0)	0<br>(0 to 0)	0<br>(0 to 0)	0<br>(0 to 0)
Croatia	Guinea worm disease	0<br>(0 to 0)	0<br>(0 to 0)	0<br>(0 to 0)	0<br>(0 to 0)	0<br>(0 to 0)	0<br>(0 to 0)
Croatia	Other neglected tropical diseases	0.0106<br>(0.0035 to 0.0141)	0.00498<br>(0.00257 to 0.00698)	0.00268<br>(0.00191 to 0.00566)	0.2<br>(0.1 to 0.3)	0.1<br>(0.1 to 0.2)	0.1<br>(0 to 0.1)
Croatia	Other infectious diseases	4.62<br>(4.17 to 5.13)	2.56<br>(2.28 to 2.91)	1.98<br>(1.68 to 2.36)	111.8<br>(101.1 to 123.7)	59.9<br>(53.2 to 69)	46.4<br>(38.8 to 57.8)
Croatia	Meningitis	2.8<br>(2.53 to 3.04)	1.03<br>(0.938 to 1.14)	0.713<br>(0.624 to 0.847)	73.8<br>(66.6 to 81.2)	25.9<br>(23.4 to 29)	17.7<br>(15.2 to 20.9)
Croatia	Pneumococcal meningitis	0.673<br>(0.57 to 0.775)	0.306<br>(0.263 to 0.357)	0.234<br>(0.19 to 0.286)	14.6<br>(12.4 to 16.8)	6.8<br>(5.8 to 7.9)	5.2<br>(4.1 to 6.3)
Croatia	H influenzae type B meningitis	0.725<br>(0.634 to 0.816)	0.261<br>(0.238 to 0.293)	0.159<br>(0.141 to 0.198)	20<br>(17 to 23.1)	7.7<br>(6.8 to 8.8)	4.7<br>(4 to 6)
Croatia	Meningococcal infection	0.759<br>(0.707 to 0.813)	0.16<br>(0.144 to 0.179)	0.106<br>(0.0932 to 0.127)	23.5<br>(21.9 to 25.3)	4.2<br>(3.7 to 4.8)	2.7<br>(2.3 to 3.3)
Croatia	Other meningitis	0.647<br>(0.572 to 0.715)	0.3<br>(0.272 to 0.335)	0.213<br>(0.185 to 0.252)	15.7<br>(13.9 to 17.9)	7.3<br>(6.5 to 8.2)	5.1<br>(4.4 to 6.1)
Croatia	Encephalitis	0.499<br>(0.427 to 0.722)	0.539<br>(0.482 to 0.61)	0.386<br>(0.33 to 0.506)	10.7<br>(9.1 to 16.4)	12.2<br>(10.9 to 15.2)	8.7<br>(7.2 to 13.7)
Croatia	Diphtheria	0.000257<br>(0.000245 to 0.000275)	0.000208<br>(0.000197 to 0.000226)	0.000172<br>(0.000149 to 0.000211)	0<br>(0 to 0)	0<br>(0 to 0)	0<br>(0 to 0)
Croatia	Whooping cough	0.0421<br>(0.0235 to 0.077)	0.0116<br>(0.0054 to 0.0195)	0.0195<br>(0.0112 to 0.0317)	1.4<br>(0.8 to 2.6)	0.5<br>(0.2 to 0.9)	1<br>(0.6 to 1.6)
Croatia	Tetanus	0.0897<br>(0.0721 to 0.108)	0.0407<br>(0.0288 to 0.0506)	0.0211<br>(0.0125 to 0.0333)	1.5<br>(1.2 to 1.7)	0.5<br>(0.4 to 0.6)	0.2<br>(0.1 to 0.3)
Croatia	Measles	0.00715<br>(0.00504 to 0.00969)	0.000846<br>(0.000608 to 0.000919)	0.000627<br>(0.000403 to 0.000738)	0.2<br>(0.2 to 0.3)	0<br>(0 to 0)	0<br>(0 to 0)
Croatia	Varicella and herpes zoster	0.159<br>(0.0993 to 0.234)	0.157<br>(0.0993 to 0.232)	0.154<br>(0.0954 to 0.23)	3<br>(1.9 to 4.4)	2.9<br>(1.9 to 4.3)	2.7<br>(1.7 to 4)
Croatia	Acute hepatitis	0.398<br>(0.308 to 0.526)	0.305<br>(0.234 to 0.403)	0.247<br>(0.179 to 0.338)	8<br>(6.1 to 10.6)	6.9<br>(5.2 to 9.2)	6<br>(4.3 to 8.3)
Croatia	Acute hepatitis A	0.173<br>(0.122 to 0.241)	0.13<br>(0.0912 to 0.184)	0.11<br>(0.0737 to 0.157)	3.9<br>(2.8 to 5.4)	3.5<br>(2.4 to 5)	3.3<br>(2.2 to 4.7)
Croatia	Acute hepatitis B	0.193<br>(0.154 to 0.257)	0.148<br>(0.115 to 0.192)	0.118<br>(0.0834 to 0.163)	3.4<br>(2.7 to 4.5)	2.7<br>(2.1 to 3.6)	2.2<br>(1.6 to 3.1)
Croatia	Acute hepatitis C	0.0047<br>(0.00259 to 0.00862)	0.0037<br>(0.00205 to 0.00682)	0.00276<br>(0.00144 to 0.00513)	0.1<br>(0.1 to 0.2)	0.1<br>(0.1 to 0.2)	0.1<br>(0 to 0.1)
Croatia	Acute hepatitis E	0.0274<br>(0.021 to 0.0361)	0.0227<br>(0.0172 to 0.0303)	0.017<br>(0.0124 to 0.0234)	0.6<br>(0.4 to 0.7)	0.5<br>(0.4 to 0.7)	0.4<br>(0.3 to 0.6)
Croatia	Other unspecified infectious diseases	0.619<br>(0.494 to 0.811)	0.479<br>(0.391 to 0.621)	0.434<br>(0.344 to 0.554)	13.2<br>(10.5 to 17.3)	10.9<br>(8.8 to 14.8)	10<br>(7.7 to 14.1)
Croatia	Maternal and neonatal disorders	49<br>(42.3 to 57.2)	30.5<br>(24.6 to 37.8)	26.4<br>(20.4 to 33.8)	1526.2<br>(1352.1 to 1720.3)	1024.7<br>(865.8 to 1225.6)	906.4<br>(736.6 to 1107.9)
Croatia	Maternal disorders	0.519<br>(0.461 to 0.605)	0.457<br>(0.421 to 0.499)	0.165<br>(0.143 to 0.193)	10.7<br>(9.4 to 12.5)	11<br>(10.1 to 12)	4.4<br>(3.7 to 5.1)
Croatia	Maternal haemorrhage	0.109<br>(0.0606 to 0.177)	0.0464<br>(0.0253 to 0.0846)	0.022<br>(0.0139 to 0.0336)	2.2<br>(1.2 to 3.6)	1.1<br>(0.6 to 2.1)	0.6<br>(0.4 to 0.9)
Croatia	Maternal sepsis and other pregnancy related infections	0.0423<br>(0.028 to 0.0643)	0.0304<br>(0.0215 to 0.042)	0.0147<br>(0.00813 to 0.0246)	0.9<br>(0.6 to 1.4)	0.7<br>(0.5 to 1)	0.4<br>(0.2 to 0.7)
Croatia	Maternal hypertensive disorders	0.066<br>(0.0441 to 0.0943)	0.0342<br>(0.024 to 0.0491)	0.0134<br>(0.00936 to 0.0187)	1.4<br>(0.9 to 2)	0.9<br>(0.6 to 1.2)	0.4<br>(0.3 to 0.5)
Croatia	Maternal obstructed labour and uterine rupture	0.0208<br>(0.0124 to 0.0342)	0.0218<br>(0.0131 to 0.0358)	0.00933<br>(0.00579 to 0.014)	0.4<br>(0.3 to 0.7)	0.5<br>(0.3 to 0.8)	0.2<br>(0.1 to 0.4)

SR Appendix Table 2. All-age DALYs counts and age standardized DALYs rates per 100,000 individuals for all causes and level 1 cause groups by location for 1990, 2007, and 2017, both sexes combined

		All-age DALYs			Age-standardized rate of DALYs per 100,000		
Croatia	Maternal abortive outcome	0.103 (0.0721 to 0.141)	0.0635 (0.0443 to 0.0879)	0.0172 (0.0117 to 0.0238)	2.1 (1.4 to 2.8)	1.4 (1 to 1.9)	0.4 (0.3 to 0.6)
Croatia	Ectopic pregnancy	0.0104 (0.00647 to 0.0158)	0.00637 (0.00397 to 0.00966)	0.00194 (0.00129 to 0.00294)	0.2 (0.1 to 0.3)	0.2 (0.1 to 0.2)	0 (0 to 0.1)
Croatia	Indirect maternal deaths	0.00752 (0.00355 to 0.015)	0.0357 (0.018 to 0.0603)	0.0081 (0.0041 to 0.0144)	0.2 (0.1 to 0.3)	0.9 (0.5 to 1.5)	0.2 (0.1 to 0.4)
Croatia	Late maternal deaths	0.0165 (0.012 to 0.0218)	0.025 (0.0185 to 0.0329)	0.00872 (0.00626 to 0.0118)	0.3 (0.2 to 0.4)	0.6 (0.5 to 0.8)	0.2 (0.2 to 0.3)
Croatia	Maternal deaths aggravated by HIV/AIDS	0.00000282 (0.00000109 to 0.00000562)	0.0000112 (0.00000431 to 0.0000221)	0.00000437 (0.00000186 to 0.00000822)	0 (0 to 0)	0 (0 to 0)	0 (0 to 0)
Croatia	Other maternal disorders	0.144 (0.108 to 0.181)	0.193 (0.157 to 0.229)	0.0699 (0.0579 to 0.0831)	3 (2.2 to 3.7)	4.7 (3.8 to 5.6)	1.8 (1.5 to 2.2)
Croatia	Neonatal disorders	48.5 (41.7 to 56.7)	30.1 (24.1 to 37.3)	26.2 (20.3 to 33.6)	1515.5 (1342.1 to 1709.7)	1013.8 (855.3 to 1213.9)	902.1 (732.3 to 1103.4)
Croatia	Neonatal preterm birth	19.3 (16.9 to 21.9)	9.06 (7.66 to 10.7)	6.66 (5.09 to 8.58)	633.2 (562.3 to 709)	328.9 (284.7 to 382.7)	243.9 (195.3 to 302.4)
Croatia	Neonatal encephalopathy due to birth asphyxia and trauma	10.7 (8.35 to 13.8)	6.77 (4.92 to 9.44)	5.73 (4.06 to 8.23)	333.8 (266.7 to 406.1)	230 (180.2 to 298.9)	207.4 (156.9 to 274.6)
Croatia	Neonatal sepsis and other neonatal infections	9.37 (4.84 to 15.3)	11.3 (7.05 to 16.3)	11.4 (6.99 to 16.5)	205.8 (111.2 to 331.3)	313.5 (209.8 to 437.8)	315 (201.7 to 449.2)
Croatia	Hemolytic disease and other neonatal jaundice	0.507 (0.425 to 0.608)	0.309 (0.255 to 0.372)	0.225 (0.179 to 0.275)	16.8 (13.9 to 20.5)	12.1 (9.9 to 14.7)	9.3 (7.4 to 11.4)
Croatia	Other neonatal disorders	8.56 (7.29 to 9.73)	2.63 (2.29 to 3.04)	2.25 (1.86 to 2.7)	325.9 (277.2 to 370.8)	129.2 (112.3 to 149.1)	126.5 (104.7 to 152.5)
Croatia	Nutritional deficiencies	6.67 (3.84 to 11)	3.75 (2.18 to 6.37)	3.38 (1.94 to 5.62)	150.9 (87.9 to 244.7)	101.4 (59.9 to 167.4)	94.2 (54.5 to 155.9)
Croatia	Protein-energy malnutrition	0.0543 (0.0224 to 0.168)	0.0324 (0.0197 to 0.086)	0.021 (0.0137 to 0.0524)	1.6 (0.6 to 5.1)	1.1 (0.6 to 3.4)	0.7 (0.4 to 2.1)
Croatia	Iodine deficiency	0.541 (0.257 to 1.05)	0.455 (0.212 to 0.89)	0.445 (0.208 to 0.862)	10.1 (4.8 to 19.7)	9.1 (4.2 to 17.6)	9 (4.2 to 17.6)
Croatia	Dietary iron deficiency	4.07 (2.3 to 6.78)	2.37 (1.33 to 4.11)	2.2 (1.21 to 3.8)	81.1 (45.7 to 132.9)	55.6 (31.4 to 96.9)	53 (29.2 to 89.5)
Croatia	Other nutritional deficiencies	0.0377 (0.0321 to 0.0534)	0.0623 (0.0569 to 0.0703)	0.0471 (0.0417 to 0.0537)	0.8 (0.7 to 1.2)	1.3 (1.2 to 1.5)	0.9 (0.8 to 1.1)
Croatia	Non-communicable diseases	1400 (1270 to 1540)	1330 (1210 to 1470)	1240 (1110 to 1390)	23384.3 (21167.2 to 25914.2)	20262.6 (18126.2 to 22816.7)	17556.8 (15372.6 to 20137.3)
Croatia	Neoplasms	276 (272 to 282)	289 (283 to 294)	271 (256 to 287)	4277.3 (4203.9 to 4366.4)	4011.7 (3942.5 to 4082.2)	3443.2 (3248.1 to 3650.6)
Croatia	Lip and oral cavity cancer	7.49 (7.09 to 7.94)	6.15 (5.86 to 6.44)	4.53 (4.09 to 5)	113.4 (107.3 to 120.4)	88.3 (84 to 92.7)	60.6 (54.6 to 67.1)
Croatia	Nasopharynx cancer	0.892 (0.83 to 0.957)	0.594 (0.557 to 0.635)	0.451 (0.406 to 0.499)	14.1 (13.2 to 15.2)	9.1 (8.6 to 9.8)	6.7 (6 to 7.4)
Croatia	Other pharynx cancer	4.91 (4.59 to 5.26)	4.23 (4 to 4.5)	3.78 (3.35 to 4.21)	73.6 (69 to 78.9)	61.6 (58.1 to 65.2)	51.2 (45.7 to 57.1)
Croatia	Oesophageal cancer	6.74 (6.39 to 7.11)	5.83 (5.57 to 6.09)	4.84 (4.39 to 5.25)	100.1 (95 to 105.3)	80.5 (77 to 84.5)	61.7 (56 to 67)
Croatia	Stomach cancer	30.2 (29 to 31.4)	19.3 (18.7 to 19.8)	15 (14 to 16)	457.6 (440 to 474.6)	256.2 (248.9 to 264.2)	182 (170.1 to 194.6)
Croatia	Colon and rectum cancer	29.6 (28.6 to 30.7)	38.6 (37.5 to 39.7)	39.5 (37 to 42.4)	451.6 (436.9 to 468)	505.5 (491.2 to 519.8)	468.3 (439 to 501.5)
Croatia	Liver cancer	4.76 (4.53 to 5.01)	9.72 (9.36 to 10.1)	9.87 (9.08 to 10.7)	71.7 (68.3 to 75.2)	130 (125.1 to 135.6)	119.6 (110 to 129.4)
Croatia	Liver cancer due to hepatitis B	1.3 (1.1 to 1.53)	2.47 (2.09 to 2.89)	2.3 (1.89 to 2.77)	19.9 (17.1 to 23.2)	35.4 (30.3 to 41)	30.3 (25.2 to 36.2)
Croatia	Liver cancer due to hepatitis C	0.849 (0.692 to 1.06)	1.73 (1.42 to 2.11)	1.83 (1.49 to 2.26)	12.6 (10.4 to 15.5)	22.2 (18.2 to 27.3)	20.9 (17 to 26.1)
Croatia	Liver cancer due to alcohol use	1.92 (1.72 to 2.13)	4.1 (3.73 to 4.46)	4.24 (3.75 to 4.74)	28.7 (25.9 to 31.7)	53.4 (48.6 to 58.3)	50.4 (44.3 to 56.6)
Croatia	Liver cancer due to NASH	0.381 (0.328 to 0.438)	0.865 (0.759 to 0.986)	0.94 (0.812 to 1.09)	5.7 (5 to 6.6)	11.2 (9.9 to 12.7)	10.8 (9.3 to 12.4)
Croatia	Liver cancer due to other causes	0.306 (0.268 to 0.352)	0.566 (0.497 to 0.642)	0.567 (0.488 to 0.656)	4.7 (4.1 to 5.3)	7.9 (7 to 9)	7.1 (6.1 to 8.3)
Croatia	Gallbladder and biliary tract cancer	5.21 (4.93 to 5.59)	5.44 (4.85 to 5.72)	4.74 (4.19 to 5.15)	78.7 (74.7 to 84.1)	69.6 (62.3 to 73.2)	54.7 (48.4 to 59.4)
Croatia	Pancreatic cancer	11.7 (11.3 to 12.2)	14 (13.6 to 14.5)	14.4 (13.5 to 15.5)	176.7 (170.1 to 183)	185.6 (179.8 to 191.9)	173.6 (161.9 to 187)
Croatia	Larynx cancer	7.48 (7.08 to 7.86)	5.68 (5.39 to 5.97)	4.32 (3.87 to 4.77)	111.1 (105.4 to 116.7)	78.9 (74.7 to 83.2)	54.8 (49.2 to 60.9)
Croatia	Tracheal, bronchus, and lung cancer	64.2 (62.1 to 66.5)	65 (63.3 to 66.7)	59.6 (55.7 to 63.9)	944.1 (914.4 to 975.9)	874.7 (852.4 to 898.7)	736.2 (689.3 to 791)
Croatia	Malignant skin melanoma	3.58 (3.12 to 5.48)	4.72 (3.34 to 5.67)	4.55 (3.02 to 5.35)	58.2 (50.6 to 88.1)	72.8 (51.3 to 86.9)	65.7 (43.2 to 76.8)
Croatia	Non-melanoma skin cancer	1.82 (1.73 to 1.91)	1.19 (1.12 to 1.25)	1.51 (1.4 to 1.63)	29.3 (27.9 to 30.7)	16.1 (15.2 to 16.9)	17.2 (16 to 18.5)
Croatia	Non-melanoma skin cancer (squamous-cell carcinoma)	1.82 (1.73 to 1.91)	1.18 (1.12 to 1.25)	1.51 (1.4 to 1.63)	29.3 (27.9 to 30.6)	16.1 (15.2 to 16.9)	17.1 (15.9 to 18.5)
Croatia	Non-melanoma skin cancer (basal-cell carcinoma)	0.0018 (0.000533 to 0.00446)	0.00174 (0.00052 to 0.00429)	0.00219 (0.000629 to 0.0057)	0 (0 to 0.1)	0 (0 to 0.1)	0 (0 to 0.1)
Croatia	Breast cancer	20.5 (19.6 to 21.4)	22.6 (21.6 to 23.6)	20 (18.2 to 22.1)	320.4 (305.8 to 334.8)	322.2 (308.3 to 336.7)	260.7 (235.8 to 289.5)

SR Appendix Table 2. All-age DALYs counts and age standardized DALYs rates per 100,000 individuals for all causes and level 1 cause groups by location for 1990, 2007, and 2017, both sexes combined

		All-age DALYs			Age-standardized rate of DALYs per 100,000		
Croatia	Cervical cancer	7.44 (6.94 to 7.93)	4.73 (4.48 to 4.99)	4.04 (3.61 to 4.52)	117.6 (110 to 125.2)	70.6 (66.3 to 74.9)	56.6 (50.3 to 63.5)
Croatia	Uterine cancer	2.88 (2.67 to 3.12)	3.04 (2.8 to 3.3)	3.46 (3.05 to 3.94)	43.2 (40.1 to 46.6)	39.6 (36.5 to 43)	40.7 (35.7 to 46.4)
Croatia	Ovarian cancer	7.4 (6.96 to 7.85)	7.6 (7.23 to 7.97)	6.62 (5.92 to 7.46)	113.8 (107.1 to 120.4)	107.6 (102.2 to 112.8)	87 (77.7 to 98)
Croatia	Prostate cancer	7.9 (5.88 to 9.66)	11.4 (8.33 to 13.4)	12.6 (8.62 to 14.4)	121.4 (89.2 to 146)	136.7 (98.8 to 158.7)	132.7 (91.3 to 151.8)
Croatia	Testicular cancer	0.695 (0.609 to 0.787)	0.681 (0.605 to 0.771)	0.575 (0.485 to 0.673)	13.6 (11.8 to 15.5)	15 (13.2 to 17.2)	13.2 (11 to 15.7)
Croatia	Kidney cancer	2.18 (1.99 to 2.68)	5.66 (5 to 6.01)	6.75 (5.75 to 7.46)	33.8 (30.9 to 41.5)	77.8 (69.6 to 82.6)	84.5 (72.6 to 93)
Croatia	Bladder cancer	5.12 (4.86 to 5.38)	6.72 (6.43 to 7.01)	7.21 (6.66 to 7.82)	77.8 (74.1 to 81.6)	84.1 (80.4 to 87.6)	80.4 (74.2 to 87.2)
Croatia	Thyroid cancer	1.27 (1.17 to 1.39)	0.988 (0.908 to 1.07)	0.832 (0.744 to 0.93)	19.6 (18 to 21.3)	13.8 (12.7 to 15)	10.7 (9.6 to 12)
Croatia	Mesothelioma	0.504 (0.427 to 0.562)	1.53 (1.44 to 1.63)	1.47 (1.34 to 1.62)	7.5 (6.4 to 8.4)	21 (19.7 to 22.3)	18.3 (16.7 to 20.2)
Croatia	Hodgkin lymphoma	1.73 (1.25 to 1.93)	0.98 (0.856 to 1.22)	0.738 (0.625 to 0.918)	30.4 (22 to 34.1)	18 (15.7 to 22.5)	13.3 (11.3 to 17.1)
Croatia	Non-Hodgkin's lymphoma	4.96 (4.72 to 5.18)	6.34 (6.07 to 6.62)	6.31 (5.84 to 6.83)	83.3 (79.6 to 87.1)	96.5 (92.5 to 100.7)	89.1 (82.2 to 96.2)
Croatia	Multiple myeloma	2.26 (2.07 to 2.88)	3.16 (2.67 to 3.49)	3.52 (2.71 to 4.02)	33.9 (31 to 43)	41.1 (35.1 to 45.8)	42.1 (33.1 to 48)
Croatia	Leukaemia	10.8 (10.4 to 11.3)	8.99 (8.63 to 9.29)	8.48 (7.87 to 9.12)	192 (183.6 to 201.1)	148.3 (142.2 to 153.9)	127.5 (118.1 to 137.1)
Croatia	Acute lymphoid leukaemia	2.03 (1.82 to 2.24)	1.17 (1.08 to 1.25)	0.922 (0.837 to 1.02)	46 (40.9 to 51.4)	29.8 (27.2 to 32.8)	24.1 (21.3 to 27.4)
Croatia	Chronic lymphoid leukaemia	1.96 (1.75 to 2.15)	2 (1.86 to 2.15)	2.37 (2.1 to 2.64)	29.3 (26.3 to 32)	25.3 (23.5 to 27.1)	26.4 (23.6 to 29.5)
Croatia	Acute myeloid leukaemia	2.64 (2.37 to 3.02)	2.94 (2.77 to 3.11)	3.28 (2.94 to 3.58)	46.8 (41.9 to 53.3)	49 (46.1 to 51.8)	50.5 (45.4 to 55.2)
Croatia	Chronic myeloid leukaemia	1.51 (1.32 to 1.72)	0.759 (0.704 to 0.826)	0.555 (0.493 to 0.628)	24.1 (21.1 to 27.3)	11.4 (10.6 to 12.4)	7.5 (6.7 to 8.4)
Croatia	Other leukaemia	2.65 (2.43 to 2.88)	2.12 (1.97 to 2.26)	1.36 (1.22 to 1.51)	45.8 (41.8 to 50.1)	32.7 (30.6 to 34.9)	19.1 (17.3 to 21.3)
Croatia	Other malignant cancers	11.1 (9.74 to 11.7)	9.79 (8.61 to 10.3)	7.47 (6.81 to 8.7)	194.3 (166.2 to 206.5)	157.3 (141.8 to 166.2)	118.2 (106.8 to 139.2)
Croatia	Other neoplasms	0.741 (0.43 to 1.29)	1.93 (0.955 to 2.81)	2.34 (0.944 to 3.81)	12.4 (7.3 to 21.5)	27.1 (13.9 to 38.2)	29.2 (12.6 to 45.7)
Croatia	Myelodysplastic, myeloproliferative, and other hematopoietic neoplasms	0.693 (0.401 to 1.21)	1.88 (0.918 to 2.75)	2.3 (0.916 to 3.76)	11.3 (6.6 to 19.4)	25.7 (13 to 36.5)	27.9 (11.7 to 44)
Croatia	Benign and in situ intestinal neoplasms	0 (0 to 0)	0 (0 to 0)	0 (0 to 0)	0 (0 to 0)	0 (0 to 0)	0 (0 to 0)
Croatia	Benign and in situ cervical and uterine neoplasms	0 (0 to 0)	0 (0 to 0)	0 (0 to 0)	0 (0 to 0)	0 (0 to 0)	0 (0 to 0)
Croatia	Other benign and in situ neoplasms	0.0484 (0.0278 to 0.0851)	0.0481 (0.0295 to 0.0606)	0.0393 (0.0216 to 0.0542)	1.2 (0.7 to 2)	1.5 (0.9 to 1.9)	1.3 (0.7 to 1.9)
Croatia	Cardiovascular diseases	494 (482 to 508)	414 (402 to 427)	352 (333 to 373)	7834.8 (7648.7 to 8046.4)	5455 (5295 to 5628.8)	4011 (3779.3 to 4257.9)
Croatia	Rheumatic heart disease	5.74 (5.46 to 6.04)	1.8 (1.71 to 1.91)	1.9 (1.75 to 2.06)	89.4 (85 to 93.6)	24.5 (23.3 to 25.9)	23.4 (21.7 to 25.3)
Croatia	Ischaemic heart disease	246 (240 to 252)	210 (205 to 215)	177 (168 to 188)	3903 (3809.1 to 3995.4)	2772.5 (2710.5 to 2839.7)	2018.3 (1913.8 to 2143.6)
Croatia	Stroke	169 (162 to 175)	140 (134 to 147)	114 (106 to 122)	2657.7 (2552.5 to 2759.6)	1801.6 (1719.1 to 1884.4)	1274.8 (1181.7 to 1370)
Croatia	Ischaemic stroke	68.4 (63.9 to 72.8)	66.6 (62.4 to 71.5)	61.9 (55.8 to 68.1)	1098.9 (1028.2 to 1165.7)	833.8 (778.5 to 893.5)	656.1 (589.8 to 723.7)
Croatia	Intracerebral hemorrhage	85.8 (82.2 to 89.4)	62.6 (59.9 to 65.6)	43.3 (40.5 to 46.6)	1328.6 (1273.8 to 1380.7)	808 (775.2 to 843.5)	502.4 (471.2 to 540.3)
Croatia	Subarachnoid hemorrhage	14.6 (12.2 to 15.7)	11.1 (10.1 to 11.8)	8.63 (7.9 to 9.44)	230.2 (194.9 to 246)	159.7 (148.2 to 169.4)	116.3 (106.7 to 127.5)
Croatia	Hypertensive heart disease	16.1 (11.6 to 17.6)	15.8 (12.2 to 17.7)	17.6 (12.1 to 19.7)	258 (182.6 to 281.1)	201.6 (155.4 to 226.5)	191.4 (132.4 to 212.9)
Croatia	Non-rheumatic valvular heart disease	3.17 (2.37 to 3.55)	3.44 (2.78 to 3.87)	5.31 (2.85 to 6.12)	49.8 (37.9 to 55.7)	44.6 (36.8 to 49.8)	59.3 (33.1 to 68)
Croatia	Non-rheumatic calcific aortic valve disease	1.13 (0.771 to 1.72)	2.21 (1.61 to 2.51)	3.72 (1.73 to 4.43)	17.9 (12.2 to 27.2)	28.2 (20.9 to 32)	40.8 (19.6 to 48.7)
Croatia	Non-rheumatic degenerative mitral valve disease	1.15 (0.847 to 1.68)	1.12 (0.894 to 1.39)	1.44 (0.919 to 2.42)	18 (13.3 to 26)	14.7 (11.8 to 18)	16.5 (10.7 to 26.7)
Croatia	Other non-rheumatic valve diseases	0.888 (0.135 to 1.52)	0.118 (0.0631 to 0.262)	0.149 (0.0941 to 0.336)	13.9 (2.2 to 23.5)	1.7 (0.9 to 3.9)	2 (1.2 to 4.3)
Croatia	Cardiomyopathy and myocarditis	9.59 (8.69 to 12.8)	16.3 (15.3 to 17.4)	11.4 (10.4 to 13.2)	165.7 (151.2 to 216.1)	249.8 (235.6 to 264.6)	146.7 (135 to 170.4)
Croatia	Myocarditis	2.51 (2.16 to 3.17)	5.77 (3.35 to 7.12)	3.18 (2.53 to 3.72)	45.3 (39.4 to 58.7)	92.6 (57 to 112.6)	40.3 (35 to 46.8)
Croatia	Alcoholic cardiomyopathy	2.24 (1.75 to 4.96)	6.15 (5.01 to 7.2)	4.57 (3.33 to 5.26)	35.1 (27.6 to 77.5)	86 (71.4 to 100.7)	58.3 (44.1 to 67)
Croatia	Other cardiomyopathy	4.84 (4.3 to 5.78)	4.35 (3.19 to 7.7)	3.6 (2.85 to 6.03)	85.3 (74.7 to 98.1)	71.2 (54.8 to 114.4)	48.2 (40.1 to 73.8)

SR Appendix Table 2. All-age DALYs counts and age standardized DALYs rates per 100,000 individuals for all causes and level 1 cause groups by location for 1990, 2007, and 2017, both sexes combined

		All-age DALYs			Age-standardized rate of DALYs per 100,000		
Croatia	Atrial fibrillation and flutter	5.1 (4.22 to 6.17)	5.3 (4.45 to 6.3)	6.45 (5.41 to 7.69)	82.4 (68.7 to 98.8)	68.2 (57.4 to 80.4)	69.8 (58.3 to 83.9)
Croatia	Aortic aneurysm	2.81 (2.65 to 2.98)	5.36 (5.08 to 5.61)	5.62 (5.18 to 6.09)	43.4 (41.1 to 45.9)	72.5 (68.9 to 75.9)	69 (63.6 to 75.1)
Croatia	Peripheral vascular disease	0.78 (0.385 to 1.27)	1.73 (0.913 to 3.32)	1.87 (0.974 to 3.38)	12.2 (6 to 20)	21.7 (11.3 to 41.8)	20 (10.6 to 35.7)
Croatia	Endocarditis	0.747 (0.641 to 0.975)	0.832 (0.73 to 1.09)	0.714 (0.598 to 1.03)	13.1 (11 to 16.7)	13.2 (11.6 to 17.1)	10.5 (8.9 to 14.5)
Croatia	Other cardiovascular and circulatory diseases	34.8 (32.4 to 37.8)	13.6 (11.7 to 15.7)	10 (8.01 to 12.4)	560.1 (522 to 605.1)	184.7 (159.7 to 215)	127.7 (102 to 157.6)
Croatia	Chronic respiratory diseases	54.5 (48.5 to 60.2)	57.4 (51.7 to 63.2)	56.7 (50.3 to 63)	879.5 (776.4 to 980)	806.2 (716 to 896.7)	731.6 (638.2 to 826.3)
Croatia	Chronic obstructive pulmonary disease	40.1 (36.1 to 44)	47.1 (43 to 51)	46.6 (42 to 51.2)	622.6 (561.3 to 680.3)	610.2 (556.2 to 663.7)	539.6 (483.4 to 595.7)
Croatia	Pneumoconiosis	0.116 (0.0963 to 0.141)	0.163 (0.139 to 0.187)	0.259 (0.224 to 0.298)	1.8 (1.5 to 2.1)	2.1 (1.8 to 2.4)	3 (2.6 to 3.4)
Croatia	Silicosis	0.0623 (0.0472 to 0.073)	0.0373 (0.0259 to 0.0615)	0.0299 (0.0151 to 0.0895)	0.9 (0.7 to 1.1)	0.5 (0.4 to 0.8)	0.4 (0.2 to 1)
Croatia	Asbestosis	0.0176 (0.0116 to 0.0289)	0.0609 (0.0342 to 0.0751)	0.174 (0.073 to 0.224)	0.3 (0.2 to 0.4)	0.8 (0.4 to 0.9)	1.9 (0.8 to 2.5)
Croatia	Coal workers pneumoconiosis	0.0104 (0.00672 to 0.0154)	0.0133 (0.00945 to 0.018)	0.0147 (0.00872 to 0.0252)	0.2 (0.1 to 0.2)	0.2 (0.1 to 0.2)	0.2 (0.1 to 0.3)
Croatia	Other pneumoconiosis	0.0261 (0.0171 to 0.0389)	0.0511 (0.0377 to 0.0641)	0.0408 (0.0245 to 0.0782)	0.4 (0.3 to 0.6)	0.7 (0.5 to 0.8)	0.5 (0.3 to 0.9)
Croatia	Asthma	12.1 (9.4 to 15.4)	7.93 (5.74 to 10.7)	7.55 (5.34 to 10.3)	217.8 (166.5 to 282.9)	155.5 (109.3 to 214.1)	150.9 (103 to 208)
Croatia	Interstitial lung disease and pulmonary sarcoidosis	1.24 (0.974 to 1.58)	1.32 (1.05 to 1.65)	1.36 (1.05 to 1.73)	19.8 (15.6 to 25.3)	19.5 (15.4 to 24.4)	18.8 (14.5 to 24.2)
Croatia	Other chronic respiratory diseases	0.984 (0.839 to 1.13)	0.94 (0.786 to 1.11)	0.931 (0.766 to 1.1)	17.5 (14.9 to 20.1)	18.8 (15.4 to 22.3)	19.4 (15.8 to 23.1)
Croatia	Digestive diseases	88.4 (82.6 to 95.3)	72.5 (67.3 to 78.6)	58.4 (52.7 to 64.7)	1429.8 (1326.5 to 1553)	1116.1 (1025.2 to 1228.2)	856.4 (759.9 to 966.7)
Croatia	Cirrhosis and other chronic liver diseases	54.2 (52.3 to 56.4)	40.4 (38.8 to 42.2)	29.2 (27.1 to 31.6)	840.2 (809 to 872.8)	600.7 (576 to 627)	410.2 (379.5 to 445.3)
Croatia	Cirrhosis and other chronic liver diseases due to hepatitis B	14.4 (12.8 to 16.1)	10.3 (9.14 to 11.5)	6.82 (5.89 to 7.74)	224.3 (200.4 to 251.1)	153.6 (136.5 to 172.3)	96.5 (83.7 to 110.4)
Croatia	Cirrhosis and other chronic liver diseases due to hepatitis C	10.8 (9.34 to 12.4)	8.11 (7.12 to 9.24)	5.97 (5.21 to 6.87)	166.5 (144.3 to 190.8)	120.7 (106.1 to 138.1)	84.1 (73.1 to 96.8)
Croatia	Cirrhosis and other chronic liver diseases due to alcohol use	22.8 (21 to 24.5)	17.1 (15.9 to 18.4)	12.6 (11.4 to 13.9)	350.2 (324.1 to 374.4)	251.5 (233.5 to 270.1)	174.3 (157.3 to 192.8)
Croatia	Cirrhosis due to NASH	3.33 (2.95 to 3.72)	2.78 (2.48 to 3.11)	2.17 (1.89 to 2.45)	50.6 (45 to 56.8)	40.7 (36.2 to 45.5)	29.9 (26.2 to 33.9)
Croatia	Cirrhosis and other chronic liver diseases due to other causes	2.91 (2.39 to 3.45)	2.16 (1.79 to 2.57)	1.67 (1.37 to 2.02)	48.7 (41 to 56.4)	34.3 (28.7 to 40.4)	25.4 (21.3 to 30.3)
Croatia	Upper digestive system diseases	14.2 (10.9 to 18.7)	12.4 (9.35 to 16.6)	11.3 (8.3 to 15.5)	244.6 (186.2 to 327.7)	203.4 (149.6 to 280.3)	183 (130 to 259.3)
Croatia	Peptic ulcer disease	6.2 (5.78 to 6.65)	5.05 (4.67 to 5.47)	3.97 (3.61 to 4.37)	98 (91.5 to 105.2)	69.9 (64.5 to 76)	49.3 (44.5 to 54.9)
Croatia	Gastritis and duodenitis	3.42 (2.2 to 5)	2.83 (1.9 to 4.02)	2.81 (1.9 to 3.94)	68.2 (43.6 to 100.4)	55.1 (36.7 to 79.3)	55.2 (36.7 to 79)
Croatia	GERD	4.56 (2.43 to 7.68)	4.49 (2.38 to 7.6)	4.52 (2.4 to 7.66)	78.3 (41.5 to 132.3)	78.5 (42 to 133.1)	78.5 (41.6 to 133.3)
Croatia	Appendicitis	0.497 (0.448 to 0.543)	0.372 (0.337 to 0.412)	0.264 (0.233 to 0.297)	8.7 (7.8 to 9.6)	6.1 (5.3 to 6.9)	4.3 (3.6 to 5.2)
Croatia	Paralytic ileus and intestinal obstruction	2.41 (1.73 to 2.77)	2.23 (1.67 to 2.52)	2.19 (1.53 to 2.56)	45.1 (33.9 to 52)	33.6 (26.5 to 37.9)	28.8 (21.8 to 33.3)
Croatia	Inguinal, femoral, and abdominal hernia	4.37 (3.28 to 5.71)	3.88 (2.9 to 5.06)	3.86 (2.84 to 5.08)	79.4 (58.4 to 104.6)	75.9 (54.8 to 101.3)	72.1 (50.4 to 97.1)
Croatia	Inflammatory bowel disease	2.06 (1.53 to 2.65)	2.68 (2.04 to 3.39)	2.24 (1.69 to 2.86)	35.7 (26.5 to 46.4)	46.4 (34.8 to 59.1)	37.6 (27.7 to 48.8)
Croatia	Vascular intestinal disorders	2.91 (2.17 to 3.17)	3.23 (2.4 to 3.54)	2.68 (2.14 to 2.94)	45.2 (34.3 to 49.1)	40 (30.1 to 43.7)	29.1 (23.5 to 31.8)
Croatia	Gallbladder and biliary diseases	1.62 (1.47 to 1.74)	1.56 (1.44 to 1.84)	1.72 (1.55 to 1.94)	25.8 (23.4 to 27.8)	20.2 (18.8 to 23.6)	18.9 (17.1 to 21.2)
Croatia	Pancreatitis	4.75 (4.29 to 5.23)	4 (3.62 to 4.45)	3.1 (2.74 to 3.58)	78.2 (70.9 to 86.2)	60.7 (54.9 to 67.6)	44 (38.5 to 51)
Croatia	Other digestive diseases	1.39 (1.13 to 1.66)	1.74 (1.52 to 1.98)	1.84 (1.6 to 2.11)	27 (21.5 to 33.3)	29.2 (24.9 to 34)	28.5 (23.9 to 33.8)
Croatia	Neurological disorders	84.4 (66.6 to 108)	90.2 (74.2 to 111)	95.2 (78.2 to 117)	1556.6 (1220.9 to 1988.2)	1549.6 (1233.8 to 1966)	1486.9 (1140.7 to 1930.7)
Croatia	Alzheimer's disease and other dementias	25.8 (24.1 to 27.6)	34.9 (32.6 to 37.5)	40.8 (37.5 to 44.3)	463.8 (433.6 to 495.3)	472.7 (442.8 to 504)	418.2 (385.5 to 452.2)
Croatia	Parkinson's disease	4.71 (3.97 to 5.19)	6.51 (5.37 to 7.18)	6.95 (5.59 to 7.76)	75.6 (63.7 to 83.1)	77.6 (64.1 to 85.5)	70.2 (56.6 to 78.4)
Croatia	Epilepsy	9.53 (4.6 to 16.7)	7.8 (4.44 to 12.6)	7.52 (3.6 to 13.4)	191.2 (93.6 to 333.4)	168.4 (95.8 to 276.4)	163.7 (77.4 to 292.2)
Croatia	Multiple sclerosis	1.6 (1.42 to 1.89)	1.72 (1.44 to 1.93)	1.59 (1.22 to 1.83)	26.5 (23.4 to 31)	29.1 (24.3 to 32.8)	26.5 (20.3 to 30.6)
Croatia	Motor neuron disease	0.966 (0.893 to 1.05)	1.06 (0.988 to 1.14)	1.09 (0.979 to 1.19)	18.8 (17 to 21)	19.5 (18 to 21)	18.4 (16.5 to 20.3)

SR Appendix Table 2. All-age DALYs counts and age standardized DALYs rates per 100,000 individuals for all causes and level 1 cause groups by location for 1990, 2007, and 2017, both sexes combined							
		All-age DALYs			Age-standardized rate of DALYs per 100,000		
Croatia	Headache disorders	39.6 (25.4 to 58.4)	35.7 (23 to 52.8)	34.8 (22.6 to 51.4)	733.6 (470.6 to 1077.1)	725 (467.6 to 1070.8)	731.8 (469.4 to 1074.9)
Croatia	Migraine	33.9 (20.9 to 51.2)	30.5 (18.8 to 46.7)	29.6 (18.5 to 44.8)	628.9 (388 to 949.6)	622.2 (381.9 to 947.1)	627.3 (387.5 to 947.6)
Croatia	Tension-type headache	5.72 (3.26 to 8.98)	5.23 (2.97 to 8.04)	5.2 (2.98 to 8.08)	104.7 (59.3 to 165.2)	102.8 (58.2 to 160.7)	104.5 (59.2 to 165.3)
Croatia	Other neurological disorders	2.21 (1.55 to 3.18)	2.5 (2 to 3.16)	2.45 (1.79 to 3.37)	47.1 (32.3 to 68.4)	57.3 (44.8 to 74.8)	58.1 (39.7 to 84.1)
Croatia	Mental disorders	77.8 (57.8 to 100)	69.5 (51.8 to 89.2)	67.4 (50.3 to 86.7)	1455.6 (1078.4 to 1874.2)	1399 (1037.6 to 1799.4)	1387 (1026.5 to 1787.7)
Croatia	Schizophrenia	7.03 (5.21 to 8.78)	6.59 (4.95 to 8.16)	6.46 (4.86 to 8.03)	122.9 (91.4 to 154.9)	123.9 (92.8 to 154.3)	124.1 (93.1 to 155.7)
Croatia	Depressive disorders	28.8 (20.2 to 38.7)	25.1 (17.9 to 33.6)	24.7 (17.5 to 32.8)	508.5 (358.2 to 688.6)	449.6 (318 to 608.8)	437.8 (310 to 587.6)
Croatia	Major depressive disorder	23.9 (16.5 to 32.2)	20.3 (14.4 to 27.4)	19.9 (13.9 to 26.6)	423 (291.7 to 575.5)	364.3 (253.9 to 493.1)	352.5 (244.4 to 473.7)
Croatia	Dysthymia	4.93 (3.3 to 7.17)	4.8 (3.26 to 7.03)	4.82 (3.26 to 6.97)	85.4 (57.3 to 124.3)	85.4 (57.6 to 124.4)	85.2 (57 to 124.2)
Croatia	Bipolar disorder	7.4 (4.67 to 11)	6.76 (4.27 to 10.1)	6.49 (4.13 to 9.6)	138.3 (86.9 to 208.2)	138.3 (86.9 to 206.8)	138.3 (88.1 to 205.2)
Croatia	Anxiety disorders	16.4 (11.6 to 21.8)	15.1 (10.7 to 19.9)	14.6 (10.4 to 19.2)	310.6 (219.3 to 413.1)	311.2 (219.3 to 414.7)	309.1 (219.8 to 410.2)
Croatia	Eating disorders	1.88 (1.2 to 2.77)	1.65 (1.05 to 2.44)	1.6 (1.02 to 2.35)	39.1 (24.9 to 57.6)	41.3 (26.2 to 60.9)	43.9 (28.3 to 64.6)
Croatia	Anorexia nervosa	0.354 (0.216 to 0.542)	0.306 (0.175 to 0.464)	0.292 (0.177 to 0.449)	7.7 (4.7 to 11.9)	8.2 (4.7 to 12.4)	8.7 (5.2 to 13.3)
Croatia	Bulimia nervosa	1.53 (0.95 to 2.32)	1.34 (0.838 to 2.02)	1.31 (0.8 to 1.95)	31.3 (19.5 to 47.3)	33.1 (20.7 to 49.8)	35.2 (21.8 to 52.5)
Croatia	Autism spectrum disorders	3.18 (2.16 to 4.38)	2.78 (1.9 to 3.81)	2.67 (1.81 to 3.65)	67.4 (45.9 to 93.3)	67.6 (45.9 to 93.4)	67.9 (45.6 to 93)
Croatia	Attention-deficit/hyperactivity disorder	0.521 (0.316 to 0.839)	0.422 (0.255 to 0.672)	0.381 (0.231 to 0.607)	11.8 (7.2 to 19.1)	11.8 (7 to 18.9)	11.8 (7.1 to 19)
Croatia	Conduct disorder	3.81 (2.3 to 6.07)	2.87 (1.73 to 4.53)	2.44 (1.46 to 3.88)	98 (59.2 to 157.1)	98.2 (59.2 to 155.8)	98.2 (58.6 to 157.2)
Croatia	Idiopathic developmental intellectual disability	0.935 (0.292 to 1.78)	0.705 (0.218 to 1.37)	0.61 (0.167 to 1.19)	20.1 (6.4 to 38.3)	17.8 (5.5 to 33.9)	16.3 (4.6 to 31.8)
Croatia	Other mental disorders	7.84 (5.2 to 10.8)	7.44 (4.92 to 10.2)	7.39 (4.95 to 10.2)	139 (92 to 192.3)	139.2 (91.3 to 192.4)	139.6 (92.3 to 193)
Croatia	Substance use disorders	27.2 (22.3 to 32.6)	28.9 (24.2 to 34.2)	25.1 (20.5 to 30.3)	482.5 (392.2 to 582.5)	572.4 (472 to 679.9)	499.9 (402.8 to 611.2)
Croatia	Alcohol use disorders	20.4 (17.1 to 24.5)	17.7 (14.6 to 21.4)	16.4 (13.4 to 20.3)	349.9 (290.8 to 425.6)	315.7 (254.1 to 391)	296.3 (234.8 to 374.4)
Croatia	Drug use disorders	6.82 (5.08 to 8.68)	11.3 (9.37 to 13.2)	8.71 (6.98 to 10.5)	132.6 (98.8 to 169.5)	256.6 (214.7 to 301.3)	203.6 (162.6 to 248.2)
Croatia	Opioid use disorders	4.47 (3.09 to 5.9)	8.11 (6.69 to 9.53)	6.18 (4.82 to 7.67)	84.8 (58.7 to 112.8)	183.3 (152.6 to 215.4)	142.5 (111.5 to 177.7)
Croatia	Cocaine use disorders	0.356 (0.224 to 0.524)	0.343 (0.22 to 0.495)	0.327 (0.213 to 0.478)	7 (4.4 to 10.4)	7.5 (4.7 to 11.3)	7.6 (4.8 to 11.3)
Croatia	Amphetamine use disorders	0.338 (0.205 to 0.525)	0.515 (0.349 to 0.694)	0.369 (0.252 to 0.509)	7.2 (4.4 to 11.3)	13 (8.8 to 17.8)	10.3 (6.8 to 14.6)
Croatia	Cannabis use disorders	0.451 (0.284 to 0.675)	0.396 (0.249 to 0.587)	0.332 (0.209 to 0.491)	9.6 (6 to 14.3)	10 (6.3 to 14.9)	9 (5.7 to 13.3)
Croatia	Other drug use disorders	1.21 (1 to 1.47)	1.9 (1.63 to 2.2)	1.5 (1.28 to 1.74)	24 (19.8 to 29.4)	42.8 (36.3 to 50.1)	34.2 (28.5 to 40.5)
Croatia	Diabetes and kidney diseases	57.6 (46.9 to 70.9)	72.1 (58.7 to 88.1)	75.2 (60.2 to 93.9)	904.5 (733.6 to 1113.3)	1005.7 (810.6 to 1241.9)	974.8 (769.1 to 1226.6)
Croatia	Diabetes mellitus	45.7 (35.6 to 58.2)	55.9 (43.8 to 71.1)	59.2 (44.8 to 76.4)	713.2 (553.3 to 905.3)	783.4 (600.7 to 1005.5)	780.5 (582.8 to 1019.1)
Croatia	Diabetes mellitus type 1	4.8 (4.35 to 5.32)	4.79 (3.96 to 5.28)	4.04 (3.29 to 4.61)	77.1 (69.9 to 85.5)	71.6 (60.1 to 80.5)	59.1 (49.1 to 68.4)
Croatia	Diabetes mellitus type 2	40.9 (30.9 to 53.3)	51.1 (39 to 66)	55.2 (41.5 to 72)	636.2 (481.1 to 825.5)	711.7 (534.3 to 929.3)	721.4 (530 to 953)
Croatia	Chronic kidney disease	11.9 (10.8 to 13)	16.2 (15 to 17.4)	16 (14.7 to 17.4)	191.1 (174.4 to 209.1)	222.2 (205.5 to 239.9)	194.2 (177.4 to 212.3)
Croatia	Chronic kidney disease due to diabetes mellitus type 1	0.676 (0.52 to 0.861)	0.764 (0.609 to 0.959)	0.638 (0.498 to 0.812)	10.8 (8.5 to 13.6)	11.7 (9.5 to 14.4)	9.4 (7.5 to 11.7)
Croatia	Chronic kidney disease due to diabetes mellitus type 2	2.36 (1.95 to 2.82)	3.43 (2.87 to 4.02)	3.61 (2.99 to 4.31)	35.8 (29.7 to 42.7)	43 (36.6 to 50.1)	40 (33.4 to 47)
Croatia	Chronic kidney disease due to hypertension	1.91 (1.61 to 2.25)	2.94 (2.54 to 3.4)	3.23 (2.73 to 3.76)	30.6 (25.8 to 35.9)	38.6 (33.5 to 44.3)	36.4 (31 to 42.1)
Croatia	Chronic kidney disease due to glomerulonephritis	2.49 (2.13 to 2.9)	3.27 (2.81 to 3.79)	3.02 (2.5 to 3.55)	40.7 (35.2 to 46.9)	47.6 (41.5 to 54.2)	39.9 (33.9 to 46.2)
Croatia	Chronic kidney disease due to other and unspecified causes	4.42 (3.78 to 5.11)	5.78 (5 to 6.62)	5.48 (4.68 to 6.31)	73.2 (63.2 to 84.2)	81.3 (71 to 91.8)	68.5 (59.2 to 78.5)
Croatia	Acute glomerulonephritis	0.0129 (0.0105 to 0.0161)	0.0079 (0.00668 to 0.00933)	0.00964 (0.0079 to 0.0115)	0.2 (0.2 to 0.2)	0.1 (0.1 to 0.2)	0.1 (0.1 to 0.2)
Croatia	Skin and subcutaneous diseases	23.2 (15.8 to 33.8)	21.4 (14.6 to 31.3)	21 (14.2 to 30.9)	483.2 (325.1 to 702.2)	492.8 (334.1 to 719.2)	496.6 (335.4 to 723.7)
Croatia	Dermatitis	5.39 (3.19 to 8.53)	4.64 (2.8 to 7.19)	4.54 (2.71 to 7)	119.5 (69.6 to 191.6)	118.9 (69.5 to 187.9)	121.9 (70.3 to 193.1)

SR Appendix Table 2. All-age DALYs counts and age standardized DALYs rates per 100,000 individuals for all causes and level 1 cause groups by location for 1990, 2007, and 2017, both sexes combined

		All-age DALYs			Age-standardized rate of DALYs per 100,000		
Croatia	Atopic dermatitis	4.15 (2.24 to 6.89)	3.47 (1.87 to 5.77)	3.39 (1.84 to 5.69)	96.3 (52 to 160.7)	95.7 (50.8 to 161)	98.7 (52.9 to 166.2)
Croatia	Contact dermatitis	1.24 (0.814 to 1.83)	1.17 (0.765 to 1.7)	1.15 (0.757 to 1.68)	23 (15.1 to 34.2)	23.1 (15 to 34.4)	23 (14.9 to 33.9)
Croatia	Seborrhoeic dermatitis	0.00536 (0.00299 to 0.00861)	0.00493 (0.00273 to 0.00792)	0.00486 (0.00268 to 0.00776)	0.1 (0.1 to 0.2)	0.1 (0.1 to 0.2)	0.1 (0.1 to 0.2)
Croatia	Psoriasis	4.35 (3.07 to 5.74)	4.2 (2.98 to 5.52)	4.15 (2.92 to 5.48)	77.7 (55 to 101.9)	78.7 (56 to 103.6)	78.2 (54.3 to 103.5)
Croatia	Bacterial skin diseases	0.256 (0.194 to 0.387)	0.326 (0.197 to 0.424)	0.296 (0.198 to 0.446)	4.8 (3.6 to 7)	5.7 (3.6 to 7.6)	4.9 (3.5 to 7.4)
Croatia	Cellulitis	0.0982 (0.0705 to 0.134)	0.104 (0.0709 to 0.141)	0.0953 (0.0672 to 0.138)	1.9 (1.3 to 2.5)	2 (1.4 to 2.7)	1.8 (1.3 to 2.6)
Croatia	Pyoderma	0.158 (0.108 to 0.267)	0.222 (0.113 to 0.308)	0.2 (0.115 to 0.309)	2.9 (2 to 4.8)	3.7 (2 to 5.2)	3.1 (1.9 to 5.1)
Croatia	Scabies	1.2 (0.664 to 1.93)	1.03 (0.57 to 1.65)	0.962 (0.53 to 1.57)	26.5 (14.7 to 43.2)	26.6 (14.8 to 43)	26.5 (14.8 to 43.1)
Croatia	Fungal skin diseases	2.38 (0.945 to 4.93)	2.42 (0.966 to 5.05)	2.55 (1.02 to 5.28)	44.8 (17.7 to 93.1)	44 (17.4 to 91.3)	43.7 (17.2 to 90.6)
Croatia	Viral skin diseases	1.77 (1.13 to 2.62)	1.36 (0.875 to 2)	1.25 (0.794 to 1.85)	45.8 (29.2 to 67.7)	45.9 (29.4 to 67.7)	45.9 (29.1 to 68)
Croatia	Acne vulgaris	1.47 (0.856 to 2.43)	1.45 (0.86 to 2.37)	1.34 (0.8 to 2.16)	33.4 (19.5 to 55.3)	40.7 (23.8 to 65.9)	42.9 (25.4 to 69.7)
Croatia	Alopecia areata	0.31 (0.197 to 0.465)	0.277 (0.178 to 0.416)	0.266 (0.168 to 0.398)	5.9 (3.8 to 8.9)	5.9 (3.8 to 9)	5.9 (3.7 to 8.9)
Croatia	Pruritus	0.523 (0.247 to 0.986)	0.51 (0.241 to 0.943)	0.518 (0.249 to 0.956)	9.7 (4.6 to 18.5)	9.7 (4.6 to 18)	9.8 (4.6 to 18.4)
Croatia	Urticaria	3.29 (2.18 to 4.59)	2.77 (1.84 to 3.9)	2.64 (1.73 to 3.73)	74.9 (49 to 106.6)	75 (49.3 to 106.8)	75 (49.6 to 105.7)
Croatia	Decubitus ulcer	0.156 (0.114 to 0.211)	0.206 (0.148 to 0.269)	0.2 (0.138 to 0.275)	2.7 (2 to 3.7)	3.1 (2.3 to 4.1)	2.7 (1.9 to 3.7)
Croatia	Other skin and subcutaneous diseases	2.1 (1.03 to 3.81)	2.19 (1.08 to 4.01)	2.29 (1.12 to 4.14)	37.4 (18.3 to 68.4)	38.6 (18.8 to 70.9)	39.2 (19.1 to 71.4)
Croatia	Sense organ diseases	43.9 (29.5 to 63.7)	47.6 (31.8 to 68.1)	51.3 (34.6 to 73.2)	738.8 (494.5 to 1067.5)	724.9 (482.4 to 1050.5)	716.3 (479.5 to 1038.4)
Croatia	Blindness and vision impairment	18 (11.6 to 26.6)	18.9 (12.3 to 27.9)	20 (13.2 to 29.3)	302.5 (193.8 to 449.5)	289.7 (186.5 to 430.5)	283.5 (185 to 420.3)
Croatia	Glaucoma	0.228 (0.147 to 0.322)	0.26 (0.17 to 0.368)	0.282 (0.183 to 0.396)	3.5 (2.3 to 4.9)	3.2 (2.1 to 4.6)	3.1 (2 to 4.3)
Croatia	Cataract	6.09 (4.09 to 8.35)	7.3 (4.94 to 9.96)	8.19 (5.48 to 11.2)	96.6 (65 to 132.5)	93.4 (63.5 to 128)	91.2 (61.6 to 125.7)
Croatia	Age-related macular degeneration	0.0576 (0.0368 to 0.0845)	0.0699 (0.0437 to 0.102)	0.0808 (0.0502 to 0.118)	0.9 (0.6 to 1.3)	0.9 (0.6 to 1.3)	0.9 (0.5 to 1.3)
Croatia	Refraction disorders	5.56 (3.54 to 8.52)	5.16 (3.28 to 7.72)	5.1 (3.23 to 7.71)	104.3 (65.9 to 159.5)	100.6 (63.6 to 153.6)	98.9 (62.8 to 151.6)
Croatia	Near vision loss	4.61 (2.22 to 8.32)	4.6 (2.21 to 8.44)	4.77 (2.28 to 8.69)	73.8 (35.5 to 132.5)	69 (33 to 124.7)	67.3 (32.5 to 122.2)
Croatia	Other vision loss	1.42 (0.942 to 1.98)	1.49 (1.01 to 2.07)	1.58 (1.06 to 2.17)	23.5 (15.6 to 32.6)	22.6 (15.3 to 31.3)	22.1 (14.9 to 30.8)
Croatia	Age-related and other hearing loss	24.1 (16.3 to 34.3)	26.7 (18 to 37.7)	29.1 (20 to 41.1)	405 (276.6 to 571)	403.5 (274.2 to 571.6)	401.1 (272.4 to 571)
Croatia	Other sense organ diseases	1.86 (1.16 to 2.72)	2.02 (1.26 to 2.94)	2.16 (1.36 to 3.09)	31.3 (19.5 to 46.1)	31.7 (19.8 to 46.5)	31.7 (19.6 to 46)
Croatia	Musculoskeletal disorders	106 (77.4 to 141)	114 (82.3 to 152)	116 (84.3 to 155)	1777.2 (1297.3 to 2378.3)	1890.1 (1362.8 to 2520.6)	1864 (1353.3 to 2534)
Croatia	Rheumatoid arthritis	1.8 (1.28 to 2.41)	2.54 (1.9 to 3.23)	2.82 (2.13 to 3.66)	28.7 (20.6 to 38.6)	37.2 (27.3 to 47.8)	39 (28.9 to 51.1)
Croatia	Osteoarthritis	5.95 (3.01 to 11.7)	7.16 (3.61 to 14.2)	7.87 (3.91 to 15.4)	91.3 (46.2 to 179.8)	96.9 (48.8 to 192.6)	97.4 (48.9 to 190.5)
Croatia	Low back pain	77.4 (55.7 to 103)	82.1 (58.5 to 109)	82.6 (59.5 to 110)	1317.5 (944 to 1761.7)	1394.5 (997.8 to 1862.7)	1374.2 (983.1 to 1862.3)
Croatia	Neck pain	18.1 (12.7 to 25.6)	18.6 (13 to 26)	18.9 (13.2 to 26.2)	296.8 (207.3 to 416.3)	297.2 (207.7 to 417.2)	296.5 (207.9 to 415.7)
Croatia	Gout	0.72 (0.483 to 1.01)	0.838 (0.556 to 1.16)	0.916 (0.621 to 1.27)	11.2 (7.5 to 15.8)	11.8 (7.8 to 16.5)	12.1 (8.2 to 16.9)
Croatia	Other musculoskeletal disorders	1.85 (1.16 to 2.72)	2.93 (2.11 to 3.96)	2.52 (1.69 to 3.58)	31.7 (19.9 to 46.8)	52.5 (37.4 to 71.4)	44.7 (29.1 to 63.7)
Croatia	Other non-communicable diseases	67.5 (54.3 to 84.6)	53.8 (41.6 to 69.2)	49.8 (37.3 to 65.1)	1564.4 (1313.9 to 1879.5)	1239.2 (1007.7 to 1529.6)	1089 (848.7 to 1378.7)
Croatia	Congenital anomalies	27.7 (23.9 to 32.1)	17.6 (14.7 to 20.7)	14 (11.2 to 16.9)	832 (732.7 to 967.3)	596.9 (518.2 to 677.1)	472.4 (391.3 to 562.3)
Croatia	Neural tube defects	0.575 (0.451 to 0.731)	0.477 (0.379 to 0.583)	0.441 (0.326 to 0.56)	15.6 (12.4 to 20.1)	16.1 (12.9 to 19.5)	15.8 (11.6 to 20.4)
Croatia	Congenital heart anomalies	10.1 (8.98 to 11.8)	3.66 (3.2 to 4.72)	2.2 (1.81 to 2.89)	347.7 (308.6 to 407.3)	156.1 (134.9 to 205.3)	104.1 (83.8 to 140.8)
Croatia	Orofacial clefts	0.145 (0.0956 to 0.208)	0.102 (0.0665 to 0.148)	0.0936 (0.0596 to 0.138)	3.3 (2.2 to 4.6)	2.5 (1.6 to 3.7)	2.4 (1.5 to 3.6)
Croatia	Down's syndrome	1.59 (1.22 to 2.16)	1.24 (1 to 1.44)	1.07 (0.756 to 1.3)	46.3 (33.1 to 65.6)	42.8 (34.2 to 50.3)	39.3 (26.6 to 50)
Croatia	Turner syndrome	0.006 (0.00296 to 0.00979)	0.00574 (0.00289 to 0.00941)	0.00493 (0.00238 to 0.00827)	0.1 (0.1 to 0.2)	0.1 (0.1 to 0.2)	0.1 (0.1 to 0.2)

SR Appendix Table 2. All-age DALYs counts and age standardized DALYs rates per 100,000 individuals for all causes and level 1 cause groups by location for 1990, 2007, and 2017, both sexes combined

		All-age DALYs			Age-standardized rate of DALYs per 100,000		
Croatia	Klinefelter syndrome	0.00154 (0.000709 to 0.00306)	0.0013 (0.000579 to 0.00256)	0.00118 (0.000519 to 0.00244)	0 (0 to 0.1)	0 (0 to 0.1)	0 (0 to 0.1)
Croatia	Other chromosomal abnormalities	1.52 (1.09 to 2.07)	1.11 (0.761 to 1.4)	0.848 (0.61 to 1.19)	50.8 (35.8 to 71.7)	45.8 (30.6 to 59.5)	38.4 (26 to 57.4)
Croatia	Congenital musculoskeletal and limb anomalies	6.66 (4.68 to 8.8)	5.58 (3.75 to 7.6)	5.44 (3.7 to 7.44)	149.2 (104.3 to 194.8)	127 (86.4 to 171.7)	126.9 (87.3 to 173.5)
Croatia	Urogenital congenital anomalies	0.361 (0.259 to 0.571)	0.287 (0.191 to 0.386)	0.195 (0.132 to 0.265)	11.7 (8.1 to 19)	11.8 (7.6 to 15.9)	8.6 (5.5 to 11.6)
Croatia	Digestive congenital anomalies	2.69 (2.08 to 3.53)	2.38 (1.76 to 2.96)	1.66 (1.22 to 2.18)	77 (61.3 to 104.6)	85.7 (59.2 to 103.4)	56.6 (42.1 to 72.3)
Croatia	Other congenital anomalies	4.1 (3.38 to 5.9)	2.78 (2.28 to 3.26)	2 (1.56 to 2.46)	130.4 (108.5 to 198)	108.8 (87.8 to 125.2)	80.2 (61.2 to 101.4)
Croatia	Urinary diseases and male infertility	6.89 (5.89 to 8.1)	6.76 (5.72 to 7.98)	7.33 (6.21 to 8.72)	109.5 (93.7 to 128.1)	94 (78.9 to 112)	90.7 (75.5 to 109.6)
Croatia	Urinary tract infections	3.21 (2.85 to 3.77)	3.62 (3.35 to 3.84)	3.91 (3.57 to 4.24)	52.1 (46.6 to 61.1)	49.1 (45.6 to 52.1)	45.8 (41.8 to 49.5)
Croatia	Urolithiasis	0.339 (0.251 to 0.445)	0.294 (0.223 to 0.382)	0.362 (0.265 to 0.486)	5.6 (4.2 to 7.4)	4.6 (3.4 to 6)	5.2 (3.8 to 6.9)
Croatia	Benign prostatic hyperplasia	2.22 (1.4 to 3.19)	2.42 (1.55 to 3.48)	2.7 (1.72 to 3.92)	32.5 (20.5 to 46.5)	32.1 (20.6 to 46.1)	33 (21 to 48.2)
Croatia	Male infertility	0.105 (0.0417 to 0.221)	0.0911 (0.0367 to 0.195)	0.0709 (0.029 to 0.147)	2.1 (0.8 to 4.4)	2.2 (0.9 to 4.6)	1.8 (0.7 to 3.8)
Croatia	Other urinary diseases	1.02 (0.508 to 1.33)	0.337 (0.247 to 0.519)	0.291 (0.198 to 0.499)	17.2 (8.9 to 22.4)	6 (4.4 to 8.4)	4.8 (3.4 to 7.2)
Croatia	Gynecological diseases	7.25 (4.92 to 10.2)	6.49 (4.44 to 9.14)	6.03 (4.1 to 8.53)	138.6 (94.8 to 194.8)	138.9 (94.8 to 194.3)	140.5 (96.1 to 193.8)
Croatia	Uterine fibroids	1.24 (0.679 to 2.12)	1.03 (0.543 to 1.77)	0.867 (0.457 to 1.53)	21.4 (11.8 to 36.5)	18.2 (9.7 to 31.3)	15.8 (8.3 to 28.3)
Croatia	Polycystic ovarian syndrome	0.251 (0.112 to 0.48)	0.22 (0.0967 to 0.427)	0.203 (0.0907 to 0.388)	5 (2.2 to 9.5)	5 (2.2 to 9.6)	5 (2.2 to 9.6)
Croatia	Female infertility	0.0848 (0.0259 to 0.191)	0.0647 (0.0199 to 0.152)	0.0744 (0.023 to 0.167)	1.7 (0.5 to 3.8)	1.5 (0.5 to 3.6)	1.9 (0.6 to 4.3)
Croatia	Endometriosis	1.96 (1.3 to 2.88)	1.83 (1.21 to 2.59)	1.84 (1.23 to 2.61)	38.7 (25.6 to 57.3)	42.5 (28.2 to 60.2)	46.7 (31.2 to 66.6)
Croatia	Genital prolapse	0.178 (0.0846 to 0.335)	0.171 (0.0862 to 0.314)	0.169 (0.0808 to 0.313)	2.7 (1.3 to 5.1)	2.4 (1.2 to 4.4)	2.3 (1.1 to 4.3)
Croatia	Premenstrual syndrome	2.54 (1.62 to 3.78)	2.21 (1.4 to 3.29)	1.99 (1.27 to 3)	50.5 (32.2 to 75.1)	49.9 (31.7 to 74.5)	49.2 (31.5 to 74.4)
Croatia	Other gynecological diseases	0.998 (0.668 to 1.41)	0.961 (0.639 to 1.41)	0.89 (0.583 to 1.32)	18.6 (12.4 to 26.3)	19.4 (12.9 to 28.5)	19.5 (12.9 to 29.1)
Croatia	Hemoglobinopathies and hemolytic anaemias	1.85 (1.41 to 2.49)	1.25 (0.982 to 1.61)	0.893 (0.696 to 1.17)	35.5 (26.9 to 48.3)	23.5 (18.3 to 31.3)	16.6 (12.3 to 23)
Croatia	Thalassemias	0.0294 (0.0208 to 0.0412)	0.0121 (0.00835 to 0.0178)	0.00682 (0.00412 to 0.0122)	0.9 (0.6 to 1.3)	0.5 (0.3 to 0.8)	0.3 (0.2 to 0.6)
Croatia	Thalassaemias trait	0.394 (0.219 to 0.649)	0.22 (0.129 to 0.347)	0.163 (0.098 to 0.26)	7.8 (4.3 to 13.1)	4.8 (2.8 to 7.7)	3.6 (2.1 to 5.9)
Croatia	Sickle cell disorders	0.114 (0.0959 to 0.137)	0.0789 (0.0665 to 0.0998)	0.0472 (0.0337 to 0.0715)	3 (2.5 to 3.6)	2.4 (2 to 3.1)	1.6 (1.1 to 2.4)
Croatia	Sickle cell trait	0.222 (0.108 to 0.398)	0.106 (0.0607 to 0.17)	0.0793 (0.0455 to 0.132)	4.5 (2.2 to 8.2)	2.4 (1.4 to 3.9)	1.9 (1.1 to 3.1)
Croatia	G6PD deficiency	0.141 (0.122 to 0.166)	0.123 (0.107 to 0.143)	0.0716 (0.0597 to 0.113)	2.7 (2.3 to 3.2)	2.3 (2 to 2.8)	1.4 (1.1 to 2.3)
Croatia	G6PD trait	0.00018 (0.0000968 to 0.000321)	0.000125 (0.0000631 to 0.000277)	0.0000736 (0.0000456 to 0.000119)	0 (0 to 0)	0 (0 to 0)	0 (0 to 0)
Croatia	Other hemoglobinopathies and hemolytic anaemias	0.945 (0.757 to 1.19)	0.707 (0.539 to 0.887)	0.525 (0.425 to 0.675)	16.6 (13.1 to 21.2)	11.1 (8.4 to 14.5)	7.9 (6 to 10.9)
Croatia	Endocrine, metabolic, blood, and immune disorders	3.46 (2.71 to 4.1)	2.59 (2.18 to 3.17)	2.41 (2.01 to 2.94)	75.5 (57.5 to 88.9)	58.4 (49.3 to 71)	53.5 (43.9 to 65.5)
Croatia	Oral disorders	18.7 (11.6 to 28.6)	18.4 (11.4 to 27.9)	18.7 (11.7 to 28.4)	311.6 (190.9 to 479.3)	293.8 (177.6 to 454.5)	286.1 (174.9 to 444.2)
Croatia	Caries of deciduous teeth	0.127 (0.0551 to 0.248)	0.0866 (0.0373 to 0.177)	0.0805 (0.0343 to 0.16)	3.9 (1.7 to 7.6)	3.8 (1.7 to 7.9)	3.8 (1.6 to 7.6)
Croatia	Caries of permanent teeth	2.1 (0.931 to 3.98)	1.89 (0.826 to 3.53)	1.77 (0.783 to 3.35)	41.4 (17.9 to 78.3)	41.6 (18.1 to 78.1)	40.8 (17.8 to 77.9)
Croatia	Periodontal disease	4.39 (1.75 to 8.95)	4.34 (1.72 to 8.82)	4.37 (1.75 to 8.87)	71.4 (28.4 to 145.8)	69.4 (27.6 to 144.3)	67.7 (26.9 to 138.3)
Croatia	Edentulism and severe tooth loss	9.33 (6.2 to 13.2)	9.52 (6.41 to 13.5)	9.97 (6.67 to 14.1)	144.1 (96.1 to 204.3)	128.1 (86 to 181.2)	122.9 (82.2 to 174.1)
Croatia	Other oral disorders	2.74 (1.71 to 4.03)	2.54 (1.59 to 3.71)	2.48 (1.56 to 3.65)	50.8 (31.7 to 75.5)	50.9 (31.9 to 75.3)	50.8 (31.8 to 75)
Croatia	Sudden infant death syndrome	1.66 (1.25 to 2.22)	0.693 (0.507 to 0.899)	0.525 (0.345 to 0.693)	61.6 (46.5 to 82.3)	33.6 (24.6 to 43.6)	29.3 (19.2 to 38.6)
Croatia	Injuries	245 (219 to 278)	171 (149 to 199)	151 (127 to 181)	4565.6 (4105.8 to 5148.6)	3205.7 (2836.4 to 3664)	2612.2 (2218.8 to 3097.6)
Croatia	Transport injuries	64.7 (59.3 to 71.1)	47.9 (42.9 to 53.7)	31.5 (27.1 to 36.6)	1265.9 (1169.8 to 1379.2)	1023.2 (932.6 to 1127.7)	652.4 (575.4 to 741.6)
Croatia	Road injuries	61.4 (56.2 to 67.6)	44.4 (39.6 to 50.1)	29.1 (24.8 to 34)	1201.8 (1109.2 to 1309.9)	952.5 (863.5 to 1052.8)	604.2 (529.1 to 690.3)
Croatia	Pedestrian road injuries	25.4 (23.4 to 27.6)	9.38 (8.15 to 10.8)	7 (5.77 to 8.57)	502.1 (464.7 to 541.1)	190.4 (168.2 to 217.7)	133.9 (111.3 to 161.5)

SR Appendix Table 2. All-age DALYs counts and age standardized DALYs rates per 100,000 individuals for all causes and level 1 cause groups by location for 1990, 2007, and 2017, both sexes combined							
		All-age DALYs			Age-standardized rate of DALYs per 100,000		
Croatia	Cyclist road injuries	3.72 (2.86 to 4.77)	4.58 (3.7 to 5.64)	3.95 (3.06 to 4.95)	66.8 (51.6 to 85.5)	87.2 (71.4 to 106.6)	72.8 (57.1 to 90.9)
Croatia	Motorcyclist road injuries	5.22 (4.59 to 5.96)	7.24 (6.52 to 8.05)	4.36 (3.74 to 4.99)	103.8 (92.1 to 117.9)	169.4 (153.9 to 185.4)	102.1 (89.1 to 115.9)
Croatia	Motor vehicle road injuries	25.9 (23.6 to 28.7)	22.9 (20.4 to 25.6)	13.5 (11.7 to 15.6)	505.5 (465.8 to 553.6)	499.1 (454.1 to 547.5)	289.9 (255.8 to 328.9)
Croatia	Other road injuries	1.15 (0.987 to 1.33)	0.33 (0.254 to 0.436)	0.303 (0.222 to 0.414)	23.5 (20.4 to 27)	6.4 (5.1 to 8.3)	5.6 (4.2 to 7.5)
Croatia	Other transport injuries	3.31 (3.04 to 3.6)	3.49 (3.22 to 3.75)	2.39 (2.15 to 2.68)	64.1 (58.9 to 69.6)	70.7 (65.3 to 76.2)	48.1 (43.4 to 54.3)
Croatia	Unintentional injuries	126 (105 to 152)	85.4 (68.9 to 107)	90.4 (71.2 to 115)	2298.4 (1941.7 to 2748.5)	1448 (1172.7 to 1797.6)	1408.4 (1104.2 to 1796.2)
Croatia	Falls	57.7 (43.7 to 75.6)	58.8 (46 to 75.6)	66.1 (50.9 to 85.1)	997.6 (755.6 to 1312.2)	928.5 (721.2 to 1201.7)	950.9 (722.7 to 1236.2)
Croatia	Drowning	6.85 (6.48 to 7.25)	3.97 (3.74 to 4.19)	2.92 (2.7 to 3.18)	144.4 (135.8 to 154)	90.3 (84.5 to 96.3)	66.7 (61.2 to 73.4)
Croatia	Fire, heat, and hot substances	4.29 (3.65 to 5.14)	3.03 (2.57 to 3.68)	2.21 (1.69 to 2.88)	80.4 (69 to 95.7)	57.9 (49.4 to 69.7)	38.7 (29.5 to 50.8)
Croatia	Poisonings	3 (2.62 to 3.48)	1.75 (1.44 to 2.15)	1.5 (1.15 to 1.94)	61.2 (53.9 to 70.8)	38.4 (31.6 to 47.1)	33.2 (25.3 to 43)
Croatia	Poisoning by carbon monoxide	1.48 (1.38 to 1.58)	0.796 (0.744 to 0.854)	0.535 (0.489 to 0.591)	30.4 (28.2 to 32.4)	17.5 (16.3 to 18.9)	11.5 (10.5 to 12.8)
Croatia	Poisoning by other means	1.52 (1.18 to 1.98)	0.952 (0.667 to 1.33)	0.966 (0.644 to 1.39)	30.8 (24 to 39.9)	20.9 (14.7 to 29.3)	21.7 (14.3 to 31.1)
Croatia	Exposure to mechanical forces	13.4 (10.3 to 17.4)	9.46 (7.28 to 12.3)	9.02 (6.61 to 12.1)	243.5 (191.2 to 313.8)	175.2 (136.5 to 225.8)	161.4 (119.6 to 213.8)
Croatia	Unintentional firearm injuries	1.79 (1.41 to 2)	0.593 (0.514 to 0.705)	0.423 (0.361 to 0.497)	37.2 (29.2 to 41.4)	13.5 (11.9 to 16)	9.5 (8.1 to 11.1)
Croatia	Other exposure to mechanical forces	11.6 (8.55 to 15.6)	8.87 (6.74 to 11.7)	8.59 (6.24 to 11.6)	206.3 (153.5 to 275.9)	161.7 (124.5 to 211.5)	151.9 (111.1 to 203.7)
Croatia	Adverse effects of medical treatment	1.8 (1.6 to 2.04)	1.71 (1.5 to 1.94)	1.47 (1.29 to 1.79)	33.2 (29.5 to 37.8)	28.6 (25.7 to 33.7)	22.9 (20.1 to 29.7)
Croatia	Animal contact	0.684 (0.529 to 0.885)	0.473 (0.373 to 0.594)	0.477 (0.374 to 0.61)	12.8 (9.9 to 16.7)	9.2 (7.2 to 11.7)	9.3 (7.2 to 12)
Croatia	Venomous animal contact	0.409 (0.295 to 0.559)	0.277 (0.205 to 0.364)	0.292 (0.217 to 0.382)	7.9 (5.7 to 10.8)	5.8 (4.2 to 7.5)	6.1 (4.4 to 8.1)
Croatia	Non-venomous animal contact	0.275 (0.214 to 0.363)	0.196 (0.156 to 0.251)	0.185 (0.144 to 0.243)	4.9 (3.8 to 6.5)	3.5 (2.7 to 4.5)	3.2 (2.4 to 4.3)
Croatia	Foreign body	3.6 (3.35 to 3.89)	2.17 (1.98 to 2.39)	1.98 (1.78 to 2.23)	79.5 (73.1 to 86.8)	46.9 (43 to 51.8)	41 (36.7 to 46.7)
Croatia	Pulmonary aspiration and foreign body in airway	3.12 (2.93 to 3.31)	1.76 (1.65 to 1.86)	1.53 (1.41 to 1.67)	70.4 (64.9 to 76.4)	38.8 (36 to 41.8)	32 (29 to 35.5)
Croatia	Foreign body in eyes	0.115 (0.0563 to 0.198)	0.124 (0.063 to 0.212)	0.125 (0.0649 to 0.21)	2.2 (1 to 3.9)	2.5 (1.2 to 4.4)	2.6 (1.2 to 4.5)
Croatia	Foreign body in other body part	0.367 (0.274 to 0.482)	0.287 (0.223 to 0.363)	0.326 (0.252 to 0.407)	6.9 (5.2 to 9)	5.6 (4.4 to 7.1)	6.4 (5 to 8)
Croatia	Environmental heat and cold exposure	2.64 (2.34 to 3)	1.52 (1.32 to 1.74)	1.34 (1.11 to 1.62)	46.2 (40.6 to 52.6)	26.4 (22.8 to 30.5)	22.9 (18.8 to 28.1)
Croatia	Exposure to forces of nature	0.00123 (0.000911 to 0.00169)	0.00181 (0.00136 to 0.00237)	0.00228 (0.00173 to 0.00297)	0 (0 to 0)	0 (0 to 0)	0 (0 to 0.1)
Croatia	Other unintentional injuries	31.9 (29.3 to 35.2)	2.44 (1.88 to 3.22)	3.41 (2.43 to 4.73)	599.7 (554.2 to 656)	46.5 (36.4 to 60.5)	61.4 (44.3 to 85.5)
Croatia	Self-harm and interpersonal violence	55 (53.4 to 56.7)	38 (36.4 to 40)	28.7 (26.7 to 30.9)	1001.2 (970.3 to 1032.2)	734.5 (703.4 to 775.8)	551.4 (511.6 to 592)
Croatia	Self-harm	44.7 (43.4 to 46)	29.8 (28.8 to 30.8)	22.4 (20.8 to 24)	798.4 (775.3 to 823.8)	566.5 (546.7 to 585.2)	428.1 (396.9 to 458.9)
Croatia	Self-harm by firearm	7.09 (5.21 to 9.94)	4.37 (3.05 to 5.91)	3.08 (2 to 4.19)	137.5 (100 to 191)	88.7 (62 to 118.8)	62.9 (41.3 to 85.5)
Croatia	Self-harm by other specified means	37.6 (34.6 to 39.5)	25.5 (23.9 to 27)	19.3 (17.7 to 21)	661 (604.9 to 699)	477.8 (446.2 to 508.8)	365.2 (334.6 to 398.5)
Croatia	Interpersonal violence	10.3 (9.42 to 11.2)	5.22 (4.7 to 5.81)	3.98 (3.44 to 4.62)	202.1 (185.7 to 219)	111 (100.7 to 122.5)	84 (73.3 to 96.4)
Croatia	Assault by firearm	3.69 (3.11 to 4.03)	1.55 (1.41 to 1.71)	0.705 (0.623 to 0.817)	73.4 (62 to 80.4)	34.8 (31.2 to 38.7)	16.6 (14.5 to 19.3)
Croatia	Assault by sharp object	2.24 (2.02 to 2.54)	0.892 (0.795 to 0.996)	0.751 (0.651 to 0.856)	42.8 (38.5 to 48.6)	18.5 (16.5 to 20.8)	15.7 (13.5 to 17.9)
Croatia	Sexual violence	0.654 (0.446 to 0.942)	0.534 (0.365 to 0.777)	0.454 (0.308 to 0.651)	12.9 (8.7 to 18.8)	12 (8.1 to 17.5)	10.8 (7.3 to 15.8)
Croatia	Assault by other means	3.7 (3.11 to 4.52)	2.24 (1.85 to 2.74)	2.06 (1.64 to 2.61)	73 (62.2 to 87.8)	45.7 (38.3 to 54.9)	40.9 (32.8 to 50.7)
Croatia	Conflict and terrorism	0 (0 to 0)	2.91 (2.04 to 4.53)	2.34 (1.62 to 3.59)	0 (0 to 0)	56.2 (38.7 to 88.3)	38.6 (26.5 to 60.3)
Croatia	Executions and police conflict	0.0295 (0.0283 to 0.0309)	0.0268 (0.0253 to 0.0291)	0.0229 (0.0209 to 0.0258)	0.7 (0.6 to 0.7)	0.7 (0.7 to 0.8)	0.6 (0.6 to 0.7)
Czech Republic	All causes	4000 (3660 to 4380)	3380 (3010 to 3810)	3400 (3000 to 3880)	32696.8 (29849.6 to 35980.3)	23724.5 (20920.8 to 27050.4)	21382.1 (18555.1 to 24812)
Czech Republic	Communicable, maternal, neonatal, and nutritional disorders	198 (177 to 221)	138 (122 to 157)	138 (120 to 157)	2387.7 (2155.8 to 2649.3)	1423.6 (1240.8 to 1625.4)	1269.1 (1078.4 to 1479.1)
Czech Republic	HIV/AIDS and sexually transmitted infections	2.54 (1.66 to 4.08)	2.84 (1.93 to 4.49)	3.28 (2.4 to 4.82)	24.1 (15.6 to 38.7)	25.1 (16.9 to 39.7)	29.1 (20.8 to 43.5)

SR Appendix Table 2. All-age DALYs counts and age standardized DALYs rates per 100,000 individuals for all causes and level 1 cause groups by location for 1990, 2007, and 2017, both sexes combined

		All-age DALYs			Age-standardized rate of DALYs per 100,000		
Czech Republic	HIV/AIDS	0.304 (0.292 to 0.317)	0.639 (0.604 to 0.678)	1.15 (1.08 to 1.24)	3 (2.9 to 3.1)	5.9 (5.6 to 6.2)	10.2 (9.6 to 10.9)
Czech Republic	HIV/AIDS - Drug-susceptible Tuberculosis	0.136 (0.128 to 0.143)	0.117 (0.0698 to 0.186)	0.124 (0.0752 to 0.193)	1.3 (1.3 to 1.4)	1.1 (0.6 to 1.7)	1.1 (0.7 to 1.7)
Czech Republic	HIV/AIDS - Multidrug-resistant Tuberculosis without extensive drug resistance	0.00265 (0.000675 to 0.0082)	0.00743 (0.00421 to 0.0121)	0.00714 (0.0031 to 0.0134)	0 (0 to 0.1)	0.1 (0 to 0.1)	0.1 (0 to 0.1)
Czech Republic	HIV/AIDS - Extensively drug-resistant Tuberculosis	0 (0 to 0)	0.00233 (0.00132 to 0.00378)	0.00405 (0.00177 to 0.00754)	0 (0 to 0)	0 (0 to 0)	0 (0 to 0.1)
Czech Republic	HIV/AIDS resulting in other diseases	0.165 (0.159 to 0.173)	0.512 (0.432 to 0.576)	1.02 (0.913 to 1.12)	1.6 (1.6 to 1.7)	4.7 (3.9 to 5.3)	9 (8.1 to 9.9)
Czech Republic	Sexually transmitted infections excluding HIV	2.24 (1.35 to 3.78)	2.2 (1.3 to 3.81)	2.13 (1.29 to 3.63)	21.1 (12.6 to 35.7)	19.3 (11.1 to 33.6)	18.9 (11 to 33.2)
Czech Republic	Syphilis	0.485 (0.36 to 0.639)	0.392 (0.276 to 0.522)	0.409 (0.289 to 0.546)	4.6 (3.3 to 6.4)	3.1 (2.2 to 4.2)	2.9 (2.1 to 4)
Czech Republic	Chlamydial infection	0.385 (0.202 to 0.716)	0.402 (0.21 to 0.758)	0.38 (0.205 to 0.712)	3.7 (1.9 to 6.9)	3.7 (1.9 to 6.9)	3.7 (1.9 to 7.2)
Czech Republic	Gonococcal infection	0.313 (0.167 to 0.606)	0.305 (0.169 to 0.575)	0.268 (0.154 to 0.484)	3.2 (1.6 to 6.2)	3 (1.6 to 5.8)	2.9 (1.6 to 5.7)
Czech Republic	Trichomoniasis	0.326 (0.129 to 0.681)	0.349 (0.14 to 0.739)	0.338 (0.136 to 0.699)	3 (1.2 to 6.4)	2.9 (1.2 to 6.3)	2.9 (1.2 to 6.1)
Czech Republic	Genital herpes	0.237 (0.0763 to 0.555)	0.267 (0.0861 to 0.639)	0.279 (0.0899 to 0.666)	2 (0.6 to 4.6)	2 (0.7 to 4.7)	2 (0.7 to 4.7)
Czech Republic	Other sexually transmitted infections	0.49 (0.286 to 0.849)	0.487 (0.289 to 0.848)	0.456 (0.268 to 0.785)	4.6 (2.7 to 8.1)	4.5 (2.6 to 7.9)	4.5 (2.6 to 7.8)
Czech Republic	Respiratory infections and tuberculosis	59 (54.6 to 64.5)	59 (55 to 64.3)	60.7 (55.6 to 66.9)	549.5 (504.3 to 607.9)	443.3 (402.9 to 499)	391.3 (348.3 to 448.8)
Czech Republic	Tuberculosis	5.01 (4.72 to 5.33)	1.81 (1.68 to 1.96)	1.33 (1.19 to 1.48)	38.7 (36.3 to 41.1)	12.1 (11.2 to 13.2)	8.1 (7.1 to 9)
Czech Republic	Latent tuberculosis infection	0 (0 to 0)	0 (0 to 0)	0 (0 to 0)	0 (0 to 0)	0 (0 to 0)	0 (0 to 0)
Czech Republic	Drug-susceptible tuberculosis	4.93 (4.62 to 5.25)	1.7 (1.57 to 1.85)	1.25 (1.09 to 1.39)	38.1 (35.6 to 40.7)	11.4 (10.5 to 12.5)	7.6 (6.6 to 8.5)
Czech Republic	Multidrug-resistant tuberculosis without extensive drug resistance	0.0777 (0.0202 to 0.228)	0.084 (0.0612 to 0.114)	0.0555 (0.0279 to 0.0957)	0.6 (0.2 to 1.8)	0.6 (0.4 to 0.7)	0.3 (0.2 to 0.6)
Czech Republic	Extensively drug-resistant tuberculosis	0 (0 to 0)	0.0258 (0.0185 to 0.0348)	0.0304 (0.0152 to 0.0523)	0 (0 to 0)	0.2 (0.1 to 0.2)	0.2 (0.1 to 0.3)
Czech Republic	Lower respiratory infections	43.9 (42.2 to 45.8)	48.4 (46.8 to 50.1)	50.6 (47 to 54.3)	405.2 (385.9 to 428.2)	337.8 (326.1 to 349.6)	290.8 (270.9 to 311.5)
Czech Republic	Upper respiratory infections	7.93 (5.12 to 12)	7.03 (4.21 to 11)	7.05 (4.29 to 11.1)	82.1 (54 to 124.4)	72.1 (42.7 to 113.2)	71.8 (42.6 to 114.8)
Czech Republic	Otitis media	2.15 (1.33 to 3.35)	1.84 (1.12 to 2.84)	1.79 (1.09 to 2.82)	23.5 (14.6 to 36.4)	21.3 (12.8 to 33.8)	20.6 (12.5 to 32.2)
Czech Republic	Enteric infections	9.82 (6.91 to 13.4)	9.95 (7.19 to 13.2)	14.3 (11.2 to 18.4)	111.2 (77.4 to 153.7)	117.2 (81.9 to 160.6)	135.8 (99.7 to 180.6)
Czech Republic	Diarrhoeal diseases	9.51 (6.57 to 13.1)	9.77 (7 to 13.1)	14.1 (11 to 18.2)	107.8 (74.6 to 149.7)	115.1 (79.9 to 158.4)	134 (98.1 to 179)
Czech Republic	Typhoid and paratyphoid	0.00343 (0.00175 to 0.00519)	0.00151 (0.00083 to 0.00217)	0.0148 (0.00241 to 0.0309)	0 (0 to 0)	0 (0 to 0)	0.1 (0 to 0.2)
Czech Republic	Typhoid fever	0.000502 (0.000366 to 0.000701)	0.000408 (0.000229 to 0.00067)	0.0114 (0.00172 to 0.0269)	0 (0 to 0)	0 (0 to 0)	0.1 (0 to 0.1)
Czech Republic	Paratyphoid fever	0.00292 (0.00125 to 0.00472)	0.0011 (0.000525 to 0.00167)	0.00349 (0.000506 to 0.00671)	0 (0 to 0)	0 (0 to 0)	0 (0 to 0)
Czech Republic	Invasive non-typhoidal salmonella	0.279 (0.146 to 0.481)	0.172 (0.0878 to 0.304)	0.167 (0.0828 to 0.295)	3.1 (1.6 to 5.7)	1.9 (0.9 to 3.7)	1.7 (0.8 to 3.3)
Czech Republic	Other intestinal infectious diseases	0.0274 (0.0134 to 0.046)	0.0106 (0.00612 to 0.0157)	0.00793 (0.00459 to 0.0117)	0.3 (0.1 to 0.6)	0.1 (0.1 to 0.2)	0.1 (0 to 0.1)
Czech Republic	Neglected tropical diseases and malaria	3.77 (1.91 to 6.41)	3.44 (1.56 to 6.35)	3.53 (1.57 to 6.79)	31.6 (16.2 to 53.8)	25 (11.5 to 46.4)	24.1 (10.8 to 45.7)
Czech Republic	Malaria	0 (0 to 0)	0 (0 to 0)	0 (0 to 0)	0 (0 to 0)	0 (0 to 0)	0 (0 to 0)
Czech Republic	Chagas disease	0 (0 to 0)	0 (0 to 0)	0 (0 to 0)	0 (0 to 0)	0 (0 to 0)	0 (0 to 0)
Czech Republic	Leishmaniasis	0 (0 to 0)	0 (0 to 0)	0 (0 to 0)	0 (0 to 0)	0 (0 to 0)	0 (0 to 0)
Czech Republic	Visceral leishmaniasis	0 (0 to 0)	0 (0 to 0)	0 (0 to 0)	0 (0 to 0)	0 (0 to 0)	0 (0 to 0)
Czech Republic	Cutaneous and mucocutaneous leishmaniasis	0 (0 to 0)	0 (0 to 0)	0 (0 to 0)	0 (0 to 0)	0 (0 to 0)	0 (0 to 0)
Czech Republic	African trypanosomiasis	0 (0 to 0)	0 (0 to 0)	0 (0 to 0)	0 (0 to 0)	0 (0 to 0)	0 (0 to 0)
Czech Republic	Schistosomiasis	0 (0 to 0)	0 (0 to 0)	0 (0 to 0)	0 (0 to 0)	0 (0 to 0)	0 (0 to 0)
Czech Republic	Cysticercosis	3.69 (1.85 to 6.33)	3.37 (1.51 to 6.28)	3.48 (1.52 to 6.73)	30.9 (15.4 to 53.2)	24.4 (10.9 to 45.7)	23.7 (10.3 to 45.3)
Czech Republic	Cystic echinococcosis	0.0651 (0.042 to 0.0903)	0.0362 (0.0246 to 0.0496)	0.0289 (0.0201 to 0.0394)	0.5 (0.4 to 0.8)	0.3 (0.2 to 0.4)	0.2 (0.1 to 0.3)
Czech Republic	Lymphatic filariasis	0 (0 to 0)	0 (0 to 0)	0 (0 to 0)	0 (0 to 0)	0 (0 to 0)	0 (0 to 0)
Czech Republic	Onchocerciasis	0 (0 to 0)	0 (0 to 0)	0 (0 to 0)	0 (0 to 0)	0 (0 to 0)	0 (0 to 0)

SR Appendix Table 2. All-age DALYs counts and age standardized DALYs rates per 100,000 individuals for all causes and level 1 cause groups by location for 1990, 2007, and 2017, both sexes combined

		All-age DALYs			Age-standardized rate of DALYs per 100,000		
Czech Republic	Trachoma	0 (0 to 0)	0 (0 to 0)	0 (0 to 0)	0 (0 to 0)	0 (0 to 0)	0 (0 to 0)
Czech Republic	Dengue	0 (0 to 0)	0 (0 to 0)	0 (0 to 0)	0 (0 to 0)	0 (0 to 0)	0 (0 to 0)
Czech Republic	Yellow fever	0 (0 to 0)	0 (0 to 0)	0 (0 to 0)	0 (0 to 0)	0 (0 to 0)	0 (0 to 0)
Czech Republic	Rabies	0.000785 (0.000592 to 0.00106)	0.000319 (0.000258 to 0.000393)	0.000343 (0.000257 to 0.000463)	0 (0 to 0)	0 (0 to 0)	0 (0 to 0)
Czech Republic	Intestinal nematode infections	0 (0 to 0)	0 (0 to 0)	0 (0 to 0)	0 (0 to 0)	0 (0 to 0)	0 (0 to 0)
Czech Republic	Ascariasis	0 (0 to 0)	0 (0 to 0)	0 (0 to 0)	0 (0 to 0)	0 (0 to 0)	0 (0 to 0)
Czech Republic	Trichuriasis	0 (0 to 0)	0 (0 to 0)	0 (0 to 0)	0 (0 to 0)	0 (0 to 0)	0 (0 to 0)
Czech Republic	Hookworm disease	0 (0 to 0)	0 (0 to 0)	0 (0 to 0)	0 (0 to 0)	0 (0 to 0)	0 (0 to 0)
Czech Republic	Food-borne trematodiases	0 (0 to 0)	0 (0 to 0)	0 (0 to 0)	0 (0 to 0)	0 (0 to 0)	0 (0 to 0)
Czech Republic	Leprosy	0 (0 to 0)	0 (0 to 0)	0 (0 to 0)	0 (0 to 0)	0 (0 to 0)	0 (0 to 0)
Czech Republic	Ebola virus disease	0 (0 to 0)	0 (0 to 0)	0 (0 to 0)	0 (0 to 0)	0 (0 to 0)	0 (0 to 0)
Czech Republic	Zika virus disease	0 (0 to 0)	0 (0 to 0)	0 (0 to 0)	0 (0 to 0)	0 (0 to 0)	0 (0 to 0)
Czech Republic	Guinea worm disease	0 (0 to 0)	0 (0 to 0)	0 (0 to 0)	0 (0 to 0)	0 (0 to 0)	0 (0 to 0)
Czech Republic	Other neglected tropical diseases	0.0128 (0.00901 to 0.0286)	0.0255 (0.00735 to 0.0346)	0.0231 (0.00644 to 0.0338)	0.2 (0.1 to 0.3)	0.3 (0.1 to 0.5)	0.3 (0.1 to 0.4)
Czech Republic	Other infectious diseases	10.3 (9.28 to 11.6)	6.51 (5.74 to 7.36)	5.95 (5.08 to 6.88)	113.9 (102.3 to 130)	65.3 (58.4 to 73.2)	55.7 (47.4 to 64.7)
Czech Republic	Meningitis	5.8 (5.26 to 6.63)	2.64 (2.42 to 2.86)	1.93 (1.71 to 2.22)	68.8 (61.2 to 81.2)	28.7 (26.2 to 31.5)	19.3 (16.7 to 22.8)
Czech Republic	Pneumococcal meningitis	1.23 (1.09 to 1.4)	0.643 (0.569 to 0.732)	0.521 (0.442 to 0.62)	12.9 (11.4 to 14.9)	6.1 (5.4 to 7)	4.6 (3.9 to 5.6)
Czech Republic	H influenzae type B meningitis	1.7 (1.53 to 1.98)	0.657 (0.601 to 0.719)	0.424 (0.372 to 0.509)	21.9 (19.2 to 26.5)	8.3 (7.4 to 9.3)	5 (4.2 to 6.2)
Czech Republic	Meningococcal infection	1.55 (1.39 to 1.78)	0.638 (0.585 to 0.692)	0.449 (0.4 to 0.521)	18.9 (16.5 to 22.5)	7.1 (6.4 to 7.9)	4.5 (3.9 to 5.5)
Czech Republic	Other meningitis	1.32 (1.19 to 1.5)	0.699 (0.624 to 0.776)	0.537 (0.462 to 0.633)	15.2 (13.5 to 17.9)	7.2 (6.5 to 8)	5.2 (4.4 to 6.2)
Czech Republic	Encephalitis	0.742 (0.572 to 1.21)	1.23 (0.95 to 1.39)	1.16 (0.906 to 1.33)	7.3 (5.6 to 12.8)	11.9 (9.6 to 13.5)	10.6 (8.7 to 12.1)
Czech Republic	Diphtheria	0.00102 (0.000776 to 0.00139)	0.000531 (0.000483 to 0.000601)	0.000453 (0.000405 to 0.000519)	0 (0 to 0)	0 (0 to 0)	0 (0 to 0)
Czech Republic	Whooping cough	0.0298 (0.0151 to 0.066)	0.0211 (0.00724 to 0.0347)	0.0259 (0.0129 to 0.0436)	0.5 (0.2 to 1)	0.4 (0.1 to 0.6)	0.5 (0.2 to 0.8)
Czech Republic	Tetanus	0.0084 (0.00642 to 0.0103)	0.000993 (0.000729 to 0.00122)	0.000802 (0.000573 to 0.0011)	0.1 (0 to 0.1)	0 (0 to 0)	0 (0 to 0)
Czech Republic	Measles	0.00974 (0.00673 to 0.0129)	0.00301 (0.00205 to 0.00334)	0.0036 (0.00231 to 0.00428)	0.1 (0.1 to 0.2)	0 (0 to 0)	0 (0 to 0)
Czech Republic	Varicella and herpes zoster	0.356 (0.233 to 0.516)	0.358 (0.227 to 0.534)	0.392 (0.246 to 0.576)	3.3 (2.2 to 4.8)	2.8 (1.8 to 4.2)	2.8 (1.8 to 4.1)
Czech Republic	Acute hepatitis	1.37 (1.14 to 1.62)	0.95 (0.772 to 1.16)	0.891 (0.709 to 1.13)	12 (9.9 to 14.4)	8 (6.3 to 10.1)	7.4 (5.7 to 9.6)
Czech Republic	Acute hepatitis A	0.438 (0.329 to 0.574)	0.347 (0.251 to 0.463)	0.313 (0.227 to 0.421)	4.4 (3.3 to 5.8)	3.6 (2.5 to 4.9)	3.4 (2.4 to 4.7)
Czech Republic	Acute hepatitis B	0.79 (0.647 to 0.92)	0.504 (0.41 to 0.608)	0.488 (0.388 to 0.608)	6.3 (5.2 to 7.4)	3.6 (2.9 to 4.4)	3.3 (2.6 to 4.1)
Czech Republic	Acute hepatitis C	0.0199 (0.0107 to 0.0351)	0.012 (0.00648 to 0.0224)	0.0103 (0.00543 to 0.0188)	0.2 (0.1 to 0.4)	0.1 (0.1 to 0.2)	0.1 (0.1 to 0.2)
Czech Republic	Acute hepatitis E	0.122 (0.0919 to 0.157)	0.086 (0.0642 to 0.114)	0.0795 (0.0596 to 0.105)	1 (0.8 to 1.3)	0.7 (0.5 to 0.9)	0.6 (0.5 to 0.8)
Czech Republic	Other unspecified infectious diseases	1.96 (1.53 to 2.4)	1.31 (1.1 to 1.62)	1.55 (1.07 to 1.9)	21.9 (16.5 to 26.1)	13.4 (11.2 to 16.6)	15 (10 to 18.8)
Czech Republic	Maternal and neonatal disorders	92.8 (82 to 105)	46.9 (38.1 to 57.3)	40.5 (31.5 to 52.1)	1346 (1213.8 to 1488.4)	641.7 (544 to 756.9)	535.6 (429.9 to 669.2)
Czech Republic	Maternal disorders	1.43 (1.26 to 1.66)	0.594 (0.528 to 0.668)	0.441 (0.38 to 0.504)	14.5 (12.6 to 17.1)	5.6 (5 to 6.3)	4.7 (4 to 5.4)
Czech Republic	Maternal haemorrhage	0.336 (0.193 to 0.555)	0.0963 (0.0608 to 0.142)	0.0746 (0.0471 to 0.113)	3.6 (2.1 to 6)	0.9 (0.6 to 1.3)	0.8 (0.5 to 1.2)
Czech Republic	Maternal sepsis and other pregnancy related infections	0.0755 (0.0482 to 0.115)	0.0373 (0.0234 to 0.0566)	0.0312 (0.0185 to 0.0478)	0.8 (0.5 to 1.1)	0.4 (0.2 to 0.5)	0.3 (0.2 to 0.5)
Czech Republic	Maternal hypertensive disorders	0.108 (0.066 to 0.169)	0.0599 (0.0401 to 0.0862)	0.0501 (0.0329 to 0.0735)	1.1 (0.7 to 1.8)	0.6 (0.4 to 0.8)	0.6 (0.4 to 0.9)
Czech Republic	Maternal obstructed labour and uterine rupture	0.0302 (0.0182 to 0.0459)	0.0315 (0.0199 to 0.0475)	0.0223 (0.014 to 0.0341)	0.3 (0.2 to 0.5)	0.3 (0.2 to 0.4)	0.2 (0.1 to 0.3)
Czech Republic	Maternal abortive outcome	0.229 (0.14 to 0.345)	0.0771 (0.0441 to 0.116)	0.0583 (0.0316 to 0.0895)	2.1 (1.3 to 3.2)	0.7 (0.4 to 1.1)	0.6 (0.3 to 0.8)
Czech Republic	Ectopic pregnancy	0.0521 (0.0243 to 0.1)	0.00736 (0.00331 to 0.0149)	0.00351 (0.0016 to 0.00733)	0.5 (0.2 to 1)	0.1 (0 to 0.1)	0 (0 to 0.1)

SR Appendix Table 2. All-age DALYs counts and age standardized DALYs rates per 100,000 individuals for all causes and level 1 cause groups by location for 1990, 2007, and 2017, both sexes combined

		All-age DALYs			Age-standardized rate of DALYs per 100,000		
Czech Republic	Indirect maternal deaths	0.209<br>(0.156 to 0.271)	0.096<br>(0.0729 to 0.122)	0.0475<br>(0.0336 to 0.0634)	2.2<br>(1.6 to 2.9)	0.9<br>(0.7 to 1.2)	0.5<br>(0.4 to 0.7)
Czech Republic	Late maternal deaths	0.0372<br>(0.0278 to 0.0496)	0.0268<br>(0.02 to 0.0348)	0.0213<br>(0.0152 to 0.0291)	0.4<br>(0.3 to 0.5)	0.3<br>(0.2 to 0.3)	0.2<br>(0.2 to 0.3)
Czech Republic	Maternal deaths aggravated by HIV/AIDS	0.00000358<br>(0.00000183 to 0.00000593)	0.00000906<br>(0.00000443 to 0.0000146)	0.00000919<br>(0.00000446 to 0.0000151)	0<br>(0 to 0)	0<br>(0 to 0)	0<br>(0 to 0)
Czech Republic	Other maternal disorders	0.348<br>(0.247 to 0.458)	0.162<br>(0.116 to 0.212)	0.133<br>(0.098 to 0.17)	3.4<br>(2.4 to 4.6)	1.5<br>(1.1 to 2)	1.4<br>(1 to 1.8)
Czech Republic	Neonatal disorders	91.4<br>(80.7 to 103)	46.3<br>(37.6 to 56.8)	40<br>(31.1 to 51.6)	1331.6<br>(1199.9 to 1474.8)	636.1<br>(538.5 to 751.4)	530.9<br>(425.1 to 664.3)
Czech Republic	Neonatal preterm birth	52.3<br>(46.9 to 58.1)	16.6<br>(13.6 to 20.1)	15<br>(11.1 to 20.2)	788.5<br>(716.5 to 869.1)	222.6<br>(187.9 to 262.4)	201.2<br>(154.4 to 264.6)
Czech Republic	Neonatal encephalopathy due to birth asphyxia and trauma	24.6<br>(20.3 to 31.5)	14.9<br>(10.5 to 21.4)	13.2<br>(8.65 to 20)	350.8<br>(300.5 to 424.7)	194.1<br>(146.5 to 263.9)	163.9<br>(114 to 235.7)
Czech Republic	Neonatal sepsis and other neonatal infections	8.26<br>(4.53 to 14.5)	8.09<br>(4.77 to 13.2)	7.85<br>(4.38 to 13.2)	89.7<br>(51.2 to 152.7)	96.5<br>(61.5 to 148.5)	89.6<br>(55 to 144.4)
Czech Republic	Hemolytic disease and other neonatal jaundice	0.693<br>(0.575 to 0.825)	0.339<br>(0.264 to 0.428)	0.307<br>(0.236 to 0.393)	9.7<br>(8.1 to 11.5)	4.1<br>(3.3 to 5.1)	3.7<br>(2.9 to 4.6)
Czech Republic	Other neonatal disorders	5.63<br>(4.87 to 6.6)	6.38<br>(5.58 to 7.17)	3.7<br>(2.86 to 4.69)	92.9<br>(80.3 to 108.9)	118.8<br>(103.8 to 133.4)	72.5<br>(55.9 to 92.2)
Czech Republic	Nutritional deficiencies	19.5<br>(12.4 to 30.7)	9.86<br>(6.08 to 15.4)	9.98<br>(6.49 to 15.3)	211.3<br>(133.9 to 334.2)	105.9<br>(64.8 to 168.5)	97.5<br>(61.9 to 151.5)
Czech Republic	Protein-energy malnutrition	0.304<br>(0.223 to 0.419)	0.405<br>(0.262 to 0.545)	1.12<br>(0.557 to 1.44)	3.6<br>(2.5 to 5.1)	3.3<br>(2.2 to 5.4)	7.1<br>(3.7 to 10)
Czech Republic	Iodine deficiency	1.36<br>(0.692 to 2.27)	0.948<br>(0.452 to 1.78)	0.953<br>(0.462 to 1.81)	12.4<br>(6.3 to 20.8)	8<br>(3.8 to 15)	7.8<br>(3.8 to 14.9)
Czech Republic	Dietary iron deficiency	11.2<br>(7.04 to 18.1)	6.62<br>(3.88 to 10.7)	6.05<br>(3.53 to 10)	106.9<br>(66.8 to 172.8)	59<br>(34.4 to 95)	54.1<br>(30.8 to 91.5)
Czech Republic	Other nutritional deficiencies	0.194<br>(0.121 to 0.247)	0.159<br>(0.106 to 0.298)	0.351<br>(0.167 to 0.896)	1.8<br>(1.2 to 2.2)	1.3<br>(0.9 to 2.2)	2.3<br>(1.3 to 5.3)
Czech Republic	Non-communicable diseases	3270<br>(3010 to 3570)	2780<br>(2500 to 3110)	2820<br>(2510 to 3190)	25547.6<br>(23397.1 to 28163.9)	18617<br>(16470.6 to 21169.5)	16824.1<br>(14691.2 to 19476.9)
Czech Republic	Neoplasms	672<br>(661 to 682)	626<br>(614 to 637)	593<br>(561 to 628)	5014.5<br>(4931.8 to 5088.8)	3817.9<br>(3748.4 to 3880.2)	3135<br>(2966.8 to 3322.2)
Czech Republic	Lip and oral cavity cancer	10.3<br>(9.84 to 10.7)	10.9<br>(10.5 to 11.4)	10.4<br>(9.49 to 11.4)	79.1<br>(75.6 to 82.6)	68.3<br>(65.7 to 71.1)	59.2<br>(53.9 to 64.9)
Czech Republic	Nasopharynx cancer	1.53<br>(1.43 to 1.63)	1.25<br>(1.18 to 1.33)	1.07<br>(0.961 to 1.18)	12<br>(11.2 to 12.8)	8.2<br>(7.7 to 8.7)	6.5<br>(5.9 to 7.3)
Czech Republic	Other pharynx cancer	4.95<br>(4.6 to 5.26)	8.09<br>(7.72 to 8.47)	7.84<br>(7.02 to 8.69)	38.1<br>(35.4 to 40.5)	50.3<br>(48 to 52.7)	45<br>(40.1 to 49.9)
Czech Republic	Oesophageal cancer	10.4<br>(9.97 to 10.7)	13.1<br>(12.6 to 13.6)	13.5<br>(12.4 to 14.8)	77.4<br>(74.4 to 80.3)	78.9<br>(75.7 to 82)	72.8<br>(66.6 to 79.8)
Czech Republic	Stomach cancer	51.4<br>(49.9 to 52.9)	28.2<br>(27.5 to 28.9)	24<br>(22.4 to 25.8)	371.3<br>(360.4 to 382)	169.2<br>(164.7 to 173.6)	124.2<br>(115.5 to 133.1)
Czech Republic	Colon and rectum cancer	96.8<br>(93.9 to 99.3)	93.1<br>(90.9 to 95.6)	82.3<br>(76.7 to 88.3)	695.6<br>(674.3 to 712.9)	543.2<br>(530.4 to 556.8)	408.8<br>(381.5 to 438.5)
Czech Republic	Liver cancer	18.2<br>(17.4 to 18.9)	18.3<br>(17.7 to 18.9)	17.8<br>(16.3 to 19.3)	131.3<br>(125.9 to 136.5)	108.8<br>(105.3 to 112.3)	89.6<br>(82.6 to 96.9)
Czech Republic	Liver cancer due to hepatitis B	3.74<br>(3 to 4.54)	3.65<br>(2.91 to 4.47)	3.01<br>(2.34 to 3.78)	28.3<br>(23 to 34.1)	23.1<br>(18.7 to 27.8)	16.8<br>(13.2 to 20.4)
Czech Republic	Liver cancer due to hepatitis C	4.64<br>(3.61 to 5.9)	4.71<br>(3.65 to 6.03)	4.79<br>(3.69 to 6.12)	32.7<br>(25.5 to 41.4)	27.1<br>(21.1 to 34.5)	23.2<br>(18.1 to 29.7)
Czech Republic	Liver cancer due to alcohol use	7.49<br>(6.63 to 8.39)	7.6<br>(6.77 to 8.55)	7.64<br>(6.62 to 8.81)	53.5<br>(47.4 to 59.8)	44.7<br>(40 to 49.9)	37.7<br>(32.7 to 43.2)
Czech Republic	Liver cancer due to NASH	1.28<br>(1.09 to 1.48)	1.35<br>(1.17 to 1.57)	1.41<br>(1.17 to 1.69)	9.1<br>(7.8 to 10.5)	7.8<br>(6.8 to 9.1)	6.9<br>(5.8 to 8.2)
Czech Republic	Liver cancer due to other causes	1.02<br>(0.883 to 1.17)	0.977<br>(0.845 to 1.12)	0.919<br>(0.774 to 1.09)	7.7<br>(6.7 to 8.8)	6.1<br>(5.3 to 7)	5<br>(4.3 to 5.9)
Czech Republic	Gallbladder and biliary tract cancer	22.6<br>(20.4 to 23.5)	17.5<br>(16.5 to 18.2)	16.2<br>(14.8 to 17.6)	159.6<br>(144.7 to 165.9)	101.5<br>(95.9 to 105.5)	78.8<br>(72 to 86.2)
Czech Republic	Pancreatic cancer	35.5<br>(34.3 to 36.5)	41.2<br>(40.2 to 42.2)	44.7<br>(41.6 to 47.7)	256.1<br>(248 to 263.6)	242.2<br>(236.5 to 247.8)	224.5<br>(209.2 to 239.7)
Czech Republic	Larynx cancer	9.67<br>(9.24 to 10.1)	7.18<br>(6.87 to 7.52)	6.26<br>(5.67 to 6.89)	73.1<br>(69.6 to 76.4)	43.5<br>(41.6 to 45.5)	34.1<br>(30.8 to 37.6)
Czech Republic	Tracheal, bronchus, and lung cancer	157<br>(153 to 162)	130<br>(127 to 134)	120<br>(112 to 129)	1157<br>(1126.7 to 1186.3)	760<br>(741.9 to 778.2)	597.7<br>(558.1 to 640.6)
Czech Republic	Malignant skin melanoma	8.59<br>(6.2 to 10.8)	9.26<br>(6.82 to 11.9)	10.1<br>(7.18 to 12.5)	66.6<br>(48.1 to 84.1)	60.5<br>(45.8 to 79.5)	59.1<br>(43.2 to 75.9)
Czech Republic	Non-melanoma skin cancer	2.88<br>(2.72 to 3.04)	2.99<br>(2.85 to 3.13)	3.14<br>(2.88 to 3.42)	21.7<br>(20.6 to 22.9)	18<br>(17.2 to 18.8)	15.7<br>(14.4 to 17.1)
Czech Republic	Non-melanoma skin cancer (squamous-cell carcinoma)	2.87<br>(2.71 to 3.04)	2.99<br>(2.85 to 3.12)	3.13<br>(2.87 to 3.41)	21.7<br>(20.5 to 22.8)	18<br>(17.1 to 18.8)	15.6<br>(14.3 to 17.1)
Czech Republic	Non-melanoma skin cancer (basal-cell carcinoma)	0.00375<br>(0.00154 to 0.00807)	0.0075<br>(0.00312 to 0.016)	0.00969<br>(0.00416 to 0.02)	0<br>(0 to 0.1)	0<br>(0 to 0.1)	0<br>(0 to 0.1)
Czech Republic	Breast cancer	44.9<br>(43.4 to 46.6)	43.6<br>(41.9 to 45.6)	38<br>(34.5 to 41.7)	337.3<br>(325.7 to 350.2)	268.2<br>(257.6 to 280.4)	207.5<br>(188 to 227.7)
Czech Republic	Cervical cancer	18.8<br>(18.1 to 19.6)	13.8<br>(13.3 to 14.4)	12.1<br>(10.9 to 13.4)	145<br>(139.1 to 151.3)	90.8<br>(87 to 94.6)	72.1<br>(64.6 to 79.7)
Czech Republic	Uterine cancer	9.65<br>(9.18 to 10.1)	8.13<br>(7.68 to 8.6)	8.06<br>(7.19 to 9.02)	69.1<br>(65.9 to 72.7)	47.6<br>(44.9 to 50.3)	40.5<br>(36.1 to 45.4)

SR Appendix Table 2. All-age DALYs counts and age standardized DALYs rates per 100,000 individuals for all causes and level 1 cause groups by location for 1990, 2007, and 2017, both sexes combined

		All-age DALYs			Age-standardized rate of DALYs per 100,000		
Czech Republic	Ovarian cancer	17.7 (17 to 18.4)	18.5 (17.7 to 19.2)	17.2 (15.5 to 19)	132.9 (127.9 to 138.5)	113.3 (108.9 to 117.9)	94.1 (84.6 to 104.4)
Czech Republic	Prostate cancer	18.1 (13.6 to 22.1)	25.4 (16.3 to 27.9)	26.9 (18.8 to 31.1)	122.7 (92.6 to 150)	141.2 (90.4 to 154.6)	122 (85.4 to 140.2)
Czech Republic	Testicular cancer	2.87 (2.59 to 3.15)	1.82 (1.63 to 2.04)	1.52 (1.3 to 1.76)	27.5 (24.8 to 30.2)	15.9 (14.1 to 17.9)	13.8 (11.6 to 16.1)
Czech Republic	Kidney cancer	13.9 (12.9 to 14.7)	25.6 (21.1 to 27)	23.4 (19.7 to 25.5)	103.9 (97.2 to 109.4)	153.1 (126.6 to 161.3)	120 (102.1 to 130.6)
Czech Republic	Bladder cancer	13.6 (13 to 14.3)	14.4 (13.8 to 15)	15.2 (13.9 to 16.5)	95.6 (91.6 to 100.2)	81.8 (78.5 to 85.2)	72 (65.9 to 78.2)
Czech Republic	Thyroid cancer	2.79 (2.62 to 2.98)	2.12 (1.96 to 2.31)	1.87 (1.66 to 2.12)	20.7 (19.3 to 22)	13.2 (12.1 to 14.4)	10.2 (9 to 11.5)
Czech Republic	Mesothelioma	1.06 (0.883 to 1.26)	1.13 (1.07 to 1.2)	1.13 (1.02 to 1.25)	8 (6.7 to 9.4)	7.1 (6.7 to 7.5)	6.3 (5.7 to 7)
Czech Republic	Hodgkin lymphoma	5.58 (4.33 to 6.19)	3.04 (2.71 to 4.09)	2.4 (2 to 3.58)	47.8 (37.5 to 53.1)	23.3 (20.5 to 32.2)	17.7 (14.3 to 27.6)
Czech Republic	Non-Hodgkin's lymphoma	13.2 (12.7 to 13.8)	13 (12.6 to 13.4)	13.3 (12.3 to 14.3)	103.5 (99.7 to 108.2)	84.9 (82.2 to 87.7)	75 (69.7 to 81.1)
Czech Republic	Multiple myeloma	6.58 (5.54 to 7.47)	7.57 (6.84 to 8.99)	8.25 (7.18 to 10)	46.8 (39.5 to 53.4)	44.3 (40.3 to 53.2)	41.2 (35.8 to 51.1)
Czech Republic	Leukaemia	27.1 (26.2 to 28.1)	23.2 (22.5 to 23.9)	21.8 (20 to 23.5)	229.6 (220.8 to 239)	166.1 (160.5 to 171.9)	132.7 (121.7 to 143.6)
Czech Republic	Acute lymphoid leukaemia	6.28 (5.88 to 6.68)	2.5 (2.34 to 2.66)	1.73 (1.55 to 1.96)	60.3 (56.1 to 64.6)	23.7 (21.9 to 26)	15.2 (13.4 to 17.6)
Czech Republic	Chronic lymphoid leukaemia	2.72 (2.51 to 2.95)	4.53 (4.26 to 4.8)	5.08 (4.48 to 5.63)	19.1 (17.6 to 20.8)	26 (24.5 to 27.6)	24.2 (21.4 to 26.8)
Czech Republic	Acute myeloid leukaemia	7.16 (6.67 to 7.7)	7.36 (6.95 to 7.72)	7.87 (7.11 to 8.63)	59.3 (55.1 to 64.1)	52.8 (49.8 to 55.6)	49.5 (44.5 to 54.6)
Czech Republic	Chronic myeloid leukaemia	2.25 (2.07 to 2.43)	1.65 (1.53 to 1.77)	1.24 (1.11 to 1.37)	18.1 (16.6 to 19.5)	11.3 (10.4 to 12.1)	7.7 (6.9 to 8.6)
Czech Republic	Other leukaemia	8.65 (8.14 to 9.11)	7.2 (6.85 to 7.58)	5.86 (5.33 to 6.48)	72.8 (68.4 to 77)	52.3 (49.3 to 55.3)	36 (32.6 to 40.2)
Czech Republic	Other malignant cancers	25 (17.8 to 27.1)	19 (17.9 to 20.3)	20.1 (18 to 22.1)	204.2 (147.6 to 221.5)	134.2 (127.4 to 146.8)	129.5 (114.3 to 147.2)
Czech Republic	Other neoplasms	2.37 (1.64 to 3.83)	3.88 (2.52 to 4.64)	4.75 (2.85 to 5.96)	18.5 (12.9 to 29.7)	25.4 (16.8 to 31)	26.5 (16.2 to 33.1)
Czech Republic	Myelodysplastic, myeloproliferative, and other hematopoietic neoplasms	2.22 (1.53 to 3.57)	3.74 (2.42 to 4.46)	4.62 (2.76 to 5.81)	16.8 (11.6 to 26.8)	23.7 (15.3 to 28.6)	24.9 (14.9 to 31)
Czech Republic	Benign and in situ intestinal neoplasms	0 (0 to 0)	0 (0 to 0)	0 (0 to 0)	0 (0 to 0)	0 (0 to 0)	0 (0 to 0)
Czech Republic	Benign and in situ cervical and uterine neoplasms	0 (0 to 0)	0 (0 to 0)	0 (0 to 0)	0 (0 to 0)	0 (0 to 0)	0 (0 to 0)
Czech Republic	Other benign and in situ neoplasms	0.15 (0.104 to 0.243)	0.138 (0.0984 to 0.187)	0.127 (0.0856 to 0.179)	1.7 (1.2 to 2.7)	1.7 (1.2 to 2.4)	1.6 (1.1 to 2.3)
Czech Republic	Cardiovascular diseases	1280 (1250 to 1320)	822 (791 to 854)	777 (726 to 826)	9320.6 (9094.4 to 9552.2)	4866.1 (4684.7 to 5062.3)	3802.5 (3544.7 to 4048.3)
Czech Republic	Rheumatic heart disease	17.6 (16.9 to 18.4)	4.98 (4.74 to 5.21)	4.99 (4.58 to 5.46)	132.8 (127.4 to 138.6)	30.7 (29.3 to 32.2)	26.1 (23.9 to 28.6)
Czech Republic	Ischaemic heart disease	755 (739 to 770)	468 (457 to 479)	434 (409 to 461)	5465.1 (5347.1 to 5576.9)	2747.1 (2686.2 to 2810.8)	2088.4 (1966.3 to 2221.6)
Czech Republic	Stroke	393 (375 to 409)	214 (202 to 226)	187 (169 to 202)	2830.5 (2708.5 to 2949.6)	1260.8 (1186.7 to 1331.4)	905.3 (817.7 to 983.9)
Czech Republic	Ischaemic stroke	233 (221 to 247)	139 (129 to 149)	126 (113 to 138)	1673 (1583 to 1771.1)	808.2 (748.8 to 866.6)	592.4 (527.5 to 652.6)
Czech Republic	Intracerebral hemorrhage	131 (125 to 137)	57.1 (54.5 to 59.8)	45 (41.6 to 48.3)	936 (895.8 to 976.5)	337.6 (321.6 to 353.3)	225.4 (207.6 to 242.6)
Czech Republic	Subarachnoid hemorrhage	29 (26.8 to 30.9)	17.8 (16.5 to 21.5)	15.3 (13.7 to 19.9)	221.4 (206.3 to 235.7)	115 (106.4 to 137.3)	87.6 (77.9 to 112.6)
Czech Republic	Hypertensive heart disease	10.4 (8 to 23.3)	17.5 (15.7 to 22.4)	20.6 (17 to 25.2)	75.4 (58.4 to 169.3)	103.7 (92.9 to 130.3)	101 (82.5 to 120.9)
Czech Republic	Non-rheumatic valvular heart disease	3.87 (3.12 to 6.31)	7.84 (5.92 to 8.91)	11.5 (6.71 to 13.4)	28.8 (23.4 to 46.2)	47 (35.4 to 53.1)	56.9 (33.1 to 65.7)
Czech Republic	Non-rheumatic calcific aortic valve disease	1.42 (0.951 to 2.96)	4.91 (3.15 to 5.68)	7.44 (3.79 to 8.86)	10.3 (6.9 to 21.6)	29 (18.6 to 33.4)	35.9 (18.4 to 42.7)
Czech Republic	Non-rheumatic degenerative mitral valve disease	2.39 (1.82 to 3.15)	2.72 (2.28 to 3.55)	3.8 (2.66 to 5.58)	18 (13.7 to 23.1)	16.5 (13.9 to 21.4)	19.1 (13.3 to 27.9)
Czech Republic	Other non-rheumatic valve diseases	0.0569 (0.0171 to 0.153)	0.211 (0.103 to 0.282)	0.306 (0.11 to 0.457)	0.5 (0.1 to 1.2)	1.5 (0.7 to 2)	1.8 (0.6 to 2.7)
Czech Republic	Cardiomyopathy and myocarditis	18.6 (17.5 to 20.3)	15.8 (14.6 to 18.8)	20.2 (18.4 to 22.4)	158.3 (147.7 to 168.4)	110 (102.4 to 126.6)	120.8 (108.5 to 132.7)
Czech Republic	Myocarditis	2.03 (1.38 to 3.84)	3.1 (2.44 to 3.49)	3.57 (2.71 to 4.2)	18.5 (12.8 to 34.5)	23.4 (18.5 to 26.5)	23.1 (17.5 to 27.1)
Czech Republic	Alcoholic cardiomyopathy	0.754 (0.536 to 1.69)	3.03 (1.62 to 3.63)	3.43 (1.48 to 4.4)	6 (4.3 to 13.3)	20.4 (10.7 to 24.7)	21.5 (9.3 to 27.8)
Czech Republic	Other cardiomyopathy	15.8 (13.1 to 17.1)	9.63 (8.59 to 13.9)	13.2 (11.9 to 15.7)	133.8 (106 to 145.1)	66.1 (59.6 to 90.6)	76.2 (68.8 to 87.5)
Czech Republic	Atrial fibrillation and flutter	12.8 (10.6 to 15.4)	16.1 (13.3 to 19.3)	19.6 (16.2 to 23.5)	92.4 (77.5 to 110.9)	92.7 (76.7 to 111.1)	91.1 (74.9 to 109.7)
Czech Republic	Aortic aneurysm	6.53 (6.15 to 6.9)	9.66 (9.26 to 10.1)	10.5 (9.69 to 11.3)	48.1 (45.3 to 50.8)	58.2 (55.9 to 60.7)	53.9 (49.9 to 58.4)

SR Appendix Table 2. All-age DALYs counts and age standardized DALYs rates per 100,000 individuals for all causes and level 1 cause groups by location for 1990, 2007, and 2017, both sexes combined

		All-age DALYs			Age-standardized rate of DALYs per 100,000		
Czech Republic	Peripheral vascular disease	2.07 (1.04 to 3.85)	4.25 (2.32 to 7.22)	4.72 (2.85 to 7.49)	14.6 (7.4 to 27.1)	24.3 (13.2 to 41.6)	21.9 (13.2 to 34.8)
Czech Republic	Endocarditis	1.92 (1.67 to 2.46)	3.2 (2.62 to 3.54)	3.7 (2.97 to 4.22)	15.3 (13.4 to 19.7)	21.4 (17.6 to 23.6)	21.8 (17.8 to 24.9)
Czech Republic	Other cardiovascular and circulatory diseases	62.6 (55 to 71.6)	60.6 (52 to 70.5)	59.9 (49.8 to 72.4)	459.2 (403.6 to 525.6)	370.2 (317.1 to 431.1)	315.2 (261.2 to 380.6)
Czech Republic	Chronic respiratory diseases	126 (114 to 138)	112 (100 to 124)	138 (123 to 152)	954.7 (857.6 to 1049.9)	717.8 (634.2 to 799.2)	751 (665.4 to 839.4)
Czech Republic	Chronic obstructive pulmonary disease	95.4 (86.6 to 104)	87.5 (79.1 to 95.9)	110 (98.9 to 122)	694.5 (629.8 to 754.1)	521.2 (470.3 to 571.1)	549.7 (492.6 to 608)
Czech Republic	Pneumoconiosis	3.71 (3.4 to 4.03)	1.26 (1.12 to 1.4)	1.02 (0.875 to 1.17)	25.8 (23.7 to 28)	7.1 (6.4 to 7.9)	4.8 (4.2 to 5.5)
Czech Republic	Silicosis	0.376 (0.315 to 0.446)	0.198 (0.164 to 0.235)	0.194 (0.152 to 0.241)	2.6 (2.2 to 3.1)	1.1 (0.9 to 1.3)	0.9 (0.7 to 1.1)
Czech Republic	Asbestosis	0.0157 (0.0108 to 0.0295)	0.0186 (0.0132 to 0.0282)	0.0229 (0.0165 to 0.0306)	0.1 (0.1 to 0.2)	0.1 (0.1 to 0.2)	0.1 (0.1 to 0.1)
Czech Republic	Coal workers pneumoconiosis	3.28 (2.99 to 3.59)	0.961 (0.853 to 1.08)	0.699 (0.591 to 0.821)	22.8 (20.8 to 24.9)	5.4 (4.8 to 6.1)	3.3 (2.8 to 3.8)
Czech Republic	Other pneumoconiosis	0.0353 (0.022 to 0.0642)	0.0788 (0.0547 to 0.0996)	0.101 (0.0599 to 0.133)	0.3 (0.2 to 0.5)	0.5 (0.3 to 0.6)	0.5 (0.3 to 0.6)
Czech Republic	Asthma	21.2 (16.4 to 27.1)	15 (10.9 to 20)	16.1 (11.4 to 22.1)	186.1 (141.3 to 240.2)	130.6 (93.4 to 177.4)	131.1 (91.8 to 178.1)
Czech Republic	Interstitial lung disease and pulmonary sarcoidosis	3.68 (2.82 to 4.78)	5.95 (4.28 to 7.43)	7.69 (4.84 to 10)	28.1 (21.6 to 36.6)	37.9 (27.6 to 47.3)	42.2 (27 to 54.9)
Czech Republic	Other chronic respiratory diseases	2.29 (1.98 to 2.58)	2.52 (2.15 to 2.89)	2.82 (2.36 to 3.3)	20.2 (17.4 to 22.8)	21 (17.8 to 24.5)	23.2 (19.3 to 27.2)
Czech Republic	Digestive diseases	154 (143 to 168)	149 (137 to 164)	143 (128 to 160)	1234.8 (1131 to 1358.2)	1040.8 (938.6 to 1162.3)	914.1 (803.6 to 1047.3)
Czech Republic	Cirrhosis and other chronic liver diseases	67.6 (64.8 to 71.4)	71.3 (68.8 to 74.1)	64.5 (59.5 to 69.9)	529.5 (507.2 to 559)	468.8 (452.1 to 488.1)	393 (361.1 to 428)
Czech Republic	Cirrhosis and other chronic liver diseases due to hepatitis B	17.4 (15.6 to 19.7)	17.6 (15.5 to 19.8)	13.8 (12 to 15.8)	137 (122.1 to 154.7)	116.2 (102.9 to 129.3)	84.6 (72.9 to 97.3)
Czech Republic	Cirrhosis and other chronic liver diseases due to hepatitis C	13.1 (11.4 to 14.9)	13.9 (12.1 to 15.9)	12.9 (11 to 14.8)	102.5 (89.2 to 117.2)	91.1 (79.8 to 104)	78.4 (67 to 90.7)
Czech Republic	Cirrhosis and other chronic liver diseases due to alcohol use	29.3 (27.2 to 31.6)	31.5 (29.2 to 33.7)	29.6 (26.6 to 32.8)	226.5 (209.9 to 244.3)	203.7 (189.4 to 218)	177.1 (158.9 to 196.1)
Czech Republic	Cirrhosis due to NASH	3.85 (3.43 to 4.29)	4.56 (4.09 to 5.07)	4.52 (4 to 5.11)	29.8 (26.5 to 33.3)	29.3 (26.3 to 32.7)	27.1 (23.8 to 30.7)
Czech Republic	Cirrhosis and other chronic liver diseases due to other causes	3.9 (3.36 to 4.55)	3.73 (3.11 to 4.47)	3.67 (3.04 to 4.38)	33.8 (29.5 to 38.8)	28.5 (24.5 to 33.3)	25.9 (21.8 to 30.3)
Czech Republic	Upper digestive system diseases	35 (27.4 to 45.2)	32.3 (24.5 to 43.3)	31 (22.8 to 42.2)	290.1 (223.8 to 384.4)	245 (181.3 to 335.6)	221.6 (157.8 to 309.4)
Czech Republic	Peptic ulcer disease	15.4 (14.5 to 16.6)	12.2 (11.5 to 13)	10.3 (9.28 to 11.3)	115.4 (108.4 to 124)	75.5 (70.8 to 80.4)	55.4 (50.1 to 61.1)
Czech Republic	Gastritis and duodenitis	10.3 (6.77 to 14.8)	9.44 (6.29 to 13.5)	9.64 (6.39 to 13.8)	96.6 (63.2 to 139.5)	91.2 (59.5 to 132)	88.1 (57.1 to 127.6)
Czech Republic	GERD	9.3 (4.97 to 15.5)	10.6 (5.64 to 17.7)	11.1 (5.9 to 18.6)	78.1 (41.6 to 131.4)	78.3 (41.8 to 132.5)	78.2 (41.8 to 132.2)
Czech Republic	Appendicitis	1.75 (1.61 to 1.91)	0.857 (0.76 to 0.96)	0.719 (0.627 to 0.828)	15.2 (13.7 to 16.6)	7 (6 to 8.3)	5.6 (4.6 to 7)
Czech Republic	Paralytic ileus and intestinal obstruction	4.72 (3.98 to 5.63)	4.9 (3.88 to 5.55)	5.18 (4.11 to 6.04)	40 (34.5 to 47.2)	33.3 (26.8 to 37.3)	29.7 (23.6 to 34.5)
Czech Republic	Inguinal, femoral, and abdominal hernia	9.72 (7.42 to 12.6)	8.8 (6.27 to 11.8)	10.1 (7.2 to 13.4)	84.2 (63.2 to 110.1)	76.9 (53.7 to 105.4)	79.9 (55.1 to 108.3)
Czech Republic	Inflammatory bowel disease	4.87 (3.82 to 6.09)	6.19 (4.7 to 7.81)	5.67 (4.16 to 7.08)	40.4 (31.5 to 50.9)	45.1 (34 to 57.3)	37.7 (27.8 to 47.4)
Czech Republic	Vascular intestinal disorders	4.31 (3.99 to 4.83)	3.78 (3.55 to 4.36)	3.55 (3.12 to 4.63)	30.7 (28.4 to 34.3)	22 (20.7 to 25.3)	17.2 (15.1 to 22.2)
Czech Republic	Gallbladder and biliary diseases	8.5 (7.05 to 9.14)	4.11 (3.86 to 4.58)	5.04 (4.54 to 5.52)	61.6 (51.1 to 66.1)	24.4 (23 to 27)	24.4 (22.1 to 26.7)
Czech Republic	Pancreatitis	12.7 (11.7 to 14.1)	11.3 (10.1 to 12.9)	10.1 (8.75 to 12)	100.4 (92.3 to 110.7)	75.8 (67.6 to 86.3)	61 (52.8 to 72.5)
Czech Republic	Other digestive diseases	4.95 (4.3 to 5.68)	5.94 (5.32 to 6.6)	7.04 (6.24 to 7.95)	42.8 (36.1 to 50.3)	42.5 (36.9 to 48.9)	44 (37.8 to 50.9)
Czech Republic	Neurological disorders	183 (147 to 230)	199 (160 to 250)	220 (181 to 270)	1569.5 (1246.2 to 2002.2)	1476 (1156.9 to 1903)	1453.7 (1131.8 to 1874.1)
Czech Republic	Alzheimer's disease and other dementias	58.9 (54.8 to 63.1)	71.6 (66.9 to 76.7)	89.3 (82.1 to 96.9)	459.5 (428.9 to 491)	426.2 (398.5 to 455.1)	403.3 (370.9 to 437.1)
Czech Republic	Parkinson's disease	10.6 (8.88 to 11.7)	12.6 (9.7 to 14)	15 (11.2 to 17)	72.9 (61.3 to 80.3)	69.4 (53.6 to 77.2)	67.1 (50.1 to 75.8)
Czech Republic	Epilepsy	19.6 (9.91 to 34.2)	16.7 (9.19 to 28.2)	17.4 (8.33 to 31.6)	187.5 (94.9 to 328.8)	154.4 (84.6 to 258.6)	155 (72.8 to 285.5)
Czech Republic	Multiple sclerosis	5.65 (3.93 to 6.58)	4.33 (3.75 to 5.05)	4.34 (3.6 to 5.17)	45.6 (32 to 52.9)	30.5 (26.1 to 36.2)	28.8 (23.6 to 35.3)
Czech Republic	Motor neuron disease	1.81 (1.67 to 1.99)	1.85 (1.71 to 1.95)	2.35 (2.13 to 2.59)	16.9 (15.2 to 19)	14.1 (13 to 15.1)	15.6 (14 to 17.5)
Czech Republic	Headache disorders	81.2 (52.5 to 119)	86.3 (56.2 to 127)	85.4 (55.4 to 126)	731.1 (472.7 to 1068.7)	729.9 (467.7 to 1073.2)	727.2 (467.7 to 1060.4)
Czech Republic	Migraine	69.5 (42.9 to 105)	73.7 (45.5 to 111)	72.7 (45.2 to 111)	626.7 (385.9 to 944.5)	625.7 (383.9 to 942.5)	623.3 (385.9 to 937.4)

SR Appendix Table 2. All-age DALYs counts and age standardized DALYs rates per 100,000 individuals for all causes and level 1 cause groups by location for 1990, 2007, and 2017, both sexes combined

		All-age DALYs			Age-standardized rate of DALYs per 100,000		
Czech Republic	Tension-type headache	11.8 (6.67 to 18.3)	12.6 (7.23 to 19.7)	12.7 (7.31 to 20)	104.3 (58.9 to 162.5)	104.2 (59 to 163.8)	103.9 (59.3 to 164.2)
Czech Republic	Other neurological disorders	5.57 (4.18 to 7.63)	5.33 (4.22 to 6.99)	6.07 (4.59 to 8.35)	56 (41.4 to 77.5)	51.5 (39.2 to 69.3)	56.7 (39.7 to 83)
Czech Republic	Mental disorders	157 (117 to 203)	161 (120 to 207)	161 (120 to 206)	1413.9 (1048.3 to 1826)	1379.7 (1025 to 1781.8)	1364.9 (1013.8 to 1758.1)
Czech Republic	Schizophrenia	14.3 (10.5 to 17.9)	16.1 (11.9 to 20)	16.5 (12.5 to 20.5)	123.3 (90.2 to 154.6)	124.5 (91.9 to 156.1)	124.9 (93.8 to 156.9)
Czech Republic	Depressive disorders	55.2 (38.7 to 74.6)	55.6 (39.4 to 74.7)	55.9 (39.4 to 74.9)	470.7 (330.5 to 637.6)	429.8 (303.1 to 582)	416 (293.2 to 559.6)
Czech Republic	Major depressive disorder	45.1 (31.3 to 60.7)	44.4 (30.7 to 59.6)	44.2 (30.6 to 59.6)	385.6 (269.5 to 519.8)	344.8 (241.2 to 466.5)	331.5 (230.9 to 452.1)
Czech Republic	Dysthymia	10.1 (6.79 to 14.6)	11.3 (7.57 to 16.5)	11.7 (7.94 to 16.9)	85.1 (56.7 to 123.7)	84.9 (57.1 to 125.2)	84.5 (57.2 to 122.9)
Czech Republic	Bipolar disorder	15.2 (9.56 to 22.8)	16.2 (10.3 to 24.3)	15.9 (9.93 to 23.7)	137.9 (86.2 to 205.1)	137.9 (86.9 to 206.9)	137.6 (85.6 to 204.4)
Czech Republic	Anxiety disorders	34.1 (23.9 to 45.5)	35.6 (25.1 to 47.2)	35.6 (25.3 to 47.3)	309.1 (217.3 to 413.2)	309.4 (217.5 to 413)	307.2 (217.4 to 407.3)
Czech Republic	Eating disorders	4.28 (2.72 to 6.29)	4.62 (2.91 to 6.8)	4.46 (2.78 to 6.57)	42.4 (26.8 to 62.4)	45.5 (28.8 to 66.9)	48.4 (30 to 70.9)
Czech Republic	Anorexia nervosa	0.826 (0.484 to 1.26)	0.855 (0.514 to 1.32)	0.78 (0.478 to 1.19)	8.4 (4.9 to 13.1)	9.2 (5.5 to 14)	9.5 (5.7 to 14.7)
Czech Republic	Bulimia nervosa	3.45 (2.17 to 5.14)	3.76 (2.33 to 5.56)	3.68 (2.23 to 5.52)	33.9 (21 to 50.4)	36.4 (22.7 to 54.1)	38.9 (23.6 to 57.7)
Czech Republic	Autism spectrum disorders	6.68 (4.54 to 9.2)	6.6 (4.46 to 9.09)	6.67 (4.56 to 9.05)	67.3 (45.7 to 93.1)	67.9 (46 to 93.9)	68 (46.1 to 92.7)
Czech Republic	Attention-deficit/hyperactivity disorder	1.12 (0.669 to 1.77)	0.99 (0.581 to 1.57)	0.939 (0.563 to 1.49)	11.8 (7 to 18.7)	11.8 (7 to 18.9)	11.8 (7.1 to 18.7)
Czech Republic	Conduct disorder	9.08 (5.46 to 14.4)	6.18 (3.73 to 9.82)	5.69 (3.41 to 8.99)	97.9 (59.1 to 154.7)	98.1 (59.1 to 155.8)	98 (58.8 to 154.8)
Czech Republic	Idiopathic developmental intellectual disability	1.47 (0.351 to 2.88)	1.5 (0.442 to 2.93)	1.28 (0.323 to 2.59)	15 (3.7 to 29.7)	15.9 (4.8 to 30.9)	13.8 (3.6 to 27.8)
Czech Republic	Other mental disorders	15.9 (10.6 to 22)	17.9 (11.9 to 24.7)	18.3 (12.2 to 25.2)	138.5 (91.5 to 191.6)	139 (92.7 to 192.5)	139.3 (92.3 to 192.5)
Czech Republic	Substance use disorders	42.2 (32.9 to 52.5)	56.8 (45.9 to 69)	57.4 (46.8 to 69.7)	385.8 (299.5 to 481)	471.8 (377 to 579.7)	495.4 (395.1 to 614.1)
Czech Republic	Alcohol use disorders	29.5 (23.2 to 37)	40.2 (32.8 to 49.5)	38.6 (31.2 to 47.5)	262.1 (204.5 to 329.9)	318.7 (256.6 to 395.1)	303.4 (239.9 to 378.7)
Czech Republic	Drug use disorders	12.6 (9.32 to 16.4)	16.6 (13 to 20.6)	18.8 (14.7 to 23.2)	123.7 (91 to 162.4)	153.1 (117.8 to 192.1)	192 (146.3 to 243.2)
Czech Republic	Opioid use disorders	6.15 (4.56 to 7.87)	7.48 (6 to 8.92)	8.05 (6.46 to 9.69)	57.9 (42.8 to 74.6)	63.9 (51.4 to 76.8)	71 (57.1 to 86.3)
Czech Republic	Cocaine use disorders	0.483 (0.299 to 0.727)	0.559 (0.357 to 0.828)	0.539 (0.351 to 0.784)	4.6 (2.7 to 6.9)	5 (3.1 to 7.5)	5.1 (3.2 to 7.5)
Czech Republic	Amphetamine use disorders	2.35 (1.3 to 3.84)	3.2 (2.13 to 4.63)	4.11 (2.71 to 5.86)	24.7 (13.3 to 40.6)	33 (21.9 to 47.4)	51.3 (33.7 to 74.6)
Czech Republic	Cannabis use disorders	1.09 (0.682 to 1.61)	1.34 (0.848 to 1.94)	0.964 (0.613 to 1.4)	11 (6.8 to 16.4)	13.8 (8.7 to 20.2)	11.3 (7.1 to 16.7)
Czech Republic	Other drug use disorders	2.57 (1.72 to 3.78)	4.04 (3.04 to 5.19)	5.13 (3.97 to 6.54)	25.5 (16.7 to 38.1)	37.4 (27.4 to 49.2)	53.2 (39.5 to 70.8)
Czech Republic	Diabetes and kidney diseases	126 (107 to 152)	132 (107 to 162)	179 (144 to 221)	948.5 (797.7 to 1139.9)	828.7 (669.8 to 1023.3)	978 (773.5 to 1221.2)
Czech Republic	Diabetes mellitus	92.9 (75.1 to 116)	102 (79.8 to 130)	149 (115 to 188)	690.1 (554.2 to 864)	639.1 (495.4 to 818.5)	812.5 (620.1 to 1041.9)
Czech Republic	Diabetes mellitus type 1	12.6 (11.3 to 17.2)	17.1 (14 to 18.4)	16.3 (12.4 to 18.2)	95.5 (86 to 127.9)	106.9 (88.6 to 115.8)	90.9 (72 to 102.1)
Czech Republic	Diabetes mellitus type 2	80.3 (62.2 to 103)	84.9 (62.9 to 112)	133 (99.5 to 171)	594.6 (456.5 to 764.8)	532.2 (389.9 to 706.7)	721.6 (534 to 945.7)
Czech Republic	Chronic kidney disease	33.2 (30.6 to 36)	29.7 (27.2 to 32.5)	30.5 (27.4 to 33.8)	255.4 (236.1 to 276.5)	189.3 (173.1 to 206.8)	165.1 (147.6 to 183.4)
Czech Republic	Chronic kidney disease due to diabetes mellitus type 1	2.22 (1.77 to 2.77)	1.71 (1.33 to 2.17)	1.53 (1.19 to 1.93)	17.5 (13.9 to 21.7)	11.5 (9.1 to 14.2)	9.4 (7.4 to 11.9)
Czech Republic	Chronic kidney disease due to diabetes mellitus type 2	6.28 (5.21 to 7.5)	6.13 (5.13 to 7.35)	6.53 (5.38 to 7.85)	44.6 (37.3 to 52.7)	35.4 (29.8 to 42.3)	31.4 (26.2 to 37.7)
Czech Republic	Chronic kidney disease due to hypertension	5.13 (4.31 to 5.96)	5.19 (4.36 to 6.09)	5.69 (4.78 to 6.69)	37.9 (32 to 44.1)	31.4 (26.5 to 36.5)	28.5 (23.9 to 33.5)
Czech Republic	Chronic kidney disease due to glomerulonephritis	6.87 (5.92 to 7.86)	5.82 (4.96 to 6.73)	5.59 (4.69 to 6.57)	54.8 (47.7 to 62.3)	39.1 (33.6 to 44.8)	33.3 (28.2 to 38.7)
Czech Republic	Chronic kidney disease due to other and unspecified causes	12.7 (10.9 to 14.7)	10.9 (9.29 to 12.5)	11.2 (9.41 to 13)	100.6 (87.7 to 115.7)	71.9 (62.1 to 82.1)	62.4 (52.8 to 72.3)
Czech Republic	Acute glomerulonephritis	0.367 (0.326 to 0.41)	0.041 (0.0356 to 0.0479)	0.0603 (0.0523 to 0.07)	3 (2.7 to 3.3)	0.3 (0.2 to 0.3)	0.4 (0.3 to 0.4)
Czech Republic	Skin and subcutaneous diseases	50.8 (34.2 to 73.8)	52.2 (35.4 to 76.6)	54.9 (37.6 to 79.2)	502 (336.4 to 732.9)	520.4 (346.7 to 754)	524.7 (353.8 to 768)
Czech Republic	Dermatitis	12.9 (7.52 to 20.2)	12 (7.08 to 18.8)	12.4 (7.36 to 19.5)	136.6 (79 to 215.3)	136.7 (76.8 to 218.2)	136.6 (78.1 to 215.7)
Czech Republic	Atopic dermatitis	10.3 (5.5 to 17.3)	9.24 (4.95 to 15.4)	9.62 (5.2 to 16)	113.5 (59.7 to 189.8)	113.7 (59.8 to 190.1)	113.6 (60.1 to 187.8)
Czech Republic	Contact dermatitis	2.58 (1.7 to 3.77)	2.74 (1.77 to 4.03)	2.79 (1.84 to 4.11)	23 (15.1 to 34)	23 (14.8 to 34.1)	22.9 (15 to 33.9)

SR Appendix Table 2. All-age DALYs counts and age standardized DALYs rates per 100,000 individuals for all causes and level 1 cause groups by location for 1990, 2007, and 2017, both sexes combined

		All-age DALYs			Age-standardized rate of DALYs per 100,000		
Czech Republic	Seborrhoeic dermatitis	0.0113 (0.00628 to 0.018)	0.0115 (0.0064 to 0.0184)	0.012 (0.00666 to 0.0192)	0.1 (0.1 to 0.2)	0.1 (0.1 to 0.2)	0.1 (0.1 to 0.2)
Czech Republic	Psoriasis	8.85 (6.23 to 11.6)	10.5 (7.45 to 13.9)	10.7 (7.56 to 14.1)	76.7 (53.5 to 100.7)	83.7 (59.3 to 110.4)	81.6 (57.2 to 108.4)
Czech Republic	Bacterial skin diseases	0.686 (0.48 to 0.916)	0.927 (0.533 to 1.19)	1.6 (0.527 to 2.33)	5.9 (4.4 to 8)	6.8 (4.4 to 9)	9.8 (4.1 to 13.7)
Czech Republic	Cellulitis	0.383 (0.263 to 0.506)	0.447 (0.285 to 0.595)	0.706 (0.287 to 1.02)	3.3 (2.3 to 4.4)	3.5 (2.4 to 4.6)	4.7 (2.3 to 6.6)
Czech Republic	Pyoderma	0.302 (0.186 to 0.445)	0.481 (0.203 to 0.654)	0.898 (0.195 to 1.38)	2.6 (1.7 to 4)	3.4 (1.7 to 4.8)	5.1 (1.5 to 7.7)
Czech Republic	Scabies	2.53 (1.4 to 4.05)	2.39 (1.32 to 3.85)	2.33 (1.28 to 3.79)	26.3 (14.4 to 42.5)	26.4 (14.7 to 42.5)	26.3 (14.6 to 42.4)
Czech Republic	Fungal skin diseases	5.08 (2.04 to 10.4)	5.52 (2.21 to 11.4)	6.11 (2.45 to 12.6)	44.8 (17.8 to 92.2)	43.7 (17.3 to 90.4)	43.4 (17.2 to 89.9)
Czech Republic	Viral skin diseases	3.8 (2.43 to 5.63)	3.03 (1.96 to 4.49)	3.2 (2.05 to 4.7)	45.8 (29.1 to 67.9)	45.8 (29.3 to 68)	45.8 (29.2 to 67.4)
Czech Republic	Acne vulgaris	3.18 (1.88 to 5.24)	3.68 (2.19 to 6.05)	3.17 (1.88 to 5.15)	32.7 (19.3 to 54.2)	42.5 (25.1 to 69.4)	44.4 (26.2 to 73.2)
Czech Republic	Alopecia areata	0.632 (0.401 to 0.952)	0.674 (0.424 to 1.02)	0.665 (0.43 to 0.996)	5.9 (3.7 to 8.9)	5.9 (3.7 to 8.9)	5.9 (3.7 to 8.9)
Czech Republic	Pruritus	1.1 (0.521 to 2.03)	1.2 (0.58 to 2.24)	1.28 (0.61 to 2.44)	9.7 (4.6 to 18.1)	9.8 (4.7 to 18.4)	9.9 (4.7 to 18.8)
Czech Republic	Urticaria	6.93 (4.64 to 9.84)	6.46 (4.28 to 9.21)	6.6 (4.42 to 9.29)	74.7 (49.2 to 105.5)	74.8 (48.7 to 106.7)	74.6 (49.6 to 106.7)
Czech Republic	Decubitus ulcer	0.522 (0.385 to 0.695)	0.661 (0.476 to 0.873)	1.01 (0.607 to 1.42)	4.1 (3.1 to 5.5)	4.3 (3.2 to 5.6)	5.5 (3.4 to 7.6)
Czech Republic	Other skin and subcutaneous diseases	4.53 (2.22 to 8.12)	5.19 (2.55 to 9.4)	5.79 (2.81 to 10.6)	38.6 (18.8 to 69.6)	39.8 (19.4 to 72.4)	40.9 (19.9 to 74.9)
Czech Republic	Sense organ diseases	88.4 (59.6 to 125)	102 (68.5 to 145)	115 (77.3 to 162)	700 (471.2 to 1000.8)	684.2 (460.7 to 983.2)	675 (451.3 to 968.4)
Czech Republic	Blindness and vision impairment	33.7 (22.1 to 48.9)	37.5 (24.8 to 54)	41.9 (28.2 to 60.1)	268.2 (175.1 to 391.4)	255.9 (168.4 to 371.9)	249.8 (163.8 to 363.4)
Czech Republic	Glaucoma	0.494 (0.33 to 0.685)	0.555 (0.366 to 0.788)	0.641 (0.418 to 0.905)	3.4 (2.3 to 4.8)	3.1 (2.1 to 4.4)	3 (1.9 to 4.2)
Czech Republic	Cataract	13 (8.76 to 17.7)	15.6 (10.4 to 21.4)	18.7 (12.6 to 25.6)	94.1 (63.6 to 127.4)	90.7 (61 to 124.4)	88.6 (59.8 to 121.5)
Czech Republic	Age-related macular degeneration	0.122 (0.0764 to 0.178)	0.157 (0.0983 to 0.232)	0.189 (0.12 to 0.27)	0.9 (0.6 to 1.3)	0.9 (0.6 to 1.3)	0.9 (0.6 to 1.3)
Czech Republic	Refraction disorders	11.4 (7.2 to 17.3)	11.6 (7.39 to 17.6)	12.1 (7.66 to 18.1)	102.2 (64.9 to 155.8)	98.3 (62.3 to 149.8)	96.2 (60.4 to 146.3)
Czech Republic	Near vision loss	5.74 (2.75 to 10.4)	6.18 (3.01 to 11.2)	6.68 (3.24 to 12.1)	44.6 (21.4 to 80.3)	40.7 (19.9 to 73.7)	39.7 (19.1 to 71.6)
Czech Republic	Other vision loss	2.94 (1.95 to 4.02)	3.32 (2.23 to 4.6)	3.69 (2.47 to 5.14)	23 (15.4 to 31.5)	22.1 (14.8 to 30.7)	21.6 (14.3 to 30.3)
Czech Republic	Age-related and other hearing loss	50.8 (34.5 to 71.7)	59.5 (40.4 to 84.1)	67.7 (46.1 to 95.6)	400.6 (273.5 to 565)	396.7 (269 to 560.2)	393.5 (266.1 to 557.6)
Czech Republic	Other sense organ diseases	3.9 (2.43 to 5.64)	4.59 (2.86 to 6.69)	5.19 (3.24 to 7.35)	31.1 (19.3 to 45.2)	31.7 (19.7 to 46.1)	31.7 (19.6 to 45.6)
Czech Republic	Musculoskeletal disorders	222 (161 to 295)	252 (183 to 338)	267 (194 to 356)	1811.9 (1308.3 to 2412.2)	1799.3 (1300.7 to 2414)	1786.8 (1295.7 to 2392)
Czech Republic	Rheumatoid arthritis	4.08 (2.89 to 5.36)	4.8 (3.35 to 6.38)	5.58 (3.92 to 7.37)	31.6 (22.4 to 41.6)	31.8 (22.2 to 42.3)	33.3 (23.3 to 44.3)
Czech Republic	Osteoarthritis	12.6 (6.3 to 24.7)	15.9 (8.02 to 31.3)	18.7 (9.35 to 36.9)	92.4 (46.2 to 181.5)	95.9 (48.3 to 188.2)	97.2 (48.6 to 192.2)
Czech Republic	Low back pain	165 (118 to 220)	185 (133 to 246)	192 (138 to 254)	1368.9 (977.4 to 1834.7)	1352.9 (967.1 to 1808.9)	1336 (958.9 to 1781.2)
Czech Republic	Neck pain	37 (26.1 to 51.5)	42.5 (29.7 to 59.8)	46.3 (32.4 to 64.4)	295.5 (206.9 to 413)	295.7 (206.8 to 411.9)	294.7 (206.5 to 409.6)
Czech Republic	Gout	1.51 (1.01 to 2.1)	1.87 (1.23 to 2.57)	2.2 (1.49 to 3.03)	11.3 (7.6 to 15.7)	11.7 (7.8 to 16)	12.1 (8.1 to 16.5)
Czech Republic	Other musculoskeletal disorders	1.45 (1.02 to 2.6)	1.55 (1.24 to 2.45)	1.98 (1.59 to 3.03)	12.3 (8.6 to 21.9)	11.4 (8.8 to 18.5)	13.5 (10.7 to 21.6)
Czech Republic	Other non-communicable diseases	160 (131 to 197)	113 (84.5 to 148)	115 (86.4 to 152)	1691.5 (1417.7 to 2027.8)	1014.3 (790.1 to 1306.6)	943.1 (725.3 to 1224.8)
Czech Republic	Congenital anomalies	60.5 (50.4 to 68.7)	29.4 (23.6 to 35.5)	26.3 (20.6 to 32.7)	843.5 (688.9 to 942.6)	376.4 (313.5 to 444.6)	316.5 (253.9 to 386.1)
Czech Republic	Neural tube defects	4.71 (3.81 to 5.91)	0.842 (0.658 to 1.04)	1.17 (0.88 to 1.5)	71 (56.8 to 90.1)	10.6 (8.5 to 13.2)	15.5 (11.3 to 20.8)
Czech Republic	Congenital heart anomalies	20.2 (15.6 to 22.5)	6.32 (5.46 to 8.03)	4.11 (3.21 to 5.25)	309.8 (237.9 to 346.8)	103.8 (89.6 to 133.5)	67.1 (51.2 to 88.3)
Czech Republic	Orofacial clefts	0.532 (0.371 to 0.734)	0.402 (0.258 to 0.571)	0.36 (0.235 to 0.529)	5.9 (4.3 to 8.1)	4.2 (2.7 to 6.1)	3.7 (2.4 to 5.5)
Czech Republic	Down's syndrome	2.39 (1.51 to 3)	1.08 (0.851 to 1.57)	1.48 (1.16 to 1.89)	32.2 (19.3 to 41.2)	13.4 (10.5 to 20.5)	18.9 (14.6 to 26.3)
Czech Republic	Turner syndrome	0.00618 (0.00308 to 0.0101)	0.00645 (0.00323 to 0.0105)	0.00595 (0.00291 to 0.0099)	0.1 (0 to 0.1)	0.1 (0 to 0.1)	0.1 (0 to 0.1)
Czech Republic	Klinefelter syndrome	0.00182 (0.000844 to 0.00355)	0.0019 (0.000878 to 0.00357)	0.00179 (0.000809 to 0.00339)	0 (0 to 0)	0 (0 to 0)	0 (0 to 0)
Czech Republic	Other chromosomal abnormalities	1.51 (0.937 to 1.96)	1.23 (0.791 to 1.58)	1.21 (0.855 to 1.65)	21.8 (12.8 to 28.2)	18.8 (11.3 to 24)	17 (11.6 to 23.9)

SR Appendix Table 2. All-age DALYs counts and age standardized DALYs rates per 100,000 individuals for all causes and level 1 cause groups by location for 1990, 2007, and 2017, both sexes combined

		All-age DALYs			Age-standardized rate of DALYs per 100,000		
Czech Republic	Congenital musculoskeletal and limb anomalies	10.7 (7.55 to 14.3)	9.81 (6.54 to 13.4)	10.1 (6.8 to 14)	110.1 (80.3 to 147.1)	94.6 (63.7 to 129.2)	94.6 (63.6 to 130)
Czech Republic	Urogenital congenital anomalies	1.16 (0.71 to 1.74)	0.697 (0.471 to 0.952)	0.58 (0.377 to 0.831)	17 (9.9 to 25.7)	10.9 (7.2 to 14.9)	9.1 (5.6 to 13.4)
Czech Republic	Digestive congenital anomalies	8.75 (6.17 to 10.9)	4.18 (3.04 to 5.55)	3.46 (2.4 to 4.79)	119.8 (79 to 147)	47.6 (36 to 61.8)	37.6 (26.5 to 51)
Czech Republic	Other congenital anomalies	10.6 (7.24 to 12.5)	4.88 (3.97 to 5.94)	3.83 (2.97 to 4.88)	155.7 (102.5 to 183.8)	72.3 (58.6 to 86.7)	53 (41.2 to 69.4)
Czech Republic	Urinary diseases and male infertility	25.5 (23 to 28.4)	14.9 (11.8 to 18.7)	16.9 (13.4 to 21.3)	189 (170.2 to 211.5)	92.8 (73.3 to 116.7)	91.9 (72.1 to 116.7)
Czech Republic	Urinary tract infections	14.9 (12.7 to 16.3)	5.46 (5.19 to 5.75)	6.34 (5.86 to 6.87)	110 (94.1 to 120.3)	34.3 (32.4 to 36.3)	33.4 (30.8 to 36.2)
Czech Republic	Urolithiasis	3.17 (2.33 to 4.77)	0.994 (0.764 to 1.27)	1.07 (0.815 to 1.37)	23.3 (17.1 to 34.5)	6.8 (5.2 to 8.7)	6.6 (4.9 to 8.5)
Czech Republic	Benign prostatic hyperplasia	5.35 (3.45 to 7.74)	7.36 (4.77 to 10.7)	8.56 (5.46 to 12.4)	38.9 (25.2 to 56.2)	42.8 (27.6 to 62.2)	44.3 (28.2 to 64.5)
Czech Republic	Male infertility	0.299 (0.118 to 0.63)	0.334 (0.13 to 0.698)	0.271 (0.108 to 0.558)	2.9 (1.2 to 6)	3.1 (1.2 to 6.4)	2.6 (1 to 5.4)
Czech Republic	Other urinary diseases	1.72 (1.34 to 2.46)	0.75 (0.611 to 0.923)	0.709 (0.572 to 0.9)	13.9 (11 to 19.4)	5.9 (4.7 to 7.3)	5 (4 to 6.1)
Czech Republic	Gynecological diseases	18.5 (12.8 to 26)	18.7 (13 to 26.4)	18.2 (12.5 to 25.8)	170.3 (118.5 to 236.5)	164.1 (114.3 to 230.7)	163 (113.3 to 228.9)
Czech Republic	Uterine fibroids	3.33 (1.92 to 5.6)	2.96 (1.64 to 5.02)	3.01 (1.64 to 5.21)	27 (15.7 to 45.2)	22.3 (12.4 to 38)	20.8 (11.3 to 36)
Czech Republic	Polycystic ovarian syndrome	0.528 (0.233 to 1.01)	0.532 (0.232 to 1.03)	0.514 (0.228 to 0.984)	5 (2.2 to 9.7)	4.9 (2.1 to 9.5)	5 (2.2 to 9.6)
Czech Republic	Female infertility	0.322 (0.112 to 0.687)	0.323 (0.111 to 0.699)	0.357 (0.129 to 0.781)	3.1 (1.1 to 6.6)	2.9 (1 to 6.4)	3.4 (1.2 to 7.4)
Czech Republic	Endometriosis	5.78 (3.74 to 8.57)	6.1 (4.06 to 8.7)	5.62 (3.7 to 8.05)	56.2 (36.4 to 83.4)	56.4 (37.4 to 80.6)	56 (36.8 to 80.4)
Czech Republic	Genital prolapse	0.637 (0.309 to 1.21)	0.575 (0.279 to 1.07)	0.694 (0.331 to 1.29)	4.7 (2.3 to 9)	3.6 (1.7 to 6.7)	3.8 (1.8 to 7.1)
Czech Republic	Premenstrual syndrome	5.35 (3.45 to 8.02)	5.27 (3.33 to 7.89)	4.97 (3.17 to 7.44)	50.8 (32.7 to 76.3)	48.9 (30.9 to 73.2)	48.2 (30.9 to 71.9)
Czech Republic	Other gynecological diseases	2.59 (1.72 to 3.62)	2.91 (1.97 to 4.11)	3.01 (2.02 to 4.3)	23.5 (15.8 to 32.9)	25.1 (16.9 to 35.1)	25.7 (17.2 to 36.3)
Czech Republic	Hemoglobinopathies and hemolytic anaemias	4.03 (3.14 to 5.43)	2.46 (1.92 to 3.26)	2.38 (1.86 to 3.11)	37.8 (29.2 to 51.1)	20.5 (15.8 to 27.2)	17.9 (13.7 to 24)
Czech Republic	Thalassemias	0.0945 (0.0595 to 0.133)	0.0338 (0.024 to 0.0479)	0.0312 (0.0215 to 0.045)	1.4 (0.9 to 2)	0.6 (0.4 to 0.9)	0.6 (0.4 to 0.8)
Czech Republic	Thalassaemias trait	1.01 (0.629 to 1.59)	0.54 (0.33 to 0.838)	0.476 (0.287 to 0.746)	9.7 (6 to 15.1)	4.8 (2.8 to 7.4)	3.8 (2.2 to 5.9)
Czech Republic	Sickle cell disorders	0.228 (0.172 to 0.278)	0.139 (0.114 to 0.168)	0.141 (0.111 to 0.169)	2.8 (2 to 3.5)	1.8 (1.5 to 2.2)	1.8 (1.4 to 2.1)
Czech Republic	Sickle cell trait	0.452 (0.269 to 0.742)	0.233 (0.134 to 0.37)	0.188 (0.109 to 0.299)	4.6 (2.7 to 7.4)	2.2 (1.3 to 3.5)	1.6 (1 to 2.6)
Czech Republic	G6PD deficiency	0.316 (0.238 to 0.381)	0.263 (0.21 to 0.315)	0.242 (0.181 to 0.294)	3.1 (2.3 to 3.7)	2.2 (1.8 to 2.7)	2 (1.5 to 2.4)
Czech Republic	G6PD trait	0.000482 (0.000298 to 0.000723)	0.000295 (0.000192 to 0.000438)	0.000239 (0.000153 to 0.000366)	0 (0 to 0)	0 (0 to 0)	0 (0 to 0)
Czech Republic	Other hemoglobinopathies and hemolytic anaemias	1.93 (1.57 to 2.46)	1.25 (1.01 to 1.57)	1.31 (1.04 to 1.66)	16.3 (13.1 to 21)	8.9 (7 to 11.5)	8.2 (6.2 to 11.1)
Czech Republic	Endocrine, metabolic, blood, and immune disorders	9.05 (7.33 to 10.6)	6.74 (5.66 to 8.2)	9.1 (6.49 to 10.9)	95.2 (74 to 111.1)	62.7 (53.1 to 75.6)	74 (54.5 to 88.4)
Czech Republic	Oral disorders	39.5 (25.1 to 59.5)	39.4 (24.8 to 59)	41.2 (26.4 to 61.4)	313.4 (197.4 to 471.7)	274 (170.8 to 411.8)	259.1 (162.2 to 393.2)
Czech Republic	Caries of deciduous teeth	0.263 (0.115 to 0.539)	0.183 (0.0801 to 0.37)	0.219 (0.0952 to 0.43)	3.8 (1.7 to 7.9)	3.8 (1.7 to 7.6)	3.8 (1.7 to 7.4)
Czech Republic	Caries of permanent teeth	4.41 (1.94 to 8.42)	4.47 (1.96 to 8.55)	4.25 (1.86 to 8.18)	41.3 (17.9 to 79)	41.3 (18.1 to 78.7)	39.6 (17.2 to 75.9)
Czech Republic	Periodontal disease	7.34 (2.87 to 15.3)	6.97 (2.76 to 14.5)	7.06 (2.81 to 14.4)	58.3 (22.8 to 121.5)	47.3 (18.8 to 97.9)	43.7 (17.5 to 90.1)
Czech Republic	Edentulism and severe tooth loss	21.8 (14.6 to 30.9)	21.7 (14.6 to 30.5)	23.6 (15.8 to 33.3)	159.2 (105.7 to 226.3)	130.9 (87.7 to 184)	121.5 (80.3 to 171.2)
Czech Republic	Other oral disorders	5.63 (3.53 to 8.28)	6.01 (3.74 to 8.82)	6.1 (3.84 to 8.94)	50.7 (31.8 to 74.8)	50.7 (31.5 to 74.7)	50.6 (31.8 to 74.1)
Czech Republic	Sudden infant death syndrome	2.6 (1.89 to 3.58)	1.28 (0.839 to 1.64)	1.07 (0.555 to 1.55)	42.4 (30.9 to 58.4)	23.9 (15.6 to 30.4)	20.6 (10.7 to 29.7)
Czech Republic	Injuries	534 (467 to 616)	464 (389 to 560)	444 (359 to 550)	4761.5 (4196.7 to 5438.5)	3684 (3131.5 to 4395.3)	3288.8 (2701.5 to 4037.1)
Czech Republic	Transport injuries	113 (104 to 125)	97.4 (86.3 to 111)	76.1 (64.8 to 89.4)	1071.9 (987.1 to 1172.2)	846.5 (761.9 to 947.1)	636.3 (553.4 to 732)
Czech Republic	Road injuries	102 (92.6 to 113)	88.3 (77.4 to 101)	69 (58.2 to 81.7)	964 (883.1 to 1056.3)	763.8 (681.9 to 861.6)	570.9 (491.9 to 662.9)
Czech Republic	Pedestrian road injuries	11.7 (10 to 14)	18.4 (16.2 to 21.1)	14.4 (12.1 to 17.1)	108 (93 to 127.4)	156.6 (139.3 to 177.1)	111.5 (94.4 to 131.6)
Czech Republic	Cyclist road injuries	7.75 (5.7 to 10.2)	14 (11.1 to 17.6)	12.5 (9.53 to 16)	67 (49.4 to 87.9)	111.1 (89 to 137.4)	92.2 (70.6 to 117.7)
Czech Republic	Motorcyclist road injuries	6.51 (5.35 to 7.95)	11.4 (9.89 to 13.2)	8.48 (7 to 10.2)	60.4 (50.6 to 72.8)	101.9 (90.4 to 115.5)	75.1 (63 to 87.3)

SR Appendix Table 2. All-age DALYs counts and age standardized DALYs rates per 100,000 individuals for all causes and level 1 cause groups by location for 1990, 2007, and 2017, both sexes combined

		All-age DALYs			Age-standardized rate of DALYs per 100,000		
Czech Republic	Motor vehicle road injuries	74.8 (70.1 to 80.5)	42.9 (38.4 to 48.3)	32.1 (27.6 to 37.4)	718.7 (677.7 to 766.2)	381.6 (345.4 to 422)	281 (246 to 320.5)
Czech Republic	Other road injuries	1.16 (0.845 to 1.6)	1.61 (1.19 to 2.18)	1.49 (1.08 to 2.06)	10 (7.3 to 13.6)	12.7 (9.5 to 16.9)	11.1 (8 to 15.2)
Czech Republic	Other transport injuries	11.3 (10.5 to 12.2)	9.16 (8.52 to 9.8)	7.15 (6.42 to 7.9)	107.8 (99.7 to 116.6)	82.7 (76.8 to 88.6)	65.3 (58.5 to 72.4)
Czech Republic	Unintentional injuries	316 (260 to 388)	286 (224 to 368)	299 (228 to 390)	2748.1 (2279.8 to 3353.2)	2163.9 (1701 to 2776.4)	2076.7 (1592 to 2713.1)
Czech Republic	Falls	200 (156 to 255)	192 (144 to 254)	199 (147 to 266)	1634.4 (1269.9 to 2094.2)	1378.8 (1023.3 to 1832.8)	1317.6 (969 to 1774.6)
Czech Republic	Drowning	15.8 (15 to 16.6)	9.64 (9.22 to 10.1)	6.99 (6.44 to 7.58)	167.6 (158 to 178.1)	93.3 (88.3 to 98.4)	63.5 (58 to 69.7)
Czech Republic	Fire, heat, and hot substances	9.22 (7.5 to 11.5)	8.28 (6.38 to 10.9)	9.23 (6.98 to 12.5)	83.1 (68.4 to 103)	66.4 (51.4 to 87)	68.3 (51.5 to 91.9)
Czech Republic	Poisonings	12.7 (11.6 to 14.1)	6.76 (5.61 to 8.26)	6.35 (5.01 to 8.09)	118.3 (107.8 to 131.5)	60.7 (50.1 to 74.8)	56.2 (44.1 to 72.1)
Czech Republic	Poisoning by carbon monoxide	7.71 (7.29 to 8.14)	3.32 (3.1 to 3.57)	2.58 (2.31 to 2.83)	71.1 (66.9 to 75.2)	29 (27 to 31.2)	21.9 (19.7 to 24.2)
Czech Republic	Poisoning by other means	4.96 (4.01 to 6.17)	3.44 (2.41 to 4.85)	3.78 (2.53 to 5.39)	47.2 (38.2 to 58.6)	31.7 (22.1 to 44.8)	34.3 (22.9 to 49.3)
Czech Republic	Exposure to mechanical forces	28.4 (21.5 to 37.6)	33.3 (24.5 to 44.9)	40.8 (29.3 to 55.9)	249 (190.3 to 326.2)	260.4 (194 to 347.9)	299.6 (216.9 to 411.2)
Czech Republic	Unintentional firearm injuries	1.75 (1.3 to 1.97)	1.81 (1.63 to 2.03)	1.35 (1.17 to 1.66)	16.4 (12.3 to 18.4)	15.3 (13.8 to 17.1)	10.9 (9.5 to 13.3)
Czech Republic	Other exposure to mechanical forces	26.7 (19.9 to 35.9)	31.5 (22.8 to 42.8)	39.4 (28.2 to 54.5)	232.6 (174.7 to 308.8)	245 (179 to 331)	288.6 (205.9 to 398)
Czech Republic	Adverse effects of medical treatment	2.41 (2.15 to 3.25)	3.91 (3.09 to 4.33)	5.86 (3.49 to 6.61)	21 (18.7 to 28.9)	28.6 (23.4 to 31.8)	36.3 (24.2 to 40.7)
Czech Republic	Animal contact	2.06 (1.62 to 2.6)	2.31 (1.73 to 3)	2.16 (1.61 to 2.84)	19 (14.9 to 24)	20 (14.8 to 26.2)	18 (13.2 to 23.7)
Czech Republic	Venomous animal contact	1.37 (1.03 to 1.79)	1.53 (1.1 to 2.05)	1.43 (1.01 to 1.95)	12.5 (9.3 to 16.4)	13.4 (9.5 to 18.1)	12.3 (8.5 to 16.8)
Czech Republic	Non-venomous animal contact	0.693 (0.526 to 0.933)	0.778 (0.579 to 1.08)	0.727 (0.534 to 1.02)	6.5 (5 to 8.6)	6.6 (4.9 to 8.9)	5.7 (4.2 to 7.9)
Czech Republic	Foreign body	17.6 (16.2 to 19)	12.7 (12 to 13.4)	9.35 (8.55 to 10.2)	205.6 (187.7 to 226.8)	120.4 (113.1 to 128.6)	81.6 (73.8 to 90)
Czech Republic	Pulmonary aspiration and foreign body in airway	16 (14.8 to 17.3)	10.9 (10.4 to 11.4)	7.58 (7.01 to 8.18)	191 (174.5 to 212.8)	105.2 (99.6 to 111.9)	67.3 (61.3 to 74)
Czech Republic	Foreign body in eyes	0.195 (0.0947 to 0.33)	0.217 (0.11 to 0.371)	0.241 (0.125 to 0.412)	1.8 (0.8 to 3.1)	1.9 (0.9 to 3.3)	2 (0.9 to 3.5)
Czech Republic	Foreign body in other body part	1.4 (1.13 to 1.75)	1.57 (1.26 to 1.93)	1.53 (1.19 to 1.91)	12.7 (10.3 to 15.8)	13.3 (10.6 to 16.3)	12.3 (9.6 to 15.5)
Czech Republic	Environmental heat and cold exposure	5 (4.25 to 5.84)	6.85 (5.98 to 7.83)	6.92 (5.9 to 8.08)	42.8 (36.1 to 50.7)	50.7 (44 to 58.3)	48.1 (40.6 to 56.6)
Czech Republic	Exposure to forces of nature	0 (0 to 0)	0.0707 (0.0695 to 0.0724)	0.00761 (0.00587 to 0.00988)	0 (0 to 0)	0.8 (0.8 to 0.8)	0.1 (0 to 0.1)
Czech Republic	Other unintentional injuries	22.9 (20.3 to 26.5)	10.5 (8.15 to 13.6)	11.9 (8.68 to 16.3)	207.3 (184.7 to 238.6)	83.9 (65.9 to 108.1)	87.5 (64.6 to 118.5)
Czech Republic	Self-harm and interpersonal violence	104 (101 to 107)	80.5 (77.7 to 83.5)	69 (64 to 74.6)	941.6 (910.8 to 969.1)	673.5 (649.5 to 698.2)	575.9 (535 to 621.1)
Czech Republic	Self-harm	87.7 (84.8 to 90.2)	66 (64.1 to 68)	55.6 (51.7 to 60)	777.7 (752.4 to 801.3)	545.2 (528.9 to 562.2)	463 (429.5 to 500.8)
Czech Republic	Self-harm by firearm	5.31 (3.71 to 8.18)	6.45 (4.2 to 8.95)	5.36 (3.29 to 7.82)	47.9 (33.3 to 73.7)	52.4 (34.1 to 73.4)	41.9 (25.9 to 62.3)
Czech Republic	Self-harm by other specified means	82.4 (78.5 to 85.1)	59.5 (56.6 to 62.4)	50.3 (46.3 to 54.6)	729.8 (694.6 to 754.6)	492.7 (468.2 to 517.3)	421.1 (387.1 to 457.2)
Czech Republic	Interpersonal violence	16.7 (15.2 to 18.3)	14.4 (12.6 to 16.5)	13.3 (11.1 to 16)	163.1 (149.2 to 178.4)	127.6 (113 to 144.2)	112.2 (95 to 132.7)
Czech Republic	Assault by firearm	1.91 (1.69 to 2.16)	1.86 (1.63 to 2.1)	1.08 (0.94 to 1.24)	18.6 (16.3 to 21.2)	17 (14.9 to 19.3)	10.2 (8.8 to 12)
Czech Republic	Assault by sharp object	4.42 (3.99 to 4.81)	2.93 (2.67 to 3.25)	2.36 (2.06 to 2.69)	42.1 (38.2 to 45.8)	25.9 (23.7 to 29)	20.5 (17.9 to 23.4)
Czech Republic	Sexual violence	1.44 (0.962 to 2.09)	1.45 (0.986 to 2.09)	1.31 (0.871 to 1.9)	13.6 (9.1 to 19.9)	13.4 (9 to 19.7)	12.4 (8.3 to 18.1)
Czech Republic	Assault by other means	8.9 (7.78 to 10.3)	8.19 (6.71 to 9.88)	8.59 (6.64 to 10.9)	88.8 (78.2 to 100.7)	71.2 (59.3 to 85)	69.1 (54.5 to 86.3)
Czech Republic	Conflict and terrorism	0.000773 (0.000473 to 0.00141)	0.00307 (0.00206 to 0.005)	0.00594 (0.00403 to 0.00955)	0 (0 to 0)	0 (0 to 0)	0 (0 to 0.1)
Czech Republic	Executions and police conflict	0.0775 (0.0719 to 0.0849)	0.0711 (0.0657 to 0.0786)	0.066 (0.0596 to 0.0743)	0.8 (0.7 to 0.9)	0.7 (0.7 to 0.8)	0.7 (0.6 to 0.8)
Hungary	All causes	4720 (4360 to 5140)	4070 (3710 to 4480)	3600 (3230 to 4010)	37271.7 (34319.9 to 40668.9)	29007.5 (26239.2 to 32220.2)	24252.5 (21337.8 to 27313.4)
Hungary	Communicable, maternal, neonatal, and nutritional disorders	273 (245 to 302)	145 (124 to 167)	125 (104 to 148)	3505.3 (3144.4 to 3847.4)	1823.3 (1586.3 to 2085.4)	1566.1 (1290.6 to 1847.1)
Hungary	HIV/AIDS and sexually transmitted infections	9.25 (8.28 to 10.9)	5.55 (4.65 to 7.02)	3.61 (2.82 to 4.99)	78.4 (69 to 93.1)	46.6 (38.2 to 60.7)	32 (24.1 to 46.4)
Hungary	HIV/AIDS	7.01 (6.67 to 7.45)	3.46 (3.29 to 3.66)	1.66 (1.57 to 1.77)	57.5 (54.7 to 61.1)	27.2 (25.9 to 28.7)	13.3 (12.6 to 14.1)
Hungary	HIV/AIDS - Drug-susceptible Tuberculosis	1.23 (0.762 to 1.88)	0.185 (0.114 to 0.286)	0.0745 (0.0456 to 0.113)	10.1 (6.3 to 15.4)	1.5 (0.9 to 2.3)	0.6 (0.4 to 0.9)

SR Appendix Table 2. All-age DALYs counts and age standardized DALYs rates per 100,000 individuals for all causes and level 1 cause groups by location for 1990, 2007, and 2017, both sexes combined

		All-age DALYs			Age-standardized rate of DALYs per 100,000		
Hungary	HIV/AIDS - Multidrug-resistant Tuberculosis without extensive drug resistance	0.00949 (0.00131 to 0.0346)	0.0183 (0.00883 to 0.0351)	0.00276 (0.000618 to 0.00781)	0.1 (0 to 0.3)	0.1 (0.1 to 0.3)	0 (0 to 0.1)
Hungary	HIV/AIDS - Extensively drug-resistant Tuberculosis	0 (0 to 0)	0.00575 (0.00274 to 0.0112)	0.00157 (0.000344 to 0.00453)	0 (0 to 0)	0 (0 to 0.1)	0 (0 to 0)
Hungary	HIV/AIDS resulting in other diseases	5.77 (5.03 to 6.38)	3.26 (3.04 to 3.47)	1.58 (1.48 to 1.69)	47.3 (41.3 to 52.3)	25.6 (24 to 27.3)	12.6 (11.9 to 13.5)
Hungary	Sexually transmitted infections excluding HIV	2.24 (1.37 to 3.76)	2.09 (1.28 to 3.54)	1.95 (1.19 to 3.3)	20.9 (12.5 to 35.1)	19.4 (11.4 to 33.3)	18.8 (10.9 to 32.7)
Hungary	Syphilis	0.309 (0.233 to 0.405)	0.199 (0.151 to 0.259)	0.195 (0.146 to 0.251)	3 (2 to 4.6)	1.7 (1.3 to 2.5)	1.6 (1.2 to 2.1)
Hungary	Chlamydial infection	0.415 (0.236 to 0.742)	0.41 (0.236 to 0.737)	0.382 (0.22 to 0.703)	3.9 (2.2 to 7.2)	4 (2.3 to 7.3)	3.9 (2.2 to 7.3)
Hungary	Gonococcal infection	0.419 (0.264 to 0.71)	0.398 (0.265 to 0.65)	0.351 (0.237 to 0.586)	4.1 (2.4 to 7.2)	3.9 (2.4 to 6.7)	3.7 (2.2 to 6.5)
Hungary	Trichomoniasis	0.337 (0.134 to 0.702)	0.339 (0.136 to 0.711)	0.323 (0.131 to 0.682)	3 (1.2 to 6.4)	3 (1.2 to 6.4)	3 (1.2 to 6.4)
Hungary	Genital herpes	0.216 (0.0681 to 0.503)	0.224 (0.0715 to 0.529)	0.225 (0.0732 to 0.531)	1.7 (0.6 to 4.1)	1.7 (0.6 to 4.1)	1.7 (0.6 to 4.1)
Hungary	Other sexually transmitted infections	0.544 (0.34 to 0.912)	0.516 (0.329 to 0.834)	0.476 (0.305 to 0.78)	5.1 (3.1 to 8.7)	5 (3.1 to 8.4)	4.9 (3 to 8.3)
Hungary	Respiratory infections and tuberculosis	63 (58.7 to 68.9)	32.9 (29.2 to 37.9)	28.1 (24.5 to 33.1)	643.1 (593.5 to 707.8)	306.3 (265.1 to 361.4)	252.6 (211.4 to 308.9)
Hungary	Tuberculosis	12.9 (12.3 to 13.6)	3.08 (2.88 to 3.32)	1.54 (1.36 to 1.72)	89.5 (84.8 to 94)	19.9 (18.5 to 21.6)	9.4 (8.1 to 10.5)
Hungary	Latent tuberculosis infection	0 (0 to 0)	0 (0 to 0)	0 (0 to 0)	0 (0 to 0)	0 (0 to 0)	0 (0 to 0)
Hungary	Drug-susceptible tuberculosis	12.9 (12.2 to 13.5)	2.8 (2.52 to 3.06)	1.47 (1.28 to 1.67)	88.9 (84 to 93.5)	18.1 (16.3 to 20)	9 (7.7 to 10.2)
Hungary	Multidrug-resistant tuberculosis without extensive drug resistance	0.0811 (0.0119 to 0.316)	0.217 (0.118 to 0.374)	0.0418 (0.0101 to 0.113)	0.6 (0.1 to 2.2)	1.4 (0.8 to 2.4)	0.2 (0.1 to 0.7)
Hungary	Extensively drug-resistant tuberculosis	0 (0 to 0)	0.0669 (0.0359 to 0.116)	0.0228 (0.00552 to 0.0624)	0 (0 to 0)	0.4 (0.2 to 0.7)	0.1 (0 to 0.4)
Hungary	Lower respiratory infections	38.3 (36.6 to 40.1)	21 (20.2 to 21.9)	18.3 (17 to 19.6)	423 (395.8 to 450.7)	190.1 (178.7 to 202.2)	148.8 (134.5 to 166.1)
Hungary	Upper respiratory infections	8.11 (5.22 to 12.2)	6.87 (4.13 to 10.8)	6.45 (3.86 to 10.1)	84.7 (55.3 to 127.1)	73.6 (44.5 to 113.6)	72.1 (42.9 to 114.1)
Hungary	Otitis media	3.59 (2.59 to 4.92)	1.93 (1.16 to 3.1)	1.76 (1.08 to 2.8)	45.9 (33.9 to 60.9)	22.7 (13.7 to 36.4)	22.3 (13.5 to 35.6)
Hungary	Enteric infections	15.6 (10.9 to 21.8)	13.6 (9.74 to 18.4)	17.6 (13.4 to 22.9)	185.9 (127.8 to 264.8)	164.1 (115 to 226.8)	192.5 (138.5 to 262.2)
Hungary	Diarrhoeal diseases	15.2 (10.5 to 21.6)	13.4 (9.54 to 18.1)	17.4 (13.2 to 22.7)	182.1 (124.1 to 261.6)	161.7 (112.4 to 224.8)	190.5 (136.5 to 260.8)
Hungary	Typhoid and paratyphoid	0.00268 (0.00104 to 0.00461)	0.000947 (0.000478 to 0.00143)	0.000744 (0.000383 to 0.00121)	0 (0 to 0)	0 (0 to 0)	0 (0 to 0)
Hungary	Typhoid fever	0.00146 (0.00023 to 0.00337)	0.000329 (0.000171 to 0.000679)	0.000237 (0.000118 to 0.000569)	0 (0 to 0)	0 (0 to 0)	0 (0 to 0)
Hungary	Paratyphoid fever	0.00122 (0.000688 to 0.0021)	0.000618 (0.000274 to 0.000904)	0.000507 (0.00025 to 0.000759)	0 (0 to 0)	0 (0 to 0)	0 (0 to 0)
Hungary	Invasive non-typhoidal salmonella	0.301 (0.159 to 0.505)	0.188 (0.0958 to 0.33)	0.154 (0.0777 to 0.271)	3.3 (1.7 to 5.7)	2.2 (1.1 to 4.1)	1.8 (0.8 to 3.4)
Hungary	Other intestinal infectious diseases	0.0357 (0.018 to 0.057)	0.0158 (0.00904 to 0.0234)	0.0106 (0.00616 to 0.0158)	0.4 (0.2 to 0.7)	0.2 (0.1 to 0.3)	0.1 (0.1 to 0.2)
Hungary	Neglected tropical diseases and malaria	4.44 (2.29 to 7.37)	3.73 (1.72 to 6.6)	3.48 (1.57 to 6.14)	35.6 (18.6 to 59.4)	28 (12.8 to 50.2)	25.8 (11.7 to 45.7)
Hungary	Malaria	0 (0 to 0)	0 (0 to 0)	0 (0 to 0)	0 (0 to 0)	0 (0 to 0)	0 (0 to 0)
Hungary	Chagas disease	0 (0 to 0)	0 (0 to 0)	0 (0 to 0)	0 (0 to 0)	0 (0 to 0)	0 (0 to 0)
Hungary	Leishmaniasis	0 (0 to 0)	0.062 (0.0103 to 0.153)	0 (0 to 0)	0 (0 to 0)	0.3 (0 to 0.7)	0 (0 to 0)
Hungary	Visceral leishmaniasis	0 (0 to 0)	0.062 (0.0103 to 0.153)	0 (0 to 0)	0 (0 to 0)	0.3 (0 to 0.7)	0 (0 to 0)
Hungary	Cutaneous and mucocutaneous leishmaniasis	0 (0 to 0)	0 (0 to 0)	0 (0 to 0)	0 (0 to 0)	0 (0 to 0)	0 (0 to 0)
Hungary	African trypanosomiasis	0 (0 to 0)	0 (0 to 0)	0 (0 to 0)	0 (0 to 0)	0 (0 to 0)	0 (0 to 0)
Hungary	Schistosomiasis	0 (0 to 0)	0 (0 to 0)	0 (0 to 0)	0 (0 to 0)	0 (0 to 0)	0 (0 to 0)
Hungary	Cysticercosis	4.26 (2.09 to 7.17)	3.53 (1.54 to 6.41)	3.38 (1.46 to 6.03)	34 (16.8 to 57.6)	26.5 (11.3 to 48.5)	24.9 (10.7 to 44.6)
Hungary	Cystic echinococcosis	0.137 (0.0914 to 0.182)	0.0802 (0.0544 to 0.109)	0.0631 (0.0431 to 0.0875)	1.1 (0.7 to 1.5)	0.6 (0.4 to 0.9)	0.5 (0.3 to 0.7)
Hungary	Lymphatic filariasis	0 (0 to 0)	0 (0 to 0)	0 (0 to 0)	0 (0 to 0)	0 (0 to 0)	0 (0 to 0)
Hungary	Onchocerciasis	0 (0 to 0)	0 (0 to 0)	0 (0 to 0)	0 (0 to 0)	0 (0 to 0)	0 (0 to 0)
Hungary	Trachoma	0 (0 to 0)	0 (0 to 0)	0 (0 to 0)	0 (0 to 0)	0 (0 to 0)	0 (0 to 0)
Hungary	Dengue	0 (0 to 0)	0 (0 to 0)	0 (0 to 0)	0 (0 to 0)	0 (0 to 0)	0 (0 to 0)

SR Appendix Table 2. All-age DALYs counts and age standardized DALYs rates per 100,000 individuals for all causes and level 1 cause groups by location for 1990, 2007, and 2017, both sexes combined							
		All-age DALYs			Age-standardized rate of DALYs per 100,000		
Hungary	Yellow fever	0<br>(0 to 0)	0<br>(0 to 0)	0<br>(0 to 0)	0<br>(0 to 0)	0<br>(0 to 0)	0<br>(0 to 0)
Hungary	Rabies	0.00223<br>(0.00183 to 0.00277)	0.00261<br>(0.00207 to 0.00333)	0.000853<br>(0.000666 to 0.0011)	0<br>(0 to 0)	0<br>(0 to 0)	0<br>(0 to 0)
Hungary	Intestinal nematode infections	0<br>(0 to 0)	0<br>(0 to 0)	0<br>(0 to 0)	0<br>(0 to 0)	0<br>(0 to 0)	0<br>(0 to 0)
Hungary	Ascariasis	0<br>(0 to 0)	0<br>(0 to 0)	0<br>(0 to 0)	0<br>(0 to 0)	0<br>(0 to 0)	0<br>(0 to 0)
Hungary	Trichuriasis	0<br>(0 to 0)	0<br>(0 to 0)	0<br>(0 to 0)	0<br>(0 to 0)	0<br>(0 to 0)	0<br>(0 to 0)
Hungary	Hookworm disease	0<br>(0 to 0)	0<br>(0 to 0)	0<br>(0 to 0)	0<br>(0 to 0)	0<br>(0 to 0)	0<br>(0 to 0)
Hungary	Food-borne trematodiases	0<br>(0 to 0)	0<br>(0 to 0)	0<br>(0 to 0)	0<br>(0 to 0)	0<br>(0 to 0)	0<br>(0 to 0)
Hungary	Leprosy	0<br>(0 to 0)	0<br>(0 to 0)	0<br>(0 to 0)	0<br>(0 to 0)	0<br>(0 to 0)	0<br>(0 to 0)
Hungary	Ebola virus disease	0<br>(0 to 0)	0<br>(0 to 0)	0<br>(0 to 0)	0<br>(0 to 0)	0<br>(0 to 0)	0<br>(0 to 0)
Hungary	Zika virus disease	0<br>(0 to 0)	0<br>(0 to 0)	0<br>(0 to 0)	0<br>(0 to 0)	0<br>(0 to 0)	0<br>(0 to 0)
Hungary	Guinea worm disease	0<br>(0 to 0)	0<br>(0 to 0)	0<br>(0 to 0)	0<br>(0 to 0)	0<br>(0 to 0)	0<br>(0 to 0)
Hungary	Other neglected tropical diseases	0.0383<br>(0.0273 to 0.0889)	0.0516<br>(0.0233 to 0.064)	0.0424<br>(0.0179 to 0.0536)	0.4<br>(0.3 to 0.9)	0.6<br>(0.2 to 0.7)	0.5<br>(0.2 to 0.6)
Hungary	Other infectious diseases	15<br>(13.1 to 16.5)	7.68<br>(6.87 to 8.58)	5.6<br>(4.85 to 6.52)	168.2<br>(142.5 to 185.3)	83.4<br>(74.5 to 93.2)	61.1<br>(52 to 71.5)
Hungary	Meningitis	8.57<br>(6.94 to 9.32)	3.31<br>(3.04 to 3.56)	2.11<br>(1.89 to 2.46)	101.7<br>(79.9 to 113.3)	38.3<br>(33.8 to 42.1)	24.5<br>(21 to 28.8)
Hungary	Pneumococcal meningitis	2.13<br>(1.75 to 2.36)	0.912<br>(0.83 to 1)	0.525<br>(0.472 to 0.63)	22.5<br>(18.2 to 25.3)	9.1<br>(8.1 to 10)	5.2<br>(4.5 to 6.1)
Hungary	H influenzae type B meningitis	2.27<br>(1.77 to 2.54)	0.762<br>(0.675 to 0.843)	0.458<br>(0.397 to 0.543)	29.9<br>(22.6 to 34.3)	10.5<br>(8.9 to 12.1)	6.5<br>(5.2 to 8)
Hungary	Meningococcal infection	1.16<br>(0.946 to 1.31)	0.418<br>(0.376 to 0.46)	0.267<br>(0.233 to 0.312)	14.3<br>(11.2 to 16.5)	5<br>(4.4 to 5.7)	3.2<br>(2.7 to 3.9)
Hungary	Other meningitis	3.01<br>(2.44 to 3.3)	1.22<br>(1.1 to 1.32)	0.856<br>(0.751 to 0.996)	35.1<br>(27.6 to 39.3)	13.7<br>(12.1 to 15.1)	9.6<br>(8.2 to 11.3)
Hungary	Encephalitis	2.13<br>(1.63 to 2.33)	0.999<br>(0.858 to 1.49)	0.755<br>(0.616 to 1.25)	23<br>(17.5 to 25.3)	10.8<br>(9.1 to 16.9)	8.3<br>(6.6 to 15)
Hungary	Diphtheria	0.000634<br>(0.000556 to 0.000746)	0.000627<br>(0.000517 to 0.000824)	0.000372<br>(0.000339 to 0.000416)	0<br>(0 to 0)	0<br>(0 to 0)	0<br>(0 to 0)
Hungary	Whooping cough	0.0339<br>(0.0172 to 0.0779)	0.0109<br>(0.00471 to 0.0294)	0.0109<br>(0.00444 to 0.0228)	0.6<br>(0.3 to 1.3)	0.2<br>(0.1 to 0.6)	0.2<br>(0.1 to 0.5)
Hungary	Tetanus	0.316<br>(0.225 to 0.367)	0.0409<br>(0.0325 to 0.049)	0.0308<br>(0.0232 to 0.0399)	2.2<br>(1.6 to 2.5)	0.2<br>(0.2 to 0.3)	0.2<br>(0.1 to 0.2)
Hungary	Measles	0.00351<br>(0.00228 to 0.00418)	0.00416<br>(0.00263 to 0.00503)	0.00147<br>(0.000925 to 0.00185)	0.1<br>(0 to 0.1)	0.1<br>(0 to 0.1)	0<br>(0 to 0)
Hungary	Varicella and herpes zoster	0.537<br>(0.354 to 0.737)	0.413<br>(0.282 to 0.593)	0.368<br>(0.231 to 0.543)	5.6<br>(3.5 to 7.5)	3.8<br>(2.7 to 5.5)	3<br>(1.9 to 4.6)
Hungary	Acute hepatitis	1.31<br>(1.08 to 1.58)	0.851<br>(0.654 to 1.11)	0.77<br>(0.578 to 1.02)	12.1<br>(9.9 to 14.7)	8.2<br>(6.1 to 11)	7.6<br>(5.6 to 10.2)
Hungary	Acute hepatitis A	0.411<br>(0.308 to 0.539)	0.29<br>(0.2 to 0.396)	0.252<br>(0.174 to 0.351)	4.2<br>(3.2 to 5.5)	3.2<br>(2.2 to 4.5)	3<br>(2 to 4.3)
Hungary	Acute hepatitis B	0.749<br>(0.619 to 0.916)	0.481<br>(0.37 to 0.642)	0.446<br>(0.33 to 0.609)	6.5<br>(5.3 to 7.9)	4.2<br>(3.2 to 5.7)	3.9<br>(2.8 to 5.5)
Hungary	Acute hepatitis C	0.0306<br>(0.017 to 0.053)	0.0128<br>(0.00704 to 0.0226)	0.00956<br>(0.00513 to 0.0174)	0.4<br>(0.2 to 0.7)	0.2<br>(0.1 to 0.3)	0.1<br>(0.1 to 0.2)
Hungary	Acute hepatitis E	0.115<br>(0.0869 to 0.154)	0.0672<br>(0.0493 to 0.0896)	0.0615<br>(0.044 to 0.0835)	1<br>(0.8 to 1.3)	0.6<br>(0.4 to 0.8)	0.5<br>(0.4 to 0.7)
Hungary	Other unspecified infectious diseases	2.14<br>(1.77 to 2.66)	2.05<br>(1.43 to 2.46)	1.56<br>(1.17 to 1.92)	23<br>(19.2 to 28)	21.8<br>(14.9 to 26)	17.2<br>(12.6 to 21.4)
Hungary	Maternal and neonatal disorders	140<br>(124 to 157)	68<br>(56.2 to 82.1)	55.7<br>(43 to 70.1)	2124.3<br>(1881.1 to 2317.3)	1051.5<br>(893.6 to 1226)	871.9<br>(689.6 to 1076.5)
Hungary	Maternal disorders	1.75<br>(1.59 to 1.92)	0.902<br>(0.818 to 1)	0.756<br>(0.662 to 0.866)	17.9<br>(16.2 to 19.8)	9.2<br>(8.3 to 10.3)	8.9<br>(7.8 to 10.2)
Hungary	Maternal haemorrhage	0.444<br>(0.303 to 0.606)	0.217<br>(0.158 to 0.285)	0.174<br>(0.123 to 0.233)	4.6<br>(3.1 to 6.4)	2.2<br>(1.6 to 2.9)	2.1<br>(1.5 to 2.8)
Hungary	Maternal sepsis and other pregnancy related infections	0.0804<br>(0.0504 to 0.126)	0.0607<br>(0.0413 to 0.0874)	0.0428<br>(0.0273 to 0.0663)	0.9<br>(0.5 to 1.4)	0.7<br>(0.4 to 0.9)	0.5<br>(0.3 to 0.8)
Hungary	Maternal hypertensive disorders	0.152<br>(0.106 to 0.217)	0.0669<br>(0.0484 to 0.0908)	0.0602<br>(0.0426 to 0.0816)	1.6<br>(1.1 to 2.3)	0.7<br>(0.5 to 1)	0.7<br>(0.5 to 1)
Hungary	Maternal obstructed labour and uterine rupture	0.0943<br>(0.0586 to 0.144)	0.0398<br>(0.0261 to 0.0575)	0.0331<br>(0.021 to 0.0494)	1<br>(0.6 to 1.4)	0.4<br>(0.3 to 0.6)	0.4<br>(0.2 to 0.6)
Hungary	Maternal abortive outcome	0.233<br>(0.152 to 0.343)	0.158<br>(0.111 to 0.214)	0.109<br>(0.075 to 0.152)	2.2<br>(1.5 to 3.4)	1.5<br>(1 to 2.1)	1.2<br>(0.8 to 1.7)
Hungary	Ectopic pregnancy	0.191<br>(0.0658 to 0.396)	0.0186<br>(0.00551 to 0.0497)	0.00672<br>(0.00209 to 0.0189)	1.9<br>(0.7 to 4)	0.2<br>(0.1 to 0.5)	0.1<br>(0 to 0.2)
Hungary	Indirect maternal deaths	0.0288<br>(0.0151 to 0.0533)	0.034<br>(0.0197 to 0.0561)	0.0556<br>(0.0335 to 0.0883)	0.3<br>(0.2 to 0.6)	0.4<br>(0.2 to 0.6)	0.7<br>(0.4 to 1.1)
Hungary	Late maternal deaths	0.0303<br>(0.0223 to 0.0404)	0.0284<br>(0.0206 to 0.0383)	0.0275<br>(0.0199 to 0.0374)	0.3<br>(0.2 to 0.4)	0.3<br>(0.2 to 0.4)	0.3<br>(0.2 to 0.5)

SR Appendix Table 2. All-age DALYs counts and age standardized DALYs rates per 100,000 individuals for all causes and level 1 cause groups by location for 1990, 2007, and 2017, both sexes combined

		All-age DALYs			Age-standardized rate of DALYs per 100,000		
Hungary	Maternal deaths aggravated by HIV/AIDS	0.0000583 (0.0000292 to 0.000102)	0.0000168 (0.00000753 to 0.0000325)	0.000014 (0.00000603 to 0.0000274)	0 (0 to 0)	0 (0 to 0)	0 (0 to 0)
Hungary	Other maternal disorders	0.493 (0.36 to 0.626)	0.279 (0.223 to 0.339)	0.247 (0.2 to 0.3)	5.1 (3.7 to 6.4)	2.9 (2.3 to 3.5)	3 (2.4 to 3.6)
Hungary	Neonatal disorders	139 (122 to 155)	67.1 (55.4 to 81.2)	54.9 (42.3 to 69.4)	2106.4 (1863.6 to 2299.5)	1042.3 (884.6 to 1217.1)	863 (681.1 to 1065.7)
Hungary	Neonatal preterm birth	66.1 (58.2 to 73.5)	27.9 (23.9 to 32.3)	20.5 (15.8 to 26)	1032.2 (912.4 to 1139.9)	469.8 (406.3 to 529.2)	357.2 (275.7 to 453.6)
Hungary	Neonatal encephalopathy due to birth asphyxia and trauma	51.4 (39 to 59.4)	15.1 (10.2 to 22.3)	14.3 (9.3 to 21.5)	806.5 (590.4 to 915.8)	197.2 (140.2 to 277.5)	190.8 (129.5 to 277)
Hungary	Neonatal sepsis and other neonatal infections	15.6 (8.93 to 25.7)	13.8 (7.61 to 22.9)	14.1 (7.9 to 23.3)	173.1 (102.3 to 275.5)	161.6 (92.3 to 261.9)	172.2 (100.4 to 276.8)
Hungary	Hemolytic disease and other neonatal jaundice	0.687 (0.57 to 0.812)	0.404 (0.315 to 0.512)	0.363 (0.278 to 0.464)	9.2 (7.8 to 10.9)	5.3 (4.3 to 6.5)	5 (3.9 to 6.3)
Hungary	Other neonatal disorders	5.01 (4.32 to 5.81)	9.81 (8.09 to 11.2)	5.7 (4.25 to 7.66)	85.3 (73.6 to 99.1)	208.5 (171.8 to 237.8)	137.7 (102.5 to 185.4)
Hungary	Nutritional deficiencies	25.2 (16.1 to 37.8)	13.1 (8.26 to 20.1)	11.4 (7.1 to 17.8)	270 (174.7 to 406.9)	143.3 (89.8 to 216.5)	130.2 (82.2 to 198.9)
Hungary	Protein-energy malnutrition	0.329 (0.177 to 0.663)	0.321 (0.207 to 0.475)	0.265 (0.158 to 0.374)	4.6 (2.4 to 9.4)	4.2 (2.9 to 6.7)	3.2 (2.2 to 5)
Hungary	Iodine deficiency	1.44 (0.742 to 2.46)	0.958 (0.464 to 1.82)	1.27 (0.633 to 2.22)	13 (6.7 to 22)	8.5 (4.1 to 16.3)	11.9 (5.8 to 20.8)
Hungary	Dietary iron deficiency	15.6 (9.75 to 24)	9.05 (5.46 to 14.3)	7.89 (4.57 to 12.8)	144 (89.9 to 221.6)	81.6 (50.3 to 126.2)	76.9 (45.7 to 121.4)
Hungary	Other nutritional deficiencies	0.382 (0.283 to 0.442)	0.357 (0.283 to 0.447)	0.282 (0.217 to 0.37)	3.9 (3.1 to 4.6)	2.9 (2.4 to 3.6)	2.1 (1.7 to 2.6)
Hungary	Non-communicable diseases	3780 (3500 to 4090)	3480 (3200 to 3800)	3100 (2790 to 3420)	28094.2 (25818.6 to 30656)	23574.5 (21449.3 to 26107.6)	19716.4 (17434.1 to 22200.5)
Hungary	Neoplasms	756 (745 to 767)	847 (836 to 862)	697 (663 to 733)	5324.4 (5231.9 to 5406.7)	5375.9 (5300.6 to 5474.6)	4078.7 (3872.2 to 4294.9)
Hungary	Lip and oral cavity cancer	20.9 (19.8 to 22)	29.5 (28.5 to 30.6)	20.2 (18.5 to 22)	151.7 (143.9 to 159.9)	194.4 (187.2 to 201.6)	126.5 (116 to 138.2)
Hungary	Nasopharynx cancer	2.2 (2.07 to 2.35)	3.55 (3.37 to 3.74)	2.46 (2.22 to 2.69)	16.8 (15.7 to 17.9)	24 (22.7 to 25.3)	16.5 (14.9 to 18.1)
Hungary	Other pharynx cancer	11.8 (11.2 to 12.6)	25.1 (24.1 to 26.2)	16.3 (14.7 to 17.9)	86.9 (82 to 92.5)	166.3 (160 to 173.2)	104.4 (94.2 to 114.8)
Hungary	Oesophageal cancer	16.6 (15.9 to 17.4)	21.4 (20.6 to 22.2)	15.6 (14.3 to 17.1)	118 (112.6 to 123.9)	135.5 (130.6 to 140.7)	93.3 (85.4 to 102.1)
Hungary	Stomach cancer	62.6 (60.2 to 65)	45.5 (44.5 to 46.5)	31.3 (29.1 to 33.4)	419.5 (403.9 to 435.6)	277.7 (271.5 to 284.2)	174.6 (162.4 to 186.8)
Hungary	Colon and rectum cancer	92.1 (89.5 to 94.6)	114 (111 to 117)	100 (93.7 to 107)	614.7 (598.2 to 631.1)	681.3 (665.6 to 697.4)	545.1 (510.9 to 582.2)
Hungary	Liver cancer	22.7 (21.9 to 23.5)	23.8 (23.1 to 24.6)	18.8 (17.5 to 20.4)	152.7 (147.5 to 157.8)	147.7 (142.8 to 152.4)	106.7 (98.8 to 115.3)
Hungary	Liver cancer due to hepatitis B	4.56 (3.72 to 5.5)	4.99 (4.04 to 6.05)	3.74 (2.88 to 4.65)	32.5 (26.8 to 38.6)	33.1 (27.1 to 40)	23.2 (18.3 to 28.6)
Hungary	Liver cancer due to hepatitis C	5.63 (4.37 to 7.11)	6.03 (4.65 to 7.59)	4.86 (3.64 to 6.27)	36.6 (28.7 to 46)	35.7 (27.5 to 45.3)	26.3 (19.7 to 34.1)
Hungary	Liver cancer due to alcohol use	9.6 (8.58 to 10.6)	9.63 (8.59 to 10.7)	7.71 (6.69 to 8.81)	63.7 (57 to 70.4)	59.1 (52.5 to 65.6)	43 (37.3 to 48.9)
Hungary	Liver cancer due to NASH	1.68 (1.44 to 1.95)	1.85 (1.59 to 2.14)	1.52 (1.29 to 1.79)	11.1 (9.5 to 12.8)	11.1 (9.5 to 12.8)	8.3 (7 to 9.7)
Hungary	Liver cancer due to other causes	1.26 (1.1 to 1.45)	1.33 (1.15 to 1.54)	0.974 (0.823 to 1.16)	8.9 (7.8 to 10.1)	8.6 (7.5 to 10)	5.9 (5 to 6.9)
Hungary	Gallbladder and biliary tract cancer	21.4 (20.5 to 22.4)	18.9 (16.2 to 19.6)	12.8 (11.9 to 13.9)	139.6 (134 to 145.8)	111.3 (94.9 to 115.5)	68.5 (63.2 to 74.2)
Hungary	Pancreatic cancer	33 (32 to 34)	45.4 (44.3 to 46.5)	40.8 (38.3 to 43.5)	221.9 (215.4 to 228.7)	276.1 (269.3 to 282.9)	226.8 (212.4 to 241.5)
Hungary	Larynx cancer	18 (17.2 to 18.8)	18.2 (17.5 to 18.9)	13.1 (12 to 14.3)	126.7 (121 to 133.2)	116.9 (112.5 to 121.8)	79.7 (72.8 to 87.4)
Hungary	Tracheal, bronchus, and lung cancer	175 (170 to 181)	239 (234 to 244)	195 (184 to 208)	1202.9 (1167.6 to 1240.3)	1492.4 (1460.7 to 1525)	1115.6 (1050 to 1187.2)
Hungary	Malignant skin melanoma	8.57 (7.01 to 12.3)	10.8 (7.72 to 13.3)	8.84 (6.7 to 11.7)	63.8 (52 to 90.8)	73.6 (53 to 91.8)	56.5 (43.1 to 75.6)
Hungary	Non-melanoma skin cancer	2.9 (2.77 to 3.01)	3.5 (3.3 to 3.74)	3.33 (3.11 to 3.55)	20.5 (19.7 to 21.4)	21.4 (20.2 to 22.8)	18.2 (16.9 to 19.4)
Hungary	Non-melanoma skin cancer (squamous-cell carcinoma)	2.89 (2.77 to 3.01)	3.49 (3.3 to 3.73)	3.33 (3.1 to 3.55)	20.5 (19.7 to 21.3)	21.4 (20.2 to 22.7)	18.1 (16.9 to 19.4)
Hungary	Non-melanoma skin cancer (basal-cell carcinoma)	0.00269 (0.00115 to 0.00568)	0.00527 (0.00149 to 0.0133)	0.00569 (0.00164 to 0.0146)	0 (0 to 0)	0 (0 to 0.1)	0 (0 to 0.1)
Hungary	Breast cancer	56.6 (54.5 to 58.9)	54.1 (52.2 to 56.3)	46.6 (42.5 to 51.1)	402 (386.7 to 418.1)	345.5 (333.4 to 359.8)	275.1 (251.7 to 301.6)
Hungary	Cervical cancer	20.2 (19.4 to 21.2)	14.2 (13.6 to 14.8)	11.8 (10.6 to 13.1)	150.8 (144.3 to 158.3)	98.9 (94.4 to 103.8)	77.7 (69.9 to 85.8)
Hungary	Uterine cancer	10.1 (9.61 to 10.7)	5.79 (5.46 to 6.14)	6.3 (5.61 to 7.04)	67.6 (64.3 to 71.2)	34.6 (32.5 to 36.7)	34 (30.4 to 38.1)
Hungary	Ovarian cancer	16.8 (16 to 17.7)	15.7 (15.1 to 16.4)	14.7 (13.2 to 16.5)	118.3 (112.8 to 124)	99.4 (95.6 to 103.7)	87.2 (78.1 to 97.7)
Hungary	Prostate cancer	20.9 (14.5 to 23.7)	20.3 (16.8 to 26.8)	21.1 (17 to 27.5)	131.8 (91.4 to 147.7)	112.4 (92.1 to 146.8)	103.6 (83.7 to 134.3)

SR Appendix Table 2. All-age DALYs counts and age standardized DALYs rates per 100,000 individuals for all causes and level 1 cause groups by location for 1990, 2007, and 2017, both sexes combined

		All-age DALYs			Age-standardized rate of DALYs per 100,000		
Hungary	Testicular cancer	3.27 (2.94 to 3.63)	2.23 (2.02 to 2.45)	1.51 (1.3 to 1.74)	31.9 (28.4 to 35.7)	20.6 (18.7 to 22.9)	15.1 (13 to 17.7)
Hungary	Kidney cancer	16.8 (15.9 to 17.6)	18.8 (18 to 19.6)	16.6 (15.2 to 18)	118.1 (111.6 to 123.8)	118.4 (113.3 to 124.1)	96 (88.3 to 104.6)
Hungary	Bladder cancer	15.5 (14.8 to 16.1)	19.2 (18.6 to 19.9)	17.2 (16 to 18.5)	100.8 (96.7 to 105.1)	111.7 (108.1 to 115.7)	90 (83.7 to 96.8)
Hungary	Thyroid cancer	3.14 (2.93 to 3.36)	2.23 (2.08 to 2.4)	1.91 (1.72 to 2.13)	21.6 (20.2 to 23.1)	14.3 (13.3 to 15.4)	11.2 (10 to 12.5)
Hungary	Mesothelioma	0.923 (0.814 to 1.06)	0.935 (0.88 to 0.999)	0.916 (0.842 to 1)	6.7 (5.9 to 7.8)	6.3 (5.9 to 6.7)	5.7 (5.3 to 6.2)
Hungary	Hodgkin lymphoma	4.59 (3.4 to 5.1)	2.4 (2 to 3.01)	1.54 (1.31 to 2.22)	38.3 (28.1 to 42.9)	19.1 (16.3 to 24.1)	12.2 (10.3 to 17.5)
Hungary	Non-Hodgkin's lymphoma	13 (12.5 to 13.6)	15.6 (15.1 to 16.1)	12.6 (11.7 to 13.6)	99.6 (95.6 to 104.2)	106.1 (102.8 to 109.7)	80.4 (74.8 to 86.4)
Hungary	Multiple myeloma	5.56 (5.08 to 6.92)	7.74 (6.04 to 8.28)	6.59 (5.57 to 8.09)	36.8 (33.7 to 46.2)	46.3 (36.2 to 49.8)	36.4 (30.7 to 44.8)
Hungary	Leukaemia	26.3 (25.3 to 27.5)	29.7 (27.3 to 30.6)	21.2 (19.8 to 22.7)	218 (208.4 to 229.6)	220.2 (207.8 to 228)	149.9 (140.2 to 160.4)
Hungary	Acute lymphoid leukaemia	4.7 (4.31 to 5.07)	2.6 (2.41 to 2.84)	2 (1.81 to 2.2)	51.6 (46.8 to 56.7)	29.6 (27.1 to 33.8)	23.7 (20.8 to 27.1)
Hungary	Chronic lymphoid leukaemia	4.95 (4.65 to 5.24)	4.71 (4.44 to 5.09)	4.85 (4.44 to 5.34)	32.5 (30.6 to 34.4)	27.6 (26 to 29.9)	25.5 (23.3 to 28)
Hungary	Acute myeloid leukaemia	7.29 (6.84 to 7.94)	8.45 (7.98 to 8.95)	8.12 (7.43 to 8.84)	60.8 (56.6 to 66.4)	64.3 (60.8 to 68.3)	58.8 (53.7 to 63.7)
Hungary	Chronic myeloid leukaemia	4.38 (4.1 to 4.64)	1.66 (1.54 to 1.83)	1.24 (1.12 to 1.38)	32.9 (30.8 to 35)	11.7 (10.8 to 12.8)	8.1 (7.3 to 9)
Hungary	Other leukaemia	4.94 (4.59 to 6.02)	12.3 (9.1 to 12.9)	4.97 (4.49 to 5.47)	40.1 (37 to 49.2)	87.1 (66 to 91.7)	33.8 (30.8 to 36.8)
Hungary	Other malignant cancers	26.5 (19.5 to 28.7)	14 (12.6 to 20.7)	14.5 (13.1 to 17.6)	211.5 (151.9 to 230)	104.5 (93.7 to 148.7)	101 (91.6 to 124.3)
Hungary	Other neoplasms	3.86 (2.85 to 5.52)	6.16 (4 to 7.35)	5.8 (3.91 to 7.96)	28.6 (20.9 to 40.8)	41.3 (26.8 to 49.5)	35.7 (23.7 to 49.6)
Hungary	Myelodysplastic, myeloproliferative, and other hematopoietic neoplasms	3.69 (2.74 to 5.35)	5.96 (3.89 to 7.1)	5.63 (3.83 to 7.73)	26.9 (19.7 to 38.4)	39 (25.4 to 47)	33.7 (22.5 to 46.8)
Hungary	Benign and in situ intestinal neoplasms	0 (0 to 0)	0 (0 to 0)	0 (0 to 0)	0 (0 to 0)	0 (0 to 0)	0 (0 to 0)
Hungary	Benign and in situ cervical and uterine neoplasms	0 (0 to 0)	0 (0 to 0)	0 (0 to 0)	0 (0 to 0)	0 (0 to 0)	0 (0 to 0)
Hungary	Other benign and in situ neoplasms	0.17 (0.0866 to 0.244)	0.203 (0.103 to 0.286)	0.168 (0.0781 to 0.265)	1.7 (1 to 2.6)	2.2 (1.3 to 2.9)	2 (1.1 to 3)
Hungary	Cardiovascular diseases	1400 (1360 to 1430)	1080 (1050 to 1110)	935 (885 to 987)	9508.5 (9281.8 to 9739.4)	6421.9 (6248.3 to 6616.1)	4949.8 (4679.7 to 5230)
Hungary	Rheumatic heart disease	21.9 (21 to 22.8)	5.76 (5.49 to 6.03)	4.78 (4.41 to 5.19)	154 (148.2 to 160.2)	36.3 (34.6 to 38)	27.1 (25.1 to 29.3)
Hungary	Ischaemic heart disease	712 (697 to 728)	587 (574 to 603)	501 (476 to 531)	4845.1 (4746.6 to 4947)	3465.8 (3386.4 to 3559.4)	2620.9 (2486.3 to 2776.2)
Hungary	Stroke	435 (415 to 454)	277 (264 to 293)	227 (208 to 244)	2930.3 (2798.8 to 3059.3)	1641.4 (1556.6 to 1736.8)	1200.2 (1100.5 to 1295.9)
Hungary	Ischaemic stroke	267 (252 to 283)	189 (177 to 201)	160 (145 to 174)	1761.3 (1660.4 to 1860.4)	1075.4 (1009.3 to 1149.4)	805.1 (726.6 to 878.5)
Hungary	Intracerebral hemorrhage	145 (139 to 150)	68.7 (66.2 to 71.8)	51.1 (47.5 to 54.9)	994.6 (959.9 to 1030.1)	429.9 (413.2 to 450.1)	292.1 (271.1 to 314.4)
Hungary	Subarachnoid hemorrhage	23 (21.4 to 24.3)	19.5 (18.2 to 20.7)	15.8 (14.5 to 17.4)	174.4 (160 to 184.5)	136.2 (125.8 to 144.7)	103 (94.4 to 112.1)
Hungary	Hypertensive heart disease	62.5 (53.1 to 76.7)	60.2 (49.1 to 70.7)	67.8 (46.2 to 75.1)	415.6 (352.8 to 511.7)	349.4 (282.3 to 410.1)	346.7 (234.7 to 383.3)
Hungary	Non-rheumatic valvular heart disease	11.7 (10.7 to 14.2)	17.8 (12.5 to 19.3)	16.6 (12.1 to 18.4)	79.8 (73.4 to 97.6)	105.5 (75.3 to 114.4)	87.5 (64.9 to 97)
Hungary	Non-rheumatic calcific aortic valve disease	6.46 (5.64 to 7.6)	10.8 (6.97 to 12)	11 (7 to 12.4)	43.6 (38.3 to 51.8)	63.3 (42 to 69.9)	56.9 (36.9 to 64.3)
Hungary	Non-rheumatic degenerative mitral valve disease	5 (4.41 to 6.64)	6.57 (4.68 to 8.55)	5.27 (4.31 to 6.92)	34.5 (30.6 to 45.1)	39.5 (28.6 to 50.8)	28.6 (23.7 to 37.3)
Hungary	Other non-rheumatic valve diseases	0.221 (0.17 to 0.363)	0.403 (0.208 to 0.528)	0.332 (0.198 to 0.434)	1.6 (1.2 to 2.7)	2.6 (1.4 to 3.4)	2 (1.2 to 2.6)
Hungary	Cardiomyopathy and myocarditis	61.6 (58.5 to 66.1)	54.5 (47 to 57.6)	44.5 (38.9 to 48.6)	458.5 (433.8 to 486.6)	366.6 (310.3 to 387.5)	275.2 (235.2 to 300.4)
Hungary	Myocarditis	2.43 (1.91 to 3.34)	2.86 (1.98 to 3.34)	2.78 (1.84 to 3.34)	21 (16 to 27.8)	21.9 (15 to 25.5)	18.9 (12.5 to 22.5)
Hungary	Alcoholic cardiomyopathy	27.9 (24.7 to 40)	39.9 (29.2 to 42.9)	31.3 (21.9 to 35.3)	205.6 (182.7 to 287.7)	268.2 (194.1 to 288.7)	196.5 (135.7 to 222.2)
Hungary	Other cardiomyopathy	31.3 (20.2 to 34.7)	11.8 (10.1 to 17.7)	10.4 (8.45 to 17.1)	231.9 (151.8 to 255.4)	76.5 (65.6 to 114.3)	59.9 (48.5 to 98.6)
Hungary	Atrial fibrillation and flutter	15 (12.4 to 18.2)	16.9 (14.1 to 20.3)	18.1 (15 to 21.7)	99.9 (82.9 to 120.2)	95.1 (79 to 114.5)	88.7 (73 to 106.9)
Hungary	Aortic aneurysm	8.17 (7.84 to 8.5)	10.1 (9.72 to 10.6)	9.19 (8.48 to 9.9)	56.1 (53.9 to 58.3)	62.7 (60.1 to 65.7)	51.3 (47.3 to 55.3)
Hungary	Peripheral vascular disease	10.1 (5.28 to 16.1)	12.9 (6.96 to 23.5)	14.2 (5.96 to 27.5)	66.1 (34.5 to 105.8)	73.7 (40.5 to 133.3)	70.7 (30.8 to 135.3)
Hungary	Endocarditis	3.55 (2.57 to 3.99)	4.13 (3.2 to 4.58)	3.71 (2.87 to 4.23)	26.1 (19.3 to 29.2)	27.6 (21.8 to 30.5)	23.1 (18.4 to 26.2)

SR Appendix Table 2. All-age DALYs counts and age standardized DALYs rates per 100,000 individuals for all causes and level 1 cause groups by location for 1990, 2007, and 2017, both sexes combined							
		All-age DALYs			Age-standardized rate of DALYs per 100,000		
Hungary	Other cardiovascular and circulatory diseases	55.7 (51.5 to 60.7)	32 (28.1 to 36.7)	28.1 (23.6 to 33.4)	377 (350 to 409.7)	197.8 (174.1 to 224.9)	158.5 (133 to 188.4)
Hungary	Chronic respiratory diseases	177 (163 to 191)	170 (156 to 183)	174 (157 to 189)	1240.8 (1135.1 to 1343.7)	1080.7 (984.3 to 1170.5)	1014.8 (911.5 to 1115.9)
Hungary	Chronic obstructive pulmonary disease	143 (132 to 153)	142 (131 to 151)	148 (135 to 161)	966.4 (894.4 to 1034.2)	856.8 (792.8 to 916)	809.5 (737.4 to 882.2)
Hungary	Pneumoconiosis	1.27 (1.15 to 1.41)	0.796 (0.707 to 0.885)	0.785 (0.687 to 0.899)	8.4 (7.6 to 9.3)	4.7 (4.2 to 5.2)	4.2 (3.7 to 4.8)
Hungary	Silicosis	0.867 (0.771 to 0.969)	0.452 (0.397 to 0.511)	0.373 (0.314 to 0.439)	5.7 (5 to 6.3)	2.6 (2.3 to 3)	2 (1.7 to 2.3)
Hungary	Asbestosis	0.0305 (0.0194 to 0.046)	0.021 (0.0143 to 0.0332)	0.0259 (0.0183 to 0.0383)	0.2 (0.1 to 0.3)	0.1 (0.1 to 0.2)	0.1 (0.1 to 0.2)
Hungary	Coal workers pneumoconiosis	0.317 (0.275 to 0.365)	0.257 (0.22 to 0.297)	0.315 (0.256 to 0.377)	2.2 (1.9 to 2.5)	1.6 (1.3 to 1.8)	1.7 (1.4 to 2)
Hungary	Other pneumoconiosis	0.0544 (0.0369 to 0.0787)	0.0654 (0.0468 to 0.0901)	0.0711 (0.0488 to 0.102)	0.4 (0.2 to 0.5)	0.4 (0.3 to 0.5)	0.4 (0.3 to 0.6)
Hungary	Asthma	22.8 (17.8 to 29)	16.4 (12.4 to 21.7)	15.1 (10.8 to 20.6)	189.5 (145.3 to 245.7)	139.5 (101.8 to 186.7)	132.1 (92.5 to 181.5)
Hungary	Interstitial lung disease and pulmonary sarcoidosis	5.5 (4.43 to 6.68)	6.1 (5.08 to 7.3)	6.13 (4.96 to 7.42)	39.1 (31.5 to 47.8)	39.7 (33.1 to 47.4)	36.9 (29.5 to 45.2)
Hungary	Other chronic respiratory diseases	4.48 (4.02 to 4.89)	4.91 (4.42 to 5.4)	3.72 (3.21 to 4.24)	37.3 (33.5 to 40.9)	39.9 (35.9 to 44.3)	32.1 (27.6 to 36.9)
Hungary	Digestive diseases	307 (292 to 324)	252 (238 to 269)	175 (159 to 193)	2334.9 (2207.5 to 2480)	1768.8 (1649.7 to 1904.1)	1161.8 (1043.8 to 1297.5)
Hungary	Cirrhosis and other chronic liver diseases	201 (195 to 206)	161 (156 to 168)	93.7 (86.8 to 101)	1510.7 (1468.7 to 1555.6)	1113 (1075.1 to 1156.9)	603.2 (558.9 to 649.3)
Hungary	Cirrhosis and other chronic liver diseases due to hepatitis B	50.7 (45.2 to 56.9)	40.2 (35.4 to 45.3)	23.1 (19.9 to 26.7)	386.5 (345.8 to 433.5)	280.2 (245.6 to 316)	149.6 (129.6 to 171.9)
Hungary	Cirrhosis and other chronic liver diseases due to hepatitis C	38.6 (33.5 to 44.2)	32.1 (27.8 to 37.3)	18.3 (15.6 to 21.1)	291.2 (250.8 to 335.1)	221.6 (190.5 to 257.4)	118.5 (101 to 137.6)
Hungary	Cirrhosis and other chronic liver diseases due to alcohol use	89 (82.9 to 94.4)	70.4 (64.9 to 76.1)	40.6 (36.5 to 45)	663.2 (617.1 to 703.6)	481.3 (445.2 to 518.7)	258 (230.8 to 285.5)
Hungary	Cirrhosis due to NASH	12.5 (11.1 to 14.1)	11.1 (9.75 to 12.5)	6.84 (5.98 to 7.75)	92.8 (82.2 to 104.1)	75.3 (66 to 84.7)	43.6 (37.9 to 49.4)
Hungary	Cirrhosis and other chronic liver diseases due to other causes	9.72 (8.15 to 11.4)	7.49 (6.05 to 9.04)	4.78 (3.84 to 5.88)	77 (65.4 to 89.4)	54.5 (44.7 to 64.7)	33.4 (27.5 to 40)
Hungary	Upper digestive system diseases	44.5 (36.1 to 56.1)	39.7 (31.4 to 51.1)	35.2 (27.1 to 46.4)	351.2 (280 to 448.7)	299.6 (232.2 to 395.3)	261.4 (193.8 to 355.2)
Hungary	Peptic ulcer disease	23 (21.8 to 24.1)	18.7 (17.5 to 19.8)	14.6 (13.3 to 16.1)	163.4 (154.9 to 172.1)	119.5 (111.7 to 126.9)	85.5 (77.4 to 94.8)
Hungary	Gastritis and duodenitis	12.1 (7.98 to 17.6)	11.3 (7.46 to 16)	10.5 (7.04 to 15.1)	112.1 (73 to 162.7)	105.5 (68.8 to 152.1)	99.7 (64.6 to 144.1)
Hungary	GERD	9.45 (5.07 to 16)	9.7 (5.2 to 16.4)	10 (5.38 to 16.8)	75.8 (40.4 to 128.6)	74.6 (40.3 to 126.8)	76.1 (40.8 to 130.4)
Hungary	Appendicitis	1.85 (1.71 to 2)	1.07 (0.972 to 1.18)	0.88 (0.777 to 0.989)	14.6 (13.4 to 15.9)	8.3 (7.3 to 9.4)	6.6 (5.7 to 7.9)
Hungary	Paralytic ileus and intestinal obstruction	4.5 (3.69 to 5.33)	4.24 (3.36 to 4.86)	3.74 (3.03 to 4.51)	36.1 (29.9 to 41.4)	29.3 (23.6 to 33)	23.4 (19.4 to 27.9)
Hungary	Inguinal, femoral, and abdominal hernia	9.04 (6.83 to 11.7)	8.24 (6.11 to 10.7)	8.87 (6.61 to 11.6)	74.3 (55.6 to 96.6)	66.7 (48.1 to 88.7)	69.3 (49.6 to 93)
Hungary	Inflammatory bowel disease	3.89 (3.24 to 4.92)	4.5 (3.64 to 5.25)	3.47 (2.89 to 4.11)	31.4 (26.1 to 39.4)	33.9 (27.1 to 40)	25.2 (20.7 to 30.4)
Hungary	Vascular intestinal disorders	8.86 (8.36 to 10.2)	8.15 (7.69 to 10.2)	7.85 (7.19 to 9.34)	59.9 (56.6 to 68.1)	48.4 (45.7 to 59.2)	41.4 (38 to 48.2)
Hungary	Gallbladder and biliary diseases	8.6 (7.48 to 9.1)	5.33 (5.02 to 5.97)	5.46 (4.96 to 5.99)	58 (50.5 to 61.2)	31.1 (29.4 to 35)	28.3 (25.8 to 31.2)
Hungary	Pancreatitis	19.3 (17.6 to 20.9)	13 (11.9 to 14.4)	9.33 (8.16 to 10.8)	150 (136.9 to 162.8)	91.7 (83.4 to 101.3)	61.1 (53.4 to 71.2)
Hungary	Other digestive diseases	5.97 (5.31 to 6.77)	6.61 (6 to 7.24)	6.27 (5.57 to 7.07)	48.7 (42.2 to 57.2)	46.7 (41.5 to 52.6)	41.9 (36.1 to 48.7)
Hungary	Neurological disorders	194 (156 to 243)	201 (164 to 247)	204 (166 to 252)	1598.5 (1257.1 to 2035.9)	1502.3 (1178 to 1912.3)	1436.7 (1104.2 to 1864.5)
Hungary	Alzheimer's disease and other dementias	61.9 (57.9 to 66.1)	76.2 (71.3 to 82.1)	84.3 (77.6 to 91.7)	444 (416 to 473.3)	425.2 (398.2 to 457.2)	390.3 (359.2 to 424.5)
Hungary	Parkinson's disease	11.3 (10.4 to 12.5)	13.2 (12.1 to 14.4)	14 (12.7 to 15.6)	72.2 (66.5 to 80)	70.5 (64.8 to 77.3)	66.3 (59.9 to 74)
Hungary	Epilepsy	22.3 (11.8 to 37.6)	17.3 (9.9 to 28.2)	14.7 (6.23 to 28.2)	212.3 (114.8 to 356.5)	167.2 (95.5 to 273.7)	146.3 (61.9 to 280.4)
Hungary	Multiple sclerosis	4.78 (3.47 to 5.54)	3.96 (3.41 to 4.52)	3.71 (3.09 to 4.29)	37.2 (27 to 43.3)	29.6 (25.5 to 34.1)	27 (22.7 to 31.3)
Hungary	Motor neuron disease	2.38 (2.21 to 2.6)	2.17 (2.02 to 2.31)	2.36 (2.15 to 2.57)	22.7 (20.6 to 25.3)	18.6 (17.2 to 20.2)	18.9 (16.8 to 21.2)
Hungary	Headache disorders	83.4 (54.1 to 123)	82.8 (53.6 to 122)	80 (51.5 to 118)	730.8 (473.4 to 1074.5)	734 (470.4 to 1076.7)	732 (473 to 1070.1)
Hungary	Migraine	71.2 (43.9 to 107)	70.7 (43.6 to 107)	68.1 (42.6 to 103)	626.5 (386.1 to 941.7)	629.3 (386.6 to 952)	627.5 (385.2 to 948.1)
Hungary	Tension-type headache	12.1 (6.9 to 18.9)	12.2 (6.92 to 19)	11.9 (6.84 to 18.5)	104.3 (59 to 162.2)	104.7 (58.4 to 164.2)	104.4 (59.6 to 163.9)
Hungary	Other neurological disorders	7.97 (6.43 to 10.1)	5.52 (4.36 to 7.15)	5.2 (3.81 to 7.2)	79.3 (63 to 101.8)	57.1 (44.1 to 76.2)	55.9 (38.5 to 82.1)

SR Appendix Table 2. All-age DALYs counts and age standardized DALYs rates per 100,000 individuals for all causes and level 1 cause groups by location for 1990, 2007, and 2017, both sexes combined

		All-age DALYs			Age-standardized rate of DALYs per 100,000		
Hungary	Mental disorders	167 (124 to 215)	157 (117 to 201)	153 (114 to 197)	1459.3 (1079.3 to 1873.7)	1391 (1030.9 to 1790.1)	1378.4 (1018.5 to 1775.3)
Hungary	Schizophrenia	14.3 (10.6 to 17.7)	14.9 (11.1 to 18.5)	14.8 (11.1 to 18.4)	119.7 (88.5 to 149.4)	121.8 (90.6 to 153.4)	122.7 (90.3 to 155)
Hungary	Depressive disorders	64.4 (45.5 to 87.5)	56.6 (39.7 to 75.8)	56.4 (39.8 to 75.3)	525 (370 to 714.9)	445.7 (313.3 to 599.2)	434 (306.8 to 585.2)
Hungary	Major depressive disorder	53.9 (37.8 to 72.7)	45.7 (31.8 to 61.3)	45.5 (31.3 to 60.9)	440 (309.1 to 595.1)	360.3 (251.6 to 487.3)	348.7 (242.9 to 471.7)
Hungary	Dysthymia	10.5 (7.08 to 15.1)	10.9 (7.38 to 15.9)	11 (7.43 to 15.8)	85 (57.4 to 123.5)	85.4 (57.4 to 125.2)	85.3 (57.4 to 123.9)
Hungary	Bipolar disorder	15.1 (9.56 to 22.5)	15 (9.44 to 22.3)	14.4 (9.02 to 21.5)	134.4 (85.2 to 200.3)	135.1 (83.8 to 200.4)	134.9 (84.6 to 200.8)
Hungary	Anxiety disorders	34.9 (24.9 to 46.5)	34.5 (24.2 to 45.8)	33.4 (23.7 to 44.1)	308.8 (218.7 to 412)	311.5 (218.8 to 416)	309.3 (217.2 to 410.3)
Hungary	Eating disorders	4.06 (2.6 to 5.94)	4.19 (2.64 to 6.22)	4.09 (2.58 to 6.09)	40.4 (25.8 to 59.6)	44.5 (28.1 to 65.5)	47.7 (30.1 to 70.6)
Hungary	Anorexia nervosa	0.723 (0.436 to 1.09)	0.722 (0.44 to 1.11)	0.704 (0.426 to 1.07)	7.5 (4.5 to 11.4)	8.3 (5 to 12.7)	9.1 (5.4 to 13.8)
Hungary	Bulimia nervosa	3.33 (2.08 to 4.94)	3.47 (2.14 to 5.22)	3.38 (2.13 to 5.08)	32.9 (20.5 to 49.1)	36.3 (22.4 to 54.2)	38.6 (24.2 to 58.2)
Hungary	Autism spectrum disorders	6.64 (4.53 to 9.13)	6.22 (4.29 to 8.59)	5.97 (4.11 to 8.19)	66.5 (45.5 to 91.3)	66.8 (45.9 to 92.4)	67 (45.7 to 91.9)
Hungary	Attention-deficit/hyperactivity disorder	1.1 (0.66 to 1.74)	0.959 (0.581 to 1.52)	0.869 (0.529 to 1.38)	11.7 (7.1 to 18.6)	11.8 (7.1 to 18.7)	11.8 (7 to 19)
Hungary	Conduct disorder	8.73 (5.24 to 13.8)	6.4 (3.83 to 10.1)	5.44 (3.21 to 8.61)	97.7 (58.8 to 153.9)	98 (58.8 to 154.8)	98 (58.1 to 155.9)
Hungary	Idiopathic developmental intellectual disability	1.68 (0.436 to 3.25)	1.56 (0.487 to 2.97)	1.2 (0.262 to 2.37)	17.2 (4.6 to 33.3)	17.2 (5.4 to 32.7)	14 (3.4 to 27.9)
Hungary	Other mental disorders	16.4 (10.9 to 22.6)	17 (11.3 to 23.5)	16.9 (11.3 to 23.3)	137.8 (91.4 to 189.7)	138.7 (91.3 to 193.4)	138.9 (93.1 to 192.3)
Hungary	Substance use disorders	72.5 (60.6 to 85.8)	62.3 (51.2 to 75.2)	52.5 (42.2 to 64.6)	615.1 (508.3 to 730.5)	528.7 (426.3 to 645.1)	461.8 (360.8 to 580)
Hungary	Alcohol use disorders	59.1 (50.2 to 69.1)	46.1 (38.4 to 55.4)	37.6 (30.8 to 46.5)	485.2 (407.6 to 572)	371.5 (305.3 to 453.6)	305.7 (242 to 383.7)
Hungary	Drug use disorders	13.5 (9.68 to 17.6)	16.3 (12.2 to 20.5)	14.9 (11 to 19)	130 (92.6 to 174.1)	157.2 (116.3 to 200.6)	156.1 (114 to 202.7)
Hungary	Opioid use disorders	7.26 (5.06 to 9.71)	9.05 (6.7 to 11.5)	8.96 (6.48 to 11.6)	66.9 (46.5 to 90.7)	82.1 (60.3 to 105.6)	86.3 (61.8 to 113.2)
Hungary	Cocaine use disorders	0.51 (0.31 to 0.756)	0.577 (0.376 to 0.833)	0.542 (0.348 to 0.778)	4.8 (2.8 to 7.2)	5.5 (3.5 to 8.1)	5.5 (3.4 to 8)
Hungary	Amphetamine use disorders	2.38 (1.31 to 3.81)	2.72 (1.67 to 4.06)	2.17 (1.27 to 3.42)	25.3 (13.6 to 40.4)	29.9 (18.2 to 44.8)	27.7 (15.6 to 44.7)
Hungary	Cannabis use disorders	0.667 (0.413 to 0.98)	0.655 (0.421 to 0.966)	0.559 (0.346 to 0.822)	6.7 (4.1 to 9.9)	7.1 (4.6 to 10.5)	6.7 (4.1 to 10)
Hungary	Other drug use disorders	2.63 (1.79 to 3.71)	3.25 (2.4 to 4.31)	2.67 (1.91 to 3.68)	26.3 (17.4 to 37.8)	32.7 (23.3 to 44.2)	29.9 (20.5 to 42.5)
Hungary	Diabetes and kidney diseases	133 (110 to 161)	159 (131 to 192)	155 (126 to 191)	933.8 (767.9 to 1140)	1009.8 (827.7 to 1231.5)	919.6 (737.3 to 1143.2)
Hungary	Diabetes mellitus	105 (84.5 to 131)	127 (102 to 158)	121 (94.5 to 154)	731.4 (584.1 to 915)	806 (639.8 to 1012.7)	722.1 (555.3 to 927.8)
Hungary	Diabetes mellitus type 1	17.3 (15.9 to 18.9)	17 (15.3 to 18.1)	12.7 (11.4 to 13.9)	122.2 (113 to 132.7)	111.9 (101.6 to 119.7)	79.2 (71.1 to 87.5)
Hungary	Diabetes mellitus type 2	88 (67.7 to 113)	110 (85.7 to 141)	108 (82.2 to 140)	609.2 (465 to 790.2)	694.1 (530.4 to 896.2)	642.9 (479 to 838.9)
Hungary	Chronic kidney disease	27 (24.6 to 29.9)	31.3 (28.7 to 34.1)	33.8 (30.6 to 37.3)	198.6 (181.9 to 218.9)	202.5 (185.5 to 221.2)	196.4 (176.8 to 217.1)
Hungary	Chronic kidney disease due to diabetes mellitus type 1	1.9 (1.53 to 2.35)	2.09 (1.67 to 2.58)	1.86 (1.45 to 2.37)	14.5 (11.6 to 17.7)	14.8 (11.8 to 18)	12.4 (9.7 to 15.5)
Hungary	Chronic kidney disease due to diabetes mellitus type 2	4.87 (4.04 to 5.77)	6.27 (5.25 to 7.48)	7.05 (5.83 to 8.36)	32.1 (26.9 to 37.8)	36.2 (30.5 to 42.9)	36.2 (30.1 to 43)
Hungary	Chronic kidney disease due to hypertension	4.17 (3.5 to 4.86)	5.53 (4.68 to 6.38)	6.47 (5.4 to 7.58)	29.2 (24.6 to 33.8)	33.2 (28.2 to 38.4)	34.1 (28.7 to 39.9)
Hungary	Chronic kidney disease due to glomerulonephritis	4.95 (4.28 to 5.64)	5.49 (4.68 to 6.34)	5.65 (4.75 to 6.59)	38.9 (33.7 to 44.3)	38.5 (33.3 to 44.1)	36.5 (31 to 42.3)
Hungary	Chronic kidney disease due to other and unspecified causes	11.1 (9.42 to 13)	11.9 (10.3 to 13.6)	12.8 (10.8 to 14.8)	83.9 (72.2 to 96.6)	79.9 (69.5 to 91.1)	77.1 (66.4 to 88.6)
Hungary	Acute glomerulonephritis	0.466 (0.42 to 0.515)	0.174 (0.157 to 0.193)	0.159 (0.136 to 0.183)	3.8 (3.4 to 4.2)	1.3 (1.2 to 1.4)	1.1 (1 to 1.2)
Hungary	Skin and subcutaneous diseases	53.2 (35.9 to 76.9)	52.9 (35.9 to 77.1)	51.7 (35.1 to 75.5)	519.6 (347.2 to 751)	542.5 (364.8 to 791.4)	541.6 (364.1 to 783.2)
Hungary	Dermatitis	15.3 (8.97 to 24.3)	14.1 (8.36 to 22.3)	13.5 (7.86 to 21.5)	160.6 (93 to 258)	163.7 (94.5 to 262)	162.5 (92.3 to 261)
Hungary	Atopic dermatitis	12.7 (7.04 to 21)	11.5 (6.27 to 19.1)	10.9 (6.01 to 18.1)	137.6 (75.9 to 229.7)	140.6 (76.3 to 235.6)	139.4 (75.7 to 232.8)
Hungary	Contact dermatitis	2.65 (1.73 to 3.87)	2.65 (1.75 to 3.91)	2.61 (1.72 to 3.83)	22.9 (15 to 33.8)	23 (15.1 to 34.1)	23 (15.1 to 33.8)
Hungary	Seborrhoeic dermatitis	0.0115 (0.00641 to 0.0184)	0.0112 (0.00619 to 0.0179)	0.011 (0.0061 to 0.0175)	0.1 (0.1 to 0.2)	0.1 (0.1 to 0.2)	0.1 (0.1 to 0.2)
Hungary	Psoriasis	8.7 (6.11 to 11.5)	9.49 (6.7 to 12.4)	9.35 (6.73 to 12.3)	72.6 (50.9 to 96.7)	78.6 (55.4 to 103.6)	77.4 (55.7 to 102.3)

SR Appendix Table 2. All-age DALYs counts and age standardized DALYs rates per 100,000 individuals for all causes and level 1 cause groups by location for 1990, 2007, and 2017, both sexes combined							
		All-age DALYs			Age-standardized rate of DALYs per 100,000		
Hungary	Bacterial skin diseases	0.953 (0.663 to 1.43)	1.51 (0.666 to 1.94)	1.53 (0.617 to 2.06)	7.7 (5.5 to 11.7)	11 (5.5 to 14.1)	10.4 (5 to 13.7)
Hungary	Cellulitis	0.447 (0.258 to 0.585)	0.354 (0.245 to 0.622)	0.349 (0.24 to 0.613)	3.7 (2.3 to 4.8)	3 (2.1 to 4.9)	2.9 (2 to 4.7)
Hungary	Pyoderma	0.505 (0.349 to 0.95)	1.15 (0.355 to 1.59)	1.18 (0.313 to 1.69)	4 (2.8 to 7.6)	8 (2.8 to 10.7)	7.6 (2.4 to 10.6)
Hungary	Scabies	2.57 (1.42 to 4.13)	2.33 (1.28 to 3.77)	2.17 (1.19 to 3.47)	26.7 (14.8 to 43.5)	26.5 (14.7 to 43.2)	26.4 (14.5 to 43.2)
Hungary	Fungal skin diseases	5.36 (2.15 to 11)	5.51 (2.21 to 11.4)	5.74 (2.29 to 11.8)	45.7 (18.3 to 94.5)	44.3 (17.6 to 91.6)	44.1 (17.6 to 91.1)
Hungary	Viral skin diseases	3.7 (2.37 to 5.53)	3.03 (1.95 to 4.49)	2.81 (1.8 to 4.21)	45.7 (28.9 to 68)	45.8 (29.3 to 67.9)	45.8 (29.2 to 68.4)
Hungary	Acne vulgaris	2.83 (1.68 to 4.59)	3.17 (1.87 to 5.23)	2.81 (1.66 to 4.47)	29.8 (17.7 to 48.4)	38.3 (22.5 to 62.4)	39.9 (23.4 to 63.5)
Hungary	Alopecia areata	0.645 (0.412 to 0.961)	0.638 (0.405 to 0.951)	0.612 (0.388 to 0.922)	5.9 (3.7 to 8.9)	5.9 (3.7 to 8.9)	5.9 (3.7 to 8.9)
Hungary	Pruritus	1.13 (0.533 to 2.09)	1.16 (0.552 to 2.14)	1.18 (0.565 to 2.2)	9.6 (4.5 to 17.9)	9.8 (4.6 to 18.3)	9.9 (4.6 to 18.6)
Hungary	Urticaria	6.92 (4.58 to 9.69)	6.28 (4.2 to 8.77)	5.97 (3.95 to 8.34)	74.5 (49.1 to 105.4)	74.8 (48.8 to 105.8)	74.8 (48.9 to 106.2)
Hungary	Decubitus ulcer	0.393 (0.281 to 0.539)	0.6 (0.407 to 0.803)	0.653 (0.445 to 0.887)	3 (2.2 to 4.1)	3.9 (2.7 to 5.3)	3.9 (2.7 to 5.2)
Hungary	Other skin and subcutaneous diseases	4.66 (2.34 to 8.39)	5.1 (2.57 to 9.22)	5.35 (2.72 to 9.57)	37.8 (18.9 to 68.5)	39.9 (20.1 to 72)	40.7 (20.5 to 73.5)
Hungary	Sense organ diseases	98.4 (66.3 to 142)	105 (70.8 to 151)	112 (75.5 to 159)	729.3 (489.3 to 1054.2)	709.5 (475.8 to 1027.7)	702 (467.7 to 1018.3)
Hungary	Blindness and vision impairment	39.7 (25.9 to 58)	40.8 (26.8 to 59.4)	43 (28.3 to 62.2)	294.9 (191.5 to 434.5)	278.7 (180.2 to 408.9)	273.2 (176.4 to 400.7)
Hungary	Glaucoma	0.576 (0.375 to 0.807)	0.592 (0.388 to 0.83)	0.639 (0.424 to 0.901)	3.7 (2.4 to 5.1)	3.3 (2.2 to 4.6)	3.1 (2.1 to 4.4)
Hungary	Cataract	14.5 (9.75 to 19.9)	16.3 (10.9 to 22.1)	18 (12.1 to 24.7)	96.3 (65 to 131.5)	92.7 (62.8 to 126.4)	90.9 (61 to 124.7)
Hungary	Age-related macular degeneration	0.135 (0.084 to 0.199)	0.16 (0.104 to 0.235)	0.181 (0.114 to 0.269)	0.9 (0.6 to 1.3)	0.9 (0.6 to 1.3)	0.9 (0.6 to 1.3)
Hungary	Refraction disorders	12.2 (7.76 to 18.6)	11.6 (7.41 to 17.7)	11.4 (7.2 to 17.1)	105.4 (66.7 to 161)	99.8 (63.2 to 152.2)	97.7 (61.8 to 149.7)
Hungary	Near vision loss	8.99 (4.24 to 16.5)	8.91 (4.36 to 16.3)	9.27 (4.46 to 16.7)	65.1 (31 to 119.6)	59.5 (29 to 108.4)	58.4 (28.2 to 105.1)
Hungary	Other vision loss	3.23 (2.18 to 4.42)	3.36 (2.27 to 4.66)	3.54 (2.39 to 4.92)	23.6 (15.8 to 32.5)	22.5 (15 to 31.4)	22.1 (14.9 to 30.5)
Hungary	Age-related and other hearing loss	54.6 (37.5 to 76.8)	59.7 (40.7 to 84.4)	64.1 (43.8 to 90.2)	403.5 (276.9 to 568.1)	399.3 (272.3 to 566.9)	397.2 (269.8 to 564.1)
Hungary	Other sense organ diseases	4.11 (2.57 to 5.95)	4.51 (2.81 to 6.52)	4.82 (3.01 to 6.95)	30.8 (19.1 to 45.4)	31.6 (19.7 to 45.9)	31.7 (19.8 to 46.1)
Hungary	Musculoskeletal disorders	253 (183 to 336)	263 (192 to 354)	273 (200 to 364)	1955.3 (1419.3 to 2614.1)	1939.8 (1398.5 to 2599.1)	1951.4 (1411.4 to 2599.8)
Hungary	Rheumatoid arthritis	6.35 (5 to 7.9)	5.88 (4.42 to 7.53)	5.46 (3.99 to 7.09)	44.7 (35 to 55.8)	39 (29.1 to 50.5)	34.5 (25 to 45.2)
Hungary	Osteoarthritis	14.4 (7.29 to 28.2)	16.4 (8.21 to 32.5)	18 (9.06 to 35.6)	97.2 (49.3 to 191)	99.6 (49.7 to 197.6)	100.4 (50.4 to 199)
Hungary	Low back pain	186 (133 to 248)	191 (138 to 256)	200 (144 to 266)	1464.1 (1047.9 to 1968.4)	1445.3 (1043.1 to 1940.3)	1475.2 (1059.5 to 1967.5)
Hungary	Neck pain	39 (27.1 to 54.8)	41.5 (28.8 to 57.9)	42.9 (30 to 59.5)	294.6 (204.7 to 410.5)	296.5 (206.6 to 413.3)	296.1 (206.7 to 411.1)
Hungary	Gout	1.7 (1.13 to 2.35)	1.88 (1.26 to 2.59)	2.04 (1.38 to 2.81)	11.8 (7.8 to 16.3)	11.9 (8 to 16.4)	12.1 (8.1 to 16.7)
Hungary	Other musculoskeletal disorders	5.25 (3.73 to 7.19)	6.31 (4.45 to 8.52)	4.58 (3.25 to 6.58)	43 (29.9 to 59.2)	47.3 (32.9 to 64.2)	33.1 (22.2 to 48.4)
Hungary	Other non-communicable diseases	169 (140 to 203)	127 (99.9 to 159)	113 (87.6 to 146)	1874.9 (1602.4 to 2219.8)	1303.6 (1076.6 to 1575.8)	1119.8 (896.9 to 1390.1)
Hungary	Congenital anomalies	78.7 (68.6 to 94.9)	43.5 (36.7 to 51.5)	34.2 (27.6 to 41.9)	1119.5 (988.4 to 1377.8)	634.7 (545.1 to 772.3)	504.2 (410.6 to 635.2)
Hungary	Neural tube defects	3.59 (2.87 to 4.28)	0.972 (0.749 to 1.21)	1.15 (0.829 to 1.53)	54.4 (42.6 to 65.7)	14.5 (10.8 to 18.2)	19.4 (13.1 to 27)
Hungary	Congenital heart anomalies	27.6 (24.9 to 34.3)	12.4 (10.8 to 14.8)	7.33 (5.79 to 9.27)	430.3 (387.3 to 539.7)	216.9 (188.9 to 268.7)	141.8 (109.8 to 189.4)
Hungary	Orofacial clefts	0.416 (0.311 to 0.546)	0.232 (0.15 to 0.342)	0.195 (0.124 to 0.287)	5.2 (4 to 6.8)	2.5 (1.6 to 3.7)	2.2 (1.4 to 3.2)
Hungary	Down's syndrome	3.99 (2.78 to 4.91)	2.48 (1.93 to 2.95)	2.18 (1.64 to 2.78)	55.3 (36.2 to 69.3)	35.4 (26.3 to 43.7)	32.8 (23.3 to 45.1)
Hungary	Turner syndrome	0.00601 (0.00295 to 0.00978)	0.00547 (0.00274 to 0.0089)	0.00518 (0.0026 to 0.00852)	0.1 (0 to 0.1)	0.1 (0 to 0.1)	0.1 (0 to 0.1)
Hungary	Klinefelter syndrome	0.00123 (0.000575 to 0.00235)	0.00116 (0.000525 to 0.00226)	0.0011 (0.000504 to 0.00214)	0 (0 to 0)	0 (0 to 0)	0 (0 to 0)
Hungary	Other chromosomal abnormalities	1.31 (0.872 to 2.58)	1.43 (0.946 to 1.99)	1.29 (0.865 to 1.85)	19.2 (13 to 39.9)	24.2 (15.7 to 35.9)	23.1 (15.2 to 37)
Hungary	Congenital musculoskeletal and limb anomalies	14.7 (10.3 to 19.4)	13.1 (8.88 to 17.7)	12.7 (8.54 to 17.3)	147.9 (105.8 to 195.2)	131.6 (88.7 to 178.3)	128.9 (88 to 175.4)
Hungary	Urogenital congenital anomalies	1.06 (0.65 to 2.23)	0.599 (0.433 to 0.876)	0.506 (0.351 to 0.73)	15.9 (9.3 to 35.7)	10.3 (7.3 to 15.9)	9.5 (6.4 to 14.2)

SR Appendix Table 2. All-age DALYs counts and age standardized DALYs rates per 100,000 individuals for all causes and level 1 cause groups by location for 1990, 2007, and 2017, both sexes combined

		All-age DALYs			Age-standardized rate of DALYs per 100,000		
Hungary	Digestive congenital anomalies	9.39 (7.85 to 12.3)	4.09 (3.07 to 5.85)	3.23 (2.33 to 4.57)	137.2 (115.8 to 184.9)	58.7 (44 to 94.6)	45.7 (32.5 to 71.4)
Hungary	Other congenital anomalies	16.6 (14.2 to 23.2)	8.18 (6.85 to 11.1)	5.66 (4.43 to 7.8)	254 (215.8 to 366.6)	140.4 (116.4 to 205.4)	100.8 (76.3 to 153.3)
Hungary	Urinary diseases and male infertility	20.1 (17.7 to 23)	14 (11.5 to 17.1)	14 (11.3 to 17.4)	140.2 (123.2 to 160)	88.4 (72.1 to 108.5)	82.1 (65.5 to 102.7)
Hungary	Urinary tract infections	8.59 (7.15 to 9.49)	4.59 (4.08 to 5.46)	4.47 (3.93 to 5.28)	61 (51 to 67.3)	29.5 (26.3 to 35)	26.1 (23 to 30.5)
Hungary	Urolithiasis	4.12 (3.53 to 5.06)	1.56 (1.25 to 1.86)	1.53 (1.23 to 1.84)	28.2 (24.1 to 34.5)	10.2 (8.1 to 12.2)	9.3 (7.5 to 11.3)
Hungary	Benign prostatic hyperplasia	5.35 (3.34 to 7.67)	5.96 (3.84 to 8.64)	6.51 (4.17 to 9.41)	35.3 (22 to 50.7)	35.4 (22.7 to 51.4)	36.3 (23.2 to 52.9)
Hungary	Male infertility	0.204 (0.0835 to 0.42)	0.207 (0.0825 to 0.434)	0.173 (0.0683 to 0.354)	2 (0.8 to 4.2)	2.1 (0.8 to 4.3)	1.8 (0.7 to 3.8)
Hungary	Other urinary diseases	1.82 (1.48 to 2.51)	1.65 (0.952 to 2.07)	1.34 (0.787 to 1.7)	13.8 (11.1 to 18.6)	11.3 (7 to 14.1)	8.5 (5.4 to 10.6)
Hungary	Gynecological diseases	17.4 (11.8 to 24.6)	15.9 (10.8 to 22.6)	15.1 (10.2 to 21.4)	157 (107.1 to 219.5)	147.6 (101 to 208)	145.5 (99.5 to 204.6)
Hungary	Uterine fibroids	3.37 (1.95 to 5.51)	2.52 (1.38 to 4.33)	2.39 (1.28 to 4.18)	26.6 (15.3 to 43.9)	19.9 (10.8 to 35)	18.2 (9.8 to 31.6)
Hungary	Polycystic ovarian syndrome	0.53 (0.236 to 1.02)	0.508 (0.225 to 0.986)	0.481 (0.215 to 0.931)	5 (2.2 to 9.7)	5 (2.2 to 9.7)	5 (2.2 to 9.8)
Hungary	Female infertility	0.178 (0.0547 to 0.408)	0.164 (0.0517 to 0.375)	0.181 (0.0575 to 0.415)	1.7 (0.5 to 3.9)	1.6 (0.5 to 3.8)	1.9 (0.6 to 4.4)
Hungary	Endometriosis	4.49 (2.97 to 6.69)	4.42 (2.87 to 6.62)	4.06 (2.62 to 6)	43.6 (29 to 64.8)	43.8 (28.4 to 64.8)	43.2 (28.3 to 63.4)
Hungary	Genital prolapse	0.641 (0.326 to 1.18)	0.529 (0.26 to 0.999)	0.576 (0.283 to 1.08)	4.4 (2.2 to 8.1)	3.4 (1.7 to 6.4)	3.4 (1.7 to 6.4)
Hungary	Premenstrual syndrome	5.43 (3.46 to 8.07)	5.09 (3.26 to 7.6)	4.71 (2.97 to 7.06)	51.4 (32.9 to 76)	50.1 (31.9 to 74.7)	49.2 (31 to 74.5)
Hungary	Other gynecological diseases	2.72 (1.85 to 3.87)	2.69 (1.8 to 3.92)	2.68 (1.79 to 3.94)	24.1 (16.4 to 34.1)	23.9 (15.9 to 35)	24.5 (16.4 to 35.9)
Hungary	Hemoglobinopathies and hemolytic anaemias	4.7 (3.56 to 6.24)	3.23 (2.5 to 4.19)	2.64 (2 to 3.51)	42 (31.9 to 56.1)	27.8 (21.2 to 36.7)	22.3 (16.8 to 30)
Hungary	Thalassemias	0.0787 (0.0524 to 0.11)	0.0424 (0.0264 to 0.0627)	0.0295 (0.0191 to 0.0438)	1.2 (0.8 to 1.7)	0.8 (0.5 to 1.2)	0.6 (0.4 to 0.9)
Hungary	Thalassaemias trait	1.1 (0.665 to 1.64)	0.676 (0.417 to 1.02)	0.561 (0.338 to 0.876)	10.1 (6.2 to 14.9)	6.3 (3.8 to 9.4)	5.1 (3.1 to 8)
Hungary	Sickle cell disorders	0.266 (0.193 to 0.333)	0.201 (0.137 to 0.261)	0.152 (0.105 to 0.197)	3.3 (2.4 to 4.1)	2.8 (1.9 to 3.6)	2.2 (1.6 to 2.9)
Hungary	Sickle cell trait	0.454 (0.265 to 0.705)	0.28 (0.17 to 0.434)	0.24 (0.144 to 0.381)	4.5 (2.6 to 6.8)	2.8 (1.7 to 4.4)	2.3 (1.4 to 3.7)
Hungary	G6PD deficiency	0.362 (0.278 to 0.427)	0.351 (0.243 to 0.421)	0.237 (0.177 to 0.288)	3.4 (2.6 to 4.1)	3.2 (2.2 to 3.8)	2.2 (1.7 to 2.7)
Hungary	G6PD trait	0.000413 (0.00027 to 0.000603)	0.000316 (0.000207 to 0.000473)	0.000271 (0.000174 to 0.000409)	0 (0 to 0)	0 (0 to 0)	0 (0 to 0)
Hungary	Other hemoglobinopathies and hemolytic anaemias	2.44 (1.94 to 3.18)	1.68 (1.31 to 2.13)	1.42 (1.1 to 1.85)	19.6 (15.4 to 25.9)	12 (9.1 to 15.8)	9.8 (7.4 to 13.2)
Hungary	Endocrine, metabolic, blood, and immune disorders	11.1 (9.47 to 14)	12.6 (9.05 to 14.6)	10.8 (7.87 to 12.7)	110.8 (94.6 to 138.1)	117.6 (86.7 to 135.6)	99.3 (75.3 to 116.5)
Hungary	Oral disorders	33.7 (21.8 to 50.2)	35.7 (23.1 to 52.6)	35.6 (23.1 to 52.9)	254.3 (162.5 to 382.3)	253.2 (159.9 to 378.3)	240.6 (152.2 to 363.3)
Hungary	Caries of deciduous teeth	0.269 (0.116 to 0.549)	0.207 (0.0899 to 0.415)	0.183 (0.0775 to 0.367)	4.1 (1.8 to 8.4)	4.2 (1.8 to 8.4)	3.9 (1.7 to 7.8)
Hungary	Caries of permanent teeth	4.28 (1.86 to 8.24)	4.11 (1.82 to 7.85)	3.82 (1.69 to 7.3)	39.6 (17.1 to 75.7)	39.5 (17.4 to 75)	38.3 (16.7 to 73.3)
Hungary	Periodontal disease	3.18 (1.25 to 6.52)	3.47 (1.39 to 7.21)	3.36 (1.32 to 6.99)	23.6 (9.3 to 48.3)	24.2 (9.7 to 50.1)	22.5 (8.9 to 46.4)
Hungary	Edentulism and severe tooth loss	20.2 (13.6 to 28.4)	22.2 (14.9 to 30.9)	22.6 (15.2 to 31.8)	136.3 (91.3 to 192.2)	134.4 (90.7 to 187.1)	125.1 (84.2 to 177)
Hungary	Other oral disorders	5.79 (3.62 to 8.6)	5.79 (3.62 to 8.47)	5.66 (3.55 to 8.34)	50.7 (31.7 to 74.6)	50.8 (31.8 to 74.7)	50.8 (31.9 to 75.4)
Hungary	Sudden infant death syndrome	3 (2.05 to 3.75)	1.64 (0.89 to 2.16)	1.09 (0.556 to 1.65)	51.1 (34.9 to 63.9)	34.3 (18.7 to 45.2)	25.8 (13.2 to 39.2)
Hungary	Injuries	671 (600 to 757)	450 (389 to 527)	383 (318 to 465)	5672.2 (5122.9 to 6351.3)	3609.8 (3154.9 to 4167.1)	2970 (2484 to 3566.7)
Hungary	Transport injuries	145 (135 to 157)	89.8 (80.9 to 99.5)	58.8 (50.6 to 68.6)	1341.5 (1258.4 to 1441)	811.4 (740.4 to 886.9)	520.3 (453.6 to 598)
Hungary	Road injuries	135 (126 to 147)	84.5 (76 to 93.7)	54.8 (47 to 64.2)	1250.5 (1171.2 to 1344.8)	763.1 (695.6 to 836.2)	483.7 (420.7 to 557.4)
Hungary	Pedestrian road injuries	35.7 (33.2 to 38.7)	16.6 (15 to 18.7)	11.8 (9.96 to 14.1)	318.5 (296.4 to 343.9)	142.7 (129.1 to 158.9)	97.2 (82.2 to 115.6)
Hungary	Cyclist road injuries	20.9 (18.3 to 24.2)	14.1 (11.8 to 16.7)	10.5 (8.43 to 12.8)	172.5 (151.1 to 198.2)	112.6 (94.7 to 133)	80.7 (65.2 to 98.7)
Hungary	Motorcyclist road injuries	14.4 (13 to 16.2)	9.68 (8.62 to 10.9)	6.37 (5.43 to 7.46)	139.9 (126.2 to 155.7)	91.4 (82.1 to 101.4)	61 (52.6 to 70.7)
Hungary	Motor vehicle road injuries	62.3 (58.2 to 66.5)	42.8 (39.3 to 47)	25 (21.7 to 29.1)	602.9 (567.5 to 639.7)	406.3 (376.9 to 438.2)	235.8 (207.3 to 270.9)
Hungary	Other road injuries	2.05 (1.72 to 2.51)	1.31 (1.03 to 1.68)	1.17 (0.889 to 1.56)	16.6 (14 to 20.2)	10.2 (8.1 to 13.1)	8.9 (6.7 to 11.9)

SR Appendix Table 2. All-age DALYs counts and age standardized DALYs rates per 100,000 individuals for all causes and level 1 cause groups by location for 1990, 2007, and 2017, both sexes combined

		All-age DALYs			Age-standardized rate of DALYs per 100,000		
Hungary	Other transport injuries	9.8 (9.09 to 10.5)	5.25 (4.78 to 5.72)	3.96 (3.47 to 4.5)	91 (84.6 to 97.7)	48.3 (44 to 52.7)	36.6 (32 to 41.5)
Hungary	Unintentional injuries	343 (284 to 417)	252 (201 to 317)	246 (190 to 318)	2755.9 (2301.7 to 3332.3)	1886.7 (1510.5 to 2371.7)	1790.7 (1383.8 to 2317.4)
Hungary	Falls	234 (187 to 294)	175 (134 to 227)	173 (130 to 227)	1771.6 (1405 to 2224.1)	1232.8 (937.2 to 1611.1)	1184.9 (884.3 to 1578)
Hungary	Drowning	17.3 (16.5 to 18.2)	8.75 (8.3 to 9.19)	5.3 (4.9 to 5.73)	177 (167.6 to 186.9)	91.4 (86 to 96.7)	58.1 (53 to 63.2)
Hungary	Fire, heat, and hot substances	15.6 (13.7 to 18.2)	9.2 (7.69 to 11.3)	7.77 (6.05 to 10.2)	133.8 (117.8 to 154.5)	74.3 (62.7 to 90.6)	61.1 (47.2 to 80)
Hungary	Poisonings	8.68 (7.67 to 10)	5.26 (4.39 to 6.45)	4.53 (3.53 to 5.94)	80.1 (70.6 to 92.2)	48.7 (40.5 to 60.1)	43 (33.1 to 57.1)
Hungary	Poisoning by carbon monoxide	3.64 (3.38 to 3.92)	2.61 (2.45 to 2.79)	1.66 (1.5 to 1.83)	33.5 (31.2 to 36.1)	23.6 (22 to 25.2)	15 (13.5 to 16.7)
Hungary	Poisoning by other means	5.04 (4.12 to 6.21)	2.65 (1.85 to 3.77)	2.87 (1.92 to 4.16)	46.6 (38.1 to 57.5)	25.1 (17.5 to 35.8)	28 (18.7 to 40.7)
Hungary	Exposure to mechanical forces	24.8 (18.9 to 32.5)	21.7 (16.1 to 29.2)	25.2 (17.9 to 35)	209.8 (162.5 to 272.5)	177.2 (132.7 to 236.2)	200.4 (144 to 276.6)
Hungary	Unintentional firearm injuries	0.845 (0.721 to 0.981)	0.643 (0.539 to 0.794)	0.632 (0.507 to 0.784)	7.8 (6.6 to 9)	5.6 (4.7 to 7)	5.4 (4.4 to 6.7)
Hungary	Other exposure to mechanical forces	23.9 (18.1 to 31.6)	21 (15.5 to 28.5)	24.6 (17.4 to 34.3)	202 (155.3 to 263.5)	171.6 (127.5 to 230)	195 (139.2 to 270.6)
Hungary	Adverse effects of medical treatment	3.86 (3.08 to 4.23)	3.07 (2.74 to 3.45)	2.72 (2.41 to 3.18)	30.4 (24.1 to 33.1)	21.8 (19.8 to 25.6)	18 (16 to 22.6)
Hungary	Animal contact	2 (1.58 to 2.51)	1.69 (1.32 to 2.14)	1.63 (1.26 to 2.05)	17.6 (13.8 to 22.1)	14.6 (11.4 to 18.7)	14 (10.7 to 18)
Hungary	Venomous animal contact	1.14 (0.826 to 1.52)	0.967 (0.704 to 1.29)	0.974 (0.708 to 1.3)	10.2 (7.3 to 13.6)	8.8 (6.3 to 11.8)	8.9 (6.3 to 12)
Hungary	Non-venomous animal contact	0.86 (0.69 to 1.09)	0.722 (0.577 to 0.929)	0.652 (0.51 to 0.854)	7.4 (6 to 9.3)	5.8 (4.7 to 7.5)	5.1 (4 to 6.7)
Hungary	Foreign body	15.1 (14.3 to 15.9)	9.47 (8.93 to 10.1)	8.1 (7.44 to 8.85)	149.9 (140.2 to 159.7)	86.2 (80.5 to 92.8)	72.1 (65.3 to 79.4)
Hungary	Pulmonary aspiration and foreign body in airway	13.8 (13.2 to 14.5)	8.36 (7.98 to 8.78)	6.89 (6.42 to 7.45)	139.2 (131 to 148)	76.6 (72.1 to 81.1)	61.7 (56.4 to 68.2)
Hungary	Foreign body in eyes	0.212 (0.104 to 0.362)	0.22 (0.111 to 0.373)	0.227 (0.117 to 0.383)	1.9 (0.9 to 3.3)	2 (0.9 to 3.4)	2.1 (1 to 3.6)
Hungary	Foreign body in other body part	1.02 (0.776 to 1.31)	0.89 (0.655 to 1.16)	0.981 (0.722 to 1.28)	8.8 (6.7 to 11.2)	7.6 (5.6 to 9.9)	8.3 (6.1 to 10.8)
Hungary	Environmental heat and cold exposure	9.47 (8.53 to 10.4)	9.29 (8.44 to 10.2)	7.64 (6.7 to 8.76)	75.7 (68.1 to 83.9)	69.1 (62.4 to 76.3)	55.7 (48.8 to 64.5)
Hungary	Exposure to forces of nature	0.000685 (0.000527 to 0.000884)	0.0106 (0.00819 to 0.0137)	0.00859 (0.00664 to 0.0109)	0 (0 to 0)	0.1 (0.1 to 0.1)	0.1 (0.1 to 0.1)
Hungary	Other unintentional injuries	12.1 (10.1 to 14.7)	8.15 (6.37 to 10.7)	10.1 (7.42 to 13.9)	110.1 (93.8 to 131.9)	70.5 (56 to 90.4)	83.5 (62.1 to 113.8)
Hungary	Self-harm and interpersonal violence	183 (178 to 187)	109 (105 to 112)	78.4 (73.6 to 83.7)	1574.8 (1534.5 to 1614.6)	911.6 (884.5 to 939.9)	658.9 (617.3 to 703)
Hungary	Self-harm	161 (157 to 165)	94.8 (92.1 to 97.7)	67.2 (62.8 to 72.1)	1366 (1330.6 to 1401)	783.2 (760.5 to 807.8)	555.8 (519.5 to 596.4)
Hungary	Self-harm by firearm	3.66 (2.52 to 5.7)	3.01 (1.84 to 4.11)	1.98 (1.17 to 2.66)	33.9 (23.2 to 52.7)	25.7 (15.4 to 34.8)	16.6 (9.6 to 22.7)
Hungary	Self-harm by other specified means	157 (152 to 161)	91.8 (88.9 to 94.7)	65.2 (61 to 70.1)	1332 (1293.9 to 1369.2)	757.6 (732.6 to 782.9)	539.2 (502.9 to 579.8)
Hungary	Interpersonal violence	21.8 (20.3 to 23.6)	13.8 (12.5 to 15.1)	11.1 (9.75 to 12.9)	207.2 (193.1 to 222.7)	127.6 (116.8 to 139.5)	102.5 (90.7 to 116.6)
Hungary	Assault by firearm	1.29 (1.09 to 1.45)	0.777 (0.683 to 0.871)	0.5 (0.429 to 0.585)	12.4 (10.2 to 14)	7.5 (6.6 to 8.4)	5 (4.3 to 5.9)
Hungary	Assault by sharp object	8.36 (6.71 to 8.96)	3.41 (3.13 to 3.7)	2.27 (2.02 to 2.59)	76.4 (62.2 to 82)	30.8 (28.3 to 33.4)	20.9 (18.6 to 23.8)
Hungary	Sexual violence	1.7 (1.14 to 2.49)	1.29 (0.879 to 1.86)	1.06 (0.722 to 1.53)	15.9 (10.5 to 23.3)	12.6 (8.6 to 18.3)	10.9 (7.4 to 15.8)
Hungary	Assault by other means	10.5 (9.32 to 12.2)	8.28 (7.3 to 9.4)	7.31 (6.16 to 8.76)	102.4 (92.1 to 117.8)	76.8 (68.9 to 86.3)	65.7 (56.1 to 77.5)
Hungary	Conflict and terrorism	0.0281 (0.0277 to 0.0285)	0.00218 (0.00148 to 0.0034)	0.00295 (0.002 to 0.00471)	0.2 (0.2 to 0.2)	0 (0 to 0)	0 (0 to 0)
Hungary	Executions and police conflict	0.149 (0.124 to 0.184)	0.0694 (0.0638 to 0.0778)	0.0592 (0.0531 to 0.0678)	1.5 (1.2 to 1.8)	0.8 (0.7 to 0.8)	0.7 (0.6 to 0.8)
Macedonia	All causes	626 (570 to 690)	635 (569 to 709)	670 (596 to 754)	33318.6 (30520.7 to 36528.3)	26362 (23604.4 to 29496.5)	23989.8 (21211.2 to 27175.6)
Macedonia	Communicable, maternal, neonatal, and nutritional disorders	118 (109 to 127)	43.8 (38.2 to 50.5)	39.1 (33.6 to 45.6)	6812.8 (6333.4 to 7315.6)	2862.3 (2550.3 to 3237.1)	2460.3 (2127.2 to 2847.9)
Macedonia	HIV/AIDS and sexually transmitted infections	0.444 (0.26 to 0.752)	0.547 (0.357 to 0.886)	0.53 (0.342 to 0.877)	21.8 (12.8 to 37)	26.5 (17.9 to 41.6)	25.2 (16.8 to 40.2)
Macedonia	HIV/AIDS	0.0313 (0.0269 to 0.0348)	0.133 (0.116 to 0.156)	0.114 (0.0917 to 0.137)	1.6 (1.4 to 1.7)	8 (6.6 to 9.8)	7.1 (5.6 to 8.9)
Macedonia	HIV/AIDS - Drug-susceptible Tuberculosis	0.0143 (0.0122 to 0.0158)	0.0318 (0.018 to 0.0496)	0.0162 (0.00891 to 0.0269)	0.7 (0.6 to 0.8)	1.9 (1.1 to 3)	1 (0.6 to 1.7)
Macedonia	HIV/AIDS - Multidrug-resistant Tuberculosis without extensive drug resistance	0.000092 (0.0000143 to 0.0003)	0.00196 (0.000706 to 0.00434)	0.000718 (0.000254 to 0.00157)	0 (0 to 0)	0.1 (0 to 0.3)	0 (0 to 0.1)
Macedonia	HIV/AIDS - Extensively drug-resistant Tuberculosis	0 (0 to 0)	0.000621 (0.00022 to 0.00139)	0.000411 (0.000147 to 0.000902)	0 (0 to 0)	0 (0 to 0.1)	0 (0 to 0.1)

SR Appendix Table 2. All-age DALYs counts and age standardized DALYs rates per 100,000 individuals for all causes and level 1 cause groups by location for 1990, 2007, and 2017, both sexes combined

		All-age DALYs			Age-standardized rate of DALYs per 100,000		
Macedonia	HIV/AIDS resulting in other diseases	0.017 (0.0146 to 0.0189)	0.0988 (0.0736 to 0.125)	0.0964 (0.0712 to 0.121)	0.8 (0.7 to 0.9)	5.9 (4.2 to 7.7)	6 (4.2 to 8)
Macedonia	Sexually transmitted infections excluding HIV	0.413 (0.228 to 0.718)	0.414 (0.223 to 0.755)	0.416 (0.229 to 0.759)	20.2 (11.2 to 35.3)	18.6 (9.9 to 33.2)	18.1 (9.8 to 32.8)
Macedonia	Syphilis	0.073 (0.038 to 0.154)	0.0457 (0.0305 to 0.073)	0.0451 (0.0317 to 0.0661)	4.1 (2 to 9)	2.5 (1.5 to 5.1)	2.2 (1.4 to 3.9)
Macedonia	Chlamydial infection	0.0771 (0.0391 to 0.149)	0.0824 (0.0426 to 0.159)	0.0831 (0.0429 to 0.16)	3.6 (1.8 to 7)	3.6 (1.9 to 7)	3.6 (1.9 to 7.1)
Macedonia	Gonococcal infection	0.0609 (0.0293 to 0.124)	0.0626 (0.0306 to 0.126)	0.058 (0.0294 to 0.114)	2.9 (1.4 to 5.9)	2.9 (1.4 to 5.9)	2.8 (1.4 to 5.5)
Macedonia	Trichomoniasis	0.0629 (0.0254 to 0.138)	0.0706 (0.0281 to 0.147)	0.0727 (0.0293 to 0.155)	3 (1.2 to 6.6)	3 (1.2 to 6.3)	2.9 (1.2 to 6.3)
Macedonia	Genital herpes	0.0429 (0.014 to 0.102)	0.0518 (0.017 to 0.124)	0.0569 (0.0177 to 0.134)	2.1 (0.7 to 4.9)	2.1 (0.7 to 4.9)	2.1 (0.7 to 4.9)
Macedonia	Other sexually transmitted infections	0.0959 (0.0546 to 0.174)	0.101 (0.0587 to 0.18)	0.1 (0.0591 to 0.174)	4.5 (2.6 to 8.2)	4.5 (2.6 to 8)	4.4 (2.6 to 7.8)
Macedonia	Respiratory infections and tuberculosis	25.8 (21.9 to 29.9)	7.68 (6.75 to 8.99)	6.77 (5.79 to 8.06)	1476.1 (1244.9 to 1717.3)	413.8 (363.4 to 481.9)	336.6 (285.3 to 400.8)
Macedonia	Tuberculosis	3.74 (3.48 to 4.03)	1.49 (1.39 to 1.6)	0.944 (0.849 to 1.04)	187.6 (174.2 to 202.9)	57.8 (53.9 to 62.1)	32.7 (29.6 to 36)
Macedonia	Latent tuberculosis infection	0 (0 to 0)	0 (0 to 0)	0 (0 to 0)	0 (0 to 0)	0 (0 to 0)	0 (0 to 0)
Macedonia	Drug-susceptible tuberculosis	3.72 (3.46 to 4.01)	1.4 (1.27 to 1.52)	0.895 (0.794 to 1)	186.6 (173.2 to 201.9)	54.3 (49.4 to 59)	31 (27.6 to 34.6)
Macedonia	Multidrug-resistant tuberculosis without extensive drug resistance	0.0198 (0.00312 to 0.0674)	0.0695 (0.0257 to 0.143)	0.0317 (0.0126 to 0.0619)	1 (0.2 to 3.4)	2.7 (1 to 5.6)	1.1 (0.4 to 2.1)
Macedonia	Extensively drug-resistant tuberculosis	0 (0 to 0)	0.0217 (0.00811 to 0.0443)	0.0177 (0.00707 to 0.035)	0 (0 to 0)	0.8 (0.3 to 1.7)	0.6 (0.2 to 1.2)
Macedonia	Lower respiratory infections	20.2 (16.5 to 24.2)	4.26 (3.83 to 5.12)	3.94 (3.47 to 4.69)	1192.5 (972.8 to 1432.6)	260.6 (229.8 to 299.2)	210.4 (180 to 246.4)
Macedonia	Upper respiratory infections	1.42 (0.828 to 2.27)	1.45 (0.849 to 2.31)	1.45 (0.837 to 2.3)	70.3 (41.2 to 112.3)	70.2 (41 to 112.8)	69.8 (40.1 to 110)
Macedonia	Otitis media	0.506 (0.306 to 0.798)	0.482 (0.286 to 0.763)	0.444 (0.269 to 0.705)	25.7 (15.6 to 40.5)	25.1 (15.1 to 39.6)	23.7 (14.3 to 37.6)
Macedonia	Enteric infections	17 (13.1 to 21)	1.99 (1.52 to 2.53)	1.91 (1.41 to 2.5)	1008.9 (775 to 1254.1)	131.7 (102.1 to 164.9)	119.5 (88.4 to 157.4)
Macedonia	Diarrhoeal diseases	16.9 (13 to 21)	1.93 (1.47 to 2.48)	1.85 (1.35 to 2.44)	1004.1 (769.4 to 1249.8)	128.5 (99 to 162)	116.3 (85.3 to 154.3)
Macedonia	Typhoid and paratyphoid	0.00259 (0.00113 to 0.00512)	0.00266 (0.00116 to 0.00526)	0.00276 (0.00122 to 0.00549)	0.1 (0.1 to 0.3)	0.1 (0.1 to 0.2)	0.1 (0.1 to 0.2)
Macedonia	Typhoid fever	0.000493 (0.000227 to 0.000927)	0.000478 (0.000222 to 0.000916)	0.00053 (0.000248 to 0.000977)	0 (0 to 0)	0 (0 to 0)	0 (0 to 0.1)
Macedonia	Paratyphoid fever	0.00209 (0.000834 to 0.00432)	0.00218 (0.000895 to 0.00445)	0.00223 (0.000903 to 0.00467)	0.1 (0 to 0.2)	0.1 (0 to 0.2)	0.1 (0 to 0.2)
Macedonia	Invasive non-typhoidal salmonella	0.0691 (0.0348 to 0.121)	0.0505 (0.0257 to 0.0879)	0.0516 (0.0263 to 0.0902)	3.6 (1.9 to 6.4)	2.8 (1.4 to 5)	2.8 (1.4 to 5.3)
Macedonia	Other intestinal infectious diseases	0.0169 (0.00309 to 0.0405)	0.00395 (0.00192 to 0.00671)	0.00324 (0.00163 to 0.00546)	1 (0.2 to 2.4)	0.2 (0.1 to 0.5)	0.2 (0.1 to 0.4)
Macedonia	Neglected tropical diseases and malaria	0.577 (0.255 to 1.56)	0.486 (0.215 to 0.911)	0.508 (0.239 to 0.878)	28.5 (12.5 to 80.4)	19.9 (8.6 to 42.8)	18.5 (8.6 to 34)
Macedonia	Malaria	0 (0 to 0)	0 (0 to 0)	0 (0 to 0)	0 (0 to 0)	0 (0 to 0)	0 (0 to 0)
Macedonia	Chagas disease	0 (0 to 0)	0 (0 to 0)	0 (0 to 0)	0 (0 to 0)	0 (0 to 0)	0 (0 to 0)
Macedonia	Leishmaniasis	0.116 (0.000412 to 1.03)	0.0434 (0.000164 to 0.429)	0.025 (0.0000824 to 0.233)	6 (0 to 53.9)	2.4 (0 to 25.5)	1.4 (0 to 13.9)
Macedonia	Visceral leishmaniasis	0.116 (0.000412 to 1.03)	0.0434 (0.000164 to 0.429)	0.025 (0.0000824 to 0.233)	6 (0 to 53.9)	2.4 (0 to 25.5)	1.4 (0 to 13.9)
Macedonia	Cutaneous and mucocutaneous leishmaniasis	0 (0 to 0)	0 (0 to 0)	0 (0 to 0)	0 (0 to 0)	0 (0 to 0)	0 (0 to 0)
Macedonia	African trypanosomiasis	0 (0 to 0)	0 (0 to 0)	0 (0 to 0)	0 (0 to 0)	0 (0 to 0)	0 (0 to 0)
Macedonia	Schistosomiasis	0 (0 to 0)	0 (0 to 0)	0 (0 to 0)	0 (0 to 0)	0 (0 to 0)	0 (0 to 0)
Macedonia	Cysticercosis	0.434 (0.215 to 0.714)	0.423 (0.192 to 0.731)	0.467 (0.219 to 0.814)	21.1 (10.6 to 34.7)	16.7 (7.5 to 28.7)	16.4 (7.6 to 28.7)
Macedonia	Cystic echinococcosis	0.0198 (0.0133 to 0.0265)	0.0154 (0.0105 to 0.0212)	0.0139 (0.00978 to 0.019)	1 (0.7 to 1.3)	0.6 (0.4 to 0.9)	0.5 (0.4 to 0.7)
Macedonia	Lymphatic filariasis	0 (0 to 0)	0 (0 to 0)	0 (0 to 0)	0 (0 to 0)	0 (0 to 0)	0 (0 to 0)
Macedonia	Onchocerciasis	0 (0 to 0)	0 (0 to 0)	0 (0 to 0)	0 (0 to 0)	0 (0 to 0)	0 (0 to 0)
Macedonia	Trachoma	0 (0 to 0)	0 (0 to 0)	0 (0 to 0)	0 (0 to 0)	0 (0 to 0)	0 (0 to 0)
Macedonia	Dengue	0 (0 to 0)	0 (0 to 0)	0 (0 to 0)	0 (0 to 0)	0 (0 to 0)	0 (0 to 0)
Macedonia	Yellow fever	0 (0 to 0)	0 (0 to 0)	0 (0 to 0)	0 (0 to 0)	0 (0 to 0)	0 (0 to 0)
Macedonia	Rabies	0.000272 (0.000185 to 0.000389)	0.000189 (0.000088 to 0.000293)	0.000135 (0.0000736 to 0.00021)	0 (0 to 0)	0 (0 to 0)	0 (0 to 0)

SR Appendix Table 2. All-age DALYs counts and age standardized DALYs rates per 100,000 individuals for all causes and level 1 cause groups by location for 1990, 2007, and 2017, both sexes combined

		All-age DALYs			Age-standardized rate of DALYs per 100,000		
Macedonia	Intestinal nematode infections	0.00186<br>(0.000393 to 0.00419)	0.000384<br>(0.000152 to 0.000737)	0.000297<br>(0.000116 to 0.000529)	0.1<br>(0 to 0.2)	0<br>(0 to 0.1)	0<br>(0 to 0)
Macedonia	Ascariasis	0.00186<br>(0.000393 to 0.00419)	0.000384<br>(0.000152 to 0.000737)	0.000297<br>(0.000116 to 0.000529)	0.1<br>(0 to 0.2)	0<br>(0 to 0.1)	0<br>(0 to 0)
Macedonia	Trichuriasis	0<br>(0 to 0)	0<br>(0 to 0)	0<br>(0 to 0)	0<br>(0 to 0)	0<br>(0 to 0)	0<br>(0 to 0)
Macedonia	Hookworm disease	0<br>(0 to 0)	0<br>(0 to 0)	0<br>(0 to 0)	0<br>(0 to 0)	0<br>(0 to 0)	0<br>(0 to 0)
Macedonia	Food-borne trematodiases	0<br>(0 to 0)	0<br>(0 to 0)	0<br>(0 to 0)	0<br>(0 to 0)	0<br>(0 to 0)	0<br>(0 to 0)
Macedonia	Leprosy	0<br>(0 to 0)	0<br>(0 to 0)	0<br>(0 to 0)	0<br>(0 to 0)	0<br>(0 to 0)	0<br>(0 to 0)
Macedonia	Ebola virus disease	0<br>(0 to 0)	0<br>(0 to 0)	0<br>(0 to 0)	0<br>(0 to 0)	0<br>(0 to 0)	0<br>(0 to 0)
Macedonia	Zika virus disease	0<br>(0 to 0)	0<br>(0 to 0)	0<br>(0 to 0)	0<br>(0 to 0)	0<br>(0 to 0)	0<br>(0 to 0)
Macedonia	Guinea worm disease	0<br>(0 to 0)	0<br>(0 to 0)	0<br>(0 to 0)	0<br>(0 to 0)	0<br>(0 to 0)	0<br>(0 to 0)
Macedonia	Other neglected tropical diseases	0.00446<br>(0.00188 to 0.00739)	0.00288<br>(0.00126 to 0.00377)	0.00212<br>(0.00126 to 0.0031)	0.3<br>(0.1 to 0.4)	0.2<br>(0.1 to 0.2)	0.1<br>(0.1 to 0.2)
Macedonia	Other infectious diseases	6.15<br>(4.7 to 8.36)	2.52<br>(1.98 to 3.53)	2.14<br>(1.64 to 2.99)	349.3<br>(263.5 to 478.8)	161.1<br>(119.4 to 248.4)	130.7<br>(92.3 to 202.9)
Macedonia	Meningitis	2.88<br>(2.15 to 3.68)	0.728<br>(0.608 to 0.846)	0.534<br>(0.446 to 0.638)	168<br>(124 to 215.7)	49.3<br>(40.7 to 59.4)	34.2<br>(27.7 to 42.1)
Macedonia	Pneumococcal meningitis	0.334<br>(0.245 to 0.447)	0.133<br>(0.105 to 0.164)	0.1<br>(0.0802 to 0.124)	18.7<br>(13.5 to 25.4)	7.4<br>(5.7 to 9.2)	5.3<br>(4.1 to 6.6)
Macedonia	H influenzae type B meningitis	1.02<br>(0.736 to 1.34)	0.213<br>(0.174 to 0.257)	0.145<br>(0.115 to 0.18)	59.8<br>(43 to 79)	15.9<br>(12.5 to 19.6)	10.6<br>(8.1 to 13.5)
Macedonia	Meningococcal infection	0.444<br>(0.31 to 0.608)	0.109<br>(0.0876 to 0.135)	0.0776<br>(0.0618 to 0.0973)	25.9<br>(17.9 to 35.7)	7.5<br>(5.8 to 9.7)	5.1<br>(3.8 to 6.7)
Macedonia	Other meningitis	1.09<br>(0.806 to 1.4)	0.274<br>(0.229 to 0.323)	0.211<br>(0.175 to 0.25)	63.6<br>(47 to 82.3)	18.6<br>(15.1 to 22.5)	13.4<br>(10.8 to 16.4)
Macedonia	Encephalitis	0.635<br>(0.409 to 0.835)	0.472<br>(0.364 to 0.528)	0.423<br>(0.363 to 0.484)	34.2<br>(21.2 to 45.9)	26.8<br>(19.6 to 30.7)	22.6<br>(18.9 to 26.6)
Macedonia	Diphtheria	0.00832<br>(0.00401 to 0.0152)	0.0023<br>(0.00141 to 0.0034)	0.0023<br>(0.00141 to 0.00351)	0.5<br>(0.2 to 0.9)	0.1<br>(0.1 to 0.2)	0.1<br>(0.1 to 0.2)
Macedonia	Whooping cough	1.07<br>(0.116 to 3.22)	0.524<br>(0.0594 to 1.54)	0.457<br>(0.048 to 1.27)	63.2<br>(6.8 to 189.4)	45.9<br>(5.2 to 134.3)	39.4<br>(4.1 to 109.5)
Macedonia	Tetanus	0.014<br>(0.0129 to 0.015)	0.0124<br>(0.0117 to 0.0135)	0.0139<br>(0.0128 to 0.0155)	0.7<br>(0.6 to 0.8)	0.6<br>(0.6 to 0.7)	0.7<br>(0.7 to 0.8)
Macedonia	Measles	0.455<br>(0.321 to 0.619)	0.000328<br>(0.000169 to 0.000554)	0.00168<br>(0.000815 to 0.00288)	26.4<br>(18.6 to 35.9)	0<br>(0 to 0)	0.1<br>(0.1 to 0.2)
Macedonia	Varicella and herpes zoster	0.142<br>(0.101 to 0.195)	0.0961<br>(0.0682 to 0.13)	0.0969<br>(0.0689 to 0.134)	7.8<br>(5.5 to 10.9)	4.9<br>(3.5 to 6.8)	4.3<br>(3.1 to 6)
Macedonia	Acute hepatitis	0.31<br>(0.227 to 0.381)	0.245<br>(0.197 to 0.296)	0.199<br>(0.161 to 0.249)	16<br>(11.5 to 19.7)	11.8<br>(9.3 to 14.3)	9.4<br>(7.5 to 11.7)
Macedonia	Acute hepatitis A	0.139<br>(0.1 to 0.181)	0.112<br>(0.0846 to 0.144)	0.09<br>(0.0667 to 0.118)	7.1<br>(5 to 9.2)	5.9<br>(4.5 to 7.6)	5.1<br>(3.8 to 6.6)
Macedonia	Acute hepatitis B	0.125<br>(0.0911 to 0.16)	0.104<br>(0.0828 to 0.123)	0.0877<br>(0.0707 to 0.113)	6.4<br>(4.6 to 8.5)	4.3<br>(3.4 to 5.2)	3.2<br>(2.6 to 4.1)
Macedonia	Acute hepatitis C	0.0228<br>(0.00827 to 0.046)	0.00624<br>(0.00317 to 0.0105)	0.00422<br>(0.00222 to 0.0071)	1.4<br>(0.5 to 2.7)	0.5<br>(0.2 to 0.8)	0.3<br>(0.2 to 0.5)
Macedonia	Acute hepatitis E	0.0225<br>(0.017 to 0.0281)	0.0229<br>(0.0177 to 0.0284)	0.0173<br>(0.0135 to 0.0219)	1.1<br>(0.8 to 1.4)	1<br>(0.8 to 1.3)	0.8<br>(0.6 to 1)
Macedonia	Other unspecified infectious diseases	0.632<br>(0.467 to 0.832)	0.435<br>(0.316 to 0.596)	0.413<br>(0.299 to 0.571)	32.6<br>(24 to 43.2)	21.7<br>(15.8 to 29.4)	19.8<br>(14.4 to 27.5)
Macedonia	Maternal and neonatal disorders	57.4<br>(49.6 to 65.4)	22.8<br>(19.9 to 26)	20.8<br>(17.4 to 24.8)	3397.8<br>(2928.5 to 3860.5)	1700.6<br>(1520.8 to 1891.7)	1492.6<br>(1258 to 1726.2)
Macedonia	Maternal disorders	0.391<br>(0.328 to 0.466)	0.174<br>(0.145 to 0.206)	0.176<br>(0.145 to 0.21)	18.5<br>(15.5 to 22)	8<br>(6.7 to 9.5)	8.2<br>(6.7 to 9.8)
Macedonia	Maternal haemorrhage	0.077<br>(0.0532 to 0.108)	0.0426<br>(0.0289 to 0.0583)	0.0415<br>(0.0291 to 0.0587)	3.6<br>(2.5 to 5.1)	2<br>(1.3 to 2.7)	1.9<br>(1.4 to 2.7)
Macedonia	Maternal sepsis and other pregnancy related infections	0.0666<br>(0.0469 to 0.0912)	0.0198<br>(0.014 to 0.0274)	0.0164<br>(0.011 to 0.024)	3.2<br>(2.2 to 4.3)	0.9<br>(0.6 to 1.3)	0.8<br>(0.5 to 1.2)
Macedonia	Maternal hypertensive disorders	0.0296<br>(0.0181 to 0.0464)	0.0151<br>(0.00996 to 0.0213)	0.0128<br>(0.00812 to 0.0185)	1.4<br>(0.9 to 2.2)	0.7<br>(0.4 to 1)	0.6<br>(0.4 to 0.9)
Macedonia	Maternal obstructed labour and uterine rupture	0.0127<br>(0.00734 to 0.0214)	0.00895<br>(0.00595 to 0.013)	0.00984<br>(0.00632 to 0.0146)	0.6<br>(0.3 to 1)	0.4<br>(0.3 to 0.6)	0.4<br>(0.3 to 0.7)
Macedonia	Maternal abortive outcome	0.0604<br>(0.0404 to 0.0852)	0.0217<br>(0.0149 to 0.0305)	0.0201<br>(0.0138 to 0.0287)	2.9<br>(1.9 to 4)	1<br>(0.7 to 1.4)	0.9<br>(0.6 to 1.3)
Macedonia	Ectopic pregnancy	0.00987<br>(0.00496 to 0.0178)	0.00387<br>(0.00184 to 0.00706)	0.00329<br>(0.00166 to 0.00584)	0.5<br>(0.2 to 0.8)	0.2<br>(0.1 to 0.3)	0.1<br>(0.1 to 0.3)
Macedonia	Indirect maternal deaths	0.0234<br>(0.0167 to 0.0321)	0.00853<br>(0.00599 to 0.012)	0.00733<br>(0.00487 to 0.0105)	1.1<br>(0.8 to 1.5)	0.4<br>(0.3 to 0.6)	0.4<br>(0.2 to 0.5)
Macedonia	Late maternal deaths	0.00641<br>(0.00444 to 0.00919)	0.00411<br>(0.00297 to 0.00566)	0.00558<br>(0.00378 to 0.00797)	0.3<br>(0.2 to 0.4)	0.2<br>(0.1 to 0.3)	0.3<br>(0.2 to 0.4)
Macedonia	Maternal deaths aggravated by HIV/AIDS	0.00000078<br>(0.00000036 to 0.00000144)	0.00000229<br>(0.00000112 to 0.00000369)	0.00000426<br>(0.00000207 to 0.0000071)	0<br>(0 to 0)	0<br>(0 to 0)	0<br>(0 to 0)
Macedonia	Other maternal disorders	0.105<br>(0.0755 to 0.138)	0.0497<br>(0.0378 to 0.063)	0.0588<br>(0.0443 to 0.0758)	5<br>(3.6 to 6.5)	2.3<br>(1.7 to 2.9)	2.7<br>(2.1 to 3.5)

SR Appendix Table 2. All-age DALYs counts and age standardized DALYs rates per 100,000 individuals for all causes and level 1 cause groups by location for 1990, 2007, and 2017, both sexes combined							
		All-age DALYs			Age-standardized rate of DALYs per 100,000		
Macedonia	Neonatal disorders	57 (49.2 to 65)	22.7 (19.7 to 25.8)	20.6 (17.2 to 24.7)	3379.4 (2909.4 to 3843.6)	1692.5 (1512.5 to 1882.8)	1484.4 (1249.8 to 1719)
Macedonia	Neonatal preterm birth	32.3 (26.7 to 39.2)	12.2 (10.8 to 13.6)	10.9 (9.02 to 13)	1933.3 (1589.3 to 2353)	1009.1 (891.3 to 1123)	877.5 (719.6 to 1051.1)
Macedonia	Neonatal encephalopathy due to birth asphyxia and trauma	7.44 (5.72 to 9.54)	4.35 (3.1 to 6.01)	4.12 (2.81 to 5.87)	427.5 (329.9 to 546.5)	273.7 (208.9 to 356.1)	250.4 (182.4 to 338.5)
Macedonia	Neonatal sepsis and other neonatal infections	2.81 (1.56 to 4.53)	3.49 (2.08 to 5.46)	3.59 (2.06 to 5.69)	141.4 (79.5 to 228)	188.2 (119 to 285.2)	190.2 (115.7 to 294.7)
Macedonia	Hemolytic disease and other neonatal jaundice	0.503 (0.382 to 0.643)	0.449 (0.334 to 0.59)	0.441 (0.321 to 0.575)	25.5 (19.6 to 32.4)	22.9 (17.2 to 29.8)	22.3 (16.2 to 29)
Macedonia	Other neonatal disorders	14 (9.36 to 17.3)	2.17 (1.75 to 3.01)	1.61 (1.18 to 2.55)	851.7 (570 to 1054.1)	198.6 (160.1 to 276.9)	144 (105.5 to 228.9)
Macedonia	Nutritional deficiencies	10.5 (6.67 to 15.3)	7.75 (4.9 to 11.6)	6.39 (4.05 to 9.96)	530.4 (337.9 to 779.3)	408.8 (258.9 to 614.5)	337.4 (214.4 to 529.6)
Macedonia	Protein-energy malnutrition	0.115 (0.0836 to 0.161)	0.02 (0.0149 to 0.04)	0.0187 (0.0146 to 0.0389)	6.8 (4.9 to 9.5)	1.3 (0.9 to 2.7)	1 (0.8 to 2.6)
Macedonia	Iodine deficiency	0.242 (0.114 to 0.469)	0.23 (0.107 to 0.448)	0.238 (0.11 to 0.467)	11.8 (5.5 to 22.8)	9.9 (4.6 to 19.4)	9.7 (4.5 to 19.1)
Macedonia	Dietary iron deficiency	6.67 (4.17 to 9.97)	6 (3.78 to 9.1)	5.16 (3.21 to 8.09)	324.8 (203.8 to 485.6)	282.7 (177 to 423.7)	244.1 (152.5 to 381.6)
Macedonia	Other nutritional deficiencies	0.0143 (0.0128 to 0.0178)	0.0159 (0.0143 to 0.02)	0.0148 (0.0131 to 0.0185)	0.7 (0.7 to 0.9)	0.7 (0.7 to 1)	0.7 (0.6 to 0.8)
Macedonia	Non-communicable diseases	442 (400 to 493)	532 (479 to 591)	567 (508 to 633)	23229.4 (21101.6 to 25736.7)	20972.5 (18847.3 to 23422.3)	19003.3 (16862.6 to 21477.4)
Macedonia	Neoplasms	68 (65.8 to 69.9)	95.8 (93.4 to 97.9)	109 (102 to 116)	3358.1 (3256.8 to 3453.1)	3526.5 (3442.5 to 3606.1)	3417.9 (3198.5 to 3650.9)
Macedonia	Lip and oral cavity cancer	0.716 (0.648 to 0.789)	0.945 (0.869 to 1.03)	1.03 (0.884 to 1.18)	35.7 (32.5 to 39.3)	34.6 (31.9 to 37.5)	32.1 (27.5 to 36.7)
Macedonia	Nasopharynx cancer	0.196 (0.178 to 0.215)	0.219 (0.201 to 0.239)	0.22 (0.188 to 0.256)	9.4 (8.5 to 10.3)	8.3 (7.6 to 9)	7.4 (6.3 to 8.5)
Macedonia	Other pharynx cancer	0.39 (0.334 to 0.535)	0.54 (0.47 to 0.665)	0.62 (0.504 to 0.779)	18.6 (16 to 25.5)	19.5 (17 to 23.7)	19.5 (15.9 to 24.6)
Macedonia	Oesophageal cancer	0.67 (0.614 to 0.733)	0.963 (0.888 to 1.05)	1.09 (0.971 to 1.23)	33 (30.4 to 36)	34.5 (31.8 to 37.4)	33.2 (29.6 to 37.3)
Macedonia	Stomach cancer	9.06 (8.44 to 9.72)	9.1 (8.68 to 9.55)	8.82 (8.04 to 9.71)	450.4 (419.8 to 482.4)	325.8 (311 to 341.8)	265.5 (242 to 291.6)
Macedonia	Colon and rectum cancer	5.48 (5.12 to 5.91)	9.84 (9.39 to 10.3)	11.5 (10.2 to 12.9)	275.5 (257 to 300.2)	353.4 (337.3 to 370.2)	343.8 (304.6 to 385.7)
Macedonia	Liver cancer	3.48 (3.2 to 3.78)	4.64 (4.35 to 4.94)	5.43 (4.78 to 6.16)	172.9 (158.4 to 186.5)	165.8 (155.6 to 176.5)	163 (143.6 to 184.3)
Macedonia	Liver cancer due to hepatitis B	0.897 (0.717 to 1.09)	1.14 (0.909 to 1.37)	1.21 (0.944 to 1.52)	43.3 (34.9 to 52.3)	41.4 (33.5 to 50)	38.1 (30.2 to 47.2)
Macedonia	Liver cancer due to hepatitis C	0.939 (0.712 to 1.21)	1.3 (1.01 to 1.65)	1.53 (1.14 to 2.01)	47.3 (36.4 to 60.3)	45.8 (35.7 to 58.3)	44.7 (33.2 to 58.1)
Macedonia	Liver cancer due to alcohol use	1.13 (0.946 to 1.32)	1.51 (1.31 to 1.72)	1.85 (1.54 to 2.23)	56.3 (47.6 to 65.6)	53.3 (46.5 to 60.7)	54.7 (45.8 to 65.6)
Macedonia	Liver cancer due to NASH	0.271 (0.225 to 0.327)	0.4 (0.338 to 0.474)	0.5 (0.413 to 0.604)	13.7 (11.5 to 16.5)	14.3 (12.1 to 16.9)	14.8 (12.2 to 17.9)
Macedonia	Liver cancer due to other causes	0.25 (0.21 to 0.297)	0.299 (0.256 to 0.351)	0.339 (0.279 to 0.408)	12.2 (10.3 to 14.4)	11 (9.5 to 12.8)	10.7 (8.9 to 12.8)
Macedonia	Gallbladder and biliary tract cancer	0.982 (0.809 to 1.13)	1.3 (0.996 to 1.44)	1.29 (1.08 to 1.6)	49.8 (41.4 to 57.8)	46.4 (35.7 to 51.4)	38.1 (32.1 to 47.3)
Macedonia	Pancreatic cancer	2.59 (2.42 to 2.77)	4.43 (4.21 to 4.68)	5.76 (5.16 to 6.5)	129 (120.7 to 137.7)	158.8 (150.5 to 167.4)	171.9 (154.6 to 193.7)
Macedonia	Larynx cancer	1.73 (1.55 to 1.94)	2.53 (2.36 to 2.73)	2.68 (2.33 to 3.11)	83.7 (75.6 to 94.1)	89.9 (83.9 to 96.8)	80.4 (70.1 to 93.4)
Macedonia	Tracheal, bronchus, and lung cancer	12.7 (11.9 to 13.5)	22 (21 to 23)	25.7 (23.3 to 28.5)	610.6 (572.3 to 647.8)	780 (746.9 to 815.5)	768.1 (696.5 to 850.2)
Macedonia	Malignant skin melanoma	1.44 (1.19 to 1.84)	1.87 (1.54 to 2.48)	2.21 (1.65 to 3.13)	71.4 (58.1 to 90)	71 (58 to 94.2)	72.9 (54.4 to 104.7)
Macedonia	Non-melanoma skin cancer	0.22 (0.204 to 0.236)	0.444 (0.415 to 0.475)	0.57 (0.495 to 0.637)	12.1 (11.3 to 13)	17.2 (16.1 to 18.3)	17.5 (15.3 to 19.5)
Macedonia	Non-melanoma skin cancer (squamous-cell carcinoma)	0.219 (0.204 to 0.236)	0.443 (0.414 to 0.474)	0.569 (0.495 to 0.635)	12.1 (11.3 to 13)	17.2 (16.1 to 18.3)	17.5 (15.3 to 19.5)
Macedonia	Non-melanoma skin cancer (basal-cell carcinoma)	0.000633 (0.000195 to 0.00154)	0.000898 (0.00027 to 0.00227)	0.00108 (0.000312 to 0.00261)	0 (0 to 0.1)	0 (0 to 0.1)	0 (0 to 0.1)
Macedonia	Breast cancer	6.28 (5.17 to 6.84)	8.33 (7.33 to 8.97)	8.78 (7.35 to 10.2)	300.9 (250.7 to 327.7)	305.4 (269.6 to 327.8)	279.4 (234.3 to 324.1)
Macedonia	Cervical cancer	2.02 (1.78 to 2.48)	2.57 (2.27 to 2.83)	2.4 (2.02 to 2.85)	96.7 (85.4 to 119.5)	95.7 (84.8 to 105.6)	80 (67.3 to 94.1)
Macedonia	Uterine cancer	0.85 (0.746 to 0.971)	1.54 (1.4 to 1.7)	1.68 (1.41 to 1.99)	41.5 (36.6 to 46.9)	55.8 (50.8 to 61.3)	51.8 (43.7 to 60.9)
Macedonia	Ovarian cancer	1.43 (1.29 to 1.6)	2.24 (2.05 to 2.45)	2.46 (2.09 to 2.89)	68.1 (61.6 to 76.2)	81.8 (75 to 89.4)	78.8 (67.4 to 91.6)
Macedonia	Prostate cancer	1.53 (1.25 to 2.59)	2.96 (2.6 to 4.32)	4.42 (3.71 to 6.07)	86.1 (70.3 to 147.6)	107.1 (94.3 to 156.1)	127.5 (107.5 to 175.2)
Macedonia	Testicular cancer	0.456 (0.386 to 0.544)	0.372 (0.323 to 0.429)	0.381 (0.316 to 0.471)	21.7 (18.4 to 25.7)	15.9 (13.7 to 18.4)	15.4 (12.6 to 19.4)
Macedonia	Kidney cancer	0.413 (0.363 to 0.616)	1.11 (0.925 to 1.21)	1.38 (1.15 to 1.57)	20.9 (18.3 to 30.9)	42.7 (34.3 to 46.1)	45.7 (37.5 to 51.9)

SR Appendix Table 2. All-age DALYs counts and age standardized DALYs rates per 100,000 individuals for all causes and level 1 cause groups by location for 1990, 2007, and 2017, both sexes combined

		All-age DALYs			Age-standardized rate of DALYs per 100,000		
Macedonia	Bladder cancer	1.48 (1.32 to 1.76)	2.4 (2.22 to 2.75)	3.06 (2.62 to 3.51)	76.8 (68.6 to 94.2)	85.6 (79.2 to 99)	89.4 (76.4 to 102.4)
Macedonia	Thyroid cancer	0.258 (0.196 to 0.295)	0.286 (0.233 to 0.32)	0.293 (0.244 to 0.345)	12.6 (9.7 to 14.3)	10.5 (8.7 to 11.7)	9.2 (7.7 to 10.8)
Macedonia	Mesothelioma	0.0467 (0.0374 to 0.0588)	0.0573 (0.0519 to 0.0627)	0.049 (0.0423 to 0.0559)	2.2 (1.8 to 2.8)	2.1 (1.9 to 2.3)	1.6 (1.4 to 1.8)
Macedonia	Hodgkin lymphoma	0.631 (0.373 to 0.797)	0.548 (0.335 to 0.661)	0.518 (0.326 to 0.637)	30.3 (18.1 to 38.1)	22.3 (13.7 to 26.9)	19.8 (12.5 to 24.5)
Macedonia	Non-Hodgkin's lymphoma	1.25 (1.15 to 1.35)	1.39 (1.31 to 1.49)	1.6 (1.4 to 1.78)	61.9 (57.1 to 67)	56.4 (53 to 60.1)	58 (50.7 to 64.5)
Macedonia	Multiple myeloma	0.33 (0.284 to 0.366)	0.499 (0.429 to 0.543)	0.61 (0.493 to 0.698)	16.2 (14.2 to 17.9)	17.9 (15.3 to 19.6)	18.5 (14.8 to 21.1)
Macedonia	Leukaemia	3.3 (2.49 to 3.71)	3.35 (2.62 to 3.66)	3.73 (2.87 to 4.25)	166.4 (127.8 to 186.9)	144.6 (110.5 to 159.9)	148.7 (111.3 to 170.3)
Macedonia	Acute lymphoid leukaemia	0.905 (0.567 to 1.13)	0.654 (0.466 to 0.788)	0.676 (0.486 to 0.847)	46.9 (29 to 58.5)	34.7 (24.6 to 41.9)	36.7 (26.3 to 46)
Macedonia	Chronic lymphoid leukaemia	0.302 (0.244 to 0.392)	0.507 (0.432 to 0.607)	0.603 (0.467 to 0.766)	15.5 (12.6 to 20.5)	18.2 (15.6 to 21.8)	17.7 (13.8 to 22.5)
Macedonia	Acute myeloid leukaemia	0.924 (0.653 to 1.09)	1.12 (0.782 to 1.31)	1.34 (0.927 to 1.67)	45.9 (32.7 to 54.4)	47 (32.5 to 55.3)	51.4 (34.3 to 63.6)
Macedonia	Chronic myeloid leukaemia	0.28 (0.217 to 0.348)	0.262 (0.215 to 0.321)	0.243 (0.189 to 0.315)	13.6 (10.6 to 16.7)	10 (8.2 to 12.3)	8.1 (6.3 to 10.5)
Macedonia	Other leukaemia	0.885 (0.657 to 1.04)	0.808 (0.578 to 0.987)	0.866 (0.561 to 1.1)	44.5 (33.4 to 52.4)	34.8 (24.4 to 42.2)	34.8 (21.6 to 43.5)
Macedonia	Other malignant cancers	4.16 (3.51 to 4.6)	4.28 (3.98 to 4.59)	4.45 (3.85 to 5.45)	208.6 (176.5 to 230.1)	171.4 (159.3 to 183.4)	159 (137.9 to 197.2)
Macedonia	Other neoplasms	0.263 (0.185 to 0.316)	0.382 (0.302 to 0.448)	0.511 (0.421 to 0.638)	13.4 (9.6 to 15.9)	15.1 (11.9 to 17.7)	17.4 (14.2 to 21.5)
Macedonia	Myelodysplastic, myeloproliferative, and other hematopoietic neoplasms	0.223 (0.157 to 0.267)	0.343 (0.271 to 0.4)	0.462 (0.381 to 0.575)	11.4 (8.2 to 13.4)	13.1 (10.3 to 15.2)	14.9 (12.2 to 18.4)
Macedonia	Benign and in situ intestinal neoplasms	0 (0 to 0)	0 (0 to 0)	0 (0 to 0)	0 (0 to 0)	0 (0 to 0)	0 (0 to 0)
Macedonia	Benign and in situ cervical and uterine neoplasms	0 (0 to 0)	0 (0 to 0)	0 (0 to 0)	0 (0 to 0)	0 (0 to 0)	0 (0 to 0)
Macedonia	Other benign and in situ neoplasms	0.0405 (0.0264 to 0.051)	0.0394 (0.0292 to 0.0475)	0.049 (0.038 to 0.0626)	2.1 (1.3 to 2.6)	2 (1.5 to 2.5)	2.5 (1.9 to 3.2)
Macedonia	Cardiovascular diseases	160 (155 to 164)	195 (189 to 200)	194 (183 to 205)	8796 (8560.2 to 9019.9)	7390.8 (7191.8 to 7588)	5880.1 (5562.7 to 6210.6)
Macedonia	Rheumatic heart disease	2.05 (1.88 to 2.23)	1.16 (1.09 to 1.24)	0.863 (0.777 to 0.963)	100.8 (93.2 to 109.4)	44.7 (41.9 to 47.7)	29.3 (26.4 to 32.7)
Macedonia	Ischaemic heart disease	66.1 (63.7 to 68.9)	77.9 (75.7 to 80.2)	75.5 (69.8 to 81.5)	3546.3 (3418.9 to 3688.5)	2933.5 (2850.7 to 3020.4)	2284.5 (2111.2 to 2462.1)
Macedonia	Stroke	68.7 (65.8 to 71.6)	89.9 (86.7 to 93)	88.5 (82.7 to 94.7)	3837.7 (3686.4 to 4001.1)	3395.6 (3277.7 to 3508.4)	2660.6 (2481 to 2850.6)
Macedonia	Ischaemic stroke	28.4 (26.5 to 30.2)	40.2 (37.7 to 42.5)	40.9 (37.6 to 44.4)	1689.1 (1576.3 to 1792.7)	1556.5 (1463.4 to 1641.7)	1215.7 (1117.8 to 1318.6)
Macedonia	Intracerebral hemorrhage	33.6 (32 to 35.3)	41.8 (40 to 43.6)	39.8 (36.5 to 43.3)	1809.1 (1727.2 to 1897.9)	1540.3 (1474.9 to 1602.5)	1193.3 (1094.5 to 1298.7)
Macedonia	Subarachnoid hemorrhage	6.7 (6.1 to 7.77)	7.91 (7.17 to 8.97)	7.77 (6.91 to 9.07)	339.5 (309.8 to 397.7)	298.8 (272.3 to 339.2)	251.7 (224.1 to 291.9)
Macedonia	Hypertensive heart disease	6.94 (5.65 to 7.59)	8.89 (6.73 to 9.63)	9.59 (7.33 to 11.2)	422.5 (336.9 to 462.4)	349.3 (258.8 to 377.9)	286.6 (217.8 to 332.2)
Macedonia	Non-rheumatic valvular heart disease	0.519 (0.413 to 0.601)	0.5 (0.408 to 0.631)	0.645 (0.518 to 0.805)	27.9 (23.3 to 32.4)	19.4 (15.9 to 24.5)	20.5 (16.7 to 25.1)
Macedonia	Non-rheumatic calcific aortic valve disease	0.182 (0.141 to 0.245)	0.222 (0.171 to 0.298)	0.314 (0.233 to 0.405)	10.1 (7.8 to 13.6)	8.6 (6.7 to 11.5)	9.9 (7.4 to 12.6)
Macedonia	Non-rheumatic degenerative mitral valve disease	0.17 (0.122 to 0.235)	0.204 (0.155 to 0.259)	0.244 (0.178 to 0.32)	9.3 (6.8 to 12.7)	7.9 (5.9 to 10)	7.6 (5.6 to 9.8)
Macedonia	Other non-rheumatic valve diseases	0.166 (0.076 to 0.228)	0.0744 (0.0533 to 0.128)	0.0874 (0.0563 to 0.162)	8.4 (4 to 11.5)	2.9 (2.1 to 5)	3 (2 to 5.3)
Macedonia	Cardiomyopathy and myocarditis	5.11 (3.81 to 6.29)	6.33 (4.46 to 8.33)	7.6 (5.15 to 10.3)	284.9 (214.5 to 347.6)	257.1 (186.3 to 330.8)	252.6 (174.8 to 338.4)
Macedonia	Myocarditis	0.315 (0.151 to 0.455)	0.275 (0.158 to 0.382)	0.319 (0.181 to 0.476)	17.1 (8.3 to 25.2)	12.6 (7.1 to 17.1)	13.2 (7 to 19.5)
Macedonia	Alcoholic cardiomyopathy	1.19 (0.78 to 1.52)	0.849 (0.548 to 1.12)	0.876 (0.553 to 1.23)	64.7 (41.8 to 87.1)	33.1 (21.3 to 45)	28 (18.1 to 39.9)
Macedonia	Other cardiomyopathy	3.6 (2.49 to 4.56)	5.2 (3.38 to 7)	6.4 (4.11 to 8.8)	203 (139.7 to 256.6)	211.4 (142.6 to 277.1)	211.3 (141.3 to 284.9)
Macedonia	Atrial fibrillation and flutter	1.68 (1.42 to 1.98)	2.26 (1.88 to 2.71)	3.03 (2.5 to 3.58)	103.3 (88.9 to 119.6)	90.1 (75.9 to 106.1)	90.5 (75.3 to 106.8)
Macedonia	Aortic aneurysm	0.543 (0.467 to 0.742)	0.975 (0.786 to 1.08)	1.16 (0.877 to 1.37)	28.4 (24.5 to 38.9)	36 (29.2 to 39.8)	35.6 (27 to 42)
Macedonia	Peripheral vascular disease	0.133 (0.0738 to 0.217)	0.202 (0.118 to 0.324)	0.265 (0.16 to 0.417)	7.6 (4.3 to 12.3)	7.6 (4.5 to 12.1)	7.7 (4.7 to 12.1)
Macedonia	Endocarditis	0.233 (0.205 to 0.267)	0.237 (0.219 to 0.257)	0.285 (0.25 to 0.324)	12.3 (10.8 to 14.3)	10.3 (9.5 to 11.2)	11.2 (9.8 to 12.8)
Macedonia	Other cardiovascular and circulatory diseases	7.82 (6.54 to 8.76)	6.41 (5.2 to 8.79)	6.28 (4.68 to 9.55)	424.3 (351.9 to 474.1)	247 (201.7 to 332.4)	201 (151.7 to 298.5)
Macedonia	Chronic respiratory diseases	21 (18.7 to 23.4)	21.5 (18.9 to 24)	22.5 (19.7 to 25.4)	1124.2 (1007.3 to 1245.3)	835.2 (734.1 to 936.2)	738.7 (640.8 to 842.2)

SR Appendix Table 2. All-age DALYs counts and age standardized DALYs rates per 100,000 individuals for all causes and level 1 cause groups by location for 1990, 2007, and 2017, both sexes combined

		All-age DALYs			Age-standardized rate of DALYs per 100,000		
Macedonia	Chronic obstructive pulmonary disease	14.6 (13.1 to 16)	15.6 (14 to 17.4)	17 (15 to 18.9)	790.6 (716 to 864.5)	587 (528.2 to 649.7)	515.5 (458.4 to 573.2)
Macedonia	Pneumoconiosis	0.092 (0.0748 to 0.113)	0.0463 (0.0367 to 0.0571)	0.0399 (0.0292 to 0.0533)	4.6 (3.8 to 5.6)	1.7 (1.3 to 2)	1.2 (0.9 to 1.6)
Macedonia	Silicosis	0.0791 (0.0627 to 0.0989)	0.0277 (0.0227 to 0.0335)	0.0157 (0.0115 to 0.0209)	3.9 (3.1 to 4.9)	1 (0.8 to 1.2)	0.5 (0.3 to 0.6)
Macedonia	Asbestosis	0.00176 (0.00104 to 0.00331)	0.00258 (0.00171 to 0.00377)	0.0032 (0.00203 to 0.00471)	0.1 (0.1 to 0.2)	0.1 (0.1 to 0.1)	0.1 (0.1 to 0.1)
Macedonia	Coal workers pneumoconiosis	0.00526 (0.0031 to 0.00841)	0.0073 (0.00432 to 0.0115)	0.00968 (0.00567 to 0.0154)	0.3 (0.2 to 0.4)	0.3 (0.2 to 0.4)	0.3 (0.2 to 0.5)
Macedonia	Other pneumoconiosis	0.00592 (0.00376 to 0.00947)	0.00872 (0.00583 to 0.0127)	0.0112 (0.00741 to 0.0164)	0.3 (0.2 to 0.5)	0.3 (0.2 to 0.5)	0.3 (0.2 to 0.5)
Macedonia	Asthma	5.46 (4.24 to 6.91)	4.78 (3.55 to 6.34)	4.4 (3.17 to 5.93)	284.9 (224 to 359.6)	206.1 (150.1 to 274.5)	180.1 (128.1 to 245.5)
Macedonia	Interstitial lung disease and pulmonary sarcoidosis	0.449 (0.34 to 0.585)	0.538 (0.409 to 0.692)	0.647 (0.491 to 0.843)	22.4 (17 to 29)	20.3 (15.4 to 26.1)	20.9 (15.8 to 27.3)
Macedonia	Other chronic respiratory diseases	0.439 (0.371 to 0.505)	0.463 (0.378 to 0.548)	0.504 (0.412 to 0.601)	21.7 (18.3 to 24.9)	20.1 (16.6 to 23.9)	21 (17 to 24.8)
Macedonia	Digestive diseases	16.4 (14.1 to 19.2)	17.7 (15.3 to 20.7)	20.1 (16.9 to 23.8)	825.5 (711.9 to 963.5)	714.8 (609.7 to 840.7)	713.2 (592.8 to 858.4)
Macedonia	Cirrhosis and other chronic liver diseases	5.14 (4.7 to 5.99)	6.32 (5.88 to 7.04)	7.44 (6.38 to 8.4)	249.7 (228.3 to 288.7)	235.3 (219 to 261)	239.9 (205.4 to 270.8)
Macedonia	Cirrhosis and other chronic liver diseases due to hepatitis B	1.43 (1.24 to 1.7)	1.77 (1.57 to 2.02)	1.85 (1.54 to 2.19)	69.1 (60.2 to 81.3)	65.4 (57.8 to 74.3)	58.9 (49.1 to 69.4)
Macedonia	Cirrhosis and other chronic liver diseases due to hepatitis C	1.06 (0.906 to 1.27)	1.34 (1.16 to 1.56)	1.63 (1.34 to 1.94)	50.9 (43.7 to 61)	49 (42.3 to 56.9)	51.5 (42.6 to 61.2)
Macedonia	Cirrhosis and other chronic liver diseases due to alcohol use	1.81 (1.6 to 2.2)	2.26 (2.04 to 2.58)	2.79 (2.38 to 3.19)	87.5 (77.2 to 105.1)	82 (74.1 to 94)	87 (74.3 to 99.2)
Macedonia	Cirrhosis due to NASH	0.325 (0.28 to 0.407)	0.47 (0.409 to 0.551)	0.628 (0.53 to 0.732)	15.6 (13.5 to 19.5)	17.1 (14.9 to 20)	19.6 (16.5 to 22.7)
Macedonia	Cirrhosis and other chronic liver diseases due to other causes	0.517 (0.436 to 0.606)	0.476 (0.403 to 0.551)	0.54 (0.433 to 0.649)	26.6 (22.2 to 31.3)	21.9 (18.1 to 25.5)	23 (17.4 to 27.5)
Macedonia	Upper digestive system diseases	5.47 (4.02 to 7.55)	5.43 (3.84 to 7.74)	5.61 (3.92 to 8.13)	273.5 (202.4 to 374.4)	228.6 (160.3 to 325.6)	216.3 (149.4 to 311.3)
Macedonia	Peptic ulcer disease	1.68 (1.48 to 1.87)	1.3 (1.15 to 1.45)	1.24 (1.03 to 1.45)	86.7 (76.7 to 96.5)	49 (43.4 to 54.4)	39.2 (32.5 to 45.9)
Macedonia	Gastritis and duodenitis	2.17 (1.39 to 3.16)	2.15 (1.38 to 3.13)	2.19 (1.41 to 3.19)	108.4 (69.4 to 157.8)	101.1 (65 to 147.3)	98.6 (63.1 to 144.3)
Macedonia	GERD	1.62 (0.872 to 2.76)	1.98 (1.05 to 3.31)	2.18 (1.16 to 3.68)	78.4 (42.1 to 132.9)	78.5 (42 to 132.5)	78.5 (41.8 to 133.4)
Macedonia	Appendicitis	0.133 (0.113 to 0.157)	0.115 (0.0977 to 0.136)	0.118 (0.0956 to 0.14)	6.8 (5.7 to 7.9)	5.1 (4.2 to 6.2)	5 (4 to 6.1)
Macedonia	Paralytic ileus and intestinal obstruction	1.15 (0.959 to 1.52)	1.11 (0.814 to 1.24)	1.44 (0.917 to 1.75)	63.9 (53 to 85.2)	48.1 (35.7 to 53.9)	50.8 (33.5 to 61.3)
Macedonia	Inguinal, femoral, and abdominal hernia	1.63 (1.15 to 2.18)	1.73 (1.21 to 2.33)	1.99 (1.38 to 2.71)	84.3 (59.6 to 112.3)	79.6 (55.8 to 107.9)	81.2 (56.4 to 112.8)
Macedonia	Inflammatory bowel disease	0.512 (0.361 to 0.69)	0.596 (0.424 to 0.832)	0.791 (0.546 to 1.08)	24.9 (17.7 to 33.8)	24 (17.1 to 33.5)	28.6 (19.7 to 39.3)
Macedonia	Vascular intestinal disorders	0.581 (0.447 to 0.653)	0.554 (0.475 to 0.633)	0.565 (0.474 to 0.677)	30.5 (24 to 34.2)	20.3 (17.7 to 23.4)	17 (14.3 to 20.2)
Macedonia	Gallbladder and biliary diseases	0.243 (0.208 to 0.273)	0.224 (0.2 to 0.252)	0.234 (0.194 to 0.272)	13.1 (11.1 to 14.6)	8.5 (7.6 to 9.5)	7.1 (5.9 to 8.3)
Macedonia	Pancreatitis	0.865 (0.743 to 1.03)	1.09 (0.937 to 1.3)	1.24 (1 to 1.52)	42.7 (36.8 to 50.7)	41.6 (35.6 to 49.5)	41.5 (33.1 to 50.6)
Macedonia	Other digestive diseases	0.689 (0.547 to 0.852)	0.514 (0.405 to 0.634)	0.633 (0.493 to 0.789)	36 (28.5 to 44.9)	23.7 (18.4 to 29.8)	25.9 (19.7 to 32.8)
Macedonia	Neurological disorders	27.8 (21 to 36.1)	32.5 (25.1 to 42.1)	37.5 (29.1 to 47.5)	1501.3 (1172.7 to 1906.2)	1405.2 (1078.9 to 1813.8)	1394.3 (1064.1 to 1796.3)
Macedonia	Alzheimer's disease and other dementias	6.18 (5.75 to 6.62)	8.77 (8.09 to 9.51)	12.2 (10.8 to 13.7)	441.1 (411 to 473)	392.4 (361.3 to 424.9)	376.7 (332.6 to 422.5)
Macedonia	Parkinson's disease	1.27 (1.12 to 1.39)	1.95 (1.72 to 2.16)	2.4 (2.06 to 2.8)	78 (68.8 to 85.6)	74 (65.3 to 82.1)	70.4 (60.6 to 82)
Macedonia	Epilepsy	3.49 (1.83 to 5.75)	3.09 (1.81 to 4.99)	3.24 (1.48 to 5.89)	173.7 (91.3 to 286.4)	142.9 (83.2 to 229.7)	145.9 (66.1 to 267.7)
Macedonia	Multiple sclerosis	0.449 (0.388 to 0.525)	0.579 (0.495 to 0.699)	0.68 (0.555 to 0.846)	21.4 (18.5 to 25)	22.5 (19.3 to 26.9)	23.9 (19.4 to 29.6)
Macedonia	Motor neuron disease	0.209 (0.0692 to 0.31)	0.0625 (0.0536 to 0.127)	0.0655 (0.0522 to 0.161)	11.1 (3.5 to 17.2)	2.4 (2.1 to 5)	2.2 (1.7 to 5.6)
Macedonia	Headache disorders	15.3 (9.91 to 22.4)	17.2 (11.2 to 25.3)	17.9 (11.6 to 26.5)	730.6 (472.5 to 1071.9)	730 (468.8 to 1065)	727.6 (463 to 1067.1)
Macedonia	Migraine	13.1 (8.1 to 20)	14.8 (9.13 to 22.3)	15.3 (9.43 to 23.1)	626.3 (387 to 951.7)	625.7 (385.2 to 945.5)	623.7 (386.4 to 940.1)
Macedonia	Tension-type headache	2.17 (1.22 to 3.41)	2.48 (1.4 to 3.86)	2.61 (1.49 to 4.06)	104.3 (58.9 to 163.6)	104.3 (58.8 to 163.7)	104 (58.9 to 161.6)
Macedonia	Other neurological disorders	0.884 (0.618 to 1.24)	0.856 (0.641 to 1.16)	0.991 (0.69 to 1.45)	45.3 (31.8 to 63.7)	41.1 (30.1 to 56.2)	47.6 (31.5 to 72.5)
Macedonia	Mental disorders	27.2 (20.2 to 35.1)	30.3 (22.5 to 39.2)	31.4 (23.5 to 40.5)	1318.7 (977.7 to 1698)	1305.6 (968.2 to 1692)	1300.6 (963 to 1682.3)
Macedonia	Schizophrenia	2.56 (1.89 to 3.24)	3.01 (2.24 to 3.73)	3.29 (2.46 to 4.11)	121.2 (89.3 to 153.2)	121.4 (90.6 to 151.6)	122.5 (91.5 to 154.6)

SR Appendix Table 2. All-age DALYs counts and age standardized DALYs rates per 100,000 individuals for all causes and level 1 cause groups by location for 1990, 2007, and 2017, both sexes combined

		All-age DALYs			Age-standardized rate of DALYs per 100,000		
Macedonia	Depressive disorders	7.74 (5.54 to 10.4)	8.91 (6.32 to 12.1)	9.53 (6.8 to 12.9)	377.3 (270.3 to 507.6)	363 (257.4 to 494.8)	357.2 (253.6 to 482.3)
Macedonia	Major depressive disorder	5.98 (4.18 to 8.14)	6.8 (4.69 to 9.29)	7.22 (5.06 to 9.68)	292.6 (205.7 to 396.5)	278.2 (193.4 to 380)	272.5 (190.6 to 368.5)
Macedonia	Dysthymia	1.76 (1.18 to 2.56)	2.11 (1.41 to 3.08)	2.31 (1.54 to 3.34)	84.8 (57.3 to 123.1)	84.9 (56.9 to 124.4)	84.6 (56.8 to 122.4)
Macedonia	Bipolar disorder	2.89 (1.82 to 4.32)	3.27 (2.06 to 4.78)	3.38 (2.14 to 4.99)	138 (86.8 to 206.2)	138 (87.2 to 201.7)	138.1 (86.6 to 204.6)
Macedonia	Anxiety disorders	6.37 (4.5 to 8.55)	7.12 (5.03 to 9.41)	7.34 (5.21 to 9.79)	308.3 (218 to 412.3)	308.9 (218.4 to 408.3)	307.4 (216.8 to 411.3)
Macedonia	Eating disorders	0.717 (0.447 to 1.06)	0.732 (0.457 to 1.08)	0.796 (0.506 to 1.17)	34 (21.2 to 50.3)	33.7 (21 to 49.4)	37.4 (23.7 to 54.9)
Macedonia	Anorexia nervosa	0.143 (0.0805 to 0.223)	0.14 (0.0795 to 0.213)	0.145 (0.0865 to 0.221)	6.8 (3.9 to 10.7)	6.7 (3.8 to 10.1)	7.4 (4.4 to 11.2)
Macedonia	Bulimia nervosa	0.574 (0.353 to 0.861)	0.592 (0.367 to 0.884)	0.651 (0.402 to 0.983)	27.1 (16.7 to 40.7)	27 (16.8 to 40.5)	30 (18.5 to 45.1)
Macedonia	Autism spectrum disorders	1.35 (0.92 to 1.85)	1.38 (0.949 to 1.92)	1.4 (0.956 to 1.92)	67.3 (45.8 to 92.4)	67.7 (46.1 to 93.9)	67.7 (46.2 to 93.8)
Macedonia	Attention-deficit/hyperactivity disorder	0.24 (0.141 to 0.379)	0.231 (0.138 to 0.368)	0.214 (0.128 to 0.341)	11.8 (6.9 to 18.6)	11.8 (7 to 18.9)	11.8 (7 to 19)
Macedonia	Conduct disorder	1.95 (1.17 to 3.08)	1.7 (1.02 to 2.69)	1.35 (0.804 to 2.1)	97.6 (58.4 to 154.3)	98 (58.8 to 155.4)	98.1 (58.6 to 153.1)
Macedonia	Idiopathic developmental intellectual disability	0.477 (0.174 to 0.873)	0.465 (0.169 to 0.853)	0.406 (0.133 to 0.762)	23.6 (8.6 to 43.4)	23 (8.3 to 42.1)	20.2 (6.5 to 37.6)
Macedonia	Other mental disorders	2.91 (1.95 to 4.02)	3.44 (2.27 to 4.75)	3.73 (2.47 to 5.2)	139.7 (93.5 to 192.8)	140.1 (91.7 to 193.5)	140.2 (92.3 to 196.3)
Macedonia	Substance use disorders	6.22 (4.49 to 8.08)	7.7 (5.79 to 9.86)	8.1 (6.03 to 10.4)	295.1 (212.9 to 382.6)	326.1 (243.9 to 415.2)	331.2 (245.6 to 424.8)
Macedonia	Alcohol use disorders	4.08 (2.96 to 5.51)	4.82 (3.51 to 6.39)	5.04 (3.69 to 6.75)	193.6 (140.2 to 261)	200.2 (144.2 to 267.6)	200.4 (143.6 to 271.9)
Macedonia	Drug use disorders	2.14 (1.52 to 2.76)	2.88 (2.19 to 3.61)	3.06 (2.28 to 3.85)	101.4 (72.1 to 131.1)	125.9 (95.8 to 157.7)	130.8 (97.5 to 165.3)
Macedonia	Opioid use disorders	1.41 (0.977 to 1.87)	1.92 (1.39 to 2.47)	2.11 (1.54 to 2.74)	66.5 (46.4 to 88.4)	82.9 (60.3 to 107)	87.7 (63.7 to 113.6)
Macedonia	Cocaine use disorders	0.114 (0.0694 to 0.171)	0.135 (0.0845 to 0.202)	0.137 (0.085 to 0.204)	5.4 (3.3 to 8.2)	5.9 (3.6 to 8.9)	5.9 (3.6 to 9)
Macedonia	Amphetamine use disorders	0.205 (0.111 to 0.34)	0.242 (0.139 to 0.379)	0.227 (0.133 to 0.359)	9.8 (5.3 to 16.3)	11.2 (6.4 to 17.6)	11 (6.4 to 17.8)
Macedonia	Cannabis use disorders	0.109 (0.0668 to 0.162)	0.104 (0.0652 to 0.156)	0.104 (0.066 to 0.152)	5.2 (3.2 to 7.7)	4.7 (3 to 7.1)	5 (3.1 to 7.3)
Macedonia	Other drug use disorders	0.305 (0.206 to 0.431)	0.479 (0.33 to 0.637)	0.48 (0.344 to 0.619)	14.5 (9.8 to 20.5)	21.2 (14.5 to 28.4)	21.2 (14.9 to 27.8)
Macedonia	Diabetes and kidney diseases	21.9 (18.6 to 26.1)	37.9 (32.5 to 44.7)	41.9 (34.7 to 50.2)	1117.8 (949.9 to 1326.6)	1392.8 (1191.5 to 1647.5)	1295.4 (1065.9 to 1553.4)
Macedonia	Diabetes mellitus	15.6 (12.8 to 19.4)	29.3 (24.4 to 35.6)	33.1 (26.5 to 40.6)	794 (649.5 to 982.6)	1071.5 (887.3 to 1306.1)	1015.1 (805.4 to 1252.1)
Macedonia	Diabetes mellitus type 1	2.01 (1.81 to 2.28)	2.83 (2.38 to 3.08)	2.67 (2.23 to 3.05)	99.3 (89.3 to 113.9)	106.3 (90.5 to 115.5)	88.8 (75 to 100.8)
Macedonia	Diabetes mellitus type 2	13.6 (10.8 to 17.4)	26.5 (21.7 to 32.6)	30.4 (23.9 to 37.7)	694.7 (554.8 to 880.8)	965.2 (785.6 to 1190.4)	926.2 (719 to 1158.6)
Macedonia	Chronic kidney disease	6.29 (5.78 to 6.85)	8.52 (7.81 to 9.23)	8.79 (7.86 to 9.68)	323.7 (297.8 to 351.9)	321.3 (295.3 to 347.7)	280.3 (250.6 to 308.3)
Macedonia	Chronic kidney disease due to diabetes mellitus type 1	0.36 (0.277 to 0.458)	0.441 (0.324 to 0.574)	0.463 (0.359 to 0.601)	17.2 (13.2 to 21.8)	16.5 (12.3 to 21.2)	15.6 (12.1 to 19.7)
Macedonia	Chronic kidney disease due to diabetes mellitus type 2	0.952 (0.782 to 1.16)	1.5 (1.23 to 1.82)	1.63 (1.33 to 1.99)	49.3 (40.5 to 59.8)	54.1 (44.6 to 65.1)	47.8 (39.5 to 58.4)
Macedonia	Chronic kidney disease due to hypertension	1 (0.836 to 1.17)	1.59 (1.33 to 1.84)	1.61 (1.32 to 1.92)	53.9 (45.2 to 62.9)	59.8 (50.4 to 68.7)	49.1 (40.6 to 58)
Macedonia	Chronic kidney disease due to glomerulonephritis	1.52 (1.32 to 1.74)	1.94 (1.66 to 2.25)	1.93 (1.64 to 2.28)	75.8 (65.2 to 86.4)	73.7 (64 to 84.6)	64.5 (54.8 to 75.3)
Macedonia	Chronic kidney disease due to other and unspecified causes	2.45 (2.12 to 2.83)	3.05 (2.65 to 3.49)	3.16 (2.68 to 3.71)	127.6 (110.6 to 147.4)	117.2 (102.5 to 132.8)	103.4 (88.3 to 120.3)
Macedonia	Acute glomerulonephritis	0.0015 (0.00116 to 0.00199)	0.00132 (0.00101 to 0.00176)	0.0014 (0.00104 to 0.00186)	0.1 (0.1 to 0.1)	0.1 (0 to 0.1)	0.1 (0 to 0.1)
Macedonia	Skin and subcutaneous diseases	9.19 (6.15 to 13.4)	9.57 (6.45 to 14.1)	9.96 (6.7 to 14.7)	464 (310.6 to 674.8)	466.7 (310.9 to 686.8)	473.5 (316.8 to 690.6)
Macedonia	Dermatitis	2.28 (1.33 to 3.6)	2.2 (1.33 to 3.42)	2.24 (1.35 to 3.51)	116 (67.1 to 184)	114.8 (67.6 to 180.5)	117.1 (68.2 to 185.5)
Macedonia	Atopic dermatitis	1.8 (0.968 to 3.03)	1.66 (0.909 to 2.74)	1.67 (0.907 to 2.79)	92.9 (50.3 to 155.7)	91.6 (49.8 to 151.8)	94 (49.9 to 158)
Macedonia	Contact dermatitis	0.473 (0.303 to 0.701)	0.539 (0.35 to 0.805)	0.567 (0.366 to 0.832)	23 (14.8 to 34.1)	23 (15 to 33.9)	23 (14.9 to 34)
Macedonia	Seborrhoeic dermatitis	0.00215 (0.00121 to 0.00348)	0.0023 (0.00129 to 0.00371)	0.00241 (0.00134 to 0.00386)	0.1 (0.1 to 0.2)	0.1 (0.1 to 0.2)	0.1 (0.1 to 0.2)
Macedonia	Psoriasis	1.38 (0.976 to 1.82)	1.67 (1.18 to 2.2)	1.82 (1.27 to 2.39)	66.9 (47.2 to 87.9)	69.2 (49.2 to 91.9)	70.1 (49.2 to 92.8)
Macedonia	Bacterial skin diseases	0.132 (0.0661 to 0.2)	0.0986 (0.0737 to 0.131)	0.118 (0.0893 to 0.155)	7.2 (3.4 to 11.1)	4.6 (3.4 to 6)	4.9 (3.6 to 6.4)
Macedonia	Cellulitis	0.0474 (0.0328 to 0.0646)	0.0471 (0.0333 to 0.0636)	0.0529 (0.038 to 0.0713)	2.4 (1.6 to 3.2)	2.1 (1.5 to 2.9)	2.2 (1.6 to 3)

SR Appendix Table 2. All-age DALYs counts and age standardized DALYs rates per 100,000 individuals for all causes and level 1 cause groups by location for 1990, 2007, and 2017, both sexes combined

		All-age DALYs			Age-standardized rate of DALYs per 100,000		
Macedonia	Pyoderma	0.0848 (0.0313 to 0.145)	0.0514 (0.0366 to 0.0736)	0.0653 (0.0441 to 0.0926)	4.8 (1.6 to 8.5)	2.5 (1.6 to 3.5)	2.7 (1.7 to 3.8)
Macedonia	Scabies	0.53 (0.291 to 0.859)	0.523 (0.29 to 0.846)	0.505 (0.278 to 0.816)	26.4 (14.6 to 42.6)	26.4 (14.7 to 42.9)	26.2 (14.4 to 42.5)
Macedonia	Fungal skin diseases	0.915 (0.363 to 1.91)	1.03 (0.413 to 2.12)	1.14 (0.458 to 2.35)	47.6 (19 to 99.4)	45.6 (18.3 to 94.3)	45 (17.9 to 93.4)
Macedonia	Viral skin diseases	0.874 (0.558 to 1.31)	0.73 (0.462 to 1.09)	0.679 (0.434 to 1)	45.7 (29.2 to 68.4)	45.7 (29 to 68.3)	45.8 (29.1 to 67.6)
Macedonia	Acne vulgaris	0.545 (0.321 to 0.884)	0.669 (0.396 to 1.09)	0.629 (0.375 to 1.01)	26.3 (15.5 to 42.6)	32.5 (19.3 to 53)	34.8 (20.4 to 55.8)
Macedonia	Alopecia areata	0.124 (0.0768 to 0.185)	0.135 (0.0856 to 0.203)	0.141 (0.0913 to 0.212)	5.9 (3.7 to 8.8)	5.9 (3.8 to 8.9)	5.9 (3.8 to 8.8)
Macedonia	Pruritus	0.191 (0.0898 to 0.358)	0.219 (0.105 to 0.409)	0.241 (0.114 to 0.452)	9.5 (4.5 to 17.8)	9.4 (4.5 to 17.7)	9.5 (4.5 to 18.2)
Macedonia	Urticaria	1.46 (0.966 to 2.08)	1.39 (0.921 to 1.96)	1.38 (0.918 to 1.94)	74.6 (49 to 106.1)	74.7 (49.2 to 106.3)	74.6 (49.4 to 106.7)
Macedonia	Decubitus ulcer	0.0495 (0.0361 to 0.066)	0.0634 (0.0461 to 0.0842)	0.0812 (0.0581 to 0.11)	2.7 (2 to 3.6)	2.7 (2 to 3.6)	2.8 (2 to 3.8)
Macedonia	Other skin and subcutaneous diseases	0.705 (0.342 to 1.27)	0.842 (0.411 to 1.55)	0.976 (0.468 to 1.79)	35.3 (17.1 to 63.9)	35.1 (17.1 to 64.7)	36.6 (17.6 to 67.5)
Macedonia	Sense organ diseases	14.5 (9.64 to 21.2)	18.7 (12.4 to 27.1)	21.8 (14.6 to 31.2)	762.2 (509.8 to 1100.3)	745.7 (499.9 to 1078.7)	733.3 (489.4 to 1058.6)
Macedonia	Blindness and vision impairment	6.02 (3.92 to 8.98)	7.47 (4.81 to 11.1)	8.54 (5.57 to 12.6)	314.1 (204.1 to 466.4)	299.2 (192.3 to 445.2)	290.3 (187.8 to 430.2)
Macedonia	Glaucoma	0.0726 (0.0466 to 0.102)	0.097 (0.0619 to 0.138)	0.118 (0.0779 to 0.165)	4 (2.6 to 5.6)	3.6 (2.3 to 5.1)	3.4 (2.3 to 4.8)
Macedonia	Cataract	1.75 (1.18 to 2.44)	2.51 (1.67 to 3.47)	3.12 (2.09 to 4.26)	99.9 (67 to 137.1)	96 (64.5 to 131.5)	93.1 (62.4 to 127.5)
Macedonia	Age-related macular degeneration	0.0158 (0.00996 to 0.0231)	0.0234 (0.0147 to 0.0348)	0.0302 (0.0193 to 0.0435)	0.9 (0.6 to 1.3)	0.9 (0.6 to 1.3)	0.9 (0.6 to 1.3)
Macedonia	Refraction disorders	2.19 (1.41 to 3.35)	2.38 (1.51 to 3.59)	2.49 (1.57 to 3.77)	109.3 (69.8 to 167.3)	104.8 (66.2 to 160.2)	101.8 (64.6 to 155.9)
Macedonia	Near vision loss	1.52 (0.719 to 2.75)	1.86 (0.882 to 3.48)	2.1 (1.02 to 3.82)	75.6 (35.8 to 137.2)	70.7 (33.5 to 131.8)	68.2 (33.2 to 124.3)
Macedonia	Other vision loss	0.47 (0.314 to 0.659)	0.591 (0.396 to 0.812)	0.688 (0.459 to 0.96)	24.4 (16.3 to 34.1)	23.3 (15.6 to 32)	22.8 (15.3 to 31.4)
Macedonia	Age-related and other hearing loss	7.91 (5.32 to 11.2)	10.4 (7.05 to 14.8)	12.3 (8.31 to 17.4)	417.5 (284.7 to 588.1)	415.4 (284.6 to 584.9)	411.8 (280.3 to 582.2)
Macedonia	Other sense organ diseases	0.6 (0.37 to 0.878)	0.778 (0.485 to 1.13)	0.915 (0.563 to 1.33)	30.7 (18.9 to 44.8)	31.1 (19.3 to 45.2)	31.3 (19.2 to 45.8)
Macedonia	Musculoskeletal disorders	33.3 (24.2 to 45.1)	41.6 (30.4 to 56.5)	47.5 (34.6 to 64.4)	1629.5 (1186 to 2195.5)	1617.6 (1175.8 to 2195.2)	1635.4 (1188.7 to 2219)
Macedonia	Rheumatoid arthritis	0.516 (0.354 to 0.703)	0.713 (0.493 to 0.96)	0.902 (0.615 to 1.21)	25.2 (17.4 to 34.3)	27 (18.7 to 36.3)	29.6 (20.2 to 39.9)
Macedonia	Osteoarthritis	1.82 (0.903 to 3.57)	2.67 (1.34 to 5.21)	3.34 (1.67 to 6.54)	93.8 (46.8 to 184.3)	98.1 (49.3 to 191.8)	100.9 (50.4 to 197.7)
Macedonia	Low back pain	24.2 (17.3 to 32.8)	29.5 (21.1 to 39.9)	33.6 (24.4 to 45.4)	1178.8 (846.4 to 1601.8)	1160.2 (833.4 to 1566.6)	1177.5 (851.7 to 1600.5)
Macedonia	Neck pain	6.07 (4.21 to 8.61)	7.77 (5.4 to 10.9)	8.74 (6.13 to 12.3)	296 (205.7 to 417.3)	296.4 (208.1 to 414.6)	295.8 (205.5 to 413.1)
Macedonia	Gout	0.243 (0.16 to 0.336)	0.335 (0.221 to 0.467)	0.411 (0.272 to 0.567)	12.4 (8.3 to 17.2)	12.4 (8.1 to 17.2)	12.8 (8.5 to 17.5)
Macedonia	Other musculoskeletal disorders	0.487 (0.245 to 0.831)	0.588 (0.289 to 0.977)	0.508 (0.234 to 0.88)	23.3 (12 to 39.5)	23.5 (11.5 to 38.5)	18.8 (8.5 to 32.2)
Macedonia	Other non-communicable diseases	36.8 (31 to 43.7)	23.6 (18.1 to 30.2)	23.3 (17.5 to 30.2)	2037.1 (1732.6 to 2382.5)	1245.6 (1001.4 to 1526.9)	1089.5 (860.1 to 1377.2)
Macedonia	Congenital anomalies	22.5 (19.3 to 26.1)	9.27 (7.82 to 10.8)	7.97 (6.55 to 9.49)	1307.5 (1119.4 to 1517.1)	639 (550.3 to 727.1)	508.7 (428.4 to 600.4)
Macedonia	Neural tube defects	1.71 (1.07 to 2.5)	0.455 (0.366 to 0.565)	0.336 (0.259 to 0.43)	102.4 (63.9 to 150.5)	36.5 (28.9 to 46.3)	25 (18.4 to 33.1)
Macedonia	Congenital heart anomalies	9.96 (5.14 to 13.2)	2.9 (2.48 to 3.4)	2.17 (1.79 to 2.57)	594.9 (303.8 to 791.4)	242.6 (204.2 to 287.3)	177.2 (145 to 212.5)
Macedonia	Orofacial clefts	0.168 (0.113 to 0.249)	0.0573 (0.0374 to 0.0825)	0.0563 (0.0367 to 0.0828)	9.6 (6.3 to 14.3)	3 (1.9 to 4.3)	2.8 (1.9 to 4.1)
Macedonia	Down's syndrome	1.1 (0.26 to 2.51)	0.507 (0.242 to 0.656)	0.397 (0.249 to 0.517)	64.6 (13.4 to 149)	38.3 (14.8 to 51.4)	27.8 (14.9 to 37.6)
Macedonia	Turner syndrome	0.0021 (0.00101 to 0.00339)	0.00207 (0.00101 to 0.00335)	0.00199 (0.00097 to 0.00325)	0.1 (0 to 0.2)	0.1 (0.1 to 0.2)	0.1 (0.1 to 0.2)
Macedonia	Klinefelter syndrome	0.000401 (0.000188 to 0.000746)	0.00041 (0.000191 to 0.000791)	0.000412 (0.000189 to 0.000794)	0 (0 to 0)	0 (0 to 0)	0 (0 to 0)
Macedonia	Other chromosomal abnormalities	0.917 (0.289 to 1.41)	0.295 (0.227 to 0.378)	0.266 (0.189 to 0.391)	54.2 (16.6 to 83.9)	22.7 (17.6 to 29.4)	19.1 (13.6 to 29)
Macedonia	Congenital musculoskeletal and limb anomalies	2.88 (2.03 to 3.83)	2.78 (1.89 to 3.73)	2.82 (1.91 to 3.87)	148.6 (106.2 to 196.3)	137.4 (95.2 to 183.3)	131.5 (90 to 179.5)
Macedonia	Urogenital congenital anomalies	0.224 (0.129 to 0.548)	0.124 (0.0907 to 0.219)	0.117 (0.0889 to 0.176)	12.9 (7.3 to 32)	9 (6.3 to 17.9)	8.4 (6.2 to 13.7)
Macedonia	Digestive congenital anomalies	2.63 (1.61 to 3.62)	1.1 (0.815 to 1.38)	0.913 (0.673 to 1.18)	152.7 (91.6 to 211.3)	76 (53.7 to 93.9)	56.9 (42.7 to 71.9)
Macedonia	Other congenital anomalies	2.87 (1.39 to 4.08)	1.05 (0.835 to 1.35)	0.9 (0.691 to 1.16)	167.5 (78.1 to 240.2)	73.6 (59.6 to 99.7)	59.9 (46 to 80.8)

SR Appendix Table 2. All-age DALYs counts and age standardized DALYs rates per 100,000 individuals for all causes and level 1 cause groups by location for 1990, 2007, and 2017, both sexes combined

		All-age DALYs			Age-standardized rate of DALYs per 100,000		
Macedonia	Urinary diseases and male infertility	1.38<br>(1 to 1.82)	1.52<br>(1.04 to 2.1)	1.79<br>(1.23 to 2.47)	70.5<br>(52.1 to 92.5)	57.4<br>(39.8 to 79.1)	56.9<br>(39.6 to 77.8)
Macedonia	Urinary tract infections	0.287<br>(0.23 to 0.335)	0.15<br>(0.127 to 0.179)	0.156<br>(0.131 to 0.187)	15.5<br>(12.1 to 18.2)	7<br>(5.9 to 8.4)	6.7<br>(5.5 to 8)
Macedonia	Urolithiasis	0.0956<br>(0.0659 to 0.132)	0.116<br>(0.0794 to 0.162)	0.132<br>(0.0889 to 0.183)	4.7<br>(3.2 to 6.4)	4.6<br>(3.1 to 6.4)	4.6<br>(3.2 to 6.4)
Macedonia	Benign prostatic hyperplasia	0.803<br>(0.506 to 1.17)	1.11<br>(0.702 to 1.64)	1.38<br>(0.876 to 2)	40.5<br>(25.5 to 58.9)	39.4<br>(24.9 to 58.1)	40.1<br>(25.5 to 58)
Macedonia	Male infertility	0.0434<br>(0.0173 to 0.0919)	0.0482<br>(0.0191 to 0.101)	0.0424<br>(0.0168 to 0.0896)	2<br>(0.8 to 4.3)	2.2<br>(0.9 to 4.5)	1.9<br>(0.7 to 3.9)
Macedonia	Other urinary diseases	0.148<br>(0.116 to 0.2)	0.0924<br>(0.0711 to 0.117)	0.0818<br>(0.0625 to 0.102)	7.7<br>(6 to 10.7)	4.4<br>(3.3 to 5.5)	3.7<br>(2.9 to 4.6)
Macedonia	Gynecological diseases	3.14<br>(2.16 to 4.41)	3.44<br>(2.34 to 4.82)	3.49<br>(2.39 to 4.95)	148.9<br>(102.8 to 209.5)	145.4<br>(99.8 to 202.3)	144.7<br>(99.2 to 204.2)
Macedonia	Uterine fibroids	0.441<br>(0.25 to 0.749)	0.512<br>(0.293 to 0.855)	0.521<br>(0.292 to 0.892)	20.9<br>(11.9 to 35.3)	19.7<br>(11.3 to 33)	18.8<br>(10.4 to 31.9)
Macedonia	Polycystic ovarian syndrome	0.108<br>(0.0473 to 0.209)	0.115<br>(0.0513 to 0.225)	0.115<br>(0.0508 to 0.223)	5.1<br>(2.2 to 9.9)	5<br>(2.2 to 9.8)	5<br>(2.2 to 9.8)
Macedonia	Female infertility	0.0359<br>(0.0108 to 0.0816)	0.0344<br>(0.0105 to 0.0797)	0.043<br>(0.0134 to 0.0974)	1.7<br>(0.5 to 3.8)	1.5<br>(0.5 to 3.6)	1.9<br>(0.6 to 4.3)
Macedonia	Endometriosis	0.924<br>(0.607 to 1.36)	0.976<br>(0.64 to 1.44)	0.98<br>(0.637 to 1.44)	43.6<br>(28.7 to 64.1)	43<br>(28.1 to 63.3)	42.6<br>(27.6 to 62.7)
Macedonia	Genital prolapse	0.0938<br>(0.0462 to 0.173)	0.091<br>(0.0447 to 0.17)	0.107<br>(0.0524 to 0.202)	4.6<br>(2.3 to 8.4)	3.3<br>(1.6 to 6.2)	3.3<br>(1.6 to 6.3)
Macedonia	Premenstrual syndrome	1.09<br>(0.691 to 1.62)	1.15<br>(0.741 to 1.72)	1.13<br>(0.718 to 1.68)	51.7<br>(32.9 to 77.2)	50<br>(32.2 to 74.4)	49.1<br>(31.3 to 73.5)
Macedonia	Other gynecological diseases	0.448<br>(0.3 to 0.631)	0.555<br>(0.375 to 0.798)	0.599<br>(0.399 to 0.883)	21.3<br>(14.3 to 30)	22.8<br>(15.4 to 32.9)	24<br>(16 to 35)
Macedonia	Hemoglobinopathies and hemolytic anaemias	1.37<br>(1.01 to 1.83)	1.13<br>(0.823 to 1.56)	1.01<br>(0.739 to 1.43)	69.1<br>(51.3 to 92)	52.3<br>(38 to 71.9)	44.4<br>(32.1 to 62.6)
Macedonia	Thalassemias	0.0354<br>(0.0217 to 0.0588)	0.00912<br>(0.00639 to 0.013)	0.00856<br>(0.00573 to 0.0128)	2<br>(1.2 to 3.4)	0.7<br>(0.5 to 1)	0.7<br>(0.5 to 1.1)
Macedonia	Thalassaemias trait	0.264<br>(0.171 to 0.392)	0.232<br>(0.152 to 0.34)	0.202<br>(0.129 to 0.306)	13.3<br>(8.6 to 19.8)	11.1<br>(7.2 to 16.2)	9<br>(5.7 to 13.5)
Macedonia	Sickle cell disorders	0.118<br>(0.0938 to 0.154)	0.0648<br>(0.049 to 0.0767)	0.0646<br>(0.0484 to 0.0784)	6.3<br>(4.9 to 8.4)	3.6<br>(2.9 to 4.3)	3.8<br>(2.9 to 4.6)
Macedonia	Sickle cell trait	0.152<br>(0.0946 to 0.235)	0.128<br>(0.0815 to 0.198)	0.103<br>(0.0643 to 0.161)	7.7<br>(4.8 to 11.8)	6.4<br>(4.1 to 9.9)	5<br>(3.1 to 7.6)
Macedonia	G6PD deficiency	0.0848<br>(0.0631 to 0.103)	0.0755<br>(0.061 to 0.0894)	0.0729<br>(0.0549 to 0.0886)	4.2<br>(3.1 to 5.1)	3.3<br>(2.6 to 3.9)	3.1<br>(2.3 to 3.8)
Macedonia	G6PD trait	0.0000742<br>(0.0000479 to 0.000108)	0.0000791<br>(0.0000527 to 0.000116)	0.0000613<br>(0.0000409 to 0.0000902)	0<br>(0 to 0)	0<br>(0 to 0)	0<br>(0 to 0)
Macedonia	Other hemoglobinopathies and hemolytic anaemias	0.71<br>(0.505 to 0.975)	0.621<br>(0.436 to 0.865)	0.563<br>(0.402 to 0.815)	35.6<br>(25.5 to 48.5)	27.1<br>(18.7 to 38.3)	22.9<br>(15.9 to 33.6)
Macedonia	Endocrine, metabolic, blood, and immune disorders	1.84<br>(1.2 to 2.39)	1.26<br>(1 to 1.59)	1.34<br>(1.05 to 1.71)	97.3<br>(61 to 127.5)	61<br>(48.6 to 76.4)	60.6<br>(47.5 to 76.5)
Macedonia	Oral disorders	5.43<br>(3.45 to 8.1)	6.64<br>(4.22 to 9.86)	7.34<br>(4.74 to 10.8)	273.3<br>(174.8 to 407.8)	258.7<br>(163.5 to 386.4)	247.6<br>(157.9 to 367.7)
Macedonia	Caries of deciduous teeth	0.0428<br>(0.0182 to 0.0837)	0.0298<br>(0.0129 to 0.0598)	0.0275<br>(0.0115 to 0.0538)	2.4<br>(1 to 4.7)	2.4<br>(1 to 4.8)	2.4<br>(1 to 4.6)
Macedonia	Caries of permanent teeth	0.541<br>(0.231 to 1.04)	0.588<br>(0.256 to 1.12)	0.572<br>(0.251 to 1.09)	26.1<br>(11.2 to 49.9)	26.1<br>(11.3 to 50.1)	25<br>(10.9 to 47.5)
Macedonia	Periodontal disease	0.792<br>(0.315 to 1.66)	0.925<br>(0.365 to 1.93)	1.01<br>(0.397 to 2.12)	38.5<br>(15.3 to 80.3)	35.1<br>(13.8 to 71.9)	33.4<br>(13.2 to 68.8)
Macedonia	Edentulism and severe tooth loss	3<br>(1.99 to 4.24)	3.91<br>(2.61 to 5.46)	4.49<br>(3 to 6.33)	155.6<br>(103.6 to 219.5)	144.3<br>(96.5 to 201.9)	136.2<br>(91.3 to 192.2)
Macedonia	Other oral disorders	1.05<br>(0.66 to 1.55)	1.19<br>(0.736 to 1.75)	1.25<br>(0.786 to 1.84)	50.7<br>(31.9 to 74.8)	50.7<br>(31.4 to 74.8)	50.6<br>(31.8 to 75)
Macedonia	Sudden infant death syndrome	1.17<br>(0.396 to 3.34)	0.351<br>(0.215 to 0.531)	0.302<br>(0.177 to 0.462)	70.6<br>(24 to 201.7)	31.8<br>(19.5 to 48.1)	26.6<br>(15.6 to 40.7)
Macedonia	Injuries	66.3<br>(57.3 to 77)	59.5<br>(50 to 71.6)	64.9<br>(52.4 to 80.3)	3276.4<br>(2840.6 to 3797.3)	2527.2<br>(2137.1 to 3026.8)	2526.3<br>(2065.2 to 3094.8)
Macedonia	Transport injuries	14.1<br>(12.7 to 15.7)	12.7<br>(11.4 to 14.2)	13<br>(11.2 to 15)	692.8<br>(626.5 to 768.8)	552.7<br>(498.2 to 614.8)	545<br>(475 to 620.3)
Macedonia	Road injuries	13.3<br>(12 to 14.8)	11.9<br>(10.7 to 13.4)	12.2<br>(10.5 to 14.1)	656.1<br>(592.5 to 727.4)	519.4<br>(468.8 to 579.7)	509.4<br>(444 to 582.5)
Macedonia	Pedestrian road injuries	4.83<br>(4.27 to 5.4)	2.61<br>(2.25 to 3.23)	2.72<br>(2.28 to 3.35)	241<br>(213.7 to 269.2)	114.4<br>(99.6 to 142.9)	113.8<br>(95.2 to 140.3)
Macedonia	Cyclist road injuries	1.16<br>(0.874 to 1.51)	1.23<br>(0.909 to 1.6)	1.5<br>(1.1 to 1.98)	56.5<br>(42.7 to 73.3)	50.3<br>(37.4 to 65.5)	56.6<br>(41.7 to 74.1)
Macedonia	Motorcyclist road injuries	1.1<br>(0.927 to 1.31)	1.11<br>(0.934 to 1.33)	1.18<br>(0.97 to 1.42)	53.5<br>(44.9 to 63)	48.4<br>(41.1 to 58.2)	50.2<br>(41.3 to 59.6)
Macedonia	Motor vehicle road injuries	5.91<br>(5.27 to 6.93)	6.81<br>(6.16 to 7.47)	6.62<br>(5.77 to 7.54)	288.1<br>(255.7 to 338.3)	299<br>(270.7 to 326.9)	282<br>(248.4 to 318.8)
Macedonia	Other road injuries	0.34<br>(0.179 to 0.452)	0.175<br>(0.127 to 0.233)	0.177<br>(0.127 to 0.237)	17<br>(8.9 to 22.5)	7.3<br>(5.4 to 9.8)	6.8<br>(4.9 to 9.1)
Macedonia	Other transport injuries	0.736<br>(0.645 to 0.843)	0.75<br>(0.661 to 0.841)	0.829<br>(0.709 to 0.953)	36.7<br>(32.1 to 41.8)	33.3<br>(29.5 to 37.3)	35.6<br>(30.6 to 41.1)
Macedonia	Unintentional injuries	41.8<br>(34.4 to 50.8)	35.6<br>(27.6 to 46)	41.9<br>(31.4 to 55.4)	2080.9<br>(1711.9 to 2520.7)	1500.9<br>(1170.3 to 1928.4)	1583.5<br>(1199.8 to 2075.3)

SR Appendix Table 2. All-age DALYs counts and age standardized DALYs rates per 100,000 individuals for all causes and level 1 cause groups by location for 1990, 2007, and 2017, both sexes combined

		All-age DALYs			Age-standardized rate of DALYs per 100,000		
Macedonia	Falls	17.6 (12.8 to 23.5)	21.3 (15.7 to 28.7)	27.1 (19.7 to 37)	873.1 (637.1 to 1167.7)	876.6 (644.4 to 1182.5)	987.2 (717.9 to 1342.5)
Macedonia	Drowning	1.73 (1.54 to 1.94)	1.3 (1.19 to 1.42)	1.16 (1.04 to 1.29)	88.3 (78.3 to 99.4)	64.6 (58.6 to 71.6)	59 (52.3 to 67.1)
Macedonia	Fire, heat, and hot substances	1.79 (1.44 to 2.2)	1.42 (1.08 to 1.86)	1.44 (1.05 to 1.96)	90.2 (72.4 to 110.3)	62.9 (47.8 to 81.7)	57.5 (42.6 to 77.4)
Macedonia	Poisonings	0.816 (0.634 to 1.05)	0.835 (0.657 to 1.06)	0.914 (0.705 to 1.22)	40.9 (31.8 to 52.7)	38.5 (30.3 to 49.1)	40.6 (31.2 to 54.1)
Macedonia	Poisoning by carbon monoxide	0.196 (0.162 to 0.25)	0.246 (0.172 to 0.291)	0.243 (0.175 to 0.298)	9.8 (8 to 12.4)	11 (7.6 to 13.1)	10.4 (7.5 to 12.8)
Macedonia	Poisoning by other means	0.62 (0.466 to 0.822)	0.589 (0.432 to 0.8)	0.671 (0.476 to 0.96)	31 (23.5 to 41.2)	27.5 (20.5 to 37.1)	30.2 (21.4 to 42.9)
Macedonia	Exposure to mechanical forces	4.11 (3.11 to 5.36)	4.99 (3.72 to 6.68)	6.01 (4.35 to 8.17)	200.5 (151.7 to 261.6)	206 (154 to 273.9)	226.4 (164.3 to 306.7)
Macedonia	Unintentional firearm injuries	0.339 (0.267 to 0.4)	0.359 (0.232 to 0.461)	0.32 (0.217 to 0.39)	16.6 (13.2 to 19.6)	15.9 (10.3 to 20.2)	13.9 (9.3 to 17)
Macedonia	Other exposure to mechanical forces	3.77 (2.79 to 5)	4.63 (3.37 to 6.28)	5.69 (4.05 to 7.83)	183.9 (136.1 to 244.2)	190.2 (138.6 to 256.4)	212.4 (151.8 to 291.9)
Macedonia	Adverse effects of medical treatment	0.564 (0.475 to 0.675)	0.505 (0.438 to 0.583)	0.536 (0.449 to 0.657)	29.8 (24.5 to 36)	22.1 (19.2 to 25.6)	20.6 (17.4 to 25.7)
Macedonia	Animal contact	0.312 (0.229 to 0.415)	0.325 (0.235 to 0.434)	0.316 (0.227 to 0.428)	15.4 (11.3 to 20.4)	14.2 (10.3 to 19)	13.1 (9.3 to 17.8)
Macedonia	Venomous animal contact	0.204 (0.142 to 0.282)	0.211 (0.145 to 0.293)	0.205 (0.14 to 0.288)	10 (7 to 13.8)	9.4 (6.4 to 13)	8.8 (6 to 12.4)
Macedonia	Non-venomous animal contact	0.108 (0.0785 to 0.148)	0.113 (0.08 to 0.162)	0.111 (0.0774 to 0.16)	5.4 (4 to 7.4)	4.8 (3.4 to 6.8)	4.3 (3 to 6.2)
Macedonia	Foreign body	1.03 (0.851 to 1.45)	0.939 (0.821 to 1.06)	0.968 (0.83 to 1.11)	55.2 (44.8 to 79.5)	47.1 (41.2 to 52.9)	46.2 (39.8 to 52.9)
Macedonia	Pulmonary aspiration and foreign body in airway	0.779 (0.627 to 1.21)	0.665 (0.587 to 0.728)	0.663 (0.577 to 0.743)	42.9 (33.9 to 67.4)	35.2 (31.2 to 39.4)	33.7 (28.9 to 38.9)
Macedonia	Foreign body in eyes	0.0415 (0.0196 to 0.0723)	0.0541 (0.0263 to 0.093)	0.0594 (0.0294 to 0.102)	2.1 (1 to 3.6)	2.4 (1.1 to 4.2)	2.5 (1.1 to 4.3)
Macedonia	Foreign body in other body part	0.207 (0.155 to 0.265)	0.219 (0.163 to 0.279)	0.245 (0.179 to 0.313)	10.2 (7.7 to 13)	9.5 (7.1 to 12)	10.1 (7.5 to 12.8)
Macedonia	Environmental heat and cold exposure	0.546 (0.409 to 0.714)	0.6 (0.448 to 0.753)	0.804 (0.618 to 1)	26.7 (20.1 to 34.8)	25.1 (18.6 to 31.5)	30.5 (23.2 to 37.9)
Macedonia	Exposure to forces of nature	0.00279 (0.00201 to 0.0039)	0.00299 (0.0022 to 0.00409)	0.00873 (0.00664 to 0.0115)	0.1 (0.1 to 0.2)	0.1 (0.1 to 0.2)	0.4 (0.3 to 0.5)
Macedonia	Other unintentional injuries	13.3 (11.9 to 14.8)	3.41 (2.77 to 4.3)	2.6 (2.01 to 3.41)	660.7 (593.8 to 735.5)	143.7 (117.7 to 180.5)	102.1 (80 to 132.5)
Macedonia	Self-harm and interpersonal violence	10.4 (9.75 to 11.5)	11.2 (10.3 to 11.9)	9.99 (9.02 to 11.1)	502.7 (470.2 to 557.9)	473.5 (438 to 504.1)	397.7 (360 to 442.7)
Macedonia	Self-harm	6.97 (6.45 to 8.29)	7.05 (6.6 to 8.23)	7.01 (6.29 to 7.97)	334.7 (309.7 to 397)	289.5 (270.3 to 339.1)	274.6 (245.7 to 315.2)
Macedonia	Self-harm by firearm	0.566 (0.344 to 0.789)	0.725 (0.383 to 0.867)	0.69 (0.327 to 0.866)	27 (16.5 to 37.6)	30.5 (16.2 to 36.5)	27.7 (13.6 to 34.9)
Macedonia	Self-harm by other specified means	6.41 (5.92 to 7.65)	6.32 (5.88 to 7.54)	6.32 (5.61 to 7.41)	307.8 (284.4 to 366.1)	259 (240.3 to 309.9)	246.8 (217.6 to 292.3)
Macedonia	Interpersonal violence	3.45 (2.63 to 3.93)	3.38 (2.3 to 3.9)	2.86 (2.24 to 3.34)	167.2 (129.5 to 190.1)	146 (100.3 to 168.5)	118.3 (93.7 to 137.7)
Macedonia	Assault by firearm	0.939 (0.615 to 1.34)	1.36 (0.526 to 1.7)	0.978 (0.469 to 1.23)	44.8 (29.5 to 64)	59.8 (23.2 to 74.7)	42.3 (20.6 to 52.9)
Macedonia	Assault by sharp object	0.429 (0.262 to 0.589)	0.404 (0.251 to 0.586)	0.381 (0.264 to 0.513)	20.6 (12.7 to 28.2)	17.2 (10.6 to 25.1)	15.6 (10.7 to 21.1)
Macedonia	Sexual violence	0.18 (0.12 to 0.261)	0.17 (0.114 to 0.246)	0.168 (0.112 to 0.241)	8.6 (5.8 to 12.5)	7.6 (5.1 to 11)	7.4 (5 to 10.7)
Macedonia	Assault by other means	1.9 (1.39 to 2.28)	1.44 (1.15 to 1.85)	1.33 (1.04 to 1.74)	93.2 (69.8 to 111.1)	61.5 (49.3 to 78.5)	53.1 (41.8 to 68.7)
Macedonia	Conflict and terrorism	0 (0 to 0)	0.756 (0.718 to 0.825)	0.0984 (0.0654 to 0.159)	0 (0 to 0)	37.2 (35.5 to 40.4)	3.9 (2.6 to 6.5)
Macedonia	Executions and police conflict	0.0143 (0.0135 to 0.0154)	0.015 (0.0139 to 0.0167)	0.0172 (0.0157 to 0.0194)	0.7 (0.7 to 0.8)	0.8 (0.7 to 0.8)	0.9 (0.8 to 1)
Montenegro	All causes	174 (156 to 194)	199 (179 to 223)	201 (180 to 227)	28149.7 (25356.1 to 31328.8)	26228.3 (23424 to 29419)	23975.9 (21174.2 to 27263.4)
Montenegro	Communicable, maternal, neonatal, and nutritional disorders	17.8 (15.7 to 19.9)	11.5 (10.1 to 13.1)	8.34 (7.07 to 9.73)	3424.1 (3038.1 to 3828.5)	2300 (2048.1 to 2596.3)	1584.3 (1351.7 to 1847)
Montenegro	HIV/AIDS and sexually transmitted infections	0.214 (0.149 to 0.319)	0.251 (0.185 to 0.355)	0.211 (0.153 to 0.311)	36 (24.8 to 53.9)	42.4 (31.5 to 58.7)	36.8 (26.7 to 53.1)
Montenegro	HIV/AIDS	0.0468 (0.0413 to 0.0523)	0.108 (0.0836 to 0.127)	0.0824 (0.0636 to 0.103)	7.6 (6.7 to 8.5)	18.9 (14.7 to 22.3)	15.8 (11.5 to 20.2)
Montenegro	HIV/AIDS - Drug-susceptible Tuberculosis	0.00505 (0.00303 to 0.00814)	0.00665 (0.00398 to 0.0104)	0.00442 (0.0027 to 0.00688)	0.8 (0.5 to 1.3)	1.2 (0.7 to 1.8)	0.8 (0.5 to 1.3)
Montenegro	HIV/AIDS - Multidrug-resistant Tuberculosis without extensive drug resistance	0.0000255 (0.00000361 to 0.000086)	0.000393 (0.000163 to 0.00078)	0.000158 (0.0000297 to 0.000498)	0 (0 to 0)	0.1 (0 to 0.1)	0 (0 to 0.1)
Montenegro	HIV/AIDS - Extensively drug-resistant Tuberculosis	0 (0 to 0)	0.000123 (0.0000493 to 0.000249)	0.0000896 (0.0000169 to 0.00028)	0 (0 to 0)	0 (0 to 0)	0 (0 to 0.1)
Montenegro	HIV/AIDS resulting in other diseases	0.0417 (0.0354 to 0.0476)	0.101 (0.076 to 0.12)	0.0777 (0.0582 to 0.0992)	6.8 (5.7 to 7.8)	17.6 (13.3 to 21.2)	14.9 (10.6 to 19.4)
Montenegro	Sexually transmitted infections excluding HIV	0.167 (0.103 to 0.272)	0.143 (0.0849 to 0.242)	0.129 (0.0743 to 0.222)	28.3 (17.3 to 46.1)	23.5 (14.1 to 40.1)	21 (12.2 to 35.9)

SR Appendix Table 2. All-age DALYs counts and age standardized DALYs rates per 100,000 individuals for all causes and level 1 cause groups by location for 1990, 2007, and 2017, both sexes combined

		All-age DALYs			Age-standardized rate of DALYs per 100,000		
Montenegro	Syphilis	0.0585 (0.0297 to 0.102)	0.0334 (0.0198 to 0.054)	0.0241 (0.016 to 0.0367)	11.7 (5.5 to 20.9)	6.9 (3.5 to 12.2)	4.7 (2.6 to 8.6)
Montenegro	Chlamydial infection	0.024 (0.0125 to 0.0452)	0.0238 (0.0125 to 0.0454)	0.0225 (0.0118 to 0.0429)	3.7 (1.9 to 6.9)	3.7 (1.9 to 7)	3.6 (1.9 to 7)
Montenegro	Gonococcal infection	0.0208 (0.0106 to 0.0402)	0.0199 (0.0106 to 0.0389)	0.0175 (0.00922 to 0.0341)	3.2 (1.6 to 6.2)	3.2 (1.7 to 6.3)	3 (1.5 to 6)
Montenegro	Trichomoniasis	0.0195 (0.00784 to 0.0418)	0.0208 (0.00844 to 0.0435)	0.0203 (0.00814 to 0.0434)	3 (1.2 to 6.4)	3 (1.2 to 6.3)	3 (1.2 to 6.3)
Montenegro	Genital herpes	0.0135 (0.00444 to 0.0318)	0.0154 (0.00493 to 0.0372)	0.016 (0.00509 to 0.0384)	2.1 (0.7 to 4.8)	2.1 (0.7 to 4.9)	2.1 (0.7 to 5)
Montenegro	Other sexually transmitted infections	0.0304 (0.0174 to 0.0531)	0.03 (0.0172 to 0.0518)	0.0282 (0.0165 to 0.0494)	4.7 (2.7 to 8.2)	4.7 (2.6 to 8.1)	4.6 (2.7 to 8.1)
Montenegro	Respiratory infections and tuberculosis	3.27 (2.84 to 3.78)	2.33 (2.04 to 2.73)	1.98 (1.7 to 2.36)	591.4 (508.3 to 691.6)	370.8 (321.8 to 441.5)	281.1 (236.8 to 341.7)
Montenegro	Tuberculosis	0.237 (0.211 to 0.268)	0.2 (0.181 to 0.221)	0.143 (0.126 to 0.164)	36.5 (32.6 to 41.3)	25.6 (23.2 to 28.5)	17 (15 to 19.5)
Montenegro	Latent tuberculosis infection	0 (0 to 0)	0 (0 to 0)	0 (0 to 0)	0 (0 to 0)	0 (0 to 0)	0 (0 to 0)
Montenegro	Drug-susceptible tuberculosis	0.236 (0.211 to 0.267)	0.188 (0.169 to 0.21)	0.137 (0.117 to 0.158)	36.4 (32.4 to 41.1)	24.2 (21.6 to 27)	16.4 (13.9 to 18.9)
Montenegro	Multidrug-resistant tuberculosis without extensive drug resistance	0.000955 (0.000149 to 0.00303)	0.00878 (0.00445 to 0.0159)	0.00384 (0.000839 to 0.0117)	0.1 (0 to 0.5)	1.1 (0.6 to 2)	0.4 (0.1 to 1.4)
Montenegro	Extensively drug-resistant tuberculosis	0 (0 to 0)	0.00269 (0.00135 to 0.00502)	0.0021 (0.000442 to 0.0062)	0 (0 to 0)	0.3 (0.2 to 0.6)	0.2 (0.1 to 0.7)
Montenegro	Lower respiratory infections	2.44 (2.07 to 2.85)	1.55 (1.41 to 1.73)	1.29 (1.14 to 1.49)	459.2 (386.2 to 543.4)	249.8 (222.6 to 279.8)	170.5 (151 to 194.7)
Montenegro	Upper respiratory infections	0.445 (0.265 to 0.713)	0.435 (0.258 to 0.694)	0.42 (0.248 to 0.665)	71.6 (42.9 to 113.8)	71.1 (42.1 to 113.7)	70.5 (41.1 to 112.3)
Montenegro	Otitis media	0.147 (0.0872 to 0.234)	0.141 (0.0852 to 0.224)	0.127 (0.0768 to 0.2)	24 (14.3 to 38.2)	24.4 (14.6 to 38.7)	23 (13.7 to 36.3)
Montenegro	Enteric infections	0.476 (0.353 to 0.622)	0.362 (0.264 to 0.481)	0.34 (0.245 to 0.459)	85.7 (63.8 to 112.3)	68.9 (50.1 to 91.2)	65.5 (46.1 to 88.4)
Montenegro	Diarrhoeal diseases	0.451 (0.331 to 0.6)	0.344 (0.247 to 0.466)	0.325 (0.231 to 0.447)	81.4 (60 to 107.5)	65.8 (46.9 to 88.6)	62.9 (43.7 to 85.8)
Montenegro	Typhoid and paratyphoid	0.00103 (0.000456 to 0.00204)	0.000931 (0.000413 to 0.00179)	0.000828 (0.000368 to 0.00163)	0.2 (0.1 to 0.3)	0.1 (0.1 to 0.3)	0.1 (0.1 to 0.2)
Montenegro	Typhoid fever	0.000197 (0.0000907 to 0.000369)	0.000169 (0.0000791 to 0.000317)	0.00016 (0.0000749 to 0.000289)	0 (0 to 0.1)	0 (0 to 0.1)	0 (0 to 0.1)
Montenegro	Paratyphoid fever	0.000836 (0.000341 to 0.00175)	0.000761 (0.000308 to 0.00154)	0.000668 (0.000273 to 0.00136)	0.1 (0.1 to 0.3)	0.1 (0 to 0.2)	0.1 (0 to 0.2)
Montenegro	Invasive non-typhoidal salmonella	0.0217 (0.0113 to 0.039)	0.0157 (0.0082 to 0.0274)	0.0132 (0.00677 to 0.0235)	3.7 (1.9 to 6.7)	2.8 (1.4 to 4.9)	2.4 (1.1 to 4.5)
Montenegro	Other intestinal infectious diseases	0.00221 (0.000708 to 0.00461)	0.00107 (0.000543 to 0.00173)	0.000664 (0.000382 to 0.000985)	0.4 (0.1 to 0.9)	0.2 (0.1 to 0.4)	0.1 (0.1 to 0.2)
Montenegro	Neglected tropical diseases and malaria	0.206 (0.104 to 0.485)	0.196 (0.0998 to 0.338)	0.216 (0.0975 to 0.449)	32.5 (16.2 to 78.4)	27 (13.5 to 49)	29.3 (12.6 to 75.9)
Montenegro	Malaria	0 (0 to 0)	0 (0 to 0)	0 (0 to 0)	0 (0 to 0)	0 (0 to 0)	0 (0 to 0)
Montenegro	Chagas disease	0 (0 to 0)	0 (0 to 0)	0 (0 to 0)	0 (0 to 0)	0 (0 to 0)	0 (0 to 0)
Montenegro	Leishmaniasis	0.0318 (0.000224 to 0.321)	0.0125 (0.000189 to 0.123)	0.0275 (0.00022 to 0.258)	5.4 (0 to 55.2)	2.3 (0 to 23.3)	5.3 (0 to 53)
Montenegro	Visceral leishmaniasis	0.0313 (0.000101 to 0.32)	0.0122 (0.0000584 to 0.123)	0.027 (0.000109 to 0.257)	5.3 (0 to 55)	2.2 (0 to 23.2)	5.2 (0 to 52.9)
Montenegro	Cutaneous and mucocutaneous leishmaniasis	0.000431 (0.0000258 to 0.0014)	0.000375 (0.0000657 to 0.00106)	0.000424 (0.0000253 to 0.00151)	0.1 (0 to 0.2)	0.1 (0 to 0.2)	0.1 (0 to 0.2)
Montenegro	African trypanosomiasis	0 (0 to 0)	0 (0 to 0)	0 (0 to 0)	0 (0 to 0)	0 (0 to 0)	0 (0 to 0)
Montenegro	Schistosomiasis	0 (0 to 0)	0 (0 to 0)	0 (0 to 0)	0 (0 to 0)	0 (0 to 0)	0 (0 to 0)
Montenegro	Cysticercosis	0.143 (0.0698 to 0.249)	0.16 (0.0718 to 0.283)	0.169 (0.0765 to 0.31)	21.9 (10.7 to 38)	21.1 (9.6 to 37.6)	21.2 (9.6 to 39.1)
Montenegro	Cystic echinococcosis	0.00518 (0.00358 to 0.00702)	0.00439 (0.00296 to 0.00603)	0.00379 (0.00262 to 0.00524)	0.8 (0.6 to 1.1)	0.6 (0.4 to 0.8)	0.5 (0.3 to 0.7)
Montenegro	Lymphatic filariasis	0 (0 to 0)	0 (0 to 0)	0 (0 to 0)	0 (0 to 0)	0 (0 to 0)	0 (0 to 0)
Montenegro	Onchocerciasis	0 (0 to 0)	0 (0 to 0)	0 (0 to 0)	0 (0 to 0)	0 (0 to 0)	0 (0 to 0)
Montenegro	Trachoma	0 (0 to 0)	0 (0 to 0)	0 (0 to 0)	0 (0 to 0)	0 (0 to 0)	0 (0 to 0)
Montenegro	Dengue	0 (0 to 0)	0 (0 to 0)	0 (0 to 0)	0 (0 to 0)	0 (0 to 0)	0 (0 to 0)
Montenegro	Yellow fever	0 (0 to 0)	0 (0 to 0)	0 (0 to 0)	0 (0 to 0)	0 (0 to 0)	0 (0 to 0)
Montenegro	Rabies	0.0000234 (0.00000513 to 0.0000397)	0.0000143 (0.00000581 to 0.0000217)	0.0000094 (0.00000329 to 0.0000153)	0 (0 to 0)	0 (0 to 0)	0 (0 to 0)
Montenegro	Intestinal nematode infections	0.000449 (0.000123 to 0.00101)	0.000132 (0.0000602 to 0.000233)	0.0000939 (0.0000432 to 0.000166)	0.1 (0 to 0.2)	0 (0 to 0)	0 (0 to 0)
Montenegro	Ascariasis	0.000449 (0.000123 to 0.00101)	0.000132 (0.0000602 to 0.000233)	0.0000939 (0.0000432 to 0.000166)	0.1 (0 to 0.2)	0 (0 to 0)	0 (0 to 0)

SR Appendix Table 2. All-age DALYs counts and age standardized DALYs rates per 100,000 individuals for all causes and level 1 cause groups by location for 1990, 2007, and 2017, both sexes combined

		All-age DALYs			Age-standardized rate of DALYs per 100,000		
Montenegro	Trichuriasis	0 (0 to 0)	0 (0 to 0)	0 (0 to 0)	0 (0 to 0)	0 (0 to 0)	0 (0 to 0)
Montenegro	Hookworm disease	0 (0 to 0)	0 (0 to 0)	0 (0 to 0)	0 (0 to 0)	0 (0 to 0)	0 (0 to 0)
Montenegro	Food-borne trematodiases	0 (0 to 0)	0 (0 to 0)	0 (0 to 0)	0 (0 to 0)	0 (0 to 0)	0 (0 to 0)
Montenegro	Leprosy	0 (0 to 0)	0 (0 to 0)	0 (0 to 0)	0 (0 to 0)	0 (0 to 0)	0 (0 to 0)
Montenegro	Ebola virus disease	0 (0 to 0)	0 (0 to 0)	0 (0 to 0)	0 (0 to 0)	0 (0 to 0)	0 (0 to 0)
Montenegro	Zika virus disease	0 (0 to 0)	0 (0 to 0)	0 (0 to 0)	0 (0 to 0)	0 (0 to 0)	0 (0 to 0)
Montenegro	Guinea worm disease	0 (0 to 0)	0 (0 to 0)	0 (0 to 0)	0 (0 to 0)	0 (0 to 0)	0 (0 to 0)
Montenegro	Other neglected tropical diseases	0.0255 (0.0148 to 0.0451)	0.0187 (0.011 to 0.0339)	0.0156 (0.00939 to 0.0268)	4.4 (2.6 to 7.9)	2.9 (1.8 to 5.4)	2.4 (1.4 to 4.3)
Montenegro	Other infectious diseases	0.937 (0.659 to 1.49)	0.583 (0.428 to 0.852)	0.438 (0.331 to 0.62)	172.1 (117.1 to 282.5)	111.6 (75.2 to 179.1)	83.4 (56.6 to 132.4)
Montenegro	Meningitis	0.272 (0.226 to 0.327)	0.136 (0.115 to 0.158)	0.0885 (0.074 to 0.106)	50.4 (41.6 to 61.4)	24.1 (20.4 to 28.2)	14.9 (12.3 to 18)
Montenegro	Pneumococcal meningitis	0.0479 (0.0377 to 0.0594)	0.0323 (0.0256 to 0.0404)	0.0229 (0.0178 to 0.0288)	8.2 (6.5 to 10.3)	5.2 (4.1 to 6.4)	3.6 (2.8 to 4.5)
Montenegro	H influenzae type B meningitis	0.1 (0.082 to 0.125)	0.0407 (0.0343 to 0.0476)	0.0224 (0.0184 to 0.0278)	19.2 (15.6 to 24.3)	7.8 (6.5 to 9.4)	4.1 (3.3 to 5.2)
Montenegro	Meningococcal infection	0.0405 (0.0321 to 0.0519)	0.0197 (0.0163 to 0.0236)	0.0123 (0.0101 to 0.0151)	7.5 (5.9 to 9.9)	3.5 (2.9 to 4.3)	2.1 (1.7 to 2.5)
Montenegro	Other meningitis	0.0832 (0.0672 to 0.102)	0.043 (0.0359 to 0.0506)	0.0309 (0.0254 to 0.0371)	15.4 (12.3 to 19.2)	7.6 (6.3 to 9.1)	5.2 (4.2 to 6.3)
Montenegro	Encephalitis	0.113 (0.0903 to 0.136)	0.0795 (0.0696 to 0.0906)	0.0697 (0.055 to 0.0823)	19.8 (15.4 to 24.2)	13.8 (11.9 to 15.9)	12.1 (9.1 to 14.7)
Montenegro	Diphtheria	0.00135 (0.000742 to 0.0022)	0.000701 (0.000435 to 0.00103)	0.000493 (0.000301 to 0.000738)	0.2 (0.1 to 0.4)	0.1 (0.1 to 0.2)	0.1 (0.1 to 0.1)
Montenegro	Whooping cough	0.288 (0.0339 to 0.856)	0.165 (0.0217 to 0.453)	0.103 (0.0142 to 0.278)	57.7 (6.8 to 171.1)	41.3 (5.4 to 113.7)	28.7 (4 to 76.9)
Montenegro	Tetanus	0.00407 (0.00379 to 0.00445)	0.00353 (0.00331 to 0.00376)	0.00327 (0.00297 to 0.0036)	0.7 (0.6 to 0.7)	0.6 (0.6 to 0.7)	0.6 (0.5 to 0.7)
Montenegro	Measles	0.000435 (0 to 0.00099)	0.0000265 (0.00000303 to 0.000104)	0.0000249 (0 to 0.000122)	0.1 (0 to 0.2)	0 (0 to 0)	0 (0 to 0)
Montenegro	Varicella and herpes zoster	0.0328 (0.0235 to 0.0448)	0.0267 (0.0187 to 0.0371)	0.0249 (0.0171 to 0.0358)	5.7 (4 to 7.8)	4.2 (2.9 to 5.8)	3.6 (2.5 to 4.9)
Montenegro	Acute hepatitis	0.129 (0.101 to 0.154)	0.0924 (0.0772 to 0.115)	0.0791 (0.0655 to 0.0994)	21.1 (16.4 to 25.4)	14.1 (11.7 to 17.3)	11.7 (9.5 to 14.5)
Montenegro	Acute hepatitis A	0.0528 (0.0415 to 0.066)	0.0352 (0.027 to 0.0455)	0.0294 (0.0224 to 0.0386)	8.7 (6.8 to 10.9)	6.2 (4.8 to 8)	5.5 (4.2 to 7.2)
Montenegro	Acute hepatitis B	0.0578 (0.0444 to 0.0715)	0.0457 (0.0377 to 0.0603)	0.0398 (0.0319 to 0.0518)	9.4 (7.1 to 11.6)	6.1 (5 to 7.9)	4.7 (3.8 to 6.1)
Montenegro	Acute hepatitis C	0.00579 (0.00252 to 0.0109)	0.00213 (0.00109 to 0.00388)	0.00126 (0.000646 to 0.00225)	1.2 (0.5 to 2.2)	0.5 (0.2 to 0.9)	0.2 (0.1 to 0.5)
Montenegro	Acute hepatitis E	0.0122 (0.00938 to 0.0154)	0.00938 (0.00737 to 0.0121)	0.00866 (0.00664 to 0.0115)	1.9 (1.5 to 2.4)	1.4 (1.1 to 1.7)	1.2 (0.9 to 1.5)
Montenegro	Other unspecified infectious diseases	0.0959 (0.0742 to 0.123)	0.0798 (0.0606 to 0.107)	0.0686 (0.0504 to 0.0929)	16.4 (12.7 to 20.8)	13.3 (10.2 to 17.8)	11.7 (8.4 to 16)
Montenegro	Maternal and neonatal disorders	11.1 (9.73 to 12.7)	6.55 (5.68 to 7.51)	4.14 (3.31 to 5.15)	2251 (1974.5 to 2588.6)	1464.8 (1301.7 to 1643.6)	902.9 (749 to 1090.9)
Montenegro	Maternal disorders	0.0748 (0.062 to 0.0889)	0.0579 (0.0484 to 0.0686)	0.0436 (0.0354 to 0.0534)	11.5 (9.6 to 13.7)	9.3 (7.7 to 11)	7.4 (5.9 to 9.1)
Montenegro	Maternal haemorrhage	0.0192 (0.0139 to 0.0258)	0.0146 (0.0106 to 0.0194)	0.0108 (0.00754 to 0.0152)	2.9 (2.1 to 4)	2.4 (1.7 to 3.2)	1.9 (1.3 to 2.6)
Montenegro	Maternal sepsis and other pregnancy related infections	0.00648 (0.00442 to 0.00961)	0.00471 (0.0032 to 0.0067)	0.00346 (0.00226 to 0.00557)	1 (0.7 to 1.5)	0.8 (0.5 to 1.1)	0.6 (0.4 to 1)
Montenegro	Maternal hypertensive disorders	0.00388 (0.00259 to 0.00557)	0.00324 (0.00224 to 0.00484)	0.00278 (0.00184 to 0.00408)	0.6 (0.4 to 0.9)	0.5 (0.4 to 0.8)	0.5 (0.3 to 0.7)
Montenegro	Maternal obstructed labour and uterine rupture	0.00366 (0.00249 to 0.00536)	0.00316 (0.00225 to 0.00442)	0.00256 (0.00167 to 0.00389)	0.6 (0.4 to 0.8)	0.5 (0.4 to 0.7)	0.4 (0.3 to 0.7)
Montenegro	Maternal abortive outcome	0.0187 (0.0143 to 0.0245)	0.0129 (0.01 to 0.0161)	0.00896 (0.00671 to 0.0118)	2.9 (2.2 to 3.7)	2 (1.6 to 2.6)	1.5 (1.1 to 2)
Montenegro	Ectopic pregnancy	0.00074 (0.000569 to 0.000949)	0.00051 (0.000399 to 0.000637)	0.000358 (0.000269 to 0.000461)	0.1 (0.1 to 0.1)	0.1 (0.1 to 0.1)	0.1 (0 to 0.1)
Montenegro	Indirect maternal deaths	0.00154 (0.000606 to 0.00345)	0.00141 (0.000536 to 0.003)	0.000904 (0.000331 to 0.00209)	0.2 (0.1 to 0.5)	0.2 (0.1 to 0.5)	0.2 (0.1 to 0.4)
Montenegro	Late maternal deaths	0.00181 (0.00126 to 0.00255)	0.00179 (0.00124 to 0.00252)	0.00167 (0.00108 to 0.00243)	0.3 (0.2 to 0.4)	0.3 (0.2 to 0.4)	0.3 (0.2 to 0.4)
Montenegro	Maternal deaths aggravated by HIV/AIDS	0.0000011 (0.000000518 to 0.00000201)	0.0000027 (0.00000131 to 0.00000436)	0.00000291 (0.00000142 to 0.00000484)	0 (0 to 0)	0 (0 to 0)	0 (0 to 0)
Montenegro	Other maternal disorders	0.0188 (0.0148 to 0.0235)	0.0156 (0.0127 to 0.0187)	0.012 (0.00953 to 0.0151)	2.9 (2.3 to 3.6)	2.5 (2 to 3)	2 (1.6 to 2.6)
Montenegro	Neonatal disorders	11 (9.65 to 12.7)	6.49 (5.62 to 7.45)	4.09 (3.27 to 5.11)	2239.5 (1963.8 to 2576.8)	1455.5 (1292.5 to 1634)	895.5 (741.1 to 1085.6)
Montenegro	Neonatal preterm birth	4.81 (4.03 to 5.69)	2.41 (2.07 to 2.79)	1.33 (1.05 to 1.66)	994.4 (828 to 1177.3)	565.6 (484.3 to 655.8)	312.5 (251 to 381.8)

SR Appendix Table 2. All-age DALYs counts and age standardized DALYs rates per 100,000 individuals for all causes and level 1 cause groups by location for 1990, 2007, and 2017, both sexes combined

		All-age DALYs			Age-standardized rate of DALYs per 100,000		
Montenegro	Neonatal encephalopathy due to birth asphyxia and trauma	3.71 (2.91 to 4.61)	2.31 (1.85 to 2.86)	1.39 (0.995 to 1.86)	759.2 (591.5 to 941.9)	526.8 (431.1 to 640.2)	306.5 (227.4 to 395.3)
Montenegro	Neonatal sepsis and other neonatal infections	1.26 (0.775 to 1.89)	1.1 (0.644 to 1.73)	1.01 (0.55 to 1.64)	216.6 (138.8 to 318.7)	195.4 (117.7 to 298.9)	180 (101.3 to 288.7)
Montenegro	Hemolytic disease and other neonatal jaundice	0.154 (0.118 to 0.196)	0.109 (0.0836 to 0.137)	0.0878 (0.065 to 0.117)	28.3 (21.8 to 37)	20.1 (15.6 to 24.9)	15.7 (11.6 to 20.5)
Montenegro	Other neonatal disorders	1.12 (0.791 to 1.48)	0.561 (0.448 to 0.701)	0.276 (0.203 to 0.429)	241.1 (169.5 to 318.7)	147.6 (117.5 to 184.9)	80.9 (59.3 to 126.2)
Montenegro	Nutritional deficiencies	1.53 (0.969 to 2.29)	1.24 (0.752 to 1.93)	1.02 (0.613 to 1.61)	255.4 (162.2 to 380.8)	214.6 (130.3 to 333.1)	185.4 (110.5 to 295.7)
Montenegro	Protein-energy malnutrition	0.0456 (0.0296 to 0.072)	0.0288 (0.0226 to 0.0423)	0.02 (0.0168 to 0.0255)	8.5 (5.5 to 13.5)	5.2 (3.7 to 8.2)	3.1 (2.5 to 4.4)
Montenegro	Iodine deficiency	0.0961 (0.0469 to 0.169)	0.0573 (0.0263 to 0.11)	0.0838 (0.0403 to 0.151)	15 (7.4 to 26.5)	8.3 (3.8 to 16)	12.3 (5.7 to 22.2)
Montenegro	Dietary iron deficiency	0.818 (0.499 to 1.25)	0.833 (0.499 to 1.28)	0.682 (0.394 to 1.08)	127.6 (77.5 to 195.6)	128.3 (78.2 to 197.9)	111.3 (64.8 to 179)
Montenegro	Other nutritional deficiencies	0.0179 (0.0145 to 0.0222)	0.0111 (0.00962 to 0.0135)	0.0089 (0.0077 to 0.0105)	3.1 (2.5 to 3.9)	1.8 (1.5 to 2.2)	1.4 (1.2 to 1.6)
Montenegro	Non-communicable diseases	132 (118 to 148)	166 (149 to 184)	172 (154 to 192)	20928.1 (18768.4 to 23429.1)	20767.5 (18548.1 to 23244.3)	19457.1 (17215.8 to 22065)
Montenegro	Neoplasms	23.1 (22.2 to 24)	30.6 (29.1 to 32)	32.3 (29.8 to 34.9)	3531.9 (3390.8 to 3666)	3617.4 (3446.7 to 3789.5)	3410.7 (3154.1 to 3689.5)
Montenegro	Lip and oral cavity cancer	0.379 (0.331 to 0.435)	0.466 (0.419 to 0.519)	0.488 (0.409 to 0.572)	56.5 (49.5 to 64.6)	55.1 (49.5 to 61.2)	51.7 (43.1 to 60.6)
Montenegro	Nasopharynx cancer	0.0313 (0.0283 to 0.0355)	0.0386 (0.0349 to 0.0424)	0.0345 (0.0303 to 0.0393)	4.7 (4.3 to 5.3)	4.8 (4.3 to 5.2)	4 (3.5 to 4.5)
Montenegro	Other pharynx cancer	0.158 (0.126 to 0.193)	0.195 (0.16 to 0.233)	0.202 (0.156 to 0.252)	23.3 (18.5 to 28.5)	23 (18.9 to 27.4)	21.4 (16.6 to 26.7)
Montenegro	Oesophageal cancer	0.414 (0.365 to 0.468)	0.548 (0.49 to 0.61)	0.587 (0.507 to 0.686)	61.8 (54.6 to 69.8)	63.2 (56.6 to 70.1)	59.9 (51.6 to 69.9)
Montenegro	Stomach cancer	1.09 (0.951 to 1.27)	1.33 (1.22 to 1.43)	1.27 (1.13 to 1.42)	166.8 (145.8 to 193.5)	152.9 (141.7 to 165)	128.4 (114.8 to 143.8)
Montenegro	Colon and rectum cancer	1.86 (1.68 to 2.04)	2.57 (2.4 to 2.76)	2.93 (2.64 to 3.27)	287.3 (260.5 to 313.6)	295.2 (276.2 to 315.9)	294.9 (265.6 to 328.3)
Montenegro	Liver cancer	0.832 (0.739 to 0.942)	1.12 (1.02 to 1.23)	1.21 (1.05 to 1.4)	127.5 (113.2 to 144)	128.3 (117.3 to 141)	124.1 (108.3 to 143)
Montenegro	Liver cancer due to hepatitis B	0.207 (0.166 to 0.258)	0.257 (0.204 to 0.316)	0.246 (0.19 to 0.311)	31.3 (25.3 to 38.8)	30.7 (24.7 to 37.4)	26.9 (21.2 to 33.5)
Montenegro	Liver cancer due to hepatitis C	0.223 (0.168 to 0.288)	0.313 (0.241 to 0.395)	0.348 (0.26 to 0.447)	34.3 (26 to 44.1)	35.2 (27.3 to 44.7)	34.4 (26.1 to 44.1)
Montenegro	Liver cancer due to alcohol use	0.28 (0.232 to 0.335)	0.387 (0.331 to 0.452)	0.439 (0.358 to 0.532)	43 (35.7 to 51.3)	43.7 (37.3 to 51)	44.3 (36.4 to 53.6)
Montenegro	Liver cancer due to NASH	0.0651 (0.0534 to 0.0798)	0.0909 (0.0765 to 0.109)	0.105 (0.0846 to 0.131)	10.1 (8.3 to 12.3)	10.3 (8.7 to 12.3)	10.6 (8.6 to 12.9)
Montenegro	Liver cancer due to other causes	0.0575 (0.0483 to 0.0682)	0.0708 (0.0605 to 0.0829)	0.074 (0.0608 to 0.09)	8.8 (7.4 to 10.4)	8.5 (7.3 to 9.9)	8 (6.7 to 9.6)
Montenegro	Gallbladder and biliary tract cancer	0.312 (0.257 to 0.366)	0.395 (0.333 to 0.461)	0.425 (0.354 to 0.501)	48.4 (39.8 to 56.7)	44.7 (37.7 to 52.1)	42.3 (35.1 to 50.1)
Montenegro	Pancreatic cancer	1.01 (0.891 to 1.12)	1.47 (1.36 to 1.58)	1.75 (1.56 to 1.97)	154.2 (136 to 172)	168.3 (155.4 to 181.1)	176.4 (157.3 to 199)
Montenegro	Larynx cancer	0.832 (0.731 to 0.96)	1.06 (0.959 to 1.16)	1.02 (0.886 to 1.18)	123.1 (108.4 to 141.3)	122.5 (111.5 to 134.9)	104.2 (91 to 120.8)
Montenegro	Tracheal, bronchus, and lung cancer	6.89 (6.34 to 7.5)	9.62 (9 to 10.2)	10.4 (9.16 to 11.8)	1027.5 (945.7 to 1113.5)	1098.8 (1029.3 to 1169.2)	1048.6 (924.5 to 1192.9)
Montenegro	Malignant skin melanoma	0.26 (0.211 to 0.335)	0.321 (0.259 to 0.404)	0.333 (0.26 to 0.43)	39.7 (32.4 to 51.2)	40.1 (32.2 to 50.4)	38.3 (29.6 to 49.5)
Montenegro	Non-melanoma skin cancer	0.111 (0.103 to 0.121)	0.155 (0.141 to 0.17)	0.174 (0.153 to 0.198)	17.6 (16.3 to 19.1)	18.3 (16.6 to 20)	17.9 (15.8 to 20.3)
Montenegro	Non-melanoma skin cancer (squamous-cell carcinoma)	0.111 (0.102 to 0.121)	0.155 (0.14 to 0.17)	0.173 (0.153 to 0.197)	17.5 (16.2 to 19)	18.2 (16.6 to 19.9)	17.8 (15.7 to 20.3)
Montenegro	Non-melanoma skin cancer (basal-cell carcinoma)	0.000207 (0.0000663 to 0.00049)	0.00028 (0.0000819 to 0.00071)	0.000314 (0.0000914 to 0.000785)	0 (0 to 0.1)	0 (0 to 0.1)	0 (0 to 0.1)
Montenegro	Breast cancer	1.96 (1.69 to 2.31)	2.81 (2.57 to 3.12)	2.78 (2.28 to 3.39)	297.8 (258.4 to 347.6)	335.7 (306.2 to 371)	302.3 (247.8 to 368.5)
Montenegro	Cervical cancer	0.65 (0.54 to 0.825)	0.816 (0.715 to 0.928)	0.699 (0.586 to 0.844)	99 (82.4 to 125.4)	100.9 (87.8 to 114.9)	80.5 (67.4 to 96.1)
Montenegro	Uterine cancer	0.203 (0.17 to 0.245)	0.277 (0.241 to 0.316)	0.283 (0.235 to 0.336)	31.1 (26.1 to 37.5)	31.9 (27.8 to 36.3)	29 (24.2 to 34.3)
Montenegro	Ovarian cancer	0.458 (0.389 to 0.535)	0.636 (0.565 to 0.706)	0.672 (0.539 to 0.82)	69.2 (59 to 80.3)	75.6 (67.3 to 83.5)	73.1 (58.7 to 88.8)
Montenegro	Prostate cancer	0.702 (0.601 to 0.913)	1.12 (0.91 to 1.35)	1.27 (1.03 to 1.56)	113 (96.6 to 147.2)	125 (100.8 to 150)	122.8 (98.9 to 149)
Montenegro	Testicular cancer	0.111 (0.0771 to 0.141)	0.0911 (0.077 to 0.107)	0.072 (0.057 to 0.0913)	17 (11.8 to 21.5)	13.8 (11.6 to 16.3)	11.1 (8.6 to 14.3)
Montenegro	Kidney cancer	0.347 (0.307 to 0.388)	0.471 (0.424 to 0.531)	0.528 (0.458 to 0.624)	53.6 (47.7 to 59.9)	55.8 (50.4 to 63)	55.7 (48.5 to 64.9)
Montenegro	Bladder cancer	0.403 (0.356 to 0.451)	0.588 (0.53 to 0.654)	0.651 (0.568 to 0.742)	63 (55.8 to 70.2)	66.4 (60 to 73.6)	64.1 (56.2 to 73)
Montenegro	Thyroid cancer	0.101 (0.0858 to 0.118)	0.13 (0.113 to 0.145)	0.137 (0.117 to 0.159)	15.5 (13.1 to 18)	15.2 (13.4 to 17)	14.5 (12.4 to 16.9)

SR Appendix Table 2. All-age DALYs counts and age standardized DALYs rates per 100,000 individuals for all causes and level 1 cause groups by location for 1990, 2007, and 2017, both sexes combined

		All-age DALYs			Age-standardized rate of DALYs per 100,000		
Montenegro	Mesothelioma	0.0227 (0.0205 to 0.0252)	0.0382 (0.0352 to 0.0417)	0.0367 (0.0307 to 0.0427)	3.4 (3.1 to 3.8)	4.5 (4.2 to 4.9)	3.9 (3.3 to 4.6)
Montenegro	Hodgkin lymphoma	0.314 (0.262 to 0.408)	0.268 (0.229 to 0.355)	0.225 (0.186 to 0.31)	48.1 (40.2 to 62.5)	36.7 (31.4 to 48.7)	29.9 (24.7 to 41.1)
Montenegro	Non-Hodgkin's lymphoma	0.377 (0.347 to 0.409)	0.433 (0.401 to 0.466)	0.456 (0.406 to 0.514)	59.4 (54.6 to 64.4)	55.7 (51.5 to 60)	54.3 (48.2 to 61)
Montenegro	Multiple myeloma	0.115 (0.103 to 0.129)	0.161 (0.145 to 0.18)	0.186 (0.16 to 0.214)	17.5 (15.8 to 19.6)	18.3 (16.5 to 20.5)	18.8 (16.1 to 21.7)
Montenegro	Leukaemia	1.15 (1.01 to 1.3)	1.19 (1.05 to 1.37)	1.17 (1.03 to 1.38)	183.7 (160.7 to 208.3)	162.8 (144.6 to 187.6)	151.9 (130.4 to 177.6)
Montenegro	Acute lymphoid leukaemia	0.349 (0.276 to 0.421)	0.262 (0.223 to 0.306)	0.238 (0.191 to 0.295)	58.4 (46.1 to 71)	45 (37.9 to 52.4)	42.1 (32.5 to 53.1)
Montenegro	Chronic lymphoid leukaemia	0.169 (0.133 to 0.215)	0.248 (0.21 to 0.309)	0.28 (0.229 to 0.349)	26.3 (20.9 to 33.6)	28.1 (24 to 34.8)	27.8 (22.6 to 34.5)
Montenegro	Acute myeloid leukaemia	0.41 (0.334 to 0.475)	0.458 (0.382 to 0.522)	0.465 (0.379 to 0.55)	64.6 (53 to 74.4)	61.9 (51.8 to 70.1)	59.8 (47.8 to 71.2)
Montenegro	Chronic myeloid leukaemia	0.129 (0.104 to 0.166)	0.132 (0.107 to 0.169)	0.112 (0.0885 to 0.151)	19.6 (15.8 to 25)	16.3 (13.2 to 20.9)	12.6 (10 to 16.9)
Montenegro	Other leukaemia	0.0905 (0.0737 to 0.124)	0.0849 (0.0708 to 0.117)	0.0778 (0.0628 to 0.107)	14.8 (12 to 20.1)	11.5 (9.5 to 15.8)	9.6 (7.7 to 13.7)
Montenegro	Other malignant cancers	0.494 (0.452 to 0.539)	0.553 (0.502 to 0.618)	0.555 (0.489 to 0.635)	78.2 (71.5 to 85.3)	73 (66.8 to 80.5)	68.4 (60.6 to 77.6)
Montenegro	Other neoplasms	0.0509 (0.0416 to 0.0629)	0.0736 (0.0573 to 0.0857)	0.0916 (0.0704 to 0.11)	8.1 (6.6 to 10)	9.2 (7.1 to 10.7)	10.2 (7.9 to 12.3)
Montenegro	Myelodysplastic, myeloproliferative, and other hematopoietic neoplasms	0.0437 (0.0353 to 0.0534)	0.0667 (0.0519 to 0.078)	0.0844 (0.0643 to 0.101)	6.9 (5.6 to 8.5)	8 (6.3 to 9.3)	9 (6.9 to 10.8)
Montenegro	Benign and in situ intestinal neoplasms	0 (0 to 0)	0 (0 to 0)	0 (0 to 0)	0 (0 to 0)	0 (0 to 0)	0 (0 to 0)
Montenegro	Benign and in situ cervical and uterine neoplasms	0 (0 to 0)	0 (0 to 0)	0 (0 to 0)	0 (0 to 0)	0 (0 to 0)	0 (0 to 0)
Montenegro	Other benign and in situ neoplasms	0.00725 (0.00588 to 0.0095)	0.00696 (0.00545 to 0.0086)	0.00714 (0.00561 to 0.00901)	1.2 (1 to 1.6)	1.2 (0.9 to 1.4)	1.2 (1 to 1.5)
Montenegro	Cardiovascular diseases	45.4 (44 to 46.9)	62.5 (59.9 to 65.3)	63.3 (59.1 to 67.9)	7309.6 (7080.3 to 7539.6)	7319.5 (7016.4 to 7647)	6424.8 (5999.3 to 6894)
Montenegro	Rheumatic heart disease	0.377 (0.335 to 0.416)	0.357 (0.326 to 0.39)	0.281 (0.246 to 0.321)	57.7 (51.4 to 63.7)	43.4 (39.7 to 47.3)	30.8 (27.1 to 35.2)
Montenegro	Ischaemic heart disease	18.6 (17.6 to 19.7)	25.4 (24.2 to 26.8)	24.9 (23 to 27.2)	2967.2 (2809 to 3137.2)	2947.4 (2805.8 to 3106)	2515.1 (2319.9 to 2743.4)
Montenegro	Stroke	20.4 (19.3 to 21.6)	28.3 (26.9 to 29.7)	28.6 (26.5 to 30.8)	3310.2 (3129.7 to 3490.1)	3317.3 (3160.9 to 3477.2)	2881.8 (2675.9 to 3102.6)
Montenegro	Ischaemic stroke	3.04 (2.68 to 3.39)	5.06 (4.61 to 5.5)	5.91 (5.28 to 6.61)	511.3 (452.4 to 568.7)	598.9 (545.5 to 650.8)	593.6 (531 to 664.6)
Montenegro	Intracerebral hemorrhage	16.8 (15.9 to 17.7)	22.5 (21.4 to 23.6)	22 (20.3 to 23.8)	2711.3 (2569.1 to 2860)	2628.3 (2507.8 to 2759.4)	2209.4 (2038.7 to 2386.7)
Montenegro	Subarachnoid hemorrhage	0.567 (0.495 to 0.646)	0.749 (0.662 to 0.852)	0.727 (0.638 to 0.836)	87.6 (76.6 to 99.7)	90.1 (79.9 to 102)	78.7 (68.9 to 90.5)
Montenegro	Hypertensive heart disease	0.202 (0.173 to 0.227)	0.282 (0.25 to 0.322)	0.329 (0.282 to 0.381)	34.1 (29.3 to 38.4)	33.9 (30.1 to 38.2)	33.5 (28.6 to 38.5)
Montenegro	Non-rheumatic valvular heart disease	0.127 (0.106 to 0.152)	0.175 (0.145 to 0.212)	0.199 (0.162 to 0.244)	20.5 (17.1 to 24.6)	21 (17.5 to 25.3)	20.7 (17 to 25.2)
Montenegro	Non-rheumatic calcific aortic valve disease	0.0593 (0.0474 to 0.0727)	0.0855 (0.0692 to 0.105)	0.104 (0.0827 to 0.13)	9.6 (7.6 to 11.8)	10.3 (8.4 to 12.6)	10.8 (8.6 to 13.4)
Montenegro	Non-rheumatic degenerative mitral valve disease	0.065 (0.0537 to 0.0785)	0.0861 (0.071 to 0.106)	0.0912 (0.0732 to 0.114)	10.5 (8.6 to 12.7)	10.3 (8.5 to 12.6)	9.4 (7.6 to 11.7)
Montenegro	Other non-rheumatic valve diseases	0.00294 (0.00179 to 0.00404)	0.00328 (0.00225 to 0.00515)	0.00369 (0.00244 to 0.00546)	0.5 (0.3 to 0.6)	0.4 (0.3 to 0.7)	0.4 (0.3 to 0.6)
Montenegro	Cardiomyopathy and myocarditis	2.25 (1.82 to 2.75)	3.19 (2.54 to 3.88)	3.59 (2.87 to 4.4)	365 (297 to 445.7)	397.3 (320.2 to 482.7)	391.2 (314.5 to 478.3)
Montenegro	Myocarditis	0.11 (0.0754 to 0.142)	0.113 (0.0791 to 0.143)	0.134 (0.0933 to 0.171)	18.9 (13 to 24.2)	15.9 (11.5 to 19.7)	16.3 (11.8 to 20.4)
Montenegro	Alcoholic cardiomyopathy	1.47 (1.13 to 1.86)	2.22 (1.69 to 2.75)	2.36 (1.82 to 2.96)	232.3 (177.6 to 290.8)	267.4 (204.7 to 330.3)	250.9 (193.1 to 315.7)
Montenegro	Other cardiomyopathy	0.662 (0.525 to 0.815)	0.854 (0.679 to 1.07)	1.1 (0.887 to 1.36)	113.7 (91.2 to 138.9)	114 (92.1 to 141.3)	124 (101.5 to 151.1)
Montenegro	Atrial fibrillation and flutter	1.02 (0.907 to 1.15)	1.53 (1.37 to 1.75)	1.95 (1.74 to 2.24)	174.2 (155.6 to 196)	181.9 (163.4 to 208)	196.5 (175.7 to 225.7)
Montenegro	Aortic aneurysm	0.96 (0.848 to 1.16)	1.37 (1.23 to 1.53)	1.42 (1.23 to 1.63)	147.9 (130.9 to 178.3)	161.1 (144.6 to 179.4)	147.2 (127.9 to 168.8)
Montenegro	Peripheral vascular disease	0.0483 (0.0286 to 0.0775)	0.0701 (0.0426 to 0.109)	0.0869 (0.0556 to 0.131)	7.9 (4.7 to 12.7)	8 (4.9 to 12.4)	8.5 (5.5 to 12.8)
Montenegro	Endocarditis	0.0556 (0.0494 to 0.0631)	0.0772 (0.0706 to 0.0842)	0.0839 (0.0735 to 0.0948)	8.9 (7.9 to 10)	9.8 (9 to 10.7)	9.5 (8.3 to 10.7)
Montenegro	Other cardiovascular and circulatory diseases	1.37 (1.11 to 1.63)	1.7 (1.44 to 2.01)	1.85 (1.54 to 2.22)	216 (175.1 to 255.9)	198.5 (167.7 to 233.3)	190.1 (159.4 to 229.2)
Montenegro	Chronic respiratory diseases	3.6 (3.05 to 4.15)	4.35 (3.71 to 4.96)	4.8 (4.09 to 5.52)	574.4 (489.5 to 662)	546.2 (463.3 to 629)	548.1 (460.5 to 635.3)
Montenegro	Chronic obstructive pulmonary disease	2.45 (2.11 to 2.81)	3.09 (2.66 to 3.5)	3.44 (2.98 to 3.91)	393.3 (339.8 to 447.8)	363.6 (312.7 to 411.2)	357.4 (309.6 to 405.8)
Montenegro	Pneumoconiosis	0.0332 (0.0272 to 0.0408)	0.0542 (0.0455 to 0.0651)	0.0404 (0.0331 to 0.0491)	5.2 (4.3 to 6.4)	6.2 (5.2 to 7.4)	4 (3.3 to 4.9)

SR Appendix Table 2. All-age DALYs counts and age standardized DALYs rates per 100,000 individuals for all causes and level 1 cause groups by location for 1990, 2007, and 2017, both sexes combined

		All-age DALYs			Age-standardized rate of DALYs per 100,000		
Montenegro	Silicosis	0.00523 (0.00337 to 0.00764)	0.00796 (0.00525 to 0.0113)	0.00619 (0.00401 to 0.00862)	0.8 (0.5 to 1.2)	0.9 (0.6 to 1.3)	0.6 (0.4 to 0.9)
Montenegro	Asbestosis	0.0188 (0.0146 to 0.0249)	0.0329 (0.0269 to 0.042)	0.0228 (0.0175 to 0.0295)	2.9 (2.3 to 3.9)	3.7 (3.1 to 4.7)	2.3 (1.7 to 2.9)
Montenegro	Coal workers pneumoconiosis	0.00288 (0.00197 to 0.00393)	0.00387 (0.00283 to 0.00505)	0.00387 (0.00264 to 0.00541)	0.4 (0.3 to 0.6)	0.4 (0.3 to 0.6)	0.4 (0.3 to 0.5)
Montenegro	Other pneumoconiosis	0.00625 (0.00442 to 0.00839)	0.00938 (0.00717 to 0.0119)	0.00759 (0.00551 to 0.0102)	1 (0.7 to 1.3)	1.1 (0.8 to 1.4)	0.8 (0.6 to 1)
Montenegro	Asthma	0.864 (0.599 to 1.19)	0.904 (0.628 to 1.24)	0.985 (0.676 to 1.38)	137.4 (95.2 to 188.7)	135.8 (93 to 187.9)	143.9 (97.9 to 201.5)
Montenegro	Interstitial lung disease and pulmonary sarcoidosis	0.128 (0.0958 to 0.167)	0.156 (0.118 to 0.2)	0.178 (0.134 to 0.233)	19.7 (14.8 to 25.7)	19 (14.3 to 24.5)	19.7 (14.8 to 25.9)
Montenegro	Other chronic respiratory diseases	0.122 (0.106 to 0.139)	0.152 (0.13 to 0.175)	0.162 (0.137 to 0.188)	18.8 (16.3 to 21.4)	21.6 (18.4 to 25)	23.1 (19.3 to 27.2)
Montenegro	Digestive diseases	5.32 (4.54 to 6.22)	5.97 (5.18 to 6.88)	6.14 (5.26 to 7.21)	829.8 (711.1 to 969.4)	780.1 (668.5 to 910.3)	744.9 (630.8 to 889)
Montenegro	Cirrhosis and other chronic liver diseases	1.35 (1.18 to 1.52)	1.58 (1.42 to 1.78)	1.69 (1.44 to 2.01)	202.4 (178 to 228.9)	195.2 (176 to 218.7)	191.4 (163.1 to 226.6)
Montenegro	Cirrhosis and other chronic liver diseases due to hepatitis B	0.376 (0.324 to 0.444)	0.434 (0.374 to 0.505)	0.405 (0.334 to 0.488)	56.5 (48.8 to 66.5)	53.7 (46.3 to 62.2)	46.1 (37.9 to 55.5)
Montenegro	Cirrhosis and other chronic liver diseases due to hepatitis C	0.278 (0.231 to 0.333)	0.33 (0.276 to 0.388)	0.357 (0.289 to 0.434)	41.6 (34.5 to 49.7)	40.3 (33.8 to 47)	40 (32.5 to 48.4)
Montenegro	Cirrhosis and other chronic liver diseases due to alcohol use	0.506 (0.435 to 0.586)	0.61 (0.541 to 0.697)	0.693 (0.583 to 0.826)	75.7 (65.2 to 86.9)	73.9 (65.7 to 84.3)	76.5 (64.7 to 91)
Montenegro	Cirrhosis due to NASH	0.0857 (0.0724 to 0.0999)	0.106 (0.0923 to 0.124)	0.126 (0.105 to 0.152)	12.8 (10.8 to 14.9)	12.8 (11.1 to 14.9)	13.9 (11.4 to 16.7)
Montenegro	Cirrhosis and other chronic liver diseases due to other causes	0.1 (0.0837 to 0.118)	0.102 (0.0845 to 0.121)	0.11 (0.0881 to 0.134)	15.9 (13.4 to 18.7)	14.6 (12.2 to 17.3)	14.9 (12 to 18.2)
Montenegro	Upper digestive system diseases	1.71 (1.23 to 2.38)	1.82 (1.32 to 2.52)	1.76 (1.28 to 2.44)	267.5 (191.8 to 369.5)	250.6 (179.9 to 351.6)	233.4 (165.2 to 329.8)
Montenegro	Peptic ulcer disease	0.478 (0.403 to 0.551)	0.54 (0.478 to 0.596)	0.493 (0.42 to 0.573)	73.8 (62.2 to 85.1)	65.4 (58.1 to 72.1)	54 (45.8 to 62.7)
Montenegro	Gastritis and duodenitis	0.721 (0.465 to 1.06)	0.69 (0.441 to 0.995)	0.659 (0.422 to 0.947)	115.2 (73.9 to 169.8)	106.7 (67.2 to 154.2)	101 (63.5 to 146.5)
Montenegro	GERD	0.514 (0.275 to 0.866)	0.588 (0.313 to 0.985)	0.612 (0.327 to 1.03)	78.4 (41.9 to 131.8)	78.5 (41.8 to 132.5)	78.4 (41.7 to 132.6)
Montenegro	Appendicitis	0.0443 (0.037 to 0.0527)	0.0421 (0.0361 to 0.0493)	0.0374 (0.0305 to 0.0445)	7 (5.8 to 8.4)	6 (5 to 7.2)	5.2 (4.2 to 6.4)
Montenegro	Paralytic ileus and intestinal obstruction	0.345 (0.291 to 0.397)	0.337 (0.276 to 0.385)	0.339 (0.262 to 0.402)	57.3 (48.7 to 66.2)	44.1 (36.5 to 50.1)	39.6 (30 to 47)
Montenegro	Inguinal, femoral, and abdominal hernia	0.425 (0.289 to 0.583)	0.487 (0.335 to 0.665)	0.537 (0.37 to 0.744)	68.9 (47.2 to 94.9)	72.8 (49.5 to 100.7)	76.1 (51.8 to 105.7)
Montenegro	Inflammatory bowel disease	0.254 (0.183 to 0.347)	0.255 (0.184 to 0.347)	0.248 (0.179 to 0.331)	39.4 (28.5 to 53)	33.9 (24.4 to 45.9)	31.3 (22.4 to 42.2)
Montenegro	Vascular intestinal disorders	0.498 (0.418 to 0.649)	0.678 (0.603 to 0.868)	0.706 (0.594 to 0.878)	78.4 (66 to 100.7)	78.2 (70 to 99.7)	71.6 (60.4 to 88.5)
Montenegro	Gallbladder and biliary diseases	0.17 (0.138 to 0.199)	0.22 (0.191 to 0.246)	0.231 (0.191 to 0.274)	26.9 (21.8 to 31.3)	25.7 (22.5 to 28.7)	23.7 (19.6 to 28)
Montenegro	Pancreatitis	0.344 (0.292 to 0.421)	0.389 (0.332 to 0.479)	0.399 (0.326 to 0.492)	52.6 (44.8 to 63.7)	49.5 (42.4 to 60.6)	47.3 (38.2 to 58.7)
Montenegro	Other digestive diseases	0.178 (0.133 to 0.229)	0.165 (0.129 to 0.203)	0.182 (0.14 to 0.226)	29.4 (21.8 to 38.1)	24 (18.3 to 29.9)	25.2 (19 to 32.1)
Montenegro	Neurological disorders	9.02 (6.94 to 11.8)	10.4 (8.18 to 13.1)	11.6 (9.25 to 14.4)	1477.8 (1152.5 to 1911.3)	1438.7 (1118.3 to 1844.3)	1465.7 (1130.7 to 1863.1)
Montenegro	Alzheimer's disease and other dementias	2.5 (2.31 to 2.69)	3.34 (3.06 to 3.62)	4.42 (4.05 to 4.81)	463 (428.8 to 497.5)	430.5 (393.1 to 468.4)	459 (421.2 to 500.1)
Montenegro	Parkinson's disease	0.454 (0.399 to 0.598)	0.678 (0.605 to 0.866)	0.808 (0.714 to 1.04)	76.9 (67.6 to 101.2)	78 (69.7 to 99.6)	78.6 (69.6 to 101)
Montenegro	Epilepsy	0.865 (0.391 to 1.55)	0.855 (0.437 to 1.47)	0.845 (0.323 to 1.69)	138 (62.5 to 247.3)	132.6 (66.8 to 229)	131.8 (50 to 267.4)
Montenegro	Multiple sclerosis	0.202 (0.176 to 0.23)	0.233 (0.206 to 0.262)	0.231 (0.19 to 0.272)	30.6 (26.6 to 34.8)	30 (26.4 to 34)	28.2 (23.1 to 33.5)
Montenegro	Motor neuron disease	0.0125 (0.00669 to 0.0144)	0.0137 (0.00829 to 0.0155)	0.0153 (0.00916 to 0.0175)	1.9 (1.1 to 2.2)	1.7 (1.1 to 1.9)	1.7 (1.1 to 2)
Montenegro	Headache disorders	4.78 (3.06 to 7.01)	5.06 (3.26 to 7.45)	5.05 (3.27 to 7.45)	732.6 (467.7 to 1074.6)	733.1 (470 to 1073.8)	731.3 (470.3 to 1075.2)
Montenegro	Migraine	4.1 (2.53 to 6.19)	4.33 (2.67 to 6.56)	4.31 (2.68 to 6.53)	628.1 (388.8 to 950.9)	628.6 (388.4 to 950.4)	627 (385.3 to 945.6)
Montenegro	Tension-type headache	0.681 (0.386 to 1.07)	0.733 (0.419 to 1.14)	0.74 (0.43 to 1.16)	104.5 (59.2 to 163.7)	104.6 (59.7 to 164.2)	104.3 (59.9 to 162.3)
Montenegro	Other neurological disorders	0.211 (0.138 to 0.316)	0.204 (0.138 to 0.301)	0.215 (0.124 to 0.363)	34.8 (22.9 to 52.1)	32.7 (21.3 to 49)	35 (18.6 to 61.5)
Montenegro	Mental disorders	8.64 (6.39 to 11.1)	9.25 (6.87 to 11.9)	9.23 (6.92 to 11.9)	1339.1 (990.6 to 1724.4)	1350.3 (998.6 to 1740.9)	1340.2 (1000.2 to 1727.6)
Montenegro	Schizophrenia	0.8 (0.593 to 1.01)	0.879 (0.654 to 1.09)	0.903 (0.668 to 1.13)	121.3 (89.6 to 152.3)	121.3 (89.7 to 152)	122.1 (89.4 to 154.6)
Montenegro	Depressive disorders	2.59 (1.85 to 3.51)	2.97 (2.09 to 4.02)	3.02 (2.12 to 4.07)	399.3 (284.8 to 542.6)	406.4 (285.4 to 552.8)	398.4 (283.8 to 542)
Montenegro	Major depressive disorder	2.04 (1.43 to 2.77)	2.34 (1.64 to 3.18)	2.36 (1.65 to 3.22)	314 (219.5 to 428)	321.1 (224.5 to 436.4)	313.4 (220.8 to 427.6)

SR Appendix Table 2. All-age DALYs counts and age standardized DALYs rates per 100,000 individuals for all causes and level 1 cause groups by location for 1990, 2007, and 2017, both sexes combined

		All-age DALYs			Age-standardized rate of DALYs per 100,000		
Montenegro	Dysthymia	0.558 (0.376 to 0.817)	0.632 (0.422 to 0.918)	0.655 (0.441 to 0.956)	85.3 (57.4 to 124.7)	85.3 (56.9 to 124.6)	85 (57 to 124.5)
Montenegro	Bipolar disorder	0.899 (0.573 to 1.35)	0.95 (0.6 to 1.42)	0.943 (0.597 to 1.41)	137.7 (87.8 to 205.6)	137.7 (87.3 to 206.1)	137.5 (86.2 to 206.4)
Montenegro	Anxiety disorders	1.99 (1.41 to 2.67)	2.11 (1.5 to 2.79)	2.1 (1.48 to 2.79)	309.2 (218.6 to 412.5)	310.6 (219 to 411.2)	308.7 (216.2 to 412.7)
Montenegro	Eating disorders	0.232 (0.146 to 0.344)	0.219 (0.14 to 0.322)	0.228 (0.144 to 0.339)	35.9 (22.6 to 53.5)	35.7 (22.9 to 52.4)	39.2 (24.8 to 58.8)
Montenegro	Anorexia nervosa	0.0455 (0.0263 to 0.0706)	0.0422 (0.0249 to 0.0647)	0.0428 (0.0257 to 0.0658)	7.1 (4.2 to 11)	7.1 (4.2 to 10.8)	7.8 (4.7 to 12)
Montenegro	Bulimia nervosa	0.186 (0.115 to 0.283)	0.177 (0.111 to 0.263)	0.185 (0.116 to 0.277)	28.8 (17.8 to 43.6)	28.6 (18 to 42.7)	31.5 (19.8 to 47.1)
Montenegro	Autism spectrum disorders	0.419 (0.284 to 0.581)	0.412 (0.28 to 0.566)	0.402 (0.275 to 0.553)	67.4 (45.6 to 93.3)	67.4 (45.6 to 92.8)	67.7 (46 to 93.3)
Montenegro	Attention-deficit/hyperactivity disorder	0.0736 (0.0442 to 0.118)	0.0674 (0.0407 to 0.108)	0.0625 (0.0377 to 0.101)	11.8 (7.1 to 19)	11.8 (7.1 to 19)	11.8 (7.1 to 19.2)
Montenegro	Conduct disorder	0.597 (0.359 to 0.955)	0.505 (0.303 to 0.803)	0.445 (0.267 to 0.698)	98.1 (59.1 to 156.6)	98.3 (58.9 to 155.9)	98.4 (59.6 to 154.6)
Montenegro	Idiopathic developmental intellectual disability	0.118 (0.0318 to 0.231)	0.13 (0.0451 to 0.242)	0.0966 (0.0238 to 0.19)	19 (5.2 to 37.2)	21.7 (7.5 to 40.4)	16.8 (4.2 to 33)
Montenegro	Other mental disorders	0.914 (0.606 to 1.28)	1.01 (0.672 to 1.39)	1.03 (0.687 to 1.41)	139.5 (92.6 to 194.9)	139.5 (92.7 to 193.2)	139.5 (92.7 to 192.3)
Montenegro	Substance use disorders	3.09 (2.46 to 3.79)	3.23 (2.59 to 3.92)	3.09 (2.5 to 3.78)	470.7 (375.6 to 577.5)	468.8 (374.3 to 571.1)	451 (361.9 to 558.1)
Montenegro	Alcohol use disorders	1.52 (1.14 to 1.97)	1.61 (1.22 to 2.08)	1.6 (1.21 to 2.07)	231 (172.8 to 299.2)	227.5 (171.1 to 294.9)	226.1 (168.5 to 295.9)
Montenegro	Drug use disorders	1.57 (1.28 to 1.87)	1.62 (1.33 to 1.9)	1.49 (1.22 to 1.76)	239.7 (195.5 to 285.1)	241.2 (197.5 to 284.9)	224.9 (183.3 to 269)
Montenegro	Opioid use disorders	1.2 (0.967 to 1.45)	1.26 (1.03 to 1.48)	1.14 (0.928 to 1.37)	182.6 (147.7 to 220.5)	186.3 (152.1 to 220.6)	170.9 (137.8 to 206.3)
Montenegro	Cocaine use disorders	0.0377 (0.0231 to 0.0554)	0.0385 (0.0242 to 0.0567)	0.0385 (0.0244 to 0.0563)	5.8 (3.5 to 8.5)	5.8 (3.5 to 8.6)	5.9 (3.7 to 8.8)
Montenegro	Amphetamine use disorders	0.0705 (0.0402 to 0.112)	0.0666 (0.038 to 0.106)	0.0622 (0.0362 to 0.0999)	10.9 (6.2 to 17.2)	10.8 (6.1 to 17.4)	11 (6.3 to 17.9)
Montenegro	Cannabis use disorders	0.0395 (0.0239 to 0.0589)	0.038 (0.024 to 0.0564)	0.0339 (0.0208 to 0.051)	6.1 (3.7 to 9.1)	6.1 (3.8 to 9.1)	5.8 (3.5 to 8.8)
Montenegro	Other drug use disorders	0.224 (0.183 to 0.276)	0.22 (0.183 to 0.264)	0.209 (0.164 to 0.259)	34.3 (28 to 42.3)	32.2 (26.6 to 39.1)	31.2 (24.3 to 39.3)
Montenegro	Diabetes and kidney diseases	7.64 (6.46 to 9.05)	10.6 (8.97 to 12.7)	11.3 (9.41 to 13.7)	1189.4 (1008.5 to 1404.8)	1274.8 (1070.7 to 1519.9)	1215 (1002.4 to 1472.5)
Montenegro	Diabetes mellitus	5.39 (4.3 to 6.72)	7.76 (6.22 to 9.69)	8.46 (6.69 to 10.7)	833.3 (666.3 to 1037)	923.3 (735.9 to 1155)	904.8 (706.3 to 1153.8)
Montenegro	Diabetes mellitus type 1	2.03 (1.86 to 2.22)	2.86 (2.66 to 3.09)	2.87 (2.57 to 3.23)	316.1 (290.1 to 345.3)	333 (310.3 to 358.6)	295.9 (264.5 to 330.4)
Montenegro	Diabetes mellitus type 2	3.36 (2.32 to 4.64)	4.9 (3.4 to 6.78)	5.58 (3.81 to 7.69)	517.2 (357.7 to 712.8)	590.3 (408.4 to 819.2)	608.8 (414.4 to 839)
Montenegro	Chronic kidney disease	2.25 (2.07 to 2.45)	2.88 (2.64 to 3.11)	2.87 (2.58 to 3.19)	355.5 (328.3 to 386.9)	351 (322.3 to 378.5)	309.9 (279.6 to 343.1)
Montenegro	Chronic kidney disease due to diabetes mellitus type 1	0.18 (0.143 to 0.226)	0.237 (0.191 to 0.292)	0.2 (0.156 to 0.261)	27.3 (21.6 to 34.2)	29.2 (23.6 to 35.9)	22.8 (18 to 29.1)
Montenegro	Chronic kidney disease due to diabetes mellitus type 2	0.348 (0.284 to 0.427)	0.479 (0.394 to 0.576)	0.529 (0.433 to 0.648)	54.8 (44.9 to 66.6)	54.3 (45.1 to 64.8)	52.3 (43.1 to 63.7)
Montenegro	Chronic kidney disease due to hypertension	0.379 (0.317 to 0.446)	0.573 (0.488 to 0.67)	0.612 (0.499 to 0.737)	61.3 (51.5 to 71.5)	68 (58.1 to 78.7)	63 (52.2 to 75.4)
Montenegro	Chronic kidney disease due to glomerulonephritis	0.577 (0.502 to 0.66)	0.702 (0.605 to 0.814)	0.644 (0.543 to 0.772)	89.9 (78.6 to 102.9)	88.3 (76.3 to 101)	73.8 (63.1 to 86.9)
Montenegro	Chronic kidney disease due to other and unspecified causes	0.762 (0.661 to 0.874)	0.887 (0.756 to 1.03)	0.881 (0.742 to 1.04)	122.2 (106.7 to 140.5)	111.2 (95.3 to 127.2)	98 (83.5 to 114.7)
Montenegro	Acute glomerulonephritis	0.00353 (0.00287 to 0.00455)	0.00323 (0.0027 to 0.00389)	0.0027 (0.00207 to 0.00353)	0.6 (0.5 to 0.7)	0.4 (0.4 to 0.5)	0.3 (0.3 to 0.4)
Montenegro	Skin and subcutaneous diseases	2.99 (1.99 to 4.37)	3 (2.02 to 4.42)	3 (2.03 to 4.37)	482.3 (321.7 to 707.4)	483.3 (323.1 to 708.1)	488.1 (327.9 to 710.3)
Montenegro	Dermatitis	0.754 (0.441 to 1.18)	0.721 (0.424 to 1.11)	0.703 (0.419 to 1.1)	123.6 (72.2 to 193.7)	123.6 (71.5 to 194.5)	124.7 (72.9 to 201)
Montenegro	Atopic dermatitis	0.604 (0.331 to 1.01)	0.56 (0.305 to 0.925)	0.541 (0.297 to 0.907)	100.5 (55 to 167.5)	100.5 (55 to 168)	101.6 (55.3 to 171)
Montenegro	Contact dermatitis	0.149 (0.0964 to 0.22)	0.16 (0.104 to 0.235)	0.162 (0.106 to 0.237)	23 (14.9 to 34.1)	23 (14.9 to 33.9)	23 (15 to 34.1)
Montenegro	Seborrhoeic dermatitis	0.000674 (0.000377 to 0.00109)	0.000694 (0.000387 to 0.00112)	0.000695 (0.000386 to 0.00111)	0.1 (0.1 to 0.2)	0.1 (0.1 to 0.2)	0.1 (0.1 to 0.2)
Montenegro	Psoriasis	0.489 (0.348 to 0.649)	0.524 (0.373 to 0.686)	0.543 (0.385 to 0.713)	75.3 (53.4 to 100.1)	73.1 (51.6 to 96.4)	73.7 (52.4 to 97.4)
Montenegro	Bacterial skin diseases	0.0249 (0.0184 to 0.0337)	0.0273 (0.0206 to 0.0371)	0.0293 (0.022 to 0.0392)	4.1 (3 to 5.5)	4 (3 to 5.4)	4 (2.9 to 5.3)
Montenegro	Cellulitis	0.0132 (0.00925 to 0.0181)	0.014 (0.00981 to 0.0193)	0.0147 (0.0105 to 0.0201)	2.1 (1.5 to 2.9)	2.1 (1.5 to 2.9)	2.1 (1.5 to 2.9)
Montenegro	Pyoderma	0.0117 (0.00825 to 0.0178)	0.0133 (0.00923 to 0.0197)	0.0146 (0.00992 to 0.0212)	2 (1.4 to 2.9)	1.9 (1.3 to 2.8)	1.9 (1.3 to 2.8)
Montenegro	Scabies	0.171 (0.0959 to 0.278)	0.161 (0.0894 to 0.261)	0.15 (0.0839 to 0.243)	27.5 (15.4 to 45)	27.3 (15 to 44.4)	26.8 (15.1 to 43.8)

SR Appendix Table 2. All-age DALYs counts and age standardized DALYs rates per 100,000 individuals for all causes and level 1 cause groups by location for 1990, 2007, and 2017, both sexes combined

		All-age DALYs			Age-standardized rate of DALYs per 100,000		
Montenegro	Fungal skin diseases	0.283 (0.112 to 0.585)	0.315 (0.126 to 0.646)	0.329 (0.132 to 0.675)	45.7 (18.2 to 94.1)	44.8 (17.7 to 92.7)	44.2 (17.6 to 91.4)
Montenegro	Viral skin diseases	0.269 (0.172 to 0.399)	0.229 (0.146 to 0.339)	0.209 (0.135 to 0.311)	45.8 (29.3 to 68.1)	45.9 (29.1 to 68.1)	45.8 (29.4 to 68.1)
Montenegro	Acne vulgaris	0.199 (0.119 to 0.321)	0.209 (0.123 to 0.336)	0.204 (0.121 to 0.33)	31.2 (18.6 to 50.3)	35.6 (20.9 to 57.4)	38.5 (22.7 to 63.2)
Montenegro	Alopecia areata	0.0383 (0.0244 to 0.0574)	0.0394 (0.025 to 0.0586)	0.0391 (0.0246 to 0.0587)	5.9 (3.8 to 8.8)	5.9 (3.8 to 8.8)	5.9 (3.7 to 8.9)
Montenegro	Pruritus	0.0609 (0.0287 to 0.116)	0.0666 (0.0322 to 0.123)	0.0696 (0.0334 to 0.128)	9.6 (4.5 to 18.3)	9.5 (4.5 to 18)	9.6 (4.6 to 18.1)
Montenegro	Urticaria	0.452 (0.299 to 0.645)	0.424 (0.279 to 0.597)	0.406 (0.27 to 0.572)	74.8 (49.4 to 106.5)	74.8 (49.2 to 105.8)	74.6 (49.2 to 106.3)
Montenegro	Decubitus ulcer	0.0163 (0.0118 to 0.0218)	0.0202 (0.0142 to 0.0269)	0.0236 (0.0168 to 0.032)	2.7 (1.9 to 3.6)	2.7 (1.9 to 3.6)	2.8 (2 to 3.7)
Montenegro	Other skin and subcutaneous diseases	0.231 (0.113 to 0.422)	0.265 (0.13 to 0.482)	0.289 (0.142 to 0.531)	36.2 (17.7 to 66.5)	36.1 (17.7 to 66)	37.4 (18.2 to 68.7)
Montenegro	Sense organ diseases	4.7 (3.14 to 6.81)	5.8 (3.88 to 8.36)	6.29 (4.22 to 9.01)	746.6 (500.1 to 1079.7)	738 (495.1 to 1064.8)	724.2 (483.7 to 1043.9)
Montenegro	Blindness and vision impairment	1.92 (1.24 to 2.85)	2.31 (1.5 to 3.4)	2.46 (1.6 to 3.6)	304 (195.9 to 451.5)	294.5 (189.7 to 436.8)	285.3 (184.2 to 425.1)
Montenegro	Glaucoma	0.0226 (0.0148 to 0.0321)	0.0303 (0.0202 to 0.0433)	0.0332 (0.0217 to 0.0469)	3.6 (2.4 to 5.2)	3.5 (2.3 to 4.9)	3.2 (2.1 to 4.5)
Montenegro	Cataract	0.598 (0.4 to 0.827)	0.814 (0.547 to 1.12)	0.918 (0.62 to 1.25)	98 (65.8 to 136)	95.1 (64.6 to 130.7)	92 (62.2 to 125.4)
Montenegro	Age-related macular degeneration	0.00557 (0.00348 to 0.00826)	0.00766 (0.00476 to 0.0112)	0.00899 (0.0058 to 0.0134)	0.9 (0.6 to 1.3)	0.9 (0.6 to 1.3)	0.9 (0.6 to 1.3)
Montenegro	Refraction disorders	0.675 (0.424 to 1.04)	0.713 (0.453 to 1.09)	0.713 (0.455 to 1.08)	106.6 (67.3 to 162.4)	103.3 (66.2 to 158.5)	100.5 (64 to 153)
Montenegro	Near vision loss	0.463 (0.221 to 0.849)	0.558 (0.267 to 1.03)	0.586 (0.283 to 1.08)	71.1 (34 to 130.7)	68.8 (32.9 to 126.7)	66.3 (31.9 to 122.5)
Montenegro	Other vision loss	0.151 (0.102 to 0.209)	0.183 (0.123 to 0.252)	0.196 (0.131 to 0.275)	23.8 (16.1 to 32.9)	23 (15.5 to 31.5)	22.3 (14.9 to 31.1)
Montenegro	Age-related and other hearing loss	2.58 (1.76 to 3.66)	3.25 (2.21 to 4.59)	3.56 (2.42 to 5.03)	411.3 (281.8 to 579.7)	412.2 (282.3 to 581)	407.4 (277.5 to 575.9)
Montenegro	Other sense organ diseases	0.198 (0.124 to 0.289)	0.244 (0.152 to 0.352)	0.268 (0.167 to 0.392)	31.2 (19.6 to 45.6)	31.4 (19.6 to 45.6)	31.5 (19.6 to 46.6)
Montenegro	Musculoskeletal disorders	11.4 (8.27 to 15.7)	13.5 (9.78 to 18.5)	14.5 (10.5 to 19.7)	1750.4 (1267.1 to 2401.7)	1752.4 (1267 to 2388.1)	1769.8 (1276.6 to 2397.9)
Montenegro	Rheumatoid arthritis	0.187 (0.134 to 0.246)	0.235 (0.168 to 0.312)	0.274 (0.191 to 0.362)	28.6 (20.4 to 37.6)	29 (20.6 to 38.5)	31 (21.6 to 40.9)
Montenegro	Osteoarthritis	0.601 (0.299 to 1.19)	0.804 (0.406 to 1.59)	0.933 (0.469 to 1.84)	93.4 (46.4 to 185.2)	93.8 (47.4 to 184.9)	96.4 (48.7 to 189.2)
Montenegro	Low back pain	8.53 (6.07 to 11.5)	9.93 (7.09 to 13.4)	10.6 (7.55 to 14.3)	1305.8 (930.1 to 1762.7)	1305 (935.2 to 1759.5)	1320.6 (929.4 to 1771.1)
Montenegro	Neck pain	1.94 (1.36 to 2.72)	2.35 (1.64 to 3.29)	2.48 (1.74 to 3.49)	297 (208.3 to 414.5)	297 (207.6 to 415.5)	296.3 (207.4 to 412.7)
Montenegro	Gout	0.0771 (0.0517 to 0.108)	0.101 (0.0671 to 0.138)	0.114 (0.0762 to 0.158)	11.9 (8 to 16.6)	12 (8 to 16.4)	12.2 (8.2 to 17)
Montenegro	Other musculoskeletal disorders	0.0897 (0.0344 to 0.168)	0.114 (0.0468 to 0.211)	0.101 (0.0421 to 0.198)	13.8 (5.4 to 25.7)	15.6 (6.2 to 28.8)	13.4 (5.1 to 26.6)
Montenegro	Other non-communicable diseases	7.12 (5.58 to 8.99)	6.46 (4.78 to 8.5)	6.07 (4.39 to 8.12)	1226.1 (975.2 to 1524.9)	998.1 (761.2 to 1282.8)	874.7 (643 to 1145.9)
Montenegro	Congenital anomalies	3.02 (2.5 to 3.62)	2.01 (1.6 to 2.44)	1.62 (1.24 to 2.03)	570.4 (472.8 to 682.7)	388.5 (317 to 461.4)	300.6 (238.4 to 371.2)
Montenegro	Neural tube defects	0.0878 (0.0641 to 0.12)	0.0487 (0.0378 to 0.0609)	0.0372 (0.0275 to 0.0487)	17 (12.1 to 24)	9.6 (7.5 to 12.1)	6.8 (5 to 8.8)
Montenegro	Congenital heart anomalies	0.901 (0.661 to 1.16)	0.396 (0.332 to 0.476)	0.198 (0.142 to 0.282)	186 (134.8 to 241.5)	96.2 (80.4 to 116.3)	50.2 (35.3 to 74.1)
Montenegro	Orofacial clefts	0.0227 (0.0151 to 0.0318)	0.0196 (0.0133 to 0.028)	0.016 (0.0104 to 0.0234)	3.9 (2.6 to 5.5)	3.4 (2.3 to 4.8)	2.8 (1.8 to 4)
Montenegro	Down's syndrome	0.283 (0.193 to 0.384)	0.164 (0.133 to 0.199)	0.121 (0.0958 to 0.157)	55.1 (36.6 to 76)	35 (27.9 to 43.2)	26.1 (19.8 to 35.4)
Montenegro	Turner syndrome	0.000541 (0.000271 to 0.000876)	0.000569 (0.000285 to 0.000925)	0.00053 (0.000261 to 0.000876)	0.1 (0 to 0.1)	0.1 (0 to 0.2)	0.1 (0 to 0.2)
Montenegro	Klinefelter syndrome	0.000126 (0.000059 to 0.000246)	0.000119 (0.000055 to 0.00023)	0.000113 (0.000053 to 0.000218)	0 (0 to 0)	0 (0 to 0)	0 (0 to 0)
Montenegro	Other chromosomal abnormalities	0.0765 (0.052 to 0.102)	0.0543 (0.0401 to 0.0715)	0.0481 (0.0324 to 0.0666)	14.8 (10.1 to 19.7)	11.4 (8.7 to 14.8)	10 (6.9 to 14)
Montenegro	Congenital musculoskeletal and limb anomalies	0.827 (0.566 to 1.13)	0.818 (0.548 to 1.11)	0.799 (0.54 to 1.09)	135.8 (93.5 to 184.9)	130 (87.9 to 177.4)	126.5 (85.2 to 172.6)
Montenegro	Urogenital congenital anomalies	0.0423 (0.0274 to 0.0603)	0.0274 (0.0204 to 0.0371)	0.0215 (0.0149 to 0.0314)	8.2 (5.2 to 12)	6.1 (4.5 to 8.1)	5 (3.3 to 7.4)
Montenegro	Digestive congenital anomalies	0.365 (0.281 to 0.467)	0.226 (0.166 to 0.298)	0.189 (0.13 to 0.258)	69 (52.9 to 88.6)	43.2 (32.8 to 56.7)	34.2 (23.7 to 46.7)
Montenegro	Other congenital anomalies	0.417 (0.303 to 0.534)	0.258 (0.202 to 0.32)	0.192 (0.138 to 0.257)	80.5 (57.6 to 105.2)	53.5 (42 to 66.1)	38.9 (27.2 to 54.5)
Montenegro	Urinary diseases and male infertility	0.376 (0.263 to 0.521)	0.473 (0.328 to 0.653)	0.525 (0.364 to 0.736)	57.5 (40.6 to 79.2)	56.8 (39.7 to 78)	56.4 (39.2 to 78.5)
Montenegro	Urinary tract infections	0.0504 (0.0432 to 0.0584)	0.0539 (0.0464 to 0.0629)	0.0578 (0.0488 to 0.0678)	8.2 (7 to 9.4)	7.3 (6.1 to 8.7)	7.2 (6 to 8.7)

SR Appendix Table 2. All-age DALYs counts and age standardized DALYs rates per 100,000 individuals for all causes and level 1 cause groups by location for 1990, 2007, and 2017, both sexes combined

		All-age DALYs			Age-standardized rate of DALYs per 100,000		
Montenegro	Urolithiasis	0.0297 (0.0205 to 0.0413)	0.0351 (0.0238 to 0.0486)	0.0376 (0.0259 to 0.0514)	4.5 (3.1 to 6.3)	4.6 (3.1 to 6.3)	4.6 (3.2 to 6.4)
Montenegro	Benign prostatic hyperplasia	0.25 (0.158 to 0.366)	0.342 (0.219 to 0.494)	0.393 (0.254 to 0.582)	37.5 (23.7 to 54.8)	38.6 (24.8 to 55.7)	39.1 (25.3 to 58)
Montenegro	Male infertility	0.0132 (0.00532 to 0.0274)	0.0128 (0.00507 to 0.0273)	0.011 (0.00434 to 0.0226)	2 (0.8 to 4.2)	2 (0.8 to 4.3)	1.8 (0.7 to 3.8)
Montenegro	Other urinary diseases	0.0331 (0.0262 to 0.0415)	0.0292 (0.0229 to 0.0362)	0.0254 (0.0199 to 0.0316)	5.3 (4.2 to 6.6)	4.3 (3.3 to 5.4)	3.6 (2.8 to 4.5)
Montenegro	Gynecological diseases	0.966 (0.65 to 1.38)	0.988 (0.676 to 1.39)	0.949 (0.639 to 1.33)	149.5 (100.4 to 213)	145.6 (100.3 to 202.7)	144.8 (98.4 to 201.2)
Montenegro	Uterine fibroids	0.153 (0.086 to 0.267)	0.146 (0.081 to 0.252)	0.142 (0.076 to 0.243)	23.8 (13.3 to 41.6)	19.3 (10.8 to 32.8)	18.4 (9.8 to 31.5)
Montenegro	Polycystic ovarian syndrome	0.0322 (0.0146 to 0.0621)	0.0328 (0.0148 to 0.0639)	0.0315 (0.014 to 0.0604)	5 (2.3 to 9.6)	5 (2.2 to 9.7)	5.1 (2.2 to 9.7)
Montenegro	Female infertility	0.011 (0.00344 to 0.0248)	0.0104 (0.0032 to 0.024)	0.0117 (0.00371 to 0.0271)	1.7 (0.5 to 3.8)	1.7 (0.5 to 3.8)	1.9 (0.6 to 4.4)
Montenegro	Endometriosis	0.278 (0.181 to 0.406)	0.281 (0.184 to 0.417)	0.264 (0.169 to 0.391)	42.8 (27.9 to 62.4)	43.8 (28.7 to 65.1)	43.2 (27.7 to 63.6)
Montenegro	Genital prolapse	0.0288 (0.0138 to 0.0536)	0.0307 (0.0148 to 0.059)	0.0334 (0.0163 to 0.0628)	4.4 (2.1 to 8.2)	3.6 (1.8 to 6.9)	3.6 (1.7 to 6.7)
Montenegro	Premenstrual syndrome	0.321 (0.205 to 0.48)	0.331 (0.214 to 0.495)	0.308 (0.195 to 0.459)	50 (31.9 to 74.8)	50.1 (32.4 to 75.2)	49.2 (31.4 to 73.5)
Montenegro	Other gynecological diseases	0.142 (0.0952 to 0.202)	0.156 (0.104 to 0.22)	0.159 (0.107 to 0.231)	21.9 (14.7 to 31.2)	22.1 (14.7 to 31.5)	23.4 (15.8 to 34.1)
Montenegro	Hemoglobinopathies and hemolytic anaemias	0.241 (0.186 to 0.314)	0.222 (0.17 to 0.291)	0.196 (0.151 to 0.258)	39.1 (30.2 to 50.6)	32.5 (24.5 to 43.4)	27.6 (20.7 to 37.5)
Montenegro	Thalassemias	0.00756 (0.00499 to 0.011)	0.00312 (0.00211 to 0.00449)	0.00243 (0.0015 to 0.00361)	1.4 (0.9 to 2.1)	0.7 (0.5 to 1.1)	0.6 (0.4 to 0.9)
Montenegro	Thalassaemias trait	0.0521 (0.0322 to 0.0796)	0.0458 (0.028 to 0.0706)	0.0374 (0.0215 to 0.059)	8.4 (5.2 to 12.8)	7.2 (4.4 to 11.1)	5.8 (3.3 to 9.3)
Montenegro	Sickle cell disorders	0.019 (0.0149 to 0.0239)	0.0125 (0.01 to 0.0152)	0.0107 (0.00815 to 0.0132)	3.4 (2.7 to 4.3)	2.4 (1.9 to 3)	2.2 (1.6 to 2.7)
Montenegro	Sickle cell trait	0.0253 (0.015 to 0.04)	0.0212 (0.0129 to 0.0326)	0.0175 (0.0102 to 0.0279)	4.1 (2.5 to 6.6)	3.4 (2.1 to 5.4)	2.8 (1.6 to 4.6)
Montenegro	G6PD deficiency	0.0228 (0.0184 to 0.0275)	0.0247 (0.0198 to 0.0295)	0.0218 (0.0164 to 0.0273)	3.6 (2.9 to 4.4)	3.6 (2.9 to 4.2)	3.1 (2.3 to 3.9)
Montenegro	G6PD trait	0.0000211 (0.0000135 to 0.0000311)	0.0000204 (0.000013 to 0.0000296)	0.0000172 (0.0000108 to 0.0000249)	0 (0 to 0)	0 (0 to 0)	0 (0 to 0)
Montenegro	Other hemoglobinopathies and hemolytic anaemias	0.114 (0.0888 to 0.149)	0.114 (0.0896 to 0.148)	0.106 (0.0839 to 0.137)	18.1 (14.2 to 23.7)	15.2 (11.6 to 20.3)	13.1 (10 to 17.6)
Montenegro	Endocrine, metabolic, blood, and immune disorders	0.403 (0.328 to 0.491)	0.374 (0.301 to 0.46)	0.36 (0.287 to 0.444)	68.1 (54.7 to 83.2)	58 (46.9 to 70.8)	53.9 (41.9 to 67.2)
Montenegro	Oral disorders	1.86 (1.17 to 2.79)	2.27 (1.44 to 3.36)	2.36 (1.5 to 3.52)	289.1 (181.3 to 432.7)	286.7 (179.3 to 428.1)	274.5 (171.8 to 411.2)
Montenegro	Caries of deciduous teeth	0.0131 (0.00553 to 0.0259)	0.01 (0.00426 to 0.0202)	0.00886 (0.00369 to 0.0172)	2.4 (1 to 4.8)	2.4 (1 to 4.8)	2.4 (1 to 4.6)
Montenegro	Caries of permanent teeth	0.165 (0.0709 to 0.314)	0.171 (0.0739 to 0.327)	0.161 (0.0695 to 0.307)	25.6 (11 to 48.8)	26 (11.2 to 49.6)	24.8 (10.7 to 47.4)
Montenegro	Periodontal disease	0.368 (0.147 to 0.759)	0.447 (0.175 to 0.921)	0.464 (0.186 to 0.958)	55.8 (22.3 to 115)	56.2 (22.1 to 116.3)	54.3 (21.7 to 113)
Montenegro	Edentulism and severe tooth loss	0.989 (0.658 to 1.4)	1.29 (0.868 to 1.84)	1.37 (0.92 to 1.95)	154.5 (102.8 to 218.2)	151.3 (100.9 to 214.6)	142.3 (95.4 to 202.7)
Montenegro	Other oral disorders	0.329 (0.205 to 0.49)	0.351 (0.219 to 0.519)	0.353 (0.221 to 0.518)	50.8 (31.7 to 75.6)	50.8 (31.8 to 75.1)	50.7 (31.7 to 75.3)
Montenegro	Sudden infant death syndrome	0.245 (0.147 to 0.392)	0.115 (0.0687 to 0.181)	0.058 (0.0233 to 0.0976)	52.3 (31.5 to 83.6)	30 (17.9 to 47.1)	16.8 (6.8 to 28.3)
Montenegro	Injuries	24.2 (21.2 to 27.8)	22.2 (18.9 to 26.3)	21.6 (17.8 to 26.2)	3797.5 (3334.7 to 4361.3)	3160.9 (2718.8 to 3717.5)	2934.5 (2443.4 to 3547.2)
Montenegro	Transport injuries	5.13 (4.61 to 5.71)	4.73 (4.19 to 5.34)	4.18 (3.6 to 4.85)	802.3 (721.1 to 890.2)	693.8 (618.1 to 778)	602.9 (524 to 694.8)
Montenegro	Road injuries	4.84 (4.36 to 5.39)	4.49 (3.97 to 5.07)	3.96 (3.41 to 4.61)	757.8 (682.8 to 842.3)	659 (587 to 738.1)	571 (496.1 to 659.2)
Montenegro	Pedestrian road injuries	1.7 (1.51 to 1.91)	1.41 (1.25 to 1.58)	1.18 (1.01 to 1.36)	269.7 (239.5 to 303.1)	205 (182.5 to 228.5)	165.9 (142.2 to 191.4)
Montenegro	Cyclist road injuries	0.442 (0.343 to 0.559)	0.473 (0.359 to 0.601)	0.523 (0.391 to 0.681)	68.2 (52.9 to 86)	64.5 (49.2 to 81.6)	68.2 (50.9 to 88.7)
Montenegro	Motorcyclist road injuries	0.725 (0.621 to 0.843)	0.61 (0.526 to 0.711)	0.508 (0.426 to 0.604)	112.5 (96.3 to 130.7)	92.6 (80.2 to 107.5)	78.9 (65.8 to 93.7)
Montenegro	Motor vehicle road injuries	1.87 (1.63 to 2.11)	1.91 (1.68 to 2.15)	1.67 (1.43 to 1.93)	291.1 (255.6 to 328.6)	284 (251 to 317)	246.6 (213.1 to 282.8)
Montenegro	Other road injuries	0.105 (0.0849 to 0.128)	0.0918 (0.0734 to 0.115)	0.0841 (0.066 to 0.106)	16.4 (13.4 to 20)	12.9 (10.3 to 16.3)	11.5 (9 to 14.5)
Montenegro	Other transport injuries	0.286 (0.242 to 0.333)	0.238 (0.207 to 0.271)	0.221 (0.187 to 0.26)	44.4 (37.7 to 51.7)	34.9 (30.3 to 39.7)	32 (26.9 to 38)
Montenegro	Unintentional injuries	14 (11.6 to 17.1)	12.7 (10 to 16)	13.4 (10.4 to 17.4)	2220.9 (1841.4 to 2693.7)	1781.2 (1428.9 to 2231.9)	1773.6 (1379.6 to 2280.1)
Montenegro	Falls	5.78 (4.26 to 7.74)	6.31 (4.59 to 8.55)	7.76 (5.6 to 10.5)	900.3 (665 to 1204.1)	849.5 (616.9 to 1151.1)	983.1 (707.5 to 1344.5)
Montenegro	Drowning	0.519 (0.457 to 0.592)	0.337 (0.308 to 0.37)	0.258 (0.228 to 0.29)	84.7 (74.5 to 97.3)	54.6 (49.5 to 60.3)	42.7 (37.6 to 48)

SR Appendix Table 2. All-age DALYs counts and age standardized DALYs rates per 100,000 individuals for all causes and level 1 cause groups by location for 1990, 2007, and 2017, both sexes combined

		All-age DALYs			Age-standardized rate of DALYs per 100,000		
Montenegro	Fire, heat, and hot substances	0.656 (0.533 to 0.791)	0.539 (0.441 to 0.674)	0.514 (0.403 to 0.663)	106.1 (86.3 to 127.3)	78 (63.9 to 96.2)	70 (55.2 to 89.7)
Montenegro	Poisonings	0.417 (0.339 to 0.498)	0.333 (0.278 to 0.41)	0.335 (0.268 to 0.426)	66.9 (54 to 80)	51 (42.6 to 63.2)	51.1 (40.6 to 65)
Montenegro	Poisoning by carbon monoxide	0.128 (0.0991 to 0.153)	0.104 (0.0905 to 0.125)	0.0968 (0.0795 to 0.119)	20.2 (15.6 to 24)	15.5 (13.4 to 18.6)	14.3 (11.6 to 17.8)
Montenegro	Poisoning by other means	0.289 (0.229 to 0.357)	0.229 (0.181 to 0.293)	0.238 (0.178 to 0.317)	46.8 (37 to 57.6)	35.5 (28.1 to 45.2)	36.8 (27.4 to 49)
Montenegro	Exposure to mechanical forces	2.7 (2.18 to 3.38)	2.44 (1.93 to 3.13)	2.19 (1.66 to 2.88)	419.8 (340.3 to 524.7)	338.9 (269.9 to 431.8)	291.8 (223.4 to 380.9)
Montenegro	Unintentional firearm injuries	0.537 (0.462 to 0.634)	0.414 (0.362 to 0.476)	0.291 (0.243 to 0.346)	84.7 (73 to 99.9)	62 (54.4 to 72.6)	43.4 (35.5 to 52.2)
Montenegro	Other exposure to mechanical forces	2.17 (1.68 to 2.84)	2.03 (1.52 to 2.7)	1.9 (1.39 to 2.58)	335.1 (260.9 to 437.8)	276.8 (210.7 to 365.4)	248.4 (184.1 to 335.9)
Montenegro	Adverse effects of medical treatment	0.349 (0.292 to 0.421)	0.322 (0.267 to 0.384)	0.3 (0.251 to 0.364)	56.8 (47 to 68.4)	46.2 (37.9 to 54.6)	40.6 (33.1 to 49)
Montenegro	Animal contact	0.219 (0.179 to 0.269)	0.201 (0.165 to 0.246)	0.164 (0.133 to 0.201)	34.8 (28.4 to 42.8)	29.4 (24 to 35.8)	23.4 (18.8 to 28.7)
Montenegro	Venomous animal contact	0.16 (0.129 to 0.199)	0.148 (0.118 to 0.181)	0.12 (0.0956 to 0.148)	25.2 (20.3 to 31.4)	21.6 (17.2 to 26.7)	17.2 (13.6 to 21.4)
Montenegro	Non-venomous animal contact	0.059 (0.0447 to 0.0805)	0.0533 (0.0397 to 0.0725)	0.0446 (0.0331 to 0.0618)	9.6 (7.3 to 13)	7.8 (5.8 to 10.7)	6.2 (4.6 to 8.7)
Montenegro	Foreign body	0.873 (0.729 to 1.03)	0.609 (0.543 to 0.681)	0.465 (0.408 to 0.53)	156.9 (129.1 to 190.5)	101.8 (89.4 to 115.7)	71.7 (62.4 to 85)
Montenegro	Pulmonary aspiration and foreign body in airway	0.445 (0.331 to 0.577)	0.239 (0.2 to 0.283)	0.171 (0.142 to 0.205)	86.2 (63 to 113.4)	46.4 (37.5 to 56.8)	29.6 (24 to 38)
Montenegro	Foreign body in eyes	0.0124 (0.00581 to 0.0217)	0.0137 (0.00671 to 0.0235)	0.0147 (0.00734 to 0.0252)	2 (0.9 to 3.4)	2 (1 to 3.6)	2.1 (1 to 3.7)
Montenegro	Foreign body in other body part	0.416 (0.356 to 0.486)	0.356 (0.312 to 0.401)	0.279 (0.243 to 0.324)	68.8 (58.6 to 81.2)	53.4 (47.1 to 60.7)	40 (34.7 to 47.1)
Montenegro	Environmental heat and cold exposure	0.136 (0.101 to 0.175)	0.139 (0.102 to 0.18)	0.161 (0.117 to 0.212)	21.2 (15.8 to 27.4)	19.2 (14.2 to 25.1)	21.4 (15.5 to 28.1)
Montenegro	Exposure to forces of nature	0 (0 to 0)	0.0000731 (0.0000562 to 0.0000938)	0.0000588 (0.0000454 to 0.0000748)	0 (0 to 0)	0 (0 to 0)	0 (0 to 0)
Montenegro	Other unintentional injuries	2.38 (2.09 to 2.69)	1.46 (1.25 to 1.71)	1.26 (1.04 to 1.54)	373.3 (330 to 422.7)	212.5 (183.9 to 248.1)	177.7 (148.5 to 216.4)
Montenegro	Self-harm and interpersonal violence	5.01 (4.63 to 5.53)	4.81 (4.42 to 5.19)	3.97 (3.56 to 4.47)	774.3 (716.9 to 857.6)	685.9 (632 to 741.6)	557.9 (499.5 to 628.3)
Montenegro	Self-harm	3.01 (2.73 to 3.45)	3.28 (2.91 to 3.56)	2.69 (2.35 to 3.07)	459.8 (417.1 to 528.3)	460.2 (407.7 to 498.3)	370.3 (323.2 to 425.7)
Montenegro	Self-harm by firearm	0.837 (0.66 to 1.27)	0.824 (0.612 to 1.09)	0.649 (0.463 to 0.841)	128.1 (100.7 to 194)	118.7 (89 to 156.7)	93 (67 to 120.4)
Montenegro	Self-harm by other specified means	2.17 (1.85 to 2.5)	2.46 (2.06 to 2.71)	2.04 (1.74 to 2.37)	331.7 (283.1 to 381.5)	341.5 (286.2 to 378.3)	277.3 (234.6 to 324.7)
Montenegro	Interpersonal violence	1.99 (1.68 to 2.34)	1.51 (1.33 to 1.9)	1.27 (1.09 to 1.61)	313.6 (264.1 to 367.5)	224.1 (196.8 to 281.9)	186.2 (159.3 to 240)
Montenegro	Assault by firearm	0.695 (0.477 to 0.951)	0.503 (0.371 to 0.716)	0.386 (0.288 to 0.535)	107.3 (73.7 to 147.2)	76.1 (56.6 to 108.7)	59.6 (45.3 to 83.5)
Montenegro	Assault by sharp object	0.369 (0.259 to 0.515)	0.277 (0.21 to 0.416)	0.235 (0.176 to 0.342)	57.4 (40.6 to 79.6)	40.6 (30.8 to 61.4)	34.4 (25.7 to 49.7)
Montenegro	Sexual violence	0.0613 (0.041 to 0.0886)	0.0609 (0.0411 to 0.0885)	0.0608 (0.0412 to 0.0876)	9.5 (6.3 to 13.8)	9.4 (6.3 to 13.7)	9.6 (6.5 to 14)
Montenegro	Assault by other means	0.865 (0.702 to 1.05)	0.671 (0.551 to 0.851)	0.59 (0.476 to 0.756)	139.4 (112.9 to 169.4)	98 (81.2 to 126)	82.6 (66.9 to 105.9)
Montenegro	Conflict and terrorism	0.001 (0.000666 to 0.00165)	0.00561 (0.00371 to 0.00924)	0.00478 (0.00319 to 0.00778)	0.2 (0.1 to 0.3)	0.9 (0.6 to 1.4)	0.7 (0.4 to 1.1)
Montenegro	Executions and police conflict	0.00444 (0.00413 to 0.00481)	0.0043 (0.00397 to 0.00474)	0.00421 (0.00379 to 0.00471)	0.7 (0.7 to 0.8)	0.7 (0.7 to 0.8)	0.7 (0.7 to 0.8)
Poland	All causes	13900 (12700 to 15300)	12900 (11600 to 14400)	12600 (11200 to 14200)	33741.3 (30820.7 to 37076.5)	26408.8 (23654.3 to 29623.5)	23039.5 (20213.5 to 26248.5)
Poland	Communicable, maternal, neonatal, and nutritional disorders	1190 (1080 to 1310)	662 (587 to 751)	606 (527 to 694)	3679.4 (3375.1 to 4028.7)	2021.8 (1800.5 to 2282.2)	1698 (1452.1 to 1970.8)
Poland	HIV/AIDS and sexually transmitted infections	12.7 (9.11 to 18.6)	16.4 (12.8 to 22.8)	14.8 (11.2 to 20.9)	33.2 (24.1 to 48.3)	42.1 (33.2 to 57)	36.8 (27.9 to 52.1)
Poland	HIV/AIDS	3.72 (3.56 to 3.88)	8.75 (8.35 to 9.33)	7.77 (7.19 to 8.71)	9.8 (9.4 to 10.2)	23.3 (22.3 to 24.7)	19.1 (17.8 to 21.2)
Poland	HIV/AIDS - Drug-susceptible Tuberculosis	1.59 (1.19 to 1.7)	1.12 (0.685 to 1.75)	0.676 (0.413 to 1.04)	4.2 (3.1 to 4.5)	3 (1.8 to 4.7)	1.7 (1 to 2.6)
Poland	HIV/AIDS - Multidrug-resistant Tuberculosis without extensive drug resistance	0.0177 (0.00508 to 0.0444)	0.0371 (0.0205 to 0.0611)	0.014 (0.00681 to 0.0262)	0 (0 to 0.1)	0.1 (0.1 to 0.2)	0 (0 to 0.1)
Poland	HIV/AIDS - Extensively drug-resistant Tuberculosis	0 (0 to 0)	0.0117 (0.00659 to 0.0194)	0.008 (0.00381 to 0.0148)	0 (0 to 0)	0 (0 to 0.1)	0 (0 to 0)
Poland	HIV/AIDS resulting in other diseases	2.12 (1.97 to 2.52)	7.59 (6.86 to 8.31)	7.07 (6.39 to 8.02)	5.6 (5.2 to 6.7)	20.2 (18.2 to 22)	17.4 (15.7 to 19.5)
Poland	Sexually transmitted infections excluding HIV	8.96 (5.41 to 14.9)	7.69 (4.23 to 13.9)	7.04 (3.78 to 12.7)	23.4 (14.2 to 38.6)	18.8 (10.3 to 33.3)	17.7 (9.5 to 31.9)
Poland	Syphilis	1.63 (0.974 to 2.75)	0.6 (0.431 to 0.939)	0.473 (0.341 to 0.656)	5.1 (2.7 to 9.3)	2 (1.1 to 3.8)	1.4 (0.9 to 2.4)
Poland	Chlamydial infection	1.57 (0.869 to 2.88)	1.55 (0.843 to 2.93)	1.44 (0.751 to 2.72)	4 (2.2 to 7.3)	3.8 (2 to 7.1)	3.7 (1.9 to 7)

SR Appendix Table 2. All-age DALYs counts and age standardized DALYs rates per 100,000 individuals for all causes and level 1 cause groups by location for 1990, 2007, and 2017, both sexes combined							
		All-age DALYs			Age-standardized rate of DALYs per 100,000		
Poland	Gonococcal infection	1.48 (0.893 to 2.64)	1.2 (0.626 to 2.4)	0.947 (0.496 to 1.84)	3.9 (2.3 to 7.2)	3 (1.5 to 6.1)	2.8 (1.4 to 5.6)
Poland	Trichomoniasis	1.22 (0.487 to 2.58)	1.33 (0.541 to 2.85)	1.29 (0.519 to 2.78)	3 (1.2 to 6.4)	3 (1.2 to 6.4)	3 (1.2 to 6.2)
Poland	Genital herpes	0.922 (0.298 to 2.16)	1.04 (0.335 to 2.43)	1.08 (0.344 to 2.59)	2.2 (0.7 to 5.1)	2.2 (0.7 to 5.1)	2.2 (0.7 to 5.2)
Poland	Other sexually transmitted infections	2.13 (1.33 to 3.57)	1.97 (1.19 to 3.39)	1.81 (1.05 to 3.08)	5.3 (3.2 to 8.9)	4.8 (2.9 to 8.4)	4.7 (2.7 to 8.1)
Poland	Respiratory infections and tuberculosis	319 (301 to 343)	237 (223 to 259)	238 (220 to 261)	836 (783.5 to 901)	520.4 (481.1 to 577.3)	452.8 (409 to 511.5)
Poland	Tuberculosis	70.1 (66.9 to 73.6)	28.3 (26.8 to 29.8)	16.7 (15 to 18.3)	160.1 (152.4 to 168.2)	54.4 (51.7 to 57.5)	29.9 (26.8 to 32.9)
Poland	Latent tuberculosis infection	0 (0 to 0)	0 (0 to 0)	0 (0 to 0)	0 (0 to 0)	0 (0 to 0)	0 (0 to 0)
Poland	Drug-susceptible tuberculosis	69.5 (66.2 to 72.9)	27.3 (25.9 to 28.8)	16.3 (14.7 to 18)	158.7 (150.8 to 166.9)	52.6 (49.8 to 55.6)	29.1 (26.2 to 32.2)
Poland	Multidrug-resistant tuberculosis without extensive drug resistance	0.627 (0.183 to 1.5)	0.73 (0.524 to 0.961)	0.271 (0.153 to 0.436)	1.4 (0.4 to 3.4)	1.4 (1 to 1.8)	0.5 (0.3 to 0.8)
Poland	Extensively drug-resistant tuberculosis	0 (0 to 0)	0.229 (0.165 to 0.304)	0.153 (0.0856 to 0.245)	0 (0 to 0)	0.4 (0.3 to 0.6)	0.3 (0.2 to 0.4)
Poland	Lower respiratory infections	211 (202 to 221)	176 (170 to 182)	190 (178 to 203)	573.6 (546.1 to 608.6)	372.9 (360.6 to 387)	331.9 (310.9 to 355)
Poland	Upper respiratory infections	27.9 (16.7 to 44.2)	25.9 (15.3 to 40.9)	25.2 (14.8 to 39.5)	72.7 (43.6 to 115.4)	70.5 (41.3 to 112.4)	69.9 (40.8 to 110.5)
Poland	Otitis media	10.6 (6.92 to 16)	7.53 (4.52 to 11.9)	6.66 (4.03 to 10.5)	29.6 (19.5 to 44.1)	22.5 (13.5 to 35.9)	21.1 (12.6 to 33.2)
Poland	Enteric infections	38.1 (28.6 to 49.6)	26.6 (18.8 to 35.7)	29.5 (21.8 to 39)	112.8 (85.7 to 145.5)	96.7 (66.8 to 132.7)	95.9 (67.3 to 131.7)
Poland	Diarrhoeal diseases	36.4 (27.1 to 48)	25.9 (18 to 35.2)	28.9 (21.2 to 38.4)	108.3 (81.7 to 140.8)	94.9 (64.8 to 131.2)	94 (65.8 to 130)
Poland	Typhoid and paratyphoid	0.224 (0.145 to 0.275)	0.018 (0.0104 to 0.037)	0.00706 (0.00376 to 0.0179)	0.6 (0.4 to 0.7)	0 (0 to 0.1)	0 (0 to 0)
Poland	Typhoid fever	0.221 (0.14 to 0.272)	0.0173 (0.00958 to 0.0365)	0.00529 (0.0025 to 0.0164)	0.6 (0.4 to 0.7)	0 (0 to 0.1)	0 (0 to 0)
Poland	Paratyphoid fever	0.00311 (0.00108 to 0.00918)	0.000755 (0.000427 to 0.00121)	0.00178 (0.000312 to 0.00373)	0 (0 to 0)	0 (0 to 0)	0 (0 to 0)
Poland	Invasive non-typhoidal salmonella	1.25 (0.665 to 2.16)	0.574 (0.294 to 0.989)	0.602 (0.299 to 1.1)	3.4 (1.8 to 6)	1.7 (0.9 to 2.9)	1.8 (0.8 to 3.5)
Poland	Other intestinal infectious diseases	0.133 (0.0663 to 0.221)	0.0551 (0.0315 to 0.0808)	0.0356 (0.0195 to 0.0527)	0.4 (0.2 to 0.7)	0.2 (0.1 to 0.3)	0.1 (0 to 0.1)
Poland	Neglected tropical diseases and malaria	12.8 (7.15 to 20.1)	13 (6.42 to 22.4)	12.5 (5.57 to 22.2)	30.3 (16.9 to 47.4)	26.6 (12.9 to 45.9)	24 (10.6 to 42.5)
Poland	Malaria	0 (0 to 0)	0 (0 to 0)	0 (0 to 0)	0 (0 to 0)	0 (0 to 0)	0 (0 to 0)
Poland	Chagas disease	0 (0 to 0)	0 (0 to 0)	0 (0 to 0)	0 (0 to 0)	0 (0 to 0)	0 (0 to 0)
Poland	Leishmaniasis	0.225 (0.0889 to 0.442)	0.253 (0.0999 to 0.518)	0 (0 to 0)	0.5 (0.2 to 0.9)	0.4 (0.2 to 0.8)	0 (0 to 0)
Poland	Visceral leishmaniasis	0.225 (0.0889 to 0.442)	0.253 (0.0999 to 0.518)	0 (0 to 0)	0.5 (0.2 to 0.9)	0.4 (0.2 to 0.8)	0 (0 to 0)
Poland	Cutaneous and mucocutaneous leishmaniasis	0 (0 to 0)	0 (0 to 0)	0 (0 to 0)	0 (0 to 0)	0 (0 to 0)	0 (0 to 0)
Poland	African trypanosomiasis	0 (0 to 0)	0 (0 to 0)	0 (0 to 0)	0 (0 to 0)	0 (0 to 0)	0 (0 to 0)
Poland	Schistosomiasis	0 (0 to 0)	0 (0 to 0)	0 (0 to 0)	0 (0 to 0)	0 (0 to 0)	0 (0 to 0)
Poland	Cysticercosis	11.7 (6.05 to 18.9)	12.3 (5.71 to 21.6)	12.1 (5.22 to 21.8)	27.6 (14.1 to 44.2)	25.1 (11.4 to 44.5)	23.2 (9.9 to 41.7)
Poland	Cystic echinococcosis	0.401 (0.286 to 0.527)	0.252 (0.175 to 0.334)	0.205 (0.147 to 0.281)	1 (0.7 to 1.3)	0.5 (0.4 to 0.7)	0.4 (0.3 to 0.6)
Poland	Lymphatic filariasis	0 (0 to 0)	0 (0 to 0)	0 (0 to 0)	0 (0 to 0)	0 (0 to 0)	0 (0 to 0)
Poland	Onchocerciasis	0 (0 to 0)	0 (0 to 0)	0 (0 to 0)	0 (0 to 0)	0 (0 to 0)	0 (0 to 0)
Poland	Trachoma	0 (0 to 0)	0 (0 to 0)	0 (0 to 0)	0 (0 to 0)	0 (0 to 0)	0 (0 to 0)
Poland	Dengue	0 (0 to 0)	0 (0 to 0)	0 (0 to 0)	0 (0 to 0)	0 (0 to 0)	0 (0 to 0)
Poland	Yellow fever	0 (0 to 0)	0 (0 to 0)	0 (0 to 0)	0 (0 to 0)	0 (0 to 0)	0 (0 to 0)
Poland	Rabies	0.00131 (0.00111 to 0.00155)	0.000615 (0.000486 to 0.000796)	0.000371 (0.000285 to 0.000484)	0 (0 to 0)	0 (0 to 0)	0 (0 to 0)
Poland	Intestinal nematode infections	0.00816 (0.00271 to 0.0164)	0.00237 (0.00114 to 0.00407)	0.00144 (0.000723 to 0.00246)	0 (0 to 0)	0 (0 to 0)	0 (0 to 0)
Poland	Ascariasis	0.00816 (0.00271 to 0.0164)	0.00237 (0.00114 to 0.00407)	0.00144 (0.000723 to 0.00246)	0 (0 to 0)	0 (0 to 0)	0 (0 to 0)
Poland	Trichuriasis	0 (0 to 0)	0 (0 to 0)	0 (0 to 0)	0 (0 to 0)	0 (0 to 0)	0 (0 to 0)
Poland	Hookworm disease	0 (0 to 0)	0 (0 to 0)	0 (0 to 0)	0 (0 to 0)	0 (0 to 0)	0 (0 to 0)

SR Appendix Table 2. All-age DALYs counts and age standardized DALYs rates per 100,000 individuals for all causes and level 1 cause groups by location for 1990, 2007, and 2017, both sexes combined

		All-age DALYs			Age-standardized rate of DALYs per 100,000		
Poland	Food-borne trematodiases	0 (0 to 0)	0 (0 to 0)	0 (0 to 0)	0 (0 to 0)	0 (0 to 0)	0 (0 to 0)
Poland	Leprosy	0 (0 to 0)	0 (0 to 0)	0 (0 to 0)	0 (0 to 0)	0 (0 to 0)	0 (0 to 0)
Poland	Ebola virus disease	0 (0 to 0)	0 (0 to 0)	0 (0 to 0)	0 (0 to 0)	0 (0 to 0)	0 (0 to 0)
Poland	Zika virus disease	0 (0 to 0)	0 (0 to 0)	0 (0 to 0)	0 (0 to 0)	0 (0 to 0)	0 (0 to 0)
Poland	Guinea worm disease	0 (0 to 0)	0 (0 to 0)	0 (0 to 0)	0 (0 to 0)	0 (0 to 0)	0 (0 to 0)
Poland	Other neglected tropical diseases	0.441 (0.175 to 0.556)	0.188 (0.122 to 0.319)	0.144 (0.0994 to 0.265)	1.2 (0.5 to 1.6)	0.5 (0.3 to 0.9)	0.4 (0.3 to 0.7)
Poland	Other infectious diseases	71.1 (53.4 to 77.6)	26.4 (23.7 to 30)	20.6 (18.1 to 24.4)	203.7 (153.4 to 223.2)	74.1 (66.6 to 85.1)	54.9 (47.4 to 68.4)
Poland	Meningitis	30.9 (23.8 to 34)	9.46 (8.71 to 10.4)	6.79 (6.18 to 7.57)	96.6 (73.4 to 107.5)	29.1 (26.3 to 32.9)	19 (16.7 to 22.1)
Poland	Pneumococcal meningitis	3.65 (2.79 to 4.06)	1.47 (1.32 to 1.64)	1.09 (0.979 to 1.23)	10.4 (8 to 11.7)	3.9 (3.5 to 4.3)	2.6 (2.4 to 3)
Poland	H influenzae type B meningitis	13.9 (10.5 to 15.5)	3.48 (3.19 to 3.93)	2.32 (2.1 to 2.68)	45.1 (33.7 to 51)	12 (10.7 to 13.9)	7.3 (6.3 to 8.9)
Poland	Meningococcal infection	3.78 (2.91 to 4.35)	1.11 (1 to 1.24)	0.778 (0.698 to 0.87)	11.9 (9 to 13.9)	3.5 (3 to 4)	2.2 (1.9 to 2.6)
Poland	Other meningitis	9.61 (7.4 to 10.7)	3.39 (3.05 to 3.76)	2.59 (2.31 to 2.92)	29.2 (22.3 to 32.8)	9.7 (8.7 to 11)	6.8 (6 to 7.9)
Poland	Encephalitis	15.8 (8.47 to 17.5)	6.35 (5.84 to 7.7)	4.76 (4.15 to 6.84)	44.7 (23.5 to 50.1)	17.9 (16.2 to 23.1)	13.3 (11.3 to 21)
Poland	Diphtheria	0.00213 (0.00209 to 0.00219)	0.00186 (0.00181 to 0.00192)	0.00159 (0.00149 to 0.00171)	0 (0 to 0)	0 (0 to 0)	0 (0 to 0)
Poland	Whooping cough	0.349 (0.196 to 0.615)	0.107 (0.0355 to 0.162)	0.0652 (0.0291 to 0.11)	1.2 (0.7 to 2.1)	0.5 (0.2 to 0.8)	0.3 (0.2 to 0.6)
Poland	Tetanus	0.682 (0.542 to 0.784)	0.126 (0.103 to 0.15)	0.0703 (0.0534 to 0.0928)	1.5 (1.2 to 1.7)	0.2 (0.2 to 0.3)	0.1 (0.1 to 0.1)
Poland	Measles	0.17 (0.104 to 0.266)	0.0272 (0.0179 to 0.0323)	0.00871 (0.00598 to 0.0108)	0.6 (0.3 to 0.9)	0.1 (0.1 to 0.1)	0 (0 to 0.1)
Poland	Varicella and herpes zoster	1.49 (0.977 to 2.16)	1.49 (0.945 to 2.19)	1.53 (0.95 to 2.28)	3.7 (2.5 to 5.3)	3.4 (2.2 to 4.9)	3.2 (2 to 4.6)
Poland	Acute hepatitis	12.1 (9.87 to 13.4)	3.31 (2.66 to 4.37)	2.77 (2.17 to 3.7)	29.1 (24 to 32.1)	7.8 (6.1 to 10.5)	6.7 (5 to 9.1)
Poland	Acute hepatitis A	3.49 (2.85 to 4.15)	1.43 (1.02 to 1.97)	1.19 (0.83 to 1.66)	9.2 (7.6 to 11)	3.9 (2.8 to 5.5)	3.6 (2.5 to 5.1)
Poland	Acute hepatitis B	6.95 (5.46 to 7.72)	1.49 (1.25 to 1.91)	1.28 (1.03 to 1.71)	15.7 (12.5 to 17.5)	3 (2.5 to 4)	2.4 (1.9 to 3.3)
Poland	Acute hepatitis C	0.239 (0.123 to 0.41)	0.0425 (0.0231 to 0.0788)	0.0302 (0.0158 to 0.0562)	0.7 (0.4 to 1.2)	0.1 (0.1 to 0.3)	0.1 (0 to 0.2)
Poland	Acute hepatitis E	1.46 (1.09 to 1.88)	0.341 (0.266 to 0.478)	0.273 (0.208 to 0.377)	3.5 (2.6 to 4.4)	0.8 (0.6 to 1.1)	0.6 (0.5 to 0.9)
Poland	Other unspecified infectious diseases	9.6 (6.99 to 11.9)	5.5 (4.51 to 6.91)	4.62 (3.69 to 6.17)	26.4 (19.4 to 32.2)	15 (12.3 to 19.7)	12.3 (9.7 to 17.7)
Poland	Maternal and neonatal disorders	617 (551 to 693)	292 (239 to 357)	249 (189 to 318)	2142 (1949.1 to 2373.2)	1112.8 (958 to 1298)	915.7 (721.5 to 1134.2)
Poland	Maternal disorders	11.3 (10.5 to 12.2)	2.42 (2.08 to 2.8)	1.62 (1.33 to 2)	28.7 (26.6 to 31.2)	5.9 (5.1 to 6.9)	4.3 (3.5 to 5.3)
Poland	Maternal haemorrhage	3.47 (1.91 to 5.28)	0.805 (0.543 to 1.13)	0.54 (0.358 to 0.79)	8.8 (5 to 13.3)	2 (1.4 to 2.8)	1.5 (1 to 2.1)
Poland	Maternal sepsis and other pregnancy related infections	3.09 (1.61 to 4.91)	0.276 (0.149 to 0.436)	0.187 (0.101 to 0.306)	7.9 (4.1 to 12.2)	0.7 (0.4 to 1.1)	0.5 (0.3 to 0.9)
Poland	Maternal hypertensive disorders	1.09 (0.531 to 2.03)	0.205 (0.126 to 0.324)	0.134 (0.0844 to 0.198)	2.8 (1.4 to 5.1)	0.5 (0.3 to 0.8)	0.4 (0.2 to 0.5)
Poland	Maternal obstructed labour and uterine rupture	0.278 (0.172 to 0.457)	0.0876 (0.0601 to 0.126)	0.063 (0.0393 to 0.0943)	0.7 (0.4 to 1.1)	0.2 (0.1 to 0.3)	0.2 (0.1 to 0.2)
Poland	Maternal abortive outcome	2.54 (1.42 to 3.95)	0.422 (0.253 to 0.627)	0.21 (0.12 to 0.325)	6.4 (3.7 to 9.7)	1 (0.6 to 1.5)	0.5 (0.3 to 0.8)
Poland	Ectopic pregnancy	0.0357 (0.0194 to 0.0582)	0.0139 (0.00828 to 0.0223)	0.007 (0.00405 to 0.0113)	0.1 (0.1 to 0.2)	0 (0 to 0.1)	0 (0 to 0)
Poland	Indirect maternal deaths	0.246 (0.0998 to 0.514)	0.0223 (0.00889 to 0.0465)	0.00748 (0.00287 to 0.0172)	0.6 (0.2 to 1.2)	0.1 (0 to 0.1)	0 (0 to 0)
Poland	Late maternal deaths	0.257 (0.19 to 0.343)	0.0717 (0.0545 to 0.0917)	0.0559 (0.0419 to 0.0734)	0.6 (0.5 to 0.9)	0.2 (0.1 to 0.2)	0.1 (0.1 to 0.2)
Poland	Maternal deaths aggravated by HIV/AIDS	0.0000893 (0.0000373 to 0.000182)	0.000158 (0.0000706 to 0.000283)	0.000099 (0.000044 to 0.000186)	0 (0 to 0)	0 (0 to 0)	0 (0 to 0)
Poland	Other maternal disorders	0.289 (0.153 to 0.564)	0.514 (0.319 to 0.73)	0.414 (0.275 to 0.57)	0.8 (0.4 to 1.5)	1.3 (0.8 to 1.8)	1.1 (0.7 to 1.5)
Poland	Neonatal disorders	606 (540 to 681)	290 (237 to 354)	247 (187 to 316)	2113.3 (1921.2 to 2343.5)	1106.9 (952.5 to 1292.1)	911.4 (717.6 to 1128.7)
Poland	Neonatal preterm birth	313 (275 to 354)	117 (105 to 133)	88.2 (70.3 to 109)	1141.2 (1003.8 to 1296.5)	515.9 (471.5 to 572.1)	400.2 (321.6 to 491)
Poland	Neonatal encephalopathy due to birth asphyxia and trauma	121 (94.1 to 154)	56.8 (40.1 to 82.9)	50.6 (32.3 to 79.6)	418.4 (329 to 524.5)	202.3 (156.1 to 276.5)	167.9 (115.1 to 251.2)
Poland	Neonatal sepsis and other neonatal infections	123 (81.3 to 178)	100 (62.8 to 147)	99.2 (58.6 to 150)	364.6 (247.8 to 515.6)	306.4 (204.1 to 434.6)	292.9 (178.1 to 436.1)

SR Appendix Table 2. All-age DALYs counts and age standardized DALYs rates per 100,000 individuals for all causes and level 1 cause groups by location for 1990, 2007, and 2017, both sexes combined							
		All-age DALYs			Age-standardized rate of DALYs per 100,000		
Poland	Hemolytic disease and other neonatal jaundice	5.44 (3.63 to 7.56)	1.8 (1.46 to 2.17)	1.34 (1.02 to 1.69)	19.5 (12.6 to 27.9)	6.7 (5.6 to 8)	4.6 (3.5 to 5.7)
Poland	Other neonatal disorders	44.4 (33.5 to 59.6)	13.9 (12.4 to 15.6)	8.03 (6.29 to 9.92)	169.7 (127.8 to 229.2)	75.5 (67.1 to 84.7)	45.8 (35.7 to 56.7)
Poland	Nutritional deficiencies	119 (72 to 179)	49.9 (30.9 to 76.2)	41 (26 to 66.1)	321.4 (194.7 to 483.9)	149.1 (89.3 to 229.4)	118 (72.6 to 189.5)
Poland	Protein-energy malnutrition	1.47 (0.733 to 3.16)	1.95 (1.19 to 2.58)	1.79 (1.27 to 2.17)	4.5 (2.1 to 9.8)	4.5 (2.8 to 7)	3.5 (2.4 to 4.7)
Poland	Iodine deficiency	4.18 (2.14 to 7.05)	2.52 (1.18 to 4.85)	3.88 (1.77 to 7.03)	10.3 (5.2 to 17.3)	5.7 (2.7 to 11.2)	9 (4 to 16.2)
Poland	Dietary iron deficiency	68.4 (42.7 to 105)	33.6 (20.3 to 52.3)	27.5 (16.4 to 45.6)	175.3 (109.3 to 269.3)	82.4 (49.7 to 130.3)	67.6 (40.8 to 110)
Poland	Other nutritional deficiencies	0.946 (0.66 to 1.23)	0.774 (0.519 to 1.52)	0.443 (0.28 to 0.985)	2.3 (1.6 to 3)	1.6 (1.1 to 2.9)	0.9 (0.6 to 1.8)
Poland	Non-communicable diseases	10800 (9850 to 11800)	10500 (9460 to 11700)	10400 (9230 to 11600)	25131.5 (22967.5 to 27609.6)	20468.1 (18344.7 to 23024.9)	17976.7 (15759.5 to 20394.2)
Poland	Neoplasms	2000 (1960 to 2020)	2260 (2220 to 2290)	2350 (2240 to 2480)	4467.5 (4387 to 4528.1)	4091.4 (4037 to 4144.6)	3675.7 (3489.8 to 3874.8)
Poland	Lip and oral cavity cancer	28.2 (27.1 to 29.4)	38.1 (36.8 to 39.4)	42 (38.1 to 46)	64 (61.4 to 66.8)	68.6 (66.4 to 71)	67.4 (61.4 to 73.4)
Poland	Nasopharynx cancer	4.44 (4.23 to 4.66)	6.88 (6.55 to 7.2)	5.68 (5.21 to 6.2)	10.2 (9.7 to 10.7)	12.8 (12.2 to 13.4)	9.8 (9 to 10.7)
Poland	Other pharynx cancer	16.7 (15.9 to 17.5)	23.6 (22.5 to 24.5)	27.7 (25 to 30.7)	37.9 (36.1 to 39.9)	42.3 (40.6 to 44)	44.6 (40.3 to 49.2)
Poland	Oesophageal cancer	41.4 (40 to 42.9)	44.7 (43.2 to 46.3)	47.5 (43.4 to 51.8)	92.2 (89 to 95.4)	79.4 (76.7 to 82.2)	73.4 (67.1 to 80)
Poland	Stomach cancer	182 (177 to 186)	141 (137 to 144)	126 (118 to 135)	399.1 (388.8 to 409)	250.3 (244.5 to 256.4)	193.8 (180.5 to 206.8)
Poland	Colon and rectum cancer	210 (204 to 216)	270 (263 to 276)	293 (274 to 312)	460.6 (448.1 to 473.4)	471 (459.9 to 481.8)	433.8 (405.8 to 463)
Poland	Liver cancer	38.1 (36.9 to 39.3)	40.5 (39 to 42)	47.5 (43.9 to 51.2)	83.7 (81.3 to 86.2)	72.5 (69.9 to 75.1)	73.2 (67.8 to 78.6)
Poland	Liver cancer due to hepatitis B	8.15 (6.59 to 9.83)	8.29 (6.74 to 9.9)	8.73 (6.89 to 10.7)	18.3 (14.9 to 21.9)	15.7 (13 to 18.7)	14.7 (11.9 to 17.8)
Poland	Liver cancer due to hepatitis C	10.6 (8.22 to 13.2)	10.8 (8.48 to 13.5)	12.9 (9.85 to 16.6)	22.9 (18 to 28.8)	18.8 (14.8 to 23.4)	18.8 (14.4 to 24.2)
Poland	Liver cancer due to alcohol use	13.9 (12.2 to 15.7)	15.7 (13.9 to 17.5)	19.3 (17 to 22)	30.4 (26.8 to 34.3)	27.8 (24.8 to 30.9)	29.4 (26 to 33.3)
Poland	Liver cancer due to NASH	2.83 (2.41 to 3.31)	3.17 (2.72 to 3.69)	3.92 (3.34 to 4.58)	6.2 (5.3 to 7.2)	5.6 (4.8 to 6.4)	5.8 (5 to 6.8)
Poland	Liver cancer due to other causes	2.6 (2.25 to 3.02)	2.51 (2.17 to 2.87)	2.74 (2.33 to 3.25)	5.8 (5 to 6.7)	4.7 (4.1 to 5.4)	4.5 (3.9 to 5.3)
Poland	Gallbladder and biliary tract cancer	46.1 (44.3 to 49.6)	47.8 (46 to 49.3)	46.5 (42.8 to 50.2)	100.6 (96.6 to 108.3)	83 (80.1 to 85.7)	68.3 (62.9 to 73.8)
Poland	Pancreatic cancer	97.1 (94.3 to 99.8)	102 (99.8 to 105)	126 (118 to 135)	213 (207.4 to 218.7)	180.9 (176.7 to 185.4)	190.6 (177.9 to 204.5)
Poland	Larynx cancer	51 (49 to 53.2)	47.7 (46 to 49.5)	41.7 (38 to 45.7)	113.8 (109.3 to 118.7)	84.6 (81.5 to 87.8)	64.4 (58.8 to 70.7)
Poland	Tracheal, bronchus, and lung cancer	480 (467 to 492)	568 (556 to 582)	599 (556 to 640)	1050.7 (1022.1 to 1076.3)	998.8 (977.3 to 1022.1)	889.6 (828 to 950.4)
Poland	Malignant skin melanoma	22.2 (17.1 to 29)	31.7 (22.8 to 39.6)	35.2 (22.8 to 41.7)	50.7 (38.9 to 65.9)	60.7 (43.9 to 76.2)	60.5 (39.7 to 72.5)
Poland	Non-melanoma skin cancer	11.8 (11.4 to 12.3)	14.2 (13.8 to 14.7)	12.3 (11.4 to 13.4)	26.7 (25.9 to 27.7)	25.4 (24.5 to 26.3)	17.7 (16.5 to 19.2)
Poland	Non-melanoma skin cancer (squamous-cell carcinoma)	11.8 (11.4 to 12.2)	14.2 (13.7 to 14.7)	12.3 (11.4 to 13.4)	26.7 (25.9 to 27.7)	25.3 (24.5 to 26.3)	17.7 (16.5 to 19.2)
Poland	Non-melanoma skin cancer (basal-cell carcinoma)	0.00397 (0.00163 to 0.00836)	0.00849 (0.0031 to 0.0201)	0.017 (0.00482 to 0.0439)	0 (0 to 0)	0 (0 to 0)	0 (0 to 0.1)
Poland	Breast cancer	129 (124 to 134)	150 (145 to 155)	151 (137 to 166)	290.8 (280.2 to 300.9)	273.4 (264.4 to 282.9)	243.7 (221.1 to 268)
Poland	Cervical cancer	81.5 (78.2 to 85.2)	71.4 (68.7 to 74)	54.1 (48.5 to 60.3)	182.9 (175.5 to 190.9)	132.6 (127.9 to 137.5)	90.7 (81.5 to 101)
Poland	Uterine cancer	21.5 (20.4 to 22.6)	25.4 (24.2 to 26.7)	31.3 (27.6 to 35)	46.7 (44.4 to 49.1)	44.8 (42.6 to 47.2)	46 (40.9 to 51.6)
Poland	Ovarian cancer	57.5 (55.2 to 60.2)	73.8 (71.3 to 76.5)	72.9 (65.7 to 81.6)	129.8 (124.6 to 136.1)	133.7 (129.2 to 138.3)	117.5 (106.2 to 131.1)
Poland	Prostate cancer	45 (36.8 to 60.4)	76 (52.6 to 85.7)	87.4 (57.3 to 100)	97.6 (79.8 to 131.3)	127.8 (88.5 to 143.5)	121.4 (79.4 to 139.2)
Poland	Testicular cancer	7.97 (7.4 to 8.61)	6.82 (6.31 to 7.36)	5.93 (5.16 to 6.82)	19.8 (18.4 to 21.5)	16.1 (14.9 to 17.4)	14.2 (12.3 to 16.4)
Poland	Kidney cancer	44.5 (38.3 to 46.8)	63.7 (56.1 to 66.4)	68.1 (60.2 to 74.2)	100.1 (86.2 to 105.3)	115.9 (102.4 to 120.7)	106.3 (94.4 to 115.8)
Poland	Bladder cancer	44.8 (43.2 to 46.4)	62.7 (60.6 to 64.7)	72.5 (66.9 to 78.4)	97.2 (93.7 to 100.7)	108.1 (104.6 to 111.3)	104.1 (95.8 to 112.6)
Poland	Thyroid cancer	10.7 (10.2 to 11.3)	7.79 (7.34 to 8.34)	7.6 (6.82 to 8.52)	23.8 (22.6 to 25)	14.3 (13.5 to 15.4)	12.2 (10.9 to 13.8)
Poland	Mesothelioma	2.43 (2.05 to 2.93)	4.91 (4.66 to 5.16)	7.16 (6.53 to 7.85)	5.5 (4.6 to 6.6)	9 (8.6 to 9.4)	11.4 (10.4 to 12.5)
Poland	Hodgkin lymphoma	19.4 (14 to 21.2)	12.2 (9.47 to 13.8)	8.53 (7.28 to 10.9)	46.3 (33.4 to 50.7)	26.3 (20.6 to 30)	17.6 (15.2 to 22.5)

SR Appendix Table 2. All-age DALYs counts and age standardized DALYs rates per 100,000 individuals for all causes and level 1 cause groups by location for 1990, 2007, and 2017, both sexes combined

		All-age DALYs			Age-standardized rate of DALYs per 100,000		
Poland	Non-Hodgkin's lymphoma	33.7 (32.6 to 34.9)	46 (44.9 to 47.2)	47.6 (44.4 to 50.8)	78.4 (75.7 to 81.2)	89.6 (87.3 to 92.1)	82.4 (77.1 to 87.7)
Poland	Multiple myeloma	13.4 (12.4 to 17.1)	26.3 (20.1 to 27.9)	29.4 (22.6 to 32.7)	29.2 (27.2 to 37.6)	46.1 (35.5 to 48.9)	44.1 (34.3 to 49.2)
Poland	Leukaemia	78.4 (76 to 81.1)	76.8 (74.4 to 78.8)	75 (69.6 to 79.8)	191.3 (184.8 to 198)	160.2 (155.7 to 164.6)	136.2 (126.5 to 144.8)
Poland	Acute lymphoid leukaemia	15.5 (13.6 to 17.5)	11.1 (10.6 to 11.7)	9.4 (8.59 to 10.3)	41.9 (36.6 to 47.5)	30.8 (29 to 32.8)	25.2 (22.7 to 27.8)
Poland	Chronic lymphoid leukaemia	9.53 (8.4 to 10.7)	15 (14.3 to 15.7)	19.3 (17.4 to 21)	20.8 (18.4 to 23.3)	26 (24.8 to 27.3)	27.9 (25.2 to 30.4)
Poland	Acute myeloid leukaemia	21.2 (18.3 to 24.3)	24.8 (23.7 to 25.8)	29.9 (27.3 to 32.3)	51.8 (44.6 to 59.7)	51.5 (49.2 to 53.8)	54.7 (49.9 to 59.2)
Poland	Chronic myeloid leukaemia	10.9 (9.81 to 12.1)	7.08 (6.72 to 7.5)	4.87 (4.4 to 5.41)	24.8 (22.3 to 27.2)	13.1 (12.4 to 13.8)	8 (7.3 to 8.9)
Poland	Other leukaemia	21.3 (19 to 23.6)	18.7 (17.3 to 19.6)	11.6 (10.4 to 12.8)	52.1 (46.5 to 57.8)	38.9 (36.6 to 40.8)	20.3 (18.4 to 22.8)
Poland	Other malignant cancers	84.3 (62.9 to 90.6)	68.4 (63.2 to 73.9)	69.4 (60.2 to 75.5)	199.3 (149 to 213.9)	138.3 (129.7 to 150.9)	127 (113.2 to 138.5)
Poland	Other neoplasms	14.6 (8.38 to 22.1)	23 (15.7 to 27.8)	27.1 (18.4 to 34.3)	33.6 (19.3 to 51.5)	44 (30.7 to 54.1)	44.9 (31 to 58.6)
Poland	Myelodysplastic, myeloproliferative, and other hematopoietic neoplasms	13.7 (7.86 to 20.5)	22.1 (15.1 to 26.6)	26.4 (17.8 to 33.2)	31.3 (17.9 to 47.3)	41.4 (28.7 to 50.7)	42.5 (29.1 to 55.1)
Poland	Benign and in situ intestinal neoplasms	0 (0 to 0)	0 (0 to 0)	0 (0 to 0)	0 (0 to 0)	0 (0 to 0)	0 (0 to 0)
Poland	Benign and in situ cervical and uterine neoplasms	0 (0 to 0)	0 (0 to 0)	0 (0 to 0)	0 (0 to 0)	0 (0 to 0)	0 (0 to 0)
Poland	Other benign and in situ neoplasms	0.857 (0.46 to 1.56)	0.836 (0.595 to 1.17)	0.754 (0.551 to 1.14)	2.3 (1.3 to 4.3)	2.6 (1.9 to 3.7)	2.4 (1.8 to 3.8)
Poland	Cardiovascular diseases	3950 (3870 to 4040)	3050 (2960 to 3140)	2780 (2620 to 2930)	8921.3 (8730 to 9118)	5398.1 (5239.7 to 5568.3)	4072.3 (3833.9 to 4298.5)
Poland	Rheumatic heart disease	99.9 (96 to 104)	29.6 (28.4 to 30.7)	22.8 (21 to 24.7)	226.7 (218.1 to 235.3)	54.7 (52.5 to 56.8)	36.6 (33.8 to 39.7)
Poland	Ischaemic heart disease	2410 (2370 to 2450)	1620 (1580 to 1660)	1420 (1350 to 1500)	5422 (5327.4 to 5517.7)	2843.3 (2781.1 to 2911.8)	2048.1 (1940.6 to 2161.9)
Poland	Stroke	892 (846 to 936)	858 (820 to 897)	740 (681 to 796)	2014.3 (1911.5 to 2108.8)	1521.1 (1452.4 to 1591.7)	1092.9 (1007.8 to 1173.2)
Poland	Ischaemic stroke	512 (477 to 545)	480 (449 to 510)	461 (415 to 504)	1152.6 (1076.5 to 1224)	827.6 (773.6 to 879.2)	643.1 (576.7 to 703.6)
Poland	Intracerebral hemorrhage	286 (274 to 299)	287 (274 to 299)	211 (196 to 226)	645.4 (618 to 673.5)	518.5 (495.4 to 541.4)	330.6 (307.3 to 354.7)
Poland	Subarachnoid hemorrhage	94.1 (86.3 to 99.6)	91.6 (82 to 97.1)	68.3 (62.4 to 76.5)	216.3 (197.2 to 229.2)	175 (155.1 to 185.6)	119.2 (109 to 131.4)
Poland	Hypertensive heart disease	77.4 (63.9 to 94.1)	75.3 (68 to 103)	70.5 (61.2 to 108)	172.4 (143.4 to 212.2)	131.3 (119.1 to 177.5)	101.5 (88.3 to 153.1)
Poland	Non-rheumatic valvular heart disease	19.7 (17 to 22.4)	24.2 (21.3 to 27.3)	30.6 (24.5 to 34.5)	44 (38.2 to 49.9)	43.2 (37.8 to 48.3)	45.2 (36.4 to 50.7)
Poland	Non-rheumatic calcific aortic valve disease	9.22 (7.57 to 11.3)	14.4 (12.6 to 16.4)	20.4 (15.5 to 23)	20.7 (17 to 25.3)	25.6 (22.3 to 29.1)	30 (22.9 to 33.7)
Poland	Non-rheumatic degenerative mitral valve disease	10 (7.29 to 11.5)	9.28 (7.27 to 10.8)	9.44 (7.4 to 11.3)	22.4 (16.4 to 25.7)	16.5 (13 to 19.2)	13.9 (11 to 16.6)
Poland	Other non-rheumatic valve diseases	0.418 (0.308 to 0.736)	0.557 (0.408 to 0.845)	0.738 (0.422 to 0.946)	1 (0.7 to 1.7)	1.1 (0.8 to 1.6)	1.3 (0.7 to 1.6)
Poland	Cardiomyopathy and myocarditis	152 (134 to 168)	178 (161 to 189)	206 (184 to 225)	356.6 (314.8 to 396)	332.8 (304.4 to 351.8)	320.5 (286.5 to 348.3)
Poland	Myocarditis	13.8 (8.17 to 17.8)	11.3 (9.01 to 14.2)	8.54 (5.95 to 15.4)	34.1 (20.3 to 43.6)	24.1 (19.1 to 28.9)	15.9 (11.8 to 25.9)
Poland	Alcoholic cardiomyopathy	18.1 (13.5 to 24.8)	46.9 (26.7 to 54.2)	58.8 (30.6 to 69.6)	41.2 (31 to 56.3)	88 (49.8 to 101)	99.9 (52.3 to 117.5)
Poland	Other cardiomyopathy	120 (105 to 133)	120 (111 to 132)	139 (124 to 153)	281.2 (246.7 to 310.6)	220.7 (205.2 to 246)	204.7 (183.9 to 227.5)
Poland	Atrial fibrillation and flutter	43.4 (36.1 to 53)	63.5 (52.6 to 76.7)	76.5 (63.4 to 91.8)	99 (83 to 120.2)	109.2 (90.6 to 131.8)	105.3 (86.9 to 127)
Poland	Aortic aneurysm	32.9 (27.8 to 39)	43 (41.3 to 44.7)	40.1 (37 to 43.4)	72.7 (61.5 to 85.8)	76.7 (73.6 to 79.8)	61.6 (56.7 to 66.6)
Poland	Peripheral vascular disease	13.1 (6.52 to 24.5)	26.8 (16.7 to 41.2)	39.2 (24.2 to 64.7)	29.3 (14.5 to 54.8)	46 (28.6 to 70.4)	54.4 (33.6 to 89.4)
Poland	Endocarditis	8.99 (6.83 to 11.7)	10.7 (9.22 to 12.5)	10.3 (8.85 to 13.2)	21.3 (16.3 to 27.7)	21.4 (18.6 to 24.7)	18.7 (16.1 to 23.7)
Poland	Other cardiovascular and circulatory diseases	206 (190 to 225)	120 (105 to 138)	119 (98.3 to 143)	463.1 (426.2 to 505.3)	218.3 (191.1 to 252)	187.5 (155.5 to 226.1)
Poland	Chronic respiratory diseases	531 (479 to 586)	496 (441 to 548)	467 (412 to 524)	1199.7 (1078.3 to 1327)	932.1 (818.3 to 1046.9)	770 (664.4 to 881.3)
Poland	Chronic obstructive pulmonary disease	369 (338 to 403)	376 (341 to 407)	356 (318 to 397)	817.6 (748.8 to 889.5)	659.4 (596.5 to 711.2)	525.9 (468.4 to 586.5)
Poland	Pneumoconiosis	4.59 (3.83 to 5.59)	2.42 (2.19 to 2.69)	1.48 (1.26 to 1.73)	10 (8.4 to 12)	4.2 (3.8 to 4.6)	2.2 (1.9 to 2.6)
Poland	Silicosis	1.28 (0.953 to 1.74)	0.475 (0.398 to 0.571)	0.255 (0.199 to 0.319)	2.8 (2.1 to 3.8)	0.8 (0.7 to 1)	0.4 (0.3 to 0.5)
Poland	Asbestosis	0.159 (0.108 to 0.244)	0.181 (0.137 to 0.259)	0.219 (0.148 to 0.296)	0.3 (0.2 to 0.5)	0.3 (0.2 to 0.5)	0.3 (0.2 to 0.4)

SR Appendix Table 2. All-age DALYs counts and age standardized DALYs rates per 100,000 individuals for all causes and level 1 cause groups by location for 1990, 2007, and 2017, both sexes combined							
		All-age DALYs			Age-standardized rate of DALYs per 100,000		
Poland	Coal workers pneumoconiosis	1.9 (1.36 to 2.49)	1.03 (0.899 to 1.17)	0.585 (0.462 to 0.714)	4.1 (3 to 5.3)	1.7 (1.5 to 2)	0.9 (0.7 to 1)
Poland	Other pneumoconiosis	1.25 (0.658 to 1.6)	0.738 (0.541 to 0.873)	0.418 (0.323 to 0.58)	2.8 (1.5 to 3.5)	1.3 (0.9 to 1.5)	0.6 (0.5 to 0.9)
Poland	Asthma	131 (102 to 166)	90.4 (65.6 to 122)	80.5 (56.6 to 111)	311.6 (242.1 to 396.4)	213.2 (152 to 290.3)	188 (129.7 to 261.6)
Poland	Interstitial lung disease and pulmonary sarcoidosis	15.5 (12.1 to 19.3)	16.9 (13.4 to 20.7)	19.3 (15.2 to 24)	35.1 (27.4 to 43.7)	31.9 (25.4 to 39.2)	32.3 (25.3 to 40.2)
Poland	Other chronic respiratory diseases	10.6 (9.02 to 12.3)	10.4 (8.86 to 11.8)	9.38 (7.82 to 11.1)	25.4 (21.7 to 29.8)	23.4 (19.8 to 27)	21.6 (17.7 to 25.8)
Poland	Digestive diseases	495 (438 to 567)	646 (585 to 719)	588 (521 to 666)	1161.8 (1021.9 to 1332.6)	1304.8 (1167.7 to 1472.3)	1098.2 (957 to 1265.6)
Poland	Cirrhosis and other chronic liver diseases	146 (138 to 155)	287 (276 to 299)	251 (231 to 273)	330.7 (312.4 to 350.8)	554.5 (531.9 to 577.4)	450.2 (413.8 to 488.7)
Poland	Cirrhosis and other chronic liver diseases due to hepatitis B	37.5 (33.5 to 42.7)	69 (61.2 to 77.2)	55.5 (48.2 to 64.2)	84.8 (75.9 to 95.9)	134.5 (120 to 150.3)	100.6 (87.4 to 115.3)
Poland	Cirrhosis and other chronic liver diseases due to hepatitis C	28.3 (24.6 to 32)	59.2 (51.8 to 68)	51 (44 to 59)	63.8 (55.7 to 72.5)	113.4 (98.9 to 129.5)	91.3 (77.9 to 106.5)
Poland	Cirrhosis and other chronic liver diseases due to alcohol use	61.1 (56.4 to 66.3)	125 (116 to 134)	113 (102 to 124)	136.8 (126.6 to 148.1)	238 (221.2 to 255.5)	198.2 (178.9 to 218.1)
Poland	Cirrhosis due to NASH	8.2 (7.36 to 9.27)	18.4 (16.4 to 20.7)	17.8 (15.5 to 20.1)	18.4 (16.5 to 20.8)	34.6 (30.9 to 38.8)	31.1 (27.1 to 35.3)
Poland	Cirrhosis and other chronic liver diseases due to other causes	10.9 (9.44 to 12.7)	15.6 (13.1 to 18.1)	14 (11.7 to 16.7)	26.9 (23.4 to 31.3)	34.1 (29.5 to 39.6)	29 (24.6 to 34.4)
Poland	Upper digestive system diseases	156 (118 to 208)	167 (124 to 227)	161 (120 to 217)	375 (280.7 to 504.7)	363.3 (265.2 to 496)	327.2 (238.4 to 451.3)
Poland	Peptic ulcer disease	50.4 (47 to 53.8)	48.7 (45.8 to 52)	51.2 (46.3 to 56.3)	113.3 (105.7 to 121.1)	90 (84.2 to 96.3)	82.8 (74.6 to 91.4)
Poland	Gastritis and duodenitis	71.4 (46.5 to 104)	80.2 (52.6 to 114)	67.9 (44.8 to 97.4)	180.3 (116.3 to 264.2)	193.4 (126.4 to 274)	162.8 (106.3 to 234)
Poland	GERD	34.6 (18.4 to 58.9)	38.5 (20.6 to 65.7)	41.4 (22.1 to 69.6)	81.4 (43.6 to 137.8)	79.9 (42.8 to 136.1)	81.6 (43.3 to 138)
Poland	Appendicitis	6.61 (6.15 to 7.14)	2.86 (2.6 to 3.17)	2.25 (1.97 to 2.54)	15.8 (14.7 to 17.2)	6.2 (5.5 to 7)	4.6 (3.9 to 5.5)
Poland	Paralytic ileus and intestinal obstruction	22.4 (20 to 26.9)	19.8 (17.3 to 25.2)	18.2 (15.3 to 25)	59.8 (53.5 to 70)	40.1 (35.4 to 50.2)	31.2 (26.3 to 42.4)
Poland	Inguinal, femoral, and abdominal hernia	39.1 (30.1 to 49.9)	29.8 (22.1 to 38.9)	30.4 (22.1 to 40.3)	93.8 (71.8 to 120.1)	69.4 (50.3 to 91.3)	64.1 (45.7 to 86.3)
Poland	Inflammatory bowel disease	16.2 (12.3 to 20.7)	22.3 (17.1 to 28.4)	20.2 (15.5 to 25.5)	37.8 (28.7 to 48.8)	45.8 (34.7 to 58.6)	38.2 (29.2 to 49.2)
Poland	Vascular intestinal disorders	19.1 (16.6 to 24.1)	22.8 (21.3 to 24.1)	20.5 (18.4 to 22.9)	42.3 (36.9 to 53.2)	39.4 (36.6 to 41.6)	29.5 (26.3 to 32.9)
Poland	Gallbladder and biliary diseases	22.2 (16.5 to 24)	10.4 (9.81 to 12.4)	11.3 (10.2 to 12.5)	49 (36.6 to 52.8)	18.3 (17.2 to 21.8)	16.1 (14.6 to 18.1)
Poland	Pancreatitis	53.3 (46.4 to 60.2)	63.4 (57.2 to 70.4)	54.2 (47 to 61.6)	121.8 (106.2 to 137.4)	125.8 (114 to 139.2)	99 (86 to 113.2)
Poland	Other digestive diseases	13.8 (10.3 to 17.9)	19.3 (16.2 to 22.8)	19.2 (15.9 to 22.9)	35.8 (26.3 to 46.7)	42.1 (34.3 to 50.9)	38 (30.4 to 46.8)
Poland	Neurological disorders	636 (503 to 805)	744 (603 to 919)	807 (655 to 991)	1557.5 (1234 to 1974)	1545.1 (1223 to 1945)	1489.2 (1155.9 to 1904.3)
Poland	Alzheimer's disease and other dementias	191 (179 to 204)	254 (238 to 271)	320 (295 to 345)	471 (441.3 to 502.4)	445.9 (418 to 475.9)	415.9 (383.4 to 448.2)
Poland	Parkinson's disease	34.1 (31.2 to 42.1)	46.8 (42.9 to 55)	52.9 (47.5 to 61.4)	76.7 (70.2 to 94.3)	76.7 (70.2 to 89.6)	71.9 (64.5 to 83.4)
Poland	Epilepsy	60.7 (30.3 to 99.8)	72 (45.1 to 111)	67.7 (34.8 to 116)	153.3 (75.7 to 253.9)	172.9 (105.2 to 271)	161.6 (79.5 to 285.3)
Poland	Multiple sclerosis	23.2 (15.5 to 27.2)	21.3 (18 to 24.2)	20 (17 to 23.4)	53.4 (35.7 to 62.7)	42.6 (36.2 to 48.5)	37.7 (31.6 to 44.5)
Poland	Motor neuron disease	6.37 (5.56 to 7.55)	9.13 (8.61 to 9.56)	10.5 (9.52 to 11.4)	15.1 (13.1 to 17.9)	18.4 (17.4 to 19.3)	18.4 (16.7 to 19.9)
Poland	Headache disorders	300 (191 to 441)	320 (208 to 471)	317 (203 to 470)	732 (468.4 to 1076.3)	733.4 (472.3 to 1074.9)	732.2 (470.6 to 1075)
Poland	Migraine	257 (159 to 389)	273 (170 to 416)	270 (166 to 410)	627.6 (389.2 to 949.1)	628.8 (388 to 953.3)	627.7 (385.7 to 951.8)
Poland	Tension-type headache	43 (24.3 to 66.9)	46.3 (26.6 to 72)	46.7 (27 to 73.2)	104.5 (59 to 163)	104.6 (59.9 to 164.1)	104.4 (59.5 to 163.5)
Poland	Other neurological disorders	21 (15.5 to 28.3)	21.2 (17 to 27.3)	19.6 (14.5 to 27.6)	56 (41 to 75.9)	55.1 (43.2 to 72.2)	51.6 (35.5 to 76.8)
Poland	Mental disorders	517 (383 to 670)	550 (412 to 707)	545 (410 to 701)	1273.8 (940.3 to 1648.8)	1289 (953.4 to 1661.2)	1281.6 (952.7 to 1654)
Poland	Schizophrenia	50.6 (38.2 to 63.3)	56.4 (42.5 to 70.2)	59.3 (43.9 to 73.7)	119.1 (89.9 to 149)	122 (91.5 to 152.5)	123.7 (91.4 to 154.8)
Poland	Depressive disorders	141 (99.7 to 191)	160 (113 to 214)	160 (114 to 215)	338.4 (239.9 to 457)	343.7 (244.3 to 463.4)	335.3 (237.8 to 450.7)
Poland	Major depressive disorder	105 (73 to 144)	119 (82.4 to 161)	118 (81.7 to 160)	253.2 (176.3 to 345.9)	258.4 (178.9 to 348.7)	250.2 (172.5 to 342.9)
Poland	Dysthymia	36 (24.3 to 52.7)	40.5 (27.3 to 58.7)	42.3 (28.7 to 61.7)	85.2 (57.6 to 124.5)	85.3 (57.5 to 124)	85.1 (57.6 to 123.6)
Poland	Bipolar disorder	55.2 (34.1 to 81.3)	59.5 (37.1 to 88.8)	58 (36.4 to 85.8)	136.3 (84 to 201.9)	136.4 (84.8 to 201.8)	136.2 (85 to 204.3)

SR Appendix Table 2. All-age DALYs counts and age standardized DALYs rates per 100,000 individuals for all causes and level 1 cause groups by location for 1990, 2007, and 2017, both sexes combined							
		All-age DALYs			Age-standardized rate of DALYs per 100,000		
Poland	Anxiety disorders	125 (88.4 to 167)	132 (94.4 to 175)	131 (92.7 to 173)	309.4 (218 to 411.8)	311.2 (219.6 to 415.9)	309.4 (218 to 409.4)
Poland	Eating disorders	13.6 (8.54 to 19.8)	15.8 (9.93 to 23.2)	16.7 (10.6 to 24.7)	34.6 (21.8 to 50.4)	41 (26 to 60.1)	46.4 (29.7 to 68.5)
Poland	Anorexia nervosa	2.66 (1.6 to 4.02)	3.19 (1.99 to 4.72)	3.13 (1.93 to 4.66)	7.1 (4.2 to 10.9)	8.6 (5.4 to 12.7)	9.6 (5.8 to 14.3)
Poland	Bulimia nervosa	10.9 (6.69 to 16.3)	12.6 (7.77 to 18.9)	13.6 (8.36 to 20.3)	27.5 (16.8 to 41.2)	32.4 (19.9 to 48.4)	36.8 (22.7 to 55.6)
Poland	Autism spectrum disorders	25.6 (17.5 to 35.5)	24.4 (16.6 to 33.5)	24 (16.3 to 32.9)	66.8 (45.6 to 92.4)	67.2 (45.5 to 92.4)	67.4 (45.5 to 93.1)
Poland	Attention-deficit/hyperactivity disorder	4.41 (2.6 to 7.13)	3.93 (2.33 to 6.26)	3.54 (2.17 to 5.54)	11.7 (6.9 to 18.8)	11.7 (6.9 to 18.7)	11.8 (7.2 to 18.8)
Poland	Conduct disorder	35.4 (21.5 to 56.1)	27 (16.1 to 42.5)	21.3 (12.8 to 33.1)	97.6 (59.3 to 153.8)	98 (58.9 to 155.6)	97.9 (59.2 to 152)
Poland	Idiopathic developmental intellectual disability	8.21 (2.51 to 15.4)	6.76 (2.16 to 12.7)	4.98 (1.17 to 9.81)	21.6 (6.6 to 40.5)	18.9 (6.1 to 35.5)	14.5 (3.6 to 28.8)
Poland	Other mental disorders	58 (38.1 to 80.4)	64.4 (42.7 to 88.7)	66.5 (43.8 to 92.3)	138.4 (90.9 to 191.6)	139.1 (92.2 to 191.2)	139.1 (91.5 to 193.7)
Poland	Substance use disorders	236 (200 to 276)	323 (280 to 372)	314 (270 to 361)	569.7 (483.3 to 667.5)	697.3 (601.6 to 808.5)	673.7 (573.9 to 783.9)
Poland	Alcohol use disorders	179 (155 to 206)	255 (226 to 290)	248 (216 to 284)	423.1 (366 to 489.1)	535.6 (474 to 612.9)	508.7 (438.7 to 584.4)
Poland	Drug use disorders	57.5 (43.6 to 73.2)	68 (51 to 86.3)	65.8 (49.8 to 82.6)	146.7 (110.3 to 187.1)	161.8 (120.7 to 207.1)	165 (124.3 to 208.2)
Poland	Opioid use disorders	30.8 (22.9 to 39.9)	37.5 (28.3 to 47.6)	39.8 (29.4 to 51.1)	76.2 (56.5 to 98.6)	86.6 (65.3 to 110)	93.7 (69.1 to 122.1)
Poland	Cocaine use disorders	4.48 (2.79 to 6.74)	4.99 (3.28 to 7.12)	4.64 (2.99 to 6.61)	11.5 (7.1 to 17.2)	11.9 (7.8 to 17.3)	12 (7.6 to 17.3)
Poland	Amphetamine use disorders	7.29 (4.2 to 11.2)	8.47 (5.23 to 12.8)	6.67 (3.91 to 10.4)	19.9 (11.3 to 31)	21.7 (13.2 to 32.9)	20.1 (11.7 to 31.8)
Poland	Cannabis use disorders	3.57 (2.18 to 5.21)	4.19 (2.64 to 6.2)	3.17 (1.97 to 4.63)	9.5 (5.8 to 14.1)	10.8 (6.7 to 16)	9.3 (5.7 to 13.6)
Poland	Other drug use disorders	11.4 (8.38 to 15.3)	12.8 (9.33 to 17.2)	11.5 (8.43 to 15.1)	29.5 (21.4 to 40.2)	30.8 (22 to 41.6)	29.9 (21.2 to 40.8)
Poland	Diabetes and kidney diseases	434 (374 to 508)	460 (385 to 552)	484 (388 to 601)	972.4 (836.1 to 1138.4)	847.1 (704.3 to 1018)	786.6 (624.1 to 985.2)
Poland	Diabetes mellitus	301 (248 to 368)	329 (261 to 415)	391 (302 to 497)	669.8 (550.3 to 820.5)	607.2 (479.8 to 770.5)	636.2 (484.3 to 816.6)
Poland	Diabetes mellitus type 1	68.3 (63.2 to 74)	41.8 (38.7 to 44.9)	35.4 (31.5 to 39)	151.9 (141.2 to 163.9)	80.4 (74.1 to 86.5)	62.6 (55.4 to 69.3)
Poland	Diabetes mellitus type 2	233 (180 to 299)	287 (221 to 371)	355 (267 to 461)	518 (398.6 to 667.4)	526.8 (403.1 to 686.5)	573.6 (424.7 to 754.7)
Poland	Chronic kidney disease	132 (124 to 141)	130 (121 to 140)	93 (82.9 to 103)	301.2 (282.1 to 321.5)	239.2 (222.5 to 256.8)	149.9 (132.5 to 167)
Poland	Chronic kidney disease due to diabetes mellitus type 1	11 (8.77 to 13.5)	9.55 (7.8 to 11.7)	6.08 (4.83 to 7.53)	25.1 (20.2 to 31)	18.3 (15.2 to 22)	11 (8.8 to 13.4)
Poland	Chronic kidney disease due to diabetes mellitus type 2	24.5 (20.6 to 29.1)	29 (25.2 to 33)	21.2 (17.7 to 24.5)	53.5 (45.3 to 63.3)	50 (43.6 to 56.9)	30.8 (25.8 to 35.7)
Poland	Chronic kidney disease due to hypertension	18.8 (15.9 to 22)	21.7 (18.7 to 25)	16.8 (14.5 to 19.6)	42.3 (35.7 to 49.3)	38.5 (33.4 to 43.9)	25.3 (21.5 to 29.3)
Poland	Chronic kidney disease due to glomerulonephritis	30.7 (26.5 to 35)	26.7 (23.3 to 30.4)	17.5 (14.7 to 20.3)	70.8 (61.6 to 80.7)	50.8 (44.7 to 57.6)	30.8 (26.1 to 35.6)
Poland	Chronic kidney disease due to other and unspecified causes	47.5 (40.8 to 54.7)	43.5 (37.8 to 49.6)	31.4 (26.3 to 37)	109.5 (95.3 to 125.4)	81.7 (71.6 to 92.4)	51.9 (43.9 to 61)
Poland	Acute glomerulonephritis	0.599 (0.501 to 0.715)	0.34 (0.304 to 0.381)	0.303 (0.259 to 0.35)	1.4 (1.2 to 1.7)	0.7 (0.6 to 0.8)	0.6 (0.5 to 0.6)
Poland	Skin and subcutaneous diseases	197 (134 to 286)	198 (134 to 289)	201 (136 to 290)	512 (343.9 to 742.5)	534.6 (355.9 to 773.2)	538 (363 to 774.4)
Poland	Dermatitis	58.3 (33.9 to 93.2)	53.3 (31.3 to 84)	51.7 (29.9 to 80.6)	155.2 (89.6 to 248.2)	161 (92.8 to 257)	156.8 (89.6 to 248.1)
Poland	Atopic dermatitis	48.9 (26.3 to 82.1)	43.2 (23.5 to 71.8)	41.5 (22.6 to 68.3)	132.2 (71.1 to 220.6)	137.9 (74.8 to 228.6)	133.7 (72.6 to 219.7)
Poland	Contact dermatitis	9.38 (6.15 to 13.8)	10.1 (6.66 to 15)	10.2 (6.65 to 14.8)	23 (15.1 to 34.2)	23 (15.3 to 34.4)	23 (15 to 33.6)
Poland	Seborrhoeic dermatitis	0.0425 (0.0237 to 0.0678)	0.0422 (0.0235 to 0.0679)	0.0432 (0.0239 to 0.0689)	0.1 (0.1 to 0.2)	0.1 (0.1 to 0.2)	0.1 (0.1 to 0.2)
Poland	Psoriasis	30.1 (21.6 to 39.5)	36.2 (25.5 to 47.4)	38.3 (27 to 50.3)	71.9 (51.5 to 95)	80.2 (55.9 to 105.1)	81.4 (57.4 to 107.8)
Poland	Bacterial skin diseases	3.67 (2.16 to 4.64)	3.46 (2.58 to 5.96)	4.56 (2.93 to 7.06)	9.3 (5.4 to 11.7)	7.6 (5.7 to 13)	8.8 (5.8 to 13.6)
Poland	Cellulitis	1.07 (0.737 to 1.44)	1.03 (0.698 to 1.62)	1.21 (0.832 to 1.99)	2.7 (1.9 to 3.6)	2.5 (1.7 to 3.7)	2.7 (1.9 to 4.2)
Poland	Pyoderma	2.6 (1.28 to 3.39)	2.42 (1.69 to 4.4)	3.35 (1.84 to 5.2)	6.6 (3.1 to 8.4)	5.1 (3.6 to 9.5)	6.2 (3.4 to 9.8)
Poland	Scabies	10 (5.59 to 16.2)	9.33 (5.14 to 15)	8.68 (4.82 to 14)	26.8 (14.9 to 43.5)	26.6 (14.7 to 43.2)	26.4 (14.5 to 43.4)
Poland	Fungal skin diseases	19 (7.64 to 39.6)	20 (8.01 to 40.9)	21.7 (8.68 to 44.5)	47.2 (18.9 to 98.1)	44.8 (17.9 to 92.7)	44.2 (17.5 to 91.2)
Poland	Viral skin diseases	16.3 (10.4 to 24.2)	12 (7.64 to 18)	11.6 (7.31 to 17.2)	45.7 (29.1 to 67.8)	45.8 (29.1 to 67.9)	45.8 (28.7 to 68.1)

SR Appendix Table 2. All-age DALYs counts and age standardized DALYs rates per 100,000 individuals for all causes and level 1 cause groups by location for 1990, 2007, and 2017, both sexes combined

		All-age DALYs			Age-standardized rate of DALYs per 100,000		
Poland	Acne vulgaris	9.98 (5.83 to 16.4)	13.3 (7.98 to 21.9)	11.6 (6.89 to 18.8)	27.5 (15.9 to 45.2)	37.2 (21.9 to 61.5)	40.9 (24.3 to 66.9)
Poland	Alopecia areata	2.4 (1.55 to 3.58)	2.47 (1.54 to 3.64)	2.47 (1.58 to 3.69)	5.9 (3.8 to 8.9)	5.9 (3.7 to 8.8)	5.9 (3.8 to 8.8)
Poland	Pruritus	3.9 (1.87 to 7.31)	4.26 (2.04 to 7.87)	4.54 (2.15 to 8.46)	9.5 (4.5 to 18)	9.7 (4.6 to 18.1)	9.8 (4.6 to 18.5)
Poland	Urticaria	27.8 (18.4 to 39.4)	24.4 (16 to 34.3)	24 (16.1 to 33.8)	74.7 (49.2 to 106.2)	74.8 (49 to 106.6)	74.9 (49.7 to 106.5)
Poland	Decubitus ulcer	1.23 (0.88 to 1.65)	1.5 (1.07 to 2.02)	1.82 (1.25 to 2.45)	3 (2.1 to 3.9)	3 (2.2 to 4)	3 (2.1 to 4.1)
Poland	Other skin and subcutaneous diseases	14.7 (7.26 to 27.1)	17.6 (8.71 to 32)	19.9 (9.78 to 36.1)	35.2 (17.4 to 65.1)	38.1 (18.7 to 69.6)	40 (19.5 to 72.8)
Poland	Sense organ diseases	328 (221 to 474)	378 (254 to 545)	425 (286 to 608)	763.3 (514.6 to 1101.1)	735.6 (490.3 to 1066.3)	720 (480.3 to 1044.7)
Poland	Blindness and vision impairment	136 (87.7 to 203)	150 (96.7 to 222)	165 (108 to 242)	316.3 (203.6 to 471)	295 (188.5 to 436)	284.2 (182.3 to 418.6)
Poland	Glaucoma	1.8 (1.19 to 2.55)	2 (1.3 to 2.81)	2.3 (1.54 to 3.22)	3.9 (2.6 to 5.6)	3.4 (2.3 to 4.8)	3.2 (2.1 to 4.4)
Poland	Cataract	44 (29.6 to 60.4)	54.4 (36.6 to 74.2)	64.2 (43 to 87.9)	99.4 (67.2 to 135.7)	94 (63.4 to 128.6)	90.6 (61.1 to 123.6)
Poland	Age-related macular degeneration	0.409 (0.262 to 0.598)	0.533 (0.339 to 0.776)	0.663 (0.422 to 0.976)	0.9 (0.6 to 1.3)	0.9 (0.6 to 1.3)	0.9 (0.6 to 1.3)
Poland	Refraction disorders	43.7 (27.9 to 67)	42.8 (27.4 to 65.3)	43.6 (27.8 to 65.9)	107.1 (67.3 to 164.4)	100.5 (64.3 to 154.1)	97.2 (61.6 to 149.4)
Poland	Near vision loss	35.3 (16.9 to 64.1)	38.5 (18.7 to 69.6)	41.2 (20 to 74.3)	80.3 (38.4 to 145.7)	73 (35.4 to 131.7)	69.9 (33.7 to 124.7)
Poland	Other vision loss	10.6 (7.15 to 14.7)	12 (8.04 to 16.6)	13.2 (8.93 to 18.5)	24.6 (16.6 to 34)	23.2 (15.5 to 32.2)	22.3 (14.9 to 31.2)
Poland	Age-related and other hearing loss	179 (122 to 251)	212 (145 to 301)	241 (164 to 340)	416.1 (285.9 to 582.3)	409 (279.3 to 579)	404 (274 to 572.6)
Poland	Other sense organ diseases	13.3 (8.3 to 19.4)	15.8 (9.83 to 22.7)	18 (11.3 to 25.9)	30.9 (19.2 to 45.1)	31.6 (19.8 to 45.8)	31.8 (19.8 to 46.3)
Poland	Musculoskeletal disorders	753 (547 to 1010)	878 (641 to 1190)	953 (696 to 1270)	1741.7 (1266.1 to 2335.6)	1753.8 (1284.5 to 2374.8)	1749.1 (1278 to 2354.7)
Poland	Rheumatoid arthritis	23.8 (18.9 to 28.8)	22.8 (17.5 to 28.9)	23.5 (17.6 to 29.7)	53.1 (42.1 to 64.7)	42.3 (32 to 53.7)	39 (28.9 to 49.6)
Poland	Osteoarthritis	40.3 (20.4 to 79.4)	53.6 (27 to 105)	65 (32.5 to 128)	89.7 (45.3 to 176.2)	95.4 (47.9 to 186.6)	99 (49.5 to 195)
Poland	Low back pain	545 (390 to 734)	632 (455 to 856)	685 (492 to 916)	1267.3 (905 to 1718.8)	1283.2 (916.6 to 1727.3)	1283.6 (916.2 to 1722.5)
Poland	Neck pain	129 (90.1 to 180)	151 (105 to 211)	162 (113 to 225)	296.1 (207.3 to 413.8)	296.9 (206.9 to 417)	296.2 (207.5 to 412)
Poland	Gout	5.05 (3.4 to 6.98)	6.55 (4.41 to 9.22)	7.59 (5.11 to 10.4)	11.3 (7.6 to 15.6)	12 (8.2 to 16.8)	12.1 (8.1 to 16.7)
Poland	Other musculoskeletal disorders	10.3 (6.95 to 16.3)	11.8 (7.18 to 18.5)	10.1 (6.3 to 16)	24.2 (16.4 to 37.8)	24.1 (14.2 to 37.7)	19.2 (11.2 to 32.2)
Poland	Other non-communicable diseases	673 (561 to 809)	481 (370 to 618)	446 (337 to 585)	1990.7 (1695.7 to 2325.9)	1339.1 (1081.6 to 1640.8)	1122.2 (891.8 to 1424.9)
Poland	Congenital anomalies	351 (299 to 391)	171 (145 to 199)	134 (107 to 162)	1206.5 (1020.2 to 1326.9)	674 (583.3 to 755.4)	504.6 (412 to 609.2)
Poland	Neural tube defects	26.5 (18.2 to 40.2)	9.25 (7.78 to 10.9)	7.42 (5.73 to 9.25)	95.2 (63.8 to 147.5)	37.8 (32.6 to 44.5)	31.2 (23.6 to 39.6)
Poland	Congenital heart anomalies	136 (106 to 161)	43.6 (38.9 to 51.3)	27.4 (22.3 to 33.2)	494.9 (382 to 592.2)	208.6 (185.9 to 242.7)	135.3 (107 to 166.9)
Poland	Orofacial clefts	1.14 (0.758 to 1.64)	0.9 (0.567 to 1.33)	0.816 (0.529 to 1.21)	3.1 (2.1 to 4.4)	2.5 (1.6 to 3.8)	2.3 (1.5 to 3.4)
Poland	Down's syndrome	6.16 (4.75 to 8.78)	4.8 (3.76 to 6.03)	4.73 (3.62 to 5.99)	18 (13.9 to 26.8)	14.5 (11.4 to 18.4)	14.7 (11.3 to 19.3)
Poland	Turner syndrome	0.029 (0.0143 to 0.0479)	0.0273 (0.0138 to 0.0443)	0.0244 (0.0121 to 0.0401)	0.1 (0 to 0.1)	0.1 (0 to 0.1)	0.1 (0 to 0.1)
Poland	Klinefelter syndrome	0.00222 (0.00101 to 0.00421)	0.00209 (0.000972 to 0.00406)	0.00205 (0.000937 to 0.00402)	0 (0 to 0)	0 (0 to 0)	0 (0 to 0)
Poland	Other chromosomal abnormalities	15.1 (8.39 to 30.7)	9.25 (6.61 to 11)	8 (5.16 to 10.6)	55.5 (30.1 to 112.5)	45.6 (31.6 to 53.9)	41.3 (25.7 to 56.4)
Poland	Congenital musculoskeletal and limb anomalies	49.1 (33.8 to 66.1)	47.7 (32.3 to 64.9)	48.1 (32.3 to 65.2)	129.2 (89.3 to 172.2)	124.9 (85.2 to 170.8)	124.4 (84.7 to 168.9)
Poland	Urogenital congenital anomalies	6.3 (2.7 to 10)	3.45 (1.81 to 4.74)	2.39 (1.24 to 3.43)	23.1 (9.3 to 37.6)	16.6 (8 to 23.2)	11.8 (5.5 to 17.9)
Poland	Digestive congenital anomalies	33.1 (24.3 to 46)	18.9 (14.7 to 23.7)	14.5 (10.6 to 18.9)	111.8 (81 to 160.6)	69.7 (56.4 to 84.3)	51.6 (38.9 to 66)
Poland	Other congenital anomalies	77.3 (50.4 to 103)	33.7 (26.3 to 38.8)	20.7 (16.7 to 25.7)	275.7 (174.4 to 374)	153.7 (114.8 to 174.5)	91.9 (72.6 to 115)
Poland	Urinary diseases and male infertility	47.8 (40 to 57)	37 (27.5 to 48.6)	53 (41.8 to 66.4)	108.8 (91.2 to 129.2)	69 (51.3 to 90.4)	84.4 (66.4 to 105.4)
Poland	Urinary tract infections	20.2 (18.7 to 22.3)	8.22 (7.67 to 8.87)	20 (18.6 to 21.4)	46.8 (43.4 to 51.6)	16.3 (15.1 to 17.7)	31.5 (29.3 to 33.8)
Poland	Urolithiasis	4.71 (3.55 to 5.8)	2.77 (2.01 to 3.64)	2.97 (2.13 to 3.91)	10.7 (8 to 13.2)	5.6 (4.1 to 7.4)	5.5 (4 to 7.4)
Poland	Benign prostatic hyperplasia	17.6 (11.2 to 25.4)	23.2 (15 to 33.2)	27.5 (17.7 to 39.2)	38.6 (24.6 to 55.6)	40.1 (25.9 to 57.4)	41.3 (26.4 to 58.9)

SR Appendix Table 2. All-age DALYs counts and age standardized DALYs rates per 100,000 individuals for all causes and level 1 cause groups by location for 1990, 2007, and 2017, both sexes combined

		All-age DALYs			Age-standardized rate of DALYs per 100,000		
Poland	Male infertility	0.939 (0.385 to 1.91)	1.07 (0.423 to 2.26)	0.923 (0.375 to 1.94)	2.3 (0.9 to 4.8)	2.7 (1.1 to 5.7)	2.3 (0.9 to 5)
Poland	Other urinary diseases	4.3 (2.95 to 5.37)	1.78 (1.34 to 2.27)	1.62 (1.16 to 2.24)	10.3 (7.3 to 12.8)	4.3 (3.2 to 5.4)	3.6 (2.6 to 4.8)
Poland	Gynecological diseases	63.9 (43.1 to 89.6)	64.9 (44.7 to 91.6)	61 (41.4 to 87.5)	157.8 (107.3 to 222.1)	150.6 (103.9 to 210.7)	147 (101 to 207.6)
Poland	Uterine fibroids	10.4 (5.98 to 17.6)	10.1 (5.6 to 17)	8.74 (4.75 to 15)	24.2 (14 to 41)	21.1 (11.7 to 35.6)	17.7 (9.6 to 30.7)
Poland	Polycystic ovarian syndrome	1.99 (0.874 to 3.85)	2.07 (0.927 to 4.01)	1.96 (0.863 to 3.87)	5 (2.2 to 9.7)	5 (2.2 to 9.7)	5.1 (2.2 to 10)
Poland	Female infertility	0.845 (0.319 to 1.77)	0.93 (0.322 to 2.04)	1.13 (0.388 to 2.51)	2.1 (0.8 to 4.4)	2.4 (0.8 to 5.2)	2.8 (1 to 6.2)
Poland	Endometriosis	17.8 (11.5 to 26.2)	17.1 (11.4 to 23.7)	16.5 (11.1 to 23.1)	44.4 (28.9 to 64.5)	41.8 (28 to 57.8)	41.8 (28.1 to 58.6)
Poland	Genital prolapse	2.77 (1.36 to 5.1)	2.28 (1.1 to 4.29)	2.58 (1.23 to 4.75)	6.2 (3 to 11.4)	4.2 (2 to 7.8)	4.2 (2 to 7.7)
Poland	Premenstrual syndrome	20 (12.8 to 30.1)	20.7 (13.4 to 31)	18.7 (11.9 to 28)	51.1 (32.5 to 76.9)	49.8 (31.9 to 74.7)	48.6 (31.1 to 72.7)
Poland	Other gynecological diseases	10.1 (6.96 to 14.3)	11.7 (7.89 to 17)	11.3 (7.47 to 16.7)	24.8 (17.2 to 35.2)	26.3 (17.7 to 38.1)	26.7 (17.6 to 39.3)
Poland	Hemoglobinopathies and hemolytic anaemias	23.4 (18.8 to 29.7)	16.7 (13.3 to 20.7)	14.4 (11.5 to 17.9)	58 (46.6 to 74.1)	37.6 (30.3 to 47.7)	29.9 (23.8 to 37.9)
Poland	Thalassemias	0.397 (0.269 to 0.63)	0.168 (0.117 to 0.26)	0.124 (0.0829 to 0.202)	1.2 (0.8 to 2)	0.8 (0.5 to 1.3)	0.6 (0.4 to 1)
Poland	Thalassaemias trait	3.92 (2.42 to 5.98)	2.83 (1.6 to 4.47)	1.99 (1.18 to 3.19)	9.9 (6 to 14.9)	7.2 (3.9 to 11.6)	5 (3 to 8.2)
Poland	Sickle cell disorders	1.72 (1.4 to 2.24)	1.19 (0.996 to 1.47)	0.971 (0.804 to 1.17)	5.1 (4.1 to 6.7)	3.9 (3.2 to 5)	3.3 (2.7 to 4.1)
Poland	Sickle cell trait	1.91 (1.13 to 2.95)	1.28 (0.737 to 2.04)	0.879 (0.532 to 1.4)	4.9 (2.9 to 7.6)	3.4 (1.9 to 5.5)	2.4 (1.4 to 3.8)
Poland	G6PD deficiency	2.08 (1.64 to 2.58)	2.3 (1.7 to 2.76)	1.97 (1.29 to 2.39)	5.2 (4.1 to 6.5)	5 (3.9 to 5.9)	4.2 (3 to 5)
Poland	G6PD trait	0.00145 (0.000927 to 0.00212)	0.00139 (0.000861 to 0.00212)	0.000999 (0.000634 to 0.00152)	0 (0 to 0)	0 (0 to 0)	0 (0 to 0)
Poland	Other hemoglobinopathies and hemolytic anaemias	13.4 (10.8 to 16.5)	8.94 (7.14 to 10.6)	8.51 (6.62 to 10.2)	31.7 (25.5 to 39.1)	17.3 (13.8 to 21)	14.4 (11.1 to 18)
Poland	Endocrine, metabolic, blood, and immune disorders	38.5 (30 to 45.7)	27.6 (23 to 34.4)	25.6 (20.7 to 32.8)	101.6 (78.9 to 120)	69.5 (57.7 to 88.3)	60.6 (48.4 to 81)
Poland	Oral disorders	140 (87.6 to 211)	158 (101 to 235)	156 (98 to 232)	321.9 (202 to 489.4)	312.7 (196.5 to 469.3)	280.8 (174.3 to 426.5)
Poland	Caries of deciduous teeth	1.23 (0.533 to 2.46)	0.683 (0.293 to 1.37)	0.652 (0.283 to 1.3)	3.7 (1.6 to 7.3)	3.6 (1.6 to 7.3)	3.2 (1.4 to 6.5)
Poland	Caries of permanent teeth	17.9 (7.79 to 34)	18 (7.88 to 33.9)	16.5 (7.31 to 31.5)	44.8 (19.5 to 84.3)	43.9 (19.1 to 82.9)	41.7 (18.2 to 78.4)
Poland	Periodontal disease	26.2 (10.3 to 53.2)	28.2 (11.2 to 59)	29.6 (11.7 to 60.7)	59.8 (23.6 to 122.5)	55.2 (21.8 to 115.6)	52.8 (20.9 to 110.8)
Poland	Edentulism and severe tooth loss	73.5 (48.7 to 103)	89.2 (60 to 125)	86.5 (58.1 to 120)	162.9 (108.1 to 229.2)	159.2 (107.1 to 222)	132.2 (88.3 to 184.9)
Poland	Other oral disorders	20.8 (13.1 to 30.8)	22.1 (13.8 to 32.5)	22.3 (13.9 to 32.8)	50.7 (32 to 74.9)	50.8 (31.8 to 75.1)	50.8 (31.7 to 74.8)
Poland	Sudden infant death syndrome	9.5 (5.91 to 16.7)	4.75 (3.58 to 6.03)	2.62 (1.81 to 3.72)	36.2 (22.5 to 63.6)	25.7 (19.4 to 32.6)	15.1 (10.5 to 21.4)
Poland	Injuries	1990 (1770 to 2260)	1770 (1550 to 2050)	1610 (1350 to 1930)	4930.4 (4402 to 5563.7)	3918.9 (3462.3 to 4495.7)	3364.8 (2865.4 to 3992.3)
Poland	Transport injuries	601 (563 to 645)	438 (400 to 483)	306 (268 to 351)	1524.6 (1433.3 to 1630)	1023.2 (942.1 to 1118)	710.1 (630.2 to 799.8)
Poland	Road injuries	543 (507 to 584)	409 (372 to 452)	283 (247 to 326)	1377.2 (1289.9 to 1475.7)	955.6 (877.3 to 1046.3)	654.2 (579.3 to 741.2)
Poland	Pedestrian road injuries	185 (170 to 201)	114 (105 to 125)	68.8 (60.7 to 79)	462.2 (427 to 501)	261.6 (242.6 to 283.9)	151.2 (134.9 to 171.2)
Poland	Cyclist road injuries	54.4 (45.2 to 64.3)	50.1 (41.2 to 60.7)	41.7 (33.1 to 51.9)	131.8 (109.9 to 156.1)	110 (90.7 to 133.1)	86.2 (69 to 108.1)
Poland	Motorcyclist road injuries	44.1 (38.2 to 50.7)	34.4 (30.1 to 39.5)	31.3 (26.5 to 37.1)	114.4 (99.2 to 131.4)	81.7 (72.5 to 93)	77 (65.7 to 90)
Poland	Motor vehicle road injuries	254 (233 to 275)	206 (189 to 226)	136 (120 to 155)	653.9 (601.1 to 706)	492.3 (456.2 to 533.5)	330.3 (296.1 to 371.4)
Poland	Other road injuries	6.02 (4.89 to 7.29)	4.62 (3.6 to 6.08)	4.55 (3.5 to 6.03)	15 (12.2 to 18)	10 (7.8 to 13)	9.5 (7.4 to 12.4)
Poland	Other transport injuries	57.9 (54.4 to 61.5)	28.9 (26.7 to 31)	23.2 (20.7 to 26)	147.4 (138.8 to 156.4)	67.6 (62.6 to 72.7)	55.9 (49.4 to 63.4)
Poland	Unintentional injuries	1060 (878 to 1280)	937 (759 to 1170)	980 (766 to 1260)	2589.8 (2167.8 to 3116.7)	2002.8 (1632 to 2490.9)	1919.7 (1502.3 to 2450.8)
Poland	Falls	600 (463 to 770)	576 (437 to 758)	665 (499 to 875)	1424.6 (1098.2 to 1831.7)	1176.6 (888.5 to 1547.9)	1238.8 (928.9 to 1631.3)
Poland	Drowning	99.4 (95.2 to 104)	56.1 (54.1 to 58.5)	37.1 (34.1 to 40.4)	265.6 (253.2 to 278.3)	142.5 (136.7 to 148.8)	93.7 (86.1 to 101.8)
Poland	Fire, heat, and hot substances	53.3 (45.9 to 62.6)	43.6 (37.3 to 52)	37.9 (30.6 to 47.4)	133.8 (116.2 to 156.3)	96.4 (83.1 to 114.7)	78.6 (63.8 to 98.3)
Poland	Poisonings	37.8 (33.9 to 42.7)	25.4 (22 to 29.8)	19.7 (15.6 to 25.2)	95.8 (86.2 to 108.3)	61 (52.7 to 71.8)	47.8 (37.6 to 61.6)

SR Appendix Table 2. All-age DALYs counts and age standardized DALYs rates per 100,000 individuals for all causes and level 1 cause groups by location for 1990, 2007, and 2017, both sexes combined

		All-age DALYs			Age-standardized rate of DALYs per 100,000		
Poland	Poisoning by carbon monoxide	26.5 (24.4 to 29)	15.2 (14.4 to 15.9)	8.52 (7.75 to 9.4)	67.2 (62 to 73.1)	35.6 (33.8 to 37.4)	20 (18.2 to 22)
Poland	Poisoning by other means	11.3 (8.19 to 15.4)	10.2 (7.21 to 14.2)	11.2 (7.5 to 16.2)	28.6 (20.8 to 39.3)	25.3 (18 to 35.3)	27.8 (18.7 to 40.7)
Poland	Exposure to mechanical forces	115 (91.2 to 145)	99.5 (76.1 to 131)	104 (75.8 to 142)	277.5 (222.4 to 349.6)	213.6 (164.5 to 279.2)	211.8 (154.8 to 287.4)
Poland	Unintentional firearm injuries	9.61 (8.45 to 10.6)	6.17 (5.59 to 7.19)	4.28 (3.65 to 4.94)	24.3 (21.4 to 26.9)	13.6 (12.3 to 15.6)	9.2 (7.8 to 10.5)
Poland	Other exposure to mechanical forces	105 (81.6 to 135)	93.4 (70.2 to 123)	100 (71.8 to 138)	253.2 (198 to 324.1)	200 (151.6 to 265.1)	202.7 (146 to 277.8)
Poland	Adverse effects of medical treatment	15.6 (12.3 to 16.9)	19 (15.1 to 20.7)	17.2 (14.9 to 20.1)	39.6 (30.9 to 42.7)	41 (33 to 44.8)	33.3 (29.6 to 40.7)
Poland	Animal contact	8.31 (6.55 to 10.4)	6.79 (5.33 to 8.57)	5.69 (4.31 to 7.32)	20.7 (16.4 to 26)	15.8 (12.3 to 20.1)	13 (9.7 to 16.9)
Poland	Venomous animal contact	5.13 (3.85 to 6.7)	4.2 (3.11 to 5.49)	3.55 (2.55 to 4.79)	12.7 (9.5 to 16.6)	10 (7.3 to 13.1)	8.4 (5.9 to 11.4)
Poland	Non-venomous animal contact	3.18 (2.49 to 4.11)	2.59 (2.02 to 3.41)	2.14 (1.62 to 2.9)	8 (6.4 to 10.3)	5.9 (4.6 to 7.7)	4.5 (3.4 to 6.1)
Poland	Foreign body	49.8 (46.5 to 53.5)	43.1 (41 to 45.7)	30.8 (28.2 to 33.5)	144.8 (133.7 to 157.5)	106.5 (100.9 to 112.9)	72.8 (66.8 to 79.9)
Poland	Pulmonary aspiration and foreign body in airway	42.9 (40.1 to 46.1)	36.9 (35.3 to 38.4)	25.1 (23.3 to 27)	127.8 (118 to 139.7)	92.5 (88.2 to 96.7)	60.4 (55.7 to 65.4)
Poland	Foreign body in eyes	0.79 (0.375 to 1.37)	0.861 (0.423 to 1.48)	0.957 (0.487 to 1.65)	2 (0.9 to 3.4)	2.1 (0.9 to 3.6)	2.2 (1 to 3.9)
Poland	Foreign body in other body part	6.09 (5.02 to 7.45)	5.31 (4.32 to 6.63)	4.71 (3.66 to 6.03)	15.1 (12.4 to 18.4)	12 (9.7 to 15)	10.2 (7.9 to 13)
Poland	Environmental heat and cold exposure	22.9 (19.9 to 26.3)	26.4 (23.7 to 29.5)	20.3 (17.3 to 23.6)	56 (48.7 to 64.2)	56.4 (50.4 to 63.1)	41.6 (35.3 to 48.3)
Poland	Exposure to forces of nature	0 (0 to 0)	0.125 (0.121 to 0.129)	0.0195 (0.015 to 0.0253)	0 (0 to 0)	0.3 (0.3 to 0.3)	0 (0 to 0.1)
Poland	Other unintentional injuries	53.2 (45.8 to 62.9)	41.4 (34.1 to 51.4)	41.9 (31.5 to 56.3)	131.3 (113.7 to 154.4)	92.7 (77 to 115.2)	88.2 (67.1 to 117.8)
Poland	Self-harm and interpersonal violence	332 (321 to 342)	397 (383 to 410)	320 (297 to 346)	816 (790.1 to 841.2)	892.9 (863.8 to 922.8)	735 (680.4 to 792.1)
Poland	Self-harm	256 (247 to 264)	338 (326 to 350)	274 (252 to 296)	627 (605.9 to 647.5)	756.4 (731 to 782.5)	628.4 (578.5 to 677.4)
Poland	Self-harm by firearm	3.94 (2.54 to 6.66)	3.73 (2.61 to 5.84)	2.6 (1.75 to 4.14)	9.7 (6.1 to 16.4)	8.3 (5.8 to 13)	5.8 (3.8 to 9.3)
Poland	Self-harm by other specified means	252 (243 to 260)	334 (323 to 346)	271 (250 to 293)	617.4 (595.8 to 638.6)	748.1 (722.6 to 774.3)	622.7 (572.2 to 671.8)
Poland	Interpersonal violence	75.5 (70.4 to 81.3)	58.6 (53.7 to 63.9)	46.5 (41.1 to 52.9)	188.1 (175.7 to 202)	135.6 (124.7 to 147.3)	105.8 (94.4 to 119.2)
Poland	Assault by firearm	6.82 (5.3 to 8.21)	3.82 (3.34 to 4.44)	2.39 (2.02 to 3.07)	16.9 (13.3 to 20.3)	9.2 (8 to 10.6)	5.7 (4.8 to 7.8)
Poland	Assault by sharp object	20 (18 to 23.5)	14.7 (13.6 to 16.4)	11.3 (10.2 to 12.7)	49.6 (44.7 to 57.7)	33.8 (31.2 to 36.9)	25.9 (23.2 to 29.2)
Poland	Sexual violence	3.91 (2.64 to 5.65)	3.9 (2.63 to 5.66)	3.78 (2.58 to 5.43)	9.8 (6.6 to 14.3)	9.6 (6.5 to 14.1)	9.6 (6.5 to 14)
Poland	Assault by other means	44.7 (39.4 to 49.7)	36.2 (31.6 to 40.7)	29 (24.4 to 34.4)	111.7 (99.1 to 124)	83.1 (73.2 to 92.7)	64.6 (54.7 to 75.8)
Poland	Conflict and terrorism	0.0561 (0.0555 to 0.0568)	0.0723 (0.0686 to 0.0791)	0.0181 (0.012 to 0.0296)	0.2 (0.2 to 0.2)	0.2 (0.2 to 0.2)	0 (0 to 0.1)
Poland	Executions and police conflict	0.291 (0.272 to 0.317)	0.285 (0.263 to 0.313)	0.264 (0.239 to 0.298)	0.8 (0.7 to 0.8)	0.8 (0.7 to 0.8)	0.7 (0.7 to 0.8)
Romania	All causes	9200 (8420 to 10100)	8090 (7350 to 8930)	7560 (6830 to 8380)	37363.3 (34279.5 to 40770.7)	30107.9 (27185 to 33375.3)	26808.1 (23876 to 30258.6)
Romania	Communicable, maternal, neonatal, and nutritional disorders	1130 (1050 to 1230)	619 (557 to 695)	486 (430 to 548)	6009.9 (5620.1 to 6477.1)	3776.3 (3422.9 to 4195.4)	2971.3 (2609.6 to 3390)
Romania	HIV/AIDS and sexually transmitted infections	23.4 (20.4 to 27.9)	14.7 (12.1 to 18.6)	14.6 (12.3 to 17.8)	123.5 (106.7 to 147.6)	81.7 (65.2 to 105.1)	88.1 (72.7 to 110.3)
Romania	HIV/AIDS	15.9 (15 to 16.8)	8.57 (8.14 to 9.06)	9.82 (9.15 to 10.8)	85.2 (80.1 to 90.4)	45.5 (43.2 to 48.1)	56.5 (52.7 to 61.8)
Romania	HIV/AIDS - Drug-susceptible Tuberculosis	2.44 (1.44 to 3.84)	2.62 (1.62 to 3.33)	1.65 (1.01 to 2.58)	13.1 (7.7 to 20.6)	14 (8.7 to 17.8)	9.6 (5.9 to 14.9)
Romania	HIV/AIDS - Multidrug-resistant Tuberculosis without extensive drug resistance	0.0683 (0.0236 to 0.164)	0.297 (0.0965 to 0.791)	0.165 (0.0823 to 0.294)	0.4 (0.1 to 0.9)	1.6 (0.5 to 4.3)	1 (0.5 to 1.7)
Romania	HIV/AIDS - Extensively drug-resistant Tuberculosis	0 (0 to 0)	0.0941 (0.0302 to 0.248)	0.0947 (0.0483 to 0.17)	0 (0 to 0)	0.5 (0.2 to 1.3)	0.5 (0.3 to 1)
Romania	HIV/AIDS resulting in other diseases	13.4 (11.7 to 14.7)	5.56 (4.8 to 6.69)	7.91 (6.71 to 9.04)	71.8 (62.5 to 79.2)	29.4 (25.4 to 35.4)	45.4 (38.3 to 52)
Romania	Sexually transmitted infections excluding HIV	7.52 (4.57 to 11.8)	6.09 (3.69 to 9.95)	4.76 (2.84 to 7.71)	38.2 (22.3 to 60.8)	36.2 (20.1 to 59.3)	31.7 (18 to 52.2)
Romania	Syphilis	3.32 (1.48 to 6.44)	2.4 (1.14 to 4.39)	1.7 (0.86 to 3.09)	20.4 (8.3 to 40.9)	19.8 (7.8 to 39)	15.8 (6.4 to 31.8)
Romania	Chlamydial infection	0.906 (0.5 to 1.69)	0.807 (0.423 to 1.54)	0.667 (0.348 to 1.29)	3.9 (2.2 to 7.3)	3.7 (1.9 to 7)	3.6 (1.9 to 7.1)
Romania	Gonococcal infection	0.865 (0.515 to 1.54)	0.62 (0.324 to 1.22)	0.435 (0.224 to 0.854)	3.8 (2.2 to 6.8)	3 (1.5 to 6)	2.8 (1.4 to 5.5)
Romania	Trichomoniasis	0.729 (0.296 to 1.52)	0.715 (0.283 to 1.51)	0.628 (0.25 to 1.31)	3 (1.2 to 6.3)	3 (1.2 to 6.4)	2.9 (1.2 to 6.2)

SR Appendix Table 2. All-age DALYs counts and age standardized DALYs rates per 100,000 individuals for all causes and level 1 cause groups by location for 1990, 2007, and 2017, both sexes combined

		All-age DALYs			Age-standardized rate of DALYs per 100,000		
Romania	Genital herpes	0.53 (0.173 to 1.25)	0.544 (0.174 to 1.27)	0.523 (0.167 to 1.25)	2.1 (0.7 to 4.9)	2.1 (0.7 to 4.8)	2.1 (0.7 to 4.9)
Romania	Other sexually transmitted infections	1.17 (0.708 to 2.01)	1 (0.584 to 1.76)	0.814 (0.472 to 1.41)	5 (3 to 8.6)	4.6 (2.6 to 8.1)	4.4 (2.5 to 7.8)
Romania	Respiratory infections and tuberculosis	563 (539 to 590)	278 (267 to 291)	218 (204 to 233)	2967.7 (2831.7 to 3125.4)	1549.7 (1473.7 to 1637.7)	1106.7 (1026 to 1201.6)
Romania	Tuberculosis	69.7 (65 to 74.2)	64.7 (61.9 to 67.5)	35.8 (33.1 to 38.9)	291.3 (267.3 to 314.2)	261.6 (250.1 to 272.8)	146.4 (134.9 to 158.7)
Romania	Latent tuberculosis infection	0 (0 to 0)	0 (0 to 0)	0 (0 to 0)	0 (0 to 0)	0 (0 to 0)	0 (0 to 0)
Romania	Drug-susceptible tuberculosis	68.2 (63.4 to 72.9)	57.8 (46 to 63.5)	31.8 (28.7 to 35)	284.8 (260.1 to 307.9)	233.5 (186 to 256.7)	130.1 (117.1 to 143.1)
Romania	Multidrug-resistant tuberculosis without extensive drug resistance	1.56 (0.576 to 3.57)	5.3 (1.9 to 14.1)	2.56 (1.5 to 3.92)	6.5 (2.4 to 14.8)	21.4 (7.7 to 57.7)	10.4 (6.1 to 15.8)
Romania	Extensively drug-resistant tuberculosis	0 (0 to 0)	1.67 (0.583 to 4.46)	1.46 (0.844 to 2.21)	0 (0 to 0)	6.7 (2.4 to 18)	5.9 (3.4 to 9)
Romania	Lower respiratory infections	469 (448 to 492)	195 (187 to 203)	166 (156 to 176)	2565.8 (2434.4 to 2705.1)	1193.3 (1127.5 to 1260.7)	868.6 (803.2 to 948.9)
Romania	Upper respiratory infections	16.9 (10.3 to 26.3)	14.2 (8.47 to 22.7)	12.6 (7.53 to 20.2)	74.7 (46.2 to 116.4)	70.9 (41.6 to 113.7)	69.8 (40.9 to 110.9)
Romania	Otitis media	7.34 (5 to 10.6)	4.36 (2.63 to 6.79)	3.53 (2.12 to 5.58)	36 (25.1 to 51)	23.9 (14.4 to 37.4)	21.9 (13 to 34.9)
Romania	Enteric infections	52.4 (42.9 to 63.2)	27.2 (20.9 to 35.2)	23.1 (17.1 to 30.2)	285.5 (235.3 to 343.9)	187.9 (146.2 to 240.9)	161.4 (119.5 to 212.5)
Romania	Diarrhoeal diseases	50.3 (41 to 61.3)	26.6 (20.3 to 34.5)	22.6 (16.8 to 29.9)	276.4 (227.5 to 336)	184.6 (143.7 to 237.8)	158.9 (116.5 to 210.4)
Romania	Typhoid and paratyphoid	0.779 (0.0469 to 1.6)	0.0327 (0.0194 to 0.0488)	0.02 (0.0143 to 0.0283)	3.1 (0.2 to 6.4)	0.1 (0.1 to 0.2)	0.1 (0.1 to 0.1)
Romania	Typhoid fever	0.0499 (0.0343 to 0.0616)	0.0231 (0.0153 to 0.036)	0.0181 (0.0124 to 0.0264)	0.2 (0.1 to 0.3)	0.1 (0.1 to 0.1)	0.1 (0 to 0.1)
Romania	Paratyphoid fever	0.729 (0.00791 to 1.55)	0.00959 (0.00153 to 0.0169)	0.00185 (0.000802 to 0.0027)	2.9 (0 to 6.2)	0 (0 to 0.1)	0 (0 to 0)
Romania	Invasive non-typhoidal salmonella	1.12 (0.62 to 1.86)	0.521 (0.279 to 0.888)	0.395 (0.207 to 0.694)	5.1 (2.8 to 8.6)	2.9 (1.5 to 5.1)	2.3 (1.1 to 4.1)
Romania	Other intestinal infectious diseases	0.169 (0.0558 to 0.302)	0.0511 (0.0247 to 0.0845)	0.0312 (0.0166 to 0.0481)	0.9 (0.3 to 1.7)	0.3 (0.1 to 0.6)	0.2 (0.1 to 0.3)
Romania	Neglected tropical diseases and malaria	11.7 (6.26 to 20.3)	8.22 (4.21 to 13.9)	7.55 (3.77 to 13)	46.6 (24.4 to 86.8)	30.7 (15.8 to 52.1)	28.5 (14.2 to 49.9)
Romania	Malaria	0 (0 to 0)	0 (0 to 0)	0 (0 to 0)	0 (0 to 0)	0 (0 to 0)	0 (0 to 0)
Romania	Chagas disease	0 (0 to 0)	0 (0 to 0)	0 (0 to 0)	0 (0 to 0)	0 (0 to 0)	0 (0 to 0)
Romania	Leishmaniasis	1.01 (0.00183 to 8.41)	0.169 (0.00033 to 1.42)	0.00694 (0.0000117 to 0.061)	4.7 (0 to 38.8)	1 (0 to 8.4)	0 (0 to 0.4)
Romania	Visceral leishmaniasis	1.01 (0.00183 to 8.41)	0.169 (0.00033 to 1.42)	0.00694 (0.0000117 to 0.061)	4.7 (0 to 38.8)	1 (0 to 8.4)	0 (0 to 0.4)
Romania	Cutaneous and mucocutaneous leishmaniasis	0 (0 to 0)	0 (0 to 0)	0 (0 to 0)	0 (0 to 0)	0 (0 to 0)	0 (0 to 0)
Romania	African trypanosomiasis	0 (0 to 0)	0 (0 to 0)	0 (0 to 0)	0 (0 to 0)	0 (0 to 0)	0 (0 to 0)
Romania	Schistosomiasis	0 (0 to 0)	0 (0 to 0)	0 (0 to 0)	0 (0 to 0)	0 (0 to 0)	0 (0 to 0)
Romania	Cysticercosis	9.3 (4.67 to 15.7)	7.38 (3.35 to 13.1)	7.01 (3.25 to 12.4)	35.9 (18.1 to 60.1)	26.9 (12.1 to 48.4)	26.1 (11.9 to 47.4)
Romania	Cystic echinococcosis	0.808 (0.608 to 1.01)	0.496 (0.366 to 0.646)	0.376 (0.27 to 0.501)	3.2 (2.4 to 4.1)	2 (1.4 to 2.6)	1.6 (1.1 to 2.1)
Romania	Lymphatic filariasis	0 (0 to 0)	0 (0 to 0)	0 (0 to 0)	0 (0 to 0)	0 (0 to 0)	0 (0 to 0)
Romania	Onchocerciasis	0 (0 to 0)	0 (0 to 0)	0 (0 to 0)	0 (0 to 0)	0 (0 to 0)	0 (0 to 0)
Romania	Trachoma	0 (0 to 0)	0 (0 to 0)	0 (0 to 0)	0 (0 to 0)	0 (0 to 0)	0 (0 to 0)
Romania	Dengue	0 (0 to 0)	0 (0 to 0)	0 (0 to 0)	0 (0 to 0)	0 (0 to 0)	0 (0 to 0)
Romania	Yellow fever	0 (0 to 0)	0 (0 to 0)	0 (0 to 0)	0 (0 to 0)	0 (0 to 0)	0 (0 to 0)
Romania	Rabies	0.175 (0.148 to 0.205)	0.066 (0.0522 to 0.0823)	0.0538 (0.0417 to 0.0699)	0.7 (0.6 to 0.9)	0.2 (0.2 to 0.3)	0.2 (0.1 to 0.2)
Romania	Intestinal nematode infections	0.0178 (0.00401 to 0.0432)	0.00333 (0.00133 to 0.00642)	0.00188 (0.000904 to 0.00319)	0.1 (0 to 0.2)	0 (0 to 0.1)	0 (0 to 0)
Romania	Ascariasis	0.0178 (0.00401 to 0.0432)	0.00333 (0.00133 to 0.00642)	0.00188 (0.000904 to 0.00319)	0.1 (0 to 0.2)	0 (0 to 0.1)	0 (0 to 0)
Romania	Trichuriasis	0 (0 to 0)	0 (0 to 0)	0 (0 to 0)	0 (0 to 0)	0 (0 to 0)	0 (0 to 0)
Romania	Hookworm disease	0 (0 to 0)	0 (0 to 0)	0 (0 to 0)	0 (0 to 0)	0 (0 to 0)	0 (0 to 0)
Romania	Food-borne trematodiases	0 (0 to 0)	0 (0 to 0)	0 (0 to 0)	0 (0 to 0)	0 (0 to 0)	0 (0 to 0)
Romania	Leprosy	0 (0 to 0)	0 (0 to 0)	0 (0 to 0)	0 (0 to 0)	0 (0 to 0)	0 (0 to 0)

SR Appendix Table 2. All-age DALYs counts and age standardized DALYs rates per 100,000 individuals for all causes and level 1 cause groups by location for 1990, 2007, and 2017, both sexes combined

		All-age DALYs			Age-standardized rate of DALYs per 100,000		
Romania	Ebola virus disease	0<br>(0 to 0)	0<br>(0 to 0)	0<br>(0 to 0)	0<br>(0 to 0)	0<br>(0 to 0)	0<br>(0 to 0)
Romania	Zika virus disease	0<br>(0 to 0)	0<br>(0 to 0)	0<br>(0 to 0)	0<br>(0 to 0)	0<br>(0 to 0)	0<br>(0 to 0)
Romania	Guinea worm disease	0<br>(0 to 0)	0<br>(0 to 0)	0<br>(0 to 0)	0<br>(0 to 0)	0<br>(0 to 0)	0<br>(0 to 0)
Romania	Other neglected tropical diseases	0.392<br>(0.1 to 0.548)	0.0993<br>(0.0602 to 0.161)	0.0983<br>(0.0452 to 0.129)	1.9<br>(0.5 to 2.7)	0.5<br>(0.3 to 0.9)	0.6<br>(0.2 to 0.7)
Romania	Other infectious diseases	80.7<br>(55.7 to 89.7)	25.3<br>(22.8 to 28.6)	21.9<br>(19.6 to 24.8)	427.5<br>(285.7 to 476.2)	154<br>(139.5 to 170.9)	136.6<br>(122.2 to 153.7)
Romania	Meningitis	32.9<br>(26.6 to 36.1)	8.02<br>(7.31 to 9.44)	4.09<br>(3.65 to 4.75)	180.5<br>(146.5 to 200.3)	53.5<br>(47.9 to 65.7)	27.4<br>(23.9 to 32.9)
Romania	Pneumococcal meningitis	4.2<br>(3.36 to 4.71)	1.39<br>(1.24 to 1.6)	0.822<br>(0.704 to 0.958)	21.3<br>(17 to 24.2)	7.7<br>(6.8 to 9.1)	4.6<br>(3.9 to 5.4)
Romania	H influenzae type B meningitis	16.2<br>(12.9 to 18)	3.33<br>(3 to 4.06)	1.51<br>(1.34 to 1.82)	91.4<br>(72.9 to 102.5)	24.6<br>(21.5 to 30.9)	11.6<br>(9.9 to 14.6)
Romania	Meningococcal infection	4.18<br>(3.49 to 4.74)	1.1<br>(0.967 to 1.28)	0.544<br>(0.474 to 0.636)	22.9<br>(19.1 to 26.3)	7.2<br>(6.2 to 8.7)	3.6<br>(3.1 to 4.3)
Romania	Other meningitis	8.28<br>(6.69 to 9.18)	2.2<br>(1.99 to 2.56)	1.2<br>(1.07 to 1.39)	44.9<br>(36 to 50.3)	14<br>(12.4 to 17)	7.6<br>(6.7 to 9.1)
Romania	Encephalitis	17<br>(7.25 to 19.4)	7.17<br>(5.17 to 7.86)	4.74<br>(4.11 to 5.35)	89.2<br>(33.2 to 102.6)	45.8<br>(28.8 to 51.4)	31.9<br>(25.1 to 36.5)
Romania	Diphtheria	0.01<br>(0.00514 to 0.02)	0.00167<br>(0.00123 to 0.00251)	0.00138<br>(0.00106 to 0.00194)	0<br>(0 to 0.1)	0<br>(0 to 0)	0<br>(0 to 0)
Romania	Whooping cough	0.405<br>(0.184 to 0.591)	0.0616<br>(0.0392 to 0.107)	0.178<br>(0.109 to 0.265)	2.4<br>(1.1 to 3.5)	0.6<br>(0.4 to 1)	1.9<br>(1.2 to 2.8)
Romania	Tetanus	0.397<br>(0.328 to 0.479)	0.103<br>(0.0863 to 0.123)	0.0447<br>(0.0345 to 0.0676)	1.7<br>(1.4 to 2.1)	0.4<br>(0.4 to 0.5)	0.2<br>(0.1 to 0.3)
Romania	Measles	0.654<br>(0.42 to 0.877)	0.0368<br>(0.0239 to 0.0471)	0.0484<br>(0.0319 to 0.0674)	3.9<br>(2.5 to 5.2)	0.3<br>(0.2 to 0.4)	0.5<br>(0.3 to 0.7)
Romania	Varicella and herpes zoster	0.871<br>(0.56 to 1.23)	0.717<br>(0.457 to 1.06)	0.693<br>(0.436 to 1.03)	3.8<br>(2.4 to 5.4)	3<br>(2 to 4.4)	2.8<br>(1.8 to 4.2)
Romania	Acute hepatitis	10.7<br>(7.99 to 12.1)	3.25<br>(2.81 to 3.82)	2.63<br>(2.22 to 3.13)	53.9<br>(39 to 61.4)	17.1<br>(14.7 to 20.1)	14.8<br>(12.3 to 17.7)
Romania	Acute hepatitis A	4.01<br>(2.81 to 4.73)	1.28<br>(1.03 to 1.6)	0.938<br>(0.727 to 1.19)	19.8<br>(13.6 to 23.5)	7.8<br>(6.2 to 9.6)	6.6<br>(5.1 to 8.3)
Romania	Acute hepatitis B	4.09<br>(3.23 to 5.01)	1.52<br>(1.3 to 1.83)	1.36<br>(1.15 to 1.64)	19.4<br>(14.5 to 24.7)	6.6<br>(5.6 to 7.8)	6.1<br>(5.1 to 7.4)
Romania	Acute hepatitis C	1.91<br>(0.97 to 2.84)	0.169<br>(0.0946 to 0.263)	0.111<br>(0.0581 to 0.179)	11.6<br>(5.9 to 17.4)	1.4<br>(0.7 to 2.2)	1<br>(0.5 to 1.7)
Romania	Acute hepatitis E	0.691<br>(0.52 to 0.847)	0.276<br>(0.224 to 0.337)	0.215<br>(0.175 to 0.268)	3.1<br>(2.3 to 3.8)	1.3<br>(1.1 to 1.6)	1.2<br>(0.9 to 1.4)
Romania	Other unspecified infectious diseases	17.8<br>(7.44 to 22.3)	5.93<br>(4.46 to 8.2)	9.44<br>(8.17 to 11.3)	92<br>(33.8 to 116.3)	33.4<br>(25.5 to 45)	57.1<br>(49.6 to 67.8)
Romania	Maternal and neonatal disorders	272<br>(239 to 312)	175<br>(149 to 206)	128<br>(104 to 156)	1571.6<br>(1395.7 to 1777.2)	1272.4<br>(1110.7 to 1454.3)	1013.2<br>(850.7 to 1195.3)
Romania	Maternal disorders	20.7<br>(19.7 to 21.9)	3.68<br>(3.29 to 4.16)	2.56<br>(2.22 to 2.96)	92.8<br>(88.3 to 98.3)	17.3<br>(15.3 to 19.6)	15.8<br>(13.5 to 18.3)
Romania	Maternal haemorrhage	4.27<br>(2.01 to 7.27)	1.08<br>(0.712 to 1.57)	0.71<br>(0.454 to 1.01)	19.1<br>(8.9 to 33.1)	5.1<br>(3.4 to 7.3)	4.4<br>(2.8 to 6.3)
Romania	Maternal sepsis and other pregnancy related infections	3.36<br>(1.77 to 5.54)	0.314<br>(0.194 to 0.473)	0.184<br>(0.112 to 0.295)	15.1<br>(7.7 to 24.7)	1.5<br>(0.9 to 2.3)	1.2<br>(0.7 to 1.9)
Romania	Maternal hypertensive disorders	1.22<br>(0.764 to 1.85)	0.324<br>(0.23 to 0.434)	0.276<br>(0.189 to 0.374)	5.5<br>(3.4 to 8.3)	1.6<br>(1.1 to 2.1)	1.8<br>(1.2 to 2.4)
Romania	Maternal obstructed labour and uterine rupture	0.45<br>(0.242 to 0.823)	0.226<br>(0.125 to 0.361)	0.173<br>(0.0927 to 0.292)	2<br>(1 to 3.7)	1.1<br>(0.6 to 1.7)	1.1<br>(0.5 to 1.8)
Romania	Maternal abortive outcome	9.93<br>(6.39 to 13.5)	0.751<br>(0.41 to 1.19)	0.417<br>(0.22 to 0.669)	44.4<br>(28 to 60.5)	3.5<br>(1.9 to 5.3)	2.5<br>(1.3 to 4.1)
Romania	Ectopic pregnancy	0.151<br>(0.0827 to 0.252)	0.0244<br>(0.0149 to 0.0383)	0.0126<br>(0.00735 to 0.0192)	0.7<br>(0.4 to 1.2)	0.1<br>(0.1 to 0.2)	0.1<br>(0 to 0.1)
Romania	Indirect maternal deaths	0.636<br>(0.315 to 1.14)	0.267<br>(0.139 to 0.425)	0.171<br>(0.0911 to 0.28)	2.9<br>(1.4 to 5.2)	1.3<br>(0.7 to 2)	1.1<br>(0.6 to 1.8)
Romania	Late maternal deaths	0.374<br>(0.272 to 0.494)	0.0902<br>(0.0675 to 0.119)	0.0791<br>(0.0575 to 0.107)	1.7<br>(1.2 to 2.2)	0.4<br>(0.3 to 0.6)	0.5<br>(0.4 to 0.7)
Romania	Maternal deaths aggravated by HIV/AIDS	0.0008<br>(0.000338 to 0.00153)	0.00131<br>(0.00064 to 0.00229)	0.00139<br>(0.000706 to 0.00238)	0<br>(0 to 0)	0<br>(0 to 0)	0<br>(0 to 0)
Romania	Other maternal disorders	0.317<br>(0.119 to 1.12)	0.601<br>(0.258 to 1.1)	0.54<br>(0.272 to 0.923)	1.4<br>(0.5 to 5)	2.8<br>(1.2 to 5.1)	3.3<br>(1.7 to 5.5)
Romania	Neonatal disorders	252<br>(218 to 291)	171<br>(145 to 202)	125<br>(101 to 154)	1478.8<br>(1306 to 1684.2)	1255.2<br>(1093.8 to 1434.8)	997.4<br>(835.8 to 1178)
Romania	Neonatal preterm birth	80.4<br>(68.8 to 95.7)	52.9<br>(46 to 61.7)	37.8<br>(31.3 to 45.1)	487.7<br>(415.6 to 579.5)	424.2<br>(372.3 to 490.7)	355.4<br>(296.1 to 415.9)
Romania	Neonatal encephalopathy due to birth asphyxia and trauma	74.9<br>(56.9 to 97.8)	54.8<br>(39 to 74.6)	47.5<br>(32.6 to 65.2)	404<br>(316.4 to 516.7)	331.5<br>(249.8 to 437.1)	309.3<br>(224 to 414.4)
Romania	Neonatal sepsis and other neonatal infections	25.2<br>(13.9 to 42.8)	24<br>(13.8 to 39.7)	21.6<br>(12.2 to 35.5)	117.7<br>(67.8 to 195.6)	129.7<br>(76.6 to 209.7)	129.8<br>(75.2 to 210.2)
Romania	Hemolytic disease and other neonatal jaundice	6.37<br>(5.13 to 7.85)	3.8<br>(3.03 to 4.7)	2.86<br>(2.21 to 3.66)	34.8<br>(27.7 to 43.5)	24<br>(19.7 to 29.1)	19.4<br>(15.3 to 24.2)
Romania	Other neonatal disorders	64.7<br>(52.8 to 79.9)	35.9<br>(31.2 to 40.9)	15.6<br>(12.7 to 18.7)	434.7<br>(354.6 to 537.3)	345.8<br>(300.1 to 394.3)	183.5<br>(149 to 221.4)

SR Appendix Table 2. All-age DALYs counts and age standardized DALYs rates per 100,000 individuals for all causes and level 1 cause groups by location for 1990, 2007, and 2017, both sexes combined

		All-age DALYs			Age-standardized rate of DALYs per 100,000		
Romania	Nutritional deficiencies	130 (84.7 to 193)	90.9 (57.9 to 139)	72.9 (47.6 to 113)	587.5 (381 to 872.9)	499.8 (313.7 to 761)	436.9 (279.9 to 673.8)
Romania	Protein-energy malnutrition	1.16 (0.928 to 1.4)	1.96 (1.39 to 2.51)	1.96 (1.26 to 2.6)	6.1 (4.8 to 7.4)	11.6 (8.2 to 15.7)	10.8 (6.6 to 15.9)
Romania	Iodine deficiency	2.38 (1.27 to 3.82)	1.22 (0.594 to 2.3)	1.78 (0.9 to 2.96)	9.7 (5.2 to 15.5)	5 (2.4 to 9.5)	8.2 (4.1 to 13.6)
Romania	Dietary iron deficiency	93.7 (60.2 to 141)	74.1 (46.3 to 116)	59.6 (38.3 to 94.8)	397.2 (258.3 to 599.8)	370 (231.9 to 575.1)	330.9 (207.6 to 503.1)
Romania	Other nutritional deficiencies	1.36 (1.15 to 1.6)	1.61 (1.37 to 2.09)	1.2 (0.949 to 1.81)	6.5 (5.6 to 7.7)	9.3 (8.1 to 11.5)	7.6 (6.1 to 10.4)
Romania	Non-communicable diseases	6740 (6190 to 7400)	6560 (6010 to 7230)	6290 (5720 to 6940)	25771.9 (23571 to 28351.7)	22525.7 (20375.3 to 25101.4)	20519.6 (18359.5 to 23062.8)
Romania	Neoplasms	970 (956 to 984)	1130 (1100 to 1150)	1190 (1130 to 1250)	3476.4 (3426.7 to 3529.4)	3708.8 (3622.5 to 3771.7)	3732.5 (3541.9 to 3915.5)
Romania	Lip and oral cavity cancer	17.4 (16.5 to 18.3)	28 (26.7 to 29.4)	29.9 (27.4 to 32.7)	61.3 (58.1 to 64.5)	93.8 (89.5 to 98.7)	97 (88.8 to 106.5)
Romania	Nasopharynx cancer	2.84 (2.67 to 3.01)	7.18 (6.79 to 7.55)	6.51 (5.94 to 7.13)	10.7 (10 to 11.3)	25.9 (24.5 to 27.3)	23.7 (21.6 to 25.9)
Romania	Other pharynx cancer	10.8 (10.2 to 11.4)	21.3 (20.1 to 22.5)	26.9 (24.4 to 29.5)	38.1 (36 to 40.4)	72.2 (68.1 to 76.3)	88.4 (80.3 to 97.1)
Romania	Oesophageal cancer	12.2 (11.6 to 12.8)	18.8 (18 to 19.7)	20.8 (19.1 to 22.7)	41.9 (40.1 to 44)	60.7 (58.1 to 63.5)	65.6 (60 to 71.7)
Romania	Stomach cancer	100 (96 to 104)	84.1 (81.6 to 86.5)	74.4 (69.9 to 79.4)	344.7 (330.8 to 358.2)	259.5 (251.9 to 267.1)	219.3 (205.8 to 233.8)
Romania	Colon and rectum cancer	84.8 (81.9 to 87.5)	116 (113 to 119)	135 (127 to 145)	293.7 (284.4 to 303.1)	354.5 (344.9 to 364.6)	390.4 (367.1 to 416.5)
Romania	Liver cancer	28.7 (27.6 to 29.8)	56.1 (54.3 to 58.1)	68.8 (63.8 to 73.9)	99.2 (95.5 to 102.9)	174.5 (168.7 to 180.8)	204.5 (190 to 219.9)
Romania	Liver cancer due to hepatitis B	6.8 (5.54 to 8.17)	11.4 (9.24 to 13.7)	13.1 (10.3 to 16.2)	24.5 (20.2 to 29)	38.3 (31.3 to 45.8)	43.1 (34.6 to 52.6)
Romania	Liver cancer due to hepatitis C	6.89 (5.2 to 9.01)	15.2 (11.8 to 19)	18.8 (14.1 to 24.1)	23.1 (17.7 to 29.8)	45.3 (35 to 56.9)	52.8 (39.7 to 67.8)
Romania	Liver cancer due to alcohol use	11.4 (10 to 12.8)	21.9 (19.3 to 24.5)	27.5 (23.8 to 31.3)	39 (34.6 to 43.6)	66.9 (59 to 75.1)	80.2 (69.6 to 91)
Romania	Liver cancer due to NASH	1.8 (1.54 to 2.1)	4.34 (3.74 to 5.08)	5.65 (4.75 to 6.7)	6.2 (5.3 to 7.1)	13.1 (11.3 to 15.2)	16.1 (13.6 to 19)
Romania	Liver cancer due to other causes	1.77 (1.53 to 2.04)	3.29 (2.84 to 3.81)	3.81 (3.23 to 4.47)	6.4 (5.6 to 7.3)	11 (9.6 to 12.6)	12.2 (10.5 to 14.2)
Romania	Gallbladder and biliary tract cancer	17.3 (15.9 to 18.1)	12.4 (11.9 to 13.4)	12.6 (11.6 to 13.6)	59 (54.6 to 61.7)	37.4 (35.8 to 40.4)	36.2 (33.3 to 39)
Romania	Pancreatic cancer	43 (41.6 to 44.5)	57.7 (55.9 to 59.4)	66.5 (62.6 to 70.6)	147.1 (142.3 to 152.1)	177 (171.7 to 182.4)	194.3 (182.5 to 206.7)
Romania	Larynx cancer	25.3 (24.2 to 26.7)	27.4 (26.1 to 28.6)	27 (24.7 to 29.6)	86.9 (83 to 91.7)	88.6 (84.3 to 92.7)	84.4 (76.9 to 92.5)
Romania	Tracheal, bronchus, and lung cancer	196 (189 to 203)	232 (225 to 240)	252 (236 to 270)	666.4 (644.1 to 687.4)	732.4 (711 to 757)	760.5 (709.5 to 813.4)
Romania	Malignant skin melanoma	8.32 (6.62 to 11.2)	10.7 (7.76 to 13.2)	11.8 (8.06 to 14.5)	31.2 (24.6 to 41.3)	37.6 (26.6 to 45.2)	40.5 (27.1 to 48.5)
Romania	Non-melanoma skin cancer	8.57 (8.24 to 8.93)	9.07 (8.59 to 9.74)	9.63 (8.98 to 10.3)	31.4 (30.2 to 32.7)	28.7 (27.3 to 30.8)	28.1 (26.2 to 30.2)
Romania	Non-melanoma skin cancer (squamous-cell carcinoma)	8.57 (8.24 to 8.93)	9.06 (8.57 to 9.73)	9.62 (8.97 to 10.3)	31.4 (30.2 to 32.7)	28.7 (27.3 to 30.7)	28.1 (26.2 to 30.2)
Romania	Non-melanoma skin cancer (basal-cell carcinoma)	0.00201 (0.000845 to 0.00417)	0.00818 (0.00228 to 0.0211)	0.00876 (0.00241 to 0.0228)	0 (0 to 0)	0 (0 to 0.1)	0 (0 to 0.1)
Romania	Breast cancer	73.8 (71 to 76.9)	82.1 (79 to 85.4)	83.6 (76.5 to 91)	264.1 (254.2 to 275.3)	268.6 (258.4 to 279.4)	261 (239.1 to 283.5)
Romania	Cervical cancer	59.4 (56.9 to 62)	62.3 (59.9 to 65.1)	51 (46.3 to 55.9)	220.6 (211.1 to 230.7)	219.4 (210.4 to 229.6)	174.3 (158.6 to 191)
Romania	Uterine cancer	13 (12.3 to 13.7)	11.2 (10.5 to 11.9)	12.3 (10.9 to 13.6)	45.1 (42.7 to 47.5)	35.3 (33 to 37.5)	36.3 (32.4 to 40.4)
Romania	Ovarian cancer	25.2 (24 to 26.5)	27.1 (25.8 to 28.5)	27.6 (24.9 to 30.3)	89.5 (85.3 to 93.9)	88.8 (84.6 to 93.2)	88 (79.5 to 96.7)
Romania	Prostate cancer	21.7 (18.1 to 29.4)	32.8 (25.5 to 41.2)	39.9 (28.5 to 46.7)	75.5 (63.8 to 103.1)	91.6 (70.5 to 114.4)	101.3 (71.9 to 118.3)
Romania	Testicular cancer	3.78 (3.45 to 4.15)	3.26 (2.95 to 3.63)	2.68 (2.34 to 3.06)	16.2 (14.8 to 17.9)	13.9 (12.5 to 15.5)	13.1 (11.3 to 15.2)
Romania	Kidney cancer	14.3 (13.5 to 15.1)	18.7 (17.8 to 20.5)	22.5 (20.7 to 24.4)	53.4 (50.1 to 56.6)	63.6 (60.4 to 68.5)	72 (66.3 to 78)
Romania	Bladder cancer	22.3 (21.3 to 23.3)	26.9 (25.8 to 28.1)	30.2 (27.9 to 32.6)	75.5 (72.3 to 78.8)	79 (75.7 to 82.4)	82.7 (76.4 to 89.2)
Romania	Thyroid cancer	4.07 (3.82 to 4.34)	3.81 (3.55 to 4.11)	4.01 (3.62 to 4.45)	14.4 (13.5 to 15.3)	12.2 (11.4 to 13.2)	12.5 (11.3 to 14)
Romania	Mesothelioma	1.49 (1.35 to 1.68)	1.78 (1.69 to 1.88)	1.84 (1.68 to 2.01)	5.4 (4.9 to 6.1)	6 (5.7 to 6.3)	6.1 (5.6 to 6.7)
Romania	Hodgkin lymphoma	8.97 (5.81 to 10.1)	5.26 (3.9 to 5.86)	3.98 (3.02 to 4.58)	36.2 (23.4 to 41.1)	21.1 (15.4 to 23.6)	17 (12.7 to 19.5)
Romania	Non-Hodgkin's lymphoma	19.4 (18.6 to 20.2)	21.8 (21.1 to 22.6)	21.5 (20.1 to 23)	78.4 (74.7 to 82.4)	84 (80.8 to 87.4)	81.2 (75.7 to 87.2)
Romania	Multiple myeloma	5.99 (5.44 to 7.73)	7.82 (6.95 to 9.6)	9.17 (7.8 to 10.7)	20.6 (18.7 to 26.4)	24.4 (21.2 to 29.3)	27.5 (22.9 to 30.9)

SR Appendix Table 2. All-age DALYs counts and age standardized DALYs rates per 100,000 individuals for all causes and level 1 cause groups by location for 1990, 2007, and 2017, both sexes combined							
		All-age DALYs			Age-standardized rate of DALYs per 100,000		
Romania	Leukaemia	44.4 (42.4 to 46.7)	39.4 (37.2 to 40.7)	36.6 (33.8 to 38.9)	187.2 (177.8 to 198.9)	162.2 (150.6 to 168.7)	145.4 (132.5 to 156.3)
Romania	Acute lymphoid leukaemia	11.5 (10.2 to 12.9)	7.92 (7.4 to 8.41)	5.92 (5.4 to 6.46)	54.2 (47.7 to 61.5)	43.5 (40 to 46.9)	32.8 (29.3 to 36.7)
Romania	Chronic lymphoid leukaemia	3.52 (3.11 to 3.97)	5.53 (5.19 to 5.9)	5.88 (5.37 to 6.48)	12.2 (10.8 to 13.7)	16.8 (15.7 to 17.9)	16.9 (15.4 to 18.6)
Romania	Acute myeloid leukaemia	11.4 (10.1 to 13.3)	11.2 (10.5 to 11.8)	12.4 (11.2 to 13.5)	47.6 (41.2 to 56.3)	45.6 (42.1 to 48.1)	49.6 (43.6 to 54.4)
Romania	Chronic myeloid leukaemia	2.77 (2.44 to 3.14)	2.39 (2.23 to 2.58)	1.65 (1.48 to 1.84)	10.3 (9.1 to 11.7)	8.2 (7.6 to 8.8)	5.6 (5 to 6.2)
Romania	Other leukaemia	15.2 (13.7 to 16.8)	12.3 (11.6 to 12.9)	10.7 (9.79 to 11.6)	63 (56.3 to 70.8)	48.1 (45 to 51.1)	40.5 (36.8 to 44)
Romania	Other malignant cancers	54.9 (51.7 to 59.3)	54.6 (47.3 to 57.5)	51.5 (44.2 to 56)	214.7 (196.9 to 228.7)	201.6 (173.7 to 212.3)	186.4 (161 to 203.4)
Romania	Other neoplasms	3.29 (2.31 to 4.58)	4.12 (3.31 to 5.58)	4.97 (3.79 to 6.85)	12.7 (8.6 to 18)	14.4 (11.2 to 18.8)	16.4 (11.9 to 21.8)
Romania	Myelodysplastic, myeloproliferative, and other hematopoietic neoplasms	2.89 (2.13 to 4.05)	3.84 (3.1 to 5.27)	4.72 (3.61 to 6.51)	10.8 (7.8 to 15.3)	12.8 (10.1 to 17)	14.8 (10.9 to 19.9)
Romania	Benign and in situ intestinal neoplasms	0 (0 to 0)	0 (0 to 0)	0 (0 to 0)	0 (0 to 0)	0 (0 to 0)	0 (0 to 0)
Romania	Benign and in situ cervical and uterine neoplasms	0 (0 to 0)	0 (0 to 0)	0 (0 to 0)	0 (0 to 0)	0 (0 to 0)	0 (0 to 0)
Romania	Other benign and in situ neoplasms	0.396 (0.187 to 0.642)	0.276 (0.182 to 0.361)	0.247 (0.167 to 0.314)	1.9 (0.8 to 3.1)	1.6 (1 to 2.1)	1.6 (1 to 2.1)
Romania	Cardiovascular diseases	2610 (2550 to 2660)	2550 (2490 to 2610)	2310 (2200 to 2410)	9602 (9400.9 to 9799.7)	7767.6 (7596.1 to 7944.1)	6264.8 (5967.4 to 6549.7)
Romania	Rheumatic heart disease	47.3 (45 to 49.4)	15.7 (15.1 to 16.4)	9.73 (9.05 to 10.5)	175.2 (167.1 to 183)	51.9 (50 to 54)	30.8 (28.5 to 33.2)
Romania	Ischaemic heart disease	1230 (1200 to 1270)	1140 (1110 to 1180)	1000 (954 to 1060)	4575.8 (4462.2 to 4695.3)	3489.3 (3417.5 to 3615.1)	2733.3 (2596.2 to 2886.1)
Romania	Stroke	889 (853 to 923)	962 (926 to 1000)	831 (776 to 882)	3219.8 (3094.3 to 3340.1)	2868.7 (2755.4 to 2990.8)	2210.7 (2063 to 2345.9)
Romania	Ischaemic stroke	429 (402 to 457)	449 (419 to 481)	421 (384 to 456)	1601.1 (1505.2 to 1697.2)	1318.2 (1232.9 to 1412.3)	1063.3 (969.8 to 1153.7)
Romania	Intracerebral hemorrhage	393 (377 to 408)	430 (414 to 451)	335 (314 to 360)	1379.2 (1324.9 to 1430.2)	1284.5 (1237 to 1344.1)	922.7 (865.9 to 989.8)
Romania	Subarachnoid hemorrhage	66.5 (62.2 to 74.2)	83.3 (68.6 to 88.7)	75.2 (60.4 to 82)	239.6 (224.6 to 267.1)	266 (226.8 to 281.8)	224.7 (190.1 to 243.1)
Romania	Hypertensive heart disease	122 (103 to 150)	171 (112 to 185)	165 (116 to 185)	443.8 (378.9 to 550.5)	504.2 (333.2 to 542.6)	424.8 (307.8 to 473.8)
Romania	Non-rheumatic valvular heart disease	14.5 (10.9 to 16.4)	14.1 (12.4 to 16.9)	14.5 (12.4 to 18)	53.2 (40.2 to 60)	43.3 (38.2 to 51.3)	39.7 (34.3 to 49.3)
Romania	Non-rheumatic calcific aortic valve disease	6.96 (5.37 to 8.21)	8.45 (7.31 to 10.1)	9.05 (7.67 to 11.3)	25.5 (19.9 to 30.2)	25.7 (22.4 to 30.5)	24.5 (21 to 30.5)
Romania	Non-rheumatic degenerative mitral valve disease	7.17 (4.88 to 8.28)	5.22 (4.42 to 6.54)	5.02 (3.99 to 6.41)	26.2 (17.8 to 30)	16.2 (13.8 to 20.2)	13.9 (11.1 to 17.6)
Romania	Other non-rheumatic valve diseases	0.39 (0.282 to 0.616)	0.399 (0.306 to 0.638)	0.4 (0.286 to 0.626)	1.5 (1.1 to 2.3)	1.4 (1 to 2.1)	1.3 (1 to 2)
Romania	Cardiomyopathy and myocarditis	150 (123 to 181)	141 (124 to 148)	152 (131 to 164)	583.2 (486.1 to 695.7)	470.1 (415.8 to 494.3)	462.6 (395.6 to 498.4)
Romania	Myocarditis	27.4 (14.5 to 41.3)	24.5 (16.3 to 29.6)	26.8 (18.2 to 33.5)	109.8 (62.3 to 158.6)	85.5 (59.8 to 101.1)	86.2 (59.5 to 107)
Romania	Alcoholic cardiomyopathy	51.8 (36.3 to 71.8)	32.4 (27.7 to 39.3)	28.8 (23.4 to 39.7)	183.4 (129.4 to 252.9)	110.9 (93.7 to 132.8)	95.8 (77.6 to 130.9)
Romania	Other cardiomyopathy	70.3 (57.8 to 82.5)	83.7 (74.6 to 90.8)	96.9 (83 to 107)	290 (241.3 to 335.1)	273.7 (245.5 to 295.1)	280.6 (239 to 310.4)
Romania	Atrial fibrillation and flutter	22.2 (18.1 to 27.2)	25.7 (21.2 to 30.4)	30.5 (25.4 to 35.9)	84.3 (69.3 to 102)	76 (63.1 to 89.7)	76.9 (64 to 91.2)
Romania	Aortic aneurysm	11.5 (10.3 to 12.9)	13.6 (13.1 to 14.1)	15 (14 to 16.1)	41.4 (37.3 to 46.2)	43.8 (42.1 to 45.6)	45.9 (42.8 to 49.2)
Romania	Peripheral vascular disease	6.25 (3.42 to 10.8)	9.1 (5.81 to 13.6)	12.7 (7.86 to 20.2)	22.2 (12.2 to 38)	26.6 (16.8 to 40)	33.3 (20.4 to 52.2)
Romania	Endocarditis	2.49 (1.98 to 2.98)	3.12 (2.8 to 3.74)	3.67 (3.1 to 4.25)	9.7 (7.8 to 11.5)	11.3 (10 to 13.3)	13 (10.9 to 14.8)
Romania	Other cardiovascular and circulatory diseases	109 (102 to 117)	58.8 (52.6 to 66.5)	66.8 (59.5 to 75.1)	393.3 (369.4 to 420.2)	182.3 (163.7 to 205.3)	193.8 (172.3 to 218)
Romania	Chronic respiratory diseases	421 (389 to 453)	272 (243 to 300)	265 (237 to 295)	1536.4 (1415.5 to 1651)	893.3 (788.4 to 993.8)	822.6 (721.6 to 927.1)
Romania	Chronic obstructive pulmonary disease	323 (301 to 342)	208 (188 to 225)	204 (184 to 226)	1159.6 (1086 to 1226.6)	634.7 (575.2 to 687.5)	575 (517 to 637.4)
Romania	Pneumoconiosis	3.59 (3.01 to 4.25)	1.65 (1.41 to 1.96)	1.25 (1 to 1.57)	12.3 (10.4 to 14.3)	5 (4.3 to 6)	3.6 (2.9 to 4.6)
Romania	Silicosis	2.5 (2.07 to 3.03)	1.14 (0.953 to 1.36)	0.816 (0.612 to 1.06)	8.4 (7 to 10.2)	3.5 (2.9 to 4.2)	2.4 (1.8 to 3.1)
Romania	Asbestosis	0.187 (0.117 to 0.282)	0.0943 (0.0696 to 0.128)	0.0835 (0.0567 to 0.125)	0.7 (0.4 to 1)	0.3 (0.2 to 0.4)	0.2 (0.2 to 0.4)
Romania	Coal workers pneumoconiosis	0.106 (0.0726 to 0.153)	0.0619 (0.0432 to 0.0882)	0.0685 (0.0463 to 0.0971)	0.4 (0.3 to 0.5)	0.2 (0.1 to 0.3)	0.2 (0.1 to 0.3)
Romania	Other pneumoconiosis	0.796 (0.538 to 1.07)	0.356 (0.281 to 0.459)	0.287 (0.21 to 0.389)	2.8 (1.9 to 3.7)	1.1 (0.9 to 1.4)	0.8 (0.6 to 1.1)

SR Appendix Table 2. All-age DALYs counts and age standardized DALYs rates per 100,000 individuals for all causes and level 1 cause groups by location for 1990, 2007, and 2017, both sexes combined							
		All-age DALYs			Age-standardized rate of DALYs per 100,000		
Romania	Asthma	62.5 (48.1 to 80.2)	41.5 (29.7 to 56.6)	38.9 (27.1 to 53.7)	244.5 (185.9 to 316.8)	179.8 (124.2 to 249.9)	174.7 (119 to 243.7)
Romania	Interstitial lung disease and pulmonary sarcoidosis	25.9 (22.1 to 31)	16.2 (12.4 to 22)	15.6 (11.1 to 22.6)	94.9 (80.8 to 112.3)	53.2 (41.4 to 70.2)	48 (34.8 to 67.4)
Romania	Other chronic respiratory diseases	6.44 (5.6 to 7.26)	4.96 (4.13 to 5.74)	4.78 (3.96 to 5.65)	25.1 (21.7 to 28.5)	20.6 (17.2 to 24.1)	21.3 (17.5 to 25.6)
Romania	Digestive diseases	475 (437 to 523)	496 (461 to 537)	454 (416 to 496)	1788.4 (1630.2 to 1991.5)	1771.4 (1625.5 to 1948)	1590.6 (1435.9 to 1765.2)
Romania	Cirrhosis and other chronic liver diseases	254 (244 to 263)	307 (297 to 317)	277 (259 to 296)	907.3 (871.4 to 940.9)	1039 (1004.4 to 1070.2)	907.3 (847.8 to 974)
Romania	Cirrhosis and other chronic liver diseases due to hepatitis B	63.1 (56.2 to 71.2)	68.9 (61.7 to 76.8)	61 (53.3 to 70)	226.2 (202.6 to 253.8)	234.3 (210.1 to 260.6)	202.1 (177.5 to 230.8)
Romania	Cirrhosis and other chronic liver diseases due to hepatitis C	48.7 (42.9 to 55.4)	64.7 (57.1 to 73.3)	57.2 (49.3 to 65.7)	172.1 (151.4 to 195.2)	218.3 (192.8 to 247.2)	187.9 (162.6 to 217)
Romania	Cirrhosis and other chronic liver diseases due to alcohol use	108 (101 to 116)	133 (124 to 142)	121 (110 to 132)	378.7 (353 to 404.6)	443 (411.5 to 473.1)	389.9 (354.6 to 426.1)
Romania	Cirrhosis due to NASH	14.2 (12.7 to 15.7)	21.2 (19 to 23.6)	20.5 (18.1 to 23.2)	49.2 (44.2 to 54.4)	70.4 (63.2 to 78.1)	65.9 (58.1 to 74.8)
Romania	Cirrhosis and other chronic liver diseases due to other causes	19.8 (17.3 to 22.7)	19.3 (16.5 to 22.6)	16.7 (13.9 to 20)	80.9 (72 to 92.4)	73 (63.2 to 83.5)	61.5 (52.5 to 71.3)
Romania	Upper digestive system diseases	103 (76.6 to 139)	83.9 (60.5 to 115)	75.6 (54 to 105)	413 (303 to 562.4)	345.2 (243.3 to 481.1)	320.6 (224.1 to 452.3)
Romania	Peptic ulcer disease	29.2 (27.2 to 31.3)	19.1 (17.5 to 20.7)	16.1 (14.4 to 18.1)	107.4 (99.9 to 114.9)	64.4 (58.7 to 70.3)	52.4 (46.6 to 59.5)
Romania	Gastritis and duodenitis	54.1 (35.6 to 78.4)	43.8 (29.1 to 62.3)	39.4 (26.4 to 55.9)	227.6 (148.3 to 330.4)	202.5 (132.1 to 292.3)	189.8 (124 to 274.1)
Romania	GERD	20.1 (10.8 to 34.1)	21 (11.2 to 35.6)	20.2 (10.7 to 34)	78 (41.9 to 132.2)	78.3 (41.8 to 131.8)	78.4 (42 to 132.1)
Romania	Appendicitis	5.05 (4.6 to 5.64)	2.11 (1.88 to 2.37)	1.74 (1.52 to 2)	21.4 (19.4 to 24.1)	9.4 (8.1 to 10.8)	8 (6.7 to 9.7)
Romania	Paralytic ileus and intestinal obstruction	17.4 (15.2 to 20.3)	14.6 (12.6 to 17.4)	14.3 (12.1 to 17.2)	77.4 (68.5 to 88.1)	55.7 (47.8 to 64.2)	49.5 (41.1 to 57.3)
Romania	Inguinal, femoral, and abdominal hernia	24 (18.1 to 30.8)	17.4 (12.5 to 22.9)	17 (12.3 to 22.6)	94.8 (70.6 to 122.8)	71.6 (50.7 to 95.4)	69.6 (48.8 to 93.5)
Romania	Inflammatory bowel disease	7.24 (5.61 to 9.21)	7.36 (5.6 to 9.43)	7.04 (5.38 to 8.95)	28.9 (22.2 to 36.5)	28.2 (21.6 to 35.9)	27.2 (20.6 to 34.8)
Romania	Vascular intestinal disorders	10.8 (9.66 to 15.1)	14.8 (13.6 to 17.7)	16 (14.1 to 18.6)	38.7 (34.6 to 53.4)	44.4 (41.2 to 52.3)	44.8 (39.5 to 50.8)
Romania	Gallbladder and biliary diseases	8.76 (7.1 to 9.3)	4.46 (4.12 to 5.91)	4.46 (3.99 to 5.81)	31.4 (25.8 to 33.2)	14 (13 to 18.2)	13.1 (11.7 to 16.4)
Romania	Pancreatitis	35.2 (31.3 to 41.4)	34.6 (32 to 37.6)	30.5 (27.4 to 34.2)	135.1 (120.2 to 158.5)	125.2 (115.9 to 135.9)	108.4 (97.3 to 121.2)
Romania	Other digestive diseases	9.13 (6.71 to 12)	9.23 (7.62 to 11.1)	10.2 (8.49 to 12.2)	40.5 (29.4 to 53.6)	38.7 (31 to 47.5)	42.1 (33.9 to 51.7)
Romania	Neurological disorders	376 (296 to 478)	393 (316 to 489)	414 (340 to 505)	1571.4 (1242.8 to 1989.4)	1495.7 (1170.3 to 1907.2)	1473.1 (1148.1 to 1886)
Romania	Alzheimer's disease and other dementias	102 (94.9 to 108)	137 (128 to 147)	175 (162 to 189)	451.4 (423 to 480.8)	437 (407.1 to 467.4)	425.1 (393.7 to 459.2)
Romania	Parkinson's disease	21.6 (19.4 to 29.4)	28.6 (25.6 to 38.8)	32.8 (28.9 to 44.1)	80.6 (72.2 to 110)	79.5 (71.2 to 108.3)	79.3 (69.9 to 107.3)
Romania	Epilepsy	52.4 (28.8 to 85.5)	37.6 (22.4 to 57.5)	33.7 (15.7 to 62.4)	225.1 (125 to 365.7)	173.1 (102.8 to 265.7)	167.9 (77.2 to 310.4)
Romania	Multiple sclerosis	7.34 (4.86 to 8.57)	5.47 (4.73 to 6.31)	4.83 (3.98 to 6.18)	28.3 (18.4 to 33.2)	20.4 (17.6 to 23.3)	18.3 (15.1 to 23)
Romania	Motor neuron disease	1.04 (0.898 to 1.21)	1.42 (1.33 to 1.51)	1.72 (1.56 to 1.86)	4.2 (3.6 to 5.1)	5.6 (5.2 to 6)	6.5 (6 to 7.1)
Romania	Headache disorders	180 (116 to 265)	174 (112 to 256)	158 (102 to 234)	726.8 (467.4 to 1066.1)	731.2 (472.5 to 1072.4)	729 (465.3 to 1072.7)
Romania	Migraine	154 (95.9 to 231)	148 (91.9 to 226)	134 (83.2 to 204)	623 (386.2 to 936.9)	626.9 (388.2 to 955.2)	624.9 (388.6 to 942.6)
Romania	Tension-type headache	26 (14.9 to 40.9)	25.3 (14.4 to 39.6)	23.4 (13.3 to 36)	103.9 (59.7 to 163.3)	104.4 (58.5 to 163.6)	104.1 (58.6 to 162.4)
Romania	Other neurological disorders	12.2 (8.34 to 17.2)	9.46 (7.18 to 12.4)	8.23 (5.33 to 12.8)	54.9 (37.2 to 78.5)	48.9 (36.5 to 65.2)	47 (29 to 76.7)
Romania	Mental disorders	319 (237 to 411)	306 (229 to 396)	282 (210 to 363)	1296.3 (960.9 to 1669)	1302.7 (969 to 1687.3)	1302.5 (967.1 to 1686.8)
Romania	Schizophrenia	29.7 (22.1 to 37.1)	30.5 (22.8 to 37.9)	29 (21.3 to 36.3)	117.7 (87.3 to 147)	119.8 (88.9 to 149.7)	121.7 (88.8 to 154)
Romania	Depressive disorders	95.9 (67.3 to 129)	96.2 (68.2 to 131)	91.3 (65.1 to 123)	374.3 (263.1 to 504)	371.6 (263.4 to 504.2)	370.4 (263.5 to 501.7)
Romania	Major depressive disorder	75.8 (52.4 to 102)	76.4 (53 to 104)	71.6 (49.9 to 97)	296.6 (205.2 to 397.1)	296.2 (205.3 to 405.8)	292.2 (204.3 to 398.5)
Romania	Dysthymia	20.1 (13.7 to 29.4)	19.8 (13.4 to 28.4)	19.7 (13.3 to 28.8)	77.7 (52.7 to 113.3)	75.4 (51.3 to 108.6)	78.2 (52.4 to 114.6)
Romania	Bipolar disorder	35.8 (22.5 to 53.4)	34.6 (21.8 to 52.1)	31 (19.4 to 46.4)	145.2 (91.4 to 216.7)	146.2 (91.8 to 219.3)	146.1 (91.7 to 219.3)
Romania	Anxiety disorders	71.8 (51.4 to 95.1)	68.5 (48.4 to 90.3)	62.6 (44.5 to 82.8)	292.1 (208.3 to 388.7)	294.5 (207.5 to 389.8)	292.6 (207.3 to 388)
Romania	Eating disorders	7.6 (4.73 to 11.2)	7.36 (4.67 to 10.8)	6.88 (4.35 to 10.1)	33.5 (20.8 to 49.6)	35.3 (22.3 to 51.8)	40.8 (25.4 to 60.6)

SR Appendix Table 2. All-age DALYs counts and age standardized DALYs rates per 100,000 individuals for all causes and level 1 cause groups by location for 1990, 2007, and 2017, both sexes combined

		All-age DALYs			Age-standardized rate of DALYs per 100,000		
Romania	Anorexia nervosa	1.57 (0.917 to 2.39)	1.43 (0.834 to 2.19)	1.3 (0.784 to 1.98)	7 (4.1 to 10.6)	7.3 (4.3 to 11.3)	8.4 (5 to 13)
Romania	Bulimia nervosa	6.03 (3.66 to 9.16)	5.93 (3.69 to 8.9)	5.58 (3.44 to 8.35)	26.6 (16.2 to 40.2)	27.9 (17.3 to 42.3)	32.4 (20.2 to 48.9)
Romania	Autism spectrum disorders	14.9 (10.1 to 20.3)	13.1 (8.85 to 18)	11.8 (8.03 to 16.3)	65.2 (44 to 89.2)	65.6 (44.2 to 90.6)	65.9 (44.7 to 91.5)
Romania	Attention-deficit/hyperactivity disorder	2.61 (1.56 to 4.14)	2.11 (1.27 to 3.33)	1.78 (1.07 to 2.85)	11.7 (7 to 18.7)	11.7 (7 to 18.8)	11.8 (7.1 to 18.9)
Romania	Conduct disorder	21.5 (13.1 to 34)	14.3 (8.5 to 22.4)	11.7 (6.99 to 18.5)	97.2 (58.8 to 153.3)	97.5 (57.7 to 153.2)	97.7 (58.4 to 154)
Romania	Idiopathic developmental intellectual disability	4.75 (1.42 to 9.04)	4.16 (1.46 to 7.66)	2.74 (0.693 to 5.36)	21.1 (6.4 to 39.9)	21.3 (7.4 to 39)	16.1 (4.2 to 31.6)
Romania	Other mental disorders	34.7 (23.2 to 47.8)	35.5 (23.7 to 49.4)	33.3 (22.1 to 45.9)	138.2 (92.2 to 190.1)	139.2 (92.6 to 194.1)	139.4 (92.1 to 193.4)
Romania	Substance use disorders	131 (110 to 155)	99.4 (80.5 to 121)	86.3 (69.7 to 104)	523.2 (436.3 to 619.1)	399.7 (318.6 to 490.9)	382 (302.2 to 469.5)
Romania	Alcohol use disorders	100 (84.6 to 119)	67 (54.4 to 82.8)	55.3 (44.3 to 69.8)	395.9 (333.3 to 470.7)	264.9 (212.1 to 330.5)	238.5 (186 to 303.5)
Romania	Drug use disorders	30.5 (24.2 to 37.8)	32.4 (25.7 to 39.9)	31 (24.7 to 37.5)	127.3 (100.6 to 158.2)	134.7 (106.1 to 167.7)	143.6 (112.1 to 176.3)
Romania	Opioid use disorders	19.5 (14.8 to 24.8)	22.6 (17.4 to 28.6)	22.2 (17.4 to 27.5)	81.3 (60.8 to 103.9)	92.6 (70.2 to 117.9)	100.4 (77 to 127.5)
Romania	Cocaine use disorders	1.63 (1.02 to 2.4)	1.58 (0.997 to 2.34)	1.37 (0.878 to 2.01)	6.8 (4.3 to 10.1)	6.9 (4.2 to 10.3)	7 (4.4 to 10.3)
Romania	Amphetamine use disorders	1.98 (1.3 to 2.86)	1.48 (0.95 to 2.19)	1.15 (0.763 to 1.69)	8.8 (5.7 to 12.6)	7.1 (4.4 to 10.6)	7 (4.4 to 10.6)
Romania	Cannabis use disorders	1.18 (0.722 to 1.73)	0.945 (0.584 to 1.41)	0.77 (0.5 to 1.14)	5.1 (3.1 to 7.5)	4.5 (2.7 to 6.7)	4.6 (2.9 to 6.9)
Romania	Other drug use disorders	6.19 (5.13 to 7.47)	5.83 (4.97 to 6.91)	5.5 (4.73 to 6.39)	25.3 (21 to 30.8)	23.7 (19.9 to 28.4)	24.6 (20.6 to 29.5)
Romania	Diabetes and kidney diseases	244 (208 to 289)	231 (190 to 282)	253 (204 to 309)	892.7 (762.2 to 1054.3)	752.5 (615.6 to 922.7)	782.1 (626.3 to 963.7)
Romania	Diabetes mellitus	148 (116 to 188)	163 (126 to 210)	177 (133 to 228)	530.2 (416.3 to 674.8)	529.6 (407.5 to 690.3)	552.6 (409.5 to 720.1)
Romania	Diabetes mellitus type 1	29 (26.6 to 32.1)	19.2 (17.6 to 20.8)	15.8 (14.2 to 17.6)	105.7 (97.2 to 116.6)	65.7 (60.2 to 71.6)	54.5 (48.4 to 61.2)
Romania	Diabetes mellitus type 2	119 (88.3 to 158)	144 (108 to 190)	161 (118 to 211)	424.5 (314.2 to 564)	463.9 (346.1 to 618.5)	498.1 (358.3 to 659.3)
Romania	Chronic kidney disease	95.8 (90.1 to 102)	67.7 (62.3 to 73.4)	75.9 (69.2 to 82.3)	359.9 (339.6 to 382.4)	222.3 (204.8 to 240.2)	229.1 (209 to 248.7)
Romania	Chronic kidney disease due to diabetes mellitus type 1	5.48 (4.32 to 6.93)	3.23 (2.57 to 4.04)	3.39 (2.71 to 4.2)	20.5 (16.2 to 25.7)	11.4 (9.2 to 14.1)	11.7 (9.4 to 14.3)
Romania	Chronic kidney disease due to diabetes mellitus type 2	12.5 (10.3 to 15.2)	10.2 (8.38 to 12.2)	12.3 (10.3 to 14.6)	43.5 (36.5 to 52.8)	30.1 (24.8 to 35.7)	33.2 (27.9 to 39)
Romania	Chronic kidney disease due to hypertension	12 (10.1 to 14.2)	9.59 (8.1 to 11.1)	11.5 (8.89 to 13.6)	45 (38 to 52.7)	29.7 (25.3 to 34.3)	32 (24.9 to 37.7)
Romania	Chronic kidney disease due to glomerulonephritis	29.3 (25.9 to 32.9)	18.5 (16.3 to 20.8)	18.7 (16 to 21.5)	111.5 (99.2 to 124.5)	63.8 (57.2 to 70.8)	60.8 (53.1 to 68.7)
Romania	Chronic kidney disease due to other and unspecified causes	36.6 (31.9 to 41.8)	26.2 (22.6 to 30.4)	29.9 (26 to 34.2)	139.4 (122 to 158.2)	87.2 (76.2 to 100.3)	91.4 (80.4 to 104)
Romania	Acute glomerulonephritis	0.643 (0.523 to 0.768)	0.169 (0.152 to 0.188)	0.102 (0.0891 to 0.118)	2.5 (2.1 to 3)	0.7 (0.6 to 0.7)	0.4 (0.3 to 0.5)
Romania	Skin and subcutaneous diseases	115 (78.1 to 166)	103 (69.9 to 149)	97 (66.3 to 141)	498 (338 to 720.5)	499.7 (335.4 to 723.8)	505.4 (339.4 to 733.1)
Romania	Dermatitis	29.5 (17.2 to 46.3)	24.8 (14.6 to 38.7)	22.6 (13.4 to 35.1)	131.9 (76 to 208.8)	132.8 (76.8 to 209.8)	132.2 (76.5 to 210.3)
Romania	Atopic dermatitis	23.8 (13 to 39.6)	19.3 (10.6 to 32.2)	17.4 (9.54 to 29.1)	108.9 (59 to 182.1)	109.8 (59.9 to 184.4)	109.1 (59.3 to 184.1)
Romania	Contact dermatitis	5.69 (3.8 to 8.29)	5.52 (3.64 to 8.15)	5.15 (3.38 to 7.52)	22.9 (15.2 to 33.4)	22.9 (14.9 to 34)	23 (14.9 to 34)
Romania	Seborrhoeic dermatitis	0.0255 (0.0144 to 0.0408)	0.0236 (0.0133 to 0.0378)	0.022 (0.0123 to 0.0353)	0.1 (0.1 to 0.2)	0.1 (0.1 to 0.2)	0.1 (0.1 to 0.2)
Romania	Psoriasis	17.7 (12.4 to 23.5)	18.3 (13 to 24.2)	17.6 (12.6 to 23.1)	69.6 (48.6 to 92.3)	73.2 (51.8 to 96.5)	73.7 (52.3 to 97.7)
Romania	Bacterial skin diseases	3.96 (1.95 to 4.93)	2.35 (1.67 to 4.68)	2.61 (1.89 to 5.13)	17.8 (8.1 to 22.8)	10 (7.4 to 18.2)	10.9 (7.7 to 19.4)
Romania	Cellulitis	1.54 (0.849 to 2.02)	1.07 (0.753 to 1.99)	1.16 (0.795 to 2.29)	6.5 (3.5 to 8.7)	4.5 (3.3 to 7.5)	4.8 (3.5 to 8.1)
Romania	Pyoderma	2.43 (1 to 3.12)	1.28 (0.85 to 2.85)	1.45 (0.867 to 2.99)	11.3 (4.1 to 15.1)	5.5 (3.7 to 11)	6.1 (3.5 to 11.2)
Romania	Scabies	6.65 (3.69 to 10.8)	5.2 (2.89 to 8.42)	4.43 (2.47 to 7.18)	29.5 (16.4 to 47.8)	27.4 (15.3 to 44.5)	26.8 (15 to 43.1)
Romania	Fungal skin diseases	11.5 (4.64 to 23.8)	11.3 (4.54 to 23.6)	11.4 (4.54 to 23.5)	47.6 (19.3 to 99.1)	45.4 (18.1 to 93.4)	44.5 (17.7 to 91.9)
Romania	Viral skin diseases	9.31 (5.94 to 13.7)	6.66 (4.29 to 9.9)	5.9 (3.81 to 8.79)	45.6 (29.1 to 67.1)	45.6 (29.4 to 67.8)	45.7 (29.2 to 68.2)
Romania	Acne vulgaris	6.2 (3.64 to 10.1)	6.47 (3.85 to 10.6)	5.38 (3.18 to 8.72)	27 (15.8 to 43.9)	34.4 (20.2 to 56.7)	37.8 (22.2 to 61.6)
Romania	Alopecia areata	1.41 (0.902 to 2.12)	1.35 (0.866 to 2.01)	1.21 (0.765 to 1.8)	5.9 (3.8 to 8.9)	5.9 (3.7 to 8.8)	5.9 (3.7 to 8.7)

SR Appendix Table 2. All-age DALYs counts and age standardized DALYs rates per 100,000 individuals for all causes and level 1 cause groups by location for 1990, 2007, and 2017, both sexes combined

		All-age DALYs			Age-standardized rate of DALYs per 100,000		
Romania	Pruritus	2.35 (1.11 to 4.41)	2.35 (1.12 to 4.37)	2.31 (1.12 to 4.28)	9.4 (4.5 to 18)	9.6 (4.5 to 18.1)	9.8 (4.6 to 18.4)
Romania	Urticaria	16.2 (10.8 to 22.9)	13.4 (8.89 to 18.7)	12.1 (7.98 to 16.9)	74 (48.8 to 105)	74.2 (49.4 to 104.5)	74.2 (49 to 104.5)
Romania	Decubitus ulcer	0.985 (0.695 to 1.54)	1.2 (0.861 to 1.81)	1.54 (1.08 to 2.23)	3.9 (2.7 to 5.9)	4.2 (3 to 6.1)	4.9 (3.4 to 7)
Romania	Other skin and subcutaneous diseases	9.18 (4.61 to 16.7)	9.66 (4.76 to 17.6)	10.1 (5 to 18.5)	35.9 (17.9 to 65.4)	37 (18.3 to 67.6)	39.1 (19.5 to 71.9)
Romania	Sense organ diseases	198 (133 to 286)	216 (145 to 311)	224 (150 to 321)	753.1 (506 to 1086.9)	736.6 (491 to 1064.2)	720.4 (479.6 to 1049.5)
Romania	Blindness and vision impairment	81.9 (53.7 to 122)	85.9 (56.3 to 127)	87.5 (57.7 to 128)	310.1 (202.3 to 462.8)	295.3 (191.8 to 440.7)	285 (183.5 to 423.5)
Romania	Glaucoma	1.13 (0.74 to 1.58)	1.23 (0.812 to 1.73)	1.27 (0.835 to 1.79)	4 (2.6 to 5.6)	3.5 (2.3 to 5)	3.2 (2.1 to 4.6)
Romania	Cataract	27.1 (18.2 to 37.1)	32.3 (21.7 to 44.1)	35.4 (23.6 to 48.5)	99.4 (67.6 to 136.2)	95.6 (64.5 to 131.3)	92.5 (61.8 to 126.5)
Romania	Age-related macular degeneration	0.252 (0.158 to 0.377)	0.309 (0.193 to 0.458)	0.345 (0.216 to 0.518)	0.9 (0.6 to 1.3)	0.9 (0.6 to 1.3)	0.9 (0.6 to 1.3)
Romania	Refraction disorders	26.8 (17.1 to 40.5)	24.8 (15.7 to 37.9)	23 (14.5 to 34.9)	107.7 (68 to 164.2)	103.2 (65.7 to 157.8)	99.6 (62.3 to 152.3)
Romania	Near vision loss	20.2 (9.79 to 37.3)	20.4 (9.79 to 37.6)	20.4 (9.89 to 37.1)	73.7 (35.6 to 136.5)	68.9 (33 to 126.7)	66.5 (32 to 120.9)
Romania	Other vision loss	6.58 (4.32 to 9.03)	6.86 (4.62 to 9.6)	6.98 (4.7 to 9.62)	24.5 (16.2 to 33.9)	23.1 (15.5 to 32.1)	22.3 (15 to 30.9)
Romania	Age-related and other hearing loss	108 (73.3 to 154)	121 (82.6 to 171)	127 (87.1 to 180)	412.3 (282.3 to 580.6)	410 (280.1 to 579.3)	403.9 (273.7 to 573.5)
Romania	Other sense organ diseases	8.17 (5.12 to 11.8)	9.01 (5.64 to 13)	9.39 (5.82 to 13.6)	30.7 (19.3 to 44.6)	31.3 (19.5 to 45.6)	31.5 (19.3 to 45.9)
Romania	Musculoskeletal disorders	482 (350 to 647)	503 (363 to 677)	501 (363 to 671)	1821.5 (1312.2 to 2451.2)	1805.4 (1301.4 to 2459.5)	1798.2 (1303 to 2451.3)
Romania	Rheumatoid arthritis	6.77 (4.62 to 9.15)	7.9 (5.42 to 10.6)	9.03 (6.3 to 12)	24.6 (16.8 to 33.3)	26.7 (18.2 to 35.7)	29.6 (20.5 to 39.5)
Romania	Osteoarthritis	25 (12.6 to 49.7)	31.1 (15.7 to 61.4)	34.6 (17.3 to 67.9)	88.1 (44.4 to 174.6)	95.7 (48.3 to 188.1)	99 (49.4 to 196.8)
Romania	Low back pain	366 (262 to 492)	372 (267 to 500)	365 (262 to 490)	1394.9 (996 to 1868.3)	1360.2 (977 to 1823.3)	1346 (963.8 to 1810)
Romania	Neck pain	78.8 (55.1 to 111)	84.3 (58.6 to 119)	84.5 (59.3 to 118)	294.3 (205.9 to 414.1)	296.4 (207 to 412.7)	296.2 (208.8 to 412.5)
Romania	Gout	3.21 (2.12 to 4.45)	3.9 (2.65 to 5.33)	4.2 (2.88 to 5.75)	11.5 (7.5 to 16)	12.4 (8.4 to 17)	12.7 (8.6 to 17.6)
Romania	Other musculoskeletal disorders	2.05 (1.22 to 4.27)	3.58 (1.56 to 6.74)	3.8 (1.69 to 7.25)	8.2 (4.8 to 17.4)	13.9 (5.6 to 26.3)	14.8 (5.6 to 28.7)
Romania	Other non-communicable diseases	403 (326 to 484)	262 (201 to 339)	220 (163 to 291)	2012.5 (1650.3 to 2345.2)	1392.2 (1140.1 to 1697.7)	1145.4 (896.4 to 1456.4)
Romania	Congenital anomalies	229 (179 to 257)	107 (89.6 to 123)	73 (59.5 to 88.1)	1320.6 (1018.4 to 1475.3)	792.1 (671.8 to 888)	574.8 (476.5 to 682.1)
Romania	Neural tube defects	19.8 (12.5 to 34.1)	4.95 (3.98 to 6.36)	3.55 (2.78 to 4.45)	120.4 (74.7 to 208.2)	40 (31.9 to 52.7)	31.6 (24.7 to 41.2)
Romania	Congenital heart anomalies	88.2 (56.7 to 107)	32.6 (24.7 to 36.5)	17.3 (13.8 to 20.9)	536.4 (343.7 to 651)	293.3 (217.4 to 330.1)	182.6 (142.1 to 225.2)
Romania	Orofacial clefts	1.11 (0.753 to 1.51)	0.669 (0.442 to 0.983)	0.584 (0.375 to 0.844)	5.6 (3.9 to 7.8)	3.6 (2.4 to 5.2)	3.4 (2.2 to 4.8)
Romania	Down's syndrome	2.93 (1.41 to 6.08)	1.47 (1.11 to 1.86)	1.23 (0.921 to 1.59)	15.8 (6.9 to 34.7)	9.3 (6.8 to 11.8)	8.6 (6.6 to 10.9)
Romania	Turner syndrome	0.0348 (0.017 to 0.0582)	0.0294 (0.0146 to 0.0477)	0.0237 (0.0117 to 0.0392)	0.2 (0.1 to 0.3)	0.2 (0.1 to 0.3)	0.1 (0.1 to 0.2)
Romania	Klinefelter syndrome	0.00618 (0.00281 to 0.0123)	0.00547 (0.00252 to 0.0108)	0.00464 (0.00209 to 0.00892)	0 (0 to 0.1)	0 (0 to 0.1)	0 (0 to 0.1)
Romania	Other chromosomal abnormalities	3.63 (2.08 to 8.91)	2.15 (1.55 to 3.58)	1.84 (1.32 to 2.74)	20.5 (11.3 to 52.8)	15.8 (11.3 to 28.9)	15 (11 to 24)
Romania	Congenital musculoskeletal and limb anomalies	29.4 (19.7 to 40.8)	26.8 (18 to 36.7)	24.8 (16.5 to 33.8)	126.2 (84.5 to 174.7)	125.3 (84 to 171.2)	125 (82.4 to 169.5)
Romania	Urogenital congenital anomalies	3.23 (2.04 to 7.67)	1.63 (1.17 to 2.93)	1.17 (0.832 to 1.78)	19.3 (12 to 47.5)	13.4 (9.3 to 25.7)	10.8 (7.3 to 17.4)
Romania	Digestive congenital anomalies	29.2 (20.4 to 42.8)	15.4 (12.2 to 18.8)	9.61 (7.33 to 12.1)	171.1 (117.9 to 254.6)	116.3 (92.5 to 143.5)	77.2 (61.6 to 94.8)
Romania	Other congenital anomalies	51.2 (29.5 to 69.5)	20.8 (16.3 to 24.6)	12.9 (10.7 to 15.4)	305 (170.4 to 417.8)	174.8 (132.8 to 207.8)	120.4 (98.3 to 144.4)
Romania	Urinary diseases and male infertility	31.9 (26.8 to 38.4)	23.3 (18 to 30.1)	24.5 (18.8 to 31.2)	115 (97.4 to 137.5)	75.8 (58.7 to 98.2)	76.5 (58.7 to 97.2)
Romania	Urinary tract infections	15.2 (14.4 to 16.2)	7.05 (6.7 to 7.45)	7.82 (7.29 to 8.37)	56.5 (53.3 to 60.4)	24.2 (22.8 to 25.8)	25.2 (23.4 to 27.3)
Romania	Urolithiasis	1.32 (0.906 to 1.81)	1.31 (0.907 to 1.82)	1.31 (0.904 to 1.81)	5 (3.5 to 6.8)	4.8 (3.3 to 6.6)	4.9 (3.3 to 6.7)
Romania	Benign prostatic hyperplasia	11.9 (7.62 to 17.4)	13.3 (8.55 to 19.2)	14.1 (9.09 to 20)	40 (25.7 to 58.4)	39.9 (25.6 to 58)	40.5 (26.2 to 57.5)
Romania	Male infertility	0.426 (0.171 to 0.886)	0.427 (0.17 to 0.899)	0.335 (0.132 to 0.711)	1.9 (0.8 to 3.9)	2 (0.8 to 4.2)	1.8 (0.7 to 3.9)
Romania	Other urinary diseases	3 (2.19 to 3.73)	1.18 (0.959 to 1.45)	0.912 (0.709 to 1.15)	11.6 (8.6 to 14.5)	5 (4 to 6.1)	4 (3.1 to 5)

SR Appendix Table 2. All-age DALYs counts and age standardized DALYs rates per 100,000 individuals for all causes and level 1 cause groups by location for 1990, 2007, and 2017, both sexes combined

		All-age DALYs			Age-standardized rate of DALYs per 100,000		
Romania	Gynecological diseases	31.7 (21.7 to 44.5)	30.1 (20.6 to 42.6)	26.3 (17.6 to 38.2)	134 (92.5 to 187.7)	130.7 (89.7 to 183.7)	127.5 (86.9 to 183.8)
Romania	Uterine fibroids	5 (2.96 to 8.22)	4.9 (2.8 to 8.42)	4.31 (2.38 to 7.47)	19.5 (11.4 to 32.3)	19.3 (11 to 32.8)	16.4 (9.1 to 27.9)
Romania	Polycystic ovarian syndrome	1.15 (0.506 to 2.21)	1.09 (0.485 to 2.11)	0.937 (0.418 to 1.8)	5 (2.2 to 9.6)	4.9 (2.2 to 9.5)	5 (2.2 to 9.6)
Romania	Female infertility	0.414 (0.137 to 0.952)	0.391 (0.126 to 0.886)	0.35 (0.113 to 0.81)	1.8 (0.6 to 4.2)	1.8 (0.6 to 4.1)	1.9 (0.6 to 4.4)
Romania	Endometriosis	7.37 (4.75 to 10.8)	6.56 (4.28 to 9.52)	5.38 (3.53 to 7.65)	32.5 (21 to 47)	29.8 (19.4 to 43.3)	29.6 (19.4 to 42.2)
Romania	Genital prolapse	1.04 (0.494 to 1.97)	0.935 (0.445 to 1.74)	1.05 (0.495 to 1.94)	3.7 (1.7 to 6.9)	3 (1.4 to 5.6)	3.2 (1.5 to 6)
Romania	Premenstrual syndrome	11.8 (7.56 to 17.6)	11.1 (7.12 to 16.5)	9.35 (6.06 to 14.1)	51.1 (32.8 to 76.2)	50.2 (32.1 to 74.6)	48.8 (31.4 to 73.4)
Romania	Other gynecological diseases	4.89 (3.33 to 6.9)	5.11 (3.41 to 7.5)	4.88 (3.19 to 7.37)	20.4 (13.8 to 28.8)	21.7 (14.5 to 31.9)	22.7 (14.8 to 33.8)
Romania	Hemoglobinopathies and hemolytic anaemias	11.5 (8.02 to 16.2)	8.45 (5.71 to 12.5)	7.09 (4.84 to 10.5)	48.3 (33.5 to 68)	38.4 (25.8 to 56.4)	33.7 (22.7 to 49.5)
Romania	Thalassemias	0.0877 (0.051 to 0.119)	0.0258 (0.0192 to 0.0332)	0.0171 (0.0132 to 0.0223)	0.5 (0.3 to 0.7)	0.2 (0.2 to 0.3)	0.2 (0.1 to 0.2)
Romania	Thalassaemias trait	1.09 (0.691 to 1.64)	0.891 (0.556 to 1.34)	0.732 (0.461 to 1.07)	4.6 (2.9 to 6.9)	4.1 (2.6 to 6.3)	3.5 (2.2 to 5.1)
Romania	Sickle cell disorders	0.416 (0.28 to 0.533)	0.206 (0.163 to 0.243)	0.156 (0.129 to 0.185)	2.1 (1.4 to 2.7)	1.3 (1.1 to 1.6)	1.2 (1 to 1.4)
Romania	Sickle cell trait	1.46 (0.916 to 2.26)	1.1 (0.667 to 1.7)	0.825 (0.498 to 1.26)	6.5 (4.1 to 10)	5.4 (3.3 to 8.3)	4.3 (2.5 to 6.6)
Romania	G6PD deficiency	0.314 (0.23 to 0.375)	0.231 (0.194 to 0.263)	0.158 (0.135 to 0.184)	1.3 (1 to 1.6)	1 (0.9 to 1.2)	0.8 (0.7 to 0.9)
Romania	G6PD trait	0.000814 (0.000542 to 0.00117)	0.000738 (0.000478 to 0.00105)	0.000563 (0.000366 to 0.000808)	0 (0 to 0)	0 (0 to 0)	0 (0 to 0)
Romania	Other hemoglobinopathies and hemolytic anaemias	8.18 (5.62 to 11.7)	6 (4.04 to 9)	5.2 (3.54 to 7.87)	33.3 (22.7 to 47.9)	26.3 (17.4 to 39.3)	23.9 (15.5 to 36.6)
Romania	Endocrine, metabolic, blood, and immune disorders	22.6 (16.6 to 28.6)	14.4 (10.7 to 19)	13.2 (10.1 to 17.1)	102.1 (71.5 to 127.6)	67.8 (50.9 to 90.6)	64.2 (48.7 to 84.3)
Romania	Oral disorders	74.4 (46 to 114)	78.6 (49.2 to 119)	74.9 (47.1 to 114)	280.2 (171.1 to 431.8)	277.5 (171.1 to 425.1)	261.4 (161 to 403.8)
Romania	Caries of deciduous teeth	0.782 (0.34 to 1.55)	0.437 (0.188 to 0.869)	0.396 (0.172 to 0.795)	4.3 (1.9 to 8.6)	3.9 (1.7 to 7.8)	3.9 (1.7 to 7.8)
Romania	Caries of permanent teeth	10.8 (4.78 to 20.4)	9.72 (4.33 to 18.2)	8.26 (3.67 to 15.6)	45 (19.9 to 84.7)	43.8 (19.4 to 81.8)	41.4 (18.3 to 77.8)
Romania	Periodontal disease	15.3 (6.16 to 31.8)	16.1 (6.41 to 33.2)	15.2 (5.98 to 30.8)	56.5 (22.5 to 117.6)	56 (22.3 to 116.6)	51.7 (20.5 to 106.2)
Romania	Edentulism and severe tooth loss	35 (23.3 to 49.5)	40.2 (26.9 to 56.7)	39.9 (26.5 to 56.4)	123.9 (82.4 to 175.5)	123.1 (82.2 to 173.7)	113.8 (76 to 161.8)
Romania	Other oral disorders	12.5 (7.77 to 18.4)	12.1 (7.6 to 17.8)	11.2 (7.02 to 16.5)	50.5 (31.5 to 75.3)	50.7 (31.8 to 74.4)	50.6 (31.7 to 74.9)
Romania	Sudden infant death syndrome	1.94 (1.11 to 4.58)	1.03 (0.748 to 1.32)	0.646 (0.44 to 0.896)	12.2 (7.1 to 28.8)	9.8 (7.2 to 12.7)	7.4 (5 to 10.2)
Romania	Injuries	1330 (1180 to 1510)	908 (789 to 1060)	782 (655 to 937)	5581.6 (4983.4 to 6291.8)	3806 (3351.6 to 4366.8)	3317.2 (2837.5 to 3909.3)
Romania	Transport injuries	320 (298 to 343)	211 (194 to 231)	157 (138 to 177)	1349.2 (1261.9 to 1441.1)	928.1 (862.5 to 1001.9)	734 (660.3 to 816.9)
Romania	Road injuries	287 (267 to 309)	186 (170 to 205)	140 (122 to 160)	1213.5 (1133.3 to 1299.3)	820.2 (757.3 to 888.8)	655.5 (585.3 to 736)
Romania	Pedestrian road injuries	113 (103 to 122)	57.8 (53.1 to 62.9)	37.7 (33.5 to 42.6)	481.4 (443.3 to 521.3)	258.2 (239.9 to 278.3)	172.8 (154.3 to 193.7)
Romania	Cyclist road injuries	22.1 (17.9 to 26.9)	19.3 (15.3 to 23.9)	18.9 (14.8 to 23.6)	88.7 (71.7 to 107.9)	78.1 (62.8 to 95.7)	78.3 (62.1 to 97.2)
Romania	Motorcyclist road injuries	25.5 (22.3 to 28.7)	18.7 (16.7 to 20.9)	14.2 (12.1 to 16.6)	108.9 (95.6 to 121.9)	85.1 (76.6 to 94.1)	73.1 (63.8 to 83.5)
Romania	Motor vehicle road injuries	118 (108 to 128)	84.9 (77.8 to 92.6)	65.2 (57.8 to 73.9)	499.2 (452.6 to 543)	377.2 (348.5 to 407.7)	316.7 (285.2 to 352.3)
Romania	Other road injuries	8.99 (7.69 to 10.3)	5.68 (4.97 to 6.59)	3.79 (3.18 to 4.57)	35.3 (30.7 to 40.2)	21.5 (18.9 to 24.9)	14.5 (12.3 to 17.5)
Romania	Other transport injuries	32.6 (30.6 to 34.6)	25.1 (23.6 to 26.9)	16.8 (15.2 to 18.6)	135.7 (127.5 to 144.1)	107.9 (101.5 to 115.2)	78.5 (71 to 87.2)
Romania	Unintentional injuries	844 (714 to 1000)	555 (454 to 682)	511 (405 to 644)	3554.5 (3060.3 to 4178.2)	2289.5 (1911.7 to 2773.8)	2070 (1669.9 to 2575.7)
Romania	Falls	431 (334 to 552)	310 (233 to 410)	311 (233 to 413)	1694.9 (1317.3 to 2159.3)	1163.2 (876.1 to 1530.7)	1155.1 (865 to 1527.5)
Romania	Drowning	94.3 (90.6 to 98.4)	46.8 (45.2 to 48.5)	29.4 (27.2 to 31.8)	428.3 (410.4 to 448.9)	240.8 (230.7 to 251.2)	160.8 (148 to 174.5)
Romania	Fire, heat, and hot substances	55.7 (49.1 to 63.8)	25.8 (22.2 to 30.8)	21.4 (17.9 to 26.1)	245.6 (218.2 to 278.8)	114.7 (100 to 134)	93.5 (79.3 to 112.1)
Romania	Poisonings	64.4 (58.1 to 71)	26 (23.8 to 28.8)	16.6 (14.5 to 19)	298.9 (268.5 to 331.4)	127.5 (116.9 to 141.2)	83.4 (73.1 to 95.3)
Romania	Poisoning by carbon monoxide	31 (28.3 to 34.1)	16.6 (15.9 to 17.4)	9.92 (9.2 to 10.7)	136.5 (124.2 to 150.6)	76.8 (72.9 to 81.1)	46.9 (43.2 to 50.8)
Romania	Poisoning by other means	33.4 (28.5 to 38.7)	9.4 (7.44 to 12)	6.68 (4.92 to 9.03)	162.4 (138.2 to 188.3)	50.7 (41.3 to 62.7)	36.5 (27.4 to 47.8)

SR Appendix Table 2. All-age DALYs counts and age standardized DALYs rates per 100,000 individuals for all causes and level 1 cause groups by location for 1990, 2007, and 2017, both sexes combined

		All-age DALYs			Age-standardized rate of DALYs per 100,000		
Romania	Exposure to mechanical forces	78.3 (62.4 to 98.2)	57.8 (44.3 to 75.9)	57.8 (42.9 to 77.9)	317.3 (256.3 to 393.7)	230.6 (179.9 to 297.9)	235.1 (176.6 to 311.7)
Romania	Unintentional firearm injuries	5.82 (5.23 to 6.57)	2.19 (1.91 to 2.68)	1.99 (1.7 to 2.43)	25.4 (22.9 to 28.6)	9.8 (8.6 to 12.1)	9.6 (8.3 to 11.8)
Romania	Other exposure to mechanical forces	72.5 (56.9 to 91.9)	55.6 (42.3 to 73.1)	55.9 (41.1 to 75.5)	291.9 (231.5 to 367.5)	220.8 (170.2 to 286.1)	225.4 (167.7 to 300.6)
Romania	Adverse effects of medical treatment	8.1 (6.54 to 8.74)	6.27 (5.71 to 7.76)	6.15 (5.48 to 7.71)	35.5 (27.5 to 38.5)	26.9 (23.8 to 31.6)	26.1 (22.8 to 31.3)
Romania	Animal contact	5.16 (4.11 to 6.41)	4.79 (3.88 to 5.88)	4.14 (3.37 to 5.05)	21.7 (17.4 to 26.8)	20.9 (16.9 to 25.6)	18.6 (15 to 22.8)
Romania	Venomous animal contact	2.56 (1.8 to 3.51)	2.35 (1.69 to 3.16)	2.04 (1.49 to 2.71)	10.6 (7.5 to 14.6)	10.4 (7.4 to 13.9)	9.5 (6.9 to 12.7)
Romania	Non-venomous animal contact	2.6 (2.15 to 3.18)	2.43 (2.02 to 2.96)	2.1 (1.75 to 2.53)	11.1 (9.2 to 13.5)	10.5 (8.8 to 12.6)	9.1 (7.6 to 10.9)
Romania	Foreign body	58.4 (54.3 to 62.8)	31.4 (29.8 to 33.1)	24.8 (22.9 to 26.9)	310.2 (285.8 to 336.6)	171.7 (160.6 to 182.9)	132.2 (120.6 to 144.7)
Romania	Pulmonary aspiration and foreign body in airway	55.3 (51.4 to 59.6)	28.7 (27.3 to 30.1)	22.1 (20.5 to 23.7)	297.3 (273.6 to 323.5)	160.1 (149.7 to 170.9)	120 (109.8 to 131.2)
Romania	Foreign body in eyes	0.489 (0.235 to 0.835)	0.52 (0.259 to 0.897)	0.553 (0.285 to 0.933)	2 (1 to 3.5)	2.2 (1 to 3.9)	2.5 (1.2 to 4.4)
Romania	Foreign body in other body part	2.61 (2.03 to 3.27)	2.19 (1.66 to 2.81)	2.17 (1.66 to 2.77)	10.9 (8.5 to 13.7)	9.3 (7.2 to 11.8)	9.7 (7.4 to 12.2)
Romania	Environmental heat and cold exposure	18.9 (16.9 to 20.9)	18.3 (16.7 to 20)	14.3 (12.7 to 16.2)	76.1 (68.4 to 84.3)	71.1 (64.9 to 78.1)	56.4 (49.7 to 64)
Romania	Exposure to forces of nature	0.627 (0.625 to 0.63)	0.594 (0.584 to 0.607)	0.387 (0.379 to 0.397)	2.8 (2.7 to 2.8)	2.9 (2.9 to 3)	2.2 (2.1 to 2.2)
Romania	Other unintentional injuries	28.8 (25 to 34)	27 (22.8 to 32.7)	24.2 (19.1 to 31.5)	123.4 (108.3 to 143.8)	119.1 (102.8 to 140.9)	106.5 (86.2 to 135)
Romania	Self-harm and interpersonal violence	165 (159 to 171)	142 (137 to 147)	114 (106 to 122)	677.8 (654.2 to 703.1)	588.3 (568 to 609)	513.2 (478.7 to 547.4)
Romania	Self-harm	102 (98.8 to 106)	107 (104 to 111)	87.9 (81.4 to 94.3)	415 (399.9 to 430.4)	440.2 (425.9 to 453.7)	392.5 (363.5 to 421.1)
Romania	Self-harm by firearm	1.29 (0.868 to 2.11)	0.883 (0.616 to 1.36)	0.632 (0.42 to 0.951)	5.4 (3.6 to 8.9)	4 (2.7 to 6.1)	3.2 (2.1 to 4.8)
Romania	Self-harm by other specified means	101 (97.5 to 105)	106 (103 to 110)	87.3 (80.7 to 93.7)	409.6 (394.5 to 424.7)	436.3 (422.2 to 450)	389.3 (360.3 to 418.9)
Romania	Interpersonal violence	61.9 (58.1 to 66.2)	34.1 (31.3 to 37.3)	26 (23.2 to 29.5)	260.8 (245 to 278)	146.6 (135.4 to 159.6)	119.3 (106.8 to 133.6)
Romania	Assault by firearm	2.42 (1.92 to 3.05)	1.2 (1.03 to 1.47)	0.846 (0.715 to 1.16)	10.5 (8.3 to 13.2)	5.4 (4.7 to 6.6)	4.2 (3.6 to 5.7)
Romania	Assault by sharp object	21.9 (20 to 24.8)	11.3 (10.6 to 12.2)	7.47 (6.78 to 8.26)	92.6 (84.8 to 104.3)	49.2 (46.1 to 52.6)	35.7 (32.2 to 39.6)
Romania	Sexual violence	3.45 (2.34 to 5.02)	2.37 (1.63 to 3.38)	2.16 (1.46 to 3.12)	14.6 (9.9 to 21.4)	10.7 (7.3 to 15.6)	11.2 (7.6 to 16.3)
Romania	Assault by other means	34.2 (30 to 38)	19.3 (16.9 to 22)	15.6 (13.2 to 18.5)	143.1 (126.2 to 158)	81.3 (72 to 91.6)	68.2 (58.4 to 80.2)
Romania	Conflict and terrorism	0.275 (0.183 to 0.443)	0.177 (0.119 to 0.278)	0.137 (0.0935 to 0.211)	1.2 (0.8 to 2)	0.7 (0.5 to 1.2)	0.5 (0.4 to 0.8)
Romania	Executions and police conflict	0.17 (0.156 to 0.189)	0.14 (0.13 to 0.153)	0.144 (0.129 to 0.165)	0.7 (0.7 to 0.8)	0.7 (0.7 to 0.8)	0.8 (0.7 to 0.9)
Serbia	All causes	3520 (3230 to 3860)	3470 (3170 to 3810)	3280 (2960 to 3660)	35076.3 (32213.1 to 38317.3)	28444.2 (25746.6 to 31681.3)	25468.4 (22682.7 to 28805.9)
Serbia	Communicable, maternal, neonatal, and nutritional disorders	349 (314 to 383)	168 (145 to 193)	145 (123 to 168)	4848.4 (4387.2 to 5311)	2332 (2039 to 2657.3)	1939.4 (1663.2 to 2232)
Serbia	HIV/AIDS and sexually transmitted infections	3.68 (1.41 to 5.85)	4.69 (3.49 to 6.97)	5.06 (3.11 to 8.53)	40.7 (15.3 to 64.3)	53.1 (40.4 to 77)	57.1 (36.2 to 93.3)
Serbia	HIV/AIDS	1.71 (0.0408 to 2.82)	3 (2.39 to 4.87)	3.51 (1.75 to 6.75)	18.7 (0.4 to 30.2)	34.6 (27.9 to 54.6)	39.4 (21 to 74.6)
Serbia	HIV/AIDS - Drug-susceptible Tuberculosis	0.701 (0.032 to 1.19)	1.08 (0.797 to 1.51)	0.75 (0.474 to 1.1)	7.7 (0.3 to 12.9)	12.6 (9.2 to 17.2)	8.5 (5.3 to 12.6)
Serbia	HIV/AIDS - Multidrug-resistant Tuberculosis without extensive drug resistance	0.00428 (0.0000236 to 0.0164)	0.0376 (0.0243 to 0.0581)	0.0183 (0.00573 to 0.0436)	0 (0 to 0.2)	0.4 (0.3 to 0.7)	0.2 (0.1 to 0.5)
Serbia	HIV/AIDS - Extensively drug-resistant Tuberculosis	0 (0 to 0)	0.0119 (0.00756 to 0.0182)	0.0104 (0.00329 to 0.0243)	0 (0 to 0)	0.1 (0.1 to 0.2)	0.1 (0 to 0.3)
Serbia	HIV/AIDS resulting in other diseases	1 (0 to 1.93)	1.86 (1.35 to 3.6)	2.73 (1.1 to 6.03)	11 (0 to 20.5)	21.4 (15.8 to 40.3)	30.6 (13.3 to 64.8)
Serbia	Sexually transmitted infections excluding HIV	1.97 (1.13 to 3.57)	1.69 (0.92 to 3.09)	1.56 (0.838 to 2.83)	22 (12.5 to 39.4)	18.5 (10.3 to 33.8)	17.7 (9.5 to 32.2)
Serbia	Syphilis	0.371 (0.171 to 0.801)	0.148 (0.0984 to 0.26)	0.122 (0.0853 to 0.196)	5.3 (2.1 to 12.3)	2.1 (1.1 to 4.6)	1.7 (1 to 3.5)
Serbia	Chlamydial infection	0.348 (0.185 to 0.672)	0.331 (0.178 to 0.644)	0.309 (0.162 to 0.599)	3.7 (2 to 7.2)	3.7 (1.9 to 7.2)	3.6 (1.9 to 7)
Serbia	Gonococcal infection	0.299 (0.166 to 0.562)	0.277 (0.148 to 0.527)	0.239 (0.127 to 0.453)	3.3 (1.8 to 6.3)	3.2 (1.7 to 6.2)	3 (1.5 to 5.8)
Serbia	Trichomoniasis	0.297 (0.12 to 0.631)	0.301 (0.122 to 0.643)	0.284 (0.115 to 0.594)	3 (1.2 to 6.4)	3 (1.2 to 6.4)	3 (1.2 to 6.3)
Serbia	Genital herpes	0.222 (0.071 to 0.528)	0.232 (0.0737 to 0.548)	0.231 (0.074 to 0.548)	2.1 (0.7 to 4.9)	2.1 (0.7 to 4.9)	2.1 (0.7 to 4.9)
Serbia	Other sexually transmitted infections	0.434 (0.256 to 0.754)	0.405 (0.237 to 0.724)	0.372 (0.219 to 0.657)	4.6 (2.7 to 8.1)	4.5 (2.6 to 8.2)	4.4 (2.5 to 7.9)

SR Appendix Table 2. All-age DALYs counts and age standardized DALYs rates per 100,000 individuals for all causes and level 1 cause groups by location for 1990, 2007, and 2017, both sexes combined

		All-age DALYs			Age-standardized rate of DALYs per 100,000		
Serbia	Respiratory infections and tuberculosis	65 (57.3 to 74.1)	37.3 (33.4 to 43.4)	37.3 (31.3 to 42.6)	799.2 (690.5 to 922.8)	371 (324.7 to 436.6)	332.2 (281.6 to 391.7)
Serbia	Tuberculosis	9.58 (8.57 to 10.6)	6.68 (6.24 to 7.11)	3.5 (3.15 to 3.85)	86.7 (78 to 96.1)	52.6 (49 to 56.1)	26.9 (24.1 to 29.9)
Serbia	Latent tuberculosis infection	0 (0 to 0)	0 (0 to 0)	0 (0 to 0)	0 (0 to 0)	0 (0 to 0)	0 (0 to 0)
Serbia	Drug-susceptible tuberculosis	9.53 (8.54 to 10.6)	6.45 (6 to 6.86)	3.39 (3.05 to 3.76)	86.3 (77.6 to 95.7)	50.7 (47.2 to 54.2)	26.1 (23.3 to 29.1)
Serbia	Multidrug-resistant tuberculosis without extensive drug resistance	0.0496 (0.00884 to 0.17)	0.181 (0.13 to 0.238)	0.0663 (0.0234 to 0.149)	0.4 (0.1 to 1.6)	1.4 (1 to 1.9)	0.5 (0.2 to 1.1)
Serbia	Extensively drug-resistant tuberculosis	0 (0 to 0)	0.0565 (0.0405 to 0.0754)	0.0368 (0.0129 to 0.085)	0 (0 to 0)	0.4 (0.3 to 0.6)	0.3 (0.1 to 0.6)
Serbia	Lower respiratory infections	46.7 (40.5 to 53.7)	22.5 (20.9 to 27.8)	26.2 (20.8 to 28.9)	617.1 (519.6 to 721.8)	223.3 (203.8 to 269.6)	211.8 (176 to 232.1)
Serbia	Upper respiratory infections	6.47 (3.79 to 10.2)	6.13 (3.62 to 9.75)	5.84 (3.43 to 9.43)	70.6 (41.5 to 111)	70.3 (40.8 to 112.7)	70 (40.8 to 112.9)
Serbia	Otitis media	2.18 (1.3 to 3.42)	2.02 (1.2 to 3.21)	1.8 (1.07 to 2.86)	24.7 (14.5 to 39)	24.8 (14.7 to 39.4)	23.5 (13.8 to 37.7)
Serbia	Enteric infections	8.67 (6.48 to 11.4)	6.63 (4.83 to 8.74)	7.3 (5.26 to 9.8)	110 (82.4 to 145)	86 (62.5 to 112.8)	95.3 (69.1 to 126.7)
Serbia	Diarrhoeal diseases	8.13 (5.94 to 10.8)	6.34 (4.55 to 8.42)	7.09 (5.03 to 9.57)	103.5 (76.1 to 138.8)	82.5 (59.2 to 110.3)	92.7 (66.2 to 123.6)
Serbia	Typhoid and paratyphoid	0.0183 (0.008 to 0.0364)	0.0153 (0.00681 to 0.0302)	0.0124 (0.00554 to 0.0247)	0.2 (0.1 to 0.4)	0.2 (0.1 to 0.3)	0.1 (0.1 to 0.3)
Serbia	Typhoid fever	0.00345 (0.0016 to 0.00645)	0.00268 (0.00128 to 0.00501)	0.00231 (0.00107 to 0.0043)	0 (0 to 0.1)	0 (0 to 0.1)	0 (0 to 0.1)
Serbia	Paratyphoid fever	0.0149 (0.00597 to 0.0308)	0.0126 (0.00522 to 0.0257)	0.0101 (0.00412 to 0.0208)	0.2 (0.1 to 0.3)	0.1 (0.1 to 0.3)	0.1 (0 to 0.2)
Serbia	Invasive non-typhoidal salmonella	0.467 (0.253 to 0.773)	0.254 (0.132 to 0.43)	0.194 (0.101 to 0.334)	5.5 (3.1 to 9.4)	3.1 (1.6 to 5.5)	2.4 (1.2 to 4.2)
Serbia	Other intestinal infectious diseases	0.0559 (0.0176 to 0.115)	0.0171 (0.00966 to 0.0259)	0.011 (0.00626 to 0.0167)	0.8 (0.2 to 1.7)	0.2 (0.1 to 0.4)	0.1 (0.1 to 0.2)
Serbia	Neglected tropical diseases and malaria	3.91 (1.86 to 9.35)	3.11 (1.57 to 5.32)	2.97 (1.37 to 5.36)	38.1 (17.7 to 101.4)	26 (13 to 43.9)	24.2 (11.1 to 43.4)
Serbia	Malaria	0 (0 to 0)	0 (0 to 0)	0 (0 to 0)	0 (0 to 0)	0 (0 to 0)	0 (0 to 0)
Serbia	Chagas disease	0 (0 to 0)	0 (0 to 0)	0 (0 to 0)	0 (0 to 0)	0 (0 to 0)	0 (0 to 0)
Serbia	Leishmaniasis	0.575 (0.0011 to 5.71)	0.032 (0.000104 to 0.291)	0.00791 (0.0000224 to 0.0803)	6.9 (0 to 70.5)	0.4 (0 to 4)	0.1 (0 to 1.1)
Serbia	Visceral leishmaniasis	0.575 (0.0011 to 5.71)	0.032 (0.000104 to 0.291)	0.00791 (0.0000224 to 0.0803)	6.9 (0 to 70.5)	0.4 (0 to 4)	0.1 (0 to 1.1)
Serbia	Cutaneous and mucocutaneous leishmaniasis	0 (0 to 0)	0 (0 to 0)	0 (0 to 0)	0 (0 to 0)	0 (0 to 0)	0 (0 to 0)
Serbia	African trypanosomiasis	0 (0 to 0)	0 (0 to 0)	0 (0 to 0)	0 (0 to 0)	0 (0 to 0)	0 (0 to 0)
Serbia	Schistosomiasis	0 (0 to 0)	0 (0 to 0)	0 (0 to 0)	0 (0 to 0)	0 (0 to 0)	0 (0 to 0)
Serbia	Cysticercosis	3.12 (1.54 to 5.36)	2.92 (1.36 to 5.15)	2.81 (1.23 to 5.18)	29 (14.3 to 49.6)	24.1 (11.2 to 42.2)	22.7 (9.9 to 41.7)
Serbia	Cystic echinococcosis	0.146 (0.1 to 0.194)	0.114 (0.0812 to 0.155)	0.0955 (0.0669 to 0.131)	1.4 (1 to 1.8)	1 (0.7 to 1.4)	0.9 (0.6 to 1.2)
Serbia	Lymphatic filariasis	0 (0 to 0)	0 (0 to 0)	0 (0 to 0)	0 (0 to 0)	0 (0 to 0)	0 (0 to 0)
Serbia	Onchocerciasis	0 (0 to 0)	0 (0 to 0)	0 (0 to 0)	0 (0 to 0)	0 (0 to 0)	0 (0 to 0)
Serbia	Trachoma	0 (0 to 0)	0 (0 to 0)	0 (0 to 0)	0 (0 to 0)	0 (0 to 0)	0 (0 to 0)
Serbia	Dengue	0 (0 to 0)	0 (0 to 0)	0 (0 to 0)	0 (0 to 0)	0 (0 to 0)	0 (0 to 0)
Serbia	Yellow fever	0 (0 to 0)	0 (0 to 0)	0 (0 to 0)	0 (0 to 0)	0 (0 to 0)	0 (0 to 0)
Serbia	Rabies	0.00186 (0.00118 to 0.00272)	0.000627 (0.000369 to 0.000849)	0.000585 (0.000381 to 0.000837)	0 (0 to 0)	0 (0 to 0)	0 (0 to 0)
Serbia	Intestinal nematode infections	0.00608 (0.00183 to 0.014)	0.00131 (0.000618 to 0.00226)	0.000743 (0.000366 to 0.00125)	0.1 (0 to 0.2)	0 (0 to 0)	0 (0 to 0)
Serbia	Ascariasis	0.00608 (0.00183 to 0.014)	0.00131 (0.000618 to 0.00226)	0.000743 (0.000366 to 0.00125)	0.1 (0 to 0.2)	0 (0 to 0)	0 (0 to 0)
Serbia	Trichuriasis	0 (0 to 0)	0 (0 to 0)	0 (0 to 0)	0 (0 to 0)	0 (0 to 0)	0 (0 to 0)
Serbia	Hookworm disease	0 (0 to 0)	0 (0 to 0)	0 (0 to 0)	0 (0 to 0)	0 (0 to 0)	0 (0 to 0)
Serbia	Food-borne trematodiases	0 (0 to 0)	0 (0 to 0)	0 (0 to 0)	0 (0 to 0)	0 (0 to 0)	0 (0 to 0)
Serbia	Leprosy	0 (0 to 0)	0 (0 to 0)	0 (0 to 0)	0 (0 to 0)	0 (0 to 0)	0 (0 to 0)
Serbia	Ebola virus disease	0 (0 to 0)	0 (0 to 0)	0 (0 to 0)	0 (0 to 0)	0 (0 to 0)	0 (0 to 0)
Serbia	Zika virus disease	0 (0 to 0)	0 (0 to 0)	0 (0 to 0)	0 (0 to 0)	0 (0 to 0)	0 (0 to 0)

SR Appendix Table 2. All-age DALYs counts and age standardized DALYs rates per 100,000 individuals for all causes and level 1 cause groups by location for 1990, 2007, and 2017, both sexes combined

		All-age DALYs			Age-standardized rate of DALYs per 100,000		
Serbia	Guinea worm disease	0 (0 to 0)	0 (0 to 0)	0 (0 to 0)	0 (0 to 0)	0 (0 to 0)	0 (0 to 0)
Serbia	Other neglected tropical diseases	0.0588 (0.0339 to 0.091)	0.0439 (0.0203 to 0.0572)	0.0536 (0.0163 to 0.0769)	0.7 (0.4 to 1.2)	0.4 (0.2 to 0.6)	0.5 (0.2 to 0.7)
Serbia	Other infectious diseases	22.9 (17.4 to 32.1)	8.5 (7.45 to 10.1)	7.42 (6.26 to 9.06)	309.8 (226 to 454.8)	103.1 (86.5 to 133.9)	90.5 (71.1 to 123)
Serbia	Meningitis	8.13 (6.62 to 9.85)	2.08 (1.82 to 2.49)	1.35 (1.16 to 1.55)	117.3 (93.7 to 144)	28.7 (24.7 to 34.2)	17.6 (15 to 20.4)
Serbia	Pneumococcal meningitis	1.1 (0.868 to 1.36)	0.422 (0.348 to 0.518)	0.293 (0.241 to 0.356)	14.5 (11.2 to 18.5)	4.9 (4.1 to 6.1)	3.3 (2.7 to 4)
Serbia	H influenzae type B meningitis	2.94 (2.31 to 3.7)	0.592 (0.51 to 0.71)	0.332 (0.278 to 0.386)	44 (33.9 to 56.1)	9.3 (7.8 to 11.4)	5.1 (4.2 to 6.1)
Serbia	Meningococcal infection	1.44 (1.14 to 1.82)	0.347 (0.3 to 0.416)	0.216 (0.184 to 0.252)	21 (16.2 to 26.8)	4.9 (4.1 to 5.9)	2.9 (2.4 to 3.4)
Serbia	Other meningitis	2.64 (2.13 to 3.22)	0.723 (0.628 to 0.866)	0.507 (0.433 to 0.578)	37.8 (29.7 to 46.7)	9.6 (8.3 to 11.5)	6.4 (5.5 to 7.4)
Serbia	Encephalitis	4.29 (3.83 to 4.9)	3.11 (2.76 to 3.35)	2.88 (2.45 to 3.21)	47.1 (40.8 to 56.3)	31.8 (29.1 to 35)	27.8 (22.9 to 33.1)
Serbia	Diphtheria	0.0335 (0.0179 to 0.0557)	0.00651 (0.00385 to 0.00988)	0.0046 (0.00292 to 0.007)	0.5 (0.2 to 0.8)	0.1 (0.1 to 0.1)	0.1 (0 to 0.1)
Serbia	Whooping cough	5.44 (0.626 to 15.2)	0.665 (0.0776 to 2.08)	0.831 (0.105 to 2.28)	84.5 (9.7 to 235.7)	13.5 (1.6 to 42.5)	18.6 (2.3 to 51.1)
Serbia	Tetanus	0.213 (0.0994 to 0.319)	0.0994 (0.057 to 0.119)	0.0685 (0.0473 to 0.0883)	2.2 (1.1 to 3.2)	0.9 (0.7 to 1)	0.7 (0.6 to 0.8)
Serbia	Measles	1.04 (0.736 to 1.45)	0.0192 (0.0121 to 0.0282)	0.0476 (0.0302 to 0.0722)	15.9 (11.2 to 22.2)	0.4 (0.2 to 0.6)	1 (0.6 to 1.5)
Serbia	Varicella and herpes zoster	0.589 (0.43 to 0.794)	0.423 (0.298 to 0.577)	0.385 (0.264 to 0.535)	6.9 (4.8 to 9.4)	4.5 (3.2 to 6.1)	3.8 (2.6 to 5.2)
Serbia	Acute hepatitis	1.19 (0.904 to 1.47)	0.782 (0.619 to 1)	0.618 (0.47 to 0.811)	13 (9.9 to 16.3)	8.6 (6.7 to 11.2)	7.2 (5.3 to 9.6)
Serbia	Acute hepatitis A	0.526 (0.397 to 0.685)	0.356 (0.258 to 0.484)	0.293 (0.205 to 0.406)	6.3 (4.8 to 8.1)	4.6 (3.3 to 6.3)	4.1 (2.9 to 5.7)
Serbia	Acute hepatitis B	0.523 (0.379 to 0.657)	0.344 (0.269 to 0.426)	0.266 (0.204 to 0.346)	5.1 (3.6 to 6.5)	3.1 (2.4 to 3.9)	2.4 (1.8 to 3.2)
Serbia	Acute hepatitis C	0.0356 (0.0164 to 0.0668)	0.0121 (0.00674 to 0.0213)	0.00801 (0.00449 to 0.0141)	0.5 (0.2 to 1)	0.2 (0.1 to 0.3)	0.1 (0.1 to 0.2)
Serbia	Acute hepatitis E	0.107 (0.0785 to 0.138)	0.0697 (0.0547 to 0.0893)	0.0509 (0.0388 to 0.0669)	1.1 (0.8 to 1.5)	0.7 (0.6 to 0.9)	0.5 (0.4 to 0.7)
Serbia	Other unspecified infectious diseases	1.96 (1.53 to 2.5)	1.32 (1.05 to 1.69)	1.24 (0.975 to 1.6)	22.5 (17.4 to 29.2)	14.6 (11.6 to 18.6)	13.8 (10.6 to 17.9)
Serbia	Maternal and neonatal disorders	215 (189 to 243)	89.3 (75.1 to 106)	70 (56.4 to 85.7)	3205.6 (2831.1 to 3602.8)	1471.5 (1276.2 to 1698.5)	1153.2 (955.9 to 1358.1)
Serbia	Maternal disorders	2.1 (1.75 to 2.53)	1.08 (0.912 to 1.28)	0.74 (0.609 to 0.884)	23.5 (19.3 to 28.2)	12.5 (10.5 to 14.9)	9 (7.4 to 10.7)
Serbia	Maternal haemorrhage	0.591 (0.355 to 0.947)	0.218 (0.13 to 0.354)	0.119 (0.0696 to 0.193)	6.5 (3.9 to 10.4)	2.5 (1.5 to 4.2)	1.5 (0.8 to 2.4)
Serbia	Maternal sepsis and other pregnancy related infections	0.241 (0.163 to 0.338)	0.0751 (0.0553 to 0.0984)	0.0433 (0.03 to 0.0603)	2.7 (1.8 to 3.8)	0.9 (0.6 to 1.2)	0.5 (0.4 to 0.8)
Serbia	Maternal hypertensive disorders	0.157 (0.0843 to 0.276)	0.0803 (0.048 to 0.127)	0.0454 (0.0275 to 0.073)	1.7 (0.9 to 3)	0.9 (0.6 to 1.5)	0.5 (0.3 to 0.9)
Serbia	Maternal obstructed labour and uterine rupture	0.125 (0.071 to 0.195)	0.0908 (0.0507 to 0.142)	0.0765 (0.039 to 0.13)	1.4 (0.8 to 2.2)	1 (0.6 to 1.7)	0.9 (0.5 to 1.6)
Serbia	Maternal abortive outcome	0.188 (0.115 to 0.289)	0.0842 (0.0552 to 0.123)	0.0591 (0.0399 to 0.0857)	2 (1.2 to 3.1)	0.9 (0.6 to 1.3)	0.6 (0.4 to 0.9)
Serbia	Ectopic pregnancy	0.09 (0.0363 to 0.193)	0.0254 (0.0103 to 0.0523)	0.0134 (0.00576 to 0.0277)	1 (0.4 to 2.1)	0.3 (0.1 to 0.6)	0.2 (0.1 to 0.3)
Serbia	Indirect maternal deaths	0.0284 (0.00922 to 0.0671)	0.0727 (0.0309 to 0.139)	0.0844 (0.038 to 0.155)	0.3 (0.1 to 0.7)	0.9 (0.4 to 1.6)	1 (0.5 to 1.9)
Serbia	Late maternal deaths	0.052 (0.0353 to 0.0711)	0.0352 (0.0269 to 0.0464)	0.0289 (0.0199 to 0.0404)	0.6 (0.4 to 0.8)	0.4 (0.3 to 0.5)	0.4 (0.2 to 0.5)
Serbia	Maternal deaths aggravated by HIV/AIDS	0.0000522 (0 to 0.00012)	0.0000738 (0.0000371 to 0.000125)	0.0000454 (0.0000218 to 0.0000776)	0 (0 to 0)	0 (0 to 0)	0 (0 to 0)
Serbia	Other maternal disorders	0.632 (0.439 to 0.851)	0.395 (0.301 to 0.5)	0.27 (0.203 to 0.349)	7.2 (4.9 to 9.7)	4.6 (3.5 to 5.9)	3.3 (2.5 to 4.2)
Serbia	Neonatal disorders	213 (187 to 241)	88.3 (74 to 105)	69.3 (55.7 to 84.9)	3182.2 (2810 to 3577.4)	1459.1 (1263.6 to 1685.8)	1144.2 (947.3 to 1349)
Serbia	Neonatal preterm birth	104 (88.5 to 119)	30.8 (26.5 to 35.2)	21.1 (16.9 to 25.5)	1630.5 (1381.7 to 1876)	605 (516.7 to 693.1)	433.5 (344.3 to 520.7)
Serbia	Neonatal encephalopathy due to birth asphyxia and trauma	60.6 (46 to 76.3)	30 (23.1 to 39.4)	25.4 (18.3 to 34.5)	882.2 (653.7 to 1113.5)	451.1 (364.1 to 562)	370.9 (280 to 486.6)
Serbia	Neonatal sepsis and other neonatal infections	27.8 (18.5 to 40)	21.4 (13.7 to 31.7)	18.6 (11.3 to 28.2)	348.5 (239.1 to 485.4)	270.4 (180.7 to 389.4)	238.2 (148.3 to 352.3)
Serbia	Hemolytic disease and other neonatal jaundice	0.701 (0.514 to 0.935)	0.329 (0.253 to 0.41)	0.282 (0.214 to 0.363)	9.9 (7 to 13.7)	4.7 (3.7 to 5.8)	4.1 (3.2 to 5.2)
Serbia	Other neonatal disorders	19.2 (13.5 to 25.7)	5.77 (4.78 to 6.9)	3.98 (3.16 to 5.16)	311.1 (217.4 to 415.6)	127.9 (105.7 to 153.9)	97.5 (76.9 to 127.4)
Serbia	Nutritional deficiencies	30 (19.1 to 45.2)	18.4 (11.3 to 28.7)	14.8 (9.38 to 22.8)	345 (223.2 to 523.5)	221.3 (134.3 to 342.9)	186.8 (115.8 to 291.3)
Serbia	Protein-energy malnutrition	0.918 (0.565 to 1.45)	0.399 (0.293 to 0.571)	0.322 (0.255 to 0.464)	13.5 (8.3 to 21.5)	5.9 (4.1 to 8.9)	4.3 (3 to 6.8)

SR Appendix Table 2. All-age DALYs counts and age standardized DALYs rates per 100,000 individuals for all causes and level 1 cause groups by location for 1990, 2007, and 2017, both sexes combined							
		All-age DALYs			Age-standardized rate of DALYs per 100,000		
Serbia	Iodine deficiency	0.97 (0.501 to 1.57)	0.416 (0.202 to 0.786)	0.725 (0.354 to 1.2)	9.7 (5 to 15.7)	4 (1.9 to 7.5)	7.3 (3.6 to 12)
Serbia	Dietary iron deficiency	17.7 (10.9 to 26.7)	13.2 (8.22 to 20.1)	10.4 (6.41 to 16.4)	179 (110.3 to 272.7)	131.9 (81.9 to 202.7)	112.2 (69.1 to 177.2)
Serbia	Other nutritional deficiencies	0.273 (0.212 to 0.347)	0.174 (0.151 to 0.207)	0.167 (0.146 to 0.198)	3.3 (2.5 to 4.3)	1.7 (1.4 to 2)	1.4 (1.2 to 1.7)
Serbia	Non-communicable diseases	2760 (2540 to 3040)	2970 (2730 to 3260)	2830 (2570 to 3130)	26066.6 (23912.8 to 28647.5)	22995 (20925.1 to 25557.4)	20750.4 (18577.6 to 23308.3)
Serbia	Neoplasms	528 (504 to 557)	604 (585 to 617)	596 (559 to 630)	4484.3 (4294.9 to 4730.5)	4411.3 (4261.3 to 4509.3)	4098.3 (3824 to 4344.6)
Serbia	Lip and oral cavity cancer	10.1 (8.74 to 11.9)	9.45 (8.94 to 10)	9.14 (8.17 to 10.3)	82.1 (71.6 to 96.6)	69.5 (65.7 to 73.5)	65.2 (58.1 to 73.7)
Serbia	Nasopharynx cancer	1.53 (1.35 to 1.76)	1.56 (1.44 to 1.69)	1.35 (1.18 to 1.53)	13.2 (11.7 to 15)	12.5 (11.6 to 13.5)	10.8 (9.4 to 12.3)
Serbia	Other pharynx cancer	6.25 (4.96 to 7.66)	7.1 (5.57 to 7.8)	6.47 (5.31 to 7.52)	50.1 (39.9 to 61)	52.4 (41.3 to 57.5)	46.6 (38.3 to 54.1)
Serbia	Oesophageal cancer	8.39 (7.53 to 9.37)	9.72 (9.21 to 10.3)	9.18 (8.27 to 10.2)	67.5 (60.9 to 75.1)	69.3 (65.8 to 73.1)	62.9 (56.8 to 70)
Serbia	Stomach cancer	35.6 (31.7 to 40.4)	33.5 (32.1 to 35)	26.8 (24.8 to 29)	298.6 (267 to 335.7)	236.9 (227.2 to 247.3)	178.1 (165 to 192.2)
Serbia	Colon and rectum cancer	58.1 (53.2 to 64.5)	73.6 (71.2 to 76.2)	75.9 (70.3 to 82.2)	492.5 (451.9 to 543.4)	510.7 (493.6 to 528.6)	492.9 (454.9 to 534.5)
Serbia	Liver cancer	17.8 (15.2 to 20.9)	20 (19 to 20.9)	19.5 (17.6 to 21.4)	147.7 (126.4 to 171.7)	137.6 (130.8 to 144.3)	126.7 (114.5 to 139.2)
Serbia	Liver cancer due to hepatitis B	3.2 (2.42 to 4.15)	3.21 (2.6 to 3.93)	2.7 (2.11 to 3.35)	26.7 (20.7 to 34.1)	23.7 (19.5 to 28.8)	19.4 (15.3 to 23.6)
Serbia	Liver cancer due to hepatitis C	6.35 (4.96 to 7.95)	7.34 (6.22 to 8.47)	7.4 (6.13 to 8.7)	52.2 (41.4 to 64.5)	49.3 (41.8 to 57)	46.4 (38.5 to 54.4)
Serbia	Liver cancer due to alcohol use	5.93 (4.76 to 7.38)	6.76 (5.95 to 7.6)	6.71 (5.76 to 7.8)	48.9 (39.8 to 59.9)	46 (40.5 to 51.5)	43.4 (37.4 to 50.5)
Serbia	Liver cancer due to NASH	1.28 (1.02 to 1.61)	1.55 (1.33 to 1.82)	1.61 (1.34 to 1.91)	10.7 (8.7 to 13.4)	10.5 (9 to 12.3)	10.2 (8.5 to 12.1)
Serbia	Liver cancer due to other causes	1.08 (0.881 to 1.34)	1.1 (0.956 to 1.27)	1.06 (0.898 to 1.25)	9.2 (7.6 to 11.1)	8.1 (7 to 9.2)	7.3 (6.2 to 8.7)
Serbia	Gallbladder and biliary tract cancer	9.71 (7.67 to 11.4)	9.8 (8.23 to 11.2)	9.76 (8.06 to 11.3)	81.6 (65 to 95.8)	66.5 (55.4 to 75.7)	61.7 (51.2 to 71.1)
Serbia	Pancreatic cancer	21.2 (18.4 to 24)	28.8 (27.7 to 29.8)	31.4 (28.9 to 34)	175.6 (152.8 to 197.9)	199.9 (192.7 to 207.1)	204.6 (187.9 to 222.2)
Serbia	Larynx cancer	13.1 (11.5 to 15.6)	12.8 (12 to 13.5)	10.4 (9.31 to 11.7)	103.6 (91.2 to 122.6)	89.6 (84.1 to 94.8)	70.1 (62.3 to 79.4)
Serbia	Tracheal, bronchus, and lung cancer	130 (120 to 143)	155 (149 to 160)	154 (142 to 166)	1033.3 (958.4 to 1131.9)	1092.3 (1055.6 to 1131.5)	1023.5 (947.7 to 1106.4)
Serbia	Malignant skin melanoma	7.74 (6.15 to 9.73)	8.06 (5.8 to 9.14)	8.29 (5.77 to 9.72)	68.6 (54.4 to 86)	64.8 (45.6 to 73.4)	64.2 (42.8 to 75.6)
Serbia	Non-melanoma skin cancer	3.38 (2.87 to 3.75)	3.99 (3.8 to 4.21)	4.36 (4 to 4.74)	30.1 (25.6 to 33.3)	28.8 (27.4 to 30.2)	28.5 (26.2 to 30.9)
Serbia	Non-melanoma skin cancer (squamous-cell carcinoma)	3.38 (2.87 to 3.75)	3.99 (3.8 to 4.2)	4.35 (4 to 4.73)	30.1 (25.6 to 33.3)	28.8 (27.4 to 30.2)	28.5 (26.2 to 30.9)
Serbia	Non-melanoma skin cancer (basal-cell carcinoma)	0.00152 (0.000645 to 0.00321)	0.00193 (0.000828 to 0.00406)	0.00445 (0.00118 to 0.0111)	0 (0 to 0)	0 (0 to 0)	0 (0 to 0.1)
Serbia	Breast cancer	43.6 (37.2 to 51)	51.8 (49 to 55.3)	51.5 (45.7 to 58.3)	368.2 (316.6 to 427.2)	388.8 (368.3 to 415.7)	366.3 (323.8 to 415.4)
Serbia	Cervical cancer	22.4 (18.9 to 27.6)	21.9 (19.1 to 23.4)	17.6 (15.2 to 19.9)	196.8 (166.4 to 240.7)	174.1 (150.7 to 186.4)	135.1 (116.3 to 153.4)
Serbia	Uterine cancer	5.25 (4.34 to 6.3)	6.59 (6.09 to 7.12)	7.8 (6.81 to 8.9)	43.5 (36.2 to 52)	46.6 (43 to 50.4)	51.4 (44.7 to 58.6)
Serbia	Ovarian cancer	11.8 (9.78 to 13.6)	13.3 (12.6 to 14.1)	15 (13.2 to 16.8)	98.2 (81.8 to 112.1)	100.2 (94.6 to 106.3)	106.7 (94 to 119.2)
Serbia	Prostate cancer	13.5 (10.9 to 18.4)	20.5 (15.5 to 23)	22.1 (16.5 to 25.4)	123.7 (99.8 to 169.4)	129.8 (98.4 to 146.3)	128.6 (96.6 to 147.5)
Serbia	Testicular cancer	2.04 (1.68 to 2.51)	1.94 (1.69 to 2.22)	1.48 (1.21 to 1.83)	21.4 (17.5 to 26.6)	20.6 (17.7 to 23.7)	16.1 (13 to 20)
Serbia	Kidney cancer	7.94 (6.6 to 9.37)	11.1 (8.85 to 11.9)	11.6 (9.29 to 13)	68.7 (57.8 to 79.8)	80.6 (63.3 to 86.3)	79.6 (62.5 to 89.8)
Serbia	Bladder cancer	10.7 (7.9 to 12.5)	13.8 (10.9 to 14.8)	15.4 (11.2 to 17.5)	91.6 (68.2 to 104.9)	91.5 (71.9 to 98.2)	95 (68.7 to 108)
Serbia	Thyroid cancer	1.53 (1.24 to 1.85)	1.84 (1.53 to 2.02)	1.83 (1.53 to 2.09)	13 (10.7 to 15.7)	13.3 (11.1 to 14.6)	12.7 (10.6 to 14.5)
Serbia	Mesothelioma	0.698 (0.588 to 0.839)	1.03 (0.948 to 1.11)	0.935 (0.81 to 1.06)	5.8 (5 to 7)	7.8 (7.2 to 8.4)	6.9 (5.9 to 7.9)
Serbia	Hodgkin lymphoma	4.1 (2.73 to 5.17)	3.15 (1.9 to 3.75)	2.45 (1.53 to 3.02)	39.5 (26.1 to 50)	28.8 (17.4 to 34.7)	22.2 (13.6 to 28.1)
Serbia	Non-Hodgkin's lymphoma	9.13 (8.03 to 10.2)	10.6 (10.1 to 11.1)	11 (10 to 12)	84 (74.9 to 93.4)	85.1 (81.3 to 89.6)	83.6 (75.2 to 92.2)
Serbia	Multiple myeloma	3.04 (2.66 to 3.45)	4.64 (3.53 to 5.15)	5.21 (3.71 to 6.03)	24.7 (21.7 to 27.9)	32.2 (24.3 to 35.8)	34.4 (24 to 39.9)
Serbia	Leukaemia	20.8 (17.2 to 23.3)	19.8 (15.4 to 21.4)	18.2 (14.1 to 20.5)	204.7 (171.3 to 228.8)	168.4 (133 to 183.1)	146.2 (113.5 to 164.8)
Serbia	Acute lymphoid leukaemia	2.7 (1.98 to 3.55)	2.3 (1.76 to 2.73)	1.97 (1.46 to 2.33)	30.6 (21.4 to 41.5)	26 (20.6 to 31.6)	22 (16.4 to 27.2)

SR Appendix Table 2. All-age DALYs counts and age standardized DALYs rates per 100,000 individuals for all causes and level 1 cause groups by location for 1990, 2007, and 2017, both sexes combined							
		All-age DALYs			Age-standardized rate of DALYs per 100,000		
Serbia	Chronic lymphoid leukaemia	1.43 (1.11 to 1.84)	2.09 (1.54 to 2.45)	2.26 (1.59 to 2.71)	12.6 (9.9 to 16)	15.2 (11.2 to 17.7)	15.3 (10.9 to 18.3)
Serbia	Acute myeloid leukaemia	4.91 (3.82 to 5.81)	5.12 (3.74 to 5.9)	5.07 (3.63 to 6)	47.5 (37.1 to 56.6)	45.4 (32 to 52.7)	44 (30.1 to 52.3)
Serbia	Chronic myeloid leukaemia	0.839 (0.654 to 1.02)	1.07 (0.742 to 1.33)	0.857 (0.625 to 1.02)	7.8 (6.1 to 9.5)	8.5 (5.9 to 10.5)	6.5 (4.7 to 7.7)
Serbia	Other leukaemia	10.9 (8.1 to 12.9)	9.18 (6.88 to 10.2)	8.05 (6.14 to 9.25)	106.2 (80.8 to 126)	73.4 (55.5 to 82.5)	58.4 (46.1 to 66.9)
Serbia	Other malignant cancers	19.4 (16.4 to 22.2)	19.1 (16 to 20.6)	17.6 (14.6 to 19.6)	181.5 (153.7 to 206.3)	157.5 (131.8 to 171.5)	138.9 (115.4 to 155.6)
Serbia	Other neoplasms	2.58 (1.92 to 3.71)	4.21 (3.13 to 4.83)	5.48 (3.71 to 6.58)	24.5 (18.9 to 34.9)	32.3 (23.9 to 37)	38.8 (26.8 to 46.1)
Serbia	Myelodysplastic, myeloproliferative, and other hematopoietic neoplasms	2.35 (1.73 to 3.41)	4 (2.95 to 4.6)	5.27 (3.52 to 6.34)	21.7 (16.5 to 31.4)	29.6 (21.7 to 33.9)	35.9 (24.4 to 42.9)
Serbia	Benign and in situ intestinal neoplasms	0 (0 to 0)	0 (0 to 0)	0 (0 to 0)	0 (0 to 0)	0 (0 to 0)	0 (0 to 0)
Serbia	Benign and in situ cervical and uterine neoplasms	0 (0 to 0)	0 (0 to 0)	0 (0 to 0)	0 (0 to 0)	0 (0 to 0)	0 (0 to 0)
Serbia	Other benign and in situ neoplasms	0.234 (0.184 to 0.333)	0.211 (0.162 to 0.263)	0.216 (0.154 to 0.272)	2.8 (2.2 to 3.8)	2.7 (2.1 to 3.4)	2.9 (2 to 3.7)
Serbia	Cardiovascular diseases	987 (948 to 1030)	1110 (1080 to 1140)	991 (936 to 1050)	9019.9 (8687 to 9371.9)	7738.3 (7527 to 7933)	6300.5 (5944.8 to 6652.5)
Serbia	Rheumatic heart disease	6.21 (5.46 to 6.89)	4.27 (4.03 to 4.53)	2.95 (2.68 to 3.26)	55.7 (49.6 to 61.4)	30.7 (28.9 to 32.5)	19.6 (17.9 to 21.8)
Serbia	Ischaemic heart disease	453 (425 to 482)	493 (479 to 509)	439 (412 to 471)	4094.2 (3866.5 to 4336.5)	3483.7 (3390.6 to 3596.9)	2805.5 (2631 to 3007.8)
Serbia	Stroke	416 (388 to 446)	466 (445 to 488)	400 (373 to 429)	3826.2 (3593.1 to 4083.9)	3179.9 (3037.8 to 3328.5)	2494.2 (2324.5 to 2676.4)
Serbia	Ischaemic stroke	223 (206 to 242)	305 (288 to 322)	282 (259 to 304)	2122 (1961 to 2288.8)	2048.9 (1937.4 to 2162.1)	1711.1 (1575.7 to 1848.6)
Serbia	Intracerebral hemorrhage	150 (133 to 165)	124 (117 to 131)	88.1 (80.6 to 96.4)	1325.9 (1190.9 to 1457.5)	857.5 (813 to 904.4)	569 (520.1 to 621.7)
Serbia	Subarachnoid hemorrhage	42.8 (33.5 to 48.7)	36.7 (32.3 to 40)	30.5 (27.2 to 34.5)	378.3 (297.4 to 429.2)	273.5 (241.6 to 297.1)	214.2 (191 to 240.5)
Serbia	Hypertensive heart disease	22.9 (19.9 to 27.6)	37.1 (28 to 40)	32.9 (28.1 to 36.7)	217.6 (188.9 to 261.6)	262 (193.8 to 282.4)	206.4 (174.2 to 230.5)
Serbia	Non-rheumatic valvular heart disease	8.99 (7.77 to 10.5)	13.1 (10.8 to 14)	13.6 (11.3 to 15.2)	80 (69.3 to 94.1)	89.9 (75.4 to 96)	85.7 (72.1 to 95.1)
Serbia	Non-rheumatic calcific aortic valve disease	2.77 (2.19 to 3.4)	4.53 (3.27 to 5.14)	5.58 (3.92 to 6.56)	25 (20 to 30.5)	30.8 (22.6 to 34.7)	34.7 (24.7 to 40.5)
Serbia	Non-rheumatic degenerative mitral valve disease	5.93 (4.98 to 7.24)	8.23 (6.75 to 9.5)	7.68 (6.56 to 9.29)	52.3 (43.7 to 64.5)	56.8 (47.4 to 65.1)	48.4 (41.8 to 58.3)
Serbia	Other non-rheumatic valve diseases	0.282 (0.193 to 0.441)	0.301 (0.198 to 0.451)	0.371 (0.208 to 0.505)	2.6 (1.8 to 4.1)	2.3 (1.5 to 3.4)	2.7 (1.5 to 3.5)
Serbia	Cardiomyopathy and myocarditis	31.5 (24.3 to 38.5)	36.7 (30.6 to 44.2)	39.1 (33.5 to 45.1)	307 (241.1 to 368.1)	283.3 (237.3 to 334.7)	271.5 (229.2 to 311.5)
Serbia	Myocarditis	3.96 (2.25 to 5.38)	2.96 (2.2 to 3.56)	2.31 (1.59 to 3.78)	44.4 (23.9 to 61.4)	28 (21.1 to 33.9)	20.4 (15.3 to 29)
Serbia	Alcoholic cardiomyopathy	5.54 (3.61 to 8.21)	5.54 (3.4 to 10.3)	4.53 (2.74 to 8.39)	51 (33.2 to 78.3)	40.7 (25.3 to 73.4)	30.9 (19.4 to 53.3)
Serbia	Other cardiomyopathy	22 (15.3 to 27.8)	28.2 (19.2 to 35.9)	32.3 (23.8 to 39.2)	211.7 (152 to 260)	214.6 (150.9 to 266.7)	220.2 (164.9 to 263.4)
Serbia	Atrial fibrillation and flutter	9.86 (8.27 to 11.7)	12.3 (10.4 to 14.7)	15.1 (13 to 17.7)	94.5 (79.9 to 111.3)	87.8 (75.1 to 104)	94.2 (81.4 to 109.6)
Serbia	Aortic aneurysm	7.82 (6.5 to 9.23)	11 (8.66 to 12)	11.6 (8.78 to 13.4)	66.8 (56.7 to 78.6)	77.2 (60.7 to 83.6)	76 (57.3 to 87.7)
Serbia	Peripheral vascular disease	0.969 (0.585 to 1.5)	1.47 (0.968 to 2.17)	2.5 (1.58 to 3.47)	8.7 (5.2 to 13.5)	9.9 (6.6 to 14.6)	15.2 (9.6 to 20.9)
Serbia	Endocarditis	1.61 (1.4 to 1.89)	1.82 (1.69 to 1.96)	1.93 (1.74 to 2.16)	16.4 (14.1 to 19.6)	15.2 (14.1 to 16.5)	14.9 (13.3 to 16.8)
Serbia	Other cardiovascular and circulatory diseases	28.5 (24.7 to 33.3)	30.6 (27.4 to 34.7)	32.7 (28.3 to 37)	252.8 (220.8 to 295.8)	218.8 (195.8 to 246.1)	217.2 (186.6 to 247.2)
Serbia	Chronic respiratory diseases	120 (107 to 134)	131 (119 to 144)	129 (116 to 142)	1084.3 (967.5 to 1208)	949.2 (854 to 1046.1)	880.4 (782.8 to 978.2)
Serbia	Chronic obstructive pulmonary disease	90.5 (80.5 to 101)	107 (97.8 to 117)	106 (96.3 to 118)	800.8 (716.8 to 894.8)	738 (671.7 to 804.6)	680.9 (615 to 751.9)
Serbia	Pneumoconiosis	0.262 (0.213 to 0.321)	0.239 (0.2 to 0.288)	0.264 (0.219 to 0.317)	2.2 (1.8 to 2.7)	1.6 (1.4 to 2)	1.7 (1.4 to 2)
Serbia	Silicosis	0.131 (0.0978 to 0.171)	0.099 (0.0816 to 0.122)	0.0779 (0.06 to 0.112)	1.1 (0.8 to 1.4)	0.6 (0.5 to 0.8)	0.5 (0.4 to 0.7)
Serbia	Asbestosis	0.0157 (0.00908 to 0.0297)	0.0203 (0.0138 to 0.0319)	0.0269 (0.0193 to 0.0365)	0.1 (0.1 to 0.3)	0.1 (0.1 to 0.2)	0.2 (0.1 to 0.2)
Serbia	Coal workers pneumoconiosis	0.0302 (0.0179 to 0.0478)	0.0408 (0.0279 to 0.059)	0.0533 (0.0359 to 0.075)	0.3 (0.2 to 0.4)	0.3 (0.2 to 0.4)	0.3 (0.2 to 0.5)
Serbia	Other pneumoconiosis	0.0854 (0.0629 to 0.113)	0.0793 (0.0639 to 0.0984)	0.106 (0.0787 to 0.13)	0.8 (0.6 to 1)	0.5 (0.4 to 0.7)	0.7 (0.5 to 0.8)
Serbia	Asthma	24.2 (19.3 to 30.2)	17.9 (14.2 to 22.4)	16.2 (12.5 to 20.9)	235.2 (186.7 to 294.1)	160.3 (123.4 to 207.3)	145.4 (106.9 to 190.7)
Serbia	Interstitial lung disease and pulmonary sarcoidosis	3.06 (2.35 to 3.88)	3.32 (2.69 to 4.11)	3.49 (2.81 to 4.3)	27.1 (21 to 34.2)	25.1 (20.3 to 31.2)	25.1 (20.1 to 31)

SR Appendix Table 2. All-age DALYs counts and age standardized DALYs rates per 100,000 individuals for all causes and level 1 cause groups by location for 1990, 2007, and 2017, both sexes combined							
		All-age DALYs			Age-standardized rate of DALYs per 100,000		
Serbia	Other chronic respiratory diseases	1.99 (1.64 to 2.33)	2.52 (2.13 to 2.9)	2.79 (2.34 to 3.21)	19.1 (16 to 22.5)	24.1 (20.3 to 28.1)	27.3 (22.7 to 32.1)
Serbia	Digestive diseases	125 (105 to 143)	121 (110 to 134)	114 (101 to 128)	1124.8 (953.2 to 1293.6)	959.7 (856.6 to 1077)	872.7 (763.7 to 998.8)
Serbia	Cirrhosis and other chronic liver diseases	47.6 (39 to 55)	42.3 (39.5 to 48.3)	36.7 (33 to 42)	397.9 (328.1 to 455.4)	324.1 (302.5 to 372.3)	274.1 (245.8 to 316.4)
Serbia	Cirrhosis and other chronic liver diseases due to hepatitis B	13 (10.3 to 15.6)	11.3 (9.89 to 13.3)	8.53 (7.27 to 10.1)	108.8 (86.6 to 130)	86.7 (76.3 to 102.9)	64.2 (54.7 to 75.7)
Serbia	Cirrhosis and other chronic liver diseases due to hepatitis C	10.3 (8.1 to 12.6)	9.08 (7.83 to 10.9)	7.94 (6.76 to 9.44)	85.1 (66.7 to 103.3)	69.2 (59.7 to 83)	59.4 (50.3 to 71.2)
Serbia	Cirrhosis and other chronic liver diseases due to alcohol use	18.1 (14.5 to 21.4)	16.6 (15.1 to 19.3)	15.2 (13.4 to 17.9)	148 (119.3 to 173.5)	124.8 (113.5 to 146.5)	111.6 (98.3 to 132.2)
Serbia	Cirrhosis due to NASH	3.08 (2.43 to 3.78)	2.93 (2.56 to 3.47)	2.79 (2.42 to 3.33)	25 (19.9 to 30.3)	21.9 (19.2 to 26.3)	20.5 (17.7 to 24.8)
Serbia	Cirrhosis and other chronic liver diseases due to other causes	3.12 (2.54 to 3.81)	2.44 (2.04 to 2.94)	2.2 (1.8 to 2.68)	31 (25.8 to 36.6)	21.4 (18.2 to 25.1)	18.4 (15.2 to 21.7)
Serbia	Upper digestive system diseases	32 (24.8 to 41.6)	32.4 (25.8 to 41.9)	29.9 (23.5 to 38.7)	300.2 (229.9 to 391.1)	265.6 (205.9 to 351.8)	238.3 (180.4 to 318)
Serbia	Peptic ulcer disease	15.2 (12.5 to 18)	15.7 (14.3 to 17.4)	14.1 (12.5 to 16)	133.6 (111.2 to 157.1)	110.9 (101.5 to 121.9)	92.8 (82.9 to 104.6)
Serbia	Gastritis and duodenitis	8.44 (5.51 to 12.2)	7.67 (5.17 to 10.9)	6.85 (4.62 to 9.6)	88.2 (56.9 to 127.7)	76.3 (50.3 to 110.2)	67.1 (44.6 to 95.9)
Serbia	GERD	8.39 (4.47 to 14.1)	8.95 (4.73 to 15.1)	8.94 (4.78 to 15)	78.3 (41.8 to 132.8)	78.4 (41.8 to 132.5)	78.4 (41.8 to 133)
Serbia	Appendicitis	1.54 (1.29 to 1.81)	1.14 (1 to 1.27)	0.919 (0.799 to 1.04)	15.2 (12.9 to 17.5)	9.7 (8.5 to 11)	7.7 (6.5 to 8.9)
Serbia	Paralytic ileus and intestinal obstruction	8.56 (6.45 to 10.1)	8.17 (4.75 to 9.47)	9.02 (4.71 to 11.1)	85.2 (68.8 to 98.4)	61.7 (39.2 to 70.4)	61.7 (34.9 to 74.7)
Serbia	Inguinal, femoral, and abdominal hernia	8.26 (5.95 to 10.8)	8.26 (6.19 to 10.8)	8.27 (6.07 to 10.8)	82.1 (59.1 to 108.4)	78.1 (56.7 to 106.4)	77.4 (55.5 to 103.9)
Serbia	Inflammatory bowel disease	3.78 (2.94 to 4.73)	4.59 (3.72 to 5.52)	5.01 (3.86 to 6.08)	35.6 (28.1 to 44.7)	38.4 (30.8 to 46.9)	40.6 (31.5 to 50.2)
Serbia	Vascular intestinal disorders	6.65 (4.5 to 8)	7.56 (4.84 to 8.73)	6.76 (4.63 to 7.76)	58.7 (39.6 to 70.1)	49.9 (32.8 to 57)	41.6 (28.9 to 47.6)
Serbia	Gallbladder and biliary diseases	2.64 (2.17 to 3.14)	3.21 (2.78 to 3.64)	3.65 (3.05 to 4.26)	24.3 (20.1 to 28.4)	22 (19.3 to 24.7)	22.8 (19.1 to 26.2)
Serbia	Pancreatitis	10.6 (7.75 to 13.2)	10.1 (7.55 to 11.5)	9.82 (7.07 to 11.7)	93.6 (69.6 to 115.7)	78.3 (59.8 to 89.3)	73.1 (54.5 to 87.4)
Serbia	Other digestive diseases	3.04 (2.41 to 3.72)	3.7 (3.19 to 4.23)	4.39 (3.79 to 5.02)	32 (25.2 to 39.7)	31.9 (26.9 to 37)	35.3 (30 to 41.3)
Serbia	Neurological disorders	156 (123 to 197)	173 (139 to 213)	186 (152 to 226)	1607.5 (1282.4 to 2027)	1553.6 (1221.1 to 1950.4)	1549.7 (1206.4 to 1953)
Serbia	Alzheimer's disease and other dementias	41.2 (37.4 to 44.9)	59.1 (55.3 to 63.3)	75.7 (69.9 to 82.2)	445.3 (404.7 to 485.5)	458.2 (429.1 to 487.9)	482.4 (445.9 to 522.6)
Serbia	Parkinson's disease	7.71 (6.67 to 8.91)	11.6 (10.2 to 12.8)	12.9 (11.2 to 14.3)	78.1 (67.5 to 90.4)	75.8 (66.1 to 82.7)	75.8 (65.8 to 84.1)
Serbia	Epilepsy	20.1 (12 to 31.6)	16.7 (10.3 to 25.6)	14.7 (7.18 to 25.8)	220.6 (135.7 to 342.3)	173.8 (106.3 to 266.7)	152.9 (72.5 to 270)
Serbia	Multiple sclerosis	4.46 (3.65 to 5.2)	4.59 (3.82 to 5.16)	4.6 (3.7 to 5.29)	40.5 (32.8 to 47.2)	40 (32.8 to 45.4)	40.1 (31.7 to 46.5)
Serbia	Motor neuron disease	0.252 (0.115 to 0.322)	0.206 (0.116 to 0.23)	0.268 (0.122 to 0.31)	2.1 (1.1 to 2.7)	1.6 (1 to 1.8)	2 (1 to 2.3)
Serbia	Headache disorders	74.3 (47.9 to 109)	73.9 (47.9 to 108)	71.2 (45.9 to 105)	730.2 (471.8 to 1069.7)	732.7 (476.5 to 1079.4)	730.7 (471.9 to 1075.2)
Serbia	Migraine	63.5 (39.5 to 96)	63 (39 to 95)	60.7 (37.9 to 91.4)	625.9 (387.4 to 945.8)	628 (388.4 to 946.3)	626.3 (388.2 to 945.1)
Serbia	Tension-type headache	10.7 (6.15 to 17)	10.8 (6.2 to 16.8)	10.6 (6.03 to 16.6)	104.3 (59.6 to 164.5)	104.7 (59 to 163.7)	104.4 (59.4 to 163.2)
Serbia	Other neurological disorders	7.77 (6.25 to 9.69)	6.77 (5.71 to 8.15)	6.27 (4.92 to 8.12)	90.6 (72.1 to 113.9)	71.5 (59.1 to 88.4)	65.6 (48.3 to 90)
Serbia	Mental disorders	138 (103 to 179)	138 (102 to 177)	132 (98.3 to 170)	1373.1 (1022.1 to 1781.9)	1354.9 (1006.5 to 1747.3)	1339.3 (989.4 to 1731.8)
Serbia	Schizophrenia	12.7 (9.34 to 15.9)	12.8 (9.55 to 15.8)	12.7 (9.5 to 15.8)	119.3 (88 to 150.4)	119.5 (89.2 to 148.8)	120.6 (90 to 150.7)
Serbia	Depressive disorders	46.2 (32.8 to 62.4)	47 (33.2 to 63.3)	45.1 (31.7 to 60.2)	440.2 (312.2 to 598)	416.9 (294.1 to 564.2)	401.5 (283.8 to 539.9)
Serbia	Major depressive disorder	37.1 (25.9 to 49.9)	37.4 (26.3 to 50.5)	35.6 (24.9 to 47.6)	355.4 (248.6 to 480.3)	331.9 (234.2 to 450.1)	316.7 (222.6 to 425.6)
Serbia	Dysthymia	9.07 (6.14 to 13.2)	9.57 (6.46 to 13.8)	9.52 (6.37 to 13.9)	84.8 (57.2 to 123.4)	85 (57.5 to 123.5)	84.8 (57 to 124.2)
Serbia	Bipolar disorder	13.6 (8.7 to 20.2)	13.6 (8.58 to 20.3)	13.1 (8.28 to 19.3)	135.3 (85.9 to 201.1)	135.3 (85.2 to 202)	135.1 (85.4 to 201.8)
Serbia	Anxiety disorders	30.7 (21.8 to 41.2)	30.9 (22 to 40.5)	29.8 (21.3 to 39.3)	308.4 (218.5 to 412.2)	310 (219.6 to 409.7)	307.9 (219 to 408.6)
Serbia	Eating disorders	3.12 (1.98 to 4.64)	2.92 (1.84 to 4.28)	3.03 (1.91 to 4.48)	34.3 (21.7 to 50.9)	34.5 (21.7 to 50.5)	38 (24 to 56.2)
Serbia	Anorexia nervosa	0.602 (0.352 to 0.925)	0.561 (0.321 to 0.875)	0.562 (0.327 to 0.874)	6.9 (4 to 10.5)	6.9 (3.9 to 10.7)	7.5 (4.4 to 11.8)
Serbia	Bulimia nervosa	2.52 (1.57 to 3.81)	2.36 (1.46 to 3.53)	2.46 (1.53 to 3.64)	27.5 (16.9 to 41.5)	27.6 (17.1 to 41.4)	30.4 (18.8 to 45.2)

SR Appendix Table 2. All-age DALYs counts and age standardized DALYs rates per 100,000 individuals for all causes and level 1 cause groups by location for 1990, 2007, and 2017, both sexes combined							
		All-age DALYs			Age-standardized rate of DALYs per 100,000		
Serbia	Autism spectrum disorders	6.09 (4.15 to 8.35)	5.8 (3.98 to 8.13)	5.6 (3.8 to 7.75)	66.3 (45.3 to 90.9)	66.8 (45.7 to 93.5)	67.4 (45.5 to 93.3)
Serbia	Attention-deficit/hyperactivity disorder	1.02 (0.608 to 1.62)	0.915 (0.541 to 1.46)	0.854 (0.521 to 1.35)	11.7 (6.9 to 18.6)	11.7 (6.9 to 18.8)	11.8 (7.2 to 18.9)
Serbia	Conduct disorder	7.97 (4.75 to 12.6)	6.61 (3.96 to 10.5)	5.89 (3.52 to 9.38)	97.5 (58.2 to 153.9)	97.9 (58.8 to 155.5)	98.6 (58.9 to 156.7)
Serbia	Idiopathic developmental intellectual disability	1.85 (0.566 to 3.57)	1.94 (0.706 to 3.55)	1.48 (0.414 to 2.85)	20.5 (6.4 to 39.5)	22.9 (8.4 to 41.8)	18.7 (5.6 to 35.5)
Serbia	Other mental disorders	14.6 (9.65 to 20.1)	15 (10 to 20.6)	14.9 (9.94 to 20.7)	139.4 (92 to 193.4)	139.3 (91.9 to 191.6)	139.6 (92.5 to 194.8)
Serbia	Substance use disorders	44.2 (35.5 to 53.9)	46.2 (37.4 to 55.9)	42.4 (33.6 to 52.2)	423.7 (339.8 to 520.9)	440.3 (352.7 to 537.2)	414.6 (327.3 to 514.6)
Serbia	Alcohol use disorders	32.8 (25.8 to 41.1)	32.1 (25.8 to 39.7)	30.1 (23.7 to 38.1)	303.6 (237 to 384)	288.8 (226.6 to 362.7)	279.7 (215.2 to 361.7)
Serbia	Drug use disorders	11.5 (8.93 to 14)	14.1 (11.4 to 17.1)	12.3 (9.64 to 15)	120.1 (93.8 to 147.1)	151.5 (123 to 183.1)	134.9 (105.7 to 166.6)
Serbia	Opioid use disorders	8.61 (6.52 to 10.9)	10.6 (8.41 to 13.2)	9.26 (6.98 to 11.7)	89.4 (68 to 113.4)	113.4 (89.4 to 140.6)	100.7 (75.8 to 128.3)
Serbia	Cocaine use disorders	0.26 (0.158 to 0.393)	0.274 (0.172 to 0.405)	0.264 (0.166 to 0.393)	2.7 (1.6 to 4.1)	2.9 (1.8 to 4.3)	3 (1.8 to 4.6)
Serbia	Amphetamine use disorders	0.426 (0.249 to 0.656)	0.505 (0.324 to 0.754)	0.421 (0.267 to 0.637)	4.8 (2.8 to 7.5)	5.9 (3.7 to 8.8)	5.3 (3.3 to 8.1)
Serbia	Cannabis use disorders	0.524 (0.317 to 0.787)	0.504 (0.319 to 0.734)	0.415 (0.265 to 0.617)	5.8 (3.5 to 8.7)	5.8 (3.7 to 8.5)	5.2 (3.3 to 7.8)
Serbia	Other drug use disorders	1.64 (1.31 to 2.01)	2.19 (1.66 to 2.6)	1.91 (1.52 to 2.29)	17.4 (13.9 to 21.5)	23.5 (17.3 to 28.3)	20.8 (16.2 to 25.4)
Serbia	Diabetes and kidney diseases	140 (119 to 165)	185 (157 to 218)	188 (160 to 221)	1227.2 (1050.3 to 1442.6)	1319.6 (1113.7 to 1570.5)	1269.3 (1064.2 to 1512.3)
Serbia	Diabetes mellitus	103 (84.2 to 126)	139 (114 to 170)	140 (114 to 171)	887.5 (726.9 to 1081.6)	993.9 (809.2 to 1223.2)	955.6 (768.2 to 1184)
Serbia	Diabetes mellitus type 1	21.2 (18.5 to 24.1)	22.2 (18.2 to 24)	20.3 (15.1 to 22.8)	183.4 (162.4 to 207.1)	161.2 (133.4 to 174.2)	138.5 (106.8 to 155)
Serbia	Diabetes mellitus type 2	81.8 (63.3 to 104)	117 (91.5 to 146)	120 (93.7 to 151)	704.1 (544.4 to 895.4)	832.7 (646.4 to 1052)	817.1 (627.9 to 1051.9)
Serbia	Chronic kidney disease	37.4 (33.7 to 40.7)	45.7 (40.4 to 49)	47.6 (41.6 to 51.7)	339.3 (307.5 to 367.7)	325.4 (292.3 to 349.2)	313.6 (278.5 to 340.8)
Serbia	Chronic kidney disease due to diabetes mellitus type 1	2.48 (1.89 to 3.21)	2.52 (1.98 to 3.23)	2.33 (1.79 to 3.04)	21.4 (16.7 to 27)	19.6 (15.5 to 24.5)	17.6 (13.8 to 22.5)
Serbia	Chronic kidney disease due to diabetes mellitus type 2	6.43 (5.22 to 7.91)	8.31 (6.77 to 10)	8.89 (7.08 to 11)	55.4 (45.2 to 66.8)	54.9 (45.2 to 65.8)	54.3 (43.5 to 66.3)
Serbia	Chronic kidney disease due to hypertension	6.84 (5.59 to 8.13)	10.4 (8.34 to 12.2)	11.9 (9.4 to 14.3)	62.4 (51.3 to 73.7)	71.2 (58 to 82.9)	74.2 (59.8 to 88.8)
Serbia	Chronic kidney disease due to glomerulonephritis	8.03 (6.82 to 9.35)	9.13 (7.7 to 10.7)	8.97 (7.2 to 10.7)	72.8 (62.7 to 84.2)	68.2 (58.4 to 78.7)	63.1 (52.2 to 73.9)
Serbia	Chronic kidney disease due to other and unspecified causes	13.6 (11.6 to 15.8)	15.4 (13.1 to 17.7)	15.5 (13 to 18.4)	127.3 (110.6 to 146.3)	111.5 (96.5 to 127.6)	104.3 (88.1 to 121.7)
Serbia	Acute glomerulonephritis	0.0514 (0.0384 to 0.07)	0.0285 (0.0249 to 0.033)	0.0151 (0.0126 to 0.0183)	0.5 (0.4 to 0.6)	0.2 (0.2 to 0.3)	0.1 (0.1 to 0.1)
Serbia	Skin and subcutaneous diseases	44.2 (30.2 to 63.7)	43.7 (29.7 to 63.6)	43.2 (29.4 to 63.2)	480 (325.6 to 696.3)	484.3 (327.8 to 698.4)	489.5 (330.7 to 716.5)
Serbia	Dermatitis	10.8 (6.3 to 17.2)	10.2 (6.07 to 15.8)	9.79 (5.84 to 15.2)	122.8 (70.8 to 196.5)	122.7 (71.6 to 195.2)	122.9 (71.7 to 195.4)
Serbia	Atopic dermatitis	8.5 (4.61 to 14.1)	7.77 (4.26 to 12.9)	7.45 (4.09 to 12.4)	99.7 (53.7 to 166.2)	99.6 (54.2 to 165.8)	99.8 (54.3 to 166.3)
Serbia	Contact dermatitis	2.32 (1.52 to 3.43)	2.37 (1.56 to 3.45)	2.33 (1.53 to 3.43)	23 (14.9 to 34)	23 (15.1 to 33.8)	23 (14.9 to 34.1)
Serbia	Seborrhoeic dermatitis	0.0102 (0.00568 to 0.0165)	0.0102 (0.00567 to 0.0164)	0.00998 (0.00552 to 0.016)	0.1 (0.1 to 0.2)	0.1 (0.1 to 0.2)	0.1 (0.1 to 0.2)
Serbia	Psoriasis	7.1 (4.93 to 9.39)	7.41 (5.24 to 9.76)	7.45 (5.27 to 9.75)	67.8 (47.2 to 89.8)	69 (49 to 90.5)	70.1 (49.8 to 91.8)
Serbia	Bacterial skin diseases	1.05 (0.79 to 1.67)	1.1 (0.722 to 1.52)	1.3 (0.761 to 1.65)	10.6 (8.2 to 18.2)	9.5 (6.7 to 13.5)	10.6 (6.7 to 13.5)
Serbia	Cellulitis	0.554 (0.387 to 0.869)	0.704 (0.424 to 0.894)	0.831 (0.465 to 1.06)	5.3 (3.8 to 8.6)	5.9 (3.7 to 7.5)	6.5 (3.9 to 8.3)
Serbia	Pyoderma	0.492 (0.347 to 0.857)	0.395 (0.266 to 0.649)	0.472 (0.264 to 0.658)	5.3 (3.7 to 9.6)	3.6 (2.6 to 6.1)	4 (2.5 to 5.8)
Serbia	Scabies	2.54 (1.41 to 4.13)	2.33 (1.3 to 3.78)	2.12 (1.17 to 3.41)	28.7 (15.9 to 47)	28.1 (15.7 to 46)	27.1 (14.9 to 44.1)
Serbia	Fungal skin diseases	4.51 (1.78 to 9.33)	4.88 (1.96 to 10.1)	4.97 (2 to 10.3)	46.2 (18.4 to 96)	45.3 (18 to 93.5)	44.6 (17.9 to 92.5)
Serbia	Viral skin diseases	3.61 (2.32 to 5.4)	3.03 (1.92 to 4.52)	2.85 (1.82 to 4.23)	45.8 (29.5 to 68.3)	45.8 (28.9 to 67.4)	45.8 (29.2 to 67.7)
Serbia	Acne vulgaris	2.45 (1.44 to 3.96)	2.65 (1.56 to 4.26)	2.53 (1.51 to 4.05)	28.2 (16.5 to 45.7)	32.9 (19.4 to 53.7)	35.8 (21.1 to 58.1)
Serbia	Alopecia areata	0.584 (0.373 to 0.881)	0.569 (0.366 to 0.853)	0.553 (0.354 to 0.839)	5.9 (3.8 to 9)	5.9 (3.8 to 8.9)	5.9 (3.8 to 9)
Serbia	Pruritus	0.957 (0.453 to 1.78)	1 (0.478 to 1.9)	1.01 (0.484 to 1.87)	9.5 (4.5 to 17.7)	9.5 (4.4 to 18.2)	9.6 (4.6 to 18.1)
Serbia	Urticaria	6.48 (4.27 to 9.15)	5.93 (3.97 to 8.34)	5.66 (3.8 to 7.93)	75 (49.6 to 106.3)	74.9 (49.1 to 106.4)	74.7 (49.8 to 105.7)

SR Appendix Table 2. All-age DALYs counts and age standardized DALYs rates per 100,000 individuals for all causes and level 1 cause groups by location for 1990, 2007, and 2017, both sexes combined

		All-age DALYs			Age-standardized rate of DALYs per 100,000		
Serbia	Decubitus ulcer	0.34 (0.258 to 0.5)	0.596 (0.449 to 0.846)	0.71 (0.504 to 0.981)	3.4 (2.6 to 4.9)	4.6 (3.5 to 6.4)	4.9 (3.5 to 6.8)
Serbia	Other skin and subcutaneous diseases	3.75 (1.85 to 6.78)	4.09 (2.02 to 7.49)	4.28 (2.12 to 7.77)	36.2 (17.8 to 65.6)	36.3 (17.8 to 66.5)	37.4 (18.4 to 68.2)
Serbia	Sense organ diseases	81.3 (54.5 to 118)	94.6 (63.4 to 136)	98.2 (65.8 to 140)	759.1 (508.7 to 1099)	746 (500.5 to 1082.2)	733.3 (490.3 to 1061.4)
Serbia	Blindness and vision impairment	33.5 (21.9 to 50.2)	37.8 (24.8 to 55.8)	38.5 (25.2 to 56.4)	312.1 (201.9 to 464.4)	300.5 (194 to 445.6)	291.2 (188.6 to 432.7)
Serbia	Glaucoma	0.425 (0.275 to 0.605)	0.534 (0.348 to 0.776)	0.552 (0.357 to 0.785)	3.7 (2.5 to 5.3)	3.5 (2.3 to 5.1)	3.3 (2.1 to 4.7)
Serbia	Cataract	10.8 (7.25 to 14.9)	14.1 (9.48 to 19.2)	15.1 (10.1 to 20.8)	99.5 (67.6 to 136)	96.5 (64.7 to 130.8)	93.4 (63.3 to 128)
Serbia	Age-related macular degeneration	0.098 (0.062 to 0.144)	0.129 (0.0814 to 0.189)	0.145 (0.0913 to 0.214)	0.9 (0.5 to 1.3)	0.9 (0.6 to 1.3)	0.9 (0.6 to 1.3)
Serbia	Refraction disorders	11 (6.97 to 16.9)	11 (7.06 to 16.6)	10.7 (6.85 to 16.2)	109.1 (69.2 to 166.6)	105.6 (66.8 to 162)	102.9 (64.9 to 156.5)
Serbia	Near vision loss	8.59 (4.13 to 15.8)	9.15 (4.37 to 16.6)	9.07 (4.34 to 16.7)	75.3 (36 to 139.1)	71 (33.8 to 129.2)	68.3 (32.7 to 125)
Serbia	Other vision loss	2.6 (1.75 to 3.62)	2.96 (1.97 to 4.1)	3.03 (2.03 to 4.23)	23.7 (16 to 32.7)	23 (15.3 to 31.8)	22.4 (14.9 to 31)
Serbia	Age-related and other hearing loss	44.4 (30.2 to 63.1)	52.9 (36.3 to 74.5)	55.6 (38 to 78.2)	416.2 (285.3 to 587.6)	414.4 (283.8 to 582.8)	410.8 (280 to 578.9)
Serbia	Other sense organ diseases	3.32 (2.1 to 4.86)	3.87 (2.41 to 5.59)	4.07 (2.51 to 5.94)	30.8 (19.3 to 44.7)	31.1 (19.4 to 44.7)	31.3 (19.2 to 46.1)
Serbia	Musculoskeletal disorders	201 (145 to 271)	220 (161 to 295)	222 (161 to 297)	1830.4 (1320.3 to 2470.3)	1840.9 (1338 to 2490.6)	1831.5 (1338.3 to 2480.7)
Serbia	Rheumatoid arthritis	3.17 (2.3 to 4.2)	3.98 (2.94 to 5.09)	4.26 (3.11 to 5.56)	27.6 (20.1 to 36.3)	30.5 (22.4 to 39)	31.6 (22.7 to 41.5)
Serbia	Osteoarthritis	11.2 (5.55 to 21.9)	14 (7.11 to 27.4)	15.3 (7.67 to 30.1)	95.6 (47.6 to 186.6)	99 (49.9 to 193.8)	101.9 (51 to 200.3)
Serbia	Low back pain	147 (104 to 199)	158 (113 to 214)	160 (115 to 216)	1352.9 (951.9 to 1831.7)	1353.4 (972.7 to 1834.9)	1355.4 (967.3 to 1831.2)
Serbia	Neck pain	33.1 (23.1 to 46.9)	36.4 (25.4 to 51)	36.5 (25.5 to 50.9)	295.7 (206.7 to 412.4)	296.4 (206.2 to 416.3)	295.9 (206.5 to 417.1)
Serbia	Gout	1.41 (0.926 to 1.93)	1.71 (1.14 to 2.38)	1.83 (1.25 to 2.51)	12.1 (8 to 16.6)	12.4 (8.3 to 17.3)	12.7 (8.7 to 17.5)
Serbia	Other musculoskeletal disorders	4.99 (3.2 to 7.24)	5.61 (3.88 to 7.9)	3.92 (2.54 to 5.8)	46.5 (30.6 to 66.7)	49.2 (34 to 68.3)	34 (21.4 to 50.6)
Serbia	Other non-communicable diseases	200 (171 to 234)	105 (81.1 to 134)	90.5 (68.5 to 118)	2652.4 (2289 to 3045)	1197 (972.1 to 1475.6)	971.4 (763.7 to 1232.8)
Serbia	Congenital anomalies	130 (112 to 149)	38.9 (31.8 to 46.6)	28.8 (22.5 to 35.7)	1951.9 (1654.8 to 2246.5)	605.6 (513.1 to 702.9)	429.8 (354.6 to 518.9)
Serbia	Neural tube defects	4.64 (3.16 to 6.88)	0.883 (0.694 to 1.11)	0.812 (0.625 to 1.04)	71.5 (47.6 to 107.4)	14.6 (11.4 to 19.1)	14.1 (10.6 to 18.8)
Serbia	Congenital heart anomalies	61.6 (32.2 to 78.4)	10.9 (9.4 to 12.8)	5.66 (4.52 to 7.16)	969.9 (499.4 to 1240.4)	220.2 (187.1 to 261.9)	122.4 (96 to 158)
Serbia	Orofacial clefts	0.403 (0.258 to 0.607)	0.268 (0.174 to 0.411)	0.224 (0.146 to 0.334)	5.1 (3.1 to 8)	3.2 (2 to 4.8)	2.7 (1.8 to 4.1)
Serbia	Down's syndrome	7.92 (2.43 to 12.6)	1.71 (1.37 to 2.12)	0.972 (0.754 to 1.34)	119.4 (33.8 to 193.8)	28.4 (22.5 to 36.5)	15.7 (11.9 to 23.6)
Serbia	Turner syndrome	0.00819 (0.00406 to 0.0138)	0.00788 (0.0039 to 0.0128)	0.00702 (0.00348 to 0.0114)	0.1 (0 to 0.2)	0.1 (0 to 0.2)	0.1 (0 to 0.2)
Serbia	Klinefelter syndrome	0.00209 (0.000948 to 0.0041)	0.00201 (0.00092 to 0.00389)	0.00187 (0.00087 to 0.00366)	0 (0 to 0)	0 (0 to 0)	0 (0 to 0)
Serbia	Other chromosomal abnormalities	3.1 (1.99 to 5.96)	1.18 (0.816 to 1.96)	1.05 (0.728 to 1.68)	46.6 (29 to 93.2)	21 (14.1 to 38.5)	19.3 (13 to 33.5)
Serbia	Congenital musculoskeletal and limb anomalies	16.6 (11.7 to 22.1)	15 (10.1 to 20.3)	14.2 (9.55 to 19.4)	187.1 (134.1 to 248.7)	165.9 (114.5 to 223.3)	159.6 (109.1 to 217.2)
Serbia	Urogenital congenital anomalies	1.05 (0.653 to 1.82)	0.374 (0.287 to 0.545)	0.311 (0.224 to 0.443)	15.7 (9.4 to 28.1)	6.4 (4.7 to 10.1)	5.4 (3.7 to 8.6)
Serbia	Digestive congenital anomalies	9.39 (4.88 to 13.4)	2.87 (2.07 to 3.68)	2.16 (1.56 to 2.87)	139.7 (67.6 to 200.8)	45.6 (29.7 to 57)	32.9 (23.5 to 42.7)
Serbia	Other congenital anomalies	25.7 (11 to 39.8)	5.68 (4.75 to 6.78)	3.38 (2.5 to 4.62)	397 (162.2 to 622.6)	100.3 (83.8 to 119.3)	57.5 (41.6 to 84)
Serbia	Urinary diseases and male infertility	9.55 (7.52 to 12)	10 (7.78 to 12.8)	9.86 (7.53 to 12.7)	85 (68.3 to 105.3)	73.1 (56.7 to 93.4)	68 (51.8 to 87.4)
Serbia	Urinary tract infections	2.97 (2.56 to 3.31)	2.39 (2.13 to 2.87)	2.2 (1.88 to 2.7)	29.2 (24.9 to 32.5)	18.3 (16.3 to 21.8)	15.8 (13.5 to 19)
Serbia	Urolithiasis	0.579 (0.426 to 0.757)	0.643 (0.484 to 0.83)	0.654 (0.488 to 0.849)	5.3 (3.8 to 6.9)	5.2 (3.8 to 6.8)	5.2 (3.8 to 6.9)
Serbia	Benign prostatic hyperplasia	4.62 (2.94 to 6.69)	5.39 (3.46 to 7.72)	5.77 (3.72 to 8.29)	36.9 (23.6 to 53.5)	36.5 (23.4 to 52.2)	37.2 (24 to 53.6)
Serbia	Male infertility	0.194 (0.0765 to 0.403)	0.183 (0.0735 to 0.385)	0.155 (0.0631 to 0.331)	2.1 (0.8 to 4.3)	2.1 (0.8 to 4.4)	1.8 (0.7 to 3.9)
Serbia	Other urinary diseases	1.18 (0.953 to 1.57)	1.41 (0.994 to 1.64)	1.08 (0.757 to 1.31)	11.6 (9.3 to 15.6)	11 (8 to 12.7)	8 (5.9 to 9.6)
Serbia	Gynecological diseases	13.2 (9.02 to 18.5)	12.8 (8.68 to 17.9)	11.9 (8.15 to 16.7)	134.8 (92.6 to 189.6)	134 (91.1 to 186.7)	131.4 (90.6 to 183.5)
Serbia	Uterine fibroids	1.96 (1.12 to 3.25)	1.93 (1.09 to 3.23)	1.69 (0.938 to 2.88)	17.9 (10.2 to 29.9)	17.4 (9.8 to 29)	15.7 (8.7 to 26.8)

SR Appendix Table 2. All-age DALYs counts and age standardized DALYs rates per 100,000 individuals for all causes and level 1 cause groups by location for 1990, 2007, and 2017, both sexes combined

		All-age DALYs			Age-standardized rate of DALYs per 100,000		
Serbia	Polycystic ovarian syndrome	0.47 (0.21 to 0.888)	0.459 (0.202 to 0.888)	0.43 (0.189 to 0.826)	4.9 (2.2 to 9.4)	5 (2.2 to 9.7)	5 (2.2 to 9.7)
Serbia	Female infertility	0.155 (0.0497 to 0.358)	0.143 (0.0453 to 0.326)	0.168 (0.0542 to 0.388)	1.7 (0.5 to 3.8)	1.6 (0.5 to 3.7)	2 (0.6 to 4.6)
Serbia	Endometriosis	3.57 (2.3 to 5.29)	3.42 (2.27 to 5.05)	3.2 (2.1 to 4.57)	38.1 (24.5 to 56.2)	38.3 (25.4 to 56.5)	37.7 (24.5 to 53.8)
Serbia	Genital prolapse	0.415 (0.204 to 0.77)	0.351 (0.175 to 0.645)	0.357 (0.179 to 0.671)	3.5 (1.7 to 6.4)	2.6 (1.3 to 4.7)	2.5 (1.3 to 4.7)
Serbia	Premenstrual syndrome	4.81 (3.07 to 7.3)	4.67 (2.98 to 6.97)	4.23 (2.7 to 6.32)	50.7 (32.4 to 77)	50.7 (32.3 to 75.2)	49.3 (31.4 to 73.7)
Serbia	Other gynecological diseases	1.79 (1.22 to 2.5)	1.83 (1.23 to 2.67)	1.78 (1.2 to 2.65)	17.9 (12.3 to 25.1)	18.4 (12.5 to 27.2)	19.2 (12.9 to 28.3)
Serbia	Hemoglobinopathies and hemolytic anaemias	4.98 (3.89 to 6.46)	3.89 (2.98 to 5.26)	3.2 (2.5 to 4.24)	51.8 (40.6 to 67.3)	36.4 (27.1 to 51)	29.1 (21.5 to 40.4)
Serbia	Thalassemias	0.14 (0.0847 to 0.228)	0.0312 (0.0211 to 0.0494)	0.0164 (0.0104 to 0.0292)	2.1 (1.3 to 3.5)	0.6 (0.4 to 1)	0.3 (0.2 to 0.6)
Serbia	Thalassaemias trait	0.954 (0.582 to 1.46)	0.778 (0.484 to 1.25)	0.624 (0.373 to 0.957)	10.2 (6.4 to 15.6)	8.4 (5.1 to 13.7)	6.8 (4 to 10.7)
Serbia	Sickle cell disorders	0.303 (0.229 to 0.439)	0.14 (0.114 to 0.192)	0.103 (0.0825 to 0.137)	4 (2.9 to 5.9)	1.9 (1.5 to 2.7)	1.4 (1.1 to 1.9)
Serbia	Sickle cell trait	0.461 (0.268 to 0.72)	0.427 (0.253 to 0.731)	0.306 (0.171 to 0.511)	5.1 (3 to 7.9)	4.8 (2.8 to 8.3)	3.5 (1.9 to 6)
Serbia	G6PD deficiency	0.393 (0.315 to 0.48)	0.334 (0.271 to 0.4)	0.252 (0.2 to 0.31)	4 (3.3 to 4.9)	3.1 (2.6 to 3.8)	2.3 (1.9 to 2.9)
Serbia	G6PD trait	0.000336 (0.000221 to 0.000491)	0.000419 (0.000249 to 0.000711)	0.000285 (0.000173 to 0.000456)	0 (0 to 0)	0 (0 to 0)	0 (0 to 0)
Serbia	Other hemoglobinopathies and hemolytic anaemias	2.73 (2.1 to 3.53)	2.17 (1.68 to 2.83)	1.9 (1.49 to 2.44)	26.4 (20.4 to 34.1)	17.6 (13.3 to 23.5)	14.7 (11.3 to 19.7)
Serbia	Endocrine, metabolic, blood, and immune disorders	9.74 (8.18 to 11.7)	7.53 (6.25 to 8.82)	6.97 (5.68 to 8.34)	110 (89 to 135.1)	77.1 (64.7 to 90.5)	69 (56.5 to 82.8)
Serbia	Oral disorders	28.3 (17.9 to 42.3)	30.6 (19.4 to 45.8)	29.1 (18.5 to 43.3)	257.6 (161.7 to 385.9)	245.4 (153.1 to 368)	227.6 (141.8 to 340.8)
Serbia	Caries of deciduous teeth	0.179 (0.0773 to 0.346)	0.142 (0.0605 to 0.28)	0.128 (0.0548 to 0.254)	2.6 (1.1 to 5)	2.7 (1.1 to 5.3)	2.6 (1.1 to 5.1)
Serbia	Caries of permanent teeth	2.68 (1.17 to 5.05)	2.67 (1.15 to 5.01)	2.39 (1.03 to 4.54)	27.5 (12 to 52)	27.9 (12 to 52.7)	26.1 (11.1 to 49.3)
Serbia	Periodontal disease	5.23 (2.08 to 10.9)	5.27 (2.08 to 10.9)	4.93 (1.92 to 10)	45.8 (18.2 to 94.5)	42.2 (16.6 to 87.1)	38.5 (15.2 to 79)
Serbia	Edentulism and severe tooth loss	15.1 (10 to 21.4)	17.3 (11.6 to 24.7)	16.6 (11.1 to 23.5)	131.1 (87.4 to 185.5)	121.7 (81.3 to 173.8)	109.8 (73.3 to 155)
Serbia	Other oral disorders	5.13 (3.22 to 7.6)	5.17 (3.24 to 7.62)	5.04 (3.15 to 7.42)	50.7 (31.8 to 74.8)	50.8 (31.7 to 75.4)	50.7 (31.8 to 74.9)
Serbia	Sudden infant death syndrome	3.82 (1.7 to 8.84)	1.14 (0.744 to 1.7)	0.665 (0.388 to 1.04)	61.2 (27.2 to 141.7)	25.5 (16.7 to 38)	16.5 (9.6 to 25.8)
Serbia	Injuries	408 (362 to 463)	331 (285 to 387)	304 (252 to 370)	4161.3 (3718.2 to 4691.5)	3117.3 (2711.7 to 3613.9)	2778.6 (2323.9 to 3356.1)
Serbia	Transport injuries	105 (94.6 to 117)	75.7 (68.8 to 84.1)	56 (48.9 to 64.6)	1113.7 (992.3 to 1226.5)	760.4 (696.4 to 834.1)	553.7 (490.9 to 630.9)
Serbia	Road injuries	96.9 (86.1 to 108)	69.1 (62.5 to 76.9)	51 (44.3 to 59.2)	1023.6 (899.9 to 1131.3)	694.8 (634.3 to 764.8)	505 (445.8 to 578.4)
Serbia	Pedestrian road injuries	27.4 (24 to 31.8)	15.6 (13.9 to 17.7)	11.5 (9.72 to 13.6)	293.6 (257.3 to 341.8)	152.3 (137 to 172.6)	106.2 (90.6 to 126.7)
Serbia	Cyclist road injuries	10.2 (8.31 to 12.4)	9.57 (7.72 to 11.6)	8.68 (6.85 to 10.8)	100.1 (81.9 to 121.7)	88.8 (72 to 107.2)	78.8 (62.3 to 97.8)
Serbia	Motorcyclist road injuries	12.9 (10.7 to 15.4)	9.11 (7.98 to 10.5)	6.29 (5.28 to 7.41)	138.4 (112.2 to 166.3)	96.4 (85.1 to 110)	68.3 (56.7 to 80.5)
Serbia	Motor vehicle road injuries	45 (38.9 to 50.3)	33.7 (30.3 to 37.2)	23.7 (20.8 to 27.1)	476.8 (412.1 to 535)	346.5 (314.6 to 378.4)	243.3 (215.8 to 275.7)
Serbia	Other road injuries	1.46 (1.07 to 1.84)	1.16 (0.892 to 1.5)	0.914 (0.692 to 1.21)	14.7 (10.6 to 18.5)	10.7 (8.4 to 13.7)	8.3 (6.4 to 10.9)
Serbia	Other transport injuries	8.6 (7.43 to 10.1)	6.65 (6.11 to 7.24)	4.98 (4.4 to 5.66)	90.2 (77.4 to 106.2)	65.5 (60.2 to 71.3)	48.8 (42.7 to 55.8)
Serbia	Unintentional injuries	193 (156 to 239)	166 (130 to 212)	180 (136 to 237)	1938.3 (1588.4 to 2378.9)	1503.8 (1186.8 to 1915.6)	1581.8 (1207.2 to 2078.8)
Serbia	Falls	94.2 (69.6 to 126)	98.3 (72.2 to 132)	117 (85.9 to 160)	898.6 (665.8 to 1202.9)	854.9 (626.5 to 1158.7)	995.6 (724.5 to 1353.6)
Serbia	Drowning	14.6 (13.1 to 16.3)	7.14 (6.7 to 7.6)	4.44 (4.03 to 4.91)	172 (152.7 to 194.9)	81.8 (75.9 to 88)	50.5 (45.6 to 56.4)
Serbia	Fire, heat, and hot substances	10.3 (7.85 to 12.7)	7.37 (5.91 to 9.31)	6.56 (4.97 to 8.62)	108.6 (80.6 to 134.4)	69.5 (55.7 to 87.5)	59.7 (45.5 to 78.9)
Serbia	Poisonings	5.31 (4.05 to 6.59)	3.84 (3.03 to 4.9)	3.77 (2.81 to 5.09)	57.7 (43.6 to 72)	40.1 (31.4 to 51.5)	39.8 (29.3 to 54)
Serbia	Poisoning by carbon monoxide	2.16 (1.53 to 2.66)	1.32 (1.15 to 1.65)	1.09 (0.907 to 1.45)	23.4 (16.2 to 29.5)	13.5 (11.8 to 16.7)	11 (9 to 15)
Serbia	Poisoning by other means	3.15 (2.27 to 4.12)	2.52 (1.8 to 3.46)	2.68 (1.82 to 3.87)	34.3 (24.6 to 44.8)	26.6 (18.9 to 36.8)	28.8 (19.5 to 41.5)
Serbia	Exposure to mechanical forces	30 (23.5 to 38.3)	24 (18.3 to 31.6)	24.6 (18 to 33.1)	292.5 (231.4 to 371.6)	217.3 (167.5 to 282.5)	220.6 (163.2 to 297.7)
Serbia	Unintentional firearm injuries	4.22 (3.42 to 5.03)	2.2 (1.93 to 2.61)	1.56 (1.33 to 1.92)	44.5 (36 to 53)	21.7 (19 to 26.9)	15.3 (12.9 to 20.1)

SR Appendix Table 2. All-age DALYs counts and age standardized DALYs rates per 100,000 individuals for all causes and level 1 cause groups by location for 1990, 2007, and 2017, both sexes combined							
		All-age DALYs			Age-standardized rate of DALYs per 100,000		
Serbia	Other exposure to mechanical forces	25.8 (19.5 to 33.7)	21.8 (16.2 to 29.2)	23 (16.6 to 31.5)	248.1 (191.1 to 322.4)	195.6 (146.6 to 260.3)	205.3 (148.5 to 279.8)
Serbia	Adverse effects of medical treatment	5.02 (3.55 to 6.18)	3.46 (2.77 to 3.91)	3 (2.54 to 3.45)	52.1 (36.4 to 63)	30.8 (25.1 to 34.4)	25.2 (21.6 to 29.5)
Serbia	Animal contact	2.18 (1.71 to 2.77)	1.88 (1.45 to 2.39)	1.62 (1.25 to 2.08)	22.3 (17.3 to 28.1)	18.3 (14.1 to 23.1)	15.9 (12.1 to 20.6)
Serbia	Venomous animal contact	1.36 (1.01 to 1.78)	1.16 (0.857 to 1.52)	1.01 (0.733 to 1.33)	13.9 (10.3 to 18.2)	11.7 (8.5 to 15.3)	10.3 (7.4 to 13.7)
Serbia	Non-venomous animal contact	0.818 (0.618 to 1.1)	0.711 (0.544 to 0.969)	0.617 (0.471 to 0.848)	8.4 (6.3 to 11.2)	6.6 (5.1 to 8.9)	5.6 (4.3 to 7.7)
[truncated: 863,917 more chars]
